# Profiling invasive *Plasmodium falciparum* merozoites using an integrated omics approach

**DOI:** 10.1038/s41598-017-17505-9

**Published:** 2017-12-07

**Authors:** Krishan Kumar, Prakash Srinivasan, Michael J. Nold, J. Kathleen Moch, Karine Reiter, Dan Sturdevant, Thomas D. Otto, R. Burke Squires, Raul Herrera, Vijayaraj Nagarajan, Julian C. Rayner, Stephen F. Porcella, Scott J. Geromanos, J. David Haynes, David L. Narum

**Affiliations:** 1 Laboratory of Malaria Immunology and Vaccinology, NIAID, NIH, Rockville, MD USA; 2 Laboratory of Malaria and Vector Research, NIAID, NIH, Rockville, MD USA; 3 Waters Corporation, Milford, MA USA; 4 Walter Reed Army Institute of Research, Silver Spring, MD USA; 5 Genomics Unit, Research Technologies Section, Rocky Mountain Laboratories, NIAID, NIH, Hamilton, MT USA; 6 Wellcome Trust Sanger Institute, Hinxton, Cambridge, CB10 1SA United Kingdom; 7 Computational Biology Section, Bioinformatics and Computational Biosciences Branch, NIAID, NIH, Bethesda, MD USA; 8 Present Address: Johns Hopkins Malaria Research Institute, Department of Molecular Microbiology and Immunology, Johns Hopkins Bloomberg School of Public Health, Baltimore, MD USA

## Abstract

The symptoms of malaria are brought about by blood-stage parasites, which are established when merozoites invade human erythrocytes. Our understanding of the molecular events that underpin erythrocyte invasion remains hampered by the short-period of time that merozoites are invasive. To address this challenge, a *Plasmodium falciparum* gamma-irradiated long-lived merozoite (LLM) line was developed and investigated. Purified LLMs invaded erythrocytes by an increase of 10–300 fold compared to wild-type (WT) merozoites. Using an integrated omics approach, we investigated the basis for the phenotypic difference. Only a few single nucleotide polymorphisms within the *P*. *falciparum* genome were identified and only marginal differences were observed in the merozoite transcriptomes. By contrast, using label-free quantitative mass-spectrometry, a significant change in protein abundance was noted, of which 200 were proteins of unknown function. We determined the relative molar abundance of over 1100 proteins in LLMs and further characterized the major merozoite surface protein complex. A unique processed MSP1 intermediate was identified in LLM but not observed in WT suggesting that delayed processing may be important for the observed phenotype. This integrated approach has demonstrated the significant role of the merozoite proteome during erythrocyte invasion, while identifying numerous unknown proteins likely to be involved in invasion.

## Introduction

Merozoites have been a focus of malaria vaccine research and development for nearly a half a century. A strong basis for this interest has focused on original work by Cohen *et al*.^1^ and reproduced by Druilhe *et al*.^2^ who demonstrated that passive transfer of IgG from individuals hyper-immune to *P*. *falciparum* to children clinically ill from *P*. *falciparum* malaria conferred protection against severe disease. Human immunoglobulins against parasite proteins including those involved in red blood cell (RBC) invasion^3^ and presumptively against members of the PfEMP1 family involved in sequestration of parasitized erythrocytes^4^ are both likely to have contributed to this clinical outcome. From the many known proteins involved in erythrocyte invasion, only a few have been assessed in human phase 1 studies examining safety and immunogenicity^5–9^ and only three candidates have been evaluated in human phase 2 biological efficacy trials^10–12^ including controlled human malaria infection studies^13,14^. Unfortunately, only limited or no significant protective benefit has been observed in these studies.

In the 1970s, it was discovered that the merozoites of *P*. *knowlesi*, a simian malaria parasite, could be purified using cell-sieving techniques and the resultant merozoites were invasive with an observed half-life of approximately twenty minutes^15,16^. This discovery aided in a number of significant observations involving ultra-structural analyses of the merozoite and mechanistic studies of erythrocyte invasion (see review Bannister *et al*.^17^). Interestingly, *P*. *knowlesi* has since been identified as the fifth malaria parasite to infect humans^18^. These early studies with *P*. *knowlesi*, and subsequent studies involving *P*. *falciparum* without purified invasive merozoites, and other apicomplexan parasites have facilitated our current understanding of erythrocyte invasion by merozoites as a well-orchestrated and rapid process (approx. 1 min.) that relies on precise signaling and multiple receptor-ligand interactions^19,20^.

In the past four decades’ efforts to study cell-sieve purified *P*. *falciparum* merozoites like those of *P*. *knowlesi* have failed to produce significant numbers of invasive merozoites. Methods for isolating *P*. *falciparum* merozoites have been reported previously but their invasive properties remained to explored^21^. Alternative strategies have been developed to evaluate merozoite invasion using drug treated *P*. *falciparum* parasites and differential centrifugation to enrich for populations of invasive merozoites that may still contain mature intra-erythrocytic parasites^22^. Boyle *et al*.^23^ used centrifugation to study how heparin-like molecules block *P*. *falciparum* merozoite invasion. Singh *et al*.^24^ used a similar technique to analyze the role that Ca+2 flux plays in merozoites during erythrocyte invasion. It may also be possible to develop methods to isolate invasive merozoites by regulating key events required for schizont maturation^25^ which may help by preventing premature activation. Enhanced molecular and structural biology tools also have improved our mechanistic understanding of known invasion ligands such as the merozoite surface protein-1 complex^26^, the erythrocyte binding proteins (EBA) EBA-175 and EBA-140^27,28^, and apical membrane antigen-1^20,29^. However, many proteins remain within the *P*. *falciparum* proteome, and in particular the merozoite proteome, with unknown functions that may be involved in erythrocyte invasion.

To identify novel proteins likely involved in invasion as well as improve our basic understanding of erythrocyte invasion, an integrated omics approach was used to study a γ-irradiated parasite line that was selected for long-lived merozoite (LLM) phenotype and that was amenable to cell-sieve purification for the isolation of viable merozoites as compared to the parental wild-type (WT) parasite line. While limited changes were observed in the open reading frames of the LLM genome and in the LLM transcriptome relative to WT, label-free quantitative proteomics identified unique changes in protein abundance of over 400 known and unknown proteins in LLM relative to WT. Using this label-free quantitative approach, we established for the first time the relative quantitation of a merozoite proteome that included over 1,100 proteins. Of interest was an observation of an apparent delayed processing of two merozoite proteins in the merozoite surface protein complex of the LLM compared to WT merozoites, which highlights the importance of protein stability and its relationship to erythrocyte invasion.

## Results

### Cell-sieving and collection of long-lived merozoites

**Figure 1:**
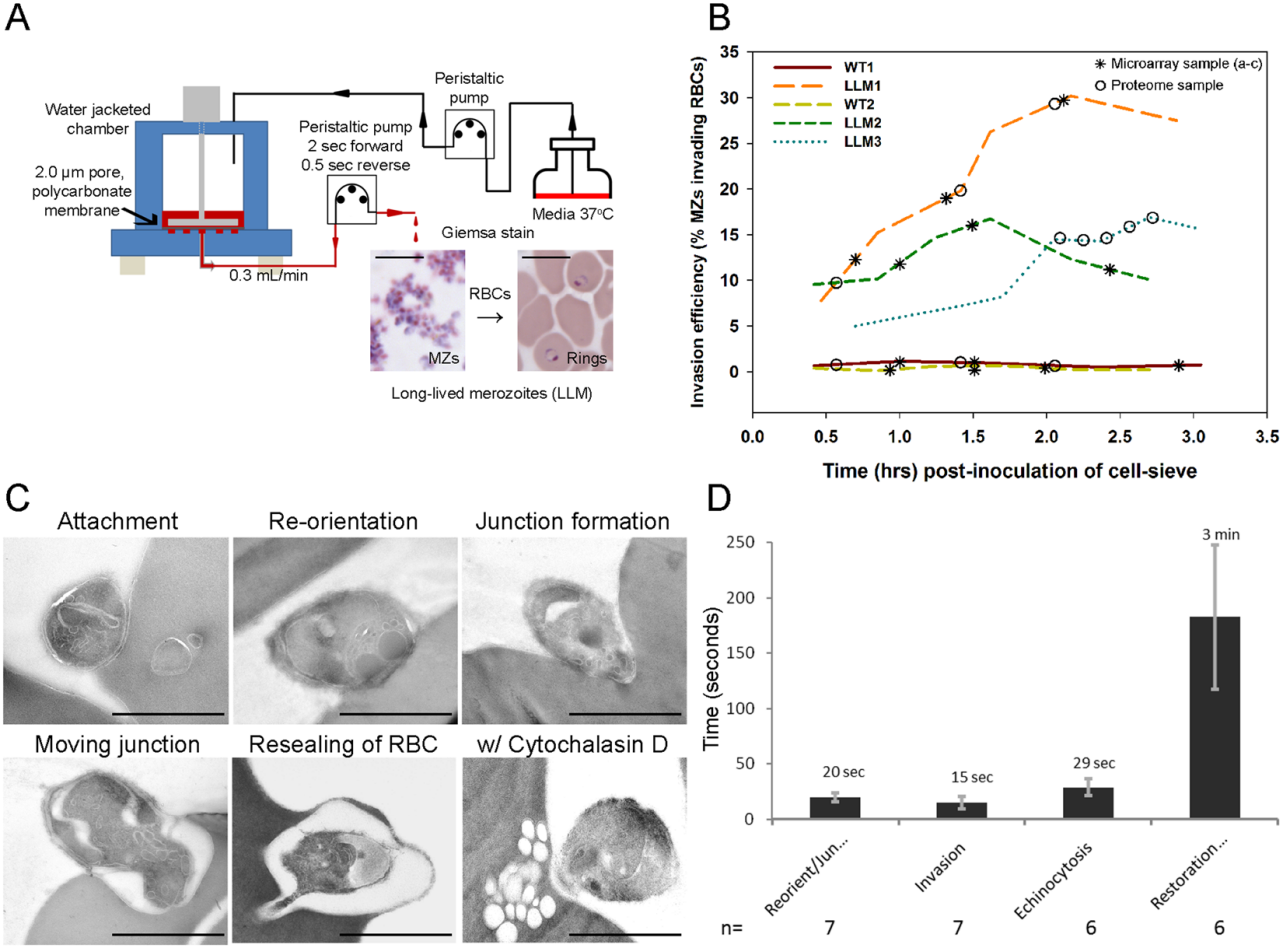
Cell-sieve purification and erythrocyte invasion of purified long-lived merozoites (LLM) and wild-type (WT) merozoites. (**A)** Schematic of cell-sieving process that yields purified, invasive merozoites. (**B**) Invasion efficiency of various lots of LLM and WT merozoites as well as the identification of the source of merozoite material used in the characterization of the transcriptome, proteome or both. (**C**) Electron microscopic observation illustrating the various stages of invasion using purified LLM merozoites. (**D**) Quantitation of the invasion dynamics using live cell imaging. The number of individual events observed for each step is indicated below (n) and the error bars represent standard deviation (SD). “Time post-inoculation of cell-sieve” refers to sequential batches of merozoites that are collected in the effluent post cell-sieving. Abbreviation “Jun”: junction formation. Scale bars, in panel A: 10 µM and panel C: 1 µm.

*P*. *falciparum* long-lived merozoites were purified using a cell-sieving apparatus as described in Fig. 1A that permitted continuous culture of purified schizonts in a temperature and gas controlled chamber overlaid on a polycarbonate membrane with 2 µm pores. A continuous forward or reverse flow of media was controlled electronically with a peristaltic pump while the culture was stirred with an overhead impeller. The net egress of media was 0.3 mL/minute. Maturing, segmented schizonts were retained by the membrane while free merozoites passed through the 2 µm pores (Fig. 1A, Giemsa stain). Merozoites were collected in the effluent at room temperature (~22 °C) in batches of pooled merozoites at approximately 10–15 minute intervals. Merozoites were collected for transcriptomic or proteomic studies as shown in Fig. 1B. On a population basis, the invasion efficiency of the LLMs ranged from 8–30% (Fig. 1B), indicating that the LLM phenotype had a prolonged viability. This was also confirmed by direct examination by electron microscopy of invading LLM (Fig. 1C). Live cell imaging of LLM invasion indicated that the invasion kinetics were similar to what was previously reported for other *P*. *falciparum* strains (Fig. 1D). Furthermore, merozoites from this parasite line were used previously to study the role of the AMA1-RON2 interaction during invasion^29^. It is worthy to note that the variation in the invasion efficiencies could reflect the differences in the maturation stage of the schizonts placed in the cell sieve despite efforts to control this by tightly synchronizing the cultures. Having established that a significant proportion of the purified merozoites from this selected line retain invasiveness, samples were collected for transcriptomic or proteomic studies to identify the molecular determinants of these invasive merozoites^30^.

### Comparison of the WT and LLM genomes

To establish whether changes in invasion efficiency in LLM parasites were due to genomic changes, we generated genome sequences of the WT and LLM parasites using Illumina Next Generation Sequencing. We first generated a genome sequence for the WT FVO line, using bioinformatic approaches to iteratively convert the 3D7 genome into a FVO genome using the reads from the WT line (see Materials and Methods). This is essential because the reference *P*. *falciparum* genome is derived from the 3D7 strain, which is of African origin and is likely to differ significantly from the FVO strain, which is of Southeast Asian origin. Mapping reads from either the WT or LLM parasites against 3D7 would introduce mapping errors, confounding our ability to call variants specific to one line or the other. Once an FVO WT genome had been generated, the LLM reads were mapped against it, and variants called (see Methods) for all open reading frames, excluding regions of high variability corresponding to genes associated with PfEMP1, rifins and stevors and non-coding regions. The γ-irradiated selected LLM parasites had only two single nucleotide polymorphisms (SNPs) in the open reading frames of those proteins evaluated in LLM versus WT. The gene ID: PF3D7_1007200, which encodes for a putative rhoGAP GTPase, was mutated at nucleotide position 1123 from AAA:TAA which introduced a stop codon at amino acid position 375 thus eliminating about 35% of the C-terminal end of the native protein. As a result, the putative rhoGAP GTPase was terminated within the carboxyl-terminus of the putative rho GTPase-activating protein domain. In the gene encoding PF3D7_1361800, a conserved protein with an unknown function that is expressed during schizogony, a gene mutation corresponding to an amino acid substitution R to I was noted at nucleotide position 7454 AGA:ATA. While uncharacterized, PF3D7_1361800 contains Armadillo like repeats found in another *P*. *falciparum* invasion associated protein that localizes to the rhoptry organelles^31^. The homologue of this gene appears to be essential for asexual growth in a genome-scale genetic screen in *P*. *berghei* ^32^. Given the timing of expression and apparent essentiality in a related *Plasmodium* species, this gene and its encoded protein warrants further investigation for a role in *P*. *falciparum* merozoite invasion. There was also one reversion (Gene ID: PF3D7_0700900, position 341 TGG:TGA) observed in the pseudogene encoding a RESA-like protein (see Supplemental Dataset S1). The open reading frame (ORF) for the WT genome sequences were used to develop a protein database for use in subsequent proteomic analysis.

### Comparison of the WT and LLM transcriptomes

**Figure 2:**
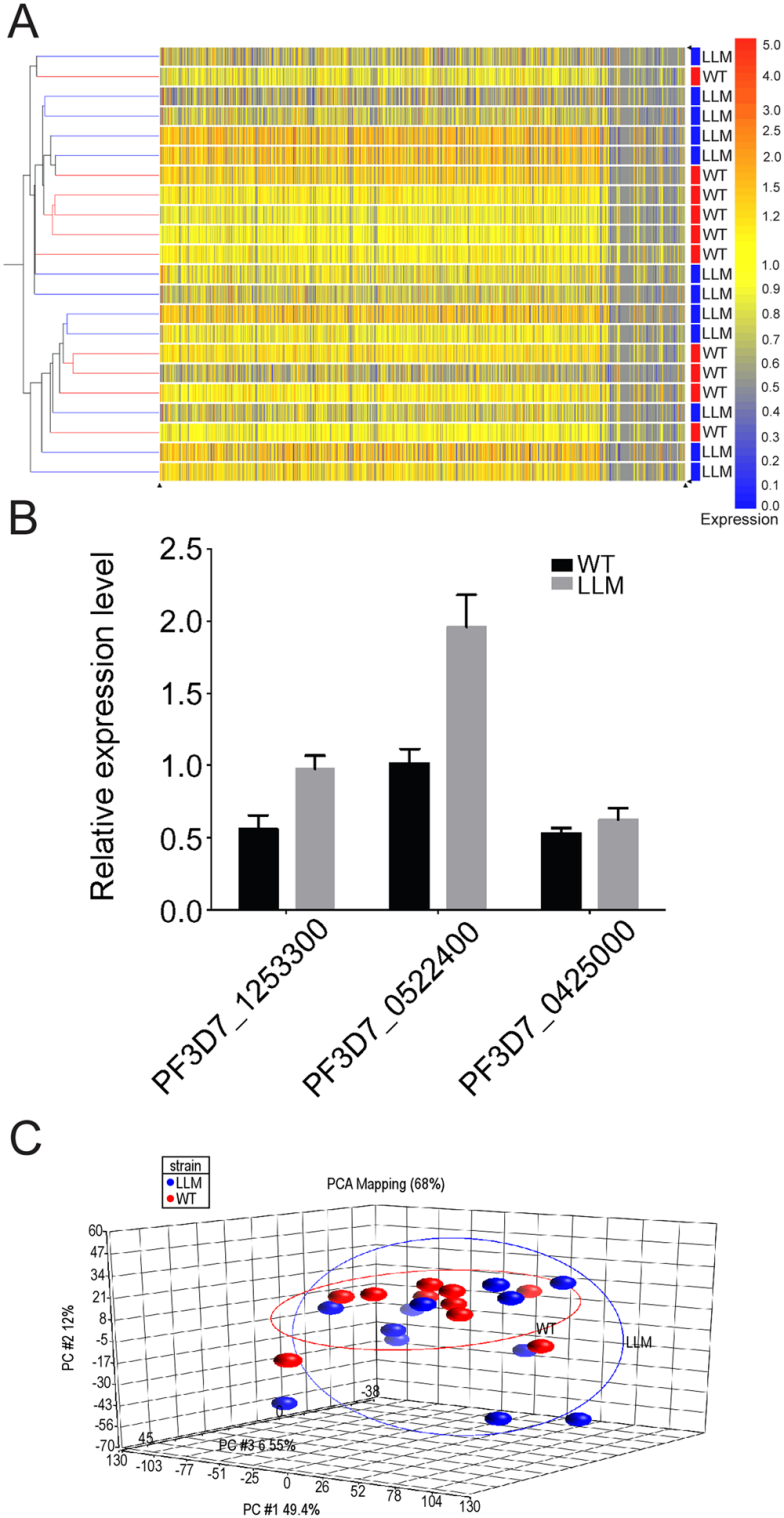
Transcriptional analysis comparing cell-sieve purified WT and invasive LLMs (10 × 12, respectively) on an Affymetrix chip. (**A**) Heat map showing transcriptional analysis of WT and LLM when evaluated using three pairs of biological replicates comprised of three or four technical replicates (includes samples shown in Fig. 1B, WT1 & 2, and LLM1 & 2). (**B**) Confirmation of transcriptional changes by QPCR for three transcripts identified in the transcriptional analysis to be significantly different (p < 0.05). (**C**) PCA showing clustering of WT and LLM biological and technical replicates. Error bars in panel B represent standard error of the mean.

The transcriptomes of WT and LLMs were compared by microarray on commercial Affymetrix chips using three or four biological replicates, which are separate unique biological samples collected from a single experiment. The samples were taken from a single experiment at the beginning, middle and end of optimal RBC invasion. In Fig. 1B, an example of the sampling times for two of the biological replicates are shown. Even though individual merozoite samples were collected at defined intervals throughout the length of each collection, the timing of the individual sample collection was not considered in the analysis. Minimal significant changes were identified as shown in the heat map comparing the individual biological and technical replicates (Fig. 2A and Supplemental Dataset S2). Only three *P*. *falciparum* mRNAs (PF3D7_0425000, PF3D7_0522400 and PF3D7_1253300) analyzed both by DNA microarray and QPCR method were significantly correlated *(*p < 0.05) (Fig. 2B). *PF3D7_0425000* codes for a stevor-like exported protein and *PF3D7_1253300* codes for a PHISTa family protein that is unlikely to be involved in invasion. *PF3D7_0522400* codes for a ~112 kDa protein containing two membrane spanning regions and is present in all *Plasmodium* species. Unfortunately, none of these proteins were identified in the proteomic studies described below. An analysis of the 3D7 sequence arrayed on the chip and WT for gene coverage demonstrated a conservation of about 97% (data not shown). The limited differences observed between WT and LLM transcriptomes (Fig. 2A and C) may suggest post-transcriptional or translational effects involved in the regulation of the observed invasion phenotype in LLM.

Due to the limited differences observed for the purified merozoites and to better understand whether transcriptional differences could be detected, a study was performed comparing WT to LLM synchronized early schizonts (n ≈ 4 nuclei) that controlled for many experimental variables including time, temperature and method. One set of biological replicates was compared with five technical replicates. The samples were analyzed on an Affymetrix GeneChip *Plasmodium/Anopheles* containing the *P*. *falciparum* genome; no proteomic study was performed in parallel due to technical issues created by hemolysis of RBCs. After establishing a reasonable correlation within the cell cycle of the WT and LLM early schizonts (3 hours) (Supplemental Fig. S2), which appears similar with respect to the age range described by Lemieux *et al*.^33^ the transcription profiles of WT and LLM early schizonts were assessed. In contrast to the observations made for merozoites, early schizonts were observed to contain transcriptional differences (Supplemental Fig. S3A and B, and numerical data in Supplemental Dataset S3). Although RNAs for many of the merozoite genes encoding proteins involved in merozoite invasion of RBCs were not transcribed during this early stage of schizont development, we could assess the transcript abundance for the merozoite surface protein-1 (MSP) protein complex (MSP1, MSP6 and MSP7), which demonstrated no significant difference for MSP7. A lower transcript abundance for MSP6 in LLM (~2-fold, p < 0.001) and a higher transcript abundance of MSP1 (~2-fold, p < 0.001) in LLM (Supplemental Dataset S3) was discovered. Further study will be required to determine if these observed changes in transcript abundance in early schizonts (n ≈ 4 nuclei) affects the levels of protein and therefore their downstream function in free merozoites. It is clear from previous studies that transcript levels are not necessarily correlative with protein translation and abundance^34,35^.

### Quantitative proteomics of WT vs LLM merozoites

**Figure 3:**
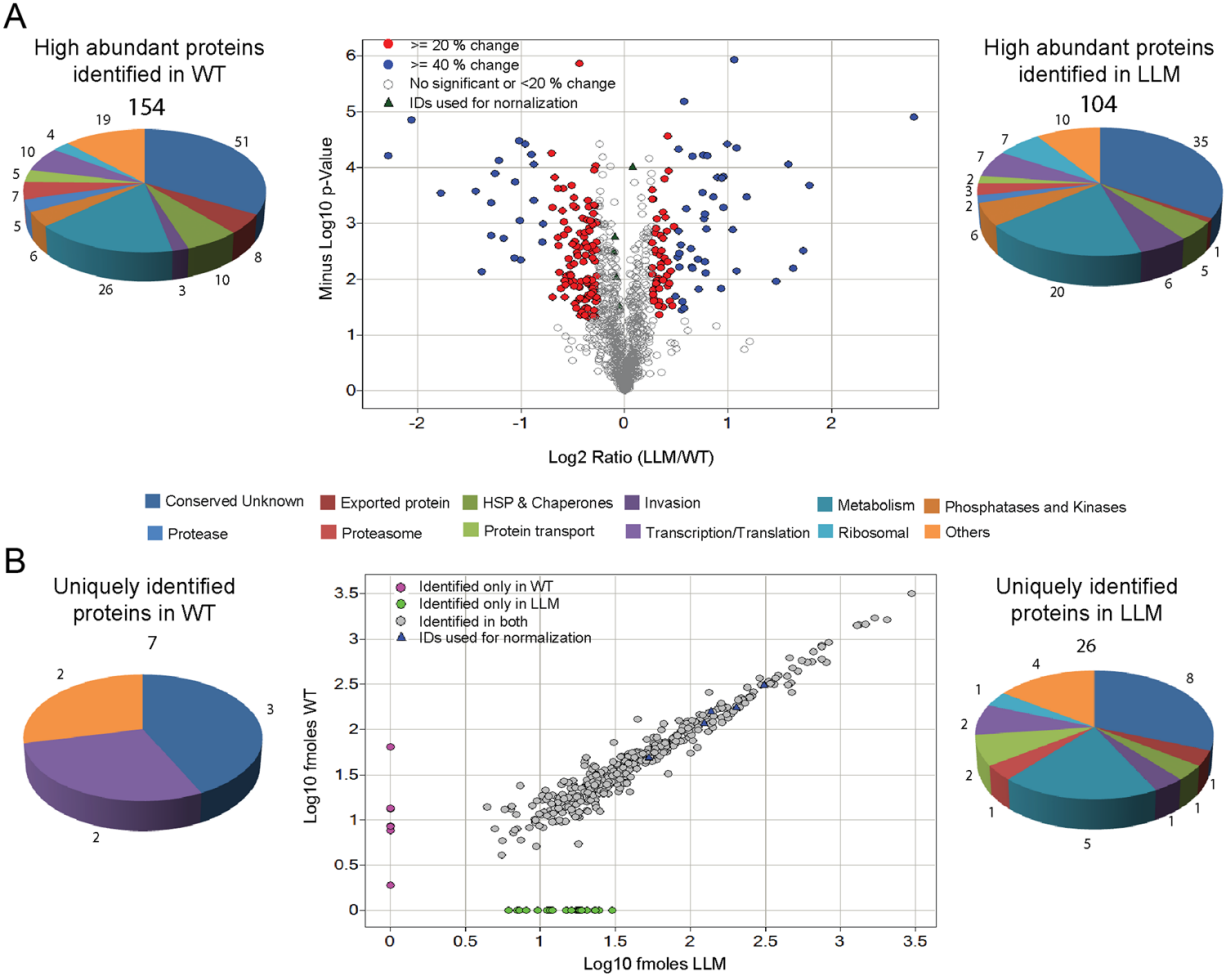
Comparative analysis of the abundance of common and unique proteins identified between WT and LLM merozoites by label-free quantitative analysis using TransOmics™ or PLGS. (**A)** Volcano plot of all quantified proteins from WT and LLM set displaying the relationship between statistical significance and percentage change of each protein. The log2 ratio (*x axis*) was plotted against the −log10 *p-*value (*y axis*). 20% and 40% changes are highlighted in red and blue color respectively. Gray circle represents either less than 20% change or not significant. Triangle represents five IDs used for normalization. A *q* value threshold of 0.2 (dashed horizontal line) and >1.5-FC (vertical dashed lines) are shown where open circles are differentially abundant proteins with small *q* values and large FCs. (**B**) Proteins uniquely identified in WT and LLM parasites by PLGS.

Next, we examined the relative protein abundance of WT merozoites vs. LLM merozoites using a label-free quantitation method, designated here as MS^E^ (multiplexed data acquisition) and we used TransOmics™ for our analysis of the protein data. Three technical replicates were identified from the single paired biological replicate using the WT or LLM merozoite invasion curves, pooled and processed as described in the Experimental Procedures (Fig. 1B). The biological replicate for WT and LLM is identified as WT1 and LLM1 with an overall invasion efficiency of about 20% for LLM compared to <1% for WT merozoites (Fig. 1B). The two proteomic data sets were normalized to five “constitutively expressed” proteins, which were (PF3D7_0406100 (V ATPase subunit B), PF3D7_0520900 (adenosylhomocysteinase), PF3D7_0624000 (hexokinase), PF3D7_1115600 (peptidyl-prolyl cis-trans isomerase) and PF3D7_1117700 (RAN GTPase)) (Supplemental Fig. S4). These five proteins also were identified to have minimal differences in their mRNA expression levels in the merozoite transcriptome data (Supplemental Dataset S2). To enhance the selectivity and sensitivity of low abundance proteins, a 5-fractional design was used to provide better separation of the tryptic peptides^36^. Protein abundance was calculated using total peptide intensity (summing all non-conflicting peptide intensities for each protein), which identified 981 proteins as present in both WT and LLM (Supplemental Dataset S4). Principal components analysis (data not shown) and a correlation analysis (Supplemental Fig. S5) of the identified proteins indicated unique clustering of the WT and LLM merozoites, suggesting there are distinct groups of differentially abundant proteins between the non-invasive and invasive merozoite populations, respectively. Using the criteria defined in TransOmics™ for protein identification, 981 proteins were identified in both WT and LLM merozoites as high confidence hits (Supplemental Dataset S4). Among these 981 proteins, based on a correlation analysis with the expression profiles, 446 proteins had significant differences in their protein abundance between WT and LLM merozoites (ANOVA *p* < 0.05). The most significantly affected proteins with a >20% or >40% change in abundance are highlighted in the volcano plot generated with log2 fold changes in protein abundance versus minus log10 *p*-values (Fig. 3A). Furthermore, protein differences of ~15% were sufficient to exert biological effects on the ability of parasites to establish infection in previous studies of malaria infection of liver cells^37^. Following these criteria, we discovered 86 proteins in higher abundance in LLM and 137 proteins in higher abundance in WT merozoites.

Two proteins of interest that were found in higher abundance in the LLM merozoites relative to WT include the kinase PKA and the phosphatase Calcineurin, two enzymes shown to play an important role during merozoite invasion^38–40^. In addition, proteins involved in motility and invasion-related functions, such as GAP50, MSP2, MSP7-like protein and EBA-175^41–44^ were found to be in higher abundance. Two other proteins, Sera 6, a cysteine protease that is activated prior to merozoite egress^45^ and plasmepsin X, an aspartic protease^46^, also were found to be significantly higher in the LLM merozoites compared to WT, raising the possibility that increased amounts of these proteases may play a role during merozoite invasion. Another group of proteins that appear to be higher in LLM merozoites relative to WT are the components that associate with the ribosomal machinery of the parasite. In contrast, the non-invasive WT merozoites relative to LLM contained higher levels of the food vacuole proteases falcilysin, falcipains 2a, 2b, 3 and plasmepsins III, IV^47^. Finally, there were several proteasome components and stress response proteins that were in higher abundance in WT merozoites in comparison to LLM, which may indicate some form of a stress response.

Data analysis using TransOmics™ relies on identification of peptides/proteins in both LLM and WT merozoites. It is conceivable that there could be proteins that are uniquely present in either LLM or WT merozoites that are missed due to the all or none factor in the comparison. Therefore, we quantitated proteins using PLGS, which is a quantitation based on the top 3 ionizing peptides for each protein identified in at least 2 out of the 3 technical replicates. Through this more inclusive process, we identified 33 unique proteins in either WT (7 proteins) or LLM (26 proteins) relative to each other (Fig. 3B and Supplemental Dataset S5). Comparative analysis of these proteins against previous published proteomics studies found in PlasmoDB indicated that 30 out of 33 proteins have been previously detected in asexual stage parasites. These observations may reflect differences between LLM and WT merozoites in proteins present that are relatively in low abundance. Interestingly, and specific to our study, 3 of the 33 proteins were identified for the first time in LLM merozoites and therefore, these proteins could represent novel proteins that function during merozoite invasion of the RBC.

### Relative protein quantitation of LLMs and implications for protein complexes

**Figure 4:**
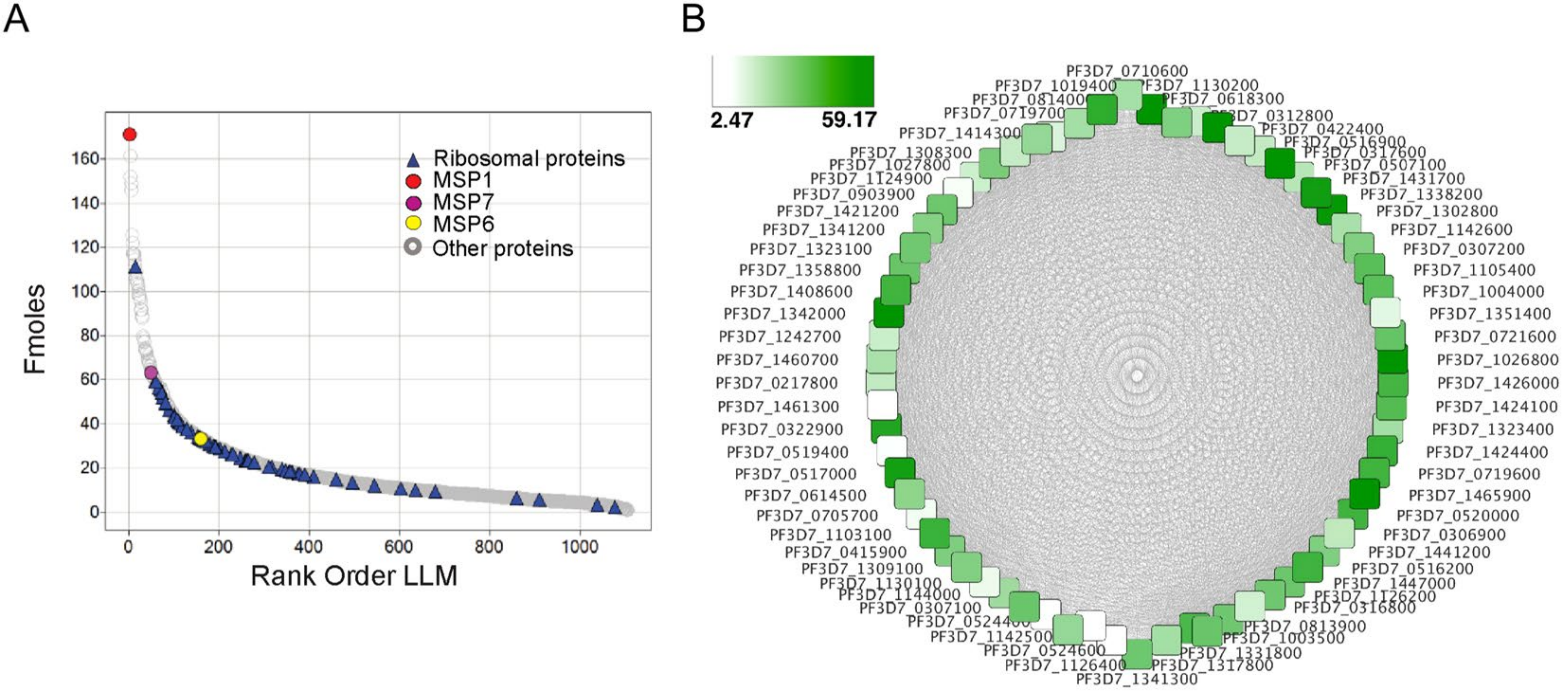
Characterization of the relative molar quantitation of the LLM proteome. (**A**) Molar abundance of all merozoite proteins identified in at least 3 out of 5 technical replicates. (**B**) Ribosomal network analysis showing statistical association between 61 ribosomal proteins.

For our first attempt at relative quantitation of the merozoite proteome, we used LLMs due to their enhanced biological activity. The individual quantity of each protein was calculated using the intensity of the top-3 most abundant peptides (Top3) using ProteinLynx Global SERVER™ (PLGS) 2.5 as described by Silva *et al*.^48^ where quantitation was performed relative to a protein standard. Quantitative proteomic profiling (Fig. 1B, LLM3) was performed with 5 technical replicates using a 10-fractional design. A total of 1,106 proteins were quantitatively identified corresponding to approximately 25,824 peptides with an average of 23 peptides per protein and an average coverage of 42% across the proteome. A minimum of 3 peptides per protein was necessary for quantitation. The dynamic range of those proteins ranged from 171 femtomoles to 862 attomoles (Fig. 4A). The most abundant protein identified was the major merozoite surface protein 1 (MSP1) whereas the least abundant protein quantified was a conserved protein designated PF3D7_1439600 (Supplemental Dataset S6) which is reported to be involved in merozoite invasion and is associated with the Inner Membrane Complex^49^. The median number of moles of protein was 19.18 femtomole.

To attempt to validate the PLGS data set, the known relationships within a ribosomal complex were used to evaluate the quantitative relationships between the 69 ribosomal proteins observed in LLMs (Fig. 4A and B)^50–52^. A complete set of protein-protein association data for ribosomes was extracted from the STRING database^53^. The association data *e.g*., protein-protein interactions between the ribosomes, curated from several sources including experimental data, text mining, co-expression analysis, co-occurrence analysis, etc. were then imported into the network visualization and analysis tool within Cytoscape^54^. The protein quantitation data were mapped onto the ribosome network and active modules (highly connected subnetworks that have relatively higher levels of combined protein quantity compared to other potential subnetworks) were identified using the Cytoscape plugin jActiveModules. Network statistics including the degree (total number of associations) of a node or protein in the network and its total number of associations were computed using Cytoscape default parameters. Correlation analysis was performed using the statistical software package R between the degree of a node - a protein - and its protein quantity. The degree sorted circle layout (Fig. 4B) showed a pattern of decreasing quantitation of the ribosomal proteins with respect to decreasing degree of the nodes, suggesting that the level of protein quantity measured in this experiment correlates with their need to form a functional association with other proteins. This initial observation between the degree - number of associations and the protein quantitation required further statistical analysis of the association between the variables ‘degree’ and ‘quantitation’. We performed this additional analysis by using Pearson’s product-moment correlation, which showed a positive correlation of 0.381183 between the variables ‘degree’ and ‘quantitation’. A linear regression analysis also showed that the correlation between the variables degree and quantitation is significant at the level of 0.05 (*p*-value 0.00123). The results of our ribosome protein data analysis suggest that the protein quantitation reported here is useful for the analyses of peptide mapping, protein processing, and protein-protein or protein-rRNA relationships.

**Figure 5:**
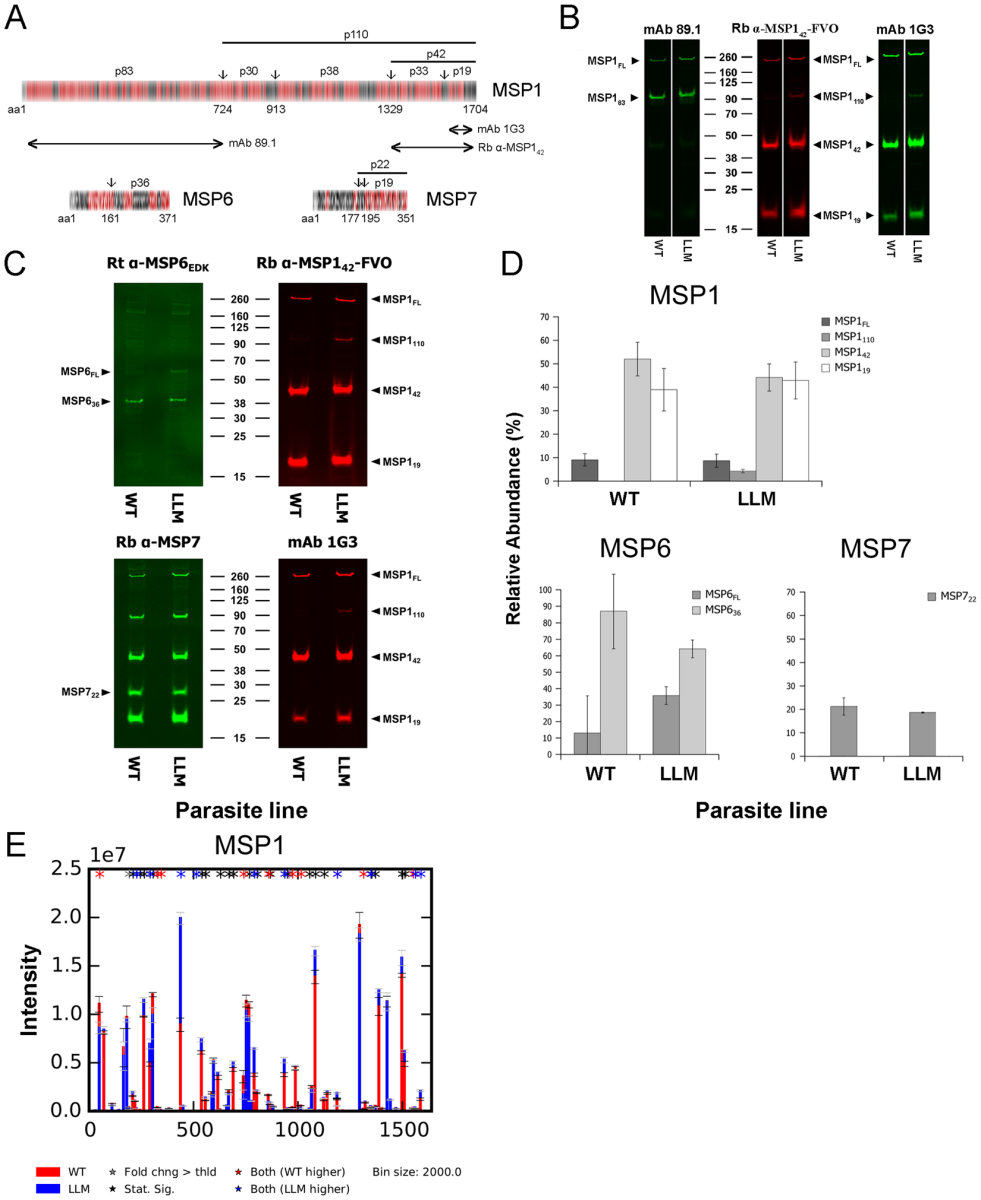
Analysis of the major merozoite surface complex: MSP1, MSP6 and MSP7. (**A**) Molecular schematics of the MSP1, MSP6, and MSP7 proteins showing peptide coverage (red colored regions) and proteolytic processing sites (arrows) and specificity of MSP1 antibodies. (**B**) Infrared immunoblot analyses of purified WT or LLM lysates showing staining for MSP1 full-length and processed fragments thereof using polyclonal rabbit (Rb) antiserum or mAbs 89.1 or 1G3. In this panel, Rb anti-MSP1 serum (red) and mAb 1G3 (green) were performed together while 89.1 was reacted alone. (**C**) Infrared immunoblot analysis of purified WT or LLM lysates showing staining for MSP1 full-length and process fragments thereof using polyclonal rabbit (Rb) antiserum or mAb 1G3 in combination with rat (Rt) anti-MSP6_EDK_ or Rb anti-MSP7 antisera, respectively. (**D**) Quantitative analysis of intact and proteolytic fragments of MSP1, MSP6, MSP7 from three independent blots, similar to those shown in panel C. Error bars represent standard deviation of three independent quantitated blots. (**E**) Peptide abundance changes in MSP1 protein between WT and LLM. Statistically significantly peptides (p-value < 0.05) are highlighted with a black asterisk above their position, peptides with fold change over the threshold (>25%) of one strain over another are denoted with a grey asterisk above their position and peptides that are both statistically significant and have a fold change above the threshold (>25%) are designated with a red asterisk (for WT) and blue asterisk (for LLM). A comparison of the WT and LLM MSP1 specific band intensities for the full-length, 42 kDa and 19 kDa fragments, noted within panels B and C, showed a variance of approximately 20%.

The presence of protein complexes in merozoites is well described^41,55–59^. However, the roles for many of these complexes remains unclear. One major complex exposed on the merozoite’s surface is a multi-protein complex composed of MSP1, MSP6 and MSP7^55,56^. MSP1 is considered to be involved with binding to protein(s) on the erythrocyte surface^26^. Each of these MSPs appear to be proteolytically processed during egress as well as during RBC invasion^60^ and specifically MSP1 processing has been shown to enable parasite egress from RBCs^60^. Whether MSP1 processing as observed in LLM merozoites affects their ability to invade RBCs prior to release remains to be seen. The proteins and/or their proteolytically processed fragments are non-covalently associated with MSP1, which is glycosylphosphatidylinositol anchored within the merozoite membrane^61^. We assessed the molar composition of the proteins in this complex as well as another leading protein, known as the apical membrane antigen-1 (AMA1). Good peptide coverage was observed for the three proteins within the MSP1 complex: 64%, 58% and 59% for MSP1, MSP6 and MSP7, respectively (Supplemental Dataset S6). The relative molar concentrations of MSP1, MSP6 and MSP7 in LLM were 171.2, 33.4 and 63.2 fmoles, respectively. The presence of each MSP1 proteolytic fragment was assessed for MSP1 p83, p38 and p42, in which each protein appeared in about an equal molar concentration: 117.64, 136.67 and 143.42 fmoles, respectively, while p30 appeared much lower at 44.22 fmoles. MSP6 and MSP7 are also proteolytically processed^56,62^ and therefore, the peptide coverage for these proteins was mapped using the LLM proteome (Fig. 5A). In general, the peptide coverage for MSP6 indicated that the amino-terminal end, which is reported to be cleaved and lost^56^, appeared to be more abundant in LLM than WT based on the peptide coverage analysis (Supplemental Fig. S6: PF3D7_1035500). In the same analysis, the amino-terminal end of MSP7 appeared to have been processed and lost, which is consistent with earlier findings^62^.

We analyzed the peptidomes of individual proteins identified in both WT and LLM merozoites using the peptide abundance data for the 446 proteins identified in the TransOmics data and flagging those peptides whose abundances were statistically significantly different between WT and LLM merozoites (Supplemental Fig. S6). MSP1 (PF3D7_0930300), an abundant merozoite surface protein involved in invasion, had the most peptides detected, and it is used as an example here (Fig. 5E, and Supplemental Fig. S6). In the carboxyl-terminal half of MSP1 corresponding to p110, of the 7 peptides with intensities >2.5 × 10^6^ and that showed statistically significant differences in peptide abundance between WT and LLM, 5 out of the 7 peptides were more abundant in LLM. This difference might be caused by more rapid or premature endogenous proteolytic processing of the carboxy-terminus of MSP1 in WT, and seemed to correspond to a relative increase in the carboxy-terminal MSP1 p110 processed product detected for LLM in immunoblots (see below and Fig. 5B and C). In contrast, at the amino-terminal end of MSP1 (p83) although a very abundant peptide (intensity 2.0 × 10^7^ in LLM, near residue 440) was increased 2.2-fold in LLM relative to WT; there was no marked difference in the immunoblots. The similarity between WT and LLM for MSP1 p83 on immunoblots might be explained if there were a post-translational modification in p83 other than endogenous non-tryptic cleavage, such as a change in phosphorylation that altered peptide recovery or detection by MS.

To address the composition and processing of the MSP1 complex using an alternative approach, quantitative Western blotting was performed using MSP1, MSP6 and MSP7 specific polyclonal and/or monoclonal antibodies (mAb). A two-color fluorescence system was used to allow a comparison between potentially variable conditions versus “a control” believed to be unchanging. First, an analysis of MSP1 using mAb 1G3, which recognizes MSP1_42_ and rabbit MSP1_42_ specific antiserum showed similar reactivity patterns and detected full-length MSP1 and the processed fragments (MSP1_42_ and MSP1_19_) (Fig. 5B). An additional band was recognized by both of the two MSP1 specific reagents in LLM at approximately 110 kDa (MSP1_110_), but not in WT merozoites, thereby indicating that this fragment is devoid of the p83 protein domain (Fig. 5B). These results held true in subsequent repetitive blots (Fig. 5C). Using MSP6 and MSP7 rat or rabbit specific antiserum, the expected protein fragments for MSP6 and MSP7 were detected (Fig. 5C, left panels, respectively). The banding intensities of MSP1_110_ and MSP6_FL_ were noted to be different between the WT and LLM lysates. To assess these differences quantitatively, the Western blots were performed independently three times using equimolar amounts of the merozoite lysates. MSP1, MSP6 and MSP7 band intensities were measured (Fig. 5D). The initial observations for the different staining intensity for MSP1_110_ and MSP6_FL_ appeared to be confirmed. No changes were observed for MSP7_22_ (Fig. 5D, lower right panel). The MSP7 antisera also was known to detect MSP1 due to its parasite derived origin.

## Discussion

The process of *P*. *falciparum* merozoite invasion of erythrocytes is a complex cascade of events that are time sensitive. Even though our understanding of particular steps in the overall processes have improved^17,20^, gaps in our knowledge remain. In an effort to further our understanding of merozoite biology, a γ-irradiated selected parasite line, identified as a long-lived merozoite (LLM) line, was developed with the aim of extending the longevity of a purified merozoite population for analysis of invasion of erythrocytes. The aim was clearly achieved and the LLM line was shown to invade erythrocytes by a 10 to 300 fold greater rate than the WT parent line (Fig. 1). Furthermore, this enhanced invasion phenotype was amenable to cell-sieve purification in a manner that is similar to what was described for the simian malaria parasite, *P*. *knowlesi* ^15,16^. A significant body of work was possible in the past due to this innate suitability of *P*. *knowlesi* merozoites to be purified and used for RBC invasion studies^17^. In a similar manner, purified *P*. *falciparum* LLM were used in the studies that demonstrated the role of RON2 binding to AMA1 for activation of merozoite invasion^29^.

Our comparative DNA sequence analysis of the ORFs coding about 80% of the genes showed limited changes between LLM and WT parasites. Three SNPs were observed: 1) a stop codon likely causing truncation of PF3D7_1007200, which is a putative rhoGAP GTPase, 2) a reversion from a FVO-like phenotype to that of 3D7 in PF3D7_0700900, a pseudogene for a RESA-like protein, and 3) an amino acid substitution from R to I in an armadillo like protein, now known as the glideosome-associated connector (GAC), that is up-regulated in schizogony and is designated as PF3D7_1361800. It is important to note that variants were called only in open reading frames, due to the difficulty of mapping reads to the highly AT-rich intergenic regions of the *P*. *falciparum* genome. We cannot rule out the presence or contribution of mutations in the 20% of the genes not sequenced or in regulatory DNA regions controlling gene transcription or translation that might account for the differences in protein expression between LLM and WT merozoites. It is also possible that epigenetic control mechanisms^63^ may have produced the LLM phenotype. However, the genomic data does make it clear that even though γ-irradiation and selection resulted in limited genomic sequence changes, genetic engineering could modify WT parasites to produce each of these individual mutations in order to examine their separate and combined effects on invasion phenotype. The truncation of PF3D7_1007200, a putative rhoGAP GTPase, deleted 213 residues of its carboxy-terminus and is likely to alter its activity and localization. In platelets, decreased RhoGAP activity increased platelet adhesion^64^. The identification of one SNP within PF3D7_1361800, the glideosome-associated connector (GAC), is worthy of further investigation, especially since GAC is thought to play a central role coordinating gliding motility and invasion^65^. It seems a remarkable coincidence that in a yeast two-hybrid assay, PF3D7_1361800 (GAC) interacted with PF3D7_1007200 (RhoGAP)^66^.

It is clearly understood that transcriptional changes occur during the 48 hour intra-erythrocytic cycle of parasite development^67,68^. These transcriptional changes, in general, correspond to the parasite’s demands for growth, which involve the need to restructure the erythrocyte cytosol during early trophozoite development and in addition, the transcription of genes involved in late schizogony that are related to merozoite egress and invasion. To our knowledge, no transcriptional profiling of purified invasive merozoites has been performed to date. We compared the transcriptomes of LLM to that of WT parasites. We discovered that in the microarray analysis, only four transcripts were identified to be significantly different in their expression patterns between the two merozoite types with our observation of an increased transcript abundance in LLM between 1.58–2.32 (p < 0.05), which were a S-adenosylmethionine-dependent methyltransferase (PF3D7_0522300), a putative thiamin-phosphate pyrophosphorylase (PF3D7_0614000), an unknown *Plasmodium* exported protein (PHISTa) (PF3D7_1253300) and a conserved protein with an unknown function (PF3D7_0522400). Whether these small changes in the transcription level of these genes are a part of the LLM phenotype remains to be determined. Furthermore, it is unclear whether the low numbers of genes that are transcriptionally different are an accurate assessment of the transcriptional environment, or due to RNA turnover, or perhaps are driven by the inherent variation introduced by our sampling methods. Finally, in our analysis of the transcriptional profiles of WT and LLM parasites during early schizogony *in vitro* (n ≈ 4 nuclei), a significant number of transcriptional differences were observed. How these transcriptional differences, if at all, impact on the observed phenotype of LLM merozoites relative to WT merozoites is unclear. However, investigation of the WT and LLM transcriptional profiles using mature schizonts (n = 8–16 merozoites/cell) appears warranted in order to understand if there are notable transcriptional differences leading up to the development of a segmented schizont. Importantly, our studies indicate that active transcription after merozoite release from schizonts may not contribute significantly to new proteins being made for invasion.

Proteomic studies of nearly all developmental stages of *P*. *falciparum* have been conducted including sporozoites, trophozoites and/or merozoites and gametocytes^69–71^. However, none of these investigations were able to assess changes in protein abundance between a non-invasive WT population of merozoites and a purified population of merozoites with a significant percentage of invasive forms. The unique property of long-lived merozoites that we describe here has enabled us to quantitate changes in protein abundance between WT and LLM merozoites. There are some known proteins present in higher abundance in LLM relative to WT merozoites. Some of these proteins are PF3D7_0918000 (PfGAP50), PF3D7_1222700 (PfGAP45), PF3D7_0501300 (skeleton-binding protein 1), and PF3D7_0616500 (TRAP-like protein), which have been shown to play a critical role in maintaining the parasite skeleton, inner membrane complex (IMC), actin myosin motor function, functional invasion of RBCs by the parasite^72–74^ and gliding locomotion of the parasite which is required for successful invasion^75^. Two previous studies have implicated a role for the TRAP/MIC2-like proteins MTRAP and PTRAMP^41,76^. Both of these proteins were in higher abundance in LLM merozoites relative to WT merozoites. Other invasion specific proteins such as the membrane skeletal protein IMC1-related (PF3D7_0304100), P38, a 6-cysteine protein (PF3D7_0508000), and the erythrocyte binding antigen-140 (PF3D7_1301600) were uniquely identified in LLM, but absent in the WT merozoites. Apart from known invasion ligands, functionally unknown or annotation deficient proteins were uniquely detected or identified in higher or lower abundance in LLM relative to WT merozoites.

The *P*. *falciparum* genome encodes approximately 5,300 proteins^77^. Here we have reported the first relative quantitation of a merozoite proteome that contains over 1,100 proteins, for which the protein molar abundance spans three orders of magnitude (Fig. 4A). Others have previously shown that the dynamic range of protein concentrations spans more than three, five and in some cases seven orders of magnitude in microbial^78^, yeast^79^ and human proteomes^80^ respectively. Before we addressed specific questions, we aimed to qualify the relative molar concentrations. Work by Hardy in 1975^50^ suggested that overall there is a relationship between the abundance of ribosomal proteins in *E*. *coli* using metabolically labeled proteins. Thus, our observation that there was a correlation between ribosomal protein abundance for the 61 ribosomal proteins within the network analysis using Cytoscape provides a basis of support for the overall relative molar concentrations established for the other 1,039 asexual proteins. Thus, this quantitated data set is likely a reasonable approximation for the first relative composition of a merozoite proteome.

Next, we evaluated the molar composition of the most abundant merozoite surface protein complex comprised of MSP1, MSP6 and MSP7 and evaluated their known processing events by quantitative Western blot and protein/peptide abundance by MS^E^. Two unique observations were made. First, using the relative molar concentrations by MS^E^, we approximated the stoichiometry for the three proteins that may form a complex as 5:1:2. MSP1 may be in a molar excess and available to form complexes with other proteins such as MSPDBL1 and MSPDBL2^81^. A second observation we made was an apparent delay in processing in LLM for MSP1 and MSP6. Peptide abundance at the p110 carboxy-terminal end of MSP1 was increased in LLM relative to WT, correlating with immunoblots that showed a native p110 form of MSP1 only in LLM. Similarly, immunoblots showed that LLM had a much greater ratio of full length to processed MSP6. These results suggest that the enhanced viability of LLM may be due, in part, to delayed proteolytic processing of merozoite proteins involved in invasion. Another difference in the peptide abundance of the p83 amino terminus of MSP1, however, was not correlated with changes on immunoblots, but may indicate a difference between LLM and WT in other post-translational modifications, such as phosphorylation (which was not studied here), which might nevertheless affect invasion. These observations open the door to further examination of the effects of the amounts and timing of various post-translational modifications (endogenous proteolysis, phosphorylation, etc.) on the ability of merozoites to maintain their invasiveness.

In summary, we have demonstrated that LLM purified by cell-sieving retained a significant proportion of invasive merozoites, which could be purified in large numbers. Using these purified long-lived merozoites (LLM) in comparison with the WT control, an integrated omics approach demonstrated only marginal changes in the open reading frame of the parasite genome as well as marginal changes in the parasite’s transcriptome. However, significant changes were observed in the proteome that may lead to the identification of the functions of unknown proteins and highlights the importance of protein stability and processing in the process of erythrocyte invasion.

## Materials and Methods

### Parasite Culture, Isolation of Merozoites and Efficiency of RBC Invasion

A line of *P*. *falciparum* FVO derived from HDGFP^82^ previously selected for prolonged survival of free merozoites through the process of γ-irradiation and selection of blood stage parasites maintained in a low hematocrit suspension culture *in vitro* was used in this study (Haynes, unpublished data). The parent WT line HDGFP did not fluoresce well or produce gametocytes, and it appeared to be more like FVO than the expected 3D7^82^ based first on restriction enzyme fingerprinting and then on sequencing. Parasite-infected RBCs were cultured as described^83–85^ and synchronized by temperature cycling^84^. RBCs were obtained from the WRAIR Clinical Trials Center (Silver Spring, MD) and sera from the Interstate Blood Bank (Memphis, TN). Briefly, the long-lived merozoite (LLM) line was generated by γ-irradiating (150 rad from a Cesium-137 source) about 4 × 10^8^ WT (HDGFP) trophozoite-infected RBC and then culturing in liquid suspension^84^ at constant 37 °C with decreasing RBC concentrations (Hcts) to exert selective pressure for long-lived merozoites by limiting contact between merozoites and RBC. Hematocrits (initially 4% Hct) were gradually adjusted downward to maintain a growth rate of at least 2-fold per cycle. The selective pressure for long lived merozoites was exerted by increasing the distance between the suspended cells at low Hct and thus increasing the time-to-contact of merozoites with RBC. After about 6 months of sub-cultures, the irradiated suspension-selected line could maintain growth rates of about 2-fold per cycle with only 0.3% Hct in suspension cultures, had twice the growth rates of WT at 1% Hct in suspension cultures, and was able to produce viable invasive merozoites as purified by cell sieving – thus the low Hct suspension-selected line was now called the LLM line. Subsequently, both WT and LLM lines were maintained by passage in suspension cultures in a temperature cycling incubator (Mid) that maintained synchronous growth on a 48-hour schedule^86^ however, 1% Hct cultures were used to maintain the LLM line, in contrast to the standard 4% Hct used to maintain the WT line. Before merozoites were purified, cultures were expanded in 4% Hct suspension cultures to allow the purification of about 1 × 10^9^ schizonts. Expansion cultures generally began as 6 large flasks of 45 ml 4% Hct per 150 cm2 flask, which was increased to 12 large flasks of 60 ml 1.5% Hct 2–3% trophozoites with the final media change about 18 hours before the Percoll-alanine purification. The growth rates for WT and LLM were similar when grown in 4% Hct suspension cultures, 8.8 (n = 5) and 10.4 (n = 3) per cycle, respectively (p = 0.07).

Merozoites from these LLM or WT lines were purified as follows. From this point on, Low Bicarbonate Medium (LBM) was used for all washes and cultures. LBM is similar to RPMI-NaOH, pH 7.4^86^ except that LBM was adjusted to 300 mOsm using NaCl and water, and the only bicarbonate was supplied by adding 10% serum. LBM was not gassed but was exposed to the ambient atmosphere. Expanded cultures of mature schizont-infected RBCs (with also about 1–3% newly invaded ring-infected RBC) were centrifuged at 400 g, resuspended in LBM, and purified on a Percoll-alanine gradient at 1200 × g for 10 min at 37 °C (Haynes, unpublished). The schizonts (generally 7–15 × 10^8^ with >90% at more than eight nuclei) were aspirated from the 40%/70% Percoll-alanine interface, washed with LBM, resuspended in 3 ml LBM and returned to culture briefly at 37 °C while schizonts were enumerated by hemocytometer. About 1 × 10^9^ schizonts in 6 ml LBM were transferred to a water-jacketed chamber (Fig. 1A) fitted with a 2 μm polycarbonate membrane and allowed to mature and rupture while being pumped out at 0.3 ml per minute and collected at room temperature (22–24 °C) into one tube every 10 minutes. Parasite culture in the gently stirred filter chamber was constantly supplied with fresh ungassed LBM as needed. For proteomics, microarrays and western blots, the collected merozoites (generally about 1 × 10^8^/ml) were passed through a 0.4 ml cushion of 5% sucrose in 1/2 strength PBS (a mixture of 1 part 10% sucrose with 1 part PBS) in a 1.5 ml tube at 1500 × g for 7 mins, followed by a PBS wash at 5000 × g for 10 mins. Merozoite pellets were stored at −80 °C until further use. Due to processing and handling times, each merozoite collection of WT or LLM was performed on independent days.

Merozoite invasion rates were evaluated by mixing merozoite samples (about 3 minutes after the termination of each 10 minute collection) with RBC in LBM followed by flow cytometry following general procedures similar to that previously described^86^. A 0.3 ml aliquot from each merozoite sample was mixed with 0.3 mL of 1% RBC (1.0 × 10^8^ RBC/mL) in a pre-warmed 1 mL NUNC tube, which was sealed and incubated at 37 °C for 1.5 hours with 35 rpm end-over-end mixing. To prevent the loss of merozoites during flow cytometry staining, PBS-EDTA-glucose^86^ GIA buffer for staining was mixed 1 part with 3 parts 10% sucrose to produce 7.5% sucrose-GIA buffer. Small aliquots (1–10 µl) of cultures, merozoites, or invasion mixtures were stained for flow cytometry by mixing with 50 µl of fluorescent HY dyes (10 µg/mL Hoechst 33342 and 2.5 µM Yo-Pro1 in 7.5% sucrose-GIA buffer) for 90 min in 5 mL tubes at 37 °C with gentle shaking. Stained samples were diluted 10-fold with 7.5% sucrose-GIA buffer and stored at room temperature (storage at 4 °C caused loss of merozoites) for up to 1 day before analyzing with a B-D LSR flow cytometer. Forward scatter was used to gate merozoites and RBC. RBC that did not fluoresce were considered uninfected. RBCs that fluoresced only with the permeant dye (Hoechst 33342) were considered invaded. RBC that fluoresced with both the permeant and impermeant dye were considered to have either adherent or adjacent merozoites. Aliquots of merozoites only were stained with HY dyes and diluted with a known number of normal RBC to aid in their enumeration by flow cytometry and used to calculate the number of merozoites per ml (generally about 1 × 10^8^/ml from about 1 to 3 hours after beginning the cell sieving). The percentage of invasive merozoites was calculated as (# invasion events / # total merozoites) × 100. When merozoite invasion was calculated by counting the numbers of merozoites by hemocytometer and the number of newly invaded RBCs (ring stage parasites) on Giemsa stained smears, it correlated well with flow cytometry.

For electron microscopy, purified LLM were mixed with pre-warmed RBCs and incubated at 37 °C for 30 seconds. Samples were fixed with ice cold fixative (2% paraformaldehyde, 0.01% glutaraldehyde in 1X PBS) and kept on ice for 30 min and subsequently processed for electron microscopy as described^29^.

Invasion dynamics of the LLM were monitored by mixing purified mature LLM schizonts with RBCs. The Dvorak chamber was placed in a 37 °C stage incubator with 5% CO_2_ and individual invasion events were recorded using a Leica SP2 confocal microscope.

### Genome sequencing

*P*. *falciparum* FVO (HDGFP) parasites identified as wild-type (WT) were cultivated as mentioned in the above section^84^. Parasite culture with 8–10% of parasitemia at schizont stage was lysed with 0.1% saponin and the genomic DNA was isolated using standard phenol/chloroform extraction and concentrated by salt precipitation^87^. PCR-free libraries^88^ with a fragment size of 450 bp were prepared and sequenced on a Illumina HiSeq. We obtained 20–32 million paired end 76 bp reads for the two lines, giving an average *Plasmodium* genome coverage of 60–90x for each line. All data is available in the European Nucleotide Archive, with accession numbers ERS038923 (WT or HDGRP) and ERS038924 (LLM or HHSS). To generate a WT parental genome, we iteratively mapped reads from the WT line against the Pf3D7 version2 reference, and corrected the differences for five iterations using iCORN^89^, transforming the Pf3D7 genome into an FVO genome. The new WT sequence was annotated by transferring the annotation from Pf3D7 using RATT^90^. Finally, reads from the WT line were mapped against the new, annotated FVO genome using SMALT (SMALT - http://www.sanger.ac.uk/science/tools/smalt-0) and bases supported by less than 10 reads were transformed to n’s. To identify genomic variants in the LLM line, we mapped the reads from these lines using SMALT. Variation was called with mpileup from the SAMtools package^91^.

### RNA extraction, DNA microarray target preparation

The purified merozoites were washed in PBS, centrifuged at 4 °C, cell pellets were flash frozen, and stored at −80 °C. RNA extractions were performed with Qiagen 96-well Rneasy system (Qiagen, Valencia, CA). Briefly, frozen merozoite pellets from parent and select lines were resuspended in 300 µL Qiagen RLT buffer containing 1% β-mercaptoethanol (BME) and passed through a QiaShredder (Qiagen, Valencia, CA) homogenization column by centrifugation at 21,000 × g for 2 minutes. All samples were brought to a final volume of 700 μL with RLT buffer containing 1% BME. Four-hundred ninety μL of 100% ethanol was added to each sample, mixed and purified using RNeasy 96 kit (Qiagen, Valencia, CA) including DNase I treatment with 27 Kunitz units of DNAse 1 (Qiagen, Valencia, CA) for 15 minutes at room temperature to remove contaminating genomic DNA. RNA concentrations and integrity were assessed by A260/280 spectrophotometry and by analysis on the Agilent 2100 Bioanalyzer.

Amplified antisense single-stranded cDNA (ss-cDNA) targets were prepared from an aliquot and used as template for the WT-Ovation RNA Amplification System (Nugen, San Carlos, CA). To monitor cDNA synthesis and amplification, each RNA sample was spiked with a mixture of four *B*. *subtilis* polyA-tailed mRNAs (*dap, lys, thr, trp* genes). Amplified ss-cDNAs were generated following the manufacturer’s recommended protocol, quantified using A260/280 spectrophotometer and analyzed on Agilent’s 2100 BioAnalyzer. The ss-cDNAs were fragmented and labeled following Nugen’s FL-Ovation cDNA Biotin Module V2 protocol (Nugen, San Carlos, CA) prior to Affymetrix DNA microarray hybridization.

### Microarray processing

Hybridization, fluidics and scanning were performed according to standard Affymetrix protocols (http://www.affymetrix.com) for the GeneChip® Plasmodium/Anopheles Genome Array. GeneChip Operating Software (GCOS v1.4, http://www.Affymetrix.com) was used to convert the image files to cell intensity data. The cell files were input into Partek Genomics Suite software (Partek, inc. St. Louis, Mo.) and quantile normalized to produce the principal components analysis (PCA) graphs. An ANOVA was performed within Partek to obtain multiple test corrected p-values using the false discovery rate (FDR) method^92^ and was combined with fold change values for each comparison of interest.

### Estimation of asexual parasite cell-cycle using microarray data

A reference dataset^68^ representing 46 individual time points of the complete *P*. *falciparum* (HB3 strain) asexual intra-erythrocytic developmental cycle and our own data set were input into Partek® Genomics Suite™ software (v6.6 Copyright © 2012 Partek Inc., St. Louis, MO, USA). All arrays were scaled to 1250. The average signal across replicates was used for reference time points represented by multiple arrays. The reference oligo identifications were mapped to Affymetrix probe sets using *P*. *falciparum* open reading frame identifications. Normalized signal intensities from mapped probe sets were used to calculate the maximum correlation coefficient across reference time points as an estimate of the specific intra-erythrocytic developmental cycle. To validate this method, a separate time series^93^ with published intra-erythrocytic developmental cycle time points of 0, 8, 16, 24, 32, 40, and 48 (http://www.ncbi.nlm.nih.gov/geo/query/acc.cgi?acc=GSE14524) demonstrated accuracy within 3 hrs.

### QPCR validation

Three *P*. *falciparum* mRNAs were selected for Q-RT-PCR validation analysis – PF3D7_1253300, PF3D7_0522400, and PF3D7_042500. Constitutively expressed reference mRNAs were selected from the DNA microarray results by ranking probe-sets based on expression level (present call) and coefficient of variation (CV). The optimal reference mRNA was selected based on three criteria: mRNA expression in all samples, low CV across treatments and gene-function. Putative glutamate-tRNA ligase mRNA was selected as a reference mRNA (PF130257). All Q-RT-PCR probe and primer sets were designed using Beacon Designer 8.12 (PREMIER Biosoft International, Palo Alto, CA). Due to low GC-content of *P*. *falciparum* genome sequence, over 30 nucleotide long oligo sequences were selected during the design to allow normal annealing and extension temperatures during QPCR (Supplemental Dataset S7). Remaining Q-RT-PCR template preparation, PCR conditions and analysis are described in detail elsewhere^94^.

### Sample Preparation for LC/MS

Samples were prepared as previously described^95^. Briefly, 4 × 10^8^ merozoites were suspended in 50 mM ammonium bicarbonate (pH 8.5) with 0.06% RapiGest™ (Waters, p/n 186001861). Samples were digested by sequencing-grade trypsin and incubated at 37 °C for overnight. Before the digestion, 5 µl of 100 mM DTT was added to reduce disulfide bonds at 60 °C for 30 minutes and then free cysteine residues were alkylated with 5 µl of 300 mM iodoacetamide at room temperature for 30 minutes in the dark. Trifluoroacetic acid (0.05% v/v) was used to quench the enzymatic reaction and degrade the RapiGest SF. The sample protein mixtures were centrifuged at 13,000 rpm for 10 min and the supernatant was transferred into another vial. The pH of the samples was adjusted to pH 10 by adding 5 µL of 1 N NH_4_OH for effective trapping on the 1st dimension column. Samples were spiked with 25 fmol/µl alcohol dehydrogenase before loading the nanoACQUITY UPLC^®^ for peptide analysis via LC/MS^E^.

### NanoUPLC/MS Configuration

Tryptic peptides from the complex samples were separated by using 2-dimensional (2D) liquid chromatography. A 2D nanoACQUITY UPLC^®^ system coupled with an online Synapt G2 or Synapt G2-S quadrupole time-of-flight (Q-TOF) mass spectrometer (Waters, Corp., Milford, MA) was operated in a positive ion, data-independent acquisition mode called alternate scanning LC-MS (LC/MS^E^) analysis. MassLynx™ 4.1 software was used to operate the nano UPLC/MS system. The 2D system was configured with three columns; i) first dimension high pH reversed phase trapping (or fractionation) column (300 µm × 50 mm XBridge™ BEH130 C18 5 µm), ii) second dimension trapping column (80 µm × 20 mm Symmetry^®^ C18 5 µm) and iii) second dimension reversed phase analytical column (75 µm × 150 mm HSS T3 1.8 µm). Twenty mM ammonium formate (pH 10), and acetonitrile were used as mobile phases A and B respectively for 1^st^ dimension fractionation at 2 µl per minute. High pH fractionation of the tryptic peptides from the complex samples was performed either in a 5 or 10 fraction format. For a five-fraction experiment, 11.1, 14.5, 17.4, 20.8, and 45.0% mobile phase B steps were used, whereas for a 10 fraction experiment, 6.9, 10.4, 12.1, 13.5, 14.7, 15.9, 17.3, 18.8, 20.9 and 45.0% steps were used. Fractionated peptides were then trapped onto the second-dimension trap column following 1:10 dilution with 2^nd^ dimension mobile phase A (0.1% formic acid, FA, in water), which also acidifies the peptides. The 2^nd^ dimension mobile phase B was acetonitrile, 0.1% FA. Each group of fractionated peptides were subsequently separated on the second-dimension analytical column with a 90 min. gradient (3–40% B). 200 fmol/µl [Glu1]-fibrinopeptide (GFP) in 25% ACN, 0.1% FA was used as the lock mass solution with a flow rate of 0.5 µl/min using the reference sprayer of the nanoLockSpray source. The mass spectrometers were operated with capillary voltage of ~3.0 kV, source temperature 130 °C, and cone voltages 35/30 V on the Synapt G2 and Synapt G2-S, respectively. The low and elevated energies used during the LC/MS^E^ experiment were 6 V and 15–35 V (ramped), with 1 second/scan. Spectra were recorded from m/z 50 to 1990, and samples were run in quadruplicate.

### MS^E^ data set analysis

The LC/MS^E^ data were processed and searched using ProteinLynx Global SERVER™ (PLGS) version 2.5 to match the spectra to peptide in the sequence database of the ORFs generated from genome sequencing of the *P*. *falciparum* WT line. Protein Identifications <250,000 Da were obtained by searching WT parasite protein database appended with alcohol dehydrogenase protein sequence. The ion detection, clustering and normalization were processed using PLGS 2.5 as described earlier^48,96^. Processed spectra for all fractions for each run were merged independently using PLGS 2.5 merge tool. For MS^E^, the search was performed using the following parameters: a minimum of five fragment ions per peptide and a minimum of nine fragment ions per protein, a minimum of one peptide match per protein and a maximum of one missed trypsin cleavage. Furthermore, we used (i) Carbamidomethylation (Cys) as fixed modification; (ii) Deamidation (NQ), Oxidation (M) and acetylation (N terminus of the protein) as variable modifications; and (iii) a protein false discovery (FDR) rate of 10%. The protein false discovery rate was determined automatically in PLGS by searching the randomized and reversed *P*. *falciparum* (WT) database. Finally, MS^E^ outputs of the separate runs were merged using Microsoft Excel. Protein identification was considered accurate when a protein (paralog) was assigned based on at least two proteotypic peptides. Proteins that were certified with one peptide were accepted at a peptide-score above 6 in addition to filtering at a FDR of 10%. Additional data analysis was performed using Spotfire Decision Site Version 9.1.2 and Microsoft Excel.

### Protein quantification using TransOmics™

For quantitative analyses, the LC/MS^E^ data acquired on a Synapt G2 mass spectrometer were processed using TransOmics™, which transformed the raw high-resolution profile data with lock-spray mass-calibration into a custom TOIP format and extracted peptide intensity features from MS^E^ data. The LC/MS^E^ chromatograms of all samples and replicates acquired datasets were aligned against a chosen reference run i.e., fraction one of each injection which was arbitrarily chosen as the best representative, due to highest resolution and therefore presumed to have the lowest number of missing data points. Retention time alignment by warping allowed the creation of a single aggregate peak map containing all peak data from all samples. After peak detection on the aggregate map, the peak intensities were determined for each detected individual peak per run. The peak intensity data were further normalized using a set of five “house-keeping” proteins (PF3D7_0406100, PF3D7_0520900, PF3D7_0624000, PF3D7_1115600 and PF3D7_1117700). The peptide identification was done by searching the MS^E^ data against WT proteome to link identified peptides to their corresponding peak features. The following search criteria were used: a minimum of five fragment ions per peptide and a minimum of seven fragment ions per protein, a minimum of one peptide match per protein and a maximum of one missed trypsin cleavage. Furthermore, carbamidomethylation (Cys) and oxidation (M) were used as fixed and variable modifications respectively with a maximum of two missed cleavages allowed for search at the precursor mass tolerance of 10 ppm and product mass tolerance of 20 ppm. The peptides were auto-validated with maximum FDR of 4%. Principal components analysis (PCA) was performed, the generated feature dataset with sum intensity of all same peptide intensities was exported and further processed in Excel. The data from the individual injections were compared by paired 2-sided *t*-test with p < 0.05.

### Comparative analysis for *P*. *falciparum* intensity measurements

Intensity measures are read from a pre-processed Excel spreadsheet. Measures for two *P*. *falciparum* strains are averaged (using numpy library method mean, http://www.numpy.org) and a standard deviation is computed (using the “std” method of the “numpy” library) along with the standard error of each set of measures (using the “sem” method of the “scipy” library (http://www.scipy.org)). A p-value is computed for each set of peptide measures and evaluated using a one-way ANOVA test where the null hypothesis that two or more groups have the same population mean (using the scipy stats package method statsf_oneway). Tryptic peptides are evaluated with preference being given to longer partially digested peptides over shorter, completely digested peptides that are fully contained within the partially digested peptide. Tryptic peptides are then further evaluated for fold change of intensity mean above a 25% difference threshold which further checked for statistical significance. All peptides not contained within another peptide have their means plotted on their respective proteins and proteins are grouped according to size, in 500 amino acid increments, and alphabetically by accession. Statistically significantly peptides (p-value <0.05) are highlighted with black asterisk above their position, peptides with fold change over the threshold of one strain over another are denoted with a grey asterisk above their position in each plot and peptides that are both statistically significant and have a fold change above the threshold are designated with a red asterisk. Reports of all peptides as well as peptides per protein summaries are produced.

### Analysis of MSP1, MSP6 and MSP7 protein expression and processing by infrared fluorescence immunoblotting

Merozoite lysates from WT and LLM parasite lines were loaded at an approximated concentration of 1 × 10^7^ parasites per lane on SDS-PAGE, 4–12% precast gels (Criterion XT, Bio-Rad, Hercules, CA) using a MES-based running buffer, and after electrophoretic separation, proteins were transferred to nitrocellulose membranes in a semi-dry blotting system (Trans-Blot Turbo, Bio-Rad) following manufacturer’s instructions. Blocking, washes and antibody dilutions were prepared essentially as described elsewhere^95^. After the membranes were blocked, separate membranes were incubated for 1 h with either anti-MSP6_EDK_ rat polyclonal or anti-MSP7 rabbit polyclonal serum at dilutions 1:500 and 1:2000, respectively. A second antibody incubation for an additional hour was performed by using anti-MSP1_42_-FVO rabbit polyclonal serum (with anti-MSP6 treated blots) or anti-MSP1_42_-FVO mouse monoclonal antibody (mAb) 1G3 (with anti-MSP7 treated blots) at 1:2000 dilution and 10 μg/ml, respectively. Bound antibodies were made detectable by incubation with a mix of IRDye 680RD goat anti-rabbit and IRDye 800CW goat anti-rat immunoglobulin secondary antibodies (LI-COR Biosciences, Lincoln, NE) at a dilution of 1:10000 each for 30 minutes (anti-MSP6 treated blots). Anti-MSP7 treated blots required a mix of IRDye 680RD goat anti-mouse and IRDye 800CW goat anti-rabbit immunoglobulin secondary antibodies. Digital band detection images were obtained by simultaneous membrane scanning at 700 and 800 nm in an Odyssey infrared imaging system (LI-COR Biosciences). Densitometric analysis was done with the help of ImageJ software package (https://imagej.nih.gov/ij/index.html) including the entire lane(s) of interest without modification and pictures/plots prepared with office spreadsheet and graphic editor software (LibreOffice Calc, Inkscape and GIMP).

### Experimental Design and Statistical Rationale

Genomic analysis of the WT and LLM lines used gDNA isolated from individual *in vitro* parasite cultures. Microarray studies used two paired biological replicates of LLM and WT merozoites and three or four technical replicates. MS^E^ studies evaluated one pair of biological replicates that demonstrated significant differences in erythrocyte invasion rates to enable optimum measurements of biological differences in protein abundance between LLM and WT parasite lines. The quantity of merozoites was determined using a hemocytometer during cell-sieve purification. The biologically paired samples were each analyzed using three technical replicates on a Waters Synapt G2 for the comparative relative molar abundance study and statistical analyses used TransOmics™ software. The sample queue alternated the technical replicates for each biological sample. One biological sample from LLM was evaluated using five technical replicates on a Waters Synapt G2 HDMI-MS and analyzed using PLGS version 2.5. The datasets generated during and/or analyzed during the current study are available as noted. The micrata discussed in this publication have been deposited, respectfully, in NCBI’s Gene Expression Omnibus^97^ and are accessible through GEO Series accession number GSE81818 (https://www.ncbi.nlm.nih.gov/geo/query/acc.cgi?acc=GSE81818) or in Chorus and are accessible at the following sites:

https://chorusproject.org/pages/dashboard.html#/projects/my/1076

https://chorusproject.org/pages/dashboard.html#/projects/my/1086.

## Electronic supplementary material

Supplemental Information

Dataset S2

Dataset S3

Dataset S4

Dataset S5

Dataset S6
