# Supplementary material for: Profiling invasive Plasmodium falciparum merozoites using an integrated omics approach
Source: Sci Rep. 2017 Dec 7;7:17146. doi: 10.1038/s41598-017-17505-9 (PMC5719419; doi:10.1038/s41598-017-17505-9)
Supplement: Supplementary file 1 — Supplemental Information [file 41598_2017_17505_MOESM1_ESM.pdf]

Profiling invasive *Plasmodium falciparum* merozoites using an integrated omics approach

Krishan Kumar[1], Prakash Srinivasan[2]*, Michael J. Nold[3], J. Kathleen Moch[4], Karine Reiter[1], Dan Sturdevant[5], Thomas D. Otto[6], R. Burke Squires[7], Raul Herrera[1], Vijayaraj Nagarajan[7], Julian C. Rayner[6], Stephen F. Porcella[5], Scott J. Geromanos[3], J. David Haynes[4], David L. Narum[1]**

From the [1]Laboratory of Malaria Immunology and Vaccinology, NIAID, NIH, Rockville, MD, USA; [2]Laboratory of Malaria and Vector Research, NIAID, NIH, Rockville, MD USA; [3]Waters Corporation, Milford, MA USA; [4]Walter Reed Army Institute of Research, Silver Spring, MD USA; [5]Genomics Unit, Research Technologies Section, Rocky Mountain Laboratories, NIAID, NIH, Hamilton, MT, USA; [6]Wellcome Trust Sanger Institute, Hinxton, Cambridge CB10 1SA, United Kingdom; [7]Computational Biology Section, Bioinformatics and Computational Biosciences Branch, NIAID, NIH, Bethesda, MD USA

*****Current address: Johns Hopkins Malaria Research Institute, Department of Molecular Microbiology and Immunology, Johns Hopkins Bloomberg School of Public Health, Baltimore, MD USA

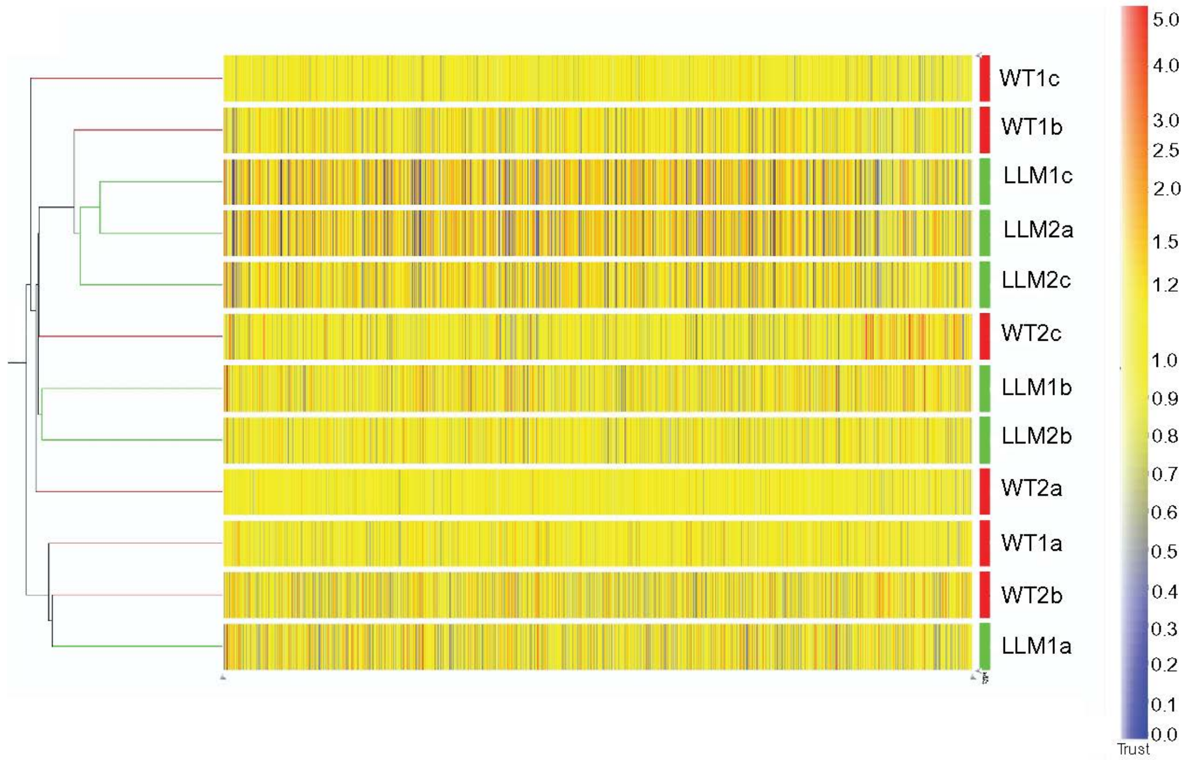

Figure S1. Transcriptional analysis comparing cell-sieve purified WT and invasive LLMs (6 x 6, respectively) on an Affymetrix chip. Heat map showing transcriptional analysis of WT and LLM when evaluated using only two pairs of biological replicates comprised of three technical replicates (see Fig. 1B, WT1 & 2, and LLM1 & 2).

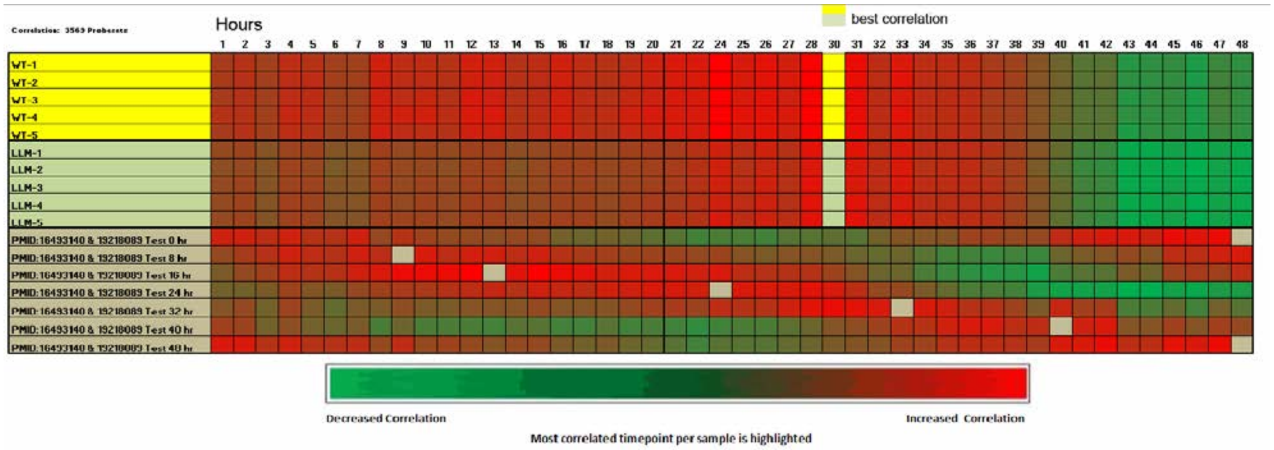

Figure S2. Analysis of paired *P. falciparum* WT and LLM young schizont samples for correlation of cell cycle that were used in the transcriptional analysis. Heat map showing correlation from transcriptional analysis of WT and LLM when evaluated using one set of biological replicates with five technical replicates. The hours (1 – 48) represent the hours of intra-erythrocytic parasite development. The yellow/gray coloration at 30 hours identifies time period with the best correlation.

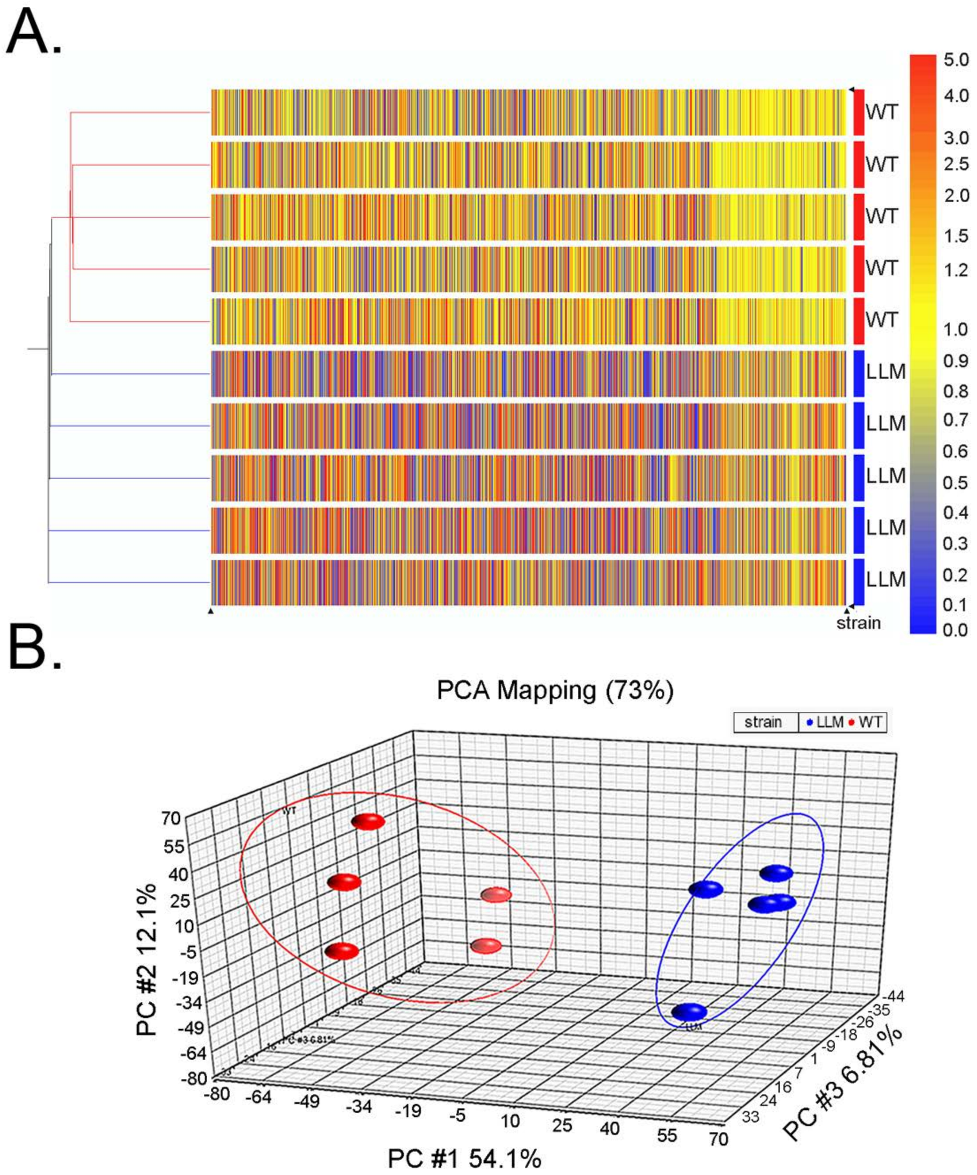

Figure S3. Transcriptional analysis comparing WT and LLMs on an Affymetrix microarray chip. A, heat map transcriptional analysis of WT and LLM schizonts (n ≈ 4) using one pair of biological replicates comprised of five technical replicates. B, PCA showing clustering of WT and LLM biological and technical replicates. Correlation in cell cycle was evaluated as shown in Supplemental Fig. S2.

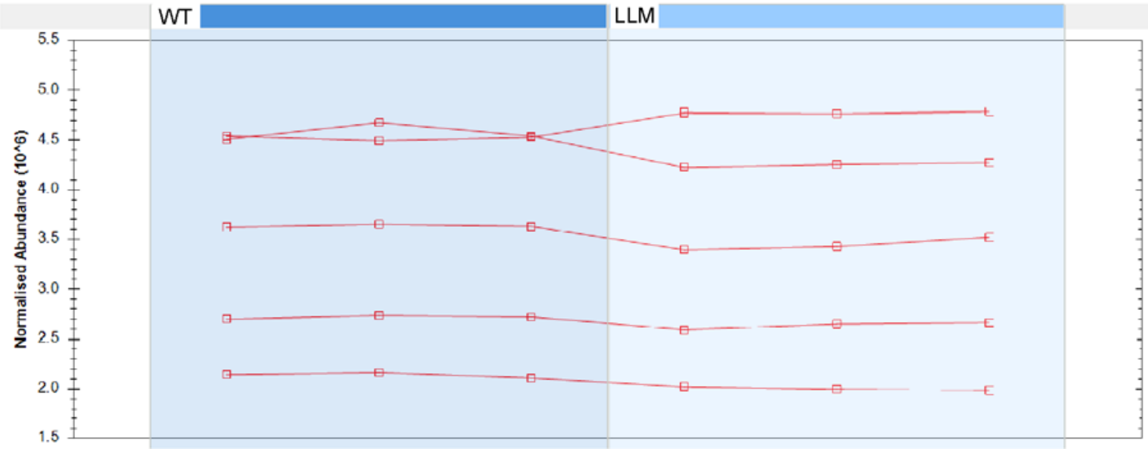

Figure S4. Normalization of protein abundance between WT and LLMs. Five house-keeping proteins were selected and are shown following normalization. Individual proteins used from top to bottom for LLM (right side panel): PF3D7_0520900 (adenosylhomocysteinase), PF3D7_1117700 (RAN GTPase)), PF3D7_0624000 (hexokinase), PF3D7_1115600 (peptidyl-prolyl cis-trans isomerase), and PF3D7_0406100 (V ATPase subunit B).

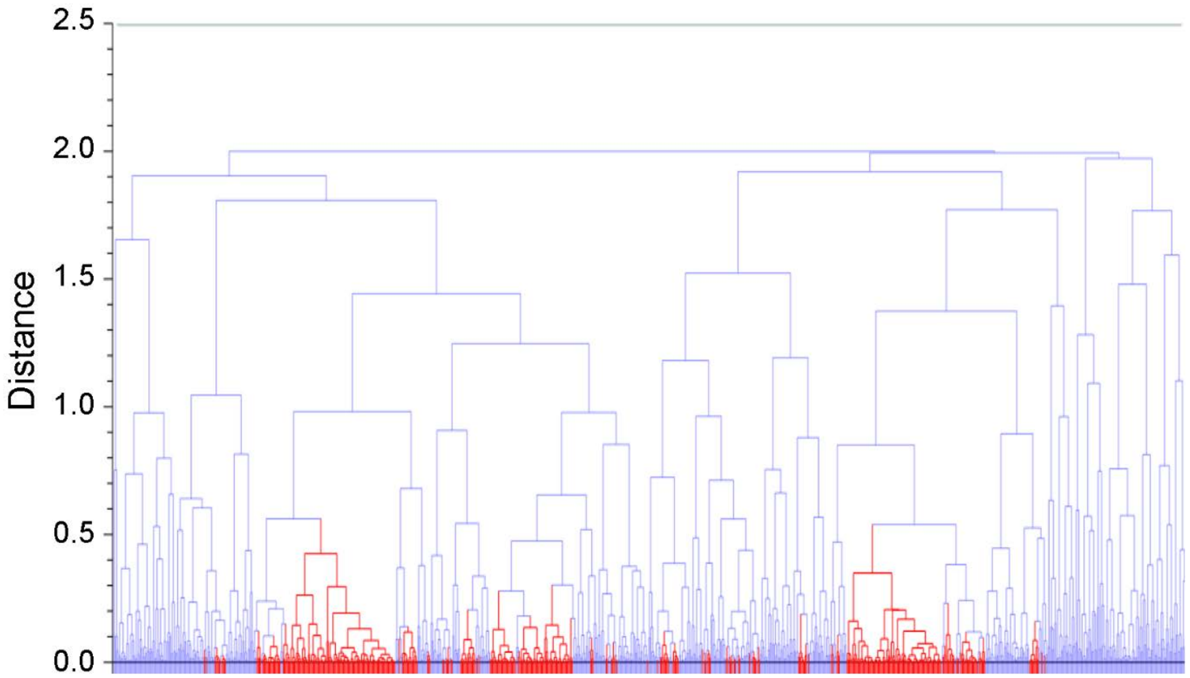

Figure S5. Cluster analysis based on proteomics abundance profiles of LLM and WT merozoites using TransOmics™. All end nodes represent data from individual protein ID, and nodes represent the clusters to which data belongs. Increasing distance represents the dissimilarity. Red color highlights proteins with a change in abundance.

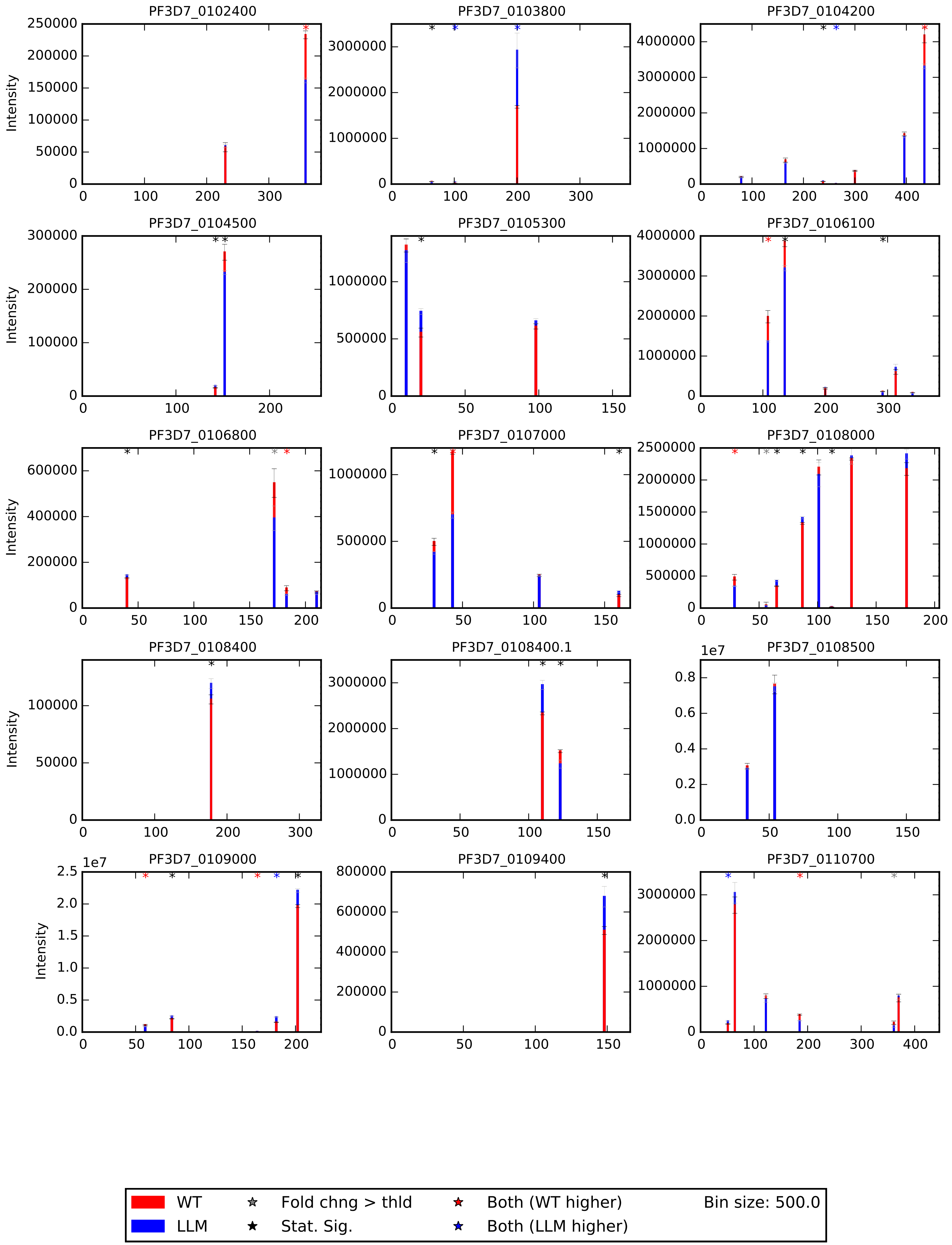
PF3D7_0102400
PF3D7_0103800
PF3D7_0104200
PF3D7_0104500
PF3D7_0105300
PF3D7_0106100
PF3D7_0106800
PF3D7_0107000
PF3D7_0108000
PF3D7_0108400
PF3D7_0108400.1
PF3D7_0108500
PF3D7_0109000
PF3D7_0109400
PF3D7_0110700
Intensity
1e7
WT
LLM
Fold chng > thld
Stat. Sig.
Both (WT higher)
Both (LLM higher)
Bin size: 500.0

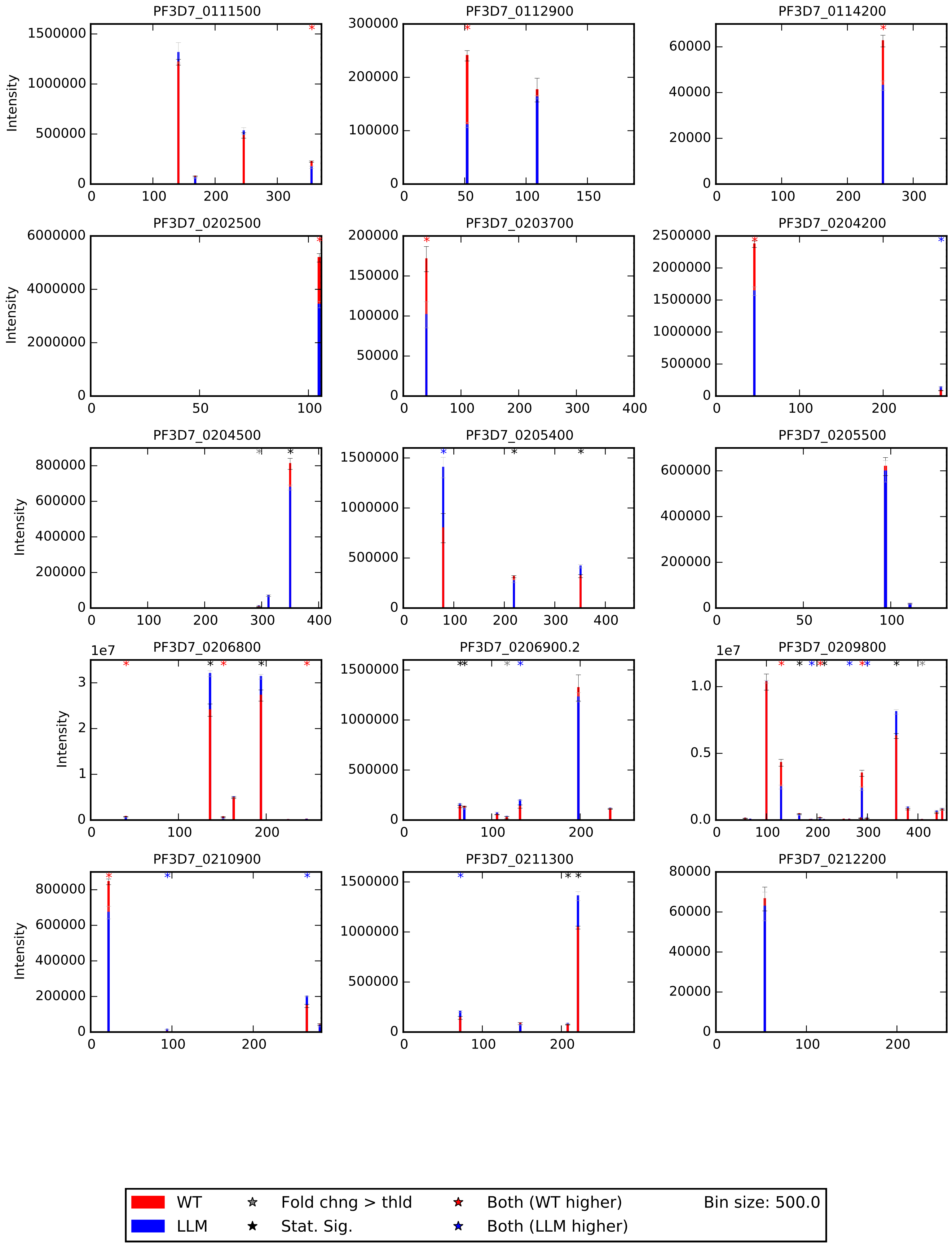
PF3D7_0111500
PF3D7_0112900
PF3D7_0114200
PF3D7_0202500
PF3D7_0203700
PF3D7_0204200
PF3D7_0204500
PF3D7_0205400
PF3D7_0205500
PF3D7_0206800
PF3D7_0206900.2
PF3D7_0209800
PF3D7_0210900
PF3D7_0211300
PF3D7_0212200
Intensity
1e7
WT
LLM
Fold chng > thld
Stat. Sig.
Both (WT higher)
Both (LLM higher)
Bin size: 500.0

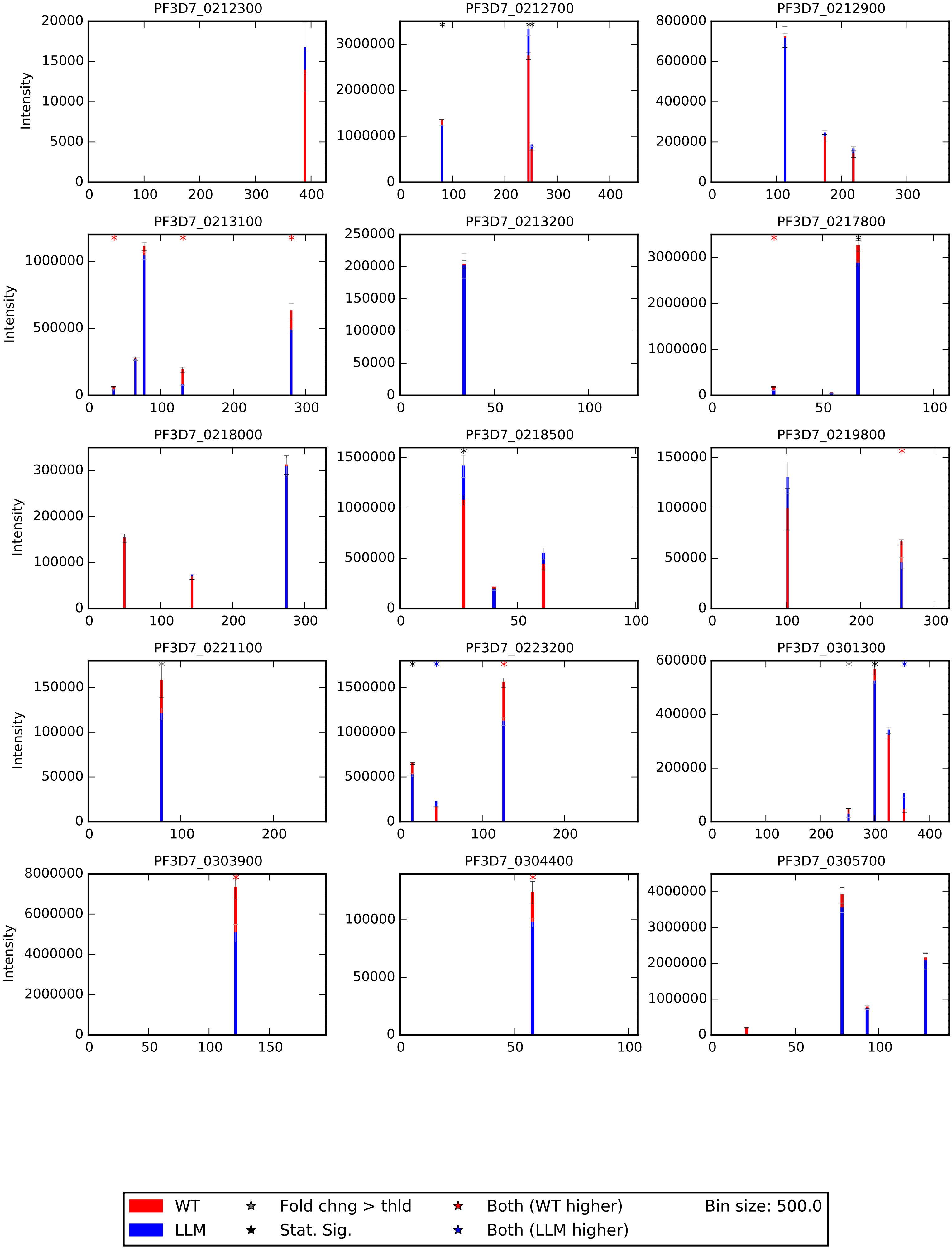
PF3D7_0212300
PF3D7_0212700
PF3D7_0212900
PF3D7_0213100
PF3D7_0213200
PF3D7_0217800
PF3D7_0218000
PF3D7_0218500
PF3D7_0219800
PF3D7_0221100
PF3D7_0223200
PF3D7_0301300
PF3D7_0303900
PF3D7_0304400
PF3D7_0305700
Intensity
WT
LLM
Fold chng > thld
Stat. Sig.
Both (WT higher)
Both (LLM higher)
Bin size: 500.0

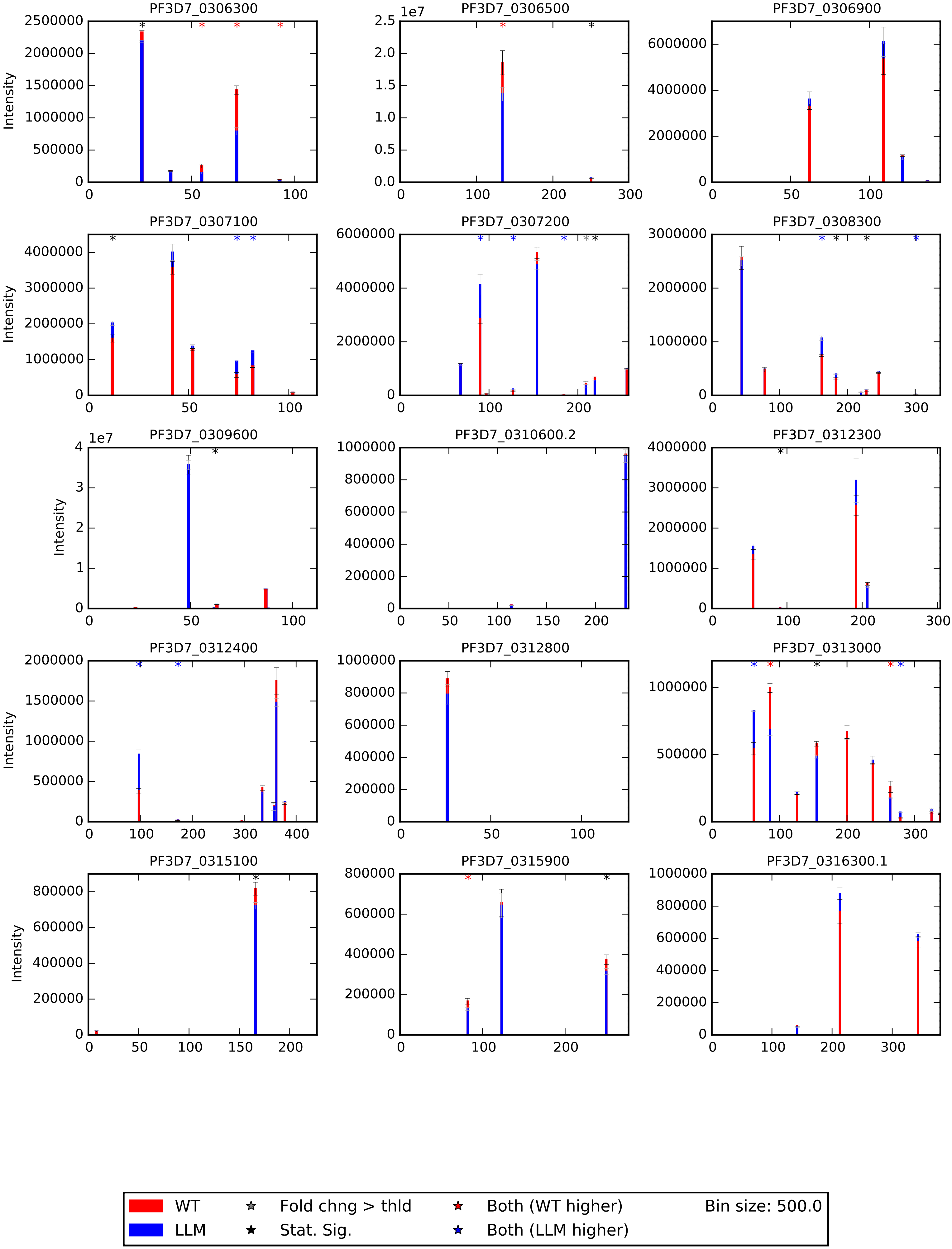
PF3D7_0306300
PF3D7_0306500
PF3D7_0306900
PF3D7_0307100
PF3D7_0307200
PF3D7_0308300
PF3D7_0309600
PF3D7_0310600.2
PF3D7_0312300
PF3D7_0312400
PF3D7_0312800
PF3D7_0313000
PF3D7_0315100
PF3D7_0315900
PF3D7_0316300.1
Intensity
1e7
WT
LLM
Fold chng > thld
Stat. Sig.
Both (WT higher)
Both (LLM higher)
Bin size: 500.0

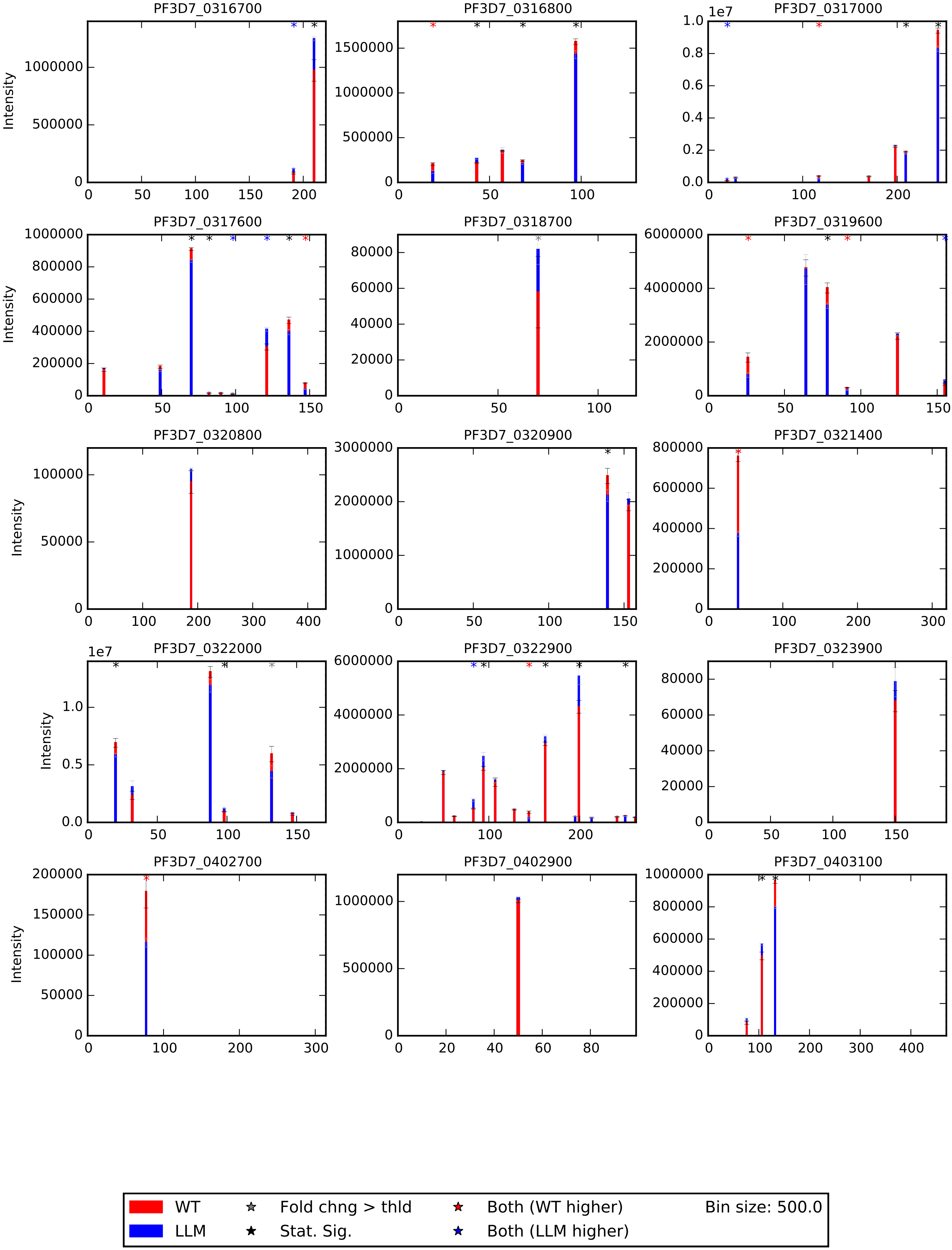
PF3D7_0316700
PF3D7_0316800
PF3D7_0317000
PF3D7_0317600
PF3D7_0318700
PF3D7_0319600
PF3D7_0320800
PF3D7_0320900
PF3D7_0321400
PF3D7_0322000
PF3D7_0322900
PF3D7_0323900
PF3D7_0402700
PF3D7_0402900
PF3D7_0403100
Intensity
1e7
WT
LLM
Fold chng > thld
Stat. Sig.
Both (WT higher)
Both (LLM higher)
Bin size: 500.0

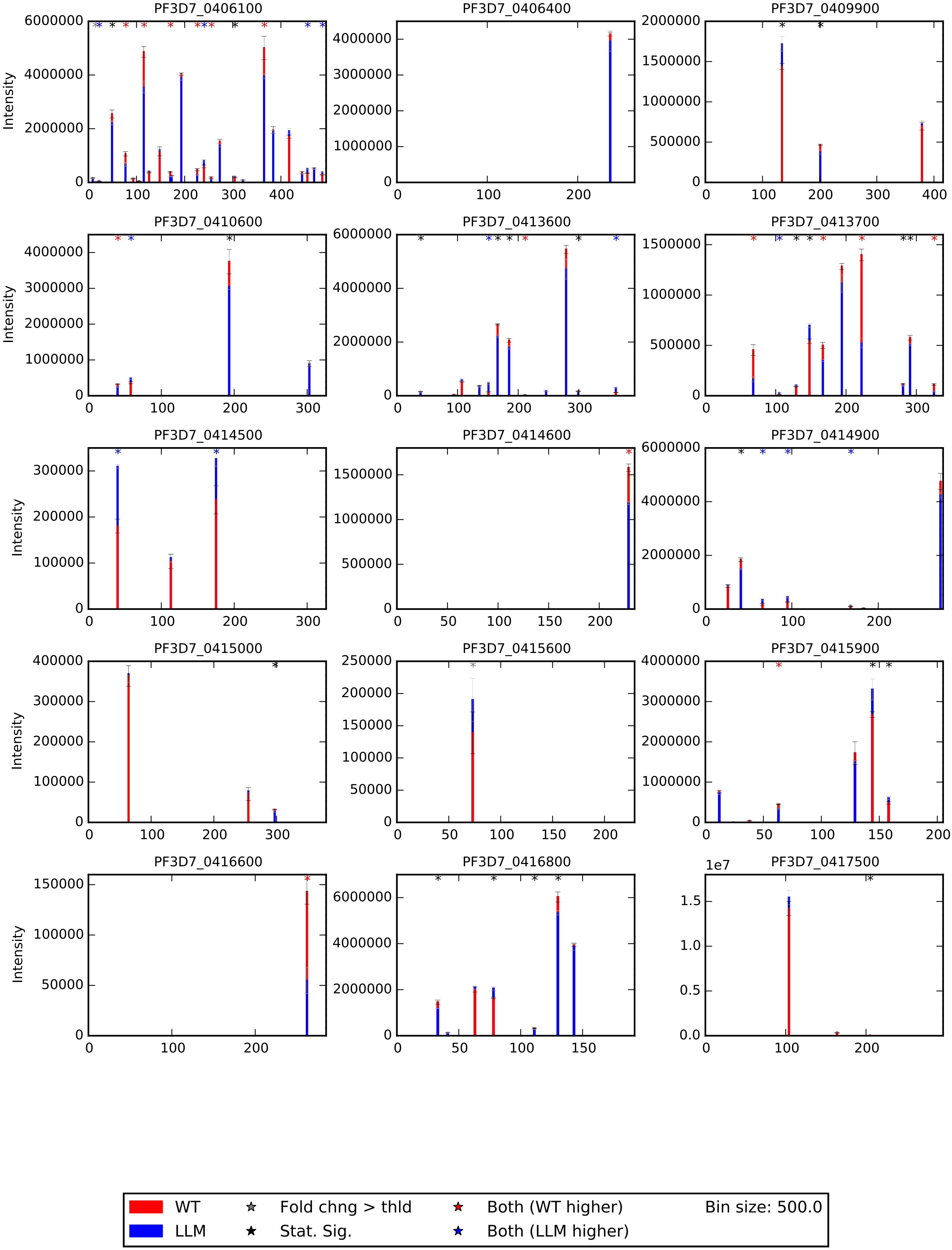
PF3D7_0406100
PF3D7_0406400
PF3D7_0409900
PF3D7_0410600
PF3D7_0413600
PF3D7_0413700
PF3D7_0414500
PF3D7_0414600
PF3D7_0414900
PF3D7_0415000
PF3D7_0415600
PF3D7_0415900
PF3D7_0416600
PF3D7_0416800
PF3D7_0417500
Intensity
1e7
WT
LLM
Fold chng > thld
Stat. Sig.
Both (WT higher)
Both (LLM higher)
Bin size: 500.0

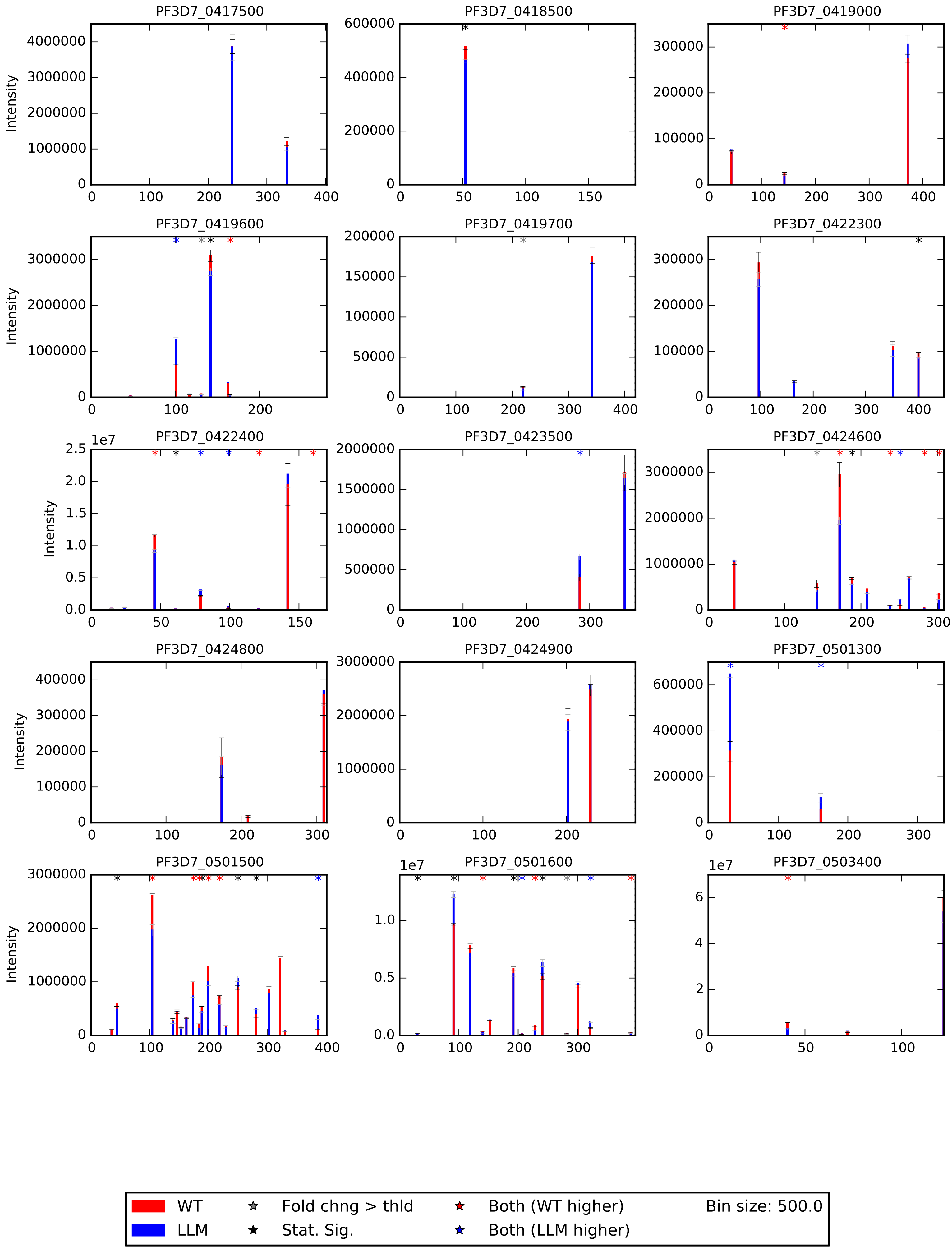
PF3D7_0417500
PF3D7_0418500
PF3D7_0419000
PF3D7_0419600
PF3D7_0419700
PF3D7_0422300
PF3D7_0422400
PF3D7_0423500
PF3D7_0424600
PF3D7_0424800
PF3D7_0424900
PF3D7_0501300
PF3D7_0501500
PF3D7_0501600
PF3D7_0503400
Intensity
1e7
WT
LLM
Fold chng > thld
Stat. Sig.
Both (WT higher)
Both (LLM higher)
Bin size: 500.0

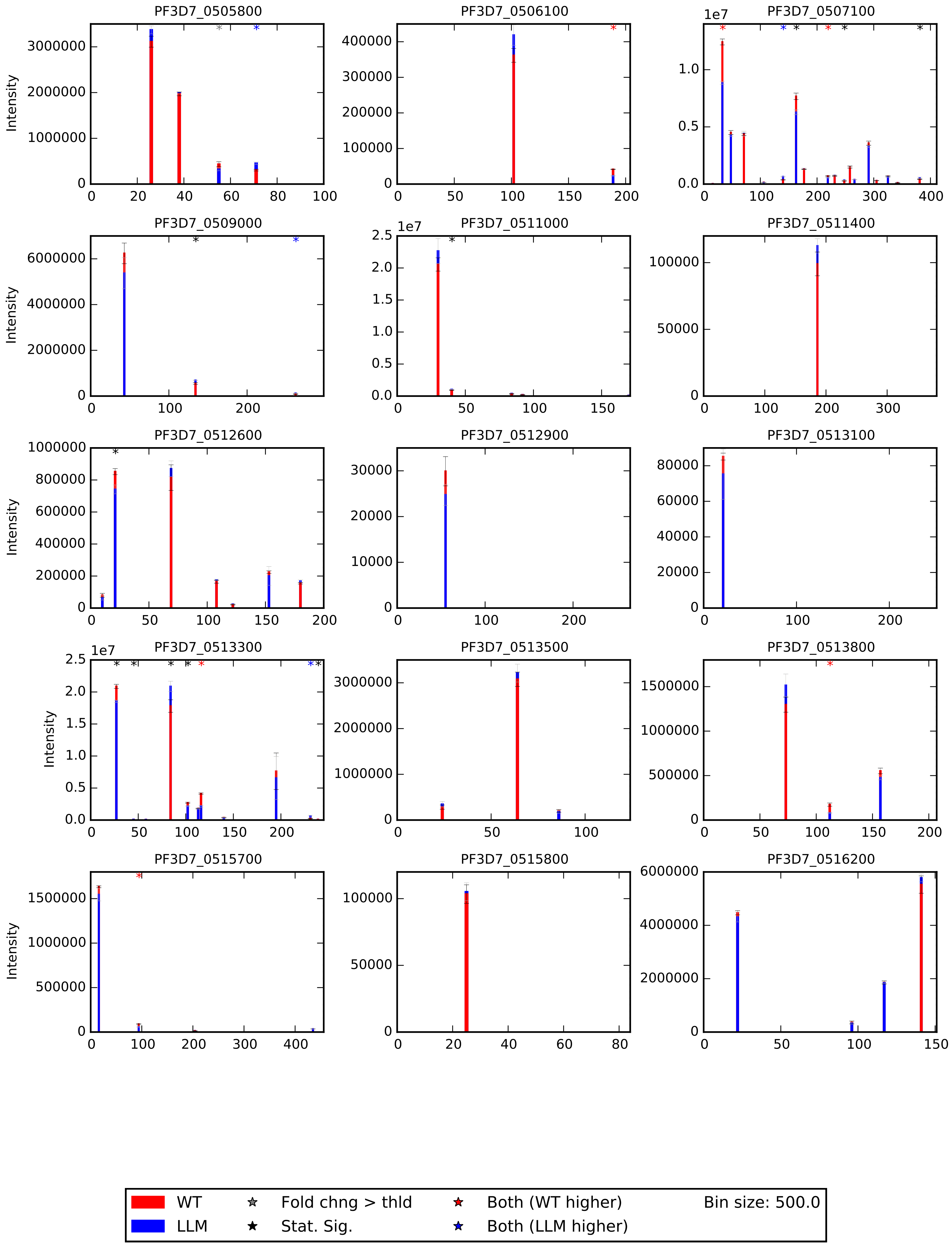
PF3D7_0505800
PF3D7_0506100
PF3D7_0507100
PF3D7_0509000
PF3D7_0511000
PF3D7_0511400
PF3D7_0512600
PF3D7_0512900
PF3D7_0513100
PF3D7_0513300
PF3D7_0513500
PF3D7_0513800
PF3D7_0515700
PF3D7_0515800
PF3D7_0516200
Intensity
1e7
WT
LLM
Fold chng > thld
Stat. Sig.
Both (WT higher)
Both (LLM higher)
Bin size: 500.0

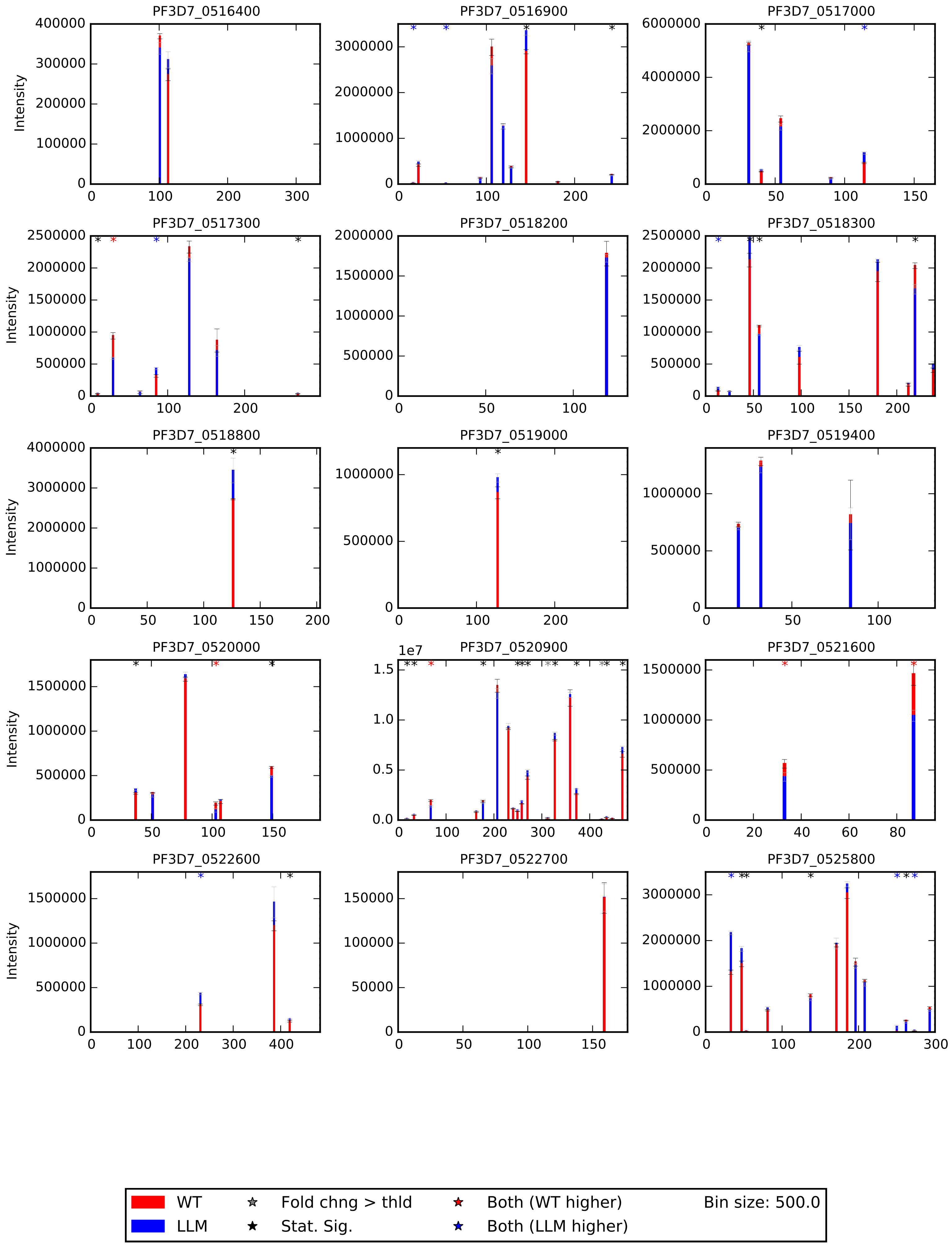
PF3D7_0516400
PF3D7_0516900
PF3D7_0517000
PF3D7_0517300
PF3D7_0518200
PF3D7_0518300
PF3D7_0518800
PF3D7_0519000
PF3D7_0519400
PF3D7_0520000
PF3D7_0520900
PF3D7_0521600
PF3D7_0522600
PF3D7_0522700
PF3D7_0525800
Intensity
1e7
WT
LLM
Fold chng > thld
Stat. Sig.
Both (WT higher)
Both (LLM higher)
Bin size: 500.0

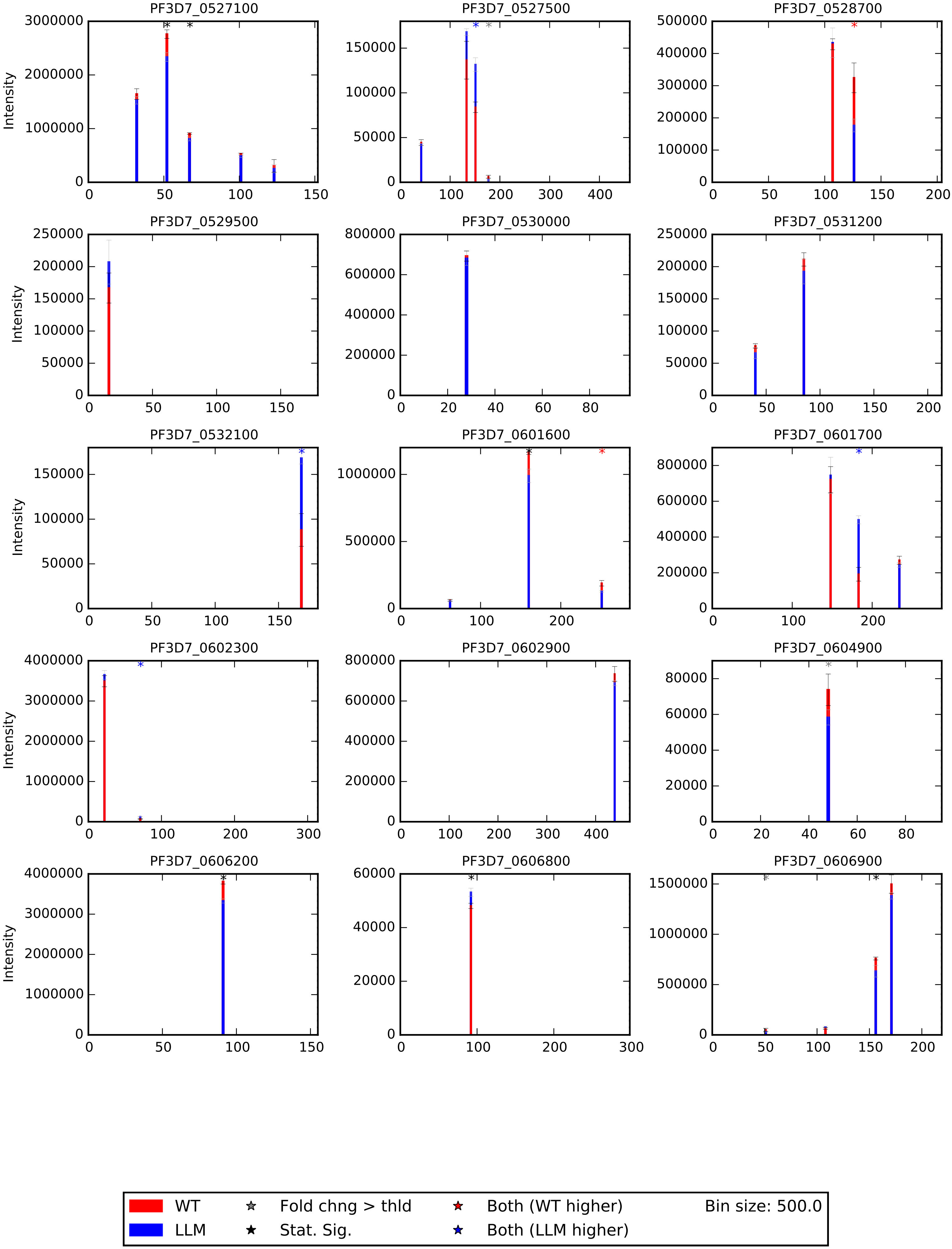
PF3D7_0527100
PF3D7_0527500
PF3D7_0528700
PF3D7_0529500
PF3D7_0530000
PF3D7_0531200
PF3D7_0532100
PF3D7_0601600
PF3D7_0601700
PF3D7_0602300
PF3D7_0602900
PF3D7_0604900
PF3D7_0606200
PF3D7_0606800
PF3D7_0606900
Intensity
WT
LLM
Fold chng > thld
Stat. Sig.
Both (WT higher)
Both (LLM higher)
Bin size: 500.0

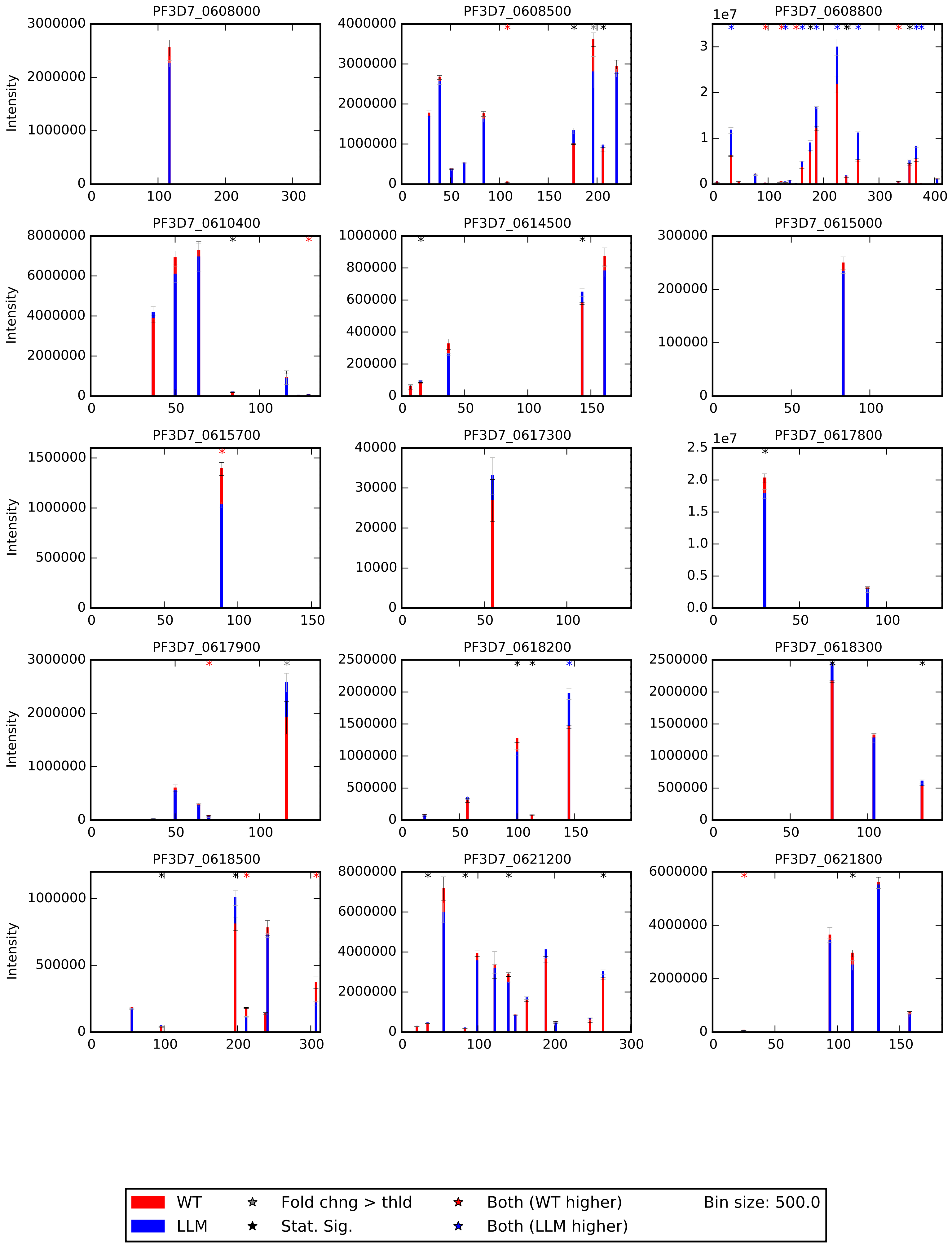
PF3D7_0608000
PF3D7_0608500
PF3D7_0608800
1e7
PF3D7_0610400
PF3D7_0614500
PF3D7_0615000
PF3D7_0615700
PF3D7_0617300
PF3D7_0617800
1e7
PF3D7_0617900
PF3D7_0618200
PF3D7_0618300
PF3D7_0618500
PF3D7_0621200
PF3D7_0621800
Intensity
WT
LLM
Fold chng > thld
Stat. Sig.
Both (WT higher)
Both (LLM higher)
Bin size: 500.0

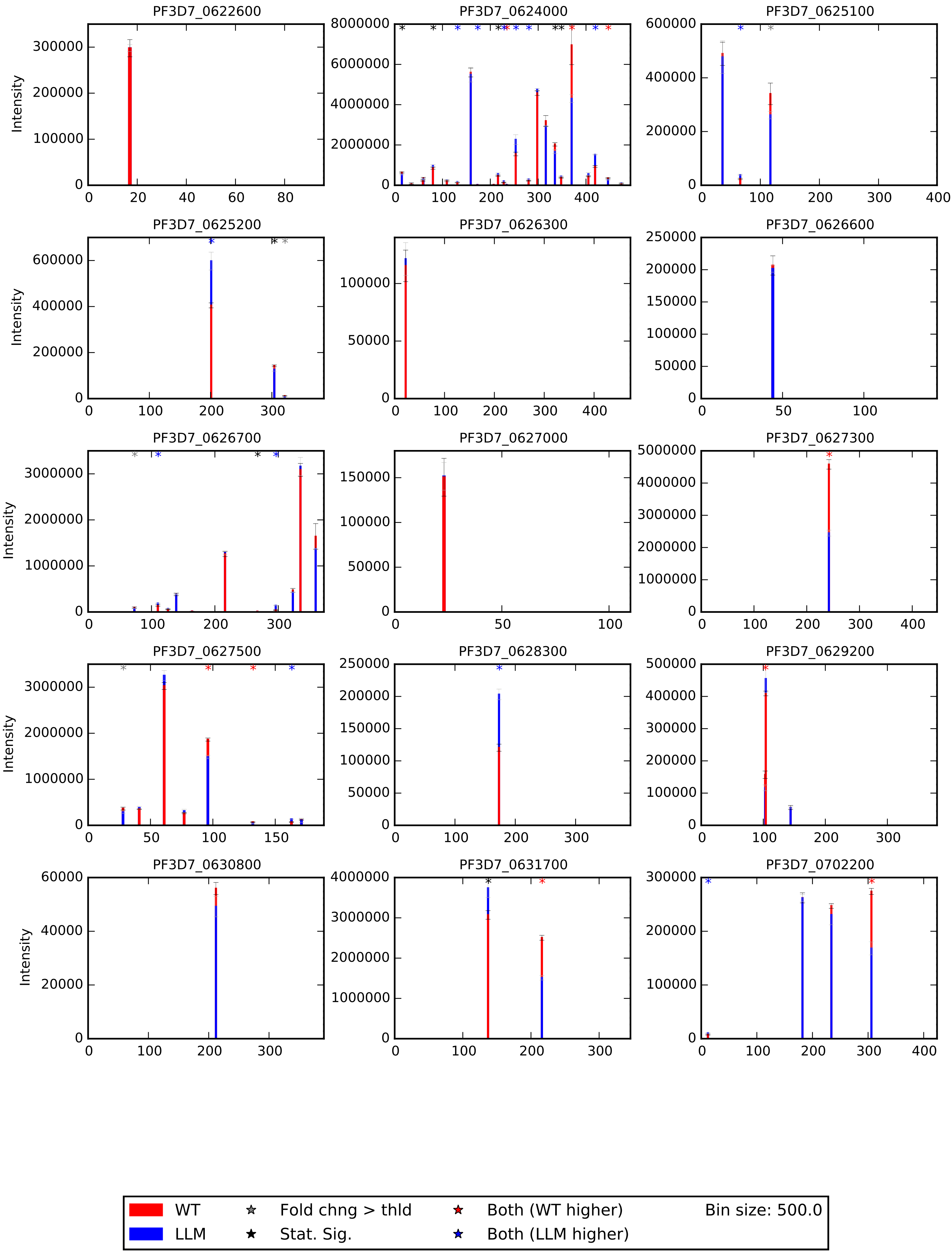

PF3D7_0622600
PF3D7_0624000
PF3D7_0625100
PF3D7_0625200
PF3D7_0626300
PF3D7_0626600
PF3D7_0626700
PF3D7_0627000
PF3D7_0627300
PF3D7_0627500
PF3D7_0628300
PF3D7_0629200
PF3D7_0630800
PF3D7_0631700
PF3D7_0702200
Intensity
WT
LLM
Fold chng > thld
Stat. Sig.
Both (WT higher)
Both (LLM higher)
Bin size: 500.0

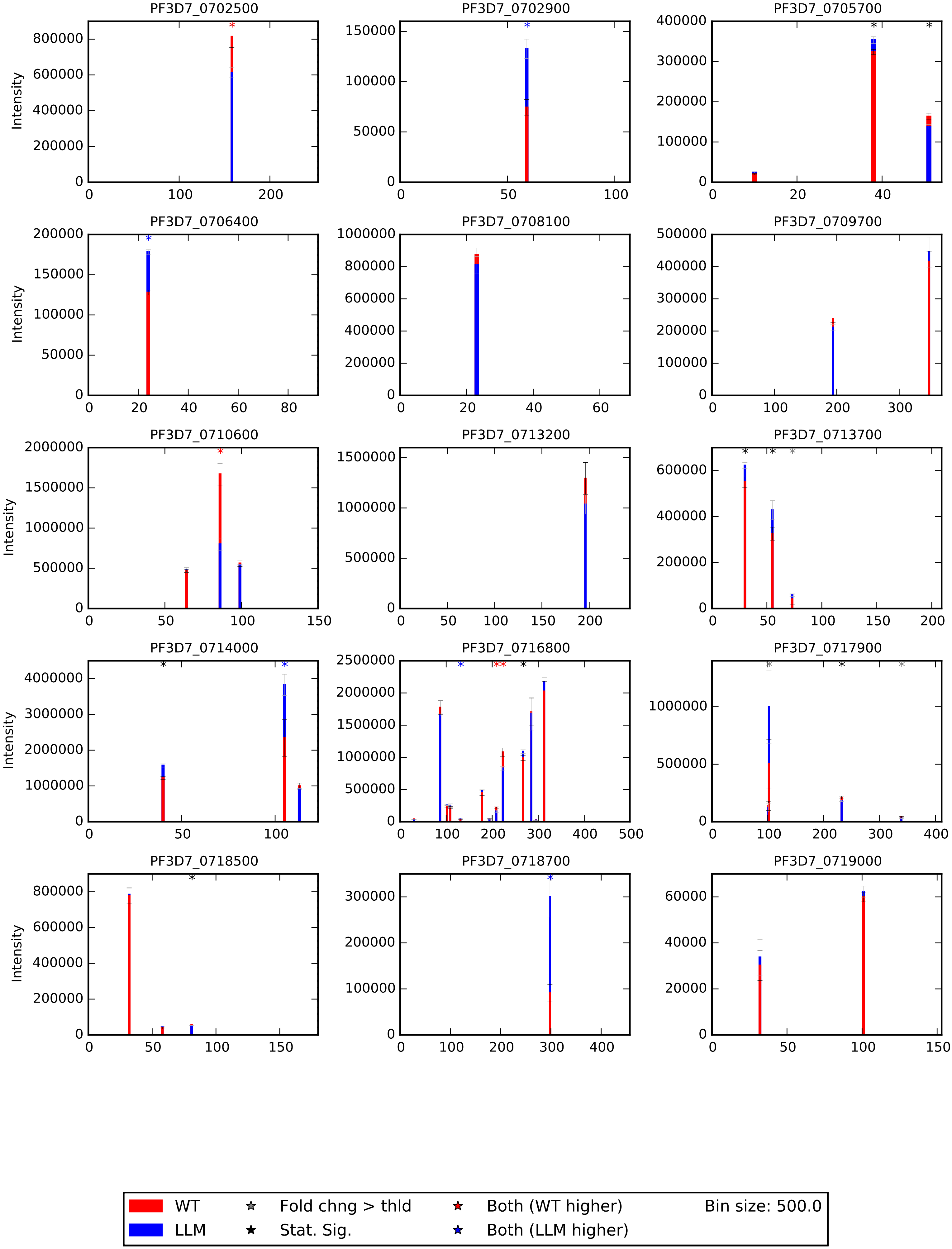

PF3D7_0702500
PF3D7_0702900
PF3D7_0705700
PF3D7_0706400
PF3D7_0708100
PF3D7_0709700
PF3D7_0710600
PF3D7_0713200
PF3D7_0713700
PF3D7_0714000
PF3D7_0716800
PF3D7_0717900
PF3D7_0718500
PF3D7_0718700
PF3D7_0719000
Intensity
WT
LLM
Fold chng > thld
Stat. Sig.
Both (WT higher)
Both (LLM higher)
Bin size: 500.0

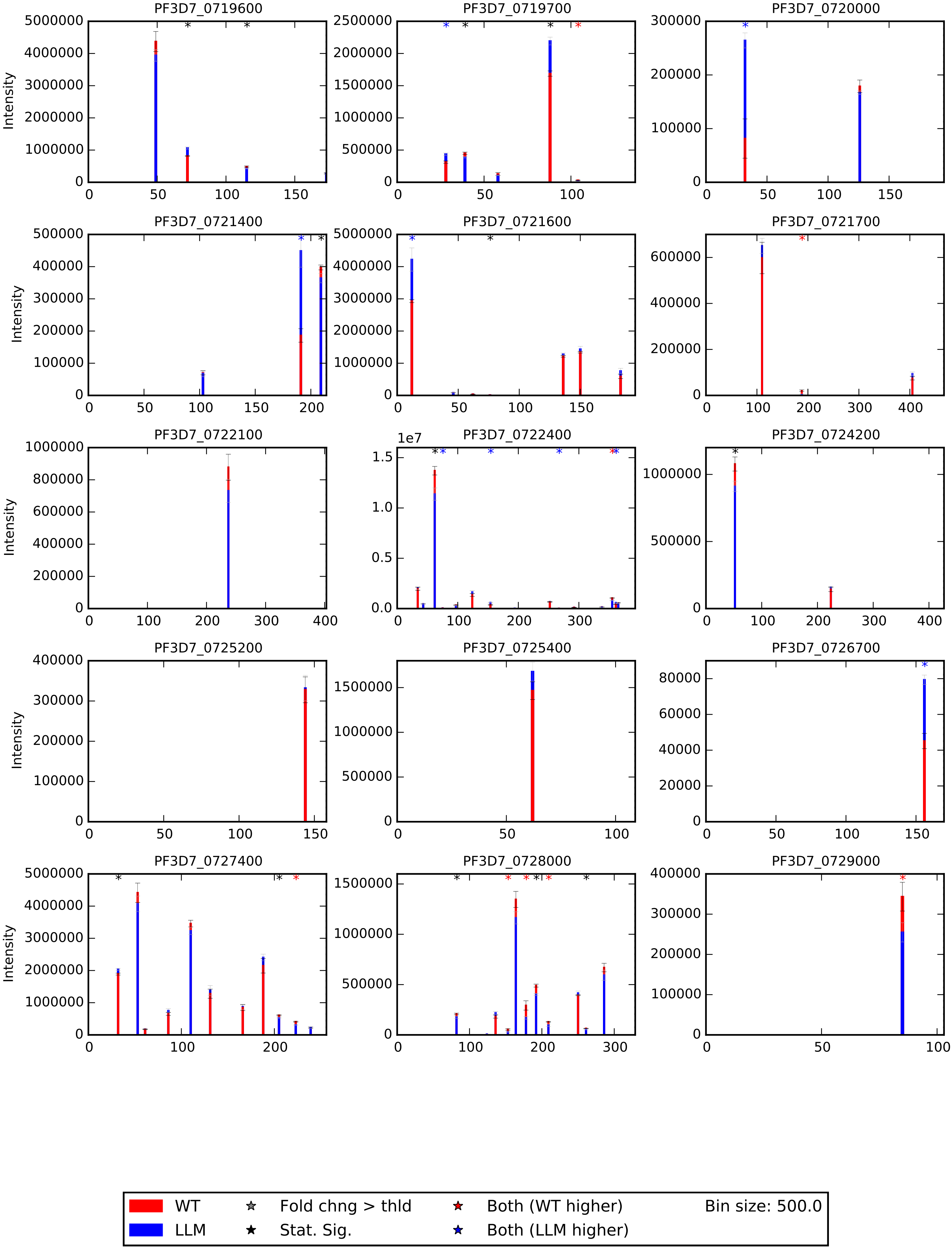

PF3D7_0719600
PF3D7_0719700
PF3D7_0720000
PF3D7_0721400
PF3D7_0721600
PF3D7_0721700
PF3D7_0722100
PF3D7_0722400
PF3D7_0724200
PF3D7_0725200
PF3D7_0725400
PF3D7_0726700
PF3D7_0727400
PF3D7_0728000
PF3D7_0729000
Intensity
1e7
WT
LLM
Fold chng > thld
Stat. Sig.
Both (WT higher)
Both (LLM higher)
Bin size: 500.0

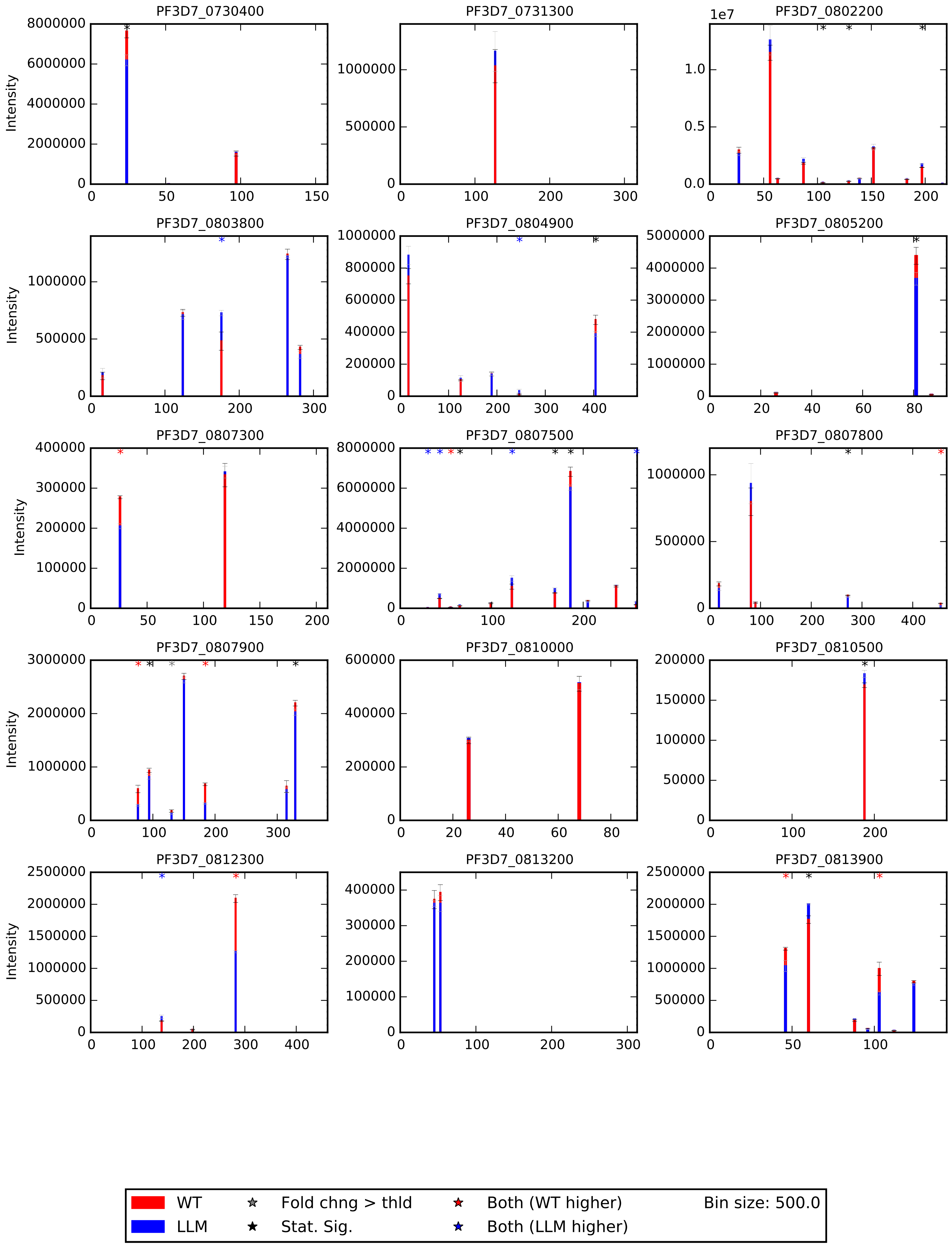

PF3D7_0730400
PF3D7_0731300
PF3D7_0802200
PF3D7_0803800
PF3D7_0804900
PF3D7_0805200
PF3D7_0807300
PF3D7_0807500
PF3D7_0807800
PF3D7_0807900
PF3D7_0810000
PF3D7_0810500
PF3D7_0812300
PF3D7_0813200
PF3D7_0813900
Intensity
1e7
WT
LLM
Fold chng > thld
Stat. Sig.
Both (WT higher)
Both (LLM higher)
Bin size: 500.0

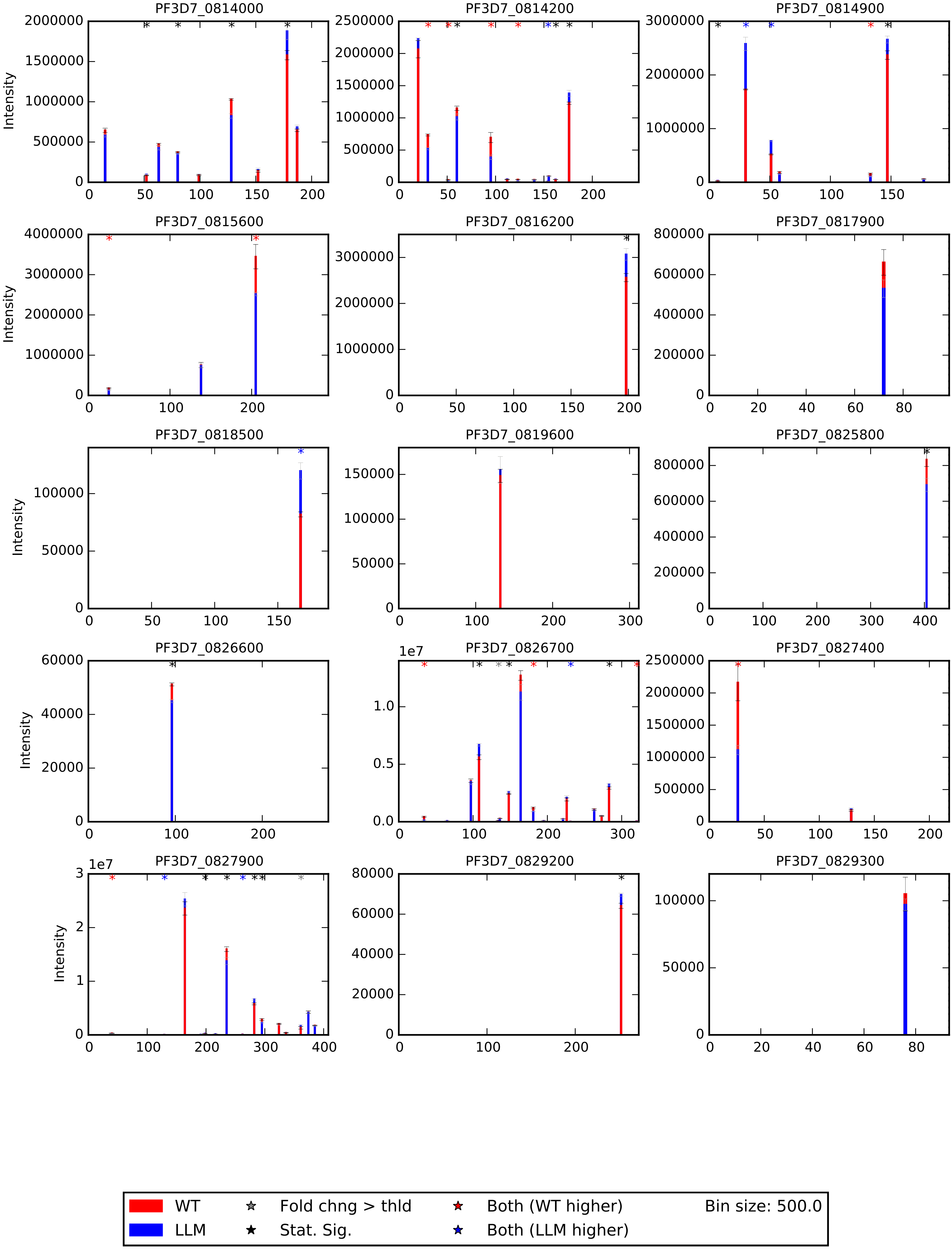
PF3D7_0814000
PF3D7_0814200
PF3D7_0814900
PF3D7_0815600
PF3D7_0816200
PF3D7_0817900
PF3D7_0818500
PF3D7_0819600
PF3D7_0825800
PF3D7_0826600
PF3D7_0826700
PF3D7_0827400
PF3D7_0827900
PF3D7_0829200
PF3D7_0829300
Intensity
1e7
WT
LLM
Fold chng > thld
Stat. Sig.
Both (WT higher)
Both (LLM higher)
Bin size: 500.0

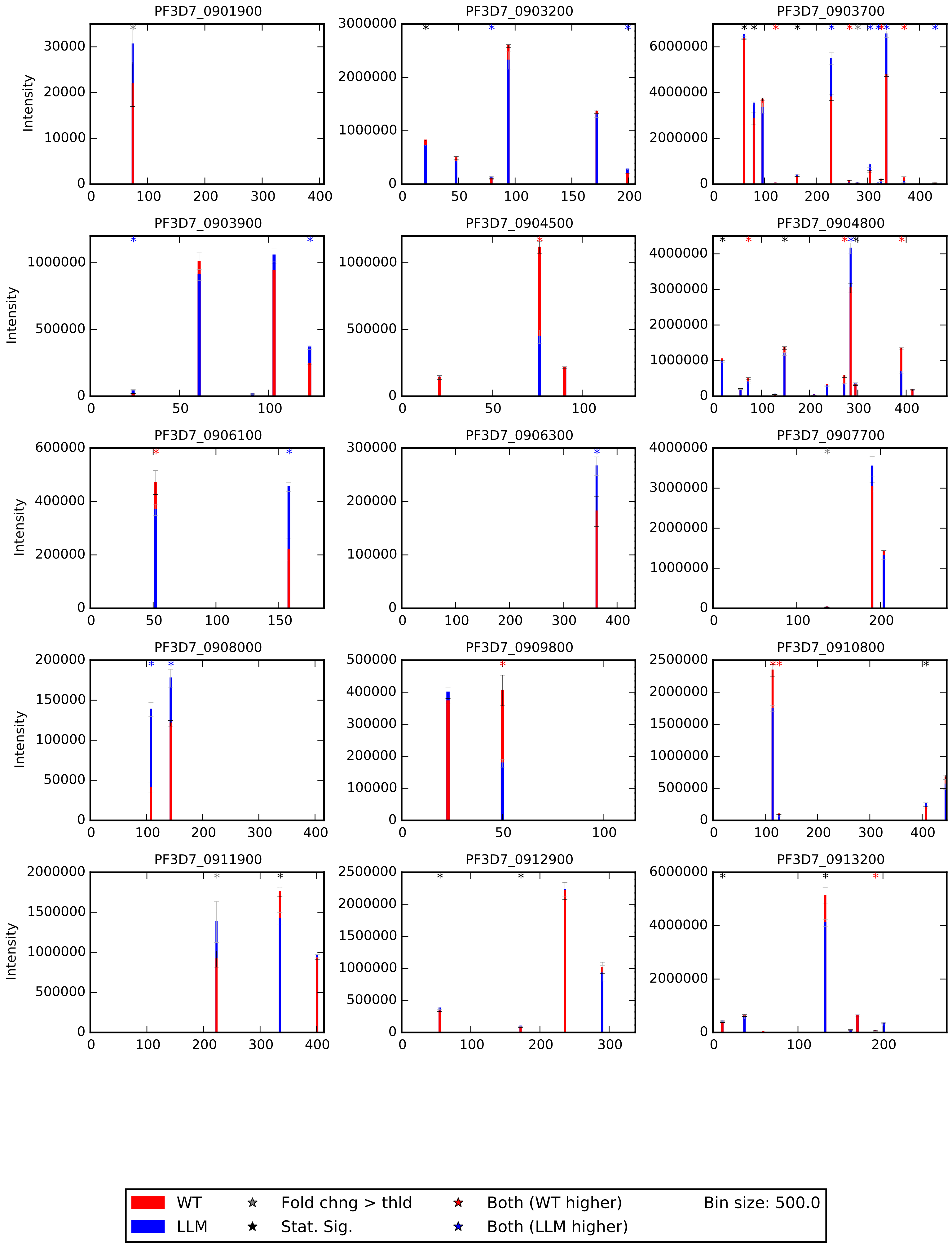
PF3D7_0901900
PF3D7_0903200
PF3D7_0903700
PF3D7_0903900
PF3D7_0904500
PF3D7_0904800
PF3D7_0906100
PF3D7_0906300
PF3D7_0907700
PF3D7_0908000
PF3D7_0909800
PF3D7_0910800
PF3D7_0911900
PF3D7_0912900
PF3D7_0913200
Intensity
WT
LLM
Fold chng > thld
Stat. Sig.
Both (WT higher)
Both (LLM higher)
Bin size: 500.0

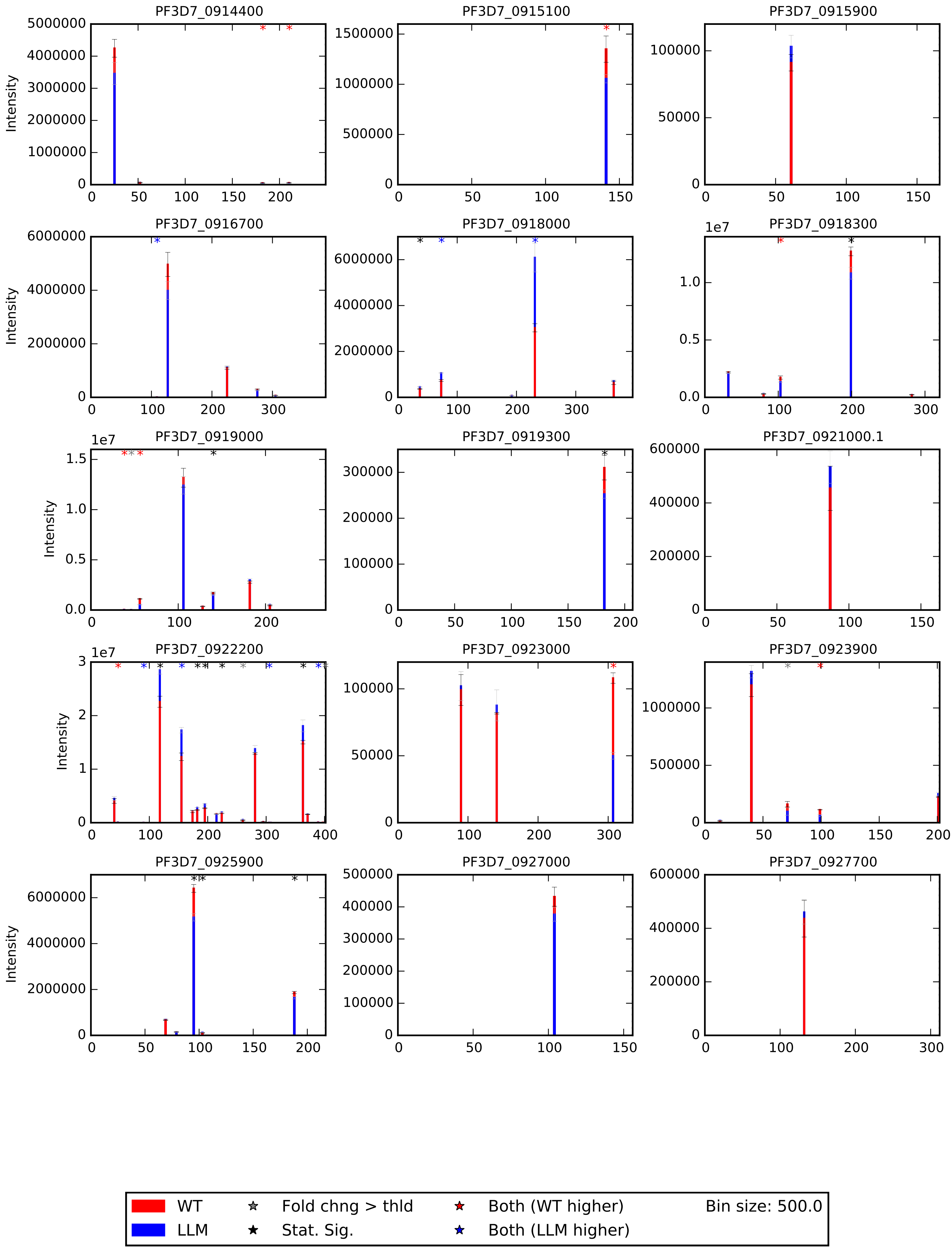
PF3D7_0914400
PF3D7_0915100
PF3D7_0915900
PF3D7_0916700
PF3D7_0918000
PF3D7_0918300
PF3D7_0919000
PF3D7_0919300
PF3D7_0921000.1
PF3D7_0922200
PF3D7_0923000
PF3D7_0923900
PF3D7_0925900
PF3D7_0927000
PF3D7_0927700
Intensity
1e7
WT
LLM
Fold chng > thld
Stat. Sig.
Both (WT higher)
Both (LLM higher)
Bin size: 500.0

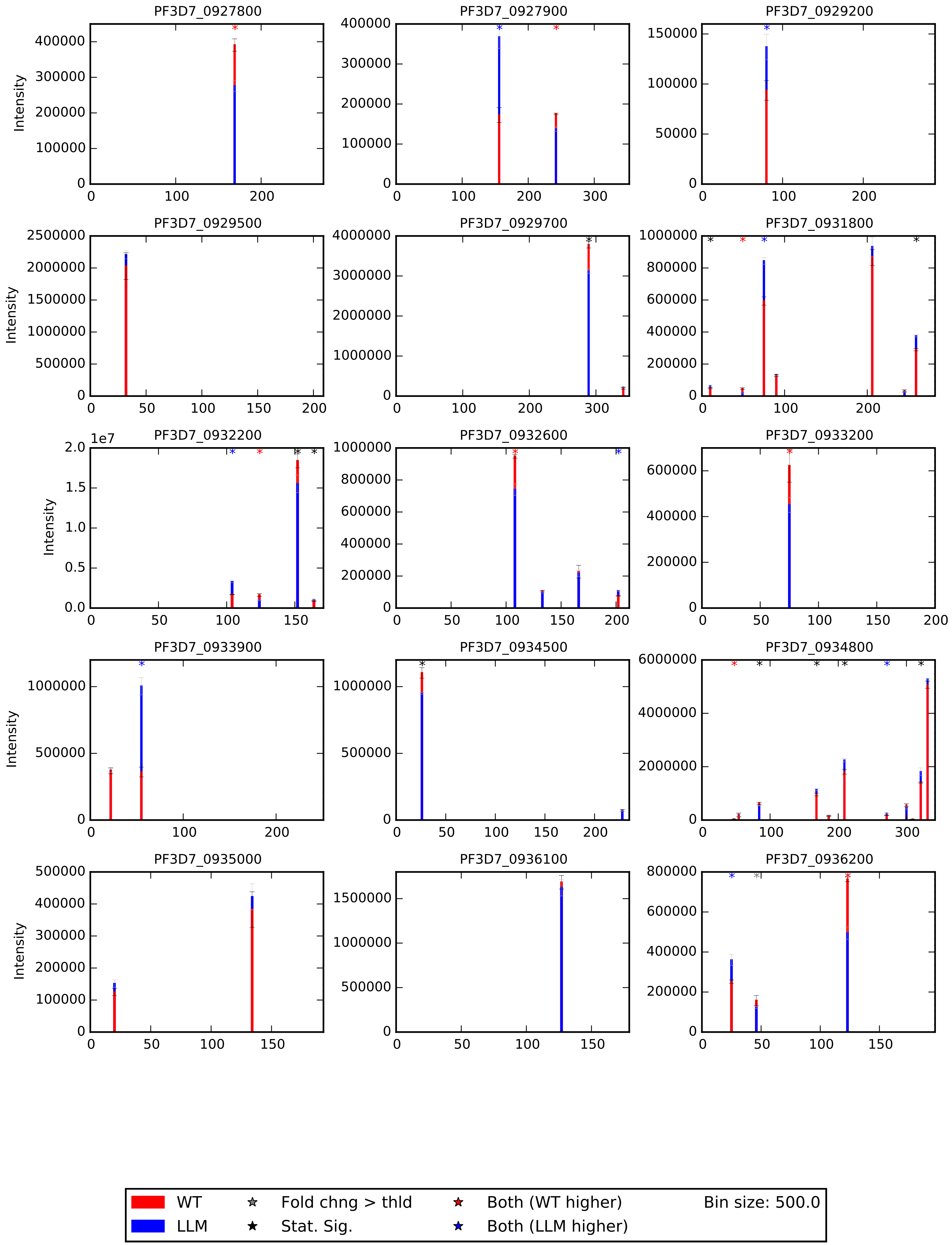
PF3D7_0927800
PF3D7_0927900
PF3D7_0929200
PF3D7_0929500
PF3D7_0929700
PF3D7_0931800
PF3D7_0932200
PF3D7_0932600
PF3D7_0933200
PF3D7_0933900
PF3D7_0934500
PF3D7_0934800
PF3D7_0935000
PF3D7_0936100
PF3D7_0936200
Intensity
1e7
WT
LLM
Fold chng > thld
Stat. Sig.
Both (WT higher)
Both (LLM higher)
Bin size: 500.0

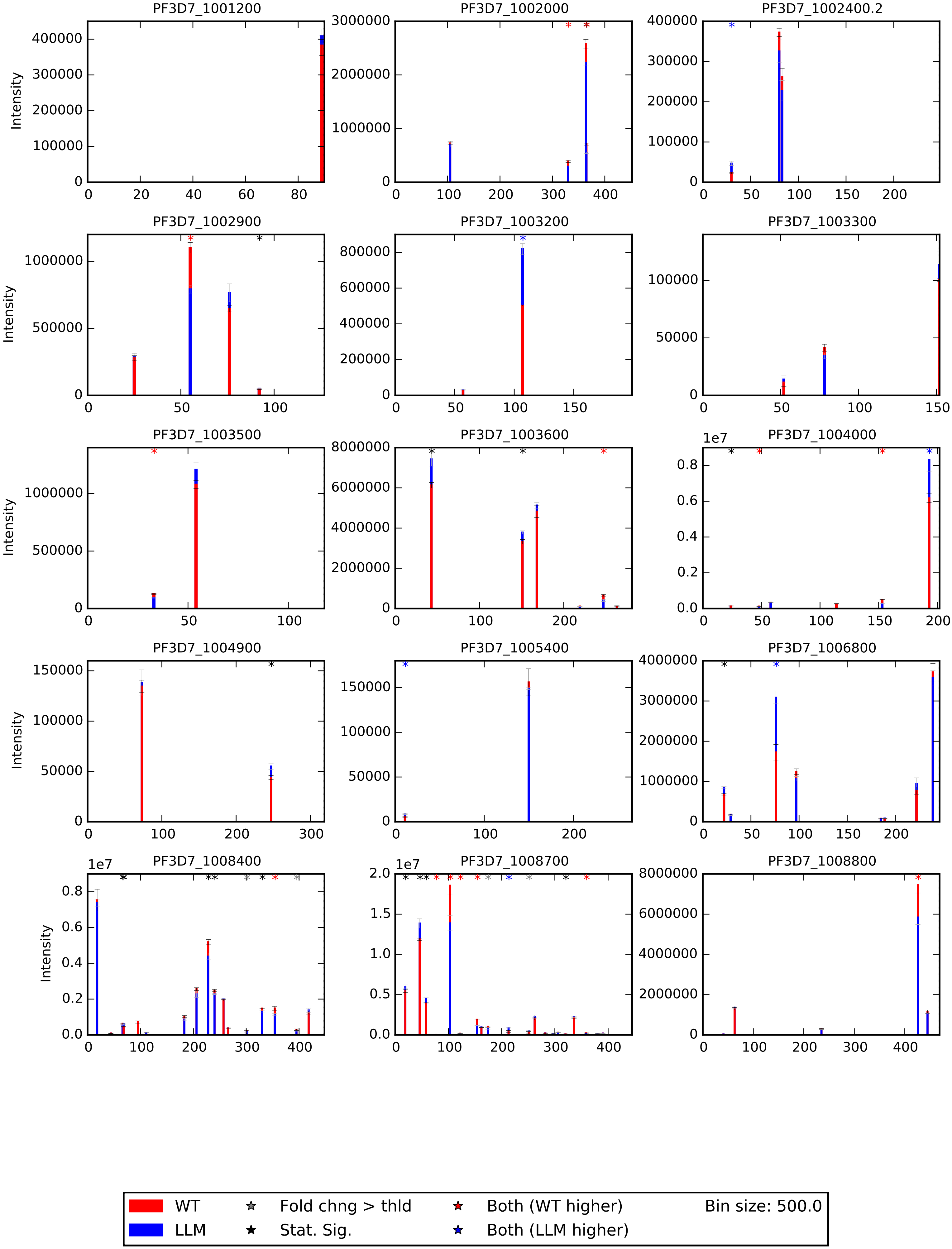
PF3D7_1001200
PF3D7_1002000
PF3D7_1002400.2
PF3D7_1002900
PF3D7_1003200
PF3D7_1003300
PF3D7_1003500
PF3D7_1003600
PF3D7_1004000
PF3D7_1004900
PF3D7_1005400
PF3D7_1006800
PF3D7_1008400
PF3D7_1008700
PF3D7_1008800
Intensity
1e7
WT
LLM
Fold chng > thld
Stat. Sig.
Both (WT higher)
Both (LLM higher)
Bin size: 500.0

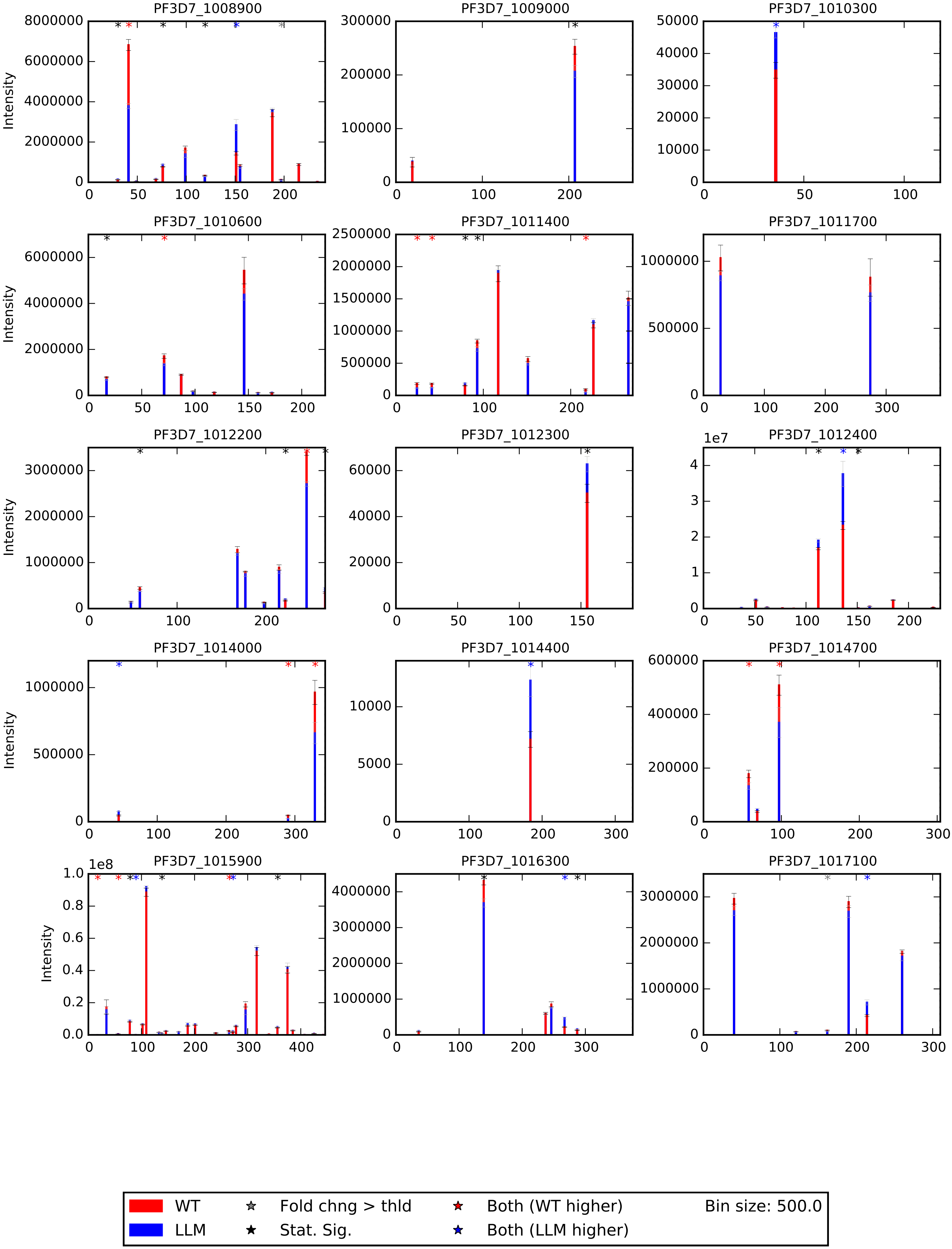
PF3D7_1008900
PF3D7_1009000
PF3D7_1010300
PF3D7_1010600
PF3D7_1011400
PF3D7_1011700
PF3D7_1012200
PF3D7_1012300
PF3D7_1012400
PF3D7_1014000
PF3D7_1014400
PF3D7_1014700
PF3D7_1015900
PF3D7_1016300
PF3D7_1017100
Intensity
1e7
1e8
WT
LLM
Fold chng > thld
Stat. Sig.
Both (WT higher)
Both (LLM higher)
Bin size: 500.0

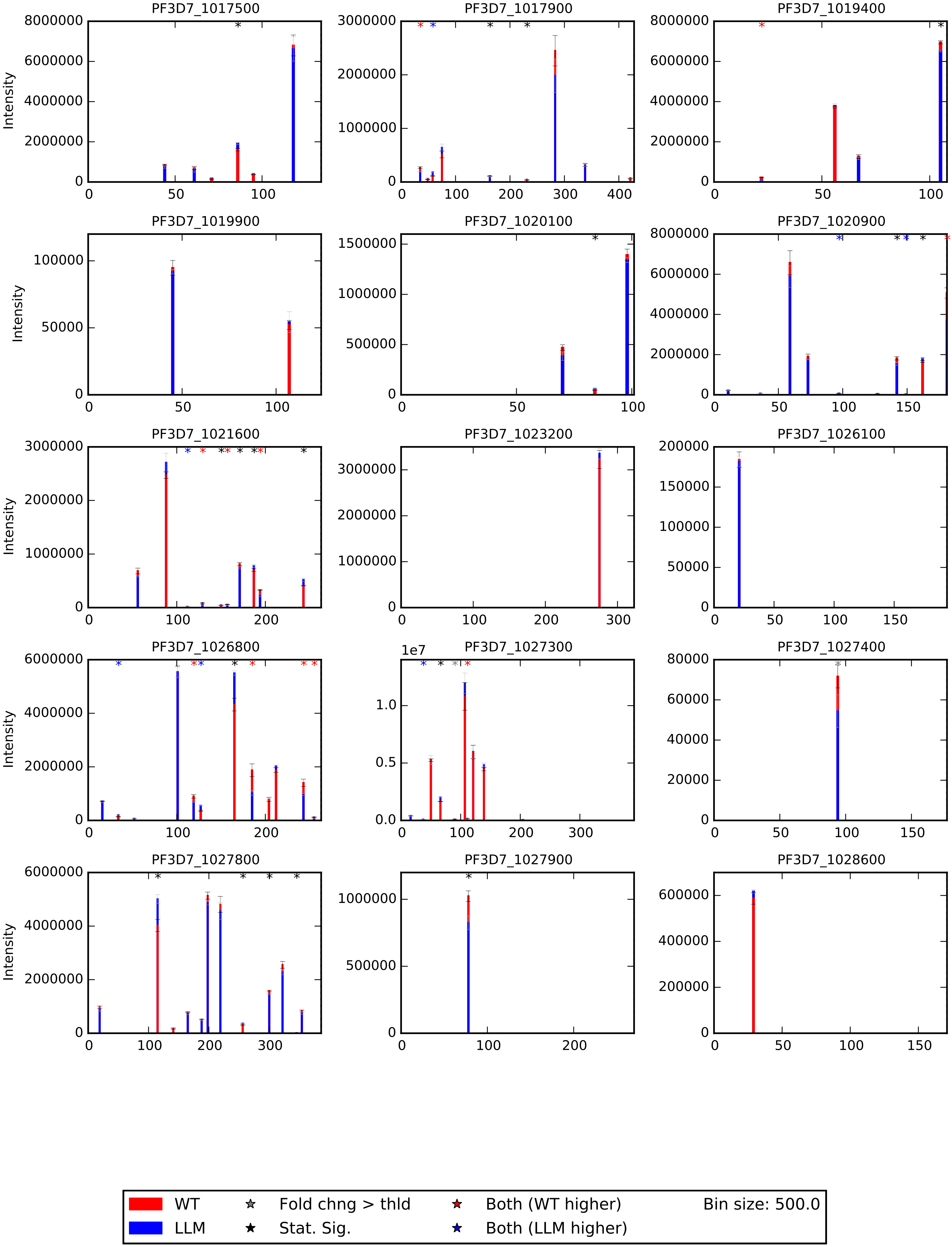
PF3D7_1017500
PF3D7_1017900
PF3D7_1019400
PF3D7_1019900
PF3D7_1020100
PF3D7_1020900
PF3D7_1021600
PF3D7_1023200
PF3D7_1026100
PF3D7_1026800
PF3D7_1027300
1e7
PF3D7_1027400
PF3D7_1027800
PF3D7_1027900
PF3D7_1028600
Intensity
WT
LLM
Fold chng > thld
Stat. Sig.
Both (WT higher)
Both (LLM higher)
Bin size: 500.0

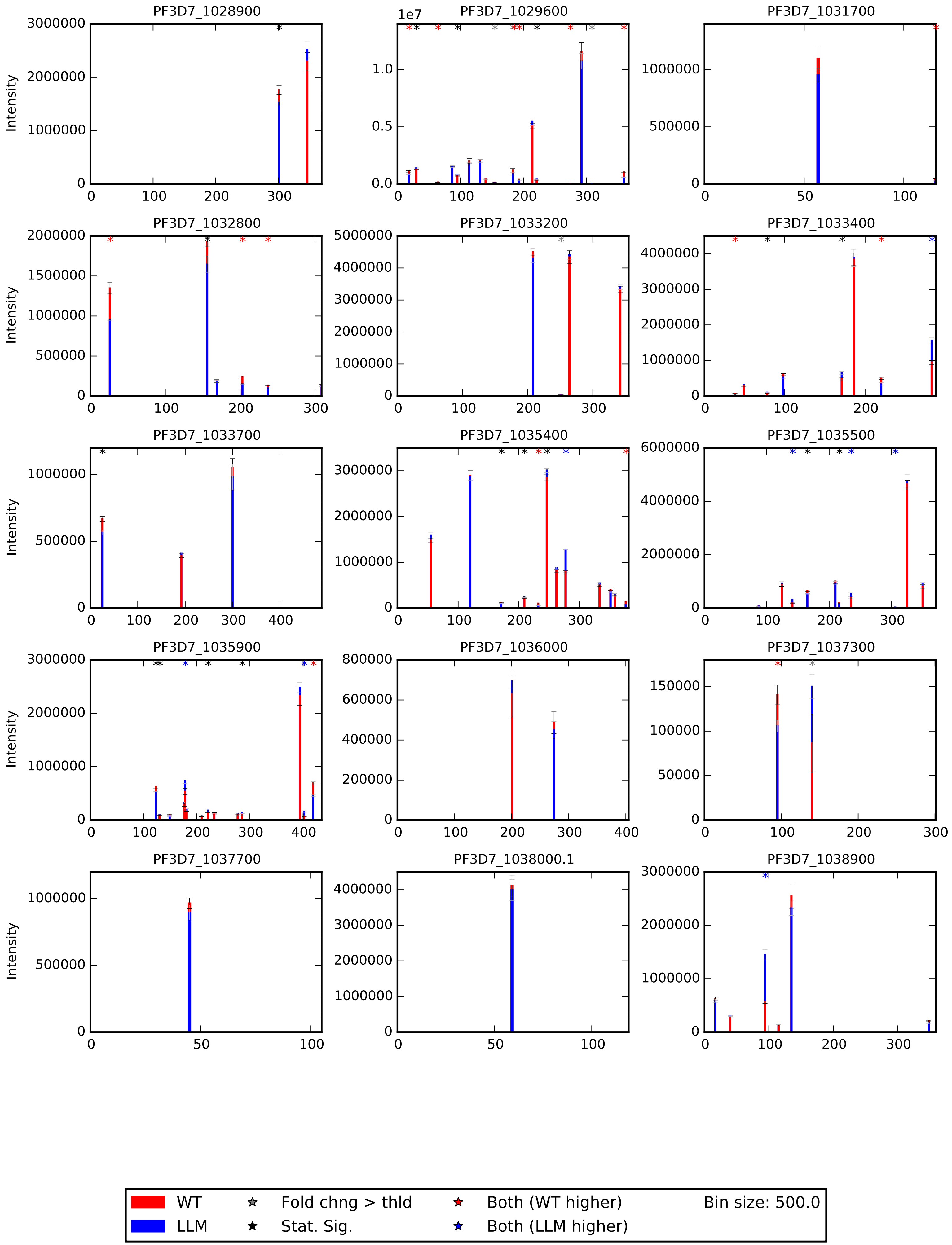
PF3D7_1028900
PF3D7_1029600
1e7
PF3D7_1031700
PF3D7_1032800
PF3D7_1033200
PF3D7_1033400
PF3D7_1033700
PF3D7_1035400
PF3D7_1035500
PF3D7_1035900
PF3D7_1036000
PF3D7_1037300
PF3D7_1037700
PF3D7_1038000.1
PF3D7_1038900
Intensity
WT
LLM
Fold chng > thld
Stat. Sig.
Both (WT higher)
Both (LLM higher)
Bin size: 500.0

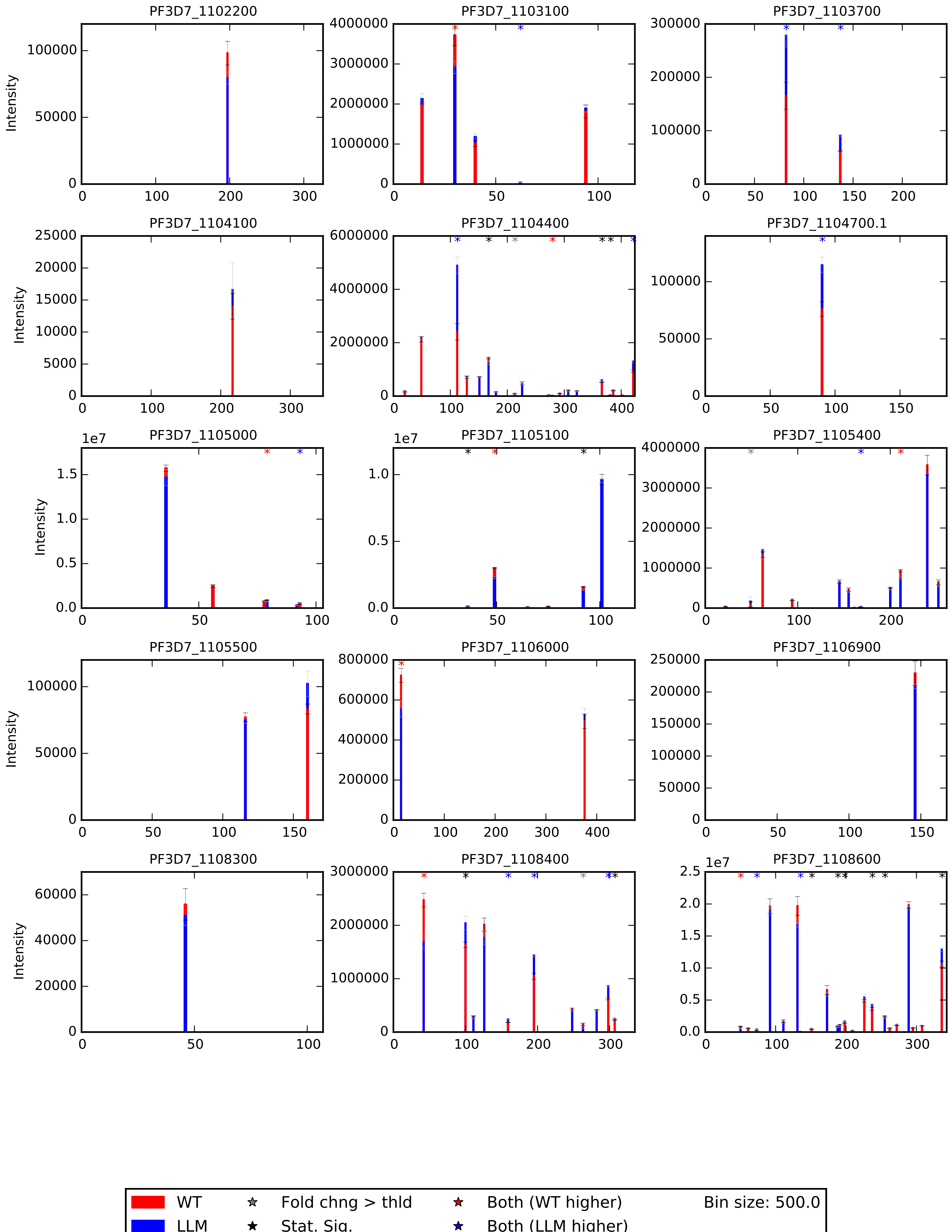
PF3D7_1102200
PF3D7_1103100
PF3D7_1103700
PF3D7_1104100
PF3D7_1104400
PF3D7_1104700.1
PF3D7_1105000
PF3D7_1105100
PF3D7_1105400
PF3D7_1105500
PF3D7_1106000
PF3D7_1106900
PF3D7_1108300
PF3D7_1108400
PF3D7_1108600
Intensity
1e7
WT
LLM
Fold chng > thld
Stat. Sig.
Both (WT higher)
Both (LLM higher)
Bin size: 500.0

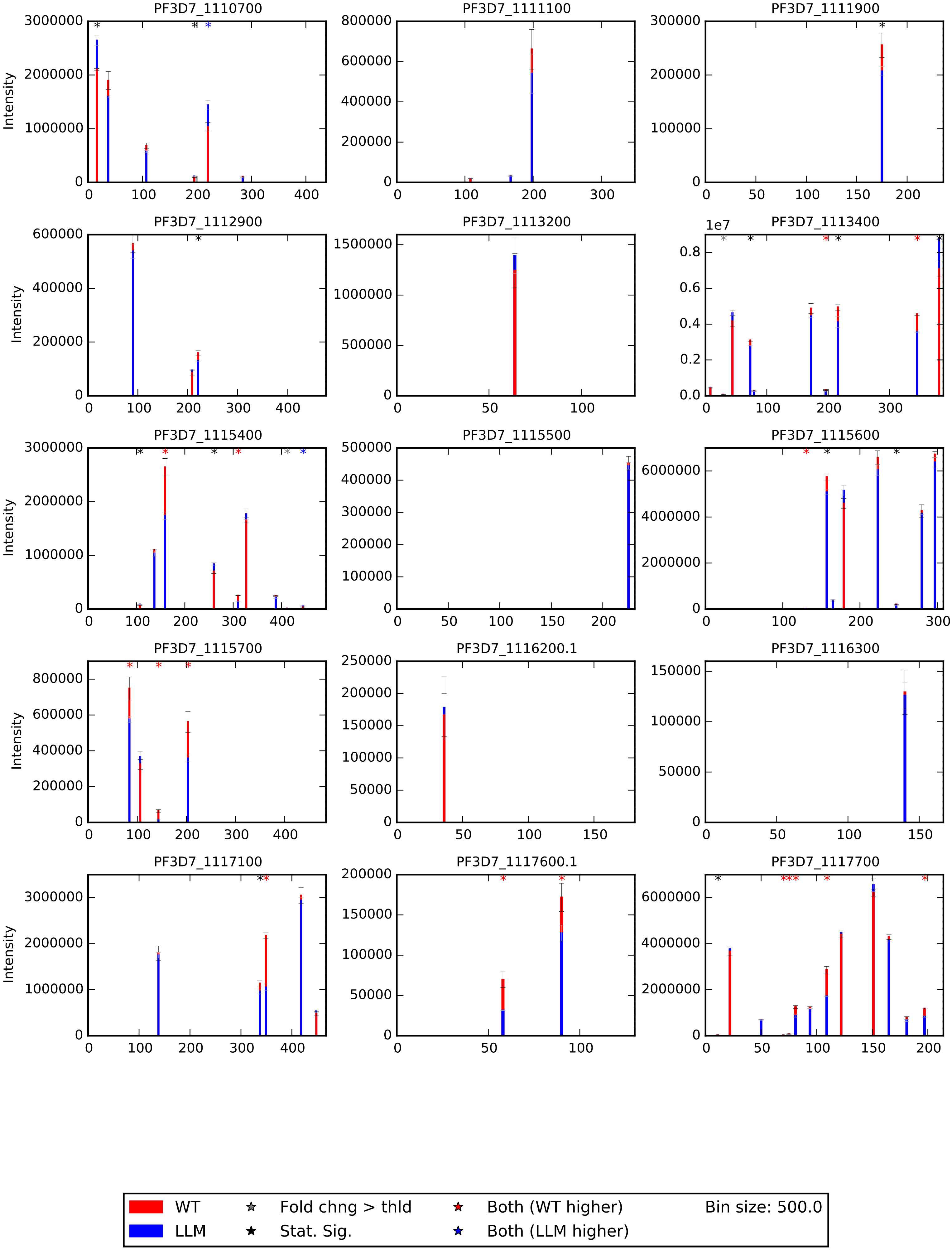
PF3D7_1110700
PF3D7_1111100
PF3D7_1111900
PF3D7_1112900
PF3D7_1113200
PF3D7_1113400
PF3D7_1115400
PF3D7_1115500
PF3D7_1115600
PF3D7_1115700
PF3D7_1116200.1
PF3D7_1116300
PF3D7_1117100
PF3D7_1117600.1
PF3D7_1117700
Intensity
1e7
WT
LLM
Fold chng > thld
Stat. Sig.
Both (WT higher)
Both (LLM higher)
Bin size: 500.0

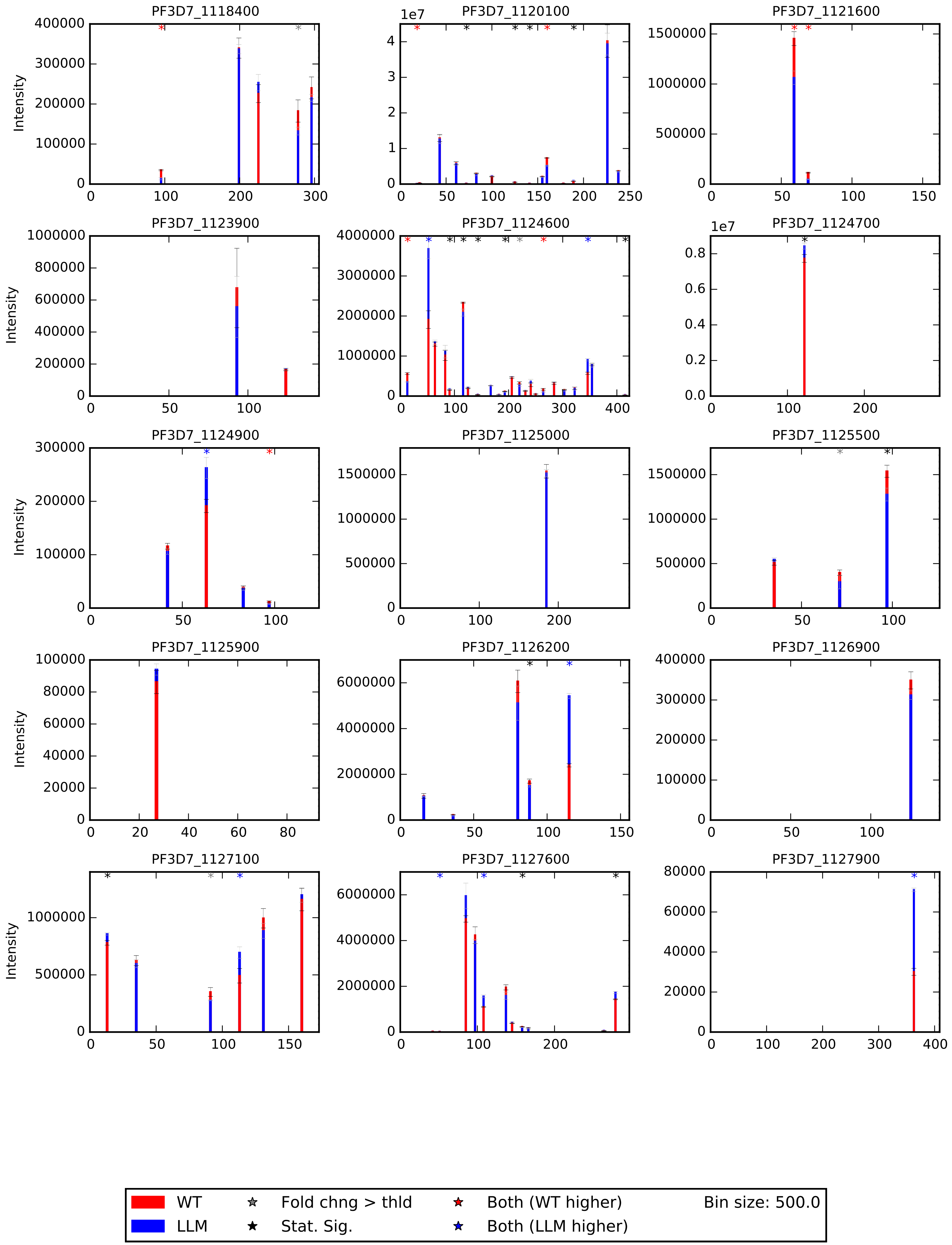

PF3D7_1118400
PF3D7_1120100
PF3D7_1121600
PF3D7_1123900
PF3D7_1124600
PF3D7_1124700
PF3D7_1124900
PF3D7_1125000
PF3D7_1125500
PF3D7_1125900
PF3D7_1126200
PF3D7_1126900
PF3D7_1127100
PF3D7_1127600
PF3D7_1127900
Intensity
1e7
WT
LLM
Fold chng > thld
Stat. Sig.
Both (WT higher)
Both (LLM higher)
Bin size: 500.0

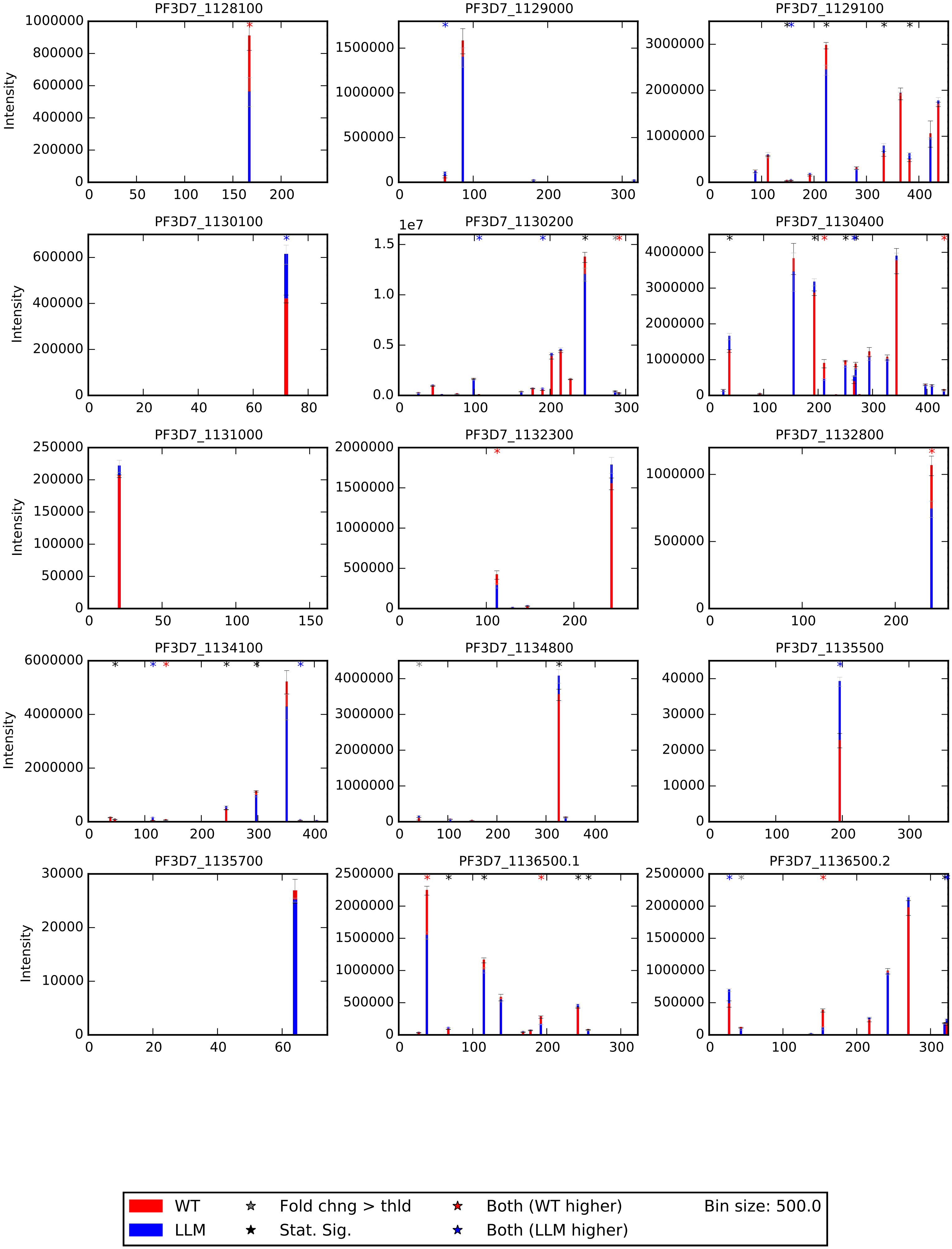
PF3D7_1128100
PF3D7_1129000
PF3D7_1129100
PF3D7_1130100
PF3D7_1130200
PF3D7_1130400
PF3D7_1131000
PF3D7_1132300
PF3D7_1132800
PF3D7_1134100
PF3D7_1134800
PF3D7_1135500
PF3D7_1135700
PF3D7_1136500.1
PF3D7_1136500.2
Intensity
1e7
WT
LLM
Fold chng > thld
Stat. Sig.
Both (WT higher)
Both (LLM higher)
Bin size: 500.0

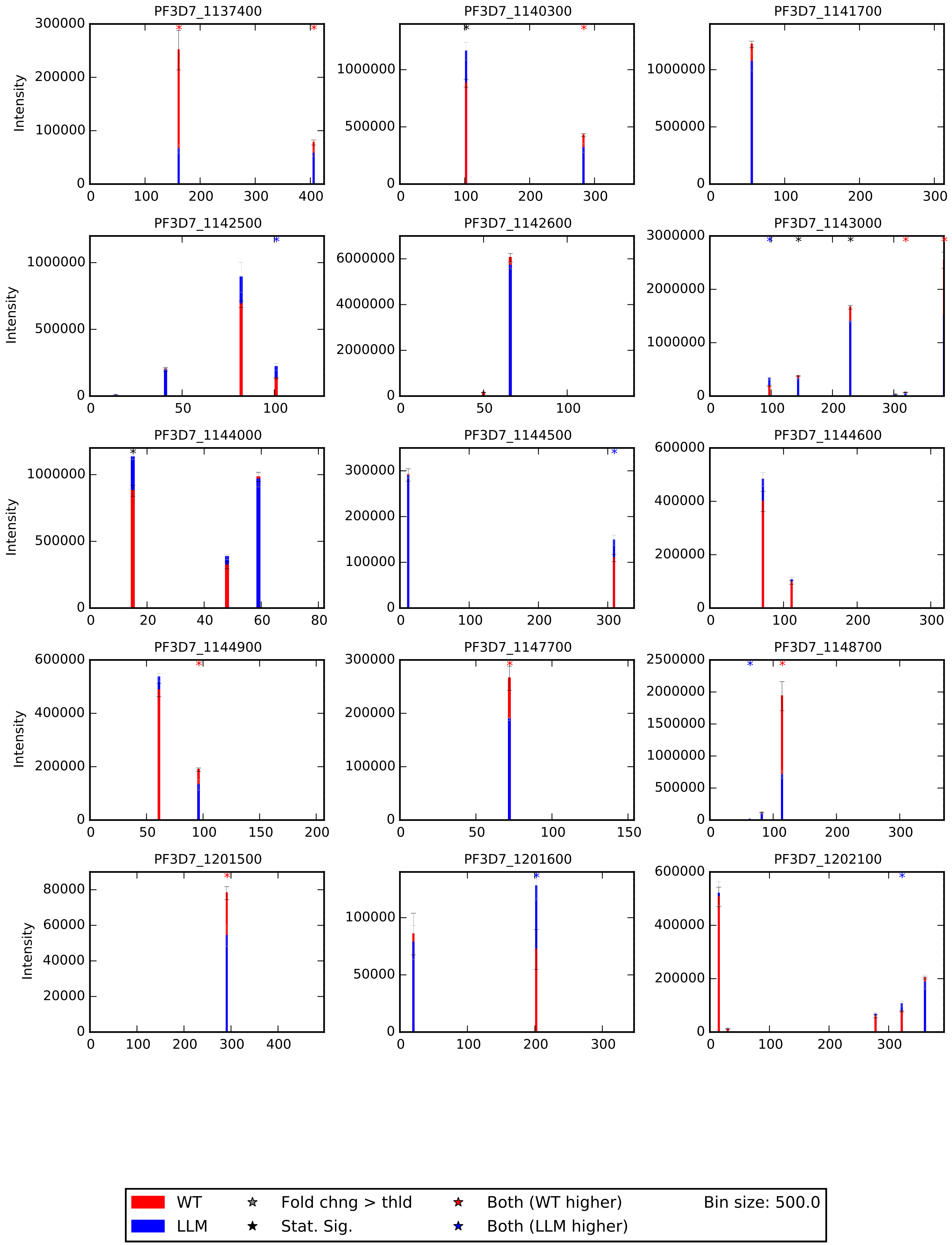
PF3D7_1137400
PF3D7_1140300
PF3D7_1141700
PF3D7_1142500
PF3D7_1142600
PF3D7_1143000
PF3D7_1144000
PF3D7_1144500
PF3D7_1144600
PF3D7_1144900
PF3D7_1147700
PF3D7_1148700
PF3D7_1201500
PF3D7_1201600
PF3D7_1202100
Intensity
WT
LLM
Fold chng > thld
Stat. Sig.
Both (WT higher)
Both (LLM higher)
Bin size: 500.0

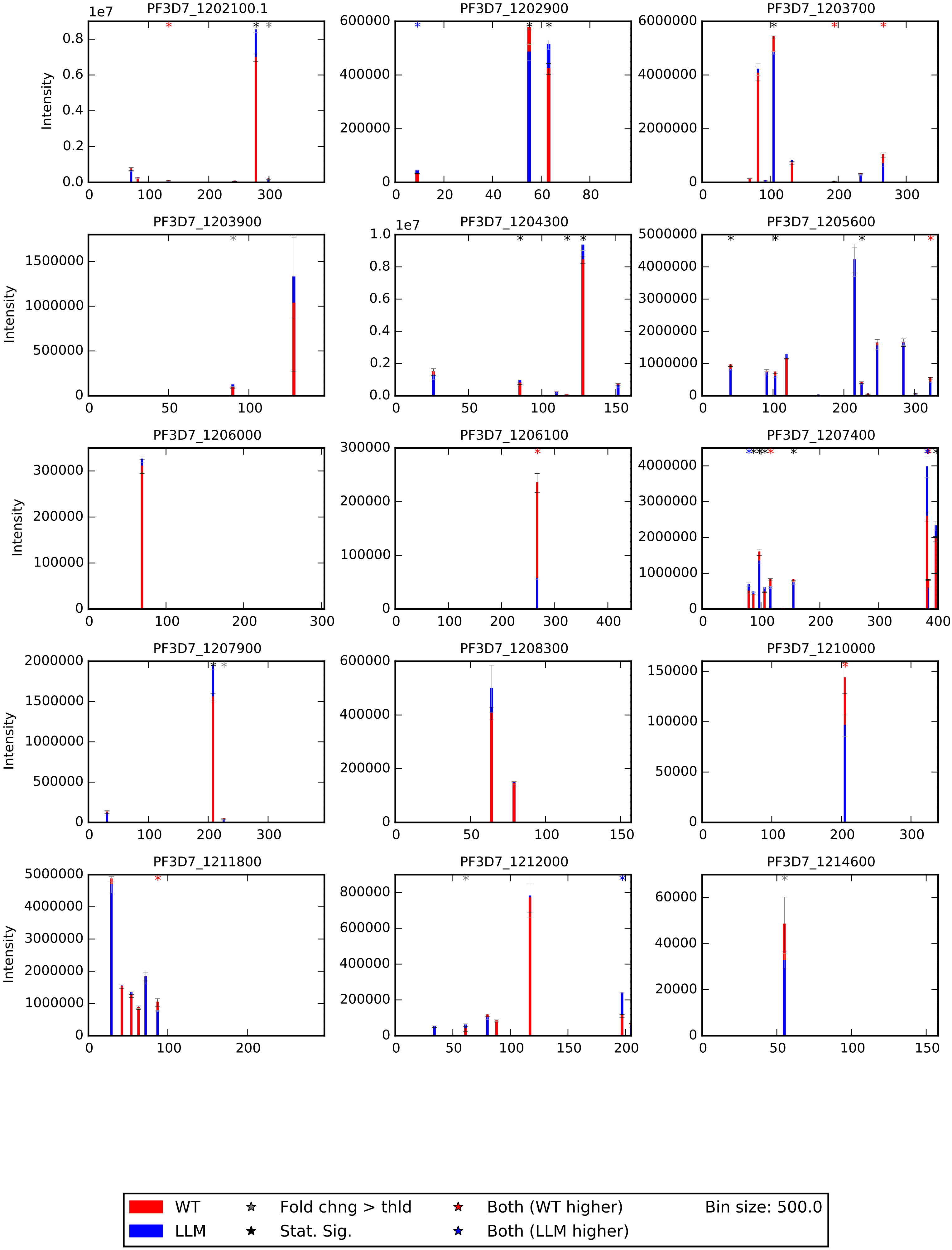

PF3D7_1202100.1
PF3D7_1202900
PF3D7_1203700
PF3D7_1203900
PF3D7_1204300
PF3D7_1205600
PF3D7_1206000
PF3D7_1206100
PF3D7_1207400
PF3D7_1207900
PF3D7_1208300
PF3D7_1210000
PF3D7_1211800
PF3D7_1212000
PF3D7_1214600
Intensity
1e7
WT
LLM
Fold chng > thld
Stat. Sig.
Both (WT higher)
Both (LLM higher)
Bin size: 500.0

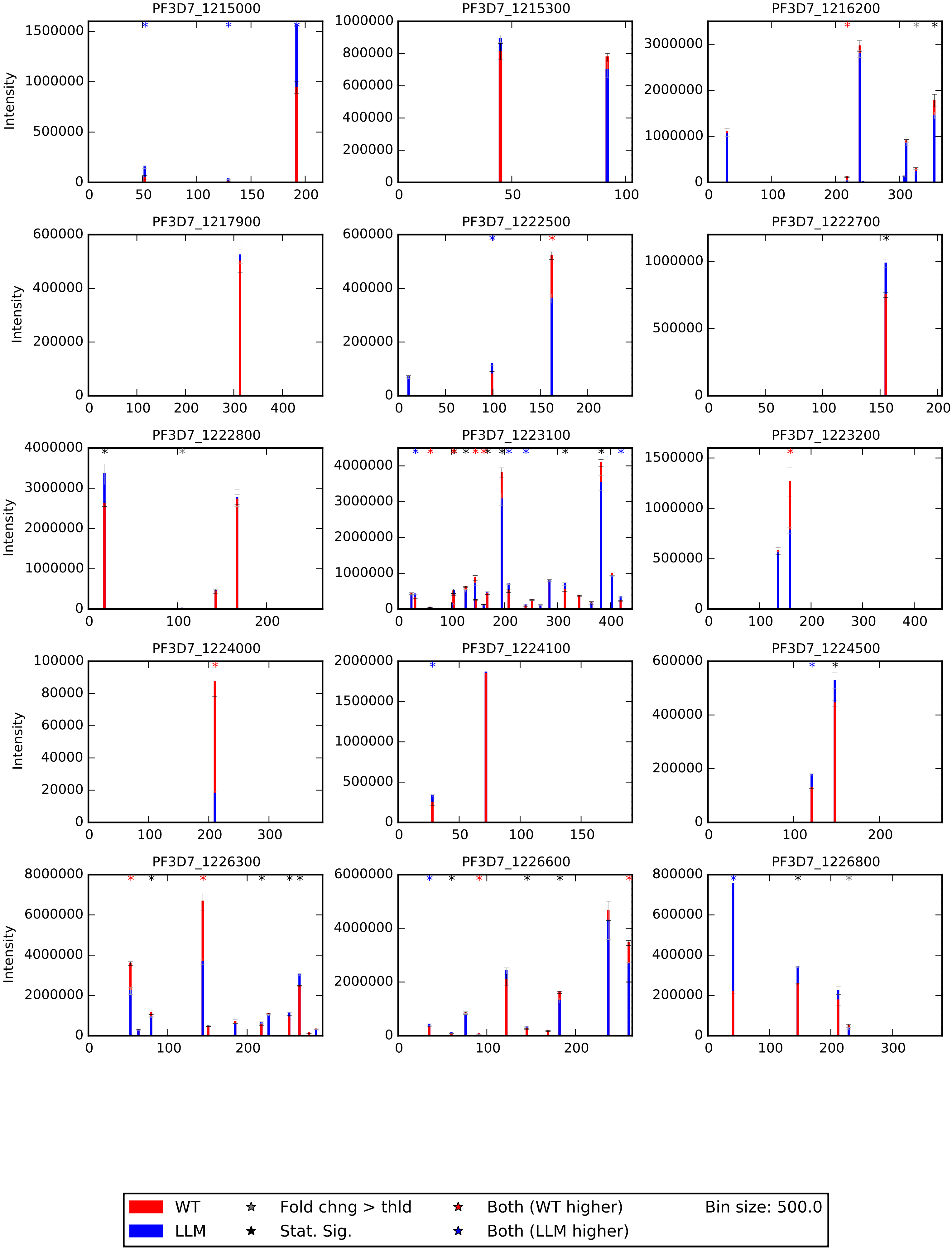
PF3D7_1215000
PF3D7_1215300
PF3D7_1216200
PF3D7_1217900
PF3D7_1222500
PF3D7_1222700
PF3D7_1222800
PF3D7_1223100
PF3D7_1223200
PF3D7_1224000
PF3D7_1224100
PF3D7_1224500
PF3D7_1226300
PF3D7_1226600
PF3D7_1226800
Intensity
WT
LLM
Fold chng > thld
Stat. Sig.
Both (WT higher)
Both (LLM higher)
Bin size: 500.0

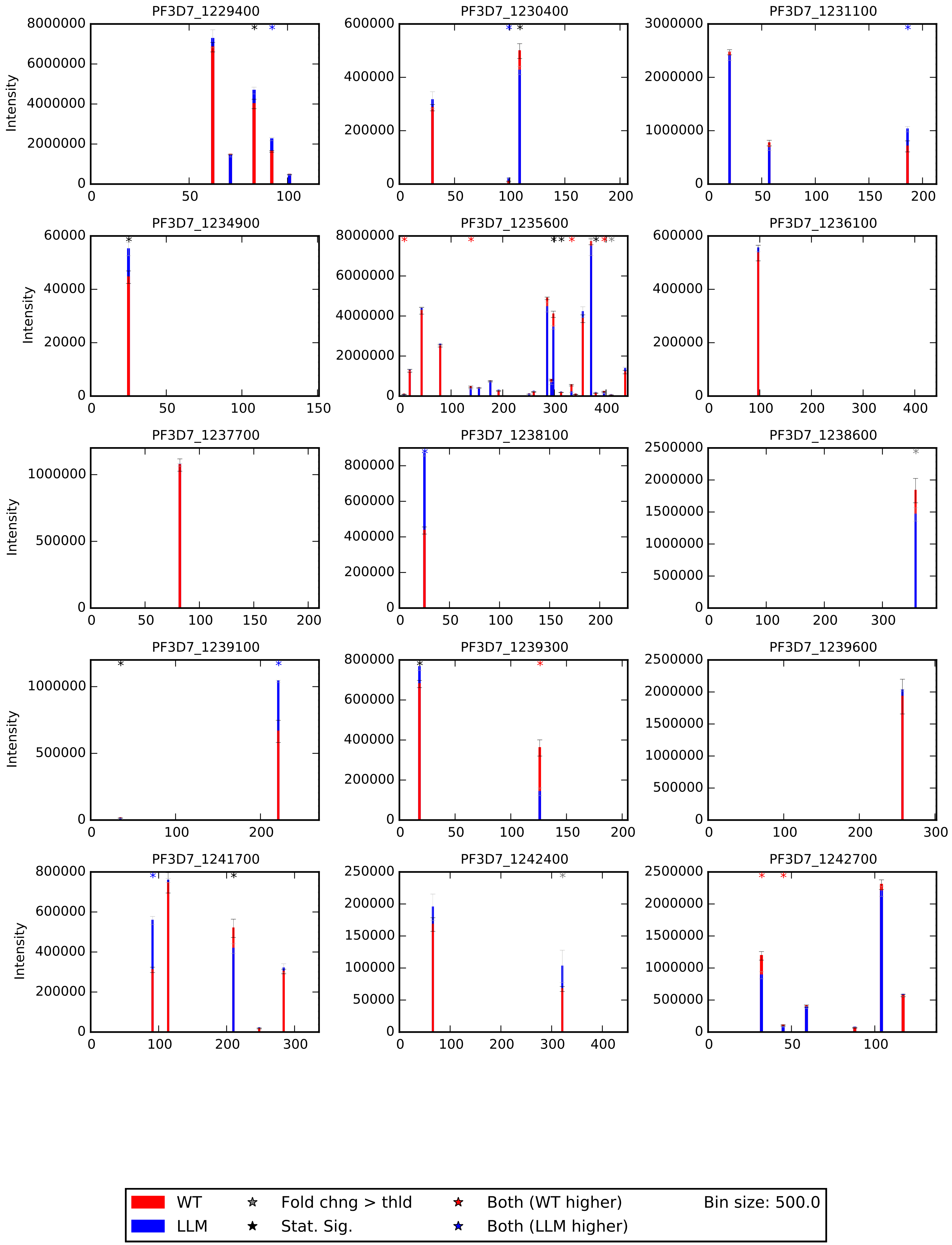
PF3D7_1229400
PF3D7_1230400
PF3D7_1231100
PF3D7_1234900
PF3D7_1235600
PF3D7_1236100
PF3D7_1237700
PF3D7_1238100
PF3D7_1238600
PF3D7_1239100
PF3D7_1239300
PF3D7_1239600
PF3D7_1241700
PF3D7_1242400
PF3D7_1242700
Intensity
WT
LLM
Fold chng > thld
Stat. Sig.
Both (WT higher)
Both (LLM higher)
Bin size: 500.0

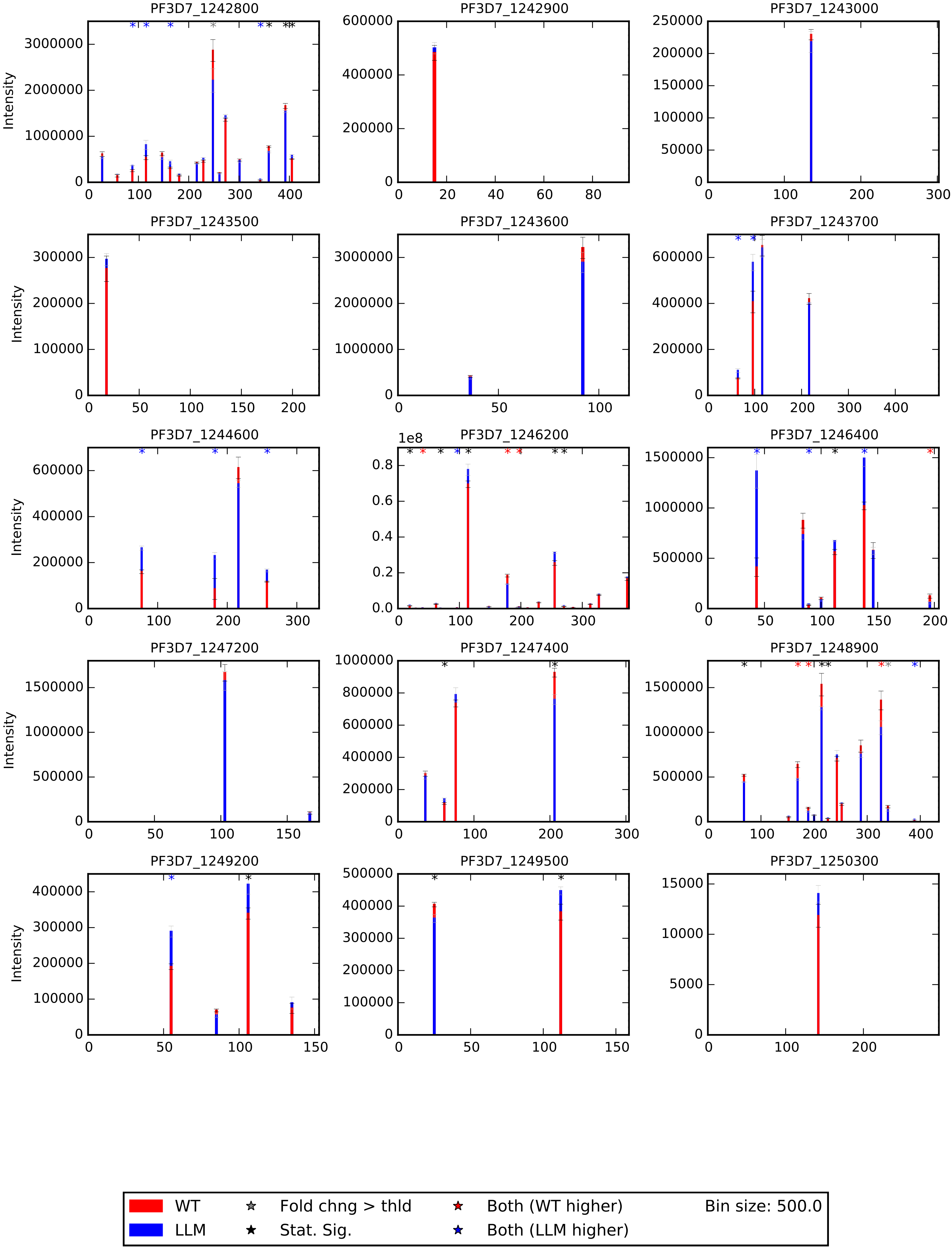
PF3D7_1242800
PF3D7_1242900
PF3D7_1243000
PF3D7_1243500
PF3D7_1243600
PF3D7_1243700
PF3D7_1244600
PF3D7_1246200
PF3D7_1246400
PF3D7_1247200
PF3D7_1247400
PF3D7_1248900
PF3D7_1249200
PF3D7_1249500
PF3D7_1250300
Intensity
1e8
WT
LLM
Fold chng > thld
Stat. Sig.
Both (WT higher)
Both (LLM higher)
Bin size: 500.0

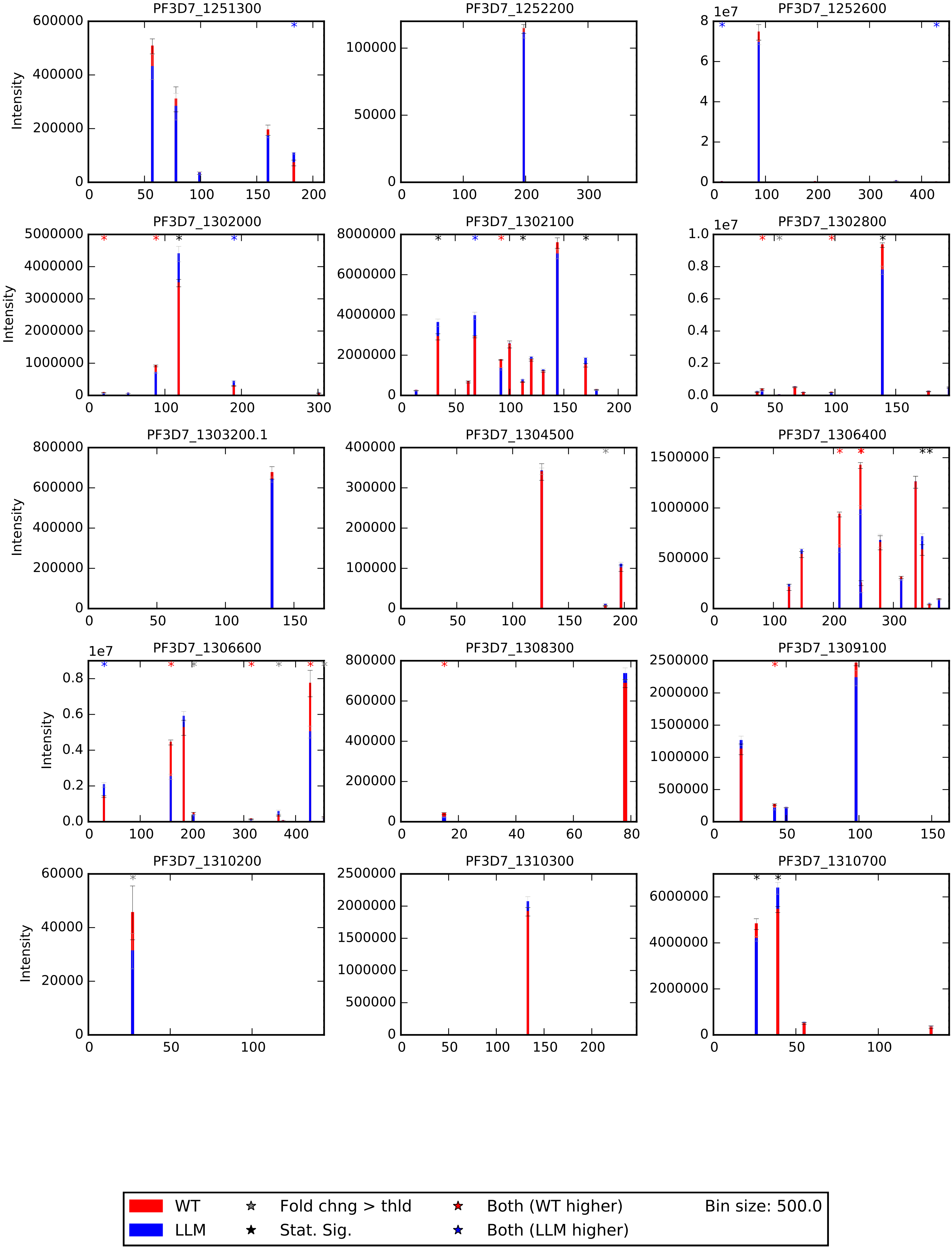
PF3D7_1251300
PF3D7_1252200
PF3D7_1252600
PF3D7_1302000
PF3D7_1302100
PF3D7_1302800
PF3D7_1303200.1
PF3D7_1304500
PF3D7_1306400
PF3D7_1306600
PF3D7_1308300
PF3D7_1309100
PF3D7_1310200
PF3D7_1310300
PF3D7_1310700
Intensity
WT
LLM
Fold chng > thld
Stat. Sig.
Both (WT higher)
Both (LLM higher)
Bin size: 500.0

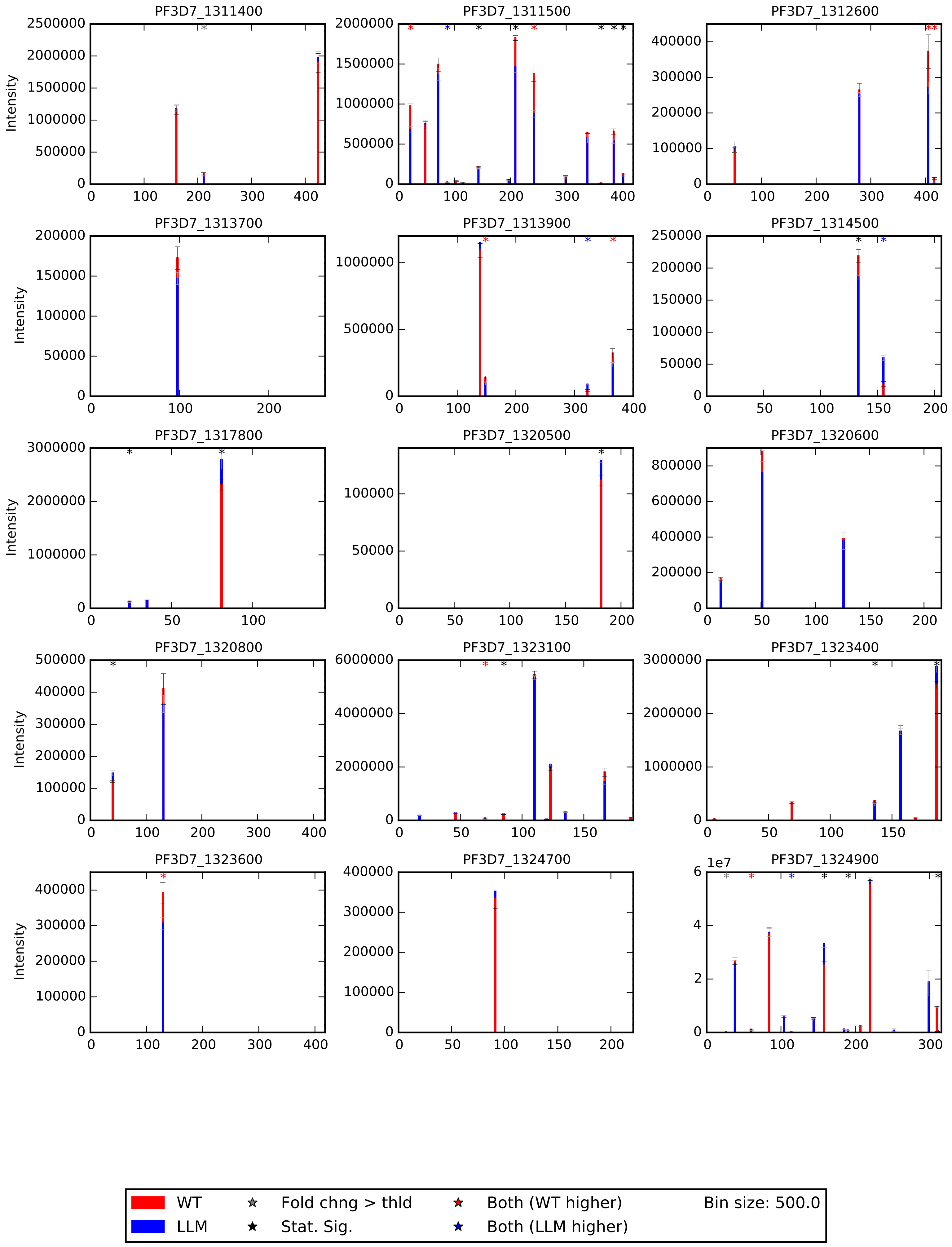
PF3D7_1311400
PF3D7_1311500
PF3D7_1312600
PF3D7_1313700
PF3D7_1313900
PF3D7_1314500
PF3D7_1317800
PF3D7_1320500
PF3D7_1320600
PF3D7_1320800
PF3D7_1323100
PF3D7_1323400
PF3D7_1323600
PF3D7_1324700
PF3D7_1324900
Intensity
WT
LLM
Fold chng > thld
Stat. Sig.
Both (WT higher)
Both (LLM higher)
Bin size: 500.0

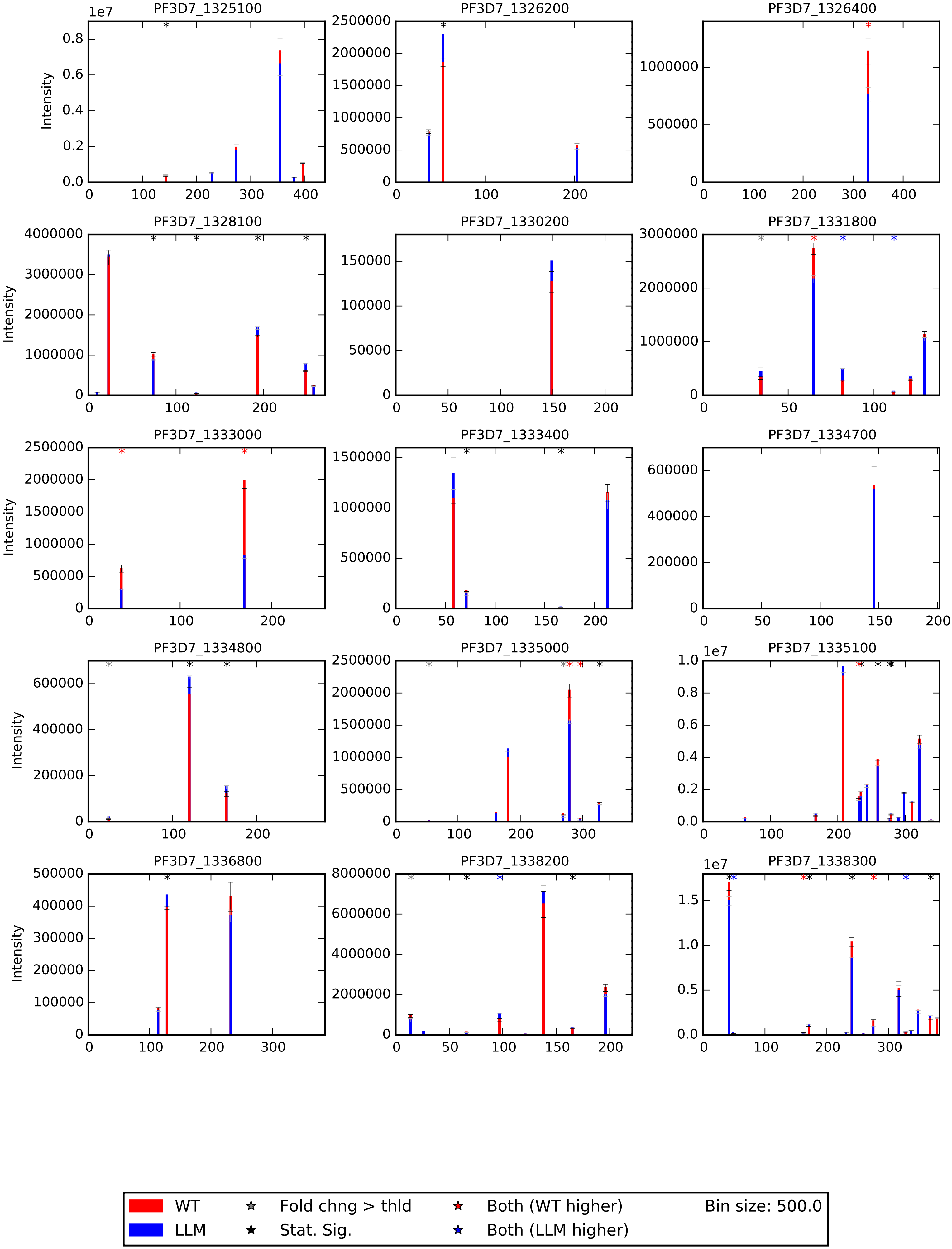
PF3D7_1325100
1e7
Intensity
PF3D7_1326200
PF3D7_1326400
PF3D7_1328100
Intensity
PF3D7_1330200
PF3D7_1331800
PF3D7_1333000
Intensity
PF3D7_1333400
PF3D7_1334700
PF3D7_1334800
Intensity
PF3D7_1335000
PF3D7_1335100
1e7
PF3D7_1336800
Intensity
PF3D7_1338200
PF3D7_1338300
1e7
WT
LLM
Fold chng > thld
Stat. Sig.
Both (WT higher)
Both (LLM higher)
Bin size: 500.0

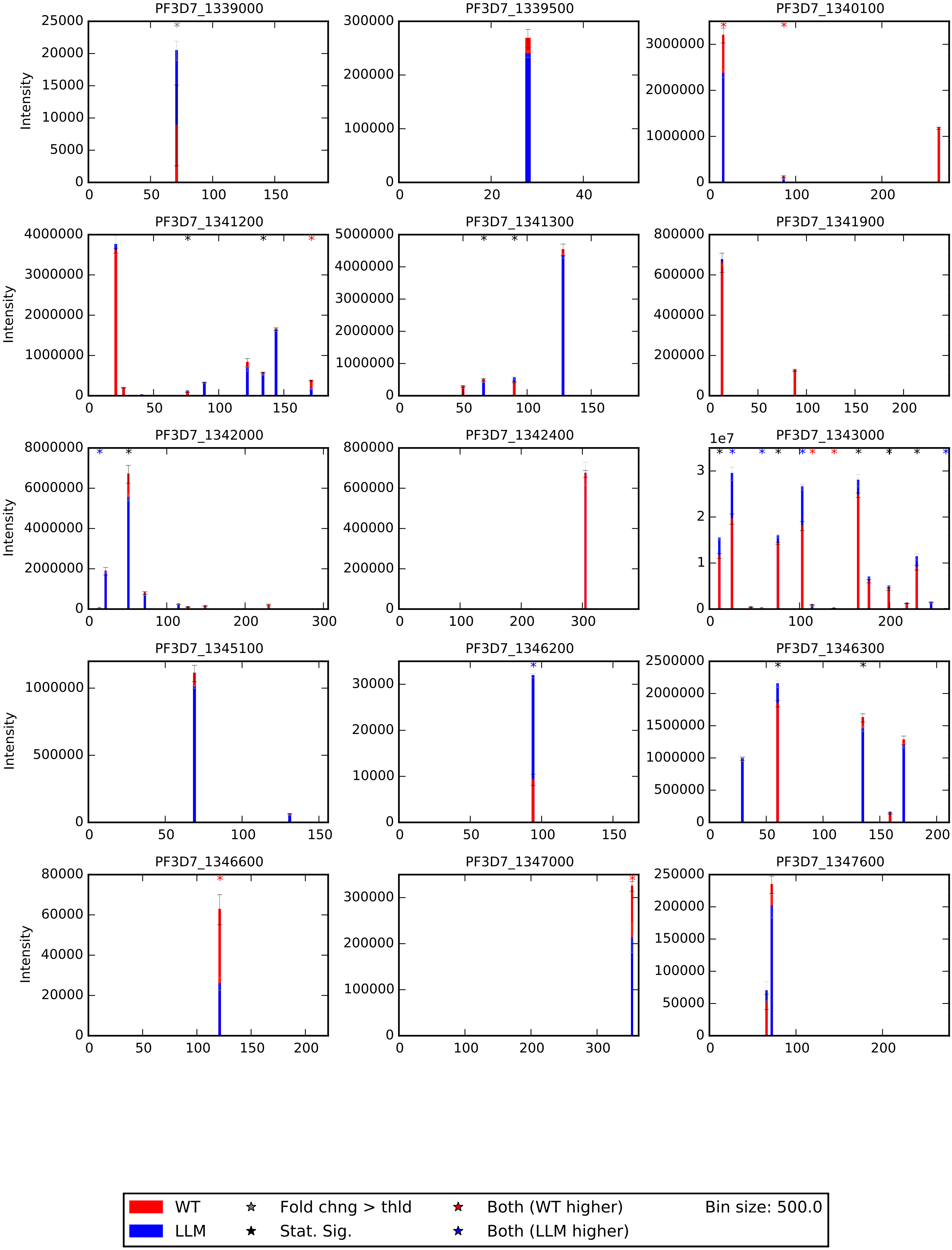
PF3D7_1339000
PF3D7_1339500
PF3D7_1340100
PF3D7_1341200
PF3D7_1341300
PF3D7_1341900
PF3D7_1342000
PF3D7_1342400
PF3D7_1343000
1e7
PF3D7_1345100
PF3D7_1346200
PF3D7_1346300
PF3D7_1346600
PF3D7_1347000
PF3D7_1347600
Intensity
WT
LLM
Fold chng > thld
Stat. Sig.
Both (WT higher)
Both (LLM higher)
Bin size: 500.0

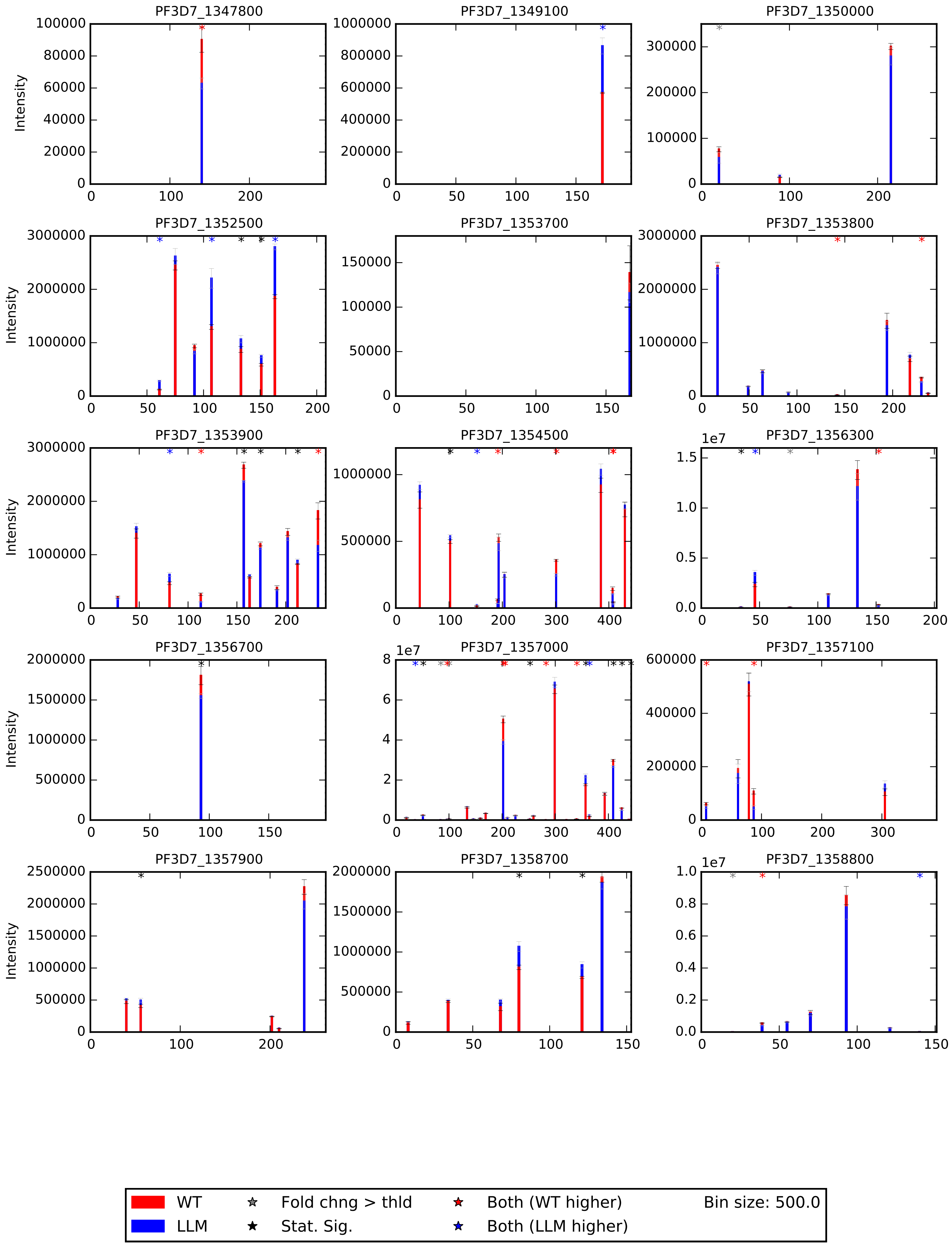

PF3D7_1347800
PF3D7_1349100
PF3D7_1350000
PF3D7_1352500
PF3D7_1353700
PF3D7_1353800
PF3D7_1353900
PF3D7_1354500
PF3D7_1356300
PF3D7_1356700
PF3D7_1357000
PF3D7_1357100
PF3D7_1357900
PF3D7_1358700
PF3D7_1358800
Intensity
1e7
WT
LLM
Fold chng > thld
Stat. Sig.
Both (WT higher)
Both (LLM higher)
Bin size: 500.0

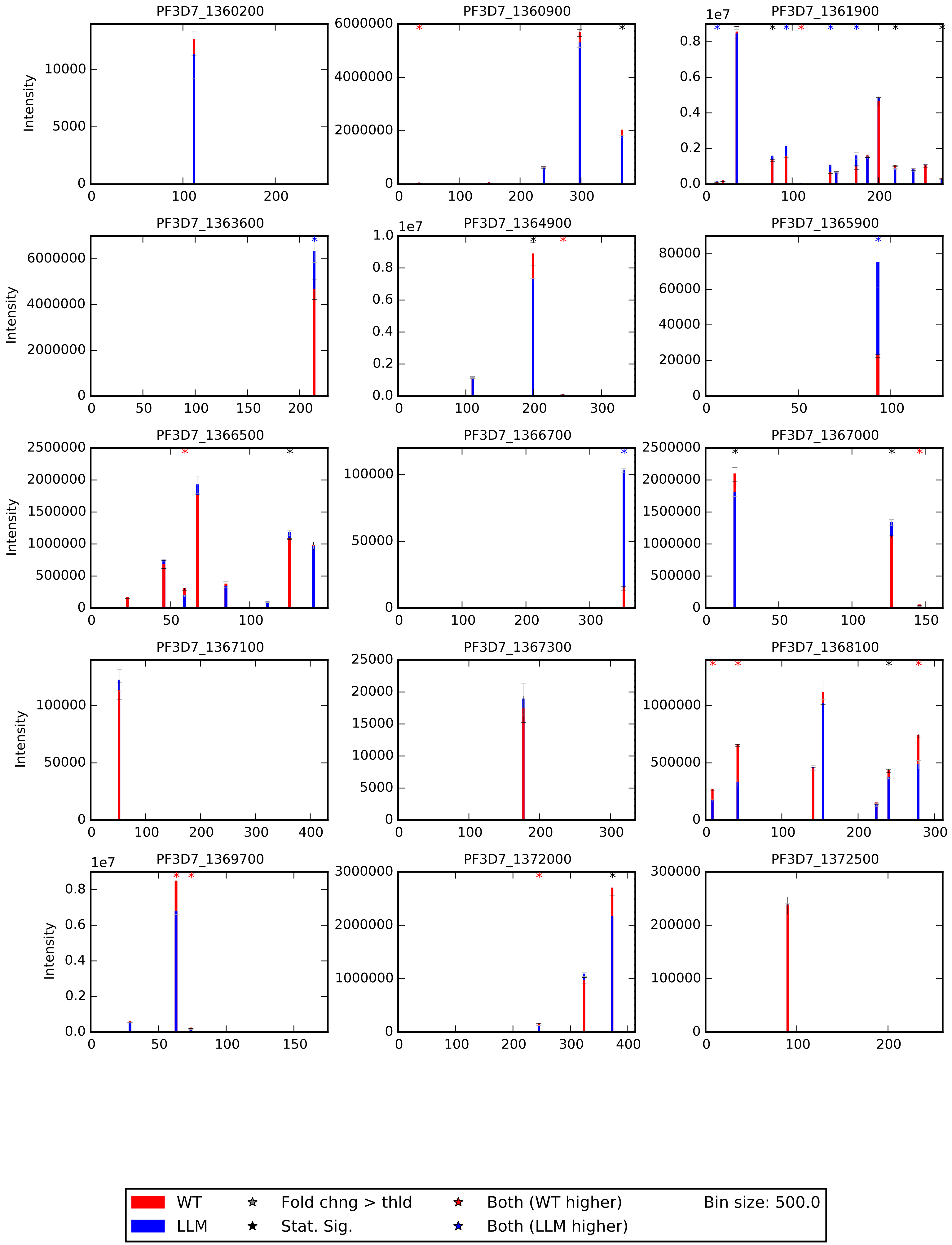

PF3D7_1360200
PF3D7_1360900
PF3D7_1361900
PF3D7_1363600
PF3D7_1364900
PF3D7_1365900
PF3D7_1366500
PF3D7_1366700
PF3D7_1367000
PF3D7_1367100
PF3D7_1367300
PF3D7_1368100
PF3D7_1369700
PF3D7_1372000
PF3D7_1372500
Intensity
WT
LLM
Fold chng > thld
Stat. Sig.
Both (WT higher)
Both (LLM higher)
Bin size: 500.0

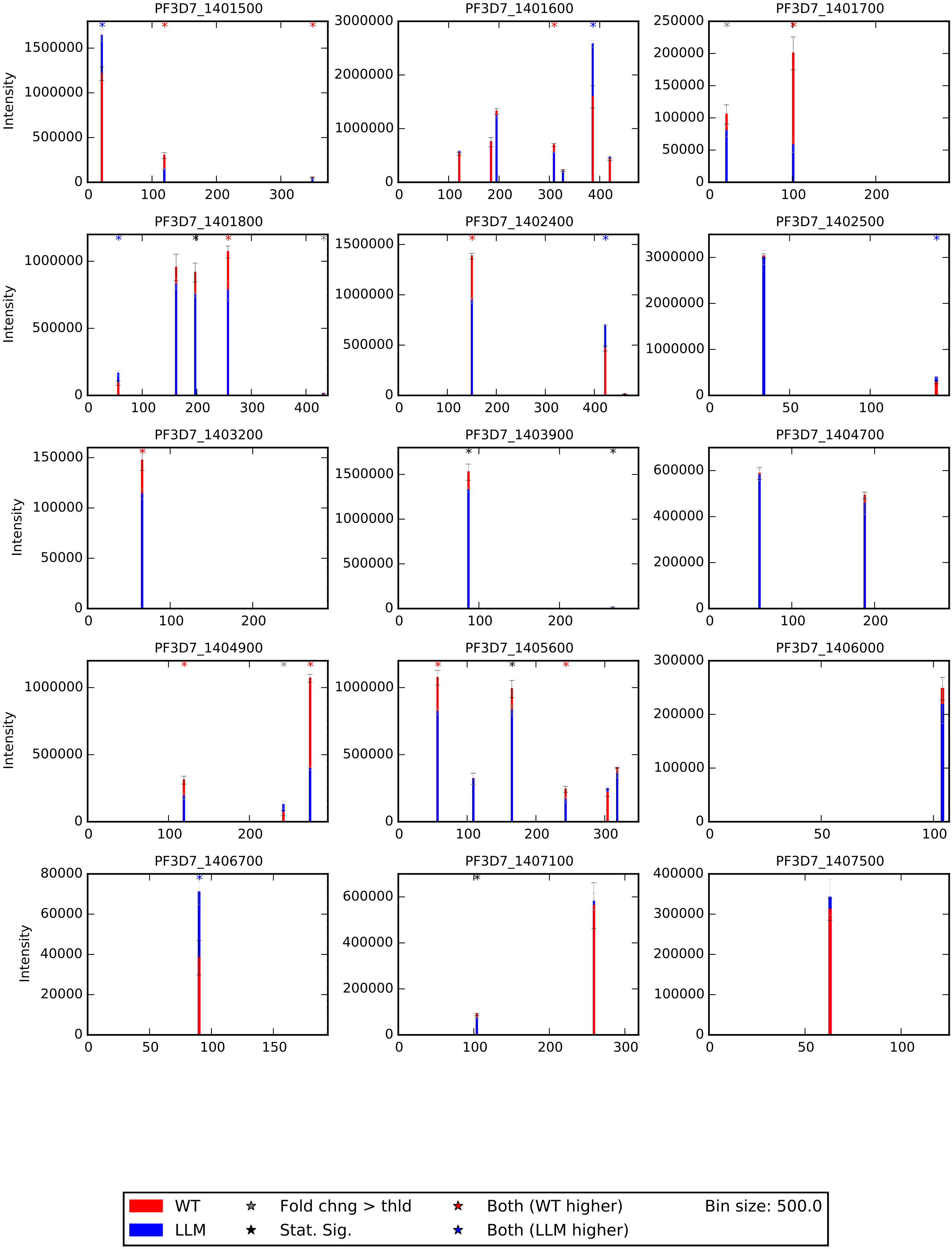
PF3D7_1401500
PF3D7_1401600
PF3D7_1401700
PF3D7_1401800
PF3D7_1402400
PF3D7_1402500
PF3D7_1403200
PF3D7_1403900
PF3D7_1404700
PF3D7_1404900
PF3D7_1405600
PF3D7_1406000
PF3D7_1406700
PF3D7_1407100
PF3D7_1407500
Intensity
WT
LLM
Fold chng > thld
Stat. Sig.
Both (WT higher)
Both (LLM higher)
Bin size: 500.0

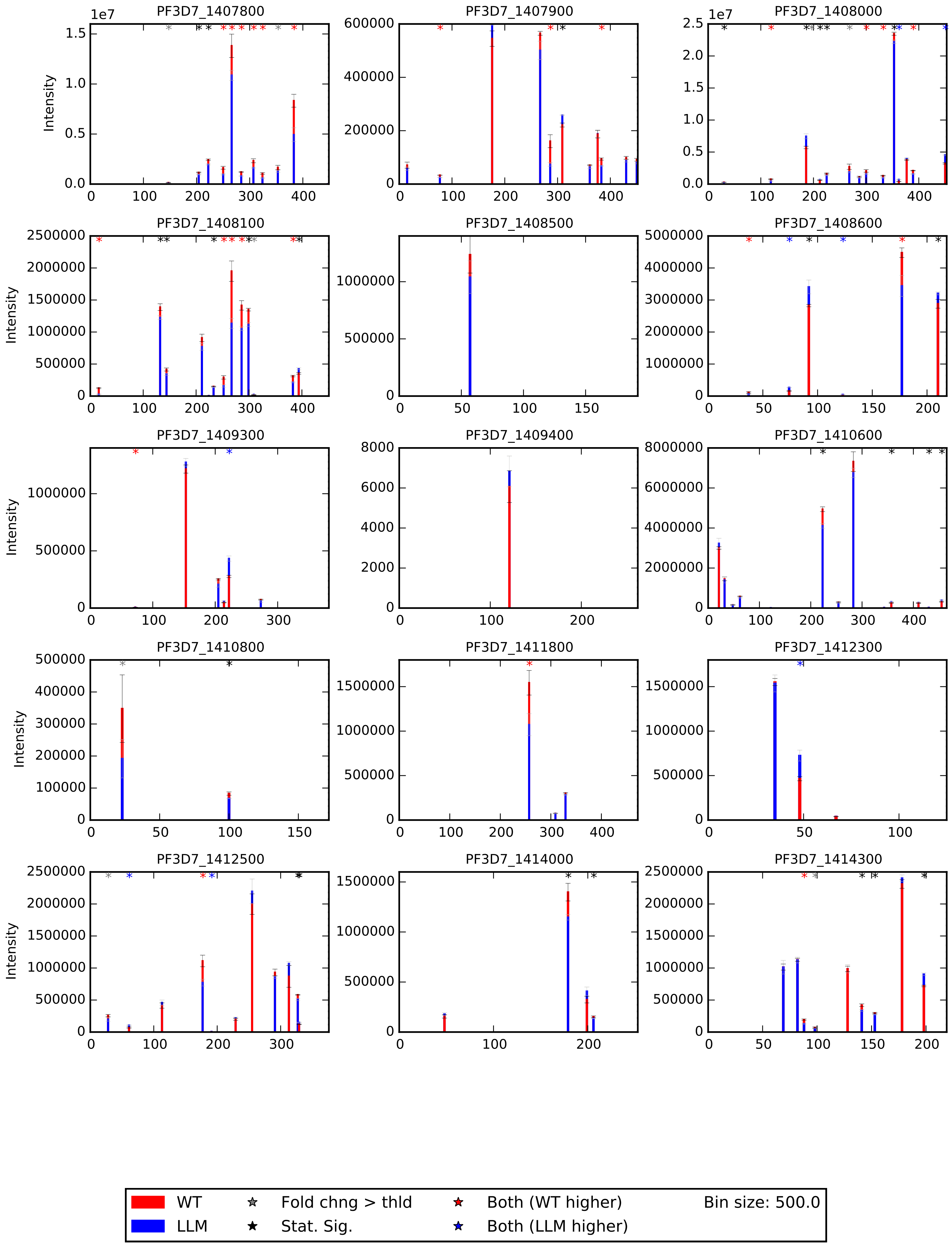

PF3D7_1407800
PF3D7_1407900
PF3D7_1408000
PF3D7_1408100
PF3D7_1408500
PF3D7_1408600
PF3D7_1409300
PF3D7_1409400
PF3D7_1410600
PF3D7_1410800
PF3D7_1411800
PF3D7_1412300
PF3D7_1412500
PF3D7_1414000
PF3D7_1414300
Intensity
1e7
WT
LLM
Fold chng > thld
Stat. Sig.
Both (WT higher)
Both (LLM higher)
Bin size: 500.0

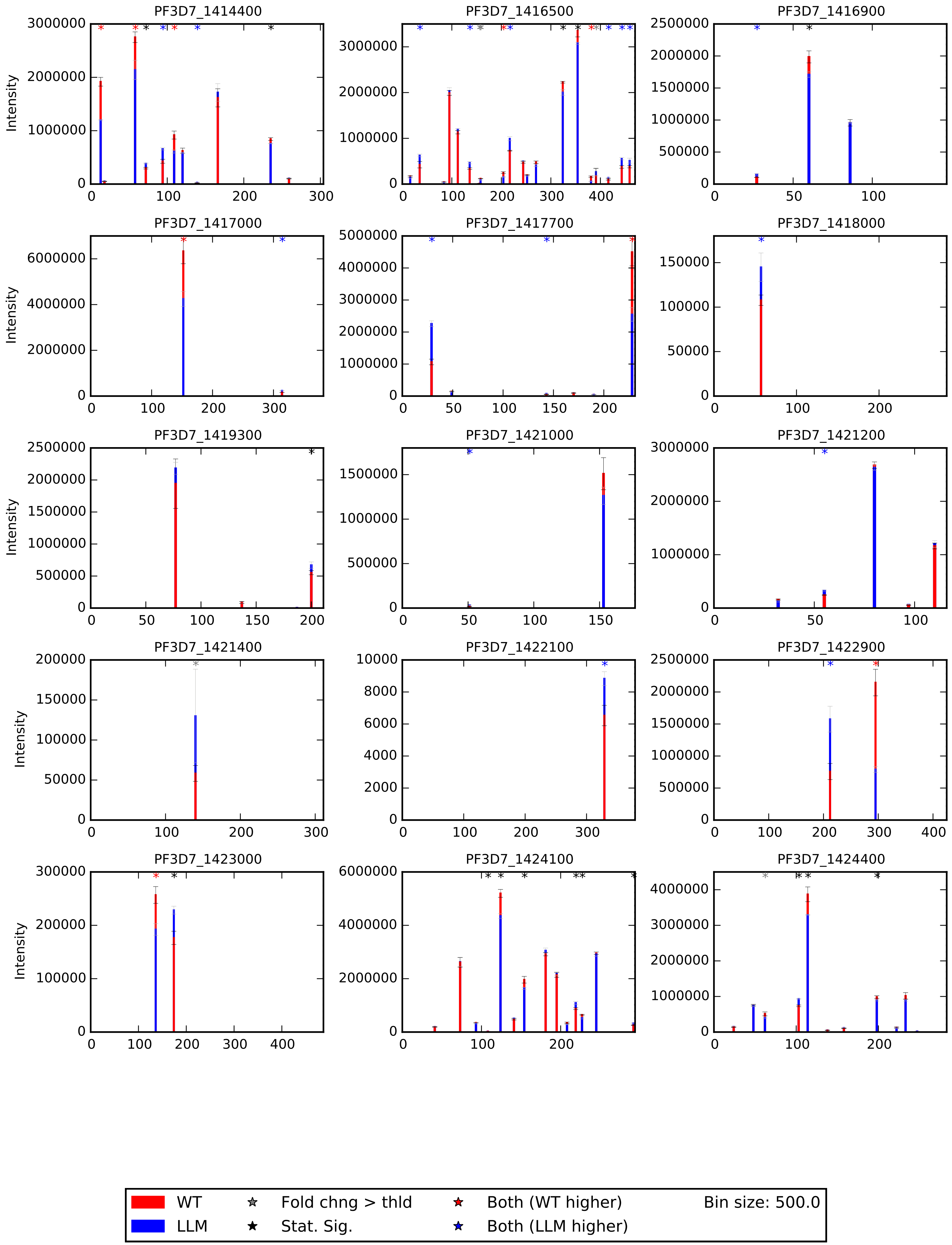
PF3D7_1414400
PF3D7_1416500
PF3D7_1416900
PF3D7_1417000
PF3D7_1417700
PF3D7_1418000
PF3D7_1419300
PF3D7_1421000
PF3D7_1421200
PF3D7_1421400
PF3D7_1422100
PF3D7_1422900
PF3D7_1423000
PF3D7_1424100
PF3D7_1424400
Intensity
WT
LLM
Fold chng > thld
Stat. Sig.
Both (WT higher)
Both (LLM higher)
Bin size: 500.0

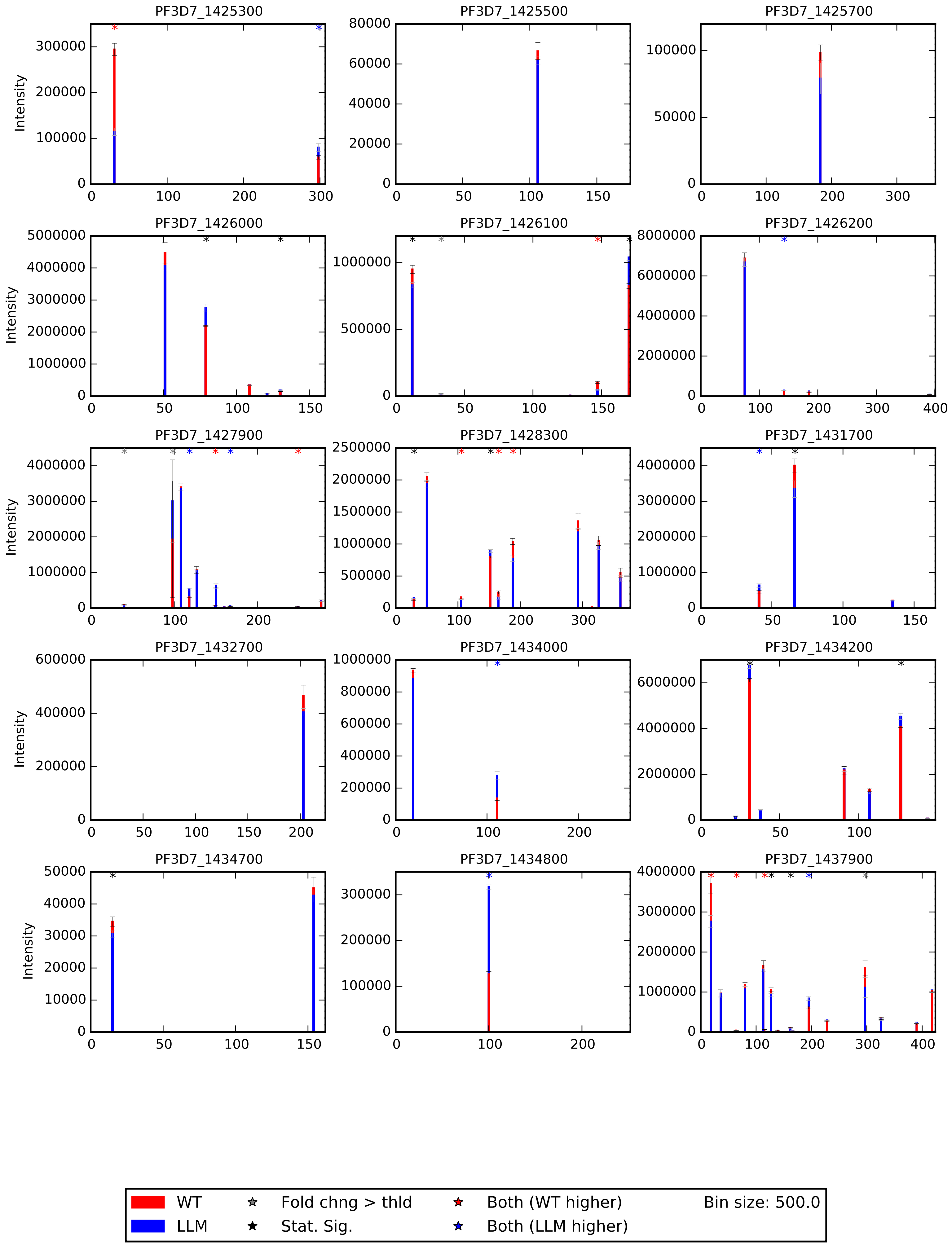
PF3D7_1425300
PF3D7_1425500
PF3D7_1425700
PF3D7_1426000
PF3D7_1426100
PF3D7_1426200
PF3D7_1427900
PF3D7_1428300
PF3D7_1431700
PF3D7_1432700
PF3D7_1434000
PF3D7_1434200
PF3D7_1434700
PF3D7_1434800
PF3D7_1437900
Intensity
WT
LLM
Fold chng > thld
Stat. Sig.
Both (WT higher)
Both (LLM higher)
Bin size: 500.0

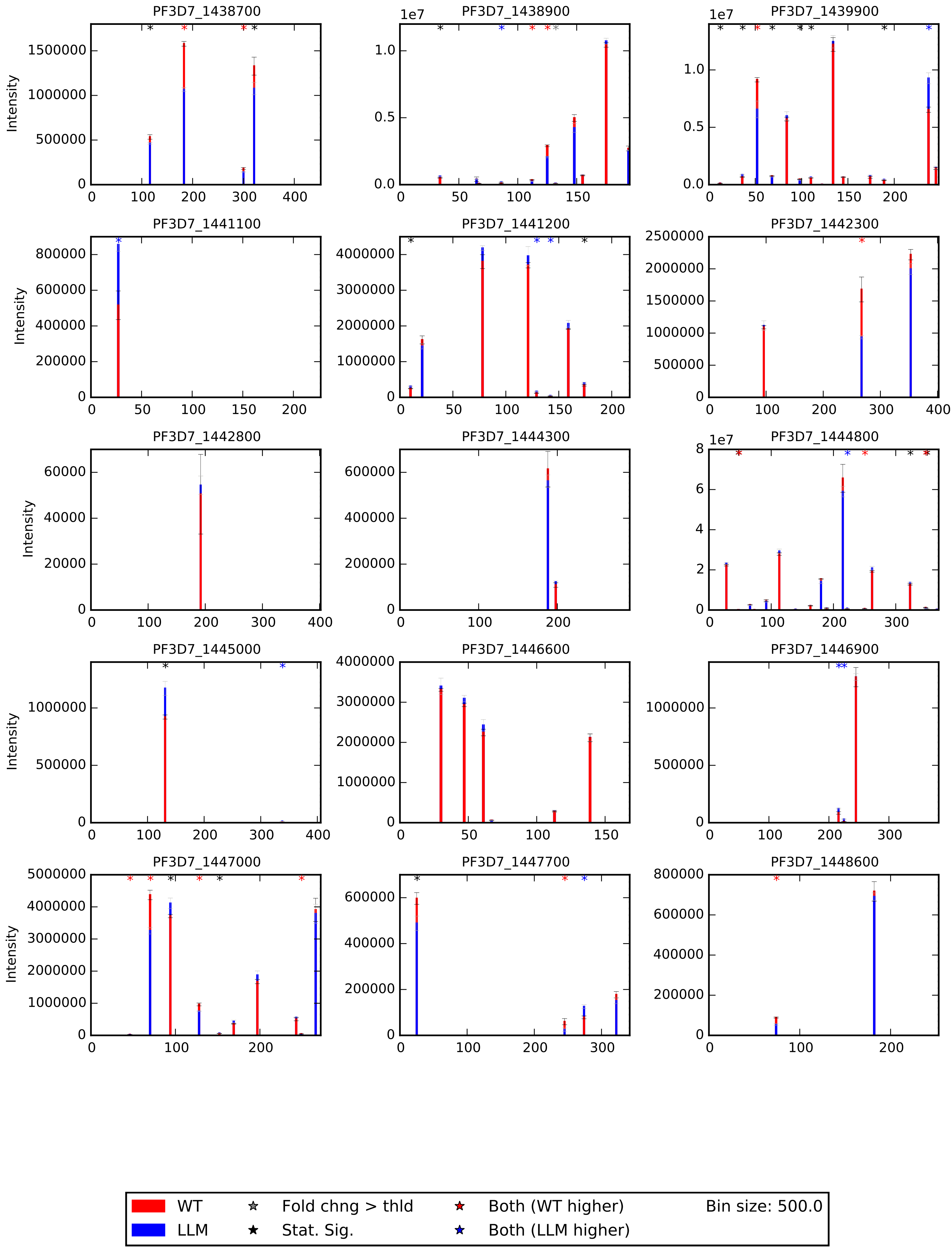

PF3D7_1438700
PF3D7_1438900
PF3D7_1439900
PF3D7_1441100
PF3D7_1441200
PF3D7_1442300
PF3D7_1442800
PF3D7_1444300
PF3D7_1444800
PF3D7_1445000
PF3D7_1446600
PF3D7_1446900
PF3D7_1447000
PF3D7_1447700
PF3D7_1448600
Intensity
1e7
WT
LLM
Fold chng > thld
Stat. Sig.
Both (WT higher)
Both (LLM higher)
Bin size: 500.0

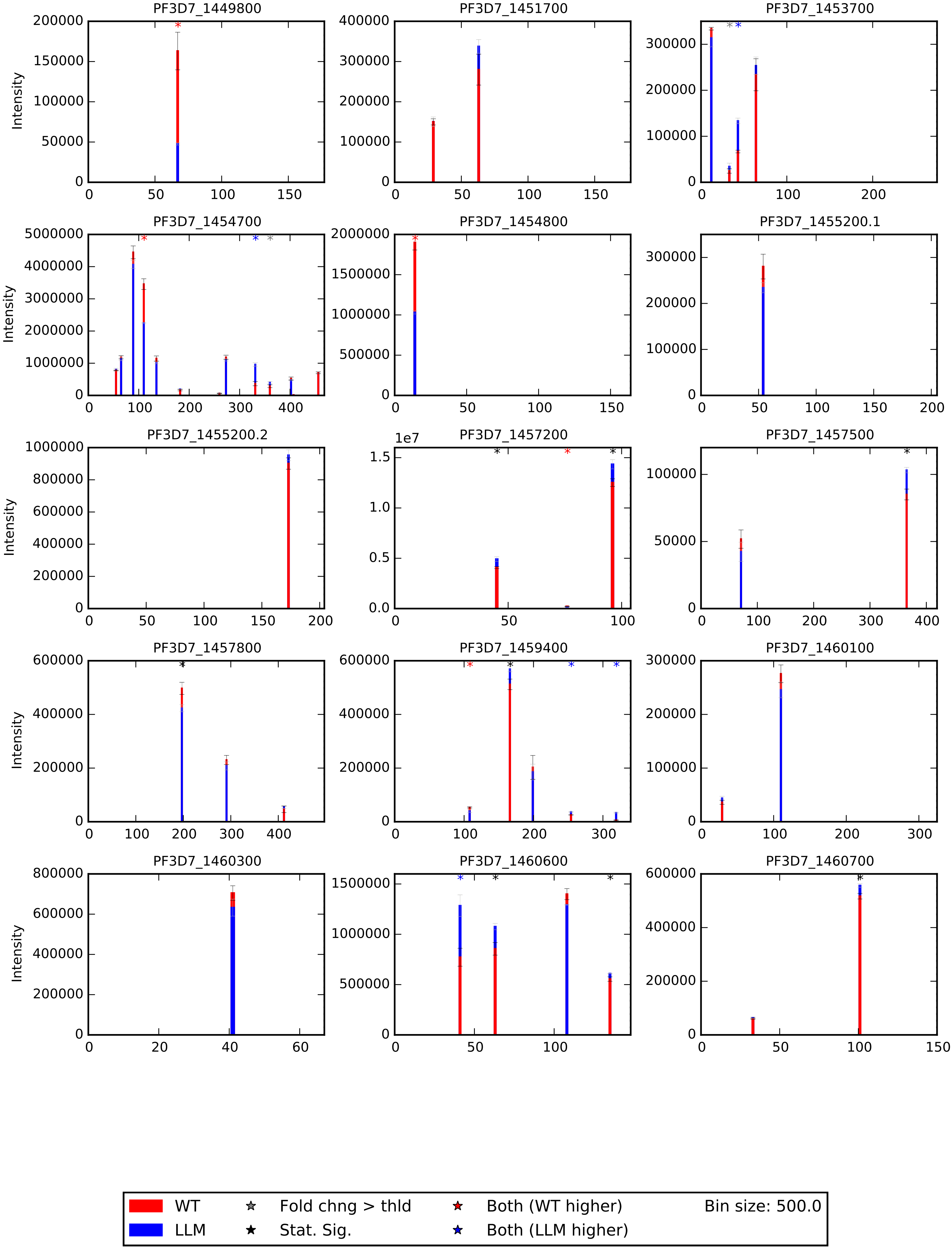
PF3D7_1449800
PF3D7_1451700
PF3D7_1453700
PF3D7_1454700
PF3D7_1454800
PF3D7_1455200.1
PF3D7_1455200.2
PF3D7_1457200
PF3D7_1457500
PF3D7_1457800
PF3D7_1459400
PF3D7_1460100
PF3D7_1460300
PF3D7_1460600
PF3D7_1460700
Intensity
1e7
WT
LLM
Fold chng > thld
Stat. Sig.
Both (WT higher)
Both (LLM higher)
Bin size: 500.0

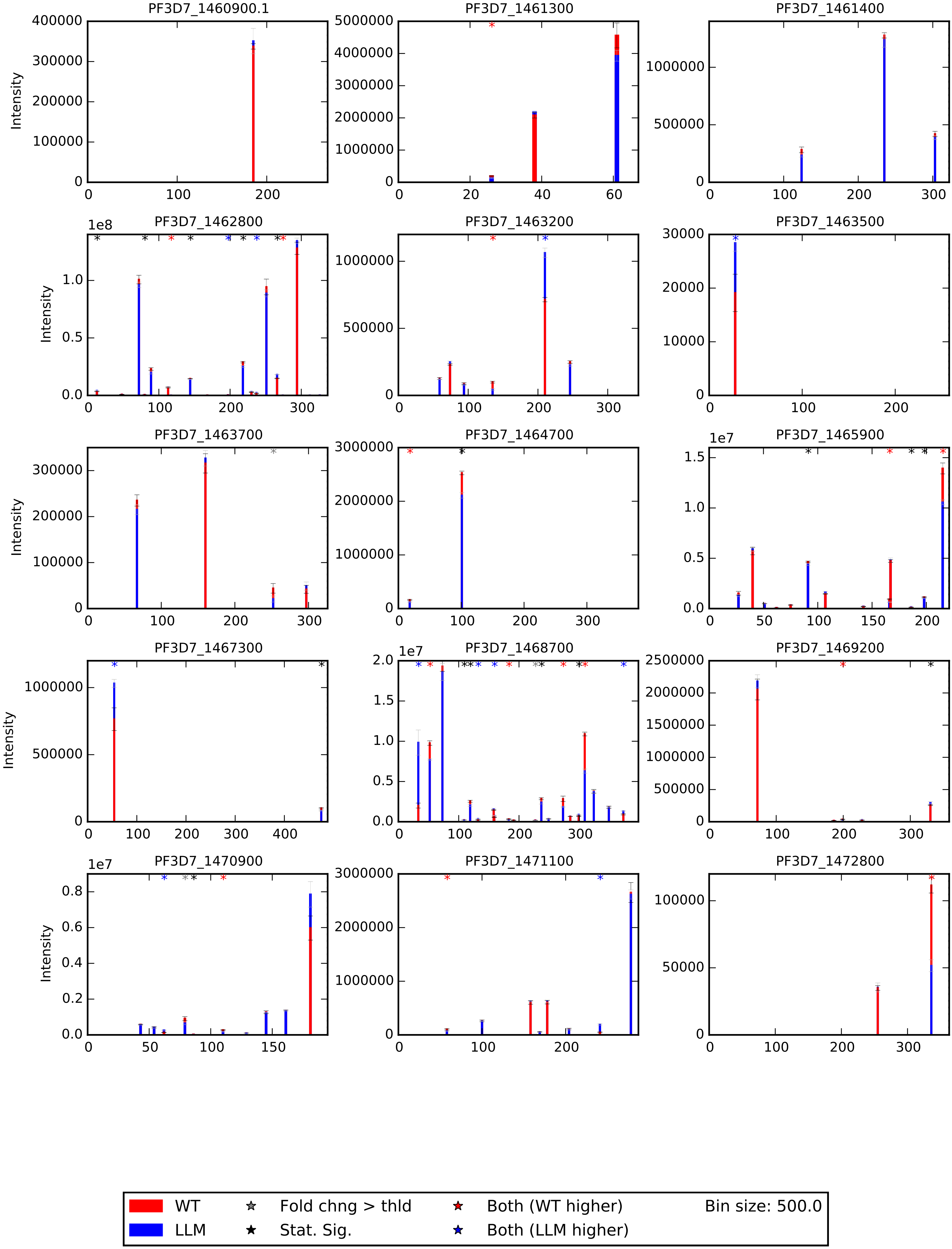
PF3D7_1460900.1
PF3D7_1461300
PF3D7_1461400
PF3D7_1462800
PF3D7_1463200
PF3D7_1463500
PF3D7_1463700
PF3D7_1464700
PF3D7_1465900
PF3D7_1467300
PF3D7_1468700
PF3D7_1469200
PF3D7_1470900
PF3D7_1471100
PF3D7_1472800
Intensity
WT
LLM
Fold chng > thld
Stat. Sig.
Both (WT higher)
Both (LLM higher)
Bin size: 500.0

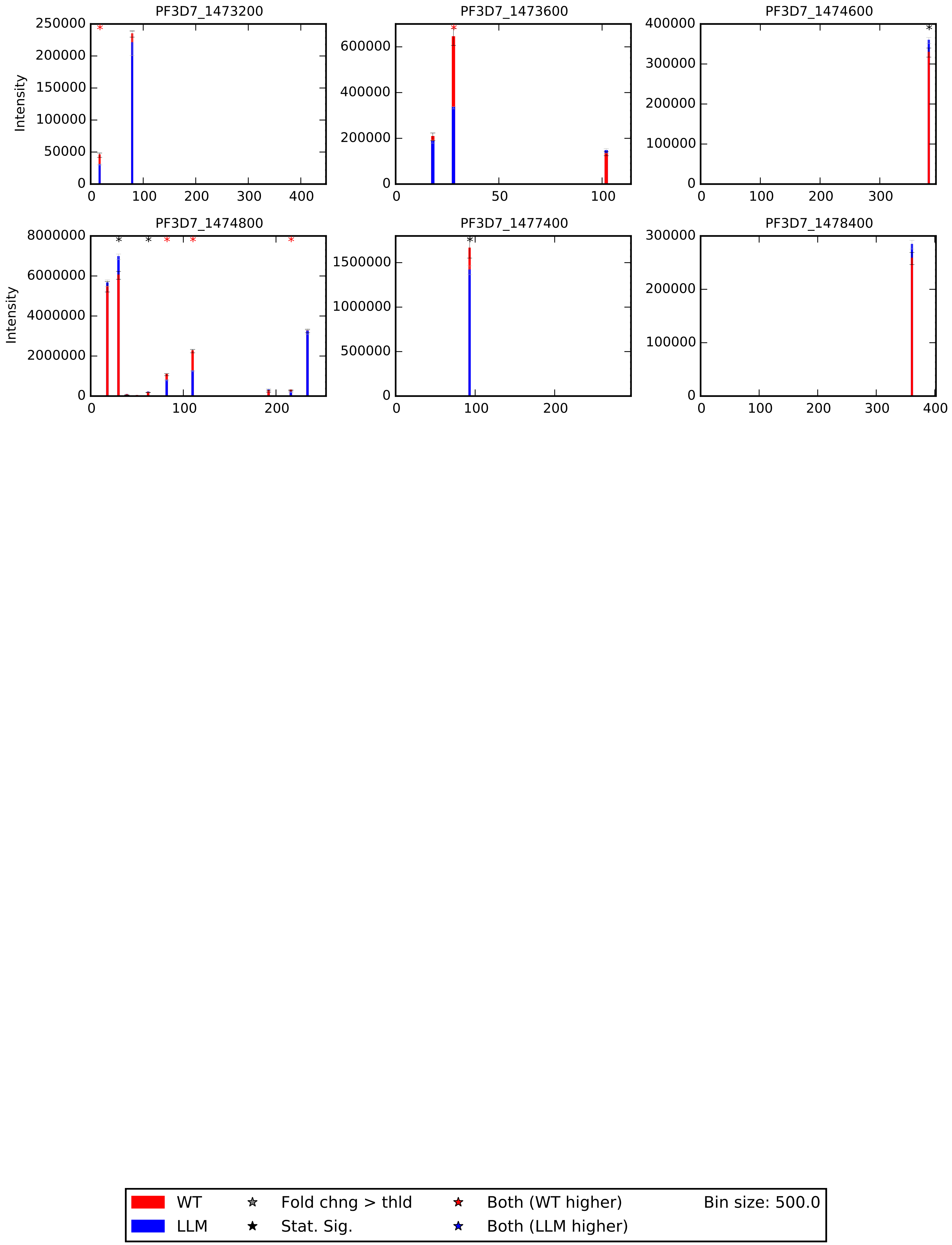
PF3D7_1473200
PF3D7_1473600
PF3D7_1474600
PF3D7_1474800
PF3D7_1477400
PF3D7_1478400
Intensity
WT
LLM
Fold chng > thld
Stat. Sig.
Both (WT higher)
Both (LLM higher)
Bin size: 500.0

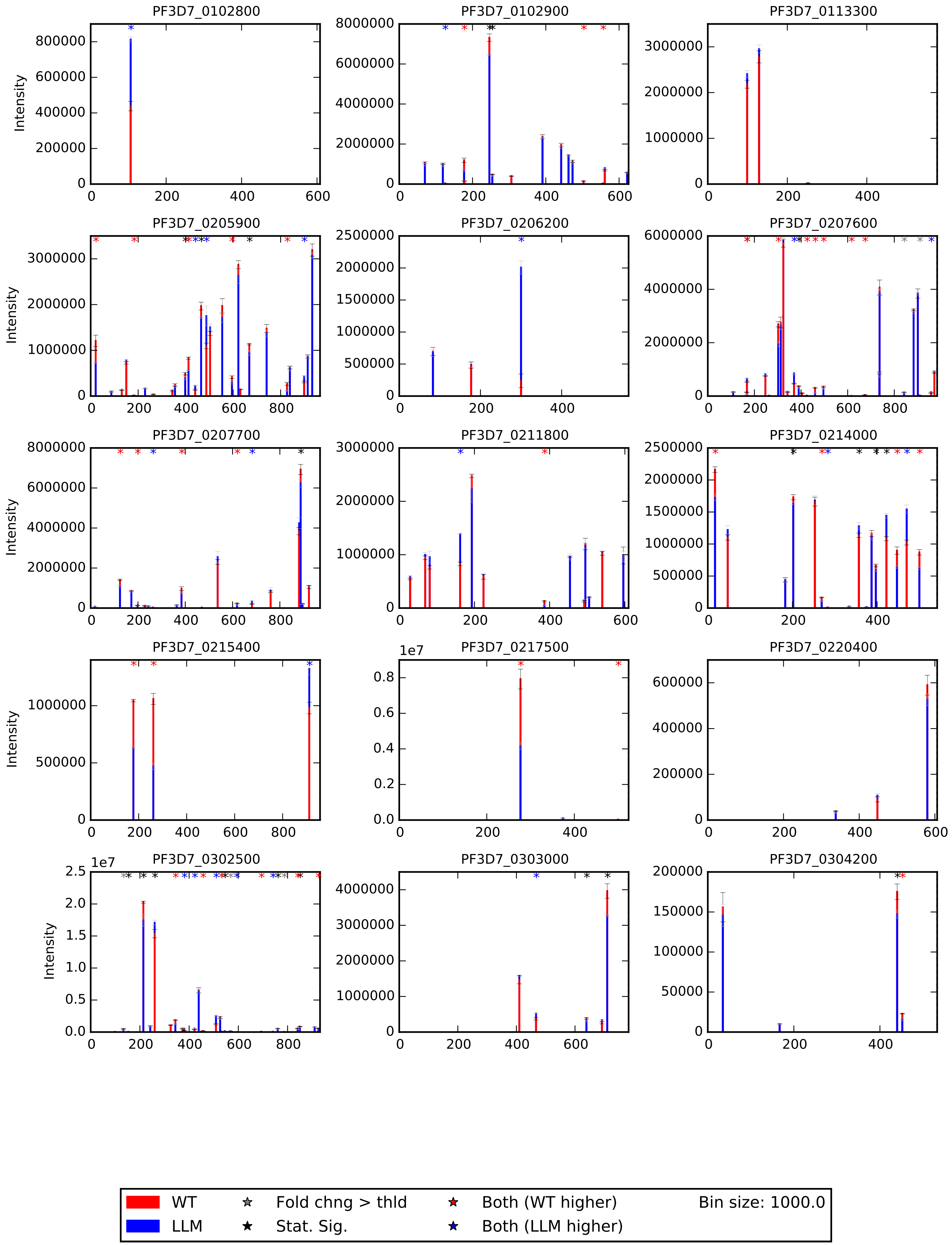
PF3D7_0102800
PF3D7_0102900
PF3D7_0113300
PF3D7_0205900
PF3D7_0206200
PF3D7_0207600
PF3D7_0207700
PF3D7_0211800
PF3D7_0214000
PF3D7_0215400
PF3D7_0217500
PF3D7_0220400
PF3D7_0302500
PF3D7_0303000
PF3D7_0304200
Intensity
1e7
WT
LLM
Fold chng > thld
Stat. Sig.
Both (WT higher)
Both (LLM higher)
Bin size: 1000.0

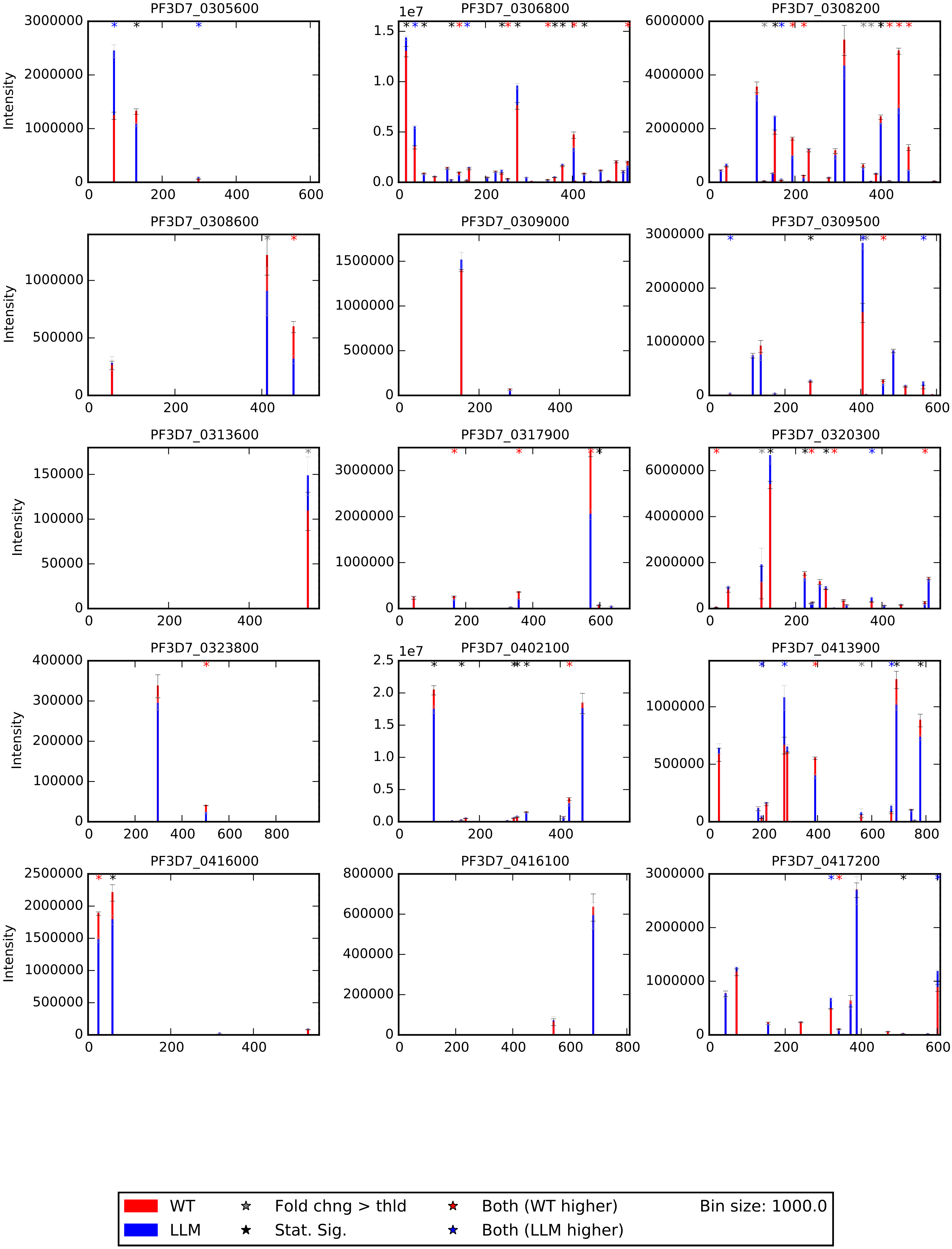
PF3D7_0305600
PF3D7_0306800
PF3D7_0308200
PF3D7_0308600
PF3D7_0309000
PF3D7_0309500
PF3D7_0313600
PF3D7_0317900
PF3D7_0320300
PF3D7_0323800
PF3D7_0402100
PF3D7_0413900
PF3D7_0416000
PF3D7_0416100
PF3D7_0417200
Intensity
1e7
WT
LLM
Fold chng > thld
Stat. Sig.
Both (WT higher)
Both (LLM higher)
Bin size: 1000.0

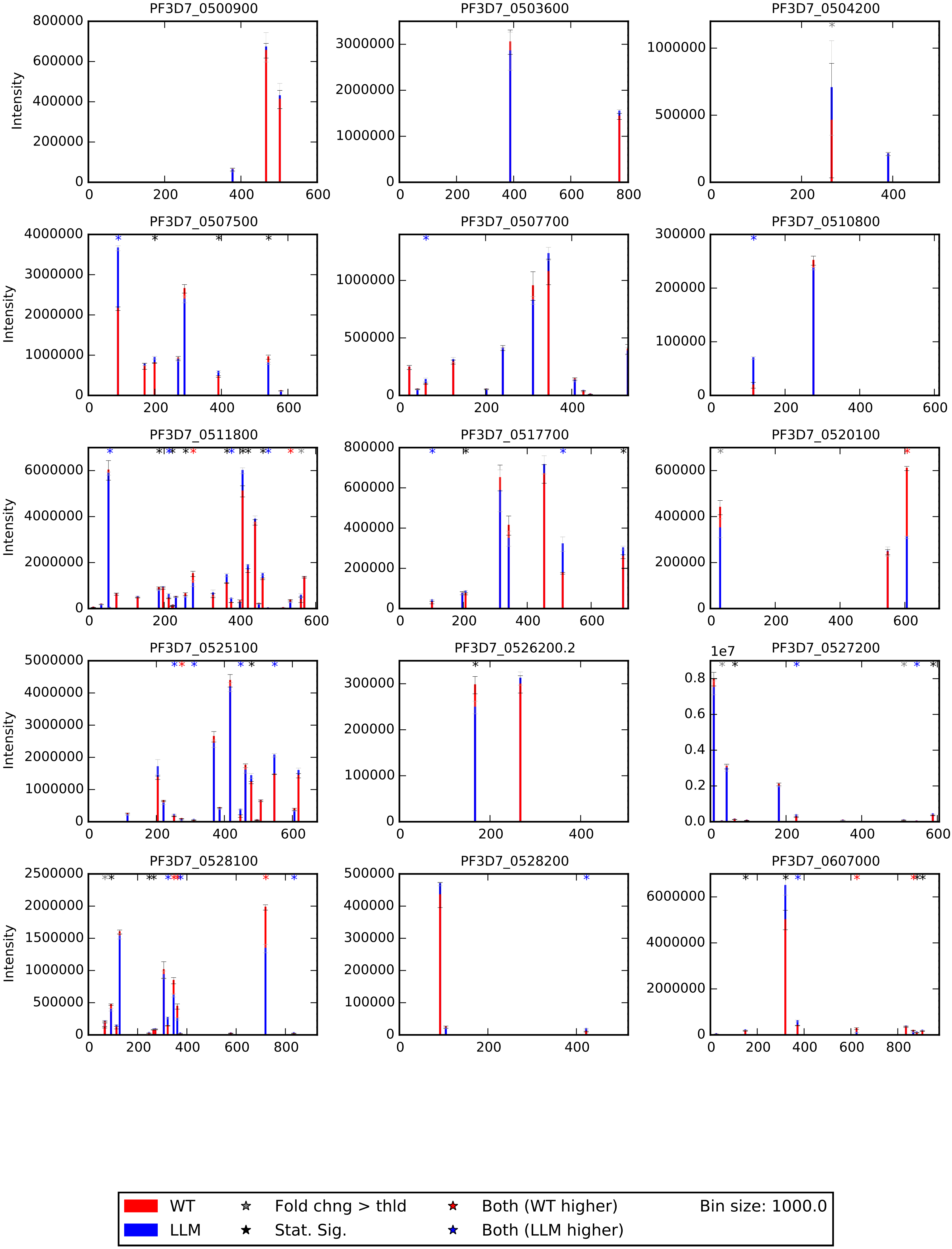
PF3D7_0500900
PF3D7_0503600
PF3D7_0504200
PF3D7_0507500
PF3D7_0507700
PF3D7_0510800
PF3D7_0511800
PF3D7_0517700
PF3D7_0520100
PF3D7_0525100
PF3D7_0526200.2
PF3D7_0527200
PF3D7_0528100
PF3D7_0528200
PF3D7_0607000
Intensity
1e7
WT
LLM
Fold chng > thld
Stat. Sig.
Both (WT higher)
Both (LLM higher)
Bin size: 1000.0

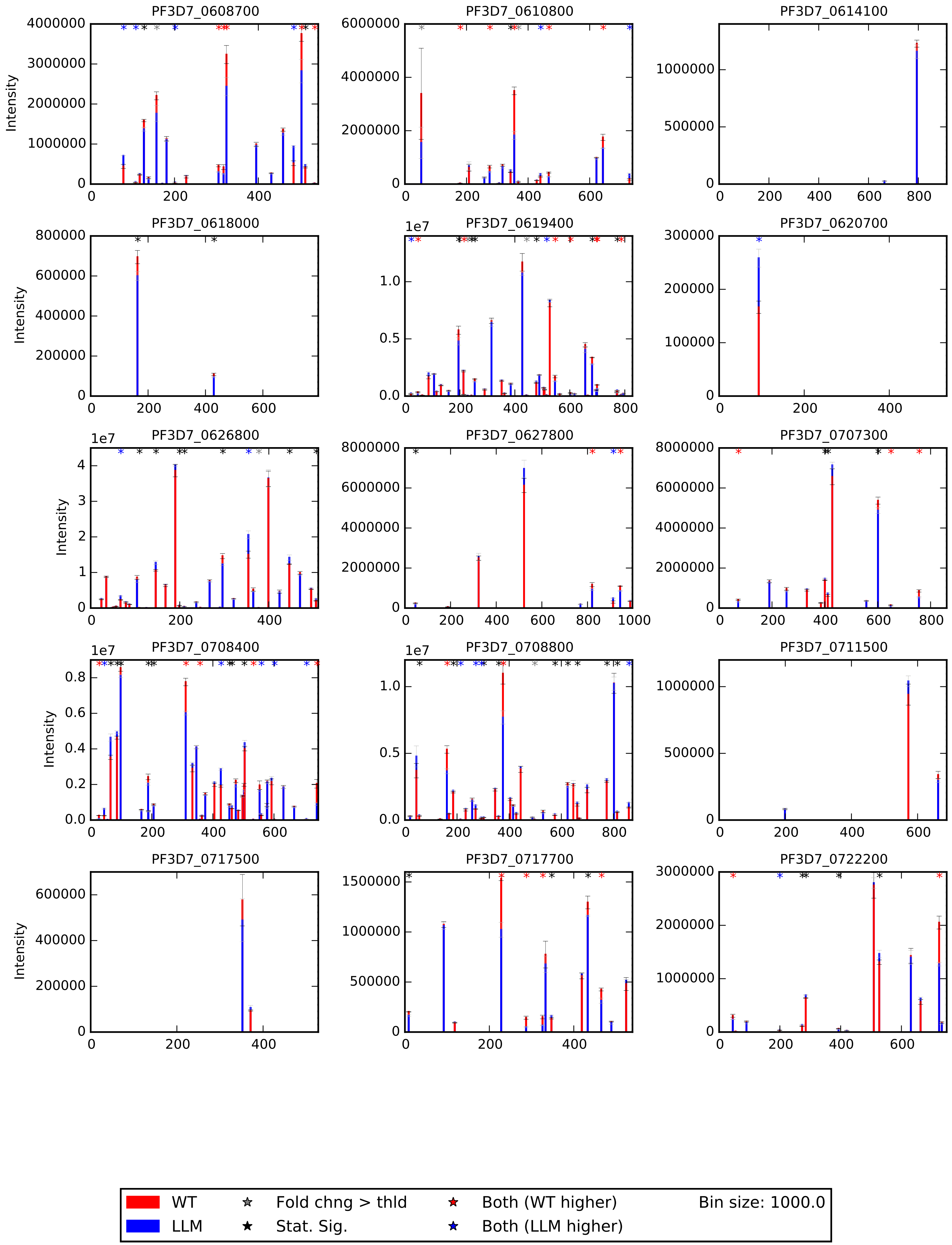
PF3D7_0608700
PF3D7_0610800
PF3D7_0614100
PF3D7_0618000
PF3D7_0619400
PF3D7_0620700
PF3D7_0626800
PF3D7_0627800
PF3D7_0707300
PF3D7_0708400
PF3D7_0708800
PF3D7_0711500
PF3D7_0717500
PF3D7_0717700
PF3D7_0722200
Intensity
1e7
WT
LLM
Fold chng > thld
Stat. Sig.
Both (WT higher)
Both (LLM higher)
Bin size: 1000.0

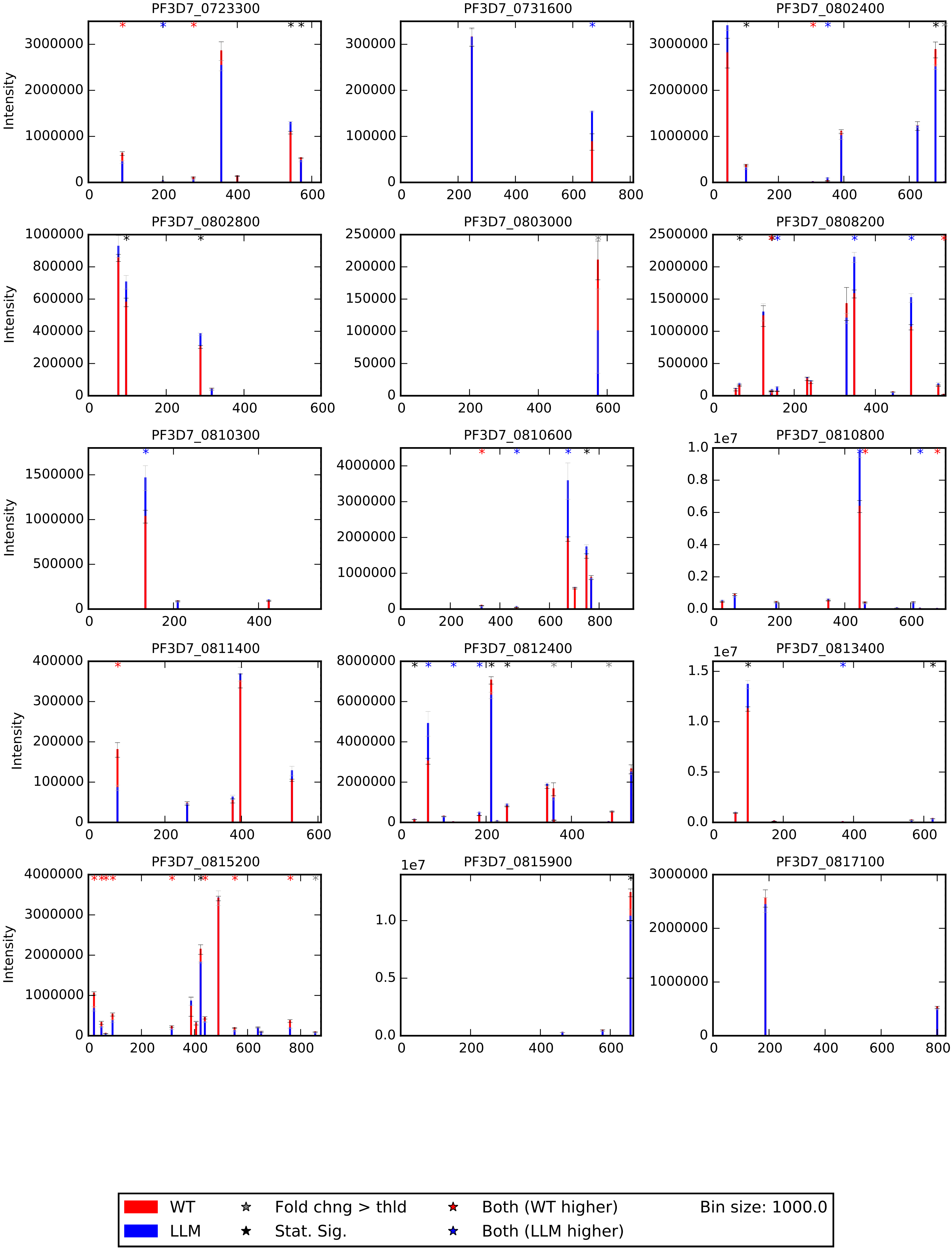
PF3D7_0723300
PF3D7_0731600
PF3D7_0802400
PF3D7_0802800
PF3D7_0803000
PF3D7_0808200
PF3D7_0810300
PF3D7_0810600
PF3D7_0810800
PF3D7_0811400
PF3D7_0812400
PF3D7_0813400
PF3D7_0815200
PF3D7_0815900
PF3D7_0817100
Intensity
1e7
WT
LLM
Fold chng > thld
Stat. Sig.
Both (WT higher)
Both (LLM higher)
Bin size: 1000.0

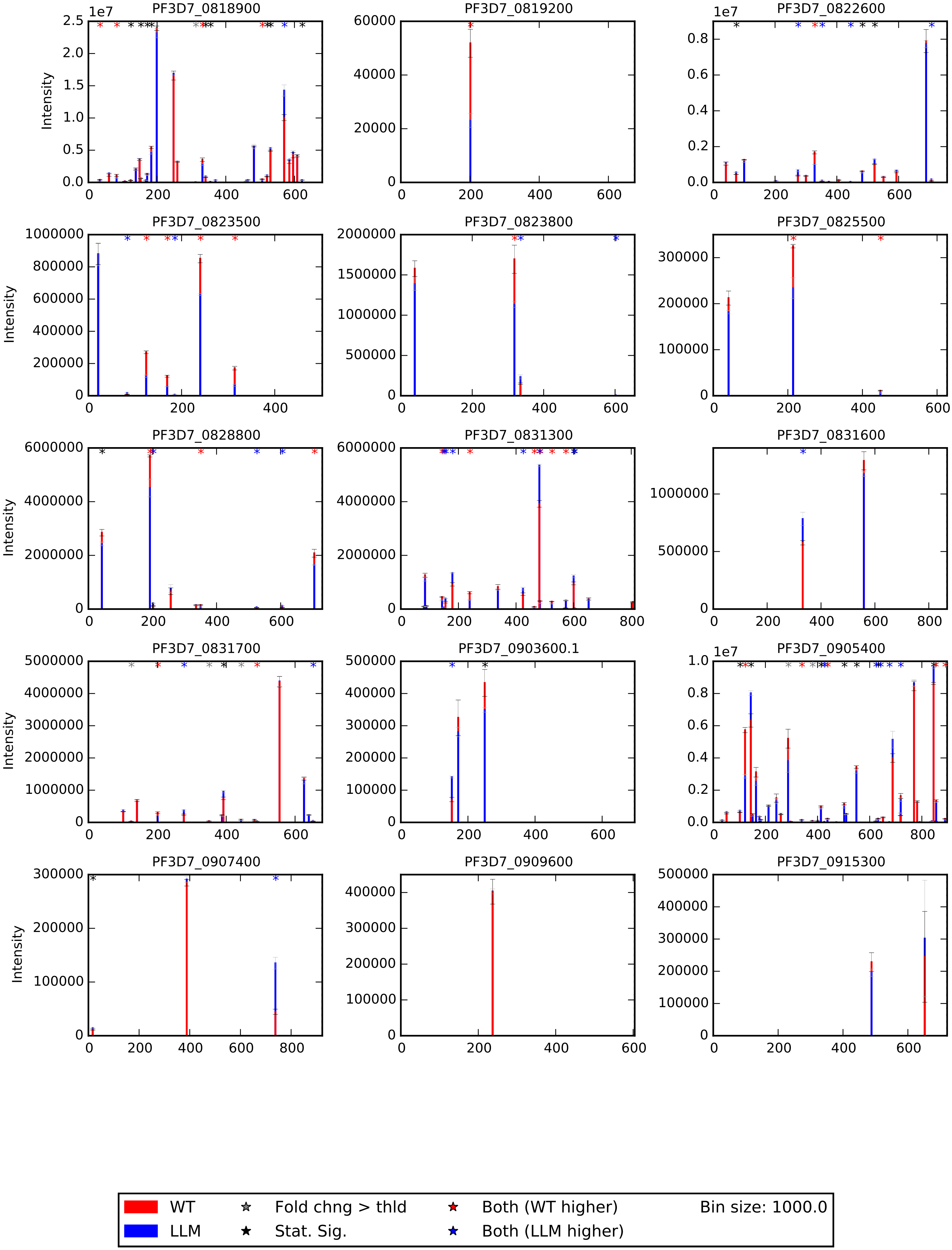
PF3D7_0818900
PF3D7_0819200
PF3D7_0822600
PF3D7_0823500
PF3D7_0823800
PF3D7_0825500
PF3D7_0828800
PF3D7_0831300
PF3D7_0831600
PF3D7_0831700
PF3D7_0903600.1
PF3D7_0905400
PF3D7_0907400
PF3D7_0909600
PF3D7_0915300
Intensity
1e7
WT
LLM
Fold chng > thld
Stat. Sig.
Both (WT higher)
Both (LLM higher)
Bin size: 1000.0

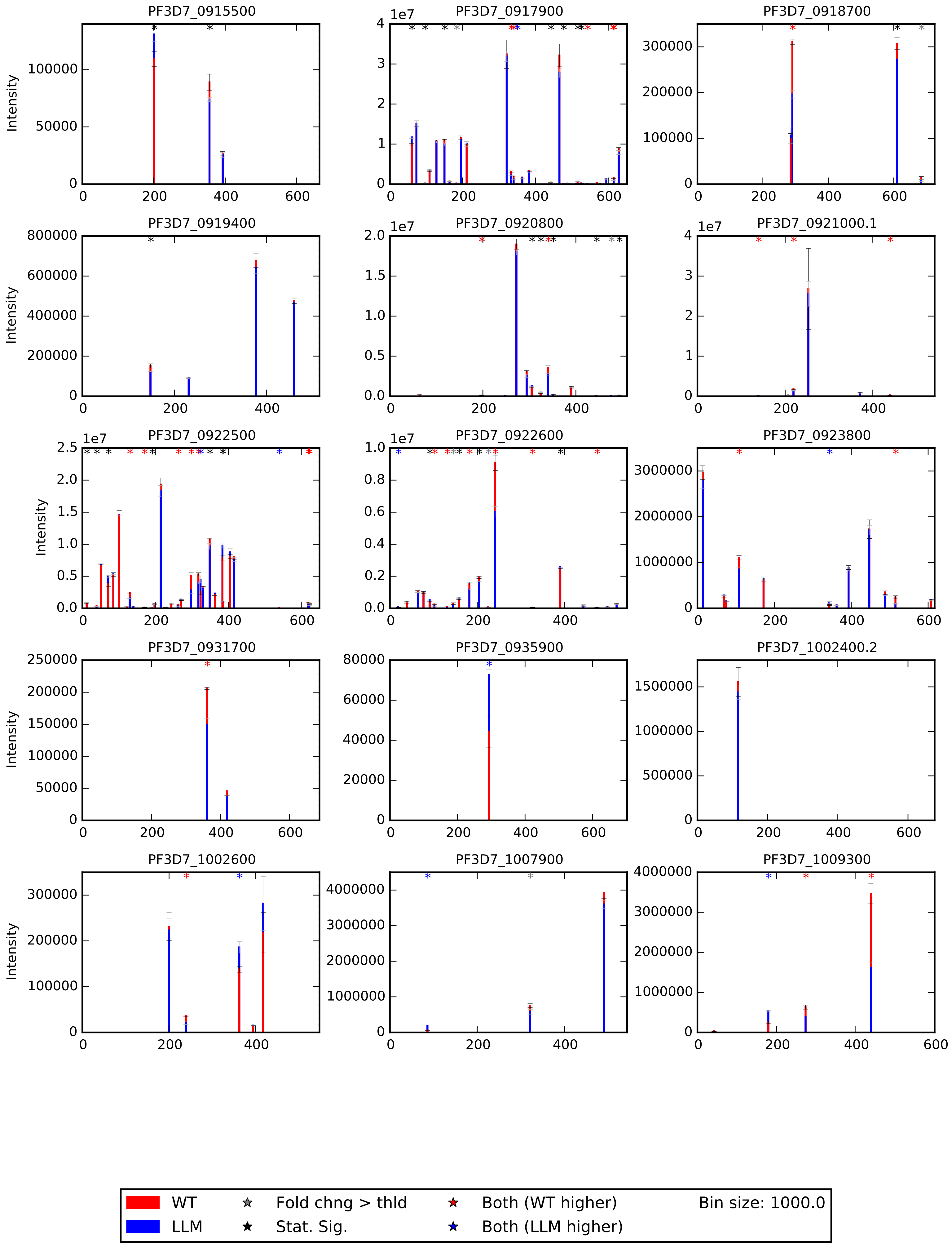

PF3D7_0915500
PF3D7_0917900
PF3D7_0918700
PF3D7_0919400
PF3D7_0920800
PF3D7_0921000.1
PF3D7_0922500
PF3D7_0922600
PF3D7_0923800
PF3D7_0931700
PF3D7_0935900
PF3D7_1002400.2
PF3D7_1002600
PF3D7_1007900
PF3D7_1009300
Intensity
1e7
WT
LLM
Fold chng > thld
Stat. Sig.
Both (WT higher)
Both (LLM higher)
Bin size: 1000.0

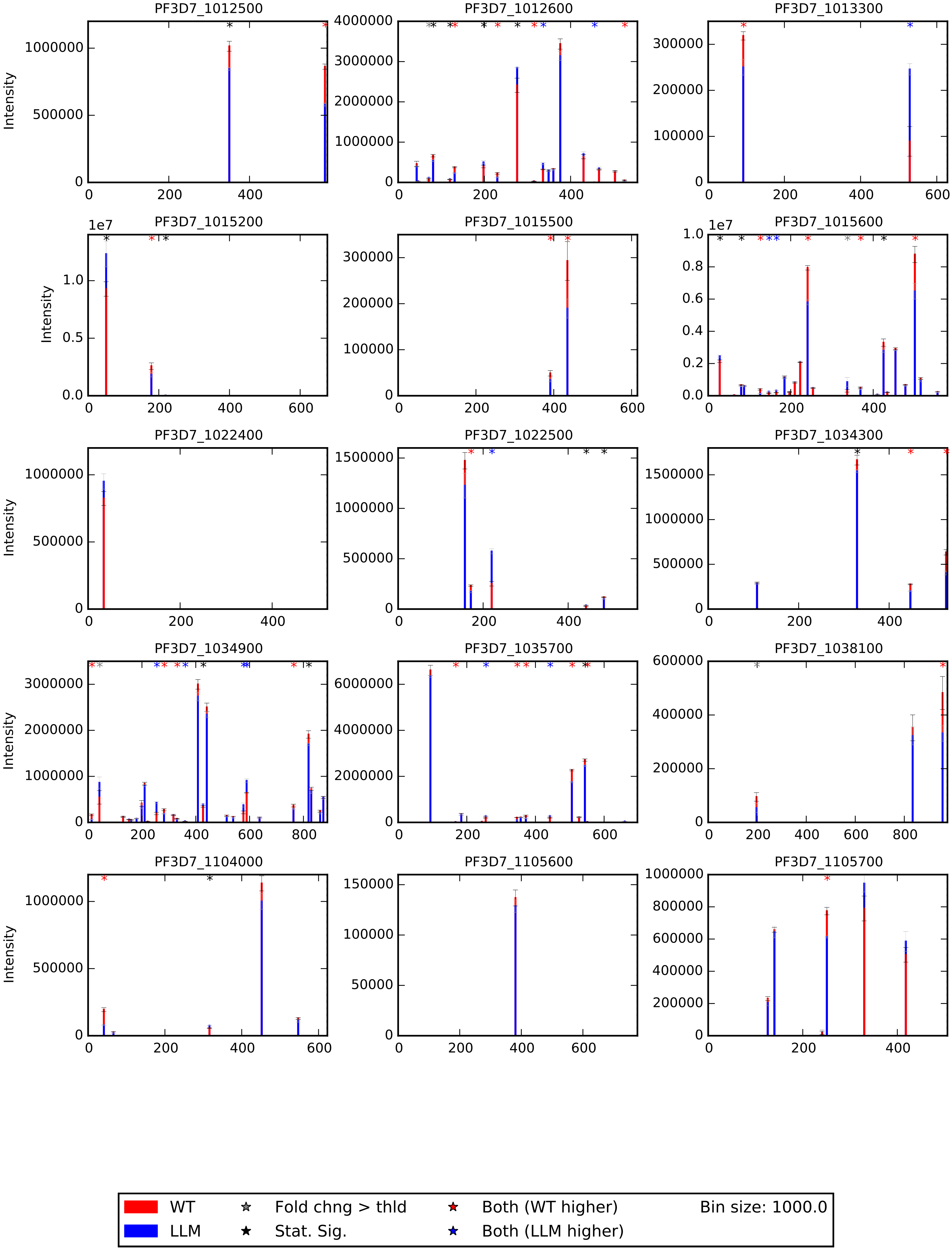

PF3D7_1012500
PF3D7_1012600
PF3D7_1013300
PF3D7_1015200
PF3D7_1015500
PF3D7_1015600
PF3D7_1022400
PF3D7_1022500
PF3D7_1034300
PF3D7_1034900
PF3D7_1035700
PF3D7_1038100
PF3D7_1104000
PF3D7_1105600
PF3D7_1105700
Intensity
WT
LLM
Fold chng > thld
Stat. Sig.
Both (WT higher)
Both (LLM higher)
Bin size: 1000.0

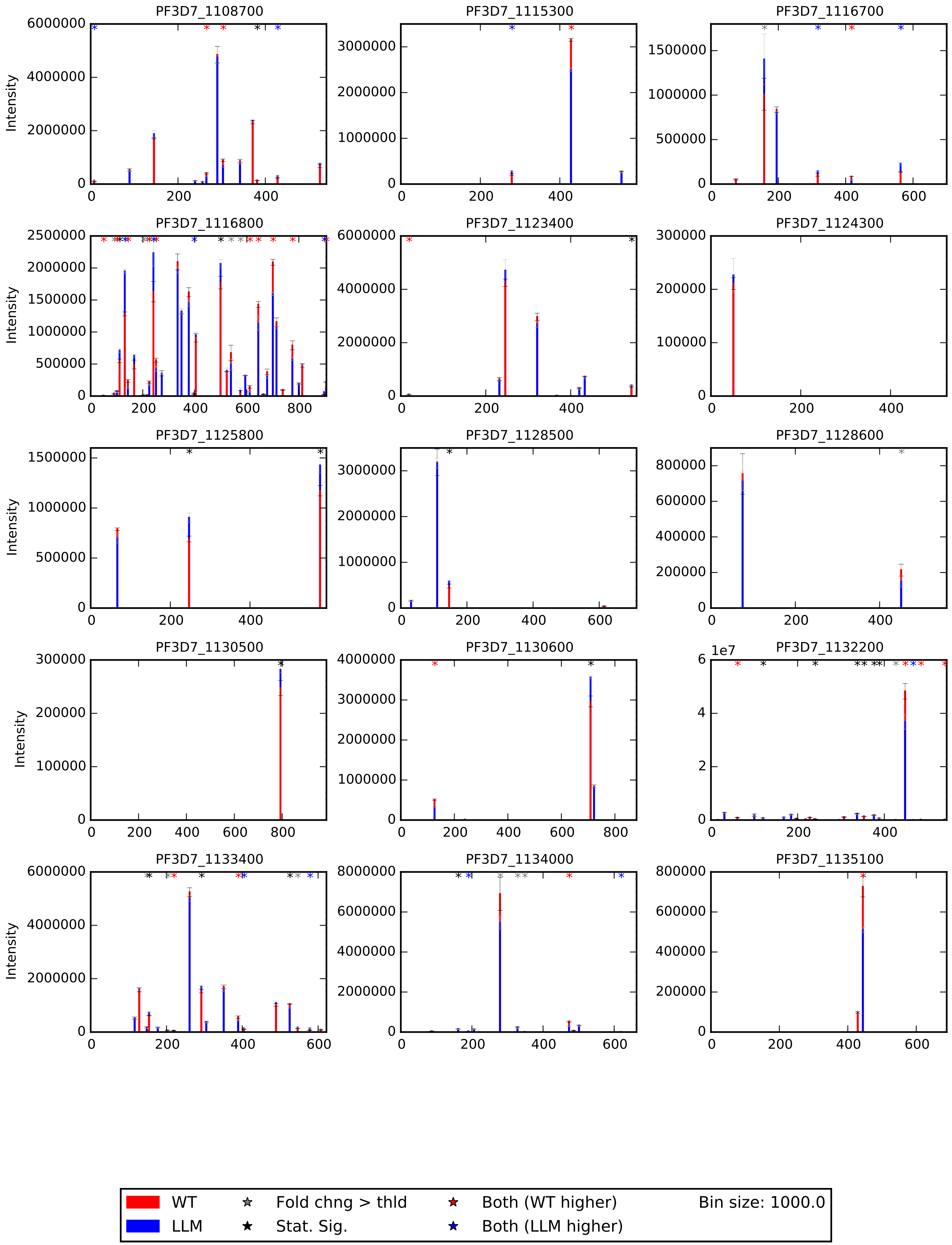
PF3D7_1108700
PF3D7_1115300
PF3D7_1116700
PF3D7_1116800
PF3D7_1123400
PF3D7_1124300
PF3D7_1125800
PF3D7_1128500
PF3D7_1128600
PF3D7_1130500
PF3D7_1130600
PF3D7_1132200
PF3D7_1133400
PF3D7_1134000
PF3D7_1135100
Intensity
1e7
WT
LLM
Fold chng > thld
Stat. Sig.
Both (WT higher)
Both (LLM higher)
Bin size: 1000.0

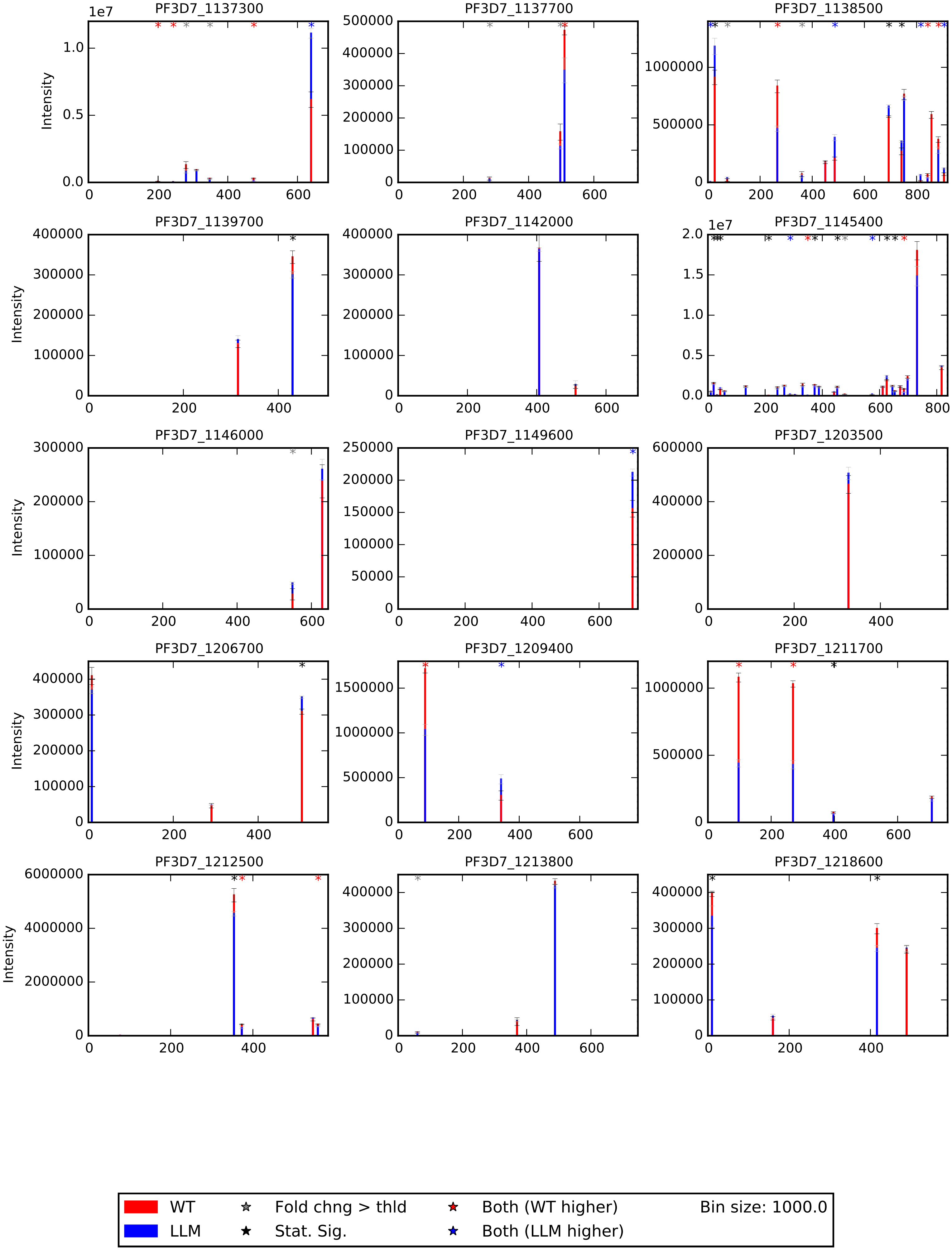

PF3D7_1137300
PF3D7_1137700
PF3D7_1138500
PF3D7_1139700
PF3D7_1142000
PF3D7_1145400
PF3D7_1146000
PF3D7_1149600
PF3D7_1203500
PF3D7_1206700
PF3D7_1209400
PF3D7_1211700
PF3D7_1212500
PF3D7_1213800
PF3D7_1218600
Intensity
1e7
WT
LLM
Fold chng > thld
Stat. Sig.
Both (WT higher)
Both (LLM higher)
Bin size: 1000.0

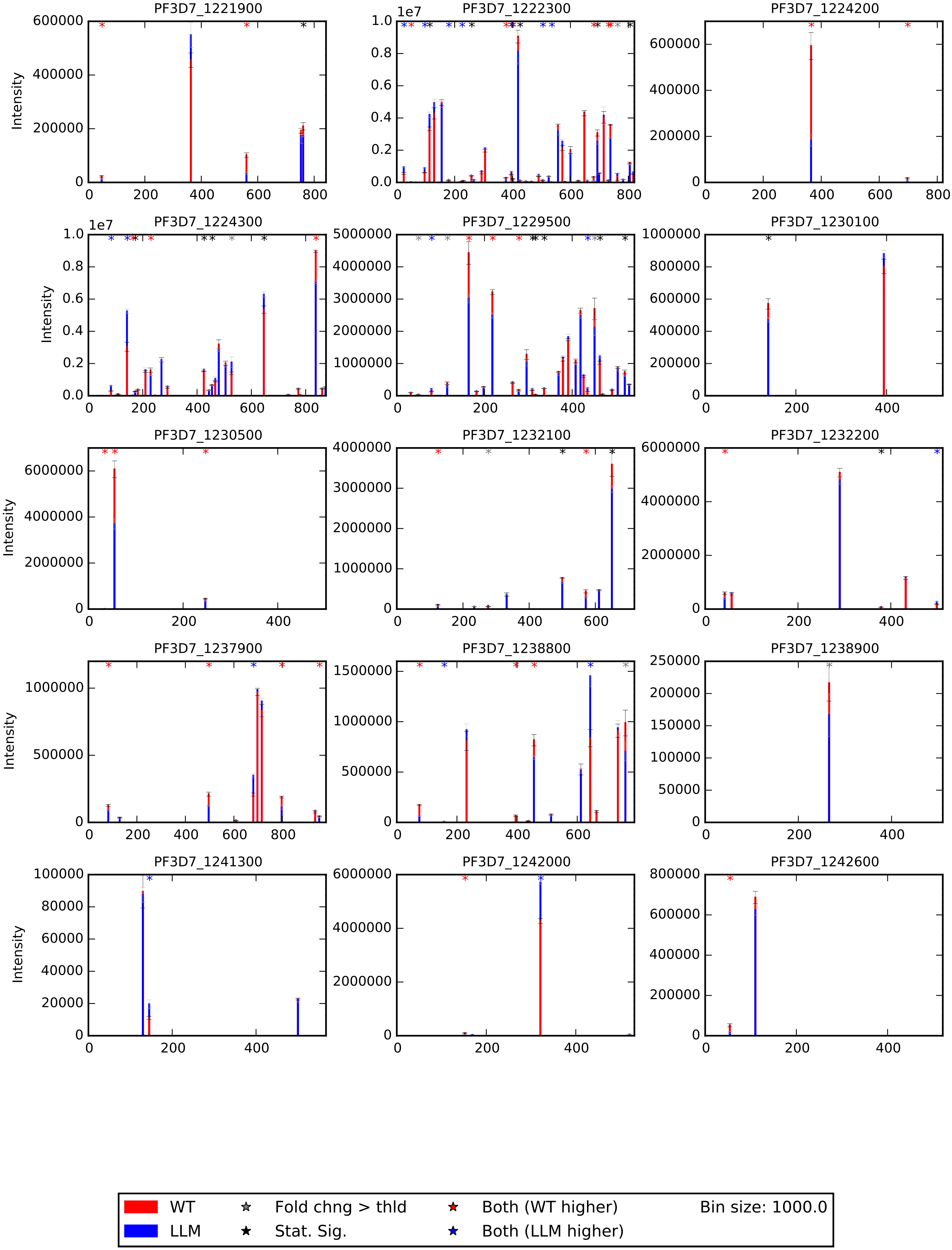
PF3D7_1221900
PF3D7_1222300
PF3D7_1224200
PF3D7_1224300
PF3D7_1229500
PF3D7_1230100
PF3D7_1230500
PF3D7_1232100
PF3D7_1232200
PF3D7_1237900
PF3D7_1238800
PF3D7_1238900
PF3D7_1241300
PF3D7_1242000
PF3D7_1242600
Intensity
WT
LLM
Fold chng > thld
Stat. Sig.
Both (WT higher)
Both (LLM higher)
Bin size: 1000.0

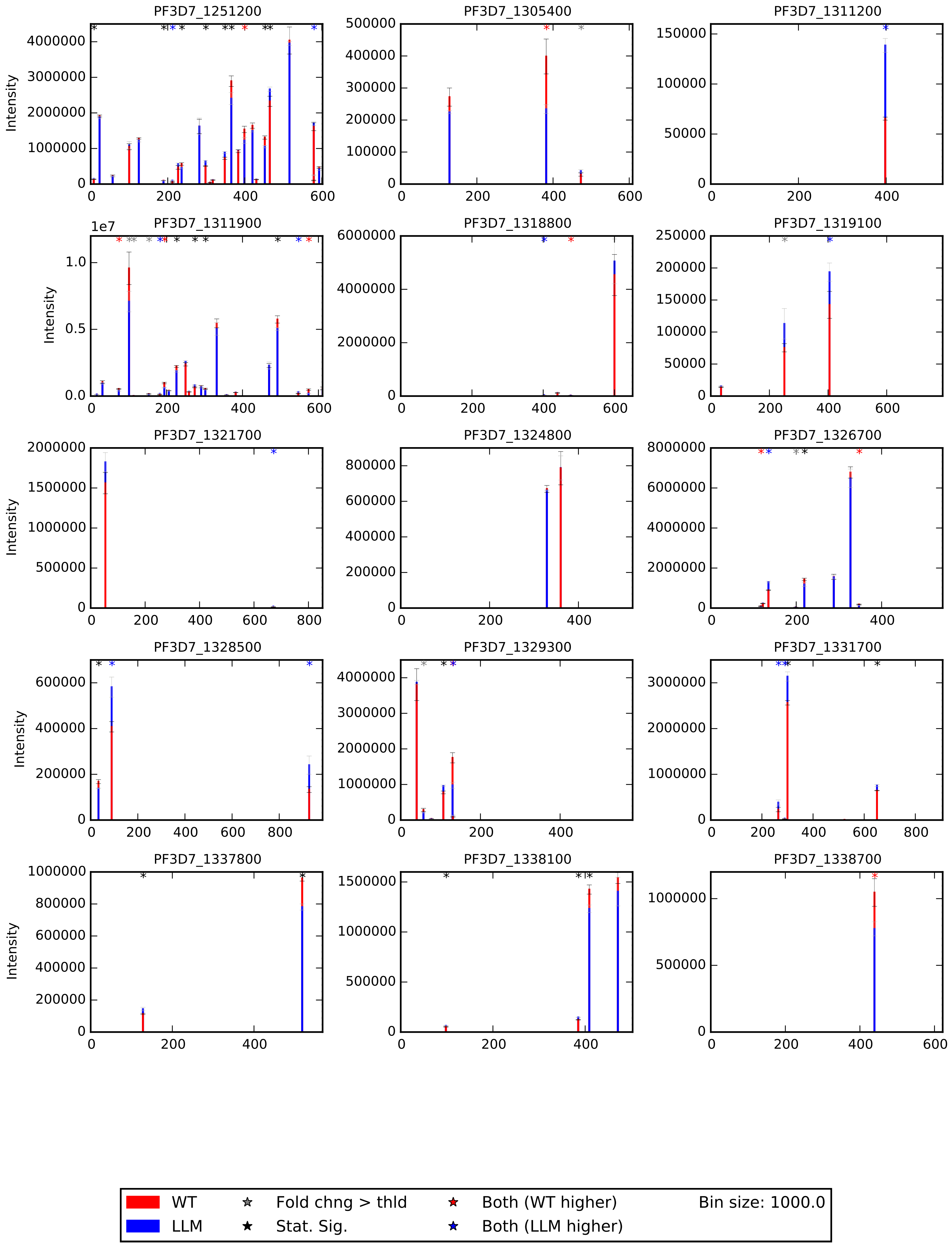
PF3D7_1251200
PF3D7_1305400
PF3D7_1311200
PF3D7_1311900
1e7
PF3D7_1318800
PF3D7_1319100
PF3D7_1321700
PF3D7_1324800
PF3D7_1326700
PF3D7_1328500
PF3D7_1329300
PF3D7_1331700
PF3D7_1337800
PF3D7_1338100
PF3D7_1338700
Intensity
WT
LLM
Fold chng > thld
Stat. Sig.
Both (WT higher)
Both (LLM higher)
Bin size: 1000.0

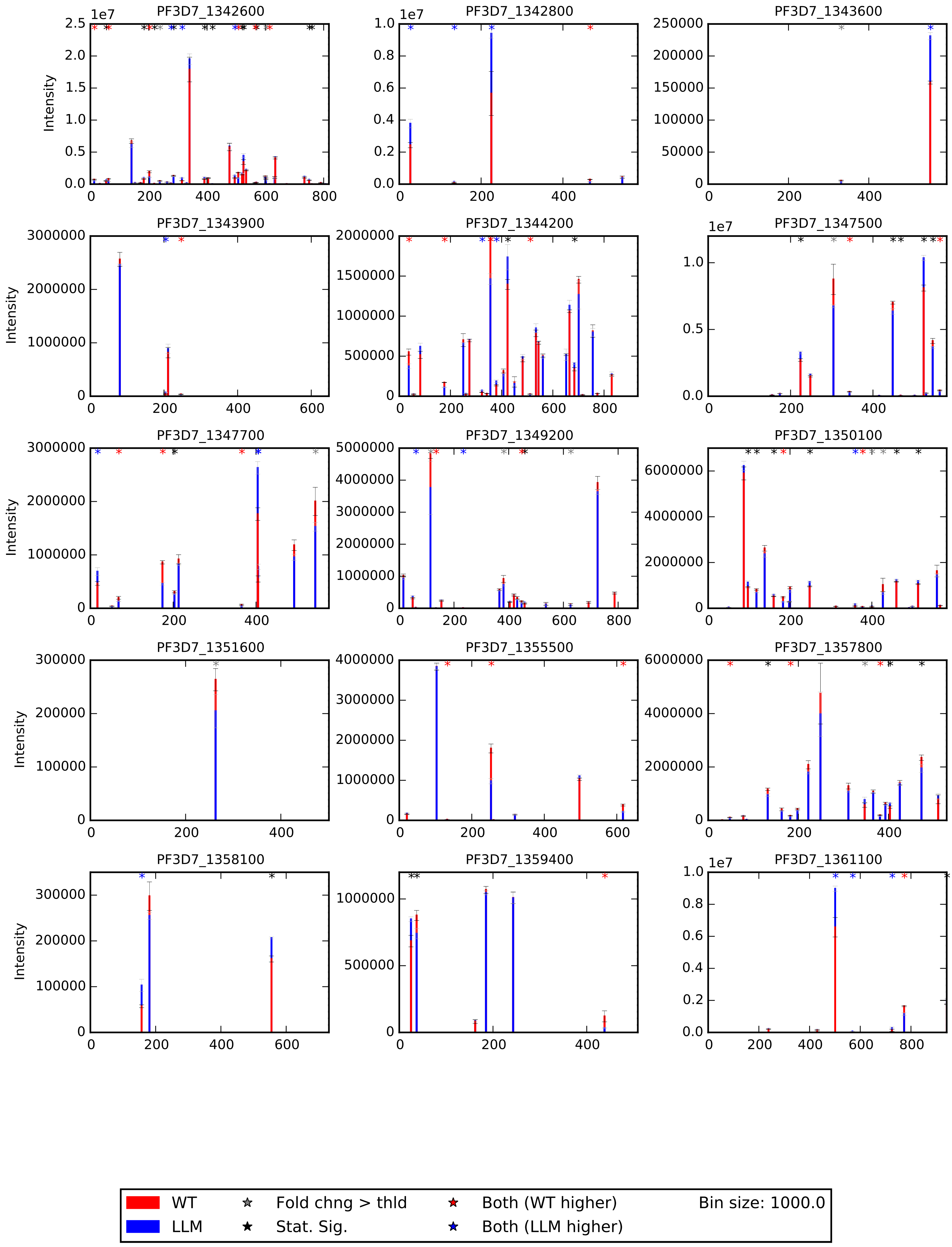

PF3D7_1342600
PF3D7_1342800
PF3D7_1343600
PF3D7_1343900
PF3D7_1344200
PF3D7_1347500
PF3D7_1347700
PF3D7_1349200
PF3D7_1350100
PF3D7_1351600
PF3D7_1355500
PF3D7_1357800
PF3D7_1358100
PF3D7_1359400
PF3D7_1361100
Intensity
WT
LLM
Fold chng > thld
Stat. Sig.
Both (WT higher)
Both (LLM higher)
Bin size: 1000.0

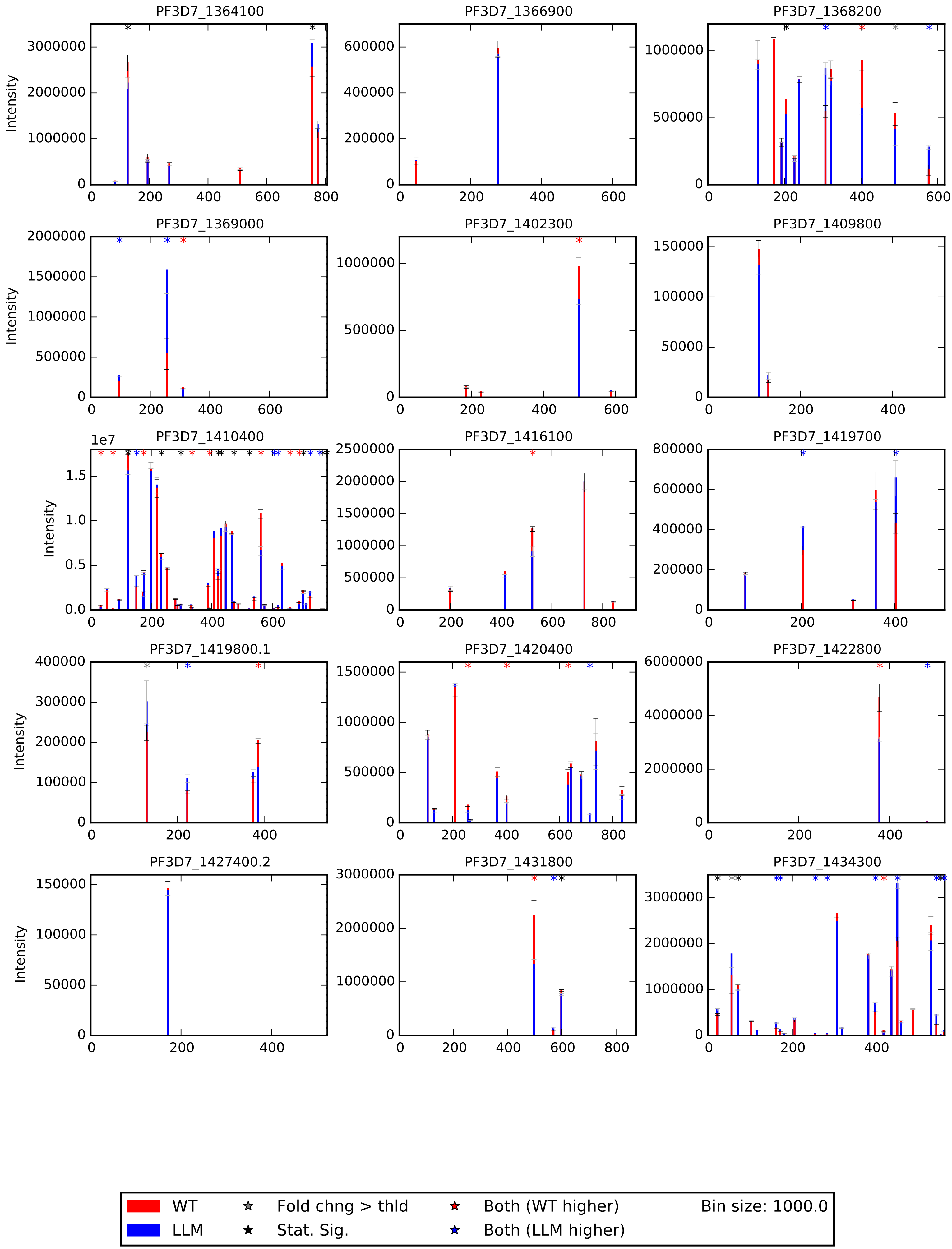
PF3D7_1364100
PF3D7_1366900
PF3D7_1368200
PF3D7_1369000
PF3D7_1402300
PF3D7_1409800
PF3D7_1410400
1e7
PF3D7_1416100
PF3D7_1419700
PF3D7_1419800.1
PF3D7_1420400
PF3D7_1422800
PF3D7_1427400.2
PF3D7_1431800
PF3D7_1434300
Intensity
WT
LLM
Fold chng > thld
Stat. Sig.
Both (WT higher)
Both (LLM higher)
Bin size: 1000.0

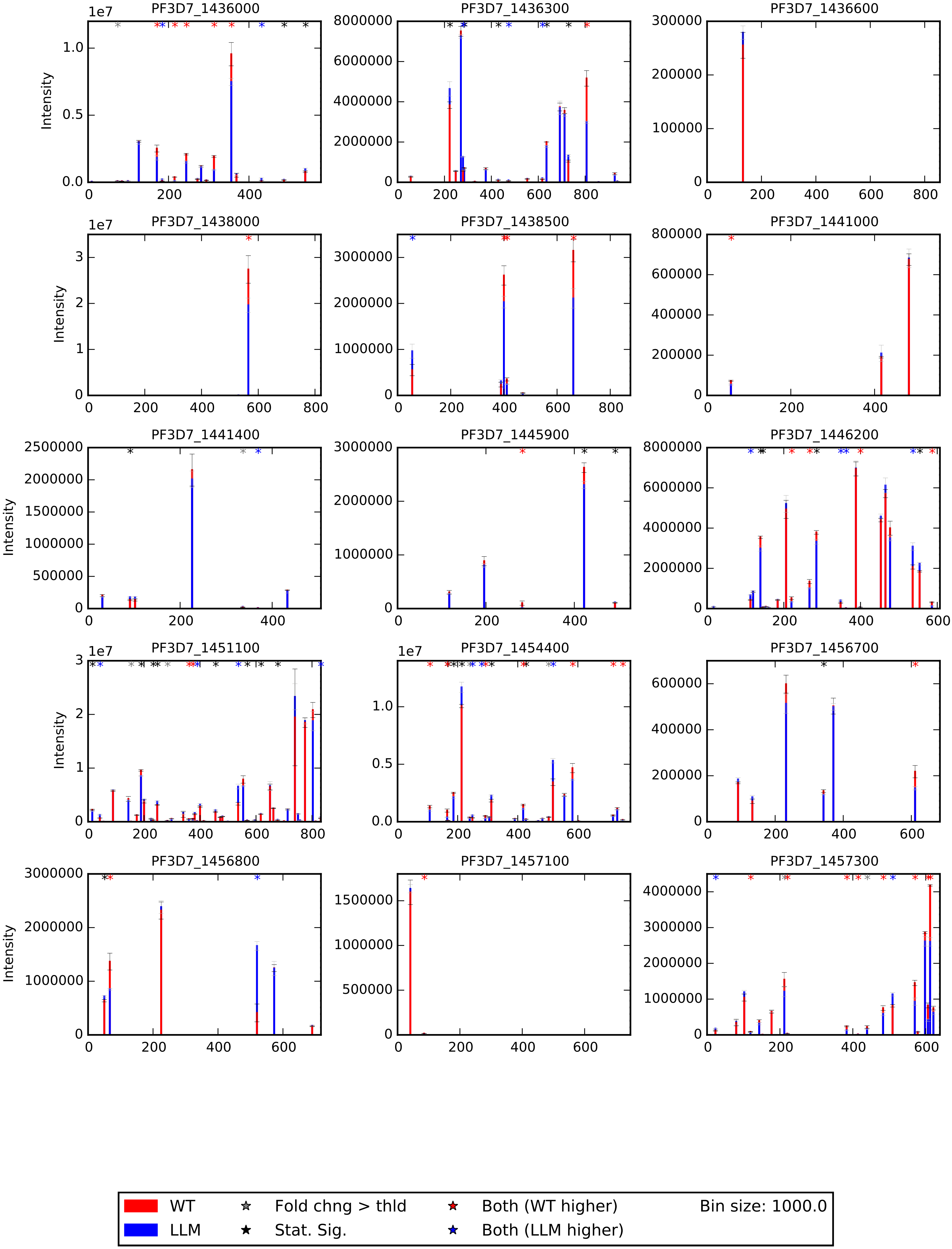
PF3D7_1436000
PF3D7_1436300
PF3D7_1436600
PF3D7_1438000
PF3D7_1438500
PF3D7_1441000
PF3D7_1441400
PF3D7_1445900
PF3D7_1446200
PF3D7_1451100
PF3D7_1454400
PF3D7_1456700
PF3D7_1456800
PF3D7_1457100
PF3D7_1457300
Intensity
1e7
WT
LLM
Fold chng > thld
Stat. Sig.
Both (WT higher)
Both (LLM higher)
Bin size: 1000.0

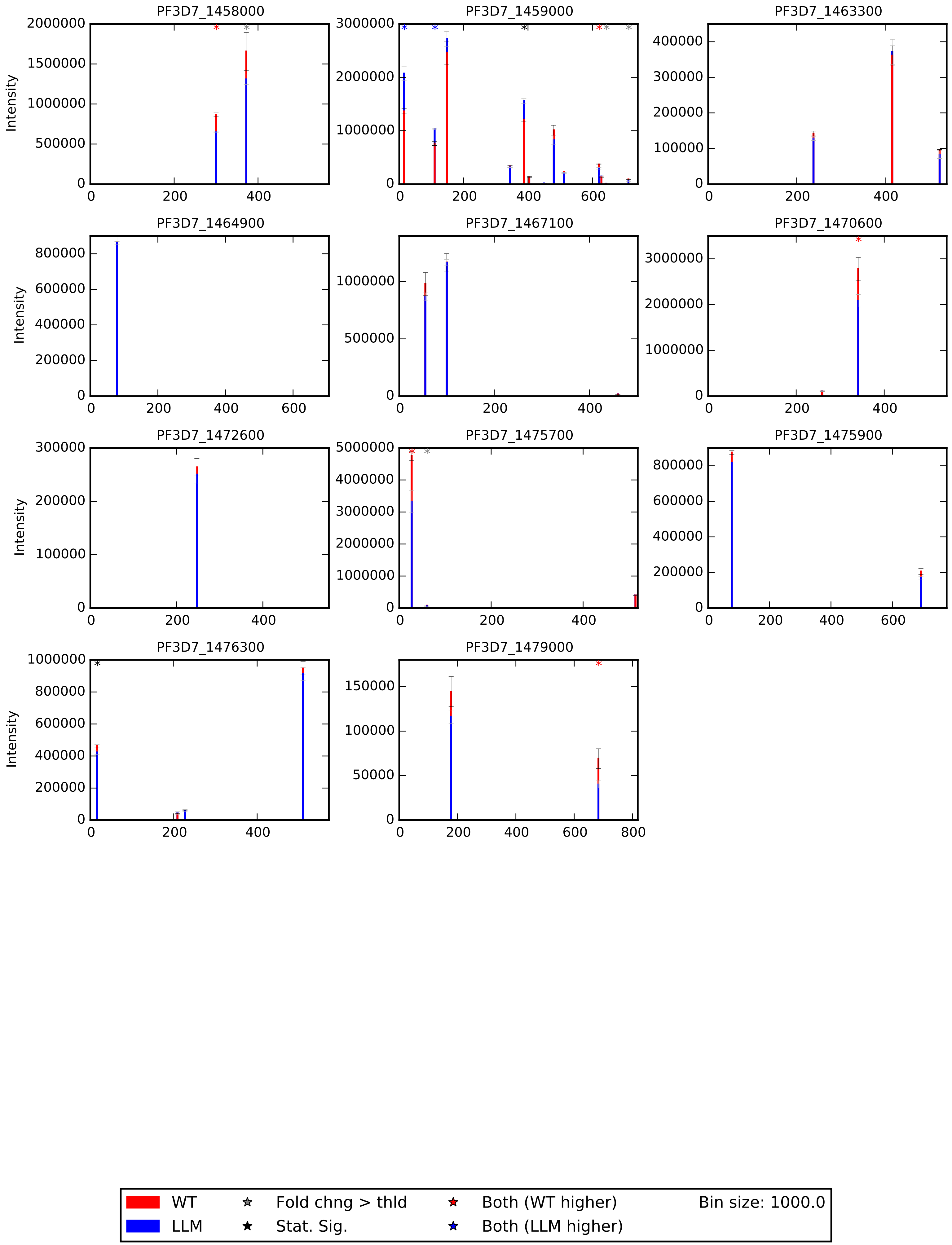
PF3D7_1458000
PF3D7_1459000
PF3D7_1463300
PF3D7_1464900
PF3D7_1467100
PF3D7_1470600
PF3D7_1472600
PF3D7_1475700
PF3D7_1475900
PF3D7_1476300
PF3D7_1479000
Intensity
WT
LLM
Fold chng > thld
Stat. Sig.
Both (WT higher)
Both (LLM higher)
Bin size: 1000.0

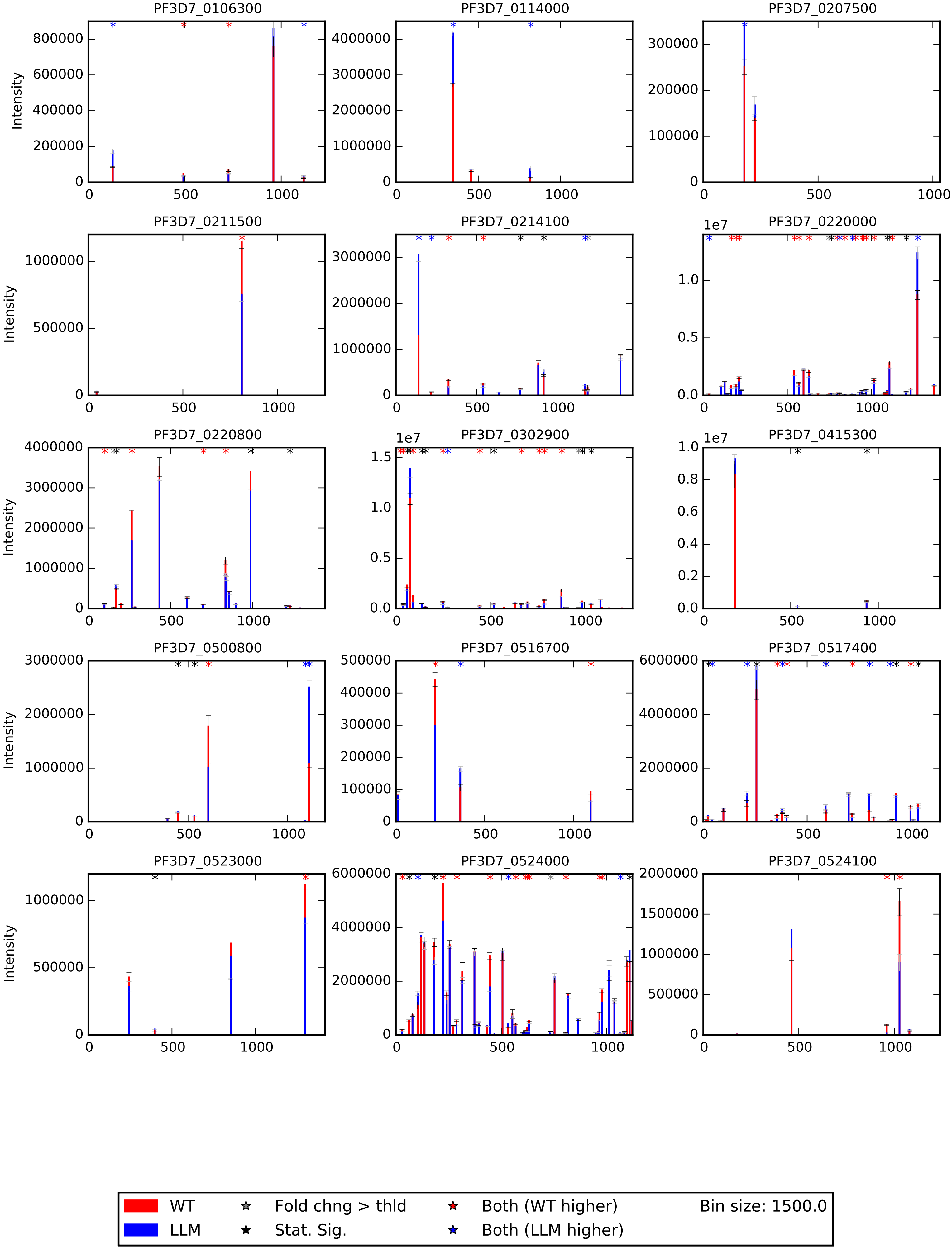

PF3D7_0106300
PF3D7_0114000
PF3D7_0207500
PF3D7_0211500
PF3D7_0214100
PF3D7_0220000
PF3D7_0220800
PF3D7_0302900
PF3D7_0415300
PF3D7_0500800
PF3D7_0516700
PF3D7_0517400
PF3D7_0523000
PF3D7_0524000
PF3D7_0524100
Intensity
1e7
WT
LLM
Fold chng > thld
Stat. Sig.
Both (WT higher)
Both (LLM higher)
Bin size: 1500.0

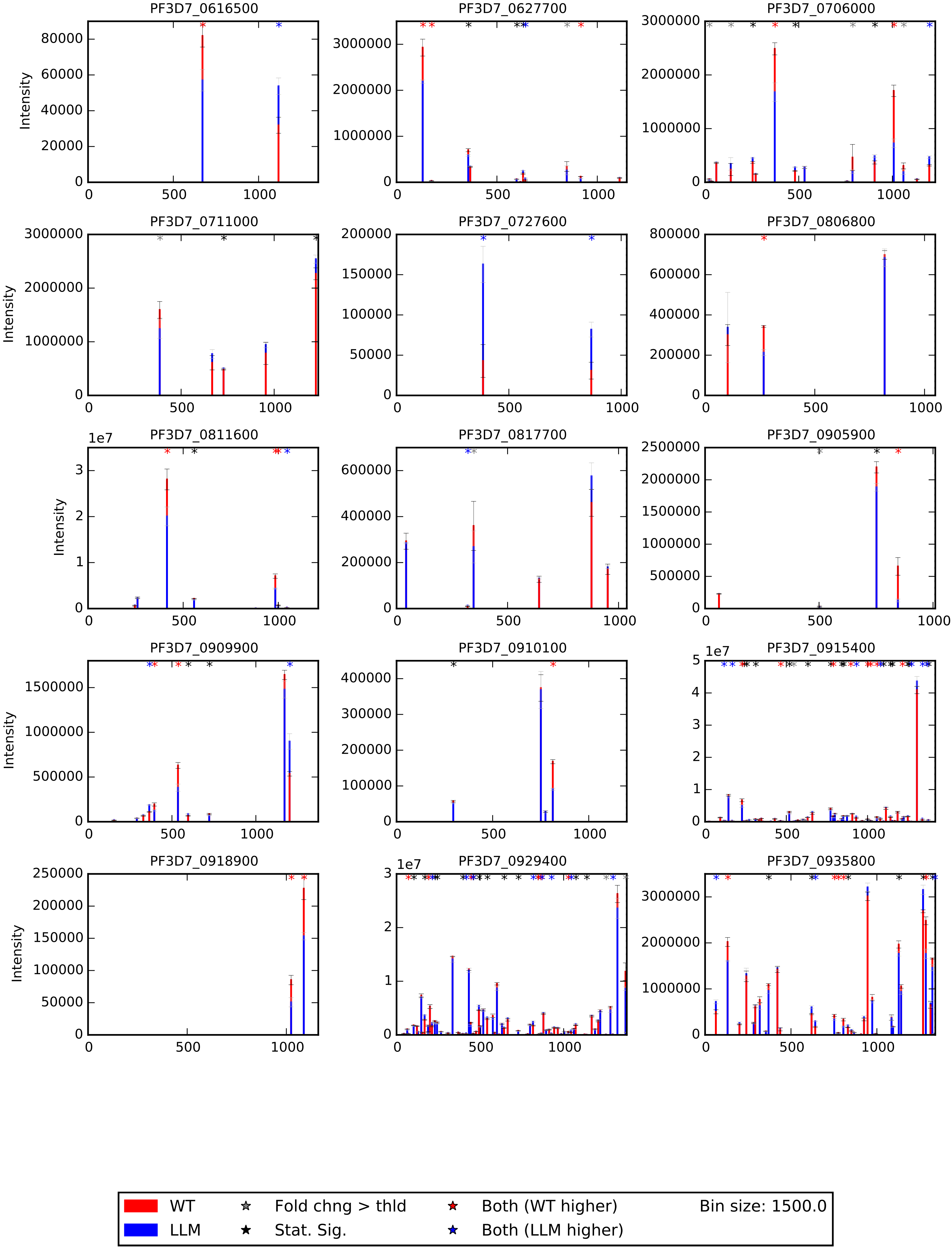
PF3D7_0616500
PF3D7_0627700
PF3D7_0706000
PF3D7_0711000
PF3D7_0727600
PF3D7_0806800
PF3D7_0811600
PF3D7_0817700
PF3D7_0905900
PF3D7_0909900
PF3D7_0910100
PF3D7_0915400
PF3D7_0918900
PF3D7_0929400
PF3D7_0935800
Intensity
1e7
WT
LLM
Fold chng > thld
Stat. Sig.
Both (WT higher)
Both (LLM higher)
Bin size: 1500.0

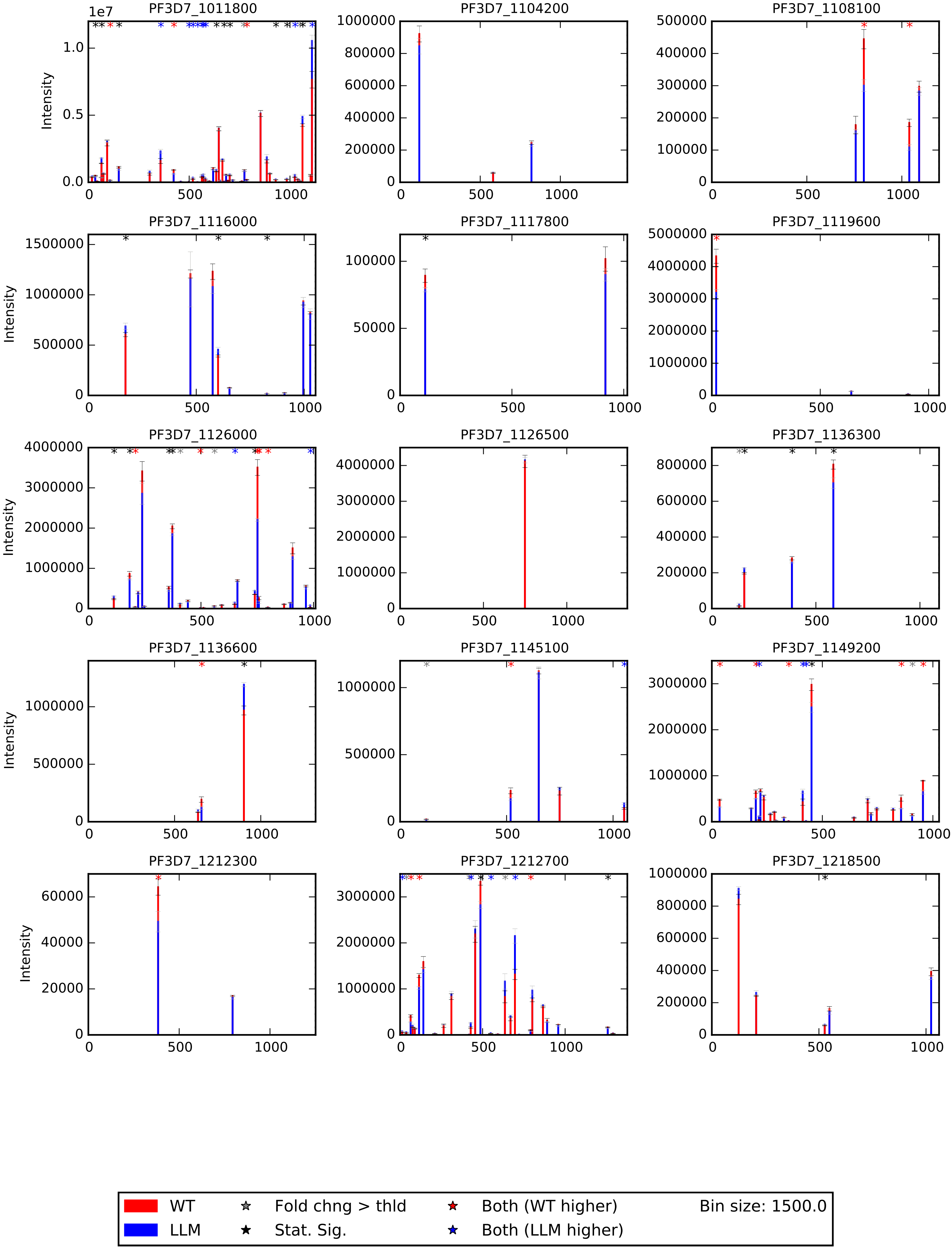

PF3D7_1011800
PF3D7_1104200
PF3D7_1108100
PF3D7_1116000
PF3D7_1117800
PF3D7_1119600
PF3D7_1126000
PF3D7_1126500
PF3D7_1136300
PF3D7_1136600
PF3D7_1145100
PF3D7_1149200
PF3D7_1212300
PF3D7_1212700
PF3D7_1218500
Intensity
1e7
WT
LLM
Fold chng > thld
Stat. Sig.
Both (WT higher)
Both (LLM higher)
Bin size: 1500.0

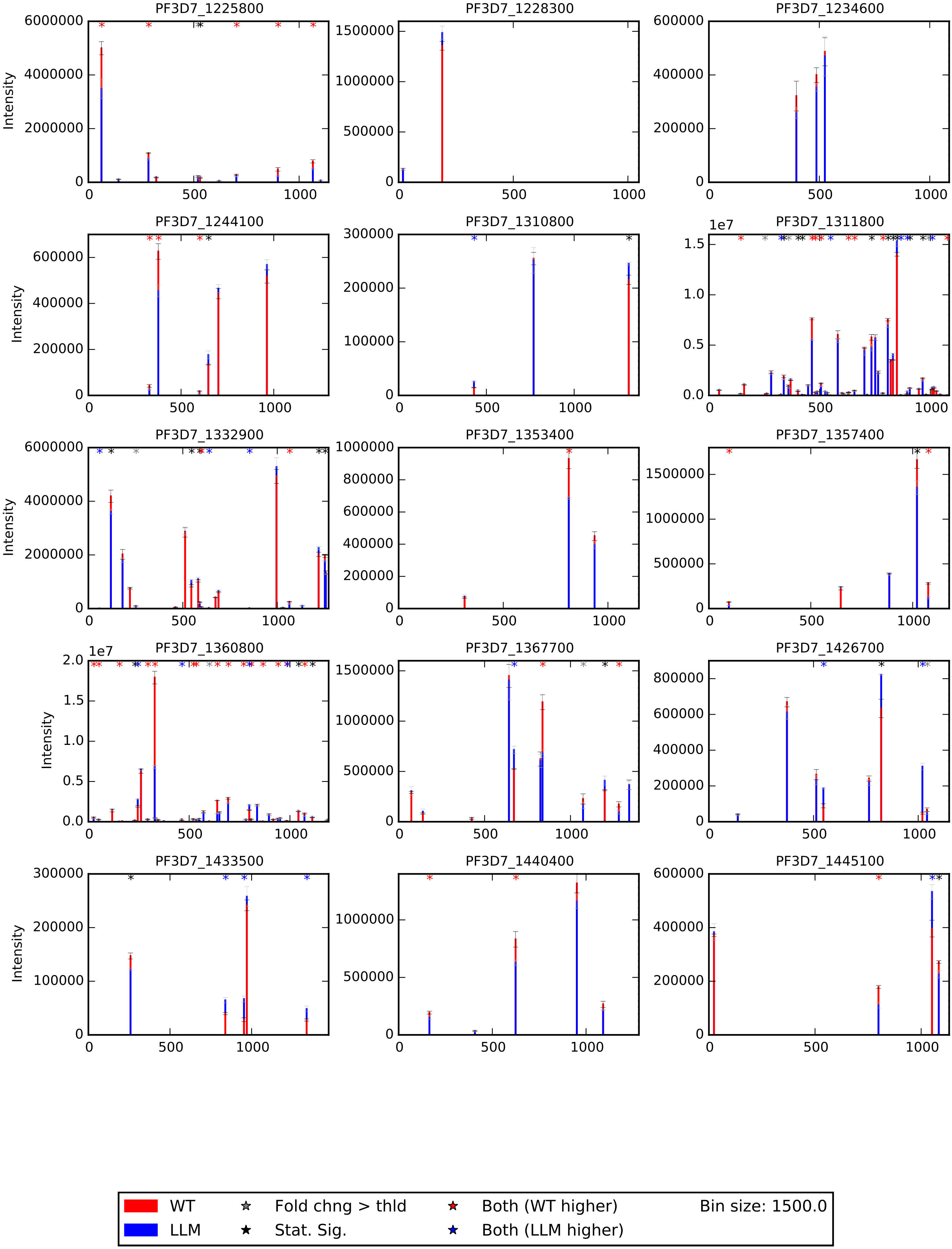
PF3D7_1225800
PF3D7_1228300
PF3D7_1234600
PF3D7_1244100
PF3D7_1310800
PF3D7_1311800
PF3D7_1332900
PF3D7_1353400
PF3D7_1357400
PF3D7_1360800
PF3D7_1367700
PF3D7_1426700
PF3D7_1433500
PF3D7_1440400
PF3D7_1445100
Intensity
1e7
WT
LLM
Fold chng > thld
Stat. Sig.
Both (WT higher)
Both (LLM higher)
Bin size: 1500.0

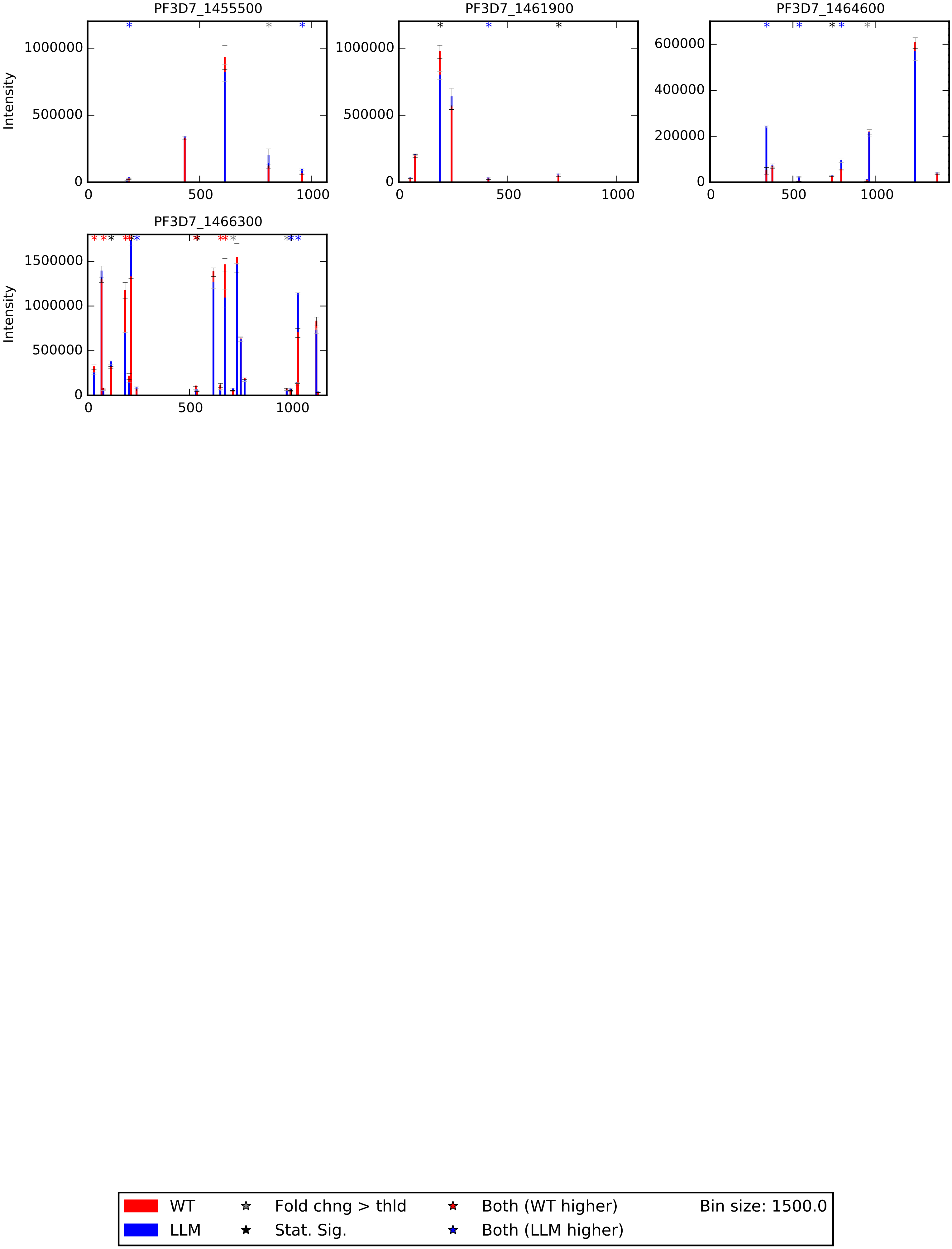
PF3D7_1455500
PF3D7_1461900
PF3D7_1464600
PF3D7_1466300
Intensity
Intensity
WT
LLM
Fold chng > thld
Stat. Sig.
Both (WT higher)
Both (LLM higher)
Bin size: 1500.0

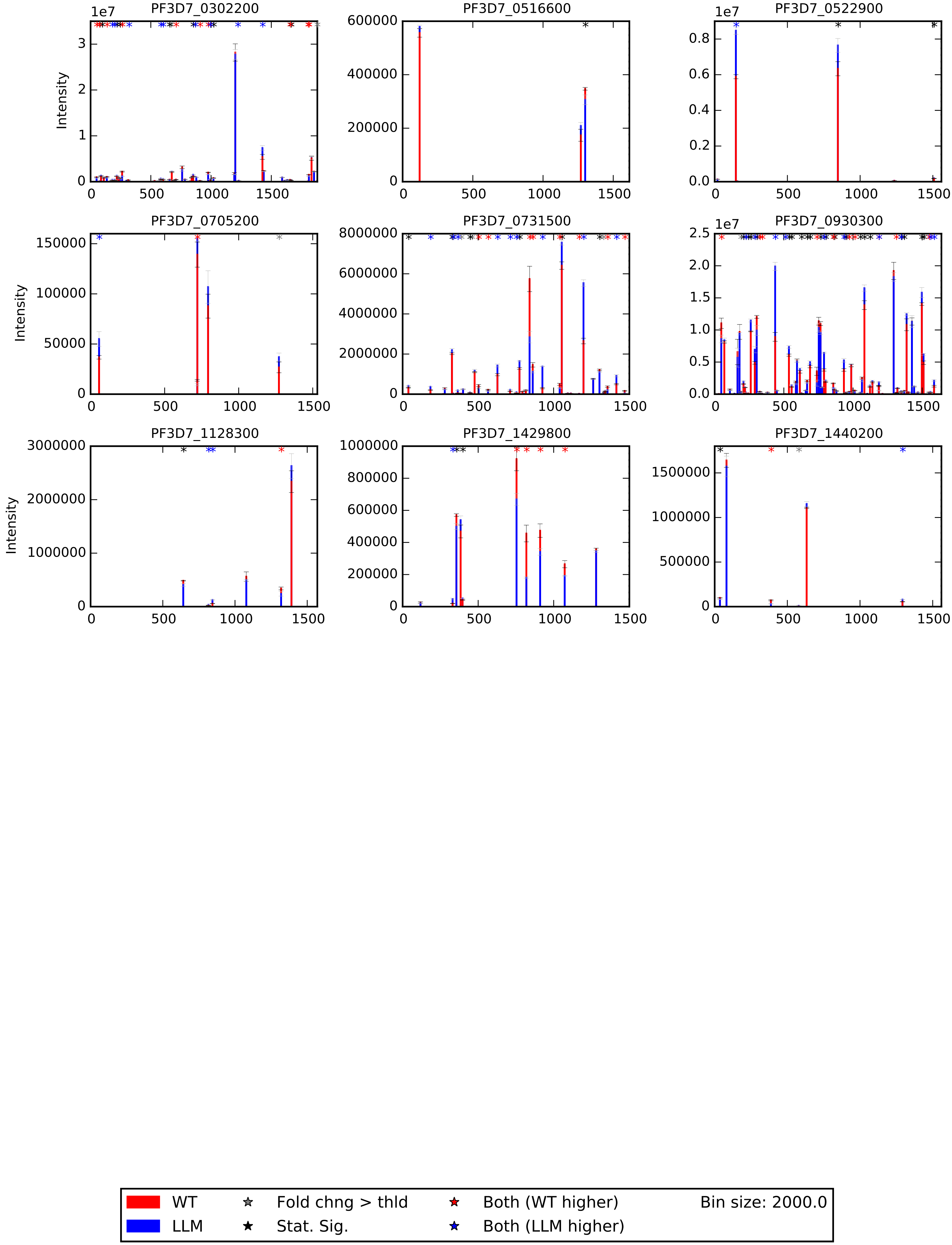

PF3D7_0302200
PF3D7_0516600
PF3D7_0522900
PF3D7_0705200
PF3D7_0731500
PF3D7_0930300
PF3D7_1128300
PF3D7_1429800
PF3D7_1440200
Intensity
1e7
WT
LLM
Fold chng > thld
Stat. Sig.
Both (WT higher)
Both (LLM higher)
Bin size: 2000.0

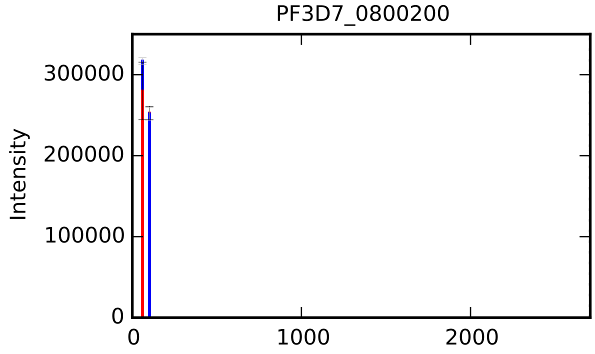
PF3D7_0800200
Intensity
300000
200000
100000
0
0
1000
2000

WT
LLM
Fold chng > thld
Stat. Sig.
Both (WT higher)
Both (LLM higher)
Bin size: 3000.0

Figure S6. Comparative analysis of peptide abundance changes for each protein identified in the TransOmics™ data set. Statistically significantly peptides (p-value < 0.05) are highlighted with a black asterisk above their position, peptides with fold change over the threshold (>25%) of one strain over another are denoted with a grey asterisk above their position in each plot and peptides that are both statistically significant and have a fold change above the threshold are designated with a red asterisk (for WT) and blue asterisk (for LLM).

Supplemental Datasets S1 and S7

Supplemental Dataset S1: WT amino acid sequence

>N.PF10_0009
MLLFAFLFNALLLSQNVNCRNNNYNIRFTQKITQQTSTKSRHLAEIERPKNPHYHNDPELKEIIDKLNEEAIK
KYQKTHNPYEQLQDLVEKKGTKTRGGYGAEPMSTIEKELLKTYEEMFGDESHIMLKSSMYTNDGNKSCECAYK
KKTSSSNKVHENYLNNLKEGCIAGVGTCAFSSALLGKFGIAAGATAAYKAAIGLLPVVSQYITSNTIGVNFFF
ISSLETAIKTATHSTLSSFIESFGVIEGAVSAGTTTFFPYAIAVVALIAVTIIVIILYVWLRKRRRKLMES*
>N.PF10_0012
LSKHNPFWIHIIVFIIN*
>N.PF10_0013
MLFSFKYIYFSSTFLLLLVLQSKTKYIQNFVNIQETLNEKCSSKYIRLISEQTSDESTSKSIKNCKCVNKRNN
KNEDNHELQSKSRSLKGTELDVTSYNKSKHTQQNNLGDSLKRTKNNITNISSKKNETYLFDENTNEYKLRNTL
RLMNERYADSIVDTMDLNDKTKDKFKKFLHLYMAKENPDKQRKLYEQIESDIEKYKKNHVICSIVDFENIYDS
FYFLKYEDRYIT*
>N.PF10_0015
MAELFEESVAFINSVPKNVNLPNDIKLSLYKYYKQSTLGNCNIKEPSRFKVTDRKKYEAWKSVENLNREDAKK
RYVELVSALFPYWQDGE*
>N.PF10_0016
MEDLFQASVNYVNSLPNNRPLSVETKLDLYKYYKQSTVGNCNIKEPSYFQYADKKKYEAWKSIENLNKEDAKK
RYIEIVTENFPNWQNKE*
>N.PF10_0017
MNRINSFLLNLSNGIFDNNNNNVNNKDKNVYSFNSNKMNNKSNKFYSSFHFMCLVVYIISLFFITLTNIYENN
TSLSIKHDKLFMRTLSEIEKENYTSIESNNEKSKLNDELNNSIIKNKVDLKTIDINDLSRQLTKDELYDVLNS
LEEIPPKRVLINLWYQSLNIAKDMKELLKELKGYVDEYLNKNNPYDCDNFINDNNSIWIDCLNDIVETLSYEE
MEYIEKFHNLLNQDITVNDIVNFIYKCINYFDELKKKLFDKYKEKFEEKIMNSQKSHEV*
>N.PF10_0018
MSVHERIRRSICRIFFFLFFLSLSYKTLLNIYLYNENPRTNGQYVNIYSRSLGELLKNPEKREEPILKVSEND
DIGKKPNLYDEDYYKKKLLIDNICKEYYKLMINAKINDSNKNNNFNDIKDFPKNDEHKDNLSSKESLEVEGEN
CSTRIKSSLELENNNSVNNLFYDDGNPEINFFINKDNLKIARYTWKVENPKAYIFALHGVTTHLRNEYLNCYG
MPEWAEKNGEENENADDTEYSISKILDYFKDHNNDHNVFKNLYDKIEIIIKDKLFSKDENETTNSNKVNDISV
IYNNVFKNLYDKIEVIIKENLFTKDENEITDVSNVNDISVIYNNIFKILYDKIESVIKEKIWNKDENETLNEK
KVDDTNIIHNNNEEENVHNINPNGESSKGLSNNTVENKDCKSINDNEVTNNNDINKKIVEEENNLHIFNDLYD
VINDNFDINIESYTLCNATNINENSELYKSLSIYRSSLEGIYFYRNIRSILNKQLFDDIGQYDGSFKKYLNDD
NVLLKDETDVNNFVDSKYYFCSICGLCEHCNCGKRTLSYKNSWIEKLNENGFTFIGIDNQSHGLSDGARNQRC
FVENFDNFVFDAVKALEMFINECKEKNELKPIIIMGTSMGGCIALRTIETIYKLNKNWKDNIKCLALISPMIS
IENQKRKLINRILYSICRYVKKYFELYEMDVLYERPKYPWIKGDTDIDPNHHSEGLKLGTAAECVFAADKCLV
HSILKYIEESDIDIIILQSKYDTTVDPTGPIDFVKKMIDLYNKNHHENSNAKEDDNESEKKELESHNETEVSN
NDQEKKNISLRDENIPNADDIQNDINGSCYNVENYVLVSGNDLTEQQKLWHSCDHGYYKNFLMKKLGKSNNLK
SKNGEDSFKRLSAHILNYGSHRLPCEPDTERSINIITDWLNNIFV*
>N.PF10_0019
MKISKILFFFVAIIAVKLFIPGYVLGGSSGSGGVKKLTDAQKKKKNIIIFSSVASVLAALIGAGVGFGIYYKN
HKSDNKDEGNDKKGSNDSKNKSQEGSTPLLTAKA*
>N.PF10_0020
MLNFRRWSSIICKELMIKINIKSNEYKLFSIYFKNDKKNTKNCLVPTVSKRITMTVLGMIYLLIKDVALIEHK
KISQLKIVDRYTRKLAEALKDDERFEKLLLSNDIYDKTGNKSNVNDHENVKEETLSDVNYDNNEDELSSHIPD
VNYDNNEDELSSHIPDVNYDIIKRLIYDDSKKYYLNEKNKEEIRNAEKKFKNILNEEKHDKDDYIIFDDGNPE
VHFFTNKDNMKIARYAWYIPNAKAHIFALHGITSHLRNEYLNYYGRPKWVNEKRNTNGGDIKNENGSTKKGKC
ESCDINELKKNVKSENASLKSASAHSIMSDTEKKKNNLKKNVHSESSVSEMNFKTHIDDTKVLLIDESDNNEN
VDSNLYYCSYCGLSDYCNCGERTLSYENSWIQKFNDNQFSFFGIDNQSHGLSDGFKDERCFVDDFENFVLDAV
QGLEIFIKELKEKNDVKPIIIMGTSMGGCIALKMFEYIHKLNKEWKSQIKSLVLISPMISVERQKSKFTNRIL
ISIGNILKGFHPLLTLDVKEGGSKYPWIKYDSEIDPFQYSEGLKVGMASECVKGADNCMKHNILKYIDKSNVD
IMILQSKYDVTVDPTGTVNFMKKMIDLYNSKIDKNSNNNKDINDTKNKKNVERTNLKGFNKNKEKKQVSADDK
DVSNSSELLDLDNEPCKHLEDYTVLEGNELEPDKHLWKSCNHGYYKNFKKKIKSKKNNKNDSDKEDKKFKHLS
AHILNYGAHSLACEPETAESVDILVNWLNNIYP*

>N.PF10_0021
MQPRPCAVFFVAVVFIFLKNASIWEEKGGIEIKARQIKILCELCIDNYQTLRNKNSEHKENDSHQQKDVNNEE
RKKKKKSKKYQDGKIDDFSNTNDVTFDNTLYDMEVVPVLRNKNHQRVLSNEQIKEKIDSLGEVVSVQDMHKIF
DLIHDNEKKKFFNMKEDIHEYSEKLSQYYDIPENIKKKCWLKASYEMEETLINKENFFFKNFYVFANIGERKR
SSLLDAIYGYQKKWKEVRYTVDETWREYIYQKFIDFDNKRKRKLRSQ*
>N.PF10_0022
MDKTNYSIKKMFTYSSSKPKILSFLGIVLICIMNLWIYKNKGTSNFHEGKVHRRTLSDNNAKKEGRSGIEKVK
SKSQHSPCSISNNAAVGYYDYLKIYNDGECEKELSESDMELLNQENDGFLEDMSDSINKQIKELENVPSKDII
RNIWFKVHCNERNKFHSLIYSIRKIHDKYFSDTNKNEELGKKVWNKCCAIITEELLKLDSIQNSQFHNLMQQE
TVTLQEFEDFVKFSVNGYKNTKKETKKICIKKLKKALNQRL*
>N.PF10_0023
MIKDHNNNEMSMNFYTYKKMINDNSTSYSSSKMEPESNRYIKGKNINIFPLLKFTVFTLLIWIVTFINNYGNY
MNDGCRMTDLSTSRFLSEPLLEFDTVFDVFKDTFLKNMGCSDEETKNIRSTMKQYFDNIDMNALSKEMKENGN
FFEQIGNNENTLERFMKKDISQLKCINVENAEEGEEIPKPYTVEEVDPNNPNKGEIGNMKFNSNFIRRSVITL
KKIGTSPTFLITMTAILLKNDNFKTALLLMVALFLKGVNFALDLKAYNDKFHFIKFK*
>N.PF10_0024
MFICIYFFYNNIVIESTPINVIIKKKKMTNLRYGNTFNFICNKYDEVIQHGCINLDNMHKNNKKGNKKKYIN
IFPIMKVLVICLLLSLVNKSNDNTYNEDIFQKDKENCGGLRYKRLLAEPIFGFDVMYDLFKDNFLEQIGYNDA
QKNELKVSMKSFFQSSNSQEYTYSRREDNIHSISKSNVTNVKSVKFEEPQDEKNLITIEEIKADNNDSKEKKN
EITNINVDEKKDLESVISSEEEKTPTSNNIRKKRKTKRSHKNKDSNTSYDNIELNKILKDDNIEVNKTLKEDN
IEVNNTLEEDNIEVNNTLKEDNIEVNNTLKEDNIEVEKLIDENRELIKKESIYKNKLKEDNVEVEDLIDENRK
LIKKESIYKNKLEEEPVEKGIKRKNYLFLILEKAKEIPPGVIVGLFSIFYVALGENRSSLILLIVIFILTVHT
YLEVKRICRFLNID*
>N.PF10_0025
MENIINKKNTNGGNSNIFVLLKYSIYTILLWIVTITYNNYTYNGYNNDGSKQVRCKRLLSEPAVEFDTIFDVF
KESFLDQMGCSDQDKDEIKNAMKIYYDNIDMNALSNEIQTNDNFFNEFGNDVNSINKIMKTDITNEQGTSADN
NTTENNVSTSQVDEQPEASTSGVERMKERSNLFANNLTEEKNDTGDIKKEENKSKPSQLDGNNHSETSSHQGE
KNNENETEKKTDQNETGTKKPSKYTMNLDSPLLKGSSEGETSSKKAQEKSVEPTKKPSKYTMNLDSPLLKGSS
GSETSSTKESNDSNGATKKPSKYTMNLDSPLLKGSSGGKTSSNKESNDSNGATKKPSKYTMNLDSPLLKGSSG
GKTSSNKESNESNEPTKKPSKYTMNLDSPLLKGSSGGETSSNKESNENNEPTKKPSKYTMNLDSPLLKGSSEG
ETSSNXXXXXXXXXXXXXXXXXXXXXXXXXXXXXXXXXXXXXXXXXXXXXXXXXXXXXXXXXXXXXXXXXXXX
XXXXXXXXXXXXXXXXXXXXXXXXXXXXXXXXXPTKKPSKYTMNLDSPLLKGSSEGETSSNEKQEESNVATKKP
SKYTLNLDSPLLKSETKSDVKRGSNKSFSLENIGKLDIGSLLAQNLELLKGFALNFQTLSLFFLVFVGQFYPK
HFQKAAIFIGMVNVFLECKRLYGRSQKKLK*
>N.PF10_0026
MQINPMEKSSFVQTRIFPPKPTIFSLEKNDDFLNTHNEGFLILSYCFAISLFMYIVLKNLYYLVSYIM*HYIY
MIYMIILTRQCLKYFI*YMYIFYFILFLHCCHSFSFLQYIFKRTRIKIIESLNNDNNVENEIEEMKRVKNLIM
EQVIHELMRKSIISKRRTRKDIETDLSQNVRNEDMNLKENEIEMTQVETLKHLIHNNLLTEFHDTKEKNLESF
IKFLQQLMTLEKNEKEFSNNILVKDLMDYIINIGKKEIQKSNTQLSQNSSQNYIPSNENDIYIFQIQVIKELL
KNNIIITHSNDGKQLDDEIITKILEESLMSQANVVEYFYIEMIKNILGNDYNELNNISYTHDDLYLQKYEKEL
IKKKLSQYAYKNMNTKQNNKYVQDKTVNKHIHKNQIEQNENTTNLIEKENTFEKTNKFDNNGMIQNNIPIQNE
TNDESMKNIIQHKELLKNHDKENKNEEIENMNQHKKEECNYNDKKNVTEHSKKSRKNFKRKCMKKRDIRHLYQ
IDKLQDKILKNLMGQDSIESDSKNNDIQQLEEQKSDIQNEKLNTSNEQEETRNTNNEHKENEVNMDQNNMKLN
IYNLNNQDIQNHYDKNVKEKLEEENILSDNIIKPEGSMAAEGSYSSYDSIYPEGSMDTEGSYSSYESIYPDGS
MAAEGSYTSYDSINPEESVTPEGNNSTSETIKEEDNMTSQNNYSSNGSYNSEEDKDNMDKIKMYDEIIQNGLI
DRINKPRTFEKKIEKCRRYSILTNRENRTYLSNWNNEKIDIKEYENKSEEELEQWKNKQWEDWKVYLEKQWSV
WLEISENDKIEWLEDKERDFRSYIKYIYNIWFKWLKKMKGELTGEQKPWMTWNEQEWETFFETRYKKQFLDEW
YDMLKYMDKILNIRKYTLWNKWKGKKLTQWLMQDWKLIEDDEWEKYEEDKSWSKRFDMKKKNEWKKWRNRIFK
EYIEWKEWIYYKGQSANVSDVSYWEEWKEKKTNELKEWLDALTKQWMLQGKWKELIRNQE*
>N.PF10_0027
MSSVLRTILIASFFYYFSIYKISPWAIIKNVDFQITYDELSRNLYIHSSGKGKCTSHNYYINDKCKIINTSKK
TNENNKAQCLYNQKCLTIAKYIFQHTGIEAVTEDIFLQPFVINNKLNILQKEKCNREVYINDKIKCLCCINDV
VKNIQGDENEKIKENVTYQYNKCKCLLNYQDIYGDSNFFNKCENFECNNGLCTIQANGQPFCSCFENYYFEKK
SNTCKKHEQNVENIHHINSNRDIIENTENDTRTIVSIKNNPPNYNNSHNYNNANNIIHNIEDLEEHNKSSNIT

TNGNTIISQRNYNSCPLNQIKNKKGICETIINYEETVCLRIDCSISTNEFQCFCTNVNGEHIKTDTFDVSYIN
ICTLNNINCNNGICNNILIKDELGCICDENYIYDYDLKVCLSYNTNTFLSLFILCMLYAIIFLIL*
>N.PF10_0028.1
MSSKVAHSESEEPVHKNDGSTLYVSNLSSKITTAKLQDIFEKYGNIEKCYVISNPITKESRNFGFVTFNNSED
AENAMNKANKMEIEGREINVEIAKRNEPHEPTPGEYKGVQNIVKRNGLRHDYYGRKYDRPYDRRRYDSRRVNP
YGRDRMRNFGYRNNGYRHDRYGRRPRNDYRNYDRRSIDNKYYRRNDYYKRNARSSDNERGPSRDDSKRKKRYD
RVRKSTSVSNSERRRYRNSPDRSPRYRRK*
>N.PF10_0028.2
MSSKVAHSESEEPVHKNDGSTLYVSNLSSKITTAKLQDIFEKYGNIEKCYVISNPITKESRNFGFVTFNNSED
AENAMNKANKMEIEGREINVEIAKRNEPHEPTPGEYKGVQIKRNGLRHDYYGRKYDRPYDRRRYDSRRVNPYG
RDRMRNFGYRNNGYRHDRYGRRPRNDYRNYDRRSIDNKYYRRNDYYKRNARSSDNERGPSRDDSKRKKRYDRV
RKSTSVSNSERRRYRNSPDRSPRYRRK*
>N.PF10_0029
MGNIIPFKTFEEKDTKTKDLINDEKYLNIDNPKDVVSTMKQHNSIKNNSSMEQNENNIKHELEKIKNENFYLF
DDSLDSNNMKHPCHAQWFLFWVFASYLNEKFSDTEKENVHAFYSNFPEQCIRGKGKNCFSDFLKIYPIRAGTR
EELMMWLQMCENYCRKKADLPVKVFNYGKLLKRWRYDDDYI*
>N.PF10_0030
MNMRKRSINYKCIYIVLFYFTIKLIIILGNNKNSNLKNIHTNNYQWKKKSVQKRSYKSKKYKLMHFIYYNNLY
KKVDVNDREYKINDDLKNIILLFGRIAEPYYPKAVEQFNHFKEPYSKYIYTNNRSNYNNNNNNNYYNININSS
SNDPSAKWKFIFRDIKKMDKNMFHERICQLKFKWPINKDENITEYDFYNYIIERCLYNINIMRSNIQKLKYLL
TKIVGNNKNKENVFLSSLSKEVEKEYMTHHVDTKKNNNHNEHHINDNNNNNNIVIIPKDKINNEPNDNKNDNN
DNNDSNNNDNNNNQHDSNFITSTSQLNSNNKIKILEKLENFNLNSIFKQHNRSYSRLLVNEVFNKEYPSKKIT
DIFLNLIFQKNVDEFTYESFIKHLNILGRKIELINCDFNSNIYFDQFHFLMKSELKKNSLSVKKKTKKKYSSK
AFKNKKENNTKNVYENKTHKKKKNSILINQPIITNNFQSIKEQQYKKLEQNDHQSNIKIHKNIHDQNNINSNN
ILQTNINSNDQNLSINIKKKVISFFNYIVNTIKKQF*
>N.PF10_0031
MFGKFISDTYNKVKSKADEVRLHVSVQAIKAKEVFGLVKPTTIEQLEVLLNYELQQIDSADMLISTYKKWIYN
ISQSDKSDCIRYYKSINKNNEKNFLSNVKQGFKNINEQIANFKDQKESGLTKKEVNDDMNEKRYHATNEDHNS
DNNNNDNNNNNNNNDDNNNNNDDNNNNNDDNNNNNNDDNNNNNNNNDNNNNDIYHFSDNSYNKKMCNNDSNNSSN
NISRCNSRDESNEKYTTYNKNIPMNINVNFSLNNIYDDHYGYNFKEYFLKSNILEKSISLILERRDIRLSLVG
PIENEDVENPRIIILDMFKLCFIGNEKLHKDILFIILTTDKLFDNHKELFLELLSILKHDYLDIYLTNLRKII
SSEVVCLKSKIQSYDTQSIPINNKLNSFSNISPQSTSSSLDFEEDQTIKNMLYIDKDKIKQTNIKNQIKDDEN
QKENIHLKNETPTQTNEITSDHLNTQDEIINEKQENIEHFPKKNICEQIEHHNKIYNVEEDNIYEIGLIENNN
NNNNNNNNHNSDNIHNSNNFNLFDKDMYNSDIMSNEKEVIESNKIHQIKILASYKLIDLHKSVEKILLYATEN
RIKRKEEIKIKLEELKKIMQVCMKDCDITLHDTEDSKVHIENVYVKELDIHTNNIQNTQDQITQMKCSIDELT
RKKNELYNEYQQICKEIHLKNQELSNIISNLSTHKKQLTQAEYNYLNKLSDTSKAKHMHQERKLYISNLDNIS
DDILKEYEEYPCINTQELIVKSNKIKKPIKMVINKHINYLKDKLYLLNILLKFYIQKIKNIMEQNINQQTDIN
SQLDHNISDKYEQKENMNNIEKKNENNLLSKPDKQEIYQKTNPNEKHIKIMKYKKSYYKVIEQLNKTWINIQE
FYDQNNEYIDQVNDTSTCTYEAIKEIYNNTKNIIRENNDIISSIE*
>N.PF10_0032
MRKKNNTKKIAEKTNETKSNNTHNKTTNYFNINEIKELYNNKNNDMYEKLFDNNNNEQCGRKKKRKKENITCE
NNNNLPSIYKKQIKQTNVICNNHNDNNKEIITQSESYHYLYNPSFIKRSKTNNEVNTLTKNKKNININKTDQC
SNNHFPYSNRIFDQTYKKSSSTYNINTRNINKKNKIINESNKIQNNCNINKNYNMNHSFNKYDFIRFVCSPNK
SKQKVNDMDSPKKMLETKECFKETNLSNTCTYQNNKIDDSICIRNKENQDNYKNCIIQNKNKASTTTQDENIT
KQSPQMKCDKNIKMDKHVQAQKKKIQNCSNEDIPLIDLNDEDSINKLSKNFFINNKKIFINDISKDKISKDSV
NKYYEYGNISPYKLGSHYMDHLSCHNKEGKNISTTTTNSNNNNNNDDNNNNNDNNNNHNNNNHNNNNHNNNHNN
NDNTIGTNIYKHPNNVNYNNNHNINDNIKINNLNDLNVHDNNYFPYDHTIHHLDDNVNNTSDKIKTKKNLSTL
KNVNIYESNVQPYSYNMFRDNNMENDKYNIYNDDNKNDLHENMNEYIKQNYKTTKSLYSYLDLSYNCSNEEIK
KAFKEKIKVCHPDKGGNIKEFLQLKFSYDILSNKKKRKMYDKYGNSILELLISDHIYDYNISSDEKTEEEIID
EEYIKIYELFVQKYHNISYLHNLLDLKITNSQYNHFQKLIYHFCNIDTNIFKNILYLYPIYSPNISTYNKKNT
TKKNENLNNSTISFQEQTSQYSQETLSENSSSSLEATNKEITIFDTIEKKNFGNLFLEINKDFDHFYKNYLIH
KMKSTNQKNEPQGINENIQKIYEHNGKDNISHNLYDYNNEMETPLKSTPMKKKNIENVENIQNVENIKNVENI
KNVENTQNVESTQNVENTQNVENIKNDAFFKDIQVSPVVYKIINEKNDKLMDDFYKWFEYFFDESENDKQPNT
QQHYETNNIDTDNQRNKNNECNNTFYEYIPIGEKRKNITNDYKKNDCTPNNHVPYIYEHVNKPNIKQTNYYVL

DEKQSIHVNLSNIYQHDNNFYDLCNETPHHSIKKSKSIKTPISYKKNIEEYQRIACSSFDKKTFHSSYNLHNK
EHKYINHPYCTPIKTNITTNKLSPYMYAEENSKNKTNHNHIYNCTLNVEEKYGFNLFKKSNHKIKDLTHDFNY
IYIGNNINNQIHVNPNNEQNTNKRKKIFFFLFIKQECINNFLKIKLLIHKMKEKKNNLKLISLFQNQIDKYMK
NMEYILLLTTHKEELIPLNDFYLLDKNIYTKRFYCIPIYIKKNNILIRPNFFHIKWIVYTLQSFIFYNFFFKK
INKYMCTYPFFNYKYTHFKNYINQHNQQQNNDENDIQTYNTNFYDYYIFFQHYIIKNKIKYVKHFPHHLMLNF
KDLPSTNMKDYKNVHIHISSIFVLTPNKIYPSVDNYMY*
>N.PF10_0033
MSQSNNKITRAQIDGIKSSELELFKSMIIPFNATVEQYNKDDESAIVQFKNGVEKKHIESLGSYKIKPLSYDS
SDKSDQSLRKRNVNFICDRIVIDEHDIINIFLNATLIAPVIFVLLSLLIFFSIF*
>N.PF10_0034
MENSIRHSVDIKSEDFVVIIALQNLQTFIMIGYTAVNKIHLTFDVEYLWALCLGTLLFIYSLISFVVIRTFVL
SKIDIGKYVLEILFFISIIVTCTLSLIIDSFKIANMQLLFFSFALTGYAYYNLITLFFFSVAAGILIQYNLNI
GSFGADIDALFFIDLLSYILQIIGGNILYFRVYELCCLLVISKKNPCKYVVASKKVKNLEKQIFSSLINSYIC
FKSKTYSHLPYPNDSKNNNNASIVTYEKKKKNSVLHHEYSHENKNSHFSSDINTTTHHNINNKNKAYLNSKCT
THYNLSLNNSKINSYNSGEKILTHNIVKKKINQKEKEKNEYFHLASSKKKKKNKTKINKISNIVQKKKSNTVN
MNIPENNYFIFPINEDNYNRIRTACDISTLKYNDDKNGNEYNISQRKKNESHVVINIANNDNNNNNNNDDDNN
NNNNDDNDNNNNNNNNDDDYSCSSSNSIYCNKNEYTSDNINHFNSSKISSKSGKNDKDSQNIEKEQTKLKNIKS
SNNLFNNEQQNGVIKKNYKTILERKDKEEDSHIIDYYQNNNNNNNNSNINLTENKINKMRNCLLIKCYIDNKN
KEADENNKMDNNNFIPADQKHKIKNNTDNVCQIEKQILCNFEDDNKIYNNCKLLTCNYNILNKTCNLYASKIF
SQSDITLYEDIIMQNEITNEQIKDISKKKKKKKKKNCTVYPINKTNNNNNNDIYNNNNNNNNNNNNNDDIYNNN
GDILSYDEDKNIVSNTYIEEQNYTTFITEDVNPYNSDDSVKLKNIKIDKNMLLSEESELYEIKKKVLGKRYEE
EKYNNNNNKSKNSIIFKKSKSAICIGDTKINKTSHNNFSNNKYFSNNKYFSNSKYSSNSKCIQNNKYHTSSKN
FSNNNSNSVTSKTFLSNYIHKTSNLDIKNSNNTASYSNINKRINMNTRQTYDILDKIIELDISNQTKKKQKKH
KRKDLSLYSNKKKIYFSSKKNGYRYKYLFMNNPKVQMLFMSLPKCLQNFFDVSKKVLFDLKKKKILMQNATWK
SKIFIPERNPLGIFKNPKIEKWYISWMDEFNKNIICKTYYIHIYLIIYIILLDLLTTYKLYTSNWIRNPLGFQ
TYVPFVLIKLLMNIIIYIFYVFISYKNINQLHYDIKKYYFSTLLLCLCKLIISLLDIYIAISSLDYFKSPYYV
YVYIHMLIIQTSILLIVRYPTHYLLYFLYIISFTSLYWSFSIHKAFMEFLFIIACVSFTIIYSYILCSRTLEI
NRRNLFSKYELPYLLYLKEIVYSLKKNQGKKYV*
>N.PF10_0034a
MILKNASTRLASSEFKSLPKPPYGTGVVFHGRMQPVKYAIKFSSYIFIIFSGFMGAMSGNFFYKKYILRKNPP
NPLRDPNDLPPERHPHAPEDD*
>N.PF10_0035
MFLYFLLFLFTLTYINNTLNIKKKTPLFKFPYFINTYITYNNSNKRRRIRTFPYFARKETNRHVIYRKRFERD
LKYALQSIFYKQGIKLNYKYHIDEDIIEGISVYKVELNSDCSVAKVFVEIMGDSIDARQGYIWIKRNTKDIRY
KLAQLIKERKRVPFLNFVLCNLSEQTQLLCEMENIKEYYSDLFKDELTNDTEE*
>N.PF10_0036
MLSIRKCNIYDLLSMQQCNSINLPENYNLRYYFYHALSWPYLSQIAEDVNGKVCGYSLGKLEEDNEYKGHLTS
VAVLKTYRKLKLAFYLILQTHQHLKDIYRANNICLHVRVSNNAALNLYYNLLNYKVNGIETQYYGNKENAYLM
EHVFAR*
>N.PF10_0037
MLYSKSNLNVGNIPNRQDKMNGSYMYIKKINSDTIIKNIKKNYNLNLFVNSQGKASLNNNIVNIRNAELGKYV
YKNGLYINNPVELNKDIIKVVHKNKYIHSISNNEESSICKTKSTNFECVNKCAHYINKDMKYINDKNISCLKN
NENNIVNNLSYLNKNIYNKIYNNNNNNNNDKLNYNNSTSNIGRSTQKNIAQYNMHPFVKYELIWDVKNDIARN
KNYFVIRKKSENDNQNIENKEEDKKRKIKNNIIKSYYKKLNIKENNKNTNKQTNDIYTIGQGKYIPINNKPIY
NLSLNNNYISYDNKECLFHNSPFFLNTQMTMNNNYKDIKFNNIDKKKNLLFQSSEKNNTSNKTVTHKSNLLNK
NVTQKGNLLNNINKNNTYNANNTNNGKVSHKLSADHMSKENLNNFNKKTEEKKEKKEEEEEIKNKKKTIKNSC
QNIQNNKDKMISQNEKSNISNLSKINHVLVLKNVNKKETNKNNNQVISDNIQNDNNIINVPSEENITKDECSK
ESNKSNDECIQESNKSNDEFSKESKDINDECTQESKDINDECSKESKDINDECTQECNKLNEKCTQECNKLNE
KCTQECNKLNEKCTQECNKLNEKCKDESNSLKSASNQEGDIVKDICKIESHVISEVCKKESEIIKEISITQRN
IIKEIHNCNNVISKKTNNNNNNKDMNMLKDIKKKKDTYIKESKEKNNNIYNISKKNKKNDYNNNNINYVPQEK
LQIINNQTKINKGIINDNKIYKKSNKIDYMSPEYISQVIKDYHNEERKQKKNIYYENVKDVKGTTLCDKKKNS
GLLNKNEKRNMFHIHTKDFYKYCKKKESYISCTYEHKAINGDSNLKKIKREDHKNKENIERDMEMSQIVNIVA
EDKLDKCHLSELIKIMKKLEKVHMKKMNIDNLILEGNCLQDFIKSVEGKNKVKHKDKVKNKGEDKVKNKGEDN
IKNKGEDNVKYKGEDNVKNKGEDNVKYKDTGENKVKDKGKANVISLSSNNYNVDNRNRKKYSEIIYNKKTNYT

KNKKNINNKCHVNDENNNVLYNVDENMMLSKKKENLIELNKSNSNKVNIINNIKNYIDVLNLTVDELIHLKNV
KDIFWYLYIKSKFVKKQNMKEFCEVIKDKMDDKIHKNYFMEYVDYESFKKFNKNNFDTFEEKIYLLENLFYLL
KSYPLDYDENRGCFYMLYPQKNKMFKNSEYDIIKNKLHHLKISKDITEHFKNNFFFLDKIFIPKDAFFFVKDS
EDIGLRDKDNNIIYIHQALVSDLKEDVNDKSIFDNLYSCSPPYKSDYFRYIQKKKIQNKIRKFKEYILYYYEF
HSVQDFFDLLILFDEEYKNLEMLNSCTDIPYNLTKRLYSEYCHKVMKIITKNEEPFKNIIDNFNNIPIKNNMN
KYNVFNNHEENCMYVSNKWCNKENDNIKRIHKDANFNITKRYISLENLENNYNQKSNINEKNDKVDIHKNLFC
SKTKDSNIWSNKFVFNKKECNVNKYMLYEKCNPLKKYNKKKNNSCTIDNSYGIDNNYGIDNNYNIDNNYNIDN
NYNIDNNYNIDNSHCVDSHFCAEDSKYEIDEMSNIYLSFFYSNDNKRKMKEITLDTFKMFVENLGKIKGLGKF
TCIDECFLGMTFCDPDIPKHLCNQKKVLPKNINYNGFMYCMRYIPYVIPDFPLSTSHFEISYNNVEIFNSRNS
FLHILKYRGNRNDIILLRNKIVLDIIEIFVYIPWNFGVDTGMYTPLVPMLPEGKRTVDMCKSSYYKNYKYYLK
RNTKYTNIKSFNMSSDNFSDSNSTSTSKNESNNYVSTMCSKEYNKRYMIGHVEEEKVGKIKERDLLHLNDNIN
NMDDNSLNSSSYNNIFFEETFLKMRKKLSYKWIPKWHMCNDIFLSRLISYDEIQICLQKIFPLYLIQTALIYM
VHISCCFLTNEEWKYFFESPFTLYKKLTPEEIAIRYIKLKGYKFNKVNNIQINKNIDIHQIFMSIPENMQCPE
IYKLAINGENTSSGYLAEPFDIYHLMFICKVFWYIFLYADNFVILRSTLFKNKPTKK*
>N.PF10_0038
MSKLMKGAIDNEKYRLRRIRIALTSKNLRAIEKVCSDIMKGAKEKNLNVSGPVRLPVKTLRITTRKSPCGEGT
NTWDRFELRIYKRLIDLYSQCEVVTQMTSINIDPVVEVEVIITDS*
>N.PF10_0039
MADSIKSSNSFQKLDNIDAKETSTVDRKWVALTAYQPVDVVTKTVEVPIIKTVEKYVPKTIIQEKIIHVPKNV
THIVEKIVEVPEVKYIEKIVEVPHIHYKNKYVPKIEIVEKVVERQKIIEKWHDKIVEVPQIKEVVRFKQIEDS
EEIIKYVPRNSKNIDWEDEYKKYTESKGLQRYSLDQNNIYQQANSFNQFNENAYNQNAFNRSYELLNKQSSVK
SQNNASGENFSQMNFYNQYSGANFEQERSIQASNFEPSGSMQLKRLSSEEIKPAGCCSAACT*
>N.PF10_0040
MRVRRLQSYLTENNLLKKSSLENIKNLTLGIDALYFLRICSDLKDILSDVSGCISPCIFHMIDKQCEYFKKWD
IKIIFIFDGITPKGHKLFSAHYHQYIDEGWLYYVNDEKKLSQKSFSEVNNICNSDISFLLFHYLKYKGYECIY
APYLAISQLAYFLEINLVDIVFGPPTLGLHNVTKIILNIVWKKNYFEWIDILYLLKMWNINKEQFIDACLLAG
TEYCLTFPYLNLSHFNNGYKQFNFGTSIEFIKQSPLICYLQHFPNDELRKSHMNAYCLCKSMLKYPLVFLCTG
HVNCFFFHSNKDQKCLQKKCDASILNDHTGHNEDGDHVDNASIKKKKKINKKDIISTSDEQNDDSDCSIYNKS
SYDKNEEDKFFNNKYNRTITNISTNDCIKLSKDGYLKGDKILDKREEDTEQNYHNSKISYDSYCEQDKKDNTI
EHPEVAKEKGKYKNSKLIYEHNSDNSKDRKDTNEDTVMEDSTKHIKDIYAKENYKENDIYKKKNHNNDKENIS
KLVPKDYIKVVGAKFPTCVYYLMSIGLLSKKLLCVLAMGEWIDYSHPIIDSFEYRDTLIDLREYRCRILGLVS
IKLNPFFYKRKVKFFDYGYYINNIMDKDNKATYLNILFNDNFLWKINKDNVSEEMNRQNVKNVDLQFVLRWHL
YSESKSISLVAKKCELYDEDMKERKASNDKEKDIYELESSNDNNRGHNNNSDDNNGGDNNNGGDNNNGGDNNN
GGDNNNGGVNTNGGDNNNGGDNNNGGDNTNSSNDTNRSDDDNTDPSNNSDYSSNDVDNYEDDDYNNYSDYCKK
SNEHTKRKKKYMHYNKHMIYKYNSYYKNILRTNNQNFHSFLCLVYFMFLENLDIFTKNCGVTLFGLLLSEVKN
KNIDSNILIIFELFKFGFLTTEALLPPDGKTYPENAYASIQNNNKLDEQDKKSVLLLSRIYSLYNSNIDHSRT
YEGLIDFDLCAFFAVVKIIKKTLRQLLQACVTNVLLTNMELIHILPENLYNPFDIHICGFFVTNHFMGVLIKY
FLLFDFDDIQNEKETLKRTSDKNMESNKMNNDIETMNDHLLKDKLENQKETEHLSKNNDFKGYDMTTNNHISI
TQPNKKNNDHINMKEINDKFYTNEQPEDKHMKVDKNQKYGINQVEHKDKRSLIELENNDKNIIYKENYNENGN
NSYVYSDEHNLSDDSKTNNYEATHKKHENNLDHKKKEEYKIDEEYCQTPINDDSQNNFNVFEKAIRKKFPSFV
NPINDLCNAINTWRDHLCLITQLEQHTNVYDLVSDLKAADNFLEKKISYIGLDKSQTYNRICLSNNK*
>N.PF10_0041
MDTKNNLYDEFGNYIGDDIDSDEEYSDYNEDGMSDGEYSKGSSNNDDNEEDNDDDNEEDNEEDDEEDNEEDND
DKSGKRNKLIENMDELQKVYDGVEVFVEEEDTQDIEEATINKINANVERISFIKKLDVEANRKNFDLVETSLP
NNNFSFKYLSELMKQTSFIRNICIAGHFHHGKTTIVDRLIEYTRDKKNNIRISNNRNKNSSTVSFFNVNNINE
ISEKSNYLSNISRKKKNINSSQIINNYNNNNIKGTPYNSKIIDHLINYTDTRLDEQARGLSIKAMPISLLFSN
RIYENIPNNILLNKKKDNVKYKSYLFNIIDTPGHVNFFDEFLCSLNICECCCLVVDVVDGCMYVTENIIKACI
YENVKIILIINCIDKLIMDLRLPPNDAYHKINYTIEEINMKIESICDLLNKSNAEKKDFLLSPLKNNVLFSSS
IYGVFFTLKSFSKIYCNIYNAYNIDIDEFSLYLWGDIYYDEENFTFVKSPLYANQKRTFVEFILNPLYKIFGY
VCSEEKEFLIPFLQSFNISLKKSDYLYNTKYLLKKINGMIFQDTTAFVDIIIDNCPSPLDNAKNKTLQIYSGS
LKTKISYDMMKCMKGDETDNLMIYIIKNYHRPECGMIDLFGRVMCGTIKKGQSVRILGEGYTLNDDEDMITRV
ITHLWIYEGRYRIEVDEIPAGNFCLIGGIDICINKTCTITNVKIKTNKNKEDHVNLNWYDDLNDDDNNIVKYK
NDKDIYNLDKSALLNDNENAEIFYPLHKKFRYLNCVNSVFKVACEPINPSELPKMLEGLRKIDKVYPLSSTKV
EESGEHIILGTGELYLDCILHDLRKLYGDLEIKVSDPVVQFNETIIETSALNCFAETPNKKNKIYMIAEPVQK

ELGDDIVQGLVHLNEDQNLNVNEYISTMDRILNKNNESKRNIYDEKDDSHDDNDDGDNMENHMNGIENKKNIF
KDKEQKDEHQINDLDKEKRKSTLNYNIDPNVISLLKNKYNWDILSIRSLWAFGPENNSPNILVDDSLFQETNK
ENLYSIKDNIIQGFSWATKEGPLIEESMKGVKVKILRADIDDDPINRGAGQIIPTARRAIYSSVLLATPRLLE
PILLTEIICSGDSVSAVYNVLSRRRGHVLKDFPKVGTPLYMVHAYIPAIESFGFETDLRTHTSGQAFCISMFD
HWHIVPGDPLDKSVILRPLEPAPIQHLAREFLLKTRRRKGLSEDVTINRFFDDPMLLNIKDEFSEYF*
>N.PF10_0042
MNPPNFINSDISFDMKKVRKDFIKSIIQDEEYKKKDDKKKEIVKTCKSYKDFCDIVNAVNMKPIKKYEPKITY
EDYIQMHFSSSSCKLDTFQKKNENIKNFFMINNYKVNRKQDISSSNNLSHKKVFNEIKDLTHVIKTYKDNYYE
YIRKNYNCDDIINIISLIKNHWDIFIGKKENMFEGYLHDPQFIINLINFLFYIFNYWTKRDFSLYFTHDELNE
INSKLHFIKGNIEKETWLNDDTLNNRWNYIKKTTL*
>N.PF10_0043
MYKKEYVIDCKGHLLGRLASLIAKELLNGQRIVAVRCEDINISGSLYRNKLKYQEFLRLRTNTNPKKGPLHLR
EPSKILWRCVRGMLPHKTYKGQLALKKLKVCVGMPYPYDKKKKYVLPSALRAFRLKKHRRYCRLGTLSSRVGW
NYDTLVKKNEVLRKQVSKAYYKKKVNNLNEKKEIKTEALNLINPEQRQVLENFGYA*
>N.PF10_0044
MSNFIKTISIFGSNKDIKNNIHLLGEKKLAYIHDNIVYIINIEKNDIYIYDIHSSPVYILRHSKLLNLSVTCN
FNNYAQANYISIWDTYQYKEQDHIEIKNIFSYDCAYHIFNECEKKRNIQQNEEKHNYILDICFVEELKCLLIL
TDDPYTSMSLWSLTNTTKPYLCIHTNKNQLTNLSILFNYNNYKNKKSLHINKSIHLDELLEDLTILTYGSSEF
YIWSFSFYLEVRINYFSPIYDNLCPCYIITCIYHICDDLFYVSTSNGKIYLFDKYIVIRYYDLFKYCILLIFL
LNNQLICVLENGYIYKINKKDQNVISHKLNINTLIQFYYNTKFNVHINTISEQNIIRSEVFLETLLVLTNNYI
ILINLCSDEIIILYELIEGDIYMSTFLYTTNKYVVCVSSFIEGGEKSLIKIFNLDNTLGKIPLVVKGKLNCIT
SNHFKEEEYVKNHSFNLKNSCERVLNSNLRNRGNKKIIYILSIKNQIVFYEEVGYMLRPFYFISYSQDRFVTC
LQFCKDKKYLFCACNDGSLYIYLIYEYDNPHFYSDHNINNLQFKDMNKNYCISLYRMFRKRMERINKIKLLDV
QKLILLVSSNNKMYLYNILENKIIHIDNNTNLYDLSHFHLYNSDDKEYIDFLMNILKNNIEEYVSFQNNKCHD
KKKSSKCMHLTNILKNINYIYKIIYFDKNQTCDNIINKDILTTDIAYINEENKNSDYITPKYMIYIFNKVVCI
INGIRYEIIKKLKENKYYGNVKQKKIYQDNNLTSSCNKMSTHIDHMVTKNKYNNNNKNNDNNNNNNNNNNNNN
NNNNNNNNNYNNIYKMVHSLHNEDYTCNIFEKHLFKRTPIFYKDILYSEINYSGHILLCSFKNIILIYSLKKK
YIHYPFQYNNISTGNTIKWEYETFPFFTYKSNYLMNEEKYKYIDFTIVLRDIKKETPSNINSQTFYNLHTFDD
NQKNNDINEKENIKSTSNQNNYITIAKEKERDIGKVLFIYNDYLNFNYFKNNICINPQKKKTHPSNIFNSSNM
FSVLTPLYSFNSGGTKKNKYKIKVPYAIDHINEKDLDEDNNKNKNKHNINNNNNNNIKRQVCRSQYVENDINI
INKNNSNDNTFYYTIVTSNSSYDIFVYIKGGCINKIEKTNRSSLKFICTTIDEDNVLEIGIPEKKVDFYDQRI
VVQINSFHNTCYLSIPMKNDKI*
>N.PF10_0045
MNEDLKLGSVVWPNFSSPFSLSCYAVNESCSTLAAGSYEGSIILFRIDENKVEYVSNKRKGKMGEGYVKKDEY
DDKSDNKISESFSSSTNHMVDETCDKKNKKEELLNVDDDKMSVDINENNENNKNNENNKNNKNNENNKNNHNS
KNNIDNINNIDNINNIDNINNIDNINNCMNNLSNNNFKRDNSKKFLKQNRKRFSNRINLLPYNILLNNSNNTC
NNSIVQLCYGLTCCNLIECVSKEILISLHVDNMIYFWSIDEGRCFKVLKNFDFLIYNLRILPDRRFLLISGEK
KVLVLDIWANSNKEIIAELNFSSNQNTQNEYNKSEKTKNVNDSKEEEDYYIGDIDDKNYSSSYGSDDNNMYNM
NSMYDYLNNNMSSDPQESNIYINSKNKNYGYNKLDRDKYNDKYKDKDINSNIQEETMNIKNMFGVSSRYINDN
NNNNNNNNKSQKYRQSNNIHTCDLKLCSIATGVVPYLKEDNQRSVIQCFKLLKRKLFSIDDKKEKDMNNKNIK
KSYEKLYEDLFINNNNSNNDTSNYNNNNNNNNNNCNNIFNDEYLKLKHKEVNDRLNNLFYCPVLISASLNNGD
IICWDLTDFLNYYKCKCRFVIRTEKTELELYNEMIEDISENCIYDELIKTRISSTNDINLKENDKNIKGKEDI
YKKHMGIYNIDPIHIANLNEFEKKTLEIASNGYSSQIAIIDNYIILLQKNRLIIYERKCLSSSNNPSDPSSNM
SSYFVPLMDLFCPDYEETQKRTSHNTHWVGIQVVRRPIWKFSSGIFQMMGFKKKSYNIMQKLSKNVCGSIIAW
TNEGNFYAYSLPLKFSSLFLSFQSNIKSFFLKKVSKDLLGSFNSAVPLIIYRENVRKSDEDIKDTTVDSMNNK
NNKNFEIKISESADMSVFSNKMYSPECIKRLNNSNEELMDNTSKKRSIEKTKGKKGDNIIKVICNDEKKNVLF
DDYKNYEHNRIEKHTKCKEDFFFFFVNKNYKRRIIVYEYLYNIWYKTSKIEKVWLLPKKKNIEHVIHMMKSKD
ADNMPRTYDNKNDYDDKNYMDKEKYINQNINTFDLNKKNNNNNNNNNNTFGDIKKSEGNICSYVIAYYNLNIY
IIISYTNGCVISTLINSYNNKLNNEKVIHNLYIPKTLYNKYRRHMFITTLYYENNFLIGGTNHGDILIWKLVN
FDLVKILKNCHLSYVCSIHKVVKENYISNNLKNKKNILDSTVVSNNKKKKKEEQDRDKKKEENLEEIVKEDMS
NFKLMEEDHWVLTCDSSNNIILLNLNPIYYDKNRSHCFCQNDPYKICITTDEEIEKYKNSKNSYFKNKENEEK
KRKKREEKKQIIYYYVHSLLRRSSGIGKENKNIYNLLKILKYKKNLGMKILRNKYYDDYYHDMFVAEKEMHLY
SLKNYGDEQKYSFDLYKNLYKSVSKIKGIQKGRINTNKNKMYDDNFDDDKDKNESNGNFIHNKNGNNQHDHDH
DHHHHNNNNNEEESISDNNNSHIKYTRKKKKRTESYIYNKEYYIIQKEKELKNMTGICFHCTCRKLEKKNEIN

YKLINNIYINTYSSLLYIILLNNYVFIYNYNTGQFIRSIYDSEFMQITNFSYDQCYLKLGVNITHLNILNRVK
IKDVSRHEIYDIIDEEYNIHDDEDDNYYDYNYDGKLSQSNYKYNHVDDIDNNNLKDPQNDYIFNKNKGSNVNY
LPPPLSPSYMSSSYMSSSYMSSSYMSSSSSYSSSFSSTYSPSICSPAILNSSTENVGKMKVSFDLLYKDNINE
EKNLKSKKEVGLKRENNMYRHNFENNCIYKYIVKTQIPIISFVLFRDSNLSKKCLPLTKLLYHFFMPKNCIQV
CKELYPFLINFPSIPFSLCIHGKESTFSFVIPMSTQIYYFSRMHSMKCKKNDIDVYITMGSKQKERYILSEKR
KKEKFFYDSLNLEYDYAYNKKFKKKYFLSDILKKKYIYLDVKMGCLKKKSFKNFREFSKYKNLTKQGSVHNNM
SNSYGERDRAKEKKVQMGNALDYNIKKGKTECNDAGDYKEDMHDLNKYEDDDERESHISLHNNAYNCMYYNNK
MGNKKKSKSNYNNNDDDKNSSKEKNNTCIDMITNKDKEIIRSDEKNVQTEEYRNRRNYHILHYNYYKDVRVNR
KNSNISPKWVLKYLDDDLSNYSSDKSSICLYIKKGNNNNKNNKNNRHSRGKNMMMMMMNMKRDDKRKKNNVHL
NKMNSKVESNTCDDKSFINNIRFEKLKVERRKKCKRNVHKNKEFFFCNNSIEIYCNSVYIKTLYSCFILRFLE
LWKRWYKIKYNEEVINNFNEYIIDNLPDGIDINLFLLSYISMYPYDKSIRLELQNFMYRKIRNMSDKLLDKYT
KTGTEILRINNKKKRIIEKNIDEISLYDTSGTYIISIIIPKVGCLYLYPFIYADEICLTLLLLLVVVKSEYLR
SCKTFYTNASMITLIIHHICYYIFVDTKYFLEKNIKFNKFKLFHFIHLLSLSFSITWDLHILIQKGIFTNNMK
NVSNVTFNLSLKKDDFIFNERLINENYYKSLKDYYVHAEINIDQNDSKMRNNSLTEINLKNYLEDYLEYINKI
SDIELEKMEANESNNNNINNMNNINNNNINNMNNNNVINSFDNIFSYISNEEKYNIRNKSYELLRITSFMDSN
SNNSSGYISRFNKKYSLIDKKNKNADNNDNMKRDTSYITFYDNNYNKLDKYNIYGHMNKDYENSNNNMNYNKY
DILDNKHDFITNAYEDKKNKRKHFFSKHYNNNHINDNDSNMENDYICRINEHNYINICHFILNIFELYTLSLN
NISFLKILINIGRLEPFLFLKTLHYISGNIHKKVSSINKILFLLIILIKNYKCIFIWYMNIIIDILLNSLDPS
NNVRILCLKLSTSLIYTLVKYYNICSFNKFTQKLAVVNNINKCIYLYDLKSAKKLKIFQGHKKYIDCIKFNTN
GTALASYSKLDLSFKLWNCTNVGLFSGFLKIQSKCTRDIQLSKIKSSYLYLSDSYIDITYKKKNEWILKREDN
AIYLIYI*
>N.PF10_0046
MNHVDDYDYFCHSCEDVKRTSDVEIIYNGEIKCKGCNNVGFVEKIDEDDPLSFHSLHTSRDVGESRSGPNSRN
DNNNNDGNNNSNNTNSRHYNYRNMNNFGYRTNIPRSRRNNHNNNNNNDDGSNNNRHNSSNSNNNNNNTRSSHV
RGEHDNFFNDFMLFRNMYQQIFNPSFRAADAEVHFTAAVGSSNNSNDINRHNNNRNNTTTPTTNNNNNNNNNN
NNNNNNNNNNNNNHMGNRNSSRNNENTNETNENETSQNDNRNRNIFGRTYSYNRNTRNPRIITRAIDITNIPF
NSPGRFNFHDLIDNMFANTFDNISLDQVLTIIMESDPSRNGPPPASEEVIKNLKVETLTEERAKELESCAICR
EEYKENDEVHRITDNERCRHVFHCSCIIPWLKERNSCPTCRFELPTDDQEYNCKREELRERLNSEMSRNNTYN
GTDNDNNNNNNDNNNDNNNNDNNNDNNNDNNNDNNNNNSNNDSNNNNNDSNNNNNDSNNNNNDNNSNNNNSRR
HNADEANYNTLGDDYNINILDSFTNEQTTSILQQVFNSFLSGSNDSYRIDRIYLSPDNYFSYSSNRNRNNIND
ENVEVIQVEETRSDYNESHDETNENNEKEEINREEGNEEEKRSKRKKTNDYDDNKDVYNNASGSNSNHSNHSN
NNNNNNKGNEKNERKEDMCNVSSGSNNNRNSSSRSNYYNNRYRYNNYRRSNRRNMDQDYFNIQDDEVEEENYY
YCTPPNETIKNNTRGQRNNDTDNVRNNNNNDNDNKNNDNKNNDNDNNNNNNTRRRNNYYDNNSYNNPSYLHEN
VFDTSNVYVISERTHSNDRSNDSNTRTISQLYLTDLSDVFANFNYSCNFNNDQYIINNEEDHTKDHDGKDEQE
GEKEKTKNVNSDHMESNINDSGVSLGGPKENKSNDKNEHQVKEKRASFEINTGKTSTTKKNEKRLYEKVNNTT
YSNASNSNNSTRRSRDSDNEKEGSRKKSKNYKNEEEYRQSINNNNNNNNNNNNNNEDRRNSNTSCSHKYNNNT
DNAPNRNPSDVVNNNESNEEQRNTTKRQSTVKKYLFDKLNWLKSYDNKDKKKNEKNQDSNNKSDENDTEHNQN
DDNATGMSNENNDDHTSHL*
>N.PF10_0047
MGKYSVSRKGEYSAERRYHYRDEKYEKYEKYEKSDNTSHGNKLDNHMKEKGGYYINEGRRHDISRSREEMIGI
IGREGHKNDNVYNNNDRRRGSLDNYERRRNEENHIGDRNRNISKGRRDYHRDALSRDRCYSNRSPRRNVSVDR
KRGNVYDRNNEDNVYEKKARYERENHSTNNNNYNNNYINKDNNKHNYKENQFSADYTNRGRNDEKMHMRRHSR
EEKEGRKAYQGYYEENRHRENNYEGHRNEEHRREAYRREGYRNEAYRHEGYRHEGYRHEGYLSRDHAHKEFHH
NENPYYKKDFSHRVPQSGAIRRERGRELRNHAYKKIGACKIFVGNISSSAREEDVRRRFEQYGDIMHMQWKKR
FAFIEYYKASHALNALEKENGKMFFGEELSVQEHQYNMNSYGYKNDNRSHFHLKVSRNYSPPNFSNESIERRN
SLRIVVKNIDEKASWQDLKDFGREVGSVSYANIVDDYHSKEKFGIIEFYNHENAKDAINILNGKSFYGRSVDV
IRYNDSDLSKRFKNKEREENYMRGVNDRYVREERAYRDKFPFDRINRTRHTFFNRGRNIRGNKYFRRDDNEDN
KSEVDDSKYKKSTRSMSMIDRDNTYDERVSHRDGHMKDDRDDDICYDRDDEEKMINGNKKRILKSSEEVGEHT
NSIINEDINKDQTDIIDEKEKGGDENISLKNYENKKENVDHDYENVIESNRGKRRSVKSKKKNENEKKGKTKD
ELYDDISEIKLQEGSVCRSTKDIDEDNYNNEETKNIKDEFLVDKKNEEEGYDDDTYTSERDKSMNEKIMKEEY
INKDDTLEKNDNIINIPNKKSRLIKKTTTKKRITRVSEESTTKVDTRESDEDDLMENEQWTDSKKICVIKNNS
PHND*
>N.PF10_0048

MSTVEELKFIWKFLFSHMNTEKSKEEICDEKKYEENIILLKKLMRTENIKFDQKYNAINILEKLKDIEDIQYE
DILNDYAKKFLTFLKGSFEKVAEEIQINDKISFRRNELIKQDLNNKLQNLNHNYNLEYINLNKCLGTEDFESQ
LNASLKFNSLKDILSKLLKEMKDYEIQYNNLSDTDKFLDMKICELENYLNNTNQNS*
>N.PF10_0049
ISFVLYKTKWHQKIKKKKKKKRE*NIIMMKICITKENFKHIFILENVDIIAGLCFMNIFLFICSGLKEDENH
GNITTNIHGTLFIISLICYSIEKIIPFSFCSDFLFIFLLSLSCTLPFLSFELKNNYNDCFNTIVFFPLYILLF
FTRYHIRLLFVLLIFYILIIFIIDLSINTSIIYSYILMFLFFTLCFNFIFYRYLYYFSRTIYISKSSPNNMLL
IFPYINEIQDLSFIKIADILIDLNNYIHMKWMYFSQYLSTINLNNPQHIKNLKITSTRFSNKFYTLKNQDIFK
NYPPYFCLKVLYHKKLYFLSSAIDKKKKKWTKIHFNRKKINLYFLLEMINIYNQYDIKNINHIVASQNKKIIN
KNINMTHQNYGTINENINMTHQNNDTINKNINMTHQNNDTINENMYISSQNYNIKEEEKINRNNDKRTNRISR
NDQKNGNLLLNKNNNKKKNTKKEKKIITQSEYDTYHQINSTKIRNEYKQNSIHDELNRNSNKIYIVKDFFSSY
NCDPSIKGKTTTNSIKKKKIKKKKIKDNLFKTNPISKNKYETNFESNYVNNLTSYNKNNIEEKKLKIDRLYGN
NLTEQITLNVDKYNKKKKIHKVINTQKNNITDKIYTFGNVNGHEKDDNISANKKNKKYITLSKHGLSYNVKEN
NVKEEFQIDNNQSNKKTNNIKEKYCDINNYYYNYTFKKNYNDTTNIQELQQKSNTKDIFPNITKCKNKKKKED
HIIYSDDYIHNNLYNIQNMMSNKNVLTNRKRTRTITNSDLYTSESSYTQSDEIYGKNIFFINFSCLFYIFMLK
IKHLIKYIEEINSVVQCVNFKQGIVPKRSILLSFLDQKTERYYIMWSNSYDLVYYVDNYIYHIILILTFHLFC
LFFKMKSFLIIFMLRFFLSLVIILIFIYPVFRTISINPRNVFIIKMNFFIISIFILFLSIFDYIWTIFIIHKN
WWIIQKESLHRLEDVYKNILFSYAQFFVVFPIYYFLTLHRLKNVWYIYIIFIFSIYIIYWHYIGTFLLGLKMN
ISLVVLLIISILFIIRPFEIVKRDIFLKCVLPYILFLDDILHFLSDEKKLKYLTTNI*
>N.PF10_0050
MGREQNYKKKTFKNESHEKKIVNKKIIFFNMIYLMVLFVVCFIGGILLGFLTTSYTSEINIIQNEDNVFQFLL
KNEKIIIGGKLNIKFRVDNKTMLSYVFNSESVKYFYYPVGQNSNCLLYNGGLEEKSANQEPLKYKSEISTPLY
NDENIFPTKLTINVDYNILHSSSKVHTLPLHIGYEISQDYKNEVQPMYNDCKRFHKIYFSVKLDQLYMSNELR
KIYNDKSYEFVFYCNCYMDDKVHAYFNAKPVYKNVILKNLRNNPKLLN*
>N.PF10_0051
MEIYYLKKKKKIKYKELFITTFLTHGGSNIISKLIISPLERIVLIKQIQPILFKNVLIEPSYTYRNIINSIRT
NQGYTSFWWGYNASICNFLSFSFLRLLFHDQIKYNLSIENRKNYFMTNFSLLYFSSCLAASIAYPFDTVRNYM
SLNHEIIKDKKKYARSVFLFIYDQIVRGKIRNLYCGYSLCLLNFIPYLLITSKLNEVFTKYFIDDNFEKNNYI
SHDASKRNNIKEEYNKLFKKTTNFFSYIALGVITGYVAQMVTYPLETYRRKYQYHVMYEQKFPNTLMHKRYLK
NNTTTQHQHIFKKVCNLYRGFTLHSFKLIPEYFIFSCFFYYVKNNIPI*
>N.PF10_0052
MRVYYSNLVKDKRKCLNFIKVEKKYGGTSLLTKIGQAFNQEHLRNMKTKLDDVKKSSSPFFGTSMEGSNNIFK
NFIDIFRKNNIIKDKLKINDSEKKEKEFYMFYVNSLMKYEGIFTYGKFQAFLKDLCDYFNIFSIKYKYKKKMN
PQFEKLKKQYEVLNSFLPYELDNDDYKIFTKESKKYIAQAANVDIKFIDELLLFHDTLKTDRTWFYRRKVLKR
NIPESFEAREMEAPYDRPVPKTYNYNIKEHSLEYDEFMKKHGRKLKFKQWNSKQHPWFRKNTSGRNRWATRPY
TKQFPYLYYSRVPSHMYLSKNKPKISH*
>N.PF10_0053
MKIYFIFILFLYLNFFILIIEILSKDIIKTRRSFPSYIKDGEKRKSNKIKRKRIKNDAYINVYRKNILDSINI
KGDQNKIKEKWLQNLKKKKAFDKISVRLSNYNDDKKEIFDITFVNNKEDRNDDNYDNKSCIKKVCNAEEYLLI
STPVYYSNDRPHIGHAYCNILCDVLYKYEKLKGVEKKKKGIIFFSGMDEHGLKIEKKCNKNKMKIIEYIDDIS
NYYKDMNKKLSVDVNLFYRTSYSFHKMFVHNVWKYLVHNNYIYKDTYKGYYNINEERYISEQELREGKYKIDN
ENIIYVEEENSYFFNILKFKDYLIDFYEKNENFIYPPYLRKQVIYTLKNELRNICISRYNTKWAIQIPNEAEG
TIYVWFDALLSYVSSMLYLHKIKKNNNNNNNNNKNNNNNNNKNNNNKNNKNNNNGINCTNGTKDVNISSTCDT
DSPICSYDDILNIVNLNANNNNTQSNILPHISSNICDEKKNEKREKNIPFNKVIGKKFWNPFIQVIGKDILNF
HAILYICLLKSLNLELPQKILCHGLIKNENIKMSKSLNNVVNPFDLLKKYNPDVLRLYFMGCGSIYEDKNYKE
QNIESFELFLRNNVGNLLYRVVSLCIENNYNMVPVIKTNDYDSNIILNEWKDNIKNKLIPYLDNMEYIQFLEL
IMTLIKNVNKFFVHNTPWNYKKDTQHFHTTIYVTLECMKYFSILMFPFIPNISLAILRNIGFDDIDEENISLD
MLETRTTKFMLNGLIKIV*
>N.PF10_0054
MGKRNFRGKKRATTLNELYELEFVKKEKKKLRKLNFVTNKNKPNDESIIKDTKHKEFNKKENKINKILLKEYE
KLKKEEEKPKGNPYYTSDHDEDQTDLENKNDQNKKLTNKQSNYETFLNILQKKKKEIFNHSLNEDIKSNHNDH
HIISKKINKQNQTTTKDNIQQNDNVKKGLLKKTNDEIYMSFLVNNINKQSNDFNLLNDIYLNNSHDIHNNNNN
TTSINHKNVNTHTQNIHNINKEEHDKNIPDRNYLNENIMNSNNIHTNNNKNCHNLSENDMNGNHFDANNNLNS
HNLSENIMNGNNFDTNNSQNNLLYNYSKTYNCISIYDNYKNDSYKLCENKMDYYFSFCQNIDEAFMEQFWNNK

KNRLRNTNEQKDNDKSNEYKNKTIEDIYVKSNLFLETNIFKSKKQIKNEYTGQIFNNVQIKPYFFSPYLEKNY
ITYYVMNILDTKPKSILDNENFYNINDNIIFHLKNYLSTFKHPVAYFNSDIYKYMQNYYNMKITKIVKDENKK
DSCDDEDVTDSYMINNVQNDESIEISKTNKTKKTKKKIINHNNNNNNNNNNNNGNVHKHKKKKKKIFRDISKI
IEHVELKSYFHYINSYVDILYTNKNVLNSHLINLLNVVHILNHLKKKRKRRKYIKKKLEKMEKSQNVQQENIE
LKEEFCDESFARPKILILCPFRYNAKEYVDLICNLIKPSETKNKNRFVLEYDIAINERKHMKEIYTKKRRTLD
YINIYQGNNDDCFRLGIKILEGDKKIHLYSPFCDSDILICSPLGLDIIIKESKENDSSQEQNEGEDNMIYDEN
SSDDYDMNNENNNLTNDMRKDKNIKKKKKNKHYNNNNNNNNNNNNNNKKKKLYEYDFLSSIEILLIDQIDIIL
MQNLLTLKNVLKFINKPLLRWGSANINRIPKYVINGFLKNYRQTIISSSIIDTNFISLIHSSINYRSFLKLFI
KNDDKSILLDLRNSLNINQYFKKIECDNILNIEESILNFFSTNVMDILTNIKQLIIFIPTYIEYLRIHELLKK
NDISFKGVNEYTNEKKILQIRKLFKYKRINILLVTGRLIYYERCTFKGANHIIFFSPPKFVFMYSELIKNLSK
DPNSSSICYYTKYHTYELERIVGQARTKQLIQEKNGKITLFK*
>N.PF10_0054a
MYEQSGEKSISSVYEEELAKAKKIMATLEDIEIDLYEELIKLNILKKNSNLEIYKRIIHEI*
>N.PF10_0055
MFWNYNFFLKNCFILSFHILIIFGISTFLNLLPTFNVKGDIKSKKKRKKEKKKKEKMKKHISEKFSKGLCCLF
DILLRNLIYNLYYAFDQIKENNYIRNVENEKTKKQICIKNLDKSKKNIEKCINLSLTVLRIMQGNKNYFIQHI
LDNYASIKSINVEKKNKNKFDDSHLMYNMYKYSFPLKEAYKNSFFYDINMDEEKQIFIKNRHIKAYTNQFNII
DTFIDKHKKYSKEDLINELNNLLHIHYLNDSTMDRENIKKENPKTKDNLIDDDLVTNDLFSEYFNNCKKSNKL
NILIKEVNLNSLKNFHNILYKNINFIRTFNEIKDDENSDMKNIELAEIFHM*
>N.PF10_0056
MVQVDSVLEKKFLSFEEGKVLVTTSLKTGRGIEILNKLINDKRYLRYVFYYLKLLCTVKHISKHKIKKSLCTL
ENVIKCKVHKIFHDICYNVKNKILIYNIKKYDKCYSNSFKKIKGLFKLEKIFKTFMKNILIDFFSKCLFSNTL
SSLRIKNKNVFKENNQGNFLSFERVYKKKVGNSNRFPFLNDCYMYHKYRYYNIYQGGSHNYIGMCPKCYLLLH
DNHNKSSENIYTKIYSFDKQNIVQPLIPYYYKNLPSIKVYQNRDKIA*
>N.PF10_0057
MDKTFDYNNIHVDNFDELYDFCISKDTNKKKIKEENYFSEDTQQKFNDDKDTCNKEVLKKNTNIKPTKKHGKN
KKSEYSPCKIIEEKRLDMLNESLEDTESYYDRKKKNSHKYKEMNNSNNNNNNNNNDDDNNSKKHIDPHGNDCI
HQGKHKNEHEQQYYSNKHMKNNSECLSDISSYTDIRSCNNVEDGIYDEYDLTNKMKYEKTNKYKNKNKNKNGY
NNKYENKNNYYKNDYSKYYSNHKDDTHNELYEEGNNYRYKNKKSKDDLKYYRCRYCEIDSIDSVVQCNNCKRW
FCNGSYGTCGSHIVTHLVRSKHKEIRLHKNSLLGETILECYNCACRNVFLLGFLPTSEEGVVVIICRDPCLSS
YISLYEKHVGPFDEHKSKSLQDDMEKEILLEDHDVDNEEINDEPNKNNKKKCENINSSNEDEKEHEEPINFCS
LNHPSDSSDYYPYYYYNHHSNYSRREKNKKHNKGSEKKHMNVCCDDNKMKDCQNNNNPNKYVDDENMIDGKDN
KKYEKKGKQNDVYKEDEKGSYINNSNINDELKKIKDWDLKKWQPVIEDRFFLEWLVNIPSNEEAERKGKLSTS
YNVNKLEELWKNKKDVYIDELNFEILNDEPNKVELKYEDAHHYQSIFSPLVQLEADYDKSIKEGQKQGNVSVR
WDIGLNKKRYAHFIYIKEESELRLVAGDELKISYTYPDGSVWCCEGHISRLHNTEEISLELRTSCTSNGPWVD
NITTGFTVEFIWKSTAYDRMQLALNEFALNSYSLSGFLYHKLLGHDISEDSLEYNKNTFHKLMHKKVMSIRNY
SAPNLAPLNHSQIDAIKRSLLSPLSLIQGPPGTGKTLTCATLVYHLVKMNMGKVLVTAPSNVAVDQLSVRIHR
SGLKVVRLCSKSRESVPSIAEYLYLHNQMKLLKTDIAEELNKLLELKEEVGELSQKDERRLKKLILFAEHEIL
IEADVICCTCVGAMDKRLKKFRFRQVLVDEATQSTEPECLVPLVTGAKQIVLVGDHCQLGPIIVCKKAANAGL
GKSLFERLVMLGITPFRLEVQYRMHPALSEFPSYVFYDGSLQNGITLKEREYPLKSFPWPNAKCPMFFYNSTG
LEEMSASGTSYLNRSEASNMEKLVRTLLQCGLKPSQIGVITPYEGQRAYITSLFQKNISFQNSSDIEVASVDA
FQGREKDFILLSCVRSNKKLGIGFLNDPRRLNVALTRAKYGLIICGNAKVLSRHHFISREKINANETITNVNS
VWINLLSQFKKKDLIVEGCLANLKPVNINIPTPTKLPSRYTNFNYFHSYDNFQKNKTYSYQDKFKSNTYKDNN
TNTTNQYNNNNNNNNNNKNDDQLFNSSHNNHYYSSYYYYSHFNKNHLPDENTNSQFNLNLSNYSYCSDRFMNN
DLYNYINKNKKKNIVNSYVNNLSQMSQFNDYPTETNHNNDDHNYLHLHDKYNDTLDDDKKSSNKCNISSSLNN
SEFSYPNNKMSSNHSIDSYNHDTNLISMSKNADNLLPYLFYYKNNQNSSSQPYMYYNQNSEQINSSKTQKGLK
NTVLNKSSTSNTMVSQKDSIIVNDQKLNCDNLKKVSENTHIN*
>N.PF10_0057a
MMDIEKCLCYYKILNVDTDASVDDIKKSYKKIVLLYHPDKNTHLCDEDKKRYTNIFRKIQEAYECLTNEVQRK
WYDRNRKRIIEGRESSEEKNSGKQKYAYSYTNINIWKYFNNTCYNGFDDTEGGFYDVYGKLFDDIIKEENEEI
KIMNNIKGCHKNGNNHKKKREDNMNSSDKNNTQQDYLNNKNNGDDNNKNNGDDNNKNNGDDNKNNGDNNYNGD
DNNFYFIKAPHFGNSSTCGKEIDEFYEYWSNFTTVKKVDYSYEYIKTYEYENRNFRRNLKKVSEKRSIKEKKE
YNENIRSLVNHIKKYDIRYINRIVELIEEKRKKVELRELKKKEEILKRKLLFEENKKKEYYEEMEEQYISSSS
SSSLNVHKYNNECKDINYPFETNKNSLPNYTKDKQNVDITEPKTQHINQKKKHFTDDDNISSVHDVKRNNNDD

QHSMDSHNEDENNERVINKIIYRCEVCRKNFKSLKQYASHEKSKKHMNNFLKNACKYAMDNMFGDENREDHIN
DPKCRDNKNKKNNDDDDNDESESIHGDNNNNNKKNKNDDDDDDNDESESIRSDYNNESESIHSDYNNDSDSIR
RNYNNDSGSIHSDDSNNNNDKDKEKNEIKEKSDSECSSQSSISKENDDILWWYKNKKKNNYKDVLTSSYNNLE
ENNVDILNEKIKTDDSSDNYKSSRKKKKKKKKNMNIKESHDNIIKNNNMNIINKVDEDNKMTSVHISNSKKLK
ENIKNLKCQICQQIFDSRNKLFQHIQEKGHSAYKNVDTNIKTNRGKKKKK*
>N.PF10_0058
MKRTHHLSNKYIFLCCYKLRNMFLWDKEKIKIYDNIIRKVENSSIISYPLNKNYIDFFYISYKLNNLIKKTKK
NLELNDEVLHTKMFLEKCLNLYYLLLSNIKLKKAHFLKKRIIEYSENNIRSLLNNKVLKMIDQKMLPYNIRIR
REESVENLFKQINDDTSRYLKKVNNVKMRHLYIPRNKINMSEEWKNKYKYFLNKKMLKIPVSKHNYEFLLCEI
NDSVIRKEVMRLLNEPYKYLNLNNDIIKILKKRYDLANILGYENWTHYCINEFTSEKNNYKDIKEFFEIIKKR
IHMDYSTINRDMELYMNKMNGSYNNHDEKKNNKILIIKNNNNNEHIYNKNIHCDDNIYGNNIYSNNIFGNNIY
SNNIYGNHSTGHYGDPFYKSKEKKMSIYDWYYYYNIMTNKIDEYTINKYFPQSYVLDNFIRIISRIYNFYYTR
VRNTELGNSWEENTIIYKIERIERKDRIWNKKENDDIEKKGNDLSASFLGYIYIIPYKNIRFKDYFRIGNVNS
LSNTYLICNGHVIINYNFVRTVPIEEKLFSTSEITSLFHEFGHAYHLLLLSYKYKLYNFNNIPLDYAEFFSHI
NEHIGNNYNIIYNLSNEKRDNSKINKHIFNSFKFDKLRLSHIYTQSIIDYYIHNINPYTFFNKNNNYKEINND
EKVLNNFYDLINSEFPYNINQFNYSLNSTSFPYHFSSLYAGSVFSYLLAEVRVRLFLNQQQLSLKNNKSLCSF
QKNFYDIISINYMDKNYYPSIIQHVLKNKNEVLQKM*
>N.PF10_0059
MSDNYQFLQFYNVYKDNRNKWGKKINKLNFNLDHYKSSRTKIFLYTLWYSIIFTYLMDFFFNLLFFFLYMPYT
NIISKTIFFLSILDCIFVIILTCRSFYAYSYDKSKSLHTLVKLFFALSVWCFIKLLIYVTHTIICFAFISNQK
HNYNGLFFYTPKNIYFNYFMEFIITINIVKSVLTLFTGQKIQYILSCIRTSTILKSKIRKDLEKQSFLFDDYT
YGTFLND*
>N.PF10_0060
MLPKIHIYIYIYIYIYFFFFFFFFFFXXXXXXXXXXXXXXXXXXXXXXXXXXXXXXXXXXXXXX
XXXXXXXXXXXXXXXXXXXXXXXXXXXXXXXXXXXXXXXXXXXXXIYIYIYIYIYIFFFFFFFF
FLASNCVNMNIHKNG*LDIFP*YVIFFFVFFTHSLSLSIYICLRFNFIFLKMILWNKKLLSVVFLLIYDTTNC
VKRSDPMKGKPKSNTSPLPHNVIKKKMEPIQMYFFAKRTKKDIQSNNKKKRKTDNFPINDNITNWKIKKKRKK
KKLKYEHENDYQKLDNVKINKRNHYTHFIRNIIYNETFKNYVEKKKRISYYDIPKNTKYKKYVSSIYKKKQGK
NTIYMIIAPKGSLHEKKTKEILGDPVKNYPWEISIPFHETITREQSPYYNFLKGEWAYLDYKRDSRRACGKIE
GGLENYDGKGLKLEKGVDRPRYKEIPYYLDHGVEWRNFNHDELIQFDIDPDGNQDQTFLETEEDLEYRQWGNW
NYSCAKETTQLNTAWRDPVLSKDLSNLIYPWNHKAHENHIIPYGYRGPSGFIPEPKLEWELAARGFFAGYFDD
PNWNRIKYYRITKKLILEWHEADKNSTKIDKIKSELFGITSRKKYTSNQIKLINEHALLRAKEILLDHILDPN
NDPDYITYYLDKHEPIDYIGGGNHKCSEEEIKNKTVVLNMCVYPVKQQHETYSSNMSIPEMAEMYHYYMTEIR
GEGRINNLSEDPVEDKFQCYELQHDRKNFPALVSLYKPLWSNRKYGEEFGDALKKEEKINIKNFYHFKRKLKL
RKKTRYDKRDTDMYNYLEDYENFDEEYDF*
>N.PF10_0061
MKKKTSKDGKVSKYKNHNKDEKYDEKSLKINKKMNCTKEKHGKNKNENTNNPLNDNKNERIHKKREAILEEVI
PAKIIDTYRNNRNQNMNKNVKNESVRSCISCFCCGWDADDEAMNELLLEEEREKLKLLEEERKRLKLLEEEEK
ERERLKLLEEEKERERLKLLEEEEEEKKRLKLLEEQNREEQKIDEIEEPSKDIIFKKKEIKPKFPLKKKANLN
FIGAAAAPNPPKEASCIGPPPQVKETNINVKEGIYLIYNAENDGQLEVHYSKSGKKGSGVMAYIMSDNIPDFY
FDKNKGKEIIFKEVSKKMQSVYINDLKPYYECYIEFMKMKKTYNGVLYFLPAFNEQPPPNLDIGFFDTKEKKL
NYVEIEKPVEFTGNYLFVIQKGLDIFKKEINVEALLSYLNSYGSLLSLS*
>N.PF10_0062
MNNKNNNSYKKDKKKKDNETMSLAAENMLENNFFKLQEMIEGSYKNSIKKSDRDQYRQYTPRMLWGNPCKFFP
TTPLSVYQSPELFEKLHLDTLFFIFYYQPGTYQQHLAAKELKKKSWKYHKKYTTWLLPDFNTIKILNEQVEHG
TYVSFDYVSTWSKQLKKNFSFEYIHLEDEITI*
>N.PF10_0063a
MNNHMGVLFLFILFLYKTYDIYKNKKEPYPFFVTSMIREEKNMDIDLSYKNDKNKIKFVLQGINHKDEYDSSL
IILLPVEENYVDKLYEEYLKKAGDNPQHNSVKNVLKNILNRYDVIDQKIFLNKFSEEINAYLNLYQNISI*
>N.PF10_0064
MLRIVWKKNIFLQHHFKRKSSFVASQLDVKKLSTKHLGSFSFDILRLCEQNKELYESYKCRVMKDIDLLDGKD
CYRILKSLEINNKINEEEELLKYILKRMCIESCRYSIKEICDICFVCSKLNIIYIPLFASLSIVFINKINLAS
PEHLTIMCLCYSKIQIKDINLFNRISVAALNILYMYDVENLINLLLSYIYLDIPVDLLLHSCIPIFIKNEKKL
DANSLTKLAYVYSNYKYKSHDINCLLKNKLPLYISSLNNIQLSELIISLDRLHIKHDPICKYYTNVNLICLDF

SLAIKVIHVLSQLKDICLELKYDEIFLCINNFVTIHGKYIKSGEIENYKYDEMLRLLYMSYGFSEQPKKGVML
NNVFGDNVHMDVNYDLNGDAPINVGNHLLINKSNDNNILPINNKNNNFSYLEANTDNVLEEHSSFSSLKIKEL
DNNEYGVSPSCDNIKNINSLKRYYFSYNTKTKLDKSSLCYLYTDIFESIYNIFMNSNNNIYEYDEKLKIYMND
ISKEIVYLKNSLNMNCLSRIFSTIGKMPIIWNLTFLPLDLIKDDFFIFYINDEKHFYEELLERYFEIYFKCDH
VEYNTMILYNIINLFSLSKYKKYCLELEKSLEHYSATLRKKGLEEEYMNEECIIYNYIEYNFKQINLWSKRKH
KGKDKNGTINNNNDNLFDHGEKVMWENCSSYNWENTINMNNKLLTLELYNIVQKFNKNIKINHKEEIYFIPIV
EYENYIAFVFLQPEDYYYSSSEKNYLCIIEQKMGYLNINSLKNGINCKNKKDTICDEQDILQADITHKPSLIL
INDVLYNPYKTFYNDTFFIKSNVLVKINFLLIKGYNIIAIPFYTWRNMSYEEKEKHVELLRKQIIIS*
>N.PF10_0065
MAAFKPYRFIIFDQQIKNNQFYKSIITQLNLSLLCKEIYLNESFSCYQYEKLFLKKLVYYSFNNLGNNLKARE
IKCSGHSSVDTGEHKLQQLINNVNKNENKNININKNININKNINKNVNINNADHNKCSDNSSDYHNFYENECM
NNLNLINDLGDRHKNHPLYNVMMKNKKKELTTQSDINKLVSFHIYDNKNEKTFKNILSLNDTYNIDHMNILCL
NNYKPIFNMLENYSINNKRISIYYPFYIINDDKNNKPQSNLYTKVADYCYNIVSDILPLNYKNINLSDIIKAI
IINTELCQNEGNERMEVLKFMDIMDIIGKN*
>N.PF10_0066
MNNSVTRKIEKQILEALKDKENDIDKEIRKYEILERKVYDENDEELEFIKNKRLEELKNKHKEKLTLLKKGHG
TYKEVLSEKEFFEICKSSKNVCCHFYRNTTWRCQYMDKKLIDLSSKYWNINFIKINAEKSPFLCERLKIWCIP
TVMLIQNGKTEHSIVGFDELGGDNFSEQTLVNVLRKWRLIETKENDD*
>N.PF10_0067
MKVQNNIIKEDPFSVSHIIGDRLLQGWTLLNSTCHMCNITPLIKQKNKEDRYCARCDLFIKIRETSHEKEEKR
NDILNEEITKDEKKDNDKDLLNEKENESSLNNDSLMYIKEIKLQNEEFNNILNKNNICTKDLVGDLLEYKNYI
KDRCGYNVAEWLNMQTVKTNQTNQTNQKKKKNQTNQTNQTNQTNQTNQTNQTNDKKEEDILNYIKNKTNEQMN
TCKHTNNLDEEYIYNVHSKDLHRKYFKNMYQNNQDEKHEDNYINVLQHVRRDLKISEENYFCPSKLHSNKYNK
LETEFFILNKTKNVLMDKLEKFTDKLSKNDNKNEELENISHLVTIVTILEKINNLKKYVMV*
>N.PF10_0068
MSMENNSPSKGCRVYVGNLPWKVTWPILKNHMKKAGDVVRVDIFEDTQGRSKGCGIVEYATYEEAQEAISSLN
DSKLEDRLIFVREDREENSGNFEKRKFNNVRKDKFYESRRRRDYDYRKEYRRDDYRRDFRRDEFRRGGGEFRR
DYRRDEFRRGGGEFRRSSKRNCTLIVYNLPPQVTWKELKDLFRKHGRVVRADLKNEDNSSKELIGVVIMENEY
EAKNAIDALNFCNFDGYILKVNYENNE*
>N.PF10_0069
MTKNKKGKNKEVINNYEEEEDYNIIDHDENAEVDVIPEPDFNSNMVYLNIYDLDSVSKVVNTVARSVGAGAFH
AGVEVYGYEYSFGYIMDGETGVTKTNARYHPYHVYRETIPMGKTPLTKEEVDLLVEVMKLQWIGDTYDILSRN
CLNYADYFCNLLDVGGIPEWVMSLQKKVTWVKSNINIAASKLKELNKAAGIPNVINYLKKKYEDSNSSDDYKG
*
>N.PF10_0070
MALFHFVNCSLATFIPYYIIYDGFKLSKNVGATKLFFLVCFYYIISQILKLFALAFCSIGLFQSMNLFNIIFQ
ESANIIDLAGIYYILSHKHTNSINLNERILSVGLSWGFYESLATNFFPFLIGGKSLEFSLKYIYRSISANTFL
FSNLSKTCLLFMWMKNTSGKTKINAVNSLLVYFTFILPLVNRIILIKEESFRSDIFIQLLFEFALTFILSIIT
KFIFSSQLNVSHEHKNATYSDDEIYQVNKDLKKKDKKKKKN*
>N.PF10_0070a
MAGSSSEISESLNGWMNNNSSIVSYVLINADGIPLKYNEDVTYEHAVKQASLFSDLLTKTKKCVKELLPQENE
FISNLRIRTKKETEYILCNHGDYSLITHQNCKDMQHKKKKIKT*
>N.PF10_0071
MLDSDAEVPQEVLAEYEKLLKRSYTENFDDLYKTDLLKVIGKDGYGSHIVLLIPCFIVAAGADPEKTLXXXXX
XXXXXXXXXXXXXXXXXXXXXXXXXXXXXXXXXXYDTLPRKYKKNLKNLYLVHSGFLSKTLLTIVTPFISPKFW
KKVEYIEKLEDLFLKLNIKASKYLKYFPYIVQRNEEVMLGGQPISPFGADLEILCQRFGKKYLHFKHIPSILI
DFLTHLKKPEIINTKDLFNLQADAATLYGIVGDIEYGEPTTDFNNIPSLVCSFKLFLNTQKHGLLGKDAFSRL
HYLKSTSASDKVIKLNLSKLYEKLSQGIKQCVLCLLHFFKTISEHASENNMTILALSKIFAPTFFRSKTPNAI
FTECISLANKCMQMIIQNPDILMNPEKYNEQSSESISSYESGSENNLTEDEDKSDESKSDESKSDESKSDDSK
SDESKSDESKSDESKSDESKSDESKSDDSKSEECKDENNKINNESDEDSNESYSSKESSSKPSSLDQTSEYSS
SILSDKVIKKKVSIKKSTSKRLSFDHSIKSEKSGDSKNSFLSEKTDEQDDSYEDSEKEEEGKKSKKENLKKEP
SSDN*
>N.PF10_0072

MNSYRSSTSKHINFINNVNLKRRKQVIEKKNILEKVHILPIKKNVYNFTKLYQKLNSLINEECILYEGFIINI
NEHDMKEKEHEINNKINNCITNEECVNYKNYNTSNVNNINDDEKETHDYNIDIEQTNTDNTHDKKETIKTKNR
DNHNISNNEKQKPTFEYKNNFHNNTDYIIINCVPSTGILSHDTLIYTDGKYVDNLRKIHILIIKDKNYKKYEK
KCQKYFGSIKKYLLKKSNQIFHEAFSFVSYSLDCFSYKLSTKDGTEKKDSDDQLTNIDSSDDVANINSCAQVD
KCNKYDQVDKCNNYDQLAKYTNYDEVANINNYREVKKNKKENVAVDKILMNYLKQYFRKNQNKLYYSGKLFLI
NNFTFLTLKIDSDVNVGFIDKNTEIILSVDTYEQYKNVHIVPMYDTLPTTYNYDLFMDYVKPYIERHYLNVYS
IHDTFFYKGVQFKIMGIDPVNIKYGKGRISCNTFIYTEGCIKPTFFDVISKESINYIRSLPFEYKPYAILNIL
QHLDSDSLLRLFPSSNINIRENHKKEEKLLEQLTKHKYIFNKNFKNNYTKNSLENNDSKNKENYHHINRKREL
HTTTSTLKSNLINEQCAVCFEYFQDYDKCIKLTCLHTYHWKCVKNWFKFNLTCPCCRYKLNF*
>N.PF10_0073
MNFVKTHIYKQEYTEDGILEIEQDTRSSKEKDAEKILKELPEFKRDNKFLSLSEQLELKKNSNNKENVSSLDN
ENNMYDILNGMDYPTEYDENYDMNNNYSFNKLQQREKEIAHEYNEAIKDCFKKKEQKDKNNLKNLPQKNINIK
DIKKKIIKQKNKKLLNPNIKAIIKTKKKNENNSSDKNDDINKNSSKHSNNKPEENVETKQTCLLHGYSDNSDS
*
>N.PF10_0073a
MVLKDKKYNRKYLKKIKERSKCIREKEEKKSKEDSHYNYDEDLPQYDSNDDFLIHDKELLSHILNSNSESSQE
NINMEQIQNTEKKNKERHIDFVISSLAFNIPLDMI*
>N.PF10_0074
MFLLCRTNLAKKIKDKIPYGVKQSQNYKDAKKQERLALEANRKLKESRGMLLDGKKNLFMCLRQNSDINWYRA
GQILKHLEIHQRAKPDITPSLREKITNIANFVKKGR*
>N.PF10_0075
METLVNERDINIKNENSKDNNKEMMIGMSHMNHDDQDLIYEKKDDIESLRLHYTDQEKLDVPSLVEICKQQLI
VILKDMCSDCSTTDEKTSFLYHLNRLRSAVTVVDLHNYIAVFGPCLSYNKLPSTWNISVCDYLKQQLNILRAA
DSQQNANNNNNNNNNNNNNNSNNNNNNNNNNNNNNNNNNNNNNNNNYLNYYDINNEYDEIMSDKKFLGINNF
THVNLSNNANGNYNNITNHANANANANPTTSHSNIKGNSHLSNANNAMNYLYSDQDNVVDDYYDCLMRTKLTE
ETPNSLKYMINLKNETLSNAEFESVLSYLKKYEMKSSKGMNKNHLEDDNGMVKYNNNNNNNNSNNNNNNNNNI
SNNISNNKDCDEYDYQEEYLKDKALYDSDMDENTNQLHNNEHHTNQHHANVHHHKHQNQHLKQLIDHNNMINN
NDNNIINNNNNYLINNMDNNNIDANNNNNNNNNNNNNINNLSHLIHNNMNNNSTLINRNHLIKSERIKKGDVG
TTTWTKSTTEGHPEYLPRIPGVRFNPKKQQWLAAWNDNTREIRRYFSVKQYGFEQARILAVKARQEAEKAGAR
CKPMFHVHGSRKAVDAAITNDLLRSEMEENFNNMHMNHNNNNNNNNNNNNQHHHQNNNSNIHHNNHHMHNMNLN
INPNHLGHNMSHNMNHNYNNNNNNMYINNNINNNNNNNNNNNNNSNSNNNNNNNNIGNDVIHRKETVKVEIKGV
KRGRGRPPKRKLSEESQMSLDDMEQTLCRNNNDNMELLECMEAYDKDCSRPMKGVSYNDRKGSWLAYWSIGKN
FQMRRFPIKKLGFEKAKELAIQCRLEAEQAGATTTENRTKRMRNLLTLNSENALEMMIDQNSIDNDDSNIHQH
MKEKGGGMNGNLRMSRMQNYAGAGISGQSVHQTNKLLNSDAGQDSENEFSPTKRTRAPRGRRMESLTARASAL
TPVEGVRFDPYSYSWFAKYLENENSKEPKISKYLLKKWGFNKAHSLAVHTVKCAYKAVPFTDEELVNIFNVDV
NNLINNQNNMINLGFHDGFGTNGLMNNQMINQMKTQLNNQMTNQMTNQMTNQMTNQMTNQMTNQMTNQMTNQV
NNQMTNQMTNQMNNQMKNQVNSHINSQVAGHMNANMFNGNNSNGTNMLGNMNMLNNMVNSNMDNNVNNNYSNN
YPGSGNNGLNNMTSCVMNNNGAEIDMDDNDNIENNDHENMMTLEKEDSINNDSIITTTTATNINSCNNNSNNA
NSLLNRLVMSNGSNGNTKLNIINNYNNNNNNNSSSGGGVMVNINNVHNNDNMNTYNNNNNNILNDGNSVVGNN
ESMIHKKNVVNCINNDVEDDLGNMNNNHNNNNNNNNNNNNNNNNNNNNNNNSNNNNNNNNNLNVFMNEHNNPTN
NRGIYGSNESINNNGLMDEGRLNFIHTAGKHVDKNNMQSGSTTVVNVLNNDENYIKSNINYTNMDNMKNNDNP
IDDDNPNNIVEMNLRVEKNILNNNNDEHIINNTEDMMSTNNKENCTLNGQSTILMNSNEENDETALDTIIQPP
IEKRNYVNMMINEQGTADNNSNVDSSNNSYSNNVYTKNLNSNYNGFNNENSNTTTNEQNDSSLYFMNVNSEIQ
TEHL*
>N.PF10_0076
MIRSTYIFSRILFRNSNEKRKNLNMTHGVFLYTHNIHSCFYKKIHTNVHSQEIKNDATINMNNNNNNNNNNNN
NSNNNNNDEKKKKKIYSLKNVFAVSILFLYGSYECINVLQHNEDIQRKVKIYPSLYNIIDKKIIPLKIKYDKM
IIHFKTYILEHIKNLQKVNDYFIQVYFLVLKYYNNLKHLIIIKITYFLDLLERCTKIIRGQESNHMVKKIKEN
AEKNINEEIDEQYNGHKNNDECNSRNNNETIYIQNHSRDKGIQSTPQKDDIEYHVDFDASEKGEEKYICNNHS
IDNTRNIILEVDDKYLQDGLRDVVDILDNPNLLHIKDVEYNKEIKPHNIHDNVNTSNYVTNLDLYEYNMNKEN
YPMFNQPTEKNIEQEKINVHKDDEIFGDKKNCEHFGDDEIYEDKKNCEHFGNHKIYEDKKNCEHFGDHKIYED
KKNCEHFGDDDNSNIIYISQDKMNKNIDCQDSTNYTLKDREKKNLHTVEQEINQKDNNDENVYVEKLDKNIYE
EMNNKYVKIDKDTYEINEKDKDKNLWYNNQFIDDVNLIQSIDVEKKCPIIYSEETKQKNNKMLRNDKEEIPDI
VNKNISNHDEPYNIESNNFVNYNMKYIKEEKYKERKKKKKYKKEKNKKGKKIRPVQKDSNTIFLRTNNNESRK

YNIIQNMLLYNDKHFNKIFDKTVKEFEVKIDKLNEEELKNKITQMFVKELVSEKYNDILLEEEKGTLKKVLII
KYKNIFLKKQKKYQKYIKRFLLKKLKKDEKILKENYEKEKEKFQKYLMNIKNEELEKEKNKIQQQYKLLQENY
FKKMNIYISHINNIKDIFTNEIKNKFNLENISNIQNKLVNLQNCIIHDISIEHILIELKKDLQKDIYLNKLFQ
ILPKNFFSHIFKPTNNNEKLKKEFYSLYEECVQQAFLNKDDNYFKQILGKMMSSIYIKYESTLHNILIRSSKN
NILKNNLLNLSYALSSIQQNKLIDALRYTNELTGNCKEIFFPFNEHIKNVILFKFYLRLVVSRIMLASKTLTL
CE*
>N.PF10_0077
MSSFNLVGVINNRTWGPDSKNEELVNYCMPDIKKYQFEPSMKFERIGKVCDFTMNSYQKNTKDINKNSNDVNA
FEEELQFQTVDLRTGQKQKGSIFNKKKIHNKQNTTQAFTQKQQEEDNLYSSRIKTAEQKKQKLLQQAKTARIN
ARHRIFTEWSIEPTPLWTVECEIMFNELPKKIIKIDNLKVEDLFFKGKSLFYDKKIENINVKNPPLLFHHTKE
GINLIPKTKEDHNLINILNNENKNKIISNEEDPNNINNIIVISTDQILSCLMSCVQSKYSWHLLIKKKGNQII
IDKDDDSIIDLLTVNENSIDAPTQDNENKINSLQALGIEAVKINQRFRKQVILKNDIAEEFEPGNFNIKNIKI
NDILYRYRKFQIPPLVQSNNKKTYTLITRGEIHSQIKGLNKSYIYVCALNEYDIKSHKNWRSQIENQKGALLA
NEIRNNTSKLQKFISQALLSGCDDVKLGFISRKNANDPDNHHILSIQSHKTKDLSTQIGLKYDNMWGIFRFVI
DNLADKPNGNYVILKDPLKPLLRLYCTHEDSN*
>N.PF10_0078
MKKKITNHKKKNENAKLNIDHDNSSDDNDKTPQIDNIFKNLIVDSYKSINDINNYMRQSNLLYNEKKSKKNKT
HPLDSKNTTDRKYNKYRRNEQNMNKVINYDDKKNYDDIENYDNIENYDNIENYDNVENYDYKLPYDEDIIYDE
KQNNSDVVIIGYKNFKKGKKKISLDSFNSELSPQSNTSPIYIKKKINEINYHFIQKKKKKKNQKNGELNKKYS
KKLSINKKKNKKYEKHKKYEKHKKYEKNKKYEKNKKYEKNKKYEKNKKNEKNKKNEKNKKREKNKSGLINNVKK
IKRNKKSLSEENLNDSLFSNDEQVNENKYILDKLLISLKNNSDANIHKKKNFYIEYEGNDDFTSFNSKENPND
YFLCMDNDEINNKLNKNKMYSSSYSTTKKKYIYKSHKGLYDNDYMFNNNIFNNKIYNNEMYNNKFNIYNHNIP
SNKNYKTNLKGRMSLQMSHDISQEALNTRNYFENNKNFEKERLKKKMRKIRELNLLNNNVEYDCIGFVCDEEY
MCEKLHFDENHVESPDRIKCIIKALKEKKLINKMVQIKCREALYDEIRECHSSTHINNIFYSLKKKLKHNNQS
VIYPFDKHDTYYTSYTGTVSIRAIGGLLNLCDVILCDKKDKFKYIDFKKSLRYNYDFYKNVNTNSIKRNYESN
NMYHKKFLRRSKSESSIYRKCTTYDTFNDFALLNHNKNVYKMNTYHEQIEKKNNNNIINSNNNNNNNNNNNNN
NINCSVYNYKNEIDVQNDIHNINNINMNNNIISEENNLDLEAADYNFGNEKDEHTNESYNEENNINQKKDYIS
QSNSSHNSYYNEIHQGDSIDYNNNNINNDSAISNKLSICEKLSMDNKLSSISNNSNHQDILPNCFKNDEENIH
KDKNRDEIINCNNTNVIETEYLNKICENDNYTNNYEENYKNNSFLNSNNSDIFNLPYRSYSSTVYSMDDCNDV
NTFTDINCGFAAIRPPGHHCSRSHPSGFCIFNNISVACKYIFKKYGIRKVFIFDWDVHHDNGTQEIFYGDKDV
LCFSIHRFDKKVEEKNKKKRHVNKKKKKKYDIKKDDDNKKDDDNKKDDDNKKDGDNKKDGDNKKDDDNKIYG
DNKIYDDNKKDDDNKIYGDNKKDDDNKIYGDNKIYGDNKISNLEKNLKYEKTSNNYKKYKRKNKKSRKRYEEN
LFYPRTGAKNELGEKEGYKFNINVPLEKGYNNCDVYYVFKYLLLPILEKFRPEFIFISCGFDASINDPLGKCN
LTHNLYQWMTFQLKHFANIFCNGRIILVLEGGYNLNYLPKCALACIKALIKKNKSTTTDKEFIKKLYQNNVNN
KETNILNDQKECISNQDSSKRFLDAHEEKKKKNDERNVNRCDNMNDAQHIHGSNNINDAQHIHGNNSMNDAQH
INGNNSMNDAQHINDNNSINDAQHINGNNSMNDAQLINGSNNTNDMYDKHYLNNHNLDNNIDEKLKSNKTYES
LVSTNCVQKNEFLNYPKNNFINKKNMSDHANFYKLKYSSYDKYENKDINYYFRRLHTNNNIVSRTKELITSGL
LHYSTYKVIKYFLNVLKGEPYHLDIELPTYNEFIKKKKLEGREINPERKIKIKINRSSEFRKNDYCFNSYEIM
NSYMQHKLNKLKHINNISNKNLQDMFIKQKNFKYTDSSTTISNISQSDLYLSNDDINYSSSCSSTCNRRKVIL
LNKLKHKINRGLNNIYNTSPNNNNNNNNNNNNNNNNYNNYNNYNNYNNYNNYNNNNNMVHTEKKTYKKKRKNQ
INDINLNAPVQMNRTQIDTNEENENLNSNKNKSNRTIKTNQNKLLSYNLSNLQNINNINNTHIIPYADLEHIN
DDNHLESHNTSNENYINNYYMNCDLTNLKYNENQVLNTFNIYTKIKKGFIFFYGSGHRNQWILPVHNKITKII
KLCSESEAFFYAWLYLSCLKKINISRTKNNYTSILDNNETIENVSIQLPFEEREHILCKQFIKFTVPCYHIFL
KRNQINSIRQKQSKQCKDDNNNNNNHNITNNTFMDYMKKTYIEWSSSKIFKNNESIQYNDKINDGTNNMIYNS
SSNEKKNNVLENDNSLYPLHINQNIQEEAEKDVEVEEEHKTAICLSNVLSSMRHPCVMDIKMGIRLYGDDCNE
ESIQKKIEKAKNRSCLSHGFHLTSLIGWSKKKKEPFFISKEDAHSIKNDDNFVQAFISYFTACDNIQLSILLI
KKILIILEEMKIFFIEQNYFAFYGTSLLFVFDSDPSKKENEDNYSSKDKIEKNELTKNISLSNENMISNDEHN
NSFYYKDKKNKLCDQDKINFEEILNFKLNIEKVFYESLSNEERNIILQNKLNQKIIKSVHVYIIDFAHASLNK
NQNDEGFLLGIISLHRIMDKTIQRIKELYLSNTDVTNDTV*
>N.PF10_0079
MMERGRSNYRRNNENNNFREGNIKNETGDDNHFWTNIDNYYFCNFDKSIISAFENHIKCTDYICNKIKKRKIN
KKLLKNINCYKSSVKIKLNGNDKNEHNNKKLVCAGTNMQYDNYMKFNENNMNNVNNVSNMNNKNNMNNMNNMN
NMNNMNNMNNMNNMINMNNMNNMINMNNVNHMNNINHMNNLNRMNNINHMNNINHMNNINHMNNINHMNNINH
MNNIKTSENYNISNNNNKCIQNNNINDKILNNQIQNVMTFDDKKSILEFMPMAKNELLLNTLHHLNLRNEHPT

NPFPNMYRNPNYFNKDENYMFLKNQGPINNLNNNVLPNNNNLNNLNTHVNNQMVENNNKTMPIPLNNLNAYTT
NNNDNNNKNNILLNNNEHNNNPVGGTNKLSDKNLEFLQIEELHNKEGNKNTNDQQVILKEEERDIQVNSNNEK
TTNEENILLNDLQKNTNNNETSNDTTPFKDVNDDINILKEENEKENVISNKINSINLAHSTIQNDNERINNED
NKNKENVTIQDNYSNNNNNNNINRGCVLNKNQNNINSMNNINNNNNNNNNMNNFNNFHLNQNVPNNDMFKQPL
LFNKPNMMNMHAYHMNNMNPSEPPFFNGPIINNNKQSVQGYINPMNHNMVNMYNSFNPNMRNDIMNKHMMITP
NNLNINDDKNMTTDIDKTGYTCPSNMLPYDNMLINNQFRVKDPHAFMRKSHGTRIRKFNENDIKDDNNDDNNN
NNNNSNNVNLDKYKRSTRSSKYDDNFFNPFLNKKYDGLGKLPYIQNKPIPNGLHISIYIPQSYDFTDEVKDQD
IKTYEDTNKNNDKVNTICDEIKNTEHTDHEKDRKSVSFEQEKSELISNEEYIYERETLARLLTEITFGDKKNL
FNPKMTSENKEKCYNTILNELKTYKSLPIILGNILPYWTKDKNIKKIKKSIEKMNKRKKKVDNPQKYPNLFYK
IKNLKRNNDKLKYIHISNINKKIKRAMMNKQNKKKQNYVFEDELNDLNQINVKQEKKDEYDRSIKTENNESSA
KLKDNMNNSKNNSQNLTNTDIKVKNENKDAIYDDGHNNIQNVEINNKNKENNYYTHNNVQGEVLHNYKIDKDD
KNENKLNLIYKNVDNEERGKNESKNVDSFDEHIVINKNNNIEEKDTSSFEHNEKITSSIINNDKNEELNKEDG
YIYKNIKGNDTIKDGENYIPQDINDDEYDDNHNDQVKVQDKCINYKENHKKKGALKKKRNLLSKFDFLSNFHF
DMPYNSSVPFNNSMFSLQRNQYKSCVYFEFVLTNINLDKYRRDILNVNYNNNNTITKRKNIYCINFKKYYKSL
QRKRKYINPTNNGINSNTDNINNIKSPINKSSSLCNNNIQNKKEEVNLTCNMVINNNIKNDSIISSLDVNKNS
YNNKLREEKRKKKKKKNFMLIKKKKYMEHVNDIFGISNINNISKANIQNYENIENINKEIFVLFSRYVHNIKQ
QKKIIGKLIGNIKSEKKKPYKYWYSIEDKMINFYINEYLKNKLNTKHTYTISKQIVDLLKLRLLINHSSLLPN
SWTEKALCNICNIGEDWEDNPIVFCDCCYTPMHSFCSGSKNIKNYNSNKKETTDDEDEQQDQHLQQKVEKDKD
KDHNNSTNVYIKNQNNFIKDCYSHDHRHGNKKKKKDDKINNKNNHKKKNKNHMTPKMNFNIMEYLMSDNYIHI
NSPQSCFNTLTIKNELHESPGNTYDSNDEFLSKHNSNENLNINHNNYKKEKSDFVNNKSDDNTNNNKNNDDDD
DNKIVHDNDHNDGNIYNNRNSSFMNSLNNHETIINKNNIDEGNINDILDKDKLYIEENKTSVQETSKSIKNHN
VTTGRKETKGTIQKKDTITSSKTNKNKELHEKNVDEEQWICPLCSYLRSQIFFIDDVNAFKIIRRLCCPRKKK
DIDLLFKSTTNEYNINTNHNNKNNHNNNSNCNNNIIDDLKREMNSFNAPEEIIVNKEILKLTHKKKSIVLLFD
TQNDIQYYKEKYNIEINDAQDFFEKIVLKYKEKYKYITLFNSIVVNKIEGSLNMYEYIYKNFQFFRQETFCIT
EYVDDSDDESDKEETNITTNVKSDKKKKDFDKGNKMIKEEKIDRRTKLYRLLQSKLHKKEDRRRKKFRNRNKL
NINKDINKNGVPNIQVEIKRRGRPPGSTKLSKLLLQNNFPIKSDNIVNDNTSCKNITNNDVTSNILTNNVITN
DNITNNIITNDIIKNDSFNNTIHNSDIYTNNNQIYNNTNIQNNTSTNTTATNITVTNTAATNIIGTNTDSTNT
ACTNITTTNTNDNNNDNNNINNNNNNNNILPNYGALIKSEQNNSLADNKPMYGMKRKKSSDYNCVEKKFNSNN
FFLHNDTKLRNHIFSTYNDEFEENNLLLKYNHNKNCSFVFKIPTCCICEFDAFYIGGGPIKRTKKKNEWCHIR
CALISNCIINNKIEINNKEKTKYKCSLCLRTNTTGIIKCNVPDCYKYYHISCATSSPKYLIELNDENKLILFC
SNHSQRKAPTEILRRYQILREKEYNKNKLEDKLNISKMFDAYLLQSYLKVCDFKLFNLLSLSSLSVFDKFVHF
NYDKFNEHVKKYNINLMDDFNNDIYQNVDHNPFLKEASNMFNNTLEVTKVGRTRRRSSNGSNFIKRDQRDDKL
EDKLEDKLEDKLEDKLDDKLEDKLEDKLEDKLEDKLEDKLEDKLDNQLDDNELESKEHSSIHLYQLSDVNQKE
EKIGESENDYEGEREFLELENSNRKKNVQIDIFDIENINRISNIKCNNIISIGNFKNIYNENLLDEDTLLKLL
SYDFLNIQKDIQEFNSNILSDKYKKLKNFENILCLYPYFNDYQLRKLSELILEKNSSTLCKINKDDDNTFISS
FNKFLSLLNNKDLMLCSVCLSKASYSDKNKEEKKLTEEKIHLSTVENNNLSNSQMTLKLRKLAYANNIKESLE
TKKERRLGSLSVLKKCTMCNVYICYFCCHRMNIDIMKNYLNNDIKLYNSFSAYNNNINKNNQDKNPFRVKRVG
RPSKYRPPIEPPPNPDNINSNNNNNNNNNNNNNDNNNDNNNDNTINNNDLKIDDKENKTNISQENITNNNSTTN
NKTQNNNYTNASSEQHTSNNHQNEVEDENVKKNNIEHVLNDRKNVQINDNIKEQSNLDNNHNSSCTKNNDIHL
NDNINYNNEKTLVENNQICKTKRTSGGIFKKSIDSSTFLNCDKKENDNEFVCPRCEYFKVKKKIGFCSYCPRL
DGFLNCFEDKGKKELLFGHPKCMEYVNTAYAKKNSVNDVKSGYNKRICSYCRIKHGVVITCSNTDCDVSFHIS
CGILLGCKMDNFFGRVDIYNPKKAYCFKHTFQYCKKSNFLNFINANKLFCFENFLYNPFNHLYNFLIGTYVFN
HLNKKCSIYLMKPIKIEEQSFTNIFEKTQLNILKKNTNMLTNDMKRSPKGNNKYNMDNRKKMSLTNNMNDSKN
LLDPSSLYNNDYSNNNNIHKYIKLSNEDDNSRTPSNTLANMPFDIQHDNINSFYSMNNMNNVHPNFGSKFNNN
PLFLANKNNMHNTNNPLLNLDHEHNKQSFMNTNNNNVPFKNNNKIFVDDNLTESAVSILSENETEELKSFQNV
SLINSRSNPYINNIIQNSFMQYVKKFQGNNTMPQPFNNNAIPNHFLPMYNMFNYPNAQYQNMQNVQNMQNNIM
NRNAKFNFIENKRKKYLYQDKNVENTRKNALSEGNIYANVDNGKLQKVDGRKKRRYHKSKNTQSINTGELTTL
TNSNSLSINNMDKFNKENISSNMNNNHNPFVKNNIMEEKNINAKNSFFNDSNTNILKEESKVNIKKCEDTVDE
EQIYCPVCKSYYEELSDGSPADGLNWIGCDKCEKWYHWICCKYSVDNPPDIENDWYCNSCLNS*
>N.PF10_0080
MTIKGLVRFINKKIPSTIKKIDDINSFKGKKFIIDGTFFIYKFMYVAWKHIINDKNRDLKYKANELATHILIR
QYIIKKSIELLKNQHEYFKSLKINTLYIIEDIGARCELQPVDYKCKKHVWKERETIRKKKNLFDILNVDSSKN
NILKSNDNINVNDTNTINNTFKKNNIVNNFLCDQKNNISNICEKKNLKKKNQMEIIVDEDGIHDLFKKNIFIK
INSKTANDIYNYLLLENIPIFITKNDAEKECAIQCSHERDIVVSDDTDALAFGAPNLIRFITNKKKRHIINKE

ELLNELNINYEQFIDFCILSGCDYSAKIPGIGPVKAHEIIKKYKTIETFLESNAFNKYKNSKLFNQKLNGTSM
SLNDYILNEFTYEQARKVFFNSY*
>N.PF10_0080a
MKLDSEGIFKIVDNNLF*
>N.PF10_0081
MGNTPGGMNNPYGFLGKKDDKDKGKDKDKEKKKLESVPISHMGKKKKKNKGTSGHSKLPNVTPNTKCRLKLLK
LERIKDYLLLEEEYITNQEQIKSSDDKNYVKLKIDDLRGSPVSVGTLEELIDENHGIIATSVGPEYYVNILSF
VDKDLLEPGCSVLLNNKTNSVVGILLDEVDPLVSVMKVEKAPLESYADIGGLESQIQEIKEAVELPLTHPELY
EDIGIKPPKGVILYGPPGTGKTLLAKAVANETSATFLRVVGSELIQKYLGDGPKLVREMFKVAEEHAPSIVFI
DEIDAVGTKRYEATSGGEREIQRTMLELLNQLDGFDSRGDVKVIMATNRIDSLDPALIRPGRIDRKIQLPNPD
TKTKRRIFQIHTSKMTMSPDVDLEEFVMSKDELSGADIKAICTEAGLLALRERRMKITQADLRKARDKALFQK
KGNIPEGLYL*
>N.PF10_0082
MSIRLLCEVIAMLLLHLIFYKVSIYYLLRHLDILNHSNKFNKNIFFITCLICMSIFSLFIFELSSLISNVIIT
YIWHIDICILLFLLYVIIPIEFINTIFDSNNYKEILSPEFHTHSHIKLLKKYYAKISSIYKESYGINKKIKKH
FFFFRGILFLTFIWVILGGLRNMIYKKNYNYVIADDNTINFKKYLLYKSNEGKNANKIYNKTIFFFFQKKHTC
FNYYFNNNMNELYFTNKKKTHPFKQFFKQIYSFFYFLCYIIIHHIILLHNFIIRELLINICALGVTTNSIVAG
ISSVYFIYDYIITFIYFLHIPKIQIQIYNIEERILINFTNYMFKKEELEKLQSNIDIHTKRLSYDNRRNIKNV
DLNKEYDKRVVLKRLNDKVDNPERDDKIYVDDKIYVDEKIHIDEKIHIDEKVHIDEKIHIDEKIHIDDKIQNN
DINTNHTNQNYCDDSKIDEEDRNKEHTYNNNFKEENCNNHQNSIYNKTFHLNDTTIDERQKYNNNDDDDDDD
DDDDDDDDDDDRRAYPHRIKETVKNVSVLLNITNKIKNICALTNASLLKSSDNEKYEKVKSNINYERSVRMNS
SNRRSSKGRVYFPYINNYKNSRRKKGNYSDHESTVINYFYKKISKNTIHSLKRSHSCPVLIKESPADNNEEMS
NNIRDIRMRKYNSLYISPSYDFHFLEMDKYFKRKNYKRKKKKYKKKIKNVKAHENVKNDENVKNDENVKNDEN
VKNGKNRKIVKNDEDKCVDSPYGQENIMLKKSEDTKNLIKPSTNEHLFFKVNKNSEDQDDTYNNEYSDSNSDN
HSNIIHSNMDDSEGEYHDLNNEDSSDDNTSSYEEDDEFLNHMLQKSLSFPVNKYNKKNKINKNENDRLEENDN
MIQVERFEKIYNNINNDDDINNDDDINNDDDINNDDDINNNDDINNNDDINNDDAKNNDDSKNNDDAKNNDDA
KNNDDDINNDDGKNDDDNNIISTKNNIHFNEKNIKHCEKSIRFNNQNIIYKESNIYNENFNNDLNKNFLGSKK
KSMDEKSLKRELQERTNNINNNNNNNINSSSNNNNNSKNYNSAYDISNGEKRKCENMKNYNKLKKEIEDIVYT
NTSMYYSLNAILSRKFEIQENTNCVLGRINVILNSIMFLTVVYKIIMSTLNIIFIRIYIRDPFSKIIEKICLF
FNIKYNIAIIYAPYISLIYISYIVAINMKKFLQQIIQISSFFSFYFKLFSNMWILLISELMGLYFVTNSLLLT
SYLPVNYNHVMNFVLGNNYDYNIFHLHSDYVFIISSAFTLIICILYMIYFYFF*
>N.PF10_0083
MLYKMNVSSDIKYQFSKTKICKHYLEQKCLNTHNCNYAHVLGELRPLPNLMNTKLCKSYKKNIPCTNPNCKYA
HKVENLQPTTDMSTYKTSLCYFWKRKKCMNEEKCRFAHGIEEIRPLKVRTQKDIHTKQKDDNNNNNNNNNNNN
NNNNNNSSSNVNGFNNNNNNSSSSSNVDGFNNNNNIFNQEDLNNLFSHLFLIENWSSQIKMNDKNEEAQRHTN
DQIVKDTSTSCNMLLQNSKSYSNEYINNKLCDEHLNANYNKQSDDYIKYNSLKINHNNNIDANLRNTLKQNEY
CNLFTSNETFCDIYEDKYNDTYISNHQDFYKDEYVNIYNHISDPISSYISSEESMNDEECFNELYKCIKEEIT
KKYENIYIY*
>N.PF10_0084
MREIVHIQAGQCGNQIGAKFWEVISDEHGIDPSGTYCGDSDLQLERVDVFYNEATGGRYVPRAILMDLEPGTM
DSVRAGPFGQLFRPDNFVFGQTGAGNNWAKGHYTEGAELIDAVLDVVRKEAEGCDCLQGFQITHSLGGGTGSG
MGTLLISKIREEYPDRIMETFSVFPSPKVSDTVVEPYNATLSVHQLVENADEVQVIDNEALYDICFRTLKLTT
PTYGDLNHLVSAAMSGVTCSLRFPGQLNSDLRKLAVNLIPFPRLHFFMIGFAPLTSRGSQQYRALTVPELTQQ
MFDAKNMMCASDPRHGRYLTACAMFRGRMSTKEVDEQMLNVQNKNSSYFVEWIPHNTKSSVCDIPPKGLKMAV
TFVGNSTAIQEMFKRVSDQFTAMFRRKAFLHWYTGEGMDEMEFTEAESNMNDLVSEYQQYQDATAEEEGEFEE
EEGDVEA*
>N.PF10_0085
MLILVETAAGYGLFKVENSKLIESDVKDIEKCFVTSEEARKNVSLYSFSKFKKFQNAFDETNKLMESKLGKGL
KKFLKKNIIKPKLNEPLALCDKTLGGLIKKKYNINVTYTNSTQEIIRGIRTHLYELLVGVNEEDSKLLKLSLS
HSLNRFKLKFSADKVDVMIVQAVGLLEDLDKEINVFSMRLKEWYGWHFPELGKVITDNKIYAKCVKLIGFRNN
AKNVNLLEETTEEIQKEIKQLAEISMGTEIEDDDLNCINELADRLLELTDYRESLATYLKYRMNSIAPNLTYL
VGDLVGAKLIAKAGSLMSLAKHPSSTLQILGSEKALFRALKTKSKTPKYGLIYHATLVGQTAPKLKGRISRSL
AAKLSLCTRVDALGNFVEPSIAITCKSHLEKRLEYITSNLQKKLSNPNNNVKAQQQGQQNKYNPNQKNDGQNN
FNSFNKRKSDFRFKREADKNNKKFIKKQKRK*

>N.PF10_0086
MNENLENFSTIDLLNELKRRYACLSKPDGRYIFLGAPGSGKGTQSLNLKKSHCYCHLSTGDLLREAAEKKTEL
GLKIKNIINEGKLVDDQMVLSLVDEKLKTPQCKKGFILDGYPRNVKQAEDLNKLLQKNQTKLDGVFYFNVPDE
VLVNRISGRLIHKPSGRIYHKIFNPPKVPFRDDVTNEPLIQREDDNEDVLKKRLTVFKSETSPLISYYKNKNL
LINLDATQPANDLEKKISQHIDG*
>N.PF10_0087
MVLYIIGLGLGDEKDITIKGKELIEKSDVVYLETYTSILFVSKDVLEETYKKSIEEVDRDFAEENCDKILDEA
KNKKVSFLVVGDPLCATTHHDIILRAKKKNIDVEIIHNTSIISAIGECGMQLYNFGQIVSIPYFEDNYKPTSY
YDKIYINLKNNFHTLCLLDIKVKERTVENIMRNKKIYEPPRFMTINDSIEQLLYCEHIHKKNIITKNTLGIAI
IQIGTDNQQIISGDLLTLKDISYNKPLHSLIICAPTLHDIEKEYFDLYHYNNMKK*
>N.PF10_0088
MSWYSLLTLLLVSSILYIQHVTCLYVEDTLKISLMNIKSGDYIKLCCDIFCSFYKYDIFKKTFFFLKKENNIT
LYNCYGVIKIYYSKKYLFYFSTYINVEQNNIIEIIDSFDKVKISRYVFFTPLFEYITTKKNNMVYYIPFGYYK
FGKLKNKMLNDKKTHNNNNNNNNNTSLIINNQKIYYTYEQHTKQINKFKEDIFKHNKYKQDRKNNKYTTVRKY
KKCTIFRKNCFTYIIPLFFYYIKSMNLKKCIPQHIKYNIHTSNNNNNNSNSNNHFSSSYYYYYYCCCYFFYYT
KKKHYILSLGHNKLLLQKIIFNKCKIFQHIIEQNEKNKLRLYMYDNIIFHNNMMKKKKQYNNFVEAEIYKFLE
ILLFSNSYKKPHILYSKDMEIIESHTVKKNNRKDIKNYNENKRIKIIVNSSNYKYEKTQSILFLLICLLFMFF
FLLFIYVIIRKKQNKKNVQNKEINKNTYNEKNLKRLAEIHVEYKKKIQTNLGSKIKDITNRDLIKLITYNKAD
SIVVCQP*
>N.PF10_0089
MIDTFEITKLCFSEHMSSHLIKMGSYNILCDVPLDMNEILEIRETIPKEINKTNVILDNNNNNNDEYSSYNSD
NRHNDGYINISNLETSPGLSKKLLLNISYIPMKIHIILISCVEGFMGLPILCKYLDFRDTHIICTKPVFTFSM
CAVECLQKQENYIPEWDDEKINIKNMDTSQIKNEEYFHTKWNFKNSLHLLSYKENVSFKQYDEILNITLCSSG
HSLGSSNFFLSTDYLNIFIVNKSSYNIKRHTSSFDSSMLNKCNFVIFTSFLLSKQLQYYVDQTDPHYNSTSCS
PQNNVDINHNEANSNNNQTCDNNNNKKIFDNNNKKKTCDNNNKKIFDNNNNNPCDNNNNNPCDNNNNNPCDNN
NNNNNNPICSNDQATHSPKSSNTTQAHNMKQLILQKIQIHIEKSYKDSLNKICSLVLKTIKNKGCVLIPVDLH
FLYFIELIELIGVIISKHLPKEEQVLIFSVLSNIQDVIHQLNLFAEWVEESRKKKCSKILNPQGPFSIDIMIK
NNRLIIGNNINDITKQFRYPCVCFIHDSSLRFFESSLLLEKWANEQNNTLILVDPFYDPIKVLYPFKIYEKKI
NVHYCPLSWDLNEFHIKDIIMSNTKNVNVTKDTSHKNINNNINNNINSNINSNINNNINSNINNNINNDINNN
KSNSINNNDTHVCVYILPETSKHIFTDIYQMSKNELPLAYQNVTIQKDNNNYNNHNNHIDIDQDTFRNILFIQ
PFEKKQIAFDKFENFNQKMIPVRFSPGETKKLERILKKVDDFFCANIKAQYTCSFIGDYIDEDHIEEFKVDEL
EGQNKYEIINDEQHDISNEQQIKLMVGSINVDVLTSNLLHSGYNKNDILVEYQQNQEKDTDTNNNVIWSITIN
SIKSKIICHDQYDIEVCTNNIEFREKVKEIFYKLVNHIIE*
>N.PF10_0090
MIKRNNFSTFKMSVNNSFNIYTNNVYVSCAEFFNEVNMINKKLLYYINDYFKLNDDIYNNDKENLDEEKRNII
RTRTNRIRKKKIGCLFPPCYSQLLINFCVLTNNYDYICIPSPIKKCKQIKEKYAFYISENNIDLILSHPFYKH
YIEEISFNLQIPYLIVYDKPDVLTHEQYLDHLKEGRINFGNKYLYKEKKEITMMDEKTNTHDEKYKSIDNIQN
DNNNNNNNNILLLKDIYIKELEKNNPCIHFQLSKQNECDRSIQFYLSSIYEQSKNLHKLLNFNKNDNIFFCIP
TNNIQYFEVLFSVLYGGGTIMFPEIKKKEFFNYNNYMKWFTKHMKKRKSHNFSVHNIILKNDFNQNDYNFIDA
NSLFEEIYNAKKPITTIVCNNYLIRDFVNFFENSDINNITKNEFINKKACSIQIVLINGNEIMPGIKEEYLEK
LKNIFINAKIYQRYIIQEIGTICLTNVTKLIEDNIKYERNIAGYVAPNINIEIDKDTSLLKIKSDHLFTQYYN
NKNITQKSFDENGYFKTKYLATLTQDNLLKINGTINDVQKISPDYYLYFKQRKDKMEKHPPGYLKRVRLHGQI
WGNFHADKRNWKRKF*
>N.PF10_0091
MGRKKRKLNIDLKPFCYYCDREFDDEKILIQHQKAKHFKCLHCNRKLDMANGLVVHMMQVHKTNLKSVPNALP
KRNDPELVIRGMNGVPTDIIEENLIKLKQKLGDKNIKRQQRVNWAQVTMAPTMEQFLKQAQMGNFSFPGFNSP
VLPTNNNILTNTLDINNKSTLPNIPLYIPNTTSNINNINNINNLLQQPIGNNIIYPPKNSTLLNMPIPPINKN
NINNHVVTNPNTFTPSNIPPNIAHKYSANINLLPTNINQHNPMYMPPGLYPPPIPPLNHPSIPPTHIPSSTYP
TKMTPIQQKRPSTKWDVPEPSEGDPSILKHNKEDSTENKNEEPHKVSQDDDLKQNEQKDNQEKNHTQNCE*
>N.PF10_0092
SFSQYIFAHIYVCSFVIIDYILIIMNIKTIDDVIYKPIMKKRRFRVELLSEFLPKNPNLLGLNIVKKSEIKIR
LRKTKGGEIFHFNDIMGTLLHELVHIVHSRHDKSFYELLDKITWEYNELYVYNKKGISGGDKVSTNIIKYNLG
RNNIMKDNIILDITKMNNVISICEENPKFMAAQAAEKRLLNNFMNNQGEIVNASLESCLTKEQRENLLNNRKK
YDDIICCLDNDIILIDTISDTYDKEYDHKSMVSQKKSDNVSFLENTKDNINNNIDYTYMQHLNDNERKQEKKE

NEHMITQNYNNQFEGKPNVIEILTPDIKKKKNKRKNLIESNNGIFDLSQNDGDDNSQVLKRKKGNQKDMKSDK
NDEVIFLK*
>N.PF10_0093
MIYTRVYCIFIILLLNNMLITHNRECSKSVQGLVVEKFNKKMNYLLKKEDFYIYRQNFNFFPKELEVQLRKKK
NKIQNETGVIDGVIDRISSISTQQNKGDNTNVDERYINVDNSNKIKGNNMNNMNNMNNVNNVNNMNNMNNMNN
MNNMNNMNNMNNMNNMNNMNNVNNVNNINNHYNKDNNKNVGKNRCNNGKKSYTTTNDHKLGIKTTYAFIKNSI
PFNFFHMVNNIYVTNKFKTNQWFSLNNKKEFGTNKEKPNFLTNYKIIKHPDKLESEDCCLNDQGFIAVADGVG
SWIKYGINPRKYPEKFLQLLQKKINENENIQIEELLNYAYINNDKEGSTTICLIIFNKNDNTVSTANIGDSQF
LIIRNNQIIYRSKPQQYEFNFPYQLGSNAVSKPNDADIAHIEVKKNDIIVVGTDGLWDNLYDSQILTIVKENN
FATLSEKIANEAFSYSKMKRWMSPFIKSYNKEFKCHKTGGKMDDITVSCAMVC*
>N.PF10_0094
MRIHPIYHSNTMDNNSIDSDNNKFAAHLKNKKALYKIYLHNTRYDIIKKVVRDSENWTEASINCNDWDVMWID
TSICEERFRKLKKFQKINHFIGMRGITRKDELCKNLKKMRKHFPQCYNFFPMTWILPNEVSDFKSYFKRKGSS
KTYIVKLKNSCQGKGIYLTKSLDDINKYESCIIQKYIHKPLLINGLKFDIRLYVLLTGCDPLRIFLHEDGLVR
FSIEKYRLPKNKNLKHINMHLTNFSINKMSDKFEYSLNPDDATIGHKRSWKVFLQKLKEQGLPMDCIMKKIEH
LIVKTISSIQPELNHYYNSAHYSDYSNSMCFEILGFDILLDYKLKPWLLEVNHSPSFSVSSKVDQKVKYAVIK
DTLNILYMHSKYRQIYIQEYLNLHKFRKLYNDLLIQNKKELKKKLTISRFHYENKNLGGYKRLYPLQDLLEYQ
NLILFVSNTWNKSIGIPYTLKQDTYEYLFQTDVTKKKLSTDSFLSQNQTYEEYHTKTMLTKKSVKDKQNILTN
KNTMQVIQTFDSSQKDFQNNLHYTKMYVNRPNVNISNCSTSC*
>N.PF10_0095
MSKDNTKRSIDFRSTYNKNNKRTKVEDDDEDEKKEGNIRNIIKLLNNYCNHDDSLEDMSDYMVSSYKNFLIEC
IYKEIKSINEEKDERNDKRDDIIELIRTCLKIIKNNHEEFKYSNIYFVCINTLFLLLNNITQNNNYHNNYYHN
NYNDVINCMIYSCFDIFFNLISTNNWTRKNLHIQIFSKLNFIHFFIISNIFKAILIQVNNTIYMLPFNHYINM
IIKNINIFELDDNIIGKKNKKEKHHEKKDDLFNTEDSNIFITSLKNRNNILQKQSDPIYDSNLFHCNDVSSDD
HMNDYFRTYTNFKRTSTYIIKEKKKKVDNSNQNNMGDINNMYIINNVYNNYSVMLKNYDISYTHQIYYDILFK
IYSTIINLIINNDLTIYIIKECVSIILQMYDISKKQHQIIFDILTVYKFYIKNKQNMLERYIIFIFYLINAIH
DEEKKRDLSKLIKPSNMYFYLLHAECSEIRICVKEYLLLHGVYNKNKEKEIIPSRDNNYNNKYNNNKYNNKYN
NYNYLLDLLFEYSFNQSHTCNMTNISNESNISIQFVKAIHLIFFHFKYIIDIHKSPNVQNDELIQQYDLFFKD
LGTDPVFKNYLENDIKMKKEKIFYKCNSIISSDKYIIIKNNIIQIENLIKSIDVYMNLLNGHNIKYVSLCIIY
FIFFFIKILKYINKIYNEDIQGNFSKKNFFFIIKNLFIQSIYVFEILIQRKTWLQINNLLIYIIKSHGKKKNE
NKRLHTFYKYNIINKKYIINYLKNISTFDESNEQPLNILIKKLYLTIINFLIPLGYKNKIQSNVLSRHIIDDS
NNNNNNNNDDEDNLDDEDNLDDVGDGDEMNIMNKGIKTKWPFFNISNFYFFNKLLLNILYFFYQNNMGENFMG
IFFFDSIFKRIKERIILEEPDIFILLKKQKIHFNEVEYDNTNIVLKYKKYISYELSSYISFCLYFLKKIILNN
DKLYDLLNCTKYKKRIINLCKLQLCLYEFFLPTKSTNNGFSLSLLVLYHIYVKIYNNKNMDRKMVHLILRLIF
YATHKSLNTMERDNIIIYKNDRNGERTMEKKNKIHNNNICNICNNCNNFNSTHLKTRFCPKENNDKLFIINKN
FQKPFEEYYLNISLHVFITGLNVLTNITKLIILKYDIDYKNNIIIKGSNILNRQKKNKKKRSSIITVYYRYLY
KLYNVLIKKTKDDFFYSTKKYVLQYIKHFIILNTVLILKYKFRNYINFKKILSFCLYNLYTDYDVSVFSIIYS
IAFSLKSYINLCSSKIKNSNRYLERVDDLLFKVVEEYKSYDIPETNLWEEYISKYVSFETLKHTYVDCPGE*
>N.PF10_0096
MIKWVSKGTGLYYEGRRIYFRCFHKGKEKMENEENSIINYRMILGLKKIDINNLSSLCRDFHNIFRINKYELL
KYAHDILPFVKYLRTSEIVMILHHYSFINYTNVHFYNNVWLQIKDKLHDMDCKELALIIYSMGKIKYTNKDMI
VFFEKEIQKWLLKFSGRDCSLILKGLQNIPCNNINNKIYDLIYEHILNIIDNLNILDICIILNTHSKCINNNK
NINNTSNINSIHSNNINIRLFEALLNKSLTFYTQLNEQCIGSLLWSISHAEIKAEKYFSLLARRLRIVMYTKL
IADGKIVNEDTKEDNKTKEDNKKKEDNKTKEDNKKKEDNKKDEDNKKEEDNKKDEDNKKEEDNKTKEDNLHNG
NVQTHIDLPQCYNNNNILDNIYTNNYEHDNYQLHNDSYNTPSDNPNDHCNPLNNHNTCHHENVIPSIIYSYGK
QRVYMKHHIDIDKNIYNHIINSYPVYDNIHNKPVEKIYMFDFSKKFLRDQNIFNNVVNNENNNNNSSNNKIIK
KYKSIKKNNIYYQDIIYIINNFLIHFLNKIHYNDLKNILFGITKIEMSINKILLNNIYELIILYTQKGLYKNY
EVINLLRSLTLIPYFNKDIWIHVLEFYKNNFLTKHTNIKNHSYLFYIFSFVKNHLQNYPFHHQLIQHINKNAN
MLQRDDIIYIIKALLNSNYYDNELVNNIFNFIQTNFKLFNIIDIVYILKYCTIHNLRNTNIFSLFALTIKEQN
KKSNRNIISLILSFYLEMDIYPKIIEDILMEAKQSKNRNYYLEEIQCDQ*
>N.PF10_0097
MCSNGVYQLKNILLRYSETGHSSRNVRFFLRYLLPEYKEENKHLNFEIHHEQYEEPKVIFEYINNTKYEISLK
DIKSKHIVDIINLYKDSAGNNDYLKHGGPKVYSNRRSIQGLWCPNIYSELNAISYLKKKKKNNIKLPKYTRES
LNLNHDVIKGYGRWGNENLFPKGFDQRYLKNIFCFPFKDSLPKKMEQNNAVESKEAEINSFYKFYKN*

>N.PF10_0098
MYDHKTFKDLLQFYDNLEKEEVLVLFFHSTLLDNNYIYELKSEKKIVFSENENIEINDSYVKYKIDKRNKDLL
ITRILIHPEWRNNNHNYNFTYKQIHTSDTYNLNILKIGNSLVLQLIDIEDPSSIHSCTINIQEYINKNITDKY
INVNNYNEYIYVDKLEKIFQTNILNYMNSKNKNKEKIHINKNNTNDIYNNKCNTYQTQNITMKPHPQNCDKNY
LLHNFNDTYFDDKNPPLLTNHILPNLNQPHPVLKPDGLLVGPNNKFFNPGKLRYDPMGPFGNEPNSDNRPFEY
QNNFPF*
>N.PF10_0099
MWEEWDDECFVRNFFNWNIFSKYFKDNEFQELEEKIKEKYSNIKLDELHKKDDNENNDNVLLILHSFIKEKKY
VDASDFLINIILRWNYYTSLKSEKELNLKAVKNRLKMNVDIYTLPDLYNNFEEYFYSYFPLFLIETQQLINNK
KENEDIIEYDVNIINDIKETYNYEFFVNISYDSIIHANIFFGDLVLLKFIPKETAKNDTQKKYTPLFDSSKKK
SDVSSNYSCSNQNDKISTDSSSVCTDLEDQKNDDTCGHTNDHTNVHTNVHTNVNTYDDTCGHTNDDTCDHTNV
HTYDHTYDHTNLEHSCKSKQNSEHVHPNHETHYDNNNNATINNKKNSPLLLSRDKIDEVTQFNSDKKDDLKDR
LKNTKESIDCKNGVAKNHKEISDENNDDIYMVRKYCVRHLLGYVVAKSRENIKIKFNLNYPNFDIIDKVRKEI
IYEIFNNEMTMHNYFIRISKLTSLISTLRLFNCLFNFRNSCILNEIVEVNTNDDEVNVHDVVIQNEQPMKKRK
RGEGPQANSEKGELMKGCKDNNKEKEKDEETDLQDVKDEDITQNRNIHMSDINLVNMNQEELHNIQLSDNITE
EYLKEKIKNYLKSYMCDDFKNKDMMNKMSNERNMTRALNNICNYDEHNHIDNKKKKKNVSSIHSNNNKNEMIQ
QNDYMLEPLDEYYKGLEYIPELLRNRFFSMYNKYQLRAINNSILNDGITLIQGPPGTGKTTTILGIISALIFY
EKDQNVQADIIQKDKSLETRKSMIDEHPYVPFNVSIKKEKKSPYVWLNYDKNDDYCYMNDNCFDAMEYEDFHH
IEENQEEEGKHIFHINKSNLNEKNDAYAIHLSIMNASNRTLKVDESVDVNHTNDENNDVYKNNENRIRNLISL
TESYYYSYVNNDNMLKKNLGGIEENKNEKSNYSYYVSESKEIVKNDCDGDGDNYNDDGDHHDDNDDNDEEPND
HIFKQNNDNLNFKFHGLKLEDREEKNMLQNMSKNMSQNMSKNISKNITKKQKKCNQLNLIKNKKILVCAPSNA
AIDEILRRLISSDLGILDENGNLFNPIVTRIGGNVSTDLLEFSLEFKEQLYFYLNKKDENKMIKSNLLKTSTI
ICSTLSSSSNTSLINNIKKFDAIIIDESSQSVEIDILIPLSFACNKIILVGDPKQLSATVFSLYAKKHKYARS
LFERLQKKYLTNERKYNLLSIQYRMHPEISAFPKKCYYKNKIRDAPHFLFTVLKELEISKYIKRKKNTKSITN
ITNIKNITNITNIKRDDENESNDEVQNKYLEHLLCNFNLVEFIKKHMGITSTSLNNIMLCQENQGLLDWFCIP
LLQHSVFYDISFSKQKKIKNSYINVEESEVVIHFIEFLHYIFMSENMTQWYKRIGIITPYATEKFFLKKILKR
FFISKGYSKNISNFIDVGTVDGFQGTEKDMIIFVCVRTKGNLKRKKKKEKNNKNISKNVNEDITTYFEEKNIL
EKENQNNELVTNTNDELILLSSSSENGNDEEIDSSNLFFSNYKRLNVALTRARFNLFIFGNCKFLKHCDEWDK
IIEHYKMNNKIIKIKRKKFYKKINNNNNNILNHMNEEELFDNNVEVINKKIENSFYNKNIIDYNFEPIYEKNN
SSFDFSKYMDMSGEELYEIQKNKINNKDNKENIIDHKIEDNNMDIINFFKELYMDDTSESLMEYDKNKDTYDM
CINKGQQVNNNNLFQDHHNVLNEMKDNNINEDNIGNIEKGNVLTEKINNNENNKYDEENNKGISLNNEHFEND
TNDVLNSDDMNTKQQKKEDNEESYEKSKINSLSNKNNNIYYLKDNNYFIDTLFNYCKSNKDLHFAVQTILPNF
SKRFLSNSLD*
>N.PF10_0100
MKLKLNLAKKKKGFEVPGLDGFKNVVNKLLGTGVERYFKIVNIAIVFLFLTIIHYIRAYNKRGSRYRSKNALY
MYYGFLVCLIGFAVSINWIYIEYVKNKKKKSNSPLDVKVVGKKNK*
>N.PF10_0101
MSEKKNNKKKKKKLILVQENGVRTNSLENYTGLGTCIFFRFHKNKKTQEKYYGHFLEGKKHGYGQYIYYNGDV
YKGTYEHGKKSGIGTYIYNIDKKRKKKRNEKIKASDDKESDDKESDDKESDDKESDDKESDDNESDDKESEDK
ESEDKESEDNESDDKERDDNESEDNESDDKESDDNESDDNERDDNERDDNENHEEDKSDSELFKKNKFSKELM
AHIKNMNKKKKEKKKIIKLPLERESCYYYGNYINGLKHNEGMMVYENGDVYIGMWKFGEKNGHGKYIYKKCKS
ILEGNWVEGYLSYGKWTLPNGIYFVGRFRQNKPTGEGTWIFNDKTQVNVFYHEINNEKSKKAKEATYVKNSKM
FLIFNSYCITSTGC*
>N.PF10_0102
MATEERVKLLVAARKGIYEDVVSLSKLQIPLGDYVQPPHNRTALWYSCRNGSLKMARLILKKGSNINHKDSKG
MSPLHICVKYGHINIAKFLIENKADIDIKDNEGQTPIFYAIIYKHYDIVKLLIENGADVQIRDNNKASVYDYA
DFNGKTQLSTYILYKSNEIIVEK*
>N.PF10_0103
MEDKVEDAGSAFVDLDKIVNDDSKQLFDFGEKKKKKKKKEVVEKVEEIIIDGTGKVFERGAVYPYDELLHRIQ
DLINKHNIDLCISKKYTIKPPQVVRVGSKKVAWINFKDICTIMNRNEEHVFHFVLAELGTEGSIAGEGQLVLK
GKYGPKHIEALLRKYITEYVTCQMCKSPNTTMEKDSRTRLFHQHCNACGAKRSVTTIKSGFHALGRGERRKAK
HTN*
>N.PF10_0105

MMNKSCFSKYIIGTRCEKNVCIYQSYINKRLINTNVQLRNPYYRKKGFWEWRRRIIHRYNEKRYIKKGIKPKF
VNNNKKEENPIKNKNDDVYWTFKVYQLKISLRNLHNLGRLIKGLHLEDAIVFLESIPQIRINNILNSLLTCKE
RIINNLNGDISRLYIDNVQIHYNTPMKYIKYHAMGHFGLVKSYRNIFTYTIKQMNIEEFYHKLFIRGNVPRTL
SHFMRLYLKQKRINTNNLIEWYPYICANSRYFFRQKLNYLNNIYQFNYYKSRHIWIQNYFNNIERRNRELQQQ
RGIITEY*
>N.PF10_0106
MDFKNAECIWDALNDLHKNDKEAYQKFIKKHMNGIQANVVIKPKYVLCVKTNVEKVIIRTSSNEYKESVCNYF
VFIYYTSKMKAPVLEDNFINNVDKINMDNIYISIAKTEGESSKDMYAEAVVHTLIYKNMENNNFKNKIILRIL
QLVNNYENKLNPDIYINTKNYKIIDLHYIPKTKHSLNSHCVNENIKKEDNNTQSIDESDIKFFNLLNQKQNKN
KNHHDHDQNKNKKEIKILQENKELLNHIESVKTTKKENDETNNIHNFIPKQIKSYHHTIIDSFLHIIVTFNNI
IYDSLQVLKLKNQIHIYVSDKYGDCMTLNFKEKLSDNVKAYFNEKLLKLTISIEILY*
>N.PF10_0107
MGNIVSCCSLDENKKYLNDDEILETFSNSEINEFKKRLKNNIQIVVLLQDGTKLPCNLQANFQEKTLCISCHQ
KVRMINFSDIRSLLYGEEQLKRVETQANLINDNCCLALHLDDSGNCIPIKFGSVKEKNLFIFIMKDYKKNS*
>N.PF10_0108
MYHSNKKKSNKTKTRHIHMIRKNFYELNCEQNDRRNRHINYTYYINMETTIFEMLLDNNIESFKNAGKLLFII
LSGISPSTLYFKRILNLIHFFFHKGGKRFYHSNIHFDIILFLNNYMKSHSISCSIYIYNLLLCNNNYKDFENY
KDIYIPKHLIASFMDHFYFVVKDFLLIIRKFLINYEQGNISNEINLEGDYEENVYDSIYGVTSNNNNNNEDNY
EDNNEDNYDNNNNMNFINDDNMYHHMNDINMNNFNNMSNPHDNDNDNPYSHNNYENFASNNSILYNNHYDDNT
FNKFNKKGMKKIQNKNFDEKIMWFKETSEDMIKNFCNILFKNTFPKNFEKKLKYLISQCTRINRALHSIYLDI
FILCNLKNVIKVLVLIDQLFCSLFPYFYFYELKLKILLFFIHKNDNQKESKKKIKKKKNNDKQVAKLMYPYVH
NNKDYVDKDCVNEDYVDKDYVDEDYVDEDYVDKDYVDKDYVDKDYVNEDYIDKDYVNEDYVNEDYVDNNYVTH
LLDHNLDITSNSAYDSSRYNRPVHHINNEDIDISSNIIKNNNKMNISNDSFINYSNEQVNDEFDCLYSSASNE
SIIPLLTEQVKNQKEEEEDDKNVYLDKFMNLYINCTEKIATSVPSISDLYDNKALKNLTYKMSNKRKIIDDQF
ILDYIKNSQNNNERNIHIEQDDKGIIIKRKDISIDNISVDEKKDDIIITKEKQHKKYYNQHVNDPLQNPEHNP
LCRKNKKQKTNTSSYPSGVIPTINNIYENTYHDIKYNLKLIVNISKCLMFASYYDPIYIKLFYSYLLPCISFD
KRIEIFLIYSYSSYKIFKPLIFLIYYNNNSPLYILNVFVKIFKRDNIIFQKFKYSYFNGIHLKNVPKKYFIFL
LLLSPFFYDDKVECLLYIYNLFYNYDRDVINDQEKDINYKHIYQDVVLHNDEEYDEEKRNINIIIIKFIEIFK
EKFNVDLYNYFDFYRIYFILFVSCVDMK*
>N.PF10_0109.1
MDFNSRKKKKKNNDLYEENINIYLEENDNYILDLEKKVQSLKLVRSNLRDEVRTSNSLLDNLSDRMENVNKKL
SGVYRRVKNILRTKGNNYMMYLILFFLFLLFFMHYLYRKNK*
>N.PF10_0109:mRNA
MDFNSRKKKKKNNDLYEENINIYLEENDNYILDLEKKVQSLKLIGSNLRDEVRTSNSLLDNLSDRMENVNKKL
SGVYRRVKNILRTKGNNYMMYLILFFLFLLFFMHYLYRKNK*
>N.PF10_0110
MICIKCGSSNSNLYTVYNKNNIRLNECNRCNKICDEYVEKNRFIIFLNILFLKPEIYRHIVFNRIKYHDKFIN
NFFLKMIIIFLIINAYLHPNFERENIKNTGILSNIFFMENNFEEYIQGTDKYKNDCSSYTLFIYKYDDKHKLY
GLYNIFNNYNMSNLVNIHKNKYLTCIVNNKFSDYNLCIVNRQFKAEDDYDKELINIFINNNGKYIKKYNTTVS
TNSKLHKDHINKNNINNDNNNNNINNNINNDNIYTSYKDNRNNKNIWYETNFFKKISGSQILYYILHFINKSI
SKIKYILKYDSIFYFKKNNTLLKNNIINLKNPEEYNDLFQIINILVYYNIHNYKIVLEPKEKENLNIDFIFKF
IKNIFFYNQFIQKKNYIQSQTNTYMNDPKKKNDLQNNCVHQSEISTFNNFTSTNNHIKNQDNNKKMCHNNNDN
LHNIYPFDDLCNEFLKNINSTLNKKMAEITKKQNKIQDTFQIKQISMYSKLLFSELDNEKYILKICNSTFSFK
KFKTVIISYITYFLFLCIFTYFFKLYQQHKYNIKITMVKYNYLFMLFVLSNYPLVIYFILKIFNYNYINIYLN
IYTIVCNIIAYHIFISTDQNYICYSIFSVLTSYLLKYLVMLKIINYI*
>N.PF10_0111
MVIASDESFMNEIDNLINDVEDERIDNDELEFCVAPVNVPRNFIKYAQTQNKKLFDFHKGTTTLAFKFKDGII
VAVDSRASMGSFISSQNVEKIIEINKNILGTMAGGAADCLYWEKYLGKIIKIYELRNNEKISVRAASTILSNI
LYQYKGYGLCCGIILSGYDHTGFNMFYVDDSGKKVEGNLFSCGSGSTYAYSILDSAYDYNLNLDQAVELARNA
IYHATFRDGGSGGKVRVFHIHKNGYDKIIEGEDVFDLHYHYTNPEQKDQYVM*
>N.PF10_0112
MGWFGKMEKCCCFPLAGGCLGGAMFHFMICITSIFSTTKDYKNMTIASNAILGCLIVLGLVLKNFIVLYIVAL
FVAFLLGIYIIIFVFLVIALFAANNMPFQHKLLTALTVLTIVLITASFLNIYISTCRVIKSGGTGWEYKSYME
IEKEKQIENKEKQNQKKKEDAMLNNDYNA*

>N.PF10_0113
MLMFMNVFKRNTGGHTIKYLRLQNRFISNLRGNGLYKKEREECVSYENGFVNISRMNSSVLCIFSNMLLKENV
QNNLLWKRIEKRSYELINDFEVNEIASFLFCLSKVRYDTNLYDNFIPLIKRKCEYFNTSNLAMLISTYSKKKR
KKNNKTEDDSIIMLLKNELKKKVHTIYNIVEISMILNALVKSNIKDNDLYAKLSNIIIDNVMYNNVHIRDLCV
ITYCYANILYNNDNLFIILSQRINQLIEQANLVDLCRILYSYMRIKKNYNHILKSCTIQIKDVMYNRSSISDV
INCIHFLPNLKEIIDNEQEEENTFFEDYYNGNKGIGNDKICDNTYNINCTNVHNMNHPILYDRTCNYHIDYFN
LYNYVIHTFNKYFISYIHMLTSKQISNILYIYSRYNILICPSTVHVFIEKIKNMQLTKELKIYILYSLSILLK
NYELKKYILKYDEKKNNTPKSYDMENDVSKSDKSHACMDDNILNSYAINNSFENNKSCDIFKKKQHNVNIYNF
YELDINKIFFMKPNPEHIIKENENIQNRKNNFLYINNYNFVKLKNNLIDCLILWEQDINDYINNHDFSLTQDV
IRRVLNIFLILNYSHASVIYSIRNYVILNYKNINEHNAYMIMYYFQQLNKLNDDDDFAEILTCKMKSLK*
>N.PF10_0114
MKIKVRTLQNNEEEINVDPDDSILDLKKKVEVVLADMPSDKQKLIFSGKILKDEDKATDILKDNDTVIVMVTR
RIINKNNQKEDINKESLSKIENNNNNNNNKSDDNINVTTSNTEEQKENKENKNDNTNDNIYNSFNNAESMLLT
GDKLKESIDNICAMGFEKEQVKKAMILAYNNPNRAIDYLTNGFPNENVNVNVNENINNESNFSNLLNSENNPL
LEENSSHPLSSNEETFRNSTFFNAIRDMALSNPQRLPELLQMIGRTDPSFLEYIRQNQTEFLAALQNYGNNIN
DHEEHSDDNLDNADDENAIQNDSFLQDVGQQVLSDPNNENINIPITPLNENEMESIKKLESLGFPKHVALEAF
IACDKNEEMAANYLFENMNDFTSE*
>N.PF10_0115
MGRKATTSSILEEKPVNKNSGVVNNMKDSLMNDKKQGGSKLNEKKGSKVTELTSNAHMNGSIQKKKEENELEN
NKKLNHKNNLEENCVIKSKDVVKGGDQKNEGQAKKKNNNNKKKKGTKENDMDKINQSGNTNDDKKKANKNNTT
EGNEKNKGSNNKKYVGNKDENMKVELMDVTKNNNSTNSGINNSSNNNNNNKKNNNNNNNNNNNNKNKNNNNNN
NIISNNNNNNNNNSDVKKDWKKEIKLKEDERKKSENDLKKKFVKLNKADNYKNINDKKEAEIKKKIEIENFIS
TINSTISRFNGQTTRPINMKLSSADMENKCKDAKSKKKKINPKNLEEIEEANTYINYLEEQLSITKNYENFKN
FQNRLISLRKICEEKLKENLKSMNETKIQEKKLRFVDKIIKMKKEKQNVDITEADIIRKDYAIPTDTYNILMK
PYNFLNRIQTKYFVYVDNNKLSLTNNNNMMNNNNNNNNNNNNTFINSYDNKTNLHIAGCKDDIEQFISYLQNV
NFNSENKNFVHLNNKIFKIMLNMYDGSFKKMEEDTNVFVHVDNDMLYYCGMKDDIENLKELIEKATNNEKNLL
NKNISKNIKLDSILARGFNKSILKEIETNTNTLIRMNYDAKTFDASATIKGSKNSDIEEAEEKLNEILKNLDS
EFVPFEEKEIFSLYKKCSYELNEIRKNLNLFIIRQDNGLSLVGKKDNITKALEILEYSKNIISNKTVKKKLTE
EEAFLFNSNYRGQIKAKTGAEVKIFNKTNHKELNISGNKNDIDKALEMIEELLKKRKCTQVDINEKVIALLLS
SKAQKIKDIEKDTCTSIQINKTSHVAQIYGHEDNIHLAKDVLENLVQSEGKEGKEGKFIPNNLYVTVEMNVET
EHIGSVIGKKGKTINKIQEDTFAKKIHIDKENKKIYIHGTQKTVDAAQKEIQKILNRSKEEMSNNNNNNNSSS
NGGAFNNSYHESRYNNRHSNNSSSAHHRKSHKSTSRSAPRSSHKSESKSKGVYINTNDEKAFPSLHDVTNMQS
KKNKKLSQSNQTTNTKKHEQAPQIDKQTNKNEEDKENQPKNEVHAETDVTKNETQEKEEQQQEKKKDKQEENQ
ELNQTEKEQEKEQEKEQEKEQEKEQEKQSNEN*
>N.PF10_0116
MIRKIIILMFTFFSNIHNEKIYHHKQRRKFLKGPLGYLNRNVIQKKHYNLYAKKFINYKEIQIQRINDYRKRS
GVDKNNINYNLRDTYNYHETHLFVRNEVLPTLAKIENENLKEKEKNKEIFRNINDYNSNFTRQTFLQFLMDLY
NIFLKIDDLFLENKTYFSILIYNGPMQLTNHLYDDIIYVSSVVENSDDVSPSEYCMEYISHLENLCEENKLSF
LAHAYVFYKNFHLSKEHLLKSICKYLNIIKKLKSSTYVADVENFEFCLNKMSRKWSRWEKDNFLASLHNATNK
MMILTKHFEKVKS*
>N.PF10_0117
MNGDHNKYVKGDISRYKEKNKLNKNIKLEKEILHINESDVEGSHKYESYESCDISVSSPRNINEEGNKKKDSK
KNKKGTKVNYLVNYNRYKPTRIYLFKKRCTYPYVKKNINQYINCNFRYYVKEKDYLFQNIDEHIKWDQIEKVD
YIIYDNTYLTCPICLEDNIISPRITKCRHIFCFFCILKYFIDEEKKIWKKCPICFEIINENDLRIVKFHYVKN
YNINEKINLCLLYTQNNKIHLKSERLYFNNNFNITNNYFIKDNKVIDYKYFVNINYMNENLTTVTQSYSEQIH
KLNLNCGVQFSKIFYIYNPLSIWIKDLCIFNFILTNNNFKFLASDHNVIQKAITNIKLKISDYLNIPIDKLNP
KLKMDQKNFDAFYLYDNLAHDVYDSIEQIKNEMKLEMELTKNQRDSNFKVPISNTSKKKNQDVTDSKDLNQIY
FYQCIDGQCIFLDPFILNLLFYEYDQDMNRLPKFLCNKEITHIESFELDEKIRKRYAILSHLPLGVNVLFVSI
NIDDLLSNRTKEHFSKEIAVQKNKHHNIMKRKMKEEKYLKLLADEEINRKEKNYWNLTSNKIVMTNTENYLSS
SITRYSKEEEEKYLHGELYEQHSYEHSNENSNVYLPGQSYKYVTQHSYEYLPEQMYKENNSLTDENLLLKRMI
DLDHLKIKKESNEHSKYMENKNSVNESSKKKKKKHAGNKSNSISPRSNVDINNSNEKNEELPKRSFLEIAKK
KCEVNDNKIQMEKKNDENLSIGSGPVSINLLDLINNKKSKKKKKNEVTQLK*
>N.PF10_0118

MNNFQRVIYASRFMRVFGKFKRAQLREKPQRKIGPFCNPVKRLVRLKEKERLFCEIGLPRMIPRAKVRESEKL
LEVIEAPKVKKEVLERRLEFLLSNESVYQQLYNKMYNGITPYQCELYMWERQMRDLRKVYRAQYLHKLSEVTN
EEREKQLKLLLQTKEDKRKRRQEIRERILQDKKRRAILKDRLCLEKKVTQSILFKRQSNRKKKNIYWLMKLQQ
KSTYLNELDFKNKIEQTNQENDPLFNRNISVSSLYKNLGYQTKEDKNENTKVFKVDKVYRKLLEDSFEFLEED
EEQYEQNFVENINDPNHVSHKQRAHILYSSFTNEEKIKLLDDKIAILTKTIEQKSFSKEVQNNDLMFYIQLKD
KLEASKQAFLEKNYLKDVQKVQ*
>N.PF10_0119
MKRFFVLFVIFLVHIWSENVDTFKCNYSKKKKNGHHIKRHITNDEEKKKEYSFLMLGKENEEENKENKENNQN
VNKDNNDNNNNEKKNEEQNHNNEKKQEEVINNNNNNVENKKEEENHNNDKKTDEQNHVNEKKQEGENNKHKKD
DTIPPEKKINKENLLEYGTHDKEGHFIPSYKTLTDEILSTNNAMEKASSFLKIACSHVMKLIEFIPESKLSSQ
YIKVDNKNIYLKDIAVECQNIYFNLEKFSMTLIVFNSKINKFIYSQEK*
>N.PF10_0120
MSLHKEICNYIVKFSSKPVQRLGYEPPKKKRSILRELYHKLIFPYYFKFIRAPYERWQFCATTKFLREHGLMY
DDMYSDKDPVIERAISLLPKDIQTRRYRRMLRGTHINYLRLFLHPSEQNYDPYIPYLAPYIEEAKFQLQEEEE
LLGYHPYDRRLYSGGTTGFGDLEPGLHFLVSIPNLYGAAIPHSKKK*
>N.PF10_0121
XXXXXXPGAGENAFDPVFVKDDDGYDLDSFMIPAHYKKYLTKVLVPNGVIKNRIEKLAYDIKKVYNNEEFHIL
CLLKGSRGFFTALLKHLSRIHNYSAVETSKPLFGEHYVRVKSYCNDQSTGTLEIVSEDLSCLKGKHVLIVEDI
IDTGKTLVKFCEYLKKFEIKTVAIACLFIKRTPLWNGFKADFVGFSIPDHFVVGYSLDYNEIFRDLDHCCLVN
DEGKKKYKATSL*
>N.PF10_0122
MENIKNEQVMKFLELWNIFKKPQYLIDETIEILKKNDEEELKCLFLKRLNFGTAGLRGKMGVGFNAMNVVTIM
QTTQGLCSYLINTYGLNLCKNRGIIFGFDGRYHSESFAHVAASVCLSKGFRVYLFAQTVATPILCYSNLKKNC
LCGVMVTASHNPKLDNGYKVYASNGAQIIPPVDKNISNCILNNLEPWKDAYEYLNENFYLKDTSLVEDIYFEM
YDSFMDDLKHEFNFNCYRNSRTKLAIVYSPMHGIGRKFVQGIMHVVGFNNLLTVPQQALPDSDFSTVSFPNPE
EKGALDMSMELADKVCSPIVVANDPDADRFACAEKFNNKWKVFSGDELGIIFAYHLMKQYEKKNIDKSKHVFL
CTVVCSRMLKKLCEKYGYKYDETLTGFKWLINKAIEYNEQNYTILYCYEEALGHALTRHVKDKCGISALGYWI
EIAVYLYENNLTFHQYLETIREEIGYFVNNNGYYIVLDSNDIVSIFNEFRSNGLYKTNLGSYKIIHIRDLTTG
YDSTTADKKSLIAPTPDSQNITIHFENTAILTIRASGTEPKVKWYAEISRDTYEQAKKEIDQLIDEVMPMFMQ
PNKYNVKKS*
>N.PF10_0123
MEGEEYDKILVLNFGSQYFHLIVKRLNNIKIFSETKDYGVELKDIKDMNIKGVILSGGPYSVTEAGSPHLKKE
VFEYFLEKKIPIFGICYGMQEIAVQMNGEVKKSKTSEYGCTDVNILRNDNINNITYCRNFGDSSSAMDLYSNY
KLMNETCCLFENIKSDITTVWMNHNDEVTKIPENFYLVSSSENCLICSIYNKEYNIYGVQYHPEVYESLDGEL
MFYNFAYNICKCKKQFDPIRYHELELKNIEKYKHDHYVIAAMSGGIDSTVAAAYTHKIFKERFFGIFIDNGLL
RKNEAENVYTFLKSTFPDMNITKIDASENFLSNLQGVTDPEQKRKIIGKLFIEEFEKAVNNIDIDINKTFLLQ
GTLYPDIIESKCSKNLSDTIKTHHNVGGLPKNLKFKLFEPFKYLFKDDVKTLSRELNLPEEITNRHPFPGPGL
AIRVIGEINKHKLNILREVDDIFINDLKQYGLYNQISQAFAVLLSSKSVGVRGDARSYDYVCVLRAVKTSSFM
TANWYQIPYDILDKITTRILSEVKGVNRILYDVSSKPPATIEFE*
>N.PF10_0124
MDMKNIYLPKGIRLPCKLKWTVDNNSMVSSNQIIAFIIEENKEVFTGEENQEVEPSIKEEYIKDENNNNKNNV
DNNNIENKKGNDILFKQDNYNEINNKQICLKDEHIVKDINSTTFNQNINNLHDDSNKTEENNSSPDNNNHNEN
NNDFIKDKNILTNDDKNKSIINDNDVQSDHKNNNVKKKEDTNVISLDSKYNEKKNVINFILNNRKNFEIKNNP
IVLRSNNNGKINILKNDDNQEYIYINNENELLCEVIDVKCNHEIIFSGICTNCLLNQEEINKSEEQKYFVTPS
FLPGQNELYINTDKAIDLEKERMRTIINKKKLCLVLDLDNTLLHASFSLLSVNVNSDIINITTDINNGILENE
INYIEELNAQVVSNASNSDDTKSNTKNKKASPLVCKLNNEKILSMNNKNKIEHENTQNYMDRNDMQNININVN
NNNNNNNNNYNNNNNYNNNNNYNNNNNYNNNSNKEDDALINEDIIYPHFCKNKNSIEMYPKIEDIKASYQNYC
EFLEKVNTINLLKHKGKYIHYEDFNDNQIKRKIEKLESSILKTNVKYQKGAYIIYYKLRPGVIEFLRTMSEKY
EIYLYTMGTLEHAKSCLFLLDPLRKFFGNRVFSRKDCLNSLKHLNKILPTYRSVSICIDDSDYIWKENSSCIK
VHGYNYFPDINFFEDIKRAPYFLTKFFTFAQSYLNFTSNIYRFINFKCSEHEEFLKYCKNNIMLYNMKNQVHF
SNVETTHNSNNYDGQIKEDVYCTTENNEVEKNELLPSMKTNTFIEPLEIKNKNEHETSLNRNNSLNINNEFVE
INDNNFIDLDKEFETDNESDLDLEDDEGDDIIDMYNINNNNNNIINSYNDTYHLDTRNDLIELFDESYNYDK
ENILEDNSNYLDKQNDIYDNKDKTLRKEIEPLQSLSYDEDNIIYDENIILQTVENGIIENNNVNLTYEENKIS
FEKEEETNENVLKNNTNLLEVNKSINKKKKKKKCKKNNQIINSDINTPFLRNDTKNYVQPIPLLKKSVHRNIS

HEDEIILKFISEENFRRYEKYVLDFLSNYFEKNENHTMKSQESSQVIEEEHVESSKCEVTNEDAPSDEDISYE
DDFEGEFVNLKDQEDEIHMFLDDDFEGEFVNLKDDKNDEDYTYMDDYMDGKYSNLKHQKDEERIYIKDEKNKQ
EIENNKNNKNNKNNKNNKNNKNTYKKNSDKNDNPRNDLKKYRMKVKKKIVKKINKNSNVKIYKSSKHVNEVNN
NTSTNNNEVKQNNEYYESFFIPKNLTEFNFKDNDKQLYYLMSLLNEIHDIFYIMLEQFKRKETNDENRDDQIY
NYFLRYPVVRTILTQYRKQVLKGFTFNIGLLSDDIKRSDFMDNIVKFGGLINNQDYNHLLTVNTFIENENIKN
VSTSNLMWLERALYTWKSTNPKYYDMKTWEKLHRNFWDVIEYEENKK*
>N.PF10_0125
MIKISSLRRKADPLLIVSKRLPSYIKKEKINLTRNSNVNKKNVKYYQIPQFVQELIKFTRKNKLWKEQNSENI
MDINNNNNNNNNNDHPYIHNIINKNKIHYVNNLLDEIPKHKNLLTSFLICEIYGCLYKLNYLKLDIIFLLFSI
LINNTINFFNLSGYVNCNFKELINITKYIYHFQNVCNSNIQNILHKKPNLTKLKIVERYLKENNKEIKSKQGY
YNYIYNYIQKYDHVDNIDILIYSLIKSKKDEKGYIHEKTKKNDIILDDENDITTKIRNMSNMSNISNMSNISN
MSNISNMSNISNMSNISNMSNISNMSNISNTSNVFFPSITNTSNYNNRLVENNNEMDSLIDYINSFYKLNDMF
NFLVNKILNYFNENSLNFDFHNLKLLFFFISKFEKFDSSLLENISNRLINEIEKMKTNPKLMDDEENSTNDPI
EKNITNHLNHNKLKRRLNRYNKKNRYINTFNKINSKEFLILPYTIGVSMNTYFNNYLIEYINIYILSLINSRV
NCDIQTFIYTLIGYKRIMVNFFILYNIFRFKEKCFQNENLLKKYKFFLNKLIYHDIQNMNQMSSNKIIKIKNH
MMDNNMESEEEKNKINHNKYINVLNSEQQKHIINHLNLDQNISVQSAKEQTLDHINNTSSKDELKMNQENIFD
LLIKQFHINLDKIMLINFDKNSLYQQYTSKDIYHFFMTYKKLFHKVYNYSIFLLDKHNINDMLTIYQHIKAQS
VNDIIINHVFIETLYNKIILNSVPKKN*
>N.PF10_0126
MVSLRLDNNRYISFNQDYGCLCMANEKGFKIYNTNPFIQTYSRDLTDRNKNGLYLAEMLYRCNILAITGNKND
KKGKWAKNVLIIWDDRQMREIAKLTFSSNIIGVRLLREIIVVILEYKLCIYRLKDIILLETLNTSKNVSGLCC
LSNIDKNIIIAYLSPIKGRVNIHIFEINSSENIHEELPYINFKTNLSIYAHDNSIGCINLSNDGKLLVTSSTK
GTIIRLFNTFDGTLLNEFRRGTKNAKILSLNISEDNNWLCLTSSRNTVHVFSIYKKKRPLRKVDIICKGKNVS
PPALLNYEKESKNKKSSLKCLLPCHPYLNSEWSFASYKLPGKKISSICAFVNDQNCIIVICSNGIIYKLRFNE
HIGGDMFKISSHSFD*
>N.PF10_0127
MSILSLFHICSPFLRSNCLEKEEIYENFICSVCLDLCDTPVITLCNHICCYKCMYYSLLHKRNCPICKQIIKH
NNLKKITGKQKKEYEEIRIKCHLCNEKIKIKDYKTHINICEYKRCKNYILGCEYYDKKSKIKSHEQSCEHRLI
SCSSCSNLFYFKNRIFMLTLKEKYQLNMRFSFTYYSFYYNLLMNTNFNRLNYLNNIKHHPTNNNSNNNYISRK
INILKNLQHSLDHILCPSVSIDTTTTTSSNHTNSLLNKENVYIEHHIPTNNVSRNSTNINFLQNQNPSSIHNR
PLNELSREIYEHNHHHNNYNNNDDDDNDNNSNRLNSSYSMEYNNISVNPLENISIESREKRKKKKTTKIKKYN
FNDLNSKPRNNALKHMDQLSDVIENDNSSNCEEFLNILPLRKNFNFKDKNSKDIITIIEELFQFDNIDLSNIY
IDNREFFLCDKSCFTNTIKKLRQELERSLVLLYFCCCVGMPVMFTLGCISYVVTKGVFKFSYIVTNTIIKLSH
KFFLKIFNI*
>N.PF10_0128
MSIISNIEIDMKFFDFYEGDKSVYFCKEYEAFLCLCDDNLYSFKIDEEKNSKYSSLYDILYPTCILESSVDID
DIKENYQKKYLNKRCISLFSSIRYYYYCDNNVFLSTDDNNIHHYVLVYNNNNIKGNDTYDDDIKNGEGVNNNN
RDIYLYERKEDIEGGGNGNAYSYSYNWIYLEKKKIWKSENIVVSCFYYKDYDDDDYKKLIVGYNNGLINVYNI
NTYKLKISYKIHNTRITNLKFYKNFIISSDVTKNIYIYDIIKRERIITCEDHMGGIIDLLFLEVPNGKNENKA
QQNEVNKDGIMINEEDDYISNDESNNDSDIGIIDNNNNNNNNNNNDKNDNHYNDEKKKLHEKNEEKLIGFISI
GNDKIMNIWDLNILNSYNEEDMNNLISNNNLSKKKKNKSNKFNKNNNLLKFNPIKQFYLSSDINHAIIIPSII
FQNENNDEKKKKKKKFIITDENIKWLILTHDNDGNLLFYNPIKGDVIYKYKENINTINPSNISKFFLLKYFLY
IFREDSSLLIYDIRYFKLLKNYICKLEGIFEILFFNPNSEQLTCDQKYSIHNKHADEDDEEEEEDMLYKTSND
EKKKEKKCVILLGDNIIRILRFKNNLISQRLLIGHEDIVSCIKLNEKNLLLFSASNDKNIFIWSLRNYYCLYI
LKDNLYPINAIDVNIKHFPSITLVSVCEDSSLRLWKCTIDEEKLKKNKSHNKKRKLEDIFDNKESIQSTLTIY
PHKVLINDIAISPNCKLVATCSKDKTVKIFETQNLKLIHILEGHKKSVQNVYFSKNDNILYSNSYECVRIWSL
NNFECLKSIQSLDFNITRVLILNDSCMINGYSNGNISIINIKNCEKLLTTNYHKDKIFCLQSYEDENKNNNII
SASINGELIFFKDISDKIITEHVFEKRKNIFYENCLENLLKEKKINESLLICLKLDKKFKFKTIVENYLNYYT
FSILNILKKYQHEYGIKQLINKDKQINNNDIIKKEQNFSQMKNLNNNTLIESSMDTQTSININEANQNIVNNT
GDYNNMDNNNFYNIFENSDFLESYIFKELSYMQKKGLSEEAMDDQNVKETEINKSTNADNKKDEKNDPSVKHT
NNNDSNDSNDNDYNSKEDDNFVLFLKKMKKKSEHSYFLYLNKLMEFITFFVVNHRCAYISNFLLNSIIKYIDH
EDICKINNYQYFFEVYNSYIPRHKNRYVKSLQKSKCFELININYYKGDIKMMS*
>N.PF10_0129

MNNENTMNNDDNTSLYHNLRKIRKPRSYDFEYVDDKTKKGRGRKKRRKRRRSSMGDINSINGNVNDTNKEENV
LIKIERTDSNIPDSNENVENKIEIKTECDINKNCYVSEKTVSTPNLPIRRYKTKKFNLEKSMNLFNTNIPLRN
NNIKIDRICEYLNYTTNTMWRYMGSTAKFRLVEDQQEKYLFLKNMKKNIIFEIIKIAMFALLKVNLNDNNIYT
LNPEEKKKNEFISKDMNSIKYTKREEYNNNNHNNNSNKINDNQNNDKTKGDDNYNTTNNNTNYHVNFHNNKSN
RNNYYAYYFYSKKPNNIENNEESNCDNNSVNKENSNGVKDASKEDETISDIKQNCNVSLKSNETNENHTNNYN
SMITIKISPACENENISYPYSHYDNSKINISRRLEEKDDILNQGVTVPEILDFVKEKYEKYYENVKQYIVTTL
NIYCALNIFKLIRRGRYTICKYENFDIFKMKYFKKYYKFFYSLKGEEEILMNVKNYLKSFYYDRNKKKKRRRR
SSTGIFGTKKRLLGDKRGSTYCLKEMDALTGTKNTIRRRRSSTKKLGRKGLSAVKKEEVTNNLAVYSYTNNNG
NVVDSNTIGDSSNIIDTNNIMNSSNIMNSSNIMDSSNIMNSSNIMDSSNIMDSSNIMDSSNIMDSSNIMNSSN
IMDSSNIIDSSNIMESNNMVDSNNNMDCSNKDNNKIDTHFNINIQNDDPNTNNNINNVIKCESSINNNVIQST
TELNNNNGLNDVKNNTPSYSNIKEDVESNLINKEENKSNILNNELLNTTNNDINKHEEEKEEEHAILNCNIVN
NNLLDLSNERIKNVCIDLSKINIKCIKVVYGMKHFFCSYDTQEISDPYFEIIKVQKKYDILRRKSIEKMNLRG
ANNPYPSSRRLLNIANNNNDNNTVKGKNNFLLRASVISSKDDPKKRNSQYGKQRKQNEEDDYAMKHEKYNYVN
FNLSYKTLRKKMICLRHYANANTNTNANTNANTNININTSTNTNINVYKKEENKTIKVKTEIKIKDKCLTPTN
ESLIQTSSINSNFSNIISVDGFSSINLESHCSQKSA*
>N.PF10_0130
MKIYRKKSTFLLIFEIFIFFCYILQNEFVKGSYESKGYPLIHLMNNNEYTGSANNFIKNNLINLLYIYKDVKF
DEEKTLGIDSFLDIEDDTKIERLIKEKMLYINKYINSRQMIKYSGFIYLYEIIEKKNFYFLLHFLMTNNNNGL
IIILPEDMYINVDEYYRINKINKLNDQNKKNKVVSQDLLEKRIMFVQSLILNMKLQQSIYFIKNNKDIEKIYE
NYQSKFAYFDLTRNVSISPISKIQNVYKMNSKSLYYFLNKNNNIHINTIFNKQQDNQTFLLTKQKTIIICADF
NVFNIFSDLPSNTTFTNSNLITIQNLIKLFYDVYNKEQVNYNILFFFTNYYYGIQDFLNSINVIFKENIEFVI
CLDNLNNSDFYIYESNKTQPQNVQAFYRILNNIVKGSIKKNVQMVNEKIKIHSKHLPSLHEYFVLNKINSFSL
SSSKKNNNMIFLNKFPMLQPKLNIQNVKNNIAIIFKSLYLYMTQKKTDQKENTQQEQSIEKNEKEKSMLTYIN
KNMSSNDDMNTIDYNLNKYNKFFIYKEDTLKLFNYFKTIINSNITDTNFLIKDYKLTTDKKQKFFYQKYAHVT
FSMAISYVFHYVHLLFVVIFLVLVYICVNYYFVPMFYKNKIKAA*
>N.PF10_0131
MNKENIEKLINIINLLDKISENKRDKNNYGEHNVNKSLSSELSKTLDSLEYRKSLFPLCDEKEKVNSNVNKII
SNFKTTKSFFYFNKSSSLNMGEDMDENMDENMGENMDENMGENMDENMGENIDKNIDKNIDTNMDENIYSNKH
LRKNIYLLSSNVKSSHIKKDNKNIMKYTNNVNINDNDIYLNTNQKNVKYNYLNVIQLNEKKYKRENSFSSST
IFSFIKLKTDNVIEQRNTSNVLRNYFSDTELLSYNNNHKSKYNLLKMLKRDELKKYEQEKFKVQNKGRQNQVE
KEELMEELEDGCIDNNTTGLLLEKIIEHLNINLKHILNNYKKEINKLCKLNDTLSGKLENALENNDEQAQEIR
DLNKKIHIIKEQKTEMNKIIEDYEFQTVNRKKLENSQQNYIKERDKLDDEIKYLKNELNMMKSTNLKIKEEKL
LLKDKFKKYKGKFHNQKEKLQNANELILELKLAQNEQITEKNLNIINNEYSKQRDIFPYEKKRKIIINRFRSD
TIMDNFERMKKKLFESNKRCYILSKTNLQLFKGIRNRKKKIYTMNREMSNLKHILLKGKEGMSRCTLLDLKEN
TAGLGTSNSNVSYNGLCKRCCKLKDENDTISEKSYNNKICNNIIDEKMLYENSSNFDLILNRKNNEYNLLKDR
YDVLYKKYIKLLTTYSCKNEDFTFIKTKTQEEMIPKKDVFHSFYVKNTKNNIEKCHSKDNKLISYIKAKVVNK
KDICKKKFKHYTESNNYRDLEFYISNQILNKLDMKDILNIRYVYSYGNKNVGKILCHNT*
>N.PF10_0132
MNLSAKIDSNGYNNNIVEKNDDKRIGRIMSDDENERKDIIYNNEKNKRTSEIKVLKNDMDNNKNDNSIQSKLS
FDIKNVLTHAYLPICLEKMKEGEYVVYKWNNNIFQKKTLKFFYLDVNNYCIRWNSKKKKLSEKKNPSLYICDII
KILDGSESLFFKKKEDDKNLSIEIISTQRNLRLTFLDIQRWKMWLFGLMYYQYKLVNKGNGKKKMKSFLYESN
KLYDNYIISGLKDINALTLSQLYIILRSLNIYLNMQILYHYFSIYKNKAVINYIGFTKILEHIFSNTHISIYF
NEYKDKKFNYIDKKKFIEFLIDIQCEGKCEEFIFNNYYQNDTNPNLTREKNTEKLEREKKNDEDDTHYNLDMM
QSSAHNNKIYNVNNQMDDISIKYKNEDDVKPKHIFNNDNKENCNKEENNYSKSENNTYFNNDNNNNNKVRNEA
IINNYDCNIIKKISNQDINQDDIKYNMDISNTLIFSDNSIIHDEDYFLILDILKSKYFYHDQNKVGNKKNTHD
NNYNNYNNNSANASRHHNLKDEKESKLNSNNNNNNNNNNNVDTCINVINKNIPNIYPIKVNVIYKLLNIIKKY
NIPFVINNDDNQYLTEIGLVYFLLSKENSIMCPEYAKVYQNMNLPLCNYWINSSHNSYLARKQIFSTSNIEQY
IYILLDGCRCVEFDCYYFNKNIVVYHGFYGYKLTSSILFCDTLIACKMFGFTTSPYPIILSLEIHCKNKHKNL
IAKILISILGNQLYIPKTTDEINNITPNNCKNKFLVKYKHFENNDSSGFYYLFEGLQSVMYDELNYMSDILDE
NDELDYDNEDMDVLQQNCQGELLHNDDVDVENFDADEDEYEMEKTFGEYIEKSIEKKYNQDSINHKQLYMKNS
DSNNNVSIEKENYEEKHNKFVEDTKKPRNILNIIKKNDNNMDNDSSDNNNNNNVKIPKNSITSCIEKKDKSKD
LEEQKKKHILKKTDNFNLKNNNLLNEYSCLKGYVFRNFYETRNYNEICSISENKFIKLIKKNENEIIKYNQKT
LTRVYPSGTRLASTNFNPLIFWNAGIQVVALNYQYNGLSMLLNKGRFLENGGKHSGYILKPELLRFNEKQDYN
TLLLDLQILSLHQINLLFSIKNKYHEKKLKKKLFQMDMIQRIQTHKKIKKKIKNWKDLQKLEKEKKNILFSDV

QSDDNKNKHISYELLLNKINDNDDVNYIHNKCLNVEKKYEDMLTEYKSFLLCSSSLSHSSSFNSCSSNTTTSN
DGNKTHSNKNNSSKYKNKNFYQTFEELKKANNLFFYLYLTISIHGYNENKYYFKTEIAKVNFYDINYCWSKPS
TFQMKISYPSLALIVFELKAYDTVKSEIIACACFPVKCLREGLRFVPLCDKYLKDIKGSGILVNLKINQEN*
>N.PF10_0133
MNTLKGNLKLLNSNINVQEKRKIWLDTIKDDPICLKGCDYKNLKSIALTFFKDKNLYNEENLVILNFFCKEKL
DDFLLNDEIMEFLQKTYLLYEYFIKEGCDKINKKQFIENISECCKRIFLKNIIKHEKEILDFLLKNLMNYINK
YSFCLIYQSLYNIYQYNQNNYNQIYYVKQKPTFEFLLCLENKKINDEVLLINNNKFSVDSLFVLNENDEKGIR
EYFEKNGNINTYDAYNERENTQEDSSYKNYNYERDKNNDIVEYVNDNKIEQRGTILRTKTCISSSYDKNILKN
VETLNKEFSCFYLFIYFRCCFENINFYLIEKFIENYYINHNNENIYKELLLFLFLIYKDKMNTLVDRILTDSI
NYLNNTYTASIMNNNDNSSSSNNNNKNNKNNNIYHKSNSSNRSYSKNYSYNHLESHKKPKRNTNENIKERNKE
LYLSDSLFYYELLINMIIYQNLNYENNYEYYLNFIKTLHDNDHFQELFLSSFFFVYISTDDVSIRNVLLTNFI
KYGSKRFSTLSKLFTYMKRIFYIIYHDVVDEMYIYNNKLFTNIVEGQCSIRRNEILDTDEKELNYLIFVKSNN
NICIDKYICLFFKEVYYIISRTNMEYFALVTHLYLYTYDYIFQKIINVIYENEMNLNDTSFDICKQVLKPFLF
LYVDIFDSFIQALKKNVYSEKVMITYSFIKKYLFKNMYEKSFLQKDLFYVIYHIYFLQYVLKYNFLNFLTNFI
TTKRNVSQYLENLLKLKEDHIKEVEATLEKNGNYENEKETLNEKGTLNEKENNHDMLEDAKKTKERNKEKDDK
VEKAQRVKRTESTYRRNKTGYYNEDDSSGDNNSNDDNSDDEEWDDYKHIKSYQNGRSKKQDNNNKKKRKKPNN
KNKSNVEKIDLTDLSCSDKENEEERYRKKKKNSKKGIKSDDNNNNNNNKSDDENDNNNENENYNQNNNLYNNN
NNTSSSFLNNEDPELLCDKLSGEEGQKNKCANNVVDAEKERIISFFEKNYLVLKYFTSYTDDMIFGHDEGDIT
SLDNYIDIKEHTKLKRISSNMKNINKSNDNNNENVNQHNNNNNNNNINSYNNSHNCIAYSSSSDDKSRFIRKL
HSFYNKKRKVDHIINELFLNNKDNVYSKILKNNLSIIKELKNLRMDILYPIFNASLVFLFDDFYFFIIQNIKL
VIEEKEKFYYNHKIFLNTSLILLHCLSKDVKIEKYLVSIFYILKLSFNKLKSIEIINEGIHQNKEQIYDKNTK
TFQNDVKYYNIKKDKDIVTSYISCLKKNNNDHEHNNKNITLNEIEENIKLYQNVFYIFLKLLQNVLKSNNLYY
NFKFIIYDILFSPYSNKKIIFSCMKCILDRAKLDEIYNYCIKMLDKITMEEKFIEELLKVEDNISLDHNNNNN
NNRVSYEYNNNLDQENDTNQNKKGYVTNNINYNIQTNNSNNFNNDEYFNHVSLRNINETYIKENNYLPLILYN
KNNLENSFSFNNIEKNIKSREISSLYINILILYYIITQKEKTNKKTTFCDHYLKFDVDNCRMIINKLICLLYS
KNDLNSSYYKYIKYLSFKTFKKIYSYLSIKEADIYIGHIKNKNTEDIINEDKKLNDIKILKHEILEYFYFISN
KEIKEYIMKNKKSNNTKQNLSTYTDEYNFFFLNNLNCNKLILKNDNFYFMINSLLFIFNMKYDKATFYKLITI
ILMEKNEYKNYVLEKIIPSIKNFFKLSYILNNVKRENKKNNLKLSNDILNHSVLLYIIRTIIILFLLIDYNHI
NKHNIIINENYISYHMFINDYIKKKSLKYYYTQLNFLLFRIFYNEKNLSYFFKMFFTTLYIISFKKKGNENMI
FFFEKIFHLNILDEHIYSSANKKNKFLKDNINLHDNHQTVANMKQFKEENEKDKNIVENFDKNGVGKHKQRTN
KSQKNTKYNKKYNTQNDIDSNTQNDIDSNTQNDIDSNTENNTDDNTENNINNINNNTDDHSEDSHSQYSSDEE
VRKNYREILRSHKKNDLELNKKCIKYKSTVRSNGSTTSSSNDSDDTFRIRINKKRKNKNSHNKKKNNKKNKKR
NSSSLDDNLITYAEQKSQRTNSSNEMKCTHLKKSAKKEYVLNQEQVKPFTDNIMEKMEKEMNIENFQFNGSLS
VKILNHLIFLCKGFHEDLELKIYNGIKSCLKDNEMDKEKLKQLLEQNIKEDEHIHNHVSSIKYKKKLELLKII
DTKIIFLTYIKDNLEKRKSLVSRMSELNLNIFKNKSMFHIDIDFI*
>N.PF10_0134
MFLLCLLLWRNIICDIKELNYEQFYKMLTTEKNNQKKNYILDKSINYKNFKSFQDFLYIKTNEDFVLYVYAKW
DTDSNNLISIFREVDRILTEHQIKLNFYIFNIDQAKDLCNFLNITSLPLILYVSSVHKKKYNTLLYKFLNSNK
DIKISNAFRYNGDMYCYDYIVEWVEAHYYFTKFVLLMKKLFSWKK*
>N.PF10_0135
MKIFYLAILYYINSLRESQANSNNFILKNNEQIKTLRRVNTKNRKHDINHILDLSFLSKEEKKENDKQNDKQN
DKENDKENDKENDKEKYNDKENDKEKYNDKHKEKNKDNNIEKENDEHKDHKNDHNTISLLDKGKDDKSKTKNE
KQDKGKNKDEFDNDDDLIDDEEVDDDVESDDLKNKSKNKSKSKQDEKKKNDKKKKNPHDTEDDEDEDLNEEDE
EENDEDEEHKKKNEKKKKDGDDDEPDDEHDEEDEEKEKKKKKEKNKEKNNKKNNNKNIKPHEDHHKNDHDHEH
HEENHKQKYKEENNKKHQEKQHEEHQNNNNHKENIKKEENHEIPILNNNPLNQMTNMAIPNVSQNILPIMDNS
IPKMEEKHTPINNEMTIKNNEDIHIPDDIKNDFMKLLKNVVSLNSKNNLYKSQHEHLKEDEKNKHSENPALYN
KLFYGQDIHNNSSFFNKIINHIKKYLMYYIIGVIVFFSLALVMQSSE*
>N.PF10_0136
MDDKSYSTFAKRKKKRVNGKINKEKFKKNIIKNDKYVKASTLNKNISKKTFLHTLFKSFIKPYNEKNTAFYSI
LCIINNIYKCTYFNEHRCFICKSLYEDTSKQLEEKKNIYYYYSSDGDDNNFPYNIYYNIKKINLNESNKTSKN
SSNSNKIIVTTNKNNAKTLNSCLLNECPHFSDSSNLEYRDEERKKNDGTTEDHNEAKENKENKESKENIKNMT
NVENCNKVKMNVTLRHISNNNKSTNINNNNNNNNNNNNNSNNNYSENQLCACPVQNFVNVKLNFCNSNQYHFL
SHEKSNFVDIYNFKVFDKINMYDKILLDLNQNEIHPNILRTGIFFNKCSITTHNHRNVDLLIALKSFIKDYTL
PPYEPINKHMKVVIDKEINYIIMCKKHSVSMGEVIRWFKNMISEHIGKNVLEETKEIITNNINNYIRTKIVIP

SINISNYVSNHIIENHDVILIYTFDYDIYLSIIKAKRNGKKFEIILVDSEPYKNSYNIKLYTKLGISVTYTLI
GGLFYNIRRCTKVLLGIDAIIHNSVYGHVGTSIICMISNLNNVDVYIVCETYKISNKILIDSFSMNNINNNLD
IYDYMYMHHYHHHSNPHKCDQKCNKNVEGGTHFNKKLNNFIHSFSDIKKKNILFNNINTKYKKPTVRYISTSG
SPFALNEKALNKKKKNNKEEWEQDEAKAVIEDKTMEDIQNVKYVENEDVKKIKDTKERKGEQLEDELKSSNKT
LFNKSNASLEDQKTENLKETEPQKLYIQYSNERITNEMKMKYESKIEHCNNYKSSSYTRHIETTKFHESTDTL
VKCQNVTNNNESNITCCTKNFKEEKTNITDVQEKYIHQMNNINDIEMEKKNSKTKEEKISLNYVKEINTVSSN
STFTTGSTKSILKQQTYLSHEIKNEKTMVKINYKNVSQTISSDNNNNNNNNNSKVINKKNKVFFNLKNGKNNF
EKSTYSLNFKNHFDIKSSNINTSTDQNIDENDKDQICCNDICHSICKSIGNVNIKNVCTDKHNDTNLFTSVFS
HINKINSNNDKSFYVANICNDVTPLKYINYIVTEVGLYTSSNKNALNAFIHNNI*
>N.PF10_0137
MTRICLVTGGTGLLGSSLREVIKNENKNIIEKENEIIVNSNENKNIITKYIFLSSEMCDLKDYDKSKLVFEKY
NFTDIIHFAAHVGGLYANKNNNLDFLINNLEISMNVIKLCHKYSISRGIFTLSTCIFPVNCSLPLTEEKIHDG
KCHQSNEGYSVSKRVLEVLVRFYREKYNYEWICIIPTNIYGKYDNFNLENAHVIPSIIHKMYLAKVNNTNVYL
LGDGSAVRQFIYNIDVNKILYYILNHYYSKNLTIIKDNIYNIIFSTNLPSNELSIKELADKIKYYLKFNNKIL
FDTTKDNGIHKKTSSNDTLMKLLDNSFTFTELDNGLKETIDWFIDEYKNVRK*
>N.PF10_0138
MNRIFYFCLFTILFWLSLVSGENVNNKNCNEKNRKAILLALLKNSLVDNKDYNNSEELKYALEHIQNSELYPK
DSKKFDKFIDEFFSYYNIHVNFTDEEKRILHISGVFKEFYVDVDNLNKDEMKEYFKKNYEKGLSLINLIVHSN
LIIQQFDHDIIDKKKVHEQNTNTNKTLEYISDNLNDLINFKNIHLNNNSTGDFIIKLYTNYVNYINPYQTNPL
PNTPHYYEHKNFHTKEHYIYDEEIVNPNMDNINTHTEEDNVYYVSATKGNQKEETEKKENHENNNAINPKYMNY
ETYYKKIFNAIFEQIDKLNKTLFEIKNKNNSETNENISESNSGNPELNNENSYSVKLSSSSPNSTNKESLIFP
YTYYNPYYMFRLTNNFKENDEGLKNENNINNNEDNQNDNMNIVLGKIHNILKDFNINENIMTNKMSAPLIMTI
ILNFFKKYMAENKFNLPMSSEVENKINKSNNKALLQQSNKDTPIHKKKEIRNKKIQTKVDVIDEKTKKKIANT
IYVNVGQSGINGFFNFFDFREKSIDSNIFDLLHVMEDMKIFDIFQTIIFIQKFTENVCASYCMNITDVLELSH
YDMIFYDKMVFHFSKDGMMIKTDKKYLYNLKEFENILNLLNINANTIALNCTCKFYVDVNYTYSEQYKMHLKG
YLHKMNEFDYINNFSASYLLNQLIIFQDKFNYIKMNGKLPIDDPKNIYNMNNVHDTAYYHNSRYFPTKDMPSL
EDNFYEHLKYPDINTIHIYYNASPVKLNEVNDLKTIIIDEIKSKIFYINSYRVGDQFFPTYSNLGKDDHDLEH
SAKNFYNISNENGDNTFNNNNNNMDNKKRKYNYNKHKDNNSRYTDNSNKNRDNSNKNRDNYNRNKDKNNTNRD
NYNRYKDNKYYYNNSDNNNYNERKRYIRKKTYNKLSYFNLPSLKSIYNNKIKGNSEEFSFDNELPEQTESFPL
NKPQDHEAFYNLKKHHTNVYEPNDEEKQNEQKLKDQIKITSDILYKDIEENKNTEDVLLIETITINNGTTSNT
IENNKDSNKEAENSNTEQNDNNNNDNNNNINNNNNNNDNKEEDMNENNNNSKVTGDSVENINEQTNNNQYPNT
EYNTRQRSINAKYLIFFFKNLHVWKTDLFCQNINYMNNYLNSIQYNKTLTFDINYDTNAVTIYFTDNITYTVK
VNLEYLVFLLEKISLITFVEDLCSLFDTDKKRNYKNLTEFLENTERINTFVRNHMMLSNEQFINKNKYAKELA
EISTSNLFYPKKDIILRSTPYNNIILDEKDIYQTIFIYMDDMLTEKMVNDTWITPYAFVVYSKSKKDMQGNNI
KIEQNKNITKYSRSAIDKYVHYEYKRISENLNRFFMESNSNAPQFNENYKEYTIIIYNDNPSMPNIVLTTTIN
VFNVSFLQSIMEMLLNIKANQQFFSYKGKFIPINAFITLENKINYIFFNYIPLENYVNNGDALDFRNP*
>N.PF10_0139
MNKRKKTKHLKVNSILRIFFFFFLISFLFSNCKLNDYIRTKYPFIQFVYSYSKKKVCTSSTDDSTCRTVVYGD
LDVSNNSVLRLKVLRSEGKGYFVTIRRDYVTISYYLKYMKDIPLKYREVVDIFNNHKYEKYTEKQIKDFTYNC
TAIKVEDANNTVGDFAPHYHEYTRGESCICPSYHLFKSDNSIKRAKLKCTYFNMLFTDSAIVYSRHCAIMDLF
YFSVYEIDYPPIFNTYIDITIQEYTYDDVSGMSLNKHDLVTKEKKYEINDSMSEIRDDYFDLWLFLRGERHGK
RTLINLSNDYVVIPSSPLDDADVIETDVMRNCGLKEDNPALKGCDYKHECNIIHPCLVKAMMLPKYLFDLSGK
TCNKLGVSLNKWRNSDGNFCGSSAGYCLSENLFKYYYIHKTSVGNRKPSKYKIKNIYGSEPQTKVYTSAKLPN
YLKDKVDSNNNKSYDINDIDNKIFYNENAAAHSHFIDYKYNGNHTVEIKFETNALEVHEIRPVSYGTITHITI
PKDCSSNQTNSKECILVVHTWNNNKTIGANFSCHVLCVDKSTQQVATHISPISKINAHIDANKNYAFYFIIKF
LINKKITSNCTAILKDADGRECSKLSFNLTSKETINVVESGIVAQPVESEAQINKYDPDVSGASTPTADKCDC
YFNLLCYILNLNTCVSYYTKLIKDYLGRFVTIAILIFLAPSLIPLLPFIIKFFISCASLPMKLFSNFSSWMEN
KKKSNNSTKQNKNYFQRKYENFKKKRTNMKKNKCTSSSVSSLTNVSSISSNNTMNSDIKKDVSFNRIKSNRYN
KENHKNKKRKTKGNHSKYSGTSMESTLTNTSPSSTPDNLSESHITSNSNKNNYSSKKKNKCNMLYKKEHSRKS
IRKKSMGISEYSS*
>N.PF10_0140
MDTGKLNEPKELIEINKINNIFLYPFSFFNLINHHIFVLHVYRMMHILSYNIMDLKDDKYDKKEIINPKRIVY
NKYIMRIKQNMYNYKMKKEGFKYIPTNNICFLLHIRDHIKISIHLKRKNIYTINKYLNNIININIMKKKGYDEEK
VKCCIIICGNEFFKKNIFFKQLENVIYKKIISICKNIKNERLITLFKNQHIVDNIIELYIYYYNKIVLLLLLL

ILGHWDILYLPFSIFVYKYINIYINVLHYTSFFMYHYYDEMKWNHFIKKLYQNLISFNNFIFFNDEYKYQISD
EKTYFFIYTLISTHIFGKGKLNKINQINNLFLHFLQYKKFPIKSYKMTYKIDNILLYISLLISSHYVTIPNFI
TIELLIIRIYLCIKKYLNQNELTAINVITPFYITPIEKKGISKLHEFYKNLIIYLNYNYICILFINAPNYKNC
SSNYVFLVNKRTPSRFNTNHVISMRAHNDFIYNTLLYHRTNTSICKDKSQGEHIYNNVFNNNNNSKLYESLVS
LSNIENFCSLYMNKLLYWSTLIKTLNFICNLTKSTIYVPIKHTNTYLSLLLFIVHIFYYIINHKECSQKLTKF
EKDLIQATCTPMLYIINNFFLIILNHIRTLINFILCINHFSIYSILILIKYKFFSFVNMKNKNKNERISLEYY
NLKEINKKMFLFNYEDIEKGKDSIKKLSIFNLEKKQKKKNIISPIKYNSKLKNYSTNNKKKIHSYKHYIKGRE
LKHINISAHFKDSNNNINANYNNNGNDMLYNRYNNNMSKYKQYCNKDIYFKKVIGKKRGKCTNIQVEKNPCYN
LIYIPSLLWIYKILNYKNNCTYNIIKHSHIFCNELMCSSDKSNNLSYRNKKSQSYSNEKKNKKSKTNDRDESS
KKETKKKDNSSGPYSDLESDSDDEVDQDKDLDDDEKQDEEEQMDDNQREDRDDKDEEDDEENEKNDKEEGKEE
DDEGDDDYDDDDYDDDDYDDDDYDDDDYDNDDYDDDDDDDNDDNDDDEGDNDDDEDDEDDDDDDDDDDDDDDD
DNDDDEDDNDDEDEDDDENDDEEDDDDDDEEEDTREETDDNICEKEDLEDDDDTYEYSDEDSTHKDRNKKRRR
KKRNNNKSKRKRKRKSKHDDYYDDNENEDSRKENDKEEESYYNKKKNLYLEKKNKKFKHNFIYLKNLFKENIC
INTLNVSNFISVSKDKLTATYTAWGKHTDIACVQVNKCALRDCSIYYFEVEILNCTNFSKIVIGMTNKNYTIN
KNPGSEYNSFGYKNDDGKKIIDGKIENYCNGYAKNDIIGCGINYFDNSAFFTKNGKYLGKACTINFKYDYYAT
VGLSTLGDRIKFHLNNFCFDIYNMIYEESEKERKIIKSIYVQKDIFSDIIKSHLIKCGYFNTYKSFMNYLEKN
KYVDDNNSIESSTNYNNGMDGNIFPNKSNTSNNSQKKVENSSNNKNEKEKNKKNNTNDMNKDEKVEMEDDNNK
ITEGNHVKMEKEEKKKNKNDINLNIKNNTMKNLTEQNPHKSATSEQLDVTPEDNKKKKDSEHENNEKNILNSI
LKNYLNVCDKFDKNKKVDDEKTETNNAIMLNEPEKIPIVIPPDNEKEKCGDNKTMGKEGDKRKEDVIDGNEKH
MEEEKLTKKEINEEKENTNEEEKEKKEVEDAKCVKEVENVKEVENVKEVENVKEGENVKEVENAKEVENVKDV
EDAKGEKEEKGLKGDAGKNELVECASNMLKGYNFSNYNYTGISLQNALWASRKNSLSNIINIRKDSSSILLNR
LRNKRSLTSKDDLNVLTSSLMNAKKDAKKNDNDINKKIHVLLKEKCSIFKNEKKKLCKKGNKCYSNDKEYMTK
YFVNLYLSEDKLNKMIDSLETRYIIRNNIMEGNIINVLNILEEEYSEMFTNEQAIFNIAMLYTQQLIEILKPN
KKYIKSLSLRKQKNNKNKKREICNNYELGKNKYNDSLCSDSSYKTYNTLSDNHFYDDIKTEYNSSDSLFCDEE
LSFVDKKNNAITSKLEKVYNGEYKQKMKDHKMKHNRDKKEYGNGNEKLNDSEKHNIKNVNNNVNNSNNNMNIL
NSNSKTTKGDMDGMVSSNTGNIHTTNDKENIGENNIDSYNKDKKKESNNEDFLKEKKMEQIIYKNICSNYDER
FSKTNNLQEDYKSHFNKLLKQYEFFNDDNPYKNECKKNNYKNINTEQNYISSSSHEDIFSDYDSNDSNFNEKF
YEYNDINFDQVYQYIYKDKIPSNNQQLKFKKDHMYLALLWIKEKLSLFNKYNLLNVRQCILDTTSLIAYHKPY
KEKLVRIFFSRNRNLLTFNSINEGILNICLNVPVYSPLEIMVKHLILCRNLLREKKGNIGIIYDCSYVCQPYK
RYMIKIKNNKKKRRYFKETSTKLKNEKNSLNGKLNDDKRLSIFQ*
>N.PF10_0141
MENNSTERYIFKPNFLGEGSYGKVYKAYDTILKKEVAIKKMKLNKISNYIDDCGINFVLLREIKIMKEIKHKN
IMSALDLYCEKDYINLVMEIMDYDLSKIINRKIFLTDSQKKCILLQILNGLNVLHKYYFMHRDLSPANIFINK
KGEVKLADFGLCTKYGYDMYSDKLFRDKYKKNLNLTSKVVTLWYRAPELLLGSNKYNSSIDMWSFGCIFAELL
LQKALFPGENEIDQLGKIFFLLGTPNENNWPEALCLPLYTEFTKATKKDFKTYFKIDDDDCIDLLTSFLKLNA
HERISAEDAMKHRYFFNDPLPCDISQLPFNDL*
>N.PF10_0142
MYSNNNIFEIYNEDAKKSCSNYEWKSLIKEGSGKCTGSSDEEELFDNFNNKTRMSYNKKERYVEIIKKICSAN
DLKHFNYYINNYEKIKNIKGIKVRGENRVWVLILCLNFIYCNLNDDIMTIQELKYQIKRNINKKKVYCKHLAK
IIYLICNKLEIKNFITNDLLPFMQKCIKNIIMKMKNLQNNIDNKERIKVKKRVNIFDDIFNFINNESNDDNMD
ILNLKRKNIDSNKYNNDNNYNNNDDNNNYKTNKFSNTNKTNNKEKHKSLLVNEIYVERRRKKNKTNENDYDNK
GRTNYNDDNTNIHMDSLENYTNGNIYNKEILEDINFFEDSNDDDYSSIFEDEDNKLKNETSFNDIIPNNIFDN
TIKSRSTYNSNIDMNEYMNDNVYNMINDNINKESKSVNNKSYSYINNNINENIPSKDNNNNNNNNNNNNMCNE
FVSSKRSVNNINRKKKRESNKEMEQNLLIQHLEKNVNLLSLYSCVIYSLFFLWNKNDNMDTNLSDKSSRCSGK
NLQYFVCSSIIITFNVFNINIKDHLICISLDVVQKIMISKKKEMLIFFHNTINEFLGFPMDPNYKDVFLYLRV
ICSNYILLQMYLFYIILNNNIMNNKNNFPFLFKRIQLYISKFFLYIQNDFLSISDVLVDHDFMFQSEDNYKNI
LQLFSENYDNINLKKFIKNMVKNYLSQEGKIIYDQYSFDDSYDIDLNNINEYLSKMLTYNLIHMEQNKTIVNI
NSIFNADNSETFYLQNKSIMNYHQHCSEVVYNEEICIEDYVNQENEKTNIVSNNNDNNNDNNNNNNEHNISPI
NNNYINSDNIKNNALNYSHKNTQNNIKKAINSDVNHNLKDSLDSCELQSFLISNINLKCDDFLKYINKQNNRN
IKNDQLPSDFFSYLPILKKINMSTINDTNMEIICYNNIKIVVQFFFFNVIKNILYNCYSSNFFSPNKLHSSLC
IRKNSQHDIGVYKEQMEEENIKEYFKSKIAKKIINKNDIINEKYISHKTFFFTCDQIYYIQNFTKNKLSKKNV
YTMLKENNIIIQNVEDLLENNLKNDKIMKPTNFLGISNTPLEDLENSTVTCSRDKNNNFVSNYNEDEKLCNMS
IPEYNKQYSNNKKIKIMLYHYDILKYNSNHEYNNCSYFKNTFVHFFDYIYNIKMMKEYNDLKAKICLNKEFNI

LFNILNKMNYSFAYRDKKLIKMNDCLYHTYHIIGIKKISSIGYIFNFKNELNDNFSKVLKTKISNILSIEDIY
FLFNRFFYFLLMYIRKFCCFSNKKDTNIPQNEAETFVNSVCNCKQDNCCYKIKTIVLINNIQ*
>N.PF10_0143
MNINKYEEFYPSILMHERQCGLSSAYIVYEKEQLEILEQEVLKAIIIKALIIEEVIKYIKCILYKIELIKYNN
NNNNNNDNIILTSYDNTNIRKNQIKNVYCPKLLNKNNNIIYDNNLIKDIDRNNFKNKLYIIQFNGNESVRRNF
FKTEQKNTISNDNIRIQGNDVIENKNEKINKSLCNIISNENKEIVLNKKNLDTLMLLEKIKTKTISFINKSSY
VVIYKSINDYNNVLQKELIDISSKCCNNKFQLYSKNINSYSIIDYVTGHINKMKEYQMRNKFLDDIIVKIKDE
LYINIDLIYKNKNNKSFLSVMENINYTIVNTYNINQDKLENFKYLHFNFFYICSKLENKSISNINKYNKVNNN
ICNIFLYFFVMIFFHLYIQNSDDKRKEDICNNFYNILKYNSYNYEKFYVFILLCIKCIVSIRNDKLINFVPHN
GRKKFSNDDNKELYNDNSTEKKMTIRTGYHFKFNINKKSIHNRNYYFINYAHHVIFNTLYYCIVKGCNIYYKI
KKSKEFKRNTYYNNNNYQWTNYFNYIQLYNKCKKINNKYRKMKIIGEIILSKNLKYENNKKNKTGSDRLSGTY
FLYNNNKKRNYFIVYYKRYIKRGYANSGNNKQKMFCFKNDNIVLLNKIILYLIDKNRKDAIKKDKLNIKEWHR
NTKLYCNSNNAEGGDGGYNNHSNSNYRNNGNNNIGNYNNNGSNFFGNNMGGNNNDNDDNDNNDENNYKPKNTC
NDDDNDNDGGNKNNSGKYNNSVNKNDNNNNNNSTDGVYGNNVDNVNNVMMNHYGDKIPISNENGYDNNSNVIH
KKMVDNKLEGINKSASNNNMINNKVMNSAGMMSSNGDMNYNLNYMNYMNNTNNINMPGSGGGSGVNRMTTVPI
NHVMNFPNSYMSGANMPSQVPQNMHFDAIENMHDMKQTPQQMKHKTSQLNSSANQGDINNNAKQVGGLPANFM
QNQMHPQYQYQQQVHMQQLVQQQNVHGYNNMMQSNNQQKFPNQPGAHKQSNASILKMPPFSSLNSGDQRSSYS
IAQKLPRHVMDTNNNVPSINNNNSSSNNNNNPRHSSSNMPSLNNPNQFNSVSLKFPYKGSSPQPLNSINNNNN
PNQPNQQNHQNQPNHMPNISAQQNMQNVNNNNNNPSVVQQMNVNHPIMQHNMIQQNQNNMHNLAPNKPNESQS
MFPLGASSNQQGNMQNIASPNKQKMQPLFQQGGQQIIAPNQKGINTASISGSISRQPNQGTGQIPISHQNIQQ
FYHNNSGQMFPQQPPFLQRVSTTPQHVPQQQVPYEWMNNPYMHHQYMLQQCQLTPQQLFMHQQKQQNNMLHHQ
QQQQQQLQHQQMQQQQLHQQQIQQQQLHQQQIQQQQLHQQQIQQQQIQQQQLQQQQLQQQQIQQHQLQQQQQQ
QQQGNIPPLSARQSKQGILQMQSQNNISHLPPNLEQHFQQQNISELQHHQQQMQNVSAQHSSISTFEPNAQQT
MYQGEMFKDSNILQGGHNLEIVDGVNEMQRTNDAFKGGNIIEASNVSLEAITEEDIFDSNYHCDICNKDITHT
IRIRCAECVDFDLCVNCFSTGKEMKSDKCEHYNYHNYIPIPKYDFPLYKLNWSAEEELLLLDGISKFGFGNWE
QVADLVNSVANITKTDRECESHYYNFYLKSNCAPLPDNKRLLIKPDGNPYEIEHVVEKDINDNDDYVLPKSKK
NNRTQIIGYWPLRGDFDIEYDNDAELLLADMEFKESDLPQQKELKLQVLEIYNSKLDERIYRKRTVIERGLLD
SKAQMQKEKKRTKEEKELYAALKPLSRFHSPQHHEYFIQLLLEEQKLRQRLTKLQEWKALGLQNIEQVQEYEI
EKNRRAKEMVKQESNSTTGIISMNNISSNNNTVTTEKLGKSLKTSKKDYKIKNKEEETDENKKLNIDTFVALD
LLNEKEVEFCKNMKLPILFFLLIKRLLIMEVSNSNLNMLKDINELKLKGYKVGQLYDFFLSFDINQKDEFLNN
GTVNTSYVKKNDGKRKKSKNNFDISNSKEIDGKEKLVVDDKDEKNDKNSSNNNNNNNNNNNNNNNNNANVDNYN
VYVDNNNSGNYSLMTLQGEVLNENEKKGKSKNEDKNKTSNLATKNSNKGCNKLKSPPKDDMNDINKDAKDIII
KNTNDMILTECVNDKNMQSIFVDNNLESENNDQLKENEVKGDELNLTEKELNEHILNTVVVDENDKKGKNEKG
KNCETQKNMDSNLLQNEVDMKSNESLFNEKKKGSIKRKSSIINNLYDKVHENNENVDILENEVKDNVTVDDIK
ENKEIPNGITETFESIENIEEEKDMTMKDIKNDNKDDTKNDCDNEKDDDDYVAQEEILEDDKKRRKKKKEKKS
EYADDKNDELNSIKINENECTSNDIEEDIKSIKSLRKKSYEKIKGFTAHLKTNSNNKKKTNDNFDEIEKETTV
SVNINDSNNRKAKKKTPEKVETNNYQAHVEPLENNSKKKNTKIRKTSEYSVTRNESKINNNSDVYNDNSITKD
DSNIDLDDKPSKSKKSISSTSTVILNKNEKGKRTKLTKEKATVDIPSNVDTKENDKKMKNFEANLKKKAKKRK
GSVSLILNPKKSLKL*
>N.PF10_0144
MYKFKFKLRNIYKIHFKPYINMNAPPNFDIHQVKKLGKLGATIGAIIGVTSFGSWFFKNSLYNVEAGKRAIKY
NRIFGLSNKIYGEGTHFLIPFFERSIIYDVRTKPRVLMSLTGSRDLQMVNITCRVLSRPNEKKLVEIYRTLGK
EYDEKVLPSIINEVLKSVVAQYNASQLITQREVVSKSVREQLVQRAKDFNILLDDASITHLSFSNEYEKAVEA
KQVAQQEAERSKYVVLKAEQEKKSTIIKAQGEAEVAKLIGLAVKDNPAFMELKKIELSREVSNIISKCQNKVM
LPTDSLLINFTK*
>N.PF10_0145
MDEKIELLINIIKKWTNKYAYKIYVKGKNEEKQEHYYNVTFFNQKEKESISNCTINTYFVITEIKLIDIKNFC
TSIQNENRYYVTFNFENENLMRTTDMNLDYEAWIDKLIKDKEKLRNNIDLSSEFMKSRFIKPLDEKGVASILD
KIKEERENTEKREKEEEEEKKQNALKKKNLKYSKKANILEKKSSYEDTYAVEKINSKTDFNIDNGNTELKNGS
TKNDENNKLNEARSFLKNILYFYFTDIDIKKKGKLKYNNYVEILKMVNYKMCISYKHMHKTTSIECEENDQEK
TNEMEDNRNNEKIVNFHFEHFKGPFLFDSDEGIYSSTDHNSSDEYSFNDNKDIKKKQIVIKDEKKYLQNLFSI
NNKEIDLIKDISIYKNNNFNGSNIFFNEYNNDDIYSELFIFNNDNAYYKYVFTDNYYDLLLYISYADEDDRGC
IFYNNYINSLTQYLYELKKNREEHFHIFNLNDLYLYKQLLYTCYENELSFIYQTFLVQFKKFDTSDSGYIHRN
HLKFILQENDHIISRQEYKLLMHIFDCNDDNYVYYNNFKEVILRLRFEGIKNSIYEKDKKLLQKYLCEQLIKN

NLKNKKKMHIFDCKNVLDFCDKLYLNKNVIHIILSSLNFDENLELDVTLFLQVSITIIMNSIKLENMQKIYNI
ITDEKEKKKEFQNGQSNINFKGKKDNKKIEKTNIPALELVERTLTKLFKVLDEKNEDYLKINDFIETLLEFNK
KKKIIDIKAICRLNINELQGFVGEINAGQQKNPFLHENIKDSTNYELYKKKKIHYASHIHKWCSKTYQIRSCE
YYSYFLNYPNELIELDDEINKKLLFCEEKKD*
>N.PF10_0146
MMFFNLNKDSNKNDDFSLESTSNNFGSKEMNIIPDVINRDTKGILNDHLVTRGDKIGEENHMNKNYCFSDSPS
NGIDEKVKSLEMDYVLKSSINKVGSNSIVLSKDEEEVFYGNNEDISVLNNNNNNNITKSHEETSVHNLSMKND
SNKIQNDKIGNMVQFNRSIVPISNDEYMKQNECHMNICKDDNINYEEKKDNYPINTISYNKNFNLDEINKNYN
NEMNKKEPFHDDVIDKEKKNFEKFSFSKGFQKDALEADDKYQINKNEYDDCIPKYNDNIYKMVEQDAGNVHSN
NRDQNSNEPMNENKQPIVRNKLLDELNYSNKKSHSSLFDDKEKMIDISLIADRAKKNIFPEMISHSNNIHTNF
EMTSTKHEMNSDYITKEEDDKYTFHTLKERIEDNKYNMDDTYKELNLEHDNNVLDEYNSRNSSDEIDQIFSKY
KIEVSNLSKYFDFHKTGKKENSEINLDNDKENNNNNNMKIKENDSKNDKENDNDFNINKRYNSNNVKDMLDYY
IKGSDNTINKVLTYVYPSNFTSNYNDYILKEEEKDEEEKEKKKKKEKLKGEETRQDYHDDYDKQSQMSLNNLN
ESSNIVINETNYITEDVLKDILNMKEEKISFEYFRNCFNDLNYDIKVSDIHLLYNCIREHQQQNNINIKNEDI
EEKKNVNNSSNTNNVDDHYITVQDIRSYIIERNSKNNNYYNEIIKMVVHFNNILLLMLNNNETEMKDKILFEL
LSLKFASIFNDILQLDNNFDNLIKHLNEIIKLILCEDSKKKNLRNIISTYLNSQKILIHKNDMLSELNKTTHK
KMKKKINNEKEITKEKKKKLNIINEKEYEKDGVNQFVDESTNEEERDSDNTALNDKTKSDNKSSKKKESNTKL
VYSCDEMKRKFRKNSKAKKNKKKRSTSHINLHNTTEINNAQTDTQNNTRNLTKDIIMNEIKNTKEICKNENLK
KEQNTTCSKKKKKRTSTPFSKEFIITSDVDSNEKSNDEKNNSVQGHNTEQVPLTNIIDNINEIKENVIQTTTK
NFEEKKEHVDENSKESQKSNEIKKISENIYIENKKKIGIINCTINNKNKENNKRVIEIKKQAVPLKRNDKSVN
SMDIKKVAQFKNNNNNNNNNKHFMKSIPNYIFKKDIEEEIERKNINFENRTYNSNHIKHQIPHINSPYFNDYM
KRFSSINNGILRQDLYPISQKNNNNTNMIQNERVNYILPSLNKNLSNSNNHNNNNINYMNNVDKYISANHEQI
PLHNMTINNEFHKMSNTNVNINKNNEIRRSMQPFQQNIYDNQHYYYYNNPHILSNNNINNINNIHEMKANNYL
RNNEQIYKNLMMKNANPKYYNTEYQYNSLLQNENKIPFISSSSKRIADQIHIHKNICDHLTSGNIYNGKKEVP
LPFDTKALIFNQNKKELFESCTNANIVEKSQKITSTSSKENIELSSEQKVNRENTILENGNEINVNREDTISE
KRDEKNVNRGDTIIENRNDKSVNINKNIIYKDDSNVIYRNNMRKATPFEGKRYSISYNRMMTPNLYTGFKRNI
NNINYKNNFLLSSNVSKCSNRYVSSKSDVHSLKKPNGKLINENMKPSKFEKDSLFTLNLEKNDTQYNENTKNE
MNKKSPYTRFTEIFTDKLKNFTLGNRKSFRSSTVHGTVSSYKNIDDDEKEKNYKGDQSFNKKNDQKEGGKEVE
NYKNDEDMENLNEKQKHEIEKQIEEQTNEEQKNNKVDDNLIKSSNLKTFIDHLNFFQSKCKANKGFMANIHEK
KQNHEYVNQKNGNDDKYDNNKMEDNMMKLNECNNESGKANNANEKENNNNNNNSNNMNKYYLSNLKKNGEKMN
KTNDIFNNIRNGLSNAINSEKGNDIKNGNNYINVSNNRNNVNVSHSHNDHMKRANYMINSNKTVNFDSEFSKN
LWNSNNNTLNTYNMNVQNFNINNKKNSNVQVLNNIGQSSFNDQHVNSQIGTLNNKMGEFFFPFQKRHSTLHSR
SSIKNTLDNDKKKIANLKIKENDMIMYDINRNANSFSNENKHIFINEKVSSMSQNNKEFHPNVCNNNKSISNN
NNTNTYMNEYNNMKRNTFFTNPFTEGEQVQNNNRNVDINNNEYLNKTKLASCGNYSSFKEKITSIHKNIVPTK
NFYLFNSQDKSNHNCILKTCSLHKNNVTEDEYENKINNEKGENLMKDTKNLYHDNENNHISSNFVNSYKQQSS
SNNMKVMQCEDENNLSIEQIMKIPGVKLGRNYIQDMDICSDWLKFNDYDKKVIEESLKNSLADKNYQNFLDTT
QFKNTFFSNM*
>N.PF10_0147
MDSKDNDVWNDYSNSLMLYERIENLYNDHLIEKSKLNEQNKELINNNMIHVQRETKNVIRNKDMIKANMYNYD
IINYYNDEFILKKKVMELSEDIMMGIDYIYDIFRLCKESNVFLSFNGGKDAVVILHLFRCAYAKYLKDMNIKR
KKPQLIYFKDEMNEFPEVYQFLNESAFMFDFHISIIKGTWKNGITNFIENVQKKYQITIIDQLKTNSIDPTIF
YSTLAFINGTRFNDTNTEKLQILNVSSRGLPPYLYVNPVFYWTYGAIWTFILYFKFNYCILYDHGYSSIGSIK
DTVKNEFLKCNDCYLPAYFLKNWNYERYNRIQPNDK*
>N.PF10_0148
MAERKVLNKYIPPDFDPDKLMESKKLLKKIEKRKNRNQNKTKKKLMNIRMMYPFTLKCNKCKTFTYVGTKFNS
RVEKLKDETYLNIPIWKFYGKCQECKNEIIFKTDPKNGDYLLIAGGTRTYDAHKEQELADDYYRNSNVKDEDK
IKNTEKQSYNAMLELKMNEQLEELQNMNKRHLDKFSSINKALNRLYEKSELENKENNFFMNNLDSEDEKAFFS
LLNKSRSSKDHIGDNLNKYNPSNNNWVNKNIEKDNIIIEEVELDDNKNNNDEVNEYISEGEGEHSDGNNINEV
NESFSDLLLKKKKNRYEKENTVKIKQGNIDTEDKPKDNQQDKTNNISFKNTISNNSSSFSNIIHKPMFNKINK
ETNFNFVIKKKENFQSLLSEYNSDNSNESDN*
>N.PF10_0149.1
MNLFLFICIALFIYLDKFHLPNSLKYNFKRSLLLKLGHQQTVLSKNSEATKSGGMFFIRNSFLYRNKNFLKNK
YIFKRLFHSKMDNANKLPKWNQPSKEGKKITNLFVNNSLTHSKVEFIPQEGNKIKWYACGPTVYDAAHLGHAR
TYVSFDIIRRILVNYFKYDVFMVINITDIDDKIIKRSVEEKIGFTELARKWEYEFWEDMKSLNVLLPTAITRV

SEYVGDIVKYIEKIIENKYAYVSEEGSVYFDIDEFKKSEKHFYARMEPLSVKDENKILEGEGDLGVISKKKKN
AYDFALWKSSKPNEPHWDSPWGKGRPGWHIECSTMASNILGDVLDIHSGGIDLRFPHHDNELAQSEAFFDHSQ
WVNYFLHSGHLHIEGLKMSKSLKNFITIKNMLTKYTSNQIRILFLLNKWDNFMNYSPNGESMVQCIEIDKSFT
NFFAIILMKIKNFDLNSCNLYWSDADNKLNLLFRQTKNKIHEHFLDNFNTPDALLAIQKLITEINIYMDKEKI
QIGLLLEIKHYINFIFDTFGLIYGDAPKGKYDKFDELLQTLGTYRRNIRINLQSNAKLIRNILKEKNKKNDLD
PVAAQEKSELLHSEFINNIKANNELLLKECDLLRDQHLLNMGILIDDRPNNEFVIKIIDDNQLQQEKNKREQE
LSKKMAGDQKKGNQNEKRE*
>N.PF10_0149:mRNA
MNLFLFICIALFIYLDKFHLPNSLKYNFKRSLLLKLGHQQTVLSKNSEATKSGGMFFIRNSFLYRNKNFLKID
KYIFKRLFHSKMDNANKLPKWNQPSKEGKKITNLFVNNSLTHSKVEFIPQEGNKIKWYACGPTVYDAAHLGHA
RTYVSFDIIRRILVNYFKYDVFMVINITDIDDKIIKRSVEEKIGFTELARKWEYEFWEDMKSLNVLLPTAITR
VSEYVGDIVKYIEKIIENKYAYVSEEGSVYFDIDEFKKSEKHFYARMEPLSVKDENKILEGEGDLGVISKKKK
NAYDFALWKSSKPNEPHWDSPWGKGRPGWHIECSTMASNILGDVLDIHSGGIDLRFPHHDNELAQSEAFFDHS
QWVNYFLHSGHLHIEGLKMSKSLKNFITIKNMLTKYTSNQIRILFLLNKWDNFMNYSPNGESMVQCIEIDKSF
TNFFAIILMKIKNFDLNSCNLYWSDADNKLNLLFRQTKNKIHEHFLDNFNTPDALLAIQKLITEINIYMDKEK
IQIGLLLEIKHYINFIFDTFGLIYGDAPKGKYDKFDELLQTLGTYRRNIRINLQSNAKLIRNILKEKNKKNDL
DPVAAQEKSELLHSEFINNIKANNELLLKECDLLRDQHLLNMGILIDDRPNNEFVIKIIDDNQLQQEKNKREQ
ELSKKMAGDQKKGNQNEKRE*
>N.PF10_0150
MANIDDIEKQIENIKINSDDNKNNVSKNKNILLNGVNLKDHEIKDNVKSVDYNNNNNENDTMNEINKHVKNDE
YCNKENSNNNNNNNNNNNLDTQINETLNLNEKFEKKNEENLCSGCKKVLIKKLSCPICLKNKIFSYFCNQECF
KGSWKEHQKIHENMNKENNEKEDHLKTIVKKHLSPENFDPTNRKYWVYDDHLKNFVNFKFTGDVRPWPLSKIN
HVPSHIERPDYAISSIPESELIYKRKSDIYVNNEEEIQRIREACILGRKTLDYAHTLVSPGVTTDEIDRKVHE
FIIKNNAYPSTLNYYKFPKSCCTSVNEIVCHGIPDYRPLKSGDIINIDISVFYKGVHSDLNETYFVGDINDVP
KEGKELVETCYFSLMEAIKKCKPGMFYKNIGTLIDAYVSKKNFSVVRSYSGHGVGKLFHSNPTVPHFKKNKAV
GIMKPGHVFTIEPMINQGHYSDVLWPDQWTSATSDGKLSAQFEHTLLITNNGVEILTKRTQDSPPLGFDTKDE
LYYN*
>N.PF10_0151
MIMKKLIENNVIKLNYENFLTKYKLNSGNEQKDLSDEELYEHFIINTFNYSKPLGCLPPNISNIKEYYLDGIN
IFEVINYVNISERMYKFENITEEDDKENEDDSKEIFSHSEPDEKHDCEIKSRKNNKNSKISNNNKKDGNNIKS
NKYRRFYRFLLYDGKSFIYAYEQEYNEIFTYLESNKYIYPKIILYNKPLIRRGAILLKKNQVIILFKGCIVSG
NDTEDNEIEDFPIISESNNINSGVHQNIHDDLYTTRNTYDNKNMTDDSHNLIRKFYYADKGMEKEEYKTYYEN
TNNCGVLSNTNRSNEYYSRSSMHNNNNYSFNEYAKHNYKYDQNHVKKNTEYYYNKDKNCYITSIDYNNNNNNN
NSINNSNNEYTGNNYNNNNKGNHYYYHNIHNNHMHDTHNSKNYIGSNRYNTDKASYNYYNNHYKNKQHYIDPQ
RNNYYTHGINHDDYSKENYLNAFEYKNHNSYKEPLRNYSNDHPRNSYNKNKYNIFNENKRNSSSKNNRNSSSK
NNRNSSNKNNRNSSKEYFKRNSNKFDNAYDNTYHDNNTRINHHSDFSRKNNDGFYRNNDNNYNNETYVKNHFS
NEAKYNAGYQRLSDNLENRNRMNCENNNNYYSTYGHSDYNVNYNNKNNRNIPLSNLNEKNNHIPSNNYYTENY
LPEKEGNRNILTNQINNYSINSVNNNFEQINPSTCNYNKNNVFPYNKEDDKNKFVEMKNVEEEYHNDNQERAR
KNSENSKKLIDLTEGFFSSEFFHKSYECNDVNKNSDIIILDD*
>N.PF10_0152
MHIPSLRFYLFLNNVPRFNIKFLKLFNSFYEFSTKKYNNDENNRNIELDKKIQLTNFKLSDSSLDYNISSDDS
DEEYILERGKLKGMENIDRCGDNNGLHMNEKYKDRENLKNEKFENFAIKTNFLKKNICYNNDTNCNMTKDGNI
NPFDKMNESSMKNKYIKREVNINNNSLIDNNLCKEKRMDTRYEKFDKMIYHGINSNDCNNIYSETNIYDTYDS
DTNNKCNNNINNNNIKNSNINNNNNNNSDDDDDDIQNYLKSMIKENQKNYCFNTNNIKSLNDELNKLEYSLKP
SINDINNMKIFLNFLQNEINKHYKNSYVTPFGSVINGFWMRNSDIDICIQIPILLNRKDQITFLKKICLLLNN
FNNGVIEQRFSAKVPIIHFYCNNREKSFELSCDISVNNILAVINSKLIQKYVAIDKRLQTMGIVLKYWSKIRN
INDRSKGFLSSFSLILMIIHFLQNVAEPKILPSLQDISIKRNEKPFYIMGVDCKFCQDDIVIQDELKRLNNSI
HNNLYVDISTLLIEFFKFYGYKYKSGIIAIRDINGYYQNFQTLKKFESYFLFVDNPFEVGKNVANIFPSNYKT
IINEMQRAYKILKNNGSWKDVCNSKENVLYS*
>N.PF10_0153
MISTLRGKIFNNGSNRNKCVSILSNIQKRNISKDIRFGSDARTAMLTGCNKLADAVSVTLGPKGRNVIIEQSF
GSPKITKDGVTVAKSIEFNNKLANLGAQMVKQVAANTNDKAGDGTTTATILARSIFQQGCKAVDSGMNPMDLL
RGINKGVEKVLEYLNSIKKDVTTTEEIFNVASISANGDKNIGQLIADTMKKVGKEGTITVTEGKTLQHELEIV
EGIKFDRGYISPYFINNSKDQKVELDKPYILIHEKKISTVKSLLPVLEHVLQNQSSLLVIAEDVDSDALATLI

VNKLRLGLKICAVKAPGFGEHRKALIHDIAVMTGAKVITEETGLKLDDPQVVSYLGKAKSINVTKDSTLIMEG
EGKKEEINERCESIRNAIKMNTSDYEKEKLQERLAKITGGVALIKVGGISEVEVNEIKDRIQDALCATKAAVE
EGIVPGGGSALLFASKELDSVQTDNYDQRVGVNIIKDACKAPIKQIAENAGHEGSVVAGNILKEKNSNIGFNA
QEGKYVDMIESGIIDPTKVVKTAISDAASIASLMTTTEVAIVDFKDSKNEESSQHMNSVNSMGDMGGMY*
>N.PF10_0153a.1
MDEDFTLVHNTNYDDILQKKIKGIEKKCEELKKDTCKDDVLKEINELIDQAIKLKENVSNIYFQYLKTSSIII
YEKKLKDLIKDIETLKQSSIKNKNEKAYTPNEFNYDDNFLLPDDVYLFKEKEEVQVNENMIDLSQYNMSFQNM
ENKRIIKGFGETGCSNLLLDNLVNCEIIILDILSSVLIQKIKQCTIWVSAVESSLMISNCQDCNILSNSKQIR
IHDAINTNFYINTISNPIIENSTKLIFHKYNLIFDELSELLQKININKDSTKWMEIMDFNWQNTQEKSPNFCI
NTEIQIYNIKIKYIKKFNGLHQEKLTHDNYVIENFPPFLKKIN*
>N.PF10_0153a.2
MDEDFTLVHNTNYDDILQKKIKGIEKKCEELKKDTCKDDVLKEINELIDQAIKLKENVSNIYFQYLKTSSIII
YEKKLKDLIKDIETLKQSSIKNKNEKAYTPNEFNYDDNFLLPDDVYLFKEKEEVQVNENMIDLSQYNMSFQNM
ENKRIIKGFGETGCSNLLLDNLVNCEIIILDILSSVLIQKIKQCTIWVSAVESSLMISNCQDCNILSNSKQFH
KTYFP*
>N.PF10_0154
MSKEQYHDQEVLLEAQNNDEILKENKFRWVMFPIKYKTFWSYYKEIESLFWTAEDYNFDKDKQYLENIDKNML
VKLFELICFYSLKDLHVYEEQALITSKMLDIIQIPEGRAFYGFQMCMENIHDEVYACIFETYIPDSKQKRVII
NKVIELDSVLKKQKWLTEIFESNIPYYNKLVLLYISKVLFNGTLNILIGYCKENSILPCLCNVHEKIHRDEYL
HGDFSVMCCNHLVNKLKYEHVLDYFKMAVDLEYQFSLEMLELNVLKIKKEQVRAFLEYLTDTMLTNLKYPKHY
NSKYPFSWPEFTKIIVNENGKEETVKKGENVYGEKQILFDEDF*
>N.PF10_0155
MAHVITRINAREILDSRGNPTVEVDLETNLGIFRAAVPSGASTGIYEALELRDNDKSRYLGKGVQKAIKNINE
IIAPKLIGMNCTEQKKIDNLMVEELDGSKNEWGWSKSKLGANAILAISMAVCRAGAAANKVSLYKYLAQLAGK
KSDQMVLPVPCLNVINGGSHAGNKLSFQEFMIVPVGAPSFKEALRYGAEVYHTLKSEIKKKYGIDATNVGDEG
GFAPNILNANEALDLLVTAIKSAGYEGKVKIAMDVAASEFYNSENKTYDLDFKTPNNDKSLVKTGAQLVDLYI
DLVKKYPIVSIEDPFDQDDWENYAKLTAAIGKDVQIVGDDLLVTNPTRITKALEKNACNALLLKVNQIGSITE
AIEACLLSQKNNWGVMVSHRSGETEDVFIADLVVALRTGQIKTGAPCRSERNAKYNQLLRIEESLGNNAVFAG
EKFRLQLN*
>N.PF10_0156
MKNKSIRKERQSGETLEDNIEEINYCIKVIPKDKINSKGNGAIRLSNLDMNRSNNGNYYNQNNNVENINDFET
ECRNYIEDILKIDEYVKLPNSINNEQDKIKWKKAHSLRNRMKEYDLITRTRLYQSNSVNNKAKLNRERYIILN
KIPRYREAFPSIMNNTSTDKIKEDIIRTYLKTHAAYFLSQNKKNSNLSLKNMGRNTYFSFRKKSDDRNNSLIN
YNKHMNDKSKSVEHNNCPNLKKKDRSYNLWKRITVDDIKNTHFTPTEKQYLKDYNIFLNDAFNNFKINQNNNK
RQLNVKNQKNIPNYDMINNKIYGHIKNNANNNLYINNQKREDAYIINPSQNNIITSPNEEQYPYFNNYSYSDK
NKNLSTYNTEGKRNIYTNDVKSNTNYQHSNNSNYSDESLNMFSNSSTSIYEKYNLNDKNFVLKRDRYALLGKI
KNNKEIEVKLVLNKV*
>N.PF10_0157
MVIFIAILDLIIVLAIYFLFFYFIVMENNICVYDCKDHSLKKMKKKKKKMRNIYKLLIVLSICIIYYFNLNF
FSVSAKNINKEKINPHGIITSSVPNDNINIIEANEKEHNRNYTTEELLNNNEEIKNEELSHSSDDKEYDKDIY
KEKFLGDDFVSNVELIKNSKFIINAVNFLIKGYKQSYTTGEKEHTSLRNGNDNPNAAEIKKLIIIKLLKCIGV
LILCYIAIRLVFSFFKKVINLIFTIIFKIFKCCCCGGK*
>N.PF10_0158
MSKNDFNNRILNNYEKQKVLEESNNYLKILSHNKNVSSNNKNNINLRKDEEEVGMTFIEEKICFDEKIEVEEV
FTLEILIKILSKIFLKGNYSLTKDDINITNCKVMIFESKYHLNNTNDEVEDFINMLFYFQNKNYLIFIIYCLN
KKNIVYLNFFNPLLSEKETIEAFNLFFMNNFFPHIILGLNNGRILLYNHEGIMCMYNKFVEYKLKNIFIENQK
DYILFLYENNIIVNSNIHLIKRALETNSKTLPYDYMFTINKNISLSHIILRCDNSYDLLKKELYTMYNKEDLM
RYINDNINTNPNTYSGNINYIITSKTITLSILNIIKPNTPNDFLSRKENFSTSEKLSSKINNFIKNIFTKKNE
NAPPSSASSALPSSVLINQVDYNMNSNNTSTNDILNSKIVHSFNDSKRQIIDICICPWNTNLLLALDNLGRIS
LFNLSTLNILYMWKSYRSAFMSFIQKQNYNCFHRQNKKKFQDTPFNQFDKGILFYLKNRSLIEIWDFKTLNKI
YSVRTYECPIFSKLFFGENNVPNKVILFNSSHHVFLMNTNFELLYLKWV*
>N.PF10_0159
MRLSKVSDIKSTGVSNYKNFNSKNSSKYSLMEVSKKNEKKNSLGAFHSKKILLIFGIIYVVLLNAYICGDKYE
KAVDYGFRESRILAEGEDTCARKEKTTLRKSKQKTSTRTVATQTKKDEENKSVVTEEQKVESDSEKQKRTKKV

VKKQINIGDTENQKEGKNVKKVIKKEKKKEESGKPEENKHANEASKKQEPKASKVSQKPSTSTRSNNEVKIRA
ASNQETLTSADPEGQIMREYAADPEYRKHLEIFYKILTNTDPNDEVERRNADNKEDLTSADPEGQIMREYASD
PEYRKHLEIFYKILTNTDPNDDVERRNADNKEDLTSADPEGQIMREYAADPEYRKHLEVFHKILTNTDPNDEV
ERQNADNNEA*
>N.PF10_0160
MTLINRSYVLFGEKLKINYINIKKKKKKFLSDYSCRKKGNCKYGMSTVHTLYFIWFVLGTLYFFLSIFDKDGM
KSVSDLVIIQRYNRCLAEFESVRNIFNWSVFTHVFEKMKNIDLLFEDDEEDTYKNEKTILSKINVKEKWNKYR
PRTNKIKRALTKIELKDNLNRFIKCLNKDRLEIVNDYNSDRSYDDIIHEDENNINNLNSKYIYNWNLGKKSLC
NMLENSDNYYINGVKYSDWKLTSIPTHGYNKEGGRVQEMFKTFVSSKEGDGSDRVGLFIKKIPVDIWVKQFEL
MNAYNGEYVLCGENYVMEATTLAFLNEYYPGITPKLYKILYEPEKKEYDIEQNTPDCMFHDLNVFNDILSARL
KCNMNGYIVIISELFGEDIYTYLTKQKKKNIFALHSYMKRKKILFECLNVLRKLHDAGLCHLDISPQNILMSY
NFEIRLCDLAKSTPIYTNNLRHLKNMNGTYLFESCVPTIGKIRSMPPECWEISRKYIRMKICEPLEQLSPITD
LDERAPFYFDVTSADKFMLGVLFIWIWNNDYLWKSSDIEQDIDFLRVSECDMDIDVFELTRTWPYELKKIIQK
LLQTEGRKNLNLHELCAHPWWFSKM*
>N.PF10_0161
MEICKNILFLNFKNLSIIKGKKRKEKITCQFPKYIGTDNNKRCIINLMLYKIFKGYILLLLWVIIILKNEYIY
EDSYIKLVSQYNHIFTRILSEINLKGEHNDKRKNSNINTGMNRPHIKTKNMDNINIKSSNVNNINANATSSNN
IMLNNFPKFNEIENNNKNDIHNNIFYSFYNNKNDNNEINDGNNILSFNDYIWKKHVQMNKTNLNSDLDIITKK
LESFTLKDDKQFGDTTINYDYCFDNRFSTSSKGDESNNMNYERNDHFVEYQNVGVDEMACGTTKGTDDLRHHN
SNTTDYSGYSTDELDEETSDENCEGYKKIEKYKRYNRYKRNKERKRTSENTTNNKNISNRNDRMGNKNISHRN
DRMGNENISHRNDRMGNEKISHRNDRMGNEKISHRNNRSNIKNGLNNEDRQNKRHRINNVQKRNGFCKEHMAY
ESDDLFKENGDPFNENSQKCYRCRNEKNHIRKNNEETNIKLKTEHKTHKESSNSSITNINSNDSTNSSINELS
SYKNRKIKNINEDKSFSEEQTKMNKYFKNNKQKNLGSSYHSQFLDDYPDEKIFANIPKKKNQNRNNISSKKMN
KEIKVEEVKYNEIVFDELEDEVLEDNLMNLKQYEYHRTLSEEEFYKMIDTLGNVLCNKDMLYIFNFINSFERK
KYLIMQEDFNIYCEWYSSVNKIPVNVKQKYMIKIAFYQTKDYLDLESTFYYNFYSFINRTYVIDKQDFILKLN
EIKTIWKNYRLEKMEYWKKYLSSKFKKYKRNAYIRRTCIKYSDLFFL*
>N.PF10_0161a
MERSKNKFPVFDEKYIHRDVEKNIGKNNFPMYIYIMLICLIFSILQDDGNYDDIMKDPIIFTHMKKSRILSEY
QCIENYNFEEKDENFSNDDIFKNDEKSEYNYSGNVVEDFNFYMDKMNETDDKWIIEKKNIKNDICIKNDPYNK
LIFGDTSPDLSESEFNEFINNLDDYVNKNEMYIIWNYVNSKEKFKYLNMQQNIKSYCENLAYNYDIPKDIERN
EWLKAYYYMTDILLINENKFHNYFYHLISYGKCDKNFFLEVIDIHINSWRNIRRSMNNLWTKKLNDTFKNHPY
VTKK*
>N.PF10_0162
MKKRKGPLFFMLEKRYITERTKNDKQQCNYKLKKESKLYQRKGIRNKCVLYKYTDLLLFLFLIFILFQASEKK
NIIYMYIIYFDIYSHNEFTKVDALKYSPKWLRLLSDVCFQNQHDYNSHNMNEPSSIGYNSRECATTINGNNFN
DNNLLNKYQGQGEVQNQRNSNKGTSSNILKESKIGNTSQSNTKCLDYDLSLLEKSFQGMSFNTHSEKGINNEE
YINNRENRNNGNSYQILKESINGNPTITSTINNNNNIGSNNNSRDYIDKMGKSAPGVHSKNMNAGGYDSGSNS
QMFVGNKYDKKNYDDNNYNNHNSNNYNSYDLNGYNHKMDGRNTSQGNFQRDLYVEENSRNYNNISYNNYSNHR
EYPYNNNNNYYYYGYENTRNNHHNIRQGNSQRISVVEGNERTCSMSSNYNTYNDPYYYSHYNNNKWNDDFHVM
EKEFEKLVYQKHGENYSDHNMYAYNNDSTYRGQYYDPSNIEDYLGTIDEAFEKVHFKGQGIRNPDFNTNFNYK
DYMTGNVKIDLVDSSDEEFENRYNSNSSTQKKKKKRSKVPDSRKHSRTLRKRDDNNDDDDNDDDVNNDDDVNN
DDICNDSEYEFPTKYNDYDENCYETEGETKGSLYSDNYNKKKKTDSMNKIAKKDSCSDLQYDKKKEENKLLTV
FESNTGVDAKWSGSKENKDDKSNNEELVSFEENYTEKEINDILSKLNEFVSLKDMFIIFNHVKRIERKKFLCM
EQYINEYIEKLIKSNNVPTYIKRKCMAKVNFHMTNDYLDLEERILQNFYTMLYRGSMSKSKFIQLINEQRDNW
LNQRRQLNIMWKDIMERRMKWYAFRLKKDKTIFEWITFLTMD*
>N.PF10_0163
MKTKNKIQNKKKNGYNLVHWNIEKRRRMSFFLICTFFNLIFLVSLLVLYKETDTKGYNNYVQQKERKIKGRVL
TEVILKNLEKYIEENYGNCVEERCYYLKEPKVNNPYECEEKKKCLREEFEKCEPCQNPKEEEKELGVDEGENK
NNIRPFEVLTEEEFNKRINILRGGIFLYEVYGLWNYLNQREKRKYTTIKQELWKVCEDLSVKYNVPHECTMKE
WKKVRKYMKEELLKKKEEDEKDFQYFVEDNIYTRWEYIAFITDKKESWKVFRHMIKEKWKDMLLENFEKYMEE
MFDKKMEKHRKKCKEVYKSREGL*
>N.PF10_0164
MKVSRHTVLLNIILIVSLLGCVLTLNLFSANDDKRALKDIDRTLEKLLRKKQIIISTAAVALAITLGGLFGSL
GYKSWKNKNKSKDKVNDGSDSEELDSSKEDKDKDE*

>N.PF10_0165
MEELKTCPFTNVIPYGLLYDKLKKEKNNDVPENYVIEEFDKLLKNYERPNVYDEIGNATFKNDEDLITFQIDL
DYTVENIFKNMIYNESGSNNSILNDIYMPYRILLSKDKNYVSVPIIRIYSLRKDGCSVLINVHNFFPYFYVEK
PDDFDNEDLIKLEMLMNENLNLNSQYKIYEKKILKIEIVKTESLMYFKKNGKKDFLKITVLLPKMVPSLKKYF
EGIVHVNNKSIGGIVYEANLPFILRYIIDHKITGSSWINCKKGHYYIRNKNKKISNCTFEIDISYEHVEPITL
ENEYQQIPKLRILSFDIECIKLDGKGFPEAKNDPIIQISSILYFQGEPIDNCTKFIFTLLECASIPGSNVIWF
NDEKTLLEAWNEFIIRIDPDFLTGYNIINFDLPYILNRGTALNLKKLKFLGRIKNVASTVKDSSFSSKQFGTH
ETKEINIFGRIQFDVYDLIKRDYKLKSYTLNYVSFEFLKEQKEDVHYSIMNDLQNESPESRKRIATYCIKDGV
LPLRLIDKLLFIYNYVEMARVTGTPFVYLLTRGQQIKVTSQLYRKCKELNYVIPSTYMKVNTNEKYEGATVLE
PIKGYYIEPISTLDFASLYPSIMIAHNLCYSTLIKSNHEVSDLQNDDITTIQGKNNLKFVKKNVKKGILPLIV
EELIEARKKVKLLIKNEKNNITKMVLNGRQLALKISANSVYGYTGASSGGQLPCLEVAVSITTLGRSMIEKTK
ERVESFYCKSNGYEHNSTVIYGDTDSVMVKFGTNNIEEAMTLGKDAAERISKEFLSPIKLEFEKVYCPYLLLN
KKRYAGLLYTNPNKHDKMDCKGIETVRRDFCILIQQMMETVLNKLLIEKNLNSAIEYTKSKIKELLTNNIDMS
LLVVTKSLGKTDYETRLPHVELAKKLKQRDSATAPNVGDRVSYIIVKGVKGQAQYERAEDPLYVLDNNLAIDY
NHYLDAIKSPLSRIFEVIMQNSDSLFSGDHTRHKTILTSSQTALSKFLKKSVRCIGCNSSIKKPPLCNHCKEN
KEFSIYMQKIKDFKNKQNEFFQLWTECQRCQGNLHVDVICMNRDCPIFYRRAKIKKDIANLQEQVTSLRMDW*
>N.PF10_0166
MKRVYTCLCFICLFFLCILKGVYNVKTQKNEGIIIDKEGKENDNGNSDRNTPNNNNRLNRMTLNKEMYEKHNK
MNNEEDNNKNINYDLSNLKDGMKKKHFGSNSSEDKTNKKSSINSVLEKYQHTKHEEKNLQNLNKNMMTHENDD
SMKSIFISEESFESAKNVCQFIVMQNDYFKNFCNITSLVYDIEKLKKNNHKIYEEILSNSYENNNGSKFKLLK
EINHTKVFKEMDNTNPKFSILFLYDKFCYGGIPLACSNLMKYDNVFDINEQSETTNKPDDNVNMEEMENYIIN
QTGMENGFEENVSDLTQE*
>N.PF10_0167
MLFKLKYLRKHNKIYDMIQLSKCKNGYVIKRSVRTSIFKDDDIYLNPQDIHNESLPKINYLNSGKLDEYVVYNR
NNIGMDTIIINSKYMNIKMINKLYKKLCDSEVNYTKRFIFLTSLYNNIFNYSYNLYDLLKILELYQKSKDIHY
LNLFKKILQNTNDLAYLIFSYKKPIISYCNGKIKGSAGFLSFLANNSASFNHSSYTYNNLNYSFLPYGGISFI
LANLRANLGFYFALTGQVIQSSDLVWCGLTKRWISDESLELMEISSESQLEVSEQDAHILLEEHFLKIPEKYT
LKNYEQVIHEHFKHNNLLTILKCLDQSRNSSDQNIKKWADETYQKIITLPPLATHLTFEILNILRNYKMELLK
KAQVNKRLWNEMIKNSYKVPTTKEQISMNELKYTIDKELFIKSLNIETNTLLNFISCPDILNGITSYLVKDTN
HAFSSTYLNNNIFQIKKDIIYYFLFYKNSYEYNIYDRPDISYSSLSVLEKYNQHYNAEGNTSHDKLFFSKQFE
RWNDDYLQEELEDINKHFL*
>N.PF10_0168.2
MFIHMYNTYCLYIMLLSYLFIGYRILRISENSPCSNVGLEIFFDYIIQIDDLKLLDSSKRTYDNFIEKIKLHE
NKELTLDIYNCRYDKIKKVKVIPGKWEGNGLLGIHISYEFLNALNEGVRILEILENSPAYQSQLIEYEDFIIG
YDKGIFRNQDEFMSYINMNNIIKENSHDKKVLFNTILYVYNYKHENIRKVQIQLNDSWGGKGLLGCNVATGYL
HKIPPCQIKDKEEKENINLKESFSSSHIKNNESESNNYKKEHMDITKVNDLEIVLLNDSVGKNTNNINNTESN
EKDSTNIIPLWKNEAEKSGREDKNYINSQDVELNHDNNIKYMKNDSNSNSHSSNGSSKTLEVDMDNNAETINY
DDKFSYELKSTLDDQPEEDMHEIKKENFQNEQVILEDIKREEPNNSQEIKNEIDTYNFKNEDIKDEIHIIKPE
PINTDNDKTSRLVESYSDYVKKMTIYSKEMNEIYESMNKNSEILNSIKMNATFNYVNDNNNNNNNNVLNVDKR
VNTEEYKNVDSQEIPILINPYENTTILNSSKMDNITKGTYINNKNDDIYSNENVQHFHEPSFNYVHDVRKNI*
>N.PF10_0168.1
MGAGQTKEIMGGYRILRISENSPCSNVGLEIFFDYIIQIDDLKLLDSSKRTYDNFIEKIKLHENKELTLDIYN
CRYDKIKKVKVIPGKWEGNGLLGIHISYEFLNALNEGVRILEILENSPAYQSQLIEYEDFIIGYDKGIFRNQD
EFMSYINMNNIIKENSHDKKVLFNTILYVYNYKHENIRKVQIQLNDSWGGKGLLGCNVATGYLHKIPPCQIKD
KEEKENINLKESFSSSHIKNNESESNNYKKEHMDITKVNDLEIVLLNDSVGKNTNNINNTESNEKDSTNIIPL
WKNEAEKSGREDKNYINSQDVELNHDNNIKYMKNDSNSNSHSSNGSSKTLEVDMDNNAETINYDDKFSYELKS
TLDDQPEEDMHEIKKENFQNEQVILEDIKREEPNNSQEIKNEIDTYNFKNEDIKDEIHIIKPEPINTDNDKTS
RLVESYSDYVKKMTIYSKEMNEIYESMNKNSEILNSIKMNATFNYVNDNNNNNNNNVLNVDKRVNTEEYKNVD
SQEIPILINPYENTTILNSSKMDNITKGTYINNKNDDIYSNENVQHFHEPSFNYVHDVRKNI*
>N.PF10_0169
MNKKKAIFLFDVDGTLTNSRKTIEQNVVDTLLELKSKKGFVLGIVGGSDYKKIREQIKYPEIFDYIFSENGVV
AHKNDEEYFAESIVNFLGEDRLKKLINYSLKYIANLDIPKKRGTFIELRNGIINISPIGRNCSQEERDEFFRY
NLKNNTIEKFRDNLSKEFEDFDLNFSMGGQISIDCFPKGWDKTFCLKHLENKFDEIYFFGDRTDKGGNDYELF
CDKRVKGYKVKNPNDTVKILRENFL*

>N.PF10_0170
MASDMEEKFREAFILFSSCSDHIEMYKFFELMNSFGIILTNDEKAALPNDINMDYWLNFAKKHYNYEQPFKHI
NNVNEQNTNVQIKIDNFLGIMKALDTRLTESDLNILLQITNPENKSTLNLKTVSQKLTESI*
>N.PF10_0171
MSDSAQVFMDNVYHLTGYLHRLLFLMKELDKKEHKINIGIERKEEVYLEKLYYAHKNKLENHIKHNYENSNTD
SHDSHNEDNNEKKHDISIENVLKKMKKKKSDLCINHDNHLNNEGDERKNVDKENNNMDMSNEFIKEEEANNI
ENIKSEGYVQSDEKKIKIKIKQDCIDIKENGNCDDNNNIDDNNNIDDNNYIDDNNIDDDDNNNVDNADNHHGK
ENKYKKKNSTISHDNDKMDEHANFNHTEENSNDGLYNNVNFKNNIISEGEEESRKEQEKKQNEKNEENEKNEK
NEENEKNEENEKNEKNETKEHPTVNPVFNNNDIKQNILKEDEKDIDFLFNDEEMNKYLNEIINDREQCMALLR
EKICINNQIAYMIKHDYEKLKKQYDKLYTEMEMNGEVLPHMLNSSKNKYTTNEYEEYYNVKNKNIYAIDYHQY
EDTYDMVNYMNKTKEEEEEQEEEEEEEEEEEKEEEKDEYEENDDIEEEEDDDDEDEKEEINKKKKKKKKKKK
ERHIKDEQNKDKYGKDKNVKDKNVKDKNVKDKNVKDKNVKDKNVKDKNVKDKNVKDKNVKDKIIKEEKEGQND
IHEYESESSKHNIPRKSLSKKHSIKYDDEYIPYISDKVKRYKKHKKNKTQNDEPSSKSNKIGIEKMDGEKNND
EVKNNDVVKNNDEVKNNDDIKNKDDIKNKDDIKNNDDIKNNDDIKNNDEVKNNDDVKNNDNIKNNDDIKNNDD
IKNIGEVKSTEGAKKNKESKLHSVKKNEDGNKSKEINQNSDMNKSNEINKNKSYEKIKSKSVDFNKNVLNTEK
IKSKSVEYNKNIINTEKAKSKSKSVEINKNSKAINKSKNNDNKNKYKTKNKSKNVNISTDHTNNNNNNNNNYK
QVIKNCSNIQTNKNNSEENKTTNHAKKNESLSSYTSSLFDINQNEETTLGNLTKNEK*
>N.PF10_0172
MKDTKLSEWKYLNLVNLIKKMELCQKDLLEYKRMEIKIGKQKEHKQSKEDKQSKEDKQNKEDKQNEEDKQNEE
DKQNKQDKQNKQDKQNKQNKQDKEQKKKKKKEVLHKKIKCNNKTKNKNTLRKNEKIILLKSIKKNNSRKGPVT
LRGKLDEEEKKKKIIKKIKNKIKKKKKTEFSKTYVLKKNIITNKNDMMNVYNNSMNDDKFFLYSKKFIKRKTR
KTFKSNKYVTSVPHHVDNKRKKQEQKNNIKKYNMNHNNILCTSSTNNKLEIKNNSNDKYFKLKKKKKKYIYI
YTHMNPKMSQKTHTFVILNSSKQKKNEKKNKKKIHLKVQGKKKKYVPNSLFHINQKKLYQNHKKIYGKNKINK
NALYKIKYVKCNRNNIPKHKIYFHNTHNHKKPSQDKHIKLKKCRALLNALNRTSCIIFTKKKKREKKYKINK*
>N.PF10_0173
MLSIHHKVYDKLNYINKLTFCHGYNIIYKKNYSSGAEYSSNISRIFYHLNEKLKNVREELRKNEDNYKKEKDK
NNNIKVDIINKSESYIKKENVNDVKDNNEISYNTKDPLEMDIMYECSSKEKKLIYIYKQYDKIICKINYDKMM
GRDLSYCMNILSKIKYFKDNIFWYKASNKLIKGYMLHRINIQSYYIIANALSNIHLLYDTKNKNIQYKNHKQI
FNKHIINESICNNKIVNNNLQYHKVPYQNIKNESSSPFRYISFEDKIILEDDKNNICKYHNIYDQIAYRFIID
IQNMNLDSISCTINLFSKMNLLKYNLLGYYFADYFVLYLINHNKNNNNNNNNKNNNKNNHYNNNNNNNKNNHY
NNNKNNHYNNNNNHYNNNNDKNDKKFISQNKSYSTIFSYLMNKNKYVDISPNKCNISKLVIILHSLVKSNVIH
IEFFYICLQYFNKYFIHLNNIDICNILYSFSRCQLAIKSNHTINKLCMRKRIMDNCFQNFVNNIIKKLNIYNE
YYSMDQINNMENMYNAYYDYFHKLKNNLLSLSSPTNFFLCINNDGKLDKLKCWNVLNKIINHIEKNEKVLNNF
TSLQISSIILSICKLKINSLNIFYALNYRVHYLWKYFSLHSIADYLYVINERNIYDEQISFLLFYQFVKIIMW
KYLQYMENKLCIASINNGIHKNKIKQNNQGIIYHLLLNGQNDHNNHNKDNNNNNAILKKKRKKKLHLLNDSDI
SMCIRIFQSFSKFDIIKNINMFLYYYHIYFPYDISTICIKYDKLLNKKKILMRDTLEMDMVIKRNTCNDEIQI
KTTNQQATICCDDNQNYNYNYNIFKSGNHFNLYNNKEGQNMYHNFLSYQQNEQDDHKNFWMNLNLYPKKSFLF
LHSYKKYIDIIMLYTLRTFFFLIFQHYHSITLDKKCLLCYFMLSVPHFLYPFKLYNKNNLLGNIYDIKYMNKD
TNNNHIDNNIYPFKLYFKKKWKHFNIYIPPSAYILLDILYNMQLDKLNEITIRQIYTCLLCLHLNIIKFCYYI
RPYNNKKNKKNNYDNNNNNNNNNNTLSLHKEFIFHLLSLNMLKKMNSFLDHVNQNRTQQNEINLTSRTHNEVY
NYLKSLIGSNMFYKKGKKINQKSEYIVGPFVLDCVIEFL*
>N.PF10_0174
MDDNTANIIGSHDNILTDPRIAQDCSAETNELLNKAEQYYKIDIINLCKGWIEDIQNKEEKLNLINTLCLVSE
GKIFVEVERSEVIRMLSKIKEEDGNIEEAANILQDVQVETFISMNKRDKTEYILEQMRLVLLRKDFIRCHVIS
RKINPSLLNTEEFADLKLKYYMYMIQYYINEESYLDVAKCYEERFHTDIVLNDRNLWIDEMKCYIIFLILSPY
DEQQNKLSNLLKMQKKKLKEIPIYQNLVQDFIEQDLIQWPLPYQQELFNFFIFDDSIFLGGQNRKDLFKKKVM
HHNIHVISNCYDQISLNRLAQLLNASIEDSENLLSELVSAKFINSKIDRLNGIIKFGQKKNPENLLNSWSLQI
NDILDLLEESSHLIQKERMLHEAKLKRIQLENKKMTLKYKMYYNILQVHSYNMFLKKFVWKNF*
>N.PF10_0175
MVIILLLAIICSYNITSYPCLNYKRRKNFYYCEIFKNEKEENSLKYPIYKYKRIKRNRSNINAFKKYNLINTL
INRNYNVNNNMKKDGFHTRRCVAQLSLFSRKKRKKTNNEKKYIFNHIHFYSYILVYFNEGLLQHWTTTPHVTK
IIFDNMYKGFRMKGGDNINIMGFYNTHNRKIHNYSLDIHRSDEKDTTDIIKKIKNKTGEKLIKDNNKKKNNNN
INSNNNNNNNNNINDISIKKDRVCCNNPHINNKGDMDEENNEDHVNKTKEEHSFESVLNKYNKELILSKSKIV
HLDFPICKNNSSNIVTMDDIFYGGFLNIYKPVKLYSMRVCEKIKKILKDYFYKLNKKKVNIKVGHGGTLDPFA

EGVLIIGIQQATKKLSDFLKCYKKYLALSIFGYETDTLDREGKIIKEEHVIHKKLKKQDILTNLQKFIGHIKQ
YPPIYSAKRFKGLRLYEYARKNISVQIKPCNVHIKDIQYYKEIDLPFLDLYIHCSGGTYIRSLIRDFANSMNT
SGTLIKLVRTEQNEFNYKNSLHYDDINLNTIKKYFIKL*
>N.PF10_0176
MVKIGKKLKGFNEGIPKLNTNINDKIIKEKEENDSEERERQREKYKNEKEHELNEMIFEHDDQQNIHEQYRKL
YLENIKDSKIYIHDDLQYYLEKNNIPTLLVLLLYRILIEKPENIIKFIIDQIYVFLFTKTETNKKKHALLKPL
ECTFQQSCEEKEKKTKKEQDENNMINSLDGDMNPSFHESINDALQKNNNLIESINYESFIKQHFFGLDNILEV
VDFLKIENTEEIYYNNLIHIIKNFQNPINNQILQRENVSYGQTISLNKALCLSETIYNNYFYN*
>N.PF10_0177a
MEKYSKVIKKNEGLLNSSSVEGLHYNKFSSDDSVYIKTNNLDKMYYAYNNNNNDRNIVRKDFMPIEKYEDIKE
DTNSNNYHNNSYYGRHHHNHILKNYKYDHYNKNCFKKYDDFNKYYFLIPNNKINAHIDEHPNYRLSNKLKGTN
INDHLNDPHIFKTSEYNYKNVIDKDTSYHMYPVHNNNIPFIHNRKSTHTIKNKNCDFNNISQNLYVSSNPYIS
LDSYDNIIYSPKIGNYPINLNENIFIGSRDMNSSSRQIYKYINNNNVDSHMNKMNEHIHASGTKNYTLQEYPH
YIYESNVNIEKVCDINDNNICGGAKKKIISYDLEEKNHKMDDSKDNIKRNINNVNYVKINHIDKEDKNKKVNY
DDDKKVSDDDDKKVSDDDDNKKVSDDDDKKVSDDDDKKVSDDDNKKVYYDDNKKVYYDDDKKINDDDNNNISK
DIRYDEKNNFMEWMSHNKLMKNIISSYNEDVNIENHGLFNLLFDIYGFNKDIFKEYLVHNKEETIKDVIKNIV
NELSNEKNNKISEKLIFLKYLFNEYGHTEYPNLISLKNFKLIFKNYKNIFSSDKIVLFIFNCLDRGKRYHITQ
TDFIIGMLACSPQINNDISDDTGKLRHQLIFRAYDLDRDGYLNDNEFLVFLYHIYELSKNLQYSKLKNDKRKL
KEFVIQEKNKIMKDKKKISYDIFYHLVLTKQIQGTTNLLRSNYDVSSVVKKYFLYTYAKNFITDTFINMDTSF
FITTKKNIKNEHLKTQNLPNHENEVHIIHNDEVICNQEDDQKGNYVTNQDDNLAQEKKETHSEDHFHDSISNN
NSYADVNACMGEMYAACDESEKNVEEHPYECVREVVHSENEQAEKCVEVKEEIKGDMNFIQSGDVEAADVEGA
GVEAADVEGADVEGADVEGADVEGADVEGADVEGVHVECADEQNNDHIPTKDKINNEDVFFDNNNDKEHIRSN
SSNEESFTKQLKDEINSSNEKDDEEKEDSFKKTENLNEEHIEKDGNNLSYLLNNNNNIVSCSGVDIKNNISER
NIIYNMNDNLYDNVAKDRYNYFEHDKKQPINNEEDINKNNNNNDNMNCNTHISYSNINKFVTNNKEYFMNNKN
NENDKKMFPSYKEQKNSTNINKSLPHFGKKEDIQDNVINKELHIEHIIEDKIERIKKLVKIYREKYITDHRLN
TLNQDIAFKIFTMFYKVCYGKKKHTYTSFFEKFQICTYNDILLLCDEVAKLFKMENSLENVTLPCKVFGDVHG
NLFDLLDFFNLYNWPMHNNKNLLLTESDLRKFEYIHNDKDIHIDKNKDMQYNDNDIKYVFLGNLINRGNYSLE
VICLLFSLKILFPKHIYLLRGNHEDRLFNYIYGFYKDIEIKMKTNMEYMGIINYQEQVISAHSYELFNRINDV
FEFFPLCVLLDKNILCIHGGIGDSVMTISDYQHIRKPILIPQNVDRYSNNKSEHVKKIIIDTLWSDPINYNDE
LDLQLLKNSSTYDIIPSRRGNITFKFGKHRLNDFLKNNQLKLIIRGHECVQEGYKYSYKRKLLTLFSAKNYCN
KYNNNASNAFIVRKNKNIIIFNQILKYQHNCNTLINNENKQVTINQLQKYNFSNDKSNDKKTSPFFNNTHLTK
KTFAVSHGYLSKCDEELNDQLNTHPSMKNCSGNNKNFNKDADKNYMNGSGENKSFNKDVDKNYMNGSGDNKNF
NNDVDKNYMNGSGDNKEDLNNSSSVPTTNINELNKNGAISICENEVLMNNKYGVVSNMSEQSRNSTENVASNN
DMDINTVNQISKSIIKKKWRDTRTNQNLNSNKMNRNNNSQLINDMGSVNIYKEVEYEEDDKIEKEIQREEQND
IQNNVQINLQNDVLKQVENINVEISNDNMHNKNDVHYISMNEINSDKIKSSHINDNVYDKNESSTFSFEHKET
YDEKGDNEKEQRNLERADSEKKNIVNDDYDFYSYDEIYINSLINSKIWEKNDDDYKIIERKDTNDELYKIEDK
LQKQVNIHNVINEFFNNEEIYQEKTDSNYNMSNENESISGDNENSKKNYYSKLSKINEMYTNKHNDGDKIDEQ
MSDINSVDLINEFTSKSLDFMMPPNLGLGNRTQLKKDISMKENNKDQRNNVTTLKSLRGDNNIKDSSNK*YIK
NMNILKCIYIYIYIQKSLEKINDS*
>N.PF10_0177b
MDVQKKKCKTYYLIAILIWFILISVVLSRGEIINKHVKKKEGNYKNYEDLDSDINKKNNYYDDKHKNKRLLSD
TNINNDNNINNDNNINNDNNINNDNNINNDNINNNNNNNSLYNNNSFYNNNSFYNNNSLYNNNYLYNNYTHPK
QQDGHKNHPEENKNYTYDDKTKFTKFNKHKNEELFTPNPPLNYEKQTLSNHQNINFDSTVEEGNKDLNNSGNI
ISQELEKKLSTDNHKEEGERKKEETNKKHDIDTNNMDDNEEKNIYEYFNKKTKDNFDGLILNIYFTPNNNLYT
KVKVGDQVLKLSLNSRLEGVYVFMKDSPACYLEDDDKRTCYNPLMSRNSTWCNNDLMCLPAILTKPYECYEDS
SLNLENKVQYPNVYYDSLKFNETHIEGSDDLEFIDLVGVQTKYIRNKKKKMDVNKMMSNENKSMDPKHVENNL
QNNNILKRDNIIIKPRVKLSENSNLLFQGVDIKLVVDMTFYNDWNLFKDTDGMFGLAGKELSCRNISAWNNIL
ENNNSIFALDINLPNDAIKTYPTPPVNSVGTLYYEYDLKKNGDNKKSSDQNINNVKDNMIDNKNVDVGVQNIN
EFETSSSVIHIGNYSKEYGNIIWSEPRERGGIFSDSFIQFTIYNLEVCNKNIFGKYSSNWQAIIDLSSQCLVL
PKMFWLSLMEYLPVNKDDDRCIRKNMNPNNNNNNNKNNDNNNNNNKNNDNNNNNNKNNDNNNGDNVDYEENSL
PRMCSVDHRNRPLPTLKFFLSDNDIVSDNNIYSSPESENKGKNEKKNEKQAIYIPLDNLIINDKKNNQNYLCV
IPDVHDGRSTENSARTTKPLIKFGTYVLNNFYVVVDQENYKVGFANKKSFHTSNEKCTQRTQCIGDQYYEPAL
NICVDPDCSIWYFYTLNDQTKKCESLSSRFYVFIIIILTLLILDIQSYYFYRKSVHVAKTSSR*
>N.PF10_0178

MDKLALEAFYVKKRKSFKNIYYKLQSLIMLTKENKRTNKMGRSFEDTLLAMLFLSSNYYIMNNNNIFLKYLNK
YYKGIKKVKLQTKKYKAEVYKVLLKRISYTIYNIFLKTMKNKERKDTPDIKYTLYNIDKNKNIQEEYLCNIFD
KYFKPLFSYIALYKGSLLIMEFYNIRYIFNKQIKFFTKIKKNNKHIIYDTFFL*
>N.PF10_0179a
MAAKHHPDLIMCRKQPGIAIGRLCEKCDGKCPICDSYVRPYTLVRICDECNYGSYQGRCIICGGTGISDAYYC
KECCLCEKDRDGCPKIVNLGSAKTDVFYENKKYEFKKQ*
>N.PF10_0179
MSPSKTMDTFYKKKRNVLTLINDEQDLYIYKDIYNNCENDIYSLYLNIINQYKNCLDGEKKLFLNKCIKNGNV
KYIEKVVKQIIEKKNVYNDIDDKSIKKSIDLLIYPCVYEINKNEFYYTTSCCSGRIVIFKEVHINNKNDNQKK
IFKNSTTSQEKKEENISHHLSNINLLNVHEKDELFLSHFKKNFILQNNEEKYTYCSDHDISSFFDVSKKKTNK
WEDIYEEKNLKENILYINKEHKKKKVRKKKKIHKKNVHILYSSHSHQHLKYDIKVIKDILKKELFQNYFDVNQ
RNDYKNISFDFMGTKKYSLQNEVNLNSSIITMSDKEGMKKKEKNNKVINILNNQNKRNIFIKFEPFIIHVKCT
TFLSALKLLKIAQYAGLKQSGILNFNKHVTVAIRGSMRLEHLLGDIHPTLQQTNLMEIIHICNNKLSKNLSQL
VHFYKCFKQFKEHEHTYQFVSINREMLSSLENNLSSNTKQKKKSNKKNTLHVKDNLQDNKKIAHHMKKKKEIN
HLYLTCSGKKNIPNGNRSIAEQDKTECKLKDEIIYENTENHINIIKKEKNIIDLNKYNIQLNEEGYIINENKN
DKFIGWKILGNNNMNVQNFFVWGHDMFMQENKIYMFGGFSKGVRNNKLKIYDIINKKHFIYDTELPSLVFHNF
VQLDDKFAFIFGGRQNPKNCTNMVWVYNIKENFWIKARRTSTLVRKNKNVLFLEKMEKNMEVKMDRMKYHMGK
KYNNDDNNSDNNNDNNNDNNNNNFLCNDENIFYFNNEEEPCPRYRHASVFVRRYIKKSKSIYIFYTYGGVNEK
NEILNDIWEGKIILNLEDKGIAHIEWNKKNCSQKTEACRINNHSMIYNKKKNFIYIVGGYQDNDKDNYTQYNE
YNNNNNNKDNSVNSDDMIGMPNLSSTVVCKKMEYLYTYDIKKDNFFYTKCLGDDNMELKAYPSDRFSHSTCLI
NHNFFMLVGGINIHRTLNDVWLFHIKTNKWNYLGTFTFQNMYVRSKVVSENNCVYIIGGGCTVFTFGSFFDVP
IWSNFSGIMKSIQHKELKNVLIFSEDREVKEGQASYNNEIKKNCGNNNDNNNNNNNNNNNNNNNKCSNNYNI
LVDKVARNEHLEKYNLCHNMLIEHKDVIKKEILVFEKKKKKKKKDEKGDDIMDVEKKNELPHIIDEEKKGTD
RDVEKSSFCLDEKSIKIGLYIIIKNKHFVKQIKVYLENKKMFDNSLKIYNPKGNQKNEENEKNEENEKNEDYN
KIMGEEKKFYVPIKKKLDDDILKNDEYFKTVLLDKIERCNDGNILYYEKVICEIYNGKYQDNIKSQNSNTFLK
KKLRILFHKFLNKKVKNYLNSSEKKMVLQGYRKYEIIGNILIFHYKYFECIMKLYKVYEGKLKKYKKEINEMI
ERRRLYRDYSYIIKNKKKHNKLVSCFLMNDIYEYKYYVKEVKKFWLAIKDIFNNKDIFNNKEIILNTNILSLK
KKKKEKKKKKKKNNNNKFYVKVKFNKKGITNKINILLYRRRKNLFDENFCRNIMKRNKNCEIIKKKRKIKCVA
LYEKVHGKLRQNKIHIVCGKNLKTVHIENKIKYKLDLTKCMFSSGNGTEKERMKNMYIVSNNDDNINNKDKNK
NNNNNNNNNNNNNDNNNLLDEKRDRVKENVVDLFCGVGYFTLPLLKCIEAQNKINNYFACDINPDSLKLLRES
IKLNNINKKNIYIIKQNSFMLSKNVQMVRKCHRIILGLLPHSQPAWKNAFFLLDNKYGGILHIHGIGQHIFDE
QVCFSSINTYDYILKKKDVNISSINKLTKLQMVEEYVSNVEDSNYVEEIKKNKDHFHNKYINHYNSNYNKKLY
LGNNIPHNLSFAQYTLIEIFKIALYDNLKNNIFWNIAISHVERVKSYAPRMYHYVVDIKCDPLIF*
>N.PF10_0180
MEDTEPDDEGEEYKIGKNIIHNNFYNQNFIKKFFFENIERKEIDYLKRDKKSSNNNKKFIVHLVPSKNSSFYN
FLQKKKNDVLKKYGQDETYKYDIHISVTGYFGCDNINVFIQTLYHFMFQYVKLYYTFKKVIFRNLYYHISLPN
KAEVESFILKKEKIKYEIKTEDKIQNVNPNIFKKQTNYYYDEEDQKHPPGYLNQKQHIVDIRKKDITYTKNNE
SEECVQNNHVPCIKETRKKNTQPTEHHVITTNDGYVIVPIHCEWLKRILENFFFIIKNNNIISYNTITNGMYT
INNTISKVHDSIKKKNINFSNNKNILSYDPYKCKLLLLTRQNCNSNISSNTTSNMLTLKKKKKQHDSTTSVGT
SNMDNTLDIISSSGKCDILINQYSEHDKKNYNNDKKICINDELTITSFGNINSHSNNLVDPSVFNKTITSMEG
DYMRNTLICNNKSSINIINNNNNNSSSNECLGGASHMEKNNLSSNKSDSSDKTNNSKKEKRKKKSNHNNHNNN
NNNNNKRTDTQRKDTLNNMKYKYNNFKVNVSDFRVKDCNHISLACNRNNKYIQKNIANMYKDLKYYFLNCSWD
LVMFECDENVFPQTNKQNKFLNEILRVKNFAVS*
>N.PF10_0181
MVHIQESSYNNQKNNDKIICIRKNENCRITYHNLYDEFFSLSETEDNNLYRTSQDMDKKNIDNCLYNYKNSST
LKRKNKNKLIKVKKECVHNNIFFSDTDLNNKKKHDPYNMNFKRHKAYSADNTSFDYNNNKKKKKKKNNNNNKF
IYLTNSFQNDVNSFSHYKRKNYKKKKEMDIEYKNKTNNDKYKEMKNMIKKEYIKFDDMQYNPNKYYDVLNNKK
DISFLLSYSNDNIYRNNHKYIYNNLEKVTDPKLGPFENINNLTTNINNKKYYQNITNEYVSHNNYNDITEDEE
NKVIKINMNPIYHSTNPSSVTTYNNLFSIKLLCHAKCYNLIKISHVFFSKQITYKWFDNYNILCAFLSPEKEV
HEFYSSVDFYKIENLNLKKNVTSSKSSKYILFIFKNGSVIIWENFNNHQKNDNFINKILLFLNSFSDELLPVY
LEQEDTIYFFQGRQHNKWNDQCVPNMKKNNKLKKKEKKNNSYLFKKKDKTKKCIHIIKNNSRQNDKNNYNKSN
ELREMKKKKKKKKLKSKLSYEQKDKKENNINTFVNNGVIYLTNKSLEQKLTASFALSQSIQLDVHEMMMDIAI
NTLFKISKEIAKKGTCIISKETISRMMNVYSSIINVNAVQDFLDIPEYFWNKVEYEHAWFEIYKYMEIPERIK
ILNKRYNYYKDFLKVIKTEVYNKKTFYTYRIVMLLLFIHVLALLLNDFLFV*

>N.PF10_0182
MRIFGSLKRWTYYSPYEYTYILCNEQKSRRKNFFSTMLYNTSELQNNMMKNLENMNGLNISYGIKKSVKYGYV
NKKMFEHYNNLIKKFYCNFTFEDIILILQSYALCKERNFEVYSLLSNKLLVIFKDMNEQEYNQQVYDNIYKYI
LASQQLNYSDYEIITMFLKIIKNNLHIYGLKKISIILHSLTKLKIYDQQLLDTSSIYIVNNFEHMKCNYVSHI
ISAYSKQINIKFLQLYKKLLQYIYENIKYMDSLSIYNTLIQIKPIIQTVDTNYMSLYKTQDDYCTLQNFKNKS
YSSSPEDNLFQKKKKNDYDKHNIYNESSNVCDKYIKEVLQNNCNSYNNNNTYDHSNVKNVHTYQSKETYPHTI
PLVDTYEKHTKCTTTNNINNDKQQKKNDENNVNISFNSSKHISNDEYCHLNDTLKINNINKEQNNLNLKKEKK
ILQNIIPLLFSKVNNCLAFLSFKQLVKLACTYKELNYFNYIYIYKRLLPYLANKIQKNKISFEECVLLLECFT
VLPYIDNNIDEIINIILTNLETVITFHYNYLCRILQCFQHLTIYNDNILSKIDCIIFKNKKAFERYCNLKDLQ
LFLHFYEKNSDEWQEMINYLKQLIEQNNNHILQSEENSIQQDTHQHDSTNNTMSNMNIYNMDKKLIIYKYNKV
QKCFNEKEKNIYEAHTNVHDDKKDEMDGTPVHMENSNDVRKKVSDYIYFNTINEQKNI*
>N.PF10_0183
MSELLIRSKSGVKLYEFKKLENLYESNIIFEYDGCIHDAIWSYDGNSFLLLHSIEGLLLISNYEEKKKRKIEK
IACIGKYKELFDDENIKLIKHVQWSPNNRFIVFFFSYEEEKHNRLGNLLVWNVKDKNILCSFKIKKKSCSNWP
VINFTNDDNYFFLQKKTDIYIYDTLKLIQDHDNITYLHNNDIPLNYIYTWNQPNVLAIYMSSYMSDDKCRFFI
VHTKNNFLGDVYIFKIKGLNNSKIVSYNVREDKEKADEMNVDGKNVNVKNVDGKNVDGKNVDEKYVNVKNVDE
KYVNVKNVDGKNVNDVGGEINVDNNVSYKNDPSCVEKIKIELLIRKSFDNLDTLTCLWSISGQYLIFLVNTND
TTNKSYGYLSNCYFCSLVSKNIHIKRINEQVAQDVKWSNIRDEFLIIEGKSDNIILLYDHDLNVKCKIYSQYK
NTIKWGPFGNMIALGGFGNLAGDINFYYKEIDNSVSIIKKYREACTVLCDWSYDGTLFMTASTYPRMKVENVF
KIYTYEGTLVNSYNFNELYDVKWKNVLPGFFKQPPKPQPNLLDSSNKKSVYKIKNLDHLLNSTQNNTIINNNN
NNNNNMNGINNNIPMNSINNNIPMNSINNNIHMNSINNNIHMNSINNINVGMSYVNNMNNKMMNNNNNNNLTN
PTSYSFDVYDENNVNVNINEVLEKDKTKTNKGMAGTTSTSLSNLTSNLLNGHRKKKTEKVKNAYDWDVDWRKK
KSMGIMNSVMNTNNVMNMNNMMNMNNVMNTNNMMNMNNVMNMNNIINTNNIINTNNIDMNQRDDYIPNEQTTQ
DDKNQTLKNDKILNSNLNSSNKNINVMLDNIKSRDNSENGIIILQNKLNNYHDKANINDNMNKDEHVEEKKKQ
KKKKKEKEKKEEKEKKEEKEKKEEKEKKEDHEKGKDKDKKEDHEKGKDKEKDKDKDINNEQYNTSEQPIQINN
NHMNDVNINDDDKLYSNSKHTKEQSKLQEMGHIKTISLDDINKNKGKDNQTNEIKKDIKDKEKNTEMVDSNMN
DEKDQDKKKKKENKNKKSKKKKNDNNNVDGVYNDNVPKDKTYNDNNNNNNNNNNNKEVEKNNSSAGSNSFSNIL
LKIIKYKNDTNNTNNTNITNNTNNTYNTNNTNDTYDNDQCHINKKNMNVELNKDLDYVKRQINVPNSKHIMNS
TNNNGTGVEHMSDMSTLNCNKKNNDMSIRISNQYNNENIKMYTNKNIPLHGDEFLKDIKNGKYNMSDMNITKD
MMINNMKNNMILNHVLKNNDMKNNTIGEDNNNNMFNTSNINDIQSINTLNNLILENALLRNELPIYQSKGKDN
ENATSRNNTNDNMNSINSMNNVHNNVASNENLINIFKKMLPHAKVNIVGNNRNSTKSLDSYNMYDTQNNSSLN
RETHQSFKRHDSFNHPYKEGNNNNNNNNNNNNNNNKNMFNEYAKEHLIKEMILKKYLMNNKEYDTINNFSLNKI
DDIQSIQSKVQINNKSNVDGVNNNNVDNVDYMNNQQYNMPQNLKNQLDNLDFFELLKCYHSLNIQQIQIKLYW
IYYKIQMFEQNNLLSCDNKDKKILYKLKEIRHILEYTNHLLKDHFIKNKEKYQSLLQQFIIILKHHDNLYLQK
REKLITDHYDKQNVVHFICNIEKFIEKNMNLTDNFISSNLNFIPSKTINNKLGRTNSVIYENKNNIKNNDHHT
RGSQKITDNYNNIDINKINHNKYVQNKYNDHNVSNNYIYHQNDANNNMNHLNQNLEFQKKFILHKMNDYFKNI
NATNEQKNIFLNKMNLTEEQKMFFLKQLNIKDEEFIEPNNTKQPFVNCLNNNNNKIKNIDEKEKKDKTNKTNK
TNQTNQTNQTNQTNQTNQTNQTNKKGGDTYIDSAYSRSSYFCINDPNADKNKENIKQNNEKTDHNEDAIGGSP
LLNLINKSHGQRVENNINSMMNNLNHINLMNNMNYFNNRADDNQGVENEQSKWLSQKNVLLNILKNQKKLKDD
NNVVVPNLNEENYMKNIKNELNKDINTFQFNNIPENISEKLKNIIHMNNKDGKDLQNHQADLINYIYQNKQMD
GVIHSYTNCNDINEQAKINLNQTKDKLKNISNLSTHSGKLEEQNNTHLQHLYSQQQRNLRNLMNTNEGDTNIP
NVTQNKNHINSNTSFLNTGNDENNNRNVNEFIRIMNNECMSNLQKKNVNNNNPLNNNNNNSNNNKTLVGMKNI
VEEKKQTIINNDTNIISNHFLTNEHQGKGDTTKTFYINKKNQNVLSQSLNNQTGVVNKGISNKHKTNNIVSNK
SLNNSLDKKNVQQSNMLDLEDTKRPDALRDKCWQYVDPKGVVQGPFYLDEMRVWSEMGYFEPMLPVRCCDSDR
FVALNKLFPPPLKPFTVVPKPQPVLQWEEELL*
>N.PF10_0184
MNNLERLKQRLEESVEKHKRRLLITREKKKQSNSAIYVNNKNVNKERSKIILNDEKSICGDNKIRTDTYPSIN
KNKLIENLRNTKLLLIKKRRSLNNMKKENNIVNKYDTKNELKNKNIQLKKNTPSIINNKKGMMKYLNKIVNPA
NQNYSIENKIRGRVNISHIKEKEKALKDEEDKGNKTNYNNNKEKIYDDNNKEKIYDDNNKEKIYDDNNNVNIY
DDNNNVNIYDDNNNNVNTFQNISKDVRDNINDLFRNFINCKINNLYVITTDTNKIPNNKIFLSETQQQFLEIK
NKQDNNEYEIKDEHSDDNNHSNYHHVQDNPYVMKKKRLDDKVKEGMDKNYMDNIIYVGKHERVSSNMIIKRNN
NNYDNNNYDDNNNYDDNNNYGDNNNYGDNNNYGDNNNYDDNNNYNNNSKSNIHNNSIYETYEKNLKKLLEIEY
KMKKKIESFEKNKDDNNIMSTKSSASSYPYGDDEDGDNITLYSHICSNIKPVKNEIQFDQSNIDIPMDAKNYI
IKRMTSSTKLKSNLTNEEGLYKADKHNDNSNNNITYCDNNNFSTHDEDCELYCIQKSIYSKDINNICHTNVSN

KHTCSENKKCIENEKIYTNNNKKNCLSSYDVENKILRKINNDDDYKYMNKQDNIIQNELIKKKKKKTHTHTHI
DLNNYSYEKNGRNKELVTMKSNQENYSSNYCYNYSYEKKEKINKRKKKIKKKKNKIKNENNKIKNENNKIKNE
NNKIKNGNNNKNKNNNNNSNSINPNQNNHVRSNISLSNSKDHNSIYYSSYIEDQYVSSYNKDDDENIYNYNSS
DHNILHGSKKSENNICYNHQIKIYNKETNEKSSCLKGLNKSSFLSNKIYSSNIHKENIKSFITNETYEEDQRI
KKKNSLENKIENGEEQKMSYYLKEKNNNCYEKFLQHSSDIIIEGGGKKKKKKLQSNEYIIIKHSKSNNNTILL
KKDKKIKENYNIHSYKYNQQNDDIYQQIQSSSSMKRETECLLNNKIKDCHRLYKDNNIHHMEHCYICSYPIQN
NEHKFILHKGEEKKGKANLDTTYKNYDNNDNYNNYDNSEYHITMTSHLIGSSHSKINKEKKKSYLSTFTNKRK
DLESYMSSHTVCSKKNIQNVGENYKNGNNDDEHKDDILCDNNIYNHCHDLGTYNPTYESEKVTPYLSEKHKVI
YKNIYSNQINIRNNERNHSEDETYIYIKKENIPFKKEKEKKDIFSYIKGMYLSPTSLPPQKKKKKEKKKSFIL
ENEEFINEESSFDIQSNNINSHFIKNKNILNIKNQTNDLENVKCYHHIKKQNNSKSQSSYFCSPAPSNSILRN
NTESYFLKNDKQNDQKVKEDGQTIKNQTTANNVQKKRNKNYVKTNSILLKKILKMKIESMKKEKEDIKEKNHF
NYNNNEKKNQDDNHNNNNNIKVIDYHHDKIIHDKNSNKIEMISTKDKTIITLINDNKQIYEKEYNNDTCTKDN
IKKEYNNDTCPKDNIKNGHNNDTCPKDNIKKEYNNDTCPNDTISNINKEYSSIYTHSHMNKESLKYNNISFYQ
NKESHDFISRKDHYIHKHEEKRSKGNKENNINNYNNICNSNNMDLKDNISPSYHTNNNIKDVVKFNQSYYCYF
KNVQKNDVYPNSEEPITKEDKTKLLIAYLKDKKKNKTQENNNTSDKYILKYNIEYFKLAQFFSQDYHLFKNYI
KHVDKKLSKNNNNDIDSLDETAFSIIKLFNSKKKNDLSTKYENKDISQKNTIFNNIEKKTNQKINIKHLHVVN
DNENIIVNNFNKSETQIINDTRKEVYSIVQNQKKSTFLRDKNGTKNYPYDHLDNYKKEIHIPTKDKYADNKNK
KAHCYKIINNGKPSLFYEDQPTKMNQNNDITPNERKGKKKQIVLKTIDLLEKYEKISAK*
>N.PF10_0185
MKYLLNLFKKTFSNNIKKMMPLNNQNKCYKHIFLNTNKIKNRKNTQNENYHMDIKTIKCINKSGQKNIYIQNK
NNLVSTITQNVINKSGENTKYIHNETKSVNVKASDCLNDSGENNKKKNTKGENIPDVCIKQNDSLKLISLGNK
KNIEYDTKIEISMNNIKCEEIRRFYLNNNNSFVNSDGLKCNILSAIHCDEKNVLFYSLVFSFTNINNNYIELI
KIHIKNEKTHETVVKNVEFIIVNNKCTEENERTFSNIKVAMKIQENNEISRYAKKNTFIQDISLDNIFYIIEY
RKWDSYIDCIAGANSNALNNSHYKRDQ*
>N.PF10_0186
MCKYALINKCDRGENCTFAHDINELRIKPDMRKTKLCKSYILGKCTDNNCIYAHSVNELREVGKPAICQLHRE
GRCIKGNQCRFAHSINDINTKLVKFYGSKVDIDVDEEIVQGKIHSKNISNINNSCINILNKSKSQMMNKIKGY
ENEEIEKNEFINIQNMKNNNSNKKDIYNFQMHVNNININDNEYTSSVNIPNDILIKHKNNNNDDIINRANLVL
HKNITGGDIYHNNNCKLNICKIYNNNNNAGNEKNEENILNISHVKKNTKLCSLNIKNEPIQQVNDFLLYNNNI
LEYKKEQKNYFISPQNIINNEYDGCNFNDMLNNNCKSLNVHKSICLDKSNMNIDEEYFDMKEKTMENEELINN
SYVKNKDNMNKINNMETRLRKNIIIQPKKKKDLTYVNTINSDNNMKHMYDMKCPKINVSNMVRFNVENELRKI
QDCNKRRPQLSSLENIITERGYLCKQNDDKYLLNVVGAINKYEDICEDICEDICEDICEDICEDICEDICEDI
CEDICEDICEDICEDMCEDICEDNNLSRKNMFITILENENKNTDDYRVNDDSNTCVINFVECINKENQNVITE
KGKTNTRGDDNSKGNNNDEIINNNTTTTKNNDNNNDNNNNNNNNNNNFMHSNDYIYCNNVVKNVTHLNTNDSF
VYSKEEYNISSDEDNLMNQQNVNKKKNINNEKEFSNEFNINYGSHSKVQQDIYNINCVNQTNGVNNIKHTNNM
YSDHGYNIEFIKNNDKQYYDENNRNSSNLVQMINKQKEKDRGDEESNRDINLKENNNDDGNLIIYYDDDDDDG
DQCYDNNGCTTNLHDESIKEKNLSDVPFEKINENLNYKNHNDDMMNPLNFCNKKTDNGFEYIENLYFQKINDY
TTNGITKDMKNNKKRNTNKLFTCKELFPKNKEYFILNKNESKHLVGYNISNDDNKIKVNMYPYKDTKLNALHN
YICVISPKNDNNNINCGEKICNDNNMLLNNSKTSNNNEGCPFSYMNIKSLSTHNDNRNYDKNINPEDLSNLTD
VKEPLQHALWMNNNANTNININSNHNPNHSNNNNYYYHSFYNNDHVDYNYNINNVNKFDNFKTYNMYPNENN
PIEVESNSEMILVHTNNNDSTNVFNQALIKNKKNENVVQVDAHQMVLNNVTELADNVSRDMCRGEIIQDETTY
QEDNKKVDDNNIYNNDIYNNDIYNDDIYNNDIYNNDLYDNNLYDNNLYDNNLYDNKLYDDDLYTSSINNPGEL
IKNGENNSVNLKNNKVINSYEEFKKYDHMNNNMTDIYQSNYPLYSTNQEKETTDSNPEKINYRLLEERKKKIY
LNNYYNMIKSVDRDNYIYNVPFVSTIINDNNVRYNNEFVNMKERKSNNVSTSTITNTNTNTNTNTNTKTNIYT
NIEKCNSFNSMELLIEKEHTLQNTMNKMNNLKNDQNNFCHMKDTQSWINNKYFNEEKDNYKILGQEEKNISTN
EDNEIVYIDTYNNNYNNNNNMNRFYDNNTLWKKQQRNMMNEKKTTMYNFNYKNMSLDNIEREDQSVCENDNYQ
KHISSYLINKQNDIIYKNHPKNNNNNNNINNNNNNNSSNNLSWNTFHKNNTFDTHTNSFNILNHHSNNNDFDN
IENIITSNMMNNLENANLQNNVTNKCFQSNEEKKKFQSHGYKNDNMLLNNTYFNNKNCFSYDSIKKNDYNLLE
TSSNHTSTFLNYDLYRTDSIYIGPNSCENNNPSPNVINKKKNTDFYKTTLAQKKKFLNYDDENIEQDNFMDIP
NNLNFSFLNKND*
>N.PF10_0187
MAKKSKKSAGDNINAKLQLVMKSGKYQFGRKSCLKALRTGKGKLVIVSSNCPSIQRSVIEYYAMLSKCGVHDY
HGDNNDLGTACGKLFRISCLVITDVGDSDIIKTNE*
>N.PF10_0188.1

MNKAMDLIDDAYFCNLIKNELRNLTDEYEKKIKKIFLFEYLYKTEGFLISLFLIPLLIGFIFLKDDDIISTHY
LGALILFLSSLLLLFTFLYYEIEKSQNNEFLNKINQAKKYLSSKSFAKGKKEDFPFNYLSNVDFIFIWRNQRW
ELLPPNLLIKQDIFLLRAGDLIPCNCIEYNITDKVYGKEYKKHEVFLPMDKNKINMNLLTKYSKDEKRVYMPS
FHLYSFVSFDNVSISMIRKYIHDNIDNNRSAKNKENLRNVILTDRIHKFYVYTWMLLFCISTILSIIYYLEIK
KIIEVPDFVIIYFLIYIVLMSITILPFFHRIFSELIYGYCSSLNMVYENYFNDIKNYEKSNISSSFDFSSTTD
DSSSYVDKKKIGLFYTLRNILSLIIRGVKIDKCYLNILSDCSVLCFLDNSGILVNTNHSIKEICIYNSLNNKK
DVSINNGVKNFPKNYNYLLTVIDIFMDNKSMYAYQRIKNIKLLHSLFLLSYYTQIPKYLRVLDEFKNNILNYM
NQCDYSYLYICMCDIGNISFPYNKFVFHMLFFCIENKSDYELYKRKNSEIKKHKKIDISKNEIPYDVNDSSLG
INIYDNLISRDNFVFIFIFHEKKKEKYHMFLKGQLEALVNKCMFYYDGKAIKKLKRRKKKVLRILNMQWISSG
IESICFAYRPLSSSEVTYLKENFSKNIYVITINKKKVYNIKNFYNEKPFPIRENEKFLSHLLSTTIFIGNSAV
KLLTNNEIQTRINDFYDAGIRFVYFSKTDQINTRNVANLLGMETNWNTSISLSLNDKPSFKNRDGKVVIPSGI
NNIKTHIKEVDDIPLRVSSYSGCNQFNTAEMIKILLENNEIITCVGNSLNCSNFELYNLCNYSISVLLPFNTI
CKDCYGKKEKTNPFENQSSKNNPLMLYSSFINSFPCNLIIEKNSLDLSQNVMELVYKLLKSSRIHKKNVSLMI
FFFYFYYSYLSFLVFIISMFFLPPFISVVDYLLFILLIIPMLSICLLRNNNNSTIMNDIPDKVISKQFLTKKM
IFFLIKWIPMLVFSIILSCYYLQLINKNFVRNYIIRGSLNTYNMSQDLKRKLLKDINYYCNDNIYLLGSFYKC
QLLIYSFYKNDSMENVNMVDTMIKQSQIFFFFYFSLFFFVSSLSNSDKFEPLYKLSCFQNSKTYFSCLFLFLV
LSFLYVIFRIYMLPLEFLKQYPDTVLITLMVIFNVVILIINEILKKVEERIKINRQKYLKVLFGTRLGMWSPK
*
>N.PF10_0188:mRNA
MNKAMDLIDDAYFCNLIKNELRNLTDEYEKKIKKIFLFEYLYKTEGFLISLFLIPLLIGFIFLKDDDIISTHY
LGALILFLSSLLLLFTFLYYEIEKSQNNEFLNKINQAKKYLSSKSFAKGKKEDFPFNYLSNVDFIFIWRNQRW
ELLPPNLLIKQDIFLLRAGDLIPCNCIEYNITDKVYGKEYKKHEVFLPMDKNKINMNLLTKYSKDEKRVYMPS
FHLYSFVSFDNVSISMIRKYIHDNIDNNRSAKNKENLRNVILTDRIHKFYVYTWMLLFCISTILSIIYYLEIK
KIIEVPDFVIIYFLIYIVLMSITILPFFHRIFSELIYGYCSSLNMVYENYFNDIKNYEKSNISSSFDFSSTTD
DSSSYVDKKKIGLFYTLRNILSLIIRGVKIDKCYLNILSDCSVLCFLDNSGILVNTNHSIKEICIYNSLNNKK
DVSINNGVKNFPKNYNYLLTVIDIFMDNKSMYAYQRIKNIKLLHSLFLLSYYTQIPKYLRVLDEFKNNILNYM
NQCDYSYLYICMCDIGNISFPYNKFVFHMLFFCIENKSDYELYKRKNSEIKKHKKIDISKNEIPYDVNDSSLG
INIYDNLISRDNFVFIFIFHEKKKEKYHMFLKGQLEALVNKCMFYYDGKAIKKLKRRKKKVLRILNMQWISSG
IESICFAYRPLSSSEVTYLKENFSKNIYVITINKKKVYNIKNFYNEKPFPIRENEKFLSHLLSTTIFIGNSAV
KLLTNNEIQTRINDFYDAGIRFVYFSKTDQINTRNVANLLGMETNWNTSISLSLNDKPSFKNRDGKVVIPSGI
NNIKTHIKEVDDIPLRVSSYSGCNQFNTAEMIKILLENNEIITCVGNSLNCSNFELYNLCNYSISVLLPFNTI
CKDCYGKKEKTNPFENQSSKNNPLMLYSSFINSFPCNLIIEKNSLDLSQNVMELVYKLLKSSRIHKKNVSLMI
FFFYFYYSYLSFLVFIISMFFLPPFISVIDYLLFILLIIPMLSICLLRNNNNSTIMNDIPDKVISKQFLTKKM
IFFLIKWIPMLVFSIILSCYYLQLINKNFVRNYIIRGSLNTYNMSQDLKRKLLKDINYYCNDNIYLLGSFYKC
QLLIYSFYKNDSMENVNMVDTMIKQSQIFFFFYFSLFFFVSSLSNSDKFEPLYKLSCFQNSKTYFSCLFLFLV
LSFLYVIFRIYMLPLEFLKQYPDTVLITLMVIFNVVILIINEILKKVEERIKINRQKYLKVLFGTRLGMWSPK
*
>N.PF10_0189
MSNNKIEFYEQSLEEGKKKELFSDLKMYMNDDNLCYVSTNKLYNIQNENEMINKNKETYNNMKVMHNKNEDII
KDKSIHLNDHESSIVDKDFFYNEYSTTIHNNIHNKNNEKNNSFYKCCNDNSDINKDKDVPIRSYGNIHMKNEE
DKDDEEDDEEEDKEDDEDEEDEKNESTTNSIDKILRNIQDDILFKSNDSKTLEHCFKYNLFEEYNYPHIHTGK
NENMYEHKESIYKKKPSCDINQPPYNNEYTHDENMYKAYIWYDKKKYLYIWNKNIKVEDMRENLLTFCKKLTS
ENFYIVRIERISYEYNNTYPEIYEDDNLKNKNLDIYNMEQEKDSTLSREGDHTIVTLPFDDKNIMYYKKIYDN
IYNYNSFDQIKNDKEYLIINNKRKVIKGKDIITYENILKENILKENTLKDNVLKENILNDNVLKDNVLKENIL
KDNIFEDNIFEDPLKFAEVLRAIEYTKEGCNLLKLTVFNIPHLRYFFVSKDLNFIKWYSSRKQDDQCKIHFND
INSLEVNYKNNKLFEHYKIDILKKLSFCIVYNNEKKKIILTCKSFCEYHYWITTIRALMFRSRKLKISRSILF
SHLNDSFLLVTQDDILNKRDNNMIIDETNIYDRKYSEHINPVFNNKCCDIITNQENEKFNNTSSCYKNKDKEN
NFYVSHEKKNRKGDVCNKLNNDDPNMIQLKSFHLYKLISFPDYNIYQIKTKFYLLKEKFYKYKIFIEQALDDF
IIKTEREHANKYNATDITQGDHELVGRNILNGQYTTIYGNNKQEKRNEDMKVNVHHPLNDHINVYKYDNMKDN
VKDNVKDNVKDNMKDNMKDNMKNNMKDNMKDNMKNKMKDNTYDNTYDNTYDNTYDNTYDNTYDNTYDNTYDNT
YDNMKDHFSTNKNICEEEHLSNYDYYDKSNILHCSKLKGKKKRYINKNNIHCLNEDNIFNIYEDIYTPNNNGE
DTDNFKLFLIIKIFNNISNKFKDIQKNIFHVVQLMDMEKLDENKNQKTNLFSFDNLLKYSTDIYKMLQQKFGQ
NENKQIIGEEKHVDILNNIQGQKIKQTNNMKKLTKMKNINNNINDNNNNNNNIYCDNLTFDHNTKKDIPSYSQ

HTHIRTNEHNYLFLSKEKKKEFGKKKKKLIHHILFQLWMCEVDLGNIDDIYTVYMNNIKNKNNNIHINNFLIN
LDTSKIFNSISSQLSATILKYINL*
>N.PF10_0190
MYKSERHKDITHSNRTKRNEQKNSDILNKVSNEKCLENNTNESLCNKMDNCINKQNNINRQNDINSQNSVGSQ
NSVGSQNNRNHKKCVNKSRQKYNKIERKKKKNASSLLGDNCVQETEDTHHEKSLNDDCLNMEQNKEIVNDHLK
EIEKKNSDNEIKADINDEKLQLERDYVIEAADQNKDALTLCVCVEDDRKKYVTKEKVNEKDMEGDNNKKDMEG
DNNKKDMEGDKDAKDIEYDQDAKDIEYDQDAKDIICDNNITLKNKIPINNLNDNNIVCNYKHRDTIEEADNNY
VVENITQVNNIDEIKLKRKRKRKNNFSEYIQEEREDNFIKDKNYNALKTLKDSKRILLNCNKYIEDNNMYKIE
DNVQNNNVCGNLLCEEKCNNYRQIDKNEKYEKNEKNDKYDKYEKYENYDKYEKYDKYEKYDKYEKNDDSRKDN
IDICNKINLPRVYDNIIQSDSNNLNKYKGSSFALIIHEISDLLSLITTVDKSKNKNKIKLVNIGHKLNTLMRS
HYERIYDCDNSLVSIINKIKKLYKCYRSMEEKYLKKTKKQNININTNTNKIEKENVAGNNDEEDPNDGINILH
DDLLKEEINNIDNNIKNDINKYTIKCNNNNEKLIQDPFFQNEFVQGIDNYINQKDENNCHDNIKDKIDTLNCY
INEKLNMDCQNIFIQKGDYKKVLNQMGEKAIYEGNEKDNHKGNNTMEHIKINDDNKTMEHIKINDENNTMEHI
KINDENNTMDNINKSIIKNIGSMESKSTAVTYKECELSLKEEKEEYIEYLKKKKKILKELDKCNSHFFFTYFH
KGELIEYLHNQINMHIVEFDKIYQLYISH*
>N.PF10_0191
MLIFFFFFFFFKYIYNIFILTCFYITLSSYYFIISFIFSTLMFFYFCTFYVISLFFLYISSFCKSIKVTQLYD
KKIKIKSFINNYLVSCRKKKYIYNNVDDKSNIGTFNLYHNIRDNNNNNNNNDNNNNLKKRDDVLFPLCNKNII
NDVQKIYDEVNNIKDKEQKINYLMEQCSSLCKENYFPPILNLNKAYRNKRIDEFNKGNKNFYINEVGKNIWYK
YVNRCDEILFMAIDIQIDEDEQRNNSIKDVHDVHDVHDVHDVHDVHDVHDDNIKTCTLIKDDKHFEKYKDIHN
DNILKNILPLDKKIDSIKNMLNHKYMKKKKCIITIDAYSNNLILYCFLYLILKHINKMYLYSFMNIQIKEITA
KLKELFDLHFNVHHIIDYIHEYIYNFLMSYHIKRKKNKSKNMKEKDIKNVFANNIIISDEENKHISKESSDMY
KKKTTITTTTTTKKKKNTMKLFTYPRIAHMLSGGVDSLMALHLLERKKFYVDNYFFNFTNADCSKNDIKYVKD
ICKNNKRNLFIININDEYFDEVLVPMLFFYADGKVPNPDIMCNQKIKYNFFLKVIKSIYKQKWNYRTKSKLCN
YDFISTGHYAMIRTNDKNNPNNIFNNNLFIKKKKKKIKNIKNKKNIKNKNNNNNIYTYNIYNLHNDNIKTNYK
KNNKYFYKLLVSNDKKKDQTFFLSSFNHIQLSKFIFPLSLYTKKNVKKYMNENNINNYNHKETKGLCLFGNID
MQTLLHKYFVNTEKDDIKNKQNEDNIFKENNILNLNNNFNQNEKKKKEKKLLVDITTTSSHLKKFRETFIPKM
NLHYKNYLINLDDQTILDINSDIHLYAIGQHKNVTNYLHNLYNKKMININGYKKKHVKNVISSFQWIVVYKKI
KRDMSTNLIHNFIYLTKNYDQDLFTHIRTKCKLHNIKWIEGKLPACIKKQFKKYNKINKKKKKINNNNNKYKT
NETFHVYNNIQESGKKKKKKKVKNIPHDEKTIFVKIRNSEQIKKAKIKFSLSNNTAYLKVKQKDTGFSPGQII
TLYFPFIIKKNNKVTYITNLNKYNNLINTNKNTIYYHCLGSATISNQFLDYNLYQHIKNIHQINDLNMSK*
>N.PF10_0193
MPSLKDEVSFENRVAETHKIRSKYPNRIPVVCERANRSNLPIIEKKKFLVPMNMLVGEFKFILHQHINQSAYG
SNMKLFRERTIYLFVNNIVPKTGLLMQDLYEMYKDEDGYLYMEYSCESCLG*
>N.PF10_0194
*KVLLISNNQTYMLHY*HNSLKKLERIRFSCNFLLRRFILNHFFYFFKE*LYSFVYNIYLLFHIYLLYVYHFN
SYILYKT*KCTIIYIYIIYIYIYIIIIQAIEIYYNVYIFELY*YNIIYIYIYIYNM*FFILYFFIFIIYVNIT
NRRR*RKSNKRRYKFFK***RSKCFRYKKR*EKS***NI*AREKKKEKR*VSIQERKKKKGNE*RRNCGKK*
>N.PF10_0195a
MSEAEEILNRIKNHKGVIGILVVNSEGLIIKSTFDQQQSDLHASLLTQLSKKARDVIRELDPQNDINFLRLRS
KKHEIMIAPDKDYTLIVVQDPYADKEKS*
>N.PF10_0195
MMIIRGELPSKGYLSPSDKKCANTFLQQEEEDEEVNKEFYNKIGDIIEYIDTSRNKIYIYIYVYLFTYFYIFV
VLLIQSENLTKRKEVTNEYDFLKKVGEVCKKSCDYYFDTNIKYHEACENFSILFKIFYDSLIYIVKKNEEADH
VQTILQQIEDNISMEKLEKEKMNILLKDKLNELEKNVFFMKNESVNLNNKIQELDYQINQHKESFDSFSTLTN
LNCEKLIVLQNQYNSLTNKRESITRSIAKRGGKKRAHG*RKRIHT*NRTFRNL*TRNMLK*HEITITSTICRE
SSELTRIL*KNN*GRIK*QYGYKKRNRRKSKYS*K*NK*NNRKNKKMQIREKKRK*KI*RITEKVW*VTKRIR
KKRK*CT*I*KEI**Y**TCKK*RIYFKSIYFTRFKKKKKFVTRRNY*L*RRNKNINIKRKRKEIK*PSI**Y
KSYRH*G*YRYIY*FCRNQFVNPRIRIYFK*NEKKN*RGRRRNMEKK*RTTK*KYQN**ITK*N**RIQ*RKR
NQFRYSSYTRKINYT**QRKTKSTKY*SFKRRNKFNGIKK**KQRRNSTFRK*NK*K*TKNRKSYITNT*RI*
TKK*NLIRNK*FLYRY*FEREETIER*Y*YIYTTD*NTI*FTKRKNRKTRKTKSR*RIQIFRSRNKKKR*FIK
LL*RIIK*TGK*YKREHYKLCTTR**TTNTRN*SL*RHKRK*KINTK*KNELFKYI**F**I*IIYNA*SV*N
WIF*CYITK*YEKKKYQISKQISKITSYY*VARKN*K*KRMSYVSSLQKNTFNTSKQYQ*KRQQ*EEQ*S*GS
YQNEKKSK**

>N.PF10_0196
MNLPVYNMESISECDILKPIDESERIELENKINEQMVELEVKKKYLEELKKRQHSVKEKNNIHSFNNVVEEYP
DPNKMLCNFLESLENNTNDNNNHHHNDKESKEQVNDLSIYSEYDYDNKKKTTKKENFTYTEQFFTHPNSMDNI
MMNKSFLFQKKKNSIPNFNFHIDLFNKQTKNVIHIQPKGINYFDKSIQVDLLENNEIYLLDNHINKKINKMEK
KENIEKMENIEKLGNIKKTSLCYSTEDKSEGDSKSHHKNKIKHTNDTSIHNDDKKKEIKGGNKNNEINKNLEE
NYNKQNGHHNYIYDENNTTFNYNDNKNNSVYVEHNDNRFNAYNNSTHLKSMNVEKKKDEILKSSSFLYFLENS
SKIVERVLGEQDLFDCTYTYDNNDMLENTQDVDKLKLMNTYYCKKYINGRGITDVRTSNIYNELFLASYGLSE
CSNCIEPEGYVLLWNVTMKNRPEYVFTSESSVCTALFNKFHPHLIIGGTYTGNVVLWDIRASLKPIQQTLLTS
QGHMHSIYCAEIIGTQNSHNLITADTDGRLCNWSLSMFTYPSNTIDLKKGNREISCTTFAFPEGDINTLYGGT
EDGTLFQAQIHGMKVGIINSDIIHDGPITSAQFHPSLDEMNDNNDLILTSSVDWTCQLLSIKNIKNPLIVFDS
FEDYIMDIKWSPTHPALFSTCSSNGKIKLWNILYDTESSFYEASLDNSSTNKLAWSHDGKRLIAADTQGCLTL
WNASSEIYQPKFEDLNKFDEHMQTLKAERIADTTSEKET*
>N.PF10_0197
MSFIYRKAASMLSKCCQGKGIKEILYEKDDPSKKKLHYLLYKTLEDLNILNEIYNMFLKKVCKNKYLGLILLS
VLVHRKKIDGGGLIKREIKKKEKDIMKYYEEYKDRFNHDEKLKEGIIKKYFIIYDEKYEDENLYKDNTKCIKN
MESIRNLENKCTLFNNDLSNNKSEHIINELMKKNIHIEMDEDVEFLYKINNKDVYKLINHYIYKENKIRIIDK
TSCLVVQAANIKRGMIIVDVCSSPGSKAIFSRTLLNKKGYLICIEKDRKRCYTLLKELLKNKEYVGIYMDENF
DDTQKITLNKKSFFHNEKENTKQNKAKNVYKNMDNNSKMDVQKNVYYIQHINKELFIKIYNCDFFNLTSQDFS
TFPNIDVVFIDPSCSSSGMPDFIYKSNMANICLHPYKDDQKNKNVINKYNTKGNNNNNNNNNNNSDNSNNSNK
YILLQSDGHKNIPYNKQDNIISHNNKFDEHKNNSFSDDVLKGNIPKVPTCFINKVNKLSEFQKNILTHAINIF
KTAKIFIYSTCSFFEEENEQVIQYVLTKYPNIHLLNAGKDKFLFTNGKYVFSNKCVRTFPLIHSCRGIFISKM
SIGPEVHNHINKEPQT*
>N.PF10_0198
MLKKFSPLSFMVLQKRYNKSEQKIFDKIKNIINSNKNLRHTDDEKDKIEIINIDNEIINKENISFKCNIINED
STKNENKKDNIDEMTNVNIYDDYFNINDEYNNVMSNDNDNVDIKSFIRETVDYYKSLQLKKKEDHLNVLNVKK
KDYSNIKEYYNKNEEIKEIVQDNNFIWNNKNYDKKNIHSGFHSDIIYYDNYDTTKSKHNQNIQNGQNIQNGQN
IQNGQNIQNNQNNQNIQNIQNNQNNQHCHNSTHEKNCGSYNFLTPAQVFFENNKEKLIQESYKYYKERENNIN
KEDYLYDENEGYYNYANMKTPIIRPNDKEGFLFNYEDDDYNNNDDEGEDDMTLEKGIMPTIEQIVCILKHEKV
KDIKVIDLDKCGRRDIGMFLILCTGNTAKHNKKVGKLISKIFIDLEIPYISNVVYCYCNKFDDWIITHCGPLK
INIVTKELRNLYDIENLFLYPHEHFDSTNFPSFFDYTPGIPPPYLVRSNASMDSYKNDDIYTKFLTEK*
>N.PF10_0199
MDDKRGKKLSSPKIFKSTKEERILKKHDETKLKWEKQNKILKDKTKKKETLGEQAEKYRAKNEINNIVEYINQ
EEKKKVNWTENLRYETQKIKFKETIHKMNITENNLSYESRKIFNKVFEKDISSKKKEKNICQNNGEEIKDDGN
YVTASYKTNKELKKEKYENILINKIKNNVEFFLSNFAVPLDSPLYIKGEKIKEEENNDMLLLSENVITKNVKD
IKNMDIYEHNNNYDDDQNKFCENKYINNTYSKKQVNYLKVNCNILEINNCGKEIFNKYIILQNCSSNIILYKI
IFRNKILLNNNNNNNNNNNNKIKLNDVLYYDVNKNFTLFIYPNSGYIKPNGSKQKILLTFIFKYFVNTFGCIT
IKYYCNNNIFKEYVYIYINSFFTINHTDERNHFEYNMKEEKLKDQDFLYNHLIDTLQSDPMKHMTNIKKENFY
IYRKKKENIYESIYDIINNIYRLKIKQICNILNFINSSYNIKLNYINIQTFLGLLNKTNNSTSFIKLNYYIKK
MLCQNKMIKLNLHKCKYKRCKYKIYKYKICKRKKKYTNTHKFYYYQYKIHKLKIVPFYKKNTTHCNNIIQFNK
YKKRDNHNISFYKRHEHILHLSNNKENKNIESFFNYMKSKFLSSLKIYTNDEHIQFFMFHFFIHEYSDFVKKG
NVNQYILSKKKKYIIRNYDHCQKQKKKDIYISNYNIITDYKYINNLYEKIYNTFNQKKEYSFHIFLYIIRTYI
NELISYYKTMNSFPHMYKNKNNITIVENAEEIEKKKKIQINDMEKHILHNNKLNKNNKDEKDEKKFEFIHFLN
YTSNQVCVDIYNPCLKYLKEKKNFFFFINFMYLQHKLCISPMFYFFYYIELIRYVYCLFVWRVRKMSNINNDN
GIYNGNNINNDNGIYNDNDINNDNDINNDNDTNNGNNICNIDHSCCCKSQDNISKSKNIFIHMDNKLLREIIK
YIYELYTSNKNNDHVNNIKECIIYLISSILMYAQQNVKNMYIFIDYMFIYNHHKNKNKTNNTTQGQNNNLDME
TFYSNIFIFFIFPYLKEYINNLGTHIYKLHLIKNEQDILLFFKNKFNYINYEENYYSLYDDNKIIDTCVIQNT
CPSKEINISSFDKINKFDDIKNVEETNIPLFFKNFKNQNDHNDHNNNIHNDDNNNIHNDDNNNIHNDDDNNIH
NDNDNNNIHNDDNNNIHNDNDNNNIHNNNNNKEQGDIWFIFNKEEFSQIIYEQYYLELNEAIKKKLKNLKCLY
KIRSKEDEENKNINMNTYKYINNLKNRVDSILETNNASNSEEKKNKKKKNKKKKKIKKNKDNDKDDNKDDNKD
DDKDDAKDDAKDDAKDDTKDDDNNNNYNNNIKNSSNVTPYDSQKKKKQKFKELKEFKECKSIKHKFINFHFIK
LFDILNIHTYIIDDKHMLNNILSIDLSKDINIEIGLCVHRIIYCLLYIFNFISLSHINILYNNYFYLKYFLFD
AYIEYNLEYIYKLYYFIVNYFYLLQYMNKNNFSVYIPLSPTFDFLKYFKEINKCKQNHYDNGRDTHKLNMIEH
MNQEKKEEGGQMIGTTYLSSNCRHKSFFSSPCESCKLCESCESYKLCKLCTSCYYNFFNFNERKEEIHNHLHT
SNTFLKREETKYICLISPNIKNDMLSYMNMKKIKEWLKLLNLLIFLNIKDVYIFGELFSLFLFFFYDSNIIRG

EICESKKKNKNNIYVKFDTNYNIQTEKGKKKKKNLHDNKKGSKGIRDNKKGNKGIRDNKKGNRRIRDNKKGNK
RIRDNKKGNRRIRDNNIDNNNNIDDNMIDMDTSNHFTKNNNHINSNNKDDHINNMKNYIDVQFCDFSAISENV
LLLLKFSLFNIFNLYEKYKINIHFPKDFIIKMKSRQCNDFTFNSCLSSFVSYPDDILLYCLLPHKNYKNMILF
SKKNFLNKYTNKLKKINMDHISKVNLFLKTNKAEDPQHNQEDHQNENKTINHTDNIMITSQNNHMTNSNNIKK
VSIINIGKLTLYHILKNINQNNKILWLCGSLEKNIKENYKNSIHIFQYIYNIQHDKKNFHSTLYCNIIHVNNF
IILNKQIYWLYYSQLSKNIYIPFIYKTYKILLQIFDRKCSQRDILSYII*
>N.PF10_0200
MKKKVDNRIKILIENNIAVGQRSMFFVVGDEGKNVVVNFYFLLNRLINRTHNILWCYKKKLDFSTSKKKRYRE
MKKKIKKGTFDQTIDNNFDMFLKNADIRFCFYKESKKVLGKTYSICILQDFSYITPNILCRCLETVIGGGLII
FLINKQNELKDLYNLTLNYHKKYTMNGICNVYNNYIHRFFLSLNKCENAMFIDDEMNILPLNENYLKIKKITS
KGSDGNGIGNNVTKLKHMEVQKEGEQSGSNINNNDDDDNDDNDNYSNSNNYSNSNSNNYSHTKSATLGGFLCP
DKNLLQEKLKFLESICEENEKKKEEEKIFLYSSKFSRNDTCTKINKNGDIHSSDNKYTRDTYETYDSVTNTST
TANTYNNYISKKTKKKDVYSFLDRNIMNLLNICLSLDQLEVLLNMCKILRNDEEKKKNIKEVLFNLLANRGRG
KSATLGLLLSLSIYFNYSNIIICSGNNEGVQTIYDFLDKGLHILGFNEFKDYEKIYDMGKIKEIVIFKNMKYL
KQRIRFFDIIEEDILNSELMIIDEAACIPIDILKKKIKGEITILSTTLNGYEGTGKTFTFKLLKQLKKKFITQ
LSYEEFKNMNFLYFDKAYIDLTLTNPIRYSYNDQVEKWLNDFLCLNCNEPTNIKDNICSPSNCELYFVNKNIF
KNYNRTSEILLKKIMTLFITSHYKNTPNDIIMILDSQQHHLFILINKNNNHYNNNNILEDQIDELDIYGVLHC
AIDGIINNGNVKKTVKLEDIIHLVKNNNTQKENPTLDTKEINDSNDQKLEKKENINQINSDNHKELLINEYEG
NLIPYIISDYFNYYFYNYIGIRIVRISIHPSMQNLNYGSEFLKKVCNYYNEYNSKQRHDNNTKQKIKINNNDS
NNCGDSNNCGDSNNCGDSNNCDDSNNCDDNVKYSENIILFNCNGKNIYFDKKLKQIDYIGTCFGLTKGLLIFW
QKNKFIPVFLKQQKNEITGEFSVLMLKHLNEDLKHIIANFYTDFVRSVTKLLPYSFKYMESFIVFNLLHNNNY
SIPLKQDDAPTIVKDHNNTDHTYNHNGHIKNNLHNNHFYYDNYEDQHLSFYNNNLINQQTIFYFFHTNDICRL
KRFVLEGRNFYEILYLLSTIANLILFKKVKIDLSFLEYTILYAVAFQKKTPKEISDEIKINVNQTNAIFRKII
HRFYNYIKDIMEKQIEKEVNAELNEKLKVKKKRSLNIELPSEEYKDELERNSLIVKKKLKKEKLSLMKEFNLE
GDIKRKEINIKTNDNLEIKKKKKKKKKKKKQETN*
>N.PF10_0407
MLYNLIILIFYLRFSKCISKNNNYGYINFGTFSNVVNNSNNLRNRKNVVFSKIEIKMPALSSTMTTGKIVKWN
KNIGDYVNLGDIIMTVESDKADMDVEAFDEGFLRVKRLEDGCEANVGDVLGVLTTEENENMDEKKYNDGDINK
TENEIKVLNPDKDKSEQIIKEDIHFVKKHINDDVNEEKIFIPFIKCKKKKAKINKWLKNENDFVKKNDLLLYV
EDDKSTIEVESPYSGIIKKLLVKEGQFVDLDKEVAIISITEEKDNEKEKIEEPFKNKEDEEINRDNILIHYIN
KIKKSEEGRKFLKNLSEQEEKTLEERLKLNYEKYNKISNDLFRSSESTKDYVLKEKENESQYEMVLPSASELM
RQNKLNPKDITNRKTPNRITYEDVDAFLNGHKNNSTNVTYCEKPKVETIEYGDPKTVDMTNIQKSIKNNMMLT
LTVPVFRVTHLIKTNELLKLYEKVKQKISMSVIINKCVSSVLLNHPLIYSTYIDKDNGKILYNKDVNIGNALG
LPDSLLTPVLKKVDKKDIYTLANEWKILVEKGKNGLLSSNDMTGSNFYISNLGMFNTYQFDAILPKNSSCILS
IGTNIGSIDNLEDLKIQKGMMMTLTCDHRHIYGSHAAAFMNDLSKFIEKDIMKIFL*
>N.PF10_0203
MGLYVSRLFNRLFQKKDVRILMVGLDAAGKTTILYKVKLGEVVTTIPTIGFNVETVEFRNISFTVWDVGGQDK
IRPLWRHYYSNTDGLIFVVDSNDRERIDDAREELHRMINEEELKDAIILVFANKQDLPNAMSAAEVTEKLHLN
TIRERNWFIQSTCATRGDGLYEGFDWLTTHLNNAK*
>N.PF10_0204
MLIFLKYVDRKFLISIVLFFCSHTFVENYNIHKKRKTFILPPRLKKSFSYKPNKVINTFEIGLKADEELDDYK
YFTEEEYIPPLPPVETWEDLILKEEIRNKKEDPKRNADDYTLTHESREKVLDIIRQTRLINHCNYEIQKNKQK
NILYFVTPKIGPYCKLMEYDKEKMESLVSKVKEKFGKKVEIVNLKLDVKMPLFEIFLQASIHTQIKIFYARGR
TLQYRLPPVFWTRCKILKENKILQEKYGGECAPGLFPQYDEYNIMQIMTECVYNDKYNFYVYDVNLEWENLCA
LTPENRELRKIVHQNVHVPTRLAVIKAFEQGVEDFRDVIREEYLIMLREQRKLKENKKYKF*
>N.PF10_0205
MEEKSENLMIKSKELCEIFLRSLKNDQGSVKYLNECKNVDICEYYGVFNLVYSNIINNDNNMVMYKDHLLFLS
FCYMNKLIEKYYNIYSNDEKINIRNSFINILFSLPINYNHILDNDILNLKVKSNEMLKTKLINEFLNQQTCLM
NSYFNKNDMYMISTYMNSTRNKISQILSIICLYNEPIYFRYLLNFLIFFVCEYINRIILYKSKENNVVMNQTN
NMFMSGSLKNKSLKDEKSGMHNDNNNNNNNNNIHINRFNNNNNNIHINRFSNNNNINDENYYFNCVLQISINF
LKEIFYNISNENYSNMLNKQQLNVFRTIIITDINIIFHLISLVFYYCLCYNKKNEFSLLIEGIKELSFFFPAH
IFFNESNSPIKFLLNILILCFKKDKSEINELNPNNNLLYDNMPLNHFEILYKNYISSNQLNDSSITHLLHNFL
EDVDVEEILLIIFLNIIEYICKINNKTFVQLNEDEFMLFLDTHFNINLNFYDIEHYNFQKIYIKMIMNLSCIP
ISNYLHKKENKLKCLHIYIINIFKNIYHPNMKILINILTICKNIFDNNKDLIYINNKYNIEESKIYNIYKIDR

ENLLNENNEKDYNNNQLIISCDDLKKLFILMFIRFIKIPMYDHNMDTNTNNNTNNNTNNNANNNANNNANSNA
NNNANYNANYNTNSNANSNANYNTNNNTNNNNNNNNNNINDNHFGVSKNNENEQNFLKISKDGKQSESILSCT
NSSMNKKLMIEFINNYISEYNEEWFHIYYKYMNEEYEENDTNNINEYDDNENILKQKIFNFIKVLLGYNHEIY
HIMIDVFNEFFKYYDTIITINNLCNETLKVQDYFIYVLLRAYFQLLSFFINTLKELKNIVTNGNKQVNENIFS
YQMNCTGEREKELKEAYIQNKINIKNNLEEKIIQMNNNNIELENNHNINIINNNNNNNNNSGSISNMSSNTYN
LDMLKREKENIELELFIIKCIEKYKGSPYYEINNKCINNLLNFFKIILQKDFMGFSKISTNFLLFEIKRLQHI
SECTYILNYNKDYAYFIMDNLMKTIIESTEDISFELKKNYLAIFTSIIMNLESFLSNDIIQKILKTFLDYKKS
TNIFKYNKEFSIFLLTLISVLKVENVNQKVPYIKLVLEDTYEFLTYFNKNINTFQKLYDLFYVHQANDTMLQV
ATFDSIHKKEDMVHSLFDSYKIVHACFSLFSYEKIDISMKTKLQRSALSQASNNNTNDHMSSSNNNNNNNSNS
SSNNKIGTHANFGDENMYYNLMNDNNALINDNEFLHLFINLFENVLLIYSLFNDMIRTNYSFEKYPKMCVEVN
YFHLTGQEKDVVCMYNMNKKKNSIDNMEHMKNMKDVNNMNNMNNMISMNYNENMKNMSGSSELLYMKNNVCTE
GKKWNYYTIRLLHENICNVIKKFIKESYFFLYNDLTNIFNNILCNSLEYIPYDYINNNILLVYVCLSEKLKYL
INNKLINQLIIQWFFKIFSVFLEKHFHYFREEYEHIKKVNSELNSEICMKEDDYSLYYDMLYKNKNYILNYLK
VCKNIFVVPNEYKNNQEHKMFFQYIYNDEKTVLLNSLLSTIEYLLNSYDCDITKKCLNFLVDSSESVTFLTFN
SPHFYFFLQIIKVLLKTFFLYNPLILTLRQKKEENEISNIILNEYTEEKIQVYIGEMNLFMKNDPLEANSFLN
VFTNAIIKISKHYFFLQKKIYNIAENISINDLLNIDSVKHFLLLFENIENSSTFNFHTILSDLLHQNNSQYFK
NLLIQYRLHKT*
>N.PF10_0206
MSLNFSDDDDVINEEVDDTQKSDKNHSNLGFMNNERKEKEKEKEKDYGMEKKKPFHNYENNPFSNMKLSREYF
ENVQEMDVNNEENYIGPDEGSEVELEGKKKKKKSVIFQLKNLFNDVSLNKLNTYIRKRRRKSQEEMNSNDHFS
RSSYEKEGEKEKNNKKKKRNENGYAYGEDNYNDNIYDNSDDKLFNDSYNNMYNDDILNDQSREDVDVDVDEED
ELQKYVREEKNKMKQIMPEKDELWELYIYMNLKKKKRKIEYNEKQYSNAMNKVFSNLENEYKSIIKEKLFGKI
IFKEYLVISDDVEYFKNNHIMNIGKNMTIPIIKKKNLLSEMKRKLILLYNKGICCNYIKEISINSGGKINEQT
NSYQNQDGSSLRINENMDYYINKIQDNNLIIEKESLNLYLNKLFDKYCIIVHKIRTHRRKDLRMNFNSFMDLI
NEVFTKLYFSIHNIFLTDEDMNINLTDIYKTIRRKYNMHDVLDKVQKLTHKKECYEHYRSYLLYKYKTTYSVC
DEAFKNHIMNDNINIFNFKKFYNTDFSKENFTIIKEEQKINTNDNDFHHFTYDQNNADEHTNIFTNDIYNEDF
FSINVNNMEAKGIQPPHRDTTTVPEKSYNAKTKDHITPVLKEEHAYNNNNNNNNKNNNNNNNNNKNNNNNNNN
DNNNNNVVEETPLEKAKRIAREKKKQLMNNRVKII*
>N.PF10_0207
MKKAIFFIYLIFFVFVKVTCLEIRRGKYNINYDYAIKSLKDNNFKRRGGEKGRRNNIYYMNINFHNIDNNFNN
EIRKRHFNENDSIKDKLNKLKKLKNILNKYKLNINKKDFDKFKNKTQEIVVDKYHYAKKYSIIIFNRLKDDMS
VYRGKLKNYYNTTTTYLKDKYFNSSIHEYLSSIPFFNKSSNISKLISYNSVNFLFLLFTILYFKSDYIYTFFK
KKYAFIGEFKNIKTDKLVKIHTLSTLLFFFYKFFVLRLLFILNSYNFFSQKLYLINNLIHILFFSYISIFPYF
LVQANWGSFHLLGYKKSKILGGLVLLQLFLIYARLIFQNNLSFIKTWTDEKFFNKEEIIQNIKDDKKTDFYVK
LLNQYDFLKRLYVGNNKIYEIVLYLHKYKYLLNIISPIHSLFYIYIAHSIYFYLNNLSFAGIYVSSFSLVLFL
IKILSNKIDMYYLTKL*
>N.PF10_0208
MIIEHVLCSFILFVCYIIKYADAYLPGMNPTTYKEGDPVIINIKNLSSRRAVTTLDYFSFPFCSSNNSKSSGE
KAPNIFKILLGDTLHDTNIETTFLNDKKCAFYCKIFIDNNVYEKYKHLILYNYNIVYSVDNLEIFREDPKRKG
FYYTGIPIGYVHERNYHLYTYYKITILYNTQGGPNSNKYYIVGFEVEPQSVDFTKNDDCVQNESTMIMGKNKF
VTFKYDIRYVKSDNSFQHRSEHYYRNLNDQSMIHWFSIINSIILVILLSFLLSTILIKALHKDLNKYNRINTN
IFETDDMDDRGWKLVHGDVFRKPRNSTFFSAFVGVGIQIMFMILVCALILLIGVYKYKQRYRYIQIMFFIWIC
ISSISGYASSILYKLFKSKHVKLTIFRTSLIYPFILFLIFFLINLVLHYEHSNTAISFSSLTSVCILWFGISV
PLICLGSYIGNKKKPIELPVRVNNIPRHIPKQPMLNTFFVSSFIVGSILFATMYTELFFLFTSLWKSNMYYLF
GFLFLVIFLLGLLSAQLSIALTYYTLSCEDYNWWWKSFVAPGSSGLFLFLYSIYYFFIKLNISTFSETFIYFA
YSFIMSYTCFIYTGTAGFLASFAFLRKIYSSIKVD*
>N.PF10_0209
MDVIKQLTYGLNLKSSLKNGDLMSLKEENLKPLKNILNIKKENESNEDEKTKKIVQSCEGIKYSQFYDNSNLI
NSYMKVNEINVEYENINRNIVPIRSFHDIIEYLKIINNERDDEKEDEKMNNINKLNHNMKPYNSDSDNQNNKE
KDMEKNKIDTLTNEQKEIISNKEKEKRKNDKSNNKTYIINKEYEKLLSTIKNTLYFKKPTCIQKMCIPSILND
YNTICISQTGSGKTCSFLIPLLIKLNNIYLEKHEREKKKNNNNNNNNNNNNNNNNNNNKNNNNNNNNLDNISDKN
NLNENLIFIKSLIIVPTNELACQIYEQALLLFESYKHINIIHLNKLNDIIENNVIDVCVCTPMILLNMIHEKG
EKAEKIEGKKKKVSLKKCFFIVFDEVDKLFEIKFLEQVNTLLKEIQNRKIQKIFTTATLPGNIKNFISTLCPN
YTVVYFGKNINTINNNIKQELLYVNNEEEKLLVLNNLIKNKEIHIPVLIFVDSIIKANMIYTNLHKSVSYIAL

LTSEKSKEERKIIFQKFQQGHIWYLICTDILSRGIDIKGIETVINYDVCYDKYNYMHRIGRACRSDRKEGKAI
TFFTSENIKYMKEIIKFVKSSGTNIPSYLENFQFKKTPKYNFAKYNKKSKKNKKNKKKNKIKTNNNKGTKKPI
KREIIKNSKEKTKKKKKNIINNK*
>N.PF10_0210
MANYAETFSAWTSICLTDHTLLGENDTEDDIRELCEESVKTCPFAAAVCVKPEFVKFINDTIKKEICPFKPKV
ACVINFPHGTDVVEKVLEDTKKVLEDGVDEIDLVINYKKVLQNVEEGLKEATELTKKVKNLMKEKVLKVIIET
GELKNDELIIKTTLAVLEGNADFVKTSSGKVPVNATPGAVKAIIEAMKQYVEKNPQKKDKIGLKVAGGVGDLN
SASYYILLARRFLSALACHPNNFRIGSSSLVPKLRKIIAQNPAV*
>N.PF10_0211
MIKALYKSIVNFLLSSFFENIEEKQLQTSLIRGKVHLQNVKIKKFFCDFIYLPYTIKYGYIRSLDLQIPLLYL
FQKASLKVSDVVIVLKEKHFSEYNIEEEINGIKGGKKNNLKLEDYNYNSNDVNQKINKGLNYLFYELYNNMSI
DIENVEIILESSEKTTNVIGLFVKKISSVKTHNCTNKIIKDFSFVDAIFYMNDEKAKYAINKIIKKEEKKKKN
KRNINEEDIYNKIKYICNNLHKKKYIISEIPSIVLSTSMYVSNSTDYYYHNSKFLFKKNSYFNITCTHCDKYL
FQCKEVKKDKSIFQNIFIKKEKIQQRNKQLYSEINCKCMKNNKNIFQKDQNTNCTYNSDHVPENSLKGTNEMN
KNIPPDLDSINVGHPKIDDDNEKNNHENNNHENNNHENNSHNDNHNNSHNDNHNDNHNNNHNDNHNLKHKRNY
NHRNGGVGQNNTGDNNLLNNTFNKHNEERTVQEKDEKKSEDCIDCNCKKNISEDLDNCLYCRINNKNQSYIEK
FHMLEENINLKENEYMTFELNVDKIDLILSFKQFYYILDIVNYYFIYSCLVNGLCIEEWLNIPEINDILIYKR
ELIKKKKKEKFDKDFIDKFEYTHSFQIISNIKKHVEEYSLDNNKRKKSIRGTGEDLSTLLNSNHNNEYNYKNE
HNRHNEHNHHNEHDNNNNNNNKVTKSQSASSHLSNVSTWNKPFFVYNKKISVNCSFNILIKKLNFCLCSGDKI
NKIIQINTDDFNFNVKSYINADFAYSLLLKDISVFFIHKDKKYPLFELFNVNEHNKKEDKKKENMKVNKRKSK
TYIFNTHLCNDAMTSFKSVSSIKNVPGNMNNITDNMKNDNYNNIYKNNEKNVENGKKCSKYVESRNLVEINNR
HNEYSKEKKNITNIKNDNINNNENNNYDNNINNKEEYAISLLTFTKYETTQAKTFIKFNKNILITLSVKEIYN
ILNMNCDYKPVDLEKKLKSYKILFYYYLLGKKYCKELYMASDKNFEDMNIHFMHNIYFNIIINKKYNLLFKMK
KGLVNSFFFTKMYLHCDLKKLTMITDLNDMQQNMDNKLVDQYTEQMKENSSYQNSQSCSNTSSVGFTNEDPTK
RRNKEKNNLNININLNLSTNDDVIINKNEEKENENKESNRTEHIKRDNSIHSNDNCSIGTHSELFKNSEYNSM
INNNNNNNNNNNNNNNNNNNNNNNNNNNNNNNNNNSFINNDNTSIDNKVSMQCRKYYQEFLKMNNLEDIKTIR
KIEKRANQNNTSNNNTINNIHNINNVHNVNNIHNVNNIHNVNNIHNVNNIHNVNNVHNINNNYYDHIYSHNNI
HINNHAIIHNNSHYNNKKTNIILNNNKYDEYNDKHSFHSENTNSYSNSNKDDYLNEDLNLYKDHMYIQNCITE
KEDNIITTIYQKRTNFELSLFILKNKNNEKIQNILSHKNKINKKYILKPLKIYIDEITYNFKNSSDKKITFFH
TNNYKNYLNFTLDDKIIFKLIFFYYHYKDYVDHIFEKFESKDESNYLNLNTIDVRSYKTLQILLDNAVHNKND
HWFNEQKDVHDKDEQNKGTIYYEQNEHDIKIDDNNEEENNDHTDNNNKNNNNNNNNNNNNNNNNNKNNNNNNN
NNKNNMVDGQIECNENVTEKEIPSNVPNSNNLTNESSNDNHNKTTIEHNNNFCEEQNKNDLIKTSFNITHCEE
ENANKNKYTEEDKKEIKGNDKKNVEMCRKDTLDYVKEDINKYKMYINENNNKKEEEYSHNNNNHHHHYHHNNM
LKDDTSNYNHVQYFHGEKKEIICFINKSKIHFNNYKKSKRMISIHLSDVLMYLFSDNNITNFLFNLKKINILN
CLSHEDSYNSFFSYIQHKKFSTHKISKKGSKLSDKRKSSNVNGIYFYNSVFKLIQNGTFLLKYSSKNNISCLS
SIHLNPNHNHYIENNNSKEKNYFLNNNNNNNKMEIEETTEFRYFNTNNFEQLNNTSGNKSIDGSNVKINNSL
DFVHKNYYNNLNNDYFNFNVPQDSNKRKDATSSPSNVSNKSGEIKSSHKKEQRKDNHMNENIRGSHRSYSTHQ
SYRTHQSYKRHANNSNIRCSSSPSERTHISKKDEIKFNKENIMTLLNDCTNNEGENHHVKITNKQIKRSKSKE
QFEDIINSNTKKTNDIEHIWDEQNNQNNKENDNVVNRVEKKSIITENYIPYKSCIEQIDDIDYNKESLQHEKN
IDGKTNIIYHNNNNNNDHINMNTTTCIPINEKNFVIQRNKKISHYYNAKEYNDSNKRRRNNYILTGEVNNIKG
YNKDDMHSSHVNNMNNSLCSSAYHNMKYEKYVDIEKYRNDDEFYEAEDDEDEENDIDNQEEEYIDMKKKENEN
ISSIEFHMINSEFIVDYLYFIYLFKWINKLYRKYEKIKQLSDFAYSNNNSTDDEESNINVSKCNYILFLQNNK
IYVPIYNYSISQYKYYNSLKGTSYVSLLPVLELHFSCSYKSMNKTMKMSINVRNLGSALVIPSINIYKHKKNK
KSNICVITFNRKDKNNESIINDLRFQINMRRNYNYEIIENEDDNSSSCNNMEIPYYEPKYTSLNTYCTVISKE
QMYSEKNKNKNNNINNNNNDNNIIMYDNNMYNYHGYHTKTGSYPKKIISFHCDPIILNFSKDILKNVLIFYNT
FCYFINNCSKNDDTGNDSNMIRDMLYYLYNQSSDILFEYFLDDIRLNCYDGNINRNVPLIVVSLFNLKFFLQK
DIISIYNFLNFYLNIDIYDSYKILYDTLLEKMEIYLTFENNRNKLNILNLTLETSYINILYTNKSKNILINFL
EPILNKNFYYFSSSYYNNLDITKNKRRKRSIYHHHNHNYHHECYENNILIKNTKGKSLVTHITSKIFNYTGLS
INVRFKQSLRNKIQNNSLNNKNKNKYLFYELQNNEHGALLNDENGEVCEVVVIIKIFNYIFEIIDIERNKNTC
DIRFLPIIYELKKFNEKKKITIKKYDINYDIYNEYSLKNKVFKTFIKLYIQTNIDEKGIVSIYFSSNLFINNS
TNSDFVLLFHPVLYYFMLFNEQSTNSNTDNNINNNNKNEDNMVDNNENNDKNNDKNNNENKDCDSVHNINNVE
SDSPVNINFFKNDNYNNNYHNNYNNNYHNNYHNNYHNNYHNNNDKGANITIINSNERYFLPLISFIPIIILYD
YLKKKNEIEENNYPDKYPMNSKNSYVTPVSMVCDLKGSFSADIKKNNFIKRFNSLNYNMNIHNGEEEEISKQN
EANDMLNYTHVDNSNNETSCRNNNEDKILSYKKRITYDYSSINSLKKLSYNNLTRASCYVNSRSSEMNLCNII

SNKGDKRNFMNNEKIIESLNLNALREINKIRTNRILKRLEKYVNEKSNSKNMKNINYIYIIKKEFYDFYIHKN
ENNNKCYFEMFNFLIKNGAKKVLNNSSIKEIICRHSFDFLKYNLYAQTLKLKTLKIICYDKYSRYLREKKKEM
EEAKTYNNNNNKNNINNNNNNNDNNNNNNNNNNNNNNNNNNMKKKQIGDIYMKNYKEEKNDIKEEQEKHDNSS
SIVVLNKFHDYYDKRDKYYFENNHRNDHYTSDLNNDDNNKYNIYDNNKYSIYDNNKYHIYDNNESNNKKDDFY
YSIPTNLDISLKCINRNERHINSLLFNHSKRKSTLSNILIKHDMIKNSVSKHILNNDYAYEYDKKEEIEYNYN
NKDNCEKNIYVLDVKNKKQNNNNNMNDDDDDNNNMNDDDDDNNNMNDDDNNNDNNNDKKKHHIIHYNNNNNIIK
KKRRKFIFTAKKQSSYSNTIICEQKNIDVNKIYSFGIYIDYIFEIHNFMHEEMKIGYCQKYYINSIPKKIKRI
KREEISPKSIKYLDTIPKYIKLFYVLNNDNYIFNFFSKKIQLYKYANTPDKIHQIKMYIEYINDENKNIIYKK
RKYKLNSLFKRKSKNKRKYKYLESKDYDKLCKEVPKFFYLNITFRKKTDHSNNTYYNENNFKHVIDSNVLQLL
IYPSFFFQNNLDNDLIIKSNGNLQKIKKKSIMFFGDVDNSKVILQIVYNNQIFLSKKIKINQVCLNKKIILTS
KNKKENIFLSMNITLNGNIYNTKTITINNRYTIINNTNRTLYVQEVNKDFKIIKKKEKKYMTYSFSLENLQNQ
NNHSNDENMEKLYIPSSNDLTNIKRNYNQKEKNEKNEKKEKNENLNKITINKRSDLTYEHENDETKIMDTCEI
SDKKYTEKGMTKICEFNKKHIKYNKHKKYNDKKYNQKENNNNNNNNNNNNTTSSNNSNSNSNSSNNRPNIISIK
PYESFYYHPINTNKCFLTFSYTNIMECKNDKNFYRRRGSYEFTHTQNEITNLFNNYLKKKIMKSQNISIINDK
EENINNNVHKNQEFYNLFYTSPIEIEKVGNYKIFHKTVHKIDVTYNNEKDATDDQPTYEDDIIQKEIKQQEKE
IEEEIKREIEELEEHREKETIIVFSNLKDSYEKEMQKKKKDRHKKNNFLNINSDHINDKNIINSYKNEQTTEM
IYIQHDYTDNIPADDEKKSSHNETILIDDLTLFNKNNKHSINERENKENILFNETVLTVDNNDTEHIKSDYVN
IKGDCINIKGDYVNIKDNWVNIKDNCVNIKDNCVNIKGDYVNIKGNCVNTKEDSHNNYQQNGMNLRHDSFCEN
YVIDFNDDDSCIHFKKNKNDINTADDINNNTVIIPIKSIKQLEGEENVHNIENKNNNSNNNNNLLCELNKTSD
NKERINTKHMFLYKEESIITEINIALNSNTNIEINISIENNPDTIIYNKTNYYLIFYQRKTNKKIVNLLKPFD
KEIFGWTDVCKPKVIKCFLIIQKNDVYVFSCNLNIVKEHNNIILNNNRRIIVLTKIENGKRVFCLEEMNIELL
DCNEKYEHKKSLAPFFSASKGFNRFREKKKKKIKKKLHKISLYNSLIKSSQMRKEKNKSKNKKKNKKQKKNQM
NQMNQTNQTNHMLQKNKQTNDNYLVKYKNEKFNKKLPLLSKKRFNIAYLKKKRKQIKKKKNNKYFHLSSFFLL
KSMDSSIHKDVEKERKKQKGKSQQAEKLFKVEKIVHNESYNESYNESYNEYHNEYHNEYHNEYHNEYHNEYHN
ENNGNINSIKYNNIIKEEENVKNKNATSNNNSSFSKKNIKLNNVSIKKDDKKKSTSKYKSLNSKKHINKFFND
NYTFRKKKKRKKSNKKNLYVNKNNEETETSDYYIEHNENKKNIELSSHDNNSFDNEKRKTSLGMTKSKGYNDL
FTVKFRKSVNENDKIDEYKENSFEDSSTYNDSFLSSSSNNENYISNNNKKHMFLNRYYDNKDDKKKYREYNLI
YNKEMKAKNNLKKPKKKIKRKLSDFIFTKIKKNQKKFKGLSRIYPMFLKSNKSDEFNVLIRNYLSDKDDSTSI
RKEYKIINDNYINNNENNNNENMNYDLKTNTYNNILHNNNYRLDKKSVFEENRKIPIKFITNLNVMLRGIKIS
LFEHTCDYVISFEINELLVTKEYMEYIDYYNVDIKINNILCYAVYKYLSACAVLYTNNDSNIKNNHLRMLMNE
LKIKRNLFLQKKRRNENKKEYSDVNIDKNNDNIKNNDHINDNDHINDNDHINDNDHINNKDHINNKDHINDNE
KEMYSSGSSRTSYTNKKKGYKYKNNYKKKKKKHEKNIYDNKKAKKKKKEKVEKKKKNLFVNNEFLICNFQYVR
KCETLFFKYFLLSLKPIVINADINSITILFYFYKTFYNFHTKKNEQGQGVQNGFNTDNYEKVHMREINMMKEQ
QQQQKKKKKKKKKKKEEEEEEEEEKKKKNKENVLQKKKTKQEMERPLKDYYYYYYSSMNILNDPDNNKRCDIQ
KNRMIEKNDSFFINVDENRKQKIQDITGDHIIDNNILSRMKNMESFIIHEEQQKNDLTLTLHNNIYNVEGEYY
NIKKSNMNYYNNIINNKKSSVQIKKKTTLIEKNDINNKYVYLQYISIDKINILLNFQSEYKKELTNHVNDLFY
KEHISAYNKLGDISNCNIYLKSLSNIHIFTNYNLLINFLQNFYYNQTIVNLSNLLISFNIIGSPTSLIAHVKN
AFNEFFYIINEDTLSKKNRYDDDKDINDYTTKYNRKNNKKNNRKNNRKNNKNNDNNNINSMYGSHYNNNYIYY
NDYYYNNNYMYRRSTLNYHYYGQGYDKYKLNRIYNNNPKLRKNNKERKNNIQQINYTTKKEDKHNFLNLIDQS
RESSFLDEKDDSLTNDDKYIMHLLNDYNSSYQNNLLSEEESFVKRNFSWNFFKKQHTNNNNNNNNNNSDDDNY
FNDSYNNIKHESSGNLNYMYTKSIKNKDLHKLVIDDNNSSMNSFLFNNKKNVIILDQVSKKKKKKLQYIKNFE
EPQKGHIYMDDDMYIDVDVDLNSYAEGKNTTKDTSKYPPHFKISYDEEKKKRKKKRLSTGSNNNRYKNDAEIL
DMNPNKKNKKKNKMLKNNNNNNNNNNNNNKNKKNKRNNKRSGCTKMFRFFKKIGLNIIYIFSKTKRICIGIYVL
FIQLILSLLFSITLILQSLINLVEKVNINNADNVFLIFNKKYERKKVVVFNNIEEYNKFNITKLFYYYISIYM
NIVNFLYYNMLYKMKKKKKDLSGEDKIFMKKLKINLKNKRKFSFFINFFLNIFNIINILLFGHIVFLLLCLLT
MNNIFQKILTACIKLVYYQRNKNYLDFLNKNSFNHMNIISYIKINQVISKYVLLKEPFKYFLSHQDFLDIKAP
KKRNYFVYTKELFFYIQENKIVFLFRKNDMKKIDIIIHTINNSFAINSYYKKKGSKTAKKSRSKNTPSNYTLN
MNM*QEKKIIALLHLYITKLTYRMIYQRIKMLYVF*IKN*L*PKETKLYI*IIKTMIILFP*K*QSKII*MLW
RIFIITPKNFLSACQKYKKASIIFIYLYVKIIIMKPKIIRKNI*IIKVQIYIKQI*M*KKEELFFQQLFVSLK
AFSYFCTFVYAHGNC*
>N.PF10_0212a
MMENKYPNELFCYINRYNINEIIENGEEKYVNEYDEDKNMSINHMNENDGICEYEIPFLLDYVDDSNKEDSEK
NSLKSYLDDGASTILSKPDELENYNKQNENEFDENNNNKNNKIDQLKEKINIIIIPNKGVINNFEEILSMANR
NDKNIEKKLNDRFYQICCKSIADINTHNLNKIKDLKKKKNNKGSLNIEHIDYGDIFLTIHDTLKSNNKIKGNN

KTNLLHDSSYEIKKKTRRGTNIYKNPFHHRGSYLTSYENQKDIIYLNNLNNIMMDKYSNCSDSRKKEYSHFNS
QEFSYDKYSMKDRMFLKNLYMKQNRLRDKRGKYHKLGDYQNIENYRKTGEHSFDCMNMSDIMHSNKMSHVNIM
DHMIYKDNNNMSKLVDTINSREKDVKNYDDNFESYNNFFKNNNDEQHICLEYDDTYNLKDTVKNIIVEEEQCD
KGVACICDKNEDVDDLFVSKKTNYSSNKKREDYEKVFLEDNLHLKQTPSKRTKINIIPDYYDNNRSNKSYKEN
EEDALFEVCGSLKNDDILYKDNKLNVINEDNIKEEDDKESVVHLDNDEDKKEEMYKDVYPNVLSCEKETIRRN
EKYNKSLNSTSSFEKIDNPSEINVESKEDTEYFDLLIKKYEDTKINVYDNESLLLDLSNELREEMAKGDSNKN
VNKVEDNDNKKENICHDNIMEDICHNNNVEDMYRNNNVEDMYRNNNVEDVCHNNNVEDVCHNNNVEDVYHNNN
VEDMYHDNNIEDVCHNNNVEDVCHNNNVEDHVNYDNEELNKKMDEMKEEKEERNEDRGIYDELLENDMCDLYN
LKMHDLHNLKSYDFGLSKDLLKKDIFIYSNNLKNDDMDDDDNNNMNDIAIGENVIYENDIHENNIDDNDMYNN
YVNGNDLYINNMQDDAMDDIVYDEEEIKSFLDKLKSDISNQMNVKNGNVEVTGNGGNEEMSYINNDENLQAFD
LLDNFHMDDYGNNYNDNEEDGDGDGDDDEQKKRKQKELHNVNGKLNLSDLNELNVDDINNNFYMSTPRKSI
DERKDTECQTDFPLLDVSRNTNRTPRRKSVEVILVEKKLKKKKQKCMDKYTDANEDSNRRYPKRNRIKTLRYW
IGERELTERNPYTGEIDVVGFSECKNLQDLSPHIIGPIEYKKIYLKNLNSNEHEENEDNNGDIIENNNGDVIE
NNNGDIIEDNNANEKNHNNLESEGKGIVYDDVNNLHVHTNSDNSAHSKKIKGAPSRFSNTNNGRKKRRRRKFI
NVVNYIKKKKKKKLIKSMDNMEVTDNFKNDMSDENKQSGDENKQTNNDIKQSDNDIKQSDDIYMNEDMNLFND
LNDNFDNNEYFINNGDKDSHAEEEMAIENIQSKSIEKDILNNEEQDNNNIFDIDNELIDMKDGNVDEMESDEK
LKTFEKLESLKSTTHLNNTDNCDVNLSEQTNEINYDEEKKVNKKTNHEKMKKKKKKKKKKKKKKKKEKKQIDI
MYKNLSRLNLNLLLPTKKKVKKSKNSFKKEEEKQKKKNKKVKKIKGINKGEKIKSNKKENKDNNNDSSTECVV
EGEKGKDLHEFNKNGNLEDEQMDVDISMNISSINCESDNKNVSKEGEEEKKDIAENKEEVDKNKEEVYMDKHE
MDLNNEEVYMDKNEMDLNNEEVYMDKHEMDLNNEEVYMDKHEMDLNNEEVYMDKHEMDLNKEEVYMDKHEMDL
NNEEVDKENEYDENILSDNIIYNENNSFGNNKNSFFNNTSPLKTEIINEEENSLNEMKEDINEYVEMENKLDT
EKIKDSEKIGGKIEVDNKMISPINRHNFYLTILEGMNKNFPRQWNKNNITLSKNQGQIYKGRKEKKRKRSYRN
DEKLLDHSILNDINISDKMDERNELLESIKSNSTINNVLEIIKYDNRKKIKKNDTNKEIIKYDNFTSKYNNKS
NDIQLNGGIYINKFKLSLDMPINKLAVSSNLGPPSSIGSTEIQPIQKVTKKKKIYIYIYIYIYIYNFNDFKMN
INVYCIRMEPHEKYSSYSHKNNLVVYIDKGEKINIIINMSKTYEKGDFFYIPRFSNFQIINDSRCDCVLYVCP
LI*
>N.PF10_0213
MTLEKSLDQEKNKTEIENTGSKSIPNDSNEGANNKVDKNDGNENVMKKNEGDKNKVYNGTADNNKVDNNKVDN
NKVDNNKGDNNKVDNNKGDNNKVDNNKVNNNKVNNNKLVNNKVFNNKFNNNNIKNNRDNNKHTKNNRADYHKV
ENTQIHISNKFQGNGKGYNMNYINKKDANHEKGYKNNYENNRSLSYFYSKKNYNEHFMPVNNVPFKKRGSDLY
NNSKKESANDYTNDNQMNNNTKNNNQLNNNIKNNNKLNNTTKNNNQSNNNTKNNTTKNNNQINKNYNKNTKLN
EQVEQREKNANLSLKNNEEKQVENVKNINKNNYSGSKNAWIKNEGTNNMNRTCKSGIHNGNKNNNNNINITNK
DDNNNNNNNNNDNNNNNNNNNNNNNNNNNNNSENVNKKKNKKKNKKKTNQVSTNAWTNDKNSNLEHIIRNNQK
KGTNPNTENDHHNENASITTAISINSNVTDTQTKKHMNYGSTNQKEQKKRVQANSKVSTNMKEDNKANIEITN
FNNANKSYSTNNNPSPNDNLNKGNVKNKKKSKKNKNNNNDSNNNNDNNNNNIMNENKKDINDNKIINNNNNNN
NNLNATKKNNTININNTIIDKNNNIENNFAFVNNLNENLEHNSMKLIGNNYPMNFENNEIKKKNITASKNVPN
KYNYDMINQGNLNYMYNPLNYYNMNKYYDMPFIDNIANNLNPMYFDNDMQRNHMMENIINYQGMYNMNNIYNT
NNNMEQNTNNNNMYNHNSYANYKHEHLSLNVYEGPLEKNNEWGKKKMNKKNNQSGMNQQNVMNQQKVMNQPNA
MNFPNNMNHPNNMNHPNNMNHLNNMNHPNNMNHLNNMNHPNNINQPNLINYQYPMNHVQHINHPDNRNKMNNP
NIPNYPNVNVSAPETKQDTKIIPNKEKKKIINNEGTVLQPLKNNVEHNKANNLLTSKKHNTNVNNKKKSKNVQ
QETPIKHLPNEEENMKQGKTKNSNNKNNNNKNNNNNNNNNNLESITKNKNNSQYISTNVENENLNKNEQKGKM
NKMKTKTQRFNKEEQNETNNFTNNNKKGSNRYQQNKKGQFKNSNMNYNSWINNQQGNDQQHLNTNVDKDKNKD
FEVNHEIHKFIDYCLKNYGNRNIMDVLNEFSSSNGMNENFDELKNIFQIQLNEENFNALAALENEAQTNKFHF
INEKNMNETIEKGIEEIFEDTNKDIIEEYDKFKKMLSESMDESSMRKIQIENKEISDNKEISDNKEISDNKEI
SDNKKISDNKEINDNKEINDNEKINDNKKINDNKDNNEKIEEKKELVENNTKNMFEENNKINITIPPGLSLPD
INRALLHSAYLDNITVVKKCLEKKADINYLDKIGRSALHYACAGGYFDICELLIKNGAKVNVSDYKNWTPLHI
AITKMHKDITELLLKNDANIHALLPHSLSPNRGKTTASMGIHFAAIKGNKEITKLLLQYGAKIDDTDLSNRSA
LHYAVYRNNCDYVKFLIYEEKANVNILDIHNRLPIHSACLGGVLENVKILVENKSYVHKKDIYNMSPLDIVQI
KKFKDIKNYLTKCMNQNFSPISSDHLKKNDEEKENEIKNIDDFSNEKNEGEQKKKNMKNYENIKKENKDIMLN
MDKQKTNDLEMFNDKFELNDEAKTMDPKEFYNDNQMIKDMLTTTISTVLNERNKNQIKRVVDALGVYISLSLL
EKTILIQNYGGIDMVNQKGKRSTGGVFFYLIKYMYKNDIISKFKYDYIIEEEKEKKKTYRKLKKKLLQDEENF
KNNETFVTKKNEENGCIEDKCVDDDKEVKGNNVGTHTTLLRKGKKNFKFIDETKNKNKYSNNNNNNENKNNEN
KNNENNNNNKNNNNENKKNNNKKNNNNNNNNNNNNNNKNNNINNNNNNNNNPQVHNKKNEKKSGCNIKTKEQN
NNKNTNNMKKQNLKNEIRNNYQMIIEDEKKNFIKNDNKVLNYDKNLLQNNNCNMIRNYNNSSQNYNNEYYNA

SMANNNNYYYNSNQYNEHNYNHNNNYNDNNFNNNNYNLNNFNNNYNEFISQNPGNAHMFNGQNNYIPIEQLAV
NIPTDDKSVKKKNKKKKNNKNSTQIIIENNKNNDTTYISKDNVEPSFVNRMQDSYGNFHNVHDIAQQKKHFYQ
MNTAPTTNSNNTNNNINGLENFKLNNNYNKEMMNYQNNVMSSYLNNTRNNAYDYMLRSKNNFPNYMNNPNMSM
LNDLNKYYGNNNQKFPLNNMEYMYYNNYSKL*
>N.PF10_0214
MNKKHNNSAHFEKFPCTTNIPYSVQNNFNLEKDTLIKNFEDKNEHINKKSFNKNLGNLIYNISKLILHNSNNN
NSDDNNINSKNSDTNHNITNNKSSNNNTTNHNTTNHSITNKNITNHNIANPNNNNPNDNPKNNYKDKNNNKNN
NKNNNKNNNKNNNKNNNKNNNKNNLNSNITCSEYNLLSTNKKNQDITQALGDHITSITMNNIESNNFLVGDEN
NGFYQILNNVDYNKKEKKKNFINNKCNSNIHMNNIENQLYNNVNNIIPMVSKNKYNLFTNNENYNQENDKYNE
KLFNNSVNFCNIPNYEQEKKKKEIEGSENYKMNDVSQLTQMNYMYRNIDYSKEKVEEESFEKNVVDFEENVPE
FMNEYKLYNEKVKHDNINNQIIKEIKKNKGNYNFKNIVITNIFLGNIPPNITEERLKNVLEIFGYIIHIEYKW
SIDKWSYAFVYFIDEKCAINAVNFLNQKKFFDNSPNHKLICFIVSKQIPHQNTMHYSKENFSLLKDGPPGANL
FLYGIPLKWTELNLIQLVNKYGHVVGLRIPYISKENDKKQGNRGFGFVSYDNKKSAIEAFEELSKMYIHGKLL
KVQLKNGEEHLLPAKLKNIYNTNKNKAKDVTNLKTAQSLVSTTDTLNTFNSLTSTEVKKKLKNNKCSNNNMKS
SVFISNKNNINNNNNNNNSTIYNKANNSNTQHLSMPDTNSSKSLLENEYKNKFPLNNSNNCDSVPSIYPKMFT
NYSDKYDLNVTTEKHFPIYASKLPQNNGANLCLSKELNTGGEFCSYTINENNSFEGKDTNKKNCGKMLKEADD
INKNETFFLIKENRENKYSNNKDNKGEQINTQNDMALYNNMESNMITYKNMEENMVPNKNMETTFSYFNMCDN
NNNNNNNNSKSNYSDSKKVTPNSNSNSNSNSSSSSNSSSSNNNHFEKRWDKNTWKKTSNIQSVEENNKHDNTF
FNFIDKMDTTRCQGGNIRNNKYSNYNYIEENNSEKNESSKLNNFKNQFGANSKNLFFFNNSNESENSNINMYN
KFFVNENNLYSVDEKNERQNIPTEKNIELTINRIRNETPSSFNEKTKNISSFLNDIWYNNGNNNIIMYNKNSN
VHDNNNNNNIIHNSNSMHYHINDIHTNMKRDNYENNNKWNNNIKGNNSNEYNYKKENNNNNNMFYYNINNNNN
NNNNNNNNNNNSEHISEIDVKQLNNIMEKKKEYLNAVSKEEQENLDDNISKNEYMNDKQNKTTQYFKNMKILF
TFLNIYAKQNSLDIDDFFNDEKNMHLFEMLINKKQFDKNDLEYLFNMLQLKNNEKEKMKKEILNHNEYFKENY
NHDKIVYTKSNTNMLNMNYNTPNRYQQGNNINTNPNVAQRNENNYYGKNNDAPYDLDINNIIYTSNNFTNLDK
YENSNFNMEIKKINDMNAYDNKFIPNVQNNVNKYM*
>N.PF10_0215a
MYEVYYLNEHDEFEKKKKKNEEIQSSLILKNHAISSEKKSKLVKHNIYEGGYTIWECTWDMLKFFHKEGFDFN
NKQVLELGCGHGLVGIKVLLDNGNVVFQELNKEVINDVLLPNIKKNLKMKLKKKKLKENKHYMKINNDKITCY
VINKPWHKLNSKLQKKKLNCFDFILGNEILYRKENYYHILKILKKNLKKGGKAYFGSKSYYFGFEDGTGSNSF
VTYVNNNEHFNFVARIIQTTTGKSVYSKDIIEVTFSPNND*
>N.PF10_0215
MKKTSDIKHIFKREDDSSKITKLIKNISPLFFMSEFLYILFLRYNDHILSTTYKEKVTPHVRENIYNIFILLY
FIKPLFAFLTDSVYFNLNNILGLIYRKIYKIYNYFEQFIQYCCSLIYKSKYLFLKIFFKRYIKNGLSKSRYLK
KKAHIGKSGYMDNFVYFPLNRKYYVIISEFITTVLLLVIYLFNEKINYYIYLTIISFISSQMLLSSCVFEGVV
VERCKRQMYFEKVFYISYVMCVKIFCSLILYYMYILKVSVFLLISKSFLIFLISCVCSEETLLLNNKRKIQRN
IIFKSGSDEVYVKNTEDILSQINILKKIFFNENLFKTIFFIILFNSAIDSKLSILQYGVQNFSWPHSLINYIP
LVSQSSKLIGISIFQLYTNKLNYKNYSMVVILFNILLKLCSFIFLYNNKNTYVSPFLFLFNIIVQNISIKILS
LPILLLCIEKAPLSLESTIINIYIFCFNLSNLISKRYFMWNIILHVSKNVFIILLLSFLTTCASLLYYFHISL
DTLNNMNSSSLYLDEKEDVNLKINKIPQKNYLRDELFLNNEEKKKQVKKVVRFKEDSIIKTQKNTNDEKKKKN
NNLFKISSDYNSDSDQWLIIDSLKSDDTRKKQGKYKSLNISNIML*
>N.PF10_0216
MWLSTFLALLIFVECVIVVYIPAYIESKLSKIKKNRFFNMENFENIASGAILALAFLHMLPEVIILSNKKNMN
LYYIFILVLVSVTFLNITDILYDHHFESTFDVNCTLACENSNNQIKNINDKEITTNYIDIKSNEVIDLELKAM
DTNINNNNNNNNNNKTDPCKTNIFFEIFKSNSFFIVLSLFIHSFIEGLLMGSLKDKNAIIIVGLSMLAHKWAE
CLIVYKNVVNKIENPLLASIYAWSFILSLPLGVFIAIFSFPSNEFVEIIFSSIACGFFLYLSFNMTKEIKITK
SNKHFISFSYFLGVGGMSTLMILFNSFESNNII*
>N.PF10_0217
MSFKPRFSKSSSCIYVGNLPGNVIEEEVYDLFGKYGRIKYIDIKPSRSSSSSYAFVHYYDLKDADYAIERRDG
YKFDGFRLRVEHSGENRRELEDRSERRRSLSEDRSNSRKRNASSDFKRELNSDDDKKSKKKRSYSASPGSAYK
SSSRELKSQDKSNDRLSESKKSYKSQSASVRYNSTEEKKSDDETVKPKRGGRGNAKNTGKNKNTKGSKKGAKT
EENHDSKSVSNDRNKSDKSEEKEKPEVVSKDEEDKKTGGAEVKPKRGRRGRKKANAADENANGNVSN*NEILL
FLKLPSFI*QNTEKNNFYF*NQIKNLKNIK*KEKHIYCICDQN*IIFYNSICFNIYVFMFIMKDLLYI*TKKK
LILLLIFYIIYICKTNIVFFFFFFHFLIFIFVQILFFKFLKQKLYLNLKINIFIYIFHNIFSYF*NKRKTLDI

I*KYFFYLINIYV*FFFFFFYLLKNVL*IILKLIQKAYFFFKEYNKINYVIYIMQRER*LHLYTCIEWN*IPL
IYCRCKFFFFLYLFH*Q*RKTI*L*
>N.PF10_0218
MEGIRYLSCNKILFKRTGSTKTLINSLRKRYVHNNFKKKTCEQVLNTVNKLHNEKRYFNFSCSFTKSSRWNIC
MNRHNSRFFSSNESNKNLYLENYANIRKHINSIDNEESVIMNILKEKTYECIKKTREQLKEIIHTYPNTPISI
CTPNNVIGGLRNTITLITDTSILEKKKGILFRGKPVEKILREFPKWDNTCEYPMAEAMLWFLLTKEVPAVDDL
KLLSRELYCRAKKIPQFVFDFIDNIPPFTHPMSQLVSTVSFLESLSLFKYKYSEGILKTDYWKYILEDAISLI
SQIQVIGAYIFKRTFINNDIRKGEGMNLDLDLDWSANFAKLIGYENKEVQDLLRLYFLLHSDHEGGNASAHVS
HLIGSTLGNPYLSYSGCVIALSGPLHGLANQECLKFLLDIKKQLGDNQLTYEFIEKYAKNLLKSGRVIPGYGH
AVLRVPDPRFLALRSFALSHFPDDPIIQILEMCYKVIPGILSATGKVKNPYPNVDCSSGSVLHHYGIKYPEYY
TVLFALSRSIGVMSQLVLSRGLMYPLERPKSVDIYNLRKICEKNYVKIEEYE*
>N.PF10_0219
MVKKDKKSKEKKEKLKLKKEKQKLKSLKSKKKKKDTLSDEDFDTICLYYENLNKKDKFGHININTTSNNTFVE
CEKPSPRSNCSITFINDEEFILFGGEYNDNNELISYNDLFKYNIVKNKWKYYFTTSKKPKPRCSHQTVYFNKK
LYIFGGELCTNTQFFHYNDFWSFDLKNNVFEEIETKNKKDDNKPSPRSGHRMILWKNYIVMFGGFFDNGKSIE
YFNDLYIYIINSNIWINLTNVYMDSLFKRLTENNSSNNDNNLSISSEKKKDLNKGKNSQILKSKFFKNFDLDS
FMPSKRSSVCLFTDMKYQKIYIYGGYSQIKNTTRNAIGFYFNDMWILNINLINEDNISVNFKKLKKSIFQPCK
RTGFSTCIYKNSLILFGGVFDKKVENNSKKIDNPNNNNNMLEESLNLKSLFFNDLYLFDMNKEHWSYLNIKDK
EETKELNKTSKANKKNHEKLEENKIGTNIKKDKFQQMEREIYYEENNNNNNNNNNKTQSKYEETSDGHVSSCF
SDDNDEDYYSNVFVYFDENGKRQIIKIEKEEKNKSSYNEKKDFDDVLKVEENNDYLNHSLDEEKNINIDKLID
NHSVFLQTKDIITTGNGNKVTKIFGDENKHCQNISNLPLNETILYVPLNNTTNSENFMYSQELNDSTNMIKVE
HINDTENVDEETCKEDSVDEDMKDNSNSDSDSNKEEKKKKFVISEEEPIGRINSHIFVLNKNLYVYGGMYEYK
NNEIILSDYWKINIFKREKWELLDKGNLDDIYLEESDMSSTISINDDDKDEKEIEDLIICSKIKKLEKKIKEL
DEGLALDIKENLNEFFLRTKDHWLKELNKISETKEIRKEAFYLCEQKYKVIKKYYNKIQKYKELLMEDDEERS
ISETISSEQEQSSN*
>N.PF10_0220
MEEKNIHMQPNINYSYRNPNMYNMNYHNPIVPPPQQQMQLFVNDWKSILSPMQTCKIKQQFDDREFVADYFMG
FKLDFNNKYLILDASTELMKFTACEKSEFCYRNCLPKICIPMNMKILSYGKEISKPDILMEKDCTCTFLCFNR
PTIKMYDFSNNNNKELIGTIKTPYRCCSYNFNLFDPSNNKIMYMDDTCCQMSILCPCPWGPFKYSNFFLRDAN
SKEKIAHLRKEVPFLKFVKRDIDNYTLDFEQVQNPEWKMMLLAFALFLDYMYYDRK*
>N.PF10_0221
MSYIKRLILFMLLFYSHVKIKKLFIKISNVNIFFAEAKKNGKKEFFLFLLNIKKNSQQKKTYHITKRNTINKS
DFLYSLLNEEGNSSKKEYKNLKDEEKYNIIQNIKKYCECTKKYKRLPTREVVIGNVKIGGNNKIAIQTMASCD
TRNVEECVYQIRKCKDLGADIVRLTVQGVQEAQASYHIKEKLLSENVNIPLVADIHFNPKIALMAADVFEKIR
VNPGNYVDGRKKWIDKVYKTKEEFDEGKLFIKEKFVPLIEKCKRLNRAIRIGTNHGSLSSRVLSYYGDTPLGM
VESAFEFSDLCIENNFYNLVFSMKASNAYVMIQSYRLLVSKQYERNMMFPIHLGVTEAGFGDNGRIKSYLGIG
SLLYDGIGDTIRISLTEDPWEELTPCKKLVENLKKRIFYNENFKEDNELKNNEMDTKNLLNFEENYRNFNNIK
KRNVEKNNNVLHEECTIGNVVTIKELEDSLQIFKDLNLEVDSNGNLKKGAKTTDMVIINDFHNITNLGKKTVD
KLMQVGINIVVQYEPHNIEFIEKMEPNNDNNNNNNNNILFYVDIKNIMNSSEKNIKLSNSKGYGLILNGKEDI
QTIKKIKELNRRPLFILLKSDNIYEHVLITRRINELLQSLNINIPYIHYVDINSNNYDDILVNSTLYAGSCLM
DLMGDGLIVNVTNDVLTNKKKIETKYDEKEEVEEEGNNKDIHRLLSRVALNSFLTLNILQDTRIRLFKTDYIA
CPSCGRTLFNIQETTKKIMKLTGHLKGVKIAVMGCIVNGIGEMADAHFGYVGSAPKKIDLYYGKELVERNIPE
EEACDKLIELIKKHNKWKDP*
>N.PF10_0222
MSWQQWWPHDPVVKTDLVDPYLVKVEKKKVYWYCSCGTSKTQPWCDGSHKGTRFKPMMYIPQTSGYRLLCGCK
QSMHLPHYDFADLWVRANRNVPKAAAFTYVALFSFGIMTSWLFHP*
>N.PF10_0223
MKMEDVEIKQALFKNENKMKGEKCTICKSKYGKFINAFLTWEIVLLIVHILIYLVIIYKFLKDGKLTSGNNML
FRSYYSAGHFTQGFLFVLLLVPFHFKKRIVKYLINTSSVQMCNNGESEIKESFDDIEKHKINNYIQDGDKEEE
EEEKKKKKSSEDIKMMINKGNNTTDKNMDDELTAINSDLNELEKEKYLNDMNIGINNENKIEANDENITNSLS
ESFINKLVFCSNTPNHKKKEIHYFFLCAKLFSLNRMNFLFVIKYVSTFILMFAYSLYNIIKRKLRYKYFYYSS
YYMNSFDFAAGLLAGGIFVLLYGIIFFCIKLYNNTIKNKNKNFHFYDNYPFINDVKCICDENIKLNIRNNPYL
KNIAINYFNSYYNYKNLLLIGQMFLVIICIFSFIFSITSIYAFELNRQ*
>N.PF10_0224

MSKKNDVYEENGEYNPKVEELLTSALEFFNIHGLAREEVINNIKKDENTIKLLNDFYEKNEECLFIFNEKDCI
KIYNSILDLYKKRKNEKFLIIYKTPQSNILYLEMNNDAENVIRNFLEIFFSNFMNKNEDINSSSLLKEMKKKF
NNFLLNLRIIYGETEEKLTLPYAPSEIKEENVNTIEEYLIHFIKCYHIILRNFMNYKKKEYINCYEFLKCLKN
KSEDINYLYNEIDKESFQEIINIILEKKKNSSYKNIIENIKKEISEHKMEILDLYTHLKPVVHFLQKLEFNKD
DDIEKKYIYITPIIHSLSISIDKSNYLIKNCKFVFNFLSKVLINSCNQNLNTPEIINSIYNCVDVLKIKLYDT
LNFLLNFLYIFKMYKSKNEKLQSLHEHNTYVIQRKTQIQVTSEKGNELDINMSPEKKKNNNINIDIISSYQNG
NNNEENLNQEDDIKNELEEEYSYFPSISLYMYKLKILIEYLKIYQTYMKLEKIEIPGEKGKNLTEEIHNISND
FNIINENFLDINHEILNEHEHIFINKYNIFESRVKELNKRLICVFVYSFDNDLKKNIKLLNSFLILRDIHVIE
VELIRHASLLCQDLKKEFILVDNLFIKNKTIIENNKKMFLEDKFQQGQCDLDGIKNVMSNNIEEEHINKKNIT
GENIISNNIQDDNTANTNIADETIPDVKAPIITNNINVNINVNINVNINVNNKMKDKLEIDKMEREIKERFED
NLKGLNINKNSPLFCEVINLFTSLLKRIEQQYFPLKKIIKVLKFENEKTEEVKHLYKNINEKISKYINDVSKE
WFKETIDVVNNFLNEYIFKKRNDLFYVNFHPYIFMFINNIKNFFLNNLSVNEDCLHIYNKANTFKKFINILNN
ITKKYNSVLTKTLDVEKELFEEKMNNIKNRLLKGVKELKWSDDNIYEYIESVDKDMSDIYNNISTLHNNIVSI
LNVTSNWVKKSIIEKRKRNEDIYTYLNNYKNNMSILKGKLNDDFKNINMNIKQSYDILKIKKNNIMWKNYLKF
LNNIIIDKLIHLTNFLFNNLYDVMKCTKRDEQLFIIRINFIENKLNLDLKLTSKKTFCIETIFYKWIDDFMNL
CSGIRRIDIKSGDYLNEILLSLSIHYNKNKIENFFQSTIKKSYEYLHSLTKYEHIWKIDIKKEFLSFKKKNEI
QLYPTYFEYNNNFQDSLKLININIPYLFPNVLAYKKLILTLKQDLNKFYHLKTYEPINFILFYSENFVEKLIN
FTKKNISLYSEYLQNFVKDVVYSINSFYENVINNLKEISIVDFFSKKGDSKKEILEKGETNTGLQKGEKNVRL
KKESIDNMDISKMDNNNNQMNDDNNQMNDFDDNDKMIDLDDNDKMIDFDDDDNNNIYDESKRYKNKNALKNEV
EEKEKEKTNVSNINKKKDIFYNLMRNLRSVKLSRKIIPLIFQKIKEIMENLKELDIIIKYNDILDNIEKNKEK
VEIQYNDIKEKILKYKNNEIKNIKLEIKDFKEELTKLKKHIFKNIPSTPKDIYNFPYEKINEFRKSFQTYKDK
QKYHNELEILFEIDITIFDELDQIDIKLTDLKHLWDLVNSIIFFIEQEKKKLWKNIDVNSNIYLIDFISLYIR
KNFFDLKHLSIYEYIIKELKKLSSILPLLVDLNNECIFDRHWNIIINISGNINNLYTKKKENNNNNNNNNQNN
YINNTDLLNTPLSNNNKTSIMSIHTFNKEEQINNDNTYDNEKETNNNIFLNKNIQIKYKELTLQGFFDLKLYK
HVDAVHDVIEQAKKEQKIENKIKEINVIWKEMNFEFFKKNNYIQITNMDMILEIVDVHTSEILFFINQKKFIL
FIQETILNTQENLKKIDEVINIWRKFLNKFERLQPIYLNSEDIHSQLPEESKMFFNIENEYKEIIHSAAEQKN
VLQVCLNEDLFYLLSKFFKNIELCEKALNDYLDQKKKAFPRFYFLSNIALLDILSNGKNPFKILPYINDVFNA
IKTIQFKEDLVQRGPVSELNEQNEENDLVKNEERSETQKDQKDQKDQKDQKDQKDQKDQKDQKDQKDQKDQKD
QKDQKDQKNVNTDGKMDGYTDDKMNGYTNDASYYGQEEDETYEGENKKCKDIKTCESKDYIAKGVYSVENEHL
EFLNELVLKGNVENYLKDLETHLKVTIRSILENAKICSENLDEQNRDETMITNYISQVVCTCNQIIVTEEINK
CFDELENGNESAFVDYKKVLIERINKLIKLVEKTDDYNIRTKLLSLIILDVHTRDVIISFIKKKISDSTSFDW
QAQLKYYWVYDKKINNYTCEIKLCDFKTKYLYEYIGNSGKLVITPLTDKCYITLTQALNLILGGAPAGPAGTG
KTETTKDLSKAIGIAIFIFNCSNQMNYFNMSQICIGLSQTGAWGCFDEFNRISIEVLSVVSTQIKCIFDAIKE
KKTMFHFIDDEIVLKKTCGFFITMNPGYAGRTELPENLKNLFRSCSMIVPDIKFICENMLMSFGFIKANKLSY
KFVELYQLCKELLQKNIHYDWGLRAVKVVLIQAGNLKRKYANFDEEIILMKALKDFNIPKITYEDIPIFLGLI
NDLFPNVNCNIFDEKDQEKENDYDEEHKQEQQTYIIQDEEKKLYKKKKNIHHNIDKYKSSITQNFTNANDNKN
HLSINKDNYETNKNLLTHYQHDICEDISQIDKTKKDVQIYDEAHNEKSDINELNQKTNIIANQNVENYINNK
EYDKDSNKIDNLKQNKFMNEEKRIIIHNFDDAIKICLKESNLQIDDNFILKVKQLKDLMDVRHCVFILGEDGC
GKSSVIDILIKSLNKINEKCLYEIINPKSIESYELYGYLTKNNEWIDGALSSIMRKMSRNISPYNENIKHKIT
LLDGNIDAEWIESMNTVMDDNKVLTLVSNERIPFTKEMHLFFEITNMKYASPATVSRGGVLFINKGDISYKLF
ISSWINLLNNNIAKTEFYYLFNIFYSQNIDMLRKQCKFAFDLSNLDIVKSICNYIDYFLYKYEKYINEVIKKI
ENKQNEEITFMKNENNREDLSNSNMKRKDKTTKDKDTKEINDIHNKDIKTHEKGSQKMDKKTNSFKDKIITND
NESKLKHQKNMNHVDDIKMNTKNLINNEEKNIINKINYNYEILKDYYNCFFMNSYMWVINNLIIDDKIMNSKN
IFSNNIKSNLKVKMGNDYCCNYIYNIYENNWKHLSEYLDEDILFLQKLKWKTSEMENEEKGYEHEVVLEGDED
DNMDDANENMDDANENMDDTNENMDETNENMDDTNENVDDIHDNMDDTHDNMDEKKKKKLIKNKKKKKKAINN
YIENMLPNNYDDIYINTIELIRIEKMIKYCLERNQAILVYGNNGTGKTKCIKNNINMNIEKFTHTIISINYYT
NSFVLQKIIENNVEKRNTRTYGPPNQKKHIFFLEDLNITAKDNCDTQQTLEFLRQLLTYKLIYDRDNLDEKKF
IHDISFIGTINNNTNKLIDKRIQNKFSIINIDDISMKTFENIYKIILKQHLLKFDDSIKCLLNNIISFSYDLY
TNITENISFNLSNLAPHYLFNLNDIHTIFYNIIKCTNPDIYNNQFKFLMIVFHEMQHAYINKLISDDHINIFT
QLFNKLVQQYFPFFKEDFEKHIDMFDSTNEPHNVEHGQDITSNKSLTFHENDNVDDDTYNNNMDNGFKNDRNV
NNNNIDGDDNGDGINVRTVEKTHLDMSNNLNNSNDKDNIMKMKKSNNINTDSYNNNNINVKVKEKNLIYDLNK
NIFTSFISIRNGLDKMYLNVKKFYVLKEVLTEKLNEYNTTHVELPLVLFDYAIIQICKICRILDFNISHLMLI
GFGGSGKQSLIKLSIFINSLNLLNISTNNNYDVNNFKSDLQEFHLKCAIKPGNVHVLLLKENDILDSFLPYIN
DLTSTGLCNDLFTKDEYLGIFSSIRNQIKYLNIGESNEDVFNYYINKIKTNLKIAITHSPISNLYRDRLIKFP

SFLSNFSFIYFLPWPYEALVNVSNKFLSDIKIKQDLKKKICEHMAYVHTSTNEMNKKYLEQKIRYNYVIPKTF
LEYIYFYKNLLNVKNFEIEKSVERLNKGLLALTSTRENVQLLQKEIEIKITNIEEKKIEVNEILNKVKEATEV
TNKEQQIVNEEKKKTEIFTKEAIEIQLKADKELSEALPIMNKAKDAVNCITKSAIQELKSLQNPPKECLDVTH
AVLIALKEIKNYSWKFAQKIMNNPTQFLSKLQKFDAENMDEETVNLLTPFIQKKFFNYEMMKTKSSACAYLAL
WLVNIVKYNEVYKKVKPLMDKLQEATNNKNKAQEKLDQLENKVKELTDSVEKLRRKMNEVNEEKNNVIKIYNE
SKDKLNRAENLVNMLSDEYSRWSDEIAIINSNKKFIYGDCLLLSSFITYLGVFSSSFRIKLWKHLWLEHIKNS
NILINNITSPIDIMVQDIQIATWKNEKLPEDIISIENALIVSTCYRWPLLIDPQLQGLKWLKAKGGNNITVLQ
FNCDHFIKKIKNVISKGGYLIIENISEEIDNVIDGLLNREFIKKGNDIYIKIDNEEMLFNYPNAINRIDKIKS
FFETNINSNTATTTTTTTNNNNNNNNNDNITINNSGASQINNTNNDNNNITIDNQNNYGNEPGDSNKNYHKQN
SQNMNKAFSSENLYKVNFFNLILQTKLSNPHFKPEVNSQCTLINFSVTCEGLEEQILAIIVNIEKPELEKQKQ
ILVKNRNEYKIILNNLEDEILYQLSTVDSKTIIDNISLINSLKTTKDTSINIQKQVADSINTENEINKTRELY
RTLANEASIVYFILILMHNINYMYQYSLDSFINLLLKSIEVVNSDSMKMLSNKNLDSQISNNNHMYDDDDNNN
NNNGEHRHLQKHDDKYYDSKIYLNDNDENNNNNNDDNNNNNNNINNVYDEHMNKLIISFRKTIYSWINRGLLE
KDKLLFNCIFVFKLLEKKKIYDKDFNMDYLNFFLKPPRGKGVTENPLKEWLSDDCWENILILSKFKEFENLSN
NIHIDAQHKFKQWCSEIQPEICKLPLEWKKLNNYSFKKLLIIRSLRPDRITVTLEKYIKSILPNSEEIMEKKN
SFVDTLESSYNFMVNSTPILFILTPGSDFIKYVEILGKKYKFYLNQNLHVVSLGQGQESIALSKLELSHKEGH
WIVLENIHLMAKFNLILENVIDKYATEGSHPNFRCFLTSEITTNIPISILERSIKLTNEAPTGFKENLKRAFT
FFSPDDYEEKDLRTKNILFSLCYFHSIIVERAKFGSQGFNIKYPFSLSDLRDSAKVLFNYLDNQNSIKVPWND
LKYIFGEIMYGGHIVNDKDMLICKTYLNYFMKEQSLEGMQLIPFSKNIQLFSPNNYSYEKILKYIDTQIIFES
SILYGLNQHAEMNFRTNESIKLLKNILKLKLKETSTFVEELTTGETKENKTSNILSEILSEIDNIFFNVEELM
KSIPDDQITPLQYFLFQECTLMNSLTSVMKNSLKELNLAIKGEINMTSKIESLMNALYKDKLPELWKNNSYSS
NRNLSSWVNNLKERIAFLTEWFNDPLLTPKVFNISLLFNPNSFFSAIKQILSRNEKCELDKIIMQIEVTNKSL
NNIHSYPKEGAYIYGLYLDGANYDVEKNTLCDSSSKQKYFLMPVIHCKPIVSMGKIDTDVYECPVYKTLSRGP
TYVTNIKLKTKESSEKWILAGVALILDIADD*
>N.PF10_0225
MGFKVKLEKRRNAINTCLCIGLDPDEKDIENFMKNEKENNYNNIKKNLKEKYINNVSIKKDILLKAPDNIIRE
EKSEEFFYFFNHFCFYIINETNKYALTFKMNFAFYIPYGSVGIDVLKNVFDYLYELNIPTILDMKINDIGNTV
KNYRKFIFEYLKSDSCTVNIYMGTNMLKDICYDEEKNKYYSAFVLVRTTNPDSAIFQKNLSLDNKQAYVIMAQ
EALNMSSYLNLEQNNEFIGFVVGANSYDEMNYIRTYFPNCYILSPGIGAQNGDLHKTLTNGYHKSYEKILINI
GRAITKNPYPQKAAQMYYDQINAILKQNMES*
>N.PF10_0226
MEGQRSLKKVNVYKRLPYLYIGMFFIGGLLEVVFCSTNYYQVYNIGESEKKLEGDVTELMKRYELRQKMKEKA
TQISEE*
>N.PF10_0227
METYLVDFIEAFTHLILYLTNVYSSEYFEKKRKFNTLVWHCTNKRVEEYIQQALYPISKFLSNKSLYKYRIIL
KNLENEILKIYTIEFEQFYKSYNIPFNYLSLEQFIWDFFTYVEMQIYENYDEEKTFEISFDFLRDCETNTTVN
DNLKDDWELISYDNLDVFEKKILKSMNISDNNEPNYICQIYVESPKNVT*
>N.PF10_0228
MITQKQKDKKRNFKILFERRIILSHQNEKHVDKSMYAFYGICLIIFSVFIILLSTMFFFFNMHPYALYYYIIT
VGTIPIYILYKYFTWIFLQLFKYS*
>N.PF10_0229
MSEDDFYNNLENFIEESKKRIIEKEKLITSENDEEIDNIKDETQEIQAYNYTSLMIPSDVSSENNSDTNENIK
QVLNLQESIDIQKIKEKKEKKKKQEVFKDIMCSDIKYEKKIKRKKKKIFENKRSSKKNKNNIRDFINLEKDII
ITKTLMYNKKKKLDDIYKYTSKKEKTINNLNKNMIKEAAHMQELLIENFKKTEDLIKKFEDISSKKKKKKNQV
QSLNIEISKLEVELEKKEEKIKELDKYKFFLNKLANCEKSDDTESTEKKKKYIDDNDDDKNEQSTINDMNKNI
KEEDTIFSKKMKKYINNSQLLIDNFNLLEEKHLQLIDDTQNAEYELEEYEKKYEDKKKAYDIKYKEIDKKIEN
INNFINEHNNKIIEYEILMKNKTDHISFNNINNKIDFICTQFSYDIINTNEIMKKLQLLENKIYSYITTLTKY
TEENSQLVYEYQREREQERRKQMRFEINLDRKGNNQNKQLMNKKVNNKNFNTNPKDKKEKRENIYTIFEKNLF
K*
>N.PF10_0230
MSFEDKNYSFSGQRKEKGVSSYKEELIKCESSDKCEYKTKIKVKIGNNQRVLLLLRHLNEEYYLILHQHKLII
GYPMTYRKIAPERSTYHYKAFYEKGDDTYENQNKRGKDRCEGYKFNDQDRLSIPIFEDFNDKTFSICCSCADT
PITFESNNSTIYGGLPRNMRLQCINDNETNRKLYKERIVSVKKSIRHLLPFYAIFRFMSDVKIFIIFHINIIK
KEKDFSTYYKLYEEKKNNLNNNISYRYVLDNSRKEIKLFDEKTFNISSSSSAFDNINIPSIYNNYLLYPFYPD

ELYNIKYDTLEYGCSYNKALKFECIENDEKKCEYTDSTCLKKSILIPITYVSDKKDVCGLIGYNDISIWKNEN
CLENIKCIKNTVESYLNTAQEITKGNEEILTKLPIINGKYPHYIFKKKNYDIKSSINKRSIQFFTDTNIDHYI
AYEYYKEDRTEFILTIMNEKNITHQETQSLEMKKLKGKNEDYMGHTNDAYSQHGENNKMHSINSDRNKNDYIP
SSYLHTIYKTFSEHINRDNGNIYDDEKKNLYDGTVFYIETLNELRIINILFSDSCNELNTDTCGVLVHIWNPS
KEAIKGRLKLQCFINKEDKYTDEKNLVYEQGVYHFLFFFKVPLETFLSFRNCTVHAYGAFQLYDMQTYINYKK
SFFRSFFYHQPKEINGIELYAQGIKKFICCQEDIHICKIKNIPQCNTKESLNLISVFISILIISLIIFIYFTG
NKKKKNTNTQENLQSTQNVSLNQNGTS*
>N.PF10_0231
MNEESICTFSSVTDDVDDDSSIRNQKILEKGGFYEEKESAKNWFAVENIKLRNKLKKLKYKYEKLQRNLNMKI
PATKKQTRQGAENLYLEIDKLLKKRRKGKTYKNSNIQTDITFLNNRFITWDSYFEDSEIQKCSSDGDAEILKK
RRLSKNINTLKFTNAFEHITKKFNNNETYKKKKEKIIYHTPDGNKDYYISIKNVNHSNNNNDNNIFKHLEEYI
NKKNAFIHNKNDGIYKNDGIYNNDGIYKNRGIYNNDGIYKNDGIYKNRGIYKNQFLYNFYTNDDVIYIKQITE
NHSNNLKDIKLKNKVHTNYNQLKETNHHYINNTDCQNEKINQINNSLLYNKQYHLIKSNHNISTSENIQKDIK
NIKNMKNIKNQHNNHFITNQVNINSDTIINKQTKKEEEENNTKKKKKKKKKNFLINLIEQIYIIIIT*VVINS
I*KKKNRHIKMYF*KILK*K*NKLVKIL*R*MKIIQIK*TKYNVLKQFLKIFKEEILNIHTAHSNF*KIVTSK
RK*IIIIVV*IMLIIK*TTQIV*PQTKYYQPKIKKKIFLIIKSFSLKICISIKMYYNHN*GISRKMKSVDIKM
KVKSIDIKMKVKSIHMTIKMKSINILIKMKSINILIKIKSINISIKIKSIHMMIIRKTKVLS*QIK*NKQITK
*HLI*KI*RKIKEKKPVILLI*TKRIPI*MKI*IIILIIIPLI*TIQILF*MKTILFLIILIKVLKI*
>N.PF10_0232
MNNNSNMREAVPHFTTDNKNNESENSIMKKNLTSNFMGQVNNNKNSTCSATYLNNTNNMNISKDKNYNSNINN
DNASISITNSLLNNDQNMSMRPQMGMNKNCGGGLHNSYQKSLLSSYNMNNMSMNHLMNNSMNRYNSLNNNNYP
HQDMNQTNMHMTNIKSALQNNNNNNNDMKGNNMKQNGYMNNNNMNYQRNDMLMRNNSNSFNMAGNFYPYMNNN
NNNNSNNSSNNNNNNNNNNSNNNNSNNNNSNNNNNSNNNSSYYGASSVMGLNSNTNNMMNNTSLNNNFNNNNN
MGAENNNKTMSLLNKTGGGSLDNNNTSHQPMKSLQSTINLNNRNINNMNNVSGNNNSNSINSNNINSNSVFNN
NNSKGVGSFISNPSMNVKGISSVCNIASYRNSTSFEKTNSIIDNNMRYMNVYNKMGYNNNVNDYHQTYDNSSA
LNDNLNNKDKKGKEEGNGDNEKNGTSNMMNNDNNNNNNNNNIGDSKLRKKRGESFNTKGIIGNNAESMIFKTY
NKNNDMDENNNKYGMNKGNIEMMKDSSANVSKNDNIMNNLSNNNIGGTTSNPYNLYPNFNLKYPMNMDMFNNN
NNNNNMSSFNMLNRNLMNSHLNNNNNNNNNNFSSMSMFNNMGDNNNNNSSSSSILYNNMNNFNLLNAYNNNDN
NNNMNMNSIGVGLNRYNAYKNMENIKNYNEALMSGNVGGAGNNNLSNLMNMNNFNNMGYLNGRSCMNDMNMRL
LNNSKFLPGNMGLSNNNYMNDHQYMNNNENLYGMMYNNMDDKNNMKYSRKGSLGAKQQQQQALSTSKGKKSAS
QKKKKSKNATATINGLQGGEEKLKKGVSGKIGDDFIMDNNGDLVNDGNNDDNDLYVNTYVKKSTTTKSSTRKS
SYYYETAEDKYNEKVRKNEKYDIFVSPNIGTIEGGRTNLRQRRKVHNYAQMEDNYFETDEEDKRVDEEKFLLR
QEKQSMGGIDRVLHHRKILDYDKINSICNNPYLYINKKNKLKGNEKNSDSNDKEKSGACDDGANIGKDNNNDV
NNEDVVKDETIKNETIKNETIKNETINNETINNETINNETINNENVKKENLADNNNNKKNEVTNVDSNKQTNN
NADNDEANAVDEFCEEEDIEDINEVEFLIKWIDKSHIHNFFCTYDYLKNFHGIKKVDNYIKKIRQSFQKRKFM
TADEIEQEKIYSEIKKQIEMDAIQAERIVMHKISDITNEQVFLVKWTSCAYDQCTEETKQTLIDHGFGKLIEE
YFDRESKIYGMKAMASVWNRGPLSVTKFDPYHETPFYLNEKKLRAYQLTGLNWIVSRMKRNLSVLLADEMGLG
KTVQTIAVVGHMLYKEKLIGPYLVIVPQSTVDNWLNEFKSWLPQANVVCYHGNAVSRELIRTHELKKVYVPNK
GYRYKFDVCITTPSILNSVSDVELLKKMPWQLMVVDEAHQLKNRQSKRFIELKQFMAESKLLLSGTPLHNNLE
ELWTLLHFLNPQQYTYYETFQKKYNEIENTSLIGEAKQKQLIQLQHELHEVILRRVKKDVEKSLPNKVERILR
VELSPIQIEYYKNILTKNYEQLAKASGGAKNSLQNICMELKKVCNHPFLCAEPLDKDEYKERLVYSSGKICLL
EKLLIRLKERGNRVLIFSQMVKMLNILSEYLTLRGFKHQRLDGTMTKEMRKKAMNHFNSKNSDDFVFLLSTKA
GGLGINLTSADTVIIYDSDWNPQNDLQAGARAHRIGQTKTVQIYRLVTKDSIEQTILERAKVKMVLDTLVVQG
LNKKQNDNVNYVGGETGGSSNGFTREELSKILKFGAQKLWEKNSSKYSPQKLDEDKNAIKGVDVDLDKILEEA
EINENANNANKELSLNSELNALQGSSNFFNSGNSLFGNTNLVGTNGATGGSGGIADDLLSSYNNITEFKYEPP
ANLKSNIYNDSTKADEVDEEENDKDFWDKTIPLEERLKLKRMKEEELLVHGPRKTRAKDMRSVLYMDHGHYYN
NTSSSHHNNNAHGNNNEDEDSDEFTIKYNDKNSKHLKKKRQRRTSTHITEKDKFRLYRSLLKFGNPSLRLDDI
QEDAKLTKVDKNVILTELNIIVETCNEIVERVSKNEQVDDNKKEKDIVVDGVVNNDDSKTKGSNNDVADVNSK
DGNKLKKGIEEENPDASLNTKDEEDNNASTTGSNNITTISCDDSNKQDDNKKKVEEEGEKDDTLINDTIGKED
EEGGTIGTVVSKRRRGSTLNSNSSSVMSHGMKTRDKASKDDDNNNNNNKNDGNNKNDDDHHKNNDDSVEDELL
LNQNDDDQLHMKNNENSNDESDGGNNKAKGIDKGKKMDAYTFFIGTNRANALDLCQRLKLFEALHNFIKEQNN
DNEYSFKLPLKKCEEVTGEDDENGKNNEKAQTDEKTEDENVKNNEVVENNNNDNNNNIPVDVMIDSSKDYLKL
KLPDSIFKHIRDLTSKDVKSWTRTDDENLIRGIYVYGFGSFNEICADAHLNLSHMKNIKCDKIKMRCVRILRM
IEEVYNNKGNEQSGGRKRRRTKNKNNANVEGVTMDGSVKKEKRKYNTNSNNNNNNNNDNNNNNNRDVADMDVD

ADVDVDVDVDVDVDVDVDVDADVEYNESSSKGHLKSGALIKVDKGNNNNNSNTTSRLSSNRKGNHYSRRSGERMK
FTHLLVDKNSASAIRKRRGYIKRGTASGVSSSNVVGGTVNGNFVDELNEEVEGEDAFLETQEDNELKERHKSN
ENNKHHHNQHYYNSKKSKKKKHANVDVSKDDKESKRKRGNNNKLNNEYDYYNYDNNEKEEDDEGGGTKFKDNE
YNDDVDDDDDYDDDDDDDEIDPNLQRLPLRFPVIEKRKAKSTILKNAKNETIVCIESSDSSSSTEDKSIRSKG
DEYDEEDEDEEEEEEDDENEDDDDDDVEDEDDDEESGECDKEEETGKRKRRRNSRYYKSSNKYIKDNEMLDIT
SVGANDEYKYEDGDNLERKKRSSKKDKKKKKNKLKEKKNKRKRKRRKIVDENEIMDKIKNLNINHMDYEQQQH
CIKKLVKKCIKKYKKLLKLIKKVILLSVENGNQVNEEENQSQVVLEHEQQEDVLSSGRGRRTRTGTRTSTRTS
TRRDMDYKKMEGGRRGGGGKTTSDVTTTNSYEKDENMMNKSMTKILPPEIFEKIEEFIIFIGKYIDKFCSICK
DMECREKLIQASWEYVSKFVIIIMDGTNNKSSTTNSKTNSNSSSSRTKGKVNNTVKMNNSEVLRDMYKQWKDK
KQVKGSDENNNNDDDDNNNNNDNVIEDEKVFIEFFYAKKASFNKNVRDRNYINNDLYSNSNELVYSKKGNKSE
EPLSKVRKNELLEENNLNINDSNESINGEVED*
>N.PF10_0233
MRKNIILVAYICFIYMRAKCEALKINNILNKSYFAIISNYNNNNNNNIKHINHINGQGKHINKSSHNKGGILN
NEVLKKPGKENKKLKENVPKDIKEHVSEISKENKFLHFENNKKISINPLFQSLCKKVSVSETCLYNIQKFYSE
VNYMNLKNKNPKDNKYFCGVLYGKYERDEFVKVEHVFFSMNKTEQGYDEKYLLHSEDRKRADQLAKMLNLEVV
GFLYAYPDIGVDLNMSNKKQKHFIKLNKKMKTLNDEENEIFIPMGGKEVLLSIMLMWQIRRQSKIVEEKNGKA
YAHMNKNKNNDSHNDDHNDGHNDGHNHNSDDTNFIEENSDQHNNGNIKKKKNETIKFKNVKKKNKTKQGKKNM
NIKPFITLSIGMNKNNESIIVEAYEMNYDLLKLIKHDMVKDINKQNSILQFEKNDKDKEIKIKTFDSEIDIMN
ELYLKCKNNILIKKIEIKKIDILFCVNNIPIFSHKGNYNYFFPYPTNTNYYYILQTFNHMIKALHNKSDIVHI
FRDFNFLFFLTNIFSIKHDLPYFCNAVKDVNNSVDIPDHYLHILQNLSNNANMLN*
>N.PF10_0234
MNRPHTSISDDNMVVLQQEDEKIDKQKDIDKKEDKKNMSTNRKEYKKKKKKYIISNKILKHIDNIDKLKYVGI
NRIDEKQKKSYLENIPGGRIKKLNKQINDNKDERKKKRTNMKKKKKKINKIKVIKKRNITNEKQIVEEKEFNE
LNEKCSDTIIENQNIIKKKNIDNNVINNSNNNQINDVKEMNIFQKDSNQIESFNKKNSLKEKNKTSNDHIIND
HIKNKKKYENNLFDEIKEAIKDDVTKQNSLNNLNLQIKAEEQLILSNYDQNKMNSQSYKKENIYISHDKQKSN
LLMNSSTELKNKNTHILQFKHAKSTHTSFLKTINNLTNEKKKKAKKKKRNKIIKKKQHINNNVNNNNNNNNN
NNNNNNNNNEFFKNLNYVKDINYTNYIKKVNLKSKEIKFSDIFKKKKKKKKKNQHHMDISKRANKNILEKKIK
EHNQQLVRKGKSKTEKGEEQKKEQLDVVDKKSSDDKKCNYDKKCNYDKQSSDDKQSSDDKKCNYDKQTSDDTQ
VKLDNQTNGDKQSIADSNKIIEETYAGEKTTIKKKTRFQMKSQIKDRNKDIKSKERHIKIKDAPEKKKKKKEN
VIIVPDEHNNVKKETNNINQNLFSDKSEHVHKNTQEMNEKRILADEILKKYIEMREEDKNKNEDINKYNKNHK
TCYPFFEGDNIVLLDIKEKKFNIDIINVFSSNKNLEDNCHDILFFFDSYVKKNKKEKMKNSTNVIRPLKKLFK
CEYFEMECIKLIIHIFDKIISKMKKEINMENITKCFERDDHSSSFISRIKKKQEIQKRINQENNTIKNNITKN
YNNYYYNDNKKNIFYNTFLNNDILYNDDYEKQKIYSILFYNSPHSYVCNEYRVFQKLRQWTDQNKLLMLREKM
KQMEKNKENQTTHKRKMKNINNFITTTNTTTTTTSSNNNNNDDDQMNCCHIYHSLTSNDNIYNNYSHYYNNEV
SSYIPLRKINIITNKIMCTILNSCCIHDKQNVMSTYKEHAPSYNFPMTKQKHWNVTLNRELLLNDKASYPLRI
SEAVGTLISK*
>N.PF10_0235
MFFKLLRGRKIYINNNKYYNKKYIYGDSFSSSPQKIMSVRNFSEYINIEEKINLPRLKLRGLPFDASEEEIKN
FFRDFQLTKQAYPIHIIKGIKNKPTGHAYVYFDDEEEARNACQAMNRKYIRDRFVEIYQDYIYNHNITPVKEH
VTTLMRDRYKKDNQ*
>N.PF10_0236
MFDNNNNMSKELKQLEKEKKNVEGNNLNLLLGDLKMMTAYEMSSEWKDTNMMNECFNNFSWFDSRILRNMQNY
LNADDVEKSKIDYAYNTLFPKPIDIKDTKLNMMALWIKSRIHYNNTFFPLQLSPYDV*
>N.PF10_0237
MKTLYHEDIIIFLSIESLEEILSVFKNIGGIKGRFHKFISFKNKSNISFFFNSSDYLFLKKEPGKIIDEKYKL
LTNNKADDKNIHENNTSEDKNIFEKQTNIDIEPRESFEKGEEKILEAISLENKQNNAKDINDCRPHISKNKNV
ENEEYNNNLKKVENKNVENEEDNNNLKKTENKNVDKISNLKYTNDEYEMIIEEVYKHLYKNETYTPMSEKKKM
VVELIIETIIYGNKIKLNIYKLNLFISIVIMTLYQIMENLGVKEKKKKKTINYFITLLKKNIQYNNSPEQNKK
IKDTQNNTSSEVNINTQNEKHITIQNQEKDNNNNVKNNNTIIKNDNKDIIKIKKTTIKNDKRKQSSNTSMNLI
DTKEENILINSTNEVKINEEKETKNDIIFFNYEEAKYIIKYMFENIFSIYNILEYMFLFPSYPVHLCFTNYCS
FVSPPDPLSLKDEIKEDEQRNEDNTFSTNAYISQVLHIPLYVLDKFYNNLNDLQEKIDNALTLM*
>N.PF10_0238
MKIGNNKICTWNICKRRISFFGVLPFELEKKGNIRKIERRTFSGWNERKFLFFSLSSDINKCVNMSEMIIRRG
NITTTKKNSGKNKSEKNESDVDNNSTSNNNNNNNNLKGSSKIVVYIKSNKLYLTYNGFLLFFFSIFAYCITKL

LIMFFEEPPAVKLVKESVLKDKKLIEEYEEVLFSRFWSGFLNDNDARIIINIKSKKQNKKGRIVSNLIKKNDQ
WIIKTLTYYNTKKTDNLKTDDLKNLQKNQQISLCPINTDSFSKKK*
>N.PF10_0240
MSFTITNIVSRRYIIYPLTKCKRRYSCTYNIKRYMNEEGERKISLHTAPIFDVEEVMNKEKLLYVIKNIQIVD
LKEKNILNNISNLLKKYINEYTIEEIYTIIHIFCKLKFTKYSLYNNFIKIIMNKKPKIDSRMLTQILIDLHKL
SSLDINVLTFFTQYYIKKETDQFSLFDLSMILYIFNKYNYNHIETVDNISKTISQYFLPYIDQDKGVLTTILL
SISTLNLNYQFYLDVMKKHVYKKYEHFEVKYLCNILYSILLRLVNTLHKDDILNIMLNDIMYILLNNINKLKN
EELKQLHISLYYLKDMKEEKYEEARKIIEKKNIKDTVTTSKIQQQIAKLFKEIGLNVEKEFLIGPYVLDFALK
KKKICIEVNGFTHYYNFNGKINAKTTLKYYILNKLKWKVLTIEYMDWKNKSKEDKIKYLETNVLEKIM*
>N.PF10_0241
MIRNILRVQSVRNDGWFVLKCRRIRRVHNKSGGDEKSVVVNKLLEKYKTLRHDDKMKLINSHLVNIKEKDVND
IIYTFCKKKEEEKEVEDEYIKRKRLKLLKVRRPAYLQQKKIKKRVHNDFTIPNEYEEKMCIEKEKELKDIEKY
TLIEDMYKSYIYSIAFILLNNQYKTKHCITNNFVHVMINTIRNEILQKVRPTLINEFSIYKNPYMLLKNAMFN
FILHQYVKNPYEESLIETYINKKIYMNILKINSARVPVEVYEPLVAFRGDVNYNYDLEEKFNDMIDISQNIIN
ILTKKNIDDKQKENNQNDSTYDHTYSKDNYMSKEEMDSIINEVLKILPENIKYHYENYPFENLKSFKSKKQKK
YIGGIKNNKPINIHEEELSMVRYPNLQSVAHSLPKDEKYRDNVIHAIKVLERSKHWDHQSKIKAINTLIQVWN
SMNSSDYYENMLDKSLPVLYTKNLLRKTKTRKDTYNKGLTYIQSLTTQKPLAMRLKKN*
>N.PF10_0242
MQIPTKFLVFFNLVILFVFLNFERVELKLNNIIYDKNNFSNNNYNYYVNNNNDENNEGKNSADNIDIIKKDDA
VDNKENEEKNSLDVFNKDDENSHHEDNAQQDENLHNEEKDDDDDDNNNIEEDLDNEDKMYFEENPKNEEDAHD
EDNAQDIVKSEEDSYDDDSKHFSDNTNVYETNTNIFVENKQDDNEEKDHEYYLKVQEGDNITVNKKEEATGFD
VDQGVSALFFDANLKKDVDEKMNKEIVFVDEEEENKNMKQEDKGDLLSTSDGNDNENNDENNGDNNDENNDDN
NDDNNDDNNDDNNDDNNDDDEDEDDDDDEDDDEDDDEEYHVNKDNREKKNKNSDNISLLGKRKISQDSIKDEL
SRNNENDYKYNIEEDGKERNEDEYREHQQNGHMISNINNTWNSIFNLFRKNNSRFINNVNYFRKEIQNEFSFR
NGLYFSPTTVGEYNKLVDTKRYAYIKLPENTLIFVITANKFFFQDLIYSNNKYLSVKRKFTFAMNYLIRNLKK
KIFFRNYQYQYLFKDDSFAYDSTRRLLDMDLLGEISRVTQTIHLGDFEKRKVSMHKLYGGWFHFLGIMVINGY
NCNIYEKNTNAEKVQVIPKHMIHEFKELVNKKHDDYHSGYVVGLWRDFPRNTFLPWRFSVELFLWDLLNVLPH
ELPHPAKLIMTYNKNMNALYSNGKQEDTDMKVVQASDGMITNNNMMIHNNNNSDFLHVQTKKIKDNNYYNMLL
NVWPEQVKNIVRKHPGFDVALVSEEDYLNNINNEDDYYVDNEQNNDIFNYDDKNNNGHGYIFLILLNKNFFVD
EFMKSSNYEQAKKNYFCDKIKEIMEDLEKLLDDLKHKYCPVDDNNNDNNNNNHDDNHDDDNHDDDNHDDDNHD
DNLPTERPVATFYNYLTDKSNYKKINPHVLGEIAGITKHLRCEDFPNISDRCVNNISIHHKYGGWFEFGGAIH
LKNMNYVEPTYEQHDDIMKETFEEVILAQANCKCQSIGLWRDIPEKDMSEYRYPLNVFVLENPKYNLLNLQDI
HPYLVVDILNKGLHAMKAYASGKMYNIVTPKDISNNNDEEDNMSNENNTTYAYLNDYHSDSTHVPDEDNEAQD
EEDEKQEIEDDEEEKEDDEEEKEDDEEEKEEETTSQPKDVDDNGPNDPFYDGTFNVLNKENVQTTSQPKDVDD
NGPNDPFYDGTFNVLNNDIKENEQDADIIAQGSSGQTQEYIKDVENNVERNNENNIKKKNIDNNDNNDDDDD
DDDDDDDSTNKNGNLLKGTISDNQVTSNFREKLFIFIILCLISVCLALLVSIAIKVYASVMKKKIGDKHDVVL
SFKDREEIPVVSGIPAPWLSS*
>N.PF10_0243
MRINDRLLFYRRNTFILNFFKKIHSERKHEHTIPLYERNSRCKYKKAKLLKRFKIQISHLNKNKKDEDIDHSF
LINTSINSQICYDKSLLLQSNINIENHTNNVVDDKEKDELISDDNNKDIHYTQKSQNYLNCKDEKVISNNNLR
NEEIEKKKKIYIISILNDVPLSFIYFKKIYFLLLNNEKDIYDLLKYMKNKNFLKLCIFMNKKKCRSYVMEKEF
LTRINHTIEYLKNESKVKENHNKTNINYVNNNVCNDNHHNNTNNINYVNNNVCNDNHHPIDSNEYDKNISSKD
QNANVFFNYYNIIYILEYIHYIGLHNLSAKKYKNNKIIKNNKIVKNNKIVKNNNNNNRWDYTYLHNIYNNILC
LILEKNILDVKQVMNILHINDKYLYFNKYFFEYVDMSFSSTFFSYNEHEITYILKYLSKILFSLSLQKFNIDN
DLYTNRLLKRYICKSIIFLDSYKEESYYSDIYHNNNNNNNNIYNLNNSYDKNQEKNKYNTNQQSVKNIVEDIS
INIDDFKKMIKGSTLLNVLNNELHKCLHEYKYYNIIDIFDFYTVFEIHNKNMIKRFINEINKFVNIMKYGYQA
KILILFVLNKKYLQLENMKTIRRLIRRIPYILNFQWPIEFIIETTIACFIFPLYKDKIFRDLFLYMKNNIKKC
IHPIFLINLLKWYSIITNSSLSYGLSKNNNNNNNNNNHYNDDGNNLYISTIINPYNNLYMSYTSTTYKNNKIN
FIIFHQIINYFKKNIDNVDICYVIHMLKHLCALNYKDESFLFFFLQHKNTFNQIFEISNKQDLVTLFHILYHY
KHFLQNKNNMYINNNIYVQSNLFIFYIEYVLFLILKKGTKTHAQNYDNNHMENKKNCIINKNVINKIEQCHQN
NIIQLNSNEISQQIIYDKSSPIYSNDKYVKTIKSSNQFNVMILKDNICNPEDETTCTSHVKMYANGNKEDDDK
DDDKDDYKDDYKDDDKDDDKDDYKDDDKDDDKDDDKDDDKDEYYDEYYDDYCDEYYDEYYDEEKNKRNKFMNI
SPHSYNKPFNTFMNIDTFIYFLKGFINLKINNIDALYFSLQYINSTNKKLFHYNHMVILFQFFSEQFITFFPL
LKKKKKKREKTNWLVQKFYYDIYEITKNNISKMCDFYIPHCTFSVLSILFIETIILNNQSNIIEIEIFQVIEN

ILIMFCNKMRGSTKLLVEQGEDKKCDSQLLHMISCILQKLYIKMNKRLPDELHKFCTHVQKNYEIRENVKDIE
KRHVQEFISFFSNILKSKKINHIINYQLYPYNLDIVIIPHDHNQNINGTLQKTKIKHDEKEKQCINTHDQLCI
ENNNKKKKKKKAIFVIDNMDNMYLKNVHITSFFHNYFVNNENINDTLKDTFLQYCDKYNQHNEHHKYIQQDNH
VHKEKNYFVHNNHPNTFLKTYEYIREWYVINNDYTISYISKDKWKQ*
>N.PF10_0244
MGTMNKAFKDMNNMYMNNPSMNMVNNNIWSSERIVLPSEEYLYYINLFNLNDKFDNQYIDNKTASSFLQNSGL
SISVLHTIWEYSDVENKGYLTLEDFFICCRLVAHAQNGNVISTEMISIQPACLPSFDIVRHKSFSDISNMEGS
LKWKLSSREKENYQRIFKSLDIKNEERIEGSILREYYMNTTNISVCELMQIWNISDMDNDGYLNLDEFFIMNK
IVEVRKERIINIPLSVPMELLQSVQNKESTFPSEDDVTFRQNADEEGTKKRESIKLSLGDILKSDISKKKEHL
GEEEEENEKLNMEYDFFEFNKDVSVKSKTPFMKGKKESIHFGDDEENDDKNGDNNKNDDNDEDDDNDDDNFHN
NDKQFFSTNFEGYDQMNNDDLNNVDHLGFNDGYDKFRQGHVVDKDLNSTNEKKKKKMKKNKKSYTNIELNHKY
KDKDKYKDKYKDKGKDKDKDKDKDKDKDDEQSEDSNYDECEASPYSEGEKKRNSKYSIFKEKKKKEKGDKNKK
KMHGKGDDDNNNTYDNNNNNTYDNNNDDDDNDNGMVDKDFHLNDKKEKKEKLKKKEKDGEKNKKKKYSEEEK
YFTRLIDFNYTDLKNYHIESKDVYKIEEINRKLEEEINKKKIDIEKKKKQVNCLAYVYEVELKKYQCLKEERR
NLEFLNLCLYKDIKYKKENIKAIKNEIKEIIDDINKINIENISLNKNYNKKENEIKCTDNKRKQLKKMIDKEK
HDLKKDEKNLIILKNMIDYLKKQKQRALKLQDNLKNRYDVTNSDHQMLIKNILHQQNYLNNITNKRLNIQKLK
NQHILLFNSLSNQSLLLDMQNTDKKFLQNNDRNILQNNENKIVQNNDKKVLQNNDILLNNLPIDNYVLCNAPS
NQHKKYYTDKKGIPNNHNDDIKHSKKNFELFEHIKNGDSVDIFSSLDDLESVKDEIENDMKHLENYISKDNSL
KSVDNSS*
>N.PF10_0245
MNFKFKTVIHNLKSKFKHEFVSDKKQLYFCNIMKNKYNVYENIGFLNYGCSIKKYLFYNRKEDQDEEFKGSFK
NGTMNGLKIWKYNNISSQKDTYNNISSHKNTYNNISSHKNTYNNVSSHKNTYNNISSHKDTYNKYYYALFNNN
IFNQKKLYIFFMAICLALNKFLNTQEENNENNNSLKKKKKKKIMNSHFFGLSNETASCCGIMAYMGNRDASKI
LIDGIEILQNRGYDSCGMSTISNKNVLKTTKYASNTTCDAIEKLKSNYLNSHKNDHIGIAHTRWATHGCKTDE
NAHPHVDYGERISIVHNGIIENYREIKTFLLKNNIPFKSNTDTEVVANLIGYFLDKKQSFQDAVLSAITQLEG
TWSFCIIHKNHPDEMVLASNGSPLHIGFKDDEIFIASEHTALFMFTNEYISLKNGEILSISKDKINDLKLLKK
VENIPEIAIQKTPHPYPHWTIKEIHEQSATLSKSLNNGGRFSSGDHLVKLGGLDPYIQDLNKIENLVLVGCGT
SYYAALFAKYLMNYLNCFNTVQVMDPIDFNISVIPKEKEGVIFISQSGETRDVIKACKLAEDLNVRKLSVVNS
VGSTIANMTGRGVYLNAGREVGVASTKCFTSEVSVLTLIALWFFQHKKNNQSSNKATSLINSLHRLPLYTGVT
IKSCENTCKTLSEKFKNTKSMLIIGNGLSYPIAQEGALKIKELAYIHCEGFTGASLKHGPYALLGGEDNIPVI
MLLFNDNTKNAMINTGEQIKSRGAHIVCLTDDENLVKHFADDIILIPNNGILTPLLAVIPLQMLAYYTSVNKG
INPDKPRCLAKTVTVS*
>N.PF10_0246a
MFKKRLNKNVFSIFKKYNSSYICFDENKQNLKKFCNIKNIELRKNVHTNKTEIFIDDKILLTNRKNVFSLKNE
DLCLLIKLEILRNKNNMDVLKTPITLCVNNLIDFVNLKEHNMLPKNYDHKYNNKGDNKYNNKGDNKYNNKGDS
EYNKCDSEYNKCDSEYNKCDSEYNNKGDNKYNNKDYSEYNKGDHFNYHNYINNNNKERILYFFEIERTLMEKK
IYENFINDLIFYTNNNEEHAFNMHKNSLHDLKKKTIHLPNCLNYINKYNINNNLYQEQKYIYNKFINLFEHIH
NIKLNRANHFETPEQNFDVQEKIQKLIKNMNNSEIFLFYKCTQYLNSFIFTYLFLNRYINYKDVYRYCNLEYI
YQFFKWGYVYDVNIMKDANTLLMLSSLRLINKMIQ*
>N.PF10_0246
MLLYFVIFIWSFLLCDIYETLNIRKSIDRKLTHLNNNIECALNISNYRNKKSKNLQFIGNNKIVKKCKNKYSN
KVHLFGFSSKEEINNKGKKSLNIEKYEYKNGNIFLYTKLFVNNEEKKFIIDLCSDHSFIFEKRLNEELLKCNN
NNNNNNIFTQNDFEQKSFPKNEGLNLQYEKKDIENIKELFHLNNTQVDIKKVVNIKHEKEKQFPFYVINNEEL
DMNFYGIIGFNFFKNYDTFVLDTIQMKILLNWNEDGINTEDPNYDNIEYEKQKGNGEQDQKEHEYYKINLYDY
FNNIKYFNIYVDNHLYKGIVDTGTLNTILLNNSNIYNTTTDYNNKDTKTSIIIESVLKNKYKVNKTNINHINI
ITKDNHKIPVELNKYIYSGNLEYINKDVILLGLDFLLNKKLIFDLKNNILIIPKKYLNNTNEFNKKYDALNIG
ITNVQENQKSQNDNGNNHMDQIKIEDKCKEIYNQLKAKELNFVRITKELENDNVYMKDCKSTNDVIHKYAIKL
LYGSNYLKKNDKNIEYEKRYNEVTEFFKKLNNEEKQNMLNKIVNSVLLLKRNDTIHDNNNNNNNNNIINHQKQ
HSSNQGYEKASDIIHKFVNYEINNNVYSLHKFKSSNVNKNNPKSEEEEYKFLCNLYETHPEYSFEMLNDFKKE
LSSKKIPYNDCNDEKDIIKKVAHSRVFNNHKNNNKRKNVIVRKYSDDGNNIHTQIIIRGNGLNNNNNTSDDQD
QNDYQYNNLDRMDDLFPNSIFSGLLKNFFIQNNNNNNNDGDGDYSSESFDQQNSSDDNQNDLFSELNNFFGFG
NLADGMFGRKKKKKVLGFKTKEPQANLENNKENADDNITQAIKQEKDVDIIYLLNKVQKLNDINLKNFILQSL
NNQNIRKILVDAVKKGYTNTYEQCKKENDNKSMYLLQMLKQSGIF*
>N.PF10_0248

MDNNNIVYIEKKTKEKKKEEKEKNKEFDSFHMKNIFLLVIHFLFYIILVHIYYTYNNENLLQCFNNSFSYILK
SPYLNIYDVEIIQNNLSNISQMCNHDKLNYSKYIDTDYFKYSFLFYNQHVNTYIDYVIIFHKLNQSEILKKEI
HISITKYFFWKEWVLYIFFNLMTCAIINIYWKKKERKENIFHVSVISLININIIYQQFIDSIEYGSTYEKFIA
LLIYIYYILLILNIFKELSKVSKIFRIPINIISNTLKHNKVLFISLSAFVVVPFVKYITSLQNYSDYIKSFDL
FEKNNIYKNEYPYYFLFSLIYNFFFIPMIIVISTASTFLLVNKNDLLLNCKLRNMPLWLKIKEYLFFFQVNKK
LNKQKMFISDEKNIKNMSILKFSNISCFGLFFIFLILKINFDQSSYDHINKKYNLLLKEYFLTDTNMEKSYDT
FTYSADYYNFLNSIVIKNIMRNKKDLQNKNLFDINLNLNDYNISIYDDFFNHFPYDVFLKFSGNNQISDEITI
ANPLLAHESNTDSTKLENYKSIIEDNKGRIKYYDAYSILYNFEYSLSLLIRTSFKVQHYGVFKKDKEIFVHEI
YEDNYNNMYNYKYILLIIMFIITIIYLILTIFMVYYIRSKIYISFFSFFFLNFFFFFLFHFMSFTPVSYFYDY
INDIKENQISEIFNNMNKQDNFRSLLFDLLKIKWYKFYANLFSYIIILTSLLNFFFLFFINHISFLIKYKKKF
LLVSIPIWISVFFMSLIGSYFPCNIQKNIFKWILLFYQIQTNQNSFPYEVLNKFIFVFFLFYLTSIFLFIYVK
KKPNIIKLKRRNYQNDKDPKQVPFNNTNICENYDILLYFKALLYSLDKTFEKVQLLKEKNKFSQMLNLQINMY
NMYEQQKMMLQYGNINAQGNYMNGQTNNVMNGQGNNYMNGQKNNVMNGQGNNYTNSQMNNYMNIQTNNCMNGQ
ENNYLNSQTNNYMNIQTNNYMNIQTNNCMSGQENNYLNSQTNNYVNIQTNNFMNGQTNNFMNDQTNNFMNDQT
NNFMNGQTNNFMNDQTNNYMNSQVHTEIPYRPNENAQTSSQIMNGKHNYDHTMVGNIQDGKGINTHAKHKLYE
IKKNDDHDIIDEDTSNNEDLERQNKNKDDEVDMIEKIENMSLFFTNILNEKYDFNFLKDENIKNNKKKTLVQW
IISGLYDKDNYKRNEEDKEKRQKNTKQLKRMIENLNEDLYYIDKIKISFTYLLFLKIKKYILIQKFNNLKEKK
GELKTTYQNKILYKSHLSKLQQNLTKQMYQLEKNVLITNNLRDGLIHIIEDEAFYKKDEQNSSEEKKEQIINE
EAQSNKGRIFEK*
>N.PF10_0250
MNYSSLKKNLVINFENFMNPYDFNEKKKRKEKSICELNTQKKKKKNADDLIEDVVKKEDMLYYTKRKKSGMEK
KELNILLEIDDILKENVNDKEKIITNNMKIINDEKKKIDEKGKLYGNKNVASQNKEIHIKEKIKKKYNNNNNN
IIKNEEYEENNMDEKKKNMDIIEVSFSVINKWRNHNELIDFVKNNLKNNEFIYVKRLTQLFLIASWENKKKMD
VLSISLNGVMINEKNETSFYELNEWKSHMNLLRSALDLRFIRKYKLYKYFFFLKNMIKSTKFKNKKFYIKENL
LFLNEAFFNGFIKIEKVLKHIGKTKLIDININNMTIDQYVELQDEYMMKFDSFLNNKIKYIINIIYIYCMTAL
NNFLIKNDITIKRKQIENEEYIDIFMNDHMNSIETFDEQIRVNTSIDLHTKEENHNVNNKIKTEKKTNTKIKT
NTNTKIKTDTNTKIKTNTNTKTQKETNTNTKNTDKKQFPKKKNNQNKNDTEINIYYNKIALSNCYSIYLKKFI
QVCQYMCDNILRNIIINTYTYFLDLLNNYFETNLEHTKDVNDLQYPNHNVHIFMDKEKYYGKKKEYNKDDDNN
KKYPNLSYHKNKHVTKNQTHGTLPYESIKQKKNSDKEKQKQRKKFLFSSITNRKEDNNTEEKIKNYKARYIVK
RAEKNSRVDDKVANGMIIKMNEINNEINNDNNNDNDNNDNNNNNTNTTTNNNSNNNNNNNNNNEVENEKVPKN
DVCLFNISISIKNNHLHIYPESELLINTMTKYINKSIENVCKREEFIKSYKIKEFFRNEIFIDNIIFHKHNIN
NNINNNNINEYKDNHADGCIDNPVDGYSYHNNNVHVDNKKFEEFINNDEVIHNLDIELKEKIKLLYDDMVDVC
QEFYEIIDTYNKYDELNIEEIDIMDVEFITNLFLSLKDLENKIKRMSKQLNIHIFKVDNTILIEYILKNINKK
WTLLINYLPTLNNKTIETLTIKLDTSLNIINKTPTNINELVSYIDFVNTFSINLVSIEEEIESIFSLFLLFKK
YYISICGNQTTKIFHMNQKMNFIKSILNSEVNYDIQYSYIFKDLNSQINNVEKNIQNFLNIMNTNFLDQQNNY
EWDDIIKDLNFVYPINHINEVFYKNYDHHITFMRENGDVHVNRYLNKDEDVLDQTTNKGDNINDGNKIYNNKT
YDEQYNKLHNCMRKDLFSINFNNNTIIINETEENKKKFYTFHILEFLRLKILEIKEDVDKYIHYQNVLKIKKN
EFVNFSLLCNMFINNSLTIYTFFQYINLNEKYRHQKIFYLNIPQFCNDINNINIFFKKINKNFVNNEIYDKCK
KFNMTYEHIIKLLKYLQPIYTNQKYFRRAIDLLNHSYDMDNHVLENINDMYTIRSSQEKNKNGHETQISQGIN
KNIPQGLLQMDHTHKNGDTHKNDEAHQNGETHQNGGTHQNDEAHQNDEAHQNDEAHQNDEAHQNDEAHQNNET
HQKHNHIDKDQYEKQNSRRHIQKKEKTFLSYNYEMIILENLFGLPFSSFLTNVEKICFEYEKEKRINDRFDRI
RTSLKKYKLDVSLINKKVYFINNINELITYIKKIMDELFFIQNDKYSKHIYNEIYKLSKQLLNFKNTLVVIKK
IQTYYINTLQKYKNNEIKKCTNDSEYFSSLINTYKYHCEEFFIYPQINKHLEKNYDIQYFYNLLKQLEELITT
VNLYILNKRKEFFSFYLLNDENFLNIYLNIKDIKTLNKYIQHIIPSIKKLIYQNEIICGVLSKDNQRIIFKEQ
IKYKKLEFLLNQINLEIKNWVLKNIQNVLQNEIIQKKDINYINNDDNNKNNKDNKKFESSLSFYPLKEDTYLI
FLKITNMQILFIILNIYFTYIMDIFIKNVQKNKYIIYILSQNVIHLVQKIYNTNLYLENEDDNVSVDNILTNE
QKTLNHINIINNKNNNKNNNDDIYNNNDDIYNNNDDIYNNNNHIYNNNNNGVYINKHLIFILLFFMNMKQKLL
SILNNDHSKNISLNHFNYLEIDKHYFKNNDYIIEFFHHNIKYKYELLNNYNYTLDIIYHEKKYSFLLYKYLSN
HFITLYSNNSYSINYTKNFSILLGFHYFLINNINLNKNDILNFISCSVSSNVSLFFYKVDKYKPHILSILNKQ
LSIINYHLQNKKKQIIINNQELNLNKRNLIYLSISKDYSPPISLYFSNNHNNINSKFNFSYTIYQSSKPFPLS
IFYLIYFSFGYLNPDLLSQATYKTYLYLKKHGELKSYVNQRKIIHIIKLSTRKNKQEEEYNVCKGIIKYVSHF
VGYEKLKDIINNICLLFNLRREYQETLAQYVDKKQKEEEIDGLNYVETLFDDENYRKKKSKKHASDWKNEVDK
IVIGLIKKTKIPFLKYQVYKATELYKLLNYIYDDKKKKIDKGQGEEKIEEINENIIETTEYIKNILIIGKNY
SGASSIIFLLYQFLKNLEGTSFCAYDICNIDKINLHYCSSEDILKYVFYKSNNMSDDTNEKLEERKEAGEYSM

LGSTIRRNNTKREQDGNNNKNKNKEINNTYINNTYINNTYNNNTYINNTYNNNDGSHNNNIFDENASPYDEHH
NLDKKKKKQNVSYSKNGTYNNPIKSMEFINLNMFKNVEENIIIKKKKMKKNLFLKYINVSDMNIVDKICNYTL
NKNIYNQINKDTHMILKDDVKNNKCFFYKPERLCSMSPYIIDKCYFVYIKNKIPYNAFIAYTVNKMNVYKFIK
NKIIKLFEDLIIEALNFFNKQKMMNNKILPFYRKREKQKEHPLKDSEKKMFLEKNLDQEKKKKKLKKKNNDGN
CKGLVIQSDEQDEQDKKNMKKNMNKNINTYININVDVKNDACTYSKQNYNEYIKNKNSSGTDNNITHKKKEPK
NEKENSFEEEDNEKSYCRQGTKEIFNNNDLNIYKLKDDDIMLQKDLLFSYNKSDIYKEYIIELLGEKNIPFYI
HINNLISMFGKFVEYFLYMLKEKKGYDKRVLETHINNIVIISFIYTFTSFMNKEIRNTFEIYILKYINNISII
PRNTSFVHIYYNIETNKLCRQEKIYRNENIIIEKNNLINNLLVTNNSFYVYEDLNILSIINFLNISTNINLPF
LIMGHTNTAKSLCVYNFLNKQKQINTYHFMFSFNYSFHYDNIIKTIKKKLDLSIPCVNQDITIFIDDIYYVPN
NNTYNTFQFLYGLIDDDYFFDIEYNKNVLFSNKKIICTMNDDNMLLDEKYTSMVTKMYKNFFVFNVSYMTIKD
IKNVYSNIIECLFLKYNKLNNVINYMLLYDKECCANFNDILNLFRNDVERIFCESTSLSYKKKVEYKEIINDI
FLKYFDKHITYKTNDLFINIYNNKLYKLENKNDIYENDHNLLSINNLKQYFFFPSKDENIRKDFFLYKTLVDH
ILTINTYLLLKRTNILFVNHQSVLINKIVTIFCYYKYYHLYFLKGHIEEIKQEIKRAYEKAIKMFNVNKQKIN
KKKIKDDINNKIEDDINNKIEDDINXXXXXXXXXXXXXXXXXXXXXXXXXXXXXXXXXXXXXXXXXXXXXXXX
XXXXXXXXXXXXXXXXXXXXXXXXXXXXXXXXXXXXXXXXXXXXXXXXXXXXXXXXXNKIEGDNKNKIEDDINNKIE
DNINNKIDDDIINKIEDNINNKIVDDYYNDTQHTRNVFLSEIFEDEEYLNLLDNVIYNGGIDILYESFINEYY
LYILHIIKHKDIIIEGNFNNYIYSFILNINHFIFYTDNFLSIYKKYKNKYRKIFKNSHIISLKLFYYETNNVN
LFNISLCIFNYIKHRHTSSFILYKNKFDISHKEGEQNVQEDILRMDNIIQKYNKKIRRKNNKINNNIGHINNI
ISYPSIYNHQHDDNIDIPSVYSSYSKLFYTHVYDNYVNTYFNIEYLTNKIKDIYTQLLKDQENFLINKKKLCK
KYMSSFFEPDVYIKINNFIKDINKTNKQIDNINKCMCSKLKIYEDKICVTKKYLKFITRDDFYYFKYKNENNY
MGLVKILYLYIKKNNDKSYIKNTFTYTYFIKQIKNIDKQHINKNIISKYENISNVEKYMNYKFVYIIHHYISS
INNYVKEKEKINKQYQNLQELKKKVTILQEHVHHIISQNLSPPSYKIYEEILFVYKQIKEKIKTQKTFLLFKS
VIITYCLSFSYKEKRKFLKLLLKENNRKEEHAENNNDLKDKNKTDEVMNNEEKDMGNSYLHDDDYKYINIKDL
LNYDNFIDREFLKKYKELNLCYDKMIMFYCNTFDYLKDNVVICIDTFLQTKKYMLKKLIVDKKNFVYIKYEYF
YKIKLSSYSTDHINICDKICDDEKNKKKYIMKNNHIDNFINKLKKFNNENCYLIFQGVDQNIYTILKQNNLNR
IYIFLNDINKINLNKINTHYHVIMCNYTKENIINLIKYYYEKEKNQYVQQNDEINQEDYQERVMNLYNECLNN
EKTKEEVVKEISEEKEKHNQHIKNSNTKDEKEFALIYVFYLLIESLKYINQQYYICNTFFLKIVKSFSRHFNI
MNNKQAEIYILNYFYFYIISLIYNKDILTVNFILSIYIDIFVNNLIKWKDIFLFFKTFTNQNKEKCIIQKYIN
LNNTNFQELYHTELDQNIQHNINIKSKYKNQVTQKDDTHNTDKNNKHNNKDASHFENNNSTSQKKKIISAQNM
NESKSFNKDDLQRNNSTQHNNKCYYANNNYDDSISNFYSYHIDSSSTSCSHSSIVDETFGVVENDLDTIDNVK
EYKYVKREEDDKEYDVHKDKDKDKDIFFNNYKESENVNDHINKKESFKKEKIKKNIYNLQNEHIIHVTQEQYR
FKQLTLNLNSFIKLFYIKKKKLKKNFYDNQNYYKEGENYEHGQENSDLFRTFDTYSEDEEEELEKYNKDDEKR
DIDSIEYYMCELIELKKHKRKIFKNLIRNIKKNKDDWDKYIKNKDRDNYDKMCIPLKEINKDELYYYKVVVDK
IIKKDMLDISIKNYISIKLKNIYINKKHIMNNLYNLINKYMNISIFPLEMEQMNKEVSNNNEIIKLENNNKIV
HTSLLDEIKNNTYFINMIDQKGRIILLFKKHIYSFIISKILLNNNNVTLVDITSEHCAVHTKVSDIIMIDINN
ERDNYKLENFMQSNLNKIFFVFYTNLNIITYSLRKISVVINLDFSMYSNEYLNFLYFVINSINNKKSQNKNEL
NSKYLFYVMLFFVLINEQSYLENIELNMDIINEFYFLIEFFNNLPILLNGKEDELVMDTHEQTVLQDDNKKNN
DNKKNNDNTNNNNNTNNNDNNNNDNNNNNNGDVKNTNANIQNNTNEKANNIYDSTNNINNKNSSDNINNDKKM
YESKNEEKYKEFFFKYFNSFLSTVFSKNMILIKNILSSFLCTFFSIKINKNVLSNINNILKISSNTFTINELT
KYIQENFHLLQKILQHNKISHIYEINYNVKSMKNIFISNSLIRNMYERKNVLYLILLSFDIFENFNYIKHGIN
TTPKLKFITEEYNTLLFYVNEMKREIHHFVKMNDTNVSLNSYYMDIMNALLIYRVPTKWNFLGNHKKENIKSL
LTYLFEKLNYLKYIINKEQRGANNVYNISMFNNPLLFFYSIYFTYAINMNTSLSNLSASFKYSEKINKKEEGI
WIEPIKIYKSEFTKKSSNNLIHNRIHKKNLVFFKITNEENKNDEYEKYMEDEENTKDEVSNDDIFEKDIDYIS
DVYKINEWNIEEKEKKFYYDTPYILIKIKKNKESLEKRKNDIYDEKNENRENDLIHFEPNFNSLFDIFKNNDH
IDSHDFLYYCPVFKYNSSTQKNDKLLFFFPLPCIYNKMIYKKLRVHMLV*
>N.PF10_0252
MGMSLNKPINNTNEANKGEVKKKKICCVCLETKKLRDECIVKLGEEQCKKFIDDHNKCLRSEGFDIK*
>N.PF10_0253
MVLFQNRKIFGNFKYTHLIINYCTPFPRYNINYKLNNVVNKFHTNSKINIYQYKNNTNHWMFLKYNNMFKRFY
NPRANINWTLIRFRHRVSQLRKKKLKYKNHSKVALRFRLTKFGWERLQSGRNASKKNVSTKKYYEKMKIKYVS
RDDVKKFKFQMPSYVLRIRDSPVDYNPNIMRARKFLPTHFG*
>N.PF10_0253b
MTKYNLIFKKNWFAKSIFGTVGFFVLLDQLARYQYNIPEYRNPNLTMWSWWLEKVNLKRLGVDKEDN*
>N.PF10_0254

MSMKGSKNMLSPLVGNIYNNVYDHKELKMKSINEQNNMGSNNYNINYVNNNNSTLSNNNHSKQNLCYNNFSSH
SPSINKDSIHEEKKNNFYEQHNYIDENATHHTMEEENYKNFKNSKNYEHTIDEDVMQNEYSNIIKDNHLKKKK
KKNIHHLNNKDINNTNLNYETNEQVLIDLPKNHLLNKLTTSIIHSINPHLISEYTHEDITKRTNKISTLKKKK
KKKKNEKEEIYESDVDNTNGEDNTNDVDNTNDEDNTNDEDNTNDGDNEQIHYHKQFDYQIQKQKNKQNKQKKE
YKEFDVYETKGYIPLSNEHKQNKSVCFLDNHVKYYTQQNNVPYEKNIIYENNLNDHQAHSYYNNTLNQFTPNS
DILNYNNYINKEYIQGIYNTNNYNIKQDSVYEPNEQVMFMSPQEEKYIYMNASPNNIIRNNDMVFYSGQLDNE
QMNININNNNNINNNDNNINSSSSVNDGCFNLHLNNMNNIFNTCTNTQNKQKTHKSFFCNSSAKNVINNENNV
KNNKTEILIYKNDKTNNYQNNNQTKQNIQSNNHYNNNNNNGNHINKFRFFTPTSNNVFVRTRSLTPNTYQKKK
KNSFFACSPLKDINLNRQKSLPPQLYTTQDITQPIYTHDISYNLSNKNNIIQNDPYNNIQNITTTLSYPLVNS
NFVHLYPYQNSNTNNQIKKIENFNVSKNYMNESSTQTSTETSSKSSKDTSLWNTQIHLLNEKIKNMKTKMENI
TKEKNEVNELCKMYKNECGRLRELLVKENYDKYKVLPICSIDYEEVNLKLEKENEKLRKQMEALGKTILASDD
INSIKKNLAKHIVHLNEENEKYRNEIKLLKKNKDINNQILFTLNKTEISSDMIDSLFMQTKNVIIQGHENINV
FYFNVKNLVQQFLEKIKYIIMENEYTKKEKLKYVVDLEDIIKKNFQEISQTVININDLRKNMKNIRAQVFDVD
RVNPECFCKPSRAILENDIKQLEEVLHKHFILLKNLRKKNLSLNLNNLYVHFNNEDDQNAYSHESFNIPSEEF
QMNKTYDNPETNINVHQKKNVSSKNLYEAQKKNNTSDVTNTKIEGVEKDKKEKESYEEKNDKMNTTSEENNIL
VGENTKVDDIFHEKLHEKIKIIEEQLKSLNNHIYCSMSESEEDDNVTKNIKKNNDLMVCKIKNNFKDKNTCPH
IRKESLSKYETKTKKENLNHKSIKKQESIHSKNSSSYSSEISSSCEKTDDMLSNEKDDFNNDDEVEKSKNKMI
ELLALLKGKHNEDLVENTKEHINSFGHDQWENNYNLKKIYNNLKAIKNTIKNTEDDTEKNILALIESQANDIK
IFGNCIDDLKSTVAQ*
>N.PF10_0257
MNHKNNIYDILRQEDKIKRKTKQLKEDQKKKENEKKDFSTFSFDINKYTSWADIVDEYDEFFYEVEKLKDQEK
NEKNNDEKKKKKKKKKNVQVSDDDSTEFDDEEDDDNDDNDDNDDNDDNDDNDDDDDNNNQENVQNYNSNKMKE
IKKKNKSKSGNKKKNKSSNNANKKKNFENLLLEYTMSNNVLNTENNNAHVNILETNNATDENCYKNNIHDNKE
NSPCNIIDVNNDDKLKKKKKKKKKEKKQKVQDGSADEKKDKDDSLNKQKEQNDDDKKNAHEVDKKSCHEINND
EKKKEVQKDTTNHVHVNEKLKLLLNNNSKKNKKKREKIDDIIAIVEKEESIKQKQKQKMLQKTKQKLKLKPQM
K*
>N.PF10_0258
MDTTTLKEADMNQMIIQLMEREKKKKKKKNIYEQKKMEPQNYSHMKKFKISPKEEKSLNKEEIKSHLINFFQE
QEKIYKDEELNTKNYIIDETKKKEKELNGYYIYCQDFKNEYNNYFNSFIKESEDIKKDLKDLQIQYMDDKILL
GNKRKMELDNMLHFYKEKLLDIKNHWDTKNFCDDNLKNIVYDILNVIN*
>N.PF10_0259
MRKCLSSIKAHSNKYSIKCHIYLREWSNNKNKRMYISSLLNYKVEETYKYSCVVPYKNDNIFTLEKQIYDEKY
SHVFIDKNIEIESDLYYLYKLLINSRTIEQIRLEVYRSYCAYENNFINDMKKINWNAYIPFSSVLKEPEISIT
SIKSRLYHTCMIKNIILNIIKRQQQLYIEKNGDENILLKKKKLISSQLSPLKINVLFKYNECKIHINLSNNMN
IRIYQAYKNQQSLKSTIIASAIFKLNLFKYINNSKQTYIIDPFCNDGTAILEILSILLTIPNGSPSINYPIFT
YPLHSPSTFYNVLNEIIISPLHNMTQLYFLGMDEKKEHIKHANRNLMKFIQTIPLKNDVTGHNDHSSVNINDP
RVHTLVGDIKNVTSNQKHNNIYTNKDDNTNYDNTNYDNTNYDNINYDNINYDNINYDNTNYDNTNYDNINYDN
MNECDDHNSSDSHIKKKTYKQLETLSTQQLKDLFDNEKKIEKISINNKSFLLNNIKFCSFNFLKLNNVIENCI
IITNIMNMEKKKILKFEKILLRSYILNAYVFSNEKYKENTQLQFRLIARFISNGQNVIFLQLFNKTKIKRYED
YEEN*
>N.PF10_0260
MELIIKKPYIEIFEKILKLFTHICENVHFKLTRNKLELSGSNNLTNELVIHIDKKFFILNDVNGDKKNIINGT
VKSKDFYNCIYNHKIVKHLRSTYSQHGSYNSHKKNDSPFLMKDDMNEEGRINNTNNDEIKSIVCDGMYPDDNN
KSYFRKDKKWNIHLSKITLKFNNINHNINNNMDGDDHDDNNNNNKLEIIIKFKKYNTYFSAILKLKTFNTPMK
NYIYKNESIIQIDPTLFLLNLKDLSNEKNIFLKNTDNSFIISSLETCDFSLNKERIKREQFFYNNKNICIPSS
KTKYFFKNKKFQDHNMSLPLNELKTIIKFCSDLNLLCLFSTKNFKENLIIYFGNIISYILEKNKNKIKKKNKN
KTFQHKQHTYMLNTNHVKYLPNISRDYKYDYTFDSSKKSHVSRNKIHDDDDKYINSNLYILSKGELNNNDSHI
YDNKNNYDNNNKSLVFYLSDYTSSDEYDSSMDEFDDQIYLPVYDNEYTKDDTNFNYNHIITGCIHFTSYFNIS
CDFNEYENDKKYEHEDVFQQPMDAFVINNNDDNDDDNDDNNDNNNDNNNNNYFDDSKKVKKIQHKEKKLSIEK
KKEDIIYDNTKSCSFNYERNNLKNIREEGKEEEEKKKKKNSSISEDIDKDNNYHVMQNIKNKDIKYDRKEDGF
SDTYGMFNHLKENKSYEENRRKKHHLVLINEKCEESEYDQNGECSHVPKDDYSYDPNNDCSYGPNNEYPYDQN
NECPYNQNNECPYDQNNNINTTFNNLVQEQEKEIICNSNIYEEFNYDTYMRQNEETQNVYKNMHEKIKKLDIT
LENLKQINKKKKKILDYQKDDVKKPFLVFSLPKSRNIKKRRKPIEYISEVNKRNKRNKRNKRSSKDLLYNDVE
NKRYDNNSVSCRNEENEITSDNFYSLNDDNEEDENNDYYDENNNNYDDENNNYDDDNNNYYDDDNNNYYDGDN

NNYYDGDNNNYYDNFPDETYYNIRHNKHGVGENMEYMNYFNNIYDKYNMSNKTNKEKTHHIKNKIYKNQLNYR
NYYMSEKDNNIQYIKGGNEKNENDEKFYNNYKHFNMSRLSHNLHDKAPLTNQYSGPKKNTRLIMKNYSSHILK
DEIKHPSVNYDNDIFNYKKCKLQKKS*
>N.PF10_0261
MDNIFINSPIIKDYYNIKDFFLHIDNEIHLTELPFPCDYNTYGHFCLSNEEINKMMCTEENRFEEYNIEKENE
DKITNYIYQTRYSRYSKYNIKMDEYTLRRNDYVKDNNVYYNPNHHKTNSITNYKNDDINKKSDNNKYFDDDIF
IFNMYKNMEERNIRKNINRYNLIKDDNNMYRNILFEHSHKNIMLREYDKTKNITFHPHGTYFLTYPFYILNNN
KKEKRKIPNMPYRILSAPELMDDFYLNLLDWSKKNIIATALCDKLYLWNNNTCTNQKLFVANKIEKEIEQKDK
GDDKKTEKKDKDKKEKRKSNNDKYYNHKCESMEILKPSEKKKNKPQKTISSLKWNINGNFLATGLSNGVVEIW
DIEKCVRIRKYKNHKSRVNTLCWNHNTLTTGGRDNKIINSDIRSKEIYYIELTKHKSEICGLEWNADGTYLAS
GSNDNSIYIWDKYTNKYLFHFKKHKAAVKAIAWCPYKNHILSSGGGSVDKKIFLWNIKTGKSINEIYTKSQVS
NIIWSINTSELISTHSHSLNQIILWNLPQLKKVTTLRGHKSRVLYAALSPDGTSIATGSPDQTIRLWNIFPKC
GDKSISLFFPLSNCYNTVR*
>N.PF10_0261a
MYYKQYYTYDTYYRPYYPQYVLYEVPREKNVFCSLTEALCSCAVLIVTTFTSLCITSTDLIIRGCDNKRN*
>N.PF10_0262
MKCINAEDKIQQDHEVLYKMFKSLRRHEDDKFINGSIKLKKKSLNEEINRFKDINDKYIFSNVKKKIKLEING
CIKIKRKGAHEEYYNQLVKKVLHGSIFIQNKNINNILDYIKYIKYIKLGGKISMVKKKKRTDKNEIISPLLSK
ENINKLRYLKTIYDVKKERNQKSPRKSSYNIERKEILDLSMNRYSHFQSEISNNYNLKNNNFYDSIKLVDNKM
GKTKGNKEKGNKGKEEDKDKDKHKDKDKDKSKEKEKDINIYKTNGSIKNTLNNNKCSFVLSPSCNDKIRHVDE
LEKKMNTFKLNIPSSIINKFNKSNNTNAVENRGSIKYNLIKERQEFMYEPIRNNKEKHNKIHLKGTINYINNN
NNNKKKNNNNNVHPTKFIFMGKKKKKIIEHEGTDDNYILGNEEKKKLLYMDQYNNETNKSHETNVLCGNKIYN
KCGNKNIFLDHSVFLNYNKKVPKHISMSCSSKNKDMKKSTYDETMLSMSDVDDIDYFMYIKEKNAIKYKDYMN
KPKTICQDKSSIEKEKSVLRIKKVEDQGDIKEKKSYMNDLKYKIDKKEPSNKENTSNIRNKKFSSVSRKLSKH
FSLSRSGATFSMSNKKEKKSNKKKRTRFNKENRYMKKYLNKIMNMSILRRHFNNVNSSVYITHQKCFDKHEEH
TKINEKKCKKGVIKTVDGKEDMNILNVNNNKEKITTKGNKNDHVGDSKDGNKNDHVGDSKEDNKNDHVGDSKE
DNKNDHVGDSKDNNKNDHVGDSKEDNKNDHVGDSKEDNTIILSKEKNIKIGIYFEETCDIKQSNDKNISTTNE
IKKMSSPVTNKLLTKKMGFPVTNKLLTKKEGMLKKFAISKEKMDITKKEGVVLSMKKSFSLKKDAEIHISNDD
NVMDKNKVNSTIKSVQDILSKQLFKSKGNPLLINKKHVPKMDSTNKMIVDKKISFKSSLFKKDENKKTNYINS
EKEEEKNVNMEKFLLNELKENYLNDERINKPGLSKEDDVDIKEPVLINEQVDLIKKEDQNICTNTEQEHEMMQ
EQKINEDEVNSKGDENTKKTENTINDKETIDIINKKNSSESVKEEIMEKEMLDEKDKNVPHYLECNNLHNMDE
NTKEIKVEKCNDYMTDNITSKNNNMIIKKINMKMLPMVTKKALPKSKALPILLKKNISIPKEKFTTNKKLSNE
SIDKTESSNIKMDEHKEEEKQIIKPSKSIHKIIKPGRHSFFNANKLLNSIEIKIGEHNSKIIEKEGPIKMFSS
STSIISDGKIDSIHKSDEEEKINDLKEDSNVNIYVKEKGKGKVFMKKGIPKLMMKFNAFSTKKMGINNLENNK
KVNTLRSIKDDKILEKTNDTLEEINGTLEKTNGTLEETSEISNIQHEEEVKNEKDATLQDDDDAGHTLKTTKA
ILMKKIPAKYKMNTVKMGDIKLPSHMNKFINKFHSVKILSTLKEFPVKKLIKKKDSNEENNSLEKIKMDKEKI
NDVDMEKEADEDVMKIEESVMNKKENDETEIEMNNNNEVDNNKVDNNEVDNNKVDNNKVDNNEIDNNKVDNNE
IDNDKVDNISQDKSEISLNKIEEPNLVAESTTIEKEPIDKIIPRSFSKSLSIPMKVPTIKENINKAKPTFWNK
PKGKMFFLTKKEKSISQAKEILRSLEKSNN*
>N.PF10_0264
MSNKKGQSPKEESIAKMLICKVHIGTKNLENKMKRYVYTRAKDGVHIINLAKTYEKLQLAARIIVAISNPADV
VVVSARPFGSRAVLKFAQYTGAQAIAGRWTPGMLTNQIIQKFTEPRLLIVTDPRTDAQSVKESAYANIPVIAL
CDSDSPLEHVDIAIPCNNKGKESIALMYWLLAQEVLYLKGVIPRSEPWNVMVDMFLWRDPEQFELKNLANEEN
TPTAPHLIENQYAAEAPYDEWTKKEEWNDNTNEDWKNPIAAEEW*
>N.PF10_0409
MEEGRKIFERFYEDINSLHLHFEYLDSTQQYCIRNMKKFIEDNKLRDDNMIIVSCDKQYHGIGTRDTYKNEDR
IWLSEEGNVFVTFLFLWNDNLIDKVKLLTQTCTVAISKTLESFYLVTQIKWINDVYVNYKKISGCLIHMYDHM
ENQKDHNIMNNNNNNKKKICLLVGIGINVNLEDQHNILNNNYTSIKKEYLQDFDTPKLIPTVEQITLKLIINV
KHAINTLCKEGFTEFLNYIISRLLYKDKKVIINQDNQYIVGYLKGLLHDGSLLLFTEDNKFINVHTGRLHVKD
DD*
>N.PF10_0265
MYRNYTGKDSNDFYVLEAREKNGEDNIYVSDDKNELNLLTSHKEVIKKNVENYSFDDLLNKDDFIYHEDVKNQ
IEIKNKNVNKYKEEENNLIINRDTKDVHNNLYDEEREIQVFIDISKLLQSKEDNDGNNGEDICNDVYDININK
IKNIEIYPNVILNDNKINDNKINDNKINDNNNNNNNNNNHYDETHLSYGSIPYEKKERNVDILHDLNNISYEY

FYKDTYINEHEHTKINPSHEDIDLFSAPKEKTSNVHISSNNNNKYNNDDDNNNNNNYYYHNNNIFVELTKTLH
EQNNKKQLNLDSTINSFQNKKEERTNLFKSPCNKINNFHSTSNNINRCISNYIKYNVDNSFIKNEKNKKEFFF
FDDDKDKVLLEKNILESYNHLNDHKNKHDENITRIINDKPSNFFNYIYNIESNKEDIKWNLKNSYYVYEKHKI
EQELSTCLENNISLDTFNYLYKKYNEDTCNIYNFNEQKSSFLKKHILQETQMTIKKNEKNNNFIQKKKQQQQQ
QQQQQQQQQQQQQQQQQQQQQQQQQQHNNNNIIGNHVNYNMGGVQDVEPYKDTLQKDEQSFLNNTGINFYID
KKIEEINDINNNTCTSTNMDMQKICLENNKECIEKNNNNSYSIISYKQNLFNNNMMKIDSSNMEHNKTQTLYD
NLNKQEDTMSSNLCTSIINYNDNKEIKEVDNVEHTMGNLERCTKKNFCMNKEKDHVERGDFMYNQYNKENENE
KENENEKENENIKNNKNNKNNCDNNINILDEKNDFFHSFIYHNNSGTSPNNKTYYRKHCTHNHCYNNINNDHV
CREDLYSDEKTKDLMYVLKNQVSKKNMTDDKKIREFFFFYEHMNNKNVGEQHHKGNEKEDIHMNNIQQSVYYK
EDENRLADNYNVMNDERSKIGSTYIEPTIFLTHNNNNDNDDDNNNDDNNNNDDNNNNNNNDNIIKNEYINNYI
LECIFNDKKVDNNYNIYDNINDMNKIHTFDSIELFKDNKDKSFYKEELYDDNDIYRDKKNLLQPFQDYFEKTN
FLRKDKIFVDNAKRNKIINMDHMNDDNINNHDDVNGDNINNHDVNIDNINNHDVNVDYINDDDVNVDYINDDD
VNVDNINDDDVNNMLQKYLEKQKKRGNIFESLNSFIYNFKNNNNINMNSSNISSTHNLLHLYDHMDVEGFHKL
DIEQKIENNRNKTIKDTKNINDIKTYEHNKHISNTRVNNMINQNDIYDDMNNCTRCVKKETMGKKNESISFFS
YNDQKEEKKKNEKDIKNDHNVDNNNNNNNNNNNNNNNNNNNNNNNNNNNNNNKCYYYLTDPFISTYPKNDMVKKE
KKIHAPKNNNAQNNTINNNKDQKKKDHNHVDGDNIILTMIWNINNISLLTGSKNNFVVSPEFKTHTKIKEFFI
LFNMNNQKDVQNKNCVDYYIDILFSYTGFCDIIFSVTCGIYRKKYTCYNISTIPWFGYKNWGVLKNCMKKDMV
SIRIDIHDIKEKNEIKEIYLNNIISQIKDKKDNTKNVNLSHDHQNDYKDSNNVFINQDKNLYQHNIAYNKEQI
KNNIIYIDDNIKKKKKESNNMSIKKKNLYEKKDNNYIFDIHKNIIHQHNKMDSILQSNKMNYINNKANFQEIN
NLKNNQNIYSFECKINKNVNHIKEENTTKNDILNNNNNIYSNNIYSNNIYSNNINNDHFKNNVSLDNHNLSYN
INKHFHKHLFVHPTQKNLSSGKNNNNILNNKYQMTTKEDIQGDDIYYTSLYGYNTLECKIKREN*
>N.PF10_0266
MDEGKVKKYTDFINSFTSFDKLGNDNKCSFMENKESLVDMIEYFCNFLIKYFYNDINMDSINIKESNLDAEQL
WYLIECLIKKKNLNNLLEFFKRYEKQIDQEKCNKKKEKKIMSPYKINRDLNELCDDNTFFKTSEEKPFSEKEK
EVNKKKKRENNMSSGDHGTKKRKEKNSSRNKESEINKKENNKNDEINHRENLEEDENQDRFFNFDEMESFLNN
EENKMLKINEREEKEMEKLNGSYEFDENEETDEEEFDLNNFEDTYEDAKKMKYADFYKDDNEDDEDNEDDEDD
DEDDEDNDDDYNDDEDNDDYNDDEDNDDYNDDEDNDDEDDEDNDDEDNDDDDNYDDDNYDEDNYDDDNYDDDN
YDDDNYDDDKAKEGSYEKKKLYDKKIEQDIYEINQFDSRLSDNEKDSLSNEFNDNKKTDDVQEKEKIEKELVA
EKHWSLKGEAYGYNRPKNSILNLNFEIPKLNTFNNNNTLENKNVGDMENTDDDDDDDEKEYASEDGHNKNNNT
NSYKKKEKKMNTLNNEIEIVVKQRIQNMLFDDVEKKTIEDIELEEFNKNDEINFDNLNFSKSKISLVDEYTQK
YQDEIMNSQSKNKENNLQKMEIMNLFKKIMHACDSLSNDYFIPKPILLNNKNNKMATLNIEDSVPMILSDKNK
KTAEEIFNPQHMKQQNELTKDEKKAIRKSKKMKKKKKILNQFKQTGSSINQLVKRNTEIINKNKKNKDQKINY
KKYGVLSKDHILNTKTGNRYNFGSEISKALKMDQNKNKK*
>N.PF10_0267
MYGGGLRFFGIRGYRFDFLGESQSLSADVIEELDKISMVSLKTIKIPDYIRIKLHNLSKQFIKKKDLEKLGRY
MCKKITSRNCVELPKVLPSKLLCYNEEERNNIERMLDKKSYKFLKTFITEYKKKVKDIDQVSISYSEDIRHKI
NISFFPEVSIAYTLHTFNGKYSVLYRILKEIKVRIPNFLPLHILHISEIPAIGIIAYTEIFEHRYEHILSIQA
SEHLLSISKYLTDHIPNIQHQINLYEDTQSSYDLIILSHMLLSLYDHNSRNIFIKNLWNKLSKGGIIIIVENG
TPTGFRMLHVLREMFICDLKYDKFHIVAPCPHESICPLALTGKDWCHFSQRVHRLSHHIYCKGSRSKNVEEEK
YSYLVIRKEQGPRTKYMNEQQTSTLHEKSFFWPRVVMPTIKAAKHVLIDVCSYPHNFQRLVVTKRSPLITNLK
TKTGHILKGYGYKNARKLLWGDLWRFTKRVTRPDARLYTPERTKQHLYRLYKKINKKKNSHSLVDNKTKEYFD
SRSIQYYGS*
>N.PF10_0268
MAQLAENTVLDESIQKVEVLNHNGETTSFYNEVEKHKENNEGIVVFTYPKANTPGCTKQAELFKEKHEEFVNN
KYVVYGLSADTAEDQLKWKEKLELPYELLCDVDKNLLKLLGLTNEEDKTIRSHLVLKNDFTVSYVKKSVSPGK
SATQVLNFLVNGDDGGDGNEEEENEENNNNEDKDNNENDEEGDVQGEGEGEGEGDEEKTADTDKEKPKKSSTS
TQKKKGSVSSTISKKSVKKSNKVVKKNVKVKKEIKKKTNKKADNKKGKNVNNKLMKSNAKGANKKGGKKNSVV
KKEDNKKKGKNNNKKNKNQNLKDLKKKNVKSGKGSVSSSNKKLPKGLKDAAKKAGKKIDKKKEQANKKNNNNK
NKNKNLSKGNNMKHNKKPAKKVVKKK*
>N.PF10_0269
MYFVIEEWKNVIIKPSQLGPRYQQYIEDMLRNSVEGQCSVKYGYVICVIRIIHSEPGRVQDGTGMIVVKVKYQ
AIVFKPFKDEVLDAIVTDVNKLGFFAQAGPLKIFISRTAIPKYFEYSEDLHYPCFSSGDYNIKPQTTVRIKLQ
GIRYDLSNMFAIATINNEYLGCIESNILQVM*
>N.PF10_0270

MTITAGKCCKYIRKYFTNGFKKNSLPYFMYCKNKPFLMNSKLVQKYKLDNVPKNVINRYVQDKRRLKNDDID*
>N.PF10_0412
MINCNRVNKKSILEAIEDFVLHIFSWFLDINVDIFKNNADKKYSIKLFDKKLINLCRTVSVVELVNEIIMKDK
NCTQREIYYKFYNIFREQNQANKHIKDVCNILGYERSSLNIHASEKGCIAGLLILKKGYEILNLSEHENGIMI
NEFLLNVDKIESMANYIIVVEKYSVYQKLCENKIWNILPSILITGKGFPDLSTRKIICELMHTFQLECVYVGD
FDPHGIKIYLNYKEGIKSNDKNNLYACKNMKWIGMCSNDIVFFPKESLISLTKKEKNILQNLKNNKKFLSTSQ
LKNEIIQMDRINYKFEIEAVQYLGMDFFIKKYLIKKILRKEWLT*
>N.PF10_0271.1
MINRKSESVSYTPRAITNRPISSNRRRGRNEITDEQKNEIKEAFDLFDTEKTGKIDYHELKVTIRALGFDIKK
ADVLDLMREYDKTNSGHIDYNDFLDIMTQKISERDPTEEIIKAFKLFDDDDTGKISLKNLRRVSRELGENLSD
DELQAMIDEFDKDMDGEISQEEFLSIMKQTSLY*
>N.PF10_0271:mRNA
MINRKSESVSYTPRAITNRPISSNRRRGRNEITDEQKNEIKEAFDLFDTEKTGKIDYHELKVAIRALGFDIKK
ADVLDLMREYDKTNSGHIDYNDFLDIMTQKISERDPTEEIIKAFKLFDDDDTGKISLKNLRRVSRELGENLSD
DELQAMIDEFDKDMDGEISQEEFLSIMKQTSLY*
>N.PF10_0272
MSHRKFERPRHGSLGFLPRKRCKRLRGKIRSFPKDDKEKPPHFTAFMGYKAGMTHIVREVDKPGSKLHKKEIV
EACTIIECAPMVVVGIVGYRETPKGLRILSTVWANHVSDEFRRRYYKNWYKSEKKAFTKSLNVPEATKNCLLK
RIEKYCTVLRAICHTQPSKTPLRMKKAHIMEVQINGGSMKEKLEFVKEMLEKNLPVSTVFNPNEMIDVISVTK
GHGTKGVVSRYGVKRLPRKTHRGLRKVACIGAWHPARVQFQIPRHGQKGYFHRTERNKKIYRIGLKTDKNSAS
TDADITEKKITPMGGFPHYGVVNEDFLLLKGCVAGTKKRPITLRKNIVPQVSRDALAQVSLKFIDTSSKIGHG
RFQTSEEKVKYYGPLKKDLKA*
>N.PF10_0273
MKEANAYEKDIRRLLPVMFYCMVLLQINVEKQYVNIDLLNEGYTKLLTFHILLLLLIWSFYKTYKVNPGNIPD
NYEWKVDPNIGRIKEREKTGELRYCIHEKKYKPDRSHYCRAIEKNVLKMDHYCPWVANCVGFYNYKFFLLSLF
YANICCLYVNINCYTSFPNFYSNPNILFNEVFYLFLEIVLASVILIIIFPFFLFHIYLTSKNYTTLEFCVTGQ
WEKGNIYDLGVEENFKQVLGDNILLWIFPLGKPKGNGLFYKTADQMDSTYK*
>N.PF10_0274
MMNFIRLSLDSYPVKYYKRTYKVTNILKVNGIGLRKWDCEKIKNFISKFDVTCMIYLIDNEDMIYIEFYRKKD
CTFVYKFFNDTLPTGSTLSTSDVSAEYANIKYEINNNSCTVYSCDYSCLLKHKDYITIINNVLDKNLSDEILE
NYKNEKWLMYTKNDEINVGYYFCRNIPNKIYKSYCISKLDSFFSLEFLERITKIFNNRQPNQITILNCHLKKA
IEYVIESSYIFDEEIAFLVLGNELPMSFLDIRNKEKLNLVIESNTLLRIKGKLRHEIIFGIPKKKKIQMQDKL
MDRKNSYLVIFRFIREDNIKNFVTQKIKNKIPQNERQEKDCVPINVNEYTSEKLEKMYVLDVYNQIALHFGHT
RYKSWKNVENIINEEKEGNIILDVGCGNGKNLSESSKYFYIGLDFSLYLLMLARKKMNTDLLLANCINIPLRS
NLADLCISIAVIHHLGTHEKRKQAVKEMVRCTKIGGRILIYVWAYEQQENVVGNRKFDSQDIFVPWYFQQQYK
TEGYDDDAEGEGEEEPEKENEKEKEESHKFKPVKKDLVKLERYYHVFKKEELYELCNSIEEVKVEKFYFDCNN
WGIILRKIF*
>N.PF10_0275
MSEYKKYNVPSHEIYDVILIGGGLFNCCLYYYLKLKNSELSIIIVEKEENLGGYIKTYTYNIDDKKILVELGP
NMFKLCNESYNLINKLNLLSSVRLLDDTLIRYIYIKNDKLYPLYLNIFSYLSFPLINISNKIKLLFKLIFKQY
KNLNSYTNDISVENYMNDNFDSQHYNFLLLPFIYGSCAGNGKISAVSFFSRNLKLFHNKVNSLYIWNEKIKSE
EKNRNIKCVNNNTFIYQHMNKKKKNFNLFTPCYNFFYISNKNMKMDVYLNVLYNYYSSNKKTEKKNVFNYICY
RIPILLLHMKQFSINIFRHISIFLKCTYQFFYPFLMLPDLAKWTTSHFDDEKKKKKNLIGKTVTFKNGLYELI
DKLQSHMNKEFIYTNHEVDFIEKAYNNLWMCNIKHENQNKTIYGKNIVLAVNSKICSNIMRNILPVNIRDILL
NFSYSSIISISLYFKKSDINIPKNFFGFLSSDKCAHILGCFHINNMFKERCHDDNKVLLTLYMGGSNHPNDIT
LDEYEIKEIISNDLKNIFDIYNDAQPNILKIHKWKDAIPFYSYNYEKLLKKVMEELNKLSYKNLYLDSGWITG
TSISDRIRSAQDLCEYIFSLTNLAS*
>N.PF10_0276
MDEENERERLRIRMSLNDLILILSVCYSVGDIFVQWNEFSKCYKPIQLWLVVSFISIVLYRLSHFLAQYLSND
NDDFIIYRRNNPPYYISFLVLFILFPFFFIWNIIGTIWIYQIIKYTPNCLPRNNHPWFIVLWIVLCYIWILLY
LFFIILSIYLEYQSRIYERTITNIQTDDIFSRWTDNIDLMRDYGIFIYRNGLRLKQIENLPFYYIKNISNESK
CSICLNDFQIDECVRTLLLCNHTFHKSCIDLWLIRSATCPNCKSPIASQGIFMNI*
>N.PF10_0277

MKVVEKQLSKKTLKVKKETEKNSTGFMEKKKINKKEKKIYKNNYNNDNIKEEEDILLNQDDFEKKKKKSLENK
ILLSKKLKEIKLQFKDDDTINNSKKNNWIERLDITSTEYYYLKKTELFGISESREKQLQNITHMNVIQALQKL
QELNIKFNRPYDFLADMLKSDIHMEKIRQKILKEHEQAEVRENNKLKRINKKFLKKCGSQKIKSHKEALEKKQ
NLLKIDQLKKTNNLQNLNVQEFFIKHSKDHDYKAGKITAKKDMKHKKKNFNNKAKKDKGIKNKQIGKKTSINN
KRKASSKNSRKSKKMKKKHKHRK*
>N.PF10_0278
MDELNKEEIVDNINNEQAKTRKGHLILKKREGVYEESSKYCLFIGSNKRSLILKNFMYDIYSIYKPLTCYMPK
AHSNLSNIIDKIDKLVDICVHNNCSFFFSVFSTKKKPSRFIIGRLYNNKILDYYVFSLISYIPLKLFPLSKEI
LYDTKPIVLIQGSYFEQNETNRYVKNILFDFFKHKNVDTFSKKSIQRLIVISAYQKNNEINADLGKKVEAGKE
NNDKNNDDDKNNDDDNKNNDDDDNKNNDDDDNKNNDDDDNKNNDDDKNNDDDKNNDDDKNNDDDKNNDDNNKNNNND
DDDDVTLNFTEDAMSTTKENLNDSFKNMDNIYNNLNTNKFNSYDKLKDKDKKNLYVISFNQYLITKEFFNINK
ENEQPPKLQEIGPRFEFTLEENYKTPEYNLFQEALRTVKKQEKTKIKNVHVDEFGHNIKKVYVQKQNFSKLHT
KHTKFVKKNKFNT*
>N.PF10_0279
MKDNQVNLFFVSGLPSDVTENEIKNYFLPDHELSHCTLHSTHCGINSCYGYVGLKSKELDIQEYVKNKKLKNK
DIFIELIEEENEEDNNINDDDNNKYDDDNNKNGDDNICDDDNKYDDDNKYDDDNKNNMYEEQNNICYNNEDVT
DEKSKKSNYIKLKKNKENKKHIKIINKNGDITKEGLSILENMNMQDVVILIIKIQELVRMDPQTAIKMLNENK
TIYYSLIHALFFFGILNVEITPLDNEEIKESHFYRMKNYFQYICMNDEKSTMQDFKEPKTNVDNQSENDCGDD
SSYEYEEMINEDIISVEENYNEDERNDNTLVNNMDNTFDSDHVICDDDNKIYNNDDNTYDDNNYDDNKYDDNK
YDDNNYDDNNYDDKNDRDNYHPNLINKKHIHVNKKTLYNNHQPYITNMKNDKKRKIQETPKTLLKDVNNNYGD
NNMKYEDIYYNMNNDTSMECNNMKGVNNHNQFIINMRMKNNKQKKDYPYKSVMQEYIKGNDLSNNNNNINGCG
NVSLSSDKRKGNLYGNNKINMNNNNNNNNNNNNNINNSNNSKNIPPKGMPKPLYSFIKNKDLNRNPINYDAKDF
VENVKENAADCGDTLLHNYNNNSNNNNSSSSNNNLYKSNKEAKNHNENPHFYNTTNYKVNNNNNNNNNKQQLY
RNNTDMRCENLPDRLINKMKHINNQRRGIKLIDRKNNPSEDNYNTNKIKIKCINIEEEKKINNKNNNNNNNNN
NNKLLTSGINSNHLTLLLKKLKISLKDIPYAEEDLVKEIINEKSILQNILISKYVDMLNWTSEQVLRVLSIRK
SLKRIGYNINGII*
>N.PF10_0280
MENLNNDNVNANTDISNKNNCEEKSDELKNNVEVFNIAEGEECNLKNDDEHVSQQGEESSYMSEMKKKSLLAE
HIAKEAKKKASVIIMKNERLQKVYKGRGVNDNENISENTEKMVEGNEIKTPIIQSEMDNNDQENSTQNEPKKK
FLIGAKPILPTCNEELLEKLKQRRV*
>N.PF10_0281
MKKTILNLYLINILFALSDVKGISTHDTCDEWSEWSACTHGISTRKCLSDSSIKDETLVCTKCDKWGEWSECK
DGRMHRKVLNCPFIKEEQECDVNNEMAEDTHMNNSYIYFNADDGDNEYEDHDDKNDDDKNYDNENDDDKNDDD
KNDDDKNDDEENYNDTEEKVKNNDIHNSSANSNNEVTNFIQIKDKIMIKHKTTNIHPVNFIQEKYTRNNKHRS
DNFSKILNNMNHINNNNYNSRSSSTSSKNARGYRGGSSNMYPHVPNYTSSSVHNSTNNERKSDEDLDNIEGDN
ITKEERIVPINNKNYDNHDEHSNIHEHDTSRNVDNEKYNSNDDLPNLSTYDYDMNNDSYKKNHMKKPMDSIKE
EQTKQENNQNNEKVSSSEKQNDDISALYEHMNTKDQEHTQHEQTNDSAHGHFEDYSKLYIASGVATLVLLGGS
ITFYFLRKEKTEKVVQEETKEENFEVMFNDDALKGKDNKAMDEEEFWALE*
>N.PF10_0282
MYVRSISKELTKRMYNPSGHRIMMMDLKNKTTNNILNSRRYIVSYTKVHRTHYLEDYYTNPEIATNMISPMSE
YIWWSWLLLQGVIVFGTLFHSNYYFTGKALPKVQGNSQLCEDSW*
>N.PF10_0283
MCDNICEDKKNYMFGRPPFLIPQNVADSPSTTVELDYPLYETAPNREIKNIIQETTINVPKVEYKNKIVEIPR
VEYRTYPLIKEVETPIYQDKYVYKNVEVPNKRLRIKPVYKEVKVPQIEYINKYIKRKYKRYKYIPKEIKVPFR
PRREIYNEIPIPRYIPENIENCVEKKKMLDTAYRGELPLFQGYDDNIINMMNPFCTYNQSDNLYNNQMCEGKR
KKEKIWNYDILNCLCINNNENGTYDIDPNNIYPSYDNRLNEQVYDHNKIYSSSPYEQLTYTPPYHIILDSDQK
KEENIFCHKVIEVTSSLLAFTGIAIIMMGKLSLHGISILMKNIKSRNNNTQVEKIINEIKDDEDTLCPPVKKN
NNMI*
>N.PF10_0283a
MNPSLSIIKYFMHSSGFIMFVYLTMYCFKIIT*NELIFQDDIFLYIYQIKNCTFINNSKYVSHEEHFKKWKHL
NVTINNDIVNIQIVSCNYLGDIYFDNITNENYMKQYPREDTNYNFNEQKEIDDYYDYFFIIII*IIIIIIIII
IYLVIYKIINTIL*ISFLFFLNKNNLYVGFNTNVDSQHVIIKKIKIIITHYLKEQNLKLYILLATLPEINDQH
IKHKIVHL*FFQLS*NYHTII*LKKEKENYVIVILININSKKFYELANNFSQ*NYEHVQVKKKKKK*EKRKIS
IIINNNNNKKNNKNNNNNNINQHMFMPKHNYHDNTQSSAYNNNSIFNKNNPFLLIDSYNDTPRDFRKITNKKS

NNIHYNINAKRIKVILCKNN**LYYVQ**KCY*EQLFL*KK*KKKKKKKWKKFIQIFLF*K*RK*KYSKK*SI
YRK**F**RRHLYE*SNCK*KKKHFVFIKISNDKKF*IKLIN*KPQKLISDISCNHGCILFEN*EIYFCLYHE
KEDKLGEENINYQLKYFDKYNRSYNNN*HDNSYISLEKLKSSKVNKINVYYCNNTYIMLSADTNLYILKYTFV
RNQRVVNFFFFFLYINNYLYKNI*IKKF*KFVNSLKIEE**IN*NVCNKLYIDNYT*YEGYIFYSILISEYLH
LQERAIY*CLIRQI*ISSNNINK*INK*IYIYIYIYVYLFIFMCV*YFFFFFFFLFLIKLNL*VHYYVLFFLL
IRL*FWMIINLNIK*NILIKIY*LKYNI*NTFI*
>N.PF10_0284.1
MIRKGVELFYFKNARWSPPSKYNIKCSRRLYHKHMINKEKIIRKGFSYEYINDDENFLFNKYNCVNSLVNYYY
VNKKFEEINLLIINKQDDGKYMDDIINILYVLCVKGNISILDRRIKYYVSYILTYVNEYMRELSDHKNKKVSK
NQHNDIHNNIHKDITTDECGYIHKDIPTDECGYIHKDITTDECGYIHKDIPTDECGYIHKDITTDECGYIHKN
ITTDECGYIHKDITTDECNKKKNGCVQMYADDNYYNSHNIYNNVNIKVENTIINNKKKFISIKSYLLFLNILK
VLDMKNDFNILLKYVSENIHFFSIDIIHRIAFNYDIKNMNNINNNNHEIFFKEILDYIYTLIDYNPSLYFNQM
KSINLCINICTNYFFEKRYITEQKKKNDNEDIIYMYNLNYIYINHIITFNNLLHEYNTTKNNIEDYKSEKEYN
NNTKKKTLCLFKKSKKIITNVSDILQKGDVSKSTIQSKSQDRKEYINLEDNLNIINEKLKHINDFYFNYYMID
TIYILYNYYTNLLRETKKKKKKKKLIMKQNYEQKEEHFLLYIPDIKNSITNILLHFINIIHINLNTNQQKIKN
KKIIQALERILKLSYEFKDKSFNYESFILNYCNMLLVSKLDLSLVDNIKILTIFKHIRKSHREGNQHNVQTQR
KQRIDKSLNTCQQKEKKKLNDNKLMYATEHCGKKNEIYDIEDNKYIEQTSLHINQSNISHIRENNLESYDINK
HLDNMIKLVCCNLKTCVHNSCGNNICMLIPLIYEFHTYMDCELKNILIVQITCNKHNINENNLINILRVFCKI
KYRNDMLDQFFYNQKKFNFYDNYLPSHKQGDKKNLYIFSCPKNFFLFFYYKSKNNLFNYKDMYYIENYVQNNF
LEMNIKDFLLLLLIYYKNKVFLLPQLLIKILSIVNNGKKLITEKKKLLFFLYLFSKNYYHLSNDNSEWIDNQN
DQNTHLIRKINVFRKSQYIQEHINHFINLINDLFYFIYNENTINYPPEQVRLLLQEKKKNKINKIIIINKKNS
ENGELAKTMKDETNKDNMSYEIYEQSESNSELNQISNHMDSIDEYKINFTILKTNLIMMKILYNIYYAYFSSS
FEKGKREGKNILNSHHEKLLNHLYICFNENYSKNVEEIKDVAPLLYYFTYFNLYSSFYFEKQMSYIMMNNIID
ERLIKYVMLSHVCVKKIIPHEIKSIIKKYVLENFAHFSGSFKILCFRLSHHFLDVPNGVALEGKKNNVGTEKN
VDISIFNELLNDLLINLDHMKPNHYLVIYSTISKNMIKNKKYYIELFYHMNKIATSYNNEQLFSILYYMYKTG
YSKPKIRKKIRNLILFRHKKRLIHLSMYIKYILPLDEFGIYHLLPIKCQNIIYDKLTVDVKSLIRPPLQHMEI
NEVKNIKVKNKKKMGETLEESSKGNEQNDAPIYIFEQMVKNY*
>N.PF10_0284:mRNA
MIRKGVELFYFKNARWSPPSKYNIKCSRRLYHKHMINKEKIIRKGFSYEYINDDENFLFNKYNCVNSLVNYYY
VNKKFEEINLLIINKQDDGKYMDDIINILYVLCVKGNISILDRRIKYYVSYILTYVNEYMRELSDHKNKKVSK
NQHNDIHNNIHKDITTDECGYIHKDIPTDECGYIHKDITTDECGYIHKDIPTDECGYIHKDITTDECGYIHKN
ITTDECGYIHKDITTDECNKKKNGCVQMYADDNYYNSHNIYNNVNIKVENTIINNKKKFISIKSYLLFLNILK
VLDMKNDFNILLKYVSENIHFFSIDIIHRIAFNYDIKNMNNINNNNHEIFFKEILDYIYTLIDYNPSLYFNQM
KSINLCINICTNYFFEKRYITEQKKKNDNEDIIYMYNLNYIYINHIITFNNLLHEYNTTKNNIEDYKSEKEYN
NNTKKKTLCLFKKSKKIITNVSDILQKGDVSKSTIQSKSQDRKEYINLEDNLNIINEKLKHINDFYFNYYMID
TIYILYNYYTNLLRETKKKKKKKKLIMKQNYEQKEEHFLLYIPDIKNSITNILLHFINIIHINLNTNQQKIKN
KKIIQALERILKLSYEFKDKSFNYESFILNYCNMLLVSKLDLSLVDNIKILTIFKHIRKSHREGNQHNVQTQR
KQRIDKSLNTCQQKEKKKLNDNKLMYATEHCGKKNEIYDIEDNKYIEQTSLHINQSNISHIRENNLESYDINK
HLDNMIKLVCCNLKTCVHNSCGNNICMLIPLIYEFHTYMDCELKNILIVQITCNKHNINENNLINILRVFCKI
KYRNDMLDQFFYNQKKFNFYDNYLPSHKQGDKKNLYIFSCPKNFFLFFYYKSKNNLFNYKDMYYIENYVQNNF
LEMNIKDFLLLLLIYYKNKVFLLPQLLIKILSIVNNGKKLITEKKKLLFFLYLFSKNYYHLSNDNSEWIDNQN
DQNTHLIRKINVFRKSQYIQEHINHFINLINDLFYFIYNENTINYPPEQVRLLLQEKKKNKINKIIIINKKNS
ENGELAKTMKDETNKDNMSYEIYEQSESNSELNQISNHMDSIDEYKINFTILKTNLIMMKILYNIYYAYFSSS
FEKGKREGKNILNSHHEKLLNHLYICFNENYSKNVEEIKDVAPLLYYFTYFNLYSSFYFEKQMSYIMMNNIID
ERLIKYVMLSHVCVKKIIPHEIKSIIKKYVLENFAHFSGSFKILCFRLSHHFLDVPNGVALEGKKNNVGTEKN
VDISIFNELLNDLLINLDHMKPNHYLVIYSTISKNMIKNKKYYIELFYHMNKIATSYNNEQLFSILYYMYKTG
YSKPKIRKKIRNLILFRHKKRLIHLSMYIKYILPLDEFGIYHLLPIKCQNIIYDKLTVDVKSLIRPPLQHMET
QINEVKNIKVKNKKKMGETLEESSKGNEQNDAPIYIFEQMVKNY*
>N.PF10_0285
MNGKNMEDEEIENVEDVDDDVIFEGIENKNDIIDLSSDDEDDEELYKNSTDINDEIIKYKEKYISYNKKFYTN
KSIYCINSFKKWIYFGSINNKCYLYNNFDDDLKNYIMSITRHNNNYNNNDYNNICSSNNYDNINFGDEKNKKI
NLRDLKHQIYSDTITNIKISNNFQYVALSIYNGDIYIYENNNHNSSEIINYNLNNMKKNINNSNCLLDICNWD
MEKKTNEYILNENMKLINILSINNDNEKRDLEYSMFCLYHEHIFISIYVNCTNIYIWDVLEGTPINIIHTIQV
PTFLNLCNYENKYYMIVGFNNGESCVYDYDIYNAKKIGKIQGKHDDNNKYSDIYKNVHGNTYTNSHNNNNNNN

NNNNCYYNDDDINDGVLCIDNNLGNEIYTCTFKNVIKIYNINNNNLINTYTNLHDDLIDYCLFNNKKYNLFAS
CSLDNKINIHDFQHNKSINQFYVNYHFNEPHTNEQTEKGINYLKWINSNLLLFTSLNGNIYIYDIRSRKCIHQ
FYSHTDTIFNVNLSLHLYQNKNILSILTASDDNSSNMHLLDISPYL*
>N.PF10_0286
MLCYTTNTKRYAILIYKYLCKNGIMYRKYNFFSFEKENQEIEIENVKHKVEKNRTLNYHHNNLRNNHQNNDHP
VDKIFKTNEEQKKEENNLTNEFYNKIMDEYKLNNDEKLDKIKNIKKMKRKESPENVDKNIKGDKNIKNEKIII
HTKHLTNNIIMDESNICTNNVNTESININVSLSTHDIKKNNKIIKKNCNSNIINCTEDKHINNMLIKQTNKNK
NDNLLTLNEKKKNDHFYKIFFSSKKVKIIKSQNHPIFIHLYKLASDRNYRQKQEKVILTNKKIILEREKNHIS
RIYTNSINNLKDFRSYDNFILLSNKLLKKLSFLYSFKNGVIAEITHKFYFDNVGLPFLAFCFYNINYEPTKKK
QMCLHHINNNIPSVQTNINMNITNNTNISNINDAFCYNLISEKNLNGSGEKNFNEMDEMASCKKIKEQLEEVQ
NGGQNGNQNGSQNEGQNEGQNEGRVQSQDQRQNQSQDQRQNQSLDQRQNQSLDQRQDQRQDQRQDQRQYQSKE
PNEDKIYTNVENDKLKNSHTCDNNKNIEYLCNGDIGTLIRSCFLLKWQCIFNIDNLNRKRKNKIKSSYKNMDI
NNIYDIDFFHPLTIRASSGYLLDIPFKNTDINEIFKYCKENKILMLKYKEDSDLYINYKNLYTEDDNKFLHLL
SKAKGVFLIMDNCNNIERTYNLIYNNYINISNNINSENIFDNIYYVNLKNDETKPLDLISTYSIFMYILRSHF
FKHIPQSSYVYVE*
>N.PF10_0287
MSDNKCEENDDFSLISVNYNACENLYNSIRNKKCVDVAVHGKVLDDDYDRNKSRIKNKKNVSFEEKLYSDIEK
KKEKFDDLSSHKSDHINISMMDKKRTDTFLRRKRSFIFNDYKYNEFMNAFKQQELKNFKNFHYLYKWKIAVSI
LLILSITFFLIGSYIYYESLNVIEININYNTGDDYKIINIPKDMKKPVYVYYKISNFYINFKTFLSDESHSLV
NEKKCSYIRTYADIYKYRCINNIQTLPEVYDDMNIDKIPKKKKKNQKCHISDLKPEEANKKIFPCGLVSAAIF
NDKIALSKNSVNYDIDKFPILHYFDFLTYMKKHKQFTNYKIWINTFSPEYKNWFHSPMTSSFIKPYGVINEDL
EAGDDYKLTFLQNVWPADEWNAKKSFQLVSLRSIGNSSFKLAYAFFLLSLLYFIMIIFILVLVKCKYYKLGKT
LTYCKLSMNKNIEKMNSRKKTNIQNINKKINSMQLEIMHKASSDPNNLAGADHSQKLCFCPLH*
>N.PF10_0288
MNLPKNEKNESLEEKEKEKEQDVDENIIIKYAVFHSCLLLLSGIQIYFICPSKKKDNKRTILYDKKKQSLCSN
ELNISKDYVEIQKNITKENIKMKHKNKSLKDHFLNDYNIYLNKPKKKILNEHTSYHIPVYKKKNNE*
>N.PF10_0289
MNCKNMDTSYEIINYLTKDELDIDLSCMDKKERYKIWKRLPKCELHCHLDVCFSVDFFLNVIRKYNIQPNMSD
EEIIDYYLFSKPGKSLDEFVEKALRLTDIYIDYTVVEDLAKHAVFNKYKEGVVLMEFRYSPSFMSFKHNLDKD
LIHEAIVKGLNEAVALLEYKIQVGLLCTGDGGLSHERMKEAAEFCIKHKKDFVGYDHAGHEVDLKPFKDIFDN
IREEGISLSVHAGEDVSIPNLNSLYTAINLLHVKRIGHGIRVSESQELIDLVKEKDILLEVCPISNVLLNNVK
SMDTHPIRMLYDAGVKVSVNSDDPGMFLTNITDNYEELYTHLNFTLADFMKMNLWAVQKSFVDPDIKNKIISK
YF*
>N.PF10_0290
MKVFSIFKNVLMLFYIIATFHKIPSEGALNNAETLDNLDDNSLLAFFLLIQLEVPYFLDGLSSKIIYEKVIDA
TMYLFKEEIIKSSHINEIKQSLVQDINNTIIGLNNILGDIRNDDEKYFKWLQLIFSNCWKIENGCINITRRNC
SLSVLTMLPKKGLPITCISCIIYALNYASHSIYLFKNFSNIENCNNENNIKLINLSNEISIIRENIIYLNSLE
KISVLKNVQSEIIVLHIKNGSGLIKLLNTQGHGAFIRIVSNSIYEVIKRSINGYIKYVNNNNNNIYKNNDYIN
NLYINMKKSITLTINGYLYKDNEKNEIFRIFDFGLFLTDIHLNKCISLLNGNNIFIQLEIGSSQTNVPFKNGN
ILNLIELEIILNSFLHFGYNYYSKVFLILKKIIYLKNYKDRDVFTYDDNNKNIYEHMAKWLRSNIKSDGNNQI
NNNLNKDIVNNDTFQPNQNNNHVNQINNIDHIELTDDQTIYCIIMVNIVPDNKCLFLKKSIIQLLNKPIYSRS
LSGFQFKDNKIINEELIEILGESYYNSSNKSKRRTNVFGSRINAREIQSAEKLIKPGLKLIHIAFHNFTSIPV
QQRNTFYSANNMFSLFQHRIHYLKEWNPKILIIKKFYPEDIIIKSYNVYSYPKNNNKYWFIISCTVIPFLLII
LFILLYKRWFPDNLICFPFLSKHRKNHNKQKRKQNYKYSCSTCRKKCFKHMYKMQKDKSHLRKRLILHKNIPK
IPNNDKNSNNLLFPLKQNDEHLTRNRQKKSTTPLFLEEIQTNDIIE*
>N.PF10_0291
MTLLRPRERVIISAIRRESRIKYKAKRMHKRFRSWAQQRVREYWLPMNVCLTSNVELLDGQYLSGCIQKAATL
RKHDYELWKGYSKRVLEISDTLTPQQIGYIFYGMGKSKFLNTLFYEKFLKCIQNSLDDFYSHPLMCIAWALNR
LLLRDEYFFKEFCKTIINKFDDIRTKDLIKINHSIAKLGIHDNNYKNFMNKNIITKFESIFAQDFRNVINDVT
LINLYDDEVKKYILTRFTNMFICSRPQHYRNAYKSAVAIRVLCPHVWDNLSNKIKSFYVKLSMRRIPQTSRTP
SPFQWEVSDCLSKLGISHRNTFLWGCYFIDIGETNDKRNCWFIDGPSCFYTGTNQYTESVKLQHRILYDLGWN
IRRVVWIDWIQFGDDTHQKIQYIKMLRQKQPLDKTLTHSPSISSQQIKEHLKKLKQYKLTKENNKQQMQIHV*
>N.PF10_0292

MNEKKNTIFKGKGYILKGVNKEFIDDRKKRDLFIDKKKKNIFNRFNPFTLPLLGKNKLCEVDEKCDMKKSKSS
DKYKEANININVLNEGEFNSYLLRNRAFRLTWTSIIEKIKHEIDIEIYKNLNDVFEEIYLYSISNNEYLPLIL
ITAGTNVADHEIIIDALSYELKIWNNNNNKKKKQHTHTNTRTQKGCMDESGNLFDYVHGEGKDYIYEKIKRDM
NKGDTNQGDHLYDKININKVNLILPGNEDSAHALMNPSHESNNQDKEIKMKKKKKKKKKKKKRKKRRIFNGNI
CNKDSSISPNSSYHNHFNNDDDNYNVNNLLNEDSYDDNDDVYVCSLNSNTSNNINSAVYNIYTQLYRDYKMRY
FLKKYKKKDGRNNYNNGVKNAYISNETRKRTRNYSNSSNSYNLNRDDIDNDCDNTCDVSNHNSFVRLSFLNKE
FREDNKVINKNMVNIDRLIDLYKKMSELNMKNISEEEIEGLSKKRVFTNKYNSDNIYNMDEMYNVMNSKDVMF
TSPKGGYKNNKGSGIYKDMNESNNSFIKEYGPLELYNKQNNYFYDGRKKVRIIILIHDCEYFNINILNGILNI
LINLRIDNKICLSVILGVSTPSFFFNRITTPDTQSKLRIKTVDILNNKLMCDRICNILLFENLLPFILNFKTV
HAIKLLLHKNNQSISHLIHILYILTKEFYDNNILSFLCIPMNYYLNICTNDDEEKEKMDSNYSAYTYVRSYQA
NLSNFFKYDIRKVHQKIISLCYSSNLYYRHLSYIKKKYSTILIKNYFQLHGPDSVVHTPRHSDDEPEFFNLKS
KKVDHMKQERNIQVVDKELSSVNNDKINENNINGDIHGDIHGDIHGDIHGDIHGDIHGDIHGDIHGDIHGDIH
GDIHGDIHSRNNKKSPFDRASYAWEFKTKTINWWYDHPFKDLIYLYENGRVRTNNKEEIIDKLLNDEENKKEI
INNMHSHINGNNNLYDIENVKEINKNLCSFTLPLNVLQLIERKYAFNIAINLINIIIKTKSEYTTSIKRSEYF
RNLFQNYEKIKWNENKNILYIEELYNIVEKDVKKCINFLIDMMTPYYFKNHQVLLNVFKEFKEYYTNIYPIVY
NLEDYLYLLKDKEYKKHQELFINDDFSKTCTYSISYSIYFSLLSRLDDMLEMLRNVIRMEEKQKKKTNQCTAF
DDNAGYIKKNICVNNLSRKREEHVEDSGCVLNVSSYKQQNEKKENEKKENEKKENEKKENDKRFFIQADDHLN
EDEDEEAEQQHNIQYNNSVNYNDPYYCAHDIGTSNKTKYFPSNNIINIHEIDNKKKRFKRYLTIENKSLKKKK
NDEHNINAEDISNCINLFIHDYIYLLLLPPISYHPLAYELILHKPDKDFTNIMTRDIKKELLTTLCYTNPYKT
GNLMCSSCCLFKDNPYNSNPENNIITNNKIYLNPTYDNISTLEDMVNLYRVYEKCNKTIDLYNLFILYLNIKV
DKYWAYSINDKNKNNNNNNNNNNNNNNNNNNNNNNNNKNNNNNNNSDWGDKEDIKSPHLCKMKNTPTENLANDILQEY
YLKFIITINTFCSFLKILKPPNVSALLNYNDKNGNNDDYYEDDEYYHTNNNTNNLGRGAKKKSSDDNNSSQES
IIQFLKEIKKSLQGCTSKKLLFGKLYYNHIIASREYFLQNLMEKNNE*
>N.PF10_0293
MSASKGRKKSDLKKIDDSFVEINKDSPKKSRSGLQEDKALLKLRACLSCRMLKSESEFYQNGCINCKFLQLAG
DRHRIHDCTTENFNGFMAITTPNKSWMAQYNDLSKYAPGFYALQVIGELPESIRDLKPNY*
>N.PF10_0294
MDCLSKINLIRKVNEELYNSIGVEDDNLSEFLIYLCEKSTSLEEFCKDVFENGGVIEQAVLKYIYNMIKKDNK
KKKDHDNEDDVNSKENIFLQNLNYNDNNLEYKKIEMKNEKMKKYHCLTLKNEDITNLVDSDENDKKEKSIKGT
KEKKESIEITSKQKKNSDKNVTTRDLSDSSYQKKRRKKKNSSGGSSKNTESSFETKDIKKDNKYESGKKRKYD
ERYYDRHSKEHHHSNKHRHSKEHRHSKEHLHSKEHHHSNKHHHSNDHHHSNDHHHSKDHHHSNKHHHRRHISS
SSDSSYYNNKKKKEKTHRDKKNEEDKSYDDYMALKLNNIFNGTINKITDFGLFVSFKTNEGYKEGLVHATDIL
PNRKRVVNMNEKFKRNMKVKVKVKGIFNQKISLNMSEVDQRTGKNLVNDDMNKEEDNYISLFDDINEDFNDLK
KKNANVFKDDPKETLKYESVIKIQSDYSKWEIQQLIKSGVVFDENIRNEYKNLKIDEKIEDEEDIIEIEVNEK
EPAFLKGQTTKAGAKLSPIQIIVNAEGSLAKAITTTSALAKERKEQKQNEQNAIYDNIPKDISRPWEDPKPNL
GERTIAEALKNIGKNYDIPEWKKNYNNNNISVGVKNTLPINEQRSKLPIYNLKNDLMKAIEKNNVLIVIGETG
SGKTTQIPQYLHEANYTEKGIVGCTQPRRVAAMSIAKRVSEEFGCILGQEVGYSIRFDDCTSNDTIIKYLTDG
MLLRETLSDTLLTKYSFIILDEAHERTISTDILFCLLKDVVRKRADFKLIVTSATLDAEKFSTYFFNSPIFTI
PGKIFPVEILHSKEPESDYVEASLITVLNIHLNEHPGDILVFLTGQDEINTACEILHERMKKLESMSPPPLII
LPIYSSLPSEMQSVIFEPAPPGCRKCILATNIAEASLTIDGIFFVIDPGFCKIKKYDSKRDMDSLIVAPISKA
NAKQRAGRAGRTGPGKCYRLYTEEAYKNEMSEMSVPEIQRINLGSIVLLLKALGINDFLHFDFMDSPSVETLI
HSLENLYYLGALDDNGYLTKLGKKMANFPMEPNLSKILLTSLNFNCTDDVVTIVSMLSVQNIFYRPQNKALLA
DKKKNKFIMPQGDLITYLNIYNKWKENSFSNYWCHENFIQSRALKRAQDVRKQMLSIFEKYNYQVKKSTSKND
ATKYVNICKSICSGYFNHVCKRDTQQGYTTLLTNQQVFIHPSSTLFNKNPLFVVYHELVLTNKEYIRDCTIIQ
PQWLIQLAPNLFIPADEKKISKIKLREKIEPLHNYYEEPNAWRLSRRKG*
>N.PF10_0295
MARVRKGKAMPVMSKNSIFVLNIIMTIGFISAIPQIFTTLMTSWREAEPIEYKYMFRGSEHSLYVDYTYYGLY
KVVYDNKHVETWTQRVQNMKRKGIEGIQQGKNSESAGSLSLWTSVCPEACRDAIVRRIEAYERVSFISLVLLC
GIVISCTIVILSVGWNLLFSKSIFILMGCFIFSFVINAGIGTYWYYETDMSWNSITKAQQYPFPRCSHCFYVF
MITTGIYALCFLSLLLLDLFNKGKQKSSHRDQLNAHNRNPMNNKAMYQPMFDNQPGMMMQRSASYSNIMPFVK
GMNNNDYSNYMQYNKMGMNMNMNMNQMPQQGFPNFRNMGSNVGPNMGSPMGSPMGPSMGSPMGPSMGSPMGPN
MGSPMSSPMGSHMGPNLNPNLNPNFFPQQSRQYSYSVSPTYQQNMPNFNNFSNRHMPSMSDLYFARQYSGMKF
GDMNNSPFDSQKPYKF*
>N.PF10_0296

MVVDKNESHTIYNKTSTTNINNTNFSYIYDEVKVIENERLQNNEWHNDKNRIDNSGKIIKNIYQGSVNYYNKI
SKKVISPKKKKKLFIENEKNISYSLRDMNNKKKNNKKVKQNNKVQCIKNNYYFDNNGKINQDKNKDTCTNNII
CGGNPCEKNRFPHYYTHNNNGANNFNKYSRTFNFYNKNEKRKYYENNAHNDTFRLTKELLDDLNKRNEAAEDD
DNDNQNFLTYVKKKIEEKKEEKKKKNKLQQSMNERANNNNDINDDKNNEMYEKNHPMGDNDNDINYNNNHENN
NHSIDNNNNNYSNDHINNDDVEYDNNNVNSKNNNTKISNSGNFDFSNNVETYKTINDNSSLFSDESTKVFVNK
KTRIKINMCENPCDIWYGLKKRNGKNIFINSSRNNNGKNKKMKKNKKNKKQNMMKMMKTNKKFKNIEKYYINK
KSGKYVKWSSNYYMKLNSTNGNISDNTIATMHIDGNYKYNNGDITSRNITKHNNTYNNNNNNNNNNNNNNINN
INNNNNNNNNNSCNIYNNFKSFHNKNIPANMQFDKINKNYLNHDYQITKKASKNKKKLNWSGKNNIYVNKYNV
RFAPIKNCKNNYFDNRTMNMNNNYYENKNGIHDENINDTIICNICGKSRENVNIDTYTDNNNNNINNHLISLL
CTDLENAHNIMTHHPIINDSDVIINKNKCNCYDNNVSQNLNKTVDNFCVQESMNNTKKNSCTSSHFENMHEIN
MNNTGEIFLNGLTNNISTVSEYNNINISNNNNNNNNNSDVCHNVVNEDNNETNINSHGNHLMCSNKNEIYENN
KKNNLDKNFCTHPNSHMNYQGNKIGLKKCASNKMYKLNTSMNNVNNCVVNNNKDYEGKYFSARYDGNTYFTRS
KTKEYAYNNFHHTNFKYNKNNYFEKNCSRNNKVISCHNYNCTKNIKSKVDECIHVNVSSICDEKNEDDKEYKI
RESSQIENNNNDNNNNDDNNDNNNNDNNNDNNNNNNNDNNNKKKNKNNKNKGYIITDYSKGYISYDQTNSYNN
QHLNNNMKDDKIYNIEEKEKAHFDDYNYMNDQNKKYKNHYYDNVKFDLSYEIEEDKKQRKKKIWKTPFGNKSI
YKNARERVQSILANRRGLEK*
>N.PF10_0297
MEERENLRYNHENNIGTKVPLKRNKKEIDSLAYSLKKNIKSNDELHNRAYYFFSPDDILDNIIFNLCQYIPKN
QHILDKNEDFDKCQSSNNFSYITSSNTDSWNDPTIFDDISFDEGKKEIIKKQKKEQKNEQKNEQKNEQKNEQK
YDQQNDQQNDQQDEQKYTNKIDIYFPINSHNKQINTCCVEWLGYVNEANGYAAIKMVGPFISQEYYTYVNRLV
YFLFMKTNYYGIENFLSHHNFVNKDVPLYMKCKNKLCIKLSHINVSNDLYL*
>N.PF10_0298
MGESDKYNHINQYLNENEEILLKELKGKYEFMDMEEIKNYKEKKLHHELTLEVQKFVNNKDVELIDKFRLFYT
YLSPLITKLKKTIYAELLYIVTVDFDTNWTIEYVKESEKNLENDKDAIIIYRCILILKYIKLEDYKNCEAEIE
NAKNMLQGVIGLNVVAHKFYNYALMCYYKALKKSDLYVKYALLYLAYTPLNDLNDYDRIDIGTYICIHSIISE
NVYNIGEIIQLPLINVCIKNNEETNWLYELIYIYNEGNIDSFNETIGKYQSNIENCILKNYQHNMLKKMTLLA
LMDLAFKKKKQREDISFHEIAQHCKIDINEVEKMLITAKSKNIITCKIDEIQKTVKITWVKPRVLNNEKIYFM
KESIDKWINNSKNLITYMEDLSVELLIS*
>N.PF10_0299
MFDNMEVDKKNGKKKYILGIEGSANKLGISIINEDMNILVNMRRTYISEIGCGFIPREISAHHKYYIIDMIKS
CLKKVNIKISDITLICYTKGPGIGSALYIGYNIAKILYSYFNIPVVGVNHCIAHIEMGIFITKLYNPIVLYVS
GSNTQIIYYNDHKKKYEIIGETLDIAIGNVIDRSARILKISNAPSPGYNVELLARKKYLLNIMKRNNNKNKNN
ITKEQEMKDNDFNPNELNDEQINDNKKMEDFTELLFFPYTIKGMDISFSGYDFYITKYFSKYMNKKSKTLNKL
EKQTTNQGCNEKRKDNQYDNKIKCNQKKQQQEKEKDTYSNINKKCKIENVSQQTIESNQNNNNMYNQNKLGKE
TFKNIEPLKDNYKNDYVKVKEDIRDNHNGTVQNINDDNNSSNNYQKCNKLFYVAGLKGNSYYEDNIIFESEHK
ENYGENDEYIDDNCSIETASDFYEKSVNEEGEGEEDVIDLTEEEKRKIQICYSLQHHIFSMLIEITERAIAFT
NSKEVIIVGGVGCNLFLQNMMKKMAKQKNIKIGFMDHSYCVDNGAMIAYTGYLEYLHAKNKDIYNFNNITIHQ
RYRTDDVFVTWK*
>N.PF10_0300
MKTEEKKSLQEKEENNCNDDNLPTNVDINDNNMNNISYINDKLKKDSYEKIKTYVVIYNIGKKKNVGCIVRSC
VAFNVHKIFIVGKRKKEINFFGNMGTYDYITIEYFDNIYELKEYLTKHDILLYACEISVNALSITTNPYEHKD
TAFLFGNEGTGIHDNVLKICDKIIYIPQYGKGTASLNVSVSCAIILQNFAVWANYEQTEIKNKKFIVQKKMSK
LQKYLNPTDDMLKQIHFKRSLRSQKKKENHIISFENFI*
>N.PF10_0301
MQVNFPEDKIDLFEKNFNLIDNNNDKKISPEEFKILIRVLGQTINEKDLDEIINKHFEDGIEECDKEQKDNDE
KNNKSINSIKKKYDIKNLISKFSNIKSNEEKTQNSLIKNKDHLNEKIINKKHINFEEFLKIFMNIYTQPISLH
ELIKYFQIFDNENKGYMDMEKLKYILMNSDEKITDEDFKFFLDSINFKNVDKIDYVILSKMIKNLC*
>N.PF10_0302
MNTYFKVLLFLFIQLYITLNKARVTENTICKYGYLIQMSNHYECKCIEGYVLINEDTCGKKVVCDKVENSFRA
CDEYAYCFDLGNKNNEKQIKCMCRTEYTLTAGVCVPNVCRDKVCGKGKCIVDPANSLTHTCSCNIGTILNQNK
LCDIQGDTPCSLKCAENEVCTLEGNYYTCKEDPSSNGGGNTVDQADTSYSVINGVTLTHVLIVCSIFIKLLI*
>N.PF10_0303

MNKLYSLFLFLFIQLSIKYNNAKVTVDTVCKRGFLIQMSGHLECKCENDLVLVNEETCEEKVLKCDEKTVNKP
CGDFSKCIKIDGNPVSYACKCNLGYDMVNNVCIPNECKNVTCGNGKCILDTSNPVKTAVCSCNIGKVPNVQDQ
NKCSKDGETKCSLKCLKENETCKAVDGIYKCDCKDGFIIDNESSICTAFSAYNILNLSIMFILFSVCFFIM*
>N.PF10_0304
MKEQIINAKSIINDCIIYVRKYFSFHDATVLLIDELINIMINNECVPLDLINQKDELHILVKNELKYEFLRIY
ESLKCTLKDINKCLKKLVQVKKQVEDYTTHNKLDILNMLQNFLKKTLIYFKQDYKLKKTLYHAMIHIDKNSDD
EINRLKLIWKETPFLYLIIQKFHLNKIITDCSQFLNKT*
>N.PF10_0306
MTEVTHCYNGNIKDGLFHGFGILIYSQHEKYEGDFVYGKREGRGKFTYADGATYEGEWVDDKIHGKGIANFVS
GNIYEGEWENGKINGFGMLCYNNGDKYEGEWLDGKMHGRGTYTYEDGDVYIGEWKNDKRHGKGCVKYKGNENK
IAETYEGDWVDGKMQGRGTYFFADGGIYEGDWVDGKMEGKGVYKYLNGNKYEGEWINDMKNGYGTLTYVNGEL
YEGYWKNDKVHGKGTLTYSKGDKYIGEWKYAKKCGEGELIYASGDKFKGQWKNDKANGYGILLYNNGNKYEGE
WLDDHRHGMGTFTCKEDGTIYSGHFQFNRKHGKGTLTFVNGHILQGIWNSGLLEKVINYELTPSSPWNDPDL*
>N.PF10_0307
MASCTGKVNFSSSGKEKGIGLESVLENAFDELISEFKKKFLHKIIKVLEEAKYNENLDIMNERRKNFFLKKYE
NDQREDEDEYENKIGNVVEKKIEGNVEREDIMIEEEKKKKKKMDSSSSSNDSTFGVYLKSIIDRNKISLLKK
LEKQKELKSVEENNKINYYNNKYKNGIIHYDENKEYHNNLNDKHVYNKNEESMNERTKLVKYTFHDKNKNKND
MYLYPQESHFKYVSERIENLYKISNKLREEKETYHNLKYDKDNENQHFKENHTEDETDHEQNYEKYNVKLKNY
VQNHNTQQMDKYQINEKNNISKLKQYNNNNDNNNNNNNNNNNNNNNNNNNNNNNNNNNNLKGNMEKNIINNHQ
KNYISYYNNSFKYPSRSHIFTERFSYHASDEHNEKSDHIYDMSPNKDKNCNNIYGILHNKDKHCNNNYDMSSN
KDKHCNNIYDMSPKKDKHCNNIYGILHNKDKHCNNIYGMSPKKDKHCNNIYGILHNKDKHCNNIYDMSPKKDK
HCNNIYDNSHLSSRGANKLPPSNNIIHEEKKQIINENINIESPQKINDLCNEKNNNLECKKEKIKLEKQFSFD
SKDSDDNYVRGKNNMYEQDDLKNNMSQRYYKKVEDFKSYQLNKKISNEKEIIKRPNNNLFFEVIRGKKRKNMQ
GFECKDCKSFYNEIYSDDDEGKCVHKNFFMKNEQQLKKKININKDVRSKNSSTDDINSVDKMNNVDKMNNANN
INNADNINNADNINNADNINNEDNINNMDNLNNADNLNNADNYPLNKKVHMNNSKKSIYYSHSLSPTCLNAHT
DTEKASMNEKKNGNMLNMDNIKKENDLEKEKEKKKKKIIQTFSRHRYHNKINDSPKNFWKFDFFK*
>N.PF10_0308.1
MSKKKRANQQKRKKKTEKKKNVKLQNEKNYNNVESERGGITYSLINDYHDTNFKKNFYIKSIRTDGNCLFRAV
SDQLYNHEENYKEIRKKVVEHLLKNEELYKNFIEYDESYKSYIERISLDGTWGGQLELQAVGEIYKVNILIYQ
ENGCILEIKNHSDDNKCIQLHYASSEHYNSVRFKNRALDNELKSILDLREILNNKEDNDSTKTFYETTENELT
DENESAQSNNIKFINKKCDENHREYYYNKLLKEEDNCYDYIERKKNGNNIFCFSDYEDDNNLTSYNVLQKIHN
NIKINRKHGRSKSMPNINDKFLYFFSKNNRNDKVESDSTIDNMNEKKSIEKKKTKKNVKRKINFVRYKSSGGM
YYYTQYDNNNNDNNNNNNNNNNNNKNEDILRSDDNNDNSSSSDSLCLDNVFSMYLFNNYVDRSKTPIVRFYFD
KLFYKYLCLSSVMHIDHINNKCDSNKYYDNIGHTNSLNNNNNNNNNINFSENYNLWNLNYPPNHPFNKNNNFL
DESNLSKIGYISHYEVKHNFIKYKKKDDEDFTNFYSEKILYNTENGCQDILYKNSYIDDNSISPNDIKNKKSF
NNNYLYDDSHVINHSDINHSYYKSGNPFYSLYMKGDASQDVNNKINTFKNNDNINGNQINVYNMSVNNNIDTH
QSTCKNNTVYIEKDIKCVNNLFNNKKEKHFNYYPYGDRIFNIIIDEYVISMESDFLCCYIGNKYNNILIRSKS
NPNGKSEYNIDENIYSTYATHIIENNNRNNEEGSNNDINTSNVNIENNNIGDSHQDNHSRNTTSTNITKDQQT
ILHLNSRDLSMKKKYQHKKFINMFSKDFYSKGLFQYLNADFILNDDKIKYIIPFFSNHNKINIFKKNIDSNDM
YLYNYLMFYFYLNKDKILNKWKCSKNLYEEFLIKEKHKYYKFIKGKKYTSSIKSRDDQGVKIISI*
>N.PF10_0308:mRNA
MSKKKRANQQKRKKKTEKKKNNYNNVESERGGITYSLINDYHDTNFKKNFYIKSIRTDGNCLFRAVSDQLYNH
EENYKEIRKKVVEHLLKNEELYKNFIEYDESYKSYIERISLDGTWGGQLELQAVGEIYKVNILIYQENGCILE
IKNHSDDNKCIQLHYASSEHYNSVRFKNRALDNELKSILDLREILNNKEDNDSTKTFYETTENELTDENESAQ
SNNIKFINKKCDENHREYYYNKLLKEEDNCYDYIERKKNGNNIFCFSDYEDDNNLTSYNVLQKIHNNIKINRK
HGRSKSMPNINDKFLYFFSKNNRNDKVESDSTIDNMNEKKSIEKKKTKKNVKRKINFVRYKSSGGMYYYTQYD
NNNNDNNNNNNNNNNNNKNEDILRSDDNNDNSSSSDSLCLDNVFSMYLFNNYVDRSKTPIVRFYFDKLFYKYL
CLSSVMHIDHINNKCDSNKYYDNIGHTNSLNNNNNNNNNINFSENYNLWNLNYPPNHPFNKNNNFLDESNLSK
IGYISHYEVKHNFIKYKKKDDEDFTNFYSEKILYNTENGCQDILYKNSYIDDNSISPNDIKNKKSFNNNYLYD
DSHVINHSDINHSYYKSGNPFYSLYMKGDASQDVNNKINTFKNNDNINGNQINVYNMSVNNNIDTHQSTCKNN
TVYIEKDIKCVNNLFNNKKEKHFNYYPYGDRIFNIIIDEYVISMESDFLCCYIGNKYNNILIRSKSNPNGKSE
YNIDENIYSTYATHIIENNNRNNEEGSNNDINTSNVNIENNNIGDSHQDNHSRNTTSTNITKDQQTILHLNSR
DLSMKKKYQHKKFINMFSKDFYSKGLFQYLNADFILNDDKIKYIIPFFSNHNKINIFKKNIDSNDMYLYNYLM
FYFYLNKDKILNKWKCSKNLYEEFLIKEKHKYYKFIKGKKYTSSIKSRDDQGVKIISI*

>N.PF10_0309
MKEPIKNEMGDREINAENVFRNNNFKSKDCLLEENNIKDLNSTCSKNMVPPITIILKEENHTSNTYEMMENNK
NIKSNDDVNNNNIKSNDDNIKSNDNDNIKSNDNDNIKNNDVNNNNNNTFKSETYIYSVNNKKSKEDKAENIQN
GNTNNNIIIYTNKKNNENNNCLNDVINKYLNDLDNQEKVLPKGEHDKCTNNINVLNNTYNINNNNYYYTIMNT
EGDILNKNYNNYFNDNNNSYIYHENNNINMNPYLNIQGYIYNRNIEEQQNGLCEQYIPYTTNNNDDHINNFIN
EVYDEDSYNIYFSNLSDNMKNENYDNNNNNNNKNNTCVTSLGNIYEYKNLSQQQQSNDENNYYSQNKKRNTYS
NNNKTNRSDIYTKENKYERGLYKTSYTKDKNNLYNNGKMENYKYDEKKKDRKMDNNNNNNKNNNNNKNEINKN
NNNNKNEINKNNNNNNNFYVERNITYHKNKKDNKFFYDDKKYKRHSEESECSIFNDDYYRVQCKLYGDEKINIP
EPLINMEQLSKLCDEELYKNICLKGYCNMTTIQKYSIPVILKKINLLACSQTGSGKTFSFLCPIISNLKKENE
ILRPHFPGSYACISPLCLILCPTRELVIQIQNEVNSLTKNMSIVCMSFYGGETMKDQVAQINEKQGDIIICTA
GRLLDLLNSCKVSLSFIKYLIFDEADEMISLGLKKQMDEIIFQKDLSPKDTRQTIFFTATFSDKLKEHIEKYM
STPYVYLNIKQKRETKNRIREIVKYVPAKSKFIELLKDIKILKGKAIIFVELRHSINNVFNFLKTKGYNVDYL
HGKMSQVI*
>N.PF10_0310
MLNEEKLGEENDLDKFNNDVNEDIIKDDVNKSKEKKKKKVQFSNNNETIYYEKDESENSYFNFTLNNFNYFEC
FYNDIYGDHFNDQIIRNNQYNENNGDEEDGDDEEDGDDEEDGDDEEDGDDEDDDDEEDDDDEDGDDQNDKGEN
IENNINDEDNKSEEINHKNNNCNNNKNNNCSNNKNTILNGINNINNNDNNNNTTNLKKKKKKKKKKNIDHSIY
NNEQVSKNMVSLKKSGNNKNNIDDLKSGKKNCLLSNIFISHDENNNLKKKKKNVGNITNDNFKREKFHQSFNE
DPSFEINNIHTAILNAEERTIHYSKDYFGYDIEPFNMKNELTQGYIDKDGNYIYNESDNDEVEEAWLKSVDEQ
DPLSTFSNNTLQAQKFQETQSKFHMTYDKLNNNILSINIFDALYSLSCLLIDEKETPIKAMIRYKNDLKVCKN
YLNQYESKIDKINSLSINSNAEQKIQSTEEHEQNGETLQTDSVGKKNLLQVRIRAKSSIQHGEENLNVVKKND
EVEKNDEVEKNDEVEKNDEVEKNDEVERNDEVERNDEVERNDMAERNDMAERNDMAEKNNDDVKVPENNLIEA
PMDKEHIMINNSEEESIGNNKKSEEQKNYNLYNDDDEKKNIVHNERLEELENLKQMYQKITLDYKTIERRFNN
LIDLTQKLTNEYKNVYFLTKREFEALCKKLEEYKENVDIHWQLKWMNGVDNNVYGPYNYYDIYNFITTGLVTV
LNPILLRRINNKNEVLENIWQMYDAVNYLIFVTNDNIKKKRKHDGLISKNKDQDSDNNEEDNENNKNTDVDGD
GDDDDDDDDNDDENDNDDENDYDLLNKKKKKKKGLIQISKKKKKKKKEHYEENNIFNDNNNNQGNSSNNEEHS
DNEDYYDNGYEF*
>N.PF10_0311
MCPMHSSSTTTTTYVQDTNTQNDTNENSSTIVRILKLAPQKVVRWDENTIDNENAQKKSSKVCCIYHKPKNFG
ESSDSESDLDSDVEQPDTQKKCNSSCKKDNPNNDKNEELELKK*
>N.PF10_0312
MSEQNLKVESNFSKEGEQQKDDAIKSNETYEEENEEISNVVNKYSDFYKNLYAFIESKSCYINLIYDCKREEE
TQVETKKKYKTTYSNYQKSSSVEKKKLDESSFNKIKEIVKEKFPKSSDKKIELYLKEIINDAPKENCSLHKIL
FVRNEQNIFVKHNLISYLFNFIDCKTLLKLKGCSKIDSEVVSLYYRKILYTLSFKNDEIKTNIHYWRNVIDYF
FYRCANLKPLDRMNCPIVLKTLEQNKNFCVINPESMDSCIYISLDYFKENLITSCTHKKPLPHTCNVKNEISG
IKNLKTEQIELNEFIQEYYKRLIINDDEIMWNVCYFRKSKEFLTILLLEFLKSMLTVLNKYNGKCIFKKTEYL
VNCGHHITHRIWIQMFYEYGFESVDADKKKNMKNYPIIQQEDGNNRTLYLTIADHYKWDA*
>N.PF10_0313a
MSIGHFIFILLILCILNVRDIKTGDLLSSNSIRQLIKNTYVLNKPKNLYHFTFFYLYNTLKCTLTGKVFEIYE
DEKYEKEIFLIKKNLENLKKQKENKNLWVFFEFNLFDSLLSSIEEIESNMKKFDIGLLEIYKELKLSDRRFKE
VTKYELNICKEEIIFDVEEIGLLLSIVPYALETLEISQKNYLREFHYILEKTLNFFNIFMSLINEHNVNIIDI
DKKEINLFLKVKDSFDFIRSNEENLFSLHDEIVLKNKYGQILIKDLFLLFKKIFFIEVDENLIYFNYIDIIPI
YIKTKNGLLKSRYYNMENYFYALIENTYKQNLQINEGIILIKILKQYKIHSDTKLAYYFDSDIIYKTILHQIY
KQTSHISNINFDFLYSQNIDSTNNLLTENDVSLSLIILINSDVDTDKLTEQFYRFSFPKDIFRGNEDINKQRI
KVMGSCVYTKESQTPIFNGFFSLGLSTYNLFKIIELLKPNKKLLYLSLEKADDNNQIRAQAIAERIVMNSKEL
FETVLLFERDDIIKYDKFDYIDKCDVCLVGYKGLYTLYFCIFIPVLVSLLICYFLYKRKIKNKQNIVDMKNKN
FINDLKSIEPSIIPLK*
>N.PF10_0313
MIVLSSYKKCLVFSFYYILLLWVLKYYMLTCQAINFKKTWSSKERRNNIIYLFNNYKRKSFIYDYSNINGKART
LFLFVHPRNYLINKKNEIETHHVPLVTSRGTYINYYSSTKKSMFFLLFVFFLKRKYDRKEKKLYHKRLGIRKI
RLNEKKKDNIYDHNHQSQHSLTNNNMPEYLEKEESEENTAKHKKEEEINQGNRQFLTETKYYKRSKYSKAGYI
KEKDHNNNVIEKHEEEKKKKKGLDIFDDFINDRYNIYYTENKEDLVEKMNEKKKKENKMSLPDFFILNYYLDK
NRKNNLDLMINEGDNHLIKGGSALTGSFSSTMKNMLQNNVLQGKAYCNNGNMDNNTKSNSSDGSSSDGSSSDG
SSSDGSSSDGSSSDGNSSDGSSSSSSNYKNTQSYSKHTEFINNYNVIGQIIGVRGLLGCLKVVSFTTFNDIRF

EPGSYRYIFMNNYNYPLPIKILDVKESQKVSFLYIKIEGINTRSDALKLKNCLICDDKRTFPDLGENQYISTD
LLNFDIHIFNDFSNISIGNVNGFLSKYDYIYSKSVQEISDDLIKIHLKKNISLEKVFNIINVAKLYNQNKNNS
INIEGTQNNSHIKNIQAIKVLINKSNTYNTHEEDIQENNIPSEPIKNDKNYYDSLDNFDGYSYKKIFKCDYCD
HIFDDIKEASIHENSHFSSDDELLYNRTKIDDSDKQKVYEVTKDQARKLKNVEYFLVPIIKEKTIRSVHYEDK
KIYLDISTIFLIDDNK*
>N.PF10_0314
MKKRNTLSTSYGRLKGRVSRYDMNSSYNLNEHKNNKNSALEEIKDYKTGETSHDNINGNNINNNNNINNNNKI
NNNKINSSNNCALVNSSNTINNNLNNINNINNISNTNHSVNNSTYQSCNNKYNKNGFVYDYNTYYTKKSSDLK
GKLKNENESMNSDGNNDVTRINSNHSNNIVLNDLNNSMNSLNIPKDNNISHVNNMNPINNMMKGINNRNDSVN
THMYNMKNLKDIRLYNHKEKKNVNEENVNVDQDNEEKNLVETQNENDKRNEKKEEDDDEDVDEAEEKEEEGMK
LLREKICFKMLKSIDIYITEIIMKSCFVTVYKMKDDELKWKRADIEGFLYIVKRSIKPYYRLIITNKKNEKHL
LQDIDTNMNLSTDQNYIFYRIINEETNVRNIYSLWFYSTEEKEQIYKVLKNIVEKAYQEKVGITQDNTNNVSN
MNNICNVNNNRNSILTNIGSMKNGSVTMEEKLEIISEQMRKMSLRPENKESHKIIQNEHIEDNNHNEANIYNN
NHNEANIYNNNHNDDNIYNNNHNNDNIYNNNHNNDNIYNNNHIGSSNFLKKSSLETNLISPNLINNSTIINQI
NNMTSLKNMKYNKMLPLKPDYNVGNDKEHILELIKNKNDIESIKRKIMSAADNTFPDAYLNNYHVGIINNMNN
MNNMNNMNNINNVNNINNMSNINNMSNINNMSDINNVNSLPSINNNNNNNNQSGNTNIYNPNINVSTKKDEKA
GRKLLYLIKGLSMVHDKNTEQNIMLNDELNNNKSPNILNKSYIDNNKNNGSHNEINSKKGGEAIMSLLGLSKN
VDTKLEDDKKKNKKKKKNNSMNNSVLSSNNNMIENVDNMIKNNDSIIKNNDSIIKNNDHMIQNNDHMIQNNDH
MVDSNNNYYISHNNVDKNVGDIVNLKNAQANLNQNIMYNNICKKKESGIGNVQKYENDLHNNSSFKYSKNLNK
YENDDIVTLYNNNNNNNNHNHTLGMSNEGEEKEMTTALLNIIKMKSSKTNVNNINELNNINELNNINKLNNMS
RHNDNNVESNVLNPYNTNSYNVLLKMKSEIEKDEKKIINMIDNNHNNKNNNYNMNNKSSFNNNINNSHNNGSV
FEETNHIVKYEKNNNKYEHTNNNISTNTSNFKNKSITLNRNTIHNIIKEILQSDEFIDLLWNKLTANQHFS*
>N.PF10_0314a
MDDYINEDADIKHRAWLLHFQQKRNNYKFNLTKKKNKKYIIKWIKEKDEENILQKWTQKKILNEKYNSEDDKD
EKINKDDMKVHTNVDATNPSRRSYRLNLQQLFNNNLTKKNYVDPASEDSSNSEIEG*
>N.PF10_0315
MTFYKKGYRKLFIHFNNNVIIKHNFSTLDINRGSLKKKQTKEKFEEVWKNIIRKKYIKKIVDIFEIEEEKKKK
KKKDYYVNVDLYDKHKKCNNHNKYRKIDENVKGIQLVDGSKKNIQNDEYTCDDNQIVDPPRTCYFVVGSEGVG
KKFIIEKAKEIFVNSPHNVQYGDCHNSEMKKNKKKKKNIFFEYDFKEMNDIINFSMKIYTLEYCLRYTLLKEL
NDDIIHNYINLKDIYDNMIYKDNINKSYNMLSRWRDFFIHIYENPQLYDYFNEKDLKEINNYQIMIKENKYNY
ETWYAFLEILLKKLKVKAFCNYFNHFSAYLYFFKMLGDYEEYDYYKKNTNNILINYTNGKFIYTYFLYILGNL
QHIYNYNFFFLFYNFHLFLLSTQPIKDFNFFVNFLKQNIYSHTYRFPMVIHAIKNLEIMKSIFMYNNIIIKWI
RDKHLVNQTKSKHIEKAQHNDLYLKKIQCNPNIKNINNKNKNNSNNNMCFSKDIQYTVPERINNNMNISSFHN
LDDNKYAESQQNFEESTFNMNNIIKNKTDKKKENIETYYYYKKYQETLSNKQLKDMHINTYNTSEEYELIINN
IIEIDDFSYDMIKSILIPHFTNNQDICNYIYKYIGGNIKLIKTICKSLYELNQQFDECEIIKQIENARKENIT
YDMDEEEEDILNSPKKTIEQIITYKKNEKEKLFLKDLCEQVLHNFILNFEQKIIQFFSLPNIEKMKINQYDKE
ANDKYNNNNDDDDDKIKQTNDNKSNHEHHIQNNKPLNFIQFYFTIFETIRYFLKKQKVFCYNIINLNNPILLG
LIDVNIIHYNYQDKYLELTNKFYHILLLNYIELKYKQYPFKLRVQYNVNYMLNYKIIEHEYKLLECKN*
>N.PF10_0316
MESAVSECNIYKKEKDKNIIYKQERKCCICMVSDFFYPNLGGIETHIFELSKNLIKKGFKVIVVTNFNNNRHG
IRWMGNGIKVYYLPFQPFLDVVSFPNIIGTLPLCRNILYREKVDIVHGHQATSALAHQFILHAKTLGIKTIYT
DHSLYSFSDKGCIHVNKLLKYCINDVDHSICVSHTNRENLVLRTESNPYKTSVIGNALDTTKFVPCISKRPKF
PRINIIVISRLTYRKGIDLIVKVIPLVCQKYPFIKFIIGGEGPKRLLLEEMREKYHLHNSVVLLGKVKQENVK
NILQTGHIFLNTSLTEAFCIAIIEAASCGLLVISTDVGGISEVLPHDMMILAKPNHIELCKAVDKALKIVQKV
DSNLFHERLTKMYSWEKVAEKTEKVYMNVLNYANPSLFNRIRKIYEINTVFSIIYIFIIMISYIGYKILKRSL
AFLIFMMTKIKMKNKSFKMSIYTT*
>N.PF10_0317
MDISGPEVWYNNLPNVTKYVITLIFLVTLLITCNLLNVVYILLDWNLIYYKYHIWRIFLNFLYVGKFSLSWVF
FMSLFAQFSSSLEKNGIFTSPGSYLYFITIQCTFLSLISILFYWPRGYPFLGNSLLFAIIYYWSRREAWSHVS
IYFFTVKGYQLPFALIFLHLIMGQSLWVDIMGLLSGHVYYFFREILPREGGPNLLDKTPKIFDKIMLKLQEFR
LNNGIRNNFSRYGYTNINNSRGTNNNNGTNRRVFIGRGVRLGDS*
>N.PF10_0318
MIVCKKIFNESTKLWKPKYLNNNIKWYQSINKEKGVNKIYTQKKLTKREKLLGNIKPFINDKYDNPECNVIAY
YLNNFLNLNILKSAYENIGCNVLYKEPYLYVESCEYFKQQNSSAVFFKNGCVVIWNMNKKNMKNFLYFCKSYI

NINENIDNYDFEELEVQNVNNKSYVSNSIIYLTMNNYRITDKISFSFGLLSAVRLNNLEKKIENKLLYENNNI
ETLKKKIKSTNLDLLSKQLFSSKITLHNLRYELNIEQDILDVPETLWELEYQKKLFLNILNIFDIKQRVDLLN
HRLTWTFDYLNSFLDYVNQKHSSRLERIIIMIIGLELILGIMQMIKTVDLGVHKKKES*
>N.PF10_0319
MKSRNNIHHHKKKNEVNNTLEEYKDIIPDIEIQIENLLKKFHITKYDYKITHFLIDIIQNESLKILKNAKHIK
KNIYYNNYNDNDDNINYYNINDEHINNNENNDNIYNVKENAHINHDTLYEEKYIQKEEGTINHINNNTDMENI
NENNSPNKCSSHMLNDLLNNFNEDSAIEKNSHIDVVNNMEDEKVNINDAEKIDNEKINDEKNDAEQINDEKND
DEKNDDEKNDDEKNDDEKIDDEKIDDEKNDDEKNDDEKIDAEQINDEQINAEQINDEQINDEQINDEKINDEQ
INDEKINDEQINDEKINDEQINDEKINDEQINDEQINDDNISNKPELSLSDIFNTIECTSNNIQEQENKDELI
NKDIPSNNKTDNKENNHIPKNNPLNCNDNNQKNLQNIGNNKNKIRDEVLIIDEESVNLAIKEYVLKTIYRKKN
VDFLYEKLEFQQKDNLMVNRNIKYPSGLPPYLPDDCSVNTILPSWDIKYNFNSSKRKIKK*
>N.PF10_0320a
MASYNVLGELTTFNEDETIITHQYSRIKKIENIEKCKKLKTLKLISNCIEKLENLENNVQLEHLELYENMIKK
IENISMLINLKVLDLSFNKIKIIENLETLVNLEELYLSSNKISKIENLQNCKKLRLLELGYNKIRMIENIEHL
TNLEELWLGKNKIEQINLPYLPKLKKLSVQHNRLTDICEKSIKNILCVEELYISYNKINHIIDTFKDLKHLKV
FDLSYNEINNISICSYLKSLEELWLNNNNIDNLEMIKKLSTIENLKTLYLEKNKIQDNLKENYRKTIIHILPQ
LKQLDALPIPSSPTLQK*
>N.PF10_0320
MDDSLNKLQDKLRKERTNAQFILNKCNFNIIERYEDSSSEEENEFENNNSKGKSDLKNNDEENNSLYNQKKDN
ILNDNNNNENTVSKNQSSTNNQAYTTYNNYKYLNNNVSFPLALHRSILNLQKNNKNEKLQKVQICENKEVTIN
KNNNNNNYQKNRINYNNIINLKKNVKYNDNNSDEHIFMNAKKIYINEKYQDDAQSKYNLTFQLNKNEVAKLEW
TNPVKDDEIRIIKNIQEIKLHEIRFDFQGKLRIQINEKLYNEKENKIIFNHFNGLYHHNEEPLNSGYTLPEIL
YLCQSSYNNQVCISLKILKHVFINMHIKLLYIYPAFQNIFNSNFMKNKYSYGFTYKRFINYINNDLKIFEKLL
IIFNYYYNKNVEINCLHALASYLFPNNIFQINDNVLFNENMDTNNKQEKYNYIFFQDYQLFSYLNFFSDLAFF
VDGYNIYFYSNKKYKIKNFKFSNVNNKNGFGHNILNKSGQKEDQNIEEDIPDDLEDQMGDIYFLDENNDIYET
KINEVDKNDVLNGNNDISNGNNDISNGNNVISNGNNDISNGNNDISNGNNDISNGNNETVHNFSNFLKRYREK
DSSMLLKEGENGINDLYINKIKRWNTKKKKQINDKNVDINASYINKNSPVDNDPIIHNDPTSKPNNKNLMSFF
EYMDTINYDPLKIRNLNFEMIKYCYMNKYNYNKILNNINNILTNNFAILEIENSCVCFLIGLLIKKKQEINIL
KNSDLIKNIEKILESKMVGTDLYQERLNKQNNNKNNLLYSHEDIFINPIHINFIYNLITLIKYIIIYNYDKEI
LNKIDILPFLMFFRTLPFFINNKNKEKDINENINVSTHKNIKTNTHTNKYINTPSNEEAYFFISTEVIKIFRL
LLYCNIYVESVDYFHDIINYVNNLVVYKKKKNEECHVYKMFLSQIYLYISLYNIMHADKELSGLLYQNKIMTT
LKNYVNNNICSSPIFDDNIKSITKWDQYEKDIPQYEKHDRLNDLSKNGSTACYTFNILCDLELLHQCYTYIYS
IFIVTKKMKDNKIDDIKKNNHLNNNIDNNMKDKEDRFINNELIKDILKSIKVVVIFIKNNLKHLLNFYIEKEE
GNKTKNNIIKTIIKEIWEECPFPFNYVIYNKRETNLYLFFLVYIQILNVILNIYYILIHMEHINEEAIMLISI
KSIFEDIEDEILIYFMMNVFKEENILQSHNIFLPLAYLFYNIYKIKCFYNTSNNNNNNNNNNDNIIKVEKRNM
NFHLLLFYSLSYSSSLYLNFECLKEIFNNPFFICDNIKNDTPIKRITLDNVDNIEAQSCHKKEVIVNIKEDLI
NIENNKTQINIYKEDIAPTYCNLQKNKKEHIYILTFLKKYISSRNNKCFLFFQSKKNLILLFVINIFKACCKE
ANQIKKEEKLNIEKNLELLLNQYFIDFLSNHMDIDIFLKIFVSIFMVDNSKSIGDYVSEERNIYVLLERIAYI
YIYKRISIASDTPQKNNIKCGKRDNTHNNNNNNNNDNNNNNNNGNNNYYYYNECDNMNGFTINLSPTLFLEKI
KNIITTFTNNNNSDIRKDENVPYNMQHLNKFRNENRSITCYNNQKEILINQNILVIIYEQIIESYKRGCFFNP
LFLSFLFFFSSSFFPNVCRELFFNDDDLIKMLVKNVFIHFYDNTHYIIFTTSTYYGQEQNNYLINITHLFPPL
YSFIKNTTEEEINLNTNLESYFKSVKKENLYTSLLHFIFYMKQAFSQK*
>N.PF10_0321
MLKHVFFCYRWRAEYGWVKSRLNFLCGASCYSSNINMDKIIDENKYEYIKNLRSQELRILSENCCVKKVDDVI
IWSEICRHSIEKYNSFKYFDALLLLSSFDKMNIVDKSLYKTFSDVFIKQISYLKPEHFILLINLYCRVNIFPR
VLFTEIFHGIIKYCNKLYPDEYVNLLTCFANLKIANKDLIKTLCKSIIKNINLFDYIHLTNIVGALRSLEITD
DIFYYVIDQKQLKELKFLTVQEIFDFIKKIKLLQYSWKLYEQDLMKEFLFKVYNFKNEKDVDQLDDPFVCLNF
LVSKGLLQGNNNTNIKKKNNNKKNNNNKKNNNNNNDNNNDNNNDNNNNDNNNNNDNNNNNNSSSDFIDDINCG
GANFLVALSKWCANQVYHYPSRSTKRPTSYQLIKLYELMKEYNIHNSDFIEKAIYRFVITRGGLENNRDKMFK
PTSYQKGRKYIFTKDPQIDHINYEKNKEHSYSHHYNLKDHTTDYEQQNMHYQTYDYNDEHNMNQQKTLSEKHK
RIKLSMEKKTQNKKETHSNSRYCNFKLRQRPKRIKNSPAPIKV*
>N.PF10_0322
MNGIFEGIEKRVVIKLKESFFKGNRNVNSFLDIPKELWEEKLKYIGCSIVSEISEDKNERRGERCRVYLLSES
SLYIFDDSLFIKTCGKTRVLFFIPFVVDLLIYHMDNVGIIEKNCVYDETFIENEKFHNIAEFIKEHFLYCFFT

HMNYRNKTKDGYFEQEYPHKSLEDEKKFFEFFFKNVQMYNTHLPMEKMHYIFFYSSDDVHMTDIASTFKFCSE
IHLFGINKYNEKNQFHDAYLNNKSLNLFTRVHEDNLKLYDSSDADKEVTTHIYSTRGTYEDTGMVNCVDVIYK
NESTLLNRNNIENIPSIENKESNNNSRCCHNNNYSGSCHNIVSVVPSERNNDHVHHRHYEDTLNRSNISAEDN
NRNAQPEKEKDEDVRRDDEENKVLIKMIDTNLYECINYNKESFLYNEFYFTPCGYSCNVSEKNNYFCVHYSPE
DSVSYVSVEVSSNLSCDRFLDFIHKQLNFYNGKYMFMINYVFCEESNNMSKMVPDDDNNNYSSGKSCVYYQDL
NKKEKEEYYRLNKKLRNDLFINSKQFYELHTFTERTVGFMRVQYFVYKLRDVVKCVEKETLLARSSSCLFMFN
NIKRNDVHDDYVTKSSNGGVIKQLTERDVDDMYEYALNFCKQNKIVVVDTNTFFDASKRKENLIKLEKVQTNE
KDEYEEKDEVYRRGNNELSSLDHLDSKNNLIHMYYEKNKCDIINKDDENSTIATNNNDNNNDSSSYDKSITIS
RSSSCNNSHLSYSSFDNNHGNEKMKDYISVDENNNNNNNNKNNNVLLTLQRNSDDENGKDKDNEKNDVSLENN
MEKNYKEEIWNYYTKNKVEVKTLEKVLNENIDTSVVCINLQKILAQYVRFKKNLPHVTPFYSVKSNNDEVVIK
FLYGLNCNFDCASIGEISKVIKLLPNLSRDRIIFANTIKSINSLIYARKENINLCTFDNLDELKKIYKYHPKC
SLILRINVDFKNYKSYMSSKYGANEYEWEEMLLYAKKHNLNIVGVSFHVGSNTKNLFDFCLAIKLCRDVFDMS
SNMGFNFYIINLGGGYPEELEYDNAKKHDKIHYCTLSLQEIKKDIQKFLNEETFLKTKYGYYSFEKISLAINM
SIDHYFSHMKDNLRVICEPGRYMVAASSTLAVKIIGKRRPTFQGIMLKDLKDHYDPLNFAQQENKKQDETKIN
HNNDNNDNNSNDNNNNNNNNNNNQKGGQGNIMNDLIITSTNDSTNKKNDHSSSQVIQNVSCTIRDKEGDNIKI
NTHTINNPNINGKENTVDGDNINIAHKNIGNNFSSSNSKLGNITNIKKKVVNINDNRYNYFSYYVSDSIYGCF
SGIIFDEYNRCPIYVIKNKNNPNQNFMNFNLYLANVFGQSCDGLDMINSITYLPECYINDWLLYEYAGAYTFV
SSSNFNGFKKCKKVYIFPESKPSLKGQPNKHW*
>N.PF10_0323
MKVGKIFFLLNILVVCHFIISCLCRNGQTTRGNLLALKAIEQDLQQKKNRKRNLILYSLGSAALIAALVVTGI
GLNMYMKKKNVDSEVQEIIDEKDEKVKEKPAEKKKTTVKIVSKRVPVKSKSSNGKSKARTVNSEVSPKLDDEK
KEDLLKFNDNDLLLAAESLKELNPKYDENTQGNDSFKNINEPRKLASFSLYDALADASEQNKNKDAESSTGQI
PTPTESSHGISDGKKDTSTNDMDPLNPYGSSKRNSSEDKPTSESKGTTPESNFDSKTPEIKEINEPIIVPSYY
PTTGPNPNTHGPPSRRISTSRSSGSSNRSSSGTSTRSKGPSSPLRDSSGRSSGRSTTPRVRKE*
>N.PF10_0324
MVKHKDYKKSDLIRILSSNISKERNKAVKLLKKFEPLPRKHLDNKFDPKNIVVHKNNVLKAFMCWRCDKVKQT
NVKVHWDTSEGMKIICTSCHSNLISLKEMEKMRKENSTNNEFLKNLSNM*
>N.PF10_0325
MHEIVDKNGKKVQKNNLNDEIKIIFTDLDGTLLNSENKVSEQNLESLIRAQEKGIKVVIATGRSIFSVESVIG
EHVKKNRISLLPGIYMNGCVTFDEKGSRVIDRIMNNDLKMEIHEFSKQINISKYAIWFCLEKTYCFEINDCIR
EYMEVEALNPDVIEDNMLEGLTVYKVLFSLPENILENTLKLCREKFSHRINVANTFQSYVELFHQHTNKFEGV
KEICKYYNISLNNALAMGDGENDIEMLSGLTHSVGVHNASEKVKNSAAYVGPSNNEHAISHVLKTFCDI*
>N.PF10_0326
MDDSEDVVGDDEYLDEFSDEGDSNEFLSDVEKNSNNSLDDKSKGGKCLDEESICEDKIKYDKSSIYNSYNNSC
DIFNLKEEVLGIHKKNICCIKLIRNGSNLIISGNDNNVRIYEFKNMNRYEKNYTKLVSLKEGSIIQSLDARDN
YILIAHGNKCFVYNKNGEYIKNSIRGDMYIKDVNKTKGHTRQINCCRFHPLNEQVFISGSLDSTIRIWDMEKE
NNTYGLNKELIHSQCIKVVNEKNLLTNNIISCEFTKDANSIIIGCSNGLIDIRNKLSNDYTYNYKSSDNYYIK
KDVCHKDSIIDILTCKKNKNYFFTRSMDNTIKYWDLRNLHLCLHTIQDVPTIYEKSNMSYFHNEKYLIIGTQQ
KKKNDSILLDEHVNYISSNVNNKNNTHDIKYRQKVEDFENSQNDYDCTEETDIRKNKYKNKEEEEKKKKKKKK
KKKCIINNNYIKEYKGDEDMNNFLTEMSSLNKIEKNIFGSIQIYDIENNFNLVYTKNYECSGIICTYYDEYIK
QLFLGTTDGRCLIYYGDNSKKGVLDYLQQKGYKRKDETNENTFFYMKNDNIYNLDNLPKEIQITQSGNVIIKK
NNLNNKKIKVNPSVQSLYQNAYERKTNVSAYAKFITDDNNNNLNYHDDIKNISNIKDDNIVEVLRNRELNKKG
DDYFMKAYKYTQPNKIIDYSSSDEQEYSNILKRPKCPQCGIKNCVCGYMKNK*
>N.PF10_0327
MRIQIKGGIWKNCEDEVLKAAVMKYGLNNWSRVASLLVRKSAKQCKARWYEWLDPSVRKTEWNKEEEEKLLHL
AKLFPTQWRTIAPIVGRTAQQCLEHYEYLLDEAEGKVYDKNKNPRHLRPGEIDPAPESKPARADPVDMDEDEK
EMLAEAKARLANTKGKKAKRKAREKQLEQARRLALLQKKRELKAAGITSLNYKRKDKNKIDHSKEILFHRKPL
KGFYDVKDEQNINDDIYENNKTNQKKSIKSMDVENINDAMEYNKNKGKRQHQHNNNEEANLLSTIENYDKQFN
ELSHLRKKRVRLNLPEPILNENEIDEIIQINKEASAFNDIIKDQNDKLPINNILPSIESSSFILNNKDKFSNLE
TDFYNNNNNKSIAFSSKLDLSIQQAAKNIISRKMNIPFIGMNNDYNEEEFERKNNIFQKSNKNVPDDLEYDDT
NNYHNNNNNNNNNTSFNSIKNSTTLYNHLDVEKNIKDVQEEIEKQKKLNEEDQQKNQKEKLTIKNKIISDIKS
FKKNISLYAHSIISYKNLKNDQSMMDNQTIQTTEYYDDNYEEKIDRAKLHIKASLANLPQETNLIELQLNEEH
PECDTDNIEKDEIEKDIQDIENEKRKNEERKEKEKFNKQNKIIRWNLPRPYFLDKINLFNNYMHNEYEDVHNL
IQREMLLLIKNDMFNYPLRNSTPVQNKVHVEDLENVYMNMAMKSINEEFEDMYGEASLNNNTKDDSNIDGCDE

KSDNIDGCDEKSDTTNKGDDTSQCSIEHSSYNHIDVWEEINKNIIFCPSKNAYRFIEDVNENDKKENYKYKCE
KLKNLILNDMEHYKKLENKYDIYTKGYQLKIKSYKKSYDTLFNSYINCINEKEALNVLHENEKIYALTRIKEE
KKENKKEIEYHKSLQKFYQDLLETNHQLKETCKQTLKVP*
>N.PF10_0328
MMLEDFNTLPLIKSIREDSLDDVLKFLENNYKDYEVNENGEAKKTIELVDKQTQATPLFYVTARKSDEESVEI
SKLIIEKYAICNPISKDLMKQTCLFYAAREGHLNLCNYLIEKGCNPNDQDNFGQTCLFYASREGKTDCVLTLI
KKGANPNLLDLNKQTCLFYACRDGRYDTVKCLLENGVNPSIKDAQRRTALTFAKGNGHNNIINLLKNVGTLSR
SSIDSNAQLGNITMLRRNSSSYIAASSPNESNLNNLTVQAENVSNVNLKTEDNQLRKKYKLQYKPLSDEDPLL
WVDAPLIKIKEFERKFPELALWPRNVSQSNVEVNNANDAFNKQWYLLANQLILSLSKYEGGHIFEKLVDARKQ
NCPDYYDVIKNPMSFSCIKTKLKKGQYAYPSEFVKDVQLIFDNCSLYNTSNSVVAITGKNIETYFNNQLIVMG
YNNFILKEKKINDMLKLVEEENIKWSKEKEDQIEEIDELENNQEDSSKKD*
>N.PF10_0329
MNKNIIQIYLFVFILLLKQHIVILKNEEFTNPYSIRKKDIKAIVNVNNKLKSINIHKLDNINKKDLLGSYNEN
YILIKLKKQDIFSKKLSTYYGEVQIGEQSENNMNVLFDTGSSQVWILNDTCKNSLCNNIHSKYKRTKSFVYKY
DKKGLPSVIEIFYLSGKIVAFEGYDTIYLGKKLKIPHTNISFATKVDIPILEEFKWDGIIGLGFQNGDSIKRG
IKPFLDILKDDKILTNKNYKNQFGYYLSDKEGYITLGGIDNRLKNTPDEEIIWTPVSTEMGYWTIQIMGIRKE
YVNNHFEENKEEEEVIVKYEAFHDGGKNSIIDTGTYLIYAPKNTMENYLKDLKINNCDEKYNLPHLIFQIKSD
EIKTIKGSAIIEIVLTPNDYVIEYVDKKNNTKECILGIQPDEQSEEDNVDGWTLGQVFLKAYYTIFDKDNLKI
GFVRSKRNVTLR*
>N.PF10_0330
MNPQTSLTRKQCDFTKLIMAGYDLELNNGSTQDFDVMFHGPNGTAYEGGIWKVHVTLPDDYPFASPSIGFMNK
LLHPNVDEASGSVCLDVINQTWTPLYSLVNVFEVFLPQLLTYPNPSDPLNSDAASLLMKDKNIYEEKVKEYVK
LYASKDLWEQQKKEKKPSNINGNISPVSELSYVDQDVQDIDLDNL*
>N.PF10_0331
MSLNIQEQQKNSAISMLNLNEYQENGNNRGNILYYSHDKIWKILIYDKEGQNILAPLLKVGNLRHHGVTLNMN
IQRERNSIPEVNAVYLIDNNKENIDKVIEDMVKNMYGSYYINFLSYVSQENFEYFASECVKNNIVSYISRITD
RYIKFISLSSSTFSLNIPYCFKILHETNDELIKDVINKITEGLISFLVTLGVVPIIRVSSNSSNPSKMIAQKL
HEKIYELLNMRYTNNYVFNSKNIQRPLLILADRQIDLSVMIQHAWTYQALIHDVFDIKLNKITIQAVNCNNNN
MNNINSINNINASYFNKKDTNNCVIKSYDIDTNDTFFKNNCHKPFPDVANNISECLNSYNEKMKNINKNEHTN
NANNANFINHSSNNNNNNITGGLMNAMNILPEMTEHKRLLDMHTNILTELIKEIKERELDKFYENEFDFECLN
DKSCIQYMNNILNSSKGNPMDKYRAFLCLYLAKKKNINIQTIDTFINQLKNLQVDTSSIQFIKQLDKYKSMNI
NINVTNNNTTTLNHSNSTTFKQQLNTYSNIFIDKGYNILQGAKNLLPKRREIKITKLVETLLENKPSSLNDQF
MYIDPKTPPNSNNNDKIFIKQENIKECIIFLIGGGNYIEVSALNDLEDKLNKKIIYGTTDFVRPENFVQELNQ
IGASLM*
>N.PF10_0331a
MMKCTYIYFFTFYIFTLVICNKETYITLNEYEKKNNFSVRVLKESSLNCGLEMDDEAEIEMRTFWVNIMPQIL
SDYQFGYAKQKFKVGDHNIAPLNKGLRGMCIGELRRIGISIGNIGKIYYEITLINYTKKYVPQNAEL*
>N.PF10_0332
MKNKVVIIYFLVYITYVILAKSKNDLKVTKAYLRYGKNDQNIQNRKREKRTHMYLNNSSLLSSKNNNMNKTFF
KYNKFIDQNNDKEYFMKTFFKNGNIIKFRYTHELWAKKKGVGSSRNGRDSNPKNLGVKVLGNNFAHAGNIIVR
QRGRTFKPGNGVKQGRDFTLIAVKSGKVHFFNRVVSIIDVSEPKPLTFKDIYQENPTIIGLSKMWMSA*
>N.PF10_0333
MSQNKLYKTNYGYNNPILLNKNERNIKTYVNQETMTNKLLTTYNRSPLQSNYEHNIPKIIHSSTIKKQGVINE
NNGDKIYKPNYRDMNNLYNSKYVFSKNTPEYINGRKQMITVYDNIKNEERNKSINYYKNKNMIKIPSSYNSNN
GNKIKSNNVKDGVLQLEKGSIQNVAKNMNTFHNKSNENGKFIKKLNNSNYKEVTSNVDEIRLLNKSLSAKISS
SIKTNYEKSNILDHKKNTNVKVSKISIENKKYNIRKKENMKWAPKYNNNNNNNNKDHKNMIIESVKVVKNPKE
INKKKTKIVQNKYKNPLPSYICLHFHDNSLNLNNGLKIFNDMITVDKNQRMKKWNGYEWDVIEKDFHFLIKAR
YDNKGNIWCINKSYEVLKIMRNKLKNFGHLGKEEIIDIGFDKKNILWCINRKGQLLKWVKTKWDNIQYKGFHK
LICFSFDKKGELWAINLKKELAIWNKKNKCWDEKYIKDNLKISCIDFDGDGKLWVVSNSGALLTYSKDQWINF
GLVCLDELICISFKKLQE*
>N.PF10_0334
MIRNNKKYIRFMQSSFCRFSNIKTKAYDIIDHHYDAVIVGAGGAGLRSALELSKNKYKVACISKLFPTRSHTV
AAQGGINAALGNMTEDDWRWHAYDTIKGSDWLGDQNAIHYMCREAPDSVLELEEFGLPFSRTKDGKIYQRAFG
GQSLKYGKGGQAYRCAAAADRTGHAMLHTLYGQSLSYNCIFFVEYFVLDLLMLNSNECIGVICINIADGKIHR

FFTPHTVIATGGYGRAYLSCTSAHACTGDGNAIVARSKLPLQDLEFVQFHPTGIYPAGCLITEGCRGEGGILR
NKEGEAFMMRYAPKAKDLASRDVVSRAMTIEINEQRGCGPNADHIYLDLTHLPYETLKERLPGIMETAKIFAG
VDVTKQYIPVLPTVHYNMGGIPTNYKTQVLTQNVNFNKQTNKSNEDIIVKGLYAAGEAASASVHGANRLGANS
LLDIVVFGKRAALTIMEIDKPNIPKINANTNIGEESIQRLDHIRFNKGSIQTSQLRKKMQICMQKHAAVFRIG
PLLQEGYKQILEICSIFKDIEITDKTLTWNTDLLETLELENLLTLASQTILAAVERKESRGAHARDDFPERDD
KNYLKHSLTWMTDRNIENTKYFTTYRDVITKPLDNEMEYVPPVKRVY*
>N.PF10_0335
MVNQKNGILSKLTGLTSTLFKLNIEDINEEVDENTNPLEKAILYGNSNVKCRKILFLKDIMKLLNHINYENFL
KYIYPLIVKLSNDDIEIQNEVCNRVGDLCAYLIQNNNHNDGCTDIMKLLFPIVEKFIKNNENKNIIETIMKSL
LILSTHINWKYRQKFILPFILSLISNDKYKYIGFILLIKLCSIFERNIFCSFLIPSIKSILRKNDHLENKIYI
SCYLYNICKMLKKEDDLFHIFLLLKNLCNDPSDTIKIINIFNSTHISLLYSKNIFFYFFVPLYNHFLRNENIY
VFYYSLINLFYFICIFEDINLIHPYYIHKMIFFFNNMFSSHTFTLNYLNETAKKKSNYFFLNNNNNNNNINEQ
NRFNHITLKYQASGIQMNNNINNNPNTKLKDMKKKNDILYHSTYEQHKQNEEPCLIIDEIEDIDNAFKIKAFS
DNVCDIDVSFNVPEAIKQDHSDGCDKLIKTGKEVAECLYVKRDMEQENLNFCMMQESYKSEHMYDQRGDMYEH
HEMLHGNYEMYEMNQTKDDYSDQKVGDLNTKLCNQLYIENHKKGSYNIKNNVDIYTHLSKINKETEINAQRCD
NEFNDIFIKGQNKEDKLESNDKYSKVSSKENEENEKINQTLITNGLKNDKTNGLSYIEDKINFICMNKLTNTK
RGHNINKDENLHMDNKKNNNLLDQNISNDVVLKNDCHFLNDNFTTVPSIFYNNNLFNDLIINNNHFSISIKQF
FEMYSNNFKYKHFVVMEKNENDGSTDSNDLFDDNNTIINIEKRNSMQARNNPDYDYNTFCIPMNDEVLQDIDN
TFIHDTNIDIDIIKSCYYNESELIRKNVAYGKMFVKRKNEDNKNKNKNLSSNNINYNNDETDDIYNVGKMDID
SIKQKFIFDSTNNVIVSHNINAVYITALHLPLLILVFKEYFFRFFSHIFYFICTYPYYIIRKTMAYNFYDILK
NMFDSEFLKIDMGKFKKRGNEKRFNKILQKRNNILYKENMEKNILSEKYMKDEKMNNNKSSGGTKKESLDIQI
NAMFNNDYATYYNMRRIQNMVIDDEKLEKEKDDNKMEEHDKDENKKEKEKENENEKEKENENEKENENENEKE
NEKEKENNIIKGELLKENIKIEDNIYSEDLLIDNEEKYESEKYEEDKTYQSSENNIYDSNNYEKHIRKLQNHI
NINLYESDFFVNEENENFFFYKQFLMKYENMYKRHIQTFKTYYKEDDSPCFFFHFFIYYFLNDSNVLVKKAIL
KNYDKLLTFFPYHIKLILISYLYNVIDYKILNYSLRKRVSKIVFKILQNVNDSKIIRDYIFPIYLKLCKDEVA
TIRTYTASYFYIILQKGCPNLYKFFKGKGKILDIEEYQKDIFVSNENGFKLKSEDFIRGKEVTLIRKIINTFA
TSKYYYDRQLFINICDGIISECPINLFLLYFLNPFINLSDDKIQVVRITWEMCVYSQLKKKGDFSNIANVYKK
LEEMYLKYGKTTVSKINPIKYNNPDSIFIVSFDLHHISN*
>N.PF10_0336
MNTCIDFHKKKKKLLIHPIHLLNYVYNYNRLFCCHLVNLPLYKKRSHLKNEKDNLKRYKNYETHLKCLNYDFL
THTNTFYNIRKKFTTFININERQNHIANIKNNIQSNHKKENNILTNNIINNNNIYMNNSHKIHGLLNKQIAQI
KNSTHTHPRSYNFDPCTKTKKIQVYYNCEMNDVIRKINYIKKFLSAGSPVDIFLIYNTDDHIKTRNIKMKKQT
KISKNKISNIEDEQQEEPHNHYDRQIFKNYESNKITKFLHTQKLNETKYSLHLYIKINFILNHIRNISIVDEI
FRHIQNKNHIILIKAYPK*
>N.PF10_0337
MGLIFSSIFSRLFSNKEVRILILGLDNAGKTTILNRLQLGEVIQTIPTIGFNVETVNYKNLKLQVWDLGGQSS
IRPYWRCYYKNTNAIIYVIDSSDSERLNSTKYEINMILKEIDLEGVLLVIFANKQDIQNALSIAQISKDLNLT
SIRDRQWAIFSTSATKNIGITEALDWLVNNIK*
>N.PF10_0338
MFLFSFNTSLIKAKIDILENYAKKNQLHKLRMDDLFEVFKLSKTDEDYKLSLHLLNVYYNFGRNLNTQQDVNL
FFIFILRTNQLNEAKDLLKYFNGWLLCPPSNKYILLCMEEFFKKQKYYDVREIFSFIRENSQIKLDSSFYGIT
IKSMLMLKNHSIEEAIIIYNDSYNMSIYLTNEIHNFVLEHNLYYYHKARSKEETSENIRSLEYYEGNIKNIII
RLINELMKNRRSVKMSSKSLSLFAWTHIYFDIKEIINKSNHTLMDVKECRSWLDIFKLSCLYNQIPECYCGPF
SELFKDILIDMKDDKDAIKALEYVNIYFKEE*
>N.PF10_0340
MMKPLNMILYPHKFFLNTVKCMLAAHIYNFNVELSEDFLYTRTFDIEKSLIINKKPLLIYENNYVSSTKAICF
LFHRLRNKSKKPFDENLSYYLGWLEWSDLLEKHIEVLNKKHIIESLDELELYLKENKNKNKNFINNDDEKDEN
KLSLADIFVYTSLKHSCIEICKESWVHINEYIEKINNLQEIQNVIKNVEEIYKYKNIYSVFISKIHDKNVNEY
LKNETFYITTAINYVNGDPHIGHAYEIVLADAIARYHKIIGRDTFFTTGADEHGLKIANQAAKNNMTPKELCD
KNVLKFQNLNKLLYVEDDYYVRTTRDSHKKIAQEVWDKCEKNGDIYLGEYEGWYNVREETYVPENEAKLMNYI
DPLNNIKLEKMKEPSYFFKMSKYQDKLIEYIQSNPDYIQPEQKRNEILQRLKEPLADLSCSRTKFTWGIQVPN
DPKHVMYVWMDALINYYSSCFIDEGKKKYWPANVHIIGKDITWFHTVIFPTILLSANIPLSKSVFSHGFVLAA
DGKKMSKSLGNVINPLEIIDKYGADAFRYHVIKETKRGCDTRFDVDNLVDMCNSDLADTIGNLVQRTLSLCQL
SNESKIPAFYPEYEIHLPIDILHFINRVEYHMKTFCVQKCCEKSVQVCKDLNKFLTDMAPWKYKDDLKNKKLH

IIRIMLEAIYFIAHYLDIFIPSLSHKIFEKLNTPKKVIADLSPWLNNLTEGVIINNDNILFKKYEVETAKIKL
QKVIIRVCKIVNIINTESSQNTTICEIEVDNDKITAVLYLPYNNNNNKLVNLLTLAILNIKPLTINNITVNAI
IPHVRKEIFTFPIEERIPTGTLIQGKNYKTLVKQRDNLTKKEVVSLDLSIINNKCFFEKNIPLVFASHEDKQV
YHSTQDNGSLTFF*
>N.PF10_0341
MNASSIKNHGIDYLIRKDVNQLEGIYGKIKTIYEDFHVHEITKKNEILHLNYVIDKNIIKNILQENEEKEDAN
IIYNITNKEEHLHILSHYLGEYNRRVFSQFLNILYEMYKLKNENETEQKDNDGDDNKEYHKKYEKCKDDEKKK
DILKEQISCNNKNNESIHTIPYCLLTNLDDLSCDIKIDNNEDIYDNKNIEEKKKVIRKNIHKIIKQYYPFLLT
ETKNVNNIDQVISHGNILLLNHYINDSINHLNTSFISSNNKNINAIQVYPSLDCLKCILSPSIFKKLKGTYKK
SRYNKTEELEYIINQHFHNINKKKLDPSQEINESHLSDSYNNNNNVKTNEHILKARLNISTCHNNDESINITY
GKKRKLDNDGYEKIETLNMNHSMNHTNENHNMYCNLNKKNDIDNTYKLQSNICDSNAPNNNNFLDNINHIKKK
KSELKKKKKYLHFNLYKENKDICEILNKFKMNLSKKNTDISYCGIKDKRAITVQKFCIHKTNKYDIYNLISNN
NNKWFCNNNVYISNLEYKRKKLSLGNLNGNHFKVIIRGVHNNVKSNFHILSENLRTKGFVNYYGHQRFGTKQI
KNYEIGISILKRNYKHSLSYVIQNTELKDEDKQNLITYINELTDHSIYENAIDKKKKKSQHTNEHIKSENSIE
KKNIYIKTDEQQNDHMCSSQMNESTTDTKKKKKKIDISKENHNISVPQEIINIINSISNHSYVEKTILNSIKN
SNNLKNAFMNLPKDIFSLFIHAIQSIVFNLLVSIRMKKFGLQIALGDLVEIYEQHHSGYSSLDESSDDNTSDN
TNNDNMLYESKIIPITENNISLYNIYDVVLPLPGDKNIVLPPNLKEEYIKVLETINLTLEDFKSEKHFFNASG
CYRKIVVKPYNFKSIFIKNELNDLNKIPIIKSDLYKLKNEDKENNTFIKEQLPNKTNQASSQIITEQQNVEDE
LIYVSNEQYHEYLIKEIPDYQTTSSIYLTCSLPKSSYITVALMELLKN*
>N.PF10_0342
MNKIWNITFCLLFQYLCIFQNKVSTNDITKEDKRNLRHRLGVDNFNIKAENNTIEGKDGKKGSEASEKETKEK
NSSNYHESSIDHNDNDNDNLLQSEEPTDSNFGVRDKTNNKKLIESTITNEKTQGKNSYHDDENTIECSHPEAL
VGGEKNRQNSFALRSNSIVTNKPITSKCGKNSSDDSNHDEIIDHHENLQIISLNAPPSPIFDQKDVSIDVVSE
HERPSKLPSDSIQVKKDNRIHLDGHQSNIGSFQLVYEGLHHGNDGVHGATEDRKKARPQKIKGPNQERVERIH
ELTMDKFEAQKENTVSGDGDKRGKVIYDENDDSLISNSLVPETSYNQDYSLYTINILNGLPDKKSNMKNIDLE
KFKDTSIHTKEEGPIVSKIHSVKEEQKDYHKYEKNILHGTKKKSEKVYDLYDYILNWVFGPMKITETNVSEQN
DSLELKQRTSWDRTNIISENEEEDDINNVEQLNEIQGEITEDEVEEEKEEIIEEEEYVDEQKMVNKKKIHDLE
KINKNEEKNVITKDIETTKYTVTHLEKYNNIKKDVPNFFKTIINLFSDYK*
>N.PF10_0343
MNRILSVSFYLFFLYLYIYKTYGKVKNTDHELSNIYGIKYYLRNGLSDKKNGKGQKYQDLEEDENDDEEDSNS
EESNNDEENKLIEGQEGPNSDGDKGPNSDGDKGPNSDGDKGPNSDGDKGPNSDGDKGPNSDGDKGPNSDGDKG
PNSDGDKGPNSDGEHSRSKNDNKKKKSKKNIINMFIGM*
>N.PF10_0344
MRNLFHITICLVTLNLFILEISAKTNTSENRNKRIGGPKLRGNVTSNIKLPSNNKGKIIRGSNDELNKNSEDV
LEQSEKSLVSENVPSGLDIDDIPKESIFIQEDQEGQTHSELNPETSEHSKDLNNNDSKNESSDIISENNKSNK
VQNHFESLSDLELLENSSQDNLDKDTISTEPFPNQKHKDLQQDLNDEPLEPFPTQIHKDYKEKNLINEEDSEP
FPRQEHKKVDNHNEEKNVFHENGSANGNQGSLKLKSFDEHLKDEKIENEPLVHENLSIPNDPIEQILNQPEQE
TNIQEQLYNEKQNVEEKQNSQIPSLDLKEPTNEDILPNHNPLENIKQSESEINHVQDHALPKENIIDKLDNQK
EHIDQSQHNINVLQENNINNHQLEPQEKPNIESFEPKNIDSEIILPENVETEEIIDDVPSPKHSNHETFEEET
SESEHEEAVSEKNAHETVEHEETVSQESNPEKADNDGNVSQNSNNELNENEFVESEKSEHEAAENEESSLEEG
HHEEIVPEQNNEESGESKLVDNDEGGFEEAHHENFSSEVSNSELNENEFVESDKSVTEPAEHEEVVSEESNPE
PAENEESSIEEGHQEEIVPEQNDEESGESGLVDNEEGDFEEPNHEEFEPDQNDSELSENELVESEKSVSEPAE
HVEIVSEKSASEPAEHVEIVSEKSVSEPAEHVESVSEQSNNEPSEKKDGPVPSKPFEEIEKVDVQPKIVDLQI
IEPNFVDSQPNPQEPVEPSFVKIEKVPSEENKHASVDPEVKEKENVSEVVEEKQNSQESVEEKQNSQESVEEI
PVNEDEFEDVHTEQLDLDHKTVDPEIVEVEEIPSELHENEVAHPEIVEIEEVFPEPNQNNEFQEINEDDKSAH
IQHEIVEVEEILPEDDKNEKVEHEIVEVEEILPEDKNEKGQHXXXXXXXXXXXXXXXXXXXXXXXXXXXXXX
XXXXXXXXXXXXXXXXXXXXXXXXXXXXXXXXXXXXXXXXXXXXXXXXXXXXXXXXXXXXXXXXXXXXXXXVE
VEEILPEDKNEKVQHEIVEVEEILPEEDKNEKGQHEIVEVEEILPEIVEIEEVPSQTNNNENIETIKPEEKKN
EFSVEEKAIPQEPVVPTLNENENVPPKPSEGESTKPDIVQIKIVQENKPNKKETPVVDGPKHVEQNIQEDDND
EEDDDDIDFEGLSRKDDEKDSSNKNKKKSSFITYISTKKFKKVSQTIVSVMINAYDGVIQVVSTIKGIAKDIV
IFFQNI*
>N.PF10_0345
MKSFINITLSLFLLHLYIYINNVASKEIVKKYNLNLRNAILNNNSQIENEEKDIKYELNEQNDENVNTPIVGN
SMEFGGFTADDEKDMEAYKKAKEASQDAEKAAEEAEKAAEQAEQASKDAEKLKESDESYTKAKEACTAASKVK

KAFETASNAKKAAESALKTNETGERNSRNNFYTTKTKEYAGKVEKDYERAKNAYQKANQAVLKAKEASSYDYI
LGWEFGGGVPEHKKEENMLSHLYVSSKDKENISKENDDVLDEKEEEAEETEEEELEEKNEEETESEISEDEEE
EEEEEEEKEEENDKKKEQEKEQSNENNDQKKDMEAQNLISKNQNNNEKNVKEAAESIMKTLAGLIKGNNQIDS
TLKDLVEELSKYFKNH*
>N.PF10_0346
MNKIYNITFLFILLNLYINENNFIRNELINEKNHNLRNGSMYNNDKILSKNEVDTNIESNENSIHESGHKIDG
EEVLKANVDDITYKKKNVDDSEIPFSGYDIQATYQFPSTSGGNNVIPLPIKQSGENQYTVTSISGIQKGANGL
TGATENITQVVQANSETNKNPTSHSNSTTTSLNNNILGWEFGGGAPQNGAAEDKKTEYLLEQIKIPSWDRNNI
PDENEQVIEDPQEDNKDEDEDEETETENLETEDDNNEEIEENEEDDIDEESVEEKEEEEEKKEEEEKKEEKKE
EKKPDNEITNEVKEEQKYSSPSDINAQNLISNKNKKNDETKKTAENIVKTLVGLFNEKNEIDSTINNLVQEMI
HLFSNN*
>N.PF10_0347
MKKIVNIIFYILYLYIYKRNLVQNENVNKSNLRKGLSTNNSENGIKSLKDEDEHINIIGDDFSAFSYGGYPIY
ETTGSLGTGVESVKAIDGESGTSMDSKPKENKISTEPGADQVSIGLVNESDSSLENDKKKKENVKKEMLGTEK
EGSPDSHDSSKEKLNLNDNSKWSDFLKNMVTFGGFGPTVVHDVSDTLSDISKDEVTQKTTKDIGSTLLDFFLP
LPTKNTNTYEKKNENKNVSNVDSKTKSNEKGRPPTYSPILDDGIEFSGGLYFNEKKSTEENKQKNVLESVNLT
SWDKEDIVKENEDVKDEKDEDDEEEEEKYENEIIKQPEDILDEEEVLEEEILEENKKDTVDTSDLEKKNIPDL
SNDNNYYSLIYKNYKDNDKSEKTAQTLITALISLLNGKNELDATIRRLKHRFMEFFTYN*
>N.PF10_0348
MKKIYSIFFSLFILNLHIYIKNIKCNDLINYNDSNLRNGLLNNSLDLTNGLNNKDNSFIDSKIEEHENKSYQN
KDNNISIVGQDVPITSVDSSKIINANDLEGNSIDDTKGLSVTNSGFDDGSAFGGGLPFSGYSPLQGNHNKCPD
ENFCKGIKNVLSCPPKNSTGRNRDWISVAVKESSTTNKGVLVPPRRTKLCLRNINKVWHRIKDEKNFKEEFVK
VALGESNALMKHYKEKNLNALTAIKYGFSDMGDIIKGTDLIDYQITKNINRALDKILGNETSNDKIKKRVDWW
EANKSAFWDAFMCGYKVHIGNKPCPEHDNMDRIPQYLRWFREWGTYVCSEYKNKFENVIELCNVRQITNQNDS
QLLEISKKDKCKGALKHYEEWVNRRRPEWKGQCDKFEKEKSKYEDTKSRTAEIYLKEICSECDCKYKDLDNTF
KEFKDNVTLLKAVIDNKKNQDSLTTTSLSTSINSVRDSSNLDQRGNITTSQGNSHRATVVQQADQTNRLDNVN
SVTQRGNNNYNNNLERGLGSGALPGTNIITEEKYSLELIKLTSKDEEDIIKHNEDVREEIEEQQEDIEEDEEE
LENEGEETKEEDDEEKNETNDTEDTDDTEDTEDIEEENKEKELSNQQQSEKKSISKVDEDSYRILSVSYKDNN
EVKNVAESIVKKLFSLFNDNNNLETIFKGLTEDMTDLFQK*
>N.PF10_0350
MKYILSISLFLILLNLYKCASISCEIDYTPSTNITSNLNSNLRSCSSNLVLSSNIDSTKLEGTYGENLNDSVS
LTNNINDHTTNESNISNVSNISNESNISNESSITNQSNLSSETNISSETNISSETNISSETNISNESSVPNES
SVNEVPQLEVPKDAVENHTESKDVLLNEKENFANGVETHVDLGSQEQYFGSSYDMNMDTEGGIKKFKNVFQSY
FNQSKGNSGTEGDGSSVFGSIFGSLLTPIDSLLEKFIGSNNTNSDSNVKNTSMGNGQNKYDNNIYLDEEDALS
DAEHYNDGSISLDEEDELSDAEHYNDGSISLDEEXXXXXXXXXXXXXXXXXXXXXXXXXXXXXXXXXXXXXXX
XXXXXXXXXXXXXXXXXXXXXXXXXXXXXXXXXXXXXXXXXXXXXXXXXXXXXXXXXXXXXXXXDGSISLDEEDE
LSDAEHYNDGGICLGEEDELSDAEHYNDGGISLDEEDELSDAEHYNDGDISLDEDELSDTENYYDGGISLDES
DDLSDPESKTKEDNYHLYYWDDFYHEYKPTYLNYHMHYTLYEPNNFYDTTNEETHNFYNTTNEESHNFYNPTH
EESHNFYTPTHDEFNVPLNYNHDYDYNYFENDNYNIQNVKDNLVKKVNDFMESDNLLVNTFKGIAGGVTSFFG
Y*
>N.PF10_0351
MLNIFNIIFLLFLINIYICEANGTLSENIESAEEIDALKTNLRNGYLNNTYFNEENNNLNIENEINNTNYNEV
TEETKEELYDINENIFPDYFFLDIFTENKEQKNEEVPMKIEVVNDGEEVKTEYVSEKNEEVENKSETEIGEEL
TEKVDEKVPEEVAEELVEKVDEEVAEELVEKVDEKVDQKVDEEVTEELIEKVDEEVTEELIEKVDEEVAEELI
EKVDEEVAEELIEKVADELVEKVAEELVEKVDEQVAEELVEKVDEQVVEEVADELVEKVDEELVEKVDEEVVE
EVADELVEKVVEEEGEKVPEEVAEEVAEEVAEELVEKVDEEVAEKVVEEEGEKVLEEVIEEVVEEVAEEVAEK
VVEEQGEKVNKNDLNDASSEEIKDSSDFKESHEELFKVFLELINKNDLVKENLKKITNNLNEMHLSTLYP*
>N.PF10_0352
MNKFLNIIFYIFLILNFSFFQSNATSKEIQKDEQKNLRNGSSINNNKNIENKNDNIETQYEASEYIEKQNDIL
NMYNDEKEKNNNNSLDTNVTKNTVIDNSNKFQSIEDNNVYNKGIFVGTGIKLNDSQTTSDNYKNERYQIDDEK
LKYGGSFDTIFSGFVNLLTPSSPTQNDGSTGRNVPPPSEPNVDTPDPPTAPAPVKVPEDAKLSSSPRPEGPRA
NNRNENNQNTDPYNHYFAWEIGGGAPTYKPENNKNDNILLEHVKITSWDKEDIIKENEDTKREVQETEDTDET
EDTDETEETEDMEDENEIVEDQLQENEDDEDNVNLEDINKNTRNDIFEEQIKLDSTQDDKAQKLISNEYKKTE
EKKSLEDHVNLLFNFLQTNNQLDPSLKDLENELTFFLNNY*

>N.PF10_0355
MIYILSIVFYIFFLHIDIYVNIYSTCFVVNEGNPNLRNNIINDDDLKGKAYNNTIDANNQNIEYNKNLEHNVN
SSHISKFLDIMDQEDKGDNENSHDIKFEEVKKNINKSLDAESNYGINEISITGNDITSGARNNGIISPDDSAF
GGGLPFSDYSHLEGNHYKCPDKNFCNGIQNVPNCPLKDFTGTKGDWASSNVRNFLTVNKGVLVPPRRKQMCFR
ININNFPKLKKTEGKFENFIYSSAGSEAKQLIKLYGNNTEKALQAMKYGFADIGNIVQGNDMIDTPTSNKTKT
YLEEVLGKHYKNVNDPKDAKTWWIQNKHRVWDAMMCGYQYEKKDNKCTGYGNIYDIPQFLRWFREWGIYVCEE
SEKNMNTLKAVCFPKQPRTEANPALTVHENEMCSSTLKKYEEWYNKRKTEWTEQSIKYNNDKINYTDIKTLSP
SEYLIEKCPECKCTKKNLQDVFELTFDGKALLEKLKKEESPVSNSVNALPEPGQITLPDPSLKQTTQQENQPV
VETPVTTAVINEHQGQTEPNKGDNNNERENHESNVGSIQEVNQGSVSEESHSKTIDPSKIDDRLELSSGSSSL
EQHSKEDVKKGSTLELVPLSLSDIEQIANESEDVLEEIEEEINTDGEIEYITEEEIKEDIEEETEEDIEEETE
EETEEETEEEADEETVKEIEDKPEQEIKNKSLEEKQIDKNTDTSEKKGFNNSEKDEKARNLISKNYKNYNELD
KNVHTLVNSIISLLEEGNGSDSTLNSLSKDITNLFKN*
>N.PF10_0356
MKHILYISFYFILVNLLIFHINGKIIKNSEKDEIIKSNLRSGSSNSRNRINEEKHEKKHVLSHNSYEKTKNNE
NNKFFDKDKELTMSNVKNVSQTNFKSLLRNLGVSENIFLKENKLNKEGKLIEHIINDDDDKKKYIKGLQEQQS
DLEQERLAKEKLQGQQSDLEQERLAKEKLQEQQSDLEQERRAKEKLQEQQSDLEQERRAKEKLQEQQSDLEQE
RLAKEKLQEQQSDLEQDRLAKEKLQEQQSDLEQERLAKEKLQGQHSDLERTKASKETLQEQQSDLEQERLAKE
KXXXXXXXXXXXXXXXXXXXXXXXXXXXXXXXXXXXXXXXXXXXXXXXXXXXXXXXXXXXXXXXXXXXXXXXX
XXXXXXXXXXXXXXXXXXXXXXXXXXXXXXKLQEQQSDLEQERRAKEKLQEQQRDLEQRKADTKKNLERKKEH
GDVLAEDLYGRLEIPAIELPSENERGYYIPHQSSLPQDNRGNSRDSKEISIIEKTNRESITTNVEGRRDIHKG
HLEEKKDGSIKPEQKEDKSADIQNHTLETVNISDVNDFQISKYEDEISAEYDASLIDEEEDDEDLDEFKPIVQ
YDNFQDEENIGIYKELEDLIEKNENLDDLDEGIEKSSEELSEEKIKKGKKYEKTKDNNFKPNDKSLYDEHIKK
YKNDKQVNKEKEKFIKSLFHIFDGDNEILQIVDELSEDITKYFMKL*
>N.PF10_0357
MFNNVDETYNNSPHCQKDLSLIESIQALTDITSGNDRLYSIIPDNISESDVQIIKDEENEILENLDDNEVMKE
LLINDIQSDVEENNLVENHLNNMENISVNYNLHDNLNMMRNVNNDININIHNNVDNNQNVHMNNNNNNAVVNN
NNVDINNNNNADVNNNNNVDINNNNNNNNNVDINNNNNVDINNEANNENDEVINEEINEEKMEENNDEHTEQI
NEETTKQNVEQSMEETAEENIEGNKEEYIEENNDEYMDESMETYEDNESEYNAEQNEVYTEQQTSSSNVEPEK
EKENEHEHENDNNVYSKKEYEYLIDTDDAEEIIVQHGDKILSLIKIKKVEKPTDQKSEKMKRRGRKKGSMQEF
IEQKKKLKGLVVMPHEMYNLYKGKGKNLPKQWEYEFEEFSDDDSDDESDFESDDEDDDYEDDDYEDDAEEEEK
DDMNEKINELADEEAEYQEDDDYEDMEDEDGDDDDLNYDFFDDENYEDDENDENNLESPEGEENPFTNNPNRR
NMQRIDIFNMILMRDMLSNLDENNVSFDDLNDIYDFMNT*
>N.PF10_0358.1
MLSRVLLWNRNSCCITFIPMRAPVKKKKTQDKKKKEVVADKDKLLETRYLPYIPPAYVVDTVSFFKDKSKLDY
LLALIFSPKNSTDTYEDRVEYQKRFEIYELLRKVKENKYQKHLEKMKEKVFESIKNLPEELYDESIKNGELFD
NSEILFGLKYENSLLKNMNNYKKKLLHAYKILLYLRYPFYLVKKKNPMLFYISESKAVSRQKQINSQKKKLKK
K*
>N.PF10_0358:mRNA
MLSRVLLWNRNSCCITFIPMRAPVKKKKTQDKKKKEVVADKDKLLETRYLPYIPPAYVVDTVSFFKDKSKLDY
LLALIFSPKNSTDTYEDRVEYQKRFEIYELLRKKEENKYQKHLEKMKEKVFESIKNLPEELYDESIKNGELFD
NSEILFGLKYENSLLKNMNNYKKKLLHAYKILLYLRYPFYLVKKKNPMLFYISESKAVSRQKQINSQKKKLKK
K*
>N.PF10_0359
MIPKNKLSDICTTLTLEKAKEDIEKECKLVEKKKQKHDDEKDEGLIVDGNEENNVGENSDEEEIRKWREKRLM
EFKKKRELKRDGVYTEVCEKDFLPCVLKNNNVVCHFYDNSFKRCDILHSHLIKLANKHLATKFIKMEAKNCLF
FMNKLNIKVLPSLCLFIDGVLIQTCVGFEDFGNNDNFKTKDLEMFLYKKKIINNMECSDSDEDI*
>N.PF10_0360
MFNRLFENFEYNFFIKRKKLKYDTKDGQYVKQNISKRNKDYYNIIFLLSLYTIQGVPIGVSSVVPLIIQDKVS
YSQLSILSLVSIPFSIKLLWAPIVDSVYNKRIGRRKSWIIPLQLFCSFTMIYFSNHVSIWLGEKDNIPNLFFL
TLFFFLLFFLMATQDIAVDGWALTMLSEENKKAASTCNILGQNIGYCLSQLSFLTLNNKNICFYIFKKYIKFM
YLIFNKSTYYRNVYEKLDLLYPSFQPFVDMKKFLKFWGTFILMLTIFTCFKKEMSDQNEEKNKLKNSNEMDNY
NISEAQKCDDKMNNAKMNYPKKINNQIRTNEHIKINDIKQTNIQKQSSEINEFANAKDTYKTLYELLFLPPMK
IFIILFLINRLPFASVEVGTNFKMLKRGITKEEFAIFNPLYIPVSIISPAIIGKIIQKLKPLDVYYFGYVLRY
FSNIILSTLLPITKYMYSNKIQSTNMKFYFYYMYIYMAQVFNHFSIDLMTVSSMSFQNIISDPKIGGTYMTFL

NTVNNLGSHWSTIFLWLLDYTDKEFCYKGKCVLVDGFYIQMILSFIVGIIINKYILSFTRKLQAFPLEDWRTK
YIGKEF*
>N.PF10_0361
MVKNTSNETMVLSLKNLSKNKWSSLSKKQLDDKSSYSPRTEKLIEIISPILDYYGYNREDVYNFISLYKFDIE
KIQLELCNIMESKEGEDEGLWKTVKSKKSKKPAEKNNKKNSFTPNNLNKKGSRNIEKLFSKGFNVKDKDVKKS
NINNSNNNNNNNNTKSAVINTGVTTTADQKMVIINNKVNHFPNTQTKNENVTLLPDMNKKSQDDKAIKKNLND
YSNKTLNIKNVSAVEKHQGEHNVNDNVTSQKSKKKKGTSTHNDIKNSTNENANNDLLKYAIQQKDGKHKKGKN
LFKTLNDVNHENFIKHKNAQGFNNDHNNINEYDEEYDEEEEIEQDDVNHQEFHINNHDYYDDEEEENEFFLHN
KRSNKNSYFNYLHKKKKYLKLEDNNNNNSNKLLADYKYTDDHYGKKDLKNKFSHASNLASGHAHFNRYNNFNY
HDKYFNKNYNRYSTTKPLNESLMAKNAKNRYSLFNNNTNNKLSLYNSANSNHINSSFNKGTQKKGLLSKKDFK
KKRTSSKNNNSIDLEYDPYTGQKLNYDMTTITEETNEFDHDVNKKVIENTTVGTNKKKTPTNINTSYASVVNG
NIMKISNLEEHNKNDVSKTKTQQQTLVKNAEEEKNDTKKSKCQLIPIKNNKTEKLWVSVITNGTQNNNEKEET
LRSPKKDANSNKELKNEKSQKENMNNNQSSNKKSVTDAGVIDSQSVNKLGEEKNKEKKKKLKKATVEDSTATK
GQHTTDVKVLNDKHANKKLNEEKKKENKNNNQANKSEQILDEKKLKKKKKKIDQEELQNNESALIKGINNKTA
LNTSVDMKKGKNKLNNNMKGLSSPVSNVEQFVIDDMMKTEEQEGKEKNDIPIYMPNLSLLGNMNKKIFFGNIN
ISGDNIENFNMSENSVMEHMKHNNMLNEVDNNKILNAENKKKINNIRKNYALEASSMGLSNNDNNISSQYYKE
ENNNNMNNRNYDVNHSDLRNQKDREFKHFDFKNKNQKREDYHNNNNINSIHNNDLFNESKNKSTYVSSNKNSH
HINNNNNNNNNNKSNNLTLPKIGASSASNSLSHQQLQTSINNTKHLLNTSTNTAFLNKSSTVATHGSQENSSA
AFMSGKNSKYLQGSFKNKNYDNNDLDDTNSIQLNQISQKFLRSHQNVVNNNISTSGINNNTTSATVISTNNNN
NTSSATNNNNNNNTSNVNITNKNTSSDINNINLMNMGMNNLNNNNMYSTYNSPPGLANQYNYSYGNLSYPYNN
MPYNGYTTQALVPQYGLMNNHYNKSSSIHNNNFNYNINYENFDESNLYAHNNNIHMNNSFVNFINGNNVKKNQ
LNGDDDNISNSGDMTITGTHQAINLTHDNSTNNVNQNATNVNNNNSSNNNNNNNNNNNNNNNNNNNNNNNNNN
GVCVDNTTNSGLGNLSLNNINNNLNTNISISCNTTDLKNKQQLNKNINDLQLQTSTQSLKQNLHTHNKNNSMS
TNQQTIQNKHTGQHQQNLLNNNNSLGQYTQNQIQPNTSQNTASSTSSSVTGKADTNNINNASQNNQNNYISKQ
LYHNSNYYNINSSSFNIPPGFNMSQDKEQNNNYLNNNITTNNNNTNVSAYRNNWNSINEKNLNHYNLNYNYNR
SNKNSFNYTSNCNSNVYNGNRDNNNNFFNYNNFSYALQTPPGLQNYYQNTQYQQQNNYNNRNYNYSAYNNNLS
MWIEKNKDRDG*
>N.PF10_0362
MNLAEKPKAFFICKFLFFYYIYKKPILPFDSLKCKISGKEIPYVCIIQILGLSLLGQRVCLYIHDFYPFFYLL
IPPEEKQNDKLEIELCSFLEEEYGKARKDPSNNVCVYNIERVKRKGIYGYNEECDDFLKISFLYPNTINYFAS
LLKKKLFKKRIWDLYEVHINYMLHFLCMKNIYGCSEIYIDKNIYFRKKFVNEINFECIEKEERWYLGEKKYNL
DNFKRNKYLEVDAPLVFTIKTINVNFSSLKRETTYDIECDIKHQHILNEKIYTYEFEKNKIKWKKEFNFDLPI
NHLDSFAKMWMKEKKRCKYMDMDLKRELFYFKSYENDLCFDTFDVLTERTKKMFQGFLEFMKRMQSEKDKVNY
EQILEYDNIENVKKYNKVINDDMKLKEDHNFNSDMNNIEVNIKQINNNDNINNCKNVVDNEEENDMLSNMISN
KKKDHMGISPNLFIQNKEQEKEKQDEREKEKQDEREKEKQDEREKEKQDEIEKEKQEEREKEKQEEREKEKQE
EREKEKQEERECFIEYVNEKGCIASFEINKKRKDVVRYSFKKEPPRINKCYAVINNLVDHISGRDNKNMKEKM
KGNIYNRIHDNIEDENKEDTSKFEYIGKENHMENKENIRKQYEQIKSDNMKKKINIKYGNIFFLEILTEIKDE
NCYSSDYNQDKIKAVFYIVREERLMNLYEDYNNCMGIIATKPFPNISYLFNESEDMNNLKGTNEVKLNESKLN
ESKLNEEELKLCKDMYHIEDVNLCTNNCRSIEKNRDNLINDKNVINKKNDDTYGSSKELCCSKNGYHTNKIIK
EITNNDIKKVKRTFFDFNINYNNVNICIVENERELIQKLINKILFYSPLSIVSYENDKYNINYINQRCLALDI
GNFYKMICKLNDQKKFLDLHNAYSRNIKGIIIESLYKLSNTYNTSFENLCKHYLNINIPSINKYTLYYWYNYN
TKRKIKANGSSTVVNKDIINNDLWNDMDEEHIYFPYRHITIKHYLRKVYFILLIYDKICFLKRKMNFCKYIHV
DLLSLINRGSQYIIESFLLKMSIKYNYVLYSPSNKEIFDQRPILHTPLILQPKSSINFFPLLVFDFQSLYPSI
LIAFNICYSTCLGTITLKRKLNQMDEEKNNEKKDENIEGSLMEKCETYKNGINDKNHIYDKNHIYDKNELSCS
NKNDLPSDEYTDMMNISEDAKYLIDLLDENINDCISSADPMIEPDIIQSNGKGLGENIPSENMITEDHDKIKY
KLDMNNKLEMGNIKHVNIMNEQSIYGNINKNKLYSNEEKSLESNFEFIKLGVMKNVPSISSRIKNLKSEDIII
TSNNTIYVKKHKRKGICPLFLEDILKTRIMLKRCMGMYEERVNKKLNERMGKLKLILNVATGYIGANFSGRMP
CVDISESIISIGRNFLLFIIEYIKENYKFVKILYGDTDSLFLLNETTDDIQSSFKLAYEILNSINNILPLPMY
LNFEKIYCPSLLLTKKRYFGFSFKNENQDNPILDLKGVESIRSDQCILVKNILIQIYFILFYFKNNCYFSYCC
CCCYLCKNFFSNLICSCKELNVHKTDPCFLFTLLKIVLSICKVRRYINQNEDKYNINNNNNNNNKSNCHHNKQ
NGNKINDISNLSLELYRKVPSFDKVKLYKEVMNWVEYYDNENLLLKTLNFLYREKYSFLIYLFNLFDEATLHK
EIEKIIQTNFEKIQNQKNNKCCFINPSNVFKSCLCVKNINENIKCDQIRCYCNVKQAMFFFYNPTYNKVDYVF
NTKLFYSHLLSHIRQTLSEYFNKIYDNQISCDNFIIYRKVKLGTYKGEMQGIRRKVSLPPQAIVARKIMRDFP
NSIITYKEKVPYIFTKKLKDDKIYSSVSHPHFIRGIYRSFRFDCGSTEQLEISEDNCNNDSEKNGDIEKNDNI

EKNDNIEKIGDIEKIGDIKKIGDIEKNENIEKNENIEKNDNIEKTDNIENFPSSEKSEYCDISENEEKSENFK
NSDMSEYCEYDKNELLYVDGMMEIEELKNRILGINIKKKMKKKKKKIKEINYDYYIQNLIIPPLKRMLDLLPF
CSLNLDEIFYRTKRKFNWNKAKLEMFSKYYKNMDNKLLKNNNVTNKGINLMNILNEDKINNKEQNGNTKKKLN
FLHNVNRTMETYKDMNNKSKIIIRLEEQGENEELKVTNKIMNSYVEINKSQNMLKKLNKICLMCANSEMEALA
CNYSVHCKIFFKRINLQENILKNKDSINRLS*
>N.PF10_0363
MNLIHICLFIIVIKCYVNCIKKNNKHIAYDKFYKQGSNTIVGSGNGSGDLLFLNNININKNVERKKNILAHSE
IGTVRNIDDINLLNHNKKNEISFTKCKQIATIGPASENFEQLEKLYLNGIDVFRLNFSHGLKSIKKYIINSIR
ILEKKYDTTIGILGDIQGPKIRIGEFEKNQINENDNNTFVELKEGDLFSFDLMNSLGNQNRVQLNYPELIKNA
KAGQIILLDDGNLKMKILENNYDTSNIQNSYIKVQVLTGGKLYSKKGFCIPNMIMPIDVLSEKDIKDILFCIN
EEVDFLGYSFVQTEYDLIFLRNIINDYYESDFYNNIKRKDRINFDQNNLQMKTFDDENDFYIKEVNDYYNNYY
LKNVQKYKQIYDVYKNQDLYINDNIQNHYNKNIVTNIGDDKNIIDEQHIHNNNNNIDNIMKNYSNLYNINKDH
NKIAIISKIEKPSAIKNIENIIKLSDGIMIARGDLGIETNLSNLPILQKKLINLCRIKYNKPVIVATQMMESM
RFLPSPTRAEVTDVATALYDGSDCVMLSAETATGQYPILTVSTQNKIIKDVENDYYYYEYTQRKNDKLKMLDH
KNNCCHENKDIQQINKYNHEYIQNNKYEQDIQNNISYFDKLIFSIRDISNNINLKSIILFSNEFNKIQKLSNL
RTKAPIIVITENKYLARKLQLTWGIYPHLSKKQNLFNHDLFSLINYGCDVSKKEGFVNSPDEYSLVTFSKNIN
SANLLYLCQPCLTN*
>N.PF10_0365
MFFNKYKKKFRNTKRKYCSSPEKNERPKKDTNIYSTNKTILKNVILKEYFKAILKKIYENFQKNIYFFEIFIY
MDLIKLIDSLDISGHYMTKDFIQFTNYFNETCYNLLTEFFKNFKANEQRCEDFSDYLEIIINNRFFIIILIPL
NTPIKTDCILNLTKSHKLENNFYNIFELINATLIYKDKKSFLYKSFYMRICECSNIYLPHQNVLVQINNLGHL
NEKNYKEICKLCFTKEYEEITNKREFKNFYILKFSFNKTIDEYNNIDEYTNFEVLFQDIPEKNESFEIGSKYN
LIVVSIPNNLTKSFVKGARVGNLWLLNYNKNNLRKIEDNIISLSENVNTLLSTESFNYLLQSQNLNDMNKIII
DILKLPKNNTCLNNICKITKLALILISISNNFLKYLTLDTLTYDIYNKVNSNKWIRRPTKENNLARIRAPHCF
FICLDEIIIIDIVKYCNYFCNIKLLNVKTDSFNILFTQKYDILVININNLNNSQINILVELMKNGLYKNKKKL
FPITTTFWVYAIKSYIRNEHEFNTYIKDNLLARFDFLFELGFEKDEDIINEILQTADDDKEINADYYFDLNNK
YPYLKKNNIYTFEFNELSDITKSIIQKYFNMASDLSTLTLYHIGISELLCISVSILLGKKECLIEHVVLGLFL
YDKFVSSTKNKLTQFDKNILHNIFNTNHRDTIPFQKLMNYFSSYLNATMNL*
>N.PF10_0366
MSSDIKTNFAADFLMGGISAAISKTVVAPIERVKMLIQTQDSIPEIKSGQVERYSGLINCFKRVSKEQGVLSL
WRGNVANVIRYFPTQAFNFAFKDYFKNIFPRYDQNTDFSKFFCVNILSGATAGAISLLIVYPLDFARTRLASD
IGKGKDRQFTGLFDCLGKIYKQTGLLSLYSGFGVSVTGIIVYRGSYFGLYDSAKALLFTNDKNTNIVLKWAVA
QSVTILAGLISYPFDTVRRRMMMMSGRKGKEEIQYKNTIDCWIKILRNEGFKGFFKGAWANVIRGAGGALVLV
FYDELQKLI*
>N.PF10_0367
MKNYLQKCNVINKITKKPLDIFFKKFLSKPRIKHAIFFSTIKNESSHVKKNNDTYDNNIFKWEEEENSKNYKN
DKNHIDDKNHINHKNHIKDKNGSLINNIKIYKQSELVRRIRKYCRKDDLCNIYRFTDEFLDLLNKQEIAHNDF
IIIIHLLSKKQFLEKDFWRNITNLNNINFIFHMNIKQIILFIYSIVNSFKYIHPQFLNIFTNNLNSVIENQKR
FLLIYREKNKIEIPKKKVQAEIQTNKEMHIIENKANEHINLINNDYNEYKKDQLNNLLCNKNINKQENKLPIY
NNKRNHLNLLDITLLCYSYSKLNDLHNLEHLNELKNNIYKLFLYFLFFYKNDFNHLILFLYSYVKLNGYIKKS
ILIKLKKNILLLNNNANIKFMFPSLYALNICSLNMLMNILHKSQFKDEHFFEEIILSVVINLNVSQPKRKKIK
SNILNGNYDNSDNINIDNLINSYGYEIQTFNNTDMYNIEDYMKEGKYIYSNNEIYNYNNNNNNNICNNNNICN
NNNICNNNNICNNNNIYNNNNYCSYNFITYFKTISSLVKHFVKNKNTYFYPQHNQIVQLKKKDFCFLLYNVNK
LNVKNYYNIYDYLKKEAYKQINILDIYNSCIVLNSLVHLNILDEKIFNKLFTHFKILFDKNIYKEKDIIDIFV
SLITYSKINYNISNEINSFLIHIYNKIEYKLKKYNFKNLLLIFCYSSYINIHFNNSILQDLCIDVLNNFEKID
IKYILIFLYLLKYKNYQMNNFFIDSLIQKVLYKLKGEKKYLNILYTYIFQIITVHSFQSLYNEKIQNMLIQML
IKNKKNFNLKEKIIISLHIYVNPILILNQSCLSFCAETFQNLLDYVYITKTKKYKIYNKMCTKGKHKDNKIYH
HYLIKTNHFKKYINVLTNDTKNIYMNDQKFYQFFLFFFKNNNVQDTSSICNIDFNIDHINIKELHFFFLYLLV
IDIIQGKNNLKHTKKIFHMLYRNMKKYSLLNEKKNIYINNQFYLYLCKYYQLNISFQNIYIKLNNDSFKYFLR
NNNKKLFYHNKTLTTYLDLNNKMKKKYIPNINVNNYSNKTHKEYNNNNNNNIILKKEKINIYKEDIIKYFPH
FEETLFNTISIYYEDLFLYMLSQNKINEENIKKNFYQSSIYYNKHNHSSSIHDNGNIIIKMKKDIFINLFYIP
YVFKPLKLTI*
>N.PF10_0368

MDKLVPIVNKLQNVLSSFISSETLSLPHIAVVGAQSVGKTSLLESLVGLSFMPKGEDIVTRTPIIIQLTNSKS
DDCYCTLTYCDYDNNRVEKHIDDFSILNEILIDVTEEITGGNKCIKETPIIIEIHKNDVLDLTLIDLPGLTKV
PVGNQPQNVEEQIVNLVNKYIKNPNCIILAVSSANIDLANSDSLKMARNVDPKHERTIGVITKCDMVEKPEIW
KKMISGSLYPLKKGFVAVVCRSQKDVEDDITIEMSIKKEEEYFSQCIDFSNQMECIMECGIKNLAKKLNNILI
EHIKNTVPFLKPKIDSLKSIEEERLLELGEPMDNMNRSEYLAVLVNYITKFSQQYQDIIDGKVFYKDRVDELK
GGARIHYIFNDWYIKSLNDFSPLEMLTDEEIRIAIRNSSGPRGALFVPESAFETLIKKLINCLKEPSLRCADQ
VYEELLKIVDNCRIADMERFTNLKSAINEQVKILLKDCLQPTKEMIKNLMLIELSYINTSHPDFLNEHFMKNV
YDKDNDYIDELDTVVHPKHNHKMLQRPKREYQETCGSNMNYSTIHHNNTYKEEMKMWKHKEDAKSFRNKEGTK
ITNINNNRENVFVLPVIPEKIIPEYSSSSKEIIEIDLIKSLINNYFNIVRKHIADAVPKAIMHFMVNTSRKTM
QKVLISNLHNGELFNLFNECSSIKVKRNNCKKNLESLNQAIKMLAEIRNQEV*
>N.PF10_0369
MMNSLKDSSNYSPNLSHTRGSRKRSDIFNENYYAKNLKRRKLKAAKKLKDSTNGEFTKKVKKQLKDYYEMRFD
KKVINLPFSTDSINIQQRGFHDYSKDMKLKKNHMNKPLWICSDGFIYLEMFNSCSKQASDFLITIAEPICRPE
LIHEFQLTIFSLYAAISVGITLDELLINLDKFSKNVLPNELISNITKSAESFGKVKLVLRENKYYIEATNKSE
LDYLLNNDTIQNARIYSTDNNNDKNMISLYNLNNRHRDNNKNDNKNNNDDNNNIDKTTSNLLNEDQKGKDCYV
TYEAPVLDTTQLGFKISESEKQLMMEEKKHANLNANENSTNSAEVYSFEVNCDKIEEVKQEALQTMQRPLLME
YDFRRDKKNPNLICSLKSHVQIRYYQEKALRKMFSNGRSRSGIIVLPCGVGKTLTGITAASTIKKSALFLTTS
AVAVEQWKKQFEDFTNIHPRHIRILTSDYKLDLWPINEAGVLISTYTMLSYSGKRSEQSLRIVNDIRRREWGL
LVFDEVQFAPAPSFRRINDIVKSHCKLGLTATLVREDLLIRDLHWIIGPKLYEANWVELQNKGFLAKALCKEI
WCSMPCSFYKYYLKSNSFIKRRLYTCNPRKLMMCEYLIKYHEQNNDKIIVFSDNIFALLHIAKTLNKPFIYGK
LSPIERIAIINKFKHDSSINTILLSKVGDNAIDIPIANVVIQISFNFASRRQEAQRLGRIIRPKNKANEKKNI
NDPDSFFYSLVSKDTIEMCYSDKRQRFLINQGYAYNVLSDNIVDFNKLNLVYKNKKIQENLLKCILASTDDGN
MDEDDDLFEDQSFKKDNTKVNKTNSNILLNKKEDSLKKIDNTTGGLLKLSSNMDVTFADKKKIPTKKFADKHI
LFRKFLSQNK*
>N.PF10_0370
MNAGYSDVVLLVQFSQKIESRTFVEYKSLKLALNGICQLYEQAIKENDPSVQRITYNMNDLFLYIDNIPKITI
LLFHTPTFSYKPHDKIWIKQKLVDHIKDQIGK*
>N.PF10_0371
MKNKNILHTDKKDKYIEPKENIKKKKEEIKKNEITKKHEINKKKLDIDTKNKTDSDYKNKRDKEGVENKYVSN
EIKYKTTDTDKKNNQLKNSILFDVHITNKDVETKGDTKISQTDVNSFIRTFFQTFLFSAVNLKIDKDVKHMLE
INLSKELSDIIFNKSSSVLINKNYFFTLVQKFFKSDEYLSIKKSLTDYTFDDILMLQNVDTNKHSIVFDTNTY
SYILEKYQNTTETFDLNKETDNNQNNFFDDTEKLNCIFIRPKKYISSNEEEIRKEINNTHTIQNRKKFKKIIY
IKRDKIKNNKHEFVECDKYTSIKNDITIRNAVQEKTLDFYSTNSIVLHDKNSQTYKAVFKNLGIQYNIEFLNK
RKEDILKSINLEKFLHKVFPMIEKSIIENIIILQKEQEQNLQSSDILSFKNIKYLNKIFIYTDVKYTTDKVVL
FTLYLSLINYLIFIIYINNYKNDNFIEGVNTDSYILIWSYNNYINPLYALISPYQISSAIVNENDYNLVIAGC
SNGLLVIWKLPPNFNGISVLNSKINEKTVNIEPYLFSSIEHSHKREVTSLIFLNDKSLILNEKKISISNQKNC
HLLVSISVDGSILIWDATNIEIAETKINMKKKDKELADELYGYKPIFKINSTRPNTEYSLGYTYFHFMELNAN
VSSFFAFSEEGEYIVGNLYGCMQKDKNYSIINKINDDYKSFKTLVCVKRNTLIKYLIITLTDTNFYLWKENEE
HPIFISPKSNEIYSCCEFSQTKISVIFIGKINGHIEIWNLSEQKNHCIYSLTISSYSLTCVSIFQNIDSNIFN
SYVHKTKEEKKNKDDNDDINYDDYDDIIDTNEYLMNMYKYSYNKIIIGDSSGCVFVYEIEDNLLNSSKEEMDE
FLLWIENRIYLNKQKLKRQIELCKEKENMMNKEEKNTNERKEQLKNKLDEEYKNSYEKYKSYLLGNKI*
>N.PF10_0415
MKKISLHNILTNKYNVNYIPKKFFSEHTIIDHIRKVKNKLDRGSLYLLQESINNNHQFIKEEKKVKFQKVINE
LNNLLTHKSYLMEEKIKILNQHNSKGYKNKEFIGLFLTGFFIAIGSISHSIFYLVSVYGIYILHKASKENNSM
IYVEKKLNENKNKLLKNKKNVEDLLTLLENDLITTK*
>N.PF10_0372.1
MGSQIQPSRFLDSAILALFAMIISASFLYMFSEFFMMSHESKLFNNKLSMILSRLFPFKTFEYNLTHILLLTI
CIIILSLKPDSVSSKYGLKDSRKPQGKDDAHKGDEDPNRLNRDKKK*
>N.PF10_0372.2
MGSQIQPSRFLDSAILALFAMIISASFLYMFSEFFMMSHESKLFNNKLSMILSRLFPFKKTFEYNLTHILLLT
ICIIILSLKPDSVSSKYGLKDSRKPQGKDDAHKGDEDPNRLNRDKKK*
>N.PF10_0373
MDEKNNKLFECDILICGTSLLNTLLSVYFSIKNYNVINIDKNNYYGDYNGSLNFCQFQNEHNELENFYYEFLP
FSNLTQIKKKELQDIVQNYMKINNNKFNIDINPKIIYNESNIVNLLVSLNAHTYISFLGIQYFYLTYKKFNEQ

QHHENSNSVNKDHTQNIQNEEKKITNDSSSTILSNNIINQNNNNDDLIFVKVPLNKSQVFLDNNLNLNEKRMI
MNFIYKNIVYDKNYTFSNFSNYAFMKKGPNQSLAEDSQKVKGSLREIKERGTQQQNNNGNNNNDDNNDNKYDN
DDNNDNKYDNNYNKYDNNNNNNININYNNINNNNINKNNFTIPPHDDENVNIINYLNTFNITDKISDYIIYGI
GLFDLDLGYCKHVHNIPEYYLSGFSKETKYVLNKNEFLKRLHILVNSLNKFKLQNLFENAFIYPSYGLNDIIY
ALSRVACLNDSIYMINRKIKHITYSNYNYTKENECHKYDNVQIYNTSNVKINEVILDNGFIIKPKFVISTGSN
INFKEMKKYVFKNECDQNNNIHIKTSRLVVLSTYSFLGQNGLAFYIHKQPKLKHQVKKNLNNSYCTIHILQLD
YSSGSCPLGFYLTYFTYLEIETENELNKQKKKYNNENNYNNIQSSGNVSFNSVPEYNNNVHFFCNNNTYPNDD
KPLNYLLLLDVLKLFIQKQQNDKYSNNDNCSFYDKNVFNEQLLKQIRHFFSTSEFNNMSQLSDINNKINTNKD
HENDEIINKREDQYEYDKNEDEINNILKENKEGSVEENMEGNMEGNMEGNMEENMEENKEGSVEENKEENKPN
DEYFMKQFNQLLKNEGIIYIAYYEYEPTVYRKDTIQLINENLDIYNNIFENIEKENLNTQGNEIKKHNNHNDN
QTISCKENNINNKKDEFNKTKNCSNHCEKKNILELKENQNIINLLFTNDVHNYPIYPLIEDISTFFYIISKFN
QQVYNCKNQTIYDTLYDFKTIFNYQNV*
>N.PF10_0373a
MFFFCNKNLLKYMRRSFITLNGVLYAKRTWQTNMKRKTYRSKKRKKYIRLGETLIRIQ*
>N.PF10_0420
MEKGNPSKSNENYKNLVDCYYLLLKKFKTKSSYVIDEFLIKENKESNGTLTIYEDVNIVSFSYLILSMIDSNY
DVINKINILNVQNANDIIKALCFYMDNVQISDILKKNTKPIRKKDTNIQKNNKTSLKKKETKPNNNAQEEKNN
DIIKKNTCNIKSLNLFNSNIDINGIIFLSKSLYDNKYSKLENLSIDCISMDMDCIKYLSLSLCNYNNLKTLSL
QYCNLNDDSIKYIIDIVIYLNSSLYSLNICGNKFTEKGMCALFESFSLNSSLNIVDASYNMFILKDSFVDALH
KIIQSETNISNISLTGNFIDDNNLSKINEAMNFNKSISILKLPNEVDTEILKQINSKLIKKKRKKKKQIKKKL
F*
>N.PF10_0374
MKKESYRFLNISINNGSDKKKKSNFFVKIKNGTDLKDTKKDRIRFHFYFYFFIVKIILLSLLIWTVIYSNNCS
NGKYKSTLYIVGNHLRFRGFRILAENEYEMKTKYKTLMVNIDDNDLGKRGNKIKPKHKKVEINLNDNNLSNNE
SKRKSRRKKVKVNLKDNLEKREGKMKRRNKKEKRRKEKDNKKMNELKLKREYKESYLLDNKSDMDVIRNDKFS
YFTLYKNNLFPNSNNYFLHMLKTYDEENKGIDENTDINENVEENEYVDKNTEGDKDGDEDKGGGESEGGDGHG
SAGDSAGGDRGGDENKDKDNEENKDKKGNKDDDDEEEEEEEEDDEEKEEKDEKDEKDEKDEKDEKDEKDEKDE
KDEKGEKDESKGEEKFLKEEKMPKKEENMQDKIERDKEFEGFKDEYFTQMEGGAYGITVEHEGQKLRETLTEG
VEGYEKVIEEMEQTKLDEFLLPTNEGKSDDFVSEMEEERLFNFVSYMEEEVKDKMFIEREQGTEEEVIHEDLS
DEKIGIEKKELETFVQDVSDEVLQDEQLIEEEKLTEEEKLTEEEKLTEEEKLTEEEIFIEEDKISPKEVEEPL
EDVLMEKMEKEYKEILHEGKSVVTQDMDEKLLSENLEDDLIPEEIIEELVDEELVEEVTAEEIVEEFVAEVEI
EEKIPKELVEEVMPEEDVDEVVPEELVEEVVPEELIEGVIPEELVEDVIPEKVLEKVIPEEIIEELVLEEVVE
EIKHEELVEEEIPEEVTEEVIPDEVVEEVTTEELTEEVIPEELIEKVVEEELVEKVVAEEIVEEVIPEELIEE
VITEKPAEEVIPEELVEEVIPEEIVEEVVPEELVEEMKPEEVVEEVVPEELVEEVIPEEVVEEIIPEEVVHEA
KHEELFEKTVPEVEVQEIIPKELVEVIPEEVLPEELIEEVIPEEIIEEVIPEEVVEEVIPEEIVQEVIPEDLM
EEVVPEEIIEEKILEEIVEEEIHEEIVEEIISEEVVEEVVPAEVVEEVIPEELVEEVIPEELVEEVIPEELVE
EVVSEELVEEVIPEEIVEEVIPEELLEEVVEEVVPEIVEEVVPEVVEEVITEEVIPEELIKEVIPEELVEEVI
PEEIVEEVIPEEIVEEVISEEVVEEVVPEVVVEEVIPEEIVEEVLPKEIVEEVISEKVVEEMIPEELVEEVIP
EEVVEEVIPEVEIEEILPEELIEELVAEEVIEEVIPKELIEEVIPEKLIEDVVPEEIVEEVLPEEVVKEVIPE
EVVEEVIPEELVEEVIPEEIVEEVLPEEVVKEVIPEVEIEEVLPEELIEELVPEEVTEEVITEEVVEEAISEV
VEEVIPEEIVKEVIPEEVVEEIITEEIAEEIIPKELVEEVIPEEVVVEIIPEKLVEEFVSEELVEELVPEEIV
EKVVPEELVEEIVPEELVEEVVPEELVEEVIPEELVEEFIPEEVVEEVLPEELVEEVISEVVEEVVPEELVEE
VIPEEVVEELVAEEVIEEVIPKELIEEAITEEVEEVIPEETVEEVIPEEVVKEVIPEVEIEEILPEELIEELV
AKEVIEEVITEEVAEEVIPEEIVQEVIPEEVVEEIITEVEIEEVLPEELIDEVVPEEVTEEVITEEVMPEEVV
KEVIPEEIAEEVIPKELVEEVIPEEVVEEIIPEVEIEEVLPEEVIEEVVPEVEIEELIPDEVVEEVLPEELVE
EVIPEEVAVEVVPEEVIEEAVPEEIVEEFIPEEQIEEVIQEEIIEQVVPEELIEEVVPEEIIEEVIPEEIVEE
VIYEEVIPEELVEEVIAEKLVKEIVPEQVREEVTLEEIVEEMIPEEFVEEVAPEVEIEEIIPEELIEEVIPEV
LVEEAVPEELIEKVIPEVLVEEIIYKEGIPEVLIEEVVPEELVEEVIPEELVEEVLPEEVVEEVVPKELVEEL
IPEELVEEVIPEELVEEVVPEELVAEVIPEELVEEVIPEELVEEVIPEELVEEEIPEELVEEVILEELVEEVV
PEELIEEVVPEELIEEVVPEELIEEVVEKVIPEEIIEEVVPEEVIEEVITEEVVEEVVPEKVIEEVITEEVVE
EVVPEEVIDEVITEEVVEEVVPEEVIEEVIIEEVVEEVVPEELVEEVIPEVLVEEEIPEELVEEVILEELVEE
VVPEELVAEVIPEEIVEEVIPEELVEEVVPVELLEEIVPVELLEEVIPEELVEEVIPEELVEEVIPEELVEEV
IHEEIVEEVIPEEVVEEVIPEEIVEEVVPEELLEEVIPELLVEEVVPEVVEEVVPVELLEEVVEEVITEEVIP
EELIKEVISEELVEELIPEEVVQEVITEEVLEEVIPEEIVEELIPEKLIGEVVPEKVVEEVVLEEVVEEVIPE

EIVEEVITEEVLAEEIVEEFVPEELVEEMLPEEVVEERVREVEIEEMVPEVLVEEVLPEVVEEVITEEVVEEM
VPEEVVEEMVPKEIVEEVLPEEVVEKVVPEELVEKVLPEELVEEVVPEELIEEVVTEEVIEEIIPEEIIEEII
PEEVVEERVSIEVVEELVPEELIEEVVAEEIFEEVVPEKLIEEVVAEELIEEMVPEVVEEVVEEIIPEEVIEE
VIPEEIREEIIPEEVVEEVVEEIIPEEVIEEVIPEELIEEEISREIVEEVIPEEVVEEMMPEEVIEEVIPEEL
IEEVVPEEMIEEVIPEELIEEVEEVVPEEMIEEVISEEVVEEVIPEELVEEVLPEVFVEEIVPKELVEEVIPE
EIVEEVIPEELVEEVIPEELVEEVISEELLEEVIPELLVEEVVPEVIEEVVPEVVEEVIPEVVEEVVTEELVE
EVIPEEVVEEMIPGVEIEEVLPEELVEEVIPEEIVEEVISEEDFEEVIPEELVEEVIPEEIVEEVIPEEVVEE
VVPEELVEEVIPEEVVEEVIPEEVVEKVVPEELVEEVIPEELVEEVIPEEVPEEVVPEELVEEVIPEEIVEEV
IPEEIVEEVMPEEVAEEVVPEELVEEEVPEELVEEVISEVLVEEVIPEEIVEEVVPEEVVEVVIPEEFIEEII
PEEVVVEVTPEELIEEMKPEELFEEVIPKELIEEIIPEELIKEVIPEEIIEEVISKEIVEEVVEEEIIEEVIP
EEIVEEVVPEELVEEKVPEEIVEEVIYEEFLEEIMPEELIEEMKPEELIEEIIPREIVEEIITEEVVEEMKPE
ELVEEMVPEEVVEEMVPEEVVEEMVPEEVVEEVITEKLIEEVVPEEIIEELIPEELVEEVIPEKLIEEMIPEE
VVEEMLLEEVVEEVVPEVEIEEMIPEQLVEEVVPEELVEKEIPEVVEEIITEEVVEEMIPELLVEEEVPEELV
EEVIPEVLVEEMVPEEIVEEVIPEELIAEFAPEKLFEKVIPEEVVEELVSEELVEEVLPEVVEEKFYQRFLKR
LYLKKLLKRLYQKKLWKRLYQKNEEVIPEELIEEVIPEEVAEEVVPEKLIEEVIPEELIKEVVPEELIEEVIT
EEVVEEAISEELVEEVVPEEVVEEVIPEVVEEIIPEELVEEVVPEELVEEVVPEELVEEVIPEELVEEVIPEV
LVEEEVPEELVEEVIPEELVEEMLPEELIEVVVPEELVEEVVPEIVEEVVPEELVEEVIPEEVVEEVLPEELV
EEVIPEELVEEVIPEELFEEVVPEELVAEVIPEEIVEEVIPEELIEEVVPEKVLEEVIPELLVEEIVPIEVVG
EIVPEEVVEKIVPMEVVEELLPEVVEEVPHEIVEEEVVKEVIPEDEKKVEIVEIIIEEKVEKIPKKKVIEKKK
EKEKIRKPKKKVEKEIKPKEVKGVKGKLKKDVKAKHLKGSKVKITEDIQKKMEDDIKEKIMEKELKEEIKDKE
YLRKVIPKEEKKEPIPKKEKAPEPKKEKAPEPKKEKAPAPKKEKVPVPKKEKVPAPKKEKVPAPKKEKPAVPK
KEKVPETKKEKEKDSQPKKEKAKETSRPPKKEKEKGK*
>N.PF10_0375
MVHNNCNKEENYFFEFQYKKKNKNEYFNLEIIIGSIINNRDKKKRETFFTFRKILIYIIFIYILYFIDYKYLL
NNHSHEQYEIYKISSNKKWRQLSESYDYFQDPLDYFENVQNRVKLFYNIDNIDELDINFLYKNILDKSKLTDK
KRINHYESSNSYMSDKANISYDDNISSINIMCCNNIKTHNNNPSNNYNIYNQNIIRYDNNGKTNLYDTNNDFF
KHKYKSNESFDTFLTNDHNENGNFQVYEISDDEIILTNIKTHDMKFEKNTNNKSKQKRFNTIDHEISHLNKDS
STNKNLLKNISILKYIYNNGKCNIKPKKIYAKCIKKIKKIKFKKIIYHILYMLGSALTTYLFLGALGPEMVNR
IGVSLIAYG*
>N.PF10_0376
MKLKNNYSIKENISKLYDKKEKGKYLFLRKCINLNRYKNKIIISAFFTFTLELFICLFCIYIFVYPNECKFRK
FSNKNTIVPFKLTLNVYRCLNENMKHFKEPYLDLSYNVDSYYNYRNTKEPYTDQIRNCDYKDETNIVKYIEDP
YINTHYHMSYEDVPNLITNYNYVHENYSMNDEDPSYMERRHDIQYNDNNILLDAYDYNQYLEPHNNNNNIYHS
SHISDPYFNMKYVNEEYIQLFYDMLYNNNEFIYDFYDFQNMDNNITELPIIDNIHEYQKKILYNMYTCHESNE
IMSCDFTDYDEEENNLSRKKLMDEQIRHRINDPMDEHKCKKININVEKHENLNDEQTCNIYQKNTTLVRQELE
DIFHVFLDKYIKMKYLQKELELIDNDITLKQYKLYLADFIGLDISNNDEKYSFTKKKESIKELINSYIKEEEL
NMMKKLLGAKNYYKKTYYDTLSITHKNIKKIKKKNNIKLGVSIGLTTVASLLTMFISYWTSPYVFRGIFYLIK
AVFLPSH*
>N.PF10_0377
MNTYLNLKKYIRNSIIISIKHNYIRLLLIFNKLFLFVLYIYIFRYTHNEHTFYNNVYEKNMLDKIWKNKVFRL
LGENSHYFKEYNNHYNTEPLLYTEESFRYIQEPTEHNHVHSHYTQQHSKEFEETTNYEQLLPYYIQEQPEISH
SFSYHIEQPLDVISQFQNNVYELSNIPNEFNFNYHNVKPREDQFYNNINTFLHHYPKEHNNIQTLQNSTKNEE
FYDKANKNIQENFYNDFVKFKDYIINDKDQNIEDINITGEDKEKLKLLFETLNEMKFYESKISALNFKYYYYK
NKSKKKKKKKSLVKMALTPIFVLLSMFTLNKVAPEVFSTAFIWLYRF*
>N.PF10_0378
MKCLKLFSFKSSKKNLHKNKLNSEYKNAYSCEDKTVKKYKRKVCETLFCKYINVLIWYILLYIIILNVCLWNS
ISSYDIEFNYIYGRKLYSTESLNREKTNTEKTIISKVNLLPNEIILSNLKNENNKREESVLEFNEQLIENLQK
YKLWNNYIAIPYVKKYNPIKYNDIDNELNEKIDNVGKNGEDIISEMNNLWLQVMNNEKSKYLSLIHRLHKYYY
NIKVKYKIPNDYHNAKWKECYEIIKMGEEDIEKRLNKMFKDCFKQKMIYLEDYRRLTVACIIAWKALSNYVQN
SCRKVMSVYFNCIKKYNKNMYIDINQFNNNNINTKKYNTQLYEGKNFGKINFCECLISAPYIDEDNSDALLSV
EDIFCIELEKGNSKMENEELNMNDERENRGNPNGNMEETKQIKLCDIKNDNPHDNTCINQNDIASNSSSDDTD
NDINDDINIFHQSKKYSRIPRYDTSNLKGYNKNSYYLDLEAEWLFNSFLEIKKLDEKIKNKKRRRNKENMNNP
LSPDNSENVFVDKTYYDILNVNPDADFVEIKNSYYKLALKYHPDKNKGDEEAKLMFQKINEAYQVLSDEERRE
QYDNYGKNATQNMFLIDGSFFFTLVFSSEKLCDYIGTLQISTFVKLVHERGMNSNDLLHSMREIQNKLSREQD

IRETELALLLRDLLQPYVDGDPNWEKRMEEEISSLIYSNYSSSILKSIGWTYKNVAKTFIKENKSFCGLGAEI
TKMKAEFRHINNCSKVTRSAIRLNSKIFKNIQDNKMLMGNLSLMNNNKITDGNYRTFDNDSMNDEGKSSSKND
VIVYDNNTTEILKEKSKIVADILDDIFTIVLCDIELTVRYAADRVLRDEGCNKEIRLKRAEGIKIVGNLMNKW
AKIKKQQMKDNKIDITDTIESALHVSRIRSRNQKD*
>N.PF10_0379
MNNLTLGDSNICGTFNKLPFIESYYNRKGILLKSYSWLVKNAIGIIVLVHGLSAHLRLQYLKLNVNVVNNDYA
TLIDADNYYIYEDSWIEEFNKNGYSVYGIDLQGHGASDGLDKLPLHINNFDDYVYDIIDYMRRINNSIVLEKS
MQKSTSNKYTIENREELLPMYLVGLSMGGNIVLRTLEILGKSNEINSNLNIKGCISLAGMISVRMVGSIDSIK
YKYFYLPVMKIFSRYFPTFRPGKKKFKFEKYPFVNDLLFYDKYRFKGRITNNLAREILVALDNLHNNIDDIPK
NIPILFIHSINDCLCWYEGTVSFYNKLQIDNKELYTLEDMDHVISMEPGNENVLKKILEWISNLYVQ*
>N.PF10_0380
MGNFCKYNYMETNRLLYMKCFKCNNSPMYTISKKDEYKKKESWNFVNSLLSRNLILFTLTFLFLNILELCKLQ
YDYPSQHIINNSIRHLSDYSSSNDNLSDEQEYFTTSDDSEEEKKHNEKINKKRKKCKKYKKRRGKLREKKKGK
SNLYHNGKNKKNVSNCKKHNINLKNNLNNNNDLLNNNYNISNDCYICEQMTSQNKTKVPYGYYEEIDYIDDDD
DDSFRNKISNEKIELVNGNSKDKGIYYKNNLGNEKKNDYRTEDQTHLGIELIEEEFKGRNGNLDEENIDYNKE
RIRENIYHGLEKIEEEIKERMEELIDDGREIENEENREYNNNDIKKRRFDGDQENITSSFERTQKSVKYDSDE
SEELSEAESNGGIPEQNGYDNEDVVSGSDENSDVEKNTEIEEANGYGSEDNSCATEEGSDSDLEENTEEGIED
GIIGLIQNIFQGSDKEDEEEKEKNNKKKNVYNWESGKKALGKLLSNSDKLSVNNIKYSEWNLERIPTLGFCKE
DDRVQEMFKASIMGNNDDSTKEVKFFIKKIPIDIWLKQYKLMNEYDGEYLLDGENFVMEAVASAYLSEHYPGL
IPKLYKVVYEPVNNNTNNNNDEKNNKKHSNDKKSKEDNDDMGYHTGDENDIFEDCNDSNSDRPIDGYDHLKKF
NDMLTKQLNNNKKGYVVFISELYGQDLFQYINNKNENKETVVSVEEKKKIMKECLKLLIKLHNAGLAHLDISP
ENILISNNSEFRLCDLAKSAPMYTYNLRHIKGDEKKSFLFQSYQPCIGKLTCMPKECWDIVKEYMRLKIKNPL
EYLKSIKNQEERKKFYFDVLSADKYMLGILYIWIWNNNYIWKRADPSKDKIFSHLLNYNMDINSIKLAEDWPK
GLKNIINXXXXXXXXXXXXXXXXXXXXXXXXXXXXXXXXXXXXXXXXXXXXXXXXXXXXXXXXXXXXXXXXXX
XXXXXXXXXXXXXXXXXXXXXXXXXXXXXXXXXXXMIYIK*
>N.PF10_0381
MEFEKISEAYQILSDKEKREKYHKEGLDVTKDMFIMDPSILFMLNYSLDQLFPYIGKYDITTIINFVTDQFTR
GNIFETLIGKSSLEKYGDLIRKMDEKEEERKNKLVLFLKDRLQEYVDVDEDTWIIKMENEIMGLLESKFSSYI
IESVGWVYENVARAFIGKEGKVMSMDEKKARKQAKHREQMNRKEAMIWSFRTVSSIGYILSGEPKNHLMIHGV
TCNMYNYNEMNNFYNYDDKLVCHYPNGYSINKMFHRIIHTFVKTMTVYFLDIIESIVRVVAETVLYDESDDIK
IRLKRARGMKKLGILMQKVARIRSEQFGEDKVDF*
>N.PF10_0382a
MEFLTNHSRINRKNMFLFKIKMMAIFLFALSLFNQNSQNSSNHQTIESLAKSDYKYNILSEPFFQELDDSTTK
YLDQLIKSGNKETNKKKKLSDTNEKNHNKNNVDNKNNNVNKKNNKNNTNNKNNTNNKNKKNNKNNKNNTKNNK
KYI*
>N.PF11_0033a
MEFLTNHSRINRKNMFLFKIKMMAIFLFALSLFNQDSQNSSNHQTIESLAKSDYKYNILSEPFFQELDDSTTK
YLDQLIKSGNKETNKKKKLSDTNEKNHNKNNVDNKNNNVNKKNNKNNTNNKNNTNNKNKKNNKNNKNNTKNNK
KYI*
>N.PF11_0034
MEFEKISEAYQILSDKEKREKYHKEGLDVTKDMFIMDPSILFMLNYSLDQLFPYIGKYDITTIINFVTDQFTR
GNIFETLIGKSSLEKYGDLIRKMDEKEEERKNKLVLFLKDRLQEYVDVDEDTWIIKMENEIMGLLESKFSSYI
IESVGWVYENVARAFIGKEGKVMSMDEKKARKQAKHREQMNRKEAMIWSFRTVSSIGYILSGEPKNHLMIHGV
TCNMYNYNEMNNFYNYDDKLVCHYPNGYSINKMFHRIIHTFVKTMTVYFLDIIESIVRVVAETVLYDESDDIK
IRLKRARGMKKLGILMQKVARIRSEQFGEDKVDF*
>N.PFB0990c
NELLSNIVKKDLQDKFNKSIITINIASRILTENNKKWYKKYIYTSIFSGNKNPQKRERKNEEENQKDNTKVDN
DNNMENEMENHIDDSIDDPMDDLMNDKWEHHNSLEDRIKEYYTLTDPSDGEENNSFFKKLKLIMNILDEVHSD
LLINNSVTDGSIFSPELVPISVLSTMTLACPPIGTVTLPYITNRINFLNRYEGQNIHTEHDLKIFK*
>N.PFC1085c
MNYFLSLFNVSLFFLLIFKYSYKVKKDLQDKFNKSIITINIASRILTENNKKWYKKYIYTSIFSGNKNPQKRE
RKNEEENQKDNTKVDNDNNMENEMENHIDDSIDDPMDDLMNDKWEHHNSLEDRIKEYYTLTDPSDGEENNSFF
KKLKLIMNILDEVHSDLLINNSVTDGSIFSPELVPISVLSTMTLACPPIGTVTLPYITNRINFLNRYEGQNIH
TEHDLKIFK*

>N.PFC1090w
MNFFHVIILDVGIVICLYLINSDIKWKKHINCCKYHFYFFSSKRCLLQHMVEEPFEKKDKSGVLLKDKNTEEG
RKKERQKPMSIKSINKKKKKNNNNNNNNNVLKNLNNEEINKQRNMTNERIRNKNKNDKGVENISSNTQMEEKN
IICKDINSNVILNQNEINDDQMVQKIKENFVKDLMKNENKEIFKQIETINSVGTMAKIKNSLYSIIFKGSNFW
KGLGIYLGTLSGAALGQLILAGILQFGTFSVMNFSIYFSAVPSFIAFSSFVGIILLSIIIVICLLVWLWPSRG
KLMGKDKTENKSDT*
>N.PFF0050c
MNFFHVIILDVGIVICLYLIIYKNSDIKWKKHINCCKYHFYFFSSKRCLLQHMVEEPFEKKDKSGVLLKDKNT
EEGRKKERQKPMSIKSINKKKKKNNNNNNNNNVLKNLNNEEINKQRNMTNERIRNKNKNDKGVENISSNTQME
EKNIICKDINSNVILNQNEINDDQMVQKIKENFVKDLMKNENKEIFKQIETINSVGTMAKIKNSLYSIIFKGS
NFWKGLGIYLGTLSGAALGQLILAGILQFGTFSVMNFSIYFSAVPSFIAFSSFVGIILLSIIIVICLLVWLWP
SRGKLMGKDKTENKSDT*
>N.PFF0055w
MNYFLSLFNVSLFFLLIFKYSYKNIVKKDLQDKFNKSIITINIASRILTENNKKWYKKYIYTSIFSGNKNPQK
RERKNEEENQKDNTKVDNDNNMENEMENHIDDSIDDPMDDLMNDKWEHHNSLEDRIKEYYTLTDPSDGEENNS
FFKKLKLIMNILDEVHSDLLINNSVTDGSIFSPELVPISVLSTMTLACPPIGTVTLPYITNRINFLNRYEGQN
IHTEHDLKIFK*
>N.MAL7P1.3
MNFFHVIILDVGIVICLYLIIYKNSDIKWKKHINCCKYHFYFFSSKRCLLQHMVEEPFEKKDKSGVLLKDKNT
EEGRKKERQKPMSIKSIIKKKKKTTTIIIIIMCNLNNEEINKQRNMTNERIRNKNKNDKGVENISSNTQMEEK
NIICKDINSNVILNQNEINDDQMVQKIKENFVKDLMKNENKEIFKQIETINSVGTMAKIKNSLYSIIFKGSNF
WKGLGIYLGTLSGAALGQLILAGILQFGTFSVMNFSIYFSAVPSFIAFSSFVGIILLSIIIVICLLVWLWPSR
GKLMGKDKTENKSDT*
>N.MAL7P1.4
MNYFLSLFNVSLFFLLIFKYSYKNIVKKDLQDKFNKSIITINIASRILTENNKKWYKKYIYTSIFSGNKNPQK
RERKNEEENQKDNTKVDNDNNMENEMENHIDDSIDDPMDDLMNDKWEHHNSLEDRIKEYYTLTDPSDGEENNS
FFKKLKLIMNILDEVHSDLLINNSVTDGSIFSPELVPISVLSTMTLACPPIGTVTLPYITNRINFLNRYEGQN
IHTEHDLKIFK*
>N.PF10_0391
NELLSNIVKKDLQDKFNKSIITINIASRILTENNKKWYKKYIYTSIFSGNKNPQKRERKNEEENQKDNTKVDN
DNNMENEMENHIDDSIDDPMDDLMNDKWEHHNSLEDRIKEYYTLTDPSDGEENNSFFKKLKLIMNILDEVHSD
LLINNSVTDGSIFSPELVPISVLSTMTLACPPIGTVTLPYITNRINFLNRYEGQNIHTEHDLKIFK*
>N.PF10_0392
EFFSCNNIRCWNCNMLIFNHL*RIVI*NGRNI*IVVNIIFTFFQVRDAYYNIWLKNRLRKKTNLVFY*KTKIL
KREEKKKDKSL*VLNQ*IKKKKKTTTIIIIIMC*KI*IMKK*INNEI*RMKEYEIKIKMIKELRIFQVIHRWK
KKI*YVKI*IRM*Y*IKTK*MTIKWFKK*KKILSRI**KMKTKKYLNR*KQLIQLVLWQKLKIHYIA*YLKGL
IFGRV*EYI*VHCQVLH*GN*F*QVFCNSEHFLL*IFQFIFQLFLHL*HSVVL*E*YY*AL*LLFVFLCGCGH
QEGN*
>N.PF11_0023
MNFFHVIILDVGIVICLYLIIYKNSDIKWKKHINCCKYHFYFFSSKRCLLQHMVEEPFEKKDKSGVLLKDKNT
EEGRKKERQKPMSIKSINKKKKKNNNNNNNNNVLKNLNNEEINKQRNMTNERIRNKNKNDKGVENISSNTQME
EKNIICKDINSNVILNQNEINDDQMVQKIKENFVKDLMKNENKEIFKQIETINSVGTMAKIKNSLYSIIFKGS
NFWKGLGIYLGTLSGAALGQLILAGILQFGTFSVMNFSIYFSAVPSFIAFSSFVGIILLSIIIVICLLVWLWP
SRGKLMGKDKTENKSDT*
>N.PF11_0024
MNYFLSLFNVSLFFLLIFKYSYKNIVKKDLQDKFNKSIITINIASRILTENNKKWYKKYIYTSIFSGNKNPQK
RERKNEEENQKDNTKVDNDNNMENEMENHIDDSIDDPMDDLMNDKWEHHNSLEDRIKEYYTLTDPSDGEENNS
FFKKLKLIMNILDEVHSDLLINNSVTDGSIFSPELVPISVLSTMTLACPPIGTVTLPYITNRINFLNRYEGQN
IHTEHDLKIFK*
>N.PF11_0035
MKNCRNILIEKIYFLLAILGTLSVVSLYLYIPNENINLSFHKYDSYIRILCEKHPDDNQFSGSLCRSDCNSNY
NKDIERESDKKKNIILENMGQNGMNKTKEHHMNLTTPLVKYEEIQNQDREKDDQNERQKRERKEMEEREERER
NERKKREEHERNEREKQENKERKEREKQERKEREEREKKERKEREEREKKERKGREESEKKERKKREESEKKE
RKKREEREKKECEQREKRLKKEKQKRDKEERKERERRERKLKKEREKQEKRESKQREKQEKQERQKREKEERK

ERKQRDKREKKEKEEREKREKKEKEEREKREKKEKEEREKREKKEKEEREKREKKEKEERDKREKKEKEEREK
QEKKEKEEREKQEKMKMENV*
>N.PF11_0036
MKCIECFENIEDFDGNLSNTSIKEIDNVIVSKKEKSLLLSNKETLFIHNYKEGISNINNESYKVLSMDNFVRN
KKETIINNIEKVYNRNVVNNIYSSDLLNKHYNKQKKHNLLHIEPSGVLYKNDEEYFKIALRYDPENNEDNKKD
ELLKQNENNDNICNVKDDDYFLKYSKFFNSKLRPKNIDEILKTLEENFLPNDDDNIIFITSGGTKVPLEKVDI
RNIDNFSTGRRGALMCEYFLKKNKKVIFLHRRGSHLPFEYHLKGVANFNNLNLVDNNIITFNLKEDEKLNLIN
NIKNYQKYKEKLFIVAFDTIFDYGYYLLEIGKLLNDNMKKRYEKYFNLNKDFNYTLVVDVYAKNILEDIVHLF
SSNNNVIYGKYNEEGRFYELLQKLILFENLRLEKYNNNNNINNNNNNNNNRCYDLSNFFKYIFDILYDMIKKG
QIRLSDESIKDILVRAKFFINNINGCMKNKSHDYILNDINHLKYNVNHKNDNKNMYMYEKINNINFCNSVHIN
GEINENVSNVGDNKSDEFLFPSHLMILCAAVSDFYIPFDDLLDFKIDSNNCPFQIEMYLTPKFYKITYKYFPF
LKHCIFKLEDQDNVLIKKSNERLEHADMLIANILTQRNMFVYIFKKEQEYFKLTKSKGEPIENNICREVCKHF
SII*
>N.PF11_0037
MSEKSLDAMPARSCPTSFWNPKMKLLHNMKAFYMSSFQRYIYVFIAFVFLMLLFNVPSIEENTYSRDILEKRF
FRNLYEDELIRKDSSKVNTREHGGESKVLHLSDIISQVLKDSGMSRSNVRSTNKVDVIFDKKGVKPNVYENIE
VEEDPNEKVIYSVEICSKQVSDSNSVDSKMKYGEQLNNDFMGNINEQEYITGKLGVRRGLKIYDRKKEIEKLK
NELKFLNKLKYENFEEYAKYKPKRKPSEINSDIAERKEEREADNAKLSIFEKEKLDIEETKKDEKVALEKSTD
NISNIKSKVVGIKEIDEEGMESISKTIDNSKTSEVVEEKKTEDVDSLNECLYFYPNDGDSMNLENVLKHIMEY
DNSENTIDDLSEEENYNQLFNLLYRAFQNKFYNIFKLHEMLYRDLTNVSIWDEYELDLYNVKENGLYTDDKDL
ELNENILSLNSKKNDVNTLRDMWNQINRNEEKKISVTIYRLNSLYTNLKNKYDVPVFHSTNELNNAYGKFLIH
MNYIKSYFNGIFNEWIKTSEGNIKEYRILIIACRLIWRKLKKNTLQSLQHIITKTFEKKIKEHESYNKYLARL
YKAKYQQEKHNYFWMKFLDEYKGDGSSSYENSITEAPYIG*
>N.PF11_0038
MEKFSNLHVGTIFYRNVKCILELFGILLHCLNLKKNCKENMKISNKKYFIFKSFAVFLFIWFLNNLKECKTAE
YLNRRNIHNGEIEIGIRRLLCEVETLVDYNNTPVYEELSEYIDSDEEENENDDAPLFDDYYMNHFKNIHYNPG
NIHKKKNCFLKLLTSLDRRIELEIVNLMKTKNESNYFMLKTEPKMRKIKRYLDRYKVFSPIIIGIITAIIIVS
KGAGTLALIPLSLIIVYSFYLWKKYKKCKYISDSFPAYKIPENFEESYVVYQ*
>N.PF11_0039
MKVTRILYFVFFIFALNFIAPNNEHNGYVEAKKKLTPAEIKKRNQKLMMYSAIASGVAVLLGASIGLGVHFSK
KKSPKKNVIRQVVKKTPA*
>N.PF11_0040
MKITKIFYFFAALLALNFIAPNYFNGYVEAKKALTPAEKKKRNQQIMLISGITSALALLIGAGVGLGIHYKNK
NNGDEKKDKAGAKTITATPKN*
>N.PF11_0041
MKKQYELITPSWVLKICRKYKHTIKKIILITNLFFVFVFISNDFSLHHVKNINKYGINNKLYQHNIETRYDRI
LTEHVKHLWKNNFSLEVNTSQKNPSSLKDHVSSNQSTEEIKKTNELITNLSKLWKDMIKNTEEEYNHNTDHMD
HKWRDDMWNKQWGKYLDAAHDNINKHLRNYNSSEDYKKDITNKWIQWASEDVEVFVKALKQEWYKNNNN*
>N.PF11_0042
MSELEASNIISPNSTQLKLQENHNQITNNVNLYEHDMDYDYLLTICVLIYLALLVLLIKICQLSCQRTCARRR
ALPYFVEADSGYVSNEEG*
>N.PF11_0043
MASIPASELPECEKQELLCTYAALILHEEKMSITSDNILKLIKNSNNTVLPYLPMLFERALKGKDIQSLLSNL
SVGSAPAAAAQVTTEKPSEDKKEAKKEEKVEEEEEEDDLGFSLFG*
>N.PF11_0044
MPNVFLSNYIFIFILLTNKFLKTFDYVESKHWNNICLKDLYDKNDIKLNKTNKLKLKYKNFMFSDLKKQKKRK
ETETATGTITQKQKHTKKKKLHGQTNGYFIKRNKIQHIFANIPYNNNFKKINQDIYTFKNVNYHPKNANKKVY
LKINNATHLETIKTYITKCIKNYRHQNEEKTQKKIRNYNNIIQYYVSRKNNNNNNNNRFIHTYNMLFINIFYN
IISKNRNEKKKKNRKTDFQKYLRKYEELYQKFTPENDAENNDINNDKNNSDLISQINKKNISNIIINEWCLRN
NNEKYNSTLMYDYISNMHNTEYQDITCVNNFIKNNEMIEKETWEHKQKPYLTYKYEFKYRDPINRKNKLYQYE
YYIPKLITYEEDISTEPKFVPSNFKSPKCYNNSPVNALTTSGFSSRNIDTFGKERNKIYSFNTPTDVKRNIFF
DDLILISSSYNQHFFPNLNFLLNLHTLQILIRDKTAIETDYMIDKFDQLKEKLIQINSPHVIDNNQNDDIDNN
NNNNNNNNCDNPYNKRIIPINNKSTNNDACTIDNISNIQHRKNQTNDQNNKSKDDEINEEISSNLTYKKTLFD
DKIQLFKNKYDKNILDDKDFVQLWKYNEKGEIVETINKIPIPKIYLNIDDPKYLSWKILQNSGKTAFKEIPTP

NRKLEAWRQQVNLKTFYKQNFDSSISLRNISKEELVDYKMKIVDNTFEKNEKVQNDISDHFDNIKMDNQQNEE
KVELNENINYYNSDNINGDNINGDNINDDNINDVNNNHNNNHNSDNHYYYGDTHKINNQNKKNVNETINISEN
TNMDDNNNNEIYSQNYKEQRIKNQDISNNAYNFNKEHIDKCKRKYKKAFYTLVVRDGIVDEYLSDDINILKNL
DNKLKKKSEINQNKENENENENENNSQSDNNTTKQHIQDEQGTEQKKSKIFVGSFFNVKDVEIEYLINKELYF
IPEHSNWYKTNTQPFIRGQIGKQSRKFDNDYPIYDYRKSDFGMAKFSSLNLASIKDCAVVYLDENIDLSDKFI
HVIFIATSKNEDIDNNNNNNDNNNDNDIYEKQSKYNVYENIPSNNKQTNSNNNSEYNNEQNNCSNKQITNDEQ
NDNEYEKEESIQKCHMEKVISSHDDTIQNCDIEKNECKENKKSKYTQIKLTEYHTHNPITNPRLVVYVKGNSK
INIYESHISLNKSNSGLVNGFSRICLEEKSNVKHTLSQELGNNVWHFHNVSVKNGLNANYKFVDVLLGSLSSR
INLQIEGEKGCKQESYGLSLLEDKQNISQYEMFHHEHPSMETNQLFKCLVSDKAHAVWRSRGRIERNAIKAKL
NTLCKSILLNFGASAVCIPTLEIIPSDIECANHGATISDLEKEPIFSLMTRGISERNAREIMMNSFVKEILDH
ISDENLKNRVYQKVLKFSQKYKSSTY*
>N.PF11_0045
MKKCISLIFVLFINKIVGVKIHLYRKYPNVENGVYQIDSRGRPIKIVDVNYNHRWEIPENGNINSYSVVRKNG
KVKKFSEGTNLIYGTTKRKLINDIKEVDEQSEEEIKNLILKNKKLNIIKNVSDVSKKEKNIKELEKKKKEKPP
IAMYIAADDIPDDKKKKQEEDDINVANELGVAIKENLESHYEITKEPSALSTDLLNMKIPTTLEKLLMDDIDE
IKWISKKPVNDKICMRDYSKQCPSMWEPITETQCSAPKDYSGPCAHIMILEPMNAKEKSSIAKDCKVNWSCIN
ESCGNGERDYLRECPENWIYNGTCEAPENYSGGCNRSMDFDTFTQNDKEQFSSTCKVVWPCKEESCERDYSIT
CPKGWSYSVKDDMCMGSNELRGLISKEEIEAISHMSYHQRIAFSTKYGIPWPCKNKCTFGYDIYACPRGWLNL
MNSGACKAPDDYIAPNNCPRITHFDYMDINEKEKFSKICNVKWLCVENSQRDYSKCPIYFEYIKEGNYKGMCK
PDEKYKGPCKEAQDILTLNLEQKYNFEETCEAQFPNLQIKDIPQTEQDSISPSTMEKLLNGTDLTNKSVTLE*
>N.PF11_0047
MFQNIQTIILDINDGEMKTGYSGECTPRYNSNLILGLPLGHNATLKHTIFPLLPNVKRDNVELLYGIKYIYDE
NNNSRYDINFDVMEKLFEGISGSKALDDNFQDHPILLTEPNKTDRKYREKFTELMFESYNVYQLFLSRRSVLS
CYGCARTSGLVLHVEKNCTNLCGIQEGYVFQKHIEEAPIGGDLIDRIYLSYLENIKNLKIYPYFSIEKNTNNL
DNKNYDIKILKCPLVTKSYYLWGSLYVVNQMKENIINDSQNITEKNKNKNNNNNNSDLYEHKNVYKLPDGQII
DDNTTESLKLIFPYIFFRKKYNQNNINKLCTNTEIFQNFDFNEFYNSLKNLKLPILHKYNYKINTTNSIIEKI
PDNSLDKTTNICYSDQIDSFFEIFDGLQDFIKKGILSFISSNINIDDVLNFLIITGESTMLHNFVGLLKSYLP
FIDTIKEKSTKLIYSKGPDRKCNCFIGASILSSLGTFPQFCMTKSEYEEYGVRNIVDKKCA*
>N.PF11_0048
MENSDSNKDLQDSKSDKSTSWVKWFNNRALSNFLVEVDNEYITDSFNLYGLKTEIPNFNHLLSIIAGDAPEDD
DDSKNSFSKDCICLYSLIHARFITTPKGLSLMKDKYIKGDFGTCPRVSCAQHNVLPIGLFDQMKIAKVHVYCP
LCQEIYKIHEDEKVYLDGSFFGTSFPHILLQTYPYYATLKTPPYCSSKIFGFNVYHNFTRTEYKLAKGEFGII
TRENFLKKNPKYFKKLRKEELQISET*
>N.PF11_0049
MNNNFNINVQMDEESVKKYEAEVNNYFAKLYTGDITVNTMLDILKGLSSSPKGSKNNDVYKCMLLVLFNECKF
FPKYPPEELDITAKLFGKLIKNNLLMDYGNSLNIALMCILEAIRKENDIKMFNFGITALEQFEEYLLCYPSFL
SSLLTMNNLRKYNPQYVTHYNDLLSILPEHIRSMPYIEPSIIIKIKQLYSMNTNKVNPTINNMQLINDEHTNY
VSLNRNNNINNNINNNINNNINNNINNNMNNYHNNNNNINENIKIGLHMDRSNYNNENITLINDNTNTNNIII
NNNNINNDIYNNNMNGYDINHDNINKRNNSINVVNVLDGILNNNINNDNLLSSINNMVPNYQQQVPNFSQHIN
NDISSNINDNIILKNKNMNNNINSNIFNTNHFPITNKNNISYNNNICNILNNENSNNNINNNENQLYLNKPYL
NSTKDIINQYNKNVNILGTNNNIKRDINSKLTHGFNQNNLETNIPNVIDNSINMGIKEHVKNILDHKKDNMNN
MNNMNNMNNMNNMNNMNNVNNVNNVNNMNNMNNMNNMNNMNNINNINNMNNMNNVQSVNIQHNNNNYNLIKAK
EQSSDIIETFYDQIQKKLPNNLNANNINGFGLGQIECLMDATELTKNIILPSSIIIGEVFSIFNTLCLFNIDE
KIKILKDFMQPDYFNWLAFYIVKSRASKEVNLHNVFLEFIDKLSYPLFIDTIISMTYDCILILFKYINELKEV
SAFRTVLKNLGSWFGFITLGRNRPLKSKILDLKVVLFEAYDKGCLVCILPMVCKILESIKLSKNFKPPNPWTT
AILCLLTEIHELPNVKTYIIFEVEVLFKNLSLNIQEFQNKTILLSKRMPPQNKKSDFVMSKNNMNNKNIMTNN
NNNNNNNNNNNNNNNNSNNNNINLNDLNIISSTNNFNLPVDLKNIYMKENRSNVNNIYNINNSINDSTKLEEL
QNISQNILSNNRINTEIDTYFNTNNQPSLNTIESHWNQNEINKKINPLINDASLVSSFNPPDLKLINSNTSQK
VVYNQNRNHYVINNDQHIPNVSNSMNRTSNINNNICDVNKNSNHKFLQSLGNAVIISPSIALFQIQPTLKRVV
PVAVDRAIREIIASILERSVSICCVTTREIICKDFCLETNEYLIRKAAHIMVASLASSLALATCKEPLRISLT
QHLRQLLQPTSTKDCNDQVLIEQVVQILCADNLELGCNLIEQAVIEKAIKDINESLEPLFLTKEIARESDISL
RESSHLSKSKKMQLEFSHILNLENPITSKHLNIYTNFINISPLKKLEAAVKALTLIYKNINDIKDSNKATPPY
QYNGNLIQNNEEINNYSKANTITHSSNIKEKIYISKDDIKINEKMNLEKYNINNKSKNEHHIANIQNNNNKEN
YNEQNVLSNNITLEVNLQLHKVLKKLELATGQLKEAINDIINLPPILFNVNKEINLSNVNKMNLYLLYSLSVD

GNIFNLIKSIPEIAALTKNKYETIISYTNKIYTFLLEITLPIQQTQVSIKSIHVTGIYLEVFLCILEKLKYKC
PLLKDHITNIIMFNKNKYDNVSKNNNNNNNNNGNNPNSQMSHINVKGELHNDNINLENKNKDIDTQNCPNDQD
QYMNMYKYNVNVTAGLIRYNLIDIEKYKKYLEKELINNKENFTNLIEFVILLFKNILIDFHIYRYNDFQNIFS
LINKFKTEGKKKKNALQNKTIYVHGWGNVNIKEAIDKLNVEAQKIKENNETVYFSDVMQFCVEYFNKEQEEDE
KEKEQEEQEEQKEQEEQKEQKGQEEQKGQKEQKGQKEQKGQKEQEEQKEQEEQKEQEEQNIEQIIKSKGEEIQ
NNEKIKEEENQNNKKKDDQTKANEDDVNRDDTYKNDIKYDENKNDNDNNEELKQINEEKVETKKKKKEKQKKA
KMLVQNNENTNNIENKQNQETIINESINKNKKEEIDIKKSLDNHSENFKRECSESLINTNKDQPCDIASQESN
ELINNSGIKKKNTKKKEKKNKKLNEECKENMDEESKEKIDEEKKEKIDEEKKEKIDEEKKEKTNEEKKDKINE
EKNNKLIKESVINKNDKNHVQFQYSNNDIKRDNLKYVQYKKKYLKILMNPKHLTTLTQIIDFCLNTNWNIIQI
KKKSKIFKDKGVGGLRVVPRPQQVTNEHEEIISFAFNQWVKLTSLNNIDNPNESYMKFFQSISNQGLLKIDSN
IDNFFTVCIYKAVEGACTVLNLYEEQKEHGTECGSANVDIKEKNNINLSLDDMNNNNNLKNEQPTEFMNNNNG
SATAEISDNKDNTINNDSNKNNNGTKKGKSNKTDETDVNSLKSEQEKKDTEDGNNTSEKKKDKKKKKDKKNNH
DINNNNNNNNNKNETCIEKTENIQENNNDTNNEFNKNKDIYIENNEEPYFDDTYSDISSCNLTFYSNISNKSE
NISDEEEYTDCVGNNENTTKGFNYKKEEILKNLTSAINQSIGINIDDIQNKIQSNIMNEVSQDNKLNDNYQID
ADKKIEDLYKSENFSMGDIKYEDVIIGVYEKMEQNKIKNDNNNDDNNDDNNNDDNNNDDNNNDDNNNNNNNNN
NNNNNKSDKNKDDHNIQTNNYSQSDIPNKSCNVNTNINDNENSNLASHANIKNQFNNNNNNLNKDNINKSTYY
TYNNNDKSLSELERNILLNDTLDTTTIDALAKMIICMMKLIDIQQISPFVLFQKVMNIFCRIIVYESRKNKSN
FNQRPYYRLFLSILIEINKYEKSFEDIYNKFILAFGYYLCILNPIRVPAFVFAWLELISHKLFLPEILKSSKG
WYIYNKLLIYLLEFLYIFLKNNYLTDSIKILYRGTLRTLLILLHDFPEFLCVYNFSFCNVIPLNCVQLRNLIL
SAFPRNIKLPHPFNPNLKIDELPEIKIPPVILNNFTFILIDYNIKKDINNYFLKRNIIYLQKIHKKLIIKNKM
KALYLKTKYNVSLINAVVLYIGMSLPSSILIFHNSSQSHPAIEIILYLTYKFDMEGRHYLFSSIANHLRYPNS
HTHYFSCLLLWIFKVSNTEIINEQITGILIERLIVHRPHPWGLLITFIELIRNPIFKFWQCSFVSVVPEIENM
FQSIAHSCLINQMEQYQNVSSMGINSNINNTNNSENENNKISVMNNISIYSNNNNNTTTTTNNNNNSSNNNSS
SGNNHYNILNNTNVALHLKNVNHLNVFNPKNVNIQNNYKNNDINSTNINNTNNKNLDNNNYNNYSISTQNMIN
NIKNFNNLNINNFPSNNMNNIKNIDNTYAKANINNYFIKNNIPSKISNHISFNTQNNTNINKHHPLNEQNNNK
EFSNMNQNIIHNFNINSHPTQTSETNIIDPNIFNINDNKSKNIPIEKDAYMKEIKRNIQNNNFINHDNNKNTN
AFNNNMVDHNVKNFLNQKKKNLTFKE*
>N.PF11_0050
MDKDTEFEFTKIENINLDSYEIPKYFEEILSEFIATLIQHKPENVYLFSINYFEEKLNSSK*
>N.PF11_0051
MPTISVYEDDLFEKLGEEIIEEKLLDVCFDFGLEVDDIEYKNDKKIYKIEVPANRYDLICVEGLCRALKNFMC
KFDDIKYDISMNNYDICIKGNQYIKVDGSVDDRRGYVVCCVLKNMNINDSVYNNIIEIQEKLHHNLGKKRSVL
AIGIHDYDKIKFPLKYKFEKKEKINFIPLNEKTNLNGMNLIDFYSKNLNLKPYLKIIKDFDKYPIIVDSNEQI
LSLPPIINCDHTKISLNTKNVFIECTAIDRNKAQIALNILCSMLSEYCVPKYSIQSFVVIYENQDFSDDQNLK
KKETQFLYPIFENKSLTCNIDYVRKLSGISHITVHEVNNLLKRMMLSCDIMDNNTFKVTIPFYRSDIMHCCDI
IEDIAIAYGYGNIKYEPPQICKKHSLNNCSELFRNVLVECGYTEVMTNALLSRDENYNCMLRTHKSYDDPNIN
LDEYNPLAAPIQIKNSKTSEYEIIRTSLIVNLLKFVSANKHRELPLRFFEIGDVSYATYNQTDTNAVNKKYLS
IIFSDKFTAGLEELHGVLEAILKEYQLFSDYKIEEKKKENISIRSDMYYKLIPKEDPSFLNERIVDIVLFPHN
LKFGVLGIIHPKVLENFSLDIPVSAIEINVETLLNVLMM*
>N.PF11_0052
MSYSNKKKEYKKFSNKNVMSFEFNSDIQNDIGVIKSYTSRISRINDDEPYSIESSEKVQEIVQEGKLKIQEIQ
KKLKNYRNITENVPQHEKVRVKLMLQRLSNSYLEAVNNFQNASKNYINKTIMNDLSTKNYNVEENMEYDSLNN
FSRKSNSNYDTNIDKYNVNIYDYNFYENNYETSYSKYNNSTEFQSVDSTIEEKTYKKKKKYKSNIFTGDKKNE
VNEYLLENNNYEQENNNYGQEKQFVSINTVDIENEILTQKNKEIKKLHKDIINIQELYKELFEQINIQGENID
NIDSQMVNTHDNIMMSGREIEIARNRYFSSLRCTIYLLFLILILVIIILVVFRII*
>N.PF11_0053
MFRNVRDYFKSSNDVNEEKKNDVGSVHMDENTENYDKESNGFVHRDFNNGINKEDKDYEELDLKKLNVSSNDI
TKEGNNMNENMMNRKLNSSVEDGNDNNHDKNVHRNIGNDTHVSYDDKRKSYLDNNEENSEQMNDNMKKRKLNE
ISYDNNEEDEFRNKKDDSMEMNDKEKGSTNMSTINDNNNNHNDDSNNHNDDSNNHNDDSNNHNDDNNNHNEDN
NNHNDDNNNHNDDNNYNSEEKPNYLQEKLEQLLAQTKRYTEKLAGQRLKMNAQMKSSKKGRCLLTEKEEDFML
LKDANEEDEAIILKQPMNINGTMKPYQLEGLNWLYQLYRFKINGILADEMGLGKTLQTISLLCYLRFNKNIKK
KSIIICPRSTLDNWYEEIKKWCTPMKAFKYYGNKDQRKELNRNLLHSDFDVLLTTYEIVIKDKSALYDIDWFF
LVIDEAHRIKNEKSVLSSSVRFLRSENRLLITGTPLHNNLKELWSLLNFLMPKIFDNSEEFDNLFNISKISTN
DNKQSEIITQLHTILKPFMLRRLKVEVEQSLPPKREIYIFVGMSKLQKKLYSDILSKNIDVLNAMTGSKNQML

NILMQLRKCCNHPYLFDGIEEPPYIEGNHLIETSGKMSLLDKLLPRLKKENSRVLLFSQMTRLLDIIDDYCRW
KNYPYLRIDGSTPGDERQVRINQFNEPNSKYFIFLLSTRAGGIGINLTTADIVILFDSDYNPQMDIQAMDRAH
RIGQKKRVIVYRFVTQNSVEEKIVERAAKKLKLDSLIIQKGKLNLNSAKENNKQELHDILNFGAPEVYKTQDI
SSISDEDIDIILADAEKRTIEIEKKLKNLENIFDLTNISLDGGLNMYNDLEKEASEESTDEEDSSGSGEETIL
EGSGNNDIVEENGVKKKKKKKKNINKIRRTIKKFLKNNKKNMTFLDLGERKSKWKVMNTACGRTNKKKMVLHG
WRAEARGGHDFQFFNVEKLDELEKIEDKWNNYMINQEKINVLIEAQNEEKDYEKIVTFNEFLDKHAKNIYQII
TLINPSLFDEHKDTKEEELLKMGTHSDVNSNDKDYKRSSFMSTDYNMKTTYLNNINSNNNNNNNINDNVGTSI
NTIDDQQYFKKKILSLFESGKKLQMIKFKDRNIKNCYTQVANFIKKNFPEKINNNDTNNNKSTTLKNKKLKKG
KQTKNVNVEKDIASVDIEKIKLQKQELMKQGFAKWNKAEFNKLMSGLIIYGTNEVEYIYEKYFSNSKKSMEDI
KAYLTVFFRKYDQIKGGVRLFDKIKRSDLQKKIIEEENDMITEFVEKQLSEGVDSIEKLQLPSNLRYDFMEKR
EVLNITEEVKTENEENVLSNEELAYYDRENKILLWLLYQQGVVQIKNIHILAPYYWPEAYNFFKYATTPFENV
EYRCRLIVDAIAELYAKKENVPLNNKDKRRI*
>N.PF11_0054
MACSDKREDVGSLENIKKCDKNEENLRNFESTFIYDWKKALKRVEEGRNCCENLSRIFSEMIRIEKDYENNLR
NLCNMFQIFDNESSGINNGIISLRKNIERRCEQIKDFINYMECEILNNTLNSTLINHKNVFDQIKVDGIENDR
IVERTRKESLKNIENCVYAYENLIDCINIFHNSRYFHPLKRIELSTSCINKYLFAKKKEYEYKNTINNINSVE
LKKEKRLKSILYSLESMDHKRISCVKDNIMKYLIFFTSYIRNIQYDINSCIDVFKDINAYKEIEEYCISHSNI
NRKKENEDLIFYNNIVSWPMLIDYFNELYNINKKHLDQNITLNGEMQVDYTNHNKKGYTNFISSFFNVNMYKN
LIQSNNIKSNRNFNNIYTNIFNEIVFFNKRENEKEDDEVEEEEENEENEDGEDDEEEDDEEKDDEEDGEEDGE
DDEEDGEDDEEDDVIVDDYVDDNNFVDDGKEEGNHKIMEKKNIVNLNKGEYKKENDKNVIDTSSMVDFFENDD
NVCLKDEDNKRKEEGNDFVYSKDKELGVDKFDKDYLKNEEHFVKKEDMKCDKQNKIKKSTNKNYDNNKNYENN
KNYENNKNYENNKNYENNKNYDNNNNDKSFIPSEKSESDIESDYSYKYIKNIYEKFENVNESNGDVNGCSIVS
PNINNNVKRMKIFFRYYMKNLFLGNFDKILDFHITSHFNIYRNRFLFCECLIDFIKKKRVFFVNVKSIVIFAK
IILILLDYCNLHLDYWSCIYILVASENFFCELEVDDINTLLSKYPFNEKVKENSKNKKRKKKILNNNIIYNVN
GKINKNININIMNEKNSLSKDIDMSDESKKNNQRKKDTFSSSSFLICDSSENVKNSQMGPQIDIENLEDDNNK
DVSRDNNKSIVNIYECNKNGSKELNDRVSVCDNNNMAKNEEIETYHNKLNDKQNEERMDVTNYNSQLNNNNDN
NSDNNNDNNNDNNNSDNCNSTIFIEKNENLCSKKKKIFLGKFLYTHNIWNNIKFWEVSILIIISEIIQEMILL
EKLRYENKELLIKNYFFFFKYFNFYNSMINFGLSICQIELLLNKIFHSFNLKNDPVSRKFFFQIIDIATNKNI
TLNYIKMDNKNINNEYKRYYLQYYNNFLNDDNNKNNIKKK*
>N.PF11_0055
MKTNIFSCVISFFILLLFCKAYNTSSLLETIKHDLQIVNNSNFNTVVNKFRNEKVFAVLFFKKSNKNIKNVIK
NYNDVASKFKGILTLCVVDCDENASLCENELSLYVPDYKSSNTHHFLIYPINPMPKFVFKDEITESNIKKYTY
LIPSKIDIIKDTQSFNVFLSKHENMPKVLIFSNKKKPNYVLNALSNSFNKKLMFAYINNENEELVAKYNVKNL
PTILLLKKNKVVDTYKGKQNYINIFDWLNVYSETFVLGGGFDISPDKTNEKPWKFELIPKFTKLSHGDICFKK
ADKGLCLIYLKEGEKLEKSEIDMLLSLKEKFKPHIDGRGINFRFMWIDIAMETHFRSLLEFKKYPSVVVFNPY
KRIRYAKLNEDLTATKENIEKLLEKISGGDAKFTMLKGQTLPEFIQDPNAAKANEKDEL*
>N.PF11_0056
MEFRRYFSSEDYIFEIKNNEQLFVCENVAKNIKPLWSLKGHRNSIEGLHLLDDERFCTVSHDNLVSLWRLENK
QNIVNIKFDDAILCSSYLKKNKYLCIGRTNMNNNINIINMDKEEIIESYKSECNSIFCINYYDDNKISYGSKD
GSICFLDLIKKKNIYRYEEIGDCINYCTSSFSDNCFYNSNINNNNDTILMNPNFHIFSTYKGNILFFDLRCML
PIYQNNNLHSNYSVNCIYCYNYFLYSGASDCLIKKYDIRYIDENKPLDIYVGHTSPIRYISFSKDFSYFTTSS
DNGSIKLWSVNKKKEENENKIYSSFNNLISPSSAFSFDLDSPVNDSLKVKKMNMKNKEMPDKFVEINKQTKNK
TIKQNKTISHNNNNNMRSSSIISDTNYYVNNLYDTTQKKKERKNIFSKKYENILLEFQKKKRTITPNTILSIS
NEKKKKVQAVSKNELFNMCKMNKTQVDKEKEKKNYKGQFFLNQSVNINKMDNILMNSKKNQSIKEMDILKNKK
RNDQLKTNIKNKLHIPSPMQYTNTKGDKTYKGKNERSYLKRNISKDTNKYLNIQSPISNKFNESDYRSFFYNN
ENVINNLYPSSQKVKIKYANLSMLNHKGRVTSMEWLGDLLFSASWDQTIKCWNVKKYLME*
>N.PF11_0057
MKGNLILSLNNAKDYLKNKNDEGNNVYDHICDIINFIVVEKPEKSYENFELISNHIKESKKCNHVRIIDEKKD
EGEREKNNLDNYILNEHIKKKKKWLHKMKKWYFENNMEKKKNNKTLLLPFLHNIFEQMKLINWAGYHVKNNLI
CYINKSMKNIIQQYKDELLYLRFWGIIKGTHNDYYILEGEVKKDITIFSKKKKNKADNSSYDEEDIKQDGQFS
DSSNVQEERKKKKRDGSDQDDESDEKSESDDNKSGDDNKSGDDNQSSDDNQSSDDNKSSDDNKSSDDNQSSGD
NKSSDDNKSSDDNKSSDDNKSEDDQNNYYEFSKTKNNIFQYKKEDYERFNKNVNKNVYWVSLNGIENWILLKS
TTPKYIEIASQMNKMLTGHMNEKINSFPNLSIKEKHYLRALISIISSSTHISPQNYFIKKKEGGKKNDGGKYK
NDDKDEEDEDEEENEDENDDNNDNNNDNNNDNNNDNNNDNNNDNNNDNNNDNNNDNNNDNNNDDNNNYDDDEIIKNK

NFNFDIQLLKNIQNWVYCKFSFLPNGHIFYPKSGNLKKKKNLTLFMNENKVLHILRNISDQKDNKNIWKIKHL
NHGDYYGKNQLHYDIIIIYNFLFYGAFTIYSNKEYFNFYIGNGIKSKHAYLHAYQPQKIQSDKSELSEIEQTD
*
>N.PF11_0058.2
MFSLFRIEDVLNIETHFKDSEIKNVIEFLLRAKYIDKVIQNVGFCVGLYDIIEIKNKEIIKGSGEIRLKVIFR
LVIFQPFENEIIEGFVKSSDSNGIIISLGFFENIRVNSANLKEPKELEKKEWYWTYEGIKFFYTKDELIRVRI
LDTYFSDPNEMNKDESIPSMSITGTVQQDGLGLVKWWK*
>N.PF11_0058.1
MFSLFRIEDVLNIETHFKDSEIKNVIEFLLRAKYIDKVIQNVGFCVGLYDIIEIKNKEIIKGSGEIRLKVIFR
LVIFQPFENEIIEGFVKSSDSNGIIISLGFFENIRVNSANLKEPKEFDREKKEWYWTYEGIKFFYTKDELIRV
RILDTYFSDPNEMNKDESIPSMSITGTVQQDGLGLVKWWK*
>N.PF11_0059
MKKWKEKRRWHINIILMFLHHFFDRINYSMRNTFLHDHYIFMKFKKNKIIGLLSIINSSVCLVISPIVGYYCD
KHKKERKKMLQFISVSYLLVNIIHYMFIRTNSLSLIIFVTAISKMLHECSHVITESIFIESIDRGKKSLIFTY
QKLISTVATMLGPFFCLILFYVYNDKWNIKNIFIIFQFSILTIVPQTIISFLWENKTVDTIKNSEEIELLTKK
NDKKYMFCFSTKHIPYIVFVSHMITLAGAGMTFKYFSLFLKSEYKVSPILMCILNIVIPVLLTVFTYISQKLS
KCIGRAQVSLFFTTIGFLLLCSLLYIQNYKDVLKVHVFRSVFQNCTNSIDKSILYDFIDTNRYTGRWMGVQSL
YYLVWSISAYFGGWLSDISSYRNTFKITTFFYLISLIIYAPLLWLVPVKEIV*
>N.PF11_0060
MKNTYHHDHNIFFEEKKKKRKRSISINSNYPNKIHVSGGNKKKTKNKIIKLENEKIEDYFFIKRFLPICEEFL
LKNEDNVFFHFFNKNKEMELAKIKIVDEKINKSNMEGKYKQTCKQIQKNYHNNRNNEQHVDSLYDDNYTYESI
ENMIMGKKKKTKKKSTKFNLYKSYIKDTKKNLKEIELPFDRAVLLNVIDKKDKTKKIIKIINKQKVLNSFGVS
WQNMMEYIISLNEHKNLMKIFNIYDDNKNFYIVMEKLYGKELFNFLVYKKQVKENICKYIISQILQAVYYLHY
HNIIHRDIKPENLMFRHKKTKNKGYMYNYELVLIDYDTCHFINNLQPTSPNVLMNKFMSPYGSTSQFCSMHNN
NNKNNKYSDHNKNKYSDHNKNNKYSDHNKNNKYSDHNKNKYRDHNKNKYRDHNKNKYSDHNKNKYSDHNNDNN
YYNNYYNNKNNNNCYYYYNAFSQNNSYLHKNTRLNNNELYTSLHADKPTCLLDHMENTPGSQKKKTTSENNKE
LEKETNKTQYEKYAQKKIYQNQKNEITNICLHNQSKDITKRIFSNTHETKEANNINKYDETNNINKDGETKNI
NKYDETNNTIKYDETNNIIKDDETNNHRTSSNKKYIKLVGTYGYIAPEIIKGFNYSILSDMWSIGIIFYILMT
GITPLPMCLMINYKNTKDILLKKEKKGINFNLLSFHNYPIAKDLCEQLLQFDPNKRISNTVCAANHPWLKYFH
ILNKTQKNKKNKKKKKKKNFMCTTRWIYQQNECKRERKEKNKKKNKHKNKNKEKKNMDNNSYCVYYDEMVNKV
DNIKSVEHMNDINSTYRLYNDSYCNARNTWHHEINNQLNVINKYIYPFTCPNQNTKQNNTYINTYNNNANLFL
HNNYYYHQNDEHIIKNNSKFLNIFHKDTYPYSSFTVQEHNEKKNNNKKKNNNKKKNNNNDINNNNDNNNNNND
NNNNNNDNNNNNNDNIFHQINKQINIHNQNMENDSILMDTYQNTVHNNKSSFIQQQHKMYPNNSSQKYSMQNY
NINNNSYINEKKGNTQGSLNTLKVKEKMYEEVKQNNIISSHFNHTNNENETHQCTNGIINMDMPYCKSGINKR
DIIQPLKDININVINFFDEYHPSNGNIFITTKHLSENTYSNEKFIELFENFVQKKKKKILSFHNSFNEFPIIN
NNNKIQINQNYIQGNLHLQNIKKDIHSINKELDIKKKNENQQNQKNQKNQKNHQNHQNYVYHQNQTNLYKPNI
MVKGEKQNENESEYEKNNEIKYKNEENNINTIQHNNHNIQIYKDKRINFMNPHKVNMYHDNMSKNERTEKIRY
DSRNEEKYIINDDHYDYEICNNYSSSDNNKYNDHNEYNTYKKHSHITTPNKRSYSFIQQNVKEQYEETHIYDI
KNDIHDNENDNKNKMHNQYASHNKEINYTKIYSDKLNNHSNIHKNREQNREQNRDSNNTFSTYTNNLSNSLNH
MNNTLLYKKKKKEKNNIIYDNLITITVSCDSAVYNKNQNNTNSSYENNINYPYMNQSNYYIANKFQKTLNPYE
VDHIPKNVPSFYQISNQLNTCETYNNKMKRNKNCTSQDF*
>N.PF11_0061
MSGRGKGGKGLGKGGAKRHRKILRDNIQGITKPAIRRLARRGGVKRISGLIYEEIRGVLKVFLENVIKDSIMY
TEHAKRKTVTAMDIVYSLKRQGRTLYGFGG*
>N.PF11_0062
MVSKKPAKAKKTGTGPDGKKKRKKSRYDSYGLYIFKVLKQVHPDTGISRKSMNIMNSFLVDTFEKIATEASRL
CKYTRRDTLSSREIQTAIRLVLPGELAKHAVSEGTKAVTKFTSK*
>N.PF11_0063
MCCTFWGLRLDNTCNNMKSVDIIKCDMCFFSHDGLFLIYTIINKIFVIQVKNLEMYRIYVESYKVDDIKLSSD
NIHFLTLIKNKGCVCIYSFYKNNLINKICDYFQTYDHSFFLSKHNNIGIVKYEKKCLTIYNMNNPEKCLINIE
NIKYQNKGYCSNYINTIFACLVENKKDVNILLLSLFNYTIIKIIKCINFFPNDILFSKSDHIIAYSNKNRSLH
IYSIDGDLLHVYKYAEDLGGINIVSINKMKNTLALGLENGYVKILCLENFKEIKFFFLDNTLQMNDKLNIYKE
NISSKNHINDILSTNIKSKRKKNEHFSFYTNVSEGIKEGEYKLKREKGSVEVATKIGITFLSFSICGNYLSVL

NEIHNNVVRIYETKNYSCIAILQQKNKVTFIQWDNILYKPRLLICTCTCYLFMWLPFECAVIKISNDFVCKEA
EWNNDGTLLLTKNDNSLALFYPRKK*
>N.PF11_0064
MRKIIPLLYSVIIFFVFKSWSYKYTVEGKEVKVIYTNDSNKNKKKNELNNFQYIFLNENEENYNISSSDLNDI
NIEEEDIYFYKSFTKIINYFKYRIPANINFTFEYNDGDIILLDKKKKGVSCKINNLFYSESGLKYLDRKNELY
VKSEAFVIKHGNQENNTNNDKNEGMNHRNNNNNNNYNNNYNNNNNYNNYYNNSFNSSVREDEFISGTALCEN
RNKNYLPKKMLCFPSSNKNNELNITFIKNEEYKELMELYMTGIKKDNTYLLKKIANYFKSILNLDYDVKNISY
YIFCKTLQCNLQNVFVLPYESEEEYKIQNIIWSYNNEQIALNNSFMYNNILGNEKIGNRNIIWRHENNNIVKH
NISKCNSIFFNEMEYYNCYKNLENVYEILLYKETNVLNKYITNKSIIKNISSVNQYTAIFYDDNDYVILLKDY
KYYISFQLKNIIDLVLFQDSINEISFYYINFESNNMLLRIYKCAINNNEVKCADISYIPYEINAKEKFIYLST
NQKKDVSSVFVSTNTKVWKIYHTKQENDNNNNNNNNNNNNNNNNNNNNNNNNNNNNNDNNLYSRKLIDSI
QNTNEEYFGHINCYGIEATYERKLFSKYLNNIPDDNKNVVGCSYLKYYINSSSKALLISFIENAHFIQRTYHV
VKNKNIIVSSGTTKLIIRVNELFDMIIMFVIFLILLLSVYLIYKILFTKKMKVRRMKHYFFDDAKNSCPSTVG
H*
>N.PF11_0065
MGKGIKKHLKRVNAPSHWMLNKMGGQYAPKTSSGPHKLLESIPLVILLRNRLKYALTFDEVKMILIQKIVKVD
NKVRTDCTFPVGLMDVIHITKSNEYFRLLYDIKGRFVPHRITNEESKYKLCKVKKILLRKGRLSIAVTHDGRS
IPYIHPDVKVNDTVRLDLETGKVLEHLKFQVGSLVMVTAGHSVGRVGVISSIDKNMGTYDIIHVKDSRNKVFA
TRLSNVFVIGDNTKPYISLPREKGIKLDIIEERRNRLKALNN*
>N.PF11_0066
MNTMLIKDNINISINEDVEKELYECFSLFDTNKCGYIDIREFYFALKSLGLNFKKEQVKNIFLDIKKDIDEKL
NFDEFFDIATKYLHTRYNDDEMDQMFSLFDPNDTGKITLQALRKVCTDIGENISDTELNNMIHFADKNNDKVI
DKNEFKKVLLCSWKNDPLSDVDSDS*
>N.PF11_0067
MIYQYIFVLCILLVHVGQCMYKIKQSGDILLSSQHNSLMNVKVVGKDNSFNLELNNMIKFPTNCIPFSTIDYN
NKKGKKPIYYIKHEFKNLIFLYKNNVNIMQMVFPDRLYMVGIAANNGLLLTLTDVDIYRIDTKKKKVLLSRIK
PGKYAHILNNRELFYTGVIGDSVLNKFFVTAAIRNNYYILHMEYNEENESIEILNVIDKVNGQYLRVLNDISI
VGDVLYITENAENIVQIIINREKNTIQEKNIYTFNNNDKIVGISASRHTLYNNNNDMVDKYVNHNMKDNQNKP
ADNMNDLSNNTMIITVDYILVNSKKMFKEKPSSEGCLYLLKITKDSVEKYTIVDLFSKDINSFYFTIYDAHIS
LDDDYKKSGIVEALHKQKRKFLNFDIPNDEDEPTNNINSNENNKNQNNNNNNINVVKIVWTNYYNCTINKGIV
DLRNVKIDEDSKEKKLPFVNVNNQFSLAPHIGASSYCSRKEKIFNDICYYDNRNNYVSLLNRSEEYNLRYQGN
FVFDGYRNYLGFDYKSFQDTHIITYPINNMLYVYMTKGFVTINLPQPYGISIDSKSSTNNQIVVYITGNDEQH
NYVNKCVIKKVEKAYDCFVVHKKKKEANELFQYISHISYGDNEEKLSTYIYVTNNKKTIYKLTKQENKWNFEK
WFNLDNPSIRVGPISTSVNYFYVQRNVLEDLIKKYPDSALLSKFINVNEQDLHITEDGYIFLTLFHTVIFTSN
NDAYGNDSGSTIHFYTSILKEKFYQSFGVDSIIYNIENDSKFSYYLS*
>N.PF11_0068
MKGIPKRPISNYIEKTNETNLYKIRKGLVNEMNVEGHIYVNEKLKTLLDEEIATYELNKNSTFLPAVMQIANV
STLPGIVKASIALPDVHAGYGFSIGNVAAFDMDNEKAIVSPGGVGFDINCGVRLIRTNLFYEDIKPKQEELTQ
LLFNHIPVGVGSQGFILCNQENLDDALCLGMDWCVKEGYSWIEDKLNCEDNGRSLYADSNFVSIRAKKRGITQ
MGTLGAGNHYAEIQIVDQIYDKKSAKLMGIEKKNQVCIMIHSGSRGLGHQIATDALIDMEKSMNKYKINVIDK
QLACTPIHSKEGQNYLKAMGSACNFAWINRSSMTFLARQAFSKIFNQSPDDLDMHVIYDVSHNIAKIEEHYID
GKIKKLLVHRKGSTRAFPPFHPLVPLDYQYCGQPILIGGTMGTYSYVLTGNEKAMQATFGSTCHGAGRALSRN
KSRNTLSYLDVLNKLKEQNISIRVASPKLIMEEAPESYKNVCDVVQTCHDAGISNKCFRLKPVAVIKG*
>N.PF11_0069
MNWKKALSLAGGAAAVVALTYMLMKDEDNHDDNDDENDKKKKKDNKLSKDSNRIIKGDSMTREDLLQLLNEML
KLQSDMKNIVKDLIVVAKNNNYDFMAVYNVAKTYNTIDPLGKYQIEMPEFDKVVESYHFDPEVKETVSKLMSS
QENYYSNMSETATLSVDKIIEIHHFMLNELYKIDPEFKKIPNKNELDPKLIALVIQSIVSAKVEEEFNLTSED
VEASIANQQYALTSNMEFARVNIQMQTIMNKFMGDHFKFMCDKEGAY*
>N.PF11_0070
MEINKNNVTFVLGIVSSILLFVTAVMLNDIDFILGSHIFFGFLFTIYTIFAFLIPNTKNSRDFLLSYATSTAC
IFVISLIFNIVVTKYNVEGNCRFSTNKCFPYILGTTSGFCSSALFLLTSILSFKLANVW*
>N.PF11_0071
MEINISNITNSSNNKKERVNIHSHIKGLGVNTNIYLHENEISLSDEKYSMFFDNSCGLVGQFKAREASLFLVD
LIKQKKLAGKCILLAGPSGSGKSALAIGISREINRKMPFVFLSGSEVYSNEIKKTEVILEAFRKSIHIKIKEE

KLVYEGEVVDMVVEENECLYSLNKAKQINAIIITLKSVKGSKTLRLAPKIHEQIAREKIKIGDVIYIETNTGH
VKRLGRCNDYAKEYDIEFDEYVSLPKGEVHKKKEVVQQISLHDIDLANANPTVGEDLASVLNSYLRPKKTEIT
EKLRVEINKTVNKFLESGLAEIIPGVLYIDEAHMLDIECFSYLNRAIESPLAPILIMATNRGICTVKGTDNIE
PHGIPVDLLDRLIIIKTFPYTLKEIVQILALRAQTEKINITEEGMNYLAKIGIQSSLRFAMLLLEPSRILATL
EGNTIIDIKHIEQADELFMDAKTSAHRVVDQSNKFVN*
>N.PF11_0072
MKNIKSCVTCLLFFYIQIWINCLFCFGYNINKNLGVYPFSLIRKKNVPQEKFKRQLIKVQNEGKENIFTQDGN
TNTIKEDKTKQTKTNIDIKNKIEKEKIEINEILKKSENILLERSFSFHKEDITIKPELLKELITQKYRKIFQR
HDKDCGSSEIQIIILTFKIFFLTEHMKKNKKDFACLRGLFKCVSKRRRLLVYLGRKDREMFEKITSYFNIKKP
LLPRTPEYYNKDLKYIHFNNTKRFKNNAEKKKKDKLKKKKVQTDKILFGN*
>N.PF11_0073
MFCSFLFLFFTFFVVQNKEIAILCSKVHLKKHIYYPRKGSHFHIYSVVDNNVNLKNKNEYNSKLKDRIKIYEQ
VHNNKCKKIYFLPLIFLKNNNKDVYIEDHKDKNIDNNNNKKDNKKDDINNNDKKNKINDTPAQNITNDKDKNY
SNNFEYIENNLLKLKNNLEVFSSYINKSDVIIVPLNYHDILNKHLIEIIPFNNIINSLDYIFLFNKNDYNKSD
ININNNNNNNNNMNKYNICPPYNNIYNIIFFIYNYNDQDNPLNISSYTPSFFTNKKDNIIHKEKFQLVHVIQK
FLNDHYNLKDQNEHKKYLNINFLFTKDNILDINKIDKDIEENKMLYYPKKNILNKETNYVNTTNNTNNTNKQE
DQNLIKYVHKNNDTFFKKYKPDTLKNLTNENYFIYNFLSSYKLDILKEYIYIAKDITIPNNNVIKHIQNINIK
KKIHNIELLLNNFKNCKLNKNINSHVNVQEIKKKQYSYLLHIILSDLLEKYDSNLEILKNYLYQVFKENIQKI
KINSDILFQFKKQINHIDQLFDYYNSKIFILPSNKNYQAQQKSYYIYYYYKIKLLQLLNQTANEIINYYISKG
LYVKNYSFNEFSFSKKYTLINYIITYIFNLIKNKVNINFNYLSPNAFGFSSYKDDISLSPKKDIMITTSEMDQ
VQKINIPRSAYTKTMLMMDKDRN*
>N.PF11_0074
MIAFISLWVLCIINVMAYCISLNKYKFIKSYNLYNKRKHQQNVLYGIPRMYKWLTSYYPTVREELINNEKQKS
VDIFYIDMNGVIHHCTHANKEKLPIYDEHELFSNILQYLKNLFYLIKPKKLIYIGVDGVSPKAKMNQQRKRRF
LSIFKINDNDNTSNLFNPNCITTGTDFMYKINLSLNKWFKILKKKKVFEFDVIFSGSDVAGEGEHKILKYIRE
NCKRDSNFKNYNHCIYGLDADLIMLSLVTHLNNIFILRDKFKISNDLHNNMLENIERTQNTFNNDEYNSFNEP
QNDNINYNEEGENIQLNEKSKTNESLVKEENNVEYENNEEINNEINNEINNEINNEINNEINNEINKEINNGI
NNGINNGINNEINNGINNEINNSEGMNNILGGYKIPFLPKVHKYTKEYYMKYESLNSNDFEILDIYKLRNSIK
TQIATYINKLKKEKNIVFSISRVVDDIVFLSFLVGNDFLPHIPNIDINEGSMNEILNSYIFYIYKYSNYITYK
DKVHIERLKIILKILSAQEFEYFKKRGINENISEFTDEQKYKKYYYLHKFGLEDPKEIQNIVKKYIEGLFWNL
HYYHFGCASWYWEYPYHYAPLCSDLLSFEKSDFFFEKGKPYSAFTHLISVLPQKDKNLLPDAYKNIYVEDEVK
SFFPENVKIDPNGKKETWEYIVHLPFINCNMINKIITEKSKTISKLKYKLRELNGREHRY*
>N.PF11_0075
MKSSSKDLCKYFCRKCNNFIYYNEACELIYKKIYPDYYKIYYNKYEFDKKTKGKKEVNPKQYDNLYRQLYNNG
LCGVNKQRNDFLNKDNINNILLFLNKGSIQCNKCNHIHMWYMK*
>N.PF11_0076
MFLSIQFIILFFALIHIKNIIALKLEIFKGRFGSPNSTSSMFNKDNKIKCRRKEVPLLFVSSNKCLKDLYLLK
HSGVSPFEEKGSFIYLKRGERNRKDKNYPFTLFIEYGTSKSANILEKDSNKYEEGNQKYTNVIMEGCKDIIDC
TKSEGLQNLMNQKVVENNKMDENIKMDENKKMDENIKMDENIKMDENIKMDENIKMDENIKMDKNIKMDKNIK
MDKNIKMNKNIKMDKNIKMDKNIKMDKNIKMDKNIKMNKNIKMDKNIKMDKNIKMDKNIKMDDNIMMRENKKV
EGKSRKGNNIRNKSLVLKRIGNRKVYNKKRKSITSRVVDIQNTNVRDYLFLPSQGRSLKLLNIIYNSWKEKNF
DNFFDVLNSDDFDHTCIENWLHKFSISLSKKYLKIKICGDHILVRNEYYEKENKTLKEYKITYSIINKCLKIL
INNIDKFCSYEITSILWAISIILIKSFKNSTYIGNNINVDNVKHNEMIINVIETFNIFLSLIINNLNKVKYNL
SIDESLWAVWSICKLLYFNISFDNYYIKLKKRISSILNKDNKNELRASNELLNNVEDSLVMYKNNNENYDKVR
NIKMIDKEFMLKDNKKDEENICYDNIYNNSSYTNYQISYMMKKFKLSSDDIVNILDVFNYLYKKLYKNISYLK
EKYYIYIPFIIFESQTLMLNNSILLLNKILYLILKNDVLLSLFSFDKIKETILPPVTKKVEDYEEKLLHYNKS
KDIFRIKTLSKIYKLIKCISKIFYPLKNQNIYNIHLHNNYKYLNSSILIKFVQACNDVLIKYIDYFVYIIYGY
KNIKSKDYSSKCNTYNEKDTSKNSMHNNNNNNNNNNNNNCEVIIQKDDTKRNVKGASNINENVFMKDNRSNDF
HNNMNDVITYNKTYDEINNMNINHNIRFKFKNEIIFIYNCLKSSLVSLIKLDLKDKYIYEWLNRNICLFQFPD
NYVDKNKDQYSEYIQAGGLQNKRNEEISNTEMNDYNMNKYINVNNDDESQYDNMKNYVEENVLSDDNEEKMNV
NDVEPEKKRKDKKEENKDDVIIINYGDELYVDNSLYNNQKIKKENEILAEQNKCEEYDKNGYINSAYKDDIML
ESLYDSKKDTIKWYDKDEEYKKNNKYIDNMDDDHNNNNNKMNESDVCLDVLALSKFTKQYSNEMKKKLIDNVE
KSLEYSLKIFQNIYNKKKGDLPVYINMNDIYIIKDKYIVSLYDEETKTFYKKNKNIEFRNIIMPKQLSIFINY
LGRNIYLIDKNKLNKIFLLAIQYINITKFNYFTSNDIIHFLQGLLNYYSVQNDNKVVNIYNNKNDINDNSNNY

TSNNKIINDYINELIINLCYIAEDTFLKWKLANVCQLVYLLTKFKHIHKNIFNKFDEFLNTIQFPQYVYQKEK
SYIRNNSTEINDHKEKNTYDTYDMNHTCEEMLSINQNNNNNNNNNNNNNNDNNNDNNSNIIKTSYSNNYTNEK
NNVVHNKKNNIYINEFKYDNLGSALWALSSLNKNILKKKYYLKFSYLFSIYFSLHMKFFLQYKLGEQQNRKCT
YLNYSNKNSHNKYNDDISFFKHNMNYFKHVLDSMTISIYINTYIRKIKKGFYFLYDVIYNNVEIIDIFSLKEL
SYVLYSLSTINKNEINNTVYRGEMGKTEKNGLLNREEKLNELNQSNELSESNESNKLSESNESNKLSESNESN
KLSESNESNELRESNKLSDSNESNQSNQSNISNISNISNEADKLGFQKNNIDNEENKSEEDFFFTFLKSNISL
CVDDLDYLYNLDYIKDEEMSNPIKTLNDINETIDKHDDIKNNEVEKSSSSFTLPKSSDILNDDNKKTNKEKKG
IVIKKDKFLIFKNERIHLSNEFLNKLLMKINEYTKLILNEKKNKEKSFINDNHNVSNASKKNRMEIIDLIKLI
YSFFILLNQKVQINQAQTINFVQVEKLIFQYNMVVKKFLHSFNYIYHIIDTYSLISNIYVLDDFTKNIIREFV
QICIKKLEEEKILDMEKKKITLSIQNLKVTN*
>N.PF11_0077
MRNKYFLLHSYNCYFNNNIKPLFLLQQAKCLRTKKKKNWESPERAGIFSQEEKKKKNHLASQQYEEYSFDMNN
KDLYNNNNNNNISYNHKNYYNNFITDKEMNKKFNHTNNDREDYNIENNNNLKHKYEEIKKKLYKKYNYHEIGS
DPQNINKYEDNYENINELNIHKMLLLGLQNIHINELNKMQINSFLTIQQGKDIIINYPDGSGKTIAYLLPILN
NLYFLHDYLEQIILDSYNEKENVTSNKNVIQKNYKFNNNFNLNNELNDYLLKYSHYKNNVFFEDNTLDISNKM
KTKKFDLLPSSFNETNKTKYIQGTGGRKSKSTGKNKLNHLNDTSLSMDNNTNELIINNISIMNKLIEIIKINN
IKLSNLNSDYTNEEELKKLDNIIKYNITNRNNNICANINDQNKCAKDRYNEESIYRYLTLNPLQINKTVVILT
INKDNISQIINLIKKLDILKRINIQTLNDVPYNDYSNNIDHMIDTQIDDVKNNMHNSHTQTHKRDNKYKSASN
KFHDDNIDKDYYVNNNIKEENYDLENLQVSKVTHIDNPVLCNDEIMWTYADILITTPDIFLNSYKNNNKISNN
IIPSIIIFDEIDMLFQNNAYRNTMMNIFQIIKKRPEIYNPHIDISSHNIDNINESIDNALIYKKRNVNGYEEY
MSQDISTNNINNINNNIHNIHNNIHDNSNNNTCYDNYSKYTRNRTNDKSDVQLNNNITNACNNVNTFYKSKNY
NNRGKDKKEDTGLPLIQLIYVSSTLPSVGPTTAGSMLTERFNNIVEIVSKDNYKIPNNIQTQWIELNKEKMIN
LYLYEDREVEHNLKDDEERKEKHENENNYVDLKDVSLSNKINKFEKSSFEHRLDILIYILKKYHERTIMYEMK
RYNNDKCDDNNKCDDNDKCDDNNKCDDNDKCDEKTATIKYFNKKKFKLIQKYPIHKTIIFVNSIKDCIKIYNF
LKKHNWPVFSFHKNLSLNSRIQNLHSFSCTQTGILITTDLISRGINIKNVDHIINFHFPLDVITYIHRLGKIN
RLHNNIMENYNNKNISTSTLDRKKKKKKDEEKNKVHEKKIYLVTNLISTSNMPLADSIRNFDYSNTSLLSLFS
RKKSFKMKNKRKMNENTSSRYINMETLKEIELEINDKKLTSLFDVDDERDKIQNTSLPDEGNKNNSYVKAPFT
VFTLEDTDDEDENMSEYINDKNDDDNTSNREHEKFNMMGKKGKNKNNNDTTNNRNNNNNNIFISSTNTYPTNN
SFHFNETEKENIQRNYNIPKNSNLNIPSWDDVQFDHKKYIIERFKQKECYLMNQVKRGKLILNNFESNNDEDE
LLF*
>N.PF11_0078
MMEKINEEQKLLDIFIKLIKLENIYELIENKLCYEYLNKDELIEKGILLNNLTIKGITKYDEKNNSYILKLVK
KKKHIDTNDLSDDENLDNFPQHMFSKGSIVHFARKRKKYILGSRNDQTLSYHINDNNKKNCDDKNNTKNNINN
TSIINDVVNIYVCSVHKIKKNQISLILRNSTELCKELNVSNMFMLCDKGYFDVCLVSSDISAQRQINAINIMK
SNLDNESDILKILFFNKSPSQSSILKRIINIFDEYDKSESKDYNDVYDNINNNICNNIIDNVGDNNNNNYFNP
CCCDKNLEKDIHTKKEDAVEEEKNMNTYFKSTHLCTHNNIKLSNDVLNNYIYKCDKINWGIKNLNINQKKAVY
SCIFSNDIFCIHGPPGTGKTTVLCELIYQLIQRNYKILVTGPSNVSVDNILRKCVEDLNIKNVVRIGIKSKVK
KELHNYCYDEKIKECDSYKLCEDIDNEIEKLKLEINKLKNKKKSDNKIFIDKKQIYNLKCEIKNLNKTRKKKR
NIFFKDLINKNNVIFSTCSSTSNYELNKFVKFSNFLFDAVCIDECCQCTEPLCYIPISLSKKNVFLFGDHKQL
SPLIKYNKHKNKLNITLFERLINKHKQNISFLLNTQYRMNNLILLWSNKIFYEQKLVSHESCKEITVDELFSS
NRPNQKEHIKSKVNKDNKENKENKENRDNDKKNKNNNNKKNYNNSKKKKNANNKGTNKNHEQEIDNHIYNQTN
DIIQNYDYCPITWIETDGFDEFLEDIDDIDLMNMESKNVDINEKKNGNSKDLKIKEKNDVDQIKCNDNNKNND
NDIINKRSSEEERKKRKESQKDHINNDLSEDSDDYNNNMLTKLNDEELMKMVKIDVDDILNLNNKSRSNKGEA
YVIYKIIEKMIKIDNINSNNICIITPYSKQTNLLRNIFYDNIYNDQHYLSTYKNIEISTVDSFQGREKEIVVF
SLVCSNYFKNIGFLKDYRRLNVAVTRAKRHLVIVGNSNTISNDDVLNQLYETVLENGKVYLVNELIDVEAVIL
NT*
>N.PF11_0079
MGNTLDSNKPKNFVTYADYKYIGKLNNKNEHHGIGIILYNSGESFYGSFINGKKEGKGIYIDKNLTRYINTWV
DNKVFGKVKVVPYNSNRVYYFYYKNYMIEKCIYFDNNINNKESHHKNNIYNNYDNNSYNNNSCDDEEKRKYPI
GVTKFKEDLSNYIHSTHIMKKNNKLFNKNDNEYNIFSSSLSYSSDSENINLLDILKKKKKNKNKKKKNKKTKN
TQILSCTQHKMYEHNMNESNFTKKDNVNCEHTDKMNISLHEKNDKKNEKKNEKKNKKKKLFKYFSNNIENLII
ENYQTWSLREVIQWLMLCNVPVKWLISFYKNNITGDKLKYININTIRNELGIIAYGHAIKILQLIKNLQVMAY
NKKFNNLIQIEEYKNYIRQKENTNKNIKKGKNIKKGKNIKKEKNKKKEKNIKKEKKKKKKETKKFNNMDKKYI
DLAIHKNVKNIQNDTFYNKHENIYNCKNQTNFIYQNDSEIKKIMNKKKVSFEYDNNEEKKKKNIIKFIKNNKS

LQNSNGEYYLINHLSKGICSDSIFYKSSQSKSSSQLSSPLSSPLSSPSSSPSSSPSPSSSPSPSSSPSSPPSP
LSYKDNFPISSSCSSLERLPSYEKKLLSSSQSNIEHIKNLPLDVLSNNNSSANIKIKKSKSKYNNDKKEQKKL
PLILNKSSSEFSPSHSYTSKSYHYNIKPSLQSSSNNSSDSSYSISSTCSSSSSYVSSLYSNRSNDILNFYRNK
IIKYCNNIYMNTKLAYSYMNGFIIPHEDLIFIHPIENYYMDNTNEKNNINNPYTKEKIMNHNFSFNTKNNTSF
IDINTNIFSSNKQQNINNFGKYKKMKSRMFKGKYMGKEVAIKILVGKIKNFKKLHQILYNLYNLRHSNLVLIM
GISIHYPFVFIIYEYMKNKCLFSYLHCIKYKHVYISTFLQRYKTLLHITQQEKIKKTNNINNNNNINHNNINN
NNINHNNINHNNINNNNINNNNINYNKDYNNKKKKEDEQHNIEHQDTFIDLPEKSNISSDDNNSTDISQIQKE
NFHFLNKKIEENKNIIYDDHTSTLSDHSIHNINKSYDNVYKNKMNIFHYQHNVLCGAYDNNDNNINDNDIYCN
NIYDNNINDNHIYCNNINDNHIYCNNIYDHHKNTSLNSKEQNTDHNIEQINECNKYASETKYNIKKSNLKNNI
ISHKNFQKCNQIQMNQPYTFPPYQKELSSYLKNEKIKRKRKVLFSYLKTHIHFNSQQINDQHNRLSVQKIMKI
ITDVTLACTYLEKEKMSPINLKPTNILLDESLNAKISDFGISKIENCLDMNIDYSYKISSNSVIKINKKEYEQ
KKAKKIKIVNKNNNDLLYLYDHNNNVYKYNTQYIDVTYNNSYPSIFYWTPPEILRGKKNKKFYSDIYAFGIIL
WEMLSNDIPYNYPFASHIMAVVGYANEELSFNNIPVSIQSLIKACVNRNKYKRPTFEHILKTISTLYQKANTK
VEDALISFMDGT*
>N.PF11_0080
MSEFYENDEDVLQMIQHIKKITKESKRKYELDIDTLVEDTKTNLINNVNDEKDNMKQEIDEKIKKLKQEITKH
EKNIKEKNKLYQKLKRELANAVNNYKNKIKEFYLQTENFMKTYENEKKELDELEDKEWKDLNIQCIEILNNTK
SNIYATKDETSEKLRKVLKSIL*
>N.PF11_0524
MVFPLTLLKCSQNQPVMVELKNGETYSGFLVFCDRFMNLHMKNIICTSKDGDKFWKISECYVRGNSIKYIRVQ
DQAIEQAIEETAEQKARNMGRGRGGRGRGRGGMNRGTYGRGRGGSMRRGGRGQ*
>N.PF11_0083
MQEEKTKKSLEREEIFKDTNHNEKCINMKREDYKTNNMYEDTDINNVSNNTFNEISGKEKNQNNIEYNENVKS
HNKTLKLSNEKNNLSSKEKENILHNGSGEINDYNKMKDKDIPGKDKNGKTDIDNDNNNNNNDKIKSNNILESE
INNIQKNRTDKKKKKNEVNDTIPSTSEGENNINKLNTNKKDTIINNKDEDKKKREPEEKNNDTFQNEQSVYVD
EVIKEYPRIFATRLPFEASKKDLEKYFSKFGKIVDIYVSRNISNNKNKGFGFVSFEKQESMNKVLKEKLHIIC
GKEIVVDVASMRDDKYKHLFHMPSENYLMKNEKKDKRFMKNRNKTNFLKYPIYNNVNNISDISKIGYHNMMYP
NVNNFPSTNYQPLAYQDFNMKASFRNVYSPVLYNPYVKPFNPTYKNSTPVVTNDYVYNLINYNIWNNVVPYNN
NLYNSKKISLPNYYYNRPYPNQSIAMKNNNNRPHNLNKNEKPRTNYTSFTRKLPGGDEWNKRGYKLFVTKLNS
ATTIEKLRNYFEKFGEIIDIYMPNDVYTNRPRGIAFVTFLDNESVKNILSDEHSKHIIDGKEVVLDLADPENK
GKNK*
>N.PF11_0084
MEKTRIESLFIYDEDIKKVYKSMTDEELQAAKVIYYYPHEINENIKVSHTSTMEGISSFINQFSKHHIDYIIT
KENLMVMNKWYKGIYIVAIVKNIYKNNKIELLMCKLLHCVLNNFISIFTLLHGHIRNFLKYKKYNTTDNINKK
HSLQTLLDDYVFTYINTINNENISIHNELESFHFFPVEKHTYVTVQNFISSLILGNKQIKNGGLFYEGYLIYS
SLEMNDIKIIYNYLVSYSGVVNNLKLTQYPFRKIASTAAINSNGGLSSFARCNSLEEKNAYLMGIKKSSVFMP
VVTLSGDKKYKLVVFIYKGILLMLLIKGSKIKDEDFDVLIDIQNKCTNKNCNNIYNLYKLDEHLSNQFKKFTN
QDDNVKFFYYNHSNNSVKYTFNNKKINNEELSLISYFHFCIKDSEHKYNHIRINKSYYSRNKETTNLSNELFN
FQRNIQNNMKDEKESNTHKRNNNTHSTLTGDDKTVRGNNKNININNNDYDDHTSCNNRSNGCKENNSLASNKN
DDIQKNVNLSNDTYKHKDQDIEIKNIDQIYNNEQDKLINKTDNNKIDNNNDNNISNDENINKKKKQNEPYNHN
KNCLNNNNIQNDKREIIDEVDKNNYKFKNNNQKKPITEKNEYDTEKKEEKPKFKLIYNEQLKKKIFDDNTDDV
KIEKIFFKEGNSPWLYAHKSLQRELFIFPEDIKTSLTKAQQEINQIVQNNFSNIYI*
>N.PF11_0086
MQSDIIVNQDLKEYSNMMKGIHHVKSCVSSLGVETNMKNEYNEKVVERNDYNNNIEDETYDNNNININNNNNN
NDNNDNKDELLNSSNDLINEKKNKKNKKNSYDDSILCNNRGSINDENLDIPNNCDTSHNNNNNSIHGDNNVFE
KRNNSQDVHSSSILKNSEDLILNKSSNNSNNENVYSNEDMYSYKENLCEQQNNDETNNMFPSKINMDNCNINN
NYSNNNSSSNSSRSYNESKKSSKDENNNNDNDNRDNNNNNNNNGNNANNGNNGNNGNNNNNNNNGNNGNNGNNG
NNNNEGNDNNDDNKNNDENKNNNKEVSDDKDKDEHDEKGSNENKNLSHNNNNNNNNNNGSSSNNNNNLNGDNKK
NETSSIKSLHKSSVLSSLNADAPEFVPSTKSLQNNMSFNNLMWNNYYQDISGGTTFNSNNLLNNMNNTPGSNN
NNNIMINNTNSLLHNMNGINNNSSNNIMNIAGSNTMSSSNNNNSVNSGVVLLGVSSGNMNNMDNNDQDKMNTT
TNNNNNTNNNNNNNNNNNSNNNICNNSLHGAKSNSNHNNIHDNNNNNNNMHAGNINNISQTIGGMSSNDGPFIN
QGMMPGITNLRSSHFMSNSSANFMNNSENSNGPFHPMDGTHAHQNFQPNGSLIAHHNFYVNHHPYILNNHPNP
LPGPQPIFAPPLPYTNTLTSTGNTTGAGGVHHDMPFAQHTSVYGSFSNNPANLIASGASISSQKSPSLSINPY
NFLSCSLPTPPPQLPHTGPLSMTSLDPHFMSTGNNSHMKLPYTNNMCPHPGRRSIYNHFSHAHISNSNNMNDN

NFDPTNSNNNNNNNNNNNNNNNNGNNNNNSNNGNNNTMAANLNYNFNTFNHPNNNNSCALVLNNDNNSTNNIHLN
QGLNSNTLNLNNTGNVICVNNPMHLNTSGNMTHHHMNNMHMNHGMIINNNIGKQKKSISMNHNNTDNNNNNNN
NNNNNNNNNNNNVKQQFNNYREGKNSLKGNMMNSNVCINNNYNHYNSPNNNNNNNNNNNNKKHSRYNSISNLN
NAYNAAYNINTTGNNVNNISSNNSKNIYNSKGVKREGYNSEDNDNTAGNYYLKNGNPIMNVANTNNTNNTNNA
NNANNANNTNNTNNANNTNNTNNALDHVNVEGSNNKSKDTNNQELKDSNENNNNNNDVENDNFNDSSFSYKGQ
KNYNSKNKSNTSINNNYNINNIQNDKIPNTHKEDYKNNRYVNNNKILEDKNSSNANKNMTNEKNLKATDNNVY
HNNKKGYNDVPNNSSHNNNNNNNNNNNNNNNNKQNNSYLNNNKYHQNHSDRRGSNIKNAESNTNKMNEKKIKND
DEKKGKEKEKEKEKEKEKENINNNSHNNNKKMSEIHTNNENNYVGNKNMNYNTLKNYKNYDKKTMEFSSWVKI
AKMNPNNNNNNNNNNNNNNNKKKEKLSVSSIYEGSNNEKNQNVEDRRNSNFINEKINTQDNKTSNPYSSKNQNK
ENDDNVLFQHKDNENNQEYDNNEDHNNNNNRRLSGNVNNENMKKRNEDEDKKRSSKASIKEKENNAHYYKDSN
NNKKLNDENKKMSYNNIVTNNRNYNTAKDNNKEKNKLKYESNKKTEMDDRRNSVGVTANAVGSTPGSSNTFSH
RQREKLKIQNVEIKQINEKIINIKNYDKKKTSVTNVHNSNDEGTKGKGAYTHNRKDEKKEDTTNNVDSKKNDM
NDPKSIDALTENENNKNDAECSDSTTVNNKKGKRTSTNNVSSNSLKNESMKSSTTNKEQVSKDIVASNNMSNT
SKSSNNGDINVIKRNNNNNNNNTGMNKGQDKGANTGESNNNNNNNNNNSSSISSSSAVPNVNESSKNTSVYAS
IKNSLWQGRISFAEMAKRKPKVETKEIKETKAESVTSEKKNNHKNNNNNNNNNNNNNSNHHHHRNSINNHHSNK
SDKSSRKEHHSRYGSSSTTREKAVSSNNTVMKEYSKDSRKKNDHKDESSVHSFNSSRLNKKDNKNDARHKSEN
KNKLYNNEHVDSYDSGVDCVSVQNKKKETDKADDISVCDTISVIEKKEDFEELKDEKNLDNYTYNEKVSDYSN
EKNNVREDLHEDLHEDLGEDLREDLHEHYVDNKEDQYESVSQKGKCSNENIKDENEIKNNDTNNNDTNNNENN
NNENNNNENNNETIVSNEDQKIKEEKTVAVNIDEVLFEEKENKKDENECSQVDRVNINNDITKVDEEKKMDEI
ITCEKNISNENKQSGDNNKLEKEKNDKNEIYEKNDKNEKNDKNEKNEIYEKNEICEKNEIYEKNEICEKNEKN
DNKLEKSSDEHMNEKISDNVKVNDEGTLLLENLEEKVQNADEIEENKEKKNIVNDEKEKAKQKKLERLYREKI
YTKIDLLEIYLGHNWGEDGDRFNFVLSQENDNLLDGDYKNSISNNNMMNNNRHSVMMDDHHNIMKNNNNNNNN
MGNNSNNNHHHHHMNEQKNMNAHNKRFNNNMNNMNNINNVNNMNSGYNNNMNNKNNNNNMIQHNHHSGHHHII
NNKQNQSNQNVNNKNNDDGWRNSIAQKNMMNNNNNNNNNNMYINNNQNNNFYDHSNNNNNNMNDMNMQNIHMNN
MNNMNNMNNMNNMNNMNTINYNNNNNNNSVSSNLEWRKSDADKLKGFFDSTRVVESADAWRNNNNNNNNNNN
SNNNNNNTGQVKTVAENSWILKQNQLKADKYTCMMRKLRGILNKLTFEKFDLLYEQIILVGITKLDEIIGLMK
LVFEKAVTQHHFVQMYVQLCKKLNVHFHNMKLEDDTTVQFKKILINQCQDSFENNLKPIEFPSHLNEEEKFEF
EQMHKNKVRGNMLFVGELVKSGIISIPIVFVCIKQLLEKRESYISIKNDTKEGNLHLEALCMFLNTVGEILDT
HEKANQEKVKELYNTLNELVNNESITFRVRCLIKDVIDNRNEKWNKKFAHKLEGPTTLSVLHDKILKETIDQN
NRTYNHNNNMNYHENKMNNNLSNMRNNLLRNVSFNMNNNNNNNNNNNNNNNNNNNNNNNNNNNNISFGNMMNNNNM
NLRNMGNMKNMDQNKNMKKLSLNLMKNKNNLQQSKMMNDDMKGTNMENNYFDIPKRESLFNKNSSFSKEDANN
ACTNNNNNNNNNTSSGTSNSFFSNLSSRLLNKEKKEGGGGNIFSRSFNLKKNNNISNFSNNDLDKKDKTNMNT
NKNEEKDNTFTNKDNVLNTKVDNTNEEPKDYTFVEEYIPKVNEILELSTTIENWEELTEKLVQLHIPPKFNEK
LHAHILKHTLKKYACNKNVERSLPYFNWLVSIVLDNKIDMESFKHCWKSFFLESDENSYEYTKEDYPLLPRLV
TNFISAVELYDKNHMLYDLSTLEQMKSVI*
>N.PF11_0087
MKQANTKEDKSQKISNSSTIDEIEEEQLYTGPLKIEQLLAKGFVKRDLELLKEGGLQTVECVAYAPMRTLCAI
KGISEQKAEKLKKACKELCNSGFCNAIDYHDARQNLIKFTTGSKQLDALLKGGIETGGITELFGEFRTGKSQL
CHTLAITCQLPIEQSGGEGKCLWIDTEGTFRPERIVAIAKRYGLHPTDCLNNIAYAKAYNCDHQTELLIDASA
MMADARFALLIVDSATALYRSEYIGRGELANRQSHLCRFLRGLQRIADIYGVAVIITNQVVAKVDAMSMFGGH
EKIPIGGNIIAHASQTRLYLRKGRGESRICKIYDSPVLPEGEAVFAITEGGIADYEEK*
>N.PF11_0088
MSNLYDILGKAVINKEKKDEFQNLILKSEGFIDDVLHEKLRERQKKRDEILQDLFDMEILIENLKLFVNMKDK
SEVETLTSLGCDSYVYADIINKNKIFIQLGYEFYLEMTLEEAIKFLKKKINLYEEKLSYWNKQISHVKAHIQI
LMRAISNLT*
>N.PF11_0089
MITHSNSINNNNNNNNNNNNNNNDDDNKIIRKDSLVMCSLDHKIENESNNKVNKKELKKKKKKKKNNNNNKTYT
SNISIINIDKEETKSNNIDDNVINVSTNNSNKIIDINKNNIVDIEEFPKKEDDILYNTSDDNYVKTKYIQPDS
FNIKDNLEDKNVYEYDKYTKENIYNDNDDMDNNNYYYKYDDVVHEEVLQIDDNKKKEASDDTRKDNIIKSKKK
LKNNSNNNNNNNNEKINNLKDIFVDQLKKKNQILTEKLRNKEYEKNQLQDKVKDIGLELYRLNKENDVLKKKE
KELDMSLEYIYEEQNKYNNENIQLSDIYNYDICSLKKEITTFSDIYKKYNNKLLELNNIKKYSEYILANTKVG
QNIMSSRVNNNETNNITHNKINKKDNLLNKMNYQINDLLNDINIKKDMILHEEREIQNKKQLIEEEKKNLLHF
KDEKNKIMNKLNNSLYQIKKRDEAISKMNTQLNIVKINILEMEKKYENLCKEYNYELENRDKLLHELNILRND
MSKLDKKKNEIHNKYINILKENQDIKNNIINEKDLLVSLRNDIHILSKNIKDKQNDIKIIKDNINKNIDKIIH

MYTEQINITHFSSKLKNDVASLNENITKKEIDKENYKNHIIRIKIQHMLQRTKMENMKKKADKLNQEYDEKNE
ILEKYNQTIKKNHYLIESRQNEVDRLNEQYDKKKKKKMGDEQYDHMKNNNNNDNNNNNNDNNNNNNDNNNNNN
NNNNNNNNNNNNNNNNGSFFVVPATLEMKIQKLNKQNQDIQKSSRLLEKEWFIKQSELIDIQNENNKMNEEICK
CKDLCLIFDQKKIELEENYKSIFEFIKKVNKNITYIRLHLEKFALKNKETIDKIEYIENDLLIWKENINNKNE
EHKNKKEYLTSQINHLKKEKENYQQNILNYENQILSLEQEIIDKKKLQQIAKEYLENKDIMQLKKDITNKNNI
FDNIKKQQNVILSNIKIALNKRNDLDNKKEFCQKNFDMGKNISFKIQHQIGLIKKSLKSLKNKKENLTNTYNE
IIQLYDNLVKDIQQEKNNLDNLNEDYNIYEAIYHILSFEKKHRFQELLKFQNALKYGLNNNSLKNADHKKYTD
IKKKCATYKKKISNIKKILQQIERPKDNYIKFIGYIQEWIVH*
>N.PF11_0090
MHIHKLKKKIKKKKEGKYLTKKHILRKLFLNEEEFRKLCIFKGIYPKDFKEIPLKYRKKFYKHKVFYTRNDFL
KLSHEKIINDFRKIKIYLKKYKKCKLTLEDFTRSKNIVANFPVYKLEHIIKERYPILSYAVDHLDDALSCIIA
YSQLPSNHKYGIKNNMVKTCEMLKDHFHYYVYKTNRIKKAFISVKGYYLQAEILKKKVTWIIPHIFTPYLDTS
IDFKLISDFIEYYIALLKFVLFKLYKLDNMLYPPKQDNDLKNEKLAHLSYDKDYSTNENNIDINMNQELQSKC
NVNTNEDLNTCQEKTKEKNHKSDNNPHEHTTNIDNNNFNNIHLQDNCDLNKNEGKNLTNNIIHKNSEADNGHV
HPDDHIDIDEHNKLKELFKNHIFYIHNDMPFDVLSIIILSCGGKISWNSRISPIHYDDNNITHEIYEKDKNTI
HLNNPENEYKRIHIQPQYIFDCLNEKNILPCSDYLTEKENLPVHLSPFIEDENFKNLVKKEEYTINKMLNQKI
KEEQYKDFSKENNNIFKSPNYKEEEEEEENDDRETANLILNNKRQAALNNQLERENEDINQLKENDTILNKQT
DQTQILKTQNLKSQEQEIQRHKIVLSKKKRKLFARIDMAQKRQKATIDKFMKKINKNKSK*
>N.PF11_0091
MLDLETNSNNNKKGMNKVEEKNMNKKWIVMGDNNENNEKIKSSYNNEDDEYMEAVQDESNMINKMDNYMNYSD
QRTQVYPMSRTTTPNVINSISMESMNDGVMYPNVSKSGLVSNEELNNLDMKGLISEDNNNNNNKNNDDSNNED
SNNDEDNINNNNNNNNNNNIIYHEQHVEEYEKTHHVSHPKDNGKYSNSEDADMVGYESANIEFEMNEKRLNLQ
ESDEKINNHMEDHMNDCENYEDEDPGHYEEDDNNKNEKLNENNNLYLNCLSDSYDENNVIKKKDATSGSLLYK
DDIIGSDAYNKKHEKTLDFNEKNHQSDCSNNNNNNNNNNNASIYLDKMCDEEAEAEENVEMDGEEDNDDGVNN
GKNKYNINCNKNNSSSYKEDVIKSLNCQYKEEDDEYQDNDYNDDMNNENIKNTLLKELLIANKNGSIDPHEIM
HPSGCGINKNKAFSVIIDNLLNLKDDDDLSIKLNRYLKNEKADMENNTINEGNNNNNNNNNNGNNNNNGSNYH
MNHCMNTGGSKKKNVNVKSHVNKNNNDSFENLKKLKVLCVDYLRILKRLTPLWGSTDRTFESHYVNLLNNVNS
NTLDNYLCCFSNIKICAVKSIFLDSIMECIEELEIIKKFKMSKEKEDGIEDKDMNDKEEMNKKILEDIEKNLT
KEIVEDIFQLNMRNPYIVNSYGNNSTNNNNNNNNKSNCSNNNNNSSSSNSSSSSLNNNNNNNNSVSCSDGESQ
YHQQGDNNKIGAHNLLYNMSGKHNNNTCNNGFNVYYDLKKGKECQHNNSCGNNRNSCNLLSSSPNNHTCGTNH
GNNNYTSCANNSSSLNYSNIFPFNCLNTNKNAISLSNNGNVGSTPVVNKNNNNNNNNNNNNSNNNNYSAVSSG
AYHKKSCNYSDENILNKLNVSIDYSSYNDFNNNYIQEAEKVVEKYKNLSKLNSKDSDNMYSTCSHSSVQDNIS
SANLNTYLQLKMLMSEYANTCVKNDKIKLKDLDNNMSDLNILKYLYSQLNGGSLLEIRIFQSCINSMEKLRML
KIIMTINLECSSASDINKFLQDYKLNDDDNYEDREGYSKYDKEQGLLLNGNDTNMNYLSSSLQNMMRKRKHYD
NYTSTNNTTTNNNNNNNINSSSSNNNVNNLNNNSLSDLNYLLYNTNVLKKLKLNLLENKKGNNINSTHNGLQN
LTGSSSSSSLVTSGVMGNNDYRKKDTNELYMDENNVIPGHMTTRKSYASNKNNLLNNIKNFKDILNLSQESLN
KKGMGNSYKNSFDHINPSTLYDFILSTNNANNIMESSVHVGTGNNNNNNNNNNNIMMNTIGSNNSSINNSNHN
NSNGLHNSSYHNLNNNTNGHHNNMNKKNSYDYNTDYNSKVSMNDSELSLNNHDNNNDVIINNNMHGGKNISNK
MGDGVINYKKGDMNNKSLSAHNANNNNNNNNNNGNGNSNKNTKNIKNNTNGTNSTTNSTNNANGNNNNNNNN
GNNNNYTGGKLKYEMPVGVNPNDDKVKGVYFSKSPRGVGKWNAYFQIANNKRLFTSFSVSKYGYNEARKLSIL
KRTEWEKEYKHHTDLKNSDGKKGKKKANSVVAATSTSSNLIKNNNNNHHHNNHHHSNKNISKGNSNSSDGSIT
YAKDNKLKDDALLYSNDDEDEDEGCYLGNNKMNHVNKNNNNNNNNNNNNNNNVLSGMLDLSNDLLDHHLTGGT
SKKKLNKLNHISSNHDYIKVEDNMNFFIERPKNDNIDTFNTVDLLRSSLAGEKINSKKNHMNNNNNNNNNNNN
NHFSSNSLGQAAINVYNNVMDSNKQSSINASRILSNTLGISNKYIRNLHFPSIDSHTVSNFLDAVADDQKISS
*THTPTK*NKKKQNKTIQSKAK*NKINICIYIYLFIYIFLDSKKFRINEIYTYIYIYIYIFYTFPKMKTQSIV
INYFILILFFVHRQ*
>N.PF11_0092
MNEEKDENLKPFSRSKYPQSLMIDMSRSYMTKQDRINALNLYIQKSSYNPQLLQDDEEEDDEGESETIGDIIL
RILSIIFPDVSPGLWFFIHFIMNLIVLCVSLSSLSEPNVHDPISEPKYNRKFIYGSMYCSFLILGINIASFSI
LMSLHAIVQKFILEKLLQPSALCAAFYNTVDPELVYLFWSSIQIIYWRTNIILKSDSNENNNNNYYILRIFGY
KDDETPFMFLKNIHWLITFPILLYIVFAVRLLFLSIISFIFELGFLMNAHDLLGKYLRKYGVLRKFNIEWFMF
VADKQENIRTLFGYELYQDERIMKQIERDDISKKFIQNTAELLLFKNLPRNCTCCKITINRNKKNTMKLLLPP
VFEAKNIIKSESSSLKNWLACHYVVNTPPLLFLLNNSIALTNKNSIRNASDIFFRQIIMSLKIYYQEKNDTYA
FTTTPIHNNNNNNNNNNNNNNNNNISMDGLSPHSNILNINTNEKNQFFLKHKKTFPSKTNPNKVIPQEKFKKKK

KNFHTYDSSDDDRQKKINLENIFHDVDTYDTKDEGTKDENLTDQDKTSHHMIYEQNIQKIQSIKLSENINKNK
KRDTSKNKSKQTSKTKKKKNIPGEHMNHMGKMSQTKKENDKNVEEQNEQKKKKKDKMDNLKVKNQMIHIEKEN
YNVNNVKIINNDITNEISDTTMNIIKQDQINEKQNYDIITKTKENLMNNSDKGNIDILPNEKYNVDKHIHNIN
VDKDIKDGCHFYDMTNSNMNDTKFNILITPSFSTIQNKKDINDTCEKDYTSNKMNKIKNNDDNNDNNNDDNND
DNNDNNNDNNNDDDNNNIIICEDEINHELKYNIKGGDKEEYSNQLENYNSIAVQINDFNIDDVLKKKYTNESI
HKMEKNSFEDNDIMIPKNNEILQNGNNEIWHNNNNIKKEDAYDDYLEDKDNKKDNFHFNEIPLLGGFLFKRNE
ESVDGNISNMNKNSCNEEDMNNNENIEYHIPFYKKKKEKSGRKLKSSGKEKHTYEINKGNKNIKNRWSLNKNT
YYYHSHLLNEEMNILERSDAINVKRYREEHNKDRYKVCKKMFRCLFCKRYKKEERLDKIRKDISSDLEDPFVM
NVKSPIQLTLSGNEYITKDMIEVFLKPEETEEFMKEFDLSGHGKIDIIMFRNAIKRAISCRKKFIKSLKGQES
ILKLVRRLMSILLSFLASVVLLFLFGVSADTIIVTGAAFITAVTVILSYMYTNFITSVIFIAFSNPYNIGDRI
RLDGGEAMYIKKIKTYTTEFETTTGKIVIYENSKLSNVKIYNESRSKNAYIDISFKVDINTPLVALKELRKSL
QCLVDSRPSDFCKTKNLYFGYSLQPGHFYEISFWIKCVEGWGNWRKVFELRTDIYDFIILQLRILSISYRLPT
QKIGFTAPLNIADGYNNNNNNNNNNIQSNYNINRNINNNMNINTQQPKINYPPHGNNNFPTHHLRYNRNKPLQ
YTSPPKEARNPLFLETAKHKREFDISSIQSNNEQHEAPNHIIYNEPVNFEDQPLKKNKLRSVYNSYKYVESTN
HNNMDTSIENGNGYMYESYLQKNEDNNDYFVYNKKKKKNNKKKKTHDFNTKKNININNNNNNNNKMNNNKNNL
FMNTFQNNVQSISSMNNYHDVYYNNDKYYKTLHGQQNLYNNNNIKNINNHENIDNHNYNKINNILYNYTNTNL
ITSDNSYSYDNKKIIKCNYVPDVSNSDDSNHTDNMKGNNIYRNNNNFNNNSNNMKVKRGDHFNPIYPYNDDIY
TAHKNKEPQNIFENLYKLKKISNTNLDEKNYFSYDSSSGYDSYDCVKHFTYLHDKKQNILLKRRKIKMA*
>N.PF11_0093
MDSDSNKNDHESLENSGLFNDENASDGNNVENFKKKMKMIKVLNVKKNKEIKKDRKDKKKRKLQRIIDDDNED
EDDVIEKENDQGKDQGKDQGKDQEDVFLFGNDDLVEEDEERHKKKRKKEKEERGKHKKKKSIKRIMENDDMHK
VHSDDSNYNNNNNNISDDNNNNNISDDYNNNNISDDDDSNNSDSSYDNTMSDHSDMKKKKKKKKKKKKKSEFI
DTIAEEGDEDEKEEDDDYDDENNINIDKMTNENKKSKKIDFDKLGLGDDDEDEDNYMNDFGNHSNISEKKKKS
HFDEILENFKSKRKRVHKISEDDGLEYCENILNQMILMHEQDLQSMKEKKPATAKLQIIDDVCKILTKPKWKP
FFMKLNIYHVLALWLMPTSKNTLPNFTIRTNLLKVIQQLPITIKSLRGSQLGKIMTYLHSHKHETEENKKLIK
NIMQNWMGPIIGINTNYKQYLKERQKRISENPEYHKKVLEKAKTLIPDSICIEKEEEQNEMKRHANIPYNSEC
SFLINVPSSIPSTSKKIIPKSKIKRLTDNMKLLKRCKKMQKVSIEGKGVAVAP*
>N.PF11_0094
MNPEENTCNNKKGGGKDQAVKHFLYQNILKKNFILFNIFKGGKNSNNEEDICDNQNDIEDMCNISRDINNNNN
NNNNNMINIDRNINDTSDDVNYTNNLYDINKYNNDCNIYENNKEKLFDNDYSGLVEENDQEVFSFFDVYNNNN
NIHYHVQNSDIILNNEDEEYILFLKYIYNDLLSFIESNKDYYLNNSNYGDMEDLIINICGTICHKAFNNIKTI
FNTYEEIKKRKNNPNPHMVDNYMVKEENINDNKYIHIKNNKNEERENHIFYQNICHNKSNVNYSSYFFIPPLE
FIVSHLNKLHKKNTYAYNDINEFVKNNGFHTYNLLPEKEKGNKSNVSEEIEFYLSCLNLHHNKGNDKKKQTNQ
IKNIYKMENSCSINLKDNSHQQNDIDEKKNVYVKKDVYVKKDIDEKDNIDNKDNIYNKDDIYKADNIDNKDDI
YKADNIYNKDDIYKADNIYKADNIYKADNIDNKDDLCEQRRDQNIYPPMQHTNSVSSYKEDNCVKTKYYTLEE
RTNIYSDKDDVSNNSFTNDQKYYEKNYKNINNGNNKEEQKKNYLYHVDENKHINYMLYNKKEHKSKIQNDNYK
DIYDHYENVCHFKSSVLTSQSSEEDSLKCKQKTKREMHSDIFFSSYPSKSKDNIDMNINNMNNNTYNNNTYNN
NTYNNTYNNTYKDEYNNTYNNNDEAGQGNGDKDGYQDGYHDGDQNGDKDGDQNGDQNGDKEGYQDIYQDEDPH
DQYHPFHKTTDQNNCVYYNVDDVYYNKSFSTHKNITIPKKKNYNEFNYLRDEDSFKQYTNYDTFFYNNFKKNI
KNEKYNLLKKEENQKGVQSIKDDFSDIYFSCNLANDKFINTQGANKYNKKNNINKLINKYDDNYNNNQYHVYD
KNDHMESLKNNRDTIDVDTNDDDTNDLDISDEDVYYMNNRGIYMSNECKREMKNFHVSDNMSDHHVDNGSDNM
NLHNDDNMSDNISLHNDDDQEEEQEYSSNINFKNNIILNDGNDYKEIINNEIYELNNVKEEDECNLIKENYDI
QSDDDDNNNININDDHKFYYRHIHSNIKDNEEGSKNILKINEEEYQKKLQNLFINKTVYENQDQRDFTKNENL
HDDYYYFKYLNNKENTTDPYHYTKGCKKTNIQVTPSPFPQFLRDIQIYNIPQKKIRLKRTSIPLKKEDDKNKE
QKYIYNKTKKKDRKKERSISVVREKVNK*
>N.PF11_0095
MRVVIQRVKGAILSVRKENIGENEKELEIISEIKNGLICFLGIHKNDTWEDALYIIRKCLNLRLWNNDNKTWD
KNVKDLNYELLIVSQFTLFGNTKKGNKPDFHLAKEPNEALIFYNKIIDEFKKQYNDDKIKIGKFGNYMNIDVT
NDGPVTIYIDTHDINLNK*
>N.PF11_0095a
MKLFDKTKINSLIPSWNYKVKPDCYNYIQRKIKFPTFNEACTFLNKLFEENKKLDHHCKYISDYNKIKIKIYT
HTSKDVTEKDIQLAQIIDDILKCHNHQIIEKNQK*
>N.PF11_0096

MSVSSINKKIYIPKFYADVNIHKPKEYYDYDNLELQWNKPNRYEIMKKIGRGKYSEVFNGYDTECNRPCAIKV
LKPVKKKKIKREIKILQNLNGGPNIIKLLDIVKDPVTKTPSLIFEYINNIDFKTLYPKFTDKDIRYYIYQILK
ALDYCHSQGIMHRDVKPHNIMIDHENRQIRLIDWGLAEFYHPGQEYNVRVASRYYKGPELLIDLQLYDYSLDI
WSLGCMLAGMIFKKEPFFCGHDNYDQLVKIAKVLGTEDLHAYLKKYNIKLKPHYLNILGEYERKPWSHFLTQS
NIDIAKDEVIDLIDKMLIYDHAKRIAPKEAMEHPYFREVREES*
>N.PF11_0097
MKYSNMKKLSFPLKERYFSATSKVFIDKNTTVICQGITGKQGTFHTTEALKYGTKMVGGVNPRKKGTTWTSYD
NKYTLPVFGTILEAKENTNCYASVLYVPPEHAKSAMIESIEAEIPLIVCITEGICQHDMLEVKSCLKMSKKTT
LIGPNCPGIIKPGECKIGIMPSHIHKKGCVGIVSRSGTLTYEGVNQTTKVGLGQSTCIGIGGDPFHGTNFLDC
IKLFLEDDQTECILLIGEIGGDSEEQVAQWLIQNNVDNGKRKKKPVFAFIAGKCAPPGKRMGHAGALISGGKG
TADGKIEALQNAGVHVILNPTKMGEDIYAVMQTLK*
>N.PF11_0098
MMKINLYKLLCFICVIFLLHKNVVRSGDNMKYNDMKGLDDLSKLNDDQVKDILGLKIDGAKERIEKLFHLIDK
NNDKEITEEELNTWSSFLKNEIFLKQVQAEMGQIDSDKDGFISLNELNDAFAQNLDAKEVEKHSEGLLKRFQI
VDKDKDGKLSINEVGLLIDPMKDEELKELEINEILEHHDVNKDGKISLDEFKQTRSDESSGVKKDDEMALDDF
NFFDTNKDGFIDKEEIIKVYFDPAHESGAINVNEIKENIFEGKKITYDLWNEKALKIAVTSLTDYGDVIRYPE
DFKLDIGKNVILPTARSRAFEDDDMDADNTEDDKDEADDASQQKSPAIDEL*
>N.PF11_0099
MNVTNVVKRRKKLSYFHSLLLIIFSFFLSCARGMDYYKRLGVKRNATKEDISKAYRQLAKEYHPDIAPDKEKD
FIEIANAYETLSDPEKRKMYDMYGEDYAQGGMGGGSPGRGEHAHGFHFDQDVVNEIFKQFAGGGGAGASGGRA
GNFHFKFTSGGPSFNHFEDEYEDIYKNEVLKINSKNIESVLNDISFSLIINFYSPTCSHCISFKKKYLKLRKK
FDGYITFAVVNCQEENMLCRKYNVKSLPQLILMRSDKTYETFYGNRTDENLTYFIKNNIPSAIIECNNQKKLD
NFLTQNIEIPKVLFFISHNDNIVMLKALSLEFKKRINIGIIYNTNYSVMKLFKKKNIKTPSLLLVDDIDSLSG
DLTQLKNFDFNILSLKLSHIVAQNRLKNNLYGHVTSYQELTKKKYESGQCHEKDSQICFFILKLLKKNYKSFD
EDIKKVANKFSSDPLKILYINIYQQPYILDSFGLSNNIQYSNGLILVAFRPKRQKFKVYDGDVNVENVHKFVD
NVVSGGIPINQNIKRSLKFVHVEQYDDEL*
>N.PF11_0100
MGNTFSSYDEKERRGLILLPENYIRYNLNEDNYKKCEENKVKKNIEKDKSLKNDLSTKNNKSIHENEHYELNE
SLINDLIKENELYIKNYKDELININQDSIKIPQTKNECLYSENNIYNCLKSMNESTFGFLNSYSRCYKYLREY
ESCVQRIKI*
>N.PF11_0101
MDNNINIDDDFNIDVDYIKKLSEKYKHVDHPLFMDELPKNLEENEDLEALYKIITSEDNPLILAHDYKEVGND
YFKDGIKYYDDAVISYNKGIDILNNYLKSLDIQNKNNSKSNNNKSNNNNNNNNNNNNNNNNNNNNNNNNNNNN
NNCDGMCTNLFHSNNINNKTNDDVFKQNVKETNNTESTGNIKNNDILKNDKNKNIKIDINDVKVLLSDLYCNR
AIIHYKKKRYIKCLDDCKNSFSFNNKKYKSLYFGILSCNNMEMYHDACKYINKFDELLKNIQDIESYINVQEY
EKLKKDIFEKYEHYLRRKKKAQDERKKIEEQEKKKINQIEDILKKRNIQTLPNVYDNNNNIIPVLYLDENMYI
HFTVFLIYYEFNIIETILDFAESQCIMDYYDIIKKRRDNQILYCYIEFPDDTFFMINNYFYMCDIMNKIKRFT
HILSIHIIENDVANINFKINKNITYL*
>N.PF11_0540a
MNRMNDLDIEEYNRNIIQELNEAAEEGEYPLSMFLHDLIEIDMESNFSYKYMIINRLVEICINSINKKMFFLA
DECMYFMRYCIDKCINLKDEIIKNSSFFNNINIIIDICSKEKVYNNDNLYIFEKTFFFFILDLNNNMDDFINL
WKIYKNELIHFYTTLIYIISITISEFYVILNNIKGEDNVDVDYIHIGCELIKLIEICLDLSLVIFNNIDEIEN
IDILENHINVMIKNFHDIPDIEIKRKIMLMMKAILKKKNICDKEWNEEINNFNIKYNLLNNVSNKQKYKNKKN
KKKNKNNDYYNYLNITKDKKHININYLKTNSYIYINNCYLFLNTYNENLNDITMNTSPSRWNKIKLSSWGYKG
SLLINFFYSYFYNTKHKLLVVISYDNMKIRSDPNYKSVFFYFKKEEISGVLSNKLRNTFKDKFLDNLEICIKL
NSKDICNNVHELMMCRINKEKLSVDEKHKKKELYYIDEEHNLPEDTFNNTMENVDNMDNDQICENDVKDNMEE
IFQNKQNKQNNHIYFNNLGRNHISLINTNNLKNEEKLERRKVSFASTKELCLLNDDISVETKTMLLEESILKH
VHSKYNNKCMNSNIIYDKHQSIHYDNNKKSLRENSGSMRNENLLFIHDNHKKEKVEPIKISYATTRDLIGKKE
CLKGKLEVENNDVKDDEKGDNNNVKDDKKGDNNNVKDDEKGDNNNDVKDEEKRDNNNNVKDDENDNMDNIKRV
YNNDIKETTLNYKNLLVNHVIAKGKQINNDKNRNEDYYTYKDNTEKKNEKEHMLNNYIHDKEKMKIIDNEDNT
NNKEDTPINIYKKKNDNIYISKECITNNNNNNNNNNNDNNENNENNENNSYYNNEGEKNTYNQNISNHLQNKD
KKKYTNSIENEALIKNNIKNDEKYINYQNYKERKILHHKNNMINKNDIESTPQLQMSYILNNSQNNYSINKDN
IIKSPIEIVRKLLNFDEKQKNIEECIENINNNNNNINDKYPKEKNKLKEDQIKHLKYDDMNSMPNDNDNVHHN
LNNLCMISKEENIITKKNTTEKYCNIFKNINNKINCNNNEDNYLINRRNEKKEKSKINKNKNNYNKEYNDEEK

KDKDHINHNISDDNFIMKNNNIKNVEGNQNRQDVVVDVGNIISINNKDEIHEHDTPRKKKQKKKFKNINLDNT
SNEYSQIVNISGYDDEPSNELIFKLESNVVSPCNIDKSCLSSKSKKRISTTMINNTTPNNNNNNNNININNNN
NNIYKDNCNYYNNTYCKETNDTYSKNPIDSPYPIKKVKYNYYYDSKLFELLKKEDNLEKLSAKYLIRAYTLIQ
YNKRYVHIQIIDYFRYIKKEILLSYDNINIKYNKLLNHIQSQHDNKLQNILIKYYKHLKEHTNQKKNLNLNKN
IPKSINRNIIKIKIKSLYDTLNIVQRTMKDKILNSTIMLNYKQSDIYTPSFSLGTIISKYSI*
>N.PF11_0540
MFIDTEAKSLYNVIEIFERKIIYLSFINEEILEKLNEHDISTIPEEFLLYFKDIIKLNKLYEHSDILKNYEDD
DKEGEDDVNYKKCVDDEEVCVNKEHQDLIDKIKKYCIRLCNIFNENASLYEILNKMKDKENHDFSKFLYIIKD
IKNIFQTKFQTSALEKIKGIENMNKLKDEEKKIQEEEKKLNEELENIRKESHKELNELEQCLKKKEKELEHLK
KSSQENLQVLLNMLPLNDTSDNLEHINMLFEKTKNIYENKIKLYQDQEISLVKKNKLLELDIENYINLIDDQI
KSKDDEIEKWTRELKKNKITEKDLDSILLRKQNEENERCFLHELTEKRNMINQKKDNINNEAAVLIQSYIRAV
KERNLFSEHEKKKKSRKKKK*
>N.PF11_0105
MSKHKVRKKHKHLRTFEEDLKNEVIVKNKIIKTKKNKDKHRKEKFRIKNNEMHIHKKKKKNDVHKILNLANEQ
NEEDLDYDINNINDDNNEEADKFLHFDDIIEENITNDNDIFFNENKNLPYNTNTEEDVDSKDIFEELNIKIMN
DSKHVSYNKMENTTQEPNPSTEDKVNLLVEKCYRTIGEDLAHYKSGKLHRALTILTKSSKWYEYLLLTKPKKW
TAHATFEITKLFSSGLKEKEVSKFYEFILLPIVLENIDKNKKLDAFLYKALIKALYKSKSWFKGILFPILHRE
CTKKQMIIIGSVIQKMSISINCVVLALDEIFSFSWNSIISYILILLFNKKYAFTKQCIDNCVNYFLRFENYQD
VLSINWHKSLLTLVHNYRGLMCDTQVEALTHLVKKKNHHQISSEIMKHLYSPTSLMNKIKDIKVNSEEYVV*
>N.PF11_0106
MGRKSTIKPATGLAGLYINFIFIMLYIKTWYL**NIFLLTVYIDIDRYRFFL*FIIIYMKFFFFFINILKLIY
IYIEKKKKKKKKIYIYIYIYIYIPTFVCMYVCILFYFLFSFFFFFFFF*
>N.PF11_0550
XXXXXXXXXXKVSFGVLGVLYFFYLKRFVIQEWDIYKVKKKERDITMNKIKIHEDNENVKYILDEERNKKKYI
RTFDYKEAR*
>N.PF11_0107
MVNNGTLNNEKFSLFSKSVILENGKHLVNVKNEKLEEYNFLNSMTNPKVTFNAKKTKGFLILQLFLYGYLNRI
SSFRSNKDKDLMYPYLKILLDKEEIYRSRILDNSMNIWNETIDIEIHYIKSIIEIQLYDMDETEGIVEDEYIG
SAFIDLMNLKFNTKYDMILKYLDNKTAMIKDPYFHKKNKKVSEENTKKKSINVVNDLNNTTNISNNNNNMNNM
NNMNNMNNMNNNDNMNNMNNNDNINNMNNLSNPRSIIQKEENINNNKSNVNKYVNDYNVRIFIKLFSKNDYSI
FFLHLYKITSLNYKYICTKNEILDNELNVNLLYENINTIKLNFDMIWLPFFCIISNFSSWKKPLYSLFFVTYF
FLSFFFSKYFISFLLLFLSLILFIIVSIIRECDRIQRHANANANYAQTLNKNIKSQKNKNLNCFYKVGTLNHN
NLNDDTNRAYNNNNNNNNESFTYWRKVRRSIYQNIQYKDYLNNKKKKKTSQKDYEEIKDNYYDGGDGDDNDDGN
DDDNDDDDNDDDDDNDDNNDDDYNDQSDESEENYEMKDNNEKKYEQNDEKKTKKQKKEITNKVDNDIMSPNDN
DNDIISQNDNYDDIDNTSESSTFTDSSLNNVSTNEYDNFSSNSISSSEDNILNVSNKSEQKQLKDDNNKNDKY
EKNISSKNIKQSHKMNKYDNIYDNKNDNNKYNYSNNSSNININNNYYTDDNMKENNLEDSNNDDSSALNYTDD
ENVDPENNLSVIRTFIRNIIDDNIINNIKYIHYFIYCFTKFSQILFFILRKFGYFLVILTLLTCGLSIYFYPM
IVRTLRCIIFLCGFVILTYNFKPMNIVYRFISCFHEYYYLERKRRRTSIYNYD*
>N.PF11_0108
MDLTSLKNEMTKNIKNDVFKNMSINNNKVKYEPFGKPPPGYIPGKGRGVTGFSGGVSRDDTTDDKDKNDYSDF
NYDEFHGYSESLFKDTEYDEEDKEADEIYDKIDSLMDIRRKSRRENKLKEEISKMRATKPTITQQFGDLKKNL
ANVTIEEWESIPTVLQYSSKQKQKKVQKNYLPAPDSLIMSRINESNIHLNFNNSASSQSGHKTPIGLGYQSSL
GVQTPLGLRTPYGLQNSLSGLKTPLSGLQNSLSGLKTPLSGLQNSLSGLKTPLSGLQTPYMRNPSSLFGMDTP
LINNNIKSNMSISGLNTPFTLSGYNTPLSASNVSGYNTPLFNNTHKLSLNDLGEARGTVLSVKLDELIDNVEG
QTVIDPKGYLTNLNASSLINDADIADINKARSLLKSVISTNPKHGPGWIAAARIEELAQRKDKAKEIIMKGCV
VCSKNEDIWLEAVRLEEKLSEVKIILAKAIKHIPTSVKLWLEAYKKEKNVDDKRKVLRKAIECIPNSVKLWKE
AISLENENNAYILLKRAVECIPQSIEMWIALARLCTYTEAQKVLNEARKKIPTSAEIWINASQLEEKQGNIKM
VDIIIKRCIENLSSKNVIFDRDKWIKFAEECEQSKFTHTCESIIRNTMHIGVETLNKKRIYKQDAQNCIHNKS
IHTARTLYNEALKIFKTKKSLWLALANLELTHGKREDVDEVLHRAVQSCPHSSVLWLMLAKQKWLNNEIDKAR
EILAESFIHNQNTEEISLAAIKLERENNEFDRARFLLKKSRVQCNTPKIWMQSVQLERLLRNYKEAKMLAHEA
LKIHKHFDKLYMIAGQIELEMYKYSNDTNININITSHKTYIKNGCDDTKSDDSKDKNINTKRTTSPKHYTTTN
NNDDDANNDSSCTSNNFHNKQNGDNKNINVNNKKEEFINITNDPYKNAQNIYEEGLKYCASSINLWICAIDLQ
IEKKNYTGARALTEKAKIKIKYLNSFNNNSHILKSKEIIETNEQNYDTQDDEYNNLNKNMDGSKSVNNTTASN
ISKSKNELEKKSSVNNNAYIKIIENYDLLWLKLIEIELCCNNKNLNPIISEALKECPSSGILWSKAIELENKN

LQNSKSVSAFNHCGNNAYVILTVAKLFWVNFKIQKARKWFYRVINLNPHFGDGWATFLAFEIDQQNEINQKDI
INKCIKAEPNRGYLWNKITKRVENWRLKYPQKLYKYIKDIYPHVLNKKISDPIWNIITDENADTSFLLTNQEN
DKEKKDQGNESVDDEKDYEKETNNNVEEDPEKDSKEQGKRKLKEKILNEKDTTKNKRKDISTNEDEKDRKKKK
K*
>N.PF11_0109
MSKKYYEKLLSDIQNQSKPVESKFGSYILQKFGWEKGKGLGKHENGDVKIIKIKKYGEHGLGYNEHEENNNGM
WWENMYNNCAKKINTDNNKNTTNSSTSNNNSKEKIVKNNQNIKYSLFVKKDTCIISSACTSLEQQQQQQQAE
DEKINKTQDVKIKNKHINKNENNIHNIKLITNKSSEKNHDEYVKRNKKKKKKKKKKHKHKHKSEDKSDDDKT
VD*
>N.PF11_0111
MSSVNEDNQMELDLKNDDVVDVEKDVITNHAENINVNSENGSNVEIKEGDEKDHIQENDKDETNVVNEVDLKD
DEVTYENNKNEENKSSNRWADMCEDCDAPLGGYLQ*Q**YEGQSC**QF**QRYE**YEAELLYGSKK*YE*L
CHVLRNIYTQPTH*YYQRRNI*FV*RVPNKKNKHFKQEGENSCCIC*VR*HRRYEQIFRIEWENDIFWK*FF*
TWYYICNNK*RQNEEFSCKSEAK*I*ENDK*K****F**YSSRK**HE*YYE*LQRKQ**********Q**S
YEQQQ*****YV*PYE*Q*PIF*****KHENGYDEKCRKYNK**YESKF*L**YQRKLFK**STSRRFKCTYE
SPTK*YVK**NE**CF*F*KPELSK**SYE*TKYC*********QY**YSKRNRTKKKINSEKKICTT*PKK
LCSQS*YFW*SQTC*Y*SRKNIK*KYYKGKRHERHK*QQ*******Q****C*KEK*KYRRYGKEKRTTRRYR
*I*KIKPSKQQYY****Y*RI**R*QKY*HV**QKCFQL*KK*QHK*KL*L**QFCKQYKK*QQ*Y**QQQQ*
*******YEENIYDE*K*CK**HEKEYEQFRK*QYVRK*KLSKFKSFG*YQQ**YG**YEQQKDE*CE**KF
KYG*Q*YN*RFFKK*YSG***KKY*C*CYT*SHIRK***AVWFKSRGRNINK**HE**LCK**NGNQW*
YERKI**L*KYTW*YGQEKCRS****HTISEERIKYNKKR**S*Y*KYYYEKY*KRR**YSTLQFRCEFV*LK
*LS***FIKYERSKNE*EEKCRK*FIE***RGFKKYG*RRRI*QHQQKYEFEGKY*KV*QQH***********
LVQL*YGKSL**LIEVCKKC*QLKQNKENYSCKNR*SYSNRKEIKWR*RK*R******HSKWRNRK*
KTLQR**IS***KIRQYQ*IRKK***LYL*IRCLRY**YYR*******RYNKY*PQIYFITNKNKEKS*EKN
NRRNRK**CRK**S*R***CKCCIRKKSKNRYSH*AKFKERGKYMGS*NSFVG*TKRKCCNE**GITL*LQKK
*VALK*SKQG**SKKRRK**EKR***KT***S*KGSL**TI*KKVRKKR***KGRKKR**QKR***KRQQ*KE
KR*K*TK*KGRK*KRRK*T**K*KRRK*KRRK*T*RK*T**K*T*RK*T*RK*T**E*KNC***KR*SC
CYH*KR*L*KKRNR*KRRK**RWKFI*L***KT*NI**T****E*L**KKK**I*CFR*KKL***KLYI
R**KKT*RFI**KF*KFT**IKL*R*L*KK***RYSI*KK*LYRL**NG**IFLQ*L*FLKT**KL*TSWFSF
ENK*L*LFK*L*IIQLL*IFIQR*LL*RYQNFIKTK**R*ARTKIRNPNYSKESYCNSCKNK*CFYKKK*IRM
FRRC*
>N.PF11_0112
MSTYKDNNNHNNHNSNMNTIDQKKFLDECIFVVKEQSFYMKQALENGSLRDTLKYASNMLCELRTSHLSPKYY
YELYMLIFNELQHLDNFISDKKKHKKKFIDIYESVQHAGNIIPRLYLLIIVGRNYIKNKDIKAKYILKDMTEL
CKGVQHPLRGLFLRYFLIQMCKDRIPDTGSEYEEAGGGDINDAFEFLLTNFYESLKLWSRMNDKVLKVPNMIQ
DDNTMNSKIKILKEKMDVKMLVGSILVRMSQLEGMTKQYYIENCLPKILLYLSNINDCLIQQYIFESIVQVFS
DECHIYSLEILLNAILKMNTSIDFKSILITLLKRLRSFIEANNKCDLPKDIDIFNLFYDHLVVYVNRTLDTYT
KVNYNDSINRSPNEDHHGYTHNDLSKGKAKDIINNNDNNNDDNNNNKDNDDNNNNNNNNNNNNNNNNNYNYNH
NNNQDRYNISKGNVQNGHIKIENMNQTNNDLNNNNIINNMGHINTNDLNKNNTGKNCTIVNTDEFVNNVVKML
QVIYEFIFLCIRIYDDDIIISKLFGLPYTIVSNVNMNNDTICEEIISIIVLPFNYLGLSALNARNMQTLLNSI
TEKHKKKLSLDIIDAIIECKKKYITYEDVEKILKYISYIFHEKDKKNKNNDEDIFNLENNNSAYTCEKICKFF
HIITNTKNIEEKYNICMLFYKYISNSTYLVHLLPTIIFTLLHVVTQITKLGQEDHFNQNDDKNNNMNTIDTID
SNNNNNNNNDNNSNCNNDNSNIDSNHTMDNEKKEDFINSSDSYNIYPNDNDKQNLNNYLHNNQNNFILDEKKI
NQYNIYVKNIFKFIHTNLLTVASQMPILTFKLFLYSAIVVNNYNSFVQTHEFLTFDNLEAICYEFITQPLIIY
EEDINISAQQFDCIVWIVGILCTHINLLDNENYNNIALKLTQHANKLLKKKDQCIGVLKCSHLYWENKKYRNS
NKVIECLQKSIKNAEIAIQSNNDNIILFTYMLDKYLYYYEAQNIDVSEETLHYLIDICQDYYNKTNDDTNFKQ
EYKKVIKYVHDKQKNSNVFQKINIDTSILRS*
>N.PF11_0113
MSRIGRFNLIVLSGTAKPSASIGQTLGPLGINMMTFFKEFNDRTKCIAKNVPIQVTLEPLNDRTYRFYLRTPT
VVWFIRRCARVPMFSSMAKHNTVGSITLAEVFHIAKCKRMDPPLINLSLKSICKYIIGTCNSMGIRVCKELND
EEKKKYFVDVNKLDNIKKDIRTRNKQQKRSKK*
>N.PF11_0114
MENKTIVIDNGSGYIKAGINSSEEPTIVFPTIVGIEKNDETKRIYTGDEAFFHESNLNIYRPIDHGHISDWDK
AQKVWDYTLNCVDPSKSIKDILLTEPPLCSISHRKKMGEIFFEYFDTLNLNLSVSGLMSLYASGLTTGLVLDI

GEGVTQCLPVFDGYIEKNSIIRSDFGGEELSMFLQKLICDIGYSMTTTKSLEYVKNIKETICFCSLNPSEDQL
RNDLAATYTLPDGDVLRDGYDSIEIAHERFYVAEALFNPQLCHRDNLSIIDIIWKSILSCPMENRKILSSSIV
LSGGSSLFPNLVERIETEVRNNAPESARSMVKVHALENRAIMAWCGAQIFSQAELRESQTGIWISKEEYEEIG
SNIFLTKARKKKKKKKKKKKRIDISISKTYLNMLEFSMDFIYSFILFIYFSFLFFGVSPLLYSIKMIFYFLS*
>N.PF11_0114a
MNLKNLYNYNNITVEYKTLVKEKKQNKEKEIQAAILIQKCFRGFVVRKKYLIFKYYIKYMQNQIQMLSCKFLL
KKMKKAKSEEQAILYLSDNATKIQKIFRGYYSRKYIHDYFKRKMQILEMDNYVKEQRNNMMLGLEERKKKQLQ
YDNTMRDKKIHQVAKNLHHLVSTKTQKGVYNLKIENIIKEQQKKLKRKDKNYDKK*
>N.PF11_0115
MSDTSSIKITRDEDIGYAEKKNKEDYPLNKKKKGNNNNNKNKRDKHNQCDDKKYELANVNNKTSLSVVENKKN
EKILIYENNDIVEYDYNYFFDEQKNNKHVVLQEEDYLSTLEHLIEKKFFPEMENLRLKEDMYKSNLLKKNNIN
DSSVNKFNSDVESYHFSNNMDNTSDHFKYIKKKKKKKDPYGNNYNDEYVDSQSESSSGYNKKENIKSNNSNNK
NNMELLKYFEEDENYSLVNYVHSDQEYIKEKKKKKYKKVILPNGKEHKINLNISLSEFQKRYTSEDNKSFEYL
LRNMKNKSIEKNFFSIDKRKKHNLKMDMIEEYTKKGIPHNIINTNKADEQDYAMMFSSSYKISNVESMKNDNP
TIYYHNTRFNNEYNNDIKRQINRIDDIREQKILNKEKDRKQQQMIEQGKFNLLKNNDNYEYVNTPLIQAGKGV
DQSPIITWGKIICTPKLVHGQKDDDDKSIHNNNDYNYNYNYNNELDNSHDPESIQKEFNLQKINNRELVAEKL
QNSRKNIKYNKDILKKKNINFLINKNFTSHISSSSFKNSVLSRYSQKRLSELARKSSLASQILTKKKK*
>N.PF11_0116
MKCIGRVGYLLNLKKEYGERADILLKRLEEDKMIQGGFITDFIQTDYINITKILLYMMKNNKVITCFDGLYVD
KEYQKYFVLYENDIFNIYEMNKTNNIKDETSNKMNSNVGYNYITNDDKIKNNIHNDMTKNDTTTVVDDHLNVI
NDINYNNNEKNSSYNNIINNIHDKNVYYKKINIQESELLNINDIIKYDGSIENVEKIIYYLNPCSILCAYFLD
IKQNEHVLDMCASPGGKSLYIVNKLFGYNISPLNRIKNVEIIYPDAVNNDKDNINNEDNNINNKHNNNNDDNN
INNNHHNNHHNNHHNNHHNNHHNNHHNNHHNNRNIYNNAKNFGVQIDCCMDVVNYNKINRNGFLVINEYNKVR
YERLKKVLDKYIPSDLINRSSNIHVTNYNGLNINSFLRFPKFHKILLDVPCSTDEHLIKQGTKELNKWSINVI
KNNSDIQLQLLINAFTLLHTGGVIIYSTCALSYLENDYVIEKFLKKYKNNIQIIDFIHEEYQTKISQYLSNYV
NNKRNHNHNIDNLHKNQSYQYINHLKENTLFLKFFEKTRYGYISLPDRSPFGILYICKIKKI*
>N.PF11_0117
MWLEKYSPQSIDELTIHKDITERLRKLSRHKDLPHIIFYGAPGGGKSTRINCLIKEIFKDEKIIRRPECITNA
ENKININVVQSNYHLELQCFELGNKDKIIVQSIIKELCSYKSSASFFSKTPMYRIFVFKDAEFLSEGAQAGLR
RTLETYIRNARVILHLEHLSKIIEPLKSRCICIRVPLPSEEEIYSVLQNICKQENVSPSFSTYEYFQTLINTH
GRNLRKCIMALEMSVYANSSKPHQSLSVAASYINELCDFVFINPTQIKMKECVTKIQSLITCQIPVNFIFETT
IKYLLRGNYDAKLKYYFLKLCSHFSYLSEASYDKSVSLIAFIVNANTAIVKYNLHGK*
>N.PF11_0118
MQSDNKIEDDKKKESGHEENKNVDKEYISDNQTNFNNDQEGVDHNNLLCKQQSDNSKEHLDFNKYDNLSKDIQ
EYYVGVPYSWEYVEDSKSWFIVETSKNYKFFYNKKSNEKTWICPEEVQKLLDKQNKEESNDIKSEEENDYNCD
NKKEDMDDILKAYKELLIEKEINEFSKYENVLATILYDSRYLNVPKEMRKEYFNKLIKEINENKKDELKILIE
NFQSLLKKKEEHFYYPFNEHDAIKVLQNHKAYDGNKTNNWVNTRNKLLKNFLEKKNKETEKKVEDKYEDALIN
YLENENPKEWIKIKNHLMKKEKYDILSYKRKNQIFEQVSENLLREIKQKKRKYKSGREQKDYEGYENDKGEYH
YDNRRKYSKKEFEDYQKEKTSEKNLFITILHEKLKCPKVDEELLKTGYNSIDNKNTFEDICLLPRDILNDERY
KNITLNDNEKFVLYKEFINNYIDSKKISFHKLLSELSINCINNTLDEIILMIDKNNKMFKDINKCHLEENYNK
WREYKIKEAKNIFRNFLMKFNYIKYDSDEYNNYKQLIDRLSQDVSYQRLNCVPKEREQIILSRIEELKEEHEK
NKNLAERLNF*
>N.PF11_0119
MNKKYEPILDNELLSSNNENENFVNSKKKDNENDKIQDTSLNNIYGKIVKIRKELEKSYNLFYSYNLIVSNNK
EHDPNSPYNNVYARSDIININNNTKNNLTLNKINALTNSFYMNVQTLKNTYSFINTNNVNNKIINNDNIIWER
RIETLSNEANSYIKTLDNIYKTNLKQSEMKNNKKSNYTKSNKRHDGDDAINYLHKENEILKQVEDHLNIFHVQ
GMNTLNMLRKQNKFLKNVRKKVIDMYNYVGLSSSLVDTIKKAHKQNLIIVIVGIILSLIFFYVIYSYFKR*
>N.PF11_0120
MKKASKNSNYHGLEKSLRRRGGKIKTIYEKYKDIDFQNIIDEKIKYVWNYINNIDDIISDEEFLKEEIKQKLE
KKQSLKKNDDFDEIIYYNKNVKNLINIQKVRKALYHFNIKVDMEDIMHMFLYYTNQDYFLKFIQNNIYSKNYV
ERKMDKYKFTSININNLYINYEMFKFIFLNIDMNIEQNGQMW*
>N.PF11_0121
MRFSHIFHSFLLFPQTKYISNRNFLLKNFSSSSKMIHTFDFEVFGKVQGVFFRKYTKLEADKLNIKGYVQNTN
VNTVIGRAESNNKETLEKFKNFLTNIGSPSSKIHKCVISNEKVMDNYCSSDFTIRR*

>N.PF11_0122
MYDNSEKNINIKVNNKKESGFNKNCKVHDNKPQLIINAKSEFNLKDENMNNISQPKKTLGNKFRSRSDAPFSR
NIIKDNFDRDNYQNHQMASNDIKICKEENETQQSNVKRHTVGINLYTTTANNNNNNNNNNNSNNNNKPFNKF
EKKKVLGYNYFTNRQKKILNQEDNNIIKNVPISIYVGTWNCEYFDFSKEYDCDKKRYSINHLNKNTQNDIYSM
KLNERFSLRSLTPLVCMETGDKTIQEKELHTNNKNHVISLNNNTSVNNNQKYYIHNQKKEINNLSSTDTADVK
SNSNDLNFYDISKINKDKNIDNFNIRNNNINDMIPKKVDNHSSNNQYYKEINLTRNDKSQLDNIKSKEYNILQ
VPKNYSDDQFSEGSSNESTKLSNNYTGNIKNQNAEQICNITNIKKKFFQTCKPTDTLIFSHWIQPYYDIYVIC
LQESISDNIINCLSMYLKEINQETYLFLPLADYKLSGYGDGAFLQMKSTTIAAWVRKSKLHPRGPVKLCASKS
IAFNKLNNSKGCVSILLNIFNQFVLFIGCHMPAKDQEIRQKSREFILTKLSEYFSNKSTSNFKDVFHHVIWMG
DFNFRVHGIHIEKVIHYLKTDNLKELLKHDEGHSAYSYDLSISFQELPINFLPTYKKNGNRPIINRSDTEWVQ
KEYKLIHNIKWYKGGKQETRIPSWTDRIFKWSCEKTKNCLVFVPNSYLSPIPEEKSIIMSSDHNPVSCCFQMF
KMKNDEDIPLTKSVWKF*
>N.PF11_0123
MFFSNNIKEINNNGLIFTTVERINIKELLKIAEENEVRKNKKDTIIDYISYLNNKKCLIKLRKLLKSHLINSQ
IVITNFCDRSYELFNSNYIFENVINNKRSYFIKNLYLSSLFSSDYITYIIKKYDQVMVSYNCKYDNKIYINNN
ILYLCINDLTKLRLINHKNVYKDKYMLNHIVSIDICNDKENKDAITYKKINHTNKCVNKNITNECININLINK
TSIQNENHGNSNNKEKIGGKLREIYENFFTNLYTYPVDLLCIYNNCDENFIRILKSILVNNKSINVQIYPLYI
KYHEYSYKLCGALFSKFSKDSEKEEGKIVTHFIYNMSVDAKNNTTINNNYNNYNNNNNYNNYNNYNNYNNYNN
YNNNNNNGPYSFMKSQYNNIMPNEINMNQVADIYNDLFSNSSINQHNNNEIIQKCYNVKDNYTNQCLNKFNHE
LNENHNIYSSRFYNDKNVNNPYVYISKNEMDDKFFYELNMEYIEKEILEKYSCDDHKIKKNENINIKINNNHD
VSSFLCNSKNHTNVINLHDIYNNNPNNNNDDKIKKKKKKIHKFNDKKFYVNYNSSSSSDISISLTQNGDYKII
NSPKEKKKKKNLKNDHISNEKKKKDEYEYETYKNKKVESVLKFLQNYKNSYYINNQHLLKKECLHDIIDNMKN
MKDFYIKQDNSDNEDAKYNNFILYFMEYIGRILLDIKLCCKQNSNMLVKKKKKFYQIQKIIFNNGLIDMKNVH
NLSNFLIHTLINKENNNDSKLHYVLTIYGQDNNFINYDRCLQEVSNQTCIHICFFSSKGDVYIMILDTQNRIL
*
>N.PF11_0124
MGVKTSEDVYNFVKDSIKIKEELDNQKKVKKSERKICELKKKPSKYELDYSKFEECINDIENEEKEERHREEH
KHDFINNKNPCSHDHSKERQLYEKSTKEKINASNLFNEEGKKAFYEKNYKLACVYFRKGLIQLDYSFPDTNEE
EKEQRKLEINLHLNMALTKFHMSKFYECISECSTVLNIDKDNIKAYYRKGQAYMSLDLYDDAKREFLKVLEIN
PNDNNTKKSLIVLRQKIIIYNKKKKLVCAKFFSSNEKDTSINEKEKYINDKSSVEKNELNMMNRNINKNNIIN
NNNTIDNNKNRIHINENKNNFFIYISNIFKKCSNFFVLNKMFLYIYISFSLFFCIVLLHFYYL*
>N.PF11_0125
MCYYETHRIPTEKGYVNVYFEKQRRLYCLVHTTNNILQAHVYTFNDFKECEYKIDSLNILENEGNENHHQTNN
NHSDSTNNEEKNNIESSNTNIFSYIKRGMYSFGNFNISVLYFLMNKHNMELQWVDNKEICQKLKDHKNSAILF
NDEQLNDKTLIAFIINIVKLKFFDIYHHRHFYTIRKISDSWFKLDSSLNKPILLPTNKDVNNHLINIVKNNKI
QKSDNYIIQVFQKVKDN*
>N.PF11_0126
YVKPLYTVIIFLCIYYSNIYYS*LKLI*YFFSYIFFYDPKKRKTKNYIVLKKNSFYSKFLFFNWEKKNIQDS*
>N.PF11_0127
MLTYMFFFFFYFLF*IMFRKDIKKIYLKNHLKKIYIVKRQNENNIIEEKKSSVLYKDFKEFILVLINIRKYEK
ALKKLKGNKASCEFSKVLQEYLDFYLSTFGYKFHTFILEYKTGDEINDPNNCYVINKLRKSDIEKIVNKEYSR
ILFKYDSIKYVLYYIKKHTLLFQTFNCHFKKLLINYLFVTLNPLNVLIFVYLKLYQNNDKTNVVSKSDDKDKI
KNKDKSDEEMNDIPLCENKNICENNNENYSENHNENYSENHNEKYSENHNENHNKNHNENHNENHNKNHNENH
NENHNDLENSQINYEIVLNKNEKSNCTNLFCDEIYWFICFFGDYIIKYNIIRCYGEIYRNKKVIQRKLRIYHW
VKKYVQNDLIQKFHCYSYSKNIKLYDKWYEHMLYIYEEKFKEYIPKLKKFIQIGNGYKSINMCHKSFSYIYDL
VLLLYFLKICFFYLNEHIHFVLHLSLYTLYKSIKIFVIYLYFNLPKSMCHYFLNIYLMFFKKKNIKPVHILSK
MKCTYKNMMSHIYAHRNFLYINAKLNSHEKRRNLPIYLRQRNLNNNLIQCEDNDKKQISKNDCTENLNKQKNV
QNCIVDSQNDQKYKISKIRLNNSQKKRYEKILNLTYFDINEYDGKDIENESQLKKFLTFHCYGSIIQLKYNNE
IFLRKKKYKDQKEKVNENNINSVEGKIKIKRKRKRKIKRIKNIKRIKNIKRIKKNEQKGINKINGFFYPRKIQ
NKKRQILLYNVLINNTKGLSIYMNKKQMDDPKHCLHRVMKKSDIIKKSNLNFDIDGEHKMYIPSGNFNFCNKK
IINKLFETILYNIIHKENHMIDVNQKEMNLFILDKKINDENEKCDYVNRSPLKEPKHYLENKCGNLNEIDLCN
EQNEDDNMVTVYKDEHNNEKIDNYENSKNIYKEEENIIEQNEIEPNDKKKNKIEEKENMDNNDIIIEEDENNN
NKDIDISVKDNMEKDTCPSDENVSVDNESDVNKLSQNVMKEKYLNENNNIKYDLKSKKNDCVGKYKIYFSDES
TSSENTTDDYEYYMKRRNRSKPKIGKNEKKKKEIVKNKKNMQLQRDKKKIHEDNVNINKTNSKNKQQQVNEH

DTNSKKDNKIISTKETNIPKDNNVEGKNIINTINEKTCNEFIKDTNESLCIKNNNTTDNIMKNKINHKNISID
EEYNLKEYEEDSINENKKSVENVKINICNDSKEGMLEEKEIIGNNKKVYKKIQTLKSKGSKKLIFNLSLSENE
ESSLIIESLDNNNQETKEMKELEEIHIDSMDEEVNIKEKSDPLKGNNNNNNNNNNNNNMIDLKEKDYHIDIHD
KGGEDVNILNEQNKLKVAYHDLYNNIGGIEKPFIDTCEIYIKENVLNNTQKKLNLIHIYHKFKLGIVYLFDHY
IIGGINLINPYNSMENERYKHFACIQSNINDMDHTKIFRYNEKELIMSFKKNSEICLLSDKNEKRIMKILCKN
GKGLNGSIFKCTINNKLFACKVQHKLHLAKKEIYFSYLLKVRKSNKINKYTKYNNVNYSGRQLFYEIYENENR
YIFPDELHYKSTSHQNSKDVEMKTYNENNKNIYNMENNHKIDKIYRDDKIYRDDKIYRDDKIYGDDKIYRDDK
IHSDDEENSKSQKKVKGEKNENIKKETLKEKRNMSILIMNTHNNVITLNELINYFIRKNYKSVNEELILFIIY
QIIVSVLQLHFLDVLHGDVKIDNILISKNEDLCNREGRRRGKKRYNAEYNKSRKGNNKYKKKEKEREQERERE
QEREREQDREMEQEKEKEKEKEKEREKEKEYSNIYKNSSYFNSLYKNNRYLGKDKLNNYNKKDSFLKTTSFPL
NLFLIDIGRGIDMKNFKKYLFYGEKNCDCYSFLNDSIYNYHIDFIGIAQIASCLLFYKHIGHTKYRYERTLND
KHNITVNNLGITYSTHNNHFNNTNGRNTCYTKRKKKGKDVANDQVKYKSSKKDKYKEIENFIKIKEKVYQEED
HMCIENNDSFMDEYYLYDNEENPKRKTMNKSNGMNKSNGMNKSNGMNKSNSMSKNKKKIKQSNNLRNINNLNS
LDDLEDMIKNKKENKCSSSLYDENHDKENIYFIDYSKILSIDNKNKEKIFYYLDKIKKQSDNYFVEKNNENKI
KNFYTKFMLKRKKYAQFWEMFFHILLNFCNIYELNHIRYNSNEEVLNNNSDDDLYVHKLINKTENYYFDFKKN
NWQEIHKSPQRNDSNISNDSNKNIINLSDRCYSNIPYENKSTNLYYIDGVENHNSKNIINNHQGHKDIILSTN
KNIPTKHPNEHIKSYENNVLINNDNNKLSISKNGNYKNTILRKDIQGHNYNSMGKNSKYFFIKNSISNFKCIR
KSLYKHKYENKTKDQNKDEMKNKNIHPPEENTQSGKLKRKIIFFENEQNKTKCRKLNNEKQYYNNEYKYNEAK
RSTRYINKLMKKKAIFILLNLKRSIERIFDEDEEKQTILLNELYNASTFF*
>N.PF11_0128
MYNFVKIFQSNFIDINKLSKFEIFLKTIFRIYSSPGRTHLLAHAADISAKYAVRKIYEYMRTDEEGIRILKEK
PLLIRQDICFNELKKLPKNTLGYKYMEFLETYKLHAHDREVSHFIKDINESYILTRYRQIHDIAHVVYNLNIS
IEAEAALKLIELIQTKLPITLLAILIAPFMTPLYRFQYIFEHNIPSNFLCPNFDYTYNDDYNYIDEMSLKQYE
YYLTDYFHVEKRESQSFYYKLYKYYFDNLNNSSHVRGSIIYGYQNKNYNDIHYDKLNNEYLYLKNNIKNYFHF
QYKPRKLLLTNLYPWAYKTAIQTNKPLHSIYVEKWFDKDIDLFRKKYNITPLPSNLNLMAGIN*
>N.PF11_0129
MNLFKGFVPNYKYQNIFNLRIKSFCFNLSTLCGKYNKSDKRKFLISSNKSIKGYDKQKEHLRKKFTHVYKKDE
GKIKNYKNFKNIKRLKDNIAEKSNIMYQNNIETSYNDEHLFLNKLNYNDDENKTQRFNDKYIMDVGHIKNMYI
QNCLKKNNILYIYTYQYLLIELLRCNKNVLTNLIHSDCIMSYIIYSFYKINTQGSNNIYDNKYANHNKGQNVD
DLLYISNPSKHKDLNILKHEERKEKKKLPYSENCVLLIVSSQEQISLIYETIVKLKIDNISTYIINNENNIDN
IRNHLFLYNMIIMNIEHIKDDYDFEIFLDHISFLVVDDIYEIYKKKKTNLLKNILKNCKKKQETDSFFQILLI
NKIFDEWLFNDIVQYLKCIKYNIYIEKKKMNQSNLLNDNVIHNNFVNTFRCVNLIQYKPKIHTHMSIYVPLND
EKKFLILFNILDNNKNKKRILIYYNKSDIYSFYLYLNSYIPCIFLSNTFKMNCTKNLVHHFNTTNDYILITND
KTINKIYDLRINLYIHYTFDDDITSYMDILSNHSPFNLKNVNENNQNVQKGDHNNEKDNNGLDIYYKNIDEQI
NNSYDIEESILFYNKKQYKTYNILNNLVNFSTYTLPTIKYMKYNFMNFLIQEIQSTLIDDNKHITEAEKIYEK
YGHTFISAALYYIQKKKLFHTNKKYKNKTQNEHFSNIHFIIEKNISINTKNQLMSFLNELLNFNKTVGDIKFF
IQNYIYSKRGYILTIPEHIYNILHKNKNDIENVEKYKHIHMYILYNNYQKDFTFSCVKKGKYKSKKAKRRLMK
RLNVKKEQIEINKLNKKLASSFKIRKRNVKNNMESIKKAMEM*
>N.PF11_0130
MSGILTNLNIESGYFNEDIKEESNESNFMYANISMLIRAYKKDRNKLILLNKKLNLIKIVGLVLNIEEKKEFI
IYTIDDTTGCIKAKLLLTYSLNSYNEKKDNIKINDLIQIFGICNTVSLNEDLTISISSINKLDSFNYLCHHHL
LVFHDYLKYQEEERRNPIEDKSRNDEDDLESTQNNENAYFNTFFY*
>N.PF11_0526
MKTLEKKLKLLNFDESFDIEKDDFYFLILKIEEEKIRLYKPKEREKINYRKEKNYIEHIIKYLKKLNINIENI
NKNNMNDLHVRTYILNNLTTLALIDEYKDITNFDDDDDDEDIQDIENGHNHLQDDEDNDDLLINFFELNYLKL
YSNDESQYIKENTFNVLIEKINEIFKHNNIPLLNINNMDRNYINISYVISALKLIKEKLKNKSKTDNTDYEHL
FSLNINVKDDELKEFVYIIRYLFNEQLKKRKIDIKEILNDIQTLTYNPVIDIKQGRLGR*
>N.PF11_0131
MKRKSVHIRRNIIKAQNTLEKFGIFKKVNILKKSRDAEKDSEKENENVKNIDKNHIKENEMIFIKKEKCDEDV
HEDEEMECVKEKRNLYYANEKDVIRIKSERCHDKEENEKRNDNIIKTEHELENDILSSSSINIKMEIGKIENN
ECISEQEKKKKNDNKNKEINKSNNNNNNNLPLHKIYEPIYKEDFISEIRSKKNPLITKILDEDLNFNLILCGP
PGSGKSSLVNVIRNKTNNCFISLFHLNNLNNELRKVYDKSVINYKISKKKSILCIKDINRLNKSQQENLLLIL
KKGYFYLLATCLFNPMNILNASLSSRCLYLYLNSYEKTELELIIKRITNKLDIQIEEDALNLIMNHSCGDARV
AINIIDFAVQRMDKQNLVIQEKSIVSQNVVGSLEEGKKDNDSIRFIKQKKCDTYNDNNNNKNNNNIKDNDDNN

SNSKDNDDNNNNNSKDNNDINNSKDNNDNNNSKDNNDDDYNNNNNEQNHINYNYSNDKLYETDPLCQNSIKKK
VIKLNHIKEILQNFPSNDDKLDHYNFISGLHKSIRAGNVKAAILYLMKSLKNGEDPIYICRRLIRIASEDIGL
ANHDVLSICINTHYACKAIGMPECQTSLIYAVMVLCKSAKSNYIYLVENTAKQICNEYNFDVPFHLRNSSNKY
IYTNQPEILSYDEHVNKYKDAQKYLPDYLENLELFPEM*
>N.PF11_0527
MKIYDSLHKTINDHLHILNDRKVLDREKKMNSLNNIYDMISNFFNSSNNEEKENFCTFFLENLYDPLNILIND
EFDECRIFVLKIYFLIEKNLNNLLLDNILFKKLKNEDNFVFICTSRLKEDENNKIIEEKEDIRLKIVQFLYTI
LSRHKNMYQQSPTIILNIFIYLEHILSALSILLKDPYPLLKKQTCQLLYELNIQKETKEEYNKIYKDILTNLL
GSLHVRKNDTREAIIKCLKIITCLKTNRNSLHNVIECFKKLCKTNISNNVILEMINCIEMWILNIKDLNNVEK
AKLIFIIFLCMNLKIVPCTIKRCYDVIENICNDLYKQNTNNRDHLKNNEHILYTFKNCGKNEKNNYEEEYNNL
LNIEKMEDIYDIFFKNINYIFTFSLNKISNSVHNFFCEIKKELYYEIISYEKSCLFDSNEELFGIITLFLLLT
HKNSFYFIKHILSFIYKSYISFKYLNYPSTPLLIDDLLFVLHENIKNQHEQNYSYQYESFLYLSKQVYVILIC
GYLMPIELCIMEIAKMIFDENDVERILTKFYLFNDKNENYKEFSSNIIETKNYYIEEMNEKFVHEKKINNKHN
NYNNINNNNEYIFLNKNFQTYLQKCYNDLRKQANQPFIIERPCSDKEKKHEFYSNDHLLLHNKNNDIGNNTNI
IINKIDERKLNSIFFQYNNVEDKNIKQTDEKNIKQTDEKNIKLNDEKNIKLNDEKNIKLNDEKNIICYPLNEY
DKNTSIIYQNKKILLIILSQILGGYYVKKYSQNNKKEHVFNEECINFVFYLIHENIIYENNDSYPYILITLKH
VIHIIEKECKKYTNILFHFLIILQTNPNFCSHSQINNLMEEIEKYGETKKIYFYNYEYYYFIKNINNIINLND
FDNFKYHIDILNILLCNISNDILNKYSKELMNFFYLIINKELNSLIKLEFLLFLNLFISRKKNVSTFFFKNGK
DILKNILLPLCTWKTGLSEAQTRKSVLFCIRELFINNVLHVDIFKNNILIQNLICVLKSQIDDTWNHENREIS
IYICSYIVKYVTNNNIILDLLDYLIKLLDDSNISIRICTTTGIHSIFQNKNLVLSKEKCEHIFPLLFLHMDDD
YEDVSKNIFYILKIAKGIDKDIFIKHAKNSSECSHHAKSYKNLLMNEV*
>N.PF11_0134
MDHKKIIFKKKGKENLNKKTFDVSPNYVKLFVIPDECLINLIDKFINNLFNEENYIHTTKKMKTNKKCIITNL
LEAQKKFFECKMIENPLFHYNINDDELNIIYNKCNRNKIHFNLFYEARRILEIVKVNYKTYDNYENIIFNNKN
KNIHKNKTFLTRYQIKNYIMKYLQNSGIKHNVQIEFKENLISAIQIFKKKNNSYYIQLQYHDMTRQKIRALCN
HEIGTHLLRMINHHYNELQKFGINNICNITEEGLAVINSMYSLKKKKNHLYLISPALKYLAVCLGNFYSFSKL
YRFLNTFVIDKNKCFKICARVKRGLTDTSLPGSVYHDQLYFIGSYSILKWFKNIDFPLLYSGNIGLSYLNTIS
KYVNTKNNLLPYFLANQKKVNAYMKFLQNVSYINGITPEKCAFIQNGNIPKNIKNIKINKNININKNININKN
INXXXXXXXXXXXXXXXXXXXXXXXXXXXXXXXXXXXXXXXXXXXXXXXXXXXXXXXXXXXXXXXXXXXXXX
XXXXXXXXXXXXXXXXXXXXXXXXXXXXXXXXXXXKKKKKKKKKKKKKNIYIYIYILYIYKFLHSPVNYLI
FSFNISIVC*
>N.PF11_0135
MENLNEHDEQNIEMSTSIIKNLDIPLLIKKQLNLRDLRRKNKDLSTKNADIRKFLNEKENYFSFKEKVYNENR
EKLNKEYEQAMNDNYDRQIILEEKIKSKEKKIKEYDLEIKRFKEQIGDMHNRIKKATHESEEKEKCLSFLSKF
KETSIEFVQLDMFDRLETIFNSREFIRKKQEKLKSKINIIKDKIKNMEEKNNLTLTNKEGEYKSLVHTSESVM
KKEKDLEEQLKENINEHNKKALLLNKIISCIDNFLSRFSTFLPELRHYNMKKDEDKTSLDKKNKPQDIMEDTQ
FIDTANFNILEEKYRKKLDSLLDYVGIYIYIIYIYIYIYFFFFFFFFFFFFFXXXXXXXXXXXXXXXXXXXXX
XXXXXXXXXXXXXXXXXXXXXXXXXXXXXXXXXXXXXXXXXXXXXXXXXXXXXXXXXXXXXXFIFIFLFIFIF
LFIFIFLFIFIFLFIFIFFIFFGIFPFCMNAHFSGVIPLI*
>N.PF11_0528
MRTYTIQHVYYFIFIENGGNMSVQKYSYGGFVNVHKFLSKSSMSIIKEIKIENNLSSVINKIKDMDKYNDNIY
EKFGNSCSIYIEKLFTETYLDKDKSITLLNELLIKEVKYLLLTNNDIISEKSRNNEKNFRKDLVNNLYEEFLQ
QEQKEGDEEERGLLYINKIDMANKLRQILDLYSMEQPEAQEIILSLIQIDTSKIIIDHKSINKNFDLILNSKN
PYSILRQFFIGNEEIINESFLRKYWFCLKNLHLFHKTYNNHVLSILGDEFKHKIIHYIQNIICNKKQISIINN
YNNEESYFLNRYYYFDSFIALVISKVHNMKVSILNENGILISIHMNTLPNKYIQLVSTKYFSNTLISNRLFDD
LTYLLVSEHFSVHKNITNFEISPTYLDNVEMNSIENNYNITNESIKISPLSSQENDDIENCINNDIKTSSEEQ
PNIQLKYYKSLKDVLSHYKKLHSFNEESIYLLLLNIIKGNLTNLSLLFSLEVLNYLDTYCDDYIENEQEIDEK
GVEYIEYINKKKKMKTNKRFKNKHKNKNLNFHFYYYDTPEVDNISNDIMENTNLSNIYDNDVLKQLYKDIDDN
NISHSSSNDIQKEMKEKNNEMKNIPQNDNKELLFNNKSNIKQPDIPKLLNNEINISLLDTLNKSTNKISNENL
YNTLYNSITHKSTQHINKTLLDPPDLSNDNITKYSTKLNSGNIFNVEHFNNLIQKVEDPTLKDEKKNEQGGKY
IFLQNKSEFINNDKDDTKNDTMIKNEIDMYRNIDSWNNIAISKNVHPYQNNNTYKNTAGTKPYDNYKHHNIDD
ATVNFLNISGNDESHKTNKKANGKSKENDILTNSKNNKDRNVKKGNEDSNISMNDLMLNSKYFKNLCKKKKMM
EKEKLKSIRELKKDLLKAKYTMFNKKSLFLNEENLKNIFESTFFDTKQMKNSIIQIISSYEIGHLSDLLNINM
SNINSHFYKLKNVFLNKKKTLHYFKLNTNQYVIHKIENKLYSVFTLAWIHYTNYLRKILDIFSTKELYLLARK

YYQLIHTNHDEYNLILLNTYGFTHVFMMYIILTYIGKNVYFITSNKSIHIKHEGLDNHNSIHIVQACNGSYII
TTNKCVSDILSKNLLNNRKIHVGYDLNLYTTLTVDKNIPDINNKTELESENINALPQKSTDHMLSSIDYGIIF
HNIQEKKDTEHFNFLTIYRNENAYNINQIPSLITAILCGKNYDRNNIKKCTKDDNIFSSFSKKLNLIIQKFYD
NNTNFKHISLLSKKNIKQNVFINFYQNEKILLKTLVAYMYNMLEDIDNLCKEKEIRHQLSFVYNIIKLYILKN
KIYDNTYDAYLYSIEEIIFKYKLYNQKLFEILKHLKIKHEDNQEYMEVLEIISLIFYGMKIRDLAKYKRENIP
EMNYEILTLDLIDIINIMMFFNNFLNKINKTDEMKLIIDKEKIIEFHIVEIKKIIETIIDLHANYFYDIIDKQ
IEYNIKDILLKKMKYVFIQYKNFIEDDLKELSFKFYDNIKNEKSFLIIQDPNSYNYPIDISYTKIENFEQLFF
LDLIEYSFLNYTSSNLNTINNNNDKKKQNQKNKNRKYSKNNILDLYEHVSTISLFYHTFNLPKEYNEKLFEEI
FTFAQSATPSKAFVKKGHLWGKQVTEKTYDKLRLVNLQRHLRYFLESNDIDQLVNYMLNYEKVKINVRNQEEK
YKEKYNSLYMCIKEQWLYKYHVKKRKHFLSNIDIKFHLNSHDIKLCLEKNENHDNSNNKVYSEKYVHLNILTN
IMLNILAYELHNIYKIEKKLHSIFSLMNIKNSDINNKIHININKKLNRKQKIALFYYSSNVLIHIIEILSQYF
KKNILYFSEKKNETIYEIYSFVIQKAKPEEKIIILKEGNVFKVYNPDNKIFKKDMNHLMETSITLACYDELMK
KNLYLENLVRTYTNKENVSQEDIQQIINNFSYEKNFENIKEMIKELLYRKDINTFKKTYLTNIMKLQIEQIKN
ETIKTKYIHILYNEIFSLLLIEPYNDIDNLLNGFFLCDKGLLAWFNISLSEAKIDSNIYDNLHIFVYTLRYMI
KKIKQMINIEFLEKQQLEGKSIQLFKKKDKDKDTKENQNNNKNKKIQDIDFYEIFPNFLNEKIKNKNNNLSYF
KWLEEFKIDKNAKLPILQDIENYKFSIMSSNDEPDYNFLSLVGLAHSYIENFERLKLGIYPTITYNCVATLNK
LYNLYFVNELMDFINLLINHILNIYNKFFYKKREFFKDNKIQLSMRRKKEFANLILYNQHFDHQIINEKYEKK
NNTMEDKEVIQNKEINKEKKENTGNNTDNNNLEYNKFEEYKGMSKKSESDIHTYHLDYLFKNLYSFFYSILDC
EKNELCFFHKNDYVRNVLKKYLKKTSFNKNMIWIIPILSYHFNKPLNVLYMKDENHIYLYKYQEESEIKFSIQ
ILVEEGNQMYLILPTYFLYINVLNIITNILIQNEHIYKRKLYNEKKNETNKSIEEQSEDSNGIENENILDHHN
RNNEKSIKNIYSSNHSCDKVLDLNLTNNILSDNYYKKMFEIILEKIHLYATKKINIYTLLISIREDISNFKYV
IYYKNFEKFMKIMDSFFLLLNSKMNINIEFSFDEIMNTPYMVVKGSINNVINFIEKENYGIFGINIKNFYTFI
LHHYDSKKYYTNTFLKLLDFLLNAIKNEIDYQNKNIQSIQPNTEMYIKKMEYIEKLKMLESYINCEHIKVIRN
MMKYVEINLHNIINFVVFKSKKNNLNYEYNHMNNIHNNNTIFSHKNNYNIKNNINKRDILYQCCLIYDYNELG
ATKLTLKYFNELEKNFVKITDFLNILEKLSNEELNYDEWYSIASKIHIDYFNNSELFYFHYMNEKLFWKDGSV
LFTDKIVKLLNKKKLYISTEDLEKLYNYKLTYNEYKHILYQSKIISKVNKNLDITYLDKENYAMSLIWSFFNT
EIVINGTHLNFDDIYYKIEEWYEKNKSKNLKDTLYLLNRNDIYHLYNSMILFHRLIEFIDRPIFDKYKAYKIT
NMLFDELMNKGIYISGDSQITKEYIQSIFDYNLWKKEIQNVKISNNNNIRSLVPIKYINYFKSSILINLLKFI
SNTKLNNRIVNIITYIIYRIFKLGNTQNDILKIIKTEGILFGSIDVFRLILIKLNIPLDVLSIWFTLLLKNLN
YITENCIIQDFIESIDFRRFFSNLFFLLHNFIEQYYFKTILKFLSGYPFVVEFIKNNEHKVKELFIHFKNFIL
LNFGTHINFFQHLYDNIEKKFISYIKYYITKFLNNLLPFSTDISTKIMLYQNDQISSLQFIEFIDEENIIPLY
YNIVSLIQHEPFIKAFMKKSLIIYSKMFSNTNGKMQLSHVQLIQNMYDRWIKRNSYKDQKNYAINKTTRDQNV
IATTNSNSSFIQKLYVPIKNRQKKNIWKGKKHSVLPPITQMDLKPYNNITYQNEDKILDEPTNVTYLNNTMKW
NNKNVPFVKNIKADNYKYDTHNNKDNEKYISKYPILNIYNKNKTNSYISDNNSNNNNNNNNDNDTNEKKTYAY
FGTSNISLLQNNNTNDKIKFSYIQLYNYRTLSCLEKLFIHGFKHVENNRETEKITTNNNPVTTCSDLYKEEWT
EELYNFSMNKLSMEERHIYSYTHDLKLIIMKGEKKNIKCEIFFKLYLNISTEYILKISRTHIVIVDFNIFRKN
NELCFLYINKSHQKIYNNNNNNNNNKNDSNNNKNDSNNKDYPSIQKNTHFCYKVEIVPFYKTNNITKYLKDVF
YTIDQKYHYDTQKKKNELGEILNDIGPNDNLYSSPFNIMYNMKNNNNINDQTHNIGETMNIKKIKDIKKKKND
FSYKNNKSRDIPRNNEMKKKNTLKKFFGKIKGKINNIKKNYYQKYKERHEIKYKLYPNLEKLQDTLKYLTDKN
IINKLLEFEYYHSNKWSSDTFGVTIQNMNGRNRNIKLILEIGDYNLTNYYEINVNDKKNILSLQKLDTFVKKI
NLKNGLMQKLKTYKKLHFLLKHLFIFKNEMKNVEYFKNERIIISDARKKESTKRNYNIENSNIIEKDNIEMKP
EHKKEQQQGQSQNIVERFMNTPSQKEEKRENDTLEYLKFTWYSHNKKILQEIILPVKSIHPFKLSNHNGNVLC
KSDSNIDNLLNFMKEQNENDIRQDAKKDIKNVHHDDPHFEEVNNYKHSNNTLDSFNEDYIKNHMNYSSSKINN
YEEKYFSDYRKYFDKIYPLNEEEKFIIRNKTHHQYILYKYRLAMLSLIIIKTNHLTNLKKMNIITNEYLKNKH
KYYKHIQYHQNELFQKKHVYIPNRQYIIKKKLECIISLDYYKPLKKALQENNVLNNFLEDLTVEITNINMLFY
KYFYTDNSNIHSSSFYLDKLKNIKQLCPIIYKLKNDEKYNLDNISKVDDTIKLNFNKKIEYDIKECEQKIGLY
GSNIAFFGKALDNSIGKKKIKNIAMFSYECERSIFLEKNLSDLDPYHKNFCINILENINDNENQNESQNEGQG
QGQGQGQGQNQSENAEQKDYYNNTYFKSINTTWFDMIYNTYTSNKTFDYKGQQKFSQFKCFGFADRDELKEYE
KKEGAKCKITILSKTLFVLQNFIDKYYILKNEEGIHNGNAYNIYVLYKNIYTVIIYMLMYETRIFKPLRIQQK
LNNIGNIYSMLLYTKKNYSNYLKLMLEKTSSLYLEIELLYNEHVFLRNIPFPNDSVYILMNEHKPFDKDTHGY
KLSENPYEISNEGDDNTRVYAFFVLNLLSFYPEIQKSLLLSSHNEDMIYSLLDINIQQINQQIQNKEHPNDQH
SFRPQHQHTNEQDKSTQGITQMVLKPLGDLYNYFFSKKEEEEKSGESKGEMKILEDSANGKEKILEVRQNGKE
KILEDSANGKEKILEVREDGKEKILEGGTEGEENMLGDSKRNEKILADELNLKYNSKEQTKDEQMDKKENIKN
FLESNKQNILLEESKKNSNNSTNFYEINDFTWMGKDQIDDIKRLIVEHGLVKYFIKGIHLMYLQVYLKDNNYL

EAYKEALNEELQKKDFYMHFIKTSPPFHFQNYNVHHLLLFSFYYGFSDLIRTRRPYVNLESTLNRTIENNKSI
YMYRTNSYANFQLAANKRVALNTLHQELAKFIYKEAIQYRYLNPYYKTYKDKENITTFIYNNNNNKDKDNVIF
KFNEEKEKELIDLIIINKKQPIYDTKDIKEIIIFVQIMVENFIYILNNYPSSFSLSKHSSYYNNFIKNVKQYF
FSYKKENNLIYLISQRDIDFNISSILHISEKSTVMNILKNIIKFIFLATTRSYDVDVFVQKILNINQNSKHIL
KQDYSQEKQKYVNLKSTYAFIQKNYNVRSIKQCFKNCLKKTKKIFNKVSNATKNTNPSVTYKRFDVHPPVQDD
LLDSVIFKKKDQFKRNNIPTKKSKKKNNDKVIKNIYIHEQPFFSKLKMRCAIMKKKLSMSMKKNKPNNNYNDY
NYFNDEYNQHICDYVPHQNVNKSSLEYEKPVNIKIHKKKEPNENKYYNMDREIINDNNYYLSTNELDDTQNRY
QGIKPVNQEKKNYIEKNIINEKINRFQKNYFDEKNNLSHSKFEQQAQKEKIINNERIRNNNEDKINRNRIMNH
RNLYNTGHINRSIDVNNTNIRHINNHNVTYNDDRQENHPNFEIRKNLYNMKNEKEKKNDNINKYSFMQFFQVN
ENIQNNNNNNNNNNTIDVIPKSTKTKFTTLRRFLGTRMNNLYSKLKSELFSFTLINSYDFLLRNSVYFYRSNM
FTKYITYYAKRNVIQSLDMLFLIVRDYELNTSLTNTYSIKHSLFNICLFKLQNISINLKNILNIIDEQKMDTI
VSRVISIIHTNYTGNISFIEDLINKINIILSKNFVLKKLFADFISYVTISSIQTFILHFHSKYKKKVLTFIIS
LLNNISGTISSILSEPFFQSYICTNFIKLGESFISEFNAENTVYGFFMDVGEINFIDIIIAYAYHFFPKAVND
FTQMS*
>N.PF11_0139
MKSLENNEMHNLCPIHYYFNGRDYNSDTIINNVNIKYFNMDNCLGNANLHMDYLNPVLNHPTKIEHGKIKILI
LDAPTNDLLPLYIKEMKNYNVTDLVRTCERTYNDGEIQDAGINVHELIFPDGDAPTEDIVSNWLNIVNNVIKN
NCAVAVHCVAGLGRAPVLASIVLIEFGMDPIDAIVFIRDRRKGAINKRQLQFLKEYRKKKKKKNCLRKCHFM*
>N.PF11_0140
MKLAKWNFSNKSDLFFSFFHLRKNRICSLNTFSFLSTKINKTLLKSEEGLTNLFFLHPLSSTTKEVNMNTYKS
STLFFNNFMLYDSKDEQSSVLRKQSIQELLFKNKKTKLALRRKRKRMGERISLRYR*
>N.PF11_0141
MVKIQKSSGMFWKRNSESSNLYNFLNGLFCIGGIYFFFIIFGYYQEKLPQLGRGSDRFYYNIFLICVLCLSNS
LCSLSAIFFKSRLNNENVMSSLKKNVDKYFIKQIMLISITYSIAMIATNYSLSHVNFPTQVLVKSGKMIPIVV
GGYFFFGKKYPYYDYISVFLITSSLVLFNLLRTKSSKEVHQTTFGILLLCISLLCDGLTGPRQDKLLSKYNVD
SVNLMFYVNIFAFIFNLLASLIIEGNKPYIFLQKYTTSYYYILAFSVSGTLGQFFVFYSLRVYGSLYTSLFTT
LRKALSTVVSVYLFGHVLKPLQWICIGVIFSTLIVQSYLKKQSKKVQSKNK*
>N.PF11_0142
MVINVSFKVTGGKEFTVAIEPDITVLDLKKICAEHVDIPVEAQRIIFKGKILKDKESLTLYGVADGNTMHLVR
SAMATKDVAQEEKESNKNENATNNDQSRPSNENNNEMGDNPLVQMLMQSGAGDMGNFNSALGGADNFNLGNFA
NMLNANGAGDFNRDTISSLLNNPLARSVLNELSNNPEMLTNLVSNNPILRNTFSQSPLMQPVLENPNLLREFM
RPEILQAGLQIENALNMNNNNNNNNNNNNSNRPGGAFRMEDILNNLNNLNNLSNPNNTTDNNNNNNNNNANPGN
NLNSFLQSPELLQAFQQVMSSNRNLGNFNFPNANMNLDFNTTNVTDNRPPEERYASQLSSLQEMGFLDNAANI
QALQETGGDVNSAVTRLLERGFN*
>N.PF11_0143
MGRILFLLKNVNKIEKRYFTNKSNIYYHNESQKKEIIVLHPILKRSKNNSNKLFQEIIYDAQEALGLAKSANF
QIAKGISMPLGGWYLKNEKQKKKNDAKNDKIDEGQVPNKELSQNNEHHHVFEKSEKEIPNEMSFTTDKSSSKY
INYDEIERKVAESILIKVNHIDNKFYFSKGKLNELSKYYLKNPTPCIFINTLLSPEQFRNLEFLFNSLLKSYQ
DELILNNKRERDNSEMYERVSDVKFDNCDSDDIIIDNSSYCLDAYNNFLDKEDEQCDDVDLEQNMNVLNEHIG
DTSCEQANDIPYEQISDTQECSKNIPMYVELFDRYSMILYILKSRAKNNLSKLQLELARANFVLNTYSEDSKS
RMKYIKYIENNVLGGSCIDYEEKYTKLNFFTVGKQNKKSNVNFSGYTSNYIKSNETYKEYEKRIINNLYSKLK
NELIKCKNNMILQNNSRKHKAIIAIVGYTNVGKTKLINYLTKSNLKARNLLFQTLDNAYKNLNISTCYSTIFV
DSIGFIQNIPYSLYESFKISLEAIKTADVIIHVIDVSHPYKDKHKKCVLETLNKIGISDEFIKNNVIEVWNKI
DKLTDNELYTLCKNKPKNALPISAKYGTNCNYLIQIIEHLINQIKDVHILNLQFPTSEAKERINFLMKNYKVV
PHSISYSDDGNTTFIKLVENKSNLKKYYEKFEIKETYKSDN*
>N.PF11_0144
MNIEDDENKEKKETCLSYIKKINMSEIINDEAIGKRAEYLWNLALSAVYINVKLSMKYITLIKKITKNNLLFD
NICCHYCNLIYIPFYNCKITHSPNQGKATYTCLLCKRKKKINLQHLSKHKNIYPLKHSDKHKPSTSNLSNVNF
FLINEIDNYEIKKNNVPLNKDDTSNTENQLITKQENKNEDILKDVSCADMQKEEHIINQDNIINQDNIINQDN
IINQDNIINQDNIINQDNIINQDNIINQDNIINQDNIINQDNIINKNDMYQNLSYLFRIDYGINKDDTQKGNE
IYDKSIVNESEKCKSVHIENNKNDDNKNMNSESKNIKNERKRKSQNNNIAQNKNQGFPNVNMNDGFLNFNKSK
KKKGKSILDIL*
>N.PF11_0145

MAQEISNLVKKYNVTWQQTMLRIYDPKETVEFYEKNFGMINIHTYHFNEYNFSLYFLITPPYDEEERKKLPEP
NTKESEKYLWNLNTVCLELTYNHNSQEKLSNGNNENDRGFGHIAFNCNDVIEQCDNLFKKNVKFHKLPHETKM
KTIGFALDPNNYWIEIVKRSNQVKWKNYKNITNFSQTMIRVKNPEKSLYFYIHILGMKLIHVKHCSDFSLYFL
KSNYACAENNKEMIEDQSNKNTNEIYDFNSLKNSYQTDEDYENFKQSWEPVLELTHNHGTEDDDNFSYHNGNT
EPRGFGHIGFLVNDLENYCKELETLNVTFKKKVTEGLMKNIAFIYDPDNYVIELIQRDTSFIAK*
>N.PF11_0146
MNENLIHKNLVKRITNKDDNNNVMGDKSSCVDGEKENEEENEKKENEVSKDKIQISYKSIFLFQIFLFLIYFL
VLLIGSFNFSFKLFQVREKHLYEFLNNIKSVPCYNSYYIKKGQNNNKKHNNNNNNNNNNNINEGKKKRNIVYK
NTDNLVHTHDKIPNLNFKYYFTRGKHYKIENNEMKEENNKEIKMFICLNKKQKLYNSDIKEILLKWKTYVHKF
LHFCLNEIKNAFFKKENKTTIQNNMKNYVLDNYRNNKDIEVYKYLYYNFLLNKNTSYKNYYVNMEHIFYMYIN
NICYYHDFNYIQTIISHNTSNTTTFINIDKNIMEDILIENNKKDITLFINHINIYLNDDIFHDLLNTLYFKIC
NKPNLINYLKKNGESNNNTLLNMDNNLSEQKHHNNFDNINYFKYYTLYNKIQEIKNNTNHFHKFINRIQKNIT
TVQVLKGLNKCLKKYIRNLTKIPFYFFMDDYYDTPCYVYSKLISNLLRNYYKGIFLYFIIFIGCVHVVFSPFP
ITLVHFRRFFIYFIIIMYRLFILYFIPSIIQYVIYKFHNFKEEYIHIFDYSDHVILFCTLLFIISLEIKAIEY
TIKHQESSSDHFHFKYNRNFCFFFLKFVLYYYYILISFFLYTSYFTSKFFHTTNEIFVAYFFSTFSIFFFFYF
FLYKNYFSFYSIGITSYMKKNNPAPSNVFHTPSCLSITGKSLKDKFNIE*
>N.PF11_0147
MLKKKKDYENADLEDCNTKEIKGYSKVGSEKNIKKKMTSIKESSSRDEEEEDANVNKNIKKFKEGNKKDNISK
NCNIVEKKNNKSKEEKINIKEAIIKNVKVPDNYEIKHLIGRGSYGYVYLAYDKNANKNVAIKKVNRMFEDLID
CKRILREITILNRLKSDYIIRLHDLIIPEDLLKFDELYIVLEIADSDLKKLFKTPIFLTEQHVKTILYNLLLG
EKFIHESGIIHRDLKPANCLLNQDCSVKICDFGLARTINSDKDIHIVNDLEEKEENEEPGPHNKNLKKQLTSH
VVTRWYRAPELILLQENYTNSIDIWSTGCIFAELLNMMKSHINNPTNRFPLFPGSSCFPLSPDHNSKKVHEKS
NRDQLNIIFNVIGTPPEEDLKCITKQEVIKYIKLFPTRDGIDLSKKYSSISKEGIDLLESMLRFNAQKRITID
KALSHPYLKDVRKENLENFSTEKIILPFDDWMVLSETQLRYIFLKEIQSFHADLIIPAKLNIHQKSFYNM*
>N.PF11_0148
MESNPKNNVKSSEENETEKNNKEENKIDMEGFQFFANICEDKLKVLLENFFSNKKYEKLQYVEEKIVMSSPEI
QDADNDSSNENDTNSCVQNKVIRSNYDKIVLKLVDDVEQFMKPYVNDRYKIIVQGIIGENKKQGIHIASKSLW
NVETDNYVSVKYVNDYIFVTVMVFLLYNE*
>N.PF11_0149.2
MSNIHTLAEYRDDYAENTPFNRNYYQSQSSFVQRSKPIDLLNLIFPHFTWKSFMVVISIIQLIVFIISVSIKP
ADFLTPSDSLLVTLGANVASRIKQGEIHRLILPIFLHANIFHAIFNIFFQLRMGFTLEKNYGIMKIIILYFVT
GMYGNILSSSITYCPIKVGASTSGMGLVGIVTSELILLWHIIRHRERVVFNIIFFSLISFFYYFTFNGSNIDH
VGHLGGLISGISLGILYNEHMDNKPRWYNHLKIGSYISLVLLAIIPTVVLFTIPRTC*
>N.PF11_0149.1
MSNIHTLAEYRDDYAENTPFNRTPDYYQSQSSFVQRSKPIDLLNLIFPHFTWKSFMVVISIIQLIVFIISVSI
KPADFLTPSDSLLVTLGANVASRIKQGEIHRLILPIFLHANIFHAIFNIFFQLRMGFTLEKNYGIMKIIILYF
VTGMYGNILSSSITYCPIKVGASTSGMGLVGIVTSELILLWHIIRHRERVVFNIIFFSLISFFYYFTFNGSNI
DHVGHLGGLISGISLGILYNEHMDNKPRWYNHLKIGSYISLVLLAIIPTVVLFTIPRTC*
>N.PF11_0152
MRINFFKEKNKIRLNKNLDFDSDIAISNANNESNNYLTANSDNEYDHYGFEKNKEYTQENNDDNKRNLEKYKK
KIERRWQLYFTFKRDIKKSYYLKSLIRKGIPDKLRPDIWPYLLDSMVLCLKYPTIYEKCLNSELESKVLSQID
LDIIRTFPHNKNYRLNSPGLVQLKNVLRAFAVYKPKINYCQSMNFIAAITLIFLKEELAFWSIVQLIDSDYSH
EKINISDYYNNEMRGLRRDIIVIEELIRVKLPDVHLRLKEFDVDLSWICSEWLLCLFCTTFPITTTLRIWDCL
FYEGDKIIFRITLALFKLNQQKLCELNSLESILLLFKETTKNMFECDKLMYIAFNEIGVLKKKTIRKLRLKAE
DIIKNAVP*
>N.PF11_0153
MLYLYTKQIWTKWLNYECLNYSKKYLKDVRLLYGFKGNHTCYFSITDKSNMNDKSNNIYVNSKNNNCNNVEEN
HYIKEYDDKTRSIIDHLKNKYDITSNSLNFDIKKTKGEKENVFDIGYTWSTFFKCEKSLYNVISKYVSTDVMD
NYLHSYLNYNEEDRYLKDCYKEDILSDNINIDNIEKRVNNILIYITSFYYKNIRDYNMISILSDKLIDLCKKI
DSINYKNSKLIFNICNVYNKVMALNDELFFILFNILNKIYVQDNKNKSIVMKDKEVLLILKTLYNQNYKNHII
VDTIISYIKYGSNLNSDIIVDTFYYLTLLSRIDHELFKKIHTYIYNNISDDEKMNSFFSSLNKTKFVELEKEN
VSSTIDNYKMNTEIVQNISFKINITPIKCIKLLYSYIVLDDENFLNFYIIEKLLVTLCDSLKDNDIIFNDEKE
CYNKKKIHEMICIIRTYLRYKKPFFYNYLPKNVKRKLKEMYIYDIGERKIKEKNFNEKVSYHLKKLRIPHIQN

VIKGGILFDILEKDKKLVWLCLSYHHYYVRTIDLTVEKLLQINLIKAMNYKISKIHYYQFSRMKAKKTRFEYI
RMCRYYSLRDRRNYDDELEGWNLPYINWYHKKNKNVHISNYFYNYTPISQVEY*
>N.PF11_0154
MNWDEKNCKNNFDLKKQLHNIFFILGLVIQNILVDNNFLEYVNINDIYKTLYHIFKTRYLNILNTHLDGSHTE
KNENNSLLRDDTDSLKKGNDNISPLINDDHISEHMNDHIIGSRKNKLLLSGENNKPLSYNNKSTNSNNNMSEN
YHETDSPVQNITPLHIESNFFYEQIKLKFENIFSHSPQREKYNNLNFIQIHNLKCLKYAITACIYSHIKISGK
SICLRNILNTTNFFISLLINNNNYSDHHHHHHKKKRSNVYINSLKDTVHNINTYEPIIKENIYQEKEESLKME
RIALFTSSFKLSNNIMKYSLLYELMFITRSPYIINKFLLSVFNDVLCIPNINNKYDDTVIVFSCILFVNHFFY
HINQKSKETKNNSWKDKNKILYINQVQKIIKLFLLLCPHNQIEQMDNIRRFTKKIVQMYSHMY*
>N.PF11_0155
MNKLNFEKKKKNGNTSECEKSQDSDDEIMVVNRKIKSKIYLTPDKKKKNEVKNNHEVKKMYTNNTFQSNDNMH
SKGTNIRSIKNLSSIHVHNKDYKLNKNVTSIIMDQKDKNDINRSSKKKHENKTAFESIMKKGINTLESNEKKV
DIYEQNVETKENESSNNEIYISDDIKKEVHKNNHNNNYSRDDNINQNKSNNNKNYYTSYGEIIHDENVKCGQN
NNTKDVNEQKKKNFYYNSETNKDDSYTLNHNISTIKDNNYINTQSSSLIKFDDFALEEINKELEKLIEEENDI
QEKLIYLTDKELDITIKLKNIKHKKNIQEIENKKKEYQGEYLT*
>N.PF11_0156
MSKDKRNSFASNSFDSSNDEKKSKNGNKIYKSKHEENSPDGDSYKINNNEKEKSKEKLKKDQKKKSKEIYNSF
NSPNSTSSDSDGNGLHLNFSNASSSSSENGFKILRTQENEDKLLEERRRKREALKEKLKNMVKENEQNNDANE
ILQNDQINKDYNNETFLLSENKNDNDIITNEIPSNPSYIDQNDAACIFAPNNDVIEDTCSSLSSDHEIIEEKQ
NKEKPEAVKECSDLYNDLKKKIDEEKAKIRSFIIKQKELHERLKMNVDDSLYVNKSKGNADTHNNLTNKKSPL
ENEEDEMQEEYDEDNDDFDMFSCVQANKKRKVEKVHITDYYTTGNNANLSDNWNDSEGYYKAMVGEVIDKRYS
VVCELVGKGVFSNVLKCYDMVNKIPVAVKVIRDNDMMKKAAEKEISILKKLNQYDKDNKRHIIRLLSSIKYKN
HLCLVFEWMWGNLRIALKKYGNGHGLNATAVHCYTKQLFIALRHMRKCRIMHADLKPDNILINEKFNALKVCD
LGSASDISENEITSYLVSRFYRAPEIILGFRYDAQIDVWSAAATVFELATGKILFPGKSNNHMIKLMMEYKGK
FSHKMIKGGQFYSQHFNENLDFLYVDRDHYSKKEVVRVISDLRPTKNITCDLLEHQYWLKGNSPKMQFLKKKI
KQLGDLLEKCLILDPSKRYTPDQALQHPYLRESIHFSKSQNE*
>N.PF11_0157
MLKFFILYFYLIFQAITCFSVNQINVGNKYSLIRNPQSNVPLKVSIIGSGNWGTVVSKIIGMNAQKLKIFHPI
VKMYVKEEIVEEEKLSDIINKKKENIKYMKGMKIPENILAISNLKEVIDDADLLIFVLPHQYLDNVLDEIVQN
NNLKKNARAISLIKGIKMVRSKPQLLSDIIEKKLDIECLALSGSNIAEELSREHFSESTIGFEKKGNEVIWQN
LFDRTYFKVNCIQDKPGVEICGALKNVIALGVGFIDGLTASYNTKSAIIRIGLEEMKKFAKMFFPNVLDETFL
DSCGLADLITTCLGGRNFKCAKEFAKRKGRDSWELIEAELLNGQKLQGIDTTKEVYDVLEYHQLKTEFPLICT
IYEISFQKKNPCSIIDVLSTKKLRNIKYK*
>N.PF11_0158
MTTLRRNNHEYHEEEELNCDENKSKDKLFHEINKLLYSSSSEKMSKKSKPKIVVSKGGSIRQSDKIKNEKKDN
NSSISSSDDGYDKNISSNNISDKNQSSNNICDKNIYSNNIYDKDIHEYNILYDNIYTYNSNSNKSEYSHNKSD
IYQLEQILMESNYNSSLKNNSISPCSLSLTSFSSRNTSSILDEYDNIIKIFIENITLDNIIYNENTYNNIFCI
SYFIYEDPLEIFSFFQGINWLPELKNRCTSVSYLEKLDNNRMEKNNYYQPETYSYYININQYVNLKNRKNEYL
YIYDDGIIYVFVSIVGVNMNKFQKYYYDNFVQCDNNIYNDHDKKENEHSLYLTNNKTNDNLSDKSHDIYSNGQ
KKKKKDKKKKKFIHQRHYKSFLNILGTCIIPIKVIDTHDEYKNKINNSTMYNIYPHDAIRSILNNYYDSLKIK
RKINIYDLINNYKHILSPIGKINISVKIKSKLLYMSNVHKYNHLMDIQHGGEEKCLMNRTNHMNKIIDEILNY
VKIYKKSNDKKNNNQDDTHIKHIKNKILSNFFLFYNEKSNINFDDYMMKSFSYIERNIDKIFNCDDEKKILKK
IKKRILNILQLYQSKETKNNKQSNNNNNNDNNYSNNNNNNNNNNNSSNHHHAVVSKEIVCTHIILFVDCISNI
KIPWKLLSHFKNDNTFFIVVYWKEEDDEDEENNMTNIISRQSRVIHLNNSNSYYYNHCNSIEVDYNSCVLEKY
QTNNQTNHIITEQCNYNINNLKEEEKNKMIKVKLNFNSCIILPYNHYNISSDINLILYHNNYPFLEFKKPFCI
LNNYNITYNAQDKFTMNLKINKNYYHSNNKINVNNLNINDTCVQLSLCKHPKHSTFFSSINYKSYYKNKTKCS
IQDTNYYNAHYSPPLFNKNGHIFIFCDDYYVNHEKYNKNENKKYSYLYPNDDIKKKMIFNKESFFLPIREGNT
YNQERDKVTNPYLKQKQKIDTYMIKDECIEKQEEDQKKKKKNISLSCILKKNILYSVVAKGDGLKGGKVNEWI
SLYVHTINKEGDNIYYGKHINIRMKIEPIGYLKSYYSFSYSNISKEIKDNNNNNIMLTQSNDANIIKNEYNNL
SLISSPMCFHSFNDIQEMINYKVEDLQNGIYQILYKVNKIGKKKLYIYCDGISLSSSPFEINICPSSPCSKLS
KVVGKGVTRCISIPYLYDINKESFFINHLEKIDRKCNGKNDDLDNMKKMYNLDDMKGTHNVDDMKGMNNADDM
KGMNNVDDMKGKNNADDMKEMNNVDDMKEMNNSDDMKEMNNVDDMKEMNNVDDMKEMNNSNDKEISNNMCDKK
DSSSNGGNSHIISIQKNEDCLKINNTNNNNNNNNNNNNDNDNNVDICFGYNSVCDTSDSNKKNTNVKYLYGEE
NNYIFKSPKYYNEINGNIIKLNEKNERTFEEFLKNMVQVVNTFDIILYDKNGERICIGNDNIKVIGKRGAYIK

NVIDNNNGTYTVQYCCCVKKDDIKKIRNINKEKLIDFYDEFLFHKNKYYDNVIINEFKKRFFNIFINCEVKVY
INEEEIYGSPFFPIIINMHEILNIYNLYDQYTYSGMLLKNFEYLLTFNNYEGCIENLCEFYETFVDTQDDLID
NKSGKSKTERFLSLFQMKNEEKINMDKKNYTSLFFNIPLNIYDMNSKYMDEMDRYKENYENEEETKKNTHSFC
NNNNNNIMMGNPSWNNYKIDDTLLIHKDNHKRYDNDNDNYTYNNCKDDSYDSLSINNYNLKNVNLSLNEWLYI
YSCKENVTQSNKKYDNSLILAYSLCNIILHHLIYLKKYKYYINCKQYENDLLQNNILIQFKKLLQEEYNKMFA
YQTNNIITYCKKIGNIKFNNLEELIKVYKNIAFELRKLKKNDLADEFDKCCENMCEELYLKNIESNLNRKEKL
LNEYEKIMNEKINKIEHIKNNELKKYENNYTIDMNKINKLCSVPKNKEVQTCDHLSYQKKNGKLSSLIELRNK
EKLINMNDKKDEKKHKSDDSIKEVVHNLDKNQKEQIFHMVREYWKNSSTYDIFSTIKNTLKNCPRLKICLEET
FNYYSCSIKNNNKMKDIQMRNIHEIKIVENLDNLKKIELQEDNFLSNHDINNFYLTYNAYISLILDMKCNSYL
IKDVDNVLWLFEKFSIEHNSFKNIYRPYGLLRIMPKYLFIPFIRELAYLNLLYVISEYVIKNQQDIKTYLTIN
HPSRLSSFHHFITYHFIPFYEQLSENQNFHNFKQQLIEINEDDDKTELDDHHKQLNDKPINLKNKNNINKHMK
DVIPLNNIYDTKSNNIYNNNNNQLSLSDIENYFNNELPLIVKHKNFSKTFPLLFDFYSNMSMNINEKKIKNDK
PQDEKTNTNLIDNQYDNSNNSNKYITTTIFIKFLREFGIIPHFFNNDRCLSFLNTLIKNNKHNKLYYEDFSKA
IILSICECVKKNILTQYNMLTSNNQFKNKLQIGKILNHNYISYEVKELIYLFGFSDLHIVKSKINIQKE*
>N.PF11_0159
MVIIKHQIDKSNVRFIHIFNDCLSSLIKVYIFNQYKFPKWKINYDIVKKNQNHIFINRVNDYFQKLIESLSRL
FWKGRKWKGTYWPSKWKWYHRHGYWATRKKLLDFDKYRPYRYHEVKGYGKKKLQFWQNHSYYRPLKQTTKFW*
>N.PF11_0535
MRITFVKIINCFSYYLLIQLCLFKSCLNSKVYEKDESPSFNTVPLSNGPLDLPSGVTINLGLRRRKYSSNEPK
NNTYLKNELNDDAKIIKDAINKTNFGKPEWDNNSVEEKLSYDFPPWLSEMISFSDKSKSTLGSLGVDNLPDLN
ECKNIKHFPKQMLCIIEGLDRIKEVYENAKDITSLTARVLAGGRLAVRALTRLKTLASSLENKQLMKSLGTPM
ATLILLQRESTSDRNRWLCGSILTLLTDLPVVSDLADVKTGSYGHVNVIMPRQSRVRNADRNILRLREGASPS
YLINGYTSIQANYTT*
>N.PF11_0160
METIFRKLRRSTSKKKTQVEIDAVDERDWAQSKTLDLDSMPFSEDLFQYSNDKIYRNKKIVRDNLKREEPSQE
DLYGYRDHIWNEDNQKYERIKNINIKNEKIGINQREYSSRTNDEITNGSTDYVNNYYVQKKKSETDDFIYHRE
NKNIEFIYNGLKNYNVGETKEYSDVDEQEGHEEEEEEIIDDENVYDENVYDENVYDENVYDENIDDENIDDEN
IDDENIDDENIYEENIYEENIYEEKINGENIDDEVVDNEFVDNEFVDNENADMEEVNMENIDVQYVDEENMYA
EELEKKYPNNENMNDVKVTLPSENKKEKNIVENEITEKKHKENDNIIVEEEEEYVEEKLDVYTIEEIDNDVEI
FNVKGKGRCMFTKKKLDPGSVIFVENPILIVTPNLNEQLWTYLNKLNDEQNFELPLKWHYAALCSITMLNDFN
YKACLDKWVPEPDKEPDNDIYNVLDKVCEKTSFVNGNKYYYYKNKLIDPKIYSRIIQVWHYNAFGHHTDNEGL
VLYNRISMLAHSCISTACWHYGENDSFVLRARINLNPGDEITISYLGDDDLYKSSNIRREKLTNWLFVCMCSR
CTHPVDNCRGFRCSSCGIGTFFIKSEYHDDIPIISKCNVCLCEITESAAYEYIEYENSYIERLQETDKNDLSD
ALAVFVQADKIFTQHWIMFQLYTILFEGYRDTSQWEKAIYYQMQRIKYAIDVIPRANYVLAWLYEELGEIHAN
SISTDILSTENDFTITFEEKKRICSHFLKSIHLLEILCGYSHDYLRDSLNKYYRIDNLTTTDAPQIEE*
>N.PF11_0161
MDYHMDYIPNEVISHQGERFVDKYVDRKILKNXXXXXXXXXXXXXXXXXXXXXXXXXXXXXXXXXXXXXXXXX
XXXXXXXXXXXXXXXXXXXXXXXXXXXXXXXXXXXXXXXXXXXXXXXXXXXXXXXXXXXXKYVDRKILKNKKS
LLVIISLSVLSVVGFILFYFTPNFRKSDLFKNSSVENNNDDYIINSLLKSPNGKKFIVSKIDEALSFYDNKMK
DINKNNNNNTSSDFKGLSLFKENKPSNNFIHNENYFINVFDHKFLMNNVEHINQFYTFIKTNNKQYNSPNEMK
ERFQVFLQNAHKVKMHNNNKKSLYKKELNRFADLTYHEFKSKYLTLRSSKPLKNSKYLLDQINYDAVIKKYKG
NENFDHAAYDWRLHSGVTPVKDQKNCGSCWAFSSIGSVESQYAIRKNKLITLSEQELVDCSFKNYGCNGGLIN
NAFEDMIELGGICTDDDYPYVSDAPNLCNIDRCTEKYGIKNYLSVPDNKLKEALRFLGPISISIAVSDDFPFY
KEGIFDGECGDELNHAVMLVGFGMKEIVNPLTKKGEKHYYYIIKNSWGQQWGERGFINIETDESGLMRKCGLG
TDAFIPLIE*
>N.PF11_0162
MEYHMEYSPNEVIKQEREVFVGKEKSGSKFKRKRSIFIVLTVSICFMFALMLFYFTRNENNKTLFTNSLSNNI
NDDYIINSLLKSESGKKFIVSKLEELISSYDKEKKMRTTGAEENNMNMNGIDDKDNKSVSFVNKKNGNLKVNN
NNQVSYSNLFDTKFLMDNLETVNLFYIFLKENNKKYETSEEMQKRFIIFSENYRKIELHNKKTNSLYKRGMNK
FGDLSPEEFRSKYLNLKTHGPFKTLSPPVSYEANYEDVIKKYKPADAKLDRIAYDWRLHGGVTPVKDQALCGS
CWAFSSVGSVESQYAIRKKALFLFSEQELVDCSVKNNGCYGGYITNAFDDMIDLGGLCSQDDYPYVSNLPETC
NLKRCNERYTIKSYVSIPDDKFKEALRYLGPISISIAASDDFAFYRGGFYDGECGAAPNHAVILVGYGMKDIY
NEDTGRMEKFYYYIIKNSWGSDWGEGGYINLETDENGYKKTCSIGTEAYVPLLE*
>N.PF11_0163

MNLFYNLINSIKRKNENKKFYIQFCKKRFHISTIYQHRKKSICLNSIQNNSSVNNNICIVKSEKQEIDKNNSD
FIQYVFKWGIGNKFRSDPENRYHPVHLKRSKEVTIRKDYFDNVNENIRYEELNEQWEVYWFENNKLNAKPFPV
KKFGINDAKREAIKFYESLKVQQRIMPKPKYESGVEGVHYDIITNCWVAFYRSNNFPVCRSFSAEYHGFETAK
QMAIERVEKHKK*
>N.PF11_0164
MKSIIYIKYLIRSHNFNTYIYIYIYIYIYIYIYLFIYLFXXXXXXXXXXXXXXXXXXXXXXXXXXXXXXXXXX
XXXXXXXXXXXXXXXXXXXXXXXXXXXXXXXXXXXXXXXXXXXXVSIILVIFFLFHKYALCAEEHEITHKTYF
DITIDDKPLGRIVFGLYGKVAPKTVENFVSICKGTVVDGKMLHYTNSIFHRIIPNFMAQGGDITNFNGTGGLS
IYGKKFEDENFKVNHSKRGLLSMANAGKNTNGSQFFILFIPTPWLDGRHVVFGEVVEGLDKLVHIEAVGTDSG
EPLKRVLVKESGELPL*
>N.PF11_0165
MDYNMDYAPHEVISHQGERFVDKYVDRKILKNKKSLLVIISLSVLSVVGFILFYFTPNFRKSDLFKNSSVENN
NDDYIINSLLKSPNGKKFIVSKIDEALSFYDSKKNDINKYNEGNNNNNADFKGLSLFKENTPSNNFIHNKDYF
INFFDNKFLMNNAEHINQFYMFIKTNNKQYNSPNEMKERFQVFLQNAHKVNMHNNNKNSLYKKELNRFADLTY
HEFKNKYLSLRSSKPLKNSKYLLDQMNYEEVIKKYRGEENFDHAAYDWRLHSGVTPVKDQKNCGSCWAFSSIG
SVESQYAIRKNKLITLSEQELVDCSFKNYGCNGGLINNAFEDMIELGGICPDGDYPYVSDAPNLCNIDRCTEK
YGIKNYLSVPDNKLKEALRFLGPISISVAVSDDFAFYKEGIFDGECGDELNHAVMLVGFGMKEIVNPLTKKGE
KHYYYIIKNSWGQQWGERGFINIETDESGLMRKCGLGTDAFIPLIE*
>N.PF11_0166
MANANYNNARKYDNRAYNNNTNNRKPVSATKPSNNGPVRSNPSGNNTTNNTNNNVNSGMAKAGMDKYQQRCNV
AYNNYDNTKSSNPNNNANNNLNNKKFNNETQIKSNPHSDTRMNNAGTTNNSSNPPSKPQASTNNNKSVNNNMK
ESDNFKKPTGGEATIKTTSNVSTNNTTNDHARKTNINNSNVSNAQKVLTPEEKQKLNRPPTDPFKEKREDQVS
QQQKQATDASIKKNEKVKNVVSEEQSINKDTKKTDTKNKAQTNDKNVSNAKNKGADNDDKTLQKKKTFTDRIK
SLFRKKSTKTKE*
>N.PF11_0167
MNNYLAFIFITILSLFIYISYIYTIQNVYLNNYSNIVRILLILFSIPFFICYYWSFAKCSIVNPGYVDDTWEI
NAEENNIPIEKRKIRNYVPNKYTICDKCDFLVRPERAHHCRTCNKCILKMDHHCPWIGTCVGEKNLKFFFLFL
IYGFFTTSYISITVIPIFINALCAKEIQENSDRINHITLLITICTALTLTLALLFMNCQYIYFISKNITAIES
SYSDMNPYDLGIYNNWKAVFDEFTWKWFFPLNVECAQKTNYLYPLNDKYMNIINTDMNDFLLDNNNENIKEDG
D*
>N.PF11_0168
MSSVRFFLCIFLVLFTFIDIVKPFKGNYGEANFSFFQATPASHIEEPQKTESTDAQSNITQNEEINNTKPLQE
NITNNQQNSNEQQNNNEQQNNNEQQNNNIQQNNIDTSISHVPNDTIKNPIDNSNISNLDKSNDTNNVIKHEEN
NQTKENNLETIKHTEPLTNQNTNEVKINDHNEHEKNTEENKIEEQYNNINHHSHDINKNQSINNNMNNENADK
NVPHLDQSAMSNEKHINEHTTNHPIESHTGENNHDKINEPIPIEHATTPTNEPIPIEHAATPTNEPIPIEHAA
TPANEPTHNEHATTTTNEPTHNEHATTTTNEPTHNEHATTTTNEPIHNEHATATTNESTHNEHATTTTNEPTH
NEHSTTPTNESTHSENATTSTNEQHSHDQNTEVHPNDKLALVPFQGIKNPIPSNESQPIISFPNEDDNHAQNE
GSINAPSEGEHNNTDNKEGPIITPLEGEQAGTAHKEDVTHKHMVGEHVPPQKTHHGPYPLKKHTMDLSLHQGN
HVPPQTHHAHIITPVGGEHAHGQGNNDTTYVTMNTDESSSSDTKGEHSNLRSYNKNMNNNHAQRDQYDSDTLN
SEGSDDAYSSMQQNFEKNGIDSFKGKGLHVSLRERIIIEIMESAKNGIDGLLKLKDSKDSGKLFMEALEKLNI
NMKDLKKDKNLISLEVYDKILSTMFKILTEMSFYEDSKFYETLGIKKDILNQSLKDIKIKMLRKLGVSYSRLP
PIIKHTEGKCAIKDIIISISSKELAQRMAIMFTKWLAPDEYGAVVDYENNVELNVLCSGAPILIQQWKYYQNM
LGFEEDKDHAYLGLIDELLVMNKRYSQNKDYVETLEKIKKSKVFKHCTKIMRIGGKVSSVPFNYENVKKPSSS
IIGSLGNLIKANISTYYKATAQRINSYFHYTEKKSKKSSPLKIISVCTLLHLTDMLYKCSDENSNGVMDLYNI
QLNTLNMKGKMVLQYLVHLKFLTQEKKNQLKEICEPQNGLIDETLTKMLILLSTDSHELLSHELENKGFDEDY
IQDEIKNINESDNNIRDKEEDDAEKMIFDDL*
>N.PF11_0168a
MREKNLNFRSESIFSGNRSFFDILENVDDEKEAKRSAHFFNSNKNILLNLFKGTNQNNNVNNILLEKKRIVSN
ARNNKCVNKECKNNELCKIELKDEFKKNDEIIIKNECNCSKKNDWIKNFCWFCSLHILDEILEKQIYKEKIHE
HVYDLIYDEDTYFDTSSMMSIPYEFARFMITQLDYSTLHEIKLNNIIKEEKYLFKKYSKKSAQNKKKKNHEYK
EEEIYKSDESNSTCSLISDDTTLNINMKNTNLDYSNYLNMRRELYNNNININSSSSNYNNNNNKVKDTSVNIR
QTKGRKKTFYDSNNVGNSGSTDLFNFKKVLSSSESILNKNDQYYIDENKKDCNNIDNNINSHKNYSNNNKYNE
ELYLGKYITNKELPDDTNKINNQVHDILSENVKYEEKSKTPFFNRRRNNSTLGTYHRQYNKSSSHSSFSCIAY
WKSKRLSDSIIHSKYFNGVKKNNEIKQKHKNDKINDKINDKENINENPLNDNVYKNYHHFDQHILNYNIYNTS

NDHKKNKAVPSHIINRFRSERYSENSTMKYEGKNYYQSFMSQSALKRCRSYTILLREKKEKKKNRRMKTCLND
EEVKTCLNDEEVKTCLNDEEVKTRLNDEEVKTRLNDEEVKTRLNDEEMKIYLNEEEIDNDKNNDILFHNKYKE
EYISKDRIYNKYKNNLNHIKEFYYLNETDKKPFPICLYNNIKSFENIKQCYRCCCFFNTKCCHCIECKEKYHM
KLKNPHYFIFQHGLTASVHDFQNIFNSLLTKYPHVFVYVTYSNQGHTFEGVDVGTERICTELNCLFKIINDKI
NISMIGHSLGGILNRSVLLNLNRKKIFKNKKLINFITFACPHIGVHENMAIMKVLSTYLGAHTIDDLNNKTTL
LLKIASVESINILKKFENIIFYGNTQSDWLVGIRTSLILPYTLFNEELILFIIEQAKNVPEIPINIFSVVHLY
MRKKKLLFFYFYQDLKNPNYLLNKRKEQSKFLDQMLQTIISSRKLLSSTNTKKFNLFTNYYTSVGLDKLKGLH
DELNKNIHNASNVYSDGSNQVVENKDEKDEEQKCDYSNVGNNNQMDNTNGLLVHLNNKDDTNNNRNITNNHDD
HNGSNNNIVQSVIIYDKNKYEYNNNYLLYTPSYVKKKNIENNVNFKGHIFPQDINTQMMPIHFRKNVTSTKKQ
GKSKNIDKNESNVIHDKEFVKYVNGFNGNKKDINNKYEEGSYYKSQTYHDMFIEKKMKENKNMSTLICRSNMN
NTDNIDNKMEYIKNDARENIKEKEHMHHNIIDVCHSNNTHLCNIPNYDEPVLYKNTDKTKSFECIYMDKYEKE
HVIKENKKMIANNDNYTCSVYQNSNCSNKNSFNNKDHIYNNTMNNDKDTYKYGTLNSNSDHKMNICDEKIKKN
KNMINDINIQNITDKHVINKNIINYKINENKTENNIPLVNNKNKILVNNNIHTYNNIHMTNINALHEKKTKFG
EYKYFEKLFFDCLKSKIINEIKTLDNNLKKKKKKKKIETNKLFSIQNTINIIKNYSFLYPSKNNNNEQKKKKN
SQSSTESSGTTNNNIDSKNDIHMNKKLEDTSHVMPNYSVGEKNSWGKEKLKKLMDIYFNEKVLKDTNVSNNMN
ECISNGNIKQMDNQQEKINESENVNENVNINDNINDNINEHEQTYIPFDDDQNEEYDYISEFFYSTSEENDMN
PNNNLSCKQHELKKDKNYITDENSKYVEFKSNKKKENRTRDKLKRNDLLKGITKYDKKQYKQILYYIYTISSD
ELLEKFFKNPELLYYEVLFYCLNRLPIQRYSIAIPIYSNAHVQIIAHPRICSEESAIVKHFVEHLIL*
>N.PF11_0169.1
MSEITIGVLSLQGDFEPHINHFIKLQIPSLNIIQVRNVHDLGLCDGLVIPGGESTTVRRCCAYENDTLYNALV
HFIHVLKKPIWGTCAGCILLSKNVENIKLYSNFGNKFSFGGLDITICRNFYGSQVKVLATFSHESYGPNIIAA
VEQNNCLGTVFHPELLPHTAFQQYFYEKVKNYKYS*
>N.PF11_0169:mRNA
MSEITIGVLSLQGDFEPHINHFIKLQIPSLNIIQVRNVHDLGLCDGLVIPGGESTTVRRCCAYENDTLYNALV
HFIHVLKKPIWGTCAGCILLSKNVENIKLYSNFGNKFSFGGLDITICRNFYGSQNDSFICSLNIISDSSAFKK
DLTAACIRAPYIREILSDEVKVLATFSHESYGPNIIAAVEQNNCLGTVFHPELLPHTAFQQYFYEKVKNYKYS
*
>N.PF11_0170
MSLSIHTNYGDIKIELFCYEVPKTCKNFLALCASGYYDNTKFHKNIKGFAIQGGDPTNTGKGGQSIYGKYFED
EFDSTLKHDKRGIVSMANKGKPNTNGSQFFITYSRQPHLNGIYPVFGKVIDGMDVLSVLENEPVGEKSRPIKD
IIIETITIHANPIAEEESVDN*
>N.PF11_0171
MNEMKVSYLNYPIYGIGSNKDYVVTSGGGGGKNYGIEDMLDINIFNEKEKKLEVVWSTTEQRGVVDSIIYVEK
YNIWLGSVKNECILFEINEETGPNILLTFVTDFNERNARQVVVKFSSKDDLILTGGEDKTLRLWKLNFVKREK
ENKDDIKGDNKKGNDNKTNEDNKKDNDKKTNEDNKKDNDNKTNEDKKKDNDNKTDDDNYIAVNNNLYLDKNKS
VEHLGDFVGHDDSIKDCDISFDEKIVCTCSSDHSLKIWDTNSFVNLHTEQMKSPQNKNDKLNFRCCKFLNHSN
SFKDFTYTLLTTAYTPRGSSYLIIWKVYYNDKKEQFTFTKDKFIWINDRPCCNIAVSTNQKFIALGFSTGALK
IYNSKFYLLAHYKKHELPITAMCFIKDDNYLLSAGADYTISTLHINSFMFRYLRKFGRFIIYFLLLFFISIIL
LDSFNVGYDLRISSLFHNKAYTNITKKKRNYNVDPKKNKVIMDMDEL*
>N.PF11_0172
MIEKSNNPFLSIDPIVERTKSNGEGLPLLSEKTKKGFDFTQIVVYLVGLSDGLTHLASLAIYYLFKDYFRLTP
YQVSLILMYPYIPFILKPVIALITDSFSIFGMRRKPYLFLFSLFQSLNFLALAFLNLSVIQASLILFFISLCA
SFCTTVAEALVVESSMGQTYSQGTNKVTEFIASKAIGSLSVAYFSGYFLEKMPREYIFIATSIFPLIISLSCL
FLKEKEYSTNRNIFNQLSDLIKFINTPIFLGPFLYIFVYMSGPDYDDAFFFFCTNKLGFRPSFMGTLRLTYGI
ASLIGIIIYRVFLKNCSLRKTLIITTLVSFPIYISPIILTEHINKFLGISNELFVLSGGFLIEAITEIQLLPL
FILTANICQPGLEASVFATILSVKNLGSLTKKGTSSFITYLMKIDSYNFDNLSLYILTCGFFLLFSLTLVPLL
PNEEHIEKLKNKETSKG*
>N.PF11_0173
MVKDENMRDELEEEIDNLRARFFVNRLFERVFCSHYLRKVMIRSKWMRYRMEVFILLLIGVLSFIIRLFSVIR
NEAIIHEYDPYFNYKLTNILKEEDFYKFWNYFDEKSWYPLGRTTGQTLFPGLMITSFLFYKICHFFGILVSMR
NICIYIGPIFSFFTCIISYFLTREIYNFSGAALLSALFVSISPSHISRTVAGSYDNESISIFLLLLCIYNWIR
CLKKGSLLSVLICSLSTYYMALSWGAYIFITNSISLFMLIIIILKKYTIKHFIIYNVYYILTTILCLNIPCIH
RSVFISIEHLATHSIYILSTFMMFLHFIMKHFHLNEIYIKHKFIQLSFIFFIIIFKYFIFTDKLSWNHRSRTL
LDPTYASKHNPIVASISEHQPTTWSSYFFDIHLILLFFPLGLYECFKKNGSIESFFLGIFTVLCLYFSSLMVR

LLLIFSPFSCILSSIGLSSLISRFVPIIRISPDTYLSSIILLHNVKRKNNKNNDNDNDNNDNDNNDNDNNDNN
NNDNNNDNYNNDNNNDSYNNNNNYNDSFTYPNIPSNGNVLDKDENVSSLNSMRRHENILEEFNTIKNDIKYMG
EYKKGIKCNNNTNIYVKKLNFNKLERRNNISILTSVSIVLMLLYYVILIILHSTWCSSIAYSESNITFYSRNK
NGDRYINDDIRQMYKWINENTEINSKIVAWWDYGYQLSVMSNRITYIDNNTWNPEQIATIGLILSTNEKEAYE
YLKMLDCDYILVSYGGYSKNSSDDLNKFLWILKITNKKYKFINPLFYYYHDKYHPLGKNATTFMTNSVLYKLS
YYNLTNNNIKGYDYIRKIQVPEIKNLYYFEEVFTSDVWGFRLYKIKDIY*
>N.PF11_0174
MAKRIFSVSFLLVLLNVLHICIKFSVADLPTHVETKNLLGKWKILRTKTSPNLTTCGSSQPNKNTYNVGITDY
KKYLLENNYEFVSELNVILSDDYVLYGDIYNTQDNEHRSKWKVLAVYDENKRVIGTWTTICDEGFEIKIGNET
YAALMHYEPNGKCGPVSDEDSLDSNGDTDCYTTSFSKIRYGWLDVENEKNEHLHGCFYAERIFDNVNEIKHLD
SFTIDKDSQNVLQTFTYDTKLNNILNSNNMLYKFGNLQKPTFTKRNNTNVQFNSELNWHRMKHHGKKKPLKKS
MLAASRQTYACPCNANEVVDNVINKGDSDNPVSPTLIQLNNNLKNTTQTGNKDTNEMDLENYEDTLNSPKREL
EINELPKNFTWGDPWNKNTREYEVTNQLLCGSCYIASQLYAFKRRIEVALTKKLDRKYLNNFDDQLSIQTVLS
CSFYDQGCNGGFPYLVSKLAKLQGIPLNVYFPYSATEETCPYNISKHPNDMNGSAKLREINAIFNSNNNMSTY
NNINNDHHQLGVYANTASSQEQHGISEENRWYAKDFNYVGGCYGCNQCNGEKIMMNEIYRNGPIVSSFEASPD
FYDYADGVYFVEDFPHARRCTIEPKNDGVYNITGWDRVNHAIVLLGWGEEEINGKLYKYWIGRNSWGNGWGKE
GYFKILRGQNFSGIESQSLFIEPDFSRGAGKILLEKMQKELGN*
>N.PF11_0175
MTRRYLKYYIFVTLLFFVQVINNVLCAPDNKQEQGKYLNRTINILNAGKNIAKSYGHNKLKPIHILSALAKSD
YGSTLFKENNVNAANLKEYIDIALEQTRAGAPLDNKSKIVNSAEVKETLALAEAAANKYKSPKVDVEHLLSGL
SNDELVNEIFNEVYLTDEAIKAILKSKFEKTKKDKDGKTGTLYIEQFGSNMNEKVRNGKLQGIYGRDEEIRAI
IESLLRYNKNSPVLVGNPGTGKTTIVEGLVYRIEKGDVPKELQGYTVISLNFRKFTSGTSYRGEFETRMKNII
KELKNKKNKIILFVDEIHLLLGAGKAEGGTDAANLLKPVLSKGEIKLIGATTIAEYRKFIESSSAFERRFEKI
LVEPPSVDMTVKILRSLKSKYENFYGINITDKALVAAAKISDRFIKDRYLPDKAIDLLNKACSFLQVQLSGKP
RIIDVTERDIERLSYEISTLEMDVDKVSKKKYNKLIKEFEEKKEQLKKYYEEYVITGERLKRKKEIEKKLNDL
KELTQNYVYSNKEPPIELQNSLKEAQQKYLELYKETVAYVEAKTHNAMNVDAVYQEHVSYIYLRDSGMPLGSL
SFESSKGALKLYNSLSKSIIGNEDIIKSLSDAVVKAATGMKDPEKPIGTFLFLGPTGVGKTELAKTLAIELFN
SKDNLIRVNMSEFTEAHSVSKITGSPPGYVGFSDSGQLTEAVREKPHSVVLFDELEKAHADVFKVLLQILGDG
YINDNHRRNIDFSNTIIIMTSNLGAELFKKKLFFDADNSGTPEYKRVMEDVRLSLIKKCKKVFKPEFVNRIDK
IGVFEPLNKKNLHKIVALRFKKLEKRLEEKNIQVSVSEKAIDYIIDQSYDPELGARPTLIFIESVIMTKFAIM
YLKKELVDDMDVFVDYNSKAKNLVINLSKT*
>N.PF11_0175a
MSYNKTYGGQFMGIGAVAKKNNNTPTVAKREFNKVEKSAESKKADKVVISIVGVRGINIPEITNGQIYFLCTA
IFDNDDVKMSMEKSNHNTSLVLGEYIVSSYHVTFDEEIVLNIPEGNNFIRCYVSCIYVTESDGTRNMKLEGIG
YTDAYVIKDCKAYVYSSCKLIKAVGAPDTEGSIKMSVKCVDEKHPSIVKEERPFNALEELKKKIMV*
>N.PF11_0176
MSEYKISSIYFSRSNKVLILMCVIFFSCSTHIFKNVFPFLFMLTGIATQHGNLNVYVKKNSWCDISSKLDRNT
EHEQYIQKEDKEKKIIKNTNTDGNKNINYDHPYNMMNLCYDELYNNGDENNEKHLLNLEHECFIKNEFTTCKI
CHNYINNNNNMYNNTYNNIYTDSYNNTCHNYYKNRNNNIRDHTRKGKYIDNNIFYYNKIHNKQNESAINKESI
SLYNNILSLIYFSSLITYIFIFFFDLSKKNYIINIFYITNLISHCIIFFILNDMQIFNSHLIQSIPNHIFVDD
HKKMFSPFSQPYLNSHNNLSIQNIFNGIASQNGYIYNILYMDKKKKKEKKIGLNNSTNERNNLKNKLDDLQK
ISILYNMDVYKSLFIFFIYPNNNIFFNPYLINMNWNIMSNISNDILYNTYEKNKDLYNNNYVIQSIIKEKNNV
PDFLFLKRNKNIYYKKYLLVLYFFLMISSICSCYIKIYQKRILYCVFYINIGVITSFLILMNSLFKLFAHSLN
YIVTNQNNLVNISDYIVLFLLIDLFGLFIIFFFSYKWYYQRNVWTHLHKIFYNHKYIAFYFNNTYYIYTVDRN
VDNILVSMDIYGDPNVNITFSEYTNLEKSIFYEYFILNRFRKNKLKIFEIHQDKIQELTTTYKQKEKGKKNYE
MNIPYRDKKYKNVEDIQSSHINNSLDINIGCYKSQRDDLNECDIPSEEKQANFYSRKPNHSYTMKPYKICIGT
LNNIITQSNIPINNTNQNINGSPINTTTTNNNNNNENNNNNENNNNNENNNNNENNNNNNHNHNQNNHHNQNI
NYPNPQNERINYPFTNEFIHNHHEYVNNIALTPKQQIIDNTILENKQNDEDINKKKLTTHSQKNLLKENLIIT
DEYFINTDTNQYMNNAQNQNNICLPKGIYLDRSEECEPKNIWNIQNESQNVLSIPDFDEQDNHKQNEQAEIIC
PNDGNYYLHNEEYNQEMYHNNVHYKNGHMDNSFNPHIHNYINNHINYDTSYHINHHNNNFKIFRNIKKFMSSF
TFVFFFFIFIYAFLLSIIHIFINYFFYIYLYVFNINIYVSNVYTIIMTGVSLIAIPFSGYIIDNIGAFLSLLL
CSSLFILIAISGSIYCYKFNLNSEVLAFLFFNLIGISQSIIPTVIISQIPTHLCVKNNEHITSAFAIFEVVSM
IIISLNNYIFGSFMVKEEYLMGLYVLFFFILLVIALITLLIISIYIKRKRYIHMYKNTNDYNLAQPLL*
>N.PF11_0177

MARDNENILEEWCLIESNPCIFYDMLKRMGATEISVEDVYSLSYFDDYINNKEIINMNHILGVDTYLGENNKT
LDKENNVVDVIELYKNNICMEDKYNKLLKHHSYIYGIIFLFNIGKHYKNNKYIEHNVPDNLFFAKQVIPNACA
TQAILSIVLNKDIELNDEIKNIKTFSLNFDSSMKGLTLSNCTFLRNIHNSYKPPIYLDKEDVHHDKKKSEDSF
HFVSYISFQDKVYLLDGLQSGPVLINADEQNKPNPNNNNNNKDNDNDNNNNNNNNNNNNNNNNNNNNNNNNNN
NNIGMNGKDWIEISREHIKKEIDEICNSQTNNDVRFNIIAVMKDKEYIIQEYINIHRIVKQRVNIKLINLGEN
IELSDEINEDEFPLLNDIPSIENLPNNVDTLYNIVNKSTLEINYLQSLLHEQKEIKKLWNKELTFKFFNFYPF
IMSSLNLMAKHKLLKDAYQKEKLKNATKS*
>N.PF11_0178
MTLIIFVVSMRMVWDIKNIYIRKRHSTKIEKKEKKEKKEKKKKKKKKKKNYWLHIYYIXXXXXXXXXXXXXXX
XXXXXXXXXXXXXXXXXXXXXXXXXXXXXXXXXXXXXXXXXXXXXXXXXXXXXXXXXXXXXXXXVKNFKLKKDIF
EDSYLKLLIENEKIEQDAPSCYFLSYLYNNIYLYYCDILFHNKNKQVHSISHIERDINFFNYENVETKKKKKI
NKSEEIIQNNIRRNNKNLIYIKGDEEKYINKKYINDVNDQNNLYNLNELYKVDKLYNIDKLHFITSHYLAYNK
MQNSILQFYIPNFNMNKISKFDKHIKGLNKTYHHSIHYNLGANTISEQSHVLQVDHAPSCKKKKYIFKYHNKI
YSKKMKRNNSNKKMKYKKKQIKSSSLNISKLLFPFFGYTTSEESVMSFSKFLPEHKIARKKKKKKKKKRQKEQ
KEQKKQKEQKEQKEQKKQKEQKKQKEQQTNNIINNDDHHDKPYTYQSKDKEEIKHNSMLIFKEIINLCHENKI
FTFKNKIKHKQNDTKKNHRVLHPERFIHSTDKDQYIKIGGKHNVDNKESFDNKQKYIKTQRNLSTRSYMNTYY
KDDINNSENCLLKRNKVQNELEKDHIYTSQGLHKKRSFSSCSDYVNKQDEYEKKKRTKKQNEFIINKNIIIKG
KEDIISHSIVDMRNKIKKRNYEGGVKNELKNESKDIYDECKENQNIYKEKQNIYKENQNIYKENQNIYKENQN
IYKEKQNIYKEKQNDILTKKKNIHFLNFMNIKKKKDDNILKMQILKGLNQTHYDPLHLEKIVNCKSGGIIYNH
QTKTKEDEEKKKKKKKKKNLYKDEQNCMEMTNIQTYKNELKSIVQKMNKHFVFKNMSVFMLNFCSLYINGEYE
EIIHLYKEEYFYINFNIFYIYIKSLIKLKKYELCLKYISYINDDNIDGNNINGNNINGNNINGNNINGDNINL
YNKIILTFLSALCFEKLNKLKLSITEYCKVVVHQIDNIIKDENKEYNKHDSDDNNKNYKNKYNNICNYNYNYN
YQCFLDPIKIHPFILICLDKVIGTHQLKIHEENTLIKYVQLHYDFNKLFHFYICKVHTMDNIQKYDIKEYMNI
HNDITFEFVKDPRSRKNNIYSIQINDKNLLYGNNKAKNQNDYNINNNIRKMNRNVEKTQNCYYNRKSELRDGC
YDPKRLMYHFPFFDEKKNKKKKKKKKGKNLYINMNNVLYYINNNENQDDYDMPKYLYSDNYYFVDDHKESDEE
INRKDIILYLQNRNEDKYTVNHNNILNNNEFHYNYFLAHYIVPQGACNHFCHKYTIKKINHFIDTAYTNKNIL
GTIKKLGVELNIIYEKCLQDYETRGNNNKRNIYNNKYIHDNKYIHDNKHIYDNNKYIHDNKHIYNNNKHIHDN
NKHIYNNNKHIYNNNKHIHNNYHHIDDDHHNNYLYKFDPVTLIDLLCIAFYCLKNYDYINCYYISKYITKERK
QYENDEAILLYITSITNLGSVFKSSQEKIKEIFLLYNERVSDIIFRKKKYYYKEEKYKYENDYIDYYMFGIIH
FLNNNFNKALLYFVKCMQLKGNFYMCYVYMLYIFLSFLTKNRKKNYKKFIFFKCLKLKPHNITPYLIYSSVVL
GIYQRYKNGMNDNINKNKNVDKDKNINKNIDIRTDNGIDIDVDKNIYIYTDEIVYNNPSDKSNITVCVEKIHF
LRDILSKALKIENDNIFVCNEYFLYNFLRKEYLQCEVFLKKIIFICKTKFYPNNINVISSILYNMSIYYSIYQ
KNIYESEKYIILLLEKNPFDIKALHVLIHIFFIKKNKNWIPLFDYSIYLEHVLLSLDNGILFYDTRKMINKQR
RNTYLFYNFFNQLEKTKHGSSLYFTYYSKIKKVRKFDTFLKKYIQERKHQEYGFEVAGGGFLCSEQTK*
>N.PF11_0179
MLGLIFKVVIFIVGYVLPIGLSLHGWKNKKYEMIEYYLKYVYFFVIFENLVTPSLGRVIYRISSFLWCILHLT
IYIILITPKLNYLNSIYDKISKINNQNNIGLYWNKYLVNPLNDKFNKIVKKLKTL*
>N.PF11_0180
MAHTIHESKGYKYGKQKLYPHFKNIRDHDVYEKSEYETDKKETPLYDDIEDNVNEKNKVNDEIKKENNNNNKY
NKYNKYNKYNNNKYYKYNDKYSNNYYNSYSNNDNDIPINNYLKRKKKKRIYSKKKKYIFKKKTLVPFEHVDNN
IQNDATNSDTYLESFVIKKKTTNIPLNNKYIENSNIELKNKSKHTLNPALSLMASRAVDGLLGGVHKHMQGPI
AISSDGNNSPLANQIVTPNVYGSPNSISPINMTNNIAPLTSNPSPTPPSPLPQSLGTSISPTTTTTTTNIAGG
APIGSPNIMSQGMVGGVATPPGVQVPNNAIPFNPMNPTNLMPLNQIGQNPAFNIHPTASNLRGDPGNVNYNEV
VSITIGIVLSLVLFCFIFGCLTKLCKPKKRRR*
>N.PF11_0181
MIILKVLFIYNGLIQIIRCYKKFAPHRLNNIIHENNKNNIGTYDIKSKALKKLYERKLIHYVSDIRNIDKILY
HNENEKEKKNRKSVYAGIDLTCKYLHLGNLVPLITLDILRNHNTDVIILLGNSTTQIGDPSFQKVERQKTLEK
DILENEENIRRTIIELFLQREICEEDMNELIKKSNIERDKEFIYESDNKGSLIILKNSLWYDKMNIIDFLKYG
EYFSINKLLRKECFLNKIKKNLTLKDLNYITLQSFDFLHLFNKFKTCIQIGGSDQWGNIQSGIELAQYISNTQ
LYGLTTNLLVYKNNIKYSKSQFNENKRLPIWIDKNYNSPYLFWNFLRNVEDQKVQSYIDMLTNLKININQEIE
TVYNPMDTNLNEILDETLDDSNKKKKNNDNNNNNNVKDINITTEHNNYSNDISLEKSYEEKINQAKKQLSDSV
TSYIFGEHTVKKIHKMKDVLKNNEFHKINNIDDIKVFPYVEITMEHINKKQINISDLLKKFDIASTNKEAKEK
ISQNCIYLNELLINDSKYSLNINNFIKLHNNYYAILRLGKRTSYSIIIK*
>N.PF11_0182.1

MFALSRYFLLSFEIPFNQIQKITARSSGPGGQSVNKAETKVQIRFNVDDAKWIPLNVKENLKKIYKNKLSKNN
DLIIESEETSSQISNYKICADKLKHILEEAEIKKYKDNLINQKKKRQQRKFNKRDYD*
>N.PF11_0182:mRNA
MFALSRYFLLSFEIPFNQIQKITARSSGPGGQSVNKAETKVQIRFNVDDAKWIPLNVKENLKKIYKNKLSKNN
DLIIESEETSSQISNYKICADKLKHILEEAENYKEKIKHTCIKDFIHLIKSDEQIKKYKDNLINQKKKRQQRK
FNKRDYD*
>N.PF11_0183
MDSQEYIPQYKLILVGDGGVGKTTFVKRHLTGEFEKKYIPTLGVEVHPLKFQTNFGKTQFNVWDTAGQEKFGG
LRDGYYIKSDCAIIMFDVSSRITYKNVPNWYRDITRVCETIPMVLVGNKVDVKDRQVKSRQIQFHRKRNLQYY
DLSARSNYNFEKPFLWLARRLSNQPNLVFVGEHAKAPEFQIDLNIVREAEKELEQAAAVAIDEEDIEN*
>N.PF11_0184
MINEDINICGNINMKDNDRDNDNNDKYNDKYNNNNNDNILMNDSNGDRRIIKLAEEDINRIAAGEVIIRPCNA
LKELVENSLDANSSSISIHLNKGGLKSLQIIDDGDGIHKEDLRIVCERFTTSKISNHKDIRNIKTFGFRGEAL
ASISHVSYLTITSKKRNSPFCYTCNYKDGKPTQDEPTVCSGKNGTIIRFDDLFYNMPARLKTMNPNDEYNKCL
EVLQKYAIHYPNVSFTCKKWLSNTVDLNTQKVGKGIGGYAGIYIIKKKRKERFDENNTTDGNLPCDNNMTCNN
LPHDNNITCNNLPHDNNITCNNIPHDNNITCNNLPHDNNITCNNLQHDNNITCNNLPHDNNITCNNLPCDNNH
TDKEATDNPDASSCMYNELEKKILEEEKYLDENYEKHLNNVRCVIQKVYGRNISKELSTIFLKEKSIPTFFKC
YGLISNPTYNGKKGCYIFFINDRLVESNIIKKSCENQYSNFLAKGNYPWIYLSLRLKYDIVDINVHPTKKEVH
FLYQEEISMLIGKKIQEFLKSFHNMRTFNITGEKLLQTKLDINSSMLEIKKEDKELSKLRQGLLHDNNNVIKR
QIDTKRVRTDFKQITLTNYFVKKENMIDDHLDNNKSVDMLYNGDHNGDAKQIDLYEHNDEYNKTYDQTNIRAN
LHMNYNNNNNKIEEKEYEILKLKGPNSVLYNTQIDKHISNKIYDRKYPCEADEISSIKKLKMICEEKEKKELT
ECLKNSIYVGPVDNMHSLIQYKEKLLLIKMPLIIKEVTYQSILNRLGKIPPFEFDPPIPLYDLLLVAVNNSYS
GFYENPNYANKNIEKVCNELEQIFYTYNEMYSDYFSIIIEDGCIVTFPACCGEYFPGQEFLPFLFLRLATQID
YSKEINCINGICYLLANFYSKITLLNDKEWTYQDDLLMIQEKEKEIQMLLNKSNKHINNSNHHNQHYDETNLD
YILGDESVVDINKHLSVSRNINLVFEKYFFPMIQLNNIMKIPTTFSNNGYIIELTSLNQLYKIFERC*
>N.PF11_0185
MEKEKNQFEVTRNLCTTSIKENLECSTNIIKNVNNFMSSPKLDNNYNYNNNILYYDGTYDKEGQNGNEMNYAS
NTNNMNNINNINNTNNNIDNNNNNDNIELFCNNYSNGFLLNEEYTKGMEYDKIIINEKYCNSPIDTLHNNLQT
NKLANVVNSIRFEDNTNIKIPVFLKNSPLYVLYPELNSYIKDTLIKNDKKQESEESRMDGMKESVVNEQNNVE
NNKIIFKNDNTQKNENYIMSNNNNNNNDDNYYYCGYNNYINNIHDEETKNINKYLSNHIDNNTIIKNKMYDYF
DYEKNNMIHILNVNNINDVLLKNEKKDPVDIILNSYKNIYVSKNKNDINMNNEMNTEMNTLNVLKEQKMPILD
HNDTDHFLSVEKNMKKKINVLKFKDNNGDDNGDDNDDDKGDDNDDDKGDGYDDDNNYYTFDNNNLYNHPDHTH
SSKLSKNPLFISFIDTKNKTAKLKTFDINNNQEVKRNQFFISEKTKKGTSKDAQVQTNEEELTKHLTENMVDS
DVEHILNIINKKTKKITENVNTQENKEISSNSHILNKNRDITLHNNQTNINSSKYDISYNLIQPSYSDKSFSN
TTNNTKGCNTSNSVVFKRNYKNNKNVQHENYKNVCNNKCTMKKNIYTTNMKMPKYNFSTPINIYNDYINRYVN
TICMTNQKSGNMNMYTRMKNNTNNMYNMNNKNNTNNMNNMNNKYNNNVYIKHHIKNHMNNSSEDIFNIKNQSL
SNINNNNYFYYQNKEEQIKNAYLPFIYHMLNIPCHDTNVNLSFLKKTYQIMEKQKNEGLSKLGIDIQKQKEET
YVQNDEHINNKVQINEHEKEDEHQYKREDEKEDEHQYKRENENENENENEHQYKHKHKQEDDREYKKSKIIKD
LLLKKLDIEKKKXXXXXXXXXXXXXXXXXXXXXXXXXXXXXXXXXXXXXXXXXXXXXXXXXXXXXXXXXXXXXX
XXXXXXXXXXXXXXXXXXXXXXXXXXXXXXXXXXXXXXXXXKKKKIYEKWKYENYLKTIYHAKGNNSLKYRNK
EYFDFLKSIYYNDKNNIHNSDYLDEHTTYNKKQQLLYSPTSLNKRNNNNSYSYENKNNYNKPINDEKSLNYDK
NTKLYKSNDHNTIFKFSNKLIPQKCEENNQIRCRSLSYKNRLQKLKLLKESIQHYKLKSNTRNVFNTDQNKQT
VKNNKHNHFIQHKDVQMIQNDISNNTNNLNYEREKNNNYEREKNNNYEREKNNNYEREKNNNYLSEHLLLNQK
QNNTNNQGQEQNNIKIKYNLKKQNMLKDNYKDTKNYELNYVTNNNTFKNNISTEIFQERNSWNNLSNTLHNET
IDNHKRNSRNYHMVGKLKGTTSNYNNYDNKYDNNNNNNNNRSQKMMNNKKNMNMNRKNLLNNINTTKNNNNFN
NHLRIKVSVKNPNEESTIRHIKNDTTTKHKSFSNKQIFNKELLNKEKAILSDTEIQFRKKNSIDQPKENLPTN
TSNTKNFLKKNNGCGGGNYLKIRPPFVNETNASTRSKSIDTNIVETKNVQHSYLTSDFKKNLSRGDNIFEKTH
DEINLENIDPNKWIQKIFGDIH*
>N.PF11_0186
MVKLSLASKYIYPNDMCELIRTVTDIIEKKHDVEVDKKQLWDIINNIYDQWINVSEIEKEEDKKDYYKLISFF
TLLLSTNWFNEDQNELEYLVNEIYFAHMGLAQSYPEEELTRAIENSLGAEVKKCIINKNDLSVEFIKGDYGDG
IFDGKIVLDVNAGLFLHTQKDVLQYTGKLPVDAYEIELAISSFDWKQRDEKKKE*
>N.PF11_0187

MIHFVLLISRQGKTRLAKWYIPLSQKEKAKIIRETSQITLNRTPKLCNFVEWREYKLVFKRYASLFFIACIDK
GDNELITLEIIHHYVEILDKYFGNVCELDLIFNFHKAYYLLDEILVTGEMQESSKKTILRIVAAQDSLMEDNK
TTKKLGALI*
>N.PF11_0188
MSFSKFMKCSTQLSRRLSNFEGKGTFNKSAFYNCTREKCSIVCLRKKMNVELKKICEISKMNKRNYSSECENY
EFKAETKKLLQIVAHSLYTDKEVFIRELISNSSDAIEKLRFLLQSGNIKASENITFHIKVSTDENNNLFIIED
SGVGMNKEEIIDNLGTIAKSGSLNFLKKLKEQKESINRNNNINKNEIDNKINNNNNIDVPIEGNEKSQEGDII
GQFGVGFYSSFVVSNKVEVFTRSYDNNSSKGYHWVSYGNGTFTLKEVDNIPKGTKIICHLKDSCKEFSNIQNV
QKIVEKFSSFINFPVYVLKKKKILQTRKDIEQNELNDHTQQNELNHHNDQNDHTQQNELNHHNELNHHVEHHK
NVVENKNDEYEKNMDNRTSDNIKPESLSKQNDYENMNQCNNEENVLNRTKELNEEHIVEEILVNKQKPLWCKD
NVTEEEHRHFFHFLNKNKSYNEDNKSYLYNMLYKTDAPLSIKSVFYIPEEAPSRLFQQSNDIEISLYCKKVLV
KKNADNIIPKWLYFVKGVIDCEDMPLNISRENMQDSSLINKLSRVVVSKILKTLEREADINEEKYLKFYKNYN
YNLKEGVLEDSNKNHYKNSLMNLLRFYSINQNKFISLKQYVNNFRNNQKNIYYFSANDKNVALNSPYMEPFKK
QNIDVLLLLEEIDEFVLMNLQTYKDAKFVSIDTSQNEDFDEAVLNTNKNDNEKKQSIFFNDEQKKELQAYFKQ
VLGSKCSDVKFSERLTTSPAVVTGFLSPTLRKVMKATMKNSDFNDNTNNSNNMNMFQNLPATLELNPSHTIVT
SIYHLKNTNQEVAKLLVQQLYDNACIAAGILEDPRSLLSKLNELLLLTARYAYHYEKKDIQENTKDQPIDVKD
NTMAIDNIVNTKENNTNDDSVMKEAQSSNL*
>N.PF11_0189
MYDYVRINSIKLEQDLEVEEYYSLDSGLRIILNKTKSPKIYGFFTLLTEAENDEGLPHTLEHLIFLGSHKYPY
KGLLDFLAYKCLSEGTNAWTAIDHTCYTIETFGIEGFCNILPIYLDFILNPTLENNMFLSEVHHIFENGTHNG
VVYSEMKSIENNCENIVERTVITNLYPSKKGGYRYETGGTLEGLRQTNNNRVREYFKKFYKLNNFAIIIFGNF
KNDVILDIIHKFEEYHLELNPSQKKQKIDINNYYNDQNMNDLIKEENDNICLNKEGKKKYIYDIKKCKELFLK
DIQSNNRPWRRPENIEKREGSLIIKKYYPCNNLNNGQVTIAWRGCPWEDVQTKLAISLLGNYLTDLTTSPVSK
RLLEDKETYCSSLDFSLEDLKENYFSIDIYDVSYKYKCHKIANAMNDHNVGTNMDTQKGITNINQNDHQNDDE
KYNQHEVNNNNDDDDHHHNDDYCKNNIKNIPCNNTQDNKNVLEPSNEKCNINVNPSNTRNDNIKMNVVADITR
NCLNEVYEKPLNMDRLKNIIKRSYLQHLRDLETSPHYLLNEILIKYFIYGSNICDLEKCLNLKKIYIQLLNED
EKYWKDILKRYILQNDYVEVRCYPSVKKAKKIESFEKKLIEMEQQKYGMEKLNKMVENIKKIKENIEKKPPKG
SIDIVESAKPRNVYIEGITVFRNFKSLMNEPNKTDEQIFANVKATITKDDNQYNQNNDNYSNNSSTQNFRLLK
KLTDDLKRIIFPIQLSNIYSNFVCIHVLINSKNIDKCLKPYLPLLSYLMFETDIVVNNKNIKCENFIEEMIRN
TINYDCNFGLGENAKNFKAGSLGNIICIQIVGLLENYEKLFDLLFLSIFKMNLTLHRLEIILKSAYQNLLQKK
NKPKTLVINLEYALRYMKNSNSGLVSIGQQELILKLIENKNNLLDVYNKLNLLKDELFNLKNIALVIDGNFSK
VHNVFSWYDKWFTIKNMQTCEYTINNPVIDFNFDSDIGNTCESFKYLDQSTKTQQHKVKQQDNVKQQDNVKQQ
DNVKQQDNVKQQDEEQNNDDINSYNYCNHMCNNKEQENESSKYISLGPNQHKSLKEYLDTQFEADDKHFEMNY
IKDKAYNGVVCGIKSTDVSYLKLTIKAPSGYDNKYYCCLLVLREFFSMTEGPLYNSIRGGGFAYECALEYNCI
LGEVSLRIYRSSDIISALREAIKILKYYCNNEMKENELSIAKSSAYYSIFNNQETVSDRASQTIFLSLKNLNL
NFYQELLSNVEKITTKDMLYVCQTYLSKIVNFKIDKKNALMGSTLSIICCSEKTEEIVNALKQKNDIINFGDI
CNVDVTQLYQFFKTYDIISALKLSSYKDNLYGNHPNMLNDDNILEEDNVLEEDNILEEDNTSDEDNTLDDDNT
SDDDNTSGDDNTSDDDNTSDECFDISCCSVHSEDDSYPGSSDHEKL*
>N.PF11_0190
MKDEQISCYYLTEKGKNKCFKNIKLITFDLDHTIWNIDALLNYADNECYKYMEQNYKRLYDYLSKEYALSMTN
LVKELLERNIDMNKDGVQILTRIRTDALKYLAKQTNYDEIKFASEIQKLWKEKKKDVHLFISPGTLEYLRELK
NRGYILGAITNGDSDVNEIKFLNEIFSFVVRSMDYNFAKPNVEIFNIAENLLKEKNINFHVDEWLHVGDDVYT
DIMGAKNKNINCAWITMFREGREISHKEWYSYLKLKYEQNDNKQTLSQYDPYADGLFAKFRKKDVVLPFDYID
IEIRHCRDLDSILA*
>N.PF11_0191
MKKLYILFECSAGYFLLKIEEWEQIGNTEELEKKILKSDIFHQMVEFCAFIPFETAERALDNLLNINEGKATT
FLLSFLEQNLPNNKNKYELGVADINLGKYLSNVGFNIIHNNNVLELFRACRQHYLKKISTYVNNIDIDIKHFN
IGLGHSYSRSKLKLDPRKQDKSIINSIGTIESLDKNINLFSMRVIEWYSWHFPELKKIVTDVCMYCKLVNLIQ
IKEKFDFDTYEDKINDITQNEDMTKNICKVANLSIGQELTEEDLTNILNFSNEVINLSNTRNILWNYLDNKLN
IVSPNLKELLGNTLSARLISHAGSLVNLAKCPSSSIQIFGSEKALFNSLKGNKKTPKYGILYNSSYISKTPIQ
LKGRMSRYLSCKSAMAARIDSFSDYPTNSYGLIFKKQLEHKIQHMVKGVKLSKNIDYINEAEQIYRNELKSFH
KDGKINKDLKNDEKKKKNKNKKNKNKRKHDEDQHSEHSHQDQDEQEEKEKEKKKKKRKKDKKKKKKDKEREKR
ENNEAQNEDDQINDDLNGDVNEELNDDVDGMTEHIEVQDEEDNERDVHDPEQNADENQEDVDQQQDSNENEED
EDQEENDNDD*

>N.PF11_0192
MVLGGKNENKVNVIKGAGSIGKYIMKENGSKIKEVGEWNNSSENENSDNNNNDREDNIKEKVDNNNKNDDNNN
NDREDNIKEKVDNNNNNDDNNNDNNNNNKSDNNNNKSDNNNNKSDNYNNKIGDVSNIPSTNTSTVSNKIQGKG
SVSYKSKEKTGDHSSNNKNTKGNNNKVRTSGKVINSLSKYSDSYALIFPNALPVKQVLWGLDPLNKVWRYCSI
VYARPKNKVLNDTNFMFPLNMNKNEIINKVNNELNNNNSVSMSEGDYDYYVHWEKFDRRLDCWLAYENLRLLD
KEPDDGLPLIKDNDNVSDHEHAGIDKEYLREHEENTKLKTINQIRFGKYLIDTWYFSPYPKEYQNIDILYICE
FCLSFFKEYSELKRHIEKCEIRHPPGNEIYREEKISIFEIDGSYFRIYCENLCFLSKLFLDHKTLKHRVNLFL
FYVITEYDEYGYHITGYFSKEKYSKNNVSCILTLPQHQKKGYGKFLINFSYFLSQTEKRTGTPERPLSDLGVA
SYMAYWYETILKVLINYEQLSIQELSEITSIETNDIISCLEEKDIFKNSLNGLDSFYYINPKQLEFILNKLKQ
TNNTQLSKNKLHWVSYDYYLALYE*
>N.PF11_0193
MKVLKTSKNNFVHPNKSNQYNCNDQPYDSLFRKNCVSFYEDFDTEAIFLLDKGSRPIYHKLKDKEWGSSNNQK
DTEKKKKKEEFEKIDEIISNAEHLNNLRNKLYSFEYQNILEPLGIYISQQEDKEENIKREMGNFYKDEYEILK
KKYIHLKENMDTILDVERIKAGLFYTKEDLQKIFEIIRKHQSKINNKNDIIKFQSSMLEKAIEHNKKNKLTII
KDKRKNEKLLEICKSYYEQNEKAQKKLRHLKYSFLEYKIALENIVSCCEKIGGDKIILMDGIKSARAQADMSI
ANDIKNKIKIEELEMNISYCEQQINYLKDDLDFKMEELRNTTLYTEEVKNEASYFKQELSKNNDLLLEVMEHF
HQMINNVDEFIYKNKDKNELITYMNKKKKYGDITKKISEKNKELNLKVKNFVPYNFSMGTELKKHEEYLKIQ
MDEMKKKEEEEKRKRREEEEEEQKKRKEEEEEEQKKRKEEEEKIEKKKKEEEEEKRKKEEQELLEEKDIKNNE
TFEEKKIRLVKELMKEVNEETLSLEKCVDIIYKANLPLTQNKLNKLKNMGDIKKDELVTFVKTLMLNEQEAFE
NMKTFFEIWDIMKTGYMHKNLIISILKQFGDNLTEEESNYIQKELNQMSESNISYVKLLKKWIYGTEE*
>N.PF11_0195
MIDHLKCNCIKYNIPDEDCEPVIKVSENKDDMLVAVSSNKRNILLYKIINREIIYSDTFYIGDPNKIIALEWS
KQNDLLIVTIDMKCIIYKKKENGKWECTNVNIPTEDLPTCACWHPHTYSFAIGFTSGIIFICSKKENQKWKIK
KINNHVASVMFIDWSSSGYILSTNSLDSTSLLVCTSGLLDDNIKNRNNIKIYKNMETFITERNIHNNDIIYKI
ESEGYIFLHSSFSFSNEKIAIIATNFESTYEKQQIIILDYFKSPANTQYVSWVGQTLQRCLFLDDDKLLVYGY
EIFPIIVEYLNDEWIISKVVLPEFNIKNLTVDFFYDQKEIQEIEQECEHLDGNEIIGEIVAHSNYILQISMLE
PYENKIYIEFITVSSDFSIVLWNFSI*
>N.PF11_0196
MNLFSPVIFPLLIFLLSYNGSYANYNIKTIKLIFESRDKESSKVISLYGEKDSFVCRSENNEYIRIQDDKTII
NNLNIYGIIHTNDLLYFEKEQWKLYYMETFDVKNESWTPSEYSTCGNSPDTFLGGPCKFAATEAYNKIKNLPK
HKELKIKLRIHFFDLWEDDSLFLQVDNKTIWTHSHRSCVSKDCLSGINICGQNIPDRLSLPVDMEFLHTSDTL
NILVGSTLKKNTNPCETSWGIDDLIIYYK*
>N.PF11_0197
MLKNINKPVIFFNVLTGLAFIYLLKNYNENTKHILNKLYSLKNWIVQYSCSFLKRYDSIQLPIIDKDIIIDLS
NEELEEKFVQTCNAVKMYRNKLKTEEWLHLYGLFKQATIGNMILSNEEDSTKNTETSSNNNDTPSNNNDTSSY
NNIENISNIDGKKKKKNKNNKNKNKNNKNNNKNNNNNMEFIEIQKRKAWKNCYNVNKNTCKYLYVQYFNKLFP
DALEKLNNDDNMKFSKTVSKMKPFKEQNKKHLENDNNDDNNICDILCQHVVMGDLASIKKNLKHNPSLINKKN
SSGLTPLHYACDRGYIDIVKYLVNQGANINVEDSYGDTPLHMAAYSEKLNVVDFLKSVGADINKTNSEGLTID
WILSQN*
>N.PF11_0198
MKQEEEDEAHEFLGNFINVIDPSDINKNGRDKKKKTKKEKKKEKREHLKEKRKKKRPEEKKRKKEKKREALLK
ILNNLNEEEKIAFLKERKLSEIKKKEEKKQFLIKSYNEGYKICFNCSFQNLMEEKEISSLAKQIFLSYHYMLK
KNVPVQFHFTHMNDNDDISSTLKKYSFDKWMVHIHKDDYWNIFNKDKIVVLSPDASEELEEIKKDEVYIISAL
VDRSVSKNLSFYQASLHGLKTRKLPLEKYIKKKKSNVLNVNTVVEILIHYIKTPNWLKVFETCIPQKKVMCFF
YDSSSSVNEDERIIKGDSEICINEKKDIQVHF*
>N.PF11_0199
MDDEEQIKEKKADNSSSFPLSEKVKKQKKKKKKKKNNNEKREKEIIEAKSNIEDIFSNKKVKENNNKKVKEDN
NKKVKEDNNKKVKEDNNKKIKEDNKLINKNEANKKNNNKISNNKKKIESERLRTPDGLPIYSMDELNMGKGGY
TKNCPFDCNCCF*
>N.PF11_0200
MAEHLARIIGTEEDRVNCPFFWKIGACRHGDQCSRSHYKPNCAQTLVIRHMYDNPPIAVAIAEGQMVEDEVLD
KAADHFEEFYEEVFDELMKYGEIEDMVVCDNIGDHIIGNVYIKYTHEDYAEKAVNELNGRFYAGKPLQIEYTP
VTDFREARCRQFVEGQCRRGGYCNFMHIKHVPRTVKRKLYRRMYKKYPEYKKRRARKDDSDDDGRRESYREGK

DKYKRDRRSSHHYSSKRKNRSDNEDDDDDEERSYKHARRENSAERREKIERWNKEREMKNMNKEDNKSNADEK
ED*
>N.PF11_0201
MFDGESKNRRINLSGKKQVIVNKDFFIEKAKKEKENNLTLIKRKNAFKKISDYIGFKKCLENRKKEINEVVSK
RINDIILLEKIVCPSIYRQALQLCVYEFSLQSSFLYSDLNCYNYYKSMDFDGIYPPVKYLLNTLKFYNKIDEN
CVDKTNGVDKENEIVDEKVKDDEKIKDDDKIKDDDKIKNDDKVKNDDKVKNDDKIKNDDKVKNDDKIKNDDNN
KKNNIPVGNNISEKHTLQFDENKNDNDNNMTEIESHFYYRHKKELDILIKHLDILLGNYYIYNYNIYSEYYKD
VKKIKNIIPTPINNNILKHHLNDHNYTNGSFSNHNNKKHISSKGNILNIESDINTNLLHIYYLYNFIINNKFI
DELVKRKITNENKKLNVFLTSRCTRSSIEMTNNNKNNMSDVDNSNRSSNVYTSVLNKKQNVKELSACLKNISD
YIILSIEGIITVIKKISIYYQNKVDVKCDNIKLMIYLCDMIYIYFYILKIKGKDMKCNNIMDIKYKIKLLINF
VKLNLEYIKEPYILFISFFKYNIYELFENTINIKNELKEWKILLIRIINNTYKDVDIMKELIYNNNYYYYYYN
YNRSYFKVPYYNMKYYLYNQKSYDEYNISGVNLFRNLLLFVHFNKNIMYDNVLNILTDVYLFLPLEYHPYIYN
NTEDINPPQKNDNSVIKIKNEENYFEKQNLSLDMGYYYNKEIIESLIYICKKYRFDKLDTLLFFLLPFRNING
KIYDAYRNYYDYMKNIEKGVYANYDKLYKSRININNNMVTNNMDHMNNNVNNNRNINISTNFKNNNKKASIHD
NINNNNNNNNNNNYMDNFENYQDKKNKGIMFYSDMNDFPFLANNQVTHYNYEDNKYNNNNNNNNNNNYLYGGH
NNNVHGNFFFSLENIIGKRDKKNLGSNSYLFNDMINNDIILRIKRILIINNIHIYLIKKIYLFWCYSCKSNDN
SFFKNVMFFPYYDNTHISIYLSSFCFLLYYYLVVNMFNQLIDINKFKMIKRNEVHHNDIRYNMIPKYDMENDQ
QHNNSNNNNNNSNNNNNNNNNNNNNNNNNNNNNVVTNFLMNKYFKRVCKLLILSLWYILKNSINCIYNELTKK
KNLNVKNQQLHYMEDIDDEDIYNDEDEEHENEIIKKEENHMDVIHSKQFTSTIYSSDYNICLLNFEKINISIG
DIMYDQNSYVVNKGHVYLNVQEDNICLIKRNIENEDKEQSVKCYNSMDEIKNIFDELDKINMYNYDNYVDRHM
FFYNKNNTNMEHLKFAHNVKSFSNDESFQYSTPFESSSFVNNNNNNNNNNYSHTHINHSYNERIMTSNHNNMS
SHNRGEFFFDNLKNKNTNTKKKINDDSCYSNVINYYLYKNDNKGLSYILPILLKKLYKLNSYIHIFDDEFFVI
KETFNLLKNRFHIIGESNNINYNNNNNNNNNNNNSNDEETTLYLEKNDIYLNNNIKFVNVLANYLLRYVPFTL
PFDDRLLIFYHFRNKNKESIRDDSTFNILERKHMLIRRTNILEDGFISLNILDNLQIKQNVRISFIDKNGNEE
TGIDGGGLFKEFIILLCREIFHENFILFQNVENHTLFPKYYNENDNLILYEFSGKIIGKAIYERILIESVFNK
VFLNNLIFDNMDINDLYFIDQNVFNSLLYVQNDENVEKLCLTFCIYEKKYPDGEKFTKEQYDKITNYFYELSK
RNHYNMYSLHTHHNNIINNNNNNNNNLFFFNQMDNLENLFFSSDDNTYNFTNMNNFFHMINNLDSMISNVNSR
NYSTFSHNNMNRNNFINIPNGINNININNIIMNDDTSNYSSTDERSAHIPPTDLEYSFSHRDFIPFHELGEQN
EHMEEELPSINIRRKNMENNDDKKNKRNNKHNIYNNKNNNITNNNLCDDEQNWECIDLIENGRNIPVTDKNKK
LYIKLYINYKYNQLIKKKTQRFSKGLSQLIPIKWFKLFSAHELKTLISGKDKCFDVDDLKRNVVYGGGYNENS
TTVLHLFEILKDFSSNEKSLFLMFVTSCSRSPLLGFQELYPKFCIYRVPDHTRLPTASTCVNLLKLPDYLSKE
VLYKNLITAINDTQGFDLS*
>N.PF11_0202
MVISQFYILSPRGDTIINRDFRGDIIKGSAEVFFRNVKLYKGDAPPVFYLNGINFTYLKSNSLYFVVTSLFNI
SPSYLIELLHRLLKIFKDFCGQITEELIRTNFILIYEIIDEIIDYGYLQNSNTEYIKNLIHNEIATNNNTVKK
FANLPNFSIKNTNTLPSNASQKPIQINDKKNEIFIDIVEKINLIMNSNGEIVYSYIDGVIQIKSYLLGNPFIK
IALNDDLYIKNIHHDNSNNIIIDDCNFNHLVNLSQFEKDKILSLYQPDGECVLMNYRINNNFKAPFKIYANVI
YNQNHTVELCIRIRLDIPSQYTCTNVFVYCNLCKHITNVHLDLNTNSDLFSAQYISNENKLLWTIKKFKGEHE
YSIRSKITLSPHYAFSKRDFGPIYILFEIPMFNLSKLRIKYLRIIENYKTSNTHRWVRYITQSSSYVYRLN*
>N.PF11_0203
MYRADIIRKKSTCLLLPSEKNAFQKKKWLLKKSLDNLLDYNNTQKECLHSLSYLKDRINNNNNNNMKNGYKEL
AVCSSSIKYNSNVDYNIFFDSLNFICEHVQNIWSNKNKHVQINENISNMFVKFLKKYNHFKNGNYKIKNRLYK
KDSFIKPSNYLQSFLLNGKCDEGEKIIKKILKDNKSLKSIKKKIMRKNEDFIWSRERNMTNLSGHNQSEHTLG
ESIINNNIINNVIKKHMSFTNSRYIGFLNNNVSKYKLNFFQFLKNMYLSKAPKGFERFENKSPNSNNFKPEEE
KPRKYDNYFFYIFFILLLFFLLFVDSNGLYNEITQNDFFYKYLSKGYVEKIKLINKDYVKAYLNSHGIKKYHM
KYVSFRVGNSDSFEKKVEAVQREMNIKRDELIEVQYVNEANILNEVKGYIPSILFFLLLIFLFQKITLKNVTN
SGMDKLFKFSKISPINKNSLKTDVKFSSVAGMKQAKEEIMEFVDFLKNPTKYEILGAKIPKGALLCGAPGTGK
TLLAKAVAGEANVPFFNISGSDFIEVFVGIGPSRVRELFAQARKHAPSIIFIDEIDAVGRKRSKGGFSGGGND
ERENTLNQMLVEMDGFHTSNDKVVVLAGTNRVDILDPAITRPGRFDRIVNISKPDINERSEIFQVHLKNLKLH
ESLDIKNISYILASLTPGFVGADIANVVNEGAIQCARRSNLLGVQIKDFELAIERVIGGLPKSSSLISPLEKK
IISYHETGHALIGWFLEFADPVLKVSIIPRNNGALGYSQHLSEEIMLFSRDAILDKIAVILGGRAAEELFIGK
ITTGAIDDLNKVTQLAYSYVSQYGMNQEIGLVSFQPNSNSEYNLYRPHSECLAHLIDNEVRSLIETQYKRVKS
ILMKNEKHVHNLANLLYEKETISYHDIVKCVGERPYPVKSAYEKFVKANPYKAISSEPLLEDKKESDNMKGDT

SNIGNVNTNDFEKGHIKENTKECTKENTKENTKDNTKENTKENTKEYTKDNTKNQYNILGNSSKDDTKQTSSK
LNEEKCKDKLGDSKKKDNKISNVKLR*
>N.PF11_0204
MIRKNMEKKKMSNRNINNNINNNISNNISNNMNSNINNNLNNNLNNNLNNNINNNINDNMNNCQNGNFYGTYN
NQTKDISLPFHKNSMNEINIISKNIDVDKGNFVKSNIFNNILNENNILYNDINKGIYDNNIIFVNEKQYDRIL
KRRLRKVKQDIDRKRKVRVYISIQKKKSYPEFFSTNTNNFGLISSHHQSPHHTLINPHSHPYQLPMNNNINTS
LYNYDWSSNDSFFKKNNENSNSLITLFENMKPIFDNKNNQNYENYISQNNFNPYPNNNITTNDEYNKYINICD
INNIHHLNNMQEINNFQHLNSLDNYNNIQTLNVYNNHINNISEFTNSISPNYNIYEHYNK*
>N.PF11_0205
MKKLINCGNRLYVGNIPGSATRQELIKIFEEYGKISDIDIKYNRNSNGTNYAFIEYENPKSAEKTIQKRNGKK
FKGYMLKVEYSIEKKNRDLNDIYRSEYRVVVKHFPRFFKNIKEFLSRAGKVLYIHKDNGLIIAEYEDKESMIK
AISTLDRTIYNSKRKVYVRVFKDIPYDYSDVNLIDYNMVFSSTKNENISPKELN*
>N.PF11_0206
MKNNLFTYENIFKNKNINIIDKIKEKRKSTQDINNVSIKEKENDNCDVNNKDTQGELSSQKGNEENIHMDKNN
INNKEVCKNDKNIDMCQSLLGKDNLEYTNNNVNGFNNTSEDTFLNFKLRKKKNDISSLFDDNTYDMEISYNEK
DEKDERNEKDEKDERNERNERNEKDEKDERNEKDEKDERNKKNENDEKDKRDDKIFDKNSFFLSPSVVMEPQE
EFSSYINSINNNEMNNNNNNNNNNSSSNYRNNIFYDNVETFNVINETQNEGNENLHIIKKGKVENYFNDCKKEF
LVDKNFNDEYYEEDINNYMSNNKVEKLIIDKEKENNEYNKINGDNFHHDNNINITEHLNNDSLIIYSHKTYFH
DPNLNDTNSQHINNENVILKEKGEEENESNNNQKDYNKIRESVSLNNNIMDNNSFLWNNKNDRMSCEYNEDRN
ICSENLKDIFNNEQEKEYMKMNIHNNNNNNNNKYIYDNINLYHKGDNVHMNVATIKDHNNNVDVISKKDHINI
PSVHKNVHVSFCFGNNGTFFYSKNSKIKYQSLINVIEIHINKKKRKMHIFNEKNNEENNIYMNSNCNYNNYCN
RNVDSNDVKHCYHNNMEKFIYCIKNFPGPFSRKSNKVDHKIEEFLKGYININKSRSVNDNIFEDIQKNCLYNF
LLNILKKPYLTNFNLKEISIEKKNDINKNMNHKGNNIISYFLDNYQEKKDIKDDIYLSDDCNEDEDIDSLSNN
EMDEFDINEYVKNDSNKMKKYDEPCNNNHINIYNPTNNNSNNIIMNNNVDKKKNVLNKDQKKCYTDVYENSKN
INDICINNNTYFFFDFINKEFIMELYKLENKNERITHDDIYLLYFHMCIYNSKKATNVCIKKELYKYFFLVLR
KYNKKKYYKMLDKYISYIKRSINNELEMKNDMKNIYKIYNYNIYDEICTEAFVFFLCILNKKNMVFKKNILVN
YWYAFYTLIFHNFIFQYKENEIQFADYKEDIINFFIYLIHLLYEKKKNAEAQFLLLLISNNPFVFSFSPNTKN
DDNYVEDVNKIHSANIGSNQMYSDNIYGNNIHSNNNMRGNNIYCNNNHPSHSNNYIRSNNYIHGNNYIRSNDL
YNRSNIEESNAVGYCGLLNSHFDIMCFQVCDIYEYMCRYEKDDFFFEQLIFYKIIYAYILLEYGILSQAQRYV
EILYYYIDVIKNDRKKNSYLLYLYDTLLEKTKYIFTTTTNKDMLSLQRKSCMSNDEVIYTYKFISNSGITTTI
PKDNMYEHMMDKNHSNNIKGYVNYNNMNDTNVNYINEYTTTNTNINVFNNSPIHNRNKTIHVQTQENINEYFN
IKTNRTVTDHLGKNNVYSNIKEEKLNSTMFDILKSNNNNSSYNENENIQSCDMLNGDNHTNRNEDSSIIHIQN
GLSVHQAINNNNNNIYHNNNNIYPNNNNMYHSNNNMYHSNNNNNMYYNNNKYYTYEHASSNVNPSSHFAAEQN
EAPYNFTINNKNNNIGTNYNFPSVNNNTHGHMYNDINNHVNTYEKSTEGTYRYNVPNNDNNNNNITFVYSQNF
TNVVEQNNNAKDTVQVQNEKGTMCGPLNVHINTHNSLYDNKNVEGNFTNNIKKDNNMNVKNNNIHINNVNVQN
SSDNNLSSSQDQQQCANENNMDLINMGKSFISGFFSNIKEKIKKTEYMQEEEEEENIFYYDYEKKRWREKGVT
SDEEKEREKQKLEKQMAMKNISPPPTGINYSSERNKNPLNMTDVRSRYVDYFN*
>N.PF11_0207
MAKKKKQIHLNIIDFQKYYQTDDLLLDTSISTEKKTVDNQKFIRKNRTLEKDEVVQNIDWRTFDNEKEKETNN
ENTSNVNKIKSPGLEKKNFKKSNDVITLGARNKNKSPNLNADDIDFTNLRNKKKEDDIDFTNLRNKKKEDDLD
FSNLRNKKKEEEDVDFSNLRNKKKEDDVDFSNVRNKKKEDDLDFSNVRNKKKEDDVNFSDVRNKKKEDALDFS
NVRNKKKEDDLDFSNVRNKNKEDDMDFSNVRNKKKEDDLDFSNVRNKKKEDDVNFXXXXXXXXXXXXXXXXXX
XXXXXXXXXXXXXXXXXXXXXXXXXXXXXXXXXXXXXXXXXXXXXXXXXXXXXXXXXXXXXXXXXXXXXXXXX
XXXXXXXXXXNKKKEDDLDFSNVRNKKKEDDLDFSNLRNKKKEESKENDTNKSEKPLYLRRLEEYRKKKKLES
QANDTAMKMHEKEQIDDIQERKEEIKEEFKEEVKEEIKEIKEEIKEVKEEIKEEIKEEIKEVKEXXXXXXXXX
XXXXXXXXXXXXXXXXXXXXXXXXXXXXXXXXXXXXXXXXXXXXXXXXXXXXXXXXXXXXXXXXXXXXXXXXX
XXXXXXXXXXXXXXXXXXXEEIKEEVKEEIKEEIKEIKEELKNDISSETTKEEKNTEHKKEETEKKKFIPKRV
IMYQQELKEKEERNLKLLEQQRKEREMRLQLIRSKTQGTSSTFIPSAKLKHLESLKEEKKKEVKTNIQPKDNN
NNNNNNNIAVLKNNKNEEQNVIKKKSIFLEIAEKTENAKIVEKTDIEEIAKKKREELYKKQLEKITKKNEEHL
KYNNIYKHDVNIIKNFYNEIKDKIIQNYYFNQDDCISLCSILKTDDCNYMESHVPFYVVISIFMLSLPQKLQN
DDYFKRASNIKNLLIYLKENSKIENHEDYILNDTLKFCDQLKYPHLSEETSLIETIFDTLLYSGVISKNSFIQ
WFQDDDSNAELKSKAMLQLIYWHKWLTEEEKDQEEEIDELDDTEEKNISDVSDIEKNVPKNFIFKKIKKKLF*
>N.PF11_0208

MTTYTLVLLRHGESTWNKENKFTGWTDVPLSEKGEEEAIAAGKYLKEKNFKFDVVYTSVLKRAICTAWNVLKT
ADLLHVPVVKTWRLNERHYGSLQGLNKSETAKKYGEEQVKIWRRSYDIPPPKLDKEDNRWPGHNVVYKNVPKD
ALPFTECLKDTVERVLPFWFDHIAPDILANKKVMVAAHGNSLRGLVKHLDNLSEADVLELNIPTGVPLVYELD
ENLKPIKHYYLLDSEELKKKMDEVANQGKAK*
>N.PF11_0209
MSAIKNAVTYYFAIVSLFSAVFLSIIGLMLLYDSDSLELHGDKSEKVKPSFICAGVYFCILVVSSIMIIRNNH
KAKKYQTKTI*
>N.PF11_0210
MFVWTHINKYTSRYINRYILKFTRYESLRLCMLNFTSTIKNENKIYSKKNFNNVLMQNIKITEDEEIICEQFF
FSKYNLPYVLKIPFSDLRLIDTCNNNHNPTILIRKDMILLRTGFLSCVIRYNELWLFEPREPLVIKATNLIKQ
NLKIIYGFKGDMSSGVEIPLNELKDEKCQENVNMYEKNIRNDLYSTYEKNISDDINDAKQKNMCSDINNIWSN
HIKIDKKRIISGNYSRLNRNNIWENDREINKKDIFNIKEKKNMNENDDICNKIKIVGKEELNNNIKEDINYLN
VENNFYRYKGNISFEFLCLDICMQLSIKEYENYLDTINITLRQKIQLQQKKEENIEINMLTNNLLREMMKIKN
KLQKLSNLLNALRSNIEKILKNETDMKNMYLTTLNKISINKIKDYSDLEILLETHLQLTDELSGELENMEEKI
THYEELIRLNLDYNRNKFILLNAKISFSTLFCSICAVITSLFGMNLKNFIEHNDYAFFIVSIFITSWSIVGIY
FTKNINTLLRFFDKYNVK*
>N.PF11_0211
MASYSKDSDLGECIKYVHLKEDKKEEDNLYSNDKDVEYFENLFDYEYDEDDEKTNFVDAYDEESTGGRRFGLT
FFILGCMTICGFRGRMVKKIAFVPPIIKGYNIENDNKFIFHNSHHEEIKELMQINNIDINYKKLKRGSTEVSG
IMLYKKPLDLNKQTILYSHGNTTDIGYMTPFLLNLVTSNNVNVFSYDYSGYGLSNKDPSEKNCYKSIKMSYDY
LTKDLNIKPENIIVYGHSLGSATSCYLINLKNVKVGGCILQSPLYSGLRLLLPLDYKKEMPWFDVFKNDKRLK
NIPLLPLFIMHGKNDRDIPYQHSEYLLKIVKKNFAKKVQKNKSQFLRNKKINHLDVHDSILRFWGVENADHND
IDEKNPELFYHKLGEFLSYCSKFNMNE*
>N.PF11_0212
MNIFGLLLLNLLINVIFILCNIKRKNKFLKRQLCSNELKHLKYKNDNMIRKGILNYKYKYKYRFFYIIIKKNY
SNKIKRKNKIKQIGVMENINNINDNNKWRCLSSSNHISSNMMGNTFYENVYINYLKDYIIDEKEKAMEKNIVK
DEEQKKDKIYFPNSNNMLNAKDIENVIHERIRKEEENLFEFLKSVNERYNLNCTLRVVGGWVRDKFLNINNDD
IDITVDNMKGAEFCNYIKEYIKEKENKNFNFGIIKINSDQSKHLETSSFNLFNFQVDIVNLRNEKYTEESRIP
EIVIGTPEEDALRRDFTVNSLFYNLKNKKVEDYTEKGIFHLKNHIISTPLEPLATFLDDPLRIVRCIRFCGFF
NFFLEKSIFNVLKNEDIKKAFTKKISKSRLSSEIVKIFSAKCKNVILSLTLLNYSSYSSKIFQLPSNYFVKDE
ELFEKLKKKDKINKGIVTPAQHYHTNDSVHDQHVDNLNDLNCVSSNNNINNKKIHLQNVENSNICNNSSNIIS
SNDLIQNKVNNVSEQSDINKEDENNQCNNVMNVQNNDKQNFEQKWLFDGLSYLKFFKEIEKNNLLKETFNNLD
YKENMNYIQLCLFLLPLKNHFLYIKNGKTEYVVEYIIRESLKFPLKYSKFCVHIFEGFTHLYNLYKTIDVLNF
LKNKNYQEEHNIQIKGQTVLCLKKIGDKWNFVFLIFYIFHKFNELNKNYITSITTNNICLSDFVAKLYQYIFK
FNLQESYNMKPFLKWPDIKHNFPNISPNQINEVYEQIIKFMAIHGENEKECIEYLKQHFTK*
>N.PF11_0213
MNKFFQILEYFKIELSINFSITFIFINILLVFFSTFLYRNIKKVNDPDSYKTSYERKEKDSTFDEEENFDILN
IFSNDPKSVENIYELENDIYNKSEESSICSNHNKNLDEKTYKINKKKVTIYLKLNEGLENIKQELHIIDRELK
NILQKEENLQLINEEENIIIEKTKSDKYDEKENKSNDNILSKKHDDENINKSDIRNTLDSELKKKYELCKENN
QLLENHHNNDNNQEDNNICEIEKQDYNNYNSDNKNHNDIFNYNYEDISLEKDKNIHHTKEITKNDTLCNTLDS
IKNNRPTEEKTKNFNYNETNIYEENKKLKDSIVNSKNGENFLLKNNEQNINNNIINVNQKQSSDHKKESFKID
TKKNDQIEQNYQNGKNEKNEENEKNGMNEQNGKNEQNGKNEQNEQNDQIEQNYQNDPNDQNDQNDQNEKNDQN
EKNDQNKKNEQNIGPKKTNEISYDSININISNNQNPKLSNVFVEDIKRILPISNNNLENSITTIKNIDDPSNS
TKQELQNSDYENMHANKKIENGKESFICDITSNTNITRNSMDIPNIMDRKKSEYDKEVDNVSIKKKSYINHNM
QRENTREDPSNNMDYTNKSTSDSNDIENENKMERCTNILGLDKRETVIKINDINKDNKHTNGIKCEDKRKSTS
IHMEDINNSTNYLNNKEYNSKGENNNNILGDDKRESTFKIYDIQKSEYNSNDLQYDNKRERTSINLDNGNKNN
VDINNYINVDDTKERISINLDNVNKNNVDINNYINDDNTKERTSINLDNGNKNNVDINNYINDDNTKERTSIN
LDNGNKNNVDINNYINVDNTKERTSINLDNGYKNNVDINNYINVDDTKERTSINLDNGNKNNVDINNCINVED
KRERYSHILNINKRESIIKINDMKKTKSDSNDIKINDNRTEMYSNIFEINEKERTSIKVDTVKENKSDSNDIE
NGNKIERHSNVLYINPTKFDPIKINDINKKGERYYDNIENEDKREIYTNNDEYMDKRESNTNHIYINKKESYL
ENAKDVIKKESPLNNVEDTKKEESYSKNIENANKEEYESNEETYVNKMEEDSNDIEDISNRKYDYKEGQYLNK
GEKYINNIDNVSIKESYYNKREKESIEISNLNKRESCTSNINNFRILENQSNMVEYENKRKSDNIEINNLNKA
DIYVNSIKNVSKRNSSYNKKERDSKKDRDSVYDENRRKSSAYQINDIYNIRDKSKEKIDMDTIQDMNKRKSYV
DNIHITNKKYSDINNAEDVIREKKYEDYNISVMYNMHNKRDQRKESNINIYNDNNMRRSTINKINDENSMHNV

KNEIKNIDNNNNVDKINVNKSNTDKIYMDKIYMDKIYMDKIYMDKRYMDKRYMDKIYMDKIYMDNEYMTDNII
KKNDKYNNNNNNNNNNSDVVEYVNERKHTSLINEHYLYERKSNVDKMKDINKGENDILYDVYNNSEKKNTKSS
LYRNPEIITQGGNDNINNMNEHISSLNKVYRISNKKSNNNYKGQDYTYIVNENSGRNSSMYNNINNSDNMNKK
ECDFINDKENINKRISEIYDLRQINHNESNIENMENIKNMENIKNMENIEHIKNMENMEHIKNIQNIEHIKNI
ENIQNIQNMEHIKNIESIQNIQNMEHIQNIENIQNIENIQNIQNIENIKTIENIKKNIENINNKSSLMVNSNN
INENIKKESDIYKIECDDDHMCDVTKIKGVDKQMSDTHKIKYDDNEKNNENKRKSGINKVFDIIKRTMDSYNI
YNLSKCKIDEDNIYMLNEKKKNNINNMNKRKSENTYNINEREKDDMNRRMTENTYNLNKEKNLSVHNIKNMQP
YNINNNIYEKTNDTIDNLNETGKNNTNDISIKGSDKNKEYKFYDNENKDNINMIIRDTINMSNLNNNISDNMQ
MAIHKEEKNIHKMNRKITYTNNLYNINESKTEYTININEQEEKSDFINNDNIKMDNEDKLYNCNERNNHIHKE
NKRNDIKYDNFKKDNIRKNMDNTKNIENMNNIDNMDNTKHIENVNNMKNIENVDNINNIDNMDNKKHIENVNN
MKNIENVNNINNIDNMDNKKHIENINNMKNMENMNNINNIENMNNMKNIENMNNMKNIENMNNINNIENINNM
KNIQNINNIENMDNMNNTKYIENMDNMNNIENMNNINNYNKIYDVNIINNKNYDSTYNELYEKDNQEENKNIC
NDKLDNSINEKTKYMKTLREYFVKSEKEIEDQKKNNMNDMYTRREEEKIKDNEDWKLYDFKNLKEKYKFQMNM
KDSEINQLQNNLIDEFKELNEVSKLKNNLKGKTYNSNIGEEMLEENKEKQHGNYVGEYFDRNDMILKERIFEY
AKHKTSIDMLKHPDDIKRNSKDLKKWKSQVITKKYADDWLSSDVLLSCFLNFIKLKKYVNITELSVKFQTTTD
DIREKLEELEEQDMINGVLDEKGNYIYLSQEEINKLCFEIQSKGKVNTHDDFVKICNKVISLSVNDKDMEKLK
EEEEKIIKAKTEIF*
>N.PF11_0214
MWIEKDIKNSTHGGCLSKQVNNDIETLKKNNTFIYNNSRFVNVHSQLKNKNDNKIKKYNTNDECIEYVESNEQ
EINGICTSSLIRIPKNVRKNISEKYFKDMKNIPTTNKINHLNNNNNNNKNDKEKQYVEGMILEKNHSQNNVNI
LHHNKMKANTFIKGEEDFLSTTFKNNYVKKRKEVNPSNVSTNKIIMEDSKKKQKNVHKNIKNNEYDTNETYLR
NNHFVQKTSHIPINKNIRDMKEYRDKLKSYGNTQYLNNISTNYNDSILNNKTTLYHEYNDIKKNDTDCEEWFN
TANNDMKLKKRNYNSFDYEQHGKDGKYNEMVKMKNDERKKKHNQPDISNNNNNDNNNNNNDNNNNNNNNNNN
NNNNNSSSSSCCYNSSNKKDYLLKMKTFIQNDIPHIIKKSVDIHLSTNIDTKIQSAIEKNIMDFIENNLFSII
EKNVNDFNSHYIYDKINDCINNEIKKNIDLVYTNVKDKVNKNINDEITKNVDNLNNSLNKNITKEIEYNMEKI
YIKVNENVDDKLNNEMLENITNVQENILKNINEELKRKMELVSDNINNKINDNMKNELIKNINLLNNKINDNI
NKEMKKNIHILSRNIDVNINDQIEKTTHILLKKTNDNLNNEIKNNIHILEKNIDLNFNEYMEKNMNKIYHQTK
NIILFDDDIKQNISHVTQQHIKQNITTHMQQNVNEVITYIHEHLLNKDKNIIDQNLKQYIEEAMSISNQNITI
NLNQQMEKILQQMYEHVDQKKENHFIDIVQNIQNNINLHTDQNIQKYMNILYENQKENIFHYITHLSKQIHSS
HFYEPIKEEITKINEHKLDLFIEKMDELYHIFMEKTNVHISDIIHNEIKMITERKSMQEENILLQNTQHLQTT
RNTEHVHNIDVIQNIKDMIELKYEHITQNMKQIEKQMETHNKKDSYNKISACISTFQKQINDAIEKNTQTLLN
INESTIKYMDHTHLQLNTTIHNKIEDIYKTNIISDNNIIKNVNDISYKLIDNINHKHEQYIKHMNHISKNLMQ
IQHVPKRFNQHISSIIQKNIIKRSNHIFVYLKRFLKNRDQKMNFIINKINKKLTYKQIYMGKKRNELNLRNRI
NCKVIGNIKKENAKKNHIKKIIQTSTKSPILFIKKENFKLNQKQKQEHMRKRKRKILRNRTCIIHQILNRNKE
NQKEKINKNYLNNSLMVDHNLHLFKYYDQQKNYYTSQKRKPLYVVNYLKNENNELIEVSEYLRKTNIKKITK*
>N.PF11_0215
MHISSKKNLEDCIFCKVTGTIVFGSVSVYSFLKFLQAPKKSGDRKFFGLISVCFSFLALYRAFTPAKPITLNT
FNKKDVKQEK*
>N.PF11_0216
MNFNEMLKSNENNIRSDKFNNYCTSFNNSAMSLNSTKQNNISLLNNNNNNNKNNNINVKVEPKAGEELEIYVE
NMLNELKTKMQSLSDNLLNKVDNMEKYLDELENTMMNYTTQNNLQ*
>N.PF11_0217
MNNHICAFRDDTRSKEADEFVRKNGFTLPLQIFQIMSFFIFIVIITLIFLMTAFNTDHILFSLFYIFFGTLII
IIFILTYVVTKTNPVDPLSFKYLNDMINEEEIKDLYQCDICGFVQPESKHCKVCNKCVSVFDHHCMWVNNCIG
KKNYKYFVGLLSTLTLFNCFVFVFCIVYFIISLKHDLIKNRWKYFYGSYNDISFYILLSSLFILNGVVFVLVI
QLFGLHIFLISKKMTTYEYILNTSHHSEAEGRNKIKTFFDWIIIDKKRSKRSQNLDQDMEKHEIERGEITLKN
Y*
>N.PF11_0218
MKGSFWNFIKSKKNDDNESKPTVAHLGEENKFYFNKELNRWVIKGEEDKIENEEKISAPPKMNTLQNANQGYR
SLVQQNKMRSAFNIYAETPGLKTIKKKNVLVQGGIVSPYMESLKNEKGSSSSNNNPSDNKTEAFGSNIFIPSN
NKVYENKVRDNKYVVENDENYINNEQKKQKTSTLENKNYNEEVKEENVLNTSYENISQLNKSTGMDVFLKKQS
YTKNDDSMFTKVYSNENIIDTPNKNNNLDKTFNDNISNENNNFGYSKNEESKIMTPIQSNMHIPVISRRETKP
TSFDNLHKFYEIKRNETKEIKSLKVVWNPNKDADNQNDVYNNNNNNNNNNNNNNIINNNNHVETVFEKKDKN
VQDSIYKVSNSTFLSPFGESKNETFPRKSMVHINNLENINIKNVQEEEIYDESNKINDPLESNNGYDEMHRSF

FIPEERESINMSKKKNNINVDHNNVNIDENNINVDDNNISVDYNNINVDYNNINVDNKNINVDNKNINVDDKN
INVDDKNINVDDNYINVDNNNINVNDSPRHDKYNDSDFSHDNYINYDKDYFAQKEKDFVEHFITILKNHLNED
NNFVNSLIKTQTEKVNSFVNENKRSLNILYNKINEFDKYKNDDNMDNVTQKHVNNNLDNNRDDICSTDLKEDD
ILNEKGKELNNLLYIINIKNKKIKSELECFITSFNNLKKLKMKNEEIISIYKTREKELIEAINVLEDRYEKKN
CLFKKKERHYKELLIQKDNDLNMEQMNYEKNVQYLKKQEILLKQELEQVQMKTKEEIITLKEELEKKKNQEWE
ILQKEQDENMKKNIDIEINKIKEELKNKYEEETKDKVEQIKQLTENDFHLKLEQYKEQIEKDKNDFINNLNIE
KNNEIEIFKNNIEKMKNEEISKMNEENQKVCTENKEKDNLLEQQKKELENIKEQWETEKQKEIEVLKNEIYSQ
NKEKEEFLKQELQNNYNQQINQLKEELNEQLEEKYKYEYEIKIQNVLNRKQEENQQKWDNEKQQIIQMYEQKL
LLQKDNIEQTLNIQNQQWQENMQLQKDKQIDMLNEKIDKLDMETKLLNEKKNELEINISLLNEENMNILNQNQ
NLEDNLKNNEDIYQKKLSILSDQNCKLEEEMKRIKEDHTKESEEIQEKFADLLEEEIDRIRKESESKVESYIK
QYEEINEEYTKKKEEYDQLLEKANQNNKELTKKYEENIQKINEYEDMIKILENKTEEMVQKKIEELTIEFEKK
KRDFIDNEKQVLLSNYEELLEENNRMKEELGIQANITIRENEEKILEITKNYEMKVKEIEKDYEDLLNQYNNI
KYENKEILEKWNIEKETKEKELKDLNNINTQLSSENNEIHLTYKNLEEEIIQLQNKYEEVKEENIFLKNEEHK
KLTDKLKNLENINKNLMDVTEKERELNLKNVNIIKCLQQQINEQTKLYKEYTDELNNEIKTLKQSKHIHTDNT
SNNNQKNNGYNDNIELIEENKKLKLQNEVISTKNDTISTVLDNLTKRLSFIESKIKEKDFGLDIIKQADHLQL
QDFK*
>N.PF11_0219
MMFKFPCFRDKKWIKEKGTNMQYPHEFLNVQFRPDFLKNYEHTKDFEKKIEHVINQIKTALFRQAIYKIQNVE
VVAMHECKDDRVLEKIQQINGYENIKLGDKKVLCDEIWTVTRCDKKFSYWIRYYEEDKNGYSLSVLPTQLKNI
YYFLKYYYF*
>N.PF11_0220
MSKSLKLMEDKNLEKNAVEAFKQKMEKKKQEYLRFNLSRNGENMKEGDEKKKEIDINNNHNFNINNHHVNDKH
INNKYINNYMNNRHIKNIHVNNDHINNIHVNNDHINNIHVSNNHINNIHVSNNHINNNHIKNNNDINRKNSNR
HKNRKVVYIETYDTYDKYILNMYDEENEIYEDEINRENVESRTPNEGMEFNRIEKREENIFIPLKILSNKYDF
NDLVEATNNFSEYNRIAKGGNGTVYKGVLKNCINVAIKVLKKNENNGFENEIIIMSRYRHNNILSLLGYAMNK
NNFYLIYEYVNLGDLRTLLFNHYYYYNSKNKENPELSYYNISCYENYLRKKKSSSSCSSTNYYTSTFYKQNIF
PYFNNSKYSPFQQNSFDNNTPLFLSFNIRINILVQIINVLCYLHTSSPIVYHRDLKSANILIDDQFNAKLGDF
GLSFVYMNNNNVFNLTGGTPGYADPYYISTHEINEQTEIYSFGALILEMLVSKSPAIHVGKNYNCIYSKNEKC
PIFYHKKKHDHDDNVFDYLVNHLNMSDYKSIYSILDYSVNFPDFLVEKLTKLSFLCLNPNIKNRPSSKLVNLI
LLEIQKECDLFMKKQQETFKRKFNCINMYLEDMERGEQKQNKKKIYNNNNINSHNNSCNSNIIQKNETKFSES
KGNNKNKKFKSEDSNKLEENYNIFLKKIKEINYNTLKKSFYNFVYPMFKNENIYNFSEHIMHKRNKKCPVEHI
KQHMENAKKVSSTMEYNELNMNDIPTPDYIRFKYNTISNLCNIKECILHRMHKDFFSKNYILNSFSFNLMNCY
FLKFISNYLKYVNMPNGSNSLLIKDGDDDDDDNEHDEEDYDDEKEEGEYEQVHLSKKKYIEDKYKEEIYKEEI
YKEEIYKEEIYKGEIYKEEIYKEEIYKEEKYKDDIHDEEKYKDDIHNEEKYKNDIHNEENKEGKPSSSFKTPI
ERLFKIYQEDIHNNMNNKLKKKKTMKEQIQQKKKNNNISNINHIDNSNNVDNNKENVNNKKNKQMNLEENNIY
NNNNNNNNADNNQNIYNKNINYHNNISNNPFKGIEKDYIDKNNMINVIPKCPLKNPNLNNIYANYANNQNDVR
YQVPFNNNDYLLNNHNFLEPNPDHINNINNNNNNNYYYYYNNNQIYMNNCYMNNKYNNMNMEHMKNYYNNGYN
NEYYNHINLSNIPYNNIQLQNGHFHNINMNNQNMIPFQNINNTLYASNQVKINNEHIINDVHNNICNNMQNIS
KNVGKSSSSDINHEINDAQNILSNSNEQYLKGNYRNNTPKISFHLNKEKNNEQDIKEACLNNKENISKNLENL
RKLHNDIQKEKDVNNIKETDEDTYCNSSSLLRDYNIHKKNMNNNDLLLSDDNFMMSPYDNINDTHILYISNFV
NFINLENNKNINDSNTCAWFNKNVFEVLIDEHKFFSSVTFLDILYEFNVFNVKRDGHVFIEHDNEYVLQGKWC
DYDPNKNNSTSVNFEENFCFEIAISEDNENNTHSVFLLKSNQLKYTDNIQNDNTITKKIWVSEYLGRRNEFLE
KILKRNISNILYECISRKHCLFLLEVLMKKINKPTSYDNKVKGNFSYFYFEYNIKVLCNSENCIFTNNHILHK
NNTYSYKLPNNILSLLVFSEDKLIKGKYHPENQINTTQKVHFPLYPFFVNINFYNEK*
>N.PF11_0222
XXXXXXXXXXXXXXXXXXXXXXXXXXXXXXXXXXXXXXXXXXXXXXXXXXXXXXXXXXXXXXXXXTYMATASADHS
IRIHNLKSLSTIKELYHKKCGHYDWVSEVFFTKRNEVLSGGLDGKLCLWNTIYCCKIQKINKMMNCSEYLEQR
KKVKEVKFKASNNVITCKEMCAHHSTISDMKFDKQNDKCITSSYDKTLKLFDIRKFRELCIYKGNHTCPITKF
LWLQNKIISADKNGCLCVFDVETSQEILNAKNTHTGNIGALNYFYLYNKTEKKMINNNPNVDHEKRIDKSLPF
SSISSNDIKKNYSLKNKNNNNRDKNNQHNKREDVNYDVIYDDNCDDNIVKKNSNRNINLIRSDNLFGQHDNYL
NLNNEKIPLIITGGQNDGILKVRDFRIFHKYVSYKKIHTASINSIITYNLNNKTYIISCSADGYCYQFEIQSI
CSSNLNNYLKKICVNEPILSARYVGNHFILVGSSFGNLFLLDFSDCSENTLTKQPKNIASINEINTNSINLSD
KFNNPLNEKKSTNNDILWAFGACKKGGINCIDCIFVYDSKNKNTTEFEMYNIITAGDDGIPCFLYIPNSYI*
>N.PF11_0223

MPKGSTFFLKTFVCATPVGILLFNEIKRIVNVRENESFNKKLFLKNFDRNILVKNKRTTVLEEEKMYGNKKKL
SFFYNSSNINKESDKEEALKYFYGKAWGYATDYEIDISLNTGDLIFIKHDLDNLNIFKKILLKINRYFQDNND
YDEIGMILKKHNISYIYIQNLLNKKENVIRYSSFLQKYRPSVVSLRRFLCDDENIKIELQENIMNSIMNDDGI
NKRYSIFLNLLYNIFKKKVSINTNENTSNHFYNDEYICKKINSNIEINNLINMLLFRSFNLFYYFINYMKNVQ
NVQWVKKKNYIILNNIYLNSSMQPNQNFNINDKKNLQSYEFPINSLTHFIFIKKDYELMKKNQKIYEKPKKEI
LKEIEKFLSFLIENEYTLMDSTEYMNEVLKNKRNITVPNNTKYNTNKFIKNLQFYINKLYSKLQTFSLINYHV
YEIYRKTHLLPFIKKDYNSISLFLSLNNFYKPINSKNIFQDTFNVPKLSNIFHVRQEEAEKLQRYFYQFNKVP
KSEI*
>N.PF11_0224
MKILSVFFLALFFIIFNKESLAEKTNKGTGSGVSSKKKNKKGSGEPLIDVHDLISDMIKKEEELVEVNKRKSK
YKLATSVLAGLLGVVSTVLLGGVGLVLYNTEKGRHPFKIGSSDPADNANPDADSESNGEPNADPQVTAQDVTP
EQPQGDDNNLVSGPEH*
>N.PF11_0225
MMEELFKILFELVKIGDEEIQNYLKERIIDEKYEIEKNGVDYFYDLVCSYADDKIKRSKIANIFNKHFKNDNI
QNGSNPNNIPNQHVLSIQNNKNLEKLEEVFNFKEYWKDKDMSNYSDPFLGVYEKQINYNTSVPISESLKISKE
KEKQKQKQLNLFKEWVKNKPKIPQPVRVHNLLHCSDVGKNKTKIDKLYDIRIDNFNISIGQRSLLSDTTLKIN
VMNKYGLIGKNGIGKSTLLAKLARYEIEEIKKDISIACIEQELCLENVTVLESVLMVDKLRHDLLLELEQLEA
RKIKLDPNEEILSNETEEKNTQKNDTKNNKHNKNNKNVENMEPIETVESIEQKILDIYEKLNSISYLEAEKEA
SKILCGLGFDSNLQKKKVNSLSGGMKMRLCLSRILFSNNDIILLDEPTNHLDIYTIQFLIDYIKKLNKTCIIV
SHDRDFLNEVCTDIIHFHNHQLTYYSGNYDQFEKTRIEHLLQQQREHDSLELKKKHVQKFIDRFRYNSKRAAL
VQSRIKLLNKLPVVNLEKEDTPFSFSFLEPFYMSNVLIKLKNVSFRNKMFTNLRVRKNKNILIGDHENETNEE
TYEDENELDNNGQPVSKTYEDKYKFKHEYLFKNASFEVDMDSRIAICGVNGSGKTTLIKIILNLITVYEGELH
VSNKANIGYYSQYHVDCLNPIYNSIQQLQYTYSNKNIKEEEAIKYFNKFNIPTNILYEPIYVLSGGQKSKLAL
AILAYKNPNVLILDEPSNHLDIESVQALIVALNMYKGGLIIISHDTYLIKHVADEIYHINNITKELVKIDYEF
DKYTQLLLNNKI*
>N.PF11_0226
MKHTKITKYLTINFFILLTLVFQKYSSCQNSLNYSKNNYGLNDQELRAMLFGLNYDPSKRNKNNKVNRDVIKN
ESSLLLRNLINEETLSEKNDKVVNDIKNMNNSTEKKINSISKGNNNIHNINENQNANVELKTDNILDNTSEQD
DINEKNNDNGDMVHKNIYNNILSDPYDINSTNAYINKSDITNLNYSSNDVINNDKVNKSYEEKNIVNNTELNK
LIESDDHSNKNDINKKTEKNETFNSSSTSDEKKQTDIKGQNKNDLNNEHIFNNNDINNNVQYKNKVNIISVDK
NNTDRDNNNLYETNNGDLKYNNDLIKEGENKRNNKLNNYKFNMNKVNDNKNFNKYTEIYNKESEPEKQNNSNN
NLGIPTLIKKEVHIKNHNTFSSNGKILENKDIDKMSDTSKKNDRNFRSNDIKNFKNNDTKNNATLSEDNKNRY
NITTNKNNEKKEYNMKKSNENEYAFNTEKTNVNNDALKEERNNYKYLNNQTDVNINNLQERDINLYNKNESDK
KLEQSFREEDIKNAYLPENKNFQKTLTNNEKNEDNKIPHIDPSNNELDKKGNYNKYEIGKIKKNNEENKQNVT
VEENINPEKIRKDHEQNIQYSKNDPITDIQNSTNAVLKKIKPTEFENYTKEELQNVSSSEVRDDNLNEINRKG
ETNMFSEKSTLKKGENDWNEYEYFKLKSNELKVLGIINKYSPKGGFSISVNCGGYDDFREIPGISNLLRHAIF
YKSEKRITTLLSELGKYSSENNSRIGESFTTYYAIGKSENIYNILTLFSQNLFYPLFDEDFIENEVREINNKY
ISMENNSLNCLKIISQFITDLKYSKFFFHGNYITLCNNVLKNGLNIKKLLYNFHKKCYQPKNMALTILLGKKG
NSHDHYNMNDIENFVIDIFEKIKNYDYVNESNNKRQKEKHIVNFKDDTFNIEKKSNYKDSRLVHNVTQNNSKD
KEEKIKFIEHINEFNNYVLDLNQKGRYIEVLKKEGWRDQIYLYWSSKISIDLYKKIEEYGSITFIHDILLDLR
KNGLYDKICVENQYAYDLKIISSCNKYYVNYGILMNLTKKGKKDLRHLMHIINVFIKEISKLFDHDSLNKGIN
KYILDYYREKALITDVNYNNDNKYIELNDLINYSNILLDHSDDSSLILSINNLIEDKNKNDFRNHIKITSLLG
SLMKNENTNIINVVDTFSIRNQSKIPYSNVTYVIGENPYMVNEGNIVNDINLILPEIKICPFNSLVNNKILFN
EKSFFCVPYNSSENFEYSESEEKFISEENKHIFKSNILYNIPCLIKSSYGYNIYFKRGLTHISKVKTDFIFYF
PSEKFTFYESVFTRIHIIILQKKIERFLSDYTTCSVNANIMHDAISYTLSIESNGYFFEEFFNKIQELLSLKE
IPSRDEYNEAYDELNIIIQTDTTSGVDKSLKIMYSLFNKYTPTNKEMYDILNAYFFYPSYNAYRTYVNEYFLR
NYVVIFIYGNIIISDLKGEENITKNNNNIFDNKKSMNYNEGDATDKNNNSNNNNVESANDSTNYYIYNENNSS
NRDTNKYTDNDYNNNNNNNNNNKDGDKYLINEKIYEGEENKKNPTTYLKKQEQFLEKQENNNKEEENKSKSLQ
ISYNGSGIEYLVKLCESFISKVTNKVIKKSESTYYTKKLINDEDIEIDMHDPGQDLSNSITVSYIIDSETLLN
NVLINIIVDLISSDFIKFVKIKYNDGYVVEVRTFFTYNGLGGLLFIIQSFDKDVEQLESDICTFVKYITFQLL
NIDISDLKKQLQNMKEHYIMNNTIFTFNQEYSSILDELITGHECFDKKYKIVQIFDELINCPNIILNKINYIL
RKSKKNIFKEYKKTNIVNIQSSNKDGTKGHDYLHLNEKCNYSYRKNMKMSNIQFSDNSELFIKKQRKKKYKYI
PSNGTTQSNNIYKKEHLFNFSNFVEIKEKGFFKYIISYFRKNNRKYLNDDNYLDFESCDEEMSKDNFQIFYNF
TNDINKIREYFRGKFTNDKEVKENCSINYEEIRKYCYDHNINKDNMIRTKIEI*

>N.PF11_0227
MKKGFLLNKNIYDIEENVINVEKNNTNKKYCKDDFEIYMHIGTGNFSDVFMVKLKNDPSKKYALKIFKKEKVN
KMNKVNDVLTEKNVMSKLNTPGHANVIKLIETFKDKENVYLLYEYADYDLWEFLKIRSVGVNEKITFNIILQM
VHALIYIHNKNIIHRDLKCENFLINKDGTIKMIDFGSSKDLDNISIKTNNEEDTINHEELSKFVLKKNNNNNS
NEDLKNANEFKNNDSLNGEEINNNDLNNTNFQKSDKNIKDQNSNKCVLETLKNNESYEFNNQILSSFKSNDYA
ADANKHNSYKKKKTFENYVGSPNFIPPEALINKCSGKARDFWSLGCTIYQLVTCTVPFDGSTEWFIYNKIKRR
ELKYPSIIPSELIDLIEKLTTMNPEERLGFNGGCEEILEHLYFQKYNYNKLNFILPEVSELEKLYTTIINKYH
IYINEKRKLRQNNNSTEENINNVEVLKKNLLNLINSDTLVCAEEEYESITLKKKIFKSINFMLEEFDKQEIKE
MEEASKWLERYQGT*
>N.PF11_0228
MNFSLIIKRYSKILNWKHDTYYASLMNKYTYFDEFMRFCNDNKEYFTKRDIVASLTYIHHMKKVDLLSPTFNN
YNDFIYKNINMFRSNISTLVHRYAVLGHTKSLLFIYENILKNNILNYDNKSISLIAWSYAKNNFYLSDLFMEI
NKILKACIKTMTLHELSLISWAYSKINRLPPLEMICVKNRVYELLNTLDEELNHVIENSATKTHLDDIKMNIN
EKSTYFEIKNENEKEYLNKYTNTDFNDHTYLDETKELISSEKHNLGNIYEDNSENRIDNLGCKKNLSQDICMI
MKSYSIMNKSDPFFFLFLFNFIIKNIQNNKMMITAQGITSIWVCLREYEIKNKKIIEYALELSRFLRLDHTVN
SSMMHEIVVSIHKLKIKDRRILYHLFYHLKRKCINMHVQHLYDIITILQDMKIYDDDLWKQFGVAVQKKAIDL
ELIQIKKLHNIFSINGKGNDRILGVLDSFIQIKEDINVYGPI*
>N.PF11_0229
MKRIIYIYLSIIYILSFVNSYLIDESINLLPLENDLVHVKLKLTVNGKDYKEFVPINILKILNYVNSLKINIK
RGVYREYYNNKYLDSYPYGFTFVVDLKKEERDKYDFSKEKEYNEFSKNEMFLLKTLLNDIWGITGVSTNLLKT
KDLIKVDNKIFAFLPDESICIESFSFIKKMYPCKNFGGLFNLINPSYLITTLSTNIGFELNDNDENFFVLYIE
YITEHNHKENITINDLINSKYNINECPLIDRTALIVKKKEEDYVSTIFEKYDNIAMVYEKINIYDILQRGKPN
IIEEYINVNVYKEESNSMITTDIRKKQSSLVYVFQNLNDKNSSNFLFVDKLPYYLSPLLHTLVIQGKDSNKND
DSKKYCDFIYFGSNAINKFDIKFSNFDMIHSLPKNGNYYYINFKYILPPKCDIIIRFEIIKIQIRSFEFDFDI
DRGILLESGIFKQKKNYISNEKNDDYIYKYTSSLLIDIVLPDTSMPFNIIAISTCVIMLFFGFIFKLTAKEQT
RYV*
>N.PF11_0230
MTPNNMYTGFCFHDEKTCEIDRIHENITAEKFYVDYILKRKPCILKSEYVIKNKLNIDLNFMKEKIENVNVHL
EKRISNSFGTGEKKKMKFHKFLSLLEKGNKKYYLNTQYVKENAYHPKDFCNSITRQMINYLPKELEIMGNLEI
YQYNIWLGNNKSTKLKTYLHHDYHDNIYVLLKGKKTFRIYSPNFAYRLKTNGKIFKVHKNGLITYWPFIRSDG
SHILDVYKKQMNNIYNEINIMNEVLNKKDPLVDNQTIKDSINKAEEKLNLLEENILNYKLKKHDFHRQPNARQ
DIPNHFCLIHTRKRKEDDNIFDKNTNIRKKYIEVNLNEGDILYLPCGWFHEVKSFSSEEQYHLAFNYWYYPPY
IKTNKSANVENKFDNPYVDKYLSERNIKLYKKIGIYKQKNSNLTEILSYYKNMKIKKIKKNKKRKNGLDLKKK
KKLRINNT*
>N.PF11_0231
MTPVTFECISVALVSLFFIKILAYIYKQKCEKDNSKVKKGKENNNNSTMNVKLDIVCDITNGFGKYYTKEVLK
KKNILGIIPVYKSYNKEYENVDKFNGFMAEFFKFLDLRNGKVILASDNNSINNYINPLSKIEDTENENDEEDI
LLASCIIYEKILIIVCVINYDMDIEKQLDYFLKNQKNDLLIVINNLFLNSNLKDEKNYKNMELLEKKCSLVPK
GDVNNLLSFFDFMNIKKGCISDLYSIIQINMKNFRYFYKLLGNTTSGTFFNGHVYDNFMSTNIYNSLMDIYDD
LISETIKKDKKKINIKKFYTFYCVKCDYKKIVKLSLMKLENNFFLFYIYNIYSYIYYQLILYVSQKNSV*
>N.PF11_0232
MENNKDNEKKGLETKNETIIEKENKEVEEEKEKEFEESEYNQSIYNENDDIIDNKKTQNVHLENNEDDNVENQ
CEVYIHEHSIEEYDNKDDITGEEKRDSLKKSPLQSEDINSNDSVYKREENMMSAEKKKDEENDDKNIKNNKRK
MSEEMITMKSTKKLKNGESNKNDDEISYDYFDKNIDLEIYMNKIKNNFNLYLEECEYQLKDLKNCYIENDKLI
KEIVNENNSTMIGNNENIDDEFETDKIKNGTKKTTKTKENGEHVEDMEEIRKKNELKIEKKRTILNNIMNIRL
IYRTVEIYLFSMRQFIYLLKEKNNFISHILDEQIIIKNNYEQVIENLLEFEADNYEDIKHIVENKILSINDML
LKNNEYDEKLKEYMSYLCTLQCLQEEISQRIDAKTDLRLLMIKKKEISDKFTKQNQRKDTSFNKLSLFKNSIQ
CIVDKYNSFDKELTHYLSSFYYKIMNHQIDLSINNMSILNNKMQFNFFFHFKEQSNINDVFQFMKYDVITKDR
NNDQNFSNRNDVHNEYISIKFYTKDNFDVSILPDVSYLNHFNIQIDISYLCKMDCLVAKANSIDYLRLYNNDC
RLLSDIYSNEDHLLLFSNNANDFLNFKYGFPYFWINALVEKPYSLSEKNELPKKEEGSTKKAENKNMDRKSLS
EIYNWHLNRTLDISVVFSKVFTRVLFRIWDIWQIRHYRNYPNVFPPFTCEKHMDALKKIQSVSLQQISTFNII
TEESYLKEEKEINNECSKDNIYACCHIELSTPYLVKAYINVPCNGKEPYFSVFLTKKKYSRRLKKLQDYLNTA
VVNKHSKVDDTQRQIILTLQLAKLREGAYKYYKSFSKNSSSIFDEEIN*
>N.PF11_0233

MQNFVDSDYKKRTSNNNETSERYINSIQNCDKDEKRILEDENVQKNLSFINNENIYNKKLSNQLKTNDNHNNS
NVNTPICSVKCLSNKNISVEDLNNEESNTQKNLEKAVNRRKKIKEILLEYNSLNLSLSSDNIDISKDKSINHN
NNNNNNNNNMNGKDQYNPITYKTNISEEKYCNTINDKISSKKEASNYKSYNKCSIKNNDTLNYNNYSNNIDNN
IHNLKSIINRDFLDKSLVSNDDEIDDYKTFLTLDSNQTIFKNKPLKVPDLSFLNNSSRTFSNTDFLHMDNNNN
NNNNREVYNEERTNFVRNKIIYEKLDGLSFCYDNDSKNFNFPPGQDDHDKSIQLQKDHKTGILYECNNKNYNM
SDKNLNIFELKKENKNNIVKNYIKINKVENSNIEQLINNHMNNSPNYILDNKKYDTDGKNYNVDNNIIKMHNT
KNLNNPTDYNRNCRIIYKNAFENFHYNNKNTNVNRDNMYYNNKDIERDVEVEKINYINKFNNSSSIVYPYELK
YKNAIISENTCKSKNKIYENLNYANIEYNKPTYNKNDFSQDEETYLNFSYMKNIEKFKELLINATRDNLNEYI
KCLQNINNKKLKHYKHYLKQVNNYNISQYFNKTFNISTPVKNMKESKLHKSDTFSSLPKSINESDELSTTTYS
ASSTNNKSINKYHKNNCNQIIIDSSCCSSSISSDSFCSNFTNKSVTYGNKMKIYKNKRKPNFNSTSMLLPSVS
KQEIISYPANINNESRLNLVYKNTKKEMYYNNNNNNKNTYYSSFNNFTPKLHKSIIRKSDFPYIKEKNSFNSL
HYINNEKRNKTYKINNNNYKHIDSMNYIQNNIYPYKNYNKNKKRNFFFSFNTDSEDYNNKYLNNSSFSMLRSH
SYENHFKHISYDNHLNDPKKNYIKYRDNNDINNNNNNNNYYNSHFGNYNHELIQHSNDEKDSNENTYNNINRN
IFFNKLESAENEKENENNNNYGKVDSKCSETSFHKNKNEIMNQHILYNNYHDVMNKNKIYEMVHANLNEYDTS
NKNYEFNKSIINNMNNMNNMNNMNNNINNNNNINNNNNNNCLSNKKDFAQNDEYLFNKSNDEEMFVNVNHGSA
TTLLKITTINNIESSIKNVTNNYDINDISIINKKQEKCDMNEENVSNVNFEENEYKNFIIHADYDLSENKNLN
DISDDNKTSTREEKNEHDSFINVRNKENLHNSLLFNTKEKDMENILDSLNYNSQNGSNNKEKLYRKNEKDNSI
ININDNTNEIKKYAQSDMPSDIKSGMKNDNKNNINNKYISNEMPSNILNNIQKEIQKDVPNFMEYHNDRPFKM
EGKDIREIMKNNKTRIENLYDNENNQSDLCLSENTSEWNYNINNDLENKKNKPPFLNMYNNMNNSEKDVSYSI
NSYKEETTDKNMIRYSFDSFNEEFENPISEPTPAASIISYISVMSPTNEVLKEKNTSNMKNEHMNNIKDLSNT
SNEMNDEIPKNDQLHYKNNHNYQNLSNNEEEKELYINRKPLNKYTDYMEQYHKNNISDEKLLNLNLFSKREDI
SNYNYNNNNNSNISVDNNITNMYKYDHVNKKDIYNIYDTLSEYSIDNRKKSINSIVTNKINSLDKKSIIINNN
NNNNNLLVHCTDLFSHHHNNNFILSKTNNSSENLRTNLKLLKDSNWMNYKKNVNSDIGHKVVNTLEIKQVKIN
TKQGVFEITAEGQVMFTFLGRSEIKDYKNVKKKNINIDISKPRKLLFIIEMDGINIYIKDVLMNIVLDFYNIF
EEELPKAHKARYEYAYNVVETLKKHTPKVSLIKKWFGIYNLMSNGSTPDFIAVLNNNIFIEKIEIKNNNVTFI
LKDQNSITLHIDDLNAVTTNINRKINEKKERSIGDEQDTVFKEFTEDNEDIKIVLMNIQKMLKKTGMSIDIII
QNWCNTLQAYSYCLSLLTKGNAEYTMKIKKTLAHITEKTELHKRNIYFSIFPLILEE*
>N.PF11_0234
MESCRMYGLEFNSFIEFDNYEGGVKLTKRLHIKNTTKNIIRFRFLFQSSTYFTYPVQEIENISPGLNKSYLIS
FLNPLNDLQTICEILNILIDDKTKDKKISIQLKATPLKCDIIIPSVLNLNEMVIKQKTKEDFVIKNQGTLNAI
IKLSHKIVSKEKKLKIKFDPSQFILKANEKKSIKIIIYSEIPQKYNEFISTSIIEIPKHIEKNIFQQEEIKNN
DVKDTNKKKEKFEKYKNDDNKKKHFMNTTINTNETEEAKNVNDIIHLLNEENINVCLIKKKTEINFLSVLPQI
NILFENNKEVFNKSILNFGKITIGQKVKKTIYLQNLTNSPIKIIARKQKEKNIFILLNEKILLKEKNKEEIII
SIDSSNPVNQYTEIIQFIACNDYCVELFLICEIVNIKLFFSKTLYLFENVKIGDNVNEKVTIRNDENADLDVD
VINTGNFINIKNKKFTIKKNSFHSLNLICNCIYPINIYKRIYFLVHLNKQIFYIDIICNFSINYKMCPLSINH
IYRYKHLIHDKETIYTSYYEKNDYIDIWDFPVEFDTYEDTAYDLLNEIMEINQKDIFIVPKELEVLEFEEKDI
MIMNKTPIEYTCVWTNGLNNKKKEHNIFEVTPVESQLLPFSYQIFRVKNIKVLKEKYIREIYECIVFPSNNKD
YRKCNSKTLLPPLFLYVTFFQFKIKYLCETDNVMNSLFFSPKQIFFLNMIEEENNYTISKFENNTDITQIIDF
TQFKNDVESIRIYPLINYVPKNSFLNVIIFYSPQKKNLVQSEIKISYLINGIEKNYISVFVSHEMNNVMLNKG
ELDINLPRVSTETESIKKVPIENMTERNVLCFLIKKEVDDIVNIHIEGKKNFSEQQEEQTDHNIKEELYKEDI
LNDIENQWENNQLTSFKEEKKIYNFYYFSLLPFEKKNLHIYAYSNTYVKKSIPLFFTYLLYINNIDMKNKIIY
LNENLKDHIKSCKKFNINVNIIKCCLKMYPKIIESNPITSGTKFNAKVRIQNEHPVKINYRTKTKIYQIDDVK
FLDEEETKEAEKNIVTEDTEDMINSCSAKYFYVSFNTNKKGRFVYRFFVIVGENENYIDIIVNVVIPYFQIID
INDFKTPTSIYWNMTSIDKINMYLKDNISNIDMEYKQNQGIENMKKLFNNFNYIDFNIGNNTLNEITSVNLIL
YNPLDISLYVEINTIKSYILPILPPYVKTHEDEIAHILYVDNTFNNFMRCLDSCEISPTKFTIKEKGTKTVTL
FYKHKYIGFHNMPLIIDIENGKVVPLNLCALTFHPNIPPIYLMNIKELNEHILGIKNECILNIDMLNDSEVDI
YYDIEKNNNFMVLNPKGIIKRKKYLSVFILLSKLSPSIINDTMIMNSYFKHLQKNIELKKIEIELTLNTTLDN
IYKDQYKKINLFNNKCINDIPDQNIKTFCSNFIPPYSYIYVQNKLFYISPSSINILYAPTNSLIERIVIIKNY
SSLKDLKFKVSNKNTLPGSILRIYPNKGIVKKEEQIILRFTFILNDVLIDIEGNIQIELMFVETEIFEMQKNE
QVKYDACDVIETNIEEVYEDTRDKTESTENKVLSLSERKKKKRFDDLTFSHAQKIFDNIQKFKYTYDNMNTDM
LQNAIRRLYGVKNDDIIEKENFKEQEKHDIQTMSKFYFYIHVKLFTCNVDNLKNKKKMFENLIETNIYSKSLY
FKKYINLPIPFEERSKSFFKRHYLDFDKLEKLNVEDEKNKDIPINEKFIYQKKDIYCCMFAEMFKSIIRNHIK
KYITYLLKINTCSIQEFENILEDDLIDIMTRNKFLNSNLPVEYDYTFLNPTIISHFFSHMFTDIIDNLMNEKI
NFKKRDD*

>N.PF11_0238
MKLNKFFDRVFLFSFIFFNIKCIQSISLKTPGLYFKNDIFYTNRNNNTYGFIGFSKKDYSFPNLNKTKVYSNK
DILKKFNRMNVLYSQDSEYGLREYKNDKSLWKRFVNKMSEVNKNVLTKLVCAGTFCLALYPVYTMLLNSKIEY
LIKNILKENFLYLNIPKSIKRYLFFISFAEFKRSPFFLSTMLIASYTLYVILKVYIEKYRESKRIKSAIEDYN
KNKNEYINTGTDSSTENDMDYYNNMDDDDYNKDFY*
>N.PF11_0239
MVLEILKNNYKGNGENYYNKNKKHQKKNKGKINVLDDDEYDNENDDKNVDSANNSELCYLYEIYKNGFNKEEN
NGIILSHKNNKKGLIYKNKINALYDNSKEMLHYRNQENEENDIFEEDIYENDNFDDNNFVEEENVEYKTYNSF
TNEEANMLNNYSRVNIKNILDSHNINKRELYNKHNNNNGDNNNANKKKNDQKNFNKDDSNIREYSKNNNITRN
LYKKNLLYFHEKYNEKDKSTYEYHIDACDNFYEYGDEYNSNHKSKGKNIFEIKDVNIDRKGTKKFQKLDKNKE
TTKDMDYDYYDEDNVDYEDDNVEGEDDNIEDEDDNIEDEDDNIEDEDDNIEVEDDNVDDEDDNVDDEDDNIDD
EDDNVDDEDDNVDDEDDNVDDEDDNVDDEDDNVDDEDDHISDEDYKDDKEDNYIKYNENDNVNDDEDNYYDEE
ENNYNNDDNYDEEIKDFNKYLKNNDKEESIKNDVNKELSHFNFNNDLKQKYIDVKGFKNENIFNFKNIIKEDN
NTLKREELFSFGNGKKLYNREENKIEVATFGMCNKNEWFEKYDVKIENCKKYNNYKNENNDNKNKNPFGFKGL
DKHIEEKEEYDKMYMDGNNYMKGKLIGNISPKIQRSHTDNNIENYISDSENMNTYNIMNEVNRVLPYNNINNN
INNNNNNNKLIYNQGDTLNSIEHTNNISKYTTVDYSNDILKTGKETYKELTKRKNEEICELLNSEDKKKHNIK
HFLDKMKSKKHIEDFFKKEDKICERSEKKEKLKIKFTDILHAGAFGHFFHRNKNGLSRSFVSSDVKRNNSFNS
INKINKKDQLKYNTLEKYETKIGAFDESKLGNVRNDKTVSYNQIECKNSINYNSKTELNIDKNKDFNFDKNKW
YKMNDNEEKIHFLKFPNGLLDNDKKKELKSNDNGIDDKLLSYVEDVKYKKYNDMSKQKNGIPKQIVNNRRNMI
NKDYYEKKYNIKVNRNNEENGVDEKMLSVHDGVFNKNDFEYGGKIKGIPNFPLNIEFIKQNGRYKEKGNEKYE
NDLEVNIDQTLSRKMLENNKLKKQNNIDEDIMVTPFYIKSKIDKVLKNNEIFEKSARATFQQFDVKNKNFLHF
SEIESLIQKLCHNLELPPVDKNILSIVYKDYDSSKNNRMNYTDFRQMYWDLLKQIKKKYYPTKNFKIKRNCII
SRKKLGGYDYSSIYNYLSFKKILGYGAFGEVHLVEDNICKLYKVVKILKKKSMKHIKINEEINVLIYLDHPNI
IKIFDVYENVDCTYIVMELCEGGELMSRIKNSESFNETYIKNIMFQILCAIAYMHSNNIAHKDLKPENILFKE
KGDDTLKIIDFGLAELINKSEGISKTAAGTVLYMAPEVFKKKFTIKCDIWSAGVIMFFLFTKSLPFGGNTYDE
VKQSIFRDEPDYKSLKSKLSQTALHMLKLMLQKDYNKRPMASVLLHHPWFQGYFDPIQISPNVLNNMKSYMKH
SNIRNIIINIMAHELSVINNHIKYINELFYKLDTNHNGSLSHREIYTVLASVGIKKWDINRILQALDINDRGN
ITYTEFMAGCYRWKNIESTFLKAAFNKIDKDEDGYISKSDIVSLVHDKVLDNNDIDNFFLSVHSIKKGIPREH
IINKISFQEFKDYMLSTF*
>N.PF11_0240
MTKFSEGHYDILNLIEVKSNIQEKNILGYSIDYTVGGLFLFGGFKIDIEENNGIICNDAYLIHIKNDKAEYET
LSIKIKPSIRCYHNSCTLLENYVIIFGGLNSEVPFVALNDLWVFNSLNKTFVEIKLKCKEKNKEKNLQNGING
TNEKGYISQTDDENCSDNKYGENQDYGSNDSDSKDGEDIDKDDSILDNSYVKNLRKYLKSNKSDELHSSDIPC
PRYFSSLDLYIIRKNNIEEEKGFKKDEIKIIKNNIIVSNSLNYEYFSLILFGGYGGYDRSSYNDLYEFNILNN
EWILRGCKGNVPSTRYGHISFIHGNNLFILGGTNAEISFNDMYSCDLKNNEWSEVDFSYSFNISKVFCRSVLV
ESIDRNIIFIFGGYNIISDEKGNRKIEYNNIELNMLKLYDTFKLEELKYNIIQNNEENIIKNNNMEEINNMNS
NNVKNEIISTKHDIYEDSNISNNITFCSITYDFMDSNLIITDNKKKIYIMNISNIIGPKYAIFNIWPKQCDIN
GNKKLLFRGKGFTNEGKILVKYKSDDINLYSEGIYINENNIYTIVPNAKNKIKNNICQVQLSINDKCYTTNSC
FLEYYYNIDPKNTLIFGSGLLEPVCLQKDNIFFLIAKNSLNEHKKIGGDKFQISIIYEQKSEQYDIKYFVYDL
NNGFYIVKYFSFLNDKQMKVKMQNVGKNNNIEQRNNNNDNNDNNNNDNNNNNDNNNNNNNNNNNNNNNNDNLN
EQLNCNVVQRKNQNDLETLEGPQNISNKNSMNNEDIKSNTNNHMEILDLNGTLKITVKLKDENIQNSPFFLKV
DNNLKDKIIYVKQFINDRLENLLRKGECLFDLIKNKNMNTNNLIQLNINIDHFLNTFPKAIHDINIIEQYILY
GLIDIDKIKNVITNLHEEDNLVNASQEEEIYIDKEIHELINNEKFKNDQTDEMGEYTATTTNNVKHTNNTNNN
DTHITDTLTHINENNDTRNNFLKVKDNHKIVDFINENKLKFLDYIDPNIMKKLRNYINLLNTEELNLKNKKDN
KKIDDSNKKDLLGIKKKRSEDKFHLDNNLEKDEKKIEVIKNLLIFYHKLKTVCLSKKEKELKDEFIKEEKESI
KKLKLNYNKFVNIKKNLEVSNVYLHNNGYDSSLKELETTKKYLIEIKDEVTKIKQISDNIIKIDNVEELNKDI
ENLNNEIHEIEKMWLFIKKKEEILNEFFFCPFKDLDVEDFDIQIKKLQNDFKKIKVDRKHNICKEETLKLKEI
IKFISVLCEIKKPFIKDRHIKEMENNINEEKEKNKEEDKINIIIDDSTLTIYFYKLNIMKYHDTIEEVIIKAY
NEKIIEETINKFEDYWDKIYFKKKEYKNNILLTYIDDICIDTIEEHQVTLQNCFSSKYFLFFSDELNLWQKKI
SNIYEVIQLLKDIEKLWIYLQNMYIYSEEVKKELPLYSKFFLTINDEYLEMLKQIIDNNIKVVDFSNEGGIIE
KLEELKVKLCKSEKPLNEYLDSKRKSFPRFFFISSTDLIDILSNGNNFKLVNTHVQKIFLSIRKFVTKNEQLT
DNEIQNEVKLNNQETITEEKNKNANENSNEIETNKYNKKEELTNNRDGDGDGDDDDNIKNDKDEKDEKEETII
KLISSYGEEICNFHEGLVLKGKVECYLNDIIDHIKYTLKYYITNLFRLKDLFNNEKEKWIDENYLAQVFILCN
TIFFVNDVENILIKKDINIYEELNKYYKNHILQLENVIKKVQKKLTIKNRIKIMCIITLDTFYRDVLEVILKN

KSSISINMFDWQSQIRMYPFFKNQKIYEQNENQTEESNLKLDNKNLDNEHQEGKQEYNNKNNDNDNNNNNNNN
NILNNELSKYTCLTHFKDLYIKIKIMDCSFNYSYDYIGNYQRLVITPLTSRIYITATQALSLYMGCAPAGPAG
TGKTETTKDLSSFFGKNCYVFNCSDQLDYKSMGNIFKGIGSTGCWCCFDEFNRLIPEVLSVCSIQFKSILDCK
RNNNNVCIIGSDEIIVKKNCAVFITMNPDYLGRSKLPESLKILFRPITVIIPDFNKICENMLMAEGYVNAKYL
SIKFTTFFELAKSLLKDKHCDWGLRSIKSVLTKAGDLKRNYPDVDENKLLYSAIHDINIAKISSSNCPIFSGL
LNDIFFSNQNDITDINDINDINENKKEKDNIEELKSDNVKEEKKTKKKHLEDNNNNKKKELFNLNNIEKELMD
ICKKNHLFGLNYFVKKIIQLNDIMNIRHCVFIMGEAGCGKTTLFNMLMEYQKKQNLKTVSIRINPKSINIDDL
YGNVHIKTREWKDGVFSKYMRNYSKKDDCDKAYIIFDGNLDSHWIENMNSVMDDNKVLTLSSNERILLKNHMN
LVFEFSDLMFATPATISRAGLVYFSVDPNDLWKNYFLSWIDKHDNFNSNIKKLFEKLMYKYVEPTFSYLSTLQ
TSIKISPMSHIQSLSALLDILLIDNNYESVEHYFIYSVIWCFGGFLGEKDNVNYKKSFDKYWKNTFKSIKVNR
KISVFDFYVENNKFKEWDEAEITNELKQNYVLQDDIFIETIESYSYKYICKLFLKSDMPILFIGKTGVGKTQL
CKKILNEEKEEFKSFYMIFNYYTTSKNVQTLMQSCLEKKSGKQFSPPYQQKLIYFIDDINMPKCDDYNTQSAI
ELLCQYIDTNSWFDLEKLNLIKILNTKLISCMNYNRGNFTINPRLIRHFFILNINFPENNTVNSIFSVLLKNH
FNNFKQDVSDLIPSILKSTISLFYNIEKTFKRTATYFYYEFNLRDIHSIVKGLLTTTPVTFQDCDKLLFLWLH
ECERVYSDKLNKKDKNKYKKIITDIIKKMYNKYEINKFVMKYDSTLLFSNFHKGSHDKTYDICKNMEELTLFL
NEELNEYNNSYNLNIVLFSDAIKHICKLIRIVDNLKAHALLLGIGGCGKTTISKFSSYISSKTFFEMDFSAHC
TDNDIKKYLQNIFHKCAMKNEDIVLFLKESKIHDTFFIYVNEYMCSNNIIDLYTKEERDYIIHNIRNIAKADG
IEQSDNNIFDYYIKKVNDNLHFILCFSPTSNNFRDKSNNFQCILNNTMIDIYDNWEADSLMCVGKNYVSNIYM
NINTGDILLDQEYINLKNKNIEKDITLGNKQIEKNYIPTISTNDGDDHYKDMDKTNMKNGDITTTINNIPIDN
NNNNNNNNNNRDNIDGNNFFKNREGNDENMKRKVYSNNFTNQNDLNTNITNNNNNNSNNNNNNIYDKNDSILK
EEEYVNLKDIITEYLKECYEDLLDISSFYYSHERSHIYITPKLYLESIKTYHIMLLKNITNINNKMNMLKNGI
TKMNETSSNVENIKNCLKDKKKISEEKMEAAEKYAIDIGNEKMVVKKESDLADIEEQNCLEIQKKVLKQQEEC
ENDIRLGIPLIEQAEEALNTLNKKNIQELKTLNKPPPGVEDITAAVMQLLATIDTTISIDKFGKIKDRSWKSA
QKMMINPEKFISLLKDYKNKIDENLVPDCNFKYVENLINLPHFNKNAIQKKSKAAAGLAEWVLNITSFYKIIQ
NILPKRILLDNTKKGLEEANEKLQIVREKVQSLKAKLSELISQYDHAIYERDLVILEEKKLKTKLELSIRLID
ALSSEEISWSKQYESLKKKKKTILTDILLSSTFVTFCGGFTKKYRNKIMTNCVDTLKRKNEIQNNIFNNMLKK
INNNDQNFINNNNNNNSSNNNSTNFGYNEDPQKKDNHNNFDISNKLNIKNEKKDNELGNDNLKKEEMIYDIFL
VNNFNLDLLINEEVLSKLSKQGLTLNSVCIENNIILENSDKFPIIIDPQMESLKWLINSHKEKSEKLIITDIN
DKILLKKIEECISFGYSIIVENADEYIDNTLYNVISKNIIKRKNNYYININDKELMFHPSFYIILHTQLSNPH
YQPEIQSACSLINFTVTPDDLEEHLLSITLENEFNHLSKKKKKLSLLKYDYMCQLSFLQSSILQKLTDAKGDI
LEDVSLIENLEKTKLLSENIAKKTEIVKNTEVHINTIINLYRPLSKRGVMYFFILQKLKNLHSFYFYSLEIFL
KIFIKCLNDSSPNRSPSKVNQEQEYYLKSEDNDFNDDYLGSTKEEEKMEDEEKMEEEKVDEEKMEEEKVDEEK
MEDEKVEEKMEEEKVDEENMEEEKVVEEKMEDEKVEEKMEEEKVVEENTEDEKAIEINTEDEKAVEKNTEDEK
AVEKNTEDEKAIEKNTEDEKAVEKNTEDEKAVEQNTEDEKVVEENTEDEKAVEKNTEDEKAVEKNTEDEKVVE
EKIEDEKGEEQKAEEENVGIEEVEKVQIDDYKVSEKKGENKNCTYEENGKIDKDKEDDLEEEEDFENDDFTNE
ETKIDKNEVEKRVNMLTDLLNIKMWMYMDKGLLERDKLIVKCLIMLHLEKLNDKISEEEEEIFINPKYKLSNN
NITSIRNKKENESMEKKLMNKSFINEELYEDCKNLENLKDFENITESFESESMSWKQWFLSEKVENEELPRKY
NNIKDFSKLLLIRVLRKDRFLIALKNYITKNIKMTNDEKNNTYALENILDEYIDNKTPVLFLLTTGYDPSKEI
EDYINKMKNNAIKKNDSNKKESNKNNIAYVNISMGQGQENIALKYLKEISKCGGYIFLQNIHLMTKWLKEFEE
ILDKIFLDAHVNFRLFLSAAIPNEKDTKLLPEKLLKKCFRINNEKSYSLKDNIKCCLDKFENGQYDDKLKTVI
LGLSYYHSLLLGRFLYGKIGFSQSYSFNDNDLEISFNIIKRYLKTYESFPLADVLFLIGEIIYGGHITDIWDR
RINKTYVKNILKEIYRNIISINEKNKNINMDFHHPNDSNNNYNDDDNNNSHKNNKNNNNNNNKDDDDESNNSN
DNEEENEEKLILKNTKHNILFDVFKFPDCSKYNINQLKKYIDEKLNKEQTYLLGLHINAEIEYMKNECSRILQ
TLQELSNKEIASTESYKKNTKIIKKEKPGDNKDNKYTHDQKKETIHKEEDDEDEKHSGSNKSTKIIYDIINHL
LNELPDKIDTNDLKIEDSQTNTFMVIALKEAEKFNKLIECINDTLIEIKLVLDGILNMNDKIQNTIKSLMLHN
IPHIWINYSYPSKKKLMPWFENFKLRIIFIKEWISKIRNNIFLPNSVWLSALFNPISFLTAIKQKFAQENKVP
IDKLKLKWQVTNITKLEDLNNKNNALYIHGLYLQGASWFINSKNDTFTFDKDNINDNVSYGNIIESVPKHIYY
SMPLIYVYCISNEQDELLKENMEYRSLDTPLYVTSDRGNTFVCSIDLNLEMEDIEDKWILAGVALFLSDD*
>N.PF11_0241
MKRNKNDDNNYSDAKSKENQNKNIKRYDDKKKNGDKKKCKNKNINKSNRGINKILIRSLRKKLKSNLNKLNEQ
HEYLEQILKNINYLKFDHENERQEYYNELKNISKELKYAYEINNDDIALLKIERDLIKSEFTKSRKSLKIFNY
HIPEECLEDEDMMGCEKEKIGKCEKRKKKKRRNNTNNSQNYINSLTPLQNFHANTLCVKCKCDHHRFSAEYLN
IKNSVVLDLFNTLSKNKYFKETNICEQSFFYQQSNRIYKQEKKKLIKNGMLPCSFLLKKKNKVQPKNNKEENQ
NQFTGDFSNLCEEKENIKNVHSIEDNNENKVTNNYELRKCDILNDHISNVQIINGDKVVNHDNMLNDNHLTNY

DNTMNNNNNDNNNNNNNNNNNNNNNNNNNNNNNNNNNNNNNNNNNNNNNNNNNNNSWSNNFCAFKYSNEKYSN
NRYGSHSNKTWNESNNVNNYISNMLCENNKNKVYWFDMNKYINKDIKKYNVRNNIHNMLHFFTSISKVYYCEV
VNKKREKILSNDDYKNNSMEKFYDYVFADDSCDVLSNDDMASEQNNNINNNNNDNNNDNNNDNNNDNNDDKNI
NKNNNNNNNNDNNNDNNNNNNNISFSNNEDYYNNESRNNYIYIYIKREYIPEYLLNIKKKIKLILLFSSNDID
NNVHNKNCPFYSKRKHDNDQKKNYDNELYDSNNIYCNYCNNCVEDNVNYYMNPEQKKKRGRLNSKKKNIQKCE
KKYKLKKKMHLNFIDYIKKEEKEKRKKQKQDEQQVKKKIQTKIEKHNDSKEDDMYDPQYENDDDDDDDGDNDL
NKKNTYPKKSCPFGFICKYKHFFNKVKKHKMKRRTVKKHIKYDHDNDYDHIGLDDNVLYNNIILNFLNKLNIE
EENEKYHYFYVHNIEYDNSKYDIVSIRKDMERILRKKEKKKITSFSPVLLLNYVNKKYSFIKIIKKEKKKEKK
NINKNKYMNNEDNTNNINEHNNNSNENVIKKKRGRGRKRKRKNPLPNEKQTEGDNNLLNNNEEEKGENQKKKK
KKKEKKNIEEKNNSTFYNNNNNNNNNSSSNNNKMKEYKNKKIYIKRKNRIHKKKRDFFFDVSFSFEGMKNNLI
KREEVEVHYLTINKYMLSFNDTNDEITLKYYLSHMQNRDFNIEIINKKMPIEILHFFDYYINRKNELDKTIKE
HTKIYKQIYTLWKEDIDISEKENEQKNIFTWGILPVRAFDHPNIFVPLPCGFKHNNKNLAYYTSCNDKKKNSD
KNLLEEKKEKKKEYMNIKYTNLTGPCINWRRNCIFVNDIKKKKFLHLSEYYPYFYSLQNIKLSLFSLQRNVVY
NNLNNILHVKQDEPLKDNYENINMNIVCKKESTIIKDPTDSTNMNLKNVDGNNVNYTNSVNDTNSVNDTIHLL
HYTNNTCDEKNSPDTFNTYDSYNIYDTFGDVDNNKHNQNKSNIPNDSKHVSRNIGKNKNSRYSFTTTTTSNCS
SIYKNNKNNSNNKEQVEEKDLYKFFTNEDILNEKNYIWNKQEIRTFLEKYILYPKNFDKISQYLECKNTKQCV
EFYYLTKNFFSLKKLLLVISESKLKRNKKCGSNELHKKNTKEEMVNKLMRKIENNYVKTEFEDMNFMNSYFIN
IRNFFKNYFVKSYKNSHYKRKKGNSHNKHNKSNSYNNYDMNKSVILENMSDGYVIPKNYNFIISANRKLCFLI
KNKDNFIYNGINSDIIEINYNNNDNQQNEYCTSNEQNNMINNNNNNNDSNSNSNSNNCDHNNNEMKIFNLEKS
GFVKNEMSLLDMKNNKGKKKKSDIYISNYSKNSVSKDTNKESSHNITDNENVIYYTNLNNSNSNPIIDQKGKY
ENKQFLISCESNEYQTEIIKTKGDDKENEEGKKQQQQQQQQQQQRRQDYPYDDQYKYTNQIEYYNGEYKGQEA
NAFISNIKNKSEKNKERETCEESLFKCFEFLKNINYKLNENTNKNSLQHSKSTCFTYDQKSKKGVKKNSMQKD
NKNVKKNKHFANLKNVTMLQKGKAVKNIPINNKNASHNNKVVNSNKSVSNNNNNNNNKSTSNSNKSLSNNNNN
NNNNKSISNNNKSTSNNNKSTSNNNKSISNNNKSISNNNKSISSNNKSISSNNKSTSNNNKSTSNNNKSTSNN
NKSINNHRKVSQKLGGSHTFCKKSISSQLVASKIKETNPKRQLQDVRVENDIPLKKKRIVKNENIEENTNEIK
KNANNANDTNDANDANDANDANDAKANKQSQIIKKKNNEGQFCTKVIKGVQNKMINQKEQEGEIINHYDNEIH
DHNDGKKMKNHINGFKKHTIMNEKEQEEFPTNHNDNDISLLKKQYNNKKYISPSIGKINGDINHNYVDSHLNN
INVKNIATIVNEDEHLYTNNNYIELVKNDDIILNGNYVNSSSDEQQTNTFDVNLHDNNNNNNNKLIENILKPY
VKEEEEELEYSKNLRKIATKWTDEEKDIYFNIFGKEGKNWDALYEALEPFGKTKDQIKNFYQNTIAKRKKSHI
IP*
>N.PF11_0242
MGLQTEKDKEKEHQKNNFKICSKKFETDELEVLKKIYKELGSRSGSSYIDKETFLQFFPLPGLWGERLFLKFN
FKNTGFIDFEEFIIGIAICCRGTKSDKINVLFDIFDLNADGFIQKSEMVAMLSNIPYIHKLKNIFFNKDNRKN
IYRDDYTKDDDNADDENEDHENGDAIIDSNVGTNSDGNIDIDSDADVEYNDSDSDDFTIDDDEDVDSLDDISD
SFSSAYEDEDEYSNNNNNNNNIENMNISSDNKNVEENNIGNCSKCIKQQKNKALNANINSKRNANGNNTSLNV
KELAKKIRKEEKRKFVKKLRELNAVHLYKDNQLSNKKEKDKERKKKNKDPNVSKNSPFFKSESEDYNSSSSNN
TDAFVHDHIKYINKYKKKSLKNEVKKNGHKYKDTTNPYISSTTDSSTSNESNYNINNDEFESCSSNKDHREVC
SSDTSDSFISIYGYLIKNRRSKNIKEKNHHSKEKDKKDQDVKGKNKADDNIKRKEKGDDEIKRKDKPLDDIKE
KEQIKHEAKNKDHKMKEEISEGKENLKYKDVEKKKDGEKNKDGEKNKDGEKNKDGEKNKDGEKKKDGEKKKDG
EKEEGKNEEKENEEENKIKKEKCFTRNDINIYKKEKKKEKKKNKLYVNEFCLPSKTARSILNDEENNKPSDKN
VDVEAIVDIMLEECEFLDNDKITLIQFKSVIHKYDFFLYIFFNCLHEDIWGLQGNVLYGRDYISNFVIKSENF
KNKEIEQDEDDAITEELYFKIRQLFIVQAPDYDCVNDDLCVNFLNTKNIEQDIKEDDEEEKEKEEKKNGADKK
DTIEKEEEEYITNNFSKDKNDTSINKKKTKNENNAKLVSFVSDIKTGEENIVDKDKIKEYEKEYSIHKDKDDK
KNANSSFESEHEDLKRQFINMKEKKKKSLKEKDELNNMKEDNKIYKKLEEYDKKSINNIENQNENQNENQNEN
QNENQNENQNENQNENQNENQNENQNENQNENQNENQNENQNENQKNNNNILKEKNSYEDDKGKIKVINNEIK
KSVHHLSDNVSDLSYDEKWLKLSSNGTNKKKELNNDNSNNFDESQRIQEYQNDNKNNVIEENNILINQKKNSH
NMKEDSINIQVDMKSNIHMNIIINKVNDMNDVNDVNNANDVNNVNDVNNVNDMNNVEGINNEQKENDFVKMNK
QNTNRNELMSKKDIVGKNNTTIINNNMTNLNDDLLNVENTENKNRYECDFNLLTNQLNDIFSKEIVEFIQASK
ISFNINDVCKERNLHKKKERLQKGRRNYLKNNKNNLNISLNYQKQKKYPFNKLFLEELEKDEKNEEINKAEEH
VTDESSFEYNQNGVFRNKVVPTKTNKNVSTINNQNNEEDMKQKEKDVNEQTVEKKEDNNILINKLCKKKSFSG
LKLQNIEKMDILEKFGLSYKKSEKTSLGCSIFHNKVDEKRGSFSKSQDGIEADVIKQGELNNEKVKKNETNDD
KAKKNETYDDKAKKNETYXXXXXXXXXXXXXXXXXXXXXXXXXXXXXXXXXXXXXXXXXXXXXXXXXXXXXX
XXXXXXXXXXXXXXXXXXXXXXXXXXXXXXXXXXXXXXXXXXXXXXXNETYDEKVKKNETYDEKVKKNEQKCE
DLKEPISNVDKISYQKSTSDKDLSKKKNSNVGALNKMDTFKKDESSSNDMASSYFSSNKKKKYDLTKKHGKDI

NLYSCPNCKGPFLMCPNCHCRYPRFCVNDNKIAMECEYCDCEHCYFYNCIYCNFDFQKCLDMIKKNSLKEGIL
YKIGKHLHQFKARYYILFDNLLYYYDKKRNLKPRGFMFLEGCYVELIAKNDNINKYGFSICHKGTKQVQKRNL
YVNTLEERDEWVQALYSSTKQNTLYNLYELHEQLGQGKFSTVYRGINKQTNSEFAIKVIDKRSVSIYEKELLR
SEISILRLLRHPNVIYLKEIINTKETLYISMELVKGGELYDFLLAETRLSEIHANKIITQLIKTVAYLHRCGI
IHRDIKPENILLTDKSRDAQIKLTDFGLSTLCAPNELLKEPCGTLAYVAPEVITLQGYNHKVDAWSIGIILYL
LLSGKLPFPINKNTEMNIQKNYVLSFKDYIWKSISSSAKDLISKLLELNVEKRISANEALEHIWVKNPTAVIN
ENSFIYKNEEINILNLQDVSVSTFNIPRYTPLHIEEEKNTEEIENKELIFNIHENNILCENNDSIDEPVIPLP
YSSAPLKEHINEKKNIQNISSPMEDISMNKQENAVYESINKETSNPMKNCSQNVRDQNDTTPHHNNENQNEQG
NLNTCIHKINECKKSSTDGHTVQINDNTNKEHDK*
>N.PF11_0243
MYIHILTSMEIKNKSENLTISNLFRTGDNRGKTNMVMDNKSKLIIDSEKKKNIWSIFTKLDDSNNKKDDDDLN
SSINLNDNCINYDENESIENFFSPRNIMEKDKKNITYSNGTLFKNTEQNYEIKNEIENISNSFKRKYNILLTE
KTDDNMLNDIISIKKMKKDQNVNCPSYNSYREFFLKSGGNKSISNSNSNSNGHSNSISHSNSHSNSHSNGHSN
NCYTLNSNDFALNNNNTNLIICANNESPFSSNSSFMNNKMIYNKIENDDISRRMNKINNIEHFKPINTLNDKN
IIMNTTSIKSTTSTISTSSSITTHAGPIRNTKYVDDNDMNSSGQNIQIYHNVEKVDSNNKNYSSYNNVNHYSN
NNNNNNNNNISKTTNVIKKKNICVPVKTLNIQHKKKLYNSTPNKKAHTFHKNDKFDILGKLGDDVFRYIISSV
KNKNLLLLNKRCCELIRSLRIKLIYDESLKSSISPDSIMKTIYLSENIEILDLSGCSHLTSHHFQVFANCNNI
KFQKTLKVLCLKNCNKISDSSLKLLLHRFKNLEVVDIRNCYKISHEGIYPLKFKHTLKKLYMGNLTSSNVTNC
HSNDSLKVLFNSKKDNIQLDTVLNNLIALEIINVKLLDDISHLYMIANNLKILNFKGCPIDDTSSNYFQYFKN
LLALNLSETNISNHLLNVVIKNSTKLKVLDISKTPEILNSTILEIPKKLKHLKKVKLAHLQNVDNFCLREFLK
YCKDLTCIDFSNCWKVNNTFCNLNGLEIAAGNKLKDIGAYQCSIDRKSCEGCFAELGCISLRVHVYNELFLFE
TSIYTDIKLLEDD*
>N.PF11_0244
MANDLGEISNKQKGYMENNEKNSEKNNEINNEVTYKIINEIKNDTVRNNMVKEGNKNINKKHNESNVNIVGNK
KYESDINMKNKSDEEKCKAFFKHEPCLKNVLRNIFLKSKSNKCYDDDNNNDDNNNDDNNNDDNNNDDNNNDDN
NSSNNNNDDNNNDDNNSSNNNNDDNNNDDNNSSNNNNDDDFYTCTNTSIDKEINNNNVKNKKQKECINNEEKI
PSEVECAICMKLLIVPVTIPCGHNFCRDCIEKAKEYKNLCPLCRSNMGDKKNINLLLGELIKQKYPLTYSKRL
EEIENLKLEQEKKVIKERINAINNSSIIPIFKAPLIFGPYFPGEVFDINIYNKRFIDLIYFISNEGTFAITSS
KEKNDEKKLYGIHVKILEQKKSNQVFYLKCVANFRVLLYNITLFTDYGNFVGLHSPLFDENISINFLTSHIIN
NTTVHTDSTNITYGNYNNEQDVGLSNNTNIGGKEKIGDLKKLLYQIEVEEDINLISYYYEQYKNITNMGELID
NNNDNNNNNDDNNNDDNNNNNNNNSNNNSNNNNNNNNITNIVKYHCCIILSKICLLCIKNQLNRFGSAGVMLFN
TKFRNIKLTSSEPSKEELEKFSYSLSCAIISRSVLKWKWFKITDTYERLESITQYFLKKKNKSILALDNSRSP
LIHRFFMLDSISSSLIILVFFLIIICIKYFIY*
>N.PF11_0245
MNEPFSFNVNAPSYYPGMKYKGAGAEEDDENNSNLDNTNNENNINMNNDVNNDLDKIKEEEDINGSEIVEDEN
VNDIEEKISKLVLNDDNDIMKDDVEELQEKVEDKKIKMAEVDPRPHLNIIFIGHVDAGKSTACGNILYILGYV
DDRTIEKYEREAKEKSRESWFLAFIMDINEEERQKGKTVEVGRAHFETKDRRFTILDAPGHKNFIPNMISGAA
QADIGVLIISARKGEFETGFERGGQTREHTLLARTLGINQLIVAINKMDDPTCNWSESRYEEIQKKITPYIKS
CGYNINKDVFFVPISGLTGQNLSEHVSDKNSKIYDPRASWYDLSKPTLFNILNSLPPPPWDENGPLRIPLLEG
YKDNGIIAIGKIESGTLYGNNMNCTLMPNKVKVKVMNVFLEDDEVPYAKPGENVRVRLFGVEEDQISKGFVLC
DSINLCSVVHEFIGRVAIVELLEHKPIITAGYFCIFHAHTACEEIQFVEMLEVIDKKSKKKKTKPKFIKSDCI
VTAHFLLSNPVCVEVYDNLPQLGRFTLRDQGKTIAIGKILELKV*
>N.PF11_0246
MNIFKRYIFFLCSFYLLYVTCSSNNNNSNNNNNNFSNINNNNSNNGIHNDNTKNRPAAGSLKNMEDLLSYFKE
HTNNNNNNNDNHVELFKNFLNTNKESSGSNINTLMKFLNSSTKNDNTGNNHLDIGKLLDTLKEFNNANKNNNV
PSGNNPNNQTTNPANNNNNNNNLNKDNYVQIFGDLLKNMNIPSNNNNNNNNNNNNNNNNNNSSNNNSSSNNNN
SSSNNNNNSSNNNNNNAKAANPMEQLTNLFSHINNNDHHDEKGDGHPLEHIMNLFGQGHNNGDAKGGNNPLEHL
TSLLGQGHNDGDGKGGNNPLEHLKKIFDQGHNDGDGKGGSNPLEHLTSLLSQGHNDGDGKGGNNPLQHLTSLL
SQGHNNGDGKGGSNPLEHLTSLLSQGHNDGDGKGGNNPAEYLKKLFGNLQGNNANGINGKNMGDLQSMLNNLL
NSEKYKKSPYTLDKTVSSGFGSRSKESMLLEYQHNKSEQEILRELQELINKNKIKNVSENNEKLKQYFSMLKG
KYNPYSYERKILNDIINEDDIKTKYNIDDNFEKNLIVNVQNDNMEYDVEMNNEEKEKKTEMFEDINELDDKDA
LSSGSINSSLMRKNKSENEKLLRGFTTNINDDNDNLHECNCNNKLNNCVLSYINYNNLEYMLQSLDIDVKGLI
RKHMLLKKNNNNNNNNNNNNNGNNLYFEKNESFLLPLPPLTTKSNVQNTSNYVLRVPRVLFLASRKSFSSSFDR
SLFHIYNIMDTQLKWNTYLWGYGFKYYPIFYPKNLHTLLHSHEKQIGSEPFDLIFVHSSFVSNYYNYYFFLKN

MPKITTLIFMNDGWDNNVKNIFLNLFPHVFFQNQVNIFEFNPLNNIDTVAEKKKLFHSLWNKVTSGTTAPSSS
MHDHVITNKHKKRKKIETKTNNVVNNEDDVLLTGDHSSSSSSSPPSNNNNNNNNNNINKYDEEDDEENKTLWA
FIPHGVNHCCSSHYYKSFIMNDTSDNNNNNNNNYNGEGEMLQDDKQNRNVQKVVNPNFYYDRARSLENQHTPAY
ELSYSLLDLFLHECSFNEKDIFNNSKRDIDILYLYNTSNNYNDYLVQKIMDYIYNNNMNNLKDKIQKYEVDLN
YWKLWGLQTTHKLIKNKMIDYTNMLQRSKVCIISSKFSGMLNKMVIDALFYGCVVISDKTHNKDINKFIVTTK
VPFEYYEISDLIYNKENMTSLANDLIDKINTTLEEVKSGKRNQMRIDVFKTILNTYTYSAVILNWIIPSIYFH
YNKEKYEIKNNYFVLPQYFEKIISKSLKITSAKREKIIANIDLSLQEDLVMNNNEVVAWGIIWFLIFSIILFY
MLKNSSLVTFLFRQHKLHR*
>N.PF11_0247
MLLTNVRFLSKCMKIKISKRKSWVKRNKKWMLPKVENSYLKQEQDFTVPHKVISTSLNKEYEKNNIKDNFKCE
DDDNNKYFKNILKELHNNNKKEKNKRNDNLLKPHEYKKLLNKDKYKKNDDKLNLKDLLRKGTKPINLNMENVD
HLKEEKDKEKKKKDIETFWYMPNPFEKKGVYGMKENSFYKSFLKDRERSLDKINEKQLNKNDQQLLKQLKKFN
NSNNNINNELLENIISINDNQNKEKKIRISPRKYWYDNNYSTPDTNTINTVKARDLRFLMMNEARLVRKGKHI
DVELWLCFMNRVIHLSGIVHVRSLLRYLQTIASVKVINKKMLNDIFCEIFKRENDMKPKHYVYLFQSCSRLKW
NDFNLIYALKNMTLCWSILRNNFLIKSANSISKLGLASNVYSKALQITLNERLHNFSGRNLKAIKAITFLEFF
NEDMIIKFISRATFYKEHFNYYTRNLQILYLYIVLFHSSIYNNLTLEQRSFLQYCSQDRNLKKINKKKHNKSM
RTLSRVDQVGNEKGEVNLGVHNSDGDNSGDDNSDGDGSDDDDSDGDGSDDDDSDDDNSDDDDSDDDNKNVKIG
NKICGGYTCMLHKEVSDFLNKLNIEHLNSINCGPFMVDIYHPSSNYIIELNAHFQYYFNSESLTTLSKWRHKF
LSQMGYKVIHISYRIWNNLHNDTQKMEYIYSVLPKVILESSSYNSKFGKM*
>N.PF11_0248
MLSYYKNVISFNNKRVIFSICSRTIFVSKRSLAPKVKPGKKGQDKKDTTSGSTTESSEKAHIFNIYNTVDEDH
EILPDHAYPKWLWKLEKPLKSYGELALMFLYGKDIENATAQDYHRFRRLHNKNLIKLNNLRLKKSKRSTVKPI
FWDL*
>N.PF11_0249
MRNLNKRKLTCVENQSCKKRSRCDVGDKNDNDIILKSENKTRKNIKIKVDKESKEKKGKNINDKEQDDVIFMN
DVDGYNTEYFKNLKKGAIIEMTLYNWMVFSGPITLRANKGINLIAAANASGKSSIACALVFGLGYNSNILSRN
KELINFIKKGEKKSFIEITLKCDEEKKYICIKRIMNIINNKVESFWFVNNKKENYEKIEKIQKEFNLNLDNLI
TFMPQENVSKFSRLNPEELFECTLLAIDKKLLDDYNYLKKKIEEKKTGENKIKTYEHEIIEEEKIINDLEKKK
SKYENLRMLLSRMKLYRVKKSLLLIEHKKKQVINIKEHIESLIKEKDTHFNTLKYYLSELEKCHKVINNLSIE
YSSKKIIIKESLNKYMKLNLTLEDMEKQIIKEEKIMDDTVQNIYENKECIKELENKKKKIKEEIYKLEEFFNE
KKDERINKILTIEQNKMNENPSIISKKIKNINTLNDDNQYHNIKEKELQNDLKKISDENKKLLMKKYNLQSEY
KSILDKLKKRKNYKNIQEEKFLNSIEFTLRERILNYKKNIKNIVETYNLLNKEYIENLKKKYDQTKLHNINEF
NDEIVDELSKQNIIYGPLCKYIKCIKPQYDYILEYFLKKYFNSFLLIQKDNKELLEVLYKKYKLSIITTNDKY
SDMNKDSINGNNIINYNDRNNNNNNNNNAIPGYPKFCQVTNEMKKRGVEYFLYELFDSPEVVKNSLINIVPIH
ISFIVKGDTLKNKSTKEINEFNKFMINEISKQLNEEVRSLFYFCEDNIHRYRISTYDKNIFIENFSHIEKKKC
KILFYMNKDVKKDIDNLMERKEICEKELEELQIQFSEFEKMKKEKNDEYNKIILERNEMNIKKKKCTLLKNEL
IKIDENLDKYMKGENIIEEKKNMILKNINLINKKKIDICDEYLYILKEHSKYDKELFYIYHKLNQWKKYLSII
KNENIENEEKHENLKNTIEIEKNKLNSYIYDINELNGLLKIQKNELSQEEINTLDTIHFNLEQVENKLKECII
QEKIYNNLDSNNNKCEEKYNILLLSIERHKENVQNKIKQIDNLKETISENEKQIHFILPQWSNQINEYIIFLN
YNFEKFMNFINPEFYGKIDLIKKNDIYEKCELYIKVKFKKNAPFLLLSISHQSGGERSLTTMLYILSIQKLTK
NGFYVLDELNQGLDHINEQKIFQLLSCLSNPIMYKQYFLHNYNYKYIHIDYCSIPQYFILTPQIIKNIVFKDI
TVHYLFNGFGVVDKQFDNFYENYIS*
>N.PF11_0250
XXXXXXXXXXXXXXXXXXXXXXXXXXXXXXXXXXXXXXXXXXXXXXXXXXXXXXXXXXXYTVKSLHKGISELVVL
AADAKPLEIISHIPLVCEDKNTPYVYVRSKMALGRACGISRSVIATSIVTKDGSPLETQITELKDLIEQMLI*
>N.PF11_0251
MKICLIPVFLSLIIYVILFLKGKKIGDLYITEHLRNKFENILLYFNIFEVQYPESYLNNEKLLGLHVRDIEKD
AYRAYPILKELKQKDYFRIFKVNLHLSCKVFLKGNEKCKEIKKCSVCECEQDEIPYNFRTNEIEIIENKMTNE
DLKKTFIESKLYKDILGIYAPSNEGFISYVDLVYNSPSFTAYEGRNIWNMIYKENCFQNEKSECEEMNSFYKI
ISGMQSNIAVLSSEYFYLKNDFLFGEIQINNKFNNDYFKNLNYDYSVTFFKEKIGLYPERIENLYFTFAILLR
SMCRLKSLFKQCKCNSGRSE*RTYWLT*TERNHCPHKFPILLCRFYINNKQI*
>N.PF11_0252
MEKSQKIRSFNLLFLEEDEEYIDDMLITMKTKKEKHDNKNSSINNNTNEKCTFIYVELKGRLRLCSKSLIFEA
YDVDEEVLKFPFKKLENVKLGIFKNEIIIKTCEVIRIKTIIHNKKTRCISQFSYERKYKKNINVLNNLYNEYY

DEYKTIDNNNIDEKIHIFEKLLNNFDDFLDLNKSYNNKHVFVDDTKKENINNTHVNSDEEKKKKNIKMQNEKN
DSIFLNTHMSDAIGNKVECDHNNNNNNKNNDDDRNQNIQTQNKNNDNKKKSQDNDKGNVCNNEPPFDRDQINY
QTKGMSINSESSTEKEKSFVYIFSCENSTMLMKKMKHIYELIIKIKTAMIINEKQMDILIKEKKSKYKSGNKE
RAEEMINKNNNIMINGNSNSNSNSNNNSNNNRNVFKSDNIKDNNYNKDIKKIDENKMNEYCTNNSVVKNKELN
LDKYIESIMDKLKENVKFDISSINIHEKIITNRIDGFWVFKVSPLLKVKGILNITNKYIYFQPHPNFTNKKEK
KWSVEKILHVFKRIITMKPNALEIIFENEKRKKYDSLYIEFLSYDDREIIIHILKRLKSECFFIEENKDFVYV
VQEKWSQGYISNYIYLDFLNCIGGRSRKDFSQYPIYPWVISSYTTNEKLEYDNKEIYRDLSKPVGCLNKNRLN
SLIEKMHDHDYFYGSHYSTLAYVVYFLIRLYPECQLKLQSGKFDTISRMFLSLESTYHTALNANSSFIELIPE
FYEDDESFLKNYLNIITNEGNINDVILPKWCSSPKEFLIFMKNALESNYVNQNINSWIDLIFGYKQNGHNAKE
CFNLFHPLTYMHSILNEKLSRDYNNKDNYERYNAEQENTFEQNFNQNIKDLITTMSSKALKTQLHEFGQCPYQ
IFKQPHVSKKEVYTFVYNEKISNFFWYYSDVINQLLREEIIKKKKKKNNINSNNNNNNKMKKKKYNHNIAKVY
GANKISMEKRYSEDGSLVHYDYEEEDVINGNMGYDMDDMEDMDDMDDMNDMDNMDNMDNMEYMDNMEYMNNME
DNTQNNTQNMFDDDDVIDANRAARRCPFPYNTSSEKQRNILVESKNLKYENINKKTKDEEYCDYFKRYNWTLH
RAETFSSCKIRGIASTSEHSCFSCNNGYIKLITHKDLLNCNIKNNNNFISLKISDDNFSCINNFENNYILGTS
TGKIVFINPKSILHIDNKKKNRTCDKNKIDIKQKGKNQNTNEEIIKNNNVLFTNYNNEDEKNIFSDDFSDEIF
NFYSNESSDFLFSSFEDIFYEKHENIYDNVNSKEDKSLFFNNNNNNNNNNNIYSQLISKKIHNDTINSISYSY
NYIATGSSDETIELINADTNFDIVQVYDNFDDKVRYVQLKNKLLFAISNDLKLFDIRVGKKNIYLNNINNFNS
YNKLFINVSNHNRGAKKNMEFARKRVYKNVHGNDNMMCGNNNIMCGNNNMMCGNNNIMCGNNNIMCGNNNIMC
GNNSMMCGNNKNNFICGPYQDNYVYVNYLNKQFLFEKKIKKNVHPNFSKNQPNIIHACLINDYNILCVDEKDY
LYFYDIRNEKWHNILSTSSSMGNIKNNNKYNSKKKRNYKIVSAACNKQRLCLVNNDGHVFYEHMNFCDNNNIL
SKTNFFPRISLITTKYITFFNHFDDINNMNNDYILFISEDAQMEIYKKGN*
>N.PF11_0253
MCNGNVEDSLKLYYDINGDNMIPNKPYDNNNKIMTEEEKSVQSNMYQTNTETTNDKKKNICPPCDDYVRAPDK
HFSQALINDMDDSNFYPYNNTNKKSNKSKIQLGDTFQKLFSPPESLICSLSFEEVRKKSKQENKFILVNIQNT
EFESLRLNRDIWNNDVIQQIIKTSFILWLRYEYDQDAALFMNTYKVHKLPYLCVLCKRTGRQLKVWNIRNFQD
PICAQSQLYEFIEMMEIKSNSRNIKDITTSSKNISTNILDTTTTTTTTTTTTTNININNNNNNYGDRDDHGDDH
MLHNINNKNIDTLKEPYRNIPPSGYDKNKLLYPETTHQILQEEKKDSIHNFNNNSYNNPNLKETESEDKKTPD
VVEEYNTINNELSQLHKLRMQRFQKKN*
>N.PF11_0254
MADNNLDKNESDSSCINASDEQKSSEEKDNLGEASVVSNVNENNSSFFTKCFSKIVNFGNNTQENNIPVAHEE
NNNEDREYNTNESNCNRYDDEEDKKVENFDNNNINNNYLKGDGEKNIHEGGDVHTDFNNEEMINAISNYNKNE
LNLNRGEIYLNDNEYSFISYIILSSEFRFYPIEETKKKKRRDTLKIDDTITITDTKRIKFDNNNNNNNKIEIR
KKENVTDIDADKGSNNHMNEKQVVNNSEDILKKEDEEEENLKKEGGDLLIKGNNVDKVLLDDIDIEKVRSYDK
IMDAEERDKLELFDEILIHIKRNFYEKILNIETNFKNLENQIEDNHSFHFRCIIEKLKNRKYDNILYIYNDIY
LYHNNLLFLSKPSSYTWMKLHELSTQITNTISDIHHKKYMQKPNRKLYHIDKEEKEKKEKKKEVLKDHEKMEY
CINEEEKLAFQLLLSKLNQDTHFELFRKFKNKAVWKTLEGGEIELDDKLTRADVFREMYNWCKFQLEMKNKQS
ELSDSESDSSKDSY*
>N.PF11_0255
MEHSSMPLWLSSFEEEIDTYIFISSRDNKLYLGILRTYDQHGNVFLTHCVEKIIVPEKNYFSDVYVGNLIIRG
DNIAYFGSVDEEKYCKMFDYTIKNDNDEEHNNMDMIKNKKEYSSSENIILVYKPINHILKFIPENNADSSIFE
N*
>N.PF11_0256
MLLVIWLNIFWFIPYYCFNYVLCVGNKNDMLFVKNKNLIYPYNDMNRIRRHKGVELSRKMVAENIQMRNMKSN
NNSNNVHENNNIDHSNNYNNNLYLSCSNKKKGKDVQSLCAIKHGNVLIKNINEEEKEKGIKKNDTDKLESKND
NEHKNEENNFVFSYDKKLNNYSEFNIYMENNNIEEYISDVNISREEICTLYEDMYLGRLFENLVAKLYYNKRV
NGFVHLYNGQEAVSTGIIKNLKNSDFVTSTYRDHVHALSKGVPAHKILNELYGNYYGSTNKGKGGSMHIYSKE
NNFIGGFGFIGEQIPIAVGLAYSILYKNEFHYNPKNTSFTSTKNKNNYIQENENMIHMNNSQNVDVVVCFLGD
GTANIGQFFESLNLASSYNLPIIFVIENNNWAIGMESSRSSSDDLMNNYSKGKAFNIDTFKVDGNDVLTIYKL
AKKKIQQIRNRTSGPIIIEAITYRAKGHSLADPDELRIKEEKTSWKKRDPILFLSSYMKKYNLVQESYFEQVK
KNTQTLLQQAELDAEQNTKKGEHIDICNVIQQNIFAPSKSTPYQSEYENYKQFDDISNDELKEYYQEVLKEIQ
RKKEKKKLDPNDKFDQKKLPLIID*
>N.PF11_0257
MEYQLREIDESEKEITQQRKKNSVTTKNFDNLSNSNSQITEMTNEQGVIPLREKDLTINVGNDRNLKVEEFMT
NFISNVFENKNALFLYCKYVLLFYGKELLNENNVESLNFEIIKGGITNILVKVEDNIHQNKYLIRLYGPKTSE

IINREREKLISNILCDKNISKKIYVFFPNGRIEEFKDGYALSREDIKNKDFQKEIAENLRILHDIQLDDTIYK
KLQALQNIQGNRSSFLWSTLWKYFNTLKEERQKKYSFNPKAYILKLIDFDMLESIITEIQELCCKKNSPVVLC
HCDLLSSNIIKTEGSSISFIDFEYSCPMERAYDIANHFNEYAGFNCEWDLTPNRSEEYHFIKHYLKTDDEQLI
NQLIDEIQPFYVCSHIVWGLWALLQGLHSVIDFDFINYGMTRLTAAFSVKFRSKLEKN*
>N.PF11_0258
MKYSTNIIKKGILSRYCSNIYGMQYIPRARYFLTFCKNEKCNTMLKKYHPFSTSKMSDNAHYKGEDMNKMKNK
ENKTNKTNEANKANETNETNETIENSASSPTEGKKKKNSLNESEEANEKKEDINYEDFNKIDLINEIKKTKRD
MEEKMVDNKVLKEKYLSVLAENENLRNRYMKEIETSKLYCISNFAKSLLDVADNLSLAIKNINEESLKTNEEI
NNIYKGIEMTETILHNIFNKYGIDKYNPINEKFNPQLHEAIFEINDSTKEKGTVATVIQHGYKIKDRILRAAK
VGVVKN*
>N.PF11_0259
MDIDFCSHHLLAYDNSIINDENEIKAKVEQNLPLIFSKINKLQKEKEDEEDIIYSVTNDNIFNMPRYSRIPKE
KKKTKWQLFAENKLRMKKNKSGLIYDKVSKGWVRRFQKKQIKLNEQKNNFVHEYKNKEDIYEDPFEKEQEEKD
IKKMKQKMRELKNKFDQKGISTEDIKYIQRQKRKRENLIDNLKMAQISSSTFGRKDKKLKKEKKLKLQNDKIT
NQKYEKRDLKDEVKQNNRLAALVLKSV*
>N.PF11_0260
MSNVKAYELRTLKKKELLDKLDELKKELSGLRISKALGNSAKNSKIHGVRKNVARVLTVYNQKRKMELRQLYK
NKKFKPYNLRKKLTKNKRLQLSPKQKAAMTLRQKKKVQNFPQRKYLVVHKE*
>N.PF11_0261
MKTRCVFFWCVFFWFVSFFYFPVFYFFHILIKCVHIKKIRTRCLPLFVCNHKQKYVTRNKSLIKNKSTYFKTF
LWGYPIYDDSPHKLRRQKIYPNFEYGELRKDQDYDYVIATNLSQNNHKVPDVGYFFNVEEMGTGKTDDIHTPI
EVVFGERDIDPKEVEKIKDVLNTFDQGNGLGICTLMGAKRGHFAFKYNGYLGHFHGIKNYLRFLINKHYPGSK
VTLISEHWIDNIDQPVCLRHSNIPLSKIGHQRRLIFGKYDYIYENDKNKKIYKGENRSNVWAFYNDPQMLT*
>N.PF11_0262
MLRIGKNKAKGSLFIKKCYYTNNSKGWLREYVYTKYRISLPNIENVKYDDIYLSCPSRDDFYVFTKKVPIFLR
YLKLITSLENRTNDFIDFTKKCENGLNVEKDVYLTKEELLDIMFINGYSTKEMNALDLSFCSTYQFHYPEISV
LFNLDEEDVYKYCLKKRSENPQTLVHLKYEKEKNMLSSYGLIFVFLYFGLNNLVLCNAWFLSKTIPFFSVFYM
LGSYFYKDIQKYINKDINLMIDENNKNKLLAEDIIYKQLRLFSKDTECTEQLISFKQYCNVLIKKYTHSYINF
QKNKIVETLEKKLKEIYNDEQNYKNSLQNILIEEIIKKIYEKIKTDKTFADSILNDGINNIQNINQNDTLINY
VKSELQNIQKMDQKNSIVTKVLEQYELKKQQYLAKYIIHTHELNQIKNIINKSKLNINNLNHIEYNELLQLFN
TINNRFGFYVNDDSISNITSSDSESKSFTQQINKFIIDTNKSFQHKKLVAFLREFQHI*
>N.PF11_0263
MVFQTIKFIVLRPRGIPQNLELDINCSDYVRNIKNKLFQEDISKALNVRFIYMGKILDDKKKLEDYLNFYNQD
ILINNKITNILYENKKGYSTKKILNDINNTHVNTNSKENASITIHVKITESFSSSRNDTFDGKNLNTSLARLS
LVMFVSLLWIYRYNYSHTFPIFSTIVLFIFTLFIGIIIFHNHIILIINILLQIIITIYQMIKYSFKKIYDHIN
KKLQNYYKNRKGKETKKIK*
>N.PF11_0264
MFVCKKCSRYVIKKIFYTFICESKYQLLFLRKGNDYKNIFFLEKGYISTHVRGDKKERTQKKKNDLDIKRLFL
DKPSTPNIKDEYEKKTNDNNNMEEYNICNNNIITNNNKIKNSSHMNTFNSNRKKVCVKDNINNNTINNNYEEQ
KENKEEYINTPKAKNNLYNLPYKNKDTHIIDKKKLDEQVVKELHNLIISIKKIDKDKKENIIKYINTLNKKYV
KNINKNIIVFFENLDIYMSVNGLLICDIGGEEIFNYINKIKLTNNIDIKKETYNDDIEKHNVNTNERFLHMDD
VTKEDNININNMFDINYIKEKKKRIYGDNIYPIVVKSEEKEEVVPKKKKKNSNNIIVHNNNIINNKCGDYNII
NCTNDLGEKTKNHQTNYSNNPKFDYQYNEEKEKEEEKESNIINVRRQMLIERSSYKKAIEEAEEFVSNLHDLK
KITEIQGLCKIYLNWISELEKRIILYKEKNKNKKKKKIFPDFIDDKLLATITVKWTIQYTFNPLKKKYSNNVS
TQSKYFEHEYSYQSLFTHIAIKIGEEINNELNFQLLEKKNHLYNYMKKNKNNSSFSHFQKYRIIKQMKQQIGK
GNHMKQHAMLGGDNQTSHLNSCANNNFDINSCANNNFDINSCANNNFGINSCGNNNFGINSCDEYYGHTNNHV
HIKHDDDNIIEWNSVKKATIGGLLLNMLIENAKMDVDINVAKNEFKNEYKYYLFLHKTAKNNAEDLYEECKYK
KELNYQKFYEEESNTLKFIILDKYKKNNSYDNNFKKNYMEKKICTYNYGTHDKNVVKQNNGDKQNNGDKHNCA
NRQELQLSDNKKESNFCQKQREKLMRKEIILYRSKRDRNKIELPVFIHSYVWKNNNWYGVIHMRECCANYLLN
NAINSHIPLNYLPMICKPKRWENFEGGMLLLKNSFIRCNIKPLFNLNVCNLNRIKNIVSEIGNVGWKINKEIL
HYIEYAYIHGITIGKIPLYKNYTLPKYINLKEQNNEEIKKYLLLKEEINRLNKCLISERPTFLQKLAVAKTFK
DNDIIYFPHNIDFRGRMYPLSPHLHHMSDDICRSLITFAEQKEIGNKGLFWLKIHLANTFGKDKLSFQKRIQW
VDQNINNIKKLTQQPFDNIEFWNMAEKPWQALAVAIDLKNALESPNASKYKSSIPIQQDGTCNGLQHYAALGK
DKYGGKAVNIIPSDEPQDIYSVVLDIVISKIKNDLMNISNGHHNNIISFSINENIKTNKKYNNNINNNINNYN

NNNHNRNSNSNVNKNELASYCFKFDLLKRKVVKQTIMTICYGVTSIGAKNQVKGKIQTMIGKELDKNIINKLS
QYIANYIFESISEIFKRAMIIKKWFNNLSKVTNELNIPITWISPIGLPCEQPYRLGNRILVNTPLQSISVISY
KNSQLHKNKQRLGFPPNFVHSLDASHLMMTAEKMVIQNNFSFAAVHDSYWAHACNVDIMNQFIRESFITLYNE
PILENIYNNFRIRLGKFATKIPSSPEQGELDISMVKDSLYFFS*
>N.PF11_0265
MKKNIFNNVFINIKFQKNLKYNKFISSKCIQNIYVTNKWNSTLCVYNQHLCSPTQLINTRKNFSTFMRNVIEQ
VKKDMKENKQYQEALKELKEKTEIDDKRIKLKKRIEENIDFFQKLKEKNVEAVKILYNDVNNFVTYCFTKYYA
LKLSKNLCIQFFVTLKNVFIKSTNKFTELSEKWDENSAFVKLDKWRQDMAIKRYKKKKEKENQHINNNINEEN
DNCILHNNDEPSNNIININKKIKKKHMNTNIMEENTTVENNETENDKSQNYELILAQESAWDKFGSKLKDMPF
LNNFFENPILGKLFGETELAAALREMKMIDKNFKLSELMYLFEYVISKHIVESYLIGDEETLRLHCGSSAFNS
LNASITERKKKKVFLDTNVLIYKNHELKGAQRMEESSPWFIFTFHTQQINCLKNKNDEIIEGKIDDIREVVYT
IALSKHPEPEKEGLLYPYIVREFAIIGNTPSW*
>N.PF11_0266
MKLVHFLMKLTNENVTIELKNGTLITGIITAVDIKMNTHMKNVKVVIKNKNIAEYNVNTKQFLSLEHVTIRGN
NIRYFILSDSLPLDSLLVEDTTPKKISKDKSFLHRDKGMSKGAKGRGRKLSKR*
>N.PF11_0266a
MVFIPVEVIFKSFPKFSKDRVKFLRRYSFLSLFLGAAFTYKAHTPDFTVRSYKPSYFYKHHLNKLKTKGIIDE
TKYEKLLNNH*
>N.PF11_0267
MTTNKFLLLKKEDSYLSKLTHETVIFGENLYKIVSKTLSSSSTDPLNQTSNENLDSISFCSELLPFYCKSEII
HQGDIFNVRFGHSCLIDKHIIYIYGGNQDIKSYNTKLIEFNLSNNLFKKLDEKNPPKPRYFAKLNLIYSTEKK
EDCLFLYGGKRGSYITNDTYVYCIKDKTWERIKVKFSPPPVFGHVSFKYKNIIFIHGGNMGNSKVNSDIWGYF
EEEKKWVKIMSKDEYYNHGSRKPSGRFFHSCSVCLANQGNDINVYIFGGINDQNKCVQDIFWCYSLNNGKWTQ
IQNSIGKIPIERYGHSSTVLNDRWFLLCGGYNLSWYQKSNLLDIYAYDINLNTWSCLNVYGMLLVTHHFYGNI
IQVDDSGYFFIFGGLRNNEASSKIYKFTPLLPSYYFKVLKNKIDDINNKVHYLENNPRRTMNPIFSKEIAEIR
YALSTISFTIVRYVQLINDINEKIKISNELVKRNYLSILEKMESNRKYYDELNNRIDVLDNSIMNKTSKTNQN
NNNNNDNNNDNNNGDILKSEDIIKSEEIVKSDETVKSDETVKNDEIIKNDEIVKNDEIIKNDEIVKNDEIIKN
KDIIKNDELIKSDEIVKNDEIIKNDEIIKSEEIIKSDEIIKNDETIKNEEHNKNIDTNKNDEKNEKQEESITN
DDLSNKHQNVIN*
>N.PF11_0268
MASPNTTQPEIILSEEKKASLTTQSTSNNTNDGNILEEQKTLTSQQQPTPVPNTERTEDNVIENLKKTDLSNK
DENTTISINTSTNTNINSNTNSNTNTNINSNINTDVNTNINTDINTNTNSNTNSNTNTNINTNANTNTNTNEK
AQDEKFQYNGDVSKAFLSPPVFHLTEIMHDERCFKKTKGHVTVEINGDICIYGGMLHDKCVENFIRYVPGINL
FEKMRLNSNDIVPRAFCSGNVITEDNKKNIIIFGGINEKDEIVDETYKFDFQARKWELIGNKICPRARYKHAS
FSFNDFLYIHGGLDVNNSLLADMWCFSKNSWTPIKQIDRIPEPRYAHSLIFSFYGNAKLVFLFGGNKKGYNAA
LGDTWIFNINTNRWKEITNSSGSKPCARWGHSSQLFDNEWMIIYGGITNGWIDNYALSDMYALNIFTFSWFEV
DISTSKNFDRGYYGSLCFLPYKKSLFVFGGTDNSEDHSDVFSMSPLVTYVSYKTLTGKIEQLNTRMKNINETS
SENENMNISEFETKITELKEDINKINFMMKAFESKFCELEKLNEQCEKLLSKNINTEELQNLEQRIRKLETSN
VLMKHDSI*
>N.PF11_0269
MDNKTEENIFENMTREEKEVLLEANTKREWESYGQWLKRKEFLLKMLNYHKEHNLQIDVEKFCKMGHMYYNVK
YLSCSYNSEVLEEMKKYEQS*
>N.PF11_0270
MKKIIMVIFFLLSKICKNNAILKSLKKNNKIVLKNIITKKQEPHLYHKLYNNIYNNIYNNNNNKKNRNRNRNK
NKNKIKIKKKENKEASFFYINNYKKKLFDEQGNTLILHRRNNWINKRDNIVENYKNVKAKKHLLKLFISKNNY
IYNTKMFSSCINNNCNTNDVEVMKKKLVVGENPEFIKKRLEKFNEIKEKRKQELEENFEELRKPITIELLDGS
IKSGESYVTTPFDIALSISKRLAEDSIVCKVTYLEKVDVELCDIEEGDDHNEEANDEVEEKSKEDNNENNNDN
KNNNDNENNNDNKNNNDNKNNNYNNYNNYNNNSHIMKSILWDLNVPLLGNCRVEFINIQSEEGQKIFWHSSAH
ILGSSLEKLFGGFLTIGPALKEGFYYDIFLNNFSINNEDYKRIEDEFNKLVKDNVPFEKVICTKEEALELFDY
NPFKLELIRSKIPDNKKTSVYRCGNFIDLCLGPHIKNTGKVKTFKVLKNSSAYWLGQKENDSLQRVYGISFQK
KSELVEYLKFLEEAKKRDHRNVGKILNLFFFEKETSPGSCFWLPHGSKIYNKLIEFIRKEYRIRKYEEVISPN
VFSCDLWKTSGHYQNYKDCMFLFNVENKEWGMKPMNCPGHCLMFKQLNVSYRSLPVRLADFGVLHRNEISGSL
SGLTRVRRFQQDDSHIFCSMEHIKQEVLNTLNFLFYVYNLFGFKYELFLSTRPKKFIGQISTWNLAEQHLKDA
LDAANVQWKINEGDGAFYGPKIDILVKDSLNRTHQCGTIQLDFQLPVRFNLQYKNKEYVTNNEKNEKNHDNDD

NVHNNNNYPINHDDLHNNEKKEFKDSQNHNEDKEHNNIHDNHSLKPNINEEGLLKKGFERPIIIHRAILGSVE
RFVAILIEHTAGKLPFWLSPRQAIVLPIGDKYNDYANYVYETLHNNMFDVDLDTSVNTLNKKIREAQLKQFNY
ILVVGEKELTTNTVTLRDRDDQNNQHVYTIQELINKFNKLLDVNSKKFNQIKEFNSNQTI*
>N.PF11_0271
MKKKFEEENKPSYILKHNNNEFKIDISYFTQLHEHKINIYKLQSNYVNLCSSTYVNKIKLGFKYKLLNRYLIE
FAHPFIHVRTIEINKKSFLKYENFDNEDEKNNMEPNDCTKTIENERNHINNINDGNKKVQKIWYIMNNYRNNY
LGVLLNFNTLEEFLKCNKDDHINYTLEPLKCYINNEKNDICKDMNLYIHDNIYDDTFWEYKENCLTVLEKINK
YVILSFFDLKKYICYYSIANPIIKPKDNYYKLIKNSTRYFFYIDSKYVYINTENRHINIIDIFYLSYKIDDYF
NNYKMFLNTNIFLLLKFDNIPLHTMNNQDYYDEYINKLYTNIECEEDQKSKKEFYQINSFYKLFEYLKLNDIS
QNSYHPMGNKSFNNHYNNNSSMLHKNYDMVILPINALSELKEDIKNSKDKILRYIKKDFFDLYICFIDINYIF
NSLSWDFRNLLYCLTLKYKLYDFQIDVLAFRDISLLRQQYVGTFKSQEGFIWSYPKVVMKRGSINNPRNYNDE
DKNNDNNNYDDKNNDNNNYDDKNNDNNNYDDSHNNNYEDSHNNNYDDSDLHKDIDMDKDKNNSFHYNHINNCL
SHQDVSFCSVTKMCKVNYNSIKDCKNDWRDDLTNEYSHDMNPIHEDIEHSSSQYENNMSVNNTYKKDNRNIKH
NHNNIYHNHLVKYILNSSLFQVTVPDKVHFIYDNESNYVDINLNGKKDDSLNKQDIHILEKKKEGDTCIINSY
LKSFSDEKKNDCIDVSSNLGFSINIRKEDNHFTTRVKYKDEEMDVLHISKGDENENNMNNATNNNINNNIIKN
CKTFCCDNKVYDVLCGWKYYEDKKKEKKSIISIINLNDFINKDTIQRISLELNIKLIKWKILKDLKFEHIKKL
KILIIGLGTLGCMVARNCVSWGIQHYTFVDNSRVSFSNISRQYLYTLEDAEKYGNIGEYKCVAAKKNLLKICP
DLNITAKVMDIPMPGHLNYLNENLEDTINELDNLINKHDVVFLLTDSKESRYFPCLMIAEKQYNSLKELQESV
NHNNNNNNNNSSSSSSSGSNKFRKGDNVLCEEENMITHEYIENIKCTKIMDKSLNNILLYEQNNNIYKSLNN
IHMYDRYQEIFYNNILTSVKRLCKMPPLGITVAISFDSFVVLRHSYLYFKGACYFCNDMHCPSDSLSYRTLDE
KCTVTRCGISNISSSIATELLLALTQHPLYFFAPHIDRDQYIYNYDNDMNQKKNSDISNIFTSCLGATPHIMN
FNLANFTIKKIFCEPFEKCMCCSERVILKYQEDKMDFIRNVIRDSSILERITNMDQLKVEENDVIILE*
>N.PF11_0272
MSLQVIDNNDFQHILRILNTNVDGKEKVIIALTAIKGIGKRMATVICKQANVDPTKRAGELTTEEIDNIVHIM
STPTQFKIPDWFLNRRKDLKEGKNIHVIANQLDSYLREDLERMKKIRLHRGLRHHWGLRVRGQHTKTTGRRGR
TVGVAKKKGA*
>N.PF11_0273.1
MKQKKMDFRKNKNNNDSNNVIIINDEINSDISLESSEEEVDNYDQHYDNFLTPKNGNGNRVLNHNHIHNLKSK
KNNIIIDLTLSDNSNKDYVEYQNVKKNCEDNFKEGDNPSNISVSTKEFIDCYKNDKVKNIKVNEKQIEQNNTK
NQESQKNIHINNTSFMKISCNEKNKNEMINEFKINEHDNISIHSVHTIEEDTVNSNEDINFNNNKNNCMNFLH
NEHNKRNYEQMMEHNIRNEKNKRHIIIENTKTHKITSPCINLEDITQPEHLNQTPQNKKKYYRNINNNVIDLD
KENEQTKDFSFYKNDHSSSNKNRKKNNPNNAYHANNAYHANHANHANPFKSNMFRNILFNKKYRIEDLYYIKL
KYDDLSNIEKKEEENVKNFYNKYYKCSFYVDKNINNICLKKYDICNYCKHCLKFLKFLIIHGNKYKGNLINIF
FIYSDIQQLIFTYKCEKKHLFNISLFHVIHNLWCPHDFCLFQCKGSYSKNYATEFFRLKELESMEKQKRLFLQ
AKVFCLFNSYGTLPVNKKNVESEYTNDIDRIIKNANNPWEVLQMNTYTKLDISDKTELKKMARKNYHKLALKV
HPDKNKNDNVSIIGDEYFNQLNAKHNVNIKWVNDKT*
>N.PF11_0274
MMNFTSPPSNDPIKKIQNLLNNSLHSIMDVVSNLSYKGEPKELVLEKYNVSDYSYFTNLLKEEENKDKRELID
NIEDEKNLIIKNNEEENNYFIKPYFEEPLKNEILDRVERMNLILNMIDESIDDLPDSIMNEEEKCEEIKKLQR
KKDEAKEELKTLYKEYDDLYNYVTDHLRYCAINMK*
>N.PF11_0275
MEGSKLYVSKEFMPLKILRDTKANEEERVDVLDKFDDEILENEYSSRLEKELILKNKNFNRDVNLLQLNYGGN
LIKNKAILSKRNVVYLSSHSSILLIDLKKKKKKRIVLSFDIDRIFYFYDKKYKEEYLIIKGMNNYIYIYRISK
NKYEFVKKFFIKNLFYINSFGNNGELCFATWEINQAKMYTNFFLLRFIFEYESYMSENYPNNLKNKNDIEKEK
YISTLLNHHIIYSYESDSECNFDNKYFPSELYMSELSDNGFEYNNNNNNNNNTLRKNPKNYDNNMNNIINDDK
QKNYNHSNNNIDMNNDYIHVLAKLKIKPILKIKYIYFSMIDLNKKLNKLVLSNNNIIIIYDLERRSYNIISFR
NYISSIKISDDNFLAVGFINGYIHVILFEELLSNNENNKNKQYEKIFNSMKPFHMYPGTDSLKTVFPYIGAYE
VDYMSDNNNKNNTNNEDNKYNTYNDYNNKYNTYNDYNNKNNTYNDCNNKYNTYNDCNNKYNTYNHYNNNNNEE
SHEVKLPPLLNIPYINFDFNKNKIINIYKFHLIKYKWHSHSVYNLEIDGRKIISWGEEAVILFYDIDKASYEF
ISHLGFPCYYIYLNKEKNLIICNSLNNSLIFINYNHRLFFYKYNGINMPLSLKNLFYHYTNVDLNIKNSMNLF
MSQINDEYDYTGNINDQTIRREFYKNEHTNNNLFNNTMYDDNINNFANCQEYSTEDSSDESSNESNDLSEVED
IISSNNKNKNEFINEETNKYDDQDNNRNEFSKHHDEDNENVDDNKKMVNIKKRKKEKRDVVNLLSFRYIEKEL
EKKLFKNSNVENNLYKKYQMCFYCDSNKGLIFCFLTSFSHLQLYNIEKDKHEKYLCSLNMTYKSRTNIEKVND
MELIFFCFNHNRNLLMTIEKRNYLMPNLLDSSSPNLTQKIYTIKFWLHDKDLEYNNIYEYAVPCNMITYDGQE

TNDVFKAIHVNINKTVDDINICADDNIYDNDNICNNDNICNNDNICNNDNIYDNDNIYDNNNICNNNNICNNN
NIYNNNNIYNNDIISGEHEKYQMIKSHPFLNMFLLFETNGNISIWCFDKSEKIFDECIYDDYVVDCVEMVTEN
IKIMDRMINEMNNGTDIHNIYKKNNNEYALGLSSLNNNNNNKSDGEENNKKNFSSNIITIIKNINYNNNIILN
GDVSSDGKIFCLCHDKFFTIWDTVTLKTLAIIKHPLYTSLNEHIFNLYKGIEVIESQYNTYILFFSFDTIFIY
SLEQFHLIYQEKFKGIIEYVKFDKYTNKYVAIGITKKSKQKNNQLIQKNYIYEFNENYLKRKKLFYSTTQSPI
LLVDFAPFNILKNKNLNQHHKSTILVSLNSKFQVHTFYINNYPNFLKLN*
>N.PF11_0276
MPKPPFSNITQKLGNLLNICKDMCRKGYNCYYIKEKENDKGSQEDVRCHIKNKINIKRNEEKAEKGETLDQME
KLLLDLTQGKYKAEKHYVYTIDGYRLNLYRIVNNNSENIINDKVVQKERINEQVFEEDKKEVFCLNHGLFESS
INYTCKGYNSLTFQIFSNNHDVWISNNRGNNFTQYVGKNIAINKLRENYTEEDLRDLGLNIKSDQIEKEMRKK
KKKKRKKKDNNINQCDNKNMYHFKILHNLFFQTIKYIYPKKNNFTYYMKNFLMRKYLCNYHMNRSNKNCFNHY
DRENIDLHKKKKIIIIKKKNNNNNNNNNNNSHLIFRPLADNNFFNIFVRNINEKKKGNIYFKATQEREDANIKR
GGTLGCDKIKEKESSEYFVDNKKLKNKSSNLTDNCDNKINKYDYNDDDNDDKDDDDDNDDNDNDNDDNDDDDN
EYTFEDMSTKDLPCIIKYIKNKTKKDKIIYVGFSQGSIQLLISSCLNEYVRKSIKRCYLMSLPIILRNKYNLE
IPMKFLLYISKYYSFVFKGKSFFQHMIPYHISTFIISNLAHIIAHHVFKYYNENIKPEEKKLFFYHTPNGSTS
KANLTRWAKAFNTCPVTNVLEKYPHECAFPITLIYGNKDTIIHVDKSIKYMNKIFDRKILKIITVPNWAHLDP
LWSDNDGIVISCILKDINRGD*
>N.PF11_0277
MTKSICELQNKNSISTELLQEIKKMENELKRKNDEYEKLNANYREIYXXXXXXXXXXXXLEKMNQDKINTIEN
LEKEMMDMKEEKKMVGEKLDLVQSQQRMIQMEIKNLNKEKEQLKEKEETLKNEKEKVKQCKEVLKIEEKQIDT
NKQVLDKEKEQIEKNKEELKLMQQNLNKEKEQIEKNKQELDKEKQIYENDKKNIMLKNEDLKKLLDEYKEEIM
NKEGHIKQIEEKILLLEDEKKNFQNKIDILDNKMQEFKNEITNEKKLNYQLKDDIDNKQNEVIKRDSIIEILY
NKLKEKENDITLFQNENLSLRNCQKELNKILDNKKNEMINIEKDLYEKLAKLLIKKKIRQRDDLHFDGHPQSS
ILKILWFGYVDICKCILKNRDGYLYKGNKNHRFICAYDTDLFLDINVENTKKGLLSFKNETRGIFQLNDEEAK
KLVYVGPFNKFKGFNFCISSNKNNLEIVKGNKCTYIFEEMYVTKKGTKLVLIKEKESGKYFSGLNKNLYLEKK
KRGVDKLDYNNVVNKLINYIANEKEENCNMIIKTSDQKLKIENEKNTCLVQKKKNSDNSYNNEEKTNLVTKRS
KTPIRKENSLHSKYDKTKTTKITNQLKKYDSKEKQNKEKSTSEKQTGKKLASEKNNEEKLTNGKINEDKLNNE
KNNEDKLNNNRELSDENLHKYKIAKEMLLLSDSNSSSTCDDENIYHLKNKYNHTEQNEFIHNNVINKSIATNY
IENINTSLFKYSKCSENYNNTNIVLTTNKEEAILFEIFNYEQIVEHDAIKLASECVFNFLLNYNKSNLHDLSN
IGSEGNSNIFSGIDDDWCILPAYL*
>N.PF11_0279
MVDSGCSILIRNLNYDTSPDKVRKIFENVGKVKDVYLPLDHYTRKPRGFGFVEYFESKYAKEAINILNHSRID
GNEIRIIIAQNRRKSPDTMKFYHNEYLYNYRHKDNRKYNNCKNYKRKYSKYYRTDERDRSRYKRRSTSSSLHK
YKKRKKKKNYSKYSTSTGSHSLTPLSTYSSTTSSIYKRSDDSEYYTKRRKKKKQKRTNKEKRIKKERSITYTN
SYSSYREDKKEQDENKQENYNKHVNTNKHRDQNNEQDDIINKENISNKQVKHKNDDDNKKKRQKRYNTPGIHS
DNSTSQRSSNKYINNKLESMENKTKRNKNFKNINNNNNKKDNSNSKKSNNSNNSNKSNKSNKSNSNSYSNNDE
SNRKSLSCDMCSVQNESIRKKEENKKYTHSHDIMTPESKSTDNQKEEVEKKCRDIYKDEENTDEGHINSNMYT
NLNKNIYSKNKFEDQTCNTNFDDIITDTKEKITEYNNKHNINYNNKLTDDEHIKEKKNALIERSVQSYDNDEI
SDESQKNAENVFFEKVQNVKNNKKKNNLENLIEDKKSDYDIYNDIIDKDSKKENYNSPSLTSSYTSKSISLSK
STSHSICNNRNEKKRIHRDYVHSYHMKKQKKRIKENSTRSKSDSENIYYSSDDSQGEKRNISVEVSRSNPKEN
KEKNKTFKYKDKNKTNIISSSYRRSKSSSMKDSRILKSYIHDTNVNAGFKELKKKVGRDKTKEKNIKDKRDDK
DKNSSYDSPYYSPRDMLSHNSFEKNNLIQKKNEDNISQGKGSYQSSKGSYYINKERSIYKHSNDKYSKRQKNK
SRRTPSKYSNTSSSYDNKKINKHNYNKKKKKKKKYYYSRNNELSRNSYTSSTYSKKRIVSIDTSTNTKDSYSS
SSYTNKKKNKNKYYNKKYYGEHKYKGYRKEAKHSNSRYE*
>N.PF11_0280
YIYIYIYIFFFFFFFFXXXXXXXXXXXXXXXXXXXXXXXXXXXXXXXXXXXXXXXXXXXXXXXXXXXXXXXXX
XXXXXXXXXXXXXXXXXXILKSFDGYMNIRLTNAEEWIHGEFKGTLGEIFLRCNNILYIREDNEKTKTDL*
>N.PF11_0281
MWNKLNDAENTDVDNHGEPRNVFFQRLKKYKTFFYLFFGFLMASLIIYAFYEYYFKKNGDFKLNCNDLNARCL
NYKFPSFKPERLHIVDVNTENNNYILRSSIPLFNGVYSEEKLLSYIKKLFEENNLSYSDNLTLHIISFLRNDL
KEGCSYTSEHCFNKKNNIFNHIIVGHQENPYDINEDEIDDKLKSMSWNTDNLINQIKDLKQKFNTMKNTIFFI
HCRRGRDRTGEFVSAYKMIEQNKDFNSIVEENEKIGKVKQQYVNMQKWLCLYLERIMKNPNVKCFNFL*
>N.PF11_0282

MHLKIVCLSDEVREMYKNHKTHHEGDSGLDLFIVKDEVLKPKSTTFVKLGIKAIALQYKSNYYYKCEKSENKK
KDDDKSNIVNTSFLLFPRSSISKTPLRLANSIGLIDAGYRGEIIAALDNTSDQEYHIKKNDKLVQLVSFTGEP
LSFELVEELDETSRGEGGFGSTSNNKY*
>N.PF11_0283
MRSCYSHKGISPGKHPGNCASYDKIGKEDIQSINNGTYTQCSLCGIIHKDPHKQKLYPLRKDQYSREG*
>N.PF11_0284
MKKHKKTPCKRKYRQRAHCNPLSDSYIKYPRNYTYVDWSLHFPLYFEKNQIKDEYITNKENSKDEHIVTDNMK
NNVENDNMNNVLYLNTNKYPVNYKMRNIFNESYENNKNVTILDIGCGYGGLLFSLSKIFNDKLILGLEIRDKI
TNYVGEKILSYRYNYHPYYQNISVIRTNVIKFLPNYIKKNQIEKIFFCFPDPHFKRHNWRRRIITIENLSLYY
HLLKNNGYIYFITDVYTLYLWVIFCFSKCPYFKVLTNKDCENDICVKLIHESSEESKKVKKNKQNMYYCVAQK
I*
>N.PF11_0285
MMERKMKKNKFILFFFFLFTIKTVHIETRKVCNVLQFGTHAITSNKENRKYFNYEVVGIIDPFDKNRVLWTRK
KRNKKFNLNIFKNIYDSFFCNINDYVYLKNSDQFIYRIFHINLKNTLKYMLKYKIISGKFNHNGDKNIMSLKE
NSKLFRFLNNINI*
>N.PF11_0286
MIIKFLIFNLLLYLFRCNTKVNFTYPQNDFLRIDSENIEEAFENNDYWLINFYAPQCVYCKFIWDKLRYIQRS
IQNENRTKIYFGEVNCDNQKGYTVCEIYGAMRVPQLKLFKGSELISTYSNDITNYNNIKSWIYYITTPLFVGV
QDENELKSYETENNIFLTCSENLPADLFDVAKLYKEECYFINITNPDMCTRMNIKTNELHVMSAYNHSTYDLD
TLDRELLKLFVNKNRFPLVMKIDHRNFFNIRSSGNNLMLLLLDMKKNPNAYILEYKQYAEKYKNHNDILFGYI
DGKYYEENLELYGTDSDYYPQIIVFSKNPREYYFEGYFNIDNVDNIIYDVKNKRIYHKFEEFTKFRXXXXXXX
XXXXXXXXXXXXXXXXXXXXXXXXXXXXXXXXXXXXXXXXXXXXXXXXXXXYIYIYIYMYVCMYIFFLFFFFF
LVF*
>N.PF11_0287
MNDNPVSSYLSKDVLDYEPTKEEIKFYHKNNLKSLRYIFCGKELDDFEKQKIRELKEFVNKLKLKEKDKEKDK
EKEVETYQTIFKNTLFDDDNYVLRFLQGNEFVFERCYNDMLRHLTWRKENLPIPLSDVQIFLDKGYCYIHGRD
KQMHPIIIINCKNIISANTKDVLKVLYYWLEFCISKLLIEGKVEQWRVIIDLTSCSVLNIPIATLKDISKNLS
CNYRSRLCKMLVLSAPLVVTGIWHMLKSIIPVVTQQKITITSSEKEKKLLDQVQANQLEKKFGGTCENATDFT
EPILP*
>N.PF11_0288
MREINVNCVGEMKYETNNNKKKDTNEKNIFLFKEREMKDNNHNMYNNDMHNNDMHNNDMHNDDTAIQEIHIVE
EKKFLIYPENENSLKFDDNIDDDKSTNYSSIDKSVHIINNSICTIKSNLKYIKSIININEATQSDYTYEDEDI
NSYKTSSDTSSFVKTYDNINEEDYTSKNSNENNFEFLLNKVINSKKEEKQLDALDIIDECISIGNLYNRKDKK
HNEINEENKKNNNINNYEFNTQDTKNGYEKLVPNNDNNIITLNNKMVSDVALNISIEQKNKKNNYITQINNIN
DDNKNIINSKQLENIPSNNKQKDTTKFSLLKKLSLNKNSVIQLQNVQDDHTPMGEKNKDSRCEGIKTYNRLGY
CVIQIDKPFTEKRNKLEDYLRIFFKNKERLTILLRERKYLSNFIIYTFFFLSYIFFGFVIIFLKRIVYHIFYD
QDL*
>N.PF11_0289
MASKKREKSCIIFNEEYSNEKENGTINSSGTFYNEEKFIKYVEINSKNIFEYDISKLDIRRVNNHIKLNNIYN
LYCYLIKNTFFKISDKNVNITDMLYVDPIISEKNKNEYNNLSKNEDEKSIIQDGKCDISNSSYDSSYSESTMS
SNNFSNSSDTPICSEISSDVSVKSNKKNNTCKLKILDVIKKFSQLKEKIKKKKKKKVQIKEQIKEQIKEKQM
EKKKKKEKKSILPSGYILKKYDAVHIKGNNNEISISQIYKNVSSNKDDDNKNIIIQLIYRNKECNKFLYNNFE
SYLNYWHVLPCIGYYNKSTINNIINKISIVNFYEFLYLYDEFPVCQTHKKKLKKLYVVEQKDEENVTNPDEFV
TSERTTDIDMLNNGYNEINLSQEMNKEMVDRNLPEKYDEDIEKNHSSVFNFNNCDKNSLMNIHNDPNKNYCIY
DEEIILNDVLQNEKKKTTPCCTFGYYYFETFLFRNNKMIPKLKKKDCIIFNRITKKEDFLFLINAKLYDTIKN
KNNIYYGKNNEYISTYKNFEFLYFFCFNCNTYYDINIIMSQIIYENEIGQKKYMHDKKKNINHKKNVNNKNNI
KNNNKTNNKNKINNKKNITNIINDKNNINNNNKINDKNNINNINENNNNHINNINENNNNNNINNINENNNNN
NNNVSLNNLHKSNIFDDYIYSNNFHSFIVEYKKLYNIKIKRQKIKQTDIYFLCMNCIKNNIFNVTHHMEYFTY
FCDLVNKYNHNFFLYNKYIHHIFNNENISFFSLYNLEEEKNKICSNNIMANQKKNKEEQQQKNKPTKKNSSSS
SCMTFSNVKKRKISDITNIMEDQNVVASEKAISDDNQNKNISKNIKKKKSSKNSNQMGEIKEEEKDILKKLNE
NNKFDENVKANDAYTNDQNVQDNNNNNSNSNSYNYERNRRTTRKKKYIHKKYDDNDEENEEYYYDDDDELFDE
KCLTNEMVNIKLQEEEEEYSFYEDMKKYQDEDIVEENQILNEELYSQDSDVYNDEFSTGGKKKKKKRRKKIKI
QKKDSIKVVHRNSGKTINNNIINDTFQSSYNNNMNSIQNTNRTNSSSSNNNISYNSNNVISHSNVSNNSSIIN
VPEKIKKNEEKKKGNPQDCYINQGDHQKDVHLNFDDLSKLEDRGDKNNLLKYNKVIEKIKQCLDEENVCDKNT

EEISKNIVQTVIDIYKNKLDIKMKLFSICSNLMRKDNSELRKKILNGNITSTNLANMDSSDLAPISLQNKRRE
HERKYFYENIYLRENLIDLKKKSNRNDEEENLYIISNILNKQQNDQDIKHIPINDPNQNLLNNFNKQYDDNIL
SNNKNTTYNTNNEHNTNQYHQDNENHHMSKENINENITSKKEEQNIIDSYTIPSMDRYNFQQTYKNLKQIYET
MPKYASSPILTFLDNSYNRVMTIIETSKNEEV*
>N.PF11_0290
MWNLINYNNENDSNANATFWSIFGYEDKPNENISINENEQKENNLKVEKNIEPELLDENTNKDIRRKKKKKN
KLKDNIDKSPFLSDISAYADYCTSDDVTDKYSLNENNDQMISSESNRRNYSNDISEKDDLTIQEEKEIIIEKE
NDLTVQEENDITIQEEKELIGEKYNDMTVQEEKELIDEKENDLTVQENKTERMVEENYNNSSLPYANEWLFDK
IKEILDPSINQESISELLTHKYNSFNEANTGIFDLLENLLVRSGSNNQENMEISKQKKGNYSYNHYNSMDESI
NEHYNFINYSIHCINKELSANVELEATKMMKIYNHSLNKYKNLINFKDDIYQDLVNEGEEISTLIFDENKKIK
KRIPKIDFLVLININSDNYNNIIESHNVQTPKLNLEEIFKINLI*
>N.PF11_0291.1
MNNSYDKSNVESSTVNEDTEHKENLSYKINEELYYSDVKHIHSSNVDEIQNDNGNVDYTNEYNMGNVEMNGGN
YMNKENINMEKKVCGEYENVNSFHNDNLINNSVNIVENENILLRNEYKESKKNPNNRMNNNIGNNGGSTTSSH
NNKKKKKNVTFSDDNMYNISYKDVNNNLINYYMDMNNVNSVNSVNSVNNMNNVNNMNNVNNMSNMNNMNNVNN
MNNMNNINNVNNINNVNNMNNIPNGYYNNEFKTGNYQIVMKHMNDHINYVNNTDINGKGRYNIISLKQNNSNN
NVNKLNNNYIEYDINNNSGMYESCENNYIYNNKNNYFNSDIGNINQDDNNVNNINNNFVVKYLKDLDKIYQLK
KHNKKLDNHKNVNNYYNMLDLLYNSCKKKKNLNDKRIRALLYIFKYIKNLEKKKEHNNSDIFDIKTLRRLNKQ
VDLYIDLLEKREYKENMHIELKQERMDTNRNNNNDKNNDKVNDHQFKHFTDSYAHNKFINNIMKKKCDLFFKS
NFNNIHVQSPEQNFYFNHSLKKVNYINNLLVYIHFIVFKYITLYSINEWNVLLFKDIDAYNVCGINNDQSLNK
TSASIVSPDSMGDVNNVIEDDNKSDHPISNSNNNNNNNDDDDVDDHVNKDDNNGEGAISPQINDQNCLEQNNS
TCEIAMMCNFHLDDYKIEKLNPLNMNENNTIDINYYEYVIEPFDFLFYDQVYNEIYNYLQMLKNQSLFQNDES
NNNNNNFSGYNNSGDDNSQYMRQLPFQYYEFMEILTKEVINVRDLIYRNKYVRYLFYYIYRNNKNNLLEGINK
IFFLEKNKVLLYKIQTKLRYNIFVTRFLESNGLSTSNEEEIEKQKKISFVEAKNKLFDSMFNEYNIYYNEYMK
NVDKYNLIKKNVFNIEEKKFTYIKKKINIYSDYFEHMKKKLAVESYFPHERFEEKIDEHQIEMNNNNDDNNGD
NNGDNNGDNNDDNNDDNNNDNNGDNNGDNNGDNNHICDVFKNINLVEYEKEKIQFDVPFNYVEFLYDKNYALI
INSEKNNSRKLKAAYIVNKNLKNKENILIITSILNIMKWKSLFFTFSNITIYKNEENIIENKVEHTNNNLIIC
IDFLPKTLNVTCDIIILDMCHFNIINQISILNELCFLNYKKKILIFNNICDNWNFISSLFLLNNPFFNKSFLD
NIQMNYSEDDKKIINNMMLDFISHFCILNKQKNVFFKDQIKEQNIINLNIKDDEQNDISKNNDNKNEDTLLQL
NNEKEIKKNKNLFFILTYMSGVQKCIYDHADEKHKRDTCIHPLLLLNEDLFLLKDFNISEKFEILKNIIKKCI
LIKKKIVICYSGDEKILKLIKILLHLYGLNDFNAITYFTLPNNNQEQKENLNNLDIYDVSIIINDHISFLKYF
KNVNITCYFLISTFTKEEDELLQLYGHEMKELYFYKKKKKEIMKANKSYRDFFYRYYLNNLISDTDEQFLMFN
IIDQKEKIYCNVNYNEMVLPTCFVSSISDPDQATQYSEHWKDIKDAHNFWNFNTFSKNKINFYLNKKNTVYDK
YENVIYYYLENPMKERNVFNGCISVALEDIIQELNRKKEMNDFDEMKKNVLLNIQQKANKKHFTSVRKVEKIL
SKFLEENKSKSFDQFLKGCEAYGQIISKCKEKLFVTFNKTYNVQFKNFEDFWCDDEKKRKEKWKKLWDMNEKN
ECEKESNLLFNVCNEKSDLTDFSKSSSFDKSKGDANLNEKKNFRIRLSSQLLEEKDLDQLYHGSKKVCLLNDS
TEKDKKTCLYNERKEE*
>N.PF11_0291:mRNA
MNNSYDKSNVESSTVNEDTEHKENLSYKINEELYYSDVKHIHSSNVDEIQNDNGNVDYTNEYNMGNVEMNGGN
YMNKENINMEKKVCGEYENVNSFHNDNLINNSVNIVENENILLRNEYKESKKNPNNRMNNNIGNNGGSTTSSH
NNKKKKKNVTFSDDNMYNISYKDVNNNLINYYMDMNNVNSVNSVNSVNNMNNVNNMNNVNNMSNMNNMNNVNN
MNNMNNINNVNNINNVNNMNNIPNGYYNNEFKTGNYQIVMKHMNDHINYVNNTDINGKGRYNIISLKQNNSNN
NVNKLNNNYIEYDINNNSGMYESCENNYIYNNKNNYFNSDIGNINQDDNNVNNINNNFVVKYLKDLDKIYQLK
KHNKKLDNHKNVNNYYNMLDLLYNSCKKKKNLNDKRIRALLYIFKYIKNLEKKKEHNNSDIFDIKTLRRLNKQ
VDLYIDLLEKREYKENMHIELKQERMDTNRNNNNDKNNDKVNDHQFKHFTDSYAHNKFINNIMKKKCDLFFKS
NFNNIHVQSPEQNFYFNHSLKKVNYINNLLVYIHFIVFKYITLYSINEWNVLLFKDIDAYNVCGINNDQSLNK
TSASIVSPDSMGDVNNVIEDDNKSDHPISNSNNNNNNNDDDDVDDHVNKDDNNGEGAISPQINDQNCLEQNNS
TCEIAMMCNFHLDDYKIEKLNPLNMNENNTIDINYYEYVIEPFDFLFYDQVYNEIYNYLQMLKNQSLFQNDES
NNNNNNFSGYNNSGDDNSQYMRQLPFQYYEFMEILTKEVINVRDLIYRNKYVRYLFYYIYRNNKNNLLEGINK
IFFLEKNKVLLYKIQTKLRYNIFVTRFLESNGLSTSNEEEIEKQKKISFVEAKNKLFDSMFNEYNIYYNEYMK
NVDKYNLIKKNVFNIEEKKFTYIKKKINIYSDYFEHMKKKLAVESYFPHERFEEKIDEHQIEMNNNNDDNNGD
NNGDNNGDNNDDNNDDNNNDNNGDNNGDNNGDNNHICDVFKNINLVEYEKEKIQFDVPFNYVEFLYDKNYALI
INSEKNNSRKLKAAYIVNKNLKNKENILIITSILNIMKWKSLFFTFSNITIYKNEENIIENKVEHTNNNLIIC
IDFLPKTLNVTCDIIILDMCHFNIINQISILNELCFLNYKKKILIFNNICDNWNFISSLFLLNNPFFNKSFLD

NIQMNYSEDDKKIINNMMLDFISHFCILNKQKNVFFKDQIKEQNIINLNIKDDEQNDISKNNDNKNEDTLLQL
NNEKEIKKNKNLFFILTYMSGVQKCIYDHADEKHKRDTCIHPLLLLNEDLFLLKDFNISEKFEILKNIIKKCI
LIKKKIVICYSGDEKILKLIKILLHLYGLNDFNAITYFTLPNNNQEQKENLNNLDIYDVSIIINDHISFLKYF
KNVNITCYFLISTFTKEEDELLQLYGHEMKELYFYKKKKKEIMKANKSYRDFFYRYYLNNLISDTDEQFLMFN
IIDQKEKIYCNVNYNEMVLPTCFVSSISDPDQATQYSEHWKDIKDAHNFWNFNTFSKNKINFYLNKKNTVYDK
YENVVSPISEESIAPPQIYYYLENPMKERNVFNGCISVALEDIIQELNRKKEMNDFDEMKKNVLLNIQQKANK
KHFTSVRKVEKILSKFLEENKSKSFDQFLKGCEAYGQIISKCKEKLFVTFNKTYNVQFKNFEDFWCDDEKKRK
EKWKKLWDMNEKNECEKESNLLFNVCNEKSDLTDFSKSSSFDKSKGDANLNEKKNFRIRLSSQLLEEKDLDQL
YHGSKKVCLLNDSTEKDKKTCLYNERKEE*
>N.PF11_0292
MACDLEKIEENEREKPNIVIQLNTLGLDELVSLTLKLEQEWKIIKKNYAILESAVVTYDKCKNAVELLNPSKY
KTKNFNLFMNELERSELIKLCNKDKTPEENINKETEEKNDKENIGDISSSKENTDINEEKKDDQQEEKQIIKE
DEKKSFDVHIPLTSLVYIPGKIVNTDNFLVRMGTNYYVERNSTQVIEYYNNKIKKINEQITKLKITIIEKKNE
IDLCKNYIQIKRREQQMNALNMPQQPTSS*
>N.PF11_0293
MDHQDLKPVIWHKTEKKTKPKDIHEARKLGIDVEVEKKYFGGKNKSSKGNLIIENKAKIEQETENFKIDRVTP
AFSRALQQARISKKLTQAQLARLVNESESVIKEYENGKAIPNNVIIQKLNKVLGVNLPSPKKK*
>N.PF11_0294
MDIENSKKHLSELQEERRRHKITLPDMFKRKVLIKEENNDTYEKNLETKNDMCYSLNRSDDEKEYLKMKFPNI
INNPIIKLYTDPYYTIHMSDKKMNTTDSINTSMDMSSYNYTTNSFMKEMINMYKPHEKLNIGILFTGQQTPGG
HNIICGVFDCLKKKNKKNTLYGFMNGFQGMLKYNFMELKNEYISMFRNSGGFDMIKSSDIQIINEDQKKKCFK
ICRQLNLNGLVIIGDVSGNKSTAILSEYFETVYKITNEKIKSQELKEKNKEETKCKQDGEKNEKEEQQNEQDD
EKNKEQQKCKQDDEKNKEQQNEQRDDEQNKEQQKCKQDDEQNKEQQNEQRDDEQINMEDNNNICEILKNYVQE
DKIYKPYNIIDNNKTCVSNENKSNNNIPYDNIQSYNMSYDNISTYDFTYDNISDISDEEINKKYPKKNKSFVK
TSIVSAPTCVYNEMKNKYIECSLGFDTTIFSYCQYISYLITHIQTYLQGYHFIKVMGNSSSHIALECFLQTKV
NIILVTEEIKKNNLTLDQIIQFIVDVIQNRYKKYHKNYGIVIIPDGILKKITQFKKLVTSIIHIKTKFIENKI
NILTDLQNILSKHLIKEQQEFFKTLPDFFQIQLLEEICSQQFHYARLSTELLLTHLVKKKLEERQINDLEFHT
HSYGKEVTCSLPTNFDCAYSYILGFSCVEMLQHKYNGYMCVIKKLKNLLLNVNDIEILGIPLCHIMKIKKKNK
NVAQINIVEQVKSAGEKKKKIEEKDNPQEIKENGKINKAEKPTKKNKTEKKDKVTKKDKTSKKDKNNTTDRVK
KSSSNIQNDNVNKKDDALTNDLCEPTDSTTNNQNKNALVIRNDPFEEFFIKRTKVNLKDDAYFIKYKKYRYMY
LYEDHYRVLTGIQYEHNFCIDYDDKSLKFRNKLNSTLSSNDYITIKPNNIFSKINFTISGLTKEYFNKKEHNE
EKSSMTQNNILQNGVILQNNNTYKFNYCKHSSMLSEIEKMLTASAIKFNTILIRHNTRITYINNNFFNKLYTI
SFIHIPKIKDFYILQDNQFHLKQNYRNVTCPKNVGVVLLSHCTPGTNNILVGLHQRLSINNFKLIGFIKGLKG
LLNNDICLINDNNLKTSINIGGFPLLGVHIQYEKKTNDDQILHIHDLIKENNISKIIKSCKNNNITNLVFIGD
EKVITLMNILNDIFINKDVNIKIVSIPISLYNSFDKNLIECSIGYHSMVRHISDIISNVQSNCLNINTYYYFI
KIHTNISSSLILSVQLQTHCNICYIGESLNNQLTSLHTVIENISLIIIERINRMKYYGVILFSSNLIYYINDF
HELCKDVDENIKNVQEIDEIVKNDILPERFQKILKKESVDLLNLLTESMKDKLLRKEKRENEDIDSNFEVMLI
NEIKKYIQNLMEKSKQNKELYCYKKHMNTISSPINVKNIKVYSDIDYILHFQTLIKVIDREINCFFPTHFDNS
LAFSHGLLAGIAVENDLLNYVTSIKHLNLNKQNWSSSLYPGIYFINNKDDKGHFNEYPSVAPVPISMKSSQMA
TMKHNANTWAYSDSYIFVGPVQYSVNPDNLGYSSYIF*
>N.PF11_0295
MENEQNNQDSENGLDYFRSMYDRYRDVFINHINDYVLEDDIKIIISKYYKLLFDYNCLGGKNNRGILVILIYE
YVKNRDINCNEWEKVACIAWCIEILQASFLVADDIMDKGETRRNKHCWYLLKDVEIKNAVNDVFLLYNAIYKL
LDVYLRNDNCYLDLITSFREATLKTIVGQHLDTNIFSDKYSHIDKDIDVNNINISQENKININMLNFKVYQNI
IIHKTAYYSFFLPIVCGMQMGGISLDNLLYKKVENIAILMGEYFQVHDDYIDTFGDSKKTGKVGSDIQNNKLT
WPLIKXXXXXXXXXXXXXXXXXXXXXXXXXXXXXXXXXXXXXXXXXXXXXXXXAINQLHHEGIEYVL
KYVMDILFTGA*
>N.PF11_0296
MDSDLIDEEFVYGQKIGKIKKKKELILDTLKSVIDPDLKKNIVELNFVRNLKINEDEKGEKYFVEFDLNLTTP
ACPVKDDLLRECKEKLGLFEWIHDINITFINFNEKRGNNKNKIENIILIYSCKGGVGKSFFSVNFSYYLKK
KGASVGILDADINGPSLPTLLPFDYEYVKFKVVNKKKDEPTKGKNGYIYNDDMNNNNNDDVDNNDMNNNIDNN
NIDNNNIHNNNIHNNNLHNNNLHNNLHNNLQNSDGKNTFCGEKKKKNSFTHEKYFITQYDNEPKDDDDDFFFN
LSLEKRKEKKKKKKPNRENLLNIFNSKNYNKLSNDDKKNSGTIEYDENLSMIEPLIYNDVKLMSYAYIKNKMN
LGFSSFRGPILNELIKEFLYHVNWGILDYLIIDMPPGTNDIHLNLFESEKIDGIIMITTPNDLSTNDVEKGIN

MCNFFNIPIVCLVINMNYFICDNCDKKHYIFNNTDMKSLKDKIRNIYEIPFHPILAKNVYTHKHNYKKDTTNV
LSIQDANIKHNEYTQQKMEHISEEHIKHFPFILAYDKNHFLIEHLENLFQQIVREIAILKYNHNLNIPNLQIY
KKNYLQLSFDTIQNKYVFSDDVLICHIKDIRRKCKCLICKENKKEKKCIQYDKNNNHNNYNFKKKISLFPPNI
YVKDIIPLGIYNVKIVWSDNHSSIYSYVYLKHLFEKKRINNNNLFCKTNNSGMYEW*
>N.PF11_0297
MENNNINLNSNKEKNLNISSTYKKCFNKDDNVHNISDNFKDDNKSVNLFLRKNEQHVDCPEIILDIENIEHTD
DKKGKLCIDSSHKKPDENIKKEHQNNEHDMLEKIHKGNHIDNIFSDNKNEINFISEENKKKNIYEQDTTYGDE
YLMSTEHNDAHINNDKDKICTCNEHNDAHINNDKEKNFTCYEHNGAHINNDKEKNFTCYEHNEEKIIIQKNVE
VSDKKKNFNTTDKEYINKINNKNCNIQDNHHDVSHEKNMLINENVRNINDTFSKTINSKFPKEEKRNEIKKKN
GNNQKTIINDDVVYDINEEHTSADEKIEKLRNDDILKSGDILKNNKILSCEEEGEEEKNELSLEEILYETTER
SKSYNRKNYGLLGILKVIKMTDPNLNILAIGSDLTTLGLNLNSPNYICTSVSSPFSENPIENEEDFIKPVSYL
NTKFQIRLSLLLKLQTETLFYIFYNLPRDVLQAYAASELYIRKWIYHIIYKKWFTPNNATSTINLEKCASWIY
FDPLTWSKKTYNNYLNVKDDIMNVEDVTKCIEQIIKLQSYYNIHQQNI*
>N.PF11_0298
MGIKIIIYIFFLSWAKWVCGSVNFTGFDNKNMIGKHVELEGRYKKEYIDRFFLEELRKHNYMNNNVILLSTSR
HYFNYRHTTNLLIAYKYLKYFGDTMDKNILLMIPFDQACDCRNIREGQIFREYELFPSSHNKETKIENINLYE
NLNIDYKNNNVRDEQIRRVLRHRYDAFTPKKNRLYNNGNNEKNLFLYMTGHGGVNFLKIQEFNIISSSEFNIY
IQELLIKNFYKYIFVIIDTCQGYSFYDDILNFVYKKKINNIFFLSSSKRNENSYSLFSSSYLSVSTVDRFTYH
FFNYLQQIHKIYEKEPSKNIKAFSLYNILNYLKTQHIMSEPTTNNSKFNSSIFLHDKNILFFNSNLLIIHKDD
VSIIYQDKQTHNHKYICLDNLSKCGHIKNNVHKKMQTLYEQTLYYNNNQQNFFSNHMSNFTDYFFTHDIYNIY
NIYNVYNIYNVYDIYNVYSFLILLLSLFFIMCSLLTYYIVFFTEKAKMT*
>N.PF11_0300
MNIRHVDGCSYFERKQDETCKDEIKKVMDNLIYERKRNFKENDNYCYNSGMSICPNIYCNIYTDLYNWFGFKN
CRNIDTESLNCCFTDVTNGGIGYYEKLIVLGGRILELYSELHFYNKYNYEKNEEIVEDLKNMKCIKEVVYHYL
YVQKRKNRVIYKNSLYLFGYSYLYTPLFFNNKNSIIKYVKACIAYAVKFNESNIFSWMPSLIEHIYYSQKLKV
SQIEEENELIQDLQEYMGYQFDYILPRIAECFSYHNIYISNSHLTGLQIIQLFSNEYFSLLADDVGICSKEKT
IWVKEETDAFLNSKCIEIFSGSMISINKFLNENNVKMDKIKNIFEMLLSSCWVLGNEKRNRIWVSALIQRLRQ
VQLLTQITTIEKWCYNIKKYNDINITNFVKDIYSFNNINGNILSPFQLHNINTYIPSKETTLKTLPYYKTMVD
KYKHKDNDKQKHNGNKIKDWNFYSILNNISNNIYKHFVPRNQGNKKSSNINKFIYTNNETVQQDVFNSADIKE
LYLKAQVPPCDNSYMSSSVQSDDMNEEEYEKYDLINHAQKKKQNGIPVKIKYVRDTLDDENSKYVIEIPKKDL
KGHEKKDHIKAVLWGNSKKGLEIKLPGTHNFIPQMKIK*
>N.PF11_0301
MDKLISNNKLKLSVVLLGGLCSLAYYHLKNKFHLSQFCFSKKWFSEFSIMWPGQAFSVEIKKILYETKSKYQN
VLVFESTTYGKVLVLDGVIQLTEKDEFAYHEMMTHIPMTVSKEPKNVLVVGGGDGGIIRELCKYKSVENIDIC
EIDETVIEVSKIYFKNISCGYEDKRVNVFIEDASKFLENVTNTYDVIIVDSSDPIGPAETLFNQNFYEKIYNA
LKPNGYCVAQCESLWIHVGTIKNMIGYAKKLFKKVEYANISIPTYPCGCIGILCCSKTDTGLTKPNKKLESKE
FADLKYYNYENHSAAFKLPAFLLKEIENI*
>N.PF11_0302
MIKIILASFLFFFYFSSSIYGNVVAPKSAEAVHTLTAKLDTENKAHQKYIDIINEVFSENMENFVFSITTSNG
FTCSINKLDVIFDNKVTGMQKVFSDENVQYIKEASLEFKNNLLEYMKGVMPSENDFSNVRKEFQKVCVKYFSE
LRRKFFQIYRDPNNKNQNEDNVDNHMNIEEISQEFFSGFNPFGTMKIRKEQNPDLSQKDSLILNENNNDSLTS
ADQKNNNMVPMENRSFSYSQQSSHVVSFDGHDEHVEQQEQHSGDNTQEDKDQLMDLPFNKDKVFSTFLKNVTL
LIEGNCTEAQQQAQEQAQEQTQEQTEEQEEEQNEKEIESDKQVQKDGEQKENDVQKKQKANAFTIQFSSSLSR
KHISTTCNASSGNVFLRKGNKPFEHFNSPNFENMMKGVFGIIQNPFENGQPTFPFFKNINSISGIGNLDIPKN
FMNDFLNNGNDQNTPISS*
>N.PF11_0303
MEDNKEENCELNKNSYPNFHLADLFYLLQLPNISINERNDLLQSLFDEIKKNHMYPYYNYICQELNLNFDEEF
FKSLKEKADEELNQIENKLSESAENFDSLDNKNDVLLKANFFCKISDKENAFKEYEETYKKGIGMGMKLDILL
TMIRICIFYNDVKNLKKYLEQARTQMEKGGDWERKNKLKIYEALNYIMIRNFAEASKILIDAASTFTATEIIS
YEKIIFYVVILGIMTEERTVLDKKILNSSVILQITSSDEDLHTYLHSFYHCDYRTFMEKTIKIAMRVKRDRYL
GRHYRYFIRNTRVRAYKQFLEPFKNVTLKNMAFAFGVSEEFIENEISSFIANGKLNCKIDKVNGSIESNQPNE
RNTMYQNTIKKGDILLNRIQKLSRVIDM*
>N.PF11_0304

MRSHSTKDPFRENFQKLMKKKKKPEQVRKYEQFRKKKKKKKNIHNITKDFIEPHKMLEKFKSIKKKKCFFILS
LNIDKKNEEKLRNSKNKIKFIYENFLEKDYQIIVKPSNKFLDTCNHGQIKPCSLKKCYNKDDIKVNFLNDMET
ELKLNVLHKNVKYNENIENKKRIEGTEIEMLKNMSYKENNISTSIRKKENGYGSTNETSENFLIKNHYMRQNK
NVYIKWIREKKEDDSFEKINYMKSNNFALNNNKIKINFDKTKVKFVHLKCPTKKLVKTHNHKNIKEHIYLKDI
FDLKNQKTKNILTNFIRRENKNEDIKENEPNNFNKTNLNNTIKRKIIFSNIYKNYLINKYKSSNEFLKTNRFN
SKGFIMNQFLNINSFTDNNTNADHTNFEINENTNNIKDVKNVRNINTCNNNSNSNNNIRDMIKKKNLYHQNEY
NRNVIDTNICDRKKIRTTYLNFLPKEKKGYLNSFLHDIYRLDRTFKLRKKNKILRKDRIVKLFVDNDNVYIKD
FKIYQNEERYKNVLSNVKDIEIMKRDILSLPENPLHLYELENYKCKINTSIIYIPALKIENEDMNLFKYKNTW
YELFFYNNQNNKDTLYNYQIIRLNFELMVSKIKDIVIIDKNTQHFNYPYIYNAYLVPPIDVYQLDNSLRIQKI
TSDNAFINNGSWTFFQKKKKRNSYDINMRRNKYYIDNCSTIKAVKKNFHLHLKNDIDSSFLKLKENQNKNQYQ
YQNKNKNDLNNNNNNNTCNHNTNTLTIDKQNNDTYMNLFQNKKQNFFNLYNNTINKSNELYKNKVSIKLLPNY
IFESNIHKKNSQEIIDNKNDVSIFYKQNKNISFYDDIIYGCSCIYMTFSNSKLEPVYPYISNNLFFLFEYYYN
YLVNMIKNVKLNKIKYKSILPKGKYLSCSNKNIETNKNIVYNNKGKMIKNVKQSTENKFMYLKRNRALANIMY
KHKFIQNVSYAARNKSSFLKIPRKTQIHNFKIRNFLNFKLYNSIFDTIREKNRSKIKKELSKEEVQVPIFLWV
IFGGKDMKSMDSLNIVYKVLRYATEIPPDEYQKYIRNLKKVQINKSTNGTLQKDFSINNMFRNVKQEKERTKY
KYEHNDLTRRDVINKHITNQNVTHHTSQKFHNLPKNIFYDTNLYNTSTEIYKRILKYYDNYKEKYKEYGPHNY
HEFLEYYKNIRKNNYYDNMYVHTPNIKKNVQNMNTFERFDQEKNNKKCLKKYGRCVKFAQMHSNNKNNCVDNT
LKRNESPRFIDKEKSSSEEMLDKIEHHLKEKNEEISEKLSEEISEGKKDGAMEDTCDKTHEETNNETDDGINK
KTYNKIYNETYDETYDIPSAQKNRGDSEWYDLYMEPSDNENSDVNTDDELYNFISYHMQGRNPSFEKKKKNNN
NNWNKEKLNYTDIYGPVIYKKKKKNQNAEYAFLLLDYPAYNNSIGHPSPLTFKTSAMSALKVAIKEIKEKNKN
NNSISINIFGYSLGCSVTLQLVLDIAKSLYNDFFEDIKKVCYEGKEIEPIKEIKNEGNLYIKLNNNNNNNNEK
INEYVYDEKKILTFQDILGDQINCSSSSIMKEYILNRKRYNNEKNINEQDKKGDDFCDNILCPIKDNNDSDMN
MITKENTFDIYTNFDNVKEKNYNIEKKITINELLNKVRERKKRKEEILKEYLKITVDKVVLVAPFTNTQQLVK
SVVNNSVLFLLSSFIMNKKCSYVHWDNMIVLKEFFKIINDFKKNKYLNDIFYNLQIDFIHGQKDTLVNYEMSM
KLYNLTNKLIFKYSINNIKTFLHIFKDDCHSSVFNSEAENKILQIMFRPLKLHPFSTINIHKIHYNLYKDIYL
LKTTYLRFIASASSKILNS*
>N.PF11_0305
MSNAEALNDNKNDEDEYKEKTLNLFDNDNFVLNNSDEEEYASNSYDNIEDDDDEEEVDNDEEGLEDPPSSDNM
SYDDLASQHEDDKYNEEETFNEDDNYNEENKYNDDEGSDEGYMKKIDGKSLGLTKRMKKIYINKKENIYHNQI
GVYKNDKLMKNEDIEDRMKYLLILLNDTKKNNIKIERSTIINELLFYYTYYYEYSKEMIKYLYYIFDIKELYL
FLEINNMPKEIHLRTNTLKITRNNLMKILKNQNVSVQEGESWNHVGITIKDVNTNVGSLNEYLYGYYIIQSSS
SLMPVLELNIQEDDMVLDMCAAPGGKCTFMCTLKKNRGVVYANDINKLRCKAIEAHASRMGINNLIVTCIDAL
KIQKHLTFQFDKILLDAPCSGTGVVNKNKTARRKTIKEIRDLAQKQKILLNNAINLLKNGGIVVYSTCSISVE
ENEQVINYILKKRDVNLLPTNINIGDPGITQYRKKQFSSKISLCKRIYLHKHNHDNFFIAKLIKRSDAVFTNK
DKNSNEKKKNKDKGKKKKKKNDNRNNISQTNLTNHQHVEYNKNEIKKNNKLTNKKKENINKHKNEKQQQTLNV
PKKNEHFKINNKEINKKNKKYKEGKKEKKNKKNKNNKKNKKNMKNIKNKV*
>N.PF11_0306
MYFFFFFFFFFFDTIYIYFILMMKNESTQKDKFNNIKEEEDVTLPNEQSDYHYSFLQRYSVELKKDLLNWYYK
YRRKLPWRGDEPPFTTSVQLYENKKQVDIRCFFGNHKRDEIKIKNSKSDNVKNNEKIKGTKIMKIKKEHNELS
LERCKRLKKEDKDEEKKYNSLSDSLKSENIKSLHDKINNNNNNNNDDDVVDKNNCNSSLLVHKEEKKHNFINN
LMYDKEHLSTKGYQIYISEIMLQQTKVHTVLNFYLKWMNKWNNIFDLAKCNLDDVLILWKGLGYYNRAKNLLE
CCKIVVDKYNGIFPNDLKLLKTLPGIGDYTSKAICIHLYNRKDICIDTNIIRIFSRITDTINYYNSGTLLKHC
EKVSEILCSGESNYSDLSQAFMDLGSSVCNNSPDCSQCPINKYCMIYLKSNKKKQHNLFNTKHPEHCNLCVND
RNVEIKYVPLAKRKKKTDKICLVLLIKQNDKKKKNNMNKNSCTKKLEKKKTASRQIKESYLEDTYMMIKNTDT
NLFSMHYLFPFILLDTYDKNDCVKHFNDLLKSLNVTNSEKDRYLYINNFKHKFSHLTYHTHIYLCTVSDWENI
TKNNEERKWVILKDIRNFTHNTFCQNIIDSYKKSMNEKINGLSEYCI*
>N.PF11_0307
MGNKLTCGEIRNDGVRGLKNAYNLELNKSNICVENSTTCENSYDEENYFYRYEDVSYVEEGEERKREYSEDEI
KEEVKIVVDNNNNNIYNYHNEIKDKNKLMKKRENQYIDKKYMINVKRILMYIKEYEEDFLCFFKKNNATSIIY
LKYIVLNYKTYIMIIEKGVIYIGELNEKNEKNGLGIIITPDQCIYIGEFEEDKITGFGLYIHYSKSKYIGYWR
RGKANRYGIFIHPDGTFYKGFWLNDKQNKNGIEYVYNNYVYIGNYCKGEKNEFGIFVWNNECMYMGNIKNNYF
FNRGIYFFNKYKIYIGKWKSNCVDGKCEIIWKDNRQFFGYHLNNLKQGLGIYKWDDGRIYFGNWLNNKQHGSG
IFIIIKNFKEYEHYIQYPILLLFKNTEKIKKNFLLNLSKENIFHKGNIGKYFHNLLNNYDNYNFYNFLFNLLY
INFYDLCSTFFDYIKKKNIIHFKTNHNFYNIIKDHICLITDHYFNNKVSNNKIAQADSSFSFLLTDAHDTEKN

RDQRYINHNNYNNHNNHNNYNIHNNYNIHNNYNNHNNHSNNHNNHSNNNNNHNNHMDDYQKYPSPNNTNMKNN
ENEMLIHSTNNQKNNTLYNQTTGCDNEKIIADKQKYENNKILKGYENYQGPEHHKISSPTHQQINRTSNVLPF
QDIQRLILYINSINLNDMSEKDINKHNPIFSYASKNILLKYGKWKNGKLKKWLYASDDSISVVNRDTINNDTN
VLSYNGKNKGGRIKHFLKNKTEGNSNNHMDNKNNMDNKKNMNNKNNMDNNNNMDNKKYMDNKKYMDNNNNMDN
KKNMNNKNNMDNKNNMNNKNNMDNKKNMNTNHKVNSNNNSNISSNISSNISSNISNNISSNISNNISSNISSN
ISRKHNSYSFSSLSSSSTKSLYNTMKKKKKKKKYINERIYDSAHEIQKDLKSKGLIPQNDNNKYIYHNKEKKK
DISYDNDHYCYHSDEDDNIYLHDEEKKNIHVPHDIKNIQNDNVEKFIKPQGINHKFSNDYNFKQHHLSFIHNI
KEYNDMQLHENRNTKKNIKKNEKKKNKLNNKLMYYKYSYITTSTSDHISTKSPNSISSLNTNVNSSIQSLEDK
RKDKTHKNTRNYLNDKRVATDISYKHIQKENKEETEITYVNNIMKENEKHQNDDEDIKNVEKINRVKNINTKE
NINQINKNEKMISCAKTGDNNMTYVNLDDMKKKNKQNMIIKKKGIIRQNKMNRKETISNESLMNQMETDKKNL
TNTRNTKNSTLPYKYKNYDLPKKGFSLIWSLKKSRRNSPLSTKEKIMYQNDDQSCDDHYDKKYVQNNEITDNQ
QKKKSFFRNFLSVNKNKKKNTH*
>N.PF11_0308
MSNKIRHYMNYNIKEKDSNKWNFYINLRDVTDLNELDDLLNEGTKCYDRYNNEHKINNINKKSLLGEIIKAYI
FCKSVKIKETCNLCLMSWISTRQCWYDCDELLLNKYFDGLLEPLVFTFKILREKVIYHFYQIPKNLYMVFINK
FKKYLKIYKDKEIILSIITSTLDILYSKVFYFKV*
>N.PF11_0309
MSDYDSLTQNKAFSIEEIYGSDITDEENKLIIEFMRNMVNERNSLPNNVNIEINVLNNNDSNNKNFCNKTHNL
LNILNKYTLKEKLDEKYLKNENKITWKSNIVEYINRLRYYIHKKYNRNVIDTFKYNSCNCSILSSLQKDNDLI
QIFSSQVFCVHLYIYGINYKQIIYYIDIIANHLNNNNNEANFIFFLWLIYLLILLDSLQALDSNVSSNLQIIK
RFCINKIENPDPNYVESKQDNFLSFLKNFYCTSKNMNNYIPSYYTVDIFYVIYIIITDIFNQK*
>N.PF11_0310
MSSKKKSTIILSHSGKEDVSVSAEKKEISKREMSSNDNVNNNEKKKGLLSYLSLKGYDMPSSLDDLLKKEEIR
LSSEQKTPFNINRSVLLFVYFMLIVLTNRLFFGWPNLSNLLFREDTYIWKCQKNEHGEYDRFDDKRYSCDEQD
KAVQTIFIFGSSAYFAFSFFNGLIVDYLGSRFSMLLGHILNLIGWVLMLMSNEHFDAYVIGGIFMSASIDLAS
FSTLNASGLFPGNENLIVNIISGAGSLSTGTMTILDLIITRYNLPFKTFMLWYMCISVSFFFLLTIFLFPKNR
YYRQYEFDNYYNNKEIDLKDYEDFDNSTKKIYDHDKKKDGHQYNNNVHSTVGVNNSLVVKKHEGGINRRELEL
KNVNSSKHVFNDLENNDSKKDEYAASTNNSSNLVKSKFNIFNSPTVKDLIKIFTCAHFLCLWIYGPLNAIYNT
FYFSVVENILSKDKNDILGYILPFSFIPCVLLGNLSDKFGVMLMFTYELIFAFSMYAFSYIKSNWAQWISVIS
NALYSACANGQLWTFISFTFSSKYHSTLIGFLNLVCGMVSFVRLSLFEWAKYTNYDFTYINLFILALIVVNIV
VVICMVFIRRVKGEKVTYGDDASSK*
>N.PF11_0311
MNIICNMKDFKESWFYKKIKPCIKKYMPKYTNEGHIIFQNPYEFSYGNCGYREKYNLSSCDLLNAINKCGIFV
GLLFIKYNYDIIFNQKEKRNDFTTTKGNNNNYENKDENNEKKNEKKNEKKNKNKNEKKNKNKNENNEKKNENK
NKNKNENNENTNENNKNNKNNSNSNNNNNYYYYNHYNMCEKIHKDLVYNYNEYIKLKNVGIIITASHNPHDEN
GIKIIGVDGKYINKTYENYLIELVNNHLRYIKKNVNCTCDDIINNTIELIVDIFKKEINLDISDDIIYENISI
LDDIIYNYNIHNKIKTNICIGFDTRNSNIHLNNIIIESLNCLNIYKCINNITYITTPCMHFLIYFLNYINEND
EKIDKQIIQQEEYTIHKKSNDLSYLKNFHIQNNKSVYYLYYSKYDQYLTNEVLTTNGTPLQQPFNISIQQHIE
KEKEKKNSHENNNFIYTHMEHIYAYNSDQIYFDYFIYSFEMLYNYITKLFHNNHMMMDMENIYLDCSNGIASL
KIDKFYPIFQILKKNICKFNCIEGEHSILNYECGAEYVVYRKQQPPKNVPPIIKHNTKFCTFDGDADRILYFFF
PQKGEEIKKKIFNDGNEKNKNNHTDININCNMIYNMIYNNNNNINCKDNNFHSTISDFSKEQMDYYDANIDTC
KNNQIAILDGPKIICLFFLCIIKMLSHIKLEELKEEIPIIDLNIIHTAYTNSAFLNYINYIKNNIIVSINIFK
YININILCTKTGMKYLDHLAQKACIGIFFEPNGHGTIYVDINKLQKWSLSLHINYDLSFIALQKYLLFFNQTV
GDAFLDFIAIELSLSILNITINEWNNFYTPFPSMYININCPKHILPKIIPHPKHEQYLIEPITLQTHINQIVN
SVDQQHGRCFVRPSGTENLLRIYAEAQTEQKMKDILDKARTCVLHYIEHIL*
>N.PF11_0312
MPKQITDIRKFLKISRKPDTTAVIIMKKKSKTKKNTVITKLKLRTKKYLYTMVFADRKKAERIENSLLPSLKR
IYYPQKKVGKTVKK*
>N.PF11_0313
MAKLSKQQKKQMYIEKLSSLIQQYSKILIVHVDNVGSNQMASVRKSLRGKATILMGKNTRIRTALKKNLQAVP
QIEKLLPLVKLNMGFVFCKDDLSEIRNIILDNKSPAPARLGVIAPIDVFIPPGPTGMDPSHTSFFQSLGISTK
IVKGQIEIQEHVHLIKQGEKVTASSATLLQKFNMKPFSYGVDVRTVYDDGVIYDAKVLDITDEDILEKFSKGV
SNVAALSRATGVITEASYPHVFVEAFKNIVALIIDSDYTFPLMENIKKMVENPEAFAAVAAPASAAKADEPKK
EEAKKVEEEEEEEEDGFMGFGMFD*

>N.PF11_0314
MNVENIFGNEEVNVEEIEKLSNSEIKTRVSLIDTEIKILKNEHARLKNEYKNLQEKIKDNVEKIHLNKMLPYL
VANVVESLDLEDEEEENEIKDEYDLYDNNLKLSHEGFRDIDDEKRGKCMVIKTSTRQTIFLPVPGLIDASELK
PGDLVGVNKDSYLIIDKLPQEYDNRVKAMEVIEKPSEDYSDIGGLDKQIEDLVEAIVLPMLHKEKFEKIGIKP
PKGVLMHGPPGTGKTLLARACASQTNATFLKLAGPQLVQMFIGDGAKMVRDAFNLAKEKAPAIIFIDELDAIG
TKRFDSELSGDREVQRTMLELLNQLDGFSTDDTVKVIAATNRPDTLDPALLRSGRLDRKIELPHPNEESRARI
LQIHSRKMNVHKDVNFEELARSTDDFNGAQLKAVCVEAGMIALRRGATEIDHEDFVEGITSVLSKKKSTLNYF
T*
>N.PF11_0315
MEKEEEKMNDGLRFRKKGELLKSENILNVMNNPMTYVIESIKYKSVFDIKNDLILMKNVIHNDIIKILNKNSK
EFMSLPANLNYIENMIPILDKEIKISKRFVKKLLNEINNINKRINDILLDKINIFYIKNILQTNIDIYELINK
IEKELRKYEKSECYNILIDIQKKIYSYNYEMNNILSSTNSCSLFIEDDIYKNNIYFLYDLAKRLINICTSIWN
IKAMIYNNYKRIKTISHKLQDFYNLLNKYGLEAKCEQQTILVTVNKFSFENLNEKLKNVIDQVEKKINDLSYI
FLKLLKNGFIQNINEHNILNQSLNYILNSCYLINFFNVFIIHFQEVYINYIEYEPFETYEDLIVYIKENILED
KNVQEIIKRINEIDEGVLFCTQGILERILEIIKNKFGDIFMLRYSDVFMERYIKTTYLFNDIKRLVGKSKYEL
FILDEKMKKYLKNFEREGYVQYVLNLLENKMNDNYKNIILFDKKYIFENNFYWLKETINLIKIFKILFTSKYY
IPECLTNYLSFIFKHFKNYITNIESFLLFLEENEEIKILKVEKKKINKFYWSPTLGYNSIGFFISDFLSLRKI
FKTSKKIYSCINKDGQYVLPNNIGDITIWMFDKILNNFINDTTLKEENVHIENNEKKNLYVNGKDHIQVHNYS
SSYIHDDIITSSSEEDNNNMNNMQSNKMNSSNLQNDKNVGFDSSNLNNKKINEQDRNYTDYSKRLNDTKNQRN
VYDENHNTSNYSKVLLNNENKCHTLNIDNVDILYKIGKENKNILREKKKKKNNSLYLYEKNNKRKIMHHIRCI
NGFLKTLTKIVINTQKIFEDFFINKMFEICSSFLIYLHSLFTVYKMTSKRIPEKPSEQVDKIVLPLISFKSFY
KDIIADIIIEDIITKIVDKISEYYLNEIKNVIDAKICEQNKRILKFNLIETFLKKDDIPFEEKIQQQIYLDVC
YYEKLCDENFMINKNTNIHMRALLNHCKMENRKIKG*
>N.PF11_0316
MEKLCPLENDCFIFPYKDNKYIKKHLKKCPLYIKNLYLHYTPFYFPYINDSNTYILKHKDVENMLNEYYILFN
NIILDTIKKKMSIPIKTFDNFLYMKYNLLLIWYIKYGKDVFFSMLENDIYKEKLHNKLNNNINKSNIVDINIE
EQNLLFYIHINKLKDIICNKRNIYNNNKERNPKYCVNEFVIQKNEISTPHIYNNNNICLEKDYNNYNTSTKEQ
HFCDSKKINDHDENCSSPNKKGGCNKMDERQNDEYKKSINMYKQHDESNKYTLPILTLKNIGNIHNYKITRDI
INYVDVFIFRYPDFFDDEERKRKGAKRKKNDNNKKNDGNNNPNDGNNKKNDGNNNQNDGNNNPNDGNNNQNDG
NNNQNDDDYKNIFDETKNKTSFKLVEKNELDKDEILSIIIYLTIYLCCKLNIMKRIKNKNFNRKDMEKIKEKD
IYSIFFFLNNLDKHDIQNINLLFLLVYFNNSFYVHFHNLLNQKEIFISSTHTNIQSLFIELGAGKANTTRWIN
FIMNNLGDILQKYNVNKGEPKNDDKKMSYINNDNNKKNDQHNILCTNEPSALLHPSQAAYEKCKMLIIEKESL
RNKKEMKDFFMQIEHNNNENILRIRTNVSDFNLNKFIHFIKDKNVQENHFLVPDIIQFYYYNDIYKKIEKEKG
KENKELNTKDEQSKYITHIKKFNSDKTSFISEEFLKNNLLCIESYLDINMKKLFTALTQGNNNLKKVYNNVNN
FLKDLDFQKVTYLTKHLCGNGSDLALRMLVNSVKDNVVENYFILAPCCHHRCEVKKIVGYKYLKELNIDKNLF
QHMVNHMSGYASCNVPEKKSIGKKIKLVIDLSRILYLLEEGLQNSYLIKYVNRHITLENYAIVFFNHQNLNLT
NFKYF*
>N.PF11_0317
MERRGKNQTNDATLNEFDDDTSEFSMNILNHPNTDVLVSPIKVVDHDFNNFINNKCLDYSIDSSLDITTSNLT
TLKSKGQQKINKSNHADQNGNNNMDEKIKKNTILVNKEEKNKKGDANVNVAHENVALQNVALQNAALQNVEEE
NNLTRNKGNFKYAENFSGSNNNHYGSRVIQKSKHTQDYRKDGSYEQEEIEDNEYINISQSKAQHEDMNNVQSI
IPHDDSIITPSNHNNLKCDVLIKDKDQSYNRNMKKYSEFNLKQQDLSISQDYLNNSILSSVGLYKENDLCFIK
YIIVSNFKSYEDENIIGPFSKFTSIIGPNGSGKSNIMDCICFALGINNKYLRVKNLRNLIYHKENEKAEDINK
RICYVKIILECNKENVEIKRTLNYKGVSNFYINDKLVDQKEYMNFLRKNRIETKTKTCLIFQGDIEDIINKKP
NELAKLFEYISGSDEYEQIYEDIKEKLKEKQINCKNYLNEKKKIEQEMKIHKIQMNDNIEHNKLKENYEYEIK
LYYLFKLYHYFKKKEKFKEDLLLYKEESLQFEQDILNQNKKKANTLEKNNLIKKKESIKIEEDIKKNYVQINQ
LKILLNEISEKKKFCNDSLQKLVSNKKLKINMQEHCNKFMENLNLQIKEQNKKLENEYKNKLKINIKFFSKYE
CVQNIIKTKIEECKMNSHIDQQDQNESNVHLNNENIKKKNDKKGKKKLNINNHDDMKISNQDVSNNNNNMMEY
YTNMLNFLKDEKILNKIHIDSTNNYENPYENYNIIEFIENIDEYKKCKETYLYLCANSNININNYTNLSCSLK
KDIKELQEECDTLNRKKQKELLEYESEKLAYDQLNERIYKLNSVIKEQDDNIQNNKMKIKMWNETILQKEKQI
EILEEQTNILNVHKNELISFEKKRDIIKNLKNTFGHDQVFDEVSNLYQVNNQIYYTAVNNVIHKYNNFLVVRN
IDIGMKCIKYLKDNKLQRMDFIPYENFVSNLKKKKINKNNITEEVYTRKNEQQKQKQKQKQGRDGGGDGDVGG
DVGGDDDVGGDDDYDYNDFQNTEHDTHLGNHKRSVSSFNNVDKVINTFKKKNIILANNCLVCDQEYKIMFDYL
IGEDTLIVNNIKDVEDIKNRFPYININMVTLNGHVISKNNNLIIDITNRYSDIEKFNNKRLNINLYNKLINEK

EECINNINECNRNIIQTNELINKQQSEYELNKKKISSILIKKNIFEKEIEAKNKVMENYEQKIDKLKNIDLKK
KFELLDTYEKELFKERNSLSMYQKDAFKNLNHKLKIDNIYEIIEHNLRDIDKINENINRIKNNIKKLNDDINE
LLDKKNEINFLEKNEESINEENIKKQLISLEKQEKESNEQIDNIQKILQKLEEDKINIYKNLNNLNQELNQLR
DNINTNFEKYEHIENKIENCQKKIQIYTQFINDLINECDINSVNIFLTNNLLKDIHDQVDINQYAQTKNKKEN
DKNYNNNNNNNNKKKKKKKKESKGKSKSKKNHMSSDPYQKRKSKLLEDSDSEQEYNTSNSNNTSCNISEEEND
IHEHDEEEQNNILLSNISFDLLSDDLKNMENDKDINNEKENMEKEIERKKKLLKLKNVNCNAEKEYEKLTSKL
KSIDVSLSEERKECNLFERNFRILQKKRSYKFLHCFNYIKNIIDNVYNNLTYNAKHHVGGQAFLDLCNFNEFN
KDDEPFYCGIKYNNMPPMKRYFEISELSGGEKSISALALIFSIQKYINNSFIILDEVDANMDPLKIQSLTRYL
NSINSQVIVISLKEKFFSKSQSLIGVYKNKHKKCSKTITLDISKYRQDVPNN*
>N.PF11_0318
MSKVIQQIFEEYNRSRIQFTQNVCDYCLKSHNIEILINTDIIILLRPLILDKVPIVQQNATLILGKLASHSEE
IALTILENDILPHLIYCLKHENKNYRKNSAYTLQCLARHNEKLAKMVGEDNCIDYLIDGLYEYDIKLKESYIN
TLCAIIKNDEELSNIVVNKGIIPLLILCLQEKDNNLIKSSINILSELSKHSIDIAKNIVDNNCLSNLIKFLDN
NDIYIKRYTCNCISNIAKHKDELTELIIENDVFPKILYLLKDNDDIVKKNCANCLKEMSKHNEDICKIITRAG
TIPFLCDFIDISKDHMKLPAILCIGFISSFSETLSLNIIFANTIGVLKKCLLQESQDYIKSATVWTLGNIGKH
STEHAKKIADENLLIILVNLYNSNDSSDDLKKKIKEALKLIIQKITDINALQPIFIKSTWPLAKYSILQFSKL
LPKNSSSKKAFIQSGCLKYLQVII*
>N.PF11_0319
MKYVLRGHKRKIQKTCEHLFKSYFSINHLCVGNFPTFVKLQEDNIFFRKKQIYFLSTCKKKLCKYDYTEESEK
EGKKKNKLINLLNYHDDNKNILTKGCLVKNERDIINILQNNIKNEKNILITPQNIVELLQAYSKYKYYNENVI
KLIILYIKYNMNKFSFIKLCLCLNYFVNLNIKNEPSFNNIISNIIMKKIKNDEYIDSYALCMVIKYLLKTNID
DINLIYMLVIITERKISEGLYNIYDVYNLILSFLNIHTSIINPRLYQKKQTKRNNINQNKKKKKKKNNNNNNN
NNNNNIHLCGQGKNYYIKKSHNPVDLNITIFNDHQMNECIHFYKNDDAHRIYEDTKKINEENIYNMLHIYINF
DNKNNNDIILLLHNLRNNLSQKYFTYKMYMNNDDKTKLYENLLKQIHFKKEIQPQYIALLLINVMKFINNSQL
SEELFLHILKNVHTHIKEVVMNTKEHEINKYNQFCNDKKTKELFIFNNNINTFCEEQKICSQNSQNEELIKKS
SFEKPHVFSYPIKKKESPYEENIKKNNKAEKKTRRHFNVQEIGLIFEFYTSMQNYIKERKKKKQSHNHHDDDD
VKFYDTHPLDMRNKDNNIIHIGMYDNKGILQSEHIFKDDINMNKTNDGKKNLTKFRYGDTCYFVDEFIRYCDV
LKLYTNILKNCLYLYSLYKQEEIKKKKKLKYSDDNINITTYCKVFKNFLEIYSTHNFLDKEIFYSFFEEFFLI
QKKEKKLDLNEICDILKIIHTYKNTYLLESLLYNNKESKILFIIQNNLIKHVTNEMNKIRNDNLYFVIMSFYY
LSFLKCYNFITYSRLINLILRNVKNEVCIKHLPYVIIGIMNFFKLYKKFKILKYKEKEEYKYDENLNKNDLYE
QDNISMFNDRYNKELEKTKINKRKEKNILIIQNNFSYSYNENRHQNLFLLPFNHDEYTNNDNKMYRLHILFIL
LNKNYCEYKIYYILNCIYMLIKLIKQKDFPTTCNYISKFYLLEIFKKLEKQMDINNNNNDNDNKKIEEKCRDK
LKIIYKEKLHITNVEKNNTHMYYYNKYDYFINYVKYIYCISFLNYYIDLNELLNNIMKKKVTNNFVYTFYTPF
NSYLISNFLYNYENMKKEKYDEIKKLYNCSYQNNDLQTYINISSLKYIDIEKSTHFNKCIEYIKKCNILNIYN
IIYIIKTYKFLCYYINQYNILLLNKFILFLYMYQNIKMSSLYELLIQYIKFLYYLEYIQKPIFLKSNKSIIKV
ILFLLKMLSKYNMNNFENNYIGMLHISLLYMSYFVKDFHSIFSFLSLKFLRHLNLFLGMSMIKNLETYDHSYN
FQIYIVNILKGVIKKKKRIMNEYNVEHTPYTIDILIK*
>N.PF11_0320
MNESSIYIYNLTRNVSINHLKEIFMNFGTLKDVQYVTYVEESMFHEKGDGKDGDKKEDNDDKEEDDDKEEDNN
KEEDNDKEEDNDKEEDNDNNDDNNDDGDDNDDNHICVHIKYENSKEAQAAKEFMDGGQIDGKIISVKYKINHK
YIKTYTKKKKEKKKKN*
>N.PF11_0321
MIKIIIGVIGYYILYSSYHIYENIKSPIFNDGNVIKNNEGDKNEQLVIPKEGKKRNSINEEEEEYINKPFKNV
LKKDDIIDYHLYFSCEEDIDINKHMQDKYLEKDERFISVYKILNGKYSWNNNVDLLDEKRKKNSFFFLFEKVH
PSFDISIPKELIDQRKDIYLHILTYVNKELYRYGSRTVVITKRKEKTTTRGKKKFLWKSLIDEEEEEEEEEEE
EEEEEEEEEEEEEEEEEEEEEEKKYQHGNYEKGGSYKVHKNISNNKPNNKNPNDKRDIKHNNNNNNNKMKKD
NFIKSNQNSLNIHKKKKKRKHKDFLFYIPKKIRFGPVIEYNDFHISKLGFFSNMHVDKDTNTYLLPIYINNDL
TPDDEYRMIKMKNSDDMMKKKRSSEKNDPYYKENVRNDNNNNNNNNNIKNNYLEIYDLSKRTYEMRNERNKKI
YEKNSSINNNVHKLEHELMDYIINIEYVPINYNYYNLLNMLKFNVSYVKKKYNFISFDMDSITTFLCCHITCS
MIIYILCIIYIIMEITYLLFDIKMWKRWNNLYTFTYNNDIVMNITLLFFILLYLRNIDYGRVIMIYYIMKMMV
LIFKIIYNYDICILNDYPYICINKKSLKEMNKEMIMDEEFEKKIKKKVNIFMIFSIILIFIYNYFYTKYDSYY
SYVIHTLGFSSYLYKFILMLPQIITNIYTRTVQRMSFPFFLFLLVNVLINDLFIIFLRMPKVHKYYLFADDFI
LFLFIIQYCIYKKENKIFGAREKLVLLKSAKKNK*
>N.PF11_0322

MIDEEKKIKIEINNINTIILDILCTEAIRKIKFILHILNELLKKKKNILHVYITNKIDHLSTHKKQRQIFQSL
QNKIELCENESHYNLIQKQIDEYINFLSETNEILCQNSPTDRNIKENRNKIIEDGNLLLNIFRKMKKKKFSSI
LNELEKFKDEQNLLINLNQNVYHKKQIINILKEEIKEQMENTKRKRINIENKIKEIEKRKKIFFVKFMLYYIY
KKEYVHANVAHYNRNMDFKENAVTKSKENMENELKAYDDINDFIKSFLNKRNDNLQNIHDELIEYYEKEKDQK
NAQLDNIKKEIEHSREIIELKKKKIIKYDEINKEREKEEKIKMKKEIENKEFLKKYNECILFLQNVGRNKIKA
YDEKMKRKKKTKKKKK*
>N.PF11_0323
MRGLKKIGCFLFENSTLPTINTFIILDIILRILKIRGNKKKREKTKKNYKKKKLKRALLLTNKTLIDLFPPDF
DFILNFLNNSLDRKDKDNDKDNDNNNDNHIDNNNDNNNENIWTVNKIKNYKNLYRNFKDDKKYNIKNNNQNEE
KNSLYINNNHICDDIYNQHIYNLKNEKQTNDTFDNLHITIEMLKDILNFIHIKYIDKENYLINLLYKLNQNKK
YNIIVINISFFFIKDDKKIKIFLDYILSSAYKNVTKELTMKNKDKNNYDHKNEFLSNSNNIIYSNIEAEMCDR
PFYKNILNIYFHYSYFINFFEYLNSVSHLSLTSHNIKTIKKKKKKKKKTNNNNDNDNNNNNYDNNYNNISQSS
NKCNIISYHHHNQKDDQINNFVEKKNKRSYFTYLLNQNHSANLHSTHTTHDKKKIKLEQEEKYEYNEYNENNN
NMNDHNQNISSYNNIFQSNNIPNNNELSNLISPQQKEENILSNQLKSHKQIDVTEIHIEDESTKGCLSMHSIK
LKKNKNKIRTKLYLFDMIPKSNIYKKMYMDIILMNFNHLYVISRNI*
>N.PF11_0324
MIFSAVSSKCLAKNVRGNIYYVSFLRINTYNYSSIFDLILRKKKERICDDNKIINDNRGKEEGYNECNIKERI
LKCEVPIYNSNNKIKKKENISYIKNDVHKDKKCSDGFELISDDNIYNNKLYILRYIKYLQINDDINKYRFISK
HIINNVYKFRYNEILFILKIFCKRKYKNLLLLQCFSEYFYWLCKLKKSNKTNISYYLYFCSRFNYIPTIKYVD
EYLDTFLCYEKWVPGMNFFSLNMIEEETNDCAKDIKYIINVLYFLNMCNLKKKKELFDTLLYMCIYNINDLTV
KNTFLLLKILITNIENDKYDISVFSTIHKNIERHINSLEDLDFHNYMNIIFYNNINPDATFFRFIHNYIDKKD
IRISSNSMLAILKIMKKYNNKDHVMFKKMARIMIDNFFNYTYDQILYVLKIFIQLNYFSQDFFNYLFKAFISS
GVNDLVWNKLPDENSKEKKKIQGIIKNKYLLNDLYIDNNNNNNNNNNNNSNYIVNRYEDVLKEKKKKVKEDE
KINYENLKTCNSYDKKVFPFDETYVGENKNNEIYVERDNLLHNDKTLQNNNHHYNNISHFDNEKRDIPNNILC
NNILNKINNKYDNFTNDPTKSFGNTSLYINIVEEKKDDKHKKINEISHLPLHLNIIKSKMKIDSSNIIPNNMP
MEDNNIYRKVTYDILYIQMHIYLFIYLGICGYRNIQALDMLSKKIQHYLYIIYNKQHDISDDNIEKKKKKNDG
IKLISFSYAINKMKEDYNNNFYLLSQEHKNILLHDDMKSIQIRNNLENRELLKDKQKIGYKENYIHMCNDNID
SKKKNKKKKEKYISNVYQDDQKNIFINQLKKSCLHFVYNKNEKLDEKLEKRKKKKKKKKLKEEKNDNMNVMKY
MNVENFRRSKSINTNNMNDTVYKNVSIIKKRNTEKIEQKKKTLAECYSGHPAFIRLHNRKLFRRYFNGMYKSE
LYLEKIKRKKTKKGNEMNKKRKGDLRLKKYRKIKTLRNTILKRYGYIFKNKYNLVSRYINDYVYNDKEKNKCL
KEETFVDMILKRNEIHIDDCLFINYNILCNISMKNLMKKKFCNFISRSNEITSDEYIIQNDYYNKKQEYYKHD
IIKWIYRYINNCNIFFKNNNNKKGQLINEYDIKKLDICIKEFNSDMKEEEEEEEEEEKKKKKKKNNNNKLLIS
LKNISLICEACTNLYYYNNNLYDVLLYHMLKIIDRFYNNIGEYVKENLICNHDKSNINMHKNFKNNNINLPGD
MSIIQDRFEENIIYNNMLCDDKSDEENKILHKEINVHNNNNNNNISNNNISNNNISNNNISNNNISNNNISNN
NISNNYISNNNNYFYFSKLHICLFKNVWKFLICHIQVNHFIKNILENDIRYNTFEKLIDIYTHKYFYYNIKNI
IYIINYINSSNIVYTNNKKYSFVYVFFINYFNISVIINKLFLFNKIIYMMSQMCYPKNVEHIKKKFNIILYDI
SYLYYIYTKTLLNFQKEIEIKNRRISTDNIKHIVSNDDIHNLFSCINFYIIFLYILKKNINEQKEYFINPTTE
RIMESNDMMITWDDLKIEKNIHPQNNLYVHTNNIVTYEKMQELKNDDHNSIMDHHYIFKIMLHCINLIRYSNI
SNDKINNNEQKKKNIYIFTLIRFYYIFKTFYRNKKLLYNIIEQNYDDFFPLFIIKILNKKKIKQIYIPQLFSM
DRCIATHNKKQFIHGLITNHFAPFFKKS*
>N.PF11_0325
MDMTYNMRNAMASSRNMEEPQYDMSTIDLTQNSMNTNYLMNGSVLTNAYNNDYIVNNRDKYTGDMISQLKIDQ
GIPTYSHTLPMNGTTLADTNDSLNIQGMMRTNMDTTGFDGYSHIMNYNNTQNTIQTNPITYRMEDYNNLNNDV
MYINPTTGEKFSAEYVNNIPTNDSDNMLYSNNFKNNSINMNTLLNSHYNNLPYPMPIYESMNNPQMYMQHMNQ
KQTVNIPLQHGEKKSAARNISTVNKRRIKTKKFFPCC*
>N.PF11_0326
MSYLKRVTNENTSNNNKKNNTNNKINNNTNNTSYSNGVKNLDSTNPEQNKNLRSVSNNMSCSRHTDICNNYKF
HNNNNKEEQTNIFGKNVTSSNDQYNKTDRNNNGRDKKNHARNRKNNRRDKKNNHGKVDGESNNNNNYNNMIKS
MCENNFGIQQNNVHNMDWFFKNENLCNMNNVRDQNGESFLCNNKGINMLQDKSYNKGIFKNDKNKIMNPDNNN
NNNNNNNNNNNNNNNNNNNNNNIYRNPYVEENNIMAHKYNLSLDQRKNNKNKNTTYINKNKVFEDHEMKNSLQ
KKKIYDETIPVGNFVQRKKNYKKDGDDIKNMMVSGPKFSYTEKVYKNDNNHNNNNINNINNNNNNNNNNNNNN
NNNNNNNNNNNNNNNCTYNAHKYSDIYNIGDEQNHIINNDHPYFLNKYKGKGKDTKKDSVRSTLPYNNICTND
EKKEMNITKKHQTHEYTQDTNKSYNVMDNMFINYFKNKNASTNNVFELEENNRGDICNIVNNIDIDKNNDHNN
NNDNNNNDDNSKDHSNDHNSNDHNNDDDSNDDDSNDDDNNNYYYYSNNYNNNYRNNKNNFKRNNFEINKEKEN

KEKIYTSVFEENNKVIIDMINNKNKDNFININEKSNEITNIHNYTDVYVDEDIDIHVDKNNNIFFDHNSNFFV
DDFNNLHVYNKHNEEYYKNIKLYNRFMENNKTGKENYDYIQEYEYEYEYEDLSNNYNMDYIYNDQIQRNNDSQ
IIHDINNYDIFSILSIPNMNNNDNNNDNNNNNNDNYYYSSSSSKNNNYYNYQDRYIPHLHHDNSHTNNNINNI
NNIIENYNPLLIYHNEWNHFLKDQKEKMKNTDNIKKHENDKYNIKSHDNNFLQEIMNKNFIYEDIKTTNKKYF
EKYISDNVVPKTHPLDYIINNDMNYVQKEDTNNNPLHDKTDKGTIQKIYDLINNHDDHINNKGNNISHNFISD
HSKMNVDNNIHVEDINQNGDDIKCSKSNKTNNIFNNNKKDDYDDDNHKCIFQNKDNTNHLDIYKQSNISNTHI
EEHGKEHLNKGDINNTKNLHASNKYDALHKECTIYIDNISYYGQDNKIDYTNNKKDSQGMEKKKDHTTNIQLN
HLLVNEKNKKTSSIIYNKGMVNINKNMICNNNNNNMCYPINNCSNEYDKKDNKDHQFNYYKWYLYHINYPNHL
DFKKAYFFIFKDDIYIYGARNNQYIIPDALYKLYNNNIEIIHTKGIIPQIYYKIYFLADMENFDKKNYYYSDD
LFYNYNENINKLYYNNSNKSYCDNPKFYFNFPNFVLCNPSFIFSNPSFYHKDNSLFRRGIFVPSINCVQNKWN
HTEKNKRNGSEYHRFKSTQRYTNNNNNNDDDTNNTTHFNGISYNKGNCEYSFNQDKEKKKNTQCFYILGCKEF
KVIDFFTIYRLDMTTFQWEKINITYNRLLNLKRDDFSITLSNNYIYLYGGVIQTGKTWQVTDELWICNLKKRT
WEHISCKEKENKTNQPCIYDNYFNKLEQRNVINKENKNIIISGKKEQDILKNDNINDNINDNINNNIYDNIYD
NINNNIYDNIYDNIYDNIKNNYKSNNDEYITHHVEKHPGARACHLSFFYKDKLFIHGGTNLKAEKDDFFFYDT
KKGIWYEIISDEYNHMKPSKRHSHTGIFLKNKLYIYGGFEKYENYNILKNDLFLYDIEKNTWTQIFTMDDFYY
LKTELINKEKHVLLYLELLVLKNMYRNYLVKLLNSSNSPKKNINLDEYNNISDQEKTLLSSPNVLECIKENDI
INAKFKQKKKQNEINQNGIYKSNIIHINHSENLSQYSNNIYYDKKSPSLNCYNNNTFNNKPKDNNFVLHEFIN
SEENVKSVNESLNIHFDKNQMNNNIKEKQQQHKYFHHNFFYLKNLNNSENIIIPYNNFRNKCFYFKNSLYFYG
GSGYNPNIKESIEKQQDYIFYDNVTQINTEVTYMDFILSYLIFNDTFFFKYIQKIQIEMLPFIQALEKNFDNR
MNEQEKHLKEENIINKICVNIQRIQDKLNNIYMNKIQSDTKKNNMNDHHDVDNIINQLYNNSNNIDGHNNANL
ENEHMNMSEKDILEKVFFYTQSKKSYELLRFLFDLYKNIYIKYLNGQDKEMVSIELLKNMGIQENNVQSNDTP
DNNYIQKNIKDESNGKSPNNFNNNFYDNKNYTLLPNMEENIFRGEQLVEECCIEEKEKKEKGYSCEILLDGNN
INKKNYEQVQRMSTIDIEGNPLKYYNSSIYDNEETRNILVELVKSYNEEKDEYMRNDKDIPNHNFMNNYINHN
HNDNIEFNEKREEDTNKNILDTNISIIINKLKNDYINIKDDINGNDHINGNNHINGNDHINGNDHINGNNHII
GNNHIIGNNHINGNNHINGNNHIIRNNHINDNNHINDSHNIYNIYNSDYLKKTSGTYVVDEMENSERNITNHL
DIENYKYNSNYCVNDNEDNFTNKNNLTSSYDSTNIGNQRIPMGRKLQIKNIFLKKREDYDNIYDNKVYDDVHK
DDRHKYNDRYNDKDNNKMYDNMYDNSPFNYPTPVEHIQIHNHLEKYNKLINETYLTDMNIRKYNIYKMIYSYS
KNVENQNKIIFEKLEELQNITNNFTELNKKNIYFTYQNNYELQLDKNDDLSYYAFNPINFENLKTNYISLKQK
LNYFNDIINIYSKKIHEQNVYIKIVQEKYEYLMNYLIKLKSSIHNDGISDDIFKFFCDDNFFNHEK*
>N.PF11_0326a
MYNFLIFFSLYSVKSDRFCYDYSNPSPIKKTTKHFTSYKTQMNNDGLFKRASLKQNEFKYDIMGITPKCNDRY
IDMKKNQLLFFRSIFLLQNKLNVIATNNINNYITNSHDTRRHSIPMLWIHDNKTVYSIDYTDEQRTRKKMKFI
NGLLG*
>N.PF11_0327
MIGGKYLNIKIFGEKRNSLGSNKTSDNTINNNNVNYIKRKKNNNKKDQEKKYNDNNYEEEYIKKRKSKPGSLY
NNIDKSKIYRDKKIIHEGWLNKWTNIIGSYRPRYFILENGLLRYSLDKYSPTKESFVLTHCKIKVCPDDQLHF
EIDTNEQGVLYLKANSPEDKHKWYISFKKAQLNYINGNNYKNKNGHHNIPDAVQTFNMSNNSLFLKNIIKNSS
KCIKREDQHLDDPIKNTNNQTEKELSNNNKEIIEKEKNVDCIIVNKIQENVKGDESNNIVKNVIYERDDKDVP
YERKDICMSHDKNYRSPLYIDNDNMSSYSKINKNANIKYKSNEYIFYKDDNVEKLSSSSSSTLDTSEFIEKMN
SYNKKYNLEDMFISSTDFEDKSPTLCLMKNIISLKEMTTDVLKGSEYYQARSILNKKLKNNIKKGVNSNIIVD
VNNNIIVDVNNNIIGDVNNNIIVDVNNNIIGDVNNNIIGDVNNNIIVDVNNNIIGDVNNNMIGDVNNNIIGDA
NDYVNNNFSNGNNLQIKSDELLSLLSQLYFSIQYAHIIIEKYIQCTELLLKEESIHSKYMNQSLKLLAKQNYY
LEKSQEINNLTHMEEEIKDKLKHFQLYQCATLSEEDEEEEDKEMYNSSKQIKQKKQQQEDLFYDCDEFIYDKN
ILSNDVFEDESKNSISLYSSEGDKNENSDKIKKVHNTNNIPDQNRTYNNEHMLTTHSTINNKNEKMNAHIDEH
YYNYINNDQENEKTFSHQTSEQRSLNIKTKSSDHDDNITTIQEDCTTDVFLKTILNNKNINETKRNTVSTEDD
IALGDVRDGLITCDVYKKDHFVCDDSVEDKDIIRKRMGIEDDHGNNEDVHGNNKDVHGNNEDVHGNNEDVHGN
NKDVHNNNKDDHNNNDNICVKDTSKNKTIYRDNHKCDICPCDNHLYDKNNLDNHLKSINFSLIPSCNSKNIKA
LNFKDIEIYTDKSIKRRKKLPSPRTEIKISMWSLLKDCIGKDLSRIGMPIYLNEPSSFLQRLAEDFQYIYLLK
YASNEKESTSRLAFVTAFTISPYASVIGRTYKPFNPLLGETYELTHRKFRFISEQVVHHPPITAYHCHNEYME
NYASIMANVHILGKSVEVTMPGSSHLILKYKKTMMSQNNLEKKINNYIHNNIHNNKVKEGNTESNMINNYNFC
GKKKHTHDTYIHTDGMSNDINSKNDTSEQIQNELLNDNMQSEENGNNCMPPIRKEFYKDRGTCKNTKDGDNND
GHNNDDNDDNNDDDNNDDDNNDGHNNDDNNDDDNDGHNNGHNNCHNNGHNNDDHLLYDQEHYTYERANMIIHN
VIFGKLWVELHGNIIIRNHNNGDFSIVKYIRKGWFEKEIHNVRGVVCDRYKNIIFFIYGKWSQEIYIAYVKDM
KKLEYDNYFFNEDGSENIYHYNKNTLNEFINTIDWQLYENNIENLNSICVWKAHKRPHNSEQYYGFNYMTVEL

NEITKEYDKDKGAAIACTDSRFRPDQRYYENGNIEVAMNEKQRLENKQRQNAKKFNNTNKYEPKWFYKHKDPI
FNDRDIYLFNNKYWITKDKNQFSDTPDIF*
>N.PF11_0328
MMNMITFMYYMVYITTTIFIFKDKYNIMNRNMKERNFFTRSFYNNKLSQIFYEDDYDFFRLVFVKFLIKVQYL
NKHVHLGDVKESFETIMLSMSMNHQNEMYMKLYEEIKKIGEDKRYKELYEHNNINIEIEKRVGKNRNRITLLS
LLPFDYELKTLNCKESYKKIQLLLDILYVSIIISYHSLFNNHERKNMLILMYENTINQWMSLNYLRKIMKYEL
SLNNDGILMYISLNKKKERTKPKEINIKLNNNKNNELRELNKDVQIMNDIKIINDDIIKNIENKFKYIINEHI
QHLYPNVIGKEKLSFLLNKLMNVYIPPVRVIGYATFNKHNLYTIDENIKINNQFTEEYFNMNKDKVVIQMMIG
NNIEGNKETAAQNDDNKNKGNYYNSTRDGYNNPSDGYNNTSDVYNNPSDGYNNPSDGYNNPSDGYNNPRDGYN
NPSDGYNNTSDGYNNPRDGYNNTSDGYNNTSDVYNNPSDGYNNTPRQHDNHTYQNNPAHDIHDFHFTIEYMYN
IFMNYIFYKDDTFLDNYKYTSFITSKYIFGDKQHNKPKEEKKYEDLSLHIFLRIKKKLKFTFSHLQKFFFKNF
AYISSLYDIHFYGGCLKYQDGDMIKVVGHFGEGIDIKLINNVLIKMNIREIIIHIKLSTKKRKAHEQNESYTY
ITIKQIKKQINNSNIFHVIYTLVDSTKLLPKHINCTTCRNLKYHYIILLITAIVPSLIIFIIFYIIYYWKDKN
WSNSKKKICTNHISQTYPCFFHFKTHSNRYIRLSDKKKKKKKKGKKKNPYTRLSKNYNEKKEDKKKIKIKNKM
KSKMKNKKKNKKKSKKKNKKKQNVDILMK*
>N.PF11_0329
MANSMEKNEKEKVDVHTNNIDLNEYKYVINNSHSSDEGEEDVSSYDENDNEEYTNDELIKEDDSNNKRNKVLD
TNKYRNNDIINHNINGRNGLINNNEDIKDISEGTMNKEDSLICDKDEENVEILNNKKNEKYNNNNNNNNCNMF
NKDDNICVKKNREGYELNHKNKNIVDNKYIYVRLKTNDCNNNIYKCRIEKNISVKKMKKGLNKILNNNNDMNY
RIIYRGRSLKDIDEIAKYNIKFNDIIYAIKVHKKKNGNDVTLDSGITSSQLSTLNDEYNEYGKYGQNDGISKL
ISNMFDNSDFIKSIMDSNKQLKKLREQNSDLNHILNDSQTLKQSFEMIKNPSLMKELMKNTDRAISNIEAIPG
GFNTLRRMYHNIQEPMYESTEKLIEKKLNKVKHYDLNSTSPPTSQAFPNPWASKNTKNKNSINNKNNYNLNNF
EKFFQTNEKTQNFKSNLSTDNNNNNNNNNNNNNNNNNNNNNNHTNSLLNNQNNLFFDKNKRGSNNTSTNNNTT
HNIMNNNNLNDLMNNSFLSNSLFPLLNKLQLPQTNKNVSNNKNVINGNNDYQNNLTDEYKNSTQHVHNLAHTN
KNQNDTLSTNRLTNNNKDNKLDETIENSSNMLNNLLMSMNKNLNMNSTSPTNNTSSDLMNTINMINSLSNLGN
YNTPYGNIVGENTTTNNSKNYAHNNNNKDNNIDSASGSIPPYYMDQSFFMEAINFLRNTTNNDIVNNNNNNNN
NSSSNNNRFNNALFNNTPLNNNFSNFMNVLQNVGAGNGYIPSGRTNNTNENQAPRTFENEIKNVEEKQEGIQD
TLKSTDILNIKNNDTNISNNINGEANDHNIYSEQLNSLKGMGFTDVEKCLKALIKSKGNVEGAIDFLLLDESN
VNES*
>N.PF11_0330
MKEDNINNDISLDEKDISRQESLFYDTSIAHRNIKPFVRPPICSVSTATDSFRCSTYSNPNYMTCPYINSYEN
CTENNKHVDGLREKNGKYKKCEEDDKKDLNYMSDNGYVDPYNIYRIEKKDDIYFSMFKNEESNIKNKESNHNN
SKYPFDGCTKDLKIYTNVYEDSKNFSSLLSKDSIYHNSISNNHMTNKIPFYNKENLKNINNNNNNYYNNNDIK
MKRNTINHDEIFKPIAYEKNGINIINKQTYDTYKKDEKYPYLSYDEKYNDMMIMLMSGLNIKDNYNNICDNNN
NKEEENPVDHKNIQDVNKNENYFKDYISDVYHNLINENINNNKEINNINKYNIFNNYIDNDNINSDDNYIYMN
RENNQDVNKNIFSFCKSNLNLKKENNNIYDTCKVFINKDYKEDILGNNNIEDILGKNNIEHILDNNNIEDILG
KNNIEYILDNNNNIEHILDNNNIEDIPYNYNQAYILDNNNNIEDTFDNYHNDNTFTNDYFYNLKKGKKVSFDI
NINKNKIIDHSSSINESYPFFLQNNIKDNKKNSLDNPCVDLNIYPNDLNEVILHDSEDKNILIDDDYILKRED
VIIDEKENKRPDVDTNKNSIIQKEENIKDNINEFILSNNITHKNDYININNIDDEKMVKGFHKKEIFAHIKEN
DDKNNNNDNNNYMDSNNYAKNNCNTCYYNYNNGWCDKSSILSENRICNYNDIQYNFSSNYSNNVIYPLHNESN
IHGENSPLAYIKDEEPLVFVNDEYDNKNKLNDMLCLDNCLCDSCVSINEELLSKEENNKKNIFENMKTYNFLC
NLLNDKQDEDFRDNNNEYITSQSCFEENNNMNHTYDNYIFDNINEEYDNTPFEENIIYPENYNNILQHKKKDK
DIIIKKKKSLAKYTLIVNVPPNTTRKDLMNVFSQFGNVDLTMVVCDKESRHPNKEWTATSGYSFVRFSTNIEA
RKTLTAATCGLIKIRGSKVRATWAKKDSYSKKEKDDIVLKIPSSILIINIQEFNCCICKIYLSYQPILFPCCF
VSSCSDCLANYIMNNINQPNFKCPNCNIILNDKIIKLDKNVKGTLALLYKYYSNIKVKCPHNGCLWIGYHYQY
LNHFISCKYNITHQIQT*
>N.PF11_0331
MSLSIYGNRESGQDVRTANVTAVQAISNILKSSLGPQGLDKMLVDNIGDVTITNDGATILKQLEVQHPAAKIL
VNLSELQDQEVGDGTTSVVLLASELLRRGNELIKMDIHPTTVICGYKLAMKESVKYIKEKLSERVSNLGKDVI
INIAKTTLSSKFISYESDYFAKMVANAIQSVKIINESGKTKYPVSSVNVIKVHGMSSLDSKLIEGYAIMSGRA
SQAMPTVIKNAKIAFLDFPLKQYRLHLGVQVNINDPKELEKIRQKEKDITKERVNKILESGANVILTTQGIDD
MPLKYFVEAGAIAVRRVNKDDLRRIAKLTNGQIRLTLSSIDGTEKFEASSLGYCDEVYEDKVGDWDLMFFKGC
RTSKSNTILLRGANDFVLDEMQRSIHDALCSVSRALESNYVVVGGGCVEVALSVYLEDFAKTLGSREQLAIAE

FAESLLVIPKILALNASYDSIDLVCKLRAYHTKSQVMNIEDSKDYKWYGLDLVNGKVANNLKNGVLEAMISKI
KSIRFATEATITILRIDDLIKLVPEERKMEEEP*
>N.PF11_0332
MDMNFGFCDDNFFEQGHKSTDKEEIEELTKRGSPEGLAKIKENEEEKALPKELCVPVTIKLLINKMMNTEELK
IHDFQISGIIVFGKVVSIKELASAIIFEICDYTGNIEAKYNKDAKNEKVKNHIESIKVNDYIKIVGVVYSPES
KSESIQISILYINKVTDWVSYFTYFTSDVVYCYLKLLKLKNICTPNGINNEEKPWSHINLDTYYNHLDDIDSD
IIKYLHTTEEKYASQQDIFNNLQKYHSEFNIKKSLTKLIEQYDLAQYEDIISIP*
>N.PF11_0333
MDIENNSKNEDPTYRSKEKIFGTKVCQQKKKKKVNALKYHLSEQAQQVKRKSIDETNEDEECSHHAIGINHTK
TMVINDKEKKNIYICNNKKICNIKSNDIKLMITKSNDIDDSILHKSNNIGTSKLEKDTKKGKTEKFGKYEKYG
NNEKCEKNEKNEKYEKNEKYGKNEKIYQNQPDFQNEKIIQIHNTNKMCKKDYIEIKTNQDEDILKNNKEKIAD
NKKYIKNISLSHINVIKDEKMKTCKHSNNHVINIFEKYDNNISCFSTFSDLKEENEKCFKNDKEKMNRQNNQR
SMNKNIYFLNDKCIKKKKINNNNNNNNNNKNNVNKENNKYDEKNKYCEKKKNCEKKKNCEKKKNCEKNINSEK
NKSHIYNLWNEKYLKKLKELEKKIRRENKIKSNIINKKKSKHTQNNILNFKIQQKDNLKKINHISSLDFSEGK
KENFEENMSGLVKNNYTDYNYYEDEQPIQLKDNNQCFYNNNNNNNQNDSSSMILRENSKESNDHNDYNDNNDN
NNNMNIPVNNKSKLRKFVYIFCNGCLVVFLGVSNNICGRMRNRVLKNFDSLTASYNALAYVTIYLTLCILYTN
SRHIRKEHWLYIYPCLKNLLFKKKKSKNNMNNNMNNNNDHNNHNNTDSNKEVESNNSYEDYFVTNDSYKKEDQ
NLLANYSIDIKQKEEESMKKKKKKKAISNFFHFNKKKIYDEPLLGRNEEKNKKTKNDNYDKSENFNNYRDERS
KGHNENQSYYQSTHNSSMKNILREDVGNHNESSNNKMCEKNQMMICNSYHVDDDNIYYKEIRKSSMDNIKDDI
TGKYNMCEKGYHNISNNNNSNNITYDDDPNNNNNNNNNNNYNSIYKYYYNIIKTKWNSLGAYKYVIMIALLDI
VANTLYFVSQLAIPLTVLLLLNQLIFIFSLILSFFILKRKYNIHHIISVIIVLIGFLFFYIPFVYKDNIKIKK
ESCVNYYLNSHYAININNIYDDDMYNRCDGGKVYNNINTVRNMNNGYNMMGDNAINIDNSSNGRNPTTNNKNN
TYNDSTYNDNDNNNNSKPPCSDPLCASPFSLVFSIFLCLISIFLTSFGGILREIFFQGYNNKYETEKKDNAYE
KVHIDDSDSYYIMSNIKTNNKKDICFTDDINNSFEYDDDIHKKNKSDLYRKEKNNVHTFNMSTSYSINDANFM
KTKKYKNTVDEFSNFKQINNKSYHNNKSVNYNKNNVTYINNILCDENKSSHINNYDYKNDIYKNDIYKNDIYK
NDIYKNDIYKNDIQYRYKQYNKRRKIKDKISVILLSFNVSLIQVIFLPLIIYFQMLFNKNKNISYLLFIKNSL
RCFYGDTIENNLNCKYSFMVYTLYIIVNALFNISVSSFYSNYSSAECFLILKSSTPITLIVLYFYNIPFISDT
DKYFSFYFLISIVIVFIGVSYFFYQSIVEEKKKKKNTQQIR*
>N.PF11_0334
MGTPDNTDETYNITLHHLERERKMRNKLDVSHNKDPFDLKLYLYNACLNFSNTDIYQEYINRLSINSYNNCDY
DQIKRSCYISNKDFLKYYNYNFYDNDFVLEMDNHTEDTSSSYHLNEMKELEELSEFVIHKMKKIKKEFVKNDI
SRKRRSEICKCTRNVLWSKEKRNTENNNSLSISYNNNNNNLLSTNFQKDNSHLFSTLIEKEKKNKNKKKNNFL
YLSHIGDIKNEESIYHNEYHITSNYLPYYFFMKKNNIFSKTCNQSREHSSISYHMDDFFLKCQSINRMKLKNS
GNSYYFCMLQNIEEAQNDPIVEMYYKYLDDVIKKKSRGNIYDNYSLCSMLDNTKKKKKKKKIKKDKMSKNKEM
YDKINIYNNTNILNTSNHSSYETEIYSNNIINIDEENTLENYYIDMEIYNNLHTSDNNKKKDITNKINEPNTL
VQINKKKKNIKRLSSVEMDIYIKTMMINEKIKSNQQRIDKRDKWNALYQFMFSCIGASLSIYFYLDLPLLRTK
FDYILTCVIIFCSYIFIGLPLLQIELSLGQLSQGCILNSLSFLKKKIKGIGLLSFIILFYLLMRNMSYSITTL
VIAVNSVWRPLPWDIKECEKINKQQTCVSDIKCRWIEWKEKDDIKTFKMYDNIQYKDLSQNGLLDKHPYRHNL
SYHNPSNDKLQLDISHVSQKEHNEDLNIFMYSKKREPVQNNSHSCVSISTMEIILFFTKNISFVWKILSLFVI
LILLYFLLKIETMSLNQSLHYILSISFIIVLLQILILYYKLEPKEYTQNENMAIKYNTCIFFSKEYFLHINFY
LIIKIICLVLFSVNCSTGINYIFSSYTNIGDNIIKQSYYIILGSFIVTITYLTYYYLCILHIHNNNNNKNNIN
VYDDEYILNYFIDYYNILPINIAHIVQEKKNIIFSFLKNNVKYHYIPHQIIIYLLVLSKYIPFSNMMCFLYFL
SSLLVLLITITIHIKVIIITLKECRKFRKINKKKFIIYIIIIYFFASLLNIFFFTSYISYWFNYNIIYNIYLF
IVFFQMISITWVYGSEYTSKKINHQKIYFYYSIHFFISILLLPIMIYPYYYNIYNKRYVFFILLTLNIVQFIS
VHIYFLYSIKLPKKTFKQKISYLYISNMNLLKRRLNNIFYGKYRKYDTQTNKENNNIKTNKIYKCHILQKLTP
MWCFLLKYFITTIFIFLLISNLLHHVYENNKLYHKLKQNNYLLSYKSQYYLHNINQSYIKYNNDEFYNLRKVH
DKNEKTEKKKSIQKNNNIQNILSNNTIHEYPKMGYGQDKDNYIYHHIYMNTLIYIIVTILFIIFLLLFSFFSY
FKHKKFNIFINSPNKLYNINDIQKKIRKPVKFLYTRSFFFFEELLPVQYIMPSYIWTHINKHLKTESFIQSLK
KEYENCENIIDKKFLFYNKAQFESYILGLICQGSRLEHFTQKKKKKRIDKNE*
>N.PF11_0336
MANRTDASAIKNFGSNPQYLISNIIRSKIYDSPYWKEKCFALTSESIIDQAINLKYVGGTYGGNRKPTRFLCL
ILKLLQIQPDKDIIYEYIKNEDFVYLRALGIFYLRLIGKSLEVYNHLEPILFDYRKIRMRLQNGTFEKIYMDV
FVDNCLILNNFLDVDFPTLTKRQVLEENNLLQRKNLEYYKALLNISSDNELFKNNEDTTHNKNKEINKNKNEY
KQRDMLERHKKINKYSSEDDHNYYHKKEGNYDKNKKHNKIYNHKYNNKYDDEERRHHSDYSSDMDRKSRKKEK

KRTHSKYDHKKKKEKRNYSSDSKHSFTSSEYSVQNKKEEHDKKKKKLFFLKKQKKKNYHSSSQSNHSYDENKK
DELSIDKWNEIRKELGMKPLK*
>N.PF11_0337
MNGLQFLLRINKNIIRRFSSLYSPNIKKNVPDYIDFETFYPKKEIKPDIMTAFTMAKQQRLEKLLENNPLVLY
KGRVIKKLSCPRIKNSGRNNVGKITVRHRGGGHAQRLRFIDFKRSRKDIYSTVLRIEYDPSRSAHIALIQYED
GVLSYILAPLLLRPGDKIIASKYANINPGNSLPLQNIPVGSIIHNIEMRPGAGGQLIRAAGTYATIISKDNEY
ATIKLKSTEIRKFPLQCWATIGQVSNLERHMRILGKAGVNRWLGKRPVVRGVAMNPSKHPHGGGTSKKHTKRP
KCSLWGICRDGYKTRSKKKPLGLIIRRNICGRLQKKYGVKS*
>N.PF11_0338
MHMLFYKSYVREFIGEFLGTFVLMFLGEGATANFHTTGLSGDWYKLCLGWGLAVFFGILVSAKLSGAHLNLAV
SIGLSSINFDLKKIPVYFFAQLLGAFVGTSTVYGLYHGFISNSKIPQFAWETSRNPSISLTGAFFNELILTGI
LLLVILVVVDENICGKFHILKLSSVVGLIILCIGITFGGNTGFALNPSRDLGSRFLSLIAYGKDTFTKDNFYF
WVPLVAPCVGSVVFCQFYDKVICPLVDLANNEKDGVDL*
>N.PF11_0339
MINIRKVLLPSCVITKSFLNNYYKRLITYYTKTHEYIKIEDGNLNNRKDMTNVKCKIGISNYGTHKLGEIVYV
DVAHNINDHVKKGDCIATIESVKSVGDVYTPVSGKIININNKIIDNVNLMNEQSEIDGWIMELETNQINEKEI
MNISEYEKMCEEEEQNEEKKIQQNEINCMEEKNKNKIFDINDMKNIENKGQGGK*
>N.PF11_0340
MCCCLCGLLIFALVALIVLLIMNGIIKLN*
>N.PF11_0341
MKENIFDTKKKNNNRKRNIIRSAKWNNKNSKIELSKKRDSSNKYKSILKYYKNENKTNKFIDKRKKNKWFHKN
RKLQKKNIFNLNDDVLFKERHISTNDFIHSDNSLKETDQENLNDNKKKGNKKYNAMLDKIEEKKLWKLKKYEI
KEKLRKFDEHFDEIQKNVLGLNGTKGGAKHSMVIENNKNKLNKVIHESKKRQNFEIHASHKGIGAEKGKQNCY
DDGDDEHFDDDDDEHLDDGDDEQLDDDDDEQLDDDDDEQLDDDDDEQLDDGDDEQLDDDDDEQLDDSDDEIYD
NQKEYSHDDEMYNDEKNVDKANYPKTTSDSQNELTNYNSYHTDNSDNEEITKLFNKETLRSKKKGSNENISKE
KLNELLEKYKIGDNINICNHFINNTEEEKQNIPIYIYIKNKEYDIKDVILLLDDYHFETQQKILYRIYYINMF
NKKGTKSIYHFSFFFSLIDYFILNIYKCLKYNIKVCELLGYYKDIIVKYCHEMKAEFYLYISFLLLIVFSKIQ
RKVKTNIFFKKKKKILQDYVILNEDNANRKIDVYIYRRILKSVDMFSSIFENYNNENIYISNIHFAVLFLTLT
VYPINNFIDDNNMSNVVENKILNPQKNLIINNNPFLDINKNNINDEKLLYKMNYLKQDINNINNYNQQKHPII
SFIIEILELLFYNHFYTNNANLLNLKDYQKYDWVFNMNTYENYHNIEACLKKLEVYYSFSSFEDVICENNKGG
KEFEHNEINNEIVNDLGIFYRKKEFKNSLILLNLYNIIMENTLEYNPSFFYLSFKILNTLLYNHITSIKEGIL
DKNKIPHVSEKEKQKIQTINNSNNNNNNNNNNNNNNNNNISNNMYDKFDLSFIIFKNIFFFLKIYIDNDINIYI
LINHVIIPSLFYLYMNFLKFIVTNHIKLDFINIINVAKNINIKEGNDFLFEEDKTYELYQKYLIILLYIFKLI
EYSQNHDIKPIIHKTTTEGNISFFTPKYANNQNPKDFIFMQNNQTKLAEMKSIKKKMKQQRKFDYNEMKKENS
YILALKAKDERDRKRRNQEKYKKIKLMAQKDVEEYNKMKTYTH*
>N.PF11_0342
MEKYFMKKIKGVFSTFKKDNPNINNNNNHNNTNNNNNNHNKSNNNNSTNSNRNDDDNINRQSTQENNSTVGNK
RIVGMTNDTYKQEKDLNYFFSENKERKEESNSNHKKKNLKNKGQKKLDSNEKDMPEFSKKMKYTKLYQKENML
SIDNYIKINKKHSLHFVKRKYTKRKLMASNIQNICNFITDKNKNENSRTKKVESLDDYKEENKNTPNKVDEEK
KMNLQYEQEHNNTNVAQETKGGKKKKKKNVDRNQNNLKEDNLNNDLLNEDILTKGQYDHDNNNKKNNSSNSSN
NNNNNSNNSMNEYNILDDSSNIQFHKKETPSKNQGKNKYIFKNVERKNTAVYDKNKNTSLNNYGQQQKKINRI
DEIFKNLIEKRKKKEMRQKYMLKKKFLMNNYKGKTNENYEHEQREGEKEEKQKENDTYGDFEELDEEIEKDGE
EKEEEEDDKNEEVEEQNEEVVEKKENVEKQKNHQNNKTSNNIYDNKSYDHHGRNTHLLKKEKKQRTKQISNKN
VPMIDESTIEKCYKQMRIFAEKFSSFKNKFDQYYNTSEHWNLKKKGKKRKNSVNTSDKENMENYFFDEDSMKK
ETNELEELNKNIQKLLNANMSNDINVDKINEIKKYTQDNHNKSLMKNINISESIYFDCENTSAKKRKCSDNAS
VSTNEENLNKLLEYATNKKNNMINSEDDIKTDQEYERDKRKKNKRKKNIYDDLQKSNENNSEEVIHLLRENEE
EEKEENKQTNMEKRKKGRKKKKTEDIQSDKVKIENVQSDKVKIENVQMENVQTENVQMENVQIENVQTENVQM
ENVQMENVQMENVQMENVHTEKAQNENIQIENVHTEKAQNEKIQTQNIQTDNVKTQNAQPDNVQTQNAQPDNV
QTQNAQPDNTQYKNEPFKIEAIENVLEEKENLSDKHLENQVEVANISNEVFETEMKNFNNSNGNMDKDINIID
IPHEDLNKYIKDELNNNDNNNNLLNIQNVEIMEYAESIKSDNIINDTKEDALPRNETLNFLKQEYVSSKEQEE
KKDEEKNILNEQELNDINEKMKQQNNVDSKLPHFAIEQRKKLIKETNRFFKMYNLHLKIEKLPINIPNDLKIL
MQKKKKIVDIINDYKIVFKTLQKYAEKEKNISEEQDGEERKLSHDNLDKQKEQQNDEHDNNNDSNNNENNNNE
NNNNDNINNDNINNNDICIDHKVTNNMVLNTDGDEQNKEQNISSQTDKGNVILTTKDETVVNKNDISSVLKEE
NEDKENKENSTHKDPDLSLDVNAETKEKVPRRRGRKKKLEKEIEVHGKRRGRKRQTEDILNDNNINDDSLYY

LSDSSKELIEKEFNKFMNIFENINRNKPNESNESNEPNESNEPNEPNEPNEPNEPNEPNEPNESNKPNESNVP
NESNVPNESNESNVPNEPNVPNEPNVPNEPNVPNENNESNEKNMKTFSNMNDTFNDQQKDEVIDDATTCTFMD
LNNCTYGDNKNNTSEYNKNDKQTNKEQYRPIDYDVEKEIQNSEKSEYFDKYENSFIHQLINDLIHNNLEEENY
IQFSNIDNEEIKYRKLKEVITNLKIKEKKIDLILSNNLSFFKYSCMYRKRQLLHEKMLGNWSYVENTINKRDD
IRNKNLPFQLLKLEQNMLENISKCYDDILLEDFSGIIITLNTNSFERETDGKIIKVSKVICGCLIEYLDNTSE
LNIKCIWAHPFLNTKSTYYILCAFLPRVILEAFLNSKGILHNDINDKDHINMVNSVKGEGLENMNSEMCEFKN
SEKKKKKKKKFLRLNELDMEKIKNDHHENDNINNSHNNIIKLEQAYFSNYSTYEYPLTHLLNFNKECAYALNY
NENLFKDNENKQDNINTCNNMKGHNNCNDNTSLSSKDSNNKFLYIIFSDIDIFPRQCYLILCSYKYMCLNMHY
DTDNYSVHYDLENMKKENVGKTTELSNSCPCKCMKSIPDDSEESYERIIGLSELYMYYMIPKREERENLGLLK
RNGWRDLICSTFLEDVHENISSILKIKTHINKVSEHISVQNRIYEKRYTPIYINKYEWCGLKLKDIRNMLNED
FNYDGNDK*
>N.PF11_0343
MSNLKNDMFISKKKRKFYYKKSSRFVRFLYSFVKWYKMERVVGPVWINKYSSMIYFLMFLFILNLSVGILILI
LSSKYIECRIPYEYKGETFTKYSIVKVTPEQCKGQKNLKELNGNINVHYEILGMQQNHYKFVSGMKKEQLNGN
IFLKKEELEECYPLITFSEGKKKKKLLHPCGIFPWNVFTDSYIFYDKEPDEVPFPTPLPLKQNVEEITIKYYR
QFYKNPSPQNVQLYKDHIYFWMEPDIQYERLQENKETNEKLLVLPQTLKYNQAGKAIENSHFINWMIPSALNY
IKRLYGKLYIPLKFPFYIYIENNFKINDTKIIVISTSQYYMRTFLIGFIFIIISIIALILCIFYLIRMNKYEN
K*
>N.PF11_0344
MRKLYCVLLLSAFEFTYMINFGRGQNYWEHPYQKSDVYHPINEHREHPKEYEYPLHQEHTYQQEDSGEDENTL
QHAYPIDHEGAEPAPQEQNLFSSIEIVERSNYMGNPWTEYMAKYDIEEVHGSGIRVDLGEDAEVAGTQYRLPS
GKCPVFGKGIIIENSNTTFLKPVATGNQDLKDGGFAFPPTNPLISPMTLNGMRDFYKNNEYVKNLDELTLCSR
HAGNMNPDNDKNSNYKYPAVYDYNDKKCHILYIAAQENNGPRYCNKDQSKRNSMFCFRPAKDKLFENYTYLSK
NVVDNWEEVCPRKNLENAKFGLWVDGNCEDIPHVNEFSANDLFECNKLVFELSASDQPKQYEQHLTDYEKIKE
GFKNKNASMIKSAFLPTGAFKADRYKSHGKGYNWGNYNRETQKCEIFNVKPTCLINNSSYIATTALSHPIEVE
HNFPCSLYKDEIKKEIERESKRIKLNDNDDEGNKKIIAPRIFISDDKDSLKCPCDPEMVSNSTCRFFVCKCVE
RRAEVTSNNEVVVKEEYKDEYADIPEHKPTYDNMKIIIASSAAVAVLATILMVYLYKRKGNAEKYDKMDQPQH
YGKSTSRNDEMLDPEASFWGEEKRASHTTPVLMEKPYY*
>N.PF11_0345
MQTNYNEIRNLFNYYIREYFYQKSKGSYFVHFKKPGILFMSLKCYKRKKMYYPCIYDWYFDNLNNVKKNKDIF
QYKNEYNTKNIYSHKICTEKENLGKNNSYVETDNIYLNKSEENLQNIYKSSNLYYNINPIYLSKICLYNLNEY
HILYSNNYYDAFTQYYYSLISYKMDVHNMKIFPNLKILISHIKNISNIINDEVIKNAYIYLIKQRNIMNEEYL
KNVFRNIYTTMLHNSSLYLFVDNHDQFIMILKIIKTDDIKSMFESAIPYPYYLIVDENINIYNLQSFNIYINS
ILNQRMQYNYPYNEKEKGSILNHYNNDKMYMTYNNNINQSKDIKIEKQTEQNEKGFNSMLDTDKGVYEKNIIK
DKNDKVYFFKFIKIFDMKPNYRFKQKK*
>N.PF11_0346
MVFLTRKILHLFSQHEKKNIKVNHCMKISGIIKWMYRYNDLNKDVLDNMNIQIINSINYIDPNKIINNLIINK
NKEINIEKKKNYYLKSLKKKIVFNSLLLKTIYCKKNFFFQLYEYNNGKKCIYDNNKHNNNINMVKNNNTREYF
DVTKTIINNNTTDKSENNLFNNDNNNFPLINNNNMLGRNLKRKKKNLFLNNKNVIFKKKNSQIFNEEIINTIS
SDTKDDLKKKSRFNLDTLLYDNNNKNNIIINYKYDHIYDNFKLFDLSYNYNSFLLNNILKNICTYIYHFKFLN
KINNEQNNGYEYISLILISKIFHYFYELNYGYKYMIHFLNGLSVFKIKKEINLENLSYEYIKSHINNKDHYNY
CHDHLFEKEICISSIINYLCEYDTGTKQERNNINEKNEHVEKKKEKKNCIYHVNNTSSYNTINNNDKNSFIHT
FVSHDKKKTYIINMKEKLLLHYNIDESFLNYFLNILGEKYNIEKLPFISYSFLMHMYSMSNHFIVHFFSLCPL
LFVKNKIHADISIYIILLNSCVFFLCSKSYMTSFSSFIINTKNEGENHLVYKENKLIQDGNLSTQRKNEISDE
HRKIFQDTDNVNIVKEKIYSFIEQIINYLYINKDILNNNQLLHILEIFSFIKYNKAMFTYIINTLTNSLSILD
KYETIYLLHSLSNYNSIYNDIKNNIYKEALKRVEEYTPQELKQLEYYMQIK*
>N.PF11_0347
MIEAPANIQLHGNEYKEECIYSSIDNFNGPFDYLNYYFLCEIVFDDFSFKSPYHCYFFLRNQFENFKNVHTNY
VQNSNSDKPHGDHIKIINQKNATELFQNEEIIYYDEIDTSDVKRKALENINNIINRLTISDLMEISLYFSENP
EWENNKLAWMEMIQRDKFRKYDKMRENLKETGMREIIFKMNNDEIDRMKNKSMIKDFLFFGVIDKKGQNYLGR
IYMIIRNDIIKNYEIYTWLLTNCNMQTDENLAADIFIEETYYEKKPINNKGSDDKKKKENSNKGGGNHKNDDH
LVFEKKMNNYTFEKKEYICFGKNETNDIVCMNPSISRFHCVLYMCEDFQVYLIDVGSKSGTKLNNCICEIHKK
YKVSNNDVISLGVSKTTFKININVEKVLEYLDKRECEIKRKMEIMNEEINYPLGNKLFFRLKISNIYYKCNEY
DILDFFKDCGQIKKIQLYNMPLKKHINQNIKNIKAYKEAIIDVFDEQTSANILNRNECFLYGRKIYIVYVPIK

NDVKQNNNKSTYENVAERYDTELTTKDTNNSIESIILTKGKGADKRSKYQKSKKYNDEKLKEEKHRGKKDKHK
KHKDEKYKDEKYKDEKYKDEKYKDEKYKDEKYKDEKYKDEKYKDEKYRDKKYKEDKYKDDKYKDDKHKDDKYK
DEKYRLKKHKEKKHRELRSSRRKSNSSKLYKRGSDISSIFSSDEERSDNRDERRNQKRHTRRSDNIKSSENRR
SKRGERYSNRRSSTHRKSTDEEYDEEIESSYRTDDVTSESDESRIEKRREHRSESKYGRRSIRKSERSSEKSS
KRGYDNKSNDKIHEKNSERSNESRYERIDSKRSGRSIRKSEKSSEKNSEKSSKKSSDKKSERRSGRKSESKSE
NRRESTRRESRRSRRYSRKSDTESDDDNNSEYSNIRDIEKYKKDKRKKYPTSCSVSNSYSESNRSYETDLKKE
RKAKKHVGSKRNS*
>N.PF11_0348
MSTYLFVYYLALLLIYIFFLCNKKGFHFCHRIILRKRTFSFKLISCNKKIKKIIRKRKGIIKKMDISDKEALM
DMSTRSAVRNKNVHLSLNNYTNVLNREHNNTKLIRSNVLSIFSYTLKYMNKKSLNKWLRNNIKATFYDTYIPK
NKPLAYIRFKSYDDLKTFEKLIENKRFYNDDSLSLIKINRLFNKQHNNVLRKKRKFNDENKLYYNKNEVKNEK
THENNTNDEDVKFLSVNNDKYGEFKSYNNSDINNYYDCNEGDRKKLKKDDLYNNNNYDNNNNNNNNNNNNNNF
NNYNIMRKQNMDDKEFLDLQSIIKINKEEKFKSIENFVTPFYKYPYQEQIIIKHNFLKKCKDQILFNLKSKWM
KQNILFGDIESNIFNEDIDLNNIQDYYDQKQNKKNQTSKDNCINKTVLTDIKTNNSITRETSLNEKMKQYNIQ
IDEPLYPNYEGTIHYRNKCEFTISYDENKNVEIGFVVGKMKTCIKDATQDKNSSEDNNCNFSENNISNNASPI
NETHSTKLRNKKLKQSYYLNPIVKNVDQCIHIHPAMKEVVQEMKTIIKESNFPVFDRVYKTGIWRLLVVRMNS
KNELMITVQTYTLDYKKKKEIKKLLINRLSKNKDDTPKYFCNFKVISIYLQEHENPNDSFDQSLNEHLWGMEY
LEETILDNKFLLTPSCFFQVNRDSCQILYKRVIDYINIKEGIKNYIFDLCCGTGTISICASNVLKSNDVHIIG
IDICESSIISANKNAKMNNINNYKFLHGRVEEFFQKEIKNIQTNNANIICIVDPPRNGLANSVLNILSAHHLI
NQIIYVSCNPITLINNVTNILFLNETLKIKNMAFVDMFPHTFHLECITNIVKKA*
>N.PF11_0349
MKVVQSIIIYYCLISLGESSNVPRNVSDKILNKSLSDTLNYNFNLDDVMGIFDKIFKNNVDDFKIEENNDIKN
LEFRKYKFNQRNYDFMYESFKDYCHNEVVNNENCIKFLDTDNILNVFHDIDSVFHSRFYKEIKEVKSYIFEII
KLLNKSDELSVKINTTFDSIKIKKNMLIKTMDERFNTAIPIISYFEGMTYNLTKENNNINFYEMMVQYGNDLN
SAIKNALVEYVYTYREVKNFQKKLEKYIEKNAKLLSKMHRKIVLSIFYMIADMNKTTEKIDNLVNKKVEEYEE
WINNENMNFSRMISNLKYNVDFNIDDINSIIDISKENLLKNNIDFINMSEYVTFQSPNKTINSIIHFLNNKYS
KNVHIIFLYNEEYIDWNFNVKEEKSEEPSSKIDLKLMNKSNFDHLDYIETTKYENILTSMKKENHSYFHLDKS
DINKLIDDLFVTGDNNNNNINGPWIKEKPMDVKEGAKEEYTDLNDEINKYTEKMEKKIDNVTDDYTFKPTIKS
YIEGVKNLFGNAIFQFNKYIYTFDKNYEDTSVNNENGDQIVDMSNKYAKDVYSEDMENGEELIDEENIEDEEN
NYKGDNKNVQEKEETEDKVDKKGNGDDKIKDMKETEGGKDEVKDGDKSDETVEGQTEDKIDLKDEDKIDLKDE
DKNDLKDEDKSDLKDEDKNDVKGEDKSDLKDEDQNDVKDEDQNDVKDEDKNDVKVEDQNDLKDEDKNEDPNKA
SVEESIKLLDEKEKSKRMKLSNMTDDINEFENEFQSGNENDYENEYENEFEDEYKMLCEHNCDGSNENKNNLL
SIFESSNLKYLWRIPIINNLYNLCCKDKPESANNENIENINVFEVDEEEILKMYDNMDSLMHEDITKWIKDNI
LYAVPDMNYILGALKSQKRYMNNISSARVGGTDSNSIHQNYFYDSFYGRDYLIMKSKGVHVETFLYPYFETLN
MELNEAGICPWGNYFSCMEKYVKEHIKNKMVEDKILFVYIGTFKEEIFKLSRSKIADDLKKTHLKQFLDNEKE
YDFFNNFMIKKYNEEYNFFENLKTHIVYHSRYKNLLMYGIDFNFHVSYFLKGDIQYLPLSYYYDSIIYFKVIF
ATSKYGYHEIVPIDDDIIKFGLLLSLDNKNKNVHYLISSEVKQDTNYDLLKNKIGVIKIYEVSYETLNMQLIN
KKYKVNVTKNMRIILEKEVIFNNNEKKLLDYIDSFYDIMNKIMKNNMNLELYGKTFFVHVQRV*
>N.PF11_0351
MASLNKKNIVKILERCVKNTLLSEKSRSLCTSKINRNRASGDIIGIDLGTTNSCVAIMEGKQGKVIENSEGFR
TTPSVVAFTNDNQRLVGIVAKRQAITNPENTVYATKRFIGRKYDEDATKKEQKNLPYKIVRASNGDAWIEAQG
KKYSPSQIGACVLEKMKETAENYLGRKVHQAVITVPAYFNDSQRQATKDAGKIAGLDVLRIINEPTAAALAFG
LEKSDGKVIAVYDLGGGTFDISILEILGGVFEVKATNGNTSLGGEDFDQRILEYFISEFKKKENIDLKNDKLA
LQRLREAAETAKIELSSKTQTEINLPFITANQTGPKHLQIKLTRAKLEELCHDLLKGTIEPCEKCIKDANVKK
EEINEIILVGGMTRMPKVTDTVKQIFQNNPSKGVNPDEAVALGAAIQGGVLKGEIKDLLLLDVIPLSLGIETL
GGVFTKLINRNTTIPTKKSQIFSTAADNQTQVSIKVFQGEREMASDNKLLGSFDLVGIPPAPRGVPQIEVTFD
VDANAIINISAIDKMTNKKQQITIQSSGGLSKEEIEKMVQEAELNREKDQLKKNLTDSKNEAETLIYSSEKQL
EDFKDKISDSDKDELRQKITVLRGKLTSEDLDSIKDATKQLQEKSWAISQEMYKNNAQQGAQQEQPNNENKAE
ENKDNA*
>N.PF11_0352
MKVYNFSFICISLIFVFIKRSLGLYTNVAEIETVDNPKRLEELLNLKKVYLVQFYATWCRVSRGFSNDFINIA
KTLKDDVKFIAIKNESILNEYKITEYPTMHLIFGNEHNNKKEVEKFDGKYKIKDVVSFIYEGVKNYRLKELNI
DTSKKSSYKKKSKGKNDGKVIILNDSNFDQNVIQYDDNVWFVFFYAPWCGHSKPIHPMFDELAKKVAHLKNAK
IAKIDATVEQRTAQTYQINHYPSFRLFPSGNKKPHTAIDYNDSRTVDDLYHFFLKYYVEKKELIQLTSQQVFD

EFCEKDVCLLALLPNKEDTEPSYFKSYVHILSNVIKDVNHLPVTLLWTQAGDQLDIVQKLNLTFGFPTVIALS
LSKNVFSILKGNYSEQSIKNFITQMMMGKSSVDNLVPFKVKNVPKFSFNASNDINTEL*
>N.PF11_0353
MDKFKICKKRILGSLKKNKFIKKKYEKFKERKDYVENDFITNSIIKYLNKEKYENKTKKVEINEFIGEDENLK
VKLYGKEDIQEEDIIKRCLFCDKDNKILEQLSYRILYDVMKKCIDHENVLMKIEELGFTADNIKDEKDENINI
CSKYKVDTFLDYIYKNLNVIKFSKNFQGLCKFIHLIKSVIFIIPYVYKVKFLFLFCSLFNIYQKEYESGKQND
TNIHNKNNKKEYSNLINIVDEDEFFTLYFELINQLINLKDNYNSYDENYKIFNYFVLRWILIFFKNKNQFFLS
QKKMSSLIKSKYVILLFSLLKNLHESSNMLGNFDISTNEKDILLSVNKNNGHMLNQSYNNMSENEPFLSQNIN
ESNYINNMYNNNMNNYSYDDNMNDINLLDKLVLNVSNKKNKEKQININKSVLNDKIYNNISFLKNMIVTFYDK
VDMNIEKEIKEVKDNMNELTQNIFINVYNILNCTHLIHKHMILLYFSFNFLKMSSYEYSYFIVVNAFNMLNNL
LTIQNFVIHPLLFLCIQEVLTFYENIIIHLYKEKGNELNEEFYIFVQKFNNKHKNIYSKLIRRIWSLYMLIIS
DDYVKNSSNKEDMKINVEQSVYGKSYLQMKHDQQMDIQLDGQMNGHIREHNNYIQNNNITGIIKLVDLDFSNE
YKSFLLNNINNTYRSSLINCLCLSYRILGLEYFLDEYFFFTHKELLAKDIFLLNKIIKDSNKLYYGGNFKFLM
NFFYPLLIFYIDLYQKEEEHTIKKKQYMVYIKNVLNHFSSSLNDCMNLYYFLSTNIEDLYVLIAKFINCNDFN
LLPNFIHFFQRLFLSTIKLNEGEKKMIFLLSNEKENKIKNTQCKYTQINIQKNIYFLNQIADNLLKLFIPRFI
SVVSYSFEQKFISNINNNNSNNNNNNINVHMVVGTFSNLIHLCLYFSKDTDFNEMLTILEQLILRNEIEKEIF
SLISLLTSIKIFIPFFSLHQINMCSVYYQKLIHMINVIYEMKSTNINKLNKKKSNRKQMKSIHVSEKFYCQNM
NTLRNSNSVNNNNNNEKDTLLTNQETINSISLKDIDNVSGSKKKNSKGRNNNEVFIKNKKGVIKSAFKKKRNQ
TLLHADKLKNIKIKKGLKEDMEKLKFVRILLFDNIAVLFKYFGNILLKKNTRLVTSQSNDDNIIHENTNNDNL
NNSDINISTSLYNNKNVYPKKDDNHLNKNELSILNGIYVDNKILEQNLPTLMRKESILQFFENITHYNINKYI
KKFYFYLNSLTFYLKECKKLDVSYTKPLFYEILPIIMKSLFFINKKVTSKLKKNELFKHLVDIGRSNIKEFLF
HVTPGLVLEEANCKKISTYILYLLIKNYKIEYEYQVQLFSICLILSNASEKNLLKVFIKYLLTCIYTFNEDIV
LNNMKELMKLINNISQNKAYVFLIEKILFKLYQLLEHDDFINYLSKSSRKIFNNMIKKNKVTKKIINDEEDVD
TFNVKDGKYKKSILNEKKILKKVKNGYISSSSSCGESSIVSDNISTQFSSDQDDEENLNEDINRLNIKINGTL
TKDKAKKMMSMKKMKKSTKYKNNNALTKFLEGLHKYRGEKETLFVKDILDELHLSKKQQQNKIRGKKNKNTKA
IKYNPYIIDLLHDLQKSTLKKKKYNNLNVSYTNQNEIKELFGDKFFMKQKKNKSKSNMKQDDYYADSDQEENV
QMNTDGKIIIRLDDDNSFSNNKKKKNHDEISQKYNDICEKQNIHNKFKLYNNKSKKNKSKNDHFITAHKNIYK
SKKGKGDIIKKNKLLPYSYVALKPVMTSQKYSAKTLKAFKSIKSNSKKKGKK*
>N.PF11_0354
MRSKSISYFLFFKKNKKKNDSCDSVIISSNKNLSIQLSKGEDDEKNEINEEKSYIKNEDVYKKEKLKKKKENK
ENNKKKDKNEVVYDYHDISNDATSEYVNNYKVYEMNTCNIKKKRESFFKKINILQKYKNYKIRKAASTFHTIG
HKTSFSGTDDEIENNQKKQKKYKIKISEWKDDKSHTFHKKNDILVFDKMDKNKKFKIDNNKNNQINIDNEERV
NKNYPMATNVQNFNIKYTSIDVTNDEYIIDSNKPEGSIMSTDKKNNKLNYNNDTYDVDKSSDINKLGNIKKNK
FDIITKTTHNINNNVNNIHNYMMYTNKENIKININHGNLNGREQNNYDEERKANVYEIFENAKKLEPNNININ
TEEHIHISEPSIPFDMKDHKNDINEKDIILKLMYNNNGIYFDDDDENHKNLLYKNKDTHVKHLNNKFNHNFII
YNDREEGVNQKHAQKKLKKKNTILNKNENEDINHNSFKRPLSNTNICYKDKDDKIKNGSNKYDILNNDYSNEH
EKNKYNDHITKNKRNQSANEVKSNNNDNHNNKKNNNFNININDSYSTNINRNQNVMINDVNDVIKDPNMQENT
QGDDEGGIINKYLINPIYNLFLRANEEIQNSNSTNNKLKMNNITKSYTNELQKTYKSMYDINDISNKRKINNK
DIRGTNLYNTKLCNNKLYNSNPYNMIPYNINTYNNNNNNKETCTSINIKHSENKYPFNKSHVNSYMKNTNHLP
HRNAITSNNRNNEEYEKEKEKDRNITNGNNNYLVEYNNSCIPPPLKKMIPIDGVRNKSINKLNNVTNTQRTSS
VSYTNKNIDENSFDMPIINGIRESKYISNNNNINGNSIGFNSSKLDNYHHQSMNVNESYPLKNMMKNNYIEHN
YDDKNNIFLVKNYEDTYSNIHNGIHENSMLKNYNLKKACTFHGYSRNHQKNMYTEENLNINQKKNYSHYHNNG
TVLKPLVNTNNVAVNEFADINLSAQKRLHSLKSMGYEDKSMENYRNKIYNNINNNNNNNNDNNIYNDNEYCQY
NNSYCFDHSDLKNMFPLNHQNSKLLTHSNNKNSFFNGINVESKHHLANPEIKTFAHNSYPILNQGLINCNPLQ
CLGYDSNQRNKHNVVYIKKNEYLNKNIGSIINVLKREGLRKISTHNGKFESFSNMDNKNVYMEGLNIQDNVNN
NNNKESCDNIKHMRTKSLNFVSRESYGEHKSLDVYQECYVKNNKLINKVNDKKYEDNNNSYLNEDDNASMQFY
EETNSNPYIVDQENNMKNYVNNVLYNNNSNYYVDSKNYDKSKENAENKSDDILNNENIHTLKDQKKKIQNNNE
FISEQADIENIRNSQEEVYEKEHEPLWVINASNEEKKSYEELIYSDMSSNRVTKNKYSDMNNVEVLLNEDNLL
TTEKYKVQLEKENKMIDMYETVEENINTIKTENTNDINEEVRNEQKRESINHINDTNINHIIDEYPNDTYNFI
KDIECVHNNENNMYNSIEQYTFYHDTRNNHLVDKNNQNFIFEEEGLNELNFEEKKVYIENNTKDDHKGDSKTS
NLTSLRNTICKSENDHNEKNENTYVVRKGEKGIKRKVSMKKRNEKLNEENYINNIYDKMDNHRQNDITKKEND
EENYILYNNVKVNYDEYIENGNKIKITEESLNVFYKENQNEEDSSTKKLNSTSKIKRANKGKTKKKNVITRVH
KTKQKIEYVTNSFNKSSKGENSEIGKIGGRSKSLLTHSKKVSERNKNKIEKINDTNSKIIKGKKSNSQSKLGK
DTKIRGKSKTGEYIKNKDLRKKSNEKNKTVMDNINTINNSSVSNLKSKKHKLKKKKKKNISMENINKNITNEF

CSMERKGTVLLSNMSIKKIDNANSCTLNEPLEENTLNYESNNNCSNSNLSKDKEKDRNILCNKYYSDEETNSL
NKMYTSNIPEISNYYKEIQAINYILSNINNPNFLNSLELNDLINIEKKFINENIYINKQIIACNVKNEKSNDE
MVEKNERKVDEEKGEDEQEIKAKENNNKEENQDNENNNKEENHDNENNNKEENQDNENNNKEENQKNENGIIY
DSRFSIIYLEHDLIYLKKNNLKVILNVLLSNVYCFFEIKLTIILLNFFISNNCQWSFSLFPLSLINKLIHKFS
LKINKKVPKYKLENMNINSPNIPYTYLFICDGSNYLCINDNSLNNEVYENKMKLNNIIGYYHYINLNRLTYYL
EKVNANFVYNHHIYE*
>N.PF11_0355
MTSIKNVTGVRHNVFRLNAHKKIIYNRLRRFNWPPEDCSTIHPIEPYGKQFFVFNAFKRDIKFDNEMSTSVTP
LLNSNNEKIASETVLCVCDFAKRPLMFLNYLDFKILQENIHKIKEGFRKLDENTEKENYLDTKFGLMLKYNIN
KTYGMQRVKKGEHNSWGMKKGVFRSRKT*
>N.PF11_0356
MYDRFLEKISIISLNLLSKINLKNSYNDENLNVIKRDKRFNTLPLLLRIEYDEINKYGDYNKRKKRNPIFISY
YKKKNVFIHLLNQEKYVPKFELLINDIIKTYRDREFLSDFSLNFLYEVVKSKNSFNLLSNNDVHGLLLILGQE
FLKCNTDTLDKEVIKEQKDNLYNNYNIFLHDENIDYNENEEENYKRKLEKLNLLLNERNMKILKISCFLLRYI
SIDNRQSKMVDDTFMFFFLMKLNFVMNMKKDIQRKHIEKDFNFNFYFYILLLYYYIYIYNTTRKIKIFDQGLI
HHYEYKPLNEFNTIYKNYENLKKNQFFNLNRYGIYNRKDEELEYYNILNNNNNYNIQYNNNNNNYNNRNMFYQ
YNYVNDKKCIFFLKRNYFINSKFLKNNFPIYFQFNDLSNINFLTNYNNFKILRKKFYENTYKYKSDIINESLK
SYVLFTLNLLFKLNNVDKTNEDMLISNVKKVDKQNSKNNLTLINSFLSAASVGKRWMLMFWFIPFSKKKENKT
NNNRNDNNNDNINNSNSVERVGKVSKNILLKNLNEKKKNCKNKIMLCELIYQSLDYITLNEQVQYNNKGYNII
ESCLTEIINHIERKYKLKYVSSTKDSNVVLDNENKKKNKNEKNTSENDENILFNIINIDNKLKNPKNIVNVEH
LLKKLNELLKLTQNSSVYVNTGLHIILRNMFINRLNSLNLDLLFLINMYSNNNYKKKFFHYDQRYIISNNDSE
KNESKNNKEMDLKELNKKQKDTHNDFLSNDINTNENIMKQYDNENITTPDSNNNNNNINGIKTLNISSLNVSP
NNKTEEDHFNLEVTKKTQGFRKRLNDNIYNELSIILRIYANCLTSKDICFDSFSLLYCMYKNIIQNILRISFN
FTYFFSAEFFFFNDNMNLNSLVNIKNQLLPSCLLFDSIRALTNMKSHYAYYTHIMKQRKKLEKKLEKDKNKKL
LLSTLNIKEIKRTKKQNKIVVTSAKEYDKKPYHKYERFLEKVDKMIKKEICYKEIKYINTPHNHSIMNLNNIL
KYIYGNTYFNNYYNNNYLIYFKNIKRKLQRKILLDEHIYMISKYNQQFYQLNDRFLPIYSYSKKYNIYRNKPL
KKYHKKNIYKNNTLEQMNQMDFHGSDKKVIDNNSMTNQNSLKNNTNEILNKKIKGREMKKRKIMKKKIKKRKE
LDLIFIHGLRGNALRTWRFSNLYHNGSDYHFYYKNKNLEKFKKKDSDLLKYNTNEKSVNNNNNNNNKNKNNVS
NNKNKNNVSNNKNKNNVSNNKNKNNVNNNNNKNNVNNNNNNNNMNNIFNNNKWNFLEMTNLNNKFFTNEKNNTL
KENNKDLSNNTNTINVKFNSMEKLEEKDNVIIYDDFRKKVRRLMKIKNNFQFIFNNHLINILMLNYKHNQNIV
PMYTKTNVINKNLFKSLFLQNKKLKHDLDIYSDELSYLIWPFYLLYPIKRNANIYVFNYHSPLYPDGNFYTKV
YYKNDNKNSTKLYTKHNDRRNKNKKVEHIKKQEDISLKNNSITNVNVEKEKNADNMYNYYMNVVNSFFNTKKD
DNNNNNNILEYNAHKEKYFYTDRMNFDEMSSFLLKKLKNTNIGKKNDIMFVAHSMGGLLTQYILLKNDHFLNK
TKCIFFYATPHFGSPLSSSAYLLKPFLSPYVYQLNDYDSKLNYLQQSFKERIKNKDLVIYSFSESEKSPLPFI
GVYTMIVPCTSAYLYYSKIFTIIKYCNHLEISKLNSEEDVKFYYLNKVMKEFSKIK*
>N.PF11_0357
MSQSKPNQEISSRNLHEKHLKTEQKKKNNNLSTLEKDKRSYSQGDNEKLICVQLNIEENNFPNDDKNLREKKY
SSNYNYEKKKKNNEINKFEGETLFNKKLEQNENTNEFNKIKEQQTKEHINEYVNGYTVGHIHKNSDKPIDYLN
VDRYAEKKYEEEKNNFLYNNIENQYTYHTLPFTTYPWENNYNMYNINDVQNGIYNNYFENEAFQNNLYCTYDN
NGMENINYGYIKYNDIKKDNRTFKNYTFLKGENINNNTICMNENNMFYKEPYPKYICKDENKYTHRTNNNNKK
KKSNINNNNVNNNNVNNNNHNNNNNYNYNINNGINNNDYYYYNSHNFKDNSNIMKGFLKEPSVLNNPIYLNNN
THNYIPYNNNIHKNDANIYNNTNSYIAPCLPYSYYYIPPFNETNIRNSNYPPTYYPFHTTNNNNNNNNNNNNN
NNNNNNNKHVYSYLYNDQQIHQINNNIHQNASNAYFINSNTLGKSSISTNYVSNNNVKGKNNSKYYRIKLCPF
LKEGFCQKGDNCSYAHSPDKLRNYISFKKTKICEMWLKNKCGNTNCVYAHGELELRAPPDFFKTKLCKFFNTS
GMCPLSDNCRHAHGQNELRKPDYRNNEHHVDTLTNKKNDKPLHYEINNKNVSNKSICTKSHNKINENKNDIKV
SKNIINNEKRGELQKKEGNKQINEGKKNTKINRKKKTNENNICNQSNEVKTLIKNNKQIVHQNKEEEKYQNKE
QENQGQIEKNKTKEHDENKTQEHDENKTQEHDENKTHVQEENETNVQTQNELHEQTSNETNVQAPNETNVQTQ
NELHEQSQIKQKNCEEKIEEEIVQNNSPNHFHSQEKKKRIPKKKKKKRNLINSSNITTNLNNNSKCNEVEERE
EDKDAEKETNPNYNILINNVRGEEGEISSPKERNIPKKDIEIIPLKNKESLNSNKDKYFSNKYDEGHLDHVPC
YDTHEKKIIKLDGKENEKEYNKIEQCEEKVNITTSSQKREKKENDIMTHQGYCENSCKINNQLNNNEMIKEKN
TVNVKDNKVLNNKFKDDEKISCIKCTFVNETHNDNSNNKNNDDRNIEKTNNYIDKNIKNINKKKIYGTYSKKE
KRLNEQHNNEASHNILTKNNENTKFIKDVNNNKCKKEKYNEKEKKILETLSHGKMEKHKKNKIVLGYKSCSCV
HNIIRNSSNNKNQNGTGNISKKETNKKEVTKTLAKSSSNCTTTTTSSNYKYKNNYMNTSFTTSNNFNKNNSFP
YNKKILKGKVFNATSKTGYIQNMRIKNMERNKYKPQNNIIVDKNLNYYPLDNSGNKIISIPNYNNPIYNVNDT

PNYFLNSYIDKNLDPISHDNMIRQMNEYNNINYNRDNISTMNYTNYSPHVKNNKILMNHNIIPMDIIPNNGNY
YNNMNNYIYDYNNPYYNIPNMGINNKNTFDNFTTINKNKNNSNISKDIKNEDISNYKPHYHIVRSKKEINIKN
DRTNKSFNYNINSNYLKKKKKKNFFNYHPINEANIYNINGYTNFDTYNYTNEQKDISSNNIIYNRSKKNFFFS
NNNNNNNNILINERNIQVPTTSYTMNNNIKENTSNNILYNNNIGNYMNNINIKWKNNHNVKDMKFYQVMDKKK
NRKKKEHSFPYVHNDMNIDQNYNKVNNKNGNYTNESIMTNINNINQNNNNSINNHNANNYYNPCNNYDNKTSF
IDAMKNISQEDVQQAFMHNMMTFQD*
>N.PF11_0358
MAEEVNRNIKRTSELINNNKVDKSFLKNINLQITDGEIKLHLKIIEALYPNMKLKVDKLINGSYNIFTKFLVQ
SHIDDFNSFINVYIKNIAENIPIMEFSPQQNNYSLLNMNKNNSESVKFFVSDIQIKNPMIKNDKGEYRADYPY
LCKLSARTYEGELLIKINRQYKDEIASTTICAGHIPIMIMSNLCNLSNLNKKELAQKGEDQSLLGGFFVVSGR
LKVIRYVIHPKYNTLLLNADNKIHINCLLNDNTVVINFLTLTRNNSYVYGFRFQNAVCSLPFHLLLMILSPIK
KKSYIFNKIKLGVENENAIKYIELFINSIFLKDTFNEKELFEKYNLSYLGRIAYLRRGIFKYNLNCYEKKAKD
ILKYCILPHIKNNSEKFETMCFMFKKLIYSNFKLITPINKDSLENHAVTTCSNLLANLLKDQVMNSLCRLYIR
YTRSFFKYFETKYNTFKLSVKQIYLRYKMFVLEEMQEDRLNQISLSKSSEDINDSSYQDTQRNLFSRCVLTPR
KLLGESWGFICPVHTPDGTPCGLLNHLAQYCHIHNLSSNEAHKLNIKLYLKKIGINVNLDDTSGIHTIYDDEN
IPIIVDSIPITYIGEKDFNRTVYKLKYAKNNNLFNLKSYFEINAYLNEPLIMNSIIINTFPGRLIRPLLNLKT
KDIEFISPSYQPYISVAINNEDVKKNNLARKLLKKKEQVVNSMKIDRTPGSIKQKFLYHQNRENLLWKLKKES
KDGSYKYEKHKIDRKLLKILDQKKNNDSDTNTDNYVSSSEYDTDSDYDYGYDSNSDGDSKSNSNFDSKSDNIS
GSDNNMSTTTYIDSDENINIDMYEQIPQKFEYMELKETSFLSFLASLTPFSDHNQSPRNIYQCQMLKQTMGIQ
SLNNVYTFTNKIYRMITPQFPLVVTRDYEFYGVDNFPSGTNAVVAILAYTGYDMEDALIINKASADRGIFRTH
IYKTEFIDLQKVGESSNFVFGNNIRYLNNNKNNNKNLAYENKGKFLNKDGLPCVQQKITEGSPLYSYINKGNE
LTTYESFRNHGEYFVDFVSIGKNNSNQVAVVKLRSTRQPQVGDKFASRHGQKGVVSRLFPQEDMPFAESGIVP
DIIFNPHGIPSRMTIGMLIESICGKAASLYGKRIDATPFRKYTQQKSFNNPWIDNCGIKGFLEKNDTIKSDLD
TCQKEKDKNKNINNNNNNKIKENKYNNSNDSNASIDSDDNTNNCNDKRDEEEKKANITYDEKIDYFAKLLLNK
GYDYYGTELLYSGIYGVPLQAHIFIGVIYYQRLRHMAYDKAQVRRTGPVCNLTHQPLKGKRKHGGIRVGEMER
DGIISHGCSFVINERFLMSSDGHECFVCPKCGLILSPIMQFNTTGKIMKGRSIGGKSKMAVCKSCDVSCKIIF
IPYVIRYLLNELICLNVTIRLNMKSVENVFDMK*GKNEALYN*YNTHICK*VDIYIYIYIYIYIYXXXXXXXX
XXXXXXXXXXXXXXXXXXXXXXXXXXXXXXXXXXXXXXXXXXXXXXXXXXXXXXXXXXXXXXXXXXXXXXXXX
XXXXXXXXXXXXXXXXXXXXMYVCMYVCMYVCMYLFIYLFIYLYIYIFLLFT*
>N.PF11_0359
MTVLSAVISTKSKILVSRQFRNISKCDLDSLTIPFHNLIERERSDHTYIETDKVRYVYQPLDNIYIFLITNIN
SNIIEDLEIIKVLSQIIQDICQGNINESTILKKCFTIIFYIDELIKNGVREIVNSNQIKTYIEMESHEEKLQT
IIRENKEKEEKERRKFIASKLEKNRQKQSKVSSNSFLSNDLINNLEYTNNIDMYKTEEQDIIDESFNTYKGMQ
LTSNKKENIRILNVVEHKVDTKPHINVNSIFDKPINIIITENIICTLSSEGTLCDLDIQGTFNLQINNHNYSK
VIVELDNEYSEKAKIHPILDKNKYNSNILELKDKSKNFRINTIYPLLKWKINHINDSYIPLNISCWPCEDNES
TLLSLEIENKRNNNDELIYDLNVNLMCPSAHKPQIISNDKGIIEHDGVLLAWKVDELKNNQSCQIEMSIQANP
ESVFPFSVEAKSNMLAHKLNVLKVYDEDTKKDIEYEIKKNITYLFTINK*
>N.PF11_0360
MNGEKLHGLFYKNCNLYVGEYILPGDIKNDDVEGGSIKNEIKKKKTHINNNDNNNNNNNNNNNGNNNNIDCNY
SKDTNNEEKKNSSKQMYTANDKEYNIEMNKDISFNQPNQENFNIIFHGEGKYIKKNEFFVGNFENNNYRKGIW
VKYKNIHNLFYFMNIHEILLKEEDNNMNDTKSCLDNFQKLLYVPLKDINIYIGEFDGNMFNGFSLYLFYPLIY
VGYFINNSMHGYGYIFYIQSPCQNNKNIYKLNHIFGPHNLYDFLFTTGIERENKTNQPTMVYNKNVVDNKNTV
DKQKTLEKKEQISSHNVDTQNINNNSKNQIHFKIYKTLEEMINQRNVDLKEKKKRNLVEEGKQSKTTCYNLIN
KIERDIYKTFKLNISKKKKINKIKKILYENEEENIHNIFNHICYENLLFKGYFYNNHFSNNKKEQILYRKLFI
HRYKNMITEKINNIQTNIMNDHNYKCDDLILENNVLFVIQKNNTNKDKKKMNYTNKDKEDNKNNNFISNKEDE
KNDIHHDFCNDDNFKIMTDNIEDEKEEKLKFELFKNIIDINLLKYIFQLPSNNNSNMEYSIDIIIDKKKIIKY
KNKIKEFQMDINLLETATLNLNGYYQLIIIKLKCKGDHTFHFITNNSNIPSVYKIKIFLISSDKSSIIKYNIF
HLTYILVYTRTLDKKTKVKLKKKKN*
>N.PF11_0361.1
MKGANFNVFSEDFYHKQNLISVLNYFFVYFIIAGACFIKIPQLRKIITKKTAVGLSFMSIYLEIFVATSLIVF
SIYEKINFILYVDVILINVQNLILVFFMWKYHKIYSKSVQILKVCFYISFILFTLYVLPKKLVPLLGLSSAPL
SCFSKLPQIYLNHKNKNTGNLSLLTYTFILCGNLARIFIILFNIKNQIYLINCGLVSFLNCTILFQIVYYWKN
TTKILMQADKIKKK*
>N.PF11_0361.3

MWKYHKIYSKSVQILKVCFYISFILFTLYVLPKKLVPLLGLSSAPLSCFSKLPQIYLNHKNKNTGNLSLLTYT
FILCGNLARIFIILFNIKNQIYLINCGLVSFLNCTILFQVK*
>N.PF11_0361.2
MWKYHKIYSKSVQILKVCFYISFILFTLYVLPKKLVPLLGLSSAPLSCFSKLPQIYLNHKNKNTGNLSLLTYT
FILCGNLARIFIILFNIKNQIYLINCGLVSFLNCTILFQIVYYWKNTTKILMQADKIKKK*
>N.PF11_0362
MNYNKLFILLGIIRNNTCKINKNLFYGILCKYHYMYEGRRKEIHTTISKGDKMNMHINVFNNIYKILLGKKLA
NKKFILLLAFSILAITLDVRYASCNGTEFIERSEEYFPKNNHYEDITVEEHSLSSSDEEDSDDGSYEITYENL
DELNSSYFQRNENHFSMAQFAANSPIEDRCFISNINIEMDYIEPENGTNSMMNNFVEEFMERIYLRIKYIRRD
DIHGRRFFNGHNYISGHNYMNRNNNNTEIYGTTQNTNVNNIDSNNEKKFIRVHNDQNNIQDNGVSNVVHNMTE
QNQLNEHNNHNPVVRAKTYNSNHGVLSDMSNPSLGLNENYGFKDELHKLSYLDANRILEERLTETCYFSLNSN
EYKNFQTCNPNFLFAAVIDGHAGGTIADVARKSLGYYLKKELIEIGVNSKRGGCRERAIVSALKKAHLNFDND
LLNQSKDYFLNGTSKYARTGACSLSVLIDERNYYISNIGDSVGLLIKKHFYLPLNRIHNASEFNEKRKLLKEH
PNEEDILVCKICTRDYKTVNNNNTYELCKTPFHLLSHHYDNCYVKGRLQPTRSFGDFHLKKKMFAYSVDGTRL
FVPEPHSFPYISAEPELRVMKKHPDDQFIVLMSDGVYEFLNHAQVINVIKTYGASPERAAKELINTVLEAAAY
SSGMTMKQLLNLDPSIRRNFYDDVSVVVIKLN*
>N.PF11_0362a
MHISIKKKFYYINEIYNNFIINIIPFYFNFISFLGTFHIYKKKKFCLLNIRNSSNIKNAYYLKT*LVYMKMII
SK*LI*VIMLS*VLGDKISSKKLTIHPILSKQKVQKI*CVYQYIIALMQNHNVLCISK*LLWSIIYT*KYRRN
LYTFIYFNEMVIDIACAKKTKKKKKSRKRYKIFKRYKRFNRKNKKIDARNYIKRYEKERKKRKSEKYINNSTH
*NI*DSNT*KDTFIKKYKSERE*R*K*NWI*YKGRKNITRSQKYIDMSNN***P**KRLSKYVKFKRTFYINI
L*VIDMI*ILML*I*II*LKIITFS*KETTNSIKNNIKVNYINYGSINNQKNNIYIYIYIYNQNIYSAIFKYK
NINLIIILTKELRKQKKIHIVYNHILNKVLNELNYLNEEKKYFKNKISVLQKVNNSKINVQIF*KDKSKK*RK
A*NGKCIVNKFTKTNHYIAFHILIMIKENIILYYVIYIYIKVQLLIQKILYYL*
>N.PF11_0363
MKSMISGISSKIVESNKAKIFLLGTRILWIITTIIYIVLFYFITNKYVSLSEDDIDEYNIKGNEEELLKDDKI
FNNYYKSYNKINAICGTFLILLCIVNIISSFYSKLNRGYMLYYKRMIGFVSLHLQLLFIAIMFLQNHENELFF
CNINKYIYWFEDYDNKTDLFINSGENLCKVHNYSYIFPACFLFFSLIEFVVLYFKIEKHFRKKFLIFYIKMGT
GIMYLALFYIFLKSKSFYNNILSEYNIFTLDDYSDPIKIIIFATQKQKMYYFQLLLVSTVSSAIVFCLLGYFD
IYTIMKSCRFSLLVNIPISVFTMIVVFSQLLFSSYILETSAFFCAYEEFIEIHKRDQKASNKMIAPSNITETD
WFCNLKSFDYIYFTNISTCLVIFGSDFIVSLYLVFSKKYAPKTS*
>N.PF11_0364
MKFFSVLFLIQFIIYIFFFKTVVKSLNLKIDLDDKRYTVLYDEKKVKFADLDLLKIMFHPNHVRHCSNSIINY
MMKLSSGTSPYILFVVKNIDITFEKPLEYNDDYKIFGAITNYGNTSIKLTVFGIGSEKKNKSYSSAIDFFNVK
KLLLDSYTSNNNYEVDENNKGIHNLLKNYNEEKSEPIKLTQQNAKELAEGKTCKIFFKAQYTLVKISKVGSKA
SVTEDFKNEYPAIDEEKLKELINLFC*
>N.PF11_0365
MIFKRFFHKYPGGSKIKCYIEKRKKKKLNISLFHQPLYSKNSKNFIDWRYSTPKSGYEYMYIYGENTIEITNL
PNNKTNEYIQERLRKSLNKYGRLKIIRCLSHRNDPYINNNICYATFYNKKDMYKCIRNINIRLPLSLQYKILK
FRSLLSNKCNDYNYFFKQDHYNYSAINIALNLFKYLEYTNCSMNIKDIYKHVFEYSFYPHKIISAGISIYKIF
KSWENFIYFFDNLFLIVKKDDDIFITAKILNDQNLSIYLNNKLIELKKKTEQNNSVYWREHSLQLPEEIENRI
NNDRPKKLKEELQLLSKTKDFYKIHDERHLFKLKLNKERKEKKKMLKKNTQQQKEEEKEKQLKKEKY*
>N.PF11_0368
MNDKDKLHKKKEKYANLNDDVEYEEIEKKMKRRKVEKKFTNTNTMEYKLNIHNNIFLKHMKENKEKYEKIDNV
EKIYRKLKADIISLNISYVQMEHYDSFFYYEKNKIDFILPELDNIINSSNIDTLKDLCDISFYPIYNPTLGKR
IIEWLTKDRNNNDKKEYTESVNMSYLFSLNILLSYLIYKNDDNSNYSCMIYLNMLHWMHNDKKILKFRLFILK
GILKRFLFCCSYFNDKEILKLKKIENDSVIDEEKLDVLKNIFLTLHNSVEYIDISNLLNINDKNCIFELIKFF
IELIKRPLIYSLHILCVNYLIKIVKSCYDFRFTYNINVELYDIGPEVNIFVKIINLILAKILSVIKDTKNDEN
MFCLEQINNINNLITLFFINILNNCVRIIIPQVLINDKLKKQYLLNNENKYNREANTNNDDKIFEDQVVVNAC
NPIFSFIEIFIINHSDKYEKNVFIELIKILIKECRKIEKRYNIMKMISDEYILFIKDGDSKRKHDKSIDTNYI
KNRKVSRKRFDSNASFKLSDEYSSSIYSETEDEDKNEDDVHKNGIRNITNNQNGINVNDGDDNNKRNNEINEE
TNEITMKESTKINKDDIISSQGEEQIKSNESPEAIDDLNEINKQPKKKVYRNNFINKLNYKRKGTFHIDNKRY
YITYESVMVRYLNLLLKYMNCERYQIRNTMLDLFLSLIKKKRTNKNESIFQSLFKLFLERLEDPNVIVKAKAL
SMLVTLTNKKYYDNSLIYKFIFNKMNEKINMYKIISSNIYSNRSNIRKVIIQYIEAIFETLIHKKLNYKIFFF

FVCNYLCTLSLDAMSSVRRQVLLTVNNLFINASHDFHITKIWIAIIFSAITDIEETIKDECVSIFLNTFIKPA
FSSSLLNINENKIKQVLKKYNITYFKINQELVEKISMKYLNENPRKYECSDMPNKVPIISLSDDTKNNKFKNE
NNIDYNNEEGKIYEDNNYSNINMDKIMEEKKNKNLKKQSDEEYDRNNDGILYLIHLCNIYFENDFNNTFICIL
KYMTKYDLLHFNIIISYLKKKKNNTLEEFIHFLNSFLYNVRYFSIKVLNTFLLDFIIQLSKEYQKKIKFENVL
FLLNRCYEIYLMNINNMNSGSNIELYNNKYKTNHLEKKDNNDHSNDNISNVVLNDNIEEKENNILIDHILSNE
DLIYEKEICTNGILKNILLKLFTILYNLLDTTEEELDESFLVKMENTIYHFNCPICFIYIILNIIRKKKKNIN
EFIEKLKNKIVYFFEHIESIELNNIFCNILITFIFLINDFKKGDTFLNMVCIKLKRIYENIDNTEMNTTSNNN
NNNNNNISGSDNNNNNIIFGSNNSSCNNNIMIKNCIYLTQSILVIDKSVSIRNDLFLNFEKDLKNVNTCMNIL
NNLIILIYYMIINFGSSCNKFVFVLLRFFKHDNSFLRYLSFYVLSKLLSEDYIKYNNQFFFGVLYLLADKNEI
IRKQSLSVFKHILMMYSNKTNIINYIIECIFVLNNFYNFKLSKHLIHIGNIFHIHNKIDRYKIYSYLLENLSN
SEKFSLQQKLINEYLIQYVYNYDNYYFEEDDDIRNENSKLKKKTILPLNDEDNEGSVLLDVLNILSCKLMKIK
IKKDFAPKLEEKNKNIRIKNVENSVKVLNDLMKNILKKNTLPILLSLRTIMFKTKSFFFKYINNLIIYLCIDY
KDSIEELINETHIRNEIIIDFQNFIYVDSILLHAEQYNHLFFDKNKNININFLEHSENIFDTQINHHQNKAKK
NKRNKKYYHLMDNNHNNNNDKKNNNNYSYYTSSDEYQQVEKVLHGISSDSDEYAQKKKKKKNNKIKK*
>N.PF11_0368a
MVQNIKVKVKKSKITERKNKSQKAKKNKEKIVVKSLRKKSKLVKINKIEEMAKRLCASTNNNLKFLNNEGKKK
E*
>N.PF11_0369
MYGGNFSIPEFILKHEKYEKERLKIKRNKYLKRNIYKDNYSFIYFIFLHNINEHVEITTNNFDLLKGQLEELK
KNTNNHIKGIILKNVIKSNNNNYSYFSKLYIPITNIYTCMPDNICSREKNKNPESKPFCDLIVSGYKNQMMNK
IYEKFKKLGNQYV*
>N.PF11_0370
MKVILKKRNGKYIDCFDLSPSTTVDQFKEIYYKKYHYYPERQKWNLDSAAGKTLISGTLNENGIKDSDILIFK
DLGVQISWRLVYVIEYLGPIIIFPFFYFCDKYVYSHTNKNKHIIQIFSLWFLLFHFIKREFESLFIHRFSNAT
MPIIRVPINCGHYWILCGVSIGYYLFHPLYKPYDIESKPYWVYGLFFVLLILEFLNLKCHLILRNLRPRGTKN
RGIPHGYGFNHISCANYFYESLIWIIFSLITNTLTGYVFSFVATTQMTIWALKKHKNYKREFPNYPRNRKAIF
PYIL*
>N.PF11_0371
MVDSEFHDVLDTTYEIVSNRILNINEKWKNKSFNNSNMKNILEERNDDGNHHIFSTHDIMKNKNVYPNDSRRI
STNNISRSESRSSKVGLYNNISDSLSKLKTIINYDKDEKRSNEKTEKCNTNIEEDMNMLKEYNTKIEDMNMLK
EYDINKKLNIKETSENEYTKNDDKYNNSTNSNGYNKEIEFLKNIAKEHSLKKIEKNINLLLKCDSDNNDNLEF
KKNNVHLNNNDYRSEDLFHDNRVNNQNNLKDIIKDYIIHDDNIMIESNNDNRNDKNCFKNTNPYNERHIIVDK
TNKNGNNNNINSNSNNFDSISNINKKISYPINMNYSNSEDKTLNQINLDMSILSSDSLKNAYSFYSLSEKNNS
NNIGMNSHKNNMNEYRDVIDEDINISKFSNISNLHDSEMNDNNEFNSLCSFNSSSKCVKDEMITQFVGNRKYV
KTMDMANDNYMKNSIEQHSINMESNHFKNQKINSGKEDANNDNLNSSHILNNNKGIGQNVSNLSDYLYSIKKQ
ESMNNLSNNEALNNINVTNNNGSSNNNKHNSNVYKTSQYVYNPNDNINNMNHQLNLSYMKNSNNLNTSNGFKK
IPKNKNIISNIDFDNNIFKSYIKENVVKNQESNINHQHVEKNYTNDEINIKNNNIENNTQNTTCNNFINTNDD
IINKKKIKEIYKKIDSISLLNDLSLNKLESLNSSIMDRYTKNNYEEKFLDDVILDDSIFATSNELLQHSNYTT
TNHIFDNNNNNNNINQKEKDLFQNDYNKETYNNNIMLSENNAENLFKISYSCNDLVLGKNNEIILDRNVENSK
TEQYEEYQNKEDIIKLYHKDDNIIIDNNYNDVNIKKDCHLKMDNQDNIKINKSQDSKKNKPINNNECNVIHNK
NFKINEIDHFKEENTLNYESKNIINTCKDNLEGDENNINNINELKNNSLQFDKNILLKNTMSLQKDYSNIKNR
KANTSNIDNINKYTSNNISNKSDIFIDNNNKSKSYNKTDIINIFSKNKSNDEDTFSKCFTYKEHLSNYNKNDE
NLFTTFSNAFHIPKINNNIKSTHNDILNISNKYYNKNNTTNKTNIDIFQNKNSLDMPETNLIKEKQQFIKTDM
SSYDNSIKNDNSRDIRENIDCSVKNEYQSFNKNMSNGNLGSMNVSPCNHMYNENNDIINISKYIEHRIRKNAE
ENTMKNNINEDTSSKDFNCFSNNEKKKNFTTNNILVPTNMNMNKIENKMNDLSYDQSHRGINNKKYNDNNEVD
NNEVDNNEVDNNEVDNIEVDNNEVDNNEVDNNEEHNNEEHNNEEHNNEEHNNEEHNNEEDNNEEDNNEEHNNE
KDNNSYNKEYNILFDKKNISNLNDNNIFSLSKNNTHSLNISSSNISPTNVRNNLNDNLYNYSFDNKHNNESKG
EFNYSSYQNEIKYSNMNSMAEMFSFGVHNNSSVFFFDNLNNYSMNENCDNSLKNFVFKDDALILKEKDEIIKN
NTIKKENIKEENIKDDIINDDMINDDMINNDMINNDMINNDMINDDMINGDMINDDMINGDMINGDMINGDMI
NGDMINGDMINDDISFENNYKNMNNLKTIPLNEVKNLIMSSNMNNNDIPYFNEYNEYHSNNNYIFNQNIVNNS
VHEILSHKEKIIKSDITNKYHFERNEIINSDAKMIVKQQNKSINSYLEDNKKKNYHNKNDNNEDDKKGFSNNI
LEGYNHMNNHKNNNHNENDNNENDNNNNNVLNSNNHNNCKGTRDKKIVRKKSTSEIASDILETCSTNYSFERS
EKAFERNEHVLKKDSLIFRKEENKDHTVRQKKNSIEEYNTPMEVKINSKYNSYFMSSKDKHVVEQNNNLRNNS
LIIHNSTNEQQNVSPNFQNYQTFDYSDDKNKPFNEKSNMGNNFQTYYNHDNNNNNNKNNNNNNNNNNNNNNKN

NNNNNNNSNSNKQNGGDDIDKTPKQNSSYRNSQTNHNINMVQDDDDNNISRIIYKEYDNTYMNRNDQNNDCKE
NYTNKHFNNNNNNKLMYIPNDVLNKSAQQQYNNNMNYINCKDNKNDNNDKNMLNIDQNQNNFHKNITMFPMYK
NEVEKNFIQVNEPFAYDNRVIAFGKYPTDSSKEIHRKGSITYVNNNFDNNIFNNNKHDKNVVNAIYNINEQKE
KEKEKNYINDKNMGKQRSQDYLRNDMNNMNNMNNMNNMNNMNNINHINNINHMNNMNDMNNMNDMNNMNNMNN
MNNINHMNNMNNMNNMNDMNKQNSKHNVLGINAHILNNIESHHEDKNNYSEQETEKYNFYKNNYVDENYQKHY
STYYAKEEIVSSNFRNNMKTLDKNFFNNRGQNGISITKNYEPNLTFKNDIYVIQKGNMKENEKHICNEQEFVQ
MQQNDVYDKNEKEIFNNNNNYYYNNYNNNINNNYNNNINNNYKCVPNNNDYQDKFDVPEYYVKNNNIIYYEDN
KFVHKKYNNQTYNNMTEQNHYDNNAGNYYLMNKEKNTNFGFCNNRKGDMISPYPVKDDHTNFSYDKMRKKNNN
NNNNNINDDCNDDWVDDYASKNKNAVNNGHIKSLPLKKFHTDYENCRMNCSVYKDSPHYELEYSDGNYVNRKY
DETDNNMRKYNTIKGHIKSANVTEGNITKGNNMKENVKIGNMTNPHIKTSNKENIIRTSNDSNKSINSDNLQM
LYDLYDEHYEILRKEKNGDVLNKIMDVMMNIQNDLKDVKYKLSKKKKNEEKEKMENDEKEAKREKEKKEERKE
KERTEERKEKERTEERKEKERKEERKEKEKKEEKNEKLSGKKRDKENEKEKEKKKDKEKKKDNEKKKENEIKK
EQQKRKENEIKKEQQKRKEEEREKEGYMVKKKEKNKVKEKERKHEDEEYYNPNYLTRNIKSAEDENILSESIF
VNRRHLDENAKKIKNLEEERKKLLKYNEKENYKSMDNSTNSIEEDELYNKQYMNRKDSNILKKKTFDENKYSN
QNNVINKTNYDNGYNNGSTKKFHMDDNSSNNNHNKKNNSMLNNNSNNNIPSIDQPPSDIIHNNHHNNNNNNNK
TSSNNYNNNHNSNDNNMSDNYYNSYQKNITSDFPKNSNSYNNYDNVEKELYTSFNFDLKNLNYEDIEDSVISK
SNRLYLFNKFFTSLFYEDNTRTHNFIMFNIYNIEQKRQTHKVSLFLEKDYSNIFPYAYNNLLFYAFDCYKFYR
HIKSKFQDTYKIYENSTSCLYINNNYETNLIKRLFMEENNYNHCKYYLNEKRILKTCTKYFMDNFLSLIDKKI
NIKNIIPYMKKNNYEFDQRNLYFFISNFNLMDKIFSDSHGFDCNKKFFEQNCPNIKNYAIFFKYIKDASHFYD
THIKNNIHDHDNNNNNNNNNNNNNNNNNNNNNKNNNNNNFNDKHNCNYRLIIVALHKGKTYNYENSSFNHFINTF
DKEIYNYYNNYDSMSFDNGSVILFNLSYAVPFYYIEYYKEF*
>N.PF11_0372
MLNFSFLFFFSYAIFYKPYLCKNSKGKYHDHLIRDFTHSKDDLINTCLEVNNGLTYYEELLKNYKYDTNCLKY
KYIGNYVLEKYQMNEFNCYEKIQKALCKLNYINKENVVVDVKNNSEEKKEKKGYHLWENNLNSKFSKCHNIKK
FFVHLINRCINNHEEDSLVHCASFDYQKRIILDGDIFCKSSFEQIKVEQNFNKNSIYGESYKNYITKTISFQN
DDFNDCLNMVNIFNNCSVVQNKLFNKTLDICLLEKNTQFCNIQLEKVNDQNYIYTCSDIILPYLLSFNMHENN
KTTYEFDDDNKYITKQKDEENQSFTNKERCHDIYNYLKILKDNKKKVLQEKKVLEQHVKNINIINKLYKILHN
ILQILKNETDKYNIDIKEFFEIMENIKSNKFIKKQYIILILNKINEEIIRSYQIMLHFPHPILNNNTINENIS
LVNYIKEHVNNFIKLYKDIASNAKLINEYIKKNDQNNNTLNDNINLNKNISNVDNMYHTDNKKNFNNLHNMNQ
INNDFNTNHTNNILKDKNVNEINIDELQKFKQIYHNINKLIQKYTKQTNNSIFNLPVPSVQDFKRDINNHNTL
IDLYTDQISLLIKNNILS*
>N.PF11_0373
MNLNKISVLLKIIIFKNTLYIFDNVCTSIHVNDLGYKVSDTINSFKYKKNYDNSVVLDKVPDTPLDNKAKLSF
QELLRKRSSDDDDDNDDEGDNDDDDDDNDNNNNNNNNNNNNMNRFYNMASIQPAAFNNISQNGNQSFTARMKQN
LSKYNPFKKSGSNNNGKVNNTNENVDEDNNDNVDDNNTKKKHRHIKHKSQGTLSNDGSNNMNNNNVNNNNVNN
NNVNNNNVNNNNVNNNNMINNNMNNNNMNNNNMNNNNMNNINVNNSNMNNNINNNMNNNNNVNASNILLGASA
LTGAAISGQNQNGINNNQNVVNNNTNNGTIQNSIMLNNSSSIFSNLFENNNKFNTSEMICSSINCTSINSQNN
DKIPLTGCSNVLYCGNCPFSSSPKDQCASIKTLNVDNTYIASGYVDSLQVKKKKIIYIYIYIYIFNFLYILLF
FFFSFFLL*LLNKNNLVNIPFTWDKTTLDFSKCLPDLNKFKLATKDLPNKGQK*GIKTFV*VSKFIFIYNKIC
GRQ*Q*SVKNIHKF*QYQYRFIKTN*TVS**M*FFITMSFKYCRN*NLQYTRNSEFGIISAIIYS*NLQCPKY
LKYYD*HNQRPWL*CGKLLFLCKN*LCQNERHYRK*NISYSYITTNKSTNNSTINSTNHSTDAY*K*SSTK*N
KKWYTT*K*YFCTIAEK*YL**RF*KRI*L*K*
>N.PF11_0374
MERLNGIVKQVISADTYVLIGAKKGGVSQERQINLACLQCPRLFMKSQNTEKVEEPLAWESREFIRKMIIGKN
VSFCLEYTYNNRQFCSVFYEEQNLGILLLEKGYATLVSNKNVKSSVYADLEPYYVQAKERKVGIFGNNIKSYV
RNIVYCYNDKNENKKVYDLFVNRRLKCVVEHIRDGANFRVYAEKEAANEKREVLVSTNNNNNNSGEKNKKNKK
NKNNNNNNNIDNTELNGMIEEDTGNSKKKMKNKNKKKTNEDENAESNLNPNEKYLTMYYFSFSLCGIIVDMFK
KEMVDNEETVKEELYAMETKRFVESRLLNRDIEIEIKHIDNNFNLYANIYYKLGNICTLLLKSGYAYINEYTI
KFVPNPIEYKKALDEAIQLRKKKWTNYTEKEIDYEKEYFSRVIEVLYGDVIIIDYKNEERRLYLASIKCEKHN
NTDIKLNTLCLMAKDCLKSQIAGEQIKIVTEYVKTPQSNSDGYIPQCSDDKGRMHFVSIYKIQKKKNKNDKNK
NHINDDVYDGLTNKWNEKKNTKGNTKTTLAHNNNNNNNSNNSTSKLNNISKKKNVKKNSKKLNTPLGNNEKGL
DYDDTDDDDDEEEEYINLNEELVAKGLAKVLNHRQDDDKASNYFRLQELEKEAEEKKLGRFNPHLEIIKINNI
SGNENALRARSFENTLNKYNNLNAYVDFIYGANKYKLYIPSQNLLINFILLGITVEKINLKDLNNMELKIKNK
NNMNGMENNEKNNILNGDADFEKNNSKKEKLEYKEIAVQAYKYTRKMLMQRMVQISIITCDKGGNFIGLLKYQ

NKDFSMHLLSLGYGVLNEVGLNNTSERNNFVKAAEEAKIQKKNIWSLEKSEYTDNLLKTENDLTAYDNIYYCS
YVEDINNISLQLKNRKEELLTLQRELNKKSNLDSSNLNEINKNTLVLAKYNDNCYYRAIVLQVNKNKKKVLVK
YIDFGNEYELDFVDIKKLNEQFNLKNYPPFSFKVSLAGLKIPTENKTDLIIYIKKFLLDKFLYVKFEKKEANL
FHVVFYDYEQFNTNKNIKSVNEEIVYNGICYVDNNSDTKIFEKLKKEEILAKKNKHVIWSYGDIDYDDEN*
>N.PF11_0375
MEEERADTFNDLTEDDIKSYMENKEFDNVIKLTKNIYDNDMNNNNVFLKARYSCLIEQNQYDKIIYEIMHCLI
KLDKKKKQKKGNSNFKKIFQENYKEIQNKNVLFELFYSMYKLRKFKKLYGCLKYFIENENKYQDFIDMLYAQV
NIILHKYNNSIHYFEVLLNNKSRQSTAHVNLNSSYFSQFMKFMYVYNFLRRSKKEKTNKNNNDKLNNVDDYQN
DDENVVESFYVDGERLDTNDMEELKNQIIRSTKNFNYEQNQNFEEIFNYTTFFIMEKNYSEAMKFIEVLEDMC
LNIESDNDNNESKDINSNEDEEITDDIKDTNSNNNDNINHNNNINHNNNNNNNNNYYDLNINFCRKEIFIQLQK
AYIFSKMKKIKESIRIYERILNQYDNVKNNKIVILIAYNNYIALMHNDNQLKLDMKQNKNIQDDYALTFHISV
EKLSEIKNFLFTNNTTDKQVQMNNNNNSNNNNNNNNSNNNNNSNNNNNNNNNNNYLTNITTHSNNIFNNMNEF
MFSTICFNECIAHLTNEQKDEFKMKLKFFSTRFSNSILLDKLIILELKNRNYMKCKSYIYNRINLMNSFENQI
KFVNAYIYLCYEKKNFKEIVKIYLMYEYLFQNNIKHYSSFFTNLFYIYICCTDYNTKEEHVDQNIVHVEKTKK
QTDQIYDNNNNNNNNIHNFTNNVSLVLDLFHKYKEIIQENTDIINYQTLYLVCKYLLYHNKEQLVQELFDYLC
VHEKNNLEFIACFTYLYTYINVSNAYTYENKLKKLVLTETYLIDVEELENTNIPFDKININNNISHMNDIQVE
KKKNKKKRKRSCKKKKLTDTNTANLNPDRWLPKHEKPEFKKLKKKKKKNETLKQKVVVEVEETKTTLKQNKLQ
NLKKRRKK*
>N.PF11_0377.2
MEIRVANKYALGKKLGSGSFGDIYVAKDIVTMEEFAVKLESTRSKHPQLLYESKLYKILGGGIGVPKVYWYGI
EGDFTIMVLDLLGPSLEDLFTLCNRKFSLKTVLMTADQMLNRIEYVHSKNFIHRDIKPDNFLIGRGKKVTLIH
IIDFGLAKKYRDSRSHTHIPYKEGKNLTGTARYASINTHLGIEQSRRDDIEALGYVLMYFLRGSLPWQGLKAI
SKKDKYDKIMEKKISTSVEVLCRNASFEFVTYLNYCRSLRFEDRPDYTYLRRLLKDLFIREGFTYDFLFDWTC
VYASEKDKKKMLENKNRFDQTADQEGRDQRNN*
>N.PF11_0377.1
MEIRVANKYALGKKLGSGSFGDIYVAKDIVTMEEFAVKLESTRSKHPQLLYESKLYKILGGGIGVPKVYWYGI
EGDFTIMVLDLLGPSLEDLFTLCNRKFSLKTVLMTADQMLNRIEYVHSKNFIHRDIKPDNFLIGRGKKVTLIH
IIDFGLAKKYRDSRSHTHIPYKEGKNLTGTARYASINTHLGIEQSRRDDIEALGYVLMYFLRGSLPWQGLKAI
SKKDKYDKIMEKKISTSVEVLCRNASFEFVTYLNYCRSLRFEDRPDYTYLRRLLKDLFIREGFTYDFLFDWTC
VYASEKDKKKMLENKNRFDQTADQEGRVKQN*
>N.PF11_0378
MDNNGLVLKVEGNELLINQVNNEGTKRNFNLYLYKLKTKNDKLRYYEINNCITSENYVKGYINENGNMVLLYD
EQFYIYENEKKKKSCLINLSDCEPVDFIYFKKHLIFIENKKISLFQAKQNGKSMVMLNLKDIPLKINFCSYKN
LFYLCTRKRLYFLKLFYVKENKTLKVKNTNMSLPIKNINDQYAYHSYRNLIHKIDQKYKEVIHVCCYSDREVT
IYKFVKGQYKNKKKCLHKLTVKEFNNDKIRGAFFFKVKLENSLKGQIKTSHYINDMTDHTDINHNNNNKKDDN
KNDDNKNDDNENDDNNNNHNNIVNNNIINNNIVNNNIVNNNIVNNNIVNNNIDDNHLNEPSDEEDIIKLYLLI
YNECGKVLIYFIDYLNEKYFKFGLTHIKNEFVVYMQLPFKPFYIYNTSDIFRKRVIKQKNIIQKKKKLSNNYN
KYLEYILNKEVYVKNHFFFECVLVEEKYATIYTIQMNYEFLSNHEEGQELVCMDSKDSNHKIMAIKEKNENCD
NNQEHGNQNKLSHMLKESKFSCYSDSDSYIDSEITWTDSQAVSEHLSFYTDTDSQIENDIDKNEKTQDNLNDD
MKINQNNKEDNHNNKTNSMELSLHTDTCLKSDEEESNVTNYLNNFKSSDIQKEDINKELDENVTYVEECTNKE
NIDMVNKYDEHVCDIYNDQTIYQNNIKFSEENSYINKEICSSSKNMNEEEVNHEMTHFETVVVSKLNLEENHI
NEVEQNKSNMITNNTNINSLQESSDICIKENNNISCNGYIDVINHKHVDNVNNTNNKIGNITQDDFNSYANKN
TMENVEEIEVNNTVPKDINMYYPTQYKNSSDGSYNSSLSMQNENMKMSVYSHSKDESSEAFYYISEDEGEYYD
LKKNMDDETADEINDDIKEEMEHDIKEEMEDEIKDEMEDEIRDEMRDELKDEIGDELKDEISDEIKDEIKDEI
KDQVENKINDDIKDEIRDELKDEVENKINDEIKDEIKDEIRDEIKNKINDEINDEINDEINDEDTIGMAIENK
PDMQELNCKIIAEKLFENEKIEQINEKDNMKNDNNDNNDSNDNDDNDNNDYNDDNKNIEKKSKKKRKKEKENI
DENITTDYESSKNIREGKKIKINEIENEEKKNVQRKEIEKQNNNISKGKKDVEDVFSDLDIESSNNSTNQLDN
KIGNKNKYNFTTMSSMIKLNISLKRYSNLVDLINVKDYKLVKKTIANLGKKYCIKLLDFLLDALFRNKYFMNR
FYLWIRNICKQHKDVLKSKKYRKIITKLSEIANGNLKNEEILNHVIDKINFTIDHIIKNQVFDNVEVLNYRDG
TIIK*
>N.PF11_0379.1
MDKLIKENINDVFLSYNDMIQDNESESSEESYKDVNNFLQQSSDSHSTCSYESENQYIFNNNSEDERTAPIIS
YVSQNYICKKERKYKKSYTASYINNMNRELSNFQWKPLGKNVPKISLINLSHKKAWDIGKEGCNGILIKNLFE
SYKKTKLNYMVLDGTNLDKFLTVYSRTYQETINGVNPSVLKIFSFFDLEEGYFLYDVKSIHLFKKGKKKNKER

SIILKDLNDSFYNAILICMNEIIKYLKICDFKNNLKSKIKSAKNDSNTSSGTSITEVDEKTYFVKSVKNEKNL
YNNNMKNVITTNKRKTLEKNNILINDIIPNNIVKKNYVSFKNKNYKNKEKIQIAHNVFSNNTEESGYKYLKDI
IKKEENELKENDELYNNSYSGYSSHTSVSVNQDMLR*
>N.PF11_0379:mRNA
MDKLIKENINDVFLSYNDMIQDNESESSEESYKDVNNFLQQSSDSHSTCSYESENQYIFNNNSEDERTAPIIS
YVSQNYICKKERKYKKSYTASYINNMNSIDNFPFKSYGHVPSISDKIKELSNFQWKPLGKNVPKISLINLSHK
KAWDIGKEGCNGILIKNLFESYKKTKLNYMVLDGTNLDKFLTVYSRTYQETINGVNPSVLKIFSFFDLEEGYF
LYDVKSIHLFKKGKKKNKERSIILKDLNDSFYNAILICMNEIIKYLKICDFKNNLKSKIKSAKNDSNTSSGTS
ITEVDEKTYFVKSVKNEKNLYNNNMKNVITTNKRKTLEKNNILINDIIPNNIVKKNYVSFKNKNYKNKEKIQI
AHNVFSNNTEESGYKYLKDIIKKEENELKENDELYNNSYSGYSSHTSVSVNQDMLR*
>N.PF11_0380
MISNKDDAYECFNLACKYMKNGNFSHAKNLFLKAKRMFPDIDITEKLKTCEEEINKSEHIGTEKTTYNNNTTT
STFNRDTTNNVHERFRKKDECLEKILRTNNYYEILGIPKNSNDEAIRGAYKKLAKLYHPDKNKDKGAEEAFKK
VSKAFQHLINKEKRYEYDNNIDEHGQHTTYRTTHYYYNDDTFTPEDLFRNFFGLNFATCNNTNFRTHINSNRS
YSNNNNTNSANNAQRNYSFVQILIFLIMFLIFFLSSHFEQPKAVYSLQKTNYFDTINYTSLNNIRFYTKRTFN
YNYPRNSHSRFQIEYEVEYKYYEHECHILTKKIKNDYYREKNKYQQKINYKDIPESCMKLKTLEEQYNNYILK
LKKR*
>N.PF11_0381
MLNIIYVVSLILIKFIFYKECNNNNNNYLSNIELYNYKLRKRNRILNNNINDRKSFLSDLEQNYKPLFDIYEL
SANFEKRRKELEKKTNGEENEIEKKKENDLEKKKENEIEKKKENDLEKEYNDVINLLELSLSSEYKELNADVS
NNDNSGHEENNKHKLNKKNSSNYKNDKSLDELIKGAILKLKQNPNIKNKNMLDYDKIFKIIKEKLINKNLASN
KIKGGDNEKLKEEKKQSDISTNVEVKKDIINDQLNKGIPTKKENKDDMINKESNKEDITNEGKSNSLNNLNTL
NNDGNIITKVYDHYTIVTNSNDILNDISIDASDISKNSIGGINIPFNENDNSSFTHQRYIVLSNNGEKKYKIV
LMTKNPKFMDMDGIYDEEEKKESLIELNQKVNKEENTNLYDGTGTLYYGKKSKKEKENTQQKGGNNPNVDINI
LNNNNNNNNNNNNNNNSNNNSNSMNDEEINYNNNNNNKESPSMFRRFINFLSFSGNENETEDTLIYHNKNDNS
YKNKKEGTGKNNDNNDPNNNNNKKILLNVDKLVDQYLLNLKNNHTSKQELILVLKGELDLHSKNMKNVINNAK
KNLEKYFKEHFKEFDKISYDISTPINFLCIFIPTVFDMNNMDLLKQALLILHNDLHEYVENWSFSSTYHTYEA
DYIKEQDSVYDRSPKKKYIKASKKLYNNKYSFLNKFLNIEPLILFAKKLNSKRSNIEKEILNFLPKELRDYST
WNLSIIRVFNAWFLAGYGNKNVKVCVVDSGADINHVDLNGNLYIPEYNEKYEMTQDFYNFMVKNPTDASGHGT
HVTGIIGGVANDLGVVGVAPNITLISLRFIDGKKYGGSFHAIKALNVCILNKAPIINASWGSSHFDVNLHLAV
ERLKYTLNGKGSVLIAASGNKSNDNDISPLYPATFTFPHVYSVASISRNFEISPFSNYGHKSVHILAPGHHIY
STIPNNSYKIFTGTSMAAPHVCGVSALVYSVCYNQGFIPQAEEVLDILTRTSIKIISTKKRTINDSLVNAEGA
VLTTLLGGLWMQMDCYFVKFNLEKGKKKHIPVVFSAYKKGVYETDIVIAIIPIDGKSKIYGEIHIPIKIVTDV
NIPNFQESPRRGKNYTIDSNEAQHDEVLSYICENALYNLYEYDSHYLLASVILFFLALLSIFVGMIYMKSRKH
SDKKCSKNLIKSNYIPEMDDGMEETQQLQQERRQYFRELFGENLEKNYDQHFVQDFGQDFRQDFKLGSTPDLK
QYSDIDLQNKIQQPERKTVKIIINNFEDRKKETKRRLLKGLNYDGENAKKHDFTNESISNSRKNFKFSNNTEM
KKNTIKSEDVKIASDDNVNKAMNQLDDMFMK*
>N.PF11_0382
MIGIKSLRNFILKNIKENINKGFFIQYIHNYSTQNEISKKSKNDYILSLDEALYLSKELGIKTTTEIQRIIMR
SPPFSPDTSPFVIENFFSSERQENDLEEFSEIDKKIMELETTKKEKEEDKKNQGDFCSNNIKISECENNYNDS
IINKILIDNFNKKRVKTFWHDNNEWLEESEGIGTNKRSCAYVYIKRGSGIIKINDKEDLYIRWPYFYNRMDVL
EPFYITNTACVFDVFIKLKGGGISGQSKAARLAVGRALLNACPHIYNELKQHDILYEDMRQKFPKMPGRKKSR
AMKQWSKR*
>N.PF11_0383
MKSHNLDLIKNQNFDKNHNFINYSELYKKYKEKYNVKSFNKLPVSAYTYPRYSKITNFDELKTELIYEKEINE
IEEEYDDDDEQNNINNYDGNIEILFPMINEKENEDIKKKKTDNIYKSEKKSLRQRIYDGEVMFYEDYIEQLAV
KDYEHFFKKPYEIKRKDEGGKNYFYGKKLEMNKEEDIYRKFYGFDDAYFLNKLQLKNKSPQKEKNKEEKQKKR
NEEKILERQCGKKLSYEDKKFILNIIEENQLQDNSIYKYYMQNKCIFDKNLPEFKKKELLCGYIPDIILPANV
RKVKHPKNCGVPLSSLHIYAQFIHELFKCPYIYNAIDVELGKYWKTNENELLNIMKSEKIKKGYKKDIYTATK
EQLEKSSAKLKNLLKNDEVSDDTRKKFTINFKMPKKEKTNLVVSKGRYII*
>N.PF11_0384
MGLSLSSPQLPANAGSNITSNNNNNNHTNENQRVPFFQKIISIIVQMLIMHMVMYFLSGGKNNMMSKSGIMDR
TNVVLKNSFEKGDIFDIYIFLSNNQSLDFEYLKNNSKLINIREKELYNHKTFSSYEKLNYTFNLNDSWKDEKY
LSVVLVPIHFYKKRNYPLIKNTTLKYEFKDKVLVENIPLTVKMVPFESEEEEKYNLMDPSNNIKKKNIEEQKK

KKRNEQLYHIKRRIDINIIYDDGKHMVKEFKMPYFKNWKVNLTSNTYTPPIFLSDFWLIEKDYYVLDDNFLKS
KDKRVNYICTYDPYMLTKKQNDIHLYITEQNMDENKKLHIEINYGTCSFIYFMFIKQISYSLNMMNETAASAA
TTTSTTTTNNNNNNNNNNNNKIFSIQNITNITANKEVHMMKKILMTTNIYMLIFSAIFILLHSIFSFFAFKNDM
QFWYKNESMEGLSALSVITAFVCDIILALYLYDSEKTSWLLLFEMFIGVALSAWKVTKAVHVSFSKKYPFIIF
KDKKNYTESMTKKYDKIAIKYVGILLIPCLIGYAIYALFYFKYKSWYSYIISVLAGTVYTFGFIMMTPQLYIN
YKLKSVEHLPWKALIYKSLNTFIDDVAFFLIDMPWMHKLSCFRDDIIFLCYIYQRCIYKVDKNRNETLTQNEK
IDNNNNANQNDQLRELTQEQNVVDSHDKKND*
>N.PF11_0385
MLKIGNVVKNKRCFLKYDVKFYSSKKKVETKKNKEQKYNLWRWGCPGDSLFSSIKVGEKNKMPEKLSEFENVN
IEKISAGCNHSAFIIDGKIFTYGLNDKGQLGRTLDGNEKSQNYSLIPKEVKTNEDIKFTNVCCGYKHTLAIDE
NNELYSWGWGGNLFKGANGLGQGNKNNINIPKRVETFKNEDSEFINISCGEQHSLALTKNGKVYGCGKGEFGR
LGKGNHGDQLFFEEIDYFVSNNIEIKDISCGHSFSAALSKNGDVYVWGRNDYGQLGIENSIGDLYSHEVYPNK
VKYFEIENIKIKYIACGDNHMIACSENNIIYFWGSRAWLEPKAITLNPLYQNSLIKKNIEKIDAGGSTYYYSM
LLSDYKLYSWGKYNSSCLALKDKKNHNEPTLVDSALFNNEKICDISCGRGRVLAKTLANN*
>N.PF11_0386
MLSFIKLLLFFFNFMIFQGKVILCVNHKSSDLSLFLYSHRIYENKKKNHVRKKLSILNLKKRLDPNDKYTVIK
RHESKIQRNLKRKYMIERYKEKRQQLKKYISEASSPIEYVYWKYKLSTLPRDSCPVRYRNRCAITGRARGYYS
FFGLCRHQARSLIQKMFFPGFVKASW*
>N.PF11_0388
MRKGKDKNDPMSTRIEKKIILDMLKENDCQTIEELKNLVLFLNNENKRLHTMNMDSLKKINILSVRVKSLEKI
SKKYDQFYASKKKYKNKLKELERKSVTLKNYTMDKENEYNKMCEEHDKYFKNLFKNLNRKIEYTFACEKENTY
DDSVSNSIYTNSNQNEDNISYNNNNINNDNISSSSISKNSNNTKNSSFNETKNINDDKIKYDDKNVYDDKNIN
DDKNIYDDKYLYDDKNINHDKYSYDDKNINDDKYLYDDKNLYDGNNIYEENNYNNNNTNGHQMKSPTYHMKNS
VQNITIYTSINKNDISKDINKINEKNTANINTSHIYQIKNDENNNNIYNNVEHIKSLCESYKNYDNDHLLYYK
KHSHSKEDIHNYPIFNNAKTSQLENSNNDFLNIMNNNIKNNISEMESSKKKKKSNLLITNEIEDKLSNIQINH
TNKINENNTIQYGQKTKGNKIGNHKTNIKEKLNDLIENIHLKNNKIENMNNMDDMNDMDNMNNMDDMDNTDNM
NNRGNMNNRDNINNRDNMNNSNNMDNKDNMNNKDNINNKDNMNNKDNINNKDNINNLNCIDNMKHDKLQKDHL
ETPPIHKSPITRKSKNNIMDILNNKMNITSLENVDTSTPKYVKYNTIHREIMNPRQHFFNQNKSSIDHIICL*
>N.PF11_0389
MATRKKERYHTTGKGNKIDFYLNLDEFENIKSITNNNEKKNDEKFSSSTYKSYSDNDNNGVSLYSKKNMLFNE
DNINVGRNNYNKTSEKHEERSNNRPMIQLNKIMSEEDNTYYKGTCCKFNDNKGKKKIDKDKDNHNIYNKKNDV
VKEYNLDELWTYLNHVHNAEDSRLKYKINEIMERKNSLNNHNNNNKEEDNDYTEKINNVNTTYNMNNKKRNHI
NKKNNDISYKNNFNDKNYFNYEEYKYIVKYILPSIDDKKLKYSWSILKENIQPQNEQLNHKDIFKFINEKTGN
EESNYVNKIVCSKLKNKMLKYNFNPSDVQLKTINYIDTIRSRACEFSKHFKDLITDNELTELFKEKEKIKKDD
LEKEIVHIINERLKDENMVYVQKKEETNKNKHKNDDNMLLSISVYNNMKRKKEGLTDFNIKAYISTLLTNKND
EVYVKDFINNLQVDYDLISNENINVKDAYDFYSRNIPPTENINNINNNMDELNKDEIERAKFLIEEIDYSVRN
NFKSHYINTNNNHKNIKLKILNDNEKSYLSLYEIFKHLDNDKDSYITKDDLIKSVNKLKLKNITNNDINILMK
YMDNEKKGYINVNDFLKNYEMEEKSMLNWIKNTNKPYFDFVSNLKNKSHERSISEKIKYNKNASIAKKYNDVI
NNYNLELDPHCPSYVIRERIRENFIAKKEDFINKHKKASRFHLTNYKNTNNLIEPLTNSDLYMNDKLRFKTTY
NMNYN*
>N.PF11_0545
MCSTYSVEPLVYDRYEYVYLKPKKAGTSAVYYPLNISWKYVAKKRSVGCFGSRKKYTLTPEAYYYPYYYYYVY
YYPSAARLVRTTRKEKVLKENNNKESEDENKEDNVGTEKKECDCSEKEKYIPTYVPLTESYYFPPSALYVPHY
SVLVP*
>N.PF11_0390
MLSENNKKVKCFIYDYNEKLKLYIKLKRYDLNNFVINNSQDAGTHIDKCVHCRGNKFYGIKTYDNLIECEKNS
YSYIVNKVLENYESIVKCCVLSFYSYNDNNNLTKLNDDYFIDNPIHVSKIDYFSSKIVKQCFNKINELYISIE
EKEKEKKKDNKKKISYSIQYSYAHTNNFFDLLEFYEDKEYFHSIKSCTLKRNNNIDNIKGKTNVNVADISELK
KFNQKNKEVKFTNIKKLKDILHTADKRMKEIDDMLRGKKIYSFFTVIAIDIYDETDDVINSTISYNKECIKGN
NNKRSKKIYFINMFYDAKKEINANDDSLKKTLFEMSNIIKYNYIDKKLKFNISNYDIISKLMYELFYEIQKVH
INIYLNFTHNEFKNNVGDLIVYFINSIDYKINNCIINSISNLKEEISKNDKCKMLNDSEKKCILPECEKIEKC
EKYEKIENVLFKNISLDEYTKSYIIKKAIQIIPDKSMDNLLLLNKTFQQHYIFYEEKEKDINENMYHLINEQN
EIMKKYEEHEKRLQDDMNKIIDEIHLVDDKNLELKKEIQKHEELQKKYITHEENKNIEDLKKLHTHNSILDEQ
YEHFLSFRKGSFVDDISWKKQESQKNIFLEQKNIAHNKSIRDNVINIINKHDEQYVKNFNEAKKYAHNKFQQS

NELKKRYNHVIDKKVELFDVLKNTTHKLNEKVNSLMNNFRNAKELKLIYPIDSSFDNFKNNYEKDNLIINNLK
GYTDLLEEFKKQDFNDKQLHWINKMNSLSKKF*
>N.PF11_0392
MNDNYNLGYNIDEKQFDGYDDYMKKKESKGYESHSDISCKIGYTDSESEGVASIKDVSYFTYGNNMNYNYKKK
KDILEKPVWKSLYDIEETVKDSLSDYEYSKNNKLDSDIESPRFGNTSEEDDVDKMEEENETYLNHSNYNNFIK
DKNEANRINYNNNNNNMDISITNEGSYKNILNKNISNNLDYDFKEELKNNMYNNLNVFKNLKDDYVEATTHFL
KPKTFKDMNGLYYDLNDEMIDIDLIKKEHQKLKETNELKNVSSINFNHLYDDDNNNNNNYNNNNNNYNNNYNN
NNSNYNNNYNNNNNNNNNNNSANVKTKNSRKDKKYKKEVSFANQNENHSYKKFQDKPKSLKNKKDTILKKIKI
YEEDELYKGQYDQMYEGEDEYSDELYDEMCEDSDDKIYYEDEKFNDMVNKKVAKDINFEWWNSKKVSKISKDI
NLKYKDSNDKMKSNDSHVKSSENILNNNNKILNRYDLPKDISYYVEEYQKRKEQAKNYHNQVENPNYTEDKNI
NDDKENYPGDNINLNNNIRSNTTTVYANSTLSNEKNMNLLNSNISEDVSSINKENNLLNTNINKSNENITIQN
CDDNNKLVDNNIYIDQEFLIKNYNVNKNKDKYDSTYIDSLKNNNFIKDIYTDNDVINTESMGIYNETNKMNEK
ISEPCIHNNTSNIYEYILTNNDMTNVGNENSNDILNKNIEKTNFENESKKLYDVYDMINDYYEKNKNEETINK
IQEKCVDKVMYDFINNNIDKETTNLDMDKKHLYLNNEKMNSTCDDKDTYLEMSKKEKINIFLKYLKMIDVNSL
SHLFQYFVDREKDEEMKKKLQFLLIGGDEKKQMEFMENYKINQNTQTLNKGLKHESVQTNNEKKIQIENIIQT
DIKDITKTLYIKNDMINKKTSIDSVFFKSLSKDSYDLYNKNKEDIKKNDTTYTKQEDMENKVDVTVNESNTET
YNEVQKINDLKVKILEKIKGCYDNYNSNNNYNDDEQTAILMLQDKNEYSKEKYMDVYNLIDENRNILSKLNDE
ENMKSNEYKNKKNRSMVTVETFASLKSFEREMNLLKSHNERLRRRIEKLYESRDRIKNEYIKMEKLKESQDRL
FIATERHIEKLHNELDNLSKKNEDMKYDLKKKNIKIIALESQIDNNLNMISNNMEKDNNNNNNNDNNNNNNNN
NNNNNNNSSSSIVNMDEIKKDEQIYNEFNNKLEEIKGKITNKTQVTIQLQSLQDQVYILKEEIKKMDSLKLEN
TQLKKKLSDMENHNFNTFDKKEKINIYTYRNNSENISENSNEHNFIVSNENTNTYTNELKCVTSNTNNSTSNS
FVTTEDNINSSENFTSNSNLYEDKVNTNKYHMNSSENSNLSLLKNENNQLYEEMFDLKEQIMIMKKKNLLLSN
RLNDYDMDNIKEDIKILQSESEKLYLEKIKILTKNLDEKKNKINLLNDLLKTSNNQTVQLNKKIVYILNEKKE
LQNKYEQCLNYLEKLKMKYDQQAQKLSHITNISYNMVPNDYYCMDDYKSLISNEQSSENLYLNSNEENKINDL
IHMEHSVNMKDEENKVKVPDDHDDVNNNKINKDKDKDKDIYVNNENHYDNNYYKKYKESTTTCGKFSDIYSYE
NVKYDVYDRRESNKIDDSNIKKEKINNISLNIENSNKTDEMVKRLEISHNNNNNNDDENNPLLRKEQNIHNNI
LNNEQKYININNIFEIDNITKDLENMNNIITSNNIKKFLSNELNEHNMMKSNMDEENNTFIQKNLLSNNNINH
NIINTNNNLKYNSFNTNLVNESYKNLDNISNTSTENFLRNIEKRYVSNKINELNKDISQYIDKKKEKIHNLYK
NNLEYNNLLEKNTNIMNNNITKTNEYTYKHINNDKHNNYTFNKEENNLKSIFKYNNNNNINEKDDIPKSIQSS
FINEDNIAYYNKEGNNMKLKNEDIIQERFSHNNIKTYEMNKNCSYDSCDNIVKINYDELNDSTQTKELNEGKS
NNGKAEAWIIDIKNNETYPYIKIDKKEKNNEDKKNKYMYNKNDNKNIMKGSSNKSMKKNNKNSNKMKHIPLSV
NNKGYNKSSINNKYENNINKNNKDKLNILVNSISKLVQSKIKQELSNKNISKDILNFEITKIKKKSKKETKNT
NHTNNKNNDNNNDNNINNFERINDNINSYNVLNNKVPYDNINIDEGNYINYGPIYAPDGSTIYTWVNNIDTNY
MYNKYFDSKKNNINHVPLLNNVPYLNNDLLINNVILNQNNMNNLENLNTNTIGSVQPFVTCPDFYANNIKSIY
LDPNLQNNNYFDNMQLLNHNNLNNNLNSINYIDQNYLSYNNLNCINGNINKDTCKDIVIGIPNNTNQNQIPTI
ELDDTILKNDVNLILNNNNVVNHSVNVEMLNNIQNVNQKLYNDIQENLHITNSLHNNNNNNNNNNNMLGGDVL
NNSYLFNINSFNPNMNAYIYNNNNNNNNNNNNNNNNCINYNLLDKKEINVKNEEINMNSVLEDTNKENEGNDMK
DNKNYIINKENNVKNNDNNIDIISNGQNINNTNANTMKEKRNNNLRDDDIYKKNMKRSNSLDFKKLDSEKKNK
SMNIEEGARKMKTNTTQLLNYSENRKKGLRDMSTYADKVLEDMKSLIPLNCNGAIKKSSTNTQKKNVSTSDNS
SLMYNQNNVNISNNIHNNFKDNKNEDSNNLSLKNLVSKDLSTNNKDIIINKSAELLPDSCNNDNNTTNEKTYQ
SYNSLDMNNNSNISITNNLGNNIKLMRSKSVTTNNHNSLIMKNDFSNSNNFYNNNYTDLNKQINNPLSHLNLN
DFSNIHYHDFNLQNTTNNKQFRKYSTNGYNNIYNELNKNVAPDIFSFNKRDQGNYFKSETRKSLSSFREALKK
EGILG*
>N.PF11_0393
MKTKVELKFLGGLESYLANKSKNYVSLEIESEEFNFENLIAYIRNHIIVDRKDVFSDFVMSDGNVKSCNVMID
DKEYSNYNLSDKGKIKPGIIVLINEYDWEILDTYTYKIKNNDKICFLSTLHGG*
>N.PF11_0394
MKIFNYICGRPLRNGGTAPLIYNPVRKWLIILMILYICLSILSYLIFLFPKASDLQCLALIDSLFNFSLSIGV
SYVMAPYYSIISCREWGTEYEWGIVAVVSAVMAIVDVLSSCYGIYVLYTITSVVFNKRIGMNNDCNSYNAVLF
FSANSILVFLHLTVATVSTVVYFLLMKGIDKQLEDNRNII*
>N.PF11_0530
MKVKKWIKKIYNSNHIHSFISSISGTLASVFFKKSLDFSTFIYDIDIITTSFIRHIMIRIIYFFLFILCNIIM
IKHYVLLMKHYSAFYATVLNFSLNFFLSALCGIIFFHEKRKLLWFFGVMLIICGLIFILKDTINEEKIKNK*
>N.PF11_0395

MSDSKKHYNESNVRYKVKYSGKIFGNNMKKLNENNNHDFTTAEAAWMEKKKKLYKDTNNQPLWDFRKIQINPT
ETDELISFPSNKICSKNYGKYSFIFKGLYEQFLRLPNIWFLLISLLEFIPQYQNLSNYMYYSKHSSFFLLLFF
ICVSIIKNIYEDSRRSNIDYQINNRLCHMLDGPNSQLKAVRWMELSVGSIIRLIENEQVPADILLLSCNNSEG
VVYIETSLLNGETNLNKKYCVNETRNETSIYAISNIRGRIVCEKPNSNMESFNGSLKLDAHPRATSLSINNVI
FKGSHIKNTEYIFGVILYTGNDTKIMKNISNNKHKLGYVNKELNSYTIIGLIFTFICVFISVLFKWTEDDKFR
NGSHFFLITVKDNICESIVKYTLLYSNIIPISILISVDLISILQSILIENDNHISTFENYETSEPSTIDDMDN
ELGDFKMDKSHTFFKKYTYFLNNRSKNFTNNRYSSTNTERASDFRKSFFGTFDKLKTIKRYFYSIKSKIKSIS
QSNTLYNKSSAGGSIIRSDKNKQTTFSKSFIDIFQRTNNNEHSQTLDNNNNNDNNNNNNICKEKYQNVNNSKN
VYVSEDSSNNKYKQNDYLQKSQEQNEIHSINNNNYNNKNDNNLQTEFSNHNFRINAYKTNRTTKDDTYNNKHI
SLNSRSKSNQKGILINSNEINRKKKKNLLQKIFPFFKKKRTNISEDLSKYVFRRSKQLNINHNNSEKYNLNDY
DEYGWGLCLNSNMHGDLGNVDFIFTDKTGTLTNNNMTFNMCSIAGKTYGSKCKNKKKIYNNPKSNNDNKLNSY
DKNIFKNKFSSEASIKKISFVRDFSNNVSSKNDLTLDDPTELISDEGNNYLRDKYEHTSDKKNDTNKNRDGAN
NSNNNNNKDVSNNKNKNNNNYNYNSNNDYIKQNGECNIYNNQNNTNNTHNNNIYYDEYNNTNEGNMKNTNVNK
DTNSKCNKKDFIINTSDIINNNNNNNNNDQKTNNLKYKSSSSWNSKAKKSSYINLNSYNKYQSFQNSSMLSSSL
SSTCSSETEEENGSQLMYYSSSNYDNLDINNDMDSTKTYSHYTEQNYYNSVKDDKDQNSEHQRYQQKKTNKQT
KNIQQNQCDQWEDNRYTQESIKNQYFFDRNYQTFLMKQNFPRPKSFRLNMNSSSVKGRCDFNDTKIYNDLNSQ
SWRGYYIDEFFKCMTLCHAVTPFIQFDYIRNKHLTYTNSRSSKGISNNMDRYNINPTTTTTNNNNNNNNINNI
NNNNNNNKQFSCDYMKNRNDIIFETSTDYYANQDNAKRKREKTLFDNVSHCGEIQEYRDNKKYRMKRSQTCSN
NRKIFSNQRTLYDYRNMNNNLKNKNSYLRNKLKSKIFLDKSYRKRKKSSGTYKGSDICKGIEKKETSRWKRFL
HMKGSNNRNIFSRINDFKSNSIIYNNQDKTNDYKNGYSNNINNNNSNNYTNEKINRSFNKRHSAVSFKQMSKE
KLIPINNVLSNRCDVESYPNEELSFSKKKKKNSEIVSFASGKSDIGFEERTDAGIAGKNMELSYNELEKYKYV
KHIDSNNKYINNNNNSIHMSHMSSNNNTFESHIYFDAIKYQSSSLDEECLIYSSSFLGYRLVLRNKNTMCIEI
DGSFNKWTIIGVNEFTNRRGKMSIVVKPDSMESGSILYVKGSDSSILSLLNLKYSKFLDKKYSRKRRNKNIQK
YKQKREEYNELKSKDINIEEFQKSKSLSLKNFKNNTPKVLFGECTLDNNNNNINKNYKYDKNDKHNNNNNNNN
NNSNYSYNYLHDSNNLDAMQNQKYSYAYEDKEDYYYTNNGECNKYKERYRRLEKQLRKFSVKGLRSMIFAFRY
LSEEETIKYKRMYDDACSSIYNKEQRLEKVAEEFERDLIYLGITGVKNGLQEKVPKTIDILNQSGIRIWMLTG
DNVEYSLHVSFLCKFLNKHTKIFHAALENSNTKKLKREGMALYELFQLEKEEKKPYENLCLLVNGRNLQTFLN
YTDLQTHFLNMACTCDVVIACRITAKQKAFLVQLIKNRLYPTPNTLAIGDGANDIAMIQEANIGVSIMTSDCI
ISAGYSDYCIKKFCYLRKLLFIYGSKHLYTISIILYWNFFKNILLILPIFFYQAYASWSCVKIYPELLYTFFS
IFWVFIPIIYYMFLQHNLNYDILYNIPLFYALSRRRYNMNCFKFLPWIFEAIFYSMIIYFFAYAALKENSHLN
NGEVITINTFGNICFIGCLLISILRLFLEGSLWSPSILITCFGCFLFVFFPSLLFICFAYLSNEYIREVFRQT
FLWAPLYVLLILWFSTCIISYIFINFTKSILFPNIYNVVNHWLFEQYQEKHNKNKYIFSLSGKNKFLKLRKLG
KKIKFKFKRNYSKKYDTNVIREHPLLHHTFKSEQDQNKCNEQFSKDPFISNSLLKSKNCKFKKDSSYSKSNIH
VDEEQTYKGLKVVLSEQVVHAKENLVSFKNEKKNKNNKNTSMNVNDNIIIKNNNINIKNNDNNNDDNDNDNNN
NNNNDNYNNNDHNKKEFKQKHIQNNYENLFNHTQVENNLIKENLSIRNDNNRRDNKNETDYIKQSDEDYEMNP
SITSLRKRECINDSKYIDNKSYDNKISNNHEEKDGFKSDDRSLINSNMMDYKNEFTENDTTYSFNDSHMFYDF
PSNSKTTKDYKVINSRIDDSIMADMKNCLNPLKNTFLVDLLPPGKRFRINEDHIYFKNNERMENIPEALDVNK
KYYHIIQNRAEYYDNDSLSEFSYTSSNSSINNKKKNENTQKEIVKVSHLINRFTLAFKDMQLESGFQIHKKNK
FYKTFTPWYRFIFLLLGVFFLYVWKLESSLSQLWNMPSDASTDVFILFLSLLLELVLLAATVTTFFSNIFIEN
FNKIISAVVILIITYHVVSYSVTHIDGVFQAVLFPLYTFVILRLPFVNAVLCNIIFLGLFIIRFNGDHFLDKK
GLAHYIPLFIGVDVFVGFVGYRLEYNQRKNFLLEYSVESSRRKQREILNTMLPPFVVDEMIYSELNEEGIPIS
LKAEDISTVTIIFCDIYDFQNIVASIEPTRLVEVLDRLFLCFDKCTEQFNCTKIETVFETYLAACGLVKREKD
EDELENNKYSNNNKNNNNNYYYNRKKKKKKNNNNNNNNNNNNNNNNNNNLNNNNNNNNNVNTSDDDGDFFEEDD
ANNHQIYNQMKGQEDAHDSIDFALSILHVSSHIKYENSKMMLKKNDSFEDANDDTHNVNDSFNNDKAENDNTN
TDNNKPTRIRVKVGIHSGRIIAGVVGSKKPQYALFGDTVNTASRMKTTGKPDYIHISEATYNLVKDDKTLIYE
KKETEIKGKGIMTTYLLTSVIGLNYPFLGEHVEKKGHFISELYEDDLSNNLNYDKTQNILGYYSNKINQNNDS
NINEGIINNNMNTFNTIDGDTSNYYVNLKDLSLNDLPNEYIKDVNTHYCEIIKLRNLKIGTAVGYQLKQKDFY
NMTFLNNGLNNDTATNFNQYIESHKDFDDIKDLRIGMNKLSISDSLYYLNENVIRNEPINEYKNKIKINSVNT
NKKSYEDAHLNAGNIIYTNNEYPNGQNIEEDDDLLLTNHYNKTNVNNNNNNNIYDVSKELIEHNEEDYKNILM
CSKRCKNCNETYCTPNDCNNNDHVYHVMYHRKLSNIKKNYLKNIRKDANIKKDIEKEELNKIHSNKKSFNFFY
LFSYIFKIFPFIGKINKEEKKNKSYKKGEQDKSSKRIIALNSEWIFLKFSDKNLEAKYRAHFYSNKSNINSIE
QALIIFLVTFVMQTLISSTVSIVFIDHKRATQTLHINYFAYWSVRSVYTFFGFVLWLLFHYRTRPEVSSLLNI
KWMIFFLNLLFISAACVFSIAYLWAISETDQTTSYTIWMTNDTIEFFFYLVILHHNTGMLFQTCILVDLLFIT
MSLTFIATSVVKTITTDSTVLLIPWYVAFNLISTYCKESIDRRTFYANESAKKTENRATELLNDMLPKHVLEE

FQQDKLKLAYTHDRLTFLFADICGFTSWANGVDASEVLTLLQKLFAKFDNDSTKYGLYKLCTIGDAYVAISEP
VTEDNKDYDPVDGTERVLEMAYSMIRIIKEIREKLYIPNLNMRIGLHYGSCVGGVIGSGRLRYDLWGIDVLTG
NLMESNGIPGKINVSETLKNFLLQQFKNRFIFKPHTTIRVIYKDVKCFIITDKKEVESSNHSKLLQNKNYLLN
KKFSTQNYLVKNIFAKKKHSSISSSKNIHSYDEKKKKKNDLFYINVNDRQSNL*
>N.PF11_0396
MGAYLSSPKTNKESLDGGNLELDPSRYGLSCMQGWRKNMEDAHICYNNLKFNEIEEDVSIYGVFDGHGGPNVS
KWISYNFRRIFIRCLKEANEEMIKKNMKRSENYKLKLIKLTLEKTFLKLDEEMLLSENQEKLKKYSVPTQEDE
EESDTRENYIYSILNDIISKNISIKAIEKDGKRCLQVVYNKDGSPVEEGNTDGSATSTLNQDQPEHNQLYEDG
NDNNNSNNNNNNNISPNLYGTNGENILRSDENYDSSNLNLGDNQDRKNYDDIENLGDNNNSSNDMLKKDDASS
DILTATTSTNDMKDEDKKNNYLVEKNEHMKKENNNCEENSEQNKQVNGMSSYNKALIEGAMGESIDEKGLLNL
NDIKTNNILIDNNNDSNNNNNNNNNNSSSNDPSMFSTAYAHHCNNNEGTIGDYLNNEENNETNNIKRLKKDIC
NDEKGNTQNDIKEEDKMRNMETYMNEPIDGVKSELTYDNLKSMEEQEKNGEIEQGINNMIDHFQSNNLLNNDN
TNGNINSYTNDDIHNNGSLQGYEQDGLIQNRNNGDEVNNSDIERLAYDEASANVIDNNINNDIHEEDEDDENN
NNDEETGEDDCNGVYSSEELRLFENYYSNDYEDNIAYSCGSTALVAVILKGYLIVANAGDSRAIVCFNGNSLG
MSTDHKPHLQTEEARIKKAGGYIANGRVDGNLNLTRAIGDLHYKRDPFLPQKDQKISAFPEITCVTLTPEDEF
LFLACDGIWDCKDGQDVVGFVKTRLEKFEEIPDDPNNSLDNTENSEHMNTNNNSDQNEKLKSEDQNNINNEQE
NILNTSNNDFQKNENLLNENNMDNINMQEKCETEEDLDLERDEDKHDAPPVIERKKYEKFNKLSQICEELCDE
CLSNNYKENDGIGCDNMTCLIVQYNPEYKIHTEKKFLNIDDIE*
>N.PF11_0397
MADEEFSDEEEVDVEEILKQAENIECVDENSIKKIGVLLKKKKTKNERDRMEYPEEPEKWVSSEVDLDEILVN
LKNLSVCTNLYKSMIDSDIFGEIIDLLNHPNNDIVIEVIDIIKEITNPSNIYELDKELNDIMIHYLNKKKLCH
FLINVLDKINEEENDEYYNAMTSILNILDNIFELENDLQNDLLKNSKLLFFLLNRINNEIKSDDSNSLYASEI
FVLLILRINQFSQNVYDDFYYIISIFNPILKYISKYKDKDPESINKKEILLNYFQALGNLLLLNKNKNVFQNT
IGFELMLKLLSERKFLCFPSLKIFAILLNDIETCNKFIEMNGLKYLFCLFMLRDIKKQNHMSVFEFEENIIII
ISNLSLFCTGTFQGRVLSKFGEKKCEKIIRLLEIRQKYHEIIIKDKKKKQKNKNQVNENLKKMNIQIDEDSKK
DLEYIELCDKGYLIYQLTDVILIALFYMNNTYICNNIFIHLYTRNIDIQSIYENILGKLINK*
>N.PF11_0398
MKNVKQNISNFRELAKQLEACIHFDSTDIEVMRDIYLKCKKDLISLEKITLEALEKGKANLFEELVQVSTEVN
RSIKLFEKFEREHKKDGTVRISEGTNQTTFSYDMKEQNKKKKKHNDRKHEKRNNRESIRKHKSEHYEDEDEYY
VDDDDDDEEENDKRREKRSKRKDSEKNKERKKEKKKSHINEEKYKDRNMNRRDTFMYKSDSKFSYNKDFDNNI
QDTNLFSSEFDEELNEYRHEKFNKHTNMKEKRKSKKDNKSNSRKGNKTKGHEHLSNMNEEDEIENEIKDDNFE
LSDENYNDNKKKIYFNENKLYNLDDSSSNDIMNSMNKRNSLEKEFLSSNKSLNLLVHISEIINVSFNLKNNVN
IFITIKSIDNKIYLKKKTKNKSIQNCSINISELFDFSFNYNKKLYLIIDIIDSIDGTYYYNSIINLNKILLRK
NKYFLPTIFTLTPKNEKAKLEQGLLYNENSTNANRYQQVPSNYIILPSNGFTNMKNMNQVSNEQVKNGVYNNK
QILTPNPFFGFKKNYNNPNLNNQNMNNFNFSSRNVNSYPNLNNFNFTSRNMNNHNLNNFKNGYLSPNLNNINY
GTRNMRVTPFNSPGLNMGRPYMFTPRQYPQMNNVNMNVNGMGYMNNVNNMNNVNSMNNVNSMNNVNSMNNVNN
MNNRNNNQFSNYFNNMPTVGNINKGNIESNCFNNTNVNNALLQNTNFNNNSNNSGAFFFDKNGYLNNSKGMSY
KREEEKVPSNYTYIIMNVFLKNKEFDHITELKKQNKLNVFKELYNGCNREKIYYLNKNKENEKKIEELDKMVM
LLELDNESYLDANKKLKEIIEGNKEIIKSIEKVLTKQNNKIEKLEKENQKVIETEKELEGNKKENNVLSEKIT
KLNSIYNENIIKLKDSSQINNNLNSKVNNLIFQLQNSQCKNEKLMLYLSFLLKHIHKLNCEQNNMLTFQKFQQ
KNSQIANSLLDKESLYHLNNYTNTYMLNKKKENEKNFIYTKENNSNKYIKEILKFVKKEKNNENYKDSKVLQD
ILNICDDDHLDDHNNNDRNNVSNNNNEDDDEDEELLHFKNEKNLLTYENNSNLIEDYNDIHDNSKTNITLKDE
NYLLLQDDSYIDDFLMNQYKKYITTSTKEKRQDDDLEEHKQNKKKGNINQNDYIYDNKKDMYLYDLNKKSFSN
EEINTDDEYNNQEQRRLRNIKYNDKSKYQYYNNDSDNTFDLNHMNKNNTYVDHAKKNIPLYDQQKNIINCSNS
TFSFNMLNNTEHDPIIISYKKIKKHKNKEINEYDNKKSDKILKKKIKEKNEKKTKSSLAQSNFFLGLKQDDDA
QKMYVHNYKKKKKKKKWNSFNKHYTFSNCEMDSYNKDTEETSNIILSDGYINDKKCLLKLNNDNKTNNKKNNK
KKYSLNWNDKINQKFSISKYGKKGLLHEGSLSQEDHTNKQQHNVNMNKTKLGLNKIKNINNLSKKTTNDYLSD
YKNFEQNFNYFVRASKNLKGTILTNNDDKLSYAININEKNYFTNLEKIINKCNKKEQNISHLDNYLNTQMGSN
FITDEEINEHKKFMRIFYTKNNKGLIFQNNLMKIYAKIYMNHSSMTNKQKTYKLDTSNVTKDISSTDNNNNNN
NNSNNNNSNNSNNNNSNNNYSNNNHSNNSNNYDSNKKNMLENNLTNDKIYMNIYVKSILYNILYIKSDLSENK
MDHIKVIKRNIKKNHNDAIEENEYINLKKNDICLLYTLCFKKAYLNHMLIFLNMDCLINDNTIIKFKLTLPFP
HLFLCKPNSLYLNNFIKSISGTTKYYCKTYYYNLVRFPTFQEFINSLKLYNSFNVFHFNSFNILYTTYPTDNK
NKILLIIVCDFIKTKNQNNTDQVKLHFISLSNTIISFAINLFKQIL*
>N.PF11_0400

MNNNNNNKYNNDIHNSINKNGSTNINNNMNMPNNIMSNIANNRGSIQSNINNIPRPMNNINNVHSNINIPSSR
NISSNINMANSRNVSSNMNMTNNVGINNNMNINNNVNRTSSINVPNNMGRTSNINLSNNMSRTNNLNMNNNIN
SANNMNMPNNMNASNNNMNISSNVNRTSNVNRTSNVNRTSNMNIPNNMNIPNNMNIPNNMNMPNNMNIPNNMN
IPNNMNIPNNMNIPNNMNMPNNMNMPNNINMPNNINITKNRSNSILNNNVNNIPNNISSISANIKPRNMSSNI
KHNLNNFNNNMVKDINVKSSIKMSNDLSNSNIHKNSIINNNNNNNISNISNNSARESVMSNINYNYNNINYNN
IMKNNIDINKKLNNDKNSNIDNAEKINKKIGNKMIIKNSMNKNSFMNTDQNNVNMYYMNDMKNTENNIYLTNS
NNNAMNKVNNIKDNSKTLQQFKENEYMENMYKREKIINDKENVYIPNKAPSNINQGNNTSVINNKHNINNMRN
MNNTNNVNNISSMNSTNNVNKMSNMNNMNNMNNLNNVSNMNNMNNMNNINSINNMNSAINVNNSNVNNSYQVD
KTVFKTINLNNINNKNRNQIYKYNNNDSNNFIMDNNINSKSNELYYNNNNNNMNNNNNNNNNINNMNKNINLL
YNEDGNFKSHNNNSGDNNINNNKQLFMKPFHNNIENTNDFKSNNEEINIRYNSNMIGINNANNNVMLKAPANM
KSTMHKVGVKKQKLKNNDTTTNNNINNNLTNIENTDNMYQLQLNKEKLNMNSTFNFMNEMDIFNDPSQSENKQ
SDYTSVRKYSTNNKIAKNNDQPNSKQPRGSKMSKINKIEKIDKIEKIDKMNKTNLELNNNMGNAMSVNNNMLY
EEIKKNNTTSFDNGINNNRIPLFNGTIPLDKNSSQHNNMKININKNSNISNSFDNSLDTDLQDDFEKLKARIE
QEFEEKKNKKKKKREKKEDKKENKKENKKEDKKDDKKDDKKEDKKEDKKEDKKEDKKDENKDDKKDDKKEDKN
KSDIQIKDDKNEVSILDDNIKDKETNLEKNDDILNLKKNEDQNILNNNHIHISKTENNNNDIKTNLETEINIT
EDCPIFKNDDKIIDNVNSNNLKSNTISDNSKNEILRNVASDETKDNNNNIDNLNNHFGSNINTNVQKDNLVTP
KIKMHYSEQPYNMINRESLENLGCDKKLIALTKIFGNVMDDSDNNMTEKEPVPKLKLFDLDTYKNMSLDTMLN
NLNLDKDVLNILKDKINNINRNINVTICSTPMEKQIDKIEKEDINAPNLINCFSEFINWCDNNLIQIKLQLYN
IAFCLLLEIYILLLTINYEYIENFKNQYLKKFSAYEPVTKFLLTCVSLTQIFEISLIRFKKKNSKHIVHMTKL
GKKSLLQYLSVYEGIPLYNIITTKIKIIEIDDTKRNFNFFYAFVSSNFFYNVKLTFPVQWNLPSIYLTNDEIV
EDENKKSLTEKEQNNYVPNENNDAYYYYKHILKTQATNRLRVTKKNIPSILYYCLNNCNDLTCAELSGYDGSL
IATAHSNNIIKLWNLKKSEMNKVMNEKRDIDEINEYMDDVRKHESYLNGKKSIPLDIDEYNNKGVSKLYGNTF
NVSSLSFGETDKILLSGNLNGDIYLYSTISNKNYVKYVGGHTPIWSIDTAFLGYFFATAEDDGNLRIYSTNKT
YPFITYKYNCPVNVCKYHYNNTLVACGYYDNYVHLYDVRINSFIKRFKNNYPSNQGVTSLSFSKNGRFLSYAG
GYTNNLNLIDLAMDKFVEVEQKENMGININSFDELKYADNIRKSIDGYLENDLMNIDERKNVSEIKKDDFSFY
EDKILNIDFSYDNNLLVTASCNNIIDFYNCSKASQEIKSTMDKKRIKLNNTKDKNRYVKLSKSFGVHYSNLIS
AKFTPENALLLFGINTLI*
>N.PF11_0402
MNNIISETNDKSSHDNMHDTYLYYPNFKMFISENNNNNKNNRIHNSNISNKSNEENFEESRKYIHREITKTLK
ANSVKINRISLDSHAENNKKEYIYNENNNINNINNNIDNKEISNNKNIIINNYSNMMKLNKYMTCNDKNIGAI
ENELDNNLCRHNENEKKYDMVNNNNIINNYDMLNNNNNILNNNNMHNNNMHNNNIIINNNNNNNNNNNMINNN
NMINNNNMLNNNSVHNKCNQVYENNNVTNNSFLNGRTDVLPNELFLEVTRNENFVQECKIKHNMDNFVFSNDE
MIYKTINGNNKLCDIYKNNNTDKNINNNNNNNNNMDKNNNNNNNMYEHEISNNINNLKNMNINLYRMNELNHA
TFIKTNQSNFIKGNNKECKKETMNKIKKVDIHNKNYNNDNINDNDNIIINNNNYYNDNIIRNITFKHVDNKND
VNESIDYNLYNEAFMDMNISYNGLMNNNLNNVSMHNNKNDDYNIMNSNYNIYMYNKNKNPKKHYSKKLFTDKN
EYSKMNIMENDCNKYKKYNNLKDNFYMNNNYKNIRTNMDYIKNKSEYFMKPDMKDKRYANNNMYDDFFTIENM
EKCDEKKDYLKNEEYNYNMNCNNRLTTNETYNSGTYNSGSYNNTSFHSANINNNSYDNICFKKSNYNNGMFSN
ATYNNYTYNNAIFSNNVYNNKTATNSIQNINIHGNNNIHGNNNVHGNNNVHGNNNFDIYIPPAPFDSADNIIS
NNKKNIKDNSFQDGKQKKIFNSLGENNNSVFNNLNKEYIHGNNMNTCLMNGVVHNKKGKEKKILFHDMEINNN
ENNMMNEKVMDSPSESNTGLLSSFDFRSHSSYNKYNDKERNNNKNKMSIKGNYKMNSILNNQMSHCIDVDDDE
TLTKSNKSHNILNNNGVIINKKECGNSNNFLPTDKHTYNKNFMDSYDNKHIDIINNNYDENGYYDGHNNYDEY
NNYDEHNMHDGNNIHDGNNIHDGNNIHDEHNMHDGNNIHDEHNIHDGNNIHDGNNIHDQHNIHDQHNIHDQHN
IHDGHNIHDQHNIHDGHNNYDDIITNHTSNNFLIGDSINFDNKIDNDNELHNGKEEIENGGEILPENKEYVIK
LFFGNLAPITTEKDMHNLFSNFGRCDSLIILKDRRSKSRGSGFVTFYNRDEAVNAIKCLNNKIILSGAHKPLE
VRFPENKEEKKIRTKLLNAAKWKGKKIAPSGCLPISTEDILNQSSLTMNSTNNISILNANDSYNMFSENENTV
VYDNKDMNHYDNTNINHLNWSEQFSELCKTTSETTSDTLNNDYFNKMEEVKMGTYNRTYRKVPHENYTDNMNI
NLMLQGNLGEFLPKFLIDNNINSGNNVVSFGKNKDINNVIQNNDKMYDNMNDNMNDNMNDNMNDNMNDNMNGD
DNNNNNYYDNNLLDMKNETIINYLSNKGKIMDNIVVNNIDNYKEKIENFNEKYYEDDLSFLNFPGTITSYDKN
HQRDKRKNCFEEYLDNNNYFIDSNLKEVIENNIYSNMDKEQIDKGIELNISPSINVIVKEEGGSLIHDKDIYK
KKKNNDDISNGYNNMNVHHLGFNLFDLIKINDDVLLKNEYINGNSLNHDMDNISSTNNKKDNNNNNNDSNNND
SNNNDSNNNDSNDSNMQSNKSIDDMIPYENLKKLNDLYNFKYNNFYEESVKLLGTHLTEDISLNNPDVNFNKY
DEYLNLYKQNEGNKKTEEQEDQTKTKDEQSNDLLSLLPHDFNKENFYLNIKNMERNNDSLSDEMLKNLINLYT
KNKSSIFTSHMFSYLNNVLCEINNALEIFNKFNANSSIKNNSDNSTKE*
>N.PF11_0403

MNNHPVINIFLNKYQGLYKNLPNKTHIILLPESQTLLNVNINLEFIKRHLFYKGPSKDIYINLLDECIEIDSK
NIYTSYGFEENKICEIIKIETNANYKFFKVIFINVPLNVQDNEENNFFSYYDIGENKERVNHFYNDSNNYKND
LNIFFNTNIKCKEFFYSQLNEFKHTYIIVKGYENFIGKKILNIVDDTLTYQNNTNQKLNDKDIKISLIKFTYA
SLYKHIWKNLLKNYENIEYKIQHKMAYLRKDISAFLKKVQLENISFFHIKVISFHIKQIEKYRDPINKIHILD
NISKIICEVVSAENNNLKEQNIAIYDINSDTLISVLVAAISFGEIQNLISHSLHLHMYIDNLSTSEKIDKLSY
IFTIFHSAIMYLCDVKNI*
>N.PF11_0404
MMSDANKRQLRTRRKKTMNSLYYNSDYSNDINIEDSKSRLSGGKKSEGSSSKSKDVISSYTKKYSLNNKNHKH
SKNIMNHKNMIYSDGYMNNMNEDNVDNEEICENHLYEENNDNNMNDDMNENNIIDEDENENTNLVNIDDENIN
DDNNNENNDNIEDMTNDMNNNVSHNNIISNDNSYNNQIDSTFLNMNNKRKDEEHINNDDEYIHKSKDKKKSGS
LVHIKNENTNKLDNSNNNNNNNNKNKKDSVSANGSNKNNCNTSSSNNRSRHVPNNNEINYAKMAEKLPHVVGV
RFDKSQNRWLSGICINGRCINRYFPVYKFGFEEARRLAIQHRKNFETANVGHLKKQQGEAKTSHLNLLNLNSQ
IPNDFKDIQGKNKLIFKYLSYDSIQKEWVVTYDYDNKVLVNKFPVDIYGYNNAYEMAVHCINKLNICNQEKLC
KNINKQEHSRGLQADLLNNFNDNNINNNNNMNDNNNNNILDNNNNLIDNNNNNNISIDNHNLNIYNNSSEHNN
MCEDLSSFKIPQNKISKLINSGQSNFLMNNQKSEMALNDILNNENMMNNLIHEKQKLNINHNNINKKLEELEN
MRKEDTMFLNDSSSYLSTNDIKVLNGIKRLKTDNSPFNKQDEEFLLNSKNLNKYSTMQNNKSYDILNDSNFNI
LENEGMFSLNDKKGINNNDMSKNRNITNSNYFKPFHDEAENNIYQLLNGKYLTNEDKSVHINLLNSVMKQSTM
NKLSNEEDLFEKKEKKRKLKNNMLEQKGDDQFINMNKRIDTNLVSNNNNINAFNNNNNNNIVNNNIVNNNIVS
NNIVSNNISNNNIINNNNNVIPFGDEHFDALSFDEYRKRINSKKSNYSNIGENEKYKKLANGLLDNGETLSKN
LNELKMYHLAGKKEKDNNMNNMNNNILYNNNNMKYYTHNEHHKNEELKRSQLNENVNHENMLNLNQMIRNILN
NNSVDNNEKIIKGINILKNMTMNDKFNLINFNNQCMNYSYNNMMKKENYVKNIYKIINKGSEDSINYDEYLTV
KLEVEDGEEKDQNGEKKKINTSEAYHEGKMNKKHKKKKKMKQNKLDEDITVNNGMINEGDNDKIEDTNKNDDI
SNNSEVTNKDIINGERNSNDSEEKEKEEKEDYNENTYNNMNTDNNTSNSNDDNEQIINNENDYILNKTNNEIN
KEINTSINEFNDIAFQLCPWKKGIQWNLLKKMWTCKLWDNKGNEITKNIHIKKKEGIDIGYKYCIKIRTNSFI
FYLSKELNKFPHIPEISYDLQNLHFVVSYKDNEKKIYSFEDGIYKSFIDCMEYLNKQKREYNENIYDISNFIK
EKNNLELDDIYKELNYFEYNRNVLSNICEETKIAYSIKPWLRGIIWEDKLKKWIVFFKDKNENLRISFFSTSD
YNDDVIMSYNKACEYFSQVKESNNFDSNSEEFAQSIKNVIKNVQFDKIIEQQNDANNNDHKKGNLQEDHDIQT
NYYNKVTQGNEGETNNNNNNNQGENMTDSQIVLNTNEIHVHNVHTLKRPEGLYNIAETQSTHFLKDNNISNEH
KNMETFYNENSYYNDDIAFLNYADSNDSLISKGMSQYLSLPSKKKKKKRTTEDIYKSEIDVLSYQNNELYSNV
KNENYDENISENNKKYGKRKMELNFLSHSENNINNINNNTYNNNDYTNDDNHSSKVYSSDCEKNTNHNKKTED
KYDQENNKYFYNKDKLLFDHENGTIPRNIKKESNNLNSEYNLDYLNSEDIKNNNHHHHNNNNNIIINGPSSDG
ETIILSQYHKNDDDEDDGNNTDNSKILRSFDKCSNENSVCINKSNEKLENILDGGVNMRLPKSEILNQASRLP
KLQGMFFDKRRNYWTVSVCGFRKSFGVRTRGVYQAYKLAAEFRNRILENNKGKYYPQRSTSMSSNYKYNTKSG
SIIIKDEKLNYSNISEMTSVDEEYDPKKKKSSFSSNNINNIDKELYSLNDKESNSSGLYYSFNVKGKTSSNIY
DYFLDKKEHDYFATNNSSNNINNNNNNNNDINNNSTNDINNNGTNDTNNKKDPKGIPNNSCYDDQYNNIENEE
EEEEREGEEAERWNISDKVNNKNNDNKIQIGNNTFGQLKYDSALESNLINFINEVGSYDFLKDAKINNNNNNN
NNNNIGGNKKNQHINNNISNSNTLNNSLNVQNISSSNSAISNNGVINNYSPTHIYEVDNLNNSQDYYLKNDEM
KNELNKVEDQKNKKDISSTNSLLKHFMNDDILINNKVKQQPFFDSHNNYNKLGHKQVKTSAYTESAIGLGFRK
NGYINDCDLVNNNNNNNNNNMHIKNNGVILNKLISSNHDSKDNSNNNIKDKDREDNNKNIYNNNNDRSIGSYA
NNLGVIENQCENSKGADENTKNFNNNKTLNNYEPENKSYNYSPVLDETLEERDVRINREVSKCKFVDGLIYDE
ANKCFRIKINGYRKAYSVIRRGVKEAYKLSIESIEQIKRQTQTNNFNNNEQSQVNTNNMTHIYNSSSENSRHG
SCYNYQHNHKSKLENSNDNPEEKYDHINNMTNVGHENKQSAEESLFPMLNVDDKYYELLKTSIIICLNDILMN
CIPQVFHLYKNINTSNDIKLEDILYTERKRKEQSLKYHIEYTQNSVGVSSLIPYLKLFSTEILNNVLPSAQSL
EIQRLIIHSLDIQTYNTLY*
>N.PF11_0405.1
MSSNEERRIPLCCEICLKNEIDVSSEMIKVAAQKWLFSLGITITLGRHDPSKIVCEKLKKYCDYIVIEPAIPI
KIVKEYNEDFKNYKSCKTNNNSNSNDIIDIKSNDDNLENHKNIDNKDNIYNVNHIDTFDHVDNNICKKLYPSH
YSVYELNRFNDLNSCDIKCSTQDNDITNNIISNSNMYDEKRKRKKITPLKIVDENKKNKSLVSSLALSQNIVH
TSNFSNDLIGKEKKNIYEESNLLFTVPIIGEKSISSIEEDPIILPNFFCVVHVVTYYKNEEHMEEKFNDVSEY
DEDQEQIGEKEKNDVPTYIQYILPHLRFHKLWDSLYYEENIKRDLLEYVSALMLFSTKKVDCNMINYNHLVLL
YGPPGTGKTSLCKALANKVCIRLSNIYTTGILIELNTHTLFSKWFSESGKQVLKLFNKIKRMISEYEENDIFI
CLLIDEVESLSADRKRSIESTDPSDTVRVVNTLLTQIDSLKYYHNTLILTTSNISEMIDEAFIDRVDLKQFIG
LPNEECIYEIYKNCIDELIEKEIIRLSTKIPNYERAKKLTKSYKDDDKEEYINGYTLLKCAKLSEGFSGRCLR
RIPFQAYAYFCQATLRFYNCTNNNQEKVLISLEEFFIALQKAIHKETINKSKLSEQKNTKS*

>N.PF11_0405.2
MSSNEERRIPLCCEICLKNEIDVSSEMIKVAAQKWLFSLGITITLGRHDPSKIVCEKLKKYCDYIVIEPAIPI
KIVKEYNEDFKNYKSCKTNNNSNSNDIIDIKSNDDNLENHKNIDNKDNIYNVNHIDTFDHVDNNICKKLYPSH
YSVYELNRFNDLNSCDIKCSTQDNDITNNIISNSNMYDEKRKRKKITPLKIVDENKKNKSLVSSLALSQNIVH
TSNFSNDLIGKEKKNIYEESNLLFTVPIIGEKSISSIEEDPIILPNFFCVVHVVTYYKNEEHMEEKFNDVSEY
DEDQEQIGEKEKNDVPTYIQYILPHLRFHKLWDSLYYEENIKRDLLEYVSALMLFSTKKVDCNMINYNHLVLL
YGPPGTGKTSLCKALANKVCIRLSNIYTTGILIELNTHTLFSKWFSESGKQVLK*
>N.PF11_0406
MKEGYAFSSTEDIDNPEKGLRKKSNRNDFKETYIWHDKNNECSKYVEKNSLGNIFMSKKIKNEEDTFEGSNEL
CRILNDTLPSNYCFKNNKQNMLEIKELDKNINIINSKDQQYCDDEAILLYNIRRMYESNTKYTKNNEKEKNNY
NISNVNSFKYNNIYKKNSENNSTTNESDIFQSVSSSKSYNDIFKSLNQEKKKNKKNKFINNYENIINMKYIHM
NNIGDIKNQPIQKYSQGFMKNEKMEVASISFKSLSDDEKNYSFIQTFNDMNEKNINENVYKKESKKNIKYNNK
QNDDNQKKLQNYKELSTNFLLSQKDIKKGSVINNEKLEKEKCKYKKNMSFVKNNMLRKNTMKKQNIIKINKLK
MTNSTFPNIHLTNTQENDTIKFFSQKNIEKSNESLKYINKSIIGRSSICSYDKLENLKNNFTEIKKNVKNAKR
NTYNTIHKKLENSSTINKYKLNNFLQDKGICDCVNKYNTFSSFENFQRNNIKVISSLHENKCQKIAVLQKEKD
SSSPNNLHIEKYTYNKEDNKNIKYMYNLKDAYCLKKVNTKSIGVQINVKKKTFNKKTNTVNIFYLDKVNKRKI
PKIKFKTIKQIYNYDFDAIMVKDGHIISRNKDPLKEMFQRFFEKV*
>N.PF11_0407
MPFYDNLFFRTKNKLKIKFDIRKRNNLHFLQKHYFSNESKYFKVGIIGSGPSALYCCKHFLKNDNIKVDIFDK
LPNPYGLIRYGVAPDHISVKNIYKTFNPVLLNKEKYRFFGNVNIGIDIQIEDLRNYYNAVIFCCGASESSLPK
EEIYKKENGIFHAKDLIYFYNNFYDDLRCKSIDTYLNTYENFSNSIIIGNGNVSLDIARILIKSYDDLIKTDI
NFNYLNIIKRHNFKHIYIIGRRGFWQSSFTNSELRELFSLKDTKVILDKKNYDLCFEIKNYDETNKMKQRQNK
LFLDMVHNYEQMKKNPTLYDKYKIIQFIFYHEIRNIHNINNHLKEIQLGLNKDIYSSQNNHTNNKNSYDINNQ
KINIKTPLLIFATGFKKDNFSLNFYNKSVDKYKDDILNKKFAIFKAGWFQTGAKGNIASHIINTKQNTTEILN
FLNTTTIYYENDITELLKRKKVHFVSMDQWNYIQSLEKNNGEQNGRCAQKFAKVKDILHILSDKRNE*
>N.PF11_0408
MYGDKLSIACYGDNKLLSKLNYFFKVDVYDLSDIKLDERIKSLRINLKYIFYNNPISEYFKNEESLLSERSLI
YIYFLSLNNIDEYKNEKEYILNRCRESLNDENEEFIIIYAYNIDDNLNELKNIKKIKSDFSHNILKSIKILSL
PILEDENYYTNKKINELYEHFQTRYCDILKICIEKKYSLIKNGYNKSLANFLTCTDIKKKTTSDKKKNNQIFK
LVDIYVDNKKEEGDNDMCTTNINMNNINSNIIINDNIPNNINKTNEITNNIFQGSNEFVDFYKKHNVFSFCNE
YVDIHFFLYQNNMCSYINSLQFCTDFDEQLYIDMYDNFLSVFMWNENLCLLYIKLNLYKKSYILYSNINKLFM
KYLNVYIKKKKCIIHSSLLFEKNYLTIARYIREKHICSLHLLEYIFFKKITILLFLNKFSYISSKALKFSQFF
YINKIFIFMHNFNRFKNNILKNSNDINSLRNIQKKIVKYKKKVDKRQSTHQTNHKGFLHISEKNKYITDHKSN
NQVKNISDDETYSNCSAMHPRRTHKIISDMDEIFINSNSIDNLIEEDEEIYEDQKIKSNKNTNTNANNINDDY
ICDNNHNNNNNNNIHSNHPMDQQKDEHDSNNAHTNTRLFSNSKEKKKNYKNHEHSQYDNYNKNKKKEHYNNYD
LNVFKSDDEKIYFLLHCCFMNKENYFAIYFYNLANLIKFLFERRGYFLYQEGYMKKIKSDKNIKKNLNFSKYL
SLQKNIMKLGYKNNFTTKKKKSKKSLKIKSKRSNSIDESYSSQISSTISDISDIKNTFKYFWYNDFCINVSNI
FKLSFVILKNILNIYHTYHSIYHNMDMKTRLYFFNMFLNYLEEKRFEKNMDKADLEDNRYFNNIKGIKDHVMN
NNNNMDDNNVDDNNVDDNNVDNNNVDNNNVDNNNVDNNNVDDNNVDNNNMNYNNIDDNNMNVHMSYNNINKDI
NNEPNNMIKKENIYQSFPIYQIKNFSKDNEKKKNSLLLNIMNKIINLCYRKYNNVSIINKFFLAILLYENKFY
KKSLKLINKIIKRSDYDFLTILCMQLLLYHNINNKFYHFCSSFFLSNRYIKIMPFKNLNINFTSFYNFQYYLF
SSNNNHSIINLEQRKNYDAHFYDALKNKVIYLLEDILNIQIDENNKNNINDEQIKNGTTSEINDIYENKHTQN
EIKKSEDKNSYNSSHIYSNADQKNLSSINKNKLIKIKTQKFCKENYFFHINKYFLKSLRKYNFQISKNQKNNH
IYIPKTNIKILIQANYNLYMFMSNMIFDNVNKEEKYESFIGGNIPDESSYDINNHNIHNKHNHNICNNDNYHI
SAGSKIFNKNIEHIELLLNFKLKKKIDNQCYIGNIKKKKKKHYKKDTRRLKKLCVQYEEKLDLFIYSAFTQN
LYIDEIILQLSIEKEYKKKNIMYVHFKNENDHCDKYIVKEGLNHIKLNIINLLESKKISWKHFHYKVKYVFLV
INNFALYHKIGVIPNKNLIQPFIKSYSKFIISNFIVIDHNKSNKNNIINTTTLGSNHCLYSNFFIDQLLSPLY
IKIETLNSILNCKMLACFLNSTSLICDNMNYIKLVINNNYNINYNNNYNNNYNNNNNLYQNQFLFSNTNMYHI
ENEDHKFIPNDFFKKNIHILINKMDEDPNYYKNEFFTSTDSILFYIEKNEEDNICIDQIKILNEDDINHLNEK
NKNIHSVPGFSNNNYINHIDHSNYFLWNNQAYFNEYKQLLNHKNFEKVYAIISFMPSKKKKMNHYNVHDYLGK
NKNIEHLTGQYEQINDNSTGTQNNNNISTLHTNHNNINNLYSIPNLCSDKITIKCIININLSNIRRKVRKTDT
VKLLNVHFMGEIVFDHFNTHYDDKHVREDINEPKNEDNISSELYLNNQIFLEKTFNLVNIFHDRINTYRSKNG
MMYELVMKLHNDNYPIYLKNLHVSILKNLNTHIKNTKIFFLNKNNDQKKYQTTYSIENQKMDIEHSDESNFTL
CSEMESKEKVISFENHDISFVEQDNYFIDQVHINTVLKGGASIFFLFEVYYNNDIQNNDSSINYRDYQNTIGN

IHITYVYKNETILKLLEKETIKEYMYKFHTLIPQFLLPINVSYNIPKTGMLHSNINININISISNNINEDIYMKY
YINTHNNHMHNSENQYNWLINGFQKKIIHISKNSSYNINLTITPLKVGLINFPPISYFLNINNNWIDITKILK
KSENFQVIVSPSLQFTPKVWQIM*
>N.PF11_0409
MKYTEITIDDGAYAKIFMHSIKYSCDDVCGILIGKYLSSNEKKKKCLITNYIPLFHTHILSPYLNLAFTLVEN
YYKDKDERIIGYFHISSDDSKNSDIENIKVCELISEKLIKNYNDAFVCLLEFSKYVNDEDNCLNIFMKNDKSN
WEKGNVVISNKNKEFLKKNISNQHYLNIYDFDDHLNSMKCDFMNPDLFNNVS*
>N.PF11_0411
MKLLYLLYPILLFYNVNVFINYKKSRLMLEMIDKYNTHFVQTTKPYYEFNVTNLTNSKKKKKKKKRENHLIGS
GENMQKKDEKNIKDFHINDYEIDGKTIHNKENKDSFKMNKNKLNDNEELFYMDNILSYKPNKKKLFTYSFSEN
EGNSEKEETLYNFKNMKNINSVQNNINKTFLYNKLKNVDYYEHGYNWDIGQCKTGKYQSPVDLPMKDLKEREL
KNISDVYLNLFDDDNYAWNNYNKPWMKGDFFYYYEYFIKKIVINRQNNIFQIKAARDGIIPFGVLFTTEQPAM
FYADQIHFHAPSEHTFQGSGNRREIEMQIFHSTNYFYDIQDDKSKYKKKYGLHIYNNLKKNSKETSKKDSSRY
HSYLMSFLMNSLSNEQLQNKYNKKKRIKKMKNQYEVISITFTSAEINASTINAFKKLPSEKFLRTIINVSSAV
HVGSDPTLVELKDALNLDALMMMLNIEDMQFLSYQGSSTLPLCDENVSWKVAKQPLPVSTETILNFYYLLKKH
TPNYSGSDNDNYRSLQNVEDNTRHYRKFSLVQVFPIQVLISSAISNIEDKKVINIIKDISPKNMSFSYYSKWD
IYFILFIFYNIVLFLF*
>N.PF11_0412
MASRRHRLFNETDLKIYIIGDEDSVVGFLLAGIGFRDGLGKKNFFIVNSKTNKSEIEEVFKEYSSKHDCGVIL
INQQISFFCRPMEISIFY*
>N.PF11_0413
MDTVSEKSQIEDVLDKILPGKDLNLVTLKSVRKDVADFLNVEESYFSENKEKKELLKTILKEKILILYNEQQN
EEKKKKKKNFQSDISEKKEKSKADLQKSESKNNNLKKKRKIIESEEETNINSDDEEEEEEYQRKKQKKQKNSN
VSTLSLLEKKKKKKRDSESSNNNDYNEEYDEDDDEQEEEEEEEEESLSKKKSKKKNSTTSPKVKNVHTIKKEK
LRKIVLDLKIGPTIFKDLNKEDDEDYNKKLEERIIQYCEKKGICSEKNKLPTPTEVYEYKKSIKLKEELDGID
PSNIIDTTTRPRRRNPNPYISQSFVASDEEEEEEEDNEDDDVDDEVVHEEGEGEGDEEENDKKKKKKLSKEKK
KKSDKNKKHDEGVDEDVDEDDGDEKDEEDDEEDEADEEDEDEEDEEDEDDDEEDDDDDDDDDDDEDEDDDEDD
KDDEDDKDDD*
>N.PF11_0414
MKKMTKIDIDCTCLDLFEEHNKSYYFNDCAILKKCIKNGILKKCDSVCIYGKENCGKTLLLTEILADLTAVKE
LKGMNCKVVYLDCDLSFNYKNYENIINKKFDKYIIKNNKNNNICTYNDLKKVYGDNSFSNIYYIQILNPGHLI
VVLNTLKNLLEGKNIFIEALFIDSLSFWNFCKYDKINFFSDNNYVKRKTSDLLDYAFTLILNLKKTFRFLFFY
TKLSIEDKFMEYTINLSINEETKKQIQDNNENIDNTDHYNFVNNNLNSLKQIFLIPQNFNDILKTHIFRKKDF
LTNNFLIEKPFLIFLIPNEKNEFTHINKKNDINSINFLLCLSSEMKNISRTHYSKFFFMIQNANTIIPL*
>N.PF11_0415
MATSETQNIKTASVNGSEENVYLKDAHMNSLHENYNVSTKNVVDTNPAEGYHTISHDENHTIDPNNIPYIIPG
ITETGMPLDPTTMQAAAALNQSLMYPNNAISTEYPQYHMFNGMNSMYGGIPQYYGGMNIPMNPYYDPYGIINP
LACSNLSIEKTKKKTKKCYCC*
>N.PF11_0416
MYKQKSLESRDINYIYEDSFVWVHINSIKKKNSLLYREIYEYYKHRNSSFCFFKGSVKRFVSKNEVEIYIFID
DNYNTSKDDFNYNIIENVKYLLPIDTNFGCSDNTQLIYLNSPNLLENIYQRYNKAYINKENRNCIYTYIGYIL
LSVNPYENFDIYDESYMKRIKDRNDLFAIPHPFSIANDAYNCLLEDKISQSIIISGESGAGKTESSKQVLKYL
TYLSYFQKGYKKKKKKMIPNECDKIEIDNMKYVDNNLKYMDDTLLYNYKRCGYTTSDNNSPQEENDTIKKAKK
NYDYDKHSYSDNNDNIKNNIDKKKKKKNNSKKNISNINLKSDDYTNIINTYSSSTYEEKIQNSNPLLECFGNA
KTIKNDNSSRFGKLMKLNYNEKGILCSASIETYLLAKSRVVDVPKGEGNYHIFYSLCQNEKLSKEFDLLPWYE
YNYLIEADKKIKNKYTYYNTYDDNIYNKNDNDNDNENENVNENVNKNTNNINDNKKERLKRNYEKDDNYLLKR
QNEKSYENTKKKKKKNDIIEDGNNINSKYKKNKYKDGDINMYQKYNKKDEPEKSFDCIQWKNLYSLDFIIKCF
NSIGVLPNEQEEIYKTLICILLLGNINFVESANDQEPIKIQNIEICKKLSQLLYIKSDNSNINKIIEILTIKR
VRETQKSYTYQQAIYNRDVISKILYQLLFEYIILCVNDSLNDSDNNIYSEWSEKDTFFENEKVIKEDESFINM
NEKKNKKRKTFKRKTKNVNIQNEYESDDTDENIRINKKINFIGILDIYGFENFSNEGMNGFEQLCINYANEIL
HAFFLKQIIHNEEKIHYEENLRIEKISYNDNSNVISLIGDTKDISIYTILEDLSLLFVSNKSNEDDNKDIFYE
KLNKNIINSAKYKNIIRNYKVANKSFIISHYAGDVLYDSRDFFNKNVDILTNDIEMFLSSCNYFISENLLKRK
RFRNWKCEDIKGETSRILKENHEKKKCDRTKQCNDVNKQCGYVNKQCGDANKQCGDANKQYDDLNKQYDDANK
QNYNDKSKKNNKRKLSVKEMISHHEKESKNTISCYNNSTNNNLYKNKVKTIFSSFKKQLEVLSNKLSKTSSKF

IRCIKPNQEKQAKLFNKNLVLSQLVMSGMVDILNLMKNGYPCRVLYDDIWIRYSGILQEDMKRYLTPKMFCKI
VLMFLEINSNEYTFGKTKIFFRFGVLSIINEILNKNEKKKNTFIQCVYKYWLHIRKRRLLNFVLFGCRFKILF
KKRRAKLLMEEGLDLYNQYLFKKKLECIEKILFFYFNVYKQRIFFLKLKSSTLIIQKNYRTYILRKKFLYMKK
QILFIQDYYLFQKYFKQRVKAANIIRKNWIMYITKYDYKYLLKCIIKIQRAFKKYMKKKYILYCLKIAHVKDS
KKKNMYHHTFTKNEIEEKKKIYPLNILKKKSFNVTDRIGSFIYVQTRKHRHTITVGDKIFLSSTNIHNRKKRK
TWDAGNNYLTSQYITTSTNSYYNENDDIKRQYQNKNSNVNTFNEHLNLGKRKNSNIEDKPIISVGNIVKNERN
KIKNVQKNINRINAKNKFRFSNLSICSGTINTDRKNINYKNDMISLSKSHKNEMKRKTYNHANNRNEFVSSYK
REKNKVSNHIKNTIYDRYIQKYKQNHMCPQKEKKKKSIYIRGNYSNISNIYSVYKPTKEEVLEEQYDFDLTDV
LIIPEDFHVTQTNIPEPDINLMNNENDILLNHFLDTNLDSKIPLIELNMFKCC*
>N.PF11_0417
MSILQKIYSNYTKEENKKSCLNDDMYSYKLANHYIKSKDNSINNYNPKNMSKRLYDDRVETFDTLENVDHFEE
EHEENSTEKWLIECSPNYYIFENSNIKKEKEDINIINKACINNLEKNNEHIYQDHNQEIYVSKLFITLLYKKY
FSKKKQKCCSPLLKIKKKKKKKRIYDHLYKNEYKNSLHLFYRSNDILNKINEKKENVTGDFCNDNIKEEDNLI
NHHNDLRYKNNNDYINIYEKEIENCNTLEENKRKEMKKNKINHEKDNKNDEKNDEKNDEKNNEMYHKSNNNYL
FIFPRLFSYSQYYREKKILRKIYKYIKIYKNKKNMKLLKQRNCNLITLSQFMKERKKKKKRKLYKNKIHNKIH
NNNNNNTIYEKYTNQNNSNNIDEKQNCSNLNILDDNTNYLENCMLENLSMENYDLDFQYIKSILNSCNIILNN
TNNIYTLKEDNKIDNKDVNIYVEQISNSSKQIKYNKIIDHIKNNNIYNDINDNINNDINVYKNNDINVYKNND
INDNINNDINIYINNNNNNNNYINNNFITHKTSRRFSIKIKRDKNNPREHVYIIYRKVHLKKKLINIINEIYK
IKNYNNVIHTNTYTLNKNAPIKPYKLYSQINSKFTTYINKIYNNKKRRKEKIDQILQHTNTTDTHIKNVTNNN
LKNIHMEDINESDVISINKKKNIDNNSFVKQNQNDLDSYKGNQKECKRSEVTNFEIKQSIEYINNDNIKDINI
KYNNNINSCNDHNKVNPLKFFLNKKKIVDVIKIRKKKDQVPLVYFYDKNNIRIGKFYIEENILQEYCKLFEKY
LNKKDKIYTVIIKKDHIIKKYINESKDMIMNKENMTEFIDSCFTLDKFFCLFSLLKIKNKIYIFLKDIKNIIL
QIKKLINHINKKLEQFEYIIYRIECKLSNMINNNNNNNDLDNNIVIKTLMNIFLIITNIYRDICKNINDQSYN
ISSNIKCNEAIIKSYLVFVKRFCKYIIKSVIHIEHYYKKIKNININKMKDINKMKDINEMKDINKMKDINKMK
DINKMKDNNTIIIKNNHNDKIYVHTFSHHNQICLFSHIHSKLIDHLKIRHNKLLIFKKILEVYLIEITNFAKY
QKKYNKNKINLFINSACPYELLLFSSLIKCNILCNVSIRNICNSLHKQTFQKKQSIYNIFLSNKIKNKILTSI
KKSVVPTNDIIVKSPKLILNNLHINKNCHMNKLKTYQVTNSNFFNMKQINNKNKKKLSYNNLNNYSNILQKQN
YYNIKHIQKKKKKKKLCKLKNIKNIFMPKKKNSCLLSNIITNNNTYIFSKLNNKVIINYKHALDTLKNIRKTI
QINSKHSQYNIQNQMNNLTNHPTNHKEKQIQMGKKETNNDFKKSNHKINCIFMTQKNKKYIVKKKIEDLQNQI
KSYELKDKKMYKSNKLNRGNHNICDQNEIEDTNKCYNNNNDDDTYKNYNNDDTYKNYNNDNTYKNYNDDDHGN
HESVKCTENKTNNEKSHSIKDNKIIQENIKKIKDFEKKYKSIQDNKNKTKYIKQQREFKLKINTRNKNIKLLQ
TKKLKNVHIKNYKKINKKNFISIIHADKKGNNTRIKFKINNENFNHPKIIEEKKKITYIKNKKCGTQLVNTTS
KLLQHNNTCNNQKIEEKNDIIYNNQKNILTCKYSNTFSTKNKQNNNYHKNHFINLLSQTNKNKVLSSNSCVKY
KLSDFSNKYKNNIFNEEKCSVLSKKDNIHTNHNHQTYDITNKISKNKYYNNNTSENYNNILEYYNKHKHISHN
DNYIYNQHEDNILYNSSNSMESINNKKGTRIKNVKFQNQYFFF*
>N.PF11_0418
MVLKRGKGNSHKSRKRKRFKDEINEDFNFEDNFRIVGEPDFNEVFEVEKIIMARYKLNCKRKKNRKDENLDDF
EFYIKWKNYDSDENTWEPFENLSPNLKGQARKVALQLTEEIDNCNKNKNEKNDNEGENIEETVVGDNINRYIE
GNIINNMENNSQQNVEQYNNKVYEQTRLVQNTDVLNPNKNKMGFIEIKPNDIDDDDEEIINIMRELEAENMKK
EKNKLRNRNFSLFNMSSNSRLSNEINSYLYENSHKMDVTYIGKKKTGNIKSILSLTNRINNDDNNNNNNNNNI
YNNNIYNKRNYNNNYNHCKYSTDLSYDGNNNSVHLNDLHHLNDEEKNNLLFLYDSDDLIKKDSRIANDIHYNN
LFLSKSIKKLRNKYLESLLNNNINNKNIFDDESLLKMNLGLSQPSNLCLNNNEKSKKCQKPHTFVNTVKNSNK
NNENIINDFNKEKDESSQNDTSFLYSTDRTSTNDYYITRKEKIKPLQKESIINNEEDNVQEHHKLLLDKNGNN
NNDDDNSTDDNNNDDNKNNNDNKNNDDNKNNDDNDNNDNNNNNNNNNNNNNNNNNNNNSNGDNNNNNNNNGDNC
NNNNDNHNNNDNNNSNNNNNNNNNHRINNKLDSFEDDNYRENYIFNYLNEKDKTDDHNSNSYDNSNSNSNMFNY
NSNINIDEQNKNIAKNNGDLKNIKRNVLNKNKGTKFLPSYNTCFKKQICNLRKNMKKKLIVNSMSNKFNISQV
NANNENNFEEFKQFCLQNGNDQTRKKRLQHEKKKSTNSKNSINNNNNNYYDYNHYFNNKKRSEDDNSFEFNDS
YFTNSANTIDQVSKLVQKYLALPSNENIMATDYLLNTTTNNNDNNKNNDNINNEDNNKNNDNINNDDNNNNDD
DNNNNDDDNNNNDDNNKNNDDNNKNNDDKNICKKGRKTNNIKKRKILVNDNNISLGNYSNNEYYDYHDVNNNS
NSNIRNMIHNKSLSDFIDEDKIFKQKSGKNIVKNLFCNKKSYSNNSAYVNRGISEYAEENIGDKNFINNQKCN
NYFLNLKELQNIYASEKYGKSKKGNKNLVSKKINDYIYNNNSGNNSSNNNSSNNNSSNNRNNNIRKSYINKLT
ESEYDSVYETLINRYCKNTTNKYSKKINNDENGNTNEPINDIKYYINDNNNNSTSNSRPSLIHDNLSENDFNK
IGDELKRKQSQITKENQKYDAHVDDDIDDDEEEEEEENTDKNNNTIRSSIQSDIIYKILNDEKSKSQEQNVHI
LNKDNINEFSEFKKKDGENDEMVFFSSDNMKSNIYVAKKNYSSESFNNKHIMKKTQHLYNMDEIKNKNKNKSK

NVSDSTNENNHTNHNNNINKYDGLNSMTINIKDILDTKNDKRDYYINVLENVLSALKENRTYNSKDLNISKEN
ENANGHIRSNSYTDTSSILLNRKIPEISGEKENYENLRNLKRRTFHNDSIEYSRTPKRVLFEINKEDHQNKKL
KNKNLIDDKVGDDDDDNNNNNNNKSDDSKTNDDNKIKKNKTVFSIYSSIKNKSRNILSNIKLGHFLTNKNKSQ
NNKTDESQNQNVVPIRIKKEKEDDELEENAENEVSNDKHNMNNDEQNNSGGVNKMRSEQDHINDDDNNINSYD
DESDVNFNNKKNDYKGKRIFSRLEILNINIVKLNYLDKLGEYIDSVDKLAIGNLDSLLKNFNILNNYKGKDKV
MIYFLNPGFQKRLLEAYSNIFLHFDIFIDNKTYNFIFNNCEYVNVLLYRAFSIKREDEYNRLRNIPSYELSKY
LYLFYELGKMTEKLNTLFKILFIPTQVKPHKYYFPSVEYFERFYIHLHYKTRADDVDVDTNQVGGEKLEEEKL
EDENVEDENVEEEKLEEEKLEEEKLEEEKLEEEKLEDEKLQEENMENKQIEDEKLEDEQIENEKLEDEQIEDE
KLEDEQIEDEKLEEENMENKEIEDEKLRNEQNLSNEYNNTANIIKKDNIFEKNINEEYNIKILEDKTEYIINN
EYLKNSPGDVSNFEDSMNEEGCNKSSIENNNIDNKNGLQNNNVEEKDENNHEKNNIYINKIDHNINNCINNGG
VLTDNDIENIDYIKKDFNNNLSTYSNSSTIIQNNNIHNEDSDDCNNNNNNNNNNNNNNSNSNSNNNNDDDNNN
NNIIKDLSLTSYEMEERLSSMSVINKTHIENEELPKEVQNNERVDTKFYDLKSKILNNNMLRKFIK*
>N.PF11_0419
MKYEKKYWNDKGKKRNEKQLNDENYRNNKKYQNDSGYNKLNNNNNKMQGEKGNYNNTPFLGNIKNKKYNYESN
TIGFDKRNNNHEYIPSFNKKSGMHQKSKNSNLYYQDKYGRENKEKSPRYNYHRNKYNKNYLDGDHINNNNENY
KSEYYNNYMDDGNNFNYMDDNNNNYNYMDDDNNNNYNYMDDNSNNYNENINYNNNMNDQGKSIPDIRQEDINI
ELNKLHRDLHNLLFTSPKNNNINNTNENNNLNLGDPVFLHNIMTKSSQDNFDEMEPLTNFNDSQNDFDKNFGN
FFNSYDMNVKYKEKNIRAVENYEKDKNEVNDYGKNYNYNVNNQNDQNDQNYEYDNDNKNRYHNLNNKGNFIKG
NTTTNNSDVLSQLSILRNIPFNNSIEEEENDKTSKNEKTAMILNDLKKCLNKNESNIYENTDNTLSGISNEGE
EYIYENHMLKNHYVDKSHKNHNNNNDNNNYNNNNNFHMYQEKNDNIVKYTNGMYNYLKNDNKSIEESSISNGS
SVEDTNENKTKKEEEYTYNDMLFPKSKIEESLRVLNTLQVDIERVCCYIKHFIEPPEPLFEVMVNVFVDKKVS
INSKMAIFYVYNHLIQELRNNLKNDLIKFNSIADKGLHIFVIPVLRHILEERGYEEMINKFFRCIGIWNDRNV
YSKIVCDQLKSLQKNPNKKMDFSMKHPGYQAHSLLSNELSKFLPINFILKMPSTNNEHKKALQHKILSALFNN
ISKETLKEFQTDDIEEASKLSDKVMRMFGQELVLINSQILELSSLITDNNNHLVKIQNALEKLNE*
>N.PF11_0420
MINKILNNINTLNDIKDDDFSHAENVFNLIEGILIHENSYWKYVNILNELTTLCYEDPVNFRSLLFSKKQKNL
EEKENKINQIVDRILIVLEKYEDIRSTFIYSISKLVLINDYNLNIRILHILCNLQNNQEYKYYIYSVFEEIIS
ATILNLKEHDTLCNIFKGLAHLPKDKFIAGGFSNCIHKVIDSLRNKLNEDALKLLSILTLDKENCIIAYDEGL
LKILTEEMNKREEENFDQVPNYMQYTYLIVSQLCNNYIKHCDILINDKMHSQFYFKKIKSIININIQELSTNN
SIFANIIMSTLNSMCKYNEKCLYELCNAYHYIDLLLICIKMENGNPYLLSASLDGLLILLSNEEIYKNNINYI
LNNSNNLKIILPLLFGQKYYNMLNIHNLENNNEDNVVQEYLYKVVEHTLDIVTFFFQKEIDKYTIKVKKQFRN
SDVNYYLLTCLEKKNTQIIIKLLKCIYFIPFTDYSLIELHKIFFILHDKSIDINDEWKEIYYYSVMIYMKVLK
NEDIKKVIECYNTEQIIISLLRIMNEFLLKDILLQKKTNDVIVNKKNMLELNEEYGTLYNKKDYYMDLNKIIV
ELLCICSHYKDLRGFLRNNLISTYFIHILTNEDKLYNELKMDYDILIERTWCCNMDNIFKTLMKNNMKKNKKV
TLRLLINIADICSGYFYQFKSNCDTNIFHLCDLEEKHWEEKKILQYFYFLNEDDKNDYIYQVNILLNHYMTDL
FTILYTFVQNDIFIELKKELQENYFPGVLKYHKLKYEKKIKKWKLKEKQIKQKIKNMNITYEDMTYQNYQDGY
KEKMNDMYNNSFNNIHHDVSSILNSLNEELAIIKEKKKKLIQKLYNNNFKQTFDISVENQIGTNIEYMFDIPK
VIKKKKSLKYLKSSKKNQGEYIDTNNLILSKKEKNYDSEQILMHTGEDMSGNIHTSPNNIKTSINSNNNIYNN
SNKYKDGVHNISDYINTNEKANDTIYNNKKNHFSDTSSNIENYSTSSSSTENSVVNKPILDKFSCELLKYNKE
NIYEHKLNNNHILLFNKRGIFINSNRGEINEAFVLYAILRIFYSIIINNINNEYYLKVKEFLLKHHVIKTFLN
VLNQCSFYDCNVYAHFFRLYSEILKMNVTAVESMNMLLFYNIFFYYCKLLSRVFINKIRNSEYILSYKEQYFF
AELSKLFYYFTQKIIYIQFSQYKEIQKWCVDCTLFHFFYKNNIILLLYLFIYIHSVHHGSIYASYIYHKKNFF
FIHTIFFYTLFTLSYLMCFSKKLKYFILYFINYKKYIHKVLFRKSIIHSLFFYNKLIYLRIVFQNQLSLDRKT
PTQIYHLSPVVYIRENSNEKKKKKKKKKKKKKKNIYIIXXXXXXXXXXXXXXXXXXXXXXXXXXXXXXXX
XXXXXXXXXXXXXXXXXXXXXXXXXXXXXXXXXXXXXXXXXXXXXXXXXXXXXXXXXXXXXXXXXVEWYFIALG
VDKYYLVYIPDNFEENITEDKDIKLIIYSERKYVDLTRICISKINDNLVIFGYINYNNNYISYESYDIFISMN
KYFQNNIVSYAQFLSGNTYETRVDLIQDNIIRNNLKHYMNTKNILISTFAYKEVSPSDYMKYKTRREKKKERE
EIRNNIEKRQDDYEDTFYSNNISDDFYSHDSLLTYSSNTEKYNNSDDNIQQNCNIQSNDEEFNIFRRNVKSKN
KRKLLFFVLTKKHFYIFKFNFKHWIFLSPFIEDEKNDLHLYVESSIDSHDEQINSKDHVIKNDKIYLNNILGI
TSNNNMVNDENIPFARKCAIRNTIKYNYSCNNTEYTNQQILSTQMSTNSIESQQRQSEIINKSEVYDERERYS
NANKLFLKHIHKYDNEYLSQIKFVNNNESIIEIQFKIKNKEEITEKKIKMILFDDYTRELWKRSLAHSLNIQL
VSSEWRRKWN*
>N.PF11_0421

MNSSKYRNYIYGEEKYPQYMNKKIYVNAPLDSYHHIYNNKYNNTRHGVIHNRNIDYDNILMSNNGICFPNHYN
MNVIKKNNIEYKNNLTSEKKRYNNRNKSYNNIREMYLYPSRIEKCRKDNLNHICRDKKKNRYTFHIGNNKNNF
ELGSNYKGIHNNHPRFAQKNMDENNKDTKCISIKTVINKPNVTKIPCINYDTKNYNIKLGWWNFGKPLKIFSN
IEKYKQNIKEKYYENRKFCKNISSTNSLNKYSNKFLCNEYFYIPITNTNYFKNKSLLNKNCKSYPVSDFHKVN
LRPITGFIQSYKKEKEKKKNLQQAIIKISKVPYVINDYKKKMYQHKHMENIKELNKKQMKFDMTKNIYDNPNL
DNTCGFMATISSKYNEENNSKGVIITCQQNNIKKKKKYYMNHKKKDNTIKGNKHVNIKLNNNTFDKYKENGHT
SHLIRDVITTYRKKKQKKKKKKYHLGNNTFEKKKKSHLGYNTFEKKKKSHLGNNTFEKKNHKNTNLDNVKKYK
IYSTYSEESGKYHITSESKISNESLDNIHKEEQLYHTYKKPNNKYMINEPIKRYRMKIKTHKKNQSNEENGKY
NENRKSSYESSYYDKKYVMKCKEIKNNQKNKSYSISSLSSQVSLNQQTNKKIDHVKNIHHKIKPTNCHKYNKY
HEYHKYNKHDNTLKTYSNINDLQKVKRHAEHNIESDHNSYTNSLYQSNYLNKEDVYDHKQNELHQKVPSRIHD
DNYNIDDDSKYFSDDKILKDNHRYKFYCDENYSCQKNHKYDSNKLKGYGYNYKNVEKNSPNYNFYRNNKLKNN
KIHESNLLEKEYDEYFHDKY*
>N.PF11_0422
MKDIKNKIHLRQRSLYSSSNGDIKNNYDYIKHHEYDDRIDEKFKKDNFVEKDENEINKINVRIKKKSLGNPTF
QENIKYKMNQNMSKKKKKNSFIKNMLKKAEDILYTNRINNLYNNNNDDEKKKKNKEDTSYKLKSLYLLNEYLN
VEPTNNVYYRRRTPHETRRRSDFNGINLKPFVRYKSLSILEKEKNKNKAIIKISNVNPYYVYNRAHTMKNPSL
YIDSTNSYIGNENLPMNLMKNNIKNNMMHKNNYNDNIFHSSHQNMFNNIYDTPNFDIYKNPEIGRRNTICSSI
SKYNYMYPQHLNFQRENYILNGYTHNIDNHNNNNNNNMISNSYPFSRCEDRNVSKYNSSNYHEKRLKKEEKNK
NKNTIFIHYNDKKNIKKNNSINNIKDYDNSSNKIRTHISIPLKKNTNHMNHKKEYNMDPNVDYNDKEHHSIKV
DTSRPINISYNNNNNSNSNNNNNSNSNSNYNYNREKKKIHNNKELYNSNDKHQSFCTNSSYTKKYSKLNKSYN
DFKEYPIHFTYRNEKKNSSKNAFLINKPLCSSNKRDNESNEEFNNSSYSNICRAREMSDQLFQIENNLSNKQI
IKNVFSNDIIRYRDKDHNNNFKRENEEDRNFKLLHNKTSKKYKNKKNKIKNNNNNNNNNNSSRSCSVIINSSR
SSSNINNSSDDTKMIKYSLSPKKNTQMEKKKYKYFEGHRNKNKTNNNISSYNNIKYEQIKDMNFPSNYKNNKI
LLKQYNTSAKDELHNDSYILNDDSLDNSINTKVKYPILKTITIISDGKNDINKKLKKQEKYIRDNIKQKKKTE
INLWSDKQIVASENSRNVNEFNIYDNKINEKNDMDIIIEENMKNDERNNIIRKKKNKSNSTQCSNINNLDIHK
NKKNLYEYSQNYLHDNFKVIENEDRKDKYTNSSHTSEILRNMKEVHNLPKRILHNDSHASHHDNSIYHGNISS
VDYKKFNSEIEELNDSKSFGRKYTGRNIKLEQLKHVKDDKYKGNNIMLHKNTYSHIYDKQKSNSYVNINNNVL
KDKEHIKKNNSLDKSFSKLNRSNVIHPRQSYKATYENKKFKEREKRTKEADKNNKKNNFVIQKYNDSNINKKE
LETSNNKLYEHILNEDIFYKTNNTLTSSFDDNSYNKGKRQLIEYSNSIHDDKYYDINEIDNYKRYNRGIIKRN
DYTDGNTTISTNNSSCTLHDDNKFNHSKHKNMELHKNMLHCKNVSQYKNMSQYKNVSQYKNVSPCKNVSQYKN
MSPCKNMSSYQNMSPCKNMSSYQNMSQFRNHLKQYKNTNYKKDNYPLVKISNGIKKKYILNNKRSGNEIYNKA
YYSENNTSSELYSNNTSEEKKRNHKDITCGKDLKQSTSHYVDNYNSSKDIKEKRFDKVKTKMNNYVDDNNYIE
TNDDVDDNKYIHTDNDVDNNNYIETDDDVDDNKYIHTDDDVDDNKYIHTDDDVDDNISLYSNNNKSVYQCTKE
DDEGKASYISNRNNYIKLNKKMEGTKDHLYNKRYNSSKISSYSKIKKSNLLNSKLKDNEYTNEIKNNIRYNQN
NISNNKIYNDNKSERDKYTERGSREVKYNTSIDNVNYKEKKNKYQSNKKCVNHEAFLSTHEQSKFFNNEIKKN
EINDINMKNSRSKSSYKKESYLDNSKDKNSSHESYENNISENVINKMDGFYKSLTPHEIKKNIYCSNISEEEK
NNYFPSQSILRCDKTKNTSTQEIDGDTINKIVNHYDEQYIKDKSDLDIRNERNEKDKVYKKCCSLKQNRMIQK
HSSLDEQNEDHDKVEIYYKKKENDMDKYIEQEKQKKKQNDNNKMNNNFISSEDVNFKNKSDITQQHNYGDKAS
THYNINEDEKKNNDINNMCVNTIYSNDDENLFQEQKKENVYCKKLKNDLIKDNYSLDIQNDKTKQTYTSHNIR
SNIYNYQLDRHTYDLQNNSKKDEDISFNKDNNITLLNENDIAFDKDKNIIENKYNKLLYSDIYKTEGNAKKLK
QNSLCKIIEEQNKNDQINNYKNCTHNKDSVYDKDEITMEKNNILDKSYYKDIRNEHSNDIYHNNNITCQSLKS
ENYNINHSNVNHNNNGKYLQGVVGINEANEYLKNQLNVLLKDNNQINKNEKNNVDENNCYNNSMDKSKHSSKE
KVYINNNDEIPIKFEKLYTSHIYNEKKGNIMMDENIDKYNDDKNKGSHFKEENFNLYRKKLSDIKNINGKNTT
NINNSEEVLLNKDLIKSMDISNPIRVDENNASTYINKNMDNNVSIINTNKNNTIHNNNYELSVDNIKREFNVD
KTSIHNVCSLDTIKGENVLKKPILNIISDDENNKNDTVKKGIENTNNINTMNGTKTNNYMVNSFTMPNISNII
HNKHMLYTNRFNEKNKMNIYNSASHQTILGEQGNYNNLFCNPNMVNNENIKRILYNKALTTANIGHTHNNNNN
NNINNNNNNILYNQMNNDQYNINKINTFDYLNKYNNMPMDISTLNKSDYGNLQNDNLNNICILPLNPNIQQHS
FNYNMNTGYIPNNNFLYNNKLCYDESGKIFIRGDKPLNNGAIIYNGNKLT*
>N.PF11_0423
MGFHIQRYIAMMGRGINPKTWKKLWVDSKNKQIIHVYNDVAEFMNNQIAQVVRVYQYRYWWWANPFGMGLIFY
LGYKTWYMVYINHKQRKVAQVVASAYGQGGQWLNPVPK*
>N.PF11_0424
MVKKENEENIAQENKEKEKPKNANDKADPLLKLKEEREKLIQWSKKLKNNNCSFMSSDKLDTEKSEKVYSSND
EYYYDEKNKLNHNDNKDNMNNHKKNNDNKNRKKENYLKNSSYNNLSDDKKDHMDPMLYQSIQKIKEIKNNNYD

NDIFDEESIYDDYNINKIRKTKKKDKNNNSQNNSFQNNSSESNNQYYIFTNRENNKDTLNNVFKSTNNQKDES
NLNYLHSDDEVFMDNSSDQKKTKNKIKINNNDNNNNNMKMMMMMKPNLYHSSNDNFINMNEMDNSSNIIKEKD
SNFISNNNYNKILNDTFLKNNSDIIIREPFFKTSKYGKQNEKNENFDEQLKGISNENNMKNSYLDNKKKIKLT
KKKKKQTINLYNNDKHDISNIYDQNNISNPYNIYSSSIPHIGFSSNVNKLSNESVQTYVPTGSFVNSKDNSTC
TTILSNNEMNTNDNNLTNSGAKLKNIQTKKMINLLENKVRTLESKKLNLNDKMKDLHDNAYMLIESKEKDNLT
IQHYETLIKNLESKYNKLFNMYQELDEHRISYVDAYREKQTKIENLCAILQIKSEENLKLTQEINIINKKNEQ
LQGQIYEYIKDVEDKETDLNKKKEECVILKNNLETVTMEKHDFKKQLEEKTKQYNDLQNNMKTIKEQNEHLKN
KFQSMGKTNDHTNNFFIPKIDNLIYILNKMLQVFKLNEQNILDYATFFKQNATVIEQKLLNNDNICNDIIQII
DKNLVNPIIDVVNQRDQQYIQKQNELKMKFDDDILKIYEQNINNVELANKHIEELKKLLKTITIKKNRIKQKA
LLLSYAQGRLNEKPILKEIKNMNIGSPLFKCKYNSYSHKPVQIYIKIVDNKYITWTKNVKGKKGFKKRKLIDI
QDVINVDYGLNSRPVYWLIEKQNQKKLQKKKINLNEFYENNPYNINPYNCFTLYTKERTYDFFSDDDEVVASW
VIGLGLLSYPYNKSPSIQSRSEFIIKRVQLKLKLYCIRNNMNYVKLWKNAIKKTQQQREVQHMEK*
>N.PF11_0425
MKNDVKKDVKDDVRNDVKDDVRNDVKGDKKNDDINCVKYHIKNKIRRYEHLYGIEYIYEKKKNRMFFLFVIIF
FSLFILYYFYLGYIRGYLSVNEFHILLFILYIFCIVVYFNNIFTEKLLLLKNIGIQIDKKDSFENYTKFICKN
EIENIFINEAIYMFEICPYLCIKLKNNDSVILFKDVVLGMNNMVNIYRDIKKIFFYNDNNILKTIKITHVKND
KGIYEIGQTDGEEEDEISDSVSYIVSEATSNHTSDKISDDISDQTDNFEEDEEVKKVIQKKYNYTKNKNYDNI
NLENNIYATNNCSSSEENIFKLLNFSSYENIKINKRSKKLRKNNSYDVYINKQLAIKIMNN*
>N.PF11_0426
MGEYVRKIPYYNNYEEKKDLLKFTFLHDKKDDEKREINKNDEVQFIIKKRENMKDILTHLPHMNTRTQGDILN
ERKNKLKEDMSTNFNTYEKRFPFLIDSVVKNPEQTRDMKDVLNNKYVDDNTIKYRISQNVVTKTMLLEERKKN
YKDELRGHNIEDNEKTINYEDIYLDPEKNYTDIVKNKNKLLTLKNYGLGQGPSNFVFSYPDIKAKGRGGYASE
IVKSNILKNKNEYSKNKIQLNIQNLQDKDIYSNREEKIFLMKNRFKYINHTKNSYFVDNYLKERPIIILRYKG
FQKKDKKNVNI*
>N.PF11_0427
MVIRFFLFVITLLGLCINMVCCNFKYSIILPTYNEKENLPYLIYMIIDELNKHEIKFEIIVIDDNSQDGTADV
YKKLQNIFKDEELLLIQRKGKLGLGSAYMEGLKNVTGDFVIIMDADLSHHPKYIYNFIKKQREKNCDIVTGTR
YKNQGGISGWSFNRIIISRVANFLAQFLLFINLSDLTGSFRLYKTNVLKELMQSINNTGYVFQMEVLVRAYKM
GKSIEEVGYVFVDRLFGKSKLETTDILQYLSGLFKLFWSI*
>N.PF11_0428
MEEEGRISYHEYKRRLKKLTEEIKKEMKKSSNKNKKSETLLNLEEKLNKLNELYNKEDDNKKVEENYNNDDNN
KDDPFHTNTLYQMNVMSKKALRNRKKMEHREYEEEQIEKSRNKEGEKEYEELSLNLKKLNKTIYSIAPDGNCL
YESIIHQLKYRIHTYQYTITDFLKLINKEQFSLDNINLKDYINTNFFNFNIFNNINPNDLSSDLLRFITSIYL
LQNKELFINFIYDSDDCEDNDTYFKYCEDIIKGVYGSEIEIKALSNILKKKITVYDVNMNISYEEDCEEELFL
CFHHKLYALGKHYNSVIDLPS*
>N.PF11_0429
MEKKSVSMQNKVKKKCIDKSKGKDTNAIKSNGVNNNKNGTYNNNDKKINNKNLKPSDANVDIKNYSDNNENDS
FCYECYHGGNLVCCDNCIRSYHLYCLSSSDKPQPNFNYWYCPLCKRRGVTVDASLKRRKKIKTIKTVEKKKDK
VKQKSEGSKYTNQINVGENYQVSNVSTFFLNSHSEKYDETSKSELVYSPYLLERMKENYLSEGQYELVIKNDY
ELAIFIKELAKNWKCQLGWHPFTPEYAFKILHHVDYNPKKAIELLKSSEFNFLEICDPPIRKYENKWRPRDKR
GQISDSPYPSSELLQSYLKRSVEFSLDEKKYHYPVNTGNKFYCEINKNNIIETNYPNERTRNSLRKEIEEDDD
EEDDYEDDDLEEEEEDDKDDEEYYEEYDK*
>N.PF11_0431
MSHYNPEQLNFVNNHRNNVKEGYFYKNNLLKEHNNKMESSKNCIMVPLINNPSHSENNFSKNNRDENINEEKS
KYQFEYQNKIIQVPELKYVDKIVYDPVIIEKVKYVPKEVIKYNIIKKPVIKNIITEKKVDVLQVKEKISFKEE
EIVEDVYNYVDKDLNTKWNESQYDNEMYKDLTKKKNYIGQNHLLPNNINKMGHINDRTYKHINNNITNLLEPF
GPQIDVEENKIIENVFVPNVEKVIEVNKKIDIPINLPVPYIVPKPKIIDVDVPVFKFNDKYVPVPVSKKIIPK
ITWTDKIYQVDCLIEKPYLVYHNIIKMVPTDSKITVREYPKGIKKINPEELYEVDNLALWMRVNADLKQEHDQ
MKNEKYETNKKKGKGETEQLDDNISSDHTCECSESYETYEKLSNEEFNSSNEETTIKSSNENILDTLPLHPGH
PLEFIHLQNKWINQDTTNIPDMYDQKYLDAHRNAVFNLTTQMPREAEVEAKQLLYIQKKLQQEETL*
>N.PF11_0432
MNKIIFFILNRKKYLYKIWGNNFCTNKIVDNIGMKRFYNSTGKEYVLKNKDIHRKKKRNLWLKKINLSELPHK
KYGNKLGSVAKKLYMNGLYQNLDRNMERYNKDEYYNDENNVSVETNGLMSSIKSQISSVDNNTIICNNLELIE
DNQNAWNIICNELKCHLPFLQPYSITVTLNYLSKINYDEYDIFKFIADNIDERWLKNFNIKDLSLLLLSYSRL

YIKYDSFINRISRELIYKISYASVDELSKIAYAYTKMKIYDYEVFLHLCNEIKYKIKNEKDDKVVCEPFFENL
KESMEGNNIAVINTVQNEKKNINKNININDNNSNNNKKNKLNNFSCEHKRLDLKSYEDVHVEIGEYGNTQKFN
DNNKTNNCKKKKKESYHTDYISKEENGVGIDYNVDDINVKEKENKKHSSLCLFLYCIGKMKHRDNKLLGIIAE
YLKLQYLNNIDISNMCYTFSLYNYYYTNDFYKSFCLKSMEIINDIEPLQRVIILTYMLKNPMLDMVHIYVIYL
KNIINEMNKNKIYKENLFLNLCINSISCDTFLNFLYELLYSNKKIISKEENQKYMTYIIDNAFIIINLILTYI
DFFIKNKNKNISSRDIAKIFNILIKIYNIKNDTLNISKYNNMKPLQDIINCLNVQIDNILNIIINDINKIHIF
DLTNIKKNLLNFKYNNNQYDEQVKTILNSLRLLVK*
>N.PF11_0433
MNDKKNSINKSGRHSFNRNLKKKNIYKDNNNVYNYSEGNKKIFKYMNDLKYSSYNNLNDGHSDKSSSNNKPSR
KKNLLKKKRNEHFNKKKMNSTNSDHHYDDEDEEDDDDDEEDDDVDDDDDDDDEEEDDVDDDEEDDDDDDDEDD
DDDDEDDEDDEDDENDDNENENDNENENDNDDDGENDQYRHKNNMNTNNNFAINKKNNHIDVDDGDYKNSYFY
NDLDKSNKKNKVNLKNYNSDYYDIYSNIYSYYDIFTEEKTDKKKFKYFNDVFSNFDKKYENNNNEYNNDKYNY
DMNNKRKVQEKYLQQPTVRTSSTIEKSNTSPHYLSQKNKNLLSKSYGREHNINDEQKISSNINEENIYNEYYD
DGMYNHKRNILKKSASANMMDNQNVKNNYINKKINKKRNENKNHKDINQNIHQDVHKNMDDDDKAYVHINYDE
KGDIHKMNNDYHNNIYNKIYGNNNNSDRNNNHNNHNNQNYYANEFSHVKENNCEYTDVDETSNVFNNLSDNYI
KNKNIRKQKRKKNKKKTTLSDNEKATSDLSSIHNKKINNNMNNMDNMKRINNMNNMKRINSMNNLQSEDLNND
PMNQQDYDKKYTYSKEKSESNISLDSSCSFKWEAHLEKEKMNDGMQKNIKFLKNPTKKRMYKLKKIVEILEYT
IYNEEKQGSKLEGKLYDYVVLTKNLKEKIINLENINTNNKNKMQSYEKDITKLKEQNEEMKLTLSTFENELSN
LINKFDKNFAKELIDTKSQNDVLKKEVERLKGEIEKKNSEIKKFENLNDLSKKLQNEYMDHAVPCDVTASNVE
NKTDANLENNQNGPFSNNNISTSFNGVYKDNATEVTNMNNNNNNNNNNNNNNICNNNNNICNNTCNNNSNNSSN
NYICDNSYHDGKTNNYTNYIEQDLFLNELPFQYGCSDSIEYNIVDEFILKIKSSRMSYDKNIEKYDDQTKSVI
YGSYISTFIIENKDICNHKKILKLLQEFCLVESKFLHIHLCLKKERCDNISQKILEKEVIRQLENNEFDKNKE
RISVISPVILLLIFKNMNIINLYKIFISCLKCDNYRWLRIIKKSLFIKFFDGTLFDTKIKITDGSFVREIKLI
NLLLNGNVPDEYIFSENMNNNNNNNNNNINRDHCINSCVVKQPLNLNQLNMVNYKMKHPLFYCSNEQLKRFVY
RMYLNFTKEPINVFNGDNIYHYVLQKKNLDVLKILKLSGKHYNYIFEKNNINKTPLDYIENEDIRVDLISGYI
LDIAGKGAENYKNSNYNIAYDLYTEALEKQIKLSESVKVGKSMNENIGKLYYNRARTLMHLNKWMDVIENCNN
CLKYIPKYVNAFDTQIQAYEYLLEYDNALLIYKNMCLKCNIKPDEKEEKLKAQINATSFQVLNINKNCSISEI
KQAFSNLSKKWHPDKLGIHISPDIKKRHNNHFKRLFKAKQMLLNEVDRIKEKKKKETNYIYPQIIEDININKN
NNNNINQTNTKSTDTNFKHMNNNNNNNNNNSNVVYNTRTNNPYYYNVQPNKSNLNNTQNINKSPSYEKDKHLNY
DQHDDKTYKYEPKKSSNHFMQSNENHIYNNSASDINKNVNNDNSNLPNVNDIRNNIFSTYKDDIDKFQGKLKS
YNNINNNNHIFNNNVEDSNKHLDNKLSNLNNDKVDESFYKKLKEEYEVLNYEELMNLKTKTFNSINNLILTEV
NLKREYQELCNKEKNNVIMNARLCILQEIQKALCVRLDKERKLKVIENIIKNRQEDQSSIYSSRNNHLNNSNN
NDNHFVKTNIDESHVHTNKSDEMNLQADRPNQQNYNALNKNKSTTTNEFSENIFNDQKEHVPLSSNMDSNKGN
TYVDEEPIDSTKNNRNDNISNKNVHKKDIQNINQNNNIVGSDQGDDIFFKNNNNNFGEGQDIQNIHHNVYNNT
PTDQNKKEYEEESSGFNNIQHDNTMKSPVDKHMDHNNVPINEWPGDAHEKELNNIFVNKIKNNNDAQSYKGIN
NKTIFLEENNDERYNQENEKEKNYTENKEYLKRNENHNNIEEESFIHLSNIKGSTTNSNIMKMGSNLNTSNNM
NIFFNQHNEEEHEKEQEREHKSEKEGEKNKNEKGGEKNKSEKGGEKNKSDDLFKTPPSCSFRKSNTINDKNMN
EVNTYLFNSNHYNDNYENIKENTNNYTNDYTNDYTNDYTNDNINNRFIPSDSQTKEDYNYDKYRKNEDINNVP
IINNENIFNNKFNTNDHSNNIHLDNSFNENFHNLKKKNNEEGNLNNDKENINSNIRNAHYDYTKSFINMKNNE
YNYDEQYNYDEKHNYDEHNNDEQYNYDENNHDEHNYEYHMHDKTNSFPNQIKKNTYHFNKKDEEFKNNDMTNF
NDEENNQIFNNKNHIHNQVDSQDDLLSSSRMNNSNLLKNNKNTDKNNFNNNNNNNIFYRDTNNNSSSNHLNYN
FTETKIKNNLNISNVMYSNDNQKVIINNDNFLNLYKENSTNNKMMNNNLESNFKNDIDRSSPSLINDNKVGDQ
KKSMFYIKEEKNNKQTYVTYDNNNDDDADEEEEEEEDNDNNKNVQEQHYINKEMTSNSEQYDNNNYDDANNNN
NNNNFRQSFSLNNIHSNHMNNYQNHNNIFNNDDQSFTYDDGDYYNLKTSNYAYENNKLRSFSIFPNDNEQINS
DIYSNDRNTSFNNLDNDINAQPKTNYSFNDNMQKNIYQKKNSQNKMFSTYEQNENVDKINDFPEDNKKKQTQD
SIQPKKGFKKFFSSDDKKKLIDQNNKKNKLKTKDKK*
>N.PF11_0434
MSLHNENSKKLRIFLNLFSTRVNLLDINTRNEIINQKRYIYFLLLNKKEEKAHICICSMLRNENIHKVLKILL
SLCKDSNLFLFFKNEKNKKDNETMRCIRNILYCSNKLMINNRAYVRNNFIHIFGKKYIDLIETNYHLIDDNIN
QLLNKYTFSSSEIGQAQNNIIIQMNKKGENINNNMMTIIDPTNIKTCICNFCKYAKREKYNDFYNNENIFFSD
HNRSLNLNKNHIKQIVELYKNKSEKVYDEEQIIILQKMEKQIISTLNEKLLNK*
>N.PF11_0435
MSTWINKKLISFSEKVIENVIDSFTNKNTPIDNIQNMQGTQLGKIINKVISSKYFFKNDDICYNKNNLDFKWY
LKKDRKKSRKIKKKQKKRKRKMIMMKRGVENVKNADSSNNDVCHDQNNNNFNDPLVSKNTNYNYLYTNNNENN

MKESTFLKIDESYLSTSYILNGKFVSGNNISDNKNDLNEKKYINIKRTNSHNDTSSLSISQNNFSKIKKKKGA
SSINSYDESSPNVSPPSMYSSENLSYNEKRHNNNSDNNNDRNMKSYNYSSSNINKNCSSSSTSSSISSSSISS
SSISSSSIISSSIISSSCSSVTCSDSSLNIYNTKRSSHGSHNQFCGSMSCYEKDKKKNRLDNKNKMKNKNILN
KKKKYKNKKMPKTIDGNDTSLLLSSSTSSCNTKVSFDNNENYGIIKEFSLCKINLFIKEAKLLFFNKNISISD
VSLYVTTIMEDKKYIGKLRKLSSRTLPMNNLIINEYINHNIKDVYTDIIINIRYKNRKKEKEDIILGRAIIPL
FLILNTYKWKIKKIKNKIRYCTKCFLWLHIFPCNNKLFNYKFFKPVEGFEEYGMLNPLYTLGFLNIQIKIIFK
RNPLFLTFLSNIRKPLFYYKLPVQFEPLYCQYYSENLYVYAKNIPLWIYKFFYIFHYKRLEMISLNCYDYICI
LIFWLFFFDLVVLSPFSLIFVHLFFCIFFISLSYKYGKFVPPYYKKKNLFYNFRPIRVSRVSRRNCDYTKRRI
ETTNFILNDQKNVEIYNREKKLDLLDDNNVDANYCKYPYCSEENNMDKLNKDGRDVNKGVDKNIIKGKNMMTR
GGGLNIYDACKMFIKGDTVMKANIINDNIVYENFIKDGIKKNDVMMDSEEDKEINAVYINNKNVYNNNNAPVS
CHDCDDPNNLSVHVHKEENNSTSNKMILPSVCSENSLKETMGNQSMENNNKINNENNNDVDSVEKTDILLNLS
NGKNNGNVTSSLCENLFVYNQDKIQRKKKVPYKNKERDNKDDLDEKKDMYICNDDSSVITSSEKGVTKERIHM
NKEKLNYNGSMECSSVCVEKNNMSYIARRIQNMMYDTKEKMKLDQIHMNKHMSGFMKLFNVKHVENEKENDID
KYHDKGESDKQVPSSVGSYKLMISQEAEFEEEEFDEKEEFDEEEEEGGQDEESKKMSRVKHIKKRENIINIEG
ENILSSDGKKSEYIIKDSMNNTEYINDIIYYNNCDNILEDNKSEYNTSMNERVMDNKQEVNKRSNNFFFSYNN
NNNNNNNNINNNNNNKNESVWRNLLGIPSSNIETVNLNSNNCTEIKNSNKKFNIIDTYGNNTLQDKSNIIDLR
KKYPYMPFVKSPFHNFYLYMNTNDNKNISIFSNNVEVPNVHVILNRFITLITWTQHVSGIFTMVYEKIKYAFN
WEFSFYTLVNILILFLICYSISFIIYMFSYIPFVFFRFLFFVTCSYFIIRSYELTEDGNRACLYYKKRKIQFL
KNRKISLAHGLFETYKWKNIIKIIKKTLKKKDTNIFKYICLTCAFKIYKLFKIIFENILLYILFILFFIKNWY
TRLLILKDIEHMQIAKLQGFKNLYFFIHNRIIKREQKNVMSNTSSNEINNRKSSVIKIVNIDDMEKNEENMNK
NDNNHDKNDDIVDVNNVHMNINNDNMNTNNEYEIIKRRNQNNMLDGKRKSVKSLMYENYKNLESYVYSSSDKE
AVSIINEDDIIDEEEEEDNHQKEKLNKDNINLDKKNINTYQDIHIDQEIQPCDDENDDKLSLSQVTDNGAMNV
NVDIFLHYYFKKRKYDLFNNFININRNHMYTYKDINLFYSNEDQKMNNINYGEYLNSDDAYSSSYDYNKRQKK
KHVK*
>N.PF11_0436
MKDEIAPNEYFRNLWENLLKSEQNNICSLIESLDNKKFQEEVWYRKSGKNKNLGGGITRILEDGNIFEKCAVN
YSCIYGAIDKESAKQMCVNHYNKEFINTTKICHSDDINILISRVLNSHNIRIINEKYKFYASGISIIAHPVNP
NVPTVHMNFRFFQIFIKTGKKKKKYNNLNNVNNNNLNNNLNNNLNNNFVKNKIDNNYKSIKHWFGGGCDLSPC
YIFPDLFTEFHNSFKLVCDKYNHLFYRHFKIWCDLYFRIKHRNINRGIGGIFFDNLLNNIIKNKKVIRSGKLK
DINKKNGNDPTINNNNNNNKDCKCYSCNNIMDKSYRMIYFFIQECIINFRKSYLHILLETVNFKYDDNMLKWQ
RVCRGRYVEFNLLYDRGTKFGIELNRYKIYRRKKKQKKLENYSSTNFIKDEIFDEQVSDYLSDEHEKIDNVFS
SLPLKCDFQYKYKIEKYSREYETLEILKYPKKWVDY*
>N.PF11_0437
MSNVSNALVWELTRKSNCFIKKNKAGKKGVFLCDPLNVNYKNTPSSSGLVKSNSTNVTLKDGKVVFSVKTSKE
SNVVNQHFKAKNMKNVEKLLQQHGSFEKAKNKEKLLKKYKRLSKLYETSHKTTN*
>N.PF11_0438
MENVKVKKEQTEVSQKKKVVKKVLKKNKKTSNKKLQAVRLYEKGVILGYKRSQRNQDPNFTLISIKNVNTKKH
AQFYVGKRVAYVYRTTKHHDGVKIKCIWGKVCRTHGNSGVIRAKFKTHIPPKAFGDRVRILMYPSNI*
>N.PF11_0439
MINNNNCVEAESNICGDIEELIDDLIYYVRTNDIVEVKKILEIDSRITSLNNIKDENDNTLLHFACANNNIDM
ILFLLYECCIDYNQYNKSGNSPLMWSIQNKHIESTREVLFFDYYLNKMKYNNLEKKKNQVFENMRYVNDNNFI
NHNFRLSPNIKKKLNIMDIYNLYDGRDVEHMNNDIIICDELLQNSNDKQLFLYKERNKINLLKKNEFNKSILS
EAFDAQNEQILHLVLNHPISVVLDNQQDQSDDDNKIKVDGDMDVNMSEHPPTKHDNNNNNNNDNNNNNINCNN
NINCNNICGSVNTKIDNNDYNTEEIKNESNIEFTTNIHEAKIIQEKTYELIINDKIKKEEYDDCNNNNNNNII
IQIREVGLNYFGDTLDESNPCNDLTGINIWESCLVASRWFSDLSLQNFFSNKNILEIGAGSGLASITIFIYSN
IYNNNKEKGIKNLIISDINNITLNNIKHNLFLNEYLLNFNNSEWKNKITVTNIDCTNINTYPRENNQITKYDC
IVASDIIYDHKLVPSIIFLLNTTLQTNGTFLYVCKKNRDGIQLLINQLENNNFTIDYFTPPQDYFKNPFLNLS
QNIFNMKFSEFDDQQNFIMMKCQKL*
>N.PF11_0440
MMKRGWILCDFFNYNKIVRCEESHDKKHKSEDSKYGEKRSNELYEESSMNNNDSLIDMIKNIPINPFIDKNEN
MNKYKYGVEKKNIERYTGVNVYDEVDEKDEKKNKPVEYPFAISNKIIFKKNNNNNNNNNKTSSSNNQINTNYS
NISSELYPEEGYKTPNKTKHFYADWERLLAYNHGLYTLKNANNNNTIINRDLHSLNTENDIRNKLNLYIDRIN
IDNPNDTCKYLGIEEYKCLLTHSFHMNTNVSNQKCVKWFNEYIQCKWDEQKLNFGYNYIENKRHKKSKAYIAA
PDYQYA*

>N.PF11_0441
MTMENHNNAKQDIDKNKVECDNKYIYTKFPMFDMHKKIKYYNNKISDINLENIKLKRCKKHLETHILEENIIF
EKNFKNYEKIISNALRLGYSCMNLCTILNKLINKDKLYVLRLLLRKRDEFKNNIIKSYIHKHMKNKIYFKTLT
NIIQNFALKNKNFVTIFLVHHIKNKIKLHFIHFYNVILYQYNNVKEKCKHISILQSNNHFISNKLYLQNIHIL
NKILALFKLSYIIKNRFRLNVQNFFVYMLEKKKKKKNYFMKEKNDNIINMKCPTNNIEDDTINTTTNYNMDKE
NLINYEYCTNKKKNFIENTFFINDNSSSTSSCILHPNDFYNYLYNEELKKYIYNDTKYNQINIYDKSIYQKLK
QKEKDKKKIYHKENSILQIQSYLDHKIVKKNIYIHSLKKELEDLFFEIPNDVKIHHNQKEQIKNVQTSCNEQI
EKIQRTLKNIRNKHFFFIIGMGPPENLQLKASDNCDLNLYKNFKFLNNMSNTSGSITNKGSKILFSIKR*
>N.PF11_0441a
MPILFYIFYIFCFPLINLSERTSKINYHNNYRFVSSEDKSMMNNFFIKLNKIKNFPNYNTLWKIKKCGYFFLS
YKNGKNNWNRISVNTITSSNNNEKITDDVLDGISFSIRDNTHIFKNETKDIPIVLLHGCYGSRKNFIFFSKLL
KSNKVITMDLRNHGDSKHTENMRFDEIENDIKNVLKKLHIKECCLIGFSLGGKASMYCALKNSSLFSHLIIMD
ILPFNYNCNKNPIKLPFNISQVTSILYHIKHEKKPRNKLEFLQYLKCELPDISNSFAQFLCMSLKENNDKNQL
TWKINIDAIYKDLPFIMNFPLNSQEYKYLNPCNFIIAKKSDLVCSIPNFDKIIKDYFPSASQIILENSTHTVY
IDEAQQCADIINGMLNK*
>N.PF11_0442
MSNPFNSSDYIKGDNEHKSGYEEMKEYTNVNKNINLNECTKDHINIKDNDGIIKNDSKECKDSHHESQDMCNV
SNKYNISANHMENIKEQNDTHQMNETLLHNKMYNEEENNILSNITEYRKNDQHEQKINDSDDVLIKESRNNYH
NNNENIQMNNINDEVIRDVLDVSNSEHDKMNVCDEGKKNEVLSYISMNNENSEKEIKMDMSINCNNAKGTNET
NKTNKTNETNGIDNNNDDNNDNNNDDNNDNINNIYSNSSCYELKYTMEEDNEIKNNSNYVKESDKNVVDTKID
NINFVNKQSEMCNESDVNNNNINDIRNNSNVLDVVNNNVSSNSRMLPNVDISSNDEEKQNCDNLNNYIINDTI
DNKQEPYNNNESSIMINTDNYSNNNSILNNENVYDTIDDEPKQICSDSDTYTYNNMNNNDVENITNDVEMYKN
DFTMINEHIVQNANYDNDKGDQLNIQDDNLCIQNEPTSFSQDNENVNASNKVIISSEMEQNVMNDELKNDHVI
NKETNEHQDENNMISHQNESEQHEICNDINEHNNNNNKSIRNQNDENDFMNKNINTTENEQIKQERTINKSND
INDTCTINKIEEHIEQKGSKDTVIYDVVNNERDTNNNNMKNGENEKNEKNEKNEKNEKNEKNMKNMKNMK
NIKNDMENIVKKISQDTVIVDVNDELLDPNINNDFIKKKVSEDTIVSAIQSERKEGKKNKRATHKINEQKVLT
PTKYFVNDMSYVGSVGKTRQSSKMVQMDPMMQMDLRIQMNPRMQMDPKVQMDSMNQMDPMNQSNQINQINQIS
QINQISQINQISQINQISQINQINQINQSNQMNHINYINQSNHINNIYHIPSSNPVEDEYKSNLMNNLNSDNM
NSYLTMNDLNGDMDCKYAQSGRKDYMRNSGKVEEQIMNMNNEKYLQWFDTIYTVCVKLDDLCCRLSGTFPHSM
NIWEREGKINMNTLKSAFYKYEDMKLNLNNNDEGNNKSVMMSPNYTASNNVDSSIDDPYMKSNYMSPSIINKN
DDIKIKRMKKYNMNHMDDSNISMYTKNAINKQINLTNNNRKNYRSNDPYINIENYTPMDNNISQMEDYLQIDN
YTPNIIDPNVCNNFINCNKNNKINQNKNNKIMNTNSVHDYVIPNDLYNVENINVMGNNTTHNEKGKRTRRKSS
DKNMKAPKLTKEEEEEMELLKLYNNNNNNNENNISNIASIQNNITNNLITNGKYSNDLFFHQNGFNNCSLYNE
ENKMNCSEHLNIVPINHNNNNIMCSNNHINMLAMNNNVINYKEENTSTTKKGKKGNRKINATKNIEKKTPKKN
STVNRSNNKVIKNESEFEYLLELSDKEGPNLDNPEDLKCDVAGVYWDKRSWIASWYDNGKRYYKSFSAKTHGF
YKSKFWAIKVRLSKVKGQTIFGKNSRKNKNNKTDADNTNGNNNNVNNTNAGNIITLNNLNNVNNLNNVNNLNN
VNNLNNVNNLNNVNNLNNVNNLNNVNNLNNINNNTNISSTNMTGSSLNSPLLNGVHMNNIHMNSINRNNINMN
NINMNNINMNNINMNNVNNMNNVNINNPNIINDNHMPYHNPIMPVNNNIPLNNL*
>N.PF11_0443
MRIHCFSFVLLYFLFVNNVNCLLKKVLHHFYCNNENCYDILGVNEKASLDEIKFSYFWLLKKVEKNHDREKKK
RIVKAFNVLVNKSTKKYYDYYLKYPNSFLNLVYLNMYIFYKLFKIICILLLIGLLLCVFQYIHNKYELKRVIQ
KSSKNKAFKKEVQNRISSQHPGFMNYDIKKKKKIEEQIEEEVVQEIVMINNQKTKKLLLADLIIVKLLFLPKQ
LWFYIIWNIKWVIKYNILNEDYDEHDKIYITRKYMNISMDKWNTLNPEEKKNYLKKELWMKAKQEEFLQEIKE
RDRLNKISSAKYKKQIRMKKKGLSFNYND*
>N.PF11_0445
MENVKYRDNFVQLGEEGPRNATTTNFYGSYYFSDKENYFDIRKFEENLEMNVIKNEENILILEIKNMDVSIAN
ALRRIMLSEVPTIAIEKVNMYQNTGIIADEILCHRLGLIPFKFDADLINYKEDNEKYNHLNCFCFKLHVKFSS
KRTGNDNYQSIYSKDLKWCPINEQQRIKFEKNPPKVVDENILITKISSGQEIELICFLQKGIGKTHAKWSPVC
TAVYKMYPHFSFNTNEQLSYEEKKDLVNICPQKVFDIEDSQQIFVKNPLSCSTCRVCIEKYPKKISFQKEKNH
FIFTIESTGCFSAADIFKKALFILRQKVINVKEALDEQLCP*
>N.PF11_0447
MPKNKGKGGKNRRRGKNDNEGEKRELLYKEEDQEYAQVLRMLGNGRLEAHCFDGVKRLCHIRGKMRKRVWINS
GDIILVSLRDYQDSKADVIANYVCIYLCMYLFIYIFLFIYLPIFISTCTF*IYS**GKKLEGSRRITRNSKNK
RNRHF**RYIYVYIIYYFI*

>N.PF11_0448
MYMEEVNSVPLSNIYLYKNNKLKLIPYLENVFIKNIFCCNYSINILLHYDDEEYNNKLIKLDNDKGYINISYG
NNNNNYNNICGYYLTRDLLLYEFYCINHMDEHKYNDRYIKDNNICNKLKNKNYFYVYNIKPFLQYDKQNENVW
HYDISKINKNEETNKYIKKRNMKYKNINTYYYGNNIFFGMNNINDICMWEEKEEHKKTKYIYYIPTNNKPEHK
HSEYYYIYNHKYNNDNSYISSFVKYLHFPKKVCIESISCGKDHTLFLSRDKNCYAMGCNQFYQINYDKDKNKR
KHFFEQPIIICITNKKKKKDIKYIAAGYTHNLICTYKNELYGWGNNIYHQIWGNKQIVIKKPTLILNRKIIK
NEICNNNKFGVLKICCGFSFSCILLKNKKCFLIGTYSNKHTIKKKKKHHNNKSYHHHNNNNNDDTNYTYEKR
PISLLQISKNKKIDDIYSTFYHIFLVENLKLSNMFPTIIHPQKKNKIVYLLFNFKIKKSLSYNIRLDNHSTKI
IKKNEKNNIKRKKKCNTLQLNILPPHGDINNENKNNDNKNKNNDNKNKNNDNKNKNNDNIHNNNHNNVQLSFR
FNYHSLKDTSNPLCLHKMFDVHTKKFKRIKHKHMLPYLINNNMINQKLYLTLYNNKSYIHFKNQYLMLSSYKG
IIKNIIPNNCTLTKKIKIKLIINKPPIYIKKKFISILFYFKGHGRKEKIQLYKGKMNKNKKYIYTNIVLPTHN
HNNKINTNNSDHHFNLCQIYFSLGYHFYKSSFPIIIIKPDILNITPNCVSINTYNHIYINMLHLSSNFHTIHV
ILYNTHLDCIYRKAYYDENTENYFFSLPLIPLTLFQKIKSEFIKFQVFASYNNVEYSQNDILLTVTRF*
>N.PF11_0449
MHDLIIYPYEKQNEYIYQERDNLINEDHIKYDENDEVNRNGISYKINIYDNNHPIDNNNNNNDGDNNDGDNND
GDNNDGDNNNDGDNNNNGDNNNNNDKDNIASQCIHTCIKENIIDDKNYDSFQKREDITNIKKNSLDFNTINEK
IFNINIKMKEPKVKTTNDTHVDYNDDNNDDSNISIRTYPKHSTSHHNYTSDDSSYSSIDDIKHILKKSKIKNL
QQNKKKYICNYISQHKKRIKEKKSNLIQKKKINKQDNYLNTNFLKINQKTKENIKRASAASIILENNFNSDHI
DEDEENSSYDENINYHTNDF*
>N.PF11_0451
MKVKIGSFENFLERLNNIKEEDIIINDENTEEMFSSFLITFYKENEGRYTIQEQEYINNLLNILINKIRDNKN
GRNFFSNLLCIHFKSITPYFINVKLKDNIYYIHLLINYILYVLNLVIYIEKENKELSLSLSIGRNIYTLTLYI
IRNIKQNRYDNFYLKIIEEGKLIYLLCKICYDKFIISYYIPEDNVVELFFVLIKIKELEKYIIECDIIKYFLS
FLLYDPQVIKKRKHLIIEIIIHMFLKNISIIKDFIKQNIIDILNNILKSIYNNIKCDHYDYYCINLLKIFYYI
IQDKNIVNLENNFFSYPKNTSYIIEHEKNNNILIFYKYINNIINKINDVIKQNMSCKDSANIHILNNNDQLNY
HFKYFLSILLCVIKSIITRIFQGKNNIQDSPSDDDKREEVYEEIIKLVNVTFEFVVNIIDKQKETSPDEEEIY
ELISYDMLICMTKINSLKYNFFEYFKGKLFDFLDCITHDEIIQNKYNDKIVIYSKNGLLDDNNINRDKINYLC
NNNKNVLSFVNKTYVKNFECLFYLCLYNEKNRIHNISINLIRNIENNIFTIYKYIHICNINTKELLNSLSLYY
LVIIFIYIKNNLYNKEKIHLLKSFIQFLIIKEIKERIFLFKNELYFLAFIKILNLSICNNLFDFKILLCEKYF
DDFIRILEESKLSMQEQILYFILEWTQIHPILKKLQIYISKNKLIFHVLFKIWKDIEYDNKLKNIKVQPEEIN
VMYRNNNQNVQFILFRIIKMLTKNFTQYIDYIINNKDLFSTYKNIIIYESKTILTIYEQIRDDMNEEHVLLHR
EEENLLLKFVNLYKEDIKKFERIFENVATFYNKKDNMELQNYYDYLRENKGIE*
>N.PF11_0452
MKKDILTKQTSDDVTNGVSKVFTEYNKNINAQEKEDNLTKKIKNMEKDKVTPNNCKDSLFNDSLFNDSLFNDS
LFNDRHCKGDKKNCRGKEDDCSNDERTENKQNIEEINNFFYIINNLIWRKNKRKVKEKEEETNEDMNKYSNEC
KENNNIYLYSILKGDNKKEKEEKSKNGKEKGKKKNFVFLALLKVPIILFSLLIYFKKNDYWSSNMYGYNNFII
KLSNFLSSYFYAYNYHDEDENTFASTSSSLSLSLYEKDNFIYIPNKKSTNTIHLKNVVGTERTDNNFLSIYRE
NMKYIRQTSLNHSIYQRSLSTECYVYFRSFLKQAISPHSLTKAIKIDKEYIYPWDVITQDDVALIIENARFYG
FLFTWFKNHRKAQKVNEEILKKEMPVLEPRFVQRSDIYTHFYKNNNNEDMLTPNFYGIHYTWLGHATGLVVID
GLKILLDPVFKIELISFKGIVRSLVNWMNVKIMGGLGERISRSPCNIANLPEDLHAVFVSHNHNDHIMEEDVR
ILCKLKKFQHVMWYIPEGSSPFFLREGCKASKIFELSWGDERWVSCWISKNQFKCRDGIWKSKNNEHQVYKYK
IIYAPTLHWSGRKDNLSDLNQSLWGSLILKGPKHRFYFSGDTAYLKKDFEEFKKIGRLHGPFHLAAISIGAYE
PNNSLKYHHIHPWESVKIWRDIRAETAIGVHWGTFRLSAEEFLQPRDDLEAALLGIGLYTLRNIDLQKVKRRH
EIMKKYNINYVCSDQNENDDLEDLKEYFYPANKQDYNDYTENFHSLFLNDLNLYYKDIDKNYIKHMYQQKKLY
ASTYSRYKRALLLKTTDKLPRSWKKLLLNLSIRFQTIPIGGSIEIISKDEKNISMTRSSEYNATTYEHYTFPK
WYNSKENEETPNQNYFSHEDLMNFQIVS*
>N.PF11_0453
MDELDSSFEGSYAHEDLKKRLLNNDLYAIKDVIVPKLRKDEYKGCSGKICVIGGSEVYSGAVYLSSISTLKIG
GDLCFVITTDENKYPLKSYSCELIVYPYLYTKKSDIKEIENSPLDKCIKYLLERIDSCVVGPGLGEIDEFTEE
CLIYILEKFLEKNIFLILDADIIQVIMTNMKIFNLIKNYKNCLLTPNINELRKMLTHLNNNIINEDVKNIDFK
HLTVYKIIQYAHALKSVLNAPKILIKGFHDVYISDHFFFVFFMKRQCLKRSGGFGDILTGIVSVFLCWASKII
KQGIELKDIISTKETFLSESFKDTVYTNTHVQNNDLLQTVAIFNASYFLKYLCKKCFRKNHRGLLASDVVNSI
PPNFYRIYDLKKKIKNNKIKNNIIKK*
>N.PF11_0454

MFNDQKVLVDIYIPRKCSATSRLIPAKEHGAVQINVGMVDANGVYNGKTETFAISGHVRQNGESDACLNRLMY
EKKLLSFQN*
>N.PF11_0560
MIANIFHKNIHINKCLNINNIFFSYFNKRGILQTKFKKKNDTSYNLPFKVSRTISGNLPVYPKIRRHGTIVTT
IVRHIYGDIKFLKII*EIFVKHQ*ENMLVI*K*KDYTH*K*
>N.PF11_0455
MSEEHSNDHIEDVVLCSQNCDETNSPKNEKDEKDFKNFSSESSDSYYKNKFRLKKNSIEIKKKSLLKSKLKND
DIQTIIKNDPILSKLNKTMTTDDEIFDLANKKENKTKFKLEKEKEKEKNEKKEKKEKKVTNDSTNNKNKNNSV
PFLNENQENKNQHNHNNKIKYELNEQTYDINNISEEILKKENHHTNIEYNEKVQDQDNTLNINQKVNKIQLNS
TKKQKQKEKTKKEIKLNEQNEIKKNNQNFNSSILNEIKKNKELSPLSLIKKHGRLINNIEEIYNSNCEQIQNV
RDEFAELKNDLNKIMNLINIGQKAVASLK*
>N.PF11_0455a
MAHGASRYKKSRAKMRWKWKKKRTRRLQKKRRKMRQRSR*
>N.PF11_0456
MELIKDDIKKKKHKKKMGRRYSYNDNIEELKKLKKILLNLDVLIDVSKIVIQKNENFDMELLNNVNDRFVEKI
YYLLKDKKKNMLPEEELVEFIFLLLKERNEYNNLEKKKKNIYINVQKNLTNCPIKNEVTTLIQKINKFYYYFK
EFLLKEKYNTKDDANKKYHHNKEDTNNYNNIPENYKNQSKHNHDYLNYHKDNIINIDINDLGYNNNDNNKESV
FYNKEIIKNNKQRNHFQGKEKKNTKDEVATTIHNILSCKDISSNQFNNYNNTLQTSDYNKDFLYKDVLMDIMS
TDSEKNMTSQKSITSEKNMTCEKNITCEKNITCEKNITCEKNITCDKNIIISKRKDNQQTFCEDKISVSSDDI
EPLISSYSEYIMRDEPTYIPDKKLLSEEENKKLEKEHCHMKNNIKHNDIAHVTNNDSINNYLYNKYYINEDNK
IMQNDSNLNHNKNEDIKKVDIENTHMINGYDPNEDILWNNNKTISSEKLCVPRTKDNEILKNKELNNYLGEAY
NDCINEETYKNMKLENCDEKKKKTNFQNVNSNFKEQHLLFCNNLQEQMKYRSDKNLKYDEKLYNNNINNNNNN
NNNNNNTNDDIKIVKPNNQHHIHNNLLHYINNKHNLLNSITLSNSLPQKNDYQINNFIHKNDTNEFKNLTINN
FQKKEKELYTLNHMNTIKSNINNIHMKDSGDTEVTHNNQSFFFNTNQIENEKKKKNNNNNNNIKTHIANFNII
HKNNLNESGKNMEHYIASQEENILFENKNNDMEELYREHSRELLEENIINKIGNNTKKKKEYDECTMSTCIDN
VVYNSHDNINGEKKDENNMEYFIKSEDESLKDFDMLLYNNRKENSEREEDKSIENIKMLGTESFYEDENNDED
IKQFDENLTYEQRKINDDNYGDMHYIDVEDDDYENVRNKNEDSSNIYDDEEIYNQKEEHDGKKIFLNRIENNA
INNLYKTYEMIQGDNDDMDDNYYLYDENEKGATKNILCEFNKKGKKGIVNKFNRDMLQKIEKNYDNNDINNDN
NNNDNNNNDNNNNDNNNNNNQKKFMNTRNDNYINNNIYLNKANPNIFNENTTNYNQKENSFNQSDFLLLKKKE
QGNSRLKKWLCKINAREKEKEKKLQQKKNESYDRELKNCTFHPTLNNNRIKREGTIKEMNDTYDDDDNKNLSE
NYDCYNKYVNDTYYGDNNKDCYNYDQEKIYDFNNNSYYKNNEQNHSFHHFNIDKKRNDNTNMKKKMNRNKILY
LKGLKSKELLLQKKIDYQNEEEKKFKKECIFHPTIKDNVKIFLSDLPNGYNKTVDRIKRGVEEKKRINNFLQY
RIPHMNNNGNIQNEKKNEGKQNNKKKTNNIPQPFSFDKGQYKVKIKPVFFERKIKISENKIACLAVREDEDPL
YIVDIFCKIHALKNENKQILYDYILDELKQESFEK*
>N.PF11_0457
MEKEKIYDRQLRLWGVKAQNRMMKSNVLVVGLSGINIELCKNLILNGINITIIDNNIVDEEDIENIFFLNEHD
MKEYMSVPIFKELKSINQLINIKSFIGHINLDTNNIIIEKELMYKDNEIIQEEKIINDCSYDICDYITNYTCV
CISCEDYPLYKLTKINEICHEKNIGFFANMCHGKYAFLFSDFGKHIIEESYYKIKNDDNKKGNDNSIEVQYCT
LSHFLKVPFSNLDKKTNDIIYYVFALILYEQDKKMNKQNKQIDEQEFLNFYNKLANKTNTQNPTELCKTYKIN
FSPSCSIMGGVTSQEIRKFISRQHESIPNFCVFDMNQNIVCTSMIK*
>N.PF11_0458
MNNSKENDSVSKLSFKNKNIKLIKVPKFVSNKWLQYNNKDIVGLLGCNNNNNNNDSEITELYVQKDDSDNVKK
LRCNKNNTVNTYILKQNVIKLKSTNKSVSNSAGTLNNKSNDYTNTNKGKNTMNNLSKDNSDKHKEFDYVVCAD
LIKTCDYTYSFLPTLDQDYSSILKERHYKTNVKKERFTIIETRNEENMDATHTLFKYYTSEDKLNNESKDKNM
KNNKRLFTDNDNLNTVSHSTSKQKAKQSKKMHVFDLDKAKISMFKIFEREGQNGVPFSFFTKSFNIPANHIKG
ILDEIAIKGKRDTDKKTVYFLKNCIG*
>N.PF11_0459
MCRFLTYHYILIFCIIIKNVFSFQPSQKVRIYHVNKIHSNNSINLNKTNISSNVKTYYSLRKNNIINFRKRKI
LAYATLKNQINNIIHGETDVTIFDKLLASISYILPTIDAIQXXXXXXXXXXXXXXXXXYLFQIERINQIYSSIP
FMSFITFMGLYFLFVKENKFKFHYFIRYHHMQSLILSMFGYALSLFYFRVFPYSYNDTDIFNLTILYSTMAIY
FGSLIIPFFSSLLGYYIEIPVLSEAIKLHIGEKKNQKEDL*
>N.PF11_0460
MKNNEEENTIRSLGCSALIELLNNNNNELYENNEDSYDENSRNKNYVKEINLFEINNSLENHSVDREQICYST
KLLNKSPYRKLSENKIDLINYNLSFNNNIKSDCFKNTYFDTVNKYNLFLSSKGKSKYKNNNNYKSYKCLMPYN

NKKEIKRNKRSVDLNFFSSEEDDINTYTLSRKKRNRNSEIVKILNAPNKNDNISDMEETKLKKKKRKLCYEKN
INETFDMGNNTYEKNNIKICNLQKKKKTSHNMLCDNIKSPDLVNNFVNKNGKDRIFIKKEENEGNEENEENGK
NEENGKNEGNEKFDKNEKNEKFDKNEKNQNIDDIEIYNNSDKCDNEKITKNKNNIKYNINIMNNISTNKYNHI
INEKNKKKCKIYDKGKLLCPDSIDENKKWHDNINLENMNSNKNIVMLEERKFTNVCNNAYTLIKNERTSEKLN
EHENYTPINCLISKRNDINMEDIKNIEIDKNVEIDKNVEIDKNVEIDKNVKIDKNVEIDKNVKIDKNVENFVF
LDFIPVTEEYLKIKETLNTYKNKKSQDNNTLCKKEDDMHIYSIPKSNLKKSAIQKEYEKDIESNTEKKKKTYH
NSEPSYIKEAYKDLKKSILRKNIKIINYDYEKNLCYILIRKEST*
>N.PF11_0461
MDEFQNSGLNKYKLVFLGEQAVGKTSIITRFMYDTFDNNYQSTIGIDFLSKTLYLDEGPVRLQLWDTAGQERF
RSLIPSYIRDSAAAIVVYDITNRQSFENTTKWIQDILNERGKDVIIALVGNKTDLGDLRKVTYEEGMQKAQEY
NTMFHETSAKAGHNIKVLFKKIASKLPNLDNTNNNEANVVDIQLTNNSNKNDKNMLSKCLC*
>N.PF11_0462
MVSTTSHFKQITCPSQVNNIKDAKKKKKKKNQNLVDIKSTNMKYTNETKRKKLQNQISNKQINEKERNDEKTK
FLNKKINEKRFTNNDTYKSILNYRNEYKPTKQKKKYCYKQSFPEEKKRNNSITNYHTKKNINHKIYEKKKINT
TTSINSHKINNTNYKIQDNKCILKKLNGEIKELNINNIHDILPNYIPYQQSIQHEYNFLNNKTACSYSYASSK
TIHERKCSKLGFIEKCTKIFKMKNDENKMHPDTNVCQKIKLPINKNLNDKQAFEGEAQLCGFEKNLTKMETIQ
GKNLFLYQSSDVKHYKSSIFPNIHSVKKNVSPIILKHSRNMILSKTKNLKFKQMSNIPIVKETRKKEKIYPRD
NNINSTTIKIPDKKNKVYDDKQEKNIFEQNMNNKQSNNFLEIYSFDEKNSTTKLEKPNHKNKCSEKDAYVKKK
MNYKVIYHSDDIVCMYKNDEDKNKKAKKECIDASSLLCKPGSVGKYLYESNSSINYSPNCDKNMNTINNNNNN
FKKLNEKSNMIENSETHDYTEKNNINIHKYDDILYYMNKAICNSPTYNIDVQYMNILNEQLYKVKN*
>N.PF11_0463
MLKSMSIKNKLQKNLLKEYKNDDEKNFVNPHEGDKASILQETRVFSSYPLNTQKCLQILTKILYLINKGDDIL
TSQECTDIFFSITKLFQSNNERLRRMIYLLIKNLPVSEKEIFIVTSSLTKDMNSANDCYRANAIRVLSKIIDF
SLATQIERYLKTAVVDRNPFVSTSALLCGLNLYNNTSSDIVKKWINEVSECINSKHPMIQFHALTLLCSIKNQ
DKLALEKIINSYTKNSHTLSSSLANCLLIKYASYLIYCTEVHSEMNNNNNILNINNNRNIFNHNIYYNKDTYS
QHYPHNNKQNINNTAISSTTNKNHHHNINNNNNNNNNNTYNNNDHSVNKNKFIHPTTKVCFDYLKNCLKNNKD
AIILFECIKCIFELAIYDQEGKNTTTVFNVDILNECIKVCQLFLLSSKIVDKFSIIRQINKLAHHRPYVVSKL
NQDIENLLYDNNKSICVLAFTTLLKTGNETSIDKLLNQINNYMTADNTTFKIKIIEEVKNLCFIYPNKCNIIL
NFLSNNLRDDESYQFKASTIDSIILIITNLPNAEETAILQLCEFIEDCEYHALLLKVIRFLLMHIPKTKNPSK
YIRYIYNRLLLENSTIRIDGMYALFYIALKCAENSKDILVLLNCLLADNDDEVRDRTNFFYYMLKEKIKELDT
SNKQISNEYEEKLQNNENINEHNNIHYSNNNLIDQLLEYDQNTNIDQLLYFISNHIEKDPKEEFSYQHVKEQI
IASNDLNNKDYVSSPFINKKNKTSYNEIPNLTNTNSISNINKYIDLTPDTLYINNMSKYIDMYNLSNINIINK
SIPLTESEAEYTVFLKKFIYNQHLVLEFTIHNTLSEQILSNLNVQMHSSENKWTILEKTTIDNLYYNTPQQLY
VILKKNIPFEKTDTHNHNNNDTNDQNNHNLQNNVDSFAVNQNFQISLHFLVKENEHDEGFPDSYSINPFSIQI
VDFINPKILRHGEFKHIWDSMHNYNAEVVSKFSLNFQNIQQAVVGLLNTLNMVACDQTDYVENNSNNHNMLLS
AQFLNDTYVLCKASLILSQQYGCLLKITCRSTNKFLSEHILKSLE*
>N.PF11_0464
MDYSNNKKNVKAMNQHQNKDSSEYNDNYYKRQYNIKNRKNEEIKVGSNLSTNNDVLQLPSVLHNHIRIVRTND
SCFKDYIVLSNLGKGTYAQVWKVKHKITNEIYAAKLLQPNQFPKESFNRIVEMFTKEIINLSICQCPGVIKLH
KVIGGKEGWILIQDYANDGTLWKENLSSNMNEAFLYFIQLLQGMWYIQEMNIVHRDLKPTNILRYDNKRIVIA
DFGWSEHIDSCNLHPTEWPGTLEINPPEVLRNTGPMTEKIDNYALGMNMILFISGRFVCRQKGIECSKVAQTI
LKTVHNLRHSKPPSRFRENLKAWDLFVKLTSSNPIERLSLQNVLDHPWVTEMLNNFSLQNSKFLWHEKVKSRW
IYLLSNNWDFFKNISSISTSTTTNNNNNNNNNNNNNNINNNNNNEGSRSIINTTHNHNNNHNNNNNNNNNNG
GNTKIIGKYNDMENQHNLSSTSTKNNKNTLIIDNEKNNKEKTNKYSRSCLKNDYELLFYMMKNNFKGKINCCS
VKKNEISKIENVQEIKNGEGKDQNELMQNNQDLDNIEHMEHSGNIDKLEIMKKSENIRMYDQTNVEQHKYNND
VDEDKYKNDVDQDKYKNDVDQDKYNNNMDNNMDIHQNNRSTKFDDMQINKNNNMGDQYLPYNNDKEIQHISTK
NKEYNSKNYIANIQKKKNITIPKNHMMNIPSATSHEINKEYKMNRQIGVVPKGYYDHNNHNINNNNNDIHNND
NIPYNNRRHAHKDHIIVNSNNCTRKCKNNRAEEQKEIFIISNSFEENAKGTYDKNKNNKKKKNSKKCDPVHKK
DMNMQFNHQEDINKYCNKYKTMNEKIFYNKNELLIHNNNNNNNNNNIYKGNENVDVYNQNADSADSIRRTKGH
TFNKQANRRTYSHIYDDNIYDDNRYFDEGKANLDTYKNDKNGMSNIKNVERNMNVNNEEDVDNDGNVYKDNKE
EEEEDEEEEEEEDEEDEEDNDDDDHHHEHEHEHEHELHNTNENIDEEEDAEEDDEDDEEDEEEDEEEDEDEDNDD
NDNGDNSDDDDDDNNDNNDDNNSGKKNSDRNIYNYHNYITYLKKNKHMNDKTNGDMKNVQDKCSNIRDTEDFI
HYNQKKSSSLFLYDIDNMKKILKNYKKQMHDTKEVLAKTTFKMSDLMSKKYKEQKCIKKKDTFTKDIVINPML
NNNRNDLNNLEKVENKEEKKINDSTYVKEEGKKCRDIKKIEHIYNYDCDKMNNESHNEYVTHYKKDRINVLSG

KNINVEDINFRWHQDKTTILSQEKWNNISRESKHFKKIDTVHNKNNVDAPIKYMNLYNNNNNKNNDMGKDLFI
KQNDKNSNEYINKYEEEYNIFLERLNYKKKKNKRDNTMNSTKCRNNINDKDMNMKKDEKKNMLPRLLLKINKI
LDDNNLDHKKEKNERNMKNKYYDDINNTKECLNKNITHDIVESEKASNYDKYNEYNKYNKYHIYNKYNDSCIN
SSYGSTYKNEDHKIELRHNPDLTKETDSLTSDKKFNFKNKTTYDCLKHNNLNISTGKRRDLYKNDLINILNEK
EEQVGDVLKGEDSYKQIDKKVDVEIYRKEKKNSLDISNNIIINNFMNNTYHHKNNSIEKRTDTPMKYYMDTLN
YFDNKNMLDMHKDKEKVLMMSNDKKKSGLNNYDMTNNDDKEDTCTKMFVNKKERVKKCSYQRDVDVDVDNDVD
DDVDNDVDDDVDNDVDDDVDNDDDVDDDVYDDDDNDVEMIKKKINNRAIYREDKNVNYDKRNVIESVNTGYDN
IFIGKKKNDDATQKNIPSYNIHTYNNNNESIKYDIKEDKKKKNVHNFNDNDMSNNLYRHGKNSFSCKNIEEAK
IYDSTKYGPNESLCSFKKEQEDKILEELINGESIDTTQTETFDDKNYNNEKKPCAYFSVHNNIKIYNKSSSIT
LDDSIKESSVKNSSCSTFDYSDLEFQYFSNKENIVDSYNTEYKYGPRELGDISRYENKSTGTKKKKNHTKQNE
VHHNNIVGKKKDVDHINNFTLDNNDIFLKMNDIQNITKV*
>N.PF11_0464a
MPCEKCEQKLKKLPTPDVNNSINRSFGSNKLLMYNNKQKFNNRKCKQCNSQLHVDGKYCSMCAYKLGKCHICG
KTITDNSAHNMSII*
>N.PF11_0465
METSMYNNLRKLINVIDELRDIGLQKYINLPRICVVGTQSSGKSSVLESIVGMDFLPRGEGIVTRRPIEFRLI
HIKEDSEIKYWAVFENEKNKKYTDFNEVREQINRLTDEIAGKNKGIIDEPIVLNIYSIKCPDLSLIDLPGITR
VPLKNSDQTDDIERLTRDMALRYVKDPRTIILAVLPANADMSTSDALQIARKVDPKGLRTIGVITKIDLMDKG
ADASKMLLNDEINLRLGYTGVVNRSTADIKKGKTISQALKDELEFFQKHPVYKKLPPALYGTNSLTDKLTKVL
LRHIKNFLPDIKIEINDKIRYINDKLYELGTNVPLDATKKTQLLWSMITDYCEIFKNTLKGKYDKRVQVFIEN
NDILCGLKVRTIFNEFLDEYVGKNVTSELTDNDIDDAICLHEGDSLPGFPSPDTFEFLILPHLKKINAPVFNC
LDRVSQTLEILSQKIANRVLNRFPKLSEQVLELSQTILIREKENTQIILENYIDAETNYLFTNDASYLIEHGS
IINAADDEHNENHNNTTNINNNNNNNNNSSSYDYGIQHRNQNKRQQSNISNSNQDTLLSTSSKYYSNMMSDQE
YNITGKAAKFVTSAQKSVMNIWNTKEKKKTRYNAQFIQEIRRRLDCYFNIVLRNVRDSVPKMIGYFLIRKLQE
KMQFELYSDLNSEQKLYDLLNEPPNVVKEREHLNNQLEILKKANQVLLKDPNITSINLDLFDANYEQDLMDYQ
KSLKNTNQFNQSSKHNPNMSQGSSANMSMYLNDGSSSMSKRNPMMHNRNMSPSSMNTNMMKQTNMLGQKMGNS
PSSYMQQGHMNDSKGKHLFEKEPMKKKVNYNPLFD*
>N.PF11_0466
MRNYGLLHFLLVLLLLNLSIKSKILNRNDHIKYNSSVMYIRRKKKTIENGTFLLNKKIRNLRNYANVYKIYNK
TQKRRKFKNLISPLKRFNNVLSFLGCHVCNKIFEVNKKNKKGYDIFYKKSRLNVVGIWKKGKRNPYNRWLYLY
RRNIGVLSNIKKKYTYYLDRIKNYIEEDNMLNFFYICKNILWKKSIFFLTIFVMINSAIISIIIPRYDNIIFS
ELSNRKFDRFGYLLCNCILIRVLNIFFCGLRNYIFMITSSYCLKSVKSLLFRIYLNKDYEYYDKVDHTIIINK
LTLEAHNFSDIIPYYINPLIRNFFSIILNFSYIFYLNKKLSLVILYCFMISSLLSMISSKLKKSRLKKINREK
IQNTKISLEALNNMNIIKLYSTELHECNKFFSSLNNILNLEKKKEQFNLLHMIINKFFVMMTYILILLKGNVL
LKNKEIDKHIFTSFFFYINNIYSYIDILDYYIDICDIVSQYNDLIKMLKNYHISGRFTSINEYVYDNMNIEQN
EKIYKEINMEHNINDVMNNGTNKLHKNIVKSEERISNFSNGLTNDRIDSIGNIQPCNKENNNLLLNYNIIHNN
MKNILHFNSNINKRNDNLILHFDNVYFKYDSNPHNYVLKNINMKIYKNTNNVIIGKSGGGKSTILKLILNMYK
CTRGKIYLYNKLINNYTRHDIFNKITYVEQDSKLLNASIKDNLTYGIINNDFDMLDLVNISKCSTSHDFICRL
RKRYETLISHKTELLTSSQKQKICIARALTRYPKILLLDESTSAIDKDNERIIFENIRKNSIFKDLSIIRITH
KKANLDIADNVFLLKDGYLTRQKKFRITNK*
>N.PF11_0467
MYVKHFSKFPNRTFFYKQKKRKYIFFTSNKYFVHNHTKINSCLYHKNIHKNYYPFYKIMSPIIYKKKITHVNN
IQSIDTKGKKKILEGYKEIDTKGKKINKINIMDNILSMKNMNRINNTNTIIYIINKRLFNNNIKYIYSNKKKK
KNDHLNYFNNNMRQIRKILFLQKYSNILQTKKYIIKSIMKHNKIKYHHHIYVLHKLSNHNYTTNLFLSLNDGH
YIKKKKEQMKIQQRDQNKIEIKKNINPPYGQYNNSHDNIYNNNNNKYDHHQNYHKPSEKMIEEKIHDKNEMNI
KRNDLLLVALSGCIPFICFGFVDNSFMIISGDLFDSSFCTYLGFSTMAAAGLGNLTSDVLGIFIGGYIEKIIV
YIGFPKINLTNKQLKMNRTRRYYYSGSAIGIAIGCLLGMIPLLFIDNNKLEERKNQEKKKKKKKKNFYYKSND
YYIKIATYISKQLPTYIHSKYAFLFIPDHNNMNFNSIYNHNIFQTSLNVGLISHVYQNKKTINYQKNDKNINT
NVRNTFKYKHNNNNNNDLPDHININTYDNTYVEKIVNDTHIIIDKNKIDIQQILAVPVLSIDGNVEAVIAVIN
SEDNLSFNHNDVVFLNMLSSHLSYDLENEEDLSDMLKLCKNIVYN*
>N.PF11_0468
MRNLKKKKIKKLCTHGNFKNYYYERYIKRKKQNVIYVKNNEEDAIEEVLICNNFLDNKLCDEEQKSDNDKYDY
QNIYYIFDHRLNHINKVHKDIFKEKVILDIGCNSGIVCFMLSLIFECNVVNGIDIDNNIINNNINLLRLFIEF
IFIYNSQSHMLELFLSNKDMSKIEMSMFKNIYSLYEKLKGSKNYDNKMNNIYNKNGLAHINALNSNNNNNNNN

NNNNNNNNNNNNNNNNNFRYFPLNIYFLCSDIFNNKFKNVNNTYDIILAFSVIKWIHLNNGDEHLILFFDRVY
FMLRKNGYFILEYNKEKKYKLRKIQKKYYKKNISLNYTHFDDIAQGKYNNNSRMVLVNKFFFFTNKGDERSKN
KNKLGMFNREICIYQKV*
>N.PF11_0469
MIKNELDYLKYIICDIFGSCCSDIVEIILLYGNKLSIYELINLSSYEFNVVRNVLLCLLIHNIVDVNIIYSKS
IECNHLSPEETRGVINVYDNNKMNQHSNNEMNQNSNNKMNQHCNNKMNQHCNNKMNQHSDNKNKYLFKVGIKK
KNIISHHKHNEELKTIPDIKNEEMQSFNFLCNSKNYEYMKRCKCDSCICKIRRVEYFVIIKNIYVLVRYPSIL
YYIHPTSIIKDKKSITSLNNFSTTSSDLQLDINTYNFFNTEMIDEFEDLTNLCDDKNGDIIKNNNNNNNNHNH
NNHNNNNHINNHNDDYLHNRNQIQDDTFFRNSNGLVEKNETLNFIKKIILIRIIKNGRMTINKCIENLNNDDY
AEVCKKDVPEVNKNYLRFLFLQLLKKRFIKKCKYFNEHECENDKRQKYDVMKNEVRKNDNYKKMYTNNMKHNS
NDIDLVQNDPIDDTSLLLYNHTNENNNNNDKNNINLNNIGCNDNVTNPEKSIHYSPYNDIYPNDYAHTTKKIY
NKNIINNKKKKDTRKDEICDNTDISKNNLKKRKIDDDIIIKEQENYMPNNIDEQQKKREVIKQLHNEDIHIK
TNKSIKNYNEYNINGIYKNNHNDNLLKLIDNKYTYFQVDNDTLSIIYLKQECFNFITHFFNLNEVSRCIFYVL
LNNVKFYYSQEENDYKETCLFLSFEYIEKCVISKLKRNKNIFVDKHVVLQNLNALIKLPDQLIQIKCNQVITY
AADFVNIKNIYKKKIISNVLENMTGLQALRIWKYLLTNSEEKINDELISENVLIPLNDVRKKLYHLLYHGYIK
AHEYNNSNNNKTYIKHSLSFSTDFYFTSNKVKENLFTIAKNIYIRKFQENNEINTLHNKSNICINETQDHNKK
KKITLTSKEYAVDYLEMALINMDKLIFIFSS*
>N.PF11_0470
MNPLLEYKRIKQALSNSSNINSFQHKKDVIINKDRNNIVRHKKINEYKYNKSLKHEKNKSKRDNIEYVKYNNN
DFVINKGDTRKNDEEEEYTLLIPEDDTINPCECPDNYCPYEDNNNWKSRTIKSNIHSNLTEISNIIENIQTNK
RKNENSTITNMKKNPIKNNQTIMKYKDKKKEINKTLSAYEKFYLEKMKVVKHKLNEVKNKSDENWDVNKELNK
YLNEVANVKYGEICHMFEKRLDALRDIEKKHLNLLVNLFDEHKEMEDVIFHMINEK*
>N.PF11_0471
MEEIEENANSNKVDKEILIQFVNHENVTTGPVINVPLSITKDNLDELINNLKRKNEDLGEQEESEDINYYSFM
INDKLPIKNSLYEAIKDNHISSEDILSIKYFPLNIFKVKKISTCTSTLPGHTSSILCLAFSPNSSHLATGSGD
NTVRLWDIYTQTPIATLNDHKSWVLVVLFSPDNKFLATAGMDSNVCIYETHTGKLLNTLTGHKKEVTTLCFEP
LHLLKEEDIENNVDHVNKRKINSNEEIDQSDNANKKIKKNDENDVIINGKDNYPSGQHKDKMMPTEGETTNSK
NNSKNNSKRNISPNNTQNNTTSTNCSYFVRSRLASAGKDGSIRINNILSNSVDQILTGHSDTITCILWSGRDT
ENSTLYSSSRDTTIKIWNVNEGTLIYNFKGHKHWVNCLTLNSERLLKNGIYNLDVIINKINIVNYIEKSKNIM
KNFFKNQHHEKIVSGSDDGTLFLIECLQNKEYKNTRLLGHQKPVIHAQFSPNGKMIVSSSFDKSIRVWSAADG
KFLAVYRGHVGPVYKVAWSIDNNFFVSCSQDSTLKLWKVSHLFQQNEITQSNIEKKQEKEQIKNQADNQKDNQ
KDNQANNQKDNQQKNQNNKKIKTLLVDLPGHADAVYAVDWSNDGKFVASGGKDKVLKLWSH*
>N.PF11_0472
MKCLIFCFSIFLLLTLSLCSTHKEQKYCNSTHRGILKYHKKKDNNEYVTVSAILKNNALELFKGSKFFEKFSL
YDIITPIVILSTDVECLTLNFVNDDSVILCCDTEESINNWWLYLTKQILCLHKGELRNENDNKTLEEEQNNII
NNNNLNEVSINITEDDLDNIPNVLIKTES*
>N.PF11_0473
MSDDIMNGDTLSEYRKIHKKNYEERIIKENELIEKQKTEELLNEKKKNEEIYRQLQIDKDREYSKQCYYEINN
FQNIHERTYNDSDIRENYTQRLINPSAYSYIDNNMTQNLLDGDMGYTFSQRIRRFFLNNNIRQCFVCTTVQLI
CNVVLIILLIAFIIIFGLS*
>N.PF11_0474
MENNTKTKLNQENVIQNFLTNLFSFIYEKLFHIIKVMSIYFNKWIDKWYIIRLNPNDFFYFIIIHVIIILSII
FLLYLHTYKKNKQNIYSVNQKANRKKTSQKKKTEDNKGEDKQIDDKYHKGRRKKQIDQQNDATYNNFRNEENQ
MININESTNRYVEPYIDLTNDHINLQENEHLIINEKRKSKNEKQKEKEKEKEKEKEKEKEKEKEKEKEKEKEK
ESTKRKKNSTNVITEHTNEYIRGDRLRKI*
>N.PF11_0476
MANNNMDDINLECENDNDNKISSEKRLVDDIIENNIEEIKKKEKDNKEKDNKEKDNKEKDNKEKDIMFNIKNG
LCDLEKTLDGEGYAYSNLTCRKKGIDFIPKSITRYIHLKYINLSHNNINDLVHLYFLPNIIFLDVSYNMLKEI
VELKKEYLKNCIYINLSHNLISHMNNIFLKNLLEFNISYNTINNINIYISNTIRILNLSNNNIKNINFKNKLN
NLLDLDISFNPIENLDFHTLMPNLVVLRINDNSTISMDKLNNLNNFKCLQSLFMQNYLYFKNISYKDVKEILL
QNSKDIHLLKFNGNRLNKKTDHIEQADVNK*
>N.PF11_0477
MPSILESNNSLNDEENNKTLRSGDINMIEPNNNIMNDNIYRLDEIHCSNNINNEKEVGNIYCDVEKKGDIKYI
NRISNKNENIEEKSNVLKDTDIDVPYYYNNNSNNGTHHCDNENYEEDNDYINNNETRKNGNDSDIDYGSNFSY

TNPDKNCNRSNSFTSNIDKESNDNNVNKKSQHVICLNVQNKDHNNNNNLKEKEYPFFGTIEKNDENVSNKKKI
SAHENISIYENGSGCMNILIGKNEHSKENKENEKNETNEKNATNETNEKNEKNETNQTNQTNQTNQTNQTNET
NQTNDIVNFDSNKNKPLDEYNHSNIGDCTSVFKNEINGNYNLEQNMDSINNVNGVAILDAEDMNISKDYDNMN
ILQNYNMMNNDISFEKNLGEKMNDKDDKNSYLEDINNKDNVNNNDNIINNDNINNNDNINNNDNINNNDNINN
NDNINNNINNNNNNNNNNNDDEIYKPGDDENSNKMYETQFNDSKIKSKISYNDNVDKLDSYNLKDFFIDNNNYN
MKEEKYSKESINNINSDEKMNERNFINDKTNKINNSNCNNNKDSDSNYINLINNGNILIDQENYPNDNTKIDE
LLKNIANGNDAYVKAHEYKRIDLLNTNYKRSIMNNINNNINNNNNNNDNNLNDPFFNYQHMNYANNNNDPSYI
YLNKDNMIFDNINKYNVSSNPDYLIKDNEMINFNQMSYINKNIKEENMNHFNEGDFYFDKGKMGSTMGNIENM
TNLEFMEYNNIMSSNNKNNNTNKKRRSKKKDSVVKEDIKTENKLYEMKDGNTNRKRRNSYMGDEKRKERKSII
YDNKMVEKNNNDNDNNNNNNINMDNNFIHTNYEYIKNNNNNNNMYIPTNLNNMNYKNYTNYTNDIDNISYMNE
SLSNNDQHYNMHNLINTNGDNFYAMNNHANNIIMNNMNIGFLKNNINDDKAMEQSEAYLNNISQAYDSHNNNN
DNNNNNNNNNNNNNNNNNNNNNNNNNNSDNNNNSSSNNNTTNDSTTTNNNTNSSTYNGTQNNTNLSNNNNSNNS
NNSFNMSSFFSKGANSNDSQRNYNFSEMNNINESSDYANLSKNTEYDVYDEYVNLGDQKNDDMSDDSNSCDDK
GNDKNGDSIDSTDKKKGSKCDSETLLPIANISRIMKRILPGSAKVAKESKDIIRECVTEFIQFLTSEASDRCT
REKRKTINGEDILYSMEKLGFNDYIEPLTEYLNKWKQLKELNNSNNYHEKKFDNIKKTEESGDLNNNNNSVEN
KNLDENAYAEENYPIMNNVYNDQNEIFESNIKNIYTNSNDCDYGNGV*
>N.PF11_0478
MNEHIKVFLRIKPNVENFTNLINEDCTIYKVQNNQLHLYEKKKSYNDMNELKTKTFNFNHIFDTHVKQNEIFD
ILGKNLVNNFMNGYNSSILAYGNTNSGKTYTLYGDRKIGEENQNYGLIYYSLKYLFDLQNISKDTDISISVVE
IYLEKIRDLGKLFQLYHISTTTNDTITYYSQFHDNIIREDEKGNTYVENITLLPIKNIEDVKEIIDLCFKYRK
TYSTKKNRVSSRSHCLLTVYQHRNIKEKELISQINFIDLAGSEKFNDIEMINKKELININNTLSILNRVIMAL
STKNKITKNMENKISGNEKVKMNMSNIPNIHIPYRDSKLTRLLKNSLNGNSFNYILICLNLNTYKIDDFINSL
IFAKRCKMIKQKIKKNKTKKYSSNSENEMSNNTSEQSSYESYDSTCDLYNSINKKNENHHDKNPFHNNLYYYK
GKKIKMKNINNHTDIMINKLFSCFTFLIRKKYNDLIKQKNSIFNNLIKLIKTEEQNKDQLKNHKNHLLNILKR
KKKQLKYNKEYLNNIIHSKKKTLKYEENKDTNKIVSHLSNSSMEQNKNLNEKNHSNFSTFSYDNTKQPHNKMN
TQMNNLTSDIHEKKYNDNTYNDNIYNDNTYNDNIYNDNIYNDNIYNDKMKQSQNIRPTFNSNITSFDKNHIKN
KNHNQKDILYEQENISNSSIYMNSSKKEDHINRIYSDIHILYQLELKELNKKKIYIQEINSINNNLYNLMNKF
ANMLRNIYLNKYSVVIKNGNKKLNLCNYYYTNINETYNINHFNKKTENLLNTNHNFKNINEDIFSIYQKINQ*
>N.PF11_0479
MSDRKEDKNDIILNKNEEEDNINNNNIILYKSFDDFKINYSYKTKNHLHENDKIKEEDDHEIKRKLIKLINTN
FYIDKCIHFKKFSKDELYKTFIYSNFLTKALILYPSLMPYVECIIEKIKKIKNENITFFPAIEQFNFSIEHAV
SSYQTGTQTFNNHPNFYTNYYQSFIKNDNIPYINQTNIFDNNIKNKYMLDDKFGSTSLYNNNNNNNNNNENNN
DKYLNTYYASPRGNQIYNLFQDINNNHHNNNINSYSNNKYFNYNMLNKNNLEETRSYPNNDYRNIINPNNLNS
NNVHMNNIYPELNNTYNNMNNTYHLNNVHSTSLQNNYNPNNNFDHSMKNIKNETNNNNNNNNNNNNNNNNNNN
NNNNNNIYSPELDTHLRVYKNINEYDIIELKICTTLNNKYICRYIGIHSGMTLYNIHRCICICLGVKKEDTDN
YLHVFILNNGNIYGSGKKCSVSIIRKIQINTDRHITFKHIIKTPLYLYKSKEDKNKNNSNNNNNNNSNNNNNS
NNNNNSNNNNNSNSNSNSNNNTNSNSNSNSNSNNNTNSNNNSNSNSNNNNSNNNTTNNSSSSNNSNNNNYYH
NNYKNEKELNNSSSLEHSSIIMNNDNIHNNINNNHINNITDLNNMNVNQSNMKENNNIIDYMNNNNNNDNYSN
NHLNNCINKLYTNNIYFTEDSQKRNPLQTYNTSKNTNNFLNVNNFTSSYNFPNINNMDSNIYNHTTCNNFNKN
INNNINDISINKHNNIFNNMNHLNHLDNHSYIQNNLYKNHMNVNTNILYNNPIMNNINNDQINNLSIPNNKNE
DNNEINHDDSNDDDSNSSHITLNKSDKNKNYFALNPKYQNHQNHNINNNIQNNLNEQIKEKNDQQNHNIKEIK
NKELLNDTISSIEDTNDNSYSKYITSSDISQNNTLNSFQHNKEISVNFMYNNIILDNNNNINDDNNNNNNYFC
IPCGYNTKEYKYNIYNTYNYPNNANHIYNNMNISYNNSAYNNNYVTYNNFHNSYHNNYILHNNFHNPYNIYDN
IQNTEQKKLYNIYQNDERQNNSFNHINTDPHKVVNSNNFLPINTFHYNNNLNHNILTESNNLNRKNENDNIPS
SYSQIHNHQICKKVEEYTYNSINQNTNNFNNNVMMLMNTSNNIPLDNNTYNSNKNKIIYKHIINDHINQKDNN
VEYENLNNSCDNTQNKETFCNQDLINSSNINNNISSYTFQNNNDFYTKKKSMQYNHDNIYKINTTSENVGSPH
TNNKTSIYNHKKGGYEQHTEQNNEQNNEQNSEQNIEQNIEQNIEQNIEQNVAQNVAQNVEQNVEQNVAQNVEQ
NVEQNVEQKAEQNSNNESIKTNTVETFKRNKNQITNSNNVISKQQHDTNNILNNININIKENINRHKINEFQW
EKSNKIDIEKNNCLTTKYDKDNDNENDNENDNTYNKNNDIVICNNHNNSSHVQKNYYNMNESMINENNIIITE
GENLMNSTEEYFTNELIKKDSLEKNKSDTKFLIKLNNEIKKEEEKKDNINIFINNNIYELKEINGNKNRSDYF
HNTKDDKENITNVSSNNHLSVPLNKYNDEDKQLIKQMNHASNMNFIYDYNYHNNYSSTNSQQLIKNNTENLHS
FKNETHSTYVKYIKSEINNMNNSIGVPTKKNDYMYTNYLNMEHIKMNNMEKEIIKKGNDNEIKGQRIQVEHDR
DVHYNTTQENNIINNQNPQTNHDGMNININSNKFMTPTTLKEKYQNNINTNEQHNKNEENKNKNVINNTSQM
INDNNVIQNDINNMNNNENENENENLYINVHTQYKSDNILSCEKNFITLKNNNHNNHNNNNNNYYYYINNDNI

HLNNSHIDIMKTNNINKDMTTNSTPHFKHNIISNDCSPNNINQNIFVDPNKYIYNNIHTNYNAYHEESLQVVG
NHNSSSLLRNINESFSNQYDNKKNLEAHHIDDDKNKEAFHNDDDKNKEAFHNVDDKNKETFHNDDDKNKEALH
NDDDKNKEALHNDDDKNVEAYHNDNYNDNYNNNYYFDGNNNMQDESFYSNNSHAEYNQSNIEYISNYDKNYSH
IQQYTNGFCYTNNNQYINNTELTNNSSYIYNNSYMNNNTYSFNKEYSDNNMCHHKNDNIHMINDVATKLNQHP
MNMYNSNNNNIIYNNNNNQIYDNNINNMYNDYYNYNNNNMYNNYYNYNNNNNMYNNYYNYSNKNFYMNEKYTE
GATNFMNIDDMKDAGNENNMHILNNNSINQTYYHSKIKNNNNNDDDDNNNNNNNNNNNNDNNDNNNIMMHNNY
QPFLYENQYNKHIHMMNQQIQKETNTSFKHITCNQKFIENNKINISNDQNVTNMPILYSMNKEQYINISNHNN
GCNYDNINSINVYENNNEYIAPKNMLYKSEEKENLYNSSSIYNQNYEQKYINYMNNASYIMNNNMNDYTNNYN
VQNFRTFKNNVFQQPLSYSNGSEAMLHASEFNQGINKENFQGEYVSNLVNSYKDNVNNVEGVLGIKKDKENDN
NEEENDEEENDEEENDEEENDEEENNEEENKEAQNNEEENNNGDNNNGDNNNNGDNNNNGDNNNNGDNNNGDN
NNNNIFYNMEGSQKICHDDITLNECLNSIDINEGEKKTFEENKSSFSMLYLFGKVKFYISIIDIIHNKTNSHD
LLWVPRCCNGSYGTFLKYNYSNMNEINKYTHDEGIDIDSINLKLMETRFSKNVASSRTTKRKRMIDIDKTVLH
YYKEHISEFFNDKNKIIKLTKKLCKYKKKRKFNDTQKKGTYKDEKDYDNYDVLPNGDEQNHENKKQEDNNNNN
DDNNNKNKNNDDDNNNNNNNNNNNNNDNNNKNNHNNDNNNNDNNNKNNHNNNNNNDNHKNNSNNKAKGKNMGKGK
KQTPNRMNNTKNVQNGKNTKNINNIKNISNMSNIESINSMTSIKSVDGENRMNNTINMNGMNKTESINNIEIT
QNMNNINSINNINGINNINGINNVNGINHTNGINHTNGINNINTMNNMNNMNNINHINNINNMNNMNNMNRIN
SLNNKNNINPINQYNDEKQNLLNSHLQFNQVNYHNNLVNGLHKNNFLSNNNYINTTDINGNNMISHNDHMNNK
LYSNINNNYYYNRANNEIPNNNSNNHNNNFNIYESKYQTMIHNNNIGQDLKQQINNYNENTSSNNNLSISQLL
EGNTNFINISNTFINTNYSNDFHQTNDLLVNHNNIDLKYLSDNINTNTYNEQ*
>N.PF11_0479a
MTRNVPLTNYEFTMDEPVKDTVIRDAKSIYKKILYVCFHQYDDENTIKKWDLWGSFIVYITLSICIFLDNEIV
DKKNTFGYFFVFFFIGHILVSLNLSLLHINIPFFQSLCIISYSLFPLILSSFLNLFISTHVLRLLFCLLSIVW
SSYNCILILARFIKSNRLLISFFPICLLQLFLASFLLIK*
>N.PF11_0480
MRKGKPRHVPMFLRSENKRIFFSFFSYFMPKNDLNFIFEHFDIGLIVLTKIHNIRKKYEEERNNMKNEEDKSD
GDDMKIFDEKLKNFFLNYKINPNIEESNDTKISKKEKNNNNKKKKRNYPLNNECKQNNVNKKVMNKSNSCSLN
KKNEKKQDIIDDKKKNKLHDMNNERDCNINKEINNDIQKMYMYNNEQGKTHNLDIQSYMPYDQQKGKENQEPS
LYKCDEILKMKSTTEDRNKKEDVSKGKKKKINNTPQSYNTPQSYNTPQSYNTPQSYNTTQNYNTTQNCNTTQN
YNTTQHNNNINENNDDDKIIIDEENCLNTIKHNEREMNNNIQKTKSCEDNQYLINYNGNNEMSYNHKHLTLLD
NKNEEQENAFILNNMNKTKNGINIKENIEHIKDNKKDEIKIERETDDIYISMKKKNETYKKFSKRMNRRKISE
LVNYHDDYCFYITDLCKKGTMLSLKKNELFNQKNDKINILSSVKYFNKPIQFNQINWNLNNLCTINNIYTKLY
DEHINDKINTTPNMQTIIKEKYSQTSSVILGNEQIQNGYQTSNLNINSNDIRIINHNKNDDISINHNNNNDIS
INHNNNNDISIINHNNNNDISINHNNNNNNNYYFMNNYMHNNINNNYYCYYMNNTNHVNNYYNNIYIQNNHDQN
NAPILQPINNHLAHINDLCYIHSEKNEYTKISKNHQMNNINPQQSNGKNQNDISNNINKNDEYYNLNEQKILC
DKNKSYIKCDIPQKCDNTQDDENSEQNQNYITNPSNGHYKIKEQMNYVQHIPDYEHDNTTNEMINTQNYTNIN
LDPYIMNNQNDNNVYLNQNYFDTEKKNKEEDINITEANTHYNNVHIYQNNSNHMNKMINLNNNTNSKSTQDYD
LYHNNMENFNNTNYNIMKEKIHINDDTSSVINNSSINSQNNLCNNKKTNNDYQNRNIKNDNSIPDSSINMNEL
KNNIQMNDYYASNIYNNNNNNNNNNNNNNFIISNNIISNYNNYMNESNVYPQINSNNYIPYIDPHLNNEPYKHT
INNQLNKNCINNNISMDDNVYDSNTISIHISVNDNEYNNSNFTNDMYNNNNNNNNNNNCNNSSNSNSNSNQVC
LYMPNHELYNSNYNITQHMPSSNDVPKIQKHIANNIIMNGHKEEHIIEKKSKEETNKTNEQVYRSINQNNTLI
LKENEIDENDINTLNQNLNIKNDMNNMDNIINLKNLNNINNIYTPYQNNILKNNEIQFLNNNKEVITKHAYTH
SSNEININVYKNIDTQINIRKNENNNDIIINKEQKDISTNNEQNQTYNYITSSVKNEYSILYNSPTDKNYKKN
LNLNISSSYNTNDHINNYHDDIDKTNKDNKYEEEKHKDINIYINNINSNGNKNHNFINSYFDLNENEKKKKIN
IFYHDNKSLKNISNEELTNTNPYKCINNTIDNNNDHKNGYIELNEINNSIMNNDEKIITAYLNNRVVQNGLSH
DILNEDVSINTHSNKENKNDNHNNNSNNSNNSSSSNNHNNNNNSNNNYANSYNVKNDLQINNNNYSYIKNEKN
VHQTFNENNILYKHYSNHVHNYITKNTIQEQKNIHSDNKYETCSLPEKKNIYEPNSQNSPNSQKLPNSQNLSN
SQNLSNSQNLPNSQNLPNSPNLPNSPNLPNCFTYVNNQTDILISNLVVQEQKLNDQKKEMRSNNNINNTKWNN
AYNNEVQHVNNNIVNEEINILPYPSKNENNLNEHNSNIYHDKNNQHDIRTYYNDSTKITNKLHIESGNNKLTN
DAIHTNNEYTINSRYPICQNYSAITAPEKMNCPQMMANHNFNNIPEYVIQNNHVNVDGNNKNDPIDNNNNNDN
DNIEIFNIQQTKQIKYNNNTTDTNNNSLIHNVENNYNTDGLDISNISYNNYTNNNMYNLNNDNINNNYMPQNY
YHINYNNIATSENNQLIQINNYNINTNINDININTHVNSYKENDKKNILYNNNINNMNNDNINHDHYKYICNS
NSNNNNNNNNNNNNNNNDHYYYYNCCRNEEYKYNDNNYINIQNKKNNIVNDNTSNIIKSQYNNTNKNMILEQHN
INMNEDNYNQINSNLCLLKNDSISTCCDNINNNNNNNNNKYSNNEINSNEYLYYKFDEQYDKKNNVSAMCDNI
KNKQHYNNYVAYNTNGSYENMSNFSSNNVYVNNKCPSYNNNNMNNMNNMNNNNCYNYHGYNVMNTKYNKAGNK

FSSVQIDTQMDNPIHVDKNKNDDILERNIKGSIINNNNDSNDDNNNDDNNNDNNNNDNASNNNNDTNNINYLM
NIKNTNNFDSTNNFDSTNNFDSTNNFNNRNNFNNRNNFEHINNINKIHHIDNENNLCREDENNHNTILTNVKN
TTNKEYINICTDHLNLLKNYNLNNYINLLKINKLNDYYYNSIINKSQDGTCNTFLQNSLNNQSNIIQNNNIYE
NNQRIQLYNYPSFETYNNNIINTWNQKNTNNSYNSYIPLSNVQCNWKNHIFNVTQNNAHINDNKEYNYQKYPY
VYNNQILSINHGKKETNTLNYLNCTGENEITYLNDLNSNEQNYNDHNKNNNPYNHDHNIIYNDVYNHMNPSYI
YNNICTPTNFIQDLIKTNKINNNSNYLNIFNNMIHLQNMHNENNHLFKKEKCDQAKPYSNTDVLCTLKNNNLN
ENKNVNIINTIPHTLNNYYNFHILLNILRRKQLYHNSFNQLISKMTKNYNINANNNNNNDNSNLNNNNNNVRY
NKRNYSISSVGFSNRSKRWTICPSYICCNSALCRFKLTCNFKFLQFNQAYNTFNIPYAIYNCYKNIMNNYHYF
TSTLCPQQTYLSKHSAQEHETIFNVYWHLLNNEKYKYIQNIILFLDSNLYTRAVWLLKKGYNMNDIEVQKAEK
KLIKLMLLVFKFTYDYKNDNNKYEYIKSFLYSLRNNHINFEIMNRFLFY*
>N.PF11_0480a
MKTKIINNLKTLVNNDIRNNLKNTDDKKELSIKLSNIIKNHIKTLTSNEYKIIVEIFLNDNKDQGVNISTRLF
YNKHTDFFFKETLINDTSYCFIVVYLIHI*
>N.PF11_0481
MNPYKNSNESKILYPPIYTLNAKKSYEESNSFNVLNASMKKYLDKNKKDTINEGEQNGDIFHFNVDNKFLWNL
DKPININMNSEKYYVENNLIRCNNKNDINLINNRNDELNLNYVPCNSFINTPNNCLNKYTNNNVNEKKDILKN
MKEPFFYFSKENKIKEDKNIISKSYVVHNLSHINNYVNPNNSTTFCNYDNVENKKTRGRSSSYVKNIGMYVHN
NFHSKEEQNNTAAYFLKKSNIKDDHMNSTNIKVISNDNNINKYNYDDNIQYLFRNNLQKMLYAKNINNSKKND
KKMYSSIIYIKNQDTQKNNKKCNNIPVIDPTDINMNEKQNDILKINKKNICNVVENNMTKNIIYEQNKTSEIK
NCNVNRIMCLKHNNEKLKYPFGKVDLKKNDHENYYKNYVNGNYNVKCKYHYNNSRKILCSNNMLNDHLVMNNK
NFLEAIKTKGNTHYDINKLYRYESKEYENKKIRFRSENKTTDKVEESYLLNKNSFSKNMMNNYNGLYSENGKI
WTSKENTNTFNGTCDMKCNIYKKNEDEYNIKKDIGIIIANNNNNNNNNKNNIYNNICVSKKYTENSKGHYVRN
DEHNYVKYYNDNITNNKKKDHYIVTNVLDNNTKNGEDINNVNIKKGMLLSTDCHYKNGLKKKSNIIYNKENNK
YVFYKDDKNVIKNIMHPNNKWDNENVYCDKIKGNKNMSPVGKILNLSNNTEKDTKEYINNMDVETSIIQQNNY
INYASSKSDGICLLNKKNNNTINHENVTHVASSILENKQNYNLHNIKMDHKMNDIISIEEEENNDNSNKNNIN
NNINNNINNNINNNINNNINNYIGNNNNNNSNSSSSNNYYNYDKPMNVKNDMETYKLQVRKYIDNIKNINYLY
SSKKKRFENNQERNVSLVIKSEPLFLKTIKTNDRNIDFMKNIKKNDNNNKCVDKDNLNYTFCNKNKKIGTSPV
IISNIGNNYNFKNDRDKNGNEESQTHFDIVYLNKLNMNAKEEKKKRKKEIGKKIKKMKHTIKLLKKKNKKNNL
LLNKIGKKNIDEVFIVDEICDNNKEIEKGEDKNNYDIESMNENIKYNDYKSNVSNIDSLIIKTMSNTIIEKPL
CDNHQKEDDVFYKGKIICPNEIYDKIDNNMEKKKSYGNNDFMSNKKEIISEKDKEKINHNILHDNNDNDNNYN
NYNNYNNNNNSDFSKSYNIKDIHCSYEEKKLNTENNWTVNTTRDDNFKFPHDHNNKEGGYIQFNKEKVIKNTY
VDNNIENSFLRTNNISNDISLPWNKDKMVKLFLNNKSKESNKEKKSDVITINTKLSRYERVLIHTCIYKLNWK
KYIDNINKGMFYWIGYNINDFDHYNYMKKKKIINRIPSIYMYTKKKTLTFLLSHLSLIFPSLFNFYPNTFVLP
ENKNIIKYILNSNNKEYYIMKPDCGSMGIGVKIINKYNDININILNGYNSYIIQKYIDNPLLMYKKKFDFRIY
ILLLPGKNYPKIYLSKVGFARLCTEEYKKKKRYICNTYIHLTNYSINKDNDKYIRKKNIHDKNNNKQLLSDVF
IYLKKNGYDIDDIWKQIKKITCLTSLAIYSYIKEKIKYNFHNNFYFYQLIGLDILLDNNGKAWLLEVNSNPSL
RIDYIDPNYANFEIQLESMFDRYVKEPVISEMFLIVYEKIYKKYIKKKNKKSNNVMVQKGKYEKCTKKNDICF
DNKKRISSKGHLNNISNNNNNNNNNRNPNGSVRSLSNFRGNRKNPKGKLSTNGKLSTNGKLSTKDKLSTKEKLS
TNGKLSIKGKEHTNKINPFKEFSLYSEDRYCIDKKNNTDLLTNEKIKNVSNGNEGMGSKLSVRNNTESNISSM
CNVSSRSSISSVTNTTNISYISKNQNRVGNIFNYTKINPSSDSEKSSIYNFSKNLSPNNYLNNIRMKNMLKKK
SKVKKNLFLKKEYMSNVDTCVDENDIGSFTLSNNINKREQISNDSLNEKNEINGIHKLKEVNSNKLSNKNLNN
NKYEDNIQNVVHDHLDDTIFDDKKIKNNFFQKILFDDMVCSKKKNIDINKYNKESNFVKIKRNDINDEYSYEY
KENIEKHHDILDCFDHKSGVSYSGEQMFDNYSDIEKGSSHIDDKKIDNIPSNEYNNDIYNYNKDNDMDDEGEL
FQSNILNKETYVQIDNHEDIQLEKFLTNDIETYKSLYRNISDNIFKKKIENLIMIRSNLYKYMNCLNVLGIRY
INNNNNNNSNDIKNIDDLYNKNISLDFKKSYTKIRKDILHPLKNDVLEKNIYINLKKETKKYYNEMKIYNNCY
ILFDFILNKYDNNLKKNKKKLEYYMDKNTFLCMCTDIKINNIIDNVYIPNNSTYNTCFDINKGSNENQIFQIV
KDLLYNQYLDRNKKNKVHKVERSSFECSVNKNVQLGNSSDITNGSIYDHKFYGVKNTQGSKKTEQRSFYGEYS
DISSRSGEILRRNEKCNSSEKMEDMLTTQMNIQKDDSIIKDDSINKDDNINKDNINNNNNNNNNDNKFLTSNF
INYNCVTFCPFTLIPNCNNVVNFNTIGLSSTKYKSKRKKKMNIYDLEYLFNRQVFFSKYINKNQGLTLIDFFL
LMQEISLLIFPYISHLCIYNTIYPYRKDFFDKLNDQENNIFSNIYCDKGNIKNDKKKKNICVYNKKVQEGQCM
NESNLTSEKIKKKKKKGDHTCEYDMSVNNKFHVHKNISHNKDDFLWHKNICSNIYNLYEYIQMCINPNVKNIC
LETFLIFIFNKYGLTCNL*
>N.PF11_0482

MVYKKHNKKGTGPTYKNSHNRENELKKKKNTKAIKKKNDTDMINNHQNTVILNQKDEHNITCGLENGKDGNLL
DMDKKMKLKEKDPILFEGERNPNTLNINSNSTNETLNINVLNKDGAILYKVQDENMNEEEKDNKICDVNVLPC
NGKNEKKNLKNEMKKNKKSTKLYANINNNENNENKTELDIYQNEIKESYKMNNKGCKKVNKNKLTTKEKKNKM
VNTNVYEHNNFNDNVMNDNVENTIYENNIDSTKMDSYQKNNKTLNEKEDKNCFIKENEGSINDINKEKEINKT
MENCNDILNGPIINEGDNDYTLLNNVKNKKREYTRKYKNKKLCAQDSEKNDLKVNEDEEKNKKKCMKRKNIEV
QEQVNLHEKNGKDIINSDNNKINNVVDDIISKTEKNVNTNAEDNVHEEKNVAKRLEKGEIKNAKRSKKMKNTS
VKNDDMISDNNNIIRNISTITTNTNNTSATTTNTSVTTTPFNHNNNNYKEDINISEDINKCLGELKDNQEKSQ
NDNTNNDSGGKKKYRRLLKNLEIDLSSKIIDHDISKRIRTWSGNLSLKEKINYFIKNNIATKTEYKYGKKPNT
LSICNQFNLYNHLTMLNDFNRLHYYRAAMRWTGHNDVSCESSSNNLVENGNNWTNTCSVNSIRSNSINMNNSN
NGDNNTLSLCYTESINSMMNTANVNTMDSMNLYKLNNMKSGNNPYIFFENNKGCYVYNKNIIEIGTGPLSLLS
INAILNGAKHVDALEVNKDASEMAKNLIEGYNLEDYIKIINCYSKVYEYKENDSHRKLKRKNLSYNFKDNSLD
EHYCRNFNYDLIISEVIGDFASQEGVADIYLDLHKKIFSYRKYQEYLTHWNSSNNSTTELSVSKMDSLKDREL
RNEGTDTKVGVTKINENDNIKYDDKNLSNINSEEDKNNKKLNVNIMNKEKKLTYHQFSSNSELYNCEEFFNMN
IKSIPYSVTTFYCPVKFPYTDNIIYKSDQYPERTIISPKNKLLQSVTLDWSNLTLNSDGKEDTTDFGILEYLY
LEQNIINQVIQKRNHIFCVKKSGPFCGFLITIDVEIRKGEHFGTKYGTCDSWYTNIVLLKNEIMLEENDLIIN
KTYTNLLNYNEHFVDKKTVLVSRPSYTFYGYILRPFNEYTNSDTISEDGNTMDGSSNSLSKQLNNLCNEDNYI
YLDDESILLLDQLTFYEKNILSTSTCKEKQEGLYDDKDMMNNICHTDEVKNMKEDFDKDTIKVEKEHIGKNSK
EDERSQKIKKNKYFVVKKRYKKKMELEDMEHKKPCNESKEVYEKDIEEITENYNLNAELYFEKLKDKILIYK
NIKYRILTFYDPVVIDYDEQATVIYKKDDIYNSKENNVNKFVNNVYQ*
>N.PF11_0483a
MFEERKNVFLSKYFNILKTNLVPKIDIARCDAPKMWRMAMGGMDPATPQAESFINFHNHTVTYMVFIMFTVLL
SLKR*
>N.PF11_0483
MEKNDSYLLRYHRNKILIHLLYELINYEKYKYTINIDFENETSYNYIENIIKLLFNYIFNLNEDYLDYDTTEK
NSINTSFSSCSSDIINEEYLNSIDHDNIFNSLFFNNMYIKEHTINDHCVNEIYKSDNYKPKHLYNNIKEQNVN
NVTQNNQVDCSHEDICEKYTEMYNNVTKLSSKQNNIEQETLPTYIQNDNKYISCDIRNNDQLLNNTVFNFYLN
CNKNSKTLKEKLKVEKQILTIYYKSFSSTIIDMLLNNPNIHIPDIIIYFLFNHVQKIEDISSYFKNQSYTFDT
IHSYIKQINNMDNKNIINKHTPLHTFEHFKQLYHKEPSENIQNENNINYLNNETEEYNEQYSDSIDIHLHKFI
NDKEIESTNNLNFNIVEFKSIHIKKEDPFMLSTYKYNFKLNKSNNLLNKFMSILNLQLEKQLHFKFCLDIFFL
KNMKLISLEASKPWIFYWCIHSIHILYNTFEIEEKIGKPTFDYIKKCVFLYLNKIKNNDGGFGGGLNQYTHIA
TTYAAVCVFIYLHDEENNFLSFLDKKKLHSYILKLKCTDGSFRVHINGEIDMRGTYCAISICSMCHILTNEVK
KNVEKYILSCQNYEGGFTSEKFQECHGGYSYCALATLCILGKVNKINLKNLTHWLMNKQSNIEGAFMGRTNKL
VDSCYSFWMGSIFFLIYEIYILKSILFNKGDITKNNPKGEYVIIEKENEIKDYSKSHELCSEKDINSNTYTEN
IKSNCFNKYLNQNNDNPNNYLCEQKDALTYHKMPNEQTKLLHHQNVKNISKESKIMEDILFFENYKNEYIQKN
VLFNENFLKLYLYLCSQNSKGGMKDKPKERIDYYHTCYALSGLSLVQNHLSSLDKKNFKDIQNKDIYNNLNKI
HILYNITVDKVYNSYNYFSPNIPLNQNKINYRTGKGAYLYLKRLLQE*
>N.PF11_0484
MIDSKKRNKSRQKKNDEIENVDNPHKEEITKRTKKEDKSTQYDISFNQNMDIFINEGYYVQSCIIKELESHFN
DKEGITNVYYIYEELKINKNISTKVYFYTWFYYFENMILKLDNFLDMILNYSYYLLNDSCNKERIFFLIETPL
KLNIHKCIDSTGHISINFNHKMFKNIEKLISEQEINNTNKKNTQKNKKDYDNTIEENCESNRYNQNINSPNKS
IDMEDDMEDDMEDDMEDYVEHNIEHNIEDDITNDIQNEKEDYIINFLKQQKIKSILYINDIFEPEHLSILFLL
HTGIKDETINCIEKTYTDGVFNDEYLNKSDYNEFGILKGYDIILLRYSYEVIYFYKEKNTSIFSDYNIQNKDD
DQNERKMFYPLNHNNNEIENLNNENEIDNFHNNNNFYYEQILNDQNKYPIMLTWKPHLRYVNNSTQIVLLDAL
NISSGMDGYIDDKNFNFDVLINDEYSYLVQYKTKVSICRLIYAVTSLFQKKLKPMVYVPYWWYNEIAFCENNL
DEPANFQLYVLKELKKDGLLKIGYRKIHSTIFTMNHKGIDINKYKKKKKIIPFLCTIFHILAIFFFFFFFFL
FFSTDIDYIIKLAMKYDAILCTNNNDIIEYYLEMNANAKVSKFISKGTFFVLTNYL*
>N.PF11_0485
MFYRTRRIFFSTAKNSNLYLSISSSSESIFRNQVIKRASFPGIEGYFTVTNNHSPLVTLLRNGIITVEFDDKE
KKQFFISDGIFIYKKSNDNNSSNNAEIVGVEIVPLEYLDKNKTIKVLQEMCAINDATDDKWRKIKTLLGKELC
SSILRVAT*
>N.PF11_0486.1
MGVLKHFFFLLFLYVNNTSAINNPQEEFMDRFDINKNHVNIKWSNSGIHGKGKFKYEIEERDVLSEGNESEKS
TICPNHVKEGTYKLGCPDYGKTFLMGFEDNKYSEEFLNEISFGFLNKKYKLPIEIPLNKSGLSMYQGLFKRCP
YNKKHYSMIKNENEYDMCFRKFYNNSNISTRIYKRGKQNRKYIYFSSHGLGGRLGANIEEPLHKYKNDEHYVT

KKMRYPEKIKNLFDCSIYSHCIGPCLYKDFNNSCFLNLPILFNHQTKECVIIGTHEEKRIHNCQSGSTDQNIQ
RCFLPVKKEKGNQWTYASSFIRTDYMTKCPPRFPLNHTMFGYFNYSTGKCETYYMNHEKRTLSFSKCIETLFN
NIKKQDDVNNSSFLWGVWTIENNANEKTNLASMDNTGSCYFLKKKPTCVLKKENHFSFTILTANSFNLNQNII
YPELKNNSSKEGSSLIHFKDPKQTNKRVLYENNKKSKRNVRTKINSVNPFTIPSTLNTNEKEEYNKNEVKNYP
NNNISYIQKVHSTFVNNRFNIHSDSSFKPIHKMIQMNIYTDNKLYNNNNQKIKDSNNNMNGMSVTQRSPSIGE
NQNGNPQQKYMERFDIPNNHIFIEWQKEGEYGNDEFKYNIISNKTAGTSQSLFHNYKDKTCPNHVYEGRAHGS
CPNYGKAIIVQNLLGEEYDKNFNLNFLNEIRTGYLNKYFKKDVEISYENSGIAMHNNMLRSCPVHENEEKLFS
VKTDYNYKMCKSKIFSNRFTMKEYDPKTRLFMYYGLYGLGGRLGANIKRDKQKEKKYEDNITLPMKNPSLIKN
LFDCSIYSYCLGPCLENSFGNKCFRNLPAYYNHLTNECVILGTHEQERTNSCRRTKEEKKKPNCQILRKTTDS
KDWTYVSSFIRPDYETKCPPRYPLKSKVFGTFDQKTGKCKSLMDKAYEVGINKFSVCLEYLFLVSPKDLYNSG
RNNYWGIWAADHSVNENNIEIANGKCYHLVVKPTCVIDKENHFSFTALTANTVDFNQSVNIRKIEELTEYGNN
DDVLKEKEINNEPIDNVNETKINRKSHVNSMERNKPSYKENEYDQMEKNVEDETYSEEFGLFEEARKTETGRI
EEESKKKEAMKRAEDARRIEEARRAEDARRVEIARRVEDARRVEIARRVEDARRIEISRRAEDAKRIEAARRD
IEVRRAELRKAEDARRIEAARRYENERRIEEARRYEDEKRIEAVKRAEEVRKDEEEAKRAEKERNNEEIRKFE
EARMAHFARRQAAIKAEEKKKADELKKKAEEKKKADELKKKAEELKKAEEKKKVEQKKREEERRNMALRRAEI
LKQIEKKRIEEVMKLYEEEKKMKAEQLKKEEEEKIKAEQLKKEEEEKKKVEQLKKKEEEEKKKAEQLKKEEEE
NKIKAEQLKKKEEEEKKKAEELKKEEEEEEKKKAEQLKKEEKKKVEQLKKKEEEEKKKAEQLKKEEEENKIKVE
QLKKEEEEEEKKKAEELKKEEEEEKKKVQQLKKEEEKKAEEIRKEKEAVIEEELKKEDEKRRMEVEKKIKDTKDN
FENIQEGNNKNTPYINKEMFDSEIKEVVITKNMQLNEADAFEKHNSENSKSSNKNADFSKEKDLLEDDIENIL
ETDENEKINKDDIERSNNNMKGNNNDIIYTKLDVEEYKKRDVNETREKIIKISKKNMCNNDFSSKYCDYMKDN
ISSGTCSNEERKSLCCSISDFCLKYFDHNSNKYYDCTKIEFADPLYRCFKKKEFSITFFYRHGLFCWSRNSTD
TPVCHWFKNDHRKVV*
>N.PF11_0486:mRNA
MGVLKHFFFLLFLYVNNTSAINNPQEEFMDRFDINKNHVNIKWSNSGIHGKGKFKYEIEERDVLSEGNESEKS
TICPNHVKEGTYKLGCPDYGKTFLMGFEDNKYSEEFLNEISFGFLNKKYKLPIEIPLNKSGLSMYQGLFKRCP
YNKKHYSMIKNENEYDMCFRKFYNNSNISTRIYKRGKQNRKYIYFSSHGLGGRLGANIEEPLHKYKNDEHYVT
KKMRYPEKIKNLFDCSIYSHCIGPCLYKDFNNSCFLNLPILFNHQTKECVIIGTHEEKRIHNCQSGSTDQNIQ
RCFLPVKKEKGNQWTYASSFIRTDYMTKCPPRFPLNHTMFGYFNYSTGKCETYYMNHEKRTLSFSKCIETLFN
NIKKQDDVNNSSFLWGVWTIENNANEKTNLASMDNTGSCYFLKKKPTCVLKKENHFSFTILTANSFNLNQNII
YPELKNNSSKEGSSLIHFKDPKQTNKRVLYENNKKSKRNVRTKINSVNPFTIPSTLNTNEKEEYNKNEVKNYP
NNNISYIQKVHSTFVNNRFNIHSDSSFKPIHKMIQMNIYTDNKLYNNNNQKIKDSNNNMNGMSVTQRSPSIGE
NQNGNPQQKYMERFDIPNNHIFIEWQKEGEYGNDEFKYNIISNKTAGTSQSLFHNYKDKTCPNHVYEGRAHGS
CPNYGKAIIVQNLLGEEYDKNFNLNFLNEIRTGYLNKYFKKDVEISYENSGIAMHNNMLRSCPVHENEEKLFS
VKTDYNYKMCKSKIFSNRFTMKEYDPKTRLFMYYGLYGLGGRLGANIKRDKQKEKKYEDNITLPMKNPSLIKN
LFDCSIYSYCLGPCLENSFGNKCFRNLPAYYNHLTNECVILGTHEQERTNSCRRTKEEKKKPNCQILRKTTDS
KDWTYVSSFIRPDYETKCPPRYPLKSKVFGTFDQKTGKCKSLMDKAYEVGINKFSVCLEYLFLVSPKDLYNSG
RNNYWGIWAADHSVNENNIEIANGKCYHLVVKPTCVIDKENHFSFTALTANTVDFNQSVNIRKIEELTEYGNN
DDVLKEKEINNEPIDNVNETKINRKSHVNSMERNKPSYKENEYDQMEKNVEDETYSEEFGLFEEARKTETGRI
EEESKKKEAMKRAEDARRIEEARRAEDARRVEIARRVEDARRVEIARRVEDARRIEISRRAEDAKRIEAARRD
IEVRRAELRKAEDARRIEAARRYENERRIEEARRYEDEKRIEAVKRAEEVRKDEEEAKRAEKERNNEEIRKFE
EARMAHFARRQAAIKAEEKKKADELKKKAEEKKKADELKKKAEELKKAEEKKKVEQKKREEERRNMALRRAEI
LKQIEKKRIEEVMKLYEEEKKMKAEQLKKEEEEKIKAEQLKKEEEEKKKVEQLKKKEEEEKKKAEQLKKEEEE
NKIKAEQLKKKEEEEKKKAEELKKEEEEEEKKKAEQLKKEEKKKVEQLKKKEEEEKKKAEQLKKEEEENKIKVE
QLKKEEEEEEKKKAEELKKEEEEEKKKVQQLKKEEEKKAEEIRKEKEAVIEEELKKEDEKRRMEVEKKIKDTKDN
FENIQEGNNKNTPYINKEMFDSEIKEVVITKNMQLNEADAFEKHNSENSKSSNKNADFSKEKDLLEDDIENIL
ETDENEKINKDDIERSNNNMKGNNNDIIYTKLDVEEYKKRDVNETREKIIKISKKNMCNNDFSSKYCDYMKDN
ISSGTCSNEERKSLCCSISDFCLKYFDHNSNKYYDCTKIEFADPLYRCFKKKEFSNMVYFAGAGIVLILLFVI
GSKMIIGKWFEEATFDEMDLNYDKIYTLAMINNEETQVSNPLSYSEENIIK*
>N.PF11_0487
MPRTCKNGQECRKYKAYENVDDKHVSIIQPILLVGMIFVFYIFYFRLFKRRYTSNESYRRKMNKKNISSKPVI
SLCLNDIVIKIIGNNAHIMENSVEPLNKLSSISELFVIAQISNDVQEKNIIDLFKKLGLFDKGLKEHRLMFCN
TSNGRASMIRQLSPLTHVDNDETVIKTLTGKIPNLVQIYENINTSDHSNFFTSLQAFTQVICAVATVEGIN*
>N.PF11_0488

MYDYTEDNSLNPYLRQRIYHIRETLRNENELPLIDQIFKYELKKNFEDINDLLHYVNGIIYKGIDSFEKLTLL
FTYDDKNDYDTTNLKQNDFIYLLRQKTKFRVKYKDENYIEYLRTNPLIFIDTLNNLLIIPGINFEYRLHNFDT
KNSKYFLKKSDTKVNSFYNPFFIRVKNTNLKKTHKIYRRTAKNSSSNKEHHIEHYFVKQNSIPLTKKLKTDDS
TNADSNNTTIPINTICEENSSKEKTSNTQYNYTNHICEKPTNQNISIHSQTKYEPKKERRKRRSLNTSTISDA
NNTDLGKNSKRNISRCLKRKASQSNSTKQSKCEEDAQQEDEETTIEEENKKMKEVEKEQMMDKEKEIEKEKIK
EESNLNEKNDQYNEDYGDNIDQEDYSSLNNYSSSTNSPKNTKSTYFFYDLINEYEIYINHAKCYIIFDIKSYY
KNLDIMKKLKKNLEELKKPNNLFKEITQKKTFKSSRDKMEFIKRFKKMIIPNFRLEKIRKQRNHLVIIELMSK
IQNSLIIKRLYKELLEKVNIEELIKNMVVLFEKCVYNMKEEEIKNFYLRMIHTYFRSKDVKEIDFRKLIQQFK
KIEEYRKNQEFYNLFQNDLNYLEKNKRECQEIDEKIHSLKYLILESILKEKQLERSVNRLLLNHEQSKYGYFY
KIDQSDENNLDTTEYGKLLENFSKEPVNFYTILNKRNLDKHAYHDIRNFNYKKKNNEEEQTKHVVNTALINNQ
ANNNENKYAINKGEQNNIPLEQAKQVNNNNYNNVKINGGDPHINNKNLNEEKHASQRIGKNEKEHIIKTNAQK
EKEKENKISNIKEKQTPKDNNANKNDNINKEYYKCKIKQMNNEKKNDEEKEKNVVLKKKKKYNTFNLFPKNNK
NDSNESYDKNYFRKEEKLLSTINLRKRLAEIDKNPYSKSDDNNNNNDNDNNNNSNNNNNDNNDNNELSGEGHL
SSTGMYKTEEYLNEIKKDIVRCICEKKNYNFINEKQEKINNEIFYKVFEQYPYSFFSKSVKNYKIILNENEEE
SELSWLTMLKKKSHNRSILPPSRDTFRDGTHFSNCRATEHTLKFFLSLLTLLRKGPIDLNLKKYLKKNIQFLN
TELFSMKLNLDKKRAILEKRLDHFNFQENSEFSFYNPLKINIRMMNLIGRGGFAEVWEVFDSINLEMYAAKIH
KIEPSMSNEIKNKIIQRAENEINIHIHCHRHIFIVKLEFFFVFGSATNLLVGMELCDIDLDKYIKYHGPINEL
LALCWIKQILLGLLYMKNLPTGKVHHCDLKPANLLIKDGIIKISDFGLAKLILPDTHQYYNGGGTLYYQPPEC
LKNKKNLLITDKIDIWSLGCILYEMLFCERPFQFNYLEKCSKELLVNKMKNGLTYPKINQKISNATLSYIQYL
LNFDYELRPSIEEALSYPIFNYFNIP*
>N.PF11_0503
MKQHITFKKFIIYTITLVYFILLLKTGKIQFNTIPYAELLHRNSRTLASAMQHSLRGDFVTQRSTCFRTEKES
KQKALNLIKNENNAEGYFNNLNFNLEYTEELNEQITSLDPKTSIQELTNIFNDLNENRRKNFHYLIEALENVS
LILAHQNDIPEKHRKSTWCKIKHDLQCDFIKEHPNIHSNFDVFIRKIQSIDEFISYIKECIHSWNNFTNEKKL
QYFPKVYETYQSDKICKENNYKKINNKTECNPKESIKNESIPKECIPNENMKKECVPNGCMKKQCVPKECMKK
ECVTNGHMKKQCVPNECMKKQCVPKECMKKECVPNGCMKKECTPNKNDKNVNRTDINNQNKTNEKLCDKKEKK
RLPSK*
>N.PF11_0504
MKVLNISFLNEKFRSINNILKNNCFFKEQYVYIKLYINKKTFNLSLLLRIILLIIMILKFSLLYNKKNVVMYF
IEEKQLYYPFNEICSRYRRIVAEEQHEIELDEESIIVLDDDINIIQEEYENMNSYDYLNLQLYNDTTMMHSHE
VNMQQDGDINIQQDGDINMQQDGDINIQDDDINIQQDGDINIQQDGDINIQYDDINIQQDGDINTEQDDDIIL
LQEDDIDLLTDEDINFLEQQHNDLLNEESANTNVQNNYSFVYDNDYNKLHELAEEYTDTLNDVISRMAYDYND
LTEDMNKEWSFNMWNIRWCKYLENMMEEVNYYLNGNFSIDDKKQYLELLLFWCKRDYKHFIDVVKKEWDKKDE
PEHYLER*
>N.PF11_0505
MEAEKKEEKQEKSVKTLMKKIPFKGPSYDEYMKVFGKALPGSVSPTMVLSFISAVVLFFVLYKSMNLIKDKKL
FKALLVFSAFYVTFFK*
>N.PF11_0506
MSNINNKDSSTEWNCKEDVGCVPPRRQNLNMERLDNENEDSVPDFMKKTFYLAAAGEGKKLREKHDESCDEFC
DAWNRSLADYKDIFQGKDMWNDGKYGEAKNHIKNAFGDMNNRKAMLNEIEKGIKDETFSRENGLDVCKSQCEE
RSRDDTEDQFLRFFAEWEEEFCDGLNKHEEQLKSCTKDINCDIKCSNFKDWLETKKDEYDIQSRVFEKKYAND
NKSKHLNYLKEGMNKCKVKNPEMVFKSGFANVAECRNLNVEGAGNKNSNNLKDLDSNSDKDGIVSESYKATKK
NGESIMDRIPKSFNKLFGYFSGSQEEEQKENDVSHRNNYDNILVDKFHRSSLLDKLDDRMFFDELNRDNIMEE
VLSKIPEPIIREAPKYVPKKPVPPQHIPRGDNVPRNIDVNGSKDEYSPETESANSKIKPTYEENDEDKSKISI
ETSEIDRDKEPFRISEEKKVVKEDVQELENIEYEFDETFDFFDEDAKRNIDDIRKDIQSQIMKSVENYNSEKE
EFKRNIETQLIESGDGMNAGNYSSALQDNSTEIPTMVLVPGVLTVFLLTIIWVLVYKHSLIDRVHGTGKTKKE
EKMKELESEEFPKEKYNIEDMEETEKENEIEKIMDENKETEQTEEGNTEEFVQEKELNQETLENEIIVDHIKE
EEDTRNVKEQESVLEESPIEELPVENNIGKINEEVEEFILNEIPLEEQVPKELPQEEIEEIVVEELPIDEHLF
SEETTVTEEDTFKGQLINEEKPVEEKSVSEEIPVEEKSVSEEXXXXXXXXXXXXXXXXXXXXXXXXXXXXXXXX
XXXXXXXXXXXXXXXXXXXXXXXXXXXXXXXXXXXXXXXXXXXXXXXXXXXXXXXXXXXXXXXXXXXXXXXNVS
EEIPVEEKNVSEEIPVEEENVSEEIPVEEENVSEEIPEGGIAIEDVPVDEETVTEEITVDEKIYDKLPNEIET
VNEEMPVEDETLTEQISSEHERVPEEIIEEKPFTEGEETESLTDEIVEEGVVTDDIPEEQIITEKVQEEEEFV
TGELSEEDIINEKVQEEDESVTEELPEEDIINEKVQEEEESAYQEIVQDGSVTKDVEYKELVNDDVRDKENFV
IEEDPFKGQLINEGLPVEEEFVTKEFPVKEESVFEELTEEDQSVTKEIPVEEHSVFKEVDEIESVTDEIVEEE

KSITNELVKEERTALEEIVEQRPDTEEVEETELVNEEVLKEEGSASEKVVKEEGSASEKVVKEEGSASEKIVE
EEGSVTEETASEEIVEDEGSVTEESSSEEIVEEEGSVIEESASEVIVEDEVSASKEIVEDEASVTEEVVEEEG
AVSDEVQVTESVEDEIINQGIVDEVIVEQEASVTDEIVKEDESVIEEIAVEETVTEVVEETKPMDEEIVDQGS
VVENVEEKKTMDEEIGDQGSVAEKVEEEELVTEEVIEREGSINEDIVKEASITEEVEQIEPVTGKVEKIESIT
DEIKEQLVPEDIKEEQLDFEEIVAQRASVNDEKVEVASITEEVEEEEKSVSEEVLEEEGSTTEKVVEEGSSTE
VVVEEQGSVSEEVLEEEGSTTEEVVEEQGSVTENLLEEESATEIVVEEGSATEVVVEEGSATEEVVEEGSVSE
EMLEEEGSATEEVVEEGLSSDNVQKSKGVIENVGEIYSVKTAKDESMNEKIPLEKSSFVDDESFKGQGPTDNV
SVEDVNSEDIINEHTPLEETKIEELPTEYITTADIHTKGETETKYNLIYEKINEEVEKAKFQEEKITENIPVE
RESVTEDIVQEPSLAQEVEQKESDTNEIEETKLANEKIIPEVSVTENVVEKEGLDTEEVLEEDESITEEIVEE
EESSSEEIVEEEEESSSEEIVEEEEESSSEEIVEEEEESSSEEIIEEVSSTEEVLEEEGSVTEEIVEEEVSTTEEV
KDIGSVSEEVLEEEGFGTEEFVGQQGSVIEEIVETESSTEKVLEDVGSNVEEIVQEEGPVAQEIVHEEVSTTE
KHDEVDRSTTEEIVEKVGSVSEEIIVEEVSASEEIVEEGSVTEEVVEEEKLINEVGETESVTEEIVQKEVSDA
EEVLGQEGSMNEEILEKESIVEEIVGPEGSVTEEIVDHGSFAEEVKEEELVTEEAVQYEGSVTEEIKEEESIT
ENEAIEESAFAEIIEEKGPNTDEIVKEEGLDTEEIVNEVSVTDEVIEEEKLVNEQIVGEERSVTEKPVEVERS
ATEDLVEEEASVTEKVSVHEGSTTEQILDESVAEEIVEEEVSVDDKIIEEEVSVDEVVEEEGSVIEEIVEEEE
SVPEEILEEELSGSEEVLEDEWVTDAFMGQEGSVIEEIEEIVDGEGSITEEIVEDGSANEKIVEEEPSRVEEV
LGKEGFVIEEIIEEGSVIEQVEDTKTVSEKSEESSAIEEVKEVKEEESISEKIVEKEESVTEEIVRQEESTTE
KIVKDVSPTEDFVEQTDSVTEKVIEQEGSNTEVAEDVEEKESASDEHEQKDVSVNAQVTYEKKSVTKEIVDEV
SRTEEIVEEEGSVTKEVEEKESVTEELVDEGSVTEELVDEGSVTEEVVEQGGSIAQEIVEEESATEEIIRDET
NVEEVLEKEGSATEEIVQVGSGTNDFVGKQGSVIEEVVEEEISTTEEKLKEEASAIEEFVEEESIREDVLEES
LVTENVVGQQESVTEEIVDGEGSFTEDIVEEEESVTEEIVVDEESVTKEIVEDEELVTEEIVEDEGSFTEEVI
EERSLIEEVEDTETVAEKEEGSVIKEIIDEKSLTEKIVEEEKSVTEEVEEKESVKEEVEEQRLVVEEEGSATE
GIVEDRLATEGIVDDILVTEEIVEDGLATDEFVEQQGSIIEEVLDDEGSVTEEIVEEEGSPNEEIVEGVSVIE
EDDNIEPVSEEIVEGSVTEEMIKEGLENEVILDEDSITEEALEKEGSVSEEIVEEMGSLTEEIVDEERSTSED
MIEEGSASEEIIQEESQVEEVVEEVSVIDEIVEEDELDTKEVVEEIEFNTEEVVEHKEEEGSVAEEIVQEEKE
GSVNEEIIEEVGSITEEMVEQDVSDNEEIVEERSVIEEAEENVWIEKEVEEEGLDNEEVIDEEDSVSEQAEEE
VYINEEILKESSDVEDVKVENELMNEEVNEETQSVAENNEEDKELDNYVVEETESVTEEVVVDEVPNSKEVQE
IESIIEEIVEDGLTTDDLVGQQGSVIEEVVEEVGSDSEEIVEEASITEEVEKKESVTEDILVEESVTGDILVE
GSVTEEVVGEEKLVSEEIVTEEGSVAQEIVEEDAPATEEIDEIESVTEEVVEEEGPVDEEIVQEEGSVTEEII
QGESKVEEVVEEQGSENEEIFVEEVSASQEIVQNESGTEEILEKVSASQEIVQDGSVTEQIIEEQKPVTEEVV
NEEESITHEIIQEESHVEKVVQQGSVAEEVVENPVSVTEEIVEKEGSVTEDIGQEGYVAEEIVEEEEFDNEEI
LEEESVAEEFVEEGFDNEEIFVEQISDSEIVKEENSVNEEVLEEEGSYTEEILEEEGSYTEEILEEEGSFTEE
ILEEEGSYNEEILEEEGSYNEEILEEEGSFTEEILEEEGSATDYFVGQGSDNEEIIEEGSATDYFVGQGSIIE
EVLEEEGLDTEKVFENEGSATEFEETESFTEVVEETESVNENILEETSINEVQKIESITEDIKEQLVPEEIKE
EQLDSEEIKEQKGSVNEEVVEEEGSVTEEIKEQEESVNEEVLEEVEETESIKEEIVEGGIATQEIIEEESDTK
EVVEEEVIDSEKLVDAGSVTGEVMPEEVSVTDEVVEEGSTTEEVLEEQKSVNEEVVEDGLTIDDFVGLQGSTT
EEVVEEDGSAIEKILEEETATEEIVEKQVSVTEDIVEKEGSVNEEIIEEASVAEEIIQGGSFTEEIVGQEESA
TEEVIDEEGLISNEIEEEEEKSVTEEMIEEVEEVSVDDEVEEVSVAEEIVEEELVDDEILPEELSATEDVIEE
VRSVTDEIVQEESVCEEILEQEVSASEEYVDDKSVTDDFVGHERSVIQDVENTESVTEEIAEVDKSVIEEAVE
KQGSVTEEKVQEGVSAIEEIEEIESVTEEIEEIESVTEEIAEEDKSVIEEAVEKQGSVTEEIVEEEELDTEEV
LEDKSVTGDVVEQEGSGKDESEAKESFTEEVDELKSVKEEDQETEYISREIEEESATEQHSEQELSINKEVVE
TESLTKDIEEEKSTTQEILEETQSVNEEIVEEERDTDEVLKEKVSPSEEVIEEQASTTEEFVEERSSTDEIVE
VEDLFTEEVKEREGSVTEEIVEEGSDTGEIVEEEGSDTEEILEEGSFNEEIVEEEGSITEEFVGQQGSVIEEI
VETESAIEERVEEESATEEVDERESVTEVVEEEVSSSDEVVEGSIEEVIENEGSVTEEILEHEVSADENFVGQ
AVSVIEEVEGTESVTEEVVEETESVSEEIVEVSPTENVVQQTDSVIEEVVEQKEGSFNEEIDIRELGDDGVEE
REKISTEEVVGQDKSATGDVEESSSTGDVEEVSSTEDEEEVSSTEGLEEVSSTEGLEEVSNTEDVEEVSSTED
VEEGSVAENVKETKSITEEVSVEEDIITDKVSVEQEVMAEASVEENILTEVPVEEEIMTEKLSVEDKALNEKI
MSEEEIVIEDGNVHEVVPAEVSVTEEIPGVEETTNNESHVKGENVVNEVVVDDNSVNDEIQFDDDSSIEIYTV
DSKDVFHKEENYDSFREEVRSDENIHIFRKKNENFVKKIENEKSSVGHVPTEYTSKENIAEEVPSHIMFKENV
TEEVPKEVKYEENTVEEVFEEVTSKENIIEEAPGDINSEENIIEEVPEVVTSEEIVVQNDVNNINTNIDHMFD
FDLDDILKIPEAQKKLNKLRTLVDVHLDVIEKMQRDEWKKNKRDFLYICLKEINKLSEDTLMKFYGSDSNKSA
NDNDTVMVIKILKDKWGTGRIVDTIANSLNKSYNSLYHNLYIEMEKDLLINKTETFNKWSKQHWNKLDNWKEE
KWFKLFKRDLKIDMRNTYESDQNDEENNEESMNELSDELIKNNTSDNMRNEQKTLEKNKEYSNLSKDLGLIEK
QKIVWKSWIVKNVNNIENWFDEMWFKNVVNELKEKNDVSNVLQENASEASVENYISDVEDSKDMTNSINDSEK

SKIVASSSKTTNEEYIILSRKDLIYNIIVMVHMMVLDQFKYDELKYAKKRFLNRSIDKFIKEKKIKDKETVLD
YYIDDIIKRIVEDTSHVNNIKEKAIDHYTSHDWFRLLRQGNKAQNSISQEVDILTEKYKKLISEEDNKKENNN
NNDDEVKPELKDNIKCEENVSFNILRRSNKKDQLPLEEKKNKNGDLNNTLTESDGKKININEAIEESKNGNED
KNSGNMEKSRKPRDRRTERKEKDQDLRVQLLQDYENIFEQIQNMENQNKDKNKKETKNILKTSIDLDKNLLRE
YKKEKNIINQSEKEFSVNEN*
>N.PF11_0508.1
MMKLFNNFTCFLSQSIRSQEKNVEEYHMSTIDEEPINYKNKNFLKSSLSRHSLVILSIIYVLFHNVFTSKWNE
PSVLQLVNTHSRVLYDTEPLDENLNYESKKVEYKCSCNCSDCPCPIQNEDSEKEKTETQEESLNKEVVEGCST
GMCPLKNNDTKKENCESCKKEKTETQEESLNKEAAEGCSTGMCPLRNNDTKKENCECCKKEKTETQEESLNKE
AAEGCSTGMCPLRNNDTKKENCESCKKDVVEGCSTGMCPLRNNDTKKENCESCKKEKNETQEESLNKLSTKNS
TNDSIKTPKKKSNNKNTKENNKKAPQKKTNDKLRRAQ*
>N.PF11_0508:mRNA
MMKLFNNFTCFLSQSIRSQEKNVEEYHMSTIDEEPINYKNKNFLKSSLSRHSLVILSIIYVLFHQNVFTSKWN
EPSVLQLVNTHSRVLYDTEPLDENLNYESKKVEYKCSCNCSDCPCPIQNEDSEKEKTETQEESLNKEVVEGCS
TGMCPLKNNDTKKENCESCKKEKTETQEESLNKEAAEGCSTGMCPLRNNDTKKENCECCKKEKTETQEESLNK
EAAEGCSTGMCPLRNNDTKKENCESCKKDVVEGCSTGMCPLRNNDTKKENCESCKKEKNETQEESLNKLSTKN
STNDSIKTPKKKSNNKNTKENNKKAPQKKTNDKLRRAQ*
>N.PF11_0509
MKPYSSYSSAFSKQYMGTKSVKAKNPTIYSFEEEKQNENMSLLKSLCSKRLVLPILGILYIILNVSFFFFSFF
K*M*IYLWLICI*ALNY*IIYIYIYIGNFGYNGSSNSGAQFTDRCSRNLYCETLPINPYADSENPIVVSQVFG
LPSEKPTFTLEGTPDIDHTNILGFNEKLMTDVNRYRYSNNYETIPHTREFNPLIVDKVLFDYNEKVDNLGRSG
GDIIKKMQTLWDEIMDINKRKYDFLKTKLQKTYSQYKVQYDMPKEVYESKWGQCLKLINQGGDNLEERLNTQF
KNWYRQKYLNLEEYRRLTVLNQIAWKALSNQIQYTCRKIMNSNISSFKHISELKSLEQRAAKAAQEEMRKRAE
KQKKKKSKRRGWLCCGGGDNETVEPQQEEPVQDVGEHQINEYGDILPSLKVSINNSAINYYDAVKDGKYLDDD
SSDALYTDEDLLFDLEKQKYMDMLDGSEDESVEDNEEEHSGEANEEEQSVEENEEELSVDENVEEQNVDESGE
QQSDDESGEHQSVNEIVEEQTVDEIVEQETVDENVEEQAVDENEEQQTVDENVEQQTIDESQVQEEISTIQEN
IEEVVSEVQQDSEVDRTLHVPDT*FYDILGVGVNADMKEISESYFKLAKQYYPPKYSVNEGMLKFKQISEAYQ
ILGDIDKRKMYNKFGYDGIKGVNFIHPTIYYMLASLEKFAFYTGSPQIVTLMKFLFEKKLTVNDLDTKSEHLS
KIMGVYQKERETYISENLISRLQPYIDSIRNWDVQIKDQIYELMGSPFDIAIIDSIGWTLQYVSMSHMKNPKK
AIKKLETRSKKNKETVAYENNKLMNILREYFGNNEQINSITYNMEYNTLSENNENGYRNILNLNHKKQKKLFE
EIISYIVNISLSDIENTVKNSAESILTVEGLDEKKLSKRIESLRMLANVIRKYILRGKKGKKYKNKDAKSLSG
NIANEINLINKELQNLKEHTQANIPEHIEENVQENMEENVEENVEENVEENVEENVEENVEENVEENAEENDE
TPQEHNEEYDE*
>N.PF11_0510
MIYIKLRLYIFWFLFLILLNLTLTDRKIFEYIRLIDIYFRILYEYNGINKLCGGKSCNKIFDQRILGEEECIY
KGDDKKKKKTKLIDMINLCKIWNKIKKVIYKDENILKSGSNLNIKENKKFIYKLNGENTGINDNQQYIDELKD
NIHSKNIYNWIEGYKSLVKMFGLSNNFSINGVKYSDWKLIPISFIEYNKKKFRVQEMFKTVITSKNDDNKNNI
SLFIKKIPVDIWLKQFEMMELYNGEYLVNAENYVMEASILAFLNEYYQGLIAPKLYKILYEENYEENNKENMF
PPYMFNEKKELNINNLHEFKNFLKERINKNVNGYIVIVSELYGQNVFEYIEKRQKENNNILSDREKKKILYEC
LKLLIKLHNVGIAHLDISLENILMTENYEFLLCDFCKSTPIYTTTLRHVKEMNHICLFESCVPKIGKISYAPP
ECIQLRKIHEKMDIKNPLSDLNYIKDIEERRKYYFDVTSADIYMLGVLFLRIWNSKPLWLIANIEEDLNFSKI
FEADMNFDKFVIAKNWPKEFKKIIQQLLHMTSRKNLSLKELSKNPWWKE*
>N.PF11_0511
MIFTNFKILSIEILVFLLLLLHIDKPKNVNLVKTDIYNGFVQENARYLAEQYINKKMDQTLQHRAQLRTAQNV
YRKKQQKKKKKKLPKSERRNAKQRALDINNYVDYLKETCNGSEKLMRLKYISYLITYMDITYKQKEKLLDLVE
QYIYSNIEHEKKKLSKFIDPYINKEDDEKSKINFQNILYEKKRIESLKYSELDLFLYTIFPYM*
>N.PF11_0512
MKQHSSYRMYFSKKYLYIRDIKAKNTTLYFFEQEKQNKNKSFLKILCSKRFLVPILGILYIILNYNFTYSGNS
TCRLQFTHRCSRNLYGKELSIKPYLNSRYPIVISQVYGLPSEKPTFNLECIPDIHYTNIVGFNEKLMNDANRY
TLLNNYEVIPHVKEFKPLIVDDELLEYNQKVHNIGRNGEDILTAMQTLWNEIMDINKKKYTVLKAKLRKKYHK
YKIQCDMPEKAYVYKWRQCLNIIRKGGHNLEQRLNKQFDTWYKQNALYLEEYRRLTVLNQIVWKALSNQIEYS
CRKIMTSDITSFIRINELEIMEQRAEKAAQEEMEKREQLKKKKNSNSVYAFLVGEIYKRMNHHSLKNVKEHEI
NEYGDVLPSLKVSINNSAINYYDAVKDGEYLDHESSDALYTEEDYWFDLEKQTYMDVINMRKEEILQSQQNQR
VNENKKENKTQENITTKKENI*VPSEKVDNELEVDKKIYLPDTRFYDILGVDINADMNNIDKSYFKLAKEHYP

INGSTIDDLKKFKEINEAYQVLGDIDNKRKYNQYGYNGIKNFNFIYPSLFYFFSCLKKYDYYTGTPHITSILK
FLFEKKLTMDDIETKSQYILRVMNEYQKEIEATISLRLIDKIQGNIDGIKSWDARIISEIKKLESHFSLPILD
SMAHIFINVSQCYIGNQEKAKKKIQNRFKKNKLKSSCAYNELRYTLREYFKTRKQVSSLSYTLENNNNKKYNQ
NKWYKNITNLDDKNKYKILYSFVKNLIKIALSDIEYTIKTVCENILTEKGIDDITIKARAESLNKLGYIIRQN
ILKGKNIKKIIKCDSRNIAANIVREINTINELLK*
>N.PF11_0513
MKSYSKLYNKYVPIFSFDNNENKCKKSVLLKYLNCRYNFVLIFGIILYIFLLNINIYDDRYGSEYIIIFNRNL
SETSSSISSGNKNERLLEINNFTNTQKKVNTYDKGLNSLFKKGEKYLEKFINNDMSKRSSKSKTDVGSKEPII
YNNIGGSKMSGTKSNVIESNVSSTITKSLERNDSNTNIQEKVPKESKNTRKDVRTKNVNTSKRNEGLNDINES
EKTRTRRASTKSNNNNNNVSYENELESVSEWFDNFLEDHMKEGVFLDGEKKTSHRKEVDDEKEHNRYDNKEKK
KNDFDKRRTKFSSSDNRYKYADTTYYDILNVSPDADSSEIKRSYYKLALEYHPDKNPGDEEAKVKFQKVNEAY
QILSDKEKRAQYDRMGMQCVEDMTLIDPSLLFMMLFSSEKLCDYIGVYDLTYMFNFIMKSMNEEHGGGLMFNM
LGLMNKFFDKFKKDQEEREFDLAVSLKYRLEGYVNGDDDWEKQMENEIEDLLESNFSGHILESVGWIYENVGK
CYILKNTTFMGWGARSAKKEYKKRDRMNDKRVFRSIFNTMGMIARFVLNPPPFMLEGQYMNYNNMGQIMNNEN
TSNSCIMCSSSNRGPYGVQNINGLGNYSNEMVVETYIRKIFDSLMSTIVTLFLSIIEGTVRTSCKMVLVELDV
DKDTLFKRAEGMKLLGQKMQKLAKRKKEKSAAKEMNSMDIIEKAIKEAKLRMESYREEN*
>N.PFA0055c
MNFFHVIILDVGIVICLYLIIYKNSDIKWKKHINCCKYHFYFFSSKRCLLQHMVEEPFEKKDKSGVLLKDKNT
EEGRKKERQKPMSIKSINKKKKKNNNNNNNNNVLKNLNNEEINKQRNMTNERIRNKNKNDKGVENISSNTQME
EKNIICKDINSNVILNQNEINDDQMVQKIKENFVKDLMKNENKEIFKQIETINSVGTMAKIKNSLYSIIFKGS
NFWKGLGIYLGTLSGAALGQLILAGILQFGTFSVMNFSIYFSAVPSFIAFSSFVGIILLSIIIVICLLVWLWP
SRGKLMGKDKTENKSDT*
>N.PFA0060w
MNYFLSLFNVSLFFLLIFKYSYKKSIITINIASRILTENNKKWYKKYIYTSIFSGNKNPQKRERKNEEENQKD
NTKVDNDNNMENEMENHIDDSIDDPMDDLMNDKWEHHNSLEDRIKEYYTLTDPSDGEENNSFFKKLKLIMNIL
DEVHSDLLINNSVTDGSIFSPELVPISVLSTMTLACPPIGTVTLPYITNRINFLNRYEGQNIHTEHDLKIFK*
>N.PFB1005w
MFCNFRFIIQEYYYYIFITLYFFSLFFLFILICLLNYLIYI**FFFSEMSVIYIYFLIKTLYKYILLVYIYLF
IIHYLRTTRNIFFKFII*NNMQWYIL*NKYIILLILY*YI*NLIIFI*YKKI*ISIIAINNIYFYIFDFIIYI
INIFYYPSLQCHV*YKMLS*LFFIFFLFIISLLIK*NAHCYRKNK*INKYIYIYIYIYIICIIYKLQNNFRHD
VSIYRIINTL**NKYELT*TYHKYYYKIVTIFYIL*L*LYYNNYIYIISFILSYYNAYN*
>N.PFC0032c
MHDIGVALSSTDIEHTLNFYKLDKDGKSIDEMKNYIYVFIKYYDTFKNDLFNKHKTIFTERIKNTQRLDM*
>N.MAL7P1.43
NENLLL*YTIILSPIKYIVNVISQIIIYLYNYIHIILFIDKCPNEPLHHYISYNKRQTNKNHIDHYENVNYTH
HLIMIMTKKRNLCCNNLIIAHNNDYENMKKECKDQCHEEIQKIVLKDKIKKELTEKLEALETNIKTEDIPTCV
CEKSLSDKVEKTCLKCGGILGGGVEPTVGFLGIVVVNQLTKATTVASIAAANKCVLKLLLII**ICFILLMLH
EIYG*LLLIQKIIILLVV*QHLLRLLKKQLEQHV*GILLALILLAMPYLIRAKYSLVQLNKLVLNLFLIKKHQ
FNELNLVR*HLQVLVIILLLLLQVL**YL*LLLWYLFIYLILRYGEK*
>N.MAL7P1.47
MI*R*QYI*WEIK**
>N.MAL7P1.50
MLYIFFICAFFLCNFFFFFFFSYLIFSFINYIILYKVLIFNIYNNN*
>N.MAL7P1.55
MEYMKLWMYVFLFFLCRVYIFYL*SLFVEYIFFIC*FYFLFAYFLCIFFFFCI*FLVL*IILYF*YIYIYNMI
ILCCILCYIIFFNMMVLLYIVVCICIVLHTYM*CYIYIYIYFNDHSS*YYLD*KYKILKKKI*YNKIIFYYLL
LILLFVLL*VLKVV*LLVLLVIILILKFNIVLFYFLLFMIFKKKKKKKHNMKNNR*IEPIYKNTY*LYLYIN
EDLSKQIDKQTYICRSYIKKTYI*MNTYNIIILIKKKTYVLKIIFFIKLLVILKYIC*KNIYHIEKKK**KYI
Y*YFFENTDTSLSLIYPKTDIYVVHVLDILIINKKQNIVIIIIIILF*FFMVLFKVFYLILLCYILIFF*FFS
IEIYYKKKXXXXXXXXXXXXXXXXXXXXXXXXXXXXXXXXXXXXXXXXXXXXXXXXXXXXXXXXXXXXXXXXX
XXXXXXXXXXXXXXXXXXXXXXXXXXXXXXXXXXXXKKKKIMRTFILTVIDILYRHITTIFVYIIYYKNKFKL
NKVIILN*
>N.PF08_0141

MDSTSTIANKIEEYLGAKSDDSKIDELLKADPSEVEYYRSGGDGDYLKNNICKITVNHSDSGKYDPCEKKLPP
YDDNDQWKCQQNSSDGSGKPENICVPPRRERLCTYNLENLKFDKIRDNNAFLADVLLTARNEGEKIVQNHPDT
NSSNVCNALERSFADLADIIRGTDQWKGTNSNLEKNLKQMFAKIRENDKVLQDKYPKDQKYTKLREAWWNANR
QKVWEVITCGARSNDLLIKRGWRTSGKSDRKKNFELCRKCGHYEKEVPTKLDYVPQFLRWLTEWIEDFYREKQ
NLIDDMERHREECTREDHKSKEGTSYCSTCKDKCKKYCECVKKWKTEWENQENKYKDLYEQNKNKTSQKNTSR
YDDYVKDFFEKLEANYSSLENYIKGDPYFAEYATKLSFILNPSDANNPSGETANHNDEACNCNESGISSVGQA
QTSGPSSNKTCITHSSIKTNKKKECKDVKLGVRENDKDLKICVIEDTSLSGVDNCCCQDLLGILQENCSDNKR
GSSSNDSCDNKNQDECQKKLEKVFASLTNGYKCDKCKSGTSRSKKKWIWKKSSGNEEGLQEEYANTIGLPPRT
QSLYLGNLPKLENVCEDVKDINFDTKEKFLAGCLIVSFHEGKNLKKRYPQNKNSGNKENLCKALEYSFADYGD
LIKGTSIWDNEYTKDLELNLQNNFGKLFGKYIKKNNTAEQDTSYSSLDELRESWWNTNKKYIWTAMKHGAEMN
ITTCNADGSVTGSGSSCDDIPTIDLIPQYLRFLQEWVENFCEQRQAKVKDVITNCKSCKESGNKCKTECKTKC
KDECEKYKKFIEACGTAGGGIGTAGSPWSKRWDQIYKRYSKHIEDAKRNRKAGTKNCGTSSTTNAAASTDENK
CVQSDIDSFFKHLIDIGLTTPSSYLSNVLDDNICGADKAPWTTYTTYTTTEKCNKERDKSKSQSSDTLVVVNV
PSPLGNTPYRYKYACQCKIPTNEETCDDRKEYMNQWSCGSARTMKRGYKNDNYELCKYNGVDVKPTTVRSNSS
KLDGNDVTFFNLFEQWNKEIQYQIEQYMTNANISCIDEKEVLDSVSDEGTPKVRGGYEDGRNNNTDQGTNCKE
KCKCYKLWIEKINDQWGKQKDNYNKFRSKQIYDANKGSQNKKVVSLSNFLFFSCWEEYIQKYFNGDWSKIKNI
GSDTFEFLIKKCGNNSAHGEEIFSEKLKNAEKKCKENESTDTNINKSETSCDLNATNYIRGCQSKTYDGKIFP
GKGGEKQWICKDTIIHGDTNGACIPPRTQNLCVGELWDKSYGGRSNIKNDTKELLKEKIKNAIHKETELLYEY
HDTGTAIISKNDKKGQKGKNDPNGLPKGFCHAVQRSFIDYKNMILGTSVNIYEHIGKLQEDIKKIIEKGTPQQ
KDKIGGVGSSTENVNAWWKGIEREMWDAVRCAITKINKKNNNSIFNGDECGVSPPTGNDEDQSVSWFKEWGEQ
FCIERLRYEQNIREACTINGKNEKKCINSKSGQGDKIQGACKRKCEKYKKYISEKKQEWDKQKTKYENKYVGK
SASDLLKENYPECISANFDFIFNDNIEYKTYYPYGDYSSICSCEQVKYYKYNNAEKKNNKSLCYEKDNDMTWS
KKYIKKLENGRSLEGVYYVPPRRQQLCLYELFPIIIKNEEGMEKAKEELLETLQIVAEREAYYLWKQYNPTGKG
IDDANKKACCAIRGSFYDLEDIIKGNDLVHDEYTKYIDSKLNEIFGSSNTNDIDTKRARTDWWENETITNGTD
RKTIRQLVWDAMQSGVRYAVEEKNENFPLCMGVEHIGIAKPQFIRWLEEWTNEFCEKYTKYFEDMKSKCDPPK
RADTCGDNSNIECKKACANYTNWLNPKRIEWNGMSNYYNKIYRKSNKESEDGKDYSMIMAPTVIDYLNKRCHG
EINGNYICCSCKNIGAYNTTSGTVNKKLQKKETECEEEKGPLDLMNEVLNKMDKKYSAHKMKCTEVYLEHVEE
QLNEIDNAIKDYKLYPLDRCFDDQTKMKVCDLIADAIGCKDKTKLDELDEWNDMDLRGTYNKHKGVLIPPRRR
QLCFSRIVRGPANLRSLNEFKEEILKGAQSEGKFLGNYYKEHKDKEKALEAMKNSFYDYEDIIKGTDMLTNIE
FKDIKIKLDRLLEKETNNTKKAEDWWKTNKKSIWNAMLCGYKKSGNKIIDPSWCTIPTTETPPQFLRWIKEWG
TNVCIQKQEHKEYVKSKCSNVTNLGAQASESNNCTSEIKKYQEWSRKRSIQWETISKRYKKYKRMDILKDVKE
PDANTYLREHCSKCPCGFNDMEEMNNNEDNEKEAFKQIKEQVKIPAELEDVIYRIKHHEYDKGNDYICNKYKN
IHDRMKKNNGNFVTDNFVKKSWEISNGVLIPPRRKNLFLYIDPSKICEYKKDPKLFKDFIYWSAFTEVERLKK
AYGGARAKVVHAMKYSFTDIGSIIKGDDMMEKNSSDKIGKILGDTDGQNEKRKKWWDMNKYHIWESMLCGYRE
AEGDTETNENCRFPDIESVPQFLRWFQEWSENFCDRRQKLYDKLNSECISAECTNGSVDNSKCTHACVNYKNY
ILTKKTEYEIQTNKYDNEFKNKNSNDKDAPDYLKEKCNDNKCECLNKHIDDKNKTWKNPYETLEDTFKSKCDC
PKPLPSPIKPDDLPPQADEPFDPTILQTTIPFGIALALGSIAFLFMKVIYIYIYICVVYVCMYVCMYVCMYVM
CICYICI*
>N.PFL0030c
MDSTSTIANKIEEYLGAKSDDSKIDELLKADPSEVEYYRSGGDGDYLKNNICKITVNHSDSGKYDPCEKKLPP
YDDNDQWKCQQNSSDGSGKPENICVPPRRERLCTYNLENLKFDKIRDNNAFLADVLLTARNEGEKIVQNHPDT
NSSNVCNALERSFADLADIIRGTDQWKGTNSNLEKNLKQMFAKIRENDKVLQDKYPKDQKYTKLREAWWNANR
QKVWEVITCGARSNDLLIKRGWRTSGKSDRKKNFELCRKCGHYEKEVPTKLDYVPQFLRWLTEWIEDFYREKQ
NLIDDMERHREECTREDHKSKEGTSYCSTCKDKCKKYCECVKKWKTEWENQENKYKDLYEQNKNKTSQKNTSR
YDDYVKDFFEKLEANYSSLENYIKGDPYFAEYATKLSFILNPSDANNPSGETANHNDEACNCNESGISSVGQA
QTSGPSSNKTCITHSSIKTNKKKECKDVKLGVRENDKDLKICVIEDTSLSGVDNCCCQDLLGILQENCSDNKR
GSSSNDSCDNKNQDECQKKLEKVFASLTNGYKCDKCKSGTSRSKKKWIWKKSSGNEEGLQEEYANTIGLPPRT
QSLYLGNLPKLENVCEDVKDINFDTKEKFLAGCLIVSFHEGKNLKKRYPQNKNSGNKENLCKALEYSFADYGD
LIKGTSIWDNEYTKDLELNLQNNFGKLFGKYIKKNNTAEQDTSYSSLDELRESWWNTNKKYIWTAMKHGAEMN
ITTCNADGSVTGSGSSCDDIPTIDLIPQYLRFLQEWVENFCEQRQAKVKDVITNCKSCKESGNKCKTECKTKC
KDECEKYKKFIEACGTAGGGIGTAGSPWSKRWDQIYKRYSKHIEDAKRNRKAGTKNCGTSSTTNAAASTDENK
CVQSDIDSFFKHLIDIGLTTPSSYLSNVLDDNICGADKAPWTTYTTYTTTEKCNKERDKSKSQSSDTLVVVNV
PSPLGNTPYRYKYACQCKIPTNEETCDDRKEYMNQWSCGSARTMKRGYKNDNYELCKYNGVDVKPTTVRSNSS
KLDGNDVTFFNLFEQWNKEIQYQIEQYMTNANISCIDEKEVLDSVSDEGTPKVRGGYEDGRNNNTDQGTNCKE

KCKCYKLWIEKINDQWGKQKDNYNKFRSKQIYDANKGSQNKKVVSLSNFLFFSCWEEYIQKYFNGDWSKIKNI
GSDTFEFLIKKCGNNSAHGEEIFSEKLKNAEKKCKENESTDTNINKSETSCDLNATNYIRGCQSKTYDGKIFP
GKGGEKQWICKDTIIHGDTNGACIPPRTQNLCVGELWDKSYGGRSNIKNDTKELLKEKIKNAIHKETELLYEY
HDTGTAIISKNDKKGQKGKNDPNGLPKGFCHAVQRSFIDYKNMILGTSVNIYEHIGKLQEDIKKIIEKGTPQQ
KDKIGGVGSSTENVNAWWKGIEREMWDAVRCAITKINKKNNNSIFNGDECGVSPPTGNDEDQSVSWFKEWGEQ
FCIERLRYEQNIREACTINGKNEKKCINSKSGQGDKIQGACKRKCEKYKKYISEKKQEWDKQKTKYENKYVGK
SASDLLKENYPECISANFDFIFNDNIEYKTYYPYGDYSSICSCEQVKYYKYNNAEKKNNKSLCYEKDNDMTWS
KKYIKKLENGRSLEGVYYVPPRRQQLCLYELFPIIIKNEEGMEKAKEELLETLQIVAEREAYYLWKQYNPTGKG
IDDANKKACCAIRGSFYDLEDIIKGNDLVHDEYTKYIDSKLNEIFGSSNTNDIDTKRARTDWWENETITNGTD
RKTIRQLVWDAMQSGVRYAVEEKNENFPLCMGVEHIGIAKPQFIRWLEEWTNEFCEKYTKYFEDMKSKCDPPK
RADTCGDNSNIECKKACANYTNWLNPKRIEWNGMSNYYNKIYRKSNKESEDGKDYSMIMAPTVIDYLNKRCHG
EINGNYICCSCKNIGAYNTTSGTVNKKLQKKETECEEEKGPLDLMNEVLNKMDKKYSAHKMKCTEVYLEHVEE
QLNEIDNAIKDYKLYPLDRCFDDQTKMKVCDLIADAIGCKDKTKLDELDEWNDMDLRGTYNKHKGVLIPPRRR
QLCFSRIVRGPANLRSLNEFKEEILKGAQSEGKFLGNYYKEHKDKEKALEAMKNSFYDYEDIIKGTDMLTNIE
FKDIKIKLDRLLEKETNNTKKAEDWWKTNKKSIWNAMLCGYKKSGNKIIDPSWCTIPTTETPPQFLRWIKEWG
TNVCIQKQEHKEYVKSKCSNVTNLGAQASESNNCTSEIKKYQEWSRKRSIQWETISKRYKKYKRMDILKDVKE
PDANTYLREHCSKCPCGFNDMEEMNNNEDNEKEAFKQIKEQVKIPAELEDVIYRIKHHEYDKGNDYICNKYKN
IHDRMKKNNGNFVTDNFVKKSWEISNGVLIPPRRKNLFLYIDPSKICEYKKDPKLFKDFIYWSAFTEVERLKK
AYGGARAKVVHAMKYSFTDIGSIIKGDDMMEKNSSDKIGKILGDTDGQNEKRKKWWDMNKYHIWESMLCGYRE
AEGDTETNENCRFPDIESVPQFLRWFQEWSENFCDRRQKLYDKLNSECISAECTNGSVDNSKCTHACVNYKNY
ILTKKTEYEIQTNKYDNEFKNKNSNDKDAPDYLKEKCNDNKCECLNKHIDDKNKTWKNPYETLEDTFKSKCDC
PKPLPSPIKPDDLPPQADEPFDPTILQTTIPFGIALALGSIAFLFMKKKPKTPVDLLRVLDIHKGDYGIPTLK
SSNRYIPYVSDTYKGKTYIYMEGDTSGDDDKYICDLSSSDITSSSESEYEELDINDIYVPSLPKYKTLIELVL
EPSKRDTFNTPSGDTFTNKFRDDEWNQLKQDFIEQYLQNIQKDFILHYSMDEKPFITQIQDRFLDSSHEEVIY
NIDWNVPENINRITNIMDDPKYSSNNMYTGTDLINDSLNGNQHMDIYDEMLKRKENELFGTNNTKNTTFNSVY
KQTHSDPIINQLNLYHKWLDKHRDICEQCKTKKDMLYKLNEVWNMERKEYLLDIPPSILDDIHKTNDETYNII
STNNIYDHPSQETPLQLLGSTNIIPSYITTEQNNGLRTNISMDIHIDEKNYNNVVATSIIGDDQVENSYNL*
>N.PFL0035c
MNITLSLFFSVIYVVYVLPCCTQLSNSKKGFAEICKSAENKNESSVYCMKDYKMKSSIYKYKHLMKFFLDKYK
LDNNKIAIVENSCGEPENYITYGNFFKKVLSFSHSLNTYEGTGVPEKIYNEEKNNGKFRLLGLYGNNSTNWLI
TDCACMISGVTTLVMHSKFSIDIIIDILNNTKLEWLCLDLDLVEGLLCRKNELPYLKKLIIILDNLTKRSEMKI
ENEEKSNGSRKSSNKQKYNESDKREDISLCALECDKEKIEKINSLKEKAKTLGLSIIVFDNMTENKIANVTVQ
NEDPNFIASIVYTSGTSGKPKGVMLSNRNLYNGVIPACDCNIIKKYTLTTHLSYLPVSHIYERVIFFITLFLG
VKINIWSRDIKFLNTDICNSKAEIILGVPKVFNRMYATIMTEINNLSRCKKWIAKQAINLRKGKNNGNFSKVV
EGITNISRKIKDKINPNMDVILNGGGKLSPEVAEGLSVLLNVKYYQGYGLTESTGPIFLQDVDDCNTESMGVA
VSPSTRYKVRTWEIYKATDTIPKGELLIKSDSMFSGYFLEKESTEHAFTNDGYFKTGDIVQINDNGSLTFLDR
SKGLVKLSQGEYIETEMINNLYSQIPFVNFCVAYGDDSMDGPLGIISVDKHKLFTFLKNDNMLKTTGVDEKNF
SEKLIDETLNDPIYVDYVKGKMMEIYKKTNLNRYNVINDIYLTSKPWDTTNYLTPTLKIRRFNVFKDFSFFID
EVKKKYEEKLSGSSTGSMNNGKSGSKSDIKGGSKDDIKSGSKDDIKSGSKDDIKSGSKADIKSGSKDDIKGGS
EEEIKNGKKYENNEEKKRVPGRQSDYIKKSVLKSENVDVSKEVKSPVRKSEEKPVSKTVQKQLRKSAEKPIGK
QIQRPLPYSEEGDKKVNEMINVIQKGPQELQVNV*
>N.PFL0040c
MYILRNMFCIKFMLYFLWLLYLLFLNIEFIKFKTFQSLVSYDRPSKCLSENSKHHVNSDNNKGNKLFGQKQFG
NINKCDVKDDELKISKDNTSKKKKICFKKEKRSNEEEYNNLEKESVEGTCNLLNILNVEKTKVFDNYESTYKH
GENNDIICMSNLKEDESKENYIYNWNLGKESLVKFLGFSDYFKINGVKYSDFELTSIPIIGENKSKGRVQEMF
KTVIPSNDGDPAKEVKLFIKRIPVEWWIKQFNLMEKYDGEYLVKAENYVMEGVALSFLSEHHPGIAPKLLKIL
YDGKNVNHDIMEEYKFKDIYEFNNMLIERINNNMDGYIVMVSELFGEDLFDFNKRFTKEKSDVRNSDEFKKEL
LYKCLRLLVRLHSAGLSHLDLTAENVLITDDYDIRLCDFAKSTPLYSDKLRHIDKKKKKKVYLFESCVPTIGK
REYTPIECWRIRKKLREKNITDPFEHVKTISMQRFRKEYYFNVSHADYFMLGVLFIWIWNCGHMWKTSFPSES
VNFTTFLENNMNLSCYPSTKSWPSDFKFIVKELMNEECRKKLNLKNLMTHPWFNET*
>N.PFL0045c
MNGENVKKQVCRTLHSMGGCNMYIRRMYRSLISLKPRGFIIGIIYILYINVLCIHEKNSISKFELDRKYSKML
CELYTSNRDYFRKKNHELNYACNNNNNIYAYKDMHLDNFDDVDNFDYFDNIDDVNNMEGVDNFGDVHKFGDYF
NFEDDDSFDDKNPFDEANYFKHECDFDKYEKSTDSKINVEDKDNNIDDKNSFALPSEHKEISDNDDIVNNEEI

ISNFDKYYKSNSLHISNDNVLMEYIKKNYGKMTMEELYVIYFYIHEIERKKYLNMECQLYLYCEKLANKYNIP
DDYKIKIWKKAYNDVHGFYLNKEKGFYKKIRKIKNGESRNTLLTINILKDTWKKFREMMNNMWSENISVKFAN
YSKKSLKKKAFIQKFYKK*
>N.PFL0050c
MYSALRSFLFRKSVVSVIGVLVALLNNECSEDGLFSSHYLNSRFSRILSSANNGNEYDILDLGIPSVFSVFSL
SKEEMMTLLKDVYGKTKKVNLSDEEKIIRDIKKYGMSCNYSAIPYIDEFGLVVYNKIDNELDADFNDLDELSE
TFKDDAYNHWVRVMKNEESKYFQNFVDLSTYYDTLKKENLILDNYKEDKLNECRHIVPLKAQKLIPGLTKIFE
DWTKNEILYKREFETLINSVRIAWKALTNYALYECKNIFIKNLDEIEDVDEEEEEEEEKEEEKESRKSKKSKK
KETETDEKKKDEKEKDEKEKDEKEKDEKEKDEKEKKVSNIKNNLRAVPQNSGSNFDEFLDVKEANEIVQDVLQ
EFVEGKISEEEDKQKYAENLDDDDEDDEEDDDDEDDEDNDDEDDEDNDDDEDDDDDEDNDDEDDEDNDDEDDE
DNDDEDDEDNDDDDEEDKGVYNLKKDETATINKDIKELEEVISVEDITNISRKITSNENNKVAILKNELSDFY
RKQCEKHKVKVEGSRGVLKSCYNLIKSELEKLQGSLNKYYLSLLMANVLTKKDAEKYIDNCLDSYDDFRKKMK
ETCEQKIIKYFNNED*
>N.PFL0055c
MQFFNKSYRSLLRILLDINNNVKEDYTYGAHVHYNKKKEKKNIRNEHNFLNSPIYISTIIYFIAICYFILLNT
CIPNNNELYGEAYYYKDKRYLCEAETINISRSNVNTPLTSNFFSLLYNTSPCILDDVFENKEEYMLKYIQEIL
GNLNKYISWDNYGLILHIDDYTPVTYNNKDIEIDKKIEMIWKKRQHRIEDIKDSWDEVMLNESSKYSNTVNEL
KKYYDKLRYQKLILKRDYDDIWNDYSKFLFIIEKHMKTELYKIFNVWYYKKALYMNEYRKLVNSCRIAWKALS
NHLKYTLKMTIIYDIEKMKHIREADFKNAIMHLYLKKDDDDDEYSNKQGNEKRKKKKKKKEGFFHNFFCFLN
DPYEIKEDEESISPNNDFNLSAVNYLECCRTARHIDKEKKTHSRKVDEETMTELGNPIDTRMSERLKELREYE
KRKKEKEQNNSKYVFDYGSFEEKSESEKSLDISKNFIFSEEVRSTNSGMHYSLDIEADEVLSKNKNKERGMNY
LLKENNYNNHSVNAVSPVNTTYYEILNVDTKAELKEIKSNYYHLSLQYHPDYNIGDRIAKLKFRLVSEAYQVL
SDDERRRIYNKQGLKATEKMFLMEPGLLFMIMFSIDEMSDYVGDLKLFYFIKEAFEKKKRIEDIESPFEDMDA
KMENDQRKREVVLALLLRERIQPYVDNNKEWMCEMEKEIKSLLESSHSNAILGSIGWTYENVATKYLSDIKSK
WRLKEPMSKYDASFRHVNRSKRTKMTKLSSRFSGMFSCSSALKSQEPSMGETSSSDNDRNEGSEGICNMDNMF
SEETLSLLEIDENKYFGFMTMHILTLILWDIEETTQYAASRVLRDEGVDENTRIKRAEALQILGKLMQKWSLN
VKSKKEIREKDIIEIMEKARAYNM*
>N.PFL0060w
MRSLKTPGYISNSARLNNKSVNSKNCTIYSFEQDEENRRNLSSRLLSTMHSSITILGVIFILILNVCLTKESS
SSLIQINFNNSRKLADVSISDIPIVKLHNSFDNVKEEFESENLEYEQHNESYYKHSPLEDLQDIMNNTSIPPL
DGKYYDTLEQWNEEKIQNKLDKLDDIHSKLDKLIIWAQIQGSERLKTYSMVYTLRRLFKSLLKEYNLKQKDKE
IE*
>N.PFL0065w
MKTIIIVTLFILILNTIIINPCTCVPNTLPIEYFDYETLKENVKIRDSNTKTQLIILSAVCASILLSLSSLWG
IRIHYNHEMNKKEINNYELNSDLLKDADFYMVE*
>N.PFL0070c
MELFVLVRIVIFITFFYILLNILHDNLFMIKLVNNYSYSYLWNIVKLKNRKILSELSDVQLEDNDIEDFIVNN
NVLYSNDFLNIIDPILFENLDNINLDEYIQNFDNIKKESSSVRNFSIDKCDLYNNHGEKKLENILEVSHDNVS
NELPRMISEKSADGILDQLFDEQSLKTSNTLSSDIMGEVNDKFFEEIIVQINEKLSEASCDETLENMYIQLCN
IMQSKGLEEKLKTDKEKNIKGNLKRYYSSRMVQETDKTGNEKCISTANMKYIIKLTLEKNKAQEADVSVSNEV
EKMTTRNVEEDTREVIKVVKEDPGNEHSKYEEGKCNMNLIKELEDQILQTVHKDEKDEILRKESKMGIENNTI
DIYNKIGADYAKGRVHLNSHISKMTFKNNSGKSKEKKIEDTKRKELEQKMGFEANKKNKERKRYRRINDLGEN
VSNDYLYFSNDYSYLKRENILYNVNTHNYKTRLKTREQRISEEREKYNIMKPSDCNTLLLSTRKRERPNIIFK
ENDKYDNTAISFVQMNNSMIPFVCSHRSKEILYTGNNGRNHFEMASFDNLYNDIRKEENDESIDESIFLLNEV
INDEMNGETNNGRNHFETTSFDNLYNYIRKEENDESIGESSFVSNDERNNETNHFEMTSFDNIYDIGNEENVE
SMGKSIFDFNDEINDGGNNERNNFETSFFDNIYNDIGNEENVESLGESIFDLNDEINDGGNNERNNFETSFFD
NTYNDIGNEEIIESVGESIFVLNDKISDEANNEMNYFEGTAFDNIYDIENEEIIESVDESIFDLNDQINDEIN
DKRNNEENNDESNEVNNEESNEVNNEENNDVYNEENNEINKEENNEVNNEEMNEVNNEVNNEEMNEVNNEENN
EENNEENNEENNEEMNEVNNEENNEVNNEENNEVNNETNKEENNKGNDEINNEGNNEENNETNKEENNKGNDE
TNKEENNKGNDEINNEGNNKGNDDMNNDMNKEENNEGNNEINKEENNKINDDMNKEENNEGNNEINKKENNKI
NDDMNKEENNEGNNEINKEENNEENNEENNETNKEENNKGNDEINNEENNEEDNEEYDYYDSDDDYEESLSSS
DEWFNDESSEEESDEEMHDYKVSSFELTNLLNRK*
>N.PFL0075w

MEETINDKVNKIEKTNNSDRKKDEEIMNKIKNYKILNNNNNNNEEEEEEEEKNIKEYPNNNIINKENHNVLIK
ENYDVTNKNDDTTKINDDNSNVKINCESSNEKREVSDLNTYSDNDVNVNINNSSNNIEKENPPKDKHYDNNIN
KISSYGDNKQNNILDDTDMSIHNNLNDTSNEKKNVIISNEDIIQNNNSKTCGNLKNYYKDLPTVIIVIGMAGS
GKTTYVGSLYNYLKVEQKKKVYTMNLDPAVKYVQYPLNIDIRDSIKYHEIMKEYKLGPNGAIMTCLNLFATRF
DKVIEILEKRKSKLHYIIVDTPGQIEVFNWSASGNIILETLSVSFPVVINYIIDTVRCERPITFMSNMLYACS
VLYKSRLPFLACFNKTDIIKHDKCIEWMTNYDTFNDDVLNDESYMASFSRSCALMINEFYEGIKTVGVSSKTN
EGFNNILKNLQILKEEYLNEYVSSIEKQMKKIKQRKEKDVKIKMEALLMEKQKEISSSKS*
>N.PFL0080c
MVCIYLFCFLLFHLFVVNTRMRILCNLLIVICEYMFPFYSFILFKFVKMLSLLLPSILSCEKKYQVYKNNGYK
FETVLDLMTSDSEIHLIRSIESDEIYISKVYDIYGINEDDLNKYMNELYIMNKLRNCENIVNIIDYIKENDTL
SFILEFCNQGDLHSDILRKKLNNEIYTESEIFNILHQILNGLNIIHQNGIIHGDLKSTNIFIKDNKIKIGDFG
ISSEQSSNNNLGTLNCLSYESIKFKKTNKLSDLFQVGCILYELATLSSPFCATNINDMISLFEDKNYKSYIIK
NISSIYSQKLVNVISKLLSLNTLERLEVITNYNLETTQHVHLENANKIISCITVQ*
>N.PFL0085c
MDVIENDIFYRDSQRNAVLFFIFSLFCNFIKIILFVFNIWVFCFSTLVTILFMFFGYFGVISDKPLNIHVSIY
ISTIYVDIILNMLITIFSFYKVYLISVNKITFFEDNIVNRYFFYCLTSVNIFFCFISTVLNIFCIYYTERFKT
FLRLSSKDDEKNKISFRLNSLRYPQGNSMNEYTNEKKKKKKKGENVMDGNFEMNSADEKNNYMSNETKYNSR
NFIYDFDHRNQDNNEKVNYLNNGDKNIV*
>N.PFL0090c
MDIKRQLVKKPDRGSFLLDHNNECTSIKQKYLKCLKENNNDHICCRDHSKEYFICRMDNNLLERQSLNDLGFI
EHEEKNESRIKNFKDVYSYNIYNENMERISRNMHDNIKSNNLLLNENNMLSKLNKNDHIKFVDINEKNDRNDF
ILLDINNKENTNKKINTDDLKNSEINDEKKIAIRRKEAEGYLAGKEYIKTLLEKKQKKTFFFLNEIFKSNNV*
>N.PFL0095c
MGSSSSSSDMFILKNHEDIKKKGLKKIKENKKISKHLKRRSYSDSSSKDSEMNSYSSKEEDDYIDEKKIKIKM
SDSICGTPTKDGFGELDVEGENNSNEINDARKYFKEGQKIITPPNGDGTRAFYESLLEENPNSIIAIKYCIEH
GILSGTKHHEALYKYYVLKKNNAFKSNFGGVRIDFKKLLEESCKDNKCSYEELIKKEILTK*
>N.PFL0100c
MYRNKLKSLCCFCENYFYTYKCSHLNNLNFIYFTTYYNFMNLNVSKDVNNYMKINGIIKKVDIKRYYNKKLNN
WSYEDNAKEFDKKDEKEYGSKYEYNNKFINEEEKKDEEKKYRYDEKIHKGLYSEDNSTSMNNRYNNNESTYKM
PKEILNRHEKIDLNSLPIDEEIINNLKIILNIEKLYMYQYIIYNEILKNSKHLLIYNKTGTGKTLSYLLPLIQ
KLINDKFDCNKKVLIITQNIYLCKQLYIYILSIYPTLNVCILSDEKESYNMNNNINNDIIVNIKRNNINPNDK
ENNDMCDEKYGIHFYLCTPNKLEYFIKNYKNKSRNDKNVMNNILNNINTIVIDEFDYIVEYRKNILNFFFKKD
LFNEIHNNNNIRDKNRFNHDIFNKSNYNIYLFTANINEVIKKKIYEHLNGFVFFDFINGIKEHIQKQVAEQHN
FNIDVLKNSENVVQLLNKENKNESLCNLNITHFVCKINSASKYKYVCYFLEEFFFHKQKISQKVYNDMVYDEY
DKLNYRNMIPMEENKFVNNSEKEGNMKRSNADKINKCIIFANTKEEVEKLYEISLLKPHAVMMHSELLTIQKN
ENINLFKVGKKNVLITTDIISRGLDIDNVIFILNYSPPTSPNDYIHRSGRTGRGKEKGICLTMYHKYEYRNVD
KIMKYTKNKFEVILCPKVEDVYKFSVDKLVENVMKIAPEKYEFFNEKSKELLKKHNTKIIAQILSILLKFDKK
SFDVSLLSGKKNYVAVLIKDPFFEVIKNRDDIINIIKVITGNKIITTIIGDVAKCDEGFIVDISSSHVNKIIS
LFNSSNFEYKSKGLEINTLIELPPIIKEKKNIIRRKKKAPWITYKLQKKKIIILGRKTEKYKRKRAEDIIKDI
NRNI*
>N.PFL0105w.1
MCVRMHFNIDLNVLKSKYKCNKIVNDDKVKRGMINKKNYVPIIFESVENSTSKEKIINVCLWGISPFTYGKIE
KDFALINARLETLHIKKSFSVLINKNRCAVVVNGYFEWMGNEGSSKKTPYYIYNGNTNNKNELKIENEELNNV
ESDKYKSENEDNDSKIKNDEDKKQGNVQVKEEIKNEIKDEAKGEVKDEAKGEVKDEAKDEIKDEAKDEAKDEA
KDEAKDEAKEEQEEDQLVDENSHKRKKVIKEEISTKKIKKELSDEDNLNNIENNQNEKKQENCSYIILAGLYT
VSNDNKKECRNTIITTSSENTNLQDIHERCPLLLSENTLSLWLDVEKKYTDIIDKVKEEHIIMSSQLKFREVQ
NWNDFTNYTKEDKKDSILKYVKKNENN*
>N.PFL0105w:mRNA
MCVRMHFNIDLNVLKSKYKCNKIVNDDKVKRGMINKKNYVPIIFESVENSTSKEKIINVCLWGISPFTYGKIE
KDFALINARLETLHIKKSFSVLINKNRCAVVVNGYFEWMGNEGSSKKTPYYIYNGNTNNKNELKIENEELNNV
ESDKYKSENEDNDSKIKNDEDKKQGNVQVKEEIKNEIKDEAKGEVKDEAKGEVKDEAKDEIKDEAKDEAKDEA
KDEAKDEAKEEQEEDQLVDENSHKRKKVIKEEISTKKIKKELSDEADNLNNIENNQNEKKQENCSYIILAGLY
TVSNDNKKECRNTIITTSSENTNLQDIHERCPLLLSENTLSLWLDVEKKYTDIIDKVKEEHIIMSSQLKFREV
QNWNDFTNYTKEDKKDSILKYVKKNENN*

>N.PFL0110c
MMKRKEVWDSRISSPVTRIKHPHEHNLSYYSKCMFGGILSCGLTHTIITPLDVTKCRIQTYPNIYKNLFQSIK
KIVKEEKVRSLTLGWTPTFVGYSLQGLCKFGFYEIFKDVYSNYLGEENAYKYKGATWLLASASAEFTADIFLC
PFEMIKVKMQTSKANTFPTKMVPSIAHMLKNRKESKFPFGSVGPLWCRQIPYTMAKFYFFEKIVQLMYDKVFT
MPKDNYSKSTQLGITFASGYLSGIICALVSHPADNLISQLGKVENKDKSLGMITKEMGMFNLFTKGICTRVLM
IGTLTGLQWWIYDTFKSVMGLGTSGSGSSAKK*
>N.PFL0115w
MNNEIEIYIKNKIIGMLNIHDHVLEECLKDTKNKNNFIYFLSSSPINTTLFFVLHKYTHEECEIDKELNINPN
DDTKNILTKDEQTQNKKDKHNQDDVEDVDSDNKKEEEQNIKNDLLEDNDVTYTNHINKSNNVAKENIKQEDES
NTLDIHTNNNDKEELKNKKKEMKYILKMSINNACIDEYIEEENEIYDNNNNHVGKKKKKKNIYKQVIYFLKIN
DKKINNINIEEYFNKTYTFGNLHPNYINNFKTFFKIFFIPFFQCLLKKNNSLEYFTNDIEQNEKIENILMKLK
NIKKKEDLNNNYYEEYQNKYLNFIEKSKYSGRQYDELINEAPENGLFLSHQGNSIQKVDINQNEKEKIYKYEH
EITNIYKDKDKNINVYTNTNTNIDIDIYNKEPPYHQYQDIHPYNYNKKYLLNSFFYLLNYYKQIIKLEQFTHN
EIVGDIIKLKDDQDNIDKYLKNICQIIKEFLLKEKKNDKKRKNDKTRVEYWKERYHRIYNVHEQINSKKFEQI
LDNLKGKNYNKELCNEIKLILLDIDNIHMESFEMIKFSKLLETSFEKLKHIHIDHMKNGIKLVIKKIKNIWLS
SKYFRKEDIIIAFFLRLSNIIFDLILYISIKVKKLNKPKKIKKIISKCIDILKFYEKEFIYVKLDIEIIDIRK
NWYFDKYIIFKDIRHLQHILATLYTVYSEIIYFTDLCLLKLKKLVNNKKIMNNIIIEINKLYQPFSDLLKNIY
LYDDERIMILMKKLNEQCLKIEKKCIKIIYIQFSNLRNTTCTYLLVEKFQNLKKSYKIDRLLIKKLYDILFLY
KKELYHYMNLFNNIENVKNMYNIKNMYNMKSMDNTKGIHKNKSGDYNIKGVNNTKDINHASNIQTNYNNVHYN
EMGCDNIYKKILFCLNIYKKIKKPILIFKTNTYITSSNEYKIIIKLYVNFCKKMFSYIVENYFMWKNNSLSQM
NKILSSNIISINKQLSFYYIINFNPNFFKTLYEYIYIESIYDFNTPIEILNIYYFKKKLYRYLYELNEIILFY
KNFTKNTSIIQQKVLIKFFQCFEQKLIYISNNINWLNLNIDHYINDLNNCKSVIENVHLSLSYSLNSIDKIIF
NMQNINVVKYIDWNCMKPLHIQPFFNYFEQYRINQVTKIIQKYREISHILIKIEQIVEKTKNGRSDIMDELYY
LYQCKIYKSLSLIIIKGLCTYETLIENNFQKIKFLHNYYKPSLYIKEDIYSDVFLNNSIQNIFKLISKLLTNL
LLSSSEFIRWLDYSCIEVPISLKEDFDVFKMKFSFYQSVSKNKIIIDRIIHIHQLIQNVFQKANKYIYSFLKY
DNIINTIIKTQTNFNKNQKHNTYPLIYYDNVLKTITNIQNKIHKEKDKKNIQFISLNISFVLNSIKTKLNHSK
KFFFNNLHFIFTTLKCSTLSEITKYXXXXXXXXXXXXXXXXXXXXXXXXXXXXXXXXXXXXXXXXXXXXXXXX
XXXXXXXXXXXXXXXXXXXXXXXXXXXXXXXXXXXXKKKKKKKKKKKINMKTS*KQLTQ*NQKTST*K*IFKK
QKKYRMESLKKYINNFKELEIEKEEIIKAQILCNMKILLFENFYLLKNTIKIYNSIYSIYNSYNTRINDFLDV
IYYKLNIKEINKSLTEVKNELTELQKNQEEAKNILAFKKIKEELYKMNIILTIVFLLQNTNMKLSNWKEIINL
HLNRKQIKNKTILENTIIINDKTINIKSITLYDMQQLQIYLYFNDIKYIIYKIKEVEKLEKNLNDIENNWKQE
SLKIKKYNDYILLEDNNHIFINIKNTFNNLHFMRNIRFAQDLIPKINYIQNKLLFIYELLSLWINTQDIWLRL
ENFFQNENFNHFSNELKNFNKINKTYIEIMKSVKKNLNLFQNCNTHFFYLLKNYNESLLFLYNETINILQDKK
FTFPDFFFLTDQELFDLLSNSDTETLKKYCHIIYQDLLTFNDIDKTIIEVVTTKNNNFNLKNGIKINGIDTYF
MLKEMIKSSLKENIMTSLKNYYNNNIILNIIINDISIVSCVVIKVILTEEVKEIMQRKNLEYTQSVLNKYKDM
LDNLINELKNMKGKDIQKTEYCIIIFLEFIEFLKKIIQENILDENHFFIYYQFKYVLNKNKEEILVTYGNYTF
KYNYDILENDNMFINLIPNNKYIFTICSNIYKSYYNMIKGKNKKSTIKYITSSLGLRTHYINAHTNDILQNNI
LGSIQQGYALVIQNVDDFNIETLSVLTNIFRIIQTCLKKKEKNIYIFNKDIIFDHSSVIFFTYKYGRKYSHKF
*KYV*RSTLK*LSRNRTPIYIYVLKQFYKYSKSFYNTMEFYGVYKFYLFQFQK*YVNGFN*YNKIM*KPKRRI
YKRSNVSSAYIYLLL**TGEC*S*QIKEFNKNILQYRN**KIIL*GSS*SFKGGVTKRIYIFKR*FVL*ISRR
YIYIK*KIK**FYLNIIRKSIHRKINNVKDI*HSI*L*TQIYIFRRPI*FTK*FERTIFI*NY*KEKR*I*NI
QFYNKSK*Y*FIYISHIVFN*FIFIKN*KT**E*YKV*NYN*V**HFLHGSLYA*QNE*SLYTRQYKHLQLF*
KQKLLYIKLYTKY*LV**KKRRR**KLY*L*KLLSREKKYSSSNIRRV*SILQKIYDTNF*LYR*KEKKKKTI
HIFKQ*******YY*SFL*I**YI**G*CEKNKTKWESKRQLNNIQFIHTTTYL***NYHK*FHRYI*K*YIV
LFKI*RRRI*RNTKK**YN*YKRIR***IKLSIYL***S*TTTM*FRKPRQKLYRKYLYNTGRPRK**KSRII
TGNLQNSYN*RKHSFYINQLSYLPFFLK*T*KI*FLSFPNKFMYT**D*C***YNK*IL*YTKGNMDYH
K*LLQHLYY*PI**RKLNILRRRNKEKN*FNQFIISRRM*YMFNRKDKKL*DCYS*RNN*QT*KN*SHNHKCV
KIYQCFKL*NFT**INQKKY*KVFSSIIVIYMYRRHTHKL**TNQPFYRVSSKSSKKFMLFRK*NP*YRKYKK
HYFVTL*SHK*T*LLSKHVILLFPHILKYR*KISTYIF*CID*SYTQQ*I*NNT**TS*LQHRRILEDKRRN*
F*TEKRKPFDIYQRKYFLVI*RFIVL*N*LYK*AGCHNKLVRGF*KYYSK*DPKKGE*KANYDITQRY*REIL
YKHRKE*FISIYKLFLKRDL*TCRKKHP*RGIFKKP**LS*FIYEHKCIC*ILFRNNHRTL*ENK*KL*NVYD
HMYE*KESCHSIKCCSIYIKYKYHNSK*NKHTRKCTR*N*LL**ITLSKKSFYSN*FYK*IL*LFLFLNL*NI
PSSSF*KKRALSNVYKNGSHIKRVEVGKYKNDNEK*YLCLSYT*KTIRFVHIQFSNL*KYL*RHSYYTYRQLG
*GRIY*LLQSVFKKYTRQSFYR*YIKHF*KKSNTQHIRRR**TQHKTNIYKKKR**GKKQNDFY*FG*LYQK*

KRRK*NK*KKKKKQQ*K*TK*R*YR*EE*K*PNRKNKRSKIKSTK*RK*****YTR*CTQ*YYY****Y**L*
*TYKTKQSL*FISYK*KNT**TK*K*SRKRGRNTKH*SK*KQKQN*K*YKQKKKKEKSNTENIFSK*KCKTRK
CTNSSKS*YPWNTT*ANS*YT***GEERKKKQTKNYKYK****K*TS*YKH*R*FK*YNKKCKT*YNTKKKPY
QTKYQKYKKRK*YHTRTK*KNKENSNERKHEHK*TKCKAY*TKNR*SK**VKSKRKRKNKQNNQYKKS**K**
V*YNHVI*P**R*YKTRTTKA*SNYKSRIRM*PKKCSTTNL*STK*IT*CR**TG*KTSSIK*NK*TK*NSE*
PRYNRKYGQFKEYK*SQTFE**NNEPTKW*S**NQTYNRY*RVQKNGKK*******SQ**SQ**SQ**SQ**S
Q**S***SW**L**YPSYT***QIIEPKQQLY*GQTT*KHNFLYDRKTKG*YY*YMHSSVLSYENLYS*E*KN
*LYKNKQM*FYRHFNYVLHFIKKQIKL*KQTIIYV*NGTKKN*RYLPKL**YKKRNRKYYFIHITNE*EYKVI
K*RY*EKFLKSYSIRRNIKFK*KNIK*NYK*YR*NDGKKK*I*KIN*RRMY*YNF*NTECRK**YQKYIKK*L
S*YYTYSYMYYDKYTYTEFISKRQ***LELF*KFIIKR*FFKNIF*F*KRKCNR*ANKKNQRIHEQR*SGL*Y
KKDL*NE*YFYISF*MDCFHNKI*RTS*RYV*YK*YYIIK*NKKRKI*YRK*YFIRTNYIK*NIYS***KSSK
NRNNR*PK*KITYYSIFTISRN*TFPTKNTKKIYIYYK*NY**L*IYHFRNFTLFILSKLFFIF*L*I*
ILFNKFMDIYNQK*LYYNKR*YKYTTNIYS*YYHFIISIK*FPKTYILRSKCTNII*SF*MASTSRYTKYWLT
MVKKKFY**SSNT*L***YNQTSRHVYNIWKNTYHTIFQFPKFIYI**KI*SII*Y**SFIT*TF*KYTKKQF
I*K*KNY*K*EYSN*FKLFNNRTINRTKHISLKRKILHKYRKKKLEYNREFKLFLVSDTNDRYFQDIEDYVNV
INFKIDKNLIINKITNIILKHEEERKVLNFNTKIKNYYETKQTLKFLDDGMIIELCSFNNVASTNNNINTINH
NNNNDNNIDNNNNNINNNNNNNNNNNNNNNKNLINAINNNALKKIEIKKLLKEYQQEIIKINDSSKNIKYLGYR
FHTIYKILLLLSQLKSIYYFNFNYLLNILNNLIKNINKKMIICNFNQLIKKFTHNCFLFILNSINKKDTLFFL
FIFYTSLIFEQQKLNSTQQQQQQNNNVKSIKIEKYTYYSLFIKINNLYNININTLNKIEWLNIKKKRQLIFIQ
KEYSNLQILINDIQNNTIEYFNWYKQTKPEENLFFLKKIQLNTLEIILFIYIMRKDRLFYYIYSQLKNYLDLT
ETLNFSLPSDLNYIDAKTFLYITEPNHSYDYILSKCNINKNKNNYSYAINHTNNKNNTTTSNNNITTTTTNSS
NIHNNSSSLKILSVGNNLNNDIQISIETSLQNGKKLLLENFHLINFNIDKFYEIYNNKKIHSNFQLILTAEKY
LSLSIIKKAIKIYAHKEQFLKTLILDFFIYVHNIYKQKLKNNFINSFLFSLSFFHTILICRCNYNNFGFDHFY
YFDNKDFETTFDSLIYYFNLIQQKNVLTHLSDAHQMDSQYFNQTSITEQHETKEVNINWQFVKDIILNVYSSK
VEHTDRNIIKSYINEYFNEYSFNEKNEFIFSVDESYKYKFLLNRSIKEYINLIKSYPFFNSCKTYGMKIEADK
KKEEEQNKNLLNNLNKICQDEILIYEKENNDKDIFFNLDSDYYSSSFLNSDSTDVLKIKELNVNENDPVITKI
NSTETNEYIINNENIHFVNINKDNSDIKTFTKMFKRSYTNNINEINYINNINNNIYNNYYYYDDLIYEHNLLN
MDFMKNIKKMNEFYELLKKLKLLFKDNLNINSEWHKNNMTTIQLCCKKETKMFKEITNNLYFDVIQIEQQLNK
NKKKLDIHTKIIMIFLSLNKIPPKWSMYFNKKIKYVNNFITYFENIKEQLYFWNITGELKFYNIQYLFYPTQF
FKALLIKYSFIYQKKIKDCILICKTNNDSDINKKENVHTQKNIYYSSDEDIEFVSNQINYSNYVHYDMHIYTD
IDIVGIYTLNNNFDCFGMKSKNNKKYDQLPIVTLSVIEKKEKNILKDKKIPLYQNKYNKKLNKNKNNKFITYL
YFKSDKHKSFIYINKIYLFIYD*
>N.PFL0120c
MNIPNPRVFLDIAIGGRNAGRMIFELFMDKLPITCENFRCLCTGETGLGYYLKPRWYKNSPIHRIVTDFMFQG
GDFNFGNGYGGESIYGQYFRNEKFIYKHSKRGILSMCQTRIKHTNNSQFFVTFKSCPWLDKRHVVLGHLEYGF
DTLSFIEEQGTLIGKPKKQVFIYNCGVIPLDKIKYKSADNSDDDYIIPDIEKPLLEKDISINENSDFNELRNM
YKYNKHF*
>N.PFL0125c
MLKVRYENFNDLRNDDEMLNISFVAHPCYYSILDLEESELFNKIVYENFCKVAEKVDIYDKNEYLSVYRRHDK
KCRELHMLKSWELLKNTYRYKMPVIFKNLKDFDLYNMEKIPFYSINYNKKKKGTKENGFVVRNKKKINIIDKG
KCFILDNHMIYSMTPEYIANNISKNILLNTHHIMKGNNILSLKEDDKYVKHQKVNDNPYYFKLRYHYNDDDIY
CKDNYSYKRKGISPIKKKRKIEPILYNNKKMDYEEKKRILIYLDPFAGAGGNCNHMNNIFTIGCDINFYRIKQ
CQHNCNFYNKNVDFILCDFFNLVTHFRKGTIDVIFFSIPWGGPKYKNKKNFELNTEIMDNISIYKCIEVSVEL
TENLVFYLPRNVCMKELYYLYGYYKELVKNKKCVNYENDDIYKYDRKGEGGKKKKKKKKKKDIKLTSNDTYYY
EHKNKLQNRSNNILLELYINRSRCNYQKKDDNSLNNFFYFFNEKCDIYDNKFIWSKVHNLFNINIDHCYDIFN
CGVREKRSFEGEIYNIKEEHVEGESLRYCIYNRNHENIETCDNKIHSSYDYSYVQDEKKEIKEKGEYSINYSY
SNDGNINYPSNRNKKKNNNKNKKNKIKNKKNIWAWHNTCMVLYLGNISTNIRNKKIINMKDVYYIDKTLSNIL
IDIEIGKSKYENFFYSYHNLFFGEKKKRFYCIYKNKKKSFNNKLYINKNELIEKYLINEENILYINIFIMKKL
YHIIEKIILLFFNNLKNMMGYEKISFLNKICIHLENIYDKILTENIDKHIDMKKYETYIYKDDEKKGQLFYKH
LVEYFNIIIRKIKKEFILLFSIYLYDISFLRYYFRHKNINIEKKKKKLSTQGLKKIHYNIIRILFNLLLYMKI
YMNIVTNNIKLEPYFNQQFIIDDMNIDHIHKNIINRCSSIYSLRKKKNRNYNFRRNNITSYVSCFKNFIKYVI
RICINLELKKNNEEERISMKNMSNFFFRSLFNVERVDILINKLNNNMNNNEEQEDIKYYKYNLYLLLIHFFTK
QFLYNSHVNTNLDYFNTILFFYFNQIYNLSKYQDGYINIFVHFLNNFLYKNFFLKIT*
>N.PFL0130c

MAKQRLTALDIRAIVTLCKKNIVGCIVTNIYNISNKIYVIKCSRKEQKLFFLVEAEKRIHITEWKREKDVMPS
SFTMKLRKHLRSRKISNIKQLGADRVIDIQFGYDEKASHLIVELYIAGNIILTDENYKILSILKSNDTVMGKK
YNVNDIYNVEEHMSVLLYKNLKGPDDKEYLKKCIMEILLYYNNINMDTLILKDGDEISNNINNVSNVHPNGIL
NNGDIEEYNKLCLKNSGTVPKKNGNNNDNKMLGTKGKEKCKNENNKNNKSNKNNKSNKNNKSKNNNDKVENAN
CSDNEKMKKVKKLKTLCDLASKLILFAHNDLIIHSFLECEVNPLDLLENYDINRLCEIFCHVIEECLSILNKL
SDETCTIKGYGFYNIENDTNKKINNNNNNNNNMCNMNKKDQDNDKKEEYNFTEFSPIILKNHEMKLNEGKIKY
ISFDDYNLCVDTYFSKLELSKYDKQQEITKSKNAITKVDKIKLDHERRIEQLEKEVLLLKKKITLIQLNDVLI
EEGIKLMRSALSTSANWEKIWEHIKIFKKQEHPIAVRIKSVNFKNCEMDYLLSDCDDRKGNKMGDDGDDNDDD
DDGDDDNNNNNKSCVKPKTFAVTINLNNSVYGNIEDYQRLRKKAEEKIRKTKMATDFAVKKVEKKKKNKDNNK
QKGKAKSSVGQIQKLRKVYWFEKFHWFISSENYLVIAGRDALQNEILFRRYFQKNDIYVHADIHGAASCIIKN
PYKDTPIPDKTLSEAGQLAICRSSAWNNKIITSAWWVYYNQVSKSAPSGEYLKTGSFVIRGKKNYLPHVKLEM
GFCVLFQIEKNEDLNVENLPLEENTIDIDDIKREDDKGDDMAHNNMINGLDNNVCDNIPANNDKNHVDNKNNV
HNYVNYLNNISVHYTNEEHGIGITEEKIKENDSVKYLVPNKFNNIYILEKNNMLNVSVIKGTNENEKNKSRDM
YIKILYNILSDDIFKTDFFHKSVLAWYNIKSKSYILKRKKNGNAFNKYIYYLTRNKKDIYNMICILYPLVIYN
ILRKMNNPTYIYKSKYVSNIYVNKFYHTFCKIIKYFFHIYNILIRKGDRCFKRESFFFEIYKYKNYIINVFFK
GLRGKRILVDSIYNFLIFLDSTLYERCKSSQVVSNIFSLSCYNKDVEKDNCGNVYKNMYMEKSIDFVNKKMLN
YIINYKENSNIENYDYDINFSKEKALFVACEVYGRPVTFKEDSDEEIVCSEKKNGNTTNEEDVNSLCSSNYSE
ISDTNKGNKKLKKKNVTFLCDDDEDKRDVSRVSRPRELTGFIKTDVNKLLKEIESDENSNIGNINSDNSNEEN
KKEKCVSFKIEANNDNVNYDNRSNDNVSRPFRSRKGTGYVKMDMNKLLKEMEEDETKDVNDDEKKKQVTFKRK
NIDSDVVKRTVSFSSEEEHICIEQPASLYMPRPVRTRKATGFVKMDMKKLLDEIGVDDMYEEKNEDVDVEQNV
KNGENRFGDKGKKVTFESYKNNDVIKKSVSFSSEEEFIPVDEPVSLKVTRPVRSRKATGFVKMDVSKLLQQID
SDERNEEMKSDDKKEKKVTFKNKDISSELEKKSTTTYTSEDEYNISSKNSESSPMVRPVRTRKPTGFVKMDVS
KLLREVEYEEEGDDGSGVRLHNNIRSNDVIKKTVSFSSEEEYIPVYQSEERVNNARPVRTRKATGFVQVDMSK
LLKEVEQELQEEEEEEEEGNDNNDDDDDDAVEGEKKKKRREQIEYDIKGEEENKNNDDDNLRKDKKVTFNDKV
GELMNKKSVSFSSEEEHIPVEGRVSLSRVRPVRTRKPTGFVNVDVSKLLRDIGSKDFKDEGEDESEEKSENEN
VDKSEEDDNDDDEEEENTSEGYSGEKKNTNYDNHNVNDKEGLNKSGDDNSYVEDIYGNNKNNMKRNKENDTLK
KNVSFEYNENENKNENKNENKNENKNENKNENKXXXXXXXXXXXXXXXXXXXXXXXXXXXXXXXXXXXXXXXX
XXXXXXXXXXXXXXXDVRIFFFFVNNMEKTK*KIIIIIIINEYLYKYV*HNIYIYIYIIKLNEIHKLTNSPNE
GDNLSFAIPMCAPYSAIQTHKYKIKLVPGNTKKGKVADSCISYFLKNATNEKEKELIKNISMDELGNCIIANC
TPDLKELKELSKNNFVQKGRKK*
>N.PFL0135w
MESDEKNVRSVTKIKKSKVRKLNIKKDKTQKKKGLSKKKKKNDEKTKLDELYKLEIKNYKNGKKYNNNDDDDN
NNISDDSDLLNNDYDDIKEISKQVNEQIKLYKHLLRIRILTQKVLTLSNQLPLLSFVTFNNNITHKDHVQNND
NDSNLSILNNIHQNEEQVKEQITNVLIILHTFLKKYFIKRNIHINENKINDIDIICDEDDKEFFQNRRKLYFE
HSTQSEQKLFSLIDTWFTYSKNMCLNFFDIIHKITKLSSIKSIKTYEQPISSQINQVMFELPSIIEKSYPQNI
SYDIIGKDLYDYLYNDKHFNLNKHIYDDEAYYKKFLLNAIQNLKDNQEDSELLKSQRQIYKIKKKYNKKENKG
KVLSNEPIPKLVNFMLPEPRDTKNDSAYEYMDNPDFINVLLSSLFQE*
>N.PFL0140w
MNIKPNDEQTEYSEILISIKSRDSIHKYVEECLHKLKTGNVKIIGRQYAIMKAFSILEALKEKNEHFDYIIEY
NNLTATTPDRRKILEIHIYLKKK*
>N.PFL0145c
MKNTGKEVRKRRKNKKDPHAPKRSLSAYMFFAKEKRAEIISKQPELSKDVATVGKMIGEAWNKLGEKEKAPFE
KKAQEDKLRYEKEKAEYANMKMKA*
>N.PFL0150w
MTPKKKIFQNFQANDNEILSPTKKGIKLNVSKLNILNFENTIITKEKTNYEYKASLNKEIDEVLNNNNIINTH
NNKKNNLNLYDYNNIKNSTHEFYIDLNEQNKQTIKYNDNKFTPINKKEKYNLDETSSSSISSSLTNISSSLTN
ISSSLTNISSSLSNSLDEKKKKKKIKKNNSTIINILNNNNNNSNIHHNNKHNIYNKYNISKKPQNKEIHTLPS
NHQIKKKSNNTYNTCQQKMKKNISKKNTHSIKNNQNDKNKEKNKEKNKEKNKEKDKNIKKDRDKDIQTKRTSH
QSQDQNNHFERRILRSYTRNNDNVKNNLKNNINNNNTLKRSSQSVRIDSDLSSAHQNKRIKYDEKNIIHRNNN
NNNNNNKTTSNNHNKNNKINNNNPSENYKKQTDTKHTNNTQNNKYNKTKTTNTFKHPSKDHTDVNTKTFKSRY
INTYTMEEVQKSIKSNTIKLVQENSCEYQDGIIYESIQINDEEYSIGEDVLIFYTPNNNTYNAKSDDKKNQNN
NNIKENIYLLKKGKISSFYKSTNSKVIEVEICFYYDESDEQRIRELEKKQTSRRCKEDFNIYLDDDTKYYNLL
GNIHFTILDANYIYKKIYVYNEIETFEEDTHARKGKNKFLCTHFIKDKEDRLCFIPNEDHWDNLVLGSSDLYY
YFANEKKLNKNKSLKLIIEKLKINDKINDTQANQKKNNKKEYMNKAQTTTNVKANTHTKTLNDHNKSKTTKNK

ESSSTSFLQDVKKKSDPHNNDFQSSLKEDQENYYINLLKNIKDPTDKAIRMMQLDVVPKYLPCREKEIKEVHG
FLESGIKQSGSNQILYISGMPGTGKTATVYSVIQLLQIKSRKKLLPSFNVFEINGMNVVHPNAAYQVFYKQLF
NKKPPNALNSFKIIDRLFNKSQKDNRDVSILIIDEIDYLITKTQKVLFTLFDWPTKINSKLILIAISNTMDLP
DRLIPRCRSRLAFGRLVFSPYKGDEIEKIIKERLENCKEIIDHTAIQLCARKVANVSGDIRKALQICRKAFEN
KRGHKIVPRDITEATNQLFDSPLTNAINYLPWAFKIFLTCLIIELRIINEFVIPYKKVVNRYKILIETSGKYI
GMCSDNELFKIMLDKLVKMGILLIRPYIPLENISKNKSKEALLGFNESSKKGNNQKITRAQVSPDIDKESGDM
GIELNVETQLIITALMKDPDCSKKLNFY*
>N.PFL0155c
MNNIDYIEDGPDLDELLKKISATYKECNKYDKYIEKDEEYIEDDDIEEEKINDKKKKNSSNNYYNKNEYSLNN
NMNKYSYDNNKNLNVIKNKKVETFKKIDDIKNIIINEKKRKNSSTHGHIKNKNDNILRHLNVRRKRIVQSSDE
SDEIEKGTSKKSIIKYSKKRKSTK*
>N.PFL0160w
MVLLSNDVFIQELNKLCGPVEGKKKTSIWITMKRVKRNDIKTLTSNKDAGDKKNTKKVNKNDNKGNKEYMCLI
RATDGKNKKISTHVYDNVISFTQNINSIIKC*
>N.PFL0165c
MEKKKKKTKNDHVHKSLENAILLSKSHRTCDDKSKNVNDKINRKNYSLFIRKEIFDHAIIYTTNKIDESNLLI
YLKHFYQHFKKENVNKNISRKYLSDEIYITLNDWIRYNELIKNQENSVHANIFTINICKSLWILLSITIQSVR
KKNNLKKQKTIFNTPFEDLHIHKYNHNDKKKKNENDNEMDKTDTYLREKKNKKEYIKEKKNTQNNYEHGIKNI
DGIKKSDGIKNIDGIKNIDNIQTNDNNQFHVWGDDWLNEEICIELVIFFIILQFLKNDNTKKMYDKSLSEDHW
PNIKESKAGALNINSVDNTNKLDNINRLDNSNKLDNINRLDSINRLDNIHCGNKNNLNCSFINKGNLCDLFLL
NGNNYKEFLRRYLLCFFCCTDLNNCVSLLNMPLYFKRYHFLLLDFIIETNNEKNVHEMYLLYNDHKILYKSKN
ILDWLLENICCNDLIDCIHDNDIKKVNEKLRGRESSSIYYVDFEQINKDIYEIKNIYGQNIYMNDDKNIVNII
NCKECNIFFFCSIEYLKINFCKDCYIICLSVEMITTLFNCINIDIHLVTRNLKVENVIDTNIYVYTETNIIIF
GDTRNIQLAPYNILNTKQKECLDKSKIIFTEKNCDLFAFPLKCKMHLLNNSFNNLINTMKAPAYFSALPMGME
KDKVNIKKEDITLQNGHNKNNDDNKNNDDNKNNDDNINNYHHHNNNNNSNIMKEKEKCQNTQKDNNHNKCISF
NSNSDVKEDDISHILRDHKKNEPLYMGNKCMENTNNLSCSYTDYVYYLLNPSNFFLIEFPNNFTYNTKKDRED
DTEYNMDNIYNKLSIPINNVNNMQDNINKYISKEEKSIYGCSFQNICLQPHMSNYINNNVQDNSKYNLDDKYV
CLYLPEVYKNAIETQDEHIINFLNFMNSINLTKIQKQKIMKILMFKLYEYIKKSKKTYRMLNNLVQREHEGSM
L*
>N.PFL0170w
MQAASMEGNFHECHIQSKDSYIKSRTHTKVLLFSYFCFFVYTWYVNIVCVYPLNFSSTYFIYYFILQGLGGIF
LGFFSDIYGRKICLNISFFILLIIFILSFITVKTSFFLFYQNIPSTNNNEKGLDLFFYTNTPHTQINAYHEKK
KNIHNNNNNNNNNNNNNNNNNNNNNNNGAYFNYKDVYTFLMNYKQNIKGNDEYIKSDNNIKDHPNNNYLYININ
NDETKDNISVENNYINNDHLDDTNNLIINNKKKKTNKKSYNKMGYSSHTNKNNNPDIHYDHNNNNNNDNSYIY
TYEDDHFVYHPNKHINYYEIYFKNKNNNNKNKSKKQKKKYYHDDIMMEANNNENLNDENYMESQISREELHQT
ILDIIKKTLEGINKKFILTNDNTFINFMNLCIDKNINYNNNNNNNNNNNNNNNNNNNNLIHMINDIYKLENNN
IPIPENSHIYRTPNHNSNNLRMKKIQIITSIINNNISSIQEICINEMINIYRIFKKMNCLKTNIEYTHNNNNN
NNNNNDDNGEGDIYGIERCLELIHKDIIKHNNNINNHNYNNYNQDNKQTSLYYQTNMSYEKMSSPHSDEDNNM
NANQKYILYFYKIIFYIITCLSGLFIKGINNILCLIITENINAKYKTKAVSLIFILESLSLIIIRSLFYFNHF
INIFLLYKINLAVCFFLNLLNIFILNKYCDKLSIPILQIQKKILQNFYTNNNWKSMEYFHADISQISSYKQKN
LSQEENKNSNMSLLLSSMENKNITSMDKVNDSHGYKNKQDIQNDGNNQNNQNNQNNQNNQNNQNNDSHNNHYN
HEKDLKLDINHRHNSNQNSNPDYSHDDNYSDSSDDDEDDDDQQKEQKDEKQEENEPRYNLEKTKSYKKQKKRN
KKKNKNKNKKKKKNSSDDYMKDLSFDELLENNFQKSKKKNMNESEKKLCANTCINYNHILNCNIIKDSEEDLF
MEKIKDSKEFIEYKQYLKNNNIFKIYIKWLYYTLYAHKYLIISLCILNFLFNFLYISFFIIYNIFIYDIKSSQ
NFYKMNDSFIILNSIILIFYIFLYFYLHRIILKILNLFGYLLITFVSFSLILFLYSTSYFHISLYDNTYIIYT
IIFYLFLFIPNVSLFLFSINYFHTIHKGLLLGVFTFIGNLGFVSYTLFRFIIQEQNMTLYNLIFSCVICIISC
IFIILFIPQINSCIDFYIVDHSYFNHFLLYGYNKKKVSPLFTSLTFPCEISHT*
>N.PFL0175c
MALIIKGNEIFENEDLRKKLKEHIEGDKLIGIPTETVYGLGGNSLSQRSLQNIFRMKNRPVSDPIISHVYNIY
QAYDQLYHINIYEKYIIYILSKYFWPGPLSIIAKAKKEAPLILTAYTQFCAVRIPDNKITREIIKICNTPIAA
PSANKFQHISPTNSLHVYDEFKNEDLLIFDDGQCDVGIESTVLKITKYRINHEDKNTDEVLKEKDVNVMKDIK
KINNINVNNSEGNNPVEIVNDEEYECECNEELAFLRAATNSKESESNMEVYEKLKNMFDLIERMNSINNDDDN
NNNDDNNNNDNNNNNNIIVDKILKYKSLYNYSIKIYRRGKYTKGDIENVLKKYDLLKDINVEMYEKIKFENLD
IFKNEYPKSGIKDYRCDNINKEDDIYKTFNNEIEKNEVCPGLLLTHYSPFVSTYLLDIISCKTIEKENKGELL

ETNNNVCKKINLNKCVLLDIGSSLLKYDKNFLKYINICYDSLEVNERIKYITKQFFLFLRQAERLAIENKADN
ILISIVNIKSCDENYLSIFDRIYRAASGKMINLFIEHKDIIAYIK*
>N.PFL0180w
MNEQKKENINIKNISNHNNSDGKEKSSIPSKNGSWYYPSQKQFYNTTKKKGYSFSQEDLNMALKIHNAVNEET
WNKIMKKEQKYFDICKEQKLIKFVGYPTKLSIKAFMLTLIGYNKPFDRHEWYIDRCGNTIKYIIDYYDGKKEK
NSAVSIYIDARPQLNHQNAIDNVKIIYIKICRFLNNLF*
>N.PFL0185c
MAANEGNQPIPPEEEKEISSLLESIKIDDDKMTDLTEEQKETLKKLKLYQKEYYDYESKFEYELFLLRQKYHD
LYGPIYDKRREALVGNGEAKIGTPNLPEFWLRALRNNNTVSHVIEDHDEEILVYLNDIRCDYIKKNKEKKEGF
ILSFYFATNPFFSNSVLTKTYHMKCVDCDNEPVLLHTEATVIDWYDNKNILKKNVVKKQHNKNSREVKTVQQT
VNRDSFFHFFTSHKVPNSNVIKQLSKHEVAQLEMIIEGDYEVALTIKERIIPYAVDYYLGIIIESESNSIVSD
VDSSYSSSENNSNYNSYESNNSAYNDENSNVDTNEYDDNEEEEEGAKSNEDPLTS*
>N.PFL0190w
MALKRITKELQDLNKDPPTNCSAGPIGDDLFFWQATIMGPGDSPYENGVYFLNIKFPPDYPFKPPKIIFTTKI
YHPNINTAGAICLDILKDQWSPALTISKVLLSISSLLTDPNADDPLVPEIAHVYKTDRTKYHQTAKAWTQKYA
Q*
>N.PFL0195w
MMHRDKLEKERIIKRNEKHVKNVVVDNLIVDKRFNNDEGNKYLRKSDAAYEDFKIREKQKLKEYIKIENKRKE
NLQRHENKWAQIDLNVQKEVEKTNRLQQMPFKSEKNKSKCSHDIINHQYINYEESMKMERKKNNSILNRRNFL
SEKINGSYNPITGCFNY*
>N.PFL0200c
MNLFPSSSEKSYKDIFDLDQNIYKRQKRKNKILNSSNDSNWSHVDGNNINYNKRKDEQDHFVEYNLFNTSSSH
DNNNNNNNDDDEKENNKNKNKEKSMIKKKKKKKNDEVPKKYKEKIYTNSSNNNCNSIKIQKKQEEKPFSLFSS
NTYSDNNINKKSIEKNEYCLFSNSSESEHCCESKKNKKESYINEKKMYSLFSSDSEYNEYIQDNNNNNNNKKK
NVSNNIFDLENNDDVHSKDEKKKKKKKKESFLKWTPHKNIKRSYNKKNYNKITYNDNNNILYKKYTNIYNSSK
KKNADKHFKQNYKHKDMKNKVNNLFKEHENSEYRLVNFEKLKKRAYRKLNFLSIVDVQKSLSDLSEERKNYKN
KKPHDIYKDTYDDNNENTYNEHKQGIYNNKQYDEKRDNIYERDILNNSICSSDSFYKSNESFFLHEKDEDSTN
LDSIENNKYNNQTLDIYNYYYQNNHEERNKDNKYLEKIVYNSTNNNCDDLYNYLFKMNDSIDEDKESTKKYNN
DEGNVGSNNIDDEDDNLNDELGKAEQRKETNQTNQTNEKNETNQTNEKNETNQIHQTNHIHQTNHIHQTNHIH
QTNHIHQTNHIHHILDDKDNLSNTRRIRQLKRRRRKKNDFLLKRNIMLYRNFKNINNEISRQNNKKGVRFKEM
IGDFIRVLNKNVLKINEQNEKKQKKKEGEYYENNINNNKSNMDGYNICDNNTNFNLFDDDTSDVESFINFECY
INSCDMMEKASCDCLFCIQHKKILNKDELYLILFKYMEDNKTEGTTIIKPYFLNIYVFTCFINFCIHYKNISI
CKADDIDENSNNSDDQCLDDHLNNMNNICKDEKGFHYNNLDKKKKKKMHFYKYSHSLYMKYNIKKMIYFIPKR
IVLTTVAICIYGVHFLNKDNEINEEIKEREKKKEKEKEECIAIINFLRWKKVRKNFYEILKLKKKRKENLMIY
KKCFFFINAIDEYCNMCNIPIDDACSYNTQKNKKNKKNVHNRMDTRLNLRNNKTNTYDNSFEYYNQAEMQKIQ
KIQKIQKVEEIQYIQDILNSNLQVNNIQFLFFDPIYVSSKKYVHFYPSHLIMLF*
>N.PFL0205w
MSKRNNTNEKIIFPLFNGKVTKSSKSSINIKSLYELHNELNHKIYTNIYTYNTLCDGIFFCRGVNNICGKKNS
GKSSMCYHICVNLFFNDLLHYFHLFYSCFFSFDHFLQKKNIHNVRDYMNTILWDDIEKEYIKQHDHFRQLLQI
IRYFVLLFKQHYGKKNYTEQIHNVDNTNNIDNTNNIDNTNNIDNTNNIDNINNIDNINNIDNTNNIDNINNIY
NTNNMYNTHNIHNTYNIHNTYNIHNTYNIHNTYTYPHTYAHITNHIFDDAYECFYTQIKHFYKRKVIYIDLDN
SFYIDRYKNMIFSSLEKIKKLMNRYTKFCSEKKGIPFLLLLEKTNKYIKEYISCNIYKNYSQDFIFFMDIFNH
YLFKYIYNISFSHVFKNLQLLKIFNFYELINVINYIYEHIQTYSSSSSYYHFNKYIPHDLGIVVIDNLNYIFK
GCPLNNNINIYYELEMILNKLSILSLEKHICILITNHENNYFTRHETFNKKNDNSFYKIISPYIYNNIHLKII
NQKIFFEYNYPKKHTKKRKQDTNQNTNNISKKKNEQIITNVADDIKHNIQQNEDCTLYNVYSSDDDQSLDQMD
VQEKEDDNSFYEKKYNLRYIKIKGKKKSTFCFFEINKYGIETMLM*
>N.PFL0210c
MSDHEMYDNIDAGASQTYPVQAGAIKKNGHVMLKEHPCKVVDYSTSKTGKHGHAKAHIVGIDIFTGRKYEDIC
PTSHNMDVPVVKRTELQLIDITEDGFVSLLYDNGDTKDDLSLPKDTEGNLDEVAKQIRNLFDNGKSVLVSVLS
ACGQEKIIAAKELSS*
>N.PFL0220c
MRIPPFLIILFFVFYKKFNVNCIIQRQDVYLDDDFKSFTYFFASSPSADFLSRIVHSDDSRFVQIKNKIDIWS
KTVDKAYAINQVSNVIMKVYVTLLLLLLLPYFVYMGIFGNTKNQTVFALSSILSYFVLLITFYLTNGILNIGF
VCSLPLVFGFLIFNLASSDCTIKSLYKYTRYIFCFIIAKLLYDVVTNLKLDDLNSFDYGISGYIYMNLLKGNY

YFILKIVHLIVLCIITLVIIKLFPNVFSSSSLQSSTSIIADKYMVSFLCALPVSAFITQIFFLICKTINPIDP
SIFFMIPSSLNFMSISTIFSLTTWIITTCVLTILRNRVESDYNHILNKLSSRLSDFL*
>N.PFL0225c
MYTIDFVNEEKYIQRVGDICGDFYVPVCLPGRYKYNVEDIENLLHLWMTKNHKIKNGEHKDLYLFSLLFKETK
FSLILTKSKYENNISSWETLWLSWKHVFKNSFDENNNKYIKLYTNNIIQFQAGCIYLLFLLYFSQHVEENHYA
LPLYLSPDIMNLCLDVTEKCEESNIFKELRFILNFMVDTNSIILCCNDELNEIYQDRYGDPLYIEKNKMSVEN
SIEINDVDINKLYDTLITDIKYISEKCDEKRRKKTLSNTSTNSLLTKLEHIKKVINKDDL*
>N.PFL0230w
MDRQVINLEEILPFSYTRDVELVPMYCFEVKKEVAKDALNIMKDYINNQLYDKYNHLKRCRKCTDSVQILIGA
CNHIPFELSQKLIEKMKQENNNDEFNDIIITKIMVSKYAPITRKQYIEWSTYWPLYFRKPDKDILILTKEEIN
KYIKLLYISIDIGKQFGTCNSGCIITYNDEIIASSGDNIKNHPLHHAPMLAIEQVSYKLRHIWLNKQTEKNNL
QKKMKIIHKQQINNRDSLNDHKHENNKSNHNILIDHIPNDQYLCTNFFAFLSHEPCYMCAMALLHSRIKCVIF
DKENKNNGALSSQEKLHCIKNLNHHFKVFKTIRG*
>N.PFL0235w
MRYRLFPPLHFLSYLKKIHIYYSPQRCEKADVARRLIIYCSSQKIKERYPMLTVTWELLAYENPPMIEVEYFN
GQKEKINIEYFSNTQKKKILDEWKYTASLDPYPEVMKPTLEKPRNDF*
>N.PFL0245w
NFSNCRKVFXXXXXXXXXXXXXXXXXXXXXXXXXXXXXXXXXXXXXXXXXXXXXXXXXXXXXXXXXXXXXXX
XXXXXXXXXXXXXXXXXXXXXXXXXXXXXXXXXXXXXINIYIYIYIYIYIYIYIYIYFISFSAYVISFVSTTY
KKINRKKMIKNIRGTEIQANNLSMATIENHIDNKQQRKDCYYSFQNYCLTPNQFNSLLFLLNGVAVITVFFIA
IVIQRTTFFPVKSNQEEIIDVSQITN*
>N.PFL0250w
MSYNDGSEEENDSNTYIHDEKKKKKKKNNKNAYALINEVNYSQEDEQEVIHNTNSEDETSNRKGNGCVYSLSD
QNVEDHIISLPEYLDLINNNNNNNNSNSYKMKKEKKKKKKKKKNQTDEENMKNKKDHIYQNNITNQQNDIKND
YKKINHHNMNNKKNKIFCQDDQNIFNINHTFQIHETVQNNLIIPPSETCLASDIIQPSDTTQSNTYLNEATAS
QNDDNNNEDSSNEMGMFKKIFYRIKKIIVDKKGTPITNENDVDNDMCELNVMENNMNNIHSNNNNISTHMDDV
IEDESNEEVFVINRNTDGYINTRENINVSTHVTRQMINLSELNPNDLLCNVSEYEEGQNINSLWNDNNNNNNN
NNNFLVGSLNALHPINHNLRNENIHNDNINNTHINYDNNNSYESPIHILSFSFKNIYNKISSYINEHITHIKE
KIKKYWLERVQEANTQLNSPRPVHTRNTTNINTNININEDENDDPSCVQILFFMGLICKFPILWIIGSIVFCI
TPSEHRKTKTWSLVNTFFALLSIIYFITTTNFRLRKPTFFVILEQNVENKNTYPKGILKYNNMIHHKHIIIDQ
SSLHKWKDLHTNKVYKTSENYFLNRNFLSSQKPDSNILLSDTIYKLLNRIQVTVTFGKGNIYSSDNIEKIKPF
FQNLRINMDPISYDKLTMTDEDIPDDFFGSGLRCERTYNNHNQNINEPQQNKEKEKWYLFWKEEEINNSHNKN
IYNISIPVGEIFFFKSEYNCRIAFLYPKSILYDQNDIPSNFVEIQKIIIKPF*
>N.PFL0255c
MNETLCDNNKAPKGGMKTSKKYSLISNQTLNQKENTIWNIVNNGRLPNYGLNEISVMNILYQISSQNLCNSEK
NVKIGERENRIYSSIVRSKYIGFGHGIGRSGNLDDVQPKSAGNSILAKLTSSFVKDLIRNFGIKSCEDVYILP
YATGMCLSTCLLYLKQIREDSQYVIISRIDHKTCYKCISFCNLKYFVVDMIYKNEELYTDLEGIEKLLEEYKE
KICCVMSATSVYAPRNSDDIIKIAHICKKYNTPHIINNAFGLQCTYICKEIQKCFESQGRVDFVVQSCDKNFL
VPVNGGIIFSSNREQMKLLKKSYPGRTPVNAYLDLFITLLEMGKDKIMNLRKERVDNFNWLKEKIQLLCLKYN
LELIKTSKNFISMAINLNHMYTHCNTDNPKVINMLGSMLFYRNVTGHRVICSPLLNRNYLLKKAREKYMNMLE
NKKGNDAMMITSNESNEENVKRDLSKKEADIYDNMYSNGFDEIKSVENDMLQNKSNIIKAYEKQIEEEFDKLN
EPNTIVIGNHKFKNFGSSYNSYPISYIAFSCVVGIERPELENFVSKLDNAIDYFIKFFKKKKEEELK*
>N.PFL0260c
MDRMKCVYSLGSAINNKNYIKDMSINGMRNLENDNYHNMYSNINKHEEGILTKVQGIPNRAVNQNGCVIKVTN
NKCNNNNNNNNNSYSYSSNNNLLYNKPFIYKKLIVFDYDDTILPTSWITSKMKLSLYDTIPPYINELFNKLSE
VVINTLNLCSIQGKLVIVTNASLEWLINSAKKYIPMVWSFIMHKNIRIISARDTLINSLIDPKDWKKVIFHQI
INEILAPYLYNTSFICFIYSVGDGNDERNACFFISQLNQYSSCIFKSLKFLSEPTCQKLIAEHELFYYFFNST
NLNASNHHNNQANNINMNNINNNNNNNMSINSLMYKNYNSIKNV*
>N.PFL0265w
MNKIHIQWNDKMINSYNKTLLLNSLRKTKNNIFYATYIKTPFLHINQRKINYLKKDNHSIYSKYYNNEPVYNT
YNPILNYDILDIIKVYGRLAEHSKYESLNININEQDVQVKENNNLFTKIFSFLKSKSTNDYNKVYWFHHFL
KCGKMNFNDFRNSLFKLKFKWPKDSIFPTYNIFLFEKGLNKNMDSPTIRKKVENYMQYQEINDNLLKSCFYCF
SNGKEYITVYDILRTFNNWKKISKHKNNDKPKGLFSFFKKEHNEEDESVDWFTFKKKIDLSTIQMYESKK*
>N.PFL0270c

MNIINYLYQKSKNIYNRNTNLYNYNNIENENLISNFVDVTNNSNLCYSKESSSQSSIFMNKSDHIYRNKKNKK
KKKIIKKDQSIIENDSMSNNSSTNYYGDFYNNNMNNYKDVIKKKKYYKGDETFVCYNINKKEKNKMNINENKK
FKGKYYNDNINVNINKYDIKSKYEDKNKCNNNIKNNLNFTKSSSNNNKINDNDLIKKNVYEFNIDNIGFDSKV
YKNRCDGVCVLNDNIYIFDKIKKCIYLYTPYNNVWYIFLNIYEEMSLRKNTLLSLCSSDIKNDINVNINKSYY
KYHNNISFINYTDMVYLNNSLFIISSNAHFMNICKINVHNMNTLFSTIVVDTFNDEINNFQSFFNLIKRKSNI
KCDRKIVNNKKDTKKENKEWDIFDDKYIYKCDDDNHSDNNSDGHSGVHSIHNNNKKKYKYKNNSLDKYSSNCF
PLDNRRNISSDSLYDDYKNIQNDLSCDNHVVSNNFYNQIECNNSKVSRKESRDLQESIKDIHQNENIGGNQFY
FNSKNEYYNNYVEYVNGMSEIYEFLQNEKNNFFKKKKRIIKARDYFSICSVHEDCYSLIYLFGGKGNTIKNND
EYVDVIYNDLYLYDFYNNKWIELYPYSENEKNKNINFEQINENSLVDDIHIKKRNDNFLLDIEKNVNTCNEKK
SYSDDICDDIFDEQKNYDNNFSLFSCNNTVPKNLEQNNDPSNVSNKSLGKNTYEQITTTIGTIGTISSTNLGN
DIFETLGEGTEFRESFDNVICSSSTTNMKNSKQKKDINYKMSQNIVSLLNNSVNTKDDNENNNNNDDNIINRN
NINDYRSDDMCNLISDSSDILDFKHINNDKCEKNGMNMYPDDTYNMNHNKINILTNEKTNIYHERNDFLCFNC
LNNKKCTYICDIIKNDIKIKRVQWLGKRAGHSCVYYKSNLYFFGGIDFYSFNSNKINLNFCNNLFIYNIETNK
CFEIIGKGEIPEKRYRHSCVLINDYMFIIGGECKNSSLPKYDIYFYDFSNSVWTEIVINSKIGSNSLYKTVWL
ENFGSIYFFGSSIIRLTKKDFQYLPYNKNKKKQENIHTNKKDYSAKNYFLSKSTFVNKTNRFQ*
>N.PFL0275w
MSISNSSEQANHKNMEDDLNLGENNNNHNFISNDDGSFIDDNNIIEDECITPKDDEENINSKRKKKTGKMNNE
ENKNIRLGRRNDQSRCDQGNTINYLQQNNNVNINSVSNALHNVINEVMNSCVAHMNNNNNNNNNMNNSNHNNNN
NNIHNIHNINNNRSTYNNLNFNMFSLNNNNTCNYNNEPNNYFLQDGVHAESNNADDDEILVQNDDGEENHVDQ
EGHGHGHRSRHRQRQRRQQEDEDNHRSINELQDDENYNNNNNNISNNNNNPNDEQNTSENEDHNNDGDNNNED
DDEENDELELEDDEHDDDDDDDNDEENDDQLNLEHLNMISSYERRNKVQNRINHQNNNNSNNNINNNNNNNNI
FVNNLHNNNQNNNSLNQLSGVHNNNENLYNDSGERYNLSNNNSSLILKNDKKKNNNNNNNNNTSKNNMNNITSN
NKSLSHINTLNSSIIDNLTQYLQKELTCPICLDYFYLPVTMNCGHTFCRYCIGHNKLNGKNCPLCRQPLGNTA
CINTIISNLVRIYNLRRKSLKIYKSIEIVNTVDEMWWNENFIKPQVSVSLFLRIFLHDMISPPIFFDDLTSCV
IDFFTVNKLWSKAKWVFNIKDCRVFSELIGYDKDDKETTSERLHAWVERYITQNPSMCMRKDEKVILKLYQDR
THRIDSHVFDSSVLPNRLPWDGGRHAKSLIHMPHSSVSLSHLLFVKAENNNIGVVDCGSTIGTMIKVNNYHVL
KEGDVIHIGDRLEVTVSIDKNTAGMPYKGYVWNKNANKVLDRHELNELIKMETNSNQDIFITEDQIPSYCENI
ESNLLIKFDFGTTKEEITPKTEYIDPKGVVLGRGPYAQSSYKKISVTNSNGYVSREHCLIYYDGSKDAGERWL
LRDTSTLGTFLKIKPFSNPIPLPIGSIFKAGQCKIEVCSHDSAQFIQYPERSISLHNTNNPPNTDNNNNNNNN
ADNRNNDNDNNNTNNNNNNNNNNNGGSNNSSGSTNNNGGNNSANNHTNNNNNNNNNNNNNSNNNSNNNNSNNNN
NNNSNNNSNNNNHSSSSNNAPNSSHANNNHSNDNNINNNYRNNHVHTCISNNSYHRNSGNTNNFSNNMHSSYN
NIINGIYNYQNNSRNLNNSSSFGRTNILQDNSHHENMSNMANQNNNFEAIVQNLFQRNVNTLGRENNPSLFSS
RNEQLRLNNNHNIMNNNNTNNNNNNNNNNNNNNNNNNNNNNNNGNNNNHCSHGDTYFYNYLRYSVNNNGRNHNN
SGSIDYLNDNEESDNNNNNNNNNSSTKERNNNRSSVNDSCIVENHQHYHQNNNNNNNDNNNDEHDHNNNNNSSG
HGNNNNNNSNSHSNSQNNNNNNNSNSNSNHNHGNGHNNYNKSHSNNHNYNHLNVNTPDYPFNVNTRFREHAYN
SSLGSCFLKNKNSEMQIPQLNCTLNSNHNNMNNFVNCNDNNYNFIPYNLDQSNSKYENNISFINNNNNNNNY
NNNYNNNNNMINMINMNSLNNQHTAALDNTNSLFNIDKFTLERRNINICANINSIFNNINRDSPCLDNRVNLA
SNETIQNAPIRNMHSLNVNKKSEKPKTNDVYKNINKNNIERKNSKNGLKKFLINNNKLDILLKKKRNHSETFL
YTNQMSTTQDEEETGPNTINTTNYAEAGGIYNEMNSLKLIYNYINNLNINNKKTNVSPTNNIIECNSDLKLEN
NANTKNLNSLICSGEFNHITENNHNNNNNNNNMNSMINNINSHNHIYNNNQNNINHFNDLHHSYNNINDPIIIG
DIKTKKLLFSDHNNVNQNSFFQSIKMNN*
>N.PFL0280c
MSEEEVIANDSSNTNVDSDVDEQRELLETRTAQELFDMGRLEFKENKNYDVAAERFSMAVEKKVKELNIEGSI
HSDLREFYLSFADALLTKEEEKNDLFEFLKKKKQVEVSDNEEAADKEEEVTDEQLAFEMFEFARKCFEKLLEE
KKDVPKRDILNYTYVFIRLGDINLLNHFFFEALKEYEKCVDLREKYKVGDENLIAPLISLSQSYMFCGKRKEA
VEYFEKVKKILLDVRQKTTPLPENTNEKIIRDTYDDVQIQINDLKRQIEEEGEEGKELGSQTIAKELVVTTKS
EFDKSVLNKEKAEVTKITVSTVNNEEHVAKKRRINLSNYKN*
>N.PFL0285w
MRFLKTLFSLNCLASVGFYKKFACKNLFFENSFIRKDLHTYFIDKRKYFHLNKKDICTNTIIIPFYKDNYSYI
FYDDKEEGIVVDPADYNIINDISKKENIKIKHVLCTHKHSDHNNGNQYYYEKNINVYGIKEYDNKYINQDISN
LTHFQINNFKINIFLSNFHSKNQVSYLIENDNNKSKKNIFFTGDFLFISGIGKNFEQDNEDLYNSINKLKLLD
KQNIYIFCGHEYTLDNLKFALTVDSTNKNLLSFYDHVVNSNKNYPTVPTLLEHEYLYNPFLRCDQNDVRKSID
LYAKKKNIKIQQESDYIVILRLMKDNFKAS*
>N.PFL0290w

MDGAIELLPNVKNDHFLENKNLINEYNKLYMHRYYHINNHTTNNDNIYNDIICNNNYNISNIHTCNSNISNHT
VLGCTSQRKSFNKYKFLLNLSKTLLSKKYLSINSSTRFEKKNVKNKDFKNKNLKNTIIKEKAKSYMLSLLNKK
ICNHLYEENKIKQSNEEKVKGPTENYDPIYNLKINNKNEDQEINEQNKSVLNTNKDAHKYVNHVNGKYLNNYN
VSESMGDNTIILGVYNNTKDIKNYELKDKTKNESINFDMRNIQLCNADIEEGERVTDRNNTNDTNSNYSKKNE
DIEDHKNDNNNDDNEDDNNDDGNYDDGNYDDGNYDDGNNNDGNNDDGNNNDGNNDDDNNDDGNNDDDNNDDDN
NDDDNNDDDNNDDDNNDDDNNDDGNNDDDNNDDDDNNNNDNNNNDNNNNDNNNNNNNNNNNNDDDNNKKNEYK
TYYEKIKELKSNVFIKEYKNSEKFVKKKILKYICKNEIIENVSNVFNYVKNIYFVIIKNEKMMFYFLYNTKDV
LNNIIYMENENKVSYNDSKFLLYFLLNEEEIYKKLFEKLNLDIEKERLISKLKKINLKFNELKYDFEKNLHKN
NIHQIYFMKKRKIFINNMKGLQFNKNSFNTSNATTISGLKMVSLKNVIPKLYNCKDYKKTNHFFFNMYENISK
ENNGVDNVNEAKKKNTSHQNISKKGEEKYLPPEEQTKKRKKKTTQKNNKKNNMNNMNNMNNMNNMNNMNNMNN
MNNMNNMNNMKHDNFTFDGNNKPIANINNNNVNTNIINNNNNDKHNNNDDNNNDDNNNNNNNNNNNNNKEEVV
KKPAIRGITSFTLFAREKRKELLDQKIYLGSSLTEQTSAVAKIWNNLSDEQKKEWAVKASKINEENFLLQKKK
KKRKFTAFSIFAREKRKEYKEKNIDMGLTLAQQNSHVSKLWKQLTAEEKNKYKVLSNTVNATIAKAYSKSESG
GTTGERGSGFKGRIKALDNMMSQHYANNNILNNDSNTKNDETVKKSNMDNININENINVSHGKNVTNIENVAN
VQNMQNIKNIQNIQNMQNMQNIQNGHINNNHISYGANSNVATMHHTNNTHMAHINYNYYDQTNIGNNNNNNYT
MNMSLPANQNITDKPAMELIPTINVLHPYANNFNGNVCNTSTNVINNNMNINNMNSMNNINNINNNHNSSSGS
SAIAPPCDIMTDDHLNSAATTTGTISTSYNGMTTNNMNNKNSNQVMIMMVNKELNQINIKNNNSNVDMHQLKG
DNNMNNNNILMDNNLVNGDVINNQNINNNINFNTFQMNYKNVEHTHINNDPYQKIDVTQVDDLILKKKLKKDE
KLKVKEKKNILRIFDKQRKQLLKEEKKMDKIKINENQYNNTTLYYPTTHLMNNNLNNNHYDISTNQYINEPNT
NHMNYYTNIDNKMVTTNVANNIMNFNNVNNVNVTNNNMGNIHMATINNIDNVNNVNHINNMNNGNTFNYSNHY
VPYNHVNHVVVNRKDKNIQMNNNNNNNNNNNNNMFVNNNNKNAPISNYNKYQHDNMNNNILKNDQGNNNMNNYN
NYTYNNIYFNGTVNHGKMPPVQNGNAIKHMDEKEININLNVPTKKLEKKNFNKIMNINEETLYKNNNELNYIQ
STLHYNQMSDNNINNKMIPAHHIHKDNMNNMNNMNNMNNMNNMNNMNNMNNMNNMNNINNINNMNNINNMNNI
NKHFDSHVLKKRANDINPNDANYFNEKLKIQNSLNDDFFDFTTTNNNCETMHSTTTQENINLNNTINNDLNMD
INSNLNLNYTTNELKKNNTYYYPYNKNNKNNDVNHIYDTHQSERNIYHKNNMNNNNHIINNDNNYSALVNTKN
FIYYPEQNFSQTIGKENQHIYYDTDLDKPERTLQDTEIVRPQNKLFFNNASHFLELVNNKIIKKKKNHKSSLN
SVFNEETCTIWDLVMKNKKKKVKKKRDKKRERDGCGNDNDKELNEMRNIEYDYNNFDLMMLNNDLNNYLKDTN
RSENNEDDIFNSILNNIDDNNIYNNSNKEVAILNNKNNVVEENRKNDNTDNLPEDDNNNCDDFLNLLNIGSSN
FSNKLNINEHGDVIYDEKKRLGANNLINENNNNNDKLGLLMEKKNEINQNLNKSQIEKYNYAYKKSKLCKWTD
EETDKFYQAIEMFGIDLMMVRALIPKFNDKQIRDKYKKEKKSNPLKIEQAIKKNKEINLDAYENEHGKIDHST
HYMYSDYSSTDEKNDNNNNAKAFTNVTGESEINILTIFDDKNENANIDDEYATNEEPSEFNVLTFF*
>N.PFL0295c
MLKNNFNPDISDDDYVSEKQIFQGSNKTILTVESIHVVKGIDKDIKKQIEEGKVFLSDISPVKNAEIEEAFLK
SLCKSSSTCYDNNNNNNKIFHHHFNDKKKDYSNENSSEEININIEENYDRFNIDKSKYYNNDNLLSYNKKNKN
NDKDDGIDDNVLIRPPSDLQEHKDDEISSQYFKSYSFVKNLSSNIFEKYTFNDLPDLLEQETINYIKKQERLL
QKKRLNANLEALRLQYIPINNDKFVTIPTDIKENILALFACIIIKHIDSFEDEDIQNHLDELCKNFLKQLADL
LVIDPIAYQCVIKPFDQRKILREVTGDKTPYVLYEKPFVDNILEKIGSLQANIDAKFKNVVEELNNEMDFWQN
DHIDDEQSDQDKEYLKNEMLLEHENLKIEDNKLYIKNNISPTKTFPEREIDDHYKEEAINDCFTINNKNEEKI
VKKINKKHDTKKGNQINDSNGCTNIGECNNDPLNVKLVDKNKLDTSKVENKDNDENKDDNDENKDDNDENKDD
NDDNKDNDDNMDDDNPNEVKECNKKKKKNKKKKKKKKIKDNPNGRKEKDKLDDLTEEDKIKSNGCYEKVKKK
NEEFGDFIKKDSNTSEQLNNSNNSLNNLENNKIRKNINEKNVKHDMINDADILSKIKSHIVNKMDIYESESYY
DESDMEKHLNHGVPIKAPEVKSVLLRDLITTYIMTGNNNARIHLFFQKFAKCLKINELLILRIEENLAADLIS
ALQASTNETAKRRNIRRMKIAAAALGGGALIAFTAGLAAPGIIAGLTALGAGGSSLTAFLASASGLAFIVSLF
GAGGAGLTGYKYSRRIANIKTFEFIMLNGNISKSLSVCVCVSGEIKSDDDITNPWLEVFPNCYCDLYCLKWEN
HLLKTLGSLIETMLSQEFAITASRIWLQYTIASTLSAALTWPLALIKYASNLDNVYLLIRERAQQAGRILADA
LSDRNTVGQRPVILIGYSVGARVIFYCLKYLYSKKLYNIVSNAIFIGLPATTSTKVWEKIRMVVTNRVINVYS
KNDWLLGFLYRYMEWKINVAGLIAVKVPNVENYDASGIIHSHLDYKRKLKDIFHLINFDM*
>N.PFL0300c
MNISYLRNFSCIYFLIIIWKFSYVYNESYSNIKWEHDLFSISDLHSDLDALKKTLLTENIIDEENNAIRENVF
VIITGDVLDPAYDDIEILYFIQNYNIKAKPLNSKIQLILGNHEVQNICLDFIGNKKYGEEYKARNKLFKKGEV
LYNYLLDLPFVIKVNDILFSHASILPYYAKRGIDYINDEGRSEIKNNCTLLKFKRITGQRFCVCCYNGPTFNR
YFSRAAEMPFRREVCKSLFKTLNKLSAKKLVNGHTIQRNRKVNEYCKGGLIMADTGISKWKYGVINYVQYFQD
GSYKVNYINSDI*
>N.PFL0305c

MKNLDINTFDNIEDIPLGSSEQDPYDFFTLSDRNVMNSDMKKNIVQWNSRYSYNQLKNKDSLIMFLVEIFRSL
FVSNCIDKNIDNVLLSIEEMFIDHYYNPQHSRLKYLIDDVGIFFTKLPITKAFHTYNKKYRITKRLYAPPTFN
EVRHILNLAQILSLEEGLDLLTFDADETLYPDGHDFNDEVLASYISCLLKKMNIAIVTAASYNNDAEKYQKRL
ENLLKYFSKHNIKDGSYKNFYVMGGESNYLFKCNEEATLYSVPENEWRHYKKFVDYDTVQEILNISEKCLEKV
IKDFGLCAQIQRKEKSIGLVPNKIPSLNIKNEQKNYMIKYEVLEEAVIRIKKEIIKNKITAPYCAFNGGQDLW
VDVGNKAEGLLILQKLLKIQKKKCCHIGDQFLHSGNDFPTRFCSLTLWVSNPQETKACLKSIMHLNIKSFIPE
VLYENQ*
>N.PFL0310c
MQSKFWAKAIDEDSGDNVTESSESEVEEKKPIVSAQAERWAAIDSSSSEEEERVIKTYEGKRLHFYETTGNSL
NENMYNNDFNLLLKDYENLYKFMIKESADCIPNFAIIYLDKLSKYVEKTFQNNIEKKNLSKNKAQTLNKLRAK
IRKCSEFYQEKLNLYNENPDDFKDDRKKDMDDDEDDEDNEEDDEEEEEDEEEQEDQDDDNEDEKDQDDDEDGK
GKKKLSSKTKSKKGKEKKEEKEDYDDDNDEEKEDEADDDNDDDDDDSDDWSYSEDDNYASDEEDDKTKNAMSK
WGLKTSTKVEKKKTVKQKKVKKESTKKEEKANRVVEDSQSAKNKGYAELLSTKNLTEDVIRERVKLVIEKRGR
KGLDKHEHINILSKLCEIAKTISTQSYIEVLEHLINLEFDVVSSVYTYMSFNIWNKVFKYVELILDILIQNEN
FYLVSINIAEEITEETTNEKEKIIKSCKTLISFLAKLDDELLKALLYIDVQTEEYRKRLGKTIHMIGLLKKGY
NYVKCLKNMPDLAIHISSRILEHMYYKPEMLFKQIWTYLMNGKEMLSDSLNNSNNIASKEDGQGKKKKRIDND
LFDEKLINNDSINPPKIIEEYVYEIFEFGTKQQKVKALLQLSYNKSLYDEYLEAKELLNVANVHELAMNSDIQ
TQILYNRNLIQLGLCAFRHGKIYEAHCCLMEICSQNKHKELIAQGISNLKNQEKTLEQERAEKRRLLSYHMHI
SIELIECVNNICAMLLEVPNLAKNTYESKKDIISRQFRRFLDIYDKQIFNNPPENNKEIIILATKHLQKGNWK
LCCEKIFSLSIWPKFPDKEKVQNILKEKIKQEAMRTYIFRYISIYESFSIDQLCVMFDLNQNVVHSILSKMMI
NQEIPAFWNESSKFILISKVNPTTLQNLALKLAENVNEVMEQNELALNMKNPKFMFMQERRTQMKEEKSNWTQ
KKGDQKYQKNYNQKKNAHYKKNYKDNNANKNYKKN*
>N.PFL0315c
MLEYLTFQQNVEKKRRKIFKKLREKSTKWKYIYQKKSIYKKCLNSRNKIVFVQGEKNKFKNIHFKGNKRRIMN
ITNIFKIYNLKNNNENNKKNTKVCSTYKDNIKNVFTHNDNKINFDKIEKNIMWKKLLIDMICFNNNLNVPYNE
SKYYSDSTSASSKNFDNHMNEKNITMYYKGKGKKKCNPINKKGNNFDYINKINYSVNIELHSLFTYVRTKLKK
KNKDSFKNTNVLNKMSLHDDNDLINNRYNKEQYAKLNNQYIPENKEKILNDIKTKPNMAPPPNRRKSIIGGVR
ENMDMYDNNNNNNYNGPYNNNYNGPYNNNYNDPYNNNNYNDPYNNNNYNDPYNNKFRPMVKSESHHIKNNVQR
EANVHIEESVERNKKKQSNNKEENKVSKEQEQLNNNESEGIDEDLFSDEFDMDDYDSDGNKKEKPYYTDRCIL
DRSTKRVSILDKLNLGLKNKGNDKNKNDENISNKDILTKKSDDNAFKLKKYESKKYVDNTNEDSNTDDESYDK
NKKMKNIKNVKDIKDIRNYHKMNSLMRNENKMPPPKKIVQNKGQTKKVDEMDTSKKEGIKDMKGNVYKNKKDA
RMKSKMNYPPKHDMNNPMDRDVNNTMNYDVNNTMNYDVNNTMNYGINNNMNYDINNNMNYDINNTMNYDVNNT
MNYNINNPMYYDINNPINYQMNNTNISNINNPINYQMNIPLDYQMIPNNNINNIHNNNNNINNSIYFQNNNKS
INYNGINHFPCNDINNISYPEQKNNINNMHIDSSNQLIEGNYNNMNRINSNNKNLILYNKGNEKYDEDFTLNY
ILQKYDDDDQFFCSNIYTQINEEDKKDELKKYTEGMNRLVEQMYFYDNFNDEYKITKENKKETTESFDIIDTS
LNIHVVDANEDNITDVNKDNQEIISKMELKIKELEKTIDEMKKKDKVDSSHHEKKKKTKKKKKTNNGRSSSFS
DESSYNKEEEEGEEESNKKHSMLNKLKKDIEEMKKNMKREEENRRIQKRGIKKKMKEMKGDIKKEENDKIDDN
VKSVQEDEKNKTKEKIENDEELDNEYAYKFNIDDIINTEQLNNIKNGILDNVYYYIYESYDQRDYDILENLAK
KHEIKGRKISYNINLPKYNFNAKRTSSAWFLNPAYENYMLEEKKKKRMSLEQNKMISSNSFEHVQFLFNENEM
VDGTIIKNGDDDDDDDDDDKSVGKISEIRDSEFSYESFISEQKKKIKKNKINLIKIKNNVVRNKSKSIIQDHN
IKEDIKENNMVGHKEDDDEKYKKYEDKEKDDINSKQIQNSQTSPSNNDNIINNIFGTINNYVQENGVLDFYNS
SNLIIKEENEKGEKKEKKVEKINEDENDNNDENILKDQIEKQKKEKELLELEIENKKKKKELEELELQLMENK
KKQFLGNKLIDDLQKDINCTKEEKKKKNEAKEEEETEKNINDQMVNEKEMDVNSKNEREIVQVHNEIKNTNNK
EEEGKKKNLLKEKEINDCLNDYINKQKKKEKKKNNWAMYGRPIVKRQNNRNINIKNDLKKLYSSKSESGFNDY
AFYAERFFEVITGYNSEPDYLSDIDNQAKNEENKNDIIHNNNIIKISKKMKENIYENSPFHTYGRPIYEKKSK
NPNNYNKIKSTTHNAILKKKRKKTLNKSISINSFTKMNSSNNKIVKRTSIKNNTIDNYNNSTIKKIIHKEQNV
EDQGYIDLKTKRKLIYDALDEINQQTQQKNLKITENITQVGKKHGQNVSNIIKNTGAMLIEKITRKGNDNNEE
DQFSEELKALEKLKKLKELKRIEELKTLEEEEKKRLEELNSLKVEEEKKKKKIWRK*KKWKK*KKSNFKERKR
KKNYYNIVEDKSKLGNWRNSKKRKN*N**KK*TSWKNKEKEKKLKKWRKKKKEKNLKK*KNNKKRKK*KNRKS
KEKKNRSN*WRK*ENLKNKEKK*N*SGWRRKKERKN*KNWKSKKKEKNVKKWKKKKKKERKKKKGKKK*KKWK
KK*KKWKKKKKKRKKKKKKWKKKKKKWKKKKKKRKKKKKKRKKKKKKKRWNY*SMKEFKKKSNF*KK*KKRNT
*NI*KKIKKIQVKKIYYITF*KKYIMLILYPII*KMIKNQLKF*KVIMRRKKMRIKSI*SKRIIYQLKK*KKR
KITKMIIIIIIIIKMIM*FVKKKKKKIYS*RKYHI*IIMIYFTKWKNKELKILKKKKIIIEIHQII*EIKDY*
KMRTLIIHT*LGIL*IKKRKKNMKIIKCRLRMKKMVICQMIICKVMIWRMIIHKVMIWRMIIHKVMIWRMIIH

QMMIWRMIIHQMMIWRMIIY*PRKGLKKL**I*KQEKKKKKNYYR*GIKI*NTTV*IKKI*SIW*KKS*IIKI
QTRTKLMIYLI*KKKLKYL*LNQKNLF*KLMIKVQEILKKHTYIENHHI*KETI*MNILKINMSHQMKTINLI
VINQIGKMICILVTIIICLIIIIYYIVIVIN*LVIVIY*VIKIITYIIKIV*YQVIISDRPIIKI*KMRI*I*
NRIMNI*IAKA*KNYLTTL*KIYI*KIRTKKINIHYLIMIIYQKIVHIFILILKKKN*IVKKNIHTI*R*IVF
MTYIKIYIIMVLNLIIMIIIIIIIINIIIIIIIIIIIYMNIKVQRTIKMMIQVLIILLKKY*NKKIIIIKEKV
TITIKMMIYYQKII*MDLTKKLNQ*K*IITLKSKKKI*YIVIMICIGII*
>N.PFL0320w
MNKYCTLKEQKKYNHLIESDKLYDIIKNKEECLLFDTSYYNIHNINNEDIFDDYDNVESIEGSIPMNSIISHN
ENSNFSFFFPTKNEFFIYLKNILIKNKRNITMLLENIPIILYEKEDIFYSPRIWFIFKMYGFKNVQILNGGLN
KWIHEEKEIIHVNNEKKIIEEHINKQTIEQNEYYISHINNLLEKHFKKNENYINKNIMNSIYYYEDIQNLIKL
KEQKQMQNYLLVDTRPNKSFSTLISINENIKVNNFIPFSINIPYNHFLNYHYENYKYVTFKNMKDIKILLEKY
DLLNDQKIIISTCNKGISACLILFLLNLFDKPFSKLILYPGSLVEYNFYKYERN*
>N.PFL0325w
MKMEKKKKKGKQEKNQKTPKKNDKKNDTLIDKVENKNNKKKINKLRKEKKIDKENKQNDDNKENIQNDDNKEN
KQNDDNKLNIQNDDNKENIQNDDNKENIQNDDNKVNIQNDDNKENIQIDNNKEKCSKENQTNKG
NKVKKVKSQKKLGNVQSVEDIKPIKEKDEDEKNSPEHMIEEKDNKINKNNNKKKSKNKNKKEKIETCQGENMK
VQTNKTQENNNIIITENITNNNIQITDTYQPNNILIKIDDSTKEKNNKDTFVKNNSSVNSSSKNVNHSTDTIQ
DNIYIELKKEKEIEINHNNVNTSLTFPSNRNKNIIHLQVTEINSEQTIGDNKNGIKKRKCVEKKIEKVFKKTN
DTNNNNNNNNNMQISTCNKNDIAEDVININNITNQNIKQNIIKKGNKTHDPIKTKQTKIILTDNDAKDKILKY
MKKANRPYSVINVYDNLHGCINKNNVQRFMDELSKENKLQCKEYGKAKVYLINQKEFKSLNTNEMNKLKMDME
LVQEQNELFKIDFNNLVKKKKKLLQDLELIQNLEEYKKKIQNIEDEIKSYEQMNKNSKFTIEEIEHIKKNHGY
LHFMWLKRKTLCIEIIKCIATLTEKDTKGVIYHLGIDVDEDVIPPNLYS*
>N.PFL0330c
MKDIIKIKAKRKNVNKINEENEEEIYQLKENNRAQNDDDDDYNNNEKNKINKIILKDENINSKNDYNNMMNPY
NVIGKSGNEEDTHIKIEEEKNKDNLYECKNDDNKFYESKINFIKKQDEANNEDNNNNNNNCYYNNYENKHLNI
FNNKNEVNNKNLSSNKILKDEYYDKNFNEDNINNLKEFIHNENKKPKTGKLYNNMNILNDEENFSGLVIKNKE
HNKKIYENSKCVNTLNEKWKLLPAYLKVKGLVKQHIESYNYFIKREIKTIMNATTNKIIKSDIDEHFYVEFLD
ITVGTPSVEENMIETKLTPQICRQRDLTYSAPIYVDVEYVKGNSIITKNNVEIGRLPVMLRSDICVLNNKSEE
ELMKLGECPYDPGGYFIVKGTERVLLMQEQLSKNRIIVEMDIKHNICATITSTTAESKSRCAIVYKNNKLYLK
HNSFIEDIGVCIILRAMGYESDQEIFQMIGSHKNYVNGILLSLYELYNENIKTNLDALLYIGKKIRPRLLAKG
FFSSMKEKQVKNEKDIIEEGLDFLSRVLLSHIQQKSKYDFRNKARCICLMIRRVLDSANNKNEIDDKDYYGNK
RLELAGQLISLLFEDLYKRFYFTLKKQIDQTLSKYMQSNYNSKLRSTGNNMNDNYPDVFRNLPKDIITRGMQT
AISTGNWNIKRFKMEKSGVSQVLSRLSFIACIGMMTRLNSQFEKGRKVSGPRALQPSQWGVLCPCDTPEGESC
GLVKNLALMTHVTNDNENNENLIEILYTLGVEDSDSLTGEEIYKEGVFFVILNGILLGVHKRPQKFMQRIRYL
RRYGKIGQFVSIYDNFLHNAIYISTDGGRLCRPLIIIENGKSKLLPQHIKALENGTINFFDLLKSSVIEWIDV
NEQNNLLIALNESDISLSTTHLEIDPLTILGVVAGLIPYPNHNQSPRNTYQCAMGKQAIGAIGYNQFVRCDTL
LYLLVYPQKPLVKSKTIEFINFEKLPAGQNAIVAVMSFCGYDIEDAIVMNKSSIDRGFGRCMSLRKHSVELKK
YFNGSNDIVLPSPLVINKLQQQRKEREIKSEIKNEKEKDGIKQEHHHNNIDSKNNKSSYNNNVSKIDNVKKDS
YGHKNISNESKDDIKKMANKDIRKYHSIDMDGVASIGYLIKEGQLYVNKFSPKNIKDHVKDIGKVDINDFKIN
EIKYKSVYPSYIDKIIFTENSEGLKLYKIIMRQTRLPELGDKFSSRHGQKGVVGLLVNQEDMPFTESGICPDL
IMNPHGFPSRMTVGKLLELVASKAAVMDGEYKYGSIFSGTPFEEIAEILFRYGFNCSSKELLYSGLTGEPLET
YIFMGPIYYQKLKHMVQDKIHARARGPRQLLTRQPTEGRSKEGGLRLGEMERDCLIAYGVSNLLLERLMLSSD
VCNVYICEDCGMMGYDLYCTFCKKCDKNIVIQMPYACKLLFQELQTMNVFPKIIVKEV*
>N.PFL0335c
MSYVNIPRDRNDPNYRYKMPKLISKIEGRGNGIRTNISNMGEIARSLKRPPMYPTKFFGCELGTMVKFEENEE
KAIVNGAHKEKDLVNILDKFIEMYVLCPHCLLPETDIVVKKGILICKCNACGNIGELNNSHKIATYMIKNPPM
ISTVGSKKKKGKEKKVEKSSKGKSEKTDKNEDNNRNGVIYSDDEENDDSDDLLDSDKSISNGKKENKKNKKKD
TSSSNNNNSNNNNNNSSSSNSKKKKEEENFVLEKECLHFGSPEIKEVIERMKTIFKESTQMNDIQYAEELRVL
QVSQCFDSKCRVFICLCSLFEDKISKELLEKNIKYIKKINDTSVTTMDIFLALEYYVNKVAINSLSIYPYILQ
VLYNNDIFESKDIIKRYDDDGKNENNHNNKSSNSNSNNIGSNNINSNNMNSNNISASNNNNNNSTNDSLKTEK
LLNNHVNDKDDNTINTQIHYDKCKCMAKHFVSWLKDNDSDSEESDDEEEKNTPNNTINSNMGNLKVKSLRIND
GNLKREHQSIFSNDYERVFVDSKSEKCDDDIFLDAKDGNVYTGDEEEEIDIDAI*
>N.PFL0340w

MAQNNKNRLLILNDVKNYYNKIFTSEVLISLQKDIKLEDLMFSYNYYSHLIYENNNVDEVYVIPSDYLNSDDD
LDYNINDNNELTCFLLRREERRNKSKANYRYSNDEMINYFDPLTSEEEFEEKIEKTFEFLNNLLLSIDVGNND
MNNNHLDGSNSSNKLHLINQICINLNVRKEDDNIYKLCNIQVDRSQINQITLIIIAYIITSIFKLSMGRKLIF
SNKNINNMLFRTLHLQNIYIQIYTMKNLKKYIVEDAEFCDIVHLKMIKVCLFSKSLCSFNRANHILLLLLREK
KNYSHDIFDFYFLRKLKYSLEHSDNIERLRLLDFITDCINVTNDENVCNLFKLKVKSSQNKTAFSKNENTNVN
NNNNNNNNSYSNNNNGINVDISQLYNTDIQLKLDKQFNRYCDESDDEDIYTDNIIFSQIHGLKDIMNKHNYKI
KDIYLMIRINLFKYLHMIYLKDDLLLKINVLEIYSKLVQNIQYCDGLHWENCYFLYNVLYDLKEKDDYILHLS
IMNSLISYSFINESVLLYLISAHSNILIKIILQYIDNKESDVEKLIVGIKAFGFFFSYKESSNLLLNLNKNAH
LVVINHISTHSNTNVLLHAINIWLKILISKPMDDIWFKNIIHSILLEKIILILKEIDDNNIQTNIFKLLHLMI
PYDIATTILNEQWIIKSLHDEFENKAYDLKISRHKFFVALYQHNKDIISNNAYADNLIRNFIEATPKRY*
>N.PFL0345w
MSLNYGLDEIIQIKCSYIIEKYKNKLKKLKEYKHALKEEYEKKNKAHYVNRYMFNKEKEQIFKDLHKYKEKYE
LETLEKIKEKYNNYFADIFLYEQEYYQCIYKVFKNLLLKKEEIDEKEKQEYAHMMLSDKNIYVEFENFANTNK
KSFSLYDIKTYEYKKNIDNNIHNADNLENLYSINVNEIYESILLDQNDDNYTHMLNTNKSFNKLKGEYDEIKH
IPQNNNNNNNNIMFTSNFDEYNYSMNKINPISIISQVKIMDPINKEDMVNNIDKNDNTNKSDIVIKYVNQNVH
QNGIDIEQNNISEKREASNDNETYIKNKKPKENESEEIITNSTEDFDVQENVEDENMKSEIKNHIRNNLKDNN
FFKKDTSYEEINIEKGDENIFPQNDDEKLQQSKFFKKSVSTTNEVLGNDIYNSHILNNSHDINSNIKYGKSEN
YINTLDLQKLHRDIVNNLEANREDKSETHKKIEELDDIIENNIDFNVSSYFSGNFDDIINKEDSLENEKSNIL
NENSLIINNDQTIEYVNNQNINSKPSSITKKENLHIYNNNDEEYQYIKMYNENNNQIQSSLFQTFNNFKNDYN
IQIERMNTFENYAQEINILENYEHIMNSLNLDNTLIQNKNNKSDENAMLNISNIHDIINYMSNHITKN*
>N.PFL0350c
MKTHSNNNNSVYSVSLSENENSVIGKSKVNLDNIKNIYKYDKYSNYINTVKNKKVEEEIDKIYKENSEIESDI
EANKISSDISVTSKNNFKETIKNSIKNGKSFITRYKNLFAKERNIVEDMKDDKNNTLINKIDKYDKNSLLIKI
KDNDSVIDSDNHSFNNLKQKNRNYSNSNIDINNSGNSDISNSSNIDNDNDNDNDNNNNNNNNNNNNNNNNNNN
NNSNEEEENDYTHTKDKKLSSYINSSKRKFYALDKLKTNRHLDLETHESYYKSNKEHSSSRQISEDIPSSVIK
NNRIKGNKDSLISDNKKVDDKMSNYEYYKNNMNPGEYLSNLKEANMKNMVIRNDENKEKSFMYNRNRIYNKGN
INNNNTENMYNNNNNNNIRYNSTTNPMDLVETNNANKIIDMNLLYNKDIDTLNSNGSAYIDANKLSVLQEKYK
NSLFYEKELANIKDINQKEGNQLKSENNNEKGYLNNLKNEVRSNYLNNLLNNRNVKDKYESEQKFNISNEKEM
QDTCKTLNYNNDINKRDNENNIINNNTKQLIIENLMNMKNNGEENKNGNKFNMNDVRNNYIDSHYKGKDILNY
DYKNVHHKRSSSSNVNIRKLETEKVNNYLNYVDDTQAKISYYQEKTRDTSNNGVSRYDKYLKLKNESNLNFLK
NKMMDLNVNEFNNRTMYRVDFMNNKNELDDNKGRNSNLDHVNNNDNMNNNNNMKNNVNMNNNNNNNNDNMNNN
NDNMINNNDNMINNNDNINNANNFNNYGEKNNSIFPNGDVSIHNNTLNSNNKQDMSNLRNFDLNELRKKHLNN
YHKISDKESIYMNNKNDEIFIDNNKIESYQLNMLNEKKNKENSLIHNNEELFSFDRNFKKKNDFKNIVCVCQA
SKQMARLQVHMEENRQMLETEKNLMKKRKDNFEKKVEMLSDKEKEIEKIHSQIKEKEMLVDKKKMDIEEKEKY
VNSIKLKYDNAQKELLDKMNECITIENKCKSKLYEYDEKFGQFNKKIKEMEEREKEIDNERRNIERKENFLNH
TKKELEEEKLNNMKEKNELEMLKKELESLEKEKKQIIECEYNNLQNKEEEIQRNERNNLIKQKELNSRMDRYN
ELLEDLNKKRKELEMEKMKLLDDVQEERIKFLNEKNNMQKEKENEINYMKEELRKERILMIEEVEKMKVIMLE
DIEKNKEKMIKNVEKENEKLKDEIEKERRNMIQNLEEEKKEFKLYLEQKYKENFENEKSGLAKKFDEENEKLQ
NEIGNEKRKLHKERDNFEEQKKIYEEEFRNKCEKYEESIQKKYDVLEEDKNKMKYLIIKEQEELENYKKNIYL
DIEEEKEKLYVQQEKLNLEKENLLVEKEQIEIELKNYKNFKEKEENDIKIKIINLSQQQEDLNKEKENIEKEK
EQIEKRKYDLDEREESLNNDKAQMEESRKIFDEQLDKIKKNKEELLNYDKELKNREIHLMEKEKEYNKKLEES
NKKKESLNSFDVELKEYSSKLQEREKKLKEKKNELQKVKDQLVNYKNSLKQKEMQLQNIEKREKELLDEQTVI
QIDRNSLEAEKKQFILTKEKHIKDTEFIQEQLKLLHEQLKNKEKSLKEKENEINLLSKLQDCRNKSGKGAIIS
LKHLENRKVSTNLSRTSNARNRGVKVKAIGKNKFDLIRRRNRKKSINFNYNNLEINNSIQYIDSMINENFKNK
GFLKYKNDNLIISDANYVSNNVDKDAASSYSMNNKKMDSKLLNNKDTNLVDKTDNVSVIYMDDSMNDKVSYSK
SGIKENRGISNYAHITKNNGNNNNNDNNDNNDNNYDNNNNNNNNNNNNNNNNNNNNNNNNNNHDNNNNMDYYN
YGGDKYNEKKDDFLNNDYQNYEKNYLKNDNKYDYDDNQNGFSTTLPRDMYEENQFYNNEEEKKRPYRREKELG
NNIHQGDKINQNNTYNNSYNSYNINNISTNIKMGKSSFNDTLNNKNSPSRLNYKNNIEHDENNIMTSLNVNNL
IESDLIDIKSNNFDYYKDNENNKYDGSMMLANYEQKAQDMTMDVIDEYNNNYIEKESNKGNASIDNINKNINC
INNDVDNINSNINNINDNIHKINSNVYGNDMSRNYANNSNEKNVKINKKNESSLNTIDNLVNKNMQSINHFSS
LMQSSKHNKMSPNKGKLNVATDKNGSNEKNNMNGKNNMTDRNNMTDRNNMNDRNNMNSKNNMNSKNNMNSKNN
MTDRNNNNYYYNNRKGKNNEYDNYHNNNDEMTRKEISNDNFLNNENDSMKKEHNFNEFLEEEYNNMNEHHHYS
DVERIAGKKKNYSNNKTLDKLYEYNNKYNEKRSNRNVDSNVQMINSKRENVQFFEGNKNIDISFIPRTPHSLN
KSIEENNGYNSDSNLLCSHNFVHNENEMLRKINSQMSNYEDHDNKYKARNNSSNIYLNNNNNNNNSNSNYVHR

NNVKPNIVKKFNRAKEEEDQEQEQEQEQDAIAHGKSGYMKNNTNVYKQEMIIENSNNMNKVNYKNNDDHKNNM
NITYLKKSHINQMSSFKEKEKEKEKEKSNFRNPENNKYSNKNNTSNTKFLSNIKSNSHNNNNNMNAPNEQKNTNM
VRIKGFMKGIQQNNKIPNDIKFNNDEYMNRKKNYENKHEINSNNIIEQLDKTLMNKSIIMNKNKKKFDKK*
>N.PFL0355c
MKDDYLNNKKESQKKELDEFQCERIKKKKHEHDDENSNDEIVKKKKIKIYNNSSNSKEEVNCKIIGDNLGKRK
KEKKKKKKILNNNLEDSNTISDDDDASSLPPRKKIKNIKKEDNHIDSEEEVLYNDDIDLEEDQESDTFENSKT
LKQIKKKLNNEGNVKKNKHIHKNQKINEDNFKIIKKKKKTIDANISRSNNINNKTKHSNNNVNKKKDKSHLKD
EKKINENSKNKDIYNNKENIKMTFNVTNRFTVLGCDWDNISSVDIFYLFESYYNFEKRKKKNIDYSKSRAVKK
VTIYTSKYGEKKLKYEEEHGPVINSDLLKKKYNKDENALYRKNNLLDYIKDEDENSENGDNDQEEENEDPVEV
HYGNNNDSEELDNQEEDSDNNSLDDMYSDSGDNNDKLKHKSKNVISKRDSYNNAGDISIYRNDEDDENEKIRL
YQIQRSRYYFALVECYNKEIVEFLYEELNDMDADFCINYLDLRIIDDNCSLDDYKIKESCDHIPENYEFHYSV
STALKHTHAQSTWEENPKRKKLLSTKFNEEQLRELDLKEYLANSSSDEENEEDEEIVQKNKNKKYNEQNERYN
KESYRKLLLGDLLNDDDFNEEKSLGDLNKSLIQNSNDDIYHSSDNFDETHSYNNEDITSFNKKQIYSKNKHTD
THFNKEHRNDESIEESEYNYFSNDENIDKKDYKSKYNEKKRSKTNKEKHDSDDSDKENEVVNVFKNFLTNKDK
KEDNRNPWEKYLDKVKEKKKMKKKAYLESLKKTDEEIKKIITKKTNKRKKDSVMKNYGEKDKGKTNDYHVVDS
RFADLYKNKDFNLDITNPNYKKTKFNEDVLKKKKSIN*
>N.PFL0360c
MCTVYIHSKNMLVGNSNINIKNDNVDMLIYSYYQLMDPLRDYERKLFWLFLYNRKRCINDVSYENITKFLLYD
DKLYMSIYNTNVLLYMGKKKYILYAITIYTYVYSIYYLLLHVFGFVKSILDSQINVQFCKGIHKIKYNEYCYS
YIKCNDNSICEEGVKDTKKKKYTIIIKQIINMYKHIEFNVTPFVEISCVRYEFYNTFTLFIMLWYSHINDHFM
KKEFTHYRYLKEIEKIIEHFIYTYEKPIFKYLKCKKVDNVNIFFSYRLNNKNKRNVYILRSICIYIIESQCFK
FFHLNNYILERNYLKKVHKFFLTLQKLLKYFNECLKYFISFLFPNKEFNIFIQFASYNIPAFYLNYIIINCYT
LLFGIILYFFKNNIKRICRYIKYYALMERLNIINVCYIRLYDKINKRSDPFVQVYSHVHTHMCEYVLEHCFDV
KHIICYSKESLYKKNINNNNNKKNKNKCGNNNYSNSKENRYNYNDKNKNDSKYILQFCSFNRNSYNNRKEQTN
NEQDDDDDDNNKKKKISKYESDDKQFLIVKRKNVKVNDNNLLKVKKKVIKKKIEKRKKGYSFNIGCEKYNNQI
IKNMKTYNKRTFYINKNEYKKVILKKLKFMNKRINNYFIKDRNIFTNKYRSKKRYFAKILKKKKYLYTYESNK
KGKKNVYLNEQSFKVKMLRSLFYIIKYKNLCDNLININYQVYDLLNKDGIKNFNYIEGNRNLNRIKEYKQFCL
NDYNDWTAYTKNEILNYISDYLYKSHKYNDFESLKKMLQRIFSCDMDTSHNNNNEIINLNNSYLDNQKKYNTS
KTSFNSSNKHWFVYKKSIKEEKKYYIPLNNVMNNKVNGCYNTNDYVNGINNDITEKMNDPNVKTLDKVVIDDN
NNNNDNNNNNNEVDKNKKSLQEKQIIYIDEHVEEDTVHMLNLQEKNLCKYFQESQIGLIRYIDCLLKRYIKNL
NHEKYFNIFYKDTYNNNNNNNNNIDNDNVLGVLYNLFTSIKIDDMIKCNYETFQIVLNNKIVDIINILDKLNT
YTTHEYNSMNCSCSVKEHENLFNQNIKYKNFLNNKYNTFDNKEVPINEYRFLESQRTNEDNQIYKNSDKVIYE
NVYDESFIFDRKVCTNNTCVSIINQSSNSDSRKLNCFIKNEIYDLGCNQRREANRNSERSYGIMYNENDMKIN
LGMLLRNDISLFNRSRNVNRINGNRNNCNRCDDNNADNNDDNNNDDNNDDDNNNDNNNNDDNNNDDNNNDDNN
NDDNNNDDNNNDDNNNDDNNNDDDNNNNNHNSNNNNNHNSNNNNNHNSNNNNNHINRDRYINHHNIISNEYIR
QQIKYTNINFNNHKNDSINENVRNKSTITQVNGLNYNTNEKDKEKINHNDNHIHTKRIIRNYSNNLDMHFMNN
NIHLDNNNNNNNNNCNYIRWENIFPYCNSCKNKYANKFEINNFDNNIMNKYEKYECTCCGNKIRNEEIYNILG
KVTSENLRNIYKSNRINNTDNILRYNRENEWMSSFFFKENNNREIYKEEYDEYKNDFFLNIKEGEKSMLNNIF
RNNSLKEKLINEENITKTNNNVYVNILQNNDIEKVGKQQDCNNSLLQLSLYRINKTNNNNDIIIHNNCNINNI
NNLNSGNNTHSSNNVVSTNNIESRNYRNDLYLNTNDRIIFHQYKNSCMINHNNHYEINKDYNKKCINECEAED
IKDTNEVKEISNDVLYNYLNDMNDRFFYNYLDDNEYHSDVRSNHISSNKDYDKKYYSNNNYHNNHNCSNHVYY
NYDSIISSPNQMYKNIQECKDINLVNDMKLYKLGTIKNKYDNIINEDIMQSYKNKYNNNNNYYKGNVENYYDI
NSEYYNNDIKIGSNILYDFFKNRNSTESTLLINHYNYKMGSINNNNDDNKNNNNNNDSSDNNNNNNNDDDNNN
YYYYYNINSDLLLHQLNNICNNRNNNISLERRLNYLKDELINKKKTNETGKKEVDDLRNLKSKRDVFSYIENC
LLNNNIEKNKNYIKLKINEIEDTKRNNQHYKNYDKYKNNYDCNNNNNNNVIFNIDENACKTSDNRSRTQPLQN
KNINKNEYFNSIKIYENINENYNVLVQNINNIRIKNEKKYPEQNSHRENNNDVINDNNSDDNRCNDNTHLMNQ
INNTNEEDSNVKIINDNVCSFSNNNIKETFKDKKKVQDILHINGQCKPCAFFYNKNKGCRNGDSCEFCHHIDH
SNLSLKQWKKQQKKMMKLGLLHTEKQNKMGKIDIANKEKQKNSINMENLNNTNNNIINNENYIRKDKGKLNEA
NNIADYTSESNDNSINKSLNNYDIFISQHKLYNDVTSMDKKEFNKQSDHIFNNYHIDGYNVDNVRSSNHISNN
SIIINKRQMMSNNKIVINNHHISNKNMIFNNNIINNNIIMYNKKKSNNSSSSCCINTMHGNNNNNNFQCSNIL
RNMTNQKIDIKDNMILKKISTNNCEMNNLNSCSFLSNSSENGSYKTDEFLNVNEKYRINDLNILHSNNKEVNN
ILKGHNIKHNNNKINDINILHSNNKEVNNILKSHNIKHNNNKINDINILHSHNEFYNIHTNNHKSDLKCVGGH
ITNDRYENSILSQIKTHEKENIRQIKHTNIEEENKKYVLPVNHNEIYLGEHNKIKSDKNETQDNDTIGKKIIY

NIEKNLLENRPLNFNKGHDYKSMINSMSTLNMNNVCYDKMKIMSMTKDIQKNVIKQTKLFPKFIRNNNNIVQD
SINVTDNNSTRDITYINDILKRELLKENVN*
>N.PFL0365c
MWEKILFFIVLIFPFFHSCEALKKGNGDFIKKNGVFFQIDRDMNEPPYSVINVFYTESLLDDNLLNQLEKERK
MGKYKTQNYFRKSLDNNKYFMEYSRKQKEQLEFLLE*
>N.PFL0370w
MSDYFTILSNIFTSTSLKKKYSSRLSTKSKKNQKRVKLIRLRNGHFRRIVDISNIDEKSIFPRSCTFASISSA
SKENERKNSSEDTKEPQENLYGKSNTSSSITIKINFDESDENKSDQDNHSIDTISDISFTQTSRKSLEIESNT
YESYREVEKEDIEEEEEEEKEEEYEEEEEEEEEYEEEEEEEEEYKEEGLKTEEEKEEEKEEDNKEVEPEEE
LKEEDDKEVEPEEEKENEQKKEEQEENNLEAPSKTLMKGVKTNIYFLSTKERIEALMCYNYISNAIIFEKGKF
LRYIFMNNVNNIIVNEHMINMLCKKEKIKYILSSNSIIIESNDFIKPLIIEFDSNISKKIFVKHLKMIDSFKL
DDKLYREYLNDLSEHERDRLKHVESFYSNAIKVHNT*
>N.PFL0375w
MKIQIIFILLLNMIHSFVIKKLKWEGEQYHKTKFQLYRTRYFRKYRTIPYIKDSGETHINELTRERVRLNKHT
ANSITLGANYLFICNLDNRLSSKDVTHFFNYFVGIDNCIAKIKKNRFTGRNMGHGILKFKKPCDATLVLLNYQ
GIKLGDKNIILTEAFKNDHLKQKKHICNVIPPSSRY*
>N.PFL0380c
MPFNKYVLLIYFILLVNINLFKKKKFNTSTRVGNCFMIYDKNIKLNKIEDIKKNNILQKEKRVFNNIILLRTN
IDKKKKNLLKLLYNSSKLKTTGCQRKYKNRKCYSYLINNRRINTVECLRKVLNKSNYKIVSKLFKKNNVKTYM
SNSNIDDSDIINLLKLNGKEKNMDNSKIGKCINTKNEEGDDELMSHKMNDMINYLNEDPMKIMNPLDITQLNF
FRDGNSQKKEKIIIIIGVTCSGKTKFSIDLSEQLMKYKIKSEIISADSMQVYQNFNVGIAKVEEEEMKDVKHH
LLDVCHPNDTFNAHKYINYTIPLIKNMNRNNKIPIIAGGTLLYIESLLWESVIDIRKEEEKKEQTYNGQIIKK
EDEYLDEHLKLNNDIKCNGERNDDMVELDKYEHKTNEELYEELKNIDEERANQLHKNDRKRVCRSLDIFYTYN
KKHSDLIKIKNHKNNNIDKMRFFPCIFYLDYNDDDLLKMKIKNRVDLMISKGLLDEAIKLKQINNDRKLSFPT
KGINQSIAYKEFDEYIKKKMDNIDDQKLFEKCKDNLIRRTYKYAKKQRRWISNRFVKVYNVELNKIDVSNNYE
KQLNNAIGIVLKFLKCQ*
>N.PFL0385c
MLLRHNSFCICIILVLCSVQRCLSDEQNINDWPIDFEYNSKSLPSIEVKLSPPEKPLPQVSAEIKILESARLK
LEEGMMQKLEDEYNKSLSMAKLKIKDTVENSLSIFNDPNILSSVISNSVKILKKKKNLRKIKETTDEEKTSDN
VSQMYERKGGPLPPPELRKHTLFLEQNYLNKTIPSVKISLTEISEPSVLIKEKIEEIEQYRTDEEVTMFETAI
SELDILTDITMLELEKQMQLQLNPFLVDKQLYIEAWRKS*RKWEKPNKGKM*IKVLKHSHHF*SKKKMKIQEI
S*M*K*AKRIIVIQL*MNWLCKCKKKGTLRKN*KDKKF*NYK*NY*KHKVK**RMLYIFLFQRL*LNIHP*SK
H*NYRL*
>N.PFL0390c
MYKFDKKKKDEETKVTHNLKKFVFKNEFKYVDATNALKNIFLKDKKNEESTKKVKPQKRIVVNKKLEDFVKHK
KNIKENVLGKENNKKELSMKIQNKGFKKYSEESNQVNYKYDIRKMSENCRGSKVYTPVNFDQKIPESRKVDMA
IVRGRKKKIYSSSEEEDIEDEEVKEDIEVVEEEVGEEVEEEVEEEVEEEVQGNIEEGDEEIVEEEVEDENEEE
YEEYEEYEEYEEYEEYEEVEECEEVEEVEDDEETGVHKKNVKYSYAIEEKVQNENVENDDIDECDKINTNREQ
NSDYTPEEQDDYDTNENQKGGQDKNKGKLENNGNVLKSDFVNLQTNENINNNSEGNDIFSLQKINVYETKKED
VHSKGDNQRNCEIENAFMILKDKIKTTNVEYLKLLNKIMEGLLEHENFYKSIKQFLKKYYVYNDIYIDEEFFH
DFLLEEEDNNYEIQYKKKTLRDTNIIEKIEDNNTDNISEGLNTQETDIYKRIEDHDIQKYKDCYNNNDQDNKN
SYKDMESKNIPLKQENMKDENEEILSVENVNKIIQKGEQEKMENEKAEDLNIYEDDSNDDEEEEDRSKKDICS
RASEEKEQKIDKEEKEIKINDKSKNEDDKEHEESKSDYYSGDSSGLSSSKTESNYEQNENDKNKEKKDNINND
SIKSKEEEKENIKSSEEESEEKSDEGSDGESEEKNDEKSKEESDNKSDDESDNESDDESDNESDDESDDKRDN
KCDDKSDVKSDVKSDVKSDVKSDNESDNESDDKSDDKSDDESDDESDYESDDKKDNKCDDKSSNRSLSESTSS
DENKNIIDDKNKNENTSESESDNDNNDESDSTHDNDIESKKKIRCDFKGDRETEGKNDINSDNENKVKNYCEN
ISKCGSQSDGGVASKNDKKSEGTNNNNGKSQNDSQSSSQSDSQSSSQSSSQSGSQRGSQSDSQNESQSDSQSD
SQSDSQSDSQSDNQSESDIQNDSSSES*
>N.PFL0395c
MQYKTFMSIFKPPKEINRFIYSNLQNIFIFKNINKCSTLNTRSQFNFSTQYRASALHTYQEHNEERECADIEL
EENVKDPFVKSTCMNSLTEQMVVNNIDNVRRNKNIYAYHKNVCDKIKDVVNELIHLRDSKNRDEQKIKLLMPQ
ILENIHSYNLYEITEILNCLYFFNIVIVKREDINRLISRLKILTFNIKEKDNVYPLVLNLLRINISKDHIDEH
IFMNINKFLNDLLRHLLLCDNIKDTLKYIYYIREIQVIYEYDFCLWINKKYIDKEHVSFLQSFNASVILRDKE

LDYIFINNIKSILYELNFNIQEINMYNYLIPIFLKEFNVIVECISDYNTFQGTLCITPYFVQRYKLFKKMNFK
VLFLYKQLIPEKKEDKIIYLKNCLYDVLK*
>N.PFL0400w
MKHIFWYFLFFFLIKIYRVNGVHIKGRCVSFINNKLNIINHKTYKSTNPLFCYKKRVKAKELRKLTTEELEKE
IIKCRLDIQKFQQQGFYDIHNYNVFYEKNARRKLAQLLTIYYQRYLDNNIRIKS*
>N.PFL0405w
MKGSLKRIKEKLKSTKKKGYYITFYDRIKQINEKEKLEDYNSLHDNNYEDYLKSFNKNDNQNNTEELKRTNFY
RALNEYPKDCYNKDFRRCCNELLIYSKNLMVILLNKDKIFSIIFKYIEHSKVKNDEQYFYKLLVILIKDLKDE
SRKYLEAILKILYNKINLNNLDLLEEIFNSYANIFRLMNKTIIKNIDKYLKLALPLLSHRNNIIKIFIADSFS
YLLKKLSLHDLILCFNKIFSFFNYIPKSKLNHYAETVSLLLLEALKVYNKNISKKKILPFLKYLICSIFLRQS
YYCNETEAMYDYINIVPMNFVEKCISNFFRDFYNFIKDDETSYEFIELFLTFLLKNYSLLYNKYYNKVITLSY
HNNNNNIDNDMDNLLNFKSVQSQLICHEIYTKCYKCETLYKELKENVSVHTYEKSNHIDYKENIIITPSFFEK
LPVHYNIIHSNNTNIKSENGGNLSTSNISEIALFINYTFPEATSFLLKKILYIWLEKSDEMKNIIIQTCLDEL
TKYIDYLKGMPLNDLSFIYVKNIYDHVFFLLNYHCINNEEICNIYISLVYKFMNIINTQWKRRKNMFSLYLLS
FLIFHIYELLNKKHMIHCEVHFKSFLKCVFISLWKYFIQMEMIDFYQNNMYIINGLNKMKKRNTGHEHVNIIL
QEDSTIKINQVYNNNNNEEETEQNMYKSNICCKDFSFMLNNLYIGILYILNDLLKYVISLNQNDNIMINEINE
KDNKTLKKKNSHNDNMYQTQSNSNIIINNNNNNNNISDVHFFNKYKMYNKNYIHHNNNKNNKKNNSFFKEIEK
ELENMLIYIFNNIQYLNIILNDISECDKNISTIEQNENKLIQIKYTIILLSSYINLFSEIICTTNLCIYNQFN
NVERISPLFYNICEISQKFKKNNYFLNICKDKNKLLDMNIYVNNIILDITNIIIILSKESGHISENLLLTYNM
FLLNCLNDWDKSDICNLHYLPKMKSIILNLYNVNYFKNNINDIFLFLKILKKFLLFYDTILITRKELIEIVKL
LMSIYAELKLYKCKVHDNMDDNTIYMIINGYDKIFSIFDMFLSLECDKYLSTYQNIYHSKIEDIVNNLDHFKK
YNIFNDKDRIFKVATKFFVSEIIMLLNVPLVTVPKAIVDRMKKYLRTFSNNHDINSNRDDFSFDFYFYVVNCI
FKNSRKKIDMIEYNINLKNMEKYNMINNSDYKNKSEEIIFQHANDDIYPSACDDKYGDNNNIINGHNISSDNN
YISVHNISSDNNYISGHNISSDNNYISVHNISSNNNYISGHNISSDNNNNINNYECPNKIDGNISINCIHENF
KKLIYSNIKDGQENIENYLNVKEKKMFILLEENYNELFFKNILISFDIIAVPYPNNLPNHLKLKMLNIYLWIM
RFLNINCSNYINEFKYFTLLNLLFDITLNINDQFHKYGDYDKMSMLLSEYKSDNNLKNENNNIKMNEIIDETK
ERKRLIYDILFCIKIFISKYLYNLHILKVPNIMKFLNILCTYNKKLNNYKNDIEEILGDQNVPITKLLLNIKK
EDENIVTPILIKIIFCMLKSNIKKKGQHKMRKNMIFYLSDISNNNYCYLFLSVIYSYINVYENKINYNNLLKY
SNKELVLIKFLNSSQEDLNKLWYYEFNVQNIDKMIHKNGNIFSYEDMFKSCYWVFQNYIIEIKKEALFETFYF
NKNFHILGQILNIMKFKLNKYFPFFLQIFLTIMHYINSYNYILKNKKVKNLFMKMCHMVHYGNNGVMAAFDEY
LNLHQKQLNNIKSKHVINSNNNNMNYMNNMNNQHLDSQNKCEDNIYYDNSNNMCNFNYVQRNNDSIICTNEKA
IQINTKRAYLKNIHKTCIEHIHFIMNNYDDMILPTLIKSKNLFTFIFYKNLKNIGRKNNFLNILFHIWSKNEA
CYDFYNKVIPNSLLILIKAINNKRIINKLNNNEWNYAVDKVYDIILRLCGYDTDTFFISNVSKEYKNAKEKKK
KKNENFNNMNNVINEHYLLLDNKISATLKKINNYNFHYNSKGMIILKPYISHIIICIQKTLLRKYKLYVTQKG
NMLKSIRKKKNFDELTIINNKELHILIELSAINKNKIYNIHIINLLITFVLINKNSLHTKNIDYIHKIKLILI
AIKKLIIHISNGSSEKSTNFITSGKHRKNTKTNLSFNNITNIKNVSKLKMDTHKKKNDINYKDVPNYSHVTNA
ENTINKKKGKSFIFSNLLYFSESYYNMLKTNMCYKLKGMLYEIIQKCYDMKCRILAAELLIMIGCIFLKIKNV
QKYLQIFKKNVDIFLEKTNNLNYNSKIVLKHIIKIPIKTQLKNKNISTNNYYSFLQTAQIFYGLNIFHTKCST
YEYDSDVQFLILLDLCNIKISYKILKDYMLNGQSKKYINCQINKDITFCSKQNDKIIYPQKEFKKFTVKHLFF
NSNLLFFEVLLRHSLFLIFNEETNDMVRDKSIQFTIFFTKLLKCLLRGYEIDNKNIINNNNNTIHDHIVRNIK
ICIHFVINLIIPKCLNALKRNINEFTKVGLQIILIASKNICPYINVLKLLNIEQFIKLVKMNYSKNNISFLSP
SRNNIITGNSYAHYYNNLINDENINEPHFKILEKLLFYDLYYNICTTLSISNSMESASANSSRNKNNQTAYIN
SKNKYNDIIVENIIDLKSENNLQGVQKLNTITPNLCFYTINKIIIPLSLNFILQKDTKKETYNKALTLQSIKL
LGLCSEKIGVKTIYVILRLLLTEMKKNLHNKIYVEKTISHVVRAYHFKEFQKLEYEQISTKLNSNSKHEDKTM
QDDKSIRSNNVHTSFNNYGNQNITRDIIKNVAPKDNNINNNNNNNNNNDDDDDDDYNNLDQKISNAYYEKKKL
NIHTNKFICDILPELKFLMFDKKLINDKKKKKSYINNLVVDYKSKDSVAKPDIILTYLVILNKINYNFEKELR
KIIYKLCYCLSSKLNMVRSEAKKTLCFISMYLGLNYFDLIIKQMSDYLTKGYHIPIFLCTTNSILESVLYEKD
KFLDKNYLTIKFNMLNECNFKRGKVKNNGLVSNKKDEVKTKDEINKNDSENVYEKTKQNSYYTEFCANIFHMI
KLEIINEIEKNTEDVNKKRIKQKTKESKKTYGSNIIRLLTNIVPESCIENNILTFLESLFSGDSFEDNEVDKY
FIFKKKYIFIVTCYFNDFIKGLEENKKINSKFILNIIYKLLIKSIYFFKGDINYDNLMAVMKNSNILYFKFNQ
INEKIINYPIFKKSFHFYVSIQTLEEKYLGHNYENKTIQYPKKRSNLEIMEGSFQGKNIYDSIKKINKYDFQV
HAQILAKVSLKLFHFIIKRSSQIFSDATNLLDTVQSNNILHDNKINGFINNTHDYMNRKDKDFYNNLYNISNF
VDFIHIVEPLLVYCFCYGKEEVFILASKCIKTIKKKKYSDFKKFGKLIILSSIDILKNLPNYHSREFDKLILS
CIDTLIYLIKGNDKNDIMSWLNSNVTIDQSYNNTTNNDIISDDENPKKKQKGEKNEKYNNNNNNKTNGIYDKE

EKEEQKKVQERKRKLCYLLDESVLSSHNYNDSDNNVSSMDEQEECKNSLRYCLLNQIYLLLESKNHIFELLIL
FKNCLLREDLSNIIRKGPVILKINMCIEQIFNIMIEESHNLKLSFLCGQIYIDFFMRFPLSQKEKKKKFFNIL
NNLNDESDESRIAVLNTIYLFLNRANSKLLKQEFYFITFASLLINFSNETNYKCRKMYVFLISLLFQQINDLD
FIYNSYKIIKQNLISGMKSSFVYSYLYILPICTSFFHKDISNYYRLVFHDISQQCDYQKKIYTKEYRRTKKEE
IMNLCLQAKGEITNGLLIYNANNYNINNDIYDNNKNKNNMIDEHNVDTQFEHKILNHFINQLLTFINKKEDEK
NKNICSNYIEEQIKKKQNSNKMNINSDIEHMNNNCIIQDQNHISLNITQYYNITFMKNQLEDLFLSIIFHLID
KIFINIKLIVKDYDDIIKHVFTSNDNITYFCNRYDIYKQVDKDIVYLIYKSLEQILSYLDINLIENIIHEKYS
HENYKDIFIKMIELKNNNNKNNYDDNNIVLTYDDNNYEKLIIYFLYFWNLIFTHGLMNKNPYIQIISMKIIIN
YISKKLTNPFFPLLLIKLYTTDKFIINIIIKKILSLLLNYTFLEYFYIYYKEVSSLLCHICNLLICFPWITNG
YNNKENEYEINHMKNYDYMKNLETLNSSLKLNNKDINIYNNNNNNNNMDNISTNEENKNIYITKNVHNNISDE
EDIYNNSSEEQEFNKVNNILKENDEDEYKKNNNSIYKNISNDYQNNKNKSYVNQNIVINKEILSYSKFFLIII
TLSRAINLHLKNKKKSFTRIYTILNTYKQIIIEFPVNLWNREHGIINNVLIPLYKIASIFKKKDFVLNENIFN
ETDTYKKFLYLSSFAWYIISLLEKKLEHNQDVFNKAFIQTRQSIHRIRFLRKKKKKLMAVANPKVFILNKMKR
RKKKQLQKKQRKILL*
>N.PFL0410w
MKKIYLSICLMIIVIFKIFKKKQHIMDKHFYDRNFYKKHNHGFTCNLFLILYIPYKRSLCYDTLFNQMIKEST
FPSKINVTYNLRYHLCPFFSSNNNITKIINNTISGVKIYNNFPFSVTGTTLKSIIIKYPNKRYLSERKYIEQN
QKKSLKKLKHDEIKKVGERKKEKKKVRYSSYNKSYLNDEKNTFDNLHSAKYISKNRIIKYKINKIKKIEENVF
KKINTKKHHINKNIKLESSGKKESKININNIIIHKNKDRHLMNNTNFVENPVTNDYIIYKQEFIRSVNTLTSL
KLYGMNLNYLEYQILTIYDSTDECSGTKLAEINILEKYDSYMITDSFKIKKVGLFLICITLTSVEYVGVLKVQ
NNLIDDFDSIINNNNISTFNCSTDAKSILIYSKERIINLNTINNEEIKPEEFINLSKYSEYLQGDISLLACCV
NSNGYVVVLDKDVIFVLYNKILKEIIIHFLKNPIAIYLYNHTIFITDAERKNIFRFVSKYILKKTSKKLTPIW
NYYTYMKLQEENGVDLGKNMEGTSELISNILKTNKLLEKADTIKRMDEFNNGESYLIKNTQLLNSLRNFPKIN
KYPISLLNENFTLNYPSGIVVVNDTIYFVDTGLHVLFAYSLQKGIITEMFGYVNHEVKSENGLNKPYSLSLFS
TNNGNTNLLFLSELTSSRILIFEINNSIKLYLIYNNNINFDIITSIVTTSYFLIICGLKQNMDIYKSYITFIK
IEELSDIDIQYNEYDTTLYYGKKFSLDPLIKSPNINEFKMEQIDKFTNKIINTGLTIHKDTGIISGRINISAE
FHINIYVKDYFKKKKLTFVNFVSLCPKGFNIENNKCIPCPIGLYYSNSKSINQCIPCENYRKHSTTLYNSAKS
KTECLCKPGYFLKDKRCVKCVAGFYKDQIGNFKCQGTCENTKMSIEVGAKNYEELKCTCKDGYFTEKKKKECI
PCPFGNYCIYNPKVKYKADIIRCKNNKLTLHRGSKSPNDCLCSQGYYHDYKMDECELCNYDQYKSHISNDPCV
PFISKSLVNQEYLINEPYFQYTNIYIKQTSNIIFAPKKGSKMFHEATLCETGYSYSMNKSICSICRYNNYCLG
LDHSSIICPKNSITIKLKSVSALDCLCIKGYGRIIINTNQSFTISCIPCPYNTFQPHHSYGKCIPCPPHTFTK
ITGATSITECIPKMGYYNISFEYIYEYSKQQMLNSIPHFIQYYHYFLKNQYEHFAKGKNNQKKMNNNNNNNKK
YTCTTHKRIAINNKSNKHKKFPRYINVEEKKYIYKDKYNIPQITNNIHGLKSPYNNIFHTNYLKNIFIFMDLF
KMNKYIYNQKSKQHRETIIYGHNNLSFVHIKNEKDFSYDEKKSKYNIKMENNNNNNNNNNNINIYSNNNLNFV
RQMNEKNKEYTKLLSTKKRNISQSTSFLNNKIIYDDYRNLIFNTSDTYNHNVSKSINIISHDINDILKIIKAR
EENYFINKNKDIKYTCYENEENETSKKRNHFMYVYTIPEIDVMSCLNSCISNIYCTGIEMNTTDDKGKTNLFL
MLYRSYGKTFIYFYKCSLYFYEDLTSYYKKENFKVIKNVEQYNNTHNKIISCVIQKNPILHLWKIYSFQKCPH
NYYCEQESLKKKKCPPNSIKTKYDGTINDCLCLPGYYLKGNIQTCIPCEKGSYKNTISNDSCIKCPINFTTLF
ETSNSVYDCVCRKGYYFSWNTNIDTNDIEKKNIYNNNINSIYRNKNEIKLIKELEEEYAFNVEKKRKNKHKFI
LHKKLNNRYRENIHKKQISLKRKIKFLNEQKEINNHLNFLHVNEILNISFFNDNQNYIYKNKNKQENDTNIEI
NPVSKQTSLHSNNISNKYMRNNFLQINEKLYINNSNNNYNEYIHKAQCTKCPRKMFCPGYWLENYENEIHHPP
IYCPKGSLILGTTIESTDIHKCICRKGYSINLSIKPNRNKNVNVNDNDNGNNNNNSNNNNNNSNGNSATLNNN
NNMCVMCKEGYYKDVIDNSVCAGLCIEFSTSFKGSISKKQCFCTVGKYMIQDEKDENKCINCSKGSICIGGFK
FKSLRELIKNQYYINLNIYDHSIPLPKKGYFATFQINHEDFKWKPLNSLHIEINNYEKGNIIITDKIILNVFH
KKLHLINIGEKNKYDQADQGIYIKKNLNNNSFIHNNNNIKYIVEESKHLRDRTMANIPNEPILVNEKLEPIIY
KNNFLMIDRIPDFHLCPISNRCMGTSSNLCFKGSEGYLCNNCSNSYDVIHFRSQCIKCRKKKTEMINLLIHKF
LFYIIIFFIMYLNYYCYIKKNYLFISILKIWYLFIICFIPYSFIMEPIENHLKNYQSYFQLYIILPMRFIAHY
FKLNCFFNNSYNEINNNITDNKVHIYIYIWYIQRFLKIAEPIFDCILFTLIFFIIYVLYISWNNEKIQIAQSV
IKKQIENIQNNSYGKHIYDFYYYYYQNNVINIIKNTNNNCNVKNWSQSYYVHDYLSKTKKKKQKKKLSKWSLT
QTKKKLIQRWKHKISRSFKHKKENSENYDYFENKENKENNSLCSESTKHDNTINNNYTTSSVEDKFIYTKKKT
TYNNNTNQSCDDENIYNVKEKLLKKDITNNFGYEHIEKEKYWTSICLSNIYNIRALGLFRYIHPYNINIWDKM
KRILSDLSCVYIIILYIYFPFIIINLLELVWCQPIQYKENPSLLILYHMPSQVCDLQNKLFLSGVLYSCFFLI
IYIILFSIYIYDTSKSIKAIGSYSNNLKSYFLFNGHNYQNRCWEIINIIKVSFIIIPFICQLYTKRNVNSKYF
ICCCIVLVLITEVACILFYSPYDKRSDNLLQKLSLTSSFFILISYLTVQVGFFFDVTILNIIPIFLFFYFHVY

VIKQIILDFAIYKNILKRKDNHISFDKKKKSNDMLNDYNFLFFFDKIEIDNKDSNTPKGIDTTVQCDEKEIKN
KDDINNFNHNKINDVNNKRKKKKKKKKKKKKDETTKSPYNEEYNLLNFVLSSNNIPSYSIYFDDKEEEIYLFQ
NNNTRGYEYKKKIINNMKYNNDIKIEQTKRISLDLLHSDILYYNIRKGTINSDISNMNTYNNIKMRKQENNYY
HQNDCNNINSDDYNNVCVQKRISNKLEHFIRIEREYDKEKHYIKNNEKNNSEDDMFFLNITHFINCFIDAINI
LYINQSYDKITVEWLSFITRFSICFIHWMKNHDENLINFVPLNDKQFELKKRNLLFYSLFTSYTEIYNLYHSI
NNSNSLAGCKKRFINSKNVSKPYKHIKKIKNKQGDLYGINNLNDTKDIGDKYQRNYKRNNYYNGNIKNEHLNN
YDLNNYDLNNYDLDDYHIYNNDIYNNDIYNNHIDNIHLDNNHLDNHKKDKPFIITSASSPSLPCFNISDDQFF
RCENDIIKLLFDETLFNNLTITLMEFYFAIYIIQFIESKKLSILINLFCEKKKFLSKERQFLKTLMSRKHIIQ
KIIDKNNLSTHMNEYIEYNYYLEYNKKLKKQREKLLNLIINKKGQIRTLKSIKNLQLMEENMVQGKNNINTHF
VKTLKKLLEHQPSHQSENNIK*
>N.PFL0415w
MRRNIIQKILFNNKNITLCNTYKRGFIQVLLKHGMNSKGIQNGFYNNKYILSAQKNSAFFSTEKSQDLSSLSK
EQIEEKILTVLKKYLPPDVEIKYDEELEKYNTKDNRAWDFLDTVEFLIDIESEFNITIPDETADNIKTVQEVI
DYIIQLNIKKT*
>N.PFL0420w
MSICISTQIEPNDETYTNNSIVDMYEKRKEERNTDMIKDTTSSSYDKEYIEKIKRLKKQKYFGNKTIGKFSSY
IYLINQIFGSGIVSIPYVFKNSGWLPSLVANILICILTIFNSLLFLRTMTMIPNNIHFNKRYEYISTVCYFLG
KKNIYFWLVQICFYASILCSNIISIVIVSLAVDHLIINMFGYTIGFVIYPNIEMRYFTNINELYYTKNYILCI
TIGYIINAIVSIYFSQSNLEDNMKVQFLSFFFLMTTILQMIYLSILKIYQYNTTNINLHLLTNNNSINKYTDI
KYPTVFGNYNFKQLISSYISSYSTITVIPCWANEMKSDVKIIKTVWLCNIFCCIIYYIFGYVLCTAYPNINND
NILNDILQTPFLNIYMKISIYIFDILTIAPGIYVYCIATRYNLINSNICSEKTAFLFGTIFPFFISCFFNSRY
IFENIFTWSSLIFSYACNFITPSIIYLIACKNIPFSEKNPLKYIHVLYDPKEYMNRKNLCQEVQKKNKRMCIQ
KDTEIKENALISTMYEGKYNDKLEHNNSNNNNNNNNNFLSIQAAEINKINNSREQKINYVDSQSNHFTVPIKT
NSQSKEGNNMENKKRDISIDNKTLQQKKKKKSVNLNTDNVIIRMNEEDMEENQHVQYEKDHDEKYDNEKNKEN
TYNNQTNYNHYKKDNINIIDDNNVGKIKHNVKNIKKTKQKKDMFEYRKYNSLFNTKKKNVSYNSQKKNESNSY
NIRRSRKHKTVMGFEKKKRRNKHEMNIIHDGYNKYQDSDSHSDNINDDDDDEDILHDKIIADLSRDIYNDIKI
IQKNYERDEYLIKCSDIFDSFLNLKLIMETEKNNNTNMRNNYDMESIYNMGNNYDMESIYNMGNNYDMKNNYS
INSHHNIHYMSNIKKQHNSVEYSNISNTLHINPKHEMKPSPFFSYPCYKSIDHSNNLYIYNSYDIKFNNMNKQ
EKNKNLGSDNGNHKNILLYSNVKDDEKEYILFDKRRVLENIIQHEKEDHNDNENNKIYIEQTNNKIGTSINNY
SDKKYIEKKKNELNELINNQNKYKHQFNNIEKEFDILISGINNKQNLTWAFDGNKKENYIGNKKNLYFVNNKN
INVSNVICTFNLNNTYRKSTSFGYFYNSNCKPYVKEGNVINKLYNMLNRMNTYGSSPLNCISDDVIIPTQTCR
KQNTDIYYYINDNERNKKGKHMYNNILSDDTEEEPFKVYLSNDIDINEDNDKIYTICDDKKGYPDFKMDDKNN
NNKTYDSNKYDSNKYDSNKYDSNIYDSNIYDSNIYDNNNNNNNNNNNKDDNIFHKKDYHARFTNHIKNNQKED
ENNKEEIIKVKSNHHNNNLEQKSTIQSQDKLLYSLNSYNKRSKVYKDINTLSVPDNVYNVIPQLNKRKGTNNY
DEDINNIYNYYKYNFLLCFNENIKNNKEYLKNYNHIRNTNIYYNNIIHNNNQSTINKKNIHKNDKIKTDIINI
TSENDIMKKNKKFKSLYINTFSNDDKKEPLINHNKNDIEELPTIKLICSEKPIYGYEDAYQIDDYVNGQINEN
IIHVYPFIYLRIKHVKITQMLLGITIMLLFIAILYDFIY*
>N.PFL0425c
MMNNYMPKSFEEILVHPQNKLSILNFFTDIIKFNNDPILSKELEYVCNNKNTEECKTLYSLKTICVILGIQGS
GKTTLIKYLCDKLNIRQCYYENEPMDYINNLRGMKERETDDDNDDDNSNKMNDNYYEHPYINFLLYSNCKIGN
TKKDKTQSGEKRNHIIQNKKYIGKNAEHKSTSVYNNNNNNINYIDDGDDNAVNRSINYNDNYNYHNYNSMLND
KFSIGGKKRIVDSNIPDGPNKFVKISNSNKINYRGTINSSLSSNNHFNNNNNNNYYYYNNRWSKEMIIEKLTE
CNNMIIQLIKLWFKLYVTYMNILKELFKYSESLGNEKKCTFPFFKQNRFYRIINKKFLLCVKIFKKINNILSV
LHNLKDTILKEFNEFIRMEKQHLINLFVEGNYNEEMIEALNKLYHTNEGINITIKESNDMSFCTNKNLHLYNK
LKVELNEEWDIRASLIEEMKDYIERKNMNKELEKYIESNNNIMYVEKENSTSITIPMINEENMNDFYIRNKYM
NKNKYNNKNKYICRDSSTCTGSDDSTCLDNNSVKSTIYETESYITNILTNHTLNKYIEHFIEINKSKKVIKEL
CVSNNLKRNFNEHYLNIIQELNKSMNDLNNINYIEIKKGKINFPHFMILPKDIHDIFLKENKEKDFSSFDNEN
ILIQLLMINNYNINFINNFNLKNIIKEVIIIHMLLIINIKDIIIINMNIVNHIKMDVINNLIMTNNLIKKKKK
SFKNDYIFGKNLLDKLTSIMLKKDNEEQNYENIIYDDINNIKEFQEKKSGKDSVTDNDKYTKENKNITKINIT
CSFENSIIMIKESLNVSPKVNNKLIKYAYEIYKLNNNIINNLIQSNTLYNNIIKSLHNEIYIKNQLINNFVDI
IDIQNEMLDFLFWILSNNSNENVMDIIMKSSLYKKVMDIDIKEKNKREKERIFYHNHFINTRAIIIDELPISE
LEYSEAFRNSCIFTMQFILNRINYCKEKIIKYKNGDMNQIKEKNETQKIYTTNQKDNNNNNNNNNNYNNNNNN
NNNNNNNNYNNNNVVCKSNMECNTNFYMLGKDNLECFKINPMIILINSYDQIKTVNSLLGFDINNSPYVHFVK
LKKIHPAYFEKILIERYYCKMIHSNKNIKDVIKNYAHNCNGDIRSCFNALDLLSRIPNVSQMNINELNNISNT

YQNDIFHFVKSVLYNNIPSDYTSRDDISPLFYLNLHTYTKDEYFSNDIDYEHIKIEENYKNYNNINISSKNNL
NHKIINHDDKKDHFVNNKMKDANLLIVNNKEEKNNVNNFIKQYDIDHIHDEQNIYISNRNYNNDKIIQNNKNI
NSYENFQILLKELDNQSDMMNIFEKFQIVSLLKENYLYFYNNLSDIAKLFTNLSIIEYSFCGLNCTNLLMRKS
HENQNGDVSRFINDHFMLTYMYFMICNSNIPKGVLYGNHISQNNKINVNINDLQRNEMNNEENGYKERVENVV
KNMNTIISIDKNNNNINNVNSNNNNNNNNNNINNISNNNYYYCTSLSSMDKKIKINNHTSIQPQSIHLKNIFF
SFKTNPMYKYYHHCSILRKELYDRYIMIVIRKLTNECINDFENIISKYCFLLRANHELFSSIFPSYILLIINY
WNGQYINECNIPKYEEIKEKNECDNTTNNEYDNKIFINKIESNNFKTALYSNKVNEIIDNFTPTCTNNENRKK
HINEIILFTETFITPKFIALFFPSYLKYLKDGTKQKKYVLNFIGNQAVSIETYFEMRNVLKLNDIAFFNYFFQ
Y*
>N.PFL0430w
MNDNISKVNSTVVELLGMSDLFKRMQNTCWLKCIPDVHDSFLSVGETSCVDRCVNKYMEIHTLVGKNLQESQI
TK*
>N.PFL0435w
MLKKLLPNIYKYKNLIEGKNVLIHFEYKRNYEANITKRSIRKLLHFFYKQVHPDLTSTLPEELKKKNSESLSI
LNSYIDILSTPEKNEENIFIERNIVFFKVFENKENKIIQGRYKHLVIKLHTLSNNLTENEKEKIIVKLIYDIK
QAIGGDQEKDKHNINHDDYKASFDDDNIYDKKYDRENKKGINLLWDNLTNYVKDTQALYQLSDEQVELINQRK
NYFYYIKKKLLEKYARIKHKKRRKLKIENIKQVANKIVLIKFPDLHQKYMKNNFQDINNSHQTYQIIQNGYDP
SLLFFHKNLNEQNKTIALQNICGLNLKDDADKWLLESCLKLLKNHPIQIPIVICDEQKEIALSSTFGYIYVPY
NFCFHDFFIFLQNNLDEARAIRKKVMNTLDISF*
>N.PFL0440c
MIKKRKKKKKEEKEKEKEKEEKYSFMNISSFNRKCNKEKYHFFNTAMNIKKKYICDDNNITLVGNNINENKNNIK
VNKNNIKENKNNIKKNKNNINENKNNINENKNDINENKNNICQKAKEQSSLLNLNIRKEKNEHEYFINDDIFI
STIKENELNNIHINKTCTLQDVEYYNVHLKNKFYKTYIHSYDNKMLNVDEEGEYDRNENINRNESIHIVNASG
YMHESIHDVSHNYEGNLTNNNNIIESDILENNIKINSNITNNNVINSSNQDNDVHVNVPSQNNYEEKKEECQQ
KERTNESINNINSDNNKNDIHNVNNNNNVNNNDSVNNNDSVNNNDSVNNNDSVNNNDNINNYDNINNYDNINN
YENINNNDNTNNNDNTNNYDNTNNNDNTNNYDNTNNYDNTNNNSDIIDSSNVSPREGENEHNSRETSNINNRG
ISFIENGHSNVSNEDDLNYYVITIRELRTNDINRRDDDNISIEDEHIYSFTKCFISKILGYDIKPDLEEYNMN
KIIRSLLFILMLFILFCTEFLFVYNNLLKIKVDSKLVMIESNYNPTFVSNFLYQFNENDLKKSLVTENIIKEE
IGKGNFMLYNNMCVIMNKLNINCNNFSDYYKINDNNENTEHLIEKSIKIFQELNNKKIFVYKMKESLFAKCND
VSNKNLNNDDNYIFIYVSEKKILKISVLFLILTIFFLCLRIALIISRILYDIILFIYMKGFRLSIESKKKYLS
KYIWSVSTLYLLEIILGDECIVWWLHDIDPIFSYHFQYFIWIISLFCFATYVLHKILKTCASRWDRTNDRENI
DNHGNHTDGNTLFGNPIIINPLNDGPEPRTTRYENSANVNLTTTTTTNNNNNNNNDNNNDNNDNNINNYHNNN
NNNNNNNVYTEFPNNTNNNINYTSISLSLKCVHDFLYGANIMFACIFLLLLNLSKVPVITIIVTSVVLFIDILN
DTYLEQVHLTSTITNTTFQRPRRKRFYIIENKKKKNFKNIFLMRINEHTYRDVTVDTINKKEKKNKVNNQIPF
NHKNNNKYWDNKNGIFNIRNIINNKYLNKKKKSLKKTVNKNNENDVQDEINNNNSNNNLPENYELNNSIIATY
EYCEICDERNKNVVLHPCMHGGFCEACIRCMIFNSLKLKDTYPCCPLCRNPIENVYKISYEDDERKVQATNIL
TIKRK*
>N.PFL0445w
MEFLSNIYDKVLSGYIFTDVFIYKDWPEKFLGKNINSKDDGNNKNENQENIKEHLEEVLGTFMKNGKISDSLW
IKDKIFIGDEGIYILGEILTICQIVEFVDSEDIENVKSNEQFKQQIIEKMKRKIIKKRNSDMIEQRINSNIVI
CNSNTSEVQQDHDKNQDNINNCNNKNNGDNINNCNNKNNCDHINNCNDKNNCDNKSTYYNKNFRDNNYNNNDD
SFYNYDTHEDNVSNQSIYKDNIKSKNKDVVSNFVEMLCEGKMNKYGFCEYDQNFNDRYHLNDSNADGKSTYPL
EKCILPFAEDYCFKYVCLLLNNGGKREVRILRFNKDNINTNKLLDTLKGTVTFLNELRYLFKSFRSQVVLKNR
INKEFCAYIKFHRGDPNFSGSKAVNEFFNSLNDKAVNSLFFGLNEEYLNLLEEAISYCPIATYAFYKNIKLLE
KLNVNTTRRTWKCVCGLQKLYKNLKRAKNKFAVEQQKEVSNASFHNMSSIYKNNVIKNQGTFPSGNEKSTDMH
IHSVDKGKYISLYKDISINNMEKKIPINNTSSNINSFYSVNKSHNSTPPGVIKVKNSNNVNNSNNTGNSQNLF
SDDEHNYNSTYINNNKNNINSFDELSCNDDKISKSQDIHILNDTYLYVTSDEEGFEQGPKSMNNTNENKSHNN
NKNNNNNNYSGNKQFCSSNRTIDDNNKTLIDEQHNTLFFHDDHCHNDNDNDDDNYKTMDGYEEKYINENVIVR
ENSVDDLRVHMVKLFKTYGGQCRIGKVKGRCEDATFQTDVPPAFGIFDGVGSWSLEGIDASKFSIGLSLACQR
EAEKLSKNLNGYAKVSYNTITRSKLLLKNSLESVKKEYADAYGSSTAIVGILDEYTGKCGISSLGDSVCMILR
REFLPGDINFERESYPKFAAESFLYYNVKGRNPSIIRKIIWKTTDQKWENGAPYQLSNLPDRSQWKDLENRGL
HSFVKILEKVDIDGDSPDMALTPPSEILCMPGDLILLMSDGVSDNLFDEEIEAYCTFAISPEEACEFGEPALY
TPAQDIAYSITNIAKRRSGDKLHCRPFFPFVGKQKDPNHIYKGNKVDDISCVAIWVVCENEESVKHIARYPNE
PSILKTDKFYTNNYFQNFNKITDICTPTKTYIKEKNKIDRVNLQQVNVHSQRRLMQQQGYDPQDSSVNNLASH

FSMNFFDSSETPIKFNTLDEGKYQDLSDIVLKDVDDEENDDHQEQDEQDKEYRDDDDHHDEDLYRYDEKTQNN
KNFKNDMGYDDMKNSPKKSNQLDMSNMDSMKTPNSKNSKHTFKDKVFDTYNFDSTPRQRVSDEHLNEHIEKIN
NMYIKTPILYMDEEFKDNKDDNVKKNKNNKSKFNKKEEANILGLHNETPVKQMNTNKNKTPTNIISQKNIMNK
FINNETPIKSFNNKETSLKVDKKITKSKRKSIFPSDKKDSPTKLGYTRKRSKKS*
>N.PFL0450c
MQKKPSTGFKLENTYRALFDNDDNNEKSYIREVVTPLGDVYIINEKTQEKFIKGTQRSDGTFRKNIRVKTDYM
PQEENCAYQVKGKLLEEQNRLLTKNTSPNVNINVNTNHNTFSHVNINRHVNLNNQQKKIPGWNPINDDQDEKL
NSSKKKKKKKKKKPNKEIAEM*
>N.PFL0455c
MNDLINEEKNVECLNLKDIRLNAEKYNWLEIFEKENSYVFCKDNVKINIIDLKGLVTIEVKLLNGISYKMCRS
NIKIDEISSFFGSSNNYSSYNDIINKTILCNYNLSVKEKIDLFNKNDKSFLKQDKDKNISKCATNNVISYNII
NNNNNNIIKGKNIKCNHMTNQNFHLMQREPFGKVEKIYTKRKEQNIFIKTDKKEDILLLHKNKLLANIANEDV
NNKICIDENVERMICTSKTILSLKENNKISRSPYDITDKKYTSNKFVYNDMGVHNVVEKKKSNRKLKNSKKLN
KLKKLKSTSYELLYFYNKTNKICNNIILNKIYYLCFFNNFFSYRTLYEYKYIYIYNSKGKKELNKRTGEKLFN
HSFERMFINMSKDNTYDKCRRSKKNIHNVICPYNNIFNGINKKEVYDNYGDNIYPYNYSTRNPYRIYQIKKDK
FVTHNLYIKKKYYNNNNNNINKKKNTSCRSYFTKYNTNKYIKNYTTNKYNIHNEPDKKYSIYHKNYRCNGNNF
LYKDLNIFKEKYVHNGSDNNINMCNYFKIYEMNNAENTYNHLDIQTFNDIKREEYNIYQPMYGIINNTYNNYH
TNKCNNMNNYIHKNINEYNSYDLNNNIYNTLTIDDNNLGNHNIYEMFCNNSYDKRLTSVSCYMQRVYNKSYMN
KTANILNKKYNNLLYSNEEMFNKSQNKKEKIKRIMYIENDMKKLYVNKGVNNNFKYQYIYEELDRFNHISPSS
YKKKNQLQNVKNEIYKKLSYLKNFQHDMNKNIPRYYFFIKHLLHDYCKKKLHIWLKVMLVKQKCGYCNEHFEN
IDILENHLKYVKTHKVFYCCQKPFPSLKYLYIHLKRKNHYGYIYYY*
>N.PFL0460w
MSLLPTAAPTPNKDNKFMTDIKKFMNQKIRVKFDGGREVVGTLIGHDAIFNLVLDKTEEYIRDPNDSFVVTDK
TRSIGLIVARGTSVALITPVEGTQEISNPFITEEK*
>N.PFL0465c
MSGIDSKNKRVICYVDENLNNNIYSESEKTFLKNNVDWNKIKLLNTSNIKPVVAGVEKKLVLDTSENKYVRQM
NNVNDKNKKNNVIFNIDGKKKDIRTHLYQDGDPIERHVKEKEDMYYDKENNLEEYKNNKSDDEIDNVDRNGIY
NINIKTNNKIYSNLQNEMHDNICNNEHKDNINNVNNINNINNINNINNIYNINNLKNNISSVFQEKFSNRYTN
LLEDDDLLMEKQDDTNINLNYQRCYQNYYNKNKKYIYNDTDDDNDKKCVKKNHVHNKDKCKNNKYFLTNDDLS
YNEDILSSSTNTCDTLDKIKLDNIYGSFIDDVKSSFVLTKNMKKSFTHYIHKQKNKEKNKDNEIHHDNNTDQN
EYSDFNDFDVSSNNNMNEQEKKNDNINNQHITKEKMTMLHYQNIYDHECKENENKSTNSIIYIEDNNSLKSEV
ILIDDTQSKCTNGNYKSPLSHEKNEDDYPETKDSYKNIFHIMKEQNKLRSLSLNRNNENVKKFGIQNVQHLFN
SNNLLNKEKNIENQEDDENEEETHEGKHTNSNSENYYENKIESDFFSNSDYPKLDEKNEDNYEQSSDEYHDIQ
KVNDDINVQVGSNHINLKEKYKGEIGDDYGINSENERGSYSDSRSASYRSESYRSESNRRSESDGRSESDGRS
EGDRRSESDRRSEGDKRSEGDKRSEGDKRSESDKKSESDKSSNDDDKRSNDGNTSESERSEVSEKSSYVSSEK
PKKRPYFYENVSSKITGFFFQVKKKINKNNDEDDDYSESRRSSESRQRSESRRSNVSRRSEVSRRSDVSKKDD
MSVKSDASRRSDVSRRSDVSRRSDASRRSDASRRSDASRRSDVSKKDDISAKSDASKKDDISAKSDVSRRSDV
SIRNDISARSDVSRKSEVSRRSNVTNKLSDRENEKNEETKLSEIDVTQVNDIHKDITNKEEDNEKIIKVRKTR
KKRIVADNNKKVAMKKPKIPKEGTKIIKKGGIKKKGKNENDREEEERKCNICNMTFINNQLMMRHVNSVHSDE
RPFECKICHKSYKRGDHLKIHLLGHKISEEKNKYQCPICKTSCKSSRELIICQKSHSEGYKKPGYTSASSKLN
QKGDSNVLSLINGKKNSRSKNVKQKQIENDNSNDNNNNNNNSKMKKNDKRDKLHNGVMENSTMNDDDNNMLQL
MDEKNITEGKEGEVQKISRKGSFSIEGRTCNICKMVFANKKLMKRHLMCVHSDDRPYKCDICFKSYKRSDHLR
NHLSSHNKTNEEKKHICLICEQSFATAKELKHHKIKHYKCPYENCSYTYSTISKMKYHLNKHKCNLVYTCPGC
SQTFVIYKDYIEHKKMCFKKKYVCLECNKIYLHLNGYNKHINKIHLKINTVFKCKIKDCNKQFCSDFSLKEHV
INFHKGIKRFFCSKCNISFGYRSSFRRHNVNMHS*
>N.PFL0470w
MVLELVVNLENHKRRIWSICWSPDGNYLASVGADKYIMIWVKKNNEKIRKTNNSNKIMKKALNIFGEKTQIKS
NIEFDIYDIIETQHEKSLRHIEFSKDGSFFVVASFDSKCSIYKKNNNDKWVYYKMLEGHEKEVKCASIHPSNK
YIVTCGRDKSIWVHAKGDVGKTDMKSKNHTNDKNGDSVKNDDSFKNDDSFKNDDSFKNDDSVKNGDSVKNDDS
AKNDDRVKNGDSVKNGDSVKNDDIYKNDDRVKNDDNYKNDDNVINDDSVKNDKNNTSDHILQDDQALTNHDIN
NNNLQNSNSLDFHFDAYLTAHTEDIKFVSWCPLSENTFISLSYDNSLKLWSKIMNEWNCIQTLNEHTSVVWCV
TFNFDGSQFATCSDDKTIRIWKSDKKLNYNLHKYPFLYERTIKDLKDTSYSKETSKNMDEEKVTGNNNTCENK
NVGQNDDTLNNNNNNNINSVNNNNNHVVGGLHQTQTCEGKELAERTNQMTEVKSRSKENINNTDINKSNSQEV
VKSKGKSENKIFKKTTNFFSKLKEIKSKKENEQKNILSNITIVNYSEQKDDLGTLSKVYIQHNFVPLYFNNGL

FKYVYNFSQVEDNTNINNDLKKKDQHNKKNNIKDHVTKNLDNDITNNIQSDQIHNNDILDKNSNHTNIEIEYK
PKREYVEEFQETSLHISEDTNSTIKQNILKKDQNVNDTNFDDWRVNNVIQGYHKRSVSYIDWNSYEDLIAASS
FDNSLKIFKKINEEWELISNVDNAHMSDVNCVVWCPQKYQDYFLLATAGDDCVINIWKYTKG*
>N.PFL0475w:mRNA
MMDTKVDQTIQPKFYVDKKLSKSFDNKLDEDIFNYPFKKESFLKSEKFSFEHTKDSLWKCIKEKAKKKSDMEY
FNCVNNLCCKFICTIRKYVKYFLYLKDSSYEIYNINLYNNNNMNIINNKNITNNKNITNNINNSFSNDYINYN
HNYNHLNNSSSSKHNNYNVNNIDEKNIKNDYNTYHNIYEQIFFKYNPSFYEYLMFTLMKKLIHYKNYIFNKTK
KINNSYNNNDIKNIDGFLIFQNINFEEIFLNTFYSSFPFKLFLHSLYMIFICFIYFVVLYFMLLKKIYTHPFI
FHLSVLKFLFDIIFFLSFILYPLFLRLKRIDKIIYSSYISSYIFVCVTFLYSFIIFKCSSYSVKMNSNTYQNN
FVFQNMLFLLINIIYICIFCFLKNYMILYSFLYNCRFSIFCILFIFLYYYLFFSLDFYRIIHLPLDNFFFPFL
CFLFFSFLFIFKIIMSLYYEYVYEKKYRILFVKKNNLIEKRITKRKNTNINNAYFTKYFSIDNTIPTSPIEDI
LNNFKHILETINIIEENPNHNLTTNIMKIKEKIKNCDNILRTKNINQVQIGKYRKFEKVYNIWCLDKMYLNYP
LNQEESKSFLSNSLNRISFNSFSNMHSLLSSKFQEHYNDIYDWNGNIENIYKANTFISIGYKLLYPLGVLEAN
FDKEKLKKFLFRICSYYNDVPYHTSLHAAQVAHFSKSMLFMLDMNHKISAIDEFCLHISSLCHDTGHPGLNNY
FLINSENNLALTYNDNSVLENYHCSLLFKTLKNPNYNIFEHYPYHIFISCKKNIIKAILSTDMKNHFEYISDF
RTSKEFIDYDNLSNDQIWQIFCLILKASDIGHSTLEWNKHLEWTLKINEEFYLQGLLEKSLNIQNSFLCDINT
MNKLALSQIDFLKHLCIPLFNELNYICKNNDVYTHCIQPIENNIERWESHKNDNQNLGLHEKYKEENLLSKLE
LIKFE*
>N.PFL0475w.1
MMDTKVDQTIQPKFYVDKKLSKSFDNKLDEDIFNYPFKKESFLKSEKFSFEHTKDSLWKCIKEKAKKKSDMEY
FNCVNNLCCKFICTIRKYVKYFLYLKDSSYEIYNINLYNNNNMNIINNKNITNNKNITNNINNSFSNDYINYN
HNYNHLNNSSSSKHNNYNVNNIDEKNIKNDYNTYHNIYEQIFFKYNPSFYEYLMFTLMKKLIHYKNYIFNKTK
KINNSYNNNDIKNIDGFLIFQNINFEEIFLNTFYSSFPFKLFLHSLYMIFICFIYFVVLYFMLLKKIYTHPFI
FHLSVLKFLFDIIFFLSFILYPLFLRLKRIDKIIYSSYISSYIFVCVTFLYSFIIFKCSSYSVKMNNFYRIIH
LPLDNFFFPFLCFLFFSFLFIFKIIMSLYYEYVYEKKYRILFVKKNNLIEKRITKRKNTNINNAYFTKYFSID
NTIPTSPIEDILNNFKHILETINIIEENPNHNLTTNIMKIKEKIKNCDNILRTKNINQVQIGKYRKFEKVYNI
WCLDKMYLNYPLNQEESKSFLSNSLNRISFNSFSNMHSLLSSKFQEHYNDIYDWNGNIENIYKANTFISIGYK
LLYPLGVLEANFDKEKLKKFLFRICSYYNDVPYHTSLHAAQVAHFSKSMLFMLDMNHKISAIDEFCLHISSLC
HDTGHPGLNNYFLINSENNLALTYNDNSVLENYHCSLLFKTLKNPNYNIFEHYPYHIFISCKKNIIKAILSTD
MKNHFEYISDFRTSKEFIDYDNLSNDQIWQIFCLILKASDIGHSTLEWNKHLEWTLKINEEFYLQGLLEKSLN
IQNSFLCDINTMNKLALSQIDFLKHLCIPLFNELNYICKNNDVYTHCIQPIENNIERWESHKNDNQNLGLHEK
YKEENLLSKLELIKFE*
>N.PFL0480w
MHLLSFLSFIIWFIHCTAKRHEYSIKKYFLNSHNFCKIKPDPFRKDTLKKRLYSSDGIKDEIIIGTRDSPLAL
KQSEKVRKKIMSYFKKMNKNINVTFKYIKTTGDNILDSKSVGLYGGKGIFTKELDEQLINGNVDLCVHSLKDV
PILLPNNIELSCFLKRDTINDAFLSIKYKSINDMNTVKSVSKTEDIHHINKKDSDHNNDTLCTIGTSSLRRRS
QIKNRYKNIYVNNIRGNINTRIEKLYNGEVDALIIAMCGIERLIKKANLKHLLKNKEQKNICQPFLLKCNNKK
CIDLCHVNIQKLNKNLIYPALGQGIIAVTSHKKNYFISSLLKNINNKKSEMMAQIERSFLYHIDGNCMMPIGG
YTNMRNEDIYLHVIINDIHGYNKYQVTQKDTLYNYKEIGPNAAIKMKEIIGTEQFNKIKAEAELHLLNNK*
>N.PFL0485w
MEENKKIMNMKEHQWRLFEYNMSKWMIENKYILDQEEKKKFYKCANYSIGSGVLNASLIYFLCKKYKNYITPV
SRFFLTFSLGVYTSMVINKIYRRKAYIEILSSKTTMTDKAKEVLNDILNINEDNIKVSPQEIKKNEDKDNINS
HNYMNDQDIPLNIHTQEKENNNLDFIPDLNNTIMKEQIDELERQDYYFLKDPNESNSVSWDEIRKRNE*
>N.PFL0490c
MLTNLSKKRFYFSLPCSRDLKNIVKLPLLEREDKYKIINIWKEKYKDNKYVISDYMDINKYEVIKNNCKNNSH
FIIPFKNNNGYITYYTQFIDSKLIFVTSLEYYNKHKSNSTPFITLHFFDEFKNKEIILSKIHIINPAISKYQA
IKIYNNILSFYYDTNYFQYVKKFNNDSRNFNYDKFFGKFKEIF*
>N.PFL0495c
MIIKRHLINYYYLINNNNNGYFLFGQNVRNVRNVRNCKNYGNALYYNNVGINNIHTYKKYSPSGGRDFFFLNN
KSGGMTCYYKSMGIYKNINIKDPNNNSNIKYGIMRKKGKNIFNNMMNNYTHKKCFRSHLFDPKKNSIIDNIKD
KFNELFMKRIGENNNNNNNNKSNNNDNNNNKNHNHKSNNVSSLKDLFNILKKERKYLGFAMFCLIASSLGQMI
FPMCISKIINMYGGKEESLKYLTNEIYKTVLLIIGISTFSFFRIYFIETSIEKITRRLRTHFFEKVLNQDVHF
FNKQKTGELINRLSNDIEISSKFLIHLSFGIRNFISALIGGICALHISPRNLFQSFLLPVSGCLLIGTTYAKF
VKKISVLKQEKLSSCIDFASEKIHNISNVRLLNGESYEKKEFINYLEDVYKIGKKHSLVKSGNHFLFFSIISL

FLLHLIYYGNYLIAHKFINTGDLFSLIMYSLFCGSGIQGMMHAIGDIQKCLGSCSKVLQIIKLPDNNFHNYWN
KTSIDFLKSNNFSITFHNVSFSYDNTKDNINANINNERNKKFALKNVSFFLPHNKSVAIVGKSGSGKTTILNL
LTKKNDLNASGEICVGNVPINKINSAVLRSIVGVITQDPFLFNLTVRENLMYPYKAYEEMLKEQIRLIEQNQG
AFINMINNENTKKEKNDDDNTINTYDSEQDINKNDMKNTYEIYTFLLKELQKVQEEIKNNLSSDKMKNIYNDL
HIDEFLKNHFNYDNMNVGVNGTSLSGGQKQRIYLAQNLIKNNKILILDEPTSSLDKLSENIINKALIKYMKDK
TTIIFTHRLDLLNYVDYIGVIEEGQIIQFDKRQSVLQKPCPILKKILTQGVSE*
>N.PFL0500w
MTLKNGYRKIIKILFIFIHIFHITIESFVTNYLCTINEATHYNNVFYKNKNRTILKDKKYLLEKKKKNLFNVQ
PSTVITPDNETSNEIIKKVKLKKKDKKIFKKYKDFLDILPKKTNEYTLMEAIEKIKTLAIHKFVESIDIYLSF
NPKKSKMTKNDNIKTFITFPHNLKKKREKKVYVVTNAKLQKIASQSGADVVGEDDLINKIKEREIRLQKRNNN
FLICTNDCIHKLTRVGKEIGRKGLMPNEKCGTLVSEFLLPKHVKLFKSENTYIFKLNKLNTLNINIGDVYMSN
DEIRENINHLFEHLENLEFFHFNFKHVKTIYLSSTMGFPFKIKKNSL*
>N.PFL0505c
MSYKNYENNKVNSTSFIVEEEKSEGNEKKIKDNILLIKKNMIYAEENLENLKNNLISKRIIEALHEEIQQIYV
KVVETEHLFREWEIRFAENPFEKQKKKYIFEKLNIHFKNEVNKLENISINVKKAANELPNIENGEMNYNNNNN
NNINGLLNNKKKGNHKFISSDDTMINNNFVLDLDKTYVNNDDFIESSTLYDYEFDQCSENDLLIENEIINDKY
EGIKKIQGQVAQAQEVFKDLANLVFTQKENIEMLNNNLYDTNVNTFKSAKELKKTYDHVKQQRFSWFLAVVTL
LIFIYFLYFKIFHFSLFS*
>N.PFL0510c
MMKKNNKNSSQGQSETLFLIKKQFFKTKMCPFQKNKNYCLNESSCHYAHSIEELKPMPDLRNTKLCDFVKKKI
PCRDINCKFAHDIDTLKPSVHLATYKSTICSFWGKGKCFNGNKCRFAHGNEDIKMNDIKMNDIKMNDIKMNDV
KLNDDAIFNDHIKFNDRIKFDDHIKFDDHIKFDDQIKFNDHIKFDDHIKFDDHIKFDDHIELLDDIKYNKNNI
YKNKCKDNNYDIKQEGTASTYSFNTCDNSINCSLETANVSSFDKSREILVLRKKNVNQENCIDDDDNNNEHDN
KIGKINEDEYLKKNEREYNNEIINNKKIIRKDIYENNYYDNNFYYDDQEKIDNIIWNGKGNMEKMDYIDNKVK
VSEDTGSTRTKHIHNEFLLNNSFDNSIEHVENIENVCINDAINNLGNIALSTFIENNEKYTKVIKYLLNENNM
LKESIKKEHKNNILLEHDKEIRHIKLDTNNSTYKNNVIKENNNYCNNICVHDVTNYMFPEDNFEKLLYSNDKK
MYSNNNINIFDDTNKMDENFYSIIKTIDDILISQNVASYRNINDTNKINNVDDNKIDDDNKIDDDNKNGEDNK
IDDDNKNGEDNKNGDDNNNKECGNITSCNLKSFENYKNIYPNFYTFNTNDNKNVHLEGYKKIGNNSCEQNFDY
TKYKAYPHNINNMNYNNYTNDIENYSSQKLFDPTMNLDVDLLNEEDTNEHINRLKNIVTQRSYNKNDETYIWN
DKRYITNGNISAPFFVNNNDMILSPHYNHNNNFNKTSKEMNKTKMTKEKNAKLNLRQYSEMGNKTMNIDKLLE
ITDEKSDILKKIKNFISNELQNNEKQNYSNKKNNLYSNISITSNGNNNNNNNNNNNNHMYRKSTLLNEHITAT
TFNGVLPDFANDIYIHDNNYHNDNDDKDNNHNYDYNYKYDCNHNNRNNDGFMNDHNIDTINSDLYKYKKDINN
DQNIWNNNHNNHNNNFNKYSYPITSHDLMNHQKNKDFFIMSKSVNLSSLN*
>N.PFL0515w
MGKLIDEKCEIKRIDDVEYIKQNIKESVNHNIECMESSDNNNNNNNNNNTTYNVNNVKIINKADDENNEKDKM
KILNNNENNMNPTIITKLKNINHEDNVISYNYTLVSYKNYNIFFISHNGKFASWVYSYNVILPMSVDQETEII
FGERNYPYLEIFCSKFMKDHAAFLKYKPIIFAISIYNMSFNDTKILTQMFDHLSNIVMETPSIS*
>N.PFL0520c
MIAEGSEKGDKSFVRVYKNKDKNKEIHFETVRKDEYICVEIPGRIKKGSSGLSAVESLGGLHKITEVFNHHKN
KYNYDETIVLRINNNDMYSSFISATCTKVNNILIKIKRTRKNNYKFEFLGFVKYFYYFNNMSDFYYIPSFYNR
YDYNTNYIHLLQKGKTSQKKKNINSQQENKHTEQGKKKKKKKKNQKNENIEDVEKQNEKNDPNQNYQQNICD
INLLNNDNFFLFNNNNIYDSTNNTQNINMANMANMVNMVNMTNMTNMTNMTNMTNMTNYYNSNQESNQIIDIE
KHNINQNNLYNNLDQNSVNNNNNICGNPCDVRNDPFYRNISMNSSKNIMNNKPTELNANTGLPEFPPYEQFSS
FPYHNEEVELLYKQLLDKKIFKDDDLSYDNNNNNNNMDRERSYSSDESEAYELHCSIHSKCINPYDYKSYESH
ITNKYIREYKHIISKFSPPINSKEEIKQGDLEFFLKRLIHTTSNDLMSNVSINTTNDMNNKNDHVGNINKYNH
NYDYNHIIPNVNNSNEDHNQMDPITIDELYNEENNLMLNKKSKNEDINNYSNKIIVTKKPVHCNPIAKYDDEH
LPINPFESALKKYVSDELYNRVKLLFEIRPIWCKDVLLEHVENISTYCLKSCFSKICFYFADGPWRRTYCKYG
YDPRKDPTSYIYQTIDFRYNLSREIKSKDIEEINKCISKKKLYIDTTITNLLKKRKKNKVSNIMVQHHNKNNP
DNLMNDNFKKREYEINYDKRNNIINSHYNVQNIINDGNNIMYGETPNSSNDFNSYLINKESHNYNIYQNEIYD
KEENIFFHKDEEINEILQFLKKSNTISLMEHFRSEAHFSVTPLKLSTMYQFIDIYDNNVMNFLLNLKTQKVCS
RENGWIDSKDLAKIRDILSVRSVTLRRAHFK*
>N.PFL0525w
MLKLNKIYKINHKNNYAALLNEEKNKINVILNEYTDIIYNNNSKRCKNKYKDMKNIHNILYSSVLKNYKNVYF
YKNMLKIVENRKNDLTLHQINIILNSLIKAQIYKYSIFNSFEEPILKHLNNIHIMINDNIYKNYNKIIEHIRT

EKKIVYIPTTTLLFPNKDIKLYFNISDTHYNSNNVHLISGQFDILINIFKSYIHFSCHNSLFNTLFFFITKNY
LFIKNKKNNLLNVWLLYIQNILSKHSIGNLEQHSIVNISNEKKINILKKKNFFKHSKLYRHQYYFNIKMKTIY
NYIIAYKKKNIKNYINKDKHNNKSYNTIFCKNYISSVLKYITILILKKDKYLLLQKKKINKNKNLYISNGYMY
KYKYSNVHISSNFAFNYLIKFHFIDNHFLLKKKYFPQMLLNDITFLQNMLIFLYNMKFKSNIFQRYVKRIAST
*
>N.PFL0530c
MSLTLIRHDEEEKRRNSVHNPTGRIGHSLHFYYDISQTKDVCEQSSMHEKEENELNDNNVNDENNNMNGYNDF
INGKYKIVLYGGGLLDYDFTKNYASEFLMNVHGDMTKCDNNENDKDRKSNIYNSTSIENYLIKTYNDTYICEE
ENEKFINWKKIKTYNVPSPRAFQASCVVNLGRNGVFLFVHGGKKKNNILADNKLHALNLTVVSSYGNNEKVTN
DDNNNNSNTNQSDSNNNNNNNNKNLCDSNNEGNYHCDQECNPYNEQSSKIAENNINQLNNSNLLNEHKISSMN
NICLSNNIEKKNKNQGNCSSSSCVDSNNHVSSSTPSLTSSSNSSDSSKLSRPPFYFKNEKDMINDTNGVYINS
RKPINDMLKTDTSSCSNYSSDYSYTSESEKIKRKWVEIKIKGKKPSKRYGHSLDFLYPHLVLFGGNEEICDED
NSFCKNDLWILNIKKCKKGKETKNGHTYCYSYFLWEEVNYQYVNPFGRSFHSTAIWYDTKKKKNNLILYGGKM
KNQNLCSRLFLLQYNGYIWSWSILPVYVNTLNEHRAYHSMICANNYIFIIGGEEYNYKYIEKMPSALYSFESK
KFQYINDFTAKACLKCFAKQDIIYSWGGFTDIPFDHNFFPNNFVLLHMNVHIASTQIKDGLSDDNDGNVSLIM
NIEENPDDNIYKRMSKRVMKIENKKNQLEKDLSYQIKLNENMNSQLKEQITQYQKIMYLLNVKNKQNAYLLDL
LKKKSGYMVDNMKGELENNNNLVSGDIQNNNLVNNNFAFNNINNNYNVYENSNVIPENTLNVMGANNMHNEIN
SNNNNNNNDNNNNNNNNDNNNNNNNIEQYRQSTTIINTPNQNMFDNNNNFNNSVDNINSYNEIIFHQNVQNYIT
PNGTTKDNIQNGLQNNMQQCSQSIDQLNVFNNEQIKNDLLNIENNISNTSGNTNNYYLNGNVNVNNNNFDFNL
NVNDYSNFVTDKLPPYSNSSDLFINNNMNFLNDASTSHVNFPLANSQVVQVNNNYIGNMEASVKHNISVENTL
NEIKAPEVELPSNQRARRKTAEKCLKLIEQDRLSRMQSESNAQ*
>N.PFL0535c
MLNSGGEHVESERRPCLKYLISLLLLLMLMLILRKQNDEEEEMNNRNKMNLRKNENYRNYTFGYMRDISYFKY
SSVIYITPNRRILPSITYIDKYTIYVNGYDNILNNFKNISLSIDQNKDDIYISSSYDNNYNIYNDDNIYNDDN
IYNNDNIFHNDDNIFHNDLCKEGDDSNKDCAIINTILVDKYLFKNMKISNCNEEIDILCFLVFVQLNKKKCYN
FDNLSYSCHINNSIKNKNLFLLNNYCYVLLASSSIHKIYEFIKLELLNKEEINDNTKNEHVQIVTHFCGNMIN
QIAMIDYVNTRILFIQRYIKHTDDDNNNNNNNTNDLNTNDLNTNDLNTNELNTNNHNNRMKGKSIELNLNEEN
KNNLYDTFNNDIKNTYETNLNFKLTYYLSIINMNTQYKDNIEYVDYIKNKLTLNLFPPIYIGYDETSNFYYII
QYENKDFLTIIFISPITLTTYYTLKITDFYSGWLLIPKYYYENKGLILLG*ISSI*R*TTRLGYKQHRT**IF
RKCY*
>N.PFL0540w
MGHTHKEYKNNEKSAIFFEWLIFFVGIIIRIIIYYYGRWQDKNFNVKFTDVDYYVFSDAAKYVLMNKSPYERY
TYRYTPLLAYIMIPNFFVHFSFGKILFSFIDILVTILINQIIKIKYTNCKNYIFYTCLWFLNPLVIIISLRGN
ADVIPCFLIIVTIFCIYKKHIFLSSIFYGLAVNFKIYTIIYALPFMLYLNKNYLLGENIFQLNEKKKKKKNDF
LLNTFFYIFRIISNFFVELFKLNYEQFLFSICSSSVFLILNCVFYIIYGYEFLYESFIYHIIRRDHRHNFSLF
FYLMYLSIEKNSKIIPLITFVPQIILVALFGFKYARTNLELSMFLQTISFIALNKVCTSQYFIWCIPFLPIIL
CAITLSKRNMFLIISSILFFIVAKLHWLWWAYYLEFRGYNTFLQLFYSSVLFVISEISICWVFMYINFKEQKT
KIT*
>N.PFL0545w
MELNKNEDINKINLDGIQVESSSKEEAIRVCTRIRPLFEKEINNGHLKAWKMNETDMHLIIDPPSMTEMLNAR
VAKKGNKIELKKNVEKSEGQKAVIQRHYAFDRCFDDYVGNDEVYKHLARDIILDTFKGINGCILAYGQTGSGK
THSVMGIPDDPGMLPRSLAEFFNGIENPSSLNENNINIGDDDGHSNESNNNNDNNNTKEFLMSVTYLEVYMER
VNDLLQEGYRGGATENLDVKEDPKRGFVVIGIQEETVTSIDEVMLLVAKAEQRRHIAQTNFNETSSRSHTIFT
VILESNQVLNDGTSINKKGELKIVDLAGNERAGRAAEGIENSKTLMEEGKAINKSLFVLSEVISKLSKRAQTI
TAGDEKKIKRIQEDLVFIPWRDSKLTRILSKSLGGNCRASVIVAVHPSHFYLDTSFSTLRFALKCKTIKKKIE
VNYLSAEQSVIMQQKELIAKLQNQLKHLATTKNVDMQLSGGGDNKYSTNRKPEDDEKILALKHELEEKVKKFQ
NFILKSEFHVNSSNYRTLRSIDEHTEKSLKYSMSISANMKNEYNWLRSLDTNEYDTKWDGFDKYDDQNAKEKF
NISKAMDYLKNKSPYKLNSSEMSDILSTGSQKDDSISLNYYNELDELEKLEKHELMKREKKRKENNKEKDRDR
EKLDARSVVSNKVYDDISKLNIIENNNFNENKDKFHLKRKETKKKQKMQKRINNMMDYMTKGTLLFKKSTKLK
NKNKKKKLTNNNININDDNINDDNINGDNINDDNINGDNINGDNINGDNINDDKINDDNINDDNINNDNINND
YINNNNNNNNIDYSFNTKKITKKYKKKGKINKTQQICRDKDMYEISSIMQNAVEEKRDKDILKGKGESPFQSF
DNNINDTSNVLIKSISSSVKFLSSKERYNNNQKRKSIISNLKEKIEEDLKKKKKIINRNVELNKQIKFIMKLM
QKLGLSAILTGTVPPGRNIENVLKLQENLKDDLRLAEYNKKSTNVLGVKKKGIYNENNDGNEIDEGYDTKNDN
MENTLLNCTLDENENDTEYNETISHVFINEMFFSFVLRIQQKYFNKGDSLNDIEEILKNEEDPYDISKKTLEE

ILGCTVANKVSDMEEKNCNSKQIKDEKNKKTIDSILNMLTPWEQKILALLYEQQKMRQDVQKIKEKFSQRIQN
INIFIKKILIDKIEVEKHLNRVLKATENYKEDLLKTSDVDSNFKFAGLMKKLEELSISLAEKNRDFNVLAEFH
LNLRKTLEHKDNLIKELKEENKLFYLVPRYNELLQQLDETEESVTIGDPKEGHISQEDRMQSRVHMNDNNTCD
VNKCDGNIGDVNKCDDNKGDVNICDDNKGDGNIYDGKYMNIATKELKKEFLLMYGRLMFSRKKLQEKEVIEHD
MRNLNKKIYTELVESISVIKNLESQNKFLEFKLNMENKKKLEKIKKLVKEKEKLNKFIHEIEKRLQNENIDFE
DVKKEILVNNNMSENNLESQDDENEHKDFIEKLENEGNSKNIKKGKKKKSKIKIKKKKEENEGRNKIKNYMKE
KRVNKNGNNQNVYDHNDDEEMYDERNGNKNDDSENQKIYYDQNINFAKDQNYNHKESTNNDYKFKNRFRNNIL
NAKIDSNLKKKKKIKIKKNISEEKIKLNSLYEDSHSGLFVGKSNKYQIDEKKKKKIIKNKIATKNIQAVDGYH
GQHIMPDKYLSSNSIINQKLMKNKKRIKSKKYIPPLDGEQKSFLDSLRSKNSVMKKKNNKKKKKTKIIDKGKT
KRNVEKNEPSEENQHQGNNNNNNNNNNNKEEKGKEMWEEDKYNSSSTDFENNEVRQMLDVEIEKKKSNQMLH*
>N.PFL0550w
MSTTNILKPKIIIESSKEQGVGEKRSSTLSAPFEKNMFSTYSYSNPISNMRSSTHNEYKYNWTKYVNSSQIPH
VDEYIKTFEGPNTTYYYETIPLKTNTNNIFEITPSQEELSRISYNPKIEIYSTCDFAVLMMDIPGVSKENLKV
ELEKGLLKVYGNKYKPHIEELEKRNEYHTKIIERLNEYYFCKIFQMPPAFSEGQNISCKLNNGELLVKILANE
LKTQKKVIDIQS*
>N.PFL0555c
MKCFYEHCTENLLECSLVKFNKTRLNCVEKNVAYEEKYLNKKKHFDSLLDLKFCTIKSRNIFIKTNKCEQNFR
EKGKYNSLSNISNDYLIDSNNIKDHNKKNKNKKKNKQTQQVINNKNDVLLKNEVEYSAYFISRQWSEEYIDED
NLEYSKRNDHKNISLFKSYVIIGKHTHRNSICLYEEIIVEILNYNVDDDGVFVQIHVNKNQKKIFLLSTKECV
IIYYKDFEKQKKRYEMEKSKRKDSIILLLTENEKTKRKKKKFKINPIKGYLLLHNKNFYVSPKRKIIKAEWFN
KSNNIIALLTYEKYCLYESYSSNFRSVFRLINVNNNPLEEVKIILPDIDILQSKISGNVDLLKKKNISNKRED
MYNKREDINNKREDINNKREDINNKREDINNKSEDMYNKSKYKDEHHNIAPSSLDKTNDKNNVTNVCLNSTKN
YVSYDNTKESEYVDNSDYYSNENPNSFLSKREENHKMEGEGERNKKKTQSDDISENNKINQKDEHNKLCNNNS
SMINKMMDSLESYDCNLDMKEEKKQNNYYDFKNNSSEYAETSNYSSSYDIFKEKKKNKTTKKQIEINGNVNEG
FDSIINYYNDDITNNNTKENDILPDDFENNEKRYLLKKMKYKNLVVDFSFGTINEQILWIETCLFLLYQNGII
LVYTPVLTKRCYISYYLLNEINEIKNKSHMIIKEQLNDEQKNIKKNKDSGDNNNINIFNNQYVEDFLEHFHYF
KYCTVEKYNSMYVYVNFFDNKQKNKQKNNKINNKNIINESVIIHNEIDHLKNSSKNNTYQYAPYIINCLKNSF
YSSINLIYYNNLVIVCVCDKFGYISFFLLNYFLTPFLISLNKFKKEENKLFSFSFAKTKKKKEPFYFNIFNIN
QNVTLQKMTLPNMMNLVNKEKYDEYYDKLKNKLTLKNIIDKARKRNGIVQSMYQRLTIKNTTKKENKTNCISS
DKSNVISSDKGNVISSDKSNVISSDQTKKETNYIKSSCHNSEVEIYNSTRENNVKNNKLEKLKHLKVKFEDDH
KRINFKNYEEENIKGHKNKYIEFYRLDENESNVECTKYHTNKKDDEKNYKKKSTKIQKDIKTQENKNDITKEL
EEDIYLSCYEDINDMYNINDYIFNDFNMEYRLLNILLFDSFYTGMNNVKSIVLSNNSLLCFNNNKVIVLNLMW
LKFLNVIFYLLYVKNYLCAKLVHEICKSGLYMKTFIFKSIINFYEFDYYNYLLLDFTKQLCYHNFVKLFKVDE
KVLLHFQKQIKKNENDKNYKNRNKYISIDNNNNNNNNNNNNNCSNNSYHSNHIYYNNAVSNNYSIFNKPKNEL
FDLFQSEVLYNVQYILFPIIVENDYNILSNNKNEVYFIFSHYINIECQRKYELFMKNYINFFTCSMNKNLYLV
YDCFKLNIIKNNQEIKKKHIKTDIHLIEKKKYNPFCLLSENMNLRNFIINTTIVNDDTLDISLYKLLFSFFMN
LGSVDSTIELIKKNLIYFKGRWNISDILFSKHFQNNQYFSKHHNINHKSLNNKQYIFDNKSITYYEHSFNSES
NKNYTHNNNDEYKQNKNHKYINKQHAKDNTHIHNDSLPNFNHQQIIVSVSNHNKNIINQYEYLYNKTNRLYTL
SSIEFIKFIFLLLDKNISTNSNTLFKKTYNNQIDNNFILNYNFPKKDDQINPDIIINDIKTFIINNDIKKHEN
SDCIILIKKLIKIKNNYIDVKDYYIKKYNVNINKSSKQINEIQYLENILKTYYFFHSLNTYYEKKFKKIKNKI
IHLIALKIISFIKTHTSIQTVSKNLVDKNSSLLKDMQLCYDNQQLIRKKFYLIKKKIIMHNLLISQQVRNTCI
PAK*
>N.PFL0560c
MDLIIELYFNKSELNDDVKTLILNWVSFFKTNWKDLFNIDKEVILKLEFFLDNKKIISKNAPPNNEVYLHELK
KNPEVVLKFMKVSLHLLLFRFLCLDKETGDITIPGDDKNEEGNKNGKGNKYGKGNKYGKGNKNEKNNKNNNDN
NIEENDNYNDKEKITKKKNINHNNSNRNNNNNIYCSNKYQMDLFSSKDEIDKFGNEDFLITKLKYDRYKESEI
FRNFFFTNKVTIYLYNWNKVDKFESLKSEKVNQLICLRGSVLRVSPIQLLITNLNFVCDKCKCIIKVEFMDGK
FEPPKKCMTNNCDGKQFLPIRESAKSLEYQKIILKENRKIVNNIYETNDTNIKNLTVTLEASKFFVNSCIPGN
YVEALGILKVISFNNSHLINGKNSLFNMYIDCLSIFPLSSKKYLNNNYFNVDKIKEIKNKIHTSFLWESYIDK
VDKRMLIYKRNNNNYLKNKLIESVHVEDDNEHADKKKKNTYLFKDKQDGSHHNILNSNKNNNKINCEENEKGT
IHKKNCNQQTNEQLNNNTDIPPYKNNERNFVGPYKYNNDQNEDISELKSGDIFFEDKYKSFENDIFRDNVINM
YGDYINEENDFNRNVHYINSSDEENENYHEKNRDNNSLHLNKEIDSKVLEFIHDMEKVGYNKFYLLISSFCPR
IIMNSYIKAGLLLSLLGGKTIYDDNNQIKRRGNIHNLLIGDPGLGKSRILQYISNIMEKSLFICSTSTTINGL
TACAVKDTTNNEYSLEGGALVLSDKGVCCIDELDKISLNDQQSFLECMENQSINITKAGIVCNLKTRCTIIAA

SNPKEGKYNYKKSIFDNIKIPFPLLSRFDLVFLLADNISEEKDYHISNYLINPNKNKNDLSEDDHTQHTDFQS
DDNSKEGSYNFSESDKEEYRNNKKKEKHGIKRKRHDYSPYIFSIKDALLYKCKQINEQNYLPLELIGVYIKYC
RKYIFPKLSEDAKKYIRKFYLHLRNLANTHNDISVPITIRQLESLIRLCQARARADLSHMVTLKHAKEVVEIY
QKTIFYPLHLKTLNFKEKPTTKKNKGKSLAALSHVFKQKIIEQAKLKHNQIYNKVKIKNKNKNNYMYVYIYIY
IYIFFFFFFFFFFFFLXXXXXXXXXXXXXXXXXXXXXXXXXXXXXXXXXXXXXXXXXXXXXXXXXXXXXX
XXXXXXXXXXXXXXXXXXXXXXXXXXXXXXXXXXXXXXXXXXEFFFCNISNKIKKKKNKVNI*
>N.PFL0565w
MSRRVNYYEVLGVPQDADLTVIKKSYRTLAMKWHPDKNPNNKAEATERFKQISEAYEVLSDPKRRRKYDLYGT
DENYMADENDEFSNFHKNFGFNDAQRIFEMFFGDSSPFGNDSFFSDVMGSSFVDKRRGRVPRSNDPFDNFFGS
SFNVSFGSSFDNFMDGGSCFTSVETSTSNGGKFKNRVVKTSTSKSTSIINGKRVTRIETVKTLPNGTVERTVT
EREEDDRGNINIRQLPAHELRRNKR*
>N.PFL0570c
MIVLLRKNNLKNTLRLFPLGCNNKLVFMRGYINEQTKGPIEKKTTDNNEKMLLNKTLEDVKVSKENSCNALFD
ECLEELYRMKKKVDKESVEYDNSPIIEELNREELKITRNIFERINKKKKHVSNLSYKDIYIDPYWNPFKDVDN
LKQEILYEFNEYSKMASIVELKKQRRAIKKSLKEQYKLYNPYSEEYNKHFNLNMDNDVKEQQMECHDKDEKYW
NPSFNKRKILNIKSPFIWRYTHLLHHFIGENGLILPRKINMTTRKQQIQIFKSICIARRMALYPYDKKPEKDD
LIPLMDPLQFLLDELIHRYIQLNDLRAQAIIKVMINRYPSLNYYKYFFYESKKEKDKNEKTAQNEQEENVSEK
KEPFSQMLQKYKKNYYENFYKY*
>N.PFL0575w
MGTEKEDDEQDIVNRSNDLKKKKENNKCDNMSNDISDSISNNISDSISTNISNNISNNISNNISNNLSNNIFN
NISNNLSNNISNNTSNNTSHNISHNICHNICQNNHDIIKKVHIKNLNIMNTHKDNNYKYKNNGLNIYNKKKKD
YNGDENLKINTRESYDIFYSYLKLSEKKSLKQSDMIKDNMSEQDFFINVFSQKLKKHNIDLIMINNKLYIYIK
FSQDIDHLIPYFKREFFFKTLIGNNIILLLAKKYEVTEVLIKSLYLKETIQKFFTSNFTIEDFLVHNNFMNKY
EIYRNIVINNFFPIQMNNKNGDHKKKKINNNNNNNNKKKYIDHEDINNFVEYVHDLEENQKYSSLPFEKKCYK
ENIKCMKNFEKSHNMNKIQYVEDFQKENTENGKILYLKEKKGINKYNNEKDMMCNHSAFDNNMVQNNNVLDNN
MCNKKNIKKKMKKNNVIKFMVDNYVINDVSNKMNHHNESLQENNNYSVLQIPNNNKQINNNIRANVILNNEFN
LDVQKFRNTEETMNKIESNKNDKEERLQFEKEKKQILKREKKLRENQEKKRKQNQEKENKKQEKIIKKKELLI
LEKEIRKKLKEEKKLKEKEEKKKEKEKFLKNEKEIKKKIKEDKKRQKEEFLRKIKEEKKKLKEEKKKLKQEII
NSIKEERKKFKELKSKFKKISKKDNHLNKDKKKTKQNKIDKSNQSNMNKKEYNNNDKKKDDINYQFLTDQINN
NTVDKKKRKKTHDISLSIKKKKKCTKNNLLLNSIDDIKSDSLSFISTFPTFNNIEKEQEDRNEINDILKLIEI
KKVVNTQEKNDYIECLHKSKSKNVKRNKDKIKQNQLLSKGNNKIITKNGEHMNDQSDPLNQINSDLLNIDDFL
INNMNTIIKKYKKGDTSNVDMSNVDISNVDISNVDFSNKDISNKDISYVDISKKNVVHTNNSLHNIDNIHYNK
NMNNSERHNESEINELILKGLKSFGDIKNLKSLQKILRIKESKGKNKIETVVHIPNNVIERSVAKKIVLNKHE
GIKNKDGVKEKVKTKKKISLFGIVKPEDIIKRGINNVYHNIEDNQKLYTDVLIIGAGISGLAASYYLNKCNAK
FLVIEGRNRIGGRAFSTILPERIINNKVLPETVVDLGANYLHCCDNADLTNDKKGKTKHEFANCTEFNNEKSM
RKEDDIDYEDNFIWNMKKKKTKKKKYKVIVKKRKYNYNKDNGNDKNKMGNFSIYDVQKEYVTNINLFKEEYSK
LINDFAYLPYFEYLNNSLQSEHNQDDENKTGDKNKISGGNKNIYRYLNSNKNISCNSNIYNNQRYYSDEDYFY
SRRIKNRRKIKLRNNMLFMIKNNIDNIRNYFKIYNKEKKDNILNFFKSLDEDIHFYNNLSDSCVHTSSVLYPK
NNCTNKSNKLLKILSFNKDTRRRREYDKSVTQLAEKLKPRVSLVCGKDNWESTFYAHWYNNENGKKIKSFKIY
RVNLLCDKIRVRAARKMRNFLFIGDSCNDEYQKDIKSEDQQKSNNNYVSDDTNEKDSDYFYNNDILKEDKQIN
NNILIDDTYHKEILFNNNHVKNIKPNENNIIRNENFEASVLYNNKDDMKLENEENGKNAENGKNEENGENGKN
AENGKNEQNEQNEQNGKNGKNEENGKNGKNEQNKNNEKTSKKHIISSKKKMSKVDMNNILSYDNHAIIYNNYY
DYGDLYNKVVRNVSSTNRNIDDEERKEKKKKEDISKTELYYNNKKRRSMWDLLMECTEEIFTEMNINEENFTM
EEWKMLMVTLQSRYGYGSDLRETSIAMSRLPFSNYLDIDMCPKYGSNNYILKNIKYYDKIKKNEQTPYISSFK
DLNTSDKIVVDGWKWLINYLSEEVQNKIFLNTVAELVYIKDKHQDKKIYHLDNDNCQINKQKSSSFLNTKKKN
NNNNNNNNIESVENNSINSDHVINQDNNSFVPQNEVSSKNKKKYINENFLINNVIKDDYKLGENNEDDYKVMI
KCKCYDTKFKNINKHFHGTSDLNNCNYKNVTIYAKYVIVALPLGCLIENDKKRKLKTCLKFEPELHPLKLRAL
QNYKMGNHNKIILRFYPYNFTWPFDSLQINCIDQKFQFLNLHAYGKIGCILVHCFPPWSCTYGYIKKEHYIIN
ECLYTLNKMFEKSGKKLPILVDYIITKWQDDNFSFGSYAYPYINCNDNDLIYLRSPHPINNPRVVFCGEYLSK
SYFQCVDGAYDTGIRAAEDIAHIGLHLKNNDTKNYKTDVYIFPQNKCPFTNIPLPPIKKKFLGFYITDGSDEA
LTDYESSSDENMNTNVDINIDINSNTNIDINNNTNSNTSSNNNNTNKYNYCNNNIPISVMKKEYEFLLYSLQS
IQNIFRFLKKQNTHKNFKQDKNINHIKNETDALKCSSYKKEIKSNTYESFTNNSLINFEDKKSNDVKSNISTL
LLNLYEYNNKSTDQIHYFDRIKSIDSYKSKYKNGSYLLELHSKDSENDSNKNDANNICTIETFNNVDKNNKGD
HINDVHNDVYNDVYNDKYTSNYNIETYSHITNNHFEYINNISNLYINKYMIKLNDAVFYNYNEIENTILNKQH

NAEYNKSYFVLLQTFSNIFKKYSDFTYFIQKELFLKIQAMKHILFNDNNFQSLQDMNVVLSQAEHKNDHIVSH
NNHMNDVDRKKAYEQNTDIINEDDKEISYFHEKMEEVNNMNKMKLSLNLIKSNMVGNNNDDDNNNNNGHIQKS
NISTDELDINKINLNSTTQNNNITNKKDVMIQTNLNIYNNIDLQNKIKNIYINNRKICYLNDYLKYVLIHLSI
SKNILDEQIMSSNNNKWNSCGFLFFLCYILQYILKHLKYDLFNSTVNTKVVIQLLCDYIYYLIFCKHDILCYK
CMNGGELNMCDSYNCNNSWHTYCLSSSEHNMNNKNDKFWLCPSCSNIDISKHVQRGCYNQNEIMENYWKRRIY
IYKIKFFLSRTRKIRKRLDLLKAHLSRSVA*
>N.PFL0580w
MIGIQEGRAFFNPTRNPNNRPENNIRIEENLPSLNEVYNYFQDFLSRYSSNTLKEGNLKRTLFENVKNNNFTL
DLKLDDIYKQQTVFENNEISATPRSERKMKKPENYSAILARALSERPLTYLPTIERVCYEVCETANILNDEDE
HLNYIQINLLNTFIRPTPIRGLLAATQERFVVVPGIIVQASKPQHKMRKITLQCRYCDHKMSIDVPLWRDKPQ
LPPYCRYSSTMKSSMGLGNAMDSQLGCNGVLEPYVILPNECTFVDIQSLKMQELPEAVPTGDMPRHLQLNVTR
YLCEKMIPGDRVYVHGVLTSYNPNPRPTRADGTNFSYLHVLGFQKYDDMSGNDLNFDVEERNELTLLAAEHNI
HEKIFKSIAPELYGMDEVKKACACLLFGGTRKRIGEETKIRGDINMLMLGDPSVAKSQILKFVNRCAPVSVYT
SGKGSSAAGLTAAVMRDSQGVFSLEGGAMVLADGGVVCIDEFDKMRDDDVVAIHEAMEQQTISISKAGITTML
NTRCSVIAAANPSFGSYDDSQDTTYQHDFKTTILSRFDIIFLLRNKQDVEKDTLLCNHIVALHASKHKSQEGE
IPLSKLTRYIQYAKREIAPLLSKEARDSLRNFYVQTRAEYRGDRRSVTKKIPITLRQLESLIRLAESFAKMEL
SQFATDKHVQMSIDLFSASTAETAKQCLIFETMSPLEQKAVKQAEDAILGRLGKGQRASRVNLFRELQLRGFD
RSALSKALAILIKKGELQERGDRSVRRT*
>N.PFL0585w
MQIFVKTLTGKTITLDVEPSDTIENVKAKIQDKEGIPPDQQRLIFAGKQLEDGRTLSDYNIQKESTLHLVLRL
RGGMQIFVKTLTGKTITLDVEPSDTIENVKAKIQDKEGIPPDQQRLIFAGXXXXXXXXXXXXXXXXXXXX
XXXXXXXXXXXXXXXXXXXXXXXXXXXXXXXXXXXXXXXXXXXXXXXXXXXXXXXXXXXXXXXXXXXXXX
XXXXXKEGIPPDQQRLIFAGKQLEDGRTLSDYNIQKESTLHLVLRLRGGFLFIYFFFFSLFLFFFILFFFFFF
KSILI*
>N.PFL0590c
MSSQNNNKQGGQDINNKKDSDDIKPSVSKEDLINSLKNDELNKNTTMDQNDMKKNENMNIKKNEVLNNSNNVE
DGDNENSKFMNKSKEGLNNINGEKNDDNNSIVKVEESPKSIGYNYYASESIENLCKEFGLESINTGLNSEQVK
INRDKYGENFIEKDEVVPVWLIFLSQYCSPVVLLLLVAAVASLALNEVVEGVAIISIVTLNACLATYMEKSSG
DAIGKLAEMASPQCTVLRNGQKVVIPSREVVVGDVVLINTGDSISADLRLFDVIELKTNESLLTGESEDIKKT
IVADNLSTPFATNLCFATTSVTSGSGKGIVISTGLDTQVGKIASQLKKSSKGSKLTPLQVALNKLGGLIGLIA
IIVLVVIISLAVIIKYRDPAHADKDPTFVIIIIGVGFAVSSIPEGLPMVVTITLSAGAKDMVKKNANVRKLPA
VETLGCCSVICSDKTGTLTEGKMTAINAVTICKNSSLSDENNKLTKTFDFYPTKGFEPCGGLFDSNELTSEKK
KEIVIAKNQNTSYDKVLYNYGNPSNKSVIVDKTRSLMFAAYLNSYDTTLSRDPKTLKWGIHGNMSEGPIVVAA
AKVGYSFINNPNHKSYLDNFQRLDDLEVTFNSSRKMKITFYKLKTVNVFENVYLDKPGKVYTHVALIKGAPDR
LLDRSTHLLEETSMKKVQVSWNSTITQEERNVLIKKNLELSQKALRVLSICIKPLTDQNIEELKKLEDADERL
KYVNYDENGGFIPMGYVASFDPPRPGVKEAIQTCREAQVKVIMITGDQKPTAVAIGKLIGLIEEKSEQVEDIN
SLAIECSELHINKNPNEPILPNDQLDEFTDKILIYSRAQPEDKITIVQSLKRKGYLVAMTGDGVNDAPALKAA
DIGVAMGINGTEVAKGASEMILIDDNFCTVVSAIDVGRTIFSNIQKFVCFLLGTNIGEIIYLSVAIVAQMPFP
LEALQILFLNLMTDGCPAVALSREPPNDDNMKTPPRPKKQPIMTKRWWFYGILPHTIFEALCVLLSLAFSLYI
CTGFYNLNGIHNLCKTVNLVDVNDANVYHEYKYFCSSYEYRISTDYVGWVTNVSFWDPQNNEAVNFWGAAKGK
VENINPLSDIVHPELRLRMQDGCSGDLTLDENRWCRPKDNKTSDGYNDELEGILKKGFEDVTAKGSKRGRTMA
FISAVWCEMLRAYTVRSWEPFYKVFNRNMWMHLACSISATLTFLSTCIPGITSILNTTCLLWWQYLLAIFWAL
LNLFLDEIVPKVIYRRKYMTIKN*
>N.PFL0595c
MFFSMFIKFILPISFICYNFGKKFNMFSYFQKIKVSEQELLSSIYDYEVKDLSGSNVSMSKFKNKVLIIFNSA
SKCGLTKNHVEQFNKLHEKYNARGLEILAFPTSQFLNQEFDNTKDICTFNEKNKIKYNMFSPIEVNGDNTHPL
FKYLKKNCDSMHDENGTLKSIGWNFGKFLVDKNGEVVNYFSPKTNPLDLEKIIIQLLQK*
>N.PFL0600w
MIYYIRKVAINFLFLYIINFCLLHYLKECNTSNYSRILKEQNGKKYIYYNLSKKKNNGIIKGGSKHNGHLFIN
NYKKKRNVKYKINKYKPCSIFSFFKKKDKNDKNDKNDKTHMKDNSNLENKEKGEIGKDKEQKEKEKEKIIILK
DNKKLDEKKKKELQKEEKKTKLTNVLKQNNKNKNNNNKNNNNKNNNNNNNNNNNNNMDDTLKKDKQTELIKKD
NLNNKNDVKKKKIDLNINNIDTNSRDILKNENKYKKHLENVKDKFIISLNQIFCSYMNSLDKKENFKYFFEKI
LQNVEKNFHTYTFIIDIEKMNNQQIKKTYMINNINCFNEIMNIIQENLYNILVFKIQYLKVKAINDIKEIISN
KKNMNDYISYSNNINKIIQIYEEQLTKLCPLNYIFTLYNNFMENNNYLLNFSYQLPLYKYKNIIDSNIEYLRT

FSNDLTKHKKKSLYNEIEKNKNYQSILQIIDNQQKQIEVLQEQLETSIDGINGKYSPFNCALAYRIPDTNLNI
STQYVKGKFNVKVNCIPDDSLHLLGSYGFVKGLPYGNLGLSLSLNF*
>N.PFL0605c
MIKCKGNTFFVLKVRFNKRLIKKCGKTYIHTINNPSVDFAYKCQNTPKEGEEFFFNKEKFVNFENVNVKNLNN
REFLYYIGYCSKNKLLNLNKINCVMKELEIKIISSINNSISTDFSQNKKNILNSKSYNQCNEKPFFDIHDLVK
VYLNICYINSYYFLKEKRENNNTIKYKNNIIEDEGNNIFLQKGYRHTQNGTVQNMKITNYDKIRNVKNECEIN
GKPFNNKIDCNTNNIDFFKNDHNVYNNSCNSTLEESKEYVCNEYVKNIFNLLSIYFLQNIDLINNHYLCRLFY
GYNKSKFSNHRYLNNLCFEIIKRIKKIRTYHLYLMLTNSYYLNYVDNVFIKILLLNIITKFSQLPCEAMCQVI
PVIPLYINSEKLIYKINLIYSKKIASFNQIGHIIILFKKMLQYNMISQKNVFLSFKHLNNFIKLKKKKNKINN
INQESRKEQEMENEKNDSDDHLINKVVENFETFEHNEDQVFEKKKNMMKKRNEDDIIINNHQFDSLFKNNNNN
IQINDKGKSKNSNCNIQTENIKENFTNDYMNKKSTVKQKSEEIIFQNDDLLFNFKIIEMHLKHDLKNIYSLLP
YEYKNFLQRIRTISCNLNKNRQGEKEIYILKKYMKMLKYKFITFTYGPYILHICDPFYKIFLQFENQWKLYPI
YEQIVQKNFEMNKMNHLKKEGFKPIFICHDTFLKCKEEQEKLEYIHSILLHTQFSKYNITLKEKQNLSIPQEL
HYIHI*
>N.PFL0610w
MEEPVNTIKKIKSNMSKICFNKSNKLNADFKDDHIEIILDNKNITPKPIYFNFQEAETKKSFDQIDELLKQFD
EKNDYTRNDTQKKIVHKEDEEQIKDILNTEKKKKIGKREFFNYINKKRDTKWTPYEKEISDTYDEIILCESET
IVLMNIPNTLNDIDEEKLKVEEKNKLYEKLLNNDESNYISKSMQTLSVFKKNKDIYVSTINKQSKSTQTNLYE
LYKLTIMNPTDISQLLHKRFVKYRNKKLKKIMDEYGDSLNINERVILKKNMWVQVVDKNVADNKQTTIIPKSF
YKSRKNNTSLFNTLFMTKNISRTFSNKSRTMRTKNRYKNMKTMKFLTSSFSEDEYLIKSIIKKKKEENDIPEE
DPYEEDNSRGIPWIINNKKKKLNKSNFIFLIKQIEKCIVQNIYVYQQMIYKNIHLNYLRQVENYDVKMFDGEN
YDALTNPNINYKKKKILIKIKIKTNMKKLCNLKKKIKDINKMKGDNIPKQPNHSNDTISIHSIKHHIHILSKK
KKEKIAKKIFINKLKNNIRKKIKKKKKKKRNKQEYDTNENNYSDFQKNKQDNDLTHKRNNNDHINVNKNIKI
TNDPITGTKTKDECSEDLLENKTSIENFKKIEDDKIFDILINDESKEDKLKKISTIIIDDVLHNKKKKPSDLL
ERDNFEKKKFEYILKRKKGFTTINVNRLKLIKEVKQDYIYKYYNNDILNDHIYNNINNNDNSIYNEYTNMLDH
INEDSNNIVLDLNNNIEHTNSIEYNNILYKRKKKKYIKNISINKLFQFHYNKLENIVTSIDTNKFYKDYISSS
YSNTLDIGSFQNSKGNIAIFSFINPNYPLKLYNVNCSVMKIKYSKNNPNLLVSALSNGHIYIYDIRQKDETPV
LKSTTIKNYYENCYEPIYDLSFKNQYTNNNSFENIFYSAHENGSVYQWNIEKELKTKEILYLKNKKNDLYQDL
SSVFVNKNLVNKPFNSSLTCIDIDYYMHHDEDFIISNIEKNDSSEFNKNENNPYVQVNENKNIQQKKDLIKKS
QNKLNHKTTPLEKDNYNNYISKEEELLNHYKNITKNIIDKKIKPTHSYYYIGTKNGIIYRCNSCYQKSYLNYY
IAHFGAVNRIKINKFDHDIFISAGEDCTVKLWNRFRNTQITTFKSKNAFSSINDIIWSPNNSTSFFCCSDDGR
IELWDFDFFSKDPLIIFYPNNLEASKITTLKMFNKEDILLCGDNLANVSMMKIKNLFNLKLDEYEQKMKLTNC
LNYLDTYDYF*
>N.PFL0615w
MNAEMKGTHLKSVECPEKSNKKYSNLKQKDNSDNEEKGTITLNKEEINYCKTFLTKNSILKDEKVINNIHNND
KNYNKVISDNKDIYDIHCETNDSIVVDKKRDMHDKNNKENCDINKKNLNNNITDIKLSDDDKETKDDANEKYN
KGDYEGALKIWERGLRSINYVLSKRDELNPERLEAFLKMHTTYCSNIAQGYMKINKYSECVKYSLLAQENDKN
NIKIYFRLAKGYFMLGEYDKSIKVLNEGIKINKDTSLINLLKLVLKKKQTHLEKEKHMMKHIFQNLKQKPLIN
DDNNTKSFFFYISSSLKFLFIFIYALFMKIYNLVLGTIMDKFCKIHKR*
>N.PFL0620c
MPDFYFLIRWLCKVIVKSVFRDVNVINPENVPLYGSVIFVGNHNNQFIDACVLIANIPRQVKFIVAEKSMRRA
VIGKLASVIGCISVKRPQDLKFKGIGHICWNKGDVKITGINTRFRLDVQIGDKLLIQNKMFPVVKIESETELL
IQEVINIECEDKMNGVPFKIIPKINQTEVYNLVTNSLKNGDTIGIFPEGGSHDRTNLLPLKPGVAIMTLCALA
DGIEDVSIIPVGLSYSKLYQLQGCATLFYGNAIIISQDLCKEYNNNNREAISKLLSKIEEGMRSCMLTSKDHE
TSRCIELCVSLYTPERMTISKNKIYNNLQLFCKMFWKFGNSKVIENLSYELKCYEKLLQANKIKDDEVWMLKQ
STSAATLKFIEHICTFIFCVIFGMTFSLLWLPLVLISIYLAERHRKAALRNSTIKIQGGDVVSSYKVLVLIVL
LPTFNIVYGLLFSIYLYHSWLKRILFVFLSMCILPICYYINLNYAVQIPSLLRQMKILLKVICGKINVWRDNE
RELISTRHELQLKVRDLVSTLGPDVSDDFLEQLYRNIPKFVVDVDTKRLIRGKDEFLPILQRSQLEYKEEIL*
>N.PFL0623c.1
MAGQSEKKRLKKASTFIIYASAFFSIFSLVYIIFLFYFRYNLITKYIFGLHCLLFFCYYYCIKSIHYGLTNGM
NYTYYTDVLILSFCINIGLFFSFKFFYIYIIIPLYAAYQIISFIFKYFLSSPFGSAEPSKRMEKMEKKKNKVV
YKTVY*
>N.PFL0623c.2

MAGQSEKKRLKKASTFIIYASAFFSIFSLVYIIFLFYFRYNLITKYIFGLHCLLFFCYYYCIKSIHYGLTNGM
NYTCFNIVLLYKYRTIFFF*
>N.PFL0625c
MQTFQKPENALKRAEELQFIGQNEDALQILHSAIGHRTFRLQGWHILQEQIMLRYIEFCLYLEKLSLVKDGLH
QYRIICQHGNIASLGKVITDFRDKAEEKVRLAKENVVLNKEKIEQEESNVDVTEKILMASLDIEITGKHERKL
QNAYRICMETYKMILEILRATPKLEKAYHETAKKAILFCKENKRVTEFKKLSDLLRNHYNLILRGKHKPEYQS
LLKIEYHLETKIIQLEAACELAMWKEASNIAEDIYNLNMHDYFYKSLKSNIMMEPELVSVIDTVKSGDKNEEE
KNKVNDVDADAKAKEDILKGVEQQDNNVENNNNNNNNNDNNNNNNNNNNNNNNNKEDEEDGKIQSGDTITK
TTDGNVGTGHNTSHSLNQSKEKLRNWIAIFYEKMADILFVYESELFHGLAWLKYCFHILNFKRNMTEKEKTYI
CNKAVLAVLSIPVIGNKKKSEDFTKQFEAHKKMSQLLGHTSVPVKESLKNALRIRNILNYADENIQKLYSLLE
NQFTPLSLCLECEVLLKELENTEYAIYTKKIKEVIFHKLILQLSRVYSYISIDYFIENICPDHFMSWNEAEKM
LVDLVYQRELEMKIDLTMRAIHFGDKQVLNSSVMIKDSLINMYNDLQNGLKMINETIAIENEVENLTMSTLTY
EEEFAYLDKSNKNINYNTATLEDIIENEVFEEKPIENEKLLKKIYEKIDEEHHKIQLLSEEHNKKRKELLKKQ
KELEQAQLKLKMEKKLLEEKLEKEKKEELARKGEKLRIKEEKHKKKTEAAEQMLKEIKKLCSTNTTKILMKGK
YMDEITIDDILNGYVDFDDIEEAQEKLRLNERNEIIKVRKMEAKKVDHYFRAVRQVELSHMNRWIALVFQEDT
EILLQEQKAAEEEQKADFEAALKEKEEYVKFSIDIEEFTKNEMKLRVEEFEKNLKAQKERLYNILKEEKIQRA
KERRDQHIRKLKAEEERKRREEEEERLLKEEMERKKKEEARKKLEEISKAQRERDLEMDLQMKRKKEESRRSE
RSHRKSSIDDDLTWRRGDKGGSQNGDDSYRRHDDHHDGTHRDRSEMRDRRDWSGRDRDRDRERDRDRDRDREH
RRDRDRDRERDRDRDRERDRDRDRERDRDRDREHRRDRDRDRERDRDRDRERDRDRDRERDRDRDREHRRDRD
RDRERDRDRDRERDRDRDRERDRDRDRERDRDRDRERERDRDRDRDRDIKRSDRDHKKDRDDKLDRDSRRRER
SGKPENEKKRENDISKRNGTTEERTKNAWNLKEDDDSKAKHETSEENTKSWRNNNKVIDNEIEKAEKYKDGEK
KDGEKNKDENSSKNKENDELANGNEENENDEVKKNDDEEASDGGDFTVFKKKKRNILRFFMKA*
>N.PFL0630w
MLKKYELKGVMNILNKKLCNNKSRNDIIQAYTYIQKRFNNGSINKEFEMKKQVEQINKVNGEVVKRKKKFSIF
RYNPTNKKRPQMETFEVDIDNCGPMVLDVLIKIKDEIDSTLSFRRSCREGICGSCAMNINGKNGLACLTEVNR
DKKEITEIQPLPNLYVMKDLVPDLTNFYNQYKSIDPWLKRKTKKEKGQKEFYQSIEDRKKLDGLYECIMCASC
STSCPSYWWNPEYYLGPATLMQAYRWIVDSRDEYTKERLMEVNDTMKLYRCHGIMNCTMCCPKGLDPAKAIKD
MKNLVQENFSEDTIKEHSQYIKSKMEKTK*
>N.PFL0635c
MNDYNNINNQLLIDTSSGMEHITIENNNNCIINNIDNIDYNNKTTNHIIKNNDSIISYKNNNMSSINNLIDID
NNGTSINNEFQQKNYNNNNIISDNKNMNYFSSFSNENTIDDNGNDTNYMNYDDNMNNSEYTNNHNNNNHNNNHN
NINSNDHNDDNLKNNNQCNSMNITDEYMSDNNISKNNSTSNILEYHINDNNNNNNINFIKKEEIIKNMDDLFI
KNVNDIRSFERGILNLTQEEFIYISNKIHFLGFRFIEIPCVKTKNEKRNTYTAISKRHLNEIKNLSCSLSNSI
YDAGKRKSKKTFDPFYSKAEFDSKGTYAQHDQKDTKDTNKKKAQTNNNVNNNNNNDYVNEKDIKNANIKEESH
NLNSTKGNNKGTNNTYHNNNSIKSQQESDKLTSTSKGVKRSASNKFDDFDNKHNNKNVHGGSTNLGTNNSTPT
HNNSNTGKGKGSKNTNSNNKKINEKESMKKRQKTNSYNEDNLMDEYEQHKKTYKKKIYRTKNPWKNYCFKILH
KLKKKEIACWFLKPVNPELDGIPNYFNVIKNPMDFETIENKLINNKYNNPFEWQHDVRQIFFNAFTYHKVKNC
VWNDAYKLAKEFDKLIKQGNDVKNILKDCTKNINSVYFNMKQYNDLIEHTNNTNNIINHNNISKTITNTSSDD
HSGSLSSSSSSSSSISSSNYDDEAYDNVSSVNYNKRDSQMHKNVHGKKSSMYMTSASTNTLKNNLNSNQIYFP
HGKNKKNEKMNKSSKYSINNNNNNNISNKKNIRDNIKNKKDVNSNTLDDDDFEPPNVGTIPEPPGIQKIGIND
KGLNNYQVNLLFKNFKRLAPNQRKAALEIIQDDLGILAENHMFDRFFTFDTDLLSIEKQKRIFLYINHMGRIN
LEHYKSSLISANKVNNSSFSKGKMGQKIVHNKSFIKHGSNNSNINAHYEKRRTNRYVSSSSNSSDSSTSNSSD
SSSFSTSSDDSDSDSDSDMDSDSYDKKRKIKRSFPNDKKKELSNKFLEANRRKTLPQYKPVNENRKSLQIRDD
VMGMHADFLGSMDSPRNTKNKHRASVSAWPEWKGQVIQQAILTQHKTNVPINKKELIAEGYDAKI*
>N.PFL0640w
MNESVEQLFDELISDFNKNDKIGEGNESFNIREKVGNKWTNEDILNNINKNDFSKYTEYSLSSLYITDERIHI
DEDNKFQDFLNVITKASDVNNIYKWIDSYYKYDSKEELDILSPKAQKKEYIFREYLEPFHLKELCLLRKYEKG
AILKSSEKSKVDVNIVSEYIVHNTYKQIMKSFGYYLSHKIYIKAHIFHNKYGNSDHIIVLILQHFKDENFKQP
LFHDRALVQIKSYSNDNKYSELTQKNLLHYAEIFKPFVSLRKLNYNFVEEFKDKIMFGI*
>N.PFL0645c
MNIKKDQTTKEVEQKSNALAEINMYENSELFSDTTMSEIDNSYKFWNEEDEHTSIFDICKEFESSEEEYEKRD
VRKKSQDYQKNNSIEINTMPLENTTQDEQTNDFEKETY*
>N.PFL0650c

MSDISDFSETEDFSEGEKKTEALTKKSKNINVSKKRIEENLESMSKNNSLISIDIVNENEKLNAQTSGTYKRS
LQTQKGSEDDNVLNIKESKTFKHSFSNSKLKGVKHNTSVNAKVADCVMSPPEFFLSHIYHDNKVFTKTYGHSM
VEYENEFFIYGGINSKNEYLDEFLTFTYGTNTFTSKKLSVNPGKRAYASMTLTYNINNNSPSLLLFGGLCGPN
ILAKDCYMYDFMEDMWSKYTFKLDSSPGTRYGHAYTYCPNSYATIIWGGVNRNNELLNSGHKFNNGEWSELKH
KGICPSGRVYSTIVWLDRTTKENIHYSFLYLFGGDLTNKGSPTDELWVYNFKKENWFLMKNSSGEAPCPRWKH
GAVIFDKNMWISGGLCSGWFSNYSIPDLYVYDIPSNCWFNCQIASKQIHNCYDYGTLNLHSQTKAFFLFGGKN
ANNDPTSNVCRFAPLCTNVSIMTMRNEIKRFSNYVLDVKNESEDTANNVSEMQKIIHTFSLDIKDFKILFEAL
TKSIQLLNDNIENINKEIELLKKKISTEDEAEKKSNVINNMPNPNDKTEEFVNEVKEPQINMENK*
>N.PFL0655w
MLYVLKRIMCKSHNYMFTFFLWIVLFFINEENIQGKVYQGQFYLKKNEKIHYLTKFSCNIGTCQFSIKMKLRD
NITAKLLNNYYKLNNKTLKEMENDKDDGNNHNNNKYDDDNNNNNNNRSNKLLHMDYDNAYYVLSNQDLEYIPQ
RITLMQDIEQRYVNKNDKCYAFGNLKNSHMFNIPFYFKYSDLVNSKTYNLKNFINNKYSMSFNAIHSELKNNK
LLQSEILNYGPILNEKNSNYAYKEIYAKTTHRVHVWFLMFDDCYDNFIRNLKLNIKTKLAYWEYLLHASKKFD
ISSLNDMDIKQDTSYNDFQNEETEKDIYSKENINNNNNNNMNNNMNEKKNKSIFYKSFNFDKITLLENPKRFL
FLEEQFTKSEINDISFFLKYYQFKKMKNFNNMYNNLYKNGYYEQVIDYIYQNEGFHIEYEVNIFQTNKSHFSY
ELLYSPILTFILTILYILLIFNYGNTIIQSITSKSHKHAMILSVAIVIFIQLLSNSLLLIHLFVYSKNGIGIE
FFKISFNILNFLAQIIMCTMLLSLSYGFTIFEASFDIILRIKVIFIIITIFHVILVILDNTYIMETSSKFFDN
DNVTGYIIFVLRISLAILYHFNINNLFTMSKITPIRKFLKKLYVYGLFYIFSFPIIFIICYIFDTYWRQRFML
FGTALLQFISTYFITKMSLTNSEYFKVSDMSASDLPGATSSWFNQKIHTY*
>N.PFL0660w
MADRKSNKNAVVKNVDMTEEMQIDAIDCANQALQKYNVEKDIAAHIKKEFDRKYDPTWHCVVGRNFGSYVTHE
TKNFIYFYIGQVAILLFKSG*
>N.PFL0665c
MASNILFEDRFVISSVDNSKFEKVSRIKAKSTGYDAELILDVHSELFKVEEKKAIYLALQDKLLNRNDEKGWE
QNENVPLNNIEYIMSGRIFKFEELSSERRTVYASFGGLMMALTTDKQFIGDLEIDMKIYLLTKNIDIERTRE*
>N.PFL0670c
MNNNTNGEIIIPQEYLEKSECLFKELKELNINFKEIKHGLAATIKDLLEMNLENSTNILKNLFLKDKKKNYFL
ICTLNNKTVDLKNLSNILKTNNLRFVDENNLNNILNIQPGCLSPLAIKNDKENIVKLYFDEEIKNMQEVIIHP
LHNYSSLYIKTQDVIKFCESFNHAPEYVQIKEDTTSKARVDKKEDVQEEMAKNEELQNNNNNNKNNSNSNNNN
NNNNNHIKDTILKGKLLSNNEVEDNKSKDSNILGITSKKIENFSDWYTQVIVKSELIEYYDISGCYILRPAAY
YIWECVQAFFNKEIKKLNVENSYFPLFVTKNKLEKEKNHIEGFSPEVAWVTKYGDSNLPEEIAIRPTSETIMY
SVFPKWIRSYRDLPLKLNQWNTVVRWEFKQPTPFIRTREFLWQEGHTAHKNEEEAVKLVFDILDLYRRWYEEY
LAVPIIKGIKSEGEKFGGANFTSTAEAFISENGRAIQAATSHYLGTNFAKMFKIEFEDENEVKQYVHQTSWGC
TTRSIGIMIMTHGDDKGLVLPPNVSKYKVVIVPIFYKTTDENAIHSYCKDIEKILKNAQINCVYDDRASYSPG
YKFNHWELRGIPIRIEVGPKDLQNNSCVIVRRDNNEKCNVKKESVLLETQQMLVDIHKNLFLKAKKKLDDSIV
QVTSFSEVMNALNKKKMVLAPWCEDIATEEEIKKETQRLSLNQTNSETTLSGAMKPLCIPLDQPPMPPNMKCF
WSGKPAKRWCLFGRSY*
>N.PFL0675c
MKKEVDFNLCYEEEKKNLIGLLIENNFDEVFEPLTSESLRFFLPMNGLCLIDYQLHFFKQNNVKKIYVVVTHH
ENELLDYIEKFQRSKKRYNDLDIEIIKMNKKVKTLGDALRDFKKSVEVYNDILLLLSDTIPIANVKDIIKAHF
TNKEKCKNQIMTLILSHCSSENKSHNDDYVLAYDNFTFKLLLFEPLKKKKHFTLTNDIFKDINIKDRKGCRYE
EKGLFKGGNKDLINNHHHHHHYVNMDDDHNVDGYNNMYNNNNNNNNNNNNRRGSLYSRGTVYHRDSVFPRDSF
YTRDSIFPRDSIYPRDSIYPRDSIYPRDSIYPRDSIYTRESIYHRSNNYHMDNLHNMECHNLIENHQDMECHN
LRTNSHGMDYRELFNNYHDIDIHHYSRNRRASFILNACSNSIIISYDLIIPNIFIITPQVLKLFEDNFDYQCI
KKDFIYNILKQEIKIEEIYVHEINGDYNYTECNNIITTLSDFRIYFKFFKMLIERNVHPFLANAYHLLPEQPK
RVLTEPGFYKHKNAIVHDTCKLYKIVLVEKFSEILDNSTIENSVIGKNCKIGKNCKIINTVIANNCIIKDNVS
ILSSFIYENVIINENVFIDECCVLGKNINIPCNIEIPKYTRLSAYYTNNVKDKLQLEKKNKEEDILLEEHRKV
REIEESQNDITNVKEEDTIDNQDTFENEKEVSDIICHQHMQREENDYVIIDEKSFDNPKEVANSNILNDQENI
KREDKEYVILDQAHAHLNASIDNEMLDHKVDEKHTDKINANQDFICGDKELPSVLSDKNEEQNNNFVHVVNDI
ENNEGIDQNTEKNNNISVDENISIGIKPSKEDIFNENENVILNIEEPFDINNYIIKTKYTDEQLRFFSLIGNT
QMNINTSYISSSEYDDSEDDEEENEESEEEEVDEENYDSGYSTTKNHSTILTDDNTSDDKGAELFNEDSVNMS
DINKHFNDINMDNEKNIFSKEISLLCKEAINKPQFMSHKILEMKSFRLSLNYTDLDVLVGIFPIIWEYINQLP
FDKESWIENFDNAKLDMLFSSFLLEDQCYYETLYDLILDYSKKQYLTSNIKNNIFGSDKLCNAFEYIYHSDIF
EFNYFNEWINKNQSDEFLKNNMRLKAFAEWLADE*

>N.PFL0680c
MFRKFKYVKNAWIGLGRTAKVVYGCFFFNIILLMGISTKRYLANKKAIDFYSNKIILSNNENDDWSCYNSAKQ
YRYECADLNEEQINHFEICEKLHIQLEKCRNKLYEYIKEDTPAMKNIPYIINKPTWMKEPLWFENIKKMKSNK
*
>N.PFL0685w
MKIKNNIKKKNDNLHHFISNHTLIFSIILLINLLIFFSFINGYFYARQKLEEKSENLELFSRKVFGDEYVESL
KKKKNTFSIINAPYDKVVILLIDSLRFDFTLYDTNYEKEFIGKEKNTDIYNNISSEKKNISNDGEKKNSLFFL
NNMINVHHILQNEKNNTLLFRFDADAPTITTSRIKSIFMGTIPNYMEVNENFSPTTSVEDNFFEQLHLNNKKV
IAIGDNTITHLMKHFSKELVYESFNVFDFYSLDIAAKKHFYEEYESNDWDIMYIHMLGVDHIGHIKTPNSKIM
GDALKDFDTFIYDIINKIKLDNLKNISTEEKEKMKIIKKKIYKTYRNGHHIQNENDHIIDNIQNENDHTIDNI
QSENDHTIDNIQNENDHTIEDIQIDNDHTIEDIENQSEQKNDDKKTLFIFFGDHGQLDTGDHGGYSLDETHSA
LFAYSPLNFISLDNDIIQNNFVLYDKDKLKKNVNTLNEENNNNENIDNYKKYHSYLKDRNKKYSYHYNVKYTK
QVNLMSTLSLLIGSTLPYGNIGNIIMDFIPNAYIKNNNKKKKNNNNNNNNNNNNNNSSLPNEQTNLYYDLLNL
HYIAELNYANLWQLNRYLNEYEKKYNIIKNEDYHFIKSSWHIIQKDKKELFFQPNKKFIKNDILLKKEKESYI
EFINEMTTLMDITQKYFYYIFNIKEKYFLILSIVLNIFLLLFLKHFYYYSKLNYYHKLIKGSKILLCMSLFLN
IIVTFNDFNKNIYLLLCICALLLYFFIFCLSIKEYKDIFRIFSHAKIIFISNNIDMIIPSIKNYHMSAKRNMN
ITNNDTYHTSHKDRKSFTNKEEKQNNTLMNIFYNIIYFLRIIRKKIVQYIIFIHLTIYNLTIGNIIFILFKLF
PKIITNSFQILRSNYFLLFVIIWSCCEMSFNYIDKERYYIHYILIVYVIFGMLKWKYHRVFNILKAFILLVLL
IINALYSHTPEYFDHGKEKIYLKESVLKSVFPISSYILSLILINSGINNLLKKRIKIIITQIWTLQYILVFLF
LNNIYHRYIQFITPPSIYFLTISTFIFILLSKNILNEKEKLQDSLDKIYEVCITLFLIIITSIPLSFIIYSNT
NLGVLFLFYMTFLFFYFILISSNCSENMIQMNDITSTWINENIHNRNDPIITKGNLENKEKCTSCNTSIKEKF
YYKLMIEKFKLLNTCSVLNEEEIMGIQIYKLIRDISYFYINETDFYILSCVLLIYSFFITGHKFILNNLPLVS
GYVGLYKYVWPISQFYIFNHIFFPFFFSLFFIIYIYNIRRIKIMNSFKQFDLYYFYVYPLMNFSFKASFLFCC
KFIISVWVSYYLNLHIMLYDYFLPKMFYLFAIHLIFIIFCLLVFLMTKHIFLR*
>N.PFL0690c
MILFKRNFHNYFANNFIKDLFKKSQHISFNDIQKKIYIGKSSLGGLGVFSLEGIKKNEIIEICPTVSICNEEI
PRNLVDYLYEGKEPSKNKAIVDIIINRKKETSNYKLLPLGYGILYNHSDIPNAYVEIHKINKNQIKQKQDVTV
SNNVMIVYAYNNIQKDDEILISYGHSWWKVSS*
>N.PFL0692c
MNLYLSLHEQYFINTIKKKLSGKEEDTILTSKHESILLSAIFWVLSSISLINKKRETIDEVLSKDFIEVIYML
VMKCLHRRKIKDEYIYKLKKETYISSNEDIKKKKKKKFIVTGFSPCNKKNVYEANIISTLSAIQILFLLNKTD
ENSISTKTLLEIYNFVIFLFDEEKGYFHFSLKSVQYKFDGDMRFMFCSLCTLYLLNKLFKERNIYVNNINNNK
CIHWIINCFNIDGGFSNLPGSESHAGTTFCAIHSLKLLKNEKGKTYFSYNPIMKKKLIRWLCERYDNFGINGR
VGKDHDVCYSWWVLSSLSSLNVNLGKAFNVNIIINFILKCQDKVNGGFSRIGQDEYNINKKCMNYFKNENFFF
KQTDQFHSFFSLCALSLIYYNIHYYMKKERLKKYELFDQISIPKHINDVLSNMANIHEAFAMPRRLIQ*
>N.PFL0695c
MEGDEKQGNSKNILDSKLLGNKKYDENSLKHNKHSLILSKQFYGIISGITVGILGVQGILGFLLFILFTLIGT
CITFFHIRKKFGSYFLKKSDLFFGDFFSGLISFILFWTLSYDIIYIF*
>N.PFL0700w
MFRISPMLLFFLFYFSSYNIYFVLTRNKNGQPSCSNVSCSNEKLNYIYDNSKFILSELQNILYDVRDQRNSHE
LENEYDKELDIQIKFPHKKDHHDLQIWDDEYEEEKQYSEPVPYITITNIEEDSDDLNKSIINDNIYNEDNSTN
RPLNEEEQIDIALSKIYELEEQMKMFKEHNGKQNFKKYLR*
>N.PFL0705c
MITKFSILNKSKIFNTILYNTIRQNNIFFNNGKFFTTQNVDEIDVTFINQDNYEKTVKAKIGDSILKVAHDNH
INIEGACEGFCACSTCHVIIDENFHDLLPEPLDNEIDMLELAPCITETSRLGCQIKLSKELDGMKIQLPPMTR
NFYVDGHVPTPH*
>N.PFL0710w
MIGKIFDNAFRIRTYNRFVCFLIGGTFAYSFSNPDMDTVYDLFPIFKQNKYAYLTFLEVNEKHSNVFEERLKD
LCRFYQKQPGYLYTKIIKRIDNINDISKYIVISTWIKSENYLLACNRMYGQILINKIQNYAKYNSYLYRIVVD
DSDYLPPI*
>N.PFL0715w
MKIIFIVYIFISTFSWKSILSYRRLKDINNMDGIPQGFSKNKQINFHELRSNANKDKEKAYTYEISEWIIMIN
PRNAKTHTGKIRSIEIYSMTYPYEQLEVFKEKLKRP*
>N.PFL0720w

MDMEIKEFDKLSGFRIQCTKDLLLKGSLSAIVGMILGIMCSLIVNCTLVEVSLSRFFSMYFGILFIVVGMIIF
WRLNANVTDEAEGRKNQLMILSGLIIFSGILCFFFDRSWLFSLSPLTKVPIYCILGISISFALTFSLIDLINY
LIGFIQGSITRPLIESVAQVYMILLFTITMGGIFGFIFGLLDVEDESSHHIRLALMKAENCCFPLGAVLGGIA
GFGNEVFRQQSEAYKIENVTEFDDEV*
>N.PFL0725w
MFLKKLCRSNFFGNSRRSFSLVTKKAYNFTAQGLNKNNEIINVDLSSFIGQKYCCLLFYPLNYTFVCPTEIIE
FNKHIKDFENKNVELLGISVDSVYSHLAWKNMPIEKGGIGNVEFTLVSDINKDISKNYNVLYDNSFALRGLFI
IDKNGCVRHQTVNDLPIGRNVQEVLRTIDSIIHVDTSGEVCPINWKKGQKAFKPTTESLIDYMNNANKNV*
>N.PFL0730w
MNISNSSKNTFISDENNVTYEDDYNETDNNKDEGDDRNTLIHVLHSYEDFQKKNMNYGKVNPYKKKETQLSKI
KKNLVRLLSSNDITIHKFNSEEIKGNKKKKMENIKFLTSRAAMWYQVEKNDDPDYDLKLESSKKKNFVYITMN
YINIFMNDLLNDKRGMTYIATFMIVLSIIITFISGFITLYNNNNNNINHYNNHNNHKYHNINSSNNNNNNTIN
SNAFFSTNEQYLKNLDEKNNWNLNMSRNNYDDINQYMNYSALQIKRGNDQENYKKYNEEIFEILEQLKKEINE
NKNIKSSEKIQNENKKDDKLNIYEIRLNIEKKINELENKIINNNKNSDSFKSNLIKEFENFKNIFHDNYEKFQ
TQFKDYTNIVNNIKSVIHNKDTFINNIQKTFTQNQVDIKNNLTSHIENEKKELLQKINELQSQVKVMEWNILK
QENLYKGMKQNILKILGQNKLNTIDNNNNNNNNNNLYDADNDSSYDDEWFGENKNILMGPPIDMDKKKKKQEN
ENNKNNKNYSNHRFSHNYMPHHISKDIHSNEIETLYTQNEFKELLNVINDIKEQINMLQEKNINSKNYLDDTF
LQMEEKILKNAEYKIKYYLEIYKKDILNEITESKVIYNEEKFKSLTLKHERLQADLLKNINNQIKIQSKLIKD
DISKSIHFMMEQKKGKHNYNNINNNNNNNIISSSNGSSNNNKMIYSDHLEIIQKKVDELYNEFILDYNEIDWA
LESLGAKIVYKMTSSPLNRNDFIEKFLNQIASFLPSEEIYGMIKPMGKDPAIVLKPTNFPGDCFSFKGNHGKI
TIHLPATIDITSISIQHVHENISNNSNATPKYFSVYGMVDLNWPEQFEENDINYDDFKNSSLYSCLHSTYGNI
IQPNEILQRWLKDNKQPNLIHIGDFYFDRKKRIATYPTKSCFPMKRIIFEFTENYGASYTCVYRLKVHGKRCI
RKFK*
>N.PFL0735w
MLYKSLKASTIIGRKCFHWWNNLPITWKVGIACSVLFPPLWNYNERRKAKEKQIFYNKSIRDYVFFDIAIENK
YVGRVLIGLYSDQVPLSTENFIQLAEGYQIKDKYIGYRNTSIHKIYPGIGLVGGNVLNDKEGLSIYGKKFPDE
NFDMEFVQDGDVALFNEGPHSNSSQFIITFSPMPILHKHNVVIGTVLKGMNIIRMIENVGTKLGHPMYNVRIV
NCGLYKGLEKDGPPFFNFTEIIEKNKEHLISKKEFEKLSKEQRQSLLNDSNKTEKKKKQAL*
>N.PFL0740c
MSSTITRKFIPLMDRILISKIVPKTTTKSGLFLPESATEPSYTGKVLAVGPGRVTSNGTKISPSVKEGDVVVL
PEYGGSSLKIDGEEFFVYRDDDIIGIIKDE*
>N.PFL0745c
MNDNKGFSKDEQTRIYKILLLTYCNKISTYQNEIKNISLNNIISSLYEGCENEHMLNDHVKSQHYFIDTLVKS
NENHEFLNTINNLIQEEVKKKNDKKDDAAHNVANTNEDNNNNNNNNNNNNNININNSGTNVGQGNNNNTTNEN
NPRNQRTIFNNYIMELMERSHMFLLIKIFFVLFLFEVNVKMYFIVSGMFILYNRGFFDLIINNLNFVSSNDSI
EQVLRRMSESRNLNNSISLNENVNEFVEGNIQNVEQTSISNDITSHQQNNIDDSFITGEDKITDSGEYVDLKK
ISDEINNEHILDYINNIDNINNNMNYYNKDICLDNKLNDDKDLMDFLNIKCSDKEDDSPMDESINEPKGEVEQ
TNKTKILKNIDNVECNNNDAYNNNNNTYNINDINNVNDINNVNDINNVNDIVKKRFLLFKKKKTEKVNESELD
SSKKKIPQDDEFDYPDTKTNEIINDPNNSYNKGKDKNVCPAKDTNEYFNNTDENVLHNGKNGINSNLRKRKNN
NLNDSSTTFNHILNNSISEFINSDDEVAPLISDPIQNNATNTEPKEEQNTTETKQNDKENNTNSNTRRKPTKL
EKYIYQSVVMFFMTLLPWWVPDVAYLEE*
>N.PFL0746c
MEDSFNLIDKNNLDEETTNIKNENMKYMESHPELKDLLNDFISSILLHTPVYIK*
>N.PFL0750w
MDKYKYLKSLRGKNIFSLKYAESFNFNSIHKNIDKHFIYTYTKRNNFVNYVNIIYKRYIKTMGENNTNDDYKK
YDNCILQNDKKKNNFYEQSLEDIQKISNDEIKNNPINNISEECGYKYDKHEPTMFGDWSHNCRVTDF*
>N.PFL0755c
MNFFKKKKNDSINKRNAFIQNSMITKNKNDNIKNDDIFKDKNVGLLQHEERSLEKDGTIYNRKELEESVNSFL
DNSYKENIRPYVEDKIQINKSNTDKTLKIDTNNNNNNNNNSNNSHSNSNSNNYGNIPKEIKQYMDNSYASDYG
MENYHEHMENQDISKNINDSSITHSKETTTILLNNEIENSIKKKESNHNHTTRHNNNNNNTSYITNNDDIIFN
TQNQSKLKKNTVDNSLQYSDKINIVKDNQKDNISNDIHNYYNSKTSIKKNENKYNQNNFMEKNYNSIKGHDMS
TLKFSTKYSHKDIHIKSKNDIKSNKDNNLSDMKDVYDNEKDEDVKKVNHLNDSIEFKKNNRRINTTQILNSSN
KYQTEANENHISHNNNIGELMNSRNSNTNYVTSLNNMNDTNITKQIDNISISNNLNTHHFNDSKRSITYSNIN
INEKSNFYEDSKKKKNIYSEYHNALNVSNKSIHINNNTSLIRSKNELMQGKNIKNSGSQNLELEETNEFKTNH

LDDLTKNEANQNETIKNQKTFTNKRNSMIDSKKKYMNNISQDMYQSNHSNKSDWSIKKSNNNNNQNILYRNDQ
NVNRTSINNIIINDDTLNVHNLENTNDMILENVDRYKKQHQNDITNSNRKCSLQNVLDNSTSYYNDDDNDDSK
NKNFHLSNNSSHQKKKDSHEIKQNNNFLNSNKNLMNSTNSDIELLLNELSESTNSLLSSQKNTQVGNSKHFLT
SQNGDNAKLSYLLQSNENINEELNNDIKEHTKEIEEKNNDMSYKTHNEDSQPFHNYKTKNMEHIKYDYIIGEN
NHYLNKSENLPRTKGKIDLNYVKYNTCVYKDDENIKRQEQRKEEHIESRHANLLYEFERGITKNKKKDELYKY
DMIENLSNKNMKYVNDIHKENNINHNMNHNNDVTNNYYFKDKEGNDILLKNNNSSGPLSNISHFTEENNFTYH
KNETQNNHNKNNISNINNINNISNIIHISNDDNFHYYTNNEEHNNHSLDFSIHCEDNMNISNNNNNSQQILQE
YNLKNSGNLNVSSINKSKGKLDKNVNYMNKLTMNNNYVNTDLQISYIENELQQKIKDFDKSLGTKFEDKCNLI
LNRIEKLLSYEWFNDGQ*
>N.PFL0760w
MINLQTLDFHLTNFEESNRCFNDDEKFGILYGMTELEEHINKEIYLWGKIEGIEYDYYITYYFNQENFFPTKK
FYYCTNDYVFHEMLKGNDDFIENVERKLPFTFFSGYPQRIFYSKDKTKGQKKKVTGGDDNDDSDDDEDSEDNE
DNEDNDDNDDNEDNDDNDNNEDNDNNDDNDNNDDTDNNDEIQSDSYNRKGKKKNGHRNNLYNDKNIDMKKKKK
YEKQNITELERLSYTVRKIDEEAFIIPYNSIKITNNLEMKFCHFTGFNILDALKLTSWVHFRYPKNLTYDKIK
NYNSFFLNNFLDSIKSDIPSDIWNIKINKQLNKISILNALYPGYIFYHILNTPFYASLYIGTGVSNYDLPFLL
P*
>N.PFL0765w
MVIWKGNVKNKILFLIFVAYFFVFVKISNGQLIKLDGQKINTNYILYVLKGLYIFGENESPYVLLGKKKDMDF
KAAHAIFENVGISTTDNKNTKYFSFEMGDTTSGNNENNNNNNNDGHNNNNNDSHNNNNNDGHNNNNNDGHNNN
YDHNNDSTLENTNLPQNSYNNNGNNGNNSSEKHKDEDEDKDKFKINLYKDNPYLRKKKEYRYSEDVDSFVTPE
LFLELIIMKEKDFNKHYLPKDHDICCYMQEEGIDGYEKYTCPGKGYLKRYVDEEHMYSLKLPVYFINDRIKDD
TNNNNNNNNNSSSSSSSSSYYNNMYNLNNGNEINHENLINHLKNKFVYNIKDTDVYALFLSNCLDSKKYELHL
HGNIHILNDYGYLPGDKISKLNLYVLSMIIYSIYLFIWSYLLIRNKNYVIKIQIWILVCVFLYLIENICLFLY
FLSYNLYAKVNNELLFISVCSSILKNVCSYLLILLGSLGWGIVIPTLDRKTFIKIKILFFFFIIFDFIKQFVD
MHLTDTQINTGYFFFCIIPVTIIYSIIYIWVFTSASQIIIQLNEDKQYEKLNMFKNFFNVLIFTLLFSVIAFI
IDIVVMLYVDNSIWNLKNYLSEGIISCLFLIILTAMFILFKPSDRLKRISHFTEIGDMDEMEDFSNFKNSIED
IS*
>N.PFL0770w
MHLSKIIFLLIILFPYHVNSRKPIKYKNVNNVFIEIYPCKAIKWERKKKKRDLIKNNKLIHNVDVNYINMITN
DNHISSEKCRDIKKKRKITSSEYGMSLEFFKKNPKKVVQNLKKRGMEKYLNVIVMLKKLINEKNENEVLRNKL
RNRRKLLSDHIKNLIFNSKKYEDIINRDITNNSDHKTNLIHVDEEKKKTEKKNDTQDILKDNQKIRYINKENQ
ATNNNINNQDNSNDVLKKNKIQIEEIKKETNQINKDIDHIEMNIINLKKKIEFNLYKLPNILLNKVPKGKTTE
DNKIIKFYKKENIIQLNNEEYDFIEPHEEIIKKYENNFIFSNISNKIGFGYNILVNDIAKLERALIDFMINTH
VNKFLYTYVKAPEIVTKSALFNTGQLPKFEEDLFKITDNYKLLNEDAYLIPTSEVSLLNLFKNSQIDYIHLPI
KLVSHSSCFRTEKNNTYGKTSKGLLREHIFQKVELINITDKKTSPFYYKKLIKQSTYILKQLNIPYRLVLLNS
IETPYSASICYDIEAWLPSQQRYVEVSSCSNCLDFQARRLNLKYKIKDSNNFCHTINGSGLAVGRVLAIILEQ
YQIKKKHKNEITKIQVPKVLRKYMNKDIIQVEYN*
>N.PFL0775w
MIINLLRRKGGTVNTLGIAISERKRYFINYNKKEFYYNMRNDMKSFNKNYYHNNNNLKYVEEKTNKKKKKKMN
DINNNCDENFKDFYFKNKTIERKVADIKIKDSHNKTLFEYIPIEGRTNKVPIAFRLFHYIFLFILSSGVILIH
VLPEINKEKYIRDIYTFQIYSLSCFLVFNGGFNSLFQLIQYAIPSNRKYKGLYNTLRFISSLIPLFCAIITTI
LSEKFPRDSLFLLTISFITLLINYYLLHIKCLIPVWLYKQYKIYISLIILNLIFLLLSEAQIYTGRNVSINVD
Y*
>N.PFL0780w
MYRNLFDKLKDGPLKISILGSGNWASAISKVVGTNAKNNYLFENEVRMWIRDEFVNGERMVDIINNKHENTKY
LKGVPLPHNIVAHSDLASVINDADLLIFIVPCQYLESVLALIKESESIKIASHAKAISLTKGFIVKKNQMKLC
SNYISDFLNIPCSALSGANIAMDVAMENFSEATIGGNDKDSLVIWQRVFDLPYFKINCVNETIEVEICGALKN
IITLACGFCDGLNLPTNSKSAIIRNGINEMILFGKVFFQKFNENILLESCGFADIITSFLAGRNAKCSAEFIK
STPKKTWEELENEILKGQKLQGTVTLKYVYHMIKEKNMTNEFPLFTVLHKISFENEDPSSLLKTFMNNKINQL
SL*
>N.PFL0785c
MVKEQGLNITNVYNRDIYNNAENNTIDESKDYSRWKIIYPNYLNKKKKVNEGRRINLKYCVSDPTVDEIALAC
KELNVSYVIEKKKYYPRDWLIEGRIRIKLPNQNNDILNKFELMKKIGLKLQTIKVNVESNVVINTNNLSKKKK
KKKNKE*

>N.PFL0790w
MFKRCILYFVISIFSLVLVVYKESRYGYNKFKYEKKYKKGCDINDDLCYNENDDNVDNAKAIGKTENDYEVVN
YDEYTNNNKKKSISLSNNKTKNKNYFDNYNNKYVDKNEINYIFKPYFEELNNNITKLDHVISLFYEFSINAKF
FILIFFKSTMNILHSYGILLISLIKILFLWFIIIQPYIKWLFIKLKKSYMKLDYQSKKYIIYMMTILLTFLYL
IYVGVFKYIINKISKIYKYFLKKIFRINNFLFKVFPYIISTLLYATIIKTIPIKCISFFIYSCFFPFPSIYSI
VIILKYVYLPTLNTCIINKVEEKYHQKNQEKNQNQTKYQSVDTSTTSKEKHEYIRTQHHNSCTTNAGSCMQIQ
DVSETNEGLTLKKKYINGKDKDKEKDKEKEKKRETSKGMLKKITKKVTGKISKKTAQEGACENDVQEGACKND
VQEGSCENDVQEGACKNDVQEGACKNDVQEGACKNNVQEGACKNNVQEGEGHSQEINHHNIDQVDEIYDSSTE
EECDHPVDYKDEKNEHLLNDQMDDGVEDYVEEYIEEQAKGENKICYKDINKNVHINHIHINDINQNNKKAMDE
KNNYCISNFSFIKKKYSTLFGLESFNLRFRYKSKRENKSELSSSQYNQIGNERFSCASSVSFLNDEEGNILSY
DVPILLEYWLFINILKFLKTAFFFHKFVKIPFLFEYFTLFVITINLSEKLHEFMFLKKYKSSILVRLLKNVLV
TTVDFLLYFLFNVRLENEKKEEEKQNNNNNNNKKEYLERSNLLIKLSKIFKEKLKMNETVKFSFTCLKSIITE
NVVESVKLPLYIKICINILIYMPQLVLLIFPSFILKIYFLYIFFLAPIFGSLKCLEEKKEIHNKIYFICYFFF
YNISSIVVNHSIFKCLPFYNLYKILITISVQTALKYIFNTLKLMS*
>N.PFL0795c
MKYPNFFILKVFYFLFFYLSFEDIYYHCINIGKPQVNTNGKNVNSNEKANNLSTEWSNRGFNFMDLLKNGYLQ
SHGNLEEVKDELANELANKIQNKIGEYLKDEKNLSVLQHLQLEDLDDLKHYVKDVSEYIGAKAGDLLDENLES
TLKPLLERKSFETFEQALHNKSTDNFVPFEENNKSGNANEETENFVDELVEDYEKTKNPDLDEQTKDIKTHSD
SD*
>N.PFL0800c
MNALRRLPVICSFLVFLVFSNVLCFRGNNGHNSSSSLYNGSQFIEQLNNSFTSAFLESQSMNKIGDDLAETIS
NELVSVLQKNSPTFLESSFDIKSEVKKHAKLMFKELIKVGLPSVEKLVAENVKPPKVDPATYGIIVPVLTSLF
NKVETAVGANVSDEIWNYNTSDVPESEENLSDDFFD*
>N.PFL0805w
MNILLKYHLSYIFFFVHLIVINIKCKEINNNLVNDEKDSINNLEEPSPFHILKKKNSNNIENHSNKINNIFKN
NYSYSYSYSLLDNSLYDNKYESDFFPQKKNNKINEEKYKGTHGTNFAIINEHMNEIHNDMLGQNEILSNELDT
TKDIIKEEHVKNKKIKNHSFYMNKENEKKKFLDNTFPENKENNKNKRNSKFIEENDLNSSGENYFENDKFIEI
EYPIKNKDIDEYEEGINRNHKEEDNKNRTYHSNGEDSIKSLNFIKYMNMDKEKKGKKKSYNRKKKENYNNDNY
NINDSDLINVLKQGDYSPDCDTCNKNINGHMNKGRGIRRRMYDRNYYKDFNLRDALPINKTHYSMLLKSDDDT
IIKEKNENDVDDIITKEKRKENLKEVYNDKKNNYLSLKYLGLGYDIIMGNPEGDPTLNVDPGFRGPVLQINLK
EISLNSYDNHKNDDNHMNDDIHKNDDNHMNSDNHKNDEYHKNDEDHNDDNYDNHADGDKRSKERKSKIQTTNE
SMKPWVIPEHSCSQSKNVEEIRNLEQYKLELLSDVKVSTPSSFPYSFSASAEFKNALKKLKVQNNVIFLMKIY
CLRYYTGIPITTTSYKFSENFKNALSKLPKYFDGLREDSKCSYEYYINKLNSPECEENVNKWMLFFKLHGTHV
AYEIYLGGKIIIKININKEEYNKMKENNINVKTFFNIYFHKMGLSSAFQKEAQKILNKFRISKHIAILGGNPG
LNVNNTSFFEKWVHSINTNSMPIRTKLLPFSFFMDDNDMIQAYKDALIFYGLTYGLQLFGQEKYNHNIISVGE
YLEKCTQKLYAGPPPGLLTCPIGTTILMGFSINLNFYKNKYLSSTNGITLCEPMKESCSGNGFEKNYSDIRIF
ALCTNKPFDFITQVVQQGEAPKISASCPGELVILFGFALMKGIGSSSANKIDIYPCRTGQNSCEAVLQNHKFK
QSMIYLACVDKTTNGLEYLQTYSKTKNLGDVISDKYKSDGYLNFSCPQNNTLVFGFSLEFHTNFQATRNNFLN
CSKYTNICEISGIGINTHLSFFRSDKHSLAIIALCRSHGANNLVE*
>N.PFL0815w
MSGCNLIWLDKATKKRSKDILCLEDIPYKDEIKYLNELKVLPYVSCIHCVNIRKKKVENAGFMFYIKYEVPLL
LNEGDNIKKSKENKRVEDIEEIFLYKDKIDYMLNLEKSINKNNIYDKNISNDKENKRKKANEKDEEMNKDDYD
NKENNNNGADNIKGQFKKGSNLIKKCIDQNIDVYDVLGVEETDDLETIKSCYKKLILLFHPDKNKGTAYLNEK
EKEKEKNKKKGKNKKKKNYMNDANNNNNNNNNNNEKDFLYFIEKYNIEKLTNDEKKNIFLKIQDSYTILSDKI
LRKQYDSSIPFDESIPTLTQLEEAKNFYNFLRPVFKRNAKWSAIKPVPDIGDENTDIKEVKYFYDFWYNFNNW
RDFSYQNEYDYEQAECREERRWMERENKKIQKKASKTENLRIIKLVDLAYNNDPRIIAENKRIKLEKLKKKEQ
AMIEKKNQQNENNNNNIQHNNNNNNIPSHKKNIDKASVKLWKHHIKSLCLTKLSNLVNTEDIQQKISIMSFDD
LCEFIYDIYVILNFTVPKNNTDTTSTLLTNIKNNTIHKKVYNPNINDPKKAFQSIGSTSTTNNNHVNNNNNNN
ISTDGKTSTGFIGHLKNVHLDYKQIQTLIDTFKKYIQDHSFILQNNDHQLNDQHNYQTDIQTGTYTQCNEQFN
KNEKENEKENENEIEKQQTHANNYSNVNTQDIRQNKFSNNILHSNNEKIYEHVKKENEEINIYSNDSTQININ
ESNEHVYEHVGKLNEDTLKYSNDSTQSNVNIYNEQNNQENELQSNKWSAQEVSLLAKALKLYPGGTRNRWVLI
SNSIKTKTVKEVIKKTKEMFENDTLKNLGRNFDETPFDHFKNQNKGVMKKIDDNLDKREYKLTKENNNQVETD
NLNGDVEKKKPWTHEEQHLLEQALIKYPTSIPIKERLKLVSHELKTRTVDEVILRMKTLRAQIMAQKSSK*
>N.PFL0820c

MLKFDWIFIWKYSQQYIVKNCYKKLNDKNILKRFYLNNNIIYKINNRKYNFFNYCKSSYYKNYALRLLQKEHI
STKRRRYFKIRKHQKKKYKKKRMGLSTIPWIPTKEKIRTYKLPRQSKLINKRFMRDNKGGRRYRLKKQR*
>N.PFL0825c.1
MPRLDIICSLEKYVVDFVITLLDEKKKKILSKGKIIDITRLFYIIQIILINIKNNIYTTLRQIFYTNPKLFIN
QRNSNKIIGKLTKIIKTSREQINIYNAPKGIIRGNILLKENKSSKWVDCMNAFELRGHLICPFGLENINISSG
VQYILIIEKETIFYKLLQSNYISTYGPTILITAKGFPDINTRQLLYEIQKRYRELKIFCLTDYDVYGLSIACT
YASKNESKLYYVYDMSIENLHWLILFTPEEGIKKNVIKNTDLTKLTLKDIRILDNLCAKLKNNNKYSAAERNN
WIENISNMKKFGVKYEIDAINDIEEHINNRIKELL*
>N.PFL0825c.2
MPRLDIICSLEKYVVDFVITLLDEKKKKILSKGKIIDITRLFYIIQIILINIKNNIYTTLRQIFYTNPKLFIN
QRNSNKIIGKLTKIIKTSREQINIYNAPKGIIRGNILLKENKSSKWVDCMNAFELRGHLICPFGLENINISSG
VQYILIIEKETIFYKLLQSNYISTYGPTILITAKGFPDINTRQLLYEIQKRYRELKIFCLTDYDVYGLSIACT
YASKNESKLYYVYDMSIENLHWLILFTPEEGIKKNVIKNTDLTKLTLKDIRILDKENISNMKKFGVKYEIDAI
NDIEEHINNRIKELL*
>N.PFL0830w
MTTFTFNSCSAEIPNYKNKNNNIVNYNKCSNLFSSKNEEDGYDKTRLIIKNIPKYMNEIDLKKHFFKMKDHNI
NFQITDIKIMKRKKIIKNKEHYESRRICFIGFLNNYHCQCFKKFFNNTYINTSKIIIEDAISPSDLKSNNSIC
NNSHFQNFNNVPIDIQKIKIKQKDKNKDKNKNKDKNKNKDKGKSIQIVKEQNCINKIVQVKKTKAGMNTTRTH
VIYMDEDNPPTLPSVQHLQNDNSEIKKKKKKKKKKKKKKKKKKKKKKKKKKRKKKNITNEDNNTQCNKNHTSD
HKNDQQIIKSSELLLDDASNTHSNNEDNALEWLKEISKKKKKKDESSDMVSNIKNDNIEHVKNVDKSDMTINM
DTQKNDTTYELDIQSESEEMNTGKLIIFNLPPINEQDIKSLCERYGPIVDVKVFKKNLIKGDTQIYLNNKSKT
KSSDDFFIKLLKGNTSNNNHNNNKKNKDDDYNKKDDNNSFIIDQSKDEQHSNNQKCNKNILYNDLTNVNVYAF
VNFMFPSSCEKAKIHLDNKIYRGKILIAKYSKEKKITNYEENIENINEKNIFIKLSNDCKTSYKKIVEIQKKK
SCQNENMWNILYTDINTSINNFCKENNCSVESVLNIKDKNIAVNVSLTETYIINKIKTWIKNEGIYLDAFEQI
YVKKKKNEDEKDITKINSNNKNENIEKNNNDNNNNNTSNEVDKDDKEIVKYRRSDDTIIIKNLSIYTNQNDI
INLFKQYGILKRVSFSPYNNICIIQYENADNAKKAFISNSYIRYKKLPLYLEWAPLNLFAKQKDIEDDNNYGM
NDKRTIKREETNEDDVTRNTDQYDHKNGDTNDNNNNNNDDGDSHNVNVNDDGDDDEDDEDDEYGEGTHASIYI
KNINFNTKEEDLKNLFDKMEGFITCNIVKSKKVILQKDKESNKISNQYNTVSSGYGFAEFKNKELAMEAIKRL
TGTRLNDHLLEMSLSHNRIRKKKNINQKKNEDEKLVLKNKKQVTKKLVVKNLAFQVNKEELRKLFSAFGNVKS
VRIPKNVYNRSRGYAFIEFMSKKESCNAIESLQHTHLYGRHLIIDFADDFIFDKNVDEYDKLKELNNQQNKDI
ITSEQAKRKSIYESQKSKQVSESKKRRLTSNLKSI*
>N.PFL0835w
MNYNKISLYIIIFCFSLSFFHTFIVIKKFNIYSKQLFPQTYNTYTHNISKKKKKKCTYNINYYYCSSLPYLED
FNIARDKKSAFAIYMNHKNDQINEQINNDVSNITNVDHIRENQDHNIFTDCNKMIEPKKKKKKKNIKMNKIRI
KVMKENKMKNKKLMKNKNTFNINQHSTSNGNNMMKEEGKKEQKKKYDEKDDDDEKDDDDDEKDDDDDEKDDDD
DEKGYDNDYDNHNDYHNDNYYGQNEFIQISSNKDYKQTLISNSQEINHNYIYDKEKRSSKTYIPNNIIQNNSN
QEDIQIDDVQTEENKKNKKKKDVELNIIRNLPLISIIGRPNVGKSTIFNRLTRKYQDGSIVLDVSSTRDKLYG
EVEWEGYKFELVDTGGLVFEQEKFSKEIKDQIFMALKESSVVIFVVDGIHGVDPRDIEICRFLRKYILLKHPQ
GKYINKKEQQMGNSNTHVNTNIKELYNNNNKEEDDVEDDVERYDKINQCHIKYNIKKETSNSTHKNVKIYPKE
DYKKLHLKVILCVNKCESYKDGFYKAQEFWSLGFGNPFPCSGIHGNGLSEILDECIKHIDKIKINEEHDDINE
QNTINISFIGKPNTGKSSILNKILNCNRFIVSPLAGTTVDSIDVLVKLKQSDRIYRLIDTAGIQKRKKNVPFN
NKTKYEYLLYNRTEKAIKRSDVCILVIDSFNGISTQDINIARKIVQENKSCIICCNKWDLIYNKNDIFNDTKN
YVLNLLKPIDFSNIIFISAKTSQRLLNIFHLAEETYKNYTKRVNTNTLNDIIKEALLLRPPIPIKNKSLNIYY
AFQSHIKPPSFVFFCNSEKSVYANYTKYLENKIREAFDIKGTPIKIYYKQKKLRKIINKDKRPDKYNLDIRAI
QKNFEKMQSIRKT*
>N.PFL0840c
MIYLEPLESNDKKEDNWENNKINEDENNDNCENIFLSAKEKRDNEINRILKNMKKEERNLCFPLLDLYFLNKN
FKWQNLKESLELPNNVNEYINLNLLKKEIFPKDIIFQNDQKKKHEKTWIYNKLIKSGLLYDKKIKDNIINKLN
NNYNIIEKSDNIKNENNHNIIVNHNFPCEWINSDDIIGTFNPIYNARDKIINDNYISSVPTIDIFYNSSKLLT
IQTIFEPINHYIYLLRLLPKFSLPLYDSGILQYVCEDPILNGKLKGLELSYRLLVHDILFYAFFSYLSDLPFW
AKPPIEILRIYNYESLYSFNDCESEHTERLVFHNIKSIIYNFSDTLNKIELLYPKNYYITIFPNIYFKNIIDF
LKSEKFKLKKMIILLKGTNYMDLQKLNYIKSIFYHLRGDKTEQISCNMNNMMDMTIGGAHIKINKAKAGDLYE
CDHDYKVEKKGTQYIIEAMKKCSKGNPRDKWDEQSNTSNKKKNKNNNKKNKNNNKKNKNNNKKNKNNNNNDND
NDNNDRYTINSCMHNHQNYTISNHFDKLKTHECYVEIANEEKGHNNVREHICTQSILNNIINSNDQVIMTKET

NDNIDTSNINNNNNNNNNNNDNSQENNNFHVYNNNNYNIHNCAHHIYCQMDHPSRENNITLAFKNENKSNSLSS
ELSNSDDDTDDENIDGFFKTKKLKTTVYDKSTFSFSKFVNKIINYEKIIDYLNNDPQRQEKEDSKIKFFVKML
LFEKRYSSYDVNNDEIDIHNDENTNEGFFQSYSSENTRNVPAGINEDGQEIFNTNSYVENIHNDVNDSVSNSS
DYISNLIQNFRRNYVSNNFLSFSRNAEKEQENNMNIETTQDNFTSITFNFN*
>N.PFL0845w
MTITLESLSEEKLQNYNNKRKYNCSMTFKRFTKKRFKNKDKNKEGKINIRTSLIKNEIIGDDNEEFIDNSFMR
TCHKFKFYKSNRTDVYNPYDRKLEKYNYERDETFCSLTDLEYIVHDDQKKKNKYFIYKNKSLNDYNSCFEKKS
KRLRWFRLKRKKKKDYVNEESIKYLNESVCSNYKEANKSNQIQNSSEYTRGCVLNTVNGIKGFSTWII*
>N.PFL0850w
MTGDNISKSKNISLNYIADYQYDYSYYNYDTEYNLDINYKYVNFCHIIEQLDILDSQKKISHESVKKKACNFE
RLKKKNEKYYSFLDLESKLNNLYDNDISSFLYENKISLDDIHRLPKIRLSKKYVELGCLGVWKLSSFKNKFNV
KNLRDIDPNTYWQTDSLAPHTITIQFIKFLKISKIYLLLNYYLDESYTPHDITIHIGNDENHLELLCETFCDM
NKYSLNEPFWFLIDFEKCNMSSYYYNYNINHLKKKNHIYCRCLKISIISSQHNGKDTRIRQLQIYGPNYFHYK
YDKMLI*
>N.PFL0855c
MYKDIEDIQDDNKNNRKNKKSEHPIEKVIIRVKRRIDDTSIPSIYVKKSNKRICNGDFYKHIDTIIPEEGQHE
KNIDCINLFLKHEKENYEMNNLYENLNIEEEKRKRKLSSLKNYEKDLKQTINKLKKYKIINENIQYINLDGNG
KGVYKIIDVDINQKEKEKKKKKKKKNDDDDDDDDDDXXXXXXXXXXXXXXXXXXXXXXXXXXXXXXXXXXXXX
XXXXXXXXXXXXXXXXXXXXXXXXXXXXXXXXXXXXXXXXXXXXXXXXXXXXXXXXXXXXXXXXDDDDDDDDD
DDDDDDDDDDDDNDNNNNNNNDNNNNNNDNNNDNNNNYFNNNNEYNKSVEKQENDDNKNYEYDLYILDEEKIQ
LHNYMDYLYDIKNNNVDSSEILVLEDIYGNNEKEYETNSFSSSISDSNSEKEMSDYPDESSSERKYDSDYQM
SDDYIQNMLNSDEFEDNIQDHFNENEDHSFDDKEDLFFNDQDDFLLHDDNIENNFNNKEYDIFDDINMNIQFN
ANINNNFGHMDYSNDQVNSQNDDDRFEKNSLKYFEHANKNMNSSGKKKKMNAHNMQNYISQNKEKGMTSYQRR
INDLNFEEKIKEELEKRLKNKNSNVLNVTLSDKLKILEQMEKEYYEE*
>N.PFL0860c
MIVESNYKAVETFDVIYEEVNLIDFEFDESIKTFFYPCPCGDIFEVTLEDLFKGENILKCPSCSLTIKILYTP
EELHKYT*
>N.PFL0865w
MATKFSVKLKIYDLSRGMVKTWSPLLIGKQIDGVWHTAVLVYNMEYFYGGGILCLEQQEFETFYNIKPVDIID
MGTTELLQTHFHEYLNGIQKNFTVDKYNIVNWNCNNFTNEVCNFLVGKNIPQYILNTPYEVMSTSKGKLILDM
MQSYQSSIAPGFDNNQNNATNQQNNQSIQNNIASQDNTLISNNIASQDNTLISNNISCKTLKLMEEKTSSIDG
NMNNIKNMSSNENDIISLKENYNIHNNENIGKKIDDSCKLPSVSLENFFKDCEIEKLLNDYIKDEKYGVDEKK
KFFSFLKTFYDNILENEDLLKNRIIYKKKHPMFFNNIHTMNEYDNILSSLSFCMGFVQNNEATNLQHFTIYID
MKKIKSTTFQQHLHLVINKEIFLKNKDPSIYENHVLYLKDISEYISNDNDKAKNNSQNGLFVFLSHMFLKNNV
DMNNTQNDISSFQVLNNIIKAGAQEEKQYILRLNKIINDHIQSLT*
>N.PFL0870w
MIDVLLNKTNYLYFTFLLFLYISSNDLCSCNLKVRNNWEHFLQVYQNNGNFKMEILEPECTIQKNLPSEEEKE
EFIDFDRKELLLHDFVVIKNSQFTDEKTLELYLSKEENNKSYLMLTFYLGDLKLMIGHNSPYEISLILNVATV
NKNMSNCKNNSYNIVLLKTTDVFNTSDLEILEGPIQFSLGKSSGAFRINVTNFFLNNTWKAFTKDKISFLIKP
EENCYTILENKINKPQLLIEKKTTFYSEWGEWSNCSMDCDHPDNVQIRERECIHPSGDCFKGDLKESRPCIIP
LPPCNELFSHKDNSTFKILMIILPIVLVISIITILYHIFYKRKGAEKELYENVAGRYMYD*
>N.PFL0875w
MNINYYNKYKKFRLILFCFIFFPYCLIVQLIGCIKLKTSYKHEPILLLKNDERIISLNKIIPYLTEEVFISKD
NIMTKINVENNICIELIKKHEILNTPIEHFFHFTNREYTIDLRNNDNIYNYTKRILNNKLDFELCNKILSFTI
PINYNICSFPSYHNYFLYINYLKDVKIELIISALEKAKFLFLKIPNTFNLNTTHANFNDNDYIIEKGILENDL
IKIDISKIIHRYICKKYYLYKSDEQINDDDLRQINNNNSNNDISDKEIKMVLMTSHTLSDIIIFPSIIQNVQA
LKHYKNIKNVVKLKKKIKKRYTEWENWSPCYNVCNNNFSYKCRYYKCTTENQEFCDQNFHMNFSECPFTPCED
AIKKENEKKEIEKDKNKSIIINHENNRDQEGPIDDETMLRYRKNKKISFFMWIINNKKLSLGLLIFFISVIVL
ICIYCYINSTLGFYNDEEYYVSKYT*
>N.PFL0880c
MYKNKKRIILKAIKFLEKKDEKDEEKITEVINEVEQIFLHCIKNDKDLCNKNIINTYIIPYMNLLLEKLLFYD
EDNNVKNNFEILSNKVDLLSRVLMFLSCIIKINKDYSYLIISYYVNLLESKFHLIKIIEHLKNIILHILKGLL
FSSNVIHNELDNIKRGIQDYVLLKCKIFNMVINFYFNPFLYNHPYKFILIKNLMLIITKEVNFYLHILKNKNH
IFQQILIQAKPTHHVINKLLNVKYLDYVLDNDSMKNNYDHIIFIWLEQNIRLFNDLLLFYIRNDKIYDHIICQ

SEGDNNQNENENQNQNENKNENENENKNNDHNNNKYNNSFIYHCKEEDQITKEHLEDTYKIFFYNKLIYYLNS
NNKHPSTLSFNNNDEYNYKYHNINNNNERDLINCKINDQNIPSFEREKNSNIQENQTNHMFDTCEKYMDYEDN
FYLSPYIIKNNDKSSSQNEENTNHNKKKFLVHMDCLINNINDLKVDSLIFLTYLMKNIFSLIRQEPFILNFFV
KNMFILIERLINIVEVYKKGDNILIKKKENEKINNQNGFDYNHHSGDDNLQHNNNNNNNDSVYTNKISYKLKV
VECLLEFIKNELYILICKKYVNAPFYYSYINKLLNVIGEKGTTEELIKKKCNTINNMYNTHIINEDKKIHILD
NNYEKFKRNILRKNNYGYNKTNKEHIDIMCYLKVKMNENDNTMKNKKEEKENMFINQIIKKLYLTNFKLDKNY
YQRFNCSKEVNIENLIEIGRRNKDMGKEKYYDHNNMCGDNNICDDNNICGDNEIYGDNKLCDDNKLCGDNKLC
DDNKLCDDKTINTNGASNHNINNKDDDTSNNLIQRFMSKFITSNKCEKDEEKKKKKMTNEKIEVIDDHNYNDN
VIIQKENDLKYKELLNVKELKYYDLYKSFFFSDMKYYNNIKDKDNILTNIDLYYFIIFSQIINNNLENQVPIL
GQNVFLNKINNLKIINNIVKRIIFNNMLKEQLKFFFLSLYIIKINSFNIYQENIPTDIFMNYSTLFKLYNNLL
FYLFIQEEKKKKQKKIKLNDEHNINQNIQCLYNFTMDDANHIMDNQTYIDNHNILGYTKVCYNYFYKLKKKLE
NAKTLFVHRSELQNKSFKIKCTYDETLNLILYILHNENLLLKHRDQYIKTFIEQILETPKLSFSFFIYLIIWL
HNIQVHVDFKGYINNTNGDKEINIKTMHHIIESDTNVDINKEQEDINFKSNDNKKKENIMMNISNDYMSPNIN
QDSDDYENDSYFNVDFSEYVNSESDNSNDTSTNNNMSSKKKNTQDVNKLLPSNNNNNDGDDNNNDDGDGDDNN
RNDVVNQLTFKNNKPINNNYNEDVFNINYYIKYNISKTTNMSNNILCFSLISNLLKKNQNIKSKKTILSIYMN
TLFNCRNDVRTLLKKLITATKGIYNNKLNYFVYTKKIFRYYHKIFILFLLYICNMYFQNLKDDIYFFSYYPFY
LWPVELMNFIHNFLLSNFNFFYNDIISLFTNYKQVGSYEKKIFSSKYYHNINKQVDLFLNNFYSSQVEDMSLE
SFMKDTDFFNNKKQEGEDNNYNSNINNYDNIECISLDIYGKNFIEEEEKECLHKNEISQRILLEEIFIFLFIK
TIDLLEDNQLWYKLKENNIFLNFLTINYVKELVNFVFTNIQNTYEKYKENFWLQFNILINEQVEDDKAKINEY
NTYIQLKEISNFVLNSIDNYLSVCVRSTIFFHLYIFIFNNCKKKEIKKLLLNKFIITLPLLKSLFHEEFFRIL
KYNNKLFSQGKEEQQQDDNIIMESNHTHDNNNNNNNNNDNNNDNNNDNNNDNNNIDKEENKNVINKDEDKNNN
IVLEESNDINNNDIKNTEENNPFQINMEIIKYISTNLYKSIPSSEENYMNFLNFCFNLYLENNNFEFIIEIIG
FLGKEQTLQIFNDIIKNEIIEENKKIEFMKSCINNIVKLPYAYILEKKKNNDNDNETYYVTNADIFYLYYNLN
KYKNIQRVMLDYFVTKVNLNTIDNDDNTKMFNDITIKDLASIVQQIAENSNTIFPIYGRFLCQITKNINILRE
FVSSIIIPLLIQKKIWTNKFIWKGCLMCISMLWPDFKHSLFYIFFMLPSDECSTLFHALEQKHSISSDLIELI
STNEQAKRMCPDYLKNLLNT*
>N.PFL0885w
MIDALYIFFINGQLLIQRNYRDTTKRTDLTQYINKYIKTKRFYENPIVEINNVFFINVNINEIVITVLTRSNS
NICLIFNFIYKFIEILKYFFNNELSGINIVNNFVLIYEICDEIIDYGYPQTLEVNILKNSLLNKVKYYSKTSR
YFQKISNELLNVNSVIEDIVHDPHIHNRTSNNKKNKSNNKIRDFYNTKSVKNKNTYDLNETNKLKYIGKETLN
RIKNKIINNNNNNKTANHFNYITGNCTWRNNNIYYKKNEIYIDILEILNVTINSNNLIYAHINGKVTLKCHLS
GMPLCELSTNNKINLLKNILAGSNTSNNNNNTSNNNNKTNQGNALRGSCGSNSLVNNKVMQNNLKKKYTLDEK
DNEEIIIDNCIFHHCVTLSKYENNKVITFTPPDGTFELMKYTITKNIQIPFHILAIYNPILEYSKNVEKKFSL
KKLTTNNKSIYGEYKNTNKYEYSVTIKSNYKGNMHASDVLIKIPIYKFSENVQVKYKSIGKTEFNNIDSLVIW
RIKKFLSSSEHNIKIHLTLENHNQIYSNMNNTQKVDDLSKVVLQVHKIKNMNTVKFLNTYKMPITLSFKIPMF
TSSGMYIRYLKVFEKSNYKIIKWIKYLTESGIYQYK*
>N.PFL0890c
MKSFIINPRQDEEIRNDIATDSSTVNGININDININDIEENIKDINTDKVKKKDENNSIEYYLFDVESYNNNK
KNEENFLDLSSIRSSYLCDNKKKKAKKKKSSYLKKQLKIYKKKYKILPEHNIDQDTNYEESVKNENSKDYKIY
ELRDIKEDKDTNDYITSKTKDIYGMKKDVYVNVIKFIILISSIFSFGSFSTTITKYIIFVKKFNYTQIVTLFE
FLIMYIILRIVIFFTKMKCSTNLTRKEYIKYIISISALLGLSVVTGNSAYSYLEIPIISVIKSSSLILIYFLS
IRFGLKEFKLSLLCSILTILTGVIMSITSLKIDSLFGIFLLIISVISSSFKWVFTNVLLRSTSMKPHIILLHV
YQVAMCIIIIPSLFIDLACMVKDYNNNILTMDKILTSLMLVGVGALTSIFLILAEFSLISYTSSVTLSIVFIG
REALILLIGSIFFGEKINLSSSIGIAISMFGTILYGYASK*
>N.PFL0895c
MMEHGNGSDKIFDLNSISREKLELVEVFYDKMKDTTNANALYLYALQIFKICNIHNELPRLVVFGQQSMGKTT
LLDFIMGGPMGYTSTDTGTKQPIVIILKPSDTNKIECYLNKKKVNIDDLHEKMKAIMLNLNESIILKELEVEI
SIPGGIYATFVDLPGIKDDSKAGSELTRKIVRNYVQNFPNDIYILVKKASDDPANWPYNLREFILKPRPLGLG
LQPKQCIVVGTRALEFLNNELTNIKTLSELYERVKKRSITDNNDNVLPLYLLELFSIPIEQKEKNDFLTNRIS
MYSKILNGRKNVLDLLLNKFENDCNDSIKKELIDCFDVEKFKQEVNSKFMNILIQQLRKVEVKLEKKKAKMDF
YNKKLEELYNKGNILSVREQVKLYIRELVNIVSNLLTGNYPILNLPKNGENFLKKYGGTLMDNLKDGNELAVE
LFEKQGLYDENFLNYLNEYLSRYSNDMDLSKSASSEFNYNMNDGLNNLNNNSIDDNNSNNRTDSYENHMDLDK
IKIENKPLLVEGVPVRFMLAKEESNMFGVVQNAQSDAKGKNILVNFYFRNNNTDEQVQVKSVEKDRLTVIKAV
ETLNGDPSFLNGLQVWYKMVRDDGWVGFDKAEIIKVFNNNSKIKDVLIRNLSKNDEIISVKIIDLYMDYVNNH

NHGHNHNNNHNNININDDDENKNDENNNNDDDILKRIAGPHTDLKILNQLAITYICKWLKYNISKLEPEKEFS
DEVLLQMMRSIHNIVDQSDWKPLIVDLLQANISGNILHLTKLASCSAAVALKRVFKAGLGEINRKIKNNYIDE
NIYLLSTNQKFIEELNQALHNFCKERAIVCAGEMKDIVFEQTYAVHFEIIEEIFDGCKLFEDNFLTPNGVKPT
MSLINKNVKQNLAYRNHQLSLTDVKINKKSRSKELIQEEVKLQFWAIKMLISVPLATKIYAHFLNNILPKNKH
LANVLDYSIDCESYLEKYIQSKLLNKEVNGVLVPIDDKELMTHYSIIDNRDNILRKIENQKRLNQYFTIVANS
IKLLKNNLSKESTLDFVTKLDFSQDKKNWHRTDSYEEED*
>N.PFL0900c
MITMECITKKIKTIFQNSIQKCFPSISEDAIVTYANLKFGHYQCNNAINIYKKYGKELNYENAQKISEFIISN
INETIFEEIKSSPQGFITVKLSKDYIETSLKKLFNGEKIDISININDIKESNENYGNVLVDFSSPNIAKEMHV
GHLRSTIIGDSICRVFEFLKINTHRVNHVGDWGTQFGMIINYIKTHYPNFKEEMPDLSNLTSLYQESKKMYDA
DKEFEKSSKENAIKLQNNDEDCKFVWNKLCESSKKEFDKLYNILDIKLEYVGESFYVPMLSTVLDLLKESKLL
TNIGDAICYQSENFKVPLFLQKSNGGYGYDSTDVAALYYRLTQLNCNCVIYVTDIGQLTHFETIFDLIKKTNW
GDKNAKLMHVGFGFVLNEDNKKFKTRSGTTVKLINLIKEGTERAKRDLLQRIEKKSEEEKSYFENVDIDQLSE
SLCVSAIKYFDLKQHRNSDYKFSYDNMLNVKGNTGIYIIYGYSRICSIFRKSTINVEDISKDELSLTSIYEIN
LGLHILKFPDIFYYILKNMLVHKLAEYMYDLTTTFTAFYENCKVLNNENEKSRLLLCSITKSLLKLCMELLGM
KPIEKL*
>N.PFL0905c
MSKNKKSIITTFTTKLQEKVKEEKKNNNKINNDVMKLINKELIIDYTTNQKVNAKLMLNKYLNVINYLMKENI
NLKKKIENIKVEYMQKNDMIDHYEKELKEKNEIIIKLKKNMSPDTLEECERTYEDNNYLMEHNDMTMDNIEKS
KYILSIKGDTENNISNNKITPYNINHTPFNKKDEQINNEDNIFLNYQNNTFNNKYIQQNDQHYMKNVCMVSDN
VKAKNTSSFNQIITPTISNDYKNYWSNKNINVEMNHEYIYNNNYNINTSNLSNNCKKNNSKENINFIKKDSQY
LLYKNDNLPNDDKYIDELDNLINNTNSVHTMITSHLNKCDIQCNNKNEENIIIYDYSKETNILSSDGEQESDI
SKNCDTQDPKHNRNNDYTIKTTQIINNKINKNNNNNNVHNNIYNGMNKSKEHINKISFMEVDKCIINKKIDND
VKIITHEHDIIRDDKNYDTSSEQNFKLLEKSTENEEDDDNYEDLENIMSTILKLRKNA*
>N.PFL0915c
MLLYLYKIWYLILLWLYTHNQYKCDLRLKPPECDVSIDTSICINNGQKILLPSAKPYGISTHITFDSLMPVDS
TGNRNHAHGKFFASSGFGGIGNSALFRQNYIYIPHSDEYFKSVDFSYTFFIYLLQDEISRKNNMEEKFCPVIH
KGIIKDKIQESSPAILINTKNGRIKIVLSTSSSTNSAGEEFLSNFKLRHHQWYHMTVVRHINHVRLFVDGILD
SSFLTEGITKTNDSPIYIGGAPYSVDSCDFPFLLDELKIYNLSIGTDQIQSEASASLSGIEPSFIYFGCFHCD
MNTAILSCPNNYHLCNKMELYIGVYNVLRKFSLDVNNIILPYSSESNLGIGICCTDI*
>N.PFL0920c
MKKEFVEYNKKYFRNIQFVYDFLDKIGMKNSLNELKKESKIDYIKDYDLYFLDRNNQDISDDENIRDEEEKDE
AENIDEDFEDIDKTFFIYTMNKMNNSILCKAIDLFDRDIKEKYVLLQNVQNVKNINHKGRDIDENRNNILYDD
NDDDNINNNNNNNNNNNDDAVNTFDGYMYRPDQNNSLTIPFMKEKKENIFLKNDNKDDESLKLYNTNNVTHKV
RMNSYNWDSYFTQDKEIYKKLNVSIVHNDISNNINDQTNKPSNILCIQYIDIYHKQNNKYINSENIIFFQRFF
PLFLLVGYANGNIILFLILYEKENKEKEQNEKNGKKNEKNEISEKNEKNEKNEKNEKNEINKKDECHNDNHIY
LYQQLEHISLGSPVMHIDINYQDNLFIASTMNGDICICQINMKEIEDIKNKLIETNDLNHDTKIKNDNPYMFL
KDVLKYHMKYSIQCLFNEDYSLFCSIANDKNLIIYEKIVANNKRNEKIDDICSNNINNNIYDDKPNYTYEKKK
IIGLPDTPTNMLWVKHNNKEIIIISMLNSNHIIFLNSSNYLIENKLYLFDLSKENNNEHEESKNEKHNILALH
YHKKKNILVICTDTSKIFVYSFKEESIIKTIYGCVLNSLSFPTIQINISGNFIYVTSEDKVHGTYILIFDIKS
GNIINTINNGFKIRCFQLLKNYICPSTFNVNSNNIDTNKKTLLILGSFDKKLHFYSN*
>N.PFL0925w
MKDFMDRNEKHVYEKYIENKNKDDNRIDNNKGNFHTNVNKNIHMYTMKNWLNKKEKENRKKSLLNMNKTYKED
DERNIYYNNNKTDENTYNNVVDDNYINNNKNKIIFQHFRNTERKTYYNGMVKGRELLYKNIKMNENKNNQISN
INLTNYLLVPDEIRHKYTKTYLFQISKRLLVMKKPWRLPKNVELQINNVYHLSNYIKVLERIASEYLNDPPIN
YYYRGLELYNKNVFANIDYLLKKVDIKEDTQTFHLKNLIKINECYYDLSTFGKSPNTIFYQSYIILDIHSNTL
TLEDEYENLFESQILNYGYGEKEYPNLKYLLSVCSSILFWLNFNKKDNFAIINYHKISSFILLIFSCAMLAID
NSLTFDQIQEILYKSSKINNNDNEDNNTNSDTSSNYFSNNIHNNSEQHKNKNIYEENNTTHGIDIHENNNNNN
NNYNINKYYIREDVKQHDDILLNNNLVRPKLLYNEKREGLDVEPNLYLPTYNYFENTNSKKLLNKDEKCSEST
LEYKNNYAPNYNENMRHTINVNRKNVLYENEFDDMKNKKSKDDKVSSTSCGMDSLNKDTNKTNNKFKDHHFWI
PFDYWKASHKRYFYYMYDLIKNKNKRVENNTFKIKSIIFTDYAPCSVSIEIYEIIYKDNSELNSDLLSESETD
VYDESEENDEIEENDESEENDESEDNDESEDNDESEDNDEIQQNDNSQHNDEIQQNDECDDKYINTHKMKGTD
TIYASNQNLKHYSFQKNGTGIEYEEKGLWVYSDREKKLVPAPRIPKDEFKRCSNDENIDNNNNNSDYNSDDDD
DEKHLCECSLNKHHGRKKKNKKKNKKKQNIKKLSVEHFSQTVYPNENNKNNINNINIINNKNNINIINNKNNI

NIINNKIYRKNFSCNVPSLETKQANILDINNTPKIDFDISHLNQRNNKKKKKIQSDQNSVVGTNYQKDISYNN
KSLIGLSHNKINSSINTLNVFNEDDSLTSINQHSEIHSRVESEFDKVEKSVTHLWSNSNFPLKNQIKKVSSVF
SPRTINSNSSCKSISFVDKLTSSKRDDFSSGRSIQTSESKESFSDNKKNIIYDHFDKKEKSSFFYNKSQIFHK
DENNMKNDFSEYLQKMKSKKKETHQIDKQQDIKQNVDEYYHLKNDKKKNNKYKLYGLLKNNYIKGKEKDTNDN
EEQIKYHINHDNDNDNYLNENMKKKKKKIKSEEKCKTNSSKCGYEYFLKCNPINYNVVSSYEKNKKKYITIDF
THDAEGNVKEHIICGDILILIGHKNIEKFHKGFSSSYSFHTGFLKKGSSEVLHLKKEDMDININYDNYIPDNM
KISIILDAATKQDCLRSYKKSLRSNNKMEELEALKNFERRKSFVLNDSAESVMNMLRDFPSEFSPSEKTYDYF
DKINSLEKMMKCAKKSDVTNENCASTENNESLKEVLKNEKQQEQKKDYEKDKHKIYDENDKHKIYDENDKNKI
YDENDKHKIYDEKDKHKIYDENDKNKIYDEKDKHKICDENNKNNIYDEKDKHKIYDENDKNKIYDEKDKNKIY
DEKDKNKIYDENDKHKIYDENNKNNIYDGNDKNKIYDENNKNNIYDKKNNVEKNNKIDNKTNVYESIVEKHYC
DHSDIEKNKGQKIDIQYSIEKNHFDSIQNDPLEEKEELMNTYKKGHSFNKVKELYEQESLDIYNKKNLDFNSR
SHYNAYSVFNEKEPTLKYNATNITQLLTSLFGFRNRDDKYSLNKKQSMCSLLTSKKSYSNIISLKDYLQRQYE
IIIKPSENITSFVKSHVIKINVPLLNFLKKITNLSNIKIQLSLKLCNNDLNKSLNFIVCTWGINILKKKSPAK
YSSSWNQIPEYRREFKKILGYHNFSSKSSLDTSKCVNKLDSVSEMDSTNYTNISYYKMLKDVKSLYDEGSKKR
IHTDECIYRIEDEEEEEKKKNKQEINNNYILSLLRKKEKNEIGKRIIDRKEKIKDEKETDYSPKDTSSEVSTS
TDMMKFTQLCTNKKENKEKEDNHVHRKITKSDIITNKKEKHDVNNIEEIQKNDTDILINNDVNKNKSDMLKRA
DNYIISNNNNNNISLKKTIGESSFFYAKLPSGELIKLKVNDSVELSTHDSMPLLLVEPIDDITDEMKKEITEW
NSLSKMNSQTSSKENTMSDNNNLHKDYLINDLEVMYNKLNKKGSIESTFSMESERSVTSRLSPSIPGETLTSL
VNVKDKKEIIVEDKREVIDEDKKIETVEEGTKESKIEGKNEIIDEEKKESKIEGKNEIIDEEKKESKVEDKKE
IIDEQKIDEKQNNVTSEENKAANDKQIKAKAIEALLKQSKAKRPPPPLPAALLKSVNVAKEKPEMKEEEIVKT
ENNTKLAFTKKGGKKIPAPPPFLLKKMGDKSKCVLKKCPEAFGLKKAAKVPEKRPLGIKLHWQLLPTHKIEGT
VFNEIKTQEVKYNLIDTKAVHKLFARVKGEKKIIKKSVDDKQKKPEEKLVTVLDRTRAQNIGILLRFPMSTQE
IVDKINSFDLTDLNVDFLQKVLHIIPSKEECDGILQKLENEKIKTEQFRDVERKLIPFVYLDKCQSKIQICLF
SLKYDKMINEINKDLDIYDKAVKEVRSSIRLRSLLKAVLKWGNYVNYGINDNEDLVALGFTLSSVLKLSEFKS
SIDSKITSLHYITVTLCMYLPNLNMNLLENDLLSVLTASKMSSESIDIIFSSLEKEITYIKGQLKTNYEEKFK
EKMVSLLEDSEQKYNTAQKQYEQTKKDVKELGIYLGEDMPKNGNLENIFIILSSIVDSFTKCYKDILANPKKF
SIMLNEESLLDDYYNVFCKGKKRPIIKMNSISTNSDTKEDDMIKNKSIPDIKIKINKSKSVSVKKNDHGKSTM
FQLRNQLMQDIQNRSILKKSLVFPNTSENESSSIQPSQQNGVDNSIAKKEAEQNIKKEEELKSDNGIKIKEVQ
TLNDESKNVNVNNTMNDVVTVENKSDEKQNKDIKVEDIKNT*
>N.PFL0930w
MSQNNPLSVCVADNLINYDIQNESFRLGNVSVEGDKYICVKENVNENTQVVVINLHNKNSTRKHMKAESVIIH
PNDPILALKGTIKNMNTIFLQVFNIETKEKICSLNLNEYMNYWKWINNDTIAIVCEKNVYHWNIDIHNTKKNK
DNNDNNNNNNNNNNNNNNNNYFNTLTKVFEKAQIFIDNNSQILYYSTDKDMKWCILCGISTQDQGKSIDGYMQ
LYSCEKKLHQTIEGFIGCFGSFVFDNLDMKPLFCFVEKKKNSTISRLHLMDIYTNKTETTIPYKIVKEINLIN
ENLNDFPIYISLNTLQGVIYIVTKCSYVYVFDEGTLSLIVKEKISEDNIFICCDSKNGEGIYAVNKKGIIFYI
TINYFHLINHLKLSNLEFKDKIIKNLCVKYGYPGCDYISAYKKCINDMDFKKASKIICLMKNTKIFEEHSNNI
AESILIMNRKNIDIRIIPPLRTQQVLNSFKSFKNTSGQLSPLLLYFSVLLEYDKLNTYESIELVKPVVLQKKK
EYLEKWIKDDKLTCSEELGDLVKVLDLRLALNIYLRCSSHNKILSTYCLLNMFNNVLSYINNFKQINFDYVGI
FITIVNYEFPQGNADNKDSAFVGDFNSGSKNETMDFFNDGAASISNDQNNSINKLNKGHINEVAIQYIKFLCE
NNISFDINKIIDYLLSKKKLQEATSILLDYLKENKAEHKNLQTKLFEFNLYNNVQVAETLFQMDIFTYYDKNK
IAYLCEEKGLYQRALENYTNINDIKRVITKSTCFQKGGNNNNNTTTTTTTTTSSNMGDGHFDMNNVSKGKISI
EWIKNYFSTLSDSVCEELLFDFMKGSKINMEVVISICVQYYDKIGIKKIINKFEENKNYEGIFYFVSNILNDL
PNLIVKGNNNDNLSNNSLSTYNNMNDEDASILLTSDICSTPQYSYETANLKLEDVHYIMFKYIEACVKINNIQ
ELDRICKDKNAKYNPEQIKNFLKDCKLSDPRPLIYVCDIHNYIEELAEYLYKNSLLKYIEVYVIKVNPNNAHK
VIGVLLDLDASEDFLLNLLNNIKNISNIGNLIEIAEKRNRLKLLLPWLESRANEGYENIELHNALAKIYIDLN
KDPENFLKNNNFYDKKMIGKYCEDLDPHLAYTAYERSNGECDEELIYITSKNGLFKLQARYLISRQSIELWKV
VLDETNKYRKNVIDQVIGSTLIESNNADEITVTVKAFIEKKLSSELIELLEKIVLHNSEFSDNKNLQNLLILT
AIKSDSKKVMEYINRLDNFSGPQIAAVAYEYKLREEAFVIYKKFNCYTSAISVLLDKILYNKNQKNKNFHEFS
SHDSTVPYYSNNNNKDNIFGSKSDNENDEYLKQFTQNNSYREKYGSYYNNNDISNSNNNNIHLHPEFNNEDKF
SSDTLHNYEDIDSTESTCDLSNYEDDLNRAIEFAQKCNNNDVWFILGKAQLKLNKIIDSIDSFVKSNNPEAYK
EVIEKCKENNFYEHLITYLNTLREQNSLKDVLVDSELLYAYAKLKKTNEMNKFINTTNSANLQLIGDRLYKEE
EYEVAKILYSNIPNNQKLTACYLKLKEYALAIEAAKKAKSLKTWKEVNFICVKYKQLKYAHTAGLQLIMHADH
LDEIIKIYEKKKYINELMNLLENGLNNERAHVGIYTELGILYAKYKPEKLMEFIRNYTNKMNTRKLIDVCENE
YLLKEAVYLYISYDEYNLAVDTIIKHSPTAYTADTFMQVIHKVTNSDIIHKVIDFYIEEHPLNLYNLLKILEN

KIDNNRLVQTLKKSNNLPLIQKYLEDIQAQNITAVNETLNEIYLQNDDYISLRNSIDEYDNFNQTNLLNKLEN
HKLPEMRRIAALLYKKNKKYKEAINLSKKEKQYKDAIEIARVSKNNVYIEDLINYFIESKNKEAFCACLIVCY
DLLKPDYILEIVWTSGFKDQAMLYFIQIISDYTNQIEHMKKQIEDMEKEKKMNKSAPNDYSAMNNQFNYSLNN
NLSIMPPQNNFMSSNSFDKYDMFNNNTHF*
>N.PFL0933w
NTKRNITTIFVITNKHTHRSVDYLRLQKKKSVAEKVEKTCLKCGYGLGGDFAPAWDLFSGLGYDAWTNYAATT
LVKISTNVGLVKVTNIATKLLGSTVNIPKFKVGKIITAGRFTDDVTFSGVLKSMDTIMCGKFDDGPHAGFCAY
VQSATKCAKLITAKYTVEEAEVTTFSDAKTGILA*GASITSILTTAIIVSIIAIVVIVLVMVIIYLILRYGRK
KKMKKKLQYIKLLKEYILRVWYS*
>N.PFL0935c
MEGDSSGDEKYAFMSDTTDVTSSESEYEELDINDIYVPGSPKYKTLIEVVLEPSGKNTTASGKNTPSDTQNDI
QNDGIPSSDTPMNKFNDDEWNQLKHDFISNMLQNTQNKEPNILHDNVDNNTHPTMSRHNIDQKPFIMSIHDRN
LYIGQEYSYDMSTNSANNDLYSGQNNLYSDVDSTSGNRDSYSDKNDPISDNHHPYSGIDLINDSLNSGNHDIY
DEILKRKENELFGTKHHPKHTNIYNVAKPARDDPITNQINLFHKWLDRHRDMCEKWDTNNKVDILNQLKEEWE
NDNSNSGNKTSGNITPTSDIPSGKLSDIPSTNKMLNSDVSIQIHIDKPNQVDDNIYLDTYPDKYTVDNINPVD
THTNPNLVGNINPVDQNSNLTFPSNPNPAYDNIYIDHNNEDLPSKVQIEMSVKNGEMAKEK*
>N.PFL0940c
MIIITSVSIFY*YICNVILNIYIYILNNLFNIINFICS*IYK*IYIHV*KF*LLFYLHIILKNKTFKIIIHF*
*LIYHIRIKNTKGNYI*IITVIYKYEYLYNS*K*MINTYISYILWIDIQKMGSLHNNKFTICFIFHIYHKGKD
VSLNLLGRVLRIILKLLICSSKNYISFFFYILFSNVIINFIKKKEILLLKDFSNLDKSYILLGLLYIYIYIYI
IIILATIF*YPTYISYFYDDLTIYKIN*FSFLMDSYYLY**HD*KLYGFFFYDLFYIIRFLFVLVTHHKYELF
YRHILMNY*LNEPQYAYSSMTHILDRP*Y**SY*YFREYSN*YCK*PLHDQYQEIYLEWM**KVFHPYYGVTY
*VVYLMVTP*AHQVLHVLYIAI*NRVSVDSIHYL*IYF*VYHH*VYHHFVYPFLMVLVPLLSMFYI*EHVIYT
YLFLHIHFRKMKK*YQ*YQT*KYFSLRLYPLEHIYTFCLYIDHFHTEHIYLVKVLSAFHTRFGLYQ*HEKDWY
AQM*FWTLHIYIYIYIYTL***SLLDVYIYYDENKNEFYGRENYNILK**TFFLFLYLYKYRKITKVLGT**C
ILFYFMFFFFFFFFFFFFFNLFIKKLFIYDVIVIV*YDIISLNYTNICFFIGI*IYILLQKKV*YNKIFKNFL
*FV*FVIKFNCIRQDKL*SRYIYLFILRTDYNIVTNNSFSLQIT*ECVK**LLKVFIILIRRFCNIYIIDVLT
YIIV*L*LKNGAQEAGGVARRWWRGRY**NKCQTFIG*HREKSTCPSTK*SCRTWWE*VKRIVVTSNIF**GN
G*HQ*SMHI*LY*SY*GYG***DMRKRKRRPFF*KPYR*I**EKNKR**WWSLCSI*EVISM*QKFGNYKH**
N**QAEVVGRGVSCSKI*RRINNT*LSKISNNIW*FSF*IMYRFSTKFCRYRRHNTRKRFVWW***EKTTIRR
EFKKNFREYI*RIVEDADEEWAEVCKRSLRTRQSKFL*ITRRLVGSK*TTSMECYYM*GGAK**IF*RKKLQW
KYVHC**M*MCYWRCSNKFRLCTSIFKMVRGMGRRLLEDWWEVNRQQVWNAITCKAEQNDKYFREKNSNGNTC
TVNKCKCATGDVLTNFDYVPQYLRWFEEWAEDFCRKRKHKLQNAKEQCRKPNGNDKYCSRNGYDCTQTIRGRN
KLVSDTECTNCSVVCTPFVNWIDNQKLEFLKQKNKYADEMQKYTNGITSSKRKKRGAGGTTTKVYDGYEKKFY
GILKTDYGDVDKFLEKLNDEDVCKKNNDDEEGGQIDFKTVNSGKNSGGDDSNKTFYRTTYCEACPWCGAQKKS
GGNGKWEAKKEDCSQTKIYNDQKKTDIPILTADKGQSDIFQKYRNFCDSVKNTANGATGGGQIKKWECYYDDS
DKSNNCVQGEWKDFQKGQKVTSYNAFFWK*
>N.PFL0945w
MIIIIVYIYIYIYIYVKSKITSAHTNLFRVIDITQNEYGMPTKPSPNRYVPYESDRYKGKRYIYVQGDTDEEK
NIFMFDTTDITFSSSESEYEEIDTYISHVPKYKTLIEVVLEPSKRDTQSGDIPSDDTPRNKFTDNEWNQLKHD
FISQYIEHVIPDVPTELPLNTQPNTLHHSMDEKPFITSIQDRFLDTGHEAVTYNINWNIPENINRTTNIMGDP
KYVSSNYMHTGVHLINNSLKCDDKKVHIYGELLKRKENELYRTNHKKKNHTIFNRVTSINNKNPLEN*
>N.PFL0950c
MSLVYRKTLNFLKGKNEDDKDVKININGENKRTCNNSVITSKYTVFNFIFLNMYEQFHKISNVYFFFIGILQV
IPQFTATNGIPTVFFPLLIVLTANAIKDAFEDWNRHKTDKIENNRMCYGIVSEEEKKIYQEKSNKKNIFKKLK
RYFFGNRKICNTENYYDEDDMCDDEITDINDYINNYEDNLENIEGTVKKRWKDIKAGDIILCRRSEFFCADIL
LLCTSHKNGIAFVETSSLDGETNLKVKEANTFLFNILGNDRNSAIDNVKNLKGFILSDKPNKDLSTMYGTIYF
EKDKKIDVENIGIQELLKKTTEEIEYRKKRLSSVDLSKSSSAIGSNLCNNNNKSDSKSDIKNYNKDDDDFDDM
DENNILKNDNYIRIPFDEKQFVLRGCKLKNTDWIMGIVIYVGRETKIQMNSSKSIKKTSKLEILTNKMTIIIW
VIQMIICLISAYYNAIIVSSSRKNRFRYLPFNLEKAKKPYIVGIISFFSWVVITGNFVPISLIVTMSFVKVVQ
AYFISCDKNMIHKVQADVPSFGEQKEIPNIPKDDISSDADVIKRMRTKKIADSSLLHIDENQTEEDNMDMKSD
CIAFKNNSSHQNINGNNKQYSSSSLGIISNAPKREISSRVISFKDSKEKNYIYFNAVPRTSSLIEELGQIEYI
FSDKTGTLTCNIMEFRKCAINGISYGKGLTEIKRNILKKKNLEIPVEPTMKFKKKTPHVNIIDNDIINHLKDP
NHFNHVNLINFFLHLAINHAVICEKDKEGVTTYSSSSPDEEALVNAAKHFDITFLYRREGKYGISIFGKIYEI

DTLATIEFTSKRKMSSVICRIPVINPDYNHPTDAKSSNMDKKKNNMDDHLVVGKNEEKEETDDLNVYPPKKEA
HSKLNNNNNNNNNNNNIRIDNLYDDKNNFNIIYNHNEGRTPEVITCKNSKNSKIMLFCKGAGSIILKKLAKRT
DVDEITIEHMETYADEGLRTLCIAQRELSEESFAEWYHLYKEASLSIKDREEKLESVAEYIENDLILQGITGI
EDKLQEGVSSTIEDLRMAGIHIWMLTGDKIETAMNIGIAANLIDNYSEQFIYTEEYIESEEALIKKIDDDILM
VEKSLNIPHYNFDDENNNVEDEKRKTFFRNNFIKNFFCAKNKSGLLLNPDKYNMLINTLNYVLVVDGSVIDLL
LSEKMERKFFYLADKCSSVICGRVSPYQKGAIVSSANRLLNKITLAIGDGANDRNMINTANIGIGIRGQEGVQ
AFNSSDYGISQFRFLKNLLLVHGRLSYRRISKLVVYMFYKNMVLIFPLFIFGSISLYSGQKIYFEFLLHLFNV
LFTAIPVVIHAVLDQDISLNTAMEKPNLYKLGIHHYYFNIRTFISWVMNSLFHGSVVFLIPLYFLSYYNIPTS
DGIPYDIWTVGCATYFLTVLIVNFKILFETYYLNILPISGIALSIFSFVLLVTAFSFMCVGSIHLLGTIVYLV
QSLRFWLVVILGLFTALLRDYVFKVYKRNFNPEIYHLLLDQENAKIGMNDVIDQLKLNEFDKDDDIRIEKSKS
LGYAFSEADPACIQLIRKQDNMI*
>N.PFL0955c
MTIPTISELKKDRIIPHVFPNDKIDLNVDLFISFKAGKEVNHGNVLDIAGTGSVPRNIKFSEEPPDGYCFVLF
MVDPDYPSRLRPDGKEYIHWVVSGIKTKELIKGTQKNCVTILPYVGPSIKKGTGLHRISFIISLIKEEDKDNI
TGLPHYKGEKYITRVKFNNYESVHNIAQINNMKIVGYNWCQIEG*
>N.PFL0957w
MDDDLEALVEKPSIDDSDECLNVRIYLIQVLFIFLVCFVIFVFFFILFFLGVF*
>N.PFL0960w
MGTLKAIIAPSVLASNISKLAEETQRMESLGAEWIHLDVMDMHFVPNLSFGPPVINNLKKYTKSIFFDVHLMV
EYPEKYVPLLKTSNQLTFHFEALNEDTERCIQLAKEIRDNNLWCGISIKPKTDVQKLVPILDTNLINTVLVMT
VEPGFGGQSFMHDMMGKVSFLRKKYKNLNIQVDGGLNIETTEISASHGANIIVAGTSIFNAEDPKYVIDTMRV
SVQKYLNN*
>N.PFL0965c
MSEEKYNYLDMHLSRKGSIYDSLIHLKPDNENKFGLDENSDICRTLSNYADYSTKKVKYFVNKRFSEIENIEK
NIEDIDYSAFKYFRTLPPKYSDENDIRNNIIFNNNNIYIYAENKNGTLNEQACISISPDNNIIYCSTKGRRYT
IGGHAGIQKNLNNEYNFLDENNFISPQNSIKTYTYDALDSSDLSSSFPKMNLIKTNDVLDDTNTNRINTANIF
NEHIIMNSCESSNLNTTKCLSKNLSVQKLFKYYKKCSQDLEHEQRICKPCAHVYNGSTCMNGDECSFCHHPDH
VLISAKKWKKLVKNNMEKLNILLHILRNPDDVNANFLNEMIKQNTKNFKKNKKINNIKNNTIINMYNQNIRHS
NSNNNTINDMNNNNINKRHNKNRIFYFPQRNTDNILRPPYNNYESNETTDMYKNNYQVRNCNFSPYHMNM*
>N.PFL0970w
MDLLKGYNDYNESDIEREDESKEISSNKNDDTEYIENEEKNDKDQNNYDLINIRPINTLNCAPDINTYDLEIK
CYKEKFQNIEKKIMFDNPEFHNILNRPQQGPSINEHYNFLKNENKNHYNGSIETTFVNKNIFDYQYNQFNVTG
IAENPALKNYYKNNYANYLVAKNINERKNMHEDLNFKKHKKKRSKNNDDNMLDKYKGPWEEKETNKKEQNEEK
QDLLNDNKKVKTDNDDNNDKKINKYILTKLEACAYEEAIKNNVIKEKNTLYNDKIISTLHLNEELDDDDNNMG
FYNKSWFQLPAEYKERDFMIEENFPPKKEIHTYKGHKMGVQKIRFFPKYGNYLLSASLDNTLKLWSVYKSKSC
IRTYKGHFKGVKDVLFDNDGSSFLSCSYDNNVIYWDTEYGKIKGIYNQKKTPYCLCLNPDDTNTFLVGGANNK
ICHIDFRTGNIELEYNEHLQAINTITLCENNKKLISTSDDKKIFIWEYGLPVVVKYISDASMFSITSVSVHPS
NNFFLCQSMNNVITVYEATGKFRLFSKKTFKGHHNIGYSINVSCSNDGKYVISGDSNGGLFIWNWKKMVNFKN
IKAHKNVCIDCVWHPFKTSMLATASWDGTIKLWE*
>N.PFL0973c
MCTYFITLIFKYILNIYIISKAINFYDLIPHVTI*PYTYLCIEMKENKKY*ILHLHFNNL*KRKKLNK*IYTH
IYIHGITMKDYFFSNNEYCN*EYN*KKKKKLEKVKIRKKNFKVELIEFNIYNTL*LIITNNELNNKYIKNKNN
RLHNSKQCNFYIK*YFFIIFL*YSKIIIYYKFVIKR*KGVYYNFNTNEEIYNYRSFIKIIIVVIKLKRKKHH*
IKKKR*KSILDKIIYIYIYMYSLNNYIKDIFIYIEETNIYLSMFHLNYIFQFSNTNQYSYIERYEFYNSFQMF
FFFFFFFFFYYIIYVFKYTNKDIGVFVNKNNYMNLYETLTKYHKQNFNIIENNKYGKKKKKKKKGNSTFYM**
HNIVLHLMKLIFKIWRII*NESIKMKLVKIKIKIKIL*IIIMCIFFN*YI**P*NMVS***TSER*RKK*SKK
KKKSANLKGKTYIKYNII*IMTTVLKGCITIRKEI*I*YEITNLLINIFIFPLIDFQIMI*ILII*NFKY*LY
*YYSKDISFISIYKYYITLYCIIFYLILLYIYGYINFFFFSSYET**KFLSKKIITI*TIPL*RLT*IMIRRT
THYYLINSHIIII*LLKGWSLCIRRKNIFKRNFYKKIIKNITK*ISIILYIIMDILKNMKK*NI*KEIVFTSF
CYY*NFEVLCYYVFN*D*
>N.PFL0975w
MDDSSSLNRKLYFLNLDKEIGAGGSETNSDDDDNNKLINYFNIKKTNDLYLKSNKKNNKQCILKDLHFNGDYL
IKKKQTKKIQKKNGHFVLPNKYDYILKKKSDIFHTDEFISKSYHLNNVHDIYYSYKEESDLCDILIKRYKENL
YVTTFNNMLILINPTDNVMAFYKLLFKPNNKNNYIINTFVRFFLEKKLQETTVEYQEVNDSEDLTNSSLDYEK

EENKSDYSSNEIKNAINYINKKHNITINNQFNFNQIKKKRKKKKLVDIKKDLCIKSNQEFVQMNTNVLKKNVI
NFNMLYNINAFFNEKEENYEENKSNHKENIYSSHVEMNNYNTHVNVYPNSFEKATSETSYKLKTYNEQNKLLF
IYGENYSNQNVINKYIFKHIIKKYQEQEDIHTSIKHKKKKKKNLYDLYKRIMNILKLFYNEDCYNFIIHFSDN
QEVVSFNIIPFMLNNTCNVTNICTKMSDVFCELLNNANMHNKDNDNVEKNEKNDQKMKDVSNRKNNIRSNDNI
KTKNSREKKNEHSNNSNEFLYNDKHNNINNSDEYNNENNFINIPYIFFFLLQSIMLNSNHTYVKPLNLHTLDI
KKIYLNYIESHEIELNESDMETTFSNFSQLGFSLDEIISINKIIFSIIFINIYIKIKQCLKMNEKETLKILQI
QLIEISDFMKLRKKKINKNNSKYGSLNNGKKCSLPQFINFNEDNYLSSGFSSLSETENIHLFNNDMTKENNKK
KNSSIDDFQGARLKTNDSSNVNVNGKKLDDNPYDSQKDVNKMENEYRKSENEVNHLGEVNKQIVSNNIKNDNN
NNNNNNNNNNNNSNEYYYTNEPINMYKKDNKKYKEPIDKKGNKYFIVFAGHILEEYISSLLNIDINLFIHILN
NNIKLKSLCSNMFYRLKIKIVKKINEYLKNVVRNELIHHSLYLYCNGGIIQNILEKNEDVNKYYKNKEKNHFN
ELINNIYNELLHSYYLKMSMFNIDSCIMHMITTLDNKQNNYEDALLCTLHNYGKKGNLYNNNINYKNDTDQII
PNFLCNNIYLGENRILHMLEKYSTKFSSVMDLHYNLYNKEKDSPSYILIKSEYYKILSLFYLAIKNITELYNK
YIINNNNKNNNSDNINNIICKCKYICNCKYKYNYSLDKQLYHIDDEKCMHDILLKYFQYLSNDSITKKKNIHG
KRKRRRKYMKYNFCITHYNDKKIEYTCNNMIFDNIKDIYIFKDIIDIIENSDMLFLRSMFFENNTLPLKSVKS
DFFLYNEMNFFINIINNTFDKFYFLVINESEKKQKDIFIPKYITNCIKHNESTTYDHYASTTSQSNLYKRFSL
TLNKSFVHNNNIKSFKSLFLHMDSVRTDNITNFSDFSKKKINKDHITFVINMFNLKNIFNYQQQYLYYCIPYI
CFIKKYILFCAYVSKEKDLIKLLNYYEEKYYKPNNSYNTHGKDTKNIIYNSSNMKDNLSNNDTNHNILNQSIH
IYKEKMYNNQNNNKNKNKNKNKNKNKNKKNIYNDTTNFIYLNKLSIEEMKDMCTLILYNFNISTNIIHFQNYL
FIKKSLYFLLENMKNEYLKYIIPFIRKIENSYISYKNKKIKFSYRNKIIVIQNYMRKYLFFHELQKKERKKNL
LTSFIIFYSYLLKHKNMEETKETLEYYKETFMSKEKKLIQHAACVYIQSWFRKIIQQRKYKAMKLEILKTHAR
EKISTAIKTYITQIKMIKNLNEVLIPNRCATLIQSCFRRYRCMVQYEKKMYLKICFLSIKRKYMAYTNVITKM
NYHYLIKNIYRKNFIKNHLKIPEAVVKIQSKYKAYVVRKQYNMLRDAIYFTQSYIHTCIERIKYEQLKKSVIT
IQVWWRNFYKLRKIFQNFPLNIYNSEYEKTKYYFLHKKNFPNYYYYILKEQKAMKLLTYNILLKQWYFFYFKK
FQKKNHLFNIIFNLKLYKNISYYYLLPWAYKINAILKMIHMKQMFKICTTNYYQNTILNIPLFETIQIFVGKT
HTILLINQSVNKDNNNNTEQIVSQGYNKNSKQFYTKYVYSIGSNDCGQLGYYDLSSSPFLYHSNVLNDKWDHV
DINEDENNKLNPINEKIEHPVKKDIYINTMDDHTKIDNLFNDHNDNSDNDNRKTTLGSLHKESMDNNSTSIFS
THKSSVDNNIINNAYNNNNKLYNNKHFSDQYNNIHISHKEEITNNKTKFKNVNDNYLRNLQHLEFQYKKKYTI
SKKNFGDMFNENVNDIIDYKIIDKEDDKYSKNITNNNKYEYDFNIDKSHKGKKIVEFTKLINETNKILNICCG
SEHTLALSENGNVYSWGNNAYGQCGQKYEKHIIRYPKIIKYFLKNNILIKNISCASYHSGYITKSNDLYISGN
FFFINLKYFQNNIYEPIFLISGCLNITCKDSFNISLRTNKNSFYLWGNNYKCILGINKKKLANLDEISIYPLN
NICPNIIVKKIACSDNFVCIITTLATTNNYTMFMWGQFSHIEKKENQENDTKSVLNTNNFFNKFRMKAKMNNT
KNDNQNNDINNNNNNNNNNNNISKNKKNMIYIAKPTPVYNEIWEQNEAVDVCCDLDEIMVLMSNLYLYGFSSV
EILGGDKKKVIANEPKVFFNHFNRKPKVEKKENDIKFEEPEIHENIKVLNPVLYMFKYFKPSYFNVKKIQCSY
NKNSLSIMNVTMGEYEIPKIKQNLEYVTPSGDIVNFPEKIKEAILEKAKKESNKWIKSNDDPYINHFLIQNSS
DSGN*
>N.PFL0980w
MSDVDEFYEYAKNNFLNFLNKNDKNENKKKREESKIKGLSDNNMSDIKNLRKEEMNDKESYDKKYTNYLLKKK
HRKEEKKNEKKKKKKNSSSDSSSSYTKQDEDKDKKKRKYEKIEKRKHEKSKKSKYDKDYKKYKINSDGKYVSY
DKEEQKKKKHSSKENEKDEIKEKKKKRKEKNDFYNDSDNSYDNNNNNNDDVNNDVHNNVNNYHNISDSNTIYT
KSYSSSSANYNYDEKQKKDIQSLCNIQEYRNNKEECDTHCTSNLKKKLEKIINERKNQMILKNSLNKNLVDCK
YCIDSNSFEKLNKLNIISISNKSYICYYNYKNIFLKDQLFISPIEHTTSFTNTTFDVIQDMRNHMKSLIAMLE
VNNQTCIFIEFNNCFNPSIELISMRKTKHTYINCYVIPMDLLEEAKIYFKKNMEEINSLYRENKQLIISDSKY
APYNVIPKNIPYVSVNFSLVDTYIQVIENNYDYINMCRCIFADLFKKDRLYKYFKNFQQYVNSVEEFKKGYEK
YDWTNYM*
>N.PFL0985c
MNKIKMKNNYIERKKYSIHNLVKLINEKKNIENSKKNELSEGTTEKKNVSIENNDVHMNSKKKKKKNRTDKEN
GHIKNEDFINSSSNDENDCTNNEENIVNDDQEELTDDKNKLCYDHEIINNGSSDDNEIKLFMFDYKECQNKKC
SCKKLYRFKKIKKVQMNKKFKGIVLTPFCEKYFSIDDKYTIENYGLSVVDCSWKSIDLLKRIKFSNQRKLPYI
IAVNSINYGKPYKLSCLESLAFCLYVCNYNKQCNDILNIYKWSVNFTNLNKELLDTYKLCNTHDEIKNAEEEF
IQKYLHEKEQNKKIDEYKIIYEDDYI*
>N.PFL0990w
MLLRVFLYFILVKIYQCFHLNYKILSKNKYHQNYVKNIHSVNYIHRFQHIKYSQNKNKLFSFFSKFFNKDTDK
FKLNKEEKEYLTRIGVNDANDYKLLTKAHEEYIDTVTDIKSKLKYKDTFFFITQKLIQKNLYDFKQKQKYNIF
GKDEYTSELSINYIKNSNIEEKFKEHILKKLNDELSNKKYTFLRIFKWKSALDIFNISTLMLPVTAIGFFLSK

MGLFSVVVNMLLTSHFLTFRKDEKRKMKISTLLLTAVPILIHSSIGFVCNGLFWEYFKFNIPNFIKKENVLSL
FINTQLYIASLIYFVNNDENEEEEDDEEYNNDYLDFNDVLNVDENT*
>N.PFL0995c
MINDYHHSNDEMHAIKNMLIEDYRGTIIKFYECIKKDIRIIIESNYNLNLDLLSQYSQNIISEIHEEKNTLRK
KNIDKITRKYLRQIDSYRLCEKDFYHNMGNEENKNEKHLYLNYHKYDSLDLRKILKNNYQQNTYGESEEPFSF
NFYEHGNIKIIDRLKELKSNISFFKFYDLIDFPTRI*
>N.PFL1000c
MNTNNNNDHLNNIYLEFSLLNNFLKELKENIKKSNNEKYTNIYDGCVEYIYSLHNKYTAWNYNTEKIDEKYID
YFVQSIILNIKNFLRNHFCLIDEDIESLKNKNKKLMEKLKSGVENAETQENYIYELEKKLIFEKEKNKCSYNL
HDKINNLLKLNEQLTNKNEQLECSSFKHKQENNKLKIELKKFWDLKEKKKKYEQNKFSYYMLNKKMSTLLKKI
NNHIPSHQNNFLNEKNRSYSYNFVHTLKKYIILRDSPYLNEFNINTYTNNYKKSINNNINDNHKMYNSYERYD
NNGNVKYGNKKYNGNEKYDNNDNNKMYYGNEKYNDDNKKYDADNNKTYDDDNNNKTYDDDDNNKTYDDDNDNN
KKCDGDDNNKKYDGDDNNKKYDDDDDDNNKKYDDDDDDNNKKYDDDDDDNNNKYDNDNDNNKKYDHDDEYFYY
NNKNYNGDNNKYCCDNKDTNLNHINSSSNPHNTKYICYESSEYIKKDDIYQGYNDTLNCHNKNINEKHTQYVN
PENSSSSNNSKDIYNINKRDKLSIHKTKHIQTYETVPSINHCDTYINDGDEEEEEEEEHTNNDHNGKRDKLYS
FTNNLSMYKMYNNSEITCTEESDHLYKNIEYINKKKTFYTNDNNKNSYLLNNIKLKNSNTLNEAYFSRRILYS
TTLLMDIRKTLSKKIK*
>N.PFL1005c
MTGSDEEFEIGDILEIKKKKNGFIYLVKWKGYSDDENTWEPESNLIHLTTFKKKMESLKTNFLSKANETNGDG
KILKNHILAPTQEDDSIKSKGRSSLAPRRKMSRKSLTNKLENKKNLSLSDNSLKKSDEEDNESVKHENHVNDG
NLLNVEDVYSVRIKNKKLEFLASLKNESPQWVEETNIRRTGHLNIKVNDFKRYVRRKKSSRGNRIVIKNLHNV
GDELYISVIHNINNKEIHSLYPSKVIEYIYPQELLNFLLSRLRYRTA*
>N.PFL1010c
MSISPLKYESKILSNLANMLKLSEKSDDEILSEINQGNENKVFEIDDDVKQVVDLNKINCTCEESKKKIEECD
KKKFVYNYNIETYGVDFFLDICNALSRSNYKPYLSEEGKKEIMNGLRVRRNSNLYKYVSFFLSKERMNKERMD
DGKKVENIYHCFKCLKSVLHICIDDEDSNNNNNNNNKMKTALYLKNNNSITEQIENKIENDILNEQNSSLEKV
NHRKGVISVSKYKSELTSITRMSNEIIMQTILEHTKDMGIKKDTTKNIQSRNDNKNDHGDEDDHRTNNINSNM
KNSNNNNNNNIIKKSKTYESVKVRNNYYYNDDHDYDNHIYQTENVIDNNNTEPTKRNIPKRTVVQKKSYVQRK
TNITNKKRISQNKPVTRNSYKKNDRINTTCDVRASKIKDTIYEKKKKKVRRSFEVNANRRKRGAGQKGVFSDE
EGMKMDNDNKIDGNILDNNKIDGNISDNNKIDDKTSDNNKIDDKTSDNNKIDDIISDNNKIDDKTSDNNKIDD
KTLNNNKIDDKTSDNNKIDDIISDHNKIDGNTPNNNKIDDNTSNNNKIDGNTPNNNKIDDNTLKDNITNNILD
DNAVPKEVDNNCENDKNVHNKESCRNDIYSSIQISNTYNNTMINSTFVEEKNIISNIMDNTNIYPYNLCNVNN
MEDPNLSNNPEKEYIKENHLCDLPNDIITSNGEDIKNKEIYKMDHPNKALLSNNTNEKDYDYNESDNASYSTD
DSKYVKYLKKKKKLQEQGKIIIDLNLFNGNSVKTTNKRDKEEKLQEKLRMKQIKKLLDEKQMVICKIEDIAKK
RNKKYVKAQVLSKDSKIERAYFSHKMKKFYNSKSSYFGCGWSSNKKEWTPFIHAPFFENQHNAIYKNRNKKLY
EEIYDTLLHGRIHPDIKVVELKDHKHPIRLCTPYNEDCYSVVYTGKKINATDDRVIFGEYTGYVANNKELSQE
KHQYMFALTFNKKVFNDRKHVVFINEVELDEEQNYSSHVVSNVMNKVKAHEKNNINNTNIYNSPHILNGNNNN
NINSNTTEETMSNRKIDKIKPVSNVIIPDYNNLNSYINKCKTNEHNKKSRDINNLIILPDNYTYAVDSSYMFN
EMSLVNHYKTCSVFNNFDFRINSEWQLVYLDGWPHIILTSIPGVEINPGEEIFADFGFEWFEKVNDICLNEFI
KNNYFYRLYKLNTSREILFNGMEDIVEKYNLLKNHITCNICMHNVNTDGNNFILCSGCNHVYHLKCVHKFNTE
VNENYEWFCSSCLQFCFNIIKQKEFVDYIEKENQKRFIQLLDDLNGDNLDTVNNEKKSDALDKLNNYNCVGNI
NNSNSIDNIDNRDGLDNPNNSNISYGVKNSNISYDANNSNISYDANNSNISYGVNNSNISYGADNSNISHSFN
NSNISHSFNNSNISHSANNSNSEDKTNNCNIEDKTNNCNIEDKTNNCNNLDKEDDINDMNKINKSNQIIIKDV
NDNNLFPVENNDKQKYLEVLENIRKLLQCKENIDDLFKNKEYVNSFLEKIDDEMNMFENKRSEDESVKLDDLK
HIFENSFELHLLIDYKKKLEDLLESTEKVDSFLNDKNVLNTMKIRKRTLSFLMENKKYIDNFIQMNEEKDYAK
INECEKKKEEDIIHNTTCRKVEKLEDRIIQNSHNNQLANPEYCSTLKGEEDKSVSLIDNKSMCERVDNHSICN
EVNSSISSTYEKTLYNKSNNIIRNLKEMKKYERECQNNNSYSNMYCSYMMKLSKKILGFPLIKDFSKGLSTLE
PSLPLNDHLKKLSVCSNCYSKHHDLAKAIICRVTKMHFEANYNDYLTDEDLFKTSSEFIQSVIRELANTVKEY
RKKELNRVYLQHATENDSMILDRVNKDMSLELNYNNVTPTRTSIHNCSCTKTNKEYNESFYKIVSPSFNNDTV
EKSNIIIQENNHIRNNEILQLGEEEVGNYECQNKEVIGSDTKEHTNERINDQTSEEKCHDDFYSKDMHILYKN
TFLPREEIDNCSIEKINDVEEKKMTHQHEENDILKDVPNDISNDVSNDVPNDMSNDVPNGMSNDVPNDISNDV
PNDISNDLSNDVSNDISNDVSNDVPNDVSNDVPNDVPNDVPNDVPNDVPNDTSITNLYEQKNDLNSYEKSCKL
NNFIPLIGVELGKTKFQREFTNGTFVGTVTEQIKDENNNNFFVVTYEDGDVEWITPSFLFQEILKQSTNNTTY

PLASTFKDIFYEDFKKDIKLNNHSYELKIEKKKRKSMFEYVPNNNVTKRQRHAFEENVLKKKNTHQEVNTSRR
SLSKLKNVVFNDSLKKTRKRTMSTS*
>N.PFL1015w.2
MKDILLLVILASFLKTFICYSPKNVITVSPPRNNISPPSIVITLNKPNIKDNYDINEKKKNLEEQNKIEDIIS
TVQHLIYNIDDYKSEMSDQYTKESLGLNTNIEILNANDENVLNLLLNSKDDTLINEKKKKKTAPIIITYPQPL
SEDEIIYYEKYKHLMENKKKTRNLLLKVAHLEKLSLIKSINKKESIKELEDTINIIEKSISKLHIIEEMLEEL
SYEPTQNEDNNTKKLKTEREYLTEKINVLNKKVEKLKLMNY*
>N.PFL1020w
MPLNVITPSEPKDPSLIYKYKKKEEYSGELEGIIVLCFLAIFFAVIFKVIEVIFVVIFILLSIYLTARNGEMN
LLSVLPLLMMLMTTSAMTWFQQKNMTKRQSNNN*
>N.PFL1025c
MACINEGNSITPRVKFKTIRKINDQKYMSEQAYLVPENDGTAVYISYELNKTLERIFQRYSTNGNMNVNEFVK
MCYDYNLLPKYKYKDILCYIFRLSKSLKVGYIEYKHFFQVLERLSAFLFRKLIMTEQEKFNVLIKHLTCQKIV
HEKATKERYSVGIQTHVKKECKGTNAVCDMRDKCCGTFVEVKDRGTMTEMDDVNYYDKKEDIKKNIDILKDNI
IYNDHNDDLNNTLNDGDNHNANILKSHASSKQYTNKMNSINSDDITNKLKEEISNALKIIDDKNMEIENIYKK
NLEQIEIEREKINQLQEEIKKLQNEKNDLIKKQENKKINDKDIKNNDELNTNKLEQKESIVLRLKRLLILSGE
QEAELIRIFSIYSIYCGGIAEYVMNKRLCANFLTTFYLLKKNGKNKNKMALDVKDAERLFNEVSYKRESLEKV
DIDEEGLTYFYFKLFLNNIGKFFYPELTERESFLEIVLNYVLNLNKNGGVKKWKDMNEQIKKKNKMRITYDSN
DLHTFDEKMVGGYTSSEKSIKHHNLEKIKTLIKNNSHMKSEQKILNDNLTTDLSFEYLESNSAKKKEKNKING
KSINARRIINVEKNTNMEKNIYVDKRKKMKKKKKFINIPNYLNEYEIYDKQNIYEVNNELIDSLKSIPEDKYH
YFKTLKPTRTKFSTFKKDSNESLYDILPLYDIQGKVWPSKMNPKKKSIPQFGME*
>N.PFL1030w
MSYQDVQEKKNEDINYDEIASSMLEKMDKHINGEKQPTIHYENLNVQEQGGAHAYYSNQDINNINSSMSFLKN
VKELNSSNILDHRTTTNDVGYPRRVMTALGPLPLPEEFKKNIPEKFVAKPIIEEREIYVSKKERKQREIEIPH
VKYEHTFENVKKQLKVNKLVPNVTQVIKEVPKEILKPVIEEKIIEVPQGVKYVEVPVEVPCLYPPKIMPKVVT
QYVERIVETIKPVVQEKIIEVPQTVIKQVPKIKTVEVPYYVPRYVEKIIEVPFKPNGEMPKIATHLPISISDS
LPPLKPTHILSAQNEKNITKHNNNMNSNNMNNMNPFQTTNFMMPLMNCFQQNPDPNTLKNSENGIPNMNMSPT
ILRPINSRDTPNNNMNPQEHIKNNITPYPNNSLNSNFTNYNMPQNLYSQIENLPHAHNLPDGFQWQYPDGVSN
NPLCNTRNMPSLVVTCPPQIGRVTCTRKDIQPDILTKTGVYEFDGFKKKGTSQSLFPPLAHHNRVPFLPAPAA
PGTPDIENIEKNM*
>N.PFL1035w
MIWNNIKNFIQSKNDIILYRTKPLYDMNIRYMKNKRRGLKEQPRKKPLDIEKKIRNPYKYEEGMHFDNLSKGE
LYLPESCKGKKLAMLCNRLYYFNINDEELLQRYAERASVIANSMSTKEMSLILNTMRKFNYRNEKLLETFSKH
IPSKLHKGVPQDISLILNAYSHFNYKDKNLINRICEEIPHKIPHFQPSHISSVISAFYKLQIRDQIIIDDLID
EIIERIDEFDPKSLTNIINSFSKLNYKNDNKYILWKKCIEAVKKLDKEFNFLEIVLITNALCKEHIIKIKNKD
NEYLHIDKYNNNNNNNNNNNNNNNNICTHLCEVIKYKIYEEKCLNVHTNAFLLCTVAHSLSKVKYYEKDLFHFI
IEYFSHENNYISLDNQHFSQLIYSSYIFNIDKPEFINMFIKVISNRIETKQLNEQALSTICYSFAKLKIRNIS
FFILLSSYIIKQKIQLSTQSLSLICYSYSKLLIKSEMLFYILSVQIFQNMNMFTKQGLSIILSSYANLKIFNV
KMFSLINKYMNLYLPNFTKNECLLICSHYDHALKKLNDPVDTNNDDTTVLVNKTTKTKKELNDFVKLLKEKIS
KIEDDEKIKNYNNKNKIVNNEEEHADHFDDENIFSIFKQNDILNDQYEDDNNNNNNNTIVNIPNPNYTLNTSD
GFIINEDIKKKEDTLKLYQKIFLNHKNVNSNILQNVSLNNEIFSQQLSSLIDKQKETNEEMNNYQAFQNNPKD
EINEKTESTSNISDVSTLSNISHTSDISNNTQLFDHLKPSSNQIYNNNHKTKSLIDLMTSNKPAILNSEKENL
IKSAEQMETEFIRDYVNQQKDSHHNSTIGRNHEKRKKKINKIKNLLSKNHQTVNDLNNLKKKWNEIYKK*
>N.PFL1040w
MYSKRFSKNTYYLPYKSKCLSNVNGSLLKEPYNLHYFLLGTNNPSCENEIHLIEYNDQFLSVRNIETYHYEGE
SEHLICLDMYENEDQKKKIILCTAGYEKCNEQKMINNDMNYNECNEYDYRNDVCSLWMGDIDNLDDNNNDDNN
NNNNDDNNNNNYDDTNNNNDNDNNNNNNDNNNNNNNHNHNNHNNHNNHNNNIDKQTNYKNQKNHSKKKKKKLT
KVLELNKGNNENFKYIKKTVVNDFNKEYNKICIIDKNSYSIFNRTKNDVNFMVSKNVNYELVDGIFDPHHENI
LTVMSNIKIYGYDIRSNMNIFSTYTNHKADLSSIDFNSNIPNVLLTSSNDGYIKLWDLRFLKDSFFTTNIHSH
WITSIHFNHFHDELLLSTSTDHLLKLHKISFPTSNFQNDNVNYELVKTYTDHEDSVYKGCWSKTDAWVFSSLS
YDGKCVVHGVPTDQKYKILL*
>N.PFL1045w
MFVRIKKLILSFSIYLIICSCVMNNKWFLFCRRIILQPNFNLCFLSYNTYRIPDIYNNYYNVIKKEKKKKRKN
FSFFSKGCYNNCSKAQKLYGENEETTYGYTKYDKKDETYINILERKADEIKNSKNCFNNKIKTNKNEYAHKLQ

PLDYTTLHLLSIELNTLLSDSIVEHITQADDKTIVLHLTKRDKEYYLYICYDNANPVISLGLKIKKLFNRFIE
DDYAEKLNPVIKYSIISNVYMKKRFVKILCIDLILKRKSQEDLDIEDILNNQYSTRKVTLLFDLHNNSCISFL
INKSTNEILISPHNYVTEKTIDKSYKEGHKYIFPQNNECKLIPDHYEFFSTFLNLFKSRKEQNFISSLMDIYE
GVSYNLLLKFYDYLNIPTHIKFEEMDKGTLLDFFNKAYIKWLRFLNLKEKTDELIFYPHYDYKLNIPSVLNFV
KSKKKKNYTIISNIPQGQFNSVKEGHQDDKNEDDDDNDSNIIKRDNNIIKRDNNNIIHINRSDNNINKSDNNN
NNIIHINRSDYYNSSDYTNIHDQEEEYNKSNHNITKNNIDTNQIIKDTLKENDKKNIKLSDSDNKHIHFETTI
ELVYYYYCKYFSVNEFYTTLKYCKDFLKKKLPQYEDMLLQYKNDREICEKAYLLHENINNLTVFNHTISKMND
WLDKKTYDALKIIENELRYNSLEDNRKKFVKKQEEDKKKEETLMKKIINTKPNTIRTPQNIYKGSLLIKINET
DLSSPFLIIGKNSKQNEKISTQILMFNDLWFHVHEYPGGHVILRNKKIKDKIITADTNLSDITIDEDIKYAAN
MAAYFSKARKMEKTLVCFTLGKYVLKDNTLNEGAVEVLKYKLIYGRPNKVASIIKDLNERNKEIEICKKKKKK
KKK*
>N.PFL1050w
MIFGFLLLNVLLCVLMKNYLCLLKNKRNVFYTIHNLSNEKKKKTHGIFHRRNNGNAIIKLFIINNTSEKKKNY
KKKNKEDFILNNKKNRKDKANVLYATQSNFFNPKQFKDLLNQDTKDKTKNTDDDDDCLDLENVENLEEIAPEL
HMKLMEYKKSEESKDKEINEKKIEDVKDITLYEGLPLLMIEGEKLEGFADTRKVKIKPEISTPFTNDDYNEYI
KSSIDEKMGNSDVDETKKNEKYDKNIKKENCSNTKEELNKFEKDIQYILNVSNEEKQKKKEMDKNEEVKKSDE
LTKEGKIERYKKVGRHYYMDSSIGKILFDRNYPGYAYTNHGENIDEMNEKSEYDSSVPSYLNPSNVHLRTGRK
REEQKKKDSGIKQKKRWNVNNTTNDIKRGNWKYYDDTVKDLIDSIDQEKLEENYYSNNYYQRSHNNTGYGGLE
KVQNNYFDVQFIGSAETYPANISVGMNFIWPINFVPLLSKQYTKRLGQPIKSPIFNLGGFDHLQLWYYPDGMN
NSMDGYCSLKLMMKPGSFLPVKIFLFAFSEYNYINTKAFYKESADYVISSSNLCKCLLKTKENLKNIENNKDY
VILGPAGNIYIGVGIYDDNYDFEEDFKKFYSINNKYKNKKNKNQEFKYKYDWDEGVHIFDNWLKKQSAVTDDK
DELLPSYYTHSELYENYKYKYVPEKNPFKFLRSKNTKQTWNRHPF*
>N.PFL1055c
MAQESTDIILSVIIDFEIYIIIFILLVYFATKKIHKIIDSNFYNDDKLFKRLSKHFYIAEVSAYFSPFRYIFQ
SAVYHSYYFLTIFILIYIFRLYSSYKKWMRQYFLSYFNFSNLNTPDINEPENIREYDGLHVTFIISFVLGNFA
SFYFYKYLCNIFKRDSRRYIDMKRTNVLKYCVELAKEAYNEFLKNQLQKKKKNSKNSKNKKNKKIPKFKFDLN
ANPETLIINSDINTYEPSQNNVGPEIKLKKKNNILFKMLNIVYINTHVLHGENTLYEDRPLKKENDSKFDSKK
LLLKLLKFLWNCIMALRKIQTTKDEVKVVDGTKEDLPNKMVDKENGTTDKENGTTDKENGMTDKESGTTDKEN
GTKDKENGTTDKDNITTDKEYEINDVKEEMSESINNTNNMNNIDNIDNLNNSYNTNSTTNEEHISPSININDI
KINNNDETDEAKYTPQGSTISNISINGNEKYADKVTDKNNILMSSLNAEELKKNQNNIINTNNNDNNNNNNNI
NNNNVGNLKNDVVNTENTTAESEYTDINLCDIKSGHHDVYVCNSHYQFKSIIEILMLNLPIYFFFKNKLYVKT
SFTIMLYILNFIIWNIITLLSMIKPSLLVYYYIINEHISQILTENINEFEKKIMLHFFYGFFCSSLIYLKYYF
KIEFKLIKIEHMNNIDRMINNSLKEAHKLKLRKMIKNYDKLKIFPLLLDYQKNCIYVEKTLLKNNKEIEELIK
LRKLKNEQLCLENINQIAKYCDKETANELLKKIKNILLNELPERLTNNLTQKLTDEIYDSMKKEHLSSEDKEN
NNSTSSSSFSPLKKGLIQRFIHGRKVNKNDEDLNNNSKY*
>N.PFL1060c
MKIFFFLLLLVFYLCGYCLSRMFRFNFIKNNTPRIKKQCQHNTTSNIKREVEKLFCSRNNNKECEVNTFYGIK
EKGFRTNRAPPYLLNFKNCRDILAYTNDIDKDCLLQIQNLARLDIIKGHITILPDVHLGKGIIIGSVFLTKDF
IIPNGVGVDIGCGVLCIKINNLKKKDLNEKIINNIYNKIKRNIPLSFDMHDQGVFDSKNIFSNLLNTYGSPNI
KGIVQPKHFKQLGTLGGGNHFIEIVYDASDDLNKTQKEELSIKDETNNIYVHNNVHDNMFNENDISQKNCAIN
TSLLNAKGNRNHISNNNSVDKKNEVLTEYNYSNVYKDISLNNKNDDLSDVYILIHSGSRNIGKSVAEFYDILA
SEESKMKRNDLAYLDLKKDNGKNYLKDMKVCLEYAKYNRIYMMKIIEKIIYEETKCTLNWDQSINIHHNFCNY
EKVMYLDNNEIKKEYMYVTRKGATSSKKNQYGIIPGNMKTGSYIVKGKGNVLSYNSCSHGCGRVLSRTKAKQF
IKQQEFIDIMKGVKCDTNNKIRDEAPQAYKNLNQVLKNQESLIYLIKKLNPIINIKGF*
>N.PFL1065c
MERNLLNKDFSVKKKYQSYNYNIYKNNNYEIAPKKLYHLLIRLGITFLTIFIVSIFIKHNKFNEKNNLFLMVT
SPDVLELFNCLGFPSNSKEIVYGKLENWGFNLSDIGDIEINKNYKDEDLSTYKYSINTYKNILLSSFEINKVN
SLIDKNNLLYMYLEILKKKEIRTFYEFLNTFMILQDLNCLPVNYKGIYINGNLFIPKNEYDIFKSDINIIKEK
NAKTLIEEEPYLYLTYHGGKKKNAIHNICKFSRDGYFLGSVLLPFEENGIFFNSISLRGLLLYQNKLYIADSF
KDNSKILMFSDSISNLSNRREYLSTFITQNYETNPLMIHPYGIKKYNDYFYVSSQNTGTVLRFNVENGQLGEP
IDLYKNTSQGLVIKFNNNDEIRGLDFDSTGKCFVSNKQVGVQIYDTNFKLIKILPVFSPISILFNSTNNHILV
GSSTTHDIKEYDVNNFELIKVIKHPLLKHVAGINIYEDSLFVVSQKKNKLLEFSLSSSLLKSITIDGFSDIGE
RVILSPT*
>N.PFL1070c

MKLNNIYSFFFLFFVLCVIQENVRRVLCDSSVEGDKGPSDDVSDSSGEKKEVKRDRDTLEEIEEGEKPTESME
SHQYQTEVTRLMDIIVNSLYTQKEVFLRELISNAADALEKIRFLSLSDESVLGEEKKLEIRISANKEKNILSI
TDTGIGMTKVDLINNLGTIAKSGTSNFLEAISKSGGDMSLIGQFGVGFYSAFLVADKVIVYTKNNDDEQYIWE
STADAKFTIYKDPRGATLKRGTRISLHLKEDATNLLNDKKLMDLISKYSQFIQFPIYLLHENVYTEEVLADIA
KDMVNDPNYDSVKVEETDDPNKKTRTVEKKVKKWTLMNEQRPIWLRSPKELKDEDYKQFFSVLSGYNDQPLYH
IHFFAEGEIEFKCLIYIPSKAPSMNDQLYSKQNSLKLYVRRVLVADEFVEFLPRYMSFVKGVVDSDDLPLNVS
REQLQQNKILKAVSKRIVRKILDTFHKLYKEGKKNKETLRSELENETDEEKKKEITKKLSEPSTYKLIYKEYR
KFLKSGCYEDDINRNKIAKLLLFKTMQYPKSISLDTYIEHMKPDQKFIYYASGDSYEYLAKIPQLQIFKKKNI
DVLFLTESVDESCIQRVQEYEGKKFKSIQKGEISFELTEEEKKKEQQMQKMYKALIDVISDTLKNQIFKVEIS
RRLVDAPCAVVSTEWGLSGQMEKLMKMNVSNSDQIKAMSGQKILEINPNHPIMIDLLKRSVTNPKDLELTNSI
KIMYQSAKLASGFDLEDTADLAQIVYDHINQKLGVDNNLKIDDLDPSIFETKKIEDENDSSKFEEEINIDDEI
QKKDNNVDNESNDKSDEL*
>N.PFL1075w
MNSPFNCNQININYSFSFNNDNTINEGTKSPCKNSFTPDGNLSYDKECVEKKSLIIKDKEEKNIYMYNNMYNV
LHNNNNEDNSCNYMCYNINKMNQNDHTSMNNKLFNDKNIVPSKENIYTMCDLKSEQEEVKNKNESVIYKNDSI
IYKNEMMINKNENVINENDMMIKQNDKLINKNERMINQNDMIINQNSIMMNQNDKMINQNGMMINQNGLMINQ
NDNMSKDEMCTYNKNDMFKKIPSQNYDTIKRESNQKFNSIEHIHNENIDNIKINEKNPKEYDNKYNHYNIHCD
NNDHMNNIDYTYYNNNITNNNSNINLNKSKEELTNNISSCDVNKKNLNQKSAYDRNMYNDNISNNNTLVHKMV
TNNPPKLSAQTYSNINELGNNKMIYHSKENLEQIKNHSNVNINSNNNNNNINSNNNINSNNNNNNNNNNNNNN
NNNNNSNNNINSNSNNNNNNNHNNNNNHNNNHNNHNNNHNNNHNNHNHNNHNNNNHNNQSCNNASDNTTLLMN
RQIKNTNQTCRQIEELKKNVEKNNNIIYNNIINNNIINNNIVLDSILNNNIMNNNIINKSFINASNNDINKNM
VDKIINQCLLNSTKKTCSTYKKDNMVNILHTNKVKNNDNKKLYTNMNNVNMDNMVTNYNMNDQNVHDMKHKVL
MVKERHEENYSHVINDTNNINYNNKINNYNNITPKGHVDNKDTYNLNSRSNSCYTRNCVNCNILINNHIDVSN
VDNISPHSPMYNPMTNLSRDDKLSLNNVNHLQEINNNEDNSIYFNNYGNYKEENISNVQNVQNVQNVQNVQNV
QNVKNVQNVQNVQNVQNLQNVQNLQNVANAKSVNSLNGDNNFVADSITHHQNNNIQNITYSECKFLDKNIYED
NKIHKEKEYSFYNNSYETKEHMQNYKNKIITNNIKSDDISPQFNNMCNLNYTPQNYDEHVVYPKDDKNKSFGN
IQIVHQQNNNHNIHMENNTDEKMFNKNAHRIKEDRGKTYYHCEYNISEAVPTHNYMNNLLINNYNGEKSNNSE
EDYISNIEIYNSGTYKKVNMNVNNMESTNHCGHDFYSNQKVYGIQREEESIIPNSYNRDMYEGCMVNGDECKN
EQVENNCDIIHNNNNNNNIDVSMYTNMSRYHKSNNDVSNMHITKDIIHSNNYSYTILNNTENDNKTITYGNYC
TAIFDPDNNTKEIYNENTKSIYDYLNTVNYKLVNEKHVQTIENQRIVHTNDEYDNNNNNNNNNNNNDNNNNNN
DYSDNDKMNTFFNDHHPMHNTLSHMNNETNYNNNIIIKDIYNTSQNTEKQFLKEQKFDDSCVNSNRFCNPNDS
SSHCILNDTCIENNGKKNKTQEIYCNNNKIHNIEDRENINNFNEVFFFLKLWLSSFNIGKSIHCKKLIYISGD
KRYIRKRNNKNKTYPFNYNEPFYFIYKPNKDMSMDHHHHNNINNNNNNNIRLNNMNGNVKNSELKNNYDFVKY
SSNLKDGDHSNMKHSQDIQNIVNTSKSVEYKNDNNCSNQSIINNNYHTIDNNMNVYKNVIHEGIEEPNYNRIV
ENESINNINMNYNRYNSIQNTYNNDENRELHINNSDDKKEGNISHIPSTLNKDSFCSFTTVSSTPVYEINKNL
NQLESYINDLYTNGNFENDNNNMGPKLSNNLCNNNITINTNNVNSNLEGNKKNMKTKNYMDTFTKITLTNESN
KNIRNNKKDKISEQMISPFIKDDIIKHKQYCINNENYNNLQMDNNKQSLQVAPHMSKENDLNDTNIFNNKNKK
NNKKKNNNNKNDQLGNHNNFSSSYTINNMNDNKKEKNIKNNENKINPVVNNIRDNVVTNISNYNDKTINNDHN
NNNNNIYNEENLDYNNMKSLCYNEELFDDDKNVPKITGVSYDRKQKIWVSHWRANCKTIHKYFSVKKYGFEKA
RELAIKCREENVNYSTTNSIINNNFTKPAYPLLNNILINNNINTSDLRDFNSSDKLNDQNHYSNSYDLYEPPK
KKKRNNIDEVRKNDNQEKKKKKRKINHNISDNMNYYLNDTNYNDSQLSQHIYHVNHNLNDIYHNDKYISTIEN
KQKRENISSNTYKDKYENFESYEKHQNYKNDKEKINNQYVLPKNNTDNFSEKNIEHIYLNTQHNYINTNQVPL
IPYEQENNNNEETQKKRKKKKQIPQMLEINKNTKNEIQNFTEKNNNQINKQMYTELLSAQLNNSIKYINTLNN
PSLNSNNYMNSTYNHMSNKKDQQPQMKDIMNYTKEKFNNNNYDNNDEYKKSHPKNYQNIQNDNNVYNPCINNK
QICSSNNNNHNNNNNHNNNNNLNNNNHNNSNNNNNNKGTYLREQHNSDNHLHCNHNYYNTIDNKNDSSYQNF
DQNKDTFNNSNNYKLSDYFKINDSSTASVATSDNHYNTMHKNELTENHLDNMDNNNNNNNNNNYYYYYYINNY
NRSMQGQNNNNITRNMNNSNNTYNEINHVYNNILSNCSYNNFHKNQNKIMQNNKDINNNNNNITTKHNYHFEK
NNKNFIVPIQNNQHFTNPEIPIKSVHLNNNQYVNNNHPTKNYQKESHLFGNSNFHMNH*
>N.PFL1080c
MNVLGLISGGKDSIQNLCYCHKNGHTIIALAHLIPYEYQNETDSFMYQSAGFELVSSIARCMEKPLIQHEIKR
KAIKLDLAYTYDPNDEVEDLFELIHKAKTQFPSINAVSCGAIKSTYQKKRLEHVCERLNLDILTYLWDRDEKE
ILQGMINDGVEAILVKIASYGLKKEHVGKSIKEMYEYLKMINEKYGLNICGEGGEYETATLDCPLFKHKIIIE
DYEIIQHTDDLVSPVFLFKPLKWKLEKK*
>N.PFL1085w

MTAKILNPFDIPVYPSSKACRYEFNFPTRKESTFDYIPGKSIETNCSKENMVQPKHLNLLKMLKKYMYEIIEE
SCFDIKSFNNISNGKRACNEVFYNLEDTIINDSRMNIKNKILQSNENVSNSCMDKKAICKKLDMLLNHRIEGN
YCQDDVSNNASLQLMQKGKEDKGNERIYCCTTKDVILHAHKPTMNYMNSYKLSSDYISKLHLFIVKKEKVLFF
PILYAEMIIRYNMKFFPLSKLYNIKNTASLMKCKRVFSYVIILHFLMNSICVTHSDDIHSSLYKNNDVMYFYV
IKGKEVGLYEITKFPMNHISYDMNIFNDSKTYIGNMNYILEKNIEEHLFDNMYFILSSISYKNVFHMNIMHIH
MKCYSNQTCMDLNKCFNFVKDIYISRKQNMCIIIGTIKIDHMFCTCSCGSTANSNMENKNSNNNNNNNNNTGS
KQSVNNSRASNNRKRINNTSSNNGNDDDEDEKYQNKRKKYFDDCNMKDEHKKEGKNKQKNKKNNKNKKNNKNN
NINNINNINNNNNNNNNNNSNNNNNNNNNNNNNNNNNNNNSFYNDCVLTNNSFKGYMEENNFILNSHSSNSLDMG
THICYNYQNKINHNITKQCNDNNNTVFHNNYSHKDNVKVLNNIYNNIITDKERESNDNDSTKQSMLNDDDADK
LDNIKLNNYIYSNMNDYFTICDPNINIDKTTLHENYLRFQPNVLEKDVNGGNKHMTMRYIDNFKHNSISNHNN
NNNNSNNNNNSNNSNNNNNSNNSNNSNRLYNISSEALNSLKKNDSLYMRLQNIISFSSGHNNTKIKVININNF
NNNANKFKNLNMDNMNAPNGVTNLNVGINNEVVKEEKTNTCLNINEQIHSHTKLKCIKNGKPRNINQKENNVV
YIESDNSHNDNNNNNNNNNNNNNNNNNNNNNNNNNNNSFENNMLLLCKSKNVKNQNRMNNLFRKEENKDTKLKS
LNYNILINPCYNKNNVINELFMNTKEKSLYGNTNKFHNTLNNNMNMNNYNNQNINNIYPLTHNISIVNHILNH
SYYNENTEQNNKNLFHNTKLVPKNCNNIMNNSDKYNYLNAYNQNYTLYNNTLNNNNSLVQPSDVNNMNCSYES
INGNMPINDQSCDTYNDEYNDTYNDTYNDTYNDTYNDTHNDTHNDTYNDEYNDTYNEINNETYNDTLNDTPND
TLYDTLNEITNDTPYITPTNACNLSYEELMYKMQNNNNIYCKKHDSNYIISNGINNHICAHKEVNYNVLNHVT
NINTYVNSKNIFSNVAVTNDTDLHACNNEQRNNISNHNINKNVVNLNNVHSINKELNNYPINSTQDYNTNYDK
NGCLGTLTCDIKNKNNKNNKNNKNNKNNKNNKNNKNKNNNDNNNNNNNNNNNNSNNNKDIIILNKNNINNANIF
NINSLKEKLLNDDISKNKEKKKKKCVIRKIKKRNKVNPKRNNNINNRNKNNGGYKCDTKRVNKINNGFMKNFK
NKYTSSCINYNMCAISDLSTNIKNKENFVIGNYPYTNGVSYITNGENNTTNNGKKNDDHRNKKKEYNSNENNN
YCNNNSNRNNNNNNNSHFGGNKNNRGDDNDEDDQEKNNKRNIYGKNGDDEEEDYDGEDDNNEENDHNNKKNIK
RKNKKSNKAYNNNDDENKCINNKDTFHLPKKHNYEYTMNNNYNMNNEYNKDDEEIKNNYEDNQDNGDIKHKSC
TNGNDVDGVKSKYHKNDHSGDNNNDINNNSDSNNNNNNNNNNNNNNNNNNNNNNNNNNNNNNNNNNNSCTNNCAT
VDDLMNLNSSNLGKFSYLTNVLSHNINNINFINNNNHKMNFLNNENVNQFFNNMNYKDYVNNMNYLEPNNYIN
HNINELNYFNKNMILNNLNYINYVNGINNDRYMNGINNSQMSYNNHFRNDIISNVLKGVRVINKESHYKNKNN
KNGNSNNNNNNNNNNNNNNNNEDEDEDDDNNQNNNNDNDGDDNNDNDNNEENNNIDDRIPFKHHNMNNVSMKNN
DGNNEDNNNNKNNNNNNNNNNNNNNNNNSNDNNNNRNGNNNIINPFFLYNKMFCMNNNANNFNNGNIINNFTNN
GMAHNHNPNMNINSFRNNNMHLFHKYGIQYKDLIPMNMFPTAYLNLGRERRSSKYENSSYNKKKDLMKRRYEL
QKAILLNVDENLKEVIDEIIRNSTILPQKGIRGRNTLDCNHPIHSVWKDTTRGHCSWRCRWWENGRRLSKNFN
VKRFGNDGALRMAITMKLKKSNPKEQMQLLKQQREYLKLCYGDNWEKKIKELQNVNNANQTVNNNNNNINKNN
IDNNNQNDNNSSKSNNSNNSNNNSNNQHYVSANADINNDATITFNNPLNNKNVPYQYLNLKDKKQTFQQYNHI
KDRENSYPYMHIKGETQQNGMKLKNDINNHHSQNQLSLSKKENNIYKNNVMDEKLLIQNLKACINESNNNNNN
NSSSSSNSNTNGSYKNFITNTNHIRNLDNNSYNANNNFNTQVNSSS*
>N.PFL1090w
MGNKCSRSKVKEPKRKDIDELAERENLKKQSEEIIEEKPEEVVEQVEETHEEPLEQEQELDEQKIEEEEEEPE
QVPKEEIDYATQENKSFEEKHLEDLERSNSDIYSESQKFDNASDKLETGTQLTLSTEATGAVQQITKLSEPAH
EESIYFTYRSVTPCDMNKLDETAKVFSRRCGCDLGERHDENACKICRKIDLSDTPLLS*
>N.PFL1095c
MDMGVEKLLNQLNELESKNRLIHIEIEELKITQNELNKKEKELIVDIENICTNLKLMEKENIQLNNDIDRVNL
ICKNVETTLHNEFVKVEIAEQKNNLSEHNEDKDKDRNVNNDLIESIDMIKNIMLNIEDSDEELNKLNEIKNKN
YLLNQISIEDLYEIYEDTKEKYQANSFKNQCDKIAIVDYKTHTYFCNPMHLLHMINLLAEKIRINKNDPNFNF
VTMSNFYGVNEKKEKEKKMENFGLSELNKIMINHYKSKKINVDK*
>N.PFL1100w
MFCYFPLKCPSRYVFKKYFDLKRRKNIFLSFSYEERNFSSNDIKCSPQLIIKCVNETYKSKSFNKIKWNCIQN
DIIKNIEKFNITEIIHLINILSRLHVGSNILIHFIPNIVSKINDIKYDKITQILICYMKANIRNKQFYYVLCS
KINNDIQKISYSFLIKLLYTLNFYSSLSNDDLLNVEKKIIRSVIDNFKSFLESNQDINNKNKKNINSIHHKKD
TLIDNTIRNHNHNHNHNCNHNCNKDISGINSKDTTLNINKNITIEQSSNQIIKSADEKNISCQYNNVYLKNYN
FILLFYSLSNYLFHHIFKEKYSCIDQKEYIENKTIIQLKGKYIEMLNKINEPKEIKALHELNIFHTLLLYKSI
INTIYISDNNMCIQKEIFIFNHIHEKINNNIIINFKKDTIHIRGKINELIKYMQILQQNIIQYMQKRKDHFKF
YFKNNILLIKNILTILQEKNKNDIKRYYLWHREKIEMIQKMLRQVEMESLGINNNNNKQKN*
>N.PFL1105c
MRNLATQILHSTYRRHLGNILFNTFLFFSNYLNFVCYLLKEIKQIFNISRYKDLYKLHNITRSNFSGIIKEKK
GINLLNIASLNDLDFLTNSIHETETEINDRLSTEIDEIDEFDDDEYNFDFIDLVQQNEMED*

>N.PFL1110c
MGNVCTWRQGKEKAGDDNSQVIKDKELQNEFKTFEQKMRSNKKNAHEGDMNNDGEDDRYKFSRGFSLSKKPSK
TKITITKTDSEILDGLDYSEMSKQVLMTLNKKNILNDDGSSDGNDTDVHSMFDRKEIERKVLDLESIHFIQKK
RLSVSAEAYGDWNKKIDNFIPKVYKKDEKEKAKIREALNESFLFNHLNKKEFEIIVNAFFDKNVEKGVNIINE
GDYGDLLYVIDQGEVEIYKTKENNKKEVLTVLKSKDVFGELALLYNSKRAATATALTKCHLWALDRESFTYII
KDMVAKKRKMYEDILSHVNILKDMDPYERCKVADCLKSKSYNDGEIIIKEGEEGDTFFILIDGNAVASKDNKV
IKTYTKGDYFGELALLKNKPRAATIKAQNFCQVVYLDRKSFKRLLGPIEDILHRNVENYKKVLNELGLDTTCI
DEN*
>N.PFL1115w
MNESENKLPLIPLKESKRFTCSDGDDDEENDTINLENNSNDLVNGFQNFLISNGSNNGSCKGTKTKKDDALIR
KENFRNLNLLKKKKNFIRKDAYGSNNLVLGGLRNNSKKLSMKRKFLWNCIDQNTNVSELSYIKDFENNNSFLH
ENNGHHMVLYDNKFRDYYPKYIKEERGDRIRNFFVYSPDIIQRWIRVIFNIIITSLIVTLIYYIFISVREDIN
KKVAIQVQNVKEESNSCRKQYNAHKCGTVNLPLLNEKCEEWAKCMKTDYKLYQDISFLSAQMLGQIINTFIVQ
FEWKSICVIAFIFLLIFIGSNYALSIGGYRSNNDYNSTYPNLNNNYNHLNNNNNNMSPYPYYPFYPIIPQPIN
YNNSGNPNMDIPIMNCMPSQNKFYRNSSFNRNAYTNSFMNNFHHNLNENDMGTHSSPRNKDFNETYNSSKKDQ
KRSSKKLGFLKYLSRD*
>N.PFL1120c
MSFKFSIVFVLYLLFLLKFNKRFIFLKSEKITSYINTQIPNYSSSPPFFLKKEAKNNIKKCYFIKREGYRNIG
TKNASNAFVLNFKSNRSTYTDNAFSCTSLCSEKKERKKVQDPYDLKAKKKEKTYELNDIKNNEKKKDDIVNAS
NDITNDKLDNINNNINESRKLIKGEYYDVEICEILSKSFLSYANFLILNRCLCDYRDGLKTVQRRIIWSMYEI
NKGIDKKGYKKCARIVGEVIGKYHPHGDKSVYDALVRLAQKHHNNNLLIKGYGNFGSVEYNAAAMRYTEAKIS
SFCYDILLDEINDENVEYIKNFDGNEREPKVLCSKIPLLLINGCSGIAVSILSSIPCHNLIDVANCCINFLIN
ENIRDDELFHIIKGPDFSTGGIIISKYDILKNIYNSGKGNFEIRSNVFFEYIKNDKKVITKHINDLSSIENSD
IDKLTKKIIIKNLPPNVKPNELIENIINLLNDKKNEHDNILLRIRDESEKEDMRIVLELKKHSQIEQIHNFLS
YLFKYTNMQISYHCNFVCIGYENTYTQFSLKSFIKLWCNNRIKFIKTNYEIKNKNLQKQLNIIDLYLIIQNKI
LDIITFFQKNQNIEQIQLYLKNNFKLNPEQIKYILSIKLQKLINIKNIDFISQRNKIMHQIKLNDEIINNVQN
IKNLIIQELIYIKNKYGIHNLNKQCIIPSTPKYKYSYINSEYHPNFSKNKQIIDTYNTVSTNDNNINHSNYTH
DLPNTDNVTKDKNVVETNVHKNIHISSDISNNIKNSDKNSSLTKNQDSDNMMPYRNTYIDSLDNNMKDIYNND
EVLILITYGGYIKKIKINEKLKNHSNNIIKLSNVKYILKENEEKLNENNKRLKFNDLQKGNEQEKYKDNEQKL
NNDIGHNINIQNNNNDNNNNNNDNNNVLLLNEEEYNSYKIKKSILVRNRDKILLTDNYNKAFLLNVYDLHLSS
YDSKGTPINQIIHSSKNITGITKFQENKKYLIVCSENGKMKVINNDVFLKRKKKGIKLFKNKKNIYFSYCNFN
DNCIVGTKNGYIIQFPLSSFKISKKNSLGNKCISLAKNDKVVDLLSYENNEKNLKNYKIIFVTKNGFGKMINL
NELKIQKKKGKGHRIMKFKKSKTRKDNKKDIEKKQINAINNKDKDKDIPSNNDDNNILHNTKVDEFLGFKLYD
MNKQNEKDILIMITDHAVLIRKNMSLLKEKNKKHSSQIYAKMNKINQLVYFDII*
>N.PFL1125w
MVDDEELRKLRFLLSNKGGRIKYFFSYIFYKLFSHIYNKKNKKRERFVNIHGRTFPNFFCDNKIKSTKYTILT
FIPLFLFYQFADFLNLFYLCVSLLQIIPIFNTGYVFTFVAPLLFIIFISLINEVVDDLKRFIKDLENNNEIYY
SLLENGNFQKIYSKDIRVGDIILIKSKQRVPADCILLRNLNKNEESNFKVEEKRLFNCFRWNKSNNVYNKINK
SYYHFNKNIDNELIDDRYNESNNNCSETSNNLLFSENEKSQKKGKKKQKKKLMLGDNNTNMNDFNFNPNDQNN
NMSNQLKKKKKRKKKNNKSKKNININETTNETANYTYVKTDKIDGETDWKIKYPINIFQNLKRLKDFFTIDIL
FILEQPKNDIYKIEGSFVIFKYNPYGDFKTTENNNHSLNDNQLLNYSFDMAKEDEGRVQDDEDVDEDDYDDDD
DEDDDDDDEDDDEDDDEEDDDEEDEEVNIPNNEKNDTQNLDDKKSVRRIFGTKNNTYSYGDDDQVQNEKKKNL
DRGYLNLEEKDTDYAYNKKDKKKEMVTFIDVEKGNNERTINLNKKKNSVNTTGNRSSYVNDGKMCNVFNNEDN
FNFYNVNYRKKLNYDNFILFNSVITSSDVICLVIYTGSDTRVNMSTQISKIKRGMIDEKLNLITLFLFIILTL
FSIYMCSVKLNNLWYLNFIRFMLLFSSVIPISLSVNLNIAKIYYTLLIQRDKEVESTIIKNSGIIENFGDIDY
IFTDKTGTLTENVMVLKVIHIGFDVIHAENEKNSIQGNNMETKQKGNYNKKMLSYNLDDMNEDVDDTSIYLAS
SKYSKHDRRNKNLRNGELASFSYEMENTSNINNNTKLRNYTKSNHHHNNISDNELYDEKNYNDDQRSLSNYSD
TSYGDVHLKNYKQNNMILNQNMGQNRNETLTKNNNNNNNNNNNNNNNNNNMSLEKKKKKVEKMVDEFLKYKSD
PLDYYNDNIDDIEYLKRHRVFQTFLSFLICNNIRTLTKESSNKEKEKTKKKKERKEKDFQKNLYYILKVNRKK
KDVQKNKKENNSDNVKSSTQNKKKKHFSSNSSEDSETFSHSDVSYQCSSPDELAFLKYATNCGFILKKKTASK
IEIKYKNIMLEYDILLHIPFSSETKRMSIFVRNVKNRNIYFFIKGADNVLIKKCHEKYKTFIYEESDHLSNIG
LRVLVHGCLNVEEQFFHNFSALYNKNKDVKGQLENILEYVEKNIKVLAITGVEDKLQEGVGKTIEMLYNSGIK
VWVLTGDKIETAICICKNANIKKKKHNIYIFRHENIKSTSNLIREFNSILHNIESYVLFFDNIIIQNCIKYIP
NAFVDFAANARAVVCCRCSPIEKKEIAILIKTIKKKKILCIGDGGNDVAMIQSADIGIGVLGKEGKQVVHDSD

IIVSKFKNIKKLILYYGNNTFLQTSSLCSFLIHRGFILTYLQFIYSYIFFSIPVSIFQGWLQIGYTTYYTTAP
FLSLLLDIKIKKNLIYLYPEIYKNKKHKRKLDLKSFFIIVWISIFQGTVVMLGALKLFNDNYNNLINISFSSL
IVLEIMNIHLEVESWHPLMISANICSFIVYIFSMFILRNYFDIMQIMSVMFWYKVILIVIFAWLPFFIIKKVK
NIITPSQFFKLA*
>N.PFL1130c
MDNRNKCVECGGISFRIEEGQYVCNECNYVCEIVIEQFEDNFDLEFDEIEDDEYVEKDIYYIIKKLYKDEHLS
NNIKKYVFKENDEFFITYQNVLIKFIETLITVHKFPMFIYDEIKKIWFYLLEKNIENMNVYVPFGTFSDSLKF
VYEVITNKKIIDTFQNIQKDQNIYKEIKTVIEKMSLSKISYYFDIQKDYVNHIINMSSFYPKKSEIGRQYNIR
LSRLYKKTSLLDNDIQNKENFQKFNQLIKSMNKNTKTDDNINNVNKKINPSYIQQNQKDTSNYFLNTIDNDIS
HISQRNEHFLSFNSFDDQKEDDYGNNVITYDHDIQENNNITTHNNNNNNNNNNNNNNNNNNNNNNNNNNNVYT
TNDIAYNNNLLKGENYFKYNDNDLDCERIYHFYSKYFINKILYARNLSKPIDKYMTSYFMNRSTNYNEVLWLI
YEHDFLYYFREELNKKCDIQFLSFYDILKTFKGEYDYLIYQAIEEEEKKKKKKKKKKKKKQNQNDTLIHNGDQ
GGNTNQVDNFNYDNNYVKLKCEQNDYDNNQTVNYNNSLMEDNNHQYDHHLDNEIMNIEMKQEQNNNISNIDEE
IEYKAFDLTEHADYIKNIREQEYKDYMNTDEYDKSNVNLDVEKNIGKNKGNEAQNITSLDNKEDVLKNVLDNY
KKYMQIKRNMSNEKRVTNNFVLPINSSSDVEIMKNLNVSDISSLNLNDKASQDMVQMFLENVNGMDDSMNGDN
INHGDDINHGDDISHVDNISHVDNISHVDNISHVGNISYVDNTNHHNNINHDNNTNHDNNINHGDININDSLC
VEYNSEYKKEYNIEHNADYFELNNLTYNEHILSEKDMDNYEKLPNRHIEEEIHIYTHNIKNKKKSILNKIHFD
NNIYKKLEYVNKLKTLKKWKIEKDYYYNGLKIKKEKFTRISKFVEECMRLEKPQDDPFFTSPYKENIYIENKY
LMDSFIFDNIYKKVLYISDKKRYRTLTIKHIYKEYINYFLPRYKSLLIDYENYKFCYNFSFDLLFYILFYCFK
RLHIYCLPHHIKNYINSDNFGLYKIFYNISESFFKQFKDNGVISFPLRNPHFSEIKKFYFIRNLKHGTPFICI
HNYEHINKYYIEEKMDKIYDDEDDNYNDENNDNYNDNYNDIYNDNNNIYGEVRKKDILYEVKFSRKIKHVPFT
IKTINSMLNKESMKYLENFNATLVNNLYGLINDLIYKFHLPITIQMYAIKFLNVVLFKYRFSKFLLNVYTNKY
DDMHIEFNNFYNYEKVYKDCFKCFGAKYKAMSLISHINKYILFLMKKEYYIKKFYIINKEYFMSDNITVNYKI
YNKINYFYKQKFYYTFYNISSSICFSRNNFYNLATGCVLMACKLYYPMFTNDFFCYENMFVKNIFESFHLNNH
YSCHLQNGTNMFNAHFKEPHTFSFSSKNKKKKKGIEKNNKEAKGIKKKKNTPITKNIDLSKRIYDSDHIVKKK
IKKLIKRKKKEKSKNTNMKTIVKRKISYIIDEDKHVQKRFKISNLNDADNNIYDKNVLVNQNITHGNHIYDQL
NNTSMVQNINYSYDLENMNEGEVQNVEDYNNDILNVADEVKNTNMYDYYEDMLKEATEDNKLLKYINKNAKYY
DENLLEDFLFNDSCDNNICETLICFDVYKENIKGISEKQKKNKNKIWDNNIPKISHIYFESSKEKYTYYFVPI
PGIFNLFSHYKKYSCLDFLKNDFERYRNNGCMSSNKNEEELFENLIYHKGDIKKIYNENVENIEDKENNDDKE
NNKGKKINKEKKKILESIKRIRAILSPSILNDDKFKIKRKFIHIKSNEYCCMDLLHNTRDIDTNKNVDMKKYK
CINELKNIMKINYYISCYPHHLNYFNWNLNYLSLCNIIHRVKEEGDSFGLENYFWSNSNVKNKEQKNITGNMY
SNVFFDNMERYKSKYILRHLYERDNIYNYKTNNGLNEEETILTSKENDVLLYNDNTNNNKGRTLHKNDNILEK
QKILYKKMFMNFDYNIFNEFLNINEDYHEGKEYYKFLNNNNNNNYYNNKYNNNNNNNKYNNKYFNNNILEDSE
NEEIYEKHESTKIKLIHLIYSKRYEKEYFTFIFYEYYMLIQCFYHYESLIDMLKSLISYTWNIYQILFLYYQL
KYDIHIYIKCMKQLLNMFEYFFVITNKKDHIDSRHFSCFYRNKYIKKKKKVIYKIKIFNFMLQMFITVYGAKY
KSYSSSTEACKCMFYEDTVIFYRAKDFNIHSIKNMNEIHSHLKYIIKFGYNYLKEVLENIFHINNIQTKINIK
MNDLFNTIVNIIKMSYHQNDYIHSYIFSYRKNMMFHIVVLNVSFFLINFESTLKLIQNKYYSYLDNYLKKFYN
MDENNVSNNVMVICVKRNTKIYDERNMNVNINQRINGYTHINTIHNNINLYEDNIFSFLTSYIHILFHLNKTQ
TIEPYLFNYLKNSTNILMKKKKIQYFLYEKKDNIIEDFINHVSKNNYEFTDNTTICKLLNCDRIINMKKKNIL
KDTCTTNQFCSIHNLSINKYTYNISPNLIIHFIVHFFKHYTIFKNVIKNSNSLFLLYYWKWITHMIHKCSDYV
NTVFINYNLAKGIYKEFYKYSLKNRKGNNKCYSIKGEEKKKKKNKKKKNKIKIVNYYEFFRNILSMENSMFEY
KNKMGICKEDQKKNENTQRNVHFIEKEGRNKSYYRNIPNKSSFYHFLVKLHNLFYSYKQKYKINIFDIYKYFM
KWIKNTKDIINTNEFDINKMIIFNLYDEIFMKRKDVILYNEAHRHEQQSGYIKKKKVKKYKNNGTYFKSFLYS
LMIDNVVKQNNQFFLIKNVNNIFDKVDKNIPDLYLKGQNDNIKKKSNRGRKRKKINYDNYINNNSNNNSNNKK
KEHYARRIYSKSQGKGEMNMPITKVNIVNRRYKKKNIYGYNILRYNKRNIIKDIEKKNDHIDTNMLNYNITYN
ENQIIDLVLKKKKKLKSILYFMEPLECVNIYESVKFYANLHSLFSCIPFNYRIYKYILKKTSKENKSKDSIIK
QKMNIEKKKANNPSYNNIRNDSITNNEYRNVDVLFPYEEQNINNMNDDIKYRNNNNNNNNGHINDRINDRIYD
EYIYLSTDEDSYKSDTIYSLKLKHLKSYNRFKKNMKQNINDLYIATKEEPYCLLNKCTSSIKSINNNIPLFHD
TYKLYIDIYLKGHISIHNNMNNYLSKCSNYNMFEWNNNLDIYDPFLFSNNMCYNFGVHNKEKNKAKKKKYNKK
YIKTNHDIDNKNICGLNNNTFYKYSEQNENSNISFNITHKRKKMSDLHICIYNDSSYFNINNMSIKSEIHKTD
KYEEYYLNYNLNRNAHFEFHSSINSFLLRLKNEYIKRKNKNLYNLIKKKNHIYKIEYIQNVIYTNKYTYFIFS
CFVYPVIQIIRVIYLFSLKYFPTLFLKTINYLNYIKVDENLVQFLYADDFEVLDSLFHKYSLSKYFGSMFHIL
RFQGYHINPFSFLGSICYIKEIKIICHFFKIYTLHAINTLHNVISLDNPRNEIEIFNLEKCINKENDYIKDDT
YICNKNDKNNASTEYNSEKAKHPKKNCILIKKYKKLSSQWDLHNIFNNNVLVVNFGDEKNNNIENKKIANFFN

SDNSCMIQKEAQNYKNKNNFNKKDRPKEKNNKNNKKKKRNNKNHDNNNDDDNNNNDDNNNNDDNNNNNTNVHM
DESLNAFFDENIDYFIDVNTNHHMQNNRNNDTRDDVLNKHLRIINKFEENIKKCENSDIININNFFKDIYKDR
KKNIKNTSKHVFNDENYELGKEIRIPRIFSYAKLCNTSFVPFGEKKKKNEGNKKHMKSHYMNNILGKSYHKDS
YLEQHQYLMANNETDGIIVLQNLKEKMNNIANEKENEKNKKNNNNNKSIYNDQISINDNQIDNSNNYMIEERC
NNQDVHLPGEFIESGSIINSNTCSNDKNVYSVDNTSINLSRVNLSFSESNIGVFESEQGKISKNNNYGDNNSC
GNKFNGKGLGKNKREKIDMLYYKCFKENAHEEKIKYFSKPSETINLYNIITKFLNEKIMLHSDSITYEKYRKK
NSVEFNHIVYREFTLHTNYYSEAYNKNVLNEFIYIFYSNNFRNNYLQKGHDDKEKKKNVIKMKETEKKKNKVD
DDNNISDMKNNKENIKSSYIMNENDELEQNHLLFNLPDVEEETIMFQDKSEISSQYNEIMNIQEPIINTPSYH
DNSIHNDASLLDNVNDMEQSYISSDYYDSSDISDDETNDDGYINCSFYLHKPVYIFDEEKDKEEMEQKMKNSN
MKTSRKRNMFYYPRTNEEHNMNCNNSYIIKNNKKKTYNYSKNHFKKLKSVFKKLIKKIECDMNIKKRCYKLYD
KNHKFLRRDYKCTKHKNEWVKFMNQYVNKKENTNDLFKGDNVLINSVYNQIYTYDNEVKNIIRIYKKQKMFLK
NMKQKSFSNKYKNEYPWIGDNIFISPNNINKFILGRIYLSFISPKYVLFKKF*LSLSKC*HSLKFFKPFKAF*
FIECHVVFADKSFWFVFGLLIV*FNIGM*
>N.PFL1135c
MEEIENAHYQNLENFNDETSEDVNDTSDDINKNNDDNNKYDDNNKYDDNNVNVLDDKNKLENEEDNNNNDNMM
IFSSILRYQRYKTKKGNIYFDFKNTNLSLNEKDMLVQSSNFYHLFDIKSLLCPHIYLNVSSHININISDYKNY
LKRILNNDNVLDDKNENVKYSSCQEFIFEAVEQAEHIEVFTKVQIVKKLFLFLNQNVAEKMKNYIDNIPIINS
NDYVLVQTNLKNDLLLGYVKGIKYNLFFEILNMKKCFHSFLNENIVIYEINEDKEGYFKTIDGKKYLKEQIVY
EKKDLLKNLDFYSMYYCDYYFSSNSIIELEKKNNNNINCVNNNINSVNNNINSVDNNINNVDNNINSVNNNNN
ISDKITHKFEYNDYNDIHLFFSFNICIKSVKNNLFLHIIYNDNNLKNGDEYLNETFPHYEELNLYNKQFDINK
MDKNLFKSNTFFINNLYYHLKCKLLCVFKFINMSDDDFMTDIHISQIKKRRYVNEQDSYMLYTFNIHTLSNEG
MVYIFFCNLYISNMFKENNFEIRVHTYLHFQLNKQNSYKKLKLIKYSSKFLYSLCFKEKSDSKKKKKKNDHHE
RDSDNNNNDSNNNNYYNSNNNSNYCGSNNIIYGHDENFIFNSCDFNTLDQIKSGNYLIKDETINENFIFEVRN
YKYTFILISSKCNIIIITLRKKKRKEEYNKKMSKYSSCSLNENVYMEGVEILNYCSSNNKNTNNIICNTIINK
YNYEINNNIKNKNKSYILFEIVCFYENGEIQKYIYTCSLKNGNFEFFLNVKEDEKTKSINCKKCFFKPILFPF
NEELLNKTKSSNDLVLYNIKLSHFKNLIYIYFQEKKIEGISLTINNECLIINYIKMVSKLLKKILQNLYNSDD
IDIVYNFQHNTKNKCNYLSQNVNIPSDNTISNVIKKNEMDNNDIKYNLFTQREVKSSFFEFKYILFGFYDLFL
MNQKNYTDLNEKKKEEYKKKNFINCFDSHSHYNFLNIIKEYINYDYIIDKCSYNISNDTQKKKKIKLNFYYFL
LIIQYFKNTYTQEKKRSFFYMVYNELLQRDKYLKHFYYTYHEIDRKTNMHLNHNDNNIDTKEAKKNEETELIL
KNVNFYDNYELINIRKYDVILFCFLFHMYNFCLKQNGLSNDEFNSHITYFLINKKMKKYKKKYRYISKKKIDT
NEKNDDNNNINVCDSKNYKGTKNFDDTTNNILNKQNESLDNLKKNMYLSKNNYDNQLSSYKNTKQNKTNINEK
YNNNNIIMNYLTWKKCLYYIYKIKKYTRKIETHEGLYISSMLYINIYLCQIFFILLNILRINNTNIYTNIHFD
IRKNNLDYFYLLILLNFYSLFYILISDQKYILHEEENDTYISKNIDNKEQMANDKNIISLLSFFNNIYEQNKE
GMLKNDQNKNNHNTKTDILYMSSSGMNISINTPFLEKYLILIYNIIKDKINIVKEDNYIMEKLFFKINCMICN
KVCVTNIYNNYYICENNHIFNKCMLTFGCIYKNHIILPTIYLLHDINDKLFPINNDHILSYNLQYELDYIYFC
SFCYNFITTQNSFYKKFFLFNQCPFCNHELNIL*
>N.PFL1140w
MVSKKTIEARKAYYNEDVVLSKEAHDFYHNLDKHGENHNLDKDNLKTIIFGSLDGIITIFAIVSGCVGAKITP
TQVIIIGIGNLFANAISMGFSEYTSSTAQRDFMLAEKKREEWEIENCPSEEKQEMIDIYMNKYKFDSEDARNL
VEITFRNKNFFLEHMMSEELGLIVTNEDKNECLKKGIIMFLSFAVFGIIPLSAYVAYTVFFGYTDYTTSFLVV
FISTLTTLFILGLFKSQFTNQKPITCALYMVLNGMIAGMVPFLLGVVLKNNISE*
>N.PFL1145w
MSNDLINGSILHCLETASLGMPLEVWKTRMCVYRNENTIKSFKNIYNKGLGQFYAGFYAKLIESGSKGAVLLL
SKEQIIKVCNDMNINKTTSGFIGGACGGICQSLVMTPCTFFITASIDKKINYKEKLIHIFKNTGITTLYKGNT
AMCLRQGTNWASRQGITEWIRNIVMERKKNRKNKNDDINNVKGMIFSGNYITTDKIKHNNNNNNNNNTSQSKS
LELTTSEELFCGIVGGALSVWNNPFDVVRVYMQNNANKNIKLTFFQSFIHLYKEGGILYLYKGVIPRCFLCIW
QTLFMVTGIKILNKYF*
>N.PFL1150c
MSRYVKYFVQAKRLDKRKRKYYKLDFLELNKELSIPHPLREQNQPKRLDLSKYLNIEVGDLVKVLYGPDKDKE
GLVLSINPKRNTVTVDGCNMKKSAWNVTDNKKGSIITQEMPIHITNVSLLDPISKKPTVVKRRYMMNGECVRI
SKISGCAMPEPVHKNILKEQNNYERFMHKKKIGPPIKDIYAEKDYKNFNLLKKIAYEIKKKRFYDMKNFFKKD
DKVENATD*
>N.PFL1155w

MYKYTSINKSDKIYETHNMEEKKKKGNNNNFSGLLNNEIDDNNKKEKLKNSISKMYSNHKNRENFNECEKEDL
VVIDEKDNNKKKKKNMTNTFEQDNNYNMNDNKRLGSFFKINDKCESINENVNNINKQSLKDSILFDNINEKEY
FNETKEENKEGNKSNDIEKINCMKVKKKTVKKNKKKINKIINNKNKISKSNDIEEQIINISKHIYKILNISKL
PKCDILKRTNRRYAETFLYLTNGYNLDIEQIIKRSLYKRMYKNNSIIKVTGIHIYSLCKHHLLPFEGTCDIEY
IPNKYIIGLSKFSRIVDVFSRRLQLQEDLTNDICNALKKYLKPLYIKVSIVAKHLCINMRGVKEHDAKTITYA
SYKAEKENPTVHSLNIDSSVENLN*
>N.PFL1160c
MDLFNCQYCSISPFDYILDYLYGDTSSKKEEKKKKSNITYDDSSSNNVSQVVSKIDEEIEKRTTNEILLLCRA
WENYVHPRAKEYWKDSQQLQNILCKIANGITKNDDPILGDDYACVFWYGDKNKNDNCPIISVKKGNDNIETIT
YVNRVLIFLYADEASFQELQKKPKKAFTMACGNINCINLTHISLDD*
>N.PFL1165w
MQVVSDLTNLKYLYDKVSLGKIINRNLKELIKNPEFCFDDEINNMNNIRNSILTEQYKGLNMNLLNLYKEYLF
YFETFKRKKLLKDKKKIYTNDIFEKNKKLEYELEEENIIILYNIGYITSYMVKEKKSLNDIKLLNKISTEAIE
IFNYMFQHMNELKLQELNDINCISCYIFLCITLGYHENMFYNTAMMKKYKRKLLAKLSFNIYTYFNEALCCLQ
GDMLPIFKQSDNFTIARNNLQYIRNPNSYLYNFVQVNRQIFISISNYHYSLKYVQLSDMEELKVTKYEEDKIG
EILCRLNFSLINVNRGIELCKKYNINVNFLSLKEKIMKLLDFFEYENKNIYFEVVPDYDNLESIKGTQVTFMK
NIEICDIYIKKNISNNLKLLFNEKAKYIYDSYNNQALPLYEYYNKHFLNLKDQYELLNISHRKDILLLLNNSI
LNIFSKKKQIYNPIHYDNHLTFLSSVENNLKDILIEADNYLTIEHNNHLEFQKNYLNVTINQESVNSYNNFLF
HLNSFKTLLHELSQNIITFKTYLEKNHEKLKICEMDVTNFFEHIINQLNISSNIHIDNLDEDYNHYKNLLSEE
NDQDLSSVNSTSDIEPISSVMSLNYSDFHNFLKTKKLLFAIHKWENNQYLFHYNTLSKYINLHLEERLYFVLT
SLYFSMNIQLTNFSNDLTFIRNGLHDQFLNSITEEKDSERLNVLLEKQKELLNAKKIKLEERITLFEKDLNQF
YNYYHEYNKLESFKTIEDFSAFMNELTEICSSLNTTYKKHEYTLKNAMLFKDDINRYIYMRESERSQIRIQQI
PNNYSRNIQNSTERYY*
>N.PFL1170w
MIATGTNMMHPSFSTASLYVGDLNEDVTEAVLYEIFNTVGHVSSIRVCRDSVTRKSLGYAYVNYHNLADAERA
LDTLNYTNIKGQPARLMWSHRDPSLRKSGTGNIFVKNLDKSIDNKALFDTFSMFGNILSCKVATDEFGKSKSY
GFVHYEDEESAKEAIEKVNGVQLGSKNVYVGPFIKKSERATNDTKFTNLYVKNFPDSVTETHLRQLFNPYGEI
TSMIVKMDNKNRKFCFINYADAESAKNAMDNLNGKKITDDGQIDETYDPKKEEATASTSGAANQTTGTDADKT
DKNDKNNKADKNEKGDSSNANNNATATGATTTDTTTTPGETTTTTANADSTGANNNNGLSPNTNTSNTTTGSS
NNSINLNENNNTAGNNNSTNNNNNSGSSMNNAGSAKKDETAASDCADTPNILYVGPHQSRARRHAILKAKFDN
LNVENKNKHQGVNLYIKNLDDGIDDIMLRELFEPFGTITSAKVMRDEKEQSKGFGFVCFASQEEANKAVTEMH
LKIINGKPLYVGLAEKREQRLSRLQQRFRMHPIRHHMNNPLNTPMQYAAPQSPQLQFSQNTLSYGRPVITAFN
QNNLISWRHQQAAQQQAVHQQAVHQQAAQQQLNFNTNLRGQINQMRLYTQNNMMNNNLNQNKPNAQLHHNQQY
VPNALAQNGQQQPNLNAAGQHNAQQLQQQGNNQLLNNNMRNMNNRANRNMGNLGNMNNQKQLPLNINNKQQNA
ASQANQMNHQAPQGAQAQQKNPQQMQQVPQGNNFKFTAQARNRMELPNKNANKVNTMNNNMNVNFNNNSTLT
AAALASAPPSMQKQVLGENLFPLVANYHPTLAGKITGMMLEMDNSELLILLENEEQLKKKIDEALVVLQKAK*
>N.PFL1175w
MHNNHIFEDDDIILTDRFLSDESSENDKREKKKYIKENKKNIENVSNVLLNNNFNKNVGMKRNNSIFNNEENK
DKNYFTNNDLIEHKNKSLLKDKSNEFSFNKHNYSNTRYDNNVTGNYFDFDNYYVSNMDNTNNPEKEIYKYDEN
IEIDGKNKKNNKRLKRSTRVWHDSDDDIDVKNEHLDTYGNYLNEEKFHSDDSNEFNYVDEKEDENLIRNIEAH
DNVFEEYIDEHVHMENISSNTNKDAISHIQADDIYIFDDEKIDMHIQKKKKWNKKNDNNIFLKYFLQKFTIHQ
IEEKKVKQLVCLPKSNLIFPVYNLNLYMLKYEDNSLNISKKISFKRKIKYVQEYKDNLYILSYDNFLRNYNLE
KGLVYKNRIHYDKLDISLPREIKFVDKNTEDENNTSSSYLFSLSFQNSGKINVYDSRSYDIIKTFEMSNKYVG
MNFHKKSNSLFALDEKGYLYNWCLNTNKLIDKLVDNYSIFPSCLNIYNDYLITGSCNGFLNLFDINNLNTPIK
SFKNLTLRVHDIVYNPSHNMLLYYTDIMKNGIKLIDLKTKYVYCNVPWFNINVKYNIYAANFFNNGNNLCFAV
SSNSFYVYNIY*
>N.PFL1180w
MSEVNVTKVIVNNPICDILDPFVFTIEFEALNKLEADLEWKIFYISAVNNEGESNQDIELDNIFLGPIERGVM
MFDYAVNPPDYKNMDIDSVLGLQAILISANYKEKEFIRIAYYMNSFYKDMELRENPPVVPQYDKICRHIFVEN
PRIVKFSIGWDSEEKDEFKEFDKEIEKIELLNCTQIKDENENNSNITNQSTQGNFVLSNNDPNNNNNNNNNPS
ITSSMIFNSNIVNNNFNQNNFLKNNELSSDLDKCEIFNANINGISRITIDNKNS*
>N.PFL1185c
MQNLSPACTFNKNEEKIKCPSSTKLGCSDGTKIIQHEINERNMMPEIPNVSLTDENDFTFNKKRHVSSIPKNN
NEYWVYPSSQQFYNSLIRKNKDIDKNYIDAVVSVHNEVNEESWKQILKYEHMHKRSCTDVTLHRFLGKFDDLS

IKARFRSIFSSMGRPFDRHDWYVNRCGTQVKYILDYYNDESINDDKNIYIDVRPAMNSFSNVWDRLRYPFYEF
YFKYVKKDELFK*
>N.PFL1190c
MGKIKKISNNSNKSKKKSETVKKKNKSNSDSKKKENDVKNLKSTPQCSSDESDVYFYNDIQRWKNTIKVPIWL
KKPKDRESLDEFYKNIIKKLLRVKNKIRAYKIQEAYLLDCLQKCMIDLNLNSVMSTENINCEIYKNLLYNFKD
KTNIENAHKENNDEIKNKNIPNKNNHVLLKPERNFISGIDNTMYSDNNNNNNREDGNNSGSSNTINDSTNTIN
ESTNTINESTNTINESNDTLNSSNKTKSCNNNNNNNIFVSNSISNCSEVNKNYTSSSFYSVNNYDTKDEEENF
INTKKDNIKDNHTNTCLKCLKEEKMRDMVEHIKNSLFKEEEKRKKSVMDDVFNKVIIPGGFFENNNFCPIFLK
LLKIDIYNTCYTVDTKVNSKLMNLFNVLNNFNENNKIDKNKNVDKEKDEFLNTKCKSPNKYKSNIDDIFFFKE
QSSLYKLSQYCIEKQVIKNNEVTSFDTVYIYNEKKVDDKEHIHDENKKNSSDDYKNNTKFKYVLDDVQKDNPS
LHYINDKKDIINNDKCNNDESQPLVDYNKDVINNPSVENYTSNENNIEPYDNTVKSYHTLKNQTTNESSISKN
NKVDSHTVINLCETIDDREVYEISSCYNENDDTNKSHFDIINDYNYDDEKKEKEKNMYHSKVNLLNDKEANIS
DIVLEKDPIIYDNINIINEKENNLINNNNNNNNNDNNNNNNNNNNNNNDNNDNNNNNNNNKNCEKKCNIPCNH
INENTLFTDSSKEENNFKGESYEQIDSLNWEYSYDSNNLNNSNNHYKEINNKSDSIYNRKRHSGSDIYNKKYF
EEDMFENNSNEDNIFKRKKANNDNILEEKKNQRSDSEENSYLKHIKNKPNNSLRFSSNDRKSYNDMFISSSNN
NDHSDDRNKNNCEKIKCGNVEYNSCDDKKNQVNSNEKNIKNNSKQHLNNEYEKFKLNVDDVNNMNENVNILKL
TKEKTTISFTSCEDSNKSIIMNKNKINNEKNILNEEKENICKYNKSLFDSDDDQIKHVTNRNKEEENKIDKNI
ESALFHFCDNDNINSMNKNNNNTGNSKLIEKDKYNNNNNIDSNIHNNNINNDDDVIIIDEDSDFTLNVNEKQD
YNKCCNKDEMRKDTILVHNNISKSNDKLKNGLTAMSMNLFDDSSDNDSVNSYDDKNINVLNETYSNKYYHTNE
KENPYGKMKDHIYEELKGNENKNYKINDKYVVDKNLNEVSILNIDLNKAHNAVLEGLMDLFGLKSNRLSRKII
VNELIIIQDYLRQKHDQFINSDNLEEEHILDNLSNVKENYITENDSIFKDTIVEQENINKNNDDNHYNGSVTN
KNNDDHKNGSSDNNIICIDLLDTEKEYKSDCDMQSFPFNNKWFDNDLLNRETDKLQEDYINHMHNKIKQMELK
LLFERIDEAIKINDIIYKNVYEEKKVEYSIFKKHLVDCKLNVNKEIIMSYSKDKNIEIISKKKYISN*
>N.PFL1195w
MLNNLPCNKKYWNSILSAQKYHFCNKWLSKRSNIKKSDICKRYFGVSGSYGYSIFSKEGNNNSNVGKNNLHGY
EEKCLINNFIKKKNFYFTKWPGKVSKLGGNYFFNKNYNMGFYHMLIEEEGKIKKDIALISSCYEKSIQNKTIE
NKMKDLTCGYSVQIVLSGKGVKCYYEDINYTPILPNYKNNIKQYYAFKNTLKDLKVVENKSDHNNNVENTQNV
KTTSNNNTQNNYRTKKVKKLFVRLGIGTKNIDITRVLTMHKKYVNINVDKTGTIVNVYGYNKKYVGSVAYRLY
KIVKMNVYTMKGGYVYLHKPKQKIPKKK*
>N.PFL1200c
MSRRNIRLPAEVSRILYVRNLPYKISADELYDIFGKYGTVRQIRKGNAEGTKGTSFVVYDDIYDAKNALDHLS
GFNVAGRYLVVLYYDPVKAQKKKEIQEKLKNEK*
>N.PFL1205c
MLNNNAKKIQLLKKILYRENIKNKQKNNLRRVFNDYINNERITFLYDKNIFIQIIEFNNKQKSLYIHSDVFKN
NKNGNDQLIIKKEGNLKSFVCHKENKHLGEKKNKINKVNIKNVYPHNNKNIYNDANMYNHNQHDISIDTSYKN
HHTHQCVKINNYISNEEIIDKKINQPNENHTRHNDPCDNMKPSTYCLEEKNYLNVNPVIIQKKKKNLEIVKKY
SDKWEKHENKKNKLIIKYEIKNTPDIINILNKCKEYNYINNNLYDDIIYNIINTRDNYLSLKSLLLISNSFIK
LNYFNETFYNYIFIHILKLCVISTHDLISILYILSNIKKLNMYNYIFLYKMNFMFLHRLYSLSIYQLYKILSC
YLKIYKTVDKLKKTNEHSSNNYTIHNKEKIYLGDNNEHVLKKYNKINSQQYHIFYKINFSQIVKNENNHNVKI
NNESIDLVFFLIESLTDKKIKINHLDTLIDISDPLNDLFFSYFKKKIGSLFLKKVHSEYITFNKEQRKILYKK
RDSIIQMYKKEQLKKNDDMSRGGVLKGGVFFKQQNDNCINITNKQGHIKNSQKILLKIISKMKRKIRARIFIP
FIKIYEEKDMNKIINKSKDIIKERDTNKLLYTFFCNTYKNMNLTFDEKSLLITLSCFSKKGPLLFNDVIYMLT
YLMEKKINYYNHEEIIDLMNSYSNMKNEKISNNVFNFLARFLFNNNKIIHLNDTHLHILLNILIKKKCFINQD
VIKYISNFIMYHKPYYKNLKNASLYLFFHYHFNSLNEQFLNNIFCTLNHKNMITLYHCNQVFSSTLLIGSNQL
KYQKGVYNILDTSILNHLKEIYFNKHVDISLNDMKNIVDFVFYFNKSITGNVLKAEYRKIKVSNDKKKKKKKK
KKKNHIIQVKEKIYNNDNIYDNIYDYNYDNIYDNIYDYNYDNNCDNNYDYNYNMMSIYTPFIAPKKCCNKKKP
VYFFSNNSFCKIYLMKFSFNIRMFILIKQIVEYLLKEKYYFKNNNQFIILMVKLLSNVYEIEKKINSLNNNYT
YLYINNKSYVKKNKIINKYFLRILNYINNNILLFLCKANERIIFLSNFYMLKNKYTFEHINYHKIIHTYLEKQ
IDKVRNKDTDISSILILFDFLSSYQIDEIMILARCIKMKGQIESSHMHEKRDLEEIHTNLMKNFLYSQKLRGR
EEKMKYEVTSIMYNEKKKKKKSSIKNIMKIFFVTLQLRKFEYISKIFKNKYEKLYMSKVLFNNIEKRMIKRKI
ENMDAKIKLNNSFRHINKYNKYVMNKFCKKWKKRKKYLILKCTHISQYIDTNNLIDLLYYTLTLTIRYYMNNI
CSQNNRIKNISSFIIYEKFIIFILNKMVLLKKPQIVNINYYLYYKMNLCIHTLKYVHVKLYKSYYYILMYWKF
IILRILQRNKTFKNYMDFIHFNNNSRTSNNAIQNIEKNEIHKFNSTYMYLLNYPSISQTSYKYKLQNDVNKNI
LQNNLHYKNTQFYKNIVHHNLYQQFSYLQNASHQLYMYFIYFQKKLKGRNMSLIFNGKYIKKEIKRRKKGRIN

RMNIINTIKLNKTSFLYNDANHKETKRKKNTSILLKNNIEILTVKKIYILHNHLIQVHVKNKSKSTNINMNTN
ENNIYVFFIYSSNNINQHVPNHEHSEKVGIQKYNHNHTKEVLRKYQKSDKSNYALKQKKEGTIIYLKRDTFLN
LYIIQHELHKKLTSPFRLIPVCSGELNKIKNRKNLYVYLTKLFV*
>N.PFL1210w
MFKYGNFIVIIFLIFLSLIIELYLSLHIQKKHIPLDNKNNSFLQFINSEHYKYKHNNKYQLNQHKKNIKDVIK
ESINLPLQFLPTTYNSLDRQKFIQQFWNCKEIYEKRNFENIKKHIYNNGNNTKQSKYIQHNNTQSCNEHNILN
KKKKKEKETYSDVMETKNSHLIKELIEKKKKLISKIDDNNNNNNRKHIFKYIKTKDYINCHNGNHLINKFENI
IYEKIKNEKYLKKNKKEILLKLYNKILNKIKIIHDGPPYANNDIHIGHILNKVIKDIYLKYFLFQNYFVLLIH
GFDTHGLPIEYNVMKLLKINHIQDLNLNSSYIHNQNESLKNEHVFFKSYINLLNKLKNQHKKKKQISFLSNIF
EKNNIISKDTIEQQKISTFKNLCKSYASYFVNEQFMSLVSYGIWGYWNYTYITFYKFYEQIQNKVFRDLLKNK
YIYISNRPIYHSYATKTVLSDSEIIYKKRVCNSFYFFYTFFKISDTFLLHLLKEFKENKAINEILEFNQDDVT
YFLENYSLVINEIIKNNKENNEGKNILNGNDLFNETNSNDSYLEGQHISNYILKLKNQIIEKIIKKVKILVLT
TQMHTIFNNKCLLIHEKYLYRIIHLKYEDEKENQFFIICDKSYNSFSDNLTKYYSKKSPIKEIKNICTFQGNS
FMSCTYKNFTNNEENNFIFVSDNEIKESFGTGIVHVAPSHGFTDYNFYYKNNKLKKIYLDISNYQEIEEKKQT
RDNNKINISCNDMKAEVSKKIFNENYFIKNNSLSLDVINENVMDENDDLKDEYTELVYKNLEKNIHFIKKYET
PSNAQSLLNSLDIMKKKTKKLNINEKDIHSLFFYSFYENILFYFPYEHSYTYDWRSHTTVQIKSLLQIYVDID
KIKNNIPFYQSIKNIHFINKHVKNNLIKTIKDRNEWCISRQKYWGVNIPLKDIELDQNQKIIFNNQIMDVWFD
SSVSYIYVLYMCKHILFHTYFNKIWKSVKNYNNTYQKGKGKYKNKINYNNKNENKLSFNIYDVYDQIMNHDEY
NIRSMENKDNILLKNILQKKKIFDTSKMYKWINKQIQKGQQKKYYSFQWNSIKDIVFSKNNKKEFFKKHFNIF
LCCEGIDQIRGWFQSFFFVFFCLNWINQRKKTRSQLLKNTDKIIDKKGVVIIKREQIKNNIKHYTLNNNNNND
NNNNNNNNNSIFSDDSLCFNNTSSIETNSKRLPSHSYLPIKNVIVHNYVVDSNNIKMSKSLNNVISPRELFFEK
EKDDIPTTSKRKDTQKPDDLNKNTDINNKGNINNNVNNNNDILMKTRKQIDLIKKKNKNKDLNKRFNADIVRL
WVCCYNFVNRNISISYEILENINKYIYLKLYNTFKFIMNNIYDLNFDGTKDNMKIKWDNIQMIDKFILYKKDN
LIKHCTKAYKNFQLQLLIKYIMNFIYTDLAIYIDYSKDRLYIHEKNSLNRTNCQKILYKILRDLIILLGPIVP
HLSEDIYYNLQLLKNKTKRKYMTTHNNNNNNNISINEGKIKSLFLRPFPKFKNYKQVNLDTLFLIKYYIHKQI
SPYFSNSLQAIVYIFSNNDNIINLIKSFLRTPDPLEQFNNYDDLRFLFNVSNIFICDNICQVEEKDKNYKTYK
IPLPNINEKNQIKNKNKNKKKNLNDFFQPNQKPIVDHMLNFDENIEHAIISIGINKTESKRCSRCWMYGTVYS
FEGEYFCPRCLNVIKSHYN*
>N.PFL1215c
MNYNEINDNTMMNTNDINHPSHLNNEKNDLSNEENYMNIYDNNNNIIIDNEVDDHLKEEYNNIGEGSPEGANN
IISDNNKIHSIHNNNNNNNNNNNNYNNNIVNNTFGMSINGNENVNNKDINNIHEEIHSNNVESFENLDDYKVNN
IIHNVNNLINNEELLMNNINNNNHINEDLSSLNLNITQDNNVNNLINPNIGINNNKKVKNKNKNKLLKHMNNN
VINETTNVSFENNKKNNINENFLAQNHNYYDNNMVNNMGYNYPTFNNNNNNNNMYNENMGFIPNNQFMHPQVY
HPKLVAENKNKGKNKINPNIYINNYNMNNDNFNNKSSNLLYKNIKMNNIEQKKKKKMIHLKTDAAQISSVEST
SEDMNYSNEYGSQKKHVEFKETNDPLILHPSTSYIRNIVIKKPSNIRNSIHNLNLYNVNSMTFDSYYTNKNGT
NIINKNGANVINKNGTNIINKNGTNIINKNGTNIINKNESNTNDRNNNIILNTDQTNSEHNNMENIINNPSHR
IYKPPVFISKYIKDMNEDELNDIYTLLNDQIIKSEENDKAINYVKNEIINLYKQNPIQLLTLQKCLSLIKANR
NIIEVMFYILENNKLINMQCDPQYYNTYINNTSYLNYQTVNNIYKHCKNESSYGNHNNYNELMSNNMYNGTSD
SYGNLINGDDTSMMGGHDGESTSSKLCSKKYNQISHINNNNNNSNNNNNSNNNNNSNNNNNSNRNHNNNNNNN
NNHNNNNNNNNNNNNHNSCYGNIYNLHNDINILCDPLSNKLEWNNNEHIQTKESIRHNSKSPNYYNNYNYKCIS
CDKICENTYYILKPTNIKRISYGVVDKCIWCSVCYNSSNYPNVLNSSNFVKVNIPYNLSNNDWNINEIEKLIE
GVCKFKNNWEQISEYIQTKTPYECIYKFISMPLSNPYFDLNNLYDINNISLNSYEQNNTLLSLLSFICNNISP
YVGAYAAKKIVDYILEKQKEENEKEKKEQIKENDNHHNGDDTNKMGHHNEEIIDHAYQKEEEKNNINEQNGEN
VNEINVNNNNNNSNNLNDDNINNNDHLTLPNENNNISNNNEIKNEGNMENNMEVNNVNNNNQINDNNSVPNNT
NDSMKKKPSTYILKDDDMKIIHDTIIKSSKKRSRELANLEEHNLKKLLKELVLLNTRKVQLKLKQHEYLQKYF
EYQNEFMVNKGKNENETK*
>N.PFL1220w
MKLDKKKDFKKKLVTCLSLLQVGDPLKIIVDETFIKLCILHKISIKEELVKLMNRQIILMTTKCISHNARNQP
GDEEASYAIRKLTHYKCNHNEENLKKNDVVDIYVKKMKLEEYSKLSKNNYFANQLTLTHNFDSLYNIKNKVYQ
CDVLKNDNVESFNNNKDKKIVRQESDLSNIHDNTTIVLSNDVDLNTNDNVTIENSNDDDTNRNNIDSLLKIND
NNMNTINHMDSLEEHNSEIHDKIKLNDSMKCIIDLVKNNNEKKFFICTNNMELRSFLRRVYIVPIIYISESGI
IKIESLSTKNLDKKKNVELKKMKMLKWERDLKMAEQKKNKLNIKKKKKKKKNRK*
>N.PFL1225c

MNKHKNSVINKNLLKNKSYVKKKKKYFTDQKFLRKFKKSRQPNEDTVKVEKIDPIKNFFFTPNHITQEEGKDK
NEEKIINSYEKKKKKNYKDYKKVGHDQLNGGNISDDNIISGDDINDDNISGDNINDDNISGDNINDDNINDDN
IKDNNIYNEKRENKKQMIGFNNTEKKKKKSVYSKEIKYSKLKESEKESYMKQKQIMIEENEKERQKKKLLRLE
KFKKLTKKTKKGQPVMKNIINHLMKKI*
>N.PFL1230w
MKITTEEMKKKKKKDHVNIKKKDLFKKKKKKKMKNNMEHEKKEKNKKFSNGVFKKENRRKDNDKKKKKNKNIK
SYNNDNYNKGDDNNNHSKRKQFHKSKYNNVDGNNEFNSYEEKDDDENSTYIYNNESDNIIDNEENINNDDDDD
KKILTTDIEITNNNKMDYVKYINMIKNEQNENEKKVNDEEDINENNILNKINNDHKDSHSNNDIYKIVLLFSP
LALTSIKNKQKTCIINVDDHMNMFMNKLENLENAIKFEKKEKEKRKLEQIIQSVKNKLENIRLDILFFTLLSL
RDSILNKKGKLQIYIYTVNGSFIFVSPLFRVPRNFTLFKKVMLNLLKRGVVYDDQKNVLLKIIFNDITSYVED
AVCIAISNKGFPTDVKKLTDKIKQTKNNYFFFISLSNSHDVTKFMDIIKKEKTKTFSYDYLVRLSDLQLSAVA
LTSKLTHFLN*
>N.PFL1235c
MKKDEKNNMDESLEEYDEENYTSELDDLPEYERELILAKRHEEYMKKKHRRMLLKNLHIDEKSKGNIHDDTNK
SIETSNKRKGKLNNKKLKLKKKLYESDEEEENDKNDKNDDDQYDNTYQSDEDRKKSNNKKKEQSKKIKGDNES
YNIDSISYVKYKKGKIKILEDDDEKNHEDEKKKTDIIDHVSVTDDSYTDSSYKNKKKSKYKKGDTNQTDKTSK
KNKNDKTNKTNKTNVSIKTKKHREDYSDDSMDDYHSTSSYEHEKSSDVHYNKKEKKIQLKVISEESHLDNTKN
KTGKGLNKILIEKEKKKKKKDLHIVDSVHKNKEDGKNVITDIFEKNEKINKDKELELPQTPERLIHLYKEEKK
IKMDIYNYMTYDIITYFQLKKTFLLDMCEHVHFSYHVIGHMIKIIDISKLNKVTNQDVQNTNNKKKIDTANEN
KKKIFFITNVIKSEPYFSTDRNTNIKFEVAHLENLAHSKFFKKIKNIMYQNKKISMDEIKSNEYYETYICDMN
NISDQKFTIDEYNHIKLFSIDLDVLKTFHLFLKEKNEDLKNFRYTEKQLQDLFEKKKQKSFYEIYTNNKSIDN
LPITRITVQREICSIQREIDKLNFDKKKTNVNDTYTLSKLNNQINELTKKINILRGNLDKARKNHAAMKSNDQ
FAQEKNMTKEKSRTNIKSTQKDDATNKFLGVKEMDISYFSKQIYDEKTNFNNMLSSKFINLPLDVHHKVVHHF
LLGTIQNNDAVFYDDPSHKIKKELDAEIDKLLGLHGNRHITLFDDIKKNYE*
>N.PFL1240c
MFLNLIKGLEKNEDKENVVNINIQKLLGDKTYEKRKKGAQELAELVKIILLKEENDEKKHIDKLLDEHKNNED
AGDFNITKKFSDMEVNKISNNLLKADEDDELNNSEYYFKTYNDEKLKKKKNVSDDIETDDDNNKGYTIINDEK
GRKMNEEYDSKNNIDGDKKSQDKAYIKDTKEYEKINKYKQNNTKKESVRNSNIMIDTKEINNSHIYGHGQEDG
KEYNGDDDHNNKNNAHDDDDDDDDDDDDEDEENYEDVNVKRYNDSDDSVDEKDKKRKKRNLENEKSNLLYQHMN
EEKYILGSEIIKKLLLDKCKENCDLDIELTKPIDNNNNNYYIRNKNSVSSVDINKEKRKKTLNTILYDNDIYL
DNFNKKYQSQKVINIINFLDNKFIQSVSSSERCGGLISLAFISISLDEKIQYYFTEILRIIISCINDADSKVR
YYVCESLYNLCKVSRKYIFHNIEDIFDCLYRIFSDTCPNVKSGGIYLDNLIKDLVCSYNNIFYIYKIIYILKD
KIYIENTNVRQLIISWLFFLQNIPTINIFEYFHFFIKDLFLMLSDENKDIQKQANQCLDIYIDKIVTSNYEQC
RTFFKHIAYVILEFCGHKNTVIKHKSLLWIYHFINILNIHFYNIFDSSPKKNNTFLIELLKKIIASTSDVCFD
IHYTARKCNELLISYVKFTYLESAPLITKLVCLIINTKIEKSELVVSASKDSTNYKNYQKKYFSRRKKLYKDL
MYDQRNNLPNGVNPSEGYNNEMNVEENNKMLDQEDNKLNNLVNKHENISNKGRNESSIIKSKHDEDNENDYSI
YKDDESYITNIGNERKVNNSLFHNDENDVEEDSLLNVNCFNNVTAEDEDFIQMNDTGKIENMRDQIGSYNKGN
NKEIINDNNKKKDKHKDINNIKYNNNKDMNSINDNNNKDMNNINDMNNINDNNNKDMNNINDNIKDDDDKNYH
NNHNNNFNNSLNNSNDNASESLSFESYNLFYKKDNYSYEGKSKPLNTQLSKEENYNCKIIYNHQKLINELKEK
KLLMGDVSKSEAYYINKIIKKKGNNINNNNNNDDDDNDNEDDDYYNDDDDDDDDDDDDNIFYDNLEKRNIYPVI
TCLQWLIEILIYKSNEIKTYYDEIIICIFKCLKNDDNKVLLLSLTVLSAMCGTVDNKFHFYEKISLNLINIFK
NDENLLIHKGKVIVQHISRCLNNKKFYAYLCYLLIKENNFTFVNKFVQVLNWVLLTSDETKYLRNVLFLKKYY
NIFSIILIAWFHNPLSAISFLLWLQKYELAYYISSYLTLLDVNSDFFHQLDNFIFLFESPVFSKQRIHLIYPK
NYPFLIKSLMILSLMLPLNTSNNILQKRLQISQLSILTSNEKMQSFFDIHNHNGIYHIEDNYDHKEIDNKIND
QDKLKKRETSQLNLYENAEEFIQLCKMGNEEQTKEYNILEKTDIEKHNEEELWNNRYNYLLTNIKKKFLKDII
TNDNHNNNTHNSVSYKNDECNEFVHIFKTVLKTHKIIKYYS*
>N.PFL1245w
MQNKTPPLKKQRTDESVKPIEFDKSSESWKRSDCLEKMTENKIDTDLYSRQLGTYGFDLMNKLVKLNILIINV
KGVGLECAKNLILSGPQSVCIYDNDICDISDIGVNFYINEKDVEDKSCRSDAVLKELQELNNYVHIYNYKGTI
EKNWLENFDVVICCDINKEDLIKYNNMIRGIDKKRIAFLSCNIYGLCGYIFVDFNKEFICYDSNGEQVKSCNV
SKISKELEGKVSFDFDKTSPFEEGDYVQFSNVEGMTEINNKIYKIKNLKKYTFEIGDTSLYSEYIKGGICTQV
KKHLKLNFYPYEYICVNPLNNENISNNEQKHNQNDNHFLDTCNNIIYENIPQPNSFIISDYAKFDMSNHLHYS
IQALKWSELQNEKGLPENSDEDALEKIYNYAVTLNNKDKEEKKSYAVEQLKKDVVYNVCRYSKSHIAPVASFF
GGLLAQEVIKFTGKYMPIYQLLYLDFFECISLNEKVDINEIKKMNCKNDNIITVFGKSFQKKLNNLNVFLVGS

GALGCEYAKLFSLLDMCTRNSEQNTNLNQNNIDNNLACCGKLTITDNDNIEVSNLNRQFLFRREHVGKSKSLV
SSEIIKKKNNNMHVQSLETKVGAENEHIFNEEFWTKQNIIVNALDNIQARQYVDNKCVWYSKPLFESGTLGTK
GNVQVIIPYLTQSYNDSYDPPEDSIPLCTLKHFPYDIVHTIEYARDIFQGLFYNTPLSIKQFLNDKEEYINKI
QEEGNNASLLENLQNVINSLKEISSQCNFDFCIKKSVELFHNNFINQINQLLYSFPLDYKLSSGEYFWVGQKK
PPQPIVFDVNNEMIQEFLLSTSNLLAQVYNIPPCFDINYIINVAKKIEVKPFEPKKVKINMDEKNLNNISISF
AEEEKIIDDFCKELLNIPTNNIKINPIEFDKDEQTNLHVNFIYAFSNLRAINYKINTCDKLKAKIVAGKIIPA
LATTTSIITGLVGIELLKYVNYYDNIQAYVKLSDEQRKKEKHDVLSYFKNAFINSALPLFLFSEPMPPLRMMD
KEYDELMKGPVKAIPNGFSSWDKIVISIKNGTIKDLIDHINEKYSIDVNLISVGNACLYNCYLPAHNKERLNK
PIHELYKQISKQDLLEDKNYIIVEASCSDQDLVDVLIPSIQFIYK*
>N.PFL1250c
MIIKKRKKKNSRNTYYIYILFILCVIQIKILLSKEGSYGNLQNLKIQANNKVKSRCMLKKSHVDNSKYAFFFH
NVKRNKKKNVCKLYFVNNNYKNNYNNNHNNVMRIKKNPRKGKTINKNIEENNCIYNLFGKKNIIKDDVTEVIN
NSNKSVNNMDNNYSNNNYSNNNNNDIIKKSSELIKHLNFKFDEECNDIMIRIGNFLEVSPHLLFKDVCVNLNK
LFTNLYTNDNANIPFGSTEHTLNVNDDVIKLNKFKIYCVLGLKVLRTFLIKYLRSIKLCIPVEVRKRYIYDFL
KVEYISKAYQEKYNLIDYKIFSNISEKLKTKLIYMYLSLNPRDVPNVLINIFKAANYNDEKELLYNYIKSSKF
TSRGGKRFARIINKEYITLYDKKKLIKKHSCEQDILWMRFFSLIKEHNNEIINKTKILRQTYRLVFSKINFFD
ENIVNLFKKKGFEIFDENISKNLNINKYIKLSTNHDNIQNHNIFTTKDKLIIGHYRKLSILNYIQNLKNFILD
YIQKKQSLHHNFYLLKKNINLPKNIYYDDTISKNTNLYHFTNINKDEQQEGVVNNTEQNLTNLDYHNKDKENV
NKINNNNNNNNNKKYDHDNNINHSNVIITPNSIQPEKTQHHKIQQSNNISLKKNETNKLSQTDINLYCDNIIN
NTIDTILDIMMQNSGFKNVKTVMGIYTSLLNNKDLKLIDFKNKF*
>N.PFL1255c
MKVLNFPFMLNKRSYFSIYNFFISLRKGGESTLKNDIKHNYIKELLTYEKEKRKMKKKITEYDGNNISNKHFN
EYLKDMEEKIKTHNDLISDDENNKKVKHLIKMNKTEGGIRIKCSTSLIYMLSLKCRYIESIWLNVAKNKKCNS
LNCLRDILLNIEWNKYIHYEDLKYIPLKIICTQSTLFDISNLKNEIYKYINEVILMSQGDQNILSKVEIIKNI
KNNINSEYSYDHLVTSKKFIDTQNDIYSDDHIIVQDNNCFTTTEKINIENNLKNIWNLLSVHIHNNICNVDIK
FTGKLSPRFYQYVNMKELKDIPNIEKYENINFSSLFYKNPKINDEKIKNIVNQTIPHWSVCEEKKKIQIDYEK
EIKMDKNNDDIYYKYNYFLDPYNKKLGVSNDNVHRNIYSSLIKKETINNNLFSLNDEQLLLKKETFEKNKNRQ
NNNYDNNNNVIHYKNPHIIGDQKENKTYNLEADLSKHFNITKHDMINYLNDLELRRMIYYNNNFFLSDDSYDS
SSCIYSLIIKKCLHNKKNINIIWDPFCSTGNLIFELLSYLLNIPLYNQDEIVPFMNLKIYNHHEFLNIYNYNM
KHLINNYSNIHIIGSDNRDMMILQCKKYLIRYALYYKKILKYIKDKNKNEITTQKDIHIPFNTKNYTNLADYT
RSNYIKKNSFLKNVLKEDLETYDGVDSSTDDMNDLFYETSPWNFSTDMDELRKVKEENIKNKNSVESIRNKNI
LNSSNHRTILKNAEKNVNTSDQCNDKINAPDMDIISISTEEDKKYNINLPFDVSFHKAHFFNLSPFIKNAIII
TKIPHFNFCKELGVNKKTFMLYEQFDQMLCSKNDWEGVYVLIRNKTFLNKSKLEWNKVLSIKDSKGRFLTLLS
WTGRKKNLYSLNNEYDKLKELEKFSEKLKFDI*
>N.PFL1260w
MLKHKYNDSCVQELFYKNNIKLIAIDIDGTLADDTGKISDENLKAIEVCKKGGIEIILASGRLHSYAMKMFTN
EQIEKYKIEKLDGVYSHGAYIHMKGYDYVYRKFSYKDLELILFSLGTYNILRNAVFLTVDSAYVINDDIKLIE
EYIYTPESEGIISDIEYVKIIDTNYKPILINKIKDIFNIGDIVSIEIYDKLYPNQDIYSDLFKVLFYELQPHY
KIYIPSSNNKIVLSPINTAKVHTTQLYAQFYRINLNNILSIGNDNNDIELLSSTGFSVAVKNSTPRALQVARC
VSTKTNNENAVANIIYRVLSGRRS*
>N.PFL1265c
MNEKIGVGKTWAQDVLLYNIKSLEREEKDFSLLYITYLLNMIYNKYKSIQPVYIYFICSQKKEKDMCIDNNKN
YKTNVHKNHDQNSLVYYTPSSIMEQYFPDLFHNVYFLCAHYTILNKEINEKDHIDYIKYNKEKQKYDEKKETF
YFIFTFFMEPHFIWEELNIPIFHEKGYVECIFDSVNNLIKLKNIFNPLHDLRQNTYDKTLIQYIFNMNKKRNK
KKFNCNNDNQINHDTNYYNEHNNVLAAGIRDNIKQNNNIYNNNGNNDNNDNNDNNDNNNNDIMSDILKDIMNG
YHLTNSNIKKKGNHMNTYHNDEHIEYSNIFSSLNNNNNNNDNDLYCYNYHIRKEEINDHVPRVQLLPFFISSN
YLYKNNKICKTRVISFNEKDIYFKNDESQNIPKAMVLYIRNKLSNKLKNIEKDVIKKEKEHVKRILIKNKRKK
IKKNSKHESFKPTKDLYKIHNDPINQKKKEANINQYIINENNNNNIVPNIQHNNIKNVLNFIQNNLQDNLQDV
KLQKLLLKYYKNYINSHYKENIYISNNDFINTLSLIQHNYKFIYKDNNQLHNTLIDPWQIGIGENNLRLMKKL
FSQRI*
>N.PFL1270w
MASSNDVHSDVEKALKGANIKLLLIDFDGTLFVDKDIKVPDVNIEAIKEAIEKGYMVSICTGRSKVGILSAFG
EENLKKMNFYGMPGVYINGTIVYDQIGYTLLDETIETDVYAEFINYLVEKNLVNQTIFHRGESNYVTEDNKYA
DFLQKMYSENRSIIIRHNEILKYRTMNKLMIVLDPSESKQVIGDLKKKFASKLTIFTTYNGHAEVTKLGHDKY

TGINYLLKHYNISNDQVLVIGDAENDIAMLSNFKYSFAVANATDSAKAHAKCVLPLAHKDGAVAYLLKKVFDL
KKK*
>N.PFL1275c
MNSNYLIEKEQNALINKKGSDEIKIKSNNEVEEKENDKKEENVAHDKSQQNKKLLKKNKRSTTISTYSPAYIS
EPIYQWEVSSSDKIFYDKINDLIIHQNVSTKALLVTDIKKNKLNYNGVYVKFDKDICEHICFCQNGSFFLYYI
VNPNRIEISDILNNHNIYLNVDINKLNEYEILSVFWIYNETIRKQDENMSFCDFVVVTNNSVEIFKISFDNLI
ITPIKKHFIKSKHCWYDEKSSYICLLSANKTNVILPYLLSNNNVIKLSKIELNILKNDNINKNDIEIITLYNE
TYCIHKDFKNGRISFRCLSSTIYFDYVLDLFYNGVIETFSLDNLFGVFNHTNQYVYLFDIKYNKKKTTIHSFD
DNNNHTINISYLTTPKQFKTQINFNHMAFEPYNLLIDNHYGNVYKVKVDYDVLMLQICQHFSNLNTSVDVLLR
RPNCKSRITEMFFLSLENDIQIEDYLSIINIINKNYRKLIEECARRGNAATITSNDSILNKTNKKIIQPLEYL
LKNIGDKTLMTERDIVLDVFHPYMIKKYHLNKKETLFNFSLNFREIEKNKRDKKKGTHINNKNDAEEYMLKYK
IKKKKKNFEENNNTELNDSNIKKENNKLVEHDNSLKQEQIIINDKNVIEHTKIYDNQKKDQVESNICNKNISV
DTSQYNNDNEYVNFLKHSEFHNMPMLLIYVLEYMKSLLSLNIIPNRILQTFIFDLCIFFKQDNFLRQLLQFYI
ISDSIEVTKRLFHYWKLTKHSWAYQFCVDMSLRLKEYEMVIHLFLSSKNYLKIINFIRNYHLIDYPISVIFQA
IENDFDSNDKYIIMNHILLSLKQWIIDSDRQPDKYPLPNLQNCEKWVTLVEDI*
>N.PFL1280w
MFQKKASSVCFFSKYHNNKYGYHIFFSSNFSIYKKALIQYSSINNHNNYNNNNNNNNNYNNNTRYRKIIVIPF
NDKDVKLEYKKIPFDKNDLDKKSKKELLNIYKHIRKKEELNKLPQDFLDNLFECTKKYVGCLLPYEFVSIYKT
LKIIKRNNKELNLLLHQQIKRIYQNFDITSISKLFQLYNFSKSRPTVLIKNLSQTFLNNINNENVKPWNIREL
ISIFSYLKIYDEKLIDEIYKKCIPIFSKYILNYQPKDVTIIFYCLVKMNKHNTTLSNVVTKHVILNIDSYEFH
QTAIVLNTFVKLGLKNNKLCKVICDKIKVKLQPQKFTNLTNNVMTREHGNNTYMYRSEEKKKKLNLYNVENVN
EDTNNRSLLNENNKEEDELNTFDELGRDNNKIVEMVHIDKKNVKEIVKVEETNIENKKSIRKKKLIGNQILEP
KDVSVILNSLIRLEYYDNQLFHCLTPYIINNVSKFSAQSLSNIIHSFSQIQINNEILLDNIVKECIKKIHKFK
NIEMSNLANSLVRLNRKDKILFTYIIDEFLYRATIGSKFLNYQFDVLSLQQLAYSFSKVGLKDDKIYTVLYRM
LIKKINERNKIVRRNNKIMNSNLIETESNKNGVANKICIKRKKKNEKEEKEEKKEDKINEKDIIEKEGILISN
KNELYKHNNNNNNCNDNFDFFSLCTFINSYGKVNVGNKNLHHFISYIIRKKKKNNEVLTNESLSFMIYGLLKL
NLKDKKIYQILLKDCIEKVDTLKPFQMVLLLYSLSKLKIYSYKFVKKCLQMLSIHINNLNLSDLSLVCYSLSN
FLYRDVIFLYKVNKIILINNYEFNPKIISQLFNSYTKLCYYHEPFYDFIFKKIIMFIHQFDEKELSNLVFSFM
YYFHMKKLHYDNIKSADPHNKNKQHVGSVPVDSGENKIGKNKCNHMNDSVNKDTKYIIMMENNNKRKGNDNKM
DDSGDINDKYNTYSDNTYSDNTYSDNTYSDNTYRDNTYSDNTYRDNTHNYNNNYINEHVHHRIDQPHDKFKDI
EHSSTKTQDNNPYNNSTCRNKNNISLGREEFYKNELNIFFNLIYILNEKYRTKISLISIYQLQVVDLYLRSFF
LNYFYFPVYLKTFFFKCRNVKLHIQDYLILSSKTHRNISRFLNIVGIRHRSEVLFGPYQLDIVIDFIEMKNGD
NNYLNILKHENNNKDDQRICNNLSNNNSNDYTIIYKSKRLEKEKIINEQDRKHKVIETVLNKNIVIEVDGISH
FYKESFSRTINSVIKDYILKKLGWNVIHIPYQEWNQCFTFKKKVLYAIEVLKNILEITKQDISLHDFINIINA
NYNNKKKKYYDTPLISNISQMKDSHNENNNNNNIQLTENKMLIYSKDKTEQISTEPTSNNRKDNYNNDLPKFY
VVSEENIFLNQIKNKNTRQQNLMKKMREDSKLKYDYNIFCKKANEAKKINHISDDLMSDYDNQEDYMGK*
>N.PFL1285c
MFECRIDGQFFKKLFETLKDICTEVNLECDENGIKMQSMDCSHVSLVDLNIVSDFFQHYRCDKNCVLGISINF
MLKILSVIKEKSTVFLFKEDNENDAVLNIGIIDEEEQSSADDSLEIQVKLINAQKEHLEIPQSEYHCQCTMKS
KKFQEFTKYLNSIGDNVSISMKKDAMILSTTGSDIKVTKQFTNDMTDISITCTKSVSQEFATRYLVMFSRASS
LSDEVLISLSPHIPISIKFNFKQQLTDLQDPSHLTFFLAPKIGDY*
>N.PFL1290w
MKFQNKRKKKHFVKNKKGNEKNKANKNIDDSEIESDDILSSQDSDNNVTFLNKKKKLNYDEEDYSDEDINNNK
NVDEENDDNEYENVFNNPEERKIYLAKKYLKDMGIESSDEDNEEEDSSDEAQSIHSAESQKSIDDKLSDDPFS
SDEESDKKKKKKKVSNVLIDNEKSQSKKHLLNLGNKIKIFYDEKLSSNVTNRNNKNEFKLKTQQCNDNNNNIN
IHNNILCDNKNLIFYYGHKKSVTCVASPDYNLSFVDQYEYKNEHNVNKYNGYNNGKFFYQNNENNNINDIHNN
NYCGEEDMSNNHNKDDIIMEPKFFENTSINTIYTGGKDACIIEWDIIKGEKVHIYKGNKNSFTGFGDKNSISH
FKSVMDIYCNKYNSFFISVGSDNLINVWDNRINKKCTNSIIGHKNIISGIVGCNSNIEELHMDHNFFTSSYDK
TIKLWDLRFFDKCINTYLGHTNNILTMNSIDQNKLITSSSDYTLRVWNTKNDNHILFNINYEIIESCCTLNNR
IFIAGTFSGHIYVFNSSYKKPICIQQNAHNSFSITSLISIPYTNIFISGSYDGYVHFWQFKNMNKISASVQKI
MTVQVNGTVNKFSFAHNYRYLYIAIGNEMRHGSWTKTKNKNGLAIIPLHFLA*
>N.PFL1295w
MSKKYVYWEKQGNDRMCGLHCINSILQGPYYSEDVLASIGKELDEKENEFLRSSSNDLVRNNSFNVLDDGFIN
ISVIIESLRRMNILLKHVYEEDLIKIISSNHQDIGYICNLQEHWFSIRKIHNTWYVLDSLKSSPLYIKDMNLK

FYFNDVIKKYHIFSVQNINPYVSLPKPDINFIPKNINQFYIPTNEISEISGVSDNFLLEDRYNLNKSENYSAF
NQINNTPNFRWPENGGRRLNDDINTTNMNNVDDDDDLKIALKLSMEEYIKNMVPPLEETSDENCINVMVKLPN
KKIHKRFSFTKTLSDLFYWIEYESAQGQDITSSLLFKNNYYLYQLYPRRKFCKYQNGSIELHTGGKTEMVHDK
CLKDLNFEKEETFMIS*
>N.PFL1300c
MFIIKCIVFVFIFCNGLLSVLGKEEKTMIRTDKNKFFQSIRNKIINFIANKKGFDRNGWMNMSIVQPKLNSIN
VRIENDHIICNEENQVLEVSLVRQNLLRYTDEGTYHEILLSTSNLSPTSQFINPLPLMKKNHTMNNITNHTGD
KLPDGFEGYLICEKKNSQALLVPKIIENVEFISAPWKVRVSIDFYRCPITNFGRVFQIIIIDHVNVLMNNQRS
FVWPIPLASFSEVYDFHLAVVDTKKGHFCELERNIETQDLNLNCPCWNRSYKFSITNALFLSGYIGMNVIQAI
AQPFLEDSNNSRKQPANSNLDDIDSHKKRITYNDLIDNTITNNNDNNINNQNQNYDDGKKKYLKKLNSITHLI
YLSITALTNTAYSMMALMQIKKNLDLFNKAYEKAPYFYPWSLKYPENYLSTHKPELLNHSNKNNINHILTSGK
SSNNFSSQTYSNSTSQYTNKQTTQKDKKKDTSSNKSNKQTNRKPTIVKQVQNYITEN*
>N.PFL1305c
MMELEEQHLIRALKYLTQTNSCELSQKECKIILNEILSQNLNDENIRDLIINIKSGIQGNKHNYVENFILSPS
MLYLLINKQLKKYDLETIFKDFFNCIQNNNEINSDDLKTFLNLCNKNMSRNSSIDFTKINLNMIKLKEPMNYN
IFINMMEQYLKKKDDNV*
>N.PFL1310c
MRRNLLKLTSINCKKNDYVSLSNNFFLIKNRSYKYITVRNKIGRNVFNEKDNCAILNRGQTHVVGIKKNICTE
NKVKQQDGEFYMNQRVPDDMDNDMDNNVDNDMDNNVDNHVDNHVDNHVDNHMDNHINNHTHKNVHDDTSYDNN
SFDRYQRKRDEIGENGFVQNRRYENYEKRKQSKTNYSYDNDYNQGSRNKRNMSYPDRYEELGSHLKDIEWDNV
KYKMERQNLYNNNYNKCNDQNNNQNSNDNLNNEQTNCLSKEDIQNELKKNNIYINKDGIIHNIINKFSDVCFH
ESILNYLNNKFSEPTAIQKITWPIALSGKDLIGVAETGSGKTLAFVLPCFMHILKHKEIIQNNNNNKQTNQNN
NLQQEQEGKTNNHHIINNYNENNDNNENNHNNNNNNNNNNNNSYDYDYDINSSERASDTYGLILLPTRELCLQ
VLDEIKSFEKNLPIKSVAVYGGVPKYYQINNLKKGADIIVATPGRLLDFLENGNINLLKCIYVVIDEADRLLD
MGFEKQLRKIMTQVNKNKQLLFLTATWPEQVRKLAYDFCSYDPVKIQIGKNELTANKNIEQNVIISSSIDMKK
KLLDWLKENYENNKILIFCDTKRNCDNLCKELRYHQYNALSIHGDKQQRERDRILNNYKTDRCNILVATDVAS
RGLDIKNISVVINYDIPNTIEDYIHRIGRTGRAGKKGKSILFFSYDYYMPQKLKFAKELIKLLNKTNQTVPPQ
LKEIAYSR*
>N.PFL1315w
MNNDNIGREPIDVVYDKRFSSRIKPKTKKKKRNENRMRLVRIQHYIYNIFTSFLCLFIVILILYASIDISINY
GPVIILYILILELGSMLISYILLQFPFFYRKLKNTFMRARNINKYSHQYRPLYYDNNSFTDENESFNISDDEK
KEKRKRKHTYSFMSSDGGRKRSSFRRFLSSYKSKKGNEFVNSKTMKNKTKKNTRRKGFLSFNWIGYKNGETNK
NRNDNIVNDVYKKYNDDNYDDNKYNDDNYNDDDNKYNDDDILNSHSEQDQRKKKKKKKEKRIVVFPRNDTGGN
SFKRESIYIDDCNMWMKKTMRKSNRPNFKLTRSLSFNINMNKDDDLMMKDSASLDNIQIFLGHRSSKFLREKN
KLQKRKTSIYNKYVPLTFASSYKDDFFKEILKSQTDGIISSSSDYSSSSEDSNKNVYKTIYINNNNNNVDKKG
KIDEEDNIVVRNNIHSNENIIIKENPYSLRNAILFHKEKRKYRKNINTLSDDDNKNFAFHSGKKKIKRKEKKR
RCFIKLKCFNKIKNLYRFFINYNMKSSKSSNNLYLFFRGFYIYTKHRFVHYLSYEPIWIVAILIRIVLWCIVW
IWAASYMERKPKNFEITEWNMKNVPSFYGYIECTFQWCGVFDYFFGLYFSNNKLKYIFSFFSLIDFITTPVSS
LIMNFFVQDNINHNYWFLILGPLRFLRLVRAESTISSCFFWLNDVKIIIIGIIILALAILFTFSGIMYILEAP
DIERDFVKPLDFVYFGVITMSTVGYGDYTPVTKAGKFLTMFIIITCISFVAAQFKRLKEAMFSPKTVMGIIPK
QDDDYILILGPVSPTQLLYICKGINNSFPNSVESIFLFTPLPVVIYRYVYGSIVKNTNIKICISGGNECFICP
SIIYDAVINSRALYILNNVDSEKYTLMYQQIFLASNNINFNNHDHQNQRISSDDILHNNIINKYSKDKTKNWK
YSDLFSEYKNEINKNNMLNLEMNININNSYSIKEKDDQECLLRFIGTYNICNSLIPITLQLSNNTYEELIKSM
NVYNYISIEELKYALLAKSVNCKGLFFLIINFFYKPKAVKSLKKYIIDLKLLMYNGMLRRKNRERLSSSNLKI
KQPSQQKSRKGNLINKISLFFKKGQNHQLDAEGISHDEKNNSNTSRTRGNNRNDHMDPQIKGKGVFEMNQKEQ
KQVMLKEGMNKNMNKNIIKDMEKNMNSNGKDNINDSINESIEKNDLFFLNSLDPNDDRNIHNLSYKNYYYMLE
KVNLNMYYYLEGLKYNIYRFQFPECMRGFLFQTASEYLYQKYSAFLIGIITINKEIFLNPVNYIIGEENKYFY
TSAFSGIILTTSLDSLIKLSSINNISKKVYEYNKRLIEEKYKSTFSKSWINSDDDHHNSDHKNFVTNTQIKET
QIDGDEVKIVLTSHDDSLHPSDRSVKNRKNSMKSIYNQKKSNLGDQKKKNRNQDDRTEHQVDDNNDSDYLQIE
SVPKKKRNMYYSLVLGICELENYISAYKDAFVDKDKPLLLVCGWPDNIHMFFKYLKRNVYNSSYRNKNNSNIH
TYDNNNNNNNNIYSKTVKNKKGKRKKMKKLNDDIKYNIIILSLHVPKFNYENDLFNYSENVVFIRGSPLNSYN
LIQAGVFYAKRIIILNSNHSLFIDKDAYRIDNEVIIIKNIVNQLFNYISKNKENYFNLLRKTFPKEYIDITIN
ERIFLNDNINNSPNINEMIKGKMSDSSYDSLDDNYSSDDDSSSSNYDDDDEDNDNDDDNDEDNVDNNNDHGGE
SLLLKKKRKKKNMIKLKKRALNVNNIFNINKNPYLICLIKNSESLEYIDGSINLSYENYNDNEKMNKIWENCG

EYIYTFELVSANIFVHEMLHNLVSFSLPISKYAIEYSVIYSLIGIDINEYSKNAQKFHKNLNLSTGYVNLVPI
PSYFYKKSFYKCFSYFLHNKLSLCIGILRYIDISSLSNKSKKIFVLSCPSRTMKIEPHDQAYVIAHRM*
>N.PFL1320w
MVDIKNIVVRRMINYYKNVKRNTSPFCMNLMFYNFHEDFILILIKTYMNILKKYESYNKTIHLLEEYYMLFNY
VILSYIPLYVYELDINNLYLFDMLLHKFNEKRKRENNMKKNVIDNNIYKKYRILNRINKNGHVEANKLHCVHH
KNVEYMNINKLNKHMNICFNDNKCDSIYSLKKQTSEDIQKNEKQNDKLILPSFQGSLKKNIYMKNNTNRRICE
TNLFYKNNNNDHLKLKKNRSPIYLCEDNLKKRKDIIKNMIKEMDNLYAHVYYWKYLTELNIKCNNNNNNNNDK
NNNLEVNYAINKGQDIYLNIYKNIYNLYKYMNKKHKQNLEKMNMKKEQLSITHNNEMENIINEKKIHKKNEEY
HKNVNDMVFKHIAEKNALCRHIEHEMNIIQQINMKEYKENIFYIFSIISKNKQNLSLPPLPVEEERDIQKEVR
PYHNEEKKKKHNNNNNNNYNNICNNNNNNNVSNINNSGCNNFNNMCENMNDLSKHKCKDIKENYIYNDKNNITM
EDKIKTYYHIEECYYHYKYIIKCFHFKYLYELIKSKIMIYNYLHFHIKKSLYNIMNVSIIFSLGEIYHIFEYD
KKKNIQFQDNFFTTLRKMQIYNYNKYYQYNMNNEKGPDKYFNSFQIYEEKKKKVTNVLEIQKDEDILKKDVNT
NEWANPTGLKKKILHLLKGGTQINKDNEHMNKFVPIYNNSLLVNNILSGYNAHDIKEQKRDDSLKCEESFYFD
ENGYFKYKKKRKGAKEFFNHNYSSTYNYINNNNTLYFMNNKMDENIYNNNNNNNNNDIKINKLTIKDKKLNCL
ILFINEDISNYTKENVYNNIFNISLTKMDFIFPTIKEQLHVVKEMYKGKKEKLFSDDLYKKSIKNICNDISEK
NESLSYGTIIIITRHKNLKIAMQGKNNFKENHQTGDNNNDNNNNKNNKNDNNNNNNNNKDIKNKEENNIYYNNHN
LDNLNYIPFDNFFFSLLNMPEQVMRTSHFSINEKMFPKKKKKKIYIDIIFHVLIPKKINRKSFKIILFSLFKI
LDLCKLYNIYCINCSFPYVHDIVYKNKRPIIYSFIYSFIMTIMKYIKKGQPEDHNNTVRIFHFIFPPLQFYEK
LTTEQMENKNDGKNQFKENINKNYDNKENNINYDYIHNVDKTKISNITYFIDKLIHDLKEKYMLIESI*
>N.PFL1325c
MKKKKKRIKKTNILYVKDGKNKLKNNKKKNKIKAKKKKKDKKEKIVYNNNMLNNITGEINIDYKEKIRHNNND
DEQVEDKKKENKNIYNNYNNLQNMNNEKDGAKYYVNTNLVKESNYEHFHNYVKVKSDINKINHVDNIKEKNRV
NTISAENVINTVHIIPKGIYDKINNYNNINNINNSKDLQHFRENNELIINEGNWKNQFFKNGKFKNCITYKNK
SRYYNNIKGNNINNNNMNNYSNNYYNNSCKKVKRLWNTDIFKTNKKRNIKYASLFNNNYHTTNIIYEKSSNKS
FKKDMMSYIEEMKNIYIPTNAEFVKVADDIKNKYYKDNIYDGNNICDGNNIYCNNNNICCNNNNICCNNNNIY
CNNNNIYDNNTCDKLKRTKQNERNIIDIFENEYINNSFLELYYNIGKDIIYLVHVMKPTLYEIYMRRSVLYNL
HLFLKEIYSDYCMIIFGSCNNDIDIYNSDIDICIYNTIQSDLINIRKLYKNMLHHPLFENVSIKKIVHAKVPI
IKCFFNYTKLSVDISFNQLSALTSTIQTQTYIQKNPLIKYILIFLKMFLYQHDLNDASKGGISSFNILLLLAQ
FLNSQSFIFYEKHNEENSNLNNNDNIHTNNSNNNNIHTNNSNNNNIHTNNSNNKNNSSDLLRGEHIKNELLFI
GEVVFKFIYYYGLFKDKNKLFYFLQNHYHTTNLLNHISFFSTNKSSKHFYAHMNDSANIFHDIFCKIQIINPV
EKENVSRISFDKIIKIRLCFKQAAFSMITLILNYINKYIFKKSDILKHHTHSYYKIFNNCIHFISLLNSLGDT
FLSKNLNNYYNFVYNNVNTLLQILNDNQEDMEKIKTDSEDINEKYISDHKSHNLEDKQISDIISCQKSQQIYT
QQNVYNKSFIQTHIGDHVHISNVTSAYNDLDKEQINTIEPHFKNEEKKETQTKSVQHSDNFIKFKEINSDNTT
YEKNKIIYTNDNMEVSTDSGLSIWNNIINIQENNNNDNDNNNDNNNDNNNNNNDNNNDQEKDNMSDIKNIKLG
NKKLIKQLHTYIKKRDLQKFKNKIKKIFKSFDNNNNNNNLKNKPSEFVKNICQHLTTLNHFMDVHDILSKRFY
YYYIYNDTFKELKNDVLLTNDNKLIQKQKKEMLKKLIKMKNYYAYNFLNLDINTNVFVQPEVQQ*
>N.PFL1330c
MYVCMMNPLLLPFKGDNKTSKKKIDIKIKSKEVYKLDLVLLINRKRKYENYNVYVYHIISLVKKKKNKVQERY
LLSLQKMKENILKNKRKIKEYKIRKKIYIPTNNIVRKYIDTYIYIIKRYYKNDDYEVLLTLLNLHHALYIFLS
HILRFYNKMFHITKKKQVDKKEKRKRRKKKKQEKKSLASNSKGYKNDKLFLWIENDICNSYCYDNNFVINNYG
DIKLRNNICFVIPNIIIIKLNELFHHINVIKYKCLFFYDNKKSDWEIFNHMEMNKQLKNNNNNNNNNKKEYLF
LFLYLYLYKFVYIKDMPFYLYNKKKKKKNPNLIFNHRLYFSLYRKKYCPYKNTCLSKKKKKKNNIKNYISINK
KYNSSDDFIFYKNFYDNNDLLYKNKKDTMKHCDYINTLVSQRKFRTVQKKFISKGCYNKIIKIKRKDILHSFF
DDVYFYVYNKDKNIFIRNIYDILKCLCTDHIFFFYRTIKALFLYNYKYYNNIYKKENISNIFLLNKRNDMLHM
YDIIYNINYYSYGVHKNTYPFINLSNKYFISFLDGQRIIIPLRNVKVFLPQEIINKHLYISSNLYDILHKNLT
PTTKRNFSLVYKSKDGNKKRKKKRKKNEQPFVKRYNKYNVKEGYNNICDGNMCDNCVDSNFDNYHHEDIFING
CKTKKRKKKTNYIGYSSCDEEECFISEEKKRMSRICPFINYMNDKIMNSYLSEAVFEQKTLEMELSILNKGGK
KGKNIIRNEKEDGMVVCPEMSEEDILPEHLINKNKNNNNNKNNKNNQNNNNNNNDNNNNNQNNINNDRWFDDE
ASTTSCDKQMICMNKEKKVLIKKKMDGVLCDELPQECIDNMLRSTCSISIPGLVEKENKDDTGENMITRSNIY
NGNKINDKSYDEYTSYDNNCKDNYINISNNNEKSVDETADCVSYKDNNKNNNDYINNNPYDDRMNEVDDLSPK
CNECTLNDSNILGYGNYECTKKNTPFDEECCKEKMNGGDLVEVEVMERHSNILINRGIKTKKCVDYSLYNTYA
KSSFLQCAEQADNHFIGNYLDRYSDENYYHLRQKIKLCLSRTFANLKTTRETCVLHFTNLIFDLYISRFNSKD
EIIKSIIDDIVTDEKQFSDVMIQLLKEYYPKIYNDFIYKEQLKNKYKILYEELKDSCDLIYHFIAIICLFISQ
KYYNYRLISIDLIARTYCKSHLEGLKKVYSYDNILIKDASPDYYSATQYLDTTFSSYSVNKNFALEIEICILK

KLNYDLSIPTPYSLLDSLLKANENLNFLKTRNDYNSTEGEILLRIASIDNTFLKYKSSTLAMAIYEILNFNIC
KDKMQKELFVFSQNNTYYDITTNMDYTPQNVFTKNEQMADKQKIEIYSEQKYDHTNDTTINNNNNKYNDGGDN
NNKYNDGHGDNNNKYNDGHGDDNNVDKGTHKNKPIQLQNKYQSSIPLDNERKEKEKKNELKLLKRQARSLVIA
EEEDRFITASKYINEKNKMFKLIKNIIIYLCSLMKKKIPKSYYKCDYDFSNKLNYYIKRFQKGKQDYMKKINN
NNNNDANNNNNNNNNENNNGNNNNSIFKDGCKQDTYYNKKKRKFQQSYSSTCTTHIRDINQDSANTKKDTQKI
ENNIYDDNNKSSYHKNKKINTYKEEHLHKQEKNTKVIQQVTANKINQNIDNSLKKNKKLYKKQEKNEKVFCET
KKKIIKRFKKYMKKLIKTKYDDIPIQYCTSYILQGKQLKVYLRKKDHHDIHMNNNNNNNNQNNNNCCGIIKLE
THNKSKNNYIIGTRTKSTDEHLIYYYNYISKLRNRLIKGLKYFLFRINKIKKRKVRLHIMNLNYLINKKKKKK
NIKKKKTIIKNMSFYKQLIKEIKILFLWIYKLTNIEIRPLIHFKDLKHITIIKKYEQNYSHYIMSKFNNSHNT
QDPSYSKKSKNKNKNKNNYNMTNNNNYENPTIVKKSNKEETKKNIKNEHVKNKYSGKNTQKCSPAITLNTSKE
NVQINKDIKNMKKKGKTKMNQEYIEEVIKCITWDDKTFYPFVKHNSLMSTNQKSSHKKEGEKSHSACEYKDNK
NELAYRENEKDVGRENKAIFFEQHMSRSNNKNMDTTPGADEKQNKKINIINDMENKKGKENKSTYESIDGYTS
QYENLYTNIQPNQYNNEYTDQYNYQRNYQYNENYNNNKFSIKKKEYETSLEKMKNTLDDTLPNNTPVLSTTDL
QKNIDKIKNTDWITINDPQVDECAKELMECFLKWKNKNYISKNWTFNEYPNCKDYYFSLVFGDLKSSETLKGI
IEMIHMKYNHLLNICKEINIQNDIL*
>N.PFL1335w
MNPLYTKSKFKTTEFANNENKGVRFINKYKTNVNNTYSKNSETSQSTQLTHKNGIMKNSNIKNKGTINYSFKY
PPKSNLKNNMKSNLKGDTKNISNTYPNKIAPFHLNKSGYNEYKNEEKKNDLMNTRRSIMVDNLRKPPLASSSN
RLISQMKKDQLFNGKNGEDPNVNDDEPLTNKYKNKTHNNNSNNNKISSSNNKLHNNNSNSSNMKKSNIYYKPQ
DNNKNKFPNKSYNVSKIFYSNQYDKDCAMSSEQFGYTYSSPVKRKYDAMDNSRNMDDKDKNEMSYGKKKFVGR
NYYLDKNMNDEEYSNCDKFGDMNMSYNVKDTTQRENNNDNRNDNRNDNRNDNRNDNRNDNRNDNWNDNWNDKC
NDDNKISNNINNLKVNVNKDGDILFHSQNINKKEINNNLPFNKRDDEDIILNRSYPRVSMSVRNDTFNSNSLG
RNYTNYERLKHSNNNNNMNNVNIGYNNVGFGNNKEESALPTDTYNDKNDMVNNNMSVKKKMMMTNYPYLEKGK
LLNGNMNIYKNEKITKSYKPTPFNNENNNNNNNNNINISSSSSSKVLYSHHSSMVRNHHNNVPHYISDTNSEK
IVMNHIDNINNKDVLNTYGIDYLSEENNNKNRRSSIFNVNTNINRNNLKKKDITNVSNVSNMSILSNRSKKKF
SCLVKNDLESEKGRGRSSVLLLSSEKKGGYGINNMMINSEGMDFKNVSNAINFYSTNDDIGNYNNDDNNNDDD
DNNNNDDDKNNNGEDENNNGEDKHNIDEDKHNNGEDKDLNKSRYNIDKNQYSDNYYDESYFNNYVESDFQRRQ
MMEKGGTYFFNNIYNNDMNNMTNKNEHLHEHLHKSILIKKKYNIDNYKKNDSEYLYFQNMIRNITKYNNEEND
MNNLYENVMNLNDYCLNFTSENEYINEILNLSNEVNNNIQDIQQILYNKKKVVQDKRHMVNKEYELTCQKLKE
CESIFCDSNDEHGEKKLKVFYEIDEKKNLLKNTYEVKKMLLNKVQYKIDKLQEYTNAVKLKTMVMSKRYKKTF
LLIEELFRLKIIKDNENQLEVCLIPKNTETDMWHRFQLDKSEITSDACDYLWNQIESFVDKDTFNNYF*
>N.PFL1340c
MVSDTKSEDSNISKKYVYGEDLENIKNDNSTTNDKINDEENIIDMKKETNENISHLNYNEACPNSIIEKGHDV
ITHNILEEKNDQCNTNIILPIVKECNDSHKDISLSRNENSIHNISENSYQHDKDHISNFNLDEVESSAQDETN
EAKSDDSKMKIKIQNLLKLIGLLKAQINQKDQEIYKMEVDFKLRNEENEKKIFKLEEIIEENESHTERTEGDN
FMMSKMKSILISQNEEIASLNEELKKKSKEIFYLNEENMTKDEKLVELKKEIESNYMHLREYKNFQNELEVDV
NNKIYSAENYVNRTNQRLIENNIDIKEKKLNIEKQKRIIKELYNELNKKEEKISELRSIIESIELNNSRDIIK
YKQNNMDLINRLSLNNSLMNSQKIEIENMHMNYKQLEEELKNKDNELKKLHQNLLSKDEENNKMVHDINRLKF
DMEVRNIDIDNVKKKIKNVKKEYAINLKKQKERYTNVINEIYKEKDEIIKNHVDELAKLSNHYNEVVLVNENI
KNEMNFLNDEILKKSCEIEKLQDRLLDYESKLLIYENNNEIKILKKNEDHLRKLLSKHIHRNEQLLNTTFLLQ
KSTLENNSLEKKIIELRAKSYRKDQEIKKLQETKSIRKSPSSNCSHNSQEYSMDLIDDLNEDNYNLMYYHNNS
IVGDQENSVYTSKKSYDKNITIGSLADTEKRSIKIKNIIPHSHNNEDPVHFALCEYLNCFKKNNLSIKINKLD
DNTYLLNDKKVVISFINGDLYVEDDASPIKLQDYLLKTVVR*
>N.PFL1345c
MIKFKIGRSNKKYEKKSNIDNNEENIKESCDDNILNDQVATDLNSEELNEKNYINEILDESSEDDGNVINSSY
EENKKTKNKEESYYYINDAIKRNYPLEYEDNKIYPKVYCYEPTNFENFKKKIKNKNELRHYECYSSTMNEFFY
KYNENYYDDILKNESFKKFAEELWSNRSNIKTDQQYQDLCVQLRKKYKISPSKHQISVALQHHYLQSLDDDKK
DMESIIELQKNSDHISDINDMDNIIAVDNTNRVDNMNKHDSSHVDETNAPSEEDEFMINKKGKDVKFVLENVN
KMIITKEMKNYKDLDKESVHFLQINKRKGVRSNSGVLVVTIITHPHKFSCKYDCHYCPNEPNQPRSYLSTEPA
ILRANQNNFDVICQFFNRTTTLVNNGHVADKIEVLVLGGTWSCYDIAYQEEFIRDVYYAANIYPVLKDRRKKL
TLKEEQEINEKSNCRIIGLTLETRPDQINKDELLRLRYYGCTRVQLGIQHIDDVILKKVNRQCTLKDCIRAIY
LLKENGFKVDIHLMPDLPYSDVYKDIHMFKYVLSSTDLQADQWKIYPCEITPFTKIEKWYNNNEYKPYFETNK
NLLISVILLVKKSIHPWIRLNRVIRDIPNPSIIAGNNITNMRQLIANEMNIRNIFCQCIRCKEVKNQEIEKKS
DSIFLRIYKYPTLGGDEYFITFQGRKKVVNHHGKKNKKNKKSKTSKKDNLNQKYNDEQMKEKDNNKKIQNKNN

VNNDNNSNNNYYSDSNGHLSSTSNVLENMETFFNQHETENNQINHSHNTQYEHSGASQNNINSEQSGASQKNK
NSEQSGANPENVHNTNVPQNKMDTEIPIDHNIYEDYDNEYSLLGFLRLRLRKKNNYCDDRPFKCLENSALIRE
LHVYGSLLKHDDFKDELNFIQHKGLGKCLVLVAEIISYFYNYKKVSIIAGVGTREYYKKLGYTKEETYVTKIL
KREEIYKNYLLNIDRIGKTILIHEYNLKHCLYLMHKEIPEPSKYKRTEITNLNSYINENILHSSDMKNYLVHK
KKCTTSSINVQRLLETETNQIVNILWNQFNKWSLDFLIKNRLIFNVSTIVFFSSTFLFTIQFFKKRNINT*
>N.PFL1350w
MFLLTHNIKLRIRKYNNRLIIFERIYRYICDDIKKEIIEHSYSKEKCDNLNLSEECKKHIDFKQKLLYDYVSK
ENNISTFVKKKEENNLSSYMCLVKDNDRSKLKIKINKKKTKEEEKEDKKFDQEKKKKKKKKEEDEILLNNKYV
EVEYMGEYKKWIKKKKNNYLKEEDNKEKEIITKKLNKNVKEQNNEHIYQNEDIYQNKHIYQNEDIYQNNKQDT
NISAHHNNNYISDEKKYNKSDLCNISQTNNNIILDIDNDLKNIYIDNSKYFKLDIERNTKKEKIKLNQYCSYN
GICSSRFVNNNVKNGLFKVNDKIVYENVEICLGVDKIELTNRGKDLLKNKITIILNKPKHYLSISNDNKSYKK
LLVRNLIRNENKYIQEEHKCMSYFIYKNVNIEKVPNLYVCGRLDANSSGLLIFTQNTLSNNYLLYRYKYNIEK
EYVIKTYNSITQENMKLLKEHFYVDGKLIYKCHIQYVDNFTLIFKIYQGFHKIIRKMCMLSNIKIKSLHRIRI
GNIHLNDLPIGKWRFLMPNEYFF*
>N.PFL1355w
MFNIFIRNFSKKAGWSWIKNRRGRKKILSISYPPKDRSNIIKLSDDSDKLIFICNGKLRNNFLSTVKNMKETK
CIIKNNNITTPCLVISKAKCHTLLELFTYDEILHLEKLSPHILKLFEEAEKGFEENKQNCILRKHEALKFRLI
K*
>N.PFL1360c
MKIDLELIKKKSEHNEGLMEDLEEISLHQLQIKKIEFINIHCKNLKILLLQNNLIEKIENLNQLKKLEYLNLA
INNITVIENLEKCESLKKLDLTLNFIDLDKIEESINNLKKNENLKEFYIMGNPCSNWTYLKYYIIFQVEQLEV
LDGCDILISDRIKAKQSFEQVLISLKKEKQINKSKENETNNLYSINNRKQIYEEIEKEELEANKCKNENNEKN
KKKDEKKELSIYNEEGEIRQCNEGHYKFMFDEYSNKKYTFLKLYLPKYLCNSLIQIDLNICYVRCIIKNKLFQ
IKLNDPILTDLSKISRKKYTGELFIKMRKKSYQKNKIVTKEYSNEENIYSYDNPTDLQQPFLLSSTMCTPASF
SSSSSSSSSSSSSSSSFFNDHYSYIVKNKNTNKKKNMLKSNLCLPHTKNSRFFLEEKRIKYFSNYLNQIPTLE
KISKRSI*
>N.PFL1365w
MIRLIEKNLRRKSLLKRGVQTYICKDKEKISSNYEELQFLDNNIKHNLYKLKCIEQKKIKSDRIVYQKILKDL
LKEKHNLDSNELVEFLYIVIYNDLYYLDISCNFFSYFYNNYIFSKKYFNNVNVNDIIHVIYSFYLFQNYHFFI
SKNNFCDLDKTHEILDNEENKKVGHILSHMKIKNLEGQLNKKNNNNNINNNIYGYDDNPYGYGDHLYGDDLNN
SRLYQKSEENNYSFINKKKYTQYYNNNTHSYFHIYNYYSSFIHHNIDYINKNHLIKLLLVLSQCINNQDISYH
LNKQKYENNQSIEKDIKTNIKNPFEHIQKNIFTLIEKFIEKIDLKKDIKKSYTPERINEKEWEKKKKRYINLI
DGSFKNYIYIDNHLNNNKLVCLFFYVLSNIFYPISPSRFFFSSSCKENIAKSHNFTANNNNNNNNNNNNNNNN
NNNNNNSNNNNNVMTHQMDNINTLLSYSNNKCDDIKVPGVYNENTNDNILVEQNGHLGIIQMFKNHYNNIEEM
SNEFYKNTDKTKSSIENFIIYKNNVLKSIKKYVFLDAIYNLDVHYKLMFFKSIYTLNFYNSPFLKHTYSIQSY
VQYNNLDNLDFYDNMDFLNTPSVYIEINQSDEKGQCPKLGRVKKKSDNIRIRKENKNCNNTDDHYEDTINDNV
KNYYEKTLKNKLIELSKEGDIFNNAYIKLLNNIENSIKNNSIEQNINTLQYMYLMRFVDNHFIILLISKLCNT
SDKLKNEHKKKMNAIMMSLCIYLSSHIDSNILWFHNTEKNLPINVFNHIYYNNKIYLSHLKKLNDFILLLYNK
QIFKRKRKELLSWTNYKCI*
>N.PFL1370w
MPSKYDDGESRLNEYEVIKKIGNGRFGEVFLVKHKRTQEFFCWKAISYRGLKEREKSQLVIEVNVMRELKHKN
IVRYIDRFLNKANQKLYILMEFCDAGDLSRNIQKCYKMFGKIEEHAIIDITRQLLHALAYCHNLKDGPNGERV
LHRDLKPQNIFLSTGIRHIGKISSQANNLNSRPIAKIGDFGLSKNIGIESMAHSCVGTPYYWSPELLLHETKS
YDDKSDMWALGCIIYELCSGKTPFHKANNFAQLISELKRGPELPIKGKSKELNLLIKNLLNLSAKERPSALQC
LGYQIIKNVSVPILSKKEHGTELTNIDSCTSNMDNKHNYAHPNDVLINKRNNERERSSSCLSRQSVITAISKD
NVKYHPPDGTNNSHLINNGKYTNNSRVGGVHADGPTKMYHIETDRFNEKEKMKREIYERERSQRKAFEMKLLE
KKRLEREKKERVEREKKGKMERIERERMQRLERERLERLEKERLDRLEKERLDRLEKERLDRLEKDRLDRLEK
DRLDRLEKERLDRLEKDRLDRLEKERLERLEKERLDRFEKERLDRLGKERLDRLGKERFERMDRLERDKLPRY
EREQYYHNDNCTTPTTSCSASNNNKRDSMENERSSSKNQGNYKSFSRMKETYDPVNNMKTYMCRNRKGNYPYD
EHNMDKDEINSLLKKKSINTISNKNTQSYSNSSTHINNNYNVVNCHGAYNNHNTLSQYSNTSVENGKYKYENK
YQGNIRNTSKDVYNENMDSAYRSPKYEKGYDDNKSVNKKKMNSNNMGNMNNMNNMNNMNNNNNNNSNNNNNSN
NSNSNYMNNNHHTNNNNSCTSNRISNMYFNDSSRRSVSAMPNVNNVSRRKSSVYLCDDNMYNQNNVEESRNFK
NLEKDREYLLEKRKMLLEKEKGIYDRMKHTSNNNNMNNMSNMNNMSNMNNMSNMNNMSNMNNMSNMNNMSNIK

NNYSNQNNISNNNYTYMAMQKYERDNGKAMDKSSEYNRRNRSMSACINDEENKNSYNVKNEQIVEDKGYALRS
RNVYTLKNLVHKKDADIYDRGLYTCR*
>N.PFL1375w
MKDICNYPIGQEIILDKLIYEKRFLKNKNKQNKIKNVDQLNYYLNNKFNYFLGHTNFMKKIFKKKKKLLKDDE
RDKHKCKNRSKEEIIINKKICGKRKNVSSECDEKKRLHKEKNVENDDTKKKYSKIKIEGNSCDIKNEQDVMIK
KQSFNDSINNCNVDIYKKEINNVVQVKREKSSEEQLQIKYKNDIKRDNTSDHICKDEREHERESEHEHEHKHE
HEHNLENNHQHNVEHKYNHSNNHKRSNNNYPGNYNHCDETSSVLSEEDEKLHSFSPLSNNSDLSNVSNMSPDM
LCFEKQFKYIYDIYKQNDKNVFLFYNPPVCKCVNKNLKEQYFQKLNDKYPCLFNCSYYVDSMKTIKYDCKKKS
DNINDKNIINNGKDEKNNNQVLNNIEDKQAHADHHNINNNYEQNIQIPKKMDIHSEQENINVSTNYGEVASYA
VEIKMYAKAFFELYFMEKYKRNEGVIKVKYDDNKKNVYDDDDNKKNVYDDDDNKKNNYNDDNMKNIYNDDNKK
NIYNDDNKKNIYNDDNKENIYNDDVKESYVEHINHNIDICKNNKKNKKEKRLCLSFLSSSSEEEALKSYFDKC
LYKIIKRRTRTCDNNLCCGLLYIPYSYIVVLLNRKIENLTYACTYLRIPSDTDIYYNNKKNVITLCTKNSQHE
FFCYYCLKQNNYVKNVIIYDVYYEGFIPFFRPILNLKSKKNQDKIQNFITYNKNINLSIVFFLTNPREKIERS
KKDGNMRDGYISNDEKGKNIPCNKKHENNEDDYRKDNTYDVFFNNNNVEDIKKMRLKICKIFNINHRNNLNNN
MSSSKRHIPHDMNPDDNIEKYFSNLCSGIEVINNMNIKMVVDKIMKIINKNDSVCNKKCIYHEYNKNRMKYKD
IQIYLCEEINSFSFDRRPLVVKKREVNLSSYVHFHKIINNIYEQKNCHQLLGLINEQLDKNIFNKAYMKDKIL
QVALSNNMFEYGSSLNKDNKDVYSDPCHNNNNKLYCSLNENKFEEDNQNILNSLDRKKDNNVNHSMVDDTNLS
NINNTCHKNNDTNNNDNNNNNNNNNNNSCDNNLSTHIEEKENIKKCTDNIFNDTFFIFNENSDLYTNEEYKKN
RIYSINNKNNINEYVKNKMMKYTSNNYLSNKISTNILQNLFNIYVCSYLMKKNNYNISHIEKKKKKKRINLSN
IYKLKYMKECYYSLFNMYKNKFNIQIKQQEEKVIHKYNWMYEDFYVNRLISQIMKKKKKKKKKKIDIKMEKEK
EKEKEKEKEKEKEKEKEKEKEKEKEKEEK*
>N.PFL1380w
MFPSVQLKKTILLRLSKILSLSSVTSRNKAQELIKNGDIKVNNQVVNQNVFIDVNSKIQVKDKPIQVDICTKL
WGIYKPKHIFCNTEKDYTYEEKIHFNKMIEEKKLLDRHNNNDNNNLLCVNNNNLNKEENYKILSSSNKMKHIN
RDNNLLNVNSYKYNYLVEKGQKNKERNFFLSELPKERSTCKHTNIITYNKLNMNIYDYIKKQNMLYEKKNHVT
NYIPEHLIIINSLNSGSEGLLLLTNDGDFAKNLKDIQNNILTTYLIKVQEDLCIDKIKLLRKGCFINNQHIYP
VNIEIIKSKHTSKWIKFTYVEKSHNYLLYLFSKYNITIRKCKRFSFGPYKYTDINTQFLMPLKIHSTINHFIT
THNSKLILSHPKGNITKQQNQNKFLYINDYLKNSIIHDSQDITK*
>N.PFL1395c
MYIEKLNLNSENEKITSLDFQAHGDMNRLAISSLHQIKIYHIPLKDKCLRNNKINIELLFVSNDHKLVYINTI
RWSFNGLFLASCDSGGTLVCYELDLNKKKIMDEIKKNNENNENNENNNNNNNNNNNNNNNNNNNNNNNVNMIS
TYSSIGNKEEWKMTKCVKIPENGEIFDLSWSRDNEHVVCGISKGVVYIFNLRRSIIVHKLFVNGTTENIKGVC
FHPSNNLIIAQSSDNITCLWKKINISGDSKNYCGNFYEKNIKTNINEDFIINGMNNMNNNMSHYTYDKKRSIQ
HNDFVTSTQPLTREYFHYIYEENMNKKYKNIDTPTIRHIYFDTFGKYASITHIPNNGRNCGILLKIKNNEKIN
RNRIFLDGHGSCIRLAKIGQKVFIDVQKKKLYSLYCQCSDNGVISFWKIFLKRLKRKSSSYMKDINNMNSVNT
VNFKNSANTVNFKNSANTMNIKNSANTMNIKNSVICLNEDKKNFNKYSVEKKKSILKRKKKMNKYARCFLIIQ
NLLEEQTCAVDLSWSEKYDQLVIGASNGSVYIIQLDVFKLRLKPFYYNIKIDISNIGPDYDMIEKEIEFKVVK
PKQIKDNYQHVDKKVKNRITPKIKYLYDEYGNIIEHNNSNNNNNNNNNNNNNNKYNIKHNNNNNNKTPDVIHI
LNCSVYSFCKQLTATDSLSKLLDERKQYSYKSNYKNRCNFYDNPWCHIKFSSIVNILLYIFMKVIKRFIKSFV
ILIYSDVYTVFHKTLPILTSTDIISQGNSNNVCRVHKYEVSHKYEVSHKDEVSHKDEVSHKYEVSHKYEVSHK
YEVSHKYEASHKYEASHKYEASHKDEASHKDEVSHKDEASRNDSPNDSRDSHNRNNDNNDNNNNNNKNNNNNN
NNNNNNNNNNKNNSNTGGGGNNNNNNNNNNNNNNNNNNNNNNNNNNNNNNNNNNNNNNDDDKKKKKKNAIFKG
EKRNSLSNDNSLDDDPNTEKKTKSVKSKKISKPIISNVTNIINSNMNKSTINSNNFSSNNNNDNNNNNNNNNC
SNSKIFKDAKTNKNSKKKTETNNELYTLKNRNDMSNDEMLTGDSIKNVVQASIENKKIPSNNNTPGNNNNLGN
NNNLGNTNNLGNINNLGNTNNLGNNNNLSNNNNLGNNNNLGNNNNMNNLNNDLKDDDYYFESFTNYLREYDTL
DSTNNQVNLRILDIIRNNYNKENNKDMYESTIPIVTQPFCNYDDINKKNNHGFALFNIEKTFNVTKVVDLSKD
KGGEKKKVNKKTDSSEKRDKPKDTNYKKRDSKSKDSVDKKKGESKQKDNNEKKRDSKGSPTNDKKRDNKTNGI
HDKRKDTKRDMKNVNKEMDIEQNKEMDIEQNKEMDNERNKEMDNEQNKDMDNERNKEMDNEQINEEIKSPSKN
KCTFENINDPNDLTKTLNKTSIIDSNSSVRKDKNKSININSNTYCNAECGMESNVYSNAESVVHSNIESITYS
NAESNVQSDVDKGTSTKNEKKEEKNKKKITTTTTKKKNNNDDNSDMEWENFNPQFTIFNTFEPNEDMFLSKEK
HDTSNDGEKTGSNNMQKKQSKRKSLDNTNDNKKHNNKNNINNNKKKNDAFFLSNEIHNNKNKNKNMNHTSILQ
SNDENNDMLNKNDHIDLLHMESQTYDNMRSSDKKYGDKNVYMKKKETDEFEQEEEHVGDKNYRGSKYNRANEK
YGDDKKLKNDEQHVDAHYMPYEHNVENNKMKNQKKRMFSNEENMNRFVKQEDGYSKKWKHDSVNTYSYDKGEH
YDINYSRKEVLGNQCIENNDDFMENFKESRNVYNIEPNKNDDTYERRSMVRQRYMENEKAQVHDIYKKYYEQE

ENNEEEHDLNQYDHPYNMKKRKYVSNFYKNDEVMLHDNIIQQDNMYNDTLQNNKNYIMQSRSNYNVEMFNKNN
RLEDCEEMRDIKKHKKNDMKKNKNNKNNKNNNNNNKEKDDNYNIKNTNEKMIKNEKDNCNNIENVHYKKIMDK
IFILDNRYENCRICCLHENALIKKKKKEKGKISQNNHIYNEYNILWQDELLGRMINYIFDNYYIYIITNNNTC
HTSLLSIYNLESKLLLDNFVIPLENVCGINIIKTFYLDNSNMYIFFYIYNINGLYYIYQFYNYSIITLLYSFS
ISELNTSVECINMSIIEKLENNISIGKKQKLYLRNYKNQFLVPKKTQIKTKLENDKIGSNIKKNNHISFIDDK
KKKKKKKNDVIKNKPNIKEIKKLIKYITFYNNQNENHILLNNMNANSFNDYNNLGLNQITLLNNKKKKKNTNG
NNNNSNNNKCSRRKSYNIKKDINKKNIFNDNNYSPSNSLLKTKMKKKIYNKKYTQLKSHSPLFGTKNYINITN
SLRPSCCNDKNQFHILRRMPISKSGDDVNLINNEKRNKKMKTKNVALGKKSYINEFFNKNIHGYDLIYDFIYR
KKSFFYSYMCIYIFLKNGHIYLIRRNLMTKDKDERFIEFNEHISQNNGITLISRLDNIYYDKSLYCDIDLRHG
KDDTEEDYLGNNICGNNICGNNICGNNICGNNICGNNICDDNACGDNIYQQYHSDIEEHTYSYDDPHDDKHNE
RRRTLRWRGYKDAKLNSPFVDDLFYEKQKLNKECNQLIFSRHLDKQHYLYRQNIHLFDNLQEIQKRGMRNVFT
YGNFFNIYNFDLKKLLLSERKIKKTKNKKEDYFQDYLQDYLQREHTDELNKDNKNSLQLLEGNEHNMNYKINK
ENVDSVQIIDDTEHFNNVEEKEKEKNKGSYFMKNYNVSKKLNNETLKEDDNIYNIHLEDHNKIEDIYNQISYN
EYEPNNLKPSRIRPKLIRTNSLPATLSSSFLTGTQSNSLINQNDHKDFFSSSYSSSNDITSDILTAPSCNLES
DEESDEESDEESEEFHDITSSNFLSDTSVDMSQDISPDMPHPIPPRVCKNSTLGIQVKKEKNKSEERDGRKGK
RKGKKKESLSNNKKNNNSKEQIKKKGYNNKMKNKSLKDKPYVDDDNVPRPFTVFNKFVYKNFPDIYNCLQTWN
KQDTDIKTNKLSKENVYVKTIKYLELQMKYSIYIMNKKAFLNYLYIYFKFLVEYLDISRIHQTFLYFIHTTLN
HCQKYFSNFDIYFQNIQSPYWSDTNSLLTMNVHFFFIVLFAYYNFLVPLYILLCRKETKKIYYSFLKFIKEMQ
NAIFYIQKIFHNHFHELCL*
>N.PFL1400c
MLNVINNHIIKYNNLHKLRKAFFYSRVVINNFEIKNNENVLLNNVQIIKDEQLLLNNCVNFLTNFEKFAIICC
ACTNPKNKVQLKKSIWFSSIGLELNDFLNYLNKDNQELKNEKIKIFEDIIKLQCPVILKDKNTVLIEAAISPK
KIVNSSKRMRHFFNISEIITCRSCSQKNKCKRFLQKYAGEPDFSDFTRLMIGFYNICKVYIKRKESDRKELRN
TIEKVNYFHYALMYMYNYLLKHKHFNYTNVEEGNKKSILKHLKEKRRQTNLIKIKGEQEKVLNIPKEFSDLII
PTKKANMKRKQKNVFEKIQKYRKKSDISEDEEKFIWVEEEDDGDKETDRNCIKIEDPSYNINNNVLNNNSECH
NLIVINNSVDELPSFRFLYLMKTGDNNITNYNIIYNKYTKMMEKKVYVNLDDQYIDEDIIKTENMLFKIPEAI
GGYTFINYIENVPNNYIFPINKNLYEGIHVYKKEEINISHLWRNLHKEYLKIRKVNFFNTFNIKNSLNLLRHK
KNKNSHDQQNEEKNDDIIELKDELTSFEEYIYNQKKEKNKQSKKDKYKKLQECVSTYNSFHDKIKDDEDERFE
YFKKLKNQNERCEENVELKDQINSVYNNMDIEDEEGRSYKHTYNIRRKLNEEQNDAKSVTVDDIQKEKKSLIE
NHKKELRERSFYICKNIKFPELTNNEGASKIKNRNKYLKDELEKYIRPPKGDKKTNISYELKEDTIKAHSLHK
GADSLKHFIKKEKKKSYKKKKKKNMSEEHI*
>N.PFL1405w
MLLYKKNTYCDNYSIYIYIYIYIFIYLFIYLYLTYFIILYTRNVLMDIIELIEKRKFKYIDKNVKLKKLQNFFFC
FLRYNNLYKGKNKYVLKIWKRLINENNYFDKYKEFSFVNFRYVCEFLLNVLSNEINTCPYEKEKEKEKEKEKK
KKYIYIYNEIDKNENNTNINKIVNIFLMQFFRVIEYHADDFLTNEQTYKYLISDEINNKIQEQNEICQNKDNT
KRVDYINSYENNEFVILKNTHIIYNNTSISSKKFETFSTNLIIFNHNYEFEILFNIIHPLIYNTYICNLIIDK
LNLCNIDINKSFLQYMLYFLYNIYDKYKNILFFYYMIYKTFKKIKKNIINHQHVLIEWKFVKYIKNKNNHIIN
NFKCLRRFLLNICDKNTITNFVNIVFSFLHEQLRQTCSNNFECNYLSYHQENNLFEFSFYVYLSLQDLFHNCN
SVFNLDIQQIKELIRNGKHGNNEQHKEDEKYKVDEKYKDEKYKEDEKYKDVEKYRRDEKYKDDEKYKDDEKYK
DDEKYKDDEVNHENVKINQNEKVHINIQNVQNEMFDIYMVLYFINKSNCNNYVKLKSILAVFIKWLVISPDSF
MQNEEKKKKKNEQDDVDENINDESINGLEKSDKNISTNINVNMYANMDAHNYCNSFLEIKDMIKNIVKKCKEG
DTDNINNNNLNDIKIRNSCEEDEENEKKKKKRKEKKNIFRKNMLKNIMHSIENNENLLDSFYLEKLNFYHINY
ITRFIYICCYNIINNEKEYLIKYDSIPYIETEYKYNYYEYSDVNYYKNYLSDNICNNIIKSNYVTIVSILIHM
NILNIHDIWSVYLLKLIENNNFFHILNFLMYKKKNVLFYLTISSFLKINYEFVLYSFPILTEQNKINEDILFS
YVTEDEHINIGTIHFYMYYYKQKLKEIFFNNINELRNNIFKEYIFNENRMLDILTFCSMNITKYIIRLPLNIL
KYIFKEIFKYLSKPIHDVKKSINDYFHSMCLRIVYYLFELIFNISTYIYYNFNDDIDINIYHKKFYIYAFFFN
LIDVYIKFVEKYNNKNNTYIPSFLNYKLIQAFLLFFKYSWFCKILVCETCDVYENSDDTLKRSHDIFEDESYN
IKNTYKYNYKYKKKYKQYMFECKNKRMFHIPPYIKHDNMRGQIFNSNEKINKNKYFINHCSDNYIMCNNINYD
DLRLCTKLHNIENFDYFKNYYSQNIEPYIITDTNKENERENSFNIILDKKKKENFNEYYSNYIIFIILKFFEN
NNIKVICYFYTLLMYCIAKLHLSNNYRFKEINYLYFLHILPWKTSLHFCIKDSHLSCFFFQKFYECVQNLLPE
IVIEHFHDRCNIPSLSFQMDALIKSFKNEKISRNSKGKVKINIPQGDNKEEHDNIYKHVYMDDPKYYNNDGGE
HNIYPNEYENKCITHDTQKNVEENNCFHELDKKNIEKMNLYEQIIYGKHIIKKIFFLLDASECHMDIITYLNN
IFDSSLLKNCLLINTFIHLYFLNSPMHECFIENNFIEMIQNIFDEQACSYYISIFQNPLNFFLKCTNKFCEVL
VHYDCLDNLISFIFTKLYNYLSLIPPKISPDDFLPLNVEDITSLQETAVAQFVMNLKREDIQKNKTEENYTIN

KDPNKEKCVDNKNVKADDNTVELNKSTNFIFEDLKFFLKKMDIFVKTFNINSLNSICESGFNSKSAFFLCFYM
TPSIFANIFYYLNNNITAIVNESFNLEENIKYYEHVHVKKTEYTLEPYNIIDKTVFYFQIVVFYLIINTNNVL
MDNSNYLENVCSFFSKCLKVSLKDYIDKLIIAITPIMLQYFYFFPSFIPSIITTLSEMPKHDNEFSKHMTKLE
NKLKIILECSYKYLNKEDSS*
>N.PFL1410c
MMRRRSVYNFDQGKHGYLMNHISWLNFVSFNWITQLLRCLKNDDFVLPCIEETSSIEHYSTNLNRNVRNIQLR
KYNKYNKYKNYCCSKYNDGNKSCSCSTKNHTNKFHKRKKEDHFLKSSGITYAVLKTFKYYLSLISFFHIIHTI
FLIFVAVCIEKYVLLIKGGSNVVTLPFGFKNSKVLFGFIVISVIFISQFFDAVLCYYDFRLRVNMEVTVMYFL
YKITLGNFNNQLINRNDIYDDHSEEGKGQHKNQCQYDENDQNDQNEQSDLRDIRHMHDKHVKHNDEESKDSTN
STTYIKNNNNQMSHINDLSITNNMSDVHILSSIKNQDQNNSNNVSSMDSNTNYMSKTTCLTCTGSEFNKEEKD
EKKELLEEAKKKDKEVFDISIYNIMFIDTPFLIYFITALIELANMIIKFIMSFYMFYYKMGSAAVLNGALLII
IMYGLMFAFEFSSSLFKLKYLKYRDTRISNMHHILKEFKLMKIFNWESIAFDYVNMFRIKEMKICKIRTYLSS
LSNYVNNISVNIVEVAIFFFYIRSELKSNKTVSFSSLITPLFVYKSLISGVSNFPNIINNLIEGAINIKRINK
YINYYLFNDDMNDYFKNSLNGMKSNTCNFQNGNTNNVNGYVDDYVDDYVDDYVNDYVDDYVDDYMDHMMEYNI
NINSKNGCSSKSRKNNKSFSKDHFSNHEQGTMQSFYKFLDTHKNKTKQKSDCIKKCSNNQLSKSNNNNVVGES
NNSLFQDRKGWYPKNKHFKDNIVINMKNCYFSSKNNDDYILKNINLTLKNNSVVIILGNVGSGKTIFFYSLLG
QFKLSCGSFYLKNYIYKYMPILYVPQFNWISLGTIRSMILFGNKYDESIYYDVIVKSELFHDIISFKKKDMRY
ISDEHSLSKGQKARICLARALYHHYIHMKHMNLYYQKNELINEKMKKISLKRDDNHTAQRSNDNTPNNNNTDN
NNTSDNNNTSNNNNTSDNNNTSDNNNTSNNNNTDNNNTSNNKNSCSKNLTEERNISYLYLFDDLFTSLDPCIS
KDIFYNLFCDKEKIQHFKKNSSFILSISEIILNSFISSNCILNNMQYDVLIYKLENSTLHYEGNLVDYIKKNN
IVVKEDIVQTNKQCEKKSLTNEQVKSMLSLNEDWNYMHRVKKKSITQKETTKNYDNNNDNNNDNNNDNNNDNN
NDNNNDNNNNNNNDNNNNNNNNVNVSKEILSCEIKTQDHYNNIHCSKTNFEKHNNSIYSKKENETRKIHSGDI
KYHKFMVLKQFKTIYSFKTIYSFNTEETNDDEYNKTYYRKYTKVIQNYDNHCLEGKKKSFRNYKSINSYNEIL
IKENMKVWEDDTYYGNDYIDEYEKIKKNVLSKLKYNYYISCDYNNSDHFDFNEELKFKGNIKLETFWWYLKKI
GRPLIIVIIIFMLLSIFTDEIKNLILFLASTILKSGDKKDEEILNQQLVYLNYFILLPSISLLTTLISFMLIA
HGIVKSAIKVHTEVLLSILYAPIHAFYSNNLGNIINKFITDVNILDNGIIKRIYKTFYTIFRFLFTLFLLIYM
VKYTIVIFPFIMLIIYFFVFNKYSKGCKEAQRGFLASHAPLCTIYSNTIIGKDVINLYKKNNYFLTLYKQKIF
DFRNYTIFKWSITIWASLYVQLIVLALTFFYIIYPHFFFKHAKQDHEINYEKEASTIGYCITFSCSLGFVIKS
LLYDYTHVEKEMCSTQRLEECSKMIKDEGYSDDNITLQNNDPTHEDNNNNNNNNEQDANLFKSVPGSYDPNDK
ENMRKYKTEIVNNTKDMYSIINKDDMLTHSINNKNNKLKKLYTSPHIDINKIKYGICFERVFVSYKKKICVDR
KNNKYEYVNEKSCLKNINIYALKNQKIGIVGKSGAGKSTMILSILGLIGTTRGRITIEGQDIKTLTLDERKNM
IGVLPQSSFVFFHWNIRTFIDPYKDFTDDEIVDAFKLIGINLSYDDLDKYIYKQQRQQQKKKNKNTHNLWKKK
SFIDLTNSISLSDECIRYLSLVRLFLNRHKYKLILIDEIPVLNFCYNTKKLTNFFTTDIKSFDYIIRTFFQNT
TVLIIAHDASTLSCCDFIYVIAKGEVVYKCSYKDVKTQTELANLLQEKQLN*
>N.PFL1415w
MKLKYHLFFLIIFIQDILCLKYEDYIKSLPEAFHLAKGDSKKDEMKKENPKEENDNNNDNKSNSNSNSNNNYK
VGMQDIVNNISNKISDDNKNKDNNSSDKYDCNDDDNNNTSNSLFHKMMCAFKKKLFKNEQVIEKDIRNVKKGV
INLKENIVKDSSNISKHTNILKDDITKNTNYLFNLFKKSFKEEGDNVNKFSSDYFNKLKNLPDSHLFFSKLLL
TLSQNDDKDKNKITHINNYYNNKMNDNTENNTNLLQNFLSFIKNFKQKDEYNNDNNIKKENDFFHSFYLHNEN
DKHKNNKDKKYSWWFTNKNDEKKNVDNNLNLLYNNSNNSFNSTFNQNQTDHENLPEQVREKKNSQEEKNKWLD
IFWKYFHKNEEEIEKKEMNEKNDLNDNNKNGVNVDDHNLNGLHNLDDKKNKFSFLNYWNDKDIPKEGSADEYS
QDNNNNKTDDQNNDDNKKFNIFMYFGKNKKNDEDMNKNKKEMNMDMDMNKDMNKDMNMDMNMNMNMNMNMNMN
MGDDTNVDVSDTLNNNLNKNEMNKVDDDPKGFVLNLVKNYYENNKNVDNINYSLLLTSGKETNENINYHPLIK
FKSCLMNCFKEINNTEKSVEKESYLSFDDYKILEKCISKCKNNNLNDTHNEDPPMKKDDTILYMEEKNKINDT
TKNNLLNDNINNLINENNESTEKETSSKWSDFFFKKNNKKNIKDSYEEDKNSNNPQDNISVLTSNETNLENLN
ILNDNLSTHLMYDINNNNNNKKNISNNNNNYKMKNFLIDSKFKYSNKEKTKNFIQDEQQDKNKNVYNKPLHFF
NYFNNSNINTDDNNNNNNNNNIYKDNLIMNNSSVNDNLLNNDDKYNSLKNINNNNNIMLNKNDIDNDENNNYI
STGFFLFLLLITFFVYLSAFTNIINQFYLSFKEKICLFIKGKYKGTFDNVYEESCESFLPKVQYKNSHNNNNN
NNDNNNFCQSYENIYHSFQENSLDMA*
>N.PFL1420w
MPCCEVITNVNLPDDNVQSTLSQIENAISDVMGKPLGYIMSNYDYQKNLRFGGSNEAYCFVRITSIGGINRSN
NSALADQITKLLVSNLNVKSRRIYVEFRDCSAQNFAFSGSLFG*
>N.PFL1425w

MLKNPGTVLVFKPNTKREEGRKTQLSNIQASRAVSDIVKTTLGPMAMLKMMLDPLGGIVITNDGNCILREVDV
AHPAAKSLIELSRSQDEEVGDGTTSVVILSGELLSVAETFLRQNIHPTIIVNCYMNALDSSLKFLEEISIDID
VNSESDLLKAIDSCLSTKFVNRYNKIVSKLALEATRCVKMDNLMGRKEIDIKRYAKVEKIPGGDITDSYVLKG
VMINKDITHPKMRRYIKNPRILLLDCTLEYKKAESQTNVEILDEKTWNELLLQEEIEVKKMCEYIIDSKCDIV
ITEKGVSDLAQHFLVKKNISVIRRVRKTDLNRLERISGATIVNRCEEIVEGDIGTKCGLFEIKKIGDDYYSFF
VECKDPHACTILLRGSTKDVLNEIERNLHDGMNVAKNILMEGKLLYGGGCTEIRVGQYLIKEAAKFNDSRKSI
TEAVASALEIIPKILAQNSGVNVVKTMNELRIKHEQEGGQEFGIDGITGDIIKVTTKNIWDLLSVKKQIYKSA
IEAASMILRIDDVVSGVGKDEKVQKTIKNEF*
>N.PFL1430c
MHIYFLVIILFYIYQKFILMKYLKKKSNVYMFYYIPSAEKQNCITYTLNSLKNISPLKKKKKNFLLLKKRDNE
NLCIDIYTTFNKKRKKRKKEKKIDQDKLFLFPLPYGNYTHYSNFVVNRKKRKHLENDKREIKNNFLHASSPGH
DDGGDDHELVLQEKRKKKKKKKKKCMNYDNMLNDENISDDQNISDENISDDQNISDENISDENISDENISDEN
ISNDENISDENISNDDNMLNGDNMLNGDNMLNGDNMLNGDNMLNGDNISNELVDNYSALNILNKYLKNKELLE
DKNYDVKNGLNKEQMLEEYNKLLQMIKDNVERKKKEWKENEKKDIYEINERVISSSRKLNIDKLKSDIEKNFK
RDEVMEKKIQDYMHLFIQNVEKERFKDIMMNEQEPKHNVNNKNNDHINSKNNDHINSKNNDNINSKKNDHINN
KKNDHINNKKNDHINNKKNDHINNKKNDIIEKQKKEKEQKNDNVTRYSEEDLQSDDKYINKSYIDEKDEYITE
EEYITEEEARMNNEILKEVYKGNFNNIDKFKFKYSKRDYENMKNEKNDKNDKNEKIDMHNTYNDTFDSNIYKE
EDKIIDALLNKIHKEKTLYHHINEKEKKLLDVYEKYKKEKKIQRREDLENLKYINKQIEEKQNKINTNRDDMN
IGSVTYEDGRRNDSDGSYNNNNLNSNEKNDTLININNNDNNKNNDNNKNNDNNKNNDNNKNNDNNDDVIIKKN
IFFNKIDEKFIEIKEEDIKEMSELQVKDELRKRGYPTFGSIDEIKTRLCDVMKIRQNNYIDIRNILKKPEDNV
LSHMRLEKLKENIKTYKENEQYGDTESKIKQLDASIEISKFINDPVHYLRIDSTHKFINQNKPKNKNDPNNNT
QNDQTNNMEIQYDTENNILKEPIINDYNFNEEISMAEKIKKTFSFENEEKLPDQIITRFNNNNNNNNNNMHGGG
MHGDGVHGDGVHGDDMCGNGMDELDEETQEKYREQVLKYGSLQETIEEFNYKHNLSLDFIGDFICKSSNAHIQ
DINKYRYKTKEEIEKLKENDFYHLFEENNLNNNFIVYKFVNTKELIKNYLTTSNIVTLIEYANIADPVDIIHY
YTPENLFMISEDYNITIDRVIDACTRLNIKLPYGGDTHLNKECFNFLTCYLSKYKQIKRKT*
>N.PFL1432w
MNKVKKVMMIKNVPIPLSFTMNRNIRRFNMVENDRKIFFAFNMLSIFLFSLPIIYMSKVNYKGCQENEILYQK
LYNSTRMNI*
>N.PFL1435c
MAFLQFKSNETLYFSNISNAKDEQSKVLSTSHPNDDQHKILKKPREGGDIYSDVYVWYFLKNVYKKMEICSFN
KMDNTYTLRRKGITYENVPADKTKPYLKDLDEIDSENNIDIIQLNEENVLQNIKNRYKKNKIYTFHASLLLVV
NPYKEIKGLYDIYQMNIYLKKLTNDHMSNEQHMKAHIYDISNMAYKNMIQKKKRQTIVVSGHSGSGKTENCKF
LFKYFHYIFFHKNGGTHNDNNNDNNNNNDDNNNDNNNNNNNIYSVNNNIYNNLCTNIYTSLKGEPDNNVEKQN
YFGLLGSYRKVRDTTNDDVKDMNGRDKIYNDSLHNERIDKLIYINNIIESMSNARTIKNNNSSRCGRINDLIF
EERENTQKGESNNMFNYCFNNIKIVILLLEINRCIRHNKGERNFHIFYQVLYGLSDKQLYERNLVRNIHHYHI
LNCDKENDKDKYSNDMSIDNINNDFDELEKSKDEKNFKLLLDGLTYIKYDSFIINQFFDVIGGILHLGQIIEE
NIKKDNYNDKLINTRNNDKNMLSYNTCNDKTNHMYSDSYKYCSECLSINLEDLQNIIKYKNINVSSETIRTHR
NKENMTSTIYTIIKIIYKNFFNKIIRDINIAHLNEKEKEEIMNEHIYMYNHNIISIFDIYGFEELSFNDFDQL
CINLANEKLNNYYINNEIEKEKIIYQDENIFCNNIEVPNYKDTLIFIDKILSNLDDITKLHNSGYKKSDDHFF
NYLLNNEKMIYLEKKNVYGFISNKDQAHTNKKQNIKKNQFFINHYAGPITYTINNWIYKNSDKIELDIENLIK
NSKNYFLNENEHNKVQGISNDTNHQQENNMSNMHAVTQEKNNYINLKKDHMSDYVEKKMDTEEKICNMDNMMI
GKTGEFVFLNEQENIWGDRCVPIIQGDEKKKEIRRNVDNKICNDTNSVYLDDKNVDLSHYDNRKKSLEISKTL
LNINLEGTKLSINKGKNTNDDYIGNNNNNNNKKNSRNVLSVSKKYMKELDNLFSHLNETDMYYIRCILPNDKM
KKNYFKRSLVYEQLKQCGSNEMIKILNSGLSHKIKKNEILTNLSKEIQKELLYCNNNDKIYYFMCFFDEKKRF
QIGSNNYVFMKSNTYTQINYYISQKYNRDEGDHNKDDEIFMDINKKRDILSSLRKMRFQRCVTVIKIYNWMNT
YFKMYIEKKKKMKNMACEYIYKIYLIYKTMMNVKKSFSYNIRKLNKCFTIKGYKFAYNKIKAVKRRGSFKTNQ
QNQYENKKEDEKKKKHVSIGDIHMKEVNIIVDKIENEKILMNDQRGGEKKKAHVTCDDIYYDSNNNNNNNNDD
DNYPSNVAKTPLEHVEPEIFVGTPDNYHYIYNNIDWVVAYVDRCIHFYNLIYTYRKVHNKYVLDYEKIEIKKL
DDSLDNEENISNEKDNAEYNTNGDMISSRAGDNICSYDSVHSKKKGCTLKIKHWEEEDHNGNNNNNNNNNNGN
NHNDGNNHNDGNNHNDGNNRNGGNNRNDDNNHNSGNNHNNGNNDKHNNGNNDKHNNGNNDKHNFVNNKMLSLL
QRKYYCTNQHALYKNMFVVIDEDLDIIVFTYPNLKTIRRFLKKKIKSLKIQSDEKNVPTTYLPKLYKTSRYEH
PLQNKDKTINKIDNVHDNTYNGKEEHINEYKEKNNFIIKKNKSYVDDFHTSTFDIFSDENLNLFMHTYKNIYV
LNNMENIITCNEKNKKINYEYKQNESQDDDVFMLVENMKRGSSNKKHIEKNYKCISKKEKKNNFYSLLKVIEK
TDYIFNDLYLNPTYKILRICFLPSSIHYVVYLAYVLINNNHYLFVTIVNYLSKPVYKYMVHILINNIVQNEEF

SQFFMKSYDQICSGHLMRKEELLASQEKNKNKDESIIINAQQKKEKKYVMCKDILNKYLSTLKIAIINNRHII
LYGSCLLSLIEIKNYNLNSSHNPYVKEYEYKYLKLIFNLYCFEHFSNVYDTYVNKLSYDLKHHLVKNDLFKKE
RKNSHLFYLNKSGENCDPNGDISKGKDRYIQKKEQVNENMDIYNNNNNNSNRMYTYEESINKYKMNKNDTEEE
YTFYIFSLFNDIYMLTNGKYKKMSSIEKMNDTNKGDDNRSLDVLFNYNEWKIIKLKNDSLDERMKICTFQGVM
NMNINYYYNKNYYKSNISLNPCIQSTIQRYNIFNEEKRKGGVIQMNYQGSIYPDVYILRELKYGEQGQKKKQA
YYYNFKDEQLLTLYDEGDCDTIDISDKAKQSYNILSCTPLNHLDTILLLMKDIDENNSYAFEVMNIVNKKKKI
LPITE*
>N.PFL1440c.1
MKNKNKLKRCLDYLKNNKEEIIENYNNTLPSTKENVLLNYLKILKKKLQEKYDIQFCEFFFDEYGIKKNLCSS
IIQIYFPFGKGNNYKEIYSLIKNYFTYEILLQRKIKTLLLPNVSLIFDWKIADCADLASIVVSSLRTKNYEAY
VVCGEAPFSICSKNDKKNICDFNLKEFKQSKREKEYIKDIKHKKKKYEIILNVNKELLKKNKFQDEKIICEND
FYIHMWILIKKSLDLKKDVFIEMSSGREYDIYDCPYKSIFYLWNEKNIYININKTGDIQTLMSQIKNNEYFVP
AFYEEIKNKSLTPIIENKFYVIRKDRFLLKYPQGSNIIFYKNCKKDDYGEFLQHDGLISMHTHYENEYYTSVD
YIFKLYKHRRDRKTAKIYIPQNFKTIEYYDDCSHEFLKKFEQRMGFYSWFTFYLNRHDGLIYYFEIKDYKLME
YFINRNDNVIYRSVKLSQKIPTIYKLQYIYIYIYICVCLYWEPLF*LFNGKEYYVTKITVKYINKVIQYTTYK
YIAC*QWSKEESIFNTRKKNNIRFLQ*LCNNKK*YEVFIF*KL*I*HMHF*I*ITFYIYIYLYLYIYFIYYIL
LDVIFT*
>N.PFL1440c:mRNA
MKNKNKLKRCLDYLKNNKEEIIENYNNTLPSTKENVLLNYLKILKKKLQEKYDIQFCEFFFDEYGIKKNLCSS
IIQIYFPFGKGNNYKEIYSLIKNYFTYEILLQRKIKTLLLPNVSLIFDWKIADCADLASIVVSSLRTKNYEAY
VVCGEAPFSICSKNDKKNICDFNLKEFKQSKREKEYIKDIKHKKKKYEIILNVNKELLKKNKFQDEKIICEND
FYIHMWILIKKSLDLKKDVFIEMSSGREYDIYDCPYKSIFYLWNEKNIYININKTGDIQTLMSQIKNNEYFVP
AFYEEIKNKSLTPIIENKFYVIRKDRFLLKYPQGSNIIFYKNCKKDDYGEFLQHDGLISMHTHYENEYYTSVD
YIFKLYKHRRDRKTAKIYIPQNFKTIEYYDDCSHEFLKKFEQRMGFYSWFTFYLNRHDGLIYYFEIKDYKLME
YFINRNDKFYLFFFFNF*I*FAYDMLFVIYRSVKLSQKIPTIYKLQYIYIYIYICVCLYWEPLF*LFNGKEYY
VTKITVKYINKVIQYTTYKYIAC*QWSKEESIFNTRKKNNIRFLQ*LCNNKK*YEVFIF*KL*I*HMHF*I*I
TFYIYIYLYLYIYFIYYILLDVIFT*
>N.PFL1445w
MKKNKIREKYIKILFDKIDDEKIQKDLEFSNYNYNFFIKHDIYYLNKKFYKVKNINDITIKKTLLLYLYKHNF
YVPKIIKRLIFKKLLLQNENNENNENNENNENNENNENNENNENFNYNIYLLLSYLKNPNIIEATQKIIDLDV
FRTRINKENEKTIYFFNLFLNYACSHFQFKYKQGLNEVLALFFYLKGKHFNMVDVYFCFESFVEKFLKEFYYD
DEFFFLQISFYLFKILIKYHDPVLSEILENNKMTPEIYASSWFLTLFASKCNMRILNLIYMIFLLEKNPFFYF
YFSLALLILHS*TSLSLLIICL*ILMRY*STIQKKKKNKIFIEIYVKEIISTFFWI*D*TVISNLFTYIIL*T
WN*LKII*TYLKVSYKKKKKKKKKKKKKKIYIYIYXXXXXXXXXXXXXXXXXXXXXXXXXXXXXXXXXXXXX
XXXXXXXXXXXXXXXXXXXXXXXXXXXXXXXXXXXXXXXXXXXXXXXXXXXXXXXHHQR*T*ERENKKKEKESM*Y
EKNMVHPKGL***RSRRKR*ISFLLFVKKKI*HELSC*L***YFIKKEN*SSEEKIKSN*FWSFRK*E*EKIK
VEPIR*ENKG**KEEGKKKKKKTKKKI*YFRSKKKKND*TKKR*TISRC**YFKGTRRL*N*FT*KFL*YF*R
*TN*YYEISS*N*I*NTFTNKPNIFTV*NK*RSQKILYR**HIM*YV*FNMLSHKK*IYYR*EKIYKE*SRRS
RQQ*RRCV*KECTKWCIHK*GFDL*THY***R**YGYI***YGYM****LYM****LYM****LYM***LYM*
RW*DECLCDQRKV*TQNRKRV*KKIRFRKK*HNKCVKCKENM*YS*K*FKGI*RDKN*YKHKFL*FICI
NKRECFERGK*FTFFF*KCIRSKKRCNNFI*R*FP*Y*VCT*IIF*FNI*IEYKKCLHN*RWYRFLS*TFY*F
IW*R*FKKR*CK**YNNGYN*IK*YT*CRLYE*I***CLYM*KEYI*RK*DKEIF*RE*ISDS*I*SMLYV**
*TF*TYEKENIR*IFF*YIQ*L*YVCRLL*FYTR*KGIYEFFSFQRFNKKKKRQGKFR*SKEERNRKRTGGR*
CIYFLPSNIFTCELY*KKKK**I**I****NS*KKRYIDIAIFS*FP*Y**ARQRCR*KYV*LYF*TRGEKQI
YKHIYE*SNGCV**F*YKERLF*FEFLS*LL**KEE*IFKCGYKS**R*GGERHVYS*****YL***YPYG**
*****YP*L*YP***YP****YI***YS***YPK**YPY**PL*L*L*SF***HYK*KVYSSSDSTKTKKRIE
R*RGSTK**KF*TM**NY***D*TCIYGK*YKRKK**R*RLLCK*****YIKCIFV***II***IFKEYK***
YTMQ**IFYI*QFLSR*KKKNVIKYKLRYI*FIFIY*YSML**FVI*K***DI*MFY*LHISTFNST*YKIQ*
YYK*FFEICKIF*NS*KKKRCKFNK*S*HIFKL**VGFKIIF*WFILLSI**EYEQYALFKSFLYIKS*YISR
KKKKYDQHIW**P**SK*IQ*FVWIEYRKSEV*RQKLCESM*RIIST*SL***F*SHI***LYL*KNKK*RIK
K**SL**NIK*FNFK*YLDKK*TRIRLM*INDI*SFFNFIWYTICMQRSYDRKTRERGRKEK*RTEKL*RQK*
RRQK**GKK**GRKL*TKKL*TKKL*TKNL*TKKL*TKKL*TKKL*TKKKA*NNI*TL**RIIE***FKNL*G
GNMQ**SFGPRNGKFFKREKRIC**GKRTIYML***V**NNNTQI*RS*KKRYII*Y*ML*IRWVMCKLE*YV
*KYISWHNIRTYK*YI*LL*ILF*FIQK*FII*N*YK**SKK*SQ*LLFL*IFHLFM***HFLFQ*FG**C*F

FFF*LP*FI*EKV*K*KKRSSQKTGQII*SRIL*KTD*CN*CLWYF*SAYYS*NNHQT*IYKNVIFLFFNKQE
YSLNGFKFRE*
>N.PFL1450c
MLQHSDHSKNDMFPHEREEIRLGKKLVVQNCGLYMDGNKNAGVVCNRITELNINSRKKTSNNKSDSSQMKNVL
NHINNKSLELIPSKRPLVSYSTYDNQQKYFETKLVPDLQGKMSICVGRKSGSATVNINIDDYDLEKTYHTWKK
ITGKNYVHNKSNLENNIFLTVANDAERSDKLPIVHNRNPPFANPDGPFEKEKLNLPKQARDNLDRTLTPLAIC
DIKNKTSQKKNIHTHKDSFLLLRPQENGITQHIKGVKRTNFPWIHSNRIKNILNYNYIHPQKENEKHLNKSGT
SQNCNYQTFLKSAIPEKSGNEERHIYDKLYDNNYEEEQIVDVDQLKNEDNNDNNNDNNNDNYYNNNYNVYNEK
DITLYDHNNYKDDENTFQRYNEKENYPHKHINHHGYINPHQHIDTLGYTKNFDKCNSLINERPTNYQDTRNYL
GNNTYNMQHNKGKCQVQNYNTNVETPQNVYLNNEHYKTNEQNIHNSHTGKCSNNSCQEINNVVHNEQEHMNGN
NNYYRVSSYENMS*
>N.PFL1455w
MKFSKKLNERAHPKYREHYIAYKDLKKFIKLITGKDTSTFTIKEVTSNFGNIRALSCTEYKTPESRFEDILNI
ELEKINNFTLHIIKEWYEDIQYYYHKLKDRLIDDIRDVESKLHALGNILIFLEDYKHINFIGFRKITKKFDKH
NDNVLNSSFYISVVIKSFFMNFDINVLIYILSVCYKYCRIIKGGISSGADEQYGNKRGTQEKEEEEKKEKKEK
KEEEKEEEKEEEKKEKKEQEKEKKNNKIHYNEHYNEHYNEHYNEHYSEHYNKHYNEHYSEHYNKHYNDQNKER
RDQINNDRGIENYDDNKWKENNWTYQYKHMKDDDISYKINENVCHEKKNDIGIKHVKYIVKAQNLILIKVEIA
KRFIFKYYNIEEHECIGDIKRFLDIISKNKIHNYLITIYFDDNHFSTYHKNVNTNLSNINNEYIRIRSYNYIS
DNQIHHEHTSLQYFNLYEPSHHHKETYLLFKDIPSSNFINIQKVNDLEVLLKEEAYNHMYNSKDDLHKTYETE
TIRPTHTFQNNKKDVNKNYFLNNTQFFLNFMQHMKNKKLTSCMRSKVNRIYFSNDKACGYIDENISYWAHNDN
KNVYAFNEENEENEESEEGEESEEGEESEEGEGNEENEGNEENEGNEENEGNEENEKNKNYQINEDYYEQYYN
HLNDKSISKNPRFKKILNKPKDFKLFKKGTNKKYAYFNYAVMDISIKEEQKNNDFLTKLNNLGLLKEIWGYSS
FLQGISLLYSNRLSTYPHWRIYTCTDLMQLSNSTENFEKGKKKKETKNKSYNKNINDNVIYNFFNEFNHDQSN
SDVLITSPFISNQSNKKKKIKKNKNNNNNNNTKDDFKKSITYTGASARIAHENISIKNTSLIYDNNLYEPLLH
QTNNTYQNTSQSLIRTIISSIKKVLLSTNNTQHNPKPKNAVVRVEPKTFFANERTLLQWLNTSVLLSTISITL
LNFSNSYGFFSGVIMAPVSIFFILYSFYIYLKRSDALVNKEPINYTDKLGPLILVLTLTFSLSTVVILNIYSR
WKTEILS*
>N.PFL1460c
MELSKYAGLSSERLDCLRNLDIRKRSIYKGSTFSYGNNIYDILHNYSDKQKENKSDGVSVAHDYKDFQRNIKF
AGLESEIKNDINIRNRRIKNDMNYRNDNLKDILQNNMNEQNINNSKYFLSDKGYVLTPKRCLARHVDDSEINK
YFSTKYAVTDSSTGRTEIMVERKPGCSNIIVQNFSEFDAYGTYKKKDERINRRDSFVHTKMIYIYIYIYIYIY
VHTFDNIYKMYYLKIKLISFLFFLKIRMFII*
>N.PFL1465c
MFIRNFVNIIGSQKSITKTIARNYFSDNSKLIIPRHGTTILCVRKNNEVCLIGDGMVSQGTMIVKGNAKKIRR
LKDNILMGFAGATADCFTLLDKFETKIDEYPNQLLRSCVELAKLWRTDRYLRHLEAVLIVADKDILLEVTGNG
DVLEPSGNVLGTGSGGPYAMAAARALYDVENLSAKDIAYKAMNIAADMCCHTNNNFICETL*
>N.PFL1470c
MEQNKIMLVYDNHDVLLVNLDNERCESKFENSIKDDEKKICMLNNGNFLILNANKKMIYQYIYNKSTAIYTKY
VRAHLNVIKLTTNERIIFGGDKIGNVYIWSAISGFLINTFQAHFGPIKDILIDQLVNVLYTYSDDNIVHVYNL
HDLFRKKKIQPMLFYQHNINSNIKQIISITPNIYDTLYTLISLTNDGTIYVWGLKSKEAVHILKTQTECCTYI
CSNYPFNTHLYVCKKNKIIRIPFLEFNKNKKNKRYKKNSSAYSHNKSDDIKLKEYGDYVQIKKIQTEQIENDE
YDENDEYDEYDKNDENDKNDYNNYNGNKNYGDINNDYSDYYNQSSDDNNNTNDEDFNDSHHLNINYLHLETNK
IIKSQNVVTNEMFLNLKDFTTFIGHKSQVLKCYVDDKKQILISLASDGIKIWDIYNCYAIKTLKYGENVINFY
IPTFKNVSYLIELPNLVLEYEDNTNIHIIDEVNEHTFQSNYKNLLYNDQNLLINMANTFASHDM*
>N.PFL1475w
MNSTRLRHIENALNNFNFMSPLDIYMRLYFKSNNIKNKDKPYISEHVYNIIKNKTLLSYLSSPSSLYTNVIKT
YFSSDXXXXXXXXXXXXXXXXXXXXXXXXXXXXXXXXXXXXXXXXXXXXXXXXXXXXXXXXXXXXX
XXXXXXXXXXXXXXXXXXXXXXXXFFFLSFRWKYEMNNEKIPAHVRYSFPKELYNQLLDCYGEKKSITLM
SILNEKAPVFLRVNNNKISRNELYKTLISKGISVEKCVNSPYGLLLTKNQILKNIHEYKKGYFEIQDEASQIV
SSKIPVHPGDKVLDYCAGSGGKTLAFSMLMENTGKIYLHDIRDQMLSQAKIRLRRAGIQNYILLNSNHILLKK
LFGYMDVVIVDAPCTGTGALRRNPEMKYKFTNNKLYDYVKTQREIFENALLYLKKNGKIVYITCSILDAENVH
QAKYFCQKHNLYLSEAPFHSLPQSKAMDGFFLATFERKE*
>N.PFL1480w

MNELVVFDTYHGRSINDCELDYYSKKLATCSSDNTVKIFDVSLSKEPVCVAELKDHSSAVWKVCWSHPKYGSL
LASCSFDKNVIIYKEVNINKYEMIYINNEHVSSVNYIEWSPHEYGLHLGCASSDGTISILSYYMNKGSNEGYW
NKYSMKAHLNGVACISWEKPYNNILLMNKNLNDNNNNNNNNNNNNNNNNNNNNNNNNNDVINSFKLVSGGYDNQV
IIWMFDNNTKEFQKIYQMNDKPHKSSIKDVAWKPNLDNSANIIASCSDEKIVILWIEDVTNNVWKNGQIIKVK
YNIHKISWSPNGTILAIACSDDNAYLYKENAEGIWEEMCNLTEENNNIQDDNSMNMTNNEQENHNENMLNNDN
NNNMNYTGNVNVDSASYMHDNTNASNLNMNYPNGSNSLINPNNNLSHQGPYQNKDTFHRSSQGNFAAYNNSTQ
QQQQYSNVNSTGSQMNNVQAGNTVSNNVISNFANNNTNQMNEPPSQMNGPPSQMNGPPSQMNGPPSQMNGPPS
QITVPPTPPAYLTMQNSKKNNTSNLTPNMNNIPPPTAPPLVVNNSHGASGPYAPSNFSQQPHGVSAPFGVPSS
SPPAQQQVGGSVTNMSVSSNINNNKSTFGGPPPPLPSTSTTPIIPAAGSSNFPKASTSSLQQQVHGPFPNVNM
SRSSFAINSQMPPSFSHPNNNNNNNNNINNNNNNSGMMPPRPSQYSNNLNLNPLSTSVPPPAFSSDTQGMQNK
NSITSNFNNNSNNSFSNFNSHMNQANLPPPNPSVMNPSKPVGSTYGSISGGYNVPHAPNMMNDSNVKPQNQQY
GYSNYNMPPRQNTHMNN*
>N.PFL1485w
MSQDNSDININDEKNEYNDEDIKEYYNEFKKREHVYLACISSLEKEVCRIHTYLNEWRSMHSYEDEEKKSSTG
KSTVNINSLRNILIDPCINLEIKELRHKIYEITKKCNIAEEKLQGCNFDAQSIAGQRLINKCKKLQEENYELG
KTIEENNLQSLSIQITKLKQHIVFYKNELRVLKELNADIDEDNELLSQQLSEITKKYTLLSEEKMELQKNNDE
LNNYIKTLKLKYNESYNDIQKDYHKNSYSNINISKKKRNHNIFSYQNDNQENNLHYENVIKGVDNLNNEYNFK
YYDYDNNSKHEYSNKYGPHSKYNKHHSNKYDKYDQHNNMYDYNNNNNSNAYEHNNKYDQYNNTYDYNNKYEPQ
RYEEYRHNKDERYNNEQDPKTEPQSNINYDKDNNSGRKRNSIDSSRDRKYRKVDKQAYKDKKYEKYKLKDKEK
HRKEKTRDKDRERDKERDKDRDKDRDKDRDKDRDKDRDKDRDKERNKDKERNKEKDRHKDRNKDKNKNKYKTK
SKMKSKRGGNYDDEEDYDSSTYSDRKKKVKNYDDEEYEDFSRGGNKYDNTVTSKNDDMEDATSEHDRYVYMH*
>N.PFL1490w
MEQYENYEEIKVLSNDVQTSIIQGEKLTNKFRNRGLTRDKRATINSVLDNRTLIILRKLKENVYNDIYGVISS
GKEAYVFNAIKYINDEELNSLKNIFIKHLEKYKNNINIKINNNENMIYNQMENIHDQCSDVHNYMSQDSERDI
NNYEQSEDTHFYNNDKYYNGFFNKNKNNKGDQSGDDTNNDIYNDIYDDLYNDIYNDIYNDDDEKQNFSKREDT
VLEEKNKSNNTYEQTFDIIKEMNDISFYDNKKSQISHIINILDKTKEKKGIALATKVYNTSILVFKKRSQYIE
GEFRFRNAYTKNTNPRKMVKQWAEKEFRNLRRILICGLRCPYPLVLRSNVIVMSMIGYIDNACPKMKDLNFDI
LKWKELYIECICILRFLFFNCKLVHADFSEYNLLYFCNHIYIIDVSQSMEHDHPYSLEFLKRDCLNITNFFKK
KIGTIVQQTQPTNLYTQRNEQNVENKTNVDINRVQTNYTNEHVEGYNVEGYNVEGYNVEGYNVEGHNVEGHNV
EGHNVEGYDDTNTIDDKTSHLLPQKDFSKLLDVPDAGFYENVEILPLKVLFDFIVSSSLPDDIVYFIENDKKK
ISLNPYEIIYLQIFGLIKNTTPIPKLNLKKIQKNRIYFEKLKRATCYYVTKFTNEKKKKLEKYSKKHSQKYAH
KDEVEEQVFLSSWIPSYLNEIKDIRTIEKDLKLLKKGKSIVNNFISNQNNDHKYQNNTKEEDPKNKKKEQNKE
QNKEQNKEQKEIYNKNEVHIDSHIFYEERNQMDVNKINDTLHISNEHSFEENIKQYDHEYKETHQEEIIIEQD
KNEENEIMSNSNTLLNIIQTNSCDHLSNSVDSDTSNIDDESDSCEQNEDEENYSSVHEQEEVKFKGIIPDGIT
RKEWCKLVKEQNREKRKHKIPKYQKKKKKKKTHIKKKK*
>N.PFL1495w
MSICQFPISNSKGICYRQRTTECVKENNGITKFFLKDLIKESSLKNNEDVTKKDIEDKEKKNVQQNEKNQSFN
VSYTWETDTISGLRFINVLNKKDKKKLGFQCIYCRDRQLFKKKSSVLYHMVTFNHGLKNSVIDELKINERTSD
DRLNFINNFNTEKQITIEKENIVEKNVTKDYNLIKLEIEFVSTMNLLTFEELKELFLFFFPEQVMSIDENIIK
ENIIEKFLLNINSLSENYSRIIEKEYHVPDYSKDLNLITLSEYLNNLRNVKLEKKTNFYCEESVDSELAINET
ITSQNKEREEKKMCANQEKNIVETSSENILSKDITEEQKVYISEENRNDKINEEDQVHIKSVEKKDNAAVVEA
KNMNNLVIEQNIEKVIVEEKKKRKRKEKINIDENKHILININDYNILRHDDQLFISLTTNKNKPQKEKTIIGK
ENFFIINNNISNDEMNKQDNNNENINTTSTSNINDNNSTKSNNNKSNFHNIYNSASSNNYYEHVKNVNGSYNS
NIHTNSNNNNLLQKINAEQYMIIDNNNNNNYYNDDENKKKHPMNILLNLNNKEKKKRSRNEGSKETKMNVSKK
VRNMKNKNNLDIHFLYEKTNENNVDNQNKNEHLYEQKLINYESIIGIDDITENNDLKEEHNSISSIPNYNIIS
TTSNTCDMQEEFYNNMNININRCLYKQTITNKNNKDDNNNNNNDNNNDNNNNDNNNNINVEETKEQGNMKNSY
NTKNNEEDTNILNNIHILNNIHNDNKIQRILQIHVDDDLKDNKKNISEKNVEESVLACSQNKSVKMVNYKDKN
MIDKSRNKSVIKKKEINNKNILKNITKINTLNSNTKGRKKKANIIETQKDNILKKNLKKNYSYKITIDKEKKK
IINYNKRNNRRKNIIKTTSSINNYYKNQHIDNIINKKKNIKLYKKKNKQTNIIHKKCYKKVNEHICTSSIKEI
KQLCNLSNENIIPHNENLSCTRSYRLSRYIKT*
>N.PFL1500w
MSPYEYLFKYIIIGDTGVGKSCLLLQFTDKRFRADHDLTIGVEFGARLINLDNKQIKLQIWDTAGQESFRSIT
RSYYRGAAGALLVYDITRRETFNHLNRWLDEVRQNSNPHMAIILVGNKCDLERREVSAEEGAQFARQNGLIFL
ETSAKTAKNVEEAFLYTARKIYDNILEDVYDLSNESYGIKYGPNSQYSRVKLDIPSMPESRSGFSCC*

>N.PFL1505c
MLWKNYSRALYKTMGRTGLKKIFVKYRRTKKNVKLLFNFVYRNYYFNKILRKSIKNFINLYVQYIYNAFANFY
HKYCNASNIKLYIYQIFFNNKIENRRKVKKKNQMKEANYEDVNNQKRKKNKKLIKSNTYKSIEDLCRTKPNDH
YIITLYNGTHLQNDHMNSSVSKNKTFKCKSNDSTTHSSDYSRNYENNDEINSSSCLENEILSFETLEYDEDEA
NEIIKNCYLYNGYVDASTYKNSDEYKFLKDHIKPEKKSYVNIDIDMSSQCYDEQVENYYEKDKEQIDQSDKEN
KNILRKRKKLKQKRIYEIYNSEITNKENMYYENYLNENYTSSYNYTSEDSNDYSCSTFTDNELEDNFIIGLYG
NNFRCSFNSDSCDNSEIS*
>N.PFL1510c
MESQDKENLKLKYKILSISSNDKHHDVINLLKNTGNEIFNPWLSEQNCTYPQEIVLQTKSAKIKYLEFLCHEY
CITKKIEIFVSNNNIDYSKVGFFRFNDNFSTNYCARELKYVYLPCTIKCAYIKLKFYNPYHNYLNKYSQIGLY
CLNIIPEEFPSNVLSETITTSHKLTSLVNKKNINYNNNLINNNEKLKKKELNKYDSNISDNNISEMNPVYSHN
WGNYRRSYSYKRDKNKINTLYEKLENKIKCFEKLKGECVVKEEFNKANELKKIVNIFIFLKNVITFLKGKKSK
YVKEENYGKAKKLKEKEIKIKIIIQNIEELKVIHNSSKKWYYEWLVLYYKELELHEKEINKIMSNENIKNIKK
ICGIVDQDIDTNDDNDIDLNNILIFIGSDNDKQREMGYDMAYKPFEFHEKTKKKNVWYNNIESLCLIIKSGIS
DNSYYIFVKSVVILEKMLIMFQHYFLKMDNNTFVENSTNEKNVKYLNFIISSLLKRLDDSNLDIVDISIKTIM
MMLHNKFTSFKNVFSIILSMLFYFFSNDTTSEVNEKIIVSLMSFYYSLINKYYTSVKEDINLKKVLEIILMFL
EVNVEAIKQTALDFFVNMYNIVENRDELFEEFLLNVSSETKSLIIKRINENEKKKSDELENFNDEKKEEIELM
FQKSNFLKDNKEFIKENTCLNKSLLKVKDSITSEKNNIDKSEQIKDLIKNTIENKEKENYTSLNKIEKDIKLV
DINSKDSLEEKSNSKKNIAEDVECEHLKADDGKKEEDEKPCLNNLGSFQKREDLINDQENKNINIKNEEKSTI
QIDNTVNKSESMEDYKKLDENEEPYVPPFTCKYCFRTDETFTEIGLEKHWIKNCPMLCACPNCFLIVELVVIH
DHFISECSHSYMYTPCEYCNKIVKQNLLDTHVMSECKGKKTEYLSCYYCSLCIESFEIDKWRDHFLSCSKNPR
LLQ*
>N.PFL1515c
MNLKWLSNIGFSNYNKKNNKQKFVRSRKRFRTITWKYTKTIGPFASFIYLFNQSIGSGLFDIPSLIDEVGWIP
VIFGNVFVCSVAVFCSLMILRAMTMIPKNKNFEQRIEYSAVIKYFMSNEKYKFVSFLYHLGNICNNICGILVI
SKIIDIFIIKLYGYTILFQIYPQIKITKCSLNLLDAMFNGYYYTSTGGYERIMISGFTIGYLINAIICIHLSS
KGLEETINYQYVVFMIFMSSFLYLSVSSTIYLLSQNYIDPSPISSIRNLNIYHKFILNRTHYEIDLIKNYHSY
NELNNFKKKLQLHKNPSFHDYPILKCITEQKKNISNDYLSELPYRKDNLYETTEIKESIQRKTENIFKMSTKK
NIHEYNNMEYIKKCPFFYQIPSHLYKEKIYIFRYSIYYIILSRCSNNIKINYFPRNPYFFTIYQIFNPKNNEN
VLCHKYKINDVNSRRNTVFKEEKNYLQYDKKEEMKTKPEDNNNNNNNNNNNNNNKIFLKNLYYMISKIEGIKT
YCMGKTFANFIDSYGFVSNIPSWGNEITDEVNVSKAIWFSSIFTSIFYFIFGLIFCLNNSFQKINTSILYIFN
FVAVAPKVITGSISMRYDLMNLDICSDNLAFFFGCVAPFLFAWIFSNGLIFSDTFNYISLICGLFCNFLSPAF
AYISACESNKYISKNPLRKYTIINRKKTVYSSSSDIRKALGNIIVHFDVNNENVKKEESENEKELNQNESNQK
KLNHNESNQKKLNHNERYKQYNSTTSLDNYVNSKDSINDSLNTSSYFKYVNGEYRLKSYSSNEDNQISQCSQN
SSTNTLSIHDNMYDNDKNYKNDNNDFSYVDKNINLNNSEIMKTNVPTDDNINNEENLKNMKKKKKKGVMFSDI
LEEYCEENNSNVKKKHTSNVMDTYINEKMLNEITFSNESKIMDEENIQLENKNISLQNKKWDDNRKDKNTKKK
LMFSDDVKIFNENSNNENKKKVKKKGVTFMDELEEDKKFDDPKKNNIEKNDNNHNSKNNEYNENTKKDEQSTV
LFLDDVKKEQNVYNSDKISFQNQPEIIYSNNIKVINENNNDLKNIILEERKEKNQEVNFLNVPKISVIKEKKN
EIDYSENKVITKVLNFTEYERNFSDSFVNFKINRLNNMTTHEEELLKKNRSFENHEQIYKRQREKLKSHYKSS
MSKVIENSNKYNNLIKDIEALNSKDINENNQCDNITIEKEKLNVKNDLDNPVFIFNTINNDSNDKTLITETLD
DEDNNNLNDENDKYNYSIKNDILYNLNIDKQDKYLINIKDSSDKNDVYVKKYNSCKDNLTLNNNNNNNNNNDG
DENICLLNSKYHSQMDLYHKRIFSSSPEDMCSKVISKNENMNSYNLSNFLREENNDTNNISHLHDFNKKKCSS
CEIIVPQKDDMMNRMQKNVINISNENVENDILNNNDLKKYEKNTLFVRQRKKLKSSFHANGDMIMGDSKEAIH
KEQINEFLRKISKESIIYKNYDNIDPIILNTSNQNDYQIKKENMTINENKNVQSNIKLQIPTINNISNTNENI
YKNEKNLSLNKTQYPKTREIIKHKTNYKFDDLSDLANDINKFELDYETNKNENYLGNINNINNNIDLHKNEDI
NSENKDCYNMNTQTNNIQIKSNENTKLNDKEQNDILEDYSSEEIEKSDVIKTIEKVYTELANKMHSENSENDV
NNYNDIIDCVDIRNFKIIDNKSPVKYYYNSFHKSKILDNKKYDHIFSNNIMIDNKTNMDHLCSVFLFGNPLER
NEEDVDKKKNTKENDESTKTTINMEQTYKIIQDKSDNIKNEKKKKISFNLSESESNELPNRAFSRNFTNSNGI
IKKIITAGKNNSNNFKGLTLRKSNINSIPDHESIDYKELNEIIDSNDKDLNKNMSDKIEIMKIRIHVYPYILQ
KYHVETTYVLLGCLTTFSFVGIITDLLFG*
>N.PFL1520w
MLYHLHSGWDVDQAILSEEERLVCIRFGHDYDPDCMKMDELLFKVVEDIKNFCVIYLVDITEVPDFNTMYELY
DPVSVMFFYRNKHMMIDLGTGNNNKINWPMNNKQEFIDIVETIFRGARKGRGLVISPKDYSTKYKY*
>N.PFL1525c

MNEKSDLHKRSIEFTDNDESKKKIKTDGEENDIMESKGKDSYSLYLEYQKGRNKKLKNNEEYIEYLKKDARRK
YLKEREKEKLDITKKLLDDELLYSTVKLEGKDLKELQFSKKIYNIAKENINLREKLQEESYYFQQDIDKNKKT
QTYENDKVDKSLYNYDEQILNKGILKFGSAISKQNIQKQNNNEFVFDDDIITTNRKSIKRESFEEKKMNESPT
KKKKKRKDDIESDNKCDKDEKYDKYGKFYKRDKHHKYDKHDKHDKYDKHDKHDKHDKHDKHDKHDKHDKNDKH
DKHDEHDKHDKHNKKYHSKYKELNTSYDNNSPTDSTYKSEEESSSSGDKNHRNNKYDSDKIKHEKKKKKKSIM
TDEERNVDSNKRSFEKKESELNENGLTDVVEFVHLESFYGMNEKEKELEKLFEDIKKRKEKKMKKIIDERKRL
PIYSYRYDILKAIKNNKILILVGETGSGKSTQLTQYLYECKYHMYGNIICTQPRRIACIAIANRVAEEMNVKI
GKEVGYVIRFQNKTSEATKIMYMTDGMFLRLLLYNPTLEDISVLIIDEAHERALHTDVILPIVKDICNFRENI
RVVISSATLDAEKISTYFNCAPIFYVPGRKYNVDIYYTINNESNYISAIVITILQIHITQGKGDILVFLPGQY
EIELVQQELENKLNELAPKYRNLVILPIYSSLPVEYQARIFEDVTEEDNMKFLEDINDKKDERNKNHKDDEND
ENGENEKNDKNEKNDKNDKNYKNEKNEKNNDKNDKNNVGNNSKMNVSQSKNKRNINEEIEELKRINTNNKIRR
KIILSTNICETSITIDNIVYVIDSGLCKQKIYNPNSGIESLVTLPCSKASVNQRTGRAGRKRDGKCFRLFTKK
SFIDLSDNSIPEIQRCEISSMILLLKSLGMDDIINFDFLDPPSPIVIIKGLELLYSLGALNDEGNLTRTGRKM
AEFPTDVKSSKMILSAAEKYNCVDEILNVASMLTHANNIFYVQKGKEKEAENIKKMFIIEGGGDFLLFLNIYK
QCEENNFSTSFCYDHFLQYHTLIKVKDVKTQLLSICEKIDIPITTCGIENHESINNIKKSIVSAFFTNAALPV
NKTELKIIKLNQVVSIYPNSVLFKKNIMEENENVCIIFYEVIKLNKSYIRHNIPVNKNLLFQIADFYFLNKTE
*
>N.PFL1526c
MLLPLNETIFLKNSFNLQNIIIPMFFSPFYFLRPRKKTQRVDRCRTKKQYKNRRHFKGFYYRWMGSY*
>N.PFL1530w
MEETTRTQVEIIKKENSFVKGNIQVDKKNEKKENCTNNNDNVGKNTNTNGNGEGRYLFSLLDRLNNFKKLKLK
TSENDASLSKENKVIDNDIQQVPEGGAEKKDHNNNKLDKMIEKESQIIEKETQIIEKETQFIERKSQIIEKEP
QIIERKSQIIEKETQIIEKESQMIENDDPRILKDNPLNEQNDNKSNNVCTLNYIKKKTSLDNDHSRIIKNNEP
KEHINQEALVRSKNPYAESKLEKIEQLFEEIKKQEDLYVDEIGRLLLLSFDNLCTLLVDQIKDVKLGSKDIEM
FLRGEIEKIKDNNNMLNRYLIKMQNEKNDMLFNMNDNMLNDIKISFYDIFEKMLNGEYKKEFEEHIKEDKNKR
IIYIILCKKQIIYILEELYKNVYKNKIKDEKYINKLYNMNKFMYILNKANNNSFNNNIVEKKNHIIVNHVENL
KRETEQVESVLENVIENVLDDEEQSFEKKEKINKIMDRNFLEDKYVEKIDRIHDSDIFENNHVLSNDTNVSLE
SNASNKTNQINKLNLNKQISNCFSKDNNNNNETQFSLSTCLTESSFMDKKMKENSSKETNITNENQNGEDYSN
DILNNNDNMNNVHCDNSTFMKIHYNDQFNNHYPITINNNNNNNDSNNNNMYNNMSNNIYHNIYHNISNNNNNI
SNNNMNNNMNNISNNNMNNNMNNISNNNMNNNMNNNMNNNMNNNMNNNMNNNMNNNTTNINNNNQFNHTHFNN
NLINQYNDQYNIPLNNHLNNQLTTQGNCNMNNELSKQLSNQFNNQVKSPLNNIVGSANNLVNEYFSDMMVYIS
WVPKSARCQYPRELPKSAAERNKLSEYIVAKEQMLYILRVMGYDEIDNIYFHPPKGSHIKIKFKSMICMNNFL
KAYKNTPDKWKEDMFRFFNIPLRSSISVRDLRIERAVPRSSAAEFKKMKKLNKNYQDLFLFKDNFNVCEEDMD
TIENMEKLDFHYDTYNKNVILQNKNDITMNKLLNEDKNSLSNMKNANINIHDENYPNNNINNINNNNKNNNII
LLDEHHHHNNNNNNNNSNNNNSNNNNNSNNNNSNNNNNAIVNNSLSIPVAGINSYYAPGVNVHGFLGSNNAVM
MNNTITNKTNNDLNNLCSSSTGAKNYMKDINNRLFVGTKIDNNTLNINTDHFNTDVMKNIHNTLTTTTTTMI
NKNENDIMNYNNLNHNNTMFASRTSSSRNNNNNININSNINVNNNNNNNNVNNNNNNINNNNGSNNMYLLKNK
SLNRNGSILSNLNFLNTNNYNNNNNDNNNNINNNNNINNNNNMLSSASVNLNNANLNNINSNTINNNTNMNST
NIMNNNILSNNILSNNNVNNTNKDFLANNFNLKKNTHYASLTSNGNNMNLLNEQFISYNSNTENINNMNSNNM
NSNNMNSNNMNSNNHHINNHHINNHPGNHNMNAHADKNVAYINPLNNNIDINSKKENNLLLANYKNSYLNNTN
SGSNTFSNCGQRVNQLKCLNTYNNKLNEELLNEKYVNNEDLKDDLKNLINIDFINDIDMEYEEKHSYNNETFL
RTSNSSLLQRHTYPKNFPSPNENTLLTVVSSTSNNGNINKMREYQTNYDKLPYNNVMNCDEQNYMMNFNQTNN
DNNNDNNNNNMFHDDFPHTNLQLNSSNNNKLLHLSSFNSSTPTNGAIVIDDFNYYQDDKFNVHNNNKKILYTN
YYNSCEMPSPNSNMKNNQNTTFNMDATINDNYTHMENIKHNNNNSININNNNNNNNNSLYRSTLNPKNFLNDLT
LNNNYTNYDDNFKDIKKNDEKMRDYNIIKVNDEDNKNFLSYPFHASSHIDTDQNFTNSYVNFF*
>N.PFL1535w
MPDTKYENLLQYDLKIYRNDILEKEKKKNDLRNILFFPSLFWFNGQFCPPIPVVKYYSNLCAFKVKDYVTCSI
HKFSVIIRRIVKNEIVHVNMFNEKKVLSCVFYINIDYILKLSNIINKYMLLDFVPLTTCLVEKENELIKYTYK
KNISLSYKLFCYNEKNEIHLFDVEKNKSLLSFSEKNQITCLTFNFLYISKKNSLYNKLFNDIYDENKIKIVDI
DNIDKQIVTKNMYCFELSQDKINNTYNTCKDNEKHLKIFKNLFYLLIYGDDQGNVYFFVPQKNVKIKKKLRNE
KINLIETNVKEYFKVQNINDLYHNIKNNHYIFVAYKECIQVVQFYFFEIYFVINFVHEKLYSMTTKFNNLGQN
YLSFSTKNYVKIYNINEMKEINCINNNIIQKRDENNEKENIILHEIDKSSENQEVEEDQEEEKKKKKHCLIAC
PVQKLHEQTGEKNINENYKEIICDDMEYKKYENNENMEEQNKDDSLIYTSNKNHKNDNNKNISDKKDLKYYTC
IHFDSADNIYISSDKGIINVYNLNEKRIIKSFHINCKIIFYLFFFILNNKEYIYIYDSEKFYCLYVIDEKKNV

YKIFTLSLWIYHIICFDNNIIFSLGNENIYNLQLKKYNNNKILNSIYSNNINICIFLINHPQLPIIAFINKKS
VFGFFYLTKVQKKRPNQEKNIIIPNIRENQHIISICWFGKPKNIIKNKDILFDYDNVNDIIEKTNNSNDVQAN
SHCVYESVSLNNNHIDIIENNVTKENKKKKRKKMNMMKMEGLKSDMNNNIYPNTFDTYLVILNEKGLYLYNII
TNEILKLNNDLLNNLYEFKGKKYMTSTIFNNEIYVFILYEKKLLFFNHNMEFDSYDINLNEKINKMYLKDNIL
FLLSNSNIYFIFIEYLLINLLNKKFNVLKNCIHDIKDDTNGVIVNSNLSNNINSDIIHADNHIEKLEYQEKEN
EKKYVSQGHELYITKLKFPQNFKILLFDFYYMTNGELYIAIFSKKKKIMIYKINLYHEDYNNRKQRNEENTIL
EIEAQLIAQFINFYIFEHINKVGSILCMKFFYNSEKKKIYLVFGGLEQFLFSWNFLKYPIIQKK*
>N.PFL1540c
MFFSFFVCFIFVIIEFIESYKKNPQISDIYFRKLNKNYHIKDDIINYNRFKYVYKIKNHPLNEIKEEVLNFLK
KKTSFYLVYNKCPLYYKNLCFKEILIRDTNETTSIKNNFYFSHDYLYIPQATSLFPYIYDLLKNNNKQNIQKC
NDTIYVRNNPYSENTPNNNIDVNNNIEKKQDNYINKEIVENNNVHIINNSNILNKQRDNFCIISNIFRKDNID
KLHFPFFNQMDIYFKITNELVHKKKQLVYFLCELLSNIFGPNYKWRIRKDSFDFTTHSLQAEVFHNNKWIEIL
GSGILKSKIIFNKKKKYEINDYLAVGIGLDRIAMIKWDIDRIRDLYLYIYNTEKNIYSVEQHNEKDKCSQHIS
NDIFNKNINEYINHNDSYNHNNVKNYFLKNKKNENQNDKHQDENILLSSPKLEGQKLRHNLLTHIKQKMHIQQ
KQKIINIPHEEVLYFSNLYNIKDEQRDLSFYSNEQWNNDDFIKNIMELKNIKNLHFLKTVYLFDIFLNEQSKK
TSYAYKFIYTPTTNIKEQHVFKNHVKELHEQIITQITKMYNIIIR*
>N.PFL1545c
MRMKRIHILLLVIFLLCLRYGYSIKKKRSPNNKNRLFINKRLKYINSKIISRRKENYVKMKMTENKVKGKDII
YGNECRNELLKGILTVSDVVKLTLGPRGRNVLLEKEYGSPLIINDGVTIAKNISLKDRKKNNGVKLMQESTNI
SNDKAGDGTSSTALMTATITKKGIEQVNRNHNPIPIQRGIQLASKMIIEKIKSLSTPIKTYKDILNIATIASN
NDVHMGQIIANAYDKLGKNAAIILDDNADINDKLEFTEGYNFDRGIINPYLLYNENKDYIEYSNVSTLITDQN
IDNIQSILPILEIFAKNKQPLCIIADDFSNEVLQTLIINKLKGAIKVVPIRAPSFGDRRKDYLKDLCIVTNSK
YISADVGLDLNNLHNNMSSFDNNYLSLLGSANTLIVKKDRTSLITKEEYKKEIDERINVLKKEYEETTSKYDK
EKLNERIAALSGGIAKILIGGNSETEQKERKFKYEDATNAVKSAIDIGYVPGGGVTYLEIIKSNFIQEIHKKI
EEDLQISSNNDEKKYLELIGNLESEMELQKMGANIVVSSLDVITKQIADNAGVNGDNVVKIILNSKDKYGFGY
DVNTNKFVNMVEKGIIDSTNVIISVIKNSCSIASMVLTTECMMVDHEKKDKGILDSSINSPNYLSKHRRTYKH
KLHDDEDTDEDDEEDEDDEDDEDDLDDDDYDDEDEEDEEDEEDEDDEDDEDSMNDGYNYDE*
>N.PFL1550w
MNSVIFRAHCFFQPLRRCFSTKKDYDVIVIGGGPGGYVCSIRCAQNKLNVLNVNEDKKLGGTCLNRGCIPSKS
LLHISHNYYEAKTRFKECGILVDNVKLDIETMHKHKNKCMGNLSDGINFLYKKNNVNHIIGHGSLVDEHTVLI
KTEKEEKKVTAERIVIATGSKPIEIPLKKLNDNNFNDADNVNDILEYDHEIIQNSDDILNFKKVPHNISIIGG
GVIGLEIGSVFSKLGSDVTVFEYNERLCGFLDADVSKVLQKTLEKIKMKFVFNTSVIGGNIENNQAALFAKNK
KTNEIKKTTSEIVLICIGRKANFDNLNLHLLNIELNKNKKIPVDEYFNVIAQPTIKAIGDAIDGPMLAHKAEE
EGYLLANILFDELKNNKKKKAHINYDLVPSVIYTHPEVATVGYNEAKCKELNMNFKSVSFPFAANSRSRTIDD
YDGLIKLIVEKDTNRILGSQIIGNNASDLILPLSIYVANNGSSKSLSKIIYAHPTFSEVIKEVALQSFDKPIH
M*
>N.PFL1555w
MVVKKLKVIDDKELKKLKTESKSCIVINDLIYDVSSFYEHPGGYDLFKEYEGLDATDAFNSIGHSENAKNLMN
SFLIGIKKNSKEYKKKEKTKIIENKIEYINYSDEEIKKEEKEPQESKPKENKPTLIKKEDKTNYPVVAGIIIL
FFV*
>N.PFL1560c
MLSLKASRADNFYYGSIEDEKDKKKKEKKEKRLNRKDYDDIKFEVPYTIICKKCNKYIYKGERFKSERRKIGF
YINSTFYSFCIVCKNCLNEIVFETNPQNCSYDIIQGARQKNEKYENILTHKGKKNINSINFEDNKKKKINPFL
LLEQNVVQNKKKGLSDCTTFENYNQSKHIITNNNKEDIFEEGKENDQKYDYDKNSVENDTYPLSQHNKNILIT
YKKNECEQRDNNSVVSNEEFNIISPQKNDEDSNTSSDDEDMNEVIKNNYKRNHILKYDFSCNSHLRKQLRNIK
KEDIKKKEQCKKKNIFIDLINDPLEDIKVKHFALLKDKYKKNITDKNNTLKNNLIKKKKNSIFDKRYLKEKKK
KIK*
>N.PFL1565c
MNTKNDCALLHICDDIFLNLIKYFSINETIKLKLVNKNIYEYLKDEKVYQLNTCLNLNNYIKFVKFVKSEKFS
AFLLSILNDEKNIYDSPYYVKRNAQYGINKKQLVPKGKGFLQKIILKGEFLRNVNFYDLSILLASHSDTLKEL
YINGLNDVNCPLFIYSTYNIIFEFFSKEVLFNNLSFNYVTKKDLQKIQKYISNQRKVPKKEKKKEKCEKCDEK
VGSQVNEKCEHEEKKILSRTKTDVDIKLFESNKLPMGVKSSLDNQLTMGTQCSVDSQFSIDTQFSLDTQFSTD
SQTSIYNKNTSVENKSYNSRQIYNEKELSLENLHIKEHLDVQEKNDKFGKVTYTNTKEYHRKISSKKIKNFTI
NKEKNNNNEDDNNNNNNNNVVCKEKKNLIIKLFDKFVKNEKKKKNLPITNKNKLHILCEHISIVYNCNKKCFE

KIKNYKQETNLSPYVNNLINLKILNLSGIQTYDMIKMFIPINMPSLNILNIYSYDYFFYFYNMLISFFSYGNG
QEIFTRYINVKKIEEDEKEKNYHIKKKKNILLEYNKRMYEVIKLMDQQLKHTCNSASSFQKIFEDEAINNYEL
HIKNNDKKVFITPSYVDYIYVNHINAPIYYADGNYIKYDTDDERATCSIERSPSWSCVRSIESYDDNRKGADI
TKMYDRKLKGEGKLKGDSKLKGDNKLIGDNKLIGDDKLIGDNKLIGDNKLKGYNKLIGNNKLIGDDKLIGDNK
LIGDNKLIGDNKLIGAKNAYDNYHFQGSEEQKSSVQNNKKKMKKLKRGGYIWPIENHKYKKNVSVEILYLFKN
ILENEEKKGRKNIICLLNNLNLEHFSLFNYSLSSPFLWLLLLIKNRNLKTFCILDICLSHLLCALTFLEAYLK
QNLFNFQGMMRRRRKRMGSIYFKFNDHDEIKFINEMYSKLINEMNQKRDIRGTDQIHHNNIQNNDINTYNMYI
NKIHINNNNNNLTNTNTNTCNTHEQNKDNYQANRKKKSKILHIVIYVYNNTKENKQFIKYIRKISRSLWRCNY
VVYYSIHYYKYKYFNKYIDIDNLYRDYIMNILLNNHRLNRYMDESKMLTY*
>N.PFL1570w
MYGLKIKHAQFNNIILSFAYKIKHLRFPKRYVFHLERKTKGNEVIVKSTNILKTKEEKVDEENNVMIKFKDNI
NNDDIEKIVDKNKYQVDNSFCSYLIKRKKFLSFFSYDIKKYEHNILYKKSDGSLLAYLYSNKIKPLIYADRIS
NLLYVNCINYVNKNKMEHNIEHNIEHNKNCNDNIHFYYKQASIKEKKNLFDILYKKYSLVFLFSDITHIDEMN
KYFIYFNKQMNISNMDNPHNNFAHTQQIYENGKILKLRDKQKPIHIFYGYLSPYNFILSNYFSTNFCMKLKNT
YFPNDNFFYINSKLNINDMNALLLQEGRQNLPSILLFDECCYVRYHIKGLYTNESAYYFFNILKNV*
>N.PFL1575w
MSYVNKSLSTCLNFLVLHLEYVKCKNLSNYKKKGKYYMIITYDNILFYEKDFYEVCFHIFFDDILQIYTCDVS
NYIHITLKDQAVTNNIGIKGMNKNVLIKQLSIAYSTFYMFQLNKNVYVPLTNETYEERSFRTGHFEKPKIDTS
IQPFIGYKKVVFDDYFFFIRKSFQNFISISCESNFYVDYRGIEISIKIDDQKNILEIEQSPDNNFFQLCRNHI
NFLTNDSKCPLIIRKNVYNKKMNLSDDLAKWSGYEIYFKNETHTVVCIIFRRTFIPPLLDKRQDIFITFKISH
ENQQEFNVSDKDLYDEVYIVSNSITPNEIHNT*NKG*MKKKIYIYIYIYIFIYLFIFIV*PSYFEYIKMFLKS
MLIILKEGEVMINSDIIEFLGEDTKVERNLEYLLNVIMNKITGLNLLDTHKSEKIKINKFLHRLSDYLIYCLH
SGFISHKYNVTDFINGCLSINKDNKLTIDSIVNFFLHLREKDYSNRYELNCLDLLKEDASNDIEIGKLLDSNK
YYINDFFLFYLHQCGYVNKYYYYKDDNNYKKIISYILKYGKNFEIKKKICKNLLIFSNDYKNKYIPLVNSMIC
FLSFNYYEKNFCLFILSTLINITNNNDELKNRLIELNISTLCNFLILLNDIDITNKIILLYINLCKEQYMCED
FINKGLLIHFLDILFRYYYIDLYIKKQMCINILCIIGHIFNCKKYYIFILNYYNGLLQLAIYIYQTTDFIQFD
KLKLIFFFKQISQHSYIIKDKICKHIVPLVIKEIYLYINKDFIYSSLHLINTLTDYKNNCLYLQKVKIFYLFN
FIKQLKILDLYQKVEQLENKLKKILNMI*
>N.PFL1583c
MTNFFCRRLLKRKPFFFFRKFRSLKTLYIKNVNKSVIMKKNIIENKIENKDKEKEKELCALNYFTKFEIFYLF
VMPFTFVSLYLILMWNIFKYICDIKDIKHFLKILRWLNGKDAYPYEYWNNKKDTAKREKSIIVAEKKFE*
>N.PFL1585c
MKTPFKNNYMNACFRKTHPSIIKNNLSEKIEQKKKYFCNILLKITQKKEHQPFQTNLDKENIYDTKIRNNKIN
YKNKTLNNLIHNVKKIATYNCKRYSTDHNILISDKNIKDNNKIKLTNSFLNQTNLKHEIVDVHKKLTENCFID
KQKEEIFLLLKSAIKPNLKGKIYFIGSCENNIWIKNSDIDCCIVVENCEDKNSYLYILKVIKSAINLIYPSLT
INIIKASVPIAKIYKEETNICDISINNTVAIVNTKFVSSICNIDERVTIINRIIKYWAKQKNINNRSQGTFSS
YALFLLTYYFFQNINNPLLPSYKSIERENAESFDINSEYFFLQDHVEMPFYTNIEDIRNKFPNLQKNKEDVSK
LLYGFFEFYSNDICKNGITLDIYNNQIIENKDMTANIYCPITKKIVNTYSINTWKKMFEKFQAAYDKLKNGDS
LNIICEETKDNTPNRKMDLKGIIFFFLFINK*
>N.PFL1590c
MIILKHILYKNNGRRFFEKTIPNKIYNFYLSGLCKFSSCCIDNLRNIGISAHIDAGKTTLTERILYYTGKIKS
IHEVRGNDGVGATMDSMELEREKGITIQSATTNCVWEINNKKYNINIIDTPGHVDFTIEVERSLRVLDSAILV
ICGVSGVQSQTLTVNRQMDRYHIPRILFINKLDRDGANVERTLHTIEKRLNLNTILLQMPIGIEQKFKGVYDL
INRKGYLFQGKNGIILNEINNKEEILSLDNSFSFEIMELLRNRILEKLADVDDEFAEIYLNNDINDIKKNDIY
SSIRKSTIKNLVTPICLGSAKNNVGVQILLNYVCNFLPSPKEINNYGYIIYSDVLEDKIKNNDICNETQNTQQ
YQSQDQNDISIINYNNQNSINNYTHKDDHHSSKNKSKKKIQLLCDNNLPMVGFLFKIQEDNMYGQMSYFRIYQ
GKIKKKEMITNMMTNKKEIVKKIMKMHSNMAKEVNEASAGDIVAICGINGSTGTTYTNGINTNLHLLNIFIPK
PVISVAVEILKKGDMTKLTKALNKFTKEDPTFYVKTDEQTKETIFEGIGELQLEIYKERLKREFNINVNLKNP
KINFKETITKPFECSYTYKKQKGGAGLYAHVHAIFETISDNYNDTTHCTFVNEVIGNDLPKNFILSIEKAFKE
QIEKGYLYNSEIINMKMRLIGGKIHEVDSNDLAFKKATINLIKENYHNFCPVLLEPIMLVEIISNYEHQSNIL
TSITKRKGLVNNIVNNLNIIYIYADIPLKHMFNYINEIRAITQGQGTYTMEFSRYEQVSKNDLDEILKQKSTA
*
>N.PFL1595w

MKDNEFVGGKLKLKKDIFKKDDKKGKKQKKDKNNKKESELIEKKKKKSSEKDYVHEDNDQNKNVKELIDGEDK
LNDILNMNLTESEKAYHLVLKKREKQRIESILKESYRDRLQKFNDNLASLSEHFDIPKVGPG*
>N.PFL1600c.1
MKEDSPNDPHINKIYAQNKTICSNAPFSNNEDHNINKNEYDQNNYYNKKNNDMLISNISEIKDRRYFSQCVDS
NLDKNYVDIGDTKCNNASVRSYSYKKEISPHHNKNIMFTYKYNLANDERNSQNLREHPINNNNYEHIINEGYY
YQNMKCVSRPTLNVSSLNMKDMNVPMIKNDKYPEGYINQTILNNNNNNNNNNNNNNNNNNNKHIIEKGKCTNG
YYSYSQPLSPMVFNNNNSGNIMLDRNTLNNSHMNMSTFNYYNNYINNNIGNEHKMDNIYIKENKHGNEDILNN
DMLSNDILNNEIVSSDIINDNNNNNNNIRDTITDGMRNYYTLGSNKMRMFNVCRNMTQTYNNGNNNNDDNNNN
DNNNNDNNYNYEHNKHNYHNRRDGYGDVLNNNKDDITKEIEKNISLLCNQPHVDNYDMDNSIGIKENTANRNY
INPLIYINEKQQNCFKGNYNENNVEMINVDMIRNNSIYNDKTTNINYEEEKSNNIKDRNDMNMNNTFIYNTTY
EPLLNRNMNIYNITNKDNYDNNQYNNCDNNQYNNHDNNQYNNHDNNYDNNQYNNHDNNQYYYNPNNQYYNNQY
FNNNYSQYHNSREHPMTNDPYNTENRQDGNHLKYSNYLNNTYNDHNYHNYHNNYHNNYHNNNNICASNNKEIY
DQNNDTINQNVPLSKNTNHSNKQTILYDKNTIPNYPYYTYNIIQDKCLKNETENKTRNSTYNSFPINTNTEQN
KSKITRKNTNINLIQTEYDTNYQNNGAYNNYMENSSNNLNEYNQMDNQINMYNQNIIHNYDHSVENGEVQANM
NRSDFVNTAIGGCENYHMLDKKNKMVDINNNLIQYESNTVLDSRNVNVYNNINENNLNKIERGNRNQDEQEEY
KDEGEDENNEVSIGRKVYDNNNNNGSNNNNDGNNNNGSNNNSSSYYYNNYPCYPQNSNINNLDTYKIETGTKI
KNDISLHIKKEKEFKNTKHILNDYGVINKCVEHDNEQLKNQILENEHEQNGKEEETNGVAFLPRDIKKVAYNT
NENGYMYNNDNVYKNEELNKNINIGVEKGYIDSSYNNVSINQTVDYKENNNYNGNIKYNYDNSDMIHNGINMN
KASILYSQSNRMNINYKMMSVQNQEDISKDENGNPAYMINNTNSINKNIAVNKDNDNNNNNNYNNNNNNVYRE
ENVSLGCFNNVEENNNINNNINNNNNNNNNNFYYNYNYNYYINDNSYTNEQVPGAKMLNHNSSHIYNNTYDMD
NLHVNNINVNNVNTNNRNINNINNINNVNMKSLDNNNNINMINTYNMAFVKHQTNNDNNNNNNNNNNNDNNND
NNNDNSYNNKSDNMTSILHNVNNVSYDNYNDIPAAINNNYINTNNMLINNKHMQQNYNCQDISNNSNYISNLK
NNNNNNFYNNNNNYMINHDHSMFNKNVQSIHMSSDNIHVTNHFDINQNDIQQNNLVINNNELNENIQMIPQNN
IYFASTSSNNNMMPNSNFMCSHSNNNNNNNNSNMTCNYTSCSNLMRPPQNNIINNNNNNINISTNELSSGYQNP
NIILSSSSIIDSNMCAVSSNLQHNNYNEDNNYKNECLNNNNNSTHNNINNNDQNYFKQENNCADEKGIHENYQ
KSVSLNINMKHTKNKKSNNNDGKKRSNKNIKEDKKNDVTDNNENNGKRNKKIRKAKTFGGTVKKGGTIINLEA
ESQRIKRTPFCIYSLNDICNHLRYGKKSANSINGMIKTSTCNCEIHNDMKFVKQYINFLIAQKEKERINKEKK
INEKEEKNDEGNKKEEESQKEEESQKEEESKNEEESKNEENNIKDNEQKRKNKIIKKKCVHGKASEKNTEDID
NTCCNNNIDGDHCEDIEFCENDESDKESNNFENILQKYKGIFCNYEYYFDLEVKYNREYFLRQLLLKQAFNDL
YNFSQCTNVTYNVRRMWHPLFNRPLGDIGRKLILKEIREHHHRDAEKTTKLLLKNGLDYGKVRFMKVNELYHY
MYALGVFEYAVKISLEFGSNIRMSTISSKIDHVNLCSNIKNGYSYCLICCSQKIRIGSIINNTINLMNYKILL
NSYAHYFYYFVIKKKLKRVENQEEYETMDQILNKDSNNLGNIIIIKQKKKKKKKKKNKKKNKNKNKNKNNNN
NNNNSDNSTRHFNNEQQIYSMNINHNSFIKKNYFHSSNNNYHLNYYTDSSYCSIVVIDNEHLHDGVYDQNSCN
LRLSYCNNTSNNQNINYNNHEHDINNDSDCSTKCGSNKIYLDNSIYNENDVFINNDGKDKGEEQGKEKSKQSK
CNKKDDVLKRYSMYLKDPKENNTKKDNKSKLDEDTQKYPITKIVDNKIFHLKNSYLIDDDRNEISSLSINYQA
KKECHIYYPNGGQVERLKNEMININIDNRNIEHIPNNNINEDKANEKNNNYKNDDDDNNDNNIKKKRSKKNKK
TDGDNVDDDDNDDDDESSNDSEYDGNKRKDDHHKDDNDDDNNNNNNNNNNNNNNNSYSNKNNNYYYYYQYDYHK
NECNRYYQYRENDYNSGNNKTNCNNNNNNIPYNYQKYYNNNIHSKKHDNNICFNNTNNYPNNINTNIYKGITH
DNEKKNVENNKILKYGNTYNETNFINNPTDISTIKKDMILYSPEKKNTDENVIKFIPDEKIIWSILKSDISPS
LTQFFFNFIIKKNQYNFFLNKSYIKYDNHMDDIYVHKTDGNTKDGYKNCQNRSYININRNRNNLSNAYNNLDY
YTHNKHLDGGNVDINDAIYTDKKKSEEKIFFQNKSWINQKSMEYINNYDNINIIQRDNKNKEKDKTNQNYIHI
NNVHKISSDNLLRTHTDIIVQHKDMSKKENEIKYYTNNHIDVNITNDKLYYNNKKLKKLFKEIKNNKLCKYIL
AKIKEIKYNKTYMENIKTNNKKRKRNITYPYFMFYQINNFLINPNPFHNSFFLNEMNYCSIMHNCTMYHKKIN
KKIKTKNNNNNNNNNNNNNNNNNNNIKSHCCSCGGGDVSNILENINKNFQHKHFLSHKHSYNEHKEEDGEGRKK
KKKIKKRIKKIFEFAQIFKKMSQKKKENKKEENLHLYNANKTCKDYRTLRHKEELHEQILFINSKKYNDEIIH
VNEYMKNEEEINLTSNCLKDNLQNVKNNDDEKTYIDNEGDNISIDYTYKKKKKIWSCFPDFEKSRRLKFIRKK
KKEKTKFKKIIRYLIQNYKIKNINQANHHKNNFHADVYFKNNDINQKGKNIKNTLRIRNNTKHIFIYLITHTN
IVKHKNKTFSI*
>N.PFL1600c:mRNA
MKEDSPNDPHINKIYAQNKTICSNAPFSNNEDHNINKNEYDQNNYYNKKNNDMLISNISEIKDRRYFSQCVDS
NLDKNYVDIGDTKCNNASVRSYSYKKEISPHHNKNIMFTYKYNLANDERNSQNLREHPINNNNYEHIINEGYY
YQNMKCVSRPTLNVSSLNMKDMNVPMIKNDKYPEGYINQTILNNNNNNNNNNNNNNNNNNNKHIIEKGKCTNG
YYSYSQPLSPMVFNNNNSGNIMLDRNTLNNSHMNMSTFNYYNNYINNNIGNEHKMDNIYIKENKHGNEDILNN
DMLSNDILNNEIVSSDIINDNNNNNNNIRDTITDGMRNYYTLGSNKMRMFNVCRNMTQTYNNGNNNNDDNNNN

DNNNNDNNYNYEHNKHNYHNRRDGYGDVLNNNKDDITKEIEKNISLLCNQPHVDNYDMDNSIGIKENTANRNY
INPLIYINEKQQNCFKGNYNENNVEMINVDMIRNNSIYNDKTTNINYEEEKSNNIKDRNDMNMNNTFIYNTTY
EPLLNRNMNIYNITNKDNYDNNQYNNCDNNQYNNHDNNQYNNHDNNYDNNQYNNHDNNQYYYNPNNQYYNNQY
FNNNYSQYHNSREHPMTNDPYNTENRQDGNHLKYSNYLNNTYNDHNYHNYHNNYHNNYHNNNNICASNNKEIY
DQNNDTINQNVPLSKNTNHSNKQTILYDKNTIPNYPYYTYNIIQDKCLKNETENKTRNSTYNSFPINTNTEQN
KSKITRKNTNINLIQTEYDTNYQNNGAYNNYMENSSNNLNEYNQMDNQINMYNQNIIHNYDHSVENGEVQANM
NRSDFVNTAIGGCENYHMLDKKNKMVDINNNLIQYESNTVLDSRNVNVYNNINENNLNKIERGNRNQDEQEEY
KDEGEDENNEVSIGRKVYDNNNNNGSNNNNDGNNNNGSNNNSSSYYYNNYPCYPQNSNINNLDTYKIETGTKI
KNDISLHIKKEKEFKNTKHILNDYGVINKCVEHDNEQLKNQILENEHEQNGKEEETNGVAFLPRDIKKVAYNT
NENGYMYNNDNVYKNEELNKNINIGVEKGYIDSSYNNVSINQTVDYKENNNYNGNIKYNYDNSDMIHNGINMN
KASILYSQSNRMNINYKMMSVQNQEDISKDENGNPAYMINNTNSINKNIAVNKDNDNNNNNNYNNNNNNVYRE
ENVSLGCFNNVEENNNINNNINNNNNNNNNNFYYNYNYNYYINDNSYTNEQVPGAKMLNHNSSHIYNNTYDMD
NLHVNNINVNNVNTNNRNINNINNINNVNMKSLDNNNNINMINTYNMAFVKHQTNNDNNNNNNNNNNNDNNND
NNNDNSYNNKSDNMTSILHNVNNVSYDNYNDIPAAINNNYINTNNMLINNKHMQQNYNCQDISNNSNYISNLK
NNNNNNFYNNNNNYMINHDHSMFNKNVQSIHMSSDNIHVTNHFDINQNDIQQNNLVINNNELNENIQMIPQNN
IYFASTSSNNNMMPNSNFMCSHSNNNNNNNSNMTCNYTSCSNLMRPPQNNIINNNNNNINISTNELSSGYQNP
NIILSSSSIIDSNMCAVSSNLQHNNYNEDNNYKNECLNNNNNSTHNNINNNDQNYFKQENNCADEKGIHENYQ
KSVSLNINMKHTKNKKSNNNDGKKRSNKNIKEDKKNDVTDNNENNGKRNKKIRKAKTFGGTVKKGGTIINLEA
ESQRIKRTPFCIYSLNDICNHLRYGKKSANSINGMIKTSTCNCEIHNDMKFVKQYINFLIAQKEKERINKEKK
INEKEEKNDEGNKKEEESQKEEESQKEEESKNEEESKNEENNIKDNEQKRKNKIIKKKCVHGKASEKNTEDID
NTCCNNNIDGDHCEDIEFCENDESDKESNNFENILQKYKGIFCNYEYYFDLEVKYNREYFLRQLLLKQAFNDL
YNFSQCTNVTYNVRRMWHPLFNRPLGDIGRKLILKEIREHHHRDAEKTTKLLLKNGLDYGKVRFMKVNELYHY
MYALGVFEYAVKISLEFGSNIRMSTISSKIDHVNLCSNIKNGYSYCLICCSQKIRIGSIINNTINLMNYKILL
NSYAHYFYYFVIKKKLKRVENQEEYETMDQILNKDSNNLGNIIIIKQKKKKKKKKKNKKKNKNKNKNKNNNN
NNNNSDNSTRHFNNEQQIYSMNINHNSFIKKNYFHSSNNNYHLNYYTDSSYCSIVVIDNEHLHDGVYDQNSCN
LRLSYCNNTSNNQNINYNNHEHDINNDSDCSTKCGSNKIYLDNSIYNENDVFISNNSKHSNNDGKDKGEEQGK
EKSKQSKCNKKDDVLKRYSMYLKDPKENNTKKDNKSKLDEDTQKYPITKIVDNKIFHLKNSYLIDDDRNEISS
LSINYQAKKECHIYYPNGGQVERLKNEMININIDNRNIEHIPNNNINEDKANEKNNNYKNDDDDNNDNNIKKK
RSKKNKKTDGDNVDDDDNDDDDESSNDSEYDGNKRKDDHHKDDNDDDNNNNNNNNNNNNNNNSYSNKNNNYYYY
YQYDYHKNECNRYYQYRENDYNSGNNKTNCNNNNNNIPYNYQKYYNNNIHSKKHDNNICFNNTNNYPNNINTN
IYKGITHDNEKKNVENNKILKYGNTYNETNFINNPTDISTIKKDMILYSPEKKNTDENVIKFIPDEKIIWSIL
KSDISPSLTQFFFNFIIKKNQYNFFLNKSYIKYDNHMDDIYVHKTDGNTKDGYKNCQNRSYININRNRNNLSN
AYNNLDYYTHNKHLDGGNVDINDAIYTDKKKSEEKIFFQNKSWINQKSMEYINNYDNINIIQRDNKNKEKDKT
NQNYIHINNVHKISSDNLLRTHTDIIVQHKDMSKKENEIKYYTNNHIDVNITNDKLYYNNKKLKKLFKEIKNN
KLCKYILAKIKEIKYNKTYMENIKTNNKKRKRNITYPYFMFYQINNFLINPNPFHNSFFLNEMNYCSIMHNCT
MYHKKINKKIKTKNNNNNNNNNNNNNNNNNNNIKSHCCSCGGGDVSNILENINKNFQHKHFLSHKHSYNEHKEE
DGEGRKKKKKIKKRIKKIFEFAQIFKKMSQKKKENKKEENLHLYNANKTCKDYRTLRHKEELHEQILFINSKK
YNDEIIHVNEYMKNEEEINLTSNCLKDNLQNVKNNDDEKTYIDNEGDNISIDYTYKKKKKIWSCFPDFEKSRR
LKFIRKKKKEKTKFKKIIRYLIQNYKIKNINQANHHKNNFHADVYFKNNDINQKGKNIKNTLRIRNNTKHIFI
YLITHTNIVKHKNKTFSI*
>N.PFL1605w
MNGSFFFSHFKEFTIRNTNYFLYEESKKSYKSCIVWYMNHYQRFGGSIRRYSNALGQLIDRKKRNLVNIEKGN
EILKSYKRVNLKKILKDGDKKKEIYENDKIVSDNIISDNNISNNNISDNNISHNNIGDNNISNNIISDNILSD
NILSNNILCDNIVNDKIKYDKGEKWINENVLKKVEGKDNENLDDFNDKDKRNNNKNIMNIENKNIYNINTNSS
TFCTHNHINDEHVKDSVDELFCTKEKKKQLMKRIIFQNNHDYMHLIERYEEYKIKQEDEFYNSSNDKNEEEYS
SYPSFIKKKEENKSNDDNNNNVNEKMCEKENFKCKEKSSNNDVHYYLYEVDKDNEESNVNMHFNFLKNNLNIS
IFPEIGKKDILESCKRKKNMQNEINKNSDIKNMFNNDIYFWTKRKVHRAIGWDLIPDNLKRNDDIIDVSELRY
GHLKGSTKEEEKEDNNREVGYKDDNNKIHKTMKQHINKDIEQNTGQDKNSNIKGKSVEEENMYMGEKKNDINV
QLDDINIQLDEINLNKDERDKKKISYIQRRVAPCNFLKKLDIYKSDATLLNDGSSTIQMNKNVDVICEEDNKE
MEKEEEKKYEETNKEPTNQMKEEKLIDIFLNNDNNLGNVKNVEYIKAEVNKGDHKLDKLDIKKEDDEYNEIVE
IDSCQNDNIKNKEKNMKYMNGSYNNMNEEDKHYESDDKTYYEGIVDNSKNVFKKMKENYDMFKKNNMSPYILE
DCEKYINYYYGIEKEERIKELRKYADMDFYEIMGNGNFGIEDYNMLIKSKILFNKEEEGFHYFNLLKKYDIRI
NIETYNSLMYTCIVQKNSKLSRLIYLQIIKDLFIPNKNTFCILIKAHILDKDIKSAFHLYRKMIKENIEVDIV
IYSTLIDGLIKNKLYKRAEQFFNYIVNYKNVVPDEILYTIMIKNCAYNREAEKCLNYYETMLSQNLRITDITL

IEIINCLSRREDYFYKVFYFYHIYLSNEMKINQRLMLYMIMACSNKGNIKRLKEILKTMNKNKIKISDEMYCY
IVRTFANNCKDKRVSLSERHNNIKYAWRIIYDLLKGSSHMKKKEKEKEKDIYIDKYIDKEKEKYIDKYIDKEK
DIYIDNYIDKQIDERHGEMISTKEHIKKNPTDDNKNIESYNKNDHISFDRVNHLYKGTELHSSTSHDCINTKI
LNSLILLYINCEYYEYAINMLKYFSYFECVPDYYTFNMLFNMLYYKMKDYGKVLCLYDYMINNTQNKPNEKIL
NLILNSAIQTKSSKNTLFILRQMFTYKIYPSPKMVKKLYHVGRYITEIQLLINSMIRQQTKDIYEVNLKENQL
IRLNIDEYELNLFKEGKTFKSKTPLDEAREQFFKRKQRMEKEKRMSKNKKSSDWLPYGQYLQSKKKGGEIYAK
RVDRPRPLAFDD*
>N.PFL1610c
MNEKKSLISQILTEAEILELGLNSSMMKYCSCPFILNKKYFVKICISYLITTFLFFCVYITLIILLLYSEKTE
LNKSILLIIYVYIFILFYIDLLISNNIKYDLIKKYNIKEVRKRIYDKYISHSKKQANTRNNDFNNKNNNVHEE
IQHNIDANKNINRDNNIERDNNIERDNNIERDNNIERDNNVDINNNVGINNNLDRDNNLDMHNNSYNHNNVDA
HEENLKLPCDKKKRKKENTQGQYIVNKKEKTSKTKNEKIFSNEKYYITEEKYMNQYHHITNNESRLNFNENFY
YNLYDLKDVNNVINIELLKYIQYENNNYSIKNKKFDIFLIVAGLINQINIFFDLVFLFYLYDYSVNLFFISLF
IYLYYFVHMCIIIKLSSNITLLLFRIYRKSMHRSFRKKIKYCKYFASYIYYKMKRAFVCILRNMIKLRTNEKI
IHERKSKIFFSKYTSYNSWSIFYNDNSCLTSEINSLPLMDETDSEYEEILRKKIYTHKTTRKKHKDNPDYEKA
LLPTHVQIIANMSSFLSYYHILNILKMKFLSAHNIYAHITFTIVFFVWKLLYTDIVLCLLKVFFLFYTDDVLS
ITLFLLISVVNIVNSYLINLLDHNLLSDINME*
>N.PFL1620w
MEPKETDNVIKDEDNVKKEEPILSIEKYLLKEFGDGKTNKKDVMRKLRKRIYNHKNSEEIYKKLKEIHGNSGI
CTRQWKEGSFAFKCYNCEGDPTCAICAKCFFASNHKNHIYRLTHTSGGCCDCGDTSWNINGSCFDHRGINEDC
LINNSILNIDIKNRVREDLECLIGTLFRDIVLKDGYFLSLVSADYIEHAFDFFKDLGITSYLFRQVICEVFTG
AKIDFLVNFHYCFYVGVQKALYSFYLSLFVHPYFKQRFAFKLAKHYKLVVRVADDRIYNDNHLNGLSVQVLTV
PEIAYTLVINNFLNDLLKLIENLCVFNNVTNCVMHKNFLRSDIEIIIRICVDLKYLLYHHEVIKIVLFNKYVI
KKILKILSLLHRMNSQVRYTKSHIIYEDLSSSYSITIENYLLRVFEPLANFCKREANANFRNLFLLYQLTIRG
IHKLDLFPFNRKKKEAENSFMNRKKLKEKKITKKRNIKEDNSTDDLTGDSDNSSTNNVCRCDTSNYDNTMFDR
SCSIPGLNNMNDDVNKSGGVSLNDDNINNDNNNTNNNTNNNTNNNNNNTNNNTNNNNNNKNCEGKANDQTNKK
KKFKHLRSLHSPLVRFFIMILNFDVIKKCLDDVYWYKYLYLRRVYEKKRIYKNRKKSPAYNTNDDNKNEVCTK
NIPCDDNKNEMXXXXXXXXXXXXXXXXXXXXXXXXXXXXXXXXXXXXXXXXXXXXXXXXXXXXXXXXXXXXXX
XXXXXXXXXXXXXXXXXXXXXXXXXXXXXXXXXXXXXXXPCDDNKNEMCTKNIPCDDNKNIPRDDNKSYNKMY
NNNKMNKELTYNDEYIINEFIRDFYSIEKHSEEIINQRKILQERYENDGNNMKITLFHFKKFFTSNDEHKNEH
LGKYIDEHIYLESNYFKWIMNNNSSSKLRECNNKYRIKYRETIQEKNIKIKCKNIYIEKKLKNSKYEFNEFLD
IKYDKEKHNIYLHPLYIDLYMRDIFEFLNLFDLKLLKNILFINIQILTFIYEIKYGYWVRNGPQMIFQVGEYD
NSLFFQNDLIGIQFAIIMMNVLPLYENQKVYLDSHVINLFHQIWNIKVQYDEPLKKLPWSKNEYNDVQNDYIH
KADDEICINEKVYDEDTNKYIDTSPNFSYNHNIQNHYMVEEHSEDKKPYYMNKIKYIKKNDEFFEEHMKMYES
MLIYNRSKKSNDNNTLPVNNKMGNSKGKNLDHDDGDHVKLLFGIDDKNVSNNNNTNNINSINSINNINNINVC
NNNNTYVVYNKVEFKSICDLLCKYSCKQLKDQENESNPLLRETEGRLTSDNINFNKLDNNNDNIQSDLLDNDKR
YYNKGSVRKESIYNEDRICEKYKKSKLEWNEYLEEFMNIEIRNPFIILYQLIFKNFRMNLNIRKINDMYVEIE
IHHIKMAYKIFSDIYTNKSTVDSRKIGHIFYFQFFYDIIIRIFNELYYLEYSNIPRKTSYEKYIERGKYHVKK
ILIQLLASKDFQYFQLENVFPKQFRHHPSFYELVNKYGYKTHMPRSNVNLIKLNKNSWYLYDAFWPLSAFKDF
QTANEKCIKEENSSYIGCSREEDKEYLNFSFRKVQNLLFYTFKNSCLVSILLIVFIINDEFQRKIEKKKKEEL
EKEEMKKHMKADVTGEDDSLLNQKGGTTNNNTCDVGVVINEEANRSSGMSQNVVTYESGINMNNDDVLGEERN
NNDDINYMDDNDDDNDDNDDDNDDDNNDDNNDNDNNDDDNNDDDNNDDDNNDDDNNDDDNDDNDEDSNLHGSS
SNMLNIINSGDNNSVTNSSNINTARNNNNTYIIVDQNGDNETLFAEILINRNPNNSINMEIVNVTDNNVGDEN
NNRNYRHLNADGLSELIRSLISENNIINNVIDESFLQNNNVGGDNSINNDDIRNRGGSIFDDNISFDNNSSSD
ELNLMDSTNSNNVRINLYGRTNGLYDLNFILDSNEIMGNLNTHNVNNTIDILIKIIKILSVAMGSAHPFIELN
SFYYFNAVNFYGTDNDDDSEKNEEIGNEENDKKYIDKKYIDKKYIDNNEEIEKKHHCDQNKIYDDVDRLSSVS
SNSYDILMNHLEESCMYTSYSDETYEKNGNVYNMREGFNVENLSYDKDILNEKKDIVEIENEKEKYIKINDHT
VSRNYGCVSLLKDEPKFSSTDGNNNNIINRCNNNNYYYDNMKHVDEKGGEGEGEDSEECQIKESYKKMSECNN
KENIIFDSINVLRKNNIKRLKNYMCKNKNCYIYYDDNNNKKKKNKKNVENQEKEFYVLNKIFVHNFINCINNI
NVNEDKCFQKVRSTILNRLKEMYSGNYDCKNNNSNNEFIELAKKKQEDLLKSMKEQQSKFSHFLEEEYSSEEN
DSLPNGGTEDFEDVDFVDDASSYLDSNSNNNSDGHEDDNKNRCTDVKTNKQEEEFSISNVNEKKKKKYMKGIT
ICEYEKIKEKNCICVLCKEGMSKNNLLAYICFSSHTNLLKKIIKKNKFSFPCKHPSIYTCGHFIHTKCLHNTR
ILKYRKLFSVKNEPTLYEFTCPLCRSIANSFVVYIPKRYISENDKYRIYDCNEEEFFLDNSNKNLSELLKISK
YSEERNYGIKRKKEREEKKKSKMIMMRGMESDNINEMASDNINEMVSDNINEMTSDNINKMTSDNINEMTSDN

INEMTSDNINKMANQMNYEQNTDGIIIKSSDIPNNVQVDIFINNNISQIYECAEQNIELLKDIKNGESTNMCN
KADNLFAIQNDNNYPYRNKESEEICEGEDFLLINKKNISCNYLNNYVTNDTLRNDNVMIHKDILLDDYFLQDD
NKFSTCSITLNDYLIARYFNITNCDYIKNDKCKSRKDICFNDNNNNYNNDDDNDDDNDDNNDDNNDDDNNNDG
DNNYDTGEESLSTLSSCSSENVSSDNHMDDSDIAEEFYFKYTHDKNKNIKMANKKNKKRNLKENHKEDFHMNN
SNTHNVSSYNFFDENKKNTIFNLNFCNLIEKDSLLNDMLAYECYEKNKKIILRNIYLNENNMYDELFEKKKND
NNVGTHNIIYSQENNDDLLNNKDDINIIYKNSNMEKQKRIRRRKKGRGTEIYFYKNTSNYVNKDYTFTYRNSN
FYYRTKNGFLSYYVSLPLSFIKEIQYFMIKNKLYNYPGNNRYNKFYLTTEVDKEEIKKEEMEMNIDMKIDPSM
FCNFEDKIKTKEEYNTVLYYEMNNDIITYVKSNFNYESINADEAVKKYEVCDFYTDGTFDSAGEKLCSEEKQD
HLITRHKIKECSHENESEQKEKRLKVGHEHEMVDGISNMGVANNNDNIYGDNIYGDNDNIYGDNDNIYGDNDN
IYGDNIYDNNNNSCVGGEDFICDNKSDDINFEFIKDLNKINYEENKKEKKEMLEIEKLNKENINGDKKDIYKF
KYPCNIEEIKDHNISMKTSLSMDVYEGIVSTNKNIYEIYNELKRKQYFYSVCDIKSLRQSIILLDGISEISWR
IFHQEHTTFSSILHHSENYKRLIDFDYNTKIDDNDEDIDIKGIGVNDLLKIYRRLEIYPDFIFNNNNVGFINR
HDLKFITKKDLLLMNRKDYERNKKERKKKNTNLYPTNSQTNNNENDDNNNNNNNNNNNNNNNNNNNNNNNNND
SRGDVNKPKNNNSFNNNSETKEGNKKKKKNHSDVLNIKYWRNINTAVDFFKIYWILYNEILINIFYKNINYIS
CNNMIEILVRNLFLYKYELNILKEENMCNLFNIQTWKNMYKICFFYVYPYRFTFNKYKKFFNKIKKIHFLNNL
SFIPLSVDVLKLFMNFFLNQYLYTPSLIKHFISISLIIISFQIMNEIFIKEISKTYVRFIFKTIQNYNKIKKY
ITRISKYMYFQKIYTNYIISHYEYVDYIERANTFIQTEETKKIRHNESSIYHNIEMVGHKQNEQKRHSLNSYD
RFHIFLFEKYVKNSIPGGENITLKYVNGRYKLYDNEQEVVRMSDLEHFEKIKNVYSKKATDILKKKNEKMKND
KEETGNEQANNCNKTLDGNKTLDGNKILDGNKTLDGNKILDGNKILDDNKLLDDNKLLDGNKTLDGNKILDDN
KILDSNNNNNNNNNGENKSWNFQNVSSYNDLIKSVLDKKNFFFIIDIVNLFKNIQTLSYLFDEETIRYVHMII
MNSKILSYDLKEIERKKNLQKKIYPNFTSYITKQKKANKLKCYNYDLLHIEKKWILYLIYKTILKYYDMLIFK
KKVQKLMLLMCEINDEYIKYIYFNGYIPINIYLSTNLIKYNEFFTNYYSYIYNKNVNDKWGDFHVLYFQNEEN
NIKKENKKNYYYYYYYYYTLFKNKHVLKRDYNHIVSNNIQPINKMNEPITSNECTSPNMSYHIESNEIFKNED
DHNLNIYNEHIMYNSHKDTNLLSSSNHMQHVQKNTLTMNLDHSNNLIINDNHKRDKEHINTKHWNKNIINSNY
FNVYNNDSSPKEQIHLEEGGGGLIDVDDDKKDNVINSDNHFKETTYNHIAYHKDQELSTELNLFKYKLYNISS
EEISNLYLMEDCNNEKGFFSCVHKNEDFEYKNINILNILKYQFELVPYINHLYYLYNIYPLLFKLKEIKNIHD
MKEKQINLCTDQFLLSLVDKDIYDCYDDVNFSDASFYSDQTIHVSEKECEHKNHNSNKYDCNNYHPFNVSHLS
YPYKDQNGDEEDTLQINSSLNILENVVDLDELIKDLKKTKKSKKKTSKNKYADKFFVSFFNTALKRDEDKGKT
NYVHYRKLRNAKNNIKLKNLFLSHQQNDTIYDNINGNICDNLYGNVNTYQNSASLIFVDPHNLTNINISNELN
NNININCSKNKKKKKPSSNDQNNTENEDMQNSSSDNPYDDSNFISYHESIDSTSSTDKYHKRKMTPMESMFLK
YTLNTSEDALSNKYNVTSKNKNKKEYLSFLKYKDANKIQRYHMLLRKNGLGRLFFYNLRKMYNYVYNLYDFIP
LYLIQNILIHYYDIYQHVNQYYYHDDKEYPTFFTQQNEDDEYEYTKFSKNFFNTNKWKKMYSKYEDKLNNETY
PEPPKKYISNKQRHKENILIYQNLQNDHMNNHYNIANNDIENKNQTNSSYLFNVHHQNCPSLSSNNLVNINST
LINDHHFVQTNNDHMNIIQHMENIPEMSECNLKTDQNNNIFNFPQDKINPDTMKKNEKMEFSVNKKIKTNHNT
TGYNKRNNDRYNYSSTPNFFGKDEYDNTSYEDYIKKKKKNFSSTSRRKRNNNINMKEFFANGRYDKLLSIKKI
FMIFYNYLRKCHFIDKKNSCKNLYKSFLKKYLKSANVYLQFIAYIIFSIYDYNSLAKKYLDYIHLYSDISKYN
IFLHILNIDEDYDKIYIYQTLQLIYHPYFYLMYKLYKYLKIDASPVLLHDFYENWEEFTKSPSLFQPSKNYFT
LIKKHFKRKCVSCNKSPKKILICLYCGSTVCLHEGDHVSGPLSQTISKCVYHTTICGGEQCLYLCLNTSSVLF
TSENRFDFMSGPYVDKNGDVDYQLKRGKNLYLSSYKLNKLFDVIINSAVDVEIYKHTLKSE*
>N.PFL1625w
MRKDLYTLDGGKISELGRLGYENFDFLSYEMNEENENNDNMISILENIHLSYLLGGCNIIGTNTFQVNLYSFK
KLGIDNGEEILNKYINIAYNSLLKYEEIKRKSKDDINVLLNPRNKNISVYDHLSSYHNIRKKFDILHEKDVSF
NIDNYLKDIKIEQNIGHNKYRKFNKNRNNYISFSNGPYSSTFADFSEYSGVMQKNKEDKNGEKKKKMKKNKEK
KEEKKNKYINDNLCNFVDEKSQGKKKKNMSEYYQLDKNIIKNKNNIDRNIKKSINYKAYYKYSLYYDIQMNIS
SVNSYGREKYNPIEIVPKMKKKIQNVNCTNKDETNIINTHNNNNNDNIFFNKKLFNYGIEYYIDVSDQEIISN
CLFKLNTFCKNSDKLHLFSLTTFSNIREVLTFYNCIKYYGSTFNNKVIINFFCNSCKYVGCSKYSFFDIVSIL
LYLDSYNKYIKAIGLNCVNIEYVYDLFYPFQKYICPYNNINTNLYHSQNIQMNNIVKDVMKDLKKNRYINDFN
FFCSPNKSLEVVSFDDQNGDVHFHNSANKTNHVYNYIEKWINVHVNGFGGCCYYNPYDISLINYKINELL*
>N.PFL1630c
MSRKLNILKAKLPKAPTGRRPIGYRGNHQHGKSLYDPVYPTTKIPTSLIPRYPIDWRNGGRFLLCVGMRRIEN
RIIKKMKDSLNFNMSECKICNDNCVKIYNRCLCAWYCKYKYRHVYKCDGLLIDYKGTIKTDKPIFTLKKEVGI
QNKKSMIEYIYNEDKHIFELNEKEDIYKKFYKKIQTCAFSEEKRIPEEFSDSENTDSDED*
>N.PFL1635w

MNFYKLKKKGEEDYDKYISNKEKKKKVDKNFLYEDEYIHIRKSRDRKSEVSINDEEYVLKKKKNSNIYESKRD
NSVMGMNNINNKIYKDKMNKHISNHKKNHNKNIHKNNHEESNSGGFIWNYMKNIYSSVTSAVKNNIFTNDKHN
NMNDISSLKKKHISNNSRLIDKNLKKNKNNRDSHFNEYKYDYVKEKFKDNISAYDKRHEQNYKKIYQHDEYRD
QDEYQDEYHDEYQDEYQDGYQDEEKKKEIDKYKKKKGNNTYYDFSENDFKFLFKSDNEDFNDNNINYENNLSR
MNIVRKDDKIKNTLYNDIAHHTDEYIYMDIKKKKKSQLINKNIHQDNIIEHDKSKQKNGLLEDTIFNHKKSHS
NLQRGVRQKEYHDNKNIFDGDNNKNNNDNKNNNNNNNNNGRHSLFNKKGRHSSNNLYNINDNKNDFFLHSSDE
ERDKKYKINLEVKKEKTKNHVILSNENYDGKQDMDNMKPSKSSVRNINTIQNDHNKEDEIMFKTHTQNHNSTE
KLYYEHIFEEINKHTNDTQHFKENTSNAVTNVIKDTNEKINNIDNHITNKNSDIQNEKDSYVEYDMSSNKCDQ
NDLLNITTPITDHDTNDLHNINSNNYSTNLNKEEVFNEEAIKDNDILKKLRYEYKNLINIIDSFIDETLNYDL
DKNSPVSKLHLDEKNDKMCNESKDHFLNNKNNFKDNDADEVYNNIDDDYKNSMKFIEDTSSEDKNKYVILKYD
EDSLIEALEKLRIDKKKKDKLIKLKEKYPEDIEKDAYDDETKKKKIDKNIFFKCSKKEYYEKAIIILNEKIEN
RVLIEKFNVPLLYSQIKCLIDTRWLNDEVINFYLSMLQEYNEQHTKNNSLTFIPKIFTFSTFFFQSLNFNGSY
NYSKVSRWTKRKQVDIFSFDLILIPLHVGGNHWTLGSIHMKDKKICLYDSLNGSNKKFFEYMRRYIVDEMKDK
KQKDLDISLWTYSKEGVSEKGIPHQENGYDCGVFTCMFAKCLSFNREFDFNQRDIKDIRLKMTYEISQGCLVF
*
>N.PFL1640c
MLSLYKRIHTLSIILKNICKSENHLLHSSYMTINRIEKRAYTNNVKRRTDDYILNILENNGVVLNKIGNFIVV
KEDMNLLYERIIFNPVNYERVIEFIQMLDKNNLRQDFDIYYYEDSLRNIKLNRDQNKKEIFINIKEILEKGKK
IKNDEKKNYLEKQNIQSLYYYNIQRYQHNKDPSSFLIDRRRLTKEEYIYQFISSILPYICFSFMLFFPHILIC
LYIPYIKKKNEKQKELCKILQLKQNIHSFKDIKPENILQIIDNNVKTVVFFYNHNIFLNMYIKSLMIDLSKIF
KYNSIPVNIVGIDVSKYNIQSNVLKDFNHNLFPLLYFILPYHYENDSAVLKIEEPLTTENILLQLRDFVHIPK
GTFDQIKDLNTISSKLKKCIFEHDIMNKNKGDILYDYGCEVAHLSCLKNNNKLSFY*
>N.PFL1645w
MSSLINSLLFNGFIKKKENKNEEKQNKKDIQNNESNSKVDEINMNNLSRRESKERINNNNENNNIDGNNKSTD
RIKISDHNKHSYHNKHSYHNKHSYHNKHSYHNKHSDHNKHSDHNKHSDHNKHSYHNKRSDHNKRSDHNKHSDH
NKHSDHKNEQVDENLPVGVNQEDLLLKVVYKNKEDLLNEDDYMNNYYFESYKKDKSMNILKNGKENIERERIK
TYYEYYNSTKYEHIHKYGKTNKFINFYNDKNILYNFKENIDTPKKQKNDYINKKETEKNMNEHYIDDILKNIP
RYNNMSSNYLLNLYNNSNNVNNLNIHNTDFIELHDIFDEKKLDNVKNKDFCFYRNIKMKKKINKNKEFYKDMN
YQEISHLQDEESEQRDMNNHKTGTIKKEHKIGNAYKMEEIKNVDEVLSNDNTKNEHNDDINGDINGDINNDDI
NDDINNDHHIYNNDDVNNDHHIYNNDDVNNDHHIYNNDHHIYNNDHHIYNNDHHIYNNDDAVYNSGGSFKEEI
NFLEEKFCSKEEGEFHISKKSSNDEFSSYEKLDIDKEFNFNDDNINNVDNMDKLFNVDIVDNLHNINNNVPHE
LENISDSFNLIENENNKNIYDKDEDNFLDDHIDNNNDNICNNYSGDNVNKSDVEFSLDSFYNKKNNNEENDES
KIQTNHINDVLTTYEKIKESDKNIVLKIKYKEEDFNSYHKDSFSSYDYDLKKNHISSTYEEKYKEKLNYSIRY
NEHDKSFNSLNEKDDSTNMDIMNNMNININNDEEGINNIYFNNNKKKSDLNNIMDVYENEQDDKNKDIHINNI
NKNDNLENHRFNDEVVNINKKKKIEKKKYEYDYFKNVYEDYKKYSLYDDMNLYGNNIINNVYIYMLLNYKYLH
NYHTKNNFNHNNNNNNNNNNNVTMILYPYNNEDYFDYVTNKKISEKDILYNEYIINELFNINIDNENDKSNML
SNSEKLKNKKIKSITNNNNNNNDNDNNINNSYYYENNMIQFVDKNNLVTFNKERNTSNNANVENKHLFAQDSI
YNNLSNFLKKNNELFFKDLDKYMNEHIFNNNNNNNNTIFLDNFSTILSKVYDDMNLQRGEENNNNTTKNNKDN
ILEYINKMLSAELQKNEVISFMLNNIFLKEKDISDVLLKSLIYRYDEYKYLKEDLFLSDSHFYKLKRNEEYVE
NTIMLQDGDNNNNNNNNNNNRRDQFSKGLYHKNKNDFLYNTYNNINNDEDNIFIQNKELRLKSFEKVLNKIDN
IIFFSNLKLDNIYKKRNPHKIENLSISYKNAYIHTRMTNDYYYNYLFCNIQGVIAYSRFHKLDFRTLIHKFVK
EKKFHLEKKMKIDMNMDLRSYEKHILLKWTERKNETLRRSYMFLKRGGRDFFTRTVHNMNNMNNIDNNDNNND
NNNDNNNNNDNNNDNNNNNNNNDNNNDNNNDNNNDNNNDNNNDNNNDNTNESMFNKDDIKSMKGSLAYDNINNK
IKKKKKNLYEIVNILYTSNFDFSEYIMCDWEIENKFDTCEIIDSNDKEQIDNPSYIYYNNSCLLLNDDGPLVV
LEYIEKDPFIFLKNGMNSKLNHYFICENDEHLYSFDYKNKLKKNVEPFGEINVIKNTINYFGVPIKLKRSKQR
ITFLENELFKSFLFTYPVYYMNARQKNKSLLNKYINEDINGVNMCKYDGNYTNRNHLNSQQDNNNINNINNSY
YCDDSRDKHKINKYMNTSHLDLDINSFEDIEEEEEKKHMDEYYNNDTDDTSFYNTDFLLIRNFSYNQNRYFLK
PLYVDVDMYREEYKGETMEKKNEYLIKEGYVINNETDMLNKERDMLNGEGDKLNVEGDKLNVQRDKTFNEEDN
IKDTYNNYNSDDVEKYNASNYNSLNKKDITIEEKEVEAYNNNNNYNNVETLNSNKSLNSEGYIKDTNSDKIDE
ENNIYYKNNMNDYPYEENMNNYVNNEYSEGIMKKNYFNCIFYNVGFLDINVDVMNPLYKRYLVEKRNYVRSWI
IKLIVKYKIKDSKKIKEILKKKLSLFINEKDINVIVKSLDINLLLNKNYMDDDEEKNFYKIKNVCIIECIYHY
LQLFKYEGIDFKTVIDNQEKLNQIISVLKIEKQRALQNINNIKNKIKYIYDKYCYKKEAKTVQFNIKKEDIQI
SSIQNYYFVGNFYYDNNMLNYFFYIKYIISYSPWNIFKDYVNTSNVSKKENQNDDNKKKKKNKKYQNIKNEKN
SMKGKKKGEKKKKKGNLLHRHDNNKNNTNGSNNSNSNSSNSSNNSNSNSNSSNNSSSGELSIQQYTSNDDTDM

SNMSNYPYHSEEHKKSSNTKEKRQKNLKVKNKKKTSHAHNASYTNSEKDEQEREINKKNIKEKENKLIHEFES
NNDEKIDLNSESTYKSDDSISSGSISNDSISNGSISNDSISNGSISNDSISSGSVSNDSISKGSASNDSISKG
SASKDSSSFDCTQSHTDDHVDRNIKKTDKRNKRNNKSNKRSKKKKDERSENINYDSDEELKKKEIQNDPKDSE
LEKNSDNNNNNNKKVGRKNYFYATYLYSHVVYNSKLHSGNISYQKNKNNNNNNNNNYCYNNNLYGNKYREQQM
KNKIFMENSNIMKRGDVINKRINMSEKRQKRQGNNTFTKGNKGYHFNMKGKYNFSYFYTKSNNLNADVQDMKK
KKKXXXXXXXXXXXXXXXXXXXXXXXXXXXXXXXXXXXXXXXXXXXXXXXXXXXXXXXXXXXXXXXXXXXX
XXXXXXXXXXXXXXXXXXXXXXXXXXXXKKKKKNYMNYKNYIEGDKRGVHDGYYKYDKEESVFEKMYKME
EYGINNKSKKKKNIENINNINNINNINNIDDGDNNNKMMMVNNIILDGDEEDDITMLNNNKIYNKDQMNRSSV
FKNMTLINHNNKNTYNDNNMFVCGKDLNKNINMSLYMKCITHFSIKDLSKTLISIGFHNKIIKNMNRNEQLNL
IKKNIKNEKILTSTNKLIHIYMIKKLISEQFKNLQQPVYLKYNKNVLYIDEKKSLVNQNDVIHQNREMKNKTK
ASNNYLYNKTYHTNNIANDNNDKGKRFNNEDNGLYYYDKERKENHSVILDIPKKTKIEKCRIKLIRKFFCESS
SENEYSPKSLSDVEGSSSFYESSVDSGLSDKMKTLHRLDDMDNIKKKKKIDNMNHRNNHLKGTYHEDKKKVYA
KDCYDEDDFIDEDEKKDLEIIREIKTKEYNIEDNENNYKLIHCLKWTRVYIRRKDDNTMRLVEYGNINTPDVY
NNNMNHNNNINNNNNINNNNNNNNKNNIPLLTPHFNNQDLKNAEQNYNMTHTFDNGTFSNNPVYTNNFQDIQK
YITNNNIDKIDNIQTLYIYGEKNINTFLKWKRFRGMLKRYFFLLKNTNNNNYDNKKNKVIQKNMQQDITNTRH
KMFAFDKRDVSKLYEENNVDVDIDGEDDGEDDGFDYFDDDTITNKNIYLGNENMKKNKKKTIKNKRYSNIIQS
NKDPAYGMEKALSIFPTLFNELKTWDNFKKIEKKKKTEEKKKIENKMHEKIKDGEKKSDLLKNKKNKKNKIKN
DDDDDDNNIYNKELYYENMSTNENLALKKQKKQKMNKTNKKKKMNDILYNNDKNKKGINKKFVNTNYSININK
NNKRFHNIISYKNKDNKKNVQKNNIKNKKTHIKNKSIVKKNKTNSESEIKKEHFHDQIIEFDSDKKKKKKKKK
NVDESSDNKKKKKKKSTSSINYIAHNKRYTLKNNNNMEEEEEDQYMSEEYISGNSNYYDDYHNDKNIKGNNNK
KRKKGKENMNNRYNSLNNNDEDEETEDIYYTHEEHGKFKKKKSNEYYYNKTNNNISENDYINTNVYNNNNNNK
NIIIDEENKKKNKKENKYKNIKEVIENFNDTLSNIMTDISKLNNYKAFLNEVNESYAPLYYTVIKKPMYINKI
IFRCKKKKYNSLNSFFEDVNLIVTNCKLYNTPTSVSAYLCEIVDNMFKDIVNKIKNNNNLQNYNNILIEHFYK
NKNLYNNTWESLQIENLWEDDQHINNLYDNTETQNNNNNKDHLINSNSISEKHRTEDVSNMSGVSIDIIKSND
IN*
>N.PFL1650w
MSLNNVKRTYECLYTHQKIQKNKKWQEGFCEIIDNYGMTKISLLNTNKCCIESILSNLINLNNIEEEIELPNY
IIQFVDIHNYVENDVPVEVTKEIKREKVKKRINSFCVEKLKSIKKKRESLSNNNTNENNYLDSIEHMSSPWGT
HNLHNDDNDKNNKNGYDKHIDENRSPNNYVDNNHSNNNHSNNNHYNNNHYNKNLCHNNKFQVPRKIDIKDVSI
NKSNLVGNSFNSNYNIKNEENEQDKNIVMEQKINLHTFKNDKPIYHNNNKLKNDNTFINIIYTNKNLFYKKKN
VKWIDGFIIEKENCFELYNLSQDKLFTREKPIMDEEYINFGNYVVYNNFLSNTKNKDKDKDEDIHNNDKKKGN
YSLLSYKNIHLNKNNKSFNIHDISKINEKKNSYDINKSISNNKRDEENDLTRDICDLTKKEYTKSYFKEYNYE
DYKYKENNYKNEDMRNKDVKNDLIRNNNITHNFINNNHSKNIISEEQKNNGNQYINNFSEDKKKTVFFYHNNN
NNNNKGDNKEIYDEIINIGKIVSLNNYSHMNIYKNISVTSMNSDIQQHADKKDDDNSYDDKKENKIYDNYKNG
SGNTQYESAINKECNKTINYTLDFCVNETTSCSKKKDKVFIEHSYKEKEFEDKGKEMYMMNDKNRYDDYTHNN
NIINNNNINNNNNINNHRNDEEGTTNFVVSNNCINPHDKKFSDEFVLHKNIGSEDKDEDDENGYCKLYKIKNI
NMFKSNNFNFHIKDIHNINKHNLFVPIYIKLLNNKIDIKNKLSNLKYYRIPNFFRNKSEYVLYFMNSILYQIY
YEVTTKILYFHDDLIQFFNDRNIYKKKIQREETIERLPQEQMINNNNNYYYNDDDNNTNGNDINTNSNDINPN
HKDNNPNSNDNNTNGNDINPNSNDINPNSNDINPNSNYKNTYEPSENCNVENSNKICNIKKLEGKNDIYTPVI
NLDDEKKYNILEDDYILFLNDCELFYSINETNKNMHTSNCMIDVFFVNEKTKYILIEKNKRKTIVNKNKYINV
NQIIIFSKNDIYLNQNDLLIFITKWNKLSNNYLEIYPLNIETFTYMNELFLKEKFASTKNYSYFLKRHKKIYF
SSCLFLIKFLEHVENLKYLFFFYSSIVQTNYSHANNYNYKYINNINHINHINHNSNINSLHNYLQTNHFINYN
FICSKIGIKREFNPMIDTQIVLNNVNVQLKREILENLKNNKMLNKKQKDIITHVVNWFKYGGPEKNEKNEKNE
KNEKNEKNEENEKNEKNEENEKNEKNEKNEENEKNEKKENLPSNNICENTHFYDDNYSRNNSLLVNGIFGSGK
STLICNIILYLDKILTHEEEEEKKKTKKSNIITKKKNDFKKDCNSDDSYNYESESKKEDDDDHHHINSQHINE
TDMKIIEHINKKQRLKKNKILLICNTNLGVDNILLKLKFDFNFYDFARVGNIYEIIFFVITNFITLNLREDKN
VEELSIYLNNISNKIDNIQSTFHNKKETNEDKLKVVNNDDVQLKLRNHHMYLKEKINNKIEKDDINDKKLLDH
IPSYMKEEHIHKIKNFDQLLNVLKKNKEFVLKNKYKNKRVIAGTCRSIHLFEKMNTIKHNINYLIVDECTQIN
ELTLLRFFMKYPIKKFIFIGDVKQLGFVLKSKNDLSRSMFSRLIENELFIKYYFVQNEKNKIVNVEDNIQNEN
TNINNNHIYCENIKSPNNLNDYTKNIDNIIDIVNNLNDQEYCTLKYLLYEKMFDLHIEKKEKYTQSEKCYNYK
NYTPYINKLIILNKQYRCHPSISEICSHLFYNNRIKNAKRTMKIKIHYDKYNAHVNLILIRSHYKEILHNSSY
INIKEARIIISICENMIDNDINPDDIGIISLYRSQSSYIRNKLNNSKKANQLKRVKVSTVDSFQGIEKEIILF
SCVMCPTQLEKKREKIKNNEEEKFVVDEHFVTKENNTIGGNNTISGNKTISGNKTISGNNIISENNTTSENIT

TSENITTGENITTGENITTGENITTGENNIMNELAVSMENDNSQLHSNMLYESFCENKNRINVALSRAKSQII
IVAHNNFIEKNKLWNYIKSKCKIYYYD*
>N.PFL1655c
MQTFLKENIINNITKSHDIKNINLYLNNIEENIIKIKNEGQSIIDDVFLAFNGSISHLSLYSYLYDHNINVGL
ECLNFIYEHSYIEEEKVIEGDAKDEEKIVDITKFDSDIMYYIVKKFKETYEENYNLTVKDLENIILEYKEKIY
EESIKLYKEKYNDGIKIYNTFLDISHFYYDGKKMKFLKKQKNDSIQNYNEYINAHTDVEFYELKYHLLSKKIK
GHPSVLFWSDAINEIHIKDKTIITSVDSIKLTNTGHQHIIGILGLNRDGELVIQGIRNEVKLFINQSCIINNG
IYCIGHIILLQGRMKLLNKTFYVTEIMHPPRNYDDEKNEEDIFYDFENENEKKEIGEYEIIVRNNDKKQNWII
MHDIYLDNPSTFEILEKMFSLYVNTYPDNELPIGFILMGDFISLKFDYNKNFNDLYIKGFEKLSVLLISKFKL
ILQNCYLIFIPGKNDPCACKNSIPKMPILPYYIKKFEQNIQSFFSCKKKIIFATNPCRIRHFNKKMIFFRQDI
LNDLIWSSSINATDDNKNNLQDILISTIVGQSHIYPIPHDNRILKRYSSFLFLYPLPNFICISDNTCNSFISY
ASDNTSDSIISNSDLSFTKKKTFTVYNVLQHDAKRYVVPI*
>N.PFL1660c
MLFLLHIYIPQNSVAYVSNVFPMTYIYPSFTFVSFLLFSYLFSHFTYILKYEKKIIKRILSSNSLHVVHNFSF
VKNDIERNIIKDDKNDENIDNINKQYMSDEEKIEKNENGDHPQLFQHNYNNLNIRHNNNQMLDFLSSVQKKES
RRFYSINLKEKNFRWSMILSIGSKKKDLELGILTNSNITGLYCSYNYKDNDELKNLKYDITLSEDLKYVECKS
IMCDNVEHSSPCLPLENYFKLINDYKIRKRSCQNKFCDYVNNMNFLNMIDKLNDRNLSICSFNTTINNEQIKG
FYFKDSFFLYKKIKTSFLYFACITESDPLHFNNKLSGFIGLANYINDKEKYHNQNKKIYSTILYSFIYKSISK
KNIFSLCFKQGGGFITFGGYDQNVLTKKEAPLSKIQLYSIDKNTNKKYVQQLNSVDVYDLLWIHYYAKNKSYY
SLFLKNVTATMDQNKMNSVINAEAKVDSCNYFLSFPADITIKLKNFVHDKCPDTDQFNDCSNLIEKGLIKLKN
KNIKDFPKIELLFEEGTVIVHPEDYIIHERYIIHIIYIYIYIYIYTIYFYIYFILFDFIYSDGVYRVLINSSG
ELKLGIPFFLNKYLIFDNENERLGVSKSECALELNDNELFGVDLSSNDDPYEEKDDGFNEGEFVSEIQNTHYF
DF*
>N.PFL1665c
MITSINVIHELLTTIYCRIFSRKRKDIYKYDVLLYLQDYYYFDVIKRVSDDIKELDDDSITIEINSNEYEVIL
NGDKSSSFFNHTFENFCIHRGYDIIKDKTYSNEYEKRIDFLHELLCDLLCLLLNENNEKKGKEKEKEKEEKKN
NMNKNMNYNKERVINKMYHNNNHLFKKFWENIQDILKEINILNENKYVEDINEENIKDILYIQKEKLIKNEDI
IIKKNVDINKITLLNNLIEKYNNEYKYRFYYIFLNNYLTIQTMFTSNMIYLYSYEIQNIIHKIIKMKINLYQP
LTIYDVLAFQYDQIMFHKSSYMNLISPVKKIKILNNIVERGGDPDEFSKKVIIKDVINANISLKAKFNKARTQ
NDNNWGNNKYKVRNYYDNNRR*
>N.PFL1670c
MKNVLRKYTCLFILINIVLTTYSKVIKRGNTNITSNYLFPNNYDNNSHTYKKRSSNQFYFLENEKNKLKELWN
KLTTRYSIKFHTNKETENEETGKNNQISFKNVFNKISIQDNMEMINNVIIKNSTVINAEILNELMKQNKLLTI
RYKDINTIINIINNYYASNNYLFSKVLTHEVVKKGKDKTLVIYVKECIVHKILVKIHSCDKEKKEGDSNWDKI
RKGFQENDNVETNLQMDHKDVKLSQHNDKSSQHNDKSSQHNDKSSQHNDISSEHNDKSSDHNDKSSDHNDKSS
DHNDKSSEQKEVSPNPHIKNFNKDIKINDKESENISSEEPKENVLLNRESVLYKDVLNYNLKEKSVSNRKVRR
FFENKMNIKENGIFVWNENIYNLLVDSNLFKYVQMKLYNDKSCNKNILEINIIENKRMILIPSLSKGFNDFLE
FCLNLSFSYLHSLTYSDKFRMKYFQNLNYKNNKHNYDFIFINDLIELEKLKKNYPSFQIMGMNINKEYKKEVH
TSSLNNLMDIISIKNDDNDDNDNETLNVSSSNINQSNSFFPNTPKYSLQVKDLYNYLYKKTLFFFFIKRMNNV
ILEIKTKFKRKDFQNFFYFLKEQRYYSSDVGSTQVCANKGDDHNNNNNNINNSNGNVSNISKWKNRFLYKCMN
LFRNDKKSVLSNKLYNLYGSKIKLSTSINLCNPNLFQTSNFLKKYFNIKNKFDLIFYFNMEKNNFPYEKENSS
NTIENNDNNEDNLNSKNKNKNENLHKDLLYFFKRVNEKSCHIKWNSINNIYLNYTLYFNKNYEINMYDLFCTF
QNMIKNKIIKIKNYCKKGKHGKKGKETKVINEFNLSNNDHVHSVYIPLYFFKLSINNMSLLLNIMFSFKKNLW
TLNNILFDVKDNENKNEKNILFHLKNILKCNIKNEQDNSKKFKEKIKNIKVTASNEHFTNNTCTIYTVNNINM
NDAYKEYFNKMNLSLTYKLIFPVIFNNYFLNLLHMKLYLFFNYNIFDKLQECHKNKNENNKTERNHKHIEDTI
NLLKGYYIKGKNKKKKEHQHNSSYGLGILLFNMNIYLNFKLHGNNFLPSLVLKSKEDNSKFNYLL*
>N.PFL1675c
MSLSKYFFLLIICSVFFFVYGFNNKFVYSSYLKDDKKKSVSFENTPNEEEEKIYGHSSFGQELIKSLSLTLKN
VPLVVISEKIKRQLEKASESLGLGNPNDEMCEINYSKLCPEDWIDMGDGISCLSPLHYKGPCEKKVIFKNASA
KSKYNFSINCNVSWPCSKICIEDFSKKCPENWIHNIENNTCEAPKNYAGKCIRQKNFDHYSQSEKKVWTQICD
INWSCYDKNYNFNILCPLNWKNNPDNKTCSAPISYMGPCSNILYLYNFSDKEKITISHICNVEWPVHEKKEIN
FNSLCPMGWNIVDIKNHICQIPLSYNGPCKHDIQNKENMNSMKYMKNINYLNNINNVVSFKNFSKKQKYDYSQ
ICNVQWPLENGNIIQNDHSFCPYNWILINEEEHICVAPIEYEGPCENIFSFKNYTAHMKFAWASSCDVIFTDR
FNAYEKLGRINDQRRNTKIFGATSKDKHISNIIQIGLKNGPMNNIGQVFNYDDEKDNMEEKKTKIIKNNNYKN

NKINNNIIINNNHIDDEINLNNLIVTNGTITDLIMLKETSDDQQLKEEIDEIIKQFKQGESPTYTKKKNYLEN
KTNPFITTEKNHHIENRKSKNKVNRNNFAFIQIGEPNKNNTYHDTLKRKEKKKYIHMNNNKPIKKEQNILYIK
KKQNIYKNKHMNNITKLYDNVRKEHKNKVPIVYNNTDNINEIKNLYENYKIEKENNPYHIYDKFNLICENKNY
NVCPIGWTYINNKKCLAPKSYISKYLRTCNSILNLNDIVKTVYDVTHDFSFVTIDKKKGEKLEKNCNLNFPCL
ECERDYVRVNCPLGWTNIGDGKCKCPSDYPLYLKNLCGIIVNFKYSSPLFKKNWSYLCKSDWPCFSTCEKDYG
KVCPIGYKIINERTEKNNEHIFICTNENWKDYKNSNNYLVNTNNYEQNRNLCHVIEIYNSILLKKEIEKKCNV
TWPCIDKCEENFYQTCPYNWIFKDNKCIAPYYYVPPKGCSHSFDILSFRNFDKFLYSNKCFAPWPCKDNCQPD
WTKPCPQNWILKKGNHKLSSLTNDRRSYNKHSLEIDNFDEDEHNIDGDNSNDIDNNDDDEHNLDTNNNNDRGD
HVNNDNDDDNENSINRNNNGVHLNSIHSDFKVGNQENVDRNNSYYNHHSDDNHNNNNNNNNIDVLKGKTHSTF
ICSPPVSYKGPCNQPYDLTNFTFLQKQDFSFRCNVQWSCGSSSHFSRHWQTEQIYDDVNFKKRNTLKFYNSFH
SSSYIKYKDAFF*
>N.PFL1680w
MPVLYHLTLQKPTAITKTVYGNFSGPRFHEIIVAKGQVLELLRSDKQGKLNVIISKDIFGIIRSISTFRLTGS
NKDYIVIGSDSGRLVILEYNNEKNDFVRVHCETYGKTGIRRIIPGEYIAVDPKGRALMICAVEKQKFVYILNR
DNKENLTISSPLEAHKSHSICHDVVGLNVGFENPMFVSIEQNYESLDKQINEELENENDDEDKSDEERKDNND
IINDKNVKDNKDNDFSLDYAKKVLCFWELDLGLNHVIKKHILPIDITAHLLIPLPGGQQGPSGVLICCENFLV
YKKVDHEDIYCAYPRRLEIGQDKNISIICWTMHRIKKFFFILIQSEYGDLYKIEVDHEDGIVKEIVCKYFDTV
PIANSISVLKSGSLFVAAEFGNHYFYQFSGIGDDNKQFMCTSNHPLGKNAIIAFKTNKLKNLYLVDQIYSLSP
ILDMKIIDAKNTHTPQIYTLCGRGPRSSLRILQHGLSIEELADNELPGKPKYIWTIKKDNLSEYDGYIVVSFE
GNTLILEIGESVEEVSDTLLLNNVTTLHINILYDNSFIQVYDTGIRHINGKVVQEWVAPKNKQIKAASSNSSQ
IVISLSGGELIYFEIDESHTLVEIFRKNLNVEVLCLSIQQIPPNRVRANFLAVGCLDNVVRLLSIEKDKYFKQ
LSTHLLPNNSSPQDICISEMNDNGNTMKERNIIFLNIGLNTGVLLRSIIDPVAGTLSNHYSKYLGAKSIKICP
VNVNKNPALLVLCEKTYLCYMHQGKFLYSPLNYDMLEYASSFYSPQCSDGYVAISSNSLRIFRFYRLGEVFSQ
NILHLTFTPRKIVPLPFPSLFYDHDSSLELERKKKIRMLAIIEADHNSYDENTQREIQKALKDIKLSDTERRK
ENDNENNNIYSNGDVDNIDVNDSANMNEEFNSNDNISLAQNHKKDVSYNKEEEQFTDNNKNDTENVIETYENN
ASLNESNDEENEDEYYYDRIGTFKAGQGKWGSCIKIINPVNLQILDKISLDMEEAALSVCACELEALHCLIVG
TTTNLSLKTKSLTSASLRVYTYDIQYKLNLLHITPIEEQPYCFCSYNGKLIASIGNKLRIYALGKKKLLKKCE
YKDIPEAIVSIKISGNRIFACDIRESVLIFFYDPNQNTLRLISDDIIPRWITCSEILDHHTIMAADKFDSVFI
LRVPEEAKQDEYGITNKCWYGGEIMNSSTKNRKLEHMMSFHIGEIVTSMQKVRLSPTSSECIIYSTIMGTIGA
FIPYDNKEELELTQHLEIILRTEKPPLCGREHIFFRSYYHPVQKIANDLERTPEDILRKLEDIRNKIL*
>N.PFL1685w
MGRSSRNRSSNSSSRSSFLSRPGSRDMNRGNNTSAQNYSGSLGQHQSNKSGGFLSNMMGTVASGMASGVGFGV
AQRAVDSILGARHVEVSHVNSNVDNQQLNQAAAINSEKNNMYKCKPFADELNQCLTRHSDISLCQNYADSLKS
CQQSM*
>N.PFL1690w
MRDIYSKYLERVKRNIHFADQKSQDTSHSKTNVIRIRPIKGYVIKTYEKNGDKVYINICFSRLIPDFHLKTFP
HLYNEEGLRIPISIGEEKKKQDRKGRKYRTYDIVLNNRVVSECLRNVKIKTIVAELVLEAVKNKYKLDLCTNL
FLFPDHKYKGNHPDDHYIKYDQQHKIREVEEEHNTFFTDKDLDAYEFMKNYIDKHDDSVEKNHVNGYGYLKEH
REAEALASKLIVTKPDWDMWFIPPKLLEDLKQGVKRFFIPYIRIFPLKGVINGLDFKPPNDKVNKEKHLVDTH
KKRQILKSFFEEYNVELDDEFLKYQDIIKTVCVFQIPLHFFALLKKTIEYDIDKYNNLIPEIIELWISDDCLY
IQFKKSPYFPDEKNPPCKSFSIRFPYYYDTSKAIAQFLYEFNLLNVIVPVSKLSSESLIFHDNKEDSDFSDKT
FIQPDEASVTSIL*
>N.PFL1695c
MPNTNKDKNVRLERHSAGKTPAYGDSSVRAGTGKYNWGNSIDSTAAESKPIDEKDPMYDSEIEKINKYLKK*
>N.PFL1700c
MVMLPLHENSSTIRKKYKKNNKFFGGETSVKNFLERYSFTNKKDNKKNEKGTKLNILKYMKLYMQKPVKHRIT
FNIIVFTLFLVIIYIFYHKIENIKTVFLISLTLYSLYFLLFSLYMLSTILNDEYGDYNTLKKYDMTNNIYMND
NELNLINDKNDVQQINNSQNNNDNYVHQMTNDHNIQKKEIKKEYEKLKYDNHNNNENNLINPYNLEQNNQTFN
QHISNEYEKNNINLIHTKNTNNDNKGDMKNEIGTNEGYEISSFDSIGIPIKEGSEGFFTVQYNSIFKISIVFT
LLILSLYIIRGEDTKLPQGDNQMTDMMNNNNNNNNNNNNNNNNSSSSNGTIIISSYAYGIITAISFLLGALCS
SIAGYNGIYVAVRANVKVAKAATYSYKKALITCFRSGAVSAIVNVALAIFGICSLLLLVNILYPTLAFSKYPL
LIVGYGFGASLVAMLYQLAGGIYTKAADIGADLVGKVEKHIPEDDPRNPAVIADLVGDNVGDCAGQCADLFES
ICAEIIASMILGGNLSENGIISEQTASYFVLFPLFVHSMDLLISTIGIYLVSVKNNNEPFSTLNLECNTKIID
KKDINYFNNINSNMHVEESAMQPLNNNNKEESDLLYAKYDCDVTSEQLENPLKAMLKAYFFTCSLGVVGFSFL

CKLLFSLDNAKNAWIYFSFCGFIGMACSYLFVILTRYYTDYSYPKVKKIAHASLSGPATNIIAGLYVGLESTF
FPVIVISISLLLSYYLGLKSNITGDHNIINGLYGTSVATMGMLSTAVFILSMSNFGPIADNAGGIVEMCKQPK
YVRVITDKLDAVGNVTKANTKGFSVGSAALACFLLFSAFLSEVSVHSKMPFSTVDIAIPEVFIGGILGSVVVF
LFAGWSLDAVGKTAEEVLKEVRRQFNEHPGILTYKEKPDYHTCVAIISKRALIETIKPGLLGVLSPIIVGLIF
KQLGILQNNQLLGAQAMASFIMFSTSTGILMALFLNNAGGAWDNAKKYIESGYYGGKNSQAHVSSVIGDTVGD
PCKDTAGPSIHVLIKLISTITMVITPLIASTTTKSVYS*
>N.PFL1705w
MRRGLGNEKRFLRRSFNRSYYYKKNEKEKIEEMNSRKNNNYEKEEEEDDEEDIDGVDEDIKEKCSKAVCDMNS
SDIEERNEKKKKNEKFIVKEKKELSVNDESFLIKNMEQNNNDKGQDKNNVICPLCVEVLDETDRNFFPCDCGY
QICLWCLYYIRDHMNNTCPACRRSYEEKNFIYNKETHEKLIKKQKSNKNKSDNNNNNNNNINRGDNNYMNNIN
LTNMNEMNNINDMNNINNNNNDDNNNNNDNNNNDNNNDNDNNNDNNNNNNNNNNNNNNNNNNNNNNYYYYISN
NYNNNYSSSGSLLYRSDTYKHPNIYNIHEYDNLLEIIKSIRVVQRNLVFVIGITATYAKKTVLKKNEHFGKYG
KILNIIINKSQAYNPQYNGPSFSAYITYSNEKEAINAIYFIDGMTLDNKILKASFGTTKYCSSFLKNASCGNE
ECFYLHELGNVIDSFSKDDIHGPKHIYHDLLYLYFKKLPDKKNDGTYDSLSNSITIKNFKLNEEDVSSLVKIE
KTETKKKLENNIEDTNVNDDIKKNNNINYTEFKYFNEWKNKNLNLEKIKQDQNKNAPPQNNKQSKWIMEDKIL
QIKKSATHDNIPIQSNDQENYDIDDDIHDNDINDMDDVDDKKSNNNNKIKAKKKKKSKSEDVLPLDMLKNNSY
IATNKNNIPTNDKKGKNYLNSVQNTSTSLIFNSYSDDKDHISKNKNNNTSKNNNNNNNNNNVMNDTDYLNELS
ENYPSINEALLDKDKKKKAKKKAEKNKAEKNKAEKNKAEKNKAEKNKAEKNKAEKNKAEINKTDTNKENTNNE
DKNNEDKNNEDKNNEDKNNEDKNNEEDRNKIITKNESEEQKKENDSTDTKKNKKKNQNNQLIENKKKEQDNIN
KNNITNNKQESNNINEKSTDDSKKFTSLISNYFSELLDKKKKKDTSKNETQEKNEEINKENVIDKKKNKNTQP
QHVIIFNNNNDNYNNNNNNNNNSNIIIGFREGQVELARRKEFIFTDDGKIKKIVEEKDKLEGKSKEDKKGDQQ
SDVHQIIDKKQGGIENQSLEESKRKAKNIQQKDNTKNAELLKCDEKKTEGNLSNTLTHYVTNQQDNFDQDIEN
NIHNQNEENNLNKNTSKGKKKIENNNKVVDTKILNIEDIIKMKSINNNDNIKLPEEIKEYIKKMKSGGIKENV
EYDIYHKFINNYFNVKEYVDGIIVVEEKEVKAMKGNDNNVKDQKKGKHEEDNKKKVLISKTEEGDTSKRKQKN
MSDKKNKKEKRKKKKDKKNDNDNEEENDNEEDQEEDEKQDIINKDGILQKENKKIKNKNKTKKKNDQEEKEEE
DEDDEDEEDDDEEEEEEIQKDNVTLIKKKKKKKEKKNISNKKNVMYHKEMEGYIMDTIMGIILNELKGKNNVK
QININKNNYDDEKNVLLKDVEIFIKNLCVAKCKSIENNKNKENILYIIEEGNDENNTSKKKEAKKKAKRNDYI
IYSNDEHTFLKSHKGIMQYILKNGNTSSNKNNKIKNVDDKNNNVDDKNNNVDDKNNNVDDKNKNGDDSNKAKI
KISEYLNDKNTFDFFKDLIISNNQDTIHTPDHIIFDKIFNEQINFVQQLCETDFKINSRNVISTNKKTDRHLY
QSNQNVHNKLKKNTQGGGNISNIWVNFDNTVQNKELNEKLNLSA*
>N.PFL1710c
MFKRVYVYKNSFWKYKKRFFSKKLVNIGILAHIDAGKTTISEDILYQSKEIKVKGNINDQNTQLDFLKQERER
GITIKSAYSCFEWNKIKVNLIDTPGHIDFSNETFISLCVLDKCIIVIDSKEGVQIQTINIFRYIKENLPIYFF
LNKMDINHIDIDSNFLSIKNRLTKKGLLITYPIYENKKLKYILDIPSMVLYSYPHINYGQTFMYNSYFLKTLL
EGSLYEKKLYSHFAMINPKPDNLSKENKNKNNINIANVLNDDIIDYIIKKREEIIEILCDLDNTIEYKYLNNI
PISYEHFKNSLSLCIDKKYIFPIFSGSALHSYGVHILLDYITHNEKGMEKGMEKGMEKGMEKGMEKGMEKGME
NEMEMEMDMEKKMEKERQMNHNKQRTNNNTNETPFLSYERINKDKYHIYQNKKCGNNSDMYHPYDITYNHNNL
IKNTDVIKNNIIFNNKKALNSLYKTIIFIFKLSNEKNGFNTFCKVFKGKLSKNTKLLNLRNKKTEIVKGIYKV
KADRYITTNVLDTNDIGMVRGFENIILCDIICEKDDEKPSHNNNDFFSEDQTTDHNKQNNEYSHSNHIENTLN
ELYHKSHKEDEKNHALHEGTYSQNNLFIGHNDLPPLSNIYKLLKDEIPNKQWLYFLKSYKKRISKNIIVCTCA
IEPKEYKKEKDLKNILKQICLEDNSILIFTDKNNKLVIGSIGILNIEVIIDKIKNDYNIDIKTSPVEIIQKEY
IQGYYENSIKKEMKVGSYISTIILGFVIKEKDEFIDISSYVQNVLKHEKISHFLSSEEGIKNNISNNKYNNNN
KYNNNNKLNISDNLDKDNNLLLYDDIRFEDNKKMYISTTNDDRQNYDEHHNINILDNMEIKESTEKDRKKNYV
YNNLKLGNSKSMYDTKGVKNWVHKYNDDHDKIYLEDNIKDHPHKQSIDDEPELLCDNDDNDDNDNDNDDDNDD
NDNDDDNDDVDEYLLNFNYDTLFENSVTVHKDVLLYIDELKKMNKKKKNVYDNILNSCIISLKNCLSNGYHTN
GNIINTEIIIKNLKIFDSSTTAVAKYACNHLYYEMIKKANIQIVNPLSLILIQTDEAYTGIIVKDLIQYRNGT
IIQIMKNKESDFKLMKIYAIIPVKFTHNYSSILRSISSGHANFLMTFCGYKKC*
>N.PFL1715w
MKNYKGEDSNDNVPKVSNSAASPSPSPSIPFNNNRGSKIGNSTYNQNFLNNNSSYNKQPNNLGASNYPYINKN
YTSPNNYVVNNNVNPSYIPMNFNNVNISPNVPYNNNNNYNYGAYPNVNNNMIHNNNINNMDGKNNYSYDNNLK
NVHMNEYTSFMGMKSYPVAPPPNINHMININQNNIHAYNNTNNNNNNNNNNNNIYMNNKFNDMKNIPPPPLPN
NNIPNVPNIPSPPSLPNNTYHFNANNQINAFAPQYNCYDKDIHMPKGEHENVINSNDDLKDGIKNERDRKFQD
NTNNIHNKGNYNNKSTTCNINVDGRERLQNDYNQNFINTGERPQNFIRDSDENKRFIKYPKLKQLLELKNDII
KKRSTPARYIRCDLRTFDLGSLDTKFDVILIDPPWKEYYDRKIHNLHVLNNINLDQDLNNDMNNEKDKFWTLE

DLANIEIEKIAEVPSFLFIWCGVTHLEDARVLLNKWGYRRCEDICWLKTNINEKNKKNKYLNEINNENSYLQR
TTEHCLVGIKGAVRRSYDIHLIHANLDTDVIIAEETEQNIYDNNKPEELYKIIEKFCLGRRKIELFGTNRNIR
NGWLTLGKHIDTTLFNKEEYTGWFEGDIAWPEATSYVGGKYMGTTNEIENLRPKSPPRNNPNA*
>N.PFL1720w
MFNNDPLQKYDKELFDLLEKEKNRQIETINLIASENLTNTAVRECLGDRISNKYSEGYPHKRYYGGNDYVDKI
EELCYKRALEAFNVSEEEWGVNVQPLSGSAANVQALYALVGVKGKIMGMHLCSGGHLTHGFFDEKKKVSITSD
LFESKLYKCNSEGYVDMESVRNLALSFQPKVIICGYTSYPRDIDYKGFREICDEVNAYLFADISHISSFVACN
LLNNPFTYADVVTTTTHKILRGPRSALIFFNKKRNPGIDQKINSSVFPSFQGGPHNNKIAAVACQLKEVNTPF
FKEYTKQVLLNSKALAECLLKRNLDLVTNGTDNHLIVVDLRKYNITGSKLQETCNAINIALNKNTIPSDVDCV
SPSGIRIGTPALTTRGCKEKDMEFIADMLLKAILLTDELQQKYGKKLVDFKKGLVNNPKIDELKKEVVQWAKN
LPFA*
>N.PFL1725w
MHKLKLFNHWKKLPMFMKFGGSPNLRMNCRYVSSSEHLNMKKNISVGNKNTEVSNKIKIGKIAQVIGAVVDVE
FEDTPPSILNALEVELDNKKLILEVAQHLGNKVVRTIAMDATDGLVRGQEVKDSGNPICVPVGKETLGRIMNV
IGEPIDECGHINHKKTLPIHRDPPLFTDQSTEPALLITGIKVVDLIAPYAKGGKIGLFGGAGVGKTVLIMELI
NNVAKKHGGYSVFAGVGERTREGNDLYHEMITTGVIKKKKIGNNEFDFNGSKAALVYGQMNEPPGARARVALT
GLTVAEYFRDEENQDVLLFIDNIYRFTQAGSEVSALLGRIPSAVGYQPTLATDLGALQERITTTKNGSITSVQ
AVYVPADDLTDPAPATTFSHLDATTVLSRSIAELGIYPAVDPLDSTSRMLTPDIVGAEQYQVARNVQQILQDY
KSLQDIIAILGIDELSEQDKLTVARARKVQRFLSQPFAVAEVFTGKPGRFVELEDTIIGFSELLKGNCDDLPE
MAFYMVGGLEEVKSKALEMAKQVS*
>N.PFL1730c
MEYTFLFYKHKHLIHLKENFEHIESYITYSLDSSFHNVNLVNDKEKDTILNHRECEYNNLTYEQIIDILQYSS
ICVSVCEKCQNGEKVNIVGIVSLDYELCINNNTNKYNFERMCDCPNINLEDFYYSLSLHIKAYNILSNLTLNN
TLIINLILNNNENYYDNLFYYLFHSIENLLFILLFYNNNNNNNIINEDLFSNGLTIDLTNLTQYEKQNKIIP
FDHVLILNKSTYVSKIFLRLTSTSDIYDLHELFKKFSQTNLEEKNHYIIYDIIDKKTDEDILITILNNKKKII
GFVSLKKYIDINILVNLYNLKEYNYLLKRDFFEDISDSLKPYKNGSKSLNKRKKKYLYIEFKQHILRTIKIKT
LEDLEKKYEISNETIYDYLYDHLIEDVDIYADYFKKKNIDIKDIVKNIYKKIYIYIYVYMYIYSFYSYSMIMQ
ITKKVPLTKLISFFSISPHLFSLGLFIHR*PMLVKSSTIILRKSKSYILILIMTKLIKKFMRKYIYIYIYIYM
IIYL**IVAR*I**N*RMKMK*KKMKTILKKS*ICLILYYSYCKDILLKIEKYFDEVDYIITTNNERGNMPFI
LNYFNRVKKKKANTLESLYILNKYTLFLQPTTDYMKLEDIEHVQRLLEKVKHKEKNKIKQYILFLQDYCKDE
VRKDNQHDHLLVQSPLELKFYELHNYYIYVSRCGNNIINITTGSIMNNMDFYYINKIYDFKNILIPNENEKMK
HFRLFFFSSIIIFKYYDKKIIHNILMLTNACSCQISTDDDVYLDIYRHFIFLNKKDNDVSIFREDNIFLRESE
KTKKNEKNRNDRKNTKQCDKKYLVLTKHMCLRKCINIDHNIVFWGTNDICLNILYKILRKNEYFFNNIILIIA
YKNKYLNTKNNVEENKSVKNCSLESALMYNKLKNIMINERIKIIYDNVYNINRKNKQIELNNKNYIYYDYLFI
CFDKQDVTTYSFNLNSFEEGKKRNFNFIETYENMNYKNIKFDFLVKEKDYEKYEHVNTFYSQKKKNYLLKKKK
KTSKQENNISSSTSDLNETNSMESYKSCDKSKVGLRNVNCKNSAGKNVPFVKKEENSLNKSGKCKKKQLRDET
HTLYNIKNKRVEKYYGDKEEDSDDEDNDDEDSDNEDNDDDDDDNYNNYNNYNNDDNNYYEEDNCEMDVSSRYT
TLSSSFLPSSEASVNDSAYNDYKDDIYNNKKEKIEELKSDEKAFNTTETNCSQNKIIITRGEKDNPNIEQNKN
EYIDKYSNVRSNNNLNECYNKNLHIKEDLKEKINANVNKKSSYLENSNKENVSKRNVFKSEEYININNENDKN
VINQYNEKNEMFNKEMVNRNIDGVFSISDPFLEKYFDKNSKYMNIVKNCVNYIIIYSNNIDILNMINFLLINN
VHTYKIIIIYPYTCNKCHGRKQKEKIKQNIFNERIYYKDKSHLKNNYLFCDYTHKNNFYHKNYVFENIKYVLN
KIFFLFHLLKIRIIYGHILAVKKSKKNRLKYVLVHLCKHRNLDLSYFNTHKFICENTLLIPCRILLCSYIFDI
NNHLHYILNKSSIVYNEKICVNHQFQDK**IHF*RR*VVCFFEQI*NKHPQHFKSRIL**YGNR*VCEQTVFK
DSN*TMLFIL*YV*VKK*RNEK*NKKTKRL***Y**NHRKGYGRNK*YNRKCPK*KDT**FRLV*KVKNF*NT
YNIFQSSSL*FLFLSF*GITW*FI*IQRFLHNAK*KNKRNA*EKKIFK*NKRQNL***KK*YDR*L*TNIS*S
LLYR*LKNKYK*RKKNEQLF*CSIF*NFKKYIYSRILL*SYN*FV*FDKFLHIFGMRCIELSQVTQTL*YANK
LFLCNFKKYQK*SSI*HFKYVK*RQGKIYLPL*VSYFQRETQKKNHYATPHKGIYTIFTKGYKRWGFV*NIYK
RPIVQRKTDVYRYN*KRG*IEFSGIY*RK**
>N.PFL1735c
MALFGELYECEEFFNEETFNIKEGEFYNYDDYRDLEESFDYSAHPEYVESNDVTLINKNNDDYDDNKSYIKNI
EIDIKENFDKISTRYLFLYINKYKEMYIKNMKGLYEVHFYDIHLKGDKKSILATDHNIIEYCMENLMNINNNP
DDNNNDTNNKSVHSSDNMHINKDERYNIYRDKKKKRKKKRVYDLFCIYAIILKYFPYSFKLWYHYIKDSIEMI
TDVYYRNKKNYKYINKIFDQCLLYMYNFKSIYIMYIQFLYIQRDVKKIRQIFNLSLQNVYLNQQNDLWECQLL
FNEKINNKVINYEYIKRYVTIYPEQIIHLFKHYVKYKMYKNAMITFFYILNSEDNFDLGNFSKYDIYQEIYKL

LNSKGSLNNDIIHLLRNNLYIFKNYESITSIYILLANNFIYDGRWNKAMDSYEEGISECYTVNDFITLFDNYI
EMLKMLIDLNIYEQEEREKDILKKTLKRKKGKKKKKKKKKKKNKKYDKEENILSDEDDDDDNDDDNDDDNDND
NDNDDDNNNNNNNSVDIDTNLIIDLYMDKINYLLDQRKTYIADIKLKNNKNNVYIWLSKIDSIINEEEKIHLY
DESLRYFEKNDYTGKLSDIYISYAYYYYNKNEYTNCINIFKLALKQNAYFKSANEIANIFCAWIEIELLERNY
KEALNIARLSIDINKKSYNTLYKSSTSILLYEDISLNNNLKNKNNYHTNFNLLSCMKLVSLIIDMEMNYGTIE
TTLNMFDFFYHSKCINVKMVLTLATYLYEKKYFNESFKVYEKALSVFHYPYVYPIYVNYINKYIQRYKDKNIS
YVRDLFKQAIYGNDNKTFIPKEFAKHIFLMYANFESNYGFIKKELSIYKEAIPFLEEPDKIKFYKIFISKVSR
AYGIQKAREAFEEAIQTLSDDSARQLCMIYIDMEYKLNEYERVRALYIYTAQYTNPLLYMDFYKDWREFEALH
GNENTFREMIRIKRSVLNIFSNSRNNMEDEKQQKANMDALENTKRKLKEIIEKEEEHQKKLKRSELYNY*
>N.PFL1740w
MSQTLYNDYKNNCYEYMKTVLYTEKIIKDNNSDQNKLLNIYEKAIKSAESMFKKMQLEVEANYMVDDKENELN
NIYNEICEYKVKLKKFKDEILENDKRKYERSSNNYNNNVNYDDRILLLSDVDLLEKGDVYINHSKILMDNTEY
ISNDVMNNLNKQRECIKKNVSNISFLSNKLDHAKIIIKNLNKKQLFNKYRLYIIFIFIILTFLLILSIKYNRY
VKKYPYIKTNDDNDHNNQNNNIDLILENQKSIQVQNQQNTNYTLDKFNNTQEAKSVFYDFEQKKKDKDINNNE
NQNKYVNINDYQNYIYKINTKHKYTDENINSNIDEYNTLIDFNEIDNNNNKNQLTKLDTYYQHTHELNKNEKV
PEEKQKDNDHIIENVIQNTDENITSSSMSSNINEYPTKSNNNTSS*
>N.PFL1745c
MNNEIVNSTIDGVLNTKLHIQNIPPHITDVHLRSLLGNVGFIKDICYFNKSKKQMNNNNFANKKIYNTALVTF
NTHEEALNVLKNIKNLIDTSGEERNIDAKFAVPNVSINNNNNNNNSNTFFQKNNMNNTNFSQGSTNYGSNYNS
ENFQGNNNMNNYNFYNNNSSNNNNNNQTNTQNNFMNRNMKNKNMNNNNNNNNSNNNMMMNMNFNNNQQMNNNP
MLNQNNFMLNNNNNYNNNAKNVNDMYRDGEMSPNHLNNNNNNINNNNNNNNNNNNNNVMFRQNNSHLAQMYQA
NDNSLEDVENVDGLSLWEMYKDKNNNIFYYNNLTKHSQWHKPVHPNKLFQYNNNEKTKQNGPSGSNLFIFHIP
SEWTDLDLFQHFCCFGNIISSKIQRDSTGRNSGFGFVSYDNVISAQHAIQFMNGYFVNNKYLKVQLKKGETVE
NTNS*
>N.PFL1750c
MAFSGFFKTKFPVDVHLYTIEGYNCKPYLFDCKLSVKKDKIIIWNNITELTFCEDKICDFLYIQLNMCYIRYI
NNDAIEKYIIVFMDISNTYYFYKYMMYCYIKGNLISYKYIDIYKVDCINRTLYKKKLFCIILQNHTKYEHIFV
ILKDDAEKKLKKYINISLLPKLNDQMNNSNGKGCEVHNADSIIRILKELYIKNEVFLIHIMNCINIQYSELEC
IYVDSTKFVLKNVFQIVEYNSFYSDGFSYRTFFLRDSDDDVWSNDEYTYYDKMKEIPVDTKPNIHNIQGIYYI
IHRALERVKTIYCRIPTGYEILRNFYHGIHNDIQSSQQNNQYVQKNNIVHQERSSSDHANRNFGQHNMHNSQE
THNDFQNILDAYQENIHLLRANLEDDHEICNIDEEMQKWEQTNERDQPTQEMNHEINNIEKKIVLINEQMCVI
ELPTYGVELSVYTIELPVHTIHLPIDSIDMSINGIKIPIHEIEIPLHKSEMSICKIEIPFCKTEIPIWKFEIP
IRKHEQRIYDIDQHNGVIDHIIREIDDEIINFNKSISDINKNHIKCDQVHSHMNQSHHNITPSNCNTDQLNFD
SDKSKMGDVQPNDQFNRFHMNNGQSNDKFDKLEIKSSNNNIVPYKNLNHKETFNNIMNILYNNIYFTNENTNL
CLDYVPLILQAIKLFNERKMENRKTLPQNEHNEHNEGVHVTALSQENNRSEEDGLEKEKDDICASAHIETNDH
VETNDHVETNDHVETNDHVETNDHIKGNDNVEKNYPFEKCRYSVNNNTQGEAQKNLFGFPNLMKNSVSAENKK
QDVDVLERYIDDYTETTIKVLRENKKRRKRRSKNSTKYEMNVEEMLKYLTCEIYLNVKNFMKEVAKQYRNDDE
LYCVISNNVLSKYATVCDFVKKKVNKSRVSMHPINKKKKTNKIQRKNTIKKNKMIQKNKYNIKNLNKDDDNNN
NNNNNERGNRNATHNKSRNIKKNLHKYNSENTSYNTSENNNTFSNMYSNCVGNKFYMKFEGGKKYKSRMTNNP
AWINEIINLSDSEESDNSEKKDDKKKKKEKQKYRMKYKHLSTSGIYSVVLRQNILPKGYKEKMERNSKELKLW
NYINYDKIYNELCIFDFYDFNNRKKLYNVKREAFIYEDEKCIPKEIHINNEEGNQLILLDERYDTNFYMMDVK
KEKLVRKWCNENIPITKLLKKMDNIYLGYNESSLFFVDTRLQRCIQNIFQYNKYASPIEHASIDNNGRITSST
SRGELKYYDGTINKQNKIKKSKNVIYCSDDMVHLTTSHKGLYSIVTCQKRIIIYDNFMDNKNFFDHVIRKCYR
YQQNSIVLKLEPIDEFIHDLGDYTFIKTVSYSNSKYLFTFAKDFCVIWNLFQGINKNVSYTINKVPEDISDIS
LYDTSDEHDALLLATEYTLTLTRII*
>N.PFL1755w
MDEDFLHDIYDLDSDFELGNINIDEDVNMKANEYMKNVKRYINEKKLKSCEIFLTELLNDFENEPFEFIIFST
IKIYCNLRLYNYRSISNDLTALGNLENSSYRFENFPDKYKKKKGTMIPFLLRLINCYYPYTLSLYFTSFDRLY
LLILHYEKILKKCIEYISIHEKKDEKKEHIKINISSSTNISNHLDFYVRKKNIVFHYICITSYVLCDLLLKKN
YIEQAIQLLRNKILYYEQNDVNTISLIGKLSLLMGCLDISMECFNKVESFISSENYENENNKYLKNNGDKLND
NNRKHILYNHSYINKNFLNLYLEEYNVALNELFKICPDFFNKNAINDYSFYCNNLSITYFYNNDLKNAIHILE
TLINENYLNTFPSLIKNLNYFYELGKIKNEQVNSINDFISNNLGEDQEILSVIPRRPSNL*
>N.PFL1760w

MNLLRVVCFCFLCIIIKIKYASSGEGIFKNRVLNPFHYQSEKRKIMKHVNSLSHIFNDFKNNLSDLENTYDTF
LKSIGVSEETHKHLINMNDENTINFIEDMNELKNKYNKFKDKIDEHLNNKKQEFDDTLKSTVKGFFYKSINEL
KGISKFVKYININARNLNKIITNLFSKATHLSDIYEENVNNINEIGKDYYKMKLKTLGNLLLSKYNSYNKKIK
LISNEKYIDNCSNIFYIEMLKDYLQNNIKQKITMIDFSTFLEHLSFMELSYFFNTFLKNYEKGYIVYVINRVK
MNYHNNKMIFIEIDKLIEIVNFIKKTYNQFKIDYIVIHIFNMYISVFINVNNILYHVNYDVFFKDQSIQLYNT
YNYGYFFINQKLNQNKYHKIKEHIYILYLGYRTVNDNYLIYNRKQMIQFKKDNAQNIIQHNKTNTTNLFLNMY
LLKLASEYNFYTDFLKLQHEYVFKFSVYNCNMILTYDSLNKFNIPNEKFTPHFKDVYILIIIYPGESLGMLCN
FFNNYLFRESIFNLKNEESNVLEIYSDVNRLTLIANNKTKHYYVNTKYEIPSESLEKSLQKIQNCLKYYANSN
YNDYFDYLNIFYFISSDEIYI*
>N.PFL1765c
MDIIKNETKEMDMYNPSVSINFEKGILKNSCDTPKDTKLEKLKREYNILTSSYDYNSGNKKERRNIWKEKVKH
NIASKNKKKMVKIKKEENNNELENKKGENLLMYVNDKNSNLNVYIDENDLFEETPNISSNTLLSYISNIFLRK
KNIYEIKKCDFIKEVNTDESLKRNFIPPVDILYDDKKVLFLFFISGNLQNFHVTSNNNYLTISGEKIPYQVQD
HTSYFSHEIKKGYFIRTYCFMKTFDKEKIYYEQNNGVIKIYVYICDDNNI*
>N.PFL1770c
MENAFYVYTKNLPDMDSRTFVKILKDAKLLNKKFTTVDADLIFAKVKSKGAKRINYDQFLEAVKCIVEKNKLN
YDKFVETLCQEASKGPILYGTKTENVRFFDDKSTFTGVHKQGGPSIIDKNKTQFSDLSEITDRSEYDIRGVKM
DVAKNV*
>N.PFL1775c
MLTFWTCLIFVLFLDIYDCFKIKNFEYPNTHPVYRNCSIKKCNSLHKGYYNSRTYQIKKNVQEPNEQTNKFDD
TYIYHTPVLLREVIYYMMKDEENSSVSSFDQNMNDNRDTKCYSNNINSINNNINSINCSNSSKSQGYILEKTW
CHLNNNMNRSSRSIKNCSFLNEKKDIEYIEECSENFNSKNTNNLCKNSYTTNYNNEQVNYYIDATLGGGGHTL
EILNKIPNCRIIGIDKDIEALYYNKIKLKRFINNNKLKLIHGDYGNILHLLHLHSLPIFNYYSGILIDLGVSS
HQLKCCDRGFSYKYNGILDMNMNKYTESQYLSKLFRNDNEHINDINDIKKNPKNSYNKLHNILNTYSLKKLKF
IIDTYGEEKKALKIAKKIIQWRKENGMILTTFQLKNIILSTCKQNYKSNNKVLSRVFQAFRIYINQELKTLKQ
LLISSHKLLKTGKKLIVITYHSLEKKCVELFIEKNKQKWTKLHQNVMPSDEEIKLNKSARSAKMFIYKKN*
>N.PFL1780w
MNVKKYMICSFLLYILVLYYEILIFLINKNVYEILEKKNVYKVCNSYIHAFLKYGFVLFYIIVSFLPQRNVYK
KRSKLNYIFAKLLLLYFIFFFIHFVINLFNNFPLNLFYLIIISFHLSEFFLSFLHNKENANYYNFLVNPNSVY
VYFFILTLFEYYLKIFFFVFLNVYQKYINNQKILHKVLLINYFFLRNYIEKDKICTYTYTNQILIGTSIKVQK
NILPNLNTIQNVYEYDKNSNIIYNSFSSKKYLSKKSPYLLSRPYNQYTQKVKQKYKSLKFINTLTYTQIKEQT
NNNQYRNVNNTKDNLIINYDNLNNEGQCIFNYTNLTNILEKYTGKKFYKNFDLNNSFLYEIISKHKHVFHTYD
IPENYEKKYNHYRFLVLLSLFFCITGMFLRIMGIINCSKSFSFYVLSSYSLEKKYMKRKHNLIKKGIYKYMRH
PCYTGWFYYSIFLQLLLSNIFCFTLSFLLSWVYFYRTIKREEKNLLECYNYEYQTYIEETRSTYIPFMTRI*
>N.PFL1785c
MASRKFSICLICDKEGFKWIPFLIKEWKIKYMYCNDEELKNCLVSTFGLVDTITNTNNNALKYIEGISNESID
LLFLALSTSTFHEFILEYVISQKKFFYIFSSNIPTWNIKKFEKYKSLINNFNPYTNIIYDVKEEFKNINKQFY
WNIYNPILNERVFHNLKTGLNELGHIYGAQLECTFIPFTYQNEEVSNILFLRTILIMSLMEFLFDKPKSILAK
CYSVNNEAASVQCVVANLLFESLNCNITISVNTFNKIFNLKVYGDNGFIEVTYSTEHNCFQMQRCLNHYEYPN
LFVEDAHQNALNELKYFIEEQVYTNKYINIHISAIYTSICLWKSNGTNIMLDNENNNNNDDNIDMIHMNKEHS
CIAALKV*
>N.PFL1790w
MHKTIRKLFSDEVCDKIENMKILLVGAGGIGSEFLKNIITIGCKNIDIIDIDTIDITNLNRQFLFKKKDVKKY
KSLVAKERALMHKKDLNINAYTFDVCTMKSSDIKKYDYVINALDNIKARKYVNKLCIMEKKVLIEAGSTGYNG
QVYPIYYNQTKCYSCEEKPKNKTYAICTIRQTPSLPEHCVAWGRLIFETFFCKNDNETLIDIKNHIEEESKKR
NMDKKEIIIFIFNYLFNDTIKELIYLKKDYTTIPIPIHFEWDENIIDLFEDESEHNQNEINIDKLNDNKNNMG
DNKYYCNNFKCEEKDREKDDNVLMLSSQNIWDKKKCIEMYIKTFLKLYKYLNINKKEEEYLIFDKDDDECINF
ITSISNIRMLNFCISQKSKFDIQSIAGNIIPAISSTNAIVASLQAFQLIHVIEYFETLKNKNNKKNNNNNKKN
NNNNNNNNENNVSYCDEEENKTDHFNIRNSKAKHVWVKSIVNGNKIFSRGNLVNAEPLEIPNPNCYICQQPTI
HIYIKNFEKMTLYNFVKDICMNELSFLYPFLDKDDRNIFDYDLFQENDDDYIQNLYASLSYWNIKHDEILLLT
DFQNNNDQLEMHLKEDPSLETEYFIQQKNVPSKKRKINYMQVEGNMDTESVKKKRHILSDEHKETNDEKQPTK
KRKQDLIKVEEIIIDDEESDNQNVVLID*
>N.PFL1795c

MQHLFLKKSKVTFCSIKLKGLLYKHLRNDGKQKNKEKQNTVNWYIYNKRKRIEEIQKVTGNEKYNELILYSFF
YNKNIKSTRDYKRIYYYCNLLKCDILTFIKKDIENLRKQKYKYFTINNRRHSFKKILYIYLNNNIKINDNDQT
HYLFELLQKHLLKTSPYNIFLFLKLCTEYKYLEKLKIFTLDTFIKKINKHMINEENKKKYEHAQKNVKYINRN
KQNPASQMSIDKKSLSKCVNVHKSDEQNEVSIKSKKYTHIQPQTSTQTLTQSPTQSSTSKPCNYHDDVDLYPL
TSLTYEYIRSNKFFTCNNKNHTCTFKNIYMFDVIHFINNNININHNDVHTNDKNECASEVSDDHIVFEQRDGF
QKGAFNNLQGDNIGRNNLQGDNLRRNNLQGDNIKNVYSRESSLKNANKKCAPLKNYLYYNNNHRFELKNKFNN
KMKDNDVSIIRRDDHIDKSEVKYFYYIQRKNNLGVYKEIYEKSDQYKMIKYYEELKKRNVKTKEEKKMSNTTN
KITKDSITQECLQNNKLRDHFKKIRDQILRIENNIKHDNLMINKNIIDQQGSVSKSGSLKNNIITNNNMYINN
NNNNIYINNNNNNNNIYINNNNNYNMFTFNNGDIKYPPLFKEHILNILLSFCINNYQDNMYKYLKYFFFSNFY
FYNPIFLFYYFSFISCTEKNLYEIYLKEFIKFLNNIIVQFHFYLLPFFLNLNYSNVFLNFFIKYWNTISSEKK
DMSIIYRLSSNHKFVKDVITYAQKQKQEKKQEQKQEKKQEQKQEKKQEQKQEQKQEQKQEQKQEKKQEQKQEQ
KQEKKQEQKQEKKQEKKQEQKQEKKQDVDTTQYVHTTQYVEKQITLFMNLFLQIYSSDAIIEKGKDTYLENLF
LYDTNKESNKEDNIIKDGDSTKEKNDFISKNKSCHHTIHNEEDEKLLIGTIFFNFMKYKGLFNKYETDIKNMN
IQKKQVFNNEIININNVIKKKKKKIHMIEGYNKSQGYEYAHDNISNNLEENKNSLNILKKNFIESTNTSTCTY
DNLNKMKITRRMEKKKKKKILIKNMSSCYNEKIYEFNMLSSYINTCIKQKEIKFINHMCDNTYNIDNIEKKEQ
QRHFIILCDEDLKYNNICIKKKDILHNFILFCKMNMFECSYYMNKIIYFYLNEYNDKELLNILISHFKLNNNN
NINSNNINSNNINSNNINSNNINSNNSNNINNIGSAINSNNINININNVSINNTKNNVCVNNLSMDIPNINTQFI
NKKKDEYYCSYDLLLVNKILNIFIYKKKFISLNNSIIISNLLAKQQLYNNNFFRFLEKNNFIYDIKNCNIHSL
VSLIYSFGKLKMKNIHLDLYKGMIKEIIYNIEKINEYGLSCLLYGLYNLIDKKKERNLTIINYDYAKYKNEVT
PSNKNVKHYHNKNYNKYYIPSNHIQKLTTKNGSYKKHVLHREGITMKQNENIVGSVSNDDTINKYDTINKYDT
INKYDTINKYDNINKYDNINKYDNICNNNNNIDKYNNVHNNNFDNKKDLISRSPYLNDEKNTNNLAKESNEIR
ILEDILIKEIIKRLIFFKNMNINSFCISISCLSKINIFPKELYEEIKDVTYKYMNSVNVTSLQHLSLALAKYY
ITNKKENIIHNIIYDIYDYLFIYKMSDISFKCACKFLYTLGLLNIRNENYILILLMVIANERGIINNVHVQES
NPNCNHHNNMYYKNEYVNKLYNQSEKYNTYNNNNNNNNNNNNIGLNNTKFMNMLNNKLFDPYHNSKLNSSLYK
SLDVLKKNEYFKSYYNSYLFFNIPKYKLNIHNILLNFSNVDNSYLIHVLDALYNLSYYSYFSIHICIIIKNIL
VKHIHDMKVSSIMSLLYSYVNLHFYDYSLYYIEKEIFLLEDINNNVNIKYDGVHNNNNDNIKYDGAHNNNHDN
IKFDYAHNNNNDNIKFDYAHNNNNDNIKYDGAHNNNNDNIKYDGVHNNNNDNIKFDYAHNNNNDNIKSDGSHN
NNNYKNKCITHFNKYNTTINNHSTNNVRYKNLESEFKKIYNCLLNNFVNLNDQEKRLYVEKLKSLISEMKNNN
KFYFVKDIYNNMNEGISTEYILSMIHDYDESKKTINNYKQNFAIPNNVHKKLNYSHDDHIIKYNNKTNINESL
NNITMLQNYKVKDKHINTTEQINKINLLHNESMHPNYQTTYKFQEIKNVVDQFGCFIKKEKSKKHQSDYTNRH
LTEEGNVNIDQHDKNMEKHIYENNDLHKLKNEDNDLHLYDHKEEGVSSSFYEKDKRGYLDKVLIEEIQKKEYY
DEFFMLKHREYYKEDILNLCLETLLKNTNYILNNYKQYKNTNIFFLILFFNIFLPYTNNDILIYLNTIQRNFA
FYNNNNNNNMRDYYFEDILKNIELLNFNDHIIHNLIRSKNLSHILIPRVNILQHDNNFLNKAIHLKSTFIKDK
TSVEKNKKNYNNYDEHNTCAKYNSTYDTSGISPTKNEHNSYNNFDEFLNNYNTHLDMKKINLEKIVPLNLLYK
FFQLFNFNINNVQLIYLLNEVYKEKDINGDVDVQRILTQGETNENVNNVNNVNNVNNVNNVNNVNNVNSVNNV
NNMNNMNNVNNVNSVNNLNNLNNMNNNMYNLNNTYRFKNISCPLIQIKNKTLIIQKKDKKIYFKNFNMIDHYF
KPARGLLINILDHHTYNSLFIYVDVYIVAYKVDILIERFNR*
>N.PFL1800w
MKEFLVKDIFNNNQMNGEKNKGGLHNIYNILKNDENKKNEKKIKEVFKYIDNILKQDFEKDVLSIPISLLKEN
DKKIIFNNLRRLFKCFSLFIGQQKYMDSLADFLFYLYKYMRKADVKRTYKLLRKSLPFESDAVEGERRRQKEK
KNENVKDVDNDKGDNHKGDDDDDDDDDDDDDDNDDDDDDDDDDDDDDGDNQITKSNKATNTPNNYTDVPQNHFAR
NQFLFNFIFYSNILIYLEKLKKEDNKNLTSDEHYFFMNLKNNILNKKYILTLSNYIPYFVLSYPSFPLFLENL
FNIFFFVRNNLITFFIKNIYKKVGELVYLRNTDINKRFTLKKYYNLLIYVYLLYKMKIIISIYINPNQYIMLN
DILNDNIIIQLYLDILRDFKNTIYFIFNIIKDKININNTKGNHCIEFCFIILYLINIVYSNHNDVLNIFLNNS
KFNKNILFMEKYKHIFSKYNKIYKILFEATKSKPYDDVKIYDQMKNILYGKKKNNFVKKSNKHKKNNRNNTPN
LENNNNNNVNSFFVPHKGKNYNYLKYIRNELLQKQVQEIQEGGEYSHVYVLLFFYLLTTRCIKIYEYTSDLQN
IIFFKKFFIYKILKEEKHRNYQHRNYQHIDDQHVNDQRIDDQHVNDQHINDQHINDQHINDQHINNTKNVTVA
KKHADINTCTNNVISENYAKYFYNTYFFNLVKNNFFLYINVDKNILNVLFNNELQMFLNNKNLNYKNLYHMLA
YNSKNRYNQLNIRLLFYKVIFKNIIKYILYIKYDDHKYEKNQNIKNLKETKINNISDNIHTSYHVEKDSLNIH
NMIYLFNKKNRNKFKSLHINEYQINKNQINDIVMKHDKNTFIILKHLDFLDLHKDKSNFLFDRNIINLIMYQL
TNETINIMKHLYTCNSKKKKNDNNMDNLNSSIYLTKNINLLIIILKSMLNLINSFFQIINMNHAGKNKSMLIY
NKQKKEAILFSEEYYNEIKNDKISTKQMLKEEIYLDYVDGFNKKCMKKKNFYIIKKMFKLYINIIKKEYNNKN
KNDNHNNNNHNNNNNNNKNDKDEFYLCTNEIIELGKVLIFVFNFTIFYIINNIKDNNTHGKMDGGHVVKNLKK
KRYTKIFCDIHNLYNLLKDKQYLTSSNELKKKLFKFYKHYHVVNQRVSSKMNDKNYFACFVQYNYLEKNKIKY

LKISKKIFIHFKSLTPAIIWRSNIEEDESNDENQIIQKNNHFILPMQDNEEENILVSGKSLLDILVDEPNNNL
AQNNYDKENDNVNEEKKKRKKSDIKKKKNHTNLFYDVTSLLKCLKINFKNQSAQSVIKSLYYLLYSLVVLSKK
VKMISKQRDIVEGFETNVSDNEESDQKDNVDDDERIHNHRNSLDDDERIHNHRNSLDDDERMQKHHNNLDDGK
RIHNHRNSLDDDERIQNHHNNLDDNYSKRNSEQSDAEESSEGSLNSDDYFEEEKKKKRKSISSIYIEDMKNLE
KHIVHILFTFKVLKDIEMKDIDKLNITIEKYLFKIITIIIKEKKNLEILRMSLKCLECYHNIESKIIQDKKKN
FVINYMFEFIQIIYHIPKLKEYYSKYLNIFFKSSFVRYININQVFNGSYNLYFESILNKFISVVDLNDYINNN
KKKINNINLQEKEYITNKFIDIIKETNKKIYVMKDENYYNYLNIIKNKDANNINMLNGYSLNELSHVIQNIYI
HFNQILTDNNAYAFNKKVNLNLLLLLKKIANMFYIKQNNIQMFKNQFIKYLSEFLNFQKRLDEDLSTQKISIN
INEFSKKLSHLVQTIKSMKISDDQKFKANHIRQISNTSTTVSVSDHIRLAKKKRKNMKRGFNIKKKRNVAK*
>N.PFL1805c
MENSLTAKYILMSDKLKNNTLFDNKLKLKYNNIDKNEMIDFDVIENFCNILKDNKCFKGVLDISYNNLNEINL
FNIMCSVYENKNIIGLNIKNNKITKMIFNKILLILEKNYLKYLNIQNTYINTQEIKNIIFTALNKNITSLKLP
HLNIENFQFLIHNLKENNSINKLYFFISYNNQGYKNCIVQDENVINNYEDYINNVKDLFKELLTVIEKKTNIQ
CVKCDADCNDKELNEIISFINSTCKKHKNNLEKVQVINDNKIQINSFEKMKLLISDINGKGKHIEKFEREEKI
VLDHDVITFLEKMLQ*
>N.PFL1810w
MITENGIIFLGTGSSSSIPKLSHAFKNILNTNNNEIKIEEYEKNLSSEDFECVDKFVEDISSKGLESIDELNN
LYLKKKYPHVKDIRCYTCYDAMSNNSKNKRNNISILLKSNDSYVLIDVGKTFRDSILRNKDKINFYKINLHSV
LISHSHTDALNGIDDLRDLQEFNKVQYDDVYYYTPKKIIDVYVNDVSYDRLRRGYDYLVHKRTENIFYSKIAA
LNIHVIKDEKYNKILSEKTTLDDIIESDGCEIKKENCIKNVEEEIYNKNNKACQTNQSNYDDGKYIDNCINIH
TYKKKDEYGFIYTQFDNDKNIRFIPFHHGKNYICIGYIIGNTNKLVYISDCSKLPNYILEYIKKMGSIDILII
DALFYKRKHYSHFSLYESINIALQIKPKQVFFIGMSCDIEHNITNLFLEKLSAKYSDITFSLAHDGLFLPFNF
*
>N.PFL1815c
MEGFDEMKMKQLQLLKEKMMKNAKAQKSEEVKKSVESVSNILNVSNVSNVSNVSNVRSVDSKDIRFSVKSGKK
IFLDTSFQNKKLMVHPKNGRNIFQTSKNTSFVSKNKNSLLKLTTEEELFDTPLSFEEVMRRKNEKKKMELQKN
NQTNISEEFINNKQGSINQNSPNKNNSHLFKIQYDNLNISQDNQNGNTTNNNNNNNNNVNVNVNVNVDNNTHI
NNLQNNSLINGNLLQNNNSSYNVDTIKNTNSLNINSFKNFFTQNISSNNEKNCNVGMLNLTFPFNNSCKDEKI
LTTKSVNGTVFNNINIVKNDDNNDDNNDDNNDEDNNNNNNNNDEDNNNNNNNNNDEDNNNNNNLLNKSTFFNK
DSIFGTALLKNDYIFSTDEKYNNKDKENNINESHNIFKNDKNNMNGTNIFSTNNFMTSEKIISNKLNNTIINN
NNQTQNNGMSQNSFTSNHNVDQVHNNTITSLNGTSAGIQKVNVDNICIPNKGPTNINNVNNVNNVNNNIFNNS
AVNNGGIFSSGKNLFGIPIARTTNNLFLQKIESNIINTNKMNNNNNNLMFNKTIDSSTNIFGKPCITNNIFNN
TLGNNHINKNNNTLFNNINTNNNMNNIISNNSFNPLITNNTLKNEDNRVVVSNINHIENVIQFNKSSENDENN
LNVSYKSVENKESTMTIGDNNNVFSFYNFKANKLTNLTNHNNNDNNNNNNDNNNNNYNNNNNNYNNNNNNDNI
VVGDHLKSNSECVQGQNMNEQNNEMVYNKQNTNIIINTNNNDNVNKEIITTMESPSENNTICKGEDHIKPQIN
VSNMDEVAKSGKEDTQHVINNNINESKEPKNIKKTFTSYFNNFIPSIFTSENKKEPKDQQNGNNDNNDNNDNN
DNNDNNENNKNNENNENNENNENNENNENNKNNENNNDKYDDVNKFENRTGDIITNDDSLTNNLCIFKKNETV
ENIPNNNDNNNDSSSNNKEEECLNNKNQLYMNTIKQDGGNSNVWNNSQNNNNFLFNNLSGNHPNNDNILASKE
VKPFLGSSEKTKNEPIELMTPNKINENSNTFFINKNVNNTNILSKGEEIENNENAEINLFNRIPKNGVFNLSS
ENYENKEEFDKFNKIYTNINCINKNINFINTYVPSNVVNINNFIEDQNSCANTYDALSKTNDMFLKKMKSDNN
NNNNNIIQTNEELQLMMRDLNKEVTHKNSNCNIENDHSNNNINEKNSLNHKESFNFTGINNDGINICSSSNNN
NNNNMYINNNISLGTNNSNNFLLKYDSLKKSGMNINMLSCDEKLKLLNETSKEITINEESDFNGIIPDHVQKI
IERREYFKNLNQSPRTEEKKIGTKKMKPFVPLEYRESFRNTFYKNENNKNNIFSLQNLGAMDIIKSSSRSNNN
QNVNSVNNMNNTNNNNNNNNNNNNNINIGQNKFKNDVFSPHDENILNNNHKQANMYSNSNNSASISSTYFNEDA
YIFKDNKINKNVINSHDDNKNGNDHMVNNFVNDEQKRGNYLLKIEKEINNETNFISNIYNNLDKNNFIYNYDS
GPKDFSVNNNDDDVYNNNDDFYNNKKKKKNSFNNTSDHDMNSLSYHENDDICSSYTQKKRKVLQNKDNFEEDN
FSQDNELSDISSKDNRCVHMNNYVKQIRNSRSLSDISLNLTKYTLESEKILCDIINDNFNMTIKVSSLNDLPY
FFDEEKIQ*
>N.PFL1820w
MGNQVLDESDVVKHSSENIEKKEKIGKKKKTKKQKKEVKKSDIVSCIAFLSRGKKKKSLQESNDEHEENEEYE
EYSSDCNHSGALSNEEKELKKKKKRKKGKNNKYDVLENIFNDEREYKKSIVHDNLISKDKKYIYEDELNIEEN
DSIVINGKIYSDMGTLEIHVFNYDEDIFNIYDDVIIDNYPLCIETIHQSYFKEKNIVAVGTLDCSIQLWDLYN
IDMLEPLYYLGDNEMKKMKKNKKRKLKTNNNIHYEKKNIKNIPEDEEPNNISKQKIKGHTDCITSLNSSKILP
SLLVSGSKDSTVKLWDLNNLNNLHTFSFHQKKINNLSFHSKDKNLLFSTSSDKTLKIYDIRQNQVALDIHLSN

IPEATIWNTHEENIILSSYIDGYINKIDIKYINTSSNKPQNNYLVNFKAFENSCTSLLSMNYKNLTLAGSEDG
VIKAYDFHNLLNEQPHCVYSKNLKKNLFYMKDNEDWPNVVFLGCDKLYDWDVFECEEIKKYFNL*
>N.PFL1825w
MGCTVSNLKCVTNVAGLASLVISLFPKLIIKNPQVLRPLLNVSWGYLFGSTFWLCFFSEVGLLRSLKNMKGVP
LPESASEAKKLLEEMKNSEGDFNRRSLDFQYFFSLATLFSGILLLSTVKLANHNLQLRLSSSVVVITSLLNSL
YLHNKVHNLKSKKESLYNDFIANPKNEKTVADLKKNKKEFHIFHGLSVLSLYVSFFGLTPYIFT*
>N.PFL1830w
MIEIILNDRLGKKIRVKCNPDDTIGDLKKLVAAQTGTRADKIRIQKWYIIYKDHITLQDYEIKDGMSLELYYN
*
>N.PFL1835w
MNKGLSKILELGFFVGSFFFIWYITGEKIFKRKKKIVKENILLILRNLCYEIYTVLCETSKTTKHVYDMLKNE
SNTTIELSRLEEVLLNNGYKKKIEDVEKNVLKRFNVSVEDFYEELKNYEKDPDVQKYLNSIKKMYNEALLGLQ
PKLPSIDEKISEDVIINLTSLIYKEKRKIYKTKIDSMLKNNEIIDIQNTLSNAQFFENLHKSTQHVEEQIIKE
NNNLVPNLFTFKYLVSYYSNDPNFLQKKKYLESKHGEKMIKILKIKTSKKKKKRAHKEDRQSSNSSVSFKSTP
SLDVYNVPHLGTLEKEEDHYSQQSKEMEHMDKDSHVDFIPHNVDANLDKRDEQIEYISESGDMQPEKEYYEGE
HITIRYEEEEDISEIYEEDRASRIYEEDRASRIYEEDRASPIYEEEHASGIYEEEHASGIYEEDHEERYRTSQ
VSERSIKKSVTSQQDDIIHSHEEDNYVSDREVIHDDSFVGDKDQIFDDNYVSEREEIKQDDDYISEKEEIKQD
DDYISEREEIKQDDDYISEREEIKQDDDYISEREEFKQDESFVGDKDQIFEDDNYVSEREEFKQDESFVGDKD
QNFDDDYVSEREDIKQDDDYISERDEIKQDDSFAGDKDQNFEDDNYVSEKEEIIYDNNNISDREQVIQDDNYV
SDKEEIIYDNNNNNNISDSEVDKENNNYVYENDEIIKDNKSMNEKDSSIHYDEEGTIKNKDIIENVKEENSDI
QMLENDIINNINNEIIDNNENEIINNQSKVENNTSMTEYEDVIDMDYKSPENLFMFDQEYNINEDHKGEINNN
DNDNSKSVSGMGEREVSNEENREDEMIRNEEVDYIKEDDEHNTDDIKEDDEHNADDIKDDEHNTDDIKDDEHN
TDDIKDDEHNADDIKDEDNDQVEDNQELNENLEGSDVPKDDEALNIEDTSEEQKETDDKIESGSVRDKKNEGS
TEKKIETKKGGLLMGIMKRKRFMSRDPKKKS*
>N.PFL1840w
MALFFTDLPNFSLRFLSMISGTLMIIAGILNVFKLFQTVVNIYVICSGILLILCDVKTFRFYRFIEFLFTVIG
RSLFILIIGSIIIHKGILNLFIGLALICISLMYITLGYYNGIPQPLMDRKKNPTNYYDSKNNGMSTCSMDTTN
EYNSN*
>N.PFL1845c
MTTEEKINNLNDDLEELNTLLLSVKRENVKAKIQECIENINGEIIKLKINKNQMPNKITETKVQLNDPIVSYN
SVQSFAWNQERNKVTIFLTVKNVHTVGEEKISTVFEERSFEIKMNDVDKKHYRFCIKKLCDKIIPNKCSFKVK
KDSVHVTLVKQENKHWENLHFKESPMSKIKPPSMDEQAEPSAMLMNMMKQLYQEGDSDMKRTIAKAWCEANEK
KSDFSVPNF*
>N.PFL1850c
MKRHISPCLQREIFLCNSQKNNKRCLVLSLNNELQFVLKAYSIQNEEPSCDVENVKDLLNDQLCYVLYNITNY
SSKYQWILILWAPEKNSILIDDIHENKEKKNNFIHIVDKEIYKYNNASKKINKLLYYSFKQNFLDIIFNNYDI
PLFEINNFVMLEKCIHDCNNKYYNYTNCPTLLNREKTKPKNDSFDIYNQYNLQKYYLLNIQLRSINNIIILNE
SSLCHDMKRLLEEPNICLNLFYIDVHNYKIISTHLNIDNIEQVHSIIKENKIFYVIYKVLDTYTFFYICNEIC
TNTKQKFLYSFFKPYLLEYLKKIKNIPIFLSIELSKTKHLIRFINADLKKNKNKNSN*
>N.PFL1855w
MSSSGKNEKKKRHKDHKRNVDNNYNERIKREKKEEFGSSSSYSTDYVNKRKRMRTPSSSYDSSLEIRKREEKK
KRRNNSYPSSNSYNSNTENDSNYRKEKIKKKKDEKELKVKVASHIRKDNNKIPDGSMSSTHSSKDKVKGKKNK
KYSYEESNSISSDKYNNKEKKGSSKRSREKKKEWSSSDNSSVYNMKRNKKDKRKGSYEGKNVYIRRSVSSRSV
SSDSNKSESNKIESNESVSSRSVSSESNKSESNESESNESYSKKSISNKKYKNKRYKNKRYKNKRYKSKRYKS
KSKSSLSSCDEKKKKGYEEFEEDDKNTEETLEINEDLIEYAKQKISNVTIDDIGKAGGVYIPPFKLERLKTEV
TNKKSVLYQKQEWMKLKKKINNIVNKVNIDNISEVCYELFECNLIRGKGLFSRALIHAQLSSPAFTNVFTCLL
CIVNSKFPNIGLLTIQRIILHFRRAYKRNDKILCFNTVKFIAHMINQRIVHEIVGLQLCSLLLQNITNDSVQV
CTYFLAEVGELYTNICRKGLDIIFDRLKDIIQEGQINIKTQYDIEKLWNYRKNNFKDFPSVHDDLNLIDEEDK
IVHEIDILDESFNSQEELNIFREVPYEQYEEEENEWKHISEELLFGDDSADEKKNKKTKKQKKKEKNESEREN
ESENESESEKLGNDDLDENNNSSEDSEAEKSDSNDDDEDENNDSDNHEEIHDMTEQYLINLRKNVYLSIMSSL
SYEECVHKLLKLNIKKGYEIEICNMLIDCCCMEKTFQKFYALQAERLCKLKKIYQENFEKCFDNSFNTAHRLE
TAKLRNCSKFFAHLLYTDAISWSIFKIIKLTEEDTTSSTRIFIKILLQELTNNLGLQAFYHKINHPAISPFLI
GLFPTNNAQNIRFCINFFTAIGLGALTSSLRKLLLT*
>N.PFL1860c

MKLNIQEKNKLLDNLKNRLFKIEKVIGDIKLYENNDDINDPLIDKDDKKEAVDENLKCYDEIKEYIENINIDN
LYKKKEELIEIIKKHKHTNSIAIHVCTIKLILQQISKDFLNDIYNQYNEFYIYIHSNNILDVYNTFEYKRNII
LSHMDFMKSYSSQIKEIIKLKDSINSYMMSETELYLERLNKIEIKNQLLFSKISSINNSLEQLAYKYGLVMKI
ASLIILKYKTKSL*
>N.PFL1865w
MENSTKKKLKLNEYSYKVVHHKSSKGGEERSHVDSNKTYNIVDNDKNRKIGFYEKLSKIYNNLNIFKKPNEVK
DREYIENRKKKKKTEIKMNDIEKTNVKGGMEGYRMNGIKSNISCDRSNDLSCNLSHFLNEEESDIRKHRKKRT
YEDISKDDNCVSKNKKEDMDNYRGPFEYQSLLKSLLYIYNFYTNKKDNCFIYNDDEDGKNSISNIMCYKDDQV
FTNNIYEDGNEYYYNDDTFKKNVYKSKDHLVIPSNDLINYKIKESQSEYNHFNNIFINVVNDKSGFFFIFNTI
YFTHLKNFYYFVDILNILNYIGKNKSIINTDEINKDIFIYEKMAMFVEYAYDIFVNHNKRASSNNNNNFGNNN
NFGNNNSCGNNNSCGNINHFGNNNSCGNINHFGNNNSCGNNNSCGNNNNFGNNKNCSNMRLHALHTKLMPSLK
KEENKETRIDYIRKELEKLKSEKSYEELLFISMNECNKKIYNEINKIPELFFDISEISEYSSNDIIISSDINN
YSSYPLFNKMDNNLLKDNKEMNIQLGIYDKLKHKLSSFTSSTLGKKKNSIGNTSNNIFVFKRNSTEDEYKKEE
ENETVSFNNETNDLLKQNMDNTHIGNDQHDDVIFVANNNIPSNHNKNKKKESIKDFVLNLFKQRNVKNDEYTP
EDINNMNNDMYNDLQKDIHNDNNYPLNENQKNLHESDEQNIDILTDEPILLNLNNEGSTNKNNSFVHDNNKIN
ECDNNGKEVDEDKSFFKLENEPTLEHETTKEHNDLSHDELGNNLSEMSGEMSGEMSGEMSGEMSGEMSGEMSG
EMSVEMSGEMSGEMSGEMSVEMSGEMSGELSDEMSDEMSDEMSDKTSDEMSNQIAVDMSDKISNQIAVDMSDN
MARTISDNRSDNMSDYVQKETLKEERRDIISQHDLENKSDKQFNDMSKEDQEITINGSVSNGKTEESNVIENG
NILNQVNKNDNLLDIYEKEQKDIKMYDREVLSNNSIVNETCQNEVLEGILKQQEINEYSNEGIMKNELKKGET
EKLEGENIEVEEEKNIEVEYQNEGIKLDEEENVKSDEDESIKSDDEENIKLDEDENIKLDEEESIKSNEEESI
KSDDEESIKSNEDESIKSDEDNQSENSIEYESGESQDYESDKSHRKKRKYNETVKHTYDVEESIEQHTCDHSS
TGSCKARNCNGINDEEIINFLKSNDKNVYRKYLKNLLVCDKKCSIENHSEEKDLFLKRVQKLCSLIKNKKFNY
KYTDYWKKKVHFKCCKLQRAYNIFIIIIFSIVKEIKNLTKELNEMSNFIFINSGIETYLNECNISLVQKRKEH
NDIINNYFNENNIINSFFFKKYFCKEEKKKKKKYISYIPLHNYKILFLCLQEIFNDKNIHDKLEKISYYIEFD
DTNILPNYCNSFYKHFKINEMYLYHIQSLYNDSVPTIPCLNMFISCMNYNYGKIHKKKSLFKYELNNTHLSTL
PYVYCCDIQLIKYFEFLKLKETCYKIIKNNKIYDNFLSHLSSYIFDDSYFIIPFFTSYGKFLIIIKTNKNIDN
LQQSMDTLKNKNSITYDVIINSFVFPNDSSFQAIIHFSHIFINTLKNLFKTRNSDEDIPDITFENLLNVDKKR
LIYHEIESNKNKRHIIMNMIFLIESFFNNTEKTRIPKEFDEGLRFLYVIRLLEFFHEKKEHQC*
>N.PFL1870c
MPRIEISKDVKLTIMSYNIQMLSIPFSVHLNSWTRRNAIIDYICSLDDIYDIDILVLNEVFTKKCYKLLTSGK
IKEKFPYHTNVIGRNYKRHDNYNDSSSSTGQKDGLSFHSSDTNINIESSRGISNYVSEQMEYIRKNKENHKNK
LSKISEDISENSNSSYNSNFDTITGKRKFFHFVNGGVIVLSKHKILHKHALIYSSGKFPDVFCSKGAIYLKCD
VMNKKVNVVATHLQAGDNKEQQKCRWKQIDELSKWVYEGIPSTFIKKFESLFFVGDFNIRYNADRLFLDKVLS
DNYLNSYVTKKSLDTTYDSFLNDYCRYIERDYNYKHKYTLDYILVANNSNVEIIVPQTSIQNYYKSLYFIKFF
LGIIPYKTTYIHHPSDHFPIYATFLILS*
>N.PFL1875w
MDNSNKDNIIKINVGGKIYMTTLNLICRYKSSYLYEIILDNYNKEEIFIDRNGNRFEYILDFLRDGVLICEND
INVLTKILIEAIYFKLFNLVKIIKKKICVLYSNIDNNISKKIFKGIFNTIEKKKKKKGYMEEACKLRKLSESI
CEFNELKCFNVKRRKKSSINISDNVINIDNTLKKEKKKEKKKKKKSYTDEEEYNEDKMDDAKYKKDLMDDNKY
KDNLMDDSKYKDNLMDDIKYKDNLMDDSKYKDNLIDDEKYKEKDGDKKEGEDKNNQNNMNKEIPDYINSGPVN
ISERKNCIEGYNHISLSDDTQYINVDNMYKERNNRNITYEEPKKIINNDNIYISNRNNSIDYNITNSFINNKE
EINDNKICTPSKNMMINKKKDNFLPFPNVKLKNVDNKYVSFSERNNIFFYNSENLSCNKQDILHDNFSRTPNK
SNIMNYQNYGNYTREANYNYNNNNNTTNLSNDILVYSEIDDVEETSPFPIVSNVNLGEQIFSTTVDF*
>N.PFL1880w
MKMDIINFKKRLYKFLENEDKVDLEIDKVDELLYFEEIKGTGKKNSSNIYRPYNYKINSEFEKTTGFLGIPAS
SKYEVFCFFCKYYYNKDLLGERKKEIRGDNDVVLGGYTFKTYAQVKNEIEIFASALYQFEEIEFNIFNDNGIY
NQFKILGIWSKNRSEWFITDLATSAINFVTVPIYDTIGLNSVIYIFKKTNMKVCCIEAEKLESLIKMKEELVD
LSILIIYDESNVKQELKQKAKNVGYKLYYYKDLIDKYKNRNIIPQTEMHDHDFHKEHNPNNAFYDKLKKEQAD
YTNNNNNNNNSNNNNNNNNNNNNNNNNNNNNNNNNIQKMNTNNRSIDERNNYLMKQIKKNKGNLNDVCTIIFT
SGSSGTPKGVMITHNSFITFLQAYLIDGNRLGLKKYDVVFSYLPLAHVYERFIEYAVCFFGHKIGYFSGNIKE
LVGDMNELKPTFLITVPRILQKIHDSVMEGLKSKSFIARNLVKTSLKNKTSAYVKNSRKFHSKIYDLILQPIR
NKFGGCIRTQVMGSSSMDKNKLIDLQMIFSSPISEGWGMTEVGVGFLQHRFDSTKGTIGGPFANVEFKVVKVQ
NMKYDPKNYPNKGELCVKGSSLMVGYFRDEELTKKCFDEDGFFLTGDVVEVNENNAYVKIIDRAKNIFKLAQG

EYIEPEKLENLYSNSIYIENIFVHGYSYENELVSVIVPNQIMLSDYAKKHNLNLPYEDLLKHDIIIKLFQEEI
LNISKTYKLNGIEKIHLFHLTSTPFSVENKQLTPTHKIVRHVILADYKKVINDLYKSRNK*
>N.PFL1885c
MEKRYQQLFKGKRIDFPLATGAASHVSLTYDEKKNPYLLCWTYIYQEKPEFTVPLKGCRIINNITEIGPCIHI
ITSNEEYQFQCRSKEEFDEMSQFFNMLGYPILGFKNVYVLNKKIGKGSFSTAYIGTNILYGNRVVVKEVDKSK
VKESNVYTEIEVLRKVMHKYIIKLISAYEQEGFVYLVLEYLKGGELFEYLNNNGPYTEQVAKKAMKRVLIALE
ALHSNGVVHRDLKMENLMLENPNDPSSLKIIDFGLASFLNSPSMNMRCGSPGYVAPEILKCASYGTKVDIFSL
GVILFNILCGYPPFRGNNVKEIFKKNMRCHISFNTKHWINKSESVKEIILWMCCKNPDDRCTALQALGHQWFL
PKLTDMHMTANINELKRNEAIVHKSNDQQDMCKKCKHFNNTQNDDIYNNNNNNNQLDPNKNHKNNYNDYKNYF
DTMLKIDDKYSENLIKDKTSMDSISLNKKDYDAYLVHSNEHDTVVLHGKCQTTKNSSSLLSYKCSRRSPPQN*
>N.PFL1890c
MEIKCDINHNLNGKCSLQNFVRNISLDNEKKVLHVFELKNIRTIYDLIFSVSFFKFSNCMTDVYNNDSVEDIS
IHKLNINWKGHKNVLCANNKLKQDNELATIVYDKDICNIKNGTIQGKNSNINDILKSSINTSKNNNNINNNNI
NNNNINNNNINNNNNNNNHLYNKDEFINNTLKNEEYNNILSNTELNKMNPSNNKEENNTTTLHPKHSNVMEINE
MNNYYEKKKKECTYNNEYVYFDIYINSDIINIFHVNDKDKEKLIKYIYDIIETNDILIMCIYLNLQNVYEKLE
KYNILNITLLKNIIEANKFEWMKNEIGISLSEFILIINFILKVKKNRIFCSNIFNQYNKNNNKTTKKKKNTKT
LCDKIHQFIEFDNWTFKNIIDNPFFQRLRSLSQLGACQYVYPGATHSRFEHSLGVGFLSAKYFTHLCNRSNLS
PNHGELKRMLRCVQIAGLCHDLGHGPFSHTFESFFMNYKKEDTDHKWNHASMSLNIAEHIIENLIDKDDVLDV
SDIKIIKKLIKGTENNKPFCGIDPIDSLITASFDIICNNKNGLDADRFDYLQRDATIAPPNGTLPSLNCNRII
GQSAVINGHITYNVKEIHPVWTVYMNRFSLFKQVYTHRKVRAMELMLCDGFRLADDIFKWSESLNDLDSFLDL
TDSSIIYDIKKKAKVCKYDEKLNHSLNLINSVIYDRNSEYTYKYISEINITEPKLIEYLKDITNEEKIARYAH
GLSPDDIIIDWNYLNYGMGANDPLDSVYFYTSDNEDEAFIAHKEYRGTHPRYFEECNVRLYCKNKKVAHLAKQ
AHLNFISNEVFTASSPLHKKQKNC*
>N.PFL1895w
MFLSFVRYTPPKTPRNVFFPWHTFCIHKSGSFLEKNRLALRVPINLTKFEIREYLRKIYNAKVIKVNTLIKIP
ERRRNLTDHRFNYYRNGPRYKKAIITLEHEVPDSVKMIQSCKNIGRNPYITKKNITYGVRSDVKIRPTRSQLW
HMGECRYSWRLPLTNLLSDYNMNLNPDLRIDDEFVQLTPDPTKPFMHSGISSETIKPNNVPKQTFPHIDLTPW
RRQVKKIYDSGTLAPPESFPVTYNGKETIEYSSENKNIKKSSSSKWKPPS*
>N.PFL1900w
MMSDKGEGDKDNMNKNGNVYDMMDNEKNEEVNYIINEVDSSNDCKNELIKMNYDNNNNDNNNNNNDISYNVLN
SNSVDTKNMENSCKNYVYDKDIENRDTYDNSNNKSNKSDDVDNKTGDISMNKNKLVNTNIYNSEENIYPCTYN
TVVENNAYLKNIDIINTIENVEDNNMKNDVCNENNNIINNLYVDNDNWEKKNYVGLTEKLNCLKLEKTNEILE
FNKTLDTSKDTQDINVVNEKLSVESNKNNVISNMNNIDSIDNINKMDNIDCIDNMNNIEKQSRKRKKNKLSDK
KNLLNNEEYDNKENYDNLHVGNNYINTKDEINKVIQNINLLKALNTNDQKVNELNMDILNKSNEHNDNNEISK
QLLDNIILLNSLSKYMNKDDINKQAKEKMKNRISKKKMTLEGTQLCKINKEQNDNMLNENNNIDDDNNDQHSK
LSDYDNNMMNPHGVLNNSSGEIISSNCSNHLNYNNNDNNNDNNNNNNIIINKDVCNNKKNKKNSKKQTILCAK
NNKGILENEDNKQLNDIRNKIYELTTNNYNSLNEANNENLQKKILLASGLLSSTDIMNPTDSEANNPLYNNFD
KDTLLLINQMKDEEKHVLRNYLMNDANNKIGMGTRIVSKRKKQGSNLVNSNNNMNNLNDLNNNYEMKHNREYV
GDNIVTTSNDNMNNNNNINISKKKKKINNIMDDKTQDLLDYQNTANLLNNVLNNNEKDVRNISMNILKNNINA
CNNDEEIIKALINNIGYSHEHNNNIVGNVKNIINLNRGKDNSLNEFSIVNDYIANIYECAQKDYLHNDKNNKN
KNNIHMDNPLASNLININREEQMNFVNRLNMLMNEADINKKGLLEAYMRKYSNININGNNNINNNSNNNNLQS
LNNQENILNNEQDNTSLSSQEKKLSYNDMLLNLKKRNVLEEFPFNFVKQKDDSAKNTMNDSSDTQVDIHMDHG
FKEGNKKQEKLNSYVQEKFNYEVEKGENVQGEEEKYKQKYLTNEDKNVNVNNDDDDNYNNSSRSNRKNCDKN
KKTNDNNMSSEEIEHNDEKHNENYFKEWYKNISDQVRKLLIESGTNKNVFTENEKNKLLLNIAKEYLLKNNVN
EKKKKKHTKNKNELNSNVENHNVFSDQYDDINNVLLNMQNGNINDITNKNIMNYEHNSTSIHPTIASTLFDIN
HENPREHIKSECSTINNNYNNNNYNNNKNYYLDYIKRLTQNNNNNENLLNIFENSKKYKKKNVSNTSLNSLNN
EINEINEILNEKFETTESFDKYENSQQNIMKNKYNSNNINNNINSNNNIHNDEEERNILCNTNYQVNIKNDVK
EEKVKERKVRKSRKNQNFVEDKKKKKNKDNNLNNGDSNNTHNKNNNTYDNNTHDNIYYDQIKTLQELHYQNAI
VNSLMNGNNIEVCNMNNSKDIFKKKKNEDNEYINYKEQNNDERKKKMKDNNKGNKVDKKKKDEEFFNHINVLK
VRDNDISHSNNNNDDDHDDNNNDNNNNIINNNNDDKFTHNINVLNNNEEKINIPYKPNEHKINIMIDESHFSG
KEKELFIKGKETLLLEGMNDGKLDDDIENIDMTRNIKKINLYNIYNNKNDDKDNILDNENREGLYLCDVMKNS
NELKRINDNFFKLHSNLASNLLSTSTSGSFGNDNYKSTPLNFDMNKLPSNNTFNNLYYMDENIITNNINVIPL
VNKNYSDISSSYDIKKKNLHKNKKKHKNHINDNNIINVDYNMKGADNINNINSNMINVKNMNSNDEKPTTSTE
NMFPSHSTNINDSNIVKVVDSHDSINNHEEGLINALNTIKADKSNGENSYSILDSNDVMIKKLMLKNNLLFEH

LQKNGKFNKDENIKNASQSSLHSNIIRNLTLTNNFQNDYFYNGVKNGSFNNVLNKEKKMMNSYDEQQLKEKIA
SLKEQNENILNKKMLFKNKSYSDNDNFLSFYLKRKVSSFSDYNKESDNNSYLTKQKRKRKNENINYDKNKEFL
VNRTNSLNIKRVRSGPLDNSQNVSQSNVHTYGTAKEIIDVDVLDENMNSNNSYNIKGGDNIISSNFTDPLKFT
ETSFDVQKESLIKESNENQTVLKEKDENLVDVKDDKTNQMSNVTTIGTSNLAESTTKNEIDIKVCTLINCPTH
NPQNYVKSNGKRGESLGDGMELKEENDDLKRIPGVYYDKNSQRWFGEHKINGVKCAQSFAVKKHGCEEAKRLA
IEWKKARIRGEVWDRFINKKKKNTNNNNNNNNNNNNNNSNNNSNNNNNSSNNNNNNNNNNSGSNSNNNSLKGS
KSNRPSVEELRLKYLSMSKNMPKVRGVWFNSTPQRMGWVGQAYKKCKRIERIFSVNKYGFEGARKLAIAFRNS
QKPANEDSDDDSWSKEDKINIKNCDENSNNYDYKNNFLVSMNTIKNNGSNIMSNDNNINNHSNNSNDNNHHMD
NNLEAQIENNTDMIMDSNMDNDIDMNIDKKNIINKHNNHDENNKDMRINLCRDAILFILHDLETILELNIPML
SKNVNMYKLCIKHHLNYLTLIKNEEQIIPYLNVFGDYIQRCILPTDLPYAELYVLIDSLIHNEILPSYDHKEN
FCEYVAVEDPGIITPSMLL*
>N.PFL1905w
MGSTDYRVLNDGDEKSLKVYFQRKNNSDEQAELFCFICLEKNFENSNLISCCSLCTACVHKKCWYTWRRNQKL
SALRSKILGLNKLNPLLCTICKTGIAKIEEENDMNWIINNNSNKEYLQDELLRIISSLLNNEINDNHMPMLHI
KYIFSFNFIFLILSIFLIILFSVVFNFTLTYVLLIALFILYEIIVLQIVLYLYIRIKYN*
>N.PFL1910c
MKNAYYFIFFCSFLIWVNTMNPGRQGNENFSEKNSSDEYEYVDSAYPGFNIHYNLEPKDWKHIELTEKNNKQF
MMDIKKEIKRMEEDKKKINYILNVQKQQFQELLFLIDHKKYNHQNINIDEMPDFLKKGTFHDDYINKLNEKQI
LQTFKDIDNYTLDNYKYIGQQNFYYHLANQWIP*
>N.PFL1915w
MNVRFFKSLSLFIFFVKGVILVVQAGKGNYANIYKKNNINIFNCMKRKYDKKLSSSVVNTNDKALYESSILKY
YYLLDKYKKPNRKKIYGYISNVSRFPLEEERKRKKLEHMNKRNIYPCYNYDAKDIVILEGLEAVRKRPGMYIG
NTDVKGLHQILFEIIDNSVDEYNNFECNTIKVVIHKDDSVTIEDNGRGIPCDVHEKTKKSALETVLTVLHSGA
KFFDDEMDDEIDNNMNNTGNTNTSEKNNRDNISNNNNNNNKRTKKNSKESAQKYKYSSGLHGVGLSVANALSS
FMKVKVFRNNKIYSIELEKGKVTKELTITNCPINKRGTQIHYKPDSSIFKSSTKHNSDLIKNRIHQLAYLNEK
LTFYFYDERSENKNSIYISKGNKSNEIIKNKKKNTSIDNENEDNKNNTSNYVSNNTLKDDVSNENENMSDNLV
NKNIEITDYNNLDFYNYEIIKHEYGLNEYITNITKNKTNLFKDNDKIISISCYHKNIYIDLRMKWLSNQYNEN
IISFVNNVNTTDGGTHVDALKYAVSRCVNYNIKKNEMIKNFVNIPGEYIREGLTAILSVKMNNPEFEGQTKTK
LGSHHLKSILESVIFEKLSEIFDFEPNLLSSIYFKALQAKLSDEEAKAARDLIRSKNNQYSSTILPGKLVDCI
SDDISRNEIFIVEGDSAAGSAKQARNREIQAILPLKGKILNVEKIKNNKRIFENSELKSLITALGLNVNYDNK
NLKNNNILSNNKKGKNSKKKSDLKNSRFESTQNNNNILNKKKDILVDNTLRYGKVIIMTDADVDGEHIRILLL
TFLYRFQKEIIENGNVYVACPPLYKITYNKFFDNSIKDIVTKQFNVTTKQSKFIIHTYSDQELNNLLKLLDKD
KIASEQKNMQNKIKNKNMSSNGEPLSEYNEQSKTNDIFVNEEGSTDSFIDDNVLFNFSKKYEIQRFKGLGEMM
ADQLWNTTMDPTVRKLIRVTVNDAIRANDLIFSLMGEDSKLRKNFILENTNSLSE*
>N.PFL1920c
MRKYNFFTKSRLFSTLTKINSVKEYHDDIIKCIEKVRVINPLVHCITNRVTTEKVANSLLAFGSSPAMIDNPK
EVEEFAKIASCTYFNLGLHTTQVENINLLEKLRKECMKDKFMLIIDPIAVGATTYRTNVIKDIILKCQPNVIK
GNIAEIYYLDKGEFLGKGVDSNNNNTHNETDVINSARNVALKYNCAVVVTSKTDYIVSPCSHYVAKINCDLKI
LTKITGSGCSVGALCAAATSVYPQNPFIACISATLIYKLAAFKAYQKEKYPGSLSHKIIDDIYYYSHNPHFLN
FQIVDIYKAA*
>N.PFL1925w
MSSKYDNQSFLSTGSDKYGEQENSVLLHDEEDEYINENKKKRNERDKKFRYYENFFNRRRSKKWNYFIIFFLG
VCLGFAIWPFFMTIITYRLFYKDNFNNTNYRTNNSSTSLKSYGNEKNKKSDNNGKVPMKDQSPNKVSPHFKPI
RFEEIAGIDESKLELLEVVDFIKNREKYHEMGARMPKGVLLVGPPGSGKTMLARAVATEANVPYIYTSGPEFI
EIYVGQGAKRIRQLFAHARSVAPSIVFIDEIDAIGGKRSSGSVNGAGQREHDQTLNQLLVEMDGFSNTVHIMV
IGATNRIDTLDSALLRPGRFDRIVYVPLPDINGRKKILEIYIKKIKSDLKLEDIEKIARLTPGFSGADLENVV
NEATILATRNNKSLVTINELYEARDKVSMGPERKSLRQSDHQRRITAYHEAGHAIVAYFLQPKTDPIHKATII
SRGNALGYVEQIPVDDRHNYFKSQMEAKLAVCMGGRTAEEIVFGKSETSSGASSDISRATEIAYKMVTEWGMS
DKLGPLNYKKRMGDGYSSNRLSAQTVSSIEVEVKSLVEKGKSLSEEILRRHRKELDNLAFALLDKETLSGEEI
KNIIDPNNSKNYSGKVEMLKNDTKKNEKKSNSASKNKSTISEDYDKNQNNVEQNYQSHDHTLVKNEENLNNMK
NDIDDNNTRDGQVNSDSFVKRKEVTDQDINGKELSEMDNKEIKENKKLDGEELNKIIIDNDKMGDMKKNIKEV
IKKMKKKQSKGSYKTYKNVLKSVSEHKELKTQKKADENKIVADNNEKSSNNSLDKNNTQGSIDEKGYKNNNNN
NNNMVKNINELFEENFPEYKKYDNENNDFVKTKENYFDNFNEVIDISHIKNVDEMKKFNIFEHNLNKLFLFDI
FKSD*

>N.PFL1930w
MQDSYRNHIREYNKDKLDSGKRKMNDVTHDLEINDVNEISMEDNILNEVVKLKNDLEKALIENKELKNKCNED
RKRLDLLSLKNNSLLNEICEEKEMLKIEYNNYKMESVKDIKRLQDELDKNVIMLNCLKDEILFKEEELRKAEI
IERLYKQESELNEEKDMKIKKLENLLNQEIYLKNDKEEKDKINKEEINEKLQLLNNDLDKKNEELNILNKKYD
NLKKTNIDIQKKICFLKEEIDYYKNNENSFIMQNIFNAENNLDDSIFKKNFMKISVLYNVLFKIVVYDQRNLF
EKEKILYSLEKENTHFLKKIVADEIYCLYLKDCISKINLTYQIDLHKQEKKYMLLNSEYDELYNKFIEEKKEN
LLLNETYDKIIFNQKSEINKLKSHISIQNKDKEILTKYIDNYITYIEDSYQKYCILNKEYKNLEEMHHDLEEK
NKSLIILVNRMEDKYPIENNNNNNINELHMDHIKLMKNNLKDKDIKIFELQNKNLELIKTIDKLNDDILKKNT
NKEYIEVESGKMKDLCFDLKDICSKLELEIKEKNFIIDDLTDKLNQSEKKNKDNQVILELSEFRIQNFNDNVK
YFTSKVNELNDKLTIKDEEITRLHDYNEKINQEKENLFYEHVHILEQLKELQMENEKLRKQNVIIKNELNKNG
IHLDEDIYMDKIICEGDVDNRKDSMMNHISKEQKEENGKYIITNNNNNNNNNNNINSSNNNNCNNNSCGGSNL
LMNSRSFQYHKSRNYTDDDKNDLIIKIDKLTDENILLIKENEEVFDKLQIVSNENIKIKNELMHTKSDAEKYV
FHLEYEKKEKLKYKMLSIQRKRQSSQIKRKFHTMMERVKDLVSDIKQIDKENKRNDMNNSFFVSSSSGSSYEK
LNQSNDDLLSANENTNTQLMLSSIGKSRKSMNNNTSIDGKHSFYSKSTKVLRLSEDMKDFNIFHVDKIKKKKK
QNNISNAFTRNETQEKILSDHIKSKKSKAKVGKGKKIYMKIRMHRFSICSNFRINLKKYKHAYKELNNTLCFL
NQKKNDNLNMIHHNIHSKNAKGLDTFYSLPNSADSKIFVDDELNEKNEEYDKLVKVGKKRRRKKIKDMNNNLN
NDFLIFESEKIINDLNYIKRKYDSKVKETLNMQKKLMNNEKELNNTNIKYENLLNEHDTLISDIKERSEKLSN
IEKNYNLLFEKYSETQDEIKMHEQKTHEIFNECNDLVEYMKKKENKIKEVDENLKKIEIKYQEEKEKNDILTK
DNISLNNLSSKYKEDIELYNKKLENMHTELKLRLSRIEELELIQKNTENEMYYLKSATYKYDINLKEAHDELN
RTSEHTMKLEKKLKECELSIDVYKEQIINRDYMLYFVNDCYKKKEYFFKHLKRRIQGFNKAFIELFENIEMKD
INRFIELHQHLEFNDNEYKKNVLTFQLNKNDNHLNNQHHQHNNNNNNNNNNIIHNGNFSSNYHNNYYNNNLDD
VMKSLDTIDELNMKYEEEKNEMLNRINEVENKLKIKMNENNLLYEKYENNMKDILIERDERVSDCLILSEDVK
KKNNEINILMKELDEKNFECNEIHMKCTFLINEVKNLEKSKSHLNDKLKDIEIHQEEQIEYLKKENNYLKEDI
YNIKNVIHNLDEEILKLKDTIHDLNDEKCCYENEIEKLRDMLEEEKKFNDMLKENEQNFIKEIKEAHLYAIKK
EEEINSFKLNYELDEKKYIDEIDKLKKKNEEFNNSIKNQRKLVSDLETKMLEYNVLLDKNTSMKMLVQDLENK
IDDLIKDNEKLCIDLKKEKLISDEKEKSILSLNEDIKLCEHKIMEYDTIMLKLKDEKEKLNQEILILNDIKKN
KDHIIEELENNLRKAVFGDIKINELEKTKLEHKIFTEQDLGFNNNDHNINDLYNSKICIFNNSYNTKSNKMNI
DELKKYSLYLKEENDLIKHELRKLKDKSSIDEHKYKEIERSVEKRDEIIHKMNNEILMSNDKIKDLEKGTKQL
NSELYAINELLAIRSDECKELSNELYELTDEKNKLIIQNEVYQKQIEYFKEQIDDKTQLLNDLDMIKLQQENN
EYDKNVLINKYNKALNRYYKICSNIIYYKKYLEKELSSKNNGTNLNDDISSLNNSFNIEDNNKMAMFGVKNNM
NDMNNMNNMNNMNNMNNMNNMNNMNNMNSSNNSIIHLDTMCNLNDTNQVTEYVNEFLKCVRLLIFKKRVFESK
LESAEKKYNNFKDKLKEEKDVINDLKKKLRQSQEYHPNNNNNNNNSNNYNNMEEQAYYENKKDANFDKNNFSM
NYKELSSQILLKVAGKNDMIEENDYMILKYENEKLIEENKYLNDQINNLFTLQSSFNKLNKIKESDEKGNVIS
NNNNNNNNNNNHDVDNHFNNSAAEYTYLKFQVNTITNELNIYKNKNEYLMKKAQNYEDIINNLQKEIIHIIDK
QKECDEEVYKMEKELEKKNEHIDNLVKKRQKDQENLDIEYSKAIKLENEFLSLNEDIFKKDEHIKEYEELVNK
LNEDIKIHMKEKDDLKEYAEDLKNKYELFEKKYNNLLHDVIENENENKSEYINKNDYINKNDYINKNDYINKN
EKKNSLSTNEKKETQSLINKLKNYDVMITDLMNEKDMLLNKYNELKNIYEEKINIEHVLHNEINELKKENEYL
NNTKTSLNKQVDDLIKENDEYNKKCIHLDNEKIDLYKKFVKVSEQLNHINSLDKYGNNKMGEIKKSNDIRNTL
KENELNEKPNVAGRISSHTNKYDQDVSSNNVHHMNNMSVKSLEDHHDDIMEKMKNDMNDLSIENDLLKVKINE
LEQLNSNIFNDNKDLINIISNIENDKKGISDSKENNINMLDMKDIHNNYENLLMENKLEKQVNENLKLEIYNL
KNKVNEYNMIINKNSHYEESNKRAKNYLYYLNDKIKSFIKIFSTNNMNYDHLKSELKKIKLNNLMIIQDNFNS
LNHSGAASSSKLSKKNISSSSNYSLEDDDDDNSSICSKMIVNFFTLYKEATAKNYDNKKSYDNYKNDIVINRS
ELQNVYKYIIKLKENYIKKCVLVDNQNIMIQELNKQIFTYSEQNKKYHDEQNEWKNKVSDIINENLIKMESFS
KIHNNLLNKLEEEINKNNKLSNELYIQNEQNKLYQENEMKIENALNRNNQIDNMDKESNSNLKKISILLDEYK
EIIETNSKLLIRNNNILLENNTLKDIIGKMDKDINKLNYQLKETNILIEEEKKENINPKELKNKYTNINHNQG
NTLNNINHKLNDTNEENSTTNILNDNKKYKNNDERINEYEEIILIIEKKNNELENNCQNLKKSNNEVYNKYNK
LVYEYDSLKKFIKDKVKNNEYIDIDLSQFEQHIKKDIYEKFGEKNNNSQNELSNINKDVILPSLISFNRCDIM
KLSDHVNSIELLNDDLLNNKELNVYISLIKTDIENMLNDHNYHNDKNDHNDHNDHNNYNHYNHYNYYNHGSGF
IPYEDNNISKINKDDLIEKLKQKDSEIKEYENKLDNMNLYVNKKEREIEKLKEIYGDQFSINKNSYISNSNNN
DNKRNSSNLSEKHLHIKEEYKELLNEYKFSLNLLNDTKNKLDTLKREKKDMEKLKELQIHDKDYIIQNLKGIN
RELENNLNEIKKSFNEKILEKENIIEKNNDLINNLNEEIIMLKSIKIKNNNFIKELELQIKNLNNEINTLNDM
LKDSEEEIRMLNHTLEEKQNECVKLRNKLEQNYLHISNEDKYDKSSSTYNNNNNNYNNNNYNYNNSDDEINET
RRRQDNQIDDLKRKSVLQSNEHYKKMNELRRDLNNSFSSYNKLKFDSEKQIEDFKEEANNLKFYINKIEEENK
KKEEKHALIEVAIKEMDKEINILKTQLEKKNVFINDIQNNLEEKKKTLEYLEEDIKIVQHIIMKYVQKLNSLK

DIEQLIDELNENELKTNEYFTFIKDIFISIHQNYMKEELNRKEQMIINGQGLIKENEVIQLEEKLKELTLDKN
RYDKDIKNQKDELRHLIDRKDSYEDELNHIINELEIKREEINQMNNKLNELKNIQNENKRKVEDIKTKEGDLK
YKEEELKYREENLKHKEEELLIKNENYMNKEKELMKSKEELIVRENDLIKNKEDLIDKTNELMLKREELQKDK
IHIDELKNDLMLKFEKLYNAQNDIKVKETKLKKIYEKLKDHNIIELFDINNRDNNSNIKNHTSNNINSDIKNH
TTNNINNINNINNINNRLSLKRLGILLGLDEIGDENELDFHFDKENESLNLDSLEYTTYFNEFKKRIKNLKNN
LLNREKDVEIYKKSYEIEKKEKEIYKIELEKLNELLHVEQMNRKNLDLELEKYKSEDTHIVKSLRESEELLNE
KNNKILELQQKLIETSYEINIMIDKNKNLIKEKDKDYEQKIDNLNSIINDYEKEIKEMNKEKLKLTRKSIEKM
IDDGEDIKKLKIDLDEAQELILDLKEKNEELNNINIELINSNKDMRLDIDVLLSNVDNLNKENILNEKKMNED
EQKYNELKIAYDQKVHEFNKLYNMLSIKMKNKFDSENDMEQNYTNDNVNMQKSRNSINNKYDINDFENDTIIA
LNEKNMIIESLNEKIAHYEEENYKKEELVNKYKNMINELSNKIHIFEDNLDFLISLNEFIKTNNFTYQNILDI
LNNSILYKKLLSKILVENQFYKYIISIRDWLFEIIKSKSYSNSNNNNNNNNIISNNSKTRESVLRMSLRKKGNF
KRTASVTSNIYNDLFLSENVLSAEKENDEIMNSFNKTLNVIDDDFNSFENICIELNNLFIEEDWNNIFIKLNI
LENQFKNIINLIIHEMKKINKKIKNNNNEKLELKKSVKILKKRLNSISNDLEKRTEEIEHLNIALNKECAEKD
ILLNQYEDIKKTYKQLNEQYNEQIILIQKNEYELKSLSDQLLLEKNENQIKTEKINKLLNSDLNHFNTSLDKS
AYQLSELNNEKKKNKILLNELEEKEKIISHLQKDFESNVEVINELKEFNEMLINKLKKEEYKKEKDNNVYDKN
SDHNYSNNNDNNNNSNNNNSSNRNSHSNNNNNNNNSWNNNMKSFHKCDIPNNEHLEDINRNELMIIISKLENEN
NILKEELEIIRNNFHILKEKKNELEDIINSHDVTLNKREIELNDILIEKEKILEQTNKYKIKCIQIIDICFNQ
DFNIIDIREKIITIFENDDEQIMDIINCHKNMLSNSNSNSNRNSNNNNNNNNNKLNILQDEESRSNIDEEKILH
MEEMSKKNEELIKKNEEIWKLNKFIENLNKEITNKNFIIISNQQDIENKNDILEKNETYINFLKDQIKLLCNN
IDENNLFDINNVNLSPTLRSFIKRNSVRPKNAYSSNDNKEDPFFDDMIYNNHMNSINKIINDQDSQLKKQRIS
EANSTSQPHYNFYDENEVKLEEMNLKIEDLNDKLKTSENKIVILKNENNELIEKCEFLKSELKNVQGTKRHLM
LCESRINILEKELEDKKNKLEAQNQTVNECVDIYVEENSENYNKILELKKLNEKYKIEINVLNDEITKLKNEI
NTYKNDLKNINATLDFYKSTHDELVNEFSKEEINNVYYIKMCEQLKQENQNFKQLLDSHEENKNELLNNMKQI
KEQLNNCIKENYEIILDLELLQMQNNILKENCNYYKEREHILINKLDENNNIKNLKIDENINEQNITEFINKL
NQQINYLQDDINSKSDNIISLKYQIKAFHYEQISKENNQPYKQNDNTIQEIVNLNNITYIQNNLFIYISLFKS
VLFIISEILFFIDPTNNLYFEILTLLKLKAQNQSYVEFDTMIKHVDISQHDCENIFQSVLKSKTILREKLQLL
QLKIC*
>N.PFL1935c
MNTNEWSTIDNTHYGKQKISNMLWSNDDILKDNVVCSGYLGLIAVLLNKDKLDNYKKEDNLKIYTNIGRLISS
CRLNYDGLICFGWNKNNELVLLFKDNIVRVYSCFCEKIFVFSLDENIKNEGISYGSICEDGIIIITEKLNIYV
NYGFSGTNCIKYPNVELKGKPYCVSHIDEDQLNDELNIEQNYFDTKPNNDILRLNIKNYITNTFEKKESTTQN
LNSKNNVLIESKENYNIKWEQMQHNQMKQQSGARTENNGEKKNATNNINNNDNYYFNNKMHKRILIKTTAYDL
NNKTDVDATEKIKTKEKQNKEKNDKKKKKNKTNNNNNNNNNNKELNIHLLITLCNGGFVLVNKYHYKYYDMDC
SHPYINMSISKSGTILAFLADNGIIKIYLINNLNKCIEETVLDNKKTIKQIVWCGDDCLAVYIPMITPSNYIQ
HMLFIGGPQNQWIPYQYRHDLFLIGDLYGVKIISKEHFEYISRIRKSTFNIFSIGSCTPSAMLFYSYEKYKNG
NICLDDEIRAFNNNLHIAIEECLNAATHEYDDNIINLLLKTCLFGKNFMKTNYDSKKYLLCCLFLRICMNVRK
PPLDIFITAAELQYITIPTFVHYLAKRKEYFLAYRICEYAGIKTDNILIEWCKEKIEKSIELTDEQLCSAITE
KIGNKKNMDYSFIAFMAAKCLRPQLATVIIQYEENKKKQIDMLLKLANYRLAIEKAILSKDIELVYMCIVNIL
NQEKLNANGEKELNTLLHVLNSNNNMGNMNSNNNNLNNNNNNNNNNNNNNSNNSNSISMHASNCFYVYCKKT
KQYNLLKEFYEKNGQHSQAAFVTLDLAFSKKNTEQKKTWLAYSAGFLTTDQMNSHIKFVHTSIMNNIDLLNYQ
KELEMKYNKKLVAGYPHKIQGLSLMKTVEYTLSIGEFLDADNIFKKFKISEPKFWRCKIHTLAKNKYFDELYN
FANYRVSPIGMDYFIECVHEYGSKQLTVKLIEKNKDLNSQYKWYTKLNMKKEAEEVLKQLNSQKITSSIFQTI
TDAISNIR*
>N.PFL1940w
MLKFNCIPYKNIGTFHDALKQHNIKFEKRNNFIFFHAFSSLRKYKSQHSEKNNILNKLFFFKKEYKSYFHKLS
NNLFLSSKRKNEVIFHKMDKTNKNVQMIKHNIITSQRRENGMEQYTAIDGKTKVNNNNNNDNSNNNNDNSNNN
KNNNNDMKANIHQDMDKVKNNMIDLELSELWTKKSLIVNFQNNICEIILNRPEKLNAINKDMINALLNIIKSL
DNDERCFMVIIRSTNSNCFSSGSDVKYVVENKEQGIQHLKQLYLYINYISQMKKNLLCIWNGYVMGGGLGISI
YSKYKVINKNAIFAMPENKIGFFPDIGCCYFFRKYFGRNIGLHLGLTSLRLNEVDLINFNVCNNYIENVDTFM
DNLNNIKATNQEDFNKKLNNLLTNKYVSKMSYNKSNNPVLTDELINNINTYYSSANTLEDLITKLKKDNNDFC
KKLLSDIYSNCYFSCMFWFSYYLYNYEKSLEEVLNNDFKITQYFLFHKNSFERGVTEVLVKKNKNFQWSKDEE
TNNADFEDIDDILMNKNLLSIKEEFTNMSE*
>N.PFL1945c

MKLFKILYLIAALLAINLIAPSVCNENVEGKKKKKGCPLRNFNFQEFRKKHHKAILISSVVSAMALLFGTACG
IGLHLNNKTFTKSILDLGKKRSASRSPHLKLK*
>N.PFL1947c
MTLSSCGTTNKTMTNIAKDILEGEVSRLKARDVIDKLKGSIFDVIFTNDV*KHISREICNITTSYYINNSSSN
NPCEGKDKDGK*LTIRKVWEPDNKNVKHNHKNVILSPKRQYMCTTNLENLVVDSVTRTGNNNASNTFLGDVLL
ASNKESQNITQYHPGVSSKDQPDVCGDVRYSFADIADIIRGTDVWSKQSQQMLLQGYLEQTFGKIKDKFKGTE
KYDSDSHHPQIL*IASRLVSSKSRIYVERNNKDCFTFCGWCYKFWNMTGLIAETSVLSEINKTISLFRSIGDD
PGRQGASLAP*PAPGVDVLGNCDTNLVPGSGGGASQHDTQSISSSSSSLSSSSSSPPPSQNLDTSRSNPSQVS
PHGPDKSYYGEHTDNQDLYKQCPHPNHQKPQNTTQVIHPHLIDNIF*STC*NSFSISLDSVPFLLFEGNKYIY
V*FVGFLGTFFIYIDIYVCFVLYISICFMYISYIFNICNYIEKRKKKKKKRNIETNLFKSK*KLKKKK**INK
HVYTFILHPYILIHIYLHMHLFFFCF*KKPKLRPTKLIRVIDIPQNDYGIPYKSSTNRYVPYTSDRYIGKTYI
YVEESPRSIDHYIDYYGDTTDITSSLESE*EDIGVNNIYVPGSPKYKTLIKVLLKSSSKTYNKQDTPDSHTSY
GGYTNDTHIYDKHDSNKLTDNEWNRLKQDFILQYFKDLPNGLNNQLPNENTIDDNMYKDIQHNDHILDVNKEE
KPFITSIHDRFLENNQQNTYNI*NGMFPKMLIALQIIWIFLNAPQKINIMEYILINDSLNCDYNEVINIYVKM
FKRKENKLYRIKYTKNTVTNTYNEIHLFHKRFERNRYMCEMWNNKNNNNKKYEILNKNERSIEKKEQYKFPYH
I*
>N.PFL1955w
MYFFYIILYYIILYYIILYYIILYXXXXXXXXXXXXXXXXXXXXXXXXXXXXXXXXXXXX
XXXXXXXXXXXXXXXXXXXXXXXXXXXXXXXXXXXXXXXNSMDSLTSNNHSPYNDKNDLY
SGIDLINDALSGNHIDIYDEMLKRKENELFGTQHHPKNITSNRVVTQTSSDDPLHNQLNLFHQWLDRHRDMCE
KWKNNHERLPKLKELWENETHSGDINSGIPSGNHVLNTDVSIQIHMDNPKPKNEFKNMDTTPNKSTMDTMLDD
LEKYNEPYYYDFYKNDIYYDVNDDDKTSMDNNNNLVDKNNPVDNNNSTYNHRNPADINKNFVHKNNQNQHPIE
KPTKIQIEMNSNNREVVEQQYPIADIWNI*
>N.PFL1960w
MDINDIYVPGSPKYKTLIEVVLEPSGNNTPTSDIPSDNTPTPQPITDDEWNQLKKDFISNMLQNTQNTEPNIL
RDNVDNNTHPTTSHHNVEEKPFIMSIHDRNLFSGEEYNYDMFNSGNNPINISDSTNSMDSLTSNNHSPYNDKN
DLYSGIDLINDALSGNHIDIYDEMLKRKENELFGTKHHPKNITSNRVVTQTSSDDPLHNQLNLFHQWLDRHRD
MCEKWKNNHERLPKLKELWENETHSGDINSGIPSGNHVLNTDVSIQIDMDNPKTKNEITNMDTNPDKSTTDTI
LDDLEKYNEPYYDVQDDIYYDVNDHDASTVDSNNMDVPSKVKLEMSVKNTQMMEEKYPIGDVRDI*
>N.PFL1975c
MFNDDQNANNYFPTKLGIDRPAIDFTSSVCNFLKNDVYKRQFERKLYVNHPIYLRRIKPLFCYSNMIDRYDGV
MSHLACSCLNKSKGMIVSLKWFNDGKRLLTGTQLSELCIWNGSYFNFEDMKRIPIGGGSVSCLEWSKNDNLFA
GNSLGQIVILSSALNLLDNYAFEGLTKNVLDISLSCCNTKLAGCADTCNPIIWDIKTRKVIKDLKCKNIDTNN
ISCLAWNPINDIVASGNRTHTISFWDIRMNKPIISLNSHKANVNKIKWNNNGIYLLSCSKDSLIKLWDIRNFK
LLYSYKNDQIQNNKFTSNYEPTYIAWNPIQNHIFSSADNKGNIKFYSTNDNKCIQTIVSAHGIDNKISSISLL
DWNPLGHILTSFGDDKLLKFWSTSSSGSIYSKEIDAKSLVHVTNSGVFPNSRYINDEYMLDVNSNQSISDSDE
NDYQQNILNNNYYKVVDKKKKKKVKKKIKK*
>N.PFL1980c
MEKQKLLNILQRELKEYGDEEMLSCFHRFYESIEANENDLSKKLFNELIQQMKKLFIIYHKRNKRDLIEKIKN
FFKEESNEVNIIDVENNDKDNDSNFNYEDDYFMNNEDININDDNNKNKVRRKGKRSKDPKDMKLPHFKNHYSF
LSTNLFDNINLHDLKSNVYEYDIFKLYNTHVEMILLEDNKDITIDDEPYFYETFEYFFNVLCLLYFLKNIYFD
LNYKTNENSYSFLFKGFEERKNKVLSYFFILKKLKIHKLMDNEVIKIFCNFFQYIYINEYLIRNIFDVSMNSF
IYTAVKNFQKKYKMDNILVPLQSLTDADYSEQQQKEKVHNDMEKIICDNTTCVNNTSSKNSISDYNICVNKST
NELIASGNNNNNNKTLGKSEISGNEIICQENITNNHKINKMNNTIITTTTTINNNSNNYNNNNNKVNRNLFSN
NYFVKNSPNHEDFLNKELNNFFISHNQNKNNKHSFIDIIYNDINYDNIYTDEIINSLFYFFNNPYLIFTFHKN
VLYYIEKKSRDSNFFHSYKNAIALIKYLNYCVTTISVIFHIFVSAGLFFNKHIMIIESNKYLNFYKLLNFDNL
LVDSDNYDQKKKNKYNPSEYNNKRNNNTYNNLSKRRSSTHMNSSPRTHEKDENIRKNTQKKKVESFDSLNDSS
SYNDDSINENELVVHNNPFDKKNREKKNYGNLEDNKFYDFNIIKYLMKYKNVVSNLNQDHFNKKDYSNESEGE
VNDFVKLDQSNINSRGSYTSSHYNKGDVTNSSYDKSNSYSDDDYNDSDTSKFQENRYKHSYKNVKYNKSSDDY
ENDSNSSSNNNNNNNTYSDDDFNSYNRNRRNTQRKSKQMKNERFQKKNKKKNKIDQEDSTMSDEYNMNNDLYS
NEYTNRSIFNDILHKAKKRKRFYDLISNINRMKNTFNVNNINKNKTIDFNYIENIDTISYFISIYNSEHIFQF
DYYFLKFIAEICTTYNNYIKSYLININNNNNNNNNNLKYRREDLDKIINQVEHFLNEVTKLIYVYTWCILYIF
GHSYSYVKYAKYFYQKKSFFLLDKKPHFIFNQFSYVTQESFDNISHQNSNSNSNINEKDDIYKIKKNNNNINN
EDHFDYRKHLYNNYNDDIPINHFFNPSNIVENLKYNYELKRNTSEFDNFNFNNLIHIYKNSYFQNIVDKLQLN

LKFIDNILFTLDNILKNKKSKKKKFLNYLCVKDQKLDDICNSIDNLKRNINKYRQTFHYCLNFNNIKNVIMLT
LEEMSMSSDKLELNKLLNEYLNKLESKGQLKKRQKKINIKIIDNNIILDTNNIKKEREREKRKSVENFLNKKK
FLKSYMDMNDISNHLIDKRRNKSIKMGTTNTNHRRSSSVANRNRSRSILSSSFLEKKEKSKKYSIKSQKKYSE
HVNTDRSYIKNNSHINNKLSICMDNNKKKNDGINKSFMINDNTNRGSINNDSYYRKNNRKYEDSVNYKNDMLE
RSRRRSHNYYSSSEDNKSDNYNINDNNDYHHNDTDTDEDNEYYSDNYKKGGNQNGNKLNDNNNNNNNNNNKKN
KNEKNKMSELIFNSEYHTERLIFFFNLLNGNNLFYYLSLHYLVKNEKIMEILHFLPLKYLLDLLILYDLKDYI
KFKTIIRVFRIIYEFQDFSPILREDIFFNKNNYCLENADFMNYEFWCTFLTVNKKDLSPKKAYDDDNYNSIFM
ALKQTAIIYNNMHDKKEDNFEDIRKRDDYSNAADDDDHEHHHDHSNNSNYYNDEKYVHMDNNSHDVKKHGFFV
DLIKKVFSLFNFSNTLKSYPVLLKELLQKLLINIIGKHSCINDNKHYKGYNSIKYYALKFVYNNLKNLTFECN
TSSYYSYTINNQTELYNISIHNFILYHIYELGMRTKELIIKSSCFKLIKYLLTEYYYYPDIFNEKCHELINSD
IKINKFYIKNVILDFVSFNIMICHLQRELTSDLNVLNNLDLFNYSIKSIANYLIGYKNNSFFFLIDKKMVKTL
LIKLTFMMPFIYKLKIPACVIRLYIRVIKSLDKEILDTIHNSNKIDLIYDSLFYIIISHFIYFFLNMNIELDE
EASNILSCNYNKGAFPNKFEKNDNYIKITNDTSEEKESGNVQEDEEQLSCRNINSFEKSIDDEGDKNNKNKAK
GKFLCIDDEFNFENNVQNEMDQNNERDEPTVERKSDDSRNDKDDNSHSDYSNDTNNSNDTNNSNNSNDTNNSN
NSNDSNNSNNSNNSNNSNNSNNSNNSNSNSPNDDLSKNQLNDDISAKNFNKLSDVLYIRKIINENSNFLENN
YFINYSLYILHKEHYSIYVFFSTFFKNALKEYNDICKSQKV*
>N.PFL1985c
MISFVKLGKTINLSPKKKFFFFFRTIKGRRRKEKYEDLENKHLYWCVNNMKKEYLDKDTDDLVEIIENDDIEK
QLSDIVGIKGNIPSNNCINENARKLYIHQQKKSDIDGTKVEEINKIFNPTLNVLKDKELEFASDADNYLIRRK
IENRTDNITNNNNNNNNNNNNSSSSSRSSSNNNNSNNFYNKSNRDAPILDIIQGKENMNNNSSKNVNTLSDNN
YNNTCVNNIYDNMKQNWNNKNDINMEKEYCKNEKNDNMDDYNFMNVDNNPNNNSLFDYESLKSKSNYQDLTHA
EYDYMNNLYLKKCDIDRKIRWFKMNNMLNPVKEAKSIINSLKLSKDEKKKNNCEQNNDPFLIKEDIKPYYQDE
GRFSNKIEKICEQNQYDEIVSRIRMKIRKEKLENSKIIKEKIPNVANHILDPIKYHVNRRRVRVEKLLHTHLE
QLLNCNNSYFRFYLLNGLSISIHHLEMKSTRSMCKIVYSLINKNVTHDMIKDKLEKVAFVLRKLLARKLQLGY
TPPLKFVPLSDQQEKNIKHLQYYKLYAKYNHPSYISDKKDQTTNLINFYNKDLAGF*
>N.PFL1990c
MNKNTLTNNIHDKNYKYSKNRKASNDEMAYITNEEINMNNSHIGKEKNVDCNKDGTIFKLVDKNNNDMLLKDE
TKEFTSKSSNKDTVFLGEKGSRYLENTITNRIDTPLNRKNISSINNNNNNNNNNNNNNGDTINFKNQVDNNSN
IYNTAIYTNYDNGLGTRDDIYNNSCINNIDNSNSRNITNYIIRSNKIIKRKKNKIIYYNTTHNNRAITQKGYD
IQEKYNSLDIVDKNKTEGVYNTDEINMECIENKLEDGYNNTNIWDKVNVSLSNCKLSEGENEKRSEEIYLNNF
VSDKNDMNNTWDINNMNNNMMYSNNLGCEEKNYLVPNENHKRWTDEYNIDEMFIKKNDIEHINPDIIINNYKN
NNIHKTTENIGNIKSNNNYINDRSTINGDKLKKLTNENIYHNEYNKNKSDDNTKYKNNRNYRNKYMNRKNNKN
KTKNNANNNKSECINDGTNCSLYDSINSYNINNNNNNNNNNNVENVTNIFKNTPFSHITMNENNIREDTSNIP
VHTNKYLLMQKNVNDIINFNIAKNNSTNENNILVDNMNDNLKEKNDMPDDIDKFLFNQNESISMDGTKYIMND
LEKCKTCMEGEKEINDVIEHISEKENIDTKKKKYSNKKKNHLPNLNSTIHTLKNNIYHNVDQKDEEKLGSNII
NNTNSDINNNKMDENEHNNGNGNSNDNDNGNGNGNILGVHISQHNNLINNMNSIISNEYTYMNEPKDENNNVP
YNYNVRNIEDSSSCRNNINNVNLFGNKTYINVPNNIVTKMFNDTNRSTTNNKNNMNNISQIYENNPSLNNSLV
ITKTNMQDSINSNMNIIHNNNNNNNNNGVYFFNGGNNCVNVNCTKNTSNEKDVFNSSVSKKTLNENMSIERPV
ILNNSQMLEAFIQGRLCLSCDSLDHPMPLCPNNSFVCPNCHNVSHRGNDCPMKCRFCLKYHMGISIMDCLKKA
RIQTEKNNSNHDKKDNHNSSCSTSNNNMNKINKNSKSNDEKVNIGPRFDISTRPDNSYGRSVYVSNLSEDISN
IQLRDTINLHLDNGFVVNIDRQDGYAFVELSNLYSTFQLVQKTVSINFKKLKIQFKKTGQFLIPDNLSQSFGN
MKNFNININMNSTNNHNNNNNNYNNNNYNNNNNNSNNNNNNNNNNNNCNNNNYNIVEYNNKKPLTGPSNTLSK
NIPNHMINKSLSNKGTTFLNIPPNRSRQYNNRSANNKGNMGANKQYNNKNNNNNNNKKKSNSNVNINHTNKSN
QINNIINNNNINNNNNNNNNINNINSNNNFNQYCSNNIMTVENSNTGKFTNTHLFNGDTFLENKNKNIYKSFH
PKYSKNTPHLFNNQQKLFHATELQKVHQNSIEPREEVKKNDIKENIKKNYACSNSFDNEINKYHIPQKENNKM
LTENNYQKNRNSYNVNTIMLDQNKKCKDNNLFGSVEYKINMENKNNFSNECNNSYDHNFIYNYNFSNDNKLPY
KCSTSNNYNCPNNNNSQNDYNTTYNHGLSKVNENTDNSSIDIPFRNHDNKIISDYMNSNTNTTASGFCPNTLF
RSNDISNKLMNQEVNIYMNISNEENRQINKSMRNETNKTTATITTTSDNIMNASFFNTMLYVKDDYVDKCDEG
NNSTNWMGYPENAINNNSNDNINIINSKNNMDNMNNIIYKNPPRNYMNNEIIVNTLNNFPHENKNISPKYINE
KENDEFEYNTHLNIKSILDDAIEINQNTIDYDQYNYYDNIPSFNNNDNNIDKYTIQNNVDQNAVTNYNTSNYN
IQMKEENNYKENDLLYYTNFDKTNIKNEEHIYINKNIWKDKFDNCYNICNDMFFGNNDIYDIFSNFNSLKMYE
NNIANDNIMENIGKHVNIDNIMNETTMDHIENDGTIDPIENDVTIDHTDNNGEDNNMAYNDTYIFNNNIRFNN
TRLNNDTFQNVEDWNKNKLSDILKKNENIMFDDNKDMTNENEQNKKDIDDLEILNIPYMKNMKDKELDAIEQD
LERHIKALWNIRKIKIVKSYDVQK*

>N.PFL1995c
MNKKLEKSFSFIRNFAEKYKNVIFHGFKKGTVKTINKKNRKKKKQNVTNIVDLKFKNPFINEEKKRKLDSFLM
DQENELDLHENYTLFNKYNMTSDNLFFNLKNDNLYNNNNNNTSGTFLKSLNIENKNGDLSDDKIKINYLDSLN
SLELKIKQEIKQNNDLLSKQKMMELNEYHYKTHNNGKKSEVRIKRTNSHLNKDSNRIYKRRKNNESQLDNVSI
KSKNKKKKKGYPNKDKSYSNSSKNINSNSNVDSKQIKGTCLIQHNEKIIKKTNQPDNENMNLNTENILLKNNE
LIKENTKNNLVFGKNIYENINDMFHNVFEVNQEHNKNDIIKTPINNLMDREHSNTSRNTNINNINNNHIDDNR
NTNESNIKEVKSNTNQKDWELFHKHINNNGTPFKEDNTFDIFFNEPLETPSIFQI*
>N.PFL2000w
MEHLKNLITGAISGAIVDAVLFPIDYIKTNIQTNNSFSIYDPRKLYNGILPTLIGTVPASAFFYCFYELSKKL
LTDYNANINKSSLYLISTSIAEITACIIRLPFEILKQNMQVSGNISTLKAIYNISKKNDLPIYLLNSYLIMVA
REIPFDCIQYFLWESLKEKGKKDFKNISEKYPTLTSALCGGIAGGIAGFLTTPVDVIKSRQIIYGKSYIETIK
DISKGGFLSFYKGCYVRASYLCFGGMIFFGCLRFFSF*
>N.PFL2005w
MEEDSFKNRLLKRNIDIWIEKYRPEFLDEVVGNPFVINTLKSIITSGNMPNLLLAGAPGTGKTTSILCLASEM
LGNQAKKAVLELNASDDRGINVIRDRIKSFAKEIISLPPGKHKIIILDEVDSMTTAAQQSLRRIMELYSDTTR
FALACNQSEKIIDALQSRCAIIRYFKLSDDQVLKRILKICDLENIKYTDDGLDALTFIADGDLRKAVNCLQST
YAGLEVINKENVLHICDIPSPERIENLLKHCVNSEWKKAHDIAYSMIKEGHTPYDISLTSSNVLRRFNIGSEV
IQIEFLKIGAMACNTMATGLTSVIQLDKLLADWCMAAKILRSKA*
>N.PFL2010c
MNDAQIKDMMNDEEEKEEIEIKENINYKINEIDDVDGGDDNDDNDDGDDDGDDDDEDEDEDNDDNDDEDNDND
EDNDDDEDNDDDEDNDEDEDNDEDEDNDDNDDNDDSDNNDDDHNDVKKSSKNKKKNNEEMFDRSNHFDNFDNI
MLDVRLRKALLYLFKYQHPTIIQKMSICKILNGHDVIISSKTGSGKTMAYLIPVVHNLIKFNLNEKDHLKFFY
KCIIICPTEELCLQIYDVTKKLCTYLKDIITVNHNVNNTFYEHPTILISTPKDLCTHIIEKKKKNNLDILMNL
KILILDEADVLHTQEFQSYLKTLTSYLPKKFNKKYQIVMASATLKRNILEKTKLFLHNPIYVSHEQKNESSFE
KKKNKTNNTNSVIMKREEAGKNNIHDEGNEGKTKYQKFTGKAFYYVYKEELIKYIYLYNLIKIKIIPYKSIIF
TTTIHDAYKIKIFLTYLNVSSSILNPNHPILIRQNIISAFNNSKFHFLICPQYEKNNMKHVKGVLGSNKMDNV
DGGDDNDDDEDDGDYNDDDDNDDDDNDDDNDDDDNDDDEDNDEDEDNDDDDGDDNDDDNDDNNDDDNDNDDQT
LNEPLSDNNSCRTYNSDDENEKTDDTTKLNEEKDFLYSRGLDFYDVKCVVNFDMPSDSETFIHRIGRTCRLNN
KGKCISFVNELNYGEKEFLQKLIEDKNICTMIKKNIQYNIVEKYRYRVESTLNKCTNKKIKLFIQKEILYQSL
KSKELKDFFNTNINEKRKINKIIKHFNKAVIPQKLIKDRNQSIFLNKSKVKNKIIQKNNTNNKNKNNNNNIKP
FALKNNNGLVITEQGYESQLTKEPEREVADPSKLPPLCGQRLRNYMYLKYIKGKKNKNGSNMNNSYNSNNKKR
KNNNKYNNRYNKYNKKGINKNHNKRNTNFNR*
>N.PFL2015w
MEIINLLHITSLSIFLLTLLGIWVYKTLIINKEKKKKKNQEENEEKKKKEKKEHSNIFFNNKEGEIYCDDEKC
YLINKENKNILLKKRDNKEIKENINVADTMSSSDMNNTMKNVINDDHINNHYKTMQENKSVNNFEGELKNDHL
QISKDNQNDNNNFICYNNKYNSVCKIKTENSLLENNSNENKSETDNENSDKKINNKMNDSTIESIFDIKGEDV
KYKDDLDNKDNVINDIKREDVKYKDDLDNKENVMNDIKREDVKYKDDLDNKDNMMNDIKREDVKYKDDLDNKD
NVVNDIKGEDVKYKDDFYNKDNVNYEEKNNHLSECVNKTQNEYPVEEHTSNEVEKNKKDIDSTNKTKYPSNRN
YYSDNYINYDTDENSNISSSKENDISDEYSSYNNDSLYNSDNSEVEKMSSSMDEDELDEYDDDDDGEDEEEEE
EENSDEDKYNDTYYNNDNNNNNSDKFVEGDNQKHIQDDILNKSVEEIKDIGNNYFKNNDYLNAIYYYNKALKK
CKDKNIKSILYSNRAACNIFLKKWNTVIEDCNKSIHLNDNFAKSYIRRSNAYEQLQKYNDASNDLNKALTIDP
NLLKNYQVKQRKLKELAEQQLNKEKEEMVGKLKDFGNLLLGKVGLSLDNFEVQKNPNNDGSFNIQFKQNK*
>N.PFL2020c
MKVTYAVRLLLPVYFLFFWIHLNIVRSDNVCKGADVIVNEFWSKLNNIRHGDVILINIKNYYCPACNRYMNIW
NDVEKKILTYEKNVSLFVFDCSCYLLVPYCRYFDVLYFPTFRLLFPVYDKINDKEYKYINPSSMIEGEKYNGD
LLLAYREVERIDNIYDFKILIEKYLCKNVNFNYNDLRICMNSSYILPEKKNKKKNEIYHFLFGYKNDKNKEEI
KDSVNVMNDDFIENNYDYEENKENVERWSNHINFNKDNIKHDIIIGILFTLKKHISLGRDIQKEYIEPFLVML
SIVSNIYNDLEEGLYDIINKLNSFTYPIKYDEWIKYTENINYINEYKLDYNNNNNNDTNLISFKLCEQNSVLC
SYWLLYHKISVYCLQHDKHNYLYYIEAITNYTKNYLNCQNCIDHFLNAQKFCYYGYCNIHSAESFIIFLWRIH
NAVTLRSMYDLLMIDNNTSTPSNNFNKKQFLNQDIVFPTLKQCKNCRNALGFTKITNHTLSTFKNGSITDQSF
DAIDSFNIKNVLHYLIHIYS*
>N.PFL2025w
MINKNPYSEKKNDISDKLNYKNINDVSIHELNCLKNNRAVYLKRGNMFFISSKEEALEVLKNKSTQMK*
>N.PFL2030w

MIKLKLQIFFVYIILNILIVLVISKNVVYNEKKLKNKNCMLPNRRHKNDLLKRQVKNRKVILFNDHNIELINL
KRKYNKRTKYKNLDAFITNLKVTHVVNHNKSIITYVKKKKLLFSNKIYNHFNYPGFDFSVLKENNNEQDKSRI
GIIKTPRGDIETPNFLFCATKGCMKSTPIDFIKKCNTQVILSNTFHLLIQPKPHIIFQLGGLHKFMNWNSPIL
TDSGGYQIFSMSFGSVSNEIKRKCAGTPQITKMSIKNKKKDNLNNEEDQVNNNFINNNCENNMYNKKGKCLSN
NNNSNNNNNNNNNSEKSVTDTNNLNKQIILKLNEKGAEYKSYYDGSIDLLSPESSIQSQYLLGSDFILVLDEC
TPYHVDKIYTEKSMHRSHRWYVRCLAEFYKSQNMKNYHEYLNDIYNKKYKTNDKWIKRDKNNQAIYGIIQGGI
YPDLRLKSCDFVYNLPFFGLCIGGCLGKDKDMMYAVIKQTMDIIHDIKKKKEKNTYKEKPIHLLGIGQIKDII
YGVKQGIDTFDCVIPSRLARHGYFLSKIKTIETIEKKLKRKLQNEYIKIKLSIFQSDNQPLEEDCACYTCQHY
SRAYLHHLYKINDNLLGTLLTIHNVYYMNHLMQDIRNSIKEGNINQIEQKYIKK*
>N.PFL2035c
MDNIRDKLERYEPDEETKILCTKYIYFLLFYNSYIFILFIYTFFTTPISSYGWLFFMNLYFCIFPVFAFMGNS
YIPSLLRFYNWMLFVSSIISIFIVLEAIFTFKKIDLYLMICYATLTCSNTFLCWHVTQSIFGGVKAPNSVLDT
LRPHTVNQNEYSNKYKKKDDGQKKFIGKGRSIKDSNYIEV*
>N.PFL2040w
MNKKKLKGKKKEAKDKKSNNKLKKLTNGELENIIKTQKEELIRINQKKHLLEKKLSEKNKEFEIFKNEYKELK
NKVNVINEDCEKFDEKIQEEDKTIKSKSIFYNFQNEEELKKLEKEKDNLKNLIDERHEETMNNILNHIEQQRL
SLDYEKNKNFEEINELNISFDVRMQNVEELFTKYLETYDIEKKEEMDDIIENYKILERNEINYIQNMYNDHVK
SIKEIHMVVLENYKKYYIDQLKENIKKIKSLKEKINELEINDKEIKNDLSVHNNEYYSMVENIKNLELKRENL
KKDLKFYSKDFVIYKNLELIYNESDVHIKNLKQFSQSSRDKITQVEKELKRISEDELNVDDYFEDIKKKNILL
KKKIESIDINLDDINKEFQSYIKENNIKDEDVQNVRKGINFCLNTYNREFDNLLYTQKKMKKKIKDTVNIYEK
KLKDINIKKETT*
>N.PFL2045w
MSKPKILISQIKNVGDIKLRNLKGIESCKKKIFGYVENMPGEKNWLKPLVGKSMQNYYFPSKYLHLDFNLKEY
LRMQTVRFKKKETNESLLKLQKCINMIIENKDMIDKFLCKIPKELFIENQSLKDFYELYLTIYPSHIYKPNLL
FDIKELNLEKERFSWFFYNKKNDRKDKITINDSENNDISYNSEGDEVHKNIKEIGNEEKNQDEDNTKNNYMQK
ENFSNYENIIFNKTKKKIKFKKIICMDQINEKTNKNNTSLINNIMSNDDNIYYLDNDLNVGLEKTIPKDNKTN
EEIEKSKNEFYNLLNENESLKKTLSIRNRFIDPLYLRRRYSYIDKLTKKKIKKEKHKTYRKHFIQHADEKKVW
PDNKGLLNKIYPNPYS*
>N.PFL2050w
MENRCLYAYKKKFGVSYPENLDDEQFEPLNNVMLNMMEQKNFVIEIKDLEEDAKKYLIDEKGDIKNKEKISLI
NTYYSDRERMLFCLLSSLIEKKEYSFEGYIICSYVIKMNSSYYSAWVYRRKCLKKLNLNLLNDLKFTKYIISD
NIKSFQSWFHRRWLIEYIYKMNYRKKNNQRNNECNYNIYIEEGKNIDNKNENLNEYNIDMENNYISSFDDEKN
FISSEEEMNSNSSYNIHDNFYNDDDGFISNCDDTDFEEGQENNRNNIYIYEELGNIINNNIFFKNSLLPNEII
NIDNFLYEELLYNNCDIFIDMKNYNSWATKTWLIDKFNILQNEYICKKHNIILHEFCFINYLLTIDIYNNSLW
VYRYFILNKLSYFHDLAKMQKEIYFCFTYANQFYDNQAIFNYFIHMVFLYIKLYQNASQIKQKNTELNIQCEK
KEETYKHDERNIFQIPLVNYIKNELIKIQHKSKFVLVFLSQLYSYNGSYDDEIECYRYLQKYDDFNEHVWKDR
IECVQKRLKI*
>N.PFL2055w
MGRVRTKTIKRAARQIVEKYYAKLTLDFQINKKITEEVAIIPSKRMKNKVAGFVTHLMKRIQKGPVRGISLKL
QEEERERRLDFVPEKSQIDVSVIYVEPDTLRMIKSLGINISNMKVHNPMINTNQQKQNRMNNQF*
>N.PFL2060c
MNEHYDVIILGTGLKECILSGLLSHYGKKILVLDRNPYYGGETASLNLTNLYNTFKPKENIPSKYGENRHWNV
DLIPKFILVGGNLVKILKKTRVTNYLEWLVVEGSYVYQHQKKGFLTSEKFIHKVPATDMEALVSPLLSLMEKN
RCKNFYQYVSEWDANKRNTWDNLDPYKLTMLEIYKHFNLCQLTIDFLGHAVALYLNDDYLKQPAYLTLERIKL
YMQSISAFGKSPFIYPLYGLGGIPEGFSRMCAINGGTFMLNKNVVDFVFDDDNKVCGIKSSDGEIAYCDKVIC
DPSYVMHLKNKIKKIGQVIRCICILSNPIPETNQTNSCQIIIPQNQLNRKSDIYINLVSFQHGVTLKGKYIAI
VSATVETNNPIKEIEKPLELLGTIEEKFVKISDLYVSTSKKPADNIFVTSSYDATSHFETATNDLLQIWENLW
GQKLNFDDLNTNADGEAPDFN*
>N.PFL2065c
MDLSSLPTDDNLDDKKRAAVLLSLQEIVQKQKENVKVMDICFNKCVSKIGPKLSSSEQKCIWDCANSYFYTNV
FLNQRLEQMTKILKSNSDYTNL*
>N.PFL2070w
MEVIYRNITNTYFDYRREIKKKKNRFKLRAYEKLDEDEDTGKENLLNENEDLEMQEQSMLPPYWIEKIEECSE
DINNMKTKLIQLQKLQKNLLNALNNDETLTENITQLSSNITFLIKNCEQKIQSVSSKDYDKDKNNIIEKLKN

NAKSSLLSQLQSLSQTFHKNQKSYIKEFKKMSNAYDDLQQYQNFNEQNELLYQQEEQQHSSVNMNKRNSDLKK
IADTVVDLHTIFKELSVMLVDQGSLLDQIDYNMEASLDKSEKGINKLKIIEKRENDKIAKKCVSYLTTLIFVL
LFLIIIKHLY*
>N.PFL2075c
MSKKRKSYENDEDVYDNKRNKKNVASSIDSYGRKVWDKEYYQKKVDEKVENDEDELILKLLPDLKKKSKLEPP
PPSERKLLEERKENLLLEKNLGKVQILTQKTPKNEQGGYYCKICDCVLKDSQTYLDHINGKNHNRMLGYSMKV
KKVTLDDVKKRLSLLKDKKQNKTEDVEKDPYEDAKKNLKDMQEDEERRIQKRKEKKMLKKLEKEKKKNENENE
NNDEESDNMNMMFKEMGLPTSFV*
>N.PFL2078w
MNATENNSVDLEPFNSILDRKYFPYFFFLFEIYSIISIFCLFDYNNVNRTKQSIKTNLILCFMASINIGLSIY
FLLLYFDVCV*
>N.PFL2080c
MESKHFYGDHMTHEKVRELLDSSNVTYEETPSFFNGNIIYSQKPTKKDNLKYRIEYLKDGTAIYYKRRLGDFK
NIFCCF*
>N.PFL2085w
MFPSFNNPYFNINKKCLQKENRIDMNPNKVEGTEVTNELYLQFQNEINIFINCINGHEKKNKEINYVETSHVM
TGSEDEFIFYLRNIQDNQEKEKNRKEKQLHSNIINYSEYNKNYTTTQHIWNGGDIEKKQKKKKKNYSSNSMKK
YNINKNNENIIFENMLNKNEEILNHKISEIIEFGKKRNDPNIYNRANKNIYTYLKNCNLKGYENGYMNISENY
NDHNNINIYNCEEEDDIWNTFMFTRNDSKDSKMIIANFINRIKDSKQEQNNEDMMLKKYTHKKKKKNSYQSIC
NNYTEHLNFKNINMENEQKISFSNYNNQSNVFKEYEKIVKKNEEILILLYKIIYKLNIFDINKKFNKKIEDVV
NIYIIKVNNIISTYKLLLQYDNIQRRKLLEYYFKKNEIMLLKNILKYIIQLMSEIKHQYSGIKKQIQKKIMEI
QKVAS*
>N.PFL2090c
MRFWFSKKKNSPEYSDNKKKNNDEIYKAILKNREAIDALEKKQVQVEKKIKQLEIEAKQKVQNNQMNSAKILL
KRKKLYEQEIENILNNRLTLEDNMINLENMHLHKIAVSALSYAANTHKKLNNEINPQKVEKIIDTIQENKDMQ
EEINQALSFNLINNVDDDEIDKELNLLKEQSLEEKLSAKVNNIPAIPNENINVPNKERQNVNFNEESDDEELK
ELIGEMT*
>N.PFL2095w
MNLAIQNLGINDPFTNENIVDKGNGKSNATNLIHIRNQQRNGRKSVTTVQGLGKTFDLKKMVRALKKEFNCNG
TIIEDIEHGSIIQLQGDKRNNVKEFLIREGICALEHIRIHGA*
>N.PFL2100w
MWYDNERIVEEEGISRLLVGRTFAYFPKYVDGKNNSISSESKENLDSIDNITYDNKEYVLITQRLGRTIIPLN
PELLAQSTFDQDIIDGYLNEIHKNVHNYSILTEYSFLMNEIPRNFYCLPQIDNLLIWDVFIMLYSTVYKNAKL
KLQIRLSHNYPNTCPEVFFITPIFHPLVNIQTGKLNLGTSLSNWDPSCHYMSLIFLYIKNLFYLQEEYNKETV
ENQEALFLLNNDKDEFIKNVQKYINQGNKKIYDHMENCMFNFNQKEEHIEIKDKLENIKTDQVCARKAEAFVH
WLINEYSMENTNGDGDTTNEDDNNINGDDNNINGDDNNINGDDNNINGDEKNINGYENNINGDENSINGYENN
INGDENSINGYENNINGDENNINGDDNNINGDDNNINGDENSINGYENNINGDDNNINGDENNINGDDNNING
DDNNINVDENNINEDENNKENIKRDDTNEEYNQNDNINVEKYSMDNYINNVSTNE*
>N.PFL2105c
MNSILYKKIKLTDDAVKNFNVIRAFKYKQSITKNISWSCDGELLLASNGNDSITLYSLIKGNNIKTLYSKKSG
VDVVRFLNNTNEVIICSTKSNNTEHKQFLRFWDIKENKYIKSLPQIGNICELNGISINYNKKLMLVNSDDGHV
KLYYFNCDSPLILYKSNFVRPVSCFDNEGHIFIASYGKKEIHFYDLLMYDRGEYNIIDLKNIMTNEEFITNLF
FTPDNKYIIISTNHNNHYKIDSITGTYICTYKYPRSENQQWDMSNTTKDIINQYTYSQTNDKLQQTLNNTNEK
NIYFQNRNDMFFMPTITPDGHYVMCGKNKRRNNRTNKIKNDKMFIYLYIYIFLLLTK**I*KIITSKYTHIYI
YLYI**YIYIYIYIYIYIYIY*NVLIIIKLIISTTFSE*
>N.PFL2110c
MILDKFKNIVNLSNSNTEKEEGKNAEINENNDPNTQLAHEPSIVEADEKTFFDEMLGAPQYTYDEFLQVLKNP
IEERGDTEDMKKHWGYPRRWKVILWDLHIQNYMNNDFNAFVDFDFGGNREECRIQRGSTMKIYAKGKTKNCLR
TPVVTNVATEQKKNMNFRNVFEYRGSYLDLENEKLRIRVWEYKQFTLNKLEGIYEEPLLSFAVGEIYNETTLY
KFIKDSRVKRCRLYFQLYFQELYDFELSFLNWSFSDLLSSSYIQAKSYNYLSNKNNVKKRYIDDTSCNIFPHM
CKNFKRFHKKKIYRKVRNYDIDKTFSFNTALSDDSDERKNKNDRTIKNLEKLFVRNLTLMQNIEENEDMSYKN
LQNINLPNPRVTITLSHTPKGHEGLNIISIEQKSIRFPIWENLGEIYFRGTLRDLDVSYLNIKVEDMSAPKSA
REIGTCQISLKGIVDYPYVMHELEAPSWLVKEAKYEGWENKLNEWKLGTVEGKVIINRVPRYRQRGDIYHIDS
KQPYLIVHIFNIDKIITVDNIKELDTYVEVSFDETSRRTRLMKKTLSPNYDSQISIPLRFNNKNDINYENLSK

KGLIYIDVWGKSEDIVYIGGISISPYEIFFNEKNVRRNKTKLEHIDLETNVKITYDTVVYRGCKKLCFLHDDQ
RMSNIHFSIWTYPDILGNSTNQKKIVAPVSFNTTMNFPIKLAEKYNKLKKLFMEVLKTIKSIPENCTDVNTST
RFYNYELINQRKEKHFLPTLITSIKSPYCAESMNAIFHYVRCIPFIHKKENIIFTPDFTLQLKGGNALDHSLL
LCSLFLGIPVLAFVCFGTLWDKQKHSWVATFEYNDEKNYGIVKFWETTTGNVYILKKRFIDHNRLKGLELKLK
ESKYKSHLRNGFLQRYENNTDINYIKEQTKRHIKQLFKNRINDIPIGGPSLPYKTIDLIFNHKNIYVNLQHSS
PLNIWYDYWKFDFWFPFSSIEYNLQPSFTIKSFTHKMEDMELDKIAKELRTNIEKNINIYRASRNLSTRWNRD
ETLEIFLQVGLELLHQLNTSRKEDVLLAKLKIEDWKKALYHKVPQSHRLLGFPYHFNTYKSKFISDKLISTLA
ILESRDRSLCLSLAVCLYSLPGNFISCYIYIITCVKITQRELRKMEIIKEKAQRMAEIKNSQKKKKSSDDQNI
DIKTMDDVEEKETFSSKNLDDTLHDEHTDIDTEKKKHEKDNYKNKKMKNTKLKDANIDNEEKDDHHHITDKKV
SKSSDLIHSFDKNDISKNTFDKEEFINNLDKDKKYSSFKKGNVVNKKVSIKNEPSINIYEDHKIYDDENISNN
IDNMENNQDEIYNLRQGKTIINEFQQVKKPQKYTINEKRDDIKTKKKRSKEKKKQDKLEFEKLIQSNAYFEQE
KKKLQEDIKKLEKDKEQFQKEKIRREEKEKQLLLEEKIKLQKEKELFENEKLERKMSYMLKINELEKKKNERN
KMEKSYKRMIQKDKEKKKKKESRDKIRRGEEEKMSADENMKEEQKMREEQKVGEEQKVGEEQKLREEQKMREE
QKMREEQKMREEQKVREEQKMREEQKMREEQKMREEQKMREEEKIREEEQMKREKKMRREEKKKRVQEPIKID
EIQVYDIIKNEKMKKKEEKEEKKRKKKKEDIEDKYKIGKEASLDENNNERQIKSKNIQEISDYEYKNIKENKN
LNKFDEKLDLNQIYSKASNFYDNQEKKRRTIKVHANRKENSDSTFSHIDDSIKNKKEYLEDSNIARYLYEKRK
HDLIGKGNYNFDEPHYGKKMNNVAYQDIKGSNLFKGFIEEPSSKKSPQKKKIVIVRKN*
>N.PFL2115c
MFIIIIFFEMFIRFNKFLKLKVSFYSLLLLLLFIIYIFFFCSPCYAYNFSKVKQTFLWPYSPPKIKRGNHLKS
SQISEKCPCNNVKKNYDVIVIGGGHSGCEASYISAKSNAMTLLVTQNKETIGEMSCNPSIGGIGKGILVKEID
ALGGLMGKVIDKSGIHFKILNMKKGLAVRGHRAQADRDLYNYYMKEYLYNTPNLDILENSVQSLLIDTYEDKN
NNNDNINDYNNNVKKKKIYGIKNKCSCEIYSHNVILTTGTFLGGICHIGKDKYIGGRIKRLINNFDLKMKKGN
IESIKTNDEPIIKQDKLKKINQNGESTINNMHNNYNEDNKKYINYEENLNIPFEKLIESSTKNIAQQLRENNF
EIKRMKTGTPPRLDINSINFHVLEREDTEKDYPFYFSFLNMNKINNNKTLPCYKTYTNLKTHELVRNNLAQLP
DYDSFDKFGNGPRYCPSIAKKVMKFSEKKKHIIWIEPEGFNNKLIYPNGLSSAYPIHIQKDIINSIKGLENAQ
IVFPAYDVEYYYVNPKCLNYTLETKNVKGLFLAGQICGTTGYEEAACQGIVAGINAALNSKKDNNKYNNNNNN
ENNNNHNINNDEVNKFILKRNESYIGVLIHDLINKGITEPYRMFTSRAEYRLFLRPDNCDIRLTPKANQLGIV
SKERIYMLRQKYSSVNKFIYVFKKILINEDNPNSTYENMNKSNTYINETKEIKKNIKHNVNQELQHIDKLNPN
NLIYSKNDNNHINTIETNIREKQFEASNNYNKQNDELLQTYNHIDNEKNILKKENKLSLNTKLFNSKNNSNIN
YTHHDNKNIKSLYNILKSGVEYPLNDLLHGIKNLHNQEQFKNYLSKHIYSLDIYKDIYTRDPSFFNSDDVLIN
IATLETACAEIKYSSYLKKQFQEIRKINDNFDLVIPRDLKYDRNNFPYLSNEEIEKLNKFKPQTLFEANKIEG
VTMSAVNYLYYHIKYERRKENKS*
>N.PFL2120w
MKKEGVDHNKLPNKEVNNFRLMTQLIELKKHKKAYKICEQILKKYPKNGETLSVKGYLLNLMDDKNKEDAFRL
IKEGIKNNLSSSFCWYLYGCLYKIYKNYDEALKCFMKSIKLNRFDFKALKEACIILLYLKRYDQFKDLRLDMY
NDSVKNVKDKAVLVFSYHLTKSYEKCYVIIKQIDNELINDNELSINEKHDLLVYLSEILLEGKKYKECLNFLN
KYQSVLLDTLWYYQMLGLLYLYQDDFKTSNLYFKKAFSMNYENINILLLILYTERDYYMHCDNNTDEKDQNLN
NCKENITNSMSTNTQGKKTLSSLFYLDYKNEHKANEKVILDNNVKILLHELILDIYGSDRNLKLLSYVEKNII
NDYICHNLDMKKYDIYSVSQFNNFVNINLIDQHNIIPSMDIDNVDGFIDKLNENIKKENKNILNNNNNNNAN
LDKSPYDYHQFNNLHFSELLGVDLPYCYKKKYEKHMDKLYYFKIKNLNEEEEECLEKYFNNLQQLYKNSNLLK
IIPLYFFNENKFSLYVEQLIRSLCFQKSLTIFNYFKPLLTFRNINIILFLLHKYIDYYDKQKDICPFIVKTDI
QVKNDIMEENTQNLKTKNNEHMENKEDLNNISDHLQNVKNLDTSTHFEKREDMEQHDEKMYNKNEDKSNGILL
SDHNNNNKDNKKSKKNDKVDEEEDDDENIPSELNDKDLEKLIEKNVEIKDLMGISSPLGNNNIKKKKDNKKNN
DNENKEIKLDLKKSCLTNEEKKEYFIICIYSFIIQLYDYINCTDKALELIEKCIKSIEQKTNKNLIYELIFIK
GLIYKRNGNYLGAYKYFEQCRNVNIGDRYINTKTIKTCLKCGLIKDAKKMASIFTNPLDNNFFKNINETQCFW
LEYNISLSYVNNHPNIHLIHYLKTEANNIYDFKNVSPCDNQHNVKNITTQNGNIPHHNDNNNNNNNNNDDDTN
KIDDMLNETPFNLKKSILLENDINSKNIFQDYSKGLHLLHMGHKQFIDIHEDQICFYYYTMRKMLFKTYRHLL
YMTGHLFSSRFYRRFGKSLIRILIDMYDFKITGKVETNKGNKKKNKTNNNNNNNNVLNQEENIFYEHIQNEPL
DNAMIYMNTFLSQPNIDISVHRLNYEIEKRKGKDYIQLINIITKMKSIFPHHNYHHKLAPVISHYLHTVPIDD
MSDNDKKEITTKLNELFNLNHSEYDVNLLKTIRKQYIEDFINFFEERKKGDLCYYQSALEVYMDCNEKIDSRF
LKKVDMNIEKNKLERCFKFLRFLIKHKEKHLSLSELIDPFKAACRKIYPLATAFE*
>N.PFL2125c
MVKIRKNVENMELYDYMKELNENVWCYFFDDSLAHETIYHSLNELEQIIISRLLFIQQVVSERAMRLWINPNY
LKKLSESIKNLVEAKILVESDMKKDNYNQYKINENFRITLLNKIYKTNTNNIFIFNNNIKEQLEREKNLYEQN

LFPHKEEIFGYAQTKWNNLLHFIASPNFNSVNNYFNSNINNDDPNKYTAYDMARRKRVGKDGGPDMGRKSLDK
MASRRSYNRRRKKKKYSDDEDEFDEEEYEGEDDDNFVDGDDSNDNSGDDDDDDDDDDDDDDDDDNDDDNDDDD
NDDDSGDDNDDDNDDDNDDDYTNDDNNIYFNDKANSHLLVKHQYDNHQLNNSTAYVENPSDDYTYKKEDDNKT
EQRKSVHFNSDIYEKAYNNLYTQQGDSNNINEMEYYMADTKTNNNKKKKKKNKHIYAYKQNELYYKSLLNMEH
SYIEEENYNNNESTFFLSNINERFMTKPSESVIEVLRRKNFLLDDPNNTKTINMSREAFSWFLKDLRSRIISL
VLEYLLIIDNGYVTNIAKDVYTKYQKKKNNAINNMNNNSSSGINNNINFDENIKSNHCEESKKSYDNNNNNNN
NNNNSSSSSSCAYITRTNKDPKKSEIYVKETLLLILSLSQCDISHPIFLENLTKAQKEFIDFGSHIGLFLKPN
DSYIFITPYALLLTINNFNSQYYLSLLSNITIEGLCEEDVRKRLSKRYFHFYLLKQDEKEETYMNKDLSSDIK
NKDEKSLILDKRKNITKKLINDSLYSDIKMNGSNNLEIGLIVQSNFKVYLYTSSLLKINILSHLCELQARTPN
MVVGILTRRSVLNAYNSDITANQIIKFLESYSHPGKNNFKSSIPMNVITQLKLWESERHRLTLEDAIVFKSFE
KDFMPHLYQQIVIWANSKNYLLYYTPWPKNNTKEFDLWIKAEKYLCCIYESKNEIIDKIKEIREKLMKKRQSG
*
>N.PFL2130w
MKITILPFFVKISLFLFFFVHKNFIYSYKINGGFLKYENYNKGGMLLRSSLEEEKNVYTSDIMLRKNDSVEKN
KRKIRYSFSPIKTNTLINIKKKIALNERRSNFVKNKKYNSMRSGVERRKIRGPPKKDIHKLLIDDINKERMKE
KIYSIKNIDNDNNDMKIIDDDNVNILPLPVEKNMEGYNCLILCKGLPFHVDNSQIIEFFKPYKIMEKYIIFMR
DKKGHFFGDILVRFINKEQKYLALKNKNYKFLLHRYIQLYNVNEEHYEEYYNIGYKNPPAYKNYVPIKNIILP
NTIEDTTNYTLHDNELMYNYNGNNNNYHLVDDHIHSFNYNQDDDEETKELREKVNVNGIMLKNLYTGRKLRGR
ITSVHSYGAFLDCNVYIKNQDNRYKKILALLHKNKLTLNVGLSSDSSSEKENKELILQKNMNIIVYVDKIIKR
ELPPEKEKINNESINNKMINRTINENPHNNNNNYNNNNYNNNNYNNKDVNSFNCSKEKDNNFIFFNLTLDSSI
TEEKINWLQNLKFKKDSINQQILLNNQKVNQENKTDMNKIHNNNNNNNNDIVHCESNNDNVLDPSYKNEIGNN
QIANIDNHNVQNYYQEDNQKKQNKQKFNSIIDKPNTNSNDNIFINNCNIIRKNRVTKVFLMNNKMCNNHYINE
KKNYFYNNTFKEKCSFGKNCNMIFQNKNIETENTNKKQDENIGGEVKKKKKEKKKKKKKLEHNKLNDNNIETV
DEFDDLFDVLINDQDGNNIEKGETIIDTDVVGNINGNINNNNDNNDNNNNNDDDNNNNDDDYYRDEMKCILKN
IYSHKETNNNKKKNSFSGEKEIKSMKNYDSNKIQKYIQTSNNNDNNDVNNNKSKPYDDEKGNVSKLEKCKSFE
RLSDREKWKSDIRREESLYYTKLFKCNEDINDYYSTNNKKEIENDNLSYDDNNEKDEMLDNNMMFNAKLNKCN
NELYYKINDNINNINYNNNNNNSFDFSSFSDMSIEQLKEEIFKRKYLLPIDVTHENLKSRLIQICICENNKLK
FDNFPVIRYYLYDVHFSIEEIKMIILTNKNFLNKSNINKCFLDSLNINELKYLLHKSMENFRLWEPDNNIKRK
ILNMNKDLLLKQNLNNTDHIDERNLLILWNDFKDFMLNFIYTIDESSDPLENIKNMNMDTIQSPRLKNDKTYN
NSNNNNNNNNNNSSCFYNNSYHNNYIYRMKKLEKENFFLKHNQPLINQIENQLNTKNHENKDAFENAWIKLNE
INKENEQDLPHRIDVKKAMAILNDRSLLKKMKKSIDDVSDEYGNRDYINKVIRYILKHKNYFNEELNEEKLKS
MSYDQLLILLDKLPLELIEELL*
>N.PFL2135c
MGKCDFFIFLPIFFFVLIWYVHYVLGQPSDVSNVRDLIKRFENSNESVNSNNNIFNRNNQLERNIHYDPNNLY
NIRQNPRHENIAGRSYVSRNPNEFSSDIMINRNNVHDEQRVRDEGRENNRRSSVPNNAHTSAKYYGSDQEIMQ
NKPVNNLHLGKSLDDFYNNLGVSSDDFEDDEQVNYNENDAFSSFSHNNQNDDTTLLYDEFEYVDSNNYTRNGS
SSSSNNNNNNNNNIGNNGKITYNENDVSYNDSNNGNSTLSNIHRDNKIKPNLSSLYGNKTFNNYSNSTFNDVS
NNYNRDDEITPFNNNRNVYVPSEYSNSLDAYVHPNNQQFSTQQYGNNMEEMNKLRRKVNFAPRDIYTVNNSLD
GHSINNNTPLNNMDAGPSYMTEGNKNVNKNININTNTNTNTNTNTNTNTNTNTNTNKSKKRSDSNFFSKNKKN
NSASTNELSRYKDVSKKIFKDNMYNRYENKNYPQSVYVFNEYQNNFFFNKILKRSSSNSIKQIFQKNTQHKNT
NNNVSVTSELQVATQHCISTNKNKLTKPVILKYIALNNKKYLNKYEYAMSYIHFTCNNKNNKKSNMCLDIKPM
LYTENDKNISDMINRLPNVYILNTIEYILTDPKTSSSYKSLIKNNIKNHHLTVTDIVLILSNNYFKNINSILV
NHYISFLNTKNETYLLKYYLSALISFVPFIKPAMKIYYDQRYINLSLYTLNLEIAKVFNGLLNVILKWSQAFK
NENNHITNQIVIQMYKNLSSFSHNRIPNLGHMFKSLEVILRYQRIYNDIKKGKEDEYEVMIGYFTKYVRNVYA
KTITS*
>N.PFL2140c
MNAAAVELINRLKKEDESNNKCFDCGISNPDWVSVNHGIFLCINCSGVHRSLGVHISIVRSIKMDIFTDEQLK
YIDKGGNKKCQTYLENYGISDFIPERKYRTKAADHYRKILRSIVHNVDPPAPLPLDEGKSLINYGRNENVNEN
NKNQYSNEEPDFISSLNTSEIIENVSATFSNLINKASSMTTNTINNLNTNDLIESTKGTIINSGSWFTEKTKK
IAENVSDNPWWAKGQSKIKDVTQNASGWISTISSTVSRTNSNFFFSNNDTNINNGNNEDINNNNTNVNSNGNT
TNNNNEQAQNNNNVNFNYNSSNANNEYLNYGMNENNSTFN*
>N.PFL2145w
MPKQRGFRPQGLIAPIQRIKYTGPTILDKINRESRPTWDDLKDRIKNQEGCKMLEEYENAKYVEELNKNREEK
IKEQEKRYIKKLKKEYKKKKKKNSDDSNTTDESSQDEDSKKKKKNKNKNKNKKYSDDTSSDETSDYDKSKKRK

KKKKKRKYKHSSSSDESMSSSDNSSSKKNNKKKKRKKNSSSSMSSSSDSNEKKRHKKQKKSDNVNPFKLSSFF
KMK*
>N.PFL2150c
MPPKKESNKKLEKEKQKIVEDKTFGLKNKNKSKSVQRYIKGVQQQVFQTKRKPEEKRKEEKEKEKLNQQKLLL
NSIYQKTEKVKKINETTSTTYDPKKSKEAQKIDIYTDIRDSKNDKENDTIDKWDIHKLNEVINIRHKNVNKTD
IICKFFLNAVENKQYGWFWVCPNGGDNCKYKHCLPQGYVLKKQEPTEAEKDETPLEDIIEEERMKYINNGTPV
TLELFKKWKEENADKYKLKKDDQKEKVEKKAKTNVLSGKELFTYDPTLFVDDDNAANTNEYDDLFYDDEDDKE
DTPEKKNCVNGEINENPNVFFFFYLEKREDEVPINSELFIDELVII*ISTK*KIKHYKNINYIYIYIYIIIFI
YI*CIYFFFFFFFFFXXXXXXXXXXXXXXXXXXXXXXXXXXXXXXXXXXXXXXXXXXXXXXXXXXXXXXXXXX
XXXXXXXXXXXXXXXXXXXXXXXXXXXXXXXXXXXXXXXXXXFFLFHFNIFLIKKCIKQKKKKNIYIYIYYIC
DYLSI*
>N.PFL2155w
MRHNSLILCNTITLFSFKNDFLRKYFNLIFYSRDYWSCRNIIKISQKKKKDIKYNNRTYCNIYNNVRKDECGF
IKAEDKTYIKNNDKINLYLGNKNINRKNLLTYIKNDKKLLLYTLCKIYEKKQFNKIPNDILNELYHDICEHVL
EKKQEKLNRRETLIFLSCINDKNYAERVHMVKSNNNNDNKNNDNYFTNKSYNKYNNIFDEKNMFLYFCTILKN
RKYNLEDKDIVNDILLFFDKKMNIMRIKNISLYLCNICYLNSKVIIKTNMLYYFVLYILKYIKYKIKKRMDFN
NIYHYVTLLTSCMHISKLKMEINQDRESYINDLNVIKMINKNEYSFDSNDFSEMCKKNIKDKKYVNDTMYSCL
IYNDNHFFHYFNIYENFEYKEESHKPIPLQIPYKKRKKKENKTKQNDDDNNNNNNNNIQTLHQINHELIYVIY
NMNYYLVNIFLKKKKKTLEQNILKKNMTIPIIIHYMFRCLNFQIFSNTLYLELQKLILSICDMNKESFGLLLL
LLAPSNIIEFLHLMSINNNLIKINEKIETCILKQFLDHLFIDNCIHLNKKDKIILYISISKILFFSKKMYMEK
MHHILTNEFINFTYRDLYCIVYALNCSKFCDLPFIESLLRNIYKFKHKYSQNKLLTIISIFYSFNLNMSIFNL
LITYTNKKDINHIRKDEEDTSEDYHINTGAKKLEDDLENKERILKELSSHIKEEASDNFLFNENDILEFNSDE
NRLTDDTSEEKFLMDEDEIKKYIKRKHKKRKS*
>N.PFL2160c
MDVKEYTHNIIDIDISNHIKIRSSNNYNNKKLKETIRQNAKKIRDITFKYWIKEKQLQSGNFVINDENINDKM
IIENKKDIINNINNDKKISYSYVDSFQTCVTNSKDEEGVLYIDDLYTYLIRQLYILFCSLEVRKKKNNVQFDF
TRIYKYTYNYKKLESHISYEYLKKYDINDDIIKNDIENFHFYAYDHNIEDPKKYCDMEENYNNANYNICIYLP
TKKEIDILFIIDQLYDYFYFVLYVPITTKEKDLIFFPFNIKNNLLVQYHFNIFIPYDYIYYTTYKKMNNFILN
YSKIVKNYELSNSLYKHNDTIILIPLIAYNKLGYRIGSGKGYYDKTLTKTRKKNDIIKQIKNIQMNDNIITNN
KDHDIQKENHLYLGTKNDTNNIICSNVKNIKHKTFNNEIEIYTQNKQKIYFNEIKIGVSFEMFLYDIDFSETT
DLILDYMINENNIYHFIL*
>N.PFL2165w
MFKKTMQQKRQSFIKNKEVYPQKSVNLGNKIKVRSKTMNSKIKVVVRKRPLSELEKKKKDSDIITVKNNCTLY
IDEPRYKVDMTKYIERHEFIVDKVFDDTVDNFTVYENTIKPLIIDLYENGCVCSCFAYGQTGSGKTYTMLGSQ
PYGQSDTPGIFQYAAGDIFTFLNIYDKDNTKGIFISFYEIYCGKLYDLLQKRKMVAALENGKKEVVVKDLKIL
RVLTKEELILKMIDGVLLRKIGVNSQNDESSRSHAILNIDLKDINKNTSLGKIAFIDLAGSERGADTVSQNKQ
TQTDGANINRSLLALKECIRAMDSDKNHIPFRDSELTKVLRDIFVGKSKSIMIANISPTISCCEQTLNTLRYS
SRVKNFKNKSTCINEEDDTNTERISILDSKGSEMNASSIENVVIKSNHLLSNNNNNKINRGKINDKIERNNIL
KNKSFDKPREGFTSTFGKYSSLNDIDKIKKNKKKGLINYKSTLYNDNTINKKHNNNNNNNDNNDNNNDNNNNN
NNDSSSMVNNMINHMINNNINNNINVNNNNNNNNNNNNSHNNHLPQPNYAFTDTSDFSSLDDMNCHLNNNDKS
IFLHKKNLRDNIKLKNRSSCDNIMNKKKNNLHLARHSVGSKLTMFSYDPQKNKDNTFFKSNINKMEDNTPKDI
LYESRNVSNMNGNVLLGLNKNTHHDISTKDENHNDNKINNGVINIINNSNVNSINNSNMNSINNSNMNSNSIY
KSNYNSNQSISDVQIRYVNEMDTSNKNNDNIFFDAISCDNNMYPNITNNNNNNNNNNNNNNNNIDVENYNNRD
GTNNSMKLYAYNSHNLFQPDNNKNTSNIQNINTNKNNQDGNVNYSMNFCHYNLNDKNYLIDLNNKEQKDKNIH
GCDNNIIQNRNDFEKKKKTNFCNNNNIVIVNNNMGNNNSPRMKYGLCGSHTSIDNMKNNEMKNNEMKDNEMKD
NHIKSNNNNSSSSSSSNNNIYNNINDDDTFQNDYCHNDNTFTIRRKNNTNINSNIYQNDDIIYTINSLNDYMS
NTLLHFKEKYTYPTLSTNEDIYNKEMEGKHIRLDDQDKYDDNDNNNVDNNNKNNVDNNVDNNNVDNNVDNNDK
NNVDNNNVDNDDDDVDFHNIKNFNNNEYLSYFQKNVDTIINNCLNSLDISSMYDDTKEILNNILLSKYKAEKD
NVIKKYINEDIKNMSLEEIDKTAQSIYEKRKVLLTKLLLLFKKNVDTQINNETSDLRKDLVMCHICNNNPDDQ
FHFYAYSRLEKDIINLIMLRQIWCESENLRLLYQFLVVEYQNKSANSVLLNVSSNNGDIILLNKKLVQDNIKN
SMDHNNIHKK*
>N.PFL2170c
MRIIKNVRYITNIKNVRYITNIKNVRYITNIKNNIQMVENAAIKYNKKGENIFHIWKYEPSMCKTYLSFDNRF
NISEQRKILYNNRISKYMNLKRNNTYKDIKYLFLYIEFDNKSVKHFFEDSLPQKSIALQILTKLNYLLILKRL

```
LNNIIPNDRTINRCINYILNNDCNANNYNQNFDDNFIIYSCIEHHTSTNKANIRANVGVHINEHININTNMCT
NTKRYNVKDVNNIKQNTYQIFNYEEKNDILINNYSNNGSIYKNVVNNTYDISNIHSPSNSYDINKESSSLCEK
NILYHMVNKYDIVIYSNINNKNIKHIYIKDIYRHISDQINYLYMFDNKLYDKNVILKLYTFYEYFNDELEEKK
KEFLFFLDRISERLLNNMNFIDLVFIFHIHIKRKYYNFLFLYILINKLHFYINNNIVNIQCNKKKLKDISKEK
LQEEYKIINKLKYHNILIDRQKNNIKYNIKWLTLFFHSLIQYFVYLNNSSHILINHKDKIIFIKHLLYNNFAQ
MFLKYLCDNYVYFTPINILSIYSSFINLNIINDETLKCVLFKYINYSNILLLKYKGDIYDYKDKEEHEHENKG
KDIDINIDKYIDKYIDKYIDKNVDKYIDKNVDKYIDKYVDKYIDKYIDKNVHKHIYNKCHKKERKNIFIYNNN
EVQTFYSSFLNIFSKFFVHFKFTNEEYTYILKSLLILFDNYLILILQLYIKNESYVLSDLCIDDKKGNENLIK
SAKDDNIDKLFYINEQAKKNPHHNNNRNNDTFCDDLKYITFINLLKIIMNSNNYTNFICSYNYNIKNINIDKN
IFCFNYDNIYNVENIEHNIKYIVNSLNSLYKITEHFINTYKINELSKLFNKTYIYNFITKFIYNKYINYNNIP
YYKKLYTYIHLSLHNYEEMYHNFYCSINRLFISNYILNLYLTNLVSVYLNKLIRNIKEEERYYLDKKEKEILQ
NEQHMNFIYLEDTKNEKIKNDKKKIEKIKNEKEKNEKENNEKENNEKEKNEKTKNISFLKNEKTKNISLLKNE
KTKNISFLKNEKTKNISFLKNKKENHIYDTFFFSQLVTNSLGAIQHMKILRYDIFLQISYILKKNIFPLSLVH
IGNIIHSYASFKIQDLVLIKYSLNKLIKTNNLIHIINDQSLGNISISLLKLNYYNEQFTRIILNNYNKIISLQ
ALINITFYLCYYNNELLECFLKNHLNYFLEKICKSNMLHNINIKAKTQLKLICYQILYIHEYTLKGNIQSKEF
SCLFNYLDNGNILHTTSYNSYIYTLKRVCNYLLIFDDKKKKQAQNNHFLKNKYILVPYQKYIKIMQSNLLYYK
YLKYHEKENKNKTNTFSSTYNMYINKNIKYDNTNQDQNNGINLFDIFNYKIMNSKINMDINGYKPKNVKQEEI
DMSYKEDDDKKNNIKKYNIKKYNIDNNQYVNQVNIINHSSYLHLLYISAFPFEVEQIYTHSELYNQVYDTIVS
LNLKRKIIKELLHYPYYVDIVIL*
>N.PFL2175w
MEERVNKIKPYELILQKELMDLDEIEGVELSPVDEKNLKEIYLSIKPTDGYLKDKKFRFVIKFKESYPITPPK
IICLSKIFHPNIDESGNVCLNVLKLEWNPIINLQMLILGLLLLLDEPSTDDPFNKIAAEVFKNDIYKFQEIND
ALYKEEQQNNK*
>N.PFL2180w
MIFSRYPLICGNKKKIEDGFFYFIKNEKKGFYASKWIRRLQLLNEKYNYKIDKEEEEIEYEEKKEDDNIKKVY
PLWNSRRSGLLGYKVGCMSIWDVWGVKHAVTVVKIDNCYVINQKTISKNGYEALELGVGNMNVIKQSKNNIGN
FIKRKLGFIHKIKEFKCTSDCFLPIHHKFSCRHFSPGQNLFISSGIKNKGFCGAMKKWNFSGKNKSHGTESKA
HRSLGSVSMGKTINIVFRNKKMNTHIGEKSLVKCSNNKLFRINSLKNLLFIKGTIGGYKNKVIKISDAKGRSF
NKFKNKIFLYYPTFIYKKNKKYKNVIDMPHSVEDPFLYNEIPLYDPPNK*
>N.PFL2185w
MKDIKSKNPEFLSTNKNNKKQKDKKTIEENKDFNNELSKYKQSFYIIKKTINIIKNHYYNKINNIDTLRIREK
KKLCYLKRKNVILEDKCTFYCEHVEEIKKIYTEQINMKNEKIKSLQDDLDIANIKSEVEIPKNTENILKLREE
QIERLSVQIDSLNELYHKXXXXXXXXXXXXXXXXXXXXXXXXXXXXXXXXXXXXXXXXXXXNKIKYFKRMFFKNV
YIYIYIYIYLFIYLFIFRFSI*IIKFKKKGIAENR*KKNLG**QIWPYKNK*INK*NKRNNK*KKELTYTKRI
NK*IYIYIYIYIYIYILCQILFFSVFFFFFFL*FYKRYNRNKKKFKLNFDVLNINKDDIKEMKDDEKEKEDIC
ISILCLLKTELEIIDDENKKKREEDDEEKEKLKVKSNNFFKKLFGSNYKHKKLGLLENISKKQFFSFYEKAFT
ENNKIKFDKKQFFFFPSNIKKKKMNKKYHHIHNELKYNIHMKEIQKKEKNNNDNKNSKWNYLTMNNIYFYNNF
NDNFDEEPDHKFCDNNKNYEGRNSIDDYVFFKKYNNINSDHIVFTNMIENNTKMREDGSNNINEEEKKK*
>N.PFL2190c
MVNKNVKGYNPNQNRGTSKGRNISKNNKIIKNKEGGKNLYTWYIDNKIDEKKKKNEDIENNKAKYFEDVAINK
NVEEYINVICRIKNNKIDINNKIDTNNKLDTNNKLDTNNNINNNNVDVIKKISYNKLVIDTFHVGNKSSLNFE
YTYDRVYDIENNNHMIFNDYIKNNIKNIFQGINCSILAYGQTNSGKTYTMLGNFDFLNNLFHLCKDYNTCHNN
ENKINKVHDKINHKDNNDNNKDVIHKDDNNKDATHNNNNIIKTNINMNNSDQHNDIHLTYDELTNCITNEPNN
VGIIPHCINYIFNYINYHKQQDKINDKIKEFTVTLSILEIYNEVIYDLISGESNLSVHMIDSNKNEFIIKNLK
EVEIENIISALYYLEQGVNNRKIAFTHMNKASSRSHLIFIIKINRYIYSTNTIRCGKLCLVDLAGSERLKQTK
ATGSIKIETTMINKSLTVLSKVINSLAIMQIKEKIEKNIKEKEKEKNKDNDKDDDKGNDKGNDKGNDKGNDKD
NDKGNDKGNDKDNDKGNHNINDNINDNINDNINDNLNDNLKDNLKDNLNDKDKYSCDNNININNNNSHINNSS
HNPHSNTCNIHIPYRDSKLTRVLSDSLGNNCKSILICTLSSKLQYLNETASTIKFAQRAKMVKAKPIVREEKV
ENKKEADTNEKEKNMKKKKKKRNEEKNDNNGDDYKEDNYDNYDDDDDDEKNNLYDNKLNIFLNFNKNHITKDI
IFFCFSLCIFSYICGFKSTGKVKYLDFFIESYKNKIDIIKEQLNKSNESNKNDINEYLMKTFDDLKVLTEEER
QRNMKLNMEKLKKCKDDNKNTINQIQKKQENEEHALTIRNLAKVDPTLISKYDIQKKYQHNNNDVYDEGVHYL
NVDDNNNDDVHADNNDETHIHDNDNDNDNDDDVLIGDILVKMKNDICNIIKFNLTSLKCNRKENDEIFEEILH
IVNSVKENNDIFLINELKNNSKDEKINKKKDAQGDKINIPDDNNNNNNNNKISFKSIKNMNIVKKYLKKDDVN
KNKNTKSILNINHKDDNKCYQDNNDNDNYITGDDKNMNNNIKNKNDILHYQVFAQKILSVTNIQNIFNYVNHN
```

YTKFISDFTTDKSTVFSNFDLKENLDRQIIEKNNTIYNFIHSATMFQHSQTLKNLNSNYFINKIQTEIYNNYP
HIIHMKYMPSINNNEPIITERQNDLIVTTPLKNILQINKEYNNTYNTHKYIPIKDIIPDHTTNQNETTCAINK
SVTFFNEHGGNKHSEEDLSNKNNIPIEENKKEINNKSITFLRQSLEGTKNKQDGSFSNIAPTNKNNKNYDDDN
NKNNKYDDDNNNNNNNNNINNDDDNICVGEKKHIRNHAHKDQSYEVQNKKDKNSKKKGSFVHNINVSDKSDNS
LEFTKLLEDKDFKKLYEYLIEKENTNDVNTFIQKEEHMNNALNELECKINSEIRRVSDHIVVDYSKNNHKNKH
NNMNHNENSSRKNSNINKNDIYNNNDNMIMVVKKKKEKNYNNLDISYLEKKRNTNLFIKKIFHIIKNIFQKKD
FKEPIEINVSDIIENELLHNKKLLEKNLDSYNIRNFFINNKKIYLLNESEYSHLRELINYKNKMLSYLNGEYK
KYKHTSQYNT*
>N.PFL2195w
MLLLKKVNSKVLSINNYLQKYLESNQFEKNLKEALNNKNYGVSNSLLYDLSISTYDVNYYKRVMTEVFKAIQE
KPTRWRRIYKGLKLCEYVMKNGCEYFISDVKDKEELIKKLTHFTHLEDLKDKGIGIRDISNNILKLLKDNKYL
KNERIEAAKYANICTNIESKPIEKKGFFSNRKKKKHKIKRTSEYSRRNINDPRNLRQSLQSKNILDQIQEERK
SIYGYTNDDDNNRFNEKRYDTVENNHNNISSYSSNVSSSSSSSSTSSSSSSSTSSSSSSSSSATSSSSLSSHR
HSSNSSHSNYKTNNNHKKISKRKKRIKDKKHSTSSSEKSSRRSNRSISNSQDEYNNNSSKLSTQSKGHSASRT
SSCVSSRTSYKHSSRLSSRSSTSSSDTNSYSSRSRGRSHMRNKHGRSKKNKREKETKKRRSTTRHK*
>N.PFL2200w
MAELLTIKNLFICSHEKSVEELIKVMDGLINKVEENNENDGDKNILCKENMNEKKKYDMIKDYMDANKNNVLH
FAVYGNNFNNVKFIVENTNLINTFNNDGQNGLIISILNKNNDISKFLLDNHINYNQVDKYNSSALLYSVITQN
YDIFNLLIEKNDIDININSYEKGNLISICIFERNIKLLKKLFNKNICPELKEKNVYPHPLIFILYSNDNHLLY
LYLTYSLYYYSKSKELFDINKTNFDYTTDDILINLQNKQSINSILKSQNMDIINFHSLLNIKDENNSSLLDIC
TQMQNEEGKNILSYYSTEQ*
>N.PFL2205w
MYIKTSILKICLYLLLVNTLILLTKCSLRILPLNDVIDGIIIHLNYKFIKYKVYAKGIHLNVNFENLGSVRHI
IESYKDLNTLYCNLNKRKEEESIDKNTLRTNNCVIFVNIKYKENLKHIYDFMEYLYINTSAVALIFISDNFLY
ENEIYNPHEDLNITIIHPSILTYFISYDRLSEYDTNNYDKYIFELYWGVNHKTDIVHINYHLDYGKYFNYTFF
IYMKNFLLDLKNHITYEIKFSIHKNFTIEPRFCFIRDSSYCISKPDYMNSNVVREVVEQQVRSLCIYELTLID
DIKSDIVQTDPSDVLQSDPQGKKKLFSEKYIYYINALFNFGFERKYCSSDFNDLTKKCSDKILNILNVSVKDV
DHCFLNNFHTYMKNMIKSKFYVYSITINDKTYKIKLNKDISIKLICSAFKNMPKRCKDYLFNELIDADYVREN
TYEELICFYFLIILLLVHIIGTFLNYLVPYYINLYEKKKNKERLL*
>N.PFL2210w
MRKKRTLKVSINEIKKYCPFVKNIQFLYNTNEKKNNLVLSVMSDLCPVGKAINEKHFIIIDNKSKINIIKILK
QANMQSKVLVQCIKNKNIEKENMSNDDLLKSGKRNNNVLFYDILEKNKNDHSFQINDNTIQKNNIIYKYINSL
DEYKLFKNNCNNNLKDLLNKLYTDKRYRIFTILNKYRINYPNVYIENNKLMLPSFYEFYQKYGYKPCIGNIRY
QLSASFEDNNKNICSFSHKNKENYLFNFWNLHIDNVSNEKTVVWCSNDYLCLSNNEKIIEVGIETLKKIGNSS
GGTRNISGSLLNHTHLEYIIAKWYNKESSLLFTSGYIANVGALETLGKLLNLIYISDEMNHASIINGIRESRC
EKFIFKHNDMNDLERILYNLRINKQYENRKIMIVFESIYSMSGHISNIEYIVQLAKKYNALTYVDEVHAVGLY
GNKGSGYLEELHLCNHIDIINGTLSKAIGSLGGFICANKYYIDVIRSYSSHFIFTTSLTPVNINTSAEAIHII
QNDMSLRKKLTQVVNKTKQKLQERGIQVLHNNSHIVVLMINSAEKCKQICDDLLKEYNIYIQPINYPTVPMGM
ERIRITPSPFHTDEQIFKLVNSLYTLFKKYQVNMFDKKNKHTLMKL*
>N.PFL2215w
MGEEDVQALVVDNGSGNVKAGVAGDDAPRSVFPSIVGRPKNPGIMVGMEEKDAFVGDEAQTKRGILTLKYPIE
HGIVTNWDDMEKIWHHTFYNELRAAPEEHPVLLTEAPLNPKGNRERMTQIMFESFNVPAMYVAIQAVLSLYSS
GRTTGIVLDSGDGVSHTVPIYEGYALPHAIMRLDLAGRDLTEYLMKILHERGYGFSTSAEKEIVRDIKEKLCY
IALNFDEEMKTSEQSSDIEKSYELPDGNIITVGNERFRCPEALFQPSFLGKEAAGIHTTTFNSIKKCDVDIRK
DLYGNIVLSGGTTMYEGIGERLTRDITTLAPSTMKIKVVAPPERKYSVWIGGSILSSLSTFQQMWITKEEYDE
SGPSIVHRKCF*
>N.PFL2220w
MAICKGKYKNAGSTSDQLLFQNCCESTEYLNLYFKDLKDYLIRYNKFEELYNYCYNVLTNVQNNSPLSRITSL
KVANLGVIINEKNVSYLVNKDILKYAYKILKRNKEGKNKKFATYFGDITNIERNILSRECEYLFRNLSHINIV
KNTKEEKYINNLKNKIEKNKISIPQKANDSLSYVLILSDFNKCKREILDCLDNEFVNHNKLDSVYSSYVMLLK
ELKEKTVKFLQNGDYHLYCNFHDEIQEEHEINIRYESYLESFTSNKSEKKELKPINKQNTLKSEGEKKNKLMN
VNSNVSSTCTENNNVDVFFPKCHNNNEISLKNNNNSNNSENKSLSKNLSSTFTTFAMSLNKKIFNDKSDQNEK
HDNEKDNLDMLKINEQGKADDIDKTKKEKTEENEIENIDNEKKKDEQIENIKKQSSNKTVAFKMDKDDPNKNQ
PNDKTKNKDNGNKEGESFNKIQNEIINEFNSFLNEENDVMYFDKINGHKYLEFKNNVSDGNSALWENLIKKSN

FYQIKEDFENYNKIMKQQEENEHDKGNEEKTLGDLLGMNKYESIKNKNIKDLKSTTLNTDDFCHIIKNFNINN
MNGHVNLENYPLNINNKDNHFVADNLVQPTNTLNVFPDTNIKLDNKQEENGALNPLNKYFTMHDNIYYIQKAI
LKNNSVLYKDDNIEISLDQYYYDLNGLVKIFLKNKKIVNCYDVDIQISNKMLFPLKMKFLNFEKTLSMNSTNC
YEIAVKCLHIYKGFPLIKVSYRMQDMFKKTIELRLPIPITKYMKKMEISKDVFNKFWSNNNFNEYKKEKIINK
SDIVDNELIVMDSCLGGALGLCYVENMIYLCGCYTNSSAGHENYFVLVGIEMIKKKLRIVCKSNNPTLSSAIL
FLIVLMHKKHET*
>N.PFL2225w
MKQECNVCYFNLPDPESTLGPYDNELNYFTWGPGFEYEPEPQRKPLSIEESFENSEESEESVADIEQLEEKVD
ESDVRIYFNEKSSGGKISIDNASYNARKLGLAPSSIDEKKIKELYGDNLTYEQYLEYLSICVHDKDNVEELIK
MFAHFDNNCTGYLTKSQMKNILTTWGDALTDQEAIDALNAFSSEDNIDYKLFCEDILQ*
>N.PFL2230c
MLKVHIIKTWKGFIKRQPFSSVLYEQKRKEYVSPPFEKNKDKFKKNTKFKEELYKYHYEEYIDCVNSRPILYL
KDEEIYEHEHEDNVETSHNNSNNNKKKKKKNTHDKRLKRNIPFIYSNEIQNLEELKKNPFVLVNVKNMKNESF
GVATFNPYSLISARIISNNTLFSININFFIEKIKNSLQWRYKLMTNHENQENDIHSSSHNVNNENIKEFINFD
EQQNKINNSTENHIHTYTYHTLNPKLCYRLINAEGDNIPGLIIDRYDDFFSIQHITLGCEMLAYNINHAILEL
LNPKAIIFRNDIKERLNEKLEIYKKVLFGKVPEQVRLIENKCFFLIDLINSPNTGWFFNRKELRKLVCNYANQ
KQVLDLFSYVGCFGIQCSKIGNAKYVICVEKNSYFVELAIKSANLNNLKYNVKMNSSQNDTNNQNISNREYNS
SIDNTNCNISFLCQDALEFLKNCKQLFDIIILDPPNLIPKSKFLESGTKRYIDLIRLAQNCVAPNGLILITFT
TKLCSYQDYINIINTSFVETNKNVKIVGQGRASPDHPINLSLYVYADFYWFLIQLDY*
>N.PFL2235w
MEEYLRNKYMKKNIKKGKEKKKGNIKIYDCEEEEEDINENDIYKHKKKKNMDQYDQIYYREEFDNYVESDENV
SDVPITITDTNTMNVIEISNKNKKKILDSLQSGHIIQTSYDKKKKKKKSKKRESDSDMSICRNKHNDKSDSDM
SICRNTKNNKNEKKTHSGSSSYTISTSRESNNSDVKLTHKPSNKHEFILRKGKYNSDEEEDKKSSYSKDSESK
KNQKTIYRDKSGKIISREKWIEEQKKEMKYKHGKYKDKKKKQEEEKKRKLEWDGGLVQKDIRQKNIEENEKLI
KKKNIINYEFDSDYDIELKMKKRKDDPMNIYLEAKEENQENKKLTCRYQSPYNRFNIQAGYRWDGVIRGNGFE
ERRLEAQKMKEQENKLTYINNTADL*
>N.PFL2240w
MNILYKKLFANANITSKRFLLFNNVILKKREGYLLQKRNLIFFKNDKNDIIKNEIEEILKRNGDDICALYEKN
KNDKYNEQKYREQEKFIHKKGKFKRLFLFIGFQSIPIIALMYLFKYIEEVKLNELNFKFDSSEDIINEAIKLI
NGSSLCFCLYFDKNVINTVYIEPLNPENAEINYERYNKEIASSSFSNSEYEIKKKNEKIMNGSGNQMIKKNNE
EIKNMNRSGQINNNENEKNKKVLNDTNVEEKNSTNNNNNNNNNNNNNNNNNNNNNNNNNNNNNNNNNNNNNNI
GGTNSSDSLKNLLYSLNKPLMQEMMCSKGSMEVPLNYMYFCISKNTDIYDYIKNKNKDISLLYSDEKKNVYAT
LTGNASIIENEDIKNIIWTDKWRYLISGDYKDNYILIKFTPSTVLLKTIGLKNEHWKSNIVRRSIINDKISWV
KI*
>N.PFL2245w
MDILNKIIKDIIYKIKIYHVKYGIHLNDFHNLLFVITLLFGLVFLLYLLLILFNIFFKKSKANNVVLLLGPCD
SGKTTFLFKLKTDKLCRTVPSMKENIAFIFLKNKKKQKCIRFVDFPGHPKLSYSLNKYFNITNVIVYMIDSSD
RQSLKFVAEKLFELFTNKVIVKKKIPFIIVCNKTDLCNSRPKQVIKEDLEREIEILKMSKYNNLDDDCIDETE
CFLGANSEFFRFEKAPCHIELCSASVKNNNVDEIIEFVEKHY*
>N.PFL2250c
MNHINFFKYFSERNREKNNIPSYTSDSTQQQKKKKDYKKYFNFLITKKNKRETHKSTNGEEDYEHILYKNKTN
VNIKEDNLKKDFDEYKEKKVNINGNVNNKKYSDKTTHINSVMTLEKNMILNNDNNTLNNNKKKKKKKKKKKK
SYSVEGTKLLKIKEYKKRKFFKIHFFLNRKNKNNDKREDPSKNIHDKRDISLEKNIINYNKYDMQFSFESVAS
NNINNNTHFKKDKKIINLKKYNNAHRDLYKNNYTNVFLCKKSFSDDEYKNKNIINNDSPCKFQKQNYDSKSKQ
GIYKANYRRFRDSCRQKLDLYKIHNRRNDVLFYGKGALKEIKKSMFDENKENAFYKDNMANCALNNMDNKNRM
ENECLYKYDEKEEIGKKRLRNSMSLSYERKKRIRPESFNYLKVIGEGSYGKVMLVKHVQNKKLYAMKILRKEN
ILSRNQLEHTKVERNVLKCVSHPFIVKMYYAFQTKQKLYFILEYCPGGELFFHLSKLREFSEETAKFYSSEII
LALEYLHDLNIIYRDLKPENVLLDELGHIRLTDFGLSKEGITETNLTKSLCGTPEYLAPEIIEQKGHGKAVDW
WSLGIMLYEMLTGELPFNNTNRNVLFESIKYQKLNYPKNLSSKAVDLLTKLFEKNPKKRLGSGGTDAQEIKKH
PFFKNINWNDVLYKKVKPPFKPKLFNQLDLQNFDKQFLNMPLKYSDQHENTNITFADPKNFVVQDFNYNYYEF
SGQQK*
>N.PFL2255w.1
MNEKNNQNSDKFIKNNNSNKEKAKKLFMQALNHESDENFLKATKFYQEAVKLYPNILNTYINDNQETCSEKID
EREEVEQPEENSYLINILSKNFYTIIKFLDFYSMHRFLFLCKSISSLISLENEYKRLCYINLINCKEKCKLYG

NSYKRLLLEYPRLRFDGVYISCVTYIRSLKDIGNIHLDPKDRDRVIYNPCVVTYFRYLLFLNESNKVLIARSE
LNKKDVIEAFRITYRKVQNWDLSLKCDELIKHLIKFHNECENNLVKMIRIGEYNYNPNEKIIEIQYPELLNDP
FKYKNIIKLRLQNYLGGNNNMLKWVSFKIVSKIKMDYSEDTLNIKNKQYKSFYFFNLKFLSHLFITKLSEN*
>N.PFL2255w:mRNA
MNEKNNQNSDKFIKNNNSNKEKAKKLFMQALNHESDENFLKATKFYQEAVKLYPNILNTYINDNQETCSEKID
EREEVEQPEENSYLINILSKNFYTIIKFLDFYSMHRFLFLCKSISSLISLENEYKRLCYINLINCKEKCKLYG
NSYKRFNELLLLEYPRLRFDGVYISCVTYIRSLKDIGNIHLDPKDRDRVIYNPCVVTYFRYLLFLNESNKVLI
ARSELNKKDVIEAFRITYRKVQNWDLSLKCDELIKHLIKFHNECENNLVKMIRIGEYNYNPNEKIIEIQYPEL
LNDPFKYKNIIKLRLQNYLGGNNNMLKWVSFKIVSKIKMDYSEDTLNIKNKQYKSFYFFNLKFLSHLFITKLS
EN*
>N.PFL2260w
MDGKNFHLIKDIKPLMNNICIQCLIIKYIEDPPDHINNLIKYHYHVADISGSIILCIPHIFIEEELEKNNINI
LNDDFEDILDGYDNMSVNMNDLANNKINEIKYNTYNINNKKRNNLKSLKYLFKVGDILNIYGAVTTWSMGKMV
IIPNTTRIRKSGETTIRTSIHRVGFFNMTVNIEPNMSNLITSTTEKKYKIEDNNTNGNCKNNDINKDIDNTNI
NSVMSNFMSINKKKSKYDIMLPNLDDDI*
>N.PFL2265c
MEKEEEKNEQNNEEKNKDINEITLLEIKRKVQIEREASKDESKQKKFRILNYTSKDSVLGNVEKDFLIYFCFI
CGYNCLISEIDLNILQKRKTDGSIIFPITKIVHKIYHKTQSQRILIKRKDDKVEIQYRILCNECKAPIGYVDN
LNEDNLYIYYYNYALLRDQMKCKMFEDI*
>N.PFL2270w
MHEKIKEEKKELNSIPKCKNKTIFHISLICIITILSRICCIINTVIWSKLIKPYKFSNNLLCEEKNGESFLWN
ILKCFSYWDGEYFLRLSLNGTEYNYEQNHAFFPTLPLIIIYIKRILTKYCIYECTNCVIYILIMLVVNNLFFL
IASIGIYVYSLIHFRNENYDIDEFKISSGKEHNCYYSYKINNVKESYRFSFFISILYAFSLGNIHASSFYNES
IFSCFSIWGFNFLQLCISSKKRGEKKNYIYEILCILCFATCSCFRSNGILFLIPVFFFNIQSCVFFQKYLQEV
PNKEKGKCETIITFKHFNNKYKLLTFIFHWMKALIEAIIIILPFVIFQLYAYNLYCTEGNELLKEENKKFYVF
FFNLFKDPNKYINIWNNKKSLLINRPWCNNIFPFIYNYIQYKYWDVQIFKSLFSPNVNILYSAPVYFISFHCI
YTFIKNNKSSYNKLSLLNNHLWGGVIHLFVLSFYILLCAHTEIILRLIISCPMFYLHYGYLLRYFEKWNYLFF
INLLYFFVGPALFGTYIAWT*
>N.PFL2275c
MTTEQEFEKVELTADGGVIKTILKKGDEGEENIPKKGNEVTVHYVGKLESTGKVFDSSFDRNVPFKFHLEQGE
VIKGWDICVSSMRKNEKCLVRIESMYGYGDEGCGESIPGNSVLLFEIELLSFREAKKSIYDYTDEEKVQSAFD
IKEEGNEFFKKNEINEAIVKYKEALDFFIHTEEWDDQILLDKKKNIEISCNLNLATCYNKNKDYPKAIDHASK
VLKIDKNNVKALYKLGVANMYFGFLEEAKENLYKAASLNPNNLDIRNSYELCVNKLKEARKKDKLTFGGMFDK
GPLYEEKKNSAN*
>N.PFL2280w
MLKFRSICTTLIGGKVYNINGKTIREEKLISEGAYSFVYMAKDLNTNRAYTIKKTICQDKEKIDMANKEINIL
KSLPPHKNIVQYYGSTIISENNYKIVIMLMEYCERGNLLNIFQKNKDKIKEIHIVNILKDIITGLCFLHNQEI
PIIHRDIKLENILCDKNGVYKICDFCSHSISNSFFPNDLSKSELSNLKEEIERDTTLIYRPPELIDLYSNFEI
SWKIDIWMVGCILYLLLFKIHPFQNNNFLSIINASFTIPYCTKYSKRIISILLMTLNKNPQKRIDTSTLLFIL
ENYADLKKWFVHIPADIKKRVNQIFEKMNELNMNNKHTDLIEVSHEKLIDVKISSQKYETNLPKSNNNFLKFF
TTSNLSGDIFKKKIEKEETKKEIKQVGNKQVSETEKEPKDKLENNPDGNKLQNDKDSGKLQNDKDSGKLQNDK
DSGKLQNDKDSGKLQNDKVSGKLQNDRDGNKLQNNHIIDYTDNKMDNDKKNKSDEISENITSSGDKQNSVPDQ
SENQSKSHSYNLIKIQSKDTLIIDHVDRDFPSDKTNTDFSFQDTLCEFEKKCMNNSSNYSNDKKVAGDIKKDD
CSVNIQVVNNNNDVCHNDMKDVNECTNLLNLDVFEYENETNDSIKKINDGDFIKQSGCDFIKQNGGDFIKPND
SNCIFADINFNNDINLNNDINLNNDINFNNDINLNNDIDFFDVIQNHDLKKKEDVLNINRNNEHDKNAITCSN
EYYNLSLFYDPWNNNTKDNHFFGDDDYNVNKSSFIFNNDLFDNYELCTNNNTEKSTHHVKNNCMDINNNIFSN
KNYDEHVDKKEKNISYDYNVKSHPIYSSYKNEKRCNLNDIKQNNSSKYIMSDKNDKFSELLDEFQKCTIK*
>N.PFL2282c
MKCSLLLNFLILLLYTFFNSFLSIKFMTNYKDNFSNISNIKKNNYNILKFFYILPEQVRKKYISYFILSCSDK
ENIKRRGKRIKKDNMEYNKYDIKEDVKKNDMCDITNDDDVISNKNHFINKSNSKLYILITSPEAARIYRYIVI
LLIHNLKNTNLKYETCNERKLNYIKKFVKDISILSIGHSCNDILKNFNKSLFYNISNKNSSIHKSPIFKIKK
KKLLQINRDISTHLNIVFTPTKANGNTFKKELRDFFFLKKNEQRNNNIYKKDKQKRNVNINQHEYINEQKNLH
NNILWISSSISTSSFDDLDIHPQNNNHIYDRINLKRINCYDTQQVTYNINDKTNKIHITKYSIICLMSTSTVL

SFYKNFGNNFSYVVCMGKSSYELLKKLNFKNIYFPENSKTKTLLNILITLYHKLNNKYKRKFHIILTRQKDKN
EEIKKVLSQQNIDIQEIPCIKTKYNFANISKLYKLLYSYINQACTQRKRTKMIKYI*
>N.PFL2285c
MIHEYRPHTVLRVIEDNKKFIYKKEHVDQDNNNNKFDNDTKIKEIYKRQNDCHYNDYPKYIEENKYKSDKEYT
NKNKRDIYIYNEKDNFEYIKNKEDLNNIIKKKKGKCDCEILCLKKREQNDYNNFFNSTKYIPTLKANKNELRY
FFHIIKNMMKKNIHNNDNIYILLINFLCKNFPSYIGLFAHLWFECKILLAEKLIIVEFFRQLKINSEIIYHFF
NCGYDSFEILLSISPHDLINIQKFNNVTWIPGHSFRLKMIFSNIKHYFKIFIQQNDDYIKKIKNYILQKRKKK
TKHINNSKFMKHYNNNNKKDSTNLKLPIHTFLSMQALHDMDINKTNINDPLSSNVKYLKFKPHDLVHQNQSII
TDYQCFPHPLWKTINSFFNTTC*
>N.PFL2290w
MNTFYIFFLFVLTTCFVKGDLPIHALMGDVSGIWKIKQTKKMSQKPEHCGGGIPNRNLDNLNPSIRNYQRFLE
NEYGNLDMMIVNLTMEKVKIINQEKPRDKWTYLAVRDYERNEIIGHWTMVYDEGFEIRLNGSKYFAFFKYERK
SNAHCPTSIEDKSYNDRDCYKTNPTQTHIGWVLNEKVKENTKEKIFYWGCFYAEKKESTPVSSFVLHNGMENK
TNLVESHDYHFEEKKKKNLPPIGRRSNYKKFRFSNLQKIYGSTQTHLRDIYGRSGERKYGCRKRDILNLKIRL
TLPKQFSWGDPFNDENFEENVDDQKDCGSCYSISSVYSLERRFEILFWKKYKKKVNMPRLSHQSILSCSPYNQ
GCDGGYPFLVGKHMYEYGIIPEQYMHYENNDYNNCIMDMGNYNHLNKQNRNIKEIYYVSDYNYINGCYECTNE
YEMMNEIILNGPIVAAINATSELLNFYNIEDKNVVYDILPKDTHQVCDVPNKGFNGWQQTNHAINIVGWGEQI
INQDKIKNDDANNNNNDNNHKLVKYWIIRNTWGKNWGYKGYLKFQRGINLAGIESQAVYIDPDFSRGYPKNIL
QSDSLE*
>N.PFL2295w
MSNFKNIIPKRTYLERGQAKHRLHLGELEKKVDYGKRREIYKKKKKIENVLKEKIMTKNPDEFHTGMVHSRVT
EDNVLVREEKVLKKEVQLKNKRQELKEQTNDLYNKLKKINKRLSNYQMNIPLRYVFNNSHELYNENEIYTLKA
ENKKLKKRGDLIQKKYNGLINMKKNLLDQIRKLDNKYITTYHKVDGYNIVTDKGKTPYRLYQPRLK*
>N.PFL2300w
MKIIYEFKKEYYEIILNENNADDNVIDLFEKNTNKCNIEIKPYDDINSDLLDFDQFKMFNIDKDDIKKKNEDK
KDEDRIKYILDKESYNNSIDKLNDNSCDESCYITDDELCKQINNPFDNTYNNKFVVKYKEYFEENSFIVKKGD
IYGADFLLYITEQKYAHSLYAVYIIYKHYILRDLIRILRVSHSIKKKVILILWNELNCFNISDTLVYIKSYKY
K*
>N.PFL2305w
MRAFFDSDIVINFTLSEEAIEKEKRIIENNKRLVRDCQRDKLLCDVKKNWDKFYNQYKTNFFKDRKWLKVEFD
HIFKEGLKNYDETIDKSEIRKNEQTKLVLEIGCGVGNSLIPLLMEYEDCNFIGIDFSKHAINFLNDKWEKLVS
INNKLKYENMTVESNMKDKYEKETNKNVELDGKFNCAFENSLNDICQKEIKNKNILLENNINDGTQNNNDNNN
DNNDNNDNNNDNNNDNNNNNNNNNDCHILEEQNCTDIYELKSYKKFGNLIKTCVVDITSSDDDNSNFICEYGSV
DIILLIYVLSAVQPDKMINVINNCYKYLKKGGYVLLRDYGLYDLTQVRFAIKKEKNISENFYVRGDKTFVYFF
KTEELHDLFCKNGQFEEVQNKYITRIVKNRKRNLEMKRIWVQSIFRKI*
>N.PFL2310w
MDRYGHNVRSDIKKQGYEDSNLPILCETCLGENPYVRIIREENGKECKICKNVFTHFRWKPGENSRYKQTVIC
MKCAKVKNVCQTCLFDLQYNLPVQVRDKFLENSIVLPENETNRNFFLEQMENDMSSTYDKMNRINMDLSKLKR
RDPYFKRNMARVCSFWRKNSCNRGDECPYLHKEIHLDKSLSNQNIKNRYTGENDILAEKILLKHNEKNNDDKN
MSNKICIQGISESVSQANIKECFKKFGDIKSIKVIPKDSKMFISYSNSQAAKKASDKYKDGLLLNGCNLTVHL
QDNPTYNNKNQQPVINYMNNNMYQNNMSPQMMYNPMYYPYYNMNNMHPSSAPYSSMLPSEAEQRK*
>N.PFL2315c
MTFLKFIYLIVVPLGIFLLLSCLLKVRFLVTFSYSFCRKKIGDTPLRIVSIILFINFLIFITESYKLKYNVRN
MYSANELITGITSDHLKLYKWRHERNWWIGLSNLCIWIMIWRSTGIINYYVKYLEQRKRQIKLL*
>N.PFL2320w
MKKLSDFYTYSKDKSISVFNFKKKEEEKKKKEETGWMKKVYLDFEKTKNDTYTYVSIKTHQMKDKFMKWNLRF
FKVNHSKYEHFPWYKRLYYIMKDYIYNILKIKEEGMDTYGGQISPNEKKISRLTSTFENFDFKNRYAQKQSED
SMIYKYRENLKKEKMNDSYLSERSQSEITRRNLSNEIVSMKGCESKLYEEMYYNNKNDDNKNDDDDNNNNYKN
RLRNLMSIGNMTNEKVYSENESKVTHSNTVDKNENYHFLNIENSPKFNIKNTWEKLSGYIS*
>N.PFL2325c
MADTNGRNSIVGQNYGAAYNQGGYNYSQNKDKENYDKSNSGQKGYYYDARTNITANGGLYDEFSLNEFSSTKI
RHGFIRKVYSILSLQLLLTFGCAALAVLYKPFNAFVLTYYSPLFIVGVLLSLPIMIALACAPHMARKYPSNYF
ILLSITLGMSLIVTLASARTNSEIFFYAFGTTAVVVIGLTIFAFQTKWDFTGWYVFLFMAFLILIVMGIIGIF
VRSKAFNLVFAGISAFILSISIIVDTQLIIGGKHKKYEFSVDDYIFATLALYMDIINLFLSILSIFSNAE*

>N.PFL2330w
MNSRTYCVIFYGISLLVYVIIIFFLNRFEKQTIPYERIKDIIKIIKHANNNIGIISNKIEDGNIQIKHQNNFL
INLNIVSGCANDNIDMFEEMLKSYIEEKIKLENDKIVSSFLLEELNNVQDIFTVTINVINKDMIKKDTIGVYS
NICNKLLSKNMMNDNINNNYYLYIIANEKKKKEKKDNLSFTLSTTNILELYYNKVEENSINVYQQYIYITWDV
LKNTFLDRGSKTKKKNFIPFVPELDLNFFLASCLYNKLNYKIGRNTTGGVEMGGVVNDVDSCYEKDCNIKHTD
DNEDINKDDNEDINKANNKDINKANNKDINKANNTYINKANNTYINKANNKYNIHNNRGKTYNPPLIAVWDFY
KDFYYPYIRPFIERLLYIYQINIYPQILSNINMYEISDNINLYIEEKEKGNARLIILDKITKFTNIFDEITFD
NVLSKPTYEVPKNINFMVVFPNGDEIYFYNNLSKTNKIETAVSIAEWGTIYINNEFTQLKDRSNYNNKNNLVN
ISNQAHVISGIFISHFRSLLGLCSNFSDCIYNIFNSDTYNIKYIETISSEIDSYISYIISEENNLSFSFFYHI
PLKNSISNYELLALIRQAYTYHIMETIQNLYKFLSISKISIYMKVPYYTLTIFNNILNNIECSLIYMQGRICG
INHIDKIVEEKFKKENDDKEKDGEELFFKAALMLAQSAYQDSLQLLGDDKISIYDILSKDFMLASFLPVVIPF
GLPIICSVFQELIKYLKKRKTKID*
>N.PFL2335w
MNVCKMKVQQKKNLKDCNLKSKNTILTSFEKKKNEKDENNLLKFSKLIDNKNKVVNSKLLKNDNYKFNERKES
LTRCSTNESLKLKNIKTLTKDFDYNNNNNNNINNNNDYNNNNNNNNNNNNNNNNNNNNNNKNNNNNNNGHSKL
FFKKNDKDIESKVREEDGKKSEKKNNDLINNKNEFKVNNKLILKSKEFKSNNNNNNNICKLNNIDDKNKKQQM
DTYSPKENEEDNSNNMKKSSSLKNIKKNQINEMEIISNSVEENHDTHSKGDKENNGVSTKNVKRMNTMSKEIN
HKNNNLKKHVSKSNSLELKKGKKGEKSKDKLSNNVSGKKGMNKEKEVVQSSSVRRNSSRYSIDYGRRDSSRRG
SVKQEKGKERRGSKNVSKKVSNSKYTRKKSKRIKEESDESDDDDEEEEEEDDEEEEEEEEEEEEEEEEEEEEE
EEEEEEEEEEEEEEEEEEEEEEEEEEDNDNSDNNDESDEVYTNRSRRSTRCSIVKRKSSLCIGKKNSKIIKKS
TSKTKNNKKKKGSVIKLVKKKEKLSKKGILKKTNKKTKGKLRKNSKLYHRKKNIKNEKDGIDEEDIDEEDIEE
EEEPSYFDEYNLSYEERKKDLEEKEKKKEIKEKYIKQIKLPKYADDESIIFINNPNYVMVLLFLTVYKDVLKM
NWNFKDIEEMFQNKETTEFGRNFILKLFFFLGRYFNSKSIFMYKYISRIFEDKEESLSRVVNFHELFPVLETK
KEIKNSGVGSKRKKDKRGKKMKIHQKEDELLYDEEVIGNEDVHIFEEEEDNEHSNDDEDEEEEDNEHSNDDED
EEEEDNEHSNDDEEKEDVHYNDEEEKKSDLNNDSTKNTVIKEEKDGLHISEGSHNLVNIVNDSMKREESCKNG
HIGDNERSGDINTNDDKNNADNNDDVDGEKIVDSKNNIENKLDNTVEGKHDINSTEGIIKSENVDDEKGLLTN
KLFLEWDDLSVISKLRIIRMLLNLVLCESNNLKKMLGDEKLNYNNGYLGKINNEKYWFIFNDTINIEYKLYLE
REEEGSFIFICDDDDMLYTIGYNFLRNSDTKFMGEALIERYNKVCRERRNRSRMLKQQKKLLCADINLNDPLL
GTKKRQHKQVEYFNFDNNEKMRRKKSDMNNNNPYSVPPTKSYQKNDRSFRLAARNAQKGSVDDLEAWKLNEGS
LGIENQGATSGNYNIANDGTNMTETNNINDNHNNDNNNNNDGVVNADGNNIYGPNNNNVIGSDGNVNNANNTT
TNNNNNNNLGTVKIPLKRGRKKKSEKKKRGRKKKILSVDGIGEIANNLSALNVDENRRKSMPNIGRGRRGTYT
KRNTKPSVSIKYGTRSHSINKYENDVMSFNEYSLNPGLSMDDFYKNMVMDPKFFHANMNMNNYMNGGFNMPNN
NNSMMNMRNGSKMGNMNNIGGYNFPYMCTPPPMYNQYYMYNGNNMYLPNGGPNNNMNMSNANNGASVVMGGNV
VSNQNTVKDVKAENMHMAKNSIMENRNSIIVNNMNNMNNINMNSMPHKNSFMGNDGINNMNSMNNNNNNNNVN
NNNNNVSNNNNNNVSNNNNNNVNFMDNMGNNNNNNNNNNNPMNKYNNYMMNRNVLNNMGGTNPLYNNMYNMKF
PYMNNDMMNYNYNINSFQNMNYDKMNKSMFKRYPNFMGGYPYNNMNPYNNFNAFNMNYQQNDQMNQLPNMQGG
YKSKLMNDNLSNYQSFNNNNVKQNHHDNNNNDNNNNKNNSEGGNKNMDDSNKNNDINKNDILDSTKLTEQPVG
GVVPNNDCITRGEDNHRIDLKGDIAEALVGNNNEKMNSSDHNLNSLKNLPNINNTYNMDHMSHMRNSVSASSI
GMINPGHMNSSHSGSIMNKDKFRDLQLNSNNNNNNNNNNNYVKVNPFDHPMKNMNPNLMDDKYPKDNNNMMMM
MMNSVYNMMGSDHFHMNNSHMMDPMGSPKMNDNMYNHNNMNFENTNTNHNNNHNNNHNNLNNLNNLNNHNNNH
NNRIQDKLPE*
>N.PFL2340w
MKYDITRRFRIFLRLVLLDKECGIEAGSLVDLKKWGREKILKNKELLSEYISYREVLNEYTKILKERKNEHIE
CNIEKVANQVGLTTKTMRNKE*
>N.PFL2345c
MAAVDFDVRSKNLSEGIKNEISEKNMENNNNNDNNKNSSALNEEQVQSGIKRYYELKIEEYESIINKKLQNKK
RLEAQRNELNARVRELCDEIQYLLEAASYVGEIVKPMGKNKVLVKINPEGKYVVDIARHINISHCTPNTRVAL
YNDSYKLHKILPSKVDPLVSLMKVEKVPDSTYEMVGGLDQQVKEVKEVIELPVKHPEIFESLGISQPKGVLLY
GPPGTGKTLLARAVAHHTDCTFIRVSGSELVQKYIGEGSRMVRELFVMAREHAPSIIFMDEIDSIGSQRIEGE
HGDSEVQRTMMELLNQLDGFESTQNIKVIMCTNRIDILDEALLRPGRIDRKIEFPNPNVEARMEILKIHSRKM
NLMRGIDMLKIATDMNNCSGAEVKAVCTEAGMFALRERRVHVTQEDFEMAVAKVMKQDAEKNFTLRKLWK*
>N.PFL2350c
MNKHIIDLIVNRRLLEKKRIIDKEIIRINLENIILHFDKYSKAYKKTHHDLLNNICIYTKIIKLDYNYLFYFY
KNVVNPLLNDKNIKTDINFLSNIIYTFKKNEKCNHILKELSDYILLNLNKNEEIVKYNTKDNNMLSNDLNTWI

NIIHVLSKYKDINIIEMLQKIIIQEENNLLGTKEYIINTNTNKKIDISYPKNNFFFCVTPYKHNVYNNIYHDK
NIFFSLKKINYKLIETLTPRTFSILLNILTKIPIEPCTKFNIFLISKIKNMLNDFSNIDLCLIFEKIYHFKGK
DENFYLLYNIIYNEILKRIQSLDLLQICLIFNNLKKNHNLEHQMNTFISLFIKKIKKYYNLYKDVTIDKDKYI
LFTKDKKCLIEMMPMFFLSYIKNSPHIFPSYNLFSTFVRYQFLTIYKYILYLKNNISLQKQNGYKKTTYNYHY
IPLNNINQTLCNFFYAYSGYMYNTLSTNGCHKHKYIYIQHFYITLNNIYTFLYFFKFLYENNSYYFKNCKHNF
LLSIHKSQLLIFFYTFWSYIFDILLYISKHPEQQTIKITNKKKNAYIYSDKPNYSIFNDIQKYLLENKKNNHH
HQTLSSFFSYIIKLSYLKKIYCILNLLDSTNLEHLMKESLKYKNTYISSIYKNIIDTLNSTQDPNTKTCYLFR
RKIIGPYTASVLVQK*
>N.PFL2355w
MEYKKTKRTNQFIFEGSKHATKRQKINVSAQCKGILLSTVSPRKMSSGIKEFINFLKLNFPEENLENGEIKIK
EAATDDHDNDSDRKTSNVEKQLNEEIRKENSDYIRFAPLRNIIKNFTFIKFNNAADKNPSEIVTQIFSMARNK
KEKYILRNICKIIPFDCICKPHISPFIKTLLPLLKENFSKGLCVQENFTITNLLKLLNVSEEKTTELANKQDE
PQEENGNEDTSKSNENKNGDSDENKNGDSDENKNGDNNENKNGHNNENKNGDNNENKNGDINENKNDETSKTN
SQNDNKEKKSENKNETVLNSSGEKKTTWGLIYKCSNTRTLTKKDVLTVLDNCIGNNYSVNLTKPDLAIIVHVT
EIMCGISIIRDYDKTRKFNISSFNEDS*
>N.PFL2360w
MDENFNKLIKKLKKKEKNNIKYNKERNFIFHIIGNNEPFNERIFNFNNIMYANKKKNSRDIENYIDKYKLVYN
NFENNINMYFNKYVEDIINSSIIQRSELQIPKRKMNKTNYTKNNIMGNLLNYEYVQYHNFYKRENEEKYFKPA
KNNNDEE*
>N.PFL2365w
MYYDRSPFKITMSSDKVELDIVNNKSNKDNINIPHEISNSNSSSYNNEKKNLFNKDIDDKISSGDADKYSKMD
DDKDVNDSIHEANDDNNKMNNYINEHNNNNNNNNNMEVCINEDIQNIPSNILENDTKTENDENMLNNNTIEHS
EEKDSDNNDKPFINNKRALICSHDDTIIKNENTKKYKVEESQECDRNGFISKVEQDNIKTEKKIKQEFNNNIK
YDNFELYCKNYDNIFEDLNNGTNLNTTNNNTHTLYNNYCYVKNEPSVDNCNIKNDKDILNDSQCNNKNEYNND
DDDNNNDDDNNNNDDNNNDDNNNDNNNNNNNNNNNNNNNNNNNSFSNNSFYNNPFYNNDDQCENETKCKSYT
HSPNINIRNTSNVSEVVKESEEKNELQDDYNNEKKNELFEEHSFVYPSIYDIYPDEWFRANKYDDWLVHKLCK
WLYNINDKLYFNIDKQEIYHVVQNEFVRIEDSKQLYINENCNTEGYNSRRNLQNKNTRMIGENHHNFLNNNND
DDTDCDMNYEMNEDTLYCSTNNNNNINNNNNNNNNYHGFDNDKYNNTHVYNRRDKFGSHDNMIKRNISQGDHR
NGKSNIRIDCKNNDDDDVDNDDVDNDDVDDVDNDDDVDDDDIDDDDDNNKYNSNNVIHTIDRARKGPNSKTLN
KKQSNEESFEEFSMVLEDDLVGGTFSKVGEHNKRENEDFYITKDILDLNNVSESQGLCFYSGIFDGHGGSNCA
RYVMNHLKTNVIAKFRQSFLITCKKKFKEKGSKLNELSVELRALYDSCIKGFDMTDKNYIELSKKYNYKDGST
ACIVLIYGPDDDGSLKVLCANCGDSGAFICHNKKPIKLSLRHKPDLQEERIRILKCGGIIANINGINRIITKH
KDRNNLNENNNNKSKEKTFLALSTSRSFGDISYKIPRKIVQCKPFISVYTIDFDLDSFLVLATDGILNVLSDE
EIIDIIWKNIHRKPEQAAEEVVNEATRRGSTDDKTCTVIFFYWRKDIFNIQPQDVNVTEINNEEDKGEDINMF
SSVF*
>N.PFL2370c
MKKIKNELIPFLHYDLKSAQSLVSFNKLLNKEYISGEIHNNLILNTNNKSGFLYFELGQNEVILGFKNTNHEL
YQKLLQIKDRKIFEIDEQEKKDNPEHIADMIKDIKNYSYIQGGAIITLHKKLLKDYIQLKLKTFKDLPDK*
>N.PFL2375c
MRAEKRCNKLKSYPFFLNHIIHFNKLTKNNCFNKYKNKSQPKYHIKSNMEKHDSPFIVVYVTTPSKEVAEKIS
YVLLEEKLVSCVNVIPGILSLYHWKGEIAKDNEVLMMIKTKKHLFDEIVKLVKSNHPYEIPEVIAVPIEYGSK
DYLDWVNNSVKQI*
>N.PFL2380c
MNIKSKFQKLKLENDVIYSSNLIINEDDIADFVSTLNIQKKNINDENCSTHNLSTVKVIHLSSLKINDWGLSI
LIPCILRSRKLTTLNLSNNCLTNDSANILSKCLKYLPHLRCLNLSNNLIKCQGAINIIEEFFQDNIKENSTSL
NIYHVDNLEENILTNNNKINQNKCHDIIQPNQKDDFIFEYKDCNNILNYKQDNYIIPKQFNEYHHICNDDKYL
SIIKNIEENEDLQEIDISDNFLGTNFIIKLSDILHKKKDKKKYKIVLKNVGINNTDICYFLPKCKDVEILNIS
ENKIMFENFPKYIEILFDVQPNLKQLYLASICGDHPDGDKNIDKNNIDKNNIDDIDYIDDEDDDNICKNNIMN
DNPNGCYNNFNNVYNIITSQNRIFTNLIKNLYKAKNLRILCLSNNNINDDNFKTFCLHIQHNKDNKIEEIDFS
YNRITDLNPLNECLKNNHTLKIINLSNNHITDEKIKTFCYENLSENLNICELSLAYNKLSNNSCIYISDALIN
QANLIKTTLKETYEFIKQNSSLTQEDLDMYINKMSDKKGDIDNEKFGHNKKEDGDNKKEDGDNKKEDGDNKKE
DGDNKKEDGDNKKEDGDNEKNGDNKKNGDNEKYDDNKKNGDNKKNGDNNNSNNNNNMNANPYKNRNNIYNNNN
KEDISNINIFKDIIINRHNEENNFFSCIGISNFYRATDLSKNLYLQETRRNCIYKINEEFNKLYNSNNIFSAN

CLKGLKFLNLSSCNINNDGISYLLKSLKTSLCTLEYLDISCCQDLSNNTYQEFTNLISYKKYKFLKTNHFVFK
NLPLTVRGIPPMLIPLYDSEDNTDGNSTGKYKHKYKHKHKYKYKHKYKYKHKYKCGYIYV*
>N.PFL2385c
MKNEDSTYKEDKYSKYSKENIQKICATPYTGSNISNTVESVYFVNYRAVVVKTKHYYVTQNSYMLFGDVFKIY
NLKGEIKKIHAFFPGDENNLSDTLIMLKTTIVAVTTSKGEALRLADEINMTCKICHKKRKATKFSIEGFLRKV
RCDTCRVKPRSVICKNINNNKKTNNSKNNTNSNNSNNNNNNNINNNNNNNNNNNNNNNNNNNNNSNNSNNKND
SLKPDECNIQSNQNKHLTKNKIIKYENIDDENIKNEKFKNMNIKKVNTKNCNITNSKAKKTDNNIQNDMNNNH
PNNNFVNNNILDNNHINNNILDNNHINNNHLNNNHLNNNHLNNNHLNNNHFNNNHLNNNQLNNHLLDNRDKIQ
SLHYKHANSNSVLKYNNSNNNYSASQNQIFFSEEENRTHFLENDPNKRDEIISMIIELIKYISYMQKALIKAS
EGNFLIENPDINVENVSKLVHSAQQLCNKLLNQNNIDGEYGNNNLCAPTSLLLHNNEKGNTIKNDVNINNISN
NNNNNNYNYNNNNYNNNNINYNNFSNNNQLKIKYTKMYQAKVNHITRTSNLNNNNNNTNNTDMFYNMNNPNDM
STTNTHMTNLSYINRNDQNKNNNMNNDTDSNIYINNKSNDNNIINNINNMNIMNNINNINNIKNINNINNIKN
INNINNINSINSINNMNHMNHINGNNNINSNNNINSNNNINSNNNINSNNNILNNTNERNKYNIENEYSKNIQ
NIQNIQNIQNVQNNQSYVIPYYYNNRHNINSSTSLDTSTNDQKNKSYIKDNKEYMYENNKKDEHEDKNQYHDH
QGKQQQQQQHHNNNNNISHTKNNYYNNKQQFLFSTLSKQYNTNVNISFANINNSLSNICKNVSKNYNLKDTVL
NMEK*
>N.PFL2390c
MEDVKEFFNHEVDLLIDKDKGNVGNNLKFIKEKYEEKLFKLLHEIKENQEMFYKNHKYKKNILKETCDNDYRN
NNISHGDIINEKEEKIDEEDDNIKINHNNDISIYNITNKSCSDNLQFFKRKRHENYNENMEELNKRQKIQKDT
NMLYISNNNNGNKYYPYDEKEYNYNMNDFIKTDANYNFMNPNEFICNIPLKEQCDLKSLFSYFTESLKKKSHH
DNIFKKTYIECLQRNNKKLLQDPIDENNLFLYKSYFYKIKKIFSLFYFDFFVCIINENISLEQGMDFLINDLF
FVNEQFFKNLIYILKSGPPNNTNNNDDNNTNNNDDDDNNNNNCDALLRCDEKTPNHYNNHIDNYNINKNVISN
YYTDNSYDINPLQTEDQGNNMNSQELYNDNVICFYNNNVLTEKYLIIPFKEIILNSLHYLIEIIDHIKEEKKE
KYNKYKDIIVHCINFLKDKNIISYKEAATIIECKNIDKLNLFNKELYKLITKLKTKNMYSISIYNLINENING
YGKIILLLQNFFTNDPIKIKENNNTKKKKKKNFKKIINKMVRKYDQKVEVQHLNNSSKVEELYIENINKTKNK
NNNININNNININNNININNNINKNNNINKNNNININNNIYNNYCEMKKLWKKHFNCYYCNDLINLKNDIFCI
AGLHNLCPRRILSILLFFYEQNIESEKQLLPLFFLYSKELITDVIILELKIRNNYITELYKEDLSKLRNMNVK
NEENENKNTGIQINENNNTSNEYNISNSNNMNHTSNSSNNNNNNNNNNNNNNNYNNNNNNNNNINLVASSNNP
NISSNNNCIFMFTENNFFTPNYFKMCSILILNDLLNFEIFYANLCPKDVLLKNMFEELYEKYYIDYDNSSSDK
VSSLFYNYIPFDINNLYNIYENVKSYTTQSKEVVKRRASSTLTSSINNVNNTNNTNILNNTNILNNTNILNNT
NILNNTNKFNNINNHIHNSYFSPASEKEYKQVTFSSSFKESFVKKNNPKYKFLNNCKSSTQILEESNPIYLYD
HIISFLLNTQDKELKKKYDDIQCLNNKDAIEYYIYDIINNKSNTFDNFIKNYLDREIKLRIENINSNYKTYPD
FFNYLYLESDPYHLILGRLFFMKNSKFLFLSALIQLNAWGLINIIIKHMETYKCNPYLNYYVHSAFTQLINYI
ILDFEKKYLQDVINKSSSSRNGKNKMKRINLGKKKNFNNENESKENDNHNDVNNKNDNHNDVNNKNDNHNDDN
NKDNHNDVNNNNNNNSYKPPCLFCNINLCGSLLCSYKSIEVIKKNKKYIESLFKNENKIKKKKKIIRNLLKRN
HKYLYSNLKQEKLMNVKNETMNNHTYNIKNETMSINYFIKSVMNIFELPESSKLYSDEEIIKNKKLKGYFKKS
IDQCTQKRKEICDTKDNCIYVLKKKKXXXXXXXXXXXXXXXXXXXXXXXXXXXXXXXXXXXXXXXXXXXXXXX
XXXXXXXXXXXXXXXXXXXXXXXXXXXXXXXXXXXXXXXXXXXXXXXXXXXXXXXKKKKSKCNDNNNDDDDNN
NNYYYNYLRKIKNIEAFMEYVLIFLQYLGYFGYIYKSLLRRILRILFFYVNSVINSNDSKMKLHPNFEKLFIK
YFFFVIINDNDNDDDDDNDSEKKKSINDDIWNILKLLPASDRYKLYFKFFDKLQKGRKDLSKLKNLKNFEIKC
KVNTKDKINKNTCVRNNLNLNVNFNDSPGKDKYENKNVNHQNNDNNNNNNDNNSSSIIISSNNSNDYSKKLFK
LAYEHKNCIITLSYINFEIIKSKLRKIIKRITSDILKDRYNPKVESMLCEFTKLINRNPFDCSDIILQQCELF
DNNMIITLSESIKNIHNFSSDIFLYKILERQQLLNVHNYQLYKQYNNLNNLELIDDSIFKPKKLINVSLVAAK
FLCKHPSVNFYPLVISIFKRIFSELHTSDHVYLKKKSYVPNQLLLLLLLKNCQQKLQKRTSISSQCDHIKNDS
NIENEENIKKENIKKDNIKNDNVNNNDHIKNDNVNNNDHIKNDNIKNDNVNNNDHIKNDNIKNDSVNNNDHIK
NNNYSYYYNVMDTNIHISNNINQTNHLSNVIKQNDLYFYYNNSNINDTQNVLTHHKDENEDTLIKYINIYPNE
YINPYDLINNEQGYDQYEQNEQNEENKKNIIEQPMLGGLNKKYPDIMTGYFFDLDYIQKLIEIYAGTNASVDV
QELNEDQLNAQCGLKLIKEEIMILQENFNLKINNTEYEYIQQIEQEEQKLKNACIKNAIHIFLNPNYIYLIFF
ILSKIKYEYLFDSNTNNLRNLSALVDKIHIVLLQFVEFLQSNIIPSLYFKYIPNILHIFQFFDISQSFHIARY
AFPFFMQTKQNEKQVHNKNKNDNNKNDNNKNENNKNDNNKNDNNKNENNKNENNKNENNNNKNNNNKNNNNND
TSVVKKDITSTIKAVHADASPEEEGNLNILKETKPKSDNHDNINCENVIGERSCNTTHDEMVKPLKKYDDPNF
HHVNEILWKMFLMPIVDKYLKEEELNGININFYLTYWRLSISDIYVPHKQYQKVLDKYENYIKKLEKYYEENK
KNDEYKWIPKQIKKLNIKKNNLKNEYEYHINHTKQIKQYLAHIVDHWVDPQKVSLNTFVAFIKCLIAPRILNS
EKDSLFCSKFIQILLELHTPLFNLCTLVYIFTKMLMPLINACTEKEALNIGIFFNDFFSYIYLLCEDIKYFQN

VSNKNPCFSYTLNFQSKDTITHNNIIQKVMKWEKTILSLLFENNNFEKSWINSKSVVIFLFRFLNTFPYSHKI
KNSIKTYLENLQNFANNQGWKDIVISINSLKTMMEKNRKSCVGEKKDVPVKSQETKQSTSRTSVHNVNADVNV
TMNPFNNSTPVHIPSNPPFAPMPKNIYNPNAYPIVQNTINPPPKPYMKDSNYNNKVPPPQEPNKYFFKRKFQF
FKYFNS*
>N.PFL2395c
MIYYSFKLRWALFFFLLVTLFKINNVFSKRKRKTYVKNVKWYPQRINHYYINGVVFPRKRRLATFFVFLGNN
GKKKNKRNYKFNIKSKLNVEGENKNEDDQKNEGDQKNEGDQKNEGDQKNEDDQKNEGDQKKNEYNNKMYNTEP
IDDKNQFISRTFEGINIFPIKQNEKDQDYLKTKLPSKEFKPKRSLGQNYLKDTNIIKKMISAIEYNVEHHLFQ
NKKRMSVRKEEDKKKKNKKKKINNNKKKKKKKKNNNNNNNNNNNQNXXXXXXXXXXXXXXXXXXXXXXXXXXX
XXXXXXXXXXXXXXXXXXXXXXXXXXXXXXXXXXXXXXXXXXXXXXXXXXXXXXXXXXXXXXXXXXXXXXXXX
XKKKKKKKKKKKKKKKKKNNNNNNNNNNNQNNNQNNNNNNNNQNNNNYNNNNYNSPNQEQCDDIKTKQYVKDN
VENNKKIDEQQNCCKESYNEDKEKNNHNCIDGIYTEDIITKKKKCNNDNITEEGENMNVQNLESLQNDGNGVI
ELGCGLGQISKYLFSKYKNMTGIEIDSRALSIISRTMPGFDFIHDDVLQINYKELSINKKTKLTIIGNLPFYI
TSQILFCLLDFHKYIEQAIVTIQYEVGERIVAKPNQKNYSILSILFHLFTYPYLLFKIPSKAFYPVPKVQAAV
MKIIFKNHESYYKHINCNLLFLKKILKYSFQQRRKKLKSSLKNLLIQYNIPKLPEQFINLRPQQLYPYQFVEL
TNLLFPLQNYPFNPNIHTKVWRKKKHGD*
>N.PFL2400w
MSEKKILQVVRPEPISVLDYVDNIVKKKKLDKELKKSSLDEWGDMKKIEKTEEIKLQWALLQHENLYTVNEYN
KLKKDEKMPEFFQVATMVNSSDKIKVGAGKESQSLHTSNRRKKKTLSALQMLERNNDLKQWCINKYTKIQKQK
NIGGKSFVRKQKKQLLKIKNGK*
>N.PFL2405c
MKLLLFFFISSIFLQLTSGKRILNFDNIIKHLKESKLLPEDIPHVLENDIIIVPPYLIYKYKGKIYHLHNNVD
VTLINHPEEDSCDKEEIWESPFPPKVPEPERPHEPEMEPQVKPEPGPLPEEVREPEPEPEAEPEKELEMEEQE
EVIEADMVLDEETGIKIPKKTEQDVVEVSKFREEYELPNVVELTPEEKEKNNILHFAGNKSTAFNLKEIINYK
KDESLMNSLSSSFDHFYTPNVEERNLSNAYNVDINDYYDLLRALHILCKDEDNKLYTINRIPKDELLFFLKHA
YVNIYNSLKKYILLNGYNFEDYIYTSENFTLDQIFKDYFFLSNDDTNENGSFNNIIESIKYIKKAIEKLNVKR
IEEKIKYFFQIYEVHAFDFKLLHYIFSRNQLLNYSEDDLIHHAVDIMNITRIDISPRVISALFMYFLNKVNIF
LVPQYVTRNRNDFTLLLEHNDLLLRTEIIYRDFLKHFFKHKTPHVHLKKNNHDNAYQLVPWSQVLFSEFNSTY
DFLNFKYMIILFHDSYHAFVDYFEGDDKLNQVLNDNKENKTIDNLDKFFNELLNLFLIENVGILSTLEEYFVS
TVKQVGRVLSDDHDIDSKEYTENYFTPEEEEQALKDFKEDEASVHHLDHYDNNMDPYYEYQGEFGSYEEEEDE
LRKFKEDEPEPWPLDESPHIEHGTLHDDELNPFQEEKKDDESFYTSEDEDTLDHSHEKEDDVSHPEHEDHNEG
YIPHTYDDDSFFTDEEDDEGDEVNEENVPLNHEEENVPLNHEEDNFPLNHEEDNFPLNHEEENVPLNHEEENV
PLNHEEENVPLNHEEENYNSFLSYQKYDERDISPHNVNQHKKEIFHHRNITPYHVNHHNQHMDQHDHHIDHHN
HHIDHHNHHIDHQDHHIDHHNHHIDHHNHHIDHHDHHIDHHNHHIDHKSNNQFLQKHQNYVRGHSSFITISEG
EENHDNRELRKKIEANLKEEWKKRFNEQQEQRERKKKAEEDEMNETIQKHDMETSKLEKKEEVDEVTQDEEFD
KQYAIDDQEELEFLRTRDSEGSESDVPKDKVKPLPDGRSPDSFYYNTAISSFHEKMEELYNTSISSSLNYVKE
INRKFDDVYKELKSKTYPKFDDLTSQTKTNCNKLFQKLNETIKDKEYQKNIQSYKNKVIDILDDIQKKANGKY
IIIQNLIIEKIDIYKGDVVRLSDRKFYKNFRKVLGKRKMKMLEDFRAQFKGAIRFIKDLTTTYMEEEYTKVLE
DIYMEKKKYYKEEYSKMRRIISSNLDYEVNKQIKEHYHKVDTISEHKFQEIRQHMRDKIENTIHELYKEMYVQ
IQIDLTNYYHQLENIHSELLQALQQNKNIPRHLNVLEKKLEITKRKKKNKPDISTSSHATDEQQVSDTLIRGA
HNHGDIIKGEDNDEVLLIEQIQSLKTKMGDNQNQVGSILEKLNNLSDQYQLLQDKLNVVEDIYKNLRNFKHYI
EKLHKESKINREKFITKVDVLSNVYSTLEYMVKFLLHDFQEWSFEKDELEKHLYELEERKKYITLEIQIRDTL
STNIQNGEGDHINNNNNNNNNVRNNLKKQVLKDLDLEISKLKGHILEVDIKKNIALEQINYLTNNTNETVPDV
IRDLMPAPRIVPVSEDIYDYITWVRDNTAVINNTLRHFVMTFDQKIYDYDDHLIFVYNIKELIYKENLADEKY
NANYYYEMNRFYLHLEEFFYILKHYVELRKIQDLGEVAIPSFEENIKTKGSLIDVHQKDKDGTQIIEYLERNV
LHIYSILRNIKNNNIHLYHYLVPDLSNILIDPEEFKKMSNGEQPELNKLHKYLLDAYTNLKKEGQVVDQIYVE
YINFHNHFEHTYDKLLWYKTILKRYIERSKKIYEKSYGTHTVSHIQLYDNKKDGHDNHHDQHHDNHHDQHHDH
HHDQHHDHHHDHDQHHNNNYNYNNISFLRSFVQLNYHNTLHERSDHIFLKSPILLQYDPEKIVESNEKRHIRV
IRDQNNFPYKLGEVKETYVLNKSIHDKFVKSKKLDEYKKYMNNKLYDIVHEHKRGKGKLYLFEKEELEKYQLI
FGGKTLIKGSLLNFLSHININLNKNLLINQINDTINEYLKKRNLSYNSIIELIKYLQKNPSVIHFIDLIKLGK
NILERSNVLYIKKEWFTLYLYTLLELLGIEVNMEHVDVHEERGLKNEHNNYISEKSYALHYLYVLRNYNKRIE
IKNQIQNYVLQFLLPEHKFDMEDYQPILYEFLKAINDSMDGDSITYNEFKKKNPEVYTTLVSVIKKGIEDFFY
TMFNKIIEEHPELKEEIYFDDNYNDEEKDRFRYANYTWERANLNEILTLYFYVLNPGLRNVIYKHFIKVNIKC
LKDYKNFDFKEIFSFLNYDDIGATFLNYLKEHELFEIPPLIIFLRFFFHEKNEELFTFNSIRYKINSYFEGKS

LDRKTKEMIARLVYSFFMRISYLSVSYFDHDIFKIINKSDLNIIILFHTIINKINYLLYCGTEKLFIERYAPT
NLFVQNRIFKIDAVKVFHHFIYSLPPYMRNVVFHFFKSNQFIDVTKKLIIKFLQHSHSLLDITNHKDGHFYQL
KNNTYKLETFLFSWKYNFTKNKNIIEPTFTKFIENKPVFKNMPLLYNRRIKYDSINEKLQYKEELDLHDMIDN
ESFHQLLDLLNKESITLEQLSFLFSRYITEHANIKDFPKLFMFKKYVTYVQDEKFFINLYEYLYNYLHKEFGL
PQPIYELYYVFIKTFIEYILLPEKLTQEEKSQNVLNYDLQNKYMNFHHFVQKLCSCLFNFYYYDEIYECQPFG
FFTKEEFSYEQQIFNILQQPFINISPIIKHNYIHDDYYMYEKDLNGNYLSSNINNNNNNNNNINNIDSNYVNR
RDKNGDYIHGTNINSDILYHFIKQYIPLVEPSDEEHLQIEVFKFKYFTLEITKFVLRHILKKLPFNYLIHMNW
NDTYIHIDHIINNLFLHFFKKNKAHLLQQNQLQGLTHKINFENNLGKDHTKDEYLSKHIPQSAQNYDRNKFSG
TINNLLKDPNNLPPTYRHIKYYMDHYILQYSLHNHMLFKYLHNIFQHIDQYKTYFPTLAELYLYIDKRVTIIN
VINNSFLYKRIHTRDVIHTFLLLCHEIGLDLYEL*
>N.PFL2410w
MKNETQHFFIVCLFDIAFFAVCVCIWLYLRKNRDENKINDNIYILNHQTHSEIKIDNKETVGVTNDDSTTEVK
KKTNRFRTFLNIKDDKIVNTEIKYYLFFLKANRNIIFSLCILGTFLVLPLYLSLPRNDINNPSFFHYISAGSI
SDINILTILYFITLIYSAISYAFIYLLWRKIRPSKKKTKKFLPQNFTIMVSRIDKKEINAYKIYEYFCNLTNN
KVVSAYLILDYSIVYHEQKKIFNATKNLKLLKENEEKKNIKRDKSKNMFFSWTSKKKKKKNASNDPLSICDKN
TNQNLKISKNNTTEGNHMHNSVSMKKSKHAFVENNNTTGNYKTKSDHNKLSSNDNIKNFNEDKKKKKHHNQNQ
NNNQNDAYSNASKGVTIQANDKNVNSDINTSIEKRIKKGTHKSNEIYKQKCSSKVKENQILGKKEKEKNIKEE
PQSDALDKDDNKEDDNNDDDNNDNHHNNDDNNDDHHNDDDDNNNDHHNNDDDNNDDHHNDDENMENIQSKRDN
DNVEEEPACFLKDTTDEELVVEKGKLRKKKMAGSNYGYKLDLEEKLMRSTNNDMLFYDLDNLSTKDNDNIDIK
YKKKKKKNIYKSESKTNFFDKIFFFLKKNKKSHWKKKLKEHLIKFYSIKNETPKKSTGVCFVSFIDTKSVHDC
IHNIPFTERNKWVISNAPPNYDIIWKNLKNDSYKVCARFIILNALLLLANTIMIISVTSIDNILKLKIKKYKA
ADPSSSNLSAILTTWLSPFIVIFVNSIIQPALISCVSMIIGFIRKSSEHTYVLQGNFIFLILNTIIIPLLSLS
PLSSIIKVMYSDEIGQWSTRLGEYLFNSSGFFAMRYLLHCCFLTCANQLLQIPQFSIRSIFKILTKKEISAWT
FDFGYWYGFNTSILALILTFSVAVPFILPLGSLYFFLRYYIDKYNLIYEICRTNLDSHGAVVRTAIKFMLFSV
AFFQLVMFTFFSRVQNKFISVGRNILFLSSSLTTLLLLCRSTEWVSTNHIKRKKGKRTFCYLCEKNVYVSNLK
DLNKLKYAYANPCESKR*
>N.PFL2415w
MLSTIFGSVCSIDLKIDADDNKKFAFLRKDKKGEKCPIFSDGEDINGTATISLKPGKKFEHYGIKLELIGQIN
ILNDKANSYDFFSISKDLEPPGFLVESKQFKWKFSAVDKQHESYFGTNVQLRYFVRLNIIKGYSGNIQKEIDF
IVQNLCIPPEINNTIKMEVGIEDCLHIEFEYDKSKYHLKDVVVGKVYFLLVRIKIKHMELDIIKMETSGVGKN
YTTETVTLSKFEIMDGSPIKSECIPVRLYLSGFDLTPTYKNIQNKFSVKYYINLIIVDEEERRYFKKQEIFLW
RKKMG*
>N.PFL2420w
MENREKRARENFPDGEIFLCEESYGKNLKEIKMFENIKSDMSLIISEQLRKRSRTTFKLKNIEKKYIYLKDIC
DLLNKSLLEGENILKKDKELINAYFEYTTSNLYYIFIKYENIICDMSEMNVHMKLKLKKIKEEELMHFKVCYE
KLKNSMNILKEKKSHIILLKEDLHFLNDEYDNIQKIMEDKKSKDLNLTLEKSYNELLIQLEKYKKELEEIKEK
CNNQMKEKKKTCINLNMIQENILDINEIIKKLNEENDLMNEDLEKLENENNEIIKKIMEDNNKIEENCHILNN
KLESIENEISDYNIKKKDLLYEYENLKNRLKNITLVCNEKKKKDENYSNIIKNKSEMLISNKKILKETDKEYQ
LLNDDFLTCKENYIKLEKVYENNQNKLEDTKKIINDYICMCEEKKVCLLNIEKSKENNEIEKIQILDLIENYE
KELIDQKNIFLKLSKILNLIHLSYEQYDEIQNEKCTMTKNEDDYTKKYEELLIKYENVKNQVKDKKEILDNKK
QEIKKVEDMLNTYNIEMISYNDKKEVMNKNEKELNEKKNKCIKKLKDEETNHNEDIIIKKTKNQLELKYEELK
NNMHTQKIEKQQQHNNFLKQGEKEKLECDLQLFISEIKNQIEKDKEEKKNIIIQKERELQIYQKRYSSLKVV*
>N.PFL2425w
MIKGVLVINNNGKPRFLRFYDESNHEKQQLITKRVYELIKNRLDRECCCFIEDEELFSSDIKVVYRHFATLYF
VFIIDSMESELGILDLIQVFVQVLDSNFENVCELDLIYNYEQINYILDEIIMGGIVLETNIDTIIDSINGSKR
LIENESSFFGD*
>N.PFL2430c
MDLHVKKNNYDQVLNINKNQGNSNYDLIEIKNNTNNNNNSNNNNNNNKLIEHNHFNNIDEQTKEKQNVFYDND
VREVNTNVTSNVKENKPSKNNTDTNVYLSINRCVNNECSKKEDYYKDVYNNISHILDHGLANKSCVPKGCLEL
DNKKDVFNKMININSNICNTNNKDIYNKITNEHNYKDLNSLNHINDEEKKSNIPNENILREKSAHCEDEKKSE
AWNYKESINYNKNQFEKKKKKSFEEKKERQKELFVAYWLNGIVDLLKRGFKNGNICGSNSVGKKIAEVLKKIV
EIYQWKNVFDLIEIIKYLGKQIIKNNKMFFVIPNIIRRVLTIIRVEHFKQLYLYNNNYMDNINMFDDKVCEEN
RGTQSPNNYCHNNNKNNCIDNNIFYDKNKVKKKNTMPIPKEGSKNIAKFDINSNTLYQREIKNFQKSFSFYFE
KPCNENTYKIPATNTLKHSIIEGIEELIAEIDTSWEEAEQRTSYDLFTENDVILTLGYTAGVEKFLKTINKKK

DGISVIVVGGDINRNGFRMSQLLSDDGVDTTYISDAAVFAVIPKVTKVVLGSVAVSSSGGTITKMGGYNIACS
AHLYSKPVIIVLPLFKLIYVPLYDPLKQNELQPGPSLIYNDEDVENLHVRIPKYDYIPEHLITLYITEMGPVD
SFQLYNITKKRYHPDDMDLSFD*
>N.PFL2435w
MEHGNFDEKFGDSILLESLKEALQQMIEEFYVEKEKGIQIYKEACMNVKKEILDNSNQLSDVHMSGQLKSYYC
RNDMWTFFFKNSLFKINKNKKMKSSSKDYKNYQPLNLRVYKNFYDKKEEFLKNCVDKNNVKFFKNFPKLYSNI
VHKENNEVESDDVFFYYDGLIKILCIEESTI*
>N.PFL2440w
MAKKIDELFSKKERYLKEIIESNCIYVKDLNHWVGTNYIARLKLKKELLKVTPKLLINKTRSRASRCRVCGFS
IEKDKLRIGYPTKDPRGDYGYISCWVHIECSKKILYSCLYIEENENVLKGYLHEYKNHNCSGEHYNKYEIYIY
NNLQWDLFFGGIENINEEELSKLKEMAKPYEIKNDLKCRNSFEMLINEENEKINSLDYLNVEKKITNYNLIIP
KELKYDLLQYQKEGIYWMINQEMSNVKGGILADEMGMGKTIQAITLILCQKLNKLKEIKKDERSDDHDKVGYK
NDDEEDEKKKKKKKSHMLHGDIYKEDILSFEERDTKHEKSIKEIDIKKSYKNSSSILYCKNSTSDDLKVKEES
DCSVILIESDDTQNEKMNHKNDVMAENKIDTENVLNVKKIKNIKKECVNNKKIKNNNNNNNNNKSNFKKNDFI
NKLKGQTLIIAPVAAVMQWKSEIEKFVDENILNVYVYHGNSKIISDEELIKYDIVITSYAVVEVNFRKIVNKH
KQPCEYCGRLYLPNNLDIHKKYFCGPTAVRTEKLKKRKKKNKDTALVAMKKFDETFVPTPRNVLLEIMANSKK
ELEDEKKKNIEISNKSLNHNNTKKNKGKKNNQIKQRNAQKDSNIDVIVLSSDSCKEKSSSSENSVYSPLSRKT
SSRIIDLSNLGFDKETMEQVIESLSEGGKKKKNVKNVKNVNSVKNVKSVKNHRDVKWNVVIKNMLENFLYNVD
MNDKCKHVLIKIYVTNTTIQNTELEKLNMGELKVVLITMGKHIFGTKVELINRILVSAKYIRKELYDENTETE
EKDTSGVHNEDGDTSMGVTNQKGKDKFCKERKIKKEDEKSSIEKKRKTVDVISVGGDVKRDMSLNKRRNRTNR
GTTMKIKGETREYASNKGKNNNDDDNNNSYYSDAKSYTSESSDESYQGDICSKKKEPKKNTKSIRRSQRKSYC
KSSLESEKKEESDNSYNTNDYNDCDDCDDCDDCDDYNNCDDNNYCDDCDDSSNSFDSYDSIIVRKSKMSKEKK
KRENMKIFDESALHQIYWNRIILDEAHRIKNRNTSTTQSILNLKCCGYRWCLTGTPLQNRISELYSLIRFIEF
YPYAYYFCSKKDCKCLLLNYEMRDNKYCYFCNHSRINHFNYFNKRILKPIQSFGYRGEGLSGMSYLKNEVLDK
ILLRRTKGERKSDINLKPLIIKIRKDKLSKEEKDFYESLYKQTSTQFNTYVNSNTVLHNYAHIFDLLSRLRQA
ADHPYLIIFGNSFLSDPSGKFIKKNTTIIPAISNDYVCGICLENVQKRNNISTKCNHNFHKSCLKQYIESFEM
ENADQQEEEKEDSNFKNSNMIKYNTYDIGQREGMNNVKSTNSCKYDIVINDDIHNNINKLETHENDNLITNKD
LLKNKKSLEKKSKSISVVKFKNKDGYINLLSNDENVKDYPLGCPVCYIPLTVDFNLLVDKEENEEDEIIICKE
ETTYINKSFINRINTQEYRSSTKIEAVYEEVQNVINNTDDKCLIFSQYCSMLDLIEYHLKKHNIVCSKLLGYM
SMISRNNILYNFNQDKQLRVLLISLKAGGEGLNLQVANRIFIVDPWWNPAAELQAIQRAHRIGQTKTVYAIRF
IIENTVEEKIIQLQNKKQLVFDSTIGDSGNAMQKLSKEDLAFLFHS*
>N.PFL2445c
MKEDWKSTIQKRIVERNLNQSKKYESIMKSYNSLVEEKNNLLAIIASLTSENMYLLNNRKLNHSFESTMMTET
NISRNEDIFLSNETKKGHNNNNNNNQNDYNNDNSLNYSRTNIITMKSPDSLSQNTITKKEFLEVIKEKGKNEE
LILLLKNSVNEKEKMLHILNKENNNLNNVICKREEELYEKKKDIFKLEELLNKKETQIKLYENNNKSLKTKIQ
LHDRKNQKYFRQYNILRFTYFKLLKQVGEMKVYNNNINQNYFSLRKEMLQKKEENEKLLNYLIHCKTSYKKEL
KKLYQEITNKQKKYILFKNNQFYVNLYLRKF*
>N.PFL2450c
MTNILLFFNILWMSTLFVSANVDKEVPTVKWGQNSSQLTLIVSIPKMEKEEIQFKENIIYVSAINKDGKHYEL
ILNLLRPIIPENCSYSVLQKGLKLKVHKQIKEPCWKRLTKEKEKQHFLIKDKILGDGNDCEEAKEIWFSYYMF
HKRKMNSPPSKNESNKKKDLVENIIENLKNEHPNFSLHEY*
>N.PFL2455w
MEKKKGKLPEQDSTIASKSKNAYYTQPALCTDTGSEGIYNSNSNIIRCMERDDSRARHNINFNSNTILSFLYN
IFCILGIICAWGTVDSFVDVISGKDMYISLICYCILLFIALSFLIYYVYFIDRDYRPCDIF*
>N.PFL2460w
MYNVPLIKNLYPDPSNNLYGDLRICSRATETCGIACSAGYIAVPWQVEGGGMIGVIRLENQVRNPPVIKLKSH
TSPILDLSFNPCYSEILASCSEDMSIRIWEIRHEDENVNEVKDPLCILNGHKKKVNILSWNPMNYFILSSTSF
DSSVNIWDIENEKKAFEINMPKKLSSLQWDIGGNLLSGTCQNKQIHIIDPRKQEICNSFLIHDGGKSTKCIWI
DGFGGEDKCILTTGFSKNNMRELKLWSLKNTTSPLTTITLDNAASPLLPHYDESVGMIYLIGKGDGNCRYYQY
SQGSIRKVDEYKSCLPFRSFGFLPKRMCDVYKCEIGRVYKNENNTDIRPISFYVPRKNSSIFQEDLYPPIIMR
DPERSTNKWIDGINLDIKRVSIKDLTEDDLLITKKFKQLPKESKSILIQDNNNPKKGSVMRQFTKKFTFRKKK
ETTEIQGEIMGETKSSIEADFEPQECKENKKGNKLNEAPKFLFACEDVEICHLKDNVDDDDYLIVNGTNEPYE
ETVIKTNENENYKENNDSSIQSIRSNSKSIEKNDDDNNNNNNDNTLQSEENEEHLKHISSIHEENNFKNFFKN
VLDNILDMKMCKSTATVL*

>N.PFL2465c
MTDDKKKGKFIVFEGLDRSGKSTQSKLLVEYLKNNNVEVKHLYFPNRETGIGQIISKYLKMENSMSNETIHLL
FSANRWEHMNEIKSLLLKGIWVVCDRYAYSGVAYSSGALNLNKTWCMNPDQGLIKPDVVFYLNVPPNYAQNRS
DYGEEIYEKVETQKKIYETYKHFAHEDYWINIDATRKIEDIHNDIVKEVTKIKVEPEEFNFLWS*
>N.PFL2470c
MKRCGLNYQMIKRYYNISVMNENIYKELNVSKYIKTWNISNYNFIIYGLMHGQICGNISSGEDCRNLIQNIKM
DYILLELCKERLNKICNDILVHSKEGNYIIKDKHVNDNNIHDSMYNKTKNLYHNQIKNQKLYHNDYNKFSYLP
RIHNGFLKNEFIPIIEECLKNNLNIFSCDRNIHIVKNRLDAKLLYDTKSYRNFFTYCTESIALRHYSYNDFIK
LYKNYYIPTQNKDQTINTQNKDQTTNTQNDLNLNLNQNLNNLIKFIKNKNIHPNKTYNHNNQTCTYLDNLNKL
NILPLLNERLKHISKPTYDVLVEEKYKYMVHNIWCFLLNNEKNIFQNNEHNKNILIVCSSNIIEQVYNELQEV
YLILFNKYKNNMSLNQVHNNTNIENLHNNQKIKHIQPIVYQNIYSSYNDYIKPHWPLILLKYYILPYLILYFL
LNTLYNLIAWIYKSNMQNTQFPSPKIIKLTI*
>N.PFL2475w
MFKKNMKKKICQDKKMSDVDNVSEPESYDKDINGKDNTNRIKNVSDRDEDDVEEMKEGTSERTSISRNNKNSE
NPKKNNNNIIDMNCLWSDLYISRPFLKVLYEQKFSNPTYIQRDVIPLALEGKSILANSETGSGKTLAFVLPIL
ERLLQSVNIKMRRNNMKGSYNITKALILLPTRELSLQCYDVIRSLTKYVTITYSLFCGGIDIKQQEYEFKKRN
DIFVCTPGRILDLLLNSSSDFINYLEIVVFDEADKLLELGFKEECLKILDVCKFKKQILFFSATLTSDIKQLA
NFSLKNPVFIQSGMSFDKNVDGKNTYNAYNTCNTYNDNDNNNIVVNNIISNSFLKISNKASFKISENLKQEFV
NIIQEKYRKASLLYLCNNIYKNHCIIFFKTKRETHLMYLLFDLLNLRCAELHGSMSQKKRIESIMKFKKAEVD
FLLTTELASRGIDIDHVLYVINYNVPSNVIKYVHRIGRTARIGKEGIASTLYLQKEKIEVKKIVKGLKKSKNL
KILKRTIAENNVLVWYKIIKENKQKLNDIIQQEKIDKEIEMSNKSIDKIKNMITFKDEIMSRPARTWFLTEKE
KKNLKKESKKNDKDKNNNNIKMNDHYENDGYDDVNHIKKKNSQKENNKKISTFNNYNNLKNKKNKKGNDEEDQ
EEQKKKLQSYRKIIRDLKLNMLHNKSNKSQNKNNRVYNKNVKNKRIKK*
>N.PFL2480w
MKKGEKKKKKTSNKGKEKEQDKKVIGKNNKGKDKEKYKNKDREKYKNKEEKNQDKVRSNDNRTSHNNNNEIFN
PQLSARELKKIQYYENMFKRMEQQVEENNMDSHVNNNNNNNNNEKKKKINQLKKSNKKEQEDYEEKKNDENEY
NKKGMQRNEDSHTNDVHMNDVHMNDSHINNSNYKYIKNDESLCNKSSSSIQNVEGTKKRETNIKSKKLNVVKS
VDETHEDIYNVVNNNKRNEEIYIEKNIMCSDKNINEKINEEDNKNDNEEEKNDHRLSDLGNVDKKDELLLEDN
KDILIKNIIYDKGNILKEQNDMMKKEICYDDFNRNIFHSNDIEHINKIKEEEFTNKNIYKRLYYDNRIKDTCK
YEYNIYNNKYIELKKSHKKYGLTNQRLIINYERGYCSDNVINYNNINKVIYKIYNNKKKNIYIHNNNSNKYNK
YNKKTLNFKRSFSISSNIGDYFYDISMVHLKNNCESSPSSSSSSMHDITYHKNLFEDDKISIVKYFMNYSDDD
NKNTYQDYLVQENNINNNVHNNGVVRDISEIGNDKNKKNENTYMNELNNLKYIKNDIYSTKNYINKTNQLLKL
YSNDKLNVLQYYDEKFKERKNNENDGSEYAQKSSIDEVHNIHYESKMYNVNFCIKKYLRNMKYVNLKRLKTIN
EKNCKLYVKGCTHFLNLNKFNDALSCLSKLQNLKGKKKKKKEKQKKT*
>N.PFL2485c
MIKGNIYLLTYFLIINFILCTNIQSISRKTKNVPLLPFFFTLPYTKRIKRSDLQSKRDCTFFTGIKPSGNIHL
GNYIGCLHPIINVEATKKCSNNLSNKNESVIKLNKIILIADLHCLTKLSNIYSLKDKVIDSVKIIISLIIDMY
KKKQSYVDVYINNVKLEDILQLIENKEPNKSNHNNENNNNNNLYEQIASHTSQFHNHIKTDNLGGDMNCVEFP
SQECHNHKNKPIKQKHYFYIFKQSDIKLHTSLYYLINSFTSINMLNSHIHIKMGGHNKSVALFSYPSLMLADI
LLYKPTYLIIGQDQIKNMEIMKKLCRKINYHFSNVAKLPKIFFSKFYSEVMNLDGHKKMSKNHLLHHDKDTSK
IIYLLDNKKTIENKIKKSKTDNYNILIYGQEDRKEINNLINIFFFFYYHQIKNKKYINRTCNNNENDNNENQN
ISKLSYIYDEHQLFINNQRTHNYLNDFTNNPNFSHYKQNNINPHFNQNIINKILHSYNNNYSKFKYDLSQLIY
NHFITTKIYYNSFNTRHDLIHSILQNGKKCVHRRASQMYKVKHKDIITYYITYFYFFLT*
>N.PFL2490c
MYGFFFNKNEENKLTKNKSSLENCSDHNINNNDVNNNDINNNDINNNDINNDDNPNNENNKLNLSVDKEKEES
NVNIKNKNNDQTFISNIDNINNMNNMNNIKSEIILKSDKFILNSYDNIIEEKKNLINECSENIVYKKDNDIHI
NNNTYNNLSIQNYANYLHKDEKKKKDISSNNMNVSNSKNDVKKKNNDTIIQENMNKLSNNISTNNKEKERKNN
HKRSDNINIDLFFSSDNSVELSDENSSLDTMEELCENIEDSDVSINFYKNMLSSKNNINDNINNEQSILNNNN
PSEEINILNNYIKNITNAYQNHSISSIDLDNKLLNILSLSFLTFVDNVIYDSHIYAQTKTYLDTTKMKLKKKH
ERNQTNQMCPQLNVTTQHGNNKKNDIMKESVQVFNNKNVDFLNIDMDKKDTQGQKEIIKINNNDDNNDNNNNN
NNNNNNNNNNNDDDDNKSYEYFHHIKEKPPDITFNPDVINMCLNNLYNEQLVNKLIHKRKKKGKGKDKGKDK
TEENHMNDTKNNSKNNSKKNNNNNNHNDNTNYSSSNDEDDVLRLLSYNSLFNKKTNQDLTYQQNDSTNKNIKV
TNNINDEKSIDEINKSLQHNIYNKLNKRKIMINQINHEIKKKNNNYVNTSTNKKPYSLEDIFEI*
>N.PFL2505c

MNKYWLYIAYVYLLLNILSKCNKIENNNNKVKNLTVYNNDIGQYFKKKDIQCKHHIEINQDSNHNEYSFLSLK
ASSIFNQYYVAKLINTLLYRGFHLNVYFHKNVAMYESFSRTNFFFYLTILNKKNVKKIVKIISKAERSSNKRK
FNVFKKYLISEFDYPNFEIDDTVKNEMNQAILVYKKAKSDSYWSVMDALKKDGLLLARTFLSVSFVQSLRGII
GLINHKLIDLCFTNAYVFNHVASFDKLIMNNIFGVIMSYVFKSLLLFFYPLIIPFRGAFAFAISAFCITQLGK
IVFAIYKNLRQLYRISYRKIYSIVLKVKLRNEPELKKYAMKLLYGDALIMITKIWKLSYVNVSEHLNGKNVYP
ILNNLFEKNLGTGFFDFSNSLFKYVMNYLEEINLLNAKSVDVEKELILNRHNFKLLLKILKITKKSLLYEESY
MKISVANLLTKFYTLILVNIEHISKLNPKEHFYNDLDNEFKHIYQDQMFELFIQKISSDIVRKPFIKRNIGRI
DKGSIELTLALIKVKLLHYKPTLNNPYSSLYFDENLKKQLNYAFKLIIIGSTSIIASLANYGERYGVLKQCPL
DIVKNLNQQCEYVSFEIKKLIFPINIFVNLLIPLFLPYDDVLVDKNIIDEVKKFFNVIIDTDDSYLQKYMKTT
INTIRKSKDLDINNVNYEEEMEKIVVQEAHKVIEEINKERRASFKFQDFVVKEDSVVLYNKSGIQYSFKFDHL
NRKEDFMRIIGSYQFKNPIGYQTSQLVFEPQNNHIGSLMVGSNKNTFDGKLSVCHMCYKEDDDSLVVDAIFSI
LWSSGIDRFSAFIFASFIGAVKQTYHQGTSWKRALSNMAPTEFYEMHKIFMDKSVYGKEKSQNYFLKNIRKYR
FQFSRGSFSRMFRTFLENSLNKINFFNSEEAIIILVMSTLYALYKNIEKFDIPLKETYKLYQQKLIESYYHVD
KYSHHYETYTLNIRRKKYNALVKSEHEKAQEASQGEGKDEAENAELKKRVEKRIKFNDIQLTMDDEVVRNTKY
LQLELQAKPQEREKIIQYLTYKYKEIPNHEMLPHLPHQCYFLLYYNYEPFLEKNLHGLEGVVSKITRKSVLSR
YLKNINLIQPEKTRFASIKLKDLINILCGTHMFLKKEHITFDEIYKYENSNDALKHLLIIVALLRIEKRTNRY
SWTTYLTLQKQLDERKRYYDTPFKYLFLKISKVRRFYGSAKKKMLLGFGKKFKGVRFLNILRYTRFFEIMEYA
ESINEFYPYCYIKFEDVLTFSNVFYKFKYSLLRYTTSKQSVRNVVNNFNLSPQTTGNNENLLLRIYRFIELII
KKKYNVDIQHVRKDDKDFNRHAYNKNEYILNDIKFNPNIEQYLRQLTGFIKLLTDMKFTNFLFLRNLYYFVKF
YAVTGDLTYSINNSSIYLCSRNYLEFILNSIIEFENFKKFMVKIKEKFDIEKYPIDPYNFQTECYSIDSVKTY
RFFLSDLDKLKSYQDIENLQNVSSFYKDYLYMGLFYENLDVRNLNVYYNFNKKKNEDHKAHGSNVFLDGGLSL
SKNMSFSDINDLSESIQNYLYLEKFLADSNIPSIPYQGFSVRRVNDGMHVTYNNRSTTPPLYKDNVLDQFELV
GKSLISEYFQKVKCSFIQYPFNLYYWLVLNPGKPNISLSSKTGLIKEDDLIPKTVEEKLKEEEEHDIKIVENI
LSEDIFDFKDKSDDDQSTTADVLSTDTDDSETDAEKTSNNSNTLHKKETPAMKYNMNIAQDEINRQNDVSKNT
TYAENNEYTSENITKPSDQNTENYGTKMPSPSFIQIDKVNQQDQQNDAHIESSKNTQENLNYDDNTNMYEENK
NDKKNKKLNNNTKEISFLERRETNPIPFSSNMIKEKNKEAHKEHYGNFNRPYNIYVPKKDIFRNFHLVVKVVH
ALISLNKFSMVSPDTILKKGIESMKKKHSLKMIKNINPFIHKMILDMNETIQKLKSDSEKAYGGPKADIISLY
KIVEFQLFNQYIIYPPLKRLTKKELKYILDVVNQGYYFYLKNVIRKLKRENLQKSKVVKIFNNFQEFSNLLDD
DGVDKLYNIFTETFKCKNIKCFDLLFQGFLTEQYNNIVYRYTHDHDAMNSYNDNIFNNKELLNKIFRRAYDNY
FITPVKNNQPKKLISLEKPSLEQPVLTLEEFAYGFHEFGNKIEKWRSMLTLFNIRHIYINICHMLLSVKHSIQ
RSHRVFMHKFGFFGIFRRKREIYIP*
>N.PFL2510w
MNFTVKYSFLVICLLCCLLSTYVSVIEGHRARPGESRKNPREIIKTFKESGKGIIQGYYPSWVSYNHNLKDLN
PNLNVVHMSFAKMDLSYDSIESIVGSPLLFKSLIGLEYIGLNEYFNDAMNLRKARPDIIMLLSLGGETYHPSS
FDSALNAVEKIANLVDELGFDGIDVDYEPNGSFDGLNDKEKADFFVQYVTKLREYMCDDKLISISQSSNGALS
CIGFNDPKKICMDDEAPYNSKYFNKPDVKKELLRAAQMASAGGAIYLMNNLKDMIDMVFVQTFNYTNSTDSTV
MKELYDSYAYYGKKYDYVIIMGFTLMFPSTPFNPNDKMLVKSIGDFVKTENKLNKRADGFGLWSLSSDNAAHN
EQLAIEYFVESLH*
>N.PFL2515c
MSDPWADYDPNIISALQEAFGDIITAKPLTNLLSGESGPAIAGGCSCNRSLMIFIALMVFNAILFWVYNRGKK
DIEKKIKKHGYY*
>N.PFL2520w
MRKGLIYRTILCYILLILIEKSHQNNIYKVNGNNENFQFSHLNFYSKNNEGLNFGRSKVSKGSNLPSEKSSDN
NNDNDYSYKYYNSSIRRKKDNKNNVHSSNLFRPHLFVTLNEKHFANNPTHNSFLSKIKVTGPKEVNNNAMSSD
KNPLSHDLRNNIIENEDPKLLDDNNLYQIDMIDENGNLKFSLYPHYVNIHVCKEMIHYSTYYNDGTENKNSNL
YKILEPLSKYMHEVKSNCLNNKNKLLNEIHNLEDPKYYKNNVQDVYDENIKNYQFLRNEFEKCLNKYDENINK
KIYEMNTDIRNMLSNTICRNNICDLKNYNLIVELYLFHINELPEKAYQNHLNNAKELLEFTSNVINDMDKRIL
GDKKTLYSINFVQSEINDIIKRYNFHLNHINKGISEIKHISTNQARLPNMNKTILMNKSFELAKTFSNFKIST
EILDMLNILLSRKKGILQELLNTLIEDIKTRLKSILYPNNLDSEYKNITSKTKITLEAAEKCFSENKWKIKDY
DMGSTLEMISVKNNGYEKLYILEKSINKLKKLLNDINNKYQTIKKAKIKLDGLVENKSADSTEQKKNLMNLLH
IIESIKENDKIQNLEHYFIDINNINLETNLLIENVQNSLNQLMKLKEDEKKKNTLINEIKSKMDNINEKLKHM
YDIIYINDIINNTVLEIEKLFDENSLVLNDYITKKKRYNINIINKDFFNNNNNNNNNNNNNNINSEHHYIHN
NLKNFFKDRTDFIDKNVNYIYENHSTQDINIMLNNTISEYNKLEFINSDIFDNISKKLKKELQDLVTLKESLM
KMNHNVLKMDPLKSLN*VLEKYEELKKNVNEYSKEENKLYDFKKQMESRLNAFITNLNNNDETLVDGKNIYDQ

FVEYKEQLLIKKRIIINNEILIINDEVKKIKDELKSYNILSYKLENDTSHDVVNSVENTPSSDVATAVSNSSS
ILSTYNSTELNKLRNFFSEKDDELNVESKVKQDENIFIEKNKIFDDIIKDIELYNKKTNAIKNLNNAINGSMN
NLSLIDSVMKNKGDIINRLSQRSYLIQTDNFIDIYEKIFLKDNLNKGLEEIENRLSNTYMNELKIEAEKQNEK
YKKLKENINTYDDTFLEKLIGDNYEWEVLKIELNGLNVNYNILQANIDTLIIKPYIDHIDHIISLIESLKHNI
ENKIKKVIPNLERLKDFIQTKFNTNDIKLDHNNLITIRIDNRDYNHMKLLEEKKEEIEKLKLKLEENMNNTKK
EMENTNISYGIKKKNLIDIYESMSSLLKSIITTDENLYKLQNVDEFRLLYELILIEEINEKIKNQIKEASVER
VEIELYKEKIILSMNNSIKEDISKLTTFKYNEIYDECITKENNIKELYTKSSSLLEESCRDKNMDIIKTNKSV
LDEYLKTSIQNNNDIKNSLASLKNMYAILQSIKLDAVTKYILDNSYKCEDHARDLEMELEKESALSKNIKLKI
EKAEEYRNKVLGSEKHESIDIYIRNIEKIKQDISVIESDIMTCIQNAYDNKTKSMLHFQNVHRGIDLIGILNK
KNQGVISKPAESGNISEEYEGQNINDYYQKCKTYSEEASDNYDEISYRKDSLLEFEKKITNILNDVLIFNMKK
TLENKKDSVNNILEGMKLTASIINEKSHVLSEKIRVLRNQKNIKDDEELLNNEKSKIAYEQFELYMGKLEYAI
PDINRLHDKAERMFSKAQFLMKPMLDMSLILENKNLEELKNKEHDYMENIEYIQDEEKHIKYELTKLIEVIQF
VDNIENLLEKYRKYYEQGNLENVYNNSNNIKDRIEETKNSLSVLVNIFSSIKNSIYLKKHSVKMEDLNSYIKK
MNDIYDEFMESYNLLQKTIIESSNDDIEYEELKQVNRNIEKFEIQLVKAEEDMKLYWNSIKNDVYNKLVYYIK
DMLLELDNRCKSVDTILNEGFDFINKCAKESNVSGDDDNVYNELNKAINKYKEIHEKSNFVCKNEAESLFGII
VKSSNIIGMKIITGLGLELKEDVDLGTMSLLNSSLHFHTASINKLYSTIESDVYDKVNDCLKSSLDIVKYSFD
IEKKKRQINSIMEEINNVHAHITIKIELANMINDSRNKISVVLNKIYVSINKIKNVKEMTCDDSSYHMIMEKG
EYDKLKEYYKNYNEEKLATLNELNTNKLKENLNSCKKLLDELQNTMKNTIEEESPKKVIEDIKKSYDEINGRI
GDTEMDAEAINVVLEELVKRQNKRKASWYTSLIGKVNTKMSFDKKRLVDRQKISQSFLDSMKLNFNMINKMIN
KINNHFDKHQINNYSLGNVEKMMNYINDSNEKGTSVIELIDNLTKKISINNSNNNNNNNNDIINFINNTKEN
LITNYENLGKKEKDLENIYLKIFFSKLEEIEKSSEKYFYISKAFNNVVKYQGQKLSDNINRMNNIKNIIEEKE
TILNNINKNFTLDTIKEFNKAYDNILFHMRDLYDLEESKYDVGRKLNIYIEYIYQIRNKSNNLINNFYDIRNT
GYIMIHETEKLKNDINEYMNKINKNINNTNKEFDITLRNIKTNNYFVNNNHEIKKVIDNFWNHLDTIKANFST
FLPQNEKLVLIKSYLNDIKNIMNELMRKEQVDVSIQTCTRNLDIELEKINKIKQNNAITDDMVENLFSLKDKY
KNEFLLTKDILKRVQDKYQEMNKIYRTLTVNTDNNENKFALKYLSNVNAFINEIKLSVSAMETFLNIIDGKTK
NLEIHRDPKLYSNNPVTLSRDGKNDIEEEEEEDEEDEKNINNSQDTTLSYHENEKARQNENTNKGQLLNEEYV
NGSKDNTKNSNNGRDRFNYNYYSNKGFDYTQIKYASGFAIGLIICSSVGFIFRKEINNSEVDFNGNVQDFHFT
KANNLREKDEIVEVPFNANDYI*
>N.PFL2525c
MNKTTSPLLNFVYPCLQNYKKSHCLGIEHKIKKGLFQKFKNSSWFFCIVFKFLLLFCIFFPTKNDIFRQHTNR
IFNQRGIKNSKPSRILEERECLMTKNKSLLKIDEDINPQSEDHDEKKHKLEKRLKEILTKYKMYQTEEYISPI
NKDDTIKEHNEQTNIESYDHHYKDQNEKYKYHNTIPHEINYFNDNQQFNHPYNLNDINPTNDIHQINNFNYSP
YNNNLNDISTYEKDNEQSLGTFNSTQIHNTNNEQTYFDPLSDEQQIKTFCSEEHENITCYNEAFFRALSDLNQ
TNNNISQEFKDYIDSYLEQKYHIMTNDELDKMNTYRIDYIKYKTLYEQLIKKSKHIKLYKKLKITFKVIFIMI
TCVITSVICPTCPVSVICALALSTYLSFF*
>N.PFL2530w
MVENELINENNLTHKRLDGTPKLDSFFNKDGLLIRTYSWTVKKAIGILVLIHGLNSHFRLQYLKHNADIISND
KAVLINSDEYYIYKDSWIERLNNEGYSVYGLDLQSHGKSDGWQNLRGNVNYFDDLVYDVIQYINKINSSVIKE
REDPKEYSYSDYNYNFKNKMPNIVRSPLYIMGLSMGGNIALRVLELIGKSKEVNDNLNVKGCISLAGMISIDE
NASKIPFKFKYFFIPLSRYVAYFFPTFRPSPNLNFEKFPYINDIINFDKNRYDKWITMRFAHQLIEATRNLRD
DMKYIPKDIPVLFIHSKDDCACYYEGVEIFFNKLGINNKELHTIYDMDHLLTMEPGNERVLDKVITWIKGLGE
ENDDNNSAQCMDDTNMLNAISHIEDEIIKRSNERVKLEKQNNDDFIAQKGNIEMFTVPLDNEKDLCNISDKEI
IENNMTHSEVEPVIN*
>N.PFL2535w
MSLFIESSFILPKGIIDSYIKFKTWNSCSYSDKEKKASQANIWTPVFSTRFAIPIIGIIYALLWNGDATFGYS
ALDMSLDEKYSRTLFETNNGNNGYVSSLTNKILNKMNKGPKKEVEEKNAEKILGFDDKNILEALDLFINNIKN
IALSSKLNSSDIYNHGNTFIDNVMSNEEIDQEIESFLNGGNYDREKLNRLWWQIMRNEELKYVSVNNKLYKVY
KALRKKYNVDNSYAENKWNECKKNIIVGRVEYQNYINKVFLDWINGDSINNDEFIKLVRTCRVTWKKLRTKMI
KALKKSLTKSFEKRHNELKSNEYIYKYKYELEEGDYADDDLLIKDDLLNNHDFQFDDDVSSVITEKTNECLKG
DHDLESVNTDNHGTELEESDSSSGSKVSNETTNSGDSDDSKKTIVLNLEGQCTIYLRRRPKNLTLSSPQRLKF
TAGGGFCGKLRN*
>N.PFL2540w
MNYSLVWKYLEREMKRYNRSVWCTLLYGRNNILIVLILNIILWNVCITNNGITLDSKYISTRYIRILCTPDII
DNVVNVKIEEEENINKENDNVIKSIKSIKYPDNVNLYNALRKKIRKNMEQNKYGFVPYSNEHLIPFYNEKDNS

INNDIDSLDGRDNLIRLKMYNIWLELMQNEQMKYYGIRHSLNKFYKNLNQKYNIKIESNDKALKECNEIICLS
AHYLQRHLNNMFNEWFKKGFVSIGEFQNIINATRHGWKVLTTNVKKKYKDILTDNLIEKMNKPYFIGDNIFNS
YLDHINVKDNTQQYNENMKKMSCIEKMPITEKEGNMEMNYNNNLNVKDKKLLYDNIVKNNSTIKNYTECLKMA
EYVDVDTSGVRVKEENNMMKRNIRRNNNIYKINNEEISNNVEKSNNIEKEDNQNINYNDGEELNETDKNEIYN
NNIEKEDNQNINYNDGKELNETDKNEIYNNNIEKGDNQNINYNDDEELNETDKNEFYNNDEENENDDIEISLE
DIFDKSTNIIKEAEEIINKRENGTSIDSCVFIDKPEDYDKQIECMNNNNTNGYLDYCNKKFCNENNSNPTYLQ
RKWKSLTHISDKDKSSNNIISSIWKYVSNKNNLLYWRKFNTY*
>N.PFL2545c
MFYYLKKISFPLALSMLVLTQYSNHEKPNTKICNQLNNMSLRNFRTLVELQSDILSMYVELEDENDAQDDLYD
KIKNIYNNDDEEINREFRSWLQKYMEVTRELKEKFSQNVE*
>N.PFL2550w
MLLKTSLKNICFLLCVVAFVYNNVGRKRVLSFNINNVYSRILSDLEERESESYNNNSNLKYNDSEFKYSFGYY
NDDTSGMSTFNGKDIISYLEEKNDNVLDDYNEDDNSPDMDTDDPVESYYDLLNVDKYGDLSELKNNFYNLSLK
YYPKMKNGKLLELNKKFEELSEAYQILSYKIRKEIYDNEGISGIEKMNIIHPLLYFNGIFIFDMMYQYIGTTE
IGYIIKIFLENNISSENIPSFREEMNENIMEYQIKREEELTELLKKRLDLHMDNDEQWKNVMENEINLLSNKS
FSNFILESIGWTYQNVANIYLEEIENVGKIYRGIYMFQANERINKNEEMFDNSRNHIHSLINSFYPYNEQINP
FLKRAQYNRTNVECITSNRENKMNSEYDALYNENVNNISDKVKYNLLNDLLISILYINVYDIEETVRNIAEVV
LRDNDVNVNTRSKRAHRMRLLGSMILQKINV*
>N.PFL2555w
MKNIINKKFDSISEYIYMNKNGMLFYRSYLFFLTWYFIGIFYISLKCTYQNKGEEKIQYDCIYSRNLCEIKKG
GFESFLNKICKYKLWNKKDDLNKKKIKKKKNKENNEDSKEQNINKNKDSCNNVTDANSMKKEDSIKVHNNNFI
SKQLTKEELYDLLYTLVEVPERKILLLLWNQTIGLYNEQLEDLLKEIYNFLPNRVYRNQKNSEGRSFYKIISR
EKIFDECVTNCREELAIKKVELTKGYYDLLNTKKKMDDIRNFIFSCIEQFEKLINNFCNKCKKDTLSLK*
>N.PFL2560c
MSRVLNFFKNLLLIYISNCLLYINNKHLLSIMHYEMYNTKLVSNLIIKRRLSETLMYNKRSQLKVSNINNYND
DHEENKNSLTEKKEMLKTSNRNENNRKSKKRRNYDVTKKKETLEDLLKEYDEEMKEIKKKKKKSFFKRAKLVL
EALDNIFIDKVTDTNIHEEYSELEEEVFENAIILSSSSMIFAIPIFSYICKRINFFDSPQ*
>N.PFL2650w
MHDIGVALSSTDIEHTLNFYKLDKDGKSIDEMKNYIYVFIKYYDTFKNDLFNKHKTIFTERIKNTQRLDM*
>N.MAL13P1.525
MHDIGVALSSTDIEHTLNFYKLDKDGKSIDEMKNYIYVFIKYYDTFKNDLFNKHKTIFTERIKNTQRLDM*
>N.PFD1220c
MLLFPFLFNTFLLSQNVNCQNNLRNKSLINNYTQKILKSRCLAETEKPHNLHYQNDPELKKIIDRLNEEAMKK
YQQSNDTYKQLHELLEKKETKSRSRNSEQEMSKIEKELLKIYEKMSGDENHIMQNLICTPMKITNQMIKMTNL
XXXXXXXXXXXXXXXXXXXXXXXXXXXXXXXXXXXXXXXXXXXXXXXXXXXXXXXXXXXXXXXXXXXXXXXXX
XXXXXXXXXXXXXXXXXXXXXXXXXXXXIQKTFMHIIYFLKYEELSFQCYMKSF*
>N.PFF0045c
MWYIL*
>N.PFL2565w
MKNVYFNRSLKFLCLTVCLILTIYSSRNRYIKIAFGKNDNIYSRNLAQLKLSNYSLLKDSKFESVTEQLTREE
LYELFDLLVQVPPRTYLLNMESQKWYM*TRNKGFIKKFKRNCT*TYI*TYG*PKSIW*ITS*NHMARMLV*L*
YDGFYFGNRTN**IL*PT**KGSN**NKKFHSFMYR*I**ITYRFICEV*KNI**
>N.PFL2570w
MSIILTFLALFVFLICVFPCITEDTHKYVYSEICGKGANENESSVYCMKDHKKKNSLYVYKHIMKFFLDKYKS
IPNEIALVEHSYGEPQNYITYDNFFRKVSSFSHTLNTYEGKGIESKKYNEKQNNGIFKLLGIYGSNSINWLAS
DLSAMMSGVTTLVMHSKFSIDVIVDILNETQLEWLCLDLDLVEGLLNHINELPHLKNLIILDTLSKNKEINLN
KEENNNDKKNKSSGNSTSVKGNDLSNMIEITCSGPLEYDKEKLKKYNELKKKCEKCGKKLMLFDDMTKTQTKK
FTIKNEDPDFVTSIVYTSGTSGKPKGVMLSNKNIYNQLFSLYNHSVRERYSFKNHLSYLPISHVFERTFAYSI
LMYGGTLNVWGKDINYFSKDIYNTKENIMGGVPKVFCRIYTNIMTEIDNLPSPKRRLVRKILSLRKSDHNGPF
SKFLESIFHISKKIKDKVNPNLEIILSGGGKLSPDIAEEFCYLLNIKYCQGYGLTETAGAILGNHADDEHFEY
IGGPIAPNTKYKVRTWETYKATDTLPKGELLIKSDSMFSGYFLEKECTKNAFTDDGYFKTGDIVQINDNGSVT
FLDRSKGLVKLSQGEYIETDMLNNLYSQITFVNFCVVYGDDSMDGPLGIISVDKYLLFISLKNDNMLEKTGVT
EKNYSEKLIDETLNETIYIDYVKGKMMEIYKKTNLNRYNVINDIYITSKQWDTTNYLTPTFKIKRFNVFKDFS

FYIDAVKKKYEEKLSGSSTGSMNNGERCEKKDDIKNGSDNENKRISKSQLNGLNECAIKSEEEDVLEEVQKAL
RKYEEKNITQGVQKQLRRSNGTSMRKAREGSLLNSNERENDINGKRYIKEIVPEEQQVNA*
>N.MAL13P1.6
MPKRKEKELFGTKHTKNITFNRVATQTYKWTIEHNEDLLDIPSSSHDDILKIKDETYNIISTNNLYSYENNDI
TPHQLGLPNIIPSGIIKHQDNGLRTNISMDTYIDETNNNNVVATSIIGDDQMENSYNC*
>N.PF13_0010
MDVQERNEKKSSLSSFHSKQIILIFGIIYVALLNVYICGDKYQQDADYSFTEGRVLAQCEFACTNNTKTSLRK
CAQTTTTSADPEGQIMKAWAADPEYRKHVNVLYQILTHTDPNDETSADPEGQIMKAWAADPEYRKHVNVLXXX
XXXXXXXXXXXXXXXXXXXXXXXXXXXXXXXXXXXXXXXXXXXXXXXXXXXXXXXXXXXXXXXXXXXXXXXXX
XXXXXXXXXXXXXXXXXXXXXXXXVVINKLLIILIGIFETYISFNITHAPKDNSLYSGIY*INDSLSRNRNID
IYDEMPKRKEKELFGTKHTKNITFNRVATQTYSDPIDS*
>N.MAL13P1.58
MIIFLHLLLNTLENDDL*IVNVGNVYKRNLGESEKNNKGSKRKRNLKHKIEGVNKTKYNKKKYKCIEPNVQEN
KYSTNNDFENANLENKSNSSISNVNYNDISKQLRRQELIDVLYWFNECSPNSDLFNLWEHTRGVNKERISALL
NEFMKGFPQDSIDFKFMICKVDKDVSYTSICLCCANEFGRVS*
>N.PF13_0073
MFSSTTNIFFSLVFLVLLLLLSIKTNILEKSSEIQGRIYNGLYSKHFRLLAEPSSHGSSKKTMKENENEEEDE
EVNEDQNEDQNDDENEDQNDDENEDQNEDEYEEEDDDEKEDEEEEDEDEENEDEEDNDKEDEDDEDNDDEEVN
EDEENEEQYCELIPILPNSSEDTLKKSKNMNIPKHNANSEYLLDKNLYDLKTFKNLNAAKKNYADSIIDNMDL
TENVKNIFKEFLYYYINKKDPNYQLKLYKQIEPDIEKYKKNHVICSIIDFRNLNSDLQYLRNPKGDFHVLSNE
EYENREKKKKEKQQKKKDKLQKKIKKEQDKIKKERIKELEKQEKMKYEKQEQEKVYLEKKELQEKHEQSQKQK
EKEIKDRRNKLLSLRH*
>N.MAL13P1.59
MKLLIEMNHIFFIYKILYMLKNNDIFTLNIENYKKKKRKNWYKISIFTYLLIFIISISFINLMTLYENRIISS
IHFNNKYASILDEYENDYYSSLERRYSKNSIRNKKDDESRNLHNNMNKNNMDNADMSKNDMYIHNKHNDSGYI
SKTESNDSLNLNYNDTTKPLTKEQLFQIINSLKEIPSDEDLENIWKHTISVAKEGLGRMIKKLYFYLDGYMDE
YEEMQKQKCFCFRKEISEDFMDEINETLSSHEKSYTYKFYKLIRKKRTVEKLKNFIFAFIDKMEKIINYLYHK
HKGIYIMKVLKLHKLI*
>N.MAL13P1.60
MKGYFNIYFLIPLIFLYNVIRINESIIGRTLYNRQDESSDISRVNSPELNNNHKTNIYDSDYEDVNNKLINSF
VENKSVKKKRSLSFINNKTKSYDIIPPSYSYRNDKFNSLSENEDNSGNTNSNNFANTSEISIGKDNKQYTFIQ
KRTHLFACGIKRKSIKWICRENSEKITVCVPDRKIQLCIANFLNSRLETMEKFKEIFLISVNTEAKLLYNKNE
GKDPSIFCNELRNSFSDFRSSFIGDDMDFGGNTDRVKGYINKKFSDYYKEKNVEKLNNIKKEWWEKNKANLWN
HMIVNHKGNISKECAIIPAEEPQINLWIKEWNENFLMEKKRLFLNIKDKCVENKKYEACFGGCRLPCSSYTSF
MKKSKTQMEVLTNLYKKKNSGVDKNNFLNDLFKKNNKNDLDDFFKNEKEYDDLCDCRYTATIIKSFLNGPAKN
DVDIASQINVNDLRGFGCNYKSNNEKSWNCTGTFTNKFPGTCEPPRRQTLCLGRTYLLHRGHEEDYKEHLLGA
SIYEAQLLKYKYKEKDENALCSIIQNSYADLADIIKGSDIIKDYYGKKMEENLNKVNKDKKRNEESLKIFREK
WWDENKENVWKVMSAVLKNKETCKDYDKFQKIPQFLRWFKEWGDDFCEKRKEKIYSFESFKVECKKKDCDENT
CKNKCSEYKKWIDLKKSEYEKQVDKYTKDKNKKMYDNIDEVKNKEANVYLKEKSKECKDVNFDDKIFNESPNE
YEDMCKKCDEIKYLNEIKYPKTKHDIYDIDTFSDTFGDGTPISINANINEQQSGKDTSNTGNSETSDSPVSHE
PESDAAINVEKLSGDESSSETRGILDINDPSVTNNVNEVHDASNTQGSVSNTSDITNGHSESSLNRTTNAQDI
KIGRSGNEQSDNQENSSHSSDNSGSLTIGQVPSEDNTQNTYDSQNPHRDTPNALASLPSDDKINEIEGFDSSR
DSENGRGDTTSNTHDVRRTNIVSERRVNSHDFIRNGMANNNAHHQYITQIENNGIIRGQEESAGNSVNYKDNP
KRSNFSSENDHKKNIQEYNSRDTKRVREEIIKLSKQNKCNNEYSMEYCTYSDERNSSPGPCSREERKKLCCQI
SDYCLKYFNFYSIEYYNCIKSEIKSPEYKCFKSEGQSSIPYFAAGGILVVIVLLLSSASRMGKSNEEYDIGES
NIEATFEENNYLNKLSRIFNQEVQETNISDYSEYNYNEKNMY*
>N.MAL13P1.61
MSFCYVRTISIALFLFIFLFLNNGNFKNNFYRKDNKYSSIKIRSFRSLAENQKVETEQSTPAKPEPTEFVNND
IHQNKNTFKKLKNNEKKKEILEEKMKDFVKLYVNKSSGECQRYRLQQHLKNYSSSNEYKKFMNKFVKFLKVHN
DLNALKSQLHFNNVRAATIIFIAGFLSIFAILLTISAVAATSKFTNNMLAIAGVGALGSALSLPGMALLFVPA
MLYVLNRKNEITNHYSERIIKQVSNF*
>N.PF13_0075
MESYVEFNIPLILKKPKTREYNKYSQKLLEGLMEYIAKLSQYKSENECKNICRNMNYWIEDMKDEFSEKILPH
FIYKEDEQVWDKYIENEYLVKLDPIIGDVCKRKPIPYIKMVRNIRKKLEQLCDKKEEIYKEFENSKLTNKEKC

KLFNEWKNEKLNELRRIKGWYKYATKDKYNEAFSINDKCSLATLFNIDFNCQNNIRRKKKKKKKKPRIKSQPP
KKSADGDNEDDEDNEDDEDDEDGEDVNHEGDDINSKVGKFPNQDRKEDPTDREKTDVNEGQNGKSLTQEKTLK
QIPSTSPNNLEQKLQQTHISSSQQKHLIKTTPTSDSTTTSSSSSVPLTKQSLHSSVPALSSTQSTTYNTTDSA
TPPFAKPHPPSLSEKENKRESEKRNSDKEFSQSYEEKAKNKSKDELKSPTPNEEKKLQVNVTKSKNQGTSENK
SPEVTTSPIPILSSRIQQEKEKLGTRGDKSKGSKIISPYNASYYLGNVLNIIEDFLLTLYSDDNTQKILNDPT
DSKNNNINNSNSQDEILKNTQIEKTQKSIIISGSPNDETYKNKQEDRTKHNGQNNVQQLYDSKPHEEPIELPS
DPIERATFIVPPQRKTKKLDPSIFGRKKIQITPPIIGVPPSILEKYPHILEKYKGFSKSIQEEEWVEGNNDAL
PVRKRDVIENELVIQTIPYDVRHIIPENNDNGKQKFQNKLNEYSLRTYDIPKNINLHLKPNEYLGNEFPKIIN
NYDYQLDHSKKYIPKYIDLDNQPNEISEPKHNPKNIDILGKKFNYPIPFNKLNNIDKNSKFTITKNDYSLYSN
NQDIIVEIPKLTNTIPKILTIPEQKITPNYTKMDIDIPHENYEYSFFRKPNKIEKNTDFEITKNTSLNTNKED
IIVEIPKIPYNIPKPSIYQQKKQQPNYTKKIVDIPLESPRNNFLKKFHKIDKISDFSVSKNPLLDSTDNENIT
KTSNRQINNPYVLTFPQTENILTYSDKISGIPNDIIHNPLFSQIFQKPDTQHNIRNPKTLSFYFDTNNINLEN
TDITEPLSDKVQIPKVQINFETQPTIIKQGGHPEDETPVCFKDPTNSETIPCTLEESLKHKDVVYTTNNGQLH
EKQVISSKPSLSYNFNPNTKSDMLISTPITLPKQLKNNQNIYASGGNILNKDIHNVQNIYKMLDLTSQYPSEG
IGSSTSQGQPIQHSNSTVGTSIGAHSYKVSPQTSTTSEIRNAGTHHHSKLSSSMGTSANTLFHSTSSVQSTIN
HDYVYANTFIPKRPYFLHNGLQQDMFQLHFPNTYSPRPLNIQNHTPEITIKNSSGSIFSGNIIRNGTTGSNMV
SMMIQIATVFMGGFMIMTVFNKRNIFIFGAKKKKKKKNALSYLDETTYVPVSEETYTESISYELLENYEDLEE
NNYDENNVDYIYPEEELISSNTREIIKENQYTQEEDNIIKDEEKHSTLIEQKKTKWKSLIELHLTILEEYKME
EWDIHKGDFLEICVQEFNNIQDFQEEKKELENETNKHILYNDDDIERQNLLWHIYIENNRYISDQWKNKKWFQ
HIKTELQNEMHNFIKLYKEDKQQQEQKKKNINITKMSIINPLMERQKDIWRKWIKKQNFIIYNYINEEWFKQV
LKLYKQHYNVSKDNLKNNKIYIIRILTGIFIIVLEEYMKDECTVAKKAFLDRGLEKMNKRDNLDINTHITDII
NSIKKNIYISNKEENNIKYKKEKWYIEMKEDWVRNEYKYLSSIKNQNIFENDLILRENKSLIDIQKDISRKHW
DDLQIKWIGEDNENDCLKIDETNFFDKKKGNYKKPKDKKKNIQTKKENQTYNNKLEKQIYVEESFNNMDDVKL
HKMKTVIEIHMELLDESKREDWELNRKQFLEICIEQFSYILETEKKNIIKLNKLQLNIDNEDIMNHMTEKQHM
FIQKMMERNKFLLEMWKRKEWFHKLKENWKFQEKYYNITQIQSNNVENFKTEIKNIILEKQKIIWKKWVAKNV
MHIEESNNESLLKDFITHNENIYFQDKKTINNIKNQNNNINENSLKKYTEKKLLTMIWINIHMLVLDELKKEE
IQESKIILLDKLIDRLKNKNDTKTNQPIIHIIQEKKENILQYKRNHINIYQGENENINFKWINEEYKNCNILP
EEIDTYDMTVKNPILDIQTDILLKNWENMQIRWIDDNNENDWLKISS*
>N.MAL13P1.62
MKIKKKIHIFMYIYMFSLLMWIMKLSINYNFYEVYKKKYKVQYIPSTRSYRALNEIEDLRDTKKPHVKIILLS
EEDKKNDEKEKIKEIIRHYRNKLDNEVKRKMYKNVNNHYFRKGKLNKT*
>N.PF13_0076
MVVLYNNKEDIAVCDDGVNKEVIKNIFMEDIESKRRKEAIVRKRSIVHFGLKLFLFSFCIWTLQYANNNYDYR
DNGNNYTLENILESRINRSLSEKQSHVTNSATEVAAQENNSTEEKKNNPPVEQQQQQQQQQQQQQQQQQQQQQ
QQQQQQQQPNELNNFENKLKDLKDCLFDKLDNAVDWENLSSNVKGYLEKVDSTIENKIKKEVQTATQQSNLMT
NLDPKTQFIKNFTKNYDLYTPPLLLMLASAMLSENLKKKIFQVIAFLLFVVLIYLYLKLKKVNKNIKKDTNDN
TTNNKSATKTNVTKTS*
>N.PF13_0011
MSKVQKDSAKPLDKFGNIYDYHYEHETHAPLSPRIRKVGDIEFHACSDYIYLLMTLSKDPEKFNYALKDRVSI
RRYVRKNQNRYNYFLIEERVQDNIVNRISDRLISYCTDKEVTEDYIKKIDDYLWVEQRVIEEVSINVDHAREV
KEKKRIMNDKKLIRMLFDTYEYVKDVKFTDDQYKDAAARISQFLIDVVDSYIIKPIPALPVTPDEPHHNNI*
>N.PF13_0012
MKVSKLVLFAHIFFIINILCQYICLNASKVNKKGKIAEEKKRKNIKNIDKAIEEHNKRKKLIYYSLIASGAIA
SVAAILGLGYYGYKKSREDDLYYNKYLEYRNGEYNIKYQDGAIASTSEFYIEPEGINKINLNKPIIENKNNVD
VSIKRYNNFVDIARLSIQKHFEHLSNDQKDSHVNNMEYMQKFVQGLQENRNISLSKYQENKAVMDLKYHLQKV
YANYLSQEEN*
>N.PF13_0012a
MNYFNVYIYVTFLLIIIKNEVVKGQRLNRRNDSKKEPKIPDEVDYITMNTFISPEENRKLAYGKVKRTNEKFK
KIEKAKHKLRDQIYVFLAGLGMLLLKQSVMLCFKIANRQMKLAKRKKKS*
>N.MAL13P1.12
MEKTQENRVVETGQSSTKDSHFFFGEGGTIVRLLIIVVDIIMLINLLVDVNISCGGYLRLWVIGAVLLSFFLF
SFIERIQRHLKENKGFFSEVVLMLICFLWTFFGTIQINKSGACQKNAPNLFWTVFTLVTIIWCSIIGLILSLM
IITIGSFFIVRSKLAKI*
>N.MAL13P1.13

MNYKLKENVPPNEDVDIYEKLKTCVINRNVEDFYEECVSTFLEKVKNIGDTNHIICHDNYSDIINFFYLNLFV
DNSEFVRVVAVSQDNNENAKICKRYIHDKNEKDVTEKSELLQELGLYLKNIKLKYSEKLKKIKNVIIDNISTC
QKCIMIYYFLQKKLVEKISSNINKKDDNVTNIIKKEIFEFNFYRIINKITCPIGYNNVDSVILELMLNGSKIF
QMDLNYILEYNKINKVNLKIFKEKFINYDNDEIYRIIKEYVDYDSLIFFCNLLFKEFKRMKNLLLQNDNYNNG
NDTSIINENISNNNNNNNNNDPLCNDNTLYNYNIQCLLPYIYIHIFILLKHKQVEETVRRVLKERIILYSLMI
NSLRYSDNFIQLIIFKIINNIFINEEEFLKDSNKKLISSIFSYWIKLSDGCIVKMINASITDESSTSHNNLSR
SSSNNTKHNSNKLYNNSCIKKTKTDDENFLKNNFTDYDIYKYMQSLICGLYLLTLIRKKKNNDHHYNNKSDNT
NQFSSLKEYNIDMLSLNHFFINMDNYIYLIGFMFSENKDTINNLFNLIFKLLYFIKYLKSYIIQNRNLKNKYF
EDTCIYSSINILYNILEVTKKFFISYFRIDEYTKNLYKNYFRDRPLTFENMAFYVIPLFINHEDYYIRIYSIK
DFDNPPVRYKHSGFSKKNLFRIFDLLINIEGYKSLSQDEEEEEKKNINQLLLLLFKCNINDNKLSSNITNLIF
SEKQKIFIKYILRLCKDEEYSIIHMKNFFDYFFVLLVKLINIIIRRAQCSLNIEKYKDFFMNIYEVVEIFMKE
LKKEKLKKHKLLFLSSGIYLCHLITNSIYKGKIHSSVPYLNVVISLIKNIEHHLNFIQDDLIYINKISKDIIS
DKKLSIHSEDEDNKDDDSKSVKDLHVLYFYNNVYSEELGKNVGNNNNMANNNNNDNNDNNINNNNNNNNGENI
EHKCVKSTYKENKNNDINCKNKQTNDKNLKSNPSDNKGTNNSEIVKKLNKYDKFMKMCLLQNKNNDEIIYNYI
NCLFYLCVSNMNKRTRKNVIKSYDLVVKYILKDEYKLHRVKKKSLLFSMPIYIIRDVENEGKRYNLIIDKYDY
ILRSYIIDFFKCKKYLPCLLLSCLSFTNTMILFLKKKNFINSTTNNNITEKEDMLDCFMLVNSKTWFYFNVLV
ECICDYFMTSQSKMDVKSQQIKEQQNEHTQKCNNKNKNNNNNNNNDDDDVHKFSWQIINLIIDSIWKILIYIFY
NIKINIFSIKVKTFSINIYGRAALLMFLQFYESWIVFIKTFLTSEKQIFISRNILLPDLFYKKFGFLSSQILS
YFHFIESNFFVDSQNEVNLRLLEKQKDVMNNILDVLIYFIKDKDPKGYVNELNSFSQFKLRIKKLTFPKKLQD
GFLSCTYDHLFKKAKELLLRVEKSKTLQMDEWPR*RSKYQLKFVANGQGR**RKCN*Y**F*YNCGKRFKNHY
*DRFKF*GCKCS*KR***RREKSIFFRTS*KNYRKKCSFKKYKESNSIE*KSCRKE*TTNF*RDERKTKRSRK
YLNEIFYYDTRIFGLGFF*FR*Y**I*KFY*RRITHTF*K*GGISQVFSTHGFGRVQV*YAQ*HDGRYKQICH
KYCG*EKNALLGSMACIIE**EQAQFG*HKTNGFNSFNSL**GQKQ*YTCR***YKYKI*QIKRHVEMY*TCY
WIS*YWIE*I**YI*Y*IN**RQFTF*GE**ENSFKIKFYYL**IPCICIM*SNDKY*GISKYIFE*KLSSF*
FNIKSCRRK*SRKRFM*YEH***LYK*L**G*K**IKKRIREINQTGETNIKNFK*I*FIK*ITNRSCEINSF
K*K*YIFNTRAPRYR*NQNSYRNSVCFICIII*KEL*KR*EKKRFVI**TN**YKEKKKDFGLFSIQFSN**N
C*EDIK*GITKFY*LNKFL*K*DKKE*YHIAKMW****EEDIVRG**FI*FL*YQ*FSW*RSTFQLF***ERK
IIQGYGIIKRFEKCR*IK**QYIK*KNESI*KTNDCS*MYTYWIIQKNP*RNPANFIRLYI*QEEKF**KSLS
CTF*KEEK*INIFHRSSRLYEIKNKRNEK*FEFE*F*KV*F*L**Y*WKVYVSEG*IY*TIFF**IYK*FG*K
IFRKFVIFI**IIITL*LEYTKIDIRKKLFRRMYDKID*DR*ADWFFLYK***RKYVI***NYI*YLIW*CFP
CY*KFRI*IFNYR*GMSVCRIKLFNTI*IESKKYYNGRRSKTITSYNLFIRL*KVWL*SFLI*AFIIM*CF*C
FIKYSISYETRNLLFPK*LFL*WFN*EC*YIIE*TIFLLFSRFGFFWML*IY*YRWNRIYDI**ILYKLC*SL
FYI*ISVIYKKYYFETSRSYQIGS*FI*ITCTF*FERYWYYLPISIASTFNKKYVRRIF*R*NPIPRSINS*C
ISGT*EAHHNIFLCKI*IGGVRK**VIT*NGNL*KSG**MEKNKRKICHI*Q***C*I*YERK*R********
************RVEIRLQ*WGTYQKMDIF*NA*YKK*ERFSSYTKVRK*YWFFKR*KKIKCCFDKS*GLFMD
NRKS*KFRNE*NMGLFNSECYHKKMLFRFKN*F*KQYHRKYHKRESQ*FFYTFGKRHK*KKI*ILRNE**FIF
*FVYNKRRTR*RRKL*RNYSR*RQII*K*K*I*IK*K*V*AKYNLE*FRGQRI*F*SSLYRK*LLV*L***RK
GKK*KL**KG*KENK*LLT*R*FVNKKKKI*PI**
>N.PF13_0013
MGENNDNINIINNVNNNYNINSDNVGGDSSNKVRLIKYEESTNKEFILKYLVNIKNDYIEKQMRALYECREVY
KDDIDEVINILTYALKNNDSVLLRHEIAYVIGQISNEKCNNILINLLSDENENIMVRHEAAEGLAAIGSESNI
PVIKKYLNDSSVEVRETCELALSSLIEKNKYAACSCINKTVPYKNNVLNNDNPKRDSNNNSNNNNNNNINNNN
SNSHSNSFSNSQDDADDDIYFHSKKKFNTIDPVVCISDSNNKKHVNDLIRDLNNEALALKNRYEALFLLRDME
TDTSLNALGEALVNDKSSAIFRHELAFVLGQVLHLNSLKYLLSSLTNVSEHEMVRHEVALALGSLGSLNLNSD
EYKIIQEQIIDTLKKYSKDECVVVAESCLVGLDYISENLNISIEVH*
>N.MAL13P1.14
MTKGNAILIDDINTTFIKDDEEKKEMVQANDGSSSSTPVSFNEPNKLSNKEKKKLEYQKRIQRKKEKQLKKVQ
KLITKTQSGSLSYLKNNIASSSVFNKLVDQKKEQNKNNIKNNYIENEAIGNKNNDVNLNNNDINVKNNDLNIM
NSDNINNTNDIYNNSNNKKKKKNKIKNINKNTADNSSESSHEEQNGKSDLYCALENISKKKLIKKLKNKNKHE
KEIEKKKLFKKIQEFKLNNTEQKNLMIPFDIKAVPKNTEHLDKLLKTYEELNIELPHYLNIIKKKIEKKRKRF
LDKMEYLKEEKNVQLTESIKSKEVDGDVKKKKNKINSLKEESESYIETNSINETYTDQCNIIIKSESSDEDYN
KMEPHGSYDDSDMDHENISSYHNSTKEKKNDINGINGINHINGINDINNNNNNNNNNSYNNAYNKDKRKVCYK
NVQVNRKEHIDKVRASLPVLDYEQELIEAVLNYDVVFVNGDTGCGKSTQVPQFLYEYGFTSNDYFIGITQPRK
IAVKSICNRLNDELNEEICGYQIRFEKSYFLKHSKIKVMTEGILLKEIMSDFILSKYSVIILDEAHERSINMD

IIIGILSIISKIRNDNYYNFKSDIIPIKIIIMSATINDNNLFQNKIFQNFTTVNIPTQKVQVIDHFLSYTPKD
YVEEAKRKIIQIHKKLPQGSILVFLTSQEEIYRLYNMLSNLKITNSEFNQQGENKIMDEVNFDIFDLSEGEQN
KDEKVSLFFRETDENEEKKILFDVSEEENSVESDIKESDIKESDYIKEGDIKESDYIKEGDIKESDYIKESNI
EESNIDESNIEESDIEESDVKESDVKESDVKESDIKESDNDVYDKELHDDDHMDDEPKKSNINTYELNNNTKK
KSSIWKGSDGSGTLKVFKLYSKLPMKDQMELFHNPKENERVCILSTNIAETSLTLPNIRYVIDCGKEKRKIYS
NLNDYSYYIIDNISKCSAIQRKGRAGRILYLLKKNKKNKKKMENEKGHVYKLYSSNYYNYFFKNDNDYPILNY
PLDSLILYLLSFNIKNVENFPFINKPDKSKFIEAKKRLIYLNCIHFVYKDIEFLFKQIDQKRCLKSSIENHIK
KFNPYNKKGGITLTGSFILLLPISTRYAKILTDVCLKSLAMKQLNTIPLAALLVSCLYLESIFSYDYKLKMKY
KKKKTTKKDKNVNMTIHQNKEENKPKNHNNLINLFLVNKKKQKHTYTSSDDDDDDDNNTLSSSNNDMSEEHIH
HIKNNINVDVLENFKEQFDNDILFYLNICTKFYFSKDRNETCQIMHLDKKKMNELLKLSNHLIKIINYKLNTN
ISLDMLQENISDISKNIIHYAVIQGFIDHLAIRSDLVHNQYTRNQNITYNSKKAYITQNMNMPIYINSSSVLY
QNRPYPKYILYNYITKNRNSYVMFDCLKIDDAELGNITNVCIFINEYEKIPPAKYDKDNDKIIVYVKPLYLPS
SHYLSITYKELNEKDLLFYNYFALFLLDGSMFLKMTKFQMYYSHTTYDVINSAQENIKNFIEALKNAQINNRS
SLIAKWKEDRDFLKNEFLSLIVKKFNKNQNDFFNNIWPPLD*
>N.PF13_0014
MDAVQKRVLKSNPSDLEKEIAQCLLDIELSSSSDIKTDAKEIKLLSCDLIEVEKLKKKTILIYIPYKIYTTYV
RKIQRKLINELEKKTKKYVVLVAKRTILKGKQKNKSFKIIPRSRTLTSVYDSILEDIVSPSEIIGKRISMKAD
GKRVFKIMLDSKERQRDNIEEKLISFAAVYKKITRRDAVFSLPPTNEK*
>N.PF13_0015
MKFSLVKKIVYPKNPITNYQILYLKNKNVNFFFRFSSSSVCIPKRVGFMKPSFKLNRKAQKYIVNVQWLKSND
TKKLEELCKKIKKDAHTFDSYELIDLIYYLSKHSNKNVYLTIEPLLFHFFLKYNKNSHTINLNGISIHRLLRV
LTEYQDIVYREWIYSISEIISRKHAHISMRDIQLILDDLALFYDFEIHKQIIPFYKTITNRINELEIKRLPFL
IHVIGRIGCNDNKLLYVLFNTLKNNIFKNELYRSDFSGLMLRGLANLKIVPQPNLLYQLYKPIKDNINCTNIK
YISWCADGFSRLNFFYPLPILVNQILKMKDKISHLELNDFSSILNCIQSYILIKHNKNTNIIIEKQICNTYQE
NRNSVHNKHLPYSNIYEQNIMQEHSNSKTTYSNISKQKSQHIEIYDQIDDNINNHMCYNNVYYTSKQNSKNNS
NIQNNNHIFNSSNQYDKNIQSTNKNLILEEQKNKNKKNDSFQNYKYSYNHTDMDFSISIEQLYDIYIKLENLI
LEKITESIYNSTPPYRVIMFELMTRLFRFYTKPIDLIITMNTHRYDTPILLKHSNSLQLILSNQQNRLMSNSN
LSFYVNKLNIVNDFFFETHHKDTHQDSQNEHKTDEPKIHNVFKKKNNDIFVNIPLPKIFKNFLHSIYFRTPAL
GGRTIMLLLIALVRLNFGQREKLEYEQKMNTNILPLMKASEIYRPLTHCIIREIIRKLQTFNSEELIICAICL
NELDALNLNKELFPLLISRFNDLKNNNYMTNDQLEQLKNLFNYWIHHKRNMIINNLKFSNAKQLKHFVRL*
>N.MAL13P1.15
MISFIKKVAKGISKGCSWCKSVSFRKYFSEDYFWTQANIGPLCIGIITAPYWISSLKNLYWSHRYEKLNKEEI
LSDRFTWLYERMLEDEVHKTLLDNLSSYNFKNNGPENMLGPSII*
>N.PF13_0016
MKNSEQVRINFNYIYSNKEIRNDVYLPSSDTFTFVEALEEEVDKISQDINIVLEMGSGSGYIILSLYEMLLSR
NKKIDMLYCVDINKKACECIKNLTYENKIFNVEIIRNNLFNNIRRCELFDIVLFNPPYVITGPDEMNKTDLTA
SYAGGKYGREIIMKFLLDIHNYLSNKGVIYLLLEKSNIPQEILNCEHVKNIYIYEEIKKKKTLNETIFIYKLM
KKK*
>N.MAL13P1.16:mRNA
MSIIYGLIAREKTVLAEYTEYGGNFSNISRLLLEIIPPHTSRKSYIYDDYVFHQLIKNGITFMAMTDKELGFL
TPYAFLEQISKIFFKNFNNTSDLITLSLDEEFKPVLKENMRVFNEYETNDVHNIKNQISNIQNIIIENIEKIL
ERREKIDILVNKTEKLYQENINFRRQAMNFNFHMWLENNRMTLYFISSIIIFIFFIWSLYNV*
>N.MAL13P1.16.1
MSIIYGLIAREKTVLAEYTEYGGNFSNISRLLLEIIPPHTSRKSYIYDEFFKNFNNTSDLITLSLDEEFKPVL
KENMRVFNEYETNDVHNIKNQISNIQNIIIENIEKILERREKIDILVNKTEKLYQENINFRRQAMNFNFHMWL
ENNRMTLYFISSIIIFIFFIWSLYNV*
>N.PF13_0017
MLLCSKNSEETYHNVINTLLDDIKNFQVDEKYYFLIKQKQLESPFFSLCKINFRDDPIIINTNNDTKEDEAFN
LIFHNKKILDKKKKKKQKEEGLIKNLTTCDNIIQDEEFIDKLSSLLKLNHDTNIIIENKKNFDTLYKYYSFYD
DTFNTSCFYIQDDYDLFLSKHVNGLYILSISKYSNILKNILSDLINMNNDKYNLKDLIKQYVQIIFDPRLINA
DTSGKRKTKTIFINDDMTILNIIYKNNIYKIKSKIKGYYYDINQNIVHNPYLLLYNTQDEGWLIIVKNKNTDL
TKFVEATDYYNKKKLCIEQYDELIKKMK*
>N.PF13_0018

MKIHLSSDEVNLLVYRYLVENGFVHTSFSFFNEANICKNPYYMSHGDKLPSGALVSFLQKALIFIYIEYHTDH
NDGKKIVCEEPFSFFRRHDCWNNEYNCLHQQERKKKKEREGDDENINDDMNEKMDDMMDEKRDDKKYDRMNDT
VNNNHANNNINDYNKNVMLNNDSHNNNDSHNNNDNHNNDSHNNNEQDGNVLKRHTPLSNVNDCKIETFNKNTD
DDLNNNNNDKSSTDHFNIMNTNDTSFDNKEENKVLKNKNNIYRNSFSNFATFNYNDSNLSFKDIKRNYASIFA
QVKTPRRNASNNKKKKINNKSKDDVIESKANTENTNNDDNNDDNNDDDNNKNNNNNNNNNENYCDVKKEEPNF
SSKKRKKCGSLKTANIKGSQNKTTDGNNNNINNSNSSSCCSNNNNESDIKNIHVNKNKNQQDNYIKDLKQLNN
EDDNNNNNNNNNNNIEGNESFQNREEENCDISLNKENNTNINNISSNCSSTTYSGSATSHKRKKKKKKKKKEQ
TNEGKKSVKGINKKDKKRNSKVESKKEQAFVKSVADKKNNKDEEDMDLSEKQILHNDDNKLNSDDNKLNSDDN
KLNSDDNKLYSDDNKLNSDDNKLYSDDNKLYSDDNKLYSDDNISNNLINDNISTAKSKEQIKAPIINTYDDIN
NNSNSTNGNMNEEICAHLKEHSHSISYISSLENSPDKLKGQKQNDTNEVKYNSLDKNNKDKEINNVESENCWL
SKVASYILPSAKKNNESIKKDEGDIPKEDGKNDNNINNNNNDDNISSSRSSSRSSGRSSSRSCGRSSSRRSNN
NVSDIHVDDYIKNDFNMNVSKILSNEINYNEFGDNICNLHNKESDSVYDKEVEKYGYNHDTLKEEKLVDCYMN
SNMEEEHLLKERERKDSDVTSFNNESYISNNTYEPFKSDYTEDKNYDKCYKYYKDDTHNIRNKDPNIFGKDIE
FFTNKNVSYTQNNVEDSCYEYNEKEKPIFLKNCKDKTSDNKFNNDIVHMEKKKKKTLF*
>N.PF13_0019
MIYVATLSSDYESRKKEDNIKGRRKLWRISIFKIILIILFLCLFKKNDVLILKDECFDEYNNSSSIWVAPFVS
MKDFVKRKCGNYIYMYIFDMKKDGIVNIESTFLETLHNNNNNYYYNDIDLNRMKYMYHDTCFISNKRNNLKRN
NFCNIANIKDFDCSCGEKKRYRILQEVKKKDSRRKRGDKKRNEEYKDDSDDNSDDNSDDNSDDNFYDDDGDDD
DDGNKNQMPTASNEVSEGIVFFCFIFISATMLQFILSKIHKFNIPVSVIWFLYGMCTYVIAKNFNMFEENTLH
KSIINARNIDSSVLYFVLLPILLYEATQDINYYAFKNFLYGGIGLAVVGVALQVGILGILFYYTFMYRQEQSP
LSSSFLLASILSSTDPVAVLSILNVVDAPSKISSMFHVESLINDGSSVLLFQFFFYLTIGYKANTSQYAVLFV
KLLFLSPVFGIGMAMITFAWINLYRKHYYNQCLATITMCYLCYFVSEYYLNLSGPLSIVCYGLFINAYGHIAL
DEIAQRKHKEIVELLALMGNSSIFIISGIISFGMMENVFKDNLYFFLYIALTYVYLLLARSVMILIFTPFLSK
IGYEVNCKEILLLIWGGLRGGIVLVLGLRIEAENKINEKLTKELAYYISGSVLLILTIQGLTFECLYRFLNIY
PPNPFRIVYLEKVLKMIDYNFYIRKKNLKNYWLFKGTNILHFSSKIVPELYWRKMNKYGEFDLRLPDIYTCLQ
DIRTCDISFWERSYDSHTFIESFDDYSCNEKRPREKNKMEKGVISNLDLKGRTMNEIDMVHDNNNNNNMRDDN
VRDDNVRDDNMCDNNMSNNNTCDKDGYTDKRNNNIQNDDNNKNYKNNKNYKNNKNNNNKVDNNDHEKENNHMY
NNTHNITNLSKLYNDEYYNKGKKCENANINGGDNKKNVYENLRDKTNFFNDLTGNEDTSDVYTYLNKHSYNNV
SNIRGGYNYYYGNNNKYNNNHNNKNNNNNIHNNIHNIHNRYRSLNVISRKISIDNSYKGEMRINVIRNNDNYY
SSYDDEDHNYTNINRSLSSKNFDHFSNNLNEFTDNVSSCSDKAFDHVKIKNLNAFNFDKIAVKDYDKLIKKMN
YREFKRTRTEKEENFNVIDNIVNSNKETFNPKYNLRLIEHATHLPKTLSDNLLMKSSIEQDDPYDKDQKKKRK
KKKKKKEKQKKKERKKNFLNFRGDDSEEDGPIDNSNPDSSEEEEFKQDKEKSIKQKNNNNNNNNNKIHNKDSS
DNDADIEEVEGESDDMSNINYDNITKEDENLLNEYKDIKKKMDDKSDYYDSREMLNLDERNKDLIMSDNFIVK
IPVDISNDDREIIKKNMKSNENIKTISVDALYNDDDKKKKISGSNNDNNDNNNDDNNNDNHNDDKNNKNDDDN
HNDNHNNDDDNNNDNHNDDDNNSSHYNKEKEANFNEYLTYQNLFALQNEDNNIINFFSNDKKYNSRKSTKKHS
SAFNISTKGTNDKSLFLLNNDIDKKKKKSLKLFNRVNNKSSCSACSGGFNFGPCTARPKKKMCEHYHEHVRKY
HADNKKRKKKYDKKNIKEGYDGNSCSSSNNIGNDDINNKKDNFEFGNENCTLPRNNTADFTPQRYDTDQTINF
ECIEEMCQKRHGGIPFYSFLKRPKIKIKNKLFNELRRARSHESYVNKEKVIKKRDYINYNTLKKRMIRKEREG
ELYIMIFNTCRELYKKLYVNGFISGECLLTLTSILDLSADFAIKKVRMNPIKAWADAFDKGRKHKRRSTRRNS
IDHRNGFEYEFHILLSKLKINKKNFFFFSPKVVNIFLVYEHCMKDLQIILSYVDVHQCTLDKGGITMKLLLGK
NLLRSYYRNIELAKNLIPHLVRKYKDVVKYCLIKIGTDMLLHLKKTIVNEQAANGLLLSQDNEKLNGIFDEQQ
VKINRYRPYLHYFLKKNILKRFIK*
>N.MAL13P1.17
MYVLNMTSDKHSNNFFSEFNIYNKKNTKKVVYRYFQNRTPKQTKYITLSLLVFSGMFLYITSKAYDRNIFYLN
NEVYKKLSKNYDVSSPMEAQNRAFMETFVKAKRKNLDLEQKPLISQIIEINDKE*
>N.MAL13P1.18
MKEIITDSEAPTYNIDSDDEAPCEYLNDIYAISEDTFCFKIIKELGNGYYSPGNGHKIKISQNVTLIIRNIIY
*KI*NIYIWSQGFF*YNTYK*NIQEHIFTYIFTYIYIWIFKNSEVKTKYDKTHNFKAEFKKGLRFKQKCVDIY
SRDPLINKYKKIKINSNDINLLMKKKKINKERKKTNTDEINIKSNIINNMNDLKNNGNLKIGDSDHLQIYNCN
NNKNKLSCDNTTHLYLTRQDINNLKYQFFTQTELCTYVIYLKEFRRVDILNDDKTIIKEIIKEGKGICTPKKN
DYVDFLLNENNKQEYIHTILDINNLKYRGLFHILQHMKKNEISKVILKGNQCNHIYYLNHDCQKNIQNHPFYI
NQQHNNVYDKIHTNHKSINIKKNTSCEEHQIINHPQKSNTNHKSIDYIDDNKTSHVHNDTTNVSLNLIKTNIE
KEQIKEFYIQLIDYKKSKIININSIININTSEKLLLYINQEKIHKTNDINKNIKKNQQFNIPTIDTESELIIK
MSLTYLDNTTKQNIYLPICYTKNNKNIKKNSAYFIYSYGSCFTSPLWFYHSLKDLKEGQQIIIPIEKNKNIFE

EKHFVYHIIYENFDTTKKSVHKNQKDHKNFQINTTHKQYNTNRNKLHNLNSDNKNGRNKIKTHNHTYNFLQAL
ISNRNQGKGFYLIKKFKKNDVNNFCNAFFKLHERYMDKNKNQKKIKKIKKIKKIKKIKEIEKIKKIKKINKLI
NFQTKMNHFNNLFLNFKRKHFHRKIIKIKMKNITLTTKNIINNKLLQICITEQNRDKNIKKKIKCTSHNNTNN
LYKQQYKKKYNNNNNTYLFEKSLNNVYFDRFFYNKDTILKIKVVKIICKKKDPWNMNISEQIENIKIYNDMGN
KFTQNKLYYAASIQYKKAFDICRFSKIYNLIFEEKKKVQDLHNNDYQNEKDNKQIEQQNYQNELLSYMEKIIT
NLSITFYKMNNYNECITYAQKACIINPQNVKAIYWKNMSYLSQNKYTQVIQNLNNPFCLNNETLLKLYNSARL
IKKKHDTNFNSLFYAMYDNKKI*
>N.MAL13P1.19
MNENNSKNVVVDKPIKNAEEEKKKVEDINSLYDILKNVSNNNDIFNNDDYILKELYPLIKNVIGDNKNICYHY
NKSFMLKRWCLKILYTLINKIKDMNLFLDIFNMCSKLIIRENEMNCLSCIKIINVILEENKNNLNMFDVDTYI
NVICLSILKNFYKKIIYKVKKGKFLKSSISIYMKKKKKKKKIERKVKVKEKEYKNIIYLQKKKKERYILNSTD
SIKVIKEIVLSVLFIIRIYPNVINNVGKYLNILCKILSIINMYDFKLESSFFIETIVEANNYYSNNKNDTNQK
NDKSNMHIIKNVFYYQYINKSQNTEHKKECKTNHLINVPSKIDKNKCSLLLCNNKSDEFDNNGNLHLMFNEIC
HDMDKNKMYDGFPFDHNHNAALVNKLECFNKSTNDCICLELEGIHRQELNEKQKKKNKKKKKKKNEDIKGDVK
YDVKGDVKDDVKDDVKGDIKYNVKGDVKDDVKYNVNHVDNFVNDQTNNSSYVNKLLYNNNCLYYEKYKWYDIK
NVINIKCCEKKKFCKHVNYNMYELDRNKHMDLLTLQNGIIYMICYLLREDKKNKFCHYKEFVIKKVIYMLINN
QHDYIEKKKEFIENIRMFLHNSFDEQMKYYISFFLNPFLYNKRNYIYINKQSLLNIYIFSLIHDMLLYYKNKN
VWNYDTINLCISNFVDVLMHKQAYIYIQNVCINMFVYIINILFMIKKRNGTFFSLYFFILNILMNKCESLKEE
IYNKIYKRIQRLRRISNNSKGQEKRKNKIKGKENTEFYTDNKKKMICNNYYSDCTYDTNRFVKRSIIKNVNIY
KRSSIISSNSICNSICSNINYLSLSRDKKCSHSKNALNLKNNKGYRKTNNNLYIIKKRNYHKMYHILIAYKYI
LYALQVCIKYNIYYIYIYFNQDIINNNSSNVLIQQGDDIKTYVLRNDDITKDDKITQKQNHSIHNTNYKFNVK
KTKNGAKYNYEKAHNICNMNIKKVENKNNNIISKTIRKEKNSKANNINSNLRLINKNNNNNNKKCKTNGSDSI
NSSSVCANSYRQNNQTQKNNSEIDEVINLQLKKCGKTFDNSNHISDEGYASLYKTKESNIPRDSIKNLNNEYI
HKEEFILNQNMNEENDNDKDVCYKKDFKDLEKDIYTPYNFFNNSNNFFFNNNDFVQNDLNYYVNYYNKENQEK
LNNIINCNNLNNTHSSDVLHLLPFFDHNGPFEYVKNLDTKKNDINISYHDKTMMENKIMKGHISDVNTNNNIN
VDYSVDMSLNNNENNINKKFMNDMSSNISMIHTLYGNKDLHHENTKIEEHKQCNDHEYFEDMYSIEDININAS
KKLFLFSYDMKDIWIKKIYKLFYNMIICLILYHHSFCYITMNIFGGKYFQNGKNIECDVNMTYGYNKKDDINK
NINEDINKNINEDINKNINEDNNNPHNYQNKHNLPYVESHNYQNKHNLPYVESHNYQNKHNLSYVESHNYQNK
HNLPYVEPNNYALKIKRQNIKSKIKSKFYECLLYIKEQEKILFYMCADIIIYLSRNNFYDFINYSIDVLYKLL
FQYPQIFYIFHVLASLKHTSVIFSKVCLNYLIKKFKNYLHIKNKLNPLLYYNHDVRIKLEGDIYKKMFKYKYM
SPYECLLSISSSSKVKKCNIIDLHEKNNKIKYYDTTCCTTQKLYDRKYEYNNVINLPYCNDEKIKSSYNNVND
CYNNKKRHKKINRNIKNLLPPLKYTNISKHYLYKKSFKQCNINMKDRNKYIKINKAKKQFHICGDFLDFPIFE
EYIIKKEIKRENGKELISPYKDLHNLYFNKCFNDDPLNELNIKDNEYMNKNDKWLNNMMICLKEIKKKRNKQF
FFHDLSKRDFLNSKNFVLNKKRINININININNGLFKDETCCLNRCTNKRIINDKKLSFLKLNKYNVTHFLSDDE
YIRKRNNHSDECYYNKNKYMYHPLYNNYNDVHENMLSHKSNLFNNAYNIYWSHNELNIRMIYFFLNYIKFYQY
ENMNFLKELDMMCKICLDLILKDYKCIELINLLKYIFETICYINISTDLMKSKKEKKQDDNNKKKQKINDEYY
NINHTEYMNKKSGHGGVIIENYLKNANDKKIPEVNKNMNDVKSDGITNLNDNNIKCDNYNSLYYSDKNVYSNI
SNSLNTYDINEKFEQDKCTIKNDNSFDRNKESYNPNVGNEKDIYSYSSIHKDDKNMCYINTSYIIHDFLKWFE
NVHFEVPKVVRTLLCELCLQLPVNYFLKDHFNIYVKSLLICLKSKNLHIISVALKCIEDIMCHTNNNSLKGII
CSSIFFDNKNKKKSYIFTKLLNLLKKNNIYYTSHQFDFNKIYFEKNNLLQYSYYDKIIYEKEMNLFQYNFMHP
YYEKILPEQARKKKKIIQINSEHVNNFYKVLRICENFIGQHGTLNNNLREMDYKNFGESFIRIGFLLKKETSI
NKNNVQIEDTQRKNVVDNYNLYDVMSKPNEDTIIMDGQKTDANEYTKDNNNNNNNLNINDLDNNNISSNNAYT
FYNITRNNNSHTFNDNNYEYKLQHLNLEETLNYCYKILRNFIKNNKSKYLYYNDHYKMNILRNNGTNYLINEM
VQDKLDSEINIKRGLRNEENVPRIFNFNNSNVLKKRRFNNNGINNRRQNKKLKGSTTKSININRNNININYDN
YDENVVDVVHLNELEEQNCLENERKSNYEHNNNNFYYKDNKKIYNIDNSNNYYQNNKMFSEKNKHKYFHGSDI
CLNCINIEQRIHCYRIVISIFIFLLNHSRCISEYYKYMIDVGLRNDDFIKNQSATSKKTKKQSVHVDMNKKDE
EGFYEKDPNVRGNKKNELLCENVKETCEENYQDDANKCIDGQNNMCLNDKIGDIIVSRNKKKKKNKYIYSFQS
CSCKKYIYKYNFLCNNYEKDNIKKLMYCFLIMVIEINHILNKKKRNCGNSFIRNKNRENIEKKISHNKMNLCA
DKDIIIITNDKLENINSHYSICNDKNWAYKLKYFVKNIIRRISLILANRYILRKDLNLIYIKNHNNEMDVSKF
IFSMCNIIEYDPCFIQMSLEGLNIIEKIIRSLLKVQAVNIKDCLKSPFFCDICECILNICRNNKMKKKIFGLL
LFIKVLRIIPPYWIEYNIYEMIDTLLTCYEKSNILIYDKIIPRLIEECFCLLLSSLFIGYVGDMNIYILKNMT
QNHENVYDEEEYIDTAKDKNMCNIESKNYYDKYNICVRNMEEDNVLRYVQDKIIRNMKGKGDLFYNFLHKREL
NKNRDDTINNNNNIYDSCGYKKVYLKCSNFWYSKDEIYTWNVLTGKEKNRRNKKEISKMGRMLYNILNMCIKC
LYKDNNNNNNNNNDNIYNDNIYNNNNIYNNNNIYNNNNIYNNNYISYYNNYNYYYYNRVFIKKCFFIISKILG

TNINDLLLIKNKLSLETLLFKYFSLSNFIKFSLKLKISYLDYLCFFLNEKPSIKFDENNISNIIIIILNIIED
IEKKDQRKYKYIYNISSNKNKNKSINNNMNSYVQGANKNNHAYNSIYLDLIYDKEQNVDYTFIKKKNNHDGDE
HRNKNIYKDNRKRKHDKSIFYNNVDKDYLKNEDLFVGSRIPVETNNNVLLNMMEKYNCVNNVDGKNIGRKDII
SCHKNKDRFDENGVVLDYGNDEEEKKDDTNIMNNFKDDKKINKLSNSYYSMYFDSKLDSTRNHNYEETINKNK
SKDMSQIKITDMFPEYKNKKDNHNEKMSLNILAQSYPVVNKRKYKSSTSLNNKRGRKGKNKNNNNNKNNDDNN
NIDDNKIDDNNNDDNKIDDNKIDDNKIDDNNYNDENDYVVGKDIKRRKGNNTMDDKDKSINNNDNNNKENDKG
NYFKDNFHTGRLNISRASTEGGYKKKSFFRNAINKITNYFHYNLKDKSTLKNDESVNVKLNVSLNVPIENKVD
QSLINNNNSNDNKNISYNNGCINKNVGHNISYNKINVVRDVKDENGKKKGNMKFISEIKSINNNNNNNYKENHK
KLGKNDDERIKDKMNEERYNDDYQDEIGKKKFNDDVDEKENDDVDEEENDDVDEEENIVIEFFIYFMNLLKIC
FEHPLYRDHLLSINIKINKKTVSLYYRCMEVLLNFLFNTDKRLCRKAEETLNNIIHVHEKDKDIIIKPQESIR
ILKRYDDNIYGLMKRGSRNIRKVDLRKSVPMCNQTSREYDMCDRNGGKEEKNEKNGQHEKYGEHEKNGEHEKY
GEHEKYGEHEKNGEREKYGHYNNTTANHKFINIIKKKNNISIDRGIIKNFQKNHNNKSVSIYKGNMNSTKLNK
KDLKIYLKGLLCNLSYVLKNKSHILKSNFIIGLSKIYCLFPDLFSSFLIEGFIKYIKLFHERIDDNPMYYKNV
EHILKIFSKFFVIPKLSYDQFKEIINLLLKLYEKDKKGYVNNENMGINYYKHYINKICNDEKKIKEKDKVVIN
DLITFQNMTGLENSFLEKGYRSNSKQSLITSYFDIDRKVSRNIKEQEINNNNNDNINIYKKEKNILETADICY
NNQNNINGFIKTAEEEKDILERYISVHNNSLCVCNLYISSLSLLCCKNYEHCYKYFLYHRNNERIFSFFILLL
DNKKLSDLRRYVCKKSYLLINLLLRYMSEMDTQKIFFFYNIFSISIVPVHDDDMRDIICCDYNKLRDFMNKKY
YDNKKEQNINQNINQNINQNINQNINHNINQNINHNINQNINQNINQNINQNINQNINQNINQNINQITYDEY
NYYINNYEKCVSSINNVGISPLRNIPFLNNICNVINIVHTLLKHDSSFIFINFYEERIKKNKLCIIEILYICI
SFMINEYMNLKNKNLLYFVNDEICKKCVKVFIIFFHYTRDLIIDIKNKIENNKDNINITKNEKQSMNTFIFNM
KNEKEDLFEENKKTKYLFHNIEYNKNLFYFRTNIFILLNFILCVPKKNIDYTYIERFFKKHAIKSLLYREKRY
IFFKIIDILRMFNLSIFPKFYETTVSFLIIPILKEYVKSNSGLEYMNRSQNVCDINKNNVDGMDSICFDDKSL
FEDMNRIGKMNDDSKVLMFLSTKLFSHATINCGGNIYMERRTSYKSDMSSPTNEYNNMGNGSTISGIHEICSN
NNKNNIHNKNFVKTYQTNDSFNYKDLNNRRGGVNNDKKNIYNTHTNKIYGPIRNLLNCSFIHNLNDLSSGFNN
NVKSSYCLFYLYYYNEENHFKAYPLYNNNYNTLFLYIEEISYVLKCVIDYIPNYNDIVIMKKLIICKIFIDIL
KKFKRQKYRDLFFKYIERENNKYIFYPLTNIKQLCINNIIKIYKHNIYEYNNNNLVSLFYYLLLGSENKQCIK
CTPHNIKELFKMIINKKKNKKILHNNNNNNNIFSTNNILKRKNMWMYLFRKYYMSYIYYGDYIIKDTIYNANN
YYEDIYNYSPLPNYKDRVIFHVDILKNCKNIFINCIKKEKNIDKKIDMIKIIFKYKFDFFNFYPYIVNIILDV
LNSKNFVISLDKINKYNTNKRINFFIKSLNIIMDFIKFNMLINCVNYLFLINEDKILLKKNPLFGDGNQEEIF
MRDITADGFEGNIIYNKLGDETKKKRTNDGIGYVGLYNDINDYVYNNICNEGHDNNDDDNDDDNNDNDDNDND
DNNNNHVHNNNREHNKNYTTHCYEKKDNMLVDLQNKIYNYFMINKKNNNIHLDHFIIPIFNILKKYDNINVDY
DLLSILFNFIICSIIKNVIEFSRYGVTNKCISILVSILKAKVNINIKYMNIYGVLACILFPSEFIKHKTKWKT
CKDKKNKYCDKQNKNVLKNNCGLNEDINNEKEINKIGYKNISNMNKKNHIYDLHNTSLKQSHVRNKNLYINCN
IYNDSIYMLNDYFNTSCVNYINIYNMNVIKCIHKILYVLILYESRENICKNLHMITCIFFLLMNNKVNNKKEK
MGLFKLLNHMMLTHNLYVSGVDNLFSNIKRKNNINEDINEDINEDINNNNIFSYIYDEKNENVDVNKKILDLH
NMLILLCIKGMKEYNKKEGKKKKNIIYYNDKIMFVINLETSVSIFKVLLNLYKERECVYKDIVKHILLKYIKS
LDRLSKYYLSHRIIRKRYKNNNINNNGDNINYCDNINYCDNINYCDNINYGDNINYGDNINYGDNINYGDNIN
SCDNINYGGNINYGGNINYCVYVLKKLLFLINANLYLLSKKYIFQFFNVLQNIIKTKRIKNKKYHNFYLFLLN
IFEYHLVKNTHTNMVNHHYKDILCNDEKSYNSFSLYNESTSKRAIKNRKKAINLFKRIKTNGSIYENYIKYIN
RRYNDKKVYIIDRYNYKPYGNYVFSMDRFIYKRKRRTLLNIFKGEIKRNSYLGEFIKKDIMIKQSGNYHIFNI
NTLYNKENDKLNHNIGIYNNRHKKDTITSLDTDDTLRKGVHKRNESYEDINYKNISADNNNNKKKKKNYLGIQ
AKIYEHNDMKKMLKRKKIHNKYKKYKKYKKYKKYNMYNVYNNLYDGSHNDIINQENNHTLTCESSCFFIRNQH
FNKHSLNKIKFNKNETLQKKSKIKRIKNYMNTFEFLYEKNIMESCLYEDIFFFRNEEHTKLRRYRYPHLCSYT
CNIKNISLTCEEKAEIIKGLIIYDYINDYSCYIKYYDILLSFIKEERKTTPKKIKNIENVMNRTIDILLPNIL
RGTFSSFPSIQYTCTQIFKKHFLCNNLFGCIYFLFNNEYYKYIIDKYFISLWINVLFSFFKKNIDVYVSSENK
TQELIPYMCYNKNERGEKIKWPNKTPERYEHNFYVNTLYEYPKRVKRKQILPYNDKMKKYNSKFSLWKMKLKK
YCMEQKLFIHKCKEKIGKAKYMFRSFLSIIHLYKEYCRNVWIELFPQLFYFFNKEEIKILNMCLIRFLNNYIQ
IYDSFKLKKNKIIRQYNSLEETHMKSNLNNSYVNNENINVLETILSSVLKINKLIFIPYEIIFYCSKHFGMYE
ICRYILQAYLYNNLKKEKAMKNKDKKCIETSNYGNNKKKSNNGYEQKEEDIIINYVNNFKELVNYLMDINYYL
NSYDNMYVLKKYYIKNVQVEKLILNNHFHFYSKIEDSYLSNFLNSQNMKLSREEINICKESWIDSLKYFNKTS
CIESYMNINNINNKNENVNNSIMLKNYFNNSNYSQNKGDDLFFFNCSYKYARNNYLNNLDDIYNLINDHIYID
DYNTMNMGLSGEKDILGNQHKYINNNNNNDNNFCSSNICSDDLHYNNCTNSKNKNEKIQKSIHDFFKCTNNTN
KICNSYKIDEKKNFFHNNYNYNYTYTNLHNTNKVDDPFCLNKINQYHEKPSREKHNSSSEIINNIINKLYEKN
CNLVGYMYDIEDNTMAPKNVKDKIFLDNNQKYSLHMSNTNKNTFLNNNFKFNIEKKHINKMNQIINNGMCNLV

NNVKDLLHYNKYESLNLLFLHSQIFEHAHRSIELIKEDVIEQKNVRKKEKERKLLSLLDNLCNICKKEQGLFF
YDNDINNLNYVDMWKNTFLKICKIILNRETEKNKTFNNLINSNYNDHYDNYDNALHFYNQNFYQQHLCNNTYG
NTCDHFVPHEYLQNDKSHILVNLIKNMRENNRLSYNYFDKMYEGFLNYNNKKMSIKNNYIYNIEIIKMNLRKL
INGNNYADVILNSGQFNNVNYNMAEKLLDKYSTMCDEAELLNDKKSNNKKEKKVKMCQDDNYLYITNMEREYF
NICINLKKLKKNKFLHKVIKKIKCYINSLNIINNFNFDTLPMVGYEKFRSEFFYMKGKILCNLNQIIIFLYKN
NFIEMNPKNNGYACTGQCINKDVIFVKSDYLNIKTPVFTKENINSDVHLLKTNSCNVQQYEPPKNDAIDNIKI
EVCHSNIRPKEKKDKLHNIDIMIDEQNNDTNVKKKRRYNKKMSINKETEDQRYVQEMKNVQDNIEITNIDCIG
KENNMEEKNNICNNPLNNNNIGKPNVDNNKCCYIIGPDNCKSSEKKNVKKKRKKKIEMKLHSDYSCKCNMKQI
KHFDSDLIEECLLSSLKIYPLENNSWIIWGDYCNKLFKESENISFCLSSIICYLMYSSLNINKGYYYMSNVLH
MLNYGTKDNSIFQVFNKYNESICPNIWLFYYPFLMNNININKKNYLYFHKIFYILMRNFPQDIFYDLRTIHIY
RKKKLYQLNEIKNKTNNNGEMSLCDGSHDILKIKNGLQINKEEHNNNISHLNNKNSEHVQDISDMCNLENGYK
EEMASTVKINLKKGKDKDNDLLRLDNINNDNINNNDSNNDNINNSISNKNNIYCYKNDEDNNACASYQTGNGI
KNNKYTYQFADIYMDGFDQLINVFKNKYSDVFLSLDYLCNEILNYNKFEAFEETYHIINKLFHNCMDIGNSEI
HINEIMQNVTNNILYKRYKKKLKKNKNMYNRKYKNVVKNMNKFWKEFKKDIKFLKKTGCIEKLNTTKMRKKKN
YIKNKNMDNFSVINEYSKNMLSGNDNSRYMLSYFIAFLKKWKYMLSIYLSQLTETQALEDCSYTLCDALHKFD
IPIELPKQRLKYIGEYLNYHYNLNTFCPSKIIKVNSSVTYNVSNNYILRKVSFLTNDGNIYYYTQQPRNYVRN
KSEHKFMLFQVLLNNIMFNFSQTKKRGLSFKINFNTQIHPNIILKEQPKDDITLHEVYNEYISVLRSDKYSMD
NIMLYHRNLVQSSLHRLLLKEIEIKLKCDKKYMNMSIDDVVQDKNDITGFTKKEGQDINKKNNHVNNNINSNN
NNNNNNDSKDECNMLLKMFSCQNDLVCETKNSNRENININIPSNNKIHLSNLEYYCSLTYEELLKHFCNEEWF
HKILKKCEKKSVRKSFKTICKMIPNNILKNYFFSIHNNNIENYSIFHDQFVKNYSILCMLNYVFFQLNTNLDN
IVISKCSGRSGYCEGRPLYKDFTMKKDINRKKLAFRLTRNIKEFIGFQGLHGSFPLCFYSSIMAIKKKKMEIK
NFLNLAISDDFFTLHVQKKNRNLMEKNKGRIKSEYIKENDNEICKKTLINEYIKCLRIDDIEKRNILKNAENY
VSTIFGRVQFLYKNKLERRKIVNTLKSNEKSNINVYEYFSDLHDITSLSELSSDNNNEYINYDEVDKKGAQNV
GTREYKRKKKENVVQQKTVKEEKPVNINMENVQDIHIDMDEENSKTKECTNNITNDDIVINSQNDNNNDRINK
LIDMTNIEGVKEIKKGENNSCNNKGKRKYKKNITNSYNNDERKNVHQLLEQRTFENIRNEKRNIQDCTEHEID
EEGMKKDENSHNNYKHVKNYDNNKYNDEEKNMKYKKIKNISVADINKSVVDLMEMSMDKKNLKNAKRTWFPWF
*
>N.MAL13P1.20
MSDEIVLNQKLEELKNLIYLDDSVFKHLDFVKVPCIPLCVLPGLSFLCARAFEEYKNGYRTDEINENDEIMVE
YFYAKRLYKNSLVKIKFPEYYDMAGALLSDATAACVGNLTPFYFELGNELCELLPQDEWPVEKLEELLLLAET
RRRVYLIKHNDQVDQTYLEGMTFMEKKMFNSFSLGSDVDKKRATLNRQIFNFFEYDI*
>N.MAL13P1.21
MKKLGVNNAGENKGNSIQNKDGTNKKTIEVNKNMRRLTFLNNESNDSIEGNKNKYDKNKVKEINDVFKNCMVA
LSHNKICTRNAFDIHIIEHLEDLINLNDEEIPEELNDEMIENGEFNLSFTRASKAIEGATKVYGYRVEAIYDQ
TYNFLTNMNLAKQFELDNDMMDDNKNTIDPLNKRMRKRKLTYLQESSTLAKSSDITVDSLSLSNISVDTFFLK
LNSTYDHSCGQKYLLPNLNLNNDLSIQFDGDIDVCEYKKRRKTLDDIGKDGVKDKEDGIHIIMKNKNDDNCDT
YDKIICNNNDINKVDEKKNNDVDICENRNKKNESCNIYVDTFNVNEHKEFFDIYKKKLFLNADILKEILYGGP
DDFNSLHICPELDYFKEELKKHKLKRTDSKDTDDVDKEDGDGNLYNDDDIGNTDKNNIINFYDENLGCNMSNN
MLMESIYNSDALNTDRKNIFPENLGMSCGNLNEEKKNNNMNSSNNNLNNCNYDNNDLYLGDYRMDDLNIENVM
QESLGFDNLNYSCDKNMNFSNNNGMLLQQSINMMDGMNFPELIKSENKELDLGNTQINNEHYLLKNNYDIHKR
HSIISEVPDDDTLWNRSVMTFENRINAIDINNELNYHYYMPNKLMMMGNFRNLIDINKNVTNNINNNNKSTIL
QNIIMQKKMKYAFDVTSIDFENLYTENNNIELSTYDLWKKEKKKYVSNALFCIDQTSYIFDTRENCVNCVNTV
TDKVMKFSKFCVYPDFLHACNKNRNKHNNNNNNINNNHMNVILNEVNNDNILNDIDPINQDDFNNNIDNFDME
NMDDMNDGHLHEGLNEAIDKYYNMDFDNIWANNDNKNNDNINNNNNLNNSTYNNMNSHNNNNNNNNNNLSLFK
KHSNFFNTALFQFNHSNTFGNVPFENVSKFVDVAKIKKILCDIVKPNEEQKSNTNDSLCEYQTEKDNQIVTYT
AEKTTTFEEIVKETTAKLNESEASSTSIHMLFVCLLYTCNDQELLLEKIPNQNNFYVRYGLPVECHVNHDDIP
MLKN*
>N.MAL13P1.22
MNFLILVVLIKMVVCKIINYVRSDYNFMLSFLRTRKIFSHKKIYFNFKVSKNFTKNSSSNSYSDILKCFSFPR
KGTNDKTKENETFEKGEVKEEEEEKDKNVKRKIINESSKNKRAKVKNENDVIKKGSLFNCVVREDEKVNDLSS
PKFNPVHFNINDLYLSEKDKEKHKFKDSLLFTFLTNAFNQIEELKGSGTGSKKNVSIILSNIFRVLIYYSPND
LIPAVYITLNKVAPDYLNVEAGVGEALILKTMSEAYSRTESSIKKDLQQIEDLGIIAENCSCKMRTIFPLPRL
TIQSVFHELKSIPNLIGSNSQQKKREVIKKLLVSAKTSEAKYIVRFLQQRLRIGVNSATVVQALSYAFILTRP
SIPDDIIKRGKEINENLINGKLDGTNNLINKSNNKTEEWLTQSGDRNCIKEENLNDELLSKDDINKCENMDSL

INEIRIRNEKINKPNIFYNLIEKVGDTRLLPLFFKELKNIYCEENNDIDIFECMEKSVKSALCELPNIEIIIQ
NLLNGDDMNTLSKKCTVKTGIPVQPMLAKPTKGVQEVLDRFNNVTFTCEYKYDGERAQIHYIDKDNIKIFSRN
LETMTEKYPDVIQIIKDQIGENVKECIIDSEVVAYDIVNNKILPFQVLTTRKRKDVDIENIKVKVCLFPFDLI
CCNGIPVIKEPLAVRRKLLYSLLKSKDGVLSYATHSEMNNIEDIDMFLQDAIENNCEGLMVKTLVENASYEPS
RRSLNWLKVKKDYVEGLSDSVDLVPIAGYYGKGKRSGVYGAFVLAAYNSETENFQTVCKAGTGFSDEILSTLY
ETLSEKIIPNKKSYYEVSDKLNPDVWFDAHYVWEVKAADLSLSPVHTAAIGIYADDKGIGLRFPRFLRLREDK
NAEQATTTQQIVDFYEAQFSSNKNKNIDYNDDTESE*
>N.MAL13P1.23.1
MSFESFVLKDEKKASNKKYDYDEIDLNDDDDDIIDNKSFDKNNYSYNIKNRLFKHYKKVHDNGDCLSRKENSI
NSSTLMNREKSSINDENDTKSRLRCSLCHKNESKIKLEGIMNENDYLIQINKLRRHVIIEIFGGKCFIREYLC
TEFLRRIKHCCHINYVKYNVGLINYRDCKQLLADNNNIASIEARLNSILVSLPPLTCVILHSSVFLVVKEDLI
RDDLIKKLCSVSKKFINSYKPDMKKSEQRPFEFCALECIFSSALEHLNAEMKLLNKDFADIKFSLKVTKYQDI
LSNLHNLKEPTNILINKINSFIKAFHDVSENHMDLKKMELTRCYFNPNINEEDNNNNNNKEHPNQDLEMLFEY
FDQELNQLHDQVKHLYDLMINLENKIISDLSLSRNNLIRMDIVISLINSGFGIGTLITGVFGMNLKIRLEEHD
YAFLYVTSLVIVLCLMTVIMSVYFFKCIRL*
>N.MAL13P1.23.2
MLTCFLFYIYEKKASNKKYDYDEIDLNDDDDDIIDNKSFDKNNYSYNIKNRLFKHYKKVHDNGDCLSRKENSI
NSSTLMNREKSSINDENDTKSRLRCSLCHKNESKIKLEGIMNENDYLIQINKLRRHVIIEIFGGKCFIREYLC
TEFLRRIKHCCHINYVKYNVGLINYRDCKQLLADNNNIASIEARLNSILVSLPPLTCVILHSSVFLVVKEDLI
RDDLIKKLCSVSKKFINSYKPDMKKSEQRPFEFCALECIFSSALEHLNAEMKLLNKDFADIKFSLKVTKYQDI
LSNLHNLKEPTNILINKINSFIKAFHDVSENHMDLKKMELTRCYFNPNINEEDNNNNNNKEHPNQDLEMLFEY
FDQELNQLHDQVKHLYDLMINLENKIISDLSLSRNNLIRMDIVISLINSGFGIGTLITGVFGMNLKIRLEEHD
YAFLYVTSLVIVLCLMTVIMSVYFFKCIRL*
>N.MAL13P1.24
MMRKYRNSIFTILLSTSLGWNINKLYRQNNLDMYGLKNDHDIIVVRHINIYDKKENENNKNNILNENNKNNIL
NENNKNNILNESNKNNILNEKELLNFLDRFTTINKKYKGFCETSVFKNSSFSSNMKNEYSDGTFNTYLVIDKW
KNHNDFEKSKKEFQNLFLYNNDIYNKKMQDFYYNFQLYRNNYETTSATNIFTKLFSMLF*
>N.PF13_0020
MDEENEVVKLLVKVGKNINKTEEQLRPFIKKIVEDNWLDSIESLKNLNDNEWYNLQLPLRLVDEIKKELLLRK
EEDTKTYEDKNKDDNNNNNNNNDNNNNNNMMVDNRYDEIIHGEENQILNCNKKLKKNLKEEKEHIINQNTFCT
NIRKENITHSNNINNHVSCDYDNKRTSSHYTDHNKSSKLINDMNVSRDNIGVYENTNKNKKEGFNYSIIKYNK
DILENMINKQTEDIKKEDIFYLSHQDDYPNNYDNYNNEYSYSEEKTRTINSINACEKLKKIPMENLKVVLPIL
CKIVKNILINPNILNTRILKSTNNIMKNKILIYQEIKNLLLCIGFVEIYIFYVMIKVDTLLLLCIYESLKNIS
KNIIKIDDSSNLNFDPFKSNIVCVDTLKKKKNLFHVNDNVDILLKQKKEQMEKLMNQPIEKNPKIYKQKKNYS
ESQESKENNKKIKKKNHHNVLDAQEKDEEDAEHTEDEEDFSHIISNIRNFCKEQTFKSKTKLELEKISNAKVY
SKTIIKILFPDDYILELSFSSGTLLRDVNEAVKGFLNDSIISKNWFLYETPGIRKFDPQKSLSFYNLMPHALL
RFKLDQQDENFVFSSFLSNESIEKYLISS*
>N.PF13_0021
MSENNLSKDNNKNKRNEALENAGMLDTFFESMYDHMNRLDQYHSDMNRMMNSLRGYYMNNDFFRFFPNRRSLT
DYDINRNRNHDLLLSKPFSSMFRRDGYSNVPAMDVLDKEKHLEIKMDVPGLNKEDVQINLDDGKLEISGEFKK
SHEQKDEQQRYYIKERCESSFYRSFTLPENVSEDEIKATFKDGVLKIDIPKKDIPDKKKKKIEID*
>N.MAL13P1.25
MLKHKYALYRCIRTILISLLIYIWLSKNVMTYNRNIKRRNNICFYIYNNKINNNIYDSTFFNNIYKKKKQNKK
NCHVNKIRNQHLVEHIYNNKDNNKDLSCENDLLKHLEILYQQFWERKLEPFKNVPKERNNINNDENKINNFSI
NNLYHYIQSTHPSALQNVKFIYQDRKKYTFTKNKISEAFINNAQVQIEPSLLKNFTNHEDQNNCDTQNEITDD
NNNNINNNNNNNIHNNHNNNYNNNNHNNNNNRCNDLVNDSILCITTNKDISKYQKICSIPNNYIINYDNIIQE
IQMFVNHFNNNYKVNYLKYIFKNNIDEEINKLDSITILLYDIYKKHNYFLNFIKNYNSSSIYLKYWDIKLTLV
VTYIHFISKYINILLYVYNFNNTLFTILHKYYIEQIKKKKNWETHASNNYNQKVHSNDLSSGHKNVNNNEKIR
DIKKNINMKKYNEINKKDMCNINDINDLYNIYNYNSYMENEIDSLYNSSDFINNIHSFIECKTSNILRNYSTF
FFYKNYEHYIQYIVNKEKLNHLPLYYKDSSFYLFNNNNIKNIILYRKHMIHEFIKMCTSQENQDEPNFQFIDK
NIIHKILFTNNKIFIELDKIINTNIHQNEQQKNNTYKFGTHTLREDQELNINELYNFIMLRFYEYDKNNGDNN
NNDRNNNRYNNNNGDNNNDDRNNNGYNNNNGDNNIYNHNNNISCNKNININEYYNQSFEKLKDGIKNTIKYVA
YNNSIFNLFENIFNYDFVMHIYSYVCTHAIKMKSPLNLYPSNDDTTNCIENKMKKKYDTQNKENTYRLKDNIV
NSSIKMKDEQKNIEELFIKECSDFNFNEYEQKEKQNENKNKNMCLIPIIDVCNHSSLSNNSTINKEELQYTEK

INDDVKNMMNHEDSVSLYDEEKKIKNNKCIDNNDAIKNNHSRQNNLTSQNNHTSQNNHTSQNNHTSQNNHTIQ
NNHTSQNNHNQPLIPSSIIQNIQSVHLVSSRNIEEGEEITISYGNVNNDLLLLEYGFIEKEKETKVYFHFDIK
IIREIIIQILGVDKLPLIMLDSLEQVKVNLFKQLNIIDDNQSVYQRFDTKNLESAHITRKKKEIFNDYLRKNK
YFNEYNIFTIKDRINKFLIDEKLQHDSRKKNKKNYLYIGSNHIVDPILLATIRIIIYDDINHLSKVNINHLTS
WDHFLSIDSEMVVIQILIKLIDEIIYEHFSHINYEYILKQGFIKYDDLKFLHNKFLQINMLHSHKSINLSQYN
NFFITIYNILKIKELKNAKDKLNQKFTQIKHLFNEQNKNIIK*
>N.PF13_0022
MMNDIVLINKKKTPSEEKNIDKNEEIKLRIYGCQLLQEAGIILKLKAVTIVTSQILFHRFYFKKSFTDFDVNI
IAPSALYLSCKLEEDFCRIYKIINTFHFLCKYENIKSKHIYFDIKNLNPEHFRINIESEEYKNMKVDIYTYEL
LILKEIGFLVHKINQHPHSFLLPYIHSLFNNLNTIHKDLTKKLAQMSWGFLNDSMRTTLCCEYQPRCIAVASI
FLAAYKLNIPLMNNTNWFKLFDVEYEDIKKICIRILELYKIGI*
>N.PF13_0023
MSVPTLSNKPETVDLLVLAPGEKKVTCTISDKGDCNIFVIKLEDHTIGNLIKIQLCQDPKVLFAAYRQPHPLQ
NAIEITIKPKGYAGVKLLSDNVNNILSQVATLKENFAKKIQKYKESNSYYEDY*
>N.PF13_0024
MENLYFSDINTTLFLRHYNNHLLLINGNKIKFYNYEENKNIVRAHNIGIYSIKEADKNEKIVYVSNNIKLKRR
LKGKKEEEDIKKNDSFKGNDLVSSNNDEPNEKNKEKEQNKVEIENNNNINDDVLPTSGYSNTSKDVIIKDEKL
NYVCIAFEEGNVWLCYQDDEYKLFNYEKLIYRKVCDIVKIELFVIKNYLNVFILYKDYTLILYSEKSQNAIKI
PIHEYTYNFCLSHNYKNLAIFSHANLYIYDIYQSDVFHILHDKDNSGSTNVNSNNNNFIEINNIFDLSKNYKY
LLNCDWHPKNHCIALCGKKNIRYLTLYNYNVYEFTSLPIHETIINSMKFITISNNITLLISLSSSDYIFSIWE
FELEYCIYKFKSDYAISYFDMSFFHNHLHISMLAEEKHLANLKLLDKNILQKIECNQSDNEDNNISLQGSFND
KNPYNHNAEYVHQQNIFADDKKNINFDNKKDNNSHEMQGIIYPNGTAHTKRKSKRNDDDIFNSFSIDHKKEEY
LYNNYVEKDKITNEYASASVQKKKRVSISINDNFNSTNVKRRKKKIFSDDDESEESFLSSYKNVEKDINAKDE
KMKENNKRHKQNIHDKNDNNYKQDEPSENAKQNINLNDDNTKDDASNYNYEESAECTMKKLHKLSSHLKTSKE
TKHNKVISNMYDDFESNQTNIQHNVQTDDKSKYGEDENIYNPKEYNLQNFINKNRDVVIDRDGNYLFDNDDDQ
RSMIGFNTYVKDKFDQLRELKKQVDHLQKQITNYTKINLDDFIVLAPGLCEEPENINDQWCMFWNDIGHITKK
KEGSKTYIYIFLFRGEEIGKKKITDIYNVTSASLSAYGFALASNPYEKSGNKNNSILCFHNLKDNIIWNKYLP
SDEYIKGVANGNNFVAVVTSNNFLRIYSTYGYIINTILLKGLPVAICAYDYLLFIITCPYIYENVTTTNINNT
YTCTLYKIYENFTNLKPSKYNTYHITILYEDVLSLPSCHYLNWVNMSNFGVPYVKDTSNYIYGLYPIFKKNNQ
LVYDWVPVFDMQILNDLENKENMLQLNENNQHILNETINKNDITSNDIHSQKDDNEDYINSNMKNATPSKLNP
LKNKTNKSSNNHRNNEGGMQFLDDEVEVDEEENDGYDDEEDGNENFDDEEDRPRINNNNNNDISNNNNNDDEY
TNNGCIYYPLYFDNFEQISVIKLQKNEIEPRSNKIESCLGYNVEKKYVVMNECNLISYEKYVNLLQKNPTLSL
DNESNNDASALLPWEQYDEMRSRIDLFCKQLFANMYHDYMNDGKNKTANDTLKSLNKLYDKWIMRMFAILKND
KKNQRLAVKVSSLFKNIKNMMLSISLVDDEYSTLHSEITNEYLKRQNKDENSKHSQENIIYYEDNKDDDQRRR
EMYEKCYTNQQNLSDDEKKNIPDIKSTSQIYINSSKSFVENKEKKENGFLDKFFTGVTKNQAVDTLFSHDKKN
TKIKKKSTLESFFTEIKEN*
>N.PF13_0025
MKLWHLLFELTILIIIKIILNSSNLSIVICFMNGNYLNKTYTKSTHNNKTYNILNINKTKDAFKLYMTKKFKY
KYRNTLEKRIKKGKIRNEIYQKLKKKHSAYIVITEKLSKLDPNQEYFKYDKNILKEYENIPDIYKNKKLGRKF
NQYDYWYSSKKKEAKYNYSRHVNFAITENKFNFKNVFSYFHILEIVHEKFKNNSFYINGLQSFINKYYDPITQ
KRIKFYDKIKEMHEMKNKKGQNINDENINDEKNDTDNNAKQKRDNISNNNNKVDIDKDNAKDISKQNNDTSLN
EELKQYEEQKERELPSKLDMFEIIKRNIDGYMYSNEVNPEDSSFTLFGSDTSCLVKKKRAPVAYNINCPFRKS
PLSSKRRRQKEKKLFDIINFNCKTRKERLMLNAVRRLSIKKLKGEDYILDATDRV*
>N.PF13_0026
MIFRFYSKLYKHQCAYKNILYNKYHIEKKLEYFYDSIKKNNYSTLYGIKKNDLLNIKKNSGEFLLLSDIFMIK
QNNLFLLNNVRRNFYYNTNIIKGIYNDSLFYYDKKYFSGRSGGLKRKKKRKEERIVNTGSAKRLEFFYPKKKR
RQRIGLIQNSRKNIVYDNIFKRFLVYHYKQGIQVFRSFSCKKKRNFEAARNKAIILSNQYNKRFNKHNMNDQQ
RKDTKNIPLNVMDSNLIAKYNNELIKQIKIIPDKNKSGYRGVFYEPSYHAYICIYNEAGVRKFQIFKIQNNDY
LEAYHLALMCRRYKLFKNYQFVFQRNRIRSGRIPLK*
>N.MAL13P1.26
MEDQFNHMNEFFQFLYSDKKKVKNDDIENIKNYMQDVILNEKEKEEEFYECVNMLFFTNNRRYIKSHISMCLY
YLSMLVYNINKEDGNDDMIKTKIYTYILKRIGDGKSLSYYDRCILCKRFFIFCSNNNNIIEELKGNKDFYLLF
SNCLTFLNSLFYTDEKIIKLNKKFKRMKKDIINKLVHNILRQNICHLDVDKSNKKLVIFNNNNNNNNNEIVS
YNTNNTSNECNENLKYFVSIFINETYRSYLIIECISLFLYTYGHLLNDEKYKMICIENFENLFFQNLLNTNKN

IPIKFIKCFKYIFEHTRNENIFDLILKQYNRYDEIALINSLSFYYYNKDLFNKIHFYKLLDIVINIKKKEKEK
NIDDSVNNMIYFICLYKLHLHNEHTGALKYLFEENKKMNIIKFNEKIIFLKTIKFLLSNEKIEDIKIIILSYK
DMIINFLKNDLNENIKLEALNMIQELCYILRNDKDIYNTFSNIICDILDKYGKNNDKFLYYILLFYIHNVNII
EVNEKFLTLIKNVFNLCVTKQILKHLYGMNMLLFLLLKDRNSNDKNLKNIDTYMNDVMDKSVYNNSGEGGEVF
YMYSMKEIRKIPLNKFFYYVYSFVLLSFFKSKEITKILSDYINNLNQNILSFLCFDKMVDRNLHDELLLNEGS
KNMINKKMSVPTNTSKNTNTKEKSKQTNNNNNNNNNNNVNNTNNIFSDKNHPMNKLNNIVFILLLSLLHYMEE
KNKKFELKKKKRKTLGTHFISLKMNDVLIKQTEDDQNNINNIYQFNYNKEYEYYVEKFEELLHNLLLEKNIYQ
LLLYTFYKYLYINWIEETYNYKNPYVLKRFLLYLYEYLLKISMTDYEKQNCYTLILLCFCHPLLFYKNINNYK
SQKKFKNKLLRRLIVPNIHLDNVIQFLLNGINYYSNDLHKSLCYNLIDMLYLWNHASGENYNNDDDDNYLDKK
EIIYSFDERKKKFQEMINKFIDMLDDKGVQSIKDDDIEICKVPWDVLYVDRNIYQPTVIVESKNIKRDKYLVS
TYGEETADLIMEEELNKKKQANTNKSVNINTNKSLGVNKNVGNNNNLLNKSLKDNKSNKNRALTKDELQIEEI
KNQNEIRKNVFEIIEKNKYILNCLQKLSSIDDIYDTKYMNLFIKKIMILLKNDLTTRCTQAYVNIIIDNLIST
NMLKCKKNITRCLYKISKYNKADESALLIFSSFNMNERISYMLTLFLFPIIFHSINMINDKQIILNILKTLDI
LLTTKTEINDVDALKCLQISYSKFDDLDVELESFLNNFNSYLLNNKNIITLLEFCITENKVRRMIILKSLYNY
VTHNLDIDDTHTNYGNSVSIKGLDVFRNDERICMYLHIYKNDNESDTQNYCNVMISKLGWENTPIEQYKLIDM
LQKECLDLQNMICETLVESTDRNKIKDLFLQLTSKYITFKEYGKIGVLKTIENLAKQYYIICIDDVEFILNFL
LGLCLEDKNDISKIKEYVIMCGTCLINSYNEYLVKYKNKNKHNKEHVISTKKNNKKKNMNEHYFDSDDDSCYE
DSYESDGICNNGQKNKNKNKKKVEQKQINKELEESFKRIYNVLNKYQDNKKSKNKNVLDLIIVIFYGCLGTNL
KKESLELLNKLIEQMLHKDTDNFTQIEISSIIPKFIENLKNGNDTCSDEMNGDETINGNETINGNIKKNNTSQ
IMIKQLNKLKGNKNNIEKTNGNINMMSLTNSNKKMNEKGTSKGGLTNRNNNKNKQNIDNCLIYLDEYDVDIFL
EKIFNLIFSTNKELRIRKGCCLLLGSVIKAHGMDFFRRYNILEKINDNIHSEDINKRQSFYLSYGCLFKVLKY
RFEPYIMKNFNLLLECYNDSVNNIKVLGINVIEDILNDIGPYGMKKMLPMIILNLKTSSIKSKNIISYLDTLY
LIICKYDILKFVDNMTLVNLVNLLCDLVSETNIKVKEICIKIFNKLEKYITNEELKNISRQILLCIYSPNDNH
LNDFLDIFLSIHFKYKIDNIGLCLLFPIFKKGINNIRLEIKKKSLQIFYFLIYLVHDHSLFIIYFNSLFSILI
LLLNDAIPEIRYLTARSIGNISQFLDMNQKLFYIQHIFHILISTSSSVEKSGVALCLSSILSKSPEQVADNFI
SKIIHMIDVKKYMQMKLKRGDVCVGKDKVDVNRKKEVGVKKSKLKRMKYNKGYTRNGGVDKEEYYNDDDNEKE
DYDDDDDDIEKEDYDDNEDGVEREDYYDDNDSVEKEDYYYDDGDDYEEDEDEDEEEDDHENNYNDDTFEIEDQ
DDYDFEGDDDEYDNEYNPRGNRKYRNREYRSNGVNDDSDDQGVEYDEEEEEEDENYNEEEDDDEEEDLFEMYS
DEESDSDYYEIINNKDNDKRLYYLNGHYLMDKNEKNEYIIKVDENLCNWKLIYDKNMISSASSKEGLIGFFIY
MPECEERYTEKFLKRILQKLILCLNDTNENVRDITLRACKILISIYSKNNTSLILKYIENKIYNNYWRIRKDC
VILLNVLIEKNLQINKEERDFELLNTLHERFYFMLSIICIMKNDKNINVRQTAYTIYKNFVNKKLLQDMCSIL
LKKITQNLSSKNNFKQLISALSLGDLVYKTDARQLESILEHMVNEFKTTKFISIKKGISLGFYEIFSKNKYDN
LIINHIHNIIHMIKELACHQNTSDKIKLLSSLLKNIDSSVLKNIINEIFDNVLETPTTSMKFFTSKHHIHFKS
IKIFLHVHTELVLDFIIHHVFKPPYDACKLKLLSYISYAKTPVSKSIFPKIIKIFINLILHNLNRLKETHEID
IKNYAFILSSFKEENKEQCKDGHVKESIKQNIKQNTNDCTYSNDNINDYKINNSLIFNENEKCQNNKSTTKNS
LYNNIEHSNVTQNRSEPFHEIDMDMKDITKCLKDFLRNINERNIDVFIEALLSELKLYSSKSDLSYNSLCRTY
ESLKLEDDKKKAQESADILKRYFIIHDKNNLNELSYLEYKEENGKIREIILSIFKYLLDIINNEDISVLVSNE
NLTPTQDNINNMDNINNMNKQKNIIKNINKKKNILCVYKKMYLLNIQRIEMYINALSNYTFVDINKNVLEISS
IIYIYLIELIKKLEDNYSYAIIFHDIINKYCTDNNLNEITHDKYEIVGLNLCKPLFSSLMNMFTNVILLSNNT
DVKIKAIDIIRKLFIYTNINISRPFMLKISGILIRILTNKFIEQAKFYIFSTFEVIIKKASDYVKPLIPQLQT
CIIKSLNNENLKNQIIHILNIISEKKLSRVDLLINDLLNNINVQINLQISITILMILSNILNNGDINIKNILI
KIINCVKPLFNHTNKDISFHSTKIYILLIFLHIPKKKEYLEPILSFKNILDTTSYYFLLHISEINNVYDILKK
ENFIDTFKEIYLHMIKEEVNSLQNICYQIFYNLTKYNYDNNDCLLFLYSIINLLKLPTFIMISIEIHRYYFKA
VKNIFKKRNDIYKENVKNFMIIMDNILLALFNNIPAFKLLGESCTKCVLEMNDENQYKNLEFLKENMESKKF
NQLLEYINRVLIKKSNVISDSD*
>N.MAL13P1.28
MRVGNNTGVVNDEKIYEDNNFIYNDKCSLVIINDKEINKTRKGRYVKRNNEIKDGSRENYNIKEDNYEDDNYS
YDITNNQSLGEENTKEKFQEFVKNEGKENHMIKKEYFKLHIGFDYSNFSMDTNYKIIKFINKGSHFLYWNDDD
IHFFDDTNLTENMKCRDDDSISNNLKWRDNNNNNKNNVRANFHSTKEYQMNEMKKNNCEEIRNSRFLYFDREH
KLLVIKYNMKNNNFVYINEEDPTYKYFLNLHNKDFLDETLIKEKKDCILIYPYTYTKIWNTLTTYINDEVINK
IEPVNKTFSSSFLKSDKDMINLHNQPYMFYSVIPKYSSKKKKDKKYVFDKNGRNNSKEKKEDNLEIDVQINDT
AEKGDDNIIDISDDNVRDKYYVDITHRFNNSKNYIKEYNTINNTNVEGEKTYFEYISIDNETNKYTPSDITIM
NMEKYWILKEIIQQEYTYIYYNNEQIEGFVKYSDKLFYILGEFQFAFILFHLGYNYQSFIQWRKLFELMTNSS

YLVTNSTNFFEEVLKAVCIQLSYLSEDFFDNSDNSFILFGIYNIYEIINNIDEKIRPKNITTLIDNIDSIVYT
KIGLHLPDLSFVYEEYQPTYVNEEF*
>N.PF13_0027
MEYKSIDFEELKKKVREEKTSEKNNENKQSSKNENYVELLRDQVNGKYLKELNNDTNNKKDVLPSSLKNNITC
KIINSFYIYNYIQLLLNEGSNKSVYILDIRKEDLFNQGHIKSSINIYNKKMMIQINKEMICKDNLKIIFYDQN
NMNNIYDDCINLYNVYFSNIKVENIYILKGGYEDFEREYCFLCIYKNVDVKGQISSHIYNSSAYINYPIKMFD
NLYLGNIIHINNIFINDFLNIKYIYDFTSTGFVIKTENKETRKNKELLYFRYNVYTKNFENTNSLNNESINYY
NFLDIHMIYKVITSMISTNQNDISHNNNNNDDDDVIYNDQNNNMALCTNQVKDNNTSFIKQNKEQLICYINST
HNNKKQNMNNQNNILIICNHGMKNPTSEKTNSISLIICMCYIMYIKKYNPNLIIAYMLKIYNNWSINSQTKSF
LESFYQSLIKCNYNLSKYYSKKYICHNKEEHINISTDNHNQTESLLNIITSDNYRKLFDKYELNKNYIYLQYD
EKYVLNIKQEHIILDINIIKQQINDQDITISYEYVIMSILFYFYNITTINYEHINEVLQIITHILNMKQNYHQ
LYLIVPYISLIIINICKILTYNTIENNSINNTNLENHDNVKYTLFHLIYKNIIICIDILINNDDISNHIINEE
FDVTCLTNQVYIKNKNIDKKFYIILLSLKYFLITLLHLYLNPHITNTKFLTVDKIFYLLKKIDTFSDYYYSVF
KININIFQSENYEAKICSADYLPLYFSDILRPFIVINNYIN*
>N.PF13_0028
MGSRNKMLLYNNALMEHKRLASSFLSVYKEKAYNYEKCQNCLSYFLSLVPLLIVGGIHLFIKMNGYYIGNLFL
TITYLFSFFFFILYYYNSYIIFVLFVFFAFLIYLCLHEFAHALVAYKYGDITMIYKGYLYLDILNYLDIFHTL
IIPLITLFITGFGIPGNLYWLQLHFIRSRFQLSFIFLSGPLSDILYILFIVFFYNLYSYFKNHKNLNVQPHSI
LFISLATAVSFLVDSFLLNLCPILGFDGWGILEPYLPYCLNNIINEEIVYTYLSYICPLLVFIYFNFIEARYL
FFTRIVNFILARILGIELGHVTNGVNTFPTLYSYLRKI*
>N.PF13_0029
MPHHLTSFVAALTGSISSGTCCGCIPFTFSPAVTPTIASTTAPLSAAIPNTVLPAAVATQSAVSSTPVLTSVM
SFFAPLLSFYKNYQNYQDFEETKVNNKEKVDKSCQCNLINSDELDFNYNKKEPSYVNENKSLEDNHISNDNHT
SDNECYIKDEILLEELRLLAHEKCSSSSEFSGTSFLDDKELEHFKNINTH*
>N.PF13_0030
MSSLEQQIKEIENNIIVKEGKIQSLLKDIEEIKNKSQLYYENLIDQQDILLKKKKKYILLELKINNLIKNNIK
TSKNIHHVNIHINCLFDELKNTKCDIKDKYNKFNNIFYFLDNYKKCDNNIILITSILNNFFRKKNEHNILLKS
LLDKNTNKKNKLVNNIKEINSNINNIISNILILDEQIISNLNKIKNVIYDNLNIYQEEKEKYQQNCEINICLN
IINKIIEKQQNDNNTFLQIYNISDNIHNNIITSPYKNDNGEKLQLELNTQEFLELENFFS*
>N.PF13_0031
MSENKDNYEERDYNFLNRKDYNSIYKKVLWPINHGIYDFDDKNLKKELCLIVTNIPVEWSKYDVVWFFREYFY
RLAINKNIFFPLIEHVYLLKNQPSAILACHDGISRETIESLSHFILKNMKNKKKIHLNIEPYYKKNNDEQTKK
DKHDIHEDKYDKNEKYGEDELDTHNNKKTGMQDENNIISKMRKISITPPRDNGMEWPDDLDLRNCYNIEMRKK
LCVFGRYKPLHWGVKEMSKFLKYYFESLKRDHENFEIPEICDMWADKGTHLVTLACKNEISRFNMLLIRACYL
NDEDAKYNMKNSLRVWIEFEKWTSYKNTVNKNFKNYKVGKNDNNKYSSSIHDHMIDKNHRRKFSQKPYYDDFQ
RYKPSYIKTSNINSFKPYNHNNFPHKDYKYRRNNDYSRNIQNNRSPSPYYSKYKKNYSKSLSSENYRMKKIKR
SKSFEERNSRVTNMDYISSQNMTQHNKKFSHSYSRNS*
>N.MAL13P1.29
MKCVLTNLSTEKNDQNDNYNNVCKLQDNAFILRKKKKNCKLKSYLKNESKIHFLFSYKENIKSSHTFNNNENV
KRTEVDKNKTKETIPSNMSNSAYLLKHDNIKLSSTLKNIKDITHMNDSFPINPPGKKKKKKEKCTLLNKIINK
DKININETKKNILLSSPINNNKNNDYLSPHDKKDQIKEITKTVVVYPPVQNEKDINNKKKDDDDDNNKKNYYN
KNYNILDNICNNTYYDDNFKDNHTIEQETSEESEILKKKKKLNDILSKYNSIKKSADKKDKSKKDIKQNYWYY
DNLLQIYEHKYLKNISKVNYFNKDEHSSERFHDDYNESNKESKGLLNYNNIINNIPSEQGNKKHHTTWNNDEH
ILLQRNIKENEKEIYSDDKDQKFLSNNTFVSIINPTNDDNKNSGDNNNNNGDNINNNDDNKNNVDNNNNNDNN
NNRDNNNDDDNINNNDDNNDNNNYYYENYINELYKIIYNNINEQNHTKPTIHIENNTFIEKNDDNNIISSPKN
ETNPSTFCHNHTDQNKNTNEQADIKKKKKFNSTLLEETLKKLSYNKNNIIDKETNFTVKEKYNVKEKKDGFYQ
TYYNDIYSNDNYIYEQMQYLYDNNLSSIYSGNETDKWIYQNDLDILQNNKNIFEKKCETKLLKIKVPPINPLN
KIIKKNINNFNTSKTICNKKEISDQSSFSNVKDKIMANRNIERIEKKKSSTHMKKNEILVKRNMLSTLREIKK
TRNFLSENNNIKPLKNEHNEEYQNINKCKEDVYGEKKNDKVYLDIKTYQHKENIFKNINKYNDIHMKNNKYEN
TKLYTFTNKSFSFNKNIQKDAQHKVKTYNTKEYKTLKNKKKDENNIKMSTSNEKQKKYILTKNEKHINNIKCK
KNDNYKIIIKKKNSLKNIHKYNINDNIRLLKHKDFCFKFPTFINYKNEIRITEKVNTYIYLCHRINSSISNMI
NFQHKYLFIHIFVYTLNLIIFFKLLKTNIFHSQNNQLLQMKGIFCILYILIVQIYIFFINNYFYFFLKKNIKN
NISDMDKINLIHVIHQLFPQYDNMIFKNNFDHQPKINQQKDKSTNLNNSDIVKQQINNLNMSLHNNDHTIYFT
SNKIGKNEDIIKKNTFDYKENALFKNNNLINTYEEYNMIHVDTYNKSNKNLNNTLNQNKGNVHFHDTIQQDIL

NHLSLNIENEKTKNKIINTTHHKINNKTDNLYGIINNNIIEKNKMYILDDKQQNNIMDQKVIDITFFILNNQS
KLNLQDFFFLKLRKYNNCFILFFTILLMYITKLFEIKDPFYFHDNKNYIFFILVNFLILLQILFCIIIEIRGL
FVTKIKNFLNKRIAYLSYELNIYLNH*
>N.MAL13P1.30
MKLFNLLTKNVNCFKKLIEENKKSKNICHIFHGRNICTNVITQEQKPKVHLMTYDMDNNNVTKDFFINTFKNV
NTYNVFNNTIYITDTIISKENDASRKLLYFKWKARKSYKKRVLNLPSTKSRRRYAQKNR*
>N.PF13_0032
MKLSLVIFCIVIIFFKENISVKIKTNDTAIFDINKVLQKKLAVLYRKGNKLNNYFFSYIEKNGKNNKCYRYIQ
EALYDPNTQIMKNEVMKSFNYSKDILKKTFHDIINKLFLYIKNVFLKKYFVFFLLYLINFNCDTLASQMNDPY
ILNNYNLELNASPSMYNLKYEEIGIPISNSTVKGWLIKPTKKSNKLFLLLHGYNSNRQACLFFLNILKNLNIH
NDTTIFIPDMKNFNERGVDDIYNILSYFKDNMGLNEVNIYTQSSTNLLVLLLSKHYKNKIANKSKEKFIKTII
PYSTDKNKNKNTENIIYIDKYIFDSPIFNLHKTINMHFNLSDIDKKLNEERMKNKQEINLHDKKSIMNYFLSF
CMWLLNNQLKGHLYDYDFNKIITENDINTSNIYILHPLNDNISLLNILSQEVKENRRFPLKNIYIFKNGKHAN
IYGSAKREYSLIVKRILKGFNILDFLYYIPLRSRFSSLPF*
>N.MAL13P1.31
MYCLNFYNVYIFNNLKRKLFNSQILKRGISDVCESKIITLTKSKEYDKFKIYRNNIYNNIYENQILHGVKVHN
VDSKGYGEIALYVTRKHSLFFLKYFLLIPEEQVNIKVLQINKKKEKISFQVLCKTKKSKYEITPQCEHFSKCG
GCMFQHIDYNFEKQLKRNLLISLCEKYNINILYSNQNYLQDDHNFVTLNEKKEQDLIEISQIKSEYINDNINN
EDQDDSENANIMEERYLNNNNYKVINKNIIYDEEDEKHEFKNNDNNIIDNEYNNITIQKENGNKKKLHNIYQK
EKNNEEKRNIYYAKLYNFIYSNDYNYRNKSSINFCVTDHLSIGFYKKNSYEICDINNCYIHDKYIQDIYTQIK
NEIKEHFIKNNIYIFNKINNNGYLKSVDIKISDYNKEKQILINFIGYTLTNTQTKKNLITIANNLALKNPMIK
GVLYNEQKNKLKQNKKEILLYGKNHIYHSYNKYIYKLGANTFFQPNQYLNQYIIRIISKIIEKYKINSSNTCL
YDLFCGIGFYSIPLANLFDHVISIDYSIDNIKSLEENINLNKIKNIRCIQMDLFNQHNLKQINLHIRRYIVNT
VKEKKIDLYDKIKEYIRLTFIQTNNENILVENIQKIQSPYSTLPRYIYEQLNNFNTNTFKENNDNIITKDIFK
NPNVHNDEYINNNNNNDMEYSNDNDMIKFKQNEFVMPSPDLIIVNPPRKGCGKVFRRWIRGLCCKHIIYVSCN
VHSQFKDISHLINLGYIIKDIIPLDTFPRTPHFETIVYLKLDCKKVHHSKEEILQFEMNEIKNKKKFKQE*
>N.PF13_0033
MDNKESIKLYVKKVIEHREIESKVKKLRLDIKELNKKYEKTEDNLKALQSVGQIIGQVLKQLEDEKFIVKASS
GPRYVVGCKSKINKSKLVIGTRVSLDMTTLTVMKRLPCEVDPLVFNMISDIDKSENSTNKVNYNQIGGLSEQI
RQMREVVELPILNPYLYKRVGIKTPKGVLLYGPPGTGKTLLARAMASNINCNFMRIVVSAIVDKYIGESARII
REMFNYAKEHQPCIIFMDEIDAIGGRRFSQGTSADREIQRTLMELLNHLDGFEELGNVKIIMATNRPDVLDPA
LVRPGRLDRKIEIPLPNETARIEILKIHANKMTKLGDIDYESVCRLCDGFNGADLRNVCTEAGMFAIRAMRDY
VIEEDFYKAARKISEGKKLEGKIEYEKI*
>N.MAL13P1.32
MIRKRSILYIITYICTNIYHCIKNEINYEYNSELNKIKNPFLIDIENKEECNFKYDNLKTLFKYIYLIECSTN
SNKLQLHFINIAKKNNKKKDIYYFYNENDKKIKSDIIQFDVEKNDKKVNYKVCPTFDKHKKNHCFYISIIIIF
QTKQINGLRELFINHILLNKEDHPIIADLLYSNNIKIIDNKLIYPVFFLVHKNKIKRYHVIDIKIERYVPSYI
YASSYIYNFVFIDKNKNNLLKEENYIYESYFYKFYCTNDNMNIYVSTCDKEKKQFYIYKINIYCYRTYKSYLN
NIYHINIVEVEDKINNPYLYNNEKDINNIKVDPSYDPVNSTTINHNNTKFINDNNKNDTNNVFIYNNPSFITP
SFHVKKHKYDIYTSSDTFLFVIKVHNNQFNNITINNKKVLSEYNIMTLKESPDDQFYDDCIYIWKRLQENNNT
DENTKKLKDEKYGRNRYKMDNHYNRSYNKNYSNDNNYNIETLNNNNNNNNNKNNAKNRFYYNRKNIPYHFFN
NIYEDDFFHHYNNQKNRNPVNIKKKLWYEFFFLSSSQKKKTKIYNHNEIKKVLYPIKNMFLLQYTYEEIYDAI
EDDNNEKKEKKQKKKQLSVVHYYFYVHKIPKNILVNNEYFIIFLINANCRHQNNICYLEKNKQSYIQIIFKKE
KGQENNYLFLSTNLLMPQENNNNNITYLNNSNNTMNPLVTLFINNQEIAKNTLYKINEIGKEKEIINIKILNN
TEKNKHTLKNKTNIILDTNLIIVREYVFYKLLLKILSYFSYFYIFFLSCLQPVQMMNPIQMIQILTFININYS
FPSLFSYFIKNMQSLSFISHFIYDLKIYRKKINMLQKNEDVQSYIIFDDPHEETQHTTNTNNIKKHAGKNSSD
GYNYNIEQDTLKKTVNNFVHNNNNKYVPGNIHKENNINENIQNVENAQNIHYVEKDENIRDIHNDNNSNNTND
ILNNSSSRNNVAWYNKNYLQYLFSLNNKKEQPENNQMTYKKLEGLKKYFINNIKNIYISKKKKKKKKKRKKK
ENEIQDMKLLHIYISTLLNIINIIFILFIFYFISKKFISKNKYLNKLHIPFLSIQNFTLFIFDIFLYPLIFSI
CNIIQFENIYHIKIYFIYIDFLVLKIICLFLLVSFVILYIVLPIPFILYIHNHIVYNYNFKKFIPKYISHING
IVNYFPFKIFLKNMLYYHKYQNESQDNQQFPPSSNKVNTSHSNINNILKIDQKKMKNMLINVAKKNINFVGKS
FLKQIIENFVHIQNEELKQNLLEDIDSTIRNSEYEKNKINGKYKNYDNIDTNHSNNLILLHKDQLDIYNNISL
YQKKINLFKCYKKLFYILEEQYDQCDHYDQHNIEDIQSIRTDIPQRVIKYNKNDSKKLKKYNTFVNPPNIYNN
CNDDIINIYTTSEINNSFYTYDYSIDYDIKKNKYIKVRIERNMLYNNKNKEQTMLFFLNVLNNINVGYTYDKI

RLFKNNIYIKKKINLKQLYILLANKKFIELFINIEQAYDNNLQNYSFLIHNLFNENMYTLVLIRFIIVLFVIY
INLCISHINIYLLSLSLLFLCIFIYRLYYISINEHLFKKCDIRKEDLLTINDQHQNDNQFIDTYKTKEQIDHS
IKTSINQKMNNHSTQTEIYQKENKYGKREKQKKNDENNKYNNDFEKISNKQHNNVQTSTSSYQQSDIIEDNKN
SRNKKLYNSKKQNKNIIKIYNFIESIFFQLFTIYVIIKNLKEKQHYSELILSMILFLVFIFYMFNEKNKYECI
PFHLLLILLTILLYIDFLVIKQIGKEIYLYIKHILMIHIYSNYYIKINIINHMKTLQHIFHIFIYNPIKSIVK
KEKDEKKNLKKTNTYTNMIRTNNNICSIFYHNNNHNKKKKYKLKQPYTNISTLKNITSNHIEIFQYEGCLHYE
CQVIKTEKNNIIFNNKKNKLYKNLYCYYTTTDIIKGLLKIDTIPIYLNDEMKKEQKRCICTIQVINGEHDSIT
KGISFFMKKKKRNNHKLYTLYYNKKYNIFITKVVSSKEQKYRNIQNNHVNEKKDRNEIYNQMNGTYITSKKNN
DNYQNHMDNINSYNKEYKIIKIDKYSYTTINENNLIINSLFIKPNKIYKLQIIFKDKSLNNITFDNIKNESKS
FIKKEDIINIKQKECTNKENTKYYNNVEDDNEKYNNMNTYILNKNNLNQGHYNIYTVICKEIGKLIFPYDMNY
IDIFEDIKVENKNKEIIYVNIHSNHICFIKKDNIIILHHPSFEKYEIYHVYLKNNKKNISDYVSNEFEIKSTQ
SGQIILSIYKDVQNNDDIIYEQKNNIEGTKENPKIMFDDNIYIYKIVNYKKTKDIFVKVQKKYNTFFSPMLDN
YDEKNMEQNKFDLSIFKTCINNMEKIDEQENKIDNTNNEFNDRYKNENIVDMVKPYDDFCKEIEYNYFIPIQI
LYKVKSRYCISLIKDLQHYFINCLNGNIGNDNNNYDNNYNNNHDNNYDNNYNKHKRKEKKKTYHDIEYNILNN
NHENVSTKEEPNILSDEKNNERKVKDPNYSNYELLQNSDKSEDSQKQSNENIIRRKKNIRNHNLFLKKDQLIK
YSYRIFKKISNIFPMRNKIQTHQNYGSHKMYSYEKKKKREVFFRYVPDYKKAINIYATSIQVYESKYHFLVSN
KGNIKLLSNFLIENLNISYIITLFNKYSLLHNQHDEININLDNIHFNIYEIYKKCSDDKKRLLSKIISYLEQL
VAYKFLCIIKYYGEKLNYLLLLDQINNDGDIYDIQRKIDYLFFNKYNFIKKEIKKNYKMKNCRNKNELNIHSE
KQCIDISFNNIKYTYENIVKKYVKQKKYMNKCFSDIKHNIYQIRKKYPTIYNYPYFPFFINKSNIIKIKYDID
QQIHYWNNCLTNFFNNIIEKSLTHINLKYNIRIMNNHILNHIKDSLLIRTKYKHIKNYNFAHIIISHHFLNPQ
DIHFFPIPPIFFFMKILPSNCIFFPNNKYNSIYNKENIEQTNKNNETHIIKNNDHIIDQSQNDKSFSTSNPCN
NNNMNENIPCTKLIPCYIQIINSDLLLYIFDNHNNKMCWKLGKKDYNIYEQSENYNEVTNSQCNEDQIKDVHT
DERHNNIQHNMNNTMQYNPCVNVNNNHFLYDKRKDTQEDQDNNYIQTYSYDKDTSSIHSLKNKRKKDKSNNFC
KDEYCTLLHDNHLNNNKLNKNLFCFTKNMNHHFSNIYNIIKNYIKNKYALFLYICNAKSIDKYNYETAPEFVN
IFIQINYTCEERLRNNLTINDHLIVNINKKKQRFLDKGIEEILLETYEILKNEREDYNIMKENKNVLIDIIPN
YTNNINEVQENSYTNKSIKKKKNKYIKNIYMNNLCYKNENSISNPDNNIYYNTDIVRTNDTNNNYCYNIELFT
YNFFKQKVTELIKYHYNYICMSEQNYVEKYAQFEKTIRRTEDEDDHISVTTMENISMISTDIMDEYTVEYTDQ
HIGKYDNLYDDKEIDDVSSIIQTFHLNENQKQYDYLLKKIERSRCSNLFNIEHDIYDYNKCIHRELIFYNRSH
ETFSKYIGDMKNMLYNGKGKLYDKFNSLIYDGEWRNCEKNGKGKLLFKYFNIWYLYEGNFCNDEIVDKGIISL
IDKEYVKNIECKKINKLCPLLIKANFCRKGQGKTTKEVPSEVIGGENMANNIENNVANDVSKNIENNVANDVS
KNIKNNVANDVSKNIKNNVANNVVNNLTNNIDKKKGKKKFTKKIMNKIQNTVMANYSYTNNVDCWSKDIIDIY
MPYLKKWNIYSFYCSSNMENINKRKDIFSNNNSKNNDNYNFKHLIGYPSFELVDGDTTENKLECYKLENDTNL
AKTHINDELGENKKQIKENYEIYNHVLNSGISSIIQKDTNNNDHMDNIDKITTTKKKTFLGNDKCNMNVHIVY
EKNLLQDEFIDSMKNINEDKLYYPIINKCIGLTKIIYADKSEYFGAVNDRGQPNTNEKYPKAFFNNEYFFYEG
EMKNFLPNGYGIFKNKEKDKNTYIGYWSNGYRQGNGCLHLKNKKYIIQGRFYKDEIKDHINIYIRDDKISKLH
LSSKRNKNCQKMKIYFKNGYIFYGYFTPDFKRHGIGILIDNHNKILYHGYYENDIIDKFCYILRHKDNTIYSG
NLNKGLKQGFGKLFYEPKINILDNQKQININPSNEHSIARKLETHDSIDINSNIHINFNIPSNNVVYIGYWDH
NNYSHFGSCNLKNGIYKGDIKHSKKEGLGIYIYKKNKTCKHKNRYILSYFKNDKIDKMGKYYTEYDKLKVYPF
DKEQIIPPLNSYEKCLKKGNTIPPHILKTDNLFINQELDSYITLPLAHMVQTIMNEIFDKSTSFINYLNASFK
FDLNYFLKK*
>N.PF13_0034
MANDSGISVITKIVESEQKNQILHDSILNKMPCYDKYGEINILSVEEVGLLKKFHDFSKKEKYEYFKENDTIV
SILFNCLQTDFNLHLMQYVLTIFYEIIRNDGSSYSYILGILHDKDMYGYLMKLCTHSDTYIADKSSFLLSGSF
CYNNNYFTEVQIKEFIIKIDFFNVSEEGKMDIYINILKIDNFRKDIYELEQFTSIIKKNLELSNNNANKQYKS
VFCVWLLTFKDYFIKQLYKNNIIAIVINLFKKCRVEKILRVSLNIIKNIMHIDDCFEIIVDNNIIQTMTVLQY
DKWRDNDIYDTIVQLLNKLDQRVKNYSNFERYCHELSNGKLKWSVLHTEKFWLENVMQFEKDEFKAIQQLADI
IKLYAHNIIQKSETGESKEEIDAVTVAVACFDIGEFARLYPNGKKICQKFKIKENIMILIATKDRDIVREALL
CAQKIMLNNWQSISNSK*
>N.MAL13P1.33
MFGKRKLIDSQEPKKKKKWKEELHDDLDDIDLECLKYVEEDVQEDDVDKNTTTNNNNNDDDINDCDNKKSTVY
SDLLDEKDKINKDKALNLSDKMELINNISNDNQCVTKKRRYDWMSSDDEDITSDEDEESLINEDIKEKTTDKI
KYDTSNNIKDKISKDLEQDISEYEHINNNKYNNNNNEDKTFIKVESKNNSIHNNNITYDILNDINKFSINPVD
IKMQDIEHIITYIYFNNIHFNCSIKNFVEFLENNINNKIIQINSDKLTNHTILYKYDEKNNDTVIINKFTHHG
KLFVHVKTYEDVVTLLKLNETLFMGRIIKSIQAYRKNDRFFILQAPSQYKYFINLAVHEKNKRKRKG*

>N.MAL13P1.34
MSTELTNNDFRRIFDNFEKEKKEKENDALLKEEKKLKRKQKYLIKKKKNEEKNENQKYRDRAEERRKGILKDV
KDESVLYNNINNTIDESKFMGGDEEHTHLVKGLDFLLLNKVRNKLIDKISSEKEKMKINNINKYITSGENVLT
NRSLSLSSTTSFINEESKYIFKYFFLFEHPHHIHFKDKINGIYENIINNMKFKNYNKNIHSVNYKYNIDMDID
QNDIPIKYIYNVDHIKNHHTYYLKNSFLNEIGACFKWHMENKKKKKSERLSRRPLTNNFSDPNLVSQVQAEDD
DIDIFKIDDKDINETDIINTNKSEIDDKNNIKTATQNEIKDIIFKDSKEDISRSIKENKDNYLNYNMKQFLSG
KNISKSNNQDKENEKNSLTKNIFKDTYDECYPGYG*
>N.MAL13P1.35
MSNNTTTHNINSINNVNNNDNINNDALNNPNDYLDKNTNFTNAHFKPSSYVNGAGKNMDSAGNIIPNINNNIQ
YHMPIPNNMPYGVNPNYNIHNNKMINNPKNMYNAPYPLNMNTIPINSYGGAEKFTHSVRIPIYPTPQNMLYNT
MNYMNSKAYIKHLKYNKVIPTDPTIPPNETLYIKNLNDRVKTDEMKKNLKDLFNTYGEIKDLIVMKSFWRKGQ
AWVVYDDKECATKALNALQGYVLFGKIMQINFSHNKSDIHAKRDGTFVERSKEPKKPKKILEREQKQKEIFEQ
MHKNYLEMQKNNFNMLNGNKEINKPEIIDLSQMDKQTLIAKAQAKANEDKNKKNEELPNNNIFSSYYQMNNIA
PVQNYPVVMPYKILFVENVVENVDTQAFNDLFKNYAGFVEARIIPQRNVAFVDFTDETTATFAMKAVQNYELQ
GSKLKISYAKR*
>N.MAL13P1.36
MEEDDVIISGTHMTIEKDKLEKMNENNFEKIYDNEYENIYRSKCSGILLKTPYYPYKYDIMNTSYKYIPKVGD
LVIGIVKSKKLDYYQMDINCNCECIIHKIDSFKYATKSSFPNLLNGTLLYMVVEKMNLENNSVVTSCINSSDV
KSWINYENYLGELVDGFVFSVNIASAKSLIGDRCYILDLIGQDIKYEIAVGHNGRIWIKANDPLEINLIHSAL
KHSFGKTRAQMNVLWKSIYNLNKKTT*
>N.PF13_0035
MDKVCKIIENMVYEFNDLKRKELFSDKEIIAIANKRRHHEYRINSPTSVLLHFILYLEFEMNLENLRTKRKNE
KKEKLIEEILQNKNLIKNHYKEFSILKNKMEEEKRFDKFKELRKLLNKNENEAKKFKNNVLKVEKKLQTLLGY
SLSEHSLIKRIINIFDVCLRKYYNNISLWLQYFNFCFIKKKYDELENAILNSLKYHIKNELIWLIYLFYFYNI
KKNIIQTRMLYLRAILFIPDSIYLNILYFHFECDVFIKLLNNYKKKIETDIHQIKAHEPNNNNNHINGTKKKN
KNDNNDTKQTVCINSTNDVLSIKTTTNSNSNSNSNSNNNNNNNNNNNNNNNNNSGDSTCNNYDSINNSNNNNYIND
NNNVIHLKDLQIDHVYGEFLKTNMDNMNIIDSNNISEIKDNAEGNHVEDYLTSFDNNIYSVIKEEDKYGLDVI
IFLIKKYMSTLSKDKSKLHIFVFLLLNTLLKIEKNLWIKMYTKQYDDFKQVVLDSINSCKFEQPFLYYYFFIE
TCVNSCYFDFFEDEEFTILKDQYYKTSMEKIENKNIYNIEKYFKYEHVKILLKQLFNMFDNELMIYFFCIILT
NLYDNISIYNNISEIFTIDINVKNDILEKNDKNKEINNTNHTNNNDNLENNILDNTDTITNNLNVDNINSTKN
ILHYMKEPNLTCHEDLEIFHFLKDDIFLCSNYKFHKINMDFIKENNINTYDLLCKLNFITHVCLMEKNHSEIS
SNNYLFSYDKTNKNKDILSSILYFFLYNPKNLVQKVSDQKAKINEIKEQNISPNFVVKKRKQAKNLLPSPFVS
KKVKRENQNKTKKNNDLNIYENDSDNKSVSDTDEETDEETDEETDEETDEETDEETDEQNDEKRNKNVEDNNN
NNTNTSNEEEKKNISTKKSENTQLMKKKEIDEKEIFIIDKLLLTLTKKIHVLVKIHIIKCILNIIVFLDNENI
KACLSMNIKQEYKNIMKMNVSGKYSNELNEMISIYKKVYTE*
>N.PF13_0036
MEEGKVGNSNEDFPEKEMNIDTNNNMKDNTHEDEKNNPSEENQLNNNNNDDDDDMNELLETFLQDIDNITSNK
EKNVEEKKLNKGDAGKEINRILEHKHSSPFEIFGIYENINMDLIKSRYRKLSILIHPDKCKIDKAAEAFHILT
RAYEELQKDEIKEQYKSVYEIAKKNIIKKHQLKKKKINEINEYLNKTEEEYEITKEIQQLINEECENLLKIQK
EKIEYAQNCKLANMKYVQEKEEERLKEEMEKEKEKKLWEQGRDERVNNWKNYKKENLKDEKEFHLYKNINKKK
QERTQQEEQKLKNVPLTYEQNNVHKKRKKN*
>N.PF13_0037
MKNFFILHLRNNYSIFLNKPLQVFTLNVKLNSHVKVNYKHELLTYEFRKITYVKKKKKKSYHNCDGRYITTLN
KTININEKYSKLKNDGLLNSTPDNHIKDNIIKEDLLYRKNNEKENEEVIKSNIDENKNVHMNDQNEQNKHCYN
KLLIESCCHIDKDVKKSLLEIFQYKEFTDVQRIVYDNIIKEKKQNDLLIQAKTGTGKTIAYLLLSINDIIKNK
ILSVHTLIIVPTRELANQIYNECKLLLTYKHNINVLTLTGGIKRRDDQLNIRRIKPDIIICTVGRLLDHFEST
YLFNTLFENLKMLIIDEADQLLSTGYENDIHRLLTYLPSNRRNILLSATLGYNLDEIRKKMCKSDYIYLNCVK
DISKHTSDQLIQYVLFHKAIDTTIILYNLLIEHMRLNQFTYKIIVFFPTARATSFYAQFFINQLKISVYEIHR
KKEMAHRQITANRFSMESVGILFTSDISSRGLNYPDITLIIQVNTPISREQYIHRVGRTARSNKKGMAIILLN
EADELFYQEIKDLNIQKLNAQNYTLKNTNLSNYLSTWMSNTQLLYLAYAYYSSILRFYKTKYTHLKINDDEII
DAVNNMLLSTGLVEQPYISRNLATTLNMQNNVKLKIRKDLDDILLL*
>N.PF13_0038
MNNDQNKRNNNNWFNNCTNEKEKKKKDKKRNTNIQIFYKSDIGIFSPCIDVEGKLCVITSTGDILRYNFVIDD
IKQNTNKSTSPTSRSLTSSPSSLSSSSSSISSSSSYRKERVTAQNELSYETQKDDDEEGEKYYDSIDDVENII

NRGDKKKTQKKEKKKTNIQEEKYISIDFSSECLCSDNDYNFYVFNPITKCIMIIDKQKKVDLYAEEYEDKEFK
GINNLLYDSKNNNLFIVDSGNIYEENTCNMYYINKDIETMISIDMDSLSYVNNICIYEKDNIRTIYACVTKEN
RIIKLIKKGNTYIKTGFLYINGNYSPLFICTDNINFVILLKDLSEEEKKGKVLEINSNAEIINSFFMDGNQFN
GICYDHNIKKYFFIEKNIIYIY*
>N.PF13_0039
MFTLISVCSYLFYTHKESLLNLIGLSENRNSRKNRRRKKKKGKLKQPFPEEKLCLSDSDMRRKKFILRTYSDV
NFNVTCTTKKERNSI*
>N.PF13_0040
MSLLFLLLFFLTECNIYWSFCVILKTKNPAFLPIQIKNPERHKPNNLKRFYINQNEGVKNRKYNNRDIYKFEG
IDEENKDGKENIDEDYLKEYEEAYHKYDDMDDDERKRRGSENKMNWFEIRKGEKEREHRYNVYDNEIIKKDLK
EIDLERVDKMNRSIGELPPYIYEGGKVYEGMPMDYKPEIYDFHKYNKYFDDLCKEKKSLIDSYQLDKKLLDDT
YYDAKRGGPKTFGFRFKQIQPVKYHEGRAYTYFFMHSHDVELSPIFLNAFRRVAIKHLKGGRVTALRIPNMKH
EFYCIVGVRENFFDLAQNLRQITFKNVPEDVDMDNYMIGKFRIKGPMIVVAGHMQLPKNVEIVNRNQYICSVA
AGSYLSMDVKIESIEEYVMPEYGSQSRNRDICKDNFIHFSSSCTPVEHFVFTGQRRGINLDTLGEINIVEMHT
DGSITPKSALLKTIDYISENFQYIENALYNNCHICDDGSLDEEFRNPEFYMDKDRYTDVPWNKYKTTSEELEE
TKNWLYRKDVHRQYNIDPESEQSKEERNLMWEKSKKMREEIQKRKEQIKKGPSASEGEMVPDEERHDCLLDWP
VDKSERNMNPPRWVIERPLEKNPHVGHEEIYDEGI*
>N.PF13_0041
MDNRGIRRRKEGQENDINIVNNADIFIRETSRNSSNNWNNNIRKYENENKNMHTNNSVRNITRKGMASDDSSN
DGKKKSVSIFNSANISSISNNCMKSFNNILNNINYNITSATTFNINNSSSNNNNNNNIYSNNYNRNHNMFNNN
NNFLQNHSKNSFSKGFGIQNNANVYNQHYNNNINQRTDNYNYNYHNNNNNNINSSNVNQQYGSRNEPTLNSHD
LEKFLNNNENVNKNHSNSSFSNNFCMNEINSLKNITNDDTYVTCLENLLRNHGNIDTNEMYKYNDKNINCLND
IIFLDVYRAYNVLTYMQEKVSVIIFTIKFIGQKLKRKHVPEEVVISLEFLNFCVKNLGLFFARFIDESFMKKI
SKLLRTTTLKKSLTKDVKSKLSKFLIVPIIHPGVATDPRLHFIKRKILFMMQLWHDSFILHQHICTAIFAEYK
SLKEKGITFPVINKAEKFFVKNADMSPSFSDDNILHEIPLNLTQINNIMKSLKAIKNMTNGQEKVKCIQIVSK
YKNDILQSINKLSDYKGNSNISLTLNSLLYINDQICIFQKDIQNENLENLQNKQHDNNNNNNNNNNTPDKRLE
NSNDNHEDNHHDYNVNNNIKKKSKKKNKNNKDKDNTQMDNLNNIIFNKYDPWNVEQKNEKTNENDNINKLFFN
NESEHFDQSKKNSSFKNLINNQNNDSLLFNNDIMFSFAENDLQNVKDISKKNENHILLDHIDSKKNNNNNNNS
NNIMQDGENIENVDDKYSFFNELNDFDYNTYNISSSQNINNLELNNNNKDCSYGKDTLSDSLNHSMNNQTVSD
ISKEKEKLDYPISNQINIVNSSSLNIINNNMCDDPKELNNECINEKNNIYMETESLNFSNFKRQELHDFNNEN
PPFTTNQIKNHNLQLNNEDNDLLNDRNDKANLKLQKNNIILTNERNKSLYLDDNNNNNNSNDTNRDNNLKVYD
NFMIDNTSYIISTDTSSYKPKSSPDIKNCSIADNEQNDKNDKNHLNYTYKEEDVKEMNVYDHMSKKDYHDLFD
TFGFNTNEEKDQKEDAHKNVPLENVISDNINKEDDHNVEYKISSESNDNNHSEYNAQKGDDNEHIYNNNNNIE
NNKSNNSKSDNHEDNNNKSVNHEDNNNKNNYDKMVNKGDAHILDPLDNNKMNEENENTPVEQILTNNEKIEDV
KNNNTNNNISIQHNENEQKSDQDEDEDENEDEDSNDINFDEIDEITKAIDDLNDNFESMDMYKYDH*
>N.PF13_0042
MNTEEKINTSNDAEDDNNECVKLKKKIENEKYDINNLKDDDVYLSADSSNSYRYDNHVNKKSIKENINEISTN
RSQSKKEKDSNEDMYNDKKRNNYKSSSESKEKGDKYCKKDKYDKHDKYDKNGKYDKHDKYDKYNKYDKHDKYD
KHNKYDKHDKYDKHDKYDKHDKYDKYNKYDKHDKYDKHNKYNRHNKYDKHDKYDKHDKYDKHDKYNKHDKHDR
HDKHDRHDKYDKQMDKIYILKKSSEHTRTSKDNSYYDSKRKYRNITSEDEEMKHHKTVDHNARKKSKSSKILV
DKYSSDVENDKKGVEEKYKDKNKNSYQTLKKNDNNINEKEYDKNRSSISHYHNKDKYNKYDKYGKQNHNIRYN
EQDIKRKSTKHYEKENFNNTHKGKNKNEKDEEQSEEEKEKKNVQKKETQNFNPSGLLAQEKIYKNGIEMKYTE
SIDAEKPDKKWRLYMFKDSNNNEPQKILHIHDKSYYLIGKEQLAVDIQLNNISISKQHAVIQFKKHESKILPF
LLDLNSTNGTYINNEKIQPNKYYELRETDIIRFGSSNREFVLLHDTCQAD*
>N.MAL13P1.37
MSNMSTSSNDEKYIKFKKENIMGALKRNNNYIKKNDLASSVNDSIENENKKTYRCYHCNRKVEDVLILSCKHL
ICLICSSIQLEENYKNFLEKFMNKKCIENNKNKKSHHIDECFDNYKDMIKNKSTYNNINDIKNVMFNNDIILD
DMYKKDKVGFITCKLCNIKNKLSLESIEILTKVGLFSHNILNVHKFFFNNRNGYEENDNNTLKYNSIEEDANI
LDNSLKDYINNDEKKKFLLLKNKYDYINNQMDDLEKYSYICNICSYNEAIIYCKDCVEYLCKECCDNIHEMDI
LRKKANLKIEKAHEYYEIEKSCFHLKKLVKAPKKFLNITQEDIDIMYNTDDESYTTYRDNNDMYKIYDNNNNN
NNIQSKNKNYCYKDDPSKKINIYNDNFENDRNIHVDIFDKLHMNDSDFDTYDEEHFYRRQKFFLDKAEKKRKN
LLLSKKLNGLVENMKNESCYESDYSSKYGTDGSIKDNKTNALLLKLKKKKKKMVQMRKENKLYNIKKGVNDL
RDIKNYDDKHIGDDDNVCRTNDAICSNESYNNNNDDKKNNNDNNDDNKDDNNNDGHNNNNNNNLVNIKYIKNV
NNINSNNLRNDTYIFSNSTTIDMISLDDEPLYSDSELKEIGITNIHTEICQKKNNKTLENINNDIINNKQFTA

IENVRCSEHYNYPIQYFCHTCLSLCFCSECAINGIHTNNCNIENINTAFITVLNNYLIQWNEIINELINDLEK
NFYESLEDIKNDWSAHLSECYYNLNTKVNYIFNNLVKKEKEIFHEFDTYIEKFKKDNLQYIELLNSKYEDIEK
TINLIRQNKFHTNPIDIIKFYTNNIDIIDKTILLNNDFKPIQDLSKIRESKIFYMDFYASQINSYLKYLQAYL
NESNMLDSK*
>N.MAL13P1.38
MEKTSFKNNQKSYSKRNSSLKKKVTNDKNDNSLLTIYSFISNYMLKEEMNYDENDNRYILLESQDIENECGLK
KVSFGEFNEKSYSLSHKRKEKNEEDIDKEEEVYEGVDQADYEEEDYEEEDYEEEDYEEDDYEEDDYEEDDYEE
DDYQDDAEEQEEEEDSAKSYNYDHNQIDRNKVNNDESVNSTYNKQNNSIRRGGKKKKNKIYNNDHFPNDEDIQ
IDHNTNEEKHSRQLWKKKKKKKRKREKKMMYTNSNSKEIYKGNNLAKYFTLSMDNINEQENIFKDEKQLSRQM
SYYNDAYYYTSEMIYKNNRRNKEKFALYLRLMSLFINIIIGIYLIIYFPHTNNDMDTFDGTFKNMHIKDREDI
KDIISKNLEINKDYHNYMKKVNLNVSILNKQKEFFENKSIGNGKASSSSNFSSSNDNNNNNNNNNNNNNNSMH
VNTFDRSINNEDSLINTNNLNNNDNNNFLRFLRNIFSRNNYEQEDNLKSSSNLINKEKANNNTNVTNTGYYNL
YKNNNNNNNIIDVEKNIYYEKDKNNNLKKKNFKNVKNNNYNSGVYYVNDKDMLKDIKTKEDVNNLIYMIDILN
LMDDEHIMTYIYNEMNKIYKYAIYSFFGELLVISIIVFSLFILKILIKDNNKFSVILTMYGIFYKIFCYIFAH
YVLFMGLYILSIYYNIYIHRDIHTHSYNFFCYHYVYNNLYIHLLMLFYALQNIVFTINDAFNCIRMVFILIKH
VIIKIYEKITCKNFITFYELKEDKSPLSLTCFNKLKNKCNMNCKCCKDKNCVDIDIDEMHDIDYEKNKLPLFP
NFNRKPYCKNLNIKTYEQSLLNMRNPIFK*
>N.PF13_0043
MENQNNEEQTNKIKETLFGLSTGIIQKAINNNVDLRNYRMRKEALETLGKCLSMFILYITDGAMEYCENEKRS
TILVRDILNSLDDSLFLDIHDELKRQLTIQEENNKTEISKVSENVQAQYASNNELNNNQKEGKPKNKDDDFDI
LLQALE*
>N.PF13_0044
MTSEFWPDLDFKTVGRLILEDGNEFVGYSVGYEGCKGNNSISCHKEYRNIINNDNSKNSNNSFCNNEENNLKD
DLLYKNSRLENEDFIVTGEVIFNTAMVGYPEALTDPSYFGQILVLTFPSIGNYGIEKVKHDETFGLVQNFESN
KIQVQGLVICEYSKQSYHYNSYITLSEWLKIYKIPCIGGIDTRALTKLLREKGSMLGKIVIYKNRQHINKLYK
EINLFDPGNIDTLKYVCNHFIRVIKLNNITYNYKNKEEFNYTNEMITNDSSMEDHDNEINGSISNFNNCPSIS
SFDKSESKNVINHTLLRDKMNLITSSEEYLKDLHNCNFSNSSDKNDSFFKLYGICEYDKYLIDLEENASFHYN
NVDEYGYYDVNKNTNILSNNKIEQNNNNENNKNNKNNNNNEVDYIKKDEDNNVNSKVFYSQYNNNAQNNEHTE
FNLNNDYSTYIRKKMKNEEFLNLVNKRKVDHKEKIIVIVDCGIKNSIIKNLIRHGMDLPLTYIIVPYYYNFNH
IDYDAVLLSNGPGDPKKCDFLIRNLKDSLTKNKIIFGICLGNQLLGISLGCDTYKMKYGNRGVNQPVIQLVDN
ICYITSQNHGYCLKKKSILKRKELAISYINANDKSIEGISHKNGRFYSVQFHPEGNNGPEDTSFLFKNFLLDI
FNKKKQYREYLGYNIIYIKKKVLLLGSGGLCIGQAGEFDYSGTQAIKSLKECGIYVILVNPNIATVQTSKGLA
DKVYFLPVNCEFVEKIIKKEKPDFILCTFGGQTALNCALMLDQKKVLKKNNCQCLGTSLESIRITENRTLFAE
KLKEINERIAPYGSAKNVNQAIDIANKIGYPILVRTTFSLGGLNSSFINNEEELIEKCNKIFLQTDNEIFIDK
SLQGWKEIEYELLRDNKNNCIAICNMENIDPLGIHTGDSIVVAPSQTLSNYEYYKFREIALKVITHLNIIGEC
NIQFGINPQTGEYCIIEVNARLSRSSALASKATGYPLAYISAKIALGYDLISLKNSITKKTTACFEPSLDYIT
TKIPRWDLNKFEFASNTMNSSMKSVGEVMSIGRTFEESIQKSLRCIDDNYLGFSNTYCIDWDEKKIIEELKNP
SPKRIDAIHQAFHLNMPMDKIHELTHIDYWFLHKFYNIYNLQNKLKTLKLEQLSFNDLKYFKKHGFSDKQIAH
YLSFNTSDNNNNNNNISSCRVTENDVMKYREKLGLFPHIKVIDTLSAEFPALTNYLYLTYQGQEHDVLPLNMK
RKKICTLNNKRNANKKKVHVKNHLYNELVDDKDTQLHKENNNNNNMNSGNVENKCKLNKESYGYNNSSNCINT
NNINIENNICHDISINKNIKVTINNSNNSISNNENVETNLNCVSERAGSHHIYGKEEKSIGSDDTNILSAQNS
NNNFSCNNENMNKANVDVNVLENDTKKREDINTTTVFMEGQNSVINNKNKENSSLLKGDEEDIVMVNLKKENN
YNSVINNVDCRKKDMDGKNINDECKTYKKNKYKDMGLNNNIVDELSNGTSHSTNDHLYLDNFNTSDEEIGNNK
NMDMYLSKQKSISNKNPGNSYYVVDSVYNNEYKINKMKELIDNENLNDEYNNNVNMNCSNYNNASAFVNGKDR
NDNLENDCIEKNMDHTYKHYNRLNNRRSTNERMMLMVNNEKESNHEKGHRRNGLNKKNKEKNMEKNKGNNKDK
KNYHYVNHKRNNEYNSNNIESKFNNYVDDINKKEYYEDENDIYYFTHSSQGNNDDLSNDNYLSSEELNTDEYD
DDYYYDEDEEDDYDDDNDDDDDDDDGEDEEDNDYYNDDGYDSYNSLSSSRISDVSSVIYSGNENIFNEKYNDI
GFKIIDNRNEKEKEKKKCFIVLGCGCYRIGSSVEFDWSAIHCVKTIRKLNHKAILINCNPETVSTDYDESDRL
YFDEITTEVIKFIYNFENSNGVIIAFGGQTSNNLVFSLYKNNVNILGSSAQSVDCCENRNKFSHLCDSLKIDQ
PKWNKFTKLSKAIQFANEVKFPVLVRPSYVLSGAAMRVVNCFEELKNFLMKAAIVSKDNPVVISKFIENAKEI
EIDCVSKNGKIINYAISEHVENAGVHSGDATLILPAQNIYVETHRKIKKISEKISKSLNISGPFNIQFICHQN
EIKIIECNLRASRTFPFISKALNLNFIDLATRILMGYDVKPINISLIDLEYTAVKAPIFSFNRLHGSDCILGV
EMKSTGEVACFGLNKYEALLKSLIATGMKLPKKSILISIKNLNNKLAFEEPFQLLFLMGFTIYATEGTYDFYS

KFLESFNVNKGSKFHQRLIKVHNKNAENISPNTTDLIMNHKVEMVINITDTLKTKVSSNGYKIRRLASDFQVP
LITNMKLCSLFIDSLYRKFSRQKERKSFYTIKSYDEYISLV*
>N.PF13_0045
MNVDLLNPDPVEESKKHKLKRLIPTPNSYFMDVKCPGCLQITTLFSHAQNVVLCGSCNIMLCQPTGGKCKLTE
GCSFRKKIE*
>N.MAL13P1.39
MLERRNIYHKKGEGGHLNKRLVTKKKSKRKGRYTKTPLNMLERNNKNIKLLITDDISRLIYKNEIREKEVISS
NDYYKKIIRSNNYVVRLHNLYNEFKLDDCLNYGLENLLKLKKNDYISKVEFLRIIGNIYFQIEKYKYSAITYM
LLCNILNRIDYDEYKVELCYNAGVVFIKCFLQYNDINFYINAKNALHYSLIYNDKKNNICYNEHIYDNTLLIL
KHHFPNKFLSKINKKKKLSNLFKEYITTTDESLAHQSSTYLSDLSIDQLKKKKKKNQKKNFIHLNNQQDGDNI
SLTSSYFYKSVSSVEDSTSRCNNYCIDYGKGKTSDKMDNSLCNKQKRISEKTDTLKDKNSSFLIMETVPSKAS
NIYLNNFLSYDNNIERENNNNINNNDININNQHNINNNNNINNQHNINNNQHNINNNHHNINNNHNINNNHCN
IKNQHDLLTFPREDRLTNLRNQETYNNNIVDDLANTCNMNPFFQSDSLKNNTNLMTNNNFQSVINKNISKVSI
IPPSNVKYNSFTNVTSNIYHNVSNNLCNNNLNNIFKKPKQNVTFNNCSNIFRSSSNNYFNDSLYKLQLQDDNS
MNVQHIENKRFLDITRKSESIINNNIISNVVNNLNVNRINDVCINAHNNIIENVSYPIHKNVLMSVQNNIPIS
QQNNFNKYLTNNNEQNRYTDMYPFFQANTNQKVASDFVHKPILHIHNNLPNNVFNKSQSYDMNNKNVGFFNYN
QGTHSQFCQPDVTNIVVPIKGMNNKLLNMSINKSSSMSMNKSASMSMNRLPNSVVKIPQLTFNNNQRLNSVNI
PQRGSLNIISSSDVINVPKNNIFTNIPEKTALHYEQNGVFHNFQHVNMNCSGQNVHMNIDIKEKHSDINIEEK
HKEFNIAQEHFNVNISEEHINVNMSEEHINVNMSEEHINVNMPDHNLKNYSNNYQKNLTNKRLSFSKSYIKGM
YLKSEESIEKENKNVYGKNEYNNLYKEHFIETIPSQDSSRKSHDYDHVISIKDDDVESKIPMNNIKVENDKVH
DNNNYSSMHKTNHQMNYTDDTFLNDLLQDNYFKKLYDRNIKKMMEERENASKNIFAKNKKKKIILNILKKVYY
RYEHNLSNEYYNIYKLKNQEKKNKFVVKNKTIVTFLEKMFRICKRFDENMNNKIQFIDTYTYKTKKLRKVQKM
KNLQNDIYNNKYENKNKCKCKYKHVYSLDNLEKYHNKLLHLPSNRKQYITSNYHINEDQSINQSNSKQLIIKS
NTSNMNNNIKFNSFKEKNRVLKRAAYELSHNPRYVCDKGNYSIISDMRKEKRQRQYIEHMNKDIYRKDVMKDE
NLNFKYFIKLYNEIRNDINDNNDNIDTINYSHMSNNCDNIHKCIYYEEKLYREKKEIEDIVHINSNKYNKKLI
RDKVKKLSSYNNPIINNISDKFIYDVEKIFLMKKYSYIIKGLRKNENIKHIYYLYQNMYDITYKLDHMNSSNF
FNISSYIIDILLLSEIKEKDILKKKINKIKYSYRKFYTSTSNINMNNIIKRIMENMRTVSGMRNYASKCEDAH
VCFNMRKKRRIIKNKDTAINIMKNNNNMKCSSTTTVNNSDSNSNNNNNNNNNNNININNISNNNICYSRDHFY
FNHKCKVNNLEKNYLVKDKDSFFTRDNKKNNTYDGKINKNKEQVIKRESISNNNKYDKKKDEENVQKNNEDVG
LSSHSTVVGTDVKKPKKENLYYKYNCAREELLYEMKKHENKYIRIETLYDYYNDNIINICDNKIRKKKKKNHD
KFILMLFYYHSNMLNDNKLYNIIFDVIRFFLYNNIITKRNAHNHITLIKIYELFILYKYKYKKILTFFFHDVE
DEHIEKNNKNEEIDYIKNICDSDNYIELIHCHLFMLRLLYKCYMNKLKNKYVYEKINLQYIINYTFTILNCLL
IKYSYNKNNVEVRRRVLFLFSEFLYINSKYYYKNILHFLEKYNIDNIIKSEKNIMKMNNLNGNDNNDNNYNDY
YNNNYYMDEGIMNSFDENTSIDFKKTQNKRQHEDFLVNSLDKRRKKKNFLIKNEQEQNYKYDEYYNEPNNNYV
LNNNSHMMYSPYQNSVHPSYHVHKNIEKKKNQNYDDKVYKDMEYEYNDVNKKNRSLCRNNIRNNNNIISNDSS
INYEYDTYEERNINELNNNGYDNINKGSTSYYNNVDYFKEIYTKIYENINVAFSSFLKCYYKCFLILNLKLYE
NNMFKKNIIRKIKKIIYFNNSLYSMLYMKKLPCNKIYNFVLNEINYNLFNKDIFNDKSSFFKILKEDIIIYQV
YKQNENYIKNIKVDCKNDYKCNMKENDIIDKKENMYSKKKMDEDIYVVSKNLYKMNNPNDIYNILITFYKCMK
MNENFDVSKKKKKYKMNKMEKNFIILRHTYIYIKKRICDEIENNNIINLFKKAKEKDENFITNIDYLHSLFDS
RFCMITLNYSLQKLIINFLYDCIYFTMKYEKKKIYFSLINNMMNIYNMIFSIFYLRLNAFSDFSTNKLNTHIY
NFNKKNIYSHDDTHIYNIFNWFSFINYRYGCSYEFINFVFTSFHVITNFLVNINISTEMSHRKYIGRKKNIIH
ISHDYLKNKQYHKNIIKNKQKNVYVEKKITNDEYIKYNNIYNKEEATNMFLLSSAHNKLDNKLEKYMKQQKRS
KRNIKPERNILNNMYIDKIYIYRIIKQIQKNVNKNGVFKIHDSYLEQKLKMDMDKHYCYITNNNNNNNNKIYK
HEKDKTKKKKQKKNKKKNYIDIYYGLMKNILLNLNMNNEQNIIHNSIKCKKKLWRDFLTNESFNKQLSNILYK
NKNSHKILNKIHTYLYFNVSTNHYINNNNFSYIRRCAKNIKLLNYQHFRFKNSKHNKNVEYYHVIKYNKDMEN
KLILNDYIKKYKRIVRKYNRLKKIKREIKCERNNMSVFTYVALINRSYNKLYTSICPTFSHILFLLISILYVE
NYNILYIKKSPDFFMINDIYNTYYIFLHISNSIILLSSSPLRNHLHFNPVLHLDKEFYKRRMITRSGKNGENE
NVENKNDVENKNDVDNQNSVRKVLYGVNKIKTNELNTINSNRKKCNFLGSDNGVHVSEGNNIFMINKIMKRNH
IHDDANNDSHKIGEHYGHVYDNYKNNKNEIDLSYDNRNVSWEHHKIYRIYLDTLLNFACMNILLITRRKHNKK
TKMKRNKYIYIFCNIIENLLQNCIFYIDLYNNSKSKNSILDMDDMYMRLNKYCKDNNMIEYYDLMKQNLIYHY
FYKYYIIDEYIHNFCIKRNELYNLEKCSLNIFYFSMNILLTIFNSDISIHLERSLYNINYFHHTNLYYDIILN
NIKKTMAFIYDSNKSSFNSIVTCVLYYLNIYRKNERLIIKNHEDKKNEKDDPNGFHFFDKSIKDEDKSNIQCE
EDKKKKNIVNVGTSSFSLKKCIFQKIQNVIGSTGNNNKNDQEKEVIYIHDEDSNKSTTPIKYDEKKNALQTIR
KYDKYESKKKFEKENNVEKINYVEKKNYVEKKNYVEKKNYVEKKNYVHEKNDVEKNQNGHFNHLKNDKKCRKK

IISSIIFDMICPYLDITNIRIQNKIQRRNHSINSLINICYSVLFHNFLFIKSVYPFDMLTKEKGEEEFDEKKV
KVTKYNYDIFNMKMLNIEVVELFLSLYIHKYMHNICLDIKKLVDYNMYMNHFFWVFKKCSIPIYHNDYIYLNY
KFNNLYYTFNDYNVLQFLINFKRSKSEGKVSVKIKKSININKKYIYIYIYIYIFIFIFIFIYIYIFFFFFFFFF
FFXXXXXXXXXXXXXXXXXXXXXXXXXXXXXXXXXXXXXXXXXXXXXXXXXXXXXXXXXXXXXXXXXXXXXXX
XXXXEFMMSFVKIFEEKICLEEEIGLDYIDPLIFNIFLDYMKDVYMTDEYYFVCFYEYTKNAYNEKKDDKMFS
LDNFDCYMDELNELNPYLIRMDTKKKLNDDIYMTYLRLVIKENINYKELCDGECKRKSVDNITKGECEYINID
ELANDKKNICIEEKTESNMFYNKLKFINKKQKVWNTKEKMKLILNNDPIRFKKFYMYYKKVTFNLINKKNINY
LDLIKVMNRKHYNHFSNIFFSSFVHIMSEIKYDYNKTYNFNILKILDKLNVGLKASFYNNKNILLYYYTFQQY
FYLYKFLEDKYYSECVLSEYLLLNIKNNKFIENELYNNDLYRMYYYIKFLYLHNILRHTDLLLNFLIYIYYKY
FYYNSHAIDHNFTHFIHLFLSIHEESYNNSLIMIEGNKTKENNIDHTNQKDIFVRNLFMSLDKCNYNKLGNNR
KNMKNITNDDTLLRNNINNITNDDILLRNNINNITNDDILLRNNINNITNDDTLLRNNINNITNDDTLLRNDV
LKKNEIIDDKKGMSIKQYSIKHKSFYKEEDYMRNDDNNNNNNNNIIYKDANNYYLVLLNSQNRYIQILFYKVL
CNFIKCKVQTLNAKNLFKNVNFYLSSFVTYSFLNTYNEDDNFYYKALNYKYSFYDFSTLKKNIKLCIYLKNLY
LKHDMFNSDILNFLHIEGYKNRKLRKAKTKIKTKIKTKEKMNEKIKMGKFYSSDNIKYDQNGCNKYEGENDVS
HNNVLENHKNSSRNIYGYTNTYPNTYVQRDYHNVKETHAVLYKNNEKLNYKRSFVEDDAINNTAVVIKKYKLN
NIDNAHDSYNDNVNISEGSVSVEEERDNRKTRKDYGNYSDDKEGCNINCNIYDMNNQYGRIKIEMEHSNDCYN
ERNNYPMNHACHVERCNMEYKEESNNIKNTKYVFDNTNCMNIKKEIYTDEDTIDSIDSLKQAEIFKNKFYKNR
KIKRVSKKYYINILNYHLKNKVHGYSIKSLDSLKLDDKSIYLIFLFLGNIFTVLYNSLLPYCGSQHKYKMIND
IKSKMEKKEKDKIVVNINENMSSDYAYKITTDSNSSSNNNNNNNNNNNNNNSGGRFSSTKDHRNNYTYEELLL
NFMLISLCYYSDSLIFLSYNIVHWDNQSEDIDTGGDEEEKNKRESKQKGDDINGGINGGINGGINGGINGGIT
DKLNDNINCVINDDYIKDIEEDSENDNNSRQGKSNIIINQDISYCVKKNLKVDEKDTFLSSNENSNKMTYAKS
IKVSNDNINDCVIKPSYVNTNNYYNFRKKAEDIFNNEKECAKMIIPLYKLLVTRLKICLYYPHYFFLACLFNY
KYDTNLSNTLLNYIKEKNFLKQGIVNYIFEEKERKSKENKIYKMNYSDKDNINNNLHSNSIYYSSDNKLYQTK
FNNFVNINRNNEKKDVKHNYISDGIYNNKENNTNNKDNQNILFSLMRKLKNTCDSFNNNVVSCKNTILKSHNN
NNSNNNNNNNSNDDIHVDNINKVNIEYIKSHPTIDNDHNNNNKNLNQYVYESNNMYINYYCDYHDHMLKGTDY
CQKTNDITSIHSNILHYNINLEEKEKNGTTIDVGINNNVVDSINVDHMNCLDFDEWEKKIEDNELNCLNYNMN
NLKVRIKNIEIRYEDVINDIIEGLQNIFENKLCSNYNSQAAYHICVYYFMRKNYSKCIYYIKIFINKGIFKIW
LNDSYNNDYYNLYCKRIRRSEFSMIKYCTILLELSKNILKKLLGKINFALSREKKKRIEDLFNNKLDGEIKKF
LLLSKNVHHKAVNNKSINNDNINCSDRLNEININNNINNNINNNINNNINNNINNNINSNMNNNNNNNVDVNL
NYNLEKERDFTQNVYREKEVIIIDEQNDNMVEAPYNDISKNEEDSDKKNIEENTNIVIDECIKDQYKAMNLSE
KHNTVLVNTTTTTKNNNNNNNNNNNNNSGSSNNNNNSGSSNNNNNSGSSNNNNNNNNYYNYYYNNNNCVVNQNEE
DISINENKWIFLGYENLEHLNDVYDILKILFESYTQIGKNLKRYNDIDALEEVTCMHGSNLSIVNIIFKMITD
HLCFIFEVLCYFTPYVLIYPLILLFTKRTIMSIKSDTNNNRNNNNNNNIVIDENNNIVIIDDNSNIYIDEKNK
IKKSTTININNEINNNININNNNNNNINNNNNNNNNNSSNTKEYLKDTIMYLKEAYVNEELIYEKSTACMDLR
ILKLFREFLANKTVTLPSNLLSRMQNVYLIICKKLLYYQKNQKDMEKNINNNMNIGNKKKKESVIKFVDNAFL
NGNKTILSSFLNVFQNMQLLKCNNYLNCEDIKILTNKLVYNSVYTDINKNKNLTRIDENKKQAENNNPMDNVY
INNIFDRIKNDLYKENFITYGNINNTQEEDDKIHNPDDNHHHMEKTKENNIFKNNLYYIKMNNMKNICLYDDY
DIFNNLKTKNEALNCVNAIVTDMNSSAKKNDNANFKKRKTIDSSLNEVRNSIRERDLRRLNREKSKIL*
>N.MAL13P1.40
MNKDGKNNIFVDDIIKNDIELKKEYINNVINYKDRIPLVEYKKEENIMDIYSKYHFINNIYINVTKRDEEDKF
DIHFVNDNDDNEIDNNDDYVINNVVEKMQRNTFLDLNKKRNVIILLSGCSGCGKSTLSCLLAMFLNIRRIIST
DIIREVLRKFKIKKEKYLRFSTYESWKLHDSEDDMNIIQLYNEKLKKNGFMNDYENNNHDDSEKEEIEKILTK
RCIENYLIQCDVLYPYIDEIIYENIKNKQSLIIEGVHLSTHVMKKLIKKYPNKIIYFLVYINDKETSIKRFAT
RTNNNKLPNNNKNEKVNKYIQNFNYISGIQSYLLQSSKDLDNTINYIENIDIYRSLQMIMNSVYAQK*
>N.MAL13P1.41
MYKRTFHRNNFLLFVYVKKRSLHINGAQEIIHERSKVNNEKNKFKNIYVYFCLNKYININSLDSKNFIRTPIH
YQKYKELVDSIKCASNVRLQNIHLIRKIINSLEVYIINYLNNLKNYHNNDLYYNIQQYNLYDDEEQEITPNSI
TTILISLYKLRYRNVNFLKLLEKYIFMNKEYFKISHIHLILYIYSYFNTINNTFINTILNIILKNKEYLTNDN
ILNIFTSLFYINCKNEKTIDILIDYIITNELNFHINIIIYILNNVKKLNYEKRNIIEYFHPQIVNKLYMLNDE
KNVDYVDPFFLKKNISNHVQDKNRHDKNMEVHNKKVYKDGSRKEKNVDINMSVKKDVNDNKYDNKKKNNNNNN
NKNNNNKNNNYSNSYSNFQINHLDNTHLDSFYGSLHNEYIVENNCTDNYIYEYYNKNDIHDKKKRKIILLLDV
IIYYKYFKEELLKVVYEYILKNLKTYDTQDIYFIISNIFNFHVYNFPYINLLNNKFLLSLCYNYIPIHKNCHS
VLFLSLKLLMEKYKYYNIFIDNILFHKIKTEILTLQERQFLNLLSQYKNSHIIKYLCDISLIYCNMLIINEQK
QNNININNINININNININNNNNDSNNNNNNNDYVGVIHTYRHIDNLKNSHTSNILHNQIHNDCIKKEKNNDIDY

TCGEIKYYEDEEEKKKKCIQNITKHIITVKYNEQNKLLLFESDILLDLFFFYVELKYYKGIFLFLKSLFVGIP
NFKEFDIFLFYKINNYHKYKENINYELDKYKHVPIYKKFNALEILQIYEYFKSCFSTYDDFIKIKKMLEEEVE
EDHKNSMYHFYFRNEIYNTFKDTHTYISNINYNMNELLENNWNCKNQNNQKVQIVNDTYDNKVTSKYILTNSK
INYISPNKHYNKYIVNKEKNKYNEETNADYKKKEISIPNSLYNNISIKYEKNKLFDINNYIYNILNNIFIYAP
VDLFALHIHYYNSLNIKFLLTNIIYKLSYLNVNYLALVISKIYKLLYKEKENEYVVFLDFFLDYLLGQERNSD
MFTYKLKDMDNKKINNLFNINANEKRTLIDIIYNNNVNKNIHDEKRIKKRIAYILYKNNNIYNSVHNDIIIDE
EFLNTFFKYVVNKKKYNSVCSFYFMTNESLYLILNSLQIKKKKEKYIECIELITNIMIFRYIQYTYKEEEGKK
NYSYQKEEYYNVAKNFSYGNDNLYVIKDIKNLQLHLNLSFDELAIKKKKKKKNYEHVQVNSKKVCKDDDDKKR
YTLEDNNNNNNNIERINKKRENIYNDNSNIENKNSYISISCDDKEEIDKNVFQDNSIMKRFIEIYYRIIKVRY
YHELAINKHIFRELCINNEYLDNHKFLSLLFFIYKYKFDEIKYIFMLQKRCFLYKELYYTHSNYRIISFLCKK
LNIHIDDNIRTTPTKQTSYDYEK*
>N.PF13_0047
MYELNDAKLERISNKDLIRKIIELQYCVINLSDSMEILKSKNKILDEENEYLQKNIENIVNDVKNESTNYLNK
TNKNKNKNDDNKNYNVDIINDECKNNCNDNYNYNQNYNQNYYTHDNIKGNISNIYTHNVIPDQHINENASLIS
NKKNNIIKTKHTIINNTHHMDEKTNDNMEDNINKNHQNDYHKNNHKHINKDITQHDHNNSTHKMNSNNDSCNI
YNNNNTDIYKNSNINDKQIIDENNTLTQTIQPKNVNNNVKNHIFNTLKDSTTKSNIYNLIMETDKLNSIFNMA
TNVLNNSFFYLSKEHDHKKNNHDTHNNYKNLNYKQSYQNKTNHQKSINLINTKNNHMKNNILCKETYLNCSGK
KDTYKTDQQQNYLKQDNEIIKREDDEKKIMNDDITKEEIKKEENNIVKDEIKNKDEEIIKGEIKNKDEGIIKE
EIKKEDKDIIKEEIKNKDEEITKEEIKKEDKDIIKEEIKNKDEEITKEEIKKEDKDIIKEEIKNKDEEITKEE
IKKEDNNITNDEINKEENNITKK*
>N.MAL13P1.42
MYAYFFRREQKMNTSLKFILCCLLLNYILSKVYSFNNLYIHKYIKFINKNNFIKANKRYDKCVNNLSKYNKFI
LNNTIQNIHTTRGVVQINDEEETEDEYFEDDDEGDDERDEENYDTIVEYEKDENRNKKNIKISGPLKKITIKT
TKVKRKKKKKKSAEGMKCKTCKKLLKPRVKFCVYCGTNVSVEKIKLKKYIEEIYLPIRKEEVSENTFRVEKGF
WKDILPKLGKYELHELGPNNWESFLKYLKSKNCSPRTMALYQSTYQQSLKYALYRDYLKSVHNFRKIKKSTIP
RRKITPLSPKEIELLLKNSSDMHRAIFALSIGIGLRPSEVLRIHWEDINFERKEIFIKGQKTKYSNTSVPMTN
FAFNELLKWWEIENKPIKGLCFYSETIKNKFNYTQTKTPLKTFKTALKGAAKRAGLEISEDGKSRRIFPYLLR
HSFATIAATSNPPVPLPVAQAIMRHSSSKMLLDTYTKAGNNIIRDGLDNFKI*
>N.PF13_0048
MPNNKLQKNLANKLPNSSKGTKLFSAQRIKQLAKDKKLLDDALLDCYGRFIALLPEFLLKDHVHLYFQIQEAY
WWYDDMWQDKYPDKLPKLSLKTFGYLICDDCPILKKYVPPSAHEQFSLNWRRYCRTIPLRGAILLNHDLRKCL
LVKGWSTDSWSFPRGKVDELEEDSVCACREIYEEIGIDIFPYIDEQVYIETHIEDQPIKLFVIPGIREDTKFQ
PKTRKEIGDIRWFDIEKLLEYKDSKRRKETFFDNKKERINYMFVCPFIPNLIKWINVLKLSIKGKVSKKNTYV
SGSSILSYKYQITGIDAHIIKKLQNADMETKDMYKSNYYFFKSDDDYYYDEEVDSLSNKDTYADVLNVNSINS
DMMKQYNTFDKGYVSMSNDSSNNNNNNNNNNNNNNNSHMLINNSVYKNEEGSNIPYGNIYNNFVPNVFNMDNM
KKDHIMDKCNEDNMNDHMMNNNMNHNIIYDKKVMSNNNIKHNNNNNIKHNNNYHNNNSNNKDAKKVINNMLVK
GNMNNNNNNNYNNNYYHYNNKYYYNNKYMYYPNYNINYGNNYNMYYNRGYHSYFNKNGDQDFYQKMYRNHIKK
IGVGHLSALRLKEVGLRSFDDQYIEKRKYFQMRVYGNNKNKLLYNAKNDIYGNHRKMMRNAYRSVDDSSVYYK
SSRNTNLDACNEKTFGENHMNGWSAEDMFKLNEQKFGIQSTYNIDNYTTPLNCNDEKKSSRYVKETLKSVNVK
GVSSNGLNGLLSASFDKSPVNKEIQYFLNNTKNMLSKGNMNSTNENVDMVITKSSNNNNINENKNKNNNDNIS
EMNKLSKCVDSNPNLNVGKEEVDHIKMVKNKNILDITKKNNNNNNNNDNVHVDNSKKVMMKKKKKSLGEMSKGI
ERNGSYYSLKGDIREKNKENVKNNKMISNVMDTNTYDNNNIKLKKNDNLSNKLKNNKNNDIFINDNKKMNSQN
MVVNKNVFSPSTSVSNICDDRDIVKKLSVSSKKNVKDKFDIFNFDDSYTDDDMDDKDVSCINGEDDINVNTLT
GKILMGLIKGSSSLNNEEEENTAKMNKQLDVKNGIDNNNNNNNNNNNNINEGVVINNKKKDTVKNNSCQRKKEI
HITSEIYKDNHKKKDVKKIHMEKANSRRNDQINNDDNKTVKDKMKRKNKTTVDNKRSENFNETEIMEKLSKHL
NSYNPFE*
>N.MAL13P1.43
MAFFSWVKKDETEIDEKIKKNLDEGNIKYSDISSISKDLELFRSLSFDNLDYHGDKCILARELIIIYMSTYKK
HIEQCKIENMEIIIKTIKRTILSVNNIICNINEQILKCFSMIRNLYNDILKLRNVYINDYCLFCIIEGILNIL
NDDKMHESKSSIWGISALLSLIITNYKKAYFIYKGLISYKCIYTIPIFINNTSQMNTPNNKIHSYILKENDEF
IGCNYSRIVAYVKLHLSVFIILNDTREIWMYLSELLNSAYRRKTYIYFPLIYAALDTTAYYSKLTFKTFFDKL
INLIKNHIIPSLEQELINKPPQPDMQKIVDYYVKKLKVEFLSNDMNSSFPEGVVVIPDERILFLGIGS*
>N.PF13_0049

MSQIKTTVKTEACSFSEYRIYPGRGQKYIARDGKVYFYLSSKFASLALQKKKAAKLRWTQTWRRNNKKTKIET
TQRRRYKKTIKVQKAVCGLTVEDIRNRKAYVQSIEAKNKAKFGTKEKEDKKKTKDDKKKNLVHFQQKKDFTKS
KMLNMAKSKMHKMMKK*
>N.MAL13P1.44
MGNCASVINHSKFKIKKKEKKNSIDDTHSQKNKYRDAINKYAQENNSRGECENYCDEYYSRRSNNTNIKLNRG
MKYSHNNNGLKKNDHFNCNNSNISSDENENNMNDGISINNIKQNNLDNVNNVDYDNLNIKEKKEESAFDKWKK
KKKKKNSDQFSELAKNNNSDHVNYKNEKREYDNNNNNNNNNNNNNNNIFSNNNCNNSSIIYDNNVFSDNYKYY
NDKCDLCNGQEKCVHRLGGLNCTHDEDDKTRKCTDENINKKLLIKNDEDSIEYSVDDINDEYENNNIINNESH
IINNGSHVINNGSHVINNGSHIINNGSHIINNGSHIINNNIMLKKNKHENLLSIKYSNMILNDIRDVDIIVVF
LFSLFLYFNANNIVDMLDRNKKDRYFLINSLNINHDVIKFPTFPKEIVESFLKNDFSLLKKYIKNKCNKLKKK
YKNVYLKKYPENNKEEKNSKKKNFKYELKKKKKEKCSFSTIVESLERDELIYRDITEIDCDKDLPDLKINFIV
MGAYCFYQKDMKPFQDKNTFFYKSPSYICDSEISVACKKGKKVDFPNQDDFTIIQTNDWILIMVFDGHGPSGH
DISNFVHVVLPLLFSYNIEKIYENPVRTMKTLFYMINCYLVNYSYCINNNINPININFIDYNLSGTTCTIILY
NFITKKIYSAHTGDSRAVMGKQNPQTNKFSAYNITEDHKPSLKLEKDRILAFGGEVKKLHGDVAYRVFVKDEM
YPGLAMSRAIGDITSSFIGVTCEPTIKILDKLEEDKFIIVATDGIWEFISSEECVQMVSKKKKKKVHIAMGGN
N*GILEKMGKNRYRRRDDLGNFIFLT*
>N.MAL13P1.45
MDDVKKRRRSRWGEVENKEENKEENKEEKKEENKEGDINKDSNNIDILLNKSSEVPINKNNNIMNMPLSSSLS
YNNLNNPLLSSNIHLNILKATEAARLAIEKVKRASRFKENNNINKNVRRLEFTPKPLMFDDQGREVDAEGNVI
NIKPVTFSTLKVNMNKIEEDNMVKKKNDIIISSDTNIGNTNNNINNDNALWFDARIQNVSKRKETKKKAFFNF
ITPGSLIKQACNKHYNNKTLMNFDLKKIIEEKKRVQSFQELSLNFLNNNINENINPNMINIGKKEEVESKMET
WDNNERYDILEKWDHVLFNLIEPTDVEKENSVDLINEFIQRQKERWETKYFIDNELNNIDNNTNIVNNNLSSH
ILCHEEHIKLNQVLKKVDVLLLLKANKKKRRKYKNNIYSLIVNNRLYSINLNCIKSYIEHPVPLNEDENYDDD
KYLIHMFLTPQERKKLRKRKRQEKEREKQDKIRIGLMPPPPPKMKLSNLMRVMGDTALAQPSKTEYAVRKQMK
ERELRHFEDNQKRKLKPEERSKKKINKWKSNSDEENDVLLIYITNLSNKKHIFKIDINAQQLHLTGVCFMTVL
YNFIIVEGKHVSIERYKRLIFRRIKWNEDVYDDEENQDNHIINTNVQNEEHAQDLYTVNNNINMNNDNNNNNN
NNITYNQQSQCSLIWNGVVKKKNFKNWKMIVAKTEMEVTDYLSQHDALHYYHIIKKHRSVLEHI*
>N.PF13_0050
MLSRMSVRTHTHNEVLTKNDSINMLKNIVKLGISLVTYLRNLFEENAYEEVCINELKLKRLLPINPQAHMIIN
WLEKGVFEAIEKEYLRILILDINDIYDNTIECYKFSFSYNNKNGDEIDISLETSNQYSNAHNNYMSNVISNNN
NNINNNNNCGTGYQQPKHNIVNYNQENKNGEISQGVSQGVSQGVSQGVSQGVSQGVSQGVSQGVSQGVSQDVS
KDISQDVSQDVCPDKNINVSQNNKTNEYPPENNLLLNSNDIYPPQGNHFMNAQNHKQDKNYYINNHNDNKKSY
INYNRLKCIKERLFNKNKEKKIINNLLFKKQAKDKTYELLRSLVLLTQTLEPLPERTYLSMKLLYYDEIVPYN
YQPPYFRNPDNNDLLKFIDIPNEDYIGKINTGHHFLSIIVNSTTNNFINQNITMSERDGVYYKHGFEKHTHHL
RKIERTQRYEKNTKYKRVVKNDENAAEVFPLTNDEDDDGEDDDDDNNDYDDDNDEYGECVKCNKFHKDDSNME
EHYNDEYNYDGQYCDDKNYCNNYPDGNSDDYYDDDYYDDDYYDDDYYDDDYYDDDYYDDDYYDDDYYDDDYYD
DDYYNDDYYDDDYYNDDYYDDDYYNDDYYDDTLNIKPTLDNPNIYNPLNKQRRRKRKTIQPRTRINQNLNTNI
NLPGNSKINSDVMLNTNINMNDHIHEESIRHIRRGKRRRIRSRRRRRRRRRPEEVGVGEHNEVRTGHVTKIRN
SSKTKNKIITRTRKREKTNILLNNKEHMSIKEDVNIKEHINLKEDMTTRLRSGTRLRIRERTASVSSNGSNKH
NNILPDGNKIITYIPNKNYGEMFNTTNNDTYLNNVQANKMIHNNIKESQYIHNGNTNMENINEHNVLISSHMN
MENIQNVIMSNHVKKKKSIHYYDNNNNNNKSDMDYNSNMDNNNNNNNNNNVMHIDHYNNSYNNNEDDLTHVNK
SSYKNHQIIDKMINAKKERRKKLLNKIRLYITRYKILSKTKIKNKFPSVSQYEIDKILDELLHDNIIKTNANS
LFKFVDKNESNEEGIININTYDKEKIHHEINENMQEQKLIIPNHDHLLNNIQDKNGGNIIYNNNNISKRQQQK
GKEHKEDEHKKEQHKKEEHKEDEHKKEEHKENEHKKEEHKEEEHKEEEHKEEEHKEEEHKEEEHKEDEHQHVQ
HQHVQHQHKQHHHKLVNNKDDQINNDIQKLYNDVHNMCIKTKYVNKDIITKGLGIYPLLAKPLLHRLIKENII
KEKFIKNKGYESNIYIPMQNTFNKKNNKDVLRCDTMSTTNSTNDEEDNEKKNKVSK*
>N.PF13_0051
MGFFKKNKRQSFNRNYEGEVQCNNIILGGTFYKHCENDLLLKNELENLVPYFNGRIFLENKEEIGKVDEILGP
INEFYFSVKLKEGIRAKSFSSDTHFFIDKSQTLPLSRFLPQEKNSQSLVKKKKKKSNRDKKKNSFNQNNKKPN
NYNFKGNNNRNDHNNRNDHNNRNDHNNRNDHNNRNDRNNRNNKNSFRNRSNSSRKFNNQRGRF*
>N.PF13_0052
MITDLLFKSQIIYNKKDILCARYILFYQHICKRGYKEAAPKVFNKKSSHTSRWIQRQISDRYVLRAKNDNYRS
RAAYKLIELDNKYLFLKKNKIILDIGCYPGSWCQVILERTKNYKNKIIGIDKKIMDPIPNVYFIQGEIGKDNM

NNIKNINYIDNMNNNSVDYKLKEILQDKKIDIILSDAAVPCIGNKIDDHLNSCELTLSITHFMEQYINIGGTY
IVKMYLGSQTNNLKTYLKGMFQLVHTTKPKASRNESREIYLVCKNFLGRKKITEDVQIKGSFSSKEGYY*
>N.PF13_0053
MNKSEKEFSLKLFKIKNGNINLLKNRITHISINNKCIHLVLCNNTLIKYNVEENELCYIDFYNKKTTNNKGEI
RSIYFDRNCYHGFICLANKEYIYIHFENNIIKNIIQLKKYNITSVIFNDYTEIKNTFPFLISTTQGEIIEINI
NNKSSKNIDYDVIFSDEKLSILDVNMINIIKCKTSLNSHKQNGHRYDDNNNDDNNDDNNNDDDNNDDNNNDDD
NNNNHNNHLNNKHVKDYQEMIKIIYFTTSNSLHEISYTFKNYKNKTDDKNDNTFTYVKLKTTSLPYNIKKQTK
VYETPLDSLCSIIKIENIRNTNYLFWLNGCSIFISKINNFKSPKYYTPDNNKKKKNNNNNNNNNNNFFFNNNM
NQDENIFSSSSNSFSDSDQDVEHIFSLSDDDYLYEMNNSNILNNAHNNDHTININNSNPIDNTHIKKQKLPRN
KLYTNNNYSHKNKDNVSNTTSIQSRNKYNKINSEFYLDNNYIIINFLDLHIFTTDNITAFSFTKSFYDSVYST
NSNLTYNFKDQENEHNEQNLNINSTNITNSTNSTNIKNNSNNNSSNNNNISTMVDMCINQSYIFLLLEEKLII
ISNVKFKKVYEQKLDTETYGQALRIIKDHSDNQVWLCTSKYIFKIIQNKKNNHIMYFNLKKKINSKQIAQSNS
YTQKIKMTNFILKNNKYDIHQYKYTNIRIDEKLISFLHKQQYFNISSFLYNNIHCYNNVIRIALYIWLIQLYI
YSIDLYAYLYKSTSYYFHTKRKNYIFNKNYMIQDTHKKQNLSDLSSDTSTGTYVNRDFLDKHNEHNEHNGIRQ
NYSCSGDCVSGSGNSLTGSGDCASGSGNSLTGSGDCVSGSGNSYSKSCDNYSWSGDFHSESHNSNYESSDKDK
VSISEKRGGEHMTNNGNRNTNNIDEIKKKLDVTKNDEEGHEISSVLLTNIRGDDKKEEGEILDPHKDKIGNDN
KGGNRTTAEDNFMNKKKNKFYFKNKNDNLLKKNKTLFLRFFDQIINKEERKEVKYHFDFGKGKKINLKALSEC
DDFYILLKIYRMKNTEIYNFLKSQKNLIMNDIETYKEIYINNDVDINNYGNDTMMNNNIYIKMKKKIYKDIYS
YKCLEVCIYIIILLKHFKNFFQLNDIIIEIFYNFNKMNFLLFYKFLCNDYNYIINYYMNNNKYNLLFNIIVLL
PQNILLDVLIEHAFALFLHKPHKYVDILIYYDNIIEDYTHIVMCMFMVIYFFKNKNDHKNIGTSKNVFKRNGN
MQYDINEQDNTNEQDNINKQDNINKQDNINKQDNTNEQDNTNEQNSVNEKDIINSQDNLTSRENIYLYNKPIN
FKYQNKHTEECIRFLEYLTNKLIEENILEKKENNEKENYIYTFQCTWEKHYIINCLLILYIEEGNNEKIKNSL
KKLKSANIHFDYLFIIRFLKEKRKEMFIPHIYILMKYFEEAVDKSLELNDYKTAKKAVILCDDEEEKKKLFIK
IIKHISKNINDNNLKEIINLVRDSNSILNLHDILPYINETIIIEYLKKDICNLLEIYNLKIKAKKEEIKENLH
TIDLLNKDIKNIQKKYVILNKNDICYICRKTIFYKKCYVFSCNHYFHSECALNIYINNKSKEELFDFYTILQQ
YKNSIITKNDKEIMICENKIDDILTEECYICGSFSLASISQPFISPNEYELRDTWNISND*
>N.PF13_0054
MKKLLNRSFEKLRFLHTSSLRKNCDDTGPLIKKKRRFFDLKLFRQHLRRQKSEEIENEKRELLKMCAKDPPKN
WDIRTFLERIEIGENIDEIEKCFKSWKEFVTITPKELFLIDCLTNEQRRKLWKYINKYNYGLYPEDSYDDFVK
NFQAPPLENENKPWTQEDDKQLDYYINYYDVNFGDPWLYISWNMKRTFDQVQNKYVELYLKKQNKKRKCELCL
TKCTTPLLMNRKFKLDPPFLFIVPSSQNFPTMNIFDYNHKECHSTNKKYEHNVYEKNNLKNDPLYPSYGSLIF
SNKFLPFVNKSCF*
>N.MAL13P1.46
MICFVQKYTKVAVQSNLLRYSLNNFTTVKLYKPSKFEGIKKKLFLKKRLFNIIHKKRANDIKGEPFYDNITQY
TQNKRKIYMNNKYNVKETKYKDIEEENTQVKQNEHVYINKNVSHNIHTNDHKTNDKSVQEKKKEHITIEEILF
KTTTFGYIELQYILSTFIYYEKENITTHDLLTLYNFVNLSEKDIYDYLSSKEIIPEQFVRTRIITNLLKFINV
NHPSLK*
>N.MAL13P1.47
MRYAFGTSYLTCFMGINKAMKKFVFKKNKYSSLHLFERNEINNFQINIMDRKYVHSIKEEKKKEESEDYYLSM
GDNIEKRYSLALYNVAKKQNKINEISNDMLFIKNHLLKDTTFQKFLHTPNIEKKEKIHFIKNECKTLNKFNII
TENFIESLFDSKRISFLPKIIEEFEFLLLKQRKEIKCVVYTANEIDNNYKQKIQDSILIKLNKKLIPIIQYKT
DPYILGGLILQIGNQVYDFSAKSKIDKIKTNFSQ*
>N.MAL13P1.48
MFHSNNMNEHINCYIKDLNFDGLGNYLFENLKHIEEIGTCICSHNLTIIINILEKCGKKIKNENLLDQLFNEI
ISEEHVDTVLLYMKNYVYFLYKIIYHFDKFQNIENNKKLCENFLTFSIYLIIYYEQNEFIEQLLLSLIHNLEN
KKKKNGYKKYILFQLEKNFYDLYCFIHLPDFIFINSFLIYSFASLSNNPNDRTKNNFNNIITNLQKINIFFEK
IHSNVYNDENTNGHLIDDVNILTQKINKDKNNYDIKYIKKTKIIHDTISNHNIKLIYNEKNHFSLLYKYINLS
FDTDTYSLDQKEYEGNYDVTNINVNNFLLLLKILSPSFFIWTATNTKFKQKEKKNKYEKNDTVTQNSNNTEIT
KQNYKYKHMYIINNTENINYLKETSDSFFFFFWYLLSLNQTSTKWNESINTLMDHIINHFFFFFQNCHPFVHG
ENERKSLSYHPFIHKNNLQTYLCNGNQIESIENLQNKEKKNFIHTSIHYNTFCEITSELLFRNKNKILLNLKN
LIIWGYISPDDTDEMNFFNIANIFIFNSNNCHLFKRHEYVHQYIHKLFSNEKHVFLFYNYTLFKKTYECKTLN
FKYMDNFSNYISSFILNIIFSLTKYYLTSSFFCGQVKIMNSTIKEKKNSEQAITLAKINKQEIKEKKKEQNKI
NSNNDINNISKNDTNLLQKGEYQNIEINHLSNLLPKDNTLNELSSNIINLYFHFNNYTYDERIEHLFNELLFI
CFKFINYPLKHVQLCCLSTIVEIITKMNIYNHIEYIDKKSDTFLTFLYDESFDKNDIIKCYMHIKCKLTKHIN

IKLVKSIEHLIQIFNLKLCNKTLCSIICKHILISFYYHPSVLPIILYKYIQIIMYLMESNSINIILDSLKCLH
ILYKINPQEIKTFNHDIIYRLHLLFNVFNQNDKNIENHVSFNSINQFEYSKDNNYISSNNITSYLKKFYFNNI
QIEKRREEMLLHIKLILYSIYVITPPDEYLSSLKIFEYYPKEYEVYSKNFEIFSRF*
>N.MAL13P1.49
MKIQYMIIIYFIILICDNFLNVNAIRKKSLRNNINKAHNHHENIQGNTLSNFEIMKEKNNVSNIQLNSSKFFD
ILASTSLMRNSGGAMSQGPVYRVVQNIHDNNENIGASLVFVIFLLLVVFILFFIFYFIFKNHVKHVKFMNS*
>N.PF13_0055
MIDPVKRDLTNLFLEKREMAKEEIDPRKDWLKRILLNNNTNDLRMFLKASELKTKEGDYCMTCKSNVKQLLYL
HTKKNYCHLCEEIFCAYCVKSIDFMKDEKEKYIKIRLCRNCFIYINELKYIINPNLSVDRKAIDLQNSFNDIS
NCYTNLCSNVPQLNGLVLLCENNKEFLDSFKSEIKQLEEKIQHDVDFLNIMKKKNNFLSDNTLILNKMGKNLL
LYLKIIRNKIIPSSIEVLNKTKELLYKK*
>N.PF13_0056
MKRNKYGRDSYEYKRVKKEDSYIHSDKKKYDEDKLSEATDQEKKSKNNEKYKHEHANNLNMKRNNSIFHNGDD
NENNIHSIYTSKEHINDNINSRNKSHVIQTKYSKGNLDTIIPPSDNYNNTSKMSVKYRKMSSTYGNLHKNVLT
SSSNQKDIKSEQNINAEKKESFVNNIIDLKKSELFIKEDKSEEEINNNLINKFYGDIKIKKTNNNIIKASYDV
IRKKINTDIKLNDILDMIKLPESENLDNDLKTPCISNQKIINDILNEGKSNVNPNSPYQFYNDLWTFPLEMLD
KYIFKNKNHDYKKWISNCTTLFVLGLPFQMNEYNLLHNLIDVYYMNKNYDGLLDIIDINKEDSLFNHLYLYSY
INQPKNKYNLLSEKLGILKFDLLFGSEYHLAKNEMLNSYLNNKYKEFYKINCNTGAMGLLYFKNEQCAMDFWM
MYNNYSSSFYEKYLKCIPDINGIKIYIEASMFYLTPDLFLTQINYSELNAVLNNIQKKKVNILQIFNDIKIYY
NESSIWNLIFCNKNIEQFFKMKKNNENVNENVNENVNTNTNTNTNTNTNTNTNTNTNTNTNTNTNTNTNTNIN
TNTNTNTNTNTNTNTHTNTNTNTNTNTNTNTNTNTNTDDHTNMNNNNINKNIDFLSNHFCISFPYVSHFKDNF
LIRIYDTYINKYLINNGFCLFACPYLDKKGGKRVYEFFKKFHLLDWFYNPNDETCYFYLFKTILNVFSIFKRN
FKTTSDYYICCSLFLKLPFGILPGIYITCLTNMRDKNIQESDYIKISDYQNISGSQNINTSQNINTSQNINTN
QNISSSQNINTSQNISTNQHISSSQNISTSQYHLDHKTVNNIENNTFAHNNENTKNTLNNLNNYPNINSQDNI
ISDIMDNNCVQNNSSDSKKNLNMSGDLVEKKKKMQQAILKKSEKLAMKNEAILINSKNDDNNEVLLINSNSKH
NHDIIQINGDIKNDNNKLTNTCFLLKKEIVKNVSYQNLDTHEEKINISEQNTKDNNLNTQIKDMNGNKKNEQE
DNKKKDITDYETNYDDTDDHSSDDDVYEEDFHDEYEKKKSKKRNKKLSLSKNNIKEKEKFVTNNISVTILTKK
RKESLKKNAKEKNINSTNIIKKTKNIVNKSKSVLQKKN*
>N.MAL13P1.50
MNNKKTNFFVKILWSIFFLYIYMIRITRCDEDVIELKNNYIEGIVKGDADLLFNCTIYLDSDTYVSPKSDGKF
LFKNVKEGEYTMYVNHPYLEFNKYYVEVKKQITRNNGTLYLVQAYELNLPFEKSNLIVNNIVFKAKRIYDFLI
PKKKFNVTYLFKSPLFLIFLFFLILLSVLPKMQKISEAANEENETEQISYKSDFLESIK*
>N.MAL13P1.51
MGCSSSTERLTSTKNINIVTSPAQQQKKNAQDTKVKIVLLGDSGVGKSSIALYLCHGRFSEKHQVTIGAAFLH
HNIELKNGATMKLHIWDTGGQERFRSMAPLYYRDAYGAVVVYDSNNVESFDSLKYWINEIKSNGPRNCCIMVV
ANKKDLPQKLNSEMVMKFCEQENVSFIECSAKTGENITTLFEKLASRIYSRFKEVLYYNNP*
>N.PF13_0058
MAFSPLCGVNCTSRPSASTINTFLTKAQHDVTLTDTDTRIFIKNIKTGVTMEQFRNSLQEFGAMQVYFYEPGS
NNDGWAWVGFENKEAALKVIEESEKMQEEMNEENLNEMQNEDNEEQGSNSFYAEDEDKKDEEEEEEEEEY*
>N.MAL13P1.52
MSGEKLQEIFWSVEELIYIHYQDEHIYEIKEKEIIESLSILYYNNNNNNNNNNNDNNVVDFKKNNEILINVLE
EENKLKNNFLENLIEEFMDLFIVIKLIKNAKHKECIRHRFIHNIFDYIKDIFSNKCINNIEDIKKEICNYIFM
EKKKHQEMEYDNNIFLHTCYIFLYEIKLLLCCYILLNIFVQYNWTGPPLNYDIKDKQIIHDEDIYFKDFIHAI
HIKNNTKFLNSSLSFLSLEGEEIYEYCELINSFSLSIIFLGLINNYNNQTDNHIFSFCEYDHFFSSEYDGNRN
KSMTSLHNDNIYNNENIHNNENIYNNGNIHNNENIHNNENIYNNENIHNNENIHNNKNIHNNENIHNNENIHN
NENIHNNGNIHNNENIHNNENIHNNHKKSNYFYPCNKLFDHLKLLYFVKSKYLWKARIFFIWQRLFTSSNDYY
YSLKINIIDKPIHIFKNIKIIPPDFELIEQDININEIYDIVDCVKENLKHVKYCNLFYNNYMSTQFKIYVLSN
FSIYLAFYNYTSAYQRILDLLSQISHFQYFFTGRMGIKRMYQKIPATILVLKTKLDQQEDNTSTILKEIEPLS
EYDILRSDYRIIDHDEHNVPISNVNQHINIDNDDSNNNNNNNICVNICNKQNASLKREETNLLSNNNTSFEKA
PHEQLCQEQNTPGDLKNTHNICNNELERQRMSNGCCNNNINEHKIYQKTDEHDNNFDLCVDDSSKEIVPEKNM
YQFKNDKMINFGEEDEIENKEKDNITSGNIDNTLEDYNKNESKEIVKISNEVEKNNSKVTWKLKDFDPDTDIL
EEPHFVDSQNNIFKVLSFQEQICLINYCFSMIRFNPHYDEIKFEKLNAVISRCLKCYDVNQKDSHLAKDKNDN
NKSNNNNNNNNNINIIGSNSTNISSNCVHPNISNNCTSYKDQRYSSQQLKYQNWLLHSCMLWFKCKGETFRFK
TVDRAAAQLNELHKECYDIKPESVERLKFIYDVYYPTTWEMKKEIGSVMIKIGSVVTAFNIFKDLKLWEEAIS

CLIQADRKEEARELLDDLLKKKKSPCLLCLYGLIEKQRALNYYIDAWEISNFKYAKAARLIGKYYYEKEMYEK
CSEYLEKALELSPLFPEIWFILGCSYMKIQNFDESIKAFTRMISMTNDNSCKSYGNLAYLYMKKGTYKAAKIC
INQAVKMNNNEWKYWDTYLKLSIIQNDIDSFCLALRMICQVNQVKQIQPWVFDYISDVIVKDKPTLIPNKNGL
AYLDKIITTMEHLSQYITELDTFWNAHSFFLFIKGKFQDSFEAKIKEIRSIESIIQKCNNEKLVDALISKIVV
AIKFLYHLIKSHDVGEKKGTFLYQLKSLIESTLNHYKNLGSENLQELSQIKNILN*
>N.PF13_0059
MNIINKILKVHNNVFSSDKKGMDFFFLRICKRYESRVKAHRYTGITAVKIHAQKTEWYLKAERDFLSERNKIP
NGYIGLWQYDDIKHLNPNIINMLHLNCGNSKQIHKYKKLNIRRLLQRRPFDVGSAPVQIGCLTEKILNLRAHI
IQRCKDHSKKRTMSILLARRQKLMKYLYKTDFELYKHTCDLLKIKCILFAIPDSRDRSRAINSAAIDADRCKF
LIRQKLWKGKYRPRPIKNSKGQTVRYTRHPIEQPPSDYGLPKEYKPQLSNSWPYGVKENYEKGNYTIPNPTAP
GIGYCPVPMLF*
>N.MAL13P1.53
MNGNQIKQLKKLYRHILNEASKFENINYNVYFSNKAKEKFREFCSDTNFESEKLKTFQNECWDYLNMLKRQTI
IHNLYHVDKPLVNK*
>N.PF13_0060
MRKHNERSINSRIQKEQKNEYARERIRNELKLDNYSRIQNVSDEKADIIKNKREEEKEKANIKREYVDEEQNM
FNMNVPGDNMSRDEFDDKRKLKEEKIKQCQLLNEKQLEEKEKEERILREKAVEYTLQNEEEYKQLKRKLDELA
ILKDRENQIKEANLIKTKNKIEEDMKINKELEEIILKEDKEDDMKKKMNYNKIMDTKNELQKQMKENIKKKKE
KYTDNLDDRAHVEQIVKNYQEEEKKKKEEDLLKQKKLLKEFQQQIELKNKQKELQKQKDKEEELKNQEYIKQK
EQKMKSYIQNKQDKNIQKQKVVNQIAYKEYLVNKEKETLDELLNKLYIEEHDFKEKQKELKENQKKLQLKNDM
LKQLEQDIQLKEQQRLAEKQEDEKRKIQLLEEKKKLDKLDQYTDQKKKEKLLQYRKEIYQTFQEKKETIKQER
LQEQVQKEKLMEEQKKYEQLIKQEKLKLLDKYSDDILQQLPEEVYQKMKSENIIIEK*
>N.MAL13P1.54
MGDRDALNISAPDVIRLILQYLKENNLIRSFYVLQEESNIKLNAISNVDKLIRDINKGDWKNVLFNITTIDLS
DETLMCFYEQLICELVEYNEKELAEKIMDECIIFKRMEKKYNDKYNKLIEIMNSKHINKNILYDGLTKDEKRN
HLCNMITNEITTCAPSRLLALIGMALKWQAHHNIIKNKKDGYFDIFRNIEKDIINTIDVYPEKILKSIKFGKE
SNVECCISSYNNDYLITGSSDGFIEIWNWITGELNLDLEYQKQNNLMIHDNPIVTLCISKDDEILLSGDSKGL
IKIWRIKTGICLKIINAHNDSLISIQFNNDQTQILTSSYDKSVKIFGLKSLKCLKEFRKHEDSVVHSAIYTLD
NSKIICATDQGKIFIYNQKTLECITSFYVYFNKSENLIFPSLNNIILINKNLEDHILVCSKSPYCYIMNMKGK
IIKTYTNTIDKDKDKDKDYDSPYFLYACISPNFKYVYCIAEDYNLYCFDYNTSKLLNQMKIHDKDILGIIHHN
NQNIMASWSLDGKLNIIQ*
>N.PF13_0061
MLKPNFSSFMTRCSMLSWNTVEKKKFASDNLRSLSLRIKSVKSIQKITKAMKMVAASKFKGDQRRLEKCSNFS
TPLVDVFNRLNKLDIHKKNEELAIIAISSDKGLCGSVNSSVSRLCKKLLENEQVNNELVDNITPNKIYLYGIG
EKIRSALSRLHKDKFQVIYNEYNKIPINFLTCSYIAERIMNNNHSNIIIIYNHFKSAISFDTQILSVFSQKQL
NKINKKELTHFEFEPEMDYIFKDIYQFYFTSILYNSIIQNLASEQSARMTAMDNASSSATDMLNALSLRYNRA
RQSKITLELIEIISGANAL*
>N.PF13_0062
MACISAIFIIDLKGKVIINRNYRGEVNVNLTEVFYNCVIDQEDNLIKPIFHVNGLTYCWVAHNNIYFLAVTRK
NSNATLIIAFLYKLIQVLKDYFKVLEEESIKDNFVITYELLDEMIDNGFPQLSEVKILREYIKNKAHQLTVNN
FKIPSALTNSVSWRSEGIKYKKNEIFLDVVESLNIIISSNGTVLRSEILGCLKMKSYLSGMPELKLGLNDKLL
FNKNLNNYPNSSNNNLNNKTKLVELEDIKFHQCVRLSKFENDRTISFIPPDGIFNLMTYRLSTHVKPLFWLDI
NITKKSLTKIEYNVKAKSQFKNKSIANNVEFHLPVPADVDSPHFQTYIGTVKYYPDKDILIWKIKQFQGQKEY
IMNAQFGLPSIVSNENKDLYYKRPVNVKFEIPYFTVSGITVRYLKIIEKSGYQALPWVRYITQNGDYQVRMS*
>N.PF13_0063
MEEETNTQSKPLDDEDINILKSYGSGPYSKTIKKVEGDISGLLVNINKLCGVRESDTGLCLPNQWDLQLDKQM
LNEEQPLQVARCTKIINADTDQTKYIINVKQIAKFVVGLGDKVAPSDIEEGMRVGVDRTKYKIQILLPPKIDP
SVTMMTVEEKPDITYNDIGGCKEQLEKLREVVEMPLLQPERFVTLGIDPPKGVLLYGPPGTGKTLTARAIANR
TDACFICVIGSELVQKYVGEGARMVRELFQMAKSKKACILFIDEVDAIGGSRGDESAHGDHEVQRTMLEIVNQ
LDGFDNRGNIKVLMATNRPDTLDSALVRPGRIDRRIEFSLPDLEGRTHIFKIHANTMNMSRDVRFELLARLCP
NSTGSDIRSVCTEAGMFAIRARRKTITEKDLLLAINKVIHGCKQFSATGKYMVYN*
>N.PF13_0064
MTGRINNHEEYIKNEKEIYNVKESANEKESDNMKNDEFVIYNDNLKDVEDINKENVNYIKSINKIECDTNNHN
NNNNNYYINNDNKFCNDHKSFASSYNSQNSDVVGTCEEGSNKCYEPSNNIHNNFILNEDYKNNTNEEYDKIES

AQNSVYCEQEINNNINESYNNINNECTQNYINNTEEITNHIILQEEKEMGNIEKEEQDNDMYKEQKIKREDTE
NKNEMDYIKDESNNIFPSDQINSVTSDQEIYITSNDKKNNLTNEKKVYISTNDKNNAVRNDKTFYLSSNDNQS
DSLPKKSIRNNVEEFIRKLMENEDMANKFNVFHIGINKIEALKALYRECKNNFNKSFREKINKLHELRKYYND
SIFERNISVDYIANTEKLNAIKKERLSRLFLYIEDIHKLKQALKVKQHNDIELENDEKKEYFYDAYEDIEDFF
IFDHALSYLNNDVETIFTECENSSFFREDSSIDNFLDEVSSKADEEYNRLNIFARFSLKNYYLFDINKNLCSI
YAFTPMIIKKFAEKINSYNLTKNEKDINNDNDAEHEKNDIDFTIDSTNNEKRNIYASCGLESNHNLDAYVQFH
CDKWQENMYDVVEVNSLELDFNRLKCNIMFDLFYILTCLNITYYKLRYIIII*
>N.MAL13P1.55
MNIGGYKKKGNLDDEFPDDFVLPPGDKVKGEKLFKKHCKQCHSIAPDNSQTNSGFTSWGPTLFNVYNRTAGMS
KGNSPFQTSPDLYTSGIIWNDVNLLKYMKNPQQFVESHIGMNFKGLSNLQERVDIVHYLKTLTYDDPYGKQIV
EKYTKKGKTSGSK*
>N.MAL13P1.56
MKLTKGCAYKYIIFTVLILANILYDNKKRCMIKKNLRISSCGIISRLLKSNSNYNSFNKNYNFTSAISELQFS
NFWNLDILQKDIFSNIHNNKNKPQSYIIHKRLMSEKGDNNNNNHQNNNGNDNKKRLGSVVNNEENTCSDKRMK
PFEEGHGITQVDKMNNNSDHLQQNGVMNLNSNNVENNNNNNSVVVKKNEPKIHYRKDYKPSGFIINNVTLNIN
IHDNETIVRSVLDMDISKHNVGEDLVFDGVGLKINEISINNKKLVEGEEYTYDNEFLTIFSKFVPKSKFAFSS
EVIIHPETNYALTGLYKSKNIIVSQCEATGFRRITFFIDRPDMMAKYDVTVTADKEKYPVLLSNGDKVNEFEI
PGGRHGARFNDPHLKPCYLFAVVAGDLKHLSATYITKYTKKKVELYVFSEEKYVSKLQWALECLKKSMAFDED
YFGLEYDLSRLNLVAVSDFNVGAMENKGLNIFNANSLLASKKNSIDFSYARILTVVGHEYFHNYTGNRVTLRD
WFQLTLKEGLTVHRENLFSEEMTKTVTTRLSHVDLLRSVQFLEDSSPLSHPIRPESYVSMENFYTTTVYDKGS
EVMRMYLTILGEEYYKKGFDIYIKKNDGNTATCEDFNYAMEQAYKMKKADNSANLNQYLLWFSQSGTPHVSFK
YNYDAEKKQYSIHVNQYTKPDENQKEKKPLFIPISVGLINPENGKEMISQTTLELTKESDTFVFNNIAVKPIP
SLFRGFSAPVYIEDNLTDEERILLLKYDSDAFVRYNSCTNIYMKQILMNYNEFLKAKNEKLESFNLTPVNAQF
IDAIKYLLEDPHADAGFKSYIVSLPQDRYIINFVSNLDTDVLADTKEYIYKQIGDKLNDVYYKMFKSLEAKAD
DLTYFNDESHVDFDQMNMRTLRNTLLSLLSKAQYPNILNEIIEHSKSPYPSNWLTSLSVSAYFDKYFELYDKT
YKLSKDDELLLQEWLKTVSRSDRKDIYEILKKLENEVLKDSKNPNDIRAVYLPFTNNLRRFHDISGKGYKLIA
EVITKTDKFNPMVATQLCEPFKLWNKLDTKRQELMLNEMNTMLQEPNISNNLKEYLLRLTNKL*
>N.PF13_0065
MTKVAVEKEEPGVVYKVAGSLVIAENMSGTRMYELAKVGWNKLVGEIIRLEGNYAYIQVYEDTSGLSVGDPVI
KTGNALSVELGPGILDNIYDGIQRPLERIANVCGDVYIYKGIDMTSLDHDKQWQFYADKKLKLNDIVTGGDIF
GFVDENKLFKEHKIMAPPNAKGRLTYIAPDGSYTLKDKIFELEYQGKKYTYGLSHLWPVRDPRPVLEKVTGDT
LLLTGQRVLDSLFPTVQGGTCAIPGAFGCGKTCVSQALSKYSNSEVIIYVGCGERGNEMAEILSDFPELTTKV
DNEDVGIMQRTCLVANTSNMPVAAREASIYTGITLCEYFRDMGYNATMMADSTSRWAEALREISGRLAEMPAD
SGYPAYLGARLASFYERAGKVKCIGSPSRIGSITIVGAVSPPGGDFSDPVTTATMSIVQAFWGLDKKLAQRKH
FPSVNWSTSFSKYVRQLEQYFDNFDQDFLSLRQKISDILQQESDLNDIVQLVGKDSLSEDQKVVMEVAKIIRE
DFLQQNAFSDYDYMCPLQKTVGMMRIICHFYAQCLRTLQEYDSRERKIGWGSIYNTLRPTINKITHMKFENPK
NSDEYFKKYFKALEEEITVGLRNLMEK*
>N.PF13_0066
MQVIFLHSLTFIIFTVLLILILPSYCFIIKDSKIINDTWKYENKENTNKIGSYRKKLRHNVSVNKDIKGNEHI
MSSYENEKYIKKLLEDYEKYKITTYSSEYTFFFPGQGEQYMSMGLDTYNNYKECKELYNDASKILGYNLMDMI
KNGPIEKLKNSEVAQPSIYTVSMASLEKLKIENNDAYMKLNLCMGYSLGEYAALTCANSLSFDDGVYLTKERG
KAMQYCSTLYNMTTVAIVGLTLDNIKKLIEDVNHKMNDDIFIVSYMTDRKFGLCGKPESMDYLNTLAKEKYKA
IFTKKLEIAGAFHSFYMFPAKETLKNVLNQITFKKLSVPVISNVDGNAYDDPVIIKDLLILQLTSPIKINTCL
QNVLKHGYKSGYELGPGTINTNLLRDISKNTKRAIPYI*
>N.PF13_0067
MDINLYIHSIEIEFITNNKRIYNNEEEDNEDDNIVYCLNIKVEDHLEDNNSKENFYETPWYMCVKMGDKEVCM
MSYEMNITHIFNLLENNEILTLYILRKNLKNDKIEVLHRNELHIKDKKYYDSENSISFDNDNIKGKIKLYLKN
AIVYNHLLKKIQSEEFLKNDFSWKSEINNIKNMNKQILFDDIKISDDIKTSDDIKISDNIKISDDIKTSDDIK
ISDDIKTSDEIKTSDNIKSNDPIQSSDHIQSNDHIKSNDNNLLSQNKYISTILDNLLNTKRTLYWVYIDDDNK
EQGPFNSYTIFNWVSNEYFEDNTLIRLHDQKEYFKLYQVIEYIEKNVLLYGEYDALFNETEKECENNYFLNNN
KNDDGMNNICKRDVLQEINKISNVQNININELKDTTNWKHIIYNEKETKGIENNLKDENKTKNIYIHNIKDDQ
NNNIYYEPIILNKDHIIENKLIEKEKKYLTFNNSHFNKIEKNGKQKNEVKMLKKEIKKIKKEMIKLKETYNKD
IYNTTYDSMDKVLYSDNISKEQNNLENTNDDIKSNQYKNNVTDNKNNACTNLKDMDTMKGKTNKNNIDNIKLN
SDIMEKDEDVNCKTDDQYAREKNEENTKNLLIQNDTEKMLTDTSLKDSTKLLSNVMRNDKEKNIHILSDVTYE

EDNKKKKFIETAMSSINEAQKCILKIRKKKMTTFLNKIMSTPIHTKTTHWSYENLQLHGKKYSSFNLKNDEKK
NIIYKNENSFDRSEANISYIHDKIKTSTTKNNSNNYHSNCSYYDFHSSLEISEEEKIERNQKIEDNKWDIIYQ
DLPNKTTNNIQIYIQDKTHTKKQRIKVHINNLVNNTKIIYIYNTTLEWFLKCIALIQHNIRKWLVKRKILRIH
NIKKKNKIKNKSYTISSGHSSSNNNYNTNDLPNDNNIKDNYIKYNPSSAENYEKTDFELEDNKLYYEQEEQKK
IKESVNKIKKLLSMQNKLKHNGNYNNFSNTNNNYIYNSNMFPREQQQNYKNGIKNKNNGIHNFVSPCNNKLKT
FNNDLNMEVETKKNEKREDLNVEYKENNIYEYNEEKEYEKKEKEKQEKDDKKNKIYEQLFKMRYINTYQDQNG
TIPIKRHNIATADKNKLLNNNNNNNNTIKRGDQLYLKKKQNLNIMHDRLNEIFNRYENQNYGPYDMRTNKNNG
DSNNNNNNNNNNNDNIITDHNNYDYPHKKEETIKNDNQYSNKTNNIQKEDFSFNRKDILLNIVNNFKFDNILI
EKKKNCKLQINTEKNSIYSVQEFLNKIKEKAPKKTSINNEDITKNVNNNKFNIIKLNDYDNSKITHLRNIEKS
PCDTNVHRMLSNKNFMKSKDF*
>N.MAL13P1.57
MKCNFIKKKKKKKKKNMLFSLFMNSFKKHNCKSFDTFLTYNHYFIIRKWISYTSYNKIHEKGLSNYLSELYKS
IENCNSGNSLFKLTNKTLTYQIHDIYIWRLIEKKFYDLQHELTPKELSCIINYFKQIKINDSKCKN*AHKNTR
FNRKSSTHNENYG**IKEDEK*F*PIKIF**NV**
>N.PF13_0068
MYAVKKVNILKPHLWNLPWRKYYENKINKIYKYSYYNEHNVYSFHTFTRYNINNEIYKDMDVKNKYYMFNQIR
NKKNRKKRERVTLNDEQKNTKLKLPIIRLEDRTTVCETPSVISKNIKKEILKVCVGKDRTRRHFKFRNKYRIR
KLLSLTHDKENIKQKNPSAFITPLTKLQHESTLPRTLDHDRFTFPHTFHYSIIWHGSSIVDNEENNICYFTAN
IKDLSLSSKEKKKFIQVLGDERVDLKSQLVCLESNFFNTYNHNAAYLGDALQLLMNKIKTL*
>N.PF13_0069
MFNFCSRLLYKGKYIPVGRRIFREKLIKGRKQKSPIILLPPYITVHELRIMLNINYETCFKAANVYKCGNVYR
WKDSEDRVFQTVNKRNVIIPYSTAAYVSKIFKYKPKLVQVELYCEQENYDLEHMNLLDIYKNTYIEEKKYYDE
FYLLSQNNEKKNTNELKNINIRGFKNETKNCNINNDISLLINNKYISSNIYTNNKKINDEKKKNYYTVISVIG
HINHGKTTLLDKITNNNLALNEAGCITQNIKPIHFEYAPFKFTFLDTPGHKVFQIFRGRAAFLSDILIIILISL
EVGAEIQTEEAIKYADKFDIPVIFVLNKADIYGSNESIVKAELKNQCRKMYDEQILKHNFSNEIDKAITISSL
TGYNLHKLINRIYFLSQHMNLPYNNINNDNINSNNIDNNLAYESMKKNFLIPQNKDDININHLDNNDNNNNNN
NNNIHLSKNNPTHDNSNLLKKYIRKSDFLLALDTYPFGMGIVVDISKNSSKGTILNVIIRNGFFIEGNYFICG
SAYGRIQKMYKFNTNFKENCTYASVGMAIQIAGIRKYGNATTDDLIFTLPQNNAFRLCQYRLMVEKLSTLQVS
GKEISVSWENDMKKNEYHAEDIYENRLEMSDKRKAIEEFGVQNEPIFNDITYEEFHKSNTQLKKEQDNYIEIQ
LEEENENQKSNIEKINKIQNVLSNDEYDDCIVIPHDNTYSFLKTSKNEEMHVSKHAQISSLNNTNEAILNDTQ
MCQQNEQMKEEMDSIYTDKINANMEKEGDIIYDQEINDNKKGYKIDSQNNSELSQINKKKNQIIKDDHNSEYD
NETNEAQVNTNNIYNINNEENSTNSNSYKEYLETYNVETTIPRRGRKNKYSKNIYKINTKSPFKDNVTNRSNY
NNDNNYNSNNNQNNDLRAPWYYEESEETWAKKVLQRNDELMETWRNKTKQRELEKQRQIFYEKQMILKNEMIK
RNLLGEEKLTEEQINAYLYDEPNKNKKYDTDINQNDQNDNDTHTDDKFNLPKKNCPVIPIIIRTNYVGIFDIF
LDEFENLQKKYNVKISVVHGGIGPITPNDVVHAEVESHFGYCCIYAFQVKVLPDSVKQAVLSNIVIKQFDVFT
DLIDDIVKRITNIKALIAHNMYVRSLKKEKTQEGM*
>N.PF13_0069a
METIISPVITLQQAPVVYTTTYRVVPQTVVYTFPNNIPVVKNIHVVPAQQLCLSYAYTSPVTVII*
>N.PF13_0070
MRNIVQKYLQRNSTKLFNRTSVFNLYKKCNFSGYKIYSDGLVHSEFSTELKTVNEVIKMPIYRILDTNGHLLD
GHEAPFKDEEVLKIYKDMVEFSIWDEIFYGIQRQGRISFYIVNEGEEGLQFGMGKALSVDDHLYCQYRETGVL
LSRGFTYTDILNQLFGTKYDEGKGRQMCICYTKKDLNIHTITTPLGSQLSHAAGCGYALKLKNQKAVAVTYCG
DGSSSEGDFYAALNFASVRQSQTMFVCKNNLYAISTSIKDQYRGDGIAPRALALGIESIRVDGNDLFACYLAT
KKLRDICIQESKPVFIEFMSYRYGHHSTSDDSSLYRPKEENEAWRQEGVHPISRIFLYLKNKNLYSEKEDQEH
RKSVKENVLKELKKYESVKRYNIVGGLFEDVYHEEDWNIKEQRENFEQFFKENKHNYDTSKFEP*
>N.PF13_0071
MKLKIIYNISIYFFVNTILYKNDNLACGHYVNNNKIITKDNEADNDNDELIIDNSKKPCNENTPVNVNFPHDM
ENASISKVFNFDENFHHDISEEVENTEDEGQELKKMLNYLWNIPNEIINRKNKVIEYISEKLNKVKLYVFPFK
KKNKDQEIKEKNIVSEVKNPRVEIKRTEKLINSPDVDINEQKLLKRYNEMKGNNDNIDKIDNIDNNDNIDKID
KNDNIDKIDNNDKNDNIDKNDNNDKNDNIDKNVNIDKNVNIDKIDNIHKNDNIHKNDNNDKIGIHHMINNKVR
TNNVSPKKKKKKINKNMNNNNNEYENSTDLKNTNMIDNKNKDLFIGNSDFFCHLNKNNDDPINIKNLVMWPIA
SRTMNNQNEITYLEDEDDEEYATTKFLRAHDILLSVDDHFPVEDLNNLKRFLLYFKEISRRLTITNNNYKNIY
DVSKHLKAFKYVENIRFTCVTNTYAECLLNFIYALDYIKYNVPLYYNKTYYVLRKISLEIIKEIEKLTMLLVR
LKHEKLYTKCNMYYKQYIELESNVKNTILEKVNEFQSILIREYLSFQKKLN*

>N.PF13_0072
MSFLLALNESKKSAEGSVASDITEEVFEKEIHEMNSLNMIMHIEDLRDKSFVDVKEKKDENKIKIKNTDEKEK
EENGEKEGEEKGGSIKNSFMKIFQKKKKKKKKKTKKIKDHNINNIYDSNCLLTNPDNVDPDKYYLSFNELKKE
NNENVNEENYSSKDKQKNIDNSKDIVQNEENMNKEQIKDNMKRKKKKIKLSSYGTNSIENVKMKKYDSNKINN
MDETYNNIYKKKQSNDLKDKKLKTKRFKNLKKKITSPKFTMSTKTKNKKNGNINENNFNFDPDDDLKKEIEKI
KIDIERIKNKGNIKRMYDDLKYILLFQNEIVDIIIEDIRKNKVRTKYEMLEDSTVKDDVNKVILNHITFDLVK
FKYINTKLKELILCVYDTKKFNGLVKRIVNNKAHEMRKIVDKNGELTVAKSMLSFLYKQLKYCIEDEIQCYYS
EVKKLNDDIHNIKRNVMNIIFKSINDVCTMPDPYQIYEMGDKINRKKGTKENYNLNDINDDEQIKCEQNFVEY
ERKQQMICPDYSSDLNLFFSLYDNIHIERKKYIKNKSVMFKNYCTDVDNVDDHNINDNINHNNNVNIYGNMKN
IKMSTYRVNNNILDKDAQFVTSSKNINESYIPNYTNKSIRSVVSQNYVSPFNMIENRHKTDDFIYGDRNSNYY
INKSYEKTSNINNNIPLNVKFLNKNAVNANYNVSNMKMKKEHYLDDLKNYYCNINIPWIGKTCTNPSSSMSKN
ILKRKSNNKIKGRVIDAPLFHSAPDNDINHTNDTYDINNIGYGNDIDNNNIFEYVGYKNSDIYNKNIYPNNDA
YSNYQHDSFNVPYPNELFNKDHVMYNNNHNMIHDENLVYNYDYKINDAQRNNYPPNNVHNNHMRNQMNNIYID
KDMLDGRFHKMSHIINNIDEKCYDYGNYKYYNLGENHNNSLNMYHNMNGNIYTERFAKQSNGTTVRCFKSEVN
IRNKDTNKYSGFEIISSKEDIKKLENLLNDKWGSFKLFEKWIKDSSKWQWIDLKLQKEYINSNIELEKLKEEK
TKYLEKCINNKIKELWCGRMKGLMNLKLCNMERKILKRFSKYHINQDIYDVVLDFERNSMCYQDLYAKLLNIE
VYSKKVDFINKYNEYNEQLDAGGDYIYKYNKDKKKVNDENNKISQKKKGYVQSLIYSFNKKSQKSINNKNNVQ
IQKEKKYNSIMKDPTTSVQYKNDHIYNIMDISNNRANAVINKIETINNKADINNNKKEIHTNLTENTNKKNNL
GDNNRIDEDTTKKDQTYDKGKKEQKNEPKQEQKKEPKQEPKQEPKQEQKYEQKQEQKHEQKHEQKHEQKHEQK
IDQKLMKKPKNIYSNAQLNNMTHIGSFSIKKNKSHFDISLQDSGGLVNSKRNSFNDSFSLFRSECNKRISDIS
LSSLKIKTRDINNEEDISFESSKIDEAEGEEYGEDGDNNDDENIEKTKRTKSNLSTFRRLFNLKRKKKDKEEK
PKKSDVFPSESFLSYFKRQPSSDNANNSMIHKKENVHNLDIDNNMDDDNNKSSVVLLNNKSLSVEGYIYDNES
IIIEKEVGEHLHIENMEDKHISLDDINNEEIYDSHSKSSINHNIYLNDINKINQIMEQTEHKNYAHNIDLIKG
VKNKPFKADDKNTIKEKNNKITEKVTDLNDSYSFLNSSRSSDEENDKGECKQSNEMSNIDNKHDNTGNDRNEH
NGDYKSNPESNNKNNLKSNNKSDQNNNIRNYSKNNSKSDNKNDSINDKIESKENNNKPCYKKQSSSHRSQKSQ
SIIEDELKKHKFEDHVINIRRKLSKEKMIDDNIDEGSIKNTSRKKENSLSRSDVSYHSKEEGNKYEKYSNLSF
FKKTDKDNKINKFFKNLGRFSFTTSKKKNNKSVQGIYTNDIILHSFSDESDLEEGYEQKNKTYNNDSEKIYSK
SFVLNSYVGKKDEASNINTSEQVDNLYSYINKLKKKKDSKELDKTQNDESVIKDNGSKVEVKKRKEKDDKDDD
KDDDDEDDDKDDDEDDDKDDDGDDDEDDDEDDDDDDDGEDDDDYNNNGDNTRINSKKEDNKISQNNYKHSNSN
NLHDKNEKKDRKNIPEDDHITNNNYSNDVNRYAENKYNNENILSPRFGYSTSNNDAFKNADKLSESNSSKDEI
SEYSLMENKNSKKDNGEENKGKVQFFKAQKDDSNNIMDKKDKKKKENIKEKNEETGKNKSSRSSSNSSLLDNI
NRDMNINHKHESSDNEYDDDDKEIIKLSDDKTDNKKISSFHSNNYDHEYNKKNAKSGISSNFIEDKKSTSIFS
KKSKMREENSSNSVEIIRLSSFKSRNDLKKKKIGGNNDKDDSLSCDNISNGYLSETKKDDYSYYNYVQSILPF
GILGNKGSNDSYSSNSIKEDEKT*
>N.MAL13P1.63
MSCMDLKQTNIHDITEKENEGKENILVESNKMGNDENRKWERFNKNNRSNTNSELYNQKNLMKMNSNHNNNCE
DKDNDWSYLRSGANTSFRNSIKNNENLIFPANNKKDNGMEQECWRSNKKFDDMNNRNNSLNNNDMNNNNNNNN
NMNNSDNSKLSFSRNFKNSFNKGMNRNNSVTGNIKGINNKNYNYMNNNQENGTNNNDMNVNDQNNYSGIFNNN
FTNLNNKAFYKNMNKNNKFNKTNNRNNNNNNNNNNNNNDDGNINYQNTNEFKDNKKNMNFKNQYNNNYKFDEN
MNNSNTMHSRNSNVEEHLRNNSIDMNNSNINNYTNQQTRFSSFMENENENENKNYHTGGMNNNIHFKNKYDNN
NSSMKNTDNNKTDTSYNMKGTINNDNNNMDYLRNINNINEYKGSAKNKFYTNYMNKNNLKFTQNNNDNMNTNE
DNNNNNNNNNNGVFSNYQNNNMNRNNSINIKRNLNNNNNINNNMNKMGSQDKNQNSNNNFYMNYNYQNRKNSM
NNNMNNNMNNNMNNNMNNNMNHNMNNNMNHNMNNNMNHNMNNNMNNINSLDSDMSPNYHAHVKMSMMNYNNNE
SNTANPNQMNFEQTNNDNMKRENNNMNNYGYDDNTVHVNNNTPSTDFFSRAVGYNNNYLNNNNNMNSAVNNNS
SNGNNMKNENSENKNVADNNDSLNNNKNNNNNINMNESINNNNTLNNNNEYNNQNNNEDEDDDDWGELGEDKY
IDINSIMKKKNIILNQLEADLNDLSKKGNDGKNKKKNKMKKDDLFVLPHTNTLLVDKKKNKKNKNKNAKNNNT
NSNNNNNNNNNNNNNNNNNSNILKKDNDANDKNSKQIVNASGVNKKKGKKTGVEANSKNVEQKGDTKTSTKVD
EEVKTDEVNNKKKDLIEDKNANNVNDVDTKPDGDDDNNNNNNNKGEEDNKEKKEDDNTVAEKPAKALYVFKKK
ENMSPIEYMERQVKSLLNKLTVENFPIITEKMCQIMESRLNTDEIQTVVNQVIDKAVLEHDWSEMYADLCQTL
KWRSPNFEMKKKTSFEIALLKKIQEQYENLPSTFESTMKEKLKSDENEEELSFVEQKQKKRLLGIVKLIGELF
QRQIVSISIVISIAHDLLIAYEEPKEYCIEAFLQLIYSTGFFIDKIEKYKNVLDTWFGRLKELQRKKMYSKRI
KFVIQDVFDLRSSEWRKKTHKDTAKGLNELRSQLETEEMMGGSIHLAQLGNIVIVGERHNLRNNESYSKYMQE
QEKLSKQNQKK*
>N.MAL13P1.64

MQILVKTLTGKRQSFNFEPSSSVFQIKMAIEEREGIDAKQIRLIYSGKQMHDDMKLSDYRVVPGSTVHMILQL
RGG*
>N.MAL13P1.66
MRSYERKKKKKTAKTSSHGRNKWTSNKSKSSYKKKKKNLVNSNYNLLNYLQREHINENIKNLGEYLNHSDVNI
CEKNIKKVDKSKNIPCVTSKDINIVNGNINKEKKIKIKQKKKRRRTNKESVCIPYVKEEKDVPILYSNKEIIL
YKKNESDIINRANINRKNIDNDNVDKNNVDKYNMNHKEKKRNCSSNTNKNNISIENKIRNNKWINKRNIFEQK
HKKIYHNMFKSEDISNQNFKNVIIQHEHSSECSNRSHEKKKKKLYYSSTLHTNFGRIKENNTLFQEYEEKQNE
IINKKYYMFHSTDITNNNANLYDNQINNNYEEEEKNEKTISNDFLDNILPVEKIIQYVDDISIEALVYILNTL
DITNSTSLIERLPHRYYKEYLMFTNNIKRIIELMENISNEKLGSFFITYKEEKDICKYINILSLEKLNNILNC
FPIDNYKEFILLFDEYKLLEILNTLSISTCLHILLNISQKKLRYIFDNINEEKLIMLINEVPLCKFYYINECI
PIQKIGTLSNNICYEKLYHLHNSLSNSKNIHHITCNLSIHIIIQLIIDLPIYKAIQILKTISLEKIAQCIMFN
KNCFNIYDILYKAFHIDKIVQIIIHLDNDVNNINILLNLANYTDIIHYINYISFNKFKSFIDKLPIPFLKKII
NDISLKKIIQFFMNNKNEKKVIICFYQLTIKNLNSVVQLLPVKNVLRLVIPSHMDIHSKETKKIMNRLDAKNM
VHLLNDLNDNEYKYICNYIPLEKIPNLLNNSTFANYEKCYIVILYLPIRKLIDTFHTLNEEKKFGILERMPYY
IKDTIICNIYDTTKKFIYLMNPIQQIFISCMLFKLNRIFGNHYKKKNINKQKNKKDDKKNTKKYITLYSIRNK
FYDIIKNINIKKYHLFLNQVTCNKIIETQNIIHNIRNKKKYEHEQIVQDLFFYFNIYQYVKKKKKEKILHIKK
NIPLNVQNHDKVRPKIFNNINSAITCISDPILSNDTSYKNSSLYNKSYITKKRNSNDLLNNIKSNNNNNMYIY
KDGIAPNNNENHYIDLSKREKILPIKTYNIKNKASTNIYYNQNSSLNNICHEKNINKDMTKHVKKKKKKKNSY
HPKSRHGLYIFLIHLIIVIKKHIQDENDRKINEKKSYDINYTTTDNNNNNNYNYNNYNNNHNNNHSNLLSNTS
STNHNDYNKFIFYHVRKIMKNIFFSVRKILQMKNRSNVIKNISIFHIMNANLLPYMINENYNNIFYDISNNIK
HTEKINHNPNISDNRNHLQEYKIFNMIQDSTEFFDANNTCVVIENEHTNIMDTSKENENIFSLKDDIPIIEDT
NNVIEFDNEQIINDICVQKNKNRNPLYYVSLLFNKPNSFPYLIKKNILRIEKNDKRYKNFFFFKTATICDTIK
NEECESNTQGDNIKDYILTTSNIFKKNRNNILNIYNKDTKIDNDIDKEESEQINSDNINILYCNTNVESHNNN
NNNKNNNKNKNNDRNNNNRKKKDIQKSKFSDLRYKWKFSHISEKYKNKIYKINNNINGEKKNVYCLKFIFVLN
NVCDLDKRNNDNINLTLNFFISKKKKKRLLNIYKQKILDSSLDIQINNEKYKNKIINFSTNISDTTCYINSNI
VNIKFNKEQKNKLEYLYSLINEEDPSNKNLFNKTKCDTYIKHDGNMNVDNVHYDYKRDDHINNDKYNYNYNYS
YNYNYDEKVSFNQLKNNTLDHNENKNNNILSEKFISNLLNKNIILLWSCKNIGYIFLNTDNSLHINIQDISHN
LYIQIDKYNYNTHKNYFFNNNNNYKKGTYIFLNRLFVKEKIHFRKLKEAFSKIIENKKQKKKGVCKSLLEENN
NENFYKCKDSENKIKYKNVNNVTNKNGSVENNNSLNKYENSTNTDILKFIHINMENNYKSEQNSAEDNMNEDI
IYFGDDKYEFFNDTNILEKKNINQKNNEISIIDKQVYHDIDIDKDNYLIQYDKNIKRKNSLIFMNNTNDTCSN
NMFYEQSKRDDPINEYYYKHHNSIVVNYFNNVIRESNKILSEKRNNINKQIREEIININEKSIHSLDSNEKEK
VHQIISNEYNNIMEHELRKIRNPYLCDMYQFLKYSYTKEEFNIILYLKKRNKKLNKIKDMSNIKMNVSKKIIN
NGNNIIDVQRNGKFVPHIFNDNHILKLKLVTLVDNELCDYKLYNNKIINNNNNKETLRFEENLRKINLSFHSS
HIFASNPFIVSREHFLLDQKTQNNSTAQIIQDNNNNNNNNNGDRKKENNIYNENEEGIINNNIFVKGINKNTS
FYNEKYNIQTQNNKDYKNEILSLSNISYNEIMIQKCIEKDEVFKNGNNLENSKNLQNEEKSPTIKEKNIPNNN
NNNNNNKNNIYDILDNNLFKGLFIKDDIKLSKRTIQDILTSSDTNNLYKNKAFSNHKEEIQSLNTEQKINKIY
IPNYSSDVVRKVSNNFSCINQSTNMPNIFSNSIGNIKRIKEKNKIMQNSNSILSDVSDIFINYNDSNKEVEKN
MNSSYFNKLIIPIPGDNVNINFFEKKETHLDDNYININSHEENNINTSHIIKKSHVVSNLNYKNISDNSLKNK
LCFEFSKTNHHHNNNNNNNVDDYINNSNNNIYINDTHKNINGIMYNLGFLNLHCLLENKNKHFYLITQGYIKA
SNYINDQMLIHTKIYNEIFHTKKYIDAKKNTSIISSSFDSVTKQNDKLYCLHKKKRMFCNICGYILKQNFLND
FFFFKIFIVTKRAIFSNHSTINIIYQSPLIKAVFHEKDNIKYKLEQTNNNQYILKIIAIKKKKIHKTFSLSKN
KHFRKCLEIELINTEKKNIENEMYERKENVLDKKIYGKQVFHKKSFDNLYNTTKDSLIINNEKYKSEVKKKKY
SSYENIKNYSQSLSNTSYSNHLSTSICKANKAILNFSILDDKNKK*
>N.MAL13P1.67
MYVKCFFYVIQILFIIFLKCHCYKIKCFNILDLNKKKYYSFREHINCEHIRNSVNRNNLSNVLLRRRTKNALV
KELYVSKLKDNYKTHTNFIRTNNIFLEEDKKIQECNINNIINNNVDIQENVEKYNILYKNQLDDINILYILLF
NTLMIYKKKYDFFMNENYIRSYYYIYKNNLGRDKKIYNTKNYFINTFSISWYNTIKPYMNNIFLEILNIIENT
LGNKIFYIDIKQELNKNRDEIINTLYNIYKGNFRKRDKKPIKLLFIGSNEYSNLCFKIILLIIKRLRNDIILD
NVITKSPRRKGRNLILKKSNVEDEAIKNNINVFYYDKLKNNIHMLQNKKMDLCISISFGEIFNCNFFKTIKSN
IFSLHPSLLPFYKGASPIQRSLLNNEILYGYSVFLTTLNIDSGNVIMKKPFWFNSNYNFNDIITILFTQGTLS
LLKNISYLANYNKDIPHKNIYNNNICEETKNNLNQNHVQNKYDSEINIHNSNLENKNNSRNNILPLNINNVLN
NYNNKMYIQNDYNINNNYAPKIKNDEKYVCFFCSTSLFIHNKIRSFINWPKAECTLFLLQNEVIKPLEIKIIK
SSYDLNNNYKFIKYDGLINTHDQHTCFDNIPRNFVYIQNDSLNILCKNNTLLKIYKLQQKNKKIVDAMSFINS
INKCSLLY*

>N.MAL13P1.68
MVYFKRFFFHKRHRILSIFNSYFFMKKQLLNNLNKILFISLNSYFLKLLFSKNTYIYYLQEKKNSYLEKKYRP
DTPYKKTESGIIYRDLIDGVYDTVEEGDTVYIHYQGKTTNDFRIIESTFKSIIPPKIIAGHYDQKHIKAIYEM
VIGMKKNTRRECIIPPYLAHPNHFPTQPLIYEIDVVRIVKKNAQNKTTLQKLEVKLEKIIDTICSYF*
>N.PF13_0077
MENYINKILFLLIFFIFHLNSTKNYKKTYRYIISFLLYFINILYAQKIWGNGKKQIRCCFIKNLYNNNRGIFR
LLDNDIKRKYNIIYLKKKNNGFANNEHAFVKNTVFIERGQNDENKINYVQGNFDENVKDIKYLSDMQKNFIYL
LERNNNLLVHAKTSSGKTTICLYYLILKFFYNCEFIFKEDIEREEYIMKNDYIDRNIYESKNKSRKYFNQNKY
RFIPYSEKYSEISDIKEYTNLMIENKCKTKLKKDEKVLILCPSKELCVQVAQNILSFMNNKNESLIKLFIDKD
YKNENKEKQNFKEEECINKMEHIQINECDDHYMHHTSKHSVNNNKDTMDIEENKLYMLEHDNLNEDNSSSKVK
KKNKINDNNNNSNNNSNNNSNNNSNNNNNNNSRSQLHHMVNLKGVRYLIGTPTCFRSYLLNLDKENLKNFLQS
IKYIFYDEIDKLLPSVSKRSLLKNKKNIKSKTAYLILETLMYINKKNLIFIGCSSTLNRELHRKIFKLLCLNK
NNAKKKIYILREKKNLLDNKENDGMTRIDNITNLIDIPIKNNEKKKNISEKKLSSSNEHIDDNNNSNNIINDE
QEEIENNEQYNLNYYLNEENAFQKYIIKVKLPSTIYHFYHLMTDETYENKIKEIHKIIETFKNQKILILIKNG
YSLIQLKKYLEMNNIFAVLLHEKLQISLRYNNDNINNMCEHYEDIKDLKSIPNDDKHTYINKYPIIISSFDSI
RGFHINNLDMVLLCNKPKNVNEYIHLCGRVGRRKKIGYSVTLENDKNINIMNNWFNNIKVYFNKLSLKSDPVI
DEKINNELENQEKNHLNYVASCILDELQENT*
>N.PF13_0078
MVDIDKEPISNENNIKYLKVYSNILFKYTEAQEKKKEYIDVALLGDNIKEEKRFFKLINIDIILIILWFFVLY
SFSYNIVEVRNFQNDLSNNINESTFYSETFYKSLSDNIKKINNDYSYESRKKDYTTFKNINNKFELASWIKNE
FVKNVSYEKYFNNNIIFGNCWRITMRLYNNNNNNNNSNDSLIKKIYNKKKLYEIYSDTNFLKEIENKQNISYP
YFDKKWNYNFSYEKSYKKIGGLYQIICENDYNKIQEMLSQDSFYTTSYPYFIPAVILTNYNIATVTLDYLLYN
PILNLISYNALKFSFLPNTKTYKEIVTLSASCNQINLYFVVSFFVFLCIFITYILKDVRTYFLVGFNLYVKTY
SCKFMIIVCVLLNMLSLGIYVLFQYKVPNLNATYENGKYKIDSLYANANSESIVNMFYDIERVILYIEWTKYI
FIFTCFITFIFSYYLIIKNYGLLIKKYNNVEKNIKKDFLYPCSILLIVVFIYLCIVSMYRYEVFNIFENENCN
MLYAYILNICLVFANFQGFNISSIINAEGNNLPYFYFIPTLFFIFTIIFSFIFFLAIKSYIKRNKKMYKWYMR
HFKDNRPLSKDNNTEKGIKIRAYKKRKSIYNKNNEYNKSERKVETNICNDERSQNEIKKEKTKEKIEKNENEK
QGETQKENIKDENIKDEYIQDEYIQDEYIKDEYIKDEDIQDEDIQDEDIQDQNIHDQNIHDQNTHNRKGNNKN
IYHNKSHHKNIHNNNTVEYNSEEDGNSKSKLSKDLPLSDLKEDTIKNNNSNTSKNSYDNSEENNIRNVEYMNE
EDGSECYNFSLNNSSDCEINVDNIDKELNYKDIIKLGKKSNTNKEVYSSNMMEENEENIFQIEKFINIFFNLR
NRFLPFSYNVKKYILKEYNKKTKNKKSEKLICFFYIFMGLLIFCSLFIINNFKKISETENLLKYQIENVSYLS
SDKLFTNMKFYHENKSLNVSINNEYLNFHKIKNKNDIIEWIKNCFPVYLENGTDLFGNNAIYHNSYKWIEIYD
LLYGKIYIRMTSREEINISDNLNNNNNNNNNNNIICNNRQDKCYAYIRNEKNILKNNLNDILSLIDDSIKELE
ISFILSDVDDHHNILVKINFHFTSTGYISKYIQFDHLFFNSFNIYQFIGVVVNFLYLIILLSLLIVIYKYYYT
NFFYFYNLCVSSLKGTIHSNNSNMTHIMNTQMENFMYRNDNNRRNLQNMDNANNFNVVNINNNNNNDNSNSYS
YGNNIMFDKYKDNNMNNEEINYYGPYYNENNFTLNNNYISNMNYHPDNFYSNANINNKTTKYNLSLLLHLKIY
LTYLFECDIIKLFIFILHILIVLFWLALCININRISYYNEILLDSYFDIHINTISFYSIMIYIFYLFLFLTIM
NMFFYLSKYVKNEVIYEALYYNRLQILKSVLLLLFVCFHFIIFHYFFYYGIDNYQNLTIYEHVIYSFLILIGM
VKMEIYLKYSTLYFFVIILPHLIFIRFLFIYTLFAPILSSYIIIKKNRSKQSNNTDNISSDKVYNIESNYGDS
SMYNMENMNTDFMYNIDNNNNNKKKNSNNNNNDIKNNNKNNNKRNLIYKEQKENDTNNQYDEEDYKAFILTRL
SSEQWKGLKEEIKEFAKMETYNILVYFQKFKNQIDSKKINFMSTILKNEYTYLNEQINNIILDLRKVELQWKF
QSKLVNSSNAYIDKINNQIDMNEEEIVESKSKLSSLKQYLQKVQMDPTKERDE*
>N.PF13_0079
MTTLQKKLFALKRKNKEDGVINKNVDPSLMYNTNNLNNYLRSNTEKNKTYNNMKSNVGVDNGKNMVNVKYAKY
SKYTHKGYPKQYIQKKQTTHNHIKRIIRKSPSWFSYQNFRNILFLKKKSTIIKDNDGLNVKIVVKKNNSINSV
TKSSNKETVLKKNTEKYIKKVPIQNKKKELPKSVHSTKNINNNIKSTNVIKNDKMINVNNGSICRSNSDSNDL
LKKNYVFKINKPEAPYIQEFLGKMKDDDSILKDKYDNVKTEREKKKKVIKKKNIIIMEKKKGNYHYYSDNSTN
ISSNKIEENMSCSFPASSQILSNNNHNNNNKNNNDICDDDNIESNSVDYKIKIYNNLMENIKPTRSYQLDISH
PLYNSLCKNELSYLSSNWHDRINFAFRNIWALSDYMYDEKSEGEREKEFFNELELFHASSNIGACGLVYSLFV
QNIEDDELNNYLKKCFVQINKENYPHIYKIYKPKFEDDLIFLFDSIVFTIVLGSTKDNINYNIARKIYNNDLK
GKMVLCDCIYNLKRKSKFCLPIANQIDFMGMRIIAEPLIPLNEQYIPIDIIKDIYEHTDMNDYMSDEDRLPSS
HVMDNTSNKMNDKRESDHFTNNEYGDNYMDMINNTCVDKNEIHMLCTNKLNSIKRKKKSKENDQFYGKIMLEI
EKYDIEFSDQLKQMSHYINYDCCILPIGISNYFELNILKNKNNVKIYKSIIDNLYFIRGTEEILPPFLLLNMD
KENYITRLRYEFIKYYYEKPLCNTTKSINIKYDEYKNHNNNIDKIKEATKECINNIDNYIIPKISNFLLNFSD

SYDITQVYHSHGININKLGYLLKKEIPDYLSEMVCRELIARTLKCIYYEHIHSFIINHLKNGTIVKNISNQEF
NNVIKGKDHGMNNYMDNNTSTTCSNNEINNINNDNNNNNNNNREYCLYSNTYIKDNDHYNLINDSNACCQSRN
KKGKMIFSCLCKKCISDWNYFPELTPHKLLIRLINLTLNINTLESLTFWNNILIPDCIKKYNINLNEYINIKD
IEIYGLLLCIEYHMGISFTNECKQNYKIKLPLTLNDMSTFSTKKDVTSFPNIYSLKNIFSSNINLNLNNILQN
NYVNDNYNNMNHLISKDNKYKYKNNLNSSSFNHHINKLYDNENINNQIDNSHDEKSFEGNNFSNNTNNQTNLK
NVYLLLEDMIDDNILSQVGDEINKYYPNKYEQNENTKKMNTYKKRGNQLFDNCNNYDKQMYFERNHFSFFYPK
SKICLPLYCTWYYKNIEKLYSNILLQEKFKIKNNLKENIVDIIGVQMSNFLSIHNISTVGTCSKIKSFIYEYN
IPCHPYCILNEKMKCINYVRSKKAIYLLLNFNINKNVLKLAKIFLHLAYIHFEHGYIEKCLKVCYYIYNNIPS
IGTIKRDILILTFQCKLKEEKIQDALLIYKLITQFSKYYDGEYNISTLWCNILLMQYYYDKANATENEGIKKR
NDIDIEDKKDCYEKYDNRNMMYNQNIMDLKRKYLINALYYSKRCYHILSSRLNDICFDFIYIFSLIMLGNILM
SLNKFAKAIKYYNLSLQYCIKGKIPTFLLVQNKCLLAECLKKNGNVYKAVELAEECLQILHTNSNTYIPNLDL
YIILKLAQMKEYIGCKDLITYYPYISLKQNSCNNNIINKKICLSDFINFEQKYLNEKNIKYRNEAINLYIILY
EKLKNKKNYAVVNAKDIFGNFYIKDDITYNNNKTEINMNNDDIERIYELIKEILKIKVISLCMNKQIMFASKL
LAIVLSKNSSPFINNLTLMKDKNRNRNILFNNKYNDDGDEDIIKNVMEKNENKNININNLIYNDILLNNINHH
HNNINGMLNYQLSHFHLNKQKKFFEKSCYTKDYDYISVEKILFDHTYFEIEDICKYCYLKCDDNINKCTNNEM
NYSLNPSIWFDILFFNVMKGTCNNKELIVLIDLFKIFLTPMQKQLILYHIKSLHNINNNDEYSEYIQYLLNRE
KTKFLHFTNVIDQNFHTSWYDNNNKPEHLSNFDKKDIIKNIDLLSNIRVTNPNLYSC*
>N.MAL13P1.69
MKEYSHIIGTGDLFKSEDRCWVLLASKSPRRIELMKLMGIKNLYICESGFEENLDKHIFSCAEDYVKENALNK
GLNVVERVWFSNNTETEDITSNGIKDLDIKVKRENNSNHSNDYNKYEIMNINSKFEPLPKIIISCDTIVTLNN
EIIEKPVNKQHAHDILKKLSSNVHSVFTAVCIFLYKKKIPITFIEKTQVYFDDLMEEDILQYINTKEPYDKAG
AYSIQGVGSQFIKKIDGCYYNVMGLPINKLSKFLTKLYIDEEFSV*
>N.MAL13P1.70
MMINQDIKIKDKEMVRTYNLLGKNYHFMLKEYDNLCSINIIKNNIINFRIVKNLLAINNLIDQVDKSLLLNIF
EENVDNDLLLVLSKNNSNFILKLLAIFYIKLSKNTKFNITNHFNRIFDINKNTSNTSGELKNIPENLFKNAII
LISLILYHLPVRLSYHIPTIIDYSKKIRKSDNLELKYFYQKCINSIIKYGKLNISECDKIFKYLKKSLNDKDF
FIQYIALKCLSNLFILHPEYSLLEYEYFINHIIDNININNYVSVVYLKYVIRFVSIIFCNCANVNYYNSKDND
GTNKKHIDDIFLFNLNIMKKKKKKYSDEDIRDEDYIDEKKKSYNDNNNNNNNNLKSDIIEDNFNNNINNNINY
NNHNNNHDEIKKKHHFFSDLLKTATEELVSKISKIKTDEKGGDVEGYMKVESIDGFVFQNYNEYNKKILRKMK
NVLFIYNFNSFLSYFKNILLNIFSDVHHDYNELKKLHFLNILEKFNYYKKESHPFLDKHDYSSYVNKKSYDKK
FEFLRNPANTNIINLTYNKETNDYINIILEYENKLMREKEFWLYSELVPLTVEDKNANKFYTTLIRNTLLKVI
FFESFKKCILFSKKFSAKSIIKIIEFFIYILEFLCSTCVKNRNIGGVFYTDNISNSFEIKYCIFYFTKIIKQL
IKQRKKDTYTVVKEKENKARFEEVNKMYPPIEETINENNDINNDDNIDSNDDDNIGSNDDDNIGSNDDDNNIS
NDDDNIGSNDDDNIGSNDDKNNDNNDKICDEKNNINGKDKMDHIHNSSDSHISKDEHNHSHSSRESEENLSRQ
NSQLSSTNITNDTHLKEGEEKNEHIYEKHSKDNKTNISEHTDNNNNNMKDANKRKKKKNQPNAKSSSSKTKYN
NFFGDYNSFLGIMPFYNKEEKKGKINKDEKKGNIHKEEKKGNIHKDEKKGNIHKDEKKNKKLKKSKSNKSSDK
KYDKNKYEQPNNNTYCEERHIRIFLENNVKKYLNLNNDTNLDINEKKKNDIILKALMNLYNKIIMNHNINVEK
YEKYERFLKLIINNMNGIYIDIDLLKHIIQLFLKVYLKFPCYIYNLITLLVNYITIINANFMTSLTLNTFDDI
EKQVSILIISSISLNKILYFSFQHYKIYHLNNLIYCIFDICKLFLSKVQTHPLKTINELRRIISFFLIYSIVF
SIVYEVKNNVCYTYQDNKMLAMIDEGADVNKNIDINISFNNMIIYEKMKNYFINYEILYNNSLFKLIQDILNV
MINEIDEEKIHYFILSYEEQLSKKKMNSHNFFIVDNMFIFIYILKNIYVMLSSHMLTFLFFDYIYNIVSILFN
FFTKINEIRIKYNSKMNKSISNNKQNLVDNEKNNNEINNENNNIFTLEDLKKHNQIKKKISREQKKRSKKKKT
VTNSFKKKITHFYFYEHGFTQICRSINIENILLLFLIYMVKVFYRINKFILFINKSYHMNEDEKMNQVSLSYN
QSENDKKFTEPNNKSNNITLEECNKYKMFLEKENTHNNMNKLFNILDAFTNVNTNDELYFDFFMSVLINFVNN
KNNFDDLIINTLIKEKCVSKSREKYYAKKKKEDKNKYIWGYSKELEILHMLTNNYDMYYDIALISFVYLFELY
LKKKKIENNTKNKKHKHVYIYFNKVMKYIYSWDINKKENKNILILCLFILDNFFNKKCFYKNNMKAKFFKSIM
PLFNLLNDNFNQQKYYLINFLIIKILTYFIGCNENIDTYIFSYIMKLYETYSSYIIKEKNTDLYIFISILIKN
VTLSYHKLKYTNCFNRKKYLKKIKKKKLNYFPGSHNIISNKLRNEYSNLENVDNEVKKICWKCQDNEKYDYIN
NSQKYDSLLLSMECITTYSNNIYSSVNKKKLTNKEHHPIHSNEQIVELNSKVRKGSTSNINMIGNLSPQTYIK
KTKDEILHLFDLYVDILKVIPHNKDIFIRVLKNFNSIIKRMKNHINVVRVEKCLDIVYAYILKRENVNSFNFR
ITRGKYIIRLYNNILNILKRIKHETGYKNKMKNNYNINHQHHGDHKHNNHRNHEYHNYQGTNISKEIDKKLL
CILEIFLISYEKKINHNIFKTFNLLFDVIDKIELETYTYTISAIIQKSIFFGNMRIMECAIICLYNLFRKNVI
IPLHENFDYYLLIIHNFYNNNNNILYNNIKALLKWRICIYGIFNINQWVKLFENVLSVKNIFIYDIIKFKSKG
SFSKFDCKNNVKKYNDQNYVKENNLLTNDTAYIENNNNINNNNNINSKCNNNNSNSSSSICYGFTFYTSLFDE

HYNFNIHINLNKEKIAQNSCMKNNKSKYFFNLFKMRKNDIIFSDNTKHLVMSLLYFFLNRISVSSHIYVHNDS
YFSNYMKKVLRDRTILKVERKLGRNKYGNVKKIDSLNIKNIKDKQHSNTYNDNNIYSSLNLEEMDKNYHHSNE
NVIQKIKYIELIIIDNSNTDDYNSVYNKNTLIDETNFTKINLEYILDNCTLYTNFELLLKIILNNINYGIKHY
RLSRLAIKILIVYLNIFKSSKIIIDEFSSTNNEIKLLTNYEINIFTSLKMYYESFNFMYLNNKRDNSLFHTNY
NNNNNNNNNNSSSSNNFNVNYYHSGNHNNNNINNDHNDNHNSSNNIITNIMDGEKKEQLQFSHFFLYPLMDIY
NLFLFLNDIKLCNSHNKFVEYFFYFAIKKPNDYLKKKQCIDMQHSGNDKLKHDKNDHMNKTDNIYKNFKDVEE
TEDKQRFLINSSLFFSEFDSCVYFLFTYKSFCYYIINNEKCQNNLSQYNLAFIVRNINYILFDSIILYLWSFD
YNKQNDDKDIVNYINHMHKFFTCVNEKSRSLSIFYFNAFPVFIQFLNIVKSFVTLNKRNYLLFKILISFLCYH
IRQYHDMLKEETKEIYLKFIYNYLILIEFSYDHDMEQTEEQQKNQKREMINQNDIKNNNDVNDEKDENSTNEK
KDLNICPKSTIDNTPNKIKKLEFFKIVLDYILTYLVNFINIKNVANILEVMQTAHDNLFIMEANEIDDNMLCN
MFTLNIYIINKFLNTNYKNEKDVIILLLWKSFLNSFVLLEKMNDLEIKENIKNLLIYLFLKKMNLLNDEKLSS
KLLKYAFQYSSIYDLNNYLKQMKNEEKEQIITILFTKDLYKLKLFYIKIIVFIHYVNACENTLDGDTQSYFSQ
EILQIFINFTRKLKTIYIYNNDIHIKHEDKNLRRQMINSSINIIQNIFTLIKEKDESLIFYFFYNLMNQENML
IPLITTKISMKNSLEYYTFLSAHSYLEDIVCYSIKIVGIFFETFLHKVDLLKKVFIAFVTYIYKMISLCDKGN
ITITKSFLVYPQEKEERKITKFLKETDYCSTYIFDNPDKFEITSNNNMTIYFKEVIEIFRNMAFKNSNLFKEM
ILNIGAEEKILIYTLMKESIDTEKQNEEQNEENTEKLDFSFVN*
>N.MAL13P1.71
MSSKIFNIILKDDVKKLENYIKSENNIDLNEYNKEGLNILLYGIERGCSECCYYLINEKNVNIFLKDRKTFEN
ALMKCMIIGNSMINVSRLLISKNIDINLKNKNGKTSLHLASENNYIKGIELLLKNNANINILDNEKNTPLMIS
IKRNNEEAAMLLIDYNADPNIKDKERNSVLHICAKEHSSNIAQYILSTNKINIENCLDNNNNSPLHIAAMENI
KTLCDLFLKYKFDPSLKNNNNETYSDILKKHEIHALLKEEEKRKNYEEKEIRKRKNYEQSMLKTDVSNFLIKY
NLNDLIPFFYKNNYIYVDDAFININEITLKKMNLTKEQRNLFFDSIDKYYNEIQEEENQRDTTHQLLIEEQNR
TKKLKFVSYIVSLIFTSIFIYSIFMSIYKRGKIFF*
>N.MAL13P1.72
MLSSRNVINTIRTNYSFFQQIRRFKKKTVENQDGKFVDSINNTNEKNRRTNYLKANSLNVKNIKEKNIILNNN
TNKLNDQKNNVSTKNIKPSGRDKNNTIYNLKYISQKSDRNNNINVSNNPNVYINKLHDYLDISNKSENEKVKI
NKLLNKLKTENLSIHIILNSLKDLCSLLLKKNSIDLDKLIVMNYVKNKNSLKRFKNYFQDSFVHIENYLTNYI
CFINEDNIFELYKYFYYLNYPLNLNTHELLQEKLYLYIDKLDNLKTIQIYYIIRSFYEHFYLKQVDSFLTLVV
NEKLQKYYEQLSRVNNKIDKDGNHIYNNDFLNNGKYINYTFNNLFNSNNLIENVDNTNSFISDHRHNNFNNFN
NFNNYMSNDSINVPNPNEKNNNNNNNMINENNEKYINDNISSLYYMCDNNLKNEEGIYKNKINVEDNEIIIIF
KYIQNYHDNMNFFYNTYITNFIQSNRTKFSIDTLLLILNIYIKNIDELFNKNILEILYYNIEDMTEDNLLLFT
EYLKKKSKMETMTNTTITNNDHNHDHNGNNYSIYYYKYNDSNIYNNNIHNNIYNNNSNNANFIIRQNGYINKI
TQEAIFNKIMNKIKKDKDLILPNNLALMFLNLHEIINIGTFNTCEGIIKPQENNKNENIMIEQNESYDNSNDQ
IVQINKGNMEKELSEKSYMNLYESTNNVTNKNMKLGENKDFNRDNNINNNNNNNINNNITMNTSNGHNSQNIQ
NNSNININKNLFMKEFINFCDNYCNNIKNFSSILDLYLIYIKNESVQYKTMNTFEKKIKINKNKLSQEDISNI
ILSLSIYPHHNTQMLNYLENLINEKLIQGKNYTPTIQYDNNSIHNNNVDEKYIHTNNTNYLIDISLALGISGR
NNLKLWNYIDVDKIILTCNKKLLLYLSYSFLLTNYVCPMSWFFIIRRIVEDVKIFNKKQCELIYEILKCTVLF
NYIDLNNFSKNILHITDSNTKKGISLSQLNYHNKKENNNFLKSFHYLLNTSYYHYKMKLLNNQYVSKVPYEDI
FVYLKLNYEKNVEFKQLYIIPYLLKDYNVIIDPLPTTPIHKSSGYIMGEIQLKHKVFQNDNYVALSFFDGMWN
EFINPNDQKDGEPTYNIKLLAEHFKSYVESHIKIKINKNYSNNNVISNNNKTTQNNTINTHEYLKFKKSNYPP
ETNNNKYLINKTNNNMHNYTNQKKYLKMKQKIINKKA*
>N.MAL13P1.73
MNEQDYLPIEIKDALISLHDEHFNYQKYLNVLKDYIYLDPIKNINTLIDYILDNINNSDIEKNICHYLSDVII
YIQDINKIVNEENNTIEEKLDENDYSNSLYYEEYRINVKDEKLKKYNVGDYRIDLIQNCTRNVNYLKLMYIIS
NVILPKNGYVLFINIFHKHEINYQYKLLSLEYFANIANKINLDRAKYIAQILPLILEKFYNTDGEPYYNDNIN
NIDILLSMCKFVNILCDESIKAQEDDKQDVALYSIVAFIMRIICYHSVYLPINRNVEKLGKYIYYDDINYNEC
LKEYSNLTCNLYKSKKHIIIKNCLSSLIWRLSIINPNLPRTIERVNIIIPNTKACVLENSTMEISILCYLILI
ERVNINAFPLVFSELYLLLNLMIRNSYNLILCVSKAKESLRDSLLIKAYHFILVCCFLSKSIYRKNKEYYTNIY
KFKWRPYDFLKLIYETIHNYNHLKFYTKHIYYSISNIMRNFQWDIFYSIYNRLIIESSSDTVRSIISSYVKDE
LFKKMIHVIESVQDIDVITDQSVTQKILKAEGANNFVQEKRINNKHYSSTNDYNNNDNNDDIKNNRDDINKEH
IPYNMTSDIQSKNSKENEIHKLGCQIKKIVHQLIGEESVLLYIDSITVALNIIKMILLNKKFTQFYKYIINID
ETSTCFIQNKMKYFYDQIKIEKSVLSYENQKSNNSITQINDNKDILHTEQNLNTTFNNINMNKLDIVLMLLSD
IEKSITEIKQNVNANTNTNTKG*
>N.PF13_0080

MNNEPTNNDKNYLKNIFNCTDKKIILFKSYLKNKLDIDSFTLGEYCFKHDEKFQRSVLSNILKEQIISKKDKN
KLHLINEIIIDTSFFKENYDFQYFLENVLLLEDLVLKKLDNKLNDEDFIFKENKKVSINNWKECYSHIKKKLN
IKGMDEKSKIYNNSILLFNSTKFSYDDINCCDSFYGLKVWDILFNYVSFDFLNYLLSNTLIFISDYFFINTNN
NFKTYVKSSYFIKIAEIQLNYQDAQNIERNIFSKKKNLYYKNTKLVKLTYQKKSIKDSTTPNLTIQKKKKKK
KKKFSKNINTNEHININININNYIYNTLNQNNEVNQYNVNHLNMDKNITYKEKESQNYTINNNLLNDQLLYYNKT
YQNNVNTHIYSNDNKTPIIANQCIDIHNRVSDPTRKNIFYHSINSLSYEASLNIFHYNNLTQHTTYIDTPNKS
QTCINSPMQHEIDEHSNNELKNQKCTQYEYVDNVCTTNKNISNDNISDKCITTKNIPLKYHINKKYKYLLKKK
YHTMYTNNDHSYGKYLYLVQCSGRILKNDFFKDMKQIQEERKKYTSNIKINSEYTNNIIINNNNNNNNNNNNN
NNNVHGFGHINNLFSSNEFPSSNISSCTNYTEKNDKLTHIRETSLLITENSSKKDKLLPEIDFFSEDRKEKSS
SVGYDIKKKNDSNIKRFHNKINRTKEEKKKKWNKIIINRNNILQHNTTNKCKTFLFNKHIIFDKIENNNIPLF
IYDLLNYIFKSDQTYFYHNNFIDEYKQKICKQIKCSTKKNDISHIITSRKENHLFHVQKLENNYKHPNINKQL
RKTKILKYVYNYFKEFINNVINTKFGKIYRKFFPRKHILNKIHKIFKIIRLQIIKKYRIINIRMNRKFIKQKV
YDTFFKNYDFLSFSFKTYKIINFMVYITKKCIPIKLLGSKHNFKIFLKNVKKFLLFNYKESFSLNQVMKNIKV
KNIFQKKISKYNIKNRILLKNIFDNNYENKILHRNNKEIITNINDNIKIYNKKNDNLNNSFKIKTTLFNKLRR
KYFNKIKKINIAIQKRHLMNRLIYFLFNYFIMPLIRRFFFLTKSEQTLHKTIFFDRKIWNHFTKISNFCLYHQ
IFRNKKLKKRNEPKMDNVQNMFNVKKKGEKIKTNKYIFIKKMKKKSTNKCINNKFSKKCIPKKKKKNLYNITR
HNNIFIKKDMEKKSKTNNLINKSIDNLYKLKEINKKSVRPYIKKFYYKIKKKYFALKKMYIHMRMAKEEKSNI
KLERAFKHFFIFAQEKEHILKYFSSHFFQNRKINYGKRFNKLIHRIKNIIIKQNSGIVKNKDKTFLHLIKNKS
NKNNNNKKNNKNNYNNNNINNNNNNNNNNINNNNNNKCKLSNSKRYNIRNNNNNKKAKNNEKNNIDDSNLEK
KKKKIYIYKIKNIIEKRNFMLKLNSINHFISKKLRINWIPKKKGLRPLINLSTLNVPEIVKQRIFEILKSKKS
SEFYFHNILNNLEREKKDKNIKKRKKYNKKNFNPVSLNNICNFSLKCLGNMRHNNNSLFKNTLTKTGEIELKL
KKWLHYLKNWFYKKKRMKKYIKNKLKNNKKIYAYICIGDFSNCYEHINHNYLFKILKNFFDNINNFEFIYLFK
RSFRLYNKNLNNSFLSYYPVNVKSFGLHYIRNLRELIIKSHLNDNHHFLLNQMFKTKSKSDLYIFADSYKSLQ
VDKRDIFMTIITVIRYYYLNIYFSIKEFKLNRKNIFYFQIFQENQMKGVYLSVRDKKRVENIKKWYLNSMKKI
NHDEILESLKNSSININNKNFMICTNHEQDTEEKGNTQNKEKHDIYIGPIYNNSFDSTTTTHSSNNYKGNNIH
VSGDYKNDGLLHKGNNSMNECYVKDIKCNNNNNNNNNNNNNNINNSYNKLNCVTNNSKNDIIKYHKTIDTDNS
KNHTYFKNKFLNFLDKKIISNIYGLPQGFSLSNILCSLYYAYLDKNEEFQNLLYSEKQINNKYFLANGTCNYF
NLNSLILRFIDDFLFITLNKKNIKIFKNLLLKKKIWGSNINSSKTKIFKIPLIYKNDLLIYNFQNKYQQKKKY
KIKNKKKIQSVRNKRIHNQLVNANKKKHTSVQKDKINKYINLIHPTIQKNDSVLSSNSIMNFERIYNKESHKS
NSSIRTDIPNSVVNDDIEYNQKSDNNSYSTNNLYNNINMTQNGDNNNVNIFKHVQNDSFQCFNSNNLYIEKDI
KENNISQINRKLCSKRNFTKKSRKINTLTYLQIDKVIKILKCKKKYIKHIKKMKYMNNFQNFKKLKKLQKFHN
ASFELKINKINKNIRRLNKLKKRKNHSINITPVTSIEWLNNSYTFDFINNSIQSTSYPWKNKCDATIRNHLHL
HNVIIDKNNKTYFMKNLVENRIVRNIISKQKKCQSLYKNKQNVYFCYKNNFSLLKSSILKFICCIKTLKKMFN
AFTNSTYNTKFILFLISYMNKMLIKNKKLKFVKLFLIQTAIEAFRYARIFNQQDSFYPCLQHFRKIKKRLINK
YKIGHNKNLLREFFFLFNFIKKELYNSWPYMFKIKN*
>N.MAL13P1.74
MLRFFFMGKRYFGSSYLTKRLKTINERALKYHIDYTKSLDILEGKNRSVMKEIFPNMKSEINDMFFLNNKEND
ENKGLLEEDMEILDINKIKDKYEYEMKNMTLEEFGNIKIIHNDIIDEEADCMLIPMVSNFIPMSGFGSFILNK
GGPELVKEIILSIKNLIKRRIDILNEHKEEYVQSSLEINGEQKFKELYEGSKTLQIGDIILSKPHNVSKKVKL
LAFLIMPYVWQGNSYISSNKLRYCFSNALKQLNELCISSIILPDISAGIYGYSPNQSSNILLEECVESLLQLR
STIPLYNINSISFVDKDYNTCKIYSEALEEVSRNYLPHKKVMPAPKYWNMVNRRLLEIPSNVIYFCRRHNKIS
FKKYHGIIRRKYKNYYSNIRPFKWRASRVLEPYPFLLYKKSGEPSSYQYGFKPNYYKNISHTLYNINKKGLVN
IRVGSRGKLQALKKISNLCLDTKPRL*
>N.PF13_0081
MVPAITRKIFVKNKISDNVTIKVKFMCNQNFGGVKFLDNNRKTDESIHFKKEDEALLRNLLKNNPDINPEYNI
TDIEGGMDNLSIDLSLVFSKHGVKDLDNNFVKDIIKVFELNGYRKMLDN*
>N.PF13_0082
MIYFKNIVLLSFLLLFFLFGIIQCTHFNIGPYEKDCIQLKAEKNNMIVGSYEFMDRKASCIISIFNRSDKKKE
PVFKSTKIQDKFEIQVPAAAVYSFCYDNRKNSDVTIMFTLRVKESHNVNDSELSTIDDVKQINEKTSELFDQF
LEVFDEQERMMEKSDLYKQFNEKMNSKLILWLEIQIILLVVLTLVHIYYIKSFFEIKTIV*
>N.PF13_0083
MKRIFRLVRRCHYSTEKRTNGPLVLVSNNQNIHFNLSLENFLLNNYNDLLKYLNINTIEKFNEPILFLWRNNR
SIIIGKNQNIWSECNLKNIKEDGVLVARRFTGGGAVYHDLGNVCFTFLNNNINTSSNFLIIILNTLKNHFNIEA
KTQGRNDITVNDQKCSGSAFKKIKDVFLHHGTILINLEKNILNKYLTPDKIKYIKHGVSSVNARTINLSEINN

NITCENLCIALIKEFTKFYEQNYKENINNIKNLENNINNSNFQNKEQININNTNENNLINNTNIIPNDITVHY
IDQNNNITKNPEFLKYYNLLKDWDWCYGKTPKFQNHIWKQFTFGKLELFFNVSNGFIKDGNIFSDCLDINLID
HLKSIFNNDIKYSKEDISIFFKKLNVENKNYLDEVRSWILQEL*
>N.MAL13P1.75
MKQYLSEQERLKLKIRNNYFEKNAIRSKIVNYLNRLTDLSIDHNVEDIKKIKQKRISSNENLDKLPEFKIEKR
PKLEEDETTVNRNKRLLQIGLFDHLRKAKDALEQEKTDEKIIMHNLQNKRVEEKIHEVKRNFEKIELDDIEIK
IIAYVKDIKMIEECIKQDEDKLMKLNLISHYEKMKNFISTNTYPTIFWCPLKFNDKTRILQQNTEEFIKKKID
AIKSTNYQVEFKDENWLDEFDKLKRIIKNKNKEYFKNKSDDDQNNIYENDIVNKSASHHNDDLEEKKCISVYN
QKSDKRSMSVYSLKSDELNKDDKKNNSPKSENDNIVEDKKYIRDEQNQNECDYKNDDNKLMVCKNEESVLEND
INENNSMEDTLQEKYYIKKDSRVMKEKNPKSPKRIKTESSDDSIKYVGDGNEKVVLKNNIVQEEEKKYMYKFF
EGEENIAKSHAGEENTGKSYEGEENTGKSYEGEENTGKSYEGEENTGKSYEGEENTGKSNEGEENTGKSYEDE
ENTGKTYEDEENTGKTYEVDEKQKGKAKRGRPKRKSKRKGKIK*
>N.PF13_0084
MDELTYMYKYKNKNEQKVIYVNNIGNFLKKIEYHDDNKICEVLKRNDLLNEDNKYLYQGKNGLLNLEKTFSHY
EIKTGDTIYMLPEPDPLPYLIYLFHQESGKVYSIELNHMDSIDMLHKQVERVLKIEEENQMLIYNGKCLHKKR
TLKEEMLTRESLVTILDKRDIPDIQNGNEEL*
>N.MAL13P1.76
MQNPQNSENKVIFVEDVVRDFDENEIFEEITGKFTWEQDVERSWNLLVENNGVLQHVSQENNENENKEKYRKN
QGSSLRKGIFRHIIILFDMSSSMKERDLKPDRINVALECVEFVKNQMVFIKYVQVKILNEINNNAETPLWMQG
MEPQLIHICFPTKKKISTQIMCSCHGKLNTDTYVCNFCNSYTCKIPSKCKVCGIHLISMHDLSHITNNLQGSP
LFIEIKNEQGNYKVCSSCNQQLYNKIYQCTKCQHIFCLECDIFIHEELNQCPFCLINDT*
>N.MAL13P1.77
MAGTLNLNDLKDEAILYYENALSYVFSNSSDENKKEIPLCVIYPNETIKVGFPIYSIKNINKNENVMNEDDNN
NNRFNEEPLITYASESKYLYPTKVRVFYGILYLLIFLEVFVTLYFKGNNNINPIKIKRRKKLKNNVICLKCIW
KYII*KIFTYKYIYNIIFLYFIFVDDINYITSGEINLGQKVIYTLMCISHFLVIMSKFKKCKSYLFDIYTSLS
LFFFILSVLTINCFSSAFISIIYIIFSFYFVIINIYIYIFIYLFIILK*
>N.PF13_0085
MYLGSIKNFEDDFLCKGEYKQVYLSLEQNENNEAIEIDICCDNNIYDILQFCREKYGIYGEYIIDSCGNVIHS
IKDIIDGDTLYLKEKKEDMNFFSKIKNNFIVNDYIVEKRIGSGGFGIVFQGVHIQTKQKVALKFIPKSNFLDV
TDVHRVFIEIQTLRGLIHNNIIKMYDVNHFQNYVCLIMEYAINGDLKNYIKNKFNGFLSEKEAHDLFLQIVKG
VYYCHSKHIVHRDLKLENILLDEKMTCKIADFGLSDFVNVDQNIKTEAGTKAYIAPEIIFNQTINYSVFKLDI
WSLGILLFIMTQGFAPFQYMEKELKNFESNTLNYANDISDDLKDLISLMLNVDPNKRPIIVEILNHRWFENYK
ES*
>N.MAL13P1.78
MCLYIIMYLFLTKRCFFLVHNNIYRKIFLSTDFLKKVISIKCRSYSSLKSIYEYDYRAEEKLPYDGLIKNVSD
KKQSLLLSDYIKFDDDKKLKHILFTNNIEDLIHHFEKNLNELKSHEYIFLLFKIYEIIFYNFYCYYEDSEILK
LENPNFIYNTFTEYMNKNLKDYSDKIKSLDDEFNDEIPFENIQKFNDEKSEHTIEMNRERDDIYDINNNKNDS
KENNNINSRCITNNTKYNENIIMNTSYHSDKNILESIYNNVVNKKERDNDKTYKFIDKNKVINNVNDIENNNP
DIYVNSANCPDTKENNTNEDNHMINIDINKYNIKILIKIYYDYITYIEDLNIKEKCWKEYVSSFFLKNKKYEN
LYCSILNTVYKKIYFFPCIDIINFWYIIKRINIYSIKDLSSLVINKLMENLKYFDEKYLVRLGYIYLNNSNKK
RNPHFISSNHSNCSNKEFIDMYINMIQNKVKNMNNNNFIKILEYVSNNNYKHIQFFKECKKEIYKRYNNFTQV
EIFKLFYLYSQNINASDKLLMNDLLKSILQICSPQEKYKNIHSHVEIICDKKHVDAKNELPYELQHNNMKTVP
LSNLLNVLWTNAKYFKDFPLLLKYSEQVILKNIKNFSSSTISMLIWAYSTVENKRREQLEFDQNIFVELKNKA
LEVFQKMTPKQLSNSLLGISTTIGRTIDNEGYVNTDFHDAVEKYLLDWDENNGKKKNRKRNKTNDKSDNKNCN
QYDNNKKDYQDGIKGNIPFRNRNFLNFFTSEDLANICYSYALVRCGSKEFHFLLQSCLLNQLNDLSPQHISKI
AYTYGTFYFYSSYSLLSSLQYEILQRIHQFCHLELSDILWCYCVNKFFDSKFWKFVLQIINFDKIYDPRCALL
YSSLSYMNIVDSSILESQNVLKIFNNLREYYWDLQVCEYPHAFANEIINTLNQENEEILSKQVDNNIKISKHN
DIYVDEKLVKENLQSVYFPSKKLSSDNSHIQNENIPTFEYVKKLIDFEGFLVDIYFEYKNEKYAVFLYTPLNT
TRDQYPLGENILKTRYMKKRKFKIIHLLYNVWKEYKHKKINLIYSQL*
>N.PF13_0086
MENYSNSSDPRTAILSEQNINTTLRMKENVNNQGDIKNIHEQNKYVDYNMYEQPENMNLIDPNNYRASLNNFR
NGEINTPSINLYNGEGYMNSNNDLMSKPHGSLNLTSIPTNDLKYYEYVNGPINLNEEGYYYNKFSMNNNLSIP
KNDNVINGMNDDMNNMMYNMNDNFNKPYLHINDEYIKKEMNNYKEINSLHNMYEYINNNNNVHLINPQYMMEN
YHSDLYHNLHNVPFNPQMYMNNFHNNENNNNNNVPLDIYHNESFPKNTHELDNREKKFSKLKQINMNKNNLPN

MDQLQAIVCLQKLEEIPSPFLNISEIKYSVSAYFNSYDDIIEKYQSKFYNCKLNQSISSYADCDLQNEIISLP
FNNEEFIYLKVIESSIYKTEVIGRLQLKVKSLSQEYPLRIPIIGDDGNSKGFLIMNFLITSSYYNLKNDILTD
KSTLTNKTKSARLRRRNQGFHFFENFTKWCCEITDHNIN*
>N.MAL13P1.79
MRGNYVRDGYKSQYRYANGSDSNSKTQNSNNRRSYYPKKGPNKDKSIIFCPHYTLRGACRFINDCKNLHHLKL
VDTFFLQRSYIDSAIIVHGPNNEIYLFTAIAPYTINVWIFVPGEGNKEIDIKHLKKIEFELTPEEESYGDEKQ
KYNNKSKQKKKKKRVLSLLYAEECIFAGLDNGLIKIMHLPSSDASTLYAHTDSIYSIVCIDGIIISSCINGEV
KFWKYDETCSRFVAIKTIETKTKIYKMIEVISPKAATNINNNNTEDNKYNRNLWVCGDSITIINLLNLEIVNN
FKCKYGNVVSVIQYEANIITAMSEGKILAYSLMGEGHFEMNTLPIYCMAGLTNHQNIPILIYGSNKALYTFSL
PEFNPFGYLKDRDSASLKFNLNDPDFIISLSGPYFIVIFSNAGNAKVWKWETKE*
>N.MAL13P1.81
MGSFTEDKKEIINLEDAYKLYNFNRDNTLSISQENDDCMPHSIILDNKSLNIFNMDSAKYQKIEYKRIFNLNS
RKFTDEPNFASAISYEDVLSDSEETKKNEEENDITQPRIHTILSFFFPFIGCLSYLINLKYPEPSLRRQYAKK
ALCVGSALSVIYSFILCSLLGQYIYQYDNKEIYGYTY*
>N.MAL13P1.82
MKMKKKNVYLYIPNIIGYIRVILALLGFIISRKNLFLFVCFYSVSQVLDALDGWTARKFNQTSVFGQILDQIT
DRLSTSLLYLLNSSVYEEYITLIGLIMIADIAGHYFHSTSCAIAGNKTHKKIEKGNKLLKLYYEKPWVMVICI
IAYESFLICAYLLRVAVKKSLIYKLSYYALICSFPLAAFKMFTNVSQGVHGVKCLVDMDCKKK*
>N.PF13_0087
MIKEGNCVVIYSDEENIDLLKLTNDGIINNKKGRFLHKDIIGKEYGSKIYDTLNKNYIYVLKRSPELIVNCLK
KKTQTLYEHDISFICLVCNALPNKKILEAGTGTGCLTYALANSVLPNGVIHTFEYNEERYKQVQQEFDDFENI
RKNITFHHQDILNYNFDDFPLEYFDSIFLDMPNPWLCVDNMKKVLKERGVFVIFLPCIEQVYKILEALEKNSF
SDIETYELINKSWKIIMNKKEKKKKNDDDYHIDNTVSFNSVNNDDKKVDNIDTLNNFYTNYRLCQKENKTHTG
YLTIAKRCLNDEHEQPEIKFENAF*
>N.PF13_0088
MFNNNNEYECLCILEEKLVKHLDVIDKLIENIYDNINNLNEYINKKYNEIKILASKKPTRINWMKCDDKTHMS
LFFDKKIGFIPSNEDALKILDSQLMLSNYRKKYEYKKNSWTKKDIDKLFETVDITLKKYACYYLIDQNLSCDE
KINKKKMIEQSEPKQIFSQIKLFFDKYNKENTHNKGNEDNVNKNINDNISKNNITHTQNCYEPIEKEQDNSNN
IFSYTKNDKNIEHNFLYFSETFWNEVSEKLSNNQNAKECQKMWLYYGCFEDDKQKKWTKDEVDKLLCLSKKYE
QRNWKCIARELNTNRSPLSCFEQYIKINKLYENKEKVKLERIAFNVLEDIQLQILVSIIGDKNWAEVKKHMES
LNSNTSRIKKRKTNLNFFEKEKQKKFLNDEISYKRRYLRLISATNNMEQ*
>N.MAL13P1.83
MDELEVAILCLYGNEHSNINKNDAQKYCENFQNSADCWKYCMSKFLESNKLEVKFFCIHVIVEKISTLKIEDM
ILIKNSLYGYIEKKYVNANEDSCVLNKIIQLYLYLIEFLYPHNMNDAFKYLINLIMLNNDINIKTIHINFFLK
LMNMFDSEYIDNVCSNKSIQTTTNIKEAIKENDLPIIIECFYYIMNMNIPESTSLSIFTLSKYVPWIDINYVV
NDKILTYIYQTLNTTNSITEASYSFLTSLIRKGMNSANKIQFIESINIICILQNTPKITDLTFDVNKNIMTKR
GELINYICLELVESIFEINKLKDYQMNMLKYNEICTKAADMLFLVLPHALDIFSVNDFYIASTVEKFFSLFFT
KFKNVIDVGSCANSIMKSSSNDYESSKNNISNNINSSINSNINNNNANSGYKISIDKLNVFINTLICTIVNKF
EYPECIPDDYDEEEEDDDEFSTFFNFRENIEKLYQRLILFDKLKAIEIIKNAIIYLNENYDNLKWNNIESKLY
AFYVTTSIYCEYKQGSTNTSNNLNNNMITNNQNKNNIIMNNSLEHFKNIKEANEVNIDYNNLLFDCLIELLKN
RKILNSTNYHININLMEIFQRLNLFFIKNPNYIEYALHIFLTNGIRSNNNKIAKKSVHIFKKFLKTNSSVISN
YIKDILQLLESYLDVPYIYPKMDINNNILNNNNMILDDNTIKYIYTFLYSNKNYNHEYQIDIYEIIGLLLLNY
DFNKFKKLSKTDSGMSIQSNNNLSNNLNLNNNDNSININNNNNNNTINSSNNNSNDGLNQNISEEMIANYKDR
IFFFKGILNKLLENLSAVKNLYLNSTKTQHDNICASFISSVIIKCIGALCKNVNINITDILLNDLDNTLGIII
SESLELFYNNYIVRDSVLFTYRILSNLFKDLSLNYTVKILPYFYNISYNMIMNKLQKSSVDNQNANTFNMITP
TSYQSVDGTTNNPMKTSNTHHEELKYLYNELNELSILVCHLISTHKEKSFDTFVNPYIYNITQIHMNIWKFIN
VQSLEMQREQNAVLSPLLLILYNISVNIPATIHSFMSFEHIALNHKQFCDIFSNDDIIKSKIADAITSILLIS
LNYKNTSDINICLYSAQTLSNMLNNATCMTNPSEVLSKYPIMQIIDTLCVTLKSLDYADPKTKRIMQEVMNIF
RLFCGFKVSANCLPNKIIESSQICLQNSLLSVFKNNPNDIAILIQAINANNQQQFRQILSNIVA*
>N.MAL13P1.84
MNKMKSTKIENPKYKLMKLIGKGTFGKVYSAIDMSTQEAVAIKRSPKWRNKVSREVDLLKKMNGSTNIVKIKS
VFYTTTKKGYRIQNIVFKYMTYSLGRYIRMKKQEKRENKYDRINPADLKNIIYQICIGIKDLHKNDFAHRDLK
PDNILIDLDSSNIKIEICDLGSAKKVQRNIISIPYICSRWYRAPELLCGSMFYTTEVDLWSLGCIIFELINLC

PLFPGKFKKDEYSEECSQIINLIEVIGSPQMSFFENIKDHTSKKNTLLIKELCELNIKPLCWDEILGNILEIE
EKELINIIDGLLKWNPNERLNIDAVLSNPYFSTLNK*
>N.PF13_0089
MNVEEYRHQVGGHCKLIKPKDSSKVYKPLIENEYIFYKKLTNFGSSSTESGPLHLLKKFIPKFYGVTEILVES
CSDDEEKQNDSSNHIKGKDEKKKKNKSRYKKYIKMDKSEKENFVNTDETYFVREQKYSEQIRMRENISNGDIH
ENIQKNMSSGELKEHKVNHMKIVDKHNESNMYGDAYGSKNDEMNKNNNEMYKNNNEMYKNNDEMNKNNDEMNK
NNDENVKKLYNENNTNATPKKFKRRKECVPHIILEDLVYGFKRPCVLDIKMGKRQRKIGASIEKKKRQVEKSF
KTTSHSLGFRLCGCQHYNKVSDTLFYKDKYWGRNLSKEHIPWAIRNWFWNGSLLYEELIPLLLEKLHSFFNCI
VELRHYRFWSSSLLWVFDGGLSDKKARSNSLDIRMIDFANTIYLQDNPSADEEYIFGLRNLIESIQILNNSIH
NIYFLPYEITTCFYSENYNMKEIKDRKVLKKSRSVVYEDNKKKKKKTVYINMEFFKKAKGKYGSNNNKQLDNN
KLVSNNKHMNNNKHLNIKKNDIKTKYIYNSDLLNTENINKFLSQNEKEEIHKNKKQNRNININKIPKKKPKKL
YIKHNKYQSFSDYAIREDIYSTPLFSFTNKLHNSVSNNPHNIKKTHNMNNFGIHCLLNNNSVSTSRVEDNAYM
EEIFNKYKNYEYNNVYDKNIGSSHNDRDPLVDTHLYDENNKILYNTCLKENDNIYYKGLENTMDKCMNNTMNN
LFHIQNYNYDKQYNNKIKTHHTSNDRTNINNEEILYVNKNISRNNNIYINNNERKKKNILLKKSYNMSMQNKN
LPYQIDECNKNKTKEEKVIYNINDNSPIFIDDKYKEDILQNKIYFDKESLENIQENQNYILNDHTDVLKYMNY
NMDNKCYDKNINININISNNNNNNILLTYQNKRKNESISDNNLHTKSDNKYDEIIQETIIKTLKIASNFYQQIKEH
INIDQQVKDQEKVPQPCSDDDNIKNNVQQGEDKIKKKEDYLLSYHNVADDHFNNNRCEDNKKDFYSFNFDESQ
KKKNSIETYKGNINFQTNNKDDNIKIKEKKKNYKKEISNYYFEKGEMENINVFPNEEENVIHEKKNKDIQVFK
TNNIHDNNVEKNNINNIKIIKNFNMLKRNINLKVLINQMIKMEIEKRTLEKQQLLLKNKQTKKRINTHTNKQI
YKEINTHTNKQTSKKNILKITNNEMTDFCDINVDIIEVQNKIKQMDIKENMLNKMIYRSEKDTLYKSSKNNIY
EQLNYNSDTILIHKDFKSCHLKMNNKNNYIQDFNMNKRCMSCNDDIISSLKNDNKIKEKKEKQNKKKIRQLFL
KKLSMLSNMKSNKKKYHDNVYYNGDPDNSNEEKNGEYINKYNDKYYNEYDNKYNIQHNDTYDDKYVNTINNIP
LRFSKYYLKEKKKKKKYLKCNQLNMIPLHKKKSIYKNEYPENNEEFNIFNMNYEKNMYTFNNIKYTNENLNYI
NNIYENNEFETYKNLILPLTETNENYKYLRRSLSEPNIYKYNRFMGTQKDSYVNKINYNNLIKNRLDKLTKVP
IYNQIYGFTSNSSNTYSSESSLDM*
>N.PF13_0090
MNVLDVLKRWFILVFFMLQRFFEFKSKITKKSFIIFGLPSSGKTSIIYFFKLGYLITTVSTLFINEENFKINL
KIEKDDTKEQNYDITFYEVGKNCSYNLIKEYSDISNDVIYIVDSVQKGNLSEARDDFIRILYEFRFIYRKCKF
LIFMNKQDSNGCLSSQEIINFFALPKDLLIRCNFISCSTLSGQGLKEGLEWLLYYNLPFYNNELNCIDRRTAT
KYLEL*
>N.PF13_0090a
MRNILFILSFFLCVVYAQIPYDVQRIYKPSNPQSVHALPGDKVASDYKFKDTKMEVEKDNNKNSLKWLAQKYY
KDVDETNKNILEELDQLSDIIGERTLYFTIF*
>N.MAL13P1.85
MNKPDMNNFLCQFDFSSLQELDPGLVDGYNLSYSKEVPFEIRMQEHESKPQEVGSLDVICVNIFVLGDELNAQ
SIKIVLTSETDLFFHFTQTVNENDFEHMQNNQKLMINFSEYLQVLIKMFNSCIKDPQSFLAIFTIKQNGIAQL
EFIKNMEYKFIELLVCQFIKSSDEITKENITYRYNVIKSKNGIMYNRLKDISILIKTKNPSLLMQLQKTASKQ
MEIFRNKKY*
>N.PF13_0091
MIPNMPNIPISSDIENVPNEQNITNETNITNGEYVSSGPNVSGGPNMPGGSNMPGGPNMPGGSNMPGGPNIPG
GPSPPSIPQIPNLPTIPGMPNILNLPNLPNLSNFPNFPGLPNIPNLPGIVPHNINNSHFMSANPMNPIGMPFM
PGLLPNMNTCDYYHKNLMPMHPGYDNYNNIMYGQPNNLGMPIPPNNMENINDMATNNPNMIKIYNKDSIANNS
QKMMNTHLMNLHNNVNANYMNNYNMEKHGWVEMVAKNGRKFYYNSITKCSKWEKPNELKTKEEIRISEKTKWK
EYSCSDGRKYWYHEEKNISVWDEPEEIKKIKLECALEDKELENKDNNKKENIKDEEDPEAETNDKSNASNNIC
DDMYNSNGEYKMNEKNKNIPFNNSSTNYVNVNNIEQINKKIDNNNNNNKSSLTWEKFDNKNDAREHLKFLFEE
KKVNPKMTWDSALKILEADNRWSSLVILTKGEKKQLFCEYISHVIKRNNENERRKRQKSREIIFQTLLNWDKL
NECTTYVEFASQFYKQEWWEWITEKERDEVFQDFMDGYKSKFKETRRKKRKQKMEILKQKFQEYATDNKNPLK
WNDVQKYFRDDEDFHSLHKIDALAAWEDFYEKYHNVEKMKLKKKIYRILRKKREAFIELLNEYYENNILNMKT
QWIFFVSKIYKDTRYTDILGHQGSSPRILFDEFIDSLQEQYLIHKSYIKKAYKEMDFTIDENITLEDFLKTFS
NVQSKYNIPDANMNFIYLSLQKKLKQKKNKEIKHINKVAKYFSKFTELKPSMSYNKVISIMKNTSKWSVVCTL
CPKEEQKLAAYNMWKTFINSQDLDNSSDSSYSNKMYNKNSRKKDISNSKEHEYYDDEDKTTSRNNKYLRDDES
DSSAQTIESLRTIENASS*
>N.MAL13P1.86

MIIKVNSVQTQKSQDGNKVHNSQDDMDNEEKSIRIYADGVYDLLHLGHMKQLEQAKHVDKNVTLIVGVTGDNE
TRKFKGQIVQTLEERTETLKHIRWVDEIISPCPWVITPEFMEEHKIDYVAHDDIPYANNQKKKKKKKSKGKSF
SFDEENEDIYAWLKRAGKFKATQRTEGVSTTDLIVRILKNYEDYIERSLQRGIHPNELNIGVTKAQSIKMKKN
LIRWGEKVTDELTKVTLTDKPLGTDFDQGIDIIRDKVHDLFKLWRYHSKKLLKDFAKSFDPMFIIIRKRYRKD
NLSAIYLSDSNYFSSSMKDELKKKKSLSSTFNNIDEYYYSADEEDTRDNSFYRVINKIANHTYYSEKENYETC
FELEHSLRDKKKSLIFKDKNMKLSPDDLLHFTNPMNSKYDHNYYYYNNTYDKLSNVTFNLNGIKKKSYDNESS
YIKTAYHDIIDEDKNLLNKEKSFNKDEKKKKKSILETDQYKNCEDQQFDNKYNHIIIGKKKSDSTYDNSLDKE
SSNEKKLSIDKGHDKGQYSNMDSSNYFHDCKTMLSEHNESIESSNNDINGKQKEHIKKGNSENQDVDPDTNPD
AVPDDDDDDDDDDNSNDESEYESSQMDSEKNKGSIKNSKNVVIYADGVYDMLHLGHMKQLEQAKKLFENTTLI
VGVTSDNETKLFKGQVVQTLEERTETLKHIRWVDEIISPCPWVVTPEFLEKYKIDYVAHDDIPYANNQKKKKK
KKSKGKSFSFDEENEDIYAWLKRAGKFKATQRTEGVSTTDLIVRILKNYEDYIERSLQRGIHPXXXXXXXXXX
XXXXXXXXXXXXXXXXXXXXXXXXXXXXXXXXXXXXXXXXXXXXXXXXXXXXXXXXXXXXXXXXXXXXXXXXX
XXXXXXXXXXXXXXXXXXSLQRGIHPNELNIGVTKAQSIKMKKNLIRWGEKVTDELTKVTLTDKPLGTDFDQG
VENLQVKFKELFKIWKNASNKLITDFTRKLEATSYLTSIQNIIDYEIENDDYASSNFDDETSS*
>N.PF13_0093
MNMNLVLFISLICLFFGCSNKLYALRNVTRKSEPMSDKSFIVNRLISYKEEIDSQIKNFRKYYELPNEMDFEL
KELWDLVNIVSESQESQMKELFKFSHSLEQSKLSLFKMLSEYENRIDQLEYNIMEKKLYDPFIEKFIYPISKY
WLKIDFNNLRSYWKYENNKNDFSIIQANNISNINIPATILLLRHHKIINGYINVDIKPLFDDVKNLKQSSSGI
IFDFVDYSNYRYIELAFINSDGIISVHQIVNGLGTKIVSKQVEADPNTFTTLTAEFLLNKINVFLNYKMIIEI
NITNQMIDINVGLFSKIGKAEFKNILSGNIDLEKLLSRKFVEFNEENISKVNMVNSYRFLNKMNIDNLGVYEI
PYNYNKKDYDNFEDNIIDNVYAVPDSDSYYDDENITGWVKTNNINNNEFENNYILNKNINLIPSSCTNYNSSK
NIFYLTEFIINNSSVHNWKITNEFGIQRIHFNNDYKKSIFKASLVYKHSTCNSVTISSYIKLEYNSEAGLIFR
YENINNMYILIISTNTKEILLKKIINDIPTIIKSKYIHNINTYQRHHLLINDQGQNGHISIFLNNIKIFSINN
EHFYKSSFYGLYVQSGYATFDTFQIEAHKKNFNAININK*
>N.MAL13P1.87
MNHNFKRSLDINNKNVDDNINKINSIINQMRLVDENLKSLFSFEETLNDHDALLLFRGRVSKRIVDYSNLITE
CDNNLTCSEYISPNLKEQYEYHLKNIDNYKRELSVWWNGRANDYHRLCMENFLNRKISDINVTSNDDDRNKLT
DINLKDTKKLMIDEINRMKNVKSELIESSQKLKKQDEIFNIFEMKIRSSAKLIYSLKKKAESDTRYVWYSFFF
FVSICVYITMRRLGLLRAMFTLIKLIVSILFYVSKLCFKIFQLFKKTNEAKYTPTVDNSKSLVLVPINNEL*
>N.MAL13P1.88
MTKGGSNHKNNKIKPIDINKKLLIIKCNDDIRKLINKESPSVDEINEIKNLLENNEVKIEKKKRNIVIPRFKI
CEDSEEEKNIKNKDTINNDEKRKDNINDDEKRKDNINNDAEKNKSLSDDNNNNNNNNNYYYDNDDEKKYSITK
FEKPSHYIRYELYKDQVTGIKLSDGSIIHYDLLKEDEIFLEGLNSYLNIHVNDESFSKLIDKFEKLTGNSDNK
EEINFKEALKAASDLKINYKSNVIKDIYTYWKNKRKKLGRPLLRMFWNNSQNSLPHYSVFRPRVKEKMTLRKH
KKKNSEIIIKMQELIQDFRRLDRILRKIKQRDEKKLLLLQLNAILFDQRINEIQDKTYVCPMWNYFKDYKIEK
IYKKFKKDKYYKNSYHHHYNNNSNNNKYYYDEKEYENYNFDANEDGYVNHNYMDEYMNDNMDEYLNNNIDAHS
HYNYRDSIYNRKINKKLKHYIIHPEQKNLLKINPSEYKNVVLIKRRGRSNRMWVDRKYVDENNINNHEINYCD
LSYAFNDFVEASLTLKKNYEEDVSNYLSGRTRIPIKLDIRKRDLKNVGDFNTSLFCNNQIHDDSLFNGMYDEL
NGERKRKRRKKKEQENLNVDENYINSNILLDNMGDQFALQANNNIHLDKTKASLNDNNNNNIIICNNNLDIIQ
KNSTTNVNVDSSNMNTTNIVTCDRTSITNDHDANNTDIKLNYFINKGNKINMNEPDYNTNLLFHKNNTEENML
NQYIQNKNNLYNINNINNINSNIKINEDNTKMVSHNILSLEHQNLKNQDFYEKYVSTNITNNYNNNNNNNIPY
PLVSRNDKYAPCIFSKEQNNKTIFHFEDMIDPLMNKDNYLFCLSKYVYVQKKILFYLEHLLNFDNYNFKSKKK
ELPNNKDMNINLKINTNDNINNDSLNNDNINKDNINCDDINNKKNDDLINNTLSELNGTCLKSKVSHKSLKHK
TAKDNNIK*
>N.PF13_0096
MERKSKKNSKSEKTESASKKKKKKDDDMSEPEEDEQKINEVEDGSLLLKSDKTENNVNNNNNNNNNDNTNYDN
QKNNLLYKKRNRVCPYLRTINRNLLDFDFEKLCSISLSNLHVYACLVCGLYFQGIGKGTHAYTHSLEKNHYVF
INLETCKTCCIPEGYEIVDASLNDIKYFLKPTYNKEQVEHLCYNSVLGKSLDGGDFLPGLVGLNNLKNTDYCN
VIIQLICSIIPLRNTLLLYECKENIAKNIIVVLSDLIKKIYNPRNFKGVVSPHEFLQAVGIESNKNFKIGTKK
YPLDFFLWLLNKIYRHSNKMLKKRKLSTLKKKTGYMKRKLEDNEPVSKEEKKNLHDKEKIYDEMNKSDNTSNK
KKEKDKWAYSNINIVDYCFDGELLIKTIKEEGNEESQENMELKFNESDNDSKDYLKNINEEEVEEEEKNKQKN
DYQNIYNNTFLNLVKKNKNDLIVKIPFRTLSLKLPNAPVFKSTTESNIIPQVSIFELLSKFDGETETFLQEHF
KIPNTLIISKLPKYLIFTIERFSKNNFFIEKNGTIVNFVIKNLDMKDYVHEDFLSLNPVTKYNLIANIFHTGT
VNQGSYKIHVLNQPTNEWYEIEDLHVISILPQLVLLPESCIQLYQRQDVKLNGDI*

>N.PF13_0095
MGTPRRRLGQQNNNNNSPFALSSSNIFGSNNEIFGSNFMHTPMSSRRTKNSKSFLNSMLNESRYLNQSNAGSQ
FIKYGHTPLAIRRIKCARADIGDVGREAFMEDVESGRLPHFIDSNLEQIKELFNQFFDEFNITNYSDVLDFTD
EDRSISEYILLHRDNLKVYLAYYGWKMIKFIETGRQNECRLNNTNYEDDDENNENSEGIRNLEHIKSFEIDLT
HIFFFNKKLYKLIIEYPSDCISEIDKIISTKYNSLLALVLEGDTRSSSSDKYPLSSTKQDYCRVRFFNKKHKD
TPRKLGPNQIETLVCVKGVIIRCSNIIPEMTMAAFKCTSKKRIGVNNYEKCNEEVYEHVIQGEVQEPVTCSNC
NNKNTFELWHNNCCFSSKQLIKLSEVTEHLKQGETPQSISIYAYDDLIDYTKPGDTVELTGILKASPVRLNPR
SRCYNSVHRTYINVIHIKKENKQKMKLTEQNDTANIILKRNEDGTVEENFEKLNEQGNLLFTTEVIQKMEQLS
KDPNIYQRLVDSIAPSIYGREDIKKGLLCQLFGGSKITDKYNNKYRSEIHILLCGDPSTAKSQLLHYVHKLSP
RGIYTSGKGSSSVGLTAFISKDSETKEYILESGAVVLSDKGICCIDEFDKMDDSARAILHEVMEQQTVTIAKA
GIVATLNARTSILASANPINSRYDKNKAVVENINLPPSLFSRFDLIYLVIDQANEDEDRKLATVLCKNFSYNP
EEEEDEDQEDQEEDEPNYITQQRARKSKGTSRKNERENYYNDGDNDDDDDISNYLNDSNDAQNKRGSWANVNI
SYDEYNNSSNKKTSKNYLIDSNTLALYIAYCRITCNPIISLESKKIIIEEYIKMRCKEGTKSPTASPRQLEGL
VRLSQSLAKMKLKRVVSPEEANEAVRLMNIATFQSLIDPLSGRIDFDQVNLGQTSQHKKKSDLIKDIIMNALV
LKNMTKDELLTHCHETIMNDPQHTTSMDRKSFEEAFYDLEKSQEITRLCSGLYKKKKKKNSTRCDKHKKWIHK
YIYVCMYVLYYSFFLLFS*YFCIIYIIAKKKKKKYIXXXXXXXXXXXXXXXXXXXXXXXXXXXXXXXXXXXXX
XXXXXXXXXXXXXXXXXXXXXXXXXXXXXXXXXXXXXXXXXXXXXXXXXXXXXXXXXXXXXXXXXKLKKKKK
KKYIYIYIIFQCISS*
>N.PF13_0097
MKERKKVSKVKESKKSLVENDNFCLNNYRNNFYMENDNKKNMKEEKEERKEIFTSRNNDSLVDVDYNKMMNDI
NKYENTILNSNNSRCNNFPNYDQIIYNNNVLSNGPVNNSVNSNNTNNNMMNNNMMNSNMMNNNRMNNNIMNSN
MMNDNMMNDNMMNDNMMNDNMMNDNMMNDNMMNDNMMNDNMMNDNMMSNNIMNNNIMNNNIMNNNIMNNNIMN
NNIMNNNIINKSNFSEVTNYSTSNKEPFIQTQNMYLKDANNNLNSCYVNTSMCDSICSNMSNNVSNNMVDKIT
VDHSCMSNNSNDHNNNSNCNYYYNNNNDYNNDYNNDYNNDYNNDYNNDYNNDYNNNYYNNTKGQEGQKQGKIG
NGKLVNMDNMFNINEHNNIYRERIDSLNTVNNTSNYKYNNYVNIINNDENNNNNNNNNNNNANYKNMENPPFKT
VTNMKYAENLLKDSNDIHVVKNDVSLFNHTCDIVSNPYNNNNNNNNNNNNCGSSNNIIHSNPLVENTSSFCSEN
HTSIYPPKNKKNNYNNNNNNNNNDNKQLCNFNNTSYNFPEQTHINCDLSNNNNYAVLTNKYNSNLNNESYNVST
CNNNNDFNHANNNNNNNYNIQNINIKDINVNIKLENYFNEVLNNNNNTKEYINNTYMNKHIDIDTQHMNVHLNN
SIKSINMDNNLEHINFPMMETQPNTPHIFNNILNNNHSNNFESDSVISNIKEITRNNITDDSSVIRNLLNNNQ
MDHKLVNNNNNNNYINNDIKKMDGGGNVNLQNMPKTMSIVTNAVTNSVTSSLGNVLENIKRPSQYMNNMSYLG
NVNKYINKNDEHLSFNKEDNIIGNNNNNNNAENTKNHNINSNDNNNDNNNDNNNDNNNDDSGNNNYNNHHLNK
HDKINNNFNSNWIRKLKSFGASSDNEDENEELPINANHNFYINNFLLNQHFNFNKNDNIRDINSKMSGGNSIS
EDNDMNHNNINSNNNNNINSHNNNMNNNNDNMNNMNNNNNNINNMNNNNNNINNMNNNNNNMNNMNNNNNNNMN
NMNNNNNNINNNNNNNNNHNNNSSNGKNMDTAMNSLNMLILPKIKGVRFDKSGRRWVASWSQNGNQKRQYFPVK
KFGQAQAKYLAIYARIQAVNCLQRKPHKPRMSKRLSEKKKLLLNMEGKEKNELGNNECNNNNNNYISKNGDED
DDNIEDSYNDDEICEDEENYVDIDHNDDEHYDIDDNDDNGDNGDNSHNDDNDDEHYDITNNDNDNNNNDNNND
NNNDNNNNNNNNNNNNNDNNNNDIKIMDQQSGDKEYVGILNNESKNVGKNIGQNVGKNVGKNIGKNVEKNIGPD
IKDEYMKSITNNDTQTKDQDIANETTKIIYPNNITDNNHIVNDNVCLIMASNNLENIPEDKKHEQNVTIHSID
EQKDQSNKFLRERKNKRMHNNYDNFYDNSFKKKLSDYNEEINKSSDEEKEVSQTNSLKYKCEEENDQNYDKQE
RKILQNDTNECEIICINLESTKQIDNLKDLSLHKEENNNNDDIYNIDNIDNVLLDEILRVQNACRKGKKKEIK
LILLNAENKMTLCFDEKDKINYMNDIDTIKNLDVNKKNLLLNKLKDYNNNNNNNNNIFLIEQHNECTISQINY
EDIESIKDDVLIEYYDCQENYNKNRNGKNTDHAKNVIDQKKKGENVFIVEEIKNCKNQISCDNLNKDKLNDTM
IHDELYHSIEFDIDKDDKKEETEYKENNKLLEKEYYSIMKSTEDNVEQVSTTQNIEITYSCGTNNSLNINNLK
NNKNDDIKKNITNNNNNNKKKINNNNNNNNSDGDGDKGSYNSYNAKQKNRRRYEYMKKLDMRKRQNNLDIMRH
VQNYHINNSKVIKKKKVLKNTLSLIKNTVSNNDDDDTYNNLCPLDVSNNDMVDSEFSLNTNFSDKNKKANPFK
GTKNKLSLFYNKNSINNIKNSMLLLNDIKRIVDSGANDSILNDNSNLSNKEDTNKVSKNSTSFLKQENIKKEK
NYELKGNSTINNNNNNNNNNNNNNSSSSNNNDNNLKGLFTGEKKTCMKSYLVNCNKIEKFIQNNMIEGYLNKKS
SIFNMNNEEENKLGNNFMKYNSSKHDEKNNVDLLNGENNNIKLYGLDSFHKPDLIKNKNVTKNKMKTIFSKKI
CSNENGKDHKGVVEDEYKVESVQQNEMLDGDQNYFTIFDDKNGEQNSSAIFYNNNNNHGNLHFPLTNQNEINL
CEEKRSNFVNPYIKNIINKDYKQNFINDNSNNCNILNHNINPINEENINYNNSIEYDKNDGNILNNNTKSTSA
YIYSTNRNNYNSTYHMDNIAYDNHKSTLDNKNCDKNIIIVDHNMNNSNNSYVLNNVRKDITIINNERASHGSK
FVLQHNMDNMENIDNMENIDNMENIDNIDNIDNTDNINNNIIGMVSNTSMDILSNNGEYISSPITNNLSYNHM
VNGNVVSEFIKNDDMINMDGVNDMNNMDNMNNMNNMDNMNNMNNMNHMNNMDNINNMNNMNNMNNMNNMNNMN
NMNNMNNMNNMNNMNNMNHVNSISDENVKLLNIENIQTELTSSNNNKLNNIYSNGYINNCINRYANNYSNESS

NNFTNNYKNNFNNVYQNPFTHNHPSDYNNEIIHMNLVQNNVVADKIRNMDNYSTYNDCYNNKEENQQRNNLRQ
NYNTNKVSNSIYKPIYM*
>N.PF13_0098
KEKHKRISRNIFLFVNIRYVLIYIYILYLCNFAGLFIILSIHLYPILSIYKAYKKYLYNKNIYV*HFVYK*IV
YNVLHDIIIIFCALRFYFISNRLISLEEIYSKNYVEEKLPIQKRVIHFLFLFIHKLDILSTHEKKKKKNITLF
*SMSNNIYIYIYIYVFFFFVEPCLLFGAPQATEEAADISLGEEKVEFLLLLNLVYYINDIIIRKHCEKFGKVK
RVLILEDNVYCKSLGICLVEFSYLDNTQNYSLYLKENLKVTVKKLDGYLEEQIKNDELYNYGGYLDYDIIEKI
KNEHMDILKNKDNLYDTLFNKSLNLNKHPLFPWFNTNLKDVLNTYIKSELKKKKKNQANNNNSEYTEKQYESN
SSDSDSDISTHVLNYIQKKNKYINYNNKT*
>N.PF13_0099
MANTWRCDGCLVVNSDDTMECVCCMQKRSLINDEKNGDVNLNGNTNNEGLLINVDTNKKKEDETVSENKMSGD
KENNISCFNNKLENEHIARIDVIGGGGFIPSIKINNNTNNKSRSIFLNNVSNSNETETTTMTNVDNNITYNSK
KNKFTDKEQKESKHSLDSSNKNVSVKSKKKKKYEKNHKGISKRVQPTRNCTKKKINYKTFKKKKINNLKNKTL
YVSILFFFLIIWI*K*IHININK*INKYIYIYIYIYIYXXXXXXXXXXXXXXXXXXXXXXXXXXXXXXXXXXXX
XXXXXXXXXXXXXXXXXXXXXXXXXXXXXXXXXXXXXXXXXXXXXXXXXXXXXXXXXXXXXXXXXXXXTCVYQI
KDAFSAFFFFFFFFK*
>N.MAL13P1.89
MGFFYSGFHKPNKKNMNDKRAKIIYVLGLFLLPSLVSYIGSNYYILNFFIDTFKPIEVPRESNFQIIKNIYHN
KKPPSKSSDYDDNTNI*
>N.MAL13P1.90
MDKKVRYQLIKHTLEELEKSKEYIKNQLKNKREQNNEKEENETYDDIFNKSNNFKNDDKKIDYETILDCLTKE
IDHYEKLLDENELNNKTDDNINNDNNKYNLFDDKHNNILNEDVEQYKICDFLEDYKENEKITFNKYKLNKKVI
VGHTTGGNSIVINNDNFLKLFKNSDIINNSFNKSNTTNMFLVNKNRNEDKLMEDNLNSNLHMDKDYLSDMSCS
TSSVISLNSFIKKEKNDRIYKNEEAYVKHKYDDNSYIYHKINYCSDCSSSSLSWHNQEYDDLFYYSNLLKEDK
HLKKQININKNKNKNKNLTKDNNKKHITDNHNCYNDGTYNLELFKNKPQNHNASPFFKYIYFKYINKDKNNFY
PVFINHKNGKKKIWNLKSLNKYHHVDTTQTNNKYELDTQLFSYKSHKYLSSVHDNSKKIKKKSSSKNVQNIDS
LKKYRFDVDEMNYSIDKIVHQIEQIHNIEKSDKKKTNYNEHFLNFIKKNKEYLKKENNFTFKKLCETEKIKLK
VKIIGTDGKIIKNRSLDNVVINKNKTFGDLALKLGHTFKLSDDKIENIKMFFDGDLCDKNMSFDNNELGLEDG
YQIDVKFPLTDDIPCTVDESNLSDDSYILFLPDSYVID*
>N.MAL13P1.91
MKENVLINENIKAHEEKGNLNDDKRDEHMISLRGLNIYYKDKLENLNTIKNIDNNKKFIKLVLEDVDLKPEEI
MKHILHLLKEKTSLHTILEINKQKYKIKYKEIIPDDHILFTQNIQNITNCKYALLGILKVSKYTKKNTFNEKL
LQSWQDVPMEF*
>N.MAL13P1.92
MEDANKPKKRTFRTFQYRGVDLDKLLDLSQDELIKLFKARQRRKFQRGISKKAKSLLKKIRKSKKNCEPGEKP
NPVPTHLRNMTIIPEMVGSIVAVHNGKQYTNVEIKPEMIGYYLGEFSITYKHTRHGKPGIGATHSSRFIPLK*
>N.MAL13P1.93
METKKKHSSGVSSSTASSATNEICLLFKLIQESHNRKYIINKLTELFLLIYKERLKRMLLYCSNVEEGRNDNN
INSDINNFDEKLKYFFRKDSIIFDIPRKQKGYNKYDEWLDVCFEKFLNLLFQLLKNDDEIFVKKSLSILFGSL
QFELKIFEKLMKTNYDNNNNNNNNNNNNSNNLYSNDNINVYDNNLNKITEDEGGKKYFPIKLYRRIIIELLNM
EHISVNTIKHICKSYICFYYDLDYFFLCIYNSLCNENKYVDSKKKKNNDPSNVDTNKKNNNNNNNNDNNVEST
KEKEQNGCNFNFPKDNHNINLLIFIILINSIKPYKKVCLNTNENNKKKKKIYLKKFKKHDLFIDDQENVSRKR
KKIYNNNNNNNNNNNNSQVHINKYYKFSEINDSHVNKNEVKKKKNNIFENYYSDDSSSIKHLSSSSNDENKLD
EEDFLKDVNLINNMNSDMDFSDDMLLENTKEKITNKIKERKNNFFINIHIDDKLYCNIYSSCWFYFITTHVHI
YTMILQLLHSIPVYVFPYTNNPYYLIDFFNISFYKSPNLYISLAALPGIFHILTELNVGNVINENNYSNINIT
KLENEKKDNLINDVMIKEEHISDEEKKEKLNISPNNSNNENVKEEDCNIEDNKSDQSNNNTISHNDDDDEEEE
DEDATKESNIFNDENKLNNNMYTDYYKRLYELITPASFYYDDTHFLKIIHLSIKNKMIPIYYILSFLKKLLRI
GCLTSYNISINILSVVYDILNYFKNELYEAMFISSSLFMIMDFKKDSFCYDNLLENFDKDKIMGMLKMNSLFL
KDTCRQDEDDPLIKKTKNESSYEFHNNINHIEGSNKGHIIINDDDEKKKKNLFNIQECIPEKYKINMNKLNKK
QLYMINHIFYEIILINNHICDNLKFYSNVYYYNFDKDSSYKSHDFYNDPSKMNWLKETSLFSSLKNFLSFKKK
RKTETTPTTTQNKIGTLFL*
>N.MAL13P1.94

MTSMKCLSTRVAAFILYLIPIIYSVLVLIFGFIEYKQKRTCNLLIICSLYAVIILLICLVGCLGVIKENATFI
RSTIILLIVNNIIMTMLITFLLIDVLHSPHVVTLHKASKFLQFMVQDKKVGLLFCSSIYSLIFFSFCFMWTIG
DYLAQLDARLCLEDLRQSHDSLQNKRKKNKHLSLGTNDEKEYLIKV*
>N.MAL13P1.95
MNIVILLLILTFSIKHSNTYKLKNTYIPINYMYHNNKNILRSQKSKLFLNFLSNNQLANSNKQTCFFKSNIKS
SISNIDNYDYIRKRYINTSNKNKLFYNITLRTNDGEKKIECNEDEYILDASERQNVELPYSCRGGSCSTCAAK
LVEGEVDNDDQSYLDEEQIKKKYILLCTCYPKSDCVIETHKEDELHYM*
>N.PF13_0100
MIKFSVLSFLTTLFLVVLKFHVVKNVKNKSCFLNTFGVSPIVTKLFPKYIDKSINRKVPITCKKSKIITFLNN
NIYEDAYDRIMNKLDLLIKESYDITDHINTFKGFLRKYLYEIKKHKSCSSQDFLNNFLIYIDTFKKYQNYEFP
NIHRYDQTLYEWSLKFWSELIDKKNSKFLGISNIKKIEEWRDKGHNIIIFSNHHIEADANIIKYFFHIQDSEQ
ISRNIIFIGGHKIRVDPLSRPFSVTANLLCIYSKKYIENPPHLKEEKTLFNHKSLNVLRNLLNKGKQIIWLAP
SGGRDRKGADGKIHISPFDPKIIQTFNVFAKRSKVKTHFIGLALNTYNICPPPNTIDVDEIEKERTCNYSPVY
LNLGQDIFDLYPNMDEGQITTYLYNYVNQLYKQIS*
>N.PF13_0101
MNENNVKEMNNKNENKFSKKGNENLYNKKSDEKLSEAASYKKSKKSSYIDNVYNKKLNLCEDKISYCDKKKKE
NINVDEEKKKVTVVEVGKNNISNVDLQKNNITNVKVQKNNITNVKAQKNNITNVKEEVENKSSMCKNVLSTKR
LKDVQKDKYNICDIKGKNNTSSPYIKTLSLQNVKKNPLSYSNDKIGKKNSQDLEVHKDAFATTKGNSLSFDGI
NMNNIKLNITDNVNDNNNDDDDNNNDDDDNNNDDDDNNNDDDDNNNHNNNHNNNHNNNHNNNHNNNYYNNTIN
EEKNEKEEVSFNISETPNAIGTNMGNYISQDEDNYNKSSFDKNDLDTAIGNAEKKGGLKQPLNIINEEINSHD
LRRNNKDIIANIEKSSKYYKEKKDSFLSTLKSKGHEKKEVYTSNLNQAKDEEIMIQTIFIEQNDNNDNVQNKI
DSIEQTIEENKNVKYNNMLSNKTGNILKKQSDILQKGICEEENYSSSSTNVATYIVNKHADSDSHNKKDYEIA
DGGIVENNIKNDKYLDDQFGEDKYLGDQFGEDKYLDNQFGEDKYLDDHIPSDELTSEIPNIMSNPHIQLSEDS
LENIKEKFDNVRSNYAEDFVDIKIYDEDSEIDDIQNNELDYFWNNKDVSNILNKESNTVIKSFEGNNEEDEKI
ILNSENCIDGVRIGPIDFPDFNIKRKNDKIIYAQMRRSQSDNLYKRKNIAHLINKRNTMDNIVNNENMEITLS
NNEYIYNNNNNNNDNNNNNNNNNLYYANNTLPEKNEEQTDDMNIADKLDIIKKKLAINNNIDIDNINYERFDM
YQVDFEKIGLNVSNLEKEEKYILLLYISEKKLEYVWNEREAFKRAHSNIKNFQVNQNKMRELKNEKRENAVLI
DTQYFKSNDMRNYETYILNKKNNKNVKYKSSNNSSTNNTNTFSNIGRSISLTSSFDMCEKKYNNSKDFSLLLE
RHKYNKMGNHQYVEKMNSDDYNDIISNDEKVQDVGFFLPFYRKLLKKYETINNVVINNDTTSNDVVGNNNICN
NNICDKRDEGENSHKYMSNEKALFEKFLKSFISLFFQDECIDQFLNYYDDMKTSKNEEDALLTHSLCIEYMCN
CFEDIYVQKDYIENDEKKFWFLHFSDIMKDIINNFIENENLVNKIRKIQIVNNILKDEVKTYSIKVAHEDIYL
NVSQNFIEDYENVIVSKDVGFFNLYILDKNYKYNYHTYTKQTYNQKMKRENSKDNINKYVDRYNQINYTNSFW
SYIFGYFNNIYNNWSQEKEDTDNSSCSNSAESLQNDIINSSKLVIQNFSNSDEEESGDISVTTDDENYPNLEE
QKCKNKNKSIIKNEQDIENTILHKRPKQNHEEKVSTDEILLKGNKKNKYRKNKYKNDDSKKIKNTKSVDTNIL
QNDEGVDTNINNKELNNYMNNEIKFNYLEKQDYILGSSTNIDDSIIESNLSNVSFHNSENSNDIKLDGFYVVN
KKEEKKQNITKEEEQSEMLNNNINIINNNNNNNNNKNNSNNNKSCSNIYKNRDCHISSYKNSKCDNRKYLGNV
ANLVSFQYKGSLPKENINNLKKINKNKNVIGCYLSSSDDSLVGVHIKSDMVQDSVDTHFVMERCRKYNKQIKK
SYVHILNANKKQYLAVNLNTYKIIYTNKYDDTIYMDEYNEQHKISTYFQLQSISDMMKNILIEDIVQSVADIL
C*
>N.MAL13P1.96
MYIEEIILDGFKSYPTKTVIGPFHPQFNAITGLNGSGKSNVLDAICFVMGINNLNLIRVNRLDELIYKQGQAG
ITKGSVTIKFNNEEKPSPLQEPYRDMKNITITRQIVLGGRNRYLLNSHNAKPKDISDFFQSLKLNINNPHFLI
MQGKITKVINMKPIELLGLIEESSGTKLYEVKRTNAIKLMVKKDQKLGEINKVLFEEIEPTLVKLKKEKEEYN
KFVSNNEEIEKYEKVEIAYKYYVAKKMMTKCEEKIEDAKSEEKILEKGIKEIDKDIEKYKIEKEKIVKETNTA
SEPMKILISQKEELEKKISQLKSEAKMENKEKAKEKRREDIKKEINNLQNKLDDYQKNNEKNNKNLKSYEDL
KKKIEILKEELNEKQLTMNCLLSAGTNNNEYTGSFREQLKNYKTNLSKAETQINNFLQNNKHLEKEIMTLKEQ
RKKYEKEYNEISKEKDIEEKKKKLCEQELDKLNKEYNNFMELDTLKTDKNILYNDMEKLQQELQVLKNIINSV
KIDYKIPSNMKSTDVLGQIYKLIKIKKEYNNTALAVHLILGGKLTYLLVQNKEHSKRLFEYNNFSSGSKRVTL
LPLEDCVISREVHEKHIEECRRNVGLNIKDKNDVIYFLDIMEYDKNLERIIQYLFNGTLICSNVDLCKKITYN
PNKKLSYTTITLEGDKFDTSGSMSGGSNKNINLFLLNYEKYKHKKEQYHDNENKLKEVSEKLKSLEKAEEKKK
IISKELQIYENNLNNIENRMETSKYGSVNKKIEEHKNEIDKGRNELSELYKEQKKLTEVIRKLEKDISEYEAN
KDKKEEDLKETIKKLKNKIKQLETEEHKKKEEIDDVLLQIENYKKQKEKETNDLSSTDEIINEIEKKIEDIEK
NINITKENLKELENKITELQSSFSSYENEMKHVVKKIEDLEKKKSENILDLKKLENTLLDLQKDLKTSSDTVK
YLYKTHVWIESYEPLFNKKYTPYDFENFRHDVIQKKIQALQNEQNKLSININRKAVQMYEQVQVDYKDLVTKK

SQVEEDKKKIQEVIADLDVKKSESLLAMYQQINEYFQAIFSTLLNNAQAKLSIVDGDLANGIEMKIAFNNNWK
ESLTELSGGQRSLLALSLILALLKVRTVPMYILDEIDAALDLNHTQNIGDMIRTQFPHSQFIIVSLKEGMFSH
ADVLFKMRFIDGISTVNRHALDIRQNTNKKEVQEVKRRRVTIHDKEPDHD*
>N.MAL13P1.97
MEYLILEEKYKNLLNKSNYENRLLKKETEILNKKLENLESAYIDTENKITEFIKDKEELEDYLYKIKRENLDL
KDEVSKLNEKIQDLKGLTKTYRKMIKNRNKELFESEILMAENINLRNNIQVVNNEKLSLESELNKKKKIINVI
KDKYKKNIGRLLEKFNQKDRHIYEFQSFIIDELNNLKEVILRENENMHFDETLMNNKFMNISFHLDILTKKLE
EKMTISIIE*
>N.MAL13P1.100
MEYLLNNTLKKCDNKLSFFLGSKYKFSSLFENSKKNFNPLRFEMKEIDDYNYRNLLKNEKAFLVYGYANYSYK
CINIFNKLIDIVKEDFENKADRNVSIYKLNVSNNNLLVKQFHIKNIPLIQLRYNNKLMQEINGNELNENNIKN
ILKRYYNYFNTLDCVNDINDFLVREKELSYNNNESFFESSKSYNNLKDESDNAYNELTFINDEKKQIITNEIK
NHFKNNILDKLHLNYNCSSYIVFKKLELLLMEKSNNIKLIKQFLNDIIFHHSTYLDINQHYSKITAKAFLHLF
DEHSISIEKLIELKNIILQNENQTDIKVDDIKLINFLEPIINFDNSKIIHYNKFQCINDFLSTSEETNDHINL
FLSSSIKKNILNTQLIDINQMNDHVKIKYLSRIYRILSIKYFHIGDIQNCLSCALHSYKLNAPLNNIDSNKSK
VLIENLILYYGAYNKDIIHFLSELQFLFTDKNFKVIKLPHTRAFKGGRPMMKRGKSGKWLWLSPDWKPRWLKK
KSKLILEEEWKCVPDKNVPFWN*
>N.MAL13P1.102
MHDTFYNNSKLSGRVTNPRTSHFSYTQKYDDALNSECKTSTITKCSSNINTPSIYNEQVIYHPTLVPRVSVIS
PYTPGIIQRKYNCLNSFPCLHAPSKIKDSTMNMKSNNPSSIRYINKSPCIYNNKEALLSDKFNRTYCNDNILL
VEKNMDHPHVDNKILMRSNMTLDNNLLLTKKNKKINNNNYSNSRSSSRDPKYVIFEKQKNMKQQYLMNEPFKK
RITKVNSHPTDMTNKINELIKEKNQEIKKLEESIQNEMNLNKIEDEHNVDNQKLKEELLDQKIKYQETLKHLK
KVETEKRNLKSNLNVRDRVLNKLEKEVSSVDHENELLLKLKENNATLDDVHHIFKYLYGNAKDLVEKNLKETQ
KKNHKNNTYKVLYNSNLFNNNNKNNGHNNFDERNSDNNINDNINSNDDNDFVLKEKNSIKNEPNNKKNKNRND
LDILKTTVVELEKEIIDLKEENKNVNFKYENTFQALSELRKDTLEYMESIVSRDMRIAYMESMLQKCEKDLSK
LRDQYTKQQIINRQQVEKFTSEIKILEKQSSHLQSMIQEKDSQIVLLKKEIVRNDILIKQYEERNKELQKELR
LMCNNLENVIEASNKKDLFMNELEKQIRENEVHRQHAYLKEVAKNRKIHADMKFKEILYDKSSKHQSDELHDL
KKELYLNQIQMQKAKEAFQILKNVPKSETKIKQKCETNDIETSSFNNFSEYKNEKESENMTFETKDSTDAGTE
YNIKNTTTKKKKKQTKLSA*
>N.PF13_0102
MSQFRDTFTKDSKKEPLLSYDDSAFIFFAGTVLICVLIPCTYVYLKNIIKRIFKNDYYNLKKSKNNSTYRSCT
CSLCKQKQEKRNKNTRIWERIGYIKIIQFFLLVLFWGLLYILINQMLNTKPMQTFDPFEILEVSIGATVGEIK
KAYRLKSLKYHPDKNPNDTSAAAKFILITKAYQALTDEISKENYEKYGNPDGPGMMKVGIGLPKLLIDEKYQL
LILSIFFLIFLVFIPATFIIYYQKQKQYGPNGVKIETLQYLTYTINENSRSKSFPEMLAATAESRDIEMKKDD
EQYIKTLMEELIEPKKRTFKIPVITKNYFLILAHMQRRHDLLSEDLKKDLEHILKFSLLITHSMIEISILRDW
FLTAQSALTFRRCLIQAFEAKNSSLLQIPHFDENIVRHVHKGKFSVKEVLDFVHQDHENRKGLVDMNPDQILD
VKSFCNTIPDIKMTAHIVVEDETHIVKGDVASVYVQIDRSNLKENEAAGYIHAPYFPQPKFEEWWIIATYKND
DRILKYVHVKNCEKIIEEKLQFLVDKVGNLSVSVFALCDSYFGCDQKVDIPFKAYSKTEIKREIFVHPEDIEL
DNEPTLFQQMLGDINKKRVSSDDEDDDDNEQGKNKNNSNNKVMKTKASADQENYVPEDDANDESDDD*
>N.PF13_0103
MSRLGNLINHCRNQNCCDDSMNCLNNHNDILLNGMCSKCCHIEHVLNMYSNELHRSYDEYNCNVCPLNSDNLT
HKRTEQISDNTYINSINYSNDIMNSDNKNKISHCSENVDSNKFSMDKKIIKYNYDCNVPFFLNSSICTKNVKF
PEFECVQKYNNYENVYDKNKTIYPIQNYEGNNEILNINNSDNPNNSNNSYMELLHENGNNTTYSFNKRGYNEM
KTQKILTSTYDEKDNLKISKKKEKCLSSNNVNIPVEIFNKCKNANDKSTEKTKPFEIKINRPHDIKSARINND
VIQNYRNQRKEEKDQSKYEKIKERSRSENECNQINDIRPYEKKLKSYSLDDIQKGKVDIPEIIFKKLPMNINA
DNIIESYKNNNKLLKFMEDCNKSTSLILKYPEIQKKGKHKKEQKTYKLPFEYSSFISNKLGSTIIYEDKFDRK
NYHPHVKNLHMSKQFEKHVFNILKNKKLMGKKLFHKSNKFKMYKYNNENGFWISNIDKQRLVCPEKNDSYITE
LIYDKCDNYYQEMDILKKCENYDNIIPSIEQFYKDNNFYYFLRKEKMYTGSIQPVIATIMKRYDIKQEHM*
>N.PF13_0104
MRGSTINDMVDSSIDYKSDYSREDKNDLSSSVKSLSSIDEINNKKRKDCTYSTFDENFFKSHCDVDEHTYQES
YMSDINKRKNVPSYLSKTRTLEKRDSCEDYKLRGDKMNSLKCDRLNSLRSDKLNSLRSDKINSLRSDKLNSLR
SDKINSLKSDRLNSLRSDKMNSLKSDKLNSLKSDKINSLRSDKLNSLKSDKLNSLKSDKINSLRSDKINSLRS
DRIRSLKHYKLKKEKRIKDERMKYIYKSYRSCPLKETEKTGIWIMDTVNEALDNLYDMNEKDSYFKNIDIIES
EMEDKNNKDIDEVNDLEDIHISCKDINEHMKIKWPDFLKLNNLIYYNHILNIYEGIRNIFNKYEKEKKKKINE

NGDEKYDEEEEDNIYDDENNIHEDILRTDEKHKYKDKHKVKNILNHNILKNKSNRLSLPKFPYKDLKDRCISK
CYKNLPEIHIINDENNMSQNIKEEGNYNDECDGDFTIQFVSPPELIRGLKERYIEFCDIIGDRLEYMEENGKN
SFKEYCMEVSRIFTSHIIDLVSLSHVPHFVYTLLDNFQGCIKNLSFEKINLRDPNRKQDHFDKESVNHIIIQP
YKCNINDDDKNYNRYDHNELFYGNNMIYNNSYNTQMHNNNMQANYSVPVDISCNNSISNYNSTMVVNNNKENN
ININGNNNDNINGNNNDNSNGNNNDNINGNNNDNNSDNNNNNINGNNNDNNNDNNNDNNNDNNNDNNNDNNND
NNNNNNSCNDYNYNYNYNYNCSYNENGYNNNKNKTFSLHPNYMFNNNSHKYDYMRNNLHNKENVISQNYHSLP
TYTRTYTNNRNKLMNSIISSEHISSKYNDIKNKCYNNNNNNLNILSRKCKTSSMNLPQLTKEDQFNNQEEDYN
FEKSSTNNSEINTNTETNMYMNNSFNITNQTENYYTSNSLPLNNSFHNMGPKISKVETNDFLECKSNYTSRYH
N*
>N.PF13_0106
MSSLINIHYDENNDEEENLIIKFADYETFKLYVLKKYDKIYKTCNYEKSVINYYSVKWINFINRKVPILLQNK
SGPCPLLCITNILLLRNQLHIDRKVKKISQKVLESKIMNILLESNKKNVTENNSSCNYRKNIIECVEILPQLK
YGLDVNCKFTNICSFEYTKGLCIFDMLNIPLYHGWVISSDDIIFYSYLKDYSYNVIINKIVRYNDYYEKKRKK
KHLLEITHDEKDIMYELEDIKEEYTPNFLQDRNKKKEKSKRKSVSPMNKNKTKNKNKNKNNNNNKNNNNNNNN
NKNNHHNDINKINKIKFNCKHNKKNYNNNDDDDDDDDDDYDNDHFGDNHNFSYPQFNGSFMKNNNLKKNSYNN
YNVPIFPDDLDQTSEDNTITKMLVEDINISDHNLNFNDIKLAKKVSLQHNNTHPCNNDFDTNNSSSISNHNNN
NNNTHNHNNNNNNNNNNNNNNNNNNNNNNNNNNNNNNNNSSSSCSNRRAFSTNIVRSSKMINNFCNMLEKKEDN
VQSCFFKENNKSNKLSSRIKKSQSFIKKEKNYNKLMLYNDLDAHNNIDAPKFPEDTLTNSNNLITNEYSEKNY
EESNIKLNNIDDDNFTNNEFFIESNKIESIINEQGFDNYTDNNTEISENFIMKNYNTEINLTPYEVHEALIIS
EFLETHKTQLTKVGLKLLHETLNPNQLVAFFRNNHFNTLFKYENKLFILAADISFLHLSCTWELFDNVENDTS
YYDNNFECLANQKTDKKDLNHSIIYSRNFDKSTARNNIYCLRSNATINNNKKKKKKKCTIM*
>N.PF13_0107
MAIYNIYMYYIYIYIFYYILYIYTKILKEELHVSHIMLRNIKYILDVMELN*IELN*IKLNKNKMAKSHLEIA
KQLKHNSIELKEYFEDLYSWQNDIKKKEEKEKEEIKLQKIYNNNNKKNNNNNNDEGLEKNTGKFYNKNKGFSY
LKRDCNSLDTYYKAWDKLNIDDIDRNAEQINKNYEHVNKNYEHVNTNYEDIHKNYEHVNTNYEDIHKNYEHVN
TNYENIHKNYEHVHNNSHNDIIFCENYKNNIPPENIPNSSQNNDNKTENERLNHKREQKNHVDEIQRDLYIEK
NKSSHDISKLNNKEQIIKHVCKAYDIYLAKNEEGKLNYQKKNYHRCLENYNDIINYIDYELKCDNIFIEIEKN
CNNELHIEVISDNYNFDKKKEELYIVRTKTLINRSLVYQRLSAHFESIDDCSSVILFYNYFLPNMKDNTTYNI
KDLGTMNIKNIIFKAYYLRGIARYKLRIYKLSLKDFKESKILSNDMNSSSTINIDNTIHVIEEIIEKSNVKKY
IRRRNEYTTTPVNHPRLKPRKLTIEIIPEEYTKGITFLDKLSEKNVHSTKKEIKIFEDEHKSNLSNDNYEAIE
YKKNNISSKKEIKNNEDSDDQNDHDNEILDRGVMQIDKDTYTNGIDKGGLQEENKKIKDKKKKSIFRNNYKEI
NNSVKENNFFRSQSIESNNSSITLSEESLYSNIENGNSPHLTSLLSHEKGLINNNINSNNNCCREQIKNKINF
ELLWNSNKIRTSFKNQIDILKTAFLEEGIFNFHLDKDMYVDILDALFKNKFLEIFQNIQDEERIKHILAINND
NNINNYNKKENNQFINYNDCIILIDILYHLTNFGKDNYIFLFIDKGERTILLNFLTFSIKNINTFIGAEKCIK
ENMLLLKSLLEMLI*
>N.PF13_0109
MIHAKENNWKKGSKRKRHFKEDDDKSRNSHICNDKNEEGNNTKESTNSLNKFIYEGSVKIKNKKNHIFYNKAQ
VFNRDMSIVLIKALEIFMKEKNKNNEKIIFRGFNIIELLSASGMRSIRYAKELEDTINHITTNDIDKYACKQI
KRNFIRNNIKKEKYTILCNDANSVMNILNVDNMYIKKRNNKKLDIGFTYINNINNCIDYFKEAFKFLNFLTNY
NRRKHNPLENVIEHDSDSTTFLSNDDEIITTKIDNSGEDETYIYEEKYDKNNIHCNEQHNRNVEDENKCLDDI
KKKDIIKKSKLIERYMFDIIDIDPYGSSIEYLESCLKYGRSNFFILITNTDMRILNGKFPDVSFYKYNSMIFS
NKVNYNNEFSIRVLFYKIKIIASKYKKCIIPFISINIDFYIRLLVHVIDDALQTKDVCIDTGMVYQCNNCSSF
HINPMAYKKDVNHLSCITENHHTKKRRKMKKNKMDLLENAANEINGDIKKDENNDDDNFKDENNDDNNNNDDN
IKDENNDDNNNNDDRNNHNNIHKSDDQNHNEAGGDANQNCKNAFTSYKYKSTKLNISKICEECGGDILIGGPI
YIGKLHNEDFLHTCISLLENLEQYNLNTIKTRERILINFRCLKQEINIPLYYNLPSLFRNFKICSFSRKLLVN
ALLNLNYEVSYFHKDPDSVKTNAPNNVFMDIFRAIIFKINSKKKNSNQLQNNKTDQNNLTNNENQNNDNSSVQ
TKKIYSIDTIKDEKLKEKLLSYQFFKKNTSFENINISNFKKETNTHKLFTLENPEPFWGPMRKHYEKN*
>N.MAL13P1.103
MENSTRKRNNLSCNNPKDAHFKDNVLNKKAVLYFDEDIVFDECNILKEDENLNLTEEEGHEELDEKGNKKVRK
RKESTQENKIGKKGKNINKVKKGENNDDKDQNEDDNKRNDKNVQRKKKKDISNNNNDDDEEKENDDDNINIYK
KGKTNIKNMDSFNSGLDSSTNSSVSARIYRSNTELKKKGSSQNIKGNKINIENFTKEEKILFKKWIDILPSTN
LEIQNILNHQLPCYNILNNIKRINKIKSIAYNENNNDEFLNSIEKSNLMFSNIKVAKREKYIIRFLCEGSSYN
LSELVNILYYVLNNKELILENFPHKKLSEKKNKKNDLAVHKVHLENNNNNNNNNMMMMMKDTCVVQDEENIKQ
INILNKNNDNQFEVHNNDNDDDKNQYEEDSNAHLTEKNIEINKNVNTDNFNLDNFSDEGYYSEGQDNQCNFHN

VTPITKEDLYSCIPILLSRKNLGDNMKKMNISKNENNEKECLWVWENDNYDIFPSYYKEIFKYFKELRSNMSK
IYKTLIKFKNSILSKDLPNIIKMSDFLNELKKKQYLESEKRCKKILLEKKKIMKKKMDIKKQEEKKKKFDESK
TVEEKKNNTLIKCDSNKIQNKVDDNKTKKQNLILSWLTFKKNQTVNTLNENEHMEQYMKYPIANVLNFNECMK
DKLLEVQSQLPIFTCDRKDAVFSNETLQNNSEYDKQHFLDSYINTCENHKKVVVDYYKVYVDNKEFYEEIPSV
DVIDGSKIVVQNEKNVRSKIWEDNEFYLKLKNADYIRSFYFYDNSWTRPFMSLLVHKVLDDNLNLLPFYYHDD
MEYENDTNEDYYEKFETIDLTTENEENETESDGSSQDMFIVPDNEINRDIELSPITIPTSQDIYFFSIFNWEW
NFEGNTKHNYNIIDKNVWKEFNFKYMENIYGCQYISWGVDNNPFKYMSDKNGLKNTLDNYDIKKLIKHSHGKM
TKKDILLEQFKQKNEHLTKADTERKFKMYLVYKKCEDNRKKWMASEEGVKLFKNEELLSKIYDIRKRKLNAMS
QRIEEAKKQKKMDKDNLKKEEKIKKKEKTIQEKLEKKESMKKDDNAKI*
>N.PF13_0112
MEKNILKKYKSFFNFVTNIKNNTEKENSIKYAQNIYLSKISKSNYNVKSNEDQIYDNKLFLKYWIRKKRPGAR
N*
>N.PF13_0114
MSTLENVKNIVNSVNVVCDISSISPKDIVKKEEKNKNKESYMLTTDTIDSNFDYIDNNECCDTTNVESTNEQK
NGSCHMIDDQKKTEEGYLLEYDNKMKTKYYNSLCNYFDNKKYILINDKENKETSKKSDNDNPMYQNNINKNIT
SDGNILKDEYEDVDDIRTKHKEKMLLCNNEFLHKYKIYGKYGNVSKRYIEESSFNELFKYDLICDVYNKKCSY
YKEKKKKNKLKFSKKNLTDSEVENYHIGQNIKKNNAKFYLLSNDFVNYEHIEEKNKKKNKNMNMNIYQYRNME
EKNVLKNIYNNNIINNNDNILNKTRMCYRNVPSFNYINNIDLLNDFSYNISENNFYKKNEKKKKLENVQDLDK
YNNHIDSKIDNEDKLDDNNKVLKNEQYIENNKDLGEIKRKCTNLKRNLNTDLMSSNNLKKKMKKDNGYSFLHL
NIYKNNNTYEFCRNNSYNTYILKTVGNSYLKHKINEKKKKMKSTIINTINANNTNNTENIPNKKPCDNMHIHM
YGSKDKMFYHDQEETVHNNNNIEDESIKENKNELIETKNYINDKELSKGTKFVMHKENVCTHIGNNISHDYIL
NDHISSDNISKEYIPYECFTNNEDVNSSNCLNKSESLNSQSSTNSAHINNMNSSGENETNCISVFNDKTVDLL
HLNLHDVVRMLENENIETLYTPEELKEILIPISGVYYSSSDEKWIYQYNNDDDNNNNNNNNNNNNIVDNNNNI
DGDNNNNNNIGDNNNNIDGVCKELNKMTSSEQTKHKSDNEKNISLNNKYSFHEGRQLALMLKYKYIKRNHTIF
KNLNDQEMFFSDINDHTNKEIYNDGNDLYDNTDNEEEHMMGSQSNKYELENDEIHKMTCEQINNMNNEENIKR
NEQKEMLKDENDKLKVKVKVNVKVMNKINDKETKIINNIHNNDINLVQKIECDEKIHFCDKEKEENNLSNNKL
KELHLNTTGHILKNEIISYNTHINSSQNINDIKDSMKKNINATNDTCNCNSLNNTINNNKTENVHNYYEDIKN
FNSSDNIYNEDKNEHVDGSTLLNHNNYDKQNRHIHLKNKKNLYRLLGINFTKDIKNKIKCLEKEHIYYEKEKK
KWIIQILEQKNNTILYFKEFDCKVFGFLYACFLSLEYKQLYLDYFLNEKKQDYLLNLIQKYFIKKRKPNRIIH
QLDLPKKKIETNKKDEEKKKKKKKKKKKKMEVNTSEMYSNVGKYLYEKDEIVNYQNKMENIKKNQYTHDDIEK
VENVLEYTYSKNYYSSTKDNINIYKKINQNFIQNWNEDKTNNEKIISIYQQNKSHDYNMHMLKNQTSKQYEDI
FGKTTYGSYKQIDKTMIPKLYEYNNMISICNNEKDIINKKYEQNLVKGSEQNILNYNIIRDDENQTIDHEIDT
SVDCEFHDKNKINNNEHVHKDIHNFCTHKNICLDINSSENVTLHDTHKYQMTNEKDDNKNDIKESDDNFINLP
NFPQNEKEITDKTLKKKRNNYSYKKPKNVKDSLAHYKNSNLSIELEEEERNNLLKLNNYVNLLNEKTDITSLA
KQTILLLLKDILNSIPHQMAPSITSRKIYDRKIHAHVHFVYQCTNIIDLVPYFFIFKNIIKQKTLPSTQSLYI
CNVLLYALFEA*
>N.MAL13P1.105
MSLVSAQEIIDGMQSPDCKIRLRYIKELRVLCEIIGNERTKNELIEFLYNLIEDDSEVLIEISKNFKFLISFI
GNVNNCNYICYLLLNFLIAYEKDIHLNAYEAFKIYINKCDPLTLTEIIYPKILELAKNDGDNYRIGICKIIPM
IIEKSIKEGQSTFVKTFIYLFLEACQDESILVKKSSCDKFGEFIFILKEYKERIDDFMNVLKMTEGKMDPHFC
IQEKGNKDIDIEIYKNVDIHDEENKSEDDKNYNRQINIIVNHKTNDKEQKQLEIESNNIINIIQNKNYDKIYN
LSKDEKYTLIKEKEFINKIWEKSQEIYYKFFSSINGLDEVQISAVSILHEILNNDINFVKNIKDLLTNICNDD
SWRVRAELAKNIYNIWKIIKNEDFSLIILLLLKDLDNHVRSIILNNLHKILFLCSNMKMNIMEEIFDDLKRDI
DNNNNNNNNNNHNNNNYNNNNVHLRISLCELLCSLPDILDKNLFIEYILPLFLLFIRIEETNLKSDLFICLHK
ISRLISFFDMKQIIIPLCNEIIKNKNWRLRYSMYYCLKFFDHYFFFQNKENIESKFLDFWNYIYIGSKDLVYS
IRMQVVETLEFLLRNKNFSFFKTGITYLLNNLKQSNNYIFRITCLQYISKLIIYFPLEYIQHNILYILDDLCK
DKISNIRFNIIKTIYYIQTYVQHVLLVINNNSYDEIINKITNMIHDKNQKYMENNENKENIINKENKHNHTYS
HLSKDQNENIYYKDNYNNDNTQNNYVPYFHSLENKYNLINNQNHDNNQFIINSCATSSLSFYDNMKNTDTNKK
SCEIILYFLADKLNYLSQDTDQDVLLASTSLKNEDFSICVNVLNIFNTLCKPIK*
>N.MAL13P1.106
MGLEFFVYLLIYYILTTQVTCDDNYLDDDNRYNPTMDIDLSSSMSDISRKMNTDSGRKVRIAKKIFKGLGAFM
ILLTSFLIVLDNITLNNELANYKKREIDLAAKLINAEHEAISSKVEYGRKVEEMQSIINKMIFHTLNHTNHN*
>N.MAL13P1.107

MYNMNIPLIITILLFLFYQHYVCSTIKPYEIDKRGLLKIYEDYKNLELSKEVVLDNEESFHNIKYLSLCNINN
KNSISGISNILCKNINFGYHIKIKGVDLLYGEIVHFGTSKFIKEINFICKYYIYDNSYEIIKCNYVIKLDEHY
DFYLGFDMEHIYEPKYKIDKNQNNNRNNNNNNDNNNNNNFIQSNNLQNNNMHSLKEQEQIVLNNVTYNENMKI
DNNTKNMSRNNNDDNNHNIFYQNDYLNYYNSHNVKMCNLRIPIYSYITNVEETLLKILIGLCIKLGYKRFMIY
SENTFVISSYFENMYISRDIFHQGFNITCRKFYNSNNIYIIKSQNSFIQNKSNHNYRSNNNIDVVRKEVPKIY
VKFFANKSYLLWRGIEQNKEIFNFKLLELKNLLKKVKYKERIYICYTKELYSYYEQDEIYIKNFMSSHKIKYR
NFRSLQNKDKKNLDIPYSYILLTFEHFCDHTIILFEECVHIIMTSKILDEKYKSIMHQFLYESFLCSQNCSYE
DDQLNIFHNDMIISTFQKFINNSNSIIDKMKKQSKSITDQIKKKEEFYNNILLPQDENIYKEFYENIMFSIEF
KSFIPLIKDELDKLSRRKNIRNDSNNSNNSNNRNNRICNILKFIKYHERLYESNSHNSIYSRLLNESILLNKL
VFENHKGDHHFDDISIINLKNNIKLNKENINTKTNNVEENIKTNEENINTKTNNVXXXXXXXXXXXXXXXXXX
XXXXXXXXXXXXXXXXXXXXXXXXXXXXXXXXXXXXXXXXXXXXXXXXXXXXXXXXXXXXXXXXXXXXXXXXX
XXXXXXXXXXTKTNNVEENIKTNEENINTKTNNVEDNIKTNEENINTKTNNVEENIKTNKENINIKGNNVEES
IKIVYNKNVENNKAQGNENFEHTNKKEIIYIMNDPIQSNTTYNSESYESISNNLDESNLNNYTYDDDDDEKYD
TEQINTFTKKYPKMMNMKIDKKNKIINNNELELKNNNKGTMHHSFIINSYGVYIYTLQKQYDYDILKKIYDEY
KKTQDNTNNSNNNNDHMKRKEFFNLLYSINKTYTFTVNNVPTKIYDILNKTNIISKDKKKDNKEHHSNMYEWG
KKNVKYFCSKEKRWPFSKKEIDFFTSDDAIYCEAQYVNEFLITVNYGEQTFDYMINDIYQKGDMFKDQNIYQK
GDMFKDQNIHRKDDDYEKNEDIHSNSHSDKNSNINFIFPEKILHKVKEEYFVKHSINESKIIGFYLLNNACVI
KYIGNKKNDINNPFLSNVLLKSVLGFCILHGFTKLKVEAESINYESGIKNKFVEILLNGYTEYEYLGLTLMNV
ARFSKDLYYIITGYTSKSNLLLSAVMKFEKNFNIYHNIENNFLKYMNKKSMNLLHELTFNCNDDYYPYKNCYD
LYPLVRKNIKNICNFETNSIYKELNLLFPDVCKIGKKIALCYEQIKKYIVCSKNSQGCKYYKFIINTFIKPRR
KTSFFINHNINVQEYLSKKAYTYYLLLCELIKNKEKQLKQEEGQNKNQNVDHAHTYFLLNNIIQYSTFFLFWN
FSTEFWKRFQYIHNNDIHNINEISYQNNNLLFCPMAYAYEFIYHLNVFYINE*
>N.PF13_0116
MVYYLIGVYFFVNVLKVYSTFINYNECDFTFDAPKLSDVYDKYNGVKDIEESARYSNYCESLESFNSVKSFWS
LKLKSLCTEKTSILDKHVFNNICSHKFETPHIRINGLSVGKELVNIEFVCAHFLYSNKREIIKCIKSNSVNNY
LDTYHIAFDLSVSEDLLKHSNDVPILLNLKDINGLSLTKKIIDDSVCRIHSSVHTIMNKYDYSLLKVLSGLCT
KLGFHNQFIITDNIHPLFNPFNKKIPYSLSSLNFNIQNTSIYYSNYEYLLYSIKLTDVEYILGSYISPSALTI
SHKNNSYVTWIGLNKDNSIYNKKFVELYNALNKVKYIERIVHCYSKYTSKYYEGTIKYFYKYLPENKKEVMQN
NPNIFIYLINSVCANIPSVHKCVEKIISLDGIYDLYFKNFMINFIYYNLSCVYNCNPLDLGKIMNNEHIWDIL
VKYFSNYYLVKYKKTKSNKDILSASMNQITPNDEYYKHFDFDYIHSGKWRTELNLEDEEAILKPSDDVVEYEF
YNYTFHSLDLGSYSFFILKSEIEEDNICKIASFIEYNGKKYVHSNKYIVDFFKKNNKRMLSHDTSNNNNNNNN
NNNDTTNGFNDNNIQSGEKNSNEHHDEEDNHHDDHFDAFNFFDDFLGDDKDQGGNFIDIFKNNNLFNKNKLLE
HLYDEDDEDYDDDDDHHHRYHRFQDKDYVFDIEKFKIPTNVGDVYPLKEVYTLKELQEVYEIHPSFNDSDDEI
RYQFLKSESFGKDIHFTVKDVPSKIKDYYKYYYEIKKRINKFDNKNDLNHYNTLKNNYKNDPNAFSKVDIKFA
CSKKGNWPIRNVSGRWISDVLCEAYFVPQLAYNHYYAEKKYKNKEMNNKDNKAIQNIFDINLYTLREHTRLIG
IEFEDQEPYRCAISYLGDENKKTILPVSATYLINIAIGFCALHGFKTVWLADSAFDINTNIYLRYTSILEQGK
TYYEQTNFEIYGQTRLVAQLEYIASGMNIDNNIITSGIYKNRYNLVSYPGVDLKFQLLMSKKVKNIIYNLKFD
CHDWTYKGCSEYYPLMKKKMKKQNMNTTNNYNHIILNGNYSDNEKEKMYECAFMHDPMFIELNKMFPKECTYG
SRIGECHKKIRKEIPCYNEKTCKYQKYIYEQFYKPHNNINLLNEHFIKVSEQLTRLSRPYYLQSIISLEHDIQ
MKKENGVNTDYDDIILFILKNSVFYVSWVTSSEYWKRAIHVQDSNLLLPPSYNENNTNNNNNKELFCPVAYSH
EFIGHMLVQYMKFPNKT*
>N.MAL13P1.111
MFKDLKPFFLCIILLLLLIYKCTHSYNIKNKNCPLNFMNSCVRINNVNKNTNISFPKELQKRPSLVYSQKNFN
LEKIKKLRNVIKEIKKDNIKEADEHEKKEREKETSAWKVILYNDDIHNFTYVTDVIVKVVGQISKAKAHTITV
EAHSTGQALILSTWKSKAEKYCQELQQNGLTVSIIHESQLKDKQKK*
>N.PF13_0117
MEYKLEFLSYLLIFKKKNERISKFDEQIKTCINIFEKSIINESDLKYLFERNILDMNPGVRSMCWKLALKHLS
LDSNKWNTELIEKKKLYEEYIKSFVINPYYSCVDNKKKEFVKETEKEPKGKNMKDEYIEYNLDRNKTYYHKDD
SLLKLQNDNNTKQMDYLEDEKYSSMDDECSEDNWLHSELFSQINKDTFRTRPELSFFNLNPQQTINSNIKILN
SLISTETFDEEEQREKKRNNLEDIEGEHIPYLVNINNVNNFNNNETIKFYNKIIGNRNDPPDNKENKSKCVDD
DKNICVHNNLPKEEMKNNKKLFPQTCKNKNVYKNDKQNISEVCDIIKPKRHYDLLCRILFIYAKIHPYVKYVQ
GMNEILAPLYFIIFNDPLCNCTLQGEADTFFCFLELMQRQKDVFCEGLDNTDDGINGKLKKFSLLLKMKEYEI
WKKLYILKIETQYYALKWILLLLTQEFDMADTIILYDHFIINNNENFILYICLVICSKLKNSLLCGNFTVNLK

LLQNIPPFDPYDIINEAKYLMNSDIKNNINITDIYNEYISQKKRNNPLQNIQYDESSLEILNEFEKPIDQNES
TLNKTANLISNIKNKFIVQNIKNYITKKKIDMIKKFDQNIDEE*
>N.MAL13P1.112
MNKKKNEENYFKVGEESLSSNCDCTNCKYEELNNMLEDIKKTFNFLYDEKKGIRRTEKCDSLKTKDDDENIKK
NELYDDSNKKDICNIYEKINNSYVNSKESFSISNTNSSMSSEGSNVSSNYDELIDDILLSDVSSLSNNDKEDI
INIDNLRCDKNFLYLIDEKKMRKDKNNIYYNCISIFSLNTYLYNDIFRYNPHLFGLYENCRNNENRCKKIGTL
CTQYNLIFLRSVYGKYQKYLFDKLKYTHTIMLDNIPWCNNLLNEIYYSALNYFYGNGGLYICWSKILFRLCYY
DFMFLSSDIIFKRKVIKFIKLIYDNKYHLYFFSLEFDLYSTQNKISNIKDLVFFMKKIFWKIYIFFLFYKNRR
FLKRRKENTKGCIKHKNGNIKSEEEIINDKHNYINCNNNIYSNNNFYHNHILNEEKKKISTMIFKNSSIFIIG
SFNIDINDNKELYEKLITLNHHGEMKDMFFYKNIHYKLQRTYNIVDLKNNTLVNGLNYCYGLTDNIFLVKSFF
LNEKDIYELMSLYNPIDIIIEKFNNFKKKKKSIDNYMIDNIFKLINKNGIYFKFQEVYNYGVDILTQKKGKEF
SDHWILSARFDIKNKNRNINLKIENKLFNFLYNVNKYFLINKKSYHKFCQVKYVKKRKSQKSNHKKVIMNNIN
HMPIQMEKYDYSRKICIITDENINSINQVETNLYHILLKCFEEECNIY*
>N.PF13_0118
MNLLIYSCFNKSICFFLRRDLYLKKKNCTKVSFFNYFNSKEWNRYEKRIRKKSIILQNDAISNKFYIKNKIFC
NKNVLLLPLEYKNNKQFQNCTIKIWKRNKNGYHIGLRNKKGSKKNNNNNNNNNKRKKINFFSHVEPIKDFLKF
TKKIVLCCFVIYGINNYIFDMTLTSGSSMYPLINQNGVILFYVCDYSLRWFNNLKKIYLSIYMNILYKCYNIL
QLHFDYEYTSYLYEIIYNKINKLKNKIKKCRHIYKRGDVVLLVSPVNEKKRVCKRIIAIENDKLFIDNFHSYV
EIPPNNIWVEGDNQMDSYDSRNYGSVHVQLIIGKVFFLLDPFKEFAFVNSERNYKPDLRRYLHISN*
>N.MAL13P1.113
MEEQNFYNKSTYSKRKKISEIEKKCEESLNEILRSASEAKEISKKAEEELQYQTEQIKHINNETDSIQENLKQ
TEYHLQGIKYWWKNIHSFLGLNSFNNNEDIKTKKAVVNNAKYNPKNNFNDNYTKNNDNYKTSLNTKTPSQRKG
TFDEKYENDLNTLSSMLDELHTRALVMGNTINEQNKMLNNVNEKMGHNIDKIRDQQKMMKEIMKK*
>N.PF13_0119
MAMKEDYYDYLFKIVLIGDSGVGKSNLLSRFTRDEFNLESKSTIGVEFATKSIQLKNNKIIKAQIWDTAGQER
YRAITSAYYRGAVGALLVYDITKKNSFENIEKWLKELRDNADSNIVILLVGNKSDLKHLRVINDNDATQYAKK
EKLAFIETSALEATNVELAFHQLLNEIYNVRQKKQATKNDDNLSIQPRGKKINVDDDNDKNETKKKNKCC*
>N.PF13_0120
MEERNVLEYAELIIDRQRDKINELEKKLEELRSSSEELQKNVIKNDEENKTLRLELNRRNDNDMILSMQMSKE
NELLEKMNFLEKQILKRDTLLEELKNLIKKRKYEYELLLEEKDNLKNTINTLRNDLYENRKNEKLKEQEVIHN
EKENEKFLNLYKNNNEELNITKRCLIQIESELNIYKKQNENQKNEIQKLYRIITCMNREKTLMPPVVLALQNE
YNIRTTLTNKKEYSAQIKDIQDEDSTYINSTKNNKKKLTTLNPIYNKINQDKGKRYDDDIQSIYDNNEGQDKK
NIIQNSDKKKGVSKLNFIQLQEQTNLYNKILQILNNLQDKNLKDDNKSNFFFFSSMLLHYCKSSSIHKSTYEI
NISSDHSNSDTAQSVDTFDNSSASHFSYNSSRRRRNHSKTINHKKKNKKYSKWDNTKEGNKLDDPLNKKDGKK
EKRKNKNYGKDNSLSESEINENSDDNIDDSDYSTNFQKNKMGTDTSSFDTETHTNSMINMNSNTTTNNEDDNY
FDKDNNKINSKESYPRGDKNGYIIHNNVNKNKHRNNKKMNLEKEGKFLLNPSNYSERFIGVTSKQMYIYNDKC
DDEPEYVLRIENLKNIKILYNNQIYALEYMSFNNMIEKHYIFIKNHDKCIRMFYALQYAGFIKTDMKNFMQRR
DYEQDNIMNNNIPYGNMYPYEQMERNINVPIENQQMKRISSKKRKEKINYQDEEEFKDQQGYSSIKELDNTTN
MIRVNIFTPNGVDKNGNNIPYLRNDILLKAYIYDNILLFTNFVEDDNKYFLKCDKYYMKYKNGKFILYFNDFK
DQHVVIPQNKDENNILCNLLYKMKWKNIYDEKNINDEKNINDEKNINDEKNINDQISNDGNIENERIKGSHMS
SHTSNRNIPGIKSDNDDSHHNSKDIYDNKSSNIYEKDKDNKKSNKDLSSSNQSNHIIASRKNDSTSNVSNISE
ENQGNNFFIKDNVLYISKNGKSFKNDLSNVNEENAFRFETNDLIVIRNDELKELTFMKNKENRNADTYVFDYP
KKEKYDQLVFELRKHNFNITSMNTYKKLKNETSSQKEENASIISSFDGKSASQLSKKGMVKDKIMENNQVVVI
EKGMLSIYKHYGNEGSVPIIKFTCDFCDVQANEQNGEIIIKDTSKDSSERIVLDCLNKTEFNRWKTALCFCGF
LKGEEHMNNTYMNLKKHIFSINIFDNYYVKNLIKVENNCVNIYPNDKFIKPLFSFDKEKVELVLMPELRKIRI
YIQRDTIYEQRYDVTMSLARDYLKIKDDILKSNFHMLNQKKTQKIKKPFILCKKNIIAIHKDKYNLKPELLLE
RKNCMVKIDKNNFSITFLIKSQVDKNLNQIKTIKLANLVNFNKWMVTLKLASFIPAAIDYEDNISFPSITFGH
TCPEAISLIKRRSSLMDFFKIKLKKKAPQAKKPKAMLAKRPPT*
>N.PF13_0121
MTKNLVFRLNKSLLHHVKSFNRTFLNHKYNSNANIEGSLKRYFSIETIKVPRLGDSITEGTINEWKKKVGDYV
KADETITIIDTDKVSVDINSKVSGGLSKIFADVGDVVLVDAPLCEIDTSVEPPEDICKTKEEVGESKNNENNY
TFNQLNRDIKDEAHIKDEVSKNEKDIFVKDPICFGNDYESINERTERRVRMLPIRKRIAERLKESQNTCALLT
TFNECDMSKAMLLRSELNDIFQKKYSCKLGFVSLFMYASTLALKKMPNVNAYIENDEIVYKNYIDISVAVATP

NGLTVPVIRNCQNKNLPQLELALSDLATKARSNKLSIDDFSGGTFTISNGGVFGSMLSTPIINMPQSAILGMH
TIKNRPVVVNNEIVIRPIMYLALTYDHRLLDGREAVQFLCAIRDYIENPNLMLIDC*
>N.PF13_0122
MSDNNATDILFVGGIDETIDEKSLYDIFSSFGDIRNIEVPLNMTTEKNRGFAFVEYVEVDDAKHALYNMNNFE
LNGKRIHVNYSKTKKADHYKPFGLTKCIIKKR*RNWKRTRKI*MMIL*
>N.PF13_0123
MKKINKKNCTNNDEKNLLIYDKSSKIGCIYFIGMSTNMYRGKIIVKKNSISEKIIRYTNLNDHLLLIKNGDFN
FKQTAILIKGITALLHRQLEVLLIDFYSMYKKLLYSNLNINKNNINGMMRKLRRNKKFKIKKNILSLHDVDYE
NNDRKDLFMKSGGNYLYKNKNIANINDILLKESNELHLNDYNFGNDDFLNEENMIYKDMLTNSSSFQYSKVNN
FYTVNMDMNNFNTNAFTQDIKTQNNINHTLIFNEKKQKNNIHNIKINNISSASLFTSKDISLKENDFSYSGMM
NSIIFFNNDINIKNNINEKYKNVNHELEENNYPKKKIINNNGINNNGMNNNGMNNNSMNNNCSNNNNGKKRIH
SEIDEEIILKDNVWHDLYKKNKKYKMSSINYVDYNFKQIDYFCLSFLNKEKQNKKYNTYNTIPFVNFYKQTKN
YISLDSRKDLYNLLDINVLQNQKSNMQPNNLKLIHTKSSLSSSLKGKTSKDDLNFEQLRQHFNENHFLDIHLG
NNNNQNIKKRKYTKNDSLIDNSYDMNYDFDQMSDLNNKIYDDESIHFDNNNNNNNNNNNRGQNKLSFQYYNNN
ITLSDNFKETLSVPSSKLVNRRSCTSSQNTYDEELQKLKKYISKLTIYNHNIIEFDNIFPSKKISDKNISLIF
YNLLVLASNDELDLIQNFPNNKILIQVY*
>N.MAL13P1.114
MNFFLFFRNIFLLNTIIFILNQYIKRKDYGFRFFIFSLKIKSSKEEFTKNVHRKATNTKEDKSAYKNEKKEIP
YRNNNKRKDKNKKGNDNKKILEKGLKDTNSNIKSSTSSNTISNVSSKLSDNSNISNTFTNIKIQKLKDKHNKK
NSDIKNVSTDKKKENDNNKNNNNKKNNKKIPIIYIPFNDIKNSPYIRGKLKLVYVKSFYEECQKELYEAKFQF
FKNNKIYISTFAVVKNTLSENIDQTEKWVTADMKILQKNSYNISVTCNDSHEKQIQDISFTKQEKEEFELMRK
SLKNVVNITYDHFLYNIDTFYNAKRKYLEYINFFFRNPHIKSNMDIKKNISSNITIQKDNILLKRLGSFNKTK
YILFRRMQRNNRIMCMVLTDPKRLENRFILYNMLRDHIIEKEGFKDVIIFETDDNNIYNYDSKLSKDIRILSN
KFEFEILMDEHAYINNPYEYFYLNKYIPVHFELQNKGIINYNNIFSWLCEHFINNSQYKYYYPHFYLSKHSDK
NDMGIRNISFYQKEDAYTFIENNKKNINKNDKCKTYDTCEKDDTYRTDKKSKLKNINRIYNTKYALSKKKNRN
HKNNEINNICSHKKEEKHMFNNYNISYNNMKYINKHNKFSFLELKEKLKSDNNEREDKKKKDESFEKNETHLM
NNDDNNIGDDEFNSEVNNDNNGSYESNFFYDMINEISNKIENSDSYLNEEDIYWDDDIFFDTNEYIDDTSTIT
TSTNNNNIRTDQNGDNKKDDSNKDIISIQKVNDSTEVNESNKIDDTKKGESYTLNNIKGYITKFKDFIQNKSE
SYVTTKNKSIDDKSSDDQISDDKINDDKSSVDQISDDXXXXXXXXXXXXXXXXXXXXXXX
XXXXXXXXXXXXXXXXXXXXXXXXXXXXXXXXXXXXXXXXXXXXXXXXXXXXSDDKINDD
KSSVDQISDDKSSDDIKLNEEQTSGDQNDLSDNNENNQETITSDDTLEKGKLDNSNMGDSDEKLKDDMKNNEH
ENLSAENVPSDENTINDKTKEGSTTLKTAMGYITKFKDFIQNKSENYMTTKDKNSEDKISDDKSSDDIKLDEK
QNAGDQNDLYDNNKDKEETISIDDALEKGKLDKSNLSDNEEKLDVSNVKNSEDENLSAENVPSDENTINDKTK
EGSTTLKTAMGYITKFKDFIQNKSENYMTTKDKTSEDKINDDKSSDDKINDDKISDDKISDDIKLNEEQTSGD
QNYLSDANKEKEKIIFSDGTLEKGNLDKSNMSDSEGKLDGNHMKNNEDGNLSAENVSSDEKKIYDKSKDDIFL
DDKLKGNHFLDDKLKGENFIVDNLSYNSNFYSRKKSFSTDNLATVKKASPGEYLSDDEQSENDSIFRSLEDSS
FDNEIGSFYQRRPEGYFSNDKMYDSDDESLPKPQTNDLLALKGKPKHEDISPSGEELQYDTCSTHSNISDVHS
EPIISQRDRNFQDVTIQNNINSNSKETQSVGDDIKTYDIHENSNKSNFVIDEDKKNGKEEDLNTYKSLSFLPK
KMVVGQIVQEWEDMFIIDWLLGDDIFCVIKLEFFDKSINKKIEMEYLKVLNGFTLVDNKKLPIIHTGKKELTV
KANIFPYYSDIRFFYHNENIDNSVVHTYNNEIFENELKSYLLLVHHLINARNSPCRNYKNVKYFYNEDTFNLS
HINFISNKMLSITYECALQVLNWEIPYGIVKKNDNCISKFVFNNLQHKINKDIYPYAIQIKKLDINNSIDFAM
DNREFIQNYVDLKYDILLKTSSKLFTLHTLGLVHSNIKADNFLIEYLNKEYYELYIYLKNFNNIVEEGKCNNF
KGSSVYMAPEKLKTYFYHIVNKFKSDVFSLGLSLSTVLIENGFFLKSAAKRKISLSLDKYALNIIKRNPMVYL
WIKSSNPFKKKRFDKSKHRSYKKVSVHIMEKLLSNKYVYKWIHNRSPSFDYAQAEKHMQLLEKHVPQQFITKE
SNIKINKIEDNKNVYNQNIQNKITYDKNMEQTSEKHNITMNKKRERVNFVDACTVHSEDTKKNQDTFNFPSKK
KHVYLLDKEKKDYDSTLGMNIEVSFSQLSTISDHSTKQNNVINKNDIKENSGDIKENSGDIKENSGDVKKNSS
DKKKNSGDIKKNSSDKKKNSGDKKKNSGDIKKNSSDKKKNSSDKKINSGDKKINSDDIKKNSDDIKKNSDDIK
KNSGDKKKNSGDIKKNSGDKKKNSGDIKKNSSDNNIKNGDNNIKNGDNNIKNGDNDIKNDDNNINNGDNDIKN
DDNNIKNGDNIKNGDNNINNGDNNNNNIEKTEPIKRKGILKKSPSDSNSENDLKDLINRIKMKYFNESCRNIK
NSIVGTIMKIFSIEYEKYRPDIFDIYSLIKKDITYIKDKDDNVKNLLTILQKIREPDMVSILKQKINGSDYIM
YKMVILKALLFYGYFTLYERLSYEFKIYEHQIFPLRENNEDTFGTIIERCMHRFNFNEWSNLLIVQHIYNNYM
SKKKYTNLYINLHILNIKPNDEKKKEMEELYDKHMTEVFKDIDYNDNVNNFLLYMKKESEDISNTLKEKNNMK
TGWDNFHINYDDIYDKHDEKDLSLIRKTILKLDNSVIRDMNINLAILLNDMFWKHKNRCKGVNYNHMKVKYAD
NTIKEFNIQKNPIIDLHKPIGSIVKGLIKNDDIMISKGHTNINTLDYIIPINIHLDVILQNSLHFVYTSTLIK

CAEKMDKKGIIYHRDITYNRKKETIKIKIGLISNSYFYLNVDISYLRYHEITLKDIIYFIYRNYKSELNKSVR
SNIEKRSFLFFDCLLVNEYNTFFYNNSTKKLIKETSFNKLISQLYNINLNDPEYIIKSTINYYNHRKSNMPLI
SLVTISHTIHERWNGIKKQTDYILQPSINNYSDEYNEELIDPSKLKFKNPSLNIKENMNRIIMRNRYEGYQYR
LVKCFDFLHKKNISYYKDQHTTNLFDQIIIPAVFIHLKNDDISKINIKLALEDRKIKFNIITDIMNLQIYENQ
IFTLTDFMYLIFRWLETYISFNDRDHCTFLTDRTQISQYDLVLSIETGKDKINILYKNNEGVIKFIFQNILKN
HQVNQYTSEFLMSAIKLNKLKLFFVLKDELKYIFPDIPEGLYFHLKESNYKIINCLKYLDGKNVKLLENNIYD
DSYVPVKIKFMNVTVIYRIKKDNFKNIIYNFTKYEVLKHYGKQLFTRFISPINDIENKFRYLFLRDLSYYIDI
PRDIKRDKDDFMVSMDSNVYNALNEKHNIYMNNYSSIIEKMITYMNVTYTEPVFIVCVVKHTVLGSKIQLLLR
EMKYNITS*
>N.MAL13P1.115
MGKGSNACKRNQARERKNVEVKGGKSQLKANKEALNVTCKVCYTVFMQTQSISQLAEHAENKHNKDVKECFPQ
KFIDT*
>N.MAL13P1.116
MNNTGLKFFEKVKKIEIKEIEKKLINIHKLVLCNLVSSNNLLEKNKENILLYWKNCCLNVTDVKANVTLFFEN
YYEYFTIYNETNILIGEYFIYLIIYFRNIKIKKNPLKVINFYIFTDTNYYKWKEKLIFHFLKNAFVHIKDKKK
ENYYEDYYNCKTLIIEYLINNYHIFNNEHINKFFYIYILKYINDKLKIAFLKRNQNYLNIYANFLITYQINAT
INFISKELLHIFFLISHNLRNILSNFPFDKDICTHVCLIYQQVKYIIIDYENIINGWLNNKTGSGTLENIGSY
KINGNKNNRDNVIYHNYNDNKEHFSSYDIPNNYSELIKNEQVNINHKGNRDIIYNDHIHDNSSNDHFINDFTF
ENLIKSNDKLKNEQNYPSIEECYERLELTKNIKNLLNDCNNIINEKKKENPFLILCKYRSILIIKYKNVKNTN
IFENFHYFLLFFKIKNLLCSSTRNPFFIFLCIESINYWLQKSEKLNYVFEQGSNICNEQMYNGKIYNDNKICS
NENNICSDDNKICSDDNNICSDDNKICSNDNNICSDDNNICSEREQMNLEELYKQNHLYMEENKISKEHISSN
NIYDTLKDNTNYMMKEMNVIYSNTCNPNDGTIKINKNNRIIKNNEEELIRYQGKRTFNIINYESYNLNEDMEK
CKQIMFHIKLDEKLLSAEEPLNINHIKLDFEILTYKEKIIFLKELYNFIINSILMILKKYCWYNIKNIWNSCI
SIYEFLLTSKKNICIKNDIYQHLTYIIFNQEKKRKYLCLSLLLNYFKNSMCEKNILEYLFYCFFRNDEYFMAS
LFELIKQCIKHIFVHSEEKYLLYYIFIKNILLTEDLFMKTFYVKKFIELFYKNDHNYVSFILNKEYFKLKDYL
DPEYGNKNEIALKDETIKLNYNFDHVQKQNYAIPKEKNNFIMLWDENYKCSNNVMNKNYFEFINIHLFEKEEL
CQLFFFNEFIEKKDDNIFLNYKIINLYKQKDIVINISEEEKKKKKLQRYGLYNFHSIENIMYLELYTLCTLRR
NQDLQIVNYKNDYFYQLNNDILLNVKTLKNLLYHSNDEVILSILTFICENKYLNICDLYLFLNFLNVTITDSS
RASRDSYRKYISLFFEKFFIFYQRMIELDKKNKNKNKEKRKTRNIQKEKKNEIEKKNEIEKENEIEKENEIEK
KNEIEKDKENDIEVEIKINVHIQIEKKKDKINNHINLIRDNKLNNISVCNSSEKRGSNDNALDTLYNESFCEH
VSQKKNYTNIYANIFTFLYGNNIFYKNRDHIHSKDAFIENSYLSCMCYLPNDKYINDQNYLFLYDSYLMNIIF
LLFKSINKDMCIESSIYSADILNIIFQYCSYFENINFLFFLFNIKTTWKELYIMSKQLFFKTVSIILLKKHKF
ISLNNLFLPLIQSHKQVDHSFFAFLLRMCFMSLNKINNNFFLLDHLNDNNKNMVFLKDHINIKFNKTEYICGT
DFFCHFIDKHMDILNIKKSNMYMINYINYLFGGNDNIYTKNINIVNSSCNNNCINKKIPSYNIHYDNINEYSN
IFLTPKNIPLYILQKFISRIKKEHIIKLTKHKDIENNCMYKIAYYINEFILYLIENTQFNKMLDMSTYYKFDE
INLCKLETYLNYDQMKLYEYIKNEDEEIRNNSYLILLKIYICNILYYVINYLNFLFLNNSKNFFFDCRGHLIF
EKENEKEATSIIIWLSIKYICSMITSIVDFCFKYNLTRDLILNEDKYMNICQNIRDNEEIKKKNKKKNKKKNK
KKKQKNNKKIEESCPKHDESTTIKQNDNNKEEKPNKSITFFNNEEVHFIIQNLLCLLLYSKHVACQQYLADCL
LNICRIIVLKSNEEMQSIPLFYIYIILHIFKNENIKKENRINHDNSCVTSSNKKDEKDKNLTKNDNLEESHKD
IEKREDFSIIQEKGLCAINKVDNEIDDNMNNNMYSKNKSYSNVDRYNNSDNAYYDENLETLNRIINKLNNILM
LSEEENMDINDILSMNFIKHGNKINIPKNLQNDNNLDNDSTSDTDNTSYYNYNFNMNRKLHMLILDSKRININ
FLRKSNNMCLALGCLTKSYRIKEQYIIYNFIIKIILNYLYNGNNQYKKVICLNIIKHIYTTIDNKTLYEYIND
IYNISLTYYKSDLFSLKNSSTSLYNILTKRLLSKLNSSYHVSSNMYYFCTGSKKRIFKNINLTVFDILNSINL
LKVFLNVFIKKCKLLTNHFINQNECKSYFDYSNFFAEFIPILIFLSNIIIFNNDDYFLLKSNKGDLASTNKIP
EFFFKNEKNNNSIYNEENLIEKKYNPDKEKYDDYNNVNVNNKNDTTNDYINSNVNIYFIFCDYLKKLFRYGNY
FVQVLVCRIIINVYLHIYLKLKKKNVIFNFLKKILINVIKSEINEKNNNHHNSSSNNNPYLLLFIEFIRRIEF
QYLIEENKKKFRKIFFQFLYLFLKSNSYTENLLILQILNILYNKINICINKNIVMLLYNKLLSYNNDMFISVL
VNELLIKITKTVSNLVCLLDLYMYNKNEVFIESLLYNWFLNIKRNEKKMFKEKENSLSNTSNYSSFKFLYENC
HNKEYPFLYILKKDDINMNVCYKNRVNNIQQYINEVGYEKLDENIYIYIYLLLFEIIDIYKNNMKIKKYCFKI
ILSAVEYISLENLVIFEKMKSYYIENIHSHYLDKYIISYSYLICLKYFNKYMKQQYCKNETMNHVSRNHFIQS
IYDYINEEKKKYIKLSLPNGNIHQIDIFNYVYFYQLIFMNFFSLLYNKNSSTYAKCFVNAFLNYGIIEYDAEQ
IKKKENIHKIDKRVLLPPASFMRYKEIKKGKKNNYKKGHIGSENIRMVKNNVSNIYEEKFLNKESIIMKNSYS
DDNNHNNNNNNNNNNYNDNDCSSAYHMLDSNLKYFKWYKQEQHKNIKKMVHNKIANQNITFDMIYIKNPLKYV
RSYNLFTSFFIVLYSCLIYMLNDESELIRYKTKISVLSFMKRNVLHLSKNVNYITCSEFFIYYIIKHYKDISS

SILIFCINQTKPCIKDEHYYKPLKLFDQEKYNLFINNISLNHLFLFYYIYNIIDIMDTGENIIKQKNVVNNFQ
QDHKRINQINTEHDIINSFYNNNIHCVKSFVQPPTEHNNNLLYQENTREYFENKEKEGVINFFTFLDTMDNQK
KEVFINNINIKNFKHNIHVRNITIYKNNFELLKYRRTWKNIMNELLSFNHTPFNCSKENNNLRNEKTFIEIII
SIIKMKYISEIKKSLDDYIHLFCFNNKVDFFDSNIIICFTNLINRIIILIINIYLFFNKQNIILHYYNSFFYD
EIKSIKGKVLSIYNKFVLRKYEINPFLLKNMKLSLHLLSSSFLKNDHFFCDIYKFLYSLKNSI*
>N.MAL13P1.117
MVQIHDAKSPFLLPLYKVYESNNIFFCKGNIITGPNIFFLLFTYIIIIISVLPIYIITYFQIDSSFCLTVALV
SLTIFFVLVLFFLTTTAFCDPGIIPKRNYVDLSLPKGRTAFTTVKINGTIIKQYWCVNCNHFKEPRSKHCYTC
NNCVTKFDHHCVWIGNCVGNRNYRRFFFFILNLSILSTIICFIFIGLFIQLCIKENGSLSFQPILYTIGEYPH
ITLYIIYSFPSSLLLINLFVYHLQMVLQNKTTYEDIQGLYSGNNPFDEGKYINLKKFLFTPVDKIQVEWKDIV
KNITHLLILICQKYINIFKTL*
>N.MAL13P1.118.1
MGKVEDFEEHNKNSNQDIEKNVGNRRRSNSNTINDQNENINKNKAIVTKKSFIMNLLRNKEKTKVEQNDDNIV
ENMNERKNSLKDMSLISDNVEKDNKVKKKNSYLKNLNILGKTKSIEFSFPSNILNNARKNIQEIDEESPLTSN
SLRTYKEEISEDNRQNQRNADNNSLNWSTSNINTECNSEFSNIEVKVLKTEKSNSVKLKDNIIDIPNDKNKKE
LQEINIKNTNKDSYKNLYLKVRNSISFNDNIKIDDEHKNDKNNIIDNYNNYDDINSSNEITIDGLNHDDNIKK
IDNINNFDNKHDDNLQDHHQNHVHHTFRSSRKSLGNIKSNNTNIKLNNDKNFDTNINKRSILEKYFYDIWKRN
ITIKKEIYSLSSKNPPNPLKSTFADACQNESSEKELLKKIPLKFNDDSIESLYVLNLNNWISSRMIIIGIVML
ILSFIIWPLTTWSLKTSTWGRETYIIILFHTLMAINTLILIFFIIIGSTELCKYSECMSYVLFSLMVALWGLW
NIAIGLTLEYNPNLSEMPTTTYELEMIYVLTYIYGFLPLVIIDIFFPSRTKYNWIIHLIFIFLNSSSIILVGS
AKPDFVPEIYVVFRILAYTTLCIFLYIGSYTSELQIRYVFYNLLVTGYKLDKIESDMKNKTSNKKISTGIEDL
INMLKECTKVILELENETDTNFNVHTKTSYCSNILEQCLSTLTKSDNLYNIDYNVLENPENKKFIEAYVSKSK
SNFAGEEVPKGVDFKLNKSFSNNDCISTDKVDLDKKQIKKFLKQINISQLTKMIQFIDNKLLSDWDFNCLTYF
DESEYPFFDINLSLICTIDHNIPINIIINFLCFVEKQYNNVPYHNTIHATMVTQKFFCLAKKLGIYDDLEYKI
KLVMFISGICHDIGHPGYNNLFFVNSLHPLSIIYNDISVLENYHASITFKILQLNQCNILKNFSEKDFRMMRS
YIIELILSTDMKHHFEIISKFRIRRENEDFDYIKNSDDLLILTKMIIKSADISHGSVSWSEHYCWCQRVLSEF
YTQGDEELKNKMPLSPLCDRTKHNEVCKSQITFLKFVVMPLFEELSHIDNNKFIKSFCLKRLNSNCIMWDTLM
KEEKTIEVYDPAAVKLKDKKKKKVDKKKKSYIDLTLFFIKNVSD*
>N.MAL13P1.118:mRNA
MGKVEDFEEHNKNSNQDIEKNVGNRRRSNSNTINDQNENINKNKAIVTKKSFIMNLLRNKEKTKVEQNDDNIV
ENMNERKNSLKDMSLISDNVEKDNKVKKKNSYLKNLNILGKTKSIEFSFPSNILNNARKNIQEIDEESPLTSN
SLRTYKEEISEDNRQNQRNADNNSLNWSTSNINTECNSEFSNIEVKVLKTEKSNSVKLKDNIIDIPNDKNKKE
LQEINIKNTNKDSYKNLYLKVRNSISFNDNIKIDDEHKNDKNNIIDNYNNYDDINSSNEITIDGLNHDDNIKK
IDNINNFDNKHDDNLQDHHQNHVHHTFRSSRKSLGNIKSNNTNIKLNNDKNFDTNINKRSILEKYFYDIWKRN
ITIKKEIYSLSSKNPPNPLKSTFADACQNESSEKELLKKIPLKFNDDSIESLYVLNLNNWISSRMIIIGIVML
ILSFIIWPLTTWSLKTSTWGRETYIIILFHTLMAINTLILIFFIIIGSTELCKYSECMSYVLFSLMVALWGLW
NIAIGLTLEYNPNLSEMPTTTYELEMIYVLTYIYGFLPLVIIDIFFPSRTKYNWIIHLIFIFLNSSSIILVGS
AKPDFVPEIYVVFRILAYTTLCIFLYIGSYTSELQIRYVFYNLLVAGYKLDKIESDMKNKTSNKKISTGIEDL
INMLKECTKVILELENETDTNFNVHTKTSYCSNILEQCLSTLTKSDNLYNIDYNVLENPENKKFIEAYVSKSK
SNFAGEEVPKGVDFKLNKSFSNNDCISTDKVDLDKKQIKKFLKQINISQLTKMIQFIDNKLLSDWDFNCLTYF
DESEYPFFDINLSLICTIDHNIPINIIINFLCFVEKQYNNVPYHNTIHATMVTQKFFCLAKKLGIYDDLEYKI
KLVMFISGICHDIGHPGYNNLFFVNSLHPLSIIYNDISVLENYHASITFKILQLNQCNILKNFSEKDFRMMRS
YIIELILSTDMKHHFEIISKFRIRRENEDFDYIKNSDDLLILTKMIIKSADISHGSVSWSEHYCWCQRVLSEF
YTQGDEELKNKMPLSPLCDRTKHNEVCKSQITFLKFVVMPLFEELSHIDNNKFIKSFCLKRLNSNCIMWDTLM
KEEKTIEVYDPAAVKLKDKKKKKVDKKKKSYIDLTLFFIKNVSD*
>N.MAL13P1.119
MFHSNNSNLYKKRDMVKNVFSLFSFTSNVDDKAIKLWPLKFIEKEEESLYIIKICHNIYKKRFFLLITHLSSL
IFMYSICLIVAKINELFSKLKFTFIFLHIFVVINILFILMLHYTHYVEKLRRWRGKLFVLHSICIFLLWCSWL
FILFNDVKDYLPIVVNVNKFLYATYTHNKINIIFCFFAYVPIFYLITIIPCRICYSFTFDLLFFILRIAIFSV
YYLITVKNYILIDNIFMIASSIVGSIFIFVIRYIIEIQRRLAFHSWNKQIKQINTLKSNLKEQKEKLSLTNIE
EIFNLINDCIGNYCNTNEHLEEKNISVVNNLKKILTILKEDNLFSPDSKFLNRKKYNHIYAYIMDVNRNKTLS
ENKEPFKDVTEEFKEESETESLMESISEERSKTKIDEKSKMYSKNDEFNVKKEMDMNLKCDNVNLDIWNTSFL
NNETLNEDIFIHIGNKLLKMYYTTNHNIPSETLYSLLYEMKNGYNNVPYHNSIHAAMVTHHCNVLVSNLNTAN
ILRDNELGALFVASLGHDIGHFGRTNIFLKNCCNFLSIIYNDKSILENYHCSYLFNILLKDENNIFKNEDPKC

LLALRQQIIELILATDMSKHIKILAQFRIKSIKIKSYIEKNIILCLKMIIKAADLSHNCVDWSEHYQWVKRLV
NEFYYEGDELFQMGYKINPLFDRNCHNNFIQIQRTFLKELVYPLIISLKTLDNTSITQDMINNVKRNYSKWTK
IEKCQIKKKKYLNELLCNIPDNWARVYYPNLNIYKTQKK*
>N.MAL13P1.120
MEENSYFEAVEKLFEIKNSKEDKINGSSSNKDELIENKELKNDNLNNNTCDSDKKKTNKREVKRKSKVINDNK
IDNENKNTKRKKKTNGDVDINIDENFDEDNDENVDKNVDKNDDEDDGEDDGEDDGEHDGEDDGEDDSDEDKHE
NIPKNKADTLKEESDVSDNHHNNRKSKNIKNSIRSKSSNSKTKKEDDDNNIHCNNSSQENEQKRKEQLLENKD
NKKEGKNTNSSKVKKRYHTSSDENDRNNDNTSSEEERTKSRKKKHKNDNSDKKNENRVVDYSSSSSSSSDASN
EYSSSDEERDHKKRKIRKTNRSRRISRSSSESNSSTDNDKRGTSRSSEEKVYSKYDKMKRRDRERERDRDRDR
DRDRDRDRDRDRDRDRDRDRDREREKDKDRDRDHDRYRDRERDRYRDRERERRKDRERERERERERERIRREN
ERERLRKERELERERREKIREERRLKEEMEEAKRDDLTVLVLNLDLKADERDIYEFFSEVAGKVRDIQCIKDQ
RSGKSKGVAYVEFYTQEAVIKALAANGMMLKNRPIKIQSSQAEKNRAAKAAKHQPIDPNDIPLKLYIGGLLGP
LSNITEQELKQLFNPFGDILDVEIHRDPYTGKSKGFGFIQFHKASEAIEALTVMNGMEVAGREIKVGYAQDSK
YLLACDNTQENILKQQQMAKNINTEEEEQDNEKIDNDDGDGGGLIAGTGSKIALMQKLQRDSIIDPNIPSRYA
TGANAIMARNSFVPSTNNINNNVTTNLVLSNMFSSNDENIGSDPDFFNDILEDVKEECSKYGKVVNIWLDTKN
IDGKIYIKYSNNDESLKSFQFLNGRYFGGSLINAYFISNDVWDMTCLPK*
>N.PF13_0124
MGENKFDGLSFFENNEFQNMNFIRGSMDNNRNEEEKGLIQKAIDLSKKGAETLQKGLKETLGKNNMNDGASMV
SNSTTAESGSMFSNFPLFNNNNQNRENETSSFFAFTTLVSYKNFPLFCILFGISVLFMILSFFTLPMIVITPR
QFGFFFTVSSICFVSSLAFLKGFSNLYHHLMEKQRLPFTTAYILSLLSTLYFTLINPLYLLALITSVIQMLAL
ISFLVSYIPGGAGAIKMLINAIYSYVKNLFRRSNTSDLPF*
>N.PF13_0125
MKVKKLVKFLSLLAVGLLSNKITKQYGFTPYSFNCNAEIIRRSSYGYNQEILNDSVNLILENTHKNKIGIIHK
SIRELLSGRYNSLNPQKVLLRISNIIKHGHINSLCDNNQSYVLIDIPHIIFNANLIGDPELQRINYLINNILN
NLDVCSSKRKYNIQLKSNVTNGCLSQSDKDIIFDTTKPSNSRSKGLQLSPSNKFFSILLKCSVDNLGSGDSTM
ECVQKELAKDNKKMSDTCSQCFKDSVSCGKSNCWLPCLFGSPCSDRCYNCGVNYCNKELIRCTGLENLPSACS
*
>N.MAL13P1.121
MKNGRLINKQAIQLILGLYVLFMFLFVNIKQCYGKNINNDEYIKGVDIYKAIVIDAGSTGTRIHIYNYHIVDD
EKGNIKIYIPSINYRTTPGLVCILNRYFSGEKEDFHNYFKNIKSFIYDNVEEQKRFNTIILFRASGGFRLLSI
NESEKYMNFLKNYFFTHFNEFLLIDDLLVNVLSGKEEAILSFVSIYALLQNFNPSPLIFTDNDINEGKQNDVN
NDNNNDDNNDHNNDDNNNDDNNNDDNNNNDHNNDHNNNNNDSDNTIGVLELGGATAQIVIKVPLSKMNDDIVN
LFNYQHKEKKKNSIIEENYKNKNIVKINLFNQDIFLYCKSYLVLGRQNAMKTYLHYILHKHKEIDQNNKFIEM
ACFPKNFKFHINNLYKTSIEEDLLEYDGNTKINDDEYIGVGIGNINMCRQEIQTILDYAQIDDLPFKIKKFIK
LYGIENFHHFAVDILNIAESFNPISLNTHMYLEKAQEVCPLTIEEIRKVVRPESNIEKAQTSCFGLIFLYEFM
RYILKIDKSILFYSTNYINKTSITWTIAVLLMEIPKIVNLIRKNEKIKSFYSDEL*
>N.MAL13P1.122
MEYKLFKNKKILNEKVNELTKKNRQRNTLDHINCIEIDDDDDDDNDNNEEPKEMNINKTNNNNNDILMKSCND
IRRNTTFYRHNVINEEQKNFEYLLSRKKKNVDSIDNVNFYDFMKNDFFNIFNNNIISEHKKTNQIVNQINNNV
DTSKNVVYNINYDEHKGEVVNLSFDKKGKETYPQVDIELYNKKMNPRYQNINEQNTCQTSDDNTTYPNVYSDN
CALNSSYTNFLRNKSLKYKKCYKQNRQDESTGAEFDYNLDESVYYDDCNNKLYKRSFLKNNKNIIEKGREEHK
QDIYNNISDICKSYIKNFDYLNKRIFHNKSKIWKLSNKKVTIHGDPKKRIERNKQEIEDHRREQDGENDQEED
NYDDYDDEDDDDDNCHIYDNYDDYLENDDYDNHNYFYHKNHDVNKIRKIQNKNCSTDFINYTSINNRNMECPN
KNADNLKNMDSFLLYMKERKIKKKKNINDHMENQLSDTLNNSNNYNKDVYDSYYDDMNFCYDKDYNMILKKGV
NSNRTKSLDRYPNNISHNYVNNISSEIIKYCKDNNITLKSDIKNIINHFNKKYANSSLTNSKISMYENGYPQR
SVDRIYDSSTNDPKSDMNGSNNNNSSSNNNNNITHITNDCDNTQTNKNKHYVNSIVNIFQKRKNVNEKKENIN
EQNCISFNTNLRNNKLNNNDDLCSNSYVNNINRNKQITNNENKKDNMLMLKGTYHGDSLNESINIEHNLKNNN
SMSDVNFVLNNNDNDKKQNSYDISEVSINCYYDDVIKCYMDYTMHGMEDETNFYLCEFCEQNIFDMNNMIKKD
KAKECMYRCNISCGRTFHKACVCYIKNNDNYICFFCLYDINFCTLCKEVLTNDSLLPCYYPLCSVSMHTKCVE
KLLLFNSHCLKQYESIILPRDINIEQQKNTQKSASDGIIEQPLNIKKKIKRRHIYRKRRRRGPRKSQITTSNK
KINKSELAGGSIINGVDMEQENDQGGNNNNNDDNNDDNNDNNDDNNDNNDDNNDNNDNNDDNNNDNNNNNNND
NNDNNNNDNNNNNNNNNNNNNNNNNDNDNNNNNNCDENFKNHLLKKDLLRDEPHHNNYDKILECNTEIKMENN
VKMSEEPIYNKLFNGKEETLNNENDEKIIVLKKFICPLHICYVCKEFDINNTESSKKELKNNLFRCIKCYKSV
HRKCMNQLKNNDNNDNDNDNDNNNNNINIYIISHKHRIICCANHMDDYKKEHMEYLKYIKEVKDICKLDEPIH

NNNNNNILSEKGFYGDENNLSISKFHNNSSYEPIKNKETCIKGKDSNYLSHDNKGITDMNNTNKMNVLNLKSC
MVNDMNTSERKKKKSCESRGSNITNKKVVFDLTDELNEKEKSPPLDNVQNKIIYGDNEIEKNVNICQKEDGGL
NLGSMNILSIGKNHMRTNNNNNNDSSCSSNNNIISVENEYILKNKNLNKSNNSLLDHNNKIKKNSTLNIKECT
DSCINVDEFINKNQNEKDISLENIDALCIKRKRNVSHPYNDTLDDTNILKDISNNKSYYVNISKKKRNVSFNY
KEEFMKGDEQFLLKGNNLESNEKNTKNKLCNNDNNNNNNKGKNTKYNTLDRKNNKNKQINDTINKEPENINHN
MQNQQLTDNFVEDNMKYKQEIYHIKLSHILSIEKGLLSLQEINIDQMNDECKKHISILCTFRQDFINYVIFLF
SKRLHKENQIEPVNGVDHKKEGNIYHAERKQEKINHNAKHNEQGSINNAKHNEQDSINNAKHNEQDSINNAKH
NEQDSINNPKHNEQDSINNAKQNEQDSINNAKHNEQDSINNAKHNEQDSINNAKHNEEDSINNAKHNEDDSLN
NAKQNEEDSLNNAKQNEEDILNNDDHRNQEELHERWKENMVYNFLANYDKKEKKYISKKEEDAIIKILSNDIV
GIMKKELKISLYDFIMLKKKTISTNENHKDNDESVVYYVEDGKHHCNDINKMKDNVNNDIIPNIKYNNNNYDDV
INTKEKESIPDASYMKVTRNQSIMNNDIYNNNIQIIEKERIMNNNKKNYYDEEKKREEYNGLFSKGKKKSFKN
NKMDLKTFFSLTNNGYKVDISILKKYSSFLKFEYISKNIYLNDKNKNLLACKSDDYKCLCQGECNLYTCYNSL
SNIQCSKSRCNLPEKIQDRKCFNRPFRKSFVKDLEIKKTEKTGYGVFCKRDIKNGELICEYVGEVLGKREFEK
RLEVYQEESKKTDMYNWYIIQINKDVYIDSGKKGSISRFINHSCSPNSVSQKWIVRGFYRIGIFALRDIPSGE
EITYNYSYNFLFNNFECLCKSPNCMNYHLLKKGESSGASNIIKETELLNNTIFNPVENFHNLHGKMQDWNIFI
EEAHTRLLYEYNKMNAFNLRLMECYSTWIFYDMNFQKNQFFSLKSKPYNVSAEFWKVLVSAFSDGEKNIINTF
NLFLPSLIKIGQLRRIQQYSYILHNIIGLEHDMWNLIDKGFADDEVCRKCKSCGNLTMCDKCFQSYHQLCGNM
HSKMYKNNELVLCRFCQKYDYKIQWIKENHGSKMKTCIEIRSKAFYKLNRDIMTLLEESVKYTQNQSLDSIHA
HNTKAFKSKKLKLRKFQYKYVKI*
>N.MAL13P1.124
MENRKVLRIGKSVVHGRTGPLRGDMEAEEQKEVRKKEESNKKDIIKENNSDNKKDEQSKVETQNTEDILNKHY
NKEDTENNSGNIFEQLLQRELAKGTLGGGVVEKNTNKLKFQIEENNIMDTADGNIDEDMEKLSKISLQERINS
TIVKYRMHAYNEILKCFNNQDNDNQKNFLIDKKIHIVNNFFKDEDSIFKYLSDNNLICQIKTSEITEAYLHII
KDIYFHNYKNEYNNEEELINSFVNEYRNKLHNMFLKIYILLIEKILINTKSFDFGCNIIIKIIEYCSYDKITL
SIITTICEYLNSIFLKANKNVSNIKGVKIKVIASILLLFYKLLHSFGPHIICVKTINKYCLKFNEMMDKAVKT
NFYSLYIEILTQVNIQEFHNSILNELSSQQRNYINKELQNKPKHTDNTTNATTLYKATYILKTNDHNNSYNFM
KNFNGNTSAGNGLDNHIVEEFDVFKDICTKQWEKRVLEGCIDKNNSTNNTNNNSNSNNNNNNSSSSKLLSNEN
LLPWKIKVEAINLLCDKMKSKYNIKKTNYLSTLLNIILKLLKSESALPVVVSTLKLLHILIEKFDKDIYNTVK
NFSFILCSKLKDSNKQVSNACMECLTKAISVYNIDLFLDDLSKNLKDKNNNTRLVTLDFLLKTFQYIDKKSIS
SLIDITKHLLNDTVANIKNTACKLYALIISYYGESVDSSFFNSIPSNKKKSILSLCVKNDKLNDANDENISND
ENNANDENNVNHINHINHGYNDDHLNSPKNQFEDSSAKKKKNVDNINKMTTLKGNNMKHTKNNISDDAEDIIS
GGKKNINTAHNNNMNKSCNINNINNVEYYLPKDMIKNLGSSNIPNKIEGLQMLNIWIKNENNIMILNFEDLIN
FIKKNCYEFKGKNVVLNNAIYDIFYEFIDIAYQYNLKNNTSFYKMLNILISLYIEKVTDKKGNMLCNNFLHKC
FMYFDNNIIIDLFLKNIDSKNTKKCEECIKILQKIFINRNKNSGVNIKNLIYFLKKFVDSKNSNLKKSSILML
QILSKNFGDKYVLSHLEGISENIRHMVKLKEDVEKFVSKHGLNYSTSILLKDMDRQHNHINDMATRNININLN
TTTDMNNNNNINSSNVRDCETFNKCDIHEGDGNNITINNQGKDTEIGRHKEKDTEIGRHKEKDTETERHREKD
TFINNTNDMNNNINQQNKKDEEMLDIIIKKRDFSLEGEEKTNKKGTVKSPINNSSKLNNIQIDYTNNYLNYNN
TNISDNTPMDISKGVNNIKENKHEKNYKNDEVKNYAHNDEEEEDDEEEKMILIIDHIKNYINRIISDNNNNNG
NNNGHNNGHNIISNSVSNIINVIEKIGKYYINPEKLDILINDNILNKIYNNNKNKCIQLLYVLIFSLKENTHI
YADILFEFIVSIIDDIDISVEQIDKLLTCMCRNLGISKYINHINTYILNEKFNNMNDDYSNQNNMNSNSNSYN
NFNITDRSKEIKKDYKRNKILIVINNINTNHILLLNESVIKKKILTFYLDLFFDDNEQIRERASIVLDNVYSK
YGRNVFFSIYEKLSAHKKECLQDILNQMKLNKDDFSFYKIFLSNNSQLKRISMNESNLSGTTPTLSKTKSVKR
LNSFTKNKTMNIDFPTNYKIKTDKLKWDKHLDHNHSNYLMKEFKSFATSELISTMFSDKPHIINRSLSFFKDY
ISNSINQSTIFSKSGFLDLLLKWTYYTLYGHKNNNEILSASINLIRIILKTIEDNYVFLNDQELVTLVNFLFD
KINASTICVDIRNKLKEIFLCLCYISNHKLYFNLLLKSLPSCDQRRLNDSLDIILKLIILYKEKCINIEKDIM
KILQVFTVHSKNKTVTIYCLKIFANIQSFYPNFYKCIENDEISFYLKSKVDEFIEKCPDFNVTLQDTENDENN
SSRNSDNTEGNSKDVEINEFVNYIKNNANQRMSIKKDVEDHVNGFNKDVEEILEIKKKIEEFEINKTNDDYIN
NQISYTSISNDLNFVMDGNNYSKTKEINNIMESNLNEKIKGLNNYKNSIIYFRFVYLTSLLYSNDIETISKVC
NLIVDNTIYVGKKSNRGNFLLKENGNYIFKNLNLFIILLKGANYALRYLFRENFESDLNSTSVHLNAILSTLL
LLDILMKKKSCVEKLSFDHFSFFFINAIVCLSIYTKVIHKHKHIYRIKDGEIARNLITRILNNLLGREFLSNH
TKLLEYVCNVGFNLGFDIINNNNILLDEMEYANEFYISINTYVKILNKIYKKIINRIFEENTTDSRFVYKILN
ILFMNLNKYDMYLCKMETDKNRKRKMDSEHDENRSDGRLGIINGMIGSNHNINNNNGGSSNNNKKICNEKNNN
NNNNNNNNKENAINLKNSNNFNNRFNQFNQKNTNNNIKSVSINHKKKKNNNDNNNNNNNNNSSNQTNFSFNQN
NNPVEQYNSFNNTNRNSNSCYGYNENEVVCNKSDYSIFDCHGTYWENLNIEENNADVFVKNNYEEIEVGVERK

NVIREYSKEEMDKINKNKMDFMNTFLFFIQLLFYGIKKNNPLILLAYINHELISKYHDNLKYYFKKMEILLNK
DLREDVPNINFSKYSELYTLPFSFDKFINEYNYLKEKESSTFQSYNSVLEVRQHFDSEVIYNNFDLSIDDVIQ
KKKNFLDSLKMININEITFNDNKYEHLKETILQYINELENKQLFCDIKNAQYLSNYNFISSMYTNNAKLGEEM
DNTNNEKQKLYTHMNNDNTNNINLDRRQEQIPLNAHISPIYYEEKNNIQFINKKKETNTNNIQNNETVVNNHF
SENFVVDQNKMKINEKDYVTHKNVEEKVREENFVHEELKEPLINDKQTFEYFCDTAYDKFSKEETQELRNIMT
DDKKEELENKSKMCKNITTNDATHISHSIYLRNCTMENIDMNKMNDEHLSIGNITKWSIPVKENIYMDTNNYT
REDAQNIEKNVHYKKTNELINNDKKNDNLIEQKKNNSDLGYRKDGALDNFNKVFSSYNNNNNNDGVTNHRNTF
VNTNTNVHLDEETCKKIENKEYNNEEQKGFKDDNLYKDLFEKEEEKNKGDINIHNKIKENEEKYIPSYNYSKV
DDKIVNVDQDIKKETNINTCILINDDDYYIRKEKKSTQNFVQNNSKDEIMEKDKDMDLLKKKNNININNINNN
NNNINNRNNSSYNNCNEKKKSNESASSVLNKEITRNDKNGNIIKNTHQTFLRSNKSNVQNLIKKYDNRRESPN
VSHKTANSIKPKTHLENTKIVNKANDTFLQVQGQRNISNTSNRSNNNHINNRNNISHINNRNNISHINNRSNI
SHINNRSNISHINNCHYSDSNDTFKTILKNASNNSYHNINSEKENIKIKGDEKSTNQMLVTKSNIGSANLVSN
VSPEPLSANTTKNNNTYLLNKKNDTYIEKSKHIYEHKNTNIVSRNNNPSDNYMQNKEYDNKSNATSNYSTKRK
NCIGISNPLKKKNTNK*
>N.PF13_0126
MVNYDDITQTLLINKIKIKYDSNSEWNKYLKYSLRNPHSFIYPDISSRINNLNNKDLEFLNSSILSQGYKLNN
IEKIKSDYTEIDVEKYILEIEKEEEKNLNKDDETLIRKIVSLFKECMYYFMCFNEKDGQFLDSSTKNSYKRDI
THNSDYELFKSLNDCIIQLHDSKKNKNQAVIMTNEFCGIFTPHYIKLTKYTDTFINLLKEHNIKYVDLFHEEI
KNNELYKKKKEHFEKLKNSSFMPLTKGEISLANKKKKKINMNTNTNTNTSTNNDIKKNTLLKNKKDEENAKRV
DKKKKKIKKEKKETSKRNSMNGLEKGDDDNCDGYIKENNKQVNDNNQMQDNNYNNNNNDNNSKLEGCIVDTIN
SCNDITDDEDSDVDTSTSYDSQKNMNTNILNKKKKKKKERNEVEEDDNSYDDNNENNNKLKNNDNNIDMCEEP
ESIKQLHKHTNSYDYGTRLKRRKKNNEKKGNEEDIKTECNNLCIVEREEEYVGMSIYQNEDICNYLNIKNEYD
KNDINENINMNAKIFTMLNKKIDSDEYIYNEMKNDFMKKMNLPNNYNYEVNVLEIINSDIKKFYRCIVKYINK
KKEHIKIYYIISNFVFFFSKMHKNNINFDDNIHKDINKNFDYKSIIIQGNMLPYNFFLFIKALHLMSIYDIIK
NFYITIEYDERKNKCLHFLNITENKFYENFKKFEGNIKSNFSYIHKFLHNMIPVYKYTILNGKLYKDLEAFTQ
DHVNENVFQLTYHENNISSDKKDMMLLLYDDNADRDNYLEKKSIYKTEGLKSINDKVSDKHINNNMFTTTTTT
TTNNNNINNNNTHVVCHPFNSSPSNYIIYDSNNLHKMVTSNLKNNCRIIDDKELPILIKDVNDHNDNFTKILL
KENRLLFLLDFYEKNSNCNNIIFNSIFFDTFISLIFIYYLNNYMKPKKQDNIKNIPLFKPSYFHMFITQILKK
KNEKKLKKNKRKSIEQEHTVDKLCNNFFKNSLNNILNYKKGSPDNIPAQQIDVRNNKDVNSQGETSNIPLLNE
TNKTVVNNNVDDINKNNHVSNSSIINSNESNPNNNNDEKNYKGHNNSTLNNLRTPIHTLQNNNTEEENNNLHE
QNNNLHVQNNNLHEQNNDLHEQNNNLHEQNNDLHEQNNDLHEQNNDLHVQNNNLHVQNNNLHVQNNNLHVQNN
NLHVVENHIIVNESFVAKNEYEDIIICIFVFINNNNSNAYSEEEKEIRELILYVLKNKEKHHTSNKIKYIFKY
RNNINDAITLSEIEIRKYEYIKIFIIGLVLNNDISNGILQIDINNLYNLKDILHQKYPSKNFDIQTKLFTFYW
IYQVYVYGSIFLKDKEIPHAIKKINDVYTLPYPLQLKSHVLWGYYLYIIQDTVESRYSEDRISSLKEQGIHVR
LCTENFEGQIIEDDINDILHMHDLDINDIKYMDKNIFMNIIFLIENGHLTYFTFINTDNIHIIQRNNYLSIQI
FKSKISFPSFNLNTPILNDSWLEDVLSKQTLLPFNDYFYKF*
>N.MAL13P1.125
MENIEKENKEENYLDRLTNLFKYLTLGVEKNNSTEKNSTHSIKYDGHKNSLQQVVYNLKLKRASELSVPHRIL
GNTSISKNDSDKERDNNTKKEENDDTRDSQIYDHNICGLIKEHQDIQTISLQQEDDKKVYFYSSSIDLEHGEI
YKIDQAHKKKKKKKKKKKEKEKEKVKEKEKEKEKEKVKXXXXXXXXXXXXXXXXXXXXXX
XXXXXXXXXXXXXXXXXXXXXXXXXXXXXXXXXXXXXXXXXXXXXXXXXXXXXXXDEHKDEH
KDEHKDEHKDEHKDTYNDKHSDKHSDKHSDKHSDKHNDKHSDKHSDKHNDKHNDKHNDKHNNKHNNKYKKRKS
KSKLYELEEQNYSKNKIKLVDSEKENILMTNLKSVSSSNNSEDIKNEPNIKQNIINNNLYTLHKNKTYISIRS
NKEKNRVIKLPKLNFTNNLESSSND*
>N.MAL13P1.126
MNNSTSCVSINVQDILFNLAKQKDERIFYFLEKDKTLINIQDGNGNSLLHWAVFLNNVYLVYYLLENNAERDI
KSHSKQTPLFWAICSNNILMIYILNKYGSNLFQEDDKGYNCLIISIQYNHILAFFYLLYLNIAITHKDYNNCT
VIDWAAYNNNLFFLRFFSIFMNNLYSLNLNTPTSTLHKAIIGNSYEACVYLIMNKHQNVYDIGTDDKKTILQF
VEDNKSKIDPRIYQFLNSKQVYRICQKKANRNIDRNRNFLYEANGTIGPYTIKKKKKLPIFLSIYTKYIENKA
ILLYPLMLLFLYVYLNITYFLYARKYINGQQLYLDLFHPFLFVIYYFVVTSNPGYLENNKNNVPNINLEGKKK
KDMNMKCRNGDNDKDTYNDVDTKDEEYKDKPLNDYDKKANNKERPKENITFQYIIKNITDEINRYERKYKLVE
FAKRIKKENIFELKNSMEINWDIPPIEFTEINMNMLCPTCFLFKNIRTKHCSLCDKCVEIFDHHCDFTLNCMG
IENARIFLLWILLNIFFSFSIIYIYGWFIFNSSLNYYNIKFYIILMAIVLSILIIYFMGAIFIRSIMNILENI

TSNEKFKIYTYKTFFTYELTMDEKKQPTVIRKFKNPFDQGMLFNFINFLTKSKTKLIKKEKQFIIIDKNIKSQ
KLTEFVDKLNKRLNEMNIE*
>N.PF13_0127
MDKYTNNCNANENIIPFCNYISEISEKCDCNEEKNMYLYGTYNGCTKKSSIEKNTIVDVCNIYERLYCGSRFF
ELIILTNEKKCMEKITCNDEKTDIFCLFEGEKIYEICSILQEIAVFLKKDKKKEKIILSFIEYNNIDEMNTYN
VHILDIYIYLYLRNYLKHTSNTEDENYNILYFYNEKERILNMEKYNYDINHFDIRNWLFNNMSFYLPSKRIDD
KCDDNNNKSGHNNNKSGHNNNKSDDNKNNNNNLLTVNKYLNVQNTSKNLSQSHNLSLFRLNSMVDNQTTKLSS
KICINKKLPNILINKNDNKNYVCNKQNCTSSIINSSTNVERENSVCYYLKCCTHENGNIFTLHQKQIYDNNFN
IYKKFIGHNNIPHIQYIIDIPKELILDNSPFIFTNKKLEYLDTNILNDECAANIPHNQLSSYNFYYVIKIQDE
NKEKLITDKVIQNIDNNLTYIHKLLKQNYNHNIISIVTQNFHVSYVFTVLKFNLQKKKKKIMNKSGKNL*
>N.MAL13P1.127
MEYLNKIYMTALGKKRDVTIDDDVDVDEDVSNENIVNRIKQKNCCKKWSTSMYNKNILINYRNDVDKDSFFII
FEKAWKLFFHDDTKGKKLLLHEENDNNIYSLPNFLIIDNLSNYNPIGTENIRSMNNDQCDNEDNIIINDDNTK
YILEDMDAKKIASNKTFPLNKKFIDIYHKHDDTYCSYDKEIDDFLILPDIIITNNETINVYREINDDDQFLNV
NNNHVKNEEHENNVDITYDKNGTYNINGTYDENGTYNINGTYDENETYGQNATNIDNIIQNVHITDNINNQNV
TTNSSNIYILNDVK*
>N.MAL13P1.128
MEKFTSLHSFFNSFFSTSLKTIFKKFRYENISKNVIENITRTGTFCRTLLPLFIPLSIYQYIRQTDKDRYSEE
LFLQYVKGEEIKKKDPHFLIENDDKNWRIKYDLYLIDKAVNS*
>N.MAL13P1.129
MEKNVNVYLFLYLIIFLEAYFCVSLFSTNIFPREYTKVVEKHLREDYGXXXXXXXXEIIRNYKDTDVFLSPSE
EAKLKVNIQKYAGDHFIKEYENLMNEDTTDSNKKLAKTMINLIKQQFIKLKVIEQEYITPNYEQYKQVAKLKP
DISDLTADTPCNTEAECKKLENMMNICTYIRGGADFAYDIFLVTTHVVTTMMAVMCACIFIGPVHICALKNFP
YTCKLPYPIFSTLFMATSAVWEVVKAATSLCRVYGDLSIMSKMA*
>N.PF13_0128
MRFLIIHIAVIVLPFVLMIDVKRENSFFLRHSPKRLYKKADYNNMYDKIIKKQQNRIYDVSSQINQDNINGQN
ISFNLTFPNYDTSIDIEDIKKILPHRYPFLLVDKVIYMQPNKTIIGLKQVSTNEPFFNGHFPQKQIMPGVLQI
EALAQLAGILCLKSDDSQKNNLFLFAGVDGVRWKKPVLPGDTLTMQANLISFKSSLGIAKLSGVGYVNGKVVI
NISEMTFALSK*
>N.PF13_0129
MKTIVSTQKVLIPEGVKVAINARKVTVSGKYGTLRRSFRHLPIDIRLNKLKKYIKVVMWFGVPDSLACIRTVC
THLKNMFTGVTKKFLYKMRLVHAHFPINSNIVDNNTRIEIRNYLGEKSVRFVKALPGVVIEKSPNVKDEIYVS
GADIENVSLTAALIHQSVLCRNKDIRKFLDGIYVSEVTTVEKDE*
>N.PF13_0130
MAQNKGSNVLIQQLLKAEEEADLVIKKAKDVRAKMLKEAEATATEELKIFRAKEKERLNKGHKEKSTAEDEAV
TKIEQNTKDEIKVYKDLFKKNKDQVAQFIYDKVYNVDLTIPDSMEKSFNN*
>N.PF13_0131
MRILKKEIGKKKEEKKRKGNNNHTHNLKSRNEKEELIQRLKRCHDNDNIFNYIDEKYKKYIIKKNRNDSSQNN
SINKEKNENYIHNEIEGSKHLGSSSFIFFESINSYELKKYKSAFSIYNILLNITKINMQKLYDENNFLNKGWS
DQEKLKELKSDRSKLILGFLKKKKEQNRYDHEQNQYDHEQNQYDHEQNQYDHKQHPSEHETYDSKFFITKNTN
NHFIDIIKTNDKNQIDDFLKTNPIVCFVHFRFTPDYYPYQKNIICYLYEIQIIKEYIKIGIGTHLINILEQLC
KNIHIHKILCTVLKSNYKAVMFYKNKCSFQMDESSPDNFYSDSHLSKECEYEILKKDIIS*
>N.PF13_0132
MTQVEKKKKNVKNVNKHKIIHKRVIKKGNRKYTNGNTKVKYRKQVVKKTINKNVSKKKLLKENVDKDEKKKKM
KISIRFKKPKTLIYARNPKCPRIVKSCHSKTLDKYGLIKYPLTSEKAMKKIEEINTLVFMCDKRANKKNIKKS
VKNLFGIECDKVNVLNTLNGDKKAYVRLSGEHDALEVANKIGIL*
>N.PF13_0133
MNNYFLRKENFFILFCFVFVSIFFVSNVTIIKCNNVENKIDNVGKKIENVGKKIGDMENKNDNVENKNDNVGN
KNDNVKNASSDLYKYKLYGDIDEYAYYFLDIDIGKPSQRISLILDTGSSSLSFPCNGCKDCGIHMEKPYNLNY
SKTSSILYCNKSNCPYGLKCIGNKCEYLQSYCEGSQIYGFYFSDIVTLPSYNNKKKISFEKLMGCHMHEESLF
LHQQATGVLGFSLTKPNGVPTFVDLLFKHTPSLKPIYSICVSEHGGELIIGGYEPDYFLSNQKEKQKMDKSDN
NSSNKGNVSIKLKNNDKNDDEENNSKDVIVSNNVEDIVWQAITRKYYYYIKIYGLDLYGTNIMDKKELDMLVD
SGSTFTHIPENIYNQINYYLDILCIHDMTNIYEINKRLKLTNESLNKPLVYFEDFKTALKNIIQNENLCIKIV
DGVQCWKSLENLPNLYITLSNNYKMIWKPSSYLYKKESFWCKGLEKQVNNKPILGLTFFKNKQVIFDLQQNQI

AFIESKCPSNLTSSRPRTFNEYREKENIFLKVSYINLYCLWLLLALTILLSLILYVRKMFYMDYFPLSDQNKS
PIQEST*
>N.PF13_0134
MKNHTVFFFFFFLVHIFYIFHIEGIYINKNSLTNDKNILICDNTINGKSIKINKKKKTTDILPSHMEHRIKNK
SKTFFIDGGKNMHVFSKKKRNVHNSNVNDGRNSNVLYAENYKINLTEKPLIEDVPRNVKGRLNEWINEFDENN
TLGFFPILLEEMSNEPSPLQVGFEDYMQFNGEHVEEIFKNMTREVVINGEKKKIFGMCFFNHKTGVLAPLAIY
AEIKDVTKVGNNLAVKGLVRGRVIIDEIISQEPCILGKISPIEDKKGLVEPEESLKIIDEIIRIHTQCSNMES
QLMEQLDQSFASMNIKLRNDLKESIDAKLEKFQIDPSDVEQRQAFIQMASFAAFDFHLMIVDRYDALMLQNSE
DRLRFVRDKIRQKQKQLAIIKNTPKDKLHEILQQVQNLKNQNMQSPNYPKNAS*
>N.MAL13P1.130
MFFTYVVRPGEAPEGRGPQFEPFWDFFMNFNLRVGFLIQFISYILLVGAITIIGKNPLGMLNFLRALPGSVGT
APMPLVLFSIGGFLMGTLLIMSFLQLTEDDSSIKQSRGYRAGTKFLLQATSMGTVSWSLSLICLMASSYYFDD
PWMEEKIGAGSSWILYFSSRLIDAFCLFLYGSGCFFLEVYHSEGAGEAWGWLCGMCFIATSFVEVLALTLFNT
PLFSSLDWFYCLFLGLSLFFSVIWGLLFEPISHRYDVKLTQSAMRNEYYKSRNAMAYYGPAVVTANGELDIEA
STENIAACKQC*
>N.PF13_0135
MEGYEEVSDLIQKFKKDTYVLNYYKRIYELNKCKSYRSIMIKDIQNKIDYKKEQEKNIKKKKKKDENDDEIIN
DDEIINEDENDKKEYYYNSEENATTLGRETSKDKIHTLHENYNDDNNNDIMKTILKSIDNKLYKYTNEVLEYF
DGQDKETLNTNYQINNCIEAHAYIDNILITNQHDIQYICKDINNIEKLTQSINNKLTNRKIILELLNTYIKII
IITPQLIRNIIYGDINQEFIKNIHILTNKIENCKHCLYDIYPSIKYSYIELEKLKKKSVDRIYFFFLEKINNI
KNKNTDIYIIQQNLLTFFELNTFLFNNNRHVYNYLLKEYIHVMNKKYFHLFKNYITNMQKKIKNNKNCTFINN
SNVGFTNNYNSQVERNVNNKISVNRNININNINININNINININDVDINNININNNNNNYNYNNYNNYYYNN
SCSNNHLDIISSNMNNVTFQNAKKTMMNFLGINPKNDNTCNTYEEPDENIFSLNCRHKVLYDMCYFNKTKEEK
KNDININDDNINDDNINDDNINDDNINDDNINDDNINDNNNDNNNENIIMNNYNYIKNYNRINNEKTFENKIN
KEYPVSLISDLDNLIYHFEEIYKSINKLFLDTGSLEYYFILNFFKDYESPDFLFLEIYSKTISLCFDFIYFYT
IQTYDVISLYCVYIMNLYYAYIMYKRNIVTLYVYIQRIQTFLWDKIYYIIQENLDSLNKKRLDEKKYVSSNFN
AKINTNELEHYQNKTCHINNLTFDKFENFTNVHSNNYQNGDNKGDNEDNNMDNENKKMDNEDKKSDNEDNDKD
KDNHYNNNISQHYYHNHNNYCMNKNIELTNNLGIYKTQANKNTHLVNIKQNDITNKHLNTNLNNQEEKKHSLH
FSTILKTQEVHSVTKKFTDFYCSVVILSNLCFHIDTYYKEKIDKQKVKVGYQEQKLKENKNQHLMNMEEKVDK
IEMDRKNYNDQKGCNILHGEADKNKLSNEQFKEDIIMENLYDKNVNFVKHNNEGIEKNKDESKITTEKRKHNN
NNICAHSNENSYLNKIYDENVTMEDKLKNDKEVNNTSISDKKNNYFLKYKKITNLISKLEGAIIHTLISIDNE
LVCPKEKLLFLINNYYYIIYILKQNKLQEKICTFEKLLKKEITTYIEYELNIYIKDIILFVNKHENIINTIKE
DIYNKNIKNNNNDDHHNYYLSHVDFISMENIAIQFTKNWKLLFKNIRNNIITSFINIDNAFNILKLLNTQILL
YFTRFYQLTKKIFSNIQPPLYIQNLPSVDVIMIQIKKDAKNVGS*
>N.PF13_0136
MQIKKEIKSNESKIQIKKEEKDKNKINGINKTSNLNNKIKQHVMKKAGNDINKKKKKKKKKKKKTMKNKIKN
EEKNNNTNNIKKTKQKNLCDTTNNLCTRKSVSTNNQSIENNNEHQFNDDDDIVVDFELIDPEEIYKENIHILL
KGSELFYNITCFDKLINIICDQQNIGKLISVSNEKDNIISFMTIININQYDEIKNLKEFLINKINKINKINNN
NNDINDNNNNNIQEFKNILLNNDKHVGLVITNRIINCPIKLIPLLYENVYEDILWSQQIEDIDPEEKKFYFFD
YLLFYTKAYKQNLNDDLIFSNYEEQYFFHKSIHYVMWNNNNIKKFFEIQKDEKKELACKEYLILFLVPMSAVR
DVITTISSITK*
>N.PF13_0137
MDNFMVRIKANNEEVNDFIKHLFTETFCNKDFADNIKLDAQKTNEKQESEKKNRYIITEKYVDNYMIPYLIRN
NESREEKRNENMNSYDEDNNNYYYNDSYNYDIFKYISCDINIRDKNSVSSEYRNNIEDDTYKNRIQFKLYASE
IYFKNMIKEKYKIKYNKYNKKYIEYNIKDKSVLLIYGDWYMCDSFMKNEKSNITIKELYDYIEENIENINEIF
NCLKGSFILFYIHYDITNINVYFFNDKFGMKSFIYFYEEKSIILTNMYGPFLNYNYNYRELNKNEFIFNDMSV
KHEINLIPTYDTFVQHDNIKNNFLVDKKKNCDYNIINNKFNNIDNKEKHDDQCSKKTINEEIAINIMPHYIYK
LILLKDNQKDKDKDKNKDTNTKTNANTKTNNNNHRQEDNIFNIHNHIMLKKINKQYCIYDNSYYQWKHPDVNK
FNILNFQDILHYFEKTNIFKNFKKQQKIQFLHTINKIIQEKNITVCQNNYLYNTNKNNIPYTKLNNVFINLFI
ILLNHIIRQKIKRFFHLTCSGKKEEKKKNDDEKKNMDGKKKHIDDENKNDNMNIPYYVYKEKKEKCIGIFFSG
GIDSTLLTILTIKNFFHFYQDGYIELVNVVFNINAIDRYTCFVSYEKIIRMFPNYDIRLILVDVYPDDLIKYE
KIIYSIISPNNKIMDFNISSALFFANLGRGFLCPRTFFESQEWRNIKEENIMNVLNVSNICTSNQHTSDNNLW
EENSLEKKICVKGKRKKEITSSSNINILYKCRICKYVMNKKCVHKCCSVCCKKLRYICINENVYNINKEINSK
GCDNADNDDDNNDDNNDGDNNNDDDNNNDDNNIDDNDGDNNNDEKDFIGMYEIQYNKENKNKNKKNIYLIVKK

ERILINFELFRQCIIHKDKLYDYKKIGYLFAEFKKELKKDKMNKKQEYKENKYHGEIIKNDKHPLCDEEYXXX
XXXXXXXXXXXXXXXXXXXXXXXXXXXXXXXXXXXXXXXXXXXXXXXXXXXXXXXXXXXXXXXXXXXXXXXXX
XXXXXXXXXXXXXXXXXXXXXXXXXPLCDEEYKKNKYHEEIIKNNKHPLCDEEYIQRDFIHQFASEKSEINEK
HNLEKIKNLFSSKGKNKKEGKKNNKNKMFINNDNINDDSNEDGDERQKKEKTKSTYNDTHLNYFNKNIYYPNN
NYDNEKEKSFYMCNHQLLIIGSGADELFGGYYRQNNFNKKLAKINKNYKMCEMIKDIRRIWIRNLYRDDRVIT
FSSFKKKYIFYPYLDMLMINFLFSLPFCIIETPMGYINTNEGNINKCHKNENLNNDDEEMEEPYNTFNTEYMN
EHTLIYEEQFNYLNTHILNECNYIYERMKTEKINKWILRMSMYFLNFKDVMFFKKKAIQFGSKSKNVKRYMKE
SLGIYPKDSEQNSSALFNEKSGRDEYILLS*
>N.MAL13P1.131
MVKSKCENNENCRKIINKKIIEKKYDKKKTNENMNYVGTKHVLNKKIINNINKKDNEVYNKINDDMIDEEYIP
NNIEEDEEKNKKKTCLKNNDNINYDIFNNISKNLNNHLSQGVGNECVKNLITQVSYSHLFTILKNILTYLPFS
INDYLIGTLELKEVIDSKYNTCFQLYEKKELMKYIYDNEATNLVNNKVYNLFRKYKIIKNKKESFNHRQIFHF
KDQNQQPILEEILIEKAKIFIYKINSFIEGREQNLSYIYEICYGLSYKTETFLLSVFIFDCYIHRINQLTNKN
HYKKVKLLVIICSILLALIKTQVFNGTSIMYLNDILYFINKMKNDKLSYTCIEISNKQMEILKLLPPNYNNYS
TYTELTYVYFINLKNCAKKYPAIKIYYIFLEHFGFLYFIYDVIYAYSMYITAVGSYMLIPPSRIVCTIILYFT
NAFYNRMTNSLLFQEKFCEEIFHLPFANDLFMWAYLFMDNFIYFFENCVVPNKHYSSIYSIFYETGRMLNIRT
VF*
>N.MAL13P1.132
MSTVNELLNYFVVNDDNDDLNKNDEKNKNKEKTVVRRYFPGKKPHYAKKGEDDDDDDEDEEEDEEAGDRNQLE
EQEKGEREFNKVINEPLIKKEHENKNIITQTIKHNNNIDNRYERLKNKSLGGDNQERIRSRRREVTIIDNKEN
EKLVEHIQENTKKVDEYEDIIKNIKEYNKNDAGDYNDDHENDDENDNEDDEEDDDEEDDEDDDEDDDEDDDED
DDEDDPASDDDYMNEGNNERIMKHEFKLKTSRRTLMENKNKEEAEKKTLQNLNEEKELIEIEKKENAIKEVLN
QDMLEEKLKSKENNVFSSEEDFEEDEQDEELNEEEYELWKIRHINRLKRDELDRKKHEILELEIKKRRKMTDK
EIIQDNKTLPNKEKKKKRKMLFMQKYYHKGGFYQDLFEEGKEEIYLRDYNEPVYEDKVDRQNLPKVLQVRRGK
FGKQGQSKYTHLLDNDTSRKDSLWNNIESNMKFKDKKKDQFDRPTYRKKN*
>N.MAL13P1.133
MNLFNILRNTSFEIDAIKINFLFFKSLIIYNVPLKNIEIRKILFHFYFIKRFLSLIFNKHANEDDNNKTEIKK
KNNKIKKNKIKKNNNNNNNNNNSSSSSSCCSNRNNDIYDNYNHHNDNKIDNYKNLKKDYIDNRHNNNHSNKYN
NEENINVSKKNKEEIFIKSIKVSINWSEVEQYNSYYNIYFKLANQNIICRILYYIFFKLLLYWFIKIFFKFYY
IKIEQVHVNIYYNYKHRHIKYCDNKKNISKSINVYNIKFYDLSVFYNTLKDTITKIEQNDSQKKKQIISRKDI
QKLSAQDNQTNNNNNMENHTYDNVQHSTYQKIYNNLCCYNTFNIFCKKIILFKENNHYNNLIHVKHPTIILDN
HKNKFFFPFFHINLKTCELKEICYLLNYINKDHFINDLIYIIERNELASYVNYKEKKNSNYEHTTTRQNENKN
YCKYFLNCSFFFNYLFIHLYLKNKLLLKNEIHKLHIIKKNCGNNKHCNNNVCYYNNKSEKKLPDRFELFFNYL
LTYSEKNVIRIGQKKKKKKYNNNNDNNNNNNNNNNIINEIYLKEEKICTSYDNPNNENKILNNKYNYNDLN
IDPKSIYDMSEHHERMIKHEEGENKMNYYDEEIFMKEDEKERNFNLKIYQNCKSSLSLIKLFVIFEIKEMLEL
YEIVQKELLLPHLYINKKEYDTTTTNNDNNKDNFLVSSLLSTDIYIQEFNLFINRNINKNIILNQNTIFIKNR
NEELCCDKSQMVDKEEIIKIEIEEIKNENKKNDIYIGIRDQRSVNQYNVENTYKDIPKENIERERNVHLNNIL
CTSVYYEEDVLYEYSLCEKINIILKIEREDSFNKKINNDKNIIVTIYAHNFLGYMLPTHVNFIYYLYSIYYLR
KKKSTHIFNCCMRNVLREKCRVNKKKLYEYKNTEYKEKDEKDEKKSSLFIIKIISKNVKLQFFCISNKYNVLN
NDCNVVCTKKGNNKNVINNNIISPLCITFSLYYFLKMNLNYKHLSYKTYCLNNHYFYFSNIKVQLLSCSNILR
DICILEKKKKNYTSCRKRSKELSNDGNTYKNLPCDMYLKISDKKIDINIINNVKIIFLNFIIYQIYIAIRRIV
YLDNIKLNYKSTKKKKFINEEKRKRKVKQNIYHHMLLKKNYFLRKTKNVPFEKNKEKKYIYDDNNGDNNGDNY
NIKDLFLLNKNAIYNSYYYPYDQQEEHNNNNNKKKKKKKKKKTESDVTIPINYKRKDPNKICTYYNFYNMKKK
KKTSLFDFSHLLKYIIHIKLKSHITFISFDNVYNSLEKLINIKKNEDLLKNQYEIYLCSNLCIYYFDLLHRYN
IKKNNFIKLFMDFYVFKIYKRLYKNKIFNMCSFKRYYNNHLEKNENNINTKQHKINNYNTIQNNEESPYFRLI
STIRNIDFSYEVLKSYHKNQHIHDINKKFDDKVYNMKKGIIYDEFIRNIKKYNTNTKIYCNHISVIYKSNDFF
NFVNNYILRDFLNIFSLKHPYEIKQKEMKYIKEMKYIKEMKYIKEMKDIKEMKYIKEMKDIKEMKDMKDIKEM
KDMKDIKDIKDIKEILNYNNMMINTYGSNKHLCNNHEYYNFVHDEVCNMFRLRNFIIFKNMIKKEHVEHTKGY
ESKGFSSYNNMYRIEKKNMKNMIISLNKYKFDFNNVLLYIPSIYNNNNKFLIFKSRNIHIKNKYIFKKEDNKI
KLYDKILVYFLNNYCFSYPTKNKVMDNFHILIQYYRNVLYKKKNPLFFLKIFLCGVLHMCPEDFNLLQQICVD
NLLNNNVEYFYDKLYYNFVHKQENQKNNKKNNNNNKKKNNNNNKKNNNNNKKEILKSILHCNDNKNFEGNQDK
NDHRNVYNIKKGKYRKDKRKTYKDMHIILHFLNFQIFIKNNYLKCNMLYIYSTYINVDIIKYINNKDTIITHI
KGFIPIVLSYLYMYPNNIIMYHKKLNKKEYINIISRKYNRYLYFDISQFNMFIKNEVKKKNISIFHEKNNLLK
QNECYEIFYKRTKDNFIGKEICNDINIKIKSLYINSDIFELYSLYIFYFVCDKNLFIYEINKLVQFERYIKRL

NRNILSSLDMKKKKKNNKKKNNNIYKNKNNNIYINKNNNIYKNKNLLIIRDKESYHDNNYFDMLIYLYKKKKM
KNIKDTDFNNYSSYIKYNNIYKINVNIKHLSFIFNFMFYEHIIQPTKLFYKQIVKKKKKKDVYNTNNKRQYKN
ELCILYKGYFYVNFIFNTWKNMVHYFVHNKKYMCINGYIFDVNNIVENKKKNVSNVNRICEQNNMSFYKDPIF
DNDINKKETNKNRHYFQNKEYSDILSVLLHLYENVNNIHNENLNIHNGNLNIHNENINIYFTIKKSNKLYLLS
PNISYVLSSNMNIQLNCQYNKDIYFVDNIKKIKFNYFNFNDIYTDHLGYRGISPAHKNNDPKDEYEDININTN
EKINTNEKINTNEKINTNAKINTNAKINTNEKINTNEKINTNIYTNICKNNPFTNVIYDIKLLYHTSDVKTCN
KNEYPLLNNVHKEYNKLDTLKTNKDCSNPFFINIKIKNLNINITLKDLLYLINKFKEINYFITYYEDIHVLLF
YKLSREMLCNQYICRIKKEKRNTSSHTIFFLNVDMYKCSFNIYKPICWYKQKEMYHKKKIVKIISNGSFSYTS
CINKKKDIDNTIFKKMNISLHTDIFFSNNNINEEFVRNIYVTFIYVKRKEQNNLFVILQNDSINVFITKNVFR
KIFYIYQCITHVNNNICNVKNKKKYNCNYNKKIYNCNYNKKIYSCNYNNRDVKNYDLTNIHINEKNNKYDTLQ
IIQNKKDLFQYNKDHIYYNSRDIYSLLNNYKEKIFFNQSEDIYNLKNDKETCLLMFYINKKKKKTKSKEYPQS
LININVDKNQKIDTKPLYIFPVLRENYIKKEDIIMYRKNNIFHFFTNVCIYNNTNYDIIFLYKNYKTLLYSKK
NNYISNFQHVEEPFCILLFYKKKLFVSKKINIYHNFQKGSYHNRFDTNHLHIKDRRQHKEIKKTFNLCENINN
NKMNRNIKDCNYSSGGIYHIPCNQGVCYKNLVSLKEDEKWKEYFSLIRCHGKEQDKKCNNDIKYTCHMPNNTH
RMFELIKRKYIKSFLNNTHISYDKNGIEFIIDMTAIQNNNWLYIHIDAHVKLLNLLPLQVFFKCRDKIQTIKS
FQKKDIFFQDITLNVSSNEFKDVNKLKIIYQKEDETRKKGDTIIINHDNKNKNNNNNDDDDNNIIPCVYRKQN
FHLNYFRFNKEIIITCHTVIFLYDVHMKNIQINDDKLFFVTKDKYSNSNNIANEMKEPYIYPLKKIKKFESFY
NEPCKYIIMENKKIEKIYSNTNIEIKIKGNNQSISNYVHTYMIICWKRQDQLSNYINDKLHMSSIFPYYDTNK
ETNDITQDRKCYNNNNNNDINNDNNGNNTNYLFNNNMLSPYNNFINQIILIHPYFILINNTNFNLVMCTSCNF
YNNNNNNNNKNYKTSEREINKNKRNYYFPRHSSHVINLISLDVDRNFFFYISNSNNINNYPFICGKVDITKEST
IILCFLNNKNIKHKENTTKHNHNNNYNMNNNNDMKYINTEKQTYHVTFIHLKISICKGFKLQGYYKKNVFPNS
MYIIISEYVNNVCKFEKYISYINNYNSNKIYVNHNIQKNNVMNIYKYIKNESQGGHIGVRKCSKKKCIQHEEY
NRNGDEYDGDEYDGDEYDGDECDGDEYDGDEYDGDEYDGDEYDGDEHNGDEYNGDEYNGDEESVKKIFIYNDT
EREILFDIFDLSEMIYIIKKTMDEAKNKNEHNNDNNNYCIRNVPFDEKETVPSIKEKEKTHDLIKTKKKMKGN
FFINIFNLYSSDENTSGEEKKNSSQEFIEYDIQNKTNVLSSKKYIDFIYKNDNSFKKYIKDNEHLFRHVKKKS
IAFFPMRKNNGIKDCFFIINTNYEEDDEKYYPKDYHDEKNDVRYNNNNCDYDNYRSYKLFHFSLKNKKNHNKR
YVIKYTRKEEKKTNYIQILLIQKKKKLFINISNMVDNLIQVKGKINMNILHKQNQDVIKTEEDISTCCNMNKT
FKCNVISPKNVCNESHIVQNLVYNKFIQIIKRYRRKKKIGIIGRNNKGYLFLNNIYRNIINNCKDDKYKHLFK
FFKKNCFLNQGMTYIVWKVLTYVHKERIKVQANIKDDTTKQNNNNNNNNRYHNNNRYHNNNRYHNNNRYHNNN
HHHHNNNRCNDFHMIKSHLFGYHTHSRNIHLYEKNNKNIFYIIKNKQEKDNKEALLPIDEYNYYVHIKNDIRL
HLFASVIIEDNYIHRNKYELGHSNDVYNFSKINYYHNKNGSSLNISSSHDTSYEHSYNIHDNIINSIESMAFL
KKKDNIDPYDDSYDKHINNFMNNKNIEIKKENTNFSSILYNQRENKKIKKNDKEDINIYKKKKKNAHIFTIIL
NNINLVINKKCNYVEKYHIFLTIKNILLLYMLSHEINKIFFKKEVDKNKNEDHTNDLFVKKKKKNYPQKDKCV
NTYDDCNHIHSLIKIEIQYFYDNNKCCFYIKNFSFFLSPIYIRLSSTHVEEIFFFYDYVCEFIKRKRWEKEKK
KKNKKENVYEDNFIFFTIEQKKSNEETKKNISENMKYQNGICKNICVGKKNCVGKKNCVGKKNCVGKKNCVCK
NICVGKNICVCNKEVIYLNLEKDTNITYPWYVKESSFCFNIKKKKKIKINNIYISTLNINLSCNKMKRNVNIY
IKKRDPPKGKDNIYNILNYFYDVIKDADFVLTSLNLKNIKENDMLNFFNYIFNFYLKEIYKNFFFYLTRMNLI
NKYLHDYYNFFHVIIDNSKIFNIDISNSIKTFKQELQKRGNQFLIPQYIKGVYEEKEYSIQENTYKYKDMDPI
DDKQNQPFDITQQKYINKETEITNEMFLSRKYKSLLLQNKRNISEHEQIYTSADALETYSDSNVYIKSKRKKK
IQNVNKELFYIHNGIKRYIFNYPTVNSNYYKYENNDDHNIINHIIMDKNIKQNQQMKNIKENEKEKEKYNYSN
FYLFNNTLKSFGKDIISSINYFIYNNDDHMIKNYEKDTLYFDLDKKHIIKYFTSTMKKIKCIKMKECLHSYEK
KDQFITYFPIYKKNTIFFNPSSIKNWIIFQNLPLIQLHNPQLSVKKEDMLILFCEDTIIHIKSKKKHCVIRKE
NIIKIEMCCIYFNITFECDKNIIFNIKNIIKKNKNNFLGLYFDILFKNKNKKRLIKKIQKNLVKILYFLYYVF
HFIYNYNKKIENMNKLKNTNLYHNINHQHTNKKKNTTSYKSKEHPFITIFVQNQKTIFSFSSITYFYHFFINI
RDQLFNTPN*
>N.PF13_0138
SAFWLKYPNNIQKHIQNIDVIDRHIDTKEKVLKMKRIIHLKYYVPGLLKKLFNVDGSGLAIEEILINLEKKNL
TINTVNYTLNPFVNITEKCEYFQKENDQNNTHYKQSTTLNINGFGYMKSLIENTIINTIREKSKQGISIMNDT
IKRTVNDNIYINNLDKEKK*
>N.MAL13P1.134
IFICSRTQSQLNQYFHELKKIEKKMGEDFSINMIIIGSRKHLCINEAFLKKYKNINELNDCCKNSKCKYKEIY
NEKMSEKWKKKNGKKKRIFYDSSCSSNSSENEDIETIDNYILITKLINNRNVDIEDIKDICKNEHIEICPYYL
SKENIKNADIILLPYICILNENIRNNLKINIKNNIVIFDESQNIIENINSCNSVSISNHHILFCKIILNEYIK
KYKNVLNNNNIIMIKQIIVYCNLLLESFTSIQQNLININKYTLLSKLDVLNLNNISNFLNNSMFCRKIKIFTE

SLMSTYFKNIKKYLHASFPIFKTTAIYILNEFTQKLIKSNLYDYVSIHYNDNYSPTIEHSENNLMNQINHSTR
TLNGNNCKDEQNIFSTDCDIDKIKNKKRIMTSHGLTPVDSKKNKLQNGNHNTKKELFMNDWDDSKDQDVNDVM
CKRSNCINNTQKCKNKNIKMSDDNFNNDDKKGFNDDTYNNLNIFFEEFFVKKRKDLFNEIEVISVSSCNNFQS
ITKDCSNVILIGGTLYPMEEFLLLFLNYNKNKIKLFSSDYIFKKQNIFARIFSTNLISSDFIDNTYKNRLKKD
HLLNLAIYIYMITFNILYGNIIFFPSYNFLNDFYNFLNKEGEYIFNRIKKKKFVFFEKKNDDTIVKNYMNNIE
NIKNIDPNLRIQNGCILFCVMNGKLSEGINFYDDLCRNVVVVGIPFIKHDSVVDKNLLRLKYYREYTKDIMNT
HDKNDNTKILIYDDVHKMCESYETKYAMKIINQCIGRSLRHVNDFSSFFFLDYRFSKKEIFQNLPSFIKTHIN
DIDNIIYKNYDDTENSYIKIKEHFQDTYANIKHFYTNQMSSCGFNFQPISQDLISNLVKDLDMLKKFHDTKNK
*
>N.PF13_0139
MNEYVKYTTSFYVFDLYKYDTSEEFLKSLQLITHEDIIIISLQNVADQNLRETYESIINKQCNQNKEYVFLHL
DYKLENDLFLLMIKRGLHTYVEDVYYKIIKISTKNMFGKIYKKVMKYYFIDYIQKVINTNNHSIQIKCNKRMD
GNNNVSTYFGNEEKKKEFNITLRKDMLLYQMDHRKYFNYLNKCFQHAKDINLKNDVNIKNKNMNKKFNHSFSH
QYNINQYEIFKKKKIQMKGTYSHDNTFNHNFAYHYNSDTILVGDRKERYAINHNICNMKNKKEIYTHNSNNIY
DNNNNKKCFNNNNIYDNNNNICDNNNNICDNNKKYDRNILHCITLMINNIRFCICFCNFDIYKYIETKSYYQY
IFYKERERKNNFYKDKENNPFYISYMNYHLNLYNIFCKQLYNHFMNKIIFETKKEDMYLFSMDIIIFIGFDKL
VLCNINENMKPEKDKSKYNNIKNKYKYLSNNSLYFDKNIIIFNKNTHHFLIQKNYKTLTREKKLNLIPIGMKS
VCDLYTLIKINIIDDFFLYHKYNKQEQHYSFINHLKSKIKKNKKKNITISVEPNIINLNVINTYEIYHTSFEI
CYDYKEIEEHDIYTYEKSHPNKNNAQLISSTFFFNNNHNTLLCDKKYYEEEKEERYSSDGKDNVCCVIFGGNK
KDKKFDEGENKTFDAGENKTFDAGENKTFDAGENKTFDVPHDYKIISDPIGEHINDDIIKNNKNHLKNISSNY
LTKQGKKYNYLFHLLHMSTRKKKVGGKKKKKKKKIYGTNILEGKEKIHNDFNKKNYIYHIQENFDCNGSPHLN
YMSDDYNKREYYRETYYKHDNILEEIKKENISKDDIYINKKSTDINDDDLYFSVLCFDAQTKSTMPINLYSFL
HLKKNTIFKYLEENIRFKEQAQDVNCKGCMVINNNDNMKRNNNNNNNNNNNNNNNNNNNNNNNNNNNNNNNNN
NNNNNNNNNNNNNNNNNNNNNNNNNNNVIINHSDYMKSIQNNKNMKKRRTKRIQNNKNRYTFATARYIYLQPY
TGIIKKNTRKKIELNIFIEHILKSQIIKDSFVLIIRIHNFDKDIFLTVKCVIQNNIITSLIHDNLSFLKNKKY
IHNRDIHTADMCSSDLDNIKNKKTIKKKYKIKKNKINYSFHHMTNMSLLRYRYNKKIIRYRKNQPENKMDNII
MLHKMNVTNNISHNMNKKTPCQIQNYSLKNDCNNILKSIINYKNMFKYDIKKKKRGSYFLSRNFTKFLFFLFF
HIDKKQKILFCNTTNEKIIDDLLYLFNLSNMNYYHLLYSFHYIFQTSKGNPDMCEYNYYSKKRFFDMVKRYIR
RNRKRDINSEHDQRDGSKNKVEMKKWFFSRGNNNNNNNNNYDNNDNYDNNNNNNYDNNNNNYDNNNNNYDNNN
NNYDNNNNRCYDNAFFIHDKQRDMKHLHNDSTVLYNITYLIRKIEKNEKIQNDISIISLLFLCEQFFYIINTN
IITSQYLSYIKNIYNNMNIKKKKKMYLIFLILVKNSFTCIYYKNIFLVFISFIKKIFIYFLQLNIQHFYVTVM
RQNDLTKNKNKKKIKNDDNIIDDGDNDDNIYINTFKQNNYQHRINYMQAYYPKNNKLKYYFDLFLDSFNFFHN
AFLIYVSSFIFSFLDIQIAIDLTHYILFYL*
>N.MAL13P1.135
MSDFSRDMSNNENSKKIALYSILIYKYESSNQPIFLTSALDLSSFPFFHRSSLKEHIYFHARLVCGRTQKGTR
EVIELESGIGHLHIYTNKENNISVLVLSTSSYPLRIAFSLIDLTHKLFAQKCRGMYEHVRQDLKEGMLIQNEL
NDLLKKYQNPSEADKLSRVQKDLDEVKDVMLKNIEDLLQRGEKLDDLMKKSQDLSNSSYQFYRQAKKNNQCCS
LY*
>N.PF13_0140
MEKNQNDKSNKNDIIHMNDKSGNYDKNNINNFIDKNDEHDMSDILHKINNEEKKYEEIKSYSECLELLYKTHA
LKLGLDNPKKLNESFGHPCDKYKTIHIAGTNGKGSVCYKIYTCLKIKKFKVGLFSSPHIFSLRERIIVNDEPI
SEKELIHLVNEVLNKAKKLYINPSFFEIITLVAFLHFLNKKVDYAIIETGIGGRLDATNILTKPEVIVITSIG
YDHLNILGDNLPIICNEKIGIFKKDANVVIGPSVAIYKNVFDKAKELNCTIHTVVPEPRGERYNEENSRIALR
TLEILNISIDYFLKSIIPIKPPLRIQYLATEQIQHIKKKFSPDNLEHNVQYPLAVILDVGHNETAIDRLCTDI
NYFHKGQNIRICISITKPRNLSVFHPFIAQFGDTLKDIFYLPSLNERTYDFEEIVEMLNNEEEIKNEIKELIL
SSSKKVGKWLAHEKQGNINEEDALKLYKRGCIPLIIKNAFLECCKDNSILLVCGTFFVFDEVLNVFDIHSDMQ
DTIFMNEPSLV*
>N.PF13_0141
MAPKAKIVLVGSGMIGGVMATLIVQKNLGDVVLFDIVKNMPHGKALDTSHTNVMAYSNCKVSGSNTYDDLAGA
DVVIVTAGFTKAPGKSDKEWNRDDLLPLNNKIMIEIGGHIKKNCPNAFIIVVTNPVDVMVQLLHQHSGVPKNK
IIGLGGVLDTSRLKYYISQKLNVCPRDVNAHIVGAHGNKMVLLKRYITVGGIPLQEFINNKLISDAELEAIFD
RTVNTALEIVNLHASPYVAPAAAIIEMAESYLKDLKKVLICSTLLEGQYGHSDIFGGTPVVLGANGVEQVIEL
QLNSEEKAKFDEAIAETKRMKALA*
>N.PF13_0142

MSTNSPKDFVESLKGRAVIVRLNNGTDYKGILACLDERMNVALEQTEEYYDGELTDKYNDAFIRGNNVFYIRA
IEED*
>N.PF13_0143
MSFFVSKKCISNWYKKKSNNFFYDKEGWYGCKVAMLVSTGIIGGYYISKEENRKDFRKVCLHMRNGLKNNRFF
DNNMLFLNNNNVFCDIGNKNKNNNYHHPLWRSEEKRPFEKKMENAILFSGTSNPLLSKNVADHLGTSLGRVNL
KRFADGEVSMQFLESIRGKDVYIIQPTCPPVNENLIELLLMISTCRRASAKKITAVIPYYGYARQDRKLSSRV
PISAADVARMIEAMGVDRVVAIDLHSGQIQGFFGPRVPVDNLEAQLIGLDYFTKKDLYKPVIVSPDAGGVYRA
RKFQDGLNHRGIGDCGIAMLIKQRTKPNEIEKMDLVGNVYDSDVIIVDDMIDTSGTLCEAAKQLKKHGARRVF
AFATHGLFSGPAIDRIEKSPLEEVVVTDTVKSNKNIDSCKKITKLSVSVLVADAIRRIHQKESLNDLFNIKS*
>N.PF13_0144
MTSVKHPKISVLGAGDIGCALAHMICEKNLGDVVLHDFRKDLPKGRALDILHTRPLNRSRINILGTNEITDIK
DSLVVVVTIEVSEREFAEFDEEDLEKQVYTSNVKLLKEVAKSLKKHCPQAFVVVTTSPVDCMAKVLQEHANIP
PHKICGMAGVLHSARLRHNLAEKLRVNPGDVQGFVIGAHGDKMVPLPRYCCVNGIPLSDFTKKGAITEKEINQ
IVEKTRNTGFELLELLPEGSVCFAPSLAIVEIIEAYLKDLKRVLVCSVLLNGHYGHKGVFAGIPVVIGGKGIE
KIIELDLNTQEKELFDDSLKHISYLFDNYKHETVVDDENKPN*
>N.PF13_0144a
MEDELIQKVKNISLHKENKNVYDEKRSLKKKIRDLENMINNKIMDLQTELYKSKEKEKVHEYDFSDILKEFKD
DVKKKMDEINDIVEKKIEEQNVKHTKLFNMLISLKNENSSINKSISLLNEKIHMIQEDIGD*
>N.MAL13P1.137
MLSRSPVNKKNKLSSLPNKYFKSEIKNENISCKKEKSGDKKWEDTNSMFCKSEDREKNIMLKNIIISKNSIKN
KICTSKNYIVQKDMNSKFASLKKNKRKINLHKTSILSMKSKITKISSLLKRNEHKVNRKSDEHIKNKNHLSRN
KIVNSYKLLYDIERMRKNKKDIMLIKNENNSKAKKKSVLIKSIIHKHYSEGAKMERKYINDCIDNECSDNHNN
ECSDDHNNKCSDNHNNECSDNHNNKCSDNHNNDSSDNHNNDSSNNHNNDSSNNHNNXXXXXXXXXXXXXXXXX
XXXXXXXXXXXXXXXXXXXXXXXXXXXXXXXXXXXXXXXXXXXXXXXXXXXXXXXXXXXXXXXXXXXXXXXXX
XXXXXXXXXXXXSNNHNNNSSNNHNNNSSNNHNYVYHYPFKRHMSYNSLPCNIKEQKKNKENEDQLDSHNILE
YNIHKKYSSLTHSNESYKNNIMIKKCNSLVLPTLNNKNEICKNEICKNEIYKNEIYKNDFSNDLHKYNHSYNS
HDKHIKLHNLFDDNKYASPIEDIKIIKENISNNYITYKKKKLQKGSDDIFHNGKVKNDTNYMMDINDYSKEFQ
NNIGNEVDVTNFSFNIATMGKEKCVNKNLAYITNDTNNMNNMENMENYDNTENAQNIQNIQNIQNIQNIQNIQ
NIQNIQNIQNIQNIQNIQNIQNIQKNNLYYEQTIEEDMLDDYEHPDDLEESLSHLSDSFNLEYLPAIPEDINL
ISSNDNFFSVYGKSIRSSSFSNNIHHKDIKNIEKDQNTYPYLEDNIKQYHEEHIYESLGSKDSQDLQKGKNLR
SHKPKYYDDEDDEDLIISMFDNDQEDKNKKKDKHSNDDTEEKIHEHKRNIDIVQKKMNSINKFEGVKEFDKVV
NNMKRYYSINHKTNKKWNLSEYCENDENMSKEIKMEEINTYNMKNIKNMNSTNNINYSYDYYDYYDGEKSNVL
SYNVKESYAHKSQKYASDLEKKKHTKNNIRRGLYLASKILKDIHEEKQKKKKIKKKENLHNYVDKNKYDNNYI
HNETYNDTYSNTIPVFYGIKKSKNILSSSYDDLYVNINKKNNTSINKNNNILCDNKYDHSDIMPSHKGKKCTD
NNYVDNEFLNMSGKNNILDYSLNSGKGYNYNYNYNYDDDDDDSSIIHVDSMDDLKMSYESISLLNNFSYTLDN
SKDIDLNNVHNIYTRINKKNDYNVNVEEGKINISLNEEKEKRNEQDYKFLEEKKDNTIIFDENNDTINGVHVN
NDDNNNDTINSVHVNNNDDNNNDTINGVHVNNYDNNNDTINSVHVNNDDHKHTQSNNIQNFSSQENTYKNTDNI
KTPCNYDKCSNEIIQKDNDFNIKYLYSSSSIYSNTSECNTKNYMKGKNKIHLLNKHINDDYYVDYFSNELDKI
IKKKNSIKEKKKDMKTNNINNNNNNNEDKNKRRNKNKNKKIYINFSDGDIQINNKKKSNSNEKCKINKENYIC
DETKNFTNIIIPKYEIMHKNNYSKKSFNNIKHNQNGEIDETNTIKDEIKDKSKHEEEEYEILHKVDNKKNDDQ
QDDHHNDDNNNKNDDQQDDRHNDDNNNNKQNDDHPDNHHNDDNNNNKQNDDHPVNHHNVNLSNDPTQRKEIYY
DYLHETGNKNIIKNRMSNSMNNMYDLKHKNYYNDQNILYNDLLEDVRHVSFENVYLKNKEKNLCKVKKKKKE
NEKILKKDKDTNLDYNNNNNNNNLLKNKSSEQNINITPNSSTKEFKLTYEHVENENEICISNSNSTLCEDMNK
QNKLSLDNCVKKEGTKIEQLLQNILCTLKNNERDNNNKIYNNNNNNNNEDDHNIPCDHNNISDNVVLSNVHMG
NNQDINENVYNIKPQEFKEEEEDISMVNTKKCDDIQENIKNDETFIEDEMSNKFTTLQYISRSIKVLKEERDN
IQNKYDILKQQNGVIQNKYDILKEQNGVIQNKFDILKDEKDKIQKQNDILTNKCNELQNQCCNNLTGLQNVTK
KYDDLQNKYNILNKLKNSLEEKNEELKKYHEHVIQERDQLIKEKESDKQEIEYLKNNVDVLKIDKSNYMIEKN
DNIDKIKKLEEEKKKLEVDINNTNDILIYKEENILYEKNKRHEERKEKEILKNELDIIKKEYDELLNKYDDNI
INEERKEKETLKNELDIIKKEYNELLNKYDENIINEEINNMKNILIKKEEEMKEIKENKILLKKDIDMYVNNI
HILEIEMKKIKEENNLLKSKNLELLEKEDINKSSIFMNHNKDIEMKKNKNSTQGYYNDNMNEYLYLNVDKNIL
KNVFDHHDKNSNTFNDSWNIFIEEKNDRFISIQENDHTNKKNYNKKNNIMNTYNDHNNKDEFKNDLNCNFLWR
HYEKILQDINHLSIDKLKIQYKDLLKEYIFLELLVIEYEKNNVALHETNKEGNKQIKEMAHYKNLCIELERSN
QELYDKLNEINNVLHIRDHNKEDNDNKNKICNRKGSSIYIEILNKTYNITKNIDIINEEIFNMKEQKNDNNIK
ISNILIYLNIISTDIMFIHTNISYINEYITKSTIYLDIHHLKNTLLYKVSTKDNINVHTSSTLCKDTNEFKKY

NNQINKTNTNIIVDDQQIFKEQEFINMKNENGNVKNIESYVNGDDIYNDNINNDNINNDNINNDDIYNDNINN
DNINNDNIYNDNIYNDNINNDNIYNDNIYNDNIYNDNINNDNINNDNINNHGEEYHHSGDIINKNILFTDMKI
KNKTDNVTNNIINKQYDEHIEDVLTFFEVSFCSDLFLILCHMCKSINEVNCSLRKNDINSCTDSLLYIKHVLH
DINNFYNNEENTSFLNVYVHLFDIFKKSVDKMLNIIQAQKDLDKIYKMFVEIYYMKKIIRFIIFSFFKFHDIY
SVFNVNTFDGVSYCAIPTMSKDDEKKVQNNNEDKDVDIVKNIHTDDNKHINNNKRVTYSHTSHICSVINKYYD
SYWDEYYTSNERERSVSNYKDKKKKKEKYNDINKKDERDIKENDREMLYSLMVKILEYNYNAHMIPNIICTYE
DKKTILNNDNKMWLFDEKRNYAKYVLKIIYEHMKLGNFLINSNEFLNTFEEKSLKNYLYKNIFFKKNYNINID
IIHQHLSMLFKQNIGINYEYYYEQFLFILNKIILNISSNNNIHYLNTTTCENIFFFDNLNIEENYINKCVHTS
PLIDLQHSNKNKSNNNINSNNIINSHNNINSHNNINSHNNINSHNNIKSHNNINSYNNINSYNNNITELRSLS
YNTSTSNTNFTTFKNNNPFNHNINYIINYPLLDIDNKYMSVLFHMWQKMELNFFFKKYYQNNKYKEIKEKNKL
SKSCNYNINNKRLTKNMWSTMNIKDCTTKKKMCPLDHIDSENEKYNMDKDIDNNQKVLHNNNNNNNINNSYNN
INNKDVNNNLSFDNKLHEEKNAENKKSSKYLNKFSSLNKMNTLFYESDHNSYDYSIDTYLKLMVFDIFKCNNE
KKEIGIDDFIFVFKQLHINIKKHVINKIWSILTGKQNLNTALKHKITISTFIKKAYTTNPSLIFYEYCKTKVQ
LKEANKNIKLLQKYNKKMLLLVTN*
>N.MAL13P1.138
MVKSVHKIIPRFYGDHLKRKCNYNKKKRYQKTIPYNFTKCTFSSNSECLKINKTTCNDNDLPSFLDLQEMRLI
GNKKISFNNVYENRVNVFLKPLITDIITDKNSDNNYDESQIILDHVQNLKKETAHEIYENIYEDKKYNQTDKI
SSTHEEDKEQIVKNNNKDDIVNKENLINERHAYVNDENETYVNCNIQNCKNNEKIKRNNTHDDDNNNNDNNDN
NNDNNNDNNDNNRNGRNYNSGVNKKRDLQFLSQDRYNILLKRKHKYIYDFEVYNISELSKYEAGEIVNVYFYN
KLHIGIGLLNRKSNIVINIIDRDISKNINDQFFIKKLYESIKRRFHFLYNIKLYEYIYSFHIRKNLKLFCKVV
NSVHDSLPGLVVYIFDKHLYIRYDNLSIQKYSYIFEKELETIFSPKNIFCKKIISKKEKRAQMGKEYILEQIK
GTDLELNYDENGYTFYNNITNISYDIFHIENKQDRQFLQNIFHESNILNVHGNVGEYIINTSFQKKKKNINTA
NYSDNISIILSDSVKNTNYINKNIELNKCDNITSLHREDILEELNNMYLNNLKFDLIIYNIKSNIVYRKNSYI
SVYGKRHIVTFKGIYSFLNYMADILQKNGLLFITVELSAVDYHKFLNIVKCVFENKKKNVSIIYENSCTIENN
ILCIDENAWYMRSVCFKLNNA*
>N.MAL13P1.139
MDSPELLKIELQRLKNDYENELSVDHVMPKTQFDYACLLICSSDLKNIKFASSLLHELLFINYNRIDCLYQLA
IAHIKLRDYKKAKNYLNALLKIDARNSNALALKSLLFDLISSDGLIGALLVALTACGLYLSFKSFKYF*
>N.MAL13P1.139a
MVKRFINISTSLPFLLKNRMYHKKKFFFIQEKHICNQHTPLHIYNATKYSKKSMNHRLFYINWMFVFLLLDFC
NGHLDI*
>N.PF13_0146
MMNDIVTLSYDVILERLGKKYSYEISLRKYYDMIKCRRNKEEHFLEASKKEEEIKGMINLHNEDIKNNKINVI
CNVPNQEVQYRRSQEKHADQKKDVDGINEKQNDFDEVILSLYTDLVDLMNYNNFFIKDCIVTFIFVDKDVYKG
KLDLYFGRIEEENKNSFNNMGNEYLNDYDDNKEKDNMIEKRNDNNIYDDINEYIFFEHYEIYGNKINNIKDIL
RNTFECLFFGMVEQYNIILKINYCHRIKEIYNYILFNEEIYKTLEHADYIEIDINKIHFDIIDYSFIFSKNLK
KFVNYLKYVKDIHSCSYFISLLIRNIAYTNKIYKYTAQLFIYNNTNNECGTIFLRFVVNNKKEIFEKIGILKR
KFTSGNILDIDISIYDHIKNIFINKYNMSFPEERIILSYVKYYYVYRLVNMKKNCDYNTNYSYIFSTNKESNI
SNTTHITCSSNSYETSDVNCQGPLYIKRKQLQKNIRKYIKCKGKNKLNTVKDKTKLSATQEIYDDDDDLNELY
KINNNQNNFKSPSDCYLVRNEYNHKTRQYKNKIIYDHDNVYYSNKCVSQDDYNYYNDTYDDDEKDGNVHNKDV
HKDVHKDVHNNIHNDVHNNIHNDVHNNIHNDVHNNIHNDVHNYYNQPNTYDRKQNYKTLRNNKRRNNNRSTKY
KKCDDNFNKEESIKKINRKNKEYNYNINKYNSDSTYKNKCITSIIYDEKENQKKERKKYTHNSRVTILNNKNC
SSRQIVTRSMKYKMDCSIETNIDSKSSLNYLDILDEKFHIKENIIIENLILDDVLEKMLTQRINVQNDNIVTL
KNRKIKPLNKNVDVKDNYVNFYSDDNEFEEEINKCIWVNEHTLKRNKRKKKKKRQKKIHKHIHKHIQSELDNI
NNYMINKYNNDIYQDIISCDQGSEHGKEDTCNNIERFYNDNVKMKNTYNYKKRPYNYATDISNNNVMMNKRRK
ICSIQNDENFALKTNYADDKKIEKPLFSESLPCDKNEGNKMKNLFYSEFLFDYNPWNDNDVNKNLRDVFYEKQ
FCKIMNENFEVYTIEDEEEANKEENNKEENYKEEDNKEENYKEEDNKEEDNKEEDNKEEVNKEEANKEEDNKE
EDNKEENYKEENYKKENYKKEDNKEEFNKEEFNKEEYKDDQFDEDPFIKDQFVEEEHIEYHDNNIIIKKLIEV
YNVENDSVAMYILNNEDESKKNPKHHQHIFNNDEKKESKSIDLFNKENDKEENNKNSLNNKEYKEDIKNKTYK
SIKNINMKYSTNVLKKYPNDILKKGMMNKNVIKEKMNNEKYKDNKINNNQNKNKKQCKNYFIDTCNNNKQNNN
SVEIINMNNIPHSKFMYNNYENDLITESVYNEIMKNLSRNMYFLNDEDNILTCVYNYKHILKNILYLENVIEK
YNIHMSVIHVSNLQNVKSNQENNNLLNENHPNKISDGNYNKNNQKDNHMEKLKQIIHHIKNKCREYKNTLYKC
KRYMSCKYIYGKQNCGLSINRQKKKKKKKKRKNK*
>N.MAL13P1.140

MDVNNSFVNMKDVMNIPVQVEGKDGDKKHNNMNDNLLNFDCSYENILNNDIERPQEIDCGMSCTSSFVDSNEK
MVNDNYIDNNYEDLNSAQGESKDINHMKFEKKDIINVPETYCKNNYDYMYNNALNLSFKKNNDQIYMNKFGNI
NENNKEDNSNNNFVLDDNNLMLNEGNIIIKNMYPVDMNNHNVGSEIHDNNEHSCILRNNGKVQNEFRHEGNLS
NDRSVYGELIDMDNMDNMNNVNNVNNMDSVNNMDNMDNMNNVNNINSVNDSKCIHALNAPGRKNIKTKDAQDY
FFNNENIHCVSKSTTYILNIGENGSTCINDIISNSNNSTSTGSKKNKRKEEKEEKEEEEEEKEKEIEVEKKRE
KMNMLGNIIPENDSTNKENHYYDLENNEIYKAMENDIINYKHIIFRLKCEVQIKNSYLENEKRKNEEYEKVIE
NYISDLNKNNKNNKNNNNNNSSSCSNVYIQNNLPGAEPLNIIEVENRQLKDTLLLKSSEIIELNNVINNLKNQ
KMIFFNQLENELDMSIKKETEYSFLLQSQTKRLDAANAYIEKQAIKIVQLKKDMENLENTYNMNLKNNEEQIK
ELLKEKEEFIEITKELEIINIDNVKKKEEIEKYKEKCSSLTDELNELLRIISINQHDKIDTFKYHNFYESENM
IMSYNNMNESLQGYYYFEKGNNLKKECYNDKDKNNENVLTIQLKRLIDENEILQKQYEELNNEKIQMVQELQL
KNVIIDKKNENYTSLLDKYNILEKDFEHIHNLCIHFEEKFKQECIHNNVNDVNNVNNVNNVNNVNDVNNVNNV
NNVNNANHVNNANHVNNANHVNNANHVNNVNNVNSVNNVNNVNNLSFDKMNSFLNKSNFSLSELDINHSQQSE
HNINHYKIYCSELKLENESLKKIVHKLKIKNNKLSKNMEYLLNDLIQTEGINEENVQIHKNTINNEHIPYMEN
THTTNVYYNNHKDDNFMEETEKKYSYQETVDILERHIKNKEIVTTDNIKLDIIKNNDIKGNHINDDIIKGNNI
NDANIKKGFNCQTGNIILPDKKDFNCQTGNIILPDKKDFNCQTDNIILPDKKDFNCQTGNIISPDKKDFNCQT
YNIILPDKKDFNCQTYNIISPDKKDFNCQTTNIISPDKKDFNCQTDNILTNTNKYLNKEINTDEKIVYDQKVD
TKDEISFMNSSVNTEEINRTEKSVNTHIENINMEQKNITHDFIQTDEINCYIKCTQTNDVQRNDKGVVTNMCG
VHDFNIKKMMIKNNMLKNKKIQVTPTNMNAYTQTMNTFYNNNNNNNKKKYIYIYNTNDEYHTDMKYSNKGNRN
YSYISSYSSNSYNHIYKKYESSSDAYIIRKGDGKKKKKKNHINNEKAIIKYSERDNMMMSQSNKKKITQIHNE
KKRKEKNIYMYNKKNVHSQGGNNSNRDVESLIEILKYSSDSMDYTNKEYDDAKMCDDNFYDDTKLCDHNFYGD
TKLCDDNFYDDTNLCDHNFYGDTKFCDDNFYDDTKLCDDNFYNKYYSRSNIPFRKKKERLLHTHTIKMKNKYV
QTSHSFLDDDFFFYNPKDIQDNINHDNDLRILKKKIREYSKDLYHTLILCENCKGFYVDMFLINIFNEYINYY
NIIMNNNNNNINRDTINNMDYYLNFDSFVSNPFTNNIYKNKNIGNNLIIEKKITTTRTNTLDPVYCVYNNMNE
NSQHSKNNNIENYAVHKKHACDKNHICSTHQNNVKEKLSSCYYDKENKTNNNNKKKKIFKMEKYIIKNIPFNI
HICTCSYVNLKYKKILIKKDESNKKKKNKLKVKNYMDTNVYKDENNNNNNNNNNVEKTNPYAINNSVCSQIFN
FIHNMRKEIRQIKYIIHTILFFKNNDPMKHMLLNNNNLNDISMCLFNYIQNENEINNVNNYVNEINECDDKYK
YTQNVYNKKVCVHYISPINVKSSNDDNINSFYNNEKSECNYNVCFDEGKRKNDNNIRDDKINGYSNNNDNNNE
HNNNNDNNNEHNDHNNEHNNEHNNEHNNEHNNEHKNYKESTDKLNFIHFYNDSNQKKENYFHNYTHEQNDHES
SSSQFFGIPKNQTSIYFYKKEKKMINFKEFMKNYEVKNIMIFFICCIFYLNELYIKYNNSLVTNIKPIDDNTK
RDIHQGEERTEIYYCKSDKINMNQVDNSNNTNCKDDDKSNNTNCKDDDKSNITNCKDEEQSNNTNCQDAEQSN
ITNCQDAEQSNNTNSSHMNHLNHEVVIPNDKLYMNTYTNNINPFDVSIFKEAEIMIEEYRTCLHENKRMNILH
DILCNIILCIKNNYDNIHNVFLLFNDDITKSDILNIKYIYKLIDNYSKIDSYIKHINNSCNEKIRNTENNFKE
IISNKEDLINIYKENIKNKIQLIEEKDLIIKDLKLEIHILLKDKEITNNLLNELTEEKNTYFNMYMKYHHEWI
LNKKEIIMKDDKDEYTYTTNDEHNNHKKINNCINQINKLNDEIILLKNKNKTLLHKRDTNKNTSSSVCSHMSS
PSPSRISSNSIFHFKQTDNQNVENADPNIIIDNNNSNTKEKKKNSLTNLILKDKNDKLKNFHIKENITSTIHM
KKIYKILFLLKEKIILFIFYIYKKIYKNAYINISYIKQFYTQIINIFINIPRYICSSYIFVKENEINYNMYIK
NNKNNKIYLHTNHSHILQLTDDNTSLNKQKINEKKKKNQNIKIKKYSHNKRNLYNHNFLENTSIQSSTSPQKK
KKNNNLQRAIGKLSDEDSSSNVSLFFDF*
>N.MAL13P1.141
MMINYFKRIQGKFIPCFINHKIVGNLHKINTNAQNDVIENINVKQESDVSYIGENIDAGNIDYKYCPYLKPLE
IKGIKRADRKLDYNSSYSFMNKQKDFTENFKTSLFTKCLYNGMKIPLPPKRYKISQYVDVRLDMFSPLIITCI
ICFPFFFTRFMWSVEHA*
>N.MAL13P1.142
MDDEVVIDSNLMKKFEICANFNNEGYITSIDLLNNIIATTNTKNIIKIYDICERKEKLTYSVQDLVINDIKLL
KKDLKENNGLSINYEIQECLFTAYEKNKKNKIYHYDLINKKVLHIYDLSDEIINNSFVLHPNTHIFIVVLKSK
ELIVYNIKNKSIIYKTNVQNNNCIVCYNKSGILYGYTGNVNTIYLCSCFGDDYNDDYFASFDISKVTANDNYC
IHIDFSYDEKKMLITTKNNSIYTLDAYTGHFLYSYNFMYENNSYISSYLSYPIFSFDSNFVLSGGQDGHLHIW
DVQGNHVIKNNIKNNILYIKWVYNRLAFVTTSNNLLIWKPSKNF*
>N.MAL13P1.550
MNENKKGTLVEKENEEKSLTINKNILFKEFNNNYDLKDTFVNIVTDYSLESEKEKSNISLFNVFKKKFKEEKY
YSRFLLLFKIPNNIEEKKFINKNLIDIINEKYQIIKGICLFVNVYSIMFLECIDTKSIFFFLKHLTTMKYISH
INVLYFSELNKQYINEEFYFYEYKKDDSKGAISNECNCVDEVWELYLNILNLSSFIKNNKDRQNIFEKNEYIK
KSFSLLQNFYTENAKRYIWNIHEFLSFFVDDFKLSVDDFSDDFLDF*
>N.PF13_0147

MVKKKDTDENISDEHSRRSEHKKEDVERHPKEKRSLSNSSQAKRSSSNCYSKEKHIERKVRRGRNSHSNNSYE
TSSEEHNDKSSSDEHRRRRRDRNRDRERDRDRGRDRERDRDRERDRDRERDRDRDRDRDRDRERRSIREERSG
RDDKYDRRKKRRHSNLNRNLTSSHERSRSRERRRRRIQADCIKKAGGFKRLADMEGHETTNVFWDGFQWVAKT
NQTNPHHLDPAIMNSTRKLRRLYFGNLPLHLGLSENAFQEIVWNEMKKRKYCNDENINPVLYVWFAKDKGNYG
FVEFATVEETERALTMDGMICKGVALKVSRPNDYSTNTVKQNQNMLLQNINKINNNNNNNNNNSNNNNNNMIN
NVINNTINNMHNYNNNPYNKPPPPPPGAPPQSLYMQNNLHSSLKNMDNIHTKYLRVIEIVSLESINHEEYSTI
LEDIKEGFHSQGLIINAILINQKYVQNTPFNIGDVIIEFESEDSVDKSIQNMSSRKYEGKFIKMDKCDQHTFD
TYVKPIIRDLYDNQ*
>N.MAL13P1.144
MSTCNISKVLSGTFVEFQVVILTLDENHFASELCDNKCKALIKICNRCMIYYIIKNIIEQRLKYITIVVNSKY
YDDMVNYINTTFQDNYKYDDKKGKHIYCIDIEPYNTNNNEDIGSIQCLLQIKNKIKSDFIVVNCDILGFVDFH
SLANLFRGENAICAILLLENNQPSNDKKKKEITDEYVNLENNVWVCIDKNSKVVSIKDSLSMKENGKMKISKV
NLLFHKNFVLKTDLLDSHVYIFKHYVLEIMEQKKNKFSSIKYDLIPYLVKIQNTSKAAEYSKGEFKYNMYNTL
IEKYEGDDEIEEGKRENLMLDIINNENVESVVCYIQPKNNGFCQRINSIPNFFKANLLFCVSRQDHLKNILPP
YCFFLLTEKNQSFKDCIISSHFDHEENILLKKSILGKNVTIKKNSSINRSILMDNITIHEKCVIQNSIICDNV
VIEENCKLIDCIIKENSVIQKNSVHEKETLPLFIS*
>N.MAL13P1.145
MISKGNKNDILNNNEENRNISFLNPYENSKISKDNSAEEIAEKYLRNKVSYKTINKNNYNININNDYYDNKNI
SECNNYVSNDNTPYYLNNKNENVNLNYVGAQNFEKKNDYMYTNLFLNTSKITKLINSCNTSMNPNETNKLYKS
KEEEGYNNYIKNQEKNNVVNTIDDLLNKYTNNTSIRKNDEKLGYFKYSMTKPYMLDAKKEITNKLRFDDTSER
GRSKYIMNDNIQYKDNNGITINNTTNNNNNNNNISNNNNNNNNNNIISNISNYNSRSNSGKRNLYEPQSHSKKK
NLIQRNKDYVHYLSKRNSILKNEMLVKKDLKKKKMSVSSYAENSFMKNKIKEDKYKRNNFFFYDTYSKNEDGK
YKEVDLINNMTIFNKKKETICEHKPTFHYNENNNPVINNMNIFEKDYDKIINNSIIYKCNKGCEHSRNSNDNS
LFDMSFLKSTYSDSSKIIDSPLSYEQDNMSIFNISNNKNKNDESVINVYDSSNLHSKSTDNVIIYYKDSNNKK
MVDVINVSNECTLESKNKTSDIYSNIYNYDNKSKTQNGDNNVYINRQMSSALFNTNTDDSKKGDYLNNTNKII
SSGLNNDGNNIRNNTYNLKYNINYNNINNDINETYNNNIINKKEGYTNYLNVRSNYSTNNINKEYVEKNYKEN
ENLSEDMNNNNIWNDGKICIKNRNNNMNRYFECKKNEDIIKDMSSNSKNNDNNNDNNNHNIYNHNNNNIYNHN
NNNNNISIDKNKETKLFPLYFSDNRLNIIYNDRAEKNYIMNSNNNFFSSEKKKTRSNLNNGDNKINCYNNNYN
YKNQNVDEEQERKKHLYNMNTSSHIRRGCLNIDKQHMNELNNINNYEDYYKYIKEFVNYIPITIKNINILLAL
SLYFLNFNINNNCTENEISDSLLTKILNKFLYCLNLKTCLLKENKINYSADKYYYIQIKENDTKNEVLCDDNK
EEKKKNEQHIDIKYNNHVKDKNNVDDKNNVDDKNNVDDKNNVDDKNNVDDNNHVDDNNHVDDNNNHHFNHENG
NSLSNNSSNSFVTFYNKRIIKYEHDEIANNLLEQNIDDRDKSRTANKYDDKKGFFVFNKNMQIISLIDIYKIV
WCLCVLKKNEIFIDLLRILYKEIEYMDVDKDLIFCVMYLFEYCNIIEIKECEINLNKNMILNITNDNNENMCM
YLHLLYICSLYNEHILYNKYHKQNIEKIILCFYDTLEYLNLYTCTHILCVLAYLNIKNDEYPYFFNKILNVFR
HNIKKLNKSILLVNILWSLCLIEVPCSEFLKDISGYIINILHYIPLSEFITISCCFGCQKIILINKYWKNVKK
TKEPNILHKYIMNISQDTINDYRQYEKDINTFYTEKKLYPYYFYRKPVCHSIIFKILAYNFLHYEQINKKKKQ
QQQEEKEQEKEQQQEEKEQQQEESNYKINYDDNKNLVSSYKKGMTNRSLSVENRHEKENSKNKKGPLQNFRFN
SSNVYGNIKNALIKNYNLQNNDVVKNDNDLTTCEKKQHLPFSTTCVDIYHNNNKKNEEHNRNINIDIDCNHTF
SYTQNNIINSFKNDWKNNINKKKNFKKFKEYYNIYDNKNNNYDFLLNSYERILFTNLIEDCCNNINKLNSIHL
IELLYTLSILHIDKSKIVKVIERNMNKNIFYEHYEKFIDKIKKKKLQNINKNVNININKNVNINDININDLDK
NFNMDDEMKNEKDDLIILNYIYNKFVKENPQFINKNDLDNHFLLIVNNIYFCKYINDDIFLNIIKFNKDLTGT
KTNINHTTTTNHDKNNDHNNKNNDHNNKNNNNNNKNNNNNNKNNDHNNNNKNNSTFFKLKNIFCGAAFKDKEC
VKQDIHHNNELTQSNQNHLINKIKDDKHHYFISQKEGSDNINNQYNQQIRNTDYNNHIHIYDKRDNNVYNNIY
NIHNTHNEQYDKSNINICQDKHISLYNNKNTHTNYSQQDDFMSNEKKKLYYQDEKNIYKDTYNNIPSSNNYNG
YLGEHNHDITSDTNNSPEIYSDNTSTKLYNLKKDLQNTFKASISYYNNKSKHNNINNDTDHKENFLPSEIYNN
IVKNRNIKNIISNYKENQIIDETNLRNNNSYEEKKKQKQKQKQNQNNVKYNYYHFNFYQIFILLLQISMISLF
LFIIIFIYTSLR*
>N.PF13_0148
MNNINYDEELRTTHFYQNIPKSQRNEPAIFLNKEKNNEHLKSNNGFDETYTFSIHNYDNVIKMNEKKELNKVL
LNYELGTQRMECFVGIDNENVLYIKEEYINHINSKNILVIINLFVCNLFYHLKNIGKNIHNLYLSFDKIVSPF
HFSFNNNFCEKCISDDNQNFTCTEHKNYDFSELSEKNSEQARRKKKKDYKEQISSHNYNKTNSEHTFMHTCKK
KEKNEYLPNVLILWCKRVKYAINIYNNNNNIKIKKKKKILEQNGQTDNNSNNLFCDEKSRYSINECINTLTDA
IFNKKNLDIFNNIHFLFPKEKSSFTNKIKHIESKRKIEKIPKEKSPVDKNTSTHINGYNNKDFLLFVDKDTLN
YNLDKDDNVIEEITDLYIINYIKCKIEDYFKNVEVKKFWNIINVQYLTNFDYKYPYNKRGNEKTFYNSNKNFY

ESIYDYIQIIRKTTVKKDIKINEISKNCDENNINKKEEKHVTIPTTPLTLEKGRILDKEFILKDMFNNPIKNY
HINEVQIYLSNYDNIFLKTIMRFYLLLNFSIELLTILYEDIDLYSIHIFYLYEKLNEYIYNEHQLCSKKGNFN
IHQIYQSEDKSGDFFEHNGNNLSHFFKNNIYEMTRMKNNNDDTFHFINSNLDDDLNYRNKNNNKTNNVNMSNN
NNNNDIINNVYNFLFHKNTNHNIFLFKEKKLDWFVFFLLKILMRKLWFDKHDDHDNYYSENNNNHHHHHNEYN
YKMSTRGKEYQKLLNSLNAEKYKEIQKEIKALIKYTLEKRKIHFEPIINYLLNIYNKYNKKRKALNNNKIVNS
KYASILKKNFLLYSLDFYTLKYFSRRDLYVNMNGFYKPRRKKYELKIPRYNNKMKEKMNRYKKYVRTFHSLII
KIQEVTNESRKTYNDNNLNSSSRDIFFKINKFILLRKSRMIYLRQLKKRVYSYYSLIPIYGNIQQTEKRRNKK
ILEDNRKRKKTKKNKKKNLTKKKKKIIKKKNKKKIKNNNNNNCSSNNSYKMKKDFFFREGVHKNFFIKILNII
KIKEKVSEIVKNKNTIYKKLVKKINERNYICIEKMNKKKIKTYEYYYNIFINLLKTVKHQDQIIMNILFSMLK
NINDHLNLFYKNICLCIPCKTSEQINVQNVMKHKIEILKGDTSLFHHSNNGNIRHINENEQEENYSHLKKGDV
GIMDISNMDMNDICVYNNNEFNSNTIDNHIYSNDVQGNSQHDNNLPGSNSVEYNPYNDKIHVDNKYRDNKYRD
NKYRDNKHKDNKHDSNKYDSNPPSSQALSTDPPSSNPPGSNAYSIDPQNNNPYSNNLYSSNLYSSNLYSSNLY
SSNFYSSNLYSSNLHSSNLHSSNLDSSNLHSSNAPKSNILHSDKFNYTLNIFNDEHIPNNSVCTKDTSNICCN
NLSSNTSCSNNDFNTNVSSIMDEKSMRLDNSNSILFDMNNKNETCNKIKKSSEYNSIFEKLNKNEYNKKDKCI
NCDSLLNEDIIRKRKMMLDNFFNIDKYYKKKNERTNYLIYDINILKDYLYIYLKKYFDDIILLFEYFFSMSSN
FFYNDFVNKTYKDTRIIESRECSNTYINICRTEEILENIFSLSSYDNFHIYNNNDCDNNSDNNNDNVLFRTRD
YNNYVFIKRMKYFINNDIIQFINKGMNLINNYVNLKIYNVEKSFRILFRLLKVNNCKDPVFLFNILWSLILKY
SKYCRKKLIKKRTINKLSNNVNKNEIEKDFIFEKKRKMFLEKFGLNYLTTSEGIILYLKYIARVCSDRNKDND
MDKDNKDNKDNDKDNDKDNDKYNDNDKECSKYGMFNIHKNNSYLYKNRFYNLLSRKNEKTNDTYDTHVLMRRK
NKKGEEVDEYKEEDNNENNNNDDNNNNNNNNNNNNNNIDHYNSDGLLKDMLNKLMQKLKGKCILQDKNKIKRV
RKGEKEGNDNLILEQLSNVYEIIHSNNLKKYDNIKLFLNIDKKKKTNRKRKFICEEENEEEDENLYIKNIDNE
SINIRKIKKIVIQFIDDNKYVFPYIFNTFDSNVVSKDMFKYEYMNLNTNFMFFHYLSQNEYSFRNSILNYVYG
YPLFCIKKKNSNNENITTYNKYDDNKDDHNKDDDNKDDDNNYDANKDDDNKDDDNNYDDNNYDDNNYDDNNYD
DNNYDDHIYDDHIYDDHIYDDHIYDSIKRGKYCDSEKNKHERNNDYNKKEASDFMNKHNFSYNPLDSFLLNNS
EGVKSKMSDFSNAKVNMGNKKSKKGKDIVHNNAAMYRNKISNNMRINRDHTINSSYDDYINNNKNYNNKYDCY
KYNSGFSNYRDDASKEMWHNINPNDKEWKRKIKKEHNKKKRKFLIKILKNTNIYNKIPLKYIHLCVIADIFKY
LNLDHIVKNIMQKLYEEKLKDIQEKYDDNNKHYIGNILLCFLLYFHSFLDLLCGLRDGHFINMSQDDHIFNIF
NNFSDLIFNNKKNYSVFSKKKKNKKKDEYKSGERKCVEQKIYMDESNISERMNRKKNKCYVYLKNRYSKRGNY
LWLLEKKEKNDLSSYVDNIWVRFIRRRNEEKIEKDDRVNISYNLVIFLNNYLDYNFSFLNNCRLDIDESKCKN
YLYLFSNEYNKKLFTHDEECCIEEENTCLYKYLNISIHNVHGNESYERSVLYILQLNDCLFKKGILKDTEDNI
FFNYELVEKRDIYDMQIGRCGNNINIHDIYKKDNYFFKIKECNCCDDVICDKAELIKLLFDIMIRYLNDMLDK
KKRCLLDAVINFKNCKNGLEDIKFTYMLIYLLNYKKKQDKKCALIIYKNLYYLLIEKLYYEQNDNINSEQMIN
YIFNLIKIARIIKIKNKHMKKLFQMLQNYIEKFNDFHFYLFNKIFLFIYEDTKTENKIFLKTYLNKYFNDRKI
ISHIFNHVWNYSKGSKFLLSQKQNQNEGTENKKDFNKIDDGISPFVYSEDVCENMHMTKSNNINNINNNNNNN
SEYSQSICYFEKEKRKERKNLINHNNISHMELIKNEEEKLQHINLMKYSNDMNEEIIKKISLKYKNLYSSDSN
SNCSSHGRVFFKTTYVKHKNYRMYEKEKRQIFIKKLKERSMLYYKKHKKINSEKKKKKHIVEDYSSDEFSYFV
NESFIHFETNLKKKNYNQKIMQLNIIGSYKDYNNIDILNNVNVNINSSSSSFGSSSLSSSSFSSSSSSSKSYKS
SRNISDVNNDLDKKRGDKKVTNNYERNDDRCNMDVNRKKKRKNNICFCINNLNGNNSENFLSLLSNADIRNVQ
KNDEKMLYVQNEEYLYRHNNKYYNNDIYKNRMGNKKDEYKIYSRLLKMPHDNSCCLIKYKAHYEFDDNRKNKV
KKEREKERKQRETQIMCLPLYISIVGGLDIFLYNYYNYICNMYINPYFYINTYYFEKYILKKYYKRENNLKYD
KNNNNNNNNNNNNTYGNSNYIRNYPHKEHTTKNYDNLLIYDYFYYNYIFNNKFTTYDSFKCVRNKRIRSECYI
KFFKYGNEGDKQILHKKSNNNEKGEIGQIEEGAYNGIKYINNISDTNVKGIVEDKKNIRNKDKDDNKKDLDTS
VSSFVLSFSYEEKDDKYEDKYEDKYEDKYEDKYEDKYEDKYEDKYEDKYEDKYKDKYKDKYEDKYEDKYNDKY
NDKYNDKERKKRNDSRNLNIYTKYYNNSKAQDKIKRKNYKSICSSDQNNNEKKNSPYYFFYMDDDLSTDDEDY
RLKKKEIDEIFILKRDLFVIDYLQNYFNFSVIRKMKNMIEDMFYNYNLNVIYVNRNVQISELDHDIGQTCEKK
NGMYINVYNNNNNNNNENNNENNNSKMYTIKNKNEEMINVSKSNELSNKQEILKKPYTNLEEKDKEKMKKNET
YFIEIIKNKKKHNINNNEEININNSHNIFHNSSIKEEIINKKKIQLSMTVINLEYWKPEKLKEEDGNIIKKMI
PNFIETYFDTINNIYQIEHTDKYLIFLYELCYIDLCINNYIFTMKLSDGLFFLFMSNLENEIIDLYKKNLFSN
NIKNMNHIYNYEYTFISSNYKHKEKYRDQTMHIIMNENIIFYDFHLIKNSNDLDKGTKKIGSKKGNVVSSKKE
TIKMKRCNINDTIRDGNNNICDDNKMCDGNNNKMCDGNNNKMCDGNNNICDDNNNICDDNKMCDDNDNNNNNN
NNNKHSILWWKQKRTSNNENDYKNGNPPVMNKKKKKRKEEGGGTNMIKIEQYFEYIPYGTKNNEEKKKKSYEK
DKEVKDILEEKHNLMTEENSYTKLNKNHSLKKKIIELNKEISINVSSNNNICNNLNNDNIDDVTKISNPLNKQ
ISKENFYYKLNFLKEYEEKEKKKIINEKNINDVYIKLFFTEDFLIQKTNIPEKFIKDMLKKLSRKKFLHKNSF
KVMNKKNNKTTQFYVYSLGNFDDPNITNFHDDKIIKGPQEELLLSPLDIQRLQENKMKENKHYDNIRNEYKHQ

KDRQMFNVHPYDSFDNDIGKGNTNIICKNKKIKTNNLLQNKVHKNSLNNTTSVYNERQIYSIVNENIKCNLYN
NNNNNNNNNSNIVEQISDVDKNIIIEKAMIKQKDDQLLHNNQMMLINNNVCYGNDDMSFNNNNPFTYDNLIYN
SVKKSSHFEGDLKIRNLNNYIHKDNSNTIINTNTSILSNNNNNNNDCNIISSNKTAVKDIICINKNNIVDDNE
QINKIETKKEQHLNKNLKEKKNKTTNLLLYINNSKKSHNSVNTNIGVNNDNQLNKQNNELVDYFLDDIVTDSD
EEKTLQKRRTFDNLNLEESYEFYESEILRITKERMNKMKLEPGKNTSTPLFLIINNLNKILTEKKLKHISQKE
VLAILNIMMKNNKIARIGGNWQPT*
>N.MAL13P1.360
MQNSVNKKIRMKLNGIDKRSSINIITNDIASQLNKKTISNNNNNNNNIKINISNNDNIHKITNDNKNDVQTSN
VRKRHSVDTIYNLKNLSKNIKINIGSCSNNHKTLTINNMNSVEKNNIEKNVNNDNIMKDNNYVNNIKDNNIYD
NNYEKNEKNIKEIDNHQKTDSVVKSDISLKNDLNRKSNTSQKSDISISSEINKSGSTSRININSMNNVTHTNR
KSTSDLLNDLTNKNNTNYIHNNNNNSSSSVMNGDKNNYNFIQRNKEVLKNNLKIFFKNENIPFSFLYHKVLTI
LKSHYNNHVSNNPTTANKGISFYHMENMLINLGFKGVDINNHLLLSYLASSKQIKVDMNEKRLCYMNPYIDIT
NMNALLNKINKQGFLYGYEINDDLINTNKEVYKWINVLLYEKKVRCIRTNNSHLRGKLKCKNIPNVCDIYAKT
KCDNCFYNLKGYLFFPLSYEHIEKERYSITNDMKMLWDEISLPSLDNILKEYKLKSTNNVFMNENLPKKKNKD
DKLSPILKKMKRIYNTHLFTADEIKNEFIHKK*
>N.MAL13P1.365
MDRYDDFIKLCEKLNKNGNKFVDKKEEKRNVKDEFFITSSKVYTKLFSACEYIDNNTLKEYGLFLNSTSCSSI
YFSKNKPKGKNKDHLLFLINNISEDIKNLEPQLQIHNDILNCLNNLLSIFVDIINKYDNNLSNYNLKLNTYTT
FYFYDVKSLKCNFDFLNKLNTQIYGSDVYINKYKDNNSELHSSLQRGKHIKVNKKNENSFFIQDGDMISPGLN
EDTHVHMNDMVVSQNNVTSNYTNDDINMYDHNNNNNNNYVNNSTRGKDINFPTHLNNNDNIKIHKSNLRKRRDL
NNNNNNNNNNNNMKNKQVHFNTYNYLDEENNPGESHNNNIFYQNSLLNNNQKLEFKKYVDLFEKEENTYIIETK
KKIAKISNLMNIFVNKIYEQNENLKMIETIIEESIDNVSQGNNYLTKIKNKKNINSLIFFFLICTSIFLFIID
FFK*
>N.MAL13P1.370
MVSHFEYSEKYFYKKYNKLKDLKNVKTKDLVTRYLLFFVFFVDKIIEKNEDENKELQKQNEEKRKELIKQNVL
NIKNKLKEKQENECNSQEETIFNEICYDNILVDEKNKNNHINYVTKHLKNNNLSDGNCKNYEKCKTNYNDKEK
NKSCNFSSICNDLTCAFYICIILFVENLKDDQNNVDYKKSVHENISSQKSSDEKDNNIETIPFVNINENKNNF
IKKDIVDNANDLSFNLIFDIHLLYISLCFYYIYLLNDDIYYLQFSLLLFLMLFTHFKNKEIIHFIYFKKDKEE
KEENKEECYITYEISVSKFLYNICKNLGVYYKEKENDIYMSIFFLTYSFVFLKYTKMKDNKKDNITNNKILMD
EIIIVQHMYEIFLDINFFSTSTFFILRTIELHLQLLKNYIDDIKVEEQGGEEKIKTKKKKKKMTKRKVLRGMI
HLYIKNRKMLIYYQMKRTKNIQNWKLIIMIICIIQIIINTNNNMNNTNNNMNNSDISFCCEKTKNNLSINENV
NRIINMFCSLYNNINECGYIFLDVHLLSIYEYLLWVCFYIVKDVYRLCCCLKKEKEKRKILEYKYMTYRNLLE
VYIIYLKYNYLNYSQVNYSINIYKNIDDNIYSKHDKMKNIFDILKIKYNSSKEKEINHMKICEEEKEINHMKI
CEEEEEKEKNDYNFLINGLHKKVEEQIEDRIFIYDYKNNTQKDITIILNNNVINISEIINTSYDTCKSLFEKK
KKKKKKNICTFENKINIYDKNIIYDFIYDNILKDKNYPRFYNEEEEEEEENENEKNNTFMVNSFYENMSFFKF
FEIKFKEDNQIIKNIIHIIHKKTNKIIIINIDTTYFEEFVNIFRFDNVPFIHFNFYICNFYSLPYHEEALKKI
KKIYKMFCKHRDNEEYVYLLQDEKIIKENMEKEDKNNKDKMDKIKELNTNDEIDKLYTTEEGKKKKKKNILEY
KHRIHYLCYNYNEILYLFEKMKKYKKKSLEYFSLFKQTTLHLDILINSMQSYFYFNFFTKTFDIYLQNYMEII
NSLSHPLKYLQNDQYVSYKREIALKCALLLKDIFICKKFNTFNENIYLNEKVKNIQTNINTNVQANGDFDDNI
RGPLTNEQKHIILQIVQFYFSFLSTYCKKEDDMINEKLIFQNNEEKQYYFDIYFYVCKTLSTIEDRFFISQAI
QHYQYILNYSLKNKMYEDESYNEYMKSFANKSIYSLTFKMKEMT*
>N.MAL13P1.155
MEKDKLINEIYTNNDDNKKKEEINKIFKNIKDSDNTKDKFMNILKLTKHINDLKEGDKINVLKSIDVEFLCLL
LRSQNEFKLFTLRLINSLIGSSTYVYLKACFPFINSIIVSYSCYVKNWNNRLKDDAQKFNKENKKGMNKNVPV
ENNWVKKEKALPIYDNKYNKYEDDDIYEDDDKYEDDDKYKDYDKYIDDDKYEDYDKYIDDDYYKLSDQDELRD
INIIYNECITFFLKTHQFLKEEEIINNFYFNSENNDLDVDVEMYFFNKRLKKKYSEEKENVINNEKDVEENNF
FVNKKKVNHDTKNKYPLKDINNTNTKRYFDNEENSSNNICDDNTSNSDLSLLDDDDNYKEQNFYFCDEKICLI
DLIFTILKENINLKELLNKKKQKKGENNDKNVENVNNVESVNNVESVNNVESVNNVESVNNVENINNVENVNN
VENINNVNNINSVYKEKNTAMYKKKCIDIAKYNMILLNEQCIYISLHFLNNIILKLNYNTYVKEYFLILNRLV
ENYNEPKLRITLMNCLTNLNIYIKNRNIENILDEKNIYKFYANIIYYFSEIKDEKEKEVLYTLYFTIYSCLHF
VYVNYDSIDILKTPLSYMSVELYLFFEDVMKYVSKKEFKNYIFSNKYFKQMIHMICTNVGNILDFLSEHYTWY
YEMDDIKNDDKGKDISSYIVENTNIMIEKKKLIKNENTKQNINNKSNNNIFINTTDKISKSRVIQLDLNNMYV
VISKIKKIMDILLEFFKDIKNIFNLENEEKKNDLLKFKKEFNEEINGLIKLFCTYLIHENVHYIDDFINLLEL
FSILINEHNFFYLLIVKYIDPNRYFNNKIFLSKLLLYILNLNMKNIVHIQILFDLYHKCTFNILKYLEIKDIK

EIKDISKFSSIIQHNTDYYSFIQMLNISIDKLNNLNFIYILCQDEYQSKMNNSSKENIKNLLLFHTHFFFYYY
FCILQQNNYDIIHEQKNKFDLYKENYYNICNNNMKKNTQQFFLALKILFSISAFLFMRFDSYVVTNILNKNEI
LFIQDCLILSLFNLKFINEHESKDNELNEMKKKKKKYFFQVLKLIYFIMYYHPYFISLLIFRLKQIKEQDNNN
VFFDLNKIKMKEEHVSICKLLNNFISNYV*
>N.MAL13P1.154
MKMDYFWVINLFVLIITLSNYTIIVSCLKKKEEIKNGDIKIITSDLNETVTLSCDDEKSIHIFDASYGNPTLD
KLLIKKNSFDAPHTLPVIQMICEGKKNCDIKVNNETFKILSLITDFHKLLIKYTCVSSNLEPFPSYKVKAKRG
DDYTWNVEFQGETLNKETYLHVNNINATCEEPLVKKTYVDINEAEYECNNMKNCVYVIFNYDGYTLCASLSYK
NATIQKNSKIYIKMFYINGNIPSNFEVFPNIQGVCEKDEIIYEMETVLNLDDIYKKCKEVDCDYFTMSTSNGI
KGASKSFRNYAWFCKGFPKYVSHEGFLFGKNYKNSKDPKKRITFMNYEGPLR*
>N.MAL13P1.153
MSNINGETWNNTDKLKRKLSEIYDTEKDKNDELKNTSKVLKNSSVEEYNIGKGNNDTCNNFYEEHMNNNEVKN
YVVQNNEVIEKSENKEPCINESNQKKKKKKNNKKKYNNMEMGKPLKQMVRINYLLQASFLMNNMNPNISREYI
KTMRRLSNKFLIKYDSKFKKLFCKKCNSVLIPSETCDVSVNPLNLQKKHKQINNKYDTKELTKESTNTNNIKP
RDEYLVSYKCKHCQHYTKLVYECNLNTCQNKEN*
>N.PF13_0161
MGIKDNINADLSDDSKKERQGLELTEEKVLEENINKIDIQEKKENKEINKGDINNDNEEIENSYIKENLDPLN
ENKDNSSANLNGLITKEHIVSSISPTDELKISQLDSDNKNLENTLIRDDKENNLNNENTHVIIDKEKNDVDVP
FIESKTWGSFSLNVNSKFPISQKKNKNAAINDAPLYIEVKGLLEVINAQDFAKKVNRKNTNLNVKAQNSLIKK
RSYSVPNKAVAKNIPKDNYNMNLKKQNYKSVSLQKNSGLNKLAITQKKPSIFIKPLSNSPKTLSKIPYSKYDV
IRKPLDLNKGGKGTFRYLEKKIPSIEDSQKINTKEIIRPNIASFKESPIGEKGKNIAAKMEKHSLKFSPKVMP
LLMKEKNVKHKAPPKGTIKHTLEYLKRAAESRMKENELYKSKKDSTLSSIAIRKNSIPPRKIETKPKLPSKFN
PLKKEPLKKTKTNLDAKKVFMKKPTSKIVQQMKIKEKEKKKKIPAKFMDPKKEKVSSKDIEQKNNLKKKESKG
FNKSKIEGIKQLEKKREEANKENKIMSINDKVIEENIVNKEEFIIDNVEPETEREKTIEDKNESELEKYEKKL
YALTESETEKEDEKLFEIKKLEKEKNIDDKENFGYKKDNNGENDKSDENDKSDENDKSDENDKSDENDKNYKN
NENNNNDNNVEKDSFNEINKIENEKQKEEEKVILKDVGNIKKESELNVSRISEVEKEEPNVDVQMEQNKFNGS
KNKQQEENLTEKVEWEKDENKMEEVVVSLNKIQNEENVSKKKEEEKLIEKIKEETMVDDIIKDDTITDDIVKD
KKKLSEIEKEENQIDKVEKEEIKIDNIEKEEKTLEVEKKDEIDIDNVTKNERIIENKKKESKIKLGEMKKKKM
DTKKNVDMKMDKKTIKKDDNTKPALNIKQNIKLPLGPKQNSKSALSSKPGVKSSLSSKPGVKSSLSSKPGVKS
ALSSKPGVKSALSSKPGAKSVLGIKSKTTMKSSATTFKSKTFVNGSLKEKKNLKNDIKTGELAKGGNSTNINK
NIPQKKSLLFMKKATTGIKKVSVSKSAIKEAEGEKKKLGKIQKESSLHNDDNNIKDMNENKIDMDNMNDMDNM
NDMDNMNDMDNMNDMDNMNNMDNMNDIDNMNGMDNINDIDSMNDTNNDYNNNDKFNVDEKKDIEEDIQKDIEQ
DLQKDMEQDIEQDIEQDIQDDMQEEDLLEKPLSKKGPSTFSKTSASALNKKKKKTLSKFKTVDVNGMKLNKNK
TLASLNSTNTKNTMIQEKLKKKKMEEKENGLNNKKLLSSRLLKLNTKSNTSAIKKISSLGDKDNNKTKKSVLG
KSGTIISKGVLEKNKGINNRPLKEAKSFKKLKSKIIKKTILNDNNPKDEEKSVKKIETSQNITKKIKTVPSQM
KLEKKMRKLKSEILHKVTGNISKIKDKKTVKKNKEMYYDDMMRNVENNNKYDNDNDNNNYDGYEGIAKRVNVG
LSERALSKIRTSKGKHNDYITPRIGANIKPNIMNVKSNASNSGSSDKVSSRSLTTPPSTSSKKSKQKEKRMED
LSIEENLKEKLINLNIQKQYSGFHMKEMEEQNIPELNISQLSAIHNREDMNKNDEERRYRSVGNIGITNLNEL
SAVLDFSSNNNLKGVTRESLRNLSLQMKMDSSNEYNITKGEQTSDVNTLYNQSTILKKEMETKENPSSWFFGN
CCTANNNEYFYERQEPVVQGSSSLFNVNNKFIPYNPNSDMLHISNNTIKLSNIYESDVFSNSMQNNLILNNQK
NSGQNMNKTMTNNNSLRLQKKRQSCSATTNYCNCI*
>N.PF13_0160
MNQYDMNVLINEDIQCEDVEKENKRKIANDLVKNYCLLKELNLLSTIKNEYKEIVHTRKNVAYNSDNIIYASK
CIMEYMELKKQNELKIKEEEEQQQMQIQVKKEKEKYSHENKKENILNDNEIVNIMNVLNKNELNLFIYAKNIM
ECDKVHNFGMFNNPLNINYLDIPEIKQNHNGTHLEELIKLLISNYNNSLNIIKMQQNVINNFIFYTHNLLTNN
KMLIQKNHKLIQTISVPKELKNNVSDKDQIKFKLPFTQENNISSLFKIQIDLYRKHIKHLYNVNDDLKKYLSV
FNDQNKYANN*
>N.MAL13P1.152
MACSRNIINRVNTKRRSNILSDRKNEYGKDDVNELITICDNKVSVVNILKKGNEQMTKIKGQNENIRKSFPSK
SNILKYYEANFLKDKKNRNNNNSTLIENKNQKDKKRQINKLGEQDSNNEELTSKEVFSKLKTKEKLQVLDEVE
KQLKEFMKDEKFNMHNDIIFLNIYSLTHKRIYSILNKKNNKEEYYSCNEILEMILKGDLHKNCLIKRKSDSQY
FTLKEKISEIHFLKYLELQYYLKKQKKEYHMKSKLENNFDKENTLYLKFSESLLGEKVKSIRNFLISPYDKDV
VEVILIPKHKNLDKFSGYHKIVSNLFITKYISVYFYHIIEKKMEKYNLIKKNGPIYIKMKLKTNYPLAIQTIF
RYIFDKTFKLHDLDFKLLVTVYIECIHFKIFSIINDITNAISEKANFNNIVQVLNMSSIFKKTPIFNDFARII

SDSAFYLFASDYHYLLDVEIYSFFLSPDNIMINEMRIFIESIKFIIVNNCHMKEQKQVFENIRFNLLNNEHIY
EIQQYIKNCFKDIMHNKYDRSNFICMRYNKPKEKNEYDNTQKKTDNNLNEVSENKLVASDNKEMPNIKKCNLS
FLKEIKKLNKVYKNINNESFEPPNWNNTEKKEISNKKLTECMNNIYNILFDNLFKKILNKEIKHRYNIWNENN
DFECINNFTNENYSIQLIKRKSKEQKYAFTYGDERLINECKLFFEIIKCKNSNLSIGIILKSNELYNSKQQED
YNKISNILTEYNDHLVIYFDFFVNDFYVCNINYKNSTLINKTKLNIYAHENKITNKDMIEYSISVINHTLNID
IKILPNNICFNYSLSILKPKTLLGDIIKKPFINIKPFFMLKDSYDSICIPCIKFKKNI*
>N.PF13_0159
MHKNICIYGGSFDPITYAHEMVLDKISNLNWIHEIWVVICRCRNDKSLTEFHHRHNMFTIIINNSSKIIKSKI
FLKDLESHSEMTPTYDLLKTQKELHPNYTFYFGLGSDLICDIFSWDEGEKLVLENAFIIIERGHFKIDESILK
KFPKYYLINIPKLSFINFISSSEARKFLTKENDINDIKKYIHPLTIDYIIKYNLYDFN*
>N.PF13_0158
MSTSKRPYKILLRKVESKEENKKDTNETKKKEEESKHYEDRHYRKNDIKHYEENEKTIKSKMHYNSRNRYYDK
YSMSYSRYDEKLNKQHSSEKKKDVLVPLLEQASINIKKKKIIIRKLPPTLTEENFFDSFSNNIKDELDYYYFV
NGSASKDSSNDIIYSRMYLSFKDDMKTDEFIRTQNGKFFYDLNGVKYKALVSYAPNQTLIKKNKPDSRNNTLE
SDEYFLKCCEEMNNPVQRVKKDIDYSELINVQTENGAILSPIVIELRSKKKK*
>N.PF13_0157
MIYKFYNCSRKLYEHTEKLKYFITKNQLVNTYQNKFQKSIYFVYKNGNNGGFNEYTVFLNNENKHYNDRKSNF
RMHSIVFANVLGLTVHVKKDENYENENINFLNNELLTTDSTVNINENVIRKNEFSFIGKNKLFGNSNDNNTKQ
SDNVLKGNNKNGSKNDKYEYYQELSEYSNMQIFSSNSHHELANEICSNLGIGLGRAYVGKFSDGEIALQIMDE
VRGRDIYIIHSTPAGGKDVHSRLMELFLFISTLRRSSAKKVTAVIPYLNYSRQTKHLDDHNYVHSLGAPEIAI
LLQACGVDSIISVDLHNARIEGFSTGKKFQPPLMNINPQSLAVEYFKKKKLRNPVVVSIDNEGAERTKEFWIR
MNKHSMNAGFTTLVSSNYNEINSREGYPVNDSYESNDFKNNRMNLHNEDNKKNNTNALNNSKIVDKKENDMYD
KEKFTIVGDIKGCDCILVDDIIDTGEKSQKVAAILKNAGARKIYLYATHAILSDGCIEKINNSCIDEVVTTNT
IHIPSNICCEKLHILSVAKLVAEGIKRAHNEQSLNALEDFSDGQIEMKV*
>N.PF13_0156a
MNFDDFVNILYEKNVFDDENSEYIKNTIFPFLVPLIKLVNDEKEKHKYFNLIYNKNEKELLSNMLEEKKETNI
EQVDIFSTDKFNTNPTELFDIQKEVIKRYIKINPILLLSEYLMEEYKSKNHNDNNNNEIKEKKSY*
>N.MAL13P1.151
MIVLTENPLEKTNNCTYSSNKVLIKRKSNTQLKVIANRITPYFKGIIRTRRFLKLVDKTGHKYLYSKNNINNN
IEKEKKIKNRSDDIKKKNLNKLDKIFKIKQNKQNKVITNRVHFYNSTLKDSYITNLQHQENSELVVHVKDKKK
RISNNKKKCKKDLFNIREITLDNLKDINTTRYFHDLHKLNNKNLQSKVKKNICNNIYDIYNNHINNKKHSAQN
YYALYDTNNKNVNNIHYVDSITNSQSLKLYPLINNNAYVNIKRDVSKKSSFHIYNKRTLTSNSLIDSIISLSN
ELNKSTVEKKNFNPDYEYDKNVLHYVNKILNDSKFNYDITPSNGNKNDIEKLKFKIKKYNNIPIFYFRKLFKY
IIEQNNNNNNNRNNSSSSYRHTNQSNIYDVFYDSDGNITYNDPFYTNRNHNDSCKYIHDDNKDISFNSFVYTN
YYSDNYFSDRTKVKKNNKRNLFHQNFRSFIPINSIKSQGNHNIDQDKRNNQKRNLKSGLKNKTIGLFHSKEVG
SFKKKNEYIINSDNIVNNNVSFHKDKHDPALNSKINNSCYHPNNYHHIKRISINSNINMYQKEKNINNTCIPK
NRINEICIKKNFNNNYKNIHWDNSKYNHINNTSQNIKTRSSIFVDSNNFSTPKEIVTNKKLKDISHPNKEVIL
KSRKEEIYKYIRNIKNEIDKNDVLNSKYNELMDGLYNPNNPENLRDSNDKSVVSILVNYPSSSIRNSVSFEGD
VNENYHNNNNNNFYGNNNYNNASNSSSNSSRSNSSCKHNNIYNNNHNKHMCIKNNVKECNDNVRNNIQNENSK
YNSFISIDANCSFNSNNIKRNDSYINISEDDNYNNSVINENNNNNNNYDNYIDFISLQKNDNRENQSYTSDLK
EQRKVHKDNITEKLEQRPSGFVINNINELKSLTSSIHSIYSSAELIKTPIESNIENNHLKNNREIFPSKDIKS
LCIFKQRNKKVRSSNNSSFLIDFRNSHTNNINMLTENQKFNNVLLNKEIQMDENQEREFSIDDCLMNCLKHNA
SNLKTNEDYERYCNEDKILNPDYKPSESRKLGEKFLQKSQKELYYSYANNGFHCDTNLNNAEVGNSCFKNDNG
YSSYNDPNMNYLHNMKINVFNNNRDFMNNNNLNSIYNNNHSVSSIYKNNYINSIHNEYYMKGNNEYITNDKFI
KYYEKYSNKDVDENEAKKLNYTDCYDFKNSLLNGLSRDDSFCSDDDLKNDENDMNFLKDSINSFDNLESTEKK
YEEILRDFNGKIPLLHKVLKPLPAKNRSNNFDICLYLLQKHGGDLNNEENICILRDKEIVEHSAIYDTKKCCI
KSNITNVTNIKLYHPKWYNKKKIIERLKEQSCYNPFTIFGSAPSLLDFDEVFDKDVYNKFVSRNSRKNHSLLR
VLAKQKVINNLNDKITDKEWPQVHAYLKKRWSKETNIELNWACDPLLYEELEWYLSTNNEYLNMTDKVADIEV
CYCPTLDPNSYYAWNDSRVIYTNLCYNKSKDEVDANNNSVNKGRYMMNKNMELKNNDKQVSFRKRASGCEHLM
FQQNIMKQKNLSKRKKKLLKKKKKMLKEKNKLLNNNNNDSGNNKGDTEIDANAYFETSENYENNSKNMYPQNN
NNNNNNYSSNDNNPYYPNIFITKRKKSVYYKKNNDDDTIPIMTVNTSLYRNNMDNRYNCNNTAYDYIKNKDHT
IKFFASPKKTKKNNSYKKSVNNSNSFYLLNSSMINNDNMYNNNFNSFDSKKSMNCNDPSSSSIYNKNMFIEKD
NIKNKNEIDARNYSGRHKFTSDINNEENVGANIEMHENIFKKNIYETNNNKYTYDVNKNYNKMNFITQDDNND
EDNNNNSYTDDNSSISGIHLKNKNDGMKSFAHMNQNQNNYEYNMKNKYINNNNYYNSDNNVVNMSIYKDIRMD

EKYKNNNNNYRNDDSESNHNCYNNYKGYNNDTKKERFNTNNFVDNYYQFNQIKVEESDEFNNFRNMDKEYTYE
SDNKSIFKNKLSTFTFSEEDESKKKYNINELKEKYVNIYKEQNINDNYTTNVFENAFDKNANNNNKYFTSKTM
GIEYFQNKQLENMPQMGNKKIIENVTSINLEKNNVPIKKTANVFPPEEDIFKLTHKNIDTKNIVEQKVKSNNT
SSIYSNKKNETDNSLNTFNSKRLSNSNISVNENRKIRRYTNKMCEENDNISFEPSENIKEQSNKKGSIETSSS
RTSDTSMSSAFKMATSVFKKLF*
>N.PF13_0156
MKLEYINILKEENGGYNFDNLKRNEILKEKGVKFPQFRKTGTTICGLVCQNAVILGADTRATEGPIVADKNCS
KLHYISKNIWCAGAGVAGDLEHTTLWLQHNVELHRLNTNTQPRVSMCVSRLTQELFKYQGYKVCAIVLGGVDV
NGPQLYGIHPHGSSCLLPFTALGSGSLNAMAVLEAKYRDNMTIEEGKNLVCEAICAGIFNDLGSGGNVDICVI
TKDSYQHIRPYKEPNMRLYHLPHPTIYPKGTTPILSEKIEYIKKFISVEDA*
>N.PF13_0155
MNHNKINYHLSVKDDEIKKHESNNKYGIYKYKNEQIYKTYDDKDTPNYINNFYDCLDTFCDNVGDKNEFEKNK
VIMKSGTTNVHKHYDNTFNKNRNSVISINNINSAYRNETDNKPYISNSVPKYLNNNLLIDENIFKTQNNDNNE
NIRKFRNISYSEIKHPNYNYLRRDAEPKCRMSNERIYSYNKDNYINPRLKDNYCNISNSPRNNRDMHLQIRDD
NCKYVEKFYTNCQIQKGKEKDAYNNNNNSSNNNHNKAGYYNYYVYNKGFNFYEETINNNNQLKQYSNIHSLKS
IDGDQYSHLNYTSKSFNDNRNMNYYQDQNKHNNNSYDATRKEGNEKQYHMYQNESIIPFLENEHSINDPIIKI
REKEITRYQNIEYLQNNEHIRNRHKGKNIPGENMMFQFDKIIEKKVNISYTHHEYVVRNKNKPIEYNKDDFYF
CKDKEDSENMMKCDLKIMNEQDVIHNINDTSMNPEQKKKIIRLQNDNNLYNEYDNYDNDMNMFINKIYKNNMY
HNERMPRFSEYQIYKNSNVMYLNKSDNKQKLMENINCHYENFNSKNNASLLPVQENRKNDNGFYMNSQKYMKN
ILKEPAQSEGSFYSYEKGYHNYNNGNKASTIKELDINMKQICKDGYDVNKKIKKNQERKECNVNYFPYKTSAY
EDKTNNTTMNMLKKVRTYSHTSSDHFLNNSYVNKNIQTTFQDHKQNVDDNSYGYIVKNVEDNYMSKLNNLSPR
KEKMNMEHLKERKEQHNRMLSSEKNCCFIDDNNETNYNRVVLLKNIDKEIEIDNISEHLIEERNNNSSLEYHN
LKQNGNDKIPKGKDNMCDISDIYNKNIYTNENKFDEFDEFAEIYDNINKDIHKSKENNNFDMYDRNKHIKNEE
DEFLEIYHNTKGLKNIEMDEFLEITDKSKKTKENNVHVDEFLEIINKNKNINESDVHVDEFFEIIEDTQKMKN
NEIELDEFFEIIESTQKMKNKENELDEFFEIVDKTQNVNNKEVPVYINLENHTKDDNINKNSVMNNMLFENVI
EKEANINIDFVKEEEKDTIKSFSRNYTYNTNAVSNEENDINLLHFYDAYETTKGDNKQHKSFMYASGKEVIIN
KDILKEMREKIFDDDKDTNYSSEENNKGDNKYNKELTEKIVERDMNLYMDRLNEEKDILKPFPRNFTCNTNVS
SNEEALINLSELCDINENAKITNEQNRSFTYASGKSIHINQDILKEMHEKLFNDDAYSDNKNKNNNNKNNNNN
NNNNNNNNNNNNNNNNKNNNNNNKNNNNNNNNNNNSYEKSAKKKNAELNTPFIYTSGKDVNINKDIKTDIEDK
IFEDDTNDSYEESVNIDNKELNTSFTYASGKAININKDKLKEMRHKLFDDDDGYNNYSFEEYKNEKNNNEKNN
NTLYTSFTYASGKEVNINKDKLKEMRHKLFDDDDGYNNYCFEENKNDHNKQVNTSFTYASGKEVNINKDKLKE
MRHKLFDNDDGYNNYGSDQKIRSEKKNILYVSHDSEKSRKVVEDKILRENERAQIDDNKNGYEKNIEEKCVRS
LSCEYRDKGSEDVDMTKSKEQELYKLDKYDDYINKKDELNKDDKNCRLLDKCTLQMNKKRKMRYDNISNSKRD
FINPRQRKQIKILEEEKNMNIYNKNNNINNNNKCKKLNLKINVRNYVKLIKDNLLNISIKKKIKDCKLTNTLI
YINENKTPFHFHWFKDDYIYFYKNKETEEYYICDINILYELFILTLKEMNLFDIYDFNWFYKKYKLISISLNR
TYRKNLKKNYKSIKKEIMNKIIRDNYLHNDIAGGLSIHEYYDNFIYEYLNAHTYINLRRKHIKLVSNGYKERI
YLNNEKEGTNHDNKNKWNNYEMEKDEFIDIKNLDDIKFNEEKDFMDKLNKMEYKELKKKNYMKMIIKSIDETE
APCPLKFMYKLLKRYLEERSKKKSILQLLKDRTISSKISMNLRVEEIIIEKKKTIKNNKNNEHNNNFNDDIYD
IHNDDNYNIVLILSDNFDYIHCFVKDDYLKNFIISEKIRKGNIIKINALDIYNNNMGEGKENHGIHTIYFLLS
SNDLIEIDKKDTLKIGFTKYKAKKIRCIQDFGSTCFFIDVIMISKSDFSYGFYDQVKKKYYLLNRNVYEKLIY
NLKNEITKMFHEENFKENNEYIRLKNELTMFLEATPFCTIQAIDYSSLERIKDTDSYEDKIGYLINSLCQIKM
FKIDEGTYESLRKGSRIQLFNVFVQKSNHNNNKFNQFLDDHVLDDFLFNTFNGKIHGDNIKRALNYHMFESRF
LKDITKINNDDITYDKYLQCIHASVLKKKHYAPIVFQALHSTYINIDEKYKSKLFEQLEDVLKLQNYIQLKTD
GSIVTKETKEIKEKTYPSFIYTNIYKIPVLSSVHFHIFKNYIKQNNRENKIYFDLIQGNLYYISGVVIHYTDV
EIKDIIDFKCAQEKKKLLNYKIFLLTSNGHLCCVNVSMTCPDFLISSCKKDYENKEREVLKKMIHVYCVEEKE
KKNNNKVEINNSNKKKNETNTYKSNSVHKNHKDLFNYLYKTENTNYEEGSSKYDINFKNFDMVLFFKDIEFIN
YDDKNDIFNFRSYTHPHFDSLKMYSNEFIKMKGNMEDLIKLNYITFEKNKIVIKLEDHCKIKVDKNNISSMHF
LYLLIYSKCKSIELTGVCVENNYLSTFLKNIWNIFVP*
>N.PF13_0154
MSLKNKVQVNDLNKTIFKENYKMKRSTKEVELMETIENLSDDIGSNNFSYDSNDSSKIHNRKNNDKSIDMNKK
DNIKNNNKQKNAPNYNKNKQEYYSNVPQIYEIYDTTPKDEGELRTSDNNGGEENSFLANTIAVVYIIIAIVII
LHLYLNEEHIVIKTITLLKLKFVDLIKSIPVFKNVMRTVSITNSKINGFLDMTMTHSKILKPYMEALKELNTE
FSILFIILILMFLTATCILHVVYGILTMKNEKDMYEKNSVVYVTNKMTVDEYEDRSFTYSELAKLHDDKDYIC
LKNKRAGEGMESWNWQIRKNKYNTYDKDICSDIELTDIDN*

>N.MAL13P1.150
MSNLIFSSLCWTVKKNNKSLLIKTKFRKDYSKTFLIKNKKGRCVINNQDITNITFHKNNYNNNDSSYHFLKCP
YDRNKFQVKKKINGYNPLLNNTLGNLYNEEYKQKWSGSLSYFCSFMKKEYENKRNVTSPTNINIDGHKYVNID
TYTHKYRYMSTFHMKDKDNNKNGKENMDGKKIIQMDEEKNIDNKNIDNNKIDNNNDDNYNVDNYNVDNYNVDN
YNVDNNKYNHNKKHPDVIKNDNIMTYKNFIKLNKNNINVPDNNKKYLKKGQHFLKINYLKNMYFLLLSKRMKE
CLYNLKCKHSYRRLMKRIKKEKDKINNIFLKYQIKKGNIKSHFNEHTYRKALMNMKKNIFTSLKRNRNKSIMD
VYYEEKKKYKLRKQKLLELQTKIRKNSQIAHSSIKRFFKKYGYVGLGTYFFVFLLTFCSSYFFVHFKYISLAD
LKYVSEKMHLNKYIDDDLHKKIDSLWGELIFAYVASKITEPIRIVITILITPYIARAIKFKKKSRLL*
>N.PF13_0153
MASGKGKENISTKDTRNDEDNTNKKYFVDVIKRLNKNDNKKYYLDEENKTEIQDAETKFKNELNEEIYNKDDY
IIYDDGNPEIQFFTNKQKLKIARYSWKAENPKAYVFALHGITSHLRNEYLNYYGRPIWAHKKDEVHDNNISSD
DNNNIPSDDNNNIPSGDNNNIPSGDNNSNNNNNNNNNANNDYKPRLDKYTNELKESEKNNNDYIIKDNLTNTI
NYGYHRGNVGEDINNDIEKTHERGKSIEVKSEVNIPKYTSTSSLNKVNGNNNIECENLRKNSLKSRESYFMNN
EPEKSVLNEGDVYESTSSLSCSSSELMDKKTLEKKLTMFLSCSSCMSNYNENTNGLNNDKKFETGSDNEMNSD
DNMMVDDDNVLLIDENYNSSNSNNNSKNNKNNNKNINNDSNNNNNYDGCNDNTVYYCSMCGSVCNYCNCGERT
LSYKNSWIEKLNENNFSFFGIDNQSHGLSEGSRNQRCFVEDFEHFISDAIQALEIFINEWKEKNEMKPIILMG
LSMGGCIALRTLETINRLNKEWKSYVKCLILVSPMISLGKQKNKISNKLLISATKFLKYFFPLLKVNVKESNA
KYAWIKHDSDIDPFQYCGPLNIKIAAECVSAADNCLKYHILKYVEESDTDIMVIQSKHDCIVDPSGSIDFMKK
MVRLYNKKEEKRLKNNNKKYSQTSTYSTKEHNEKLATVHAVENNNDDNNDNNNDDNNEDNNDDNNDNNNNNNN
NDNNDIVTRFQNDLISKDKENTTINSFMNKPFINSAKDNTINNNETDDHQNEQTLNDGTLTNNNTMMMMTIND
DNNNENSEIINKNNNNNNKNNNNNNNNNKNNNNNNNNNNNYSSKGSIQRTNYQNPIHDEDKIKIQKLMQSYEK
ADYTQANLYNSEDYSNQTCNKFFENSYIKLSKSTLKNEKELWNPFDHGHYKNFILKKNNKNCNFSKEEDKYKK
LSIYILKYGCHTLPGEPDSKSSANILVNWLNNIFH*
>N.MAL13P1.149
MEDEILKALTNRELKKTILDKAKNIISQRKKDITEKKKNIVTNEEDIITPLALCDKEENVGNTNVAFFNLQDF
CLKYSNTCKINDLNYENIEYELIISFISTFNNELKYQLKSHNMNKKEHKEVDDMIHNMVKIFMDKLIYYCTNP
IIFLMQRNDSNSLFDKEIKNHINIFIKQVEQHSDTNILIKNCMNELLGKIFYFIPDPNDMASWISRQALVDLL
KIIIDENENRNELCIIYSSDLIEKNYENLDFFQVDNVTVLIEIIQYAIIFLVEKKYEVKSFPLGNEEWFEHFK
RLLISLKNCKWKIFLNQLIVMETFLYDKKREHLKKSFNELYKHQIELSSIKGNAYSKWFKIEMPLSVSLFK*
>N.PF13_0152
MGNLMISFLKKDTQSITLEELAKIIKKCKHVVALTGSGTSAESNIPSFRGSSNSIWSKYDPRIYGTIWGFWKY
PEKIWEVIRDISSDYEIEINNGHVALSTLESLGYLKSVVTQNVDGLHEASGNTKVISLHGNVFEAVCCTCNKI
VKLNKIMLQKTSHFMHQLPPECPCGGIFKPNIILFGEVVSSDLLKEAEEEIAKCDLLLVIGTSSTVSTATNLC
HFACKKKKKIVEINISKTYITNKMSDYHVCAKFSELTKVANILKGSSEKNKKIM*
>N.PF13_0151
MTDYKVASTCIEELKEICSELLNAKEEEVFNKLSLYDEFEEKIKKIQPIITRIRIRRNETNEEKKIYGEKMIK
NVDILLERFDILYNIYEEELTIFKENYEIEKNRKIEKMLLEENEKKKNEKELLNRGRIKTQIEQEEILRRNLE
KENLLKKEQEEYDNKMYRIETMKTIIKEKCNFLYDEISNACNKYETIKYIYTQLNGNNVNFNNIFTNIINDNY
NDNENEILLNNSIYFIDCIYMIYKNNEFKLFKEALKNLIEYLEELIKNIDNQQLKLINLMNKTFQKNILSKKG
ILFLFILIGFVLKKPDEIKPLLKNINTDINYENIYIYLEEPNITTNYDQWKLWFQHIQKCLTILCTFFRHIHK
FSDVPDDEKIKFIFLYLKEKFSNEQNVSTLGT*
>N.PF13_0150
MMKKKNIDIEELKRLIEESSMKKRFVKDIKRNCEIKSIRFGIMSKEDIIKYSEVKIMNREMYKNNSGIPYPYG
VLDLKLGAHKSNSVCETCNKKLINCSGHFGYIELNYPVFHIGYYKYIIHILYCICKYCSSLLLSKEKIDFYCN
LKKKSTDDSFYKKHLFKRILNNCKKVNKCYICGNPQGVIKKIIKPSLDQFMKLKHILKVKENGKMIIKEEDLN
SLYVLKLFKNINPYHVKLLNIENPEKLIITALLVPPNTIRPSVIIDEHGTAEDDLTCILSEITQLNNTIYNQC
TNGYQTNQFLGNVEFLQLQITRFINSDSPAVSQLLATQNISKPGRGICQRLKGKEGRFRCNLSGKRVDFSSRT
VISPDPNISIDEVVIPKIIAMRLTYPETVNKYNIDKLKMLIKNGCNKWPGANYIIKKSKKGIDPYSDISTSYN
NNSNNISSIGCSNIFNVVNNYINNNCKNVRYNIKDVNNNVLLKDMCDINNMNNDINNNINNIYKNTSETNLCN
VNNHNNNNNIYCNNQTQDNEEERKNSQFNKISLKYANKNHVIQNLNIGDVVERHICDGDIVLFNRQPSLHRMS
IMCHKAKIMDFKTFRFNECVCSPYNADFDGDEMNLHVPQTEEARAEALYLMNVKHNLITPKNGEVIIALTQDF
LSASYIITNKDTFLDRDTFCLLCSYFSDASLYIELPIPAILKPKELWTGKQLISVLIKPNKKENTIINFEIQE
REYSNKFGDLKHLCLNDSYVCFYKSELICGSLGKKVLGSSKYGLFYYLIHHNSSHIALKIMNRLSKLTSRYFS
NKGMTIGIDDVRPSQTLTEKKKDLLLKGYEKVNNEIILYNEKKMQIQPGCTLEETLEIKVKSILDDLRNDAGK

TCNQYLHYLNKPLIMFNSGAKGALINIAQMIACVGQQNVAGQRIQNGFINRTLPHFHFHCKDSESRGFVQNSF
YTGLSPTEFFFHTMSGREGLVDTAVKTAETGYMQRRLMKALEDLSIHYDYSVRSCDKQIVQFIYGDDALNPSY
IDNNNTYLDQFDKVFDHIVSISSSHLLLSYKNKIPYLPHVQHQNKTSNMNNIYNNMNNINNNDSNRSIIYNND
SNMNNINNNDSNMNSIHNNNSNMNNIHNNDSNRSIIHNNDSNNNNNYKDCTHNPYICNESLIIRNIMNRLIYQ
NIAQEDLFIPLEHDEFLVNKIMESYTDQECNYEDIIRSLDLNKNVSYIHNDQGKHLSLQMCAEEHITINNTNN
DNTYVEQIEMKELSKNKTKEKQSFKGTIRDMHEDSEEQMNKFITKKAKFFIEKKKGKMHECNDDIEYNNTQYD
NIQYNNISCNYIKSQNLENTHHQVNNDLSFIKNNVILPPKEYHSIFHFVNDYRNVVEIKNLMDKKKIFLNNSE
KNVVQSKYNRMSKNLKKKIEIINNIYRNEKKKLNRWKTKMDNDDNYWSSDDDSIIAKKIIKIKNKEKRKYHPK
EEKENFDRNNYKMITDNNNNDNNNNNNNDNNNNDNNNNNNNNSNNNNYYYNLHDDVNNLGVTNYNTNIYPNDC
NGIYEKETNNNELTTNSNMCDKNNDFSDEFFNNINENDLLYDNKYYRQIFKNVIGFVSVFEYVESYKQHYILF
PYEIIKWTSFLLEYLTEIIPTNIFLHTKLSKKEKPTHQKNTGKMKIYIEEIKKWLFIKAINIYKYFSFKKSIE
LIKKKDYFNYIIKNYDISHRYIIHDYSFINLKQLYLFIFFNIYKYFKYISTPGDAVGSISAQSIGEPGTQMTL
KTFHFAGVASMNVTLGVPRIKEIINASNSIQTPILNIPLEVNDNYNFALMMKSKLEKTTIRDICMYIKEDYTS
RGVFLSVKFNEELIQKLFLNINAYNIKDIILKQSHINKIKINKIHINVINKYKLHISLKNDEFIFFQMESLKK
GLLDLLIYGDKDIKRCIIKKEDIEVTDNEDEICDDMDEYYNVSQGTELYERKCNSKEENKNAIRVKKEEIDDN
LEKEENIIYVSEKDSVNQLKSEKKKDINDDNNNNDDNNNNDDNNNNNDDDNKINDTIFNDDIDSDRNNLKENG
SKLENVGEHIIERLSYKMKEKNVKKEHIKKEPNLINLDTINLDTLNFDEINVHNINNEKIEFYDEHLNICQGN
KKHIQKKKKKKTVYSILVEGNSLNYVLGLEGVDFKHIISNHVINVFQVLGIEAARITIINEIKKCVEAYSIDI
DIRHIMLLADIMAFTGDILGINRFGIQKARQSTLMLASFEETNEHLFVSSFFKNVDEINNISESIIVGKNIPI
GTGAFQLLYDYKFEKETKNLTLLEKAERETAMNY*
>N.MAL13P1.148
MEGANKCVIGTKIFIRDKNKVWASAEIINEDEDNIIVKTEDDDIIKLKETDEFYLRNLDLFDSSGLSAPSDLT
KLTHLHEASILHSLNLRFDIDEIYTFTGPILIAVNPFKIIKDLYSDNMLAKHVQPIKSKSPHIFATSNSAYLG
MCNNNKSQTILISGESGAGKTESTKYVMKFLACAGSDIKKRSLIESQVLESNPLLEAFGNARTLRNNNSSRFG
KYIELQFSVDHKNYIKGKLCGAKILTYLLEKVRVCDQQEGERNYHIFYQLCKAVKEASIVDNSIMSNNENDDV
ENYVNYKCDEDKNKDHIENNICEEPTQKKKKWYHFPSTSKFRNLENVEPMKIDFTDFKEHVHFRYLTKSSVYE
LNEVNELEEFESTVHAMQTIGISNTEQYQIFKILEGILYIGNILFNNDETKEEATILEVSNDDLRKAAYFLDI
DENDLINSLCYKTIVANNEHYKKPVNSNVANDIRDALSRAIYGCLFLKVVERTNESIGYIKDINLFCGVLDIF
GFESFPVNSFEQLCINYTNECLQQFFNNFIFKCEEKLYMEEGIRWDPLDFPDNKDCVDILESKPYGVFCMLDE
ECYIPSGRDKSFCSKIISKHTSSSNNNQSNKRFKSIKTDSSSFIIVHFAGQVVYNSTGFLEKNKDQLSLDAQK
LLLHSKNTYVSHLFETYLRRNTDKRKFVTVSSEFKEQLNLLMTRIKETEPHFIRCIKPNAQNVPDVFDRISVN
EQLKYGGVLQAIKVSRAGYPVRLSHAECVQDYKILLNKEDRHKLEMYNKDNKSWSYKANFILSSLYVTKEIQE
YINNLKLLKDKKKEEENKFFGKLSSKKNKKMQDTTTTMNEVSNNVQINNKDETNNNNNNMKDIVYDPVCGDAN
EEEEKNNLFIWSVGKNLCFFKSDAYNILSTLRSDLRSLKAIVIQKNYKTYTQKKLYELMKRKIIIIQRWFRNR
LNIIRKEKLKRQEAEKLICSYIYTYIIRKRFLHKRNCCILIQSHIRRYLMVRFYKTYRQNYYASKIQATWKAY
KEHLFYKKLLKSTKKIQLKWKSILARKQLRRLKMESKEVGSLLSRNQVLMKELKKEKNEKIEIESKLLKASAK
IDKLIKKIDNLEKDNKNNEKVIKDLLEKVSLLSYKQSNECISPRHVSKKNENIKQKKNNKELNKDIIMSLKRE
DTEHVDSMEIMSIQGNNTHDDPNNVRNSYSTDTNLTNYLNNNKSNNNKDHNNDHNNNNNDDGDGEDIYMDNNL
TFTQEKIGDLKIRNTIRNEKNEEKYNLLNKIKKLEIQNKEYIKQNVTLNDKYNKLVSLLGQIKDTNNNVYNDV
LINNINNYHSNGMSNKLLLNNYNNYHNNNNNNNNFFKNLTNLSSCNSNSLIEPKIENNINYDNKSSFKKNDGI
VDILVCGPRNVGKTSLLEDLFVRLGDEDNLGLLRKNKKLVNNDLSCYNYYNTYIITHKCSQIKIVDCGYSSNK
NVEESLFNYIKNSICIIVVFDATNKESINPAVHLLQEASLINVKKTTKLYLFENIFNEKINLNFNKNDVSYAV
RVSKACNASYVKALDIYDIVNNYANKITLHSNYFLDNTHNNCNYNNNYNYNYNHVTSVPPNQHSEHNNNNYYH
NNKEKDNDKDKDKDIYVKGLAHLNHMNKSKRNTHSYMSSYHEESLKDSDDAALSSFGNKSNDLIKSKRGDIGK
NKYNNNNKNNNSSNNNNNNIYSVVNTLKAICGIQNSNKKNQQIQLLRESMPLNNYIYGNKKYNSDLGKGLQPV
YEITLKGNIPITYLCMGQDNINKNYTLLSVGCKDGVIYIYKCFRTSLELKNDYFYYYNDDSSMYNMNNNMDKN
VNNVYNNMDKNMNSNTTLNIQNNDNNNNNNNVGCDDNVKKSSSILSLKNDSNKTYDESSTCTKLLSKLSGHKK
AITCLVFSFSEDKIISSSIDRTIKIWEVSSGFLLKVFSDSSATLSVLLFPTNLDIFLCSNCTSLLRIVNLNSG
QVYQKIKVESEIRALEMDDTCLNIFAGSKNGTIYVLEVIYNERVEIKFRFLFSLSPITCIKFIPRHSVKTSPA
IIVNSCDNHIGIIECVYGNKGILTTLYVKHRIRINHALLPIRNCYSRFGGGWVMSGSEDGNIYICSLLPQSNY
KLIFLKHHKAPVMAVVVNNIDTLMVSGDSKGNIVFWRRSFV*
>N.MAL13P1.147
MSTNINEEKCLEGDDIKYEKPDTHKNFWKSFGLNNEAGKLLYHLYGESNKIKPNILSSKHDGDKNKNDKKKED
AKLNTTYRKPQIHYPSLKKKAVKENPIDTIKHRKPLSKILEETQNYDCIKDIPISIGMNRETEKNKLHNIFVE

EKCLMVPPSCAPMILTQEEKKEIIEKAQQRYIYINEENKSREEKHIQALRNYKLELIQELNEKRKLLQDIINE
DKNIQGNISINQSDKRVSNKGNSYNIIQIKNDIEQCQKSIKKIEDNLNTYE*
>N.PF13_0149
MLDILQEKNKIYDFKESYELNTPIENEVTENNYVDIQNERHIIWRKNTPFLYSSLLKHKLDWPSLTVEFLGGD
NSFKSKLNYFTNKVLLGTHTSNQDLEYVYIGEIKCPIFSIKEDVLQYENYSGFISNKKKKKGHPLPSFEVKAK
LLHPGEVIRATNLPSNSFFIVTQTSNGNALLFDYTKHPSFPSDMSTCYPQMILKGHTNEGSGLCWNVNRVYDS
YKNPNIFDGYMEKVLYTGKEDDSIKDIYSVKDMYSLKDTFSLKDTNSVEDIASSKDIQSVRGAYSIKELEAHN
DLDIVNDLDTEESNDSISNSNTFGTEVNTTNLLLASCSVDGSICLWDINKGTKSNDVPRTYGLNKSGKTADYN
IKIYENTPTLSPLCTWYNKNTKTAFNDIFFHPKFSNALGVCDDNGYMSIYDIRKKNFFTKAEICFNDYNEPMN
TFSFDNFSEYIFSCGYSDGLISVWDMRYNKESLLKLKYHTQGINRIKFGMISSGIFASCSDDGTACIWDISRN
NNTQILPLQKTEDDIYNNPNPVPKQLLFVHGGHIGSIYDLAWANSNTFTIATVGVDNSIHVWHLNEQFLFQ*
>N.MAL13P1.146
MRLPNKNDGHKTVPQKEKGWNNLYETVVNISSKSSIKYDEYTLLNKDLNKLQTCNRFSFTLTSTGAVTKEQLE
VSRKLIRLCNLRDTYIKKFQDIDTCLVESKSLNKNYKSKNIDDYESSEPIYNPYNVKILKNCNAFINFVDGIF
FVHWDPHTDEGPSSRDMCVESNKLANHRNIKSAEDYLSSIQEIMNVVQDPACKSFCFQRLKYLEKKFDFHIMF
NGPLELSETRDIKHRDFYNIRKVDVHVHHSACMQQKELLRFIREKYRTEPNTVVYINEKREMLTLKSIFDEEL
KSTAYESTIDTLGVNALGNCFHRFDLFNEKYNPFGQKLLRDIFLKTDNYIEGRYLAEITKKQIKNLERSKYQH
VEWRISIYGKNKNEWLKISKWVLNNQLSSIRVRWMIQVPRLYHIYKKMKLINTFADFLSNIFSPCFEAIKNPE
ENKEIFIFLHQIVGWDSVDDESIISNYTLKGGELPTPDKYVSEHNPPYSYYAYYMYINIRMLNEFMISRNMRP
MAFRPHCGEIGNMSHLACMFLLADRINHGINLRKSPVLLYLYYLKQIGLALSPLSNNALFLHIDKNPFKRFFK
IGLNVTLSTDDPLMFHFTDEPLLEEYSICAHTWKLSTVDLCEIARASVIQSGYEPAFKKHWLGDEDGFFNFQN
DPNKTNLSNTRMVYRRNTLEEEIKNIERLASYSSNN*
>N.PF13_0162
MDNLYYNKYTSEIYEKKKYPFKNNIHEKENVLKDANTYDYIKNYVLDVIVNYIINDSIGPKYKYHCESFNHNK
NNDISNLNVRSPQNDFLISKVLSKKYNNIPKMDLEKNNNVIVQSKLEFFSYNKILSLLRKYEYEEFSTLKDDF
IRYLLNNAHLYNNKLHMILSLIILSFYNAKKNMGCYFLLDVLMKTINYYKTYNKLKTCLILFSPIIEMHKRHK
QYVNLSEIVLNIDDDKNDAHKDHPGNSSNNNNNNNNNNNNNESCCKNFLLKFESTKELINIIRKNRDINSSTTA
TSNTLSTVSKKKRKDAYYFHKKKLNFKDRHLKDDQNIRNKDMKELNFLNNELTKKKLLQYIKKKKKVKKNKCH
DITHQNYDNTFQVHFNNSDQSQGRQRKFLQGVVVSYIKYNNSKFNLSCYISMKDYNFNSSIRICYVPFYNNYY
KNKNIWYRLNMKIFLYCWWYVEEVCFSDCTMNTLEYVSNDEEVRRLEKINYYKEKYTKIINEERNKLERNKIV
LKNDNKWTSDQYDKKNDNINIFNNNNIFHNNNISHNNNTSHNNKIFHNNNIYHNNNIYHNNNIYHNNNIFNNN
NSSERCPYRRREKKWNKIDPSNNPYSLLFTSYHKKLRGYHNNNYRYNSSSFISNFNDDGMSTKFPNSRDVEPG
YNSDNNILYRNNKRKRRKKKRLNVCKNEKHYCKYLSDDDMTYDNYCNYFYESVESVNVSSYLYYFKLINKKKL
NMKINQMEQKLEFNYDVFLISIYDSNEKKRNMMYNGYYNSEIFNNKEENYDYEDSNDDNYNENDDYNNNNNNN
NNDSYDIIENEDMLPHHPCLNYDHFNFITCYEKIIKYKPITTKYILCCKEAFFCSTEEYEDDEMNPNINNNMN
NMNNNNNNSNSNSNSFYNHNNNYSHMADGRRKGMKRFAEGNLNKEHHGYGEEEEGYDEYHNENIGNNRVNGLLSN
RMMKQEIDDDMENHRDNNMHNNNNNNNSNNNNNNNSNNNNSNNNNSNNNSNVRGEVMNGYNYNNDLHLNNIYVK
DEMKDINYNDGDDIHDMEEGKDFESIIDEHNEYNEEIDVKYEISTENNESTTLHLPMNDMDVGKGGSMTDHGL
NSNSNFLCTSSTNENGLDMNVLDSKMKNGTMHNNSNNSNNNSNSNNNNNNNNSNNNNNNSNNSLKHKVQQKNNN
EGKEKVKRRRKNPFSRSVVGMSPASNNDMSKAYGDMMKDGSNNFMYKNFVVPKKPNNFSYLNYKEVKEYLKDL
TILKSGANWKTYKMEDLPAELYKNIRPINQRVTGFKTVLLVDELFEKLWEFPNTDMIKVRTVTRGDQFIGDIR
IDFYYRQSERTLCRACGRHILKQKFATEHYQRNINCWKEVMWRLGIPELHYYNSVRNDFFDSRSNNMENNNEQ
ALLDRTLMLYNQELIKKATDFVNEELKYYDENVVSKPRKSKVNSCFELDILLLDDNNIIIPSVKNEVDEPYFE
IAAIYPNNGFPDENYSRNFCYIKITFNKLGGMVLRDLFTLECEFLVDWGDNLLIQAKKYTLHEVYNNSESYDI
NLRNLYDQGFAYLRCNVPQKPNGKVNLKVRIPKTVWHYWEAVAEESLKINYEFLDKHNVHRQLLFCSYLDNIN
KNFTAYVKNSSKSGGSNNNSGNNNNNNISNNNNNNNSNNNGNVNGGNHHLMSNLMTNNNMNLTNNNNNNNNN
NNNNNNNNNNNNNHMFELMNSNLANFAKMEDDFMPRNSYNNMDKKSKNNGMPPPPPDLMNSNNKMMPYNFNNF
SKGPPGGVVGAPDLSNMSNMNNRLMYNQNNNPNNVNNNNNNNNNMYNNMDYAAVLNSMPYSNYHAFNMNHAMQN
DSDTNYSKNFDQFSGHPELNY*
>N.PF13_0163
MEYIIKNEKEHSIVKNAEVNVFNIDNNKINNIYNNVTSELSTNNFHVLYHNDTIKKKEEGKGKEEEEKKKKNG
DMIDQEDNYEIKLNIINDDNEHNEETIIKNDITYNNSNNNNNNNGNNNNNNGNNNNNNGNNNNNNGNNNVCNN
SVCNNSSQKDMSMNISKNKHKLTRNEEDNVLKKRKKKTKKKNIYMNFQREETNNLNHVNIQMKNEIKKNIQKK
MNQNDYLLCTINEFLKQGLKNECIPLFQRLQQNLFFLVMLANQPEDNLDDAHSNYSSE*

>N.MAL13P1.156
MKLLNFPLSRYDVLITRFFLFASFLMVLFMCENFLKKSYMTSINIRNNKLCSFIHKNFENLEKYLTCDFVAYD
LGMNDWKSLKKKIIKNGEGEYNDMKDPSYMEKKHFEIEKSSHKIKSRQNEMNEQKSTLMNRPRKSKYNNTNKN
NKVKGNLNKKKKKKKNKKKAAQKYYTNKMGYSIGTNNNNNNNNNSNNNSNNNSNNNNNNNNNINIDSRRDKYK
EVDKYYHESFNYIVDTNNPSVDEQVLKKALMVFKKLDINKNKYIDYIEFEKNVNILSRMNEINKNILTYLFDM
FDIDKDKKLNYTEFMSLNSYDFNYIKLVHIIFDEDHVVDKRIVLEYLEIYITEFLETVIEEEKHKYLRQHNLI
QYYTNLFYINNKKKWDINEDDKLQIVEFHNFQLSVLIEIDHLSNFIQIDYNIDGYIDPSELLYYINEDKHMFN
KFKKYIKKIKNKNTNNNNNNNNNNSNNNNMDVFKFMKEELNIPEGLFLNIKYLYYSFDINNDMLLNFDEYKDQ
VTTFSVLDSAPDIVYAT*
>N.MAL13P1.157
MTSCRFVQLLSIWDKYKSKFLKSNFSTSNKFEYSSPTALLNYNNCTPLSTNKHEKNKIHKNGNHLNKKETSCN
NNDTFPCNNEEFFEMIKKNIKKNKNEYRLLNIYCKELMYRINDSNISANIITKTFSFLSYTDYKNDHRISEIL
NRCILRLNEFDLIHLSSLVISLSKMNFRSVYFLEQVQKKFIREEEYYFQKHSPHYICLFINSWVKLNCRRKKN
YVTNYRNDHHNNIIDHITDNINNKHDYQGDHINNNNGNNNNGNNNNGNNNNGNNNHCNNNHINNNHCNYLSHI
DHRESTENVQIINFLLSKISHRKVEEYSLIGISLLLYNISILKLYKYYYLFSLFKNHIIKTKSHLNVIQIVNI
LTAYNNVCLLKGTFLIELLECINVQIDSCYFHHLVRLMDISFCFVGNHNKNMLDIYNKDNIDIYNKNMIDLNN
KNMIDINNKNMIDINNKXXXXXXXXXXXXXXXXXXXXXXXXXXXXXXXXXXXXXXXXXXXXXXXXXXXXXXXX
XXXXXXXXXXXXXXXXXXXXXXXXXXXXXXXXXXXXXXXXXXXXXNMIDINNKNMIDINNKNMIDIYNKDNID
IYNKNTIHIHNKNTIDIYNNTSVDEFTQICHFITNLFNQLVKNTKYCKKKDVILLCGTLSKYKKWIDKNFTNT
SSPLSSINMNCLFYAIRNNVKNFISLYNHVELSILLYCYSLYEINDLFLFHKIKLRSLKIFITFNQITFSYLF
KAFENMKINDLIFEKACLHKILQINKFSCPKSLLFTLSALSSLHKKKKKNPIIQDIWQVIFRNFQDIFINNFY
EITNINKKDGNIIQNRNLSIPSYLLYIEEDNMDSYSMVTCPKNETFCSFQNKTEIKTEIKKKRSNLIKGDILC
MLVHFFSKIKEFDLNYKLIIILINEYENYKNILSTHNCISLLNSMGTLCAHMKNEKSNIFWRNYYLFINQLTK
KILVSVDLYEKKYYLINFLSACGKIFQSYISIIYKINEEQKKFCFYLNNILDILITPLNKYINEFSLGDIGIL
FETFNKENYLQEFIYTAPKEKDDHIKCSFVNKIINRLIDLLNHINEKDYKNVIIIMNNIIKMNNFDKTIYNLI
IKNYILQSFYYESIPMLSQSIFYICINVLFRDIIINTQQIYLLKYLFKKLFEQFINQEIFLFSTHNECLLISD
KNINNNEYNNMMIDTIFIKSLIYLCQSIFIILFHISNIKEISYEYKIKSKVIKEENNELLNSITFLRLYLTIF
MLTEKCIQEGYHFKKIKNFTSFDNSIITHEIHNIVKHMPIKSYYTSLLFYKLLLIKT*
>N.MAL13P1.158
MNEKEKTLLHFVKNINDVGRRKVMKKNLLLLGYGNINKYSFNQNDKISTYHSFLDLEKLCKNKYVKSILYQKA
SSVLFKNLNKSCNFLNEKWNETFMNCIKKSLFYNPLNIMTLFNLIYYYNIKMDVKNSLFYSYLLHIILMKIWK
KGFYRTNIASQNNKKKKLYIKKKVDKQKNVDKKKNVDTPKNVDTPKNVDTPKNVDTKKHLDTYNNICINKNTL
RYILFSLYNNKCRGECLKDDYKINLIKSIINKWEIIPFINDKLLLILMLENLQFLYSNNYKLVSVQKVIYYTN
LTLKILKLNKIYNYIKGNYLFLTTNMYLIKAHCLLMLNCIDKVKKNKNKIEYSKQQKEFHNNHMNEYLLYQED
IDNKNNYMFNYDDILSDRNHEKDIYNMVELNHKEIEQMLHELFRNNIKSENEEITTQLTKNVKDKKYMNGKNI
ENIFNLMDINNIEYYEKRFISFLINEKDINKEVCKMNDYFVHPYVNVKNTLSILNECIQFCLNNRKYKFLSQF
FIWRARIFFHLAKFHKYVLSDCCKASLFNIYASAYLSDETIEREKKNYSNIIIKNNINNNNNNNNNNNNDNIC
VSHSCSNHNYNHNLYNFYNDQDIYSCYNLMITSLKKMNKLHPFDLYIAYMVNKNTDKLKNMSIQKNNYYQHPK
KETFINERKHQYAWSRSYQDQMVLNFIKQNKKKIPFII*
>N.MAL13P1.159:mRNA
MPVSHHEGCGCKNSDEVLKGGEFLLKYINIDKVTALNEKTHGSCRKILKSYDNRLSPDNCESDVDHELIINIP
FNSPCKISSLFLIGGEEGTYPRKIKIFSNREDIDFGNINDFKCVQELELSQDFHGSIEYPLKVTSLFNVSYLT
LYFYENYGADTTKIFYIGLKGVGTNYIRKAVETVYEASPNLSDHKIEGSSKAAHFRFDAF*
>N.MAL13P1.159.1
MPVSHHEGCGCKNSDEVLKGGEFLLKYINIDKVTALNEKTHGSCRKILKSYDNRLSPDNCESDVDHELISSLF
LIGGEEGTYPRKIKIFSNREDIDFGNINDFKCVQELELSQDFHGSIEYPLKVTSLFNVSYLTLYFYENYGADT
TKIFYIGLKGVGTNYIRKAVETVYEASPNLSDHKIEGSSKAAHFRFDAF*
>N.MAL13P1.160
MPSTQIFIVLNFCVCFFFFFVIDLCINKNIFITNYNNLFINNIEQLERRKGKKKLCIHFIRTNNKNYYQKTFC
NNLYNKKYIVLKAKKFDQKELKRRKNYQYAMKHMYDNNGRRIKDINKEKKSLKKKKLFDYDGFKVDKIFTHLS
EEDMQELREEELKRKSGEILHNNKYIDNYGVETETDIDNI*
>N.MAL13P1.161
MNIPFINIIFLFFFFGVHIINLYEGKNISDHHKIWNNHYTYDITNNQNKYKYIYRNTRKHKLLNIKTFDFFPL
EKNHVTIISRREKKKYYSFIKFKTLRRLINKWFLLNNDENIIHPLKNCLWKITVYNFFLQKKDSFYVHLYEDG

RVKTSNNLEGRWYFNNYYLTWVIEYEDRRVFYTAELFWNQEKSKMIKGIIYQETKKNNSFIPSYMFRKILGSF
SGKIENQ*
>N.MAL13P1.162
MFLIKKRFLSFYNIISTSPILNKKFVYVKNNYIFQKRHFSNKNFYDILNIKKDSNKNEIKQAYRKLALKYHPD
RNPNNRKESEQKFREITEAYETLSDDNKKKMYDSQLNNGFYSNNFNNNYYNTTSNSNHMNYNYQSKRMTDEEI
ENVFKNVFGNMNLNDIFKSNIFNENSFSSRTMGSDIFSNFGSSASYGTPRNENIKQTNIKTEIIPRGNKIIEK
TTKIITYKDGNVKQEIIEREISNNSKGS*
>N.MAL13P1.163.1
MKGVNDFFRKVNDAEKMKRYLSDHSSSIKIYCFFLLLVFIFYHLFSDGDFSFLLTLSSVISMFSFLMVFLKIE
MNKSCAGVSLKMMECYVVLNTSRLISIVPFEGYLPYDKSGDWLYQLVEAVSLFINCCIVYLCRYKYKNTYDST
NDIFNNLFLIIPAFVIAIFVHPSLNSFLPADVAWSFALYLESVCVLPQLSMFQKEGKVAAFTTHFLASQAFSK
VLSFLFWIVSHKELNSSDNIIKSYVGFWVVIMQIVQLVLMGDFIYHYIRCLSKGVSFDNLLNENV*
>N.MAL13P1.163.2
MKGVNDFFRKVNDAEKMKRYLSDHSSSIKIYCFFLLLVFIFYHLFSDGDFSFLLTLSSVISMFSFLMVFLKIE
MNKSCAGVSLKMMECYVVLNTSRLISIVPFEGYLPYDKSGDWLYQLVEAVSLFINCCIVYLCRYKYKNTYDST
NDIFNNLFLIIPAFVIAIFVHPSLNSFLPADVAWSFALYLESVCVLPQLSMFQKEGKVAAFTTHFLASQAFSK
CHTKN*
>N.PF13_0164
MEEDKLTHKEKRKDIEKYTNEGDGNMNTISSNIKEEEKGSLKDDIIKFCEDNLNNEKEKNINKKMEENVEKDC
CHINNRQYDNIENNPCCEEKDLNKSEEKKDIQENSYNNNNNNNNEYEEFIDMWINLSYTSVDNINICEKVDDL
NINHKEKEKDKTKMEKFRRRQLIKDDPKEQNDDVGTSEEMDIKEDDSDSSSMEDVIISNEDEVTIEREGQRNI
SSDCSTQQCLDIYMRDDPKLCQDKEVDKNDKDDKDDKDDKNDKNDKDDKDDKESVKKKKKKKKEKNKKNKDKN
DKNDKNDKKDKQYKQKKEQNIKGLLCLNSLDKSFIQVPLNCILNTFRNIQKEIEKNFMIITLFIEKKLKDLSD
DVSIEKLNTMLEKLQALKNKVNESNFLLNKYIKRLVSRLKYIYFEGDIQVQNLKYDFRFQNYEKRINWLINEY
LCRYGYFDTVSIFCKRYNLEDYSDKDVYKEYIYIINELMKYNTQPALDYCQKYKSQLKKIDSNIESELHLQYV
IHLIFDNKYLEAIEYIKKTISQPHGCLASDLKFLITHIALHNKKKKKLKKFNDNRWKRVINLFKKVYSDISGL
TTNPLLELLIKAGISVIKTDHCGSKTSTKCPTCIKELKNIINKLPNIQKTKSFLVCPYTNEVMDEHNPPFTTP
TGYVFSEKAISLFLKAEETFECPITNEKYRMDEFSRLFI*
>N.MAL13P1.164
MLKSKCIFLNSFTKSDKLKHTFERLNLMMEKRNMNNLLYNKAVECHIYIRRDRDTKETNKKLNNMYNKYSCKC
NITNKCSNKNKVYNIYKRQNNLVDNKDKFFCIFRQNFAIGIFERKKPHMNIGTIGHVDHGKTTLTAAITKVCS
DLNRGVFKSYEEIDKTPEEQKRGITINATHVEYETEKRHYSHIDCPGHLDYIKNMITGTSQMDGSILVVSAYD
GLMPQTKEHVLLSRQIGIEKMIVYLNKIDMCEDQELVDLVELEIRELLSFHKYDGDNIPFIKGSALKALNGDQ
SEYGVPSILKLLDACDNYIEEPKRKTDLPFLMSIDDVLQISGKGTVATGKVEQGTLKLNDQVEILGIKEKSIK
TVITGIEMFRKILDTAQAGDQIGIMLKNVKRNDITRGMVVTKATNIKTFKKFESDIYVLKNEEGGRKNPFSSY
YRPQAYIRTADVNCAVILNEDTQVANPGDNVKCVIELMYPLALTYGLRFSLREGGKTVASGVITKLL*
>N.MAL13P1.165
MNECNTKKIQNGDKTKRKKYTYLFLSILICFVIRIFIFFLLNILEGSTYEYINKLINVDLNIQHIKKYDESRE
LKENINNINKKGKHMEKIYDDERNNKNNLYNINNNYYNKIDIKIIKTNKYIKELELYFQKNKRYNNLRSFLLK
NDYMHYLFNTDIYNLKYLYDSYVLRILKKDMYSSLVIRINPIFLKILHFLLFKPIKINIDVLIKICNNYEFRF
FLFICIIDILIAIFLFLIIDKLNNWNTYFLYIISNNYTTPHFNIINPLLLINIYLNNPINILANSFLSLDNFK
LLIITANFYLTLSRIQNVSNQQKQNVYKNKYKRICSFFNLLLILFHNAILLYITSFHFILVVIGINNFIIYIE
EDFLKRYHIKQNVQFCKLAHMLFKNMFLLLITLIIYALFIVISYFQNSHSLSFLNNTVINEYKILLLLPNLGN
FWYIFSTMFRDYYYSFLFLFHFHIFLYPVPLFFRLVKTPLIYLKVLIAIALVFQPNITINDIVYSLLLLVIDY
ERTIYAIPFIKLLLILLSNLCLCFVTLNLWLRKNTGNANYVFFNQLVVFNITTFFIINSMKFYIRVQTPKSQL
NEGKCISILNKKKSKFNFLHLFKKKLA*
>N.PF13_0165
FFIFFFFFSLFFYYFQFLYIKYNDSNVDNKATESTAVDSEAKMEISSKVSEKKKTKKNEDKKKKKKDDTSES
SDSSSDSDDDSEEDNKIEIKNENKKENKKENKKENKNENKNENKNENKNENKNESKKENKKEVKKDDSSSDSD
SSSDSDDSDDSDSDEVEEKKEKESDKVVKPSNNTHKVVEKKKAETKSDSDSDSDSDSDSDSDSDSDSDDDDDD
DEEKDEKKETGKNKEEVTKKKEGAKANSDSDSDSDSDSDSDSDSDSDSDSDSDEAKEQKEDDKLKTNKGKSTH
NLEVTEEKNDLNGVEGKNKRKNINVEENKKGKNKKQKLGNNVETENMNENNMNFQDDGNFGNEKGEYILTMLN
ISRDTTDVDIRSFLKDYLDTDYSEDTIYIHINMYNNSAYIYCCEEKAKNKLLSMNNQKFNGVPVNFLDNPHID
KKIILHHTSKDTSQQDIRNMFEQYGYVVEVFFPKTYPKVFVRFDNLQSCIKALEQDGCLLKDKYIQVTLALNN

SMQDNNKRNSMHNNNGMNKNRGGGNKGFFGKKFSSNSNNYNNNNNNGNNYNNNNNNNYNNNNNNNNGNNFNKN
NKKKFSKPFNNFKNKKSFN*
>N.PF13_0166
MSKWNMLRSRIVWPEEFVFAIENEGYDNKFGYSYVIKYNGNIVHNNCKKHLNKVDKLLNVTHRMCENLEDENM
GNNSDTFNDENNDEEINKANQILLRLNDKKKYIKEKLSNIKTIDEKFDIIIKNEPCYIFRIDMPNLNVEPDYL
LEYLKREYYNENHKDSDEQKVGHHRRLSNYDRANNKINSNNKINSNKNNNYYYRANEGEPYKTYLYNIKLMEC
EDTIMDNREFFFSEAKQSGLNINILINEGKYIKNKLYEYKEYCVNNYNENEEDDEKNKENNDNNGNAYGHMKN
QQSKEFIDKKNIRTQNKSIIERDNKKEINYEEISVLPYSDIIILKNKNVHTESNIAGFLTPFLLNGNIKKHIS
KNVQPYIKYHFNNQLIYKNLCNIIKLMCYLQDCNICHGNIKPSNLFISNDGLHILLGNFIPRLKLQNFYFYVI
HKKKNIPKYISPEIFFYLNKKIAIVDTKGKKKKPKHIEKYFYKNDIFCLGLCFYYIIMMNEDILYYINDQYIF
QSKVEMMQAYITKPELFSLVRSMLIYDYKQRPDWSALAQIIQTEKR*
>N.MAL13P1.166
LQADKYILDELCITSNKRKLLQLGIHRKKYFNFSNQLIYISLSGQIYTYPSYEGVLQADKYILDELCITKFGE
SSAITDIKLLYKKIYKNDYDPSKYTKQNFEHYYIPPSIIKAYNELGIHKLYKEQAECLCKIFLNDDIENKIKN
GIYMNHGNNEEFVSNENTHCKSNVEVNTDISMNLDFFCDMNIVDMKNIKYTPGKKEDIQIDEFTDLIKSDITF
GKKKNVDICNHMDNKMKVYTNEYTNEYTNEYTNEYTNEYTNEYTNECSNEDTNEYNLNNSHSKYDTLVNVENS
SYKKRSQICYWSNNNNNNNKFYNNFLFKIPTGMGKTLIYDILIIRQVLYKGFRVILTLPTLSLINEKYEYYDK
LFGDQTVSLNIKKFNSNDFTGYSYSLSTDLALCTFEQANTILSIIIKNNLKFNYLFILDEIHYINNQKRGFHI
ESLLTKIKYIQKNFSHIYNIKVYGFSATISNIDQASWLDANVYESKTKLQRIKYLYKIDNSLYKDINKNEVER
TLENCNTLDPNHLGYLISEEFILKKNVLIFCPSKNKSEQTASFISSTLPYYLNKRDYKINMELQEKRFKLLND
LKGLTIKVANVEKMIMNGIFYHHSGLNINEKSLIENSFRNNILFCLCCTTTLSVGINMNIHTIIIRSLKLGKS
FITKDEITQMAGRCGRSQKKLYDSTSSCKNFDESTNKSYMTPDRRYTDSSGLPPILDYDYDCDGKVIIFVSNV
EKHYLEKILYDEEEMSKLKTTLNNIQMCKFLLDFIHLKLIKTKKDMFDFLYLYSIKFFKAKRVEGNTNDDDQN
NGINMNINNNDKCIHNNNDKCCSNNNNNDKCCSNNNNDKCIHNNNDKCIHNNNDKCINNNNDKCIHNNNDKCC
IHNNNKCSNNMNEKILIDVKQIFQYLFENKLITIPYEQEKNYYEHVFNKIFHINFCDINKIFTFQYINQNINP
NILMKYNITQKINIFKKLYHNYKGKNITTDKQTSFFTLPFITILLIFKDIEFNKNLFQNLYVEFIKYIFLMNI
NLQQYDLFVYDILKDDDPIACTELFSYVQSASSIMEFIFNYSLVQYIYVKGFLPDVLLMIFAFCINSEINLKI
HFDIYEQILMSHNKNIKNVFQFFGLNVEKLKNFELKNVDDLFDASANILKGXXXXXXXXXXXXXXXXXXXXXX
XXXXXXXXXXXXXXXXXYRLKLKEIKTVYLRCIFNLSYNCRILKNFKNSLDIFCIILENLLAKMKTKNFPFI*
>N.PF13_0167
MYNDEDDSVEENPGNLPSYEDDFLFNTTGKDHEEEVKGKEKKRGDNNNNNNKNYDNNYDYKYDDIYDIITNSI
KESEMKNEMKISDININNDNTSTLVINYRKKKRTKLNDSSYYSLKLPKFIDVCLDIKKKKNNNINDAKVNEDD
INKNNHIYLTGKEEKNNFNKYFFDEDDHITLLNKKKNGSIVQWIFDNHLYEQMKYQKKEKEKEKEKKKKKKKK
KKKKKHNRYYKCENIDTYEKMEEPKKDSLHLSSTKQKLNNNNNNNNNNNNNYYYVDDEDSNDEDIFHVKDLLD
KYDIEYLSDSDYITTSSNESDDEKNILLKNLEHLETNSFIVEYDNGQHFFFINDKTYMLEQDKEINYLIECTD
ENIMPIHAKLKNKFYIKSLTKEDKVHPSDLKLQKSHVFYSLNNHKI*
>N.MAL13P1.167
MDFIKEQYNSLVLDLRKTFRNKRDGLSHILNVICLLLNALMIWKLLVVFTGCESPVVVVLSGSMEPGYYRGDT
LALYHPPKIHAGDVVVYQINGRDIPIVHRILSLHTSKDNKFHLLSKGDNNNIDDRGLYDPHQYWLENEHVLGL
SVGYTPYIGILTIWINEYPVVKWAIVSIMLIMILMGYE*
>N.PF13_0168
MKKLNLLIGCISLYSIIFYTAKISCTVQVKNGGIENLASSSGLLKNVLKHSPRISEEEIKESEIKIIKNAHEE
EKKILDKYGKCLKDYTLPCPNYWKRRTDKYGKNVCIANEEYNGFCEQVQIFDEFSEHEKMSYESSCNVEWGCK
GSSKEICESGKRDYNVPCPEGFLVQNDNSCKADISVYRGMCNNETINFTHLTSSEKENWSIACEAYWPCYTDC
ISEEYISDCPKNWKQVNKYDCIPDKNYKGPCRNIKNFKYFTLSMKKDFEEKCKTKFECNNICEKNYEQECPLN
WKVEKGYCLAPDTFDLCKRKKISIENMTRKEKENIEKECFVSWPCINNKNTNKPNCQINWLADCPFGWDRKKE
DKRDQKKDEYVCILPTEKQIYTEENKFNSLNKEKCTNIFLKKNSDEVIKREIASVCNTPWPCLNNEKIYISHN
VDHEQKEENEKLNGPLTNEGKIYKNGKYHIVEEGNFSLSDIMK*
>N.PF13_0169
MNVETIQDIDIKGKNVFEKREKNKSNGVGKSICFYEDFIKLREKILLKEKEILEKRNKCLSKNDDNCYVGNKN
KVIYRTFDEFYKYKKSCEENEHKRQKEALHLLNKRNKNNNKQIIESIRNNEKYSYIKFKKERQGTVKYLKEYF
QKMQEDNLKKKKKNDVLNKLERKPYMIKKDVTKGMVYRKKEKDDDIINGDIINDDIINDDIINDDIINGDIIN
DDIINDDIINDDIINGDIINGDIINGDITNDDIINGDIINGDIINGDIINDDITNDNITNDNITNDNITNDNT
PNDYITNDNTPNDYITNYLSIDNSENLQFDNMNEKEIKLLQELINDMKKNCKNDEDYISNVIEDLYNNNQIYI

IDKIYNILKNVDKEILQESKLNAINYDASHLFLNNKEMLMNGDILQKNKKVPSMENIDINNMDNCKNQNESIQ
KINDYKRTKKNQAQEILDNMNEYMYYNKCNNYVNCNSDFHEVLWEKQNDDNNKKNDDDNNKKNDNDNNKKNDD
NYNEYILTLNNKEDVNINNENNSKLFVEDDNLENNKNGDYGDKDINITKKKNSKLIKKFKQKSNKNIKDANYT
NKFDWFYNTMNDIYKEQIVNNQQEDINSNLYEESKDIFSDEDSEKNDMFLWLKNKDIENVLCEKRATMDENY*
>N.MAL13P1.168
MSATSKCMLIYNLVCCCLWTVILFTSLQYVFNKEKYPITTFWSNYKNIITITQSLAIFEIFFTIIGIINSVVS
IVTIQVFSRLFVVYLIFNFLPNTNKWILSCLIAWAIIDIIRYLFYSLNILNLRFNILASLRKKLPLILYPIGI
TSEIVCTLASLNNIYATPFLRTYPYSMPNNINFQIDIYYFCIVVLILYIPGSILLYATAVRKSKQKIPIPEKK
SDGANKKKI*
>N.PF13_0170
MEKIYSKNKLDYLSIIFNFYSWYKRHFKGTLLINENSNIKNTTLIELLGNKKLLLNEINACEAILKLNEHHEN
YDSSSGTCNVNNINQPNNNNNNKENINDNNNNNKENNINPCSTNSFNINSFSTNNYNNFNNPCCSFSCNIYDY
YDILKIEELKDFLKDFILRNKKINKEFFDTIEEDMTMWKKIFFFGQKKRTPEFLDFLLFYILKKENIHFFEEE
TRYINLHNWFLKNKIYDISNDKKEDNTPSACNTNFIQQIIEEDLKNKKHTHVITRFPPEPNGYLHLGHAKSIC
LNFGLSNKYGGRTHLRFDDTNPVTEEIRYIESIKEDVKWLGYDWKEHLYFASNYFEQLYEWAKILIKQGDAYV
DDQSIEEIRKNRGNLKDPGVDSPYRNRTVEENLQLFENMKNGLYKEGEKVLRAKINMKSGNMNLRDPILYRIM
YKIHPKTKNKWVIYPMYDYAHGQSDSIEKITHSICTLEFETHRPLYEWFQEKLHIFKTRQIEFARLNVTYMVM
SKRKLLTLVNEKYVKDWDDPRMPTISGMRRRGYSPDAIKDFCNKVGIAKRENMIPIDLLEFCVREDMDKKAIR
LFAILKPLKVIITNYNDAPDYEHIHNLVASNHPKMEQMGFRNLKFEKEIFIDHDDFQEIPQDNFFRLAPNRTV
RLRYAFCITCNEVIKDDQGKVIELRCTYDPSSKSGLCTNQKKDNVNRQGGDGDNKQGGGGDNKKVKATIHWLC
AKNSLQAEFRMYDKLFTKPNPESNEDTELVQKITSTAHLENNTNNQGNGKDEYASDTLLQDEHLEDESKNAGW
RKYINTNSLIIHKGLVENYSTRFKIGDPIQFERVGFFTKDKDTTNELPVFNLTVPLVDNTMLKKKKEDLLQKE
LDKLKREKIAAERKLKKEQKKIREQKKKEQN*
>N.PF13_0171
MKRGRAGTLKNKMRITLSLPVGALINCCDNSGGKNLYIIAVQGFGSCLNRLPAASLGDMVLATVKKGKPDLRK
KVLNAIICRQSKAWRRHEGYYIYFEDNAGVIVNPKGEMKGSAITGPVARECAELWPKLSSAASAIV*
>N.MAL13P1.169
MPYVDKTEEFFKIIEKLSNDNINIRKNRSIVQDTQVGELASKITDLLQSGYQKLQQLERCVKQKGIFNDKTSE
IEELTYEVKQTITDVTNELDLLVQYSCNLNISNPQSKTHIDNIISSLKNRLFDFTKKFKDVLQIRSEHIKKQV
NRRKMYSYTSNEATFNNDNYKFTPLGDIDIESGQQQVLKQPSKHSYLHSRADAMENIQKVIGDLAQMFQKVAT
MVTQQDEMIKRIDEDIDISLTNTREGQNYLLTYFNRLTSTRTLILQIFACIFILIVFFVIFS*
>N.PF13_0172
MIDYVLKNPFIVSLVISLFSVVLEFYTSLGKFLRAYFFSFFCGIIMNIICEFQRDDKIYDTGFFLKQTLSYSL
CISIMLTFSFIKKKVNNIPFLNKSPICTTEFYKNTYNYKGIKNISMYNFIIESRLLHFGIAFFFGAISTIIGV
VMSYILIKHIFIKNEIDIDSYYLKNIACCLVSTYIGGFINFIEVSDLLNLNTTLRNSVFILDDMFTNIFLMML
PLCKNYGKYFYCYNDNPLFSINRKEQEGLEKQEEYIKNKIYNSLILNYDADEEQKLINKKSLFTSYGSFTNHI
NNINYKERHYIESPYFFIYNRIHLIICDFFAIMLIFFLTRKILNDFEYLYKHISFYINMDKIKTPFLLIITFI
YIFTIDYILFIMNKILNNNHIINFVMDIYHRSLEYYSTILKLTTIYYLSLSAISINIKNVFNVAKPLVLFITC
ILIIHVLCTFIFSYIYNSVSKSEHAFIYIDEVLLAINANIGGPTTAALMSEMIGRSDLIFASLFWGVIGYLIA
TPIAIQINSIL*
>N.PF13_0173
MKKKKKSGEDDVWDESLNDFMELNKNSCSLTRDTIHGRLRPEPEIIENILEELENKIRHYFNLYQIEKEKRTC
SDKNNYYLQKELYKAYATLDKNKLYTKKLEDKLNSIKENECNLIDTVRRIDLLYIQLDTLVQSFAGVCTQVIV
EINENKDNVMNTIDIKRKKGETIKMLLDYIYPCRTLDPRLNSLYGMLYYQSIGLLKDESFVGPPIPPIPPVVP
SESNGWLPPCGNNNNNKMMITSDNYVNILSDHRFSDLIRMVHSGQSAVINYPDIIKNMEDAEKVPHNENKNNI
NINSNLNSNNINMNDKGSSYESLIVQDLTGFDVHIGIIEINCHMNDPKIVCVVRYDDESVNSAIQDVQRVSKP
KSPDQVMSTGEYIFNVDNKIHLNKLPQKKTGDVPSFMIDIHDVNAKELLGVCSCSFINEKTLIKNSIWDIYSK
KNNSTPDMIIGKIYVTISPYPSKSILPAQLFKNVKHTQYNTTGRMVSQGVVSKSNVDMSKKMNDIKISTKGKV
GFQKSAVLSKVIVEKTPLESKMKTSAPIQRGKRVSFKTFSLKLESSGSKEKADNNTEEESKKQREKEENVADN
NNRNVDDKKKEGDKNVEETNKISSTFSVKNFIKNFANKIESNKIEKENVSFTLKRKTENEEIKDDKNLTEENK
VEKKEPEDENKNKDNIVKKEISKAKDGILKIKLPALAKKEPSIVTKEMAKSNSIEQKLDDEKKMISMGKSIEM
KGKGLPASNENNNENKNDNNGNNNNSADGKGIIGKPKIPLPKIKMELKKENLNKILLNKTIAKTSTDETNMSN
TKNEEGTKPKFSIFKKDTPLNKKEDSAKVSKFAKISKTVLNKNDMEQNKIAEGSKANDVAMKKETTAKGEIVS

EKKKTSKGSMFSFFKLRHSKVENEGDTTKVGEVQKGVSETQKKEVGNKAIEKDVSKMIKLPIQKKEAPSITSQ
DSVESEELKSRIQEAKSKTLLIDKSKIISKLSKNVLVKKEIKKFVPKLKNFA*
>N.PF13_0174
MNKSTGSMSSNLSQVFVLTIIGKGDIPLYEADLSINSKRDISEHLTQFIIHQSLDSLDEIVWKSSSMFLKNID
SFNNYSVSAYCTPGHMKFLLLYKNRNEGGTNLTNTNIYIPSDDHIKSFFETVHENYIKVLLNPLYEPNGIITS
SLFDQNVHLAAKKYLHQ*
>N.MAL13P1.170
MEDDYYFFLFQEEESEQVKEAKQDLHNALKKNREKIKNVSNNKPMEKASNIIWLGQDDDNLKNEYKKHNEGKH
NRDRKKSAEDINILSSDKRNIINRRNDEFTKKYEQDKYHSYDGRHDNDDDVDSFPFNDIQNKKRKKETSYSKN
EMEYGYCEHKDSFDKYNNKKKIRKNDDMYHEPNNNNNNNNNKYNYYGSNNNNHDNVEHKKLNNKEENITDPNV
KYLSNLNNQNNLDDKEKKNKLLDSKGRSNNSHIITVSSNSSLSSDDSTSTNYLPSSKKRKNINETCYSNKYSK
DSNNIIIEGSGYNMKKKSLSRNKSYKEEKRKNSSFLLNDANVEHGIHNKEYEARDRNTTNNVLKSNIHKNILK
KFKINYKNLSSMEKEYYNYMIKKLDINYDDEKKVLYTDSLFNEKFSYMVSKENKNFNFLGYLEYSCFYILEWL
TPSKEEKLLKLKSLIKLELLVKSLFPKSKIEVFGSYTSGLSLPSSDIDVCIMNIKENELDCLYILAYALIKLN
IASDIRIIKDARVKILKYIDKELGVQIDICINQKSSKETTDFIITQMKKYIYLRPLVLLLKFFLHSRNLNETY
IGGIGSFLLSCMVLHFLQMHTSTFDNNHFNNTYLIKLLIEFFYFYSIDFKLHDACIVVRGLGHVLPRRLRKEY
EYNDQRLCFENPIDTSIDIGRNTYRIKYILYLFSYTYCNFISLISKLRKHSNNFHFPYIPNVDEQSHTKNKKN
GKPLENKNCKINNNTKKNYIYGSKIKSNCIYPLFYGNIFNPDNIIFTKRYKKNFPSNDWDIRHFDFIFTKQEI
NNMFNMSCEDLNSYVNMDYMIMNTSSMLYANIDKIFSHSFDVYNNIFKYSTSHDI*
>N.PF13_0175
XXXXXXXXXXXXXXXXXXXXXXXXXXXXXXXXXXXXXXXXXXXXXXXXXXXXXXXXXXXXXXXKKKKKKKKKN
IYIYIYIYIYKISKYLRIYLQSSFFFFFLFFFFFLQMSTKYIIEHLDELEEWCIHEYIHICETVRDENVIFTK
FCENFSELTNGKYKPACYEKSIDELKNELLSGNICLLDMKAKNKLTCNDKNKIDFLLFGGILGNVPSDDRTSI
LRNFNFPISRNLGPIQMTTNTAVLVCYIILNDNVELENIPYVDDPEIFLKNNKESITLPFRFVSKYYYTKCEQ
DKNVPVLPEKFKEYLIKLGDQQFDDMSSFLENEDN*
>N.PF13_0176
MFFSFYHSSFSSLSSFFFSFFSLLLYMLVLLQIFPKNYYEAGTLSIRVIRKNTFNHSYNNIKNIHYIHCGYPN
IFNNCRNYYMNNFYNNNLSAPFKNKNNYMHIFFKIVEGNKKKKREHPFFKYLFHMNMLEKSGDKKNTVISKIT
ESCQYGDIRKYMNMKKESLEEGEKNKSGEEYNKHVEQNNIKDEKIHFKDGTFIKSKEEEIENEKGGTICLKKE
IQKNVLEINNNTYNNDTKYLSNPKGVTNKSKQSKKEIEKKKKKNNNNNNNNNNNNKIKSEKSANDTKNIPPIP
KNTEDRWNDYNKIKEYAKISNVYLGAHISASGGVQNAPINSFNISGLAFALFLKNQRKWESAALTNENIKQFE
ENCKKYNFDKNFILPHGSYLINLANPDKEKRDKSYLSFLDDIKRCEQLNIKLYNFHPGSTVGQCTVDEGIKNV
ADCINKVHKETNNVIIVLENSAGQKNSVGSKFEHLRDIINLVHDKDRIGVCLDTCHTFAAGYNIKTFENFDNV
MKQFDDIVNVKYLKAVHLNDSKSDIGSGLDRHENIGKGKLTMDTFKYIMKSKYFKNIPIILETPDITNDESIY
KYEIQNLYKLYFEK*
>N.PF13_0177
MIICARTKKPSLRFCKLFRRYYIRKFLKNKIKEDLIKKKRYKYKERENVDGTRLYNEEQINSYSDQSNNITFE
ELGVEDWLIKISKSVHILYPTKIQQLCLPLIIQGKNVIGSSETGSGKTICYCWSILQELNKNVYGIFSLILLP
TRELVFQIIEQFHLYGSKIGVMILSCIGGFSLIEQRKSVMTKPHIIVGTPGRISDILESSIDIQNCFKRLRFL
VLDEADLLLQKCFEDKLQNILNNLPKNYANERKTLFFSSTITNSLQLLIDTFPYNNLILVNVNKKQKPPKNLD
QRYIYVEEIAHITYLIYILKNKVNNLSGIIFTANSYKCELVYTVLNMLGIDNVDAMHSSKDQKNRFATLAKFK
NGLCKILVATDIISRGIDIPKISFVINFDFPNDTVQYIHRVGRTARANRKGLAISFIDKKDVNSFNQVKNIMK
DKLKPYTLNKKEVLENMFKIGRVIKKAEIMLEEKKDIKRENERLKHYIFHNE*
>N.PF13_0178
MAIRVQFENSNEVGVFSRLTNSYALVALGGSENFSSVFESELSQHIPLVYTTIGGTRVIGRVCVGNRKGLLVS
SICTDQELLHLRNCLPENVKIKRIEERLSALGNCITTNDYVGLIHTDIDKETEEIIQDVLDIEVFRTSIAGNL
LVGTYSYFTNNGGLLHAMTSSQEIEELSELLQIPLITGTINRGSDLIGSGLVANDWSAFCGMDTTAIELSIIE
KVFKLNNITDNNMEDTFKYKSSIIQTMI*
>N.PF13_0179
MLRFVNESFLKVSNKELVHINIHISRNNLFFNKRIIRRVDNIIRVDIDRLSYYISGYRVNKLLSFVNFNKRIH
TLRLEEMVTLTFTGISDNPNIVEEEERILKYWEDIDAFNLSNELSKNKKPYIFYDGPPFATGLPHYGHLLAGI
IKDCVTRYFYQSNFYVERRFGWDCHGLPVEYEIEKENNINKREDILNMGIDVYNEKCRGIVLKYSKEWVATVQ
RIGRWIDFKNDYKTMDKSFMESVWWVFSELYKNNYIYKSFKVMPYSCKCNTPISNFELNLNYKDTPDPSIIIS
FILCSDFPKVKNVYNIQDDIQPLVQKYSVLYDCYMKSEKCDTSNEKHADEDIYSDHINVNKNNNKNNNSNNNN

NNNTNCNDNNNINLNYNNNCYAQHCEVFAWTTTPWTLPSNLALCVNEHFIYLRVYHIESQRMFIFAECRLEWI
MKELKWNVENIKIVNRFLGSELKELKYKPLFTNFYEKHNFKERAYKIIADDFVSDDAGTGIVHCAPSYGEDDY
RVCCKYGIIDPESNILIDPLDWNGYFTNEVEELKDMYIKDADNVIKKKLKENNRLMSNNTIVHSYPFCWRSDT
PLIYRAIPAWFVRVSNSTNKLVENNETTYWIPAHVKEKKFHNWIKDAKDWCISRNRYWGTPIPIWCDEKMETV
ICVESIKHLEELSGIKNINDLHRHYIDNIEIKNPKGDTYPKLKRIPEVFDCWFESGSMPYAKVHYPFSTETND
FHKIFPADFIAEGLDQTRGWFYTLLVISTLLFNKAPFKNLICNGLVLASDGKKMSKRLKNYPDPLYILNKYGA
DSLRLYLINSVAVRAENLKFQEKGVNEIVKSFILPYYHSFRFFSQEVTRYETTNKMQFLFNTDYIYKNDNIMD
QWIFSSLQSLINSVHTEMKAYKLYNVLPKLLNFIENLTNWYIRLNRDRMRGMLGKENTHQSLNVLCRTLYLFT
IIMAPFTPFISEYIYQFLKNIKYTNNQNMEHNENNETTNIKEEDLNRNDIHKSVHFIMLPQVDDKYIIKYEFI
ELIEHMKDVILLGRNLREKRKTPNKKPLKSLTILHKNESFFKHFDRISNYIKEELNILNVEYSNDISCLNFSA
IPNFKKLGVKLGYNIKNIQNKIKNMNAEDIKLFQQNKQIIIDNILLEQDDIIIQMNHNIQNDNTEAISNNYIT
ILMDFTADQQLENMASAREICNHIQKIRKNLSLNQNSPVLMHIYIYDQTFKNHMLNENEYIKKCLRRNLNIID
LEKDVQNLTDKFFEEKITVNEKEVLVIFTNQ*
>N.PF13_0180
MKIIFLLNIVILFLTNVLTKKHVISNTLNFINVNPKQFNNSTKRLKATEYKIDNKVIRGPLTPLNEYILIQKD
EAGDTTDSGVFIGDTLKKNQYIGKVLSVGAGAINTKNGERIPIDIQVGDVVIFNPNDGNKVKYNDKECLLISN
EEVLGKINDSNEINPLNITPLYDRVLIKLINPNVNSDSLIIIPESKNNDKVTDGQVVAIGNGIYDENNQKVPI
DLRIDDYIKFSPFSNESCEFTYQNAKYTFVKARYVMAKY*
>N.PF13_0181
MRREDKTNTCFYCLNIIKERCTYINNLWRTYKMCISCKNNLKSCICCEKKIMNNNNISSLTSCCGSSRGKLCE
ECRSIPLICCNKNVVNIIGDILCFLDLYLHINISQEFITFQNIYTFNKIQYNEKNHIYTFQLNHMKYNKIETS
TYITLNHREKNNMKGGEKKMGNMKYNINNKRKKKRTNFYLLHKNDESQIKDNVTISPKMKMKMKMKIKIKNVK
KVHKVEKTNQTNQTNQTNQTNQTNQTNQTNQTNQTNQTNFINHMNQTNQTNQTNQTNHMNHINKPDNININNITTN
NNQRKYFSSFFKSFKSLYTKKRNNIYISFNTDKNRLIPSINIIEAEKNDIIKNDNINNVNNIQETFLINNNIK
KNKEMMNKTNISKQTNISNNNHKKYDTFNNNIPIHNTSRISRFYTNTSAAFFDYIKKIKKQQQKYKKNIKRNK
KDKDNEKIETKKKKRKFIFYENILLSMYEKRNEQKIKDNEKNNLPNKRNMDKYKLNLVLYFMDKQNFNGVYRR
LLQNELKYCDIIYLNYIIKKKCLNKYGNDKSNEYIYNKNTHGSNTHSNNTHGNNTHSNNTYSNNTHSNNTYSN
NTHGNNTHRNNTHGSNIYNNYNTYDDNICEKTVSSCGCVTVSGKKLFKLHEQNKRIHNYKYKNINLKYLNISN
ENIKLIDNVSLTSVIPKISFYFYLAHELMHAYIWLTKMEKSHNYKHIYMIVEKLYNNNYFEVQNEMHYNDYHN
NSSFYISRELEEALCIYVSIKFLKYIQTDPYYNIQKSFYEKQLIQYYIKKYEQSTSSMYGSNYRFLKKILKNY
QIIHILHIINDIYFSKFYPIVTLNLLQAVISFIDFKI*
>N.PF13_0182
MFLSIYYFSMLCILPFIYKKTMNILCIHIVYCFIACLLLCNINQVFTLHVLNYNNGYLYKNLSFSIPLYKNLF
QQKTTCKKRKQLLHIKSRGSTYNECSKNCDAYFELNKTNKNITKRIGNIPNVLRKEEKKGRHIINNTKESNNE
NCRYKEEKNIIINENGDKSFYQGDDIEEDLFNSNLNILESEKKYSRQIYTHGYNEEKKIRGSKIIIIGLNGIS
CEICKNLSLCGVHEIGIYDNNLLTYEDIDNLYLCNKKLVNEQIKSISCVDNIQKLNESCKIKAITTNLYDNIL
NYDIVVTVNQKTNFNIKLNNYCRENKKKFICVNTCGLFGRVFIDYGDFYYTNNIDTNTTYDNHNNIISDLYKI
NNLEFLKNNTIVLSYLPFYNMQLNESDKVKLLLNNNSGKNIQLDNLIIDRICRKNHKIYLSISKNKCNNSIFK
IFNHIFQPFFINQLFPYFFNSFFYTYPLNLISYLFKFNKNKFLYNNYSHFFNIKKFVQENKLNYSIRLIKLPE
QFRLKYISLEEYIKKQKKKTKIYNIYNMFNIYNNVFNIYYIYIYIIKNTLKLIKENIRFVLKNIYRKLYGIRK
NEHEYKNVDKLKDEEICFMIYDEIMKNKNNKELSDDDIKKFTYLCKKEKKDISNEIINQFCSCAHIELSPFSF
FWGALLSQQILKGITHKFKPIHQMFYYDRRDLFPFSNISKMYYGKYMHEKNFFGEEFHKFLKKLNILLIGSGA
LGCEFLKLLAISGVSSNMDKISNQIHNNKQNNDFNNIKNKDKLDSTSNLLHTNQNTNVYVPSNMEKKNNHVIL
QNKENNIQGCVQVVDYDYIEESNLSRQFLFRTKDINKLKCQIACENIKMINDDINCDFLKMKVDDTIFDNKDF
LLKRYLLFTNKKNEIVKKQKTKNIRENPLICILCLDNLKSRILMDKFCLLNSIPLIESGIEGLKASSQVVYPF
CSETYSSDSNNNTSSSNLFDEEKSNSCTITSFPRNHKHIIEFAKSVYNNMFYENVVKMNKFINDPLYFLGELC
KYENILDVNNLLHFFKLTKLFFHFNLHSNIQTLWNSIFVHNILNLLKNSKQEEIVQYFDSLHKLPQPIYFNKD
NKYHINFFKYAMNNYKKVFKKFMKIKDEMNEDIFYYKKEDILFTNQQDDSSLSFEKVITNLIEKNNIHMDVRK
IFYFLDVIKKNATIQLYELLKKELLELFNNPLFLLSLKYVQNKKLAVNIMRDKTKIPSTLNSGDKLDHRVDTV
GEMELNTIRRNIPGNISNKKNNNNNNNNYNNYNNYNNYNNYDNNNVDVDLFCPLIYNLESNKDDINFIYSVTN
IRCENYNFKKLNMFDFLKISNNIIPSIVTIVSMISALAFFEMYKIVFFLLNKNKKSKNRSTSYEQKYNVHEKE
LLKDLNTKKSDHKSELKKVLRHDISNVNVNNNFTESNRSCSNMNNIEMLPYKKLNIYIYNNQIIVRNNDNKNL
FYFFNANYEKLKEKSKVIYNQYVNLEDDFITISELKNVDTWTYDNPIFVKKNIFFSIWNYLYIDIFCKNKNVA
KDTCPLGCSGINPSHLLDNNNNNNNNNIYYNTFNILSKEKQNNMKIYINKINEHIEYILNLIKNYKEKENYEN

NKTYNKEESYNVLVSMLKKICDEYINVIINNIDILSSYVNKKKKNIKENRTTFDDTLQFVLHLKKTLCNIKDK
IESINKNNVNPNINIKNNDHNKYISDNIKKLDNKDEREPRISNILFMLNNIKKEIENVKENILKYIDRNNLDV
NTYTNNITLNELVESIEIVFNVNIQTICVQDKIIYSKFNIPSFKLYNHKYLYDILCDIFKNDKKFQARELPRT
FILSIYAYDKLTNKEVVLPDVQVNVHNHADG*
>N.MAL13P1.171
MKKILWLCLVIIFEIIGYNGLEIYIKMKGKKLKCIKERINKDTLVISKFKTNHKNALVSLYIYDVDVNEKNFH
TQPKLPIFETVNKHDIKTAFTTFYSSSYSFCAYNNSDKLIEVYFEIKYGTEARDYAQVAKTEHLNQGTLYLKQ
ILDQMNIFHVNLKRIRSDEDKEKKAHDKLNDTLMWFSFLNIIIIVIAAIVQDFYFKKFFTSKKII*
>N.MAL13P1.172
MNETLKSSSDRNMINQNKANVVDELVKRKDEICSWCFDTLYYELKNEKYDYVPPILQKLHKGGFQIPIFVKWM
KLYDKKDMGSYDYDAYELKGCIGCLADVDILEISYYAIQSSLHDTRFLPITLKDLPYLIVSITYLYNFEDCKH
VYDWVIGKHGIKINFTINQKKYSATFLPEVAIQHNFNHETTVKKLIRKANYRGEINEELLNIIQVQRYEGISS
SLTYQDYQKFKSSQNKRNA*
>N.PF13_0183
MNLINKGFTYNIFHHKWLLKKCNCRGIASYKNIEDSLYNIKEKKKHVNILSLVISNNNLCYCLLKNQIIKKIG
LINIKKDIYSYEENDFMKNKADYTLATQQRSEQKQKQKQKQQNTKVEKTKILERQKKNLRYNCQKEENPLSFN
IKEILTMLNFIKLYSNGKGHTENEQDENNYVQHDIKNDNLQYDDKNDNLQYDDKNNNLQYDDKNNNLQYDDKN
KNLQYDDKNDNLQYDIKNNNLQYDNKNQNFFHISKCSIKKDGENNLPEQIRQNMKNIEKKTYDEKSEKKEEWI
IGIEKVNDKYEKNKLKEKILSILVYFLQSIFDCKILFFCPKEARNHFSNKCNYELNNREETYKYIKNKIKNFP
CIQNDDNNINCLFSDSYVISFYTYRYYMYELVKNNRTIFNYLEKQVRKNKNFHYILQTLQKTKNKKNKTDLTD
LLRDKINKIIDVETYKLVDKFMF*
>N.PF13_0184
MNCSGYENKTEEDTCKGDNNVRYNSKGGISKDLLYRNNDEKKISVNLTDFNFYEIEKKELTYIEISPCKSYMS
ICDSFCNIYIYKILINDFLFLSKLDIYSTSTTIIKSIIWIKKKKKEKGKHFNFFNIDINDYILGISTICGSII
FYDINSKNIIYKIITNASISSCKLNNSLEYFGICNLNGYFYLFNIYKNEEGKIYNCFDKFYDTYIKNNKYGMN
HNYGVNNNIEYITNKRMRLSTDIDHLVDQYDERENITIDNNNNDNKNNDDNKNNDDDDDVSNLINHVTTDNFN
NMNHLNDSYAEENDMFNIYIKNKFKCKEKLVCIYFMDHLKKRSYLLEKMNERIRRKEEHKKIMKKRKSIDGKF
SGSNYDDKDGKYGEDGKYGEDGKDGEDGKDGKDGKDDDDDDDDDDDDDEDGEDDHDDDRTNRIYNGDKFPSHRP
DDLKKNERKLKRHIKSIEESFYNYNHYVLLGSENSRIYKYNLSNNKYIGEFVSMNKNCIIWDVIYIYKTDEVI
CVDNNGSLIIFDNENYSIKYHFNNHNYKCISLSKSFNEDYLFSAGVDRHIIKYTLRSMGKNFKINNSYYKNGN
YNSQHDNNDKNMISYNEQNDHDDDINHPINNNINIIQYMNNPKELFFYNKIKKSRKTNKKWYFTCKQSAHIAD
IKKMIILNNNFILTISDDLTFCFYRIENNKKIYYSIRNIVSNTSMLYFSSNLKNILCVYSKQINIYYNNNYDV
EKNINEKKQTYEKDKSDNKQIESTNMSFIKTSNYKHIANITFEKNEFINICKTNKKFNKIACLTNKKFSIYNF
NMTNMKIYNYDISKLNFFRVYDFDFLNNDTLIVSMIRLNYDDMSKNMNSYNISQNLNHTNMNNYINIEQLHIR
DDYIITGRNNEQNYILSNYKNNNNNNNNNNNNNNNKYHNNNNKYNKNNYFINNNYYNINQNLSMYQKYFSYHL
VIYNVKKKKIKEELKVNNILYNFKTYNQKRLILCSDFMKNIYIFFKNSKKSSLKNCVILDEYNLFQHKNNSIW
LFYFVVENFLFIVTLNNSIFIYTIDFNMKKIYFLKRTSIKNFHFNHFNEILLMNLNKEYKGTCENDSSSETEE
KSNTNKANIETSADGLCNKTDNNIHGDEIDNISKNVKQSTHNTQLGYLKYNYCLILKSLNKIELVSLNIFDDS
HIEIKVSNAENLLKYNNNTFNQYYISSLNFKYNFLNIKQMYYQNKNIYTMIKNELKKTCTQNDEHMLNDKEHT
WSYEQHPKNYEHTNNKNIHYDVINLSFKKYIELKKKNIINVHFIYTRENNIIVFICVPKNIDNTLIHVADTKK
YTMSM*
>N.PF13_0185
MVRTKKNIPNHNPLNAFNRDKSFKTNKTLPNRTVHHGISSKTTNINRPSVNRGGINEVAQKNLHRTNIRKPHR
YRPGVLALKEIRAYQASTQLLIPKIPFVRVVKEITRLFELPDEQFRYTPEALLALQTASEAYLVSLFEDAYLC
SLHANRVTLMPKDIHLARRIRGRD*
>N.PF13_0186
MPISLNSFKEEELISLEKGDFERVLYKGSDIEFIYNKLNLGEGKLYILEKRLLWINENANKKNVTNFKELCTN
NIYLNHYEKNRNFYLHLLNEVNNISIDSSNIALHAITSDKKICDNSCVYIQLNTDISDHLENWKNDEPVQRII
KSEQTNDDEENSDTDNDLPYDEISTPEILLVSKNSTDNDLIFRNMSNMDNSNNEDEEMDEEEDEQEEEEKEVE
GDVVEDEENEDEENK*
>N.PF13_0187
MYWGVINKCSYSSKGTEYEIFENEKGLEKFHEFKVNKIIYQKDYKNYIKLYKLCNPEYSKLTIEKGNLFNFSI
EWIFKSNNIVVRVQNGECKKIKTFTYTLSLLQEKLNAYNILSKERNKEKEKKKKEKNNNNNNNNNNNNNNIAI
KSYDINNLKDIYNDEKKLLTRTVEENGNKYKSKINYVKLIKIWDTYLFFKEKKQLEEKKVYFEILKKSFLKAE

FFEKNKKKDFEFFENILTSLLLKPSIFINVLNSLHFIFFVSPVSLQVKQKNDTSKENVNHLINFQVRKYILNY
LFNIYYEKYIGLKNLYKTFDLHKSYKLKFCRKENDTFKFVSLDNIDVEKRGILLIKYYKNNKETYKKKSHDED
KLNTYNNDMHNNDMNNNNTHVDDIHNNNTHVGNIHNNNTHVGNIHNNNTHVGNIHNNNTHVGDIHNNNTHVDD
IHNNNNTHVDDKEASSYLIGVVKNIRKIQSFCVLYMDIKKYKYNEEHPNMSSTLYEDIDINDEYYECFLNNIN
HINQSSHSNINTIIKQRELKNEDDKNNTLLFEAVPLTIQLTTISKRIKLSMINIFDRKDFLNNNITRVLLNGD
NYMETEKRDEKVEQNKENKENKENQIDRENQIDRENQINKEKLKFNNLNNIFFKNSINYNYLIEEAINKFLEN
REREIQQLNIVDKDMIKKDQIIYKLYKSLKNDISQFNKMCNMYKKFDIYQKSVLYDIVINEKRVPIHLVHGRT
G*WKKRTNILHNLYANIRKKEQRICRY**THICRKYKK*ID*LEFMFE*RSYKYVRTTKILYIYRYNLPSF*N
KR*KNQTFIYR*GIKFIRI**PNMFKFEL*FYICIW**KTVNIS*FNK*KKEIRYKLFKYI*EIKKI*TYKKS
FSFNTI*INFPHVFIYIILFL*SEINCF*KYYG*LF*RK*KNI*YIYGI*KSTNFIY*YIS*KF**TNI*TKS
*LFIYKSL*SSNHIKISRNTYVYNKSKKCSCSYTLY*PKNIYTKYVRK*LQR*YYNIESKYETRK*SF*IYKI
SECYE*FM*PFSKEPSFFNTTKWTFYRNI*************QK*Y*YE*ISYYVE*K**NTRNRLYIRT**Y
KHIYI*Q****YS*KDK*PFI*YIQY*NTINS*FRK*RRK*SFHLQKCTYH**LSRL*K*YYYYKYCKIK**K
PTWILK**KTYECIINSNEKKNYYYREF*YIKNEFLLERIYIVP*FF*F*
>N.PF13_0188
MPVIPECCICRLSLKNNLCVEKNCGNIFHYNCMIKWMELQKSCPLCKSSCCKKNLLCIHYEINEDNNIKIDEN
ILNKSKDELYEELSKYEADLIKTQNENEKYALEILTLTNKNKILSETVSKSNVKINEGKIEKEKLKDLKDEYL
KDKILLTAKIEEYEKELKKYKLIENFLEDLNKEDLNRINLLFGFNILSISEQKNVILNYIKNCMTKEKKNEQI
IKELNEQIVERDNQIQNFKEKLYKYKLNEEINDSNENKLSNDEKDTKPIKIKNKIIRRVATLESKRNRNRFRY
SHNNNIISSNNTNLSKSKTSNDNILSNNNLDFINFIDNKINNSNSLESIQTTPKRKKFHPIEIDLLRNKSVDK
LFSNLIDKNFEQQKKENFELYSNKTEPCPTQNNQSTPKAQLLPRSSLKVHENIKTSRHRKRKSLNSQSSKITD
FFKKTMN*
>N.PF13_0189
MKETLNNLNLEDIANVKIKAYKNENILSNNEKKRKENFYNVYILTYIKHIVRIKSNLALHGGLYSNYFEKYEE
ESISNIMFENLLTNDENTKNRNKNFEKINRIQQNENDKKNILQDDLNNIHNNDNNNKKDLNYSIKELLKKLIK
SYKIRENKIFLVTGMLGCGKTTCVSLAIEYFNKFLQIERNMKNKNYDTDQNKKNKNVLFLTEDEPINKKIYKK
TPYINKSYHQDMNSSNSTPRDNKKIKINSNEPLFNFTPKKKRKTDSDESPDENDQEERLYNDLSNDTNSNNDT
KYHHRHYNNNNNNNNNYYYNNNNYNNNMESNTFNTYHTFGHQKKGNYCDNKNNNEFVNIKTFEQQNKKDFSSD
VEQINNNIRNIFENNDLSPLRKNANIRNKIQLEQLFKDENEKHERYFRSLSNDDENLDFNMKYKKIVDEINKE
ENEETFIYIIRISAYLYRDDLQCIKSILSQLHNYIYTDEKLESNLLLNDYINKLENLLIKINNERKQALLIIE
NVEKFCLQNKQNLLYTLFDLLHKKNIYINIICLTNVLDITQTLEKRIKSRFTFEMIHISPILNIEDISKLVYK
ILYVNLDDFHLIKKYYSVYFTYPDLIKIQRFLQIYNSTMQKEIADSNLLKQWSYDINYGLDIRHIYHTCVDAI
LSICEYKHILCPNQNKFESNTILDLPTSQTNNSKQITNTDSQTIQQNTNSDHHDNKNMTQHSFCDNQMGFLTK
KEKRNNLEFLKERPGKLDATPIKRHYHKPQEDDDDNEEEVDEEDDEEAENQKNYNNNNNNNNTDDAFFFNMNM
DMSPRKLKENIKLKENVKLKENINIKNKMESQYILNNVQSKFIRRHYIKKVLRDLCTIEYMILCAFTRLYKKE
ILPKTLYVIILEINKFKHAYPQEQIIGFSLDAYRRSFFRLVDLDIIVLSSYSLNHLNIKNFSNDLFGLHEPTK
FPCYDIFLENIDTYNLNHNLIQWVKHNTEVMQ*
>N.PF13_0190
MVDDKNKDKNCSKKDEKNNKIDEDKEFDEKVKNMSFDDKVRKILDLKLEGNNDYKNKNYEKAINKYNEGMKYM
KKIENKNEKIKELEIALYLNLSICYSNVEKYNEAYDNITKVLSLDKTNAKGMFRLCQIEFNRCNFDVAKDKIQ
EFIKIYPDNIEAKKLLKNIILKQNEHNKKQKEAFSKIFEKASGLYDDREKEIMRKKKEKYEKEMKERKEKNED
IIKFEEWEILDNEKRIKEDEEKRKEKEKEREKQNEKEKQKQKEMEKKNKNKNDSLMSSSTTNNNSSYLDIDEE
DQKIINETKKMGYCYFKKDIKEDDKKFFEKNIPKKIDTTNYENSNTLYNNQNNKAISSWNAAGTTYEEKDMTK
WAKNKIEECLKNIHFKNDDDSDFLNLNNQNGNNFSSVNYPDILPIQYLSKIQLNDVKNLSVDAQIVVIRGTKR
HIFELSCNLDITIFINITELHVHKVLVEIKELSSELEAGKTWKNFITIKKNDKLKIPDNLFNDIYDFVIEKVQ
QQIKNFTQEYNKM*
>N.PF13_0191
MQSEFFICVTFFFVLLHYISCNKPTRNISVKSNKDKDELNNIKEKLDLINNSIKDKVIENFKEDIELLKKKVD
DLEKRKSDNTLGKRQKKEDDDDEEETDEDDDEDSDEDDEEQEELNVEPKEREDEQEETDDEQKETEDEQKETE
DEQKETEDEQKESDDEQKETEDEQKETEDEQKETEDEASEEYSDNEEDDEEDDEEENEENNDNETNEENEDND
ENEEEIEVTDVEFVGQSTNKNVRNNMIRNSNKDIKSSSQNSSIKAQNSSTKIGNTPTKLSTQNTKSNSTSNQL
ITQLQSEKSSSKVDNNKNNTNEIKYMDKLCDDVLTELKEKDNVDNNMNHSKYNNLKKEFSTFTMNQNECDLIK
KLIITFSQENVEMKRESIKEIFLKALDDKKYREVFKNFMYGVYSYAKRHNYLDIEKMEKNERAYKKLFENTLN
LLDTI*

>N.MAL13P1.173
MKGRIISFSFFLFCMVHFVFCDKNTFPKEIIEYEKDLELAKIKEKLQNLNDIIVDKLIESFKDNVDVLKVIIQ
ELEKEKENKLAKKKREISNNDFLNNKNSNKFKGKGFFTSTWKQIKGNSEENSENQQKSSKSQSLQSNEPNKLN
QENISPVQTTITNTNVDNTISVIKYLDNAYDEVLKEMNLSKNSDNEKYRSRFDNFKQGFENLILNQNEYELIK
RLILAFSNQEESGTNKKNHIVNMLKKALEEEKFSDEFKNFIYGIYAYAKKHNYLRLIDSNRDIYKNVFENATN
LLDTLQMKLKRVPSQ*
>N.PF13_0192
MTVQYNSILFLLEYLKDSQKKKKKKKMKSKKIICSSCLFLIFLSVIFCSEPDTNSFDENVKKNEVFNALNEHL
ESISNIVKVNIMDALSNNPSLIKKTYEAVEINDDDYVLEYVGDSTGKYGEGSIFYDESKKYNYRKYLDIENEL
RNMKGEDDDDFDEDDDDFDEDDDDFDEDDEDDEDDEDDEDDVDDEDDLDVEDNVDDEYDDEHNHNYNDDKLSE
NPEKYSNYNKNIHEDKKKDNLNEPHFKQTHYIYSSNHDNNETSRFPKKNVPKYDEKLNNEFKTYLRRPEKKEE
TKEYPNNGCSVVQISIVTNEDFLKKVRERNKKRNNKKRTNIYDSDEESESSEETSKDPYSSGPYTVDHKNENC
FPYKQNSDIHPNINNDNNDNNENNDNNENNDNNIPVNNKKNSHTPHVPKDTKNNIEKNHSNYNYKPINQENLS
MYFNNNNKKKNKKNSQKNNQKNNQKNNQKHNQDLYNIKKNNDLPQYEESNKTYKKIFSNEHEPRSMITTSRYE
NKISNDNNNNNYNLNEQERKLIKNMIDIFIYTYKLNYTNSKSISKLFKNNLLKKNFRTYFTNYIYTLFNYGKT
YNFLTPYNKDNDHMYRQLFDEAVQMMDLLINKMDLALNPTKL*
>N.PF13_0193
MIKGLIFYLFICFFVFFVHAVSSQEQPNHTDIYTNEDYKLLEELEKYLDNLKNTFTHKFLENFKGDIELLKKN
IQNFEKNNYNNVTGQNVDDYDKNGDNNNHNDLEQNQLLQSNIKSFFGQSRTTNTVDVSASKVTQKSDETKADE
KAEQNAEANASSSASSEEQSSSLQNTNDQSFGNTNNPKMDRKNADEQKRDAGLSNLKNIDKLCDELLIYLYKK
NEVNTNNYLRKYDEFKNKFDQFLLNVNKYELLKKLILNFFKDNLKDHRAQYKFYIALMKALENEKFSQEFKKV
MYGLYS*
>N.PF13_0194
MKRTRHITVFIFSAFLFLLNYATTSQSVNYDNEKKQINNLKERLENISSDIPEDLKNHLKDNLNFFNNSLEEA
QKSLLDQPNISTSTQGSENNTSGVSTNNNLTNPNDPTQLNEQECQSIKKFIKELLSAATNDEHVNTPFSTTFK
NALEKDEFRDSITKLTEALCGHTKDNHNNLRGTSQNDSDYLHSVYDKALSHLDTLQ*
>N.MAL13P1.174
MKGQAIYFVFVFLYFLNCVGCNSKNNNKRTSYNKKKENLKNFDNIVLDDFYSAFDSQDNYESKLKKNEDDIIG
QGIFSLISKKNQEKEKSLKGESEDNTKLQVTKVQGAQVDQAVEPLEKSPEDENKEIPTLDSPQNGNPTSYTSN
LSTPPLKRMDEVFDDVLKHLNKEDKVVTDENKNKYNEFKKEFDIFTMNVSEYEIMKNLLITFSKKIDENNQIQ
TKIENIFNKVLKDNKYKEQFKNFIYGLYSFAKRHNYLIVNKTNDTTLHKDLFENALNLINTI*
>N.MAL13P1.175
MDNFTMNENEYEIVKKLVNMVFPKEIIDTNK*
>N.PF13_0196
MKRQIILFTSLFIFSLNLTLSYEKSNNRLGYYNEENADNKEKNKITFMFNDIKVGMQNDVEENLDNGKNVLED
EHLVIGQEGQTDNEGPHTETEPERSVESGGTGDSVSNHESELPRDTETREVVTTVQPGTQLGESGAKETLESQ
TSPAPQGTSGLQGEKVPGALSSVSQTSSPDVSSRSEQPQTMPESPRPEGTSTESQPRGSTESDASNKNGSQTN
VRTISNSSNSITSPQTTQPSNNQNNTVSTTSEVKYLDLLYDELLTNSEGKHQVHFGENHKKYNIFRKHYDNFV
INQKEYDIIKKLLNSIFRDNNENEKMKNLVSIFQKALNDKKFHDQFKNFIYGIYGFAKRHSYLRNEKIQNDSE
YKEFFENAVDLLNTL*
>N.PF13_0197
MKSNIIFYFSFFFVYLYYVSCNQSTHSTPVNNEEDQEELYIKNKKLEKLKNIVSGDFVGNYKNNEELLNKKIE
ELQNSKEKNVHVLINGNSIIDEIEKNEENDDNEENNDDDNTYELDMNDDTFLGQNNDSHFENVDDDAVENEQE
DENKEKSESFPLFQNLGLFGKNVLSKVKAQSETDTQSKNEQEISTQGKEVQKPAQGGESTFRKDLDKKLYNLG
DVFNHVVDISNKENKINLDEYDKKYTDFKKEYEDFVLNSKEYDIIKNLIIMFGQEDNKSKNGKTDIVSEAKHM
TEIFIKLFKDKEYHEQFKNYIYGVYSYAKQNSHLSEKKIKPEEEYKKFLEYSFNLLNTM*
>N.PF13_0197a
MFLWRKKNRFIYLIHSLENATTYNYDKEEYDKEEYDKKLSIYKIYDRFL*IFRKEKK*Y*E*IK*YYY*CF*N
DESIKL*K*YICNTKYYLKKI*SLFITLK*GCSSTFNDTYLYKAKNLLQSTYNVIK*I*NEMENNQSIYSAMC
IYDHLTVVMKRYNFHLNNMNYGKEHITQLLQNKNQSTSTFNLNNQNICSTKYIINKLFE*
>N.MAL13P1.176
MKITLFCSISFCNIIFFFLELSHEHFVGQSSNTHGASSVTDFNFSEEKNLKSFEGKNNNNDNYASINRLYRKK
PYMKRSLINLENDLFRLEPISYIQRYYKKNINRSDIFHNKKERGSKVYSNVSSFHSFIQEGKEEVEVFSIWGS
NSVLDHIDVLRDNGTVVFSVQPYYLDIYTCKEAILFTTSFYKDLDKSSITKINEDIEKFNEEIIKNEEQCLVG

GKTDFDNLLIVLENAEKANVRKTLFDNTFNDYKNKKSSFYNCLKNKKNDYDKKINNIKNEITKLLKNIEGTGK
MCKTESYVMNNNLYLLRVNEVKSTPIDLYLNRAKELLESSSKLVNPIKMKLGDNKNMYSIGYIHDEIKDIIKR
YNFHLKHIEKGKEYIKRITQANNIADKMKKDELIKKIFESSKHFASFKYSNEMISKLDSLFIKNEQILNNLFN
NIFNIFKKKYETYVDMKTIESKYTTVMTLSEHLLEYAMDVLKANPQKPIDPKANLDSEVVKLQIKINEKSNEL
DNAISQVNTLIIIMKSFYDIIISEKASMDEMEKKELSLNNYIEKTDYILQTYGIFKSKSNIINNNSKNISSKY
IIIEGLKNDIDELNSLISYFKDSQETLIKDDELKKNMKTDYLNNVKYIEENVTHINEIILLKDSITQRIADID
ELNSLNLININDFINEKNISQEKVSYNLNKLYKGSFEELESELSHFLDTKYLFHEKKSVNELQTILNTSNNEC
AKLNFMKSDNNNNNNNNSNIINLLKTELSHLLSLKENIIKKLLNHIEQNIQNSSNKYTITYTDINNRMEDYKE
EIESLEVYKHTIGNIQKEYILHLYENDKNALAVHNTSMQILQYKDAIQNIKNKISDDIKILKKYKEMNQDLLN
YYEILDKKLKDNTYIKEMHTASLVQITQYIPYEDKTISELEQEFNNNNQKLDNILQDINAMNLNINILQTLNI
GINACNTNNKNVEHLLNKKIELKNILNDQMKIIKNDDIIQDNEKENFSNVLKKEEEKLEKELDDIKFNNLKMD
IHKLLNSYDHTKQNIESNLKINLDSFEKEKDSWVHFKSTIDSLYVEYNICNQKTHNTIKQQKNDIIELIYKRI
KDINQEIIEKVDNYYSLSDKALTKLKSIHFNIDKEKYKNPKSQENIKLLEDRVMILEKKIKEDKDALIQIKNL
SHDHFVNADNEKKKQKEKEEDDEQTHYSKKRKVMGDIYKDIKKNLDELNNKNLIDITLNEANKIESEYEKILI
DDICEQITNEAKKSDTIKEKIESYKKDIDYVDVDVSKTRNDHHLNGDKIHDSFFYEDTLNYKAYFDKLKDLYE
NINKLTNESNGLKSDAHNNNTQVDKLKEINLQVFSNLGNIIKYVEKLENTLHELKDMYEFLETIDINKILKSI
HNSMKKSEEYSNETKKIFEQSVNITNQFIEDVEILKTSINPNYESLNDDQIDDNIKSLVLKKEEISEKRKQVN
KYITDIESNKEQSDLHLRYASRSIYVIDLFIKHEIINPSDGKNFDIIKVKEMINKTKQVSNEAMEYANKMDEK
NKDIIKIENELYNLINNNIRSLKGVKYEKVRKQARNAIDDINNIHSNIKTILTKSKERLDEIKKQPNIKREGD
VLNNDKNKIAYITIQINNGRIESNLLNILNMKHNIDTILNKAMDYMNDVSKSDQIVINIDSLNMNDIYNKDKD
LLINILKEKQNMEAEYKKMNEMYNYVNETEKEIIKHKKNYEIRIMEHIKKETNEKKKKFMESNNKSLTTLMDS
FRSMFYNEYINDYNINENFEKHQNILNEIYNGFNESYNIINTKMTEIINDNLDYNEIKEIKEVAQTEYDKLNK
KVDELKNYLNNIKEQEGHRLIDYIKEKIFNLYIKCSEQQNIIDDSYNYITVKKQYIKTIEDVKFLLDSLNTIE
EKNKSVANLEICTSKEDIKNLLKHVIKLANFSGIIVMSDTNTEITPENPLEDNDLLNLQLYFERKHEITSTLE
NDSDLELDHLGSNSDESIDNLKVYNDIIELHTYSTQILKYLDNIQKLKGDCNDLVKDCKELRELSTALYDLKI
QITSVINRENDISNNIDIVSNKLNEIDAIQYNFEKYKEIFDNVEEYKTLDDTKNAYIVKKAEILKNVDINKTK
EDLDIYFNDLDELEKSLTLSSNEMEIKTIVQNSYNSFSDINKNINDIDKEMKTLIPMLDELLNEGHNIDISLY
NFIIRNIQIKIGNDIKNIREQENDTNICFEYIQNNYNFIKSDISIFNKYDDHIKVDNYISNNIDVVNKHNSLL
SEHVINATNIIENIMTSIVEINEDTEMNSLEETQDKLLELYENFKKEKNIINNNYKIVHFNKLKEIENSLETY
NSISTNFNKINETQNIDILKNEFNNIKTKINDKVKELVHVDSTLTLESIQTFNNLYGDLMSNIQDVYKYEDIN
NVELKKVKLYIENITNLLGRINTFIKELDKYQDENNGIDKYIEINKENNSYIIKLKEKANNLKENFSKLLQNI
KRNETELYNINNIKDDIMNTGKSVNNIKQKFSSNLPLKEKLFQMEEMLLNINNIMNETKRISNTDVYTNITLQ
DIENNKNKENNNMNIETIDKLIDHIKIHNEKIQAEILIIDDAKRKVKEITDNINKAFNEITENYNNENNGVIK
SAKNIVDEATYLNNELDKFLLKLNELLSHNNNDIKDLGDEKLILKEEEERKERERLEKAKQEEERKERERIEK
EKQEKERLEREKQEQLQKEEELKRQEQEKQQQRNIQELEEQKKPEIINEALVKGDKILEGSDQRNMELSKPNV
SMDNTNNSPISNSEITESDDIDNSENIHTSHMSDIESTQTSHRSNTHGQQISDIVEDQITHPSNIGGEKITHN
DEISITGERNNISDVNDYSESSNIFENGDSTINTSTRNTSSTHDESHISPISNAYDHVVSDNKKSMDENIKDK
LKIDESITTDEQIRLDDNSNIVRIDSTDQRDASSHGSSNRDDDEISHVGSDIHMDSVDIHDSIDTDENADHRH
NVNSVDSLSSSDYTDTQKDFSSIIKDGGNKEGHAENESKEYESQTEQTHEEGIMNPNKYSISEVDGIKLNEEA
KHKITEKLVDIYPSTYRTLDEPMETHGPNEKFHMFGSPYVTEEDYTEKHDYDKHEDFNNERYSNHNKMDDFVY
TAGGVVCCVLFFASITFFSMDRSNKDECDFDMCEEVYNNDHLSNYADKEEIIEIVFDENEEKYF*
>N.PF13_0198
MFLLISEKELYEFCTIVLISISLDDNVRDFSNSSKSLKYISKSSFVLFISTFFKISAFFTIYAFFVSSNVLYS
STLSKISLYFSKLYCIASISFNLLDTISILFEISFSLLITLVICIFKSYNAVDNSRNSLQSFTKSLQSPLSFI
LSKYLRICVEYVCNSIISL*TFKLSIDSSELLPK*SNSKSESFSNVDVISCFLSKYNCKFNKSLSSKGFSGVI
SVFVSDITIIPEKFANFITCLSKFFISSLLVQISRFATDLFFSSIVFNESNKNFTSSIVLIYCFLTVI*L*ES
SIIFCCSEHFIYKLNIFSFI*SINRCPSCSLILFK*FFNSSTFLLSLSYSVCATSLISFISL*SKLSFIISVI
FVLIILYDSLNPLYISFNIF*CFSKFSFIL*SFIYSL*NIDLNESIKVVNDLLLDSINFFFFSFVSFFICSII
LIS*FFLCFIISFSVSLT*LYISFIFLYSASIFCFSFKIFIKRSLSLLYISFIFKESILITIWSDFDTSFI*S
IALFKIVSMLCFIFNIFNKLDSILPLFICIVI*AILFLSLFKTSPSLLMLGCFLISSNRSLDLVKIVLILECI
LFISSIAFLACFLTFSYFTPFNERMLLLIKL*SSFSIFIMSLFFSSILLAYSIASFETCLVLFIISLTFIISK
FFPSLGFIISCFIKRSITYILLDAYRKCKSDCSLLDSISVMYLFTCFLFSDISSFFRTSDFILSSI*SSFKLS
*FGLIDVFNISTSSIN*LVIFTDCSNIFFVSLLYSSDFFMLL*ILFNILLISIVSRNSYISLSSCNVFSSFST
YLIIFPKLLNTCKLISFSLSTCVLLL*ASLFNPFDSFVNLFIFSYKSFNLSKYAL*FNVSS*KKESCILSPFK

**SFLVLETSTST*SISFLYDSIFSLIVSLFFASFVICSQISSINIFSYSDSILFASFKVISIKFLLFNSSRF
FLISLYISPITFLFLL*CVCSSSSSFSFCFFFSLSAFTK*SCDKFLICIKASLSSLIFFSSIITLSSNNLIFS
*LLGFLYFSLSILK*IDLSLVKALSDRE**LSTFSIIS*FISFIRL*ISSMISFFCCLIVL*VF*LHMLYSTY
KLSIVLLK*TQLSFSFSKESKFILRLLSIFCFVWSYEFNNL*MSIFKLLNLISSNSFSNFSSSFFKTFEKFSF
SLS*IISSFFIIFI*SFNIFFNSIFLLSKCSTFLLFVLHAFIPIFKVWRIFIFKFIALISCNILSSF*LLLLN
SCSSSLIVLSS*GIY*VI*TKEAVCISLIYVLSFNFLSRIS**FNKS*FISLYFFKIFISSEILFFIFCIASL
YCNICIDVLCTAKAFLSFSYKCNIYSFCIFPMVCLYTSKLSISSL*SSILLLISVYVIVYLFDEF*IFCSI*F
KSFFIIFSLRLNR*LNSVFNKLIILLLLLLLLLLSDFIKFNLAHSLFDVFKIV*SSFTLFFS*NKYFVSKK*D
SSDSNSSKLPLYNLLRLYDTFSCDIFFSFIKSFIFIKFKLFNSSISAIR*VIESFNNIISFI*VTFSSIYFTL
LR*SVFMFFFNSSSFINVSCESLKYDIRLFNSSISFFNPSIIIYLELIFLLLLFIILLLDLKIPYVCNI*SVF
SI*LFKDNSFFSISSIEAFSDIIIS*NDFIIIISVFT*LIALSNSFDFSFIFICNFTTSESRFAFGSIGF*GL
AFKTSIAYSNKCSDRVITVVYLDSIVFISTYVSYFFLNILNILLNKLLSICSFFINNESNLLIISLLYLKLAK
CFEDSKIFFISSSFFILSAILFACVILFIYSFPFSICFK*KLYLFIISLISSCIYPIEYMFLLSPNFIFIGLT
NLLLDSNSSFARFKYKSIGVLLTSFTLNRYKLLFITYDSVLHIFPVPSIFFNNFVISFFILFIFLS*SFFLFF
KQL*KLDFLFL*SLNVLSNNVFLTFAFSAFSKTISKLSKSVFPPTKHCSSFLIISSLNFSISSLIFVIELLSR
SL*NDVVNNMASLHVYISR*YG*TEKTTVPLSLRTSI*SKTLLLPHIEKTSTSSLPS*IKEWNEDTFEYTLEP
LSFLL*KISDLFIFFL*YL*I*DIGSNLKRSFSKFISDLFIYGFFLYKRLIEA*LSLLLFFPSKLFKFFSSLK
LKSVTEDAPWVLLDCPTKCS*LNSECETKYKNINI*IYKYIYIYIYIYIYIHALVVNIID*HNVEHCTAFVKI
*KYKIIKI*TTSKHNLFSNVYIILPKKKNIILQKDMLQNSVIFII*IAIL*SHAFINN*KKKKKNQAFYVIRI
YMIIYFNTILYLIIYIYINELNLIILQL*YIYTFKF**HEKETIYIHTHIELSKEQKRKKREQLFYQFKIIHI
KIY*HIQHIYTHI****YVLLDTHLLSQNKKNKYNLHFKKKKXXXXXXXXXXXXXXXXXXXXXXXXXXXXXXX
XXXXXXXXXXXXXXXXXXXXXXXXXXXXXXXXXXXXXXXXXXXXXXXXXXXXXXXXXXXXXXXXXXXXXXEEE
ERKERERLEKAKQEEERKERERIEKEKQEKERLEREKQEQLKKEALKKQEQERQEQQQKEEALKRQEQERLQK
EEELKRQEQERLEREKQEQLQKEEELKRQEQERLQKEEALKRQEQERLQKEEELKRQEQERLERKKIELAERE
QHIKSKLESDMVKIIKDELTKEKDEIIKNKDIKLRHSLEQKWLKHLQNILSLKIDSLLNKNDEVIKDNETQLK
TNILNSLKNQLYLNLKRELNEIIKEYEENQKKILHSNQLVNDSLEQKTNRLVDIKPTKHGDIYTNKLSDNETE
MLITSKEKKDETESTKRSGTDHTNSSESTTDDNTNDRNFSRSKNLSVAIYTAGSVALCVLIFSSIGLLLIKTN
SGDNNSNEINEAFEPNDDVLFKEKDEIIEITFNDNDSTI*
>N.MAL13P1.177
MDDVFAIFQKKNKTKKRSSITDNTENKNKRKSLFHLNRKNPRRAHNEEDTFNSYEPLNKLLNEKKINNDLENI
VVVDFPPLPLKNTEVCLSFYYIQYLRNQLLYGNQNIQIDENTRMIAEALLDKIENNIYELENKEYSEDKYKND
IKILVYKSIYDRYYKIITTFLTYSDDIPLPSNLYEYTLNNGAYIRSRKNNDFNCRENIHNIYSYLDNVEVGEN
DLSINDEKIYIDNIDISKATILNKALINIDIDIEYLPLKINSSYYYNNLQYAKLFLNDAVNMSLYDKALGELV
VVKALVDIPNVEIEFIEGFDIKEMKAGERQWIPIYIAISLSSYAYVDVEFPFWFYIKNFINIKEQEYKNLNEL
FDLPSPYFFEICYMFLDQKIFSKATPIETVGQKSYFKYMAKVAGYVEDIKHCREEKIIKHLEEQDVHSNYIYI
SNLQYSETYLVNLSLYSFWKYDKNLNEKTNTIDFTSYLLEPFIKEENEDNVLMDL*
>N.MAL13P1.178
MNDETKVFNKCFKKISNCYTLNKKGEKENSTGNQFVLHNCIQTLLSDPIVVLYTDNNYICTGSLTGKVCLYKI
TIKENKETNTNISYVYNNNNNITDDEKKCINDLNNREELKKENYSKSTIDDVVISKKELNNHNNINNNNNNNN
NNNNNNNSEIIKKYHILCMFRNNSIKSIYIDKNNIYICYESCIDVFCVRSYQFIQKINLDVTNNKQVIYYENY
ILFNNSKSLFIYDITSNFSSYFYKNFLKNIVIFDFNINYLLCYKNNFGKCHIRVLQIFADAQKCEHELIFCVD
FSSKFISHGKFLDDEKIIVAKNGKRLIIFDFKKAIDMYRIRKLKKKILDIHKSVENMLFILVENQIFIFNLST
FVFYKTVQLPKGLFYFKWSYFIRYKNRKIIFSSDKGVYYIDYSIEENV*
>N.PF13_0199
MNTKKEDYNNDKLYFKLYEYISRIDKFSLNDINLQNEKKKRKYINKQKKKDVYKNLNHETISNFNKNVVNTKR
TDDNTFKEKNEHIIKNDNINYDNQNTYGYIQDYDEYKNYLSNDSLSVYSSFDEEENEIDSYITENEQYEIDQK
NKEDRKNNNLPIMLSDQKEIKNNHLHNNNITDNILHNLSEEKSSFHISSNNEPTNDSYNNRENKEILQIKEKN
NICQYKFKDIEKNNNIDIFMEIENYYFIYKMNRKIEEYNSVNEMKIIKSERNEKLSEMFKKIHSILTHYKEIK
SELKNLIKNKQLIYKNFYSIFLHYYYRYSEVKMIKLNKSKIEKRLKYYTNIERFEMILDKMEKNVYAYFIDIY
KTSMIKNTQVGKNQECVVAHEVEGEYTSDNEETDEDLIDEMDEEDDYDEAYDIYDNINDNINDNGDDNIYDNI
NNNMDDDIYDNIYDNMYDNMYDNMYDDVDDNIYDNVDDNIVNNTDNNNDNNKDNNNDNNNDNNDDNNDDDNND
NIMLGNINKHINNIIDNHINDHSKEETLDDISQEDQQKIDLSLANEKKQNSKISDTNKKNVTKTKNQIKDKKK
NSFFGINKSTLENNESACSSHPFNQLFLKNMECNFLNNIDDDNFKREIYKKLLNINKIDINKIDINKIDINKI
DINKIDKNKEKRKRKRHINNKLNEKNEIYSMLHFSEKAIKFFKKNNTYFNAKGKILKYQSIIKKILKYIINLF

RDVLNNTDLNINMTKEDRGQYLYDIQYENKGTDEINDTIINRNSLKSQKKNINNNDIMSCDEYINRNWEKSPI
SNNEIKPFCNTQNENYSINQTGNKKENALFEENIENAEKMTENFITYEDIKKTNIYENNINDNNIYKISTINN
LIKNHSLLQTNDEKEIDKIYNNVNIIYFNKYIDEFEYYKKYKIKCYCMKDIINFIYKKSLNDENNLYIDDYNS
LESIYVSTRLKILNNNILKNFEYFFDKDICKYIKNVCLLAIYISKLESDLFLYIFNNNLNSSINIILNSIGVC
IYDNINEKIYELNNMKIIRKIIQIIYVDIIDIYTDDSYNIIHDYLKRICTTLKERLLYIIEMYISYYTKNINY
NSTIPYMCFQSIKKKFTNIINGFLYEDYIVSSNYEIINHETKKNKDKRLNDPQDNFHDQNIHSDHMNHIEKKI
DNISSGNIYNSNDKDMEISENDDYSSESIESENNYKEHEYNINILNENNVYKSFSFNNIQFNKGTIFSLRGID
MNIIGTILILKTINFIIEENTFIDLFKECLENTFNSITHVYKQYIKKHDDMFNSNIFLIKNLSFLLYLFYKVT
NDNEFLNTYLHKHLIDHLLFNVTHKESKKNDQKYKTGKDNNNNNNNNEITGVENEISIFNFIKRVYTVSSNTD
VQNKILNMFNESLYNFIITTISKVCAPLINILSNEYNDYENENDIKEVLYNWIDGKKKEKEEELNNDNSRKDL
FSFLKDLDFEQLKEYTKKINEKKSHELSNKNMDDIKNELQSFRQKIYFLFPKIYFYIKLFICSNTDRYSENNF
FYVLFDYIIGVLKYKIIYMLSEVYFLLECKYSKKISNIFEENYINDIFLFLNSNEQYSCVFSDIHQYYDMKKN
YNFL*
>N.PF13_0200
MCDFLGTKLTNYKLHDIKKEFSKKRKHYDEYDFYVYYKNLLASEKRKKERKEQKKRKHSNEQEKEDEFKPCHY
ISSIYKRVKNIERKNIYDYIDEDDNIYEDIVTNRNYGEEHNFTDDITFTFFNENLSYTLLRNNNYIDGIPIGV
NIKIKKDIQKKQSDNNQIYSHIENNENKEINQKKFKNEDGNNCKERKGNNKIIGPQMPHIFEEIRKQMKNEEH
HNFSEDNIEHYENKYVSILSYQNYMISIKRKEQKEYDKYIQLTYTPKNNFEGAFYVKNKSDIKEIKNNEFKLL
THDFLHNKMIHNYDVEDCESKYKQNEYRQNKYVQNGHHNKFYDEKINDDNKYSNNYVKSEDSISLDDKSYDAG
FGTLKRREKIKEDTHFVLYKKNSLIKEQDIYTYYKKYYESLFAKINIDKTHKNKNSENFKEIYVLYNKLLNLN
KDETLTPNELINLYVPKKSWIQFNNSQNKNTDNMTNQIYELKNQINFNNDLKKKNRFYFFCKMNENKNVKNKK
NIFSIKEMDEFKGLIKKFKTYNPEFYVNEIINEDLNTNFQICDYEFSKVLCEKLNIKNKDHKDNEQNDEKRES
KIIDDFLKIPQFVFESIFC*
>N.PF13_0201
MNHLGNVKYLVIVFLIFFDLFLVNGRDVQNNIVDEIKYREEVCNDEVDLYLLMDCSGSIRRHNWVNHAVPLAM
KLIQQLNLNENAIHLYLNIFSNNAREIIRLHSDASKNKEKALIIIKSLLSTNLPYGRTNLSDALLQVRKHLND
RINRENANQLVVILTDGIPDSIQDSLKESRKLNDRGVKIAVFGIGQGINVAFNRFLVGCHPSDGKCNLYADSA
WENVKNVIGPFMKAVCVEVEKTASCGVWDEWSPCSVTCGKGTRSRKREILHEGCTSEIQEQCEEERCPPKREP
LDVPHEPEDDQPRPRGDNFAVEKPKENIIDNNPQEPSPNPEEGKGENPNGFDLDENPENPPNPDIPEQEPNIP
EDSEKEVPSDVPKNPEDDREENFDIPKKPENKHDNQNNLPNDKSDRSIPYSPLPPKVLDNERKQSDPQSQDNN
GNRHVPNSEDRETRPHGRNNENRSYNRKYNDTPKHPEREEHEKPDNNKKKGGSDNKYKIAGGIAGGLALLACA
GLAYKFVVPGAATPYAGEPAPFDETLGEEDKDLDEPEQFRLPEENEWN*
>N.MAL13P1.179
MDKGNIGLNNSVNDKKNILLNGGNDLSKNLSYLNSNVNTNLNLSQNMNVSNENNTKTLLPQININIVNTTQNK
SQEELKNSVFNNELQNISQNENMTFENKLNFNSSLKIRKSLTEQLCEEISMKLGKIFLCKSYLETFIYEKNPY
VMNFIYLKDDKSESKNRVLNSLLLGSFCNLLWNDNIYTHEYLISKEILSKYIKALEKNPDKNNYCIVPIYDMD
KIKMILCDQEKHIKILKKKKLTIKKNNENLLFILNIIKNNFQEIKIKAQRIIQRILFRLDILDKLFILKNNSV
FFSNQYEYNKIKLMEILDFFNSFNIDNQLQKINQNITDIIKEKKNLQKFKYYDNEYTTSLKNVLEKNEEDITQ
LKSTTSEICMNLEENKECLYHLNAFEGEANFDIM*
>N.PF13_0202
MMMFRPHNVIKNIINSDKKVFLSTINPLNKENKNVIDIYDNYEVLKNMKEKNTLKSSLKKFREIVKKETKRKG
YNFYSGWPPVNRIKVTDDEKLGLFGRKMFIYNRIKGIRETETNLFICCKNKNIICFNKNEFISLLKNMEVIKH
QLEEFRKKL*
>N.MAL13P1.180
MKEYTGNRSVPTTIQNKKKSITEEINKSLHTQDSKNNQNNYEICLSDVSVSTISNSSGIDSDLYEEVIKKIKK
EKKRKRKESKRDIYKEKVKYDKKESNSNDENKNDRGNEKDRKNNSDIENNSDSEYINDKEKDRKKRKHIDIDK
KKRNKESHHHVNKKKDKNISTRKKRKYTSSLSSESYERRKDKKKKKEKYRNSESSYKSMVDENIPKDPFNPLL
LAYEKKEKKKYRKNDEHNDLNDRWKHDRYENTSDDEDINIEKAYENSKPYFDPHEFPKILWKSKAGGVCVPQI
SDDKK*
>N.MAL13P1.181
MDKLYGDYDKVYEKYRNNNLYRRRNEKEEFNKVDKKNKMELKKEKDNNKKDKKMNKKMKKENGDISDISQNIY
TDISYDYVDAIRKNKKGRKKKDNLRDLSFNEYKIRKKLSIEINKKIYSDSENVHVHHNTFVNKNQGINENNYV
PTKKKKLKYDDQKVKKMLNKKDRTKFVLNFFFVLYYIFYFFHYVIDLQLNNIYNIVLNTFTIVIITSLLLYIY

FLKARNKKLKKIILYINAYINLIYCFNIYNTLFYWLHIYLNFTLSKRIYLKYKTLYKNISFVFSYFSFDEIFF
FLIIFYGFFSFTSTILTLTLFYYIYNFINYNF*
>N.MAL13P1.182
MIEDNIDENCQIYLNPKNWLKEFENTKINENDINEVLMNYFCVHRMYDVAKEFQKESNVKPDMPINTVKIRYL
IQNEIMNNKIEEAIEHINNLDKGILKKHKDLVFFLKKQQLLKLILNNNINEAIIYSQQELASYVKEKPSLINE
IDDVMMLMAYQDFNNEEAKNLIQKIEKKKNTLKRIDDIILNYYNVDSESTLEYMVKNVFFTQNVLSSKYPCSV
PKIKNLKSGYIEYYEKYKKKKRKSSSKKYRSKDKDKVDKHDKKNVDNIVKDFDVTNDEEYRSV*
>N.MAL13P1.183
MWHRGVLSLLCNSCRYVIHKWHVPLLGVDCNANPRHKQVLSNPPPRSRGIPKYLEKYIFYKQAPRNPRYKSQF
ITMYTAGRKYRQQIKKTA*
>N.PF13_0203
MVPKNILFIFVILFHLIGYLKNENKCFCQFTDQPEGEHEDEHMNDPSPTILEKQAAPEPKADDGNINNNNNNN
GNFDFFNNYNFNGGDDDDDDDDDDDDDDESIDTTNNDNDDNDDDDDDDDDDDDDDDDEDDDDDEDEINKRKPGN
KKKKPRKKKPIHF*
>N.PF13_0204
MDKDIVVNEKFDFLMLNIAKECGDINNLMEHFFSFLLRKTDFITNSHNVDECENVILKTLRKYYKKKDEYLMK
LKNEYKKMDEEKKKQYEKKKKQEENEQNNKMQQINIDNNKKDKYTKTIEANTSNRNSYDQVNNVNKTINNNKN
NQRVIDLDDENYMNDNVMEMVNQNKNNLNDSLNHMHQNKHLHDEKNDQDNEDDDDDGEPPKGNGGKTDKYVWT
QSINTLDMYIDTKDNIKTKDIKIDITYKKLYVKVKNQVLIDGEFFKQIKPEESIWTLEDNKIIHIFIEKLNGM
EWWNTVIKGDAEIDVKKIVPENSRMEDLDAETRSVVEKMIYDQKQKAMNLPTSDEQKKYEIFEKFKQMHPEMD
FSKANINYGNSSSSNNMFFGR*
>N.PF13_0205
MDKLKTIYLDSALSIIKGALCIILQIPTSRTTESVKKKANNVGVITVKSILSEPTIHQYDDIKKLIKNKLQEC
VPFYNYNMNRSFAEKIYGDCIYDNYGLSKEINEINLIILEEWNINCNKNRVLKNTGLIKEITINQFKYSTNKE
SLEVHFAVSPKYTFEELSTMYKNEKGLYEFLLSPIIKIICNENDKKLLDNMNEECTYLNAEDILPKNKVLPPS
GIENIDYERSKDVTPWDVNINNEEGINYNKLIKEFGCSKITENHIKRIEKLTNSKAHHFIRRGIFFSHRDLDF
LLNYYEQHKCFYIYTGRGPSSLSMHLGHLIPFYFCKYLQEAFNVPLVIQLSDDEKYLFNQNYSLEYINTLTNE
NVKDIISVGLNPELTFIFKNTEYAGYLYPTVLSIHKKTTLNQSMNVFGFNHSDNIGKISYPSFQIAPCFSQCF
PNFLGKNIPCLVPQGIDQDPYFRLSRDIAVKMALHKPVVVHSVFMPGLQGVNSKMSSTKKKKDDNGKSNSTFD
HNNSVIFLTDTPEQIKNKINKYAFSGGGTTIQEHREKGGNLDKDISYQYLRYLLEDDNKLNEIGEKYKKGEML
SGEIKKILIDVLTELVLKHQEKKKSLTDEEISYFFDPNKPSLQKFKNM*
>N.MAL13P1.184
MNHISYKLRGIKCYRTHLRGTIFKRNYFWEKVNRESGLYKIWNKLNLKNLVKKSCNKLIIHDKKEKIYNNNNN
NNNNNIINENIPSSCFYDYVGSTDILNDDEKNKIDKILEDINRRNKEIGGYLLELNGIKNNGDGIIKCSYACI
RSCDNILSKICKEENIFKIINMVDTVSNNLCKLGDVLELLRNLHNNKNVIGKAHEALEKLTNYIDKINIDTDI
YNFLKKKYNENIHLLNHEHKEVLHNMIVSMETQGVHIKDQKERERYLELQSQEKYFAFHASSNYSDEFKGIYI
EKNKLLKYINQKCLKDYEDKLIPYIKNNQIKVEKKYPFNEYIYILQDSSFLMTILKNVNDVEIINQVYTLLRE
PNHTFLNNILILQYYRNRLINYRNFKNYNEYSLKDCILNEPRRVNYFLKNFLHKILPYFFKELQFIESYISLI
TLRKKEKKSVNTIKKNLENYNHKDEIPKLNASNIYYYMNEIKKVRLKNIEAEMKNNLSLYDVIKFVITLLKNS
YSLEMINVNPLKNELWDENIIKFEIKRGHYVYGYIYMDLFERENKNNSIAQYTVRCSKNMNYSLKYKWLEENA
DQCSFVYTGIVKDEYFKNDDKTKCAHNEKFKEKINNNNNNNNNNNNYYYYHNGDNTYEYANSNKNINLNNNHE
NNSLLNSSYRQTTSTFLVCNFNVNICDKEKDNNYESYDNDDNLFLNKKISFLLEKICMSIDKVNIFLHEFGHT
LHCILSSTYLQHLSGNRSGVDFSEFSSHFFEEYLNCYDALLLLYNNKKNKKNEEYMKSLLSNYMKNKNIICYY
SIVQLTIQSIIDQIFYAFSSNSNNMIDRKESIEKQIKSYFEGIYYKDIHILDFFPQIHFSKTTHLVHYPSNYY
SYLYCSVLAKYIWNMTFKDNLFNTEKADKIVNFLQKGSVDSSLRNIISLVENDQSKIDYYTENPHKIPLNDFL
DYYQENKEDKYTSFLNSI*
>N.MAL13P1.185
MNRIDISNFDFLYVIGKGTYGIVYKALDKKENNFVAIKKIINLCDENYGISKCILRELTILQKIKHKNIINLK
YVFYGKDIEDKLKGENLENSCLYLAFEYCDIDLFNLIKKHNLNIKEIKYIIFELLLALSYFHSNNYIHRDIKP
ENIFITSEGEIKLGDLGMSVEKSDHMTPTVVTLWYRAPEILLKSTNYDQKVDIWSLGCLFMELIQGRPLFPGK
NDCTQLELIYLLLGDKDKLTTVDKERKDMFPYFEINMLKDAIDDEHTLDLISKMLIYDPNYRISSKEALKHPC
FQDIEQVKFSYNF*
>N.MAL13P1.186

MIFNYVFFKNFVPVVLYILLIIYINLNGMNNKNQIKTEKIYIKKLNRLSRKNSLCSSKNKIACLFDIGNDDNR
NTTYGYNVNVKNDDINSLLKNNYSNKLYMDKRKNINNVISTNKISGSISNICSRNQKENEQKRNKQRCLTQCH
TYNMSHEQDKLANDNNRNNKKNFNLLCINYFNLKRMKNSLLNKDNFFYCKEKKLSFLHKAYKKKNCTFQNYSL
KRKSNRASHKLFSGEFDDYTNNNALYELEKKEYITLNNNNKNNDNKNNDNKNNDNNDYNNNNSCNNLGERSNH
YDNYGGDNNNPCNNNNDKYDIGKYFKQINTFINIDEYKTIYGDEIYKEIYELYVERNIPEYYERKYFSEDIKK
SVLFDIDKYNDVEFEKAIKEEFINNGVYINNIDNTYYKKENILIMKKILHYFPLLKLINNPSDLKKLKKQYLP
LLAHELKIFLFFIVNITGGHFSSVLSSLEIQLLLLYIFNQPYDNVIYDIGHQAYVHKILTGRKLLFLSLRNKK
GISGFLNIFESIYDKFGAGHSSTSLSAIQGYYEAEWQVKDKEKYGNGDIEISDNANVTNNERIFQKGIHNDNN
INNNINNNNYINPSDVVGRENTNVPNVRNDNHNVDKVHIAIIGDGGLTGGMALEALNYISFLNSKILIIYNDN
GQVSLPTNAVSISGNRPIGSISDHLHYFVSNIEANAGDNKLSKNAKENNIFENLNYDYIGVVNGNNTEELFKV
LNNIKENKLKRATVLHVRTKKSNDFINSKSPISILHSIKKNEIFPFDTTILNGNIHKENKIEEEKNVSSSTKY
DVNNKNNKNNDNSEIIKYEDMFSKETFTDIYTNEMLKYLKKDRNIIFLSPAMLGGSGLVKISERYPNNVYDVG
IAEQHSVTFAAAMAMNKKLKIQLCIYSTFLQRAYDQIIHDLNLQNIPLKVIIGRSGLVGEDGATHQGIYDLSY
LGTLNNAYIISPSNQVDLKRALRFAYLDKDHSVYIRIPRMNILSDKYMKGYLNIHMKNESKNIDVNVDINDDV
DKYSEEYMDDDNFIKSFIGKSRIIKMDNENNNTNEHYSSRGDTQTKKKKVCIFNMGSMLFNVINAIKEIEKEQ
YISHNYSFSIVDMIFLNPLDKNMIDHVIKQNKHQYLITYEDNTIGGFSTHFNNYLIENNYITKHNLYVHNIYL
SNEPIEHASFKDQQEVVKMDKCSLVNRIKNYLKNNPT*
>N.PF13_0208
MRNRRKKNGKNKQKGKRKNDRLGKIKRKKVVDIKEEKGNDNEKRLEVSFYGYRNIYLKDIHNFIVNLRLGKYI
FKNELFDIKNNDCLNNLIMVIVPNLSNYCVKDISTDNVFNKLQRNNNFRKIHSESCLKKYYNNIILKALSVSV
LKQRKKNYTQQIFNLNKYLLTKEQMALNKYPTENMDEYINMNDIEYVKNDKNIRDIKSYLTETMNSINVLHMN
NDCSVKENKKNKINMSHVMNNINNDQYNFSNMECNNENHINNGTIENEQNNFERQNIFTQEHIQIYADILEKL
IKASHTKKYENNTTTDCNMANMESSKKHISSENINNENGEKRKKKKNEQNDDNLNHLNLGNLVSHKINEMYNE
NRNMDEKIETNNNNDNHSDNHSDNHSDNHNDNHSDNHNDNHSDNHNGNHNNDMGFRGENMDKCNDIKKIIDEN
NSCDEENLIDETNYGEFDLDNIYSIDCEMCETTNHQRELTKITVVDAYMNIIYDSYVIPDNKITNYLTLYSGI
NESTLENVTTKLKDVQEHLKKFLNKKSILIGHSLENDLHALKIAHNYVIDTSIIYCNSGYYPKPSLFQLSKKH
LNIIMKRENGHNSIDDAKISMFLALKKMSEFDSNEFSTFYEALPTFFNKDNFVQVKNSILCEEDICYKYEKSL
CIFDSKNKYIEERIPKNFLKNSFYCVCENDEECTENLIMNIKNENKLKNYILILRDYENLCNDKIFNCIQNVH
NSNFKIEDSEKIFEIPSRVETNEVLKKISANIEKIYSNMSKNDVLILLSFNNNYLAEEKVKETYFLAKDIFYS
NADMEEKINNIKDKINSLQKIINKKKNNNEVINYSFYEFQDLLLNYDKFVLNYLIENKNNDKNIYIINSLTNL
KNILNINSKKKEFNGWFSLLLKN*
>N.PF13_0209
MDENVKYANFLRSKIVKYFNLKNDELDINYIISILNCKSYDLYDSIFLLNESFYEQNKNTKCSSLSNVKKFTQ
DLVNSKKKFFSSSDANLDSPDKNFLLSNSNDIKTVGTCFQDIFENDKTINLAVNTYQNNISSHIKKNEIKPNE
HFTNDFKQVLLESKRKNNNGKNKKIFDDNNKHINKNEYNDEVKNNIFQNDIIKQQNNLIKNHFINEHFINDHS
MLDIEKTNEQLKLEKDIKNQKNQKNQNSEQKSNDNKREDNYFSFFNAKENNYSLKEAINFIENENNVVKYNNE
QNVKEKKKKNLQICICNGQNHKIYANCLYCGKIYCTKIKYKNCIFCENQLYKSHIINNLFFQSPQNNNNNNTK
KLISSVKSSNNFLYKYYFNTNNSYLKKAFELRDKMLKNSINEEQTRVIDDSIDWFEDDIKNEFNNQSIHFSHY
DDEIKNEIINKYHEIFGKRFNEINIDIDLVNMKITENKDFLKIKEFNDYLNEKEKEYQTYLEHKKQQDINFNN
ARNYLSAKEKKNLTYINDLKKIFFKENAVKNDTKLNNVAQSYKCDKPTNYKSNKKYKCNVISIADEEQEDTPS
*
>N.PF13_0210
MKPNDNLENNMNLSIGKIDLSNIYMDILHIHETLVNHIQTIVTQVYSLIEKTLCDHIERNKLDIYMFEEKYKD
NNIIDGDKDKMIIHSLNNLNNNYFGNDIKNKFILEKVNECILDNENDKKNKIKISSKKKYFNELLNFGNKNKN
KNVQIKTITSSICTYKNGVDDEHENDNDDDDNYCDDNDCDDNDCDDNDCDDNDCDDNDCDDDNNNDNDDNYEC
INIDDNNLINSIKIRNDENNLLFNNRYTRNHHDNNSVNCFMNINKKTKEVYNNECNNNYFREEQKNNLYDRKK
QKYKKKAEDVVHKVMNNKCIQNEIYENKIKLQKHIALLELIKYVILYSNTNIKKENTEIIKKREINEINSYQN
GNVTNKRYKKGLHKNGIHENDLHENDLHENDLHKNDLHKNDIHKNDIHKNDIHKNDLRTNDLHKNDIHKNDLR
TNDLHKNDLHKNDLRTNDLHKNDLHKNDLHKNDLHKNDIHKNDIHTNNTCLNDTHMSINPYDNHYTEKTKTDL
NHSEYDKGIKDEHTNSNINLSNVKLKYINIITYYMDLLSPFNKCYSEKNGTTNEDQKNLKMVCKKLDQIVLNY
ESIIDQILKKGNLNYSDINKLYQAHNHNNMEEYSSSQLNDENQCENKRVQNNMCISIDNKINMKNKSIPCECT
KCLEEKNTNKFMDTANNEKNKNNDINKNNDINKNNNINNVNNNSSNSYKNRRHHNTYIKCCHDSHLYSREIND
QGHDKNKCSHNINNSTPESIVQNNKNISDINLVNNTNISINCNKLLNENCIKSSCDKSNFLNTPNIVVPKYSN
IRPITQKDSLSNDLLRNNNLREKYHPQNDSNYYKEIRNTNCNNDVEKNIKDNILHTKKQSSHLNNVDYDSDNK

LKNDYSEGINNFDQNNNQKNNMIPLFNDDHIVNYRNISSSNHHNINNIPNYFIPLNNNDEKKKFLDIETYKHK
NIKMNNNDKHIYNNMNNNDKDIYNNMNNNDKDIYNNTNNNDKHIYNNTNIHEKDNDVIYHEIVTRCCRNNTCT
CFINNNDSVDVNINLKLKSEDNSLVLSPSSCSNDKSFINDFNENKDDMSFIYNQINIKNDKQEYVICEPTKST
QHINEKNVPINTFNNTNCHFVKNRNKEVITTTYNDNINIYDQMKKENMNQFDDQTVPTSSKIICSDQHHVERI
NNKSINQNCLNTLTDKNIYYNDTIKKYENRKMTTDNIRYSNICALNNGQNSCKPNVILNQQIFHKVDEHGNFI
YNMRDHENIQDEKLLSDQLHLDSLYKTSDGRNKERFGTTPLYNHGYNNKFVDNRNNNYSDEESTYVNEIHNYI
NKDINYNNEKNECNHHMVNYNNEKNESNHHMVNYKNDMDNYNHHMVNYNNEXXXXXXXXXXXXXXXXXXXXXX
XXXXXXXXXXXXXXXXXXXXXXXXXXXXXXXXXXXXXXXXXXXXXXXXXXXXXXXXXXXXXXXXXXXXXXXXX
XXXXXXMVNYNNEKNESNHHVVNYKNDNKEYNHDMINYNNDMNNYQHDMNNYNNDNNDYNHYMNNYQHDVNNY
QHGVYNYQHDVNNYQHGVYNYQHDVNNYQHDVNNYQQDVNNYQHDVNNYQHDVNNYQHDVNNYNHCVNNYNHE
GSNDSTDNKYNGNNNYNGHNNHHGDSDHNADNNYNDNNYLNPSGRNNNSSNNDGGNDDESTDDNDENDKEEDE
EKDEENEEKDEENEEENDEENDEENYKKDNEEDYNQHKNCNDQFMKIEGDNIFDNSTSNSLHNICYSYKNRYN
DSHAYLMSGENEIMAHNKIYRPQEKDIPITQDNSFVFQNKNNGNQYDAQMSDFYNFHLNSMNSINDTNSINNM
NDINNMNGINNMNGINNMNNINNLNNINNMNNMNNLNNINNLNNMNSINNIHNMNNMNSINYTNNINNTNSVN
NMNNINNMNNMVTINNMNSINNMNNIQNVNNLYNFYNENQNNISVPNIHGEFMSEKTNEEKISTNDLDTYENL
NKDMINDLSINMSLNNYNNEMMNNEICSSYFDMCNNMDESYDIMNNNNSTNSSNNNNNNKKNENENENNENNN
NINNNNNDNNNNNNDNNNNNNDNNNNNNNNNNLFECDKCNVDYDIMNFTNNTYMDIQKILQNDDILKQEFANI
EECFKIHTSSINYMKVKGKGECCHKNQQIMYEHYDAGKHSNNNFIRNNDDMYKNLSMDEFFLNPNNIEANIIN
NDIINENINSDIEKIMDIMTSNNNNEREDNKNHSRQEDDKGRQQTKGKEKCKEKDKYKDKEFDIYKEINNDKM
ISEHMGIYGEQINLKKKEFTNFSSSSSFNMSNNNNNNNNNSNNNNNNNNNNNYNNNNNNNNNNHTFDISTKNK
DKKRKIDSLGNTYEHIISSNESKIESINEQSTSRKFHKINEDKRKKYQKLDIRTLNKNVQKNKEICTNCYIHY
DNFKSSYILTFINRKQKKQRKIFPVEPNEKDEAYIVQIIKYIEKLKEQDKIFGINKNEEKQENKNNCLNEGEQ
QQGVLNINTNINNCNDNIMKENNMKENNMKDNIMKDNIMKGNEVAHHDSMNDHKVINKDESNENNMNNNNLNN
NDNIFNNDNIFNNDNIFNNDTIYNNNIYNNDTIYNNDNIYNNNIYNNDNIYNNSNIYNNNNIYNNSNIYNNNN
IYNNNDNNNNCSNNNVNMMNQFPYGNNTEYILNNKLTFNNNDLNQDKIDLINDMSRTNENIKNNNMNNFVQNF
NEQFFVQQTNFNYINDNINNISPINNMNNINGLKKHMNRTYSRSISMYEDINIPHNVDYINYSIENDNVYDEN
YNIVLNQCASNNANVTMFFESNDNKDNSTKPSGDVYMEDMNMPNNMNNMNSMNSMNSVNSINSINSVNSINNG
NNINNLNIMNNINTLSCNINNNSFANNLYDNNIISHNMKNIMYPYINNNITETTNTFINEKNQANRINNIPYT
NFHNIQNNNDADTMKNFHNIQNNNDADTMKNFHNTQNNNDADTMKNFHNIQNNNDGDTMKNFHNVMNEYNINV
NNKTNYEDLESVDKNISRENNM*
>N.MAL13P1.188
MFNVSSLIYFGNNTSIKKSIAGDVYIYTSKKKKKNQYSEPSLIHDINQNKEINNYHDNNNHDDNIGEYNCNEN
NKWERYYCFIKANIFYIYKLRGDYKPHVIFLLEGNEIKNVNYYIAIKNKIIQELDEFQIGENEDLIQIKIIQE
NKEDTYIFYIRDIGTYKKWMNILKRSNFLYIYNSINTINEENEEIKKQLENTKRLNDIEIKNKDIHIKELKCQ
INSLKDDVEDIRTKNKRLQIAAEVNVKSADEYLQKKMNEMEFMQNEIGIKMEENKNLREKIQKTTEECYKKSK
VLNELEIEKNSLEKKMKDIMLTLEEACSDPERLTLINYNTNERYQKIVLNNKNLKKEINKMNERFYELEDRYK
EKIKTIKEIVEIGDIFDYLHKLILLCQTKIKYYEQAYKYNEEEEKEIINSIQYMIKETKISEAHARICYITHR
SKILEERLTYYTTHKIPSDYFYFACTTLRRLGWIFGEIEILSSLNHNQNNVYSIYSYVDNMNVIPMREQIFFN
EGLEGRNNYKIVNDVDIYTIPRKMCPYQQMQLCENKYNYIKIKLNDLKKDFNIFKKKTKPQNASIISDLQWAD
MKKNAYQRLERNILLIEKKVEDEDDLQVHEMY*
>N.PF13_0211
MKETEVEDMDTNRKDGKIKKKEKIVNMKNEEVKSTTKSTLADSDEDYSIITLCTKCLSKKLEDNKNRIILDSK
AFKDNRLKGRCSVSSNEDPLDNKLNLSPYFDRSQIIQEIILMNNDELSDVYEIDRYKLGKGSYGNVVKAVSKR
TGQQRAIKIIEKKKIHNIERLKREILIMKQMDHPNIIKLYEVYEDNEKLYLVLELCDGGELFDKIVKYGSFSE
YEAYKIMKQIFSALYYCHSKNIMHRDLKPENILYVDNTEDSPIQIIDWGFASKCMNNHNLKSVVGTPYYIAPE
ILRGKYDKRCDIWSSGVIMYILLCGYPPFNGKNNDEILKKVEKGEFVFDSNYWARVSDDAKDLICQCLNYNYK
ERIDVEQVLKHRWFKKFKSNNLIINKTLNKTLIEKFKEFHKLCKIKKLAVTCIAYQLNEKDIGKLKKTFEAFD
HNGDGVLTISEIFQCLKVNDNEFDRELYFLLKQLDTDGNGLIDYTEFLAACLDHSIFQQDVICRNAFNVFDLD
GDGVITKDELFKILSFSAVQVSFSKEIIENLIKEVDSNNDGFIDYDEFYKMMTGVKE*
>N.PF13_0212
MDDSDSSVEETIVMLNIRQLNNDEEKIKNLNRLRKKKGEKIYMESDHGYDNKKKNEEQNVDEITKNEEQNNDE
ITGNKNNYKNDCNDNNSCNTDFHNDIKNNTKKDMDNENNNMDNKICIDNIPLEINNITLNKLFDECPQCIINK
KYIFKGIHTSSIGTNLFFKEPENHKNLTNDTKSDFSSNENININKQNANKITRDDFSTYQGYSTKIISFEIDY
*

>N.MAL13P1.189
MDIITSSIFVAFILLIFISSWIFYNYFVNYHESTILAALTFIISLSTCFILVLFIPIDIYLVSNGNLEISHLE
ITQKVISKFYHSMFWVLIFEAYVLVPFSYFYLKNKKSYKNEFDDNVVPFENTIESLKKTIYFILLLIVLSIIG
LIYRPGHKLAMEKGKELEYISDLFDVKHTGESAIIFLMGCVVLMGVSFWATYTSYGIACLPLSLLQQRNIDHD
KKEIENRFMSLKEKEIMIKNKYNINCEIKETDKDEMLKIEKMKRVLSKYNYKLQEIEKLSESWLSYLIGIVFT
FRLLTGMIFLAFSFIIYISLLASITDKYFNSICAYKCGFVLEQINTIFNLLDTLLIFFSKFFPLDILIIASLA
IYIFCCSLYGIVNVGIRICFIPLYKLKPKKSSPETLLVLCFLIIHIILVLIMTLLTIAPNYITYGIQKIKLND
NLGYIKCSLKTDKHICKMSVLSVFFNKIFFGIPYFANSYFFSNWLFIIMFTLSFFYRLFFNKISYLDNIDESE
LNKESFDENMKLLPLEKLT*
>N.MAL13P1.190
MKDKHKKVEYAEKDVEIKEIEKDDGDNKVFVNNLLTSVNYINNAIVHKDNRHMLRMLKYIKNIRLSIKNDKNM
SMPIIIYLIKRIFKENYPIYNILNKYMNIYEEGLYKEISDFYNINEKTYMNCLPELEVFFYLLILLYLLDEKC
YDEGMELSTIIINRINKINRRSLDYINAKVYFYYSWIHELGGKLSQVRQKLLFIYRNACLHRDIMTQTVVVNL
ILRDYIKHNLYDLAVRFISKTSFPENLSSNAQYARYLYYIGKILAVQLDYSEAHRKITQALRKAPQHTQSAKG
FKLAATKMEIVVELLMGDIPDRSIFSNKIMYKKLIPYKHVVSAVRNGDINKFAQVMNNYTDLFIHDGVYLLIK
RIHHNVIKTALRIINLSYSRISINDIGKKIGIESSLDVVGITAKAIHDGVIEGTIDYENQYVESKSNSDIYIT
GDPMKTFHKRIAFCLQLYSDAIKAMQYPDENEKKENEEAKERKMRQQEELAQAEEGDLGDDNDLL*
>N.PF13_0213
MTNTSNELKHYNVKGKKKVLVPVNAKKTINKKYFGRKVASKKKYVVQRKLRKSIEVGKVAIILTGKHMGKRCI
ITKILNSGLLAVVGPYEINGVPLKRVDSRYLVVTSTNIFNFENIAKLKDDFLNYAQDIDDDSFIKTLEIKKKQ
KKLLKNKNEALFMNNVIDKIKEIRKEDPKVQKLEGIQKDIGSLLKPEILKNKVFAHYLKSKFTLRNDMVLHKM
KF*
>N.PF13_0214
MPNFELGKDNKTADFLKHSPLGRLPVLVTSHGSIFESNAVCKYLCSIHREGDYLGKGSFEEAQVNMWVDFNTY
ELEIPMTCYMNNKSCEKSLKHIEDTLKCYNNHLLYNQYMVGNSITIADIFISVILYFSLNSGALNESIVLKYK
NLYRLYDTISNQKQFKYVFACDQNKKKNTQDKSTGAKKKQGNNKKDDNNNNNNNDADNQHADLLSDDLAEKKQ
PKKTNPLDLLPPSNFSLDEWKYKFSNEKDLLNNAMPHFWKTYDPNGFSLYYMKYDKLEDECQISFVACNMAGG
FLQRLENNFSKYSFAVVTVLGENKSYDIEGVWLFRGTDIPFEMKDHPSFEYHIFKKLDINNTQDKKIVEEYWC
SKETVDNRPLVDRKVWK*
>N.MAL13P1.191
MEGDINKLTSVETEYLEKSGKKRRQKSIIECINNYNEETVENHRNQNILDEINSYNDISLTKERSNEVMINMT
DGEMSEEDLDTLEVIRQLSSIKLDSDEESKDKIIYNKKEKKKKLKNKKNKKKNKENKNTYIFDTSDIECVEDA
IIYDESKDTEFPDLHELFSEYNGKYFFNKLSSVHVNWSNKMKLCAGICIFKKSGYCCIRLSLPLLKLRKIKEY
RETLLHEMIHAFLFLTRSNRKHDGHGPEFKKHMYRINRATGLHITIYHTFHDEVNFYRNHIWRCTGVCRKYPP
HFGFVKRSMNRPPGPKEKWWRRHSSYCCGNFVKIEETEENKKNEMVNMNKAGNNISHITNVIQKRNNKRDKDT
FEKRQDNNNLFEDMINDEIIILDNENNNRNKKEVDDEMDIINLIKNLFSDNKDKILSFPDITVDYHKAFEGKN
YFEID*
>N.MAL13P1.192
MLLKRDLFYIKKNYVMSFSFFPHCKIKKNDKAYQSAYNLMFYYNNKRYNTNINLTKRENNLFEEELTKVFKEY
PILKRNNISEYEEILIKEDDTNKKYKIGDIISLDDHSFGVVLQINQNNTIIGKIYEKNIKNKHINNNIIDIDI
NINEKKNVYSPYEYLKYLFSKHNKLNIFHHDNKEIYHKRSIKKQLHSNIFLIDIFNKIKYGQKICVIGETDTP
KNVMLLSLIYENLLINTICKNENLFIICSNMHKSELKFFFQKLHELFRQMLNKSGKKKKNNNNNNNNNKEIDN
IKKVNMKIENMNENKDLYEMNILEKLYSENNIYEEDDIKIPNDILWINSTTHYDTQFSCYLAPILTTYNLNEY
KKKYKNIIMVFYNVTTYNEIVTDLQSYMNIYMKNYYNNKKIEEKESKIKNIWKDDFFITSLPLSAHSIISKYL
SYTMYPHYKEEQIEATTKKNKCIYDSTSFQNSTIGAHEKNDMSYTLDSYKYYDYINLKGNINIDNLPKNNISN
SVTSFCFINKAQHELNRINDYALSLCDNYILLIGNKYDINPEMDINSFLNIEIMENNQIWYIIKKDIQHIFNK
REEYIKLIESKRKMNIYIDHWEEENFVHYNNIYYIIVYKNFKIFNLNSFQLLVLLRSLIYSNFTNPHISKDHI
HIFYQQFFDYYYQNFKYFSILHDEYYKNLKNFKQKQGAQEFINKVDTVIKYIKPNFKNEKNFI*
>N.MAL13P1.193
MFFFMYSKEKKKYKNVLLIFFLYIIFFVSKSYSGKTLACNYSLIPFHIKTKERLPLNFIKNDLYEIKRKQRRK
EIYSSNITKTFENTIPEEYIKYNIPEEPIYPYISVPNNLYTKTYNENNNDNVSDKQEKDKNCPKLTHGIKKMS
YEEYKKWNEEKMKESKDLPEPTIDDYIEDVEYEKYIHPALIKNVDVNNPRYSLYFLKDKIKNDTDQCATLNCF
DKNDLEDDLTEYDAAFNGIGPWPSTEELIKHQNNIEYDKTDMEMEYNITYDKTGYYQYKEKLINEQMKGIINK
KDINKNVSETLHKRATTIQKDPNNDKEENNKNCMYKTESKNNNNSNNVLTNDDLFDLLHKEKNITTDDIRKLY

YKKEIKSIKEAKEEIVKWDERKTVSRSKWTLSKDEINLFPPYIKKLYYEKYEKYRKEKDEEINNIRKNSAGSQ
MFMDMKDAYDNKENLTSWPSDHMNDKMNNISDIQIPSVKVGRLNELTKLYGNNPSSDDIIDDMRNNINNKSIN
NMIHNISDHMKNDPTFGQEINLKESQNKYTNFRLLEDNVLYTRAKSPINEILYEWDDPLNCLWRKRTEEVIRD
VIMYDYPFKELRRPSNLDLYDVTWYAGKIDIFVKVEEGKNYKITLFDLKQLVKKIAERLKVLEIDDEIVILPF
FELVVSSLPKKNILICRRDWNNNIGKEVVVFFKDNILQPVEGILLGSPSVFHVIINLNNQKILNLVINNIDKI
ILKNTEDELKDNIILKAAISQNENANINEQNRKNINENANLENDIKKIDDQDIYDQGDNTFDNKYDKQKTKNE
FKDIEFDELDKLRNANKTSKDKRVNVMKNINNIDQVQDDITNVINEEDDQDEEEAEDNDEYDDDDMDNDVDSD
VDNDVDNDVDNDVDNDVDNDIDSDIDSDMDSDMDSDMDSDMDSDMDSDMDNDMDNDIDSYDNDYNDYSSGEKI
KK*NKV*FIHIFLQYFNIYIYIYIYIYVCIYLFIYLLXXXXXXXXXXXXXXXXXXXXXXXXXXXXXXXXXXXX
XXXXXXXXXXXXXXXXXXXXXXXXXXXXXXXXXXXXXXXXXXXXXXXXXXXXXXXXXXXXXXXXXYIYIYIYI
YICMYLFIYLFIDLFFCKFKKYIFYHYILDKKNYEQVITKKKKKKKKKRL*
>N.MAL13P1.194
MEKLKGAHLVCTSDNKIENTKLNSKKKYSNLVRIFKEEVDYLKKKKQEDIIFEQKKKELLKMLPLIRKESSCE
LNNLTHSFSNKTDSSDDMCFYNKTPHRTEEDNFKENNNYMKTSYSTKKRVYTPCNYDKTDIYSDKEKEDKISL
YTTPKRCKRRNITKKESSNVKSRGSNSKKKKNLCDELVNIRTPVKSKNEYLIEKETYESITKDKWLMNESNMN
ELPENIKDEYVSYLCSPLKRSERLIKSKINKMLNENIEQIKQEQMASNSDTHKNSCTNSDILNNDDNSSIHCD
FIPEEEEKCWDEFYTQSIDKTRPFTGITTELAVDIITEMLRSKIKKIKIKNKEFFSPISENDTIMEIGHGNHP
LAVQMFEKWGTVGRYVGIEFSGEASKEALKCEKLKNLYLMRKVEFIKILSMKYYKDNYLNGVVAESNISPVNT
KTDFIELYTFKYIFAKSTLDYITCRMDDIGNSCDWEEDLQISPTVVEMFNSLADSLQNCKKAKNNSYIIFVEP
SNSSKFRDHILTIFKVIYTATFKYGSSAKYLRLYKLLNHNKACGYMLEKRNEVYESFEEMRHEFLKLILKSSI
TKNIDEVDWFLPTQVPKRWLSRNPADIEYLVKLDRSHF*
>N.MAL13P1.195
MNYRFVFLLSVIILNVIQKGCCSSWLNQEDKKATFSFSGNLKLDIKNDKENKLNDFENNKNVERLNEGVTKIM
QKEKELIESNKHVKDVVDEVGIVIKKKYNINELPPSGLTKDEEEDVKEDSAFLSDDLEEAAENFSLSGEKSDK
ENEQHENEIKDIRKYQAEIINSMNENTIYKKFDTNEFEKMKQKMEDHSEICYPDYSMPCPLHFFRTSTGCVPL
RTYEGPCNKIQNKLIYLYDEQKASWAEICEVNWPCMPLECSYGRDYNSVCPINWIDIGKGLCRNIYKNEKCAG
DINFSNMSFEEKKSMEKKCGIIWKCKSITYTTNFDDICPLHWENIGNYKCKAPQDYKGPCPNISNLKKYNTQE
KKENIENVCLVNWPYSIKVNEYQRDYNVDCPIGWSMMENGFCRAPENYKKSLKCKEEVSFDDMNADQKHSYSL
ACNVDFPFKDKQDCKRNYSFACPLGWIPSKKINYCKAPLNYKSSICKNVSKFKNILDNQKNYYLKFCDIDWPC
EGEIQNSMIYTQISVDHLEEHGKKKYTRGPVDSETGLII*
>N.MAL13P1.196
MKLSSCITYVSILCNLIIVGRFLYSSFLFLFSENILNYFERNNPFIHRKKYLIHKRTQPILHSSKYRHPYEGS
FKKKVIYDNNYFGIRKEINLVHYNKFSFYEYLFLLLFYKKGIHKIFDYVYVYYYKTNIFDSLYNYNNEYVKNN
LLFAFAKDIKKYFLIFNSNIKKLDVQLAILIKKKRRKKKKRIYEQKHLASYSSKLERTTNGNKYINTYDGNEE
ENIFVHEEINTAYGNYEGKPVLNSKYCLIKKIKNILKKKTLRKSNINKLDVIHLPIDDESVENKNNVELNNYV
CKSCIIDIEEKSLYALKIREEEKEKDSNNISLYKEKSRHIQIYYDYEDMQEYIMKKKKKNEKNEKNEKKKKKK
IMMRNFNKSISGTKPIINTPYYEPLKNNYQMINFTKRLFLSNPMKIQEKGFELNRYYPRRVNYPMKEKLGTGS
YGEIWYAININKNSQYKDVVLKKFLITKDEETSELNAMREVYFGEILKNCDNISRFIEYFKEYEINETVNKKK
EEYIYFWLVFVNEGYSLSKHLFDTSSSTLGLVTPSALWWSIKKQNIGMLVLKDLLRQILNGIYIAHKKGITHR
DIKMENIFVSSTTPFTVRIGDWGSAVEYKNEHFSFIPSKDEETNGYQPPESLFGHMKNNFMRLPYYDMWSIGI
VFLQFILGTKNPLEVKDKESERRLKKIFSKYPIDILKEAIFLQSLSELCLTPWVKKSTHNLIRLQKKKKNKII
HECDRYDITINDVHDVSHNNNINPYNRPNHIYRKHFFSTKSSYNSLYTNNNNNFIMNKLKYYITNDLINIKKQ
SFHNIYNIISDKYKSLISLPSSPLCSDGMCLHKYDQAYDKKNITQPNDTGLICLNKYKLFFSENQKNILPNYT
CDDEKFQNILKDRDPSGVGLPNKNARDLLRQLLNFDYESRITAEQALNHPWFQEN*
>N.MAL13P1.197
MDNFKIIYASIIRFNDKIPIFTYHIKVDSQLRIELVKSLNVYFRSTSIAAFPGGTDVFPFDDGIIYSLYNKNL
TLIYIIITQKEYIENKYAFFFLQECMNELEKKIDFTNLMRLPKNKYSRTFTQYYQQLIKKYNAEVCSPIERLD
KKIIRILSTVCRGIETTLKNKQGLTKLESRMNSLSFNARLVSCVFIS*
>N.PF13_0215
MKMELLNIKNNDNIPLGSMRYTDLSYGCESELSSNEESPFFSVGDFTNELHSSFDDISDYILVDESDLKRDRS
ENTTGSESNVMIASMRVNDKRVGNEKIKKELKSFSYSEESKTCSDNTHNEDGSKKQNFSRTLGTEGRKRMLKL
LRRAYKYNDECKIIMKNKNPPLSISFLPYFNLSMLWQLAEDFGVYNEALKIHRECIKKRNASVKLKASAKASA
NYNLQDITKVSKNSKTEYASDIASNADMNTAEDYDMTNGSINEDGQGKRKRKKISF*
>N.PF13_0216

MGKIDHIAKNTLNEIVKNGNHNMSSFYDKKLIYISKPTHGNFVYWKIRNFISLFKKKTKRCARYLDLNHDFSR
AALVGVPKTNFDPKNNIFYIQMDNEEWAGIGSKMMLNISMNILFYLHISFWIYFIYYRMLVNNKFNVFSKWES
KE*
>N.MAL13P1.200
MNFFKFFKFMHTPKSYFSIYNEYLNSYKKKINKIPFYIRRTVLI*RPIICLFF*
>N.MAL13P1.201
MHFCSKRGQFNFLKLYKNNIKTVKNNIKKEDDYLFLNKIKHKEKKKICFKNIFLRVCVINLSIFSFTLYNYLK
KNDENYFDKNDICIIDHIVMNDLVSTFGVNNISIYLKNKLYDKLLLNIQDEVYISKENSLLVLMEAIYKNKKY
FHQQKKKIQNNIKNKNDNIHHEHNQQKDYYMKQRSDDINNNRNDKNISMDIDMYNKNNGNNVNNAPYEPFIYF
LFENIKKNNINYSKKKILSSILFYYIKYSEDDLYLSYDQIWQLVYNPNLFYNLHNRGEIISYLLLKILRNQKC
TEEIYTNEKNIIHTYIDMENQKENHIEKKQNYSTDMQNQEQHMNKFENTIIFFLLNQDVEEKKEKSAIHLMDK
INGEKKYVKEQNGKTNKLTHENNNNNNNNNNNIDNIKNNYNNNGIKFVELPIYKFYKSFKKNEENYYKQQGLA
QIQKYLNKINSTYFLGKKKNNNNNNNNNLIVKEKYNNIMNKIYWKFLENTVLYTFIFSFVLHNLKSKEYNIKT
YLYMFKDINKSIYTNVLINSSILIQKSILNNMNEKKKTNKFILTSLMFNLFNTFTLSLSIYRCKYALVPLLFS
QVIKDNYFL*
>N.MAL13P1.202
MDPYESPSEFLNKKKQKSQKKGNSLIISNEEKNKCLEEENNKMENEKKLKFIDIFDEENELGISNDIIINNNK
INTNKELVINNYDKINNPRDINKQHYLINTSKEIAPKKKSFFNISKRKGNKDHEHSFHHIHEKEKIYLDTPYN
NNNKKNNVKDIICDDKQINCYNKDIICDDKQINCYNKDIICDNKHINCYNEDIICDDKQINCYNEDIICDDDG
NSPDDERNNIIYNNHYDDNDYYPYDINYLQNKVCEVKILLDASLEKEKTLCEEKKIMENNYNIAYKSMEEMKN
EIINITNIHNEEIIKLKESYERRLDLLKQEIDEKDIFFKKKIETQEQENVTQIKKCEELYIEEKKKKEHLEDV
IKRLEKEKEDVIKRLEKEKEDVIKGFEKEKEDVIKGFEKEKEDIIKGFEKEKEDIIKGFENEKEDIIKRLENE
KEDIIKRLENEKEDIIKRLENEKEDIIKKMEQQNEDIILTMNKEKEQEANQRINEYKNLIESYKKDKEKYCNN
EKVWAHNMNESKEKINNLINTLKEYEEENKNLQNIINQKNDTITNIVNDILLVKNVYEDLIKEKDMHEIDKNN
LIHKIKNHENDMLSLQNNIDKLKKSNNNLNEDLRKKEKIIITLKNENMYLKKENEIFNNNFLSIKGEDKDTKL
KIYSTDVTDSTNDQDDKTNENHKRDDTNNISDDNYIKHQETNIIEEKKIIKKDLLNSKILKFGKYLNYNNIIT
KDDSSDYVEEKSTRNKDLSKKKKKKDKDLKKYLKYYDISNGGKRNQNNDIDLIMEKKLYNGPTSNNTKEDIIK
IGNYKDNKDHNNDHNNDHNNDHNNDHNNDHNNDHNNDHNNVHNDHNDHNNNGQYYMLCKVEEKKKADPSFSFS
SSSSFSSYPSHSSYPYSSYSSICSKEKNNNIVHININKKNNPKEDEKGTRRRRHKSCNNYYKTRYDSNSPIKN
SKNNSNNLHLHSSSLNASYSHTSSYNKHKNMNHIINEESKMRNETVSRKQNKQIKSAENKYDKGLLNKIYYKN
EEKNIQFNFSNIIQQINEKDKVENNNIYPNQNIDRMKYKNDIDYVDNYMDSHQTSHINLKKKENNSYNHNIQS
HDIYRKELLSNKYLYDKNLYNYKKTYNIFSFSNNHIGEHNEIKKRIAYNNNNNNNNDNKKKDLSLLLRNEENI
KTQHPLNNIKKNILHENIKYVHNLDKNVLDINNFENDEPVEKIQLNSSLIRHKMREKGFHMDYEEKKENDFMN
SDEQRYKNMKYYFSTILKKKPCYNKLNIQTNDDLDILSHENRNKLVGDTFEDLKKKIKDQKENRGFIISDYDI
CRNEDCVDTMSDTIKYNKDNNMNSINSINSMNSINNKDNILTSQFSSSNVLDKSMNHFVHPDDTNNNEKKISN
VNSYLLKNYKKKSNMYGLFIINERLKCIYNNIENKRKSISNLPLYIPKVQETKKCNNSFHLLRNEHINNQDTK
ENNGKVISKDNLPKNNMNINHMNNIPEEDKYTNVNINNYNKRIFKKDMENIMRKDNMYKNMGSNYICSNKTNN
INNIDNNIDNNIDNNIDNNIDNNIDNNIDNSSPPFMNNKNNEQGKKLQIYEYIQNHNYIKNEQDVIISNDKHL
ENETVCVDILEDNINKRNQEKIKDIEHKKLYKIYDKSDMGKNKQIATLNNGNDDNNTNNNNNKSYYEKECSEE
NNMYSPNSNVKNNYIGDHNDIDLEYDEMLYKKYIQKLKINKINKYKEDNNTNNNNINEQDILQHNFDNFLLLY
KNKKKKKKEMINLYSNKSNILENIKNQMNDEHRDIKNDIFNEEIQWNEKKKKQGDNTYTEKLKLLSNDYPDER
NHISIKNKKDLQKKKNLIDYYNKETNGVDKKIFFKKIINSNDIIKKMMLENEKDHFLFNIKRV*
>N.PF13_0217
MNDKEAKKEAIEIIYEISNILNVNLDKETIVILIQLCEYGVSPKILSHIIIQLKKEREKFLDNLNNMNNLNK*
>N.PF13_0218
MGNSLSLCLLRETSKDVFRYFESLYIKYFKSYNYINYITLKYNKKDDKMHRKKVITCADINEGPKNNRIKKEN
YIFRAFLDMTKHEKTLLSLAMVFLIINAYTNINYPRIMGECIEASNHMNNNIFNNIHNNNNDNIFQSNDNVIY
NTFCNINICNNVINFFFDKISFIKNYFVDRNIIKTIIFYFPYFICGGIASYMRIYFTNQCIKNIEIRLKKQVH
DKILMNNEEEFKEYKTSDYLVNCTFIEIKYSAKELITCITQTFRYMNSVVGGLISMSCISLYLTKLCLFIIPI
YGLSVLCLLKSLKRIKIETTTMEEKQIARFSDSLQKKNIISLFGNESYEHRYFCKQLHVLNNLYDKYITCESI
FYSFLNIGTNVVICTILCLGRLELTTQHITHGQLVSFIVYSTMLGLGVGGMLKLKKDINLLQISLQKIYEILD
LTKSETQTDINNYEQGKLTIQDKQVNLSKNKQSGNSMGKYKPDNDDDNKEDNKEDSKDYNKDDNKDDNNDNNN
DNNNDNNNDNNNDNNNDHHNDHNNDNNNVVMNELPFKYPHKSNYIPNNIKGSIRFENVSFSYNHYDKERKKSV
LKNINLEIKASEKVAIIGKSGSGKSTLWKLLTCNFSYEGNIFIDNLNIKNIHNHFLKRNILSVSEQECCIFNR

TIYENLIYGLVPLYIKNNNNNNNNNYYYQYLSKCIFQNMNPGNNIHKKILNNIQSTKYNSQCYQKNKHNITSN
FITQSYRTQDEDPHNISNHKHIDNTSRNIYQNYYTDFLNSYDEYKLNIINSSINVLCEELDLKQFINSMPHNI
HSNIQYNNMSSGQKQRLSIIRSLMKDTPIYIFDEITSFLDEGNIHKLHKLIDLLIPNKTIIYITHSIHILNKM
DKIIILDDGKIRAIGTYEQIKNDTLFRDIFSLHKPAV*
>N.MAL13P1.203
MKKGFFLVICLCSLILIRTYSALSLKGIKEGDPPRLMYDNNKVNHGNTLKVSKDELMKRIVQDVSYDINNFIN
DQYENNNNNNNNNNNNNNNNNMRRDKTSENVKELQNDDTVYIKSLQNKPLLKMNNKEKNNKIEMKDSKKGNYN
KYKIKHNNKSYDGGISNSREQNNDENYMREEQRGNMSFKLNNLNKKKSRIKNNKNGKKENNIDINNNMIKINN
IKKDIPKIDNDNNNNNNNNNNNNDNNNNNNVDGDDNTTIQNKGPTNISSTNEHKNINTNNQNKQKLHSENHHKN
KLTETSHNGDVIEIRIISDPGFVKSNHKKTEKNKKYDMKEKDLLRIKNGKRNKELMGVPDKMSKRREVEREKD
HIKGNSKNKNKKKKKNKNFTIKTNDESKKNKISYIQMYTDKNGTDEYSPKLNDDETFDEDMYEGNDEIDSNMS
IKKINEDKNLLEFGDEGVKDIGSTENEMIKNKKNIIKNSRKVYNLNLYNCDLIDDWDLNHEYIDEEGKLIKLS
GYVFQNIVSTDSMPTVNITDWKLRGSCSYDNYICGALNYMNNIYSKGDRVIFDGKIYEATAEVYETPKVMENL
WIEKTKDCYDF*
>N.MAL13P1.204
MSSLNYIEDSINCNIRIDGRTLLTYRSIEINKNILVSGDGSSSVINEENNVICGIKLSLITPDVDTYDEGNVN
LQIDCPASVAANRIKKDHLQTMTTIIYNLCIKNNINKKILCILPGKFAWSVDINVMVLNAGGGLLDIISIAIY
VALKDTFFPIVKPKKEIEEENTFHEISNKDYQLEIVENEHVNFPYENIPICISIGEINNKYIYDMSKVEEELV
QNIFVVAITSKGKCVAFHKLYGISMEISSILNITENSSKISHTLFDKIHQALDKIHANM*
>N.PF13_0218a
MIIIIIILLLLLYHIWNKNKNYIINFYYVNSFICFNKTKKYRDVKKELGEYHKYYSDN*
>N.PF13_0219
MSEIVEGHNMDENGEETKVEKKVKKKKDKKKVNKEDKKKVNKEDKKKVNKEDKKKDKKKDKKKDKKKVNKKDK
KKVNKKDKEKDNKLNRNTHTELDETSIQNNLDSPYDDDSESLENFFKENCIDDASSDDSLKKMEECIMTNDEN
GNKLKYINIEYVDSFLQSMNKKTRLNKVIKILSMYRDALDFFIEDELKNDEIPLIEKSDDSEGNDKIINKKKQ
KKKKKNKKINKNKKKYAMNLDTSFFVVFNVLYNIDTMFYNVMNEGNLKMCVLDIEKIKSNEIFIDEEEKGEKK
KNKMNDDDNDVNNDDDNNNNNNNNNNNNNNNNNNNNNNNNNNNNNDSFNYGGGSKYGNNNLGKNINNNHRNNIQDD
TYDLNNMLNYIHIKKYKPLIIYFFNNLAQNLKKMKNNDIYTGILKILKKKEILRWVVILDYGKIFLKKICKLF
VVSTNSDIYFFHYILIQNIFQLYNERKKIIFMNLCNISKDKKEEEDIKKTYNMERIIYRIYQNFFQSYILHYG
LYYNDILQINHRNFKENCLLELFTYLSHDVAYTIVFRYIQIIIQKIREENNKTFRLEKNKKKDTDENNKKEKK
YFNHENNKNKGILHLKNFHIHSNYMMLLIKLLTKIIKIYDNLHNLIYGVTILIISILKSKINHMKYIPINLQL
ITLLIRTMEHQKKYIPLFSYISCIINGLKSYKHIRTITKNQTIRNEIENFDINTSLEINESLLCDFQISHQIY
DKIHIVLYDYLGLMVCHISFPEFFVAIESYFKKYYAECQIQTFKNKIKNLLTHAKNSIQIILNKRKNINIYNI
YDQFMFFQKDNLPLYTQRQIVLENYENSYLNKIKAKLSGLQHIKNQNNQNDDAYGYHHHSRDQDTNHNNKEIK
NFKNKDRKRKAQKENLKEDSQTNHHINKKKKNKNKNKNKNKNTNINQSTKLPTEDNVEDFSMSSDEQ*
>N.PF13_0220
MYFSESYQKIAGRKNYKKNALDSVSSQGIFDNIKNNIYNIDKTDYKILNHYNYKKDQTSTNLSKNKQKDHFFI
RSNNINNNNINNNNNNKGKDMCVNPPYEKNILYYINNKNLGRYKNSCDHPTVHNILKNNKSNIEIHQYIPSLT
STIFDLLYVNSNKIEKNIYPQQNNNKNMSHSQKFNEYIPMNGFRSIYHNNNMNNGNGYYTNTFNKQNSNLYTH
QNNMYGRNNLMFDEDSIPYGMYDQSNITGTNTMTCGKNIPCDERMMYRYNTMEGVGPCHNIHPMINTRMNYST
PLSLPQYNPNIRNYLTHEGYPPQYYDYNSNLYSRNKRVLKQSYDADNIDNEKNHKKELSRKNISKIKKGDIDI
NIETSSSDKRGVVIDLNIGV*
>N.PF13_0221
MCEKSQKSQNSPEQNGDNVELFRKKNIEVTCITNNTTKKYIKKKTNKHLLYNNKEKHINNEESKILSLQKKQK
QKKNKKNVYRFDYIKYYERVRKWKEKRYGHDYEKKKTITRQEKELPHHTKNHHQKKKNKIVQKNDINKNITIY
KNYIGSNDQIITCEEGYKDVVNCVQQYKHVEKNNKKENKIYQHSYNINNEHYLNNYTPYIEPPNRNNNNNNNN
MRGVSIINNIYNNLNNIDILKSSETILLQDQNKINFSNKTIISSFQDMESFQNLNCEDDEDKYNDIDIIPMSN
DTHIHHPLMINDLTHFCNKYNYVSLKNKMIFYPYNYDNNKYKRFSLPYFRFTSIGRNGTIQKPKSDEYINKQK
KKSKDIDYFKNQYDEKHFDLKKTFVNNYLDENKIKEYHKKKTNLLKKKFIKTSIYKERIDIKKLENNFFQNNS
IEYRDNINTPTLQIVNNYGSKDLNMEHINNPTYNNLKYVQHIQQKINKDNNIYKMEHLNKMNDSYNYTHTYHH
NKNDNLINNAGNYTYECITPTFIISKNKNKNKKNKNIKKIKNIKKIKNIKNIKNKISNNNNNNIYYYNNYYYN
KVNRNIYVNKKKTSQYFKNFKFKGKNLKYMSNNKIFKNVNLDIHKYKRNIYEYKKNKNKKKKNNKIKRLSITP
SILPLKNKKKYNFSYDIYKQVDLQNNLLNINKHKKLLHVDEKDLTKNFDIFLCLFMKYEKNESQLLALIHDED
LTKYSYFYQTYIKPILDSYKKCKWEKIKNSNINEQKKNERKDCTHYNNITLNKIVLQIFIPSIKKNYLKYLNI

LYYKNTNILDIHTSEELLKEIIKIEKQINNRRRKSYENNTNCVSLISNNNYEKKIKLIPKPQWANKYNLKVLL
KRQEHFNPFTIFGTSVKEVNLEEIFTLDVYNNYITNDDRYKNKILYELYVGNYIHNLYEKITYDEWNDVLDIL
KKHWNNFITLECNMILDPLLLEEILWYIDNNKMYKDKSKDMYTYENCFCPTPDPKKEINFNDFKNFSKSMSNK
YTFQKINKFKKYSLNEESIYKDDHLLLKYDDVYSRWGVSSNPKKKKNKKSNNHNHNNNNIREYDTIDNNYTYN
INCNNFTNNKHTLEKQNMLLHIPRPSSAIYYDINFFQNKVKKNIFTKISKCENTSYWNMLHYNNYSLRRSKSK
KWISQNYFDNFFSNYYLYNFGTIEGTYNYTI*
>N.PF13_0222
MFIAVVGCTHGELDLIYSTLEKIEEENKIKVDLLICCGDFQSVRYNVDNECLNVPAKYKKEQNDFVDYFTGKK
KAKILTIFVGGNHEAMNVLKQLYYGGWVAPNIYYLGYSSVHNINNFRICSLSGIYKKYSFFKKYYESYPYTDI
TKVSAYHIRKYEIEKLKLLKNNVDIVVTHDWPNNIEKHGDVHDLLRRKYHFQSDVYNNTLGNPHTEILLNKLK
PYFWFASHLHVKYSALYIHNDQKQYTRFLSLDKAQEYKHFIQILNIVKKKDSSIHLNFDHVPKVLLPEPGSKM
DIQNDAQPNHDLENCPNTKTNTCNNNDHHNDDSINLDYDHEKALYELDRNMQLDQEKNDEKNVDKSADKNVCN
KDISLEDKNQHNNNNNNNNDDDDDGVDIQADTSTNVADQNNNSVPTNLKENEEESLNDQNENKDEETSQDENIT
DEKKKKKFYLCYDIEWLAIVKANHHLISASCDKDYNLETPTYPTKEDFDFVENKLKELDNKITIKGKDYYCVN
GYNTPNYKNLQEQRQLFLKRFELEELSIYTESELNFFAEEMKTLEKMNTDIHNEEDKNECTIEA*
>N.MAL13P1.205
MYIYIYFFFFXXXXXXXXXXXXXXXXXXXXXXXXXXXXXXXXXXXXXXXXXXXXXXXXXX
XXXXXXXXXXXXXXXXXXXXXXXXXXXXXXKKKKKIYIYIYIYIYIIYLYIYLYIYIFIF
YVILCKTHLLSRYIRGSLPSVAKATIGVEFATRTIPLAVGGTVKAQIWDTAGQERYRSITSAHYRRSAGAILV
YDITKKKTFLSISKWLEEIRQNADKDIVIMLVGNKVDLTEEDETKRKVTYEQGANFARENNLFFAEASAVSKL
NVKHIFENLLQEIYNNRLKNNNRSFSNRSVATCESAIQLTKARSVIKLNEVYDNQSEDNNMNKVKCC*
>N.PF13_0223
MSNVGSFFHNVKRNILSIMLWAPLANWGFVIAGCNDLKKEPVYISEKMTTVLTIYSLLFMRYALAIKPKNYLL
FSCHATNTLVQSILLYKKLKYETEHQKIKITAK*
>N.MAL13P1.206
MVTGPDMLWLVITSGIACFFMAFVTGANDIANTFSTSIGSKAISIKKALIVAFFFEALGASLLGGTVTDSIRS
KIINFQVFYDTPEFLMLGMCCALMGATVWLAVATRAGLPVSTTHSIIGALLGFGLATGNMKSIKWEKINNIVI
SWLAAPILAGTCSAIAFTVLRMLILRKKNSFEIIKKMYWFLIFLITLPFSVFLIFHNPIVINTQCKMKKDGKV
IVSSPCYIEDWSAAHSFYASIICILLSSLLTAIGSFVIYIIYNKRINNYNLKKKIFCEELADIEKNPQNNFCA
INNSSLNSVASNETQLTQAHNNTSNGTKQNQVGNGTKSNNNNVLVLPGDKNVKSQQDDSKTNGTQKTGSVEAH
HVVHIKDNGSEDKSHENMLKKNLDKVTNMNENNNNSNKNNNSNKNNNSNNGNSNEGNGKGGDPKNMENVIIEN
FDPQTEIVFSSLQIISAILGVVAQSANDTANAIGPFAAVFNTYNNGIRGKIKVQWYILLFGGLSMSLGLSIMG
YRVIKTVGMKLIKITPARGFTIELISGLVVLFFSICGIPLSSTHCAVSSVIGLGLVEAKMNADNKRHARKSMD
KDIIQVDKDKSFTLTEKIKYPFSFLNTSCVNLRLFRTVFLSWILTVVFSGNKNK*INK*INIYIYIYIYIYIC
VYIYIYNIYIYIYIYIYVFFYT*
>N.PF13_0224
MVKKIDNSLNTNIHQYHIVGRAIPTAKDKNPNVYRMCIFAKNDTNAKSRFWYFMKKINKLKKSNGELLACEQI
KERFPLRVKNYGVLLRYDSRTGTHNMYKEFRDTTKEGAIAQLYSEMAGRHRARASSINIIRISEISSSLVRRP
HIKQLLKRRLRFPALHLPTLQKEYRKKFASKRPSTYRM*
>N.MAL13P1.209
MGIALKNVGRIKKHGRKHLVSKNPYLRLLVKLYNFLARRTNANFNKIIAKRLIMPKRYRPPLSLSKLQYHMAN
HPNDIAVVVGSITDDKRLFSLKQLKVCALRFTETARKRIEDAGGECLTFDQLALKYPTGKKCVLLRGPTKART
AEKHFGKAPGKPKSKARPYVRSKGRKFEKARGRRKSRAYKK*
>N.PF13_0225
MANSIYDKSNNSKSPFSIIKSDDKKLSKDTTLITERSVNGITTENMQHVKEKPLNEVLYNVSYNDSCKYNFPN
NNIAESFMDARDTNNYENIILKKGLSENQHLYSTPNNNNNNNNINNMDNINNMNPHHNNNYFNVYTLDNKMFM
KKSKSNMNDPNSNITYNMINNDGIWNMKEYPSNIENYDYVDIPNFPYQTNNQLYSANNIVPPLYGRNYKIQEN
GDSINGYNTNEYIKHGTHVGRIKPFNVLGACQLPSLCTPSYTTSNNNNNNNNNNSNNNNNNILFGPFENKTLN
MPYILNDQNKEYIYPYLNKGMNEITNDYNMMSTNKFISPNYEYLMTNPRSPYYMLNYINEKKLDIFKKITLSV
GTSLDDMINIMIAMLETSYKSMQINRNKNKVNLYDLYPFYNPDQNYTRNERPTLKTGSAFLDKVNSTLDATLL
EKHKNLPKVLGRILIPKTQKTGSIVMDGINKILDGLLDEPISYHQYDYFSDKFEEEGKIKGAI*
>N.MAL13P1.210
MIYNDILTLCANKLRGLIDSKIFIWLLIFFRIFNCLFVVTSFYPDEYFQSVEIAHFWAYGYGHMSWEWEPCVA
LRSVITPFIYYVLFLFLKLINIDHPVCVLYIPKLCHGICAALCDLGIYKLLIYWYVELYNDAWINEDNIKRNE

KDENNGNNNNNNNNNNNNNNNNNNNYYYHNNILYNTNDIISTILCCHFFCWFYFYSICRTSSHSFECLFNIWGV
YFLSQNYYPLKNQSNKIEKIDLLLQNDVIIQKGKKHLNEWTNLKERRNDHHFDTYENNFIYHKGTQNCKQYDK
NMVDQNVCGQNMVDHIIQNRNNLCRTHFYSSKFNKIQEAKNLLFSLFFSSLSVIFRPNALVFWLSLYILYIIK
NIFEKQNKINYKEIFKIGITYTFFFLTIIIIIDSYYFGHITFPFWNFFVYNFLSGNNKYFGGHSFFFYFVCVI
PSIYLTLTPFLFYGYYIIYNNILNKVKYKTINIYMYILKRIDWIVYLVTHLEILSLSFSKHKEHKIVIGYIPF
LTIFVGYALYIIKLHYKKYNGKNGKNIYNNNKIQYGNITIKGRNKYIFLISSSLFTNISFLLQFLCILFFCLI
HNRSPEHVASYFRNLETKDDQNIYIFITNCYDIPLYSHIHRKFNIGFLDCSPYDTSNDEATKNWRKRIYEDKF
KEQFFNIFQEKKNNNHHINSTYGDTITPYIIPDKSFYWFGHHHFNKKNNFQYIYQNINLSCLNYRFHIPLQGQ
LPTYIVTTTIELTHLQLFLSTYNYKLETKPFFSYFMISEAKGIVPVYHYIFKRVPS*
>N.MAL13P1.211
MDMYSLYHSNIENQLAKELGYITKTREVNQINDDDKNYYKECNYKLFFFIIQLQQKIKDLEKENEKLKYLLKK
YNRESIHNDNKTIKRALLVVSFVIFIIIFLLF*
>N.PF13_0226
MDEKSEINYDEGADPSYSMHMSESHSAYEEVNKTSSDSEFVDDDYSKVISENNLESDNVKYNPDLYNLGKARQ
YLCPKVQEYGIPGQVSPYHFPYNEEDYLSNVDTEYDENGFPIRTNIDDNDNNEDNKKLWSWTNDGKLKLLCSQ
PIIPVSVVQGILRRDKIILIPQVEVTDVVIPKVYNQNIKHDVPTLNIEIMKSNVNIPNVKYVDKEIIIPIITG
YTHKFVPKWDIHEVPRPVVKYIGEQKIVEVEVPEIKYIDKFVEREVIVDTVEKRVPKIIEVPKYVDEVKYVWK
PIEKIVYVQKLIPKFDVNLECPPPLIVPYPVQRIKQISPVMVKKNNTEIPDYNYYDLNSPEGSLPIPEEYIHH
YSRKQKMNKGLLSTCCDKKIVTEEEKNVQFVSVPLSNEHESVCVDEEIKKNINMSQENINTQLNNNMDNFIYD
SDIKKNKDKMNSSINGKQQLYINS*
>N.PF13_0227
MGALDESTPVPSRITLQLMKQKKKSAFQGYSLLKKKSDALFIHFRDVLKDIVKTKTKVGEEMRNASFSLAKSV
WAAGDFKGQIIEGIKRPVVTLSLSTNNVAGVKLPIFQVNIDPTVDVLGNLGVAAGGQVINNTRENYLQCLNML
VKLASMQVAFFSLDEEIKMTNRRVNALNNIVLPRLDGGINYIIKELDEIEREEFYRLKKIKEKKSDKLKDSNI
DTDADGDYNASKRQYNYACTQKDDDIIF*
>N.PF13_0228
MKLNISNPLNNVQKSIEIDDEKKLLPFMEKRIGNAVPGDSIGEEFTGYVFRITGGNDKQGFPMIQGVLTNNRV
RLLFKKGMKCYRPRKKGERKRKSVRGCIVGQDLSALNLTLVKKGVNEIPGLTDKAVGKKLGPKRASKIRKLFN
LDKSDDVRKYVIGRAITKNGKTKFIKPKIQRLVTEKRLLRKRNLLQAKEKRRLEKKQQLKEYKQLLNKYRSEL
NQQHDVETTKKKKVKKSLSKTNKTASKSKLNTKQEQKDKTEKKQNKTNNIKNDKSEKKEQAKKKTKTNENTQQ
TKQNKPDKKNKAKK*
>N.PF13_0229
MHFRRCIRKYCINKKIVNPFEHVQRTLNEKNYYYYDINELHDSRIKSLPYSIRVLLESAVRNCDNLKVSEKNV
ETILGWKENCKKKKEVPFMPTRVLLQDLTGVPCIVDLATMRDTAEKLGCDAERINPLIPVDLVIDHSVQVDYS
RREDALELNEKKEYERNLERFKFLKWGMNSFKNMLILPPGSGIVHQINLEYLAHCVFNKDNLLYPDSVVGTDS
HTTMINGLGILGWGVGGIEAEATMLGLPISMTLPEVVGINVVGKLSDYLLSTDIVLYITSFLRKEVGVVNKYV
EFFGTGLKDLKLADRATISNMAPEYGATVGFFPVDDITLEYLLQTGRDKEKVELIREYLMKNSMFNTYKDNVE
YTDVYTLDLSKLNLSLSGPKRPHDNVLLSELHKDFTMCLESPIGFKGYNIAKEEREKKISFVCSKDGKEYVLS
QGSVVLCAITSCTNTSNSSSMIAAGLLAKKAIEEFGLKSLPYIKSSLSPGSKTVQKYLEAGGLLKYLEQLGFY
NVGYGCMTCIGNSGHLDEEVEEVINKNDLIVSSVLSGNRNFEGRVHPLIKANYLASPVLVVLFSIIGNVNVDL
SNYTFNYKGKKINALDLIPRKEEIEEYESTYIKPEMYTEIYKNIKYVNKYWNDIQIKKNKLYEWDKNSTYIHK
PPYFENMTLEIEKIQDIKDAHVLLFLGDSITTDHISPAGMIHKTSEAYKFLKTKNIKDQDLNTYGARRGNDEV
MIRGTFANIRLINKLCPDKGPNTIHIPTNQLMSVYQAAMKYKQDNIDVIIIAGKEYGCGSSRDWAAKGPNLLG
VKAVIAESYERIHRSNLIGMSVLPLQFINNQSPQYYNMDGTEKFTILLNDGNIKAQQTIKVQMNQKGKIIIFD
VLCRIDTEIEERYFRNGGILKYVLRSLVNEANK*
>N.PF13_0230
MKSFRLCYRKWGRLVNNYNKHNNHNNHNNHNKHYYHNKNDNTCSIYNQFLIKFYLYNNSKINIDVYIKLISSL
NINKDEYNKCNNILNSSNFRDPNLILYKYFQYVDKKNSNEKIIKLLSLLYSKNLREYRDKNTYLLNSSIHVNH
IFFKHILQYQDRLNNNNKKNDASYYMNNKNIYSMYTDFFLFTLDFYLYYLSKNSNMLNINELNIISEIYSNVN
ITINKESFENVEEKEKERISNKYHHHTNDKNELYDKNIYSDDKNICFNYYMCKEKKDNIHNDKYKDPHTYNNK
ICLLFIYISCSIVSQTYKHIMKKENNVEKKRKKMKSLDNFIIKNINECKHILMDNLINKCINDQYIRYIILRR
KYCSTYLNINVLKKYLNSIKYLNIINIKKSFYYICYLYYNSYIFNKSIYYCSLIFYLLQKYNLHYTYLYQILL
IKFNLFLLKDRKIYNDYNKHILLYGINKFVDTIIISRIKKYDRINTIKNMSSLFKISHNFYININYGKEYVDNK
IIHDLLFNKVYIIQNKQHKNKKKIKKITNLHLLQLTYFEYVILNLFKYLYKKKPWLNHKSSNNKFNTSNINYV

HTCDQFQTLYKNQKYTNTIFHKSSIYVSENYLSNIILLTIYVETSMPILFSYLQKLYIFPKVRMKNYVKYKCA
KQLNILMRKNHRKCNVYKKYVFLKNNKNGNKHEYIKGKLYNIYRNPLCNDKYLNIFMYKRLQRKEQKVGNNKD
NNNDNNNNDNNNNDNNHRNNNQHNNNQHISNFLCSIYNLFKSKILENNKLDPYYSDKYKKKYIDQQNLFNNIR
NNKNNKIKTSLTHYYISSNIKNIYNDNNIYNEKNILTFFCDIYFNNHIIEIDGPKHFFLYYNYEYDNEHFLSP
FILKNNDIFNYKYVFPFFFNSTNKIIKNIGSDYYIYNDKSIKKNFFLYTSGYFIKHINYKEEHITDYKYLYNL
IFKKKSDLHII*
>N.PF13_0231
MLKLKRVMKTVSNKNFRFSIRNFSLCKKIYYNGPKDTYINSGFELKNEDTEKLIDLVKESLKLKLLSCSYCSE
EIAKHYLELAILEHKNLLLNESKDHYIKSYEIYKKIHGDISIMCANIETYLGVVYKDLGDLKKSEDFLQLSLS
NKKMVIKEKKYLLIDTLNNLGSLYQHKKEYNTSIEYFEECIRILMSSEIELNKNEQITLCYYNLSFSYIGLND
LNSSITCLIRSYNTANETFGPDHNLTLRIKDLYERLKRESSLKE*
>N.PF13_0232
MEFVSNDESADDIIQDESNEGEVELTDADFYDLTVINDKIDEEIIEDDEEEADNDDQENDNVQEVYNIDDEDN
DIHNDKLLLDQQRDNDVNEEEEEEEEEEEEEEEEEEEEEEEEEEEEEEEEEEEEEEEDEDDDDDDDDDDDDDD
DDDDDYDDEYDEDDFNEATVSWIEWFCQLKQNLFLVEVDEDFIRDEFNLIGLQTKVPHFKKLLKIILDEDDDD
DDDDDDDYDDEDDEINRDSEEMYKNKDMHEQNAACLYGLIHSRFILTSKGLALMREKYKSGIYGTCPSIYCEN
AKLLPTAISEIPKFLSPLLYCPRCCETYYPSKNSLLNQLDGCYFGTSFASFFALSFNIASDKKKVYYTPQICG
FTINRNIRETLYMDVNKDNTESSEECQ*
>N.MAL13P1.212
MKMKIPICFLIILVLLKCVLSYNLNNDLSKNNNFSLNTYVRKDDVEDDSKNEIVDNIQKMVDDFSDDIGFVKT
SMREVLLDTEASLEEVSDHVVQSISKYSLTIEEKLNLFDGLLEEFIENNKGLISNLSKRQQKLKGDKIKKVCD
LILKKLKKLENVNKLIKYKIILKYGNKDNKKEMIQTLKNEEGLSDDFKNNLSNYETEQNNDDIKEIELVNFIS
TNYDKFVVNLEDLNKELLKDLNMALS*
>N.PF13_0233
MAVTNEEIKTASKIVRRVSNVEAFDKSGSVFKGYQIWTDISPTIENDPNIMFVKCVVQQGSKKEKLTVVQIDP
PGTGTPYDIDPTHAWNCNSQVDPMSFGDIGLLNHTNVPCVLDFLKHRYLKNQIYTTAVPLIVAINPYKDLGNT
TNEWIRRYRDTADHTKLPPHVFTCAREALSNLHGVNKSQTIIVSGESGAGKTEATKQIMRYFASSKSGNMDLR
IQTAIMAANPVLEAFGNAKTIRNNNSSRFGRFMQLVISHEGGIRYGSVVAFLLEKSRIITQDDNERSYHIFYQ
FLKGANSTMKSKFGLKGVTEYKLLNPNSTEVSGVDDVKDFEEVIESLKNMELSESDIEVIFSIVAGILTLGNV
RLIEKQEAGLSDAAAIMDEDMSVFNKACELMYLDPELIKREILIKVTVAGGTKIEGRWNKNDAEVLKSSLCKA
MYEKLFLWIIRHLNSRIEPEGGFKTFMGMLDIFGFEVFKNNSLEQLFINITNEMLQKNFVDIVFERESKLYKD
EGISTAELKYTSNKEVINVLCEKGKSVLSYLEDQCLAPGGTDEKFVSSCATNLKENNKFTPAKVASNKNFIIQ
HTIGPIQYCAESFLLKNKDVLRGDLVEVIKDSPNPIVQQLFEGQVIEKGKIAKGSLIGSQFLNQLTSLMNLIN
STEPHFIRCIKPNENKKPLEWCEPKILIQLHALSILEALVLRQLGYSYRRTFEEFLYQYKFVDIAAAEDSSVE
NQNKCVNILKLSGLSESMYKIGKSMVFLKQEGAKILTKIQREKLVEWENCVSVIEAAILKHKYKQKVNKNIPS
LLRVQAHIRKKMVAQ*
>N.MAL13P1.213
MYIREQDEDISTEPVVYICGECGIDTVIPPNASLRCKNCGSKIFFKKRSRRVMQYEAR*
>N.PF13_0234
MVEKSSDYDLAEKMEDMKKIVIEEVRRNLIYKKPIGPIMSSKDILTLSQEQESKFNEEVHELGLHVNSIHHNS
TPAFLYEMALKYEGNSFITSTGALCCISGEKTGRSPSDKRIVQEKSSEDDIWWGNVNIPIKEKSYEINKSRAI
DYLNLQPNLYVIDAYAGWDERCRIKVRVITSRAYHALYMLNMLIPPKNAEEIQNFVPDFIIYNAGEFPSNRLT
DGMSSKTSVILNFGSMNMVILGTQYAGEMKKGILTLFMYKMPKEGKLPLHSSCNIGKKNDVTLFFGLSGTGKT
TLSADANRYLIGDDEHVWTDDGIFNIEGGCYAKCKGLSKRQEPEIYKAIKFGAILENVVMDPVTREVDYNNCT
ITENTRCAYPLSYIENAKIPAYIHTHPQNIILLTCDAFGVIPPLCKLDVYQMMYHFVSGYTSKMAGTEDNILK
PTATFSSCYAAPFLALHPMIYAQMLADKYQKHKPNVWLLNTGWIYGSYGSDNGIRIPLKYTRLLVDYIHENKL
NNIQYKKTPIFNFNIPEHLEGIPDEVIDPLIGWKDKEDYLTNLQILAKEFINNFSLYLDKAGPEILSGGPNL*
>N.PF13_0235
MQNKSEFNSTNSKEFYQDEYDNVPCHRNEYINNQHKNVDYTNNNYVHEMSLINNTENLQNSNNSNLISVKNEL
TQIYNNFPFTYEDVDITCKEKKNDQIKMYEKENVNIYNEQNEQNEQNDQNDQNEQNEQYEQNEQNEQNEQYEQ
NEQNEQSEQNNPFFLGSYKKENDIIPCDNNIIPCNNESYNLHINKRSTNEPLPKDEYLYNDKNYEISNKNNNN
NNNNNNNNNNNNYSNNNYSNNNFSNNLYVNKHDMVNYYMKDNNEKLLNPSNFNNIYHHNEYPDNNNNCNVNSF
LKNNVGENNKYNNYIVHDNMFDNINNVNCVNNNIHSESYNKPTIYKNVMNYHNKNNYDMVTYSNNNCNISNNN
YEDIISPYNTYNDANCKFQNFNNYNMPMHDNYSIKKLYHNNNFYYNNNINNNHYQNNYHHNNIYNNIDTAQNI

NIPFLNNYNENINTGLYNNMINNNMNTQYNATPMLNQMFLNYNNDEHVKNIHNENNLSSYKNIPIVQNNTFIN
NEESHFIEDKKLYYNNMIDSNNMDILNYKHPIYNNMNNSVQRKEKNVKTIPSYMNNLSDQHISDKDNIVWKQT
YNNINIVGDKKENIDEIYDMNINISALNKEKSNINLEKYKTDCYNSNMMGPINMDNKMNGENIMSGDNKMNGG
NIMSGDNIMSGENIMNGENIMNGDNKMNGENIMSDDNNYNSPWDVNFPNSLYNNNCISNKSKYTNRSNSYELS
LFKDNKKNNINSNSNSNNNNNNNNSNNNKYDDMLNKKMLYSIYLQSLLNNLNNEIYNDNEHDDDISTSVFYKDY
LKRNNINNQLILYNKKRNISNRFFNNIPNINNYFSYLNYYIESLRLLYNKLLRNRNLILILAKEIFSKNKKLD
KTKKNIYTSNNEKYSYIRQLLSVLLPQPPIYPPFEIWFFMSKKCASQLHKLHLTIYMVYQKIIYNFSNILLQI
NNDMNITKPNKIQKDINESNDYLNLYDHNENEKKNIKYDDKHEESYNQRLYNLKKTNLDTNQNDSNSQIQLDK
PIISNTLPTTDEKMIISLNSHNNDDRIINNMDTNMLIQINKKLEYIITSINNINKIIMNKKNVQNILQGHHNI
NEKMYNMHPINPIQTSNLRTFESISDNIISCDDENNINIIEDEENFKNTKENKNETTNEKTNETTNEKTNETT
NETTNETTNENTNDIDYINIDKELSNNISTNKQIHINNNQIIAYNNLEYNSLFNKWNNIKSDIHNYIYEENPF
NILDNIKYELKDVYNQMMNLKKTDNILTINDKLLHNLLKLDNSTHQMDIPNISYDNKWKGSSNNHNNDLNNNN
NNYYHNYYLNNNNFNENKNVLEELLSNNNCFNRINNQFKNKDNFKEPNLMTHNDIADDISICSNEDVNDIIYN
INRTDDILKRLMYLSNNYYNNMIEYDNYILHSYQHNDHILNELNNNTVTYYNNIKKKKNFNNQLLPRTKRNED
FIFNNNHIVTPNNIYNNNNNNNKLADSEIMENVMQTCNQNNYQFNRTESNDNLKNIPQENNFLLPNSNMEKFG
ELINVDMNNIENNINMFNNMYNNVNNNINGNNINGNNINGNNIIGNNINGNNINGNNINGNNINNNNINSNNI
NSNNINSNNINSNNINSNIYNNMYNMNYKNNVISSNNNYINNINNEQHSNPMNYNTYGYDLSNNVNHLFYNNN
IMDGRYNISNNNIIQNENIKGIYPHIYKSNHMVNVETNNFDIYDNVPNKYTSMDNKDFVGEYISDREERYYDI
NILNDENNINKNIINNNINDMNVYDNNSIHSNNNNNFDYIKNIDNNNNNINDRVFDSRNSYECSFPLLTNGPY
YYENENNTSSNILCNQDVNLTNNNFINYESNISNNYNQLISDNNNEGNINMNKNFNVFNNNVLINNCTINDNN
VDNNKMIDSNMIDGNMIDSNMIDSNMNGNNMNENNIIENNMNGNNIIENNMNGNNIIDNNMIDNNLIDNNLID
NNMIDNNMMDNNMYDHINNFTINSPMIDMNSFEEDPKNYNMIHINETDIINLTNEDLINNSENYYKHDNTSSY
LLNYQNCKNFELKNVSNVEIEQTNIMDSNNTYNKITEENETNSSHILLEEKINKTNDMCKNDTEINDHSLAIL
LKKNPSEKKKIMNNHHNNNKKNNKKKKDDLHEHNNKEKLQNCHNEYLDSNDYNSDNNTNKDNVFVINNIKNT
NCNINQNNDIISKNVDNSSKNNDISNEKDNINKMKLKKMLEITNKLIGKKYRGICYDPTRNGWSTFVYKDGVR
YKKFFSSFKYGNLLAKKKCIEWRLKNLNPSSHAYSFSLKAKEEFNEVLNDNYKDVGILYDNKKDSNNNNNIKN
NGNQDDDGNRDILYVNAFLNIYNDENVKEKCFTDDNDNVKDNEKNTKEIKIIKNKIPPPKKKTVQNIRTNEKC
NEEKEIYCGDNTNFNVTNNHNNNNNSNNNSNNNSNGNNSNGNNSNGNNSNMLDERQHSYYNDTINEKKKKIIN
KNTINILHDDIEQTYSKNNNLCDNNLSNLLNEKKNNALNTGKRKNDRENYTNNHMNTDSFLLNNSGDNLMDKN
KNCKLFSNTLENDETYSCDTQNVPDDVLKNNEYKRKKMCTVSEAKIIKDTNDFVDMMYTLVERNNTFDNNSDL
KKDQMDNKKEQEDVEKDVKENEKNDIKEYDEHKDVHVDVEEGLKKTTRNHLININNCDTYNHENNNNQNYLIH
NSTNNTKTKNKSCIDNIDLTLNNINCEIPLIEDNYLYDKDKIMNDINTYENIEDINGKHHNSCNFMNVDYKKD
KHVTSYNDNLKNNEEDIFFNNSNKNNFVLHNNNSNKIKNDENYFAYKDKLLKYMNTCKYTNDEEKHIFLQFLK
IKTEWVLVELNNLEEKYHHYFRNKIESFYKNYLLKVKNNKKQNENIKELEIIFENKLNTPWMCMIFPYYFISY
FNYKIFCILKCTKKKRVRKNTNLIKDKILTSKLKGVNFIKYKKAWCFTYVDVDDKKKKKIFPVNDYGFVESKA
LSILFRKSFFFHLNKIHNFLKNILYNKNNISNTMINQGNTYNNINEYIDHYKKYEEFLVCYGKIIYFNEMKNI
YICSENKQNALNYMPKEIKRQICNKMCSHKEYNMDNSMDNSMDNNIDNNIDNNIDNNIDNNIDNNIDNNIDNN
LCKNLCNKIDPINLLITTRYYFFKKSKIMNFPKGLVYLSGYFLWLVAFLNNEKQEIIISFSVRKYSYDIAKAK
CLECYYCLLHKYKFKPLNISGVIDIVLESDIECKNYNLLDYSSEDIMSLEYLFLYFSPSSYVLHNNTLYKKIS
RKNQELHKQYDKLFPGQYVHLNTYQTYEITNNDLIDNNIKCANAEMVSNIYNDKLYQDDKNKFISPRNEQNIL
GTMIYNNQEEYHDVNMDQINKNKEEPITNITKNISNDLSPLIYDHQNITYKKNDKKSIPLCFSDDEEAKEIND
KIKKKKDINLNCMSNLHNEISKKSANINITNHNINVQMNDSMNGHLVDEKIHMDNKNNTNKIDNNINNIHNNN
NSCSSCCFYVKHVNRESDNGNSENLFTSKTFQDTYKQTYINNDYYKNILNKNICFFFDNNIQDIYLKKYYDEL
FNEKSNEDENIKNEHILFKRIDKKEENIGIFILLNCQWLSDSFVNNVNQIETKYADIYSFKNYLNTCEEVYNW
KHKKNFIKDCAEISKKFPRIVGVHYDSYATAWVVNCSFNKKRHDKKFSVKTFGFLQARKLAIEYRERWIQLKA
FHRLNNLKKKTEKL*
>N.MAL13P1.214
MTLIENLNSDKTFLENNQYTDEGVKVYEFIFGENYISSGGLEATKKILSDIELNENSKVLDIGSGLGGGCMYI
NEKYGAHTHGIDICSNIVNMANERVSGNNKIIFEANDILTKEFPENNFDLIYSRDAILHLSLENKNKLFQKCY
KWLKPTGTLLITDYCATEKENWDDEFKEYVKQRKYTLITVEEYADTLTACNFKNVVSKDLSDYWNQLLEVEHK
YLHENKEEFLKLFSEKKFISLDDGWSRKIKDSKRKMQRWGYFKATKN*
>N.MAL13P1.215
MRKEEMNRNFQNASCKEDNYIKAWLKQTENPEFYKNLNDLDDQSYTNEFVKVVDFNNFNSNFINNMMEEPFGD
EFSKQKKRKAYSLFYSNEIGPNDEENYDLKNIYTSFDDSDVKYLKSLDKTPSGNMKDRVKGRYKDEHMSIKKL

KRENICNENNEPIFPNNIMMDNSPYYKFRHSNKKPSYGSESTNEFFNNYDFETPIKILSSKKKSYDFKNRKDN
STNTTKENSSRENVSSNNSETKRTINFSNKTDDFNDIPFNSDNEYISSPEYMDHEQNTKYINKKQREVNFLGQ
KLEYKVVGNVINCSKNHPQFTLF*
>N.PF13_0236
MPYLIWFSSHNKYDDCKISELNSLLEIYGYGKEYDKKGKEKGKEKENDREKKEEYNMNQIELFETVKKSCDRK
NREWEEKFKISRMITNKFRNEVFLKINISEEDLWVNVISRSVLIKGVIELWSEGNSYNEILKELLLKKDLFEK
TLKDKKWCFCFNSYGKIINQEEKVDKMNFFKILLEPYTNIDLMNPQVQIGLIEEYEQESNHSHILKRVYFGKC
IALRRNQYMNNINKLKNDHKIIGKPKIAWWTSYALNKRPILGPTTTDNDLAFIMCNIAKIKKGDIVLDPFVGS
GGLLITSSIFGAICIGNDIDIRLLKGYKLSYLNPHMKHKSNNKSIFENFLYYNLNIPEIIVSDNSNPTWNFFH
KPWVDAIITDPPYGNRATVRICVNNNVEMNNVQNRDILPICVENEQSINEYNNNNLCSSEEKLKKKEKNISNA
KTITYNCTSAVKDLLNIASNVLVDNGMLVFLFPVQLDTIQEEINILKHSDFYLISYDLQTFTPITGRLIVSMQ
RKARKV*
>N.PF13_0237
MNNIKDKRIKKQIQKRTKLYNTKKEEKNMEISSTKLKEIRMNAQSDIIDDDMLTPRAFDRTKKSMGAKGFVMN
ISENNESKNEDLKKDEIINNNLNNKMLKNNQSNENNGFNNNNDNNNDNNNNNNNNRYNGILNNDERHNYEYNH
NKNMMSPNKRNIPDCNNSTTDNSRYINCDNDNYNYSMKQNVSSFMNAGWQTPKKSNVGSYYTGIDHPSTLKKY
KTPCEKDMLEPDESLIGNKCITINNYVNPPKYINNLVTPSKFPLSGENDDFMSYEDFKGLFKHNACLISEYEY
NNDKSSTSLLDNCSKQYLEELNDNNQNNAWPKKKDNYSSPQKSNTILQCSNNIISSNNNNNNNNNNNYGNFNN
NKDSPRSKKKRFDAHKNISTRKILNIVVSNGKRKEDHNEDSYNNSIHNNIMKKRKVNSSSEKSDPCDLDSIDY
EVNPVLKTDIKKDKNNNLEIKNDVAKNTNVEIDNSAEKNKEGEKNKHVQININNNKIALNDDESNNKSVNEMI
KEKHNPLKIKYEGYSSDLNVIFNKETGTPYQLIAMYRNLLVVISRRNQKNEPVFFQIVKPEVRVLNNSISFTQ
ELIKQLAWIAPNLIQLNKVTITKEVFESNQRYYPEFVTGKKIEDIQIREHTDNCDNLYKYSNEEKLEMLKRII
INWVNKKHNEFLENLNPNFYLAKYSQLKKWHSNFNYEEIVFPSVTLEENKVLAHLARTPQESNADDFIVIQDS
PIKCATNDLRKNGTFLQDMEKEKTRTPLGKNRRRGSTYLGAIGAYNNSNNNNNQTEDSPSKKKETKNMKNIRE
EANKKKIIKEIKMEIDKEFDELLDQKKKLENALWIAEIIHDSFIVQGTHTVIEIQTFAKKLSDKYKSNKDFLM
DQNYIAQIIELLPQYINDIHIKPGMMNKNKKNLSVKSKKNLYNFLEPLSNEINTLNKKYQELKNNKENKLIDL
WKNYNVQYELTENIKKYFSNPGSVSLADI*
>N.MAL13P1.216
MTSSNYFKNDSKVYKESDKISNYNYDSDVIEIISLNDNDTINSNTQNSSYFSKDVKKNIEYNKNKEMSSDIEI
IEDIGNYKYEKIKLEKLKEHQLNSKKAEFIRNKGVYFEYYLGCIETESIILSSEVCEIKDDKNVIISYEISPK
STKRSYSPMYTLKINNNYDISNDYIRIIGRNIPMNNKKVKKKKVSNNKKKNSASSNDGYNEQNYDTNKIISAD
QKFVEYDKCKSEVLKDIYEGYSCIRIKHNNRDISKLKSNLSKTLSVLLVLNAIRIEIKLEKISQSELKFGGSL
KTFVHVILYPDAFKIDSHKFHEYNSLIKYSVNNMFKELKIIPLRLAKNIDEETLETYNVNKDELLKSVSPLKS
IIINYNNKNDQVMNKSGETSTGFCTPHKALGYIESKKTIDITNLSGTKEEEEIIDSLSENEIEEKNEEATNLV
FVEEKFRGGYLLEDFEEIYPNEYYFKPMLKTYQAEGVWWMYTRENPPEYTKKLERQKSNINEIIIDDVIKIPT
QKNDIVIKSREEIYKNVNNQRKVINIVDITNICKNENYEQKFKGKLENDKLEIKDENIKCDLKVKGETENFET
NIKNEHLYIKTEGKEEISNDTIKCKETMDNVKLEESSDYENADDDIRNREPLNPLWEEHAFIPNIKIYEKDKL
VCILKYFYVNKLTGAFSLTYPQFVPPFRGGILADEMGLGKTIQSIGLITHDIYHNKLYSKNNNLENKKNFTYL
IENTIKGVHFKRGGTLVIAPLALIYQWKEEIERHTKEGFVTCYIYYGTSKDISSEELSGYSVVLTTYSTLVSE
YKNTLNKKFSNKAENNNKSTYTNKESFFKDVSSSTTKKKMNNFFMKTVLNSGNKNNDNIFFFDKKKTNTMLYN
MKEYPLYNITWRRIIIDEAHVIKNKNSIQSVAVWKLRGERNWCLTGTPIQNSIFDIFPLFRFLGIKPYGTIEW
WNKEIVDYVNKNKLNLALDVVRKISSPILLRRTKKSKTKNGNSIIISLPKKNIHLEKLEFSLEEEDFYRAIFYR
SKTKFDTYMHDGNVLSHYSHVLQLLLRLRQCCSHPLLLFSKPFFEEWNKDDMNDALKNKTDKEDNGDDLKKSE
YMNNTDVKKVFKSENNKMDEEIEYNNPSFSKVDHSIENNIGNNLIYNFILGSTKSKNIDDDYAKELEILKNGN
VMQCCICLEDATYPLISKCLHIMCKKCADDYFHLTQIADKKCPQCDNYISLKSLKTLQENKSPLDELLKKMKK
DNFVYSTKLKKLFEHVQNDMQNELHIVVFSQWIGFLKIIEKLLNLYNIANKIYDGSLTFEQRKATLNWFNIQK
GKIYQPGVGFIKPSYVIPVENFAGKVLLCSLKAGGVGLNLTVSSKVYLMDLWWNPAIEDQAFERVHRIGQLKE
VNIYKFVLEKTVEERILQIHQSKQYTANQILTQEGNKLNTEMNMVPQKLGMDDFILMFKDWNTDE*
>N.MAL13P1.217
MCNLFLNRKIILCTLYLVCLKFGHVLNNGIVKHIHFYKNVNRDEKKRKNLYRQPLYILKYKIYNKKKKFKLNN
WYNCDKKRPERKLEPYELNIRNDKKFPKYSKKPVSPSSNLNNFSPFKNIINSFQPVDHNIKNKLREEGFQFES
ERKETNMFPSLDEIIKNGDPSTNMHFYRRKKLKSLLYSDPLKFVKGRKVVEKYNRSVPEEKRLNYDMFRCDIF
YVHSDGTIMNIEEDRPPFDKKLEESDRYKEPRYIISTAXXXXXXXXXXLVISSL*
>N.MAL13P1.218

MQKVFELLEQYNQDLLLEYFKKFPSFDFNNLNANDLEHFLQNLNKIKNEDKEKKEYLISPPEIIDIDSIYNCS
KPPNGSIFINTYKKENLCNELNEIGIEIIKKNQVSVLFLAGGLGSRLGLNKPKVLLEVTPLTNKTFLQFFFEQ
IIFLQEYCSLYEKKEHLNKINDNHKLVNSDNFKKGINNNIHINKNNDMYSNNNNNNNNIDYNKSEHSKSSITN
LNNIKGTNYNKCKINLDKHQFKNQEKKETLIYIYIMTSNFTHDNIVTYLQNNNFFGLKKEQVIFFKQCDNFST
DMNYNLLLSSPEIFLENPGGNGCIFKALDRYNIIDHMIKQNIKYTQIISIDNILNKIADPILIGFSSSFNCDI
ANKAVQREDEESMGVFCLKEKVKNKINKKYNKKNKDNIFKNDNNTFSVCEYTELNECILNNKELFKYGNICHH
IITVDFLKHIVKNRIYNKLKLHKIIRKKQYTDIPSLINDNNEHLINSKVFCYEYFIFDIFKYARNILSLEVNR
QKEFYPIKNKNNEYGILNAQKALSNLHKSWLQYKNINIIDNKDEEKNFCEISPLVSYDGTFFFELPQKRDINL
PYILQRNINS*
>N.PF13_0238
MEGEKVKTKANSISNFSMTYDRESGGNSNSDDKSGSSSENDSNSFMNLTSDKNEKTENNSFLLNNSSYGNVKD
SLLESIDMSVLDSNFDSKKDFLPSNLSRTFNNMSKDNIGNKYLNKLLNKKKDTITNENNNINHNNNNNNNNLT
ANNITNNLINNNMNSPSIMNTNKKENFLDAANLINDDSGLNNLKKFSTVNNVNDTYEKKIIETELSDASDFEN
MVGDLRITFINWLKKTQMNFIREKDKLFKDKKELEMERVRLYKELENRKNIEEQKLHDERKKLDIDISNGYKQ
IKKEKEEHRKRFDEERLRFLQEIDKIKLVLYLEKEKYYQEYKNFENDKKKIVDANIATETMIDINVGGAIFET
SRHTLTQQKDSFIEKLLSGRHHVTRDKQGRIFLDRDSELFRIILNFLRNPLTIPIPKDLSESEALLKEAEFYG
IKFLPFPLVFCIGGFDGVEYLNSMELLDISQQCWRMCTPMSTKKAYFGSAVLNNFLYVFGGNNYDYKALFETE
VYDRLRDVWYVSSNLNIPRRNNCGVTSNGRIYCIGGYDGSSIIPNVEAYDHRMKAWVEVAPLNTPRSSAMCVA
FDNKIYVIGGTNGERLNSIEVYEEKMNKWEQFPYALLEARSSGAAFNYLNQIYVVGGIDNEHNILDSVEQYQP
FNKRWQFLNGVPEKKMNFGAATLSDSYIITGGENGEVLNSCHFFSPDTNEWQLGPSLLVPRFGHSVLIANI*
>N.MAL13P1.380
MFQKFFSDILYKILGNFIYGLNDEELALSDLLSGKLELTNIHLKQSVIDLIDIPCRLNFGCVGYFKINLPLLY
FMKNPINIYMEDVIIVLSTIPSKYFDDKLYKEKYIENKKNLLLSSEYRAIVCSAEGGVIWQMILSLINNINIT
IKNVHIRIEDFTSNPSNCFSLGIKIEELFLNMPKEGLPFNRDGYYTKKKELVSNTMINMITVKRLAFYMDSLD
MKKITNKKYFNKFRDIFINYCNMDERNYPEINMKSNYTNDSKNIKKVHMNCEIQNMNNFKRNRKNKKNKSKHN
NHKNNNKINNNTKKLFKKKKNKRSNQQNLESFLNIIKSIKKSSYYVCKNDNNINKMDKINQRILLLNDTISAK
KKKNKIINNNIKMKNTHNEQQNKDNNCSISISNFYKFNPFSSKKEEKVDMPILIDDIKLDNDISWIACITLFA
DSLENSFIKEKKEELLKKQRNKKKNNTNDKNAQPEKDDDGFYQTCKTIDENKNIKDHNHSFNNKLNKNNEQIK
SNNYEKEKMGSHTFIDKDNNNNNKNNPSFNSDISNTRKLTSNYYLNKAVSFLSMKSFTKIFQKKKLSKNMHRH
KNKENDFSSNDQNISDDYFTTKEKMSDIDFNSEDIYSSKYKKKRKNEMKKKDFNFKKNGYSKYEKKDEEKYYD
VKDKNQSDEHISTFHHSKNTDKNYFSFSDNDKNNKKYMSQNKKKGKKKTYIEKQEDYGKNYKYKTRNEYDKTN
NESYKNQNFNDSAHNLISNYEKCIQLNDKRKEIKMEQYRIMNYIKKNEIKEKIRNKKRKKIHSVYLMYEKTYK
INKSKLNYFYIFLRLTKNIKHEYIINPNYFEGYVEIFLSLIPTDHGERPFSRCFSSWGIQRRSQNEFDECLPK
ISVFVTLKNMKIIFSDKQMSNLIKWLNLNFVTYSTWKAGILSQFEKPKATPNEEMCYINKWVLKLLDTHSSKE
EKEDAEKYCELFENTHSIHIINLLRTKAFSKLQELRKDMETKMKPNNNNNNNNNTYDMVNDTNQYNEIGKKE
NEVKQVANESINVLKNLMKNEKAFNKIKTSFRDKNFTIDFCFDICIINSTILLTIETFNKINTRKVNFVKTYA
LLLQCIHYHNQISNTQEFKNSMDIELYPLTLLLIINNLDNKYIKPEINVLYILTKSDKKDKYKYKDKKFLFNM
ENFDILKRYKYIRNTYLYRNDNQKNSNEDRQKIEKEQELYNHENENENNHNKQRDTLKLHNLKQKFKYLMEEN
FFFSKRQNNMYCNELCDITSLSDPSIYLRYDSQSYTILEKPDCHLFLHNCAYSIFNINGEIANSFVNDYFHFV
DNRSVLFKRKTNNFSFKKKEYFLELGAKYCNDIMDNYTKHTSFFIDIELEKMLSFIITKSGQKAEIQGYSLNL
NPVKVVSQLSKRSESYNIDNSKKNIYDSFFVQFEGINIKRILNWHEAAKHYKTYIDDNFIKPFHVLFHPINKD
IYTNRNQTPDKFFFNLNRKKNIPQLNENGNSNSNGNSNGNSNGNSNGNSNGNSNGNSNGNSNKNSNKNSNENS
NENSNKDNKISYQNDNIYNNNSNNMHKRSSSYKNLNNETINYNNDEKKKSEIYNLIYNTRKSLSSYTHSESVS
KNHIYDNNKNIYIKKKKMNNKKKKSKNKKNKKTKLNNIRSSSCPPPKNINIYFDSSNKSDILYNDRKTTLYSL
NYMYEQNRQSTITYNNCDIGCKACDVSGNCCNISNICCCNFTRDYYNSYLLKSWGNKCEHKKKNITHSDNDLY
KKYEKQWNDITNCTIYDNNDIDYNKKKNNNDNSNYVLSMSGNTFFTIHLCHIKFNPTHPMFIVKLNEEKLCIN
ICDYDMEFFFLIFSQIFDHYSQQELACKKKNIIFLKNKINSISHLNDKYYYNTYHKNEVKKLGISKDITNELY
TIAKHPSHMYDGEFINLKKKNKKLKKQKRPKKQKIKNKKYKHKNDDLINNHEIKSINGKRKHDELEQNEVNFS
ILKKTNKRNYSLQGQRNKEDIKKYNMINTQDTNELPIDIKEKEYKETRKEMKTKSKTFCNKSNSYRKNTFVNY
SSDNNTRTTKELSLFKLKNDIFKYRQSAEKKYEDKNINVDQYINKKDTKTDILLYNKKDDVSLKSKKLLFPNL
SIDRIKNSIKFKNSLDSTYVSNGSQYSDTIYVHKTEYKNGNKNKNEHVNEHINKNEHKRKKNKQKKKKNKLKN
KKKHINKTKHNTNILYQKYQMFDETCNSNNMQYGYHNDVLIPHDNYSFSSDDDSWVDLLENYDSDESLDDYEK
RNVKNMLIDIDNIKKRFSNNNFIITCSFSFSIKNVFIRLWKRKSPMKNQNFKTTEWENLNSVQLLDNISNIEY
NKDNRNDNIYKSKTKNIKTEYDEYKLLDKNSYYNLTSVVKENEINRFHKKERHNIIEDDDNISLCNKNFIILN

EHLQKNMFNNIQYGMPLYTIILSDIFFVAKVEKNNCAHVLYSMNNIDIKDETNITLLSMNSIYHSDLKSMEKK
TFPNSYKYKNNIPDNIYINKRKKNSSMNSKGDMEKIETEDTFFSFDYSINNLDLPDLEKTHLVLMYFGSLFKN
NHTFKFILNKTKLKIFWEVVDEFLSWIINTSDLFPQLNTIQYDIIKKLDDSENKHLDYLHKIQKINYDILNVK
KLSSSFSTNVGFKIPFHILINHYNINYEIYSGRKNLGNYNENMLDFYNVDVEPIEEYYIITNEQTCRSYNNVC
FMNNPEDMNNMIKEILFNENYFFNYTFNKLNKNKNISDILYKGNNTNQNVVNFYNDISNDNVIINNNNNNNNI
QNCNLNNNNIDNYNLENQKSYIKIESSFLKDNNVTIKSNEELLSNEYLFEPSTNNAKIINENNISIDNLYEEN
ECSDCFHMMNLNYTSYTKELCKTKNYPYIHVDIEINYFEFWISLDPIRIPQKRKRHCGMYMNKKLYYHERRKE
RQKKNRKRDKRKHTIINNNRFNKEKSFCTYYSTSKVLDEEKLNNTTKLCKKIKYYKNLNTDKPYVLATKGCLK
SVLFILLYNRQEINNEVDNTYEFINDDSSYDPLYDYLLNNEKDKIKGNNYYISKMSSYNSENYIKSQIKNKER
QSNHNLKNKLMEVLQYSNLNNIINIEEFLNSKFICFLNNMSSYLPCVVNMNIFLSQITSAIAKPVTKNSFLKA
YIDWNNVPYNNILLQPFRANFQFELKLPNPEILLERLLGINKITSLKVMKNNNIIYKNEAEGIDINCSFEPIV
LNVDSNTLTLLNQLYNIIFDFSNYILKLCAPKNKDANELLMINSFNDNLYDNFTEYMMSDDFYFNLNESSLLS
TSLSRSTSLSLSSFELNNKYKLKNIQSNNELLYTSKNTHKFQMNNEDDDNMLVNFKLNYNNHNPVNKINNIDE
NIIPIDAEAPMVEHIEQSELQVKKNNQEKVYSETEKNKEGFDGVSIHDEKKKLNVSDRNIPTEDNKKRSLNKP
LILHHKYNTFMNNTNEPILNTDCNMKANNQTYLLESDLSNILLFNFVENVKVSVHLNFDVILLQIWDSNTAYN
RCSFNFVIEYINIRFNTTNIYEMNLQNDKLELYNIKENNTYDEISKNKKDPNDNINYRNKNENFDQSNRTLLQ
RVSETKNIQDSNIDKSHLIHSGITNDACSNMNSDINKKKYTYGKSAVNFLTNENQLYVNNIKKKYRSVAGDIN
INMNKETNDNMLTKKKELDYFESIDNNMMNCNIEKDKILDLSRKEKIDIYNSTKENTKIVNSNLENNINNNSN
YSNNKNLFSKLFNRNNFVKNLKQFKEEIMKIVKKLFCRKKLDEILEIIEEKGNELETENEMLGRRKKHEHFKN
IFLKNELMSNDLKKNNDKKKSKDIECTIEFLIYCENFNKKENSYQTFIEPICTEIIIMKKELNTPIHIYYYFS
WININWNFNFLDNILSLCCSIIFSMITQRTRLLLGRHMNDIDKRKNRKKIIQGHTKNEKMEDGSLKNPHVNTN
TTHPTEYDDNNTERKNLSEIDDTQTSMYLPNYYGALNDEQYKDLALITNENILFRYNKFDVLHTVNKSLDTYI
FGELILEKLLRNYQYSCQYPGDYLSKYENEFNVDKVNSPSQQLLHIKLFNYIKKKKRLEMKNICKLNNLLGQP
IAVCTIIEEKKNIQYKNIKMTKSQMKYENNKYNSKEIYNNENTIYDYNKRNILLNSINAITNSNRKKYNSFNP
FEDYKCLQISDMNHKKLGKCQNHSEEFGNSDNSSLNDTKDILKYQWKIISNNESLDLPIHENGKVKFFLIRFR
LLNYIYDIPSNILNTEKENEDVIRLIIPERRLPPNINEQVEQELYNQEILEMEQENDTMDSNSHTIYPTNYLF
KKYVTGKNDTVWCPSNIPQPRNHLFIYFRTSSRISHEEKQEYDFFLSSVVAIKNNTDFPLYIFKSVPGNVNKS
SIFKEYFHKYNTKPSPPPTYTLKVDKKIEYLIENQNKDKNDFSNGNNENEQDNNIEKRNNIFYSTDQIIINNN
NNNNNYNNNYNNNYNNNYNNNYNYCFDPNKDHNSYSTRQHNEKLSKQKTKQNKNKNHKKNNNSNDIYEKKDNK
QGNSKNYSNNHQNNNKDQEEGKKKNINSSNESYLHNRKMIKNIFPLMKLSSMEHYISGDIKFSSLAPLKYKRK
KKKKKKKNKLKYELQKEYIKKKIKELYLKSIWKKTQRKKINKYKNDINKINQKIKEYSYEEHAPYRNIMSIND
PFFNFIKQNSSSSQKLSNNSLNISDVTNITLSSLSSTFFSIHSSQSNNSTYEDSLYDYDKDNCLEQALLDEYF
KKQNDDDVLLDIIEISNNSNKLIPIPLYWLVAENSFIWLSIQNEEIYKNKDLYNFLNEEMNADYLCTKKTLDN
ILNYQSPFVADTLKLFKPYLIKLKNHLLKEDNLFSKNKKDENETFMTNYNTNYMNLKEVYFTSSVVSVYNRNY
VLNRKDAHNFFQISIEHVLMINNGLPKTMYINCEVLKSKLKKKNTIKKNISHYKSFPSIEKSKQYKDSENRED
SYVIESSCNKTNEEYSSYYNDDKNDNDSKYYDDGKHDDDSKYDDNSKYDDNSKYDNYNNNHHNNNNNNNDNNN
FMYNEGSDMFSQNSHEVKRDKGVDTFYDIHDELTLEEKQNMKKKKITLVVDKFNQPLNEDTNKDHIYIVDKYS
DNNRTNNKRVFIKEANIDRNVFIYKNTKKNENDYIDDNEKSKNYMDDNNFIVNIKKEENKDIYEIKINPGQSI
KLPFLLHNITISFDGYHSRNIFINYPHKKGREKIMLERQISPDISSFFTFDYEESNENNDEHSSNKSTMNYVN
NKNISENTNKNYHHFDSSLSLTDKTKNQLTYKNRNHQKNDDYPLSKYSDYHKRKTTKYEEYQKQLSIWAIFND
FYETGKNRYQDISLEKIYHGTYSISCTFFASACIINRLSYPIKLKRLNNENVVVIEPYIRTLLPCEFSMRRNK
VVISHETLKDIKNWKNYPLKKYMIKRTKLIKTRNFIKKDNQIIVHSTKCDDGKQIKHKWDNKQLIRGDKNVSI
KKNKSNEEIILRKQNETTNEINNEKIKSDIIKNDGSYQYDRKEEDHYHVDDNNDDDEIEYNKKNIPYNKNTLC
GYKYKKKRRFRFFFYRNNINKPLESEDFRITDLSVTRLTCSNKNKNIPQLKYSTYMSIAPMPFFRTTVMEILP
YIVIINNTKMNIVFQEFIKNYTYFKYNKLQPGDYVEFHPQSKKKVMLKMCGIYQNTFDHLIDDYLQKELYLEN
FEKKNEKLIDDIKADKHYDKKNMEKKKKYSTNKESKKKKKNNIANNYIWNISKRITALKDMENVYDSNNSAKY
NDNEKNYINNKMKNKNKKNKYKNNKNKKNNNENNENNYSTSSKNSSNSTIENYSEEENSNGPFNSSKHNNIKK
HKYMNEYTFLYWSEVLDLNRVSMIYFRHPVISETNFKIEEKKLQKLNSILRKYGKNDDAYINILKELIKKNNQ
ITYKYSCCSMEITTFKGCKFVRFQDIDVVPYYLINLTKYNIKLRQVGIYEHSEILQKIPHKKKTSFNEIFKNY
AFNFSFYNPYKDPKLKVTLLSNNKKNDYYNNNSKYKKEKKRTLLKKIPNKLKHNLKHELNYLSFLTKNYITAK
EDAKILNDKIFQLKRFSQQIEKLNDEKMNIIDLGNNNNITLLKLNYKNSLIYILCTKRTYNGKTIYLFENYDD
LISNILNFNTISFNSKCNSKLLKSFSLFRDTIKSKNKYSRKDTYKKKKKHIKYMKTFIHNPNVKYLLNFFKKK
SSASSIENKLKKNIKQKKKNKINKVYKNSRTNKRTTHIITELYQKNMFNQEEETKKKNKNIILNMNIFAHLIF
KGIGISISNHNLEEILYFSMEYILIIYKQMIDRDLFHINIGWLQIDNHTKNTDYKNVLLPIVDLQKQTNKNNA

KEINEEKKYFINESNIALYNSISNNKRDDKFNKINKHNTFLHNRYDTISGHIISGDINSRNLKSNNFNSFNTF
SRNINSCNTFSCNINSCNINSCNINSCNINSCNTFSRDTFPYSFRYSNNFSKSSTLNMINSFYSNRNKSYNSF
IDNDVLIVTNLNNPLHNSYKVFIPQFFYSEYNSVIYISIIKLKRGNMKNFLELSDVQIKVSPLSLNIDSYFII
EMLKIFDEFLKIIEFDLSNYNSLNLEKNIELTDRDFDSLEYKKDIGPIEELIKYHILKDYYLYEQIINFKFNQ
KVDGIRNQTNNKSHNYKELENYNKIKKNSQDSIFNTNNLYDKSNLDVIEFKEYINMLNEYENIKKNKAISKDI
RNKDMLSKMHCTKNNILKKGCTKKSRRTKNIKFYNKNDDDNNNKVNDNKKDYKIDTQINEYIKDNNNNNNNN
NNNTDNKQYINIKKNIININKSCLINNKENMYYDTKKYSDIDKKKKSDDNEDTQVELLDKEKIEYIQRCDKYY
KNSLNNRKKILRKIQNINIKNNLISFDKIFISKLEINEMKLIINIKNKSLSYDKKSEIMESNVMNALVVLIMN
IPNISDAQLHFDKEVKKNICGSLYELIFNEIMNDYITPGMNQMVNVLGAIDFIGNPLIIYKHWKKGYETFIHD
LKKSLDSCSFPLLFCILFLNTLGKFGKSVLSGILEGINRLCSSWSTCFERFSRNSDNYSIVTNSNLFQHEILD
QPSNCAEGIYYGCQSFINILTISFVNIIYKPFIAIQKSKEFKKKTEKGKYKIFFLSLIVLSYGIFSGLSSFFF
GFFNSVFSFFTFLFIGTLNQIQTLTMKSVVRPKKMSIIKEYSKFLNYEYPLSFSSHVINEKKRKKELLKKNIV
AIILLYTINDNTPNNIKSYLWVSNNELGYCQKDKLLWSLYINCIIKVDILLIEMDNGNFKRYSISSRKENKYL
NNMNNKEKNDANKHTKNSMNNNSNNNNNNNYNINEKLTWNEKNKEQNNQINNNRSTWKIKNLFTPQPFRQLSD
FKPSYYIRILYKSLYKNKKEKRKTNISNLFLYKKSDSNNRLKRIMRKSTYEKYFENEFKEEQKVHRYHTTSKD
MPEEPSAKISHINDERKKREKKNNKINIYDNLYPDKTKNIKHHNTKHYKKETKKKHLNKNKNNITSYIDDQNN
HMKLWKQNKKHKTKNYKYNNEHNTEYEHVNIKKLFKYNNHKKKKNKYLYISKLVKCENKEVALHAFSLLMSFL
MHKSPYYLKTTN*
>N.MAL13P1.385
MKIGDVLHDYKLYDNTKKKSSEMVINENDNKERLLEEFEIRSKVRKVCLGIPTQDIDVKNILRLLKEPICLFG
EDSYDKRKRLKNILITKYDRLIIKKKIEEEDDVEEFKNILKRYYIDFSDLYPSGLSEANKINEVHDKHKLKDV
HDTKEEQNVHMKTVREEDKDILKEKCYTEGTKDLKKSRIEITIKTLPRIFLYKEMINKFQNGYSKKEYENYIT
SFNEHIKKESDLYVSQLGDDRPLTMGKFSPDNSVFAISSFNSYINIFNYRNDDYNLIKTLKNGHEEKINCIEW
NYPNNYSYYSTMNYKDLSKHDLLLASCSSDKSFCIWKPFYDEYDDTNDNINDNINENINENINENINENINEN
INDNISDNTSDTISDNINDNINDNISDSISDNISDNKNNNSHKVDKYNSNKNKLSAQNKNYLLCKVNAHDDRI
NKICFHPLNKYVLTCSDDETIKMFDIETQQELFYQEGHNTTVYSIAFNPYGNLYISGDSKGGLMLWDIRTGKN
VEQIKMAHNNSIMNINFNPFLANMFCTCSTDNTIKIFDLRKFQISCNILAHNKIVTDALFEPTYGRYIVSSSF
DTFIKIWDSVNFYCTKILCNNNNKVRNVDIAPDGSFISSTSFDRTWKLYKNKEYTQDNILSHFM*
>N.MAL13P1.390
MQKYISYFANEFCHYKIKDGSSLFLQFSFPSFLDELFIQKSNRKFGTYGAVTRRRQELRQKFYEEKYKHNPNN
IPKYSKVKKGSRGGWIKNPLKEVTKNCGNNFKYEEKIKEEILSEEEECIKNMIKMRKELMNKSNIDKDYNELI
NNDSKNINISNNILNFNLFSICDNFIDKKKEIIHKIESPQYFVDKHIKTLVDTYSNINELHENYNANNNYPKT
PDYLKDIHDKKKKKKKQTNQKNGSGKEIENNFINVKMEHNCIMDKNEKEKEDNLKCHNEEGSNVLIDNMLINS
NNNTNVGNPLFNSFIETDELYKQHVIPIPLEEKGKNDENHVIEKFECDPNNDNLKNLYMNEHDWKKLYIEDLL
NYTDENDIDIGCLDIFSTLNTIDRNRKLGSYFSKNNASFILYNNCIIPSKYKDGTLNEYLHTRNKCSLFDKSY
QLIIKLSGNDCFYICNQFISSDIYDVNNYDAFYTCIIDNKAYILDTCYVLKAEKDITLITSGYYKKGLYEFLS
DYILFCKDSGMDVHIQVDINKRILSLQGPLSNMIFNDILEYYDWNNKDKSIGYLNNVIIKKHNNKNDQKKICT
DVNTLYFKSDDHNPIKSSYFQIPYMSFKKFNVIKNGAILNDNKQNINKSLDIYEIICIRIGDTGEDGYEFIVD
NNISNLFVDIFLNHKIVKLAGAYALDILRMESGFPLYGTDIIKNTSPITASLAWTLKYKKIKEKSIFGFQNLL
KEYSMKPKFLRVGILSNQLIFKTCKILSYPYKRPIGYITSCTWSPIYKKRIAQGYIKRDFAKNNEQVLISIPT
DIPQDFSKKKKYKILRSRSAHKFTLAQVCALPFVEHKY*
>N.MAL13P1.395
MGEGYSKSTINHGNLSEEEVQNIRKKFNLNKKELNETISTFEFIYAYPHILRPYIALVLPSFHEVLRKKNRHN
KGKNSSTSNNTNNYGINYAINILFSKSNKKISNEISLQDIINILSYLHNVKSRIIIRILFLSFLRYSISNNND
TIKLNILEENDLENENNNEELNITKIVEANFKREDCCMATDTYEPNIYDDPQNNNDDDINDDNSLDKKRNSFD
LVYSDSNVNTINKNNKKERNYKFIKDNIFIVEKNKITSQYPQNYIVKDLISIEEAIHHLFTYMYIEQLYLLCP
SNMLFKLSNVNQLAEKDFPINNIKMKCYDDSIINRQNSWDLAFFFKELSCSLETNLDFKNIVIGFRLYANSLE
NNKNSIYSLVIEYINSTFTNMSTNYSNVTWKHFMNKENVLTEGINKDNKKKKKLSNAAETDIQLEHEQQKEVD
KTNTFIIDENEIPNINDICINSIELIHSIYTSMKKNEIAKKDNKNKLLENDAYKQSASIIELQNSSKGWRGLF
LNESSKILTDEIAFSLRQCSPCFSNNVWYRLYASWKQGTSFSRFMSCLFHYPSPIVIVIKTNDNQILGGVCTT
PLKDSHLYHGCSNDFLFSAYPVFRIIRTNQFGTNYVYLNSKNSFYPKGLGFGGKPECFRLFLSDEFKDSYCTE
SDFTYKSGHLYFPQQKNKKGKKCNYTYASSDSKDGDNQDDKDKREELEPEGEQQAQEQEQELEQDDEEDDLDS
YSFLYKLSISEVEAWGCGDEKALQEQKLIIQNEEACKQERRSTDKSKIVQNSFDKEFLLPKVFSVGKYEELKP
ST*

>N.MAL13P1.540
MRPRFFLFLLFIIYIYNSLRIKCSSLLGIDFGNEYIKVSIVSPGKGFNILLNNQSKRKITNSISFANKFRTYD
EESKIYSTKYPQLTLLNSNNILGYNLFDSLKNKENFVIENYDENNEEFYSDINNYDFSNDFGSKYYSYDYVVD
HKRGTINIKLKDNMVISSEEVTANILGYIKKLAYTHLNIDYKVKRNINLNIGCVISVPCNFSQRKKQALINAS
KIAGLELLGIINGVTAAAIHNVHDIPLNTTKLTMYLDIGSKNINVGIATISFVEKDKVRSRSVQVYACESLEN
NSGNKIDMLLAENLRKKFEEKYNVSIENDKKAMRKLIVAANKAKLLLSAKKSADVFIESLYNNKSLNESVSRQ
DFEELIQEVIENMKIPINKALEKGGFQLKDIEALELIGSGWRVPKILNEVTEFFNPLKVGMHLNSDEAVTMGS
LYIAAYNSANFRLKDLDYKDIVSNEYHILVNTDEEENNTTNEEKVNIKKELVNYNSRYPHNKNVILTYKDNLK
FSVYENGKIINEYVLGNLDNAIKSKYEHLGTPKLNLKFHLDKFGILSLDKVLVVYEEQKDGAGDTKDNKKEGD
EENNNNNNNEEINKDDDTNNNKSDDEQNKGDENKSNDENKENEENKQNGEKKKNDIIKHNIPIEFQTRNIKPL
PLTFEEIKEKKEILKNLDEHDIDIFLKSEKKNTLESFIYETRSKMKQDIYKQVTKEETRNEYLNKLEEYEDWL
YTEKDEPLENVSNKIHELQDIYNPIKERAEELQVRDKIIEETNKKIQEMIEKIKDLSEKKPWAAETIKMVKDS
LDKEVQWWNHAQEEQKKLDNYTAPFFKHKDVQLKFKSIQMLIKTLDKLKKPVEKKEDKKNTDNQNENTSKQDA
GADKNHNTTENQNEQSAQNQNNENNDDNQNNEHDANQSSNDEQNKNDGASDQKDEL*
>N.MAL13P1.405
MLRVETKTWTRDSHDLFDYEAQQVNKKSFLISTAIKLFRSKAQVSCVADSPECLPNTTQDYLLSVRPQEDKYV
ITPAEHSLSNQYNIKKLWIIVKDLPEKYYALHENDIIKLGRFRLKVRQFIESVDTLNTLKLDDCPSKKCETIL
DSSNIQCRICLIEGNQENDPLICPCDCKGSIKYAHLMCLRKWINGRLNLNDQLFSGSVFIKDICCELCKSKYP
KSIKQNEELVQLVKIPNLKTPLIVLDNIIGQTSKGVHLISFADKKYLKLGRGHESDVRIPDVSISRYHATIKY
EKGLFKLEDHNSKFGTLVALRKAREIFPKDTMSLQVGRSVVHFKMDEEIPFKSGIIDIIENKEVLDSSEAPTN
DKSTVNSAPPVNTTTNTDETNNRNNFERNQFLDITNASNSLQNSYSPYRVDNLMSLLNYQNEYRIFGIRNCYN
LMENRNNQQLIPQMNFVSGANIDMNNDEINNDQNLLHHRDAHIHANVDEVAHNDCEETQQPRDISNQSNDDNN
DDEEENHVNDQNDDDEDNHDNDHNNNNNNGGGSSSGSSDNNNNNNNRGGNNNNQYYGNNNNNNNNNNNNSNNN
NNNNNNSNNSNNSNNNNYNCNDRTYYSALSNKSKNNTIKNYNKFNNIHIGNLPLDLSNIYESNNNQKDFVPNS
DFIKNNSMPYSIRKNKLSYNKDLLKETKKYKDVDETNEKNLSNNENKYEKEARSNHNKNEIYTNYNMDENNSS
FFFSNMKTPSLVDFCKYDNSSPLSYNNNKTKIDESDKNGSLIKEYKNDNKFNKNSSSSNIYHKNNYLTINSNN
HVDDIVKKDQHFINSKTLEDNDEYNTNDQRTTRSSLNCNKMNISNDRKDIAPLNNFSSCISNESNINAHNTYE
LYNSEIIHYVYPNQNVQGDNNCTTPRKYMKYENNRTDDKNTKFNEINTHNVDKKLFKDTPTRKYSCTNMNNMN
EDNYLNNEYNNNNNKNNNLSGPVNCRERNTTYNNTIENCSCDNKMVKGNKLKKNYKSNSCDEMNTCNKKMKKN
MTKEIIQNKDNILNVEERLERSIYNNNNNEIDLNEKSSNNLYQNFKSAVFKKRMIGSPYTNINTYKNDIPNES
FNFNLKEKSYNELETYHSLLNVNMNSNNILNKFNDHKKYSNNNINNNNRTYQFNNEHPTRISTSKFNIVHQLN
GKEDYICTSNYALHEPGDVHNKNEDSNHDVEHITSSDCRGTSIGQHVVLLNEGIKNNDKIFSDDNMENDMNEK
YKNVKNNKHRLYSYDTLSFKRHMNNIKIDNNDDYSKNKCNKGSLKNKNIGKHFNFNFEKDSRDLTLNHVDTSS
SHLDHFVSESKKEMKFIHRSISMVTIQDEDNEIVNNFREENNKNLNIYNGSKLKNKFSCEDDKGTLLKYKNIT
STNYDYKYNNLFCTKSNNTSNHNIYINQKKKRCTAKY*
>N.MAL13P1.219a
MNTKNDVNENIILNCKNLKRNIKRKIENFYDENYVDTLNYLNKRKNYGIGLKKDIFEKSKNESIKIDTPLLYN
NNKRKLQLDYSDVKKQKINNNITSNVYQNHNVIINNLSNENLNNIYNHSEVKDSNQLLNPLSSEIIKNQGNNN
IIYNHNERENIKQFSIIPFEEQKNTNTILVNCDENNNENYNIKKNVLNILYQKDIFYNKLKNQIQKYKPLEIK
CMDISLLLNKYKHESNNNSFNYHNHDNIMNTKDDMTKTEGGIIKKQDNITNTQNDITNTQDDITNTQDDIINT
RDGIINTQHDIINTQGDIINKQDDIINRQDNIINKQDHNNINNVKYNENIISSLHNIQQEPIYVYNKNETNME
SCNMNNSTSYNNNNNTSTTNYGKIFINDYQINNYDNNIFHKKDNNYINKSCQNIKHCNNTIDNSNINPYKYFS
NTNNINNLNDNMSHYIYENIINFNNLNNTNQINNNVYISNFNINNNNDNIT*
>N.MAL13P1.219
MYIFKKCIIIFFILKNCMYVYIYIYIYYYFFLLNKNVKKE*FIMKA*VIMSNQGDFNTYIYIPGYNEKYFNMI
KMNI*KKEIKKRKIIVNKYFINTLIYIYIYIYFFFFFFFYL**NVMRVSRKGANYVLTY**YK*RGNTQTYIL
STI*IK*TY*RK*Y*KRF*RTFAYYYIRFA*FEKIYD*FLY**NKRGIEKSKQVKKK*TN*TNIYIYIYIYFF
FFLAFIYFFEMK*TCIKVKRTQNTFVLIP*KKINKNYI*IH*KKK*IRY*MNLILKIHI*
>N.MAL13P1.220
MHFGIPSLFYLYILFSIIMRIKCVITKNLKKTKKRTCSYIPHGNMEKGIILNYIEKPNPAYLKRGKNKNKNKN
KKGDIYKLRNVEILLYANRYVHEGNENFSSTTKKLLLTPKVGNKMPEGKKPDWFHVAAPTVAKYNKLKDDIKK
LNLHTVCEEAQCPNIGECWNIGTATIMLLGDTCTRGCKFCSIKTSSNPLPPDINEPFNTAKAICEWNIDYVVL
TSVDRDDLPDGGASHFAKTVELVKFSRPDILIECLVSDFQGNIDSVRKLAFSGLDVYAHNIETVKRLQKYVRD
KRANYDQSLFVLKTAKEINPQLYTKTSIMLGLGETKEEVIQTMYDARKNNIDVITFGQYLRPTKNHLSIVQYI

SPQMFEYYKEEGLKMGFKYIASGPLVRSSYKAGEYFIKNLVNQRNKDKKNNLKIKYKKLKIKNKI*KIKN*N*
NIKKKKKKKKKKKKKKKKKKKKKKXXXXXXXXXXXXXXXXXXXXXXXXXXXXXXXXXXXXXXXXXXXXXXXXX
XXXXXXXXXXXXXXXXXXXXXXXXXXXXXXXXXXXXXXXXXXXXXXXXXXXXXYV*
>N.PF13_0239
MFLASYFDRKDKVNEKSETEIEEIEYKKNNLEKVLKNALYLHKEKNVKLKDEADKKERIKEYLSSFLKKKIKD
EHKMNLSYFIYDENEKRNISKYSVDNFNEREEDQRGIYNLLKENKSLNNISKDMLILANYLQHDKLKNIDIIS
VYNIIDKYIDKYKLEIDEIKKELDKEADKEIKKKQNNLDTIYNIKKKLGNIHIVEEENSNNTVEYMLNEMKII
KKLIKQKEYIICVKNTLISLDYAKKCAVNRKYDESLNIILDIVNILHIFKKNFKKQVIKNIEFFIPLLAEYYI
NKSKLLLSSIHWGNNISYVSTNSLEDVINDMDMSEDNEEIYDDMCDDNEDIYDDMCDDNDIMSDNICDNTCDD
NNEKGDGTNNYYQDFENTDINYSTTFINAEKTKNVAQQKEKKRRLLYYNRHMNKFHDKINNSVIFDKEYMGLI
KKESLEFINILICCNYIEKINICITEEKEENENILFVKQKYYSSSFYVEELASSIINFFRSFFQNDKSPLFKF
DKPEWGLKYLFYQSIISNYILKMLLNYAHMENFKSNNTILKQTLEYMFTYNNKYKEILRNINNIENLDRNPTS
VNYKKETIAMSSVSSDVQYERDHTKLFPEHNNDNNNNNNNDNNYNDNYYRNNNYFHPMSDQIKNSSHSYLLDN
NFNIKDRWPSKNTHIYYNTYNCMKDEDKQILFEDISNYKMVLEKLNYEIITECRLYILSRITYFIHLYTFDEN
NKDDIKKSFLNFMQHIFLIYKKWWLYDRHNCKHLLDDFYNNTYIKLNPMENERTNQHNNCNNNNNNYYDDDDN
NNNNDNNYNNNNNNNNDNNLNGINHENNINNYNVDNNTIQHLQNNELLKNRNLQLSSEENIIHMNEKTNDGVY
IRDFFMLNEKEFLIDILNDMSNKKCCIKLKNNIILDENDCINEYSDIFIQLLKKIKKRIWCFRNNHEFMEEYM
NNVVKKLLIIVKDEFRFHWNNISDLIGDSEITCLLYISFHRINNFLNIYQYKKYLEEIKQNFDILEKKMFHNF
LDAFYHFISIRIYNLFSVSNVFHEYIINNLYKIKKNLPDIIFIQFLNRILYKLDKTILKYLLNQNISFLQNEL
YFQTFINNSYILLQQIEELNCKNKKEKLNNNTGIFIYNMNLLREILKLMTDNIDTLKDKINEIKCSYAFLKNK
NNWIDQVTRITNSLLKDYSSSDDNKSSHHDYKENWENKCKNTEISIIKIKMLLLRRPDIKTIAEKSIVVKEFI
EY*
>N.MAL13P1.221
MIEIFCTAIVVITILIVGVFVYMIIRTKKKKLKLDNMFYINSKYKIDLDKIMTKMKNKSVINIDDVDDEELLA
ILYTSKQFEKILKNNEDSKYLENKVFCSVFLEPSTRTRCSFDAAILKLGSKVLNITDMNSTSFYKGETVEDAF
KILSTYVDGIIYRDPSKKNVDIAVSSSSKPIINAGNGTGEHPTQSLLDFYTIHNYFPFILDRNINKKLNIAFV
GDLKNGRTVHSLSKLLSRYNVSFNFVSCKSLNIPKDIVNTITYNLKKNNFYSDDSIKYFDNLEEGLEDVHIIY
MTRIQKERFTDVDEYNQYKNAFILSNKTLENTRDDTKILHPLPRVNEIKVEVDSNPKSVYFTQAENGLYVRMA
LLYLIFSSTS*
>N.MAL13P1.222
MIDVPYEFMNIKNYEFLYNNLKIRNDMIRNNDYNINNYIYDVYISKLIFVMVNIYNEHSMNNNNNNSNEYIIN
NTQNMYMIKNIYSSNSYEDGILNNKEYNHASLIKSKRDNKLLCYNKCIRRHKKIKPYERSNKENCYMNVLNLV
NNIRNLRISKIKRKKKRINIIMRRKKKRKRKKKRSMIILNNDKNYSINRNNCSNNNYMDNKIYNILLKNNKII
YMKYDDTINGEESKNKTKLFDMLKYLSSIKYKSNNNILCPLIKINTDKNEYNKKNIINYLNFDRTIYFIFNQI
KKLIKKLKNIFISRNIIYYRYFKNITYTNICKYKYYISNYVISFYYNYLHNDYFHSQNCFLKCFNNKYSYRKF
LKIFIQLFLDKILCIRKYIIKRKKNKNDSNSKKKLKNKINSINIKTKKCKRKTLIDILNTLSLSVNNKNYVVH
NKYNPNKNYNHFIKSYVRKISHSYFSILSKRLLNYYINDMNRYYKQYEQVEAINMKNNYYNTYMPTFDSTNNS
CSKYKSDKTSIIFNRKNKIQQNNEQNYCKIKLPNKRKELNSGDNHEIVMCNKINYKKFYKNPFTYNNNNNNII
NDTYRYLFNERILNKECIYLNDQNEKRSDNYSDYSSDKQGDINVKNNNADNNNNNNNSNNNNNNNSNNNDNHN
NDNNNNDNNSNGNNNNNNNNNNNNNNNNNNNNNNNNNNNNSNNDNGDNNRNNDNDDNDEGKKKGDDHVVKTDEE
DKDDDDNDNDDEDVRIEPKDRNENDSENINHNDEDNIIHINDENNADVLYEQNSDGILNVSINKDKQLNKNMN
DENGINNMNNMNNMNNMNNMNNMNNMNNMNNMNNMNNMNNMNNMNNMNNMNNINEHENYYRLIKEIEFLE
RKENMKECINPNINDYVDRNKNHNKEEDMIDEEEYKIKLCNDENVKIKSEKMEIKIKEEHINISNINQNLTHN
NTSPIINHPLKLPLLPEMSHPNVFSSHSPRSNNISSNSSSSSTSSSNSGGSNNSSSSSSCGGSPKNIPNQLND
PDFEEACDIRLMNSSENLKFSQSPPTSSLHASSLHLTDVNELVLSPNQQLYNNSPIKSPTNTPIRSPSSPIFE
TNYNEENIEKVNSNYLISSPYRNDIFSEFDMLGSLFESLDELENKSDYTESSSNDNCKYGNDFSLDKYNSDIK
KGTIEKDEIIYNFKKDNFKKVFHAWCNKGKHEINYDNKKFNSVMEWINYIEELMSHELGITKDDKKNDDIRGD
MELSVDIFSNQDNYSVDEQNHVNQNIESDENILINKNISTNINHQNEDDFISNSNDPFNFINEHNNNNMVENN
TVLNDKNIILHNNDIVEDTTTSNSMSPLKSGFSDITSCVNNSNDTVNLFSEYNNAEDHVTYSNHHKEKQQDED
TYFNDQHIINNKIPWNHSEHNFVWNNNDIDENDEKNDHLKKKNEILQQNSDFNNISDINTNDFDINSVEDKGV
INIQENSKDNITSSNYIEVTDPWKK*
>N.MAL13P1.224
MEHSENIKKRYVNHSDMKLEKFKKAAYEVIEYKEKIIRMLCAILEVQGITTVNIKEYISSSNEPSLDSFLDNN
QYESKGYKKKTSSRKRTLINVPDFRTLKKNKELNLFSNPDKMNEELIKEKNFSDSVLSSYRMSLDHKKLQNKM

IDNMLFRDISGGKKRTFVEEVEDNEEKNNKSAQKCIDGNDKNFIESRKKALENFQQNENNYDCTYKTIKKKNI
SGCNTTTKLKDVNSSEECQLCLMPNSDSLHGNFPPTFYKLTCDHIFHLMCLYETVIRRECRKTCCICHNELSE
NDKNEIINKVKLEKKENEKKSKLLFKVMKLQAENKDYPRKDICS*
>N.MAL13P1.225
MKKYIFFFLFSFINFFFVYDVTCTKEVTSTNDDPLTPLNRFDKYYLRMFKKVPRLQQNGSNIINGVNMKNTVI
VLYFFAKWCQACTMQSTEMGLQKYYGKRIYLLKVDLDKNESLARKFSVKSLPTIILLKNKTILARKDHFVSSN
DLIALIKKHL*
>N.PF13_0241
MLIYHKFCKYKKKNEDLFINIIYNKHNSLFGNLNKSGKKYIHTCTALKRKDRVRCERKIKLKEKFKIIVYASL
YFFTCDYLYHEFILKNSKNEKNEAKKREQNIEVVRLNNGIINKGGDNNNINNSNNNIYKQNDIVKKDQSYFFL
YSNPFVNFFIDSIKKLNIFINSNTIHNENEDKQGDKNLKKSDRKNDEKENNSYEIKITRGSRNKNIYENSNMD
KEKLYKGDYHNNDNYKKHNNNNNNNMTNIFTNHIYNKNYFFNKCNLFLLCNGLIFITWRISEVLINKRLFHFM
CRNFICSYNNIKQKYIHTIITSSISHITFPHFLFNMWAFYTITNTLLSPEIGEKKKKFYFFNYKSNVLQQKMN
DQDIINICVLSSICSTIPYILLNKKNQILGASGSIMGLVYILSTLKPNEVFISLFPLPYLKLTALQLCHLSIL
TNIFFLFFRKKKVNHFNIAWSAHMFGLLGGALYNIYQRKIKNNNNYYPFIQLSIKNGYIDYLNSYLDLIDMLK
CFCLQTKMFFSLDIKTMQSLNRKINSIKSHTAQRRIHFQIKKMKNLEFLSKRNQLQ*
>N.PF13_0241a
MNNKLSFPYETYEIKKFAFELPKDYHPNVKYGFFRNALFGQHTFYYIKSILKVSTKVCAGFLLFYYPLDKLHC
NIRRFKNNLKK*
>N.MAL13P1.226
MMHILKNKRNKIIFSSDDEESNDDSEQINTDNISKNVQDNANPDLDPNKNVQENFEENTDNNIEDSHILNKEN
IQYKSDDITAASQIIKNDMDEINISENSLHEKSKKKKKKLKKKLAEDDDDNNNNNERFPNSNENISTNKLKKK
NFIINNNVKKQDIHDNIGYSHSNNSHETDKVNDEDKEENYDVKDSNKKKKKKKKKEITQKEIDKNKNRAKYED
SKNDENYKNSDDDINNIYDEKHQNSDDKNDDDSLNDNPDNDYGANDDKENNRTFDETENNDFKGKRRKTKNSK
LMEDINPNEDKKKLKSIQISFSKKEYNIEAYLMECLKVLIPLNMRDIFLGSENIILDSMNTGDIKNMEIFWFN
KLTKNRQKSIIECYTSLNKQKKKGKVEDNNSIVFNSNSVSNMENNGIHLSHLNNEKDVNEKNNENVENNMYNI
DNNIDNIMKKGGEDHSLNIPREDEIFDNNKEVIIVEHLVHICNNQIKLIKEYISYIKKNIYDLSSISENNNKI
KELKILEPLKRREYFFYLYIAVSLKYVEERIANVYCVPAVISPDIRGFNLHPMFTHINKKLEEKSNKKENIGN
PWEKLKLLKKGNNKKNDGHTRNELNKGKLKETGTMNKSEMDDNLFENNINMKDTQEDMKNIDDTETSDKKELD
DLEKKKKALEEMKRKKKEKDHKKTFNIYSLLESAAQFYGEGPKYCNPNMHEFYKSNNNQLVYIKPPTNVEEKN
LLINFFLQFPSLNKKLEYNKKRIYAENYGLIKIIRNAENLPINFELNTEPWKNAEYEELWNEQNKLNNNQIWN
MSNIINYDNLLEYNQFSTTKTDFLNHTDVSRSVLRKNEFDDNDYQKSCAISEFEKSMARSEYERGEDKKEYSR
EEYIHLDSIHIGENKMEPKKNNQLDDTDMKKDIKPQENNNMDNDILPKVEPKSEQIRINMDNIKEEHHDKSDQ
NNISHNVSYGDVKKTEMREKKKPEIITKKLKKSITHDDMKQMKLTSFLSKEKINKNTNVLKKEEESTNNIDNM
KNVYNVCSENNMNDNNSGEHIVKDELNDESNLKDPADNMDYIIKIKRKKKKVLDDEQMNHFTNDNAHKEADVM
ESNSLTDNKENTERSNTFYINNTKQSTKKNPFLKESRNKEDDLDSENLEEAKEKKKIKEEKKRIMDDKMGYVF
ELEAEESEDENLEDPEERKKIEQFKKEQYEENEYEDDQSNSEDLKEFINNEEYNSDNDIVKLKHAEEMEKLEE
DLFYKKFTYQGKQFNDELTNKEKLELEREKQLLRKKKLLLNCSIGNVKYSDFESDTSTDSDDSKGDFKNPLYE
PYNESEHTDILKKNNNSFEYLYNDNVFQKGECLNEIVDEKKKKKILEKMDKVIYTKEVKTNEGKKIVIKKKRK
IRFHQNLSDVTVTEEEKLDEYEKTKKKQKKQNANLIEHNKVQNNTNNIMNHNNKASIKWNNNIKDISELDTPT
NSEVFKGFRKVQEK*
>N.MAL13P1.227
MFNIMRPIILFNILIGYYFFIVINNVVGNLPKKNSYYNIPNYKVKNVSNIKNNKYNYFIIDTYNNMKRKNNSI
YEHNFNNFNRKNKTYLFIDNKKRRRKFFYSKIEENKNLQGKELKPSRTVEKHIKTKYNLGNANYRIQKELNNF
LKNPPINCTIDVHPSNIRIWIVQYVGLENTIYANEVYKIKIIFPDNYPLKPPIVYFLQKPPKHTHVYSNGDIC
LSVLGDDYNPSLSISGLILSIISMLSSAKEKKLPIDNYTHADAKPGSSQNNFLYHDDKC*
>N.MAL13P1.228
MEKIEKNFDETSETHAESATRPFGVDGYSSESFSITQPNVKEYYTYSSIDPREYGSNSNYSMRYGCTGNTLIC
KTQNPKVFIRRVDRKRKEANKKSNSLNGSCYKEFSPYGVLPNSSVHGSNIGDYNYSMPTSNSVQYNNIYTPDK
ANTMSYTYSFDELNYPLSQQMLNNNNSHLRIPLCVQESNRNNNVCNIMNHVTPLNKNPYQLNYTIPKMVEAKC
DAISKRIINYIKLILRYIYKVLKLAFQKLKSDLNTKEIYYDSSMPPIEDIDIHCEVCRAKYGNILLEKKHKDC
ISFFEGFDENRTVFSKMWDVVDNWLEPTINNNKIEYMQVHPRKNEQVYYQMENFPKDENGKIVLPQFSNYVKD
NTVLI*
>N.PF13_0242

MGKHIRILKNQYLQFMSKRCIQSKAAFNICGKINVENPIVELDGDEMTRIIWKDIKEKLILPYVNLKIKYFDL
SIENRDKTNDQVTIEAAEEIKKTSVGIKCATITPDAARVKEFNLKEMWKSPNGTIRNILDGTVFRTPILIKNI
PKLVPNWKKPIVIGRHAYADQYKQKSLKIEKSGKFEIVFTPDDNSQVLRETVFHFKSPGVCLGMYNTEESIRN
FALSCFQYALDLKMPVYMSTKSTILKIYDGLFKDIFDEIYEKQFKKSFEQHNLWYEHKLIDDMVAQVLKSEGG
FLWACKNYDGDIQSDAVAQGYGSLGLMSSVLLCPDGVTCVSEAAHGTVTRHYRAYQKGEKTSTNPIASIFAWT
KGLEHRAKLDKNDNLKQFCYALEKACIETVEDGLMSKDLAGCIKGIKNVTEKDYIFTQDLINAINEKLKLKIL
QNQSKNDPQATSYKLKNDNWNFYAPQEHST*
>N.PF13_0243
MEDVQVDKNVKKKDDFSLDVNNKVNDFNKLSYFPNENNKSVSNNLINKRELGNIERGDVNKMYDIKKDVLLTS
GEEEDDDDDDDEEDEDDDDDDDDDDDDDDDDDYEDDDDDDDDEDEYEDDDEHFNDYNYNNNKNNTSKINKKSSK
YHKDDNSSSLNCLSDEDNLYYSMNEGYRTNYIMESLFDKNSFIYFTVPYNKYNYTNYFINWNKISFRMYASLN
SVMYRNIRKRKLGSISDLNYNMKNPLICIMNNFLYFLLLSEYLNFNELFKLMPLCKSSYNIFNHTFCINVHIN
PYKQKISDIIKFMRKNKEYQFNILKNINNQRRDRSSFYEQKEKLFNDEKKFNMILSHELRNEKNNVFSDTIVH
NMNDKNEEQKENPDNKNNPLCNKNIIGNIKKSTNYDIIKCSDISEVFFTNNILNMRYLFSLYEYIRLYLSKCN
REKVQFCILQQKFKYNRKHNNISFDNISPYANKVSSFEFKVKMKKYIQRRWNIKSNYIHEAYFCHKKEKSRNN
NTKEDNLKNDSNNNNDNHNDNNNNNNNNDNNNNNDNNNDNSTEYYSGGVIKIIENRYSHLYSSFSSFITLDKE
GYVNMWKINNMDSVLPTSLYKSKLYVNDKKDLTLKDGICLGEDKFLIVDMKSLYHVSLEDKEDQPLNKINVDL
KKDECVIKSIIQNGRNKCTVGLNKTICQRIDLEKECIESSKSISDDFQKMCCYDDNTNIVLSKKNILIDDRRL
GNYVCYINYNLIDYENSKKYYKTTNFFCDIDLTGITLTSLNSNNSMFIFDLRYKYPSTCLYNDNIIIETMYTK
ENEKLKYLFNMKSNYNSKYYCMNKKYSAYHDNIILCPPCNVGNIKKNNIQYKNKYNNMEQINDTSLFVKNKIL
SDESFIYNITKDKNKKQFYNLPTSYLNIWRWCNKRQFKSLFHYYDDLTKNRKFHINYDANNNFYEHLIFDSIY
KFVDTPNHLFVTYEDSNYIHSFMAPSYGLHMTSSFNETPNYMHPTNL*
>N.MAL13P1.229
MEDILDNIEIKNSLQKQINEVMDENEKQYNEIKAYIENEKKQIEIMKNKTKEKKISINRLNHEIEANTVLVTE
LKESVMEEEKKLEEYPKLISEINNKLNLILKNFDSSKLEYNTVKLHKDYIQGEWKKKLETLYKLLGFEIILED
DKIVIEFSNIQDSEPQKKYRATLVLKNGIYEVIETNPRINKFEDFVDSLNKGLPFTTFCCLLRRSFKELK*
>N.MAL13P1.230
MKDEIEEGDEKKENFQNRQREIETNEYKTNHENNNNMDEIRKDNNISNNINENLKRDDSSDYNVKENIPMVHK
DSIIKEDISSKNDLLLDDKNNFVKNMITPEIQNEQMIDKDMLNNIKLYSKKFEEKRKKDILNCYNECNNNNNN
KATNKKDYSSYLDNNKKVVKEKLKPDIRDIFYKSQEKEIFEYIPDSSNYYVCHNEPKELYKDIVNLNKNILDN
ININIYSSPLDTYLSNMMTEYSEKEHEHILKEENSSIILNDEEKIKKYKQEDIEIKEFIDISTYNEPKHSYEF
YKNKILKIYDNGVQVEIQPDKLKYNNSTYYEYNQGMKSFLEDPLCYLQKLKCEVDDISSYIIEIGNIHTTNEA
LNNEGNNNNNILNDEEIKENDKLIKHILHDREPQEVLVELLSLKKDITNILNDESLKKYFQHEYNNKEIYNEG
NEKCDTYDKNDMNYPVNINSNNVDIHTLFETLQNCDDLFSNNTNIQSCSENQIDNTKSVQKFDIYTVKNKKEE
RHIHVTDLLTMEKKITRIEKVLGIDKMSMLPYDDLNHAILDIYNKLSLLDNNKLENVKKKVQILQSEFLNLKK
YKKELLNISKERGNYEETIDELFKILDIWKKTHHIIPNVLSRLYQLKKIHDNADSFSSRINDLENEQIKLSDT
LNIAESNIKLINSKMDESIELLKNTLTKIECQNSTYNK*
>N.MAL13P1.231
MVRFLNLIKPVMFLLPEVQSPDRKLPFKEKLLWTAVSLFVFLICCQIPLYGIVTSKSSDPFYWMRVILASNRG
TLMELGISPIVTSGMVMQLLAGSKIIDVDQSLKEDRTLFQGAQKLLGLLITLGEAIAYVISGIYGNISEIGTG
HAIIIILQLFFAGVVVILLDELLQKGYGLGSGISLFIATNICETIMWKSFSPTTINTDKGIEFEGAIISLIYC
LFTESNKISALKKAFYRTHAPNVTNLLATILVFLIVIYLQGFRVDLSVKYQSVRGQQGTYPIKLFYTSNIPII
LQTALVSNLYFFSQILYKRFKNSILVNLLGQWQEVESSGTSIPIGGLAYYISPPNSFADITNDPFHTLVYISF
VLVACAFFSKTWIEVSGSSAKDVAKQLRDQQIGMRGFRDTPTSLTRVFNRYIPTAAAFGGMCIGALTILADFL
GALGSGTGILLAVTIIYQFYEMLVKEQEKAASLF*
>N.MAL13P1.232
MEETLYEKHFFDKYIKMDIPNNYLDASRFRIIPDNQEVYVHKYDNRCFIIEVLCYEVMNDNDKSKYYFNDLAK
ENGSIENMIIVNNNSLSHVQRNYNLIVGKQKIKKQNENDYENVFLYIGIFPYKEYNADILITWNVPKDNFNIH
PELDIFNNMLKSFRILDYQLFV*
>N.MAL13P1.233
MPGSTKSDTKLENEIRISYKSDALDYVYKAIVLFETYDEIILSGVGKAISSVVNVAEMVKRRAKGLHQFTKLY
EKEHVIKREDNSGLKKNAKNEDKKSGDEEEEEEEEEEDEENNKNKEANNRTVEFITTVPCMKIILSKNEDKID
KNEIGYQKPLDEKEVNVMTPEQILKEKSYRRRYRRGGRGGDRFRSYRRNYYETSMWNNRRYEKRN*
>N.MAL13P1.234

MFESLVEKLLNKFLAPYVEGIERNLHLGVWSGNIVLENLKLKPQITEILDLSFKIIHGNIGRINIQIPWSSLG
KNPVCVLIKNVHIYIKPRCYKKSEEVIIEELRKAKMHRLQLLEEEISLIKLQKNNEKSSEKSTLIFKLLNKII
NNIQIDIQDILIHFEDPDKNFSIGFILKSSSVKNCQNNDEMSTQANSNAENKTLNHIIEFKGLCIYSNSDIKS
RTKKKIRKKNKKRSKDMVEILEDDKNKDNVKNSSNGKNISEMKYEKSELSLSNEKFPNMNKTDTEDMEIFDIS
SKIKDSVYNNLDSNDNNINSNDNNINNNDNNLNNNHNKLNNNDNNINNNDNNINNNNNNLNNNDNNLNNNDNN
LNNNDNNLNNNDQNKDTHDYSKLSSCDSSTQDDYLKTTLNRISSFLKEEENIILHNYNFEYLIKPFDLVLPVE
QSSNKKELKAKLEISDKWEGITLTKTQITKIIEIMNEANKSRNQTNKLLLKHAWTVRLDIESLRNETKNEFIN
LYNKVLGEEYNISNTELSAQEINRLQILYDVVGVRHLAKWRLHCRTTLEKFIEEKNLKKKFLYDSIYKQQSWW
SWVTGNKKEIENKVQNILNSEQDIINENELFMIQEAMTNDDNYDVVMPSKYDFQFKLANFSINVYDDCKKRII
DNRNVNNHHNNHNNNNNNNNIYIRGVSNKSEVTNNRQHIKEDKLNFNDIIKDELSINSSCETLLTSSINLSSTD
SKLNKRRVRYERVNILSINFYQIYSSLSLQSVVDHNDHDNFQWKFIIELQNFIAKHKNKVFMEFRNNKNSFPY
NMNTSGSLYKNSIFYSLLYSQTVCAYLEINHLVTEKGNTLSTILRLNPLELYLSPLLIKSILSFTFPLLDIIN
KSHKTAMKKYKSKNVSLSVEKQKQKEETSPGVIKKKESISGTRKRSNKNKKSHHEIGSIKKDVNNEENDTIKQ
DDHKNMNADSNKNNDNNYDNNDECDDNNDECGEYNDEDDEDKNNDNEDNNNDDDEEDEDDDDEDDEEALLQIK
KMEQSELLEGLKERGENVYNRAVQHLPELFEFYIHICGPILHFDNLTNGIVELHLGNLVAKTEHPCTYNKFNL
IFEFNETQITCLKIGSGYMKKDEKCKKDIIFNENQKDLHKSTKAYKKMDKSNIYLEINEEGLFSNNAMDNTEM
DDLKDSYFNSIKEKVLTGEKFYILQPIPVKIYVEYDLKILKTNIILDGIFFQINPDAISIILAVPTSITRYLT
GVYSKNKNENEFNKNKKKTSVEKKEGDIMKGDDITRASMSNIDSGVQKRNSITDIIKGNKDNDNVVEMNNKGM
PKEEESFLYDIDFLIKNSSFSIKNKKNCEILKYEACDISYKNYLQKKRKIIKMEIEQLWICDPSNKQPIFFTL
TKDINSKDIFLFRSLSTYLEEKSHNNNSIEQKEIDMESDNHINVGIDDVLEEDHFMMGNNNNINNNSSHIRKN
SFNIGSNAFGSLERFESAKVMDDNVYTNGKHKGSIKNVMIDNSIINNEQLINNRAEGGKYMSTNRFSASGINQ
FNKYVGMNIDRKNEETKLNGKKENDENKNVEEEDDDFMDAIEEKQLSISLQILQEHNEKNIEETHINFIISDI
ELHWKYKTIKQIFKTMKEYKKTLQYGIEKDMTYIKNKLKNEKDLKNYKMFISENTLRSVQETLKNVKDSLNIL
DMESAKDKDDIINNVQEKEMDKDNVKMNAEGIHLMNPYYARDEDLNMIADNKLLFNLRNEKNNEEININNANT
VNDNNGINNNDDINNNDDMINNDDIINNHNYNILNDNILSKGLNDTLPKLHGQQNSYPKYFFNCFIKSASLAF
WKKKKIFSRIQVSNIFYENKIYINFDQKMFLNIEKGIMSMNNKNIISNNINDYKYDLFISKDRKRFGSSDIEN
EDRNMLSHLKYGKKEGNLNAMQNDDDKEKEEYTAEDTFSERRNSRDILNNINLDNLSIEIKKKKKTMHKESKE
DLFIGKIKVYNDKRNYNICLLCEISRMYYIFYLKDLRLFLEYLDDGILNVFISKSYKKVVQAAQTKYFLFNFT
FMDPIVIIPEDKNMIYNYKGEVKNVSRYFKSSTSNNNTKENEDTNDIAKRSSKEYILSNEKKDTKTEACNNYN
NVNNKNIHNTNFDCDMKSNYDKNSIGRNTYNSNNIPYIESYLQFHLSKLKLKNSYTLKCTEEIRVAQRRQMIL
KKKKYKGRSEKEKINKNKYENESFIRSDIVSKEQLRPLERKRNSVNNDLVKLDFTLYIDILDIESKACENGGS
SNIEGEILHKVNLGFCLINARNGIFIYLNGNDLCLDLTVFQLAFLLDIINENFCYKGYFPMCFICDKDVSLDS
MINSEWFKKGYMNFNNFNNRKEMKGKTNDNKKEDIMNNINNINNINNNNNNNINNINNNNNNNNNNKVNNYEI
GDGAINNNVYDSDRTIDKEMDHGKNKLDMSMNQEKKSDSLTNDKLIIKDSKENVTKKTGLKLYVYINFESLKI
KTAFDTNTPVAIITFQYISMSFRLVLLDFYYVYFFDLHGNSLYIDDARKNSINYYKRVAYCCIENEKKKFKRE
NKKAHELYKNVYNEEAYNDTLNKSNVYEYNNVERNSYQINRENKIYDNNGSHFYNNKDVQDEYDEKVQKLHNN
SSNNISVTTQSDIKDYKNLLIYMFENNVFLNEKKKRKKQKGIKIKINSFIEDLLLNIELDDAYICFFFLIFID
IYKFLTTGFNLSTLHLYPKPSPYIVSYKNENKKKKIKGHEYVENSESKSIHRKKKVVCSKTEEEKHIKEQISI
RCTRSQYKNTSNENKNKNINNNNNNNNSNNKSSINCKENTSINIEENVFKRERIHYDEEIIEQNVQLINEEDN
EKHWVQKKKKIIRRSSVLEQSKYIKGKLKEQKNKIITNINEVLKLDNKPFHVSFKVNNGNFIIFTDVEKVSHP
IIMWSNNFVFSFSLFNKCIVFRKIYAIDSKVKRINYVSSSHVHYKYDTIKKRNNKSNHNNKNNNNNKNNNNNK
NNNKNNNNNKDNNKNNSNDNNNINMDNNNKNNNNKIFDPKNISNPLHNYHKKKILLCDNLNVMGEAVYESVDT
RTEDYKLKSSVIRKDNNSPFITVFELDINIGNFDIRLSNDDVEILLKASSTLFGDVPSSFTNIVVGPVIPPLT
FIHKKIKNKFMQNNIYYISTNIKEETELFCGSDLQITSINILKKRRKKIIRKKEKKDARNRKEMERNDHNKIK
NIRNDEINMNEKKSIVDCHIEEKKLDQKEYNEFSNKDEIYTDSSNMMEYVYYKKISDVSSSTNTLSYSNTSLS
ELHGEKNILYKRGMKYDDISDINNNSSSNNNNINNNNNNNQTNSYRDIKINIKLHNVMCTFIDNIRNSIVPIL
RMIFSMNISINLYTDECSYNISDLNSKLEYFNNCIGEWEPFLEKCNISLDIHNIFPNDEYDEDKEANKSPISI
IKINSIKALWFNITPQLINLLFLFVPVFSEKVSNGLRKNAKKEIDHYNDKDNKYVSECIKNDILMEDMNNKLN
VRNTSINKDIETSEQMNSLNDFSRCDSGSVELEYKNMSVKSKSIFEDCSSVFNSFPCDTDGKEANDFHLDDFL
KKEENRSTYYNRIDNSVIYYVNLTSEYFYAFLMPESKIEEIMKRKKSVTMKNVYKSVNEADGTNMTIKSYKWD
EKKKKVEKGFIQRENVTKANGMKGNDIKQNGIKQNEIKENIMMEHTLISSDDNSTHNEGNEDYISEEDLFNMI
PDVYAKIITTNELISLDRLLINEIENNSLIEKKCLYLYLIPIPPTNVVNIVHDMFSNISKRDIVLDLMITKNP
KKTIENLLIYNQHKEHIRIRRNSINSIWTENMMYDDNNKKKHIELYLKKMRMKAEEDGGNKVGTDNIHLLEDQ
NMYLLNKEMWNDYKNVKGIIKGEGKYTNSTISKTDHTSRSNNMDNNVKGNDNINGNHSDRESEKRQDMNQNNV

NENILNVMVEGNNEVLDNEQRVDKSLNLYTKGCENDDANEDVNDEEEGSYDSSSDSCEDYESTDDTHSIDERD
NLNYEYYFKYNKKKKNDSSSIFSSNKFVGPFLRNTECVIINLIKNSCTSLTTSLKNSNVIHLLKPGDFVFDEK
YKYNFSSKKTNEIMENYSITDGIMADDDITNGSINNNNNNNMCNKGIYDNTPTKQMVDKYVSKNSMSTNKNNK
SSILNKENIDKINEWERRNEEMIKQTYIACKYRLKNNYLPQTSLVENVCEIISPMPNYKILFMSSTVRIINKC
GIPLEFCFFDGSRNPILLTSLENRTIPINTLYPNHSDSFKNYKVSNSLNINPNIKLRQKNNNLSFTVILNHEY
LLSAPECVFCGPSHVYMSFKPINVITAENEYYDIIAGGPTTNIANNNNNNNSSSSSHNYYGNKSNINDLLKKD
KGGSNDLIDPMSINIENGWSDIFSSDISQGTYVKKCKYKDMNSFMSNINKSNGSGNNFFYFLVKIENKISALP
AEKNLKIITIYPHVSVVNAIPAFVDIIITSDVVEEKQNDYMYEERNKIDALKNKRAKLLKQCEDINSYLNKMN
SSKYGGSKIYSDSSSRVKGNKDNSNSSNSNNSEHVYMQELNKLEREIYHIDEEIKYKKKELSSPLVKGYVNRR
LNPFSIFYIYEIKKYTCLNLKMKIGNSQCEWSEKLFLVEDDEESVTRFSLHFKKYASVEVEIIKNFSGYFNSL
SSILGNKQLYIFSLPRCFIDRTGLGIKAINSNKYYPVINGITLLGDHSQIDLLLPHKTYTQPNNNVNSGNNNV
EDDTYNYYSKGDMKRSNRMYDKRGDHRKDYNDTNESYGNVDDVNKNDSRYVSKNAIIIDYNDIFNDFNYPSPV
NNSVILFKATLPPIGSYTETNVICKNFFYTFCLNTEKIKTTNIPYIISRIITVVPQFIISNKLNFPLLIKQYQ
NDQIQGVRANDTSPLYFTKKSSILLFQFKCLDPCRTNNEEKNHMLLDPKNVIKSNGNKYYKLNKNIYWSSVIY
PSENFVGTNYMVINTGNQEKSDVYAITCIPDRGTKNIIIEKLENKKKGFIAYNNSSIAKYLKIRTFHDDTRHM
KIHDEENYMEKNMFLSNFLKPTDMEHYFNIEPNKYSYLGWVNPFIYVTRNVQIEIVLEDLKIIPKSPFVLKFA
IYNYSQKTYYINYYNITFVICIEYIEDLITIKLSHKLNISSNLLDRSSYYYMMNNKSNITDEIAYSNSSHNNI
MFSKHMNSCDKSVTSNNDIKLGLNKKKKYMEMENQVNIEKYNIINGKDISNERNIIDKNSENSLSLSKNSVYA
QKQMIIDHDEYHYVKYIDEKDGGTGSYKNNSISSSTNNYYKNESVKMISNTYKNVHIIINVTQIGVSIISNIL
KEEVFFIELSKLCALFYMKNEEEVIDIKITDVQIDCQLESCEKCVLLANRGISSSNNNNSSSSSNNMNNMISG
GDMSGSNNVYQNYRNENKSFTINESDLLRFKKTFDRNSNDKNNHMNPLYDNNKNKGTTTTTTTSGSNNNNNYN
NNSSSVLNKNMNANEEKIFLNIYVERSFISHNDVIFKKIQVSLDDVEIEMDAETLNGINLLTAEYIESISIVQ
KKNLLYEEIQKWTILPVYVNYKSPEIPLAINIQYMQIDKFTLIVWCSFLLDKMHMMSDLLRIGLRILMVSGKL
ELLGAPVTLNQEIFNNIRVSIKSFYALLKDKYSHSILACLGFIVGYSSLINIPKIPLEIGRNTIGLAVYAVDN
VSVGIGSFLSNLTFDSEYINRRQKERTFKTNTNMKEGLISAVKNIGEGVLSLSNIVTKPIEGAQKEGFGGFFK
GIGKGVAGSLVKPLDKVGQAVSDVTRGIKAEVSKPIGGHKYKTKRHRKPRMLWGEYGKLKEYNINEAELRECL
GLKFSKNIMKCLTVHKQENHPPSHYALLLYPKVIIYANLYANTNAQKNDFLLSEKKTDIVIWSIKIEDITEIR
ASSHGLIVRTNTSTNTVYKIPCNNAILINKIYRELHNSKNSINSTVILGANNSALYKYL*
>N.PF13_0245
MMEGNIYKEKKEQGYMKGSNELLKKQAHHNESRFKKFCGDTNIKVDVPNINESLKFLKHDVDNSILNFQYNTM
ILNEKIERLNDIERQVCIPMHLPISYSINIEENEDINNWINKENMYQNQKQKQVEKITSEENMKEINKINDDV
YEINNINYIGDMNEYASVNNYSIDEHDTLLKIINNKNLSENLKDISSNIPYVSCFEKDDIDLLNITIPEYIKN
MKNKKNKLNNNTKDKIFIEQSSDDLLLEKIDSANSKKKYLNYNHFFRHPILKNEKIKNIYPIVPYISVWENKY
IQGIMDIESDTEQITTSGNQTNKSNDNKFYGLLHLIQKSRDKHIYSLYKKKIKDEKEGTSSYRINKNPIIINL
YENDDNIKNDDNIKNDDNIKNDDNIKNDDNIKNDDNIKNDDNKNNNEEDAEFENNTNKIRKKKKISLSKFLIK
KHIIKLRKMEKLKEYLNNENIKNMSLDQMNILNNHEEDNDDINNVSSFNKSDININNKNISEDHNEKRDDVYI
DKETHTNDMSNKNTKKKEESKQYFKYIRDYKSPPFTINENDQLSYAIALSKKKNLAFLFPTMSKKIIFSKMGQ
QKRKNYILLKE*
>N.PF13_0246
MKLSCSFSLCRFSCNTSFKVFNRKYGNICTKGLFNSIPKNKRKEMFFFFNDKKNYFNALYNKKNDKKDLMNEM
DELYDNILTCNWNDSFNFVLNVPIWEGILNTIDEKVKAYEYDEQIIKKKKELNQLLDILFILEDIRDHINELL
EQSSRSKGLAGAHIMCSFEIQNMNEHIELLKKKYEELLLTYPLYKYQIDLVLGRGLALLRQRYVFDWKYMHEY
FF*
>N.PF13_0247
MMLYISAKKAQVAFILYIVLVLRIISGNNDFCKPSSLNSEISGFIGYKCNFSNEGVHNLKPDMRERRSIFCTI
HSYFIYDKIRLIIPKKSSSPEFKILPEKCFQKVYTDYENRVETDISELGLIEYEIEENDTNPNYNERTITISP
FSPKDIEFFCFCDNTEKVISSIEGRSAMVHVRVLKYPHNILFTNLTNDLFTYLPKTYNESNFVSNVLEVELND
GELFVLACELINKKCFQEGKEKALYKSNKIIYHKKLTIFKAPFYVTSKDVNTECTCKFKNNNYKIVLKPKYEK
KVIHGCNFSSNDSSKHTFTDSLDISLVDDNAHISCNVHLSEPKYNHLVGLNCPGDIIPDCFFQVYQPESEELE
PSNIVYLDSQINIGDIEYYEDAEGDDKIKLFGIVGSIPKTTSFTCICKKDKKSAYMTVTIDSAYYGFLAKTFI
FLIVAILLYI*
>N.PF13_0248
MCMGRMISIINIILFYFFLWVKKSISEILSSTQYVCDFYFNPLTNVKPTVVGSSKIYEEVGCTINNPTLGDHI
VLICPKKNNGDFSNIEIVPTNCFESHLYSAYKNDSSAYHLEKLDIDKKYAINSSFSDFYLKILVIPNEYKSHK

TIYCRCDNSKTEKNIPGQDKILKGKLGLVKIILRNQYNNIIELEKTKHIIHNKKDTYKYDIKLKESDILMFYM
KEETNVESGNCEEILNIKINILSNNNVVLKMPSIFINNINCMLSSQDQNNEKYYINLKADKTKHIDGCDFTKP
KGKGIYKNGFIINDIPNEEERICTVHLWNKKNQTIAGIKCPYKLIPPYCFKHVLYEKEIDSQKTYKTFLLSDV
LDTPNIEYYGNNKEGMYMLALPTKPEKTNKIRCICEQGGKKAVMELHIASTSTKYISMFLIFFLIVIFYMYVS
I*
>N.PF13_0249
MSRSSLIFENNNDNNEKKKRKLFQDEYNYQSCYPLSYLNVYNNNNNNNHNSNNNSNNDNNRIYDYAYSQSYNY
TYDQTYNDVYNKIYNDSKNIAQNVLNVQVNEEPYDKSLNNSHTTYPFSNIINSNQLNSNILNNNEMIDMRNFY
ENGNFVNPMIENKDQNQFNEPNKNNLNNTMANVNNINNINNIQFDKSLLINCIDVLLNFMNYNNLKDTITEET
LHENLINLRFYILRLNGGKVSQKKITEYFHKM*
>N.PF13_0250
MNIENKPQIIEHINYCLDNTIYDLKWVHGKSNIIAVGEMLDKKGYIHIYNLDRGKFTCISKTNLDKGVKTIAP
FFSSTGTYTIACGSFDGNILLYDINNMSEEYYKIRKHTKLINKIDCKNYKNNNIIVSASRDGSVKIFDIRTKQ
EVVSLEPPKNSSYIPDCWCVETGNNYVEHNAYSNMEEENLNICAGYDNGDIKFFDLRTMRLEHEVNVNNGVCA
VNYDRKDTKKNKLICSTLEGNIYIFNLDVYNEVSGYSYSKDKIISGTCWGTPFLPQNRDIFATLGGDGNLGVY
KYVYPEKNSIFDEKIGCKKGIVGELNKLNDLNVSTQPIISFDWCKDKLGLSVMASLDQTIKIYIITKLNLY*
>N.PF13_0251
MARLKVLNVAEKPSVASSIAEILSKGRPNKIKSCSKYNPVFTFDYKIKNDIWYMYVTSVTGHLTDQKFDDRYK
NWHNTDPQELFDAEITVYVEKDKKNIENNLKKYSKECNMLILWLDCDREGEHICFEVINACRITNRKLIIHRA
QFSAVTEKDIIHAINNLKEPNKNLAYSVDVRREIDLRMGSIFTRFMTIRYIELVKHETSIISYGPCQFPTLGF
VVNRYLDIKNFKNEYYWSIKMKYVYNNDEINPMDDENNYDDHDDEQMDSDSDESDSYYTNNDDSNNYSDETDD
YYGDEKKKKKKKKTKKKKKKKNSTKKKKKKKKNKINNNNKKNKHNNVVDFTWSRIRLFDHLAVILIYEELLKN
PLCKITNVYESETRKYKPYPLNTLQMTKLVSIYFKISSKECMMLAEKLYNKGYISYPRTETNYFPDSMNLHKI
INELRKNDNFGWYANKLCEEHKYQKPRKGKMNDKAHPPIHPVKNMNKSLKVEEKEWKLYEFICKHFLAVCSND
AIGYNTKVTAKIQEEQFFCKGLKIKEKNYLEIYTYEKWNDKIIPSFQVDDEFYPTSLLIEEGITQPPKYLSES
NLLTLMDKFSIGTDATMHEHIENIQKRNYVIKNSKSLFIPTNLGIALVQSYKKFKDIGIDLTDPSLRAKMEKD
MSLVASGVKQKNEIIRNYIDIMKYIYQEIYNRIDVLDKNIHYYLNNPDQLSYT*
>N.PF13_0252
MSTGKESSKAYADIESRGDYKDDGKKGSTLSSKQHFMLSLTFILIGLSSLNVWNTALGLNINFKYNTFQITGL
VCSSIVALFVEIPKIMLPFLLGGLSILCAGFQISHSFFTDTQFDTYCLVAFIVIGVVAGLAQTIAFNIGSTME
DNMGGYMSAGIGISGVFIFVINLLLDQFVSPEKHYGVNKAKLLYLYIICELCLILAIVFCVCNLDLTNKNNKK
DEENKENNATLSYMELFKDSYKAILTMFLVNWLTLQLFPGVGHKKWQESHNISDYNVTIIVGMFQVFDFLSRY
PPNLTHIKIFKNFTFSLNKLLVANSLRLLFIPWFILNACVDHPFFKNIVQQCVCMAMLAFTNGWFNTVPFLVF
VKELKKAKKKKEIEIISTFLVIAMFVGLFCGIWTTYIYNLFNIVLPKPDLPPIDVTQ*
>N.MAL13P1.235
MIRIKEETIVNDKYSRNALINYDEKDCIHNKLGSSFLKNYTFYYLKNNVLNKDSFDLALQNYKHFFENNKTLC
ILSILNVLIILFPLFLYLIFSIVIILPVFFLLCSLFFCMLPLIYPLIKDKIQIKIKEEIIFCTSITAGSFFFF
ISILFIYLIPFINFFLYPFNAIFMPLVSTLILPIILFINILACVIYLFQYTEVTS*
>N.MAL13P1.236
MASEKYKNPFNRNKLNKIEEYNKENEMMKLHNNRELETREAGRRGCFNLNDFIYNKENSRVLKKFILFVLLII
LSPVVLIVLYKYFFIFILSKNNALLCSLYIVILYIICLTLLYAYLAFQEDENYSKNQNRNYERKMK*
>N.MAL13P1.237
IRTNIYYYIYSVFI*M*KKKKKKNIYCYNYIYIFLYIT*KGYINHQFDIILYHH*IQKKKKKRVTFFL*HKNK
NKK**IK*I*INKYIYIYIYIYLYLYILIPFVYIVDIYYLLYTYY*YLL*LLFIWNFFFFFLKMENDKKHNQK
QNNVDENEFPNSKVLLVSVKRTRRFLERTARELLAGGTRYIILSGLGDALPLCVQLQSSLQSKNAANVVKIET
SYSYFNSNYSYTPGLKIYMEKHPEFKGSRISPGYVSFHEKTDSFTPIYDENPNEYICSLNAGDNNLYVGGEGI
NGAFSELLSSHNQEVDKYESLFKVIKIKL*NNKIIIMIKNNNNNNNNNTKKYQDAILIL*DVYKYILHIFIYY
LHII*FHLFILHIYI*FYFTLFFIILFIILFFFFFL*ELLTKAVNENGEKPDEEVKSVLYDNVDKKYPDVKLA
LCRIRNSLKKGSDHSTGSVFIVTFKKNFPHKKEKNMGMVYVVGPKGKNYNSVEEFLDEVQETAENLMTTLCDY
NGLVKREEIKHVRMNTCRICLFSGSIFKHPNASKLDVAKAILNGLAVGYRHGPSPRLNFAYDENVFKDAWVET
TGLQVFNHNEQ*
>N.MAL13P1.237a
MKNTFFFVLSFFLYITILDITLTSLIQKNILKEKVDKEYMKVFLFVNNSQKYCEKDNIILAYNSEKLYNLNKV
LNFCEAKKNCDFISYTPKRILRNSLYYKDEEPLKNTGANWICKGDTWISSRQKKDWITAIKEEHIRNQMKNFD

SIFLNITGECQKENIIMKVTKFITPKEAAHECQRIMNCKFIILNYNKQLGSKSLNDDKAIFCSHTPINRISKL
GFMIAGKNSSNMIHYTNNKKEKNTGIVNTGMGGTITPDDYYYNYKKGDIVQASLKEHI*
>N.PF13_0253
MSNQFLVDTIYNHGYMRKFLSILRSVKKNDKFQYIMKLCENTNIEEDEELYKVFINELHNISNTSSTRRKKND
SSCNESTNNTGNNKENGSNNTHFEDMKDINSSISNNSEEINEFEIDSSTSTQEKTKETRIYVDGIFDLSHSGH
FNAMRQAKKLGDIVVVGINSDEDALNSKGVKPIYTQEERGALIAGCKWVDEVIIGTKYNVDMDLLEKYNCDYA
AHGTDLAYDKNGTCCYEEVRKFNKLKIFERSYGISTTTIINHLLQAVNNSNYYSSSNNNNNNNNNNNNNNTLV
NSNNNNNNNDTNSVSTNEISDINNETNYVYNTNTNSEQLDNFNKNKDNPNILEITEEQIYNSELGISDDNKTK
VSEQQHDIDTLPKNLLNRNRCHITTSQIYQFIDNNELIKKKKNKKVVYVDGSFDIFHIGHLRILENAKKLGDY
LLVGMHSDEVVQKMKGKYFPVVSLLERTLNVLAMKVVDDVVIGAPWVITESFIKRFHIDVVVRGTIVDYIYSN
NEIDPYDIPKKLNIYQELSSESNITTYEIIQRIEKNKKYLMRNMSKRNKKEESIWETSNTYAINN*
>N.MAL13P1.238
MKNILNNDEVKNIIEKNEGYYSIIELNDVLYLNNKLYTKIECLQNLHNLKTLYLNNNALEKIEGLECCINLIA
LYLNCNQIKRIENLNNLIRLRILNLEDNNIFVIENLECLDCLEDLNLSNNCLGSKGSPNTSILHKNKKIYILN
LSNNQINEDILDNLLMLENLSILYLMNNPILLKYNNYRKLFIYKLKSLTFLDYKPIKTDERXXXXXXXXXXXX
XXXXXXXXXXXXXXXXXXXXXXXXXXXXXXXXXXXXXXXXXXXXXXXXXXXXXXXXXXXXXXXXXXXXXXXXX
IINK*
>N.MAL13P1.239
MRVSNLIVFLKNGKVNKRRKQNPPCFLFLGNRYESTRVVSKKEDASKNETKYNINNKECSENKNKYESTEICP
YFEEKKNTNINKNDSYIKMSKELNENLKDPNVSRKTYDILKYYDDNKNIIRTTRYILKEHINYNIYIKELNMF
YNYFYNAYPFFITKYVNYIKEQILLNYNDNMKINTFFSNYKNNENIKTFLSLFHYFNDKNNLFVNNNDNNNNN
NNNIQKVILKSLHCPNNVTQFLRGLKDNIYFINNKNKKDLLQIKEIFCEKILECIDNINDINKKIEILLYLSN
ICDNDKTYDSLYIIHINKKIEQIYEELNKKNKKTVEDQYMMFLLYKEYLSSKNEKYGKMEIKNFLSQELSFYN
FLKNENINICLLSLIYRDLSFLNNYEIKYVLMNLVYSKISNSEFFLYSSKEDFLNNKKIKSHVLFNEQNNSIN
INENNISKMENYKLNDDDNNHNNNNNMLNSGIRNFFANSLLRNNFFASLKNEDMTHSSNILNYYSHNVNNTNI
HNYVFYNLLTSIIQLNESQKKSFDVILLNKMKLFLLHNQKHNHYHLIPYILKIKENVDYMNNFIFMNNIFLIK
YVLKYKHIFSNNLYLYILNVFCNTVQMIKKCDHFTDINKKNMNLLNDCKKIINYFINEIYYSINLYTLKEFIQ
FVNLLNKFPSNFTFEIKNNIYLQNGNKMENINKCNNNNNNNNNIMDITNILHFNNEYISNKIDNNNNNNIKMG
SNIIDKNLNIESDIYDNKNIINSKISDKQNNVLYSSILNKNNFKTKKKNTHSIINKDDFIILISLSIFNEINE
EFIFKIKEKEKDILKNNNIQHQYQIYEYIIENHFIPFVNNTSNYLHLLNQKYDENTREQIMNYFIIFTYINQL
EISEIYSLVNVMHKINKFQSFIDFYISKHVQKILNEKWEHIIYTKRSGKKKELVNYEFDENYLDEYISRVNIE
KSINKLENINSVENTNNVDKTNNVDKTNNIDKTNNVNKTNNVDKTNNVNKTNSVSENGPVLSSHFEQINLNTH
FMYTSNFEKYYYDYDMNTSIINLFILLNEKSQTRNMLATFLPYIDYKKINIFKINLNKFFSSSFINNEANKEK
ENIIKVEGLNERYKDKLKKINNLNTFSLDNNNDTICNNNNYYNSSCGNYTNILNGLFGDSFNINKNEHNQNIN
NNYEEYTLNHPVIYNIVQSRLKYNINLKNYLENNEKDTSNEEIKQFMKQEMLSLQNITSLINYINNIKENDPN
YDINHNIRNNTNNFNNTNCVNNNDNNNYSSMHMDLLNLALKNLIFCKNDIVDIKSYRMISNVLLNINNIYQKI
YISSKYSYLNILIMYYIKRMETFLPFFNLQDYLQILQIYIKYNLSKIYMKHLYILNNELNKMNIKNINYPFVH
IILYFYFKLNYINETFVFKLLNHYISSLNQQQHYMDSSIIRNLIKTNDVLINLCVKNKDVLYFNYMILQKYGN
FKDNLELEENVEMDQKNNAVLEQNNMDHINEHITSAIKINDNSLSTNDTPSSFQSKEQKEQNINKNHLENNTN
NNNNNNNNNNNNNENSHYMEQNNENTINYNSYTMHYKNLEVNQNESTTYEEIDNSPYSTSDINYNSNKINKIN
NANNNVQKNIVTSKFELSLNQKLNIIYFLCKFHFYNETIKKFYLQVINECINNKNSNINDDDYCKLYEIYVQV
ILNFYFLSFNKNNKYINYILSNLPCYYWYKREEDKLNFFISSKEYNNIYHILKLVNLPFLVPTLTEIYFIHFF
NDSKKDNYNNHIPNNCLHYYQKYNLLIDDIKNKNIAILCVPEENELRDGNNSKRILMNESYYIYENIKKTYAT
SLLFLSEWKGLSTEGKCDYILKVIHSAVNN*
>N.MAL13P1.240
MSLNGVDLHTTDFINEKEEWMKKYENIELEYGALKKKFEEFSIDYKNKNEEIKNFATIKKDMILRYAYMKNEN
EFLKTQLNILILEKEEEEIKKRELIEINEKQKREIDGYKKIFEEVKSYSISIKRKNEETEKKICTLVDYDISL
KKDNILLNEVIKTSEKLNNINNNVNKLLQVEIDDTKKRLDMANKKIEDFYQSFNILKNNYLKYKQDNDKKMML
LIQCNDDLKKELEEKKNIQMYNDQCKIRMDDFNRSTILLEYYEIKNKVNNVNKTILWNKINMLYCHLYDERRK
DKNDEINIFIENRKVDSNDATNKQPNNKNDIYKNDIYKNDIYNDIYKNDIYNDNYNNRSENNNFHALIDQLKN
KILKEETNEDIHNLFFYNLSLDDYKKEIKNIQKYYEEHKYVEENIDILIIQNNFHISYILKVLNFNIKIMNDI
IDCYNISQILLKYIYNTYLEKTIEHIYNYMSIDVIQEKCSIRFGVFFFITLSNFLLCIIIYMKLIRNLGQGTY
LKLIENEEIIQLFCFSKYILEKYMDKIKMKLFSSNTDYITLYMISHRLKELYNSVYCSNIIKNKNNTNDKVIN
NNNVSLHMKYTNDDTFKNNTIHNIDNKNITSLELFCFLNFTLATSILLIIDTDIILECACDVDLQIQIVKKCK

DMIKTIKVPKKITNLSNIPLNKYKMSFKNYIEHITNFQQVVTQNINPMEDIIKREKISIEYINNLSTQILNEL
NIIVGECCTMYSINIHDEQNKDKNKCDLYPLQICDIYIKIYNKIVSIKNKNIIQKTDIVQKDDIIKNLKNEIV
LLQNKIKSIKNKMNALIIKEEKSNKINMDLDILKKEKEEYIQIINNLRKIQNNNNNEISYITKQYNDTKNKYL
ELLKNSESKKKKKYLNNNKNIDEYPNIDNIYYMKKVINNLYYENFLNKINKYYYLYDQMDDYYYHNYDKEYTN
IKGGITNKIKKKKKDTSKEESNDIDVMDRKKDIDRKEDIFRIDVNGSIDSFGCSTWNNNFFHINNIHNYTFEG
FIRNNLINQQFYNQCEDNILFDDIYNCEVIKNRLSQNKKEYFKNKIFQYTFTNTYEFNDINNKNIHILDIIER
YKKLKNEILIEILNTSNDDQIMSIKQSNLYKKKKKNYIHLYHKITELKEMIKQYYHNYNITLFNQNNDSLNKI
MNITIEQNVENEEKDDVSNSLGNDNEMKSYDKYKGEDKNYNDEQTLQINKNKSLTKSHIREDKIILGDDSLGH
LIQNIFNI*
>N.MAL13P1.241
MILLQLTVVGYLNTGKTSLINSIMNNEIFTNYSHTPLPMIYYKVHKDKNRAFCVEIEDTSVDVDINNFTNMMR
KEITTNSKNMKNPVFSYFENPCIPFQAHDPYNSISYGRMAYFLVFDLTNPSTFEYVKMIYLNMSSIYEKYYTL
KPFISLVGNKSDLADENCALVREAENFSNEHMVQLWLTSAYTGKNVKKLFLHTINMVYNNTNLWKYDIEESGS
ESSES*
>N.MAL13P1.242
MWINKIGNKSASFINHKHFHPGNIKNLERVWLAEENEKKRKEEEEKYLKKREEQYKLYELKKQLRKNEEENKN
DVHNILYDINNELKKENSFKKKILHNSISNETTPNENIVNKILIRSKYDEDIFLKNHRSIYGSYYDKEKNKWG
FKCCTNIDKNSKCTNNTNCSYNLNNNLIKKDLTKSKNKKKKKKKLIVDNSITAVLNKI*
>N.MAL13P1.243
MDFIKHLSDNDKIRNICILAHVDHGKTTLVDNLISSNKIISEKNIGKIKYLDSREDEQKRQITMKSSSILLKH
IYNKDYLKDMLIENKDKNKKNNNLNNNNGIYNEHAVERNIDNKIKDNIKSNDIKSNDIQSNDKQSNDKQSNDK
QSNDKQSNDIQSNDIQSNDKQSNDIKSSDNIINSDNINSGQNIPKEEKNNMDTFSINIIDTPGHVDFSSEVST
CIRICDGALILVDCIEGLCSQTKIVLRQSWKEMIKTILVINKIDKLITNQNMDSISAYEHINNIIEQVNAYIY
QLYIEENMDNENVETKNELEKYSYSPLKGNVLLCSSIHCWCIDMDIFCYSFCKKMNIDTSNSDKIKKYMWNQY
YFNVKEKKILKIPNETYTKNHNNNNNNNNNNNNNNNNNKKNSYGNYNNTNDNDYNKTKKKKKNLFSLVVLDFLW
KIYDITIINRDDEQIKKLCRELNICDSFINKNQQNNLENNTYILTYIMSRFLNLSRSIFNACIEIFPSPKNID
ENRLFKIYPSLYNDEIYKHIINCSTQKFTIIYISKYICANLQNNTLVGFKGFYDKNTFLSICRIYSGMLYENM
ILYICGKSTKTIIKKIFICMGGDLIPIRYAYAGNIVALYLSIMYDDISNNIGHNYLENDEITRDLNNINNKRE
ESHFMRDNVHINCNNSVEKNMKETSNHKNDIEQNNSSNYSNNNNNNLLYNKNVEHALNRNNINMEHNNTGDLQ
YLSNTLMNLIKNKRNNKTEHNIFLMNNDGIFLNKNITLSNYKNADSFILPFTDTCSTILHTIIEPRNIQDMNK
FLYGLILLYTCDTSIDIDFNEKGEYILKFCGEIHMQKCLSDFVNIYSNIEIKTSDANISIREGIHENYIKLKR
KKNKIPDNLKILYDYYLKIQSTVGVTECSQKNQDNDINNNMLCKDTFKNQNVINVMNNSIDDNNMDKNNYMIS
ENLFKNVPINKDNHFLNILFNYNNNTICHKLNNDAFYLFVSVLNMPESLLNFFDKNYSNIQTILENRSISPQF
LNYNKNDLSSINENFMYKQCLINLEKYVNEICFSDKMYSDNMTDEVMKEDIQEENKDNMTSINENKDSTEKLN
MLKKNKNYHLELWDICVQNGSITLLYLKKYYNKKNNNEYLQNNILTNDKYRKVINDRSFIDTYLSDNNSDINI
YLNNICLGFKLASKYGPIAQEPISAFIQFFQISRRIKICSGNVIYDIQFSHWNKLNEDIFLLNDTSTFIYDED
FDVKLMENTATDIVNYIRRAKVEKKKKKXXXXXXXXXXXXXXXXXXXXXXXXXXXXXXXX
XXXXXXXXXXXXXXXXXXXXXXXXXXXXXXXXXXXXXXXXXXXXD*
>N.PF13_0254
MFRSIVPTTVGLNNCKYVHVNIDEEYDMCNINKDVKIYNAAQDVSNINCKSITHLGSIYNIKIDSLEKCKSPE
EIAIKILKSQNWENKRKYTNDDEYYYKKKKRISNNIMEPSYINDSSYSLIDHCDNMYERNNYVNERNNYMNEG
SNYMNQGNYYMNERNIYTNEYNNQLINKYAINNNMNNYMNNYNYYNNIPYNTFSQNQKEVYHNVSQNNYDQCQ
NYNQTENNNEILYDTHVDHDLIKQMNLLQLAYKSRFKKSNNKVKINETLLYNNNNEALQNIIKCSMNKENNDQ
YYDNLHYNDNMKDNYLLNQGKILFNNDSTEKKGMYNVQQTNEQSLNENNYSHDMNYNINLPINQSLNGPTSGQ
INEPINEHINEHINEHINEHINEHINEHINEHISEPINEHINEHINEPINEPINEHINEPINEPINEHINETI
EELINDPINDPINDPINDPINDQMFTQNFNEHLIPVNLKSSHDYDKIKTCTSNICGDDNTIRCQEKDNNILEY
AKNNNNIYIDQPNKEDQQFEYHLETFLDLNEDDDIINNKQKMKRIINYIEEYIRKGNIKNMERHKFFFNDNKI
KLIFDKLLANCKNILNIEISYRKNKIVETYTSEDDISMNDINNKDNVDMKDINMDYINYNDQVDNDSMEKISD
KECSNFVPLKKKRNKHKIQEHNINNINNINNINNINNINNINNINNINNINNINNINNINNINNINNTMMNKL
IFGHYNILLYLLLLHFDVFKNKNMNIDNINDNILLNLKIHMQNVIIKLYLNEVKNRVIEFYNIHETYLIEIYN
IYEKCFFYLYDIYMYIEKYETVLVNLIDTCMLTFQIHHNVQGIIKHSINLLLRVFKNNNSKKMKKYIIDNLLM
NINNVNFKYNKLNFKNFQNNSTYVHLVHFFLLKVAETFSFYEGDMKEKFLDSKHRDAEKKKNDHKKGKDDKSD
GRGNEYEDDNVDDDDSDYNVNSDYNVNSDYNVNSDYNVNSDYNVNSDYNVNSDYNVNSDKKKYRNDQHKENMN
NTTSTSNLSEDSFCLSLFSSENDYKNNIVNNNCKSENDITGYSKKKNSKKKKKQFYNIDKEERKKYSLRNKKK

LNRYDYHNLNNTKKKIYNTRTKNKKIVSSSFNNTCKKEKSIFTNKNNNYKKAKFNIIKNGSNINIHQVEDSQF
MCSLHNFMMRKNFKRLNNIINYILIEMIENFLFEDNKNGRIILQHFILDLIKCCDNPRFNISSQFIKNIIHIF
FDIINKKYDNNIKETCLYLLRYIFKYIFNIYNYINNFSYHMLYYFNYTIKTNKFIQPLFYDFNNDSFYEKDKI
LEDNLKNKTKAIVHNNNEHKKKNIKKGTNINTQYKINNDKKSIEIKMSQNNKTFNHTKCCNELKYEERILKCM
QCQSFYHISCIQKNNNHILFKKDTYEINEYVEPNLFFINKEKNNILQNGDIFDKREDEKEEENNNNKSNLSQD
KNGNKKGKGEKKGIYNNNYYNINNRDKPKYMSKVVDYNCDICKIKNLIKIFIIRESQINNSFLNLMFKKKKEK
NKGFYCYDKFLNSLKIFIVYYYYILIYLNKDKINEEIDKDNNYVYSYILLLYNNFVHEQIKKDNEIVSNTTTK
KKQILQKKKKNKINKINNNNNNNNMRYISNENNTDDERKKKIFNSINDMLIYEFKNVKKNEEMCYSFSCTHNFD
IYINHSWKMYLYFFFYDFFCIAFNQLFYILYENSNKNLRYYSISIISNIINDNYLYLKSKQTQHILLSCLTDN
YLKVREYTLYIFYNFCKHFFDIENLIIYQKEITTLFDDELKNKKYVCHQKNDELCNRLKETSQEKNNNKKNCT
LNNNNNNNNNNQEEFNEYNNEQIHDKPYYAVNQQNRDHKNILDLNEYMTNNIVESIRRCSKDIKISVRIISVK
ILKYIIYFNIYIKRYNGSIYKKNIQNYDNPMKDNHSNCADNNNNNKYNNNKYNNNKYNNNCDVEHVHNNYYEQ
KYLKNEQNTSCPNSQQTNHENITQNCNAENISILNDLIERYVSTYDNDNMKNTVMEIFIEIFFMNIFKTIKMK
NIYSHFKNENIQIHSSNNKQIVHTENYVYYESFTKDIESDYSQSLQKEEEEGEEAYEQQEQRSDNKYIKIEKD
IYTLFIHLLYVMKNKHNINIVNKMLSYLDKNIKKYQELFVVIDKILEDKNLTQADYNNNNNNKTQRKGKQIDN
YKYAKCTDTNFYNNSIYDYNYNVDNEINTEMETQSERQEEDDENEDVEEEENNNVKQNDNKKKKKKKKGKNNN
IKKKFDKQKKNQNICNTGDYENNNYIAYNNHMLNIKNNVYNIYINNSYKHLDILNKKNITYILEVWIYNLICL
FIKTRRNSLLENETRKIMNILNIIIEIIPQLFIKYVNFFYPYIYNNDVSKDVCELLSSIFPYCKLDLQLKLQF
KKVTFNNSLLYHHNIFISRSYIQLLCTLHTYIFCNFVYFKTYIEECFIKLYKYKLCFLINNTVVQLNNFVREF
YLMKAEQKEEIKNNLISLYNKHVQNVNQNNLRNDTTMNDEEKGKESNNNLEYKKNECNENKNESNKYIDKNLN
NLSNPEHFFIYNIIDITYEEFIYHLDLYKKHIFDIFSITHNDIININKYAWLISIMCEFINMNKIIINDLYNI
NETIHIQVQNEHKEKETTNKQNIKKVTKNDKNDKDNININNNNNINSNSYSYSCGYGEPILSEKFNIRCPNNN
MKYIKLSDIVKNVQVYINAILYFEFIYNKQNDYIKRWNIVKLKNEIISEIIQKKININKIEKCNDIFDYKFYN
VSLILLNILIDLFYISNDIKCKCFFLHCLCKILSNYHNSHFINDLKLRNVFFYCIDAFSNAALQKSFLYSLLQ
LIKTYEQFLRNKNNIHIAQNRKNKTNENNYSTYKKMTQLVPNEKQQLNKKKIKINNNKNKYFSSDERTTCDEK
EYSSFPISCINPQSYSSASPNLCESNDNETASHSGKNNTKKEKKKKKKKMGQDEKMNTKELYEENETSVVDIK
QENELVKGTHKKKQTKEKIDPNNYSINEKKLKKNHVKCQKNYGQENIKSYENNKNDVNGANYVNNQLVDVHNI
TNKTNVDKNQNKDIIHVLLVASLFSQKIIDLLMVNKYMYIDCTQDELNFIKCLGIQILSHLYKEGFIQSSKIS
PSIFCLCFSPDIKLKSRSEKVINCIIEKENYNFLNNLAECLQYLLFYIIENNYMYNNRESNNINNLFLFNKIE
SSFFKTLLDIYENLTSKKLKIKYLKIILKEIENTCHFILYDKIKKFIRETNGKYNNNNNNNNNNNNNNSSRHK
VHSDQNKNDDDIYDCNNESTIDHSNNNNNNNNVYYNNTNIYNNQDLSKNVMIHIYPINQIYSYIINTYIILPN
NKTIKKNGKSKKIKEITTSSLSSYSSSYSSNSMYNKDDELKNKKNNENNKNKKKNKRSKHKDLYYKEKTQSSK
DDSSVTENIYDNDNYQIRDCKDIMEKIRNKYNYLLKDYYMKVDEYIYINYFLFLYLQILINLISYLNFSTYNE
IAILIHDINKLYCIISSTIHMDIDADDFSRTHEESFFTFNEHMKQYTSVNDINVENEDYMSINLNLNKVKCFV
MAILNNLTNNLIKHYDIDTSRLNELIEEDNIKENKIEDEKREEPKNNIFNYMEYIYECIEIFIEDNNTIVEKY
SKCHLNIHNLNNDYINKLIKMEKIKNLYKRKNKRRQLNKDV*
>N.MAL13P1.244
MKLDENNDKKNEEVEQKNISQRMKKLCSILNSPIIDINELKNILWGGISDEVPFIVRARCWKLALGYLPLNTD
DTQKVLKKKRDEYENLKKQYYNKMKLSEDELKILRQIKVDIPRTKSCYNIFINKKIQQLSEHVLFIYSVRHPA
CGYVQGINDLITPFLIVFIRPFILKKEINSDDIDNMIDEDLKNVEADLYFCLSKLLEQIQDNYTFGQPGIQRA
IIKVKEIVKRIDNSLFNHIYNNNIDFIQFSFRWVNCLLLREFPINISVRLLDTYISDICDIFTDFHPYICAVF
LVHWSKHLQKMDFQQMLLFMQRFPTQNWKIQDIESILSEAFVLKNAFQSSPKHFS*
>N.PF13_0255
MSFKEKLNKRKKNDEIIFEGYGKIQQEIKTNDAPRGSKQENLSEKLPFFLNKKKKTTDYDFMVNISEEDKDVR
SVSRTIKLYLNINNDIRLRLSLLYVSEQYELFKSYLIALKDMMKSIIVIKVTRKGNIKKKRLSFNSYNMSIIG
NWSKKVLLYDEITQINIGSCSTKELKIYENKFQDYNNRENYVVIRTLYRDYSFLFLTDYDIFMKLKNSSAIEK
LKFILNNKNNPALLNDKDMKNNGANQKKDNINMRSKTNNFIDFKRRTVYEQYVNHKDDNNTNEDMDLYDTSRG
FKFFFFKVLRSIKTKTIDMENDNIKAIMKPNNIIYFHKYNTLNHKINSFFLYCQIQLDICGPEIWFTSKFDDI
LFTHKS*
>N.MAL13P1.245
MEKELYVDSYIGFKGNISNNLILIRKEKEIDKKYYKEQLKYIEEEEYLENEKKKKLKSVYNLKNIDINHNYYE
LKREELKYNFFMIYAIGTNIIKENILDNNRTIFKSDESEINKLYVDKNKKYICCCKESKLISNIYIYDFENKK
KPIILSLHKFRTVDISISNDGKYLLSSGGHDDKYLILTQIENQKFIYKCLSDYPYTNISFFHKTNNFVIAKLN
SIKICYYDFSKKLISEEEVNTSIYKRQFVCLELKENDEYAYLGTTTGDVLVVNIKSKVLEKIIPENYLFGNGL

NVIKILDDNTIMTGSGDGYISLIDIEEKKVLRKTKLYGSINSIEMRNKSTLFISIYENIIYVMDLINNNHYVL
LLIHNHYVNDLCFPFNYNYIMYTCSYNSVMAWQFYDKKAIYLKNIDKGKFIYYDKKFLNIPTEKRKKLCKLNL
MHESKINNSLENKKKKNSLQNYEKINKNLICHSIKITNDGNYIALSIHNNIYLLTSKYMKIITVILNSHYDFC
NVLIFYNKYDLITAGNNGDIKFWKFDKGKYLNVNTINYHSSIINQIILYADKYLCSCSEDGLITIYNIQESTL
FKKIIDINNTRYKQISLNNQYDILLCCGNNNIFMYYDLIKYDISKNFYYSPFHNVLSLDIDKKGNYFITGSDD
YKIRLYEFKSCTCLYIGVAHNNVIHKCLFTFDNMYIVSASKDESIKKIK*
>N.MAL13P1.246
MIEEITKSLFPYDAKFFKGLFDKYMKEEDYSKDLKCLQNSRKLIEEIISKENGLNFYIDENYDENFLKKCKLP
YFKKLLKYIKYMEEQKQDDVKNKNVENEDLIDEDNKNEDIQGDKKTPKKNVSDYFIFNKKINMINSYRIHYYC
SNIIQSIPSLTYLSVIKIYLQNYPLKIFLTCIYFYTFVTYVCDRDNVIDFLEALFFSLMVTIGCICLTIVHYL
KENRINSITSKFNHIKRNYICRNFIKHNVKNKKMLMDKFESKKTMEIKKYSKYFFLKSFLNRVNSELYDYEHF
KDKSNIDYIPFEVNENVFNFDSDNLNNDELEKEKTNKKNDKNNNKNDKNNKKNDNTNKKNDENNKKSDKTGME
GNEDLLIKNIKMEDKNEIVQEYNGNKVNVEKIFLDKEKNTNKMKGNITNEEKINDEENINDKRNTYKSRDSVN
NNNNNDKKVNINNFIKMNDNENVLSKNLDKDTLNKESNIIIFIDERKKNDLLNYIFFENNFYFINGKNLLVGD
IVYLFKGDIIPADGILLKSNNMIVDESNLMNTLEKKKKRKISLDEYIYEMEKMKKKKISVDELKKTKMNYFEM
LNLKMKMKIKKFSEDINIKKNSNIEKNNDTYIMNDIENMENVKKSIDILCDSKKAYNEGKDENINKMTLHNNN
NNNNISCGMYDNNNLCEKYDDVCPLLLSESSIIEGSGIMITTCVGKYKQMFYYNSIKRIESNTRIENLINRYS
KNMVILIIFSCSICILFIFIHFLINLIENDMQTFAYNLLMFLLNTIILEILKYLLLSIDQMPLLLQNCLALNS
SNILKENFMINTKNTFENILFSNTIFIDINRYIKYKCVYFFFNTQYTYSFVNTYFLYDTLRNPSPSFKIFNRD
NDKHIMNNLFFHLLIECILTTSNMYQYNEYFLDIDISLINFMKNFQINVEDYFVKPHNILQLKYVKGKAVFAF
VLLNEEKEKNLYIQNGKVHGKQPNYNDVKRKVCEGKVMRIFIKGHVDFVLSKCKTYLDGDTQKGDIEKLKEKV
KNIKDGEVDTILCFAYKDVILNENNMEDDLYIKKRDNEYNLKYKEIDDNNYTCISILVLEKYLSKYLYFDYSI
LEANNLNAKFFTKNNLESTNKLFDNFGKHLESIKAYDATKINEIKSFDLSSDEDEINNFNKKGEKGKDNEEKR
KGKEEKEDEHSEEDIIYLKNELNATNLMEYNNIESYELRNDKNEISKKKKKLKSEIWNLRKYDESSTTNLYND
RYTQENECNEIRRKQYDFLLSHNIFYNCNKKELECLLSTCNMYGKNTLVINDDKYISTPNKNNNKNNTMNINN
NNKNNNNDNNNSSSCCSSSSSSNIYNCSSILICDNKCTNDTKEKCDMVILNKSLYDYIKLKYISNCILMNIKL
HIEYNIIFYISLVLFSIISTLVNGLEFLSTVHILYIYIIKNIFFHYISCYRKCNYSVVGHDPNNKYSYNFFEE
KDINNIICSILSKITILLVVFLFGHLFIPENKWIFITDEIRENFEFSEFSFLIDRQQSNYFHTVRSSLRFKKN
LENLKIQNNIDIKNDYRTLNEWEYHISPSRHGTIVFNVFFLLFFFSYIYLYIKTFSKEFQNYSQQFKIEKYNY
LYFIKKKKDHVFLEKDLMKRLMHDVKKEIVISDEKADGNKKHNKINNNNNNNKINNNSNMKKNHDDDTNIYNE
TNYTNKQDSKILKKISSSLYNIKNELYKKREMINNSIFMESIFENYKIFFLFLFIMIIHICAIQYGSFFFHFH
VNGLTFIQWVFCFLCCLLDLLIYTFISYFGFFQVSSDFFKMFQYLYEPREKNIFDSLNEYRKSSTSHRYKYDL
KE*
>N.PF13_0256
MEDNYASDSSNKKKENVKFSKFVTSLSFMKKNTEHHENAKKKIKYSNDNHMWIVNEYEEAAKAYLKKESPQKS
KKHVSLNCLGRKTYNNYNNHVNLYNIEIIKFINSVKRNKPISSLKE*
>N.MAL13P1.247
MKILYICDSRFHLLNTKEKNYLYKYWSYSLQDILNGTEARDIHFWLVCISLIDYIDLITFSDLFNYGKLKSAI
DKFSDYFQMDNENLIKENKIYVDPLLAFLKNADNTSKIKECDKIIILSCNIYLWENKISELQIIGDVFYTFMC
KFYFFNVKNKTIDTTNSLEHIQTNLKKYHNFFFKNMELNKIDILQISKEILSINFSVFFTLNLKNELLFKCEG
CPLINELSYSAFVGLNNYPVEFDIRAKALKEGISQHLIYGIPIFIYTTIHYETNLISLESLSSQLFENKQVLI
LRSNINPTDLNMHDKDVRFLWVASPMIKEDQSIILCLHGLSTLENYSLSIEESYACQRTDDEKKYINDMKLKE
IFNFLILIEEFNPFDYSNQRLEYSFKFSKSSLSNILQKTNRTKKSKKLYTKLKNNICITDNDSQKLRKDTDIL
LVKEKQDLKKKKKKNEITDPRFLLLTNMNKAFEKNII*
>N.MAL13P1.248
MKDSINYDFEDNKNELVIIVNEKNEYQELKTRKIMRKDNLWHRSTSILVFTKIGQEYFIYIHKRSKIKDYCPS
YYSIGFGGVLSRNEDFLQNAVKELHEESGILKKPEQLIDLGLIKCDTEYAKAFVQCYVTFIQPDFQTVPQLNE
VEFITRVSMNDFDKFLEEEKITEISKSVYNNFRDKVKKSKLDEFYKTLSS*
>N.PF13_0257
MIENTKVNIFYGNNKYPFICKSILNVYKNKNKKILEKTETTVNVDENIKFIKDANVEELKIEFLTNAPNNNNN
NNNNNNNNNNNNNNNNNNNNNNNNINKGNMNHCGKNQEEEKKNLSKSNNIEKVPYICSSDKIFAKILDHLLKT
DLYNISEKIDPKNNKVVNIYVQTQYEEWLDFFRSRNVNKDIVFICEHLNKHLHLNTFVVSEYLTLSDIFIYFQ
MYKYFHVSSTQYAKYSKMYKNINRWHKLIDSLIYFEDAEWKKNLKCLICDNKMNFDDRKDNLKNNNKSMNNTD
DVKCKKKTVTTSYSGKLENAVIGNVVTRFPPEPSGYLHVGHAKAAFLNNYYAQMYEGKMLLRFDDTNPVLEDI

KYEKSIIEDLENLGLKYEKISYSSDHFDLLEKYCIDMIKMNKAYADDTGVEDMRNQRGEGIESINRNNSIEKN
LELFNEMRKGTEIGQKNCIRAKINMQSKNKCMRDPVMYRCIVDVPHHKHQFKYKCYPTYDFACPIIDSIEGVT
HALRTNEYSDRIEQYNWFISTLNLRKVYIYEFSRLAFVKTVMSKRKLKWFVENNVVDSWVDPRFPTIKGILRR
GLTKEALFQFILEQGPSKAGNLMQWDKLWSINKQIIDPIIPRYAAVDKNSSILLILTDLTDQVIQKERDLHMK
NKSLGTCNMYYNNKYLIELEDAQTLLENEEITLIKLGNIIIKNIEKENGKIKQINALSNFHGDFKTTKKKIHW
LPYLPQQLITCTLYEYDHLITVDKFENDNKDDWTNFINFNSKHETLVYAEPSISSLKVSDKFQFERRGYFILD
KIDPHQHLHLIKIPDGKSKNMSIITTKVDPKQLAGTKDTKDTKDAKDTKNTKDTKNTKDTKDTKNTKPN*
>N.PF13_0258
MSEHSSGVSRKLFCNSDKLFYDNFRNLDDRLNLNNNFNENRYEEIYNKKYLRNNRRFKTEPPKHLYENESEQS
YSSLFHNILRSISKNIRNDEKEESSDNNNCDDDNDNNNDDDDNNNQSDSIPNNSENFNYDDNSCCEISPYDEI
SKKTKRVHNKIHKNEDGEYYKNVLYNNNNLSKENQINNICSSINKTQRTLKRIDTNINETLNNVKYIKMVNSD
IISDVFLKNNVKSLIVYDTGGIDIELIENENPEFLYHTYTGNRISNIYYEYDDCIKNKDTYKNEEKKKKKNTF
NSSLLYRENYIFKKEHELNYCYKNKDDNKKKIKNNPSEKNQIMNYSSDICSNSDSKKLFTKNKKQENVINKNI
YYNNPSDYNILNNEVNYNKNTVHNTNLSKDTCEHTYKNVSNKYDIQDHNTNSSAEKYYNMNYKSSLLNKNDKY
IENTSSERNKGYRSYSSMSSMLSLNAYEKENRKKYYYIGETINNIRNGWGMLIYNDRVIFEGEYKMNNAIGYF
IKYNEYSTEIGYRSPISVKSVIIMSDNDNFIVQNRSENLDTSKNKEQKSGNNSWNVYKYDNDNSDHNDDSDDN
GDSDDNGDSDDNGDSDDNGDSDYNNENIYNNENILNNENIYNNENILNNENILNNENILNNENIYNNENIYNN
ENIYNNENIHNNENIHNNENIHNNENIHNNENIYNHHSNDSSGENIRDSFSKNNIKNISKDTYKINNNENINN
LDKNVPIYDNSSNSINNCSYYINSKYANKSSSNKGNKMISKHSTLKNSSCINNDIKNIKFSENPEENAKELKK
FNNLTSYVRRNSKHKTSPARSINYLKPPVMNLIRNNIYFNRNTLGKNDKTTFCTSNIHSSNKKMREHKTTHFL
SPMNIIVTSLDTDDEDNISDDNGKTDVISSCTQYEEERKKDEQNIKTEEVQYIETYMKDNDTRVLYELKINKN
DDILSNHKTNESIFEKKDFKKYEEIYNNDYDHQNIIKTVCSKNDNNIDNVKNMKSVDIYDTNKKNYNRNYMNN
LDNANNFDEPTNNTFEKYVKKNEGEELIFNDDQNKLHIDTFEKYKYLICENINNDKFVIKNNQITTFEKFLKN
QQNFEINNKWRDTLNKIKSQNNSMRQFMQPKINNYNLCKYYVCSNNIEQIKKGALWNNTRLMGDIIKTKRENN
NILNGDILNGDIINGDILNNDILNKDILNNDSLNNDSLNNDSLNNDILNNDIPNSDVSSNVHNISSEYLNNND
KENIKFCKWGRNIFARRLRGATFPETHDDRQKHCFLFIDDYLTSNENKFDIINQNVKLRASDYNKWSVNMLYN
FLKMIGLKKEAYLFKINKIRGYHILKLTDKELKKLNINNSYVRKFVLSVFRFLTNSIDNADPLSLNYNSNKKF
SFNNIENICNTHITILNKIGGGSYAQVFRAKYKTIHVACKIFLYKPKDLAEESYCESYISTPRSSHRYIFPKI
NKNINGEDYKNTDMNNEKRKDQNIFTDNFHMDESSSMQHVPHVDDINYIEQEDNIENNTKYKNKKNNNDINNS
SDTLQRNKKIKKMANLEIFRYFPTPVKYRNYEAKILYSLRECKHVIKLIGVCSLREGEESLILQHCPGGSLEK
YIYKDEKKKNSPYAKSLTRPKLVKIFQQVAEGMYNIHTNQCFHRDLKLSNILLDENQNAVISDFGLSTNFSSN
DSPTAYAIYGNIFYAAPEVLKGEGFFKESDVWSFAVSLWEALTKKIAYDGISASEVFCKISSGELFLPIPKDI
PMELSELLKSMLEYDFTKRPLFNVIAKKLEQIRIQAEDKLHLDIMSFFDG*
>N.PF13_0259
MVELTEEEAINFLNIALGEAEKSLQKELKEMPIFCLLINEEKKIISSSYNYTNESKNGSRHSELIAIDKYIYD
GNYEQMKNLNLIKCYNNNENSIEKSIQDYFVSLDEKKNSELKINNYENINDKYMNEKYNKIQERINNLKKCCI
VVTCEPCIMCVYALKLIGIKNIYFCCLNERFGGCGSVLSLHKKYEDINVHYIEHPECSEKSINLMKAFYKAGN
PSAPEEKRKRPIS*
>N.MAL13P1.249
MLHLQRSTISDIENIEYILKKKDYSGIKTNQDIKKIFKDLGSAFRIFGESLKKIHNDGNTIICNIKKKKENDK
EKLDENVYLVDCFTNVISVIKHMYEENILASDEIEKNIVNELEKEEKERIEDINDIYNEENNLEDNVQNKDKY
INIKNVNNDNNDNNENDDNNENNENDDNNENNENNDNNLCFDEYLKNDICIENIPIIHFENYLKKCLEKNEFI
NEYVNHPQFINYFSELPKLDLKLEKEIHSFKEIMDKFAKNASKHLINKSHERIQAIYKKKKEILSFLYENIDE
LKHNKKNIISKKDMVLNNYHNANHNANHNANNNANNNANHNANHNANHNANHNANHNANHNTNHNTNHSSNHS
SNQNKSVKGLNNISKKKRRTNNKLQEESLYLEKFEIREKYRIEKIYSSFRCFIKLLALKHIRINKVIYETSKE
ISSYDSLIDYQQWLSIILKKNINIGNLRECIQNNNILTKEEEFLLMPSETNNSNNTYLMNLLEKVQNSAPHFL
KGELIFNENKKEKLKIKEDNDINKMNEADDMCLEKNENSIKTHQDDMLNTELNKKENNNNNNRTISNTEEKYN
IKSNIHKNDVINSHRDSQFPDIFYIPIIIKQDDIDEINSVCNHLNDQPYKDHVHLFLDNKYKNGDTNKYKNGD
TNKYKNGDTNKYKNGDTNKYKNGDTYKYKNGDTNIDLDIDRTIYSNADYCSKLNMNTYFKKDMDRYNGKDIVT
FYKNRQKCYEKYILQFNDLNMLSYIYKNKNIKIDNNDNYIHNNIIDDSSILFDYKNFQGFFYEESHNFYPYKI
PSCISNHFSIFQKMNSLHNNKSYNIHQYDIDENDYKMMSYESLLLVYYFLSTQVNCYFLYDIGSRNKINKYMY
KEICDKYSDVLNDLFMKDEYYLNNEDKKKNVDNNNNILFLPKNRYENDEEKKKKSIYLSSSEPIHNTKNSSSV
DYRNEEKGGQKKKKKTEKYDKCNIFNNNKRENVKGKKKEMNDYFVYIDYIYNVKLQNLLLIKRLISIFRNKLY
LNSRNNDLITMLTIPNHYMNYGYKYSFHVLDIRFRIMRLLESLQFHDLFCTYDQNEKCKKGDSNNKKNINSNN

KKKNINSNNKKKYINSNNNNNNNYYYNNCNDELYIYLSWVRRQLHLLYMSLYFKFKRFMIFTPYLYNLYKRYN
NNNNNIMSDEEDEDNIFKENEDYFTYINKCKEENNLFVRYIRNVQEKIKRIHHVILTRKGNVNCNIEHIIMNE
HMMEYIDFSCLNYDEKIVFFSNLLLKNLIKSFRIVLLLKSLIKENRWNIGKVDERLHLTNLIKYFTKIECYDN
EIKNVIYNSYKHVHMCNTNDDTIKKNTSIEVEKNYKLEKMKSEDSSVIYATNNNMCGINNMCITNNIYEKSKR
DYSKYICNNNMNMCYSNNNNNVGNSFEIVHDKGKEKKIRNNDISICKNIDNDELFKSIFRNECIIKKKKNIQK
RVFYKLNVETVLSSCWFLDRILIHLFLYYYSSCMIDKNSKEKIFFDAPYIMQAIILVIHYLLAGNDLTLRNKK
REKINKKYIQERGFVCNKYKESAPIINITNTTSTNQNDSNNNNIDNNNNNNNNNDSCSNNYGDHNKQYYVQDM
YNIYNDGQESTLIPMSTFILYFVIHLFYSFFDKNLLTLYDNKEYKSYELFLKRKNDEKKKEDNLDDLLNQSST
NNMTNEIITVQMASRFFFLIKNFEQFLSFRSVDEEKFLNNSYKNSSIKNFKMKKRDFYSDHGDNYSCADQHLK
KSKNKKCVQEHKNNDNHNNNNNHNNYHNIYDMEDKKQNKKKKKKFLFCTNIPETTNNTFNDTFIMNENFVKDI
ETRKIKEEEYKNDVIYLRRYYNIRKFFNYDHFENLISCYVPNILSYYLNEHTYSKDYILYDTITLNNQVHIIG
NYKKSMLCDKEEKKEIHFKMLKGLYEEESSYEDSRNSYYFLFGSLMKENDKKRYNEYMDNKNYSNSKENDFNS
TSSKISTFSSDTNNSSSCISYNSSSSSIESISSNKKNSKYNYNNNYHYNHFNDDIYNKIDMKKNNIPSNCNNL
IIKYKKNNELSYISLIEISRIEKVMIDIFIMRIISCDFRNYFFLIFCNYRKFIDMYSLPYILDVFIMLNKFFT
INNNSIIDKYNNNNNMPNIYNQYSSGCEAEKQKKECYQNVDMINSSQNHNVLKHEQNKQRGGKNYGKISPFEL
FCNIKIDEEEDNEIYVQQSDEKREDLIRLQDINNIKETQGKDIYKIEHSNRNNIENDTNTQYNKYICGEQFNK
NKNMYYIDCMNDAMKYVDKENNKVNMDQHMNNRKNEKFDKNRDLHNGDMYINTFNEHDNSTTSGYERMSTYFK
YFIFSSAKNIFYNILCGIKDDMTLNDTYGDEEKVLVHEKDDNKIVEKNYTNDNYKEKSNMQKCSDDYGDDYGD
NHDNNHVNNLDNNHDNNHDNNHHNNHDNNHDNNHHNNHDNNHGDGLDNPINDKRDSTKNQKIEKNMNDDKKKK
KKLCNVIISNSMLNEYVNIINKYINEIIMEILFFTNIWKHYLSLEEHPRIVLFAANKTLYYEVLKFIKNNKKK
TDEESFKQSLSIIVSLYNFNLLNKEIVLQTYNYETRKLMKNDIRKNNSILNDEHNEYYYMGSKNTKEYKLRNG
SKVFNEKIKNKVLNYYENRNSVRFNINENINHTVEKEIDNEKNKLNKVKLSSLDDNQNNSICNIKNSNEDDMF
INLSLIISNSNDPCMVKLMFKLEKIFIKFIDKKLQENENLLSKCLKNEKLIPLNAPDIMYNSTTVDIFTIISH
ILEALFEYKCPVEWIITPFLEFLNNFINDYCENYKKRYTSFIISVLNQYADKYFNDLLNLNEQQKIQWDKYSI
DHKKKKYKKTENNDSQFNENFTIFKRNDNDITQNHQDVEEEQDQIFDNNIHEEYKDADEITKRIRHKNKKKTN
VFYKLFDYEEIDSIIKNKIDEILTDKNEESISKTSNNYQQQNLNQNIYHKKNHKVTQNGNDGQTTNDDYDNHN
ECKNKTDGSYMSDDLINIIDEINFLFNDSDLSNSLVITWNLYFFSEQLPLIKKKFEKYILQYVCTRTTNVESI
SQIFDEHLHFNITQDKLQKNFYTKTIFDVLDMSINNIKNTLNNTLYFIFKVLSIRIICYEFQKEIFYNLYEPF
HVNNIQSIVSIFPNTIEKFFKQLPKIYFKSILTVFIELFIKMWMLVIIEKGFSNYHFSDQHIHIMKKDNQFLK
LYLENNHIESTHNFLSKKYHINEYIDTFLDSLYGNRDLFEEILNEQKHKKHKNIVSSAYSKGMAIISGVNQHI
QEKTKWME*
>N.MAL13P1.250
MNTSLSNNENENNFFIKSFFESLSNFIKNIKIEIKDTFELLNYPCVYQNSKKKFSVSENEDYKNSLTTNVISK
IDKKNIENFRVYQKELHNILQNIKTFNEKYKLDVPLLSIMENLIILHLINEYKINNIVKKIQEHNIPMPELNS
DLPDYLIPTLELEENQNESNLNVSISEINNLSINNNAVKHILENAQLKVDFSKLVKLNGENHLNSSSINPSSE
LNEKSDELTLYKKDDKIHNLTHDIKEEINNERNEDQYLNRSHNNEDEKHTLMTSKKEEKVNLIQNIGLSEETL
KLLNLLPKKRKKDQN*
>N.PF13_0260
MIFDFALNNCCERNQYITFINISVCAIINIICWKWKILEPFKLLTVFLHEFSHASACWLTGGKVKAIEVNKDH
GGSTRTVGGNQYLILPAGYIGSCFYGMFFILMAYLSKWTLLISTVFLCFLLLLVLVVYAKNMFLRFLCLLFLS
ITISIWVLCEYYKDKVYYWPLIIIMTFIGVLNEMYSIVDIFEDLITRSTPDSDSYKYAKLTKCSSKLCGVLWL
LINFFFIILTIYLIGAIQVKNFDTELYHKVGIQIKK*
>N.PF13_0261
MKYGQVVVGPAGSGKSNYCKMMKEFMKIKKRNCYVVNLDSACEEYYYERKKKPINTTYNIEKELNDYYDTIYD
IDIRNYVEVNNLMEEQNLGPNCALLRSVEILYENSYLLEDELNNYDDDDNYFIIDTPGQIELYTHTDYFKKIL
NIFSEQNIRLVIVFLIDISFISSNTKLLSAYLTSLSTMINFELPHINILTKCDLLISKNYYHEFNNFKNRNNF
FYQKQLYKKINQKLIYYKKQGTYQKNNAEENDLYNNNHNLRHMKNNYHNLNLTNQEHNQNNDEDDKSFLEEIE
FINNTKSFKDEFVLWSDVENTSVSSYSSFNSNQKENNNSIYNSSDEQKSVISDECEESIYKKNYEKLNDILSL
DPHDIIITANKCMSKKYYKLNNAFANIIEDFNLVSFLPLNIYDDDNVDFIINSIDMIIQYGEDKDVNDNYDMF
S*
>N.MAL13P1.251
MKFIFLYLLILYKIILSYCYLLKRNKVLYGHINPSSCIFFIVQHNSLKNIAYNNNNNNNFVYKKKKFSNNRNK
KLCDSNDDEKKQPFINNSNIFRNKYNYIPTFDEVKKDIEIFKKDYPYYFTEHSILELIRIENNIVVINIEGQF
FEDINVVFSEVTKYLLNKYMGILGVHPYNIRNLNIRGNEM*

>N.MAL13P1.252
MVNPPFDINYFLSSCTNLEKSYEHLLKEKKLYELKLNQALISLQEINEKYEEERKNNLDVSLRISEQTHKIIK
NENIFEDVQRQNEELKEKIEEINKEKELEKQLMEDKIKILKIQKEKENEEYEKQMRILKNELLKLNSVIVELD
LDISSKDKEINNLSSYLKSCKEKHDKVVLEYKEKINDLIKQNEEREKKNKEALENIRNINDNLALNNLRNKLK
LLQEDYNDLEKEYLYLKKSLSKTSTKKHLITKRIQGKSICNNKISYKI*
>N.PF13_0262
MTSKSFLLSFLKYKHVNTYIFEKSFSKILKNTKKHIDCHLKSCFVTMNEKKEHVLEGEKNKRVVNASKDKKKE
EEGEVDPRLYFENRSKFIQDQKDKGINPYPHKFERTISIPEFIEKYKDLGNGEHLEDTILNITGRIMRVSASG
QKLRFFDLVGDGEKIQVLANYSFHNHEKGNFAECYDKIRRGDIVGIVGFPGKSKKGELSIFPKETILLSACLH
MLPMKYGLKDTEIRYRQRYLDLLINESSRHTFVTRTKIINFLRNFLNERGFFEVETPMMNLIAGGANARPFIT
HHNDLDLDLYLRIATELPLKMLIVGGIDKVYEIGKVFRNEGIDNTHNPEFTSCEFYWAYADYNDLIKWSEDFF
SQLVYHLFGTYKISYNKDGPENQPIEIDFTPPYPKVSIVEEIEKVTNTILEQPFDSNETIEKMINIIKEHKIE
LPNPPTAAKLLDQLASHFIENKYNDKPFFIVEHPQIMSPLAKYHRTKPGLTERLEMFICGKEVLNAYTELNDP
FKQKECFKLQQKDREKGDTEAAQLDSAFCTSLEYGLPPTGGLGLGIDRITMFLTNKNSIKDVILFPTMRPAN*
>N.MAL13P1.253
MATTNKKLQKIMTQPINQIFRFFTNKTVVQIWLYDKPDMRIEGIILGFDEYMNMVLDQTKEISVKKNTKKELG
KILLKGDTITLIMEVKNEET*
>N.PF13_0263
MSNKIKEKKKLKKKKGKNDPEQKKLQKYMNELWGFSSSENEEEKKYENENKDGNKYDTERKSKNIKNNDNLIN
INNNNNNNKMECILNENHKKKDYPADKDLNILVNHKKKKNNNNNNINSTNNDNNLSLGIGNNKKIQENHKKKN
NNHNNNHNNNNHNNNHNNNHNNNNKNTLFTKNSTMKNDLNDKNLYKEKLKEKKIFMDTVHEIRKMTLPYLDKF
QRKNVQNFQIKTLGGKFDKSSKVHYTELMSRKKSIKKYIQKRKEKDKILGVQTQTGNYIDMQDVFRKNKKKYK
SKKKEKLF*
>N.PF13_0264
MKNEEYKRLISLWGIEHQEILMNSSIFFLGSNILTLEICKGLILSGVRNLIIIDDGLVDIENLRNYKLYYNLC
NTNDYKCNVIKEYLKRINKNNVHINCIITNPRKYFYNLLKERKEIDKYKCNDKKTNVDIVICNLNIKDNIYIE
KLCSQYNINIITCNYTNMIGYLNVCIKEHNHFYIHNNHNHNNNNNYSFLFYHYISLSLLNIPEINEYVTKLDY
TSFKHNSMTNKIFFLIKCYKDIYNIQQEKKIIIKKNKINKINNNNNNNNKSYSNICYSYIDSHEIINCNHILL
FIKNKLLLTNLSFPNFQNITHEFLALHNILCLIKKYIIQSNIHNYENKLSISKNHISFFLIVYKSFIQKNNYL
PYLYDDINFEDQNINTIVIRKKQHDEQKIQHIINKKKIKYSFYKPFSITYFRYFFSHFHFIKHITKDEIKNND
ELLNHWQKFVCLYKYNMEKKEKQKKENKDNNIYIDHTHDNINNNNIYIDHTHDNNNNNNNNNNNIYIDHTHDN
INNNNNIYIDHTHDNINNNNNNNIYIDHTYDKNCNHFHNNFHNDHYNFPCANTHYNHTIQIKKKERKIKNSTPI
LLCNNKNNDILYFFKNTLTHHIKETIKKGTTNVFKNENINTIYTSIYQEKEIYFVKSLLLHSHRNTSTLKETI
QNINNSLLKYNKTNNFPSNICLVLLMSGFITQEILKIASLYLKPHINYYFFEL*
>N.PF13_0265
MKRKNIHVFFYLLILSFILYNKIINVLNFPINNGINKKDDSKIKRGKDEDSNSNSSSSSISTAVFYLSDERIY
INEQHFTNIKLLQNILTEHIKSNEHKKELEELGLLNIKSLLYEDFKQEIYNSMKLIFQFIKQNFYYLCKYINE
EKKRNKLSANNYLSDDQIYSVIQNKIINTNVDKNKKFGDLLNYIIKLKKIFNISFSMNLEKEIRPIILITHVN
PANNSVHININNNMTEESIDEMKKNGSLKKDKYVEEISESILNPIKTNSLFVKISNSLKWPLHFYRNLCYKNN
YKVASENWVSYFYDHNETLCHVETFGTGDCLFLSLQYLLQNNGIKKNNFVENKNVKESKFIPWYIEKVKQSNK
VNYDVTDLRYITTFYVIKYFPGFSSDNDIDENNINDKLDLLINLELTNYYLEKDLIYEVIKNKGPIVNQQENV
KETIQEDKNVEEGDKIKNDDKNVTPCVDDERGLLTNKCNTTGKNNNNDNNNDNNKNNDNNKNKNNDNNKNKNN
DNNKNKNNDNQIDDDLTGPYNNHINQHNNKVEEKHLVHFNDDQNHINNLNKEAEGSTEHSNSNHDYSSEKENA
YNPNGSFSSFSSFSSFSSFSSFSSFSSFSSDEDTSYASKKPNSLSCSENTENTKMDSSLLTKGDNHNDYNDDD
INKDKENILNSDENFNPENDLYSNKEITIYKVNNNEVSKSSKNNRRYKSEPHYRIVYKNNKSSNTTNNYISPH
FMNEENMDENNKNVTFSNNDNNNNNGDKKQKSINNFNNVDEKKESYDNDVSSSKSMSEGTKKKTTKQQGIINY
YEKRRCINNNSRSKSLSCKGDKLSSNEKHAYNRFENNSINMNAKNKFKDDNNDNNKNYSNDTIYGNEKQKSGT
TSKSIFQKIKSMCYDNHDDTDDYAEILDEVQDLGYRLYELIFIKLISLSKNKLKLNELMKLSKNDLKKLQGTD
HKVRVIASNMFSKKLSEGTVLPFYTFLNIKNKLNNPFSNMDFSNKDYYIVVMKTTFNKDIKRKKDGLVTRSLI
LNHRGDSISYWSIKNNDFHFTSDNKVIQTETIKEKASAFFYERTRLGHVHWGDETDYDAFQKMFNIGLITFMN
NNTKLFFPKGTFKEYPMHFLIYYYSGIHFEPGVHVTLNGKTETCHSSYKTGDIPNSFMEIPEK*
>N.MAL13P1.254
MNRIYLFVFLFVCCFVSSVIGMNVVIPRNKESRTKRVIKGIKKKYKGFTERYRKSFHLKDEDVIGAYSMQAYY
FFDKNIEHMFKWRLFYNFCKKSPSPCNYFKLRKEFKRIYEGSTFTLKKYVSEVNIIFNQFIKEYSTVRFNVDL

KQNHIANLSYTPNLTTQIMKSLTSLINDQLMENELKNPTWKLSFNYLTGTKVLIITVPSAVPGLNFFIYYYIT
TFKYYYSKLFGTLGNIYFRFQGSIKKHNVGYFSIERRYERKT*
>N.MAL13P1.255
MIFVKHIFLLYIISLFLNNLNTLCNSLKSPKKKHIGLLYKLSKRNNNKNVAIKKKLFVFPLCFIKVYPFIFKN
KVAFQNVEDNYVYTLKRMERNKWRMQDERKVIQNEQNVCNNTNKGFSDLDGNSLDAARYSKNIVENMNHTGYK
KDSDHNNENINKKDSDHNNENINKKDSDHNNENINKKDSDHNNDTINKKYSDHNNDSINKKDSDNNNDTINKK
DSDHNNDNINKKDNDHNNNINACNNNGKVSSKGKKRIIETDMYGLPKEKLNIKRQTNKNIKSKYGYCKIKTYN
ETINVNYRKKKILSILEGTLKNKRLYSPDTYTRPMMSKVKESIFNILTHLNILNGYNINVLDVFTGSGNLGIE
CISRDVKNVTFVDLSLNSCRTVFENLRLCNIYDTNKKIIRADAMELLQNPYKFNVHEKYNLGFFTPPYEQIIY
SDLIHNISNSKLFEEDGLIFIEYPKEINLLPKKVGNLIGLRNRKFGRTYYTLYVINSTGKYIPYQNENEFYPL
HYNRKQRRQEKYIE*
>N.PF13_0266
MDDFLIELKKISEQYNNLLLEKKKIEEKIKKMNENIKNYEHKKEEYLKQVDKKKNEMLMNKNKHIIRCQKMEE
LNKKLNDIILRKQNIDDNEEKCMIEKQMNTIKNTFILLKNIQKLNDIIGKKNNYYKLLNEKIHNNNFTNKKNL
NILNAFLNKSTVNIHQMDKSFSNSINYFKIIETKDMMKKLNLLFSNTHTDLSLEKQQFVFVLLMNVLKKIIQS
EESIIEDVKSCNECIHNFLYNNYILQQ*
>N.PF13_0267
MFEENEKEQLLYYERNKESEKCQKVWESIICKGLGYSKEEIRRDVERSRREKERLLSILYGSVFTGPKYLLYS
KKWRGKTLNEIINEDREKNLNKNNFKNMDLDSTYFCELSFGYMDTAEENDDKNKKEITKQMEKKNDKVPNIRK
GGNTNNSQNKRKTYHGEKNANTTNKDLDEFYSKLNYLEKRSYKNRYRNDNNNKKNNNNNNNNNNSNSNNNYYY
YYSNGRSSYTNELSNEKNKYNEENSDEGNVIKYKYLPTGVFYSRVSKSFIANWIDDKTKKQVKIPYKISEYGI
EKCMILAILSRNLRLSNLSNVLKYYDELTDSQKEQMLHAIRTTQKSEKLFEDIINRNGNKKNENNIINNYSNI
HNDTSINNNNNNNNNIGNTIGTQHKNKNKQNANDQKNIPMKKKLIEKKKVKQSYVNEEKLPTGVYFYQGSYVA
NWWETNQKKQFKVPFKISEYGIARAKNLAIISRLIRSSSIPDINLILTQMENDHNLSDMDYTAISELAYKYIE
NMAKKE*
>N.MAL13P1.256
MKIVEFRLAMPLTIEEYKVCQLYFVAKASLEDAENNINSNCEKGDENNDSKKGIVILKNESYINEDGTCGQYT
YKRINLINKLPKWLLNFIDPKYCIIDEKSWNAYPYLKTVYESSGFPKAKIQVESAHFNGYDTEENALNLSEEA
LSLRKVIYVDIVNDKISYKDYNESEDPSLFYSDKAKRGKLEKNWKENHSIIMTCYKVFTINIPYFGIFCSKLE
NWIISALRDNILKYHRKAFCWIDEWIDLTIDDIRNLERDVQKKLNKFWNDSQDENVTDDILDEENNNKNENVK
DQYVIHHNNNEENNDTLSNDIIDSLTFKNNQKDDIQRNMSNGNNMMNNDKICNNNNNNIYYNGNIHNNECTNK
QNLSQGNDQGRKEISKDLPNGTYITSSLNMDNDNLDFICTKYNEAYDDNDKENNKDMKKKNININININDMQGEK
QIIQNENTLNNKMINVYGDNNINNINNIARNKSVITKSTSLLFPNDLTIKNNLNGNHNNVIDNIEKKKKKKKK
KKEKKMESENYFFFKKNEENKEFGEYLYRLNEGMFYSWKLRYFVIKNNKLCYYVNHSKNELKGEIDLLNAQIQ
WIGEYKGRNSVFVINSLSKNVNYLSSENEIQTKKCMIDIQMATLMNSESVKKREIKQENGKIVSKGVQNGDNK
EDVDDDKEDDDNDDDEDEDTEDDNNDDDNDDDNDDDNDDDNDDDNDDDNDNNNDDDNDDDNDDDNDNNNDDDN
YVDNNHYNNNDDNHINYNNKDTPNYHTHKNKRIVDNKKSQDMPKNKNDGTINNYNLPYSKESNKEIDINNHHY
NNYICVNDKDEIVHSKLILSDNDLSQYHNEENKTISNNSHYISTLQNNTDQFNGIFVTNYDHIFNKKMEKETC
SSNKKMNKLKKRKKKKGLKMKTQDDKNKFVESAVNNYWDNKMDKNFNKKNYITSENFNDKYEGYSDNNKNNYD
MSYINDDNNKEKKKKSKKKRACQKSEQMNEKRNILNYFYKKDDLYNTYNNNYVDKLYMNIDPCGLYDIKNIMS
TLLNVETKNEHVDTFKNIESHITKLPIFHNNKMNNKKKNKHLYLFYFIFNIISIMSCMYSFISLYLYFKIYFL
SFFLLFFCICIIYIYISNHKPINHMYKCSLNIPYNINYVINFLTSHNKYHPNEINKKVFKTKNDNIQYVYSTF
NIYCKSILFKIFPCHIFFKPRKILCTQFTQICNSDNNILKDKEGGLRKYIILQYTNKNTKAFLKSLEFNEDIN
KLDMNIYKNVIGLKRNDKEEEFFKHGQIKIEDVNNENFNNEQMKNKYNDEEKMDISKSLKSDFLEKDQSQVIT
SEEDSEMKSSCREKNNNIKMGIPKMKNNSNYNKNDDNNNDDNNNDDNNDGNNNNDENDDNNYGNNNNNNNNNN
SVDKCKGGNTNEPCHYVDDTKKWSSDLNQNSEYHIYNNLNYISAYCYKHIKKLLDNVIKKIICSILLKYKYVY
KYFKKYYYNKYVNVDGCDIFLIQEKKDNTCELIFYTYYNYNSFFFNESINYTRCNNIREKLLTVDIPKYFKHE
NIFYCDKYYVEEKNETLLFLEKKKIEKEICKNIFFNINSNETYNKNILLYIYELTYKGYFNEKFLSSIHNIDD
TFFIRNISKVFLYIIQNINLYDVKQFKNISEFIKNKKNKIDYTDEYFISNIIHIINTLPLLHYTFSTSLIFPK
KNEKIECSYEQSNIDITLKNDNPITFFIIAENYKNKTKITSELILKTLCTFDQIIIYMQHQMQITNKSNYNIT
FNYPHIILRNLLHGHMNFSFAHKMVIKDTLSNTAEIRFIDKDKSNGELFGVIKRNELITDFLSGNIFDKIILN
NDKQYNSVKDIKINVIHKENTKNLIKSFFR*
>N.MAL13P1.257

MKNTVVRIKAELENVKRLFCDDEYLWIFNIRDSTSSLTRDNIQFRKTDILEIPNSRGTANFMIKWTEYPKYST
INFVNTKNSCSYEEVNNNEWRDFASFECRGIELIDFFPSNNFIVEDTKGKLYYDVNLSDQNWCDYNEEHEMCV
GIYNLEYEVN*
>N.MAL13P1.258
MNLLVICIYYLFFLNIEKNISCSCSTTKNKSTFVSLWDKFRQLFKDKEIEKEKNIITYNFIKHYNDEKYVIEE
NKKNRYFTGYEINHINRLIYMNSFKTYILSAYIPYIYMSVRDIYIVLKKIKELNFNTVYTFLFWPENEYLEDE
YDMNNSKLFYLLNFCASNGLFVILDIGPYINNIYNSNIPTYILFNKKLNDYIRNKYIERKGDLYKYFLSPWEN
IWKRKRNSLIKETRNLNRQIIEKNINNTKSFYKKNNYTYNNIYSLYYFNRVIKWYNYILPQLKKYMNINNGPI
IYLNIDKTFDNYYMYIIIDLKRKKIFNNICNAQHYMERSNVSYYNNKIIDHDNISSINKSSSSEFFLNCVISF
FRRIRRSYIMTLGYLLYIKFNKYLRVIYEYDLGLLFINEINKLVQKHLTKVNILTTNYPYINDEFINSYAGNN
CYNHFLKNNWFDNECINLNKPCIWSQVWTGAKYSIHNVNSSSILKRKYDDHILYTSPYVNIENKVDKISSPHP
DHDKANDKKGNQPTEKKDKQKNIDNKKNNNNNKNNNNNNNNKNNNNNNNNNNYYSVDKGGSVQNKEELSHGKD
DGITNNYKDSKKCYKDKKINMDDINSNVKKRRIQINKNNNYYNNNYIGHIRNFKDLTFNIVVFIAKGGVFLNI
FPYYSGNNINNIHSYIEHYSKETGQPLDLYFNHKEPLYSHIKRIFKILYKYGNYLLKDEYYINPIKISNNIEL
YDYDIIKIICNYNIKGSTFVKIGHVNYNINSFSCIIYHEYKKKIIYDTSYNYSYEYSYINKKETYKPIDKYLY
SLNTINTGPLMCIKEKQQKIIKPSNKNKNKLSKNINIYKKIFTYLFPNNFFHSVYEINVLNHFYLTMDFTKFQ
WYILSFKNTMNYVKLFISNYSLYIYIYADNKFIYGGFNEYKFIEIMNCKHIYIICVNLGLGFPKEQVRTEFFN
YINFNHLKYNYQENNFNSSSHTSINSDHNMNDQGGPYFNMNKSASSKNKRGIQETGKGNSKNKMDHDNRKEVV
NSDKDEKHLKEQKDPKVMNKLDKNHPNGNNNNNKKNYNNSKNYNKDKNFNNNVSSNGYTIFDDLYDYYKNKTE
QNHNIYNNFMYDEKNYNMHNYYDEDSTNTYIFIVTRNEYELFNCVGLNGEIIKNEKHMNEYKKMYNFLDYSFV
KTYLMNTQSSNTNDVSEQNKNKNKNKNKNKKDNKGNNNNHDDDDDDNDNNKVISKDTNNNDKNYIQSNDNIQI
NNQPNQINRDYQNYHNNNFKSLLTNKKIEMKSITKAFLNLCTSGFFSFIFNKIKYYCKTGIFKYLSTFNKVNK
NNTSTPLTWYTLLYFIKNIDFLRSKYSLKLSTYDKKTKNIDGLFRGFVYINNHFLGSFWITDDVDSYEKEINE
DEKLKNGKKIKKEKSKFLNNENLIHIPLTRYMRIPTDWLVEGTNIVILFDEFGGNPYKVEIVREILHGGVYRL
TKKEKYVNWILFLFILSIIVFITYFFFKALHNYFKTKEEKERNKQNYQNIIENIITHNIYINSSYDDITEETN
TENIQNVSDFLDS*
>N.MAL13P1.259
MKEMCYIVPGELGLLIQVFMGCISLSILFTKYIFEKPKRLFVIFLKDLIIISCGSITLHLVNICLCILLFRYD
ILLYIYNIHMDECSIYFIHIIIDITLGLYLQHRIFALYKFIKVRNGYLQNSSISHIYKPVDALSHYSSFLNFM
NNSEDDNKVGPSKELTNVINIINKDKNNNIFYKGYNNNDKEKEDIYRNYFKTYHDYEEEVTLEANIHEQEKKN
IYDLNFLNNKTHTDICASSKYENIQHSDKQIITYEKEKSMYDFNFYKNIGEKYEDLNFLPSIILWIFVILTTK
IITLIFFLVLFPLFNFFVIYTIDFIKDTKVKLLLVMIILPFFFNFIMYFFIDTMIQRKHKYKKWHNDDSI*
>N.PF13_0268
MVKYAKKIRNLGKCAKGAGVDLRVHFKNTYEAARAIRRMKLLEAKKYLNDVIEKKRCVPFRKYNGGVGRTNQA
KEFNHTQGRWPAKSCKFLLNILDNVQANAESRNLDVSKLRLIHVMVNKARPGRRRTYKAHGRINPFMSSPCHI
QIIAREINKPAKKSLLSNAEKQKKLPFKVTLKKLIKLNISNQNRLKNKSVKKQQTVQ*
>N.PF13_0269
MNVILSIDQSTQSTKVFFYDEELNIVHSNNLNHEQKCLKPGWYEHDPIEIMTNLYNLMNEGIKVLKDKYTSVI
IKCIGITNQRETVIIWDRITGKPLYNAIVWLDTRVEELVTEFSAKYNNNDIQKKTGTYFNTYFSAFKILWLIQ
NNPEIKQKIDDGTAVIGNINTWLIFNLTKGNCYTDVTNASRTLLMDINTLQWDEKMCKIFNITNMSVLPEIKS
NCSNFGLVKSEHVPDYLNIPITGCIGDQQSACIGQAIFDEGEAKCTYGTGVFLLINTGEKVVYSTCGLITTIC
YKFNDNDKPKYALEGSIGTAGSGVSWLLKNKLIDDPSEASDIMEKCENTTGVIFVPAFSGLYAPRWRSDARAS
IYGMTFNTERSHIVRALLEGIAFQLNEIVDSLTSDMGIEMLHVLRCDGGMTKNKPFMQFNSDIINTKIEVSKY
KEVTSLGAAVLAGLEVKIWDSLDSVKSLLRRSDAVFHSKMDDKKRKKKTSEWNKAVERTLIQL*
>N.MAL13P1.260
MLKNTTKCDNIIKREQNRVLLQKKLSKDSVTSTKFWDSSEEREGSSNTSSNVSFKILEESNITKNLNEDKSYS
FECDKEETLRKDYKKLNKKNSNKINKSNSLNQTNEEIIRDKTSVQNKKIHHEVLEEQEKDNSIKLNKNKVGIY
FDNTTNSINNNIQKDNSDTLGTESYSTHKSNLNEVLNVSGDNSKHLLAPISSNDIIKKEDSNIYYNLNENSTY
YYPNNNSKNYINKFLNNYNTPNVVDSFSYCGTNFHSGNISTSYNDRYLNGYSNNYMINNSINSSINSSINNSI
NSSINNSINNSIDNSINNSINNSISHFNTSYPNSNNNNNNNNLNYSMSNNFNSYPNNILNYNLNNQFINAYPI
NNLNYNSTSHFNMYPNKNMLINDTLNNTNSYINNMKYSGNNIMSEQNNILNETEFIGSYHNSSAPIISNQENN
ISNLDNTSAYVNDNTTYGNISGRNMYSGVINYENITDDNKMIKSDNNVVPTINTFYSSKNIGESTNGNVVVRK
NYVDNTYDNNSNEEHSIENKEIYKSKSVSVSENETIKNDNVSVSVSEKVSSHTDYSRAKHSSHVVDKRIVHEG
FDTIKIPKYREVEIVEKIVEVPVVHKVNKYVNKYEIKEVEKVVKKHINKYVETKIEVPELHYQDKIVEVPELQ

EVIKIIEKPEIKERIIYKNKIETKIIPKYIEVPVVKIVNRYEEYDDVGEVVKAVPVKKIVEIPNEIIKKVKVP
VKKIIERPNYVPVIKYRDIPIEKIRYVPKIETVELVKKIPKIIDVPVPVKVPKIKVIDKPFYVNKYVDKPVVI
PVSKTIKPVYKYEGKKIIEIPIHKPYLVTHDTVVNKNVENNMNSGRYSVYAKRLDLNSFDPIKRNELFNLVNK
DNINFQRSMSVDDFVNNNSYVDGKEKYTDKLNENSVFNNYNGIGNKDNLDNMNKIKFTQSLNNNNNMSTTKHS
NMEVSSQGYGYINDGKENMYNNYNNMNNLNIKGSEDASFHTKKSIFDNLNNDNINNINNNNNINKNYEENRKS
SYGTSIHNTPNILPNRHNSMYPSNGNHMGEFISLKSFQNKKYSNSTIVDSEKAMNYTYGNFLSPTGVYNNGLY
MENKDKNNMVYENNKKDMNTINYNNNNNNNNNNNNNIYYSANNKSPNNIHKLNMSNSMMKTNTFGNYPNIYRN
SYMENKPEDMYLNRYTTKSNIINNNNNNNNNNNNSNNSNNSNYFYNGRNSSINNAFAYKQYYDSNKNGNNQHR
LSHPSNMVHNINNSRTRSPSTCSADGISAYVVEYVGDENIMYNNGNTPFSSNGLLNNLGKTMEGSNYSFNGVP
VNQK*
>N.PF13_0270.1
MKVLLYNILFVLYSICALCVKERNNAFSFIGFPKRGEGRYLFGRNQMKSQDIEKNNKELSNKMDTQDSKQYIT
YLYHSSICQYCSKVTSMLKNNDNVEIIKFKENNKIEDFGKFTKPIVVLLKNINKENSLERSIFYEELKRKGKK
VQVPALEVNNIILFESDEIIIFYKKLLQKVSNDDKSSLQNRGNIKNDQKKK***L***L********Q*****
L**R*WL*LPY*
>N.PF13_0270:mRNA
MKVLLYNILFVLYSICALCVKERNNGFPKRGEGRYLFGRNQMKSQDIEKNNKELSNKMDTQDSKQYITYLYHS
SICQYCSKVTSMLKNNDNVEIIKFKENNKIEDFGKFTKPIVVLLKNINKENSLERSIFYEELKRKGKKVQVPA
LEVNNIILFESDEIIIFYKKLLQKVSNDDKSSLQNRGNIKNDQKKK***L***L********Q*******L**
**R*WL*LPY*
>N.MAL13P1.261
MKQKKKKNTTQEKKNKEEEEEQEQNEKAENKIEDIKNKEDDEITTEINKYNKQKINMIEQNKDDAKKNLSNLL
SLYNSSEDEAEEQVNNEYNDQKEKGKRNSKNNQQDDQTCDDKICDDKICDDKICDDKICDDKICDDKICDDKI
CDDKICDDKICDDKICDDKICDDNNNEDTYQNNESNSIIKNKKNIKDKQIGCKQNTSYNICTTNNINNEIHKY
PQKQYNHIYTNQSNISHNIHNTYYSQNAYQNENYYYQYLNYPHVANFNMMMNTTNPVLYNNNNNNNNNNIYDK
SKKNNFNNLGFSKNKEMNIDINKIPVVDQREILREWKPNIIKEEKKSQKYTIKTKVYNPVNNTFDKAIIHGKN
QKRKHQINWLAKEAVEKEYEILQKTNNNKRRTTSDKYGW*
>N.MAL13P1.262
MFMKFIYAWSIYILIYTKCNSYILRKKYFDNLFFVNYDTVPLYDYNRRCVIYDKANKRWKLRNQRIYKKEQND
EREKGKVNRFTRCYLLNNNEFRQYDKKLKKRRKEKEGILFSKFDKMFKESFNEDDKLKKSELISILACICTNI
VIEFLKLKEEKKTEDFTYTFDYSVMNVLKMVNFEYDVNEELKKQKKKKKMNKTYTSLYGEIKGGGNKKIDDYN
DNIHMKKNEDPTINNSLFNEERIGANDKIEMFEETKEINIDINNKNTKEENELLDMYIQKLEFNTLEMNEIKT
YIDLFFGKNTYEKVFVKNNININKIFDVLHTALFDKTQERENVMQLQQFVDNQEKKLNEKKINVRNINTFNDY
LDKYVSLEEEKNINDLDNILYDDEKYESEQERTSEKKCMRKKKKXXXXXXXXXXXXXXXXXXXXXXXXXXXXX
XXXXXXXXXXXXXXXXXXXXXXXXXXXXXXXXXXXXXXXXXXXXXXXXXXXXXXXXXXXXXXXXXXXXXXXXX
KKKKGTKALDRVNMDIDNDGDNNNDDDDNNNDDNNNDDNNNNGDDDNSEDNDNSGDYYDNNGEDNDNNGEDND
NNGDYYDNNGDDNDNNGDDNDNNGDNNDNNGDDNNNNYNNIDKDNTNNSNSNKHRKHDNYYNELSNDQHNKDN
HNPNNHNMNRKTQHNNSHEEENRYINKIHVKNIEEDRNFSKNNNEDKKKNHEEEKKFLSGLSYALIKGCNIQM
EHKEEKMNEEKNECCTTHYDDKKLESCHDNQNHIILLNFIYDNKYNIEFRTDVGVVAYDLLKNEFVEDNIEEC
LIKDIKLNHVFNKDIINIFENIIKNLNLNVICSLPYKKRFYEKQKLIKDINNKINFHNLNDEMLHMLLYIYFI
IWGEDLHFYELKKVHIEPIKKEDINKSMNTNNYVDNKNENILSAHNFFVNFNYNDNDDIIIDSDTYNNNLSKN
KNNLFDSTFFENDYISLNLKIFINNKKEINNYDELKNAFNIYKCNLSEYIQNFLKSINEKDLNFVTPEYFVKN
YIYNTNDEFINITEILYKCKVQKKYISLYPEQTNSMERNKKTHFKLYYSCPTSAPLVNTKIKMLQNKYGFIKN
FKSNIFKINKKNNNNIIKYYHDCNYNKNDYDCNKNDYDYNKNDYDYNYNNSETPVGRYSFFLFSKNKKKYETV
NKKEKSKYYQTINGNNNISTTCDFLSNEQFDEEIENFEHIDNILGHHNYDNFNEEEIENTNTKQDPSNKINHY
EKSKMDKSNGPTDNNNLTCERTKSSNNNPYLQKMFEKQKYMNNILSLTNMNNNIKQNVHVLSYEDAYSNIKND
LKNKSKDKKKKKRNQNDDNDYNDDDNDYNDDDNDDVNNDDDNNNDNNDNNNNYYYHNNNMDEQNNILEQIDEE
KIEKQLTSLLQNKDNYDMIKENEEKKKNKELEKLNNEKEKISTFFKNMNVEIKLNKNVCVGCGAIFQSKDMNK
FGFLKNDIYEKIINKDEDLDKKNILSEIYEENKKTNTNISDINIIYKNDTINELYKLKEMNIKKMSEDDNKKI
QYDEDHNVNNNNNNNNIDNNFLKVQNNINKNEMDEDIMDQKQYICKRCFDLKHKNKITNNLIINYTNNNEINV
QDFEKYVINIFKKKCFIIYIVDVLDLYVYSNLKKLFNLYKKLHNDKSKLEGFYFCVNKIDLLSNYKEFTVKNY
IYNFLKSNKINVLFKNIFLVSAKTGYNVKKLIYTVFMRSKNILRNTKKKNKQNLDEEEEDTEEDEDMDSLQCG
NDKQEKKKKKKKKKKKKKVKHFFLKICINSNNDDIKCDDKNNHTNNKIDDLNDYDLIKYRKELFSNDNKNNFL
NEQLDENNYNDNDLKNYYSKNVNIYIVGNANSGKSSLINYLLKNVKNEKKNFLISHSIIPGTTLKNIKIKLN

KNITINDTPGIISNNSILSYLNFEELKYVVCNKLKNKITSIYINENDYIFIGGLLYIHILNIKKYYSIMSFFL
SEKLPIIKRKNFSKDPSMFIKQKIKTGFLYPPFKEERFDEINNFKKYYFNINNQSVDIKNSSYDIHIQGMGYI
TFYSFENIEFNLYTLKNVDVMSRPSIMPYHKKFGKLNFTKKLK*
>N.PF13_0271
MRSFYNKCFTNKLISAHSLFCQHVSTLKNNKKECFEDVKRSNRISDEFYSSFLYHSYIENLKSTKEYVYFIYL
QDIYYNINIFKNKGRLYISNNGLCNDKLKDNNNNNNNNINSNNFGNSYNAYTKKKSFYHSFNDLNEAKEIRKK
RKNEKSEKNIVEYLLCNIINPNENYHIQNIYNHNLNNNVSIEIHKKNDSVMYHYNNEKKTKTSKMTTDIKLKQ
INNVYIKYVPYKNKIITLACPNKYYPTLYRYFSSHTNKKEDAEENDKNKNININNVDNRYKEDFMYGCENKND
SEKHKKTTNNNFGDEFEKLKVKELDKILNKLEMLYDNKNNKMNINVKILGYIFKNFLLKNKELKRKIFCSLFF
LLCSKMAIIYTPILLSTFIENVNLQKSLSNNIDIYSTNKSSVLLLCAYVFSRVLSSTMNELRNSVFSNISQKI
STFVSKLFFYKIHNLNLTYILSKKNGELSFIFNRGCKSITNLLNVMVFQIIPIIIEFILYLYILTYKIHYTVS
LVTCFNMFLYVLFTTLITKRRTIIRKHMNKAEQNTFNIFLDSIQNVEQVKYYTNEIHELKKFIKEQKKYEKEA
INVQKSLSFLNFGQQIILNTNLFLCMYLTYLNIANDIFPFSYLILVNTLLFQLAMPLNMFGTIYRETKLSLVD
IESMIKILVKKIKSPDYGNQMLIKNGNIKFENVYFKYPLNEDINGLEQNYQAKEKDPNIINNINNININNININ
NINNINSINNIYTFESNVKNISSTNMITSSINKLKKWYLDKVKDNKKNVQYNNNDVKKTNPNITKNLNKEIKE
NINTHLKNHKIINNSPNYNNNYLFQNFTCNIENGEKVAIIGKSGSGKSSLIKLLLKFYEVNSGNIYIDNKNID
DIDLYTLRKNISVVPQDTILFNNTISYNIKYGNFQCTDKQMIQASIKAELHDKIMKMENKYDTIVGERGTKLS
IGEKQRICIARCFLKDSKIIVLDEHASNLDNENKKAIEKALTKLCMGKTTFIITHVMENLKHMDKIIFFCGKN
IYVGSHKNLMDDNHFYREYYDSKNNKML*
>N.MAL13P1.264
MSFFPPVELIRKKNKLIKVNNDYSFEFNVLKSGKEKASIKSISICSIYIAIAYNNTIIFNNLKGEQLDKKYQC
RENVSKLKFRDEKMLGVGLENGEIELIGMFCYDRIKTFKGHKSSINDLIFSNNFQSLYTCSRDFTIKIWNIWQ
GICEHTIDYHMDNITSLILYNNNDTDYLISSSYDGFIYFYDIIKKKHTNKLEIQEPIECIYIFEKEYLILSVR
NVIKFYSITNFKYIKDIVISTKTIFYINSFKKYIVASSLDMSIYFIDPFYKNTEEIKIVCIVNYSNHPKSFEI
YNDIIALGEIDGTWSIELYHKKEKLHKNKKKSKFNKIYYEDEKFVYTDNNVNLMIKKFKYNDALMYIIKNDPQ
STMSLLDYFSKHKVLTAACRTFCVDRAISILIFLRKKFVVDVLMFEFFFSFFSANKWILTTKDKRILEELHLL
KKGFLNVKRYMKYYNHLKDIADCLRN*
>N.MAL13P1.265
MNFCLKNYFSSIKHCRKKLYEFCDEKEYIYKLTHEYIYKHIELIKYNKDILNEKENESYIYIKNNINVNKYVG
KIINHLKVNKGNSGLIYFLPTVVDIYFFNYFQLNYKYKLLFVYNILNINNVKCFVSTHQSIEKKKTHFLNNFC
KILLYNDIYSYINQVHIIYIKKKKEQEQKEKGKGKGKGNKRELQKEKDKSYNLLYQNEAKNRDCQNNNIIPFY
KKNILDHLLSHVQNAKDLCIFLFIVRNINAIHSFCYGKLLDKVLKNLYLFIINGNVLINTHINNNNNNKNNHY
KDTHKNGYYNNYNNIHNNYNANNNLLYNINSLKMRDIIYIFYQLIYLKNNNKYIFILIKILNIINKKFFYINK
KIYNVIYTKYILDILHILYLSNTDIEQLLFLVNKNIYIQQFFIPLILTHFYLKSYSNHNNFPSLIYFNKLFKK
NYPYKNNILLPVDIVPVQCIISYLQNFKKVETQNYFNTFYQMLCIKFLLKRGKTKKRKKKKHDDYNDHNDQNI
KYLFNYTNGFYHPLNINYFIFYKYIINKLINHSFNLYVSKVEEKNKCLKESIHYLFVKYQDNYIYEFTKSKYF
YYIYLKEVIKRKKKKKKKMDSKRKYVKMLIC*
>N.MAL13P1.266
MNDFSEEYDFMQRIHKVEGYNECNINNGFEGTKNDDLLFGKDKCEKEFYLKSKYDKYSNVLNGFGKDDKYLYL
KKLKNEKEHRKRTEGSSLGRSAVDYDGKKSNINELTKIHEIVYYIHANKRNVYDYLNKQTDLENNYYQIEEKP
QNLTNDIVKENEEYIYLNENAYYKLKINKEDKDDTGGGGGGGGSRGGGGGGFINGVTHNMGNMKIRYAVDENG
NNIMGDIIEGDNFLLNIANSLFTFKDYIKNVIYFLSKIFHYNFLDYTYIKNYSLTNYFDKNVKDYMKYLKKSE
DTRFLSQEFILRKKFIMPHFIYNCVDCNYNTNDNGIIILSSIYPFIHSYDYNQKLKDLRNQEKELDNILNNER
KRRRKTFFSFVNKFRNFVVNKIYSDKNNVNNMTSRIRNQSEYSEFLKKFESSINEEGELIQDIIFPGKAQMIF
INFKQLENNFTNMISNVDVKNERVQVSNLILGYEKKEHIKYTDILISNILKNIKKKYPDMVINDENPVNFLLE
QKDAIPKKLSAESGNDFKNNININLNTNGINNNNNNNNNNNNNNNNNNYYGDNNSYHNNSVNYNMHNFIPSTNT
SSYTNKNFLPSEMSSDVYFNKKDLYKLSDLKDYICNSPSHLMKEKKSSCEYVTCVCFDNNSNNCNLINNFVLG
YNTGKLEYIYGKIIYSNISHCDLLIDYYSKKNKKFSYELNKRIFLNGSVIKISFAPNGFFCLILTSRTLYLCN
FFYFSIYEITNNCYNSQYVNFLWLNNNSYVIFNDKGHIHLYEKNLKSDGFQGFSVIKTFFINQEIFFNSICEY
HLYSNSIYLYTGEIEKRKRKKSSGHLFKKKNHINNNNKNMKKKRTSEYFYKYDCNLIIIDLNSDVHKNISNYK
GKEFLNMYLKDNMNDIDNNMKEIKMLEKLIDNEKTFEDEDIPFDKLTYINIKDFNNYISTIKTLGNNLYMNNE
SGDEHMLEYDPTKADENFSVKKLFVNYIKDVKRYLYNLKNDNIVNILLPRYYSSTDDYNYNNFLNIRFFTIEK
SDNKHLIALDTETDFVYIYKIRKNVYLDDDTLDNLKNRRLVGGIGSGFHFNNPLQMFYSTYMKKESLLKDEWR
IYNDYIYINTKKPQKFIKYNLLYIIKNQRKIFFPLHVYVINTKQNENEYFLFIKWATIKNSFIYSSYSSLNKV

SKMKEYFKRDDEKLLKVLENPKNIFLYNINENDYPTSYLYNNNNNNNNNITSNRNNINNNRLLSRRNKLIDKD
YLSENDESYLLGNKYKDKLNKQHDPYRHSYRQYLGPEDYINKTPMKNIPRNFFTSPVSPNNLNFNNMNMMNNN
TYNIDPSRYSDNDVYHNNTAFYMNNKAHPTYQNYLQEKKNNEADSKWSYFKKMNIFRKDNNNDMDNFHDADED
YYNRQRAMYNSDKDPRYISNKNRNNLNIDQDHIINDMRSPHFYSTSNHMRYNRLQDGTDGSSTIENNDINNMN
NMDFVNDMNHINNYPKNNYDNNYLKYDSYKNIRLDTNNTDNNNNNNNNNNNNNNNNDKDKDYNTNIAYNNKNFM
NNDMYQISSDHNSYLNDIERTNNNIGYIYKNNKDFNNVDHMYNEKNKYKTDNQKDKESSYRKYI*
>N.PF13_0272
MAIFKKSIIIFLFFTFFNYCFSQDVIELNDSNFESLTQLSTGNTTGSWFIKFYAPWCSHCKAMSKTWAQLATE
LKGKINVAKIDVTLNSKTRKRFKIEGFPTLLYFKNGKMYDYKNHDRSLEAFKNFVLETYKNAKASEPPKPLNY
MDILKDFLNETFQNIDRIYKYAFPSLAVLVSVSFLTGSIFSLILLKCCCMKSGASKVAKKKD*
>N.MAL13P1.267
MFINLVLIKFFTLFKKDIYENSHIIQKSNNNPLHSFHIESEQNILKNLIPINTLKENNNKKIIYSYNILDGFK
YKIIIKRKTDNINNIYKIVVSLDHPNIIKLIQYAESDYYFISVSEFFSAYMTQYPSLQYMMRTKLEKSFIRKL
TPHSFLIKKINNDLVIKLDDIHNIFSMIIKNCHQFLIFIYLFIFIFLLLLVYFLVCGEFPLYYYKKNEKKICI
SKKLIKYYNNPFNIIIFYLFDRWKNFSYKGRDFIKKILLGNMSSIISLREALDHEWFKENIRQNTYINFEMLR
SIYNFWKKNTFKRYILNNLAKLIINEDIYICQSLFFYLDILQQGSIKYEQYFIIMKKLGLLDGDINKTIAFQF
FKKVDYNNEGIITKKKLYKFYNLKTKNEINSYAKKKFTFEEFYNYIRKE*
>N.PF13_0273
MRKTSKSKKRKASDKSSSRKNSSQDTFDTNSSINEKAQDGFNDEPSNLEKAEESTNNSYKVNNNNINNDDDDD
IKFLIEHVDFQQFCDIKKKGSVPYKNKKNDENINNNNNNNNDNMNDDMEYNNSPLNIIGKTYDYLRRKNFKSV
HLKKLENYQYFEKILWPYYNMYDNFYELNNNSIFFHSNDELNNKKNVHLKHLLSIVIFLCYKYEEKGKHEIWE
QLIYNKIDEFEKLVYNEKNGLNRNSTTFDIDEKYNLYKEFLIEKFNNLFFNTLKLINYNFVISEFLSSDLKGN
NDQILLDNLKRYRKKLFKLNFLEKCYILQFFINIYSSIDKKFLFKLCVDLCNPFIWIYINDTYLNTFLFKTNE
DAKVLYNNILNVMKRKYDNYENYDILSIYTSNCAVDTQEQEHNIPNHDKKKRKRSSTKINNDIQNEENKFCYD
ENVILFINKNAFFYNLINYFLLVLNYVERNNFDELHDTDILSDTSNDENIFDKMNIFENDDDDDDIYTFDDNK
KIGTQLLEKLKIINENITEEENTISYLEDMDILLKCENKINDEKYNKNINMLINIDLQNKEENFENDYKYVSS
EDDIYEYNEYKYNNNNNNNNNEHYKKEEILSLFSKYEKKVKDMNNKEYMYRKNKIQKKDTCTQNNNKYILLFL
EKCIEFFIDLLSQLYSRRILFIFLKYFNVFIYCKISKLNKDKRQFKELVKLFKYYMEMYIDNFSGNPLTYLEI
NNEHYKNFESFQIFCYKYYKDHNILKNYYLKSVYVMDKKKEMWENLTKLDHHVLSFLCQNLNYVINIDTYIDN
DESMSEPKKKLSLTSDTDVNMDKNINDNINDNIKDNIKDNIKDNINDYNNNNNNSCSSRGKKGRKGKNKENEE
KVETKRENNEEKNREQFKCYNENKYNYLFNHLNIFHEKKKIFYIILLIENLSFKKNIFEEIEKKCDYPNEMDL
FDNILIYSNEDVKTNNEKKKKKKYILYTKPLFKLNLQYLCINDYIFRIYNLFKLQSYYDIKKNILEYVYETNP
KNKKVNESSILKYVYEIDDDGKIDYHNMNEKEYKKNYYNNEETNNYQSSNTIDNVNNMRKKRKSNNEIEENDI
ILYNDNHIHKNTYNEYQLKSIIIDINKIENTYFANERRMSNKIDSFKIMNVDDDNKKVTAEIIIDLRYKQYER
IKNEWNMIRSSDILFLVSVKNYTNLYKNKLNVINDNDMKKLLGINYVRGCEVIKYSNIGNDDEDINNEKCTLK
KITVYLDYIQYQKDILNNPDIYESFNLLIRRKQKENNFYYILNNIKTLILNPDDAIIPHWVHDIFLGYCDNSI
KYYDETLHNAYHPDKDEQNVEDESDSDNNNDEYQTDSNDNNNDNNSDDNNDNNSDDNNNNNDEDETHSDNNTD
NAPLNDHNNSNTSDVLSNVDEAQSSKSLIKNKISYNKSLFKFMKTNVDDINYLNTFLNIGHILKTTNVGYMCV
DLSYMNILNDNNFNVHDFLNEYIEPLYLSYVPIKYEHEKDCMKKNVLEKNIIESLFYHICVINKYKIDDTSFV
SKFVANIDIVYECNNFFVFQFEFKEKKYFLIFNNFLSKQQNQKNVVQQFEQNQKEDVSTKKKKIKKNNNNDNN
NDDKDGNSEANLANNLQTNNNKCVTIKDHNKIKNIYHKIIEDSIKYLRIKDGIYRLQNKIYEDPNYPLEGLLI
HTQKKNSTNVKYINNEKKKEIIKKNIYYTERQIECIKSGIYKGITIIEGVPGSGKTSILNKIINILFNNKKNE
KILICTHSNSCLNYIFNLLVKENVIHQKYLCRVGMGEVDSENFINEYDIMNDKLNKQYNKKDEYMNTYELNNM
YNNYDDENFNFSKYGRINYMLDLRQKLLNDLNLLSESYNNQKIYNCLTANQYYERYVKKRIYLYFQFVYLFKN
KFNNEENKMDEFFNLYFSLSAEDYDIYNLFLCPKIYVQDHEDNINGLLWKNYNIEENSNCKKIKCMDLLKDDE
PYFYLIHPKNQLKVLNLSIYNILFPFKKYLNLKLQRISLQMYLENKSKLYEAEKGEYNLQVLDKIVETNLISD
ENHNTNGVINVEYDDKNNNNNIINNNNNNNNDNVDSLSDNYGHNVQNSAHIAKINDLENNDKNDKNNNILKKN
NNTNPYINDDLDENHFNIQFQQGEDDLYVSKYYLSKLVKNFEHLKDCRAFEVLRNQRERCTYIIAKLARVIAM
TCTHASINRSKIAKLQFYFDNIIIDECTQITENDTFLPLLLQENRYYKSKLKRIIFVGDSNQLPPIIKNKYIK
DFANYEQSLYKRFLRLDLPSIYLNEQGRMRSEICNIYKYFYSKYNIEISNLECIYKDNFVKSFNPGFTYTYQF
IHVPSEEYSPIPYFYQNLLEAEMTVAIYMYMRLIGYPNDVITILTTYNGQKELILDILKKNCMYNKLIGMPKK
VTTVDKYQGKQNDYIILSLVRSRSIGYMKNIKRLIVAFSRARFGLYVLGNYNLYKKAYEFKKPLYFFKKNKLQ
LSLHINEHYLNTTYRHIENTSNNSSLIINDLNHFYTIIYSLLNYQLEQPKK*
>N.PF13_0274

MEITCDNQNSNKNEKEKNIISSTYSNDKDKLIDSVYEERDIIQHNTNDDNSLMNDDIKFYDVNGIDDIKEDDE
NIFLFNEEEYCEQFLMRSDQNVSQYEEVYNSYTDDDLEKFFEDKENNDNCKNKNSVENKIHTLRTDNYNDDIV
INKDDIKSSFENNTLIYKDSQSSYIDEKNNINGTFEITNSIKENNENENENENKNDDGTIYSKNEKKYFEENS
SHIIEQFTLNEDCRDTKELSNEQHTILSLNSIETNNFIDNKPPCGDDQKEDSENIFFSEGEHENLTKGEEEKE
GIDNEKYEQNEKYEQNEQNEESGNHKNNNINEIINTSQGEHNKENMNHMNTNQKYIVDNKNEVDYELSDKNIE
KSNLIKSDLNNVEEHNNSQYINEKVKNNESNINNDLHIKDDIKNIEFDYDEDDEDLKSEKFNKKLDILNYFEN
NENQDNVNNSKFLLKKKKVNEDQTNTLWYKHKRHFFIFTFSGKPVFTRYGNEENLTSFFGTLLAILSKVETFN
FNDINSDNNKSNKKTNGLSPKKNTLKYIISNNTKVVFLDKEVLYLVCISKVNESINYIMNILNYIYSQIISLL
TKSIDKSFQIKPSFDIRYLLDGADLLMCNLISSCSKNLYSLLDGFEPLPLKPDYRNKVHNLISSFKINNVLLS
FLIIDDKIIGLSLSKYTLNSMDIIILINMITSMKSFKNAESWTPICLPIYNPNLFLYAYINYIKKKICCVYIC
SHASSRDFFHLSRHTSMIESTLISTGCYDEIVKASNNAPFVLPQIPGIDIIHLCYYIPNLKQYYSSKISNDKI
KRIFRVYQKCDDIMKDCKLPTQIYIESEYEKFYCIKTNLYHLYLSVPFYVIINDENINEILKVIATYHKEIFI
NNIKQITS*
>N.MAL13P1.268
MRVFWVVLCYLDTLVNILNIKIQVISLLPNIKNYPFPFNFDYSKIEWNRKLAELSTYTDDKIKNTSGSNIKEV
NHQYNRGEIKIPEGVVGHGYESIRELFPELVDELIVRNYDKELKREKKLYKITYDVLKKFERNVKVYNNDLLQ
NMNAVLRPLYKSIVHNDIRLLRLMCGDNMDLNINTIGQLMVDGKTVSNYLTNISFETNMEYIADDIKSSINKQ
ITLNHGKLLHLSSEHMKLQRYVKLLHFIVVRIPLVYFRSANNFFLMFQINAMIILEERLNTVLNLNYLEHLNL
GRNYYTVNVDTVNFLLNDMLRITKSTIDTEVHRAFVKMNKVLNGDLKCLLAIFLYILNDAMSENTKSIQSLEG
ETQDDINNHPILSNLHKILLDEKNNIKLLKEKFEMFVQNVFNFDINEPSVRNLQQKLYEFRKGEIMSVFYTIV
HQLITMRQIIKYKEFVDDLQKKGQKYRSLYRKIIDRFVVPQSSGILTFHTDEYDLLLRDFLHIREKGNIHFNL
LGGNRTLRTLLQLCLFEINKLKIINKLYIELMEAIDILHEASVEDPNEKIMSKKKIVFYIYVLNKFNNRWV*
>N.MAL13P1.269
MELNTYNNINSTVKIFQTTSTSALNHDKQIVEKINMDHVVQLFYILTIIILKYVFKSVNKDIKKRRRKKKSYK
ELKYVNTNEVDAIGKKDDNGEYANKHKMLPQKNEHDFKYTRVRILHELFKEIEHKCKDPKKDDHIKNKEKEEN
EKFKLNEEHCAEEKEKNKHKCSHKEKEKDKNKDYYKDNIKDKDSDKEKDSDKDSDKGKGKGKDSDKGKDKDSD
KGIDSDKGIDSDKGKDSDKCKDKDSDKCKDNNKDKDSDKCKDNNKDKDSDKEKDKSKDKDIDKEKKKEKTKEK
EIILQEGTEEKRLIEKGVEKFQSGMKKIKEWMEGIDKPNDDIPNLLVTVNKEIEVPQGKVQEFEEGIELFEEG
VKLLKGNTVLEEDGNEINEIEIVFEKPNITDEEKRKKKKQLVENWISDINKMVDTEIHKIISEIENDNEDMSH
SLEENNEIYEISSQDVKLTKSKADNNMSEVIQINEKTYNQDVNVNINENLNNNSNINLFNNLSKNTISQDPLH
NHLSENRNSEEDLNKSLSAYANNGKDVNKMDGHKLNIYERKIKAWNWKKNTDTEWEQFMLLEKEKKKKFIGKK
MKDWEEWFPTIYYNWLAYVNNLEEDFMHDKWEFWLNWKKEDWEKLIDSEWTKQMEIKFKEFMRTAENSLKQKQ
FKRWSIWKSNKWNGLNGWKKREWNFEGD*MWINQYSAVQNENKLKWQELSIQEEIEWLDWVKEKESYMTNKQL
IEWDTWKEEKRNWFEKYINLIKKDWLDHNPWKNWKVQRIKMFQPHESEELEELYDIEHNGKLAVLFEY*
>N.PF13_0275
MKTYNSLNNIMGFQGEHNSTVPSYNKSSMEKSSNIRNNRGRVYSFHFLVKIFACSLFIWTLYVSHNGNVSTNV
DVVNTTQGLSKGRILTQGDHHKETEDVNLKAHHTGERTSYTEVKHRRSSSNSHVESNTFQNKEETEVNADSAE
QQNYDEEPSQKGKDVKKMLKKADNFIETKLVSALHPKKNHLLKLAILAFPIFMEHVSNIFKRRGFKYIGSAMH
LVCLVFYVYIFKVIVNHLLTNKEDRFAHMESHGKHAFDDNKSKKKPKDNKRYGSGSHDHEEEVVEA*
>N.PF13_0276
MQPCPYDVYNQINHVGTHWAQHLGEHLHHLAHMHQHTPHVHHHIPHVHTLAHDRPCMPITAFFCRHHEHCSSH
LMLIFLLLAFFLVVVYRLYNEVVNSAKTVRIVNITPVNEEHKAEASKEQSKSTSDSSTSTQQTL*
>N.PF13_0277
MDFFIIIFILLLSFFFFDYNFCSRGLVFYIGNKSLKLLKKCNNLEKVYRSTIYLIFPFLQHIFVHIFYYYIEK
KKEYLNNLIFEQSKEHYDIKGQKEFEACNFKKEDIFNYIIETSIKIKNYVYNKALHIFFFIVEYTNIYENINL
NCHINTREVLNNNNFGVVIIDYYLPNTSIKKKKKKKKDQTYNYINDSEKIDLYDLVNIKQEKKIKFVLIDNK
WKHILGLGKIEDPNVQHLYLQDTVLIQYLQFSWIFKNHFFKKLYNDRDVSTHNYYEIKKNSMQQNDRDGVKEQ
YKKTCLGEEELIVDGNTCNNNHLNDHVNIKDEEYFNNKNYMNDQEEIIIRSLKKWNINKENVCSNKEENFNLI
TNNDKLMENTIYKNDNKDLYIETQKMKEDHVYNNSINLKKTKINEIEQNNLNTQNNNCYSNKEKYYINQEKKK
QQLKYIKNENMIRDKLKMQLNRWKYKNIKGIIVILPEIFYNYINMNEMENKIPIYYFLKKDIKIDTFTVIAKL
LGYICIHIHININFGFSIPFIFKSKTTNILNISNNFNLHMNKNLFNIYNENGTSNIKGTHTNISSDDKKLKCS
KHISKQDIKNNINLCHKSVSNLNDHEKENKNKQDKMNIYSYINDHINIYNNDNNDNNSIHNNLYNKQNDNIKD
TINYMDQNDKNNCHDMFLNNNYNEQSNEYHNHQQCVNNINDENRYQNYAHNNNNNNYNYIHTDYTNSKLNSIV
ENKFNMDDNYTFLFSTPFSFFSDNYNDLNIAMNHFKAIFKNYNFVFLCFNDGTNIMLNYFLEKIYKKKKNKTP

GGMTTLISTAGVAENIKNTFNLYNTNAYKCHINNYKKKHVHDTKGDTYDNEKKKHSKLYPINKSEEDKKSKCT
LHKANNKSNIMSLFFGEKKKKKYLEHEQMNQSQRFKKITKKWDIHKILSDIKGNDKSSFINQDKKINERKYNE
IDKNDSDDSNASYDDEKILSHVVKEQMHSGEDEKEELGEPKEKGSKSCQEEEEEDEEEEDEDEEEEEDQGVNN
YDNYVDGVDRDVENYDNYIDDVNRDVENYDKGIANVDHHLNDVHKDLDDHKKVEEVIADIEKEDKNVERKNVN
LKGAQNKTEYQQELVKKLDTDQNINIDKKTMNQINNSEFSNNVENVQDKQKNKIKKEEMFTKNTKKNMKIKMK
DNKNNDMGKLRNIKEKLKKKKMETLLNLSKLNIFHKNSEQISENYIKEEDNLYRNDYNTNESKIWSIYESHVS
NDPRGSASTYKGNSSINKFANMSLSNFASFIKKHKNNNSNHSLVGSFKDNLEIKEIISKTNCEKNLNKYIDLN
KENVMEIDMKEFVEPNIKHDVEKNVYKDINKNTNNEIVKTHNIVDEKNLNTQIQKYVNYDNEKNIKEDVERNV
ENNCERHEMKNSEIKKIKLNILEKFRKKEIRKCDNMDDAPRISNKFRYSFFKIRNRFKHNFNACDNKTNQEKK
NMERDKMMINSFIKNKNAMVSDANSTSTADSNFDDYGKKHTKEICILINFSYNNMFDIFNYPDDFIETKKRKF
SIVHKFFYSLNIFFNSFSTLIKQRYFFRCTSELSNFLKRSLFYDFYNNLLCKISTDDQDRNNFLLDINNSSNE
YDVHLKRLSDITYICNSEKPDKYRDLLENKSNITSIYDDKKNVSRTNMSTYINKDMECTWHDNNQNKKSEKNF
ENIKNENIQEIDKIDLKYNNINNNNNNNNISENNNIRSNIGNEIYNNVSDMNKKNSSNNVNNPMEYNKDYTYN
NSSIRDNDNNINVEIYKKNSLNTIEYFENNLSFFNKIHKMNKKKDEYKCDEKLYCKTKNIKSNNELYDVKNSY
KLNNNNNNNNMIFNETYNNNTFFKNIYSDSFESKIRVYFNIKSLYEKWKIYFEDKQIDISIQDKFLYNLSFIY
NVYNYLILIYIYTYYNEESYLAFCNNQKFYQFIEKIHCYFNEKKNNHILYKNVRNSESFNTSIIPRFLNYHDY
KLLKVFFFMLQNSSNKFISKNIHLFNFPVVFIFSSDSKNFNFNHFDIIKISKNNNIIYLLYKRGNEGLFLSGL
KPYIWIYRVLFDFVESLFLSSFDN*
>N.PF13_0278
MSSNYRNYKNNHDSYCKENDYRNNNNKEKNKIKNNKRDVFERERNQSVIDNNKNMFEKNKYNSRDYMDKKYDN
KYNKKYDDKYDKKCDDKYDKNYDERYNKNYDDKYDKNYDERYNKNYDDKYDKKYDKRYDDKYDKKYQDKKDNE
YHKGKRKYDERKHSYVSQEKDYNRRNNSETYDKYKECNIEKNNQHTKIMNHYNNNNSLYNKIEYRKKRSFAKS
KEDERNKSEEDLSEDDKNKDYSSASESNFYKYKKRKNNTYEYKDDKDYTSYDNKFRKIRNIDDILEMKPNILL
SRFIFIYKLVDNISEDEIDELIRNISINNAFSLPVNIYINKLSFFSIKDELFVKENLEFLKNNSYFNIIQQKI
QSNFLLENRINDDQCCIIEFPSDEASGKLFSLYEKDNCIEIKNNISYIFPLFKLKNKGKNVEEKTGSNKVSDW
YCSACNFLNFSRRTACHFCKAPKTSDAKLVDKETSTISTFIKNNINHQENNLYLINNKNLYNNMHVDKGTYNH
MLSDPLNMQKVYVYNNMEDNYENILNDTYKDANNNISNNNNNNNNDNDYNNNNNNNNNNSKNNNYNNNYNSNY
NRGNENNHLKLSNNNIFFSYNPFHKFNEDSQNYENINKEIICDDQNTNMLILKNMDGNILIKDFIQFLNVTFD
KNDVSCIYLFNDIKGSSKKKGFCFIEFYNINMAKKVMNNMEKNYYLNFQDNYLKLDYVYEKEKQYFFNCIQMA
KLDISKSSATVVKNNIPYFNFFVNYFEAVVHMNIHCYTYFLMWSSQIIILKKGKPELSEFFFDYNSQYYYHPL
YQLYFDNNTKYYMSLSKGYYIWEDGLKCLLRVYLDNLGENVYERENYDKKFSLMDASKNKEHEETHQQARIND
DHKYDNISNNNIINGHMLEQKLSNYKIEKENEKKNNNENVILNKISSFVEKAKEIALASKKNIEQMNMNDNNL
SILEKKNKEIIKKHFTTDSADDEDEENDNDNDNDEHDDDHDNEKEDNDDELNNVSIKNKDNISDINIIEKQSN
DDHNNKQRIDNSSYYDYKKNVKLSDNISNNINNNIPYQNNNNDMKKGYTNVSNNSFNNSNIYNNNNEHINNND
EKDVISEQSEKNINICFICLRKFLNEEMLQRHIDVSNLHKKNVEILSDPVLSN*
>N.PF13_0279
MIIKMEKNEENTKKDDIIKDEEVVKNHLILIIDVNLLIWCEGVQIKFENNVIRTLKLHEFLKSVFQFIRFYCI
MCNSERICIISTCSNNCKIIYENYISFAKNNLTEKDFCSDTYDKLVDFINENKKEKKSESALSSALTIALCYN
NRIRNLYENINTRLFLLDISKSHYYTNQYTQLMNIAYNAKRNNIIIDVFSLNYKTQILEQICNITNGLYLDNT
IFQSINPYGNIQDILTQTIIFWFLPSVNTRKYFSNTYLNEDTNIAVCTCHNKQIDIAYICSCCLAIYCSEKNL
QTNKDRLSCAICKTRFTKSLLRNKHVSDLDFSFM*
>N.PF13_0280
MNIFRLIGDILHLVSMYILIMKLKKSKNCIGISCRMQELYLIVFLCRYIDLFFVFVSFYNTVMKITFILTIAY
TIYLIRLKLPISQTYNRKVDNFKSEKYLIPPCLVLSLLTCKTYNLYNILWSFSIWLESVAILPQLVLLEKQRE
VENITSHYVITMGLYRAFYILNWIYRYFFDDKPYINVVGWIGGLIQTLLYIDFFYYFALAKWYGKKLVLPFNG
EV*
>N.PF13_0281
MMNWFRKKDSNENKKVKSEYDEYVTPPPFGNYLVSEPKKPKSLKNDKTAITEFKGFTPPPKFEFKEDISDNKY
EEDFSKYTSNNIIDSSFYDDKKKLSDVNLSHRTRACFESIKMGVKMGTMVGGIFGSLTGIYASFAHKNLFILP
VSVLGGAVSFGFFLGCGMIVRC*
>N.PF13_0282
MARRYDSRTTTFSPEGRLYQVEYALEAINNASITIGLITKDGVILGADKVFISKLIDKANNYEKIYKIDKHIF
CGVAGLNADANILINQSRLYAQRYLYNYNEVQPVSQLVVQICDIKQSYTQYGGLRPYGVSFLIGGYDTKDGYQ

LYHTDPSGNYSGWFATAIGTNNLTASSVLKQEWKNDMTLEEGLLLALKTLAKSTDTEIPKSEKIELAYLTNKD
GEVYQKYLTEKEIEELIKLYTQKYIKE*
>N.MAL13P1.270
MSYDRAITVFSPDGHLLQVEHALEAVKKGGCAVAIKSSNFAVLAVEKKNIPKLQNPKTTEKLIKLDEHNCLAF
AGLNADARVLVNKTRLECQRYYLNMDEPAPVDYIAKYVAKVQQKFTHRGGVRPFGIATLIAGFKNNKEICIYQ
TEPSGIYAAWKAQAIGKNAKIVQEFLEKNYQENMEQKDCIFLALKAIFEVVELSSKNVEVALLTEKDLTFIEE
QEINSMVELIDQERTKNNEQNE*
>N.PF13_0283
MHRLIYVQNHYTYDKKNNNIKIIEKEKDKHLYVIKSIPLKGKQLNVRNDTKFYNKYNINNSLNYERKKRVEYY
SNNKPNHLNSKNNKFILYNTNNSKNERNMSYGKEIYFKNNNKCYINVKNSFNNERNDIKQMQKNSYINYLNKL
KKNGNKYYDTCQNNIYGKETQVDNIYRTNMSTSSKKYMNNMNSFGKYTINNIIKNNIYGTNNNINYNIQQFNI
NCNEKNCFHKLNKSGLIKTYSFNNYKNSYATYKQKQIIIKNKSLIQKKGENNYIYRNRHENTNYSPCYYMKCQ
QNKSNLSYYKKCCYLKDSIDKLKTIYTNNKKYYYNKNHNNINLNKNKSSIKSTQNYSPLFHRSIHKFELKKNL
SNIRNWEYFYGVDNNKKKKKKKKKKFFFCSTFLYDNIKSNMFPNKTDHTNDSKKDMCPKSVSLIVKKENHIIR
NQDNDDDNIIPNNNYFINLLVNGEKEAHLLIEKAYKNRDDLMKHMNEKIDDEINEFRIKEHMKYELSYKQMEE
EIKNYELFIQNELEYMELQTKQITLNVEDIADYVIRHIIHVNLTIGCNLLKGLVPVQNLLNIKLSSEPKKYIN
NRKKKKIF*
>N.PF13_0284
MRNMNDKNNVHNNYYYLKLENVNNGWRNKELKNDITNENIYQFIGKKKKNYSSSEMSKNSYSTCNTNNNIYIN
YNNNNNMYNNYNNNIYKNLNNRNINYKGYKFKQNYHTNAYVNISKNRNYYYPSESKKLTNEYNKYDFDNLEKR
ECKIINDESYKIEETTDMENDMLYIHNDSLPSHQPTINNNTQELYHINNYNNNIYFYEPCEKDNYLDSSIKDI
YFDIYSNDTSENIKNETNDIINVDQKGNKYEENNINISNNTHEYKTFVNDDKQLLLDHIKRLEYQNNIFLNNM
LNMYYVCIDYIKLQDDKIKEHEQVINSQKEFISNLKKQLSSNDNTNVAYNININHINKYKHVTK*
>N.PF13_0285
MTNYSKIPLKKYYLESHGKYLCIVNVENNVKNILKINYSENINITKEQECITSKNTIIENDNKNKFWFYGCLG
IIKAENINFLVIVSEAEVVCNIFNRSIYRIKRISFVQLNCEKMNKNTNELYEYEICYLGSNGNLTSLNNIKKD
ENKKFFCNKNKIQTKNYFDNLFQSTNFLLENCVYKYNRFYYNETYIHKFKNCTLKEDVLKIITYFLHAFNKGP
FYFSYYYNLTISLQNQYMNELDKKNDMNKKKKVYLKISEHASKKLNIYSNKNNNNDNNNNDNDNNDNDNNNNV
NKIQFNEINDEYTWNWKILDTFKNVDAYGFVVFLIHGYINTNIFHVEDNKKISLYLISRKCKNRSGVRFWCRG
SNENGDVANFVETEQIVVCKNKERINIFSYIIVRGSIPVLWKQQPTLSIRPAIHVCPNMSENKRILNLHMKKL
QTNYGKISITNLINKKFGEKYLGECFENCLSDCNVEHNFTWFDFHSEFKKLNFENLDYMLEKVVSDLNKFSYF
SFSIPIGNDKNLMNDYDVLNYNNFHFNLWQEAQIYSHQNGVFRVNCIDCLDRTNVFQSFLSKYVLYLQLKSVD
IKLQQRDNFPFYFFHNKYDEISYRRIWINNANAISLIYSGAGALKNDITQNGKRTISGLFNDLYCIIVRYVNN
NFLDGYNNDCINMAINEKIKFIHNFNINKGNNNPLIQVLVEFIIIFSTAICTSPVQNFIKSIYFCSHNCTISM
FSRSINYIFLLLKNNFNLFLFPYNHAKYLSFISFLAKTSGVLSTSLLIFLFFCVYVFTQRRRVISSPKLDANS
*
>N.PF13_0286
MGKKKKVGKERIDKYYKLAKTAGYRARSAFKLIQIAQKFNVFKNANILIDLCAAPGGWLQVAYKNMSKQSTII
GVDLVPIRKIDDNVITIKSDITTSDCIKKIKNIIKMDKADVILNDGAPNVGTTYSYDSFNQNVLVLNSIKIAY
IFLKKNGIFITKVFRNEEYISLIWVLEKLFGDVKHIKPRSSREISSEIYLVGLNFLGKKVDKKLFDYTYIFSD
QFKKNSNKSSLLETKQESYLDNNENYFSDEESNDEEKVGEKIDKKKKGLSTILKEKKKKNRQGYDVGDDYRA
TDICTFIKSDNYVDLLIKNNKFTFDKNYKNNSDPMIKTIYECIYKNECTTKEIFFLCNDLKVLGKSDLYNLIK
WRYKIKKSVLNQTSKKNNLDFDNHDVEQTTQELSDVKESSQIDDFNTIVDKNISENELDNTSDEATEKDEKDQ
VDEIEEFSAYIEKKKKKEQKKKEKKLKKELEKKKKSNRGHQLDFDENEIHFNKDILKLLNKQKFEDHLKILNN
DQKDDLEIDHYEDTSSETEKLEHLNQLKNSDLDKLEYLVDLDYEKQKMKEKKLNEEKSNEKLSRRKRAMDFKN
EELMKIQKMMELKNEQLLMEKKLREYLSDEDDDEDDDDDDDDDKEQEPKQQSDKHGDHQYDNKKSNSDNDVDS
LETTQDIFKENKHIKSMVDKIIRLRRSVKNEEEKSNVNHFFDQNIFSNIFSQLDEEDIDAEFDSHIKNKKNVQ
KKDDSDKDGSISNDDNESIKEIDEHNLPKIPLPDKLAKKEKKKKLKEKYGNNEVKMKNSSFSIVKTDENAQNL
NNYFSNLVKDEDELAFIKCIGEKLIHKKSRMDLIDDSFNRYSYMEDESNLPDWFLEEEKKYRRPVIPIDKKIL
DEYKGKINKITKMPIKKVIEAKMRNRKREITKMKKLEAKIGKIEKNEDDPFLKQKAITNLLKKNKSGKKRDKS
YIVCTGKGSKMAKKKKNKKGKTMVKYVDKRLKKDKKAKKRIEKKKKNISRTKYSKSKPFKFRRKI*
>N.MAL13P1.271

MYNSWFEIVRSISPYNWAMLGIALSLFLSIMGAAWGIFICGTSIVGASVKSPRIISKNLISIIFCEALGMYGV
ITAVFLQIKFSGLSTEVHPPLVLTNKTDPLIMNTIRGGWALFASGLTAGLSNLVSGVSVGITGSSCAIGDAHS
SDLFVRMLMIEICASVIGLYGLIVAIVSIGDIQLT*
>N.PF13_0287
MNIFDHQIKNVDKGNVVAILGAQWGDEGKGKIIDMLSEYSDITCRFNGGANAGHTISVNDKKYALHLLPCGVL
YDNNISVLGNGMVIHVKSLMEEIESVGGKLLDRLYLSNKAHILFDIHQIIDSIQETKKLKEGKQIGTTKRGIG
PCYSTKASRIGIRLGTLKNFENFKNMYSKLIDHLMDLYNITEYDKEKELNLFYNYHLKLRDRIVDVISFMNTN
LENNKKVLIEGANAAMLDIDFGTYPYVTSSCTTVGGVFSGLGIHHKKLNLVVGVVKSYLTRVGCGPFLTELNN
DVGQYLREKGHEYGTTTKRPRRCGWLDIPMLLYVKCINSIDMINLTKLDVLSGLEEILLCVNFKNKKTGELLE
KGCYPVEEEISEEYEPVYEKFSGWKEDISTCNEFDELPENAKKYILAIEKYLKTPIVWIGVGPNRKNMIVKKN
FNLN*
>N.MAL13P1.272
MDKKKSKKEPIKKKEYNKVKHVKFRRIKKINFKEEKIRTDKRKINIEKIRKRQKKFDTLYKKKEYTKKLNGQK
NEYMNLKNELVNEKFDTERQCVFIIRNDVDCIHNESKLLLKELKLINKYDGIILINTEENMKKLYVIKPYICY
GYIKKYNFYNLMEKRLYIRDQDQIKRCDSNKMIEQIFSKEGIYSFRSFCDYIFECKDNADIITKNYIYPFDFS
FLNTKITFDFLQFKQDFKGFLKNEINEILEKII*
>N.PF13_0288
MPWEENEMVVLHFTIHDVYDNIENKISLEEKPYIKFYWKNKKYKNYLKYFTDEINWFSEFFLPYKIGDYIENL
IVQIWANSYILKTKKKVAYNYININDVERKRKINGKTELIGKRKGLKIIYSLQLIRYSLYEFMKNTQMLILDK
ISIYKMIQIHRKNQNISVHRYHNNHHLFDKYIISLFEKENYIKKNTALKLKTIKEHKKKKKNSNIKSNVKNEH
IQIETNNKNEGRKKIIITDQINYRKNKTEGNIKNNPQKNNSKNTSVLDSKKNLKSQKLNKKLNKSNQTKQNIT
DNKKSNNNSTHSKKKKKIMTNNLQLKNTSHTKVLGNHKGSIEKKEPNNIFNIQKQNSFLDLGYKIHRSSNKRN
FKILNFKMVQSNYHDNEKFNNNISHEKGLNKFKNTDTKIYVY*
>N.PF13_0289
MEKIYVKIYELSGLEDKDNFSCYIKIYWQNKKYKSCILQKNPYKFNEIFLLPIDIKNNVKDEKNNILSIEVWS
SGILNNNKIAYTFFELDHIRRERISSEKINLIDVVKKCTLQISVHIINNNQDILFCNIKDIFGNNKNDKEIHD
AILKYGGNERHIIKELRKEKEIGQYNNIYFNDYVNVLNTDPSQNYIYNDMPKITPNNIYNNMNNDQTNHTYLK
APNSLYNNENTIYSSNVHYSTYMNNSPTYKNSNNMNHVTNMYASNDLHNSNHFKPHSNAYSTINYDNNNYIYP
QNHTNIYNRASPGSDQTLYFSPCNQKKALLIGINYYGTKYELNGCTNDTLRMKDLLVTKYKFYDSSNNIVRLI
DNEANPNYRPTRRNILSALMWLTRDNKPGDILFFLFSGHGSQEKDHNHIEKDGYNESILPSDFETEGVIIDDE
LHKYLIQPLNEGVKLIAVVDSCNSGSSIDLAYKYKLKSKKWKEDKNPFHVICDVTQFSGCKDKEVSYEVNTGQ
IAPGGSLVTAMVQILKNNMNTPSIITYEYLLHNIHAHVKQHSNQTVTFMSSQKFNMNRLFDFEHIIKNKNNQL
GQIINKYIEKNKSKNKNKLKHELKNLFFF*
>N.PF13_0290
MSSDIKDLIRKEKERRKLMREENRIKEKEKKKDDNDDEKKKEKELNVKNCKNGDKLFLNIQKNYENTDKQIKV
EEDEKNNNIQVSKKNYYDIIKEKFTFNRESKKVSFDEPKAPVNKLNKNNDLQKAFLEYGDDTESDNSFNENIN
YNDKNIKSKVHNNINNDEDNNLPNDFFDSLNNENNIDEKLPKNLQHSDNEKNQKYLTTKSDKTTQHYSMNQYD
NIDDKKILEEGLASLTFNKEKTNKHTTSNYLNNDNNYNNNNKNEHVEILETYEIIEPNKDDLKFDENDDFHKK
VLNKRRKIEKNNLNDLLSLEYDDVDVDSNEDNDKSEKDVLTESNYTTKDTNIIKEEQEKEMKKNLNDLHINSN
KKDKNNLHNSNNIINIINNNLNDVELYKLKDSAYYEELDYLYKLLIQKKKNILGNKYNEEKEINDEKEDVILQ
ELNDFHKKNNNNNNNNNNNYDAEQKNNFNILEIYEILNMKKPQVENDLLYTRINTKKDTNNIITTQKENIPKG
FFDDEEKDILIRENISLSQLNLKIKELKNEMNRILSEDKNTEQMYEEKKNNFIDYLYDDKFDNKEHFLKEIKK
KKKNKNVKINKFKNKIMERKEQKIKNNPKKVMKMGNSQMDFDEMLFDWRKKKIL*
>N.PF13_0291
MSAIFNESELSGLDAHSAFGNNETSSFQKKKRRIDENSQMSVNDLVDDGEEEEDDDDDEEEPSYVQDQNMAKV
FEKFLKTFSEKKSEEDDDDGDSIWKDSLNFDFPSNLEIAKDSHYVLLLFSILQNSYSRNKVLIVDMKHVLMWE
PTDKNRFDIGSQLYMYIKRHFLRILDIFEKKVQLLAESINPIKTKEVGKICLRFYNKKNPIHSLRSLRCEMLG
EMISVRGQVTRTSDVRPELTLAAFKCNECGNIINGVKQQFRYTQPSKCPSASCSNMSDWSLVLEQSYFVDWQK
IRLQEIAQESPPGSMPRNMDVILRNDIVDSVHAGDRIIVTGCLIVVPDIPTLMKPGDVPRSVARQLLKKNENS
LVSQGLTGIKGVGVQDLNHKLCIYACQIEKLNSSKKDNNFDEQTQVDINCEEILNCDDLKWLRDIAMHPNTID
ILAECIAPKIWGNLEIKKGALLMMTGGVQKITSNCKLRGDINMCIVGDPGTAKSEILKYVESFAPRAIFTSGK
GSTAAGLTAAVHRDPDQGDTVLEAGALMYADQGICCIDEFDKMDEKDRVAIHEAMEQQTISITKAGIQATLNA
RASVLAACNPKYGRYDTLKTFAQNVNIPAPLLSRFDLFYTMLDCIDIDKDTSIANHLVSMHCGEEAEKHLRAN
AGKLDSVKLEIYLELSKRVKPLLTDEAKYKLIQYYVSFRNIEYSPGAQRSMRMTVRQLESLIRLSEAVAKLKF

SHFVDVKHVEIACSIFKASMKKISNEKEINLDEEFDKVNNSLMGNKVNKLDQETENTEQNKKITTIKASEYQY
ISAIIFEIIKEYEFNNNNESITQDQLIETYLQVYAKAESSEQVDEWIYKLKKIINRLINQDIKLLSETNEDDP
EKVIIRIHPNYAGPIVEGVSNKNTYGYNTFKNYQTQEKAPEDDVDFQEEIDNF*
>N.PF13_0292
MTIIKNRCCLFLNYLNKKSTCILINNNKHGNLNHQKKFIRTSKLSLNVLKRIKSEKNINIKELNKYLTDDNKM
NLDIFKKLIYKAKNNNENEQESVENLLLILVLFNKYYPYFENNFYSNLCLDINKNLLNSIKFKIHYLYTSKML
IHTMNILTNLNLVDNVLLEGYMNKCFYFIKNDEYNIEDLIHYLSILNKISIIQKNLYCKKLQSFNWSLIKIIC
DKLTYNFDHLFMLSDKNNHFSFFIKKNINEQIKNLSNDINNKVNRTYHKINYNEKKSSVIQNKTNVNNILDTP
LISQKSYNIIQKNQNKINQEYYNIICNHNVYINNLNRGQNHMKLYNIQEQQENNNCNNNMKNYDHNNNSLYSN
TLTIVEKKLLILDTLLSISRSLKDLNYVHLKLTNEIVNKLKNEFLNISDDEDITPYIDNNSVNKIFYIMQCLL
YLKLDYHMFYKSVLNMFMNYLPFSNNLIMLFFLLAKNKLFPSKTIHIFDSVFSKNIKQKVYDTNSLIILLESY
SIHKYRQSDIIGDILSYFMYSNIEHNIYDKNIDNAKRQLSNNNSNIFLNKKNTFDHNLEMSLYPQDDNIVIKN
KNNSYDEKVTKGENKNDPTSFIFSSSHNIIKNNEMDNLIIPDDNQNIKLINKNNNKNNNNTLYNNLYNNQLCQ
EKLKNIDIKMNNYFTSINISDKVKIFYSLFKLDIYDEGIIYHINNILNNERIHEINFKLLIKLLLGLCYFSFE
KVHIYNLIITNLIKYDIILDNVYLTQLKICELAIRTQHVPNVYNHLNKESIEYLNCIKNKQKTIEYHVKSDLQ
KNVKNILLTFNLTTLEEVTIGPYNVDFVEEDESFFKIPKNYILHQNKHLMKHKHYTNDNNNNSINFQTYVKQN
DVNNVTNKFENKHKKLIIEVNGEHHFYKNTKSYISLSKFKHKLLSDLGYVVINIPYFDWAILNTDFDKKAYIK
KLINDHCDIQIRNIINLNQKNEHINKKELDEISKTIQKSKEKTDLLTNIENLRKKNKIKFLKKKIKHI*
>N.PF13_0293
MFKIEYKEDRGKCVVAVNQIRAGYCLVESHPEIFIPLCVKYMAPRIIDQNNKKNNFKIINTCFYCLEKFNKCI
CCPNCKYVVYCSDMCLERAWKSHREECDIFRSNIFDRYCPSITMRLVINCYLNHFNFYDYCGSSTDISKEKYE
RLKYPAYVVAVALMSKRKKIFHNFDNNESILKNIIEKFIKISKNSLQIIDNELEPAGLAIYKKPVPFFNHSCL
SNCVTVFRNQKLYIRTLMDVYPGEELTISYIDIAFDRCTRLSICMDQYFFTCSCKLCKVNIASECHNIFNTEI
VCTNSENCKKFLGYMEIVLISELERKQCYINKSSFKTYPVLRKSNIDNVWRCMLCKAEVGDNIIKGLVDKEKE
TFKETVYLETLFNEKYTYDNKSVLQSLNKIKSKIDYLTNYYHHAKYSLQKMRAKILYISIQLHEFKLAYNIAN
QYLKSIEVSYGKYSPIYGYYIFLTGKLALFLDLKNEGLGLIHKAKKNIIKTYGPDSLIYKDLEKFLYTNKY*
>N.MAL13P1.274
MVIVTEKINNNVKIEDNNINRFEEDMDKNVDEDNETIYKINEEKVNICNNIEKEHNQKEDKENVMLNNHNVEE
EEDIKKTDEVLMNDEKIKNDFHLEIGEYSKNHKCDDPLNNNKTDHCDKERKDICLELLKTCDALKNIGNKYFK
ENNYIISLRYYTEAIDLIKKSFEQCPNNVTDIDNENNTNNLHDDVEVDDEDKELFKEYYNKSAISKKSDFISI
KETDLHIYYTNRSFCHIKLENYGTAIEDIDEAIKINPYYAKAYYRKGCSYLLLSDLKRASECFQKVLKLTKDK
NSELKLKQCKKLIFEQQFQKAIELEQKMPYYETLVLDSLKIENMEAPIYDRNNLNLDFLKKVADYISIPNNKL
NKKCVCAIVLDVIKLLKELPTLVYLNLEEDETLTICGDVHGQYYDLLNIMKINGYPSEKNSYLFNGDFVDRGS
FSVEVIIFLYLAKLTFPNNVYLTRGNHETDNMNKIYGFLGELQEKYDEKMHVLFSDSFKFLPLAYVLNKNIFI
CHGGIPSKTDTTLEDIEKIDRNKEPLDEGVMTDLLWSDPNEEKGFKPSKRGIGFSFGTDITENFLKINNLSLI
IRSHEVRDEGYSLEQNGQLYTVFSAPNYCDIMKNKGAFLKFKGNSIKPECVTFTEVEHPNVPSLKYAHNLYQN
I*
>N.PF13_0295
MNNGSNKKFVIVHYIKGEDSNQNKSNSKKKKEKKTGYVTIEISRVDDRKFANKKRKNQLMLDKIRRNDFLSRS
AKNISKKKIRDNTWTNTLIDKLQGATTTINQKITSFFKICIFNSKELKMKNDMIGRDSFKYFNTEDNEQFINS
ANLMNAMNVKMDNMLSQHININKDNFNFNNFNMHFNGNSNINNKFMKDNIFNGNNLNNKFANKKMIDASEIWK
KDIPANNIMNYVDKRKVDANNNPDMQNIKNFYISVINKKSNVKNVRKVSLMEVCKDPYFFISQNFLSDLESYL
ALNHCLLYIKKNKGELSQIHHNFFSVLINVDDVHFLEENVSTSILIHTIKRLNSLFKIPIDDITSVEFCLYLK
NSEENEPFIYTEKDIYKYSILIFLSSKNGNFIEFPFNGLRIMTVIGNCLIYQCHDNKNTNKHIFNFNISDEKL
FFLKINLKENIHLYKMFQGQKPVTPSNMDEVCKQNKITELYTNVFKNKNTLPFEQFMKTKPNETNTINIDSMK
NHYSLVKKNMECINKRLMELNINRMRSLFIKAKNARSNNPYPNPMQFKKRA*
>N.MAL13P1.275
MSVNSCEKMNENINKLIDINDENINYNNTNNINNNINGNMNMMNDNHNNTINNINNNMINNNITMNSNNNNNN
NNNNNSNSNNNIDGINVVYNNNRNVGYNKDLDTTYTKKRILNTNRSLKNTYHNNNYNKNNHQNFSINKNNKNN
KNNKNFRNKNIQYNKTNMKQNKGNMNMNMNINMIDNNMMENNQENIKNIYNSGDNINVEHSYNGNNNDILNNM
YYEENEKSDDYSSLSNNQNLVNKVANNRNVYENMRLINNTNNGNNSSMYNNTNNEDNNEINVYNNNMKSVYSN
EDMDTFNDNNNILQNDDLNMNNGMGKMNLNRNINTHNNNNNNIKKRRIGKYENNAFYNKNKVKKNLHNTLFND
SLKRYNNNSINLKENNNKKNDNILDFHSNEKDQIDQQQEQKENINEELNINEDGSNINKNMTFIKKDNYNKMG
KYNNRNSYFNNNNNNKNNNNNSNNNNNNNNNNNNNKPFNNMTFSLNKYLNPYVNYNKTNINSRNINSSYHMGN

KNKLLNKNRNMKNNANPHGSSNNNNNNNNNNNTYYNSTYKNANERNHTNNNCRDDYSRNNLNNINFITQNFNM
KLLSDYTKHTQNDQNNLDVDTHVNTNPNFKNFRPNNRNQFYNNQNYIECLSEVSEISDEETNDLSNDDNMAKN
QNVDNDQYNNKWNNLKNNQGFKSLPHAESALYTNNFNDNNNLELINGLYKLANFNKNLKNENQDIYSQSKVLN
NNQPTNNNIIHNMNNDIYSNDNNFNYNNYTSNIIEKKIEDQGKLNNDYVEGEEENNMDMTNKNILNFIKPDDM
YINSYIPYPPEKYNNIYDLHEIKILSPHILKTLFQKEANKTYHSSLKDGKLILLLDLDNTLLQATSFAKFNME
LPLENFVDDNGEAELYKFYLPQYNFFYYLKFRPYVRQFLQILSLYYELAIYTNATREYADVVIAILDPDRTIF
SDRIVARCSSTDRDENKYFSRIYPNVDPKYVIAFDDRKDVWIDIPQSHILKAEHYNFFELSKYDIISHFKEPT
TARKKFVDMDMHLHYMIKFFLKLHKNFFENPLETDVGKLIDKMMGSTLSNVGVYFTGFRKNSKNIQNVLSADC
EERQKEIALELGATIFTNYDEPGVTHIIAAKNCTDNLIKSKKSDYDHILKVHTLWLYHCRGTLEKRHSSNFDA
DNLCKIYSNKPPLHPKKDHWFFGPKEDFKKQEDNKDCVKIENLKIRTFLGTGEYTNDAVIFSPFEQINIKWIE
KEVTLRQNFDTSYNAMVQNIQKEGNKNQINDDNDDENNSYINIEDVSGKIPTI*
>N.PF13_0296
MSTFDRFNIHAQLEHLQSKYQGSGHADTSRWEWLTNIHRDTLASHVGHYSRLAYFAVVENEPIAKIRYRCLQN
MSLPIVPKKKKKN*
>N.PF13_0297
MSQKNKKVYDTQNEQENTNSSSNSSSNSDKEIQKDKEEYKKGENNFSILNVEEVNIIELSREELYKVCAELQQ
KELEALKYIYVLDSELNIKYENDEDKLTIVEINLNDQNISNNHINFTFELPKDYPLGSISIINVTVKNFTPDT
NNYINEEVYRYLQRYNEQRECILPVIYKVNEIIENIIQYKEEPGEKSLSSSTEGKSDFDQEQTFNEWDNKNKQ
RKADIVCFPELSYSMNFSKRPNILARRLCYSHHILSLVKRSCIIKWAKQLKIGGYSKIGYPGIIVCEGPKDEV
DFYINSLNKLRWKHFDCRGMEDIELDENENLDDARVLPTTVRELDAKSMRTLSDICTQCGLRDLFLTSMKIYN
NNDKKGSVKEKNKNGEENKPGNTKDILKEKGKKKKKRSSK*
>N.PF13_0298
MHNNNKKPSLSKRGQENTLSISFIKSNNNGKNEYKSKGLFSPMDNINNINTLNNECFERISKNSWYDTSCLKR
NKKTDDIFLFCIETKKNDNSHTNRSYDKSENVSNDLVNTFSTLNEKRNNNIIMPINDLKTYINDEWSNENGKK
KNKKINKGNCNYVNYNNNLEPYKSISINNVKRKYKNKSIIKKSYNLFKPCCHNKKEHIKNGNSLTLNAPTNVK
RDMFEKCNNEFDNLLTTKCVKNQILMNNKMSLNNKDIRNVFPKYERTNSLNYYNNYDVYKINKNPNSVVKNEN
IYMKRYNIPQMYNDDNIYETYDENILVKDVQETKQKKININNGQSTNNNIIDHKKKTTLISQNNKNANIHSYF
NNNNNYPYYNIDGHDTDSTFYEKKKKEYNVNSFLPSNTSYNSKGKKIKDDNYIYINNNTTLKKNHFDEKAKKR
SLIRNPLKISHSTSKSIILKNIMNTYTNEENINSNTVLSNMKPEQEYINNYSKLCLEDKSLFYTNNETKISFT
IDFKIYPPILMKNITQLNIYIENKMIKNIYISEFPQTFSVILQHCIKINNEKAYIKIKNKIIHMKETNVIKFI
FFKNEKVIGCSYISITNLLNLGLEGGVFLLVEEYKNEYNKKKKYMELKNSEKQKDELPKKNSSNNFHKIPINM
QEVHIMKSKIFLNYKINCPKFLINTISPSDIITTKDKIKYLEDMIREIYYSFQSYNINHTTRKGNVLTCLNDR
SIRKQNQNQIIHSENNNNHNRNNNNNVNFSINTLSISNNTNEQKDNTQLMKGNEPKCYHMHINKIKENDEDII
EHNKDTMCYQEKLKMLGILNKKDDDNLSESKIDERTHTYISNEQNDLICVVNNIDENYNLQYDENVYNDITNS
NDYSDKNKNNNNNIINNYNRNHFNKNIVEQEKILIPIDHFNVQENNDNVNLKEIKENIKRDNILHEVEKVNSN
IENKILKTDIKNNNNIDHTSNLYYKKDDNIIKPEDDKLYNDIEEKLDIKKYISIIYKRKKHMNIIMKRKDNEN
YKIKKKIYTTNSIFYKKYIDKEKKIKKLKNIIEKYKKELQLSRTQVEKLKYNNHNTSILYKVFYKRIMKGNSN
DSIFDNIEKNKYKSNDTFVKNKRGHILRKTCSLNVIGGFLKSKEKFIENNKNIDNNKNFDNNNNNIDNNINVV
NELVKNDNYHKNFNSLINSNKDNVIFSKITTNVMLNNNKTKPLGHKMYSHEALSAYTNKYDNDIHYYSDCTYE
KMIDIKKNKKQHHRYNKTFTKIKHNTTNIISSTLDIIGTNKNYEHTEKQLEILLKKNETLKEQINKLKTNDQR
FLYNIRTSINKRHTTNNNKKYNIKRNIDEHFNYSTENIYNICVNKTHNPIVNIENTKKNRYSNNTRFHYPLNL
IKQYSYDYTSD*
>N.PF13_0299
MKSKDKESSKKKTWTDEKKEPNTNNTNRGNKNGKNINTSSKKDNDQLNKNIKEERSKGQIKNERNNKIEEIKN
AYKMKENNDYKETINNKLNKENELSIHNKEINANKDINYLMGDSSIHEKKRIFFEVYKKYYFIEMTEYHKSRE
NCTNNFLNFLERLTEKIIDELKHGTYNITNLNKFFKEFLKNDTTYFKNLQFGNTRMNNLTQSFLNITNDEYFT
NNHLLESYRKRESNLSDKSDKPNINEQNNDVMNNNDLKENNSHSKEKDHHDNNKKNKKNKNKNNKKETGNNNN
NNNNKSNSSSSSNNNSNNSNSSSSSSVQDTSFNNKSKNVEKVCNEKDIHFLSRNNSKGWTNKINEIVDNIIDV
NSDECNLSHEMIDEWVKYGLMNYLKVMNNMKINCDLIDNKIYQKKIVQLRDIYAKEQETLIEGLKKKKNDFLK
QLDICKMNWKSFENSYSNSEQIRSDGIKDSKRIKCSWLCQRKYLKNVKDLLKIQYEYLDIVYLSVKSFFQLVE
WKKNSIRDILCSYIHLYKSVLNFYLNNMNILYQSILEEKIIDKNQIDNLNSATIMDDDLIKQDRPFHFDENCN
NIPTLNNALNLIRKSILFYNKDINVKSILCIFTSKISGYLTSVGFFNKCTEGLIVITWDKFVHFFTDVEDASP
QWSYFIGDIEIKMVKCKKNIQKNLNDNIPNNKKEEQNIGEPLDDKNKEKSAELSHDNNINKKSEIKNVKDQDN
DGKNKTNANDNSNNLPIDNSSNIEPEEMEIQMKEKKKTLLLSGWSFTFKCETQHLTNVCFQLINEHLYSEYFE

EETCFYKFPKIIDNGEIKNDLQYNQEMDNFYKIQDDEHFINQKLNSYNIKQIKKHQNEYSNIKNEKNNVSKTN
LNNNLNNDNVDLKKTQVNSKKTTNISNDKRKIGLFKSLKNSKKEDINDEDDDEEDDDDDDDDDDDDDDDDDD
DDDDDDDEDDEDD*
>N.PF13_0300
MGDYLKTGSVNYDLEILKKKPEKKLSIDKQNLYLQGYGRQWGEKLVYSVGLAYGSGLLLGGGCGLINGIMKGG
KTKKLFLNSVLNSTSVIGPSVANQMASLTMIFYALNNMVKLITKNDEIYNSSIAGFLAGCIYKSSSNYKILGS
YSIMSSAVFSCIDYGFKKGYI*
>N.PF13_0301
MASASRSSKELLRLQKELKDIEKENVDEIDAHMKDTNIFEWVGFIKGPSGTPYEGGHFILDITIPNDYPYNPP
KIKFNTKIWHPNISSQTGAICLDVLKNEWSPALTIRTALLSIQALLSDPQPDDPQDAEVAKMYKENYSLYLKT
ASVWTKTFATVPKLEPREDIIKKITEMGFSEDQAKKALIKANWNETLALNTLLENS*
>N.PF13_0302
MNRKRYSINTSIHNENMNVNMRSSSGSFSEKSDFRDLKYNACMFLQGELMRQFLMDEDIWLFMQNVKMKLKED
PNEMDIWLDTQLHMARLNSGHSFNSDIPPSLHTKTKFENSHLKSDKEYEKMFDIYNENDKTENIYYNKKKSKN
QYSNYPINEPENVNFNTKEEHVDEMDSQIFLKKILGDEYKEMKETNVNVNLKIDESYCNANNVEKSNNDNLKN
NINLCDNIFSNNKVQPPFLKHSMDKQKILDSITNKINYIKNKKKQLEERSSSIINHINDNKIEDDVSTINPLS
VYDLQYDNLLNDVSNKNVDIIDNRTNTLYNTVNISLNMNGNNNNNYDDDIIYTNKNSVINNIDKDINQEVIHS
NINFFDKTNGLSENNKTCEYYVQSNSVKVEDIKENVTKKEGSSNIDNADSYTKKSSYNENQDYNLYNDDKEKM
IEELDEHKNNFNKYNKNIEENVKNNYNSINVDGVNKLPIDEYKIENMKKDNISIPKMTISIGELIDEDTDIKL
QKIFNQCNNKMDLNTFENEIVVNFLKIGRYMSHTLFSRVEKGSDDFMTYENFKKFFSKRFIDGEKDPSYYNDE
RYIFMFSKKNKDYYNHSYGSKCMTNENTHNNDDRDILKESEISNVVENSQKGDHIDVSYRKCSIINFFNAIKE
ENKNYIEFKDFDIYIREILKRNKSLQFLLEHTEFLERYIESVIVRIYYYIDINDTNRIYLNDLRKHDLAHIWC
FLDDSVKVQHIKNYFSYSHFYVYYCTFCQTSSCKDMLIDDNDLYRFDNHSLNDFIVNRIWSRISMKLGGPNQK
YMCLNDWIYFIMNYEDMTTNRSIEFWFKLIDLDADCVIRDHEIQVFFHIQTERLKSHYLEEPKFEDWMCQMND
AIQPSIEGNFTLSDFKKNKKYASKFFGCLVSLSKLLAWESRDVHKELQIETEFPCWTPWEIFCKTKYDELCYI
ENESYYDENFDNYDNYDKYDNYDKYDGYDKYDIYDNYDKYNNYDNYDAKNLYIIDSD*
>N.MAL13P1.276
MSIFKIPGLYSKIYGSIFRRKFSGPSPLYKTPLRHIWAIFFYIDIFILYILVFSFFFSIIVRNECAEIFSTVF
FTTINIIFSFLIDFFFLLIKDEEVVSEDEIIVSTCLTILLCIVKIFLNLYSIYFCINVYGHIVESKERHIKET
CASYMGKGLLLHYSVINCFSLVETYIESFLTLISIISFKAQVQSYQIII*
>N.PF13_0303
MTHFFKFKKSNDENVTQPKLVTKAIISSDFIKKLPKYEDKRTIIYYWGKRVINENDETPEHLPTIYTELSNVK
IREISCNRACALFLSNTGNMYSFGKGLHGELGQGTMVVYNAQPSLIPTVTNIKQIACGYNHSLCLSHDNILYA
WGHGNYNGLKCNYDRFTPTILNIHDYGDVIETTLNALSHLITDYEKSEKKKKTCTQIYAKYNQSIAVCDNNSS
IYLWGETYNNYLKYIPTFFYKFQLDNKIKQIAMGKNFAVVLLVNGELYGWGDGTYGELCKSSFDDEGGCYFIY
PEPLILRDANNKKLPNINKVSAGARHVLLITEDEHVWSFGDKISGQCGISGFQNIVTTPKFVELNENNQKAKK
IFCGERHSACINKFNQLYMWGHSAHHKLIFTASSDFLLNQNVQPGISIQSGLKQKFNKATLIYSMLHQKVTNA
YLGDDFTIVVVGGETFEKSKQGDQHSDKTFYSVQEENAFISI*
>N.MAL13P1.277
MNYYKILGVTQNACKKTIREAYLKKVKLYHPDLNKSPDATTKFKQIQEAYQALYNNDKSNTSFYNENSYTKNY
RTEDMKKGQGFTNDFSDFHREFYEEVHRMREHERNEQNRRYSNNNYSYSYDIFNKYPREFFYINLIFKLFPLF
VVPFLFLFVVYKQYILKSHFKEKPILIYDAYGRAFLVDIHGRKFRASEFDKY*
>N.MAL13P1.278
MKDYYMDKIKKNINEIKKYQNINILEKTNETNNAFFYENKNVTLKYNEEQKYKKTEDIISNISTDKKDIKNNN
CNNISNDEYVRIENGPKNNYVSYNNNKIINNDNEKYDNIYNNYGSVIHKLNVQDMNNIPNQNYNNNVVISTKK
NKNDVIYNIPYNTNSFIDTKQNMNINNDVGTKYYLDNNKNGYIMNENINYDIENKMTNTLNDINKCVHVNRND
FSYYLNIKDGNKIEDDNATYTHYINNSNIHQYDNTTNNTFYNKKCDEQYYDKCESGTRINKINEKYINEHFNN
NGSDGDNIYFHKLLSHKNSNEKKYVFQEKIEKRNNDKYKYCYNDKDKDISSYMVNKENYNGNKDLDILNNYYQ
MKNNLLSNKNNIYIINSNNQKRDLSLQNKEDEKIDVVKENCVQTFDNKKEKKKIYIYQQSFNNNNNNNKHFDL
NENECVHKNDEQIIRSTYIQDNTQHIYNDMCEKQIHYEIKENNQNNVLINDNAVHAEEDICVKNSCPISLLSS
HDIILSKKVKEYFSGSENINSQSYQCDELKKENFIGKHIINKNEINDNRPIEFAQVEKNNLENKYLHYPNVFY
TNDKINDKTLQEEVNENCIKKKIICDKIKGIEKLKEPTNSILLNKYNKYDLCEENKMGNNNSHVIKNIYNKEK
KSFNEEDKGIIQNENANLYYNYNKQFPNSEENFHNEHKKQYNIPRNNTLRLLDEQKENYQKNDKKNDKKNDKK
DNENEEECKFPSFSNFMSHEEKYKNDNIMPYINKASNDSGNSNELTNLNKGQAHKNSIYEKVNIDNDKVKKKN

LHSINDKKIKINKTFMNEKDMKGNNRKKYNTEKRDNIKRNENDNEKKKLYINGNKNIFLSNKNSEKVHLDNYD
DNNLGRKKRSCTINDNINLSEKKQLEKELDVYLKIEGFLLSSSAKSSISLPSNLSNTYWGQKNLNEETNLSNV
LKKQFSSNNERKRSTILTTEYSNKIVNYEISSSSIGTYDNYEIQQVFDEMLNNRCTEREYSKKVHLEYMDDKD
NAFSSSNRMTDMSKKISKIFNKNGSINDKINDNNKENIRDNNNDNTYDNNNDNTYDNNNDNTYDNNNDNTYNN
NHYNNINSKNSCDKVVKNQNKQLDHCISKKHNSLLIDLMKKKNRKRMRSSQNNFQKKLDKVFDLVVLPKSEKE
RNLIDKIIYCLDPSYVNNTLDEETKWKQKLKYMQEKFLESILCISYPNDLWTESTFEAYLESLNFNSLLDDTF
IKYEKDLNINLFHPEEEIFSDPGNIGEGGFGIVTKMRFLTFPQYYAIKKISKDHIIKSQAAGQAYLEAKYHSV
LSHVNIIKMYGCMQDEEYIYHVLEFCSKGSIYSISKNFKKRRIPDELAYKYFCHVVNGLYYLNQMGIFHRDIK
MENVLVDHKDNAKLSDFGLSAMILGKKSHSSLCGTLVYFSPEITSGTGYDWRSDIWSLGVLLYEMLVGDVPFD
GTKTQIVESIFSCNLKFPDFVNPLAIDLIKKTLVVDVNKRIRLCELSSDPWMQEMWKLSFQKNLNTNQNINHL
KKEDKNDFNFISNLIKKQCFINSSFNASLNFDKLKKGKKAYKHFIHSNSKDDMYSLIVETQQRLADYLELDDS
YMSDKIDSTSDTISEYDSNKYTSSFYNLSNKEKKSKSLSGTAIRNEFGKKNKNKLNEYNTISDPVICKVVKET
HLNKSFKKKEGELFKNEKITESKDLKKKKKKKNECDITNRENIYNSIKEKFSDSNKDNTFVNKNDDKIKDVLS
NGMTIKYSDTYSQINDQLDITKMDTIDNDNFEKKLKEKNQNDSIEPNNIYMEKKTIVDMKNGDSISNIYNNNL
KKEEDIKENVEVYNNMNEKIYCENILKNGTSEKQHTHIMEKTDKNSNKELKDVFEKIVDTSCEKTNVDDNIDK
CHVLRNIPTFYMSSFSGSLENINKMTNEKIDIDTKNSEEDLKMEEKLKRVNTKVSTNNNEYIMNKIKSKEINN
NNIDENLTNIKYEKNHINNDVSQNNSVEEINKSYIPICVHKECSTDLIKQNKCSKINLKLKDTYIDEKIRKDQ
HVGDLKFTDGKGEEQKNDTQINLKNDELIKIANVEEKKNEKKNNDQNKLSSDVNTEILRKYISDVKNYLLIKP
SLNKNNNNNNNNNNNNNNEYINEEKKDNINEKNDISKDKKIQSNNETQKEPLFSKLLNKAFGKKYTHEKNYKN
DNVVGDIHKCSDSHILYKGIGECNKNGNIIKECENKNVEKIDENTSMYDKYIEKINKLYLNIKNKKFIYDLEN
MKNGGSDENFNKCNDNNHNNTDHIICNNNNVDMNENCVYKGLNYKMDKSKDLHNNNNNDNILNEYNCNYNYIH
GYKVDTSKCYNDTNKKNEKHYYINNPYFHEETHNNNINNLYNSNEYHKNVQNTSQKNIPRLGNTLYNKFNYEK
YIDDKLVDLEEKRICKANSCSLYKENKKKEQTLVELKQEIVGDNKLISRTRITPDFISVDKKYMNSSNSDNGD
FVIGQKNKLNCKNGEEGQDDYTNNFILSSSNSDFSNERKYKNAEKYKSVKSNNALLNNNSNEYVQYGKGNKQN
SFSYIENNMISNNNMDKYEKIEFNNKNKKGSCNNENNIISFKPLSFNNKVTFMALENYNDMDNMHNVNKNENM
SDIQINHKNLFNGNKRISYELSNLESIKKINKNDISNNLNYIKEDSPSFNSTTKGNKHSSFTYNARNKVNNVV
KNNKRSVSTNRRYLIINNNNNNNNNMKVGNPNMYKKGLSYKVPPCNTNYRELNKNNLPSETQKRTLLRDYIKN
NDKMYTKDKQKMNIKCDLKYNQKSIIKEKQKINIKHNLKSDAKGTGKKSCSHKLQNAEKKSNDKISRNIKVSN
CLNKSLKVKNLNNLQKGINVLQKNDKHTEKEQNDIVDYKLNLLDNKIDTYKNTKKEENIDEKSYLNDDSSVIN
RKINHHYNDKSALNKNKEIEVYNKREDNNINENQNEYDNKNLLIYQQNDDPNVVQVEKTQNDFILNNKKVNNT
IMKRHNSNSINMKEKMSRINETKNINENINNSKNISEMSETNNKMDNMETRKNTISLQDVIKNNLNNLQKNRK
MLLPNNLKNSKVKQYLKSKIEKIKMQNDYNLLYNTMDSQNGINKKSKILDIYNKECNSKDILNLQNVHHTKRS
TSEFLQNPNSKAFNNTNQDLQNVKTVRNVTYTSTNNLGQNMKPTILPTYINKDQIQKHKSDERKKNIIRNNEK
ISRSKSEGIKHLRLSSSDSDLSCYLDSFKNTFQDKINNMTNKIKGDENRDEIPLQKINGNYIEKNIIQKRECF
NSNINKNTVSNNKNKFNTKNKLCTNVESNKNLNFSNVKSKIDTNIHKKSNNMEENILKNKKNSNVTFNEYNRR
TKSAKIFNKSDSLLNTTINTYKNSSITNNMKISKHVVKNKVISSSKRDNSVKLNAKFSEYKRSPMKGKDSLYI
ESEDIKYEEELQKKLLLLNKDDTYKKEEKKYASKNDDKENVHLIIENYVDRNDKLDAVKEVEEMDKNRKENDN
KKGIDDNKKGIDDDEKKINDNEKKINNDEKKINDNEKKRGDNEKKRSDNEKKRSDNEKRINDDEKKINDNNNN
KVYCDYNNKVYYNNNNYSDEKYNDNCDYYCRDDTHELDKHNKNNVKNIHYTTNRDGNNNNLEEKDKKMNNTHE
KYYKTLPSRTKYYKDSDTIFDEEELNIIEGHIMKHVSFENLEGVISSSDTRNNSNKIKRNYTFSNIRYKNHTK
NTLSVERNYSTLPNMNIDQMNTYGKLPKEENNKNSTAHVFIKNGYYLEKQNIKQNNNTKDIMKDKKNICNQSI
QTYLNGGRNLNNINNINNINNIRNKENTKTTISPYQYNKRLQSEQNVTIRSSYNRQISQTMNSQYNKRNNSHV
KNKNVINIISAKIPAMSNIQKDTLLTQNKNVTKPVSKKYIRTYTLNTYGMNNLSNKSTECMKQINNTYNNNNN
NNERIEKNNSTLFMVNHHKIHSLNNNTENINMINKQQRLGTEFIKSNNREKLNGNINLKRNVSATILNKTT*
>N.MAL13P1.279
MEKYHGLEKIGEGTYGVVYKAQNNYGETFALKKIRLEKEDEGIPSTTIREISILKELKHSNIVKLYDVIHTKK
RLVLVFEHLDQDLKKLLDVCEGGLESVTAKSFLLQLLNGIAYCHDRRVLHRDLKPQNLLINREGELKIADFGL
ARAFGIPVRKYTHEVVTLWYRAPDVLMGSKKYSTTIDIWSVGCIFAEMVNGTPLFPGVSEADQLMRIFRILGT
PNSKNWPNVTELPKYDPNFTVYEPLPWESFLKGLDESGIDLLSKMLKLDPNQRITAKQALEHAYFKENN*
>N.PF13_0304
MGKEKTHINLVVIGHVDSGKSTTTGHIIYKLGGIDRRTIEKFEKESAEMGKGSFKYAWVLDKLKAERERGITI
DIALWKFETPRYFFTVIDAPGHKDFIKNMITGTSQADVALLVVPAEVGGFEGAFSKEGQTKEHALLAFTLGVK
QIVVGVNKMDTVKYSEDRYEEIKKEVKDYLKKVGYQADKVDFIPISGFEGDNLIEKSDKTPWYKGRTLIEALD
TMEPPKRPYDKPLRIPLQGVYKIGGIGTVPVGRVETGILKAGMVLNFAPSAVVSECKSVEMHKEVLEEARPGD

NIGFNVKNVSVKEIKRGYVASDTKNEPAKGCSKFTAQVIILNHPGEIKNGYTPVLDCHTSHISCKFLNIDSKI
DKRSGKVVEENPKAIKSGDSALVSLEPKKPMVVETFTEYPPLGRFAIRDMRQTIAVGIIKSVEKKEPGAVTAK
APAKK*
>N.PF13_0305
MTWSIVF*KFFYILFFFLSFFFLF*FFFFFNFLKYILL*FLKLY*NKISLNLQNTFLHFY*YIIRKSFFLI**
IYF*KNFNIFEYHKIIYIIFNKYITDLYK*I*IEKKKIYIYIYIHIY*YTKLFKKKKN*NYFKMGKEKTHINL
VVIGHVDSGKSTTXXXXXXXXXXXXXXXXXXXXXXXXXXXXXXXXXXXXXXXXXXXXXXXXXXXXXXXXXXX
XXXXXXXXXXXXXXXXXXXXXXXXXXXXXXXXXXXXXXXXXXVSVKEIKRGYVASDTKNEPAKGCSKFTAQVII
LNHPGEIKNGYTPVLDCHTSHISCKFLNIDSKIDKRSGKVVEENPKAIKSGDSALVSLEPKKPMVVETFTEYP
PLGRFAIRDMRQTIAVGIIKSVEKKEPGAVTAKAPAKK*
>N.MAL13P1.281
MIFIYFFFVTLIIINLNTIESKYLFGYSYNGNPKCPINILYDKKYIYSFILNNNKRNTYNSRIKCAKKSSIIP
NEIVEGKVRLRFAPSPTGFLHVGGCRTFLYNYILSKQMNGSLILRLEDTDIKRNTKDSLDEIIKDLNWLNLTW
DEGPDKVGEYGPYKQSEKIQLYKKIAHQFVNEGKAYFCFCSKEELQEIKEKSKMMKKKYIYNRKCRDMNNEQI
KMKLEQNIAYTIRFKSPLKRKIILKDILKGDIIDEVLEDFIILRSNELPTYNFSVSVDDYLMKITHVIRGVEH
ISNTFKQILILETLNADIPHYAHIPVITTEEKKKISKRNNEYLIRNLREEGFKPECVVNYMTTLGWGSISKKE
IYTMSELIDTFNIHKLNKSSVVFDIKKLKWMNKKYMLEQDNETYVREAEEYMINNNILSSNEYKEFVELCVDI
FKYEVHNYSELKVCILNALKYEHHVNDPLNTDDIYLKQVSFLLYDWFRKNDVKDNTEYLEMKMIDDFDSLIDD
IVKSTNLKKNQVLLKIRLLLTFQSKGIPFIYLIKLWASARKHNIPNYFSLKKRFLHLKNLFKF*
>N.PF13_0306
MESSEVKKYSSKFEIKGICMNSENCEKVCKISLKAIKENKFEKDIACQIKTKCENDEILNKDNLNDENYLNVI
DNLKNQNIGSWQCIVGQNFAFSINYQFNCMIYFQHRSTKLSILIYKSL*
>N.PF13_0307
MLVKKGQKKNTNIKTKVNYKEKEICNDVTDNEINRRGKNGTNAGNNSKDKISVLSKGRDKKDVRNIDKVNNES
SEIKNNEENVVNTYDKDIDHNNDYKDHNNNNNNDDDNKNNDDNKNNDDNKNNDDNKNNDDKSDGIIEGGFVNK
DNYNNDNIDIKKENDENNDNNTNNNNDKIEHNVYKNNSNNNSNISYYYFNNLKNNIMQSKSRLRKKVITDDSS
NDERDILKIKDSLKEELRYKNNIIDEKDNELSSYENNSNDEIKMEKKKKKKFRNLSNICNKDLDVTPVHRKRI
RKVNENINYDNISNDTLTFKKIDKNNIIHSLNTIDVKDLSNRDDMLRKGIIDIDNLCIDDCILQESFYEKRKN
RKDSKIKSSDDLSYNSNSDYINSYNKNSFNKNQGSYFSDSEYKTPNTKKSNEYELHLKIFNEQNLDTDYFNFN
TISMKKSIRFYIEFIALSLLSPKFQYNRNYYKISEGAKQIEKDLKEKYSSLHKKLVKKESLQECCNDSNSIIY
RRKKAKKKAKKNKNEDEDDDYFVSNDNEVEEYGRNNKLDKKKKKKKKNLKKLCIYISDSELSQPPNQIVTNLD
KNKSNDKFLKKNKKKKYVLSDSSYISDDVTLNEKKNNSKEEVDLTLASTSKKEQIDILDIDEKEYKSSCKSND
IHKSHNYDKFDDFDKNSKTINVNEDKGEEYAEKEEFDPEDQYEEDEYDEEDDLYEEEVYYNEDDDLSEEEDYY
NEEEDDYIEEDDDYNEEEDDYNEEDDDYYDDTGEYMEDFVVEDDIFYDEDNIKKRFRNKKKSNHPLKSDENVK
VKLIEKINDGFMNNLIKGEMLKVYEFIRNEEKLSNYEFLKMICKINKKKSQNYYEQCIQKIENKILSKRDQFE
SHPFQNNFKNILKNYANILIFYLEHNKMYCCCCNRKLSYACPVFFIKPFYNSTDLWNNNFYNFMKVNNFEWLG
YIYFSGTSRSSLEILSDYKLDENKMKKIKESNKLFIKNQNEKRKKTLLRSNSSNVSTCTIMNSEEYIFNHLKE
FEEMNGYRYIGSIDDKRLKKRKRSFKYNYDFHLKGGEIYNESSLNLVMKDICDKFCYSSENYIEKDILVLQLG
SYCVSTVYYWHVFHHYKFFFVKYIYMKLFDVYSKNKDIFKEPLILAYILSKKLHKQLYRDFKILMNIDISQIQ
NKLNECDLFIK*
>N.PF13_0308
MEVFNFGKYNGKTFEEVFEKHKSYVTWVKSLESPSGSLLDFKNYVLQKEGKKNSSNMANNNHYNNEKNSNNNY
SNNMYENNVRSPNIYNEQYTNNNKMKENYDRNANMNNNEICTNNNRINENNVKSPHYYNNEKNINNYSHECNR
SKIAYKHNYSENRNSYNNFEKSSVDTNYTNEEKKKFHNMNSSEYRNYEEKVMSDINNAYNQPEEKKEFDIIVA
FEIFSDDSFKIVQKDNNNKKFVSFRNFISKDLFKIISELNPTLKKINNYTCITFEAEKYEYVLNNLKEKCTIL
GGVHTIPNFLLKCFKNYTKFSEPQRISEITANILTNTMCSYTKQHYDKLDHLLGEKLSVELRNFQREGVFFGL
KKNGRVLIGDEMGLGKTLQALALMAFYKDDWPFIVVCPSSIRFQWKDQALRWLSHLIREEHICVVKNGKTDIP
SNTKMIIISYELITKNDKYQDKYKSIICDESHYLKNSLSKRTKVITPIIKNAKRCVLLSGTPALNKPSELYEQ
ISSIMPNFFNYHEFCDRYCFKDKNLYTKKIEYVGCKHTEELHLFLTNTIMIRRLKKDVLKELPDKLRSKIPVE
IPQKELSEILNYYKKLESKKNINFHDIDNIHLSNWSNSKSEDGDDENLSISHLFKITGYAKVKAIKEYISYLI
DADIKFLLFCHHKLVMDEVETFLKEQKCSYIRVDGLTPMEKREIYIKNFQNDDNVKIALLSITACGMGLNLTA
ANTVVFGELFWVPGQIIQAEDRAHRIGTAHDVVNIHYLIAQNTIDEIVWKIINRKWNTLTTALNGIEDSLNVK
EVNKFDKFMVDLTNDTNKSYPTSLVTTPKVRRKSSEHGTFHNSSPKKKNRDIRDFFTANKCNESVEKSWNNNI
NKRSYHDSPMNDSPNTTLKFLSKKYKTEIN*

>N.MAL13P1.282
MPKFNFFPHVNFLAYIKKISIRYNPCGTYNDNCRKLIHLIENKQKKDKFLNLDYKLELIDYKDEPLIEIEMQN
SKVFKFNPENYDLMEIQRVIDNEQQQLHHNFMKNNLLENNEDSFRL*
>N.PF13_0309
MGVEKNISMSLKLSEVFCTDENNIKEYNKGKNNNIDIIENVEDYKEKSKQILNKYKITEFNKKIKSDQNIYEK
KNKSIDDEYINDNRVNNGNIKEEHQDENIYTEKNYKNIIPSKESNLLIANGTTGIIVHNGIMCLSMKGIQPFI
ITSVKNSFCVYDPHKLRKAFLSEYFLEDIKNLYSTNNFVYVIFKRQVYKINDNGNKKIFSDHHKYNIINILIT
SDYLLTYSTKEIIIWNDNNDRNDFTQSDTNSELSDNEEKKDEKHVKTDNNTKGNPNSINKNNYTNDNDRNLDN
NKNDPSNNKRNVKNNNKKNNIQENMPKKSDYFYKSINLFDNSSDVEIKSVIHPEGYINKVIITTNENKIYLYN
INKEKIIHEYKAINILCLNEEKYIKIITPTLKNEELCIITSSNELYILDIEKDQIVYTKNIHMNDDFVSCVSF
YTYQFNDEMNYIILIGTENGKLLMINLKNNQYFIRRDVHDYVKTILVSGQGYIYTCGYDNKIHLLNINKNNFT
LDIIKTRNSCIGIVNNIKYLDDENHKLIVSANMENTKEGNLFIISPHNPEQNKDFSLNKKLNILNTTIIDFDI
NINRHYDWNNILICFENSDRIYLASSYKKTISNEFLILPNSYSNKRQNKEKVNNEPFLKNEENVEEDNNADLE
NEEYKIYNEYETYQMLHLKKQKYATSVLISSCGHIGIVGYNDGEIHSFNIQSTTYRNEYKLNKYSISSKAHIN
GNILKLYLYGINYFVSASSSKEDTCLRVWNIYTSDLIYVYNIKNEYTNSVEDIHIVSFYHYNILTAVCLSNNH
TLILDIEQKCITRKFHFAFLVTNIIFSKDNRLIFFALQNNTLLIYDIISNTFVDYLLFKNSVTSMVYDDIYLY
TAHKNSYNFVYSFTNKNLFNKYNYYVSDYKSFHAIPIEVFSDTEDEKSYNIMNIKDLINMNEQSEDNLNNNIN
EKLSKHDEPINESEKNDYVNIMETYTSSENQINKNLITLSGFNISKIAYLIFLDKIKDKCKVEENVKRNDEIP
FFLSTKLDKNIEYKDDKEEQFLQNVTNNLENKTSEQESQQQDEQEENQESNVESKEIVKSKHIGKNSKIQIPS
SKLQEILSKNEESYIKVLKYFKSLSPSGIHYNILCLSTKEELENMMNFFIYHVKTNDNVDLIQAYLFIFLKAH
GKKIMKSKEKKLKNTTKVLLQEIQGCWSNINFLFENIIFFIKFLTNIQLE*
>N.MAL13P1.283
MAEVAKNKNTEKLNRNEKQNDVRLTNILAAKAVADVTRTSLGPKGMDKMIEDGKGGVIITNDGATILKEMAVA
HPTAKMIVELSKAQDVEAGDGTTSVVVMCGSFLNVCKSLLDKNIHCQKISESFFEASLKSEEILREMSIPIDL
NDKNMLIQNAITSLNSKVVSYNSSLLAPIAVDVILKITDINKDTNVDLNNVRIVKKLGGTIEDTEIVDGLIFT
GNKISKKAQGLKNLTQAKIGLIQFCLSLPKTDMDNTVVVKDYNSMDRLLREERLIIGKMIKKIASTGCNLLII
QKSILRDAVNDLALDFLAKAKIMVIKDIDREDIEFISKTCNCIPVASLDYFTSDKLGYAENVTTESVGYGEIV
KITGVESKNTISVLLRASNNLMLDEAERSLHDALCVVRSLIKEKAVLPGGAAPEMELSQKLYQWANTLKGSKQ
ICVKAFSDALELIPYTLAENAGLSPLHIVTELRNKHAEGHKYHGINIRTGTISNMIDENVIQPLLVTSTAIKL
ATETVMMILKIDDTVICR*
>N.MAL13P1.284
MENIKLGFMGLGQMGSALAHGIANANIIKKENLFYYGPSKKNTTLNYMSSNEELARHCDIIVCAVKPDIAGSV
LNNIKPYLSSKLLISICGGLNIGKLEEMVGSENKIVWVMPNTPCLVGEGSFIYCSNKNVNSTDKKYVNDIFNS
CGIIHEIKEKDMDIATAISGCGPAYVYLFIESLIDAGVKNGLSRELSKNLVLQTIKGSVEMVKKSDQPVQQLK
DNIVSPGGITAVGLYSLEKNSFKYTVMNAVEAACEKSKAMGSK*
>N.MAL13P1.285
MVKNVYIYFVYLVYLFHMFSLSLRMQKNIFKYITPLSNRFNLTKIRKLKLFRKEKQKKQVRVFNHVVNNYIGL
DLFFTKLNNITSNHEKLNYINFLQQIVEDKAYEHLLFSNNYMRIIIYILNEKCLVIKKNEEKYDDLDYEIIYR
ILLIFKNLTRYKSFYHVILNDYTHKNKKISLLYTLMNILNEKNDFNNYSNNNFDKYKEYNSIFNYFTSFFKNA
EDTKNYMTQDSSTELDSKMKNEEQEENEKINEINKMKNKIYDIGRNILLKLKDEKLKVENKINSQYVRGSNHF
TNETSTDLKSTKDQEDILLEEKKKSDDEYVDKDNYNTTENNFKQEHMNITHKNQNNQDHLYDDTPQNDNISNV
DDNKNINGIGKISNVIDPYNKENEITKNDTKELFLKKQSDILFVPFYFNKNTKDNILTSIIINFLNKENIIVS
KKKIKNVKGKGNIIDHIGPDYNCNNYFDIYENFVEDDKDILKYEKNNNINTLNNNNSVQNVNKEYKSYEDDQF
EYEIEDEEEYSNDDDFNEKKEEDKKIYERKLDENDINIIFNEYKYESSKLKIYNENKTNSINMGLMILIRKNN
VHTNTFVNMHHHNNNDIEKSINNKNQDSTNSMKNIEISSDKEYNKINDTNQIYEDIYVKSHDQIDRKETDNYV
LNGFIKSNLDLLKPFNINDEDDTTTTTTTTTTNNNNNNNNYSNNNNYSNNNNMITSECLDISNNTCIDIKSPY
LNFVLLKNYIESLFTHINLNDYFTNKLLKLLYEILIENKNSIIYNLYYYTILKDENIKKVTHILSKNVKNNLN
KANTTLLLRIFYIMCFQKSFYINTAMHYKTNEKYAYLYKNIAKYIQDIKEGQFIKQLQKQEDIIECLKNLSHF
FENKYKNQNSLYISDVNKDQYLYEKKIFKKKLNNIYNRHFICEYKDLIIIRKTNIILKSLGVNMYDLYNDIIF
LDREKKTHEYLMKENYIKKNIINYINDVFFNIFNKFKNLFTYQYKEEPPLSVTPNKTTNSNNLFNENRNQTNK
LKTNDNVSKDDNQIKPMGNKNNISSLDTKSDQNINNNNSNNTSNLNSNMNSHRNNFKNDSDFISSSNIGHVKK
ENRTVIYNLNNKEYISFDKFKNIVIDLKKKKKRKLRILCLDGGGIRGLLSIEILKYINSNLNKNIFEYFDIIC
GTSTGAIISILIGLEKAHLNEIEFLYNLLINKIFQKDTYAVRNTRYLLKHSYYDSNILNNILNSFFKNIKMFH
YNSDFYTPYVFTVSTQMNITPLQPVILKNYNGNLYKIRESSVMENNKNINYNNENNDESDGENNNTPYNYTNG

SYDNNENITQTNNSINSVYDTINDNNLDNNSNNDIHINQNISNVSIYKSFYNIFVKYVLRCTTAAPGFFNFFS
FDNNIYADGAICFNNPTLLSLNELKIIFYNYINRKKTSFMDKLKCYFFHKNKSNDNEKNKTKDVINMNDYIDC
IVSIGTGKFKPKVINELNENKSYDTFLRWDVLLKQIVYSITNTELTHDICNNLLDKNKYFRFNCFINNIKLDE
TSPEIIMKLKQIGKRYFEDHKYNQQKLIRLINILEDKEELKKYEEKKRKYIWNDLYNNSIKSKISNFIFNNNK
LNSDSSFMNNSFLLSKNNHINNSQTENRKDSLKKNSLNDTKNNDNIDTNNNNNNKKKKLIPPYLNFNFNLMEE
GDKFDLNNIEEILDMIYKNNNSFTKNNTSSSSSSSGFINYFTKLFSNNNLFLEKLESVNKSNIFNRNKNEKNN
YVNVIPNTGIRILLNEIYFILLKKNLYFHTDEYNNLTYMNNNDDDLNIDRMITYDNKNTPNISSTKGTDTDIN
LKNENQTGPNQTGPNQTGPNQTGPNQTGPNQTGPNQTGPNQTGPNQTGPNQTGQNQTNDNQSKKSDEYIKDNK
NKTMEDDLNLSCITDHEGTDKNINDGTFFSENQNDEQKNYSDNIHNTSDNLIDDELIHSDNTVNLTNNPNINK
NRNSKFKTDSNQGNYEFFNSEHNSNNKKITILDLLRCFFFKKET*
>N.PF13_0310
MAKKKNNYLFVDTNNIKFTENSESEDIDVEEYDDDDDVDDSNIDKDDLSNSDDNDNDDDDDEDVSEEDDNDDN
EDENINNNLDEKNKKDNKDDYVKISWEKDKKNYYQYESENSSSDDDEEENKERLKEAIYLNKKEKENLEEDDF
DLYNIYTNENKDNQNVKENIIKKIINDMNNELKEKKEEITLENKDKQEEIKQLVMSEQQEYLILLKELSLNIE
KVFNEIKENQKLFEFKDINEKNVPSSDINKNTLLYLKKKNETMLTYIIYITYYVFLKVMNSYTPTHPVLDKLI
YMNTIISKTNELDNKIKFKIQQLNKSADKTFKKLNGMNGEELQEETQEDKYEDDEEERDINDNYEEYDDKDDD
KDDDEEEEDDDEEDDDEEDDDEEDDNDEEEDDDDEEEDDDGDDEEEEEDEEEGYNEQKKDLKNKKYKISKSLI
TEYTDSHIREKEKEEKKKKREKMKNEKNIFVKEIKDMISSRPEKIEEENYLKKMEEKYMDFDEKILNKKMKEL
SKKKNKMRNLTNVGMTTNDLLKFVELPEMNQQNDQKNNKFDEKKIFRSNINKIKQIKKNKLEHNPNDDFVSYK
KFDKNKQKTFNTNEYKDQQNNTYGKKKIHDEIDDEHIKNMLNFKDMKKEKRKKFLDERNKQIRKKLVDEENEE
VDRRMPNQKIMQNKGLVRKRKSTDGNARVHNRFKYMKKMKVYNSQHAKLKVHDNNYSGEKRGINPYLKKSVDI
K*
>N.MAL13P1.286
MGKALFNDDDKFMKSLNKSRDSYNLKKQYFKRDHYIHSEEEYMKNKIQKNQIIKNGIYIKSKEFVPKRAEEIL
CDQNENIIGKIINVKSIFSNTKKNIETKDLNKLIDNYEEEIKKRSKNLELFMNKNKNENNYNDKWDVINNFNI
QMNSIGNNMNGFENDLLYELYLNKKKKKKSYDSLVXXXXXXXXXXXXXXXXXXXXXXXXXXXXXXXXXXXXXX
XXXXXXXXXXXXXXXXXXXXXXXXXXXXXXXXXXXXXXXXXXXXXXXXXXXXXXXXXXXXXXXXFENDLLYELY
LNKKKKKKSYDSLVFFKRFFNCLNGYIYFLNKCEKNYNKLKIKKSLLYLRNCKDHILLIKAILKFVKNQDQNI
KKDEQNNNNTNNNNNNNINKMLKNSNADKQKISPYNKNIDRVMRKEEHTSFNTHTNNNDNNNNDNNNDNNDKN
NNNNNNNNNNNNNNNNDNNNDKCFSDEVIIEDLRNLLNIKNISSYPNNNITFIKEMKNKYNELLNKLKDIVDI
IFNKMFEFKNNNIKIYKYIYLRSNDNLTNADMKEQKFCYNTFWYLAFILNVHVKYLNKIKKSIIPYFFKLMVI
IYCIIKHIKETYILNEISQMDDVNSISYENIKNVVSKLINKKDNLDESIYYESMNDFDKFRQYIFCADNKNII
QEDIIKTNDSHIKSEQNDFHNINLYNYICENDDSICVDLDRLFTNLISLINNNINILNETNELKKKNIGKENL
TNDENKTKYNIFILLSNCLINFTNLMELLFIFNNMGMSCFDMHIEKNVNSYDLTNIFNFYFDNQNEDVISDQR
YFYKYDESSTCEENINHVTDKLKLKYDEVIQRGTKEYIRNPDYDNEKKDSANNKNVNMQISNNTFALYILNKE
LNENIFSFFHKINLKRNNLNDIYNVYYIYMWRQKQESFNFLLKHIFKKNFIYYINYIYTILGDIFVSKNVDDY
ILIDDNVDKYLFNCFFDMYTYEIKNRNDVKYESILNYIDEIHAILKKNIQGRIEKNMQQKNVKIRTTNKGEEI
KMIIITNDDIKKHKKDNMNNNDDNNYNFSSYDEIENEDNIEKNKNNLKIIVQPNKLGCIQIHRNILHVVIFIY
RMVSYSYDILFKMKEEEKNKGISNVKLQNNKNKNKNIYFTQEQLMFIIHLNIILLSYKIVKSIYNIFLSYSLF
IFRFCSLSNLKDEQVYLLCIINTCIFLKRILQNINNLYMNYKDMFHFYIFSDDILLQPLNTSKRYFPNSDKND
YRDDDIKENYSDHDIDDDIKENHSDHDIDDDIKENHSDHDIDDDIKENHSDHDIDDDNDDDMSISDDNNDDDD
DDKCSISYNNYIFEEENFKNKDLNKLNNMDNSNKNSNNNNNYYYSNSNDDADKSSNPLNPISFEENKHLGVGG
GKKKKKKKIVHIKKKKVCKNNNENILFIDKISILSKLHLLVDRININIDSFQNILIKLHKQNISKLLNKDIIL
MEEIYLINTLEIINIIFEFFQIQDVYKLIHRSLLLRIMDYFFYLINKNIHNFIYEKKKINELERNHLYENFVL
IQNKLSFVMINCIGDQFEENDDSVHEENYFLKHIDEMCLNLTNLKRSKIMFLLLFCKVKHILNFKKIILDMLD
KDVIDLYLSNNPYVYDDENYDTILNEFM*
>N.PF13_0312
MNIIVNFIYIYILFLSYYKKGNCFIHNKINYEKSRSFLTNSTKIILKKNGFKYYHLKHKKIIKPIYSLKSSIN
NIWNGIKSIKVKKLLGIFKSEELKRHFHNYNLSYASYIFNKCRLDRVLISINVLLYLYLNRIDKNEERKIYFH
KGNLIQEQDEQKCEKYKCNYNDVYKNRNYKTFLTSIFIHKNILHLYFNMSSLISIYRLISNVYTNTQIFLIFL
LSGFFSNLISYYNHFKKKNEEIYLNDIIDQNYINKKIFNFKNNKIICGSSSAIYALYGMHITHVIFFYFKNHY
VINTSFLYNFFYSFISSLLLENVSHFNHIVGFLCGFVFSFLIIAFEKN*
>N.MAL13P1.287

MADNNNNYNNNINMDRLSEIFKSTLSLMKNTVHGLAQRNETIYNLLYMSDSMQNKNDNYNEMLSNEMTFNKNM
IIKWVIIAIILYLIFMLFSYVFVNYVLFYAVIFFVCVLIIKYFVYHFNNNEIEVLENYQSFEDNTI*
>N.PF13_0313
MSNEYTNKEKLDVMDDTIEVRSMCINCEKEGLNKIVKINIPYFKNVLIHSFECEFCNYKNNVIQDLNQIKDKG
VKISMKINNKELLDRQLIKSEYGVLKIPEIDFEIPKETQKGSINTIEGFLHTALNNLTIYLKEIKNMYNEANT
ITANEANEINEANEANETNEANEANKSNLTNEANKSNVTNLTNEANKSNVTNLTNLTNKSNDFKLNSGENPEQ
KDNIVSSIDEEKNNNNDENHNENNLGANTNGTCQQITIENYIKMIENTVQNLSKFVILKEFPFTIQIIDPSGL
SSLEHYEDDLKYKIVHIEYYNRTKEELNELGFYEEYFEENEKTNDINSNMIGTNTNEQQNIKKENFDFIKKYI
HMNDNNNNNIHGSNNNTNTTMKYKTLSNEEEAKLIESFASNCPCCNHMGMNNFCEINIPGFKKCLILSFVCPN
CNFKTSEIKSSGEINPKGKKITLTVNNKNDLNRFVIKSETASINIPVVELTSDYGTLGGTLTTVEGLIMKIIE
SLEEKFKFLLGDSNINTHQYENENTPNAVNNNVDTTSYKIRELIKKLYKLCKTEEFCPYDLIIDDIASNSYIS
SDVVGEDQNLNEEEYERTYEQNDMLGITSMQTENY*
>N.PF13_0314
MKMSVTLRQHFWKTKLCPLHMENRCNEGSNCDYAHSIEDLRSIPDLKRTKLCYKLLKGEKCFNKKCNYAHNQD
ELKSAQNLFAYKSSMCKFVANKRCLNGATCRFAHSVDELRIPRIPDILLEKKDNNQTDGGNKDLLNIGIDNNN
NNMLNELNSSHKKTRNRNNFNNNLDMLINNFNEMHLINNDYMKNYNNSLNIHNKGENNYDNIINMNNYNDNGN
TFVDHVNDNFCDKIFNSHLDNNMNIHMNNHVNSNINNHVNNNMNNHMNNNMNNHMNNNNNNNNNNNMNNHINNN
INNHMNNNINNHTNNNMNNYINHNMNNHYSNYSYGTYDNRSMQKNCELKYQDDMKKKEKNKYKNKGKLLKNEK
NNPNYIGKQNNSSTEKNKKTTKNCIMNSTSSSLKNTEDKSYDSNTTISSSLNYVDEDKNKTNEDSFASPTFEK
VDKNQYYAKTEENINMNHMGKSNNKDDSKDETNKLEIKKNEKLNIKDIYDENKNIYICDSNKQNNNMNNIKDK
KKKNNIMASQTKEGENKINCTEYENFTNSCLYKKININQNIPNYYNYPNYHSYPNYDNYPNIYNYPNIHNYHN
FQNLSNIKSYNPYFNYIQTNDKISKADTCVDNQYSQYINPNEYVFYDHQNNIQYAPPNYYGNYLYYYAAPYGY
GPVGLPEKVVHNNSPNEKIIFNDNRGEEYTKNEEISIKEAETEIEEDKSDDTNNKVEDSDELNSVSPKCQEYT
SKEQIYENNIYKESKHEFDIYEEKVLQVDTEEDNINEQDISVIKNYKKVNEKCSMEENVKGEDSKICSLDDIK
AEDTIKNNENYYITNANENVKTYSIENIMNEEFTNKSITLNTENPNTQNKDETQNNEEVQISSTTLHKNEEEI
KTKDMKNYHKENKSKRKKKISKLVSLKDFKKKNIRNNNIIENGKNTKLNPVPSSVYSYIGNTINAQGVNYDTT
YMKPLSNDMDIYMNNLYDINPLNNDYINYNLNNRNYGYYYCSYPNPSIFNDEVYLN*
>N.MAL13P1.288
MGLHILPKNIMHLVNMIICIICPALKTYNLLINKKEKATEDTLQYIHFLTYWILYSFYTYFESAFIVKLMNVV
PFYGELKIMFFFWLYSDTFQGAGYLYFKFIEKHYSIIDKKICDLIQNKIPKNVSNFFYFEKSNKKIHNKIKSI
VSKKDLY*
>N.PF13_0316
MGRMYGKGKGISSSTLPYKRKQPSWLKQKPSEIEDAIIKLAKKGQTPSQIGATLRDNYGIPQVKSVTGNKILR
ILRAQGIATTIPEDLYFLIKKAVSMRKHLEKNKKDKDCKFRLILTESKIHRISRYYKRKKLLPSNWKYQSSTA
SALIA*
>N.MAL13P1.294
MGLLEKIKEIEAEMARTQKNKATEYHLGQLKAKLAKYRSQLLEAPKAGKKGEGFDVQRQGDARICLIGFPSVG
KSTLLSKITSTTSEVADYEFTTLTCKPGIISYKDSKIQLLDLPGIIQGASEGRGRGRQVIAVAKSCDMIMMIL
DATRDDSQKIKLENELKSVGIRINQEPQRITLTRKKMGGVVINSTVPLTKLDNKLIMSILHQYKIHNCNLLFN
EDASVDDLIDVIEGNRKYIKCIYVYNKIDMLPIEDINKIALCDNTVVISSSKSWNLDVLKEYIFQKLEIIRVY
TKVRKEKPDFTNPITLTRQRGTQTVEAVLNQIHKDMIKDFKFALVWGRSTKHNPQRVDLHHKLADEDVIQIAK
NS*
>N.MAL13P1.293
MLREQLKNLKAQKNIQVFEDDSYDKISRKKYSLFRNLYKNFILSSLHIESVKLCFENLKGLNKSFEEFEKLFC
LEDGDMVDRLYNEDEKGNNEIYLKHREFLTKEENKYIFEKIKLFLKLCSLYCDRKEIKILLEFLIYKYDINVK
CSQDILLCLLPILFNKINLKNILEILYIQKDCVFVFFNNCRHDDLVEYIDKAILIQNIKKNINIYKIIFNYII
DLIGCIHMIQDIEIYMTYINFFISISEDIIHSYINIPISLMEFYFNILLSIIKIGKHAFHMFNENRVMKKKMN
ITQNNNPMSDNIYINQINISSKFLDKFIKLFEMAIQKKSKEITNFHVLKNNILTIFEKEFIVMHDMVITNTSC
SDFIKSLILLLNIIYKYSYNIHFDNYINTKQIKYISEEYEEHEIVQQSSEKNGSNMIENIQNDFHTTNGLNDC
IIANKYINIQQNRTKRSGEIINSMKDNVDKNTNYIIDLIHFVKDNKYNLFDNEINNILQKEEMILNFVNLFCD
VKNVYEITDNLIKIIFIKCYYLKINISNLEYIFYNIPFKKNNKNDIFNIILIINLISYYIFLIKKEHEQDEKN
KDKQFMPSEHNEVKTIKDELKPNTNFIQRLIKKCVHNNNSNNNNNLYVNKIINPNHFFDIIKYYMNENNELFK
KKIIFFETYSKIDQGLILLLNVGKNKMNHIPLYNILYKSLDVDTTYPYLINEVLRNIKFIYDYKMKVTKMNAL
IEPAVLSNDIPTDHMNNNNNDNNNNNINNNNINNNNINNNNNNNNNNNNNIHSNNDACLDEKYEQMRKEYLTL

GLINKGKQFLKSKKHIESLYNKVIFLLKENRIEFIDNLKYYEKELLYFFPSKKYMYKILFKSFKKLKLEKFIQ
IDKERNTLFLKFYIKVFMVKMKKLQEKYKKDLMEDKFFRKFINFNFSFFFIIYDYITNSKSYPEHSYFSRYIA
LENFKLIKKFFLYLSKLRNIEYQSFEEFFQKEIKDHRLNNIKIIIKYSNPFKSLILYRIINYFTMVDLYKVKG
ITFINTDQNGNIFKINASNNSNNNNNNNNNYNILTIEGQNELPLLNNTNKNYNNKNEIFAIENIENNLNQNKKE
SENITYDEIMIRKKELKIFLMKVFKILLNNINDTISIKSDMENANLYMNILKAYQKNILLFVNYDISLTTYLY
YKYLNIFYVSNTYSVFILDTLNYYFFLYRTNHYKKLIGDNNICSAIVFELYKLLLKDENTKIDKKEFYQFIIT
SILLHSIPDKSNINRVAYMWKYLHRKLRSKPFMMEYDIMNQLFLQKKTINDLGNFVFVNENQNTFDVEINNTT
EKQHIKKDNIIINNVDLHHTDYNTSNNINSEELHINFNEYDFIKSCNDYHFDYRCFVVSAKNVPTRNNVYKEV
MKYYLFIFSYFNKHFFDIFISNFYIFQLQKCILYEFLNSNIILIFLKRIMRRKSYPYKITQIYKSVLSRNLDK
IIKQEHIYLFLHLIDYYINVLENNFSKKKKKKKTEKKKNISINSDIQKRNHTINHTNEKVCKDKEDNNIIPIT
NNNIPLNDEKEKKYINQKLFRTVHSINMDIYLTKLRNELNNKKIPKIKYTNLLNEEYDENMFFDSLKGFFKYH
QNLFEDIYFIKENDIKHICELIINIIINSKCYLFLIQLLCIKNFSISSLYKKNVLQAVEDLEITNMVFFKKLF
LNKKTQNYNSLLLMCKNVKNLKEKNKIILFIEKQMDKKNNYTNYNKIIKILHILSETTDDTNQHNTTLYHEYV
NTHISIFKKISYYLNICDSIFIKLIKYITNISTLIYQSTYLIAYHFSLHKNNFFSNIVNFFQKNLNYLYENIF
LHLEPLSRILDKIIKKKIYLYVIYEKKQMYTDYSNSKKRKGNHDVIKENKLLDQLKGNHIHDNNDDVSIVKEN
DDNSDHLDTPVEQAKKRKLQNNNNKNNCDDNNKNNCDNGISMYEHNSYNISVDQKNINFDNHNKIYDHMINNN
SKNSFNENNFMNYYHLHIFAFICTTINTIFKNEVDIKKFTVLTNNIIFLFNKCYSSYVVAICLINLIIKIPLD
KLYDCKNYILEIFNIYNYDDVDYCINNQLLLLLALYLCPENYKKNILVEELKTEQEQNIDNHTKYNNNMQISY
NENNKNLVYPYYVYSKLKYIKPHLKKVTNIHNLCLKLISLISIIHNVNEKAIKSISYFIYFSTFQNFILCLTL
AQDKLISRKLNLLYKKFSFQNTDTFVLTHILNNFKIEYDLNTQGKINNDQIEDNINTLQHYNTYYEKKNAINN
QMQKLMKTDQADIEKYSYINLSKFENSYLYKFLFHLNKLKDYMKFNDGFQKYKKFKQDKNNNENRSNASCDDI
LEDKYFVYDIFCDILNCEPDKSIQLFKRNVFLFYERLMDKLYHLNFATNKNLLKEIIIFINQKLNKEMDCKYI
FDAVMYSLCNKKEIMISPLNELYINNIFNKSLFNIKLDDSMKIFIISKINQMLTKLFNQYYQVGKYERNKINK
FDKNNNYSQKNVTHMEANQNDDNKKKHVEQEENSSLNNSNNNNNNNNNNINSSSSSRTRKQHNVELKTNQHKV
YEEIDIIYEEYKKYNEQFGSDILIESVNQKDTTMNNTFDHDIYISDINSIDSDYNFDEGDDSYNSKSKDQSID
MVNLNNRDIINLHNNNHTLDTLNNINDLCTVDNMVNIFNDEEIKNIKFINKMKDLKKLFGIDSYYMNNILKCI
ATLLINFPIITKNKMIELFHVMFLNNKKYILISTKNLLVEYFYSLSNEKKINNLRKIITNPLKKLDYNHLFFI
FSNNHMNICLKHLIKYYQKQKKIYFFKNNDDTKYDYNGSVIMNNCLFYYQHLIVYENLFQITKKKGKLGFNFL
FALIKNYIKNCKAHITDLYNKHKQNIKYLNSEEFHCYGENIYNINSLTVDLRAEEQNIVTSLLYTSTKMKVSS
LSKLFDNLISCFENKIFESTSSNILDNYKSTYEKRIYYIFFYRLYKNFGSVLNSSNKYLYDLLNNIKFTLISC
QKNSQDIFQVGKNHTYNKYMQNGQVEKIKNKLGDQFDDNTYYDNINETLLNKNNEELQDRVEEKGLILNNKDK
NNNNNNNNNNNLKSKWYWFDLGYPTLMCLYEILKSEKNNQTNMNPIFFNTCFDDVLNYFNIFQYLPRIHINDQ
FENNVNEKILTNSEHFDMKMVSIILLDMLKLILCRYYILYLNDPEKIQIMTMRLSLKNSDNHINSYNITICII
LTLKYIYENIGYNQLLFSVKDLYQIFSELNNHPDDEIEYISKQWFNTIRKNSEVGNI*
>N.MAL13P1.292
MVHDKYHKIALIDADYYIINYSSTITTYIQHICNNLLNNNRNDVNIKKETNEQHNINNKNDDILYKKKQIICI
FMLQIFLNNLDKNLNLFDYLIIVINKYFHLVQKYKLTNTYGNIKTEDNTGICKNKKDTKIDVDKLEEGRQSHT
EISLSDYANKEIAINNNINNKFSTPTTTKEAKKTTSINSNNNNNNNNNNKKDQEKIDCINISNLNNLSKDNII
TTQDDQYKHLIHNLIENNNYINIDNIIKSHLCFVNIKGNEHDKSNKNKSSSYLQINNIIYEEKLCNNINHSEI
DIDNNLIMSDENDFINSTNEDIKKKFPDMINNKKLLIDYNKINIKLKHFDDIININDLIKGYKVYEDVGSIYE
IKNLFRILILSKLHIGVYNLLYFLKKKNIFMLFYSSNKNLTNLLFKYNKIYKYYQNYYNVIDSLDELKKYEKD
CHKFLIFSNRPCFISHAKRHGIFYTAIGNKKNDDNNKNDDNNKNENNNDSNNIDKKNYYYNRKTSNCDDIDWT
NEIYEQIKSDECIENFSNEYNNVVYPFIKNCNLTLDILSWRMIYIFKKYLYIYGKVVKGFGRGSKYLNLPTAN
IFHPNFISADIMPGIYFGICKLRDKIFKSVISIGYNPYFKNKHMTIEAFLYYKTDDLFYDENIHLIIIGIIRS
ESNFAYFSHLIQAIQFDCELARIVLNKLKNNETFQQCKYFLENIK*
>N.MAL13P1.290
MDRKKPKAPPSSYLIFCNYERENAKNTLLQKCDKETIRITDIQKELSNKWKNLPEDERKKYEEQAQILKSKYN
EELLEWKNHSNEYISGDLVINNTAKFPVMKIQKIMQLNGNVKKVNNEAINIFQKAVMMFLIELVNKTLEFKDE
KNTARNITSFDILSCIQREGIKYKFLEDCIYLLKEERGQTFFDEEDVQEETLFDLCEEDKKEYNYDVEEKKIS
KLKKKVQGSNKIKDKAGINKNIYADITTFFKKI*
>N.MAL13P1.289
MTFLKLTNRKGERHVQKYFYKLNANNRINKIVREVYLRNDISCGIEKCNICENNKKKLLDGERSILILDLNTL
IKYMDFLYDCDIDNILIPLSIYDYIKSFNKILYKKLRNLCYDVDEESIIEGSSSSSSFNHNKRFCVFVNTFCK
FTYVEDNKDDTNGFNKELQEIIKIVIWLKHHNNKLNIKVISNNKLLQEDCYNNDIPCSTLFEYVNELMKGNDY

KKKNKEGKKFNMDTLKMYFEEDILNEHNDSKADHTESTCDQKTYHTLNNLSLNSNNNNKRPLYEPHLSKSDMI
QKLRENKIIKGVFSVICVNKLALVKFSDVDEIVIRGTKYMNRAIHNDVVAVEIIDEPNEDLYEEDYLEEQISP
EEEEEEIEEEVEVEIEEVGEKVKIEINNMNKEKLQNSYIKNEDVKEKENVIDIYNKNNDIVQKDMINLYEKKM
NITYDKQINENIYNNINNRHISEKKKLYGKVVGIISRCRKEYGGIIKEYSNNLNNIKKKLMFFKAFNNKIPYI
MIKAQLEDELRNKRVIVTIDNWDIYSRNPLGRCISVLGNCDDIETETKLIYNEYNISTREFSENVYKCLPPNN
WVIPDEEYEKRNDFRNVLTFSIDPPGCQDIDDALSVEFLQQDGNIEKNYIYRIGVHIADVSYFVKQNSPIDLE
ASKRCTTVYLINQRVDMLPKLLTTNLCSLVEGEERLTYSCIFCFNKDFDIIDISVKKCIIKSNKSFSYEQAQN
VIDDKNDQSDTAKSLRLLNKIAKHLKQKWSNDGALELRGNTEVLFEFEANDFSKAKNLKPYVCYETNKLIEAF
MLLANRSVARIIFQNFKAASVLRRHPPPKHEYLKELNDYLKTIQVFDFKYNTSKDLSHSINNINLKNDNILSN
ILKVMVTKCMNEAVFISGYNVHNNDMLRHYGLAADIYTFFTSPIRRYADIMVHRILNHIYQIEELDKKYLDII
YLNKQVALLNDKYRNARFASRASVDFFSYLYIKKIGNQITNAVITNLKKNGIQIFLIDYFTEGICYLKRKDGF
LFDEKKKRFIKIDKYKNELFHLSFYDKIQVHMQVDNYDIKCQNHFIFIKKI*
>N.PF13_0315
MDDHENIDINDNSSDANTANYNQDKNENTSEVNDYSKEDIEGDENLKSSKGKQENNTKSSSDKDENTIDYGNH
DFPCNPAPPVPIKLFIGRVPKNIEEDQLRPIFEEYGIVNEVVIIRDKITNVHKSSAFVKMASISEADNAIRLL
NNQKTLDAQLGSLQVKYASGELNKLGFPQNIESGVDQAKLFIGSLPKNITEDNIKEMFSPYGTVEEVFIMKDN
STGLGKGCSFVKFSYKEQALYAIKSLNGKKTLEGCTRPVEVRFAEPKSSKQPQIPLTLQPMQNPPHAMAPQPS
ISSPNNINFGNNFSVNNNYPRQVGPWKEYYSGEGRPYYYNEQTNTTQWEMPKEFETLFMNNSANMHNLSESSG
PPGANLFIFHVPNEWQQTDLIQAFSPFGELLSARIATEKNTGRNRGFAFVSYDSLESAAAAISQMNGFMALNK
KLKVTVKKGEEDEMKKYVNQNGVNSFQQVPRVQKAIPAQPASMQPNLAYHQNPQAQNFFYSNSNSYRCGPY*
>N.MAL13P1.410
MNFVKTYYKNCMDACTPAKYSIDQNEINLADISTRLNLKIFDFYNSTPSNISMMDLYENNEDDKKGIKQKTKN
KTKLTKKKNKRKNDCTHIDNFKILDNVFLTHVSETCNMNKKNHRNNQTNLDKENKEYYKKKGDIYIENAFPNI
LSNKIYDGTMNISYKDYNKMKETSKNKYTILLKEPTRDQNIISYKFKQEDKIKEGKKKGKMKKISNKKSRKKK
EANMLYENVNVLKIGKNNSYEDTYALNSPNANIIPSSKIYIPRINLSRFN*
>N.MAL13P1.295
MDGNYKNEDEEKGDLENYGANNFILNKMNSLDSKTRTVNNEMRNKDEEKLYYHSSNISNIMYNNYRNLNNDLL
KRSDKFNKLVDSNYNSIFHNRSADNHSVNQNDHEHINTSNIYNNMNIYNNGYIYNKKSAAKNDSMSNNNLVRN
YYDGLYPFLNNYKEEECSMNYSNVQEYSEQYKMNNNINIDSSSLCENNTSFQCSNINKNKNSNTTFPNSEENK
TNKNHSNNIMNHLFHMKPHCNLKDKVDIHNENYNNEENNSSNSTLPTSENSSNFFNTEHVNNNEMNINDGNVV
LGNVSNDRASDGVKKKKNKENKKDNTNNNRECNDKDTNNVTNNNESNQDITLETSSNEKGNNSSKKNKKKNKK
KKNINKKGNSNNNNNENNENIDNNENIDSNNNNQLINENKNSGAIINNDDNNNNNNNNKWKENQNNITNHPNS
VNSSSTKICSNIYSVNDIGQSNLISERYCTNNKYFNSTFFNYVHNKNNSKDTFNTYERNNVLNSKIENNIEKI
PHINNEYTNKNPKNCLLYKNEETNYNDNNIKDYINSMNFKKNDFNISTDMNNTNVSNNSINEMKNGCLINDNN
IGTNNILNTNNIVRNEKEENYTNSKYYNHFKNINVLPNHIHDQHKILMKDEKNISDNLNINNSEYGIDSNIRM
DVNMFHIQKTLDFTIPKNLNDNLKACPTNISNMKNDWCNSNHKPEEMNLNDYNFNNNYKGNINLSRTNENVNG
DDMKSINIPLEMYNMKNNINTKQNNNEITNEYNYYIKDNMKRNVINKNTHIKNSGLENIMPHVSYKINENYYL
QMNNQDNNFIPLPHNKSYNNIVSSNNNAMVYPVDLNFLNNNNNNTLKNNLNHFINMNASRNQAYKNNNDNEND
YLMKGGYNNFQCSNENNNEQHITVDDTCMDKEILFSHQNMREKNTIHEDKNMITTNSNTRGLMNYMNHLNDLT
CNNLMDNNYIGENENFNDKGNSNVCVSLNKDMIYTNDVEIIQNIPYKKEIYDSIQEDDYFNDKAKQPDQLLSY
GDIYTNDANNNDGATTTTTDNASEKNVINHNNIYDTNLNVEHNKGEDSSNTKNNNSKNFNNNNKKKNKNKNKN
KNKDKHKKNNNQENSVNSKSSNSNNTINENIEESNINDSNNIITKSEKEQNKKKQNKNEQVKLNNNNNDNNKI
NVKEEDQITHKINNINPPSSDINSRKNDEKYVNGSNGLENSNVFENFNEFDYNEKLNRMVNNVVHEDYNMNDF
KDNTVECKRDMYTQNCNTEPLNLYDIKFGNNNYENEILCNKGTSKNKKFIKFPSCNLFNNKLNDFINNSFGDN
NIFPVKEYNNFVNYSEAFNNKKNENKYFGNFDNPNDFNICWNDESSTGVNRNINTFRSGTDDEEQVFKTTKNN
INNMNNMNNINNMNNINNMNNMNNMNNVNNMNNMNNINNMNNVNNMNNINNMNNVNSNNSNNNIDERNSTHRT
ISMNLNKNMKKSHSLNNDKRDEFYMQMENSMLDENEEDLNLLSSRERENRMNNLILNYNNKNNLNNDYILGSQ
EYINDLKNEFPTHTNSRFRIFKTNDENVQYPSGNTEPIHGIMNSNEKGVFNVDEEYNVYESMEKIESYVGNLK
RFEKEKRVYGMNNTNMKMNDDFKAYGLDNNMNDVNNINGVNNINGVNNINGVNNTMKNVLEHNRMKEQENIYN
SNMNGTENNMDMMLNNNEDMVGYSNRFVGENSMNEKISELQNIINSLKFKNKQLEKELNDVKNMHLKKKQSVI
LNSLGNKNDDINTYSSCKDDMNNSDDEESVDNSNKYVQNILNDKEKYNYDTKISIQKFHLAYTNNSIGKLKIA
AQRDISGSFMGPKGIHVKTIKSSLHISVYKSAKDVWFPGFADSHVFLLKGNIFGILRACQLLYHYVKSKMSSS
KCCIYLVAPFECVQKLLADGCKRMAIIKEECGADVRLGNLYVQVHEGFTERLMEIRGNEVSVDCALEKLVIFM
QSCFSVQSYDYEILKYPCRSVLNLQ*

>N.MAL13P1.296
MKNLKLYRCKILTDIYVENKELEEYLNILYYLDHSNKYFIDNLKIFIFDEFYNIEDDNEKEGKDINPNDNCIN
DKEVQINSTSEEKKLLNNKILEYKKSFENKMIYNKVNNEMKYYEIFIFYNNKFYEDIIYNSIGLEIYECPCNI
NNMEHSKEILILSSHDDNKNVQDIKAVHNEKFIDNEKFIDNEKFIDNEKYMNNMNAYCPYNNKTENKKIKKIL
SSIQINDINNFSGTLLKFPMKINIEKKHMCDKIIMATIDCMLFMDNIKNIDIEMNDSCIYNFEKSKKLINISS
SHSEEENIDLCKEISYTSVEYTHEGLCTEYILYNKSLNNDNNKNDNDFSKNDEIIFIYTTSDMYEIQKYYDKK
KQHIEFINLFNNNIFFIKCIDTLPIYIKSYDSNIIDKIKNYLFSLNTISELLCNAYIVFLLLLRELNKFNNVY
DYSSYFPQLYILEPSKFDKISFLLYDKLFDEEKRLKLFLNKYNMQWLHFNECLFISRQLQCLFDDSTLYEIMK
YSQVKNIFYFDDKLKHALYYYLNELFEQKKNYNLIIKIVRKSLRYIDDIPDKLVSMCKNDNQEEKLKYNFGKK
GTSNNNNNNNNNNSGHNNTINNSIHNNHCNSNNNDICIYGAFRKNENRFFFDIIIDILKYMKYEKCLNILSYM
LQLNDESIDNCLRNYYCFPNEMKKLKKIGELIYPIDEVYYYKYNQETYPINNEEYKMNDNLQYERKQMSDYID
KENLISIDFMNFIGKKNLNKLKVLGLKIIEEDYKFNDNINDILFDLKKLYSSTNIYENKLKILNKIISRMEKN
IHYLKDSHHILEKLKDTFFLPVYKNKTLNIDNQVNMKTTKDNVTNLIGSEKGLCHDKMIIKIDDYHMIQDDIT
NDHMVNDHIENDDITNDDITNDNITHGNIKNNDLVNVNTVNDDVTNDDVTNDDIKNDDVTNDDIKNDYIKNDD
IKKDDIKNNDIKNNDLTHNNILNNDTTNYNFNNYMYEDSEYNCSYNNNYSEKICTDVYNSTPNFLITNNNLKN
LVHISPTFSNKSIINNNSNNNIRNCYIPSSSLLNNKNKNNGYNNFVKIADSYDMKYYNLLFLIKKCYSTENIV
MSSELCELLDIYRKPSIKDILSHMNLLIRNFKNNYMQNMFKVYDYIYEYFENYILENKLNGNISTILKNNKDS
PKNNINSNNIDSNNINSSHINSSHINSSHINSNNINSSHINSSHINSNSQAQNTIFSNLSSGIYIEELQIEEH
ETFIHFLQNNNYLYIKNKLWNPKDVYYHLKNVSFNIFLCDLPKLYKIKYPNMLKLSGIQTKLFHSKIISILNK
IKNKVEMKYKKMVECNKLDKKTNKLCKKQFNNNYPNYITHNQNINEKKDQVLLQLTGLLRMSYMNTINLIDSS
ICDNIIDTDHMNKDELEYNEENNISSGTKKGNLSTEKKKLTCKCFDNIYLPNNKYILIKKQECIYIKKATSHH
NNNINNINHINNINNINYVRNDDNIVHKDIKECIIHLLDITKAQKEMKQNKQNKQNEQNKQNEQNINHQPNTT
YNVNSNDNNNNNNKKHINSLNNKKCAKIIKNKIEIFKKNQELSFKKINQYNIKSSGEKIKSQALNKVNTLTDS
INKKKNKDTDPSLIRYNERKKETSEIKKKKIPLPIKKNEKLNLSSLQSCKNDIIKKKNRINDYNGNINNQACS
LSGKKIRKKVYSIENISTHKKVEKQRNELNETRLFKDIKNLIYFLDDIGCFDIKFILDKRIFNNTYEDIPKRF
LKRACLHLYIKFKKEIYTNLLINKNEMNKYMNIYNFSNMKTYTCDESNISESLKSCNDLICLYLFKHFSYILD
YLLLLNGNNISEWYRLKKEECKNHNIKEKNFTDILKENDINEKDEQTNSHHFISTEENQIYKQVDNKNTSQIM
DTKIQDCEEKYVIYKRKNISLNEKDKILMKNILLYDRFDNIGKVRKSKLNLSSRKNRNNNIVVKERNQNRGKK
KEAIKMKILDDNIIEMNQDPNIRNNEKMNESENMNSNDEQINDISNFKNEKKNINEEENNDEKIICIRMYNKN
KLPLLKIVEKAFLECKKFNENFLIFSKNIIKIDFIKIFEKDERAEIITNLGISMSENHFYLRQKFYNSVKSLY
PSNLSGAGDNINVNNNDDKNDSNKNINSNNNSNSNNNSNSNCSISYNNNHNENYNNDSLNTKEQKLEIFLQCQ
IYNGNKSSRWIIGLLENTCISICFEHYQIFRKYYLFHNLNIISNIESLPMHIKINVSNNNFSNLKKTFKDNEN
VSCIVKNVSILYIHFLKLAYRNTVKKILKHDTSKILYKHKDKWIKKKKTNIGKNNNNNELSTISQCIGYKNCS
YNNNNDNDIKKNNYIYKIDKCDIKDFNEIYKIIKNKYLNFAPKKYHKDDLFYNVVKNVVLNCYELQILPCLQK
KDTLYALYWDNIYDCINIYPFCKYVNHITFFLLDLGLKVLLPYYEIDNLIHIHKLVRSSLNETNHLNNKNLKD
SKTQINHDIKNGFTFSKDIYNNNAFCSSIHMLNNENVIRNVPNISPSYLRNKIKNHISIYVCFVKECLCEMRG
SSVMYLFDYLFSDIKKNDSKDTIKNIYIKNIPLFAILNCDILYLKGKIQSYLYDKLGRIKKDYLIKNMFEQNE
SKYDDNGSKYDDNGSKYDDNGSKYEYNVSKYDDNGSKYDDNGSKYENNGSKYENNGSKYENNGSKYEDNGSKY
NKSFDQKIDEMDYYNGYTIYTKNNNSNNNNIDINNTYIINNKSNNNDNLINVKREVNIISAAAYNSHDDIHFD
YIKTYNIDNKNYLNYVFDPRNNRNISLFCDDFVKIKLNHFDTENFLNFDNIHLIKYDEDQKIYCSKYFYMFDL
SKVMFINKHLYSSSILSLLIETECILKLTFTNLIDILEYNLYPNIFHYCSTYKIKNKENNNKISDNAKSTNAT
FLNDYDHSNITKELDVKHINTLKINFNENINNAYDYNNKIEEEIEYFKKISIYDNMMKIPYDINIIIYNRLLL
DMVNNIYKEYSDMNFLEQLKDFRLLITIDTLNNNENPRYMYIHNINDIKNIININFIDTNVEEVLYSIGYKKI
HNSLSNDSIFNQYFLNNYEDILLSLYRNMSNINWSDLNNTHIHTLLFAFLSNIKFNSNNISYLKPLPIFHSLI
DSQFVNIISKHKNYIVIGTNYEDLFLSIITQIFQKDNHYNEDNMLFQRDNTYQPIKKNYMNKYYINNSTSNGK
NVQDSNNIPAVLSNMNMGNQNVDEIKKNDNHMYDDHFNYNIISSRIPKNEKTNSYILNSNEEINSTLIDNSYN
IITNIMNSNSNNDNIYNIIMPNTDEHKLNSKYIFLHHSNINMDYWKYINIKYWYDYDIILYFFLNLLKNCNNG
HLFENIILFIYKEYERIYKKRFVENDTSSNQLNNKDIMNDTNNMNDTNNINNMNDTNNINNMNNMNNIKNSIH
SEIPYTDFLINIISELKEIPLKFKGNLKCSQLYNFNNDVYREFIYINKLYNFSKLQNVPYEQKFLESLNFHYI
PNIMEINNFLLILINILKQKGNNIMDKSSMEMIFNNINLDFSRYDTNYEEEYLNRKMSVLLKYINENLKHLLS
LQDENFTYLINSLIGLLMHDQKFVEIIKTYDILIIYYTQRDKEIGDIINNRNKSKEINETTNDGDKDFINVKS
EYMYEKKPNINDIFKE*
>N.MAL13P1.297

MLFFFFFVHNMGNTVNKKTVVTNENVKVKTIIPQKKYNILILGLNGSGKTTLLYHNFIPEWTNITSYMEPTIS
YHYEEIKWINGRLGFWDLSGNPLMRNIWPLIYRNVKVNAILYIINIMDISDECISENNSLISLLLNDECLQTS
CIVLVFNTFNEVHNIQENLKNDMLIKYKIEDLINHYGNRIHYLFVDCKNCKMDKGWIQLMQQISYYF*
>N.MAL13P1.298
MDVQKNKRKNSDNNNCNKLKYINNNTKSSNTTNFKKLRDISKENILFLYDKKVEHLNNRKDNSNDDAELKKKN
EKIERMSKFNNNENEKIKSNKNKHSINMSTLSSFEEDENNNKKNDIIVSSEYAEVSKNKNMLIMGNKNKEVIK
HNKNNLNHIFEEVCSVDNNNIKDNIPMLSKRLSDPNKKENETYNIHQNNYENVYNINQFNNNNIKMRHVLNNV
KSLKNWTDDNDEDEKEKEIDHFDLSTDSRNQNSNNMNSNNYIHDVNKHKLTNKTNSFKFEMDEDKCNKMDDDF
SKRKILKKRSVYNDEDSDGSFIKEINDVNLDIKNDNICCSSFKKSKKNENHVRIDKNNVNDNMKELHSNSMGY
IDIRKKRKKELSKELKNMDWLIYTIRYMDYRRLRRRFSGIRKPISKKIVFIITIFCLILISWKYFNISYENNK
FSFSFEIQTSLLFFVEAILLTIGIIIFAKFQTRLSLHWPIYASYIFIICAVILLLFFENDKLDKNSRGEHILM
LYGIVQLSLIIIVKIIIFSGPLLAIYGFFCPCTEHCRILKKKISTNKLNITISRYNDFAGMCGNNVCCLCLCA
YRSFYRLVNCFYNKFSNFKFKMITETNDEAPFSHQLRYKGKTDIYGRPHGYGEWIEDHSYGEKLRGFWYHGYP
VGPFISQEIGSGSLFVNTRVGFAACVGKDWSDVRYGVACTECSISGHFFNDFPLTHFFNSKIEKNVYGRLPTN
RYDIVIKDILKENYEDILCYDLKWCFNMLKVNSGTTNETCSNNCMISLDHVTMSLKVHNYKRIDTVKRKNDIL
DEINIKLVHVPHEKKKTKKIHKRDKFKKLEEKWKQVENDENALYINQNKVYTTIRNVNSSSNNREMNVPVQEN
YHSYYKNKFSDKYLDSVDDDIYLYKMNKTLQPTEIHKASYWYESSDSKNQTKQKEDKSRDKSRDKSRDKSRDK
SKDKNKDKNKDKNKSINNNIEAPFNSSNGMIGHHYDDDFTCNILQLPKNGYENFEITKKNYELDNINSITHRT
NVHTVHDKNSNSYMNPLERNVINNKKNIPNSNNMNEAGTRNNCSYVSYPLCINECKGRMDNSVNSNVDEFVVD
IKGYEEIYQRNNKKKKTVLNNNIKNIYNNYHPNSGCTRQNFRNYEKYLPNVKNVSKKDASDTEENKDQNLDDH
SIFSQNEYPAFLENKKENIYDNMNCLLFEKQNVVGIKREESSEKSCDKNMIMCNKKGIMDMPNSEDETEMDKL
SKHKKETSKNKELLKKKKFCNKMIEENSNINEMYENYDMDNKEHYEYDIDKNDYEAIPIRKEILKSMENEHKH
EHKHEHNSNDKVVENKNIFMSDIFKSFIKSGVQRNMKKNSNIILSSDEIDGISVKRSNVKRRKNVIKKSSYRL
LGSLKENNRRKSSVFDKSTKKLSMNKMKNSKIKSFVNKYSKQGKKSSIFITSGNRKWDKLLRSKKYKKNYKNE
RIIGTIGNKMRNLNESYGNFNTPTISPTNIKRNKTIAQVFAEEDEWEQFRHQHKQKIVIDGWQSLSLKQNLNF
MPEEILIYIHGYNVKLNHGCSQLAHLVSFSKLPAYIQPFVFHWEGAMWGAFSALSYPVAKKRTEMTILGNSFR
TFIKELINSGIKNVHIISHSCGSRLFFNGFRSCVEDNLFYNVLKNKESEIKKGDNVKSKGIERSTTGKDEYVS
DDDNDDSDHINDNNMNGSNNMHSSNNLTNSNKINNINGINNINGINNINNINNINNINNINNINNIDNINSSN
SINHSNGIGRKENTTSNKNKGNVKTNTDKKNKKQKKQIIVKTVILLNPDYPLDTFLEKDFFMLRSHCNHIVMY
GDTRDQALTYSETWNREKCLGKRIFKLKLPLYKIHHYEDYLNINSDGNKFMKKRCKVEQYKVCDSVLFPISSY
ADEENKNKLKDKDNKDQEKKNKGESKCINKAFKTDKNVLNSYTLDLNDVGEGEGGVSCVSEDVSQNYLNENFL
KTLEFSDTSLKTFKSKKKFRFSTAFKKIKRKWLFKKQRTNIYFNENTLSKRYSSMKFKKKSFKMSNQKFKKND
TVYISFDKYAWLDMDVIDTTFVETNVDFLKHSFYQVKREIIDDIREVLISNIRAHERVSRLDRRRGNVFVLRV
APAGVGSLHR*
>N.PF13_0317
MNVSRVLLNNSKILKRNIEFKEIFTPRWFLECPNYSRMPLWRRFFEGQYTNGSFLFFGNAWTSMFAFAFMLWY
SRIFDPPPLERIDKYWLNSPKFRILSAFYNQGKRPGVKISLMTYEARYFYRGMDHPFTINEIKDLWFKLKENY
LIESVPAIQYPYVFRQYNNISSPSDLHVHLH*
>N.PF13_0318
MRENDNTEQQISDMTTSKDEKSNNKKPHLRKAAGIVWKDPTLDEWPENDFRIFCGNLGNEVSSDILANAFRKY
KSFNMAKVIRDKRNNKTKGYGFVSLSDPQDMLDALKTMNNKFIGNRPITVKRSRWKDREMNSQKNKDFDNFLK
NSQLPTKKFRKFKKIVNNNAKDIHERLINKDTLN*
>N.MAL13P1.299
MDALILDEQIRIYALLPIFVIVVLVCIIKSNLAQMIHPGPKTDMEKLSQNNYLSRFDLLKSNNGLLSPLAFIS
RKLFYNKPEVGYFNDLPEQANPFDALLKQDPSDLFGMMKNQIPFLVLQLGLGFLINLFFSGYLVAKIPFPLTY
KFKSTLQMGMDIELLDMKFVSSLSWYFLVMFGSSGLISIIDYFVLQDKDRSQNNAMDNLMATHNPLAKPPGNM
NNIPDLRKFFNKKKEELDNVKYEFLLENIECELLQKWE*
>N.MAL13P1.300
MSTFLFNKKDVILYFAFVFFSFFINYNNSHIFNNDDRKLKTLFNRICHKIFSSEHIGNVNRRNGIVESCTFGG
SGKEKKRNSSTFECINHKLLKKSKILFYIWSNNLQKIRVNEIMEKEKIFIDLNEEYIYLKKKERKEKTHELNY
IDLSTFPTYCSFYKLKVQDLLYYDKKINYYSKRYFIKTDHLNSDPLNENATIFIWGNKYENLQNSKNKIYKNF
HNILYYYITILMNNILNHTFYFVFNMNKEKNYENFIIYKNKYIYPFKNIQLNYDKHKDINNIISLKNSNVQNN
NETITDFYLVEICHSLKGITFKQNHNILKKKLNNIYFNIEFVTYIFQFPPVFYHIIFIIFFSLLITYVFYKIF
IKYKTYI*

>N.PF13_0319
MNEQNINNNNCDINDEGTSEINDENIIGLCIKNILDSTLEKNIHCENFLVTKNIALNNFIKANNVSEDNIKVS
DTVDDDAIPYDIIYNEDILNIKNDDDIINEDINYTKELKNYNTNNSNIYSFNNNESNEINKKKVEKKRKRNYL
NINDYNFDYDEEDFVTSSNEVFLKTHNDNDNTSETSKIINMDDDIIKNLIMPPVVPFNDMFKKFNKEISDIYI
KNKNNNIEDLNIFLASQLLCLEEKEDTFINENNFINSNFDSFILKKLKIINDPDCNNFSNQNKDLQKLFMYLM
SWYFSGYYSGKMSVLKELYRQ*
>N.MAL13P1.301
MKTQTLSLMNINGKRKFLGTNNKIYRKVIINPTSEDDIQKFCRNYFRIYNFSLYNFIRRLISFDAILVYSLFL
TVYIFSEINHGETKKYLFIDTAISLFFNIILLIVIESLFELKKLKDVKNANSQYYLRIVPKMSYFEKVMTKDI
KVGNIIRIFQGDEFPADVVILYVKNNANAIVDSFKIDGLFRKSIKYAVDKYKIDKDYLKMLSEINGVIRCELP
NKNIFCFQGNFKLDKHPRSLLLNYENFALQSSVLKGAEYIDAVVVYTGADTKKNLNIPQKIEENKTFCIKMNN
IVYYLIFMYFVFVVLSIVIKTIFFHKKNSFQNSRDSFLSMLEDFVGLYILVLPIMMYSEKSLIYIIQSLRIEN
DLRMRNTDSEKPKVFNKNKNDSLGNVDLLATSRNGVLVKRKELLVSCVINNVMYGKKDIICSRTNFKLPTLNI
LDSERKNVSNLLNLDERIFKDPENIFFPTRDFYSFLKLFENKISSIYNPYSSSLSNLLKEKYKNYVNEEILNK
NVKLTSFVKSQLTIGYNQICEDDELSYNCYEIKEDSQKENIQSVKIEDFILGLCGCNRIIIYNEKSLDISMNE
YKSDNFMETYSKFETENEEYHENDHDEYHNMEHSDDENINSIEYEDICLYNIIRNTGFSIYCYKNTLFLYNLM
KECKVYFLTCYHDFLRSNKFSMCILKCGYSINNEKEGGILYVRGYDFNILPYISKEKNNIKKIKNVIKIYTLN
YLKVIILCKKQISNEDIAKYIILKSISKKLSFKFYDLIKLFFLYDLEVIGIIGLKNQLREGVKETFNDVINFD
IKSWIFANECSKDTYLTALQCNLIVSSSNLFLINYYNLKNTHEEGANILFHNFISSLYKLKSNSYAVVINDES
IKNIMTNVESMKIFLCIAMRATVVLFCKLQNETKGKIIRTLYALTSPKLTVLGIGTTLNDAYLLKYSSISVFL
SLNEHVNILYNISDYVLQEFKFISELLILGRLNRFSLCKVFLWIIYLKITVVSFYFFHNFDNYFSGSSASSIL
YTQTTFALLHYFLIIAFSTYEIDLPYKFVRRVPYIYQLSRRKYFLNNNIILLTIIEAILISLTSYYILRLNVF
HLITHREFTFHIFILNVFITTEKILLLSKTWHIYFFIMAVLIIGILLIYVNIFTLVDCIKNGKCEFSLFQMEN
IYFWTSLFPILYINFIFDKLMKYIKNRIYPDISDYLRKYFLRRICCHNNDKFLSQRKIKGINKFVNFEKNDIL
LKYIPTPKIYKIKDDPTYYNKSKRSKFLYDTFRKVIDINVKYRNQQLNLEYKTYEKGNKLKLRIIVILLFLIY
IIIFSSQTIIDINTKSNIHYITMFYIIYFVLACVLLIYIRIRNKATSTFFYFLSRFLLICGFCIELYDNISND
ILNVLITYSFTVSYIFFMSFKILEALLVCISILLLTFWVYYEKNKNMIDICTHFCSNPYLSINNLDHMNISCL
CKKQIVIFLISLLSFTLICLSMKYYEIFYLKKKFLFRYKQKVNLAKQIEILHTMLPNFLVEYLLISDPKNDGI
MVGKNISGEDRGIISVIFCDIDDFQNMVSTLQPHVLVETLDNLYLYFDKCIKYFNCIKIETVFESYLAASGLS
EKKNNALDKIMYDTKCAIKLAIAQLSAKYYISYKVLDTREHFSDNSTSYDKYINKNISLKIGIHTGKAISGVI
GSVKPQYALFGDTVNTASRMKSTSLPDHIHVSYDTYKYLKEDNTFIWKERKVFIKGKGKMKTYLLVDILDDVK
RKGESLNYYSSSNLLLSQLGSEAVSIYEEREDIKEGSMDIIKESSRDIIKEDSRDIIKEISTNISKSSSRNIS
KSSSRSISDIKEGQIIDKEDLIFKINRMKNKIDSRYSKRIDKESRDKISDKTNHVLDEVVKHSDIHLLNYEIN
NKRCKKMKGDTNNENKLIGDIFNMYDKKIKYIYKKNYKSKSMENISFIKHYRNTKYKKSDYLLLDNKGESKKF
KRNTSYVLESPLHLIGDIVDNNIKRKKKKEIKTIVSDDMFTSPVNIKEYNYNEQERKKEIVGNLSYDKTKKI
FPFIKFTKEGRIKKKKIEKKEKKENNNNFLYNDDYSSYSSPKYGDNENNFVIKYIRERKDFQKKFDHPNFNFS
KFLHNYNPMKNKNKNKKNNKNVRRNEYPNYTSSSKDGVSYNFLSDSLFSSDNEYSSDNEYSSDSEKYYKKRFK
KNKKIIKFDDLFTKIYIKKKRLLQMNNYDVKGKGKKLKNKGMERNKTKYKNVNEITKMKYFVNNENRDHEVNK
EDISKSMQKYFLHISKHKKEQIEDKKKTHKYFHKNVECVYPYAGNNINHNFSRNEKRKYSINLYDHLDEQEKI
KGKKKYFNKDKELIGSINKQTERKPKKKNKKNIENKKDKKKIRMITNKTKEKHSNSIISVEEQNMNHNNSLKK
KEVNFTGKNEEYLNRANTNCSLGIKEMEEDVYEFHSNNIYYNNQTSYSDDINNTTKLKGMGNNTNDISKNKGK
NKLGKKISFFSMNNKYHESEIMNEEDNKNMLNLTQSQIINKDKYNYFTHCPSLKKKKSVFTKINNLFKSYFKS
IDVHEKFGFSKKFKFHSKDSDDIKGNNNKISKNRYNNNNNNNNSNYSNIDSGKYSHNNKKNHHHNNNKYHHHN
NNKYHHHNNNKYHHQNNNYEKHHHSNNSRVMLSKGEKTEKNENVDYAYQFDNYDKKLLKKLTSNLQLNKKNVK
NFNMFYYKFNDEELEEEYTRNYYREIINIDLTKKLIIIFIFTEIFLSLCNIIELSFYEKKLRYNDSIVIIWLI
RSIYLFIITYIWIILKTKLKEYKNNSSKMMWTIFILNIFLCSWGIILIDLSCIHYSILLGNKNERALFFMKDA
SELIICIQLIFIKNMLFKHKFFFFVFFYIFLIYSFSKLFSIHTCQTHICCSIILFISINILYFWYSEYLDRIQ
FLVKRKRNRMEKISQDFLTKILPRQVLEEYQNDNLQLTYKHEKIAFLFADIVGFTKWSKTVSPKEVLKLLQKL
ISKIDKDTIKLGLYKLFTIGDAYVATSQPNSSITDESEALEGILNILKLAKLILHNINTIKIQFNKHDFNMRI
GLHYGSCVGGIIGSVRIRYDMWGLDVLIANKIESNGIPGEIICSEQFRHFFIQNEPQAKLNFWYYKSISINDQ
DIKLYVIEDKNYEEDYDPKVIDYTTLLKLRQKKGLRS*
>N.PF13_0321
MIEYQDVNRRVNELLKRNFLQSKSGTFVGSPEMFFNNIVLDVLTDFNNIHVNEISSIQDYDIMLKESTKTISN
INKIVNMTNDDKNITSSTDSKNSSSSNFNKNITSSNFNKNIASSNFNKNITSSNFNKNITSSNFNKNITPSTT

NNIHNELDLSAVALLEYYSKKNNIPKDLSYINSQLSPFELKPVENYINISNSLDENNILILDTLKKKNVDTSG
VSIENQLDSITKSFNNIIQGDNKNDNNNNNNNNNNNNNNNNDGNNNATTINNNNNNNITNNSNYYNLSSCSDY
NSNYDNDNLIPFDVFNINEAHLSLDQTQQIIPSDTINNTENNNTSTNNNNNNNNKNFYLKFEERFNMNNDRIIT
NKLPDENNFQALNELNKELRNAHNLSIINYDNNNNNNNNNNNNNNNNNNNNKLNKKKKKKKNSITTNINKKNNNK
INRSNNINNSYSSFRRIDSICNNFNFSMIKYSNKHNNNNNNNNSNNINSNNINNNNNKRKNTNNKTIYQYINPN
NTNQHFIKRLRKNKRISKKDKNLKENQKKKIKRK*
>N.MAL13P1.302
MTYNVNNTNMAEYLNKLRVYDLNQLCRKFLLPQHGKKVAIIERILQYITDVEREEQIYEFILATKPSIFEIIN
DKRMGTNILHDSSKLFFSNSNYVRNDNNINVNCNNIDKLAYASSDPPSTKKAASSTARKGKVKIYEEDNEFST
CVCGGMSKNMSSKNGIVKCIECKKSQHVSCYIPNTFINKDLSNYEILCIACRVKDMDPFYPMKKVLWMKNIST
NTEKLMINASDIKQWRNENKDVIVFCINLEPQNLKNTAPIKQEWPKTFNLKVNGNITEKIFEPSWEHKRRDSP
LKITHTLKAGINSIDIISTNYDIPKLFVVTFALCKYESEQVIIENVILRSSLNFKDAKDRIVNILSTKHDSDE
VMCMEVNRKVSLHCPFSLDRILIPCRGIMCSHIKCFDLKSFIDVTKKTKAFNNRWKCPICSFYLRPKNLVIDT
FITYILSQVPKDIKEIELSKQAEIIFNKNNQEPKVIKQLDDVDTLDLQKKHIDIKNEYIMGKSYNEQKNNNSF
NNDEIIILDSDTDDNNSTYNINCDKYNMEKNNNIIQGILYIN*
>N.PF13_0322
MNLTKLMKVFGYINIITNCVQSFTNRADKKRYNVFAKSFINTINTNLYTFKAVMSKTPEWIHEKSPKHNSYDI
IEKRYNEEFKMTYTVYQHKKAKTQVISLGTNDPLDVEQAFAFYVKTLTHSGKGIPHILEHSVLSGSKNYNYKN
SIGLLEKGTLHTHLNAYTFNDRTVYMAGSMNNKDFFNIMGVYMDSVFQPNVLENKYIFETEGWTYEVEKLKED
EKGKAEIPQMKDYKVSFNGIVYNEMKGALSSPLEDLYHEEMKYMFPDNVHSNNSGGDPKEITNLTYEEFKEFY
YKNYNPKKVKVFFFSKNNPTELLNFVDQYLGQLDYSKYRDDAVESVEYQTYKKGPFYIKKKYGDHSEEKENLV
SVAWLLNPKVDKTNNHNNNHSNNQSSENNGYSNGSHSSDLSLENPTDYFVLLIINNLLIHTPESVLYKALTDC
GLGNNVIDRGLNDSLVQYIFSIGLKGIKRNNEKIKNFDKVHYEVEDVIMNALKKVVKEGFNKSAVEASINNIE
FILKEANLKTSKSIDFVFEMTSKLNYNRDPLLIFEFEKYLNIVKNKIKNEPMYLEKFVEKHFINNAHRSVILL
EGDENYAQEQENLEKQELKKRIENFNEQEKEQVIKNFEELSKYKNAEESPEHLNKFPIISISDLNKKTLEVPV
NVYFTNINENNNIMETYNKLKTNEHMLKDNMDVFLKKYVLKNDKHNTNNNNNNNNMDYSFTETKYEGNVPILV
YEMPTTGIVYLQFVFSLDHLTVDELAYLNLFKTLILENKTNKRSSEDFVILREKNIGSMSANVALYSKDDHLN
VTDKYNAQALFNLEMHVLSHKCNDALNIALEAVKESDFSNKKKVIDILKRKINGMKTTFSEKGYAILMKYVKA
HLNSKHYAHNIIYGYENYLKLQEQLELAENDFKTLENILVRIRNKIFNKKNLMVSVTSDYGALKHLFVNSNES
LKNLVSYFEENDKYINDMQNKVNDPTVMGWNEEIKSKKLFDEEKVKKEFFVLPTFVNSVSMSGILFKPGEYLD
PSFTVIVAALKNSYLWDTVRGLNGAYGVFADIEYDGSVVFLSARDPNLEKTLATFRESAKGLRKMADTMTEND
LLRYIINTIGTIDKPRRGIELSKLSFLRLISNESEQDRVEFRKRIMNTKKEDFYKFADLLESKVNEFEKNIVI
ITTKEKANEYIANVDGEFKKVLIE*
>N.MAL13P1.303.2
MDLESTMEWNKNFQTNSIFVYNFPNEWMENDIKKNFMIFGTINNIIIDKDINIYAFIQYNDTEASQKAIEVMN
GKEINGKLLKVTSRKIVDECIDMNTNKLDSQQKSQSSNDNKKTTLFVFYLPPHWNDQDLFDKFKTFGNLESAT
VAKKNDKTSKGYGFVVYTDPHSAALAISNMNKVEVYTGKRLKVLLKSSSNETNKRKIKPGCTIFVFYLPNDWS
DKDLKRHFSHYGNILGATIKRETNGKSRGYGFINFENQQSAINAVAGMNGFNAGNKYLKVSIKKGEEQYLAPQ
YLNIDRMNNSYNSPPPPPPPPMSCHGNESSNYANNEMYSIQNTMPNNRYNSRVSTNDVHQSFINTQYGHFPYNF
FKNNEQGPYMQRSYNKTYNNMGKT*
>N.PF13_0323
MTIKNKYGYLKLGIEIEYHKIPQCEENNIDPDFFACKLFNDNTKNIYNELTTNESLNNSSMINGNGKRCSYEI
DPLFGNIYEDKNIWEKNIYGLMSTWINPDDENENSSLYSMDALNKELQWSTYISISKLIINVPNIKCDNYARY
INSCINNYNMLSLIMRIPIVIKKKKPFHKESYNNNMTCDIINGWNLWAKFISYCNFNYSHLGLALELSNINNI
DLSNIQLDVWKSEPVKLIIIPLDAFLLDTKTGYPYLPKKLKDFLIYFFRKNIEVLLITNEDINKMNNHFIHLK
NEYCNEYQHNICNNEMDSYDINDNNNNNNNNYNISNSIHTKTNNNYYLKCCIYYIKRLFMSIENFDNDSLFDS
FYWDYLQIPLQPLKDNLSSQTYEVFEKDRKKYEQYELATSKYLSNWKKGKKINNKNKQSNKNKNNNNNNNMEE
QQNDHKITIFVVGAGRGPLVDTTLSALQKNEITDYEIYAIEKNDSAIIILNNRVQTEEWKNVKVIHSDIRYLD
IPKKADIIISELLGSFGDNELFPECMDGIKKFLKDDGISIPMNCVSYLEPISCSALYHKVMENNISGGNESFY
VVNMYSYTKISQESSKECFFFQVPPIHTKQDNSHNYRYKNINFKIKMDTYIHGFLCYFKSQLYDDVYISIEPK
THTPNLHSWFPLYIPINKIQFLKKGQNLSFSIWRLTDHQKIWYEWCINEPITTSIHNYNARYFSIGR*
>N.PF13_0324
MQPYDYNRGLNNNNTANYNNQNNANAPINNPFLHNNDIGNNNNMKTNEGPYNAPTYYNPGQHQQQQQQQQQQG
PPLHQGYQHSGVYGMNQGNYKTNNIGENMYNQDGHNNTSYINQGQPYRNVTSQFIPVSSNNTLKAGGNMLGYD

NMGNINHVQPIINDTYQEFLQFNAFSHFVKSSVSYMPANTTLKQKAYVPLGFVIQPLAPIPDGYPELASVNFG
NSTVVRCKKCRTYINPFVRFEAGGKKWNCNMCYNINDTPQFYFVPLDEKGKRKDLFQRPELCTGSVEFIAPSD
YMIRPPQPPVYLFLIDVTVTSVNSGLLDVVCSTIKSLLPKNNDSTENNNQNDNNNNNNNNNNSKNLKSFDSRT
LIGIMTFDSTIHFYNLNSNLKQTQMMVVPDIQDIFIPLPEDILVNVHECQKVIDVLLDNLPGMWRNNKISDCC
AGNALKAAFMVLKKVGGKLLFFLSSVPNIGDLTVSVNRDNKDKSKYKNIYSSSSSGNNVVDSKLREVELLNPC
NNLYAELAQNITQYQIAVDLFACPLYNLDLASIYPLIKNSGGSLYYYPQFNVHQYNDKLRQELLFALTTETAW
ESVMRIRISRGWKITNWYGNYQFRGADLLALPNCHSGQNFSIIVDLEENVVQDSIVYVQSALLYTNSNGERRI
RLHTYALPITQNIKTITDSINPQVVVSLLAHQSIDISKKGKIADGRNLIQNLCSQVLSSQLLQSECARLLSLY
ILGMLKSIAFRDSGDVPPDLRIYHWYRLENIPVESVEANFYPRMFSLHNLEKHHGHLDENNNIVFPDALNLTC
ENMTQDGCYIVEDGETIVMWIGRSINPQWIYAVFGVQTIDQLNTEYAENHLGSTGNPFGVQILNIINALRKIR
TPCYMKLLVVKQGDPLEYKFFSYLIEDRSQHMMLSLKEFLAKICPKFPQFTPSMTTNPLATQHGR*
>N.MAL13P1.304
MNNNNNNNNNNSNNNNNSNNNNNGNSNNNFFSGKGNALSAYQNKILNIKSNNNNAHHFVNKNVPTYSPPNIIM
ANKKGGNFNNTSGNIINRYNVENNNHRNTYHPSNNNTRNSVNFLNKNILYGNNNNNNNNNNNNNINITNISNN
NNNINITNISNNNKQPISSNQHPYQQKQSHHHNNSINYNEYMDEKNMNTSQSIFKNMTIQRNSQQFNTSDFVN
NINIMNAPHINEHSNIYKRNSLNIVNNAHIISNNVNIQSNRNSNISFPQNMNANIGGLKNSNHNLNNIEMKYN
TLNNNMNSINKNTNITNVGTLNIQMKNNPMNVNINQNNYNTDFYVNENKVNSKNKENNNNHINIEKMNYIKSN
VYLDNTLVQVNSNNNYNMDKNILNNNNNTYIINDKKNSTVNNNITNMDNNLVPGVMSSMNIPDDIKKRKKKER
KKNENIYNNRNKSSINTEEHNNNIIDVANQNSEHFLQNNKQYGNITNIQNNNLSHDMNNYSINNSTTSDVIGI
VELYKNSLSSKAVNKKKSKLIKDVIDDNKKRNKKEKKKTIPNNDSIINDMNKNKNVELLNETQIFDNKNYDKN
NDIHNNIYNSNDNNLIHNKNNVNNDHTNIKEANNNNNRKSEHSEKNKDVHNYYYANNYQCITDEKNNKQYILW
NNRTIEVTFVWLFITKEFNENRKKYTAFLPYLKHFYPNRLKDLIEQLEKYSLLKFNYIMHSSYNMQEEYNKNK
EPNNINSNDNNNKNDDNNNNNNKNVDGNNNNNNNINSNDKEVLMNGMLLSDKSTLNSNKQIDNTLINNINSGF
NNIIKNMSIDDNTIRSIMDNIENITKGKKKGRKKKQTLENNGDNIKEDIKSSKKDKKKDNINDNNNDNNNDNN
NDNNNDNNNDNNNNNNNNNNNNNNNNNNNNHNNHNDNKNNQGDSKNEQEKKKKTRQYRKKSKITNDDNNEKIKQ
DNINSNNPKNDLKNNEIICSEEKNMKEDNIPDDTHYKEKRRNTFNLFNLDEGTINMDLFNLSLLENDDALNKK
ENDMVSKSNIPSSFSSPPKETNNKNDIDKEQSDKHNNVQEFQNLNMNNEKSKDLYFNKNDIDNNDNKDKIINE
TSSGTFMQNLKETFYEKTKAMFSNLLSDTKISKDDELNNEVDQNCVKTSSGILNKEENNKKEDDEKHFDDNTN
EQKKNVDNGEYNEMTAEPGRKKRKKDVLERKKKNLNKEIIKSEKRIRKYRTKKMLLKEAMEKGISNNIVESNI
TANNNNDNNKNNDNDNDNNNNNNNNDNIINNNNNNGDMFSNSYDNSYIKENKYNKKLCFPQNNLLSDFRSEPIII
QQDKRKIIKINTINKIKRKYKKFRFCINKVFKKKSINDIIALNENIHKNKDLLTLFKKKDLANLKKKNLSFFM
DTLKLEKIDMLIMKRIQMCLEKIKNTLLLTCTINNVQEIVNILKKAFEKRLYLMWPLIEFSNKYRLDQYFHLL
GKNKNHINSSFKDTKLFVHQNISSLILYFNQRSMDDKWVEYLKSQMKPKRRRRKTKMKEQFLEDKPIDYLNTM
NSQHSNNFIGENFSEIETVESKANEYAFVGYNQKRLLTQITPYDYRVVLNSNFCNKFFTPNWREQQSIFIDNL
HFDMVPDTDEIKKHFENVYIRYMEYDEEKLRSKSDTKSKEHKKKDKKYKMLFKKKEGKGKPGRKKKIKLEIEN
VSNEIKIKKPRKKYERVKPRKSKNAMMNEEKSGNSEKQINNVLNVTNIENKHKSKKGRKPKESNLNNLNINED
INVAKASPDTLHRASLEFMNPNLFT*
>N.MAL13P1.305
MYENKIVPLQMDRRKYLQSCVENMSITFVLFGTINIFFNLLINLINIGRINYEHFFKLFSITSGLWIVIILVQ
ITSYLFSSGFFENLATIFLHDNERNKGDIWNIKSIGMFLINIITLGWLSIFSFYGIFSYIINSFDLINNFMLS
TTILNFSVNLLFVLNQFLYFLFKVHEIRNIEFHNCLIHFLPGFSRMLESIVLTVKSKIMDNLSKNKNFVKAKN
KKRFIDHNSYPYENLFLIN*
>N.PF13_0326
MVSGVKVSDECVYEFNKLKIKHIHKYIIYRIENYEEVIVDFLEQDNSLKSYKDIIIDIRNNLKTTECRYIIAD
MPIPTPEGVLRNRIYFIFWSPDLAKSKEKMLYASSKEYLVRKINGIFKSLEITCDLEDFEDELRTIILNT*
>N.MAL13P1.306.1
MEKTEEHECNNIPCEIYDKDHLDFRDDFLLCSTCPNISPYHKEGYLSKKFSNSWCGFKKRWFVLRNNKLYYYK
NEKSLRPSGVLDLDIINMNLYYENGSTAKTKTDDCFLYCIQSRENKNHKKIQSYLTLNPKNTDIKFHLKGDDT
EIYNWYQLLNNALCHKKVLNQPEFIFTDCNFWKIHRISVDVFESIADTGDIVLFRSDVTVARLQRIFTRGEYD
HIGMILRNDRNNLFILEALSNTGIILTPWKLFKENKWNEIYSRLTLRRLTWDNSEENLQKLLSFLKDAIGKKY
SLKIIDFIAPQFDDSSGYFCSELIGECWKVMGVIPEDTCCSYIFPSNFSEKLEDKIKLQSGCQLNNELLIDFS
L*
>N.MAL13P1.306.2

MEKTEEHECNNIPCEIYDKDHLDFRDDFLLCSTCPNISPYHKEGYLSKKFSNSWCGFKKRWFVLRNNKLYYYK
NEKSLRPSGVLDLDIINMNLYIAFNHEKIKIIKKSNHI*
>N.MAL13P1.307
MISGNIKKYSHFALKNGRIFNFFFKTYRKVTHCYKNNFQISSILLKFPPRNKRYFSNNDSNINKLPLHWENAE
DIADMLFEEYRNIDPLTLRFEQLENMIIDTVVKKNKRQLNGHCNEGVLENIQMNWLEKYNEEND*
>N.PF13_0327
MFKFNSKLYIGLRNINQIINKKNVNYIKPNVLGNMPFKYKEFATVNKSDHSDKIKKTNNNAHTKNNHEHESHT
KGFYHHIDHHHGDPRAHLNEDGTRKPEYDFNNFHWDDYWANTPKQNIVIVNGQKMIKGDETKPMEYLFNVSQQ
NIPFWARTRLNVWGNYNMVLKVEFLFFWIPTLIIFSIAIPCFTMLYMLDEIVHTTMTVKVIGRQWYWIYEVES
PPDDDDNHE*
>N.MAL13P1.308
MPKIQVKRGGDPGVSTAVSSIMQHENFKRMLMFGLRSLSDFCNPTSKAYKENASDALDRGVVVSIKNAVINYK
DDDDILFCSSRVLLSMSDYCMSEKDTNALKKLITDGGVDGIVEIVKSFPSDPDTLKNCMAFIKNMNDSNYQIE
GREVGIALLNVFTSKTYTNKLTNGIVLALCIISKSTSGSKGLNDEGAHHKLLDYCLNINSLNDDTAEIVESVF
DIIKNMSSNGYVDPTIIEKSVIILDKFKSYPRVISKGSDAMKCAVGPEELSKCLNVLKKSAQGSKEQDAALEL
LSSLSYISSITDKVVESGGIPVLIELINSGLQQYESNPEKISRLVAGASRMLGRISNNPPHAAIVVEYGGIAT
LCTAISYFPNDVECSKAICNALTPFVSRSNYVSEINNYSLFASLLPILYASLESVELAKASMECIASASMINE
FHEQMVNNQAIEILSTCVQYHLTEMDYLLNCFTAYFRLSDYITTVEPINQYGGVDGIANALLAVSSNSKIVEI
GLKLINKMLTTSDSVNYLSNKQIVDSVLTVMLENENKEVIIQEGTKIMEKLATESDCQRHITNLETIFNSSET
NQEEAYKTLAAISGLSRIESLKNILESKGADTSIFNGIKIWIESARFIEQTKLIKAGLKTIKTLKLNASATLH
DVLGSIVDLMCLSQVKRIAESDEPDENILITSAECINYLTEVNKINSAEIVEACLENIFKLMKKYSESRLTQI
NLISAMNNILLSSNKIGVDILINKGYIKHIITYLQKVPMYVDVQIIGFTVLANLVKISPDSVEGIKKLNALIP
LQTALRTHAKNMKLKTTCAPLLSVLMPLDSLTNEIQDIIKLCNKSMNDKNLSKLHEYLVALNELLLTPEACKI
ASRSNIGEFFNDLTAWLKKNPTVYDSSSKDDYEISGRSLFDAVISEIAHASTNISQTRLGLVHLTKCNMVSSL
VQLHDILKLPGDNYTEEAVSNILEALSLLLKYDITNAEIGFNSGLIKKLCAGINYFSHSDSVINKTFGCLACM
CTSKNRVGQLISCPEYEGLIKLIVELIGDSEKNKLSRGSAIKAVYELLKTEDEDIIKDISCKTSIVDNLYKIM
GEYQADLSIVQDCSRCLAIIVDYVNIEEIMKVDKYTPMKVLLECLNKSKNDELTVLEMLTVLVKLCNSDDKMK
LKELGAIDVISDITMIHSENEEISRLGGVMYSYMGADEQVKKLMKLILNVKKEDSDAVQKIDNFTSKLEMFLR
APLENPSDALQYTEVTLQVLNSYLASEVDNSSLQTNIALVTKRLVDRVKHDSEDPLGSWAVASAGTLNQYIDM
ISNKIGLSNFKFVSPVYGVLAACVMNPYTKQLVMDKLPLLIDNTYEILEQNKNRPSVVQGVFEFLEQVVNDEE
GSKLLYKKYNGSMGNLIDQTITVMNLNRANDSVYLPGIKLLSAICQTASVSGYGNDMNTSNIIESCELLLNLG
SEKDRTVEFLNLIDTMILGNLLDEKVASEALKKINSLVAEENLSKYTEENRVEIIKSYAGLVKDCAATGLFAH
IRGIEKVEILNNLGKCMEYEQNDEVTIAVLDALSQISESDPSIATKILTSSLPIIMENNRDNILNDAATAEAF
LQALQNLVLHEGVGRQLINNTELQNLLKDLEQALDERKEELGNDYVEDMKLKISNICNAINGDKPKEKKCKDV
YDVLTDYKKSNLIIDVLQEPSLEEDMDFVLERLKVYNQDNLLPTTATGTDNSYGHMAIEMFCENEVNINELIK
RNFHVSAFHALSKQSNENVIHYSCRSICAFTKNPKGLEAVKDIKDFANIISKSVGDLSKENTLDKEIKEDFLI
HRVLLIDRTAHNRNVYDKTNAVHYLIDIWNQYDNGDYSVLLLRHVFRSMRKIVSDAHVQTLLKAGVLVRLINI
INDIETDKIIFPDVLFLIGSLSVVKEIKIQIGELKGIDACVNLLLRSINVEKMEPTITNCCLALANMCIDHKG
NSSIFCNLKGPDVNVKILKLYRSNFDVTNGASVLLCNILFRNEEMKKQYGINGAPAELVECLKSYDGSDDKNA
VRCIESLFKAISNLSLYTVNVKYFLEAQIQVSYESWLRNLNESFPDAQLETGLRTLSNLVMENDEENMKNFGV
TLIPVLNVLKQNREDTKVIFLLLDILCSLCRLNANAKIFAENNGIETTINVIQLYDYDINLLSLAIHLLSNQC
KIESSLPLLVNADAFSILISCMEAETDEFEMTELVVSSLRCVRRLIQSEELAYEFCNCGGVPSMANIICKSIK
KSIVMLEVLRVLLCVLYYTQNVEGVTNEYPEEEEELYNARLGGWYNISMDKEMIDSIIQAVLTCAYDVNHQKQ
LRLQKVSLGLLAYFAYHRLGIISMTASGFDSLTRELLNNFGGDAVIMQLLAICIDNIAMYSVEVYDTTITRDI
IKCFKSALSKMNNKKEDKQLWQKVELTLEAMNSADDPLEAFKNTLLIFDFNLSEFDKDPYVNGVHDLASNIKD
CLRKGGHSKIYYQSDQRLLFKWKASQDLNTLEWTIGDDTERVFKISVVRIKNISKGLSHPILISANKREPRKV
SAKVTLCIYGPPTEDFPEGLELPIKTKTQKERDAFVDLIVLWRDAASYNY*
>N.PF13_0328
MLEAKLNNASILKKLFECIKDLVNDANVDADESGLKLQALDGNHVSLVSLHLLDSGFSHYRCDRERVLGVNIA
SLNKVFKLCGANESVVISSKDDEDNLNFVFENNKEDKVTNFSLKLMSIELDSLNIPDCEEGFDAEVELSSKEL
TNIFRNLSEFSDTVFIEIDSNCIKFTTKGIVGDAEVALKPRDSTSEDDIGVTIKSKKKIKQSFAIKYLNLFSK
SNILADVVVLGLSDSRPIEFKYEIKDTSPDSDTLKIGFVKFFLAPKMDDDMDNKD*
>N.PF13_0329

MAISRSKIRYLFPNATLQSYYPNSEMFKVPKAGSAPRIYNGLDPRKVHSYPWINIFKTRRIKETGYQYGNWAG
PSIHSMTLDELATFFSNKDHFKYFSIFQLIKATYGQFQFLFLFLGSIVLTISPILLFTLYMQKFEPLEVTIDP
EEYYKHFRWHYYGGEIDHHAFSQYLEARRAVRYRNAEINPVDWIPPQYRNVEE*
>N.MAL13P1.309
MNQYIDNDISVSNKEELIYYIKILNHLGSYDETVTLIKSVNVENYNFNYTESLSVGFAFKNALNVKRKEKTIL
ENIITNEKSSEREKICAELLKSKLNTDIRSIEKNTYAVLKNKCISRTTDDKILMLYWHILGDMSRYCADTFHG
TDKEKMHEKSMKSYSYALHYANKMKIPPSSPKMLELLVSWTVLHKDMNKDINYSIELAAEAFRNAIQNMHLLE
NDDECSKTIRILGILRDNINKWCEISGRKNVNALFEINGENLDKYKDIMNSTHT*
>N.PF13_0330
MKLEEVKDIQKIERIGAHSHIRGLGLNDCLDARYCSEGMIGQMSARKAAGIVLRMIKEGRISGRAILLAGQPG
TGKTAIAMGIAKALGEDTPFTHISGSEVYSLEMSKTEALTQAFRRSIGVRVKEESEVIEGEVVEIEIEKFNER
DINNKNKKLGKMILKTTEMETLYDLGSKMIEALQKENITAGDVICIDKGTGKITKIGKSFARSKDYDAMDPNT
LFVQCPEGELQKRKEVVHTVTLHDIDAINSRTQGFLALFSGDTGEIKNEIREHIDMKINEWQEDEKAEIVPGV
LFIDEVHMLDIECFSYLNRALESEQSPIVIMATNRGITHIRGTDYKAPHGIPLDLLDRTLIIPTYPYKHQDIL
KILEQRAEEEDVDIDEYAKELLCKIASESSLRYALHLITLANLVSKKRKATEVTVQDVRRVYNLFIDVKRSTQ
YLIEYQNEFMFSELPKEELSVKEEDSSEEKRELHEKNSENSSTSN*
>N.PF13_0331
MEDKIRKYDVFCIMGLAILCGIFSEFLSWLFVYRNEKFKKLNEEVKVLYEEVQKEKDDGLLSKLDKKKDKKKK
ASAEELYIEKTKEMTTLKTKSNFITGLIFMCVMPVLFSLFEGLTIAVLPFKPIFPFTLLTRTGLQSKNVYHCS
STFIYTLTLMLTRQNIQKYFGYAPPAGMFGDYKMPDEQADVWK*
>N.MAL13P1.310
MGCINSKVKEKRKIKKRKKEECNLIENVEGNNVGNKNVSSYVMKEKKNNEKDDENNNIDCNNNDNNNDNNMNC
KNHYIYNDNNLVKNNIDNSKERFYYKKNIFKNISNKCSDKKLLTSSDKENITYVIKSSTNNKSIINNNNDNNN
NNNLFLFKDNNENKSNKSYYYNEVYTNIEPNKYDICKSRSMDLENVKINCNLIEDKKYKIKNYENKKRNDKLL
KNSFTNIIHTISNTLLNRRSYTKNKYKKQKKVQSAHDVFTSYRNNKDDNIFDHHFKHYEDENDFYMLYLKNNY
KNKKDQQKLFSNMRKNKDLLMYYHDFKNIILKRNSFLYIYEYYKYNCDVFNLLSLMPSHISENKKASKILFES
SFIGKYIILPWIDNDPDIKNNLYLKYVQNYLDMKEHIKEMNHSHYHNNPNQQLSYCNLFNNTINNTNHHDYNN
NKKKKKMKCAHTNKTQSNFSYTYLLCSHPYEDRKKKTGIVLNKMNKKNLFLRKKKEEKKNKYVTTNKTNLSLH
QVFKNNLRKNLIYNNIKDNHKTKIKNKHITNELKESLINSEDFYKIDTTLNNMLNQGDKIKSINHIEDIGTNE
CTNKISHNMYNIYDKKEKNINVKSYDNTHSTNTIIMSNDNIKKNDLHYINEHYNEKCDDNIYDYYITSSIDAF
KSRPSSSSHLNENHDKKSENFNLNLIYQKKKENNNKKKENNDNKKKENNNNKKKENNNNKKKENNNKKKENNN
KNNNNNNNNHISINNIVKPSFIFEANYVEEGNHRDTQHNNLEYANAEINNKIYDVHTYRNYNIEKEEKHYSHYV
ENEKEGMGKSKERKGYLKCNTSKYDKIKNNNYINDVYNNNASVKDIQKFKQKDKETDKLNDKWNDKSYDKSYD
KCNDKCNDKCNDKYNDKSYDKSYDKSNYKQKSKTNESKNIKLEWEKCLLDTCDHKYIVNEDCIKKNYMCCKKC
DKKRCIFHYVNGFKLYGWLNYKWTDPDGFLELSIRQKRKFDSWKRLSELYENPIVMSPYLYNNCIRQGFVADC
SFLSSLTVLIEYEKKHKIPVISSIITPCSWNYLCINKTWPIFNPCGMYICRLHCNGIQRKIIIDDYVPVKNNN
SLLVAYSNNQKELWVTLLEKAFVKLMGGSYSMFGSNPGSDLYYLTGWIPVTISLKSKISSSPPSFDKHSTHSL
IRDERIDELVYETNEVMINKKHGYVNSVEFVEDTKYNHNNNNNNKNNTDHDQYMCDDNICFNNYNFCYKDDYE
VRNKLQKQFLKEELATMDFAKYMNGSVDNYSDLIKRGGHKTLGINCEEGKYEPYEQYEQYEEYDEKWDSVWNN
IYDGIKKGKCVVCLGTLELKDAAPSGLDYPEGVSISTGIVTRHAYSILNIENYNGDKLLYIKNPWGCIRWKGR
YSHNDYETWTKELQKKLNYSLEKAISKDDGCFWIPWKDVIKYFSHIYICWNSVIFPYQFEIHTKWENNSLLNS
SILLDDTHLVAYNPQFALHLNVRKQDLSEDGLYYIGKKPIEVWILLSRHVRERKTDVSQKYLALHVHAGNDRI
ICPLFPIKQGIYSNGECTFIKLIIGGKDDLTSINMMYDSKKEKNKSNTQNVENCKNVENIINNTVNMNNMYNV
DNVDNVDNVNNVDNMYNMYNVDNAITNVQKENGTYNKEKYNNNIVTSKDRTNVNNEKQNIENDPDVLRNIDYV
LIVSQYSQKEEFNFTIKVFSHTHLSLYEIPPLLPDNYSSLYFKGMWTNKSAGGCSNNLWSYFRNPHIRLYVPE
CTRFYIFLECSQEHSVNLRIFKGNTSSPRSLKKGDIISSGPYKAGCCYIECTLESGIYCLLPSLYRANVTGNY
QICVHYPNNKEKPILYFIPYSYIVPPFSFFKYELVHLYSLKNYSVILFHTTCMTLLSLRITFFQNMDIKGIPF
VNIYKRISDSRKIADDCENGNILKNNSFISCITYNNSIYKLIQKCNIHGNTGNDVLPLSTCAYDYYIKFNSVL
IPLVELEKEDSSYLLLITTNIKDDILYNHEAHFISDKQIFVDKFYPPQ*
>N.MAL13P1.311
MTSHISYNKIREKRNIRRVLSVYNFCSLNRFSWYRQDVRLDNVIYRKNYEYIYYNCIIRKCSRNYISTRNNIY
IKNKIYDIIPYLCKGKDVKNITSNIIFYILQHLSTKNVVNSNEYKDNIKKIYFNLLKCYHKIFEYNNEYGEYI
FSLFNDDIIISVSPSLRKKQKNIIQDILLNSLHFFFQNNITYKNKLNINLMCEIINYPKFLYVLHSINYDMNI
LKQNISVQNVDYLFSQYINKTNIYITTAIQFASFFKNVNMNIFTPFKKHGTFNYFLLLKRIVNKQLKNTLFLL

LNDIECHRLRQEMLLHLLSSDDTTGMCFNEWSHLAAKKYLLMNKYENDLKENIRDQEINVNILKRSIDYVKDY
DDDNDKVNNNDNDNDNDLYMEYFNLPEDVKNVKYIKCVDDFNNMVITIKEEVNNHWENNIYNKKDMVNYTNEN
YNDNILTYEYINETLRKEKKRYYIGIDIEWDSYKKKKNTVSVLSISTNNKIYIIDLYYIDYNYKFMIYTFFKW
LLENPFIYKLFFNFPSDIKIMSSYFQNISHINIYNNIIDLNNNIYIYTRKEEGTPSYKNYNILYFETLNRDMI
QSNDVHLFKELVHSTPYNFNKNLMNKIKKNNNNINIPNKQMFKLYVKSLNDLCIKILNKKLNKKFQLANWNIR
PLNQEQIIYACIDSYVLIKIEEMLIEKGYMSTCDSNNNQMMNLFLQKYKFKDSTWE*
>N.MAL13P1.312
MESLDERWNNQVDNFHIKVNKLRCDMNEYKKGCYNTYNLRNNKNDVFPHLQNNNMKRRELINMNNNYEIKNYL
KKQHNQTYILKDNKYVNVDDITFPVSKNISNKVIKTNDEKILENKCCFKLNVRNKRKNEKVIKINEKRWLIAT
KHVKIERVNKNGVEQSEEKNKKDKNRYIYLEGEEGNLHLYPMYSDSEVGFENISHIEDESLSKVKKENQDDDD
IMTTKHLAQWSSDIVHKYLEEAIEKSKSIFQNCNMESRQEEIFKRIKL*
>N.MAL13P1.313
MENYSVYSCNYQMKDENGNSVPQSNTQNELHGMNNTSNMNNQNIIPQNNHYYGNDVNYPKPNYSNNNYNYMAY
HKMSCLNYVYPYNPYRNKNYPYGNLPNIYYPDGYYANYCYPNYYYPIYYYPNYYYPNNYYAYNNFPHNNFRHN
NIPHNNFRHNNIPHNNFLPNNFPHNNIPHNNFPPNNFPHNNYANNNTYYTLGYHETYSERKYIDNNVPNLPRN
YSYDNMNRTWNCNNISSMNNMIISTNRESNYMVYTEDTINTTHNMNNYVNNYRSCEINADKNNGSNEQCISDV
RETEYGQNKDVHVFEESVRDYTNNYIKERKKNIFLYNNNENNYKEILNEYESNMGNMDNMRNMDNMNNMDNMS
NMNSVDSRRENVRITNDVIEIVSELEDNTENNNLMDDNTESIINIDDNSENIYDSDHYTENVYNSDNNSENVY
NSDHYTENIINIDDNSENNNLMDDNTENIYDSDHYSHNAYNSDVNSINILNIEDNIENMNYSDDISEKNCNSN
GNTEHISITNDYTENMSNMDNYTENMSNMDNYTENVSNIDNYTENMSNIDNYTENMSNIDNYTENMSNTDDYI
ENITDTDNNKRNETNDTKDINNSFNNNTNENNYTKITTYHNKGNVKSYPLDKLHKHVKSIILENQVYKHNNES
EHAYIDNKKNSLINSNIDDCTEPYSDVNKTYQYKNESIKNEIGTDMCNHTNDEKYVNEDNVGNLKKKKKNKKK
KNKKKNKNAQDCTTSEEVKNNVERNNEEEEIKKSNHINDTTKNEKDTHSDNNMKSVPNNKNKNKNQNKNMCTNM
DHINMMNNIDCINMNKKNYMYNINNMNYMPNVNSKYENYRNYPYYPRASNLPYNNETMIRSSNAPSVQPFKKI
INDNSMNSSNNHFREGSNVNSCTEENTGANNKEKIFVKSCDENIMKNYQHPENINTGEKNVSVQGGEECNENI
ESMNNNTKNVPEIFNTKKRKYKNKVGIKNMNDKEKTDNVNSKNYDNNGKNNIKDNGKYEIINSSNVKDVHQVD
KINKKETLNCSDISIKLKNNMNSDKDEKDDDDDEKDDDDDEKDDDDDEKDDDDGEKYDDDDENDDDENDDNED
DNEDDDENDDDEDDDENDDDEDDDENDDKDDDDNDDDDYNDDDYNDDDYNDDDYNDDDYNEDDYNDDDYNDDD
YNDDDYNEDDYNDDDYNNNNLKNNYSLENKNISENKKTSNIKNMFDSKKKIKKNKKFKNVQNCRSQYNNNNHC
DKCNNNIFTGMNMKNMNSVNYIKNRKNMHNNNCRNTNCNVMNMEKNKKNIKYKNHTYNMINVNNTNNIKNTYP
MNTNFNKNLFYGFGYNKCEYIPIIYDYHICYNKKENLSLKNDMKKKKKLVGKSSNTNENVKYIPQSKWNDRHY
NKYYIHYNGKTKNEKFRHNCFYKNFDILNGNKRSDKNFSYNTKSNIYNIQDNINNRYNNNDTFKVYKDIIITL
NGPHNYPENTIIYYLYTRENSSLNNNMRNSYENNIHKKNNDLFKNDKPRSSQNNIFRLYNRNNNSNYRIFQNV
NNHNRSNIISSNTVNDTTQIKNEFNSKEYDKTSDCDKNNTPLKNGCNQGFQDNNRHKNKQNKQDKQNKQNKQN
KQNKKVKKIKKNKKNKKIKKNKRNKKIKRNKKNIQNNSLEDKIEIYESDKCNHNGVSKCDINNLEVSVETNKP
KSKSYNYNSNNNSSHIQMSNNERNNSKGKSTRIENTIIRFDPFVDKKKKNKNKNNNKNKNNNKNKNKNNNKNK
NKNKNKKKNKNKNNNKNNNKNKNKNNNKNKNKNNNNN*
>N.MAL13P1.315
MDRLIQINNTLFIDLNNNNEKIDGREKFNLFDINEDEYILNKDGNNIEIEYKFEIKRCVNIFVNLLKDQNVEY
DIYKKMLDSNINIIKMIQAINNYGLKTEEFHSTRLMMIIYLKNYVYEFINTYLKDIISTYEKNKYKKEINNFN
NLIYTKNDKIVEKEDNGFYSNEPRHVLNKNEENKNIMTSTQCVENSFSFISFLRKGIFDYSSLIENIENKSKS
ITIEEWIYLKERIFSLIKNACVMNTNLSNNINERPMNFFEEINKNMYKSYKKYNSIIEMKNFNECLYFIDIFF
NILLVDYPYKWDNLLEDLFNNFNFISIIKLDDFSNILNKDYNTTINYVMKIINNMNISNSEIQYMENEEKCNM
YKMVIHYSKFYLLMLLINKILKKYIPATSELDSYSNIYYTYFSSNINTSNKNLLEKENDFHMLNIPCENLLVG
YKDIIKLYFAFFNRFLYYLLHQERNIYIIFIIQMIVKCIYKLLLCDLCILSEEVINEHELLFINIMKVLEINL
SEYVNKIYFMNIEKSSNGESISVSKIFEEHSYENINEVMNNDEDNNIDRLFLKILLKSKKWCLKILNIFLYRS
IHFDEMNDTWKIFLKHFEDKYIIFLEKITLLILKIMMNTTYSDDERNNINGLFSYIKYVINYTDYIKYADKSL
SLALNYVCMIISLDHLKDFIYQNVLSSIFLNCLFIHIDNITHIECIDNFQVNETRQKEENKVCDNLKFEKYII
CFEKHPIFRNYLLSCYDDSSCKSILKKYINYIIYLFCELLCKYFYDIIFYLDEQIEENLKLYLLYQNVYMNVL
YPEMYIKKDMNFLDNEQISINTYKKSNLFFNAEISINTGNHNIYINNIIDYISEIIRNHMKENKIDYNFDFSN
FHLLNNDTFNNMIYIIKNLPLYKYTEEDLIFKDVYSIKNEHNLNYSFIIMNDINLYKYFSLYMFLIYYKYRYV
RGTLKNIITKMNDEYLFLTLEHVSDDDNRDTCNKEGKKDNNSNKYYNDDKNGGTYCNSNYFDFDFGKNTENNL
SMEDLIQEYFIHKERYKDAFCKDNNDMEKFEYILDNLKNKDKNFFLKYMMVNILHPYSNNLKRKLMWMIKEYI
HVATFNDNVIQFFFYSCFINLFYFEKHIKLNSFETLIHLMYIYGIPSKILYNDFREVLIFLFFQLLILFIKKE

SDSIDSLVYEDNRSVILFSNYEKMNLLRLLKGIMVKEVVDNGERNDENNDNNNKHNKHNNDNNINHCVFNNFL
EFLYNKNNCFSDNFINDVICKNFYFYFYLEKLEENNLNRFLKFISLFYQNEINNNIFLFINLLNESLLDIIDI
RIPGKDEEYNFLRILKFFSFFLEHYKNTCSTQDVLIVKILQNENFIIYFKSIILNIIKKNSKKKSKGMLIGKE
KHCYILHDEILNQIYYILYIFTKKSLFNYKIFWFYFKYAVACISNDLNFFRIEEYMNIEEKLYNMPIYIFQFF
NNLIRRDILYVFVSLGIHEPFESNDFPLSLIEILRKVSLLYLVVSDENMNEQTFCYNSIVEINGLYLYNNILS
YLVMLLIRHNIFFTYPLWFELNRSYEIIQEGNVLRDLDECVSNNSFKKYGHAMEGNDVGSKMCLVNDGLGEAS
KEFGFYDIKMVEGGEKKKLRDVEYVNDMDEKKNVEYTLPNERNELKGNPNESCKSRGHSDVCEEILFYREMKT
YFIERKKSGYDIKGEKRNNEIGVGIDDVNKSTLDSDDDIYDNTNENRNYNIKNNMFNNNDVNINNKEMELLNN
IEDTKIAELMNTLYFNIFLLKDKKEEHIRKLLKILKYVNDNFLFYNCQNEYQIYTMNLMIQKKRYIYQSFSMY
VFLDIQYFFDILVKSNLFDRVIDDWINDIFYIYQNSDDLKYFILAFSNMLIYLCLYESIKKKRDEKNNKFGEN
NMNENICTKEEKDSMEVWNEMEDEYLLPFFFFLKNKRNGINSDYINGDYIKGDNINNSSNKLFEEILKKDMIR
FDNINEYATNKLREKISKIINALVNIVLILPIPYEEEMKVFNKNLLRRLAPICGERENIKDNNLLKLNSSNSN
IINGNKSALNNIQFDDLIQNILFMHLNNYEQKFYERNNKEEKQMNKSYYYNVVHINKFDELKLHGKKVSIHYY
FQNKSNEEFIDKLTSECNCNYFKISFYLKKAFSLLTQEGFTNDDLINIIGCSDKYYNFINIISMDI*
>N.MAL13P1.316
MSCNIKKRSCDMNKSEGDEKYCSKTKNKSVCRYISNEGNNNMLKRRKCNVVGEKEKYDEQIFMEIKNLYTNDY
KKDDNITNDMNTEYNNYNDMNTEYNNYNDMNREYDNYNDRLCISFNDVLACDGNNKDDNFCSNILYEEMYKKE
DENNVYTNEHNNLCTSNILSNEKKNIIFKKRTKDNLYEKVYYKSYKNSSKKNKIRRLLNEEYVRKFSHETDVY
NTKDYFINKEKKKIYNYYSVDKIFEIPSDYKNKDEGYNNIESIKSNKCNKNNEGNINNTIERNLKKCKNDMEK
EKDDVFFLKSSDNNKLCEVLLDKENHLDEEEKCKKENINDNNLNGNIIKYMNTSVIENKSNLYNKECNIFNKN
DINFDEYKVSIYSMDKDKKKYFTHNNNNNNNNQHHNNYIKSSYNSSSKRSGTIESVENVKHIRDMKDNIYEKK
KKIKSIHNKILRLIKEDNKKCVQNVINDNKNMYEKSDKHIKYEQNGDNKKKELYNNDSSMDDKDYKKNEDVVE
INQRDNGNYVTDTKWNNKKNKSFNICNNNYVDKRCEDNIDDDKRYYDDKRYYDNKKCDDNKKCDDNKKCDDNI
DDEHIILEEEIKKRNIIMDEQIKECPIFMDKKSEKEKIKCCTKKYKKVNLSHTFLNNIKLIYERKDLKENKKK
YILIVNKKCLEKYSYFNTIVNTYYIGKEWIRMDKYINYKLSKILFCNNIIKQINKYNFKKILKALDFYGYSYD
TIFLKNLCNKIIINEWLKNDIFFENILKSKRFLNVALFYDLIYNFLYKNNIFLKYFFSFSGGRFIKTFIDYNK
NKALKLINKIIKNIKDLNPCTNSVIDFLCGFYSNDENEMKKYSKSSRRNNLYAIKNKDINIPNKINDSRKKSS
DTYLYMKNKKYNKKILRNILNNVWLKTDMFIKESIYYGANFSLQNNNLRKKKKNIHNIEKIENFLYHNSDRTY
SSMCTKSPNEKFYKYDIYKNIDDEYLDDHSSIHDENDLEHVINNFYSSNELNINFNEHMSESFNQCIYNQSEN
VNSDNNIPDDSEQYGDDYIGSHGNHNKTFQSFEGIPNMAFDLYTQDNQNRPRGDKFISLCFKEEETCNTNKYV
KDISFLGIGVRSKENKINDFNCFNINLKLMIRNNLLSKIKCNISKNGKKISPFFPVWPNIPDFFGYKMFKNIK
KDFKRMQKNIKLKEKQNSLDIINNKKKYKNIISEVYEKVGTFQKRKFNEEEYTLHNNNNNNNNDSSNNSSNNN
NNSNNSSNIELSNINNTKSINIDEIKLKDLHYILNIKIYPIRTLVLYILYNSFFKDYNNEFIEFFCNSINNEI
REWLLLFLLPNFIHKSIHKVTYPLKLTKNIFSESSEFYEDFFSNEYLKMNDIEKIIIYTKNCNEEHSDICPFL
FCYLWLKSNDYKIDKWISSKINEILFTFPYANFLLSKYFSNFLLFIQLFRTSLDVDHMYLCKSHFYVSLKHNI
SFIHESLVNVLFSTTIYHP*
>N.MAL13P1.317
MPLFKVKGEGTNIAYHEQIYTYKNVEKYDEDIIDIDKKFLKIPNKNLEILKRINIYSKYDKNEETNNGLIKKR
KRIRNKIYSNNLIDDIKYMDILICGDKNSGKTTFLNCIGCIENNCLGNKCEYYEKDNKKDETKNKYHNNNYNN
NKKIDVKKLENNISYNNFNNNEKYSNNIFNIKKKNQQVIWNDNKLELLSYIPIIFSKLINRNYEQFRKSFKKN
FLDTDVCEMSLFITRDDLSFINYEFEISNDFCTNHDFNYIKINFCEYGEDFFHKIKKYYQIYYYKKNKYILHE
EMEKKKKKKKKNQNLPLFHSPRIMNMIECALFRIREIKYINFFINLKKSFILVKCTTRLYNIMIKKRYFKTS
VLKIGSCRNIKRTINNILFYKRNIHILNKYVDIKSDNKQFFASYIKYKQNKNNNNNNNNNDKRKKKKKNLFVT
INEEWILKTFENINIIKNLNNYNMKDILFLASRSFNFEKLSKKYYIFFNITYNMNIINNIINKTKKYNLQILY
YLINIFVYKNWKIYKYKEKKNKTNMITEMIFKYYPNKKKNIYNTTRENNIFIIYNTTRENNIYNIYNTTRENN
IFNIYNIYNTTHRNDIYKIYNIHNRSHKKNILYISRKQADMVNFLKKDKLEIHNFFKMFYDEYIYKNQNKSYV
KLLEGEHFDLCFLFMKLYFYYYFYINNEKEENTTLYRKNEPKLNFQNFIYLKQIEIVRKNDGITYNNICVPSC
VYSFINLLKMYYKNYPFFTLKKNNILFLVHIILLSIAHYKLMRKSCDFLFPSNKWTVNYICYFDKAVIINSIV
SLLEKKNEHGEHNKCEVNKNIKNQQDHTNKYIFSHKNGEKKKKFHAIWSCAHYKKYKRRRNILQGIVKNIDIN
NIPPYFIINNIKTNWLYIKKVMVNLNICSYHNQYMYIYPNISYDIILYFNDNNKHLFSYNMNEKNNFPKISFK
PIYKNTYRHIKNKDDDKKKIKKKNILHFPFNHKVLKYFSDTFQMTNDDKENELLLQYLYMNFSKELANLLRYI
NKEKYKNKHKRINRFVRNNIFILLNCVMDIYYIMEYTYPGRKKKKKKKVKLNEKKKKYIYITINNSNDTYINE
ENIQNKSFIEKEEKKITFHIYKNDPMFNSIYSLWEDLKINQTQKLLINTYLEHLREHLNFSIYA*
>N.PF13_0333

MCAKEYSASQETIEDDLFENDKCKIRNSIINNIINLQLKDLIKDFNNEQEINDGIECINEKINQLNNFINKIA
KAKYEYFYKYIDRLSHAKKRKEWNSYDLEQIEKTYYKNLESLIDKRNDIFTLIKEKKFLIEIERVLKLYKIIN
YEITILYNEIRKDNKKYDSFDFLYNKEETDIDECRKEYIKIIEIDEENFNYFTSDMYFSNFFKYLKKLNNLRK
CIHYNNLQNIPIISYEVKKMNYCEMYIINIFINYLYHNFFQDEDYEEKVQVCLKAFILLSIEKQCINKIIQKK
INKISSYINNIKKKNENIKENFYFLLENCKNCIDQIYFLNQMFKRQNSYFLHIQKKKKNIYITEKQKERTYDK
DYYEHHNINENGNDNEKDIQKNTTNEKPEKKNIYQGNNIYQYNIDIYNDFYIPFVNVLKDIKLNEYIPKDNML
YILNLIEEFLYIIRNKYDINELEKDMLLNLFNKEYEEITFNSINKLANLVELLFEKKNDQNAYMRNDNIYVMD
NYDHQSYNFPTLYDIKNYVVTLYKELYTHICYPHIFRKIIVSCNNSLLLLSNNLNNLKQNINLNIDIDMNTKY
ININYAHKSFQYNLELFLISRKLLRYLFINLNKLKRRINHIKKNTYKNFKVYKNSEHVQENEQLANFVHNKIE
QDNETKEDLIVLSFKESFDDVFTFQDHLYNNFTNNILYMENENDEMDNNYDDIDYFLKNDEENKEDKEEVDKK
NEGDILQNQINDMEKKYTVKDEEMKLNNFEINYISDIKFNSFNNLNDSYDLYTSDENYEITSFFIKEEINKEK
KKKKRFYDLIELEKGICELNHAVNSCIYEYFEYFEKKTYTYLKNNFSNLNINDIYLLFDELCNYFHENIVQHI
PYYERIQLVNKLINRILVYLISLSSYYLIHDMDTYIFKYYNSLLKIASVESLSVLNYEYTLCQLYKKAVDIKN
KMESYENIPTFFVPIIFVILHGGKNIVNNLNISEEKFINLMVDHLKNPKNSVDKKGGQNQNLVCMLRKYSKSL
LL*
>N.PF13_0334
MVKRNLPQFELPNKNWNIFEDSLLYRIVKDKEDKIYKKIFSFDLDNTLILSRGFFKPAQNEKDYIFYSDIIEF
LKKKHEENYKIMIFSNQKGVSSGKLSLSNLINRVDDIIEKIGLPLECYLALKNDKYRKPRIGMYLFATQNYKQ
NLDEVIYVGDNANRIYDEKFKQEFINHLKYIYSKNNVNININDIYKRLKKDYTDTDLKFALNINAKFYTPEDL
FLNIKNHISTDFLFNPKYFNKELNEQENINKKNNHFYEICMKDYHSQNENINNKKNNTNNNIIPNNQYTTNEK
YIPPNDQQKLIILIGPPGCGKTFLCTNYFSTYIRINMEEYKTISKCIQVLKEKLLQRKNVIIDNVNSLIKNRL
LYINEAKKINQNIEIRLIYFNYSKELIIHLNTFKLITDKTNKFNDVPTIAIHSFYKYVQEPSKDENVDHIIVL
KDENFVPTKFESEEQKKLFFMYLN*
>N.MAL13P1.318
MFRIRELKPLYNLQKHFMSSYLNFNRYTVKRKTYIAAVENKYTYIILLNNITHLGEKGEIIKVRRGHARNLIK
DRRAVYATYENVDHYADKGKYKRKEKMNIKKGVIIEEDFEKYFTYLKNIDITIYLDVYKYTNNVSYNLYDFFN
YLSKNYEIDLTYKNLHKINYYKNTENYHNNKGEQIYSDITNVNDLIIQNNVLFKYTGIYHIYYYLFMPNMKFL
NDIIFRISSLQEYELLKQEKNTKKAEIVYRI*
>N.PF13_0335
MADKEYELRGFLVGHKKGIRCLSTIYSDLFEELKDRNFLFEYIISADYNGTIFVWKRFENKKGVEDVKKISNE
KKDECNELLYDDNFKLYKKIEIHEKIVYSLCYSKYIGIKELQKICDEHDEEKYIHIEDHLHIYSGGYDKNIYL
FSLNGFIELVLEGHKNSVCSIVEKSKDILISGDWNGQVIIWKIEMNKLNENCSFNNVTKNKNDNNNNNNNNNN
NNNKYDNNYKLNGQFNKGDVINKNNSLDSNKYRYSILKIIDNHKHAVYVTCINDFILTLSQDNILKMWDVEGN
KINEIQDIHKDSVRDIILFNNNNNVITFSNDENIHIYDMNFVLLKQYKGHNGFIFYVCVNEKDKIMISCGDDK
TIKFWCIQDIYEYMEIFLKNKSLDDHISFFKNKNNQTYNCLQTINLTDTMWNVKLLRNHDIVSACNDSYIRIY
TNKKDKQLSRNIIQMIDNKYSKKEDILNEKNQNEYVHDISNIQNVVGKEGDIKVFKNQNKYEAYKYEKNQWIL
IGDVIDDVNTNKKFYIGDDIFEQGYYDELFSIDIGSDHIKILPYNKKDNIHLIAEKFCKRENISISHIKSIVD
FINQNCNVNNNNNNNNNNSVHNNQNDNLIKKKKKFNTILNVITIQQAALDKILDKIKEFNSILLQQNNQYKLN
NDEINSISNIIKIYKTNIKTSYLFNTTDINVTTKIFNWSSDHIFPIIDLFRILVLNKNCDFLYNNKYSFNAFK
LVYDCISYYINNYDNQNHTNKLDSLLLCSLRFYLNMFYLSTPRYFMFKKYNFVLKQINQIQSKNNNIILLLFR
IFFNYIITLNENNDKELRKILFEQIHNFSKFISDPDTLYTYSLCFHTSSEIYTKQTYEFINKYNVIDFVQNKS
NELKNQQNESNEKTFKNISLILEDLKNIL*
>N.PF13_0336
MLDKPSCVISSYVRKILIRSTLLKNEYKILEYSFIRKCIYIRNENSPKKFLNIDIRNFVNIINNISDKGVHKE
IINIPSVRNIKSGEKKEKDTDKIKHIKENNINNNLISNNINDNLYYKQDKFIGSKKLPINYIKKINNNNNKNN
NKNNYNNNNIIFKYHSSFHNYNIKKYTIKTFHNLVFNINEECTMENKFLGKYEDNNILYEKDVKNNYDEVNSE
YEENTLIKSDLRKEKSKYIFKEFSVFSFNILANSLVDYKYDNNGYNIMQWMNRKKWIHQNIMNKLSDIICLQE
IEESYFIELKNELEKLHFKGLFLKKKKDTCKDGICIFYNTKVFELLFFDEVIYDKSCLLKKWHVGLIIALRNI
ISKRIDHFEEFHENNMNKKNYKNECDRILQNSNTHMNNNKNNNNNNNNKNNCNIFNMCDDIVIVSNTHLIFDS
YKGDVKLYQICYMTYRLILMMKKCINYIKKRKKGNDNTNSDTSSSSCYNYKNDLSLCPKDDTFLKPCIIVCGD
FNITPNSLLYYFIVNRFINLKHINLKYLSGQYLMFNKQYYFYNYLKGIEITKMFDDNIVNDLKYGCSNKLPEQ
IKKEPLFLIFKDEQILNEEYLLNLINKSNTNKNEKNNNNNNNNNNNNFDCYMDNYNSFEDASYNIKNKDTQNE
IYHNYRKRSNDMLSIKGKIKNINNINNINNNNDNNEKKGSYVIQINEKERNVHMNLNEEHNNNILDNEEEDFI

LYYPLYFKSIYNKSTDNIVEKSCYKYDSIDTLNNLENNLMLCQIPFTVFHGKQKGCVDYIFYSYKNLKQVSYT
NLPTFDKLAKYGCLPNQKYASSDHLYLHATLIRTIEE*
>N.PF13_0337
MEESILIKNFHKLDNVCNSLIDILKNLKELLFHLDPLNKYEQDNDDSSLFLDMKDSKNYIIILSDIIHKNVNK
IMMILYEFIKTIPPSYTYYSSFSYKDYVILQEMKEIKKYNDENNQIKNDIIYDRNNVTTSMEAHNADNNQSNI
HHEHTKNDNIYNNNDVFERYSENSNDHHINNNYDNNMSHVINPQNINEYFYYSFLIDIEYSNLLHMKKYEEEI
KEYLSCII*
>N.MAL13P1.319
MIARIYKYIITLFYFLLYIFILFHCLNSLIIKYNVKVEGLRLVSRMKRGKILKGCFPKDGILHNHYISTLFIK
NDGEIYNKYIGGKKEKVRKRKNDIGGRVRFRRYGKDSRNPHLAYEYINKIIENKKYEITKLLEENCDENNPLQ
IRMKYLQHTMNNKLSESLKRSNNDEKHRLSLIADMKRKIFCSIEKEKKKEKDHKTKEGLYNNINVGYDNLQDE
KYIYEDILKNEKEFDDDDIMKNNEEKKKNINNNYIYQHNFLNLSNPGNVSLLLHEIGFDVLIVNIDELSTQGN
INDLKDIIKSTRTLPRNKRPAIVVDDIIIHPIQIALAVENQADGVILNLSYLKNDMEEMLQYCVNVGTQAIVE
VHDYNDIYFATQCGAYILMINEYDFYNNIYEYNHAIKAINYTIPEIITIAKVNTNEVNYIEKLGSLGYDSICL
EKKLIDDDLQQFVTSCKGWSAPHKTLLYLNRNNYLKEFLTYDKNGQTGENHKDITENLKNLYDDKNISNNYSH
TLLKRYEKEHIDGEDITNDEAMNGAINHDISQDINHDINHDISKDINHDISQDINHDIKRYNVLNKDNPMKYK
TEGNKNNYAEKKESFENKCIDNKNIKHTNMNKKEEILSKEEKQIVQNFKNEKKRELMLLSQMKEIIKEVDDQC
KNYDTNTDEQNQKKEEKLESLLENFTKLDKNFLKGFFSDEEINNIENTMKNAVEQKNKIQSGEINIDHKNLTG
YPMGTLQNSPMNNLDIYNLTKEYFGETQNSDAHTENIFDSDQNKLLDESTEDSSRGLDSS*
>N.MAL13P1.320
MLNLFVFLILFIIRFIVIRGSKYRYENNSELLIERIKRGKEALDDILKAKMKAEEEYQKNKKRIKSQNKNSYK
NKKEDDEEEKDYDEDDNNNIYVQEIKCYEYVLEQLNNLNVYNCNEINENNKSLLALAKTKCLFVKSIRSFPDE
NSGCILNPKKLNKLQLYLYNNNLFNEFSNQNFSRTLNLNELRKIEKIINPCYYENDQTDENYVNLLNDISNIK
EKQRNNYEHINYTYNNDMDNLDGTNYMSEKNNYLKKKLCENLKYKIVTNCTSNKNMSDTAFQIYHSELNHIDD
ICFYIQSNEWNKRTEENINRLAQTSLYISKQMTTNLENMKLIENAQIKQIENTNRFDNFLKGLKNDFSEIIQI
LLKIKYHHESITKFLRAFKMIVMYLLIIILVLFITSRSYAYSRTTLIYFYKMFTVNEYVYILLNINEHFISYS
IKGIRYTFVIIGIKVFISSIITYKEPAKVIEEELKVIKKIVQKNSQQYQNKIKEIENGNNNILYNDIDQKTIS
IINLWSCFNENMDSLYEDDEDFNLSNYNSDDESSSSHTSLASEHFTLNEIDE*
>N.MAL13P1.321
MENRECEIRIFNGTSCCFYRYMHSVKHGVWLINPPECLKSKGEEKCIAKFTKTMGNFYGILQYCVVINSDEYI
LNARFDIPLLGDNTATSIIGLNIKNEDKQILSQQNYFLVKYSFDNTYNSKFYINIVETEEGHRFIEYHKKKLK
KGLLRVLHIRTLEEKEKLRKENMNNNLRINNCSNIISNNNNDNNNNNNYYYNNEDNLTNDKLIHNYHRDNDIL
DILPLSFIINIKNYEWKKRLRKAHKSLYIIIINFTRQTLNLTDCRGMKSVELNEGNWIELPNEKISSLRSCEF
GCNSDGMFSSISGFCKYNFMNESCYLNILWDINSVNSKIKCNIKNTSKYTIIKNVELYNECTAVFHILEYYYY
PPIKILECRALTNNVINNYNINKDNIDKNINSIYQCSINVIRQFCQYLLNNNTSQENNSNDEDFMNSSNLQKK
FVYNDDDDDLKFINNNMSSFPKINKNAISLYTNKGNEIISSKKKKKNQKNPYYNSKVPIASEDIYKKGEKNIY
NSEYENDNDNNKDSCHFNEEENEELEEDGDFLNIDHLKSSTIQEKNLEILKKRKACSNNMNINRKNIFTNINI
TCGRDFFCYMHGNNYSNNIPINSYLYISWNIGNVIYRNLYNSSENIIINAHSLLSSHNINDDVILKLNIDENN
RRLYPSNLIHSLLMNTESNIYINNEYVILDIILSIFHIISTNLSPIIEKILDAEYGTRWIMDSKIPKSNIWEK
SKGKNELDIEGIIHIITTFWIDIFEKRIKNIEIIQNLQKASIFWSNQEIDQFDQDFVKKLIESSSLLLKLFGD
YNTAKSIEKLYYNKYSIFNDFCVD*
>N.PF13_0338
MFAVNLKICIFLSLVSFLLQCKNTLANVTFEQKVQTNLSHDKNGDIVYGHREFKGGIVYGHREFKGGIYAFLG
YDNCTIEVNKTVNGIDWNEKKDVKVSGNNNIAVVYSIFTSEEKMILIFKCDNKFYITKYGKEFKWSDPKVIDV
SNVIGTNTTPAVYSGSLLSMNNDFEKYILVCENHSQNYINVVDQEYMREIRLLGKCMLSFDEGNNWKNEVMNL
YSDEGYTKINTLRLSDYGGKILVKGTNAQNLNQTIRSIILLCSNLHDWKLFCGLPTIRFRKDISVENLTYLNT
YHLAIVKNEDKLQLAFTYDLFETFDPQYLNTEFNGVSHYFVLAPDEMVYLFYHGNEKKNYVIKIKTVPRKIGC
ELNTNDTVNTIYTYTYKYIYNNKLSPKTCKVPSSHLKYSSDGLYKLFEVRLPKDIKVTENCFRYSFLSDLNNK
YHTTIIKTRVINKLEDYVEVQFHFPIYYTKFLYNYKSTYCVLSNNYRIVVEFDYIRNHIDLDFPFDTDTVKLY
SNESVTHAFRNNTEKTHVHKFPKGTYMTSYFSYEKEYVISNYIEEPFSTTFTILTQTQMNVHFMAGGQKYKYE
GIDLTDSSPNYELSLNSLSDSQNVDIFVSKFDNNKTIGFVCPVKSSYDGLNCFDNVYIKNKTLVKIEYLFGEN
DIFVVPQRRIYKTEGTAMESLLYLNNNNVKKLIDDKSIIHFYCECNVNNNVIKVNYYISPFYDENSIKQEINK
KDQEIPMINKTIPQDEKDILFNNEKVVPLSNEPQEIIQPPIQEKLNTTDPSKAYIYGANIIFIAIISIISLSI
SSFI*

>N.PF13_0339
MNVEDNSLKHFNNLKKHEDHVSNKSISSNTKSGKISKKKKAKKNKNNDNVDEENDSNESFNDKSNLSNNSSES
FLSISDDTSSYASSFYRTESKHDRTYKTPKKYNKKLRNNSLDINSGESDKDMSSDFKSSNKECKQKYLNNILN
LNNCNCSNIHTPFENHFENSKNEKIKYNILHECNKNGDVYNSHIDNVNNLSEHSSNKLNYDYNKNSSKNNDKN
SSYEKDGAKNYGMSHRGSNALNQHEDYVNVMNKVNNYVLNNNKKNERDVKNDTNKDAINNNNIDHQYIDDTTT
NNNNNSNNNNNNNNNNNNNSDDDLYSVISGSDIGNMQLNDNDIYNKTNIKLNNIRHKDNKKFNSVTSRGFHKV
GQDNTFNSVNSNKIKKMKCLDEIINCQNENREKRFSTNNISYNNVGIHNNMSCNMQNDYEFKKLITSTINTCY
PKIKSNNDSDDILNNMERLSQIVITGENESVKKEEGDSSNNSLYDDVDNILNIRNKIYIETRERNLLSEKKKK
TYSLDTSGKINTLTKNDNKEKYNITVNNNKNINENIGINDVQYDDQNNSRNFMNKSSLMSNEKLQFLKQYEER
LSRNDKYLYEYGYYLNKHMIEINDNINRLSNNNSDYAQRNVCTKNIINLIPSLHNIYENKNLCYIKDCPNFSE
VAKIIPQWKYSFEALNTRAHEDTRDMKNILKRIENMKKDLDTLHKDLKEDKNEFERIKFETIQHDDMIRTMEN
KTKLHIKGVLKLDNISFKLKRIHNIPRNNLHNYNISETVHRLLKFVDNNNNNNNSNSSNNNNNDSIMFNNMNI
LNLTDTFPEHSNIDILRFQHLDNNLNYVNNPDEECSFIMSLLNEELKQMLMEKKEKLQNIIRRTQLLHILDIN
TYKDHINNTLNKHSILINQIKANLQNNLILLYNIYDMYIKGNFRIIKIEDIYVKSFLSEDIRKKHIIKTFNDN
KKNREKYFMTVNFTRNKPIAHPFFIYHCLSEKFVKNFVSFVLDPLSEQNYKNFMQYCSIPGFNSVLFSQRVIE
LYRKEEHFSKKERLCDIISAYRSICLSQYNNAMNAYNNFISNNDHNVLNNTNIINSSNIFSNTNTLNNNNNNN
NIINNINIINNNNYINNNILSHNNMTPIVNHKITKSKEDNEYLISLRTASDQCFKSIIENHNLDEYFFLNSKN
SFNLIQHIPDSITNMCNTYAAICYEGINNNQGIILGIKPYDNINKINNVPTFVNMSSALNTNNIFNTNALTAN
NIFNKNNVFGTNTSTNLFGTNKNSTLNPIPAISTGGTFNSNIFSSGTSNIFGTNNNTSTNVFNNNMTNNVSTN
IFGTTNTQPSSMFNTGGTNNSLIGTTTNTSFSALNNKNTLNNMSTMNNMNTMNNMNTLNNNIISSSSNIFNKD
KLFGSSGTPNIFNNNNTLSNTANTFGSTTNTSNIFSKNIANSTSGNLFGNTGTTNNMFNTSNNNLFNNNNNNN
NNNNNNNSFRNSLYSSNNNSLNISNSLINNNNNINMMGSSGFPNRSTNTSNLFGLTTTTNNNNNMTNNILNKS
SQNVSSNLFGNNNNSSNNIFSSMNTSSQGNKINTYDNKNLFSSNLNNNTSSMLKDNNFASWGNNKNQMMNNNS
FVLNNLNTSTFGSNKFGINTQNNNSNTLFNNNPSGVIGVGNNYANIIGNNMSNANNNNNNVSSSSSSLFSSNN
MFNTNSNNNMFGSKNGLLGTSNNNNVSNNLLLNNNTSNNNIFNNKLFQNTNNNFISGTNTNITKNTFSVNTNM
PNNNIFQNNNFSVNKNISQISNFNNISSNNNNNNIFQQKNTTSNFNSVFNSSNKNTLFSR*
>N.MAL13P1.322
MIAKSYVYSDSSSDGNKKNNDKIQTGFLYNEKKNEKTLEFKRRKVNKINKKLTTDDVKSNIFKKIDTFEEASF
KKDKVSKRYLNNNEKEKYYKDRDKNKIKKNNIKDKHVNQKYNIYNSSTSPDNSDIDNLYNHKENKNNHKYNKS
DYSEDSINISINIKNDESNKYNLSNKQHHNRYSNKTFEKDEKQKKDYYKKNNDKILDEIWYTKEDEFVDSFYN
MDIEATYEKEKKMINNLKTRINSNTGKKVNQKNLDNNLWELNKLKQGGVTSTYNKLQLEKINEANVTNEIKKI
VLTRTVNPPFIDKFKSYNKRFVNIEQRNNFDDQKSEKKKSDNINNDKYYSNKPIQSTSYSTVVKDETCDFVKA
AKKGSEFLKYFKSENEKSKARDRYWEISNSKLGELLKLYKNKKKNKNDNTSKEDYDNISYNSSENNKEDDNGS
DVFDYKKDKIYSSLFNIENNNKDKKNTLKDKEELLKLKESLPIYKSKHELLDAVYNNNIIIIVGETGSGKTTQ
IVQYLYEEGYHRNGIICCTQPRRVAAVSVAYRVSYEMNVDIGSLVGYTIRFEDNTTKDTKIRYVTDGILLRET
LNDKELDKYSVIIMDEAHERSINTDVLLGILKNICLKRNDLKLIVTSATIDAKKFSAFFGNAPIYNIQGRTFK
VHIEYLRTPCNDYIECAVQKAIQIHVSDNNYDNNFGDILIFMTGQEDINATCYLLSERFYEVYESYKESKNNK
KDTINKIKNILNEDNNNNNNNNDSNIKKKVDGDNNTNDHMIYPFYIFPIYSQLSSEQQSKIFKKYDLRKIIVS
TNIAETSLTLDGIKYVIDTGYCKLKVYNQTIGMDVLQVTPISQANANQRSGRAGRTGAGICYRLYTENTFLCD
LYQNNIPEIQRSNLSNVVLLLKSLHVQNLFEFDFIDVPSKESIINSLHELWVLGAINNEGNLTDIGRKMVQFP
LDPPLSKIVIYSQNFQCTKEILIIVSMLSSPSIFLESKENNESIESKKEKFTVPESDHLTLLNIYLQWRSHNY
SYSWCTKNFIQYKALNKAKEVYSQLIDIIKTLNIKNVSCDNKWELIRKTICSGYFHNAAKLKSFSEYINLRTN
VSCHVHPNSSLYNIGYTPDYVIYQEIVFTTKEYMRNVTTVDPEWLCELGPLFFYMKNV*
>N.MAL13P1.323
MDFAYKQEAEEDCLISLDYILDKYDLYRDNIINNVELRKLLDRFIFQYNSIDECEKKLLELLSNNEKYGMDKI
EKYYLFITNIIKEICHIELIPIIYNKILQEKDKKDNTFIVNRQEFLKNENKKESEEDYKIISGSIYKKYILVL
IKILYHSYKLSYNYQKNLITSISYICILMSKYGTVNLPHESIPELIYRIFWKYFSQNFNIINETFFTQHNDEI
VLSSSCSSSMSSLSDDSSDLSSGFLSSSEDLFRKNDLKAIAQRMGISLDSEKDVSNEISCGGTPKEPGEKKVR
RRRRRTKKNATGDTNITGDDNKEDINNKEEGVEPGAAQLIKNENAMQDQGTVVKKRGKRRKRTILTEENNNNN
DDNKNVNVINNDDNKNVNVINNDDNKNVNVIINDDGNKNVNVINNDDGNKNVNVIINDGNKNVKIECDTNTQN
KDASVNNNVIHAKSNINEEDIKTYIRIKNEAEKDNKMNTPVLVNTENKTKVEENVDTLWNKLDINDENVFFVN
IILNIYINLFNIKNMEVFLFYHENEITYKKFLLDNIEVIVNQIKSMMNKVKNDMSVCIIHFLNLILFILIYLN
DLARKNKKKGQMNHIKSNPYNDIKSNECINLKNGDKNNIMRNYKTSIISNNNNDNVINNDNMINNDNIINNDN
MINNDNMLNNDNMLNNDNMLNNDNMLNNDNLLNNDNMLNNDNLLNNDNMLNNDNMLNNDNMLNNDNM

LNNDNMLNNNNMIYNNNVRKSVSGKTKKDTSIIEKADCLLRDTQKSGNIGRYISNSSSEILKKNISSGIIHKD
LSNVSRKKENVTNSYNSSVESKLNKNFSYSNDTVSVNSSIFNNNIINNKTFKKKGILSNHSNSSYMNNSDINS
ISYNKNNITSFDNNNNNNNVNYEYCSNVDLCSNSFNDNNNNNNNIMSEINYNRINHEGTFHSIENNNITSNSY
NIHNNSDDNFIHNYNNIMTKGSVDIDNKIYNSKNNIHNNMNNNINNNYVGAFNNYNNIPYNDVSNNSSLGNKY
NSDEDDLYVLYDENSFINNFDYVKNIIEKKRKGLYYVIVDTINNLYNTTFLEILDNLLKCVEIFFNDENVVIV
NKIFSYMLESINYCNEINKYKIYMFILSKIDKFIKIKRYNEDNNSTLNNYNCYFLLHLILNIFKTSKKERNIW
TILFEVHLSKTKRKRSSKRNNSSFNMIKVNVSNNSNNIMINKSISNNNNNIVLSSNDVDKNNNNNNNNNNISY
NNNMYLSSFLKNNKKLKIIKSEDITTCNKKKVQEIDTNLYDGKNFILNNIINFLLECCISNGCIIRYMSLRVF
YKMTKLFILYIKNRKLEYEELILKNYLIKSVYQNFFVCIFNFLKEPDTNIYVYVKLRNLCINLLYICSKLMSS
NFLSDFYERVICSNLHSVKRFYKTVEENTTSGNNNDNNDNNNDNDNKNDNDNDNKNDNDNDNNNDNDNNNDND
NIINNNNNNDNNKILLEEKTQIQNQGHLDKEHNKNVDENSPCDNHNHLKSAYSLFIKNSRDLYLFTLMVIYKK
YRCIENFKILKKILKLSTEEINIIMNNLIISAHHNNSNNNNNNNNNNNNNNNNNNNNNNNNCNKDGGVVRTRR
KEINKVDKLYIKNINIDDFIPKEYYIKLKNKGDDKNKEKNDLKEIVYKKLYFINIFFFRELKTHDCYDNTFVY
YIYNCIYCFFHIYINRFAFFIIFIKQEHMNLHDYIYSDICNKEAQMDTTSDDFKNVQEFISQMLALLKSYLHV
VILIEDHIIKTNLENLLKKKKGIRGRNKGKDERDNEDIKLYQEDVKISHFNIHTNDNKNKMFDETNKLYDEDD
EQIEFTNLRNFKKLIIIEIQRFYLLFVKLLKMLLLLNRQNGYSITCFEYFFFKLMKLIDNDIIDIYTKRYIFK
FLKYFTIKKKFTDIVITKINFLYSKLKEKKKKICHMLITKEIKNNDMLYPYLNKCIEENCVNDDLWKINDEMC
SLSKSELIKKDREIDEEDVEKNLKDGIVENVNDIDDKVKVKRRRRRRQTVDGIINKTSIDDNVDNNEIKMEL
SNNNVDKTNNQTCNNQKENGKHESIEENEQEINNKLINETIKKDVGISNSEPFNMNNNIKDEKLYDQNGNNNE
TITINTDEQKESKLISINNNQKIDVEQNDVSRRTKNERKKSSKFLLNDQNGNNIQPNDENQIQDGNNNNNNNI
KRRRRTRKRKANDKSGENHTLNESLKKDGNTNNDILEEEDKKCTLSVYEKKDTNDLMWLDSYVNFYVYTNKKK
KNNYDNNNNNICDDGFNTVELLNEKTNHYISNISLCCENVIEELKKFNFNKIYNLETFKELKLEKKFYENIEL
YLLSIFVFEKNKNICDIYSFKENYKHINDLIEYCKMCNIEKGLEIIVKCLFTYLREIKLDKKSYVYNMIKLLT
HSFLYIPFRRYTATPFIFISFCSLFRNSSRTFMNKIIIHNFLIICYSYIMLIKKQQVDLKRQMLIDKRKKRKE
KKGSQHMKHDLENENLLDKNMCTIMQSNVIEKTKGEDTNNNNINNNNINNNNNNINNNNNIYNNNVINIKVEN
IPFSSYGGEEKVNRDKKKKKNNNNINEIREDLIKDVGECINNDNLINDKKPSKGKKRTSTTTEGTQIVRRRRR
RSNSSTKEIDIDINNNIKLLKSLQNEEKDLSSTNNNIKKDNITNLLNNNMETYNIQIQNEIEIDKKNNKKYLE
DNLFLHLINFYINFSCSVYFHYLTHYEKNFDILKNIIFLGLNKVLEKKENKKLSCFVLSLLYILMCLLLKVKI
KEEYILTLNKHKSNRCSMKRKEKHEENNGFVTNNISKYNMRSDNIQTSTVSMNCLNNDNMEIHHNINDNNNNN
NNNNNNNNNNNINNNNSNNNNNNNNNNNNNNNNNNNNNNNNNYYNKCAPNYYACNNYHTNNDIRKFFVKYLIN
LIDEKECKNIYLINDKKYYKYINKIIDIKSEETLNYFKSYFPNKIYKNKMDKYIEKIFVLNKKKKLFFMSSFN
LKKNIRKINYYNSIYSNKFLFNLLSEPILFEKDNNLLINTDFKKNMNHDNNMMRYNLLDYIIYTCFYIIFCKI
KLIKLMKIIYKINKNDCEYVPMNTYIYNRKKYSIWQFYLSIEILHLIFSNIKYVPFNSIKLILDIFLNKIYIS
ICTHDIKEKKNLNKLQNIFYDFFCQTFYTYPETFTHLPHFIYQYQKTLHLLNLLSNSVVCKKKKQTPSYYSLI
LEIMKTCFPKIDSDDVNEENGKKKVEEKQGIEKSQRMLRSNKANKT*
>N.PF13_0340
MIICNNNVNHFWKNIKKNIRLDGRTFEDSRNVCINFLKDYGHVEISIGFTKVICKITSEIVKPHDRKLKEGIL
KINLDIDNFIEDNENSDNMSDECLEIKNIIDRVLKTSNIINFESLCIIPGKKVWCICINIMVIENDGNLTDAC
YLSAYCGLVHFKNHQVRVIKNGDIIIDKEEINYSPLSILNSPIVTTFAYYEEHDVCLIDPCLHEEEFMSSKIS
IALNKNGNLISLLKPGGIPLACSHIMESIEIAKKRILTILKILEDTLEEDKNIRSYLTKRNLHVKYSSAPVTI
RYDNSFKLKPDISLERYTKNTLIFEDTIKKIEQYIQYNQVQECLNNMNVKLEENDQEEIETCTLKGEGNLNNP
NFIDPMRNTNLLNPTDITIIKRQEFVDNHYDANNNKYNTQANKTFKKELIDFSNLLTQNHKKSNNLSNNQKDI
SEQHDNSQESSDDASTIRSDESSDIDFSVAINQNIKKKK*
>N.MAL13P1.324
MPRLVIFYFLIFVFMKINCINYNSFSKRKINYHLIRDKQENIKYKYLREDEKLNLSRRNRTKRTKETNGGTQR
IKRIKRIKRIKRIKRIKRNNLSNIFYNKNDYMIKKINVPRTYFIKNMGNSSLKGCIYKEKKTIFNIYNIYDDK
NIMNPKNFGNEDGNLLNKLGNDYSDKDSYNKNMNDKKQIVLDNKNEKMYIENMKKNLSRTDEVTYKYVEEMEL
KKINIKGVDIQYRLYNLEEGIYIVDKENYEKIKKLWDKDELKKAKENFEDTFDIKKQGIKDEDWIMLPFEYEE
NKNIKLVKADFDNPTYDYILTYYDKERNYYWEYKRRNYYKMFKNTVHETPPYKSDLEEDKRYYGKEIIIPKKK
LDNDIFRQPMLSDVMTSGCNREPLGFESWRFVKYPYGNLIESQKYSKLYCVKKNDKDKPSMKYHYLGNSNLAV
SEICLGTMNFGNYVNEKLAHELFDYAFEEFQVNFFDTAEIYPLPASENYYGHSEEILGNWLEAKGKANRHKFV
IATKICGRTDKLPWMKKYKIRTEQKNILNKRNADIYNDNNYKKDHYITNSKSNEYSHRNNNHMLDKEDNKNGY
DKLKELKEKEELYLKKDYDKINKFEEYEKERLDNSANLITLNKENIINSVDNCLKRLKTSYIDLLQLHWPDRY
YPDQSSGDFSHVLYDYNKYYDDFIPFIEQLQALDELKKKGKIREWGLSNETPFGLLKFYELCKHLHISPPVSV

QLEYNLLCRNDVEKGFPEICRPQNTNISILAYSPLCAGILTGKYLEYTDYTTKGRMQKFPSYMKRLRGSIATY
IIRELYYLSQKYYFPNLTVAALKWVYTRSFITSTIIGVSDFLQLRENLYSLTNEVLFTDKLEREINALHWKFR
DPIRIIQ*
>N.MAL13P1.325
MVSKKEARFYLSNHLDNINSAIFYEENKGLLKLWDIIHDTSITKWNVNNFIFVKPYRINNDHIVTPIKHDRDI
AIYDIKIKQENRYKQVENVELIYINSESNNNNNNNCFGYNKVGAINKNKLSYTKIQMMIPNFLETFFLNNMNN
TVLCYHINNNNYVVSINNNVLYHIIIGKDQKFYTKNVIQKKKKKFNNIVIL*
>N.PF13_0341
MEDPVTRFYKCRKTCCEMLEDRGYIMTPREKLENFSTFKEMFEDNDRQRSKMGISTTHKNDSNNRIIVYFADE
VKKTGVKPLRELTERMEEKSIQRAILVTQNILTPFARDAIKEAAPRHIIENFLDTELLVNITKHELVPRHIPL
TSDEKKNLLQRYKIMENKLPRIQDVDPVCRYFGLSKGQVVKIIRPSETAGRYVTYRLVV*
>N.MAL13P1.326
MDVQDFLNCNKLKISKEKISNLNKSKIGILITNLGSPEKLTYWSLYKYLSEFLTDPRVVKLNRFLWLPILYTF
VLPFRSGKVLSKYKSIWIKDGSPLCVNTHNQCLALKKILNEKYNNKVVISYGMRYGERSIKKGLEYLQKENIN
KLLVLPLYPQSAECTVSSTLDCIGKNLKNWSNVPELRFISGYCLKDIFINTMKENIENYWELYGKSKKLIISY
HSLPIRNVIQGDLYPFFCIESTKKLVKSLNLNKDDYILVFQSKIKGQQWVKPCIEDTIIRLAKQGYKQIDIVS
PSFSSDCLETLEEIKIHYQQLFRKYSNGNLRYINCLNDTTIGIKLIMNLIEQNIIGWV*
>N.PF13_0342
MKINLSFNKNKENKNRNKNGDTDIDNDTRNNKSYKENNPMTRNKIETIFSMSDDEETINYENTHNKIIQQKKE
NQLNDKIKEYIKEHEEPALTTHEKKEDKKERIDEKNNKKKIQYLGYKSNELTKLNNPKTDDSYDYPFEEKEIY
DNLDEKERKKKTIEHNDKDGHEYYKKDHKNHKKYTRSHENDNEVNAYNKDCDVRNKTIDKKTYMKKYDKNIQD
YEKKKKKSENSDDDVNRSYLCIDDIFKKKENIDGKKKSKYMEALINSAKRRELEKEFLIQKKQKIDTEKEEKV
FVTSAYKKKLMDRELIKKDMDLEVEEKKVDKSSNYNLNLFLKNINAPSNYNRYNRR*
>N.MAL13P1.327
MIRLNKTYSYWHYKAWQRATAGYLRTFLKNNLANKEMIGYVINDKHPKSIRVACDRYMYVVRYKKTFRYTKKI
WAHDEKSEAKVGDVVRIQPLGYRIGPWKNYILVKILYKENKD*
>N.MAL13P1.580
MFYPGMFREKPKDIISDKWKIILRVSGFLVSCYVICFHGDLFDIPENNKLS*
>N.PF13_0343
MKIKELLYNELIIIKITEFLTILEIQNMIISLRINVKRNIYFMRECLSLMNLDTDGYDHNVSNKNTPNKNKNK
NTSTTEPLLLSSSVNLTTDIDDYVHNATSYVGDQHFLQQEGLNLITYDDEYDEYIFNDYNEGGNNVDNVNNFD
NVNNIDNVNNFDNDNNMYNYSNHDIYNNTSYYNTRADENFDENFMYNTKYSHYEDTLNDMTHLMSTYTDNNIN
YYETNKTNFGALYEIKKYLNFFEQEKEGHVQKCFNNVWIYIHLKNELIDIKKNLETYFMRIKTNTQVNRNKRI
RIDIFQLFKYNHTYYSFYDMPWVSIYYDFFLNSICTLCNIKLDHRSICLFSEKFNLIQTNDKLLKYINSMSDD
IVSNRKNEKKRDHQKVDMLFGEDVEREKTKKKTDMINNDNKNDSNNYDNNNDSNNYDNNNDSNNYDNKNDSNN
YDNKNDSNNYDNMYNLHDDYNQHQETLAEIKLNEDIFSCKNKINTYDEEFIFIRKTHIFCEECTKIIDYKMNI
NSLLESIKKDYELLKKIRIIYKMIDIPSDLFCFCNYFFFKEKYITFFKNVSNDLQNLRKALKKKLTNNFILNF
SINFYKFVISSLILCDEKKLFSQENIFLFGFYIKYRNILKLFSSPRCTHIYFSFNIIYQKIVKFQSFFKHKHF
SKLSKVLHFDVITILEKLKHSKTKLLYKNIAVYLYQHLNDKILRKYNYDEAYAYFFQCLQRYHFS*
>N.PF13_0344
MYQVVKEYLNNYQRYIYYGVFIVVGYFLHDLINYAQREGVRIGRIRKIFDLFSKVLKKLYIFFNFYERRIITL
ENELFYKNYDKEVIDRHGKLLNIYDIPHDSLYKIFNTKILIIGLGGLGSPVCLYLSKFGFKEIGLVDGDKVEK
SNLHRQIIHKEKYIGLNKCISAKLFLKDMDVHVSDCIKCYPFFLDKLNGINIIKEYDIIIDCTDNISTRFLIN
DLCILYKKKLIFASALGIYGQVNVYNLNNNTSSCYRCLKSFNNHSQNNDCDENGILSTVTGVIGLLQANEVIK
LSIGLDQDVLTNFLTYNSFSNKLPFESLNINYKNKNCLCSMKNFKELYNFILSHNYDNINNTNNTNNTNNTYN
TNNTNNTYNTYNTNYNNNYNNNNNSSSNKINNANNIYCNNLICFNQTNETYIYQIDTFQFIDILNNNYSSLSF
PVNQICILDVRKINNTNIYGLRNSVKWSFYEIIETFNKLQHNKHELLQIIIDKLNISKSKKNILIIVVCRRGI
DSLKITKHFNNLFLYNDKLDSNKKEATYNNNDISLYNTKNKKFNKTLLNDAHIIAYNLKGGYLQLQKKIFKNL
PFL*
>N.PF13_0345
MKNIFKYKNRLTFFRKGFSTTNKPKVEIKKTILYDSHKKNNAIFKIQHGFYLPDEYKDITLITSNLHTRTNCS
LFDYTYRPILKISGEDKINFIEKYVGSDIKGLWENECRISLLLNDKGGIIDDIMIILREKYLLLYLNIQCKEK
VYKYLKDKLLENGKLQVQIEEFTSHSSICIQGSKSSDVLKELIDYNNESVETNLDNCSFMSSTLTKINKIDNC
ILNRYTCTGEDGFDILIPNKYVNDLYNLILKNELVKPGGLAVQNTLRLESGFCEYGKDINEDITPIESNYKWS

LGQRRLKELNFNGAHIIMDQIKNGTKIKRVGILINTNIVPKENTKIYSHENAEQIIGYITSSVFSPVLQKPIC
MGYVKSEYAHINNLIKVDCLNKLEIAQITKLPFVPLSIYKL*
>N.MAL13P1.328
METLNDKNNSTYSFFFKNLSVTGFYEENALFMTVKELFDNSVDALYNNDKEDNVETKKDLEEEKKKKKKKKVE
IIVEEYDKSLSYYKITCRDNGKGSKIKDLEKFSEIFLTSKDNCSTSGKFGIGLKTILLYSLKTAYGFLHIKVK
VEENKIWDFMLVMDKNLNHTFIQNFKEYIDTNWNWSVEISLILKINNKFIHDQRIYTYIKLVLLWKRDINIKC
IIDNMDEFIYTYEEHANEYEFTLLHSILNYNCKNITFQKEALASFNFKVCIYINVLFSNNFITNPSVAHIFLI
RYVNSMPLFGNTGNDCSIVKDFKNFLRLYGPQYGMDLLTLDNLNQVELNDTNDSEHMNYLKDAFQTFYVKKSE
FCKWNVIIIGIDIRGCDISYANLNKNCIKEGEYLSNIIKKCLLNLFTRVKEEYPDEFESISDYHIRQALDIYG
VQLASSLSKIILNGRDEFKNKILFLLNEKKKKDQTSVVTCEENNVDKKNQIKENELTDEIYHHIREKVLSDEK
AYEHMGVKNNSSDKSNESDEDDEEEEIECEGEGEDECEEERMINEINDIM*
>N.MAL13P1.329
MAHIENHKKKKYEYLPKDYDTIFNPNEKETELLNQHYNSGINYNYDTSVKHGDNNDNVYNNNNYNDYLSNNPS
NFQYMKKNKKNKTNMEKIKLINKIPLHQQMNNANEYDHININYDSNDECLETHYDFENKYNNDKNLENRILIE
SEDFISSRRNSTRSKMVIAGKIVLAILFFILVVFLFTRFKNFLNIIHIVIEWVGKQGSWSILLFILLFTFLSP
LFMSVEIMCVGSGLIFSGVYGKALGIFVAVFSVAVGYVLGMSLCFFISRYLIHNYIYKKLMGYPIYMAFNQAI
NTNGLSFVLLIRLSPILPASVVSYILGVTSLKYKHFAIGSISALPSIFLFVYIGVLLQDISNLSELENHWTNL
IILFVGFLIGVIAIVYISVITKRRLNNLNIMNASLSTTNADIE*
>N.MAL13P1.330
MKAFVNKIQFRKSSESLKEDDEFFLERKQLNSLHSYFSHSLLYLERLEDAIKNINIMISDLNRSLMLFFEQEV
HLDIVKHVSYILDSFSDVREKTTSRIAKSRVLVNNTLDKIKKLQTLCIKKKNLGSSIDHYEKKIRKLQMVPSP
NQKHLDKIIRNQHKLNIVKTDFLQHHEYIKKGFDIVLENKTNKVLFDARKFI*INTYIYIYLYIYIYLYIYMC
TYLKFKYQDVKSDKFPVEYNFFLEEEDRMNIFADPKKFEHRNPFNQEFPRLENKNYKHSTSENHTNTIEQTNE
KKENGFFKIKSLLGVNDKSVQKVKSLSSPNITPIFPQ*
>N.PF13_0346
XXXXXXXXXXXXXXXXXXXXXXXXXXXXXXXXXXXXXXXXXXXXXXXXXXXXXXXXXXXXXXXXESTLHLVLRL
RGGAIEPSLAQLAQKYNCQKLICRKCYARLHPRATNCRNKKCGRTNQLRPKKKLK*
>N.PF13_0347
MSSLANIQNANNEESPNNTEGEKLVKCATYINNEKVYECSSGISSVDKINNINNERVDIFMNNNNLTEHIKSI
MGHVNDYFTKKINDEKKN*
>N.MAL13P1.331
MERKKAMPLIKSEDELSKCIEILRVKRQEIHEQIIKEEDKERMQKEIAMLNDKLQEVCHSIAKKLQTRLEYDR
TIKETSAAYTKIRESAKSLLHILKREESHLGNKLYSGTSDEDLRSLRELRNIQN*
>N.MAL13P1.332
MSNVNKEKRLLLLITRVNMLINLLQTRLAYPLIYPFNTTALNNEKSLNLYLEKLRKEGNFDEQTFMKSLGFIT
PSIICLTKLVYILKAGYSLCKYTGVNNKMIYIERKNNNKKVNLLNEHEKKLHLLKPQVWRLLKPNDEMNTKHL
KWRK*
>N.MAL13P1.333
MYFNKDRKDKDEEVKKNVVVNKSMMVQKNKKYVKNMKQSSYNMKYVNKFYKRNSNISTFNNNNFTFMNFNNLK
VENKNSQHVYFNRNSNFNNNIKDNIRSSIYSNKQNYFDQDNINYNTLINSQTCVCSIHIDKTDDDISKQQNII
NNVHLYNDLNHSSKNSYPIFMEQINFTDNIVLLTGILILPYNDKIFNNLEFEGKTNPIPAECNLSIYVNVVNQ
YDSHYFKQLYKLSYNFKNRLLFAATNNDTIDSSLLIDQIIIKGNFKNLSLIVLGQIENVPEALRYFKENLYDN
NMRSSTNKETLDVENVDFEKDEIYAPHKNVDIKTYNLNSLIKENISLDILNYQCNIEQNFIQTGYRNTHLYKL
SNVLEEQFLSNNIQHMSQLKDDKNDEISNNQEEKHYRESKNVSENIQEESITKEHISSDKENSPNNNNNNDLK
KHKKRNEKSVLTNTSVNNNLLNNDNNEKENVSHNEHTDIEKKNIMKEPFVPPSNRLIIQLLNELTSMYKNEKE
IYKNNIPIFKKIIKFGAMWIFYIYKNIQNEKDAFFLNYYKYIQIKLHDILGALFILRRCALYPELSKYIIHLN
FNTNILYYDSTCPHVLINLIDMLHIHDIYFLSSWKIKTAILKTIIAFVSNAYVMNLFCNYVDENNDTLYKRLL
ELSSHRKLKRYNIKRLLGIILSKVKLFEEISKWEESIMNIITNRKTYEPNLFNKEICCKLIYELKNVHKELFQ
HTCYYKSKNSCYDALYENEIFVFDEKNNKKIFYIHKIHNYIISYLEYKQFFNLFLYILKEFEIHIKNSLFLNS
YDICFITAMNDFVFYFLNCSGGINVLMKYNISLYEVFYRNICLIINKKMKEINMSINEKKENEMNEANNNEDN
YSDENNIPNENMNIIKENNINDYDDNMNDQLDDNLNNNMYFQGTNNLNNPMDDIIYDNNYNTRNEDNELTCVY
DDPYSENYTHNNENKNDNKSGIHINNNDDNNDNNNNNNNNNDNNNEGYFFSGQGTKETNDLLNNEENKIKESN
SYFLNSNYTTDNNNNNNNNNNMKDEKDVVYNVDDINMEFIYNEKNFFYDNIKSVYTYNDILRNKDISDELYLN
GYNYKDMKNIRNYCSYILCSLKIFCKTNILIYNFFNSGKEISLTYIKFFNVLCNYPLSKLAIFNNNNFYNFFH

HIIYLFYYFIYLEKIYEHNWKKNNKKKKNDIIKKNNQTEQNERETPQGIDHNDDDDNNNNNNNNNNINHCVNN
INILDPSNNSQQDNKQNNNTNEFILVSIRYLLNIIYNLIFYDDSGYFIFHFGYSIFVCLEEIQHITGEIKKEK
DHSYGTLNNKETKKIFLKHYISHDKLFYKTYFDLYYNLRGLYLDLYKYEKKKFNEMDKNKKKEAYKDSIFNIL
NKYNKSVNKRNNNPTQEHININENNRNHDGNKNNDFVEKIKQDLFDNEDIILNLFPLKSKYNPNIKKIIECCK
IDKDKYSLRRSKELNNSITKLNNDIYINNSTRNCILLGNTNYEFVELLEELDDTSTENNINKKLDTYPFLPLH
LLYYYMNLCSNVNYFDNIYDPYNEKQTHAVDRTNESNLEYDDDNNDNNNNEDDDYDDDEEEEEEEDEDVDDTY
DDDNNNYNNYYDEENKYNNENFDYYDKDGQNKKNHAHKPKNNHNKKVLKKRKRYDYQSKEPFIINNDGFFYKG
FEYHNYLCIKNNKLDEVNKPIKEYSNVHTFLEKEKKKKKKNLLTSQVNYNNIYYTNVDPNERYTENIFLISNY
INKYDVENSIKNIGSALYCENNLDFLIKLISKSIIFLNMNIFKLALMNFNKFSGRNLYKILKSRLKTLFFVKS
ILNVLFNFFYNLTHDNNEFITLKTYRNNELFYSLLYLLNKLLSFNDWLGSNNITEYNHHSSIYILRYNIIYIL
RIFYLWFNKTSDNQSFLFRSVLKYTRLIPSYSTSGMLLLLTFLDFKYIFPSYVYSFYISKDLKNICYEGTNSA
INVKEEEEDEEEDQGTKNREDELILEDSELDDLDYINDDDDDDDNNNDDDNNDDDNNNDDNNNDDDNNNDDD
NNNDDDNNNNDDNNNNDDIKLPKGKRKNSKKKEPIKKTTINKRGGRKPKNKVEDIKKEDNEQVNNHEHDTEEM
NKEEETNECDNKKGIRGRRNSTKSKRNEDTENLKEETIQNNKEEVHIDNNLIEEKKVDLKINDNICNTINEEY
TIIGLPNFNKLDIYENNYETFVLYSNILNRNNGTRLFSDEEEEEEEEDEEEVEEIEERKKKKRVGKKEEINEE
VENIKQEKEYYGERKQQFGKPNQDYEYEEKRNAMERELEARTESHILPSQEEKDDLYSERGSVLNIYKNKEYN
SYGYNNIYMDKKNDYQNNMLLRKDKELEKIILYYKLNNPFYLNEKEKSECLYLCCDIDITKQIMNENYCEHFR
VFNLNYSSEDYEEVFFQDYKKRGFYMRKNYISLLGNLSMNFKNIKDEMDTLTNQKEKSNNNGINSMMNNSLED
NVLNKNFLGNHFKGVSFLTSKRILNNNSNNNIRTYGKNNGINISNDYLNNTSHRENKNVSREHLKYEQSILLK
TDERKGDKSLSSNNKNNLSKKIKRIEYITRTCPYSIKQFLQLNDDNCEEVENNNININGSSSNNNNNNNNNN
NNNNNNNNSYKNEGNFNYYFNETKEDDHEDVILNKLRREEYLYDQHISVNNLNEEERSKLFLIRKELKNKEYT
NFEDFCDYINFIIRISKSNNIIARCLSVQCCVIFVNHRIPIFTILYDHIYKIITKLKDFNFLSANHKKDYRSS
SIVMNKINYFSFYSYNSTQLNNVYYTFNEEIYLLKELSNMFSFLLDIIKICFFNNYKNNYNKLIKLLYLPSEK
LLIILYNFINNLKKIFPQELIRFIIKISSTIFHGYAYVFFLFFHKHSIFYNNTNENMQSKKAKDNLEGTKKKR
DINNSDDHLVYDEDDLFYNHDNLFYHKDHFFKEREYVKKVQKQSTKECKEASYYYSKSGSVNSLQYNEKELFS
CPNYMKYIKYFIQYYFELINTKNIVDRNNKEILYKYTDYVDLKTVQYCLINIYEIIKNKIFYFFTFFKVEKNL
INLFFKYIHNYKDHRKKIPCIFYKARGKNIIISMNKEKQNKNNADHNNKHHETNSDSMNHHHHRDNNEEIYNN
VKYYNDEIVFFHVYDFCVRLNKHINIGNLLDFFSFYFDIIEETNNKIIELYEYVYIIDIILNIIITIINYNYN
SLFILFQLVNTEFPPLLMKYIKKEIKRVHKNKAIEIKKINILHQKGKIKENKCDNMSTDNLSQMGSRETKNIT
TDYCNVVNKKVVKFLEDFKKDFLNNMLQSLNNLYDFVISKMDGKLGNHNKVCEDPSNDNINEDNTKEDNTNGN
NTKEDNTNGNNTNEHNTNGNNTNEHNTNEHNTNQNNTNNQGDDHYGDDHYGDDHYGDNNYHDNFHINLNSSLD
ILSKYILNMIKKIKFIKDAVLNLDMCFIPYISFKDFLMFASYNLNELNNLYSKEKMKKHAHIHNNEMGYLYDE
QGPPAKKIRKNDDTDNEMEKNAFDDEEENNIPINEVNKMRNEENESTTFDNSTLIHKNDCNNSDYNIFNIFCN
NKKGYLFFFHNMFNNINLMINNKFLYNKMVDVFFSKNILNKIIKGYESHQHLDNNYLLTIKNINSNNIIPSVN
KKLYDYCSHFFSDIQSKIYLNENDEDIKSFIVFNIAQDNKTKEIDENHLLYNYIKILKDIEENQKLEKKKVQF
SNLKNINTKAKIYKKSDKNNFEDPFRNRKTLSSRAPSKHVDDYEASNVAQNNNSFVEDKADIHNNNNNNNNN
TKVLTNNITNEQKESNYVILNKNKNDNINNTHEENHITTNSLTNEFDNSKIGTTKDLSNGLNNTYQNEDIYNK
NNYDNYDNDDDDNDDNYYNYHDGENEYDNYEDVYNNNNYNYYDEEENFQEGEDQYNNYDHNNSNYMMNANMEK
ANVNANVNANVNANANANIYSYNSENNNIPGKQINNITNNNKNNYNNNNINCKNDNVFLSNDHNMDNHINNDQ
NYTTNAYENNQAYNNNNNNIETNAQNKNNKYEDCTCYQFNLLGWKEFSEDGHKNDLDVCKIVNKPFLLRDPRI
KMKFLKILERHNNIKDIFKKLGVDIK*
>N.PF13_0348
MYDISSEQINIQNKFLKNLDLYCILKFIITNNIEIYNEESYNVLINLVKSNKIITVEESKRIFSNNKKLFKRY
MYKKNKRRPCQINNTHNIDDDNNNNNIDDNNNNNNNILKNYSPSEIHTDCANICNDDKKMDVNQNLYNKEIYTN
DSLFSKDLNNKILFEFFQFPESTYVSEYINNLCKEKLIHEPLNINLSLVKFDKKKKKSKKRKTCKRNNIGEMQ
TSTNKYNSDSGIGVTAKTNTDINKNINRNVNRNITMMGNANTNTNANTNTNANVNTNVNTNTNTNAEINTESF
DREEHNKFYKISSKCFSRVLNEDDVSLPNIYYIKGVSNDNINVHINYNINNKMNNNIFKNENNTLVESFKTDL
LYSIEKNKNDIYAHNCSNSDEDSIYNYFSDNTNIDDHNKMNNVIRERVNNIGKNYYSTEKYNNNNNDNNNNND
NNNNNDNNNNNDNNNNINNNNNKKKNNNNNNNNNNNYNNICNSSSNNKMNDYKHMIFNNNDNTIFKHKNSLSSL
KLSMYNYKIFKKDTTFNSTHNLFSYALTFKHFYDIHNVLNRDPNGLRKIKVILKKDIGTISISPFYINFDSIR
IFDNVCKTFNITDYNYKKQNIYNFMNLCSTNNQSLYILKNIFLKDLYKQQKIYQNDHHNNKKKIYRKRNALRK
KKKKEKENILEILDEEKRINVLYFINRFLYSNKTPLSYLFRNYKTTLLNQQINVNQKNEHTHKCIHSNQVKQE
NEFVLYSDELTIDYFLFLMIFEKKVEQNFSNYLINKINECKKFKTTDNTYTNTDNKTHVSKDVLVHFDDDIFL
KKSFDVSTNISRDVSNKMSVDMSVDVSDDLEKESTKDDMILDNKQIEDSSDNNNNNNNNNNNNNDSSNNNSSNN

NSNCVDRSDNKSYNDKDDSNSLKNKKSGDQLSACTEIYEKKNKLTNEQNCCDNSSSEKNISNMKPNYIIDKDY
NNNEDNTILSKQTYLIESKNIDKDKNNDINNDINNNCSNNNNNNNCDDSNCLKSIENRNNNRDIKNNKIEGES
TDSLYTSSSEDEYNDRHDNIKKIKKRTDIENEIILLKNIRPFPTIYWLINKNICGYISHLEKINIIKNIENFI
NHPGEEFKLLRYYLIYDHLKYIVIRLRHIHKRILIFFYNYFLNSEYFISQHKQNNHLSNIDLYKNNPFSQIFE
ITPIIQSYYEKYPIDFNSICEILRKINTLRIKGIGGISNFLTLKCIHLYFASHLSYPNTVGYIIEEYFKS*
>N.PF13_0349
MEKSFIMIKPDGVQRGLVGTIIKRFEKKGYKLIAIKMLNPTEEILKEHYKELSDQPFFKNLVAYISKGPVVAM
VWEGVDMVKQGRKLIGETNPLTSNTGTIRGDFCLEVSKNVIHGSDSVASANKEINIWFKAEELTQWKHHMKEW
ICS*
>N.PF13_0350
MIDVINIFNKGGLLLWSYNFYEIDDTTIRTIVKHVLIEEKYEEFSKKHNNYHAKWKLINDLDLVILIVYQGIQ
NSTYLENLFSQVKKNFIKILPKNKDVFDYDLPIDFEEHFLQIIENLDEHKNLNNNTKKDDSKKNNKHNTKNNK
SDDETSSDNQNNSNSTVEDDDDENTVKNKKNVKVKKTAREWELSQKVTKRDIRKLDYSKNEDNDNNNNNNKNN
EMYNNNNNNNNNNNNNNDNIYNNNNSKYEGDYDDSSDGAIESKNNILNKLNDSLFKMFSYNSKIEEEDIEKIL
HEIKNKLLSKNVAIGICDTLIDRMKENLIGKKKTLFALNVKKTVSNVLSETIQSILIPKDSVDVLRSAIESKS
MGKLYSIVFLGVNGVGKSTNLAKVCYYLKNKGNLKIMIAACDTFRAGAVEQLRIHANCLNVFLYEKGYGKDAS
AIAKEAILYAKKENYDVILIDTAGRMQDNEPLMRSLGKLILYNNPDLILFVGEALVGNDAIDQLKKFNQALTD
ATCNSTNKRTIDGIILTKFDTVDDKVGTALSMVYLTGKPIVFVGVGQKYTHLKKFNVNMVVKALS*
>N.PF13_0351
MDEQIKGYLNNFKNKVGKTILISSGKNNHKIDINYVKEVLIKSNIKRHIEINNKFVELFKRLNKNINCLSELH
NILCLEKNKHIDKYENLLSKEYFFEKKKKEESYINEKYCFIENIIKSMRTILLKCDEILDIFKELYLFFYLYL
YKFACNINDFVGYLYNHKDVEENKKLKLLSMMNNKSNSNNYKNNDNTCNNNHNNYKNNDNICNNNHNNCNILF
DNPCCFNKNLLCEIFCLNKICEEELAHFYPQKLFLSKFYNNKSINKFLKFILLKEKEEFNFIYLKEVELFFLL
TCVLYYMERDMNLKVHMNEVIMKHINKNITGEKKEKINKNNKYLFDINNTLDEQIFKNYSKTIFHNPYMNKMK
KISYII*
>N.MAL13P1.335
KFKIKKIKIKYICIYLYMHKNIYIFIIIIFWILKRKIEHSKNIYIYICIYVCMYLFISFFIRIKSIGLFVLTY
GDGLPKVSRK*KKKKKKKKKKKNIYIYIYIYIYXXXXXXXXXXXXXXXXXXXXXXXXXXXXXXXXXXXXXXXXX
XXXXXXXXXXXXXXXXXXXXXXXXXYSLMC*AVT*LV**PVFFL*SILTSNFMIGKSQIK*SQTKRISYFLLL
INYVEKYLQIVVPYYY*YSYHLLQIL*I*MYFF*KLRFSYIM*I**L*LEHLP*VLFLARHVRNFIAFLRKE*
LLKEPFIYFSKLLYFHWSSF*PFDGRIL*FQTNPI*QELIWSGYSSHQLCHQYYSFFMLMRV*
>N.MAL13P1.336
EVIKEEENIKKEDGVTKKVGENTEDNDISLANNVSLGKHLLIGSSLKKGISVSVRGRTMNLGMNAKQAGSAWK
KKETENVEEEGEEKEKKIDDESFPDLLESTEKMKSELSKEKDKKKKQLKDGKKVEDNVNVFNSGFDRGGKTTD
GNSTEFNDKKKYNMYDGNKKNDNAFNKKWYNNQQNDMNNNNQNNQNNMNNNNNNNNNGHIFDTNESEEVTGYIL
PGFKGKHMVGFTCFLKRGTQNPVPPPPAPPLTNNQGENNIPTDNRNNENKKLMNTNIKMNQQVDNNNNNNNNN
LGSAMNTPMNNMNKGGPRNNVNNSEHMREHSNHTVNRFAPMGQYANSKNNNMNNVHNVNNVNNVNNVNNVNNV
NNMNNMNNMNNMNNNMNNMNNMNNNMNNMNNMNSMNNMNNMNSMNNMNNMNNMNNNNNNNKFNENNILYKGTNY
NRNVKNLGQNNNEGNTNMKHGHNNNRGQGNNNNNNNNNFNRRNDKNDNRNFRRKDIDLEINWRNTANPTQEEN
NNNMNHNNNYNNNNNNNNNNNNNNNNNNNNTNHGKNFRNFNNLNNMKNNNSSNNKMMGMNHMQQKGMNSSTGG
HKYSFNKNDDQKNNNKMYKNNMGNANNNNNNNAVNTNFNNNNNNGNFHMNNNKSINSMDVKKTRMKDIRNLND
PPKVNNNEA*
>N.MAL13P1.337
MKNDKIKLVSFEGDEFIVDKNTASMSTVIMNILEVMTAEEDTIPLPNIKTPILKKIIEYMEYHINNPADEIPK
PLITSNLQDVVSSWDFDFVNTDKETLYELIEASNYLDIKPLLDLTCGKIASMMKDKTTEEIRAEFDIVNDFTR
EEEKQIREENRWCGDI*
>N.MAL13P1.338
MSAIGMPQHILILFQARPMLEFYKPIKKKKPKEYSGLSDFLNYFEEGEAPPKIKVESFKERKEKKKKEKMAYN
ELILKEKRKEYDPFKNEDLTSDPKKTLFIGRLSYEVSEQKLKKEFESYGKIKTVKIIYDKNLKPRGYAFIEFE
HTKSMNDAYKLADGKKIENRRILVDIERGRTIKNWIPRRLGGGKGPARGSEEKKKIIHNINWTALINKDKYRN
DKKRSDELYKNVPLYNERNNDDDEDDGISSTMRDYKHHRSDDHRRGSKRDRRHRSRRSESSDRHHHKHRRRDR
SRDDRDRNYYDRHGRHDKHDRHDKYDRNDKYDRHDKYDRHDKYDKHDRYDRHDKYDRHNRHDKYDRHDRHDKY
DRHDRHDKYDRHDKHHNSDNPYDNSTKDDIRKENEINDIQHNDYENNREHNEGFVYESITSIEDCDD*
>N.MAL13P1.339

MSEENNIRKGPSFVFHDIKKKRMREIILMCNKKKQKKMLLKSLNIKFIYEEKNKKRVKIHVDNFLPVSINSDM
EIIKKYKEILYDDNYCNIYDINKLKIGKMKKILKDFFYDIFLFCVSKNLTIKEISTFLSIKKYIFYKFIYKCQ
NIINLFLEYKQIMLSHCINRVPYFIKVFSFSSLHILLKYSIRSFFKNYSFYKYIFTPNYKICFLCIHKNSYYS
EIEKEDTSQICFDDVSTCQEINILGSEEYLRGLVTSSVGFIDNYDDKNRGNNFLEIFQKHMNIFDIKKDDVHF
KINKYEQRIDIEKAFSHIKNNILQKYDERRREKKKDNDKLIRRVQNMYDDVENKIMSSLKKILDITNYDEERE
KC*
>N.MAL13P1.340
MYRVIFYVATFVLILNFVLRIPQICSCIPITNDYVSELFDMDLFDNKERTKIKNEIYDMINETHKTYACCKTC
IKIIQLLYQIIKQEKKKEKYIDIIIEDILEINLCNPYLWNKHFYYNIALYTEYFLDNCKYTLNIIKDNIEKYI
YKSYNNQNDLYIYICLPINPICKTAIMEQQKDLNHNINIQTVFFMYSQYLVSSENFIITTKGLPYKIIENKIS
KEIKIKQNSYVIIQSQIKVIHSKVLQDDFSYGKYRLIKISKLEEKIQELFLKMREGYLFVFFFYTKYHHEGVI
LPSNSILDVKIKIHQVKDHIKDIHIEKRKKELINDIDGFLLYS*
>N.PF13_0352
MKGFNISSKKKWSSFSHKENNNNNNNNFHRYYNKNNNKSFGWKNTKKRGSKFDGTYRNDDENDVERSYEDIYDY
IINISYILNEKYEKYLIYEMRDENINKGNKAFHHINLDNTCYYIEDDICILLKVIEEIKNNEIKIFDHRKGSK
IMEKLIYYCFFLLKYNEKNVKNEKISMQCIEAYNNLLRVVSRYFMKLTLSNYGSHVIQSLLCLFPYFNKYEYI
YIDKLIIQNVEYNKMYDYFKNISDLVCDHLFEIIFDRCGTHVLRSFLYSLGGYLYINISNITFRKSKINKNRI
KNKLHNNTNSELKYVDKNFQSNNFYEYIHKIVERISNEIEKPNISLKYKNLLYQYVYYDKNNKNENEKENNNN
NINNNNNCSNNCSNNIMEQNNTKNVIPNIDKTHNNMNNSDNLKYIAESNSQHFICPLLYNTYSVPALVILFEL
LNDKNIECSSLISQIFLINKTKLYYIEKTKEKDDVDNNIVSVHVESPLKQLLDILIVLDNSSIMIEKLLKIKN
EETFYIFNMYILKNVNKLICENIYSNMVLCNYFTNEYISEEMFDLLIKNIHIEQLIKYKTFNILKCLFFLAQW
YKRNCRYLFNELIKNFHLNDEHKKGNSRKFMWICILCMCNYDDLPSLVRDIYINKGDDNCTDINSKEQKDILY
NKNNLGKNINNYNFYNYLKIDINGYYILSYLLSFPKESITNLTNSFKYFCNFIKTVNINEPSSIDVENYQSFM
ENFSRKTEEVIPVDCFTKHEEGMHVDKNVLHNNDNIKTKMNNSKPFYRKNIQLRGNILLYFSCDKNLSILCEK
IVHTFNLVNEKYLRRFITLFQNQYNNIAKHYIGAHTIVTFFKLGDDNIKKNILDSLIQNDINVYNNYIKNFMK
LKEYQKTKTLSTNNKKYLKAKKLFEDILMSKENGQNQNDINHKKEHDKTFIEFERIHKISKHLDSDLNSNDKD
TTVPIYDEQSNDDHDNNNNNNNNNILKEETNDDEANKENDQTYDNVTKSYKSSSNEKEDIVDNDNGFMNIITDY
IKNTKTKSRKRKKEKDQIDNVLKRK*
>N.PF13_0353
MKRSFRSMLRSIFMRSLYFLTSNISSIIIFCVSISFLGYFGKELHNNNKLFNLYSKKHEYEELDTKEKSQEKP
FLNGKNQSFKLYKIIKLTPTVKIFIFSYPDEYEYLGLGICKHIKFNASNIEGKIKGKWNNNDDKEKNLKQISR
SYTPVYIDKKKKHVHFIIRVYYPDDEYIDGGKMSIQLNKLNNNDEIDINGPFGLLEYKGNNELLHLSKSVKIK
KHIVMIAGGTGMTPFFRLINHLLLTKEKELPSDPVYITFIYANRNENEILLKSIFDDYENRFENFKRVYSVDK
CLNTNQMGNFENIGFINEELLRKYVLKYEKLNIEVKNKDTLILLCGPPPMTSSIKSILKDQIHMENIIVF*
>N.MAL13P1.341
MPKSKRNVKISLTKVKKKVNKKEMKDLKLLEIKKMIQIPNVYVYVLDIRTYSNNNLKVAIEHFKPNGKFFIGK
NKLMKLALGINENNEVKPNMSKISKLLIGNRILLITKDGPLSVLKFFNEFQPEEYIKHGNISPQDITLKCGEV
LNVPVSMQKDLQKRKLNFDIVDQKIILKENKVLAEKDKLISLENSKILRMLNMKIAFFDITVLGYWYLDKFVS
LMN*
>N.PF13_0354
MIVYSFSFLIFLYFCLYLNIKESIILGNCKIILSRPFKEEKKKDIYFFFNSGKNLLKRKKSTFDLLPLHSNK
RRRINLFINNDKKKIKSNSNNNNSNNNSNNNNSNNNNNNSSSIKRECGEPFFSFYSYENILTVHNNKILKDQ
NNNYVLLHLCKKIKKKYKKEKNKKREYCNHYKFMYLNSIKRKDIFIKLNKINNKSHFYKDIHKSNKCIKTFIY
SSSHNFHNSNYSSLNAFSFKSLFTKNTYNRSFLNFLKYTTKNNLKEKKNIKDFFSFSIKLGKDKNINLDNLKK
GYYKDINTNNRSNHNKHNNNKYNYNNNNCSSNHFCSNHFCSNHFCINMDMNDKLNNRNNWSNTNNPKDDNIKN
TNCSTNDEGQKKYMSAEEVRNNFINYFHKKNHTIIESSSVVPYNDNTLLFTNAGMNQFKKIFLGNADKNSDLG
KLKRAVDTQKCIRAGGKHNDLDDVGKDVYHHTFFEMLGNWSFGDYFKEESISFAWDLLTNVYKINPDRLYVTY
FGGDENLSTCPADHETKKIWMKYISEDRILPFGLKENFWEMAETGPCGPCSEIHYDRIGNRDASSLVNKDDPS
VLEIWNIVFMQYNKDENKNMNKLPFPCIDTGMGLERITSILQNVDSNYDTDLFQPIFKQIKELFPYLPNYEGK
INEQDVDKIDTAYRVISDHIRCVTVAISDGCLPSNEGRNYVIRRIIRRAIRFGKQVFNIKSNVLWFYKLVDSV
CFILGNTFKDLQNPDKINFIKNTIKQEEMVFNKTLEKGVEQFHKIIKKNTKNNIFSGKDAFDLYTSFGFPIDL
IQIMCEEKSFNLDLQEFNDLFKKHQLVSDTNNFKINKFFDIPVEKSHELKNVHNINPTVDHFKYEWNNNCAND
GDNKDIKFETYVQVIYDGNNFLDNFTLPSNNDEQVLEKEKSIEENKKKYALILKETNFYYENGGQIYDTGIIQ
NDNMKFQVLNVQKINEYVLHIGVFLKGYVSKNDKVQTIVDFDRRKLVACNHTATHMLNFMLRKVLTEKYTNSG

KSSKTHEGNNEKHEMSENDINSNNNYDNNKEEKGFSIFTCEQKGSLVDDEKLRFDFSFIENINIDAITKIENE
INKLIKEELNVTVKTMDLDESKKIKGIRAIFEEDYADKVNVVFINKDVNNILNNMNIDYTYLHSIELCGGTHI
GNTKLIRKFLVTSEESIGKGIYRITAVTNKKADEIEKTFNDLYDKYKHVLQEPNENKLADVQNYKRTLKENKF
LPYIKKYHILQELEQIEKTIVEKTKNMQKELFNKATNIGKDYAVQDKNNILLDIKFFDEIKGNQKVLEKIVQS
YSKNNKNLSYFFIICDENNTYCVLEVKEALKNKNVQADLFMKEIMKSVLGHSGGSKNKAFGSVEKDKGMVIKE
HAEEMLKNINK*
>N.PF13_0355
MKLNTLISFFSFVCLFFRVLSCEGIHEKKKINLKIELIDKDVLLSDDHLKKEKVIMQDGRKIKIKNFERIKEN
VLERAIIVKELDKLKYINTKKEAITLETLDDDNDDDENNEVSSNDENNNDENNSNENNSNENNSNENNSNENN
IDDSQQVDEEQHNMKVISSEESVENDIHMLRKVYQRDSENGSTTYCYMRYTFNFNLEKLNENSRSQLFKGLDK
TLDYLSFLPKNEDVTDTESILDNEYKMLDNTLGDTDPIVSNVELKNYNFDIISTSEYADTIDEIINFLNTRKD
QLNISTSLKYKLMEAKNNLNKYNNKDMLVNNPRDSNLYLYEWLQHLNKAFLEKKKALELLNDTKDIKENYNDK
LFANSVEQLFYCYNLFDVSNDSVDSFFLNNMGNKKESEEVNINIHKPLSRELLSKNIDDNADDELTKCKSFLS
LITPNELFLNDDLGISHNDMEKADIDMNQEEDEDEEYENNKNNDMLLNNLNVQNRDKLVKSMGEEDGVEDSKN
YEMNNNEMDSLDVSSLRKNKSYLENKEAFSQLTQEIKNDEMTEDYDYSYDIDSEEENKEGTYEDSDEDSEEEN
KEGTYEDSDEESDEESDEESDEESDEESDEESDEESDEESDEESDEESDEESDDEIDEESDEECDEESDDEID
EESDEESDEESDEESDEESDEEFDEENDYDNDDDNDDDSDEEFDEEDDYDNDEENDYYNDEDDEESDEELDEE
VYDNDYENLHYKGKDNYSNYSLKSALSDLAKSGTNHLIDKATNSISSALKKNFNKKKITDKSNKLFDKISKGL
NNSKKIINKTKKDIKKGINKGAKLIKDTKKDVKKHVKNTKEKGKNLTRKAKKVVKKGNDIVKENAQIGLNKVK
KGTKNGINKVKKGAKNGINKVKKGTKSGINKVKEGAKNGINKVKKGTKNGINKVKEGTKDGINKVKEGTKNGI
NKVKEGTKNGINKVKDGAENGINIVKEGVNNGKNKIKEGSKDGINKLKNEVDKGVNKVKEYAQNGVNQVKNGT
INALEDAIKEISKPTGNLRFKEKESINKVNTSPNKVTNKKGKTPLNNTKSDEKKKSNGRKMSLISLTEVSNKK
SLNKNKIKKEINKINKEYKKLKKMENKMKKELNNPIPSDKKIISEFDDFEKSNEVKKNKNI*
>N.MAL13P1.342
MRKYLCKVSFNVIILIILFCHIIIARNNKTIKWIDVEKDGKIFNHIVRYFIKDDKILKIYTINNNIIMENIDD
DYYMNNKESLLKNNDKNDDNFFNNIVNNKVPLSFLIEMFYGYGKKEILSYSGMKTFKLMCNKICNLEERNVVN
KCPYYLFRNLIYNFIPENAFIKKDGNNERVNNNNMYIKNKKEIPFKCSSYIFFLDNILSKNQKKKEECIYRMN
HVNISYTYVEYEIKDTFENIFFDVIKYISTIMEANRNIMNLQNYDNKEYKMFIELNNIILEKESKLKHVYFYN
TLEEYIQSLDGINKIFAEDIIKEIMDIYSNKNNDNVDNGKSFFRTNKFIINRRSNDINYNNNNNNNNNNDIFM
LYNDNISNENSIFPNVQFVSNINDYEKRGANKNEHDILKVTMESNMNDIKNDEEIDMLVLLNKYFFDFYINVR
RMNEEYNEYFDVTYKYGEIKNVQNKNKDNQLLRKKENEEKKRKRVYTSFIKLKEKYMNEKINNGSNITNEYIS
GETINDTYVNMKHSNGELLNPQNSTDKIILNSMNVLKNITDKIKKDNNFSHVNKINEIDIPTVAEEERKIDDF
YEYEKTTMTKIHENKENIYRMINEDLDILLKPAQDEYIKVAKNIKNKLLQEYEQILKEQISDDEI*
>N.MAL13P1.342a
MLKKDLFLFFLFFCFFLQQKNILCSYKECSLLTDDIRILSYCKNESKCFIKNFNNSEYSSITCICKKYLNESF
FAGPDCSIRVPYHYKTMKNNEVHNTSWIEDLLKLNTWKNEENRVGKLCINPQCS*
>N.MAL13P1.343
MAGIPSSLRELFYSFSDGNGMNNETLADTSEQVYISPLALLKILKHGRAGVPMEVMGLMLGEIVDEYTIRIVD
VFAMPQSGNSVSVEAVDPVYQTNMLEELKKTGRHEMVVGWYHSHPGFGCWLSGTDVNTQKSFEQLNPRTIGVV
VDPIQSVKGKVVIDCFRLINPHILMLGQEPRQTTSNIGYLTKPTLTALVHGLNRNYYSIVINYRKNELEKNML
LNLHKDMWTNPLKLNDFHEQKKSSDETLEDIKKLTTLYNKNLRNEMKKTSEEILLENIGKIDAKKRIQNSVET
LLNESILTCIGTMANTLFF*
>N.MAL13P1.344
MKKKNKEDLYKENKLEASKLRIAIVSSDKCKPKKCHLECKKNCPIVKTGKFCIEVDHASKIAYISETLCIGCG
ICVKKCPFTSISIINLPKDINKDVVHRYGPNTFKLHRLPIPKLGQILGLVGTNGIGKSTALKILSSKLKPNLG
KFNNPPEWRDILSFFRGNELQIFFTKLLEEKLSPIIKPQNVDLIPKQIKGNILEIINKKDKFNQKDKYIAELD
LEHLLDRNVEDLSGGELQRFALLMSIIGQSTNVYMFDEPSSYLDIKQRISMAKIIHKLVKHDNYIIVVEHDLS
ILDYLSDYVCCLWGKAGAYGVVTCPFSVREGINIFLDGFVPTDNLRIREESLNFKLATDQDATDEDKKRLHFY
NYPTMVKTLNSFSLTIDKGHFSESEIFVLLGQNGSGKSTFIRLFAGLIKPDNLESLSFLESLSVSYKPQQIQA
KFTGTVRQLLMSKLKGLYNDPYFNNEIIKPLKIESILDNQVLTLSGGELQKVAIIVTLAKNTNIYLIDEPSAY
LDSEQRIIVSKIIKRFILNTNKTAFIVEHDFIMATYLADHVIVFDGQAGVNTVANTPQTLAAGMNKFLKIIDV
TFRRDPTNYRPRINKYDSVKDKEQKLNGTYFIIDE*
>N.MAL13P1.345

MEVEWASKILSQLLIKEKCLKEKDVYALMGKFGDVNKIIHHMNEKLEPLGFIVNNELIKGEEYIILNSERMRN
PSNKDSTRENEPFVNVKEEYLFNSKLKKNEISLYYLIIEYIISNNKNLKIDIGDTTYKSLCVQLNIKNAHTQK
NILDKFIFYNWIEKKSGELTLGIRFFTDLIKFFPLNNLDKCSLCNNAIIVENKRCSHCLSVYHPHCFNHLAEK
LSLCFVCKNAL*
>N.PF13_0356
MKIKNKKKEILEKKKKENSKFLRYVKNNQKYEKFQKAILLNNKNQRLNKKIHPNIKIKNNNNNNNNINENSIN
LKKSDEYVIEQSTFYKAYNYIFDRGEKEEPLNEQQENLFYDSYLIYCNFDLSSIYNIGKHYNFSIELRHSYYS
SVLILVNDNSEKWKKLAIENKLRKIRKIMTYDKFEAVYNKDDLINEITSKFDLYIFDASIRSKKYSHVISKIK
KHNKNFTTLQLNEDNFVEDVDKATRKAFADLNKGSTHSIPIGFLNLGKEKLYDNIQQATKRMLEFYEQKNVSV
VSINLRYMSMTIPLYIHALKNIIHPGYY*
>N.PF13_0357
MAHTFLKRCGCKEYDLFLKNKGKACCYFNFYKRESVHNNVVSNINNETASNENDKNDFSKNINRAFDNTCVNS
YNNEKRKDLILHKSFSLLQNCNVKETIDILNIYIKLKNENIEFLKNICLNLLMNKEKIRHMEVLILLKKFSVI
KCKDYYLFCYFKQILMDNMHVLNFEELVDIYFCYTNLNYFDYNFFFLIEKRIFHNFHKIDIKRLIMLIQCFNK
KRIISRNYMTILLYSISKNMNNMNIFQLCVLFSFFKKFHINNTILISTLIHRFNYQVTINEAPKYISMFHNFL
SYVKRKCVENYKVIEENKELLHMIQNGNEIFINKKNVGYENGQQILNNKLKEENNTMDNVHDDSPTNLVLFSK
ELKEIENNKYVVQCDELDKIREKTNDIKYNRYNMNILYIIQNSVKNIEMITRKHIKNMNVMSLCLLSCSISHI
NKNKYMLEQIAESLGKNSTKLCPSLVSSFLLSFSKCGHKHGPLIYYSLDFFYKYYNFFDMNNIGLFCKALHLF
CIKENEFIDKLNNYMINNKNTNNNISNIIYDNMSDENHDVVSINNYIKNDDHVEKSIGSDGINNLCYYIMKKL
DNDMSKLYYEDSNDYCHKDLLNNEKEKNVDKKKIINNYNKIKDHKYVNFSYIDYENIKRKNYYKNNLYNINNN
IGSKYNIYINNIVYILEYYAFLLVNNINILKLLCNIFINSELNYILYSRIFYAFYILQFNDNIIYKLIHKFIL
HQPLYEVMYKEKHIIKIIEALKYYDDNKKEDKNSDIYFYVLPKKTFSFIFNFSKYFLTYIFLRKNENFLKSYL
YPIKTLPFYNDESIYIHVSCNLFNKRL*
>N.PF13_0358
MEKSLDLSAFNKSDRDKILKKINKAEYEDTMNTYNSIVERCFNECITSFRSKELDNNENNCILNCVKKFSIFS
QRIGMKFTQNLNNEMQKKT*
>N.PF13_0359
MDITNKNEEEKKKNKNKILVCGLISGILTKTLFAPFDRIKLFYQIQPMFNQFKKEEKTNLEKEKHNYHNKKVA
ILNHNIILKNTTNVENKLKSNNQQSLCLTKNDDKTKLRFHLVKRQNVTFLNFKKKINSLKGINKSKNQINKLN
KINHPHNNNNNNYSYNYYRMIQNQNIKHDFFSTYNKQVGTKNINKYIPSYYNIQSSPHKMCKKILLNYYPNVN
HIHNNIKHMKHMEHIKHIKLVNKNYKNLYRNRNNNITYNFGKQIKYIQRSYIKNPEASIKYRNIIQSFFFIIK
EEGVLGLWKGNFVNTIRGGIVYSAKFGTNDIIKDKLKKAKIKQKNVYNNNNNNNNMDIQNSCQNNSNISTNNT
FNYYESITAGYISGIIQKTLSYPLDLLSIRMALGVNEKYLTNNNPILYKKKSIMEMIYEIIQKEGFSGFYKGY
IPTLLTGVPYVTLQMLFFDLYKNIFQTYSSNHYNNIPTLALYSSLAGSLSNLTSLIIVFPGDTVRKRMMNNGI
DNKNYIYKNTLHCIKNIYYREGIRNFYHGLFPSMLKCIPSGAIQFMSYEILKHLISKN*
>N.MAL13P1.346
MYPLYYEKKIVKKLIEQDSLIILADGFNELNILAIFIFYYQNRCLWDEQYINNDNIFFDLFNLNKYNKTYDNG
HIKVVQDDGQEKNIQNESTTKYTLNKNKIQNDNEVEYYIDDSDNSDESKKYDKSKKYDKSKKYDKSKKYDKFD
VTTCRKNIIDNLFDNYTYKESNNELPNNNNEIILLDHPEEKEKTEIKENDIDINKKNDNCNNMCNINKSQNKH
LKDMCTQNKLIFILNVTPKEYNIFLKYQLSLYEQINEIDERYNDKIKINYFKTEYIRDQKSHERIEMYIKRGV
YFISSNVLLIDLLTFKIIPEIIDGIFICKNHRLIYNMKEIFIIELYRKRNKFGFIKGISNNKKLINHQHIVNL
AQKLFMKRIYCYPRFHKHIHISLNNKFLQPHIYEINLDIPNVLLKIEENILNILHYLNLEIKKNYNFHDFDLN
PLLYSENAEAYVLNYIKTKTLNYNTKKLLKEIITLVNMLCNLFIYDSLIFYNYLNNLKEADKECIWLYCNEAN
EIFYLSRERNNQFLNQMDILVNQNDINNTQNIINVIQYDNNYFHKSYQVKKESNQLYNWVYELVYNRDIEKNQ
YEKIIEQKERIKNKNLMKKKFILNKKKHNESNKKPLNHNKHTEDRTSIILNNNINIKHSKNGNSNSNTTENNN
KRRKIIKLQKFNDNNSNNENMYNLIKKEKDHIIQIKSSDTEDNSVNNKNGIIKSDEHIVINDHKECKYQMDKI
NLDQRNDNNIMHMYKNQTKINKSMDKIEYPIVIIVDNFYMQKEFYNLLLHTNDDKVKNTDSITNPNNNHNNNN
SGDNNFECDKHLNILIKDEKENKITDNYITDDSVSSQESFNNNNDNNVNMSNNANLFNLQNNPFNIPYDMIKN
KTRNLKYIKPHIYILCINKNYENIYTKDNFVQTLHQKIDAINKKHDIEPNREDEIKLNEQSEVDGKSKKNAKK
KKKEKKKDHHNKQQTNDEQNILKNDDHLGDDNFFQINEYCGNLDFLEIFLMTIKPYRIILTTLDLNIFRNVEI
YCARLFQYSIYQLHEEKYFKYIMKYEKDNFIYVKNNENNINKTDDNKNDDNKNDKNDDNKNNNNSNNNNNNDY
NDNNNKKKNKCYIHNNNHSSSSINTNSTYYNKDHQNNLIKTEMKDDNNTFQGKKKKKKKNESIYEPIDVFNKL
KIWNDMCNNVEVFLIFFKNNIHYNKYLNKVKVEKSNWINFLENRNNLRFELDRNVFNKNEELFKNVINSYLKF
QQRFKDNKKNITNFNQSLCDEFKSFQDVKIDEYAVENNNVLLDNHYNNFISMEEEEMFIKNYQNKMNKNISSF

TFDEKIIQQIQEILHKFSIDSFNLNFILYSIFNNTKPIVIVDIRELKSDLTYKLYKSKMHIIPYSLLVGDYIL
TKDICVERKSIIDLIQSLNNNRLYNQINQMSKYYQTYVLLIEFNNKNLFYFASLNDKYSVYTKLIMICIQFPK
LKILWSPFSLFTVKLFWSLKVNASQPDIFKSLHIDMTLQKNVREEYIISKNKVKKAKEVDDQNNSTGTKQIKD
KEIQEDNNRKNDELVVQTNNHLEVQTNDHLEVQTNDHLEVEKNDHLEVQTNDHLEVQKNDHVEVQKNDHVEVQ
KNDEENVENKQETYKYETIYDLFDKNLYHLQNIENDQEIKKVENVTNWNAIEILKSLPGVNEKNMHYLINNIK
SLRHLCEQTLEQLENYMSKSNAKLLYDFLNENIS*
>N.MAL13P1.347
MALNPTLIQGNEFLFPANKGSEVTYLRREDVKLKLYLPDRTIKEDGMIFLTSSRLVFVKNEHSKTNPNFAGVE
FPLSLIEKPKFEQPVFGLNYLSGIIKPLIDHPNSLKSACKWNLVFLNGQCSSFLNIFFKVFEAAKKNRPLVGL
NEFNEQFFSNSNAYVDPNDPTFLYINEPINENIYTPQNLSQNHYIPLGSYPNDNMNPSNNNINTCNNSGNTLR
QEENIPIYKRPFVPKNNNPTNMMTYNNSNYVNTCDHINNRAMGNYNQNVFVPNNYNNPNNNTQNYDSINNSLT
HYNNSSEGRTLYNNNNTNMGHYNNTNNFRNYDQGNNRNVTAQMNYHMYNNMSNNNIPNNNMPNNNVPNNNMPN
NNVPNNNMPNNNVSTLHHRMNALHPQPNNHHYNPAYNEHLNNSYNQQNNNTLYNEQNNAEQNSSNTYNYNKNS
ETYNKNN*
>N.MAL13P1.348
MGFSENPKFCLLINKLIKKSKVIGVFLSIIGVIFFLVFNKFNKNAELDARTFTQFVGNSVLNKKNEKFYNDTN
SYFMNYTYEGKEDIIKLIYDYIRKNILVNVENEMVKIKLTDRIEQNILISNVGCKYCNNMESLVVVINFDFKE
RKYFHSVIIGLTLMEHFSKCNYMSKDVTFLFTNKELLYSLGVQEFIQKYFYNNTNRIGKKIIRSSTIIEFDSI
YPSYIKINYEGLNGMLPNQDLILLLTNELHFYSIPIKMELTHGSIFDMALEKNYENGHIYFLRENIPAFTATG
GSKVPIRNKMINLFNLTKALQSYLRSQSNTHEGFCHSSNFYFFNTFRRHIPISIYCYSVYLICAYSIMKLFKS
TIFRSYINFLTGFYTYLITILIISLPIYLISTNKKFYELLNFEENYIPSCYEWHPDNFDKYIKIANIWWNVLF
FSIFGAFFFNLFISFLVNKKRKVIPKKNDQNESFDGYKKVEKVERILILEKIKELQNEIMKRKGITNNHNNIK
NYNIYTNENIYNNNINNINNNNNIYENLYDNGEVKKNILVKPKIINSDDEDFLLEKKNSEFIKKIEKQIEILE
EKLEFLSNDENVKYIFYNNSIAPFNTMMIYMNIFYFILVALLSSLYNWSYSVLFSLLFVIPISILHNLKTKPV
RIFKKIILSLFILCMFIYMYPNDNHLWNIRQKLTNLFRNNISKCCKYLDKHKILQSKYFPESLQFICSNRLFD
SFYSNKYFLDNLNIKFSYVLDIQNGFLLTLYNLARNHFCIGTATYPLICFTLFPIVFYIVFLFFC*
>N.PF13_0360
MVKRNNMNDEEKVLYKFIENKILLKEFCDNYVSIKKNEEALKEEYNKVKLENLQLENEKKKYVEIIKNIDLKK
EKEIDSSMFEYDTKIKKLEKQIEFLNDSLNTEKENYNHKVKEYMCLQVEYEKCKEYLNRDKNKNVIDIIHNNN
NNNQNDMNQNNVNQNNVNQNNINQNNINQNNVNQNNINILNKIYININEKIKDLLQQIKEIKTCRYYIEQNMK
GYQSLLNDYLKKITDLNLKYDECYKLYKQTQYKNLIYKWQKKLFICEIELLKNNNQILTNQIILFKKNIKKVD
EEKKEYYTLLKKAEKENFIFLHKLKKRNYTKMLQDLENTNTNANTNTNTNANTNTNTNTNTNTNKKTNTNANT
NTSTTFSCSSKDQLVQKTKEHILQNKIHNDIKYEQHITYKKDMIHDKQLINDNNIIHNNNNNIKHDEHTKLEQ
GISPHSKESSKDKTTCIINTTSKENVSDIKQNITNKQIINVNNINSKMLFVSNKVLLEKETKIRKGMLFFSDN
DTDKRKRKKIYIHYSL*
>N.MAL13P1.349
MEKESNEEKKKETQKNVFYNFNNVIVDQNYDVNKRNLNFVNSKANGGDTGCLKTNSNIFNKSNKMKKKKKNNN
NINSDNNNDNNNDNDNNCNSNSNINYYNYKMRNNQNMNTSLSNNRRKNYYNMSNMGNSQNKYEIKNKRINKNK
TKFGSNVNSNVTQSMNTYEGIINNENVVTLNKDDYNSMRIPNKNNKKNINNNMTTSSIGDVNRYNNNNNNNNN
NNNNCNNKDEGINYDSLNKNKKNNINYESHVKYYQNYNIKQTTNNYVKPINKLNLTYTNKLYKNVNNKNVMKN
NISTEMNNPNFNDINKKNKNYFQNSNNLNKINPINITSNINMKKNVNMSISNMSNSNMSNSNLSNFNVSNSNV
SNSNVSNFNVSNFNVSNFNVSNTHEPNFNMTYSHVPNINQPLIKDISHIKDLCKNNIVHKKVNSKNNRTKNYM
KRLQNVPLYGKDNDCKRRKGLYNLNNKNSNCSVSSYLDQCVKENSSSSKEKNINNDKKSNMKYDNKRMECIGN
NTCEGYFHSGVYDKEQHYLRVIERKIKNICDILEYILYVDDKDCMDLLYFLIISLERIKKILGCLKNPYMNYD
IFSIVLNLYSCLCVKLFSENNILVIMNLKCFVLMFEILNLMDMQSFNLSYVLFLYVNYLKNNKNGIDILEKKN
NIKYKKQVVENDKKMYKKLLNSFLNIFSYCSENTNVLVRVQAIKSLYYVLRFLYDKKKYNYNGEDTIKKEDIN
TQCNTYDENLKQGTIFLFEENLIKESLSIKKVSIIKGFVNSRMIYLQKFNSNFFENNFDLIDMKNKNNINTTN
NVNNMDNMNNINNINTTNNVNNINNVNNLNNINNSNITSNTNNKKKYDESENSLHNILLLKNFETVFNCVPND
GWITNKIDDEDENMLFYILHIFELFVEIYEDKDINEIIIKNIVFFLNHTDLKIFSIVTRIIIKISQINPINRI
FFNKYISVLMKNFDKDRRRNIFLMLSYCKYEKSGIEVLLGILTNLCLYDFNYKTNTYYLEEDTEENDLNIEQI
RTLRRLKNRRYKKTLINSDNIKNTNVQNDIETYSNTSEDEIFLIDKKIYNIEEWYYIYPIIIILSLNYSKYIQ
SFFFNKFNKTYIQNYYNITPYFNIFDWSNKFCYYLSKHIFTSHMEKSSFFIEQGLDIFHLLDSTLFHNELQNY
RNSHKLFFLVHKSTYNGIDNGRQVECAKNFMKTEKSSNDISLDLLFYRTYAFLEYPNYVKLNLSYKENCYIYI
YMLSLLNFFFNKEMNIFEENCFFLKRKIWELLHDQTNLKSNSFLDYLGIFKDINFIRIHDTKYLTKLFDFINS

NNITHLNRKDQSGLNRNCKNNEILFSNIKYDDDLLYSDNYKKESEHNRMSCVEELLLSFIINKENTKKLKNVL
HYDYQDKGNDSINNKSVKNHMNILEKKKIRKLIYFEESYMKYNENICDIINHNIISKIYYILYLKNFCFDEKG
MKIINADTFFSNYLCKDLLIELILVPMLYRSNTTIELFYTYPIKDMITKSYFSNMFTKYFGRTSANTCSVGKI
NNCTIINNEKELFNDTIHCVYDPSGVTSQNDNIDMNQIYRIQNEENKSSITSIIDFNANSREENGVLRNSPND
RALYKNKVRDIKILNNQKNNVYGLLSSAEENIKYVKQDRERNRKSNFPTCLEKDSYLKFLEVKIKIKNSNMGD
VETMNIINNMKEKKKKNKNKKYEKGFIEYEENMQNKFDIYFYHKRLININHQINKNLIKIYHLFYRNYYCNLN
MSEILHIILNKVYEENDVLYNQLKHFVLNFSFDYHGNDTKMSSKSFYDICYNNKLEKINKYLSQNRNGYEYSN
LIFELHKIGINKDDHKKKSVYSINVSINKILKLIIKYLQNEDYINYEKCINFPYLFLSILHIKIKLSYELILY
DLFDNEDNDFNTLLKKLPMYINYFFHNLYVYIEYEDMFIIKEEETKKEKEKKKKKKHNYNYHEYDISYNRKKH
LCKKRNMLLPSQNNNNLLMYNFMVENILSEDYSLTHLKSLNKTKKTETKNSLFAGRKKSKGTSQNIKVDCEFH
LDEKYLKENIIHKNKKEITNQTNKDMQHFINKSHSMKIYTNEWNITSCMPLCRSQDNNSNTYRYIIKMECKKN
VGNNFDFSSTLPIKCNINLFYYYIENDIFYFIPLSKRKNVYLHPLRQ*
>N.MAL13P1.350
MKCPSFSCKSKYYHHTSKYNYLSDDDDLNLFLKDTQKKKNFNYLLEKAVNNIIYETKKNCDISSDVEQIEKLN
YENVEESENESLDDIDIILKKSKSENMFLKNKNNQNIYLPPKNIKHRIYSTNQEENQNVEKKKIKNKIIASQN
DKIIESQNDKIIENFKNVLCNDINQYFKDIFKNLRNNNLSTNTPLNENIEIQNKIIQQNKNFNISNETSEEII
SNTFKNNSNNYSSIMHNNVNSYNNSEYNISIKDTYVPKNLQPSNDNKAKSNTYRKNKYTKKNNKFTSIHLNNN
NNSNNKLYTHHMNHKKKKKKKKKNSQEKISNHQHDNIMDQKKNFLENKKNIHNKYFANSYIDNNTVKSFQLIS
NVQIDGISKDLINSMDNLNNWESSKDNCNETPRDDFTFNHFQKNKNNNILNKLRF*
>N.MAL13P1.351
MNEEEKKKKSYDEILLNLCLTKDDKIEAVLNKLVPLLMDEIFLCEVSLRNKLVEIISNCILRCKSIEGIKLPV
SIMIEGYFKNININNSSRILYNSTLMILLDICFINICDDEKIKYQELIIENSDKLCVNKEMCMFLVIKFIESL
EILNNGRNKKIMRDYLYNDYSYDMNYKIMNFVKYINEFLLIPIYCTNFNSLRFVDIHTNKIYKEKFLYKKGYS
IRNHIKMKKNTLYFLNTFINNCNLTYSSYIICSYEKYDEIKDYALSLLKSKRIYINYNDSFFIDLIFSIFLYF
DNFVYKMDYIITLFILFKNGSRLLCSEKKYLDLILMYIFYFLFFHDSDFMNINFQEKFSRFEKLFNDKSQFED
YISKNVYSVIKLEDEKLIKSIFDIIIAILDNITKYEYIEQYNDSIFLFIFMYLRKLNEQYNEENMNNSYINNN
NNENSDSDDINDNVTNNNSSLCMNNEHSNIYTCSSQLSSLIEKIFEINIKYDNINTIVLNKSFILTYIFFDKL
RKYKKCDNDSYILYNNIIKVLDILEKYYMHILKLNMSKLYIVYKKGETINELHNMMEYLILSEISDKKEKKLN
NNNNNNNMKQNIYNNTLYICSMNNWKNILIENIINILDSFIYYSKNNVLISTVLKWCVNIFLADNNSPICCAF
IYYLLLYENSSDVILSNLSKDYLNLHKDIKLSFDEYIYFVSTRLFNMRNKDVIYFLIEKDQYDEENKKEIINS
IENVSETNNNNSNINSINNKYDKYHNIDEFVGYNIINYNVDMLKENIPLNDMKSLIEYSNNNYSHFNLLHLYN
LIKYIKNLKLDKFSHIESIYFTFFIMDLYLLNIIKLENYLDNDNYIITYCIIFNLLIKKIKKFFRNNEKKNKD
NKMEYSCTNISLLDSEVCIIYHNLYILMKARFNLIIQNLLYIKNVNETKNKIKSENGDSNRNGSSYNSLRLLK
YTSKFLTKVYICTNLKNDELLADLLNFLHSTNFQEIHNVDNQLIIKICFNLLYFGTIIKKEKTFDEKYYGILE
KVTKYIIYIYNTYVKENIPFENSLLKYCIKFLYLSFFSERFLFKWNNVMKKLIDSEIRDINIENQCVYSYIVF
IFISIEKYIKCCTNTKDINNKYVCNIGLLKNVLKLLSCFITLKDDKINNMLKENIKDCIMYYDMLPIHKIYGF
YISHCFLKLDHMSVNKLCYDWLDYILLSDNLLEDLSKNNKVELGEKYKCLCIIMFYIIYYNPFLDFINEHAFK
IRDLFIYYIKLKSDIYSEYSFLGLSVLFFSLSIQHHMNINNIYMDHGNMDYLDSLKHDFLLLNEKKNIESKMF
ENKKGFNIINNVVDTNNMEYINLIDRFNMDNFCMSNLIKKKKKDDEKKNMNFAIMENDTSYFINELSCFYEDR
IKENICPDKMSYNNLYNMKNKRVTYDTLQNFVEGILNSFKDCNIDISFYKNFLKNNKDKEFDENFINEIKKFE
TRKDHILKNIYEYITNERNMYLINQYINLSKYCHNNIYLFLFMQYSELLIYKNYKKMKYFFLTKLDNITNMDN
VINPMNNINIIDNNHHVVCEEIFEPIYEKLKKNIDLHMQRDNINNLKVCNNNINFDNYIYNVTDWKKVNKKIH
DIIKYMYKKVFIYDIMIEKNDMIKFKFFENICLKDVSKTMIKMRTDFFKMKNEFEDSIYGINLLIKELGNFYL
INNEIINESILIFLSEFLKKINIKEIFCIIIDYFLCFICRIIDTHMMNLKMCISFLNTFKSVCMKFCSNVLNE
KENNEADTKRVMGFEKYNMLTTHNNNNNNNIYNINNLSNNINKCNTNNKNMMCNNSCCNIKDTDERCICSILL
GWYEKIEGNKKLELFITDCINSCILSSDTSFCELRNIQDLLFFYIKNSEKIEIDYNDLKIEENRKDEVTIEYK
EKEIYFSKMLRYNNLVKQKNAIEKLVSHYKNVLNIEEDIRNILNNILKNGSIMNSKYILSTLCFIIMKLIDKY
ETLSVDIYMNVHLCIISFELLSTYISKNINGNYLIYMNELLYYLIHKVPFYYYTKFVYFHLLKDYFDINLNFK
DIQNVRNYSSSVVLYLLKKHYLLDLHEKHIFRDNKSVENIIMYEQIILLKNVNEKIKDLLQKKEYNLNEFLIY
IKGENVLKDEPSHNNNINNINNIDENYKYICDSFLNICDKLDKVQLLGCNFIFDMKRRKEGLEDDGNYLYIEN
FILKRMKDKIYENKHNINMMKDRDVEVFVTSMINFMNIKIKNILNMEIEKKKLFNEENKLLLKSLESKMLVRS
FILRNVQKNDDNNYYKDIYDILQKRNMFHFYKFFNDYVEEFLHFLDSKFLKDKKGCFMSIYVFIQSFVDIYKE
DNYDELDAENIKNLINLYRKIKKVMDEKEYVQSFDNYYILLSLLKCINLLLLLIYNSTKKNNKINDHNNNNNN
NKMDYSTTSRDSSTCPHYIHAFENNNLSFNMNEIYKTFEELLCIFVKVQEYEIFQHMYIFLFNIPSFLIKNFN

FAKLYECIVQLMDAYFRKKDIHSDEIIIYDNYFLLHFSKLALCVFSYFLIKKDRNKWLHFYNFEYLNKYIKMG
KLNDYTFCLDNTQKENYSNRNNVKDYHYIFLLTYIKRNEYKMEIDKSLSNSKENIVKNEIQKNNDINILNEYQ
EYIYDNNILEVNRDSYLNIQENFFKYLTKLLFILFEYCKENNTKHNISLNMYNDISNSNDNNHMIINSNNNIL
GLIKTFFYFMFYNKIYLFEIGNIDIWERIYNYIYLLIIKCRSRKVFDTLMDVLNILLCIYNSYSYENENDRNI
YTNIKIKELTKFRNNKENKENNEYDYIFYNDDNEDNYMTGTINFLFIAFLKFSEKSKLFVFPDYIMKNLKYAI
LLNYPHFLL*
>N.MAL13P1.352
MEDIEFHFIKNEIIKIGDKLIDSEDIFELCKKISKSKTKQCIDTILECIFYMPVKIGIYATILGIITKNNEKL
KLDVVLKLNHNIEVFIKDKPLQCLLWFRCIISLSCCHIYDFKYCLDLFNKLYELCVELYKEKKYIKGDNILYM
LLSNFFYISKNIYDENKGVLDNLLHNSFDLIEKRQECIDNIDIDNNDITTIEYIYIYINSIIHSDNFNDRLYY
LKNALNKYLENNLKSVATHRFYQKEIFQEIFLKKNSELDDHVWNIESITTNILERNKLSDQNNEDNHDVNNNN
EDPSGGNLKTNLISLPSIIEKCININHFVNYDCVEYISSLQIDFRKKKKKAYKNMHDIWLIQEHVLDILELYK
DSVEFSSKVLGIYVQLQSKLYNNILIECIVNKLLCSFNEKNYYFYISLINRLLLINKNLGTVIIRCIKYVLRK
IGTLDNESFYLFMELCLCMLTFFSYEHKLLKSKENFFKRKYNDKNKILKVQGENMQNNVEEITGGVNESEGQK
NKKGKKRTRDEIGDNDDNDNDDNNDDNNNDDNNDDNNDDNNDDNNNDDNNDDNNNDDNNNDDNNNDDELNEGY
EKQFPFNRNNKKNVVILKKNEQTLNELKMLDSKIPFCIEADVKYNSDEYSDDEYLCEDNNNDNNNNNNNNNDK
DDYTFEESEIDLDENNNDNNDYMFDYETFNEELEKLDLCENLKQFTKLMYCVIKKKHCRKWCRNFYFKSCSLV
YKNELENLIPRCIINYCNNFKCVIDTKHDEFHEYLIFNSMIKYTYDEINNIQDRSLLLKKNENHTKDDAIKKF
NDSMNILIQGFMNFLNGKCFLNNVPLFFANLSQNIMNIISNISNNHLDGNMFNSYVFENDFLKDSISWNSYDL
FILFFKSLILFDSSNVSSLKQAFKNHSIILLNYKDAQNFSSDDDKRVFEMDLINIVYSHFNNSVLLNIVISLL
IENLIVDEFSVMHFIFEKLDDANLDEYYVVRLLYDGINNLIILKEANDIEKNKLRRKQTRNENEEQINELENK
KNELLQKIFLLCNKSIFMLCDKMMKLKKSNNSYMSKELLKESLVFLRTYLSYIDVDQFLQLCHENNFESELLE
LATIFKYASLKSKNKEN*
>N.PF13_0361
MKIILKITLLFSVINNLSICFRIYGGNNKGNLFLHSNKRNNILNRNKYNTNKKCKSILYNKDIENSDISSEEK
EALIREQIKKEKEEYEKIKQENKDFINNNFEYISNNIPINVEKRYAYEKFKYSHELLTYIFFVINLFKHEQFK
NKLYKEYLQKQASKENKNVISSHKNDKEHDISVLNNTENDMVHIQKQRNHNLISNFIHRFGDKFKFFNTPEKI
QREDLANMFKMEQEDKEKFFKKIKIKNEKTNEYYFVIYYCNWKYECMALYNAFHNILNNNYNNINFYEILHKQ
DEEKEKSELDDDEQEIGSNKKGKKKIGDENDQQEEDTDENDLTKKDIEQIKQMYIKDLQSNKVMDMESLLNEE
YNINEENNSERNDKEKGETKGSNKEGTTPTDNTTEETETKTKTFQEIMEEEKKKLDEEFFLHGYSKYEDEEQI
KKEEKLIKESKRIIKEEKLNRTRLNSKKDFDNILNDFYISMDNYKRYYNYSNNDDNNDIINEDTNEDTNEDTN
KDTEIEDQRKRKLTKNNDNHIHATDINISNSNQDINNKNVDHDENNKTKVNINVIFVRISNSTVIGKTKNIKK
KIKKKWIYSHEKEIKFYELILSLMLNENIYYKNIPHMDIFSLSYNKQNLFEFFNSIQTNLKHSFIHKNNVYDI
YNHIHNGALDIYNFNIYDKSTYGNRLHNDDMNVQNDDINLQNDNMNIHNDDMNLQNDNMNIHNDDVVMEELDI
NDEIHDMENYGVIYDNLVDDTIQNDINNNEEYMYNNNMDNTIDTTPINTKNHNKKKKSKYETLFKQKYINVLV
NNIRNKISIEKISSISNVDNYSNYTVDTFKHISLNNIFKKNSTHEHFKIDTNYVYYLNHKIPKDIFPFILQLS
LAFKYEHTHILINAPKKVEFIYRNV*
>N.PF13_0362
MRITIDMINEAEQSRNPVLENTISLRGNKISVIENLSITKDYFECIDLSDNEIIKLNNIPYLKKLKTLILCNN
KIARIDADIFDNLPNLNSLVLTNNKLEKLSDLKSLFKAKNLIRLSLLENSVCKLEHYREYLIYNIPSLRYLDF
EKIKTKDRELANSTMENFNENDDENEESS*
>N.MAL13P1.413
MAEQAAVQPESVPTVGTVPQADVPTEGMDVPFGFFDKNTLKKLMFIFMRDVDNYARNWFTNFMHAQTEDDDQT
DGEGKHAYLLNHKRTWFEQFKASLSEALDGKNSVFLLLFLFFGFVFCLLYHAFLYHSIKSEHKAKKLHLEQEE
NDDDYHHYHHAPHFYPFFDPEYMHDHDHDHEHDHTIKPAHAHELDHGHDHGHDHGHDHGHDHGHDHGHDHGHG
GHVCTCKNKANKKPGEPCDCQKAKLEQEKKDN*
>N.MAL13P1.461
MFLGYNKLYLVIIFIYLQILSLKTYDYNKYSKNIAVNLIPLQTLTKMYKYNSQNEDIIRNNLLTKEFNKYQYL
LYELSHDKLKKNKNIKNNYKKCSNYSNNVFKKFNKLFSSTTKNIFKFCKKLFSFSPQILHSSKDNNTSDLLIY
NLLFFIFFGIPFILILTIIII*
>N.PF13_0115
MNVPLNIKFLFFLFCLHCVSSKDQTSEKVKSQKENDSLQVHKKKSGELNNNKSGILRSTYKRIVGYTQKKLED
LFHVNGEYIKKEEDEKEKVDQNKDMLKSGLMIIDQNKDGEELENLIDPIKNIKDSNNLENYYDDNYNNIKFDV
GNKSNHLKYMKTRNSDSDERFSNYYFIDNNLFLRDNKEQEKCFFKSIIRNKDVDISIKDFEKNKFIDTYSLYE

CGKKIKEMKWICTDNQFKSNNLCAPIRRIQLCIVNIILFSENENEYIYKNDSINNKFKENILKAVKLESNLLV
QKHNNEYNSKLCDDIRWSFLDYGDIIIGRDLIYKNNTDYIKEQFKKIFNNEYNNNELNDELNNELNDEKNIKL
RKEWWEKYKEDIWEEMTKEHNDKFIEKCKYFAKDEPQIVRWIEEWSKQFLDEKNYMLFTLRNTYNEMNIIHEN
NCKQYNKWVQNRKKEWTFLSNEFNKIFPERNVQIHISNIFKEYKENNVDIIFGTLNYEYNNFCKEKPELVSAA
KYNLKAPNAKSPRIYKSKEHEESSVFGCKTKISKVKKKWNCYSNNKVTKPEGVCGPPRRQQLCLGYIFLIRDG
NEEGLKDHINKAANYEAMHLKEKYENAGGDKICNAILGSYADIGDIVRGLDVWRDINTNKLSEKFQKIFMGGG
NSRKKQNDNNERNKWWEKQRNLIWSSMVKHIPKGKTCKRHNNFEKIPQFLRWLKEWGDEFCEEMGTEVKQLEK
ICENKNCSEKKCKNACSSYEKWIKERKNEYNLQSKKFDSDKKLNKKNNLYNKFEDSKAYLRSESKQCSNIEFN
DETFTFPNKYKEACMVCENPSSSKALKPIKTNVFPIEESKKSELSSLTDKSKNTPNSSGGGNYGDRQISKRDD
VHHDGPKEVKSGEKEVPKIDAAVKTENEFTSNRNDIEGKEKSKGDHSSPVHSKDIKNEEPQRVVSENLPKIEE
KMESSDSIPITHIEAEKGQSSNSSDNDPAVVSGRESKDVNLHTSERIKENEEGVIKTDDSSKSIEISKIPSDQ
NNHSDLSQNANEDSNQGNKETINPPSTEKNLKEIHYKTSDSDDHGSKIKSEIEPKELTEESPLTDKKTESAAI
GDKNHESVKSADIFQSEIHNSDNRDRIVSESVVQDSSGSSMSTESIRTDNKDFKTSEDIAPSINGHEKIGSSA
DDRGSEDKSIIDKDSENFENNKSSHSDIKQSDNEGSTDYESLTEESPKGDLESVSPSSIDMDLKPNKSSPVTS
FDHVDSPNISELQSASQNADSYQGEKPS*NILRTDGDVSEKEKISVSPPNVSVTYDEGDKRQGISDDSSIHHE
IDPEKNLHYESFISEGGLEEGDIEKEKGKEDGSLIPISPEKINDFGKRENIVDPSVSERVDDNRSDISNVVSE
EQESSIISSRNGTEGINNSEELKSEEHTSVDVNNRDEHNKQENLVSSSTQQESEREKREKKENADSSHESELS
SISEVGETIRRNDAEASENDKGEDILQSEEQQIVTEKTKLIEPTVNILQPSTPLGEIHKEKSEEIDDKDNTGG
EVTHTDANTFQYRSSESVEVITNKPDEMEMTTKPPSQYIEKEIGEIDSTKNQDNDEQSNSIIPLINKNNEEDG
VSIPSTRNVMESGSFVSRNEQIIEEKDDKHITDDTTINPSENGLKGYGEMPNDSIKSVTITESPLRDVEQMIE
PIDGKGNEKNNIIGEPQESTTEIRKQMDGPISNVNIPEELHPVAEGSKLEEAKERSMDDADKGTITEDITVVE
DPNGIGEHQNLKEVHEQASELNTYNSLDGRTNVEVKERLDENPGSIPNDRITTEHIELDKEKEIHEPNELDAH
NGEQEEMIRNEVSDNRMDEQISRDNETRQLNQDHESDSKDEIIDKREMENLEENPNSSSDSLENPEGKEKGKI
EHTHSSEELDSVRDETYKYKGIENQITETEIESVEQQDTNIPGNSKETEVDNSRADMEEEKDVKIKQIITEES
EEELEISKDTTTSHSEKPNIEEQSVNIVDSKNEINVQIEKNVQNEQNEGDPIILLEEQNKNIAILENQKNEYN
NPSQLSHKERTLLEVDDLEGSMDTDCLTSELNKNKCDSIQIIPEASNTDNKLNKDITENKDDFSEIEKSVGEI
HENGKDLLNKESAESDDVPVQNKIEHDSENAGVIDQYEYRTDYEENEKVNFVGLPGKPEEERSDVTRGSVPGS
VDAFEKKLHEPLDSNEKNKEELNVSKEIEYPVGLDILGIDSESSDSVTIYKKPTDTFVENVHVLSRDIKSLFE
NEKTVGLNEQKKETEKNIAGGMIDVLNNKNIDDDQSVRLSTSLYHKGLDDDQSGRLSTALYDKGLDDDQHYLH
DNSYNNNINNIKNEVQCKENCGRNENVSSFVDTTTLRDVKQNKEHTEETKLIENTKIDNTEVDDGVDITSDNR
SSFISVDNYDEKKKETIINEKKNDPKPSRKEEKDVKHDDSENDNNNLSSNIKIKERIENIYNYIKKLFNLTDI
NNYTEKKDMPRYNLNNFINTKSYTLFENSKESEYHGNELLQSENIPEHHNKSNNIKVNISYFNGKWILMLLFF
FIFLIIFIIKRYKSSEKDTLANNDVDFMIKKNNDNYRSDKEQLEKNNSMFYS*
>N.tmp3
MKKKENTQKLLNFVYYKIYLTFIVGLLYIFLLNILINHGGSKNDVRFTNIRCVRIFSENIKNINEISKKIYLY
NIKNEKDDIICRDSLDNINEINNKINYTSVKGEDIIILKEGKYKNGCSDINFLDRKDVNKNDDQSFRNYHKTN
NNKDENVNMKSYVYNWELGQKSLIKMLDYADNFYFNGVKYSDWKLTSMRRFNLNNNVLKDHKTYKSIINSKKG
NDMKKVKLFIKKIPIDIWVEQFNLMKKYEGEYLIDKENYVMEAVSLAFLNEYYPGITPKFYKILYESDKNNMN
EKNCKKYKFQDLNELNDILTKKLENNINGNIVLISEFFGENVFDYIKRKKNTLFVVSDISNEDKKKILYNSLN
LLMRLHNAGLTHLDLSPDNMLISPKNYEMRLCDLSQSTPIYTNKLRHKEKLNSIKPFESFEPCIGKIEYIPPE
CWKIVWKYKMNNIKNPIEYLKNISNQEERKKYYYDVSCADKYMLGIFFIWMWNNGFIWKCSDPIQDKIFEIFM
KSNMDLNKFIMTKSWPHELNNLINKLLHMEHRKTVKLSDLSRHPWWSSKN*
>N.tmp4
MYFLYIHLFLFVLLPNIYLSSSHHIPPSKYGHNKYSRSYVPISSTPIKPLPEYYTFEFIRDQLKHIKEIKDEI
IRNKLKNHALMNKIDIQDLMEKEKKKKSNVLKGTLEKYNKYKLLLDKDEEEKRKKNLLLLENDEENTEYFDEV
EDKEEEKKNKKKKKYKTDDNQIKKDENTFHRRYKDIREMNKRLNDKEKLNQLNKKDSYRKYIKKELSEIKLLK
DQIIQDKIEKRRLKTDKLIDYELTKLLLKEEKRQFELSDEYKEQQKKKKAKMLKLKKRKEKYNRIKLSIKKSI
KKQLRNIKLAIYKMLEKIFDRDMWDKVFYFIYRVTFIEIIFRKLDLWYDKAEDSVMENETGGYAFLFACLIGT
IGLLYYASTIFSIVSAVAYLILLIFLAGFVLGTVFSIYFIITEKSPYAEKKRSKRTAFIKSLYENNEDKKLDN
SE*
>N.MAL13P1.465
MIFFLSKKSSFGFLLCGLFLLLNNNVFQKWSNIRNNKNNGLILTNRCMAESFTKEFSKSILSRINALTELKNY
KSSVLKSDNHVDNNKNDLIKWYVSGLSYLPSVSTVIPGIGGEVSVSAHSNKIPNDNHKYGFKWEDLMKKSEVG
IPSYMVESLKDVGPQVINGIIDKLRSKNNENLDYYPCTLAEFTALGVLFSLLPSLEVELNETSE*

>N.MAL13P1.470
MNLKNLGFKSFFSDDKKNKSTLFSFSFRNIYMLICIISVMYVSFMNVHMNDENNTSDSKSVSLVERNLSEYYI
GRYGALRNYYGPIIGVNKKHYSGKHNKDTSYDALEEDSNDSEEDSENDSEDESEVESENESENESEDQSENDT
ENDTENETEQESEEQSQNESDGESENESEEESDDEPVKKNAPKNKEDTKNEEKKDLRQGKGDIIRKTLNNTNP
NKDKFNDSNNKFKAPLKNLNYNDITRQLNSYEISDVIKGLKENIPNEDLVNVWIQTLGVCKSGGHDMISNLKN
YEKSCLSEYKKIILPHYGGPPREKNPEEYWEKCLYDFEVKLLNASYEYNSKFYELLNKKDKLFKIKKFLFSCL
DEFSNLKKQLYKEYKNELYDNIMRASGKKYSILKIIYIYIYLSKRDSD*
>N.MAL13P1.475
MDTKDFLKRLFLRYSVGIFLGLFYIVLLNICKYDGKARLSKSFMSRCPRSLLGLEDSKEYFADDMNDDMVYES
SVYLTDDTLKHIMYDNYARLSNYVNSEDTHLDDELNEMSYDENVETSKGEYDELYDLDELADNLNEENNMKSK
MDETEIYDVGKLKETFIDYYNDRKKADSKLIYVGKSLRHLLFENYMDVPKNVDKLLIEEESNDNISTKIDEEE
KNRFNDVIILKEEEKPLLRGISNNEVQNDIYEKNKKYKKESEDIELFRQDDFSELLDDILLLQLDEENDSNWK
KILKDYMNDTVQIDVETIFGDDSFRKDDEDKINISYVKEYKPKDDNVKKILDDILSYKNIKDINKLINYFRKY
SGVSNKLIVECVNYLLYHLNTEEANNKSLDVLIEELMQCDENNIFKKNKKLFKYIKNEGIKKEYELDFNIEKN
DKETNVYSNEKDFSINEDISSLKSYNMCVKKCSDLWLNVMKNERGKFNHIITDLYRFYRTLIKKYKVSGNFAF
NEWTEVNSNIRLYMKNAEIYLNTLFNQWINNNRLNIGEFKMLVMINRFLWRKIKKDLYDTNKKNISKPFQDKI
NEENTKKKKVTDKYKKIYQEKNKNI*
>N.MAL13P1.480
MVSFSKNKILSAAVFASVLLLDNNNSEFNNNLFSKNAKGLNSNKRLLHESQAHAGDAHHAHHVADAHHAHHAA
NAHHAANAHHAANAHHAADANHGFHFNLHDNNSHTLHHAKANACFDDSHHDDAHHDGAHHDDAHHDGAHHDDA
HHDGAHHDGAHHDGAHHNATTHHLHH*
>N.tmp5
MKNVYFNRSLKFLCLTVCLILTIYSSRNEIHKNSLGFGKNDNIYSRNLAQLKLSNYSLLKDSKFESVTEQLTR
EELYELFDLLVQVPPRTYLLNIWNHKNGICRQGTKDLLKNLRGIAPKLTYRHTGNPNQYGKSPPKITWQGCYD
CNMMVSTLETEQTNRFYNLLNKKAPIDEIKSFIRSCIDEFDKLHTDLYVKYEKIFSEQK*
>N.MAL13P1.485
MSIILTFLALFVFLICVFPCITEDTHKYVYSEICGKGANENESSVYCMKDHKKKNSLYVYKHIMKFFLDKYKS
IPNEIALVEHSYGEPQNYITYDNFFRKVSSFSHTLNTYEGKGIESKKYNEKQNNGIFKLLGIYGSNSINWLAS
DLSAMMSGVTTLVMHSKFSIDVIVDILNETQLEWLCLDLDLVEGLLNHINELPHLKNLIILDTLSKNKEINLN
KEENNNDKKNKSSGNSTSVKGNDLSNMIEITCSGPLEYDKEKLKKYNELKKKCEKCGKKLMLFDDMTKTQTKK
FTIKNEDPDFVTSIVYTSGTSGKPKGVMLSNKNIYNQLFSLYNHSVRERYSFKNHLSYLPISHVFERTFAYSI
LMYGGTLNVWGKDINYFSKDIYNTKENIMGGVPKVFCRIYTNIMTEIDNLPSPKRRLVRKILSLRKSDHNGPF
SKFLESIFHISKKIKDKVNPNLEIILSGGGKLSPDIAEEFCYLLNIKYCQGYGLTETAGAILGNHADDEHFEY
IGGPIAPNTKYKVRTWETYKATDTLPKGELLIKSDSMFSGYFLEKECTKNAFTDDGYFKTGDIVQINDNGSVT
FLDRSKGLVKLSQGEYIETDMLNNLYSQITFVNFCVVYGDDSMDGPLGIISVDKYLLFISLKNDNMLEKTGVT
EKNYSEKLIDETLNETIYIDYVKGKMMEIYKKTNLNRYNVINDIYITSKQWDTTNYLTPTFKIKRFNVFKDFS
FYIDAVKKKYEEKLSGSSTGSMNNGERCEKKDDIKNGSDNENKRISKSQLNGLNECAIKSEEEDVLEEVQKAL
RKYEEKNITQGVQKQLRRSNGTSMRKAREGSLLNSNERENDINGKRYIKEIVPEEQQVNA*
>N.MAL13P1.490
MLLFPFLFNTFLLSQNVNCQNNLRNKSLINNYTQKILKSRCLAETEKPHNLHYQNDPELKKIIDRLNEEAMKK
YQQSNDTYKQLHELLEKKETKSRSRNSEQEMSKIEKELLKIYEKMSGDENHIMQNLICTPMKITNQMIKMTNL
XXXXXXXXXXXXXXXXXXXXXXXXXXXXXXXXXXXXXXXXXXXXXXXXXXXXXXXXXXXXXXXXXXXXXXXXX
XXXXXXXXXXXXXXXXXXXXXXXXXXXNTKNIYAHNLFLKI*
>N.MAL13P1.495
MKKGRRKIIQIIIFYYKCELYVSNYDNDPDLESVEENFDIQTSQRFEEYDERMKEKQQKCKEQCEKDIQKIIL
KDQIEKSLTEKVEKDYLKCGCGLRGVAVSVGIIGPVAVNEWTKTATAMAIDFAIQECIKEGIQVVIERIKLFD
FISSYLTVDWEMFINGSNYNTASGLIEAANSAMFSKGNSCTSPTGYFWRVCEAISNAKASFSSVAVEGEQAAF
NTTKGIQVTMLSAVEAPSANMYSAIGYSVLAILIILLVMVIIYLVLRYRRKKK*
>N.PFL2575c
MNFINFKIYIFFILFHTFILSNTNIPRTNNNKNNNQKNVTNDTTPLGTIKDIIALDYIRDLLKHIELIKDEII
KDKLKKIKLLKNNKNLIKKIENLEKGLIATRLLCKNYIKNELKEIKIIKDQIVNEKIQQYRGKKEKLIDTELN
KLFLQEKMKKDKKKYEQYMKKQLQNTLLQKGILKTNGIILKILQKINNLFI*
>N.PFL2580w

MKEIMKRYNERTTILKEYDERKKEKEKKYKEKCDKDIKEIILKDKIQKQLTKQLSALEKVTHTDALFKGRN*K
ML**I*ENIRSYLIIMEYFS*Y*YV*MYIFFSTEGKIDGNIKTMKDDLINIGYIGPINFGYLGNKDGKYYIEM
AHLFSSIMSSCNALFQKYIDNDVTFNGSSFKKQVFFSIYALWEILEHIVMPAVDMFFFKSSDNEITSVQGVNN
GDVCKHVTRVHVTHENACADLNYITIFYNSFIAIYDME*
>N.PFL2585c
MKMKHVYFKVLLLFSLPLNILVTLHHVDKKTNDYRPYTLTYTKLENTITSSRLLCECDTYAATNYDNDPEMKK
VMQQFDEHTTQRLLEYNEHMIKKRKECKEQCDKDIRKIILNDKIEKQLKEKFSSALQTDIGANYLPKCTCEKS
LSDKVEKTCLKCGGLLGGGITPTVGFFSTIVTYLLTDAAAKQAGILAAKKVSVEAGIQTFIQQLSDMSNLHVL
IGHKLVNLITPKTVGNIMELSKTIHETLQSTCFAAEPRINDVMTILCSTDSYIFDAQYLQQQVANIATKAAEE
GAKAGALASQNAEANTWSLGTISNFFFTNSAGIAFTTIVLIAIILLITYLILHYRKKKENEEKTTIYKIIKGI
DMFYYVDVYR*
>N.PF14_0016
MKVSNILFFFFAIIAVKLVYPGYVAAGSNNNAGAPTGKKLTDIEKKKRNKNIVLYSSLASALALLVGTGVGQG
LGFRVQGSGFRVQGLGFRV*
>N.PF14_0017
MVENELLYENDVSNSGLDGKPRLHSFFNKDGLLLRTYSWTVKKAIGIFLLIHGLNGHVRLQYLRQNVEVISND
KAILKDQDNYYVYKDSWIEKLNDKGYSVYGIDLQGHGLSEGWENLKANINNFDDLVYDVIQYLEEINRTVCLD
YERNKYLRAIREKNTDMKALKKDKIPIYIMGLSMGGNVVLRTLELLGKSGDYKNLNIKGCISLSGMICLEELS
SKASMKYKYFLVPFSKFISYIFPKCRINQNFNFEMFPFVNDIINFDKHRSKKWITFKFGHQILRSITNLRKDI
QYIPKDIPILFVHSIHDCACYYGGVVTFYDQLDNDKKELYTIYDMDHLLTMEPGNEKVLEKVLDWISGSFNNK
RRRRRTSS*
>N.PF14_0018
MVSYNEYKLCLNNPRLDMKRKNYKYNVHEGIIRKGAKWICSMNKAFHSRFFICICVLLHMMVFGVCIYKVEGS
VHVTEPNSSSSRSLYENKGVTYESNVEVHDNFLNKLYMSSNVENNGIKEDNVFGNTDLGGMESHEKFKEKIRR
SSTLSQGNYSNFKDGFGYEYSELADSMTDEEIDKEVDKLMEYTNNNKEKVYSLWWQIMRNERVKYVMIMKNLY
NFFLKLKKKYNVDNSFSEKQWNICKKRVKKNRLNYEKSINTTFLNWCNSLLLRKDYYRKLIKANRLKWKELSI
KTEKACRHNMLQAFEEKVKAQSTKLDLDYVTEHIRKNKKKKKITKDGDGCYSTGPNGQYNECMKTSDYIDAQD
SDALVEDEVTEEENISDESVKKEEEEEEEEKGVDVAEDEDALKDSTASGDSSTSRYTVLSADSAEYMESDILS
VLDVPDVNGPASARSVYVQAYTSEPKEMLYRPKIRYVTI*
>N.PF14_0019
MYVIKIYHIIIFYVCLHNFLKTSLNIDYEKDVECSLYLINKTNYNKFYIKEKRELGERESSQYLQNLQNLQSL
QSLQSSQNLQSSQNLPNSPNLQNLHISKFSRKRTFDSSLTLDELYELKNIIFFERRILDDLVVDVQNSIDDGK
HIIFLKDFVKNKMRLHIRLYGLNYSFNGINNMGNNLLNQMEEINSNFPLTHESVNIMYISYKTELDISMDLLD
CVLRQRDEQKRNEYLCNMNDIRLTIYVRFNSEGINIKEEIINKMIIRIKARIHHIIIYHENLPSYRYRH*
>N.PF14_0020
MESKICDPIGLDKNKFRKVEEENEENSKIVENGQMTYNDDLEMFTTIQLNQEIDIRKNPFPLHMINQKNDIPL
CAQEFSKLTDPLYIKKICLEKVHDWSRCNEDDVCVNQILSGLTNQLFEVSIKEDTAIEYRITRRHVLFRIYGK
DVDALYNPLSEFEVYKTMSKYRIAPLLLNTFDGGRIEEWLYGDPLSIDDLKNKSILVGIANVLGKFHTLSRKR
HLPEHWDKTPCVFKMMDRWRLAVSNYKNLDKVTLDINKYIQESHKFLKFIKIYTQIENIANDIVFCHNDLQEN
NIMNTNKCLRLIDFEYSGYNFLSADIANFFIETTIDYSYNAYPFFIINKKNYISYESRILFVTTYLSKYLDDS
TAASDQDIIDQFLEAIEVQALGLHLIWAFWSIIRGYQTKSYNEFDFFLYAKERLKMYDEQKQYLMSKNIIKDY
DD*
>N.PF14_0021
MEEKTKNVELITSDFIENEILEKYDIFSKAYSSSDNLSFVDSEEDQSAKNLDDSKKKNILSLTVHNIINIYPN
NERNREFKKYKEKKIEEKRKKQYEEEKEIIEQKKKIYEEKRKIKFEKYKVEKMKKKLIEREKYNKENIEYNYI
TIFNYFNYLMNNRKHYDIYFSFHDLYISPMRLYYFINMKIDEIYYLDMSRKWLSDNYVSIILQLCSCKKVKML
NLSYNKISYKSIKLFCNFFKENKFLFYLNLENNDLTNQGNIGEVINELFDVLKNNKSIKILNVANTNMKEDNA
ESICSMLEMNKMITDINIENNNFTFDQNYRIISYLRRNKKIWIEQAEHEKAENEKMEKEENYMHTYLMSVESS
IIEIENRERRRSLNKMMYINMWKDEIREKKLKEEEIHETLEKEHEKRIKTLQRKKKKKKKK*
>N.PF14_0022
MSSNNNLNEEEEILFFYEDPKSFDEKNLYEVNRAPWFYHLINVLGLTKDNFMEIKHMVEDKKNNILNLINYKS
YTDNYLKKNDTMKNMLCAYLYSCKTSLYEYSKNINSEQNIHFVFTFGNITADLDSVCSSIIYSFFLFIWYSLK
TNLIGKKKDDPLMFFIPVINIKKNDMKLKILIIWWLEKCGIENPEELLIFNDDLNLLEVLKNDTKYDTCLVDF
NKSENNCVYNINNIKSIIDHHILNEEMKNKKITKSVFPIYVCSCMVIIAYFYKYSSEFLGISLLNRDIMWLIY

GTMLKDSNNFPKDDFRKRWIQSDLNIYLSMKKYFRIPDIMDIYITQKFNNIRFSIDLKKFGIENLLFVDYKDY
NYDIQGKKFTIRICSLDFSVESILSHENVDTLVNKMCELCEENKFAAFILMGSYMINYVFHKDIGLLFFNEDI
TKDKLLTALIANQDINLCEKGFKKITCKNESRNIDLFQINNTSYSRKRLECFLSYYFNSL*
>N.PF14_0023
MTNHCCFRCSISCEDTQDEDYLFFDEHFLKDFNEADDKKINEECNNKKNLPNLVNDKKKFLQYIQNEADTKNR
PLKDIYKDIYMNFYLCKKCYGLHNIDIILFYNENSKFHHELMKYVDDIHIIIDEKVKNNVINNMKYIIESYTN
NIDIIFFLLYLYVNNILNGTFVFNIQSYNEYYIKIKKFIILHNLIESYQPYLNHRIHKMFSYFLMYVFMFFCY
NDIFKTLNLDQDKIKLLIILYENASLVFTNENYKNKKNNNNNNKIYYNQHINDQTLDGINFFSASQRAKDSFV
CEHRNGYNKKRKKNNQYQCQCQYQNQCQYQCQCQYQNIYKCDENNIICTHNYDNYHDNHNSCKDEIEGLNQNF
KQMDDREQSLINTKTSENPTQGNEQERQVFNECKTYHMYISFHNLCICGYYNKYNKEISQTKWLINNISNSVL
SIEECISNIFNNIFSFSTATFIGSGREDKDARMMNIGRPFVYVLKDTKFSFLNFFLFFSNLSNKQNGIKNYNT
INVNTIQEYNQFLLQEKKKKKYQHILKNDKQKNIIPSHDNNNCNDHPNDPNKNKNLQYNDIYPLIKEKKENNS
LSIFDMKKNIFMKIFLHVQNSKQISYNNNIEDISKGTIHNTYNLNNFFKLPLSYSFNKELEVLFSKVFSFKYS
SNYIYSLNITNDSETPMLTDIKKEKEKEKEQEQEQEQINKHKDNNNMYNNSYNIEHFAEIKLNNLSFSTNYPL
VKKLMKYGEERKKEYKCIIYHSSKMNKDTIQKINDQISNHEPQTLTYVLKIIQKTPIRVLHRRSLINRERKIF
QIKLIYLHKHFSLLYLLAESGLYIKEFVNGDKGRTIPNLKQFFGDDTYVNILNLDVATLIYD*
>N.PF14_0024
MYSGFIRFSQAKCAGNHIYLGKWHDFNQWKYLAPRNLLNIIYPVEKTNSIHIGKEENDIFTYLNSSYHGNIIT
IDDINTSHSKNNQKIKLRGSAFHGPERTDVFRLTLYNRLKQKQPQRIVPSIDQMKDANKLEVSIVWFLWSFVI
FYCGMCMFGSNFLIKNKSNSFPWMPKRTDGSRGEGPMFWFLE*
>N.PF14_0025
MDNFEEIERGYKEIEDEIIKTIEDLCGGNYLKIKDEVIMPHMSDELINKIKILNKHINDKSNEELFEKRVTNE
KVMQINDKLIYLLCDYYINKKEIDKLIEFTTSNENYFNILPQAKTAKLIRNIVEKISKKIRNISTLYIIFKKY
MNWAYEKKRNFLRCRIEVKIIILYIIKQKYKTALSLIDRLLKEVKKVDDKTLLLELYIVQTKIYMLLKNSTKM
KASLTFAKNIANTINTAIYINSEIDLLSGILFIYEKDYKSAYIYLYECYETLYTYIYNSHNNTMDFLSKKHND
FYSILIHNIINTSTISSQNKTKSISQISPFLLSFYTFYEYYDTNNSILSLDEMNIISNNLNLIYSDVICNISS
SYEELEQDKMYCITNPIELNYELIQKLSIDNYGNLIETKMDTKAKTLNRENENIDLKNDNNENNNNNNNNNNN
NNNNNNSSSSSFCSSFFIFPHNNNAKAVGNFGLNLNIENVKIIIPLKYMLLCKVLEENNRKEINNILCENNKL
NYVSHKEIQILLDISKSYENRSLDIFEKIIKNSLFLINIDKVIYNYLKELYELLLEKNILKIIEAYSCIDLHY
IGQKLNLDINKIISKLSEMILDKKLNGTLDQNVGILILYDDMPETKMYQNVLDIINNLTESVDILYEKAQLTI
*
>N.PF14_0026
MTDYWVSSKKHYCETCNVWISGHKVNIKNHEKSARHIENFKRLINESFKRKEKETQEKEFLEKELRRLDDIEK
KYLLDINKKDENDNKSYDSYVYKHTNDNKNNNNIYNNNNIYNNNNNICNNNIYNNKYILMIHEDSGSLVFFNT
LKKELLYDKPADFYEPLPEYQTFSEQYGWYKYLDNNSNNFYYFNIYNSKSIWEYSSHNIDYLINYLKKCEETG
LASFNQNYMNKSGNLVFHYNNENIKYDNMNKRVTKNDITLLGEFGNNKIKDEEMKLKNVYDKKNKIQLNIPLD
RKNDKSRFNKEKDILESATTKEDDIKNVFNKSDKKYEKENGNVNKLNDEKKEKLENVALKNEDKNKGENLNEK
QKNEMSFISNDETSKPGEWKIVEGNPINNISNENIEEIFYNIKSKEEREKDNLEEIKNNIRYEYSSFNEFYVA
KKELDNEDLFLNKEFEFVDKPIYKKVIDKNANRKVDFAKRNIKAIKNKKKIT*
>N.PF14_0027
MKILINIPYDESLCLESSNINNIKNVKEQIFELKGIPYELQKLYKNGRHLEDEELLEIDESDYAYTLNLNFGL
LGGAKKKKKKVYKKPKKEKHKKKKVKLAVLKFYKVGDDGKVFRLKRQCDNCAPGTLMASHFDRDYCGRCHLTI
MKK*
>N.PF14_0028
MYIQLNRNKKKKKQFEDDKLNFKHMYHSN*HFFYFLFFFFFS*I*KEEETDLLFFF*F*F*F*F*F*FLSS*N
FIGKSLNKSN*NRKFWMNGKA*RKNLS*PNRKKKKSK*IKR*RR*NKMKICYLIFLEKRK*PICI*EIYPPRL
LKNISVKNSESSVK*TA*K*CILVKMKIKRKQG*VVLFVLKILKMRRMQKML*MVLKCAEILFV*GGVRLYQN
LVIIQKMRYLIIIWINIIHITMLM*IRK*L**YPMIKKQNG**IY*RNM*LKKDIVLKRP*KKRRRITLSFIS
FLNLQIYFIIINGGYFLLLRGIVIKTGEQIRSIYFLMVIYMYHQR*KKKIKVFS*NEKGGEIKDVILTRRKRI
NL*IF*VH*IRRG*VYVVL*YFVQDIVITVWTL*KLFLTF*QM*NMIY*KRLISYIYYQTFYIIVVTNFFRRG
HTENI*KMSYLGFFIF*ENILKKLIVK*RESYL*IV**IYSICGIAGPYTIPFL*MVYYAYYVI*RLII*
>N.PF14_0029
MENIKKNEENNNVYFNKNKIDYLEHNDILFFQEKNKNKIGLGRVVNFKIELVLNDIHFYYPCCETSANHCLKI
LNNIDNKEYVKKLINKLNKNNKISTNTKNKEDNNKNDDTKNSRNKENKKEYSILHYFNKRIINNITKSSILLL

KKNNNINYNNVQSKDQDIKNQKNNTLLHKENYSYFKKNSNDNNSNPNFEHHNNHNQKCSHNYNYPYHMYNIKN
TGDTNQTSTDIIQDQRKKERRIFYFIQSLETYKYNIQYKRIFKHYKIPYNKHWENIKTIYCKNMQNKIVFVLA
MKIFQIKKLYPNNIHFQNVMINNLMKIYKSEKDLHVDYLILNYIKKLDVRVILQNCVLISNDEFNEILSKYNK
KDHPTEKSAHEKKNATLLGSKRASKHQLNENASDGITKKDFSQDERDEENKEDDEDKDDEDKDDEDKDDEDKD
DDEDDDEDDDDDEDDYEYSIEHSDEHSDEYSVEHSDEHSDEHSDEHSVEHSDEHSNEHSDEHSDEHSDEQLDD
KLFRNKYKIENVKDIKNEEKKPYDTLQNHSDHLNDSEQMNTLHMKKKKRNNPMYNDQENINKYNNEFLNNNEN
IIESRTKESNFFISKNVVKKRKIIEGQTYKYELKGNILAKFKSPHGIIYRGHIKNIFRNKNNILSYFLILPSF
FFMNNSFYFSCLNIDIDILKIFEIIKILDKSKINQIDSIDFSKYKNLKQVHENDLKINKTQDNIFHEKYSTQD
TCIRKVDSEQINLQAYDKKEEPSNEQNEEQAKKNNNLVRKKRKKDINGEHINGDDINGEHINGDDINGDDING
DDINGDDINEKHMNNILRENTNLFLKNDNNQRGVNNDDHPNNNNNNNNYQNKEEPNLKDKARNKIESHLFFLR
RRLFDVNRNKTDNKKNRTKSIFSISNEKKNTYFSKITNFFKKIEPKKNNLNCIYEKENVNQENIKPVNDKSDH
INLTEQFVDKSEEIKNISNNDNNNNNNNKGDEISDKEQDTSFKSLKKCKNINKEVENKNDENNNKNYKNSKNN
KNNKTEYDNHESFVKTEYNSSDYNDYIISVEREWKRRETFNSVEGTPTPTSINNNNNDNINNNDNINNNDNNN
NNDNININNNNNNRGNVKKEGNNNRYNPKNDDIKYNYINNYPNNENNSYMKQIQNIIEEEKTKKVEILYKYCK
NNINILLYIYVMHIYINKLCKNIIVKILKPNIKYGNKTKYDHKDFILSPLIGYSYSKFYGLKFNYMNIQLRSD
GKIDRRYKLAKEHLKKEQEEKNKKIKKIKKIKKIKKIKNKYNQLNCNIKETDTELNLSNDTLIYDKRLKVKKN
KTIKSKNHIMAKSKRHVVQKSKRHIIKKNTKLNYKQTKKNGFSTQQYHNDSSKENFLSHNYHVNEDTDNHKKN
DAEYMKKSYNQNINFYLWGMFNWDYRKTLRVSLNSISNKKINNFDIYNFFFYNYDNISNLNEINKDLGILIDI
YKSIKNILYQNLSLYGDLLLLNVPNNMTRTHTDEELICFWLKTLNQLYLIRDGFSNYFKNF*
>N.PF14_0029a
MKKLYPTNAVIFYSHKICGHFSMFKHNIIRLYSNIEKILPQEEKKKNKNLSHIPYYVLNNKNQTDNEKYNFEI
LMKNVKNKAEEYNLIKIKEIQNKILIHLNKFNINELVATLILSYKYNLLNQLILNNIIDIIFHNSGFLNSKHV
FILLLITKNIYSKKNDYSYSIQHQQKLPQNDMNKSGEKEEHVINSKWKNKFFHEVIQNMLYDDNINDHKEEIK
EDNSNEYNINNKNDNLYDIPCIPLANEEKIYPHNIIKKKKKNLNKSNNYQTYEKKNSYNNIQVDNKSISNMDV
ISQKLNNKINNNNNDNIYQNVVYNDDYNVDTLNYMQNKQNEQDIFCELNNKQDIFCELNNKQDIILNDKISEL
KKSHEKIREILLHNYNHILFNFTNNIYTNLLLLNYMYDENIISYDEFFNIIYSINNEYNNDTFEQNDLKNYQP
KSSYSIHIINSYIQHESYHIYSIYDIYIYTHIKLLKNIFKQNKYMKNKYISEHIQSNNPIQDIVYTNKKNFIH
TFDKFFQNILTAKQNIDYIKLFYLNICSHEIFKLIIINNEHITNVKIYYHILKKIPLSYIIAQLHIKKGQATN
QFYYQLLNILYYLQEKQKIRKSNEGIFYEDLHALNIEQMNRVYISYLNSLNDYTIHDLLIKNNMHNINTTHNI
NTTNNINTTNNINTTNNINTTNNIKTPHNINTPHNINNVSTFTQTHNNILPPNTSVIKNKPNQINMNYKDIYE
QCNKPNVFFIDRHRYFFKYFIMDLTFNIINYLNYMDIYDKLFIYKYLIILNYKNEYIINIIHDHLYNFFIRKL
HYEDIQHIFYFLQYVLSSLIYSPSIFFFHIINNIDIATFQKLLNIIKKEKIFEDHTKKIYDHINIIINQNYKT
LHKNKCKMNNSFYYYKKRIDHFQSVLYDFLFL*
>N.PF14_0030
MESKNNVLQVESVGVKLQKNENSKQTYTSKLKKD*KEVQQE**RWFIQYLNK*STQ**CQM*K*KT**R*NRY
NTIS*SKKKDKPTSQYIPYRYKKKNAKK*TTK*TKK*TKK*TK*TKK*TKK*TKK*TKKEVS****K*RK*NY
KNKK**LLS*PTCKKRRYLYTMCRTEKRRK**HKYIL****CF*YIIQ*TRISYEYKPIKRYK*S*YI*YTYY
RTLQKNHKTRT*RKKKEKNTRNKSKAH*KNNNEKCLYSSCS**VHKILRKY*TSYRQNCRRCIL*STQKNIRR
T*IRKN*KKY*FL*KYKK*KL*ISSSVSRK**SICQRNEKKN*RQSPT*EECRNYHDEKYGTHRSSKICSLYF
SKKFRYIFR**LYA**CSEKYKLDHRTIHNRTYHLFDECTKRNCSSHNGRYDRTGAFSTIGHL*
>N.PF14_0031a
MLQGRIKKFKVPVDNNIDNFIKDINIRKNEYIYACDLVDDDNINLRSENVISLFDYLNESYSNVLLLLCQEGF
DSILNYMKVCNNLSDIENEKIFGCLTILIEKGVDIFKNYYKCLEKCHKKDEFYNDSEKMEIIINENNSIMNII
IECLESNYKEYSINEKKRNLTYFEINEIIISFFNALTFIYINIFNSYYNSNFSKQNNNNNNNNNEIIINVQRG
RKKQKRDSDEGNNIPLKNINSLLNNIILLVSNINFDLLYDNINISILDIYLHFFLNIYTLNQENTTIFNMSTT
YHEEISIIIGNLLKLYMKKDTHKVIGSAEKSVIKSSGNFLNHMEYKKETSRVVDNKKKKKRGKHMDDEEEDDD
DDKGDKGGSGNNNNNNNNNNNIDNDNYNDNNNDNYNDNYNDNYDDNYDDNKIGEKDNSHSKKREDEEDENFYS
FSFSYIFLNVCKKCTSVNICDCILRVKNTEIPKVIMKEFIDHIKENSIIYLNLSVQTHMQNEILNMLNCLEYF
SKNLSVHLIFYLKDLTDMLGIDIYYIRKSIFEIFKNFIILSKGSEEMKVNHIKDSELSTNNRRKEKNGRRDIK
RCTGYEDEKNNIGKGNNSYNFLNDSDDSDDSDDNDDDNIDCDDDDNIDCDDDDNIDCDDDDNSDLFSDDDTDF
FSDRNINDNVCNKRGEKSKKKKEKEIKNKIKNNNKRSECNNNNKYNISKKEKKKKKYFNLKRNENKICNNKEM
DHLLKSSSLHINENSISTYKDIINKLVWEDLYFLQLCFEECLKNRSYIMHVLICRQYDSKLHVRCYLLKIFYE
LIENNFIPLNYYNNICVICSERINDKSPLVRQRAFALLSCIANDVVKTKYMIPLDTKRIKKDLYYLDRRKNLL
YKNDTSRADINGTVHGDILPMKRIKNVKGKKKINLSDSSTDSEDMDEIIEKDKNYNNRHNNGHNNIHNNGHNN

GHNNIHNNIHNNRHNNILDRVNIRSDLTNKCDIIAGESSDFHLKDNIQNEEDRNKYSNQLDLLIRMYNEVLFI
SLIVDECVDLCFKLLYSTIETDQKSAIKFIILAHICGNNKAGEDMNKVWSLIFSNNNIIVEIIINEFVNVNIS
SEDYRISAFRLINVTTNSKLKDISCIEKIIECLLLQEKASTLSVGKLLDELFNILFVDKFSDNKNMIIKEGAL
ILMKILCYSIYTVNIKKGKKDQHFLFTNKKKHLILLFINEYKDNLHFINLLILILKYNKTNKIIQSLLIILFN
LIFDKMVDNDVLDDGMMCKGGESKFVNGEDNINNNNNNNDNNDRDNNSFYNNSKGEFKEHCLYEDKNTWFKCC
QSIVESMYVHFDDFLLIFTTKIRNMLHFVLNNNMCKPYLVNKEISGDNNNKISCLTLTKFIFLCGHLGLYTYI
YAEKIQNKLKKIVSKNDSNYGMCTKEEKDREYFDYVVEHMIVCNNLLGKKVMPLIFFIINNPKEFFNECIIND
DKKVRYCFNKEQVGYKEDDTLYMNYEVYLLILVCLLTLCKFAIISQTFCQKIMSNNSSIIKLIVSIITEEENQ
LFLRSGKVANGSTDLSGDMSMNILSLCDSGMNIEDTSRNKNNNNNNNNNNNNNNNNNNNYYSGNVKSEHMNEV
DMKGGEKIGQKIYDKMYNNNNVTYLNNKETNRDSKTFQYDVFKSCIMYKTISNIRKMLLISYADLLYRHPNLL
EPYNKYIFKVLNDEDINMRRTAVSVFTHLFMTDTVKAKSILLVHMMYLTIDSDEKISSGSKSFFYELDKKSPI
TLVNNICDMISVLVRNDRKLEYEMNKKILLFLLTFLKKSKYNETLVEKVFKKMKEVNINRTDDLHLYMQVFLN
IHIDEKILAKINKCFPLIRYIIRENEYVHDNFILICKKAAEKKRGRPTEDGQQQNNTNISNSNNNNNNNDHNN
NNDKMETEKNDNKMRELAEDILGKIESTTNKTRHMFSFESEIKSFSKEETKSKNEERK*
>N.PF14_0031b
MTFLMAKNYVFFFYLCSSLLYHRYPFKSKFVYDNKNLIPNLFFFKGKYIEGSNILSWNEETYGYYKYNKMNEY
GKFIGKDIKNIFMSKNQCLVNHIERREMNTSFLLYNIISQINEIIYLFKYNTIKNIKNIFKIVLVKDFFLMYR
ENENKNYRGKELKEEKHKSSVIFVEREVNERGKMEHFNIKLKQKEWFRNVRILNEEKCFLNCGKGVCKNAHGY
EYCNCPQGFSSNPEKNFECEEHCKVNNGGCDKDAICVPILSEEEEKNNVVKGVGVICKCKNGNTKYNGYYCG*
>N.PF14_0031
MKIQNYLKQMKNEKNIFYYKEEGNDKIYKYDDIEKNTYRRKENKTDIYLKKDLLFQKCILYKLRCLNNTSSWL
LELSKCEKNKEDHINYNKKLIDNIKFRKPFSKYKKIISLFYKKKYKEAYNLLCQISTRNSSQLLKSNLLWKDN
FFVKNKYNLNGLIERIDFINKGKEVCEENHKKDEKVKGHKTRINDRANITILDMLQRKGKYKKEDKIKEIKRK
YHDDIIINPKYMKYENKINRRCNNFVLYKKCSGFEYDFINIILHNNDYNTFPTQESNKCFVDIHNKILNKNKN
NIYDDTSEGDMCYIYKKNEQFINHTKNRCINKKVKIKKRNTDKYHINENMRFYLYIKMLCLYMHSHSYSIYNE
KNVRKQYLHINIEHMRRKEILLYIIQYMKNMRKKFIVFDTHLYLLLSVAYYDLGKYHKSVLYIKKSIKKDYFN
ITAWIFFNNLICIEKFIDNHEIEGKPKEIYRNQHNYDNYSTISINCNDKKKKKKKNIYIYVKKKYFINNHNKL
DKLVNHLFENEFFEFSKNEYNKINIMNHHNFYTYINPITNMNEDFFFKNNYFVNQIFEEGMMNKEGNEKIVGH
MDRCYKNCKNYIKNVHHVENVKNKKTIFNDVFIKYTKYLREHSFKNNFMTLFGFAHYCSLNNKRYQDSICIYK
YLIKILRGKNNLYISGELAKLYYYCGQEKKSLKYFNKLKIINKENAILRKQMMNIYFIYYYSQYGQSKNCKNQ
IIFLGPNNKFEFYDKDKKHIMCMSMKKYKAFKRLIIPKYFSDEFIYVELLANIYFLNNSTFDLFLLAHSYQKK
HKKKNYDEKLFYILGKYYSLNKNYEKSITFFKKGIKKDKYHIYSYISLAQEYFLFKNNVNISIYILLKVLFIY
FNNTYVWYSLGQCLEYKKHYIFCIFSYEKALFFKEDISIYYFLSASYLKRGDIYNYIYVLIRGWNFKKNVLFS
SMIFSINLNILNNEYYELIRKYDISNLLLSDEKKKECIEHIIVHHYYKKTNNNCLLWCLIYLKGYLKKITKNL
HHDNIYISEFLKDNSYEMFVKNNFFFGGEDNNIKKKKFKEYHKTNLFFKNFNLIYYKNTSYLYFIQCLRNSPT
VFFNAVTYLANYCFYNKQFKYSFKLLEILWDVKGSISSNYLEYIFLIEKMLRKEKDRNNFLKKKNS*
>N.PF14_0032
MDILLCTSKEGRIEKRRNKILNKIKEDNNGTEKKLSEIKNNFEENDKENEDNQYSLVYNFEYTNNEHTENYAN
IKNDIISRLSEKKNDYLHELSNKRNNEEKHFLQNIKKYSTDTLTKNNALNFDQYIDYKNDFPEHTYVSSFQYN
MMDVTNLALKEKETYKKKVDKYFSLLKYKDEQIKKVCEINSKNVTTFISFVRNQMEDILNKTRDDLNEKRKLL
LNNNKEILDDFLRMRNLNSNTFVEDLESEKILNEKIKKEYETKHLDLKLKNELLENNLNNNIRHLENINHILM
DKEKKLYSRKLLEQKNSYNLKLINFYKLEINKLRESLLSVKTYYYNYKIKSKKNINELINQYERIKYQFIELQ
KRQKSYEKNFQKKYAKAWDLQRKEAMIYIEKLISANKIIHEQIFLKDFQETNYKYLIQENIHLVTSHKDDENT
VIEMNIKKVTPEQIEKVKKLLLQECLFLIDETNIKDEEEKIRKILNYIGVHTKEDLELLTELFYIDADEENYL
INKFNKNEDENNIPYNSEYTLDIILKYYQEKEKENTCRISKDKQKYKNRLNISLKIILDRKKQEKEYWYNLSN
LTPDHMITLWKTFSIYVEKYYHILKERASIIQNIFDEEKLMKQNINKINRMKKELS*
>N.PF14_0810
MEKNHQILKYANPFAISKELKSYMLKVMDEYKKHFEKSFIKNCNLDDNINVIRDLILDEQNNYNEDIFLKIQK
SFHMYDFFNLSLLFPIKVFKKTDKTLIQLISEDTNLSDLELIQAVLKNIENNNMDKLNFFYVENIIFELLDEI
IRQVICEYPKRGFALLLITNELQKNINYYKNLIDIIEHNNEMNTQGFKEMNEKNNLILQDLLVEENKLKEKLI
DVQTRLELLKXXXXXXXXXXXXXXXXXXXXXXXXXXXXXXXXXXXXXXXXXXXXXXXXXXXXXXXXXXXXXXX
XXKKKKKKKYIYIYIYIYIYIYNTLFKL*
>N.PF14_0033

MDDRKENKKNADYEIFLRDRKLKYEKIRKRNALHNKFILPIINFIKGIINFIKTVINIXXXXXXXXXXXXXXX
XXXXXXXXXXXXXXXXXXXXXXXXXXXXXXXXXXXXXXXXXXXXXXXXXXXXXXXXXXXXXXXXXXXXXXXX
XXXXXXXXFFFFSS*
>N.PF14_0034
MDKQYLHTLEEYFLSVKNKGYLEQYLINPKLSRWDFIIFFVFFVLITLLRLVISGVQKEVIRRNSILYGLSRN
SILDRVNRKYDISKSGSVYKWKENFWFALWHSFSFTYNLILLFVMSGYLNNKNGWIKMCLKERTGKWFFLVTD
EEYKENKRGWPFMYINNYVYYFYILQISYWFSCLFYLNYEIRRKDYYVFVLHHLSTIILLTYSHVLNFWRVGL
LILFVHDIVDIVLYLSKLLNYTNLKNRIFLTFFYILFVLYYFFFRIFLYFYYIVLPLSNTKIIRSYTDGFISS
HKDVPGGLVLLIFLWTLMYIYIYISINT*IN*XXXXXXXXXXXXXXXXXXXXXXXXXXXXXXXXXXXXXXXX
XXXXXXXXXXXXXXXXXYIYIYIYIYIYI*
>N.PF14_0035
MAPDTLLNKYISKFQKINDKLLGLVNKYRYAYFKALEVINHYEELVKRNGELFDDKELIEIYKKRRQCEECIL
EEEKEIRKKLDREKDNNIMSTNYTKDYNINIERDNLKEENVHVVMNDESVEQIKDNNPMIDHNTSCIVDINEK
NKSNYVDLGTLDFSAYKEDSQGCIQYYENKEDVQEQKQGKSQEVESKRHMRVNSSRYESQYGIKNRNILKQSI
YVKQNNNSNNMKSVSGSRYVPNKSVINIDKSFKVTHNRNYENLSKKSIYNNNNINNNNNINNNNNNNSNNNNL
VNKIGVGGINKKGGEKKMDVHKSTYIGSNNKLLNNMNRSIYIGAKKNINSNNINININKNSNIHNNIHNFKNPYD
NNTSLLSTHMNTVVINKEKDMNDTGNNKNVTNAYTQNSLYIKKGKSFNVSNNSLYINKNICQSSVYNKSNTII
YNAKKRKTNEKEKEEDKDGYKNNEIVCTNKNKESNEDNNSDERNKNKHLTNNIINKENNKIYIKNDISKNISY
IYDNLNTYIPITLEHIKNLIEWDDADYEDIKKYTFGNILHLKDTNYNILEGIDLECDKTKCMEKICEKTNEDV
PTYKNMYPIYKGDNMKGELYIKDEKCNIQNINNKSDDKKKCYLTKYKNVHIYKSDISSCPNEYKNNVDESNEQ
QEDSFVRERDNANLDGSNQVHIKKINDNNSNMSTEYTCEQISLKNNKNDGERNHVNKLIIDKVPINKLIKEPS
DDISLKYKNIIFNINDEIKKKVNKEKNEEINEHVESLSKVCNKPRNIKNVPFPSSLKKNILLESQEKKKNPIN
NRCPLHLDINVDVKYITLNTIDIDETINKGVCPYKDMEDNYISSEEHIKDIIIYSDILKTEKETFLYNPNDIE
KYDNNDDSNNNVRKIPDTIIHFKSSNEIKNDIIKLVGISIFDCIHKSYEPTKSLINDENLEIWFTKREKNKND
DMINNMRGNIVQLNYGQDKELVFEKKKKKKKKNIINKRISSMGYFSQAVVIMLCSLKRQNEYKSLKKIIASII
LCTCDSSCLEKFLHTIPDENSKNYTLWKETLNKLTSYFGPNKCDEIVERLMIIEEDEYMNKSKKECREKNDYY
EGDNIKSNNNIYNIYNNNDEKDIIKHCKLDNDMEFFDDHMNNLSFPFECNEEHNKKYNDIQEDEEIYEDEQFC
LFICRIKNVHKRFGYLLLIDNFDFVYEDLLKNIRNKLSSIELILTKHILLKQLFCNILYICNWLNKPRKYEWF
EWNMVVKKLRKLYGYSENGRISKERCIMLILAKHTGEIFTNKELYFLKKISTFYIKDLYDKCIDFINTYLEIK
NDIDTEEFLDSCCIHNLKNEIKDNNTSYNHSSKYFLHDKFLGIVKKFVQKNYPKILYIIWNIVLLIKQYLVII
LWFNDIKPFFPLFNYPNDDNKQNKFLQDFFVNFVHFFENYNKYIDILKKENLNEKIKNTSNDIFCDTSNININ
IPSSENEHNTLTNNYDIYTNNRLSHQSTYNAETKKRKSLTNTIDYACLLKRKSAEHKETMEISKPSNAGNFSD
VEFEPSD*
>N.PF14_0036
MVLIKVENHEGKNVNANKFEPFFFVLFGDIQYGMIRGNHGWYEERELLKSAIEKTNKLKPPFVVALGDLTNKF
PLDPIQTNQITDLKNDFKLLDKDIDLYVFCGNHDVGNKPSMEGMEYFEEQWGDSYYSFVYNNCAFIVLNSPIL
YDETHVKEMKEEQLKWLEKTLEKLHSLNVKHKFLLLHHALMYDDIYEGENIGLIYGDKFHNYSEKNEFHLKKE
PRLFIYELMKKYKVTHVFCAHLHANRENDIDHNIKQITISAVGMQAKDDKSGIFIVQVTEDKVDYKYYPFEYV
PNYIKA*
>N.PF14_0037
MKVLGKDEFITKFETFLVIKKELEYSRNIDKHLYNKYLINKDDNFKEIYEKYFMKLISDKILYKSIHKYIMDT
CPHVIPKNIYHKNDNESKDIIQDISNKIVQFFKNIDIYDFDKKEKIQMIDIHCVNIVDLYVIMNYADKKCEEY
KIEQILNMLKDVHITNNNS*
>N.PF14_0038
MSRPEPQVQVPEGDYKKGAKLFKAKCAQCHTINKGGAVKQGPNLHGFYGRKSGDSDFPYSDANKNSGIIWSDK
HLFEYLLNPKLYIPGTKMIFAGIKKEKERADLIEYLKKASSE*
>N.PF14_0039
MANSSDNKPEANETVHENAATKNDKENDKTENKTSEVNGKNKTHTINNNVNHLSSEHEKKYVQETYSFTFDKS
LFAAPKFHPYFMMKNYTVHYMDPIIKKAKAKKHKCCCN*
>N.PF14_0040
MKNFTCFFLLTLLLTLAYGKGVRNKSENRTPYVHYHNNSNIKNYSAVDIFSPKKTGKECIKCLPDNFCECECS
CKNKTGFSMKYRHASKGSKRYRKKMTKTSSRSRQCNDLPLEKSEQETEPELEPEVEPEVEPEVEPEVEPEVEP
EVEPEVEPEVEPEVDPEVDPETDQGTGSQNNITECYSSCKSVTGVQTEECSCSCSC*
>N.PF14_0041

MPPIIYKSPLPVRLKAPKGRITPLNGPDIKIGKSLIAACPKRIRGEKLAEMKQTIRKYLGKKKEYLIDVHATY
SVTKKPDGTKMGQGKGIIDHFVARVPSGKTIFSIPTISPFNTLGFDDPVYRVLKKAAAKVAIPCVFRTQNNLF
RVHNIKYISQQKVKNDQLKHFNQFKSKLFQKKDEV*
>N.PF14_0042
MVEKEDSYITNKKVEENKMNKIIHIKDEQINKKKNKKNKKNKKEGKSVTKEMNETSNLGLYSNICEEKKHKKK
KKEKEKDKDKDKLDALNKNSKKKNKKNDKSNKKEGKKLENNDEMKDDVIISDHEDDIIYEHDDIIYEHDDIIY
EHDDIIRDHDDIIRDHDDIIRDHDDDILDDNNNTQLKNNDIIINNNNKVITPFDYERLIASEKNKSAIWISYI
AFYLEQNNLEEARKVAERALKTIDIHEIDEKLNIYLCYINMECLYGDKLNDIFKRALLCNNEKSIYLHTIHIL
KKNKKLTQLKDLCEEAIKKFKYSKKIWSCYLQILHNTFKDEEYAHNILLKSLHSLPKKKHLNMIINAARFEYK
YSNKERGKTYFEKLIQEYPKRSDLWFTYLDIHINSLTKNENKEKIKLNLKELQFIRNIFERFLSYKFKPRVMK
IIFTKWLLFEKSQGNMHSQKTVQEKAYKYVESLNALV*
>N.PF14_0788.1
MESMKNRPLKESLKFTNKMHLALIKRYFMSLIFIMLSYIMIEIIYISKESKSPMNYFIYNLDLIVFDEFYETE
FLKALYFYSAIQKNKRDEEYLISIKSKRKLKNFINKKEIDISGINYLLWDFTNLSHNDLLKFVTPSSANQNVE
EEIILDHTIKNINELRYFETKIYESKDFIFHINIKRYIERTIKNIIILKIFIIIFSFIILFYFTCELNVLLFP
IESILKKLKLMKSNPTLALEMQEELLNHELKNILINTKLKRKSIKENYEILKMEENLMKLGTLMLLGFGEAGA
KIISKNINEQERVNLLINGEIVYSVFSFCDIRNFTEITEVLKEKIMIFINLIAEIIHECCDFYGGTINKNIGD
AFLLVWKYQKKEYSNKKMNMFKSPNNNYDEYSEKENINRICDLAFLSTVQTLIKLRKSEKIHIFLNNENMDEL
IKNNILELSFGLHFGWAIEGAIGSSYKIDLSYLSENVNIASRLQDISKIYKNNIVISGDFYDNMSEKFKNSLR
KIDRVTLKGCRNPLNLYTFDICLNKITKKVNMENFDAKPHFDVKLLKVFDDIKKKAERKKRKKEVLNLSYNLY
EEYAKNDDIKFIKIHYPKDYLEQFKIALESYLIGKWNESKNILEYLKRNNIFEDEILNQLWNFLSMNNFIAPS
DWCGYRKFLQKS*
>N.PF14_0788.2
MGIFIFDHIFKRFFLSSFKQNIFISILLFGCLIEIIFNFLMDSTYIFYFMFFDIISFMLLFFDIFLFEKYFFD
FFIYFTNSLYTWKKIDKDNIIYLIQLFKSLRIIKIYRFVINFIKKHTKEKYKHRNEWNFEKMESMKNRPLKES
LKFTNKMHLALIKRYFMSLIFIMLSYIMIEIIYISKESKSPMNYFIYNLDLIVFDEFYETEFLKALYFYSAIQ
KNKRDEEYLISIKSKRKLKNFINKKEIDISGINYLLWDFTNLSHNDLLKFVTPSSANQNVEEEIILDHTIKNI
NELRYFETKIYESKDFIFHINIKRYIERTIKNIIILKIFIIIFSFIILFYFTCELNVLLFPIESILKKLKLMK
SNPTLALEMQEELLNHELKNILINTKLKRKSIKENYEILKMEENLMKLGTLMLLGFGEAGAKIISKNINEQER
VNLLINGEIVYSVFSFCDIRNFTEITEVLKEKIMIFINLIAEIIHECCDFYGGTINKNIGDAFLLVWKYQKKE
YSNKKMNMFKSPNNNYDEYSEKENINRICDLAFLSTVQTLIKLRKSEKIHIFLNNENMDELIKNNILELSFGL
HFGWAIEGAIGSSYKIDLSYLSENVNIASRLQDISKIYKNNIVISGDFYDNMSEKFKNSLRKIDRVTLKGCRN
PLNLYTFDICLNKITKKVNMENFDAKPHFDVKLLKVFDDIKKKAERKKRKKEVLNLSYNLYEEYAKNDDIKFI
KIHYPKDYLEQFKIALESYLIGKWNESKNILEYLKRNNIFEDEILNQLWNFLSMNNFIAPSDWCGYRKFLQKS
*
>N.PF14_0788:mRNA
MLLNIFFIVYFYKIYISLYISYVVFLNKCKXXXXXXXXXXXXXXXXXXXXXXXYMS*YTYKQIFQILQMEI*FYI
RYFYFHSLIWMSYRNNF*FFNGFNIYLLFYVF*YYIVHAVIL*YLSFRKIFF*LFYLLYK*LIYMEKN*QR*Y
NIPNTII*VLANN*DISLRN*FYKKTY*GKIQAQK*VEF*KNGKYEK*TIEGKFKVHK*NALSSYQKIFHVFN
FYNVILYNDRNNIYLKGEQKPYELFHL*FGSYSI**IL*NGIFKSSILL*CYSEK*KR*RIFNKY*IKKEIEK
FY*QERNRYIRN*LSAMGFYKLIT**SIKICYSFKC*PKC*RRNYFGPYN*KYK*IEIL*NKDI*IQRFYISY
KYKEIYRKNY*KYYNIENIYYNIFFYNIVLFYM*IKCLIISYRKHIEKIKTYEIQSDLSFGNARRIIEP*VKK
YINKHKIEEEKH*RKL*NFKNGRKLNEIRNINVIRFW*SRSENYLKKY*RTGKSKFTDQWRNRLFCFFLLRHK
KFYRNNRSFKRKNYDFHKFDC*NNT*VL*FLWGNNKQKYW*CIFISLEISKKRIFE*ENEYV*VSKQ*L**IF
RKRKYKQDM*FGFSIYSTNIN*TQKVRKNTYIPK**KYG*VN*EQYSGVKFWTSFWMGYRRSNRK*L*N*FII
FIRKCKYC*SITRYF*NIQKQYCYIRRFL*QYVGEI*KFS*EN**GYS*RM*EPIKFIYF*YMS**DYEESQY
GKF*RQASF*RKTFKGF**YKKEGRTKKKKKRSS*SFI*SL*RICKK*RHKIHKDTLS*RLFGTIQNCP*VIF
NREME*IKKYIRVFKKK*YF*R*NS*SAVELFEYEQFYCTQ*LVWI*
>N.PF14_0044
MIKVLLAVLFILIKLENIIGQDEKSVKNICVCDFTDKLNFLPLEKTKILCELKPQYGEDIKIIANKEYEINCM
NNSKVFCPLKDTFINNTNIKLYSPKLHFEIKDITHKGKNAALYYLKIDEEASDIFFSCSIKPKQVSGLLEGEV
RVNLKKHINEEYSIFNEEEDVHVCDFSKGNLDITPSAGFYLKNSRNVSCIYRVIPNKLFLIKLPKLDIVTEKL
LPSIVNCLSEFSFINFTLKHVQEGDNYISFNVIFGEFKKHFNLTCSLDLSDFKQEPCNLGKTANITFIFSK*
>N.PF14_0045

MYIYIYTSTNEDFINTFELFCLFVFVTWALFLNNNGILYPVHCLKSAGDTYVDTLRGSNFNNEFIDLLSTRDI
CNTIKNYITREQIKCAISYDVLNISNIINSLEKTLLRDHNIIINGKEDILKKYLNTFLTLYIKRQSEDVNNFI
KKFSNSKLDVVINYRYYEEKFLSEYEEIDSLKKGFSNHISEIKDILNNISSSNNYKKKSGVIYFPDVKETLYS
KIQILKEILCKLRNKVSFMYNINFALDEFKENFDKQVYNFKGKEGLNDFVRITSDITNPTSTFTNVVDINKFN
VNTQMDEIQNGTLSKEHLNDFRRNIINLDVHIRDPSSFCVNQVENPTEMNINNPCNNNDGNNINCKDLVEEDA
MLNHFSFLLYHLEKMTCILIFSLKNKISSARDDIQKDINKMESELINVSNEINRLDIVVDVPQHHILNYHNKN
ENKVFNLMNIKNEYYEYLGGHNKINNNFDDFDNTLMLLERKTNWIKDQNIMTYGDREDIHEAISSTLEMIDKL
KDMYVTENFNLLITYENLYKEINLFLYNKQYNISEEAYIYAWNSLENFKGKKLLTEGIDRLLGSVMSISYVIK
YVKVANKHLNPQICFNLNKLSNAFMNIEEKLVLYRNQFYRLNHDITTLKLFKNNIEKLGYAYRDNMNKVHINM
DTYNIATENLQHEIDNILENISDVLYEDMLINELKTMRATWKNFVYVKYDYFKQNNKLIEEFDENKLIIFPPQ
FGLMKQSEQTNIRNDNNNNNNNNNNNNNNNNNNNNNNNKDNSVASLGSSILTKTSSPDNYLIGQNFIKSPYYN
LLYEFATEKINCAKTPEERIKSFGEIYRTIDRVSSVIKENRKNLRSKYDNMRIEILKLIDYRKHTFEETKDVH
KELIRLEGFILNTLDNLFAKRMTLSVQMKNSVEMLKGSLYKDKLPDYCKAVEMFIPKYFLTMTRWRNFLLEYR
KIMPSRVISKFYST*
>N.PF14_0046
MLSVNKVTALLFFYILLIKGYYGRTESKNILNTVKNDVKNGNYQNMMNFPDMDKIKELINQNKVEEARELFNE
MQKQFMKEKMEANNNMNNKKPAKKAMDNSNLFSNLINPNDLQEMIKFYMYLQNILNSKNETSEKKMVKSFLRK
TIQKMKDNENSEEKKSIDDIIKDTEGHNEMLEKLAGLMKINKEKLKDEQVKNKLNQILIGMLKFIDYTNNSDI
TNSLMDEIQIEQVQAEDGTIKPKVQVNISNSKAHLDMLQKATNFMGINIDQEELKKLTVNNKWYESFLNNLLN
SSDEL*
>N.PF14_0047
MDDDKIKSIYFSLKNKSKKEWAELMEQLTKDQKRLIFYIRKKNKHSLHKIKNIKTEKSNEKSKTKSNRPYYD
HILIDYTNVNYDLNLKNYIARILRLYKLEDNFEFIKALHLYMNILCYIIFKKFSYEYKKEQKNKNLLLFLKKL
FPFEFHNFEKKYNMRWGNDASSLSENVGNKNIPNVEEINSMDDVEENINEKNSNERKEQSVNSNNICKAQNEI
FAENKSVHNNISNVYEEQNASCNKNDFISENNKTKTSQLDYKINCDNNDVHIKMEKDNDDSFNSELDNITILS
DLNKYVKKELHKEFFKNKDITTIMQNLFDNKRYGYRIRFRSILSKSLNEIEYKKYCDQREKLFKYKRKNFLRW
ISQFTNIESIDMYIINFFIFLFLDRLYLILETYLRLNYVYSNKKTTQEYVIDHIKQFHNFDFILNKLTSSHGH
NISSTNKTNNVISEGDELDNNTGTIDSLLHNFNELYKNLTKPNISNFYLSLDLIYNTDVYSFKITNKITFEKL
VKIDISSYINETNVYKIIQFDKDKNTDPWKTLTNFALLQNRKLNLPKFDCFSIFLIVRFKYYLEEFKENSNID
YIYKIVKEEYDKVEAYLKRDQFDEEDFEYKHLVPIYLDLKKELKKVNEYIKFYGNLISFVNFIQKYANPSTNI
VKTEENGEEKIFDLSFFLSSHLNDNVKKE*
>N.PF14_0048
MVYSSNDDIPLDNRKDVIFNNAEKKNINFDKRRSCDYEYDEEENDKIINDERIMSGGEEKIYMSNIEGKEKNN
LNDSNILDKGRSRSSSNNVFKNIDKENDRKKNDDIKDKEESNNIIEEKDYYVMDKQMNISNTHKIDVENEKIK
NIHFSMNNEESNIDDENKKENIFDIKKSISSYGNIKNTSDSHKKGSVHKSETEKKDMNPIYDDNNYVWDKNDE
NMKYGKNHINNNNNNNNVKNNDNNMYYENDEKFYKREYISTDEHMKVDKEIVEIKDEKKEQKKPSILNKEENG
KKNKDHVIHNVEEAPGYEYSHNIIYHELYVELLHVNKNLQNEIKNLKKIIEMQKILIKSKEEVFLDHLNEKNK
MSNKKLMNHNYFLNFFNKKKKKSKEYKTSFKEEYEEEEDDEDIGYVVDRKKQTYFDVNKNYHGGKRELEEEIH
NVSEEHFDKKGSYLECEKIYNDDEDMFEYDMDVIYEKEENKNNKYIKLPSIDNNDFSEMTDRDIDSLYAYNNN
NNNNNNNNNNNNNNNNNRNHNKNNNNSNNNSNIINIEKNDVYFFNDEKEESNYSRKYEFEQSMLYNIEVEDES
CNNSMTIQEITCLTFWYEEILPLVNNDKKKNLLINRMITNYMPNSIKGYFWEMCIKNKLNLTEYFVQILIKNT
NFIQAYVYTNNRQYHNHVSRYFKSLIELRNNNLHIKEETIENDQDIEQKHDDNNIIFDSNKFHINDVHDKFDE
QNDNIFYETQNMFDHKNKDPSDNKNDNPSDNKKDNPSDNKKDNPSDNKKDNPYDNKKDNPSDNKKDNPYDNKK
DNPSDNKKDNPYDNKKDNPSDNKKDNPYDNKKDNPYDNKKDNPYDNKKDENFCDDKNVIIYDNNKDKMSNMPN
SNANINLLQKFSVQKFFYQILIDLDRTMYIITKNKEYFAKYNIQTDNVLSSVNLSDMKKKLNTLLQMYVLFKP
ELGYVQGMSYIALVFLLYSNLEKAFVHFANFMERKDIYNLYSFNKEEIKIYIYTIKQILTKRNIEVYKEIVKH
YNIDNIFIQWIYTIFLTCLPFNIYIRLFDIYTFNEKIIYEVIICIFTYFNKYHPTDNVDIIIKDLSSFAFNSY
IKEEKFWNLLKKIKIKKRKILYYKDKYFNKMNSKDDDCSIEK*
>N.PF14_0049
MILSNEKIPQVTDKWGDMYYYFYIFYKNQCIYSIDLKNNNTQEKKKKSNKNETEKLLLGSIYAINYLCSNIQP
NKKLKNLYKSISNSNPKLINTNIQTQNNINTQENIHVGNFNCFNTPFYKLHYVETLTAYKFVLITHKNIPNLS
NFLKDIYKTIFIDLIIILNPVYKIGDEIKDKMFDEKILEKIKRLYVG*
>N.PF14_0050

MAIDPESFYGKRKNCKSTFEENIEKYKLLRKIKTHDEGCDRKNIKKRYFYKQHEVLSYYDDLINDKNISGYTD
IIIYTEEMENGKRCFILESFYSFLKYYCFYAMSLNEIYFVKNENINKENTINEQIQHDIDNKNSINGNPDMHL
YELILTNEKRWLYFDIEYDIINNYENKESILFIFLIEFCLFIYSNFNIKICLNDILILDSSTNKKVSFHIIIK
NIHTLNNDYYEYLIDYCNFYISQNEKEQKSGNPFYDKYKNKQYKRNHNKQNEKNKPKEHIQEYEQNYLLFDDE
NSIKHFVDLFLNHISDHIKYCENCFLVNHTTVYIECEDLVELNNNTSFVDTNEIYNNNKESQNFSDEKENNHS
EENNHCDENNHCDENNHCDENNHCDKNNNCEENNHCDDDLQKIINDFSEVTDIQHINFNSTSIPFIQNMNRNN
CEEKKTKGYFHLYKNSLVDIITHYINYLEKLKEGEKSYCNYIVLLYAFKREDNSDQNLVYDMNMLNIKKNNFV
SKNDLILNDCDNTNNNYEENNSLLINEKENIIIEDDKYMEELIVLFENTPYNYEHNEYKKKNNNKIHTLKCII
DSSVYSKNRNFRLIFSSKKNKKNKLLLSTKNVKKYQKTDINDIILKSLVTFYFKSDVNYKINEGRFCNKNLLN
NTMNYYFENLYEKKQINNDINDININNTHNINNNNNNNNIHNIIINNNNIHYINSDNIKNVDFKFNHMNSSNN
SFKLLKHKNICIPKNNTFDKYVIQNLEHFHNILKILFFWNFELYKNFRKNKIYLNCFQNEIKKDYFYKIVRIY
NNMAFENYIKEDNSNMKKYNETNEHTFEEKFSLENSKKICQLSNISEKENESKDKNNEPNQNQKEKNDKNEPT
YNTKCDLKIEDKNYININNNVQLKGSNKYNTICTKKKNIYEYDLDSYNNLFLKSENFYIDLLNEYKNVFSKEI
RQDDLSLLIKYFVHSFTSNNEEYIISLKDNKFCKNKNRSHKSNHIYIIYNYKKKLFVQKCFDHECAHYISEIY
YL*
>N.PF14_0051
MFFMWLYKYKVYIYVIFFVLVENTMSMKKNLMFTCFNKKKFIYVKKNVLFLNNKYISNLVYTKEDDIYKINEI
SNVIGRNRKKKNIIINNDEDNDNIDSKNNELGDNIISSNRYMNNVFFTNNKNTSLEVRNNNNEDGGDDNNNNN
NNNNNNILDVSYNHDKYNLCDNELQLYKNKCKIPLYWIKKLDNLKNMNAINCIEYLKDDNLLYFDNYKNKGLL
KFLNDEKRKYNNCIILSRVGDFYETYGLDSIFLIEFLNIKKMNNRLSCGFIKSSINKALNILTNNNLNVCIYE
EINEKSLKMKKRYLSQIVTPEFPIYLNNIQYYNKDEDILQNGNNNNNNSNNMNSYNNMDKCLDNGNMNALSFF
SSYDLDDYFVIKEIVCIYIESKNIFSLSKINLSLKTISIYDNITFDVLNVYLKNTNFLKVYIHQHINTSFTKK
ITELFKIENYYLFNNFKSSFYFHMFILDKLKKQIHVKGLFRLIKNKNVFKIKSADCKKEEKKIDSNISDYMNY
EENNKISNRHINNVNNNIEDNQNVEINKNVEDNNIEKIKNKNIIDNNFEYSFSSYCTPLNIFTSYNMGIYKQN
NHYENKSNFLFYNIIDINNNNSNSINISESLEFFKNIFLFYPPFEVTKHIRYINEYIKKNVKDLIIPNVKPFK
NNIIISLLSNLKADHHILKKILVNIDAVLNCIRNYDFSLLVSIFNVLNHQNSFKLNIIKFYELLMNIQKIMKD
NLDLGLFSKFSYQSDIQAFNDFVIYHENDVHNIINEKLITEENKELEKSRTDLLNTIISNYSECDKTKNAYKD
INLLNKIIKIDNANDIIGIRKNIQKKKNKNEQISDFFHPLNKKSDVMKNLYVTQDVQNKIKTYLFSIYKKKKK
INEIIHNINIQLSSSVHILSFVSNFLQIIQALYNHTINSLKKGWSLPICKHLHLSYENMSFNNIDDKLAYIQK
KVYDDKDEIKEYTLTKNVNKNDPSNYFINDQKNSNEFVKLSPCDEKKILENIDKDSAEEIKKMYKEKNYVNDS
ITYIIGAKPYNIIKHNLIKYDFFLKKKNFILLTGKNMSGKTTLSFTILSILFLSNLXXXXXXXXXXXXXX
XXXXXXXXXXXXXXXXXXXXXXXXXXXXXXXXXXXXXXXXXXXXXXXXXXXXXXXXXXXXXXXXIISAVS
DYLKNYCGIIITHNYDLLNKICQSENIVFKRISKDINYLKEQDNKIVGKLEDGICKNSEALETCRYTNIDPHV
LNLLNVYEKKYKFIHNLSNTLYTKFLEYIQYRKDGKCLNDFFDQYINDIIKQKSDKHEIQYNTPSNMENNYNS
YSSNDQKNYISENHKYDYNYDIDYIKDKELKNIINSNNNIQEFLNNYDKTRIHNDNPTNHKPDQYDNSLVYDD
KLNVIIKKIEEASNKKVIKIGMNEEVPIYYKNKSIVYILCIFSKDENDPYFYIGISDNISERIKCHTRNLLNN
KNLLKNPKKNNLLNYKYDWTKFYILLFHVDNKMVASKYEKDLSNLLKNNYNILSK*
>N.PF14_0052
MIGITIITGFLGAGKTTLLKNLLNESIEKDKKIAIIHNEFTEKNNNIDKIVFKDINDIYNIPKIKEDDKNNVN
NNLKKDIQKNKNELRIINEIDSEEGFIYELNNGCLCCSNKSNFVKLIENILSLKTKYDYIFVEVSGVYDNIQI
NNLLWLDELNKSKIYLDSIIHIVDSYHFIKYYNKQEIKQNTKDTLSNEINHEHEKKKKNEANNVYHNEKNNES
NNVYHNEKNNESNNVYHNEKQNEANNFGPNEQTPEYEQIMVSDVIILNKIDKLNEHNINELKIFIHKLNPIGT
IYTTSYCYVPIENITNLKCYEKKNIKNIIINNTKVENVVSFHYNDFHNMTFHFDHDISSLIQLSDLLNKMKNK
FIKQKDTNILKQIVTILKNKNIFSYKKINHILATLLWNSKLQIYRGKGVFVAFNDDIYNNKHKIKLNIYYYQS
VGDLYEINHVMTDIHTFFQTYVTKNIKENEIIHTNNNNNNNNNINIISNNINNENTQTEFNIHNINYDHEEHT
CDDLLEFENYISDDSSCSENNEYNINNILNNMHIFTSRFLFIGKNINMEDIKNKLDDCLYK*
>N.PF14_0053
MADVINISRIPIFSKQEREFSDLQKGKEINEKILNKESDRFTLYPILYPDVWDFYKKAEASFWTAEEIDLSSD
LKDFEKLNENEKHFIKHVLAFFAASDGIVLENLASKFLREVQITEAKKFYSFQIAVENIHSETYSLLIDNYIK
DEKERLNLFHAIENIPAVKNKALWAAKWINDTNSFAERIVANACVEGILFSGSFCAIFWFKKQNKLHGLTFSN
ELISRDEGLHTDFNCLIYSLLDNKLPEQMVQNIVKEAVEVERSFICESLPCDLIGMNSRLMSQYIEFVADRLL
ECLGCSKIFHSKNPFNWMDLISLQGKTNFFEKRVADYQKSGVMAQRKDQVFCLNTEF*
>N.PF14_0054

MIIDNTISRNDRINIPKNEHVRDDNTGNNNVVNIEKEKDIINANNSNNMNIVKGENNFSKNVNEHTTSVKRIN
VEMNNENKKNNNEKVCNNVNETINGIIMKNKNNNNICNDDNNKENDNNKNNDDNNKDNDNNKNNDNNKNNDNN
KNNDNNKDNDNNYIEPTSTDGHIKEEGGKQNDVVNDLSENNQNVDNNQSGDNNQNVDNNQSGDNNQNVDNNQN
VDNSQNVDNSQNVENNESANSNPDNIDDGVESFNSFLLEEDINLSRRAYRNFHICSIFIHLSLLLMVILLMGT
LSYDFFKSYISKKEIVTIYICAMLLGMLCIHICLNLYISYKLLMQVQVSKSLKSIESKIHLIVVMYFSFCFYL
YLFERGKYAINSTFSVTIILAIIYYFLPIFLYIILRLIFFVVILILVFLKRKSPTPKRILKKLKIMKYIEYRK
YCEENFLTTNYFYSDNFINDQNKCVQKNEDMSNIQPNGIGQCVKNEELTTNMVSEIPINNNEISDMKNISTNN
NTLGNIPNLKDINNSNNVDNVNIINSDNNVVINIESPTNILNKNGENKMKNMSEENIKKEGEKIYNNNYSIYN
DPNHVNDVININIDNDQIRNDNKTGVFGYFQKVLKKKKNGSANGNEINEKNEIIEIPPENNVYINIETSDLVC
SICCVEYANDDDICILPCNYLHYYHKDCIFTWLKKNNDCPLCRKIIEKI*
>N.PF14_0055
MNQNGEINTKNNEGKNKEQNKDGDKKKVKKLNYDKDELLENDVTKVAGEFDEEKEGNQEMKSFEKMTEKSKKK
KDSKKMKNMKKMKKKKKLEKMKNLINSTNNSNNEKDENDFIETFKKKNKKNIKKDKKKTKDTDYEDTVVSSEI
HDVITGAGDNSNIYINEEIDESDDEYNINTLGNFDLKHYEDFDIIGYDIEGKKIYKSDENLIDDFLNSQRDEN
HWRKIKDVKNNRVVQLTDADLQIIKSIRENKVATILNDANYIYENDKEEYKLYPSGIINQKKNEENGSVHERK
KISKIMNYLRNKEKYPERYKDKKENDEDYIYDLWNNRIYDEFNNINEISLPHILPGHKYSYNPPPELMLSSSE
KRKLLKNNKDAFIPQNFKNIRNIEYYKKTYFDLYQRCLDIYLCSRTLKNVLHINKEDLLPKLPSKKSLKPYPE
CAFVHYVSNVEEDEKTVDDINKKKMNKNISSYLYPIDINENDHCVYTIYHNKLYIFDILTSYNIATIDLLHYF
KSVVPLKTKKKSIEEDIHNNIIIKVNKAYSILAISYTNVVFILHYESYVPPLKSKCETFVDNIYDTTNSDVKR
KKPKNGEGNINKANKSKNDALEEESILEGDINDKDGSYHGNNNSDDDGDDNFDDDDENFDEDDYENFDEDDDN
FQSDESYDAGTNNNTENTNNDDISKKKKQVHINRNMVYYKTKGLLSVYHSNFKNSEDYCYSKDINVKWMLLKS
NHKKIKYCVAIKHEGEIKDISWNNNGNYLSVTCMRNIGQYNDCYLHHMKTMKSIKLIKKYKHKRGNIISTMFF
PRSPYFMVAFENNITIYNLKATNKKERIFKKLRGVQHITSMDVHTNESYILVSDSLGNVFIFDLDLSCNPYKK
FHLQECPLKKVQFHKSYNLFYSLGDNGKINLFYSKFFQDYITNPVLLPIKEISNESKIVDMVWSEKKPWFFTY
TQNNFSVLYT*
>N.PF14_0056
MNVIDDQAIDKHKVFVRNIKESDVPLITKEFERICNKHFFFSNNSYTSKNAICHFKSAKDAEDFISKYNNIKL
CNSYIKSEYALKKKYEDKKLISIVYNRKKIKYDNSLKIYTDCNVDNIVILNFLKTLYVNNTNLIYPLVEQYTK
SESNSNVYNNINSSTYKNNDKIGDNNNSNKFIDDIEIVSDKKNIKFEDIIINIEKEMKHSRNNEKNKNNNTRL
IDKNASKKIKIVYVIEFLNIKIAKMFFTLINKTVFQNFLKDQNVKNTHDKNNNNNNNNNNNKNNNNNNNNDNI
YFRYFFHELCQTKKNDKKVILQNLSRSCHVENIQKLFKHIEPNAKIFFPKKNNKKQGYAIVSFASLYNAQKAL
QLNKTKLCGNIITIQTINNNNDKSIGKEEEHNKYIKENEVNVKREHEEEKYSKYAIKNKNDDNEGDDDEGDDD
NEGDDDEGDDDEGDDDEGDDDNEGDDDNEGDDDNEGDDDNEGDDNNDHSDNNDHSDNNDHSDNNDHSDYNDDN
GLDKSFEKINNFQKKNNDDIIRPNDEKQKKKKNLNDVEEGKTLFITNIPPETTDDEIRNYIINNINKDYIYIK
TCQSNNKNLSVFVKLKYKQDADTFLKKIGEYEYDENNDENDNTNNDENNNDNSISDDEENIIDKFYKNKKNKK
EKMKKILLKENKNKHNINSEILFFKNTYLMIKRAVNRDLIKEKRKNYIKSTDQENENNLKKKTKQNNIHLIND
NNVNNENLSENIIKRNNNLMEKKKELLKNKNFFINPCRIYIRNYPAVLEQNTFRQLITKYFTPIIMKKYNMKK
KEAFKKASQIIKKIKIIKDTDTTNSKDLNLSKEKSSKLKNKNIILNTNSNNNNVASKNVKNYICFVDINKHEN
AKKMIQLLQNKNIYELINEIIYKKKFQTIKKNKNIIYVDYCIEDIRMIHIKKIKEEKFLNHVKQQNLDKNINN
KIIDKKKKNKKKNKVSRGKRQREKKRLLKMKNECTNILNVINNATLTKQIINQENNTKNEITDKKDKDKHTKL
KKLNKTNKSIDEKKENKTKKNVINKKKLDKQKEAKAKKKTKKDDVKLIRKDVLNFLNKINK*
>N.PF14_0057
MEDNNLNNVLKNDEVPAKSVEGWIIIITNIHGEARDDYIKEVFERFGQIKNLHLNIDRRTGFLKGYAFLEYEN
FVDAKRAIDEMDGTMLLNQEIHVDWAFVQEKSKT*
>N.PF14_0058
MIYNDKNEDWKDRENRLVDIIKKEYYYNKNEECDEYNIGGGIIGKDKYGYNDCYKGDNIILIITEYIKKDCIL
DNYILEYKKLYDNLDYYIHYIEKKSDIDYLIDNYFIKKKRDITSIINDIEKYKKLNGDMNDIEMTTYTISVIL
KLNKHILSAFPMLKNVIEALKNDNHNENFYYINNRIGIIEKWKNKIFNMSCKKKTYKSIAHSFLQTNNEIMDK
QKNNENFDKCDQYMNPCYINKTYKNEHNNYYEYNYVHNKNVNDTYIVDNNNMKNYYDDNIYNNSIYNNNIYNN
NNIYNNNNIYNNNNIYNNNNMYEPNHMYQGDFSDDTINVMNSTNKNKNSNKLIQKINAPYENNIVNYEDKNST
FFYEINITTNKNLIRLKSVLLLIEIVMSFYSFFIRWEKQRDKEKKNISDEREERDINYIKDNINLNSDDNIYH
MNDINNINKRKDLCTHNDNINKYIKDTEYQHGEKEKSKSTYDEINYQLFYDLISGKVKRKKNIKNEENHEKKK
KEVNMQKVKRKKYKERKKKMNNSNDKPDNFFPYIKYECHESENTVSSNNYENIIGTNKGISSDNNNNNNNNNN
NNNNMSSNSSTNNKSPNSDLNNISSYSGSNNLSSNSDSNNLLSNSDSNNLSSNSDNNNVSSNSDSNNLLSNSD

SNNLLSNSDNNNVSSNSDNNNVSSNSETKIKLSHKDMYNCYNNYSYASSYSNNDNSFIARDINNCNDKGENVN
PLHYNDSIDAPNKFLNGKICYGQHNKNIYYISKENNIYIDTEKKNKINKNNNNNNNNNKNNKIIINDKDLNKM
MSYIKNILIYVIDECDTYFNIEFLYTLHTFELYYLFHIFKKLLLKKKDIEDDLIIMLTYCAYKIENKEIIEYC
FWRLVSIFESECIPNSWILLDDKTNNQLKKRLDELKFNFKDMKKRNKSIKKKAKIKNYSDYRIDIFYETFNIF
DTEEIRDVSSQININEGNLYFKNNIIDKDIFINFINKTKREFSYYNDIPKGYIINELQRIRKFNEFYSCCYIL
KNDKKEKILMGFKKRGQNKVYIYKYDKNIKKKFKNIKTSFNVSGFLGVLVCNFTGMKIKIYDNGISEKYANFF
PNFERNNVISIRFESNIISELPRHFICNIYKENKNIKLIYENKCPIWNEEKEIYELPFYGRVKMASAKNLQLI
LKKCIFNESKKNDFLNKNINDIIEYVSASQIEHKQNFHEKEKEKEKDKDNDKDNDKDNDKDNDKDNDNNDNIY
NDIYKDNNTNNYHNNHDNMPIKQKYSEYNHTKCENKNSDSGNKKVKKHNDEYINSFIKKKFSFDLIKNNLKKK
KEEIKYEIINNDEDEIFLIFGKNSKDYFTLDFRHPLSTFEAFSVAISSLLKKKAVS*
>N.PF14_0059
MNIIESNKNDEKEKNTEGNEETDNSNLKRKNEGFLIRSFKRIKHKIKGDDDNNDDDNNDNDNNDDENYDNDDE
YENKDIVIDDDNIEKNEKVNKNDDNKNDDDKNDDDKNEEPLKLNGQSYIDESLKNDMNENYNYKYNKKSKKVI
SEDYENEDVDEKKKEERKTEDIHMNKKIVTKKSSKYELKNIHKNVSKERTDYIKEKEDNINKRSDREKKYIRK
NVKEKRIQYKKGKKDRRDFEEDDDDDDEDDEEEDDDENNDEMKGFIVDSDEEDEEEDYSDDDSSDDDSSDDDS
SDDDSSDDDSSDDDSDDDSNDDADHDSKKYSEDDDIIKEKKKKKKMKKKKKKKKKKKKYDYFENKSLDDEDLE
LIAENTGIQVIRNEKDTKHRRLKKIKDSNDEDDSYDHSSINENKNNELKDKDDFIDDSEISLRKKKGDSNNNL
FNYADNLSDTRENEKHALQEGDEDLEEEEEGFGVTSDNIYKNDSYIHNVITQTFGDINAVLDIINASPSKKKK
KYDYEDDEIESDYDDEEDQDSKDIIVDDEYPSRKKRKNKKKMVSFFDDINESMKRIGMDDDDSDYEMDENEKY
DDQDGLEEIEEDYDEYAEDAYGEEDDEEDDEDADDEEDDEEDDEEEEEDDDDDNDIYEQEYNKIYNHEYDDFD
EDEHASTNDLGEFYKEHEEAKEDIDIDHTDKGVLKVDQDSTQKEHNINVEENHEEDKNNILSIEDESQLVKES
KEKKDLFHDDDDDNENKIEKKEKPKKKKSKKKKKRLDLIVSDNWKKIAEPDEALNQLLTKKDDLIRNTDMPER
FFNIYKKRQKKLNKKMLLIESKWILHKLKEKYPTYFTYKNFEKIIEETYNKTYDINVNDFLNDSFLLKKCILT
LHMLIKYNNEIPYIVFHKSYLIFPPFNINMLWDIYELDQSWYKTFIKIRKLRCRLKRLSKANNIHMDIFDILN
KYNEMYYEDVNIYYYYLKNNLLNTYNNNNNNNNNSNSNSNSSSNNNNNNQNILSLTNNDNDKDIFGEDINNDK
ESKENNIYMKDDLFGEDILADEKKKENNMNVEFENNILNMNKLKIENDEKEDNKNNLYNKFNERNIPGLQFLE
YVKKNRFNIWDEYLLTTEEFYENICYLYDLIRNEKIYPSELNYDDHEKNKYNHNKDDSDNDDYFNKGDKKKIN
KKENLKIFKYNCSKIKNIVSNVPEIYINDLNVESQIEDWCKSFFSREISNNKEIFKTLICYYSKLLASHPCIK
YIFRFFFLKYASITTVSTNLGEMNVDICNNDYFVYRLYRLPIYYLLTNEHRNQFLLERNEDKYNNRRDTKNSY
HHSNYSHNYTNDNDNNNNNNNNNCPINNDVLPSFQVSHDPCYIHNYNLNFFNTQLDESNIHIDVKKRELESYYH
MHKYKHIYLEILKNKENKLIDIILHPILPHETVPWKSEEYNEREKIKKKKKKNERDIKNSFEKEKNKLIKAEQ
LDSEWINNILRQLSYAYCYNDMENNNIFVYIQKKILNNFLCVELLPLFKKNLENLLLSSAQNWLMVYIHQSFY
LTLNVKPIKIFKNDIIRDNLEKIKKKGKKKSIKNKDNNNNDDDDDNSGDNNDDNNDDNNDDNYDDDDTSYDDN
NYGDDEEYSSESKSRASSTSKYEEGMLKMDYNHKKDKKKYYSDDSYHSEMSDYYKKEDMDDKHNEKKKKKDKN
KIKDKNKNNNNNNNNNNNNNNNNNNNNNNNNNENSDEDNSHNNSNKDHNNKDNINNDIMNNDKKKKDDIDNDEE
KEEKKNQSDDKNNVKYSNKTDLFHSTYSSDEYNSEVQNDRKNESKNKKKKSNHMYNEESEKYKKNINYDNYSD
KSGKSYVSNKNDEDKKKQSLLKIMKKNHNVFDVISIVCEYVQYNVRVHLMACDIYGDILKYIYLDNIYIDIIK
KEKIPVEKERIAHDLKILKTFIKRIKPDVIVVGIRDIPSYLVYSYIVNMFNKNNMDKSNMKEENDDIYHKDKH
GHKYDDHKYEDHKYERNEENLINPTNNKYHIIPESLYIPNLLTNSLKYSKELVDKYTREALMCLSLCHYVQNP
LGVVISLFDEDTKSMLIKVCLHDLQNYICNYKLQYLFERVMIDVVNKIGCDINFLRKQKKKHLENSLNYICGL
GLRKREELMKLLHNTNLNTREDLLTLSNNKKFIGKCVYRNCSSFIRIISNGDDFVEALDNTRIHPSNCYDIIY
DMLKNTVEKKNSFMKNKDLYELVNYLIDKKRLIKHIEIKDYARKYYNSANNNNNNNNNNNDNNNNNNDDNNND
DDNNNDDDNNNNNDNNNNGNGDHNNNSNNNKGGDGGDNNNKIINNNYTKKNNYNKKKNIYLYPYLKFIKNELL
EPYKDYRYIYDKKKEEELFYLIINEKKENLVVGSEVACKINYINKNNSYIGISILPYNIRGFISDSKEFLRQL
RNYYNERMNYINSNMEILNSFNNKNNFMNHNNNLKDAQYINKSVIGEVIKGRISAINYNLNNKFETNFYVVVT
DENIRKIKKQVFLQNMSYPFSYENLSPLIELDINYDNNNFIQTHFKKLNDNEDFDDFVEDEKNLFIKNKNKYY
QNKQFINHPNFKLWNYQEVYTYLKQNNISIGESIIYPSNEINKLYVIIKTCDNPFYIVKFTIYEKNVQQHNNI
TNRNIHQPIQQIYLINNEQFKSIDQIVTNFCDNLKKNLLELYNHPKCKKRKSFEQVQKELSQEMILKPDNIMW
SIIPPIMPEIKNNNNKGDKNAIQDFNPLRFTLMVIPPINHQQHLYSNIHSTYEPKDFIVLHDSIYVDHKSFKL
WTRTEKNFKYLITWWKEKGYWNRQNERLEYMNEKKKKMEEYNKKIKSIKN*
>N.PF14_0060
MIYFLLFICIVSLLKTIEMSSPSASIVGHRGCGSSTAGGISRYPENSLFSFKKALDENVDGVELDVWLTKDNQ
VIVLHGTEDGLLGHTLLCNKDCENKNIEELNLDEIQKYHFKEPWILNHGKTFNENNYELNNNQKKYSSLTEQE
KLEKENEYENFDKAYINLEEHEDIEKLINEKFLNNFYNLNNKNNYEEPINDTEYIEKEKKLFEEYNLDTNINT

EEDYINSIKCSHCKDIYKKYMMPKTHYHLKKKQLLFKFISMFYHVPLLENLLNLYKNKLTYDIELKGNKEDLG
LYILNILQNYKDYKFKFSSFNWLLQYNDIQEKIHKQENNNSFNYEAYPFYNVDRIDLLKVLRNNKLNIPVALL
FSDDEIMPDINTILYTMKYYNAQWAHFSFRLKKNSIVVHSNPSNITIPIEDFIKIFHNNNKKIMIYWGTEDND
KADDIFSYLKLDADSLCPNDIVLAKYVLQGIKENDTI*
>N.PF14_0061
MKLTYYYYAFCIIKVIASLRLDSYKKQNVFFLKAFFKNVINIKKERKKNLVNSFFYVGRRNKIPIKYSNNEFY
NIHNENYDNTKYYNNNNNTNDYDKKDNNVGKRKYSRKNMLYMNSSEKDNFLNENILKKKSSEKEIEQSLTPLN
MDWVKVMNLIYSSNDIDATTLAFNAAMSAVEKKGCLSTMLDLIGTMKSKNIKPDLVSYKLVLSLCDKYHLVDT
AEILFEEMIESDKINPNYEIYAIMISCYAKTGNGYKAIELFEKLRNDPFVEEMRSLNITNTNDNKENSNDLQT
SIIHNNMEDNNNNNNNNNNNDNNIYDDKFKHISNKIKNVENCSGKIQYSEYANVIYACNISNLYEQGIKYFEEL
LKSGKYMPSIFVFENIFDLLSKNGDYEKSLEYYNNLKNDPNFKKNINVNILNNLLKALSIHNKINVAEDIWNN
EFDELLLTPNNLSYQILLKIYSHIDNYEKAFKLFKEMQVNKLLNNKNILPFIYTIESTKNCGIYNYAIYVLRV
AKLLNFKANDLLMLYNNTMISCINSKKYDVIISLYAELINMQQKDTSFQININTLTFVLLAFKELNMKQDFIN
LKNIIIQRNYKLPPLCSKIFSETENY*
>N.PF14_0062
MMYTFEKKEEERYLTKAIYAYGINKNIYNPFYILDDNSIFYCVGTNGVLHSIIEKKQKFLLSHESSYGIICLG
ISFDKKLLALGEKSVNKPYISIFSNGTHKLIKRLTLDISDNGSRIMNICFSSKNKYIYCITNGNTKSLFLCYD
WLKEKLMFSKIFPFSLFSNICEICINPHNSSYIALLSIGILKNVIDIDPINDEEKTEECVSMDNDKKKKDRKK
EDVKNKDNYNVNCNNNNNNIDINSIDNMDDTSSNNEVRNIFLYHNVDKNLDDIVIKNKKLEKIDNNFTNCCWL
NDGILLLINKNNYIIFYDVKKEKLKIYNKHVSSKEIIKVISMTKGFAVFDNYFIYVYEKSSDLSMSLCYLLKY
YVRFNYNILYNSYCLFSPCEKMLYFLEKDGNIKRYDVAKRKVAFVNNEKVNYESLRNKTNMKEDLKTTKKEFD
DTNLINNNNNNNKNNNYDKINCTSYIHNSCNGSFSGDENGKEEDHFDLETVVYNIGSSKINDFDVCLKHPLII
LCYDDNMIRILNYRKKEIVLENSFNNEPLHLSIHCSGHLLMVAFTDKLRIFHILYNKLKIKKEFFLKNCSCCK
YSNGGNFMAVSKISTIYIYKTYTYELLYVLKSHVNYISDIIWSSNDFSIFSIGQDGYFFEYSLYNNGNKMIEV
LQKEKKFLCIDLEFLDDMNKKNVNGEGNNLDKKQKKYDNEYKSLNNLTSNEIKNIYISSDDKTIKQFCNSKME
CVLECEYIINKILLYKNKFLIATYHNGYFSRIRFYTLPLSGIYLEIPCHILNCVNLKFDINMELLFSCSKDGS
IYIFSIEKVENAFIFSQNMKNNHVDTNKNIYPNGNKYNLHDQNDTINLYQHNNNDNKTNETHADDMKKKRDIL
NNDDIDKINKIENKKKNVHMNNGEDNNNNNRDDDLKNGSENMKNNYNNSNNSHIFNSPIQHNDGGGHHDHLTS
PKELCLSDLNNDSFKNDDNKNVNLSKEQMKLLQNNILLYDLSNKINYSLNEEEKDNEEILIDFFYVQKKNKEY
LELEKKINNLKNQMDLEMKNKESIYKNELKKLKKEKNLEIKNLMKINKSLVKEKEKMENTCKESLYELEEKHT
YFVNQLNSQFYLTNKISEEKFLKVQEEFNLYKKNSTTEILELKNQHQIQIKELQREKDEQVQKKEDYLQNCIL
KYDNLQKEKEQYIKQMEEEVDEEILMITKKYENEIDQLKKDKYDLMGKFKLYEHIESELKENIQEEKDKFIKN
NITAKRLHENIDNLKVDLSNLKDNIARKEKEIESKNSEIMNLNKKNEELEKVKIVLTQKIKNLESNLSPKESE
IKVMREKIDEMAKSFENNHKKRVNLQIEINEYKMKIKFLHDDLLNYNKTIMNYEKILKNLQDHIKECYLHLHD
KKIFNSSFLNLYNKFHKVNDVQNYDAKNVFSEYIRQKEYLENMIEVLKDKLHKETEAFRNEKIKMMNENSLLL
KEINDLKMDLNFLKSECHEAQLQNRKMKFLKKRSESKERKKEKKEKKENKDNNEEKNIDIK*
>N.PF14_0063
MNVLYIFIAVLILNGILNIHVSKKKTSFLNNTYPINKYKTINIKRHYRKVQNRNNKLYVSLFDEYDEKCIKAL
IMAREVAKNDNENEILLKHLLIAIIRIDSNLVQNILKNFHISLTNFLDKFHVAINKISKSYTNSNNGHNNIYE
QSGSAQNNLNEQEKKNISLTNEEIDMKNILNEEDENQVENRKQNENGNVNGNGNVNENVNENINENEKLLNDF
INKHLKDMEEKINILKNLNNEEDNASLDDINNKDYIINDISSSNNITKENNNNNNNNNDKGNFNNLDTNNDSG
NLSNNVPKNESHNVLHNHTYKINNNNKMMNQEILGNPNFDIKFSENCKLVLHNAVLEAKRKRKIFVNIIDILL
SIINIAQEKKHYDFLKYLEELNININDLKSLLLSYDEKNYDGNNIYDTNANNTNYNTHNRNIPLNNNNNNNNV
SNKGYNNQRLNNMNNNEQANHIINSLNNEYLNNGRDYKYNEDHPFSSNNKFLNPSTSSASSISFMKDCLIDMV
HEAQEKGDDHFFGRKKEIKRIIEILGRKKKSNPLLIGESGVGKTAIIEYLSYLILKDNVPYHLKNCRIFQLNL
GNIVAGTKYRGEFEEKMKHLLSNMNKKKKNILFIDEIHVIVGAGSGEGSLDASNLLKPFLSSDNLQCIGTTTF
QEYSKFIENDKALRRRFNCVTINPFTSKETYKLLKKIKYNYEKYHNIYYTDDSLKSIVSLTEDYLPTANFPDK
AIDILDEAGVYQKIKYEKFMKQKLRAERLRKIRIHMNTQENNNNNNNNISNNNNDYIISDEDQHIHNNNYGYN
NYDVINQQKEFSNDEDINKLNDDDTNLRKTYKQEIDNKIKNDENGNNNIKTDTNGNILINNVESLQNDTYDET
RNLIENVHMKYVTSDVIENIVSKKSSITYIKKNKKEEEKILKLKEKLNKIIIGQEKVIDILSKYLFKAITNIK
DPNKPIGTLLLCGSSGVGKTLCAQVISKYLFNEDNLIVINMSEYIDKHSVSKLFGSYPGYVGYKEGGELTESV
KKKPFSIILFDEIEKAHSEVLHVLLQILDNGLLTDSKGNKVSFKNTFIFMTTNVGSDIITDYFKLYNNNYSNL
GFKYYIKKKKNENDINQSKQEEQYLVHTSNGNIENEQNKKYMDPTKQSNENNYESTINHTHTADTKYNNITTD
NNNNNNNNNNNNDDHFEIFEEKLRTNKWYDELKPDIEEELKKKFLPEFLNRIDEKIIFRQFLKRDIINILQNM

IDDLKKRIKKRKNINLIIDKNVINYICSDENNIYDMNFGARSIRRALYKYIEDPIAAFLISNIHEPNDSIYVQ
LTNDKKIKVQLIKAPVQQFSS*
>N.PF14_0064
MSGKLEDIGELVLLIGDFHSPIRNLGLPDCFKELLKTDKIKHILCTGNVGCNENLELLKNIADSVHITKGDMD
DNFDFPEDITLCIGDFKISLIHGHQIIPWGDMNALLQWQKKYDSDIIISGHTHKNSIVQYEGKYFINPGSVTG
AFQPWLSEPTPTFILMAVAKSNIVLYVYEEKNGKTNVEMSELHKSTVI*
>N.PF14_0065
MWFTTRQDGLDDNCHTNRGPCFQISGFFGTTLRIGFFLEFIALTFLFMSYWSNGGKGLFSYDLKNINDEYRLD
QTFRNSITLWTGVYLIGAIFIMSFQVLLADDTCWARGYRAGSKILRLASFLDTISATLQFIFYLYISKFYTRK
WYVHFNEGGSEWVFFIFVRLVHAFSCLLYGLAAYLLEVYHDEGAGDLHAYINGVMFVFAGLTEIFVIFCNSGC
YSNLLLWLALGAVSLWSYYFEPEVNHVSPALHETELTNDVEQQVEKFSRYTPYPQDTNQNAYYPA*
>N.PF14_0066
MEKSKRYISLIKMMERKKFEKYRLKQIMDNIYKGKIIEINKMKNIPTEIRRELKNIFHNNILSIKPIKELKYD
RAYKVLFQCKDNEKIEATSLDFGSHKSLCISSQIGCSFGCKFCATGQIGIKRQLDIDEITDQLLYFQSKGVDI
KNISFMGMGEPLANPYVFDSIQFFNDNNLFSISNRRINISTVGLLPGIKKLNNIFPQVNLAFSLHSPFTEERD
QLVPINKLFPFNEVFDLLDERIAKTGRRVWISYILIKNLNDSKDHAEALSDHICKRPNNIRYLYNVCLIPYNK
AKNVDENFHRLDDAEKILQFEKILKKNGISFFYRNSFGYSIDAACGQLYADYEPKKRKEKIESKNMSLLL*
>N.PF14_0067
MIKFKDILIFINIYILILNAYCKEWCKATFEYGKSDYAECISEGDSISRYMIETIPILSKDVDLYSNVSLVLS
NGYGSKTKEIIIGNENDGLLNKIFTVRSDIGNPEYIHIKLNSQNKNWKCKKITVWKDYKYWVFDCIGSLNDKK
PESTYFLSGNKIYIAYVQTGKDIEAATTGTIEIILLGNNKRSNTKVLHEGFSAGGLKKIKFQASDVGELEDII
LINNSLNDPWYCDFVKIKSDNNNKIYIFNVKSWIGAPYDKNVKVNIKSDTIEGTSKDIDCHIRAIDLINTNNI
NKLLQNKVQIFKVRCPQNCHNSEFAIIEGSSIHPSSTSICAAAIHDGSISPSGGEIIVTVASELNHYYTIKEK
IFNELEALDFSAKADEKNFTFFTYHLDSIDDIISNVRIVDSFGKLSSLGRLEIRLKNKNSWGTVCVKGPNFEF
NDDAAKRACKDLGFPNGIHIKENCANLNGQNYCAGYKYPFASSGILCTGHEKDISKCNADDFSHCVDHHDDVI
IQCLHYSSNELINDGAIRLVDINGAPSNNGIGRLQIYYHGVFGSVCSEGWVKEAENIVCHELGYNGLKGNGFS
HHSCTDISGENLCGPDTEKINAVNIKCKGDEKLLRNCPHETHDDIYCSHDEDVIVGCVGGDNNSHGEEQNKKH
LINLEKKKFHPKIELTCFDKMLSKSNLSVATTGDVFLASCPEKCDEEVGIVKGTFLYTYDSPICKSAIHSGVL
PNNIAEDIVLSIAHTHNNFIGTKRNNIESHDFKGTSKSFTISIPTMSLLREERKSNPKSEDEMINKNEIDLTY
DHSVFNNKLDHHITSSMKPTFQWIAPTGFVGFNGKENDYIDCTNLPNEKYIRSLSNFTFIVYFTLNGGEGTWR
TILSHSLCEGISISINEDNELIIEQNCNPHLLKSKFKPKFGQTYHISLVFNKINKTLYLYINGKKVITEKNTY
NFTLSGDLIIGRSNQTTKDYFIGNIHLVEIYKYTLSEQEIKESLNSSLSLEYLNMNILEQMSNTKKDKKKNQK
NKKGVQKTIDGRDCVTPCKSKNMINKDLQINTQQINLKCQDNLLSEQFNGKIGSQFLVSCIENCTKSKYFIKG
TNNYYTPDSSICKAAIHAGIYKPNMGNKNNTFVIRIVEGLLEYKSSRGHFGILSKSEKQSQLRSFSVLSENEE
NIFTCSTDGQFILNLSVGEKRTINCPSNCNIIKDKIYGTNIYSPTSVLCKAAIHSGALSNQGGLVEIIVGTGQ
EEFKGSTQNNVESFSSNNHSRSITFNKHMEL*
>N.PF14_0068
MTDSFRGGSGNFKRNSNNFGKGNNNAVRKGSNWKGFNGGRGGGGGGRGGGGGGRGGGGGGRGGGGGRGGGGGR
GNKDRKSFKKDNKSGKVIVVPHRFPGVYLLKGKSDILVTKNLVPGESVYGEKRYEVMTEDEKIEYRVWNPFRS
KLGACLMGGVGNMPIKPGSKVLYLGAANGTSVSHVSDMVGDEGVVYAVEFSHRSGRDLTNMSKKRSNIVPIVE
DARQPTKYRMLVDMVDVVFADVAQPDQARIVAMNAHMFLKTGGWFIISIKANCVDSTAKPEVVFASEMEKLKK
ENCKPKEKLTLEPFHRHHAIVLGMYR*
>N.PF14_0069
MAKYKKKEENDEKHDNGEFKNIKNKLEDLKNKTQISDEKNENIKKSVDPYNDLKLHILNILDETRKCIREKES
MQKIHGNNMEVIKRGNIIYNNMKNVETYFTKLEDILNKQLKQKYNFTKEELLDKNETFELLKRQMYECKKLSN
YDEIKNTYVVNFNDIKNKPKLNKKDTSDISHDEDDLVVINRWKERDKQFNEDILKIGEVIDKIGANTVILTQK
AEEQNEIILNLHEQTEKTQDNVKEVNVEIKKVMKKHSQVTWCCRITLVIIFLMLVVLTSNIISNKFLKKL*
>N.PF14_0070
MDKNNNTENTTTCAPTEKKDPHQNDLNNKEVYNYYYYNNLNDGMGNNRIGYNNIPCENNYDGNRNIQNYYNYN
YSYINNKDHVAYGTPGQYMMNYNFMDTGYYHMNNNNNMFNNTLNSFVNKTGDIISPYHAHNIHPNSQLTMKYK
LSGNIITNNSFHSSNTNYTNEEKKNCLEMTNTTTYNVNTLLRNNILSSEYFKSLIPIKTFKEVVDEIHSYADH
VEPYCIGSNRAPSTLFCCLYKFFTMQLSEKQLKSLIENKDSCYIRACGFLYLRYVHSPANLWMWFEPYLLEED
EFSISCDKRRKVTIGEYVQSLLSDDKYFNTVLPRLPIKIKNVYGARLMIIDDHRRRLKKNKENISKFLKGEPV
LAYINGEWEKGEIGGIVNHGKDKFFVRLREIDGNEKLVNIGYVKLEGKDSHKEKDIQKHSRERSTSISERKR

RRRRRNSSSKSTSRRKSRHSRSSSRRKRKRRQRDESRSSSRTRRRSHNRSHKRSSSYEKDYSIDKYSSRSHSR
SRRRKHSSKHSSHYKSHNNHRDRSINKYNRPSEEDELYKSKKRRNVSISRSSSSSTRYRKEKYEKTEDELISR
FKKLESQKALATGKDYARRPTSYKSSLSLKVDNIPVRRRSRSRSPRRIDNRVVVQVVHTDNTNNDNNTSENMK
LKELMKKYNKDDTNDEPKMNNSNLEEMDIMTLG*
>N.PF14_0071
MKRYFLHNDKFYNRKKAKIECEFNNELKKECLTPNFYDYQASSKNVDDEKDDINVRGFNLFHLEENGKRRKIN
ILNIFDNNIKKKNETDIKYMNNQNNNNHLVRNKNEEKVCQYNDPDLCNENIDCYMNNHINHKNLYKSKDKLDN
DNLSYDKMEYGRKEKWTNIKNKYYEKINNLLKDMHMIKLARNNGKIKMLTSLKK*TNIDIFTTIYEGECTKKK
KKKKKKKKKIYLWVYPMNIDTYININIKYIXXXXXXXXXXXXXXXXXXXXXXXXXXXXXXXXXXXXXXXXXXX
XXXXXXXXXXXXXXXXXXXXXXXXXXXXXXXXXXXXXXXXXXXXXXXXXXXXXXXXXXXXXXXXXXXXINK*
>N.PF14_0072
MRLLTHNFLKCNETQCTGGYPLTIKLDMDSQENIKIIDQDINVEFVKNVLSKVDYDVLYNTAKQFGINLLASY
NSDHLEDEEFLNSVHHALFKVHIMEGSLVCPKCNISFPIKDGIPNMLTGNEK*
>N.PF14_0073
MKEYSYENYYPKDKNDIRNISKEKDRMIVLHNILKDCIIAKVKNDYGDDEKYFINLKDYPEKKDDIKNKNIYL
YKISNDNLKTCSNYFDSINQKVLVPFLENSNSIIDKKDCKIDHICYNSTNESEGKNNDIIKYKERPDHMLSNE
SVHIKRSVNMLHNNIIKRLKDNTRNDNNNNNNNNNKYVENKFYKSKGIYNEEKEKKQHKKSNTKKEMKKSNFS
FVRKESSSKLKYCNNINNSKKKKIVNSKGTINGKEKIENKRGSTKGSKKNMSTYFFKTNDKNDIKLRNRNHSN
NSQKRSRNNNQINENPDFEFLFNTKVVKQENIGKDCNILSNELVFSGNIMKVKKQKRKKIDIKNRNNNNKTVE
RNISNGTNKIINPYKMEKNKKDLKNNENIKNIKNIENIKNIKYIKNNKNNKNNFITIGQRNISTNHNKINEEC
FNKNCKDEHSTNTESSYEISIQGEVNLNFDLSDKEGTEMKSNNNIIFMCNDNYVKKRYINNSNDINNIKSKEE
KNKNNCISQQNGKMEKEKMKENFLFHNDYKNDQSNMCRHNKETEEKKVDHIYQNGKCMKSSSSFEVKHLYKKI
NDIFDVNIKNKKKYIHIEKCDNIIKKYCPIHPDEHVNMYMNNVKEKRNKQNSSEPVKGINNIALNVCTRTNNE
NNIFDKSMITQKKEKKKKNFHEYNIENNHLNNEMHKIDDNTEYKKKKQISLLEKSNIKHNNLRIHKNTDKGNN
NKWISGNIINNEYKTTKDIYYNNLYKINRVKLEKEDNNNGRIRFKRGTFNETYDNIYHHKKCYDLVLDELLKV
TRKKKNKKHYHEYDKMHNHEKNILTNNSNNNNNNHNNNRFDINKMKEPDEKRKNDKSSITCDNENIRIDYKRS
AMETIRNQKIHQVTNQLNVTKYESKLIELEEKMIKLKELLKKEDIENEQLKSFLILQDEEINALKEELKILSE
QNNKKKKISKTKKDKSHQNTTFYKNLIKSKPSKFMEIILKNVKEKKQEKKTCNFKELDKNNDEMIMKDPIIFD
NKNNINPIVKNVNSLSTSCNSVVHSCYPYDKKNIENEENEKNEENEKNEENEKNEENEKNQKQANNHNETAIF
NDMPPNIKNNQIDMNNTFSYICCDMRKRRSLDSSTGFIIKEKNDENASHKLKTMFCPMYKNEWRINKNDEMRK
EYHPHETSNGQIHNYVTNKYPIKNDNTKINLLKVNISEGKKERIASVNFNKCEDDNKNDIENMNERTSNIQTM
NKIENQIKNKIDCNNMENLHKYNKESNTNDAYHNLNLCPSTIFRKKNNNKKNNVKICQEKINPHIVHINYISN
DNFGQMRKSGKNKYFLRKVKEEKINEEKKENIFSKFFSYIKKKSICNEKKDHNKNLRRYNSHVHLRTKNYIYK
SPDNKRSQSFINLFKNCTAQNGSKNYTDYVLTDNNFNSINTYQINDKEKTEIKKDSPIFVPLNKIEENNIISN
NHMKISENVNHPEQLYLKGKFVPKYKINNEHDIKKKRKSFVCKKVIKKGIIKSNQIYNFKINNGITKNMNLYS
QNVKEKKEQLNKENMKHMDILNIRGKRKKNGNINMHKLYNKDQQVKSVNSEEIHFEKYEKIQENGKLLNDRNY
DKNIISIIDGENIGHDSQKRIKHKEDNKIINNKKYISNVSSMSNVSSMSNVSNVSNVSNVSNVSNMSNVSNIS
NMSNVSNISNISNISDDIMISPNQKAHYINSSNNNNKKKTFYLNNPSDNSTNTNINLLKPSIRKMYGPPEGST
CSNDHKKYLWINDEEEKNRTNQDKHKKKKEKKCHYDHNDDNSLENSSKININDFLKNQEIQGKFNLHLTNANK
KLNDSSQNVIKKIVLNCKCKKLKEEKKKNKSIKNFNNENKHINDMKNVEIIKNKVSHLDNKKKKNIINNNNII
HENKMNDNINYYLFKNDKIKKKNIYIHDQDIPQEYISKQKDSSCKKYINNMEKQNINYDFQISSYDNYSNKKN
CHMYENIIPIESNQQNNVLNSNGYHINHEPKIHFLENDESTIIVEYPNVNYYIKCP*
>N.PF14_0074
MENEYATGAVRPFQAAESNERYQDPQNYELSKKAVIFTPIYYFDGNSWTALERLLSLKKTIFHDNRLVTLCPV
ENNITPIELEASISGKYDIKVYRHCEYILCIEGEQKILIKIPVTKNIITWNSEQRLPLLPKTWKPTIFLLNES
NIFLRFIPDKCLVISQVSYSDSYKVNCINFSEGFCCCHPINNLALLYGEYQQNQESKIMKLPKLPISNGKYNY
FIHFFTWGTMFVPKYFELSRGPLCNFKKNIIALLIIPPKIHISIELHSSSPVVCSMEYKKDFLITARKPNITD
IEIYTIIQDQLIKYDFSYDLRLNKENASISHLNIPIGFKISNEEKEKKKKNSSHICKWTFIETKDQRTLNRSG
NSSSEHIMSQDLACIFDAEKGIYYSTDYGIRYCKAFKQLKV*
>N.PF14_0075
MALTVKEEEFSNTLIKNASAFDRLKLGNLKNLKIQKKLQFLYLILFVLITGVFFFFLIGNFYSHRKLYQVIKN
TKHTTIGFKIDRPHDKVLSSVLKNKLSTYVKESFKFFKSGYAQKGYLGSENDSIELDDVANLMFYGEGQIGTN
KQPFMFIFDTGSANLWVPSVNCDSIGCSTKHLYDASASKSYEKDGTKVEISYGSGTVRGYFSKDVISLGDLSL
PYKFIEVTDADDLEPIYSGSEFDGILGLGWKDLSIGSIDPVVVELKKQNKIDNALFTFYLPVHDKHVGYLTIG

GIESDFYEGPLTYEKLNHDLYWQIDLDIHFGKYVMQKANAVVDSGTSTITAPTSFLNKFFRDMNVIKVPFLPL
YVTTCDNDDLPTLEFHSRNNKYTLEPEFYMDPLSDIDPALCMLYILPVDIDDNTFILGDPFMRKYFTVFDYEK
ESVGFAVAKNL*
>N.PF14_0076
MALSIKEDFSSAFAKNESAVNSSTFNNNMKTWKIQKRFQILYVFFFLLITGALFYYLIDNVLFPKNKKINEIM
NTSKHVIIGFSIENSHDRIMKTVKQHRLKNYIKESLKFFKTGLTQKPHLGNAGDSVTLNDVANVMYYGEAQIG
DNKQKFAFIFDTGSANLWVPSAQCNTIGCKTKNLYDSNKSKTYEKDGTKVEMNYVSGTVSGFFSKDIVTIANL
SFPYKFIEVTDTNGFEPAYTLGQFDGIVGLGWKDLSIGSVDPVVVELKNQNKIEQAVFTFYLPFDDKHKGYLT
IGGIEDRFYEGQLTYEKLNHDLYWQVDLDLHFGNLTVEKATAIVDSGTSSITAPTEFLNKFFEGLDVVKIPFL
PLYITTCNNPKLPTLEFRSATNVYTLEPEYYLQQIFDFGISLCMVSIIPVDLNKNTFILGDPFMRKYFTVFDY
DNHTVGFALAKKKL*
>N.PF14_0077
MDITVREHDFKHGFIKSNSTFDGLNIDNSKNKKKIQKGFQILYVLLFCSVMCGLFYYVYENVWLQRDNEMNEI
LKNSEHLTIGFKVENAHDRILKTIKTHKLKNYIKESVNFLNSGLTKTNYLGSSNDNIELVDFQNIMFYGDAEV
GDNQQPFTFILDTGSANLWVPSVKCTTAGCLTKHLYDSSKSRTYEKDGTKVEMNYVSGTVSGFFSKDLVTVGN
LSLPYKFIEVIDTNGFEPTYTASTFDGILGLGWKDLSIGSVDPIVVELKNQNKIENALFTFYLPVHDKHTGFL
TIGGIEERFYEGPLTYEKLNHDLYWQITLDAHVGNIMLEKANCIVDSGTSAITVPTDFLNKMLQNLDVIKVPF
LPFYVTLCNNSKLPTFEFTSENGKYTLEPEYYLQHIEDVGPGLCMLNIIGLDFPVPTFILGDPFMRKYFTVFD
YDNHSVGIALAKKNL*
>N.PF14_0078
MNLTIKEEDFTNTFMKNEESFNTFRVTKVKRWNAKRLFKILFVTVFIVLAGGFSYYIFENFVFQKNRKINHII
KTSKYSTVGFNIENSYDRLMKTIKEHKLKNYIKESVKLFNKGLTKKSYLGSEFDNVELKDLANVLSFGEAKLG
DNGQKFNFLFHTASSNVWVPSIKCTSESCESKNHYDSSKSKTYEKDDTPVKLTSKAGTISGIFSKDLVTIGKL
SVPYKFIEMTEIVRFEPFYSESDVDGVFGLGWKDLSIGSIDPYIVELKTQNKIEQAVYSIYLPPENKNKGYLT
IGGIEERFFDGPLNYEKLNHDLMWQVDLDVHFGNVSSKKANVILDSATSVITVPTEFFNQFVESASVFKVPFL
SLYVTTCGNTKLPTLEYRSPNKVYTLEPKQYLEPLENIFSALCMLNIVPIDLEKNTFVLGDPFMRKYFTVYDY
DNHTVGFALAKNL*
>N.PF14_0079
MYIKMMNTTGEIRNLCLNILNSVKKKCDEDIESNDDKKNLCENNSDIVEKMKGNYEYKDDNNKDDNNKDDNNK
DGTNKDDNNKDGTNKDDNNTDDNNKDGNNKDDNNINNNNNIDDNNIYDNLMETKGDKKKYYKSKKNDHHQYKD
INKLNKESSLNNKKEEKYNSTCAIINNKGKAISKGNGKYPNELIKKEYNKKKNEKNKKSIDENKKQNDENKKQ
KCENKKKCGESKKKSEDNKKSNTENKKDVKYNYYSRTNYFVPNEMNGNIFKNNYTHLENKNEFGNYIPTYISN
DNKNMNRSNRNENNLYLLNAGDEEKSSYVYNYEEKLNISCRFFIQESNPYELDIRMLKENCLTVFELLYREKK
KWCSLYISTMNGPMSNFNYHLFKILCSNDEIYMYFFLFKKFIFSCYIYFNLVSIQIYSNFSNIEESETSYGFK
NLINTNVQGTNNKIDYISADIHHHNNNNNNMSSNSHSVSKKKKKDITYDINEQGENKIEDDILKKKYNKYISP
NFFSNSTNISSACINSYNMNNLNEEKSYVSNTFTNNHNNLCDTTCDRRNNIYLKNMNNFSSTKDEDEKLLNFI
YDPSKHLCGNRINYCSSYFHFFDKNQEDIKKKLQLFINTHSPTMVNITEKWNNFYENKNKIYSFVEKYKDIKV
SSIDHFRKDTTEKFISTFIETFSYVNRVSWKDQNGVVEHNNCNDNDKEKEQNGPLKQDNNDNNKDNNKDSNNG
NNNVNNNNDNNNDNNNNNDNNNDNNNNNDNNNDNNDKDEKKKKKKKNQSNYYQQHNICDDHKSLYDDVKKKDEE
QNCNTQHDEKKEEKSDTLLENHDNQENIVKRTDEIINEENDDYDKKKKKNFWNIFELIKNDTYNNDNNNDNNN
NNNNNNNNNDNNYVERKENSSISKILDEKNNLSNNDPIDKSVEKDIFTDQYKTNKNGSIIQESDNDNYNTTRN
EEIHEDEDEEDDDEKEDVEKEDVEKEDVEKEDVEKEDVEKEDVEKEDVEKEDNEEQKEHQIVESSEKKHKIILNNNTH
NGDISHNDNNTLYNVGIGMFNNNCSDNSNTKDSLSLMISHKSLTMDLNESMEDEKKEQSKDYNEDKNNNNNNN
NNDNLCSNSFDNIHVKIKKEIVVNDDQNDDQNDDQNGDQNGDQNDDQNGDKKNMSGGNFQNDPSDKCNNDNPS
FNNKKNYFPNYIILNNLNILITDLNELYDYVENGIDKNLLRQKNIPIDYCLKGDESLPLGVLPMEYEQNDKHH
HHHHHHHHHHNVFVVEPTTCSNELCKRYNNLSNIKNKENEHGKCNSNCVSRKCYTIHHNDKEMVEKNEENKKD
ECCNFDMMSTLDYQRFCRVCSGIEYENFERKIDYTKDNNYKVPEVFINESDFFCNCNNMNVLTTNNPNYSFNN
TKGSNVYDVDYNNKNFFNDKSYNDNNNNYYINKFNDIHKMNEVIAQHCFYNDEYKTQVDDKKRKLGVHIDKRN
NNNIIQNDDMNNDYYNAYVTKAKTKFWGFDNNKSLETNKNAALNNRELRNSKKNKLLTKNNQSETNKTNNKTT
KSASTIEGKSPRVKKLEKFTNVDQEELREVFGPTGVSGVYFEKSRSSWTAQYKVSGGKRRAKRFLVTKNMSYE
EIENVKQQCIAYRKQMEREYIKEFLNEENKKKINNSENDPLNTDVVSTGVKKVKRKRKLKNIYEG*
>N.PF14_0080
MDNITKKNISSPEIIKSEEINKENIIRNKEKIILEKYEKDRHNIVEKIRNGLITKNNLLNKKIITTENFLIDK
TYPSDIQKDVESPYIEYIKTSCTPYV*

>N.PF14_0081
MIEQKIHKRYKIVKKDDVNHRYTINDIEVYFPYELYDCQYNYMLSVLSALKKRENAILESPTGTGKTLCLLCA
SISYVVDILEKKGHFNENINISENNKNISLEFGKSNESVPKKVVANEFPKIIYASRTHSQLKQVIKELKNVYF
IKNNEKYKLLTTILGSRDQLCVHNINYNYKGTLLNNMCKRTRKNGECIYHNGLKYLYKLKHLFTTPMDVETLS
EIGKGNSVGQNIHFCPFYATREIQNECHIILLPYNYLFEESTRKILKLNLENSIIIIDEGHNIENVAEEAVSF
KLKDTDLNLFLEALKATLTILEKVNEIDKEIKDKINIDELFILNRSINALITWLHNLQLEKNEKKKIKEKHKI
YQGKQIFEIFEKNSINITKDNFDNFNNLLTAMIELLNKYTNDFKLSQMDVKNINRFIASIDHVKKAFGILFSD
IVKNCIEYFHLYINEEKINNTDTFNNYNNNYGGSSNIDSMNFINSGKNYDGMNRFIKKKIAYDNNVSNNNNNN
NTNYYDTFGSNNIYNNNTTNTFNNNTVCKNISLLCFSATASLCGIIKEKINSIIVTSGTLSPIEPFSKQLSGN
YFSFKHILENDHVIKSHQLFVGCMTHYNNQILLSTYENRANENYIRSLGNCIFDIIVCIPYGVLIFFSSYSSM
TETVNSWKKMKIYDKINTYKTIFVEPNKATDLKDILDQYEILIKKKRKGAILMGVCRGKISEGIDFKDDCCRA
VIICGLPYGNVYDSKIIFKKEFLDNFKYQKTDERPNSSNITSNISKGNKWYNEEAMRSINQSIGRVIRHKNDY
GAIFFLDSRFSNNQRIKEISKWVRTHFRIYRDVEQIQSDIDKFFELFKGLNNPLQLQKDQNINQKSNNTKIMN
IYNDNIHDMNNSNTISEGGVTIKENNESIDNTNNNSSTKLIGKNFQDCFIKSDKNKKTNKHDMPQNKFSFNPT
KKMKNQPKILSMIQNITKGKNPEKKSIYIDNKNENDIENQNNPNMNNNNNNNNINTLNNKEESNVNNKCSSLK
LDNAKLLIASFREILSKDIYDKFFELIKETKKKSNEKKYEILINNILELITQNFIRGNNEKKNINMSDEKQKH
ILFNDIMNAQELIDIWKLLIKLINNFFPVQEKEKCFFILQKRFKESTTDFDELEKYVVYNYRLEKIEII*
>N.PF14_0082
MDDKKENLNENISQEIVNINNNIVVEKESNKATSFLNNINNNNNNNISDELQNEINKMIKRNVTYLNIKENDK
NYLKTKKVNINKDNVKNKPIRRTYNKFKKVSTKKNNKLKQLTKAGKDLENKMLEEISERRKKNKIQKEKKKAQ
KLEGELKVGNMTVIKDISKLRKRDKKTKNKIYKLSSEVINKIMKKK*
>N.PF14_0083
MGISRDGRHKLRLTGGKKKIHKKKRKYELGRPPSNTKLGSRQVHVVRGRGRNYKYRAIKLDSGSFSWPTFGIS
KNTRIIDVVYNASNNELVRTKTLVKNCIVVIDSHPFTTWYENTFGTTLGKKKKEKKEEDNKEENKQEVENNEE
AAKDETTKTYGVIKKIGKSKNIDPLLLEQFKQGRVLACISSRPGQCGKADGYIIEGDELLFYKRKMDKKKRN*
>N.PF14_0084
MNNYIWNNTYSLEFLSEDFYDLNISTKDNVYEIIKKKKDENEVESLSINISSHICMFVQLIDNIIGKLSYSND
IINDTLNYKKLSDVFNFIKNQLYNCDYSLFLRIYSIYHTYNHRKNGYLDVNNLYNFILDIINKIENLNENHFL
ILPGGFYLQKEKMLVKILYIIHKKNNLYSFAVLNNSIYGTQYHSFKIDDIDNLGILLRDVVLCIKNIQKNYLT
NSLFWLCLYRLYIFPNADNVDILYQILLPYLNNSFLTNNWNICDNNTINVVSDENGSKDHSNGLMNTNNNINN
SNNNNNFNNNNNYNNNFNNYNNNNNYNNIYNNNTPFDMNFSNFNNNLNNNINNVQANLGNGPNYMNTSGNNFN
GFDNFNNINNLNDFDVWNNFNFNNSDKFPNDLNYFNDFDLNVFQNNNNNNNVMMASQNIPVQFSEQARYNDNK
NVRKKKIREKYEKYGGIYVNLYKDKFTSFSMSLYYSMIYMLKVMNIEEEDIEVFKIFIYYSIILKLCDDLIRY
EYIERRKNEMGNVRSSQHDNSNNNNNEVNNNNNNNNVNNFYGSQVNNYNNNFGHNNVSSIYSNNNNSELHKSE
NKNDPNRIDDIFFVFLDMSVRKLCVLFHLYFCKMKVDVYDKSKMIRSNVLNEIMKKILFVKYMYNKIELKIYS
DLTLNVDIIKNRNYVNSIKFPLYIKFEEELFKSYRKVEIKKDINMPISFYISRRNVESFNDLYVILYETNLIT
NILINQKSYIKHCYILAFSFISHVLNNVIPSTKHFIYLSKYAKLKRGLQLSIFYEIYRMYNNIYFISCSLHEN
KEIVMYKIINSSMLLFLYDFLLRVKCVDVCSIFSIHYNGFTNDSSYYMNQKRRKKQKKYIKSKNDRKGSVVNK
SKYNDKSSNNYDNKYNKFKDRYKDSMIHNNVNNISMSSYNRNSDINNSSSNNEYNDDSFVYDNIKRLKKCRNF
NDNDSYSSLDELAHASEGNIRLSGYCIDISVLECFSNFFLIINPKLNIIRSDVIEYFYKINEKKINKNSQLFL
FEKSNDINRSEIEILENMSLDLGFDRKKNDIIEYYCGECILFKNLVPEFYHLRDMLFIFKSYMCPYIEKLPQV
KVYKYDDINLKFEYKNKLIISVFNKKIDICGYMCPPKDTKFNFFSYIYQFIQTQENKKDNCILTYSNPNMYTT
DIYIENELDVLYVNDLPYFHPSLDLRDVEILLQYLTVPYIRIPLILEFFCDKNRIKSFESFDIQQLILCILFE
PYIFKNKYKNVEEDNFIPSKDRNIFATKYGLLINELIHSPKNVLCSVYSYLEIFFSFDNNNSINQKVIFIFII
IISSYVNSYIKLVLETNKEIRRNLNNIENIINKNKLIKKRKSYREKSYPHKDIIRPSEEEKEKKKRTISNYGN
CDIKKDNNGINNNSEEHINGIHFDGNERTYYKYRKHIEKRERKRMFNFLNKLKRKLIKNDLYCSDDVLMNLEK
YYKMIKKKLQNDVLGLLLKNIQMCIQKNDITTGSIFYGCILLIYRDIKFRELNKNNVKHILSSIFFILTYYNF
SAETNYSDLKNIYGLGSLFVNNNDGTNNSNIVGETRYSLQKSTKLCTKENSYNETDSFSLLIKTFSLCECDLF
ISIHKYRNMLLKYLEKNELDRNIIFEHTIHICACVHDNTHNDNNNNNNNNNNNNIGGSVKKNVYYNNRMNKHN
ANVFLNFRTNDNIIRKWLPVHKFKHVGIYTIEDIKNEKNKRKYLSNTNEYLEQEDNLGRHTKMTKNYYDNEYV
KDNNYHDMNRNYYFTSSSSSSYDMDDDFSSSEEDSSSYNDEISLSFCEDNMNEDINEDEKKKKMTGKEEQIIV
QYERNNMDNMSYDIMRMEKNEKKKKYKNYNKYSKGMKLKKKLKYDNYIDNNNNNNNSDMKKINPLHDTESINS
NVMNNYLSLPSFFKNSYYNKDDNNISYNNKVKRRKNLITRKDSILSNNKYISYIKNIIKEKNILINLQLFTFS
YKDNNIITLNDNITKFKNYRDVFLNGSKNFMSIERRRRGYRMKRHMKVLITQSYQNFCSFNIIGKKHNLEVWR

NSSRITYHNLMSYPFNKSKIFYLKSSSLLNNIKTVNNNNNLNNINNININNNAQNNDHNNNNTVGRDESLMIT
FNNFLLNEVKCGVLYEQENNDIYIKLIWKYNMNELERLTNENNIKSGNNKNTGEDNKLLENDMKDEEHRKMLK
RKRKKELYDLKSFNINYETQNIEGLFNFKYEENQYDSSLINTTSESSYSNCSSYNSVSKRMLNNINDGDENKS
DISSKSFSSSVLMMMMMNNKSSSNLSSSDDNIIISNNDNMNKDNIINNNDMNEGVYYNVDINSKEVDPIYKVN
NESVQINYNNKKNEDYINIGNNNLMKKKKKKKYITDGKCDSLLYTNDDTFNEGNKKNIINIDLNKEKKKKKKK
DSISKDIKKKHNYKNLMNLNIEEHGLREIIYRKDVDCIFIYNIFFHMNRFIKKLYYCSDIKKIYFSTSINRCK
NLFYHFENNNVRLCYYNNSEGYEKYHVNISRNIYNNIQVQNYMSPNYLSTFVPSILLKNYNFWYNNNDNSLIG
EIKHNKKNKKNKNFIIYIKLFNDKGENQYDTINSNKSVGNNNNNNINNNENNIYVNESEKRMNTIFNLMNNSG
EKKRNMPIYPYGVIQKLNIFNSRKMNEQKITSLKVNEEYKALTLINILSVENNNVYKDFYNILNRFDHITYIL
MYTLNVPILFNDNTFTQDSNKNRYINDDEINCMNINVDIIEIPRLNLTLKRNNIDIKINTNEKKSIDYVDNYN
EKSGLGRNKNTTNNSCMNNNINKKEGCKYYFEELNMFIYNGNIQKYNYIYNVIKKMNNTVLLQNHKNDFFFLV
SLLNKPFFLNISTLYKEFNNKNIHLCTLLEKHINVLLKNYMYVSIYYHNFNINTTNGNKYFDLLNEDYNNNGN
GRNGCSLQYIVLSIHSSKLFLNFRNLYSCFFFIVYKYLNKEHEEVIEYCNNLSICKEDINEINYLLNILNYCD
YSIYSDVDSCACRLKIFLTLSKQIKIILQKKRYRINSKKKNSWDVNNYYNIFNYVKSLWNIEKNDDQDDSSNN
ELYNTNDYYINKRAYKDKYYMFDKNKGRSHSTMGHRNIYTSKNKMKNKHRNDKNYYDKDDTRNNVHTSDDSNN
SSMYNTLSNISSSSDDNNVSSEKDIKRKELDAEEKINEDGDEEDDDHNDINNNNNNNNNNNSNNNNVDTLKIS
NKKKRKKLKTKLVGRSGDEKSKNRTKEHFNNNKKKKNNVNNNNVGGSKNFMGNQNVYYNDDNNMDSIIVYWDI
FKDIVFYINNLLYIKAKCKLTIDEEIFLYQNCNINIHKHKLIYNRYIILNSIVESKLSSKNGNPLFFYNILKK
KQIENQKKMRNYEICKNKKRNIFENLYNNKNEECKKSNIHDIDGKDSPMDNSNLGYGNNNYSSNMLYDNTNNS
CFINKKENNISFLNKEEEEERKKKKEENKYKLLNLDSIKDNLLHMKEMIITNRIEQKHADLNNLVVSINVPKI
ETVKYINNIDENIFMNINLYDNIGNIINNISYKKLEDKNNVEALNYLNNLINTIGIHNFFVIYNFFINNLNIK
IVKGENTFLYANLLSRFLYFYNYNISQYSKIIYIFIFIINHQEIISMLPKIDDQKLKKNYLNFFKTNDSLFNF
FNDIFKFVQSYQTDKKKKIDKNIIPLKINNNINISVRDPMKYRLIININVDDYLCEKRIIRPFEIKNGFYVDS
ELLNELCFSPFKKLYKYLRKIRNNNQIDSVLHVGIDTKLNHDKKDIGTVDKEKKNIYMEKTCVNIETAFKNND
SNVMFDDNINKKKQEEMIIPTMHENNVKKISNMNEKMDNEDKKRNKILNFDFENSNFSCQNSYSKNFIRNMKN
DYNKYLDYQKKKIDYEFIGFSVEEIKMTFNNIKKLKEYVNMFVCIKDILLEIFMGDNKLLECINEYIDDSMYI
NKDFIKKENDNNNNNNNNNNKNNNNYNSSSSSSNFYSFDKNNNLNDYSLSSDEDINLLANNYLYKFGILGKAE
KEINLKILISCFMSKYAYEYLMLINPNLNRSHSEKYINIILLYMLIMNRKIFLHECINNVINILNIFKVYNLK
LKILKQREKAYKSRTSLASTNSSILNNNNDSNNNSNNNSNIHMNSEHVNVLNKILNIQKKIINKAINTIKLGT
KQLTQLTTTERHYFSLRNISNNKKVLYKNLEYNSDSSDVSSCYFSSDNNDLDDKENTMNIMEMNNGINHHNNY
NNNSNNNNNNNNNNNCSNNQDEGNDKVNNSSCYYIKYGEKDFNVNYYYTYDPRLLIFEYTNNLIIRRKQVKMI
KKLKRDFIKKKKQYCYQIIMGGGKTTVLSPLYFLIFSNYFLIFNMCPHPLIHFTMNSFRSRFSSIIKKNIFYF
NFSRYNNITTDLYISLCSALENKYILVTSPTCIKSLFLKLISTIQELNSIKTFDMLKQKAKTHITDALTKFLG
FQNEETKGINSGVSNTMSLAKTSINNNNYNNINNNNNNNITMGTQFSKNGSMSSIPSDYNMLKKQLDLCVRIF
DILNKSILLIDEVDNVLHPLKSELHWPYGINRDIDLVNVVRNFTDIFMNNPYLKFLYNNKDDENVDIKNVKIS
ITNEMKANDNLIYNIKHLKENEEREREQNKLLINYNTDHMLKNEYNMYNKMEDMIGRHKNSIDLGEKHIIMNN
MNYSKYKSPLYNKKKEDLYNDHHDDNIINNKSTGCVVTSGDIVKALNNTEKDISFTHIDLSLFRYKLCWILID
FFFYPICKKLTVPMNVHFNINADYINDLLLVFNKEVDIGVKNHDISFIPHFHIINYKWYDNIVWILARYAYYF
LLSYKFNGLSQKIVESYLCIPLCIGYNDIVSNKGRNNNGNMNYATMRRLLNIKKIHNKNYNMNINNVHMNNMN
ESDICFSNDYHLKIFEKINQLEFVFCKMLNLCRLYINGILPFVFTKINTVHYGILSVEQINKCKIIMSKSRYF
LAVPYIGKDTPSSMSEYTNVDVVVALTILSYRYQGMRYVDFMLSLKNILYNYSLEKDNEHIQKDTSDDSSDTT
LNNFITDESSSESSHSTKALSEDSTSRKRNISERRDNISQSLISKSIDNVNDSENTEESLNEENGQEDYSSNI
ESDASSSSSYYSSSDSRNDVSLINLGKSNDRVISSTCVKDMSIEEEAKINGDIQKEVNHDIFIHEENNKKKQN
NNNNNNNNSKNNEKLNKRKKKKKKKKKHINNKKNNEHTFKNDEKKYTNRRKRNLYGSNNDDNNKTNQRYVEIK
SMYTLYKEIIESSGAKILKTKKYINNSNFFDYDNTVKRREEKKKSKLGNYVDNIDKINLNDSETLYNLYNLIK
MNPLLISYFLLGIILEKYIKHTEKQLSSNSQDFTNNILFKYVLGYSGTSNDILPEEICKCKYEKRTIGSILNI
INDTNIVNFIFLPSKFSPIQILKSLGYSKIKFNSLIDTGYLINNISPKKVAYYILQFLDYIDCCIYIDNGKHM
CMLPMEKRRLKKRRRKIKRNKNGKNIQKMECDNEEMIINPDLLMKGYQSKNEQNETETNIVKENDLYKKKVKK
SKKSKKERKSKKWNSKIEDNLNFSVEIIPLQDCYVPIKRRFIYFDHINTFGQDIKQYINANSLVTVNIGNDIS
SFSQGVFRLRSIGMNQKIFFLIPLCLVKLMNINTNLFNELIYSSFMKKHDTNDDKNNNNSSNNNNNNKTSVMG
KRNKINIMKKFQRSKKVIIKMCNKNYSDDSKSGSDSDPILFSSTDSSDSNNSNNSNNSNNSSNSSYDNNNNME
NYMKDVELFSTYINNNNEKYILNNDYIDEENMLYMMDCYNSIFLDNEVKENVLKELTHFLILNNINNNVKQCE
LLCVHGIVNLIKKKSIKTLIDNYINIGVISYKEYLDIFNTSIDFDIQTQVPSKNNSLLCMVQLIEKFHKIFTA
QDELYIQDIIYKYFCLHYDFSNKTIYMYFDAFFPKKYFLKMNVSQVILDLFNKLKKIKKDNLILNKKKILMKT

SDNNCGIYNNYNNDDDDDNNSNIDNHLFSMENEQEKEKEKEKEKEKEKEKEKEKEKEKEKEKEEEKIVDDPKKKN
IKYKKDEYKLYHWTVDDINIDLYGMPNKDHINNKSTKNFMNNKINETFKKLRDDGPLFIPLNKMCVNDIYLNF
PSYIYVSKNFINNENINTKRVKKLKNVLVVIECVYNNTKGYDINMDNKNTINNVSNMDNMNSMNNTCSINYMN
NNNCSINYMNNNYDVYGERDILKNIWNILNESDNIEEMLNILDINAFKYSYDLSNVIRNYLDKNRNVNNNSFI
NFTNILNELELYFCNGNKKYIIVSLQEAETLRYHIHKRSNIQKIYEAFFRSTVHVNNKEQIKCMYNDKEEDQN
VDQYVDQNVDQYVDQYVDQKNHEHFKTSKLCVQDNINNNYKEKENMNIFDIIEEKNNNKKNHISNNNVVDVDI
GKNISNDIDTYIKDNMNDDNLLNDPWGLQENIPNINLIGDKSNDSIDNFFSGNMDKINNKNIVEHNNIDKIDN
KIEKKENTYNKNILDIMNIKDCGQNMLIACNNININIYNYNFWFTPIEISDKNNLRKYLSRQIQIVYSVCKFF
NSDRKFIDAHVNLLLQYLNFNPPKHRFNYFNELYLIRRLMKENQNSGLKNIEKNFYAPKKLQLEILEKKLFEG
NGDIYAFKVIQERVRQSLKGKNLTLQKIFEKDLNYICTMEDIRIVFDYLNIRDKNCINILFDVLGRNITLHEL
CLFLNPVENRRDKKYNRLEKNKRMNGNMLQNDMNYINNNILQTQNIGIQNKGNNNNNNNNYIDHQHNNLIRDI
EKHLKNFRFSLTEHTHMKLVWSGKIAHQRIHHDMTHENNKNEMCSMNNINPSSYNFAKNESHHNNMNNNKNSF
SNNNNNNNNNQYDYVKKSSTNDLISDIFYTFQNKTHNRDEINLLSNTNQQNNINKMSYNEDMFYIYEAENVDP
KDRGIMSKNKHKIVCGHYASNKMYDINNNNNNINNNCSNNIYNSINAFPIIEMTDITINSMYTSTSGNDMLNL
LFPYPKKYLLLWHEDYSLKNVNECLHIWKPILSSSAHFSNNIFVGHVATVGPQSPPLNKIRAIPKVLVKNVQS
KKYSTFFADNSFAIDNSGVFRTLNIHFFNGLEFKNYEEFSFRNFSLSIISKMKKIKSVNEDIFNLWGDENNEI
EQDEDDESSSEWEIIAQPF*
>N.PF14_0085
MSKYIKSNSTILNSKKNIPYDIKSNSLIDNNLDDDTKKQYDQAYDEIKKNIIKLEKTFLDNVLLQVKKKNIAG
NKNTVEKSAINELNKYKKNIELLKSKVSVEDNTDKIVKLENKIKENEEILQKLINDKEGLENISKQQLKAIDE
ICLNETQISIQTKYEEIRKLKEDLMKWKFTYNENENLLKKKQEQLIHIDNQLKKKIFTIKNSKNNNMSSGNSN
KNLSNIHIQTLKTQIDMINDNIANDTQMYDEKIQQIQNNINSAQNDIEFLNSQIEEIKKNIENISLDIKKMRK
QKKEK*
>N.PF14_0086
MNNQYYDCYVEKKEYEKSFWESLGKPKYISAPMVDLSELPFRLLCRKYNCDLTFTPMLHSKNFVEHEKYRKGY
FKSCDMDKPVIAQFCGNDSKILLEAINFIKDDVNAVDINLGCPQQIAKKGNYGAFLLHKHDEVVNLISDITNN
CVIPITCKIRKIDNDYQKTLNLCYDLQSRGIKMITVHGRTKEEKGINIKQCDYEIIRIIKERLNIPIIANGSI
EHFEDIKKCLNYTKADAVMCAEILLEKPYFFSNKNIDAVNIVNEYFELYLKYESNTKYLKGHLFKFLYKYFQV
HTDLRDLLNNCHTLNDYINFKNVLNERKNMGTLTETSYSWYRRYRKDI*
>N.PF14_0087
MNKKGNKRPLGESLNVPLNSNILNEDNEDKKYVYPSIGNFPIIINNDMLIYGYGKYLLFFCLKDMKFIKIIDD
HQSAIRSLDKFKEGNFFLTTGDDKTIIIYDKNWCKYMKILHKKKIVKAYFLKEVQESEHFKILFIDKYGDIYI
YKPPENLTNKNNNKIQINDKDDNYETVQLLYLYDSLNDVEAKDEDIFFMKEFDQEFHEDILLKDSDDDEKNKD
SVNYDKYEQNDNCVDKCVDNCDDNYVDNCDDNYVDNCDDNYVDNCDDNYVDNCVDNYDDNYENILNEKNKAIN
INSIKKKLEIHYAECFKNENLLYPIHTCNSSVISLYYDDKFLIIGDRDEKIRIIKNKKIHKIYNFYLNHKLFI
TCVELINEHIFCSTSADCYVHLWNIKKKEVVDSLYLDMNFLCKYVQLSSLFPNKSNMENYKFLISILYFHKKS
FSLFAIIENVKGFLIIPLKEQKENNNDNNNLMAFDEEKIYFYELKEHVLSFTFLSLNNQKDVLLFVDREKGYL
HKINLNDDNSLSKDIKIFPHSFFNSADMIDIGLINYWKHTTIEGIIN*
>N.PF14_0088
MKYKKRLFVILFITFYSTYICNILCVLLKESSYHNINHIPKYKSIKNSKNTYKKYTMNNELNLSINKNKNKRK
KRKSRSYFITNNYILHFKPFGYNKNIIKYNHISIVNNKTNYEIYNLNKNKQKLYSNDLGKEQDKKKKNTSKNK
TETRNTQTNKADKSILSTEKKTSKTKSTLRKNKNNAKKKDNITIQANNEENKEIKQLQFETTENKDTQKKKKT
YKKKETDNIENADKMEETDKKKETEKNKKKAQNKKTAKNKKTAKDKKTAENKKIAKNKKTAQNKKTAKNKKTA
KNKKTAKNKKTAKNQETTKKQETPPNEETDKKKKPTLRKNKINESKETLNLQLINKNEENQNGKAPTDNENQK
PISKENEKIKEQEIELAELIKRERNYGDEDDDEENLEEYYDELQNDNHTQDMSDNEIVEEEEKRKKMKSIEEE
KEKEKLERQKKIDFIKKVEHLLEEDQKNHVLYEKRPDIDKLKFGDKPPPGSEDLMNTILKNVNEDHNKNEIDK
IEQMNNLKKKHLFYELYRISNSSSFKAHKIYTHPDLIYGMKYRKLGESNLCVSELCIGTNMYENDNFISRDDV
NVLLNMAFYEYGINFFDICEYDPFPFDPDSYRKGQNRNMNLFLKDKKRENIIINLRMCSSRNVNKLEYGDYYL
SWIMEGLKDDTPTFYNIEERLDKILKNLNTNYVDILTVDIPERYLPNNEKGEDTYIWGFENLNTEQNKNSISI
EEQFDILEKLILKGKVRHIAVSNESVWGIYQWCNLAKKKKKKYMKIVCVQNLYNLLHKNEVESSGLVEMILKE
NYNVPLVPYGLLAGGILTGKYLDPERYHTMGPEKVLGDDQLDHDLNEARGNIPEDYGYLSYGPKNGRCNKYPN
LYKSHRCVWAQDATGEYLKLARSHGMSLSQLSLSYVYSRPFVGSSIIGPRTLGQLKDSIFALNYPIYQHTEWD
IHEIFLRYRGCTMDGNTILNSLEDPNKTSQNVFYKTANIPILSGGTFWKNYPLPDIMKRYEYTDLKEEHDRIK
STFGLNDEPNDANFISSRMWVEREKRKGEYFAVKESNIFKWNKLKIYKGAYTKLSEKEKQVYDTSDFHFTFKN

NTISVTPTDEEIKNFYDNEKKIKKIITQNINDHQELFQYKEELGQDLPAPFNNLDIDLIYKQLLNRHNINPLD
KKELDSIYEYVKLTPSELKTQDFWYIYKYNPFDTSFAFRTGTEP*
>N.PF14_0089.1
MIKSKFFCKSLKFFMHPYSKIKNEKSFIDLNFIKGYSNFIKKKCVPYDKKKYIFCNKNRLISNVGNKINKKES
EEQYDSDDDNDKLVLNDDEDDEKKQVHINNKTEATNITNINKNIENIKNDMSNLNNMNDSNQKIKMKNKGKYD
SDVIIMYDHFFQLPQNFLSSKLSEEEIEYINTGIYNPDFDNYINNIVMKKKK*
>N.PF14_0089.2
MIKNPYSKIKNEKSFIDLNFIKGYSNFIKKKCVPYDKKKYIFCNKNRLISNVGNKINKKESEEQYDSDDDNDK
LVLNDDEDDEKKQVHINNKTEATNITNINKNIENIKNDMSNLNNMNDSNQKIKMKNKGKYDSDVIIMYDHFFQ
LPQNFLSSKLSEEEIEYINTGIYNPDFDNYINNIVMKKKK*
>N.PF14_0090
MVFITISDDNNIITSLDVHEDTEIWTITNIIENDFSLNMNINELTYNGNAVDKFDTIKKLNIKEGDLLFVRKK
ISADIMNDNVNNMSALNNILSTNNNVGNIGNIGNNLNNENVQNLLNNPAFKTLLDQFKVYQENEYIKKESEIL
LEMKNDKSKMAVLKLQDEPLYNAIFSQNLEEIKKIVKEKYETEKKEKEKEQQMYENALKNPLSEDSQKFIYEN
IYKNEINNNLALAQEHFPEAFGVVFMLYIPVEINKNTVHAFVDSGAQSSIMSKKCAQKCNILRLMDKRFTGIA
KGVGTKTILGKIHMIDIKIGNYFYAVSLTIIEDYDIDFIFGLDLLKRHQCLIDFKQNALIIEDNKIPFLSEKD
VISISTQSIDIDANNDL*
>N.PF14_0092
MSSEYQGLLNSKDREDESNGAHLAEKVEKGGEQIENTLMKLNVRYQTLFFSSGVMTVFCGTISLLESLRYFYF
TNFVVSTFLITMGLIMMILDIPGTPRWASKHRIMIRKYIKFLTRLTGKSVWFFFLGSMSCLNLWPHSKHVSLF
RSFWVILCSSFILTVAVVGFLIALRKSLRLEKLKKTIKLVSKGAYIDCYRKYSVADPDHGMQFEEFNRMCSDH
TNGYIYFDFLDLFIIFNALDEHQKCSINEREFLEWINGPVTYL*
>N.PF14_0093
MKDYNDNIHRPNPHNLYKNISLLCPPKMKGNSENLSTNNNPNLTGKNIKVSNRHDKMKDIHIHKLPDVENISS
DMNPKSSKMFSFKNINHNKEENEIHLKDKYSFEKREDDMYNMSVSNMHKVPIHKQNIYSKNNYNTSVCLQQNK
KETNNISYINNIHNTMCITNNKNDHSVEHKINIYNNKYNYNNTFLCNKKLCTQKIIQYIGKNQNTKHPLHSLY
HTNVVGMNKFNSSNNLSDQINILNNNIQHINSTFNNLRQNNIYKNNDSIELFINNNLKSGDTNKYATFYKNVK
ISEKNNMYKQKEDKKQINNKNPYIFTCQKYFMRPSNVLHNIQNCGIHKKKKKKKKNHQIKHRSFHNIYKQINI
INDQIDLINNKINKNSKDQNRAHILEISPLSLPSYIEYKNKNNNIFSSYFAHPNNKTHNFKKIKMIKKNISNP
FNKEEPCGEKKNSPQVNDSKSFRYFDHSTCNNNYHNKEYMKKEQKSHLHSNHNINHEKGSDPSYNLNTKNTKN
VTNTNDQTCIYEDHTFEKAVENNKVMYLKNKPIQSKLLKLNNQILNNTDQKKYEKCIDHGDTCNNKDEKNIKC
TYKIDDILCSYNNGNNIKYEREVIKITNKYRNALEKWRYKFVHKNKTTKRNITSSNQKGKCNFNIFKINKHIN
KKKNKKIKKIESNKYGNMYNFVDKKVNNNQGNKKKNEKKNEKKNDKINDTINDKINHKINHKKNDKINHKKND
KINHKKNDKINNKINHKKNMIPNQKPINNMIKYNIKNKKKIYNDTNDHADNRNIHSHIHKKVPNKKDNNINHM
YYDNNNNINQPISYIHTAYDNVCHQINNDDHCIPLSCVIKNRKTRRSRKNKNLFNNKKNYTNEP*
>N.PF14_0094
MNYCTLLTICFFFCLFLTNVVYEYVHVLNSKKRKSRLEENNDKKKLFKFFLHILILKQIIFESFINLAIKMSI
LLHVVKRYLTYNKKNYIGYIKNEEDHDIKKVDNNNNKNKNNYYLNNNFNDKKEKEKDTKDEKKNKKKNKINYT
NNDNEIIGNNNNTNMNEIKIYNNNFINNDYVNYNNILKNNTNHLENKKNVKVKTIYFIRHSESVWNSVFNKEM
STKQFLKMFMVILYELVFLFSKKSALIDSPLSNTGIIQSIELSNFLLEGRTDFNDEFCEETFNHIEYINDKNK
TNDNNIKNKDEYIINNNNYNNIDLYKLSKKKLKKNKNSISNYNKKQENNDILTTNNNDYSHDNSNDNHKIHEE
QDNILPNVKNYITYDNDHDNYSNKGNYNYRNDEYNHEEIINLNLKDHIDILNNKKYDSVLLCSDLRRTISSCF
ISFYNRINKNNEDIYVLNSLQEISRNPDCVPLMKYYNKYVTTDIEKFLHKDVEKLFRKNIKFNKNFTRNSFLD
TLDYIFNHEKNIFIIFGHSLWFLHFFKYFLKESHKAKTNKLKNASVIVFNIYKYENDDEESTTSFKVEEVIQN
DDINQDQNTTNQQQEQYNTQQEHYNTQQEHYNTQQEHYNTQQEQYNHDINNMSDTEYLTHDDLTENSDYLSSN
DDYEEDVTKADEQENYYENHNDDIVLHHVDNLQHIRHRRNSDNSYEEEPSNTTKAKKKLKLKNPKRKHKLHKK
NKKNKIFVNEKVKYEVDQNSIRVLYKGFDEK*
>N.PF14_0095
MNERLMKGKKDIYIYFLNKRYYNPPSKRKWSKQNDKYKTRRPLAEDSNVHKINIKGEYWKQKHVNKQFVVEHG
NQDMINERTSLNFFGYPNISLYNKLGGSLYIEQMDQVTLCIMIHKCIKENICDSFIWKNLLKRCTDICHKMDG
NILSYIFKYTSKTEYYNHYFYLTMLGNISSNLYSFNIKNCSNILFGMNNNNNYYNEEIFNKIIEHASLLILNR
NDIDINNILSIISSFTCFNNENKDHIIQNKNYISISKNVNNSYLLFDCISKTFHRYDLDLSEYDNVCSSLLVY
CFLDRINLFFQERSRKVVNMLMSHLNNNIEKLNIKHISIFSYATTIFNKNLLTDNLNNYILPRIEKDCYLLDN
NYLSILFYSLKDYIWEKKERNKRIYNIIYNNLLNDIHLASLDSICLITYSFLKCGKNKELQYRENDSKQKGNQ

SKDEINKSSDMCNNYTDDNVSNKNSFDCDYIKYDNNFTKQNINLMNMDKGKKQNNNIIVRKKNKEKNDSDSLT
LSYSFNLFNDFLYGKIKYNIKHFNMFQLLLIIKGLYESRMLINNKMEELKLIILKEIQLNVYNVPIHLLSYFI
KMLTQYVINNKELIICLKNVSIIYLHIYKKLYLVDSDYTLNDKTCIIQEYYNNIGNSDIKKKEKDDLWFIYNE
KKKEINSLSDKYRINMNLFKKQFKYLNENLDIKKEEIEKVFSFSQNYFFNYYLKEKELANFFYFCTIMEKENA
IIYFNQIKAIVKEKIINDELKFSPKSILYIFKSLDYMNMIDVGNQFFNLLLKQLFDKIFSVDISLCYEFLLIF
YKNKISNYYFLKKAKHKLDMNR*
>N.PF14_0096
MSYLSNMSNMNNMNNSTQQYNNENSESPYGEARMSEQCSYQMNSPYNPAPSIPIKLFVSSIPKNLTEEDIKSI
FQEYGNIQDVVFIKDKKPNVNRSNVFVRMESIFYGQKAIKDLHGKRTLCETLGPLIVKFAIGELEKYGVNMNN
ANENEAKLFVGSLPKEITEEELRELFNRYGNVTEVYIMKNSNGISKRCAFVNYAYKEQGIFAIQNMNGKIATE
NAEKPIEVRFAETKNQLQEKHLFNRSLMNPLNNNMNNMDNMNNMDNMENMDNFDNNNNNNNSSYMQSHAFKNM
NMDNFNRYSMNMNFNMQNNNNNQRNNNNNNNGNNNNDNNNNNNINNNNSNGNNNNNSSSNNNSPWKQYYSKEE
GRPYYHNEITGETQWQKPRKIEQSFINPINMNEITGPVGANIFIFHIPNEWIQTDLLSAFSPFGNIISAHIAT
ERDTGRNRGFAFVSYDNVDSAINAVKYMNGFLAHKKKLKVTIKKGEEQYVQALINQRNKLNSNDARRNFMENT
TNA*
>N.PF14_0097
MPRKNSKTMTNKCANSNSNDFIEDKVLDDKKRKKSTESSTVDEKGDIIKRRSKSKSLDDSNGGIENMDKVKVS
NNNRKKKSETMINNDNNNDNDDIKSMNHMNSMTHTNSTNHTNNMDNMDNMDNVNNMDDVNNMNNVNNMKNNIN
DELYKLGSFEYEEECKKRNIRNSECKRRRNLKDMKYFKDIKPLKDFVNLKNSNNNNNTNQHNSGIFSNFTRFY
GYNNKNSFNSNRSKQSTCSNSNLQNNMNLVNSESFESKLQTFKVRSQWTFILILLYFIILAAGHFYCSILVLI
LVTTLYKEIISLKSIENKDKKLPEIFYIRWYWFFLTIMTLGIPWVIPKLKHQIPLYKFLLTYHSINMFILAFV
GFVWFILSLRKFSLKYQFSQIGIILLSSLFIVTQSLMHIANIYSGMIWFIVPVSSVVINDIFAYVFGILFGKT
RLIQLSPKKTVEGYVGSSIITIVWGILITYFLQRYKFFICPQKYITFQPFVSWNYIDCDINPIFQQKVYEVPK
QISQILSIKNIYYSKMIFHGLMLSLFAAFLAPFGGFFASGFKRALKIKDFGKSIPGHGGVTDRFDCQIFIGMF
TYIYMKTFVKIKGGIYSYDLIIESIQKLDHKEIIRLFNQLKNIVDKKRRKQCTDNNTNDIHTRKDKRSNIDKP
CTDKNKIPNK*
>N.PF14_0098
MKEENILYYVDNNVKSLQRNYEICLEKNIQELIIYYGIKLLKKYGKKNSIYKWNLYEEILKACIELKLYEYVD
IYFKKLNDRFGHLNGKKIEILKGMVYELKDKNDEALCIYKNYIYNDPSDLLIRAKIVKLKKKVEGNINNVIQI
LNDHLKEFPVDVEAWHELGEIYLKNCYYTYALYCFEEILIHAPKNLYYILSCAELHYTISQYELSSKYFCLSL
KLQKNNLRALWGIVLVNLSRYGNKKGKNLNDNVDVILTKKCIDRLYNLYDKNTNFLYRNIILQYLKEIGESFV
*
>N.PF14_0099
MYTQYDIEYEYVTCTKGYTFIIYSPNLFENVNAKSSDSTSPYTEKNKEEDEQQEEQQQDEQERDEQEQDEINN
ENIIVLFLHGLNGNSYQFENVFYTLIHFNYKFISIDFYGHGNSSLLRNVNKFTEKLFIEQIYDVLKKKNIFNS
NFVVIGFSMGCIIAAHLSVDNKLKIKKYCLISAAGLAKPRHRFLHFLLKHNIRLCLRVAKRYSHLFVNEETFK
NEYNDIHNNAYYSHNRCSILKENHEKFIETFLKVLTGTKIQDSKKYYSAFLKKNSDVLFIYGRDDDVTPCAYT
IKFLEKQKKYLNNVKLIIFPECSHLVLTEKSYELAQHIIYFLQNKYIYNVERNSDDI*
>N.PF14_0100
MDSVDDEKPIIKYIIVTGGNMSGLGKGTAMSSMAVLLLTKNILLTTIKIDPYLNIDAGTMSPYEHGEVYVLED
GGEVDLDLGNYERFLNIRLTYKNNITSGKIYEEVIKKERKGEYLGKTVQVVPHVTDAIQKWIKDVIDENIKKM
KKEYNIDSFNKIPCMCLIEVGGTVGDIESAVYLEALQQLINNLNNDDVCLCHLSYVPIIGNLREQKTKPTQHS
VKILREAGLKPDFIFCRCEEPLTQEAIKKIALFSQVKNEHVISLHDTSNVYKVPLILDKQNVCMNVLKKLNLE
NVVLNNKLQISPYSFNIWKQLADRYESSSEQVVIGIVGKYTASNDTYLSIISSLVHACLECGFKLVIKYINSS
HLSLKQKKQKKNMDWKKKARKYSYENFHENSSTEKIIFVDDTTSDEDYDKKRRIKYEKAWETLKSVDGVLVPG
GFGTRGIEGKYLSSKYCRLHNIPYLGICLGMQIAVIDVAREYLNVNANSEEFEDTTEMQYTSHENNIADSDNY
NKDDNNNDNNQHAKKNPNVTNCTYNEKEKKEKKDKKENIINLENLENVENVENVENVENVNKTSVHINEICSQ
DTTASCISIKTNSSHNPKELASYTERQKHDNINNLKKEIHFDDNISTAIINDDINIENSIYSKYQDTYMTDKN
TYDDTDPMFDGKEYLKSQSLILREKCIEDIPKKLSEESIKEKIKLIGIESYYKSIEEIDNNNVIISMSEFKGD
DNKGGTMRLGVKQSKIIDKDSLTYKAYDEELYIYERHRHRYEINPKYVPLLEAVGLTFVAKDIHSVRMEICEI
KNLDFYVGVQFHPEFTSRPFKSNPLFLAFVLASKKKLKDRLNKYGNKLCSGILYK*
>N.PF14_0101
MKNMRNDRLSGTPWDNVKTLKNCQVDLLCSYDLHKGVEHKLISFALNLLCVSKKYELIFISYGQYIYVYEMNK
FINNSHFFMDKNENNKYKFQYYFNNEEIKKNNSVFKNMNKISYIYEELKKEFYLKKMNIPCPSLILSPHLYSK

GYVNIKCEENEAMNKSILISVGWSEDTNVYYVEKIVESINTKQKIKKVLENNIYDFFQKYENIYNDQYVPSPL
FSNISKDPYYFLKKLKIDEHHFLLNILYNNDNDNYNNDNIIEPYNNHYLNSKSKNKLDTLMCNPFIINEEYLA
NYSNDIVDMIKFSKISNYFDYSKNKIKEKRNMLFYKYHENRKRKALSLVSKEKKKKKKKKKKLDNSNDKIDQI
INVETINNFPNNIEHIDQNNKIYDIHDGDIMDPNSDDDIINHIQNDIVNNDRLGDGNMNHVNKSNERKKKKKV
NIMENHTYNEKEKKRQKIDILKIKKKNNMYNEDVSKTSHTGVLKMENDKMYNEMSETQREKKKEKCIKRKKQK
CKFRISDIYSSLESEEEELEEELRDEEKHDEKHDEKHNDNKNNDKNNDKNNISCKDLKNSKMKKRKKIRIKRN
YKSTYKNLHCNNYLDYGEKEVDQLDFYNKLRKKVYIGYNLRNNLELNEKNDYINTFRMKNHFINEYMKSSSDD
EINDDNINEENFSLNLNDTNMDMINRKEEKNRFYTRDYSSSDTSNYYPIHSHDDNYYDSYHNVNTYNLHRKKY
HYKPFENIKKFKNKNPYFYPQRKYEIHVKEYKGKNKKRQLKRLNKNIKNYMLYTKQLNKLKEERKAFINFCKS
YIKNKYYNDYSRRLETFLDPYNKIKYHNIWKGFNIFTFNLFNVDYNDKILKSVQIYIQPDIVFNNKMYIKSDT
STWAISFNFKKNLLAIGSNTHNIHIYNLNNFYYFRKRYDYDEAIDYFRNMFIHNNIKVGELFENDKGCAFLYE
DIYYRNVAQTNSPNDNNRVQDYNNKEQNILHEYSETKENKMKNKNLSHDCTKGKKSVLPADNIINNILIRKTT
IPVDLSLFFIFRKEYLKSVLITISQNFPVDSLLLILKQAYIGKEFYNIQWDIYYNMKKFMLRIKKMYSNIFLR
RCNVPLNLFDSNTLQVNSHNLLKNGYIKSLYQYFLLYSHYEDAYNKYNITKEQIKKKEKRNNIRKKKLKTLLN
TYHGMNKNSLNVICKKYDNLRMYAEHAKDFLSQYIYNSKTKNESELILFNDDLFIQYSSDEEIMNDNNIIYRK
YKNYSLLRIEKCYADYAFSKLCRMNNNNNNNNINKVSSFFEKKCLFSRSYIYCYRKANIVLKKISNCFRDFIK
ARHNNNFSFKHQLFSNDDAIFIIQFGNEIKKKKKKKVCKISRNYKQAPLKYIKKLKRVIKYIQINNMSPNDLR
LCSRNYININNNEKEFFKKKLLVICELRDHNNNLLDIIHLSNGLKEEYSDLLEANASYKLLNYEGFSRSIDYD
DDNVDDDNVDDDNVDDDNVDDDNVNADNVYVDNVNVDNVDADNVDVDNVNVDNVNVDNVNVDNVNVDNVDVDN
VNVDNVNVNNEGDNNISVNNVDALYNIGVHDDDDDDDDDDDDDDDDDDDDDYDDDDYDDDDEDDDDDNYKRKNNF
RIFKRRYLNKNNYLLEKKRKELTSTSLMKENHLSGIINNTIDMEMNYMTYYKGHMNKLKKDKRRSNKDICVRK
YNTSYDINDESFSYSSLSCSSSSFSYDDNNMVDNNLPYKTNYSFVNIPIAHGVPYTNTPLNNSSLQRHNTILR
NRINSSIENSNNNNNTIPSTQHFRLFINNPLSNNLTAVESNVPNRRSNSVGHNLYTYINNMLYNPNNTLNFNR
DMNVVNMMGRTFDISRIGNTRNTNAQNYQNNMDNRNADCNNENQTMNEQNEGYYLNVEVIPGSMHLQDNVQNG
QSVNIQNGQSVNIQNGQSVNIQNDQDINDQNNKDINVQNDKDINIQNGQSVNGQNDEDINDQNNKDINVQNGQ
SLNVQNDLHANVVSSNAQNTNTDNSNNNIINNVINIRHTNEQNNVNNMNQNNNNNNNNNNNNNSDNINMISNI
SNEMRNVPNTNRQPLDIEYRFLDMIYNNILNSRNERRNITTSLNTRNLPFNRNNVIISRRNNVSNIPTQDISE
DMIRNMIAISIGNMDVLNEKKPLNNFVKGIAKNSNSKINKKRMIFNLIPLNWKNVCTKKKYDVKIKYIYTLSD
CADKILQKYKKNDQLDLDFVVICEGLCEPSMQNTQKRKENKLQDDKRGENKIENDEMKDHKIDDNIKENDEMK
DHXXXXXXXXXXXXXXXXXXXXXXXXXXXXXXXXXXXXXXXXXXXXXXXXXXXXXXXXXXXXXXXXXXXXXX
XXXXXXXXXXXXXXXXXXXXXXXXXXXXXXDDNIKENDEMKDHKIDDNIIENYEMKDHKIDDNIKENDEMKD
HKIDDNIIKNDEMKDHKIDDNIKENDNIKDNFILEEQNLESQRTLIMINKEDDIRDDKKKNEESEQNIEEYFC
SKNNESSENNLNNSYACFMELNNNKMNDNNCIYDEHIRYNETEKENIKNTIPGKENEINTIMLDKEYNNSIIE
NKENISSKSSSYDMYDSYSISSNGSSDSNLSSDMNEFNFFSSSEECSECEYNTLCLHNSSNINSYSVYKFLKK
DNSILFVNNCNVKIKGGEGIIKEYHLKGYKRNKNMNIKNMHSRNKNKKYNRKTKLIPLKINDMEYKYIVNENN
DIFISEPFFSIKSKYLEKILLQEGYYKFEENEMNEIQYVNERITKGNTYVNTKDIITSFNSNEKKDQNIYCSK
MKYNEGKNNINENTNENVNSCNSFVNMKNIGDEKKEMLDISVLPKKIFNYNISAFIQYMSKKVCNYLFDEHHL
KYFNNVSKKKRNFFIDNVYKKKNIYEGTKWNSDIYWTLSNVSFYTADWYDILYRLFFDASSRFYEMLLKHHTH
NIPCIKFSPDDNFLLSCSVDKSISLWYPNNVKNDKLENNYNIYDNLFPYYKNEKKKNSKKKMTKHNKTSSSYN
DNFNHIFQELDGSYETYDIFMDDNNLMCNNIIEMNINKKIVKVKNEMKKKKKKCSFEKLYNETKRWYINKEKE
LVEQKRSNILCKQELKHMGWSGDFIIKKNVKKMDFFVDIFFNIPSNIYKDNFICSQYFPYTNKPLNNFLNIFE
KYSLFELFKLSFLNIQRKDEKIGNIPSNTNMSNLVNYLLKMNSKKLKYMKENNIDNIVNLLTQGELCINRNKV
KNEKEGEKDYMDNENKDERKRYKRKTLKRIYVAMKYACRHMLKKYLLFRPNNYKEKNYNTKKNMISSEEIFLN
NNKVPLLTDYDFNFSSDTDDEHISYKKNKTKDINNNKLPDSFTYEHNELKKKFHNLHYQQNISDEEKSSSSND
EFSSSSFHYISSSTRTYREGKNLSKDINESDEEYIKKKKIKESLFYKYINKIRREFNSNYVLYRNEKNVMLKN
KWMYFLQKLLNQYIHMKCFKKVYAHIKNKLLLNAIRKYKKHNNHKLYFNFHVTTNYKESKILKEVLQNYSTYF
DKTNKTKFDNNKNDYKMVNKKFMSYLTLIKKKKLFKKTRRGEQKLNNLNVFQPLCEHINNNNNNNNNNNNCDN
NSFINNHNYCYNDNRGYTLILKNNVSNFHNIIHNIINISNHNKIIKHSKYLKKYHPHDNNQNIPLCKKKNSKN
HQNNHMEEYNFYREKLCTDHEKKKKKKNTFNNIFNDFNKDIRKGITFYSASDSLNSDINEFSYKGKVKRMIKR
KKKIKKLYVALSSKKEKTNLYNNNNNNNNMEDSFMIKNDNIYIDKKKMGDNNHSYRNVTLHKHDEKNNNINYN
RFHPFNNVRCLKKENIKEHILMKEKKKFNICFLNLNKGNYQINYFSNIGIMKKFYKFCGKGLILVNVQYINND
TEKGKKENIHINNNIKNLNLKENVDVLNKRHKKNIIFQTDIYICSTYKGNKNIKKKKSRTIIFIIKVNVEELK
IYLCRKKIFEEKYINNSFYIHTKVVQIIYFLTLQNCKIYSSHNNNNNNNKTEILNVHNYNVNYNMDYSNIDEK
SKQKLLKYYLSNDEYMFFQFLTSNLFEKNNRYKNIKPKEIYKNIVKIRNYIFNVKEDDKYEQYMNLLSTCEES

NIEIFKDLYNQCRNNKYQSISFNIFFSLLSNYKKNILMLNVSTLRFLYSHMKSEHTNNTNQEKKSQKKKKSSK
KKKKQKKNICCINKDDYIYNNIINKEQKKELNKYLILCADKCNIYLLKFKFTKITRNLFTTKFLPLSIQKIPK
EEYETFKQAFATSRISLLKIIYERSCIILANQYSNKIFIYFVQKCMYTNTYFLIPYFILPLYTLSIGRKLTPN
FNRDKLNCHKKYIINNINYNLKRLLDGKSLEESKDNDFCIAGLDVIYVNKRDDTFELTVYVLLLSNIIFCYKV
NFEYY*
>N.PF14_0102
MSFYLGSLVIIFHVLFRNVADGINVNGDNNYGKTIINNDFNFDDYNYWTPINKKEFLNSYEDEFSSESFLENK
SSVDDGNINLTDTSTSNKSSKKGHGRSRVRSASAAAILEEDDSKDDMEFKASPSVVKTSTPSGTQTSGLKSSS
PSSTKSSSPSNVKSASPHGESNSSEESTTKSSKRSASVAGIVGADEEAPPAPKNTLTPLEELYPTNVNLFNYK
YSLNNMEENINILKNEGDLVAQKEEFEYDENMEKAKQDKKKALEKIGKESDEAPFMFSENKFLENQVKERNVA
GSFSRFFSKLNPFKKDEVIEKTEVSKKTFSGIGFNLTDKEAKVLGVGATYQEYPETMLYNCPNNSNLFDTIES
LQGRIIDIKKRESMISTTFEQQKECLKNMGVLDLELNDTQCKFGTCIGSFGEHHLRLYEFENDLFKFHPNIDY
LTLADGYKLQKNHIYELSHVNFCLLNPKTLEEFLKKKEIKDLMGGDDLIKYKENFDNFMSISITCHIESLIYD
DIEASQDIAAVLKIAKSKLHVITSGLSYKARKLVYKIYSEIQKNPDELYEKLTWIYDNIYMIKRYYTAYALEG
VCSYLEHDKSQMYTELHIYNKIVDSVRYYSSCFKNVIVYNAIISGIHEKIKHFLKLVPRHNFLLDYHFNSIFE
KEIKPAKKYSTSHIYFDPTVASYAYYNLDRRTMVTIINDYFEAKKKELTVIVSRMKTDMLSIQNEESKIPNDK
SANSKLATRLMKKFKAEIRDFFKEMRIQYAKLINIRYRSHLKKNYFAFKRLD*
>N.PF14_0103
MNKVQILKEHVVNGNFFLSRMFSSTINLLKSSNLNKAYKNKIIEERENFIKINNTGYKIVTHKNKSLFLNDNI
FIYNLNQNTNENLSNGNEERKVLHFWNRSPGARYPKKANNGARPDCRSVRKIRKRLKTGK*
>N.PF14_0104
MNINEKDKLAEQNLETLDVTKLTPLSEDVISRQATINLGTIGHVAHGKSTLVHAISGVHTVRFKHEKERNITI
KLGYANAKIYKCTNPDCLPPECYKSYESSKEDNPICPRKDCNHEMKLLRHVSFVDCPGHDILMATMLNGAAVM
DAALLLVAGNESCPQPQTSEHLAAVEIMRLKHILILQNKVELIKEEQALKQQEEIRNFVSGTAADSAPIIPIS
AVLKYNIDVVCEYIVTQISIPKRDFISSPHMIVIRSFDVNKPGEDIETLQGGVAGGSILHGVLKVGDKIEIRP
GIISKDDKGEITCRPIISQILSMFAENNNLKYAVPGGLIGVGTKIDPILTRADRLVGQVIGHLNKLPDCFAEI
EISYYLLRRLLGVKSQDGEKNTKVAKLKNGEFLMINIGSTSIGCRVTGIKTELAKLELTGPVCTKIGDKIALS
RRVDKHWRLIGWGQINKGKPLELQEPI*
>N.PF14_0105
MSQLYLVCVICCLVYYVRCINVQIGNQKYSLCSNKIKKDLLDFYNTLGVSKSDKNLKDNIKKVNLYTKFRKRL
YKDRLSIYANVSIKEKGEEVVPVKVVDEKENKENEEKTNILDQEIFTNFMQYNKNNFFCIDELGLDISIPFYK
NSGFGANVVYQLPEMVYVNAYTNIYNTIIKGEFNSIDILKRISFFQFFDNFYLNAHYDFKSEKALLNLESKLY
EYYNKHGDNISFNNKIIVERNMHNEYDGSVCLSLKYNNNVFTPIFNFKDKTYEYVYDNLSANRKFSVKVDKER
NVHFNYLSFDRVDESNKYFLFFNFIFSNPMQSIITLKREFIF*
>N.PF14_0106
MFNYESILINEDVVSEMTIEDAKKLKPYWNVQIANFKKSSKEPMFTLLQMAILLNKKDIVGYLLARRGLDINA
LSRNNQTALMIACDKKVPLDWIEAILKRGGDLGINIKDDYEQTALDKCNFNSKAYHLLLKYGAIEKKNCVKGK
GATTKSSKFGESLWLNVCGCGSRYYK*
>N.PF14_0107
MVGALFQPINQVKFTNVAIVKYKYKGKKFEIACYKNKIIDWKNGTELNIDDVLQSHLIFTNISKGEIAKKSEL
NSCFQSDDNYKICKTILEKGTLQISNKERAILKEKIYKDMIEILHEMSVNPQTGYPISTNMIESMVKNIGYSI
NIDDSAKKQALKVFDTLSKEYDDIIQRAFMRIQIIYDDIIKDELINLLNTNNVIIEEENIMNNMKREDNVKSK
DNETDSYYNNNLNDEKDDHINVVDSNLEFKENNKSDNTNTIHNVIHSDIKLQDIVEENNHITMHYETYVNEEI
QINYIKDRENESMPHKINDNVNDSVSSSMNNNTHNSLDDNNISNEDKIKKTIESNTNIVEEKHRKSEKNEKDD
TSLDHMSDRYKISGKKKNKSEKEKEKEKEKEKKDRHIETYKTVFLCYPCVYRSIEDFTKSHKKNCSNKILNNN
VKIVTSNKKKETNVNRDTETQSCENKNNDDKNKNKKNKKNKNNKNSKNNKNNNDDDNSMTNEMSEEDNIKSSN
KKDRKNKSKNKKDQINDQSILNTKDDLKNNNKFNYEENQNKNYICNNDNISNCQKELNSNNNNKLNSVDVIMC
TSCLTKIEKSNYKLHCRSDFHVYNVKRKYKKLPPISLEEYREIDFDVSHFHVDM*
>N.PF14_0108.2
MEIKLNTKFDEILLIVCDKHVIENHEKFFVDKYENVMCVECLYKFLFKKYPLYKKYLLFKSINNLFCIKECEE
FRYHFSFYLLDHTLFLSETFSYIEGISQCEEKKEERKIYSSNYNNENIKNNNNNNNNNNNSIYDDDDDYYYDY
NYIYLVNSELIKLTTNACKEFLYKCSKELCNIFKDAIYTCVLVRLKNFSSRNKKRNFFFLLDFLKSLIDNVEI
IFITLKDESTIIEELMKLFIQNDEMINSKLSNNNYGYIYEYTGYTNKCIEKKKQNNNQHYNSELLLNLQIIYK
LWHDNLLWQINNNLLKRFFIYLITKFKYTNKNTDLCTYYIQFIYLITLQYNVLNYFFHQIIFDSSPNSNIKAF

NIENNNRNKSKSSNKSKSSNKSKNSSSNKSKNSSSNKSKSSSSNKSKSSSSNNNLYSVIIESNDINTSNTNSS
NNNSVEHNSNYCHTSSTCITKIKTKNTLAPNILSIYNSEQENKKNEFTKEQKKINRCFQTNIYPEDIEDNQDV
KDENSIQENEEKYKQICEVNKTPNEYMNRHVKKNFYYHDKEEYNSPTSNNSSIKSNIFFIPKHDLIYSDDEKN
VTHYPLKKKDISNLGEYEMESVLYNNDNMSIRSEGTLKNKFSSHEKFLNVSKFSTESKYTILSRLSNHFSFTK
NSVLKEKDNFVFSNCDNTNDNVSCVESSHTSYSLSNIDEKKKKKRKKKKINFNKLEILENSHIFIILCDNLQE
TLLNHFNSDIQMKCLDIFYTFCSFDDNIVNIILTNTSLVEWVFDYISNTKYENLREKALKLLVTFFFNNTLFS
NTHTHYAIDVLINIIMRYVNKDNITLSNKVIYNNDMNNKLVHKQFMNNNSKNTVYNNNSYDHSSNKFKNNFFT
ISYIIKALNMLINISHASIKISHIFKIINIFSCLIKSPSCAPTSIDDIILLFESILLKNNNNYEISYGGKLEE
SICTQEHDSIFCKLKSKEKNEQVNKILHMNEECNIYINTFNGIQKIKQDNFLHKKKKNITIKKNNITKSDYKV
NNNDKGDPIAATLGNNLLNDIINKVYITNIFILFKILKTALNIIKTVNIYKKDIYIEIIYNEDKNYIQNITIS
IMKLLFCLVYILNRNEEFFIDLNIINENDKLDNENNFESIAFIKLMLSFFIELDIFMENIYLQNSNIINQEKK
LYNIQFSYLTCIFIIHNIYEKRQIFNNVSWNILFSSFFNHFYNLLARVLYHVDHITETYLKGCEQINKSENFI
NIDNRNNETIIMSMSPKRDGSKEDEQKKEKINNHHNNDNNNNNNNNNNNISINEQHKLIVDKFVIYKKRMRKE
LLSNRPFTLFFQYVSRKNYSEECHRILKLLIDEEETIIHQRDDLKDILIDIIKKNDFYFSNVLLFNFNDNEVI
IETVIYIFYLSLKYDKGFLEKKLNKSKKINYVQHLFVNNNYNNNVNPLFIFMSLTYSYYFIKKDKIYIAFQHI
QNQLFKINLYLWKDIKKLKNIYLAFDCIFSLKVDNNNYNHFFPFIIYIIKLELSLYASGEISEVDNKLYLSIS
KNRELVNMIFEHIEMDNILSDQIIYIYYLIIKLRKYSVTSCNKNISLYKMMNTVIRYMNTVENKTDEINNIFS
FFFLFFENLEKYSEKDIFNIITLLQKLSLYVKYSLEKFFSTQKIYENNDNNNNCYSINDENKMEYSNNHNNNN
KNNNDIIYNILKFENSFFYFLLNLIFYCRKYNQIQNINVLSSSISLIHLLIYSIKNTNTHFRSLSFYILSLLI
LPFPKSPQNTSPLLIKLCTSFDKNIEEILSLHNNTSNTNQENKSDKCIVRKNLFYPLLTHESMEIRLSSLSLL
LCLLLTNEWDNIQNDLLLATINVLLLSTNINLKVKSFCFNYIYSFRPFFLRCILHLNRKQEVIIHRLFFLLMV
VKIKPLWFDITTCSEKILKIIMNVVTRKNMDLFIFNCIISVAHETFIKDKKNYVNNNYNNYTNKENKNVIENL
EEYLETYKKNIKKNSDMDIYNISSFNKFKCENINYYVVLSVLNTTTILLYK*
>N.PF14_0108.1
MEIKLNTKFDEILLIVCDKHVIENHEKFFVDKYENVMCVECLYKFLFKKYPLYKKYLLFKSINNLFCIKECEE
FRYHFSFYLLDHTLFLSETFSYIEGISQCEEKKEERKIYSSNYNNENIKNNNNNNNNNNSIYDDDDDYYYDY
NYIYLVNSELIKLTTNACKEFLYKCSKELCNIFKDAIYTCVLVRLKNFSSRNKKRNFFFLLDFLKSLIDNVEI
IFITLKDESTIIEELMKLFIQNDEMINSKLSNNNYGYIYEYTGYTNKCIEKKKQNNNQHYNSELLLNLQIIYK
LWHDNLLWQINNNLLKRFFIYLITKFKYTNKNTDLCTYYIQFIYLITLQYNVLNYFFHQIIFDSSPNSNIKAF
NIENNNRNKSKSSNKSKSSNKSKNSSSNKSKNSSSNKSKSSSSNKSKSSSSNNNLYSVIIESNDINTSNTNSS
NNNSVEHNSNYCHTSSTCITKIKTKNTLAPNILSIYNSEQENKKNEFTKEQKKINRCFQTNIYPEDIEDNQDV
KDENSIQENEEKYKQICEVNKTPNEYMNRHVKKNFYYHDKEEYNSPTSNNSSIKSNIFFIPKHDLIYSDDEKN
VTHYPLKKKDISNLGEYEMESVLYNNDNMSIRSEGTLKNKFSSHEKFLNVSKFSTESKYTILSRLSNHFSFTK
NSVLKEKDNFVFSNCDNTNDNVSCVESSHTSYSLSNIDEKKKKKRKKKKINFNKLEILENSHIFIILCDNLQE
TLLNHFNSDIQMKCLDIFYTFCSFDDNIVNIILTNTSLVEWVFDYISNTKYENLREKALKLLVTFFFNNTLFS
NTHTHYAIDVLINIIMRYVNKDNITLSNKVIYNNDMNNKLVHKQFMNNNSKNTVYNNNSYDHSSNKFKNNFFT
ISYIIKALNMLINISHASIKISHIFKIINIFSCLIKSPSCAPTSIDDIILLFESILLKNNNNYEISYGGKLEE
SICTQEHDSIFCKLKSKEKNEQVNKILHMNEECNIYINTFNGIQKIKQDNFLHKKKKNITIKKNNITKSDYKV
NNNDKGDPIAATLGNNLLNDIINKVYITNIFILFKILKTALNIIKTVNIYKKDIYIEIIYNEDKNYIQNITIS
IMKLLFCLVYILNRNEEFFIDLNIINENDKLDNENNFESIAFIKLMLSFFIELDIFMENIYLQNSNIINQEKK
LYNIQFSYLTCIFIIHNIYEKRQIFNNVSWNILFSSFFNHFYNLLARVLYHVDHITETYLKGCEQINKSENFI
NIDNRNNETIIMSMSPKRDGSKEDEQKKEKINNHHNNDNNNNNNNNNNNISINEQHKLIVDKFVIYKKRMRKE
LLSNRPFTLFFQYVSRKNYSEECHRILKLLIDEEETIIHQRDDLKDILIDIIKKNDFYFSNVLLFNFNDNEVI
IETVIYIFYLSLKYDKGFLEKKLNKSKKINYVQHLFVNNNYNNNVNPLFIFMSLTYSYYFIKKDKIYIAFQHI
QNQLFKINLYLWKDIKKLKNIYLAFDCIFSLKVDNNNYNHFFPFIIYIIKLELSLYASGEISEVDNKLYLSIS
KNRELVNMIFEHIEMDNILSDQIIYIYYLIIKLRKYSVTSCNKNISLYKMMNTVIRYMNTVENKTDEINNIFS
FFFLFFENLEKYSEKDIFNIITLLQKLSLYVKYSLEKFFSTQKIYENNDNNNNCYSINDENKMEYSNNHNNNN
KNNNDIIYNILKFENSFFYFLLNLIFYCRKYNQIQNINVLSSSISLIHLLIYSIKNTNTHFRSLSFYILSLLI
LPFPKSPQNTSPLLIKLCTSFDKNIEEILSLHNNTSNTNQENKSDKCIVRKNLFYPLLTHESMEIRLSSLSLL
LCLLLTNEVVLLEEEVFLFFLFLINCSFLSEWDNIQNDLLLATINVLLLSTNINLKVKSFCFNYIYSFRPFFL
RCILHLNRKQEVIIHRLFFLLMVVKIKPLWFDITTCSEKILKIIMNVVTRKNMDLFIFNCIISVAHETFIKDK
KNYVNNNYNNYTNKENKNVIENLEEYLETYKKNIKKNSDMDIYNISSFNKFKCENINYYVVLSVLNTTTILLY
K*
>N.PF14_0778.1

MFIIKSNIQSDNTLDNSAKKEHNKINKFLISLIVIINVLSLLFTIILAINFYYCSFNVTSFLFLTTQTCLWTY
LAILNVYDQKYIFSLSSFLGIHLYTQCYQNYDKLFLSDELQEYHFMSVALSHLPFLYICYDIVIFQYDSVFIK
STLDYTYNEKSLIVLNFLRGLFCSFCIAVIFIISLILNIQNTFRTFPMLKLSNIKTKKYLLSVFLFYLIDSQL
RIITFLFIIETYEVNPFKIFVFIFSQIMTICVCIAFDTHIFKNIIVGISSILVSPLSIILHSTNRNYSNERVV
RLSKLLINFRFFEFFLILFYGSLYYDDDEIGNTFILYLQLISLILLFFLIIVYKKVLSYKRKFEENMDDFNVD
MYNNINDN*
>N.PF14_0778.2
MFIIKSNIQSDNTLDNSAKKEHNKINKFLISLIVIINVLSLLFTIILAINFYYCSFNVTSFLFLTTQTCLWTY
LAILNVYDQKYIFSLSSFLGIHLYTQCYQNYDKLFLSDELQEYHFMSVALSHLPFLYICYDIVIFQYDSVFIK
STLDYTYNEKSLIVLNFLRGLFCSFCIAVIFIISLILNIQNTFRTFPMLKLSNIKTKKYLLSVFLFYLIDSQL
RIITFLFIIETYEVNPFKIFVFIFSQIMTICVCIAFDTHIFKNIIVGISSILVSPLSIILHSTNRNYSNERVV
RLSKLLINFRFFEFFLILFYGSLYYDDDEIGNTFILYLHIQKSVII*
>N.PF14_0110
MVKKKTSVNVKSILETKNKREKKDEDDDEDNKTNKLYNNLYKRNKSIRINILDKNENNGEGISLSSEKQKVSF
KKKNNNYDINESDGVGDYGDDECEEESEIENEDIINNKNNTNNINNINITNNINNTNNFDNTNNIDNTNNIGN
TNNIQINNILERNSKEKSLYKEELTTNIQNGENNTVQFFNFKKYDYNPDRKKTRDDLNDDNENSCSSFTRRGK
YNKTKTIKKSNKLKLTNTHENLLSDHHDYTSLKVNYKINKKSNYDKTKKRPFLSITFNEKQNRPVNRSSVCTD
NAKFLSFLNDYERKTYLIKNRKRFNEKKIKKLQTNIKKREKYDTAETLNNYIVNIDEDNKYEENLPSEKIPKI
DTTPRKHFPFPMKTDTCMFDYKKDRDDIQKLKSEERLKNYATLVEEQKKFLRDKSKSKDIEKHKKKKCFLLPK
KKLRKRKEKIIIFLNHNSSAYKNGPFNDHFYKTVYKNDLENLDEEKLVIDYSKDNTFKKAIHRIFPQFSIFSL
TLYVTFIQWVIFILLISVKSDFPLTPSNDSLKNFGSNFPHNIFKNAEFYRLFTSLFLHSNFNHICANTYVQLT
VGFLLEYLYGTYVVFLVYVFTGIYGIILSSPLTYCYSTTESSSSSSGIIGIFFSEILMMTNFNVDKISISVHL
FCFFLLLLFLKFSLNTVSINIYSHFFGFLGGFLIGIILKRNQLKYFLRNNFLIQILCFVFLVISLGAAIFIST
YVVQNCPN*
>N.PF14_0111
MDIFSLASNLQQIAIGGVNYVNNKLLKKSLNYETNYITYIVIGENEKTPYDINMFFLPFELSTKLRFKDFKKY
FPFKGNIIFRFKIALCDMINVINEGIINNSPLLLREKNIHHNIPHNNNIISDENEIKNILKQDNFNYVWIDIT
NDEAFIPTFNGSVIVKVLFVNHENYKEYNNIYFKNYNYSHKTYHINESHLCSYIPLKYENKIKKNSSTDDNIY
NDDILIHNKRNHCETIKRSYDHDNLVNFSYTNSSDEFQRVNSDEHCKKGSDKNLNLQQNDLHKSNNYYQNGVY
TYNKYASNEHINISNNVNNNNNNNNNNNKLYDEQNTKQEDNSKEYNYQYNYNNSNIYNYHDSHQVLERRSNSDN
LNTLHEENKIINNSQSNYIPSIHISSLNCNEIDPHNKYISTKGAIHNSRSNLLTNQTKNIQNDFNNKTQMFKE
NENITNKINNRLQELKEYRDHEQAKFKEKVVINNQIKKQINKWCKNSDNTYKDIKVLLTTLNEVLWKNAQWKN
VYMADLISNPNVVKSAYKNAILLCHPDKNRNTTTEQELRAEMIFQALNNSYKDKRNL*
>N.PF14_0112
MLLYKFYFLYFLLVHLSLCIRYRNQNKTDSYLKTNYKLLKKRKKYENRRYKFPKNRKGNNNNIYDAINNNNTN
NIYNNDTYKNRIFFRNEKNDENFIKTNKYSYYEKRNKIKLNSATSTFVSKYYKININDVYNYLHRKKYEFIET
DIKITLKYCPFCPPHKYKYDNMYKHEIFKNTGNSYCHRCGYKGSFYDFKLKMGDLITSNFESTVVHNNNFYEE
EEEKITLNDVKVYNMNLLYSKEAENARNYLMNVRKLSIDTLKKFLIGFSVMEFQSFESSGKFEKHECIIFPFI
KKVDEINMIETNGINSNMNKMNNNNNNNNNNNNNNNNNINDNYEVVRIKIRSLKDKGYMRLYPKNVRNEMKLFF
FGDHLIKNSEEIVLTEGEIDAMTISQETKYPAISLPNGSKSLPIYLLPYLERFKKIHLWLDFDKAGKSSVFNF
VNKIGLGRTNVITDANVHYLNPDVFKRRQKSRLTKKSLLLTSMASNAMEILQKDKEENMHNIYDTTNNDYMDN
KILSNNLKSISSDKIKKKEEIDLFNGQKISSNNINVNILKNKKNETDNITNKENKSDNNLKEGMEKKEIQNEI
SVIEDNNNNKNNIIENNNDDMSEKIKVEKSIEDNISYFVDNNIMYIPNNIIIKDANDCLKHNIDIRFFIETSE
KVKHSQILNFNDLRQRILEELKYPDRINGVKSKTIPSLNKYLYGLRMGELSIWTGSTGVGKTTLLSQLSLDYC
IQGVSTLWGSFEINNVKLGKVMLNQFCGKNLEKNIELFDIYADKFELLPLKFLKFHGSTNIDQVIDAMDYAVY
AYDVKHIIIDNLQFMLNINKFSDIYELQNIAIDKFRSFSTNKNVHITLVVHPRKEDNNLLSISSVFGSVKSTQ
EADNVFIIQRHVSKTNETVFFIDIKKNRFKGSLGKIPYLYNKENMTIKEISINNFNEHVVSNTYLPSNKFSSS
SLQNNTTNNNFIQNDNLNFTLCDEYDYMKQLADEYESKHAFKKGYRSKLDPNLRVNNIDMIKTEIIDNSDMNN
NKNVTLYVDSLENIKTISTDDKTNDDKRDVNEIKSIKNNERKNTLKIDGNKSLGSTQNLNYENENKNNNNNNN
NNKSNQEMEKNNIDDKSSTGNNKNINGNSKGNNNINNNKNKNSSSSSSSSSNYNNNEGIKNILNTSAQNNIP
FKDTISSYTLTNEGLIKLCEELKDEEAEKLKNRVVVLSMRNCIIDNNSSIKDIRTFIKTNKLNIKTAGKNLKK
MDIFISILQNIPKEYITIKSGQKYGPNKGLPNDKSKNKIKEPHNNNMLEYNKVGSNIGDGQNTSSSCMNINKI
YSEEENNIYINNNNNNMNKEPQTLLPNDRNDSNSHSYNNINYTMVKNGNEGNINDYLNRNYENNVDNHHNDEI
TKKYIKDNIINVDDNIIKKKDIFKLKNENNEITEFAFEYFESKKKFDDDIESRFFIINDNNYNENINLIYKDI

KYCGLDIETTGLEVFDENIRLIQIAVENYPVIIYDMFNINKKDILDGLRKVLENKNIIKIIQNGKFDAKFLLH
NNFKIENIFDTYIASKLLDKNKNMYGFKLNNIVEKYLNVILDKQQQNSVWNNSLLNNNQLFYAARDSSCLLKL
YKKLKEEIKKENLHIVNDIENKCILPICDMELNGIKVDLENLQKSTNEILNELNIEKDNLKKKLKDENINVNS
QQQVLKALQKNNVRDISNKLIENTSDSNLKNFLNHEEIISLRNYRRLYKLYSAFYLKLPLHINTKTNKIHTTF
NQLKTFSGRFSSEKPNLQQIPRQKNIREIFIPNDNNIFIIADFKQIELKIAAEITNDEIMLKAYNNNIDLHTL
TASIITKKNIPDINKEDRHIAKAINFGLIYGMNYVNLKNYANTYYGLNMSLDQCLYFYNSFFEHYKGIYKWHN
QVKQKRALQYSTLSNRKVIFPYFSFTKALNYPVQGTCADILKLALVDLYDNLKDINGKIILCVHDEIIIEVNK
KFQEEALKILVQSMENSASYFLKKVKCEVSVKIAENWGSKD*
>N.PF14_0113
MKLFMKKGHNLSRKEIRKLARKQIKKNKLLYSKKKRKNSFLKKEKKQVSFSNNDQIIINKNHESNTNQFLSDS
DKNLIKKNKSVKNSKKTKKIKLNDNSNKSFNYFNNKRNYLEEQEKDDYLLSYLSKKLKIKNEDGSNKKNEDKL
IKELEKDGFDTSLLKLADIIFNESQNFLTKKKIQKGKDDEYNEDGEYNEDDEYNEDGEYNEDDEYNVDDEYNE
DDEYNVDDEYNENDEYNEDGEDNLSQDNDKVNNKNVDNILSGKENEQPYNKLQDDNIDNVKDKKEIKKKKKKD
KKKKKKKKKLDKHSSDNNINNDNPIVEIKQKKKKKVSFNYENDIKKMEKFLMSSLNKTSEFNIKSIIDDICKY
FHDIDNVKLKLCYNDILIKQISTYFKNVNTTDIHICMCVVIICVLNNLLCENILYDFLKDLTAIFKYYYEDNV
NLMKIVERENEMNNKIETQSINSVNDTYLLNRKNENNMLSNPQGDNYKISPKEHEKYQDFKILFRNLLKCFSL
FYALSYIEFDFIIDIINILCEHMSINNVDNIIIVLKICGMKLKEEDDNIHLKHISEYLKKQIEQYIECNNILL
EKSKLRFLIKDIEDLENGKMKFHFLNKFEFLFSVLKEYENKYVYKKTMISFSFIKVFNTISVENMHDEKKRGK
QKNKNKNKNICNDLHTNISNNQFHDIENKTKINKLNYLIDQEQFNEVHFNKLLKKYKIQGILPKKIFLIIKYS
LDVDECVHNLLALLKKKKNIPHVIQTIIQIILYNKNYKESYAKLLSNLSHVNNRVYTFSLKTIFINYIKNISN
MDIKNVLFLSKLFTYLLKEKLINFLIFKHIKIEEMKNQEKTNDEINQDTNANSNIFFFLKTIFILISLDDHKE
NKLKNKNVWTNILHILYNQKLSTSLIYSFKNIIKKYIFDEVKNIHKVYPKFNMKYIDHFYKFLEKIQILQ*
>N.PF14_0114
MISFIFFIFTIYYESIVGSVLFSRWRVGKRIMFLNNMKGTKFKRRKRENSRSTLYSMQKMIKKNEFHDRCVIK
VKAGNGGDGICCFTVFSQKKNKKYASGGRGGNGGDIYVIGNKKIDNFLNVKLKSFYNAENGGKGLNNNQVGYN
GKDEYIYVPINTIIYNEQKEFINFIYMNNQKVLIAKGGKGGKGNYSYRTKSLKIPFVCQYGEKTKEKKIYLKK
ILFTDLGIIGYPNVGKSTLLNKITKANVKIANYSYTSKFPNVGMFKRVEDDSDIRKDNEFISFDGTPSEETIP
EIGYSEEDTMVEKYNKDDKLENMNNENDSLLNNNNDNNNDNNNDNYDNNNNNNNNNNNNCSDIMYNNNNMDDY
NLLDDHDQYNFIEDIYNDNIPNEEEEEKKNPYLSHTLNRQKKKNPSIEKKRTHFSVIDFPGIIKDLDKKINNI
SYKYLEHLKYSKILVYMFDINNDNIMEIYKNIKNVLVQYDNIFLNKKEVVVLNKIDIYDNKENIQTFINNLKE
QLKIHNIYYLSALTGENVEKTINDIITLHLNNNEDLVNTFLKTLPKVLDMEHLENSDNYKPFHYKIHKYNEKV
YIIKGDYLENQANIFNFTKTDSAQVFMKLLNDLNVNIKLKHMGAKEGDKIIISNYSYDFYE*
>N.PF14_0115
MQADKTKKGKNFKIRYSSKCKSDKTWKDNIRNRKKENSKKYEKNNLMHDRNIHKHIYIDYLELSKKYTFLKIF
LHKNEKSTNVYYNFEQSIAVYFLSKSILKEYYDLNFYMPYIKNDEIQNNIILGNNSFNTNEFIYSINELKDIN
SLKNIILLSYKNYDAYLLNLKNDSNTNLSCRQYDDYTITYNNNINEQRNDIYCKNTFECNKKNENIILQNDQG
GKFLCPCVPGRVNYIHILADLTDMNELNNLKNNLNSIKNNDKYIPVTNINVNININNTNKVQNNMFLLYGNII
KVLDIGVGANCIYPLLGNNIYKWSFLGTDINIDSLKYSFINILINNKENDIQLKYQTNKRNIFQNIINNSDLF
FFSMCNPPYYTFIEEVNKNPYRMLEANVDEVVYYLGDQKGINIDNNNNDKENNKKNKKKNNKKNKKNSNDHNN
HVDNMHGTNIFIDNIHTDDIQTHNINLDRTISSELGPINTENQKEQKVEREEENNITKIKMENSNHCCNNKNM
SNQINDGNITSFKISINEEEEEEEHNLNNTPYINSTPNYNNNGGEYKFIMNMLEESISYFFNVIWFTTLVSKF
KNVKLIKKEIIYSMRLYHEYKKNQIYFLNCIIKENLYFNQTFVFKNIQKKIPPVYIAQYRIFESYSGNITRWI
ICWSYYNPEQIDALKKLYYEKAK*
>N.PF14_0116
MYLFFLTAYIYVLIYIIFNNILVSFYSCYILLNKIDVVPLLIKNNKGIYKTTNIFSSLDRLNKKINIKGKKEK
ICSLQKLYWISNEGKYMPFQHKKKKKYKILQNVNNEEYLEEGGETEKKKKKKKKKKLIKGNNEVIKGNKDIN
KENNEEYNKENNEEHNKDYNKTRIVKRKVKKISKDVLQNIEENKCLNEKEKHKKELENEEFDLLNYFRMEENS
LKSQINPEKSKDIIRSEHFKQMMSDLNIKEDDVLQMLKRSEKDVTHFINKNLTLDEIQKKAESDDEHNEKLFD
IYMNNKSINFLENINFKRIGKKYKDIVKEVIDIFIDKNFNFNELIKYIDEDEQKNNCIYILKENILFKDLDNA
TLFEIIKKSILIAYLNITFSNDIHNYDWNAQIENYITTSLNTQYYRINEISYSNNSININIKYIKNDKDNVNQ
HEEIEYNIKDSIRQYEHNNNLNILSNFNIFIYFT*
>N.PF14_0117
MSNIFVIFCSLLILIFTKRCSGWCDEGHMLVSAIAYNFLNDDEKTVLDHIFKNYKEDNDFNDPVLGSVWPDHI
KYFNYNYPNKMRRIDGLELMNKWHYVNIPYNPTNIKLNMFQKEYYKRTDNAITILKSIFKSLKNVKKKENHGT

FFSYNFLIRYFIHIFGDIHQPLHSLSFYNKNFPEGDRGGTDIFVMYNNKVENLHYLCDSVFRARNQKWPHLNS
DMINKEAQKLMKLYPKEYFADRLKQSEFNNYSYIDFIIIETFDLAVEYVYSNFPHDTLDQKTTYVLNDHAVIN
IKKMLTEQIVLAGYRLTHYLKIIIQNVPTDLLNTK*
>N.PF14_0119
MIILFLFCISVFINIIASWSDEPHMLISYIAYINLNDGEKEILNRIFQNGNDAIFDNPITASIWADKIKPNNH
KRTFHSSNFRRNELLDIFNEWHYVQLNYNPMKIYIAPYHLRAHKGKHNAMGILKHIYRILIEVRQKMGHGTYY
SYNFYLRFFIHIFSDLHQPLHAINFFNSNYPNGDRGGTDISVNYKGSINKLHYLCDNIFKTRKKQWPNINMTN
IERDARYLMSTYPPESFGNKLFLPHDKIKYIDDIAHESHDIAVQNIYSFFPLTDLKRSEQYSINQHFVINTKK
LLNSQMVLAGYRLSAYLKDIIANIPPDL*
>N.PF14_0120
MNVLNQIDECFSDYLKIYGPKEIDSHWDEGVVVNKYITLGKAKDNNENKDNINKGDDNNNNNNNIKEEINKKN
DLSEEKNGNYKNYSNDKENDNSTYMAKNKKQKENLFICNDLVEKCICLNNLESLEEIEDASLVRWTCMIQQII
SPESYLGIYKLKNKENGKIIMKSSKYKDYIDAEDNWEIVEENEKRGMGEYYEFNINKYYENGDNKMDQTVDIL
KNNQDVDKKIDEHNKKDEKSDPNKICSNEQNDITNNNTYDNMELYENDGNFNKYWKRYLFFCTNIPGNKSAWT
KELYDYSSSYVNREVFLKSKSDYENGIYLKDVKDPMNNEDVNNSVASSTDISNYMNSNNNNNNSINNIYSSRE
YICSSNELNETQNNNNNKDKNEYKKIDTNDKINNNNSNNNSNNNSKSNHNNNYYFINSTDLDMNSNISSNICS
YNISPVNTSNKIRCVIKIYDDDSQYNGKNDKEFLKLNDVIEIIGIYRKHQIKDFEDYKKNYNFYFYYDQHFLK
YPCIHIFQYKKINYFNPLNNCILFKNDLSIIISQGPFNDIQKLRHHLIMYISSAFNNDMLIAHYFFFYLCGSY
IKESKLKLGKISLNIFNILYNNEIEKLNDHISSDNVNPDQMKKLQNKIPNDIDLEKDANQLKVTNNNCFLNSN
NKVLVKEADKKGIAPHAKKINKMIKNLIPLYRYIPLILQKLNTEYLVSVMNNQYGELKKGKLQLANNTYLTFD
ECLLDVGNLNNISIKNFQCIERLITSQEIPFIFNMDIIFQTEHNILILSKKKSMYSHYVDIAIPICHYNKIKH
NLTDTNNEQKNENIENVKGSTNNDEPSNSHDQKNEQTKGIFSSEFYNNINNNYKPNEKELMQFRRYINYILSK
NHSAKIPEDITNYITDSFVLLRQKNKDINQFVLNSWICMSRILAFSDGHNEINRDHWDYIMKLENERRLRLNN
LNKIYI*
>N.PF14_0121
MNEQNISDIKENLNFIVNEDEEFLNIFNYKKIKCYKTPNYIYIGQVKDNDDMVTLKNINNAGNKDVIKKSQTK
LLGAQKDKVDKNKSKKNYNFIKNGYGVLISIKKNIDGEDIIINKFIGNWINDKRNGLGFNIDIYKNIYYGYYE
NNLKNGWGYYKWSRKGSTYEGNWIDNKMNGKGLYINKKCIYEGDFYNNLFINSKCEWIDILEIEMKNMYKNNF
ITNVSNDKGSNDIYLIILPFNFIKINFKNIASYIKYNYNKIPFLICTKKFIEQNKGFDITKFIFLNYYLNSSE
NLNEVNMDSLIFDTTENEKGKKISKNYNDSIMSEDKDSNSSLDVQTNVNKMYSDDKSKRKYSNESKDLRISSH
SSDSKEKNNSTSSTNSNNKFSDTNDSIYNSDCNDNYDSEPSNYSEISLPSMTSKESHTSIFSQPDDIKRYNSY
ESQINNIPDYADTSCISSSNNTQTRPSIFGSKEEGNNNIEEYKDHSYIHTEEKEEEKKKKNEINKLINKYLNI
YIDTDTDTHTGKDRDSSSSEYSLISSDMENFKNINELSKMNIKNDKELVNKNTEFEKINTIPESNVLYDDIHI
ITKKKISRDISKIRININHLNTLRNSNISCIYRIRKKIKNSLKMKYPFIINFNIKKNKYQNVNEHKICLNTCT
IPEWWNLELYFGDSENKICHDLFDPLYFEYNKGYKKIMNIGNIEKYDKEQEDIVSDIFNLKMFLSTDLLIDNT
NDIPQLKNIIKDKFDKISYINNLFFIIIDQ*
>N.PF14_0122
MDMLNPQFEEIGKEFVNHYFQLFNSGRNELAALYKDISMMSFENDQCRGTSQIIERLNKLPPTVVHKCLSLDI
QPTPNNGILILVCGDIIIEENKPLKFCRSFHLFPLPSGGYFIFNDLFRFCIS*
>N.PF14_0123
MEEEKLSDRYVSFQNNPKQLNPSKKFFEWIFNYPLFNEFRNSSVSNHFSSLCINEREAIIEKSKAILKRKNYK
KENIHFNIKRQDASPKVLMKYEINQNDYSLINKNKFYVNIIKKSEKIYKCYENVIKEEKRKKRVFEKIKQKYL
FRKKKQDLINYVHSPPNDIDTNVNKKRDTISKEEFNPFFNFFENDKNKNIYHSNINFTMKTHIQFNKYFYKLI
INKLSYYKIKKDLYVNIVNIVNEITKYYPTYGYDILNNIKIKYFPYKNINHSFYIYGLVFSNCSIYFDDLEIK
NPKILLLNAERKIKYDVLESYFNHNYNYTYLFWKNLSNKNINIILVYGELDYYIKKLLLSKKIYFFTSIKKKN
MSRLSNLLCKNILSYENIKNFDVSYIARANYFKIQKYKKNVKNIFLCCNNKFLTICIFGKQDIRDDNITSEML
KKRKGNKNECYVYSYDENNSYDNTCLINERNRYDYLESMHNNVDVLLKKEIEKENILDNFINVYYKNKKDDYI
IDYYFVNKEKKINLYEKDTSIQQNKKWVLINHLKNIVIIKKVKKILQFCILLTFHIYKQMYLNNYMNRSFISL
PERNIKKYTNVSNDVFYQNLNLLIISSSFFFYYNNEIGKKKKNNNKNNNNNENDNNNNNNNNNNNNNNNNNNN
CKRKKKKMTPINLMSNLFHLINIPNPLRRERMKALSSIIKSNNIIKQQIYNNYEHCFFNNSEKNKIINIVNYF
LFNSFMKNCDITYEYLYYYLKIINSIYLNYNNKNIILDEELWLNQVQIFNIQSNPKNPIGIEKKMNEKIEDVI
KQNILYYCYNFESAKNIEMIIYFICPNYMYINNIYNNINMYMFDNRHKNSKRLFYNFFFNHKYVFDISLQQFV
YIIHSLSSKLLCPFDMCKNYLCYHQMCIQIFLRRVLILFKKEKNEESEEGLFLKIICEKCNSVEERDMNINIS
FIEFILCIIYSENYTNLYCNHGVNNSYVIHFRNIKISLHMYENNIYKSIVRKRKGAIDLYDKTQSIMKYEKN

KKHKNIYKNIYKNMYKNNYDLVGNIYCLNKSKGTNHIINNVKRNIKLYIKKNIKNIIYNDIFYYFPCKCLTNI
LNNYSNKNEVENENLYSNFNLYNIIENFPFFNSSMLPLYSSFKFLIYNCAIHYYRSKNKDMYKNKKKHHRRKK
KKKKKKKNYNNSNSDSNSNSNSNKYVKSMKNSQNTNPIDSCSPLYFKNICDKCKFIKIKNFCALLFFNILFIM
KRIFYNYYIMINFIITLLNKNMFIKRNQVYRLIKMYSSYDPMESNYLLIKQGDNTKERLKKNLSKEDISKMNL
LEVSNTTNIIDTMKKTYIHSNNTLELDNNILNINNILNGKISTTMFDNNKTDVLYDHIYENNNIDKSQNYNYY
NRNIIKETECNNMKKTKEIFINEYFIYKMFKKQMTKYKKYLINVFTNFYYYAIIELMYNKSLSLDIEIFFSIY
IIILERINQSILNVIENMENYKKKGNIENVHNTILTSQFTKKEYHMPIFFYEHINEEENEIISKDRTNIKTNS
LLQYNEGVYNNRNFIMKNKRKKEITLLFNIMNCYNNFNNTLHLKKENNQIIDNTNSVINGEYDILKLYTKERN
YLYNIESCRKKKNIYMYNLYNNDENETNNQNLFYHYIGRIIDRGRYIIFEDMKKKRKKKRKKKNEQEKNKEHE
KEKEKYQEQQEEHKEEATRGCYFFVFNPNDTSNIIFHSLLSDVYKNKLKVIYEKESKKKGGLKIKHTNNKMKT
DSYVTTYDNNNNNDNNNNKNDNSNGIKMDAYHTTYDYNNNNKIKMDAYHTTYDNNNNNIYFYDDDDNLIVRRK
RNIHDSESEIKNISFILGNTLNCYNINTYIKRCINNLCLNLKNDDIDYYINCKNEDILTINLNETICVYIFFP
LQFHYLRKLLCDKEEDFMKSLKKSNFINFDHKKRHFVKTYDDRFIIKEINKYEFKSFINKYKEFFQYFSDIVL
KNKKSLLCYMYGLYQIEIKKKNKKNMKTYIILENVKIENPNSKILIFDIKGARKKKNLQKLIKQKRKTSFPFF
FFRRNESYSLNTCKITEDKYMSDDCSSGISENLKTFRKYMNREKHLKDTILQERKDDECSMIYNKNAYLEFLS
LDNTKEIKNKISSNIKDMSDESLSFIPNCEYNNSEDYVVHLMENNNIGQKNEEIQEGMQEKMKEGIQEKIQEG
MQEKMKEGMQEKMQEGIQEKIQEGMQDKIQDKIQDKIQDKIQDKIQDTIQDKIQDKIQDTIQDKIQDKI
QDKIQDKIQDTIQDTIQDTIQDTIQDKIQDKIQDKIQDKIQDKIQDKIQNKIHHEIEQYHNINVINEEETNIE
IRKDERSPCIFINRLNMEGLERKPREDMNTKYKNDDINNNDVKDYGKDKSKNKLLSNKNKVKQINQEKINKMF
KRIRKKIIRGIINVCTIPRIISPIKLYNFTMKNTHKHKKKQKKNSKYIYIYKNNNRLKHDLSYHMDNNLQRCK
NENVFIKEILEDTKMYILLNRSIFKMMNRKKMLREDYRNNLRNVYLYGPMVSLEYLVTKRFCLQNKNKNIKDM
KDICINDTNNMISNNNKYKSEEGNVDNKKENMDDSMYYYYYYYHSFVGNGVGNNMNVDNSSNSIEKYNNFTID
NYNALKNYNVLFDDNFRDYIKSKVINLEYNDYKYLMDSLNEDTNFLCSQDIMDYSLLIHIDIHNYEIIFKIID
YLRPYTWDKSVENFSKSVLYLTKGYRPTIIQSEYYKKRFLSNIKKYVFYYVPIYSFKKKVVFKLSRGQNREFS
IIQLNKKNPYQKYFSYNLFNLILRYYNNFYLYKKYYISSTISCDLKYNQNWYYIPISKDIYCKKNIYIFLSYF
MKEYYIQYIQNNNKYQHNHFMLTNLINIMKSTIYENDIKSNTLDSTNKYEDKIKNIIECIDKFVYKKGNHIND
HICGDICDDICDDICDDICDDICGDICDDICGEICLNNNQKDRKEHFYDLIIPYSSQEQYNNIYDINNKNMEI
LYPYYNNHYVEKHSKLGPYIILNTDSYFYNQKYYKKMKRQFYKRICDLQISILKKLQKNKFHMEKNISYYIQN
NVYIEMTNVDINYTNMNFLDVFNLYNICEKEKNDHFCKRKSKSKRRTNTYMYVPSYFLFSLHKS*
>N.PF14_0124
MSEEAVALVVDNGSGMVKSGLAGDDAPKCVFPSIVGRPKMPNIMIGMEQKECYVGDEAQNKRGILTLKYPIEH
GIVTNWDDMEKIWHHTFYNELRVSPEEHPVLLTEAPLNPKTNREKMTQIMFETFDVPAMYVSIQAILSLYASG
RTTGIVLDSGDGVSHTVPIYEGYVLPHAINRIDMAGRDLTYHMMKLFTERGHTFTTTAEREIVRDIKEKLCYI
AMDYDEELKRSEEHSDEIEEIYELPDGNLITVGSERFRCPEALFNPTLIGRECPGLHITAYQSIMKCDIDIRK
ELYNNIVLSGGTTMYNNIGERLTKEMTNLAPSSMKIKVIAPPERKYSVWIGGSILSSLSTFQQMWITKEEYED
SGPSIVHRKCF*
>N.PF14_0125
MVDHVSFKEVNKIRSDDECDADSHNEGDNIEDAKASVFVKSSLIPEKTDVVKGLNFDKEVDLHEFINNYKYMG
FQATNLGISIDEINKMIYYKYKDENIKSEPNNENNLNCNNVSEDLNKDQENHLYHYEKKKKSCIIWLSFTSNM
ISSGLREIFVYLAKNKFIDVVVTTAGGIEEDIIKCFSNTYIGDFNLNGKKLRKKGWNRIGNLIVPNDNYCKFE
DWLQPILNKMLHEQNEKNEQMFLKKLEKRKKKYNNNKNKNDNNNDNDNVWGNEKNDQNENQYNQGQESFKKDS
NIYTNDVSNKKNHINNYINNYDSDSDDQCDMYYLSPSEFINTLGKEINDESSLIYWCYKNDIPIFCPGLTDGS
LGDNLFFHNYGKKIKNNLILDIVKDIKKINSLAMNCEKSGIIILGGGLPKHHVCNANLMRNGADFAVYVNTAS
EYDGSDSGANTTEALSWGKIKYGQTNNHVKVFGDATILFPLMVLNSFYLYDQKRKKDM*
>N.PF14_0126
MNINFYVYDDEADNEIISSNFNVYCSDEFLKIHNIEKNDNAIIYHEIMRQDEEEGQEKGQEIGQEKDQEKDQK
KDQNKDQKKEQARNINHINNIHININKIDDEYVEKEPKPNVQNFSVLCCFKNYNELNIKKKLENNGLCINKYI
LKSLNLDIKEIEEKNIYKSCKDQNNNMKKIGIFKVSIIKPQLFIPIKEIKLHVQEIFNTSYEYLKSYQYENID
LLQSFWKNIKSNNMEKYINLSLLNTCLFVNNFLKITILGVHSYITVGQIILTKDKKTKMKNNNNNNKNKTVRN
MNAHDKQENNKNGDNKNKNGDNNNKNGDNNNKNGDNKNKNGDNKNKNGDNNNNIVVPFIDQNTKIVIISNDDI
KNVVDDKHIINKYDKEKTNINSDHMKKLNLKKMVVNKKEKKGLNKLGGYNKIKEDIYYYILIPLIYKHIYDAY
HIDIHKGILLHGPPGCGKTYIALLIKEELSLLKKKFCTITQNKTSKQLNNTKNDNNYNNDDNIIIPEIEILKS
TDLIDYNNTGYKINELFLRCHKRYQEENKCTIIFIDEIEILCKKREENNNMNIYTSVLLNNMDGIKKHTHTIF
IGATNYINKIDLALRRSGRFDKEIEVNLPNLKDRISIFQKKLNLIKHNIGKKKIHKLADLCQSFTCSDINSLI

NISMFLNLKENKIISRDIFKTANVHTIDDTNDIKNDQTSACLEDCIEGKIGTNEMHNTKENKYINMNNNNDND
NNNDNNNDNNNGDNNNYYLTPYDKNHLYNNSLINTQSNSCHEKKMMNYNKNNLYLLRYKHLLKALKYVKPSGM
KELYIDIPKTRFKDIGGYKFVKRCINECLIYPKKYKNIYDKYNIESPKGILLYGPPGCSKTLFAKAIASEIHM
NFISVKGPEIFSKYVGESEKSIRNIFKKARENHPCVIFFDEIDSIAVNRNNNQNFVSNRVLCQLLNEIDGIKN
RLNVIILAATNRPDLIDPALMRPGRFDRIIYVPLPNYSSRFAILKKNLKFFKIHNLIEYDKKETINDLNHNEI
KRECIIQDIQYNDHPKNSNQKINNHITKSSYDHIHNYTFNKNNMNIKTNEDSSLYKHYKYTDIKFIKLCHVLA
KKTKKYSGAEIVNICREASICALRQTLKKRNIKINPKIKIKIKNNETNNTQNNIAHSEKNKYPFDKEPLIGLS
KKHFLRVLKKIKPQTSESLINFYKNYKEQNKV*
>N.PF14_0127
MNDDKKDFVGRDLYQLIRNAKDKIKIDYKFWYTQPVPKINDEFDENVNEPFISDNKVEDVRKEEYKLPSGYAW
CVCDITKENDRSDIYNLLTDNYVEDDDNVFRFNYSSEFLLWALSSPNYVKNWHIGVKYESTNKLVGFISAIPI
DMCVNKNIIKMAEVNFLCVHKSLRSKRLAPVLIKEITRRINLESIWQAIYTAGVYLPKPISTARYFHRSINVK
KLIEIGFSCLNTRLTMSRAIKLYRIDDTLNIKNLRLMKKKDIDGLQKLLNEHLKQYNLHAIFSKEDVAHWFTP
IDQVIYTYVNEENGEIKDLISFYSLPSKVLGNNKYNILNAAFSFYNITTTTTFKNLIQDAICLAKRNNFDVFN
ALEVMDNYSVFQDLKFGEGDGSLKYYLYNWKCASCHPSKIGIVLL*
>N.PF14_0128.2
MKTLKEALTNVLSSLNIAEKKEILNVLYHILQKIIENPSRAKFRSLKKDNKTFVNKLLQFKESDELLRSLGFE
EEPNRNSGFYKGACKHDI*
>N.PF14_0128.1
MKTLKEALTNVLSSLNIAEKKEILNVLYHILQKIIENPSRAKFRSLKKDNKTFVNKLLQFKESDELLRSLGFE
EETEIWFFPVKHDHKLSSNLTEIRAFIKEHVNTIYNNQDSIFETGTASSSAHLPTDEKNVTENTSNLGDTCNN
SEGLTLGGLSKRRLEKERLELLSQKENTIKLLQEHADKWIIQITGAENTLYSNETFQMQFKFTEKYPIESPEV
IFLGQPPIHPHIYSNGHICLSILYDHWSPVLSVNSICLSIISMLSSCKKKRKPLDDILYCSTGPRISPKNMKW
MFHDDKV*
>N.PF14_0129
MVTKVEQKKKKKKTKVKAYDIDESKKVFLYAYEYQLKNEKINWKRAEELKITKHSGSSMRSHYINTIKHEISL
YLEMYPEEVAKLFVKVKQWKKEEKKKKLLAFQNNIVTQRSNISLNRITKCKSTNYNKFNSLVVSKSFSMKYKH
IHTNNNNRSEKNNSAIKLNKSNNKEKNKHVHKKKIVHDSIDKNKSVNKKIGNDSINKNKNDNKKNVNEKINKN
RNGTSNSTTKMSLKKNKGKHEKDTSNVMNITTRSSGDTTSNNNNTNSTNKSNIVTKNKKVNKKKKKKESKNDI
KVIKSTIEKTNKNINNSFNIDRMEEAVDNNNKCNEKDNKDIFIGSQTNENIKKNVQFVKEEDILKIKLEQIKI
NQKKKNISKNNDNWKKLLLQTNYTNDERESKVIAENKKPYTNYVTNNAVPTLNMNKINTINNVKNVNNVNNSF
ESYKSNISNNDIKENKSNVITSIFGYIYKKIFR*
>N.PF14_0130
MSTVTKKNKSYLESLSEEDVLTLIKKCYRDKLIYGYVQWKLLDRKIFSKRFSVNTFKNIWRIINKSFDDYYGK
YENDLSQVEVEAQEVLWKNKEEKKKRKADKEKFIQIYSSKLSVSLSNISLLRKFSKVNSSLHFRNSNSNYYSP
LQNNLNNSYGNMLKSVCNDEEKNKENYVKKDVLKSDIHNNSNINSNVDYIKKNINYIKTQSDLLDLYFLIISL
IHDNKRFLLLRKIDEIYEKNILKCYNNINNVYNNYILNIIRMIYEMCAISNIMHNNKTSQEQLNKNISKYMNK
KTNSNVSYNIYSSYSTNLRSYGHEIYNKSNKHTHHNHFINSKCIDNKQNIIIIETNEKIYKFDKMHLNYDIFY
DIYKNNLTLKNNQVITFFLPLPKNKPNHLTYKNNNFYYFDDIIYLNKNLSDDVYNYIYILQKYFLSFKFWYFY
LRYQGIDKMKKLFNNELSNLFFYLANYDKINNKSSNISFYQNKGTNKQKKKNYLLLFEYNIKTLINLCLHILK
IYLNKEIKINISYFQIIKILNLCLNPTRNKKIMRHYVVHMKFVHSIFMKLIKIKEKKKIKKK*
>N.PF14_0131
MSKVSDNIKNVDDQRKHFDEKEKKKLFLYGYEQQLLYGRINWKLAEKKKITKHSITTMKNYWNNSIANCLPQF
IENYSRDVERLNEMKKQNKMKNIKKGKIKKNDKTKKTATPKVQFTKQRSNISMNRVIKGASLDYKKLSTNTFR
NKQIVVNSILKKKKTMNKIIDKKNKLVSLKTKNVHLKKDIHKPNKTKLVQGLPKKDKKKDNTYTTKGLLKEDN
KKAINKGKKEDKGKKDDKKDDKLKNKINKKDLKYDNKSQKLDKHKKEEKYEKLKTDKFKNDKGKNDKGEDKNV
DKTKKDADKKKEKNEEKIKLDDKNKKDKIKKDKKDEKKDEKKDEKKDEKRDEKKDEKRDEKKDEKKDEKKDEK
KDEKKDEKKDDKKDKIKKDKKEDKKDKKSEKKDDKKTKKNIIKNSKGKK*
>N.PF14_0132a
YIYIYIYVYIYICIYLYIYFFFFFFFFFIECKQKEIKYIYIFFFLSFNMYICLNKMVIFFFFYIFFKH*
>N.PF14_0132
MFFNHVKSYIILFILFCSYYINGRGVNRIYKNCGRKKLGNIFCCANIICVKERVKKIKHIDRWEYKRKKRKNI
NIFLYIPIKKDINKMKYYNFRNIVINDFFFFINKYVCASKIINIHSKCRKKFVLFKKDKGSDKSNNALKKKDK
KTFSEEEIEELRKKAKEKSQANRSISEESKRKKSIEKGMKRKRYIKNQKQVKKKKSTSDEEEKKKKELKDKDD

TKENVDDGDDIYLRKSKESKDKIQKDEDLNSYIGTIDDFEKISNVTKNIINEKEVEYLQKIIDCDDALLKYDK
REKNVKLNIEDTIFDEGNYMEHYINNISKFRYDIYNLHNKNEFDRLTKYLNYQYVEEIPITFPTDKNYHIKLK
KYFYESVCDLINKYKHRFQNYTLQQENMSDSHDKMGTIVQNDATNISMEKRNINLYNDTKGISQNNMVLENFI
RENEKEKMETQTHSTINKIEEGVTNPYDKSEENIMKKYMTFDLYNVLCDVKEEYEYMFDNGGIEKFSFDKNRI
KNKEKLIFSGKKKRALAQVFLERGNNNLIINNRDGYQYLQYQILNLNKIFSPLLNLCLHRHFNIVAKVEGGGL
SGQSVAIFHALVKYIVYNFSLKIKPFFRSLNFMTVDTRKVERKKYGLKKARKKKQYSKRNKGVYINLFSFYIL
QLNICTYIYIYI*YVIIIIYLFFFFFFFFCLYLILITLILYNIYIYIYIYIYIYIYMCIIKFNILFF*
>N.PF14_0133
MKRRKWLNITWAVTIYIFFGMINVSSYNEGRKRKNKMYSFVINGYNEYKNKYSFLRKPKNVVHMENNNFDMDR
LSLLKNLEEESKIITEGEGWDKTHPLLEIKDLHAIEIEGEKEILKGINLEIYLGEKHTIMGRNGSGKSTLAKV
IAGHPYYKITKGIIKYKGLDLINLPVNVRSLCGIFLAFQYPVELPMVKNNEFLRAALNSHRRHRNEEEISVSE
FNLMMIEEIKKVGLSSEFLDRPVNYGFSGGEKKRNEILQMLILKPTFCILDETDSGLDVDSFKLTSNVITNFS
NDNNSFLIVTHYKKLLELLKPNFIHIMHQGKIIESGDFSLVDKIENKGYSQFLKE*
>N.PF14_0134
MDENHKEHYFQYKSPNRFDSNKKKTINISGELKMQKLNSIKKKIPSYNKGYVLENKKKIYVSNCGAPDIFDIS
TYYANEKKEKEKQQCLNILNKSKQTKKNEHNNKMCNIKKEHIVDNRPCKYAHIKNKNKLKYYKYTNNEILFKI
KYCNGKKKNHYKDNLNKIHSHNNKKVKNKLNNQTYKCKQTNIISAHVHSNNNIKLQNFNHKSSTKKKNLYNKE
KILKKKENVPTCFIPINKKCPKENEKIYNMDVIKNFKYLDTKMKNYINEQIKYYGQNWRPEYITDLYFFIKKN
YHKMDSSDKFKRTKETSYCPYKYKISQNKEKQKHQMLNKVLSPNSCNKNNDYLLIKYSLVLLNLMDTYNIIDE
NYDKINTGNNVHQSLNKNNLIKKLNEFKLDERKYIFPFYQVYTYLKNQQHIFEKILKQKKIIKYKINHKKTTN
KENNDNFHWKELIQIELKIIRFLNNFQQIIKKKNEEKEHNNNLPNIKYYKNIPSNICKLIKIVNEVTRILKIK
LNK*
>N.PF14_0135
MCNKYRLHLLNQINPTTNTSSYKQHQLNNDLYKLKKKMEKNKVLFNKDDKMDILSMDTLKLADEIKTKIIAQQ
LHTVLVLYLQILKEKKIEEKNINDDIGQYIENVIISALQLYSLIEKQTKDRKKCYIKKICQLCNTLSNILIEY
IKNIKGIQVQHNSSKINENNQKLLIDSLENSNKLSKEKSTRSIESSKEKNENVFEGTSGKLNTVSYPCGCVHS
QDMKIKEISLENEFSKGYRLMCLYSAQELNEQINYNKNIINNSFKTFQKKYCNVKLRDRRELIYKFQKILENK
NIQFYDIYKGNVKSNIVSNQVNNNAQEFHTDLYDGCSYNSNHIIKNVEEKGIRHLINILLHTNKSITTFEINS
QGKIDAIIQFLQKNEKKKDIILLNEYEYFFTNTDDYTQKEKNKNRRKYRDRENVTCNNRMVKRTYKNVDDNNN
NLKEENENIYFYVGNKKKIVFDIKKKQKIVYEIDEKNSLLTKKKFLKDENNKDILLKVYDENDWSDENMEFCY
ITEKKKEKEKIVIKKNKHGKKKTVYTNKNNELDNNVKNFIFYKKNIKNVKEINFYDDEEVILSLLKQNEERKKL
EDTKDNYDIKNMENTISKDDNTLVCEKNKIPSIWNKAKNKTCTNDRDMYILEHKYVENKNDEINVESLDTYTN
IITNRKEFLLKKHYLDNQQDISSFLKIDDYYYFKKNGNDSDTNKFNKYDNDYKMYETNVKGNMDDTKKCIGNK
FDKMRNKKLDNIIAINDDDNNNNNNNYMNNVCLESYIDLYLEPKKKNEKEKKKKKKNYLINRMNTLISGDGKK
NISEEYNMNDKEKIKIEDFLFNEYLKKVSVRSQSLKKKKEKKHEHEHEHEHEQEHEHEHEQEHKHEHEHEHEH
EQVHEQEHNKIKKIIYDTKIDMKKEEDTQRKIENNHNIISNEKNDILQIHNIVKKDNKHMEINKCSKNNETHN
NLPMSNDQKNNILDKNNKMIDKNLLKKKSSLQMNSFNSDKKLYDNDISNVKEKKIYNERKVSHENSMENQRYE
DDKMGRKYLSEGFSAYVHVALCENTIISKKCIISCNINTKMLKIETKEKKFIIDMNKLHVQEIPTTYDNLAII
KLIIKKKNNNNSNCLLVESKNLGALQHLGDEIGWNYDKTGYSMDHKFNDEKEKKERRKTDDEHINNKPQLIDN
STVGFFDKIKLNKFLGKKKTEEKDI*
>N.PF14_0136
MEQISLVKENVEEKKKKVHCSIPKFILENKLLEKAIKKCLPENYNFEVYKCIDIILREKYKRIALQLPEGLLV
WGLYLSEIFYFFCESVEEVIILGDVTYGGCCIDDFTSGKLNCDLIIHYGHSCLIPLTVTKIRCIYVFVDIKLN
STHLVDTIKKNFDKNDIILLLGTIQFSCIVHNVHNILKKENYFHTFLPIPQVLPLTKGEVLGCTSPNLYEFLY
EHIIKYERDKKYSELEKEKGEEGKNKETQEKRDNINNIILVNKMKESLNEHDIRKECQIFLKKKNVKIIFIAD
GRFHLESVMIHNPDFLFYRYNPFDKIITEEKYDYKLFYDIRKNEIKKCVNCKSIGIILSTLGRQGNVNILTNI
INIMKKKNISFFILLLSEIFNEKLQLFQNVDLFIQIGCPRLSIDWGNYNLKPLLNTYEAYVLLNSVPYKDIYP
MDYYSHKGNIWTNYAAGVGFYNEKNLTTKEIIRRRIQMRKSKIAIHYHQ*
>N.PF14_0137
MEEIILHMIIIAPLTKWLIGSNRRWNQGKREYGIYIALLVLFCVGIYELRKPNQNLYEVLNLNAYASKTDIQQ
SFRKMSRIYHPDKNKEPDSLDRFNKIREAYEVLSNDKKKYTYDRFGDFGDSEITSFFYVEIIIIAMFQFAISF
IFGFLYTYGKDNEKYRILICLYIALNFCMELILRFSPESTVFLSFIPILSHYTPFERIHAFRVLVPLIMNAIL
LVDIYYIEEDTEVYVSTFCEYVFENNSKSIKNYDDAVLFCARLVDGKMNNASNFSWREEKSSLDITNMYELDD

DKVFDKQYDKDDIFYSLLYNIIENNKNQIDLKIPKKELCRRFDWSRWYTTAILEKNTEEKNFVESNATKGIIF
SSVLYFIGLVSHLVSK*
>N.PF14_0138
MDSAKIHLQINAGKCIYDGKMVKPDKRKGKLVLYKIYDNLYNFQWINRENNEIEDNLILTKSISLERVEQCKT
GRVYLLRNKTRGEISFYWMQDYDDSKDEIFVKQFNSIITNELANIDAGSKPSNTNDYLSELSGLIISQTRNMN
KDNTKKDIYFKDIFKGEYFSKLLEIPEALEELKIHMPEGYKTKEDIIDVINSRALNPNLKALDMSLPTHLNFV
LISLNLPPHEGEVNDPMEYIVDALEKKYTKEENN*
>N.PF14_0139
MIETYIYWNFRGSRGNGNILRISLPCSYNTIKKKIMELTNIHIENNLDILLYHNNKILSEYESIHHEMLIEIQ
RSSIDVVRDMLQKSNDLFLEKNKSKTEANKNNGITQRTIQNHQKINNRMINDYNNKISKFNNNNNNNKTTGYF
HNNNRPWNIYNRNNIIKDEDKNIYGENNLITNQQQTEEAAEELEEDEEMKIHLAMEKNNVYKSNYNNNKIRLN
YYKRDRNNATHFMQNKLKNTSHNIIKNNKNVIANNNITSSSKVDGEYSTTYHQKNNYLPNTYTHGGTYNKFKN
HITNNHNNIHANNMDEETINKKVSPDYICHMCGKKGHSIKNCTMSSFNNNNKKIKVPTGIPTNFLTKIKTEDI
YKYDQIYILKDGSYGILKDVEDVSGSAYLYRSVEDKINIYLGVHNNKNNNNNNNNNNNNNSNNSSNNNYYNNS
DVPNEPQNNEKISNLYKCLLCRKLYTEPVTLHCCAETYCKACLYKYNKKKMKDTSNISSSYHNKNNNYTNQES
NKAIGAGAGSASASASGGQLMKCPSCSKYINSDELIVNTNIKNVIDTIVKNQKINQVNDEMGNKPSANTFVEI
NNNMNLMDDHNNNINNNNNINNNNNINNNNNINNYNNSNNSNGVAGADEHNNINSHNNQNNTHNFTNNSSNDL
GYNHNQNIFNLSHKDNIHNIKQNEFNHVNFNPLITFSFNLYELKKEHDFAAAYIAQYKLKKKKKKKRQLNINL
SVLNKKIC*
>N.PF14_0140
MSGAAIGVENRCLMKNKNNKNNSCEQVNVDINYIIKDIYGYKNCKKRCIQFIEDDTICNDDIDLRKMKNKKIK
VENMWYKRYDSNDMNNNNNNDNDNNDNDNNNNNNDNNNDDESQHNNSNNNDVDNNTFKRYNNYNEKYEENDNM
EKEVYHTCSIQSPERSAVHNNNNNNNNSNNYYYMTYQMNDEHIKNSNNIKIHNNVNINMSSNNVDNNDDANSI
DSNDDSNNMDNSNDTHNVNNKDDTNNKYNHVNHNYYHKKRNKQQPILSSNEPEDKYVLSKKTESLMDTILFNI
HSCSELNQAKKIMLPLLNDFIQNNFYKYIKFYEDNQNSSKHNVQTLQKEKKVLISAVKIQYQKILQLQKLIEN
QKSDIKKKTEELNQIKAKVHQYFYDINNPNKRIFSILSPDVY*
>N.PF14_0141
MGRRPARCYRYCKNKPYPKSRYCRGVPDPKIRIYDMGRKKADVNEFSGVVHLVSYEYEQISSEALEAARISAN
KYMITNCGKDNFHLRVRIHPFHVLRINKMLSCAGADRLQTGMRGAFGKPNGVVARVDIGQVLLSIRTKENFVS
KACEALRRAKYKFPGRQKVFVSNKWGFTPFSKDEYQQYKKKGRIISDGVSCKFIREKGPLDKIYKDINTVLES
*
>N.PF14_0142
MALEIDIDNVISKLIEVRGTRPGKNVNLTENEIKILCLSSREIFLNQPILLELEAPIKICGDIHGQFYDLLRL
FEYGGFPPDANYLFLGDYVDRGKQSLETICLLLAYKIKYPENFFLLRGNHECASINRIYGFYDECKRRYSVKL
WKTFIDCFNCLPVAAIIDEKIFCMHGGLSPELNNMEQIRKITRPTDVPDNGLLCDLLWSDPEKEINGWGENDR
GVSFTFGQDVVHNFLRKHELDLICRAHQVVEDGYEFFAKRQLVTLFSAPNYCGEFDNAGAMMSVDETLMCSFQ
ILKPVEKKKAAN*
>N.PF14_0143
MNQYDQWNRRIRTIWYCSSLYVEYKNALRKSKKMPVEKKNEYWEKKHEEFATKMLNNIYELRGWWVKVGQFLS
TQENIMPVAYIEKFTKLQDMMPTSSFDKIENILKKELGNIYEMFEYIDKEPLASASIGQVHKAKLKKYDKHIC
DIDSRKHKDYNVIIKIQHEGIDQFLSSDISTLKKVSWAFGLIDKNFYFTDFIDEWQDSASRELNYKYELYHQL
LAYNSFKKSGIPVKIPKIYCAHTTSKVLVMEYIKGFKITDTELLKKYNVDTYKMVYKIIDYFAYQIHNDGFFH
GDPHPGNILVMMEEKKKRKRKKKYNKDKINNSKMKMKNENIYMNKRKKNHKKSNRNSKKKLRSCKKKKLEINK
EREKGEEKKKNMYNNNDINYCENKNEDIITNNPKLQNNSSDMKNIIKFTSYSKSVSSSNASLMDHFVDSRYNI
DKMRAPNNFSNPFHKSFSFVDYSLNQDVDSDKMNQEDYEYILKENMSDGLDNMKNCLSRSHEYISCDQSRSGV
TSIATSRKTQNRDSNKASENSIMESRETTMFSFVNNMEELVKMKSETKTDLQIKGNDKKVVKGSKCDNKNDQN
VNNDNINNDNINNDKINNDNINNDNINNNNIVNNNNNISNGEKEKNKSGPIYKSKFKLSDLKKRKNAKYNFVP
VIIDWGLIKQLDSVMKFAFCKLVYNISCMNVLNIIEAFEDMGFCFKEDFTYDPEIYIENLKTYFLSKLEESKS
KGNEGEKEFGSANTIDEANKSNKNMEILKNIEKKDVMDKNPISDVPKDIIFFMRVASLLHGLCTQLNVKINYL
NIFSRRAKEALEKIYIPINNSIHTIPIDKCPNTFVEKRIHQFIKTLYEKKKILGCQVAIIHKKKLVVNTCVGI
TSTTDKRPITKHSLFNGYSLNKIILNIALYHLICNSVNEQTSGESILLGMDKKKDKLGEKISQKIQEKISEKI
KIKSSAKDKNQSDEKIKMKINEKDKDKDKDKGKGKEKNKNNNKQNNDRDKNNNEENNNNEDNNKDNNNNNNNN
NNNNNKLLNEMDQSKHNANEKTSNMKNNNKLYEEEDVEDEVADDNKTDNENNYITEKLNEFNKIENDYFSANV
NENIIKCEEFGESKDIISPLSVNSFISFDSYNSYNNNVSWISSMSRNSYKSMIERIEDDVEYKEINDDLPNDS

KVSCMSEYKKDDKYKGRNYIHDHNNDDVHHHNNHNNHNNHNHHHNHHHNHHHNHHHHHHHDNNINKSNKDTES
NPNKSINIYPNISYNKMDTLIQKKQSYIDEKNFIKSIESKQIRNFKNIINDHICNYWDGFICNNKKNITIKDI
LTLKCFIKKPFHDKITLSKFIDYDQMINMIENSKHYKSSDKTSKYGEYLYLIDTYIISELIHNISGLHYYEYI
YKYIVKPLNLTEEMFVPIPSELLKNQKYEDSKKKNVNNQNNVNTTGGLSNNNLDKTKKYIKDKKTNVIDKKSI
VMDKWPNMMKDYTTGALKNNEYKSTSFIGGKKFKKSSTISNFIDDINDDMNFYIMNAELLKFENINLKNTISN
KKKFDSNQKVLSKKRSISVDVYYSKKKIKDGSKKEKKKNKDKKDQKESKTKMDFEKRDIAEMKKVDRNEQKDQ
TNQQKDQTNEQKNQPSQQNEQMYDDIVETYEDIIETYEDKIELNHDNDDSNIKTNDVYHDIIEEKEDNEYYNN
IKNEYENKRNNNKIEISKDSLNKNPSNIECKMNILINNELIDNENARKLDSSNLLESTLRNKESFSFKNKFLN
HVDNLKLKTKKINDSIKNMYENNENIFLKNFFLSSKKYDNIENNLDHRNVEANCVDNNMCESNSLNNSCYNNS
FYNSFYNSHSNSKNSSKIRNYNNMDKENNNVNSDNINFNPNDNKKKNSYNNNNNNNNNNIMVSSSPYIYNYNN
KYILKENYDKKFNMNKFNKVMLSNQKEPVHKNNKKDNLFLFEERGKRSHSYSDCRNESHHVMKNSYMENINNI
NNYSNSSMNYLNYYISNKKSVNYMNANGVVINYDDNNNNVNNVNNVNNMNNINNNETNNTNKMSFFTNTILNT
FQDKNNKTKKKKEVKCLSNEDKYFYNLEERRKYYKYNFDKNIRKLFNNVDTKTSGEESANSLLKEKYNKYHKR
NKKYKKLFEYIKQLKDHIKKKSDHIREANEAFIRSIKQKQEDENKKKEENKKKEENKTTDGGKTTDEGKIIEE
GKIIEEGKTTDEGKTTDECKTTDECKTTDECKTTDECKTTDEGKTTDEGKTIEECKIIEDNKKGSEDYNKNEE
NKSKEEKTPNDDKYEFVQDTEELIKKVTKKDQELLKKLIEYKYQILKKQNNNLMKKKNKENIYMNDYSDNIEM
FSDTETDSTDYYDEDDADYDEHMVEYNNYHDESNQNTKDYIFYDKKKTRRELKELRRFNERKKRLETYLIMNK
LYNQNKKGVISKEYHTINKKTDVYWRLAFARRNINVLENVTQEDVNNQLEMLKSNGKVQKKGAAHNKENENEE
KNKNNKNNYIHNSSNNIKNDQEKSSLNDNKNFDNKLKMYVDNFNIKQYNKYISLFQLMQSKPYILDPLIYDSK
KILDKFIPINGRFTAKALCKLFAFVNNEFFFPSYIINKIRKKYTIDKSVEALILTGAMSRTWGMGFQLFECEY
NANINDEFNLYKKKNKYKKKNVESRKKKKINKKKKKIVGYGQSDFSGCLAISFPEIDLSITILLSDVFKGADV
CHLLLEFILKLYGLKPQWKVPVKMSELVKVF*
>N.PF14_0144
MITSTYHPGEKIENEFLKEKIRSKINEMLKWKRRGFPGCNPVSLTNHNIKNLFTKEYLICEKTDGVRYFLFIA
SNTTFLIDRNYEIFKNDMHIPTIEDLSKKQQLTLLDGELVEDIIYNEKTGVEEKKIVYLIYDGLYIQRKDITN
LSYFERLTNVYNYVITPLKKYKKSQKNKKNKKNNLQTNHENESLYIELDEKDNIKKRKSNLNNMLTEEENVLIS
HKKNDHPHINNKNMNAVNVNGVDVNGVNINQDFNNHNENNNLLMNQGILIDENNNGIQNIGTNDNINSLNNCN
LLLYKREEHREEKEYEEEEDERSYSSDDTASTIHEEEIPFEIYLKDFYPIEKICELIKIMKKLPHYSDGIIFT
PLHSPYITGNFYQLLKWKPLNLNTVDFGIETIYDEYNIPSKFELFISINGVRTSYKCYLAEYGDVYKELLQLA
ISNKISHYIIECYYVSKNIFSICKGENGREQKVEGGWIAQKIRFDKNIPNDISTLNKVIQSILDNITIDSLIK
EISRNRKAK*
>N.PF14_0145
MSFVKGAIHVSILNNYMDFYKESTVDKIETSFSIRNNNESNTKNNEKRKSSLCIMNDEEKMKNKISKCIKNNN
LYKNKKKRRKEKNIISNKSRSKKFLKFNIKGKDKKNNINSNIYNNANNNKLKIKNKNNNNNNNNKINSCCRNV
YKGSTNKRNSKLLLNGEIKHDEIFHTNIDNSFICMNKNDMNLIRKCKENNHPIKQMNTKIKKKKYNKKNVLIS
KQINKEIQMLKSNNSYIMYHMNEKRNIEKEKKKKKCKKIVKSILYNRKRSHYYISNFLKTCMKIKNNKIQKKL
KKYVYLKDIYSTINMSNKQTCNNLNEQNKSQFFFDDLQKVRQVNKCDNKLYKMNFKSLKHQTNGKKNNIKEND
NNNNFYNEDNSIAEILSDDYIILNKNKSNKMENKIYNNVINHRHSSEDIYLNHSKSKENNNNLVNSYVMKENN
MEDKELMNDNEKKEGQLILKNVNKKIQLCNESKLDNEKKNKGDNIKLGNTKNKDNIYKYLNVLSINKLIKGET
YLEKRTKKSDNSNEHKNDNIYDDKKIKYKSVENDTSELLDKNIDNSNFFDEANEKKKKLKKKKMNTCVNKNVM
DYVNEDEYYLEERVKKKRKKKKKDNKKYNIKNKNESEEIFEDVSSTNSCIDSNNSGTFIYDEEICKHVINKGN
IISNNNNNNNNNNYIVLTGNEKNMNAEEISEYITKQGLNFKEYLIWKEIKIKESKELIHLNLDSSFSVSKGAG
LYNYGQNICFFNSIIQAIIRIPYICKDLLNKLHSINCEKRKKKTFCFYCLFEQFACNIISKKSVIKNVLIPYI
KKYMCNSYHIGYQEDVHEYLRYFLNSLEKTSFFSSIYIQKMFTGVSKNVTICMNCNNVSLKYEQYYELSLDIS
SSNNLEDALKHFLSKEMLAGENGYYCEKCKKKKKATKQCVINKLPRVLTIQIKRFFMNSNFDIVKNHKNISYP
LYLDMKYYVNNYDLFENNFNNNVISLYEKMNKTNCVGSTTNGPNKKNNKIYHQNNSCNIKSNNHFVHTNYNNK
GQNILTPEKLTQQNIIPEHSFSKNKVNYENDFFLNNMNKKRECSNDDIISNINKNENIFFNNKDKEYMINEKY
MNTNQCIKLRGGNHNINIHVLKIIENILIDLKESMCQKQIQNKLTSKVLRNMIREKKKLLIKQLKKIKFSKLY
KDISLRISKDIDMLYYYFKVNRENDQFKKIIYKYKIDYYEYYVGSTNINADFINYNHNFDSPAKDHHNNNPHQ
NNDENNNRHSNVNFKEGNAKNSNKKNNYFSFELTGLIKHIGSGTDYGHYIALTKSNNNIYLQCDDNHISYINK
KDILNCVKNAYVFIYTCINPEFIDFYNKYVDVLEKKNFDINLPVFEKRVKFKERITMPKEKFINKSLCY*
>N.PF14_0146

MGKNSRLETWLQYRVRVTISDTRYFVGTFLSYDRHMNIVLVDAEEFRKVKSQENSLKEIKRVVGLILIRGENI
VSFTAEQAPINKKSMGTVINKGIATGRGIPLNNYVPMQNNFNNPLGNPMGNMPTGMVLNTGTNKNLNPAINPN
IRLPNMGINNQRPIMPPISMQINQNPPHNNANQAKGLPPGVPQLPFPPNVNPPAE*
>N.PF14_0147
MLALHWNLLTFRRKANYKRCAYYFYRTSTNKKRNSYENDLKRVKEVKCVVLLNKPLFPGLTYVLNSDKNVVKY
LGVHNDYKEKKNIFVGLFFHKEKTIETHLETNNTFYNKNCNTYKPPDFLNEKRIKVDKNILKKDIDICTNIND
IYSYGCVGVIKEILYDEDNLNSICDEKDYNNINESNDIMKKHHFNENNKKEKDNYLLKNHIDMTNMNRSNDLL
IHNKLKEKNNENLNLNMHKSIYRIVVETMAKIKIEKWNEVEKVGYIDIIRNEDYDINNKEIKIYYLEIIDKIK
KIICMNSCNNREYNILLKYYNTKNLYNLIYFVGNISLSKNNIIQNLLERNILKDQLKICIQILCDDIYLLEMK
QKLNEQINEKFENDKKNMIIKEQINILKKQQQLLLQTQRQEQNMEEYEDGNNTIKYDHDLLCDEFRNKLMLIR
TYISDEAYRIINQDINKFMLYNENSNEYSSTYQYLSTSLNIPFNKYKMLNDNIFLCEQILNKNHYGLKNVKKY
ILEYLALYILNKNIKSKILLLVGSPGTGKTSICKSISECLNIPYYIINMNNIHNMNELIGHRKTYVNSYQGQI
INSLIATKIMNPIVILDEFDKVAFINTNIYNMFLNIFDKEQNEHFKDQYINFEVDISKIFFICTANSVENIPD
VLLDRVEIIHIHAYTNMEKIDIYKNYLKHKIENETKITSLYLNISNDVLLYILDNYTQENGLRQFYNILYNIY
KKRAYMLIKGYNKKVDINLSNISEFVGADQLIVKKNYHNNVHININKENIMMGNDKSEKYERNERNEKNEKNE
RNEKNEKYEKNEKYEKNDKSEKNEKYEKNKNYEKTCGGSVKSLAFTENGGQVIIIEVSSLSDKIKSHNYYNYF
PLYYNDDYMISTHEEKLKDNKTEKYNELSKKNNKILEGENRWELLNNIEMVHPINELFSISNKSYKNIYNMNY
EEDDKKSLTSSFFITEDGYVKEKERAQQVSFMNNQQVLNSYNEKNKNIYNHDNIVQNNSNKYIKKENKIYTYN
NSPYELHPIHNNNNNNNNNNNKNNLNHNITITGNVGRIMQESILIAYTYSMKLLNTIMLKFESKPLHINLSDG
DLKKDGPSAGINFVTCILSYYLNIPVDNTLCMTGEINLNGYVLKIGGLYEKLNVARNFGIRTLIIPKENSNEY
QSLPQHVKQNIRVLYVHHYSQIFNFIFNNKK*
>N.PF14_0148
MNNPTIQKTIDQFFKVKRKSSILSGEIEKKRKKVILEEVEEKSLEGSLKEENVNILKTKKLMNNDEDIEKMGT
ISNISMSTSTIDNEINNNVKQNVCEQGYMEEIKKLMHIEWYELLKDELKKNYFKNMYLKIKEERKTKVIYPPE
QLVFNAFLKTPLSNIKVVIVGQDPYHQKDQAMGLCFSVPIGVKIPPSLKNILKEMKQKSNHGNLISWSEQGVF
LLNTSLTVEENKPASHKNYGWETFTDTVINIINRQKEKIIFMLWGNFAIKKCKNIDINKHFILKAGHPSPLSI
KHFENCNHFAKCNKILAQHNLTPIKWELPQ*
>N.PF14_0149
MGNAMYAKSVYSSEGSEIFSENLSKEKRSEKSIHKMEYINDKEYKEDEYILNVYNKENKNIQDMNNEKINSGM
KLKYDMNKNNLYVILSKKKRVKRFNPSTCLLRNCIYYVNCKINDKYFPILLELKNNPKCAICLIDESCNIIKE
ILIKDIKTIESSINSINLFLKINPEYSIQNKFNLIRFILKDNNDKISFMDNIKMMYGIEILEYGIVKYNKIRT
QEYEEIYVYEKNLLNKENNQNLKHIMDNVTYNEYYNISDLITENENTHEKKMNQIIKNLIQLGNKYNPIIILG
DMEEGNIIRVKEINALSNRTTNHYSSKDKQISFQQNYNSYDDFTLVEWYLSTKIGSNKTFRKEPIYSSNKLLL
KSFMIGHFIKVKLSKNTFIKNKKTFVTSISIKGPVTINDNTAKQILHYLSNVNHSIQIFLSSDDIYNIFFSSI
DPKINVLGLFIFYPCTLFLMRSGIRFAITLNGKSYSVDYLWNSFYMTKKDILFDKSPDFIPSYEDTQDIHLYF
ITASTNGQNVKSIIRTNSTDEKNYIYSTVFFYKHQKKIRSLNEFLRDSLSGSYNNLKKRFSNIFMEINANDLS
KIESMN*
>N.PF14_0150
MEEKKYYENLKNLTHATFCFENEDHTLGNCLRCILLQKEGVEFAGYTVPHPTQPEINVRIQTTGKPAIDILKE
SLDDLGNMCDIMLNKFNEALKQS*
>N.PF14_0151
MVEINKGDHRNVLNGNQLCFVKLLINNLVAGSVIGKHGSIITSIENKTGCSLKLSPNNSYFPNTQERVLVLCG
KIDQIKDALLIILDKIKEVANQHSHEKQNMMNGAAKYTCRIVVPKSAVSAIIGKGGQQIKQLQDSTGSKIQIS
SREDGLNERIITIIGSFESIRDTALKVANSIQKDPNLKDLLNVIYNNESNNYNSRNNNHNFINQLPLNGYVLP
QRYNVFQHEPYVDINMINSLMRHSRDLFNLPCEISIQIPDEFIGAVIGKNGSRLTNIMNSTGAQIRISRKGEL
VPGTTNRKVRIIGSVAAVHAAHVLLLQRLESAYMQLRTMQIKCDP*
>N.PF14_0152
MDVKNLRSEYILEEENNVFEDYKSYISDSVENKSIDFKNDLSSSCSKNINEEYDKEEEEAEEGLKGNVYENKN
EDKKEEKLNDRTNEQCDNINVNKIKTTNLINLKVEDNKLEERNYNDDNNKINKNTTTNKMLQDLEDKNYSIKE
KMKEDPCYVPKESGYFLHDNRFENNEKKLYDKKVKNYKNQYTEEKRWKHDLFYDYKYDEYVKRDNKNHNKTYH
NYNNNNNNNKFYVSKNKYDNNKKNDYNFSYKNYKERYYYDKGNRIKHNNSITYYKHYNDIRTKVEIKRETNIK
NDNKNEKVNFLTLDNKTNNNTQKKKHLKETLLKKYDDHMEKITEKENQNKSTSSKKEKQIILNGDIVIETKKE
EEYENIKKGEEKCIKDNIIHEQEEKNINDVSSKNMVASINDVSSKNMVASINDVPSKNMVASINDVPSKNVVA
SIKDVPTNENHEQKKNEESNKNGTNYKTKKLLNEKNKEEEIYKNHNRENHKNFGNSYKSQNYYNNNSLNDKYT

SYKIDGTRYVFLKDEPNHSNKLLYNKITLKKHYTYEDNYNNKKKLYAYHTFNGSEQTRRKGYSINKNKENSKN
LDYKRMHTRDITLLIIIQIKFIVDQINFMEQLRLRTQRNIGLHREIIEMISISKESYKKEYNNIYDEEDNKSD
KYRNDRDVRDRRKGFRNVDREKAHIYKSEDKNEHIREGGNEQKREDKYDRKREDKYDRKREDNYDRKRDDNYD
RKRDDKHDRKKDDKYDRKRDNNYDRKKDDKYDRKRDDNYDHKKDDKYDYKKDDKYDYKKDDKYEHKVEDINEH
KVEDINEHKVDDINEHKGNNTNEHKGNNTNEHKGNGTNEHKGNNTNDHKGNNTNEHKGNYTNEHKGNDTNEHK
GNDTKELQNESRNHNRDERRKENIDEHKNDNKNGYKIKNNHYNKKDSDSNMFMKKGNMYNMRYNRYTENSSEK
EKVEKNNRNGLDKYNNSRNTSKKYFNKSKDNKYYHNNKYVNNNTDIHKNYRTNKNSKENYYNEKDENKNNIIG
THEKDNKDLNDINDSNIRNNINKKNEKNNIKNGKMNKAFYNNDVNIDNMGNINNKENNDLMDKNKNGDNKNIN
KDSMKEKSYNEKLSNGMHTTMNGDINNKNNRNYTLQNKNNATDFIDTKNDNNNNMVVMKDDVNNSYSKQYSFK
GSYTHMKKKNPKEDMFNDTLNNNMYEYKNNMNYNKIFDYNNITNMNHHNQHQHHHLHPHQHHHHQQQQQQQQ
QQTHHHPHTHTHTHTHQMYHDNRKNTSINNDKNNYTNEKVCCNYLNNNVYNNYCYDNNFMDNNNYYFDDNMYQ
RNNNNYINENHAFYNDNRYINHMNDHFINANYMNNNYINERNYLNVPPHNYMNEPNYMNDHNFVNDHTYQADG
NFIANNYINENTAYINENYIHDATYISNNYMNGTNNYFYENMYPAYENENYYYADNKFSKQ*
>N.PF14_0153
MLRNVAKSSSDNPIEDDKLKNKNEIFKKRIHKEIKRLKESKVIDDINVYITFKEFNDSNDDQIHSNNNYNGNN
CCKYVHLLQTFDEKYFLEETFFVNNFIKHIIILKKNNNISDSYKEENVYISNELIKYYTYKNTTCPFYNIFEI
LNFEKFFNSFKSFLNEINNFLDIFEIFKKDFMKEQNVFYKEKYKIQEKKKISENYYEEKDNVEYFTNIFFEII
YKDFISFLSKIQREIINNIYSCDEYKYENENFYKKFFIFFNKYYNIKESIVYSFFIFHDYPFSKPLINFEHFP
FSIFKKKRQQNLMGEKYNKKNILTTLLKEQKWMPKITLENLFEESKKFVDKLFFYYQYKIYLFYYYLNKHKIF
EQFFQNDFRISVDNYNVIYSYIFRVDKKLISNKMKGKIIVPKKNYSNLLYSLNNCECINPNIVFYKFFYIYKK
LKSKNYETHDQFSVLRAQRKKRYQYFMYLFFIIFIFFIPQIIKNIYIYNTDETETYISEPSHIQELNKNVDNY
VLFDGYVNLIRTSINYFDKKKDDHRYMDNSTIYGNIKNVKGNEQDLYREGVIYDNINNNNMKNMDNMNNMKNM
DNMKNMDNMDNMDNMNNEENITNEKNEQNTNDALLLNNNKPENDMISNIKKKKGEKHKNMFLIISSLFELLFF
SLGIIYNLHLLRKKYDQSNFIVNICSSLLILYNPILFCSYKNNMIVISIGLLIWSINFMLLKKIFLCIVVYFL
SIYFNIINIIYFFSFFFMYVYINSRYIIKRGNQIKSQFKSKLRILKYAFIYLIIFMIISHVLIYIFFDKEKQN
EDVFTNLFINPIKYWYHKYFLKVYFNNNNNNNNENVHGHNMWAPMKFIISSNIYVNNYIIKYTPFFITFLFNY
FFIINTIIKFYSSLMFSSIIFLFMSYTYFSCNYLLIFIIIILLLFINILGTSSVLLNILLSSYIIIANNHFHI
YTFGLTILYFIFHIYLMFPSNNLFQNINYELKICSELIKNLYYFFLSNILHLYEAHIKHIILSFVIIFYPSVL
NNMSNEKTIQNIIQKKNIQKKIILCLYSHIHHDYFFIPLSCFSFCLFVMLGILKLYYSLAYIKIAVYIFQFFT
ISSLFTILMLFYIKRKSFRKLDFLSIPDSSSKRTFPLERSKKL*
>N.PF14_0154
MVYKNFDDEVGKRKIFLNNRNCEDYSINEGIKIKMEDWRSENKYNNMHNDKYKHDRDILNKYSYQIDNVKKKD
EREYGNYKMNDYDRRGTNLYGVTKDYDDNMEIYRERCETKMNETYTKPTDEYIEGSKRRISQRDIKELLNKYN
KKDEYYHENKYITNDKRHTYHNFNRKYDSLNQTNNSMLDSENKRRNNYSHDDLTRDKIRYHSKNSRHYNLDKS
QNSVNMNYINNNDINNLKIFNRKEKDKGRYNRINENVEYCKKNFNNINLHNEVSSTFDSDEYEKYNIRNHERK
LSPKKKNQYINYHNNDNNIFENSISPLRRYASLNKSMGYKNSFNDNLNTPCYNKEYENNKRKTELKDYEHNKL
NDTYERDKKKSLYDTFFNITEKLESIKKGKEQNGSLQKNEDKEIFQEEEKNEYYSNENNNNTIKKKCNLLYHK
NDILHDMPNYENMHNSYIYNNRDSFRNKEDKEQNDNNYQEEAPFDFFIKKYPSMSKKNNIKRNDINVENIYKG
VTCFDDILKYNSEDSITCEDDKHILKNLNNNINNNINKNISSHINSNINKNINSNINKKDTNENYQEKIQENL
PMDEEFTNIKKPNANLDDTSLSSNNLKSTQLHMSAISHGSNDYSTLYDYTNYSKHPTKESYSDENLYNVSKYE
QYMLDKKLNKSSMEEKKKKQYEYNLFQSMPTRNCRNEISYNNNNNMNDYYKINKTYNIYDMDNMNEYHNFYKD
KHLRNKNELVDLSKLKYDIDRDIFLQQHNEVLQSNIFKNKDMDENINSDNNNNNNIKLSTSRFGKINKSNDNY
IMNDTNLFSDNQRYFTNNNPYVNNYYYKNNNHNGDDKNLHMMNKLKYFDQEHFINEVNPKYFNSNINKINHYN
YNGKNNNKNNINQYENENDNINIYNKKYNSLPYEEDEKYTSNITNMVENDSDYIIKNIGKKYILNEDSDQLNK
LLELRKNNKKKKMRIQNNQKYSYQDHFYFDDEVNDKYISKTLNENDMNTSYNSFNSTYNKRNNMSDNCNDNIF
NFLKKNRSYTRPMNIDDFNMDDEDQTEQDKYNDDDYDDDDNIKKKFKSKKNADLTYNEIFFLIFIYFIKIICM
FGKYVIQVILFISFFIYIYFKKKPYKSICTTVLVSFPVLLFCLSFIILSYRSFNTEYFDKDI*
>N.PF14_0155
MHLKGLMDYNYFLGLEVLQNFDIVNYLIAGIVLLALILAVFNEDASAGSPRLSWVLGEFLPIQHSIFALNSNV
EKKLFRKRSSKIYTFITCLYNLFVELRNSITEGNFIMLVKGWFLKVLNKIILYKNLLLLKYRSQSKRHLFYML
VKKKYNLPIQKEENEMQSYLDAKRELIRLNRWSFMWRVSNVKNEFLLCEKRKARPISSYSYLDFIREPLVQNN
AIQAAMEWSTGNHGPRMLGGNSQILRDLEKVVGNFFGRNDSILAVCGFLACMSGIVAVATHEDIILYDSRTHA
CVKMGIQICGAKAYSFKHNDYNHLEVLLIKYRYKYRNCWVCIESVYSMDGDIPHLPTFKKLCIQHKAKLYVDE
AHGLGVLGKTGRGIEEHFNMPGTADIIVGTFSKSIGGVGGYIVASDEVIEFLDFHCIGNVFSAPLPAYCAGGA

LKAFELIDTQPWRIQKLKFNTKYLRNGLKTGMGHWPKDYPESYKYIIEGDDATSVIPVIFPNDPDRLFKICNL
LLKMNWMISAVVYPACPLKYPRFRVTATSAYTIEYMNEFISDIVRATVRVKPSPLDTQLII*
>N.PF14_0156
MSSYFLNNCLKLFKNNIVTMKVNKGFYNNIINNNKSTKIIKNVSDGISKKSHGKRNISTSKVQKGNKMNMILY
KKHGQHLLKNPGILDKIIYAAKIKSSDIVLEIGCGTGNLTVKLLPLAKKVITIDIDSRMISEVKKRCLYEGYN
NLEVYEGDAIKTVFPKFDVCTANIPYKISSPLIFKLISHRPLFKCAVLMFQKEFAERMLANVGDSNYSRLTIN
VKLFCKVTKVCNVNRSSFNPPPKVDSVIVKLIPKESSFLTNFDEWDNLLRICFSRKRKTLHAIFKRNAVLNML
EHNYKNWCTLNKQVPVNFPFKKYCLDVLEHLDMCEKRSINLDENDFLKLLLEFNKKGIHFFNISNVKNNDMTN
IFLDDDMNDSDQNVDD*
>N.PF14_0157
MGRRPLDKNLKKEKNVKVIKKRKRKKGSDSDDDDDDIEIANTNVPINKNVSTKFKQREKVDINLILAQDEEDA
FKTKTYCWTKVNAGNTSLVKRKLCTVCGFEGKYKCLKCFEHKPTSFVRYTCSLNCKKIHDESSCCKSKMLGTW
*
>N.PF14_0158
MNVQKIVTFCFAISYLLYCHIIWNGCYNIYSSSEDEVYNLLYYRKKAPKTVKLEIRNKLKIKDIQKVYVKKTP
CPVLPRPIKSIFHLNWFRKSPDLIPISMQLYSMFDEKNNFLGFVLLCSAIFTIIEWIYLVYLVGSLLWDGVIK
PIFKILYMGISVYLIIRFSVLFLPYTKSYFSQENYSYLSSFILHSYNLITKISKEFGNVFNKVIQENSVVEKE
L*
>N.PF14_0159
MEKGVEKTQIIDYDGNVIEDLKEWMIDNKLNDLGFSYNVIAVLGSQSSGKSTLLNNLFKTSFDVMNTKQGHSQ
TTKGLWLSYDKFDDETNNSSSFFKLKKKNKPTLILDVEGTDSKERGDNRLTFEHRSALFCLALADCVIVNLWY
HSLGNFTASNYGLLKTVMEVNLELFQQEKNSPKTILLFTVRDWFEEFAPIEVVRNKILDEYINKIWKEMKKPK
EAEKLNINNFFIIEVVGLSHGIIKKEDFLKDVNNLRDKWINNLRPSKYSRNIPSDGFAQYCNNIWNTIVKQSQ
LDIPSQKEMLSTFRCQEIKNNVINNINKEIKEKSIESHNKVIENFKEWAEKNIIQKCLDDYFKDASRYKENIC
LRTSQELLDYLFTQLQAIVDNNLQYIQRTLCTKFFNELSNMYKICTIEKNTFSFSRDSNLKTVKDNNKNTSHE
DIKRDLLNNNQDKCVHLWSNFLYNADKLEYFTFCNFYENYEKSNIEIKTNMKNDDNLKNDAHNNITTHQFNYK
PTLSVLSTSIYKDSNRIRNIQCGILLNKTRQTIKNSFKNMDTYLLTTKNTEEYWNNTVQLVLKLQDNINTHLT
KCFINLKGTNHNNLNIYNDDMMYNNDDMVFNNNDDDNNNNNNNNNNYYYYNKNDDANLSKDENYNNKFSFSLT
NEKGIFQNINNNNEGSDEICYTNALKKIDLIKNKQVYKSTINEDINNKLQNKKYIHELKNYYLDEIMDVLKNK
LDEISENLATIIIQRFESVFNYDEYEQPRQWRDISMAELKKIFLKSKNYAFLIIDILQKNIKVELIDDYLPNN
FIKDEIIEKGKIKAKRRIQEICRDAQYIQETGSKMSLKNVPVVFWIILLLFGWNEILFFIRMFFKLNVILPLF
FAAAFIVSTFVYNGNTQALSYINKIIFYMAKNSYNFFKHIQAISNPPPKNVQKQE*
>N.PF14_0160
MVYNNGSLRGRKKIEEKYIPMNRKSTSLKDPSRTKNNNYIKTLDTYINEEDSPTKILSKGKKNKNENIKKRIN
EKDNDTDREDAASLNVDYNKNVMKKYNTRGTFIEKVSSSERNIEHNNNIIRKLGSKDYSKARVQTMTTNIGNH
NALKKINPKVPSITSRTKSYTKVMNENKYNNNNSNNSNNNNINSMYYSKKLNNKKYSTEHTQSIDNNNITSKR
LNNKKTATDKAQSTSRYCISNSVKKSYSTKGYNTDRSQTMSRYTLQNKKDSNTNTNRKGYSTHRGQIIQDNSN
INNQAEKKKRNMSLKKKYIKESSNSTITGSNEKKNSNNILKKNSTCDLNYLNKEYNNNNNMHEHAENVYNIND
NEGCSSMQIINDEDVQKNVPEFYTINLGSETKNMGENYIFKNRDKKIKPNIYNERNNMDLMEHKLNDDMSYNF
ADHIKDIECYINQLNESNKCNIYKECDPSKLISPVYDNVNDNILENNQQLNEYEKHNDILTTSYLKYYSHTNN
NNHYYNDEDVHLDLGKTNEGFSNVVKNMDNIPWNETNKKESFSNIKKKIDHTDDRHIAYVDNSSMKHTMNDKD
QYVKNVPYNNTMEEVLLLNEKRGNILGSLISKNHDAKRATSNNNNINNNKYYYYDNKYSEHGGDSVSSNIGYP
HGYKNNNIYNYVNNIKDLSSPLYCNTKTKRAHKFHTDNIMSDQLNKYEDNNKNDGKFFSGNISSKQKGKNNLT
NYTTHSSEYRIKSIESNLTKDYRKSLTYNDIPNFLDYNINNTNVQNSDFGDSHYINRNVSYKNSMNSNDDIQN
SNNNNNNNNNNMVNNPNEYLVYTNEKHYVRINNKLYEKTRDNTYIEVDSNSNFDIINDINSSHNSPLDNIKDN
TLYHNTNKKEYSDYVSSYSNINKYEDINLPNNIHVSKDGKYLFKNYSVSQNVSDDNSVQDSLFSRTERSNSQN
RYDKNGVNHIEQNYYNNSNRRDDIYYYHNQLKKLERLTQQNIKSGKYQRNIPQNDDNDHNDDDSEIKEVKKDK
TKIDMNKHLHLNDKKIFNQVKRENTGVHMNASKTIKNMKEVTYRNINNDTNYNNNNVKINYLYSDELGKNKNR
DTPLKTKQHGDSLDNKTELKKKIYNDIINNDIINNNIINNDIIKSGAINNNIVNNNLINNYNADNRNVSNSIF
TQHINNINNNFYNSDDERINIIDNNKRMDMTKDESDRFRNNYFVELEKRKKNLGKTNNTTPISLSKNETINNM
MINKRNFFNNYISDKVRDKILKREISCSVNTVRSSKTNNKTNSNYSSNNNNDNNNNNNNSNPFILDNSIRKSN
IIITTSNNAKLINNTIISKGINNNILNINSNKNKSLNSAKINALGLLSSRNDLNYIMNNNRTSNNHINQHLLY
NNRNYSVIKIPNVHKEKRSSKIPHNINIKSNLNLEPNIKTYTNIKTPINIKAYTNIKAPTNIKAPINIKASTN
IKAPTNVKAPTNVKAPTNVNAPTNIKAPTNVNAPTNVNAPTNANAPTYVNTPTNIKRQEFSNVTLNYKGISKF

DINEDKNTTIIPPLHRQSNSNFPFKNTKLNLNSDMYTEKELIKCKSINNSDIYNYLRNTYNKTPSKFYRTVSY
RNMSLKKNKSMLENRKRKTEKKEENLMSTFNYTSEVDSKYIVKKAVVVGCNYVSEERSRLYGSVNDAYVFCRA
LVKYFDFLPENILLLTDSLPSNAYIYEDFDINRKKYINVDEEENIKNNEPLKKKNIFNLFNTNALYTTLKKTN
EEELNCNSCKDVEIKNVDISSEKMNFNLWPTRVNILKAVNWLVRDSIPFGSYVFYFAGKSVQVDNMSGWEGEG
YDEAFLCSDPFNKISEHNVITAVQLKDLLLSINESAQMTIILDCSGGQTILDPAGTENSLSYIKGCKQKGIWP
ITNPTNKVHKAIYDITILNNTSMKKYFCRSRYSKLIEVESTSAMIDPLLQSISSLPVAPKAYCLCAATWEQIS
IEGLFPIIEFARVSQLKKPESYKTGEGHYQNMNKKNIRAEKSNRNGPNGLINKNNNLSKKTNYEKNFNFTLNM
MKMFFSNTNNNENKLDEKEKINRLGFNENDSDYIDDNYNSDDNNNNNNNYYYNYDGEKGFKNFQENLNNVGQK
DVIKNKKFKDNYILVSHGVFTYCLIEAIIEFKEKELKYNILEKKNEQFIPMTLKNLINVIQQKMQNIKYNKLK
KINQKPEFTIHPGANATNNNYFVHYSKNIHFQNYKCNFINADLSPFLNVNKAWEEINRTTLRNRKSLSLSSTL
INTASSKYFTQKNEQFKNSYSLKY*
>N.PF14_0161
MKSIINTFLPYYKHNNNLTLNVTLKNKVRFLFFYLDRFVYSPILQILLRNQNVKEQNVIKKIYKLKILNKLPL
QFKKDKNDEKHHVKNQKYINKNSFECKEKMNDKIRNIKNDKINYYDHIEINALWPERISYVQPFEVKKNLEKD
KFNVIYIGHMSILIQTSKYNILIDPVLSNRIGFCNILGVKRIIKPGINFEHLPSIDFILLSNNRYDTMDKTTL
KNIILRDNSIVIGGMNIRRYLLKRNFPVVYPLNWFNKLSFENISFYYLPTLTNSHRFLVDKNVYLPGSFLIHD
SSTNATIFYSGHSGYSNHFLQIKNYVNNVVLKRNNHIDLSILPIGIYKPDSLYNNFHMSPREALQSHFDLGSK
QSLGIGTDVFCLGGEEYKEATKELTHILDSYQKKNNNKITFVTLQPGENFII*
>N.PF14_0162
MSSNIYNIKELNKDTILLKTRKLKQLCENAENINSQHVTCLIEILNYFFEKYDDCFSSSVVWEVVDKIIILEN
TIEERKNINVDLDTSIKFCEYIFSKLILYTNYQNDNLTTFFSFNCLRRHLCSLISSFLNFNNSSMRKKIESSF
TNELISLTKKLFETIKIENDIELKVSYTEFLWRSFRRIDISTFNLKNFRIGLVSVEKLRNFKINKFDEECINW
LKEECYIDNKIVMENGNFVINTSKKSVTKNFLLCYVGMCSIFLYYDNEEENEKCKIEIPYYHITSINIKNKNL
LELECKAIVDQTNIFYLWGEDIKNSIDHEIISKFKLSMDIKRGSKEIKNLLNKLMDTLNIKLKNEKKKREQII
LKSIKYNKKYNEDQNHGTVDEEEDKSFDKINEPLHYFNDISLERNISEKNIKNKSRKRTDTLKQEDEKKRKSI
CNEEIVKYNKSLEDINYINEYSNKKDTKKFIEDDKFRMIKRKNQICAEKDSDTEMIIEKIISCHKEKKEKFKI
NLKKNFSDSLNEIDNMIKSLIEKQKIRREELNKKYAKKKKNIIFNTEKEINLLCKEMNILIESMRKINVNKSD
VKKIYDEGKSIFSTNEILNKSQIFMKKIKDNLERMNTDIVGKEKEYNKRKKLCFKALATAYE*
>N.PF14_0164
MSALKDKTGRFVVLDKNASNYESLVDQEMNNVYERVMKLDPNQVEFLQAFHEILYSLKPLFMEEPKYLPIIET
LSEPERAIQFRVCWLDDNGVQRKNRCFRVQYNSALGPYKGGLRFHPSVNLSIVKFLGFEQIFKNSLTGLSMGG
GKGGSDFDPKGKSDNEILKFCQAFMNELYRHIGPCTDVPAGDIGVGGREIGYLYGQYKKIVNSFNGTLTGKNV
KWGGSNLRVEATGYGLVYFVLEVLKSLNIPVEKQTAVVSGSGNVALYCVQKLLHLNVKVLTLSDSNGYVYEPN
GFTHENLEFLIDLKEEKKGRIKEYLNHSSTAKYFPNEKPWGVPCTLAFPCATQNEINLEDAKLLQKNGCILVG
EGANMPSTVDAINLFKSNNIIYCPSKAANAGGVAISGLEMSQNFQFSHWTRETVDEKLKEIMRNIFIACSENA
LKYTKNKYDLQAGANIAGFLKVAESYIEQGCF*
>N.PF14_0165
MEKSSKLYLLQEYMKEYNRATTVPHDSKNKDNDKYLSLYNDINNEDQYILNPAIIEYKNINEMKNSVDMNNYL
STKNLLNETSKFDDNKEEIYLRNGSLDMEHIKYGNIQDINIQDVDIEDGYIKDLYISNEQFENKSYPNDISRK
VLIKESKKDITQDYNLKDIKETEKNRTMNKSSSYKQYNMNNCTRKNSSFNYNVTDNICHGNEKYKMSDNKQIC
EIIKKKEQLIIDEICTMVKNANKKIKNQVEEYKNKNVSVINRKDNTIQNSDINNTQNILHRNEDIEEYKLNEN
DIHNTVKITKEVYSSNSFSSNSDTTLSYESVNNKKNKEEIRNHSNNVEGLRDLTKEGTLIYKSEKEYTKNDII
KNMNYLNMECKLNPLNNNDDTSDINTNNNSKYLYPENNSHKLDIFNNSMHIRNIINNNNLEDFKNIDTLNNPR
IFNYKKNVNKNGINHTKNDVCNNIQTIHLSNIEETQCDESFMDIVEKRKNTYDLSDTYKKDQIKDKELQIGCF
LKEENNNNKTDILKNPLKEEIRHLKLKSKNSIVKEQTCINDISNQIEEHFYSTYISKDALIGAPTNDEKYNNN
NNNYYYYNIPKNLERTKETIETCNKYCNEDIQERHINKSLDENNKQSLSAKEQINKENIPMPYKNEQLISNIK
NIEIPHCEENSIDEKKYFNVNEKDFFENFKEHIENDYFDYSIMEENRKCNIDTNFYYDNKFIDKNKIVTHEFG
SDKEKYIEQNEPSIYNKSSNIIKNNDYNEKVKYMMSSINASCKSLKRTSLYNEKKNEKIEPSLLLNLTETNTL
ENKLNDSFESQVVEKVSKDVKLENKVSSNYKQNKNNSNDNNNNNNHNNKKKKYANICTNIYNNYDQKGYKENI
NIKLTSLNHKKRSNTPSIMIADVLKIIDKNSEENYKERKKFVSPYNNKNTDDENNCSYNNIKFGNIKNEKSNK
IGYPKNSNRKVVNIKSHYISKYNNMFLYMDTDKNKKKNEENKTYKKINVPCHKKIYECEKRDHSKEIIKSNCK
KDDKKKQCSFNGNNKKEKLFEQKLPNFGGQEIPIWGGIEVPNWEEEYKPLGEKIREPYVEEKKEERIRNKIFN
VNSNMNKIHYPSPNKLKKMNSIHNTQETEKLRKLKNNNGNKCTYEKGEEKNKNFCSQSRNNSLTSFRNKNDKN
IKKNVLTKKDSILNQDKINDCKDDIKKMEKKKIYMNSQMNQKKIESDKKKKNKFEKYGKYGKNEKYRGKYHKY

PLDIEASYRCKNLYLKINVDTNNEQILKYIPNKISKRNMEFKLIITKFIDGFIKCTESKIYKSSNIILQNIET
DISYNITIHAYNTVVPSLWLYASISFYLTKFHLENFKLKTPCHFTLAKNTDFLNSITNNDNEKKIVPLKNEKN
ITQKIIFHNINEKKKMKNNELTNNCTNMLKDIEEHDNQHFYKNNSTCSDEDNSDESIVPIKNLHIQNNLEFKG
IKNQKNGNINISTRNNVTLNNNINNLNNKYNNTIDGKTEMNSQIHNTIHEDEEIKSTSSYEELLLSFHDLNKS
EIRNKFTQVKYENCNNVDERKITPTFETPSGIEYPSFCKYDYNSTSNQKIEVINKVCMEEVKDKKKENYIVYK
KRKDNKELKNTTILNHNPRNLNKQSFKFSEFSLKKKNVNLNTKTQKNINIKNDNNNNNNNNNNENNDINDIDI
SNMSSNELTIKEITDSSSFILSSSCLSKENFKNDNKKKNIELIKSNKDDSTENSHQCEEKNINNIQEGKQTLR
DILLNQLKEKNGTYDDDDKGYIQKKIHKNDNINVNCNINKYDIDKNKNISINNNTVNNNTINNNTVNNKYIKK
EHNSFINYKNNEMKNIKLDKKKKGDESIKNYEKNLQDIYIKNNNYCNNYMKKSKEETDENCPVKKILPSKDNT
SKLDTNVHNNMCDNIYNMLNKKDDGKENKRDSIPDYVSMADSIFHLDDCKDTQSNNSHIQELQDECVEKIDMI
DKNDIYYSSSTNKKNDEIKNRNDYFKNHNICNRGVQNGNKNLCVNNKANEFNNLHKKENNILNKDTSAINSIG
NNLYKNNRSVKNINDIIIKRSHTPEIVTKVLKIEETINDNTLNVVHKKETHHKQNKGTDYISNEFKENKKEGK
SNSLKRYLNKTCDNLNVTSRINMLNKTSPTISFLNKTSMNNKNVDKEDNYKKCSDTIKGKSYNSEDLLYKGNF
YKNVRRAYKIENKQPFDNKKKDKKGFKVDDNNINKNRALIYDNNNNNVYTHNVNSKNYINIPNTSLNTSYGNI
NSINIDEENAKYYNSNNKQNIMNDSKYDKQNLQYINSNKNKNTLQFDGNYIKNGSINQSEENYIIKKKKQPNY
IGTDNGENNINNITSTIKNVNNYLDKLKNVKHNNNNNNNIKNIYSGNTILNNNPYNYMINNNKLNYMQNRNPI
NIINNNDNSYCYVNNNTMSSNNNNISYIYQQYNNPIKKEDNVYNTNSNKHIPYMLNRANPFIQPNYSNKKNIR
NDDYFVQSAQPHINTYTNNNEESFANLNNNVYNKFNMGTCQEENIAYNNLCRDQNKMYLLNQYNMRYNQNINQ
THNELSNETKNNINNFIEENNIDKEKNIFYNKQNVRSNNDLNKKVSTPLSDVPKMIGEDNSYVIYKMNDEKNK
KNYFHNNIRNINNHIMKNVDNNIHKNQYIYKPHVNPKYITPQISNINMKNNLNHMVKRINHIYPKNQNTHINN
IHINNNINNIKNVNYLNEETERKKFIQMNKEYLKNIPMDKNKNKIYYPYTNVTDMGQHYEKQINASKLIKTVI
NKDSKNNVEHKEYQLPLSYYNVTQNDTSNENNLRCLNINQIKQNNHSSYDENNIMYYTSQKENYIKQNYNNFQ
HINDMKSININEKPINVYANNNLLYKKKIEKENEIKKSSNINIEENNILDDTNNISHMNNNVFLKSENCINNN
IRKFSPSFGNINNEDIKNNNYKKEEKLQIYTNSQTFQLNIQLDEYTCKEIAFTYRDVLDEKVIAFINNNKLKK
TFLNPIKEKMKYMMQNGIVKWYISITEFL*
>N.PF14_0805
MKVNKSLLFIFHILILFYQIYMVLSSSVRKDEMNIMDFFPSNSLLYPLDFQKNWQASEPIPLNIHYSVPAYGD
KDMLNSLEYHNDLEIYNNEKEEIKRRIISEQKRMEDNLWNKIQLLKIKQKRIENQNYLR*
>N.PF14_0166
MKKKKRPYIILLFLLFPLNIFLSFSFIRYNSISFKNNQSNKNNFFFVPNECIGTICNNRKKKKHFSLYNHGKE
FHERVKKLHILSDALNIDAYPSSFIKRNIKIDDLKKKFEHLKDGERDDKIYVIYGRVTVKRNNGMFMNIQDDE
GNIQIYLDENLPIKGKKNDNKKINNNKIEEVPNKYHMENNTVSSILSNNNKTNNNDYVINDQSYNEKYETYNN
QIMARKIIELGDFVAVKGFVRKTLRGEITLHVKDIYMLSKSLLPLPDKYKGMKDVEYKYRKRYLDFLTNNDQK
DKIKTRYDIIQEIRKYLLKRNFLEVDTPILQLIPGGATAKPFETYLKSLNLILYLRISPELFLKKLIVSGISE
QIFELSKCFRNEGLSSIHNPEFTMLEIYKSYTNYKYMMNFVEKIIKHLFKKFPYPSNINSSNSNTKEKKENIN
INNKWKKISFIKILKDYTSINFLKLSFDEAYDEANKLNIHFDQPKEQLNWGLIVEEVFKKKVEPYLPHEPIHI
YHLPSDTSPLAKTLNKNSRLSERFETYIGQMEIANGYSEEANALIQEKKFLSQFALKNMIKTNEKNMGIPTTF
SYDQYKSNHNETDQSIKEVPNKDNTTNIYDENNKSDNNQIDYDYVTALAHGLPPTGGLGIGIDRLCMLFTNTN
TIKNIVSFPIIKPHGKD*
>N.PF14_0167
METKSNVESPNISEPKESKLTYEQIEKDRVQLVSKIEELYQDVVEHKLVLEALENVPSDRRCYRMVGEVLVER
TVGEIKPALMDHKNKVEQVIAECQKQLDQKNNEISNFIKSAKAGNLPGGIINGNPSEDTNNLNDKEKKSDGII
F*
>N.PF14_0168
MSIPFHSSCFSKAPKYALKQVHENIGIPVFHKTPKIVEIPEIREITKFVESVKVVDVPIEQVRIVPKLKVREV
EKIRHVPGPIEYIDIPQERIIHKVYKEIREKIHEIPQIEDIEIEVPIYVPTPIGPPKDIHVNIPLPYDIPQFY
YTPDNRYSHILPCDILPHGTQMPDINFEEKSFNDYSEDKNICMTHNDNIIHSNRRMEYNKNENNICNNNTFDN
NMNDKNIYDSNIFDNTTYYNNTIDNNIYDDNTYGNNMYDNITTYYEDQENNIHSKEKDKYINNNYNNKYINDY
YKTIPTKGILKKENISTSPGIKEQYYYSYSKHNSKNAGKAHHYDKDTYDDMSTWDNYAENMNYIYEDDENENE
YKNRKKRVTELIVKKKK*
>N.PF14_0169
MKFNVKNNLEEQIKSINNNNIPYKYYNSPNNINIHNYLCDKSIKYKCPSSSYLSLNELLKFVDSINNIKDEKI
NYMDPSVEYVKTELKERMPIQNERYQEKYNISENILNNRILRNEKIAFRQPESYHHDEHIQKVSHPNNNDKKN

INNHNNEDKKNPKELYHINSSLKENLSLKQEITHSPKKSVPSQPPTQHNIILHNLLQCRTNLSKLNNINDPEK
LISTKNIKLLSLHRPNTIEDIKNLNLTGFGEDKIKKYGYEILKVFHSSYQDKYN*
>N.PF14_0170
MRMDYHDKYNYFMNIKQHFFFKKKIKEFNVKLGGDNVTNKSLLNISEKQPKFENEENEDSNNINENFEYVDEK
KKTKLNSKNSMNNKFNNNNMMNDNNIINCNDNPIFIEESIPKLTHNITTDIQNIGSNEDTQDNILSSYESSCT
NDTYCSSYYIKDNNLKNEFSDSCSNNIYNSNKEELNDFMDNENMNNVHEKKKECTHNIEDGNAINEPLYVEHH
IRRNNDNFDHINYNEHGINNVMNPVNNMMSIQMNYDSSIINTDKKKKCEHINNTIEELDNLCLSENEHIQNIE
ANNINNKKTNDSVTNKISGAKANKGNNSNNNNNKSNNNKTSHNNKSNNNNKDDGSKNNNNNNNNNSNNNNNNN
NYGNNNSKKNNNNNNDNNDNNNNNNNNNNNNGDNNKNNNNDEDDDDNDNNGDNKKNKKDKGNHKDTSNKNNNT
DKINDSHMVKSDKKKKNTKNNTNNKNNSSISNTINMNGSCIVNNNNNNNITNPINKNNELYNKLYNMNPPNTY
DNHENIMNPEDVVNIKHMDMKIYPNFGNMNNMNIMNNANMMNNVNMINNVNNMNNMNSINNMNSMNSMNNMNN
INYINDMVDTNGVGNFFNMPNNGFINICNNNGNNNNTNNTNNNNNNNNNNNNNNNNNNNNNNNNYMSNINYVHN
PNYIPNGNINKLNIKTNIHSSENDMMNNNFNPNNGMFTSSYSEINMMREQDDLNNMNNGLFMNYNNNMMMMQH
GNNGDIFFNEPNKDKLYLNNESLNNYHDEKNNSNTNIIFLSGTMNPKFEESNITPKNGLHYEMLNNKEIMNIG
ELKVEENDLMHGMPRNDWMYNNNNNNNNNNINHMNHINNMNHINNMNHINNMNHINNMSPINNMSHINNMSIH
EEQQNNFKNHFENKINEESRSHKKNKKSVCDINNNKSNDNNNEYVEDEKHMNQNLEDSLNYNEASTLNLLNIS
FNEIFRDKKDLIDKVITKKFKYKLSKILKDINQDNIKSKAKKIKRLIENEKKCDKCEQEEDDAKNVSSFNKNN
NETSEPSSSAKVEKIDEDSLSCKSTPIDDNKKIDKKEHHSNSQIKDKKKGAGSNNEIVNNTLEGATKQGKDKE
QEEKKKKNNKKNEKNEKNEKNEKNEKIEKNEKNEKIEKNEKNEMNEGNEKNSILSQTVSLNGDAEQNINDINK
NAPQKNSLQSDIYDEFSHQKISNDDICCINEDDVKLNNEKQQGKSSKKNKNNINNKEGSSTFLDEKKKSQLTE
NEENNMKSVDDNNNNNKNNDNIVKGGNNVTLLKNKKKGSVTYDLKNNVEIGRYGKIQENNETKTDDDQPIGEN
SLFDNNFEDYTYYTDYTDDPIMSDDISDDYEDGEDEEEESDDRNDDNKYITGINDRYKEIKIEDDVQIRRDVF
FFNLLKSVIKKKTSEDMLIYMNFLNKIKMEKIFDNIIQLSCEIFKTLLNFIEITKDSSTYRLLLKNLGAWIGI
ITIGRNKPLMSKYMNIKQLILYAYDNGYLIVTFPALCKILESIKNSKIFRPPNPWTVSILNLLGELHEAQSLK
TILIFEIEILFNYFKINVFDYYNKCNIIKSRNLPVNSNDLFFRNSFNEHVSVNINSINENNNSMISVNEITPT
NYTKSFNTNIIYSENLININNDRTNRGGLGLSINKDVRFNMLNNYNTTQSALATSAATSNNNNINNNNSSSNN
NNNNNNNNNNNNNNNNNNNSNDFLSFKGANCSLLINNRKEDEGKENNNTRDLNNNNNNINININNAHIMNHLNQN
HAHLSNGKNIVNNLICNSSSDIMLLNNNYRNTFSMNPNLNNVNNKIFKNIDLYKSTNKNILNTNDVHQKSSSV
SKKLTNPNIMKDNHLNTISNYLSNDPNINHMYTKSGSVNNNNNINNNNNNNINNNNNINNNNNNTFILNNSMD
MYQVDNNIYNNMYVNKNMDISNMMNNNENFTKCNNNSNEEMCVKDVKFSNKKKDKLNEIDFKNNIPKSSNNYN
INKTKFSVNNKNETSYKLMDMKNGKLVHNKEMIYYENVMKDSYASSSPSMNNINVKGSYMDSSLFKNMALNNN
LEKLKMNEYNNNNNNNNINNNNSGSNNNQYSNNKNNMMLPPSVVCSPYIVGNNEMSMKNHMMYQENANNPMNN
NNNNNNININININNNNYNNNININNNSVVFNEILNDEILNILKKNLIISPNIQLIKIHPKFKALIYLAFDSSIK
EVITSIVDRFVLIGCITTRELVKKDFANESKETIMHKVSLLMATSISSSLVLVSCKKPLKNMLIQNLRNIIEQ
NFPERNPNLIIVDDFIKTLINDNINLLYLIIEQVAIEKSSIEIEEIMKPIYASRKYARINKLNINDNTTNRYY
NALPSFLKAHNITLKHIQVYKNFMNLKIFKKLQQKLLSNNNNMEQGNQEYENENAQMNYPNKRNMDNNPSNQF
DQKVGADFNDYNNNVKRESQIEYHNNNNNNINNGNNQDNGLIKIIKRDDAPKDDNNNNNNNNNEKTNSKFMDD
NKFYHDNNIMDNNEDNNHNLVTSHHKNLSKINTVNNNGLNFIPCKNNSIKENSNFHIGNYNYINSNNNVINFM
NNNNLYNSCNFNYMYNNKNSFNFNYNKSDTNFNTNRYVNVDNVNTGNNMRMYNSSRNMVDTQVGNMQVIQNNN
NSSGNMDTGIYDHIHDKNNGNSIENSNGKICNIKNISNINNVNSINNRRQKKSEYEMNMDNGNNSNIQVGGNI
NHFEDKTFRGLTEHNDNDSGFNKNGLFNNNVCNNMNGYIPNMNYMHNKDVLNNQGNMMNNEQYMMIINNTIMK
KGIIKNNLFNELNNHYIGTNSNIMNSNDLNKMNAIYKNMISNNENILNNNIIEKLVMNNDMCNGTFTTNSNNI
SNINNMNNINNMNNMNNINNMNNNMNNNINNTCINKLMNKDNIMQLDSEINNKNIKNNDNIHLNKGAMDNNQK
FIHKDSKINTPNSIHGNNIECNENNNMYLKKLKNIDLSCKKKFSFFISEIFLKENIKKINIKEIISLFEFCML
SIKEYIKYIMTYVPLIPTKNTFNDYCKIKTSFFKMERIISTDEPSLLLLSCLSSNHDIFILCKIIVYVCENCT
NKNEVYKIIGTKLYKHIQETIKDENSVIYSNKINDIIILNNENINEEENNNTTNSYNNNNNIGDDKKNVSSST
YCFNNKKKYICSLSIDNNEYNINKKKNLYSTCTVINEFNVLLELQLCILDMLCKKNKILKKLITSYILSSPSN
NVSHISLFIRYDIVHYTNLDKSMSKYLKNTTHINIIQFCTELIYNLIIQKKYLKCEHLKKSIKKIVKIDKILS
NDNKYIHIPHAEKMQLKNVQDRLIQMIKQIKRERKYSKRNTQEQGVQQENNEQVEKGPPDLTVQNIEKDKKCG
KEEIKLLRTLTEYICITKEQKRNNNNNNNNNNNNNNNNNNKCDLDGVTNKIHNEEVKKKDNKNEKEVEQQNKLI
NMQNSKEMVSNNKNKKEHYLLSKLKIEYDNNNVDENTMFLLEKLSYENKIVDSEEIIEKEVVKNINHTKNVNI
ENQNKIISKDKLDTIYPNVNDNINNKYVTNVEMDNLNIMNKEEDINNLSDHSILSYKSLTSTSQDNNIDTASS
LSSKSFSASYDEDEHSNISSYHFSDCSSTISDTSVISTFSLSYYNFDENISKENNEHNKNEHNKSEHNKNEHN
INEHNKNEHNKSEHNINEHNKNEHNKSEHNKNEHNKNEHNKKDELNKCDDNSNNILSEKDKYSNKKYLNKKSF

YLYDVFNIEPIKYKYLPSPPIINEEKGVIIACLFAEWYFIYKNYLNILKMSISNDMQDVVNQQHKVKCINFFR
KLSKNGFLNMDEETDQFLSHSINISVNIALYNLEKDNMLQEELFERIQNKENNENELMDDDMKEKNDVKNVYT
PLYDDEKKDMNKEQKNDNHAKVQLMDLPKNEIDKNDEENLKECPSVSELLNYNYIDSWTKMIITMLKLVDIEK
ISPIFILQKVLNLICRVIHKKYESEKKHFNQRPYFRLLHCLLIDINEQCIYSNDLLIYLNCFSEIFLLLSPLR
IPAFCFSWVELISSKYFMPKFLSNICGWDNYKKLLIHLFTFLKCFLKKIQVSKAIESLYIGTLRILLILLHDF
PEFLASYYVTLCSIFPINFIQLRNLVLSAFPRNIKLPDPFMSDVKIDLLPEIKIVPKILTNISFLLFKNRLKF
LLDEYFHKKDYNLLFQIKDKLLLPKNKIYQSFIKYDVDIINSLTLYVGTYICKAKVNNLFKFNNNCDPINLTK
YSDDSTDDNTAILQELILENEQDNLNKGKKKNNKKCNSRTISKKEQSKLKNEKNNLSKKYVELMKDEYNEPNQ
ANEEEPKTSTKLNSNINGSIESRKEKNKSVIVVKNNLSYTLFLFLSKELDMEGRYLLLSNIVNHIRYPNSHTH
YFSCLILFIFSQSNDIIIQEQIIRVLLERILAHRPHPWGLLITFIELIKNTKFNFWECPFVHEAAEIKKLFQS
VAQACLGRV*
>N.PF14_0171
MSKEMKNFEDNQKKIEINSTIQEGKEEENKTLDEIKNVPVAINSNKINKKDNKYIKKKNSKKTKNNFIHIKNK
NVDDLYSYQNKSKYKILNYFNNFKYNFSIKRYFKILVNVSLLNYLPLNLRNISNKVYIFGLCLNLKNPDEMKI
FLILCKSKILFTYRSNFLIRINDNNLYRSNNIMLVDNNVSQSNIFTYDNTTYNNSNITINNNNNNNSNITINN
NNNNNNNSSNITINNNISNLCINNNNHTSTSNTTNSSDNSLPILCQEYEHIDQQDYLIYISKKKKKKKKLKKK
KIYYKQRIINFTDIPEHFLKKIYFDDKECFYINFKKSSKGHNDTYTYQKQHSQEIKENYSLQNNKKKKSKEEI
YGDTCTYTTSESNKTIIYLENSKKCNSVDNKEIKNKNISKDDMHYYREQKKKIIKNKSENNTCDDDLECFNNP
DNDRNDPNEEMKKKKKKIKINNNNNNIESEATIKNINIIKTKKNSLKILHSRSKKHSVLKIKKKKKNNIIKNN
NINIKEYLKYVNQNITKSQIYEKDKKYICMSDNGWGCMIRVIQMILANILIHFNISNRYVYFHNVNDYILYKN
YINKLTTCTNKENKIIQIEEKTMNKDKEESIYLNYKNKDSTKEMVKHEKNNQCESNECNLNKNASQNNKESDT
LMIHPQSNIINGNNNNINSNNNNNNNNNNNNNNNSNNNNNSNNNNNNSSSSTTFSNSNTYLVPDSFKQEYNKSS
IKTDFLQSDKQITLNDTNINESQGSDIYNIDNVENITNKYNSSDLHHCENYNNFLLNNKNNNNDSNSEIMHIN
NSLIYSILSEFRDLEQAKYSIQNIIYEMIKYKKIDDNQIEHFVHDWLGPTSSAIIISNLINKKKVRFVKKNKM
KNNFRGTNIHMDKNIYIEKEQKELTNYTNNNKQIYKLLNQKENINISKSDDNIKNDKKKYNKLLFLKEKKKKK
YTFFSVAFETGVIYNNKVLKFFQIKQDIFIIIWICLKLGIDSLNVSKYKKSLLSCFLLKQFQGISSGNTNTSA
HYFYSANDNGLFYLDPHIKCQNAFIDFNENISSQFFMHKVKFLPWEYLNSSLSLIFVVQSKDDYFNLIQNLKL
IDSSLFEIYHEEPQYVVYKNELNYDTDDSGLVVL*
>N.PF14_0173
MEDLESKAIENIHENLKKCIDRKIPKNTNENEEESSKDMFFIYNSLKEYIEQLKHTNNFIQKENDYLRKKNEE
LLKEFNEKNLINGNSNKSQKYGTYINEINQLNNENEFIKYKFQNMLRKNISLERSNVELNNQLLNKDNISQYL
KSQSRIHNNKYIKDIRKKQSKSNKSLENIKGYNNVIKMDTTTTTTTSTNNNNNNNTDENIRDSIKLSENIKGI
NNKITNKNIHTYENINTYENIHTYENNNECQNIHIKHNNVTNIQSFNFNEKFKSICTCSMLLHDILNILNQNI
KYDEPGIFQKSLKNIKIEKDELIMQNKLLKEKILQIETFILRDPFLVEKLCPIHENINNNFLLKDKNKLYQNN
KNISLYRNLCYLENKNFNLRKELFHYKQKILFYKTYVNDMRDKLKHNIIQLYNQYHQNKNENTEIMTSVDETF
NTNNQQVNTYNQYNNKHISYSKKNICDNNTKNNSLNQNYDFIKNQKSFFGLNNSDFNNILKTNNSKNDHNEKI
IMQTSTKLNELNVEKKNIEKLRISIWIHKDIPLVDLNIHNKNDMYYFEIKELIHKRKIELDKKLKGDINVNIN
TSHHNINSGCNYVKGREEDNSKNKSTLSGVIKNNILENTDNLTYINKSNNSKPEHINNYNYYINKNIMYTYIK
YKYMYDFYVQNKNILKKEMNKEEFNEFVKKYFLLYKKNNKTNQHFNNTSNDILKHYNQSIPCVNRRNEHSINL
NEDKNINQKETNDEDTKNDDINMNVEDYHDITLGDSHQNFIYNIEEENIKQDLPEETCKKEDNINDYFNEKKN
KYQDNIKCTSLKETGKDVHKIQENIMNKKKRKQKEKKKIKEKEKEKEKEKEKVKKKDKDKDKEKEKEKEKEKE
IYVNKKIYNSFDITNDTYKNTEISKKNDEESISKTKNEYEQYNNNENNKNTEERNHNKKHENFERFYGVEEYP
RRHDSSLEVLKKNIKDLLKKKNKDEKYAFQDDKKDINVSEGKKDGNMQEQKVFANKYNNNKIEDNKEKVDDKL
LKEENNIKQNSNINKNVIESEQVKDSFNIIENVRKYEEYIDDKNDRINTHTIDEKNVISIEEDNNGKQIMKQE
SRELNDNEKREDNKVNKNLINNDNINNYSIEENKTNHNDHNDHNGHNDHNDDNCINSFKSYIHNYNSKNEKIS
TE*SIIINNNDEEKYIEDKINFRNTNFFMKSGSEYSEQDFFNNNKYSKVNIQTFKEIIYYYDINVYQFYMNLI
LYSKIDLIKMYKNKIFSVQINSADILQYALNTYMNFNNNTSDIEEEFIINKKDGTIKKLNEKEYKTVIEYICN
NSNNTCLKTIYHKINFITSESYEEFKNRFVECGGVVNLINLFYDEDLNNFDKIFEVSFVNFCDIIGISRDSCS
YHFNCMDKKFKAKGYIFLRNILRYLNVEEEMKEEYHKYNFISMHLKNLIYIYITENFHNLNEFFNAVIKQNKL
YISLNEFIYFFSANEKMTKYNFAQEDVVNLYYSIIFYVYYTNKNKILQTVNYFHKGKKYEGAYIKNKSHNNDQ
YVGGQKDNAIINLYMTNQNNKNNNKSINNNNDNKSINNNNDNKSINNNNDNKSINNNNDNKSINNNNDNKRIN
NNNIHDNNKSIMQDDEYCFVEYDLSLCKEFFTLEDFDFAIEVKDLYFYFEGSECPLSKIKRRIIEIYGSVKKG
IFINDQVCNSSDDSMNSDSDINEKDIEENTYEIFKKKKYKHIDKITLYEKKIKNTLENDNSDDIKINKISKKT
FMCLMYNIGIQIDYCLTLWGNFEPFLEDHLLDYKMFSAFLNGQIKCSNHENEENIQNIKNKINDIEEDKEISH

RLNHNNRLKGTICSNLFMNIEEMNNGNLLTADEIEVTCQILNNISPFYYLDKNERKMFIKQLKKKCYKKNYDL
NLLKDHDNTNKNKQNTSNDKNDYFKNEIIILINGILRNNKDGKCFINSVNVIYKYDIYNYIAHTNIAIINIDN
SIYTHEFMNLIDKKRNISEEFMSIVNKIPILKNFPNMKKISLSMNVKKSMFEKNKIVIRKHDDPLQFFILKEG
KINIFYHTSKTKNNPINTINENQFFGEVSLIFNTLRTCDAIVESNTACCYYISKEYFLSLLNKDIVTEFVEYI
RKTYTKGIESIISNYVNNNEDIEQINKYRKVQNSKKNKINKNNNNNNNNDDGNNTVTYDEDKIDEGQNLSSDE
KKKTSIIYHKENKNEQDVKKSSSSPLSITNSFNNINKNKKYISNSFNKKNYYTNDFSNKTLNNKLSNTSNNLS
TDYISLDIEYIDKNNIQSFYSDLDKLLLNIFNAEQKYFTEEIETNLRKIKSFKKILKQMKDKETFLKNIKYEK
CPKGKYIILEGDYCEYFYFIKQGQISIQKFNQHKNMYDEFVTYNENQYFGHQLILNNIKAFFIAVAITDTELY
TLEASLFVEFLSPFMDMLNIINDINQIDVLSENVKNDFQKKKQDKNYILKVLKFLKKVNIIQKLDNDTLYNAS
KNFTLQFFKKGQAIIKYQDKPDFFYIIKKGIVTVKTDFKEDTNQCVTNQKGVEEKINEEENILTDKINKTKSF
YLYKYDYFGELSIINEQLRTTRCEAHTNCFLLAIDKISFIKNFSILFNDLLQEADIKYTRSYSLPPWLKSMYG
NNFNTNMNSLSIKIPDSASSFSSISEDSSDVFSFEKEINEKLKEEKKKKEKENEKENEKEKEKKKKRQKPKSI
*LSNKER*TKRYNREKY*TYKRTKGKKF**YPNL*YKQF**FKKYF*RI*YVQYR**KRNEKN*GGKGAKN**
N*DEGIESY*KDKIERREKDKINYEKEGRRD*YEE***YY*F*RGV**SR*YKK*CYKG*ARYSNKYICYYYI
*****************************L*YFKLSEPGGKNRSRKKEKKKFS**MVK*K*S*CDKRK*RS*
YI*R*Q*RFK**YNKK*IHKYRQ*RKRRK*KRRK**ICY*FS*EIFKYLKKKN**L*FI*T*YKQTFGNIN*V
H*F*I*FYISFL*RYR*I*HWICKI**FLRLHDFFKYRRFIWISRKFRKCV*TFM**KEYIDINRFI*KYL*G
*RNR*I*IKFKINRSLR**YLSF*KYRIFKRGRCFM*LY*L*NCM*ESWIK*CKYKEYMG*N*YNE*RIYSYG
NCS*YNEW*VIY*RSIYCI*FKEISFKSSG*FFSRKRESY*NEY*YDGNKF*SNI*RTYNTK*RSS*NIYK*M
YTYS*IIRK*CIF*ISNTRTKIFFHYING*KNYELW*CSN*TK***STNYMYL*W*SQFNNI*FIWYRSSNRV
C*K**SIWM**SY**NSIKL*SKNKFRYGNCMDIRTKCI*RKYKTYVRRKTKKLYSYFTSS*KCSYIKIFI*R
RTCSNIICNEIRNVPS*SYYYKSKYIR**ILYYM*RISYC*KKYRR**R*NSFI*FKKN*LFW*IGSHQKY*K
NS*CYLRYRSYFIIS**FRI*ETSSFTF**IPK*SQSKL*KSTNEKKNKHTI*NG*S*Q*HKYSFTWLI*IKK
*NSYHTG****R*T*I**YYAQVT*G*HKFHS*RKRS**SKG*WTKSTTR***EKER*RKI*KK*G*R*GKR*
K*RKE*KN*K*
>N.PF14_0174
MEEKKMDYKIEPERKEAAIDTSNWPLLLKNYDKLNIRTSHFTPLPMGNSPYSRNIEEYLKYGIINLDKPSNPS
SHEVVSWIRKILRCEKTGHSGTLDPKVTGVLLVCLNRATRLVKSQQESGKEYVCVCKFHSKPKSIEEVKLVLN
NFQGAIFQRPPLICAVKRQLRVRTIYDSKLLDYDETNNVCVFWVKCQAGTYIRTLCEHIGLLLGVGAHMQELR
RVKSGNMTEYDNMCTLHDILDAQYIYDTTGDESYLRKIITPLEKLLINFPRIVIKDSAVNAICYGAKLTIPGV
LRFDNNIDVNTEIVIMTTKGEAVALAIAQMTSTVIATVDHGVVSLTKRVIMDRDTYDVKWGFGNRSMEKKKLI
LAGLLDKYGKPNEKTPLSWIKSEGYAPKLIGTNTTNYNLDIQNHNTVNENNNNNKIEKNNLTTSTINTNINDQ
ISEGNKNQNENAEKSDSSDLKKKKKRKT*
>N.PF14_0175
MNSNKNFVNKLYRYYRKNDNYNKKRIFIYENTVYYTPDDCVFCTQLNCTIKKRWDKKKKKKLKSAEKEEYFKD
YKYFETENNDNTDEYYKNDEEENEASDLMITYCDRHVSLINILVNMFKIYNKKKTYLNVTLNINYVFLQIIYN
IILYFVYLYIYYTKYIYYILRTYLFLLLYNSFHIIRENCYYPNIFLYNYYRNRIKKNILQQWKMKKKKKKKR
KKKKHSLFLCKKNMRDTKSNHFVRNYSYKIIKDSYTNHVDDVIIKFLLYMFYEKFINKFNKYINIHHHYHNVY
KKIYYYKIKEGMLPFLCDHIFKCNYNLFYIFNRYNFQFNAYNILLNCKEKNSEQIFIFFNKKFLIKKDYVMNY
LIYVINNIREKINKIKIIYDDTKKCYFIFYKNKCTSCLITHNILNVPIEGIKKKSDNIYFEKPKTNIKNEDVN
CKYNKIFYKYYISVINDMLCISKNNMIIKNIDSINKNHKRLIKISNYNITNVCMFLLLPEIFCKYYKKGDKKK
KKNKNVKKIIFKERRVNIKSYKILLKCEILTHFNFFNVINCDRYIYQNNLYNNLVGLKKGSYMKTCTNDETNR
NDGDVNNKNGYNNNEHSNSSNERSNNNGDNNNNNHNNNNHNNNHHNNGSNNHNGNNNSNNDDNDKDDDKKGND
KKNEDKDDENKSDDDNDDDKKNGDGLNRSLDTKKRKEKSKNKNKMKPDNTLNENVKELLFDKEGKSYTCTNND
LSLVNNTICESIKKRKSNRGEKRNYNNEKNDKNSLNNVIAENAYKIIKNGRLYKPTEPVNIFRSHNKFMLKEM
MWMSIDYYEEKRWKKNVSKKFGYLMSNHFEEKKKNDKYFISSQISNDIKMFWFFMLNEIRPDLVPVDLDHKIK
NKNHLKNDFLNSFRKNLQIEEYNLEMDDNNQYSFNQHDGINEESSVQNKSEKNSFSINTSIDDNWGLMNKMSH
QNGKNNNHNDYNNKCEDNKNNDYNNNKCDDNNNDDYNNKCDDNNHNDYNNKCDVNKLNNISIEHTNCINSNKY
NLLNISTHKEINMIDNNTNDISTVCCENNNNNNNNNERNNHINVMKKKNIIDYENYDQIVMNIEENVNKELLK
KHKIYKKDYYTVENVHINHIQYDDNNLYYNDDLYNYYQSKNDDGYLDLSNHLYIYCLLFISISLIEINEHINF
SRPDNHLPNDVDNALLEDEQNNSNNNNYNNYNNYNNDIFVNYMNAYNNGELFCNNTQRDNIKFPQYGTFKKIK
NEFEDFRELKKKKKTDMLKNNKDGLLSNVDNINLDIIRKGNTEFKENKIVLNNENNVCYPNAKRNSIMDKDIY
ENEKDINNCENNILIGKQENIKNYEENKMIETYYDEMLLKKKSINQLRLISEEMLLSKRENDNGNDYKNKNFI
DQYDEENYNGYDKLNKYVNKRILSIDNNNNNNNIENKLDSFVVHKNMNNSNIYNILSVPSLNNTDVKDLVVIP

YSCDNNNNSNNNNSNSNNNSNSNNNSNSNIKTKESVSTTNHNNSSNNIIYENIKNDYINEIKEKNVNVYLHNM
ELYGGIPYPFEYKYIDDFYDDINLPLMNLAEYDKYTFFLYLFYMKNSSYILKKQMKKDKRKRKKEFTDVLLTK
KRTSKRKINKLENEKKIEQIEDAEKVLSSEKVVDGENILKVINDQNNLSNEVNIIKDVTISGTKINSKNNDKS
DDKNDDKNDGKNDKNDEKDDNKTGEKGDNKIGEKDDNKINEKDDNKINDKEIVGTVLRTSTKNKTKNKLKNKD
GYVIKKEHLGDLEERQQKKIKKKEMETIKKWKEKKDEWELEETNFLLILTKTYMNYINMDEIIKREYGISLNS
CTALGDIKNKNNMNNMNNMNNMNNMNNMNNMNNMNNIDETDNGNNINIENNVYNSNNGNINNVVEIKNANNSK
TKEHEEINNDNSNVENKNVTNDFIKKYKGHEYIMSFNKFENNINNKLKNYTDFINWNIISVALVSYNKISHIY
EKKRGPNECKEKFLSLIKDDIYKITKLQNLYHDYYLNTIKNNNSMKRKKKKCITKVKFLPLFSSNNVNKLIGM
FNKYMNEKLKKYELKKMDMNNNNKIETLKVKNIIIKNEKIEEKKHDEEINILNKNDDKINEYKDELNVKDEDI
HLIKKVKKKSSIIKKKKKGFFHNLFSTFSGKKSRESSEEDTDCGRDSFFSCSDVEISATKLDDSCISFSNNKN
KSNKKKRAKKNIKLGEEENESECNNEGECNNEGECNNEGECNNEGECNNEGECNNEGEGNNESEIASTKKELL
DINNKINDENDNHNFNKKRSSISYYFDDYFDEHFEDYLDDYFEDEWDDYVDENFVDNSDEFFEEDVNEFKKIN
NIDENTTNNNSTDIIKKNEKNNIEHIKELNNTYVDENSHIEKKEPTICVNIKNSTNENNNILINNDTFKSKDE
KKDTYNKEVCVNSISQLIIYNKCKDDEYKYDFNYILKNRDKIKKWLNCICKNKKNIKEFMNIINNDYFVKNKY
FHRLIESVVNNVSKEQSSFTYSNNIIINSLNKEPDYELTYRENSNDRNINNPLDNEFIKMIRKIMEYDKKRKL
KSIQKLKNISNNYSYNTINEFYISKPNESYNTVNEFVQKILNYVPYVDDKKKIIIKKIRKRFQCKNLITQILL
ADYILDKLKNFPINESIESSKNNNKTIDMLLSETRLKAYSKYINEHFNDNYDPVKIETSFLKMFSSENDELYK
KNILNNNNNKEVTHINIKSEEVALNKSIQETTNTVLKDTIIESNEISCIVDKTVDIHNEQNDKKHDTSPADIN
NKDYIGYMHVNKKELVSMQNSSNPSDIIQQCVNKVEVYNKDVCHTNGYMNINDDNKMNNFVNNNITHINNNVN
NNNNIVNHLNNNNNNNNANNINNLNVNHLNNNISQINNNYNNSNMSSIYIASDQNEHFNKMTVMEKQDPMNNT
KQRSHSSYHTSFPMQNNNTPLYNKLEGDQIKLSKYNSLESNKNNIDINSTNNNATKTKDHVIKANNKGSTRGK
RQTLSLVKPHKESEHVQDSSVINQPVKNVSSKGEEIILGNNRQKSQPIKNVDITNKMTKYKTSVSSSILSSDK
LKNNNILNTVQNESAYLSSKFNSNVQTLNNNVNNNVNINVNNMNSNISNNMNSNINNNMNSNMCNNMNSNMSN
NMNSNMHVMDQGSFFTSNMVNREVNNLLNEEQRINTNVVSSHYNSVVSSTQFINDRGNYTNRIQKILSNNKYS
LTPMKNNITQENMNNCIVNHQTPNKNIQEYYIPNASPNKLNKSISNVDIMKSNTSSILYPQAHNSVSSNFNNV
SETLQNVNVLHASKKYHMNNSNDICTNINNNNNNNNNNNNNNNNNNVHHNSMNQNNMNTINMNNINMNNINMNN
ITMNNINMNNINMNNTNNNVNNINMNNINSNVNNINNSMNNINISNNINYNISNNYNNSVSMYNIPNKEDDKF
ISPLHSSMQNNKYMPINRNNSSMSYNKTPICDSVELPIKQMSGNISNNCNASMQLEVSSNSSSKKLQHTIGSN
TINTDKKSLLMYIENRARTGSSSINKINDDMFSIKSSTPSNYPPSYTTHQQFISESENKSNLYHDQMLNQDVI
MDNKNVQNFTYAPCSNSLNNNLKNTNTMNNETRSNIISNYKQVKYSNISVNNTPNNVIMQTQNMTVQNNAKMS
LNGSTTHPIVNRTNMENIDLIKLANDPLNIHRNGIHMGNIKNDNIEEKKNVSSNIRSIADNSQRLQNIHLGNQ
QNDIQHMNLDNINNNNNNNNLKMKKKSKNNININNNPNNNLNNNGQIDNAYLNVSPTISNSLQQTNMYQSDMK
NSIIYNSSIQEKDPNNRNYNTQGNNVQTMNQSNGNVYNVNVPNRKQSNLNQYVNPMNFLIQVLNKCNSNTNGQ
VKASASNANVNYANVNSTSLNQPNINNVGMNQPNINNVGMNQPNINNVSMNQPNINNVNMNQPNINNVSMNQP
NINNFSMNQSNTNNFNMNKPSTSSFSMNQPSTNNFSMNKSSTNNFSINQTSANNFSMHQPSTNNFSMNKPSTN
NFSTNKPSTNNFSMNQPNINNISMNQPNTNNVHMNNLNMNQSGMNHTNMTSPIFNQSNSSKQSVNENAFVPPA
YHTSNIIQKNATSVYNDNFNNPNVVNVGLKNTMTKNIIQRVPQKKGYSTHDMNQQERLQQERLQQERLQQERL
QQERLQQERLQQERLQQERLQQKWEQQKIQQELYQKSHNDKESETNNSSSYQELNNHTLQENTIAKDSMRQHI
FLKTRQQENIPKELQKMHSHQLQTEKMKTQQLSAHSLQQQMYPQQMTSQVHSSFHAQKIKQQLQQQMNHQQIN
KHQMNQQQMNKQQMNQQQINQQQMNQQQINQQQINQQQINQQQINQQQINQQQINQQQINQQQINQQSMSKYP
YQAQEIKKHMDQLQQNVSIVTQQKMQPIYSVKQNMPFNEENEMKRNTRKITLPMTHQIPNVPNIQQQINNKMD
ERMSTENINENNLYNMMNRKLSTSINKFNNHDQYYPYDSYQQQQLQGNNINVMDIRNVDKNENNIFLLKNNQS
RDIQNNCTNIMMNNNIPSSYITPGQAYKLNSNQNINHTQQKK*
>N.PF14_0176
MNVEDITTEQLENATKTFVSIESAAQISRRHVRQKLEEIFNLKENDLYKRKKEINDIVNRVLTNLLEEENKKR
NEELKIKKEQENNDNVNDHEDKPTADVDTNKNKMITKEENTKNKNVKKDNNSDDQTVCSQTDAVDKNSSKKRK
STGNDVVSVRKKQANIMTKDYFLKHAKSILCNMSENINLTLEPRIFSTGSCGWHIMDKIYLLVGDRNVLCQFC
INCSVIGSKQWD*
>N.PF14_0177
MEDKKKLEEDLESNKYDIDEEDLLEDEGRLNEEERQAELEGESLFEEDEGFIFGADDEKKEMQKLRNLGLDND
DYDDDFIDDELDYEDNLKARRAAERHMQMQRKQEGKYEKNKFWKTLEDQLEGDDEEEDIFDKVAEKVAKRREN
LHLTAEETDIPDLSNLESAKICLSVNPKDVVFDERYQQAADTCFRYFLHKFSLKDSMGLNIDESNTELHEEEH
MNSSHQYYIDKIEKMILNDKHTLIVSAKHLIQFHCENLVQWIEFKPEQILEVLHECLMVEAYRISPKLYKGRI
CKVVLRDWPYSTQLRNLRCTELNTLIKVTGVCIKRGYVLPKLRVMYLKCNSCDTTLSEVPIYFADGKKPVLPR

RCPHCQSATFSVDRIKTAYTDYQKITLQESPCSVPAGRAPRQREVVVTGDLVDKVKPGEEVEVLGIYKTKYDI
GLNIKYGFPILQTEIEANNIERKEDIQLSELTEDDIKDILKLSKDPNIRERIITSIAPAIWGHKDIKTSIAYA
LFGGVQKGGDKSFSKNNETNNFGVQNRDILNNFKGGHTIRGDINVLLLGDPGLGKSQVLQYVHKTNLRTVYTT
GKGASAVGLTAGVRKDHTTNEWTLEGGALVLADEGICIIDEFDKMTDKDRVSIHEAMEQQSISISKAGIVTTL
RARCAVIAAANPIYGRYDPSLTFKENVDLSDPILSRFDLITVLRDIPNVDEDFYLAEYVVTNHQLSHPKLENT
QNYQKIIENLKNVIVSSSAYEPIPQDLLQKYIIYARTNCKPSLSDVPYAEISAKLSNFYSRVRQKASASGGYP
LTLRHIESIIRIAEANAKMRLSHQIYSKDVDYAIATLLESYVSCQRFAVAKQLSKEFARYRALFRGGYEVLRE
LLRRTVQQMIQRQNLKNASAKDFQNDSESGTESEAEILNPNNVFLPLHIFMKTAMQNKFSEYQVVNWMKSKVF
NEHYAVIKRDGVEGIISKKFKV*
>N.PF14_0177b
MKEKCNEAKLKYYKCLNKSNRNPGKCKSFESELRQCSKITGESYCINEINNLMECSRSPDPSMCSKEFVLFRE
CNRPDGPHILIEYKKKKKITHAYFIFYENV*
>N.PF14_0178
MWGFSNFNNLWPRDFLNVSEPFQEEYTCYPVSFIGKDDMENGNKIILPQTALNALARRHISWPMLFEVSNPYT
DKRTHSGVLEFISDEGTCHMPYWMMQQLNLKEGDIVRVTSVSLPKGTFVKLKPCSKDFMELSNHRAVLETALR
NYATLTIGDNIVIHYLGKTYEIKIVDLKPAFACTIIETDVEVEFEEPIDHSEPVQYVTEIVPEEENKFKGKGQ
RTDGKSCHKALKKEIIKPKVVENLEPWKDKLAQGIRTKCTEYEDLLKKGRIPGIVGKFTERKN*
>N.PF14_0179
MKLSVLYLVVSLYFFDRKIVCNKKNGKSKSNNHNYFNNTTKHIDKQVIYNLPENDNLFHVLKYLIHGENKNIL
YDVDESLYTKIENKEYITKEDLLRTLALIEKKNVDCNDNIKKVKIAKLLIDINDLLRTNYIILDDYKEINKND
LTKNGFLLKLQNSLNKRDMVNSKRVNQDIILRNQYLEKNYKLCSISSDMLIISTPRNLNISDTSIKEIKHPSS
INIMNEIFCDNVISSNVSNISPLQLNKSIGLYKPLLDLDIKSRIDEYRKKIITYYQDSMNEIFYLIRDEFNEI
KKKFYLYIHPSKMPEAKPNNDIFKKEDMEGNEIITYVSNKIENKENIKNKEEYILKSNNNNVIKISSKRTKKV
INIPIKKKKLSNDIKENVNSSTKSDNNNIISNETVDACTKIESENGNKKKDDNVKNINRIYHIFLKKKNNEKK
CDISKQTLYNNSFKENDVIKTFTHNDNILKKIIIIKDVSREKPTIKEIIHQQPLPVENSELYNISEHIQNKCN
CCFYSIPLIHIYHPNGSMHCLSCSQYEKNVNRKCPIYEKYITFNYNDTCLLCVPSSYITNNCFCNLIHSKGLG
EKTNDVPKKLNMMPHFSSIIDVYGNKNIDGYNNFENDNDLLVYSNNDEPILKKNHKERPNKKCSNRYKLKCPV
GNGSSDMFSGQSISIFPIIGDDINRGHYENCPYYDEEDVMCYNTITNKKININNEDDNYEKILYDKDKIVYLN
SSSFDTINIKIEGEDLNMNDRNEENDLLDKESIEYINEINEKEYYDSIEACKDYRYHTFEESIKNVNNRECYK
NVYYSTVHKKEDNIDKKCSHDIESRNKRVNEYEDECENDNNNNNNNNDDDDGEERNYFLFDLRFIYKTGFFTK
RKTIINSYLKTNKIDITYNKEKILYLFKNLFEKTFKDINYQIKESLVHMFPIVQNSEIQGFDIDFGKVKLHYA
TSINMFKYSVFDLINMKIYFISPDKTYILLEDIIREIREEKENSNNDGSRRKNKIINKYSHEMDITNIEWSTE
NSKTNTTNSESKTTTTRENTIVIKEKIGTHDEKKKKCDNTKNNRNNNDDNDDNDNDNNNDNNNDNNNNNNNNN
NNNNNNNNNNNNNNNNNNSNSNSCDNNNKRDNDSFEHVDIQKNDTITTNIDCNIIHNNTIDSSNKNRIKYNL
NILKFLLKYKDLLSKEDINMISDKNNLVTINKNDKTNIGDEIIEITIRKNEKRNIENNCTYTNNSIINNIDNN
ISESININGGEGINAVIETVDKYLDKYFSVHYMPIYYMENMKISKNIIYVTSDVNSDLDMLKMSILGICNITN
KSIDNFNFFLVRNEAELYDPNKCQKIPEHIKNVQDLYNYSKMLDMKIIIAPVYAEDIDIIPKSFDYLFKLYKD
GDDEKGVTTDSRDNDCQQKGNVDTQERNKTENDTTRITLSNIKHNNIIKNNNKNNNNNNNNSHNNNHSYYNHE
LSLFNDAESNKPNDNTTTSNDKNYYFFNYDNKKSLILNDRENDENIEYLSEEISIYEDEHIYDEGNLYNSFVI
YIDNPFKRINIENAPILKGYTLNLFINDIMNKLIHIPSTFINTQSYKKGMCVMSICYCFNYYSLNIYTNNIIL
LNFDKDVTLYEVIKEFKSRENEYLLIKNKMNEECQDVDNLSNIKLPHTIDLIDSKYIHLEFPLLYIDEHSNFF
QDKITLMNVPENLTVYELCKSMKNILNESLISFNLSTINDIKIYTIRGRQWEMVLDNLLVFDLKINYEDIFTI
LIDNNLLTKNYYELLSNIIIDFRDNNMYIKKLDIYIDYIFQPYTIYNIPIYITPNNLKYILLQYTNTFLSNSL
LDEFFFTYNKRHTTINVVPEVRNWEHVEDSDVYIYEENDINDKKKEQKRNISFIYVLNMLSTFYKIKFNSSKL
LESVMGEIHQLCGKRFNENLWLDIKKSTKNEKDIEIYLENSSKKVLIKNVPLDLLVWKLINIIMRGSFNIPIE
DKEKLYNLFMLVPKNKDKIKDKNVDYYFLSTPGYTVEEMLRVFEKKKKPKKKGKEKNSANDNNNNNNNNDNNN
DNYNDNYNNDNNINSNNNYGDMKQTYYKILLIKAYPQHLHGIQNNERFNEYLTFNLEALPSVIDVKNPTTCLS
FYVNYKNENKMTHIINVPENISTDMLLKTILSDNFCRWKKLPNEEKMKFSLHLNKKEITNIKDYINNGDIAQL
KTFILINNKKRRVQEKYIDLDNTTELGSNVNNISKEELKEIQLYDNVLREILNKNYIDYNNISLSGYTITVYV
FDGNKYEPLTIYNVPLNITNKDIINFLLIKSNHINITKLIDEFLLLNEKLYNTYIVKNKNKNMLEEQNVLFIK
KLNEEEEVFQLNESKFYLVHNPLFNPLDNLLQNIVHKKYDFKSEKIDIHSVGEKFEILTLNILINNDINNMHN
NNVNNNYVNNNNINNNNINNNNINNNNINNNNINNNNFSFGKKKFVITVLNIPYNMYITEFLRYSIGYQRKWI
YKFVLFYIIKGSEKYILNDGNDIIRHGKTVSEIFQICKSGDLCTLHIDINFNEADNNLENDIFNEEINAKEMK
NVDVDSHADVDVDSHADVDVDLDANVYLDANADVEGNINKFVNSLHNDNENRFIVLKELSKKIYTDDYENKKI

DMSIICFEYNAGLYERNLFGSSRICNIPKNYTVGDVLRYVKNKIIEDTNISMLNDLQLKIIHNDNYIDIPKDM
NIEYAINYLFIYTPFIVYPSDDILKYNNIIDLILYNTIIDIKENSFVKKDIPIYVKKGNIFKNVSLKYIPINI
KEDELLTYLLHYLTDNHEVVSFIRDITCICVYPLCDSIYIFHEKKQLSLIPSISYHIQLKNVYYISTVETYEN
FYNILGHMELNLNKFLHIQNDGNNYKKKEAYNNIYYMNNVDELIHIDVNIIFPSIQKIIRVKNLNVNIKMEEL
LYKIFGCITNKIYKWGELFRNFVGKKEKINIVNNDIKNNSNNINSINNMNSINNMNNINNMNSINNINNINSI
NNINNCDNIDRIDNKVLKDYLCEEQNIITFIFCSHDNSIHDYLTSRMIYLPAIINYQTNYITFYSSINGFANN
ETTFHGFPNYLSPETMLTMLQYNFFHVIGKNYLKLYGSLYDKGTDEDIIYNTNDIRKSENKDIYNKNLIAMRG
AIKFILRIKNKGKKKSLDTIQNLINNELNIACENVENDVDMEECNDFLSSLNNSSIFWKNGILGFMANSIVTR
DNMNNSLILPYVDFEINEKMFLQNILYHINISPYLYKLFELTHIDKKCNTYKKKLKNNVAHIQCLLSYGFSIY
FDLHYDSTFDKYKGFTLGDNSFDITQIHSFQNLHEQNFVDLILYNHDNTFITIKNVYRDMTVATFLNDLSKTR
CHILGLTYNQISTYYKLTYEIYNETHDISPNLSIADVHNIVISFSKSSILLKIRKIDITNDISHNNNNNNDSD
FIINSDNIDYINALPDLYLSPYPPVIDLSQNNFQVNIYMHSKFIPNDYSLRKDIVPHITINNVPSNVFILSLK
ELLKTIFIQHMEKSQTTTFQLNLYDFEVEFFILTFNRFTSKFHKMNSNVLSNLTISNFYSTYIHGGLFLNIDK
IKIDIPNLLNDFIDHFSKVVIDFSHHNIVKQ*
>N.PF14_0180
MSETNKQNDNKKNKITRESLLDDQFSENPSSKFVLNKEEKKHEEINPNISQHKVNKKKPLIDRSLIGKFSVEK
YKSFLSKIEKSNEDIKNKDAESVRIDKDILNSECDNNNNNNESCVYMDVSVGIYDVCNENISDDKLRDMNITI
ADVKNQDEDEQDGKDLIEEI*
>N.PF14_0181
MRIVDKQIKESFLLADRNFDGHISSNELLYALRFLGVESDYSLMENKSGATYSMNDYVKIAKKHLGAHTPKER
ITNSLKKMDKNNNGTISVDALVHLVMTMSDILTENDYRKFKKFVDPESRNIIPLHVFVEKILS*
>N.PF14_0183b
MINNKGNVGKGKRIFNKNYGSISKKKKKRNDKLKKDVSNNKDERLELQKRIDETLGLIVEENKRKQYEEKINN
DLEKNENVKNFLKSDKTNNNTYDEDELKNNKNKKYKNNLTIIDKNVLTSAEFKTLPISKRTLRALNENNFIYM
TNIQYVSLPIVLLNKHIYAQAQTGTGKTLCFCIPLIEKMYRNSIDNYNKILGGIIITPTRELVFQIFEVLNML
NKYHKLNICCAIGGKNEEKEKSIFSYANIIVCTTGRLLYHLENNYYCNLDYLSTLIIDEIDKLIDKSFYDNLK
NILLYKPKENCQICLFSATICKFLNVILNTFHIKDYEYVSINDNDKYIESNNVKQIYIECDIYEKINYLYTFL
FSKKNKKIIVFFSTCKQVRFMYEVFKKIKVGVMKFLQLHGKLKQTSRLNTYHFFSKKKNFVCLFTTDIACRGL
DFSSIDWVIHFDFPDNIETFIHRSGRTGRFTNMGNSLIFLTKQIDNKKLFLNVLKDNNIFIKEKFIKKQKLFD
IKNKIHSLNAAFVDIKHLAKKALIIYLRHLYIVMKFKDIKKKIQLNELAYSYGLIEFSQDMINELNINDEKEV
QVKKKKSRLEKFLEILKNKKALKNKQNHEKKEEEKYNNTQDEKEYDNDNDNEQGHEIYDEAHENYMCEIKKKK
RVKDISHIDQSQSNENPFIHSDIQSDQDDLFIQKRVEIKDDTPIMLPTKKRKKRVKVAKTLSNSILYDDSGNE
IKDDNVKFKILVNELNQNEDHDMEQDTHYDDDDDNNNNNDKYDQKTYIESLKEKIQRDNEKMKISKLRKKKDF
IADRKKHEEDSSSTDNTNFSDIQDKDDLEYNESSEGDNGNYNESDDFVKNKKKISEKCDPTDDISDLENKVMM
FL*
>N.PF14_0184
MTLNNLKKVNTRNTHLTTLDNNNENTKCDIININSIQKNKNNNKINTETNSRCTTYRRSVRLLLLNKEKNTNL
TNNIVHSPSSDKIKNNNNNNNNNNKNKKTNNNIKNNNHVVFNTKGHHIIKTSENEQSNKTILKGSNEKLSIIR
NNGHITNFVKKKNDQDYSTKLLNNKTTSQKTSIKNDSKNDSKNDSKNDSKNDSKNDSKNDHKNDHKNDHKNDH
KNDRKTCSKSIDKKKRNDEKEKKDIEEIIVDNNRNNKNVKKKNDIDQIYLNNEDIKKIDDEYLFLQRKFKKQK
TDNAVLSKKKKSKMFNKMVKEYIKQNMENNIYVADNKFNLYSYGTGNALYTLERINYNNDKTNEDHTRKLNVI
SEIIDVKKQKFIIGSSYRKCDLVIKGNIENEHCELICRCIEKNINRTNDKNRQINKYNLFIINKSSNNSSVFL
NDIPVDIDQVKDDDYIYLGMDEKNNKNTAKYIYKIKYNKLANIFNVNNMNNLNPNKENSLKHLSKITEKVGTT
SKILSNNFVLSNQITTIKNTQIQQQQLLLKIQQIKEEEEEEKEKEKKEEEEEEKKKKKKEELELELEIGEKH
EEKKEKFKEKEESDISIVLFLIIPKKKEHINEPIYYINNFNTLTNTLNSTPKKFLDNTKTPGKNISSVNKTIK
EDSPNTRFNESIKKINRICQSRISPLGIKDSIKKDEKNKGVLKSSSNSKELPVTLLNACVELCKEINRSITKE
NNKMNSSFPKSTPILNHSNLTPSNGLSYFNKNEENVNEQIDKEHKKNEKMELANIYNTFNTNFHSSNTPRVDN
KKGEYLQMESHNNMNVHVDEEIQYTSKKGNNLCEDAEEKINKDQSTLEVPKNLEYKQKEEQNNIKNQTDCTKH
NETLLNKDIEEIEKIKANEDTENNNNNNNNNNNCKNNLDEVIIINRNNELKGDTINIHEKYTTEMYHFNSKSN
YDELYLKEKNCINNEFQDNLLVTPKKNDMFLNINNNMEYYDCKEEKVFNIVHHNNNDDIVKTSDTNDNLRKEA
EKNLYSLLYSKNYDDLLSNHDYVICLKNYEGIEQYVKVIGQIKVKRNAFFTDIKHEIDLKMLDNIIEIKTLDK
INYFNTNNISNPKYSIHLNAFTDKIDENKFHELSALHLDYIDHTNFSHMYTSDSFELKKMLFVKYYEN*
>N.PF14_0185

MNEEEKKKYPFDELKLDESIIFSLYIQKYFYCANIQKKTIPPLIKKENVLLHSQTGSGKTLCFVIPILQYLIH
HRNKLCPNDEKFIKRNDLFNKKNVNDTVSYNKDQIERIYETVENINIEKKNAYRDVNVNIDIRNFLLYTHNML
RNTNEEDISKKVEINQNDNKVMTYKDKIISDDQVNIIKKDEINIQKNNNQEEIIRKNDLQNITFATTCNSNYQ
DDKQETNKEKHEQKIEEKYNEQNIQTNNQTKKPHNNNNNNNNSKKKTQKLLNIQAIIITPTRELCIQIYNLIN
KFLFFLRFYISHTLNNNMDNNVLTKENFFINCLLFRGAVKISEDIKIIENEILKNNRYQIIISTPGKLSSLLS
EYNNKYFNTNELKYFVLDEGDKLLEDSYIRYMENIIKNIQAYDYTTCICSATCLQEENVYNLFQVKKKNFIKC
INTNNQDNSSTVINTYQMPDRIQNYYIILRNIDKALFLFKFLNTMVNDNETVIVFFPTCLCVEFFFHLFKNIF
NTKKINKQNESKNKKNKNKIYKGIHNDNVDNINVDNISVDNISVDNINVDNHIDYIFQLNSKYNNIFSDADMA
NFVKLICYMNKYIGEKKNNFNFLKIHRKMKDKKRVATYNKIINTCVSNKGTKKKENRNINSERKIIFCTDIIS
RGINMDIHWVINYDAANKNMTYIHRSGRTGRFDKTGKNIILLNKEEKEYIYFLKNKNVHVVNFKKTDMFQHMI
NYEKTLLKSEHIINNDALRIIEINHNEKKKNNITINKNSFFNLYPQILYDKKLIGKHQNGDLQIQKGEHKKNK
NNLLILNKKLVMFFLKYITFFVIENREIYLLSTKAFLSYIEFYKNHQLKFIFSFNKLNLTHLCYAFSLIKIPK
FKEKSKIKNFQNINIQSFQIPYKNEEKEKKRQEHLKEKQIDIITQEQKKENQIKMQKKKKRKTIVQRKHEQRE
LEENEIDSLFYEENLYKKLKKKKITKDEYDRLLNMDDIDKMFSSTSKTSKCTNLTNNTHFFKSNRKKSKKRMK
TYNIKIKKNKRKKKKRS*
>N.PF14_0186
MMKKLILFVLFQVYIICIKNVKSVHHKKFFTINHVNVPIKVNTNQHRKLGYIDLKRKNNDERKRNPFDAKLSN
QKCPLRKVTFKGLMKATGISIPLLFGSLMLYNKIINDNSNLSAPEKVMGRYLYKHNNKLLLSNVLDNERSHNI
FYYIINNIFNNIRMFINFYLNIKKVKSKDILKEYTILFFHSYNVDRFLQSRNIKTYSERLKEMYKQINKDNNV
NVIYIPLDNKLMFNIKHFSNMNNWYSVLFHDKEHILKLIKKYNIMNIPTMVLLDRNMNIINDNINYLLLYDNQ
GFPYKHINNFTYIKHVYDKNNNQYNLKNLNSDYIFFYFNNSKDNKQDIQTLLKIKKKLAQKNIKLDIIFIRDI
ERKITQNEKSEKNEKNENNMLDTTNQKDQEGISKNNNKLKVDEKDKNKLRINDNNNNNNNNNNKHGVDNNDTM
HNNQNNNMPMEDYLDDVYYLGNLENNVIYKLLLYDIFDVTFKPISILVNKKGNLLNKYINVSKKENEITTFIL
SNHKSLNEKVNEKNYLFKYNNISSMNNLNAFSPIFILFSDKLDLDLLKQYNELIEEYNKNRKGKKINFYFLPN
QEKKYDPLKKLCSINNTTEIVILDLFNQKLYKENGNNINIIKKLENGKYIIDKEKFFNFLNKYYDDELYASTI
ILTKESTS*
>N.PF14_0187
MGDNIVLYYFDARGKAELIRLIFAYLGIEYTDKRFGVNGDAFVEFKNFKKEKDTPFEQVPILQIGDLILAQSQ
AIVRYLSKKYNICGESELNEFYADMIFCGVQDIHYKFNNTNLFKQNETTFLNEDLPKWSGYFEKLLKKNHTNN
NNDKYYFVGNNLTYADLAVFNLYDDIETKYPSSLKNFPLLKAHNEFISNLPNIKNYITNRKESVY*
>N.PF14_0188
MKNYNSNSSRNKYKGENNNYNNHYNSFNYYNNYNNNNNNNLSSYYKKKNKYGKNAYENDYVKTKDKPKNIEEN
VYKKYGSYKKEDNNHLKTLNDKDNNILEDVDKINNITFGYTANSESDFNYLMNNNLIENEKKLFKMSTLEKNN
LNCSEFLKSQENKLIDENMNLMRNKVEKEKLNMNDNVKYISNDKNMSYVKNVKNFKNVPILKNERYIKYDEQN
DHSFVSNNNNNNNNNNNNNNNNNNNINRINNIHNNNRPMDKNDTNNKESDDLFCFNKRMSNLTNLINKDLFFS
NENEDMNDKLQDNSNDGLYVEEDHDNDNYQGSSLNVEPFNFTITENIKDISNISKKNYINIVKKTFEEKEEML
YNNNNNNMMSYEHIREKQQNHEYKKLDSSEVSKSYLLSNFEQTHNYPYDGKYKKYENYGNLESLPNDEYRNIL
NVQHLNSNYNKWEQEKDILYEESESSNEKFMNNDNIQMNEYHFNNNNNNNNNISINDLSEYINAMSGNNVNKLN
YINNISNCYNNINDIYDMSNIMNNMDNMNNMDNENNMNNMKNVNSMNNMDNMNSMNNMDNMNSMNNMDNVNNM
GNFKDIDNMKNINICNINSLSNIKNLREMLNSCYMNDMNEDDDYIHKSNKITSLLNEYKLNKNELDDDFILNM
NQKNGFNDISENYNINDEKYNDLISLLNKYKLEEYKDMNELFNDDSIVNKEKTKINELIYNNNNTFVNNNDSF
VNMRNNNTTFQMMNHHKNELKFDDYILRSKLDEGDFYLNNNLKNIGKEKKNMEMEKRQEEEEVFVISENGDLN
LNKSADLNVLNMLNEYYLNDKSRDFMLIESNKDMISELRDKELRNEYHHREKMDDKNDDMNNMNNMNNMNNVN
NMNNMNNVNNMNNMNNVNSMNNMNNIYSNINNVQLNVRNNFYNAIPSAENKEEIYYKDKIMQNYMNIDGKKNN
INDKSMSKNNNNVNNMNSMNHLNNRHNVMNDMKNKNKVSTNFPNHNHPININRQNRSYENNNVGNKKNINISM
NIMNDNVSKNVSMGYLEKYDQKNYYYKNYERTKNKNNNVNHINNKGNGQNFVKMEYDKSNSYNNNNNLNILIN
NNNNNSYFHMNTLEFNNSKEYNMSDENFIMRLKRSNMLKFTNFLKTNYKDSNIIDLNEPLYNYYRFMNKINAN
IKQNNYLIKYKNIMNQSDKDKLLKIQLSYMIINPSIQKNSGKWNFKNEDKDKEINTIMNSDHSDHNDEKHLNH
KNYDNYNKRFDDNVDNDDEDQGLKNLKKYENIMQLFKEGNVFNNLENKECSPILENNNINTLQYEQDFNNIYY
FNPLDYNKNIPINENHYLDININKLLNFSKLKDEKNNQNDVIKKDTILSNDPYDNNIFNKDINHYAHILKDGK
EVGDNENLLKMQTSEKSMLQHKNNEQEDLLKKDNYLNKLMIEKKKNQDLCKYENINKLGKFVFATVHEPRNLI
TLIKNKDIDDHNVIIEKVRKLIESEIYHEEKNHIVNNDASIKKKKDNVINTYNHIYNVDKKSNDNLCKDLTYD
GKGENGKNISFTCKKKYNNIIIKKMLENLWDLYIDIKNIYKDIDKCPYTHIETINKYNEEIKKKKNNMFQFIF
LNKISDNTITSSIKSIVDNNHMFLKKENITNLDIIINKYIYLYVTEILKSDMEEQLSTESYFPNMDSLIKSLN

FVKWKEIKDHMNVNKNVSTNTNINVDKNGLSKNNDDSSIGLSNTNHILSNDNSEKGKENQKKKEKTKKKTETD
TDTDTETKKNISNEENSGDVKNNICNLFLYNFPYQNNIKELNSYNMFGKILLYNNKYNYFNFDKYNNYDIFLP
ISKEEKKKKGFFFFKGGNNNNKICTSTKDNDDDYNNGNKNDDSNNNINNIKVHNHIDILDIDKDQINEELIRI
KCSYYIFVHSLLRYKGASVYKKIFKVIKKKKRRTLIYTFFTNFFLLYYILYHADQCFDNKLEYEEFLKLQLLH
INSNINTIHKLLKNEKEQNKVIKYQTNISNNENNQSLLNIRYKLYSTNIYTSLYIYLIDISLSYLFTPNMGIS
YMQDILSLFLFYNYICLYLTKILLYKSGGALITILLVKIIPYVKEFDRNLIICFLYSVLHSIFYIMCNIFLYN
LEKEKKIFQGSEVDLYVKVLSSLTNEIEFYKIIYKSVCSYSHQDTFYEEEIMAMLRDLSKDDIVKVIMSHLQK
*
>N.PF14_0189
MDIDILEEKIKNLEDELKKFEKEEACKIEKIEKDIDSLDKSVKDLRKKKVNKLSLSVNKCFEKKFNENKNSRK
KKKKFLKGVKKDILLELKLWQNEKKKSNNTFLNNTYQIKNMFEEITNEIKHTFVSLNTKRQYLFKCLFDKIAN
FKNNAILYYNKYIIIQKEIDSLFESINNNTITDIYILNQNSLLFQKELVNMFQRFYYQMENSIME*
>N.PF14_0190
MELFKEISQEESNKDSKENDNINEEIDIEQLKDKVKDLELQLEYEIKQHNVEIAEREETIKILETRIQELEHY
KNDITEKDEAIINMSEKLLILSNKYDILVKESKLQEEELKHFKNKKKYRNDKTNEYILGLRNQNDEIKKEHEL
LNEKYAQLVKEYAILQNSCKVLNEQLENNQKNMELLIKEKSNNLDEKTAIKILNIGSKELIHKRVHNKNVDEL
SNLKLELEYKDMILNKLMRKQVSEGKTIKVEYTNNENKFENEKKLGEMYQKYLDSVCDNDNLKKEINKLHNKI
NDMKVEEEEHLKTISILRDYINKMYKEDVNINQFDDLINNILKDNTSLNEELSKQKRKNMADIYYFTKELQTV
KKDKFKIIEKFDSKKVTNIPHLYEPLNLTLSKENNKREISDDDDDNKGPKGDLNFLKKNSDINCSESNIKKPL
KTNDLVHIYMQDYMNNVQNKA*
>N.PF14_0191
MKYVPPHLRVHNDEKPEAPVENVVEDLKEKVVELKKEEKEIDTDSSTEGDEFDIVLNSVVISQISKNKDLRGE
LLGYYIKNNDDKKKKKKKKKKKSKNNNNDDNEKNGNQDEEDGDDEEDEDEDDDDDDEDDDDEEDDDDEDDDDE
EDDDEEDDENDNEKKNKKKNKKNKKKNKKKDNKKPNVVDINQSFCVSNIEDICIDKIMKLTNKYELPKQRYNE
LLNNLTLYFNKNKYEEEGEKYINEVINYYEDYVNIMSELNFETTSFGFYININDIRKKEVLVTLLLCSLINKN
SFVLCFSEKYGKLIFSAYKIDVDVIFEIKNQIKNSTFNQFNDILNVELKKNGLNSKNILQPVPIVIKNSVISS
IFLKVLKNKNKNYISSLDYLNTLEVDSLENHVHETCKSLELLKTEQEKLIKYKKDVFKQLQTQKVYQEKKKLE
KEKNKIVHDNPDTNDEDINMQNLFKNIPQPSLLFPYVLTMSNNVLNDNINNLCSHAIAKSYLYYKNAKP*
>N.PF14_0192:mRNA
MVYDLIVIGGGSGGMAAARRAARHNAKVALVEKSRLGGTCVNVGCVPKKIMFNAASVHDILENSRHYGFDTKF
SFNLPLLVERRDKYIQRLNNIYRQNLSKDKVDLYEGTASFLSENRILIKGTKDNNNKDNGPLNEEILEGRNIL
IAVGNKPVFPPVKGIENTISSDEFFNIKESKKIGIVGSGYIAVELINVIKRLGIDSYIFARGNRILRKFDESV
INVLENDMKKNNINIVTFADVVEIKKVSDKNLSIHLSDGRIYEHFDHVIYCVGRSPDTENLNLEKLNVETNNN
YIVVDENQRTSVNNIYAVGDCCMVKKSKEIEDLNLLKLYNEETYLNKKENVTEDIFYNVQLTPVAINAGRLLA
DRLFLKKTRKTNYKLIPTVIFSHPPIGTIGLSEEAAIQIYGKENVKIYESKFTNLFFSVYDIEPELKEKTYIK
LVCVGKDELIKGLHIIGLNADEIVQGFAVALKMNATKKDFDETIPIHPTAAEEFLTLQPWMK*
>N.PF14_0192.1
MYKHRYFHFFFFFFFFLVSTKIIRSFTFLNNNTNLSNPVYFKKNANMVYDLIVIGGGSGGMAAARRAARHNAK
VALVEKSRLGGTCVNVGCVPKKIMFNAASVHDILENSRHYGFDTKFSFNLPLLVERRDKYIQRLNNIYRQNLS
KDKVDLYEGTASFLSENRILIKGTKDNNNKDNGPLNEEILEGRNILIAVGNKPVFPPVKGIENTISSDEFFNI
KESKKIGIVGSGYIAVELINVIKRLGIDSYIFARGNRILRKFDESVINVLENDMKKNNINIVTFADVVEIKKV
SDKNLSIHLSDGRIYEHFDHVIYCVGRSPDTENLNLEKLNVETNNNYIVVDENQRTSVNNIYAVGDCCMVKKS
KEIEDLNLLKLYNEETYLNKKENVTEDIFYNVQLTPVAINAGRLLADRLFLKKTRKTNYKLIPTVIFSHPPIG
TIGLSEEAAIQIYGKENVKIYESKFTNLFFSVYDIEPELKEKTYIKLVCVGKDELIKGLHIIGLNADEIVQGF
AVALKMNATKKDFDETIPIHPTAAEEFLTLQPWMK*
>N.PF14_0193
MIRNKKKRKRNIYDLYIEMQKENYEDEEKRISYIDDGTITLEKHVKMRLEEQTNLLSIKGIDRVDIKQKKGKH
SIICIHYIKNMCMKNLFCNYLHQLIYSRIPTCKNYMKYNYCADRVRGCCMFRHTLDNSNNNLYYNESKDEYLD
DALKFLHEKNICVNYLLGFCSLGYGCRKIHKTKSRRYINIISTLPKFFLDSILVNKHLYTHLYNNPKKLSNDM
NKLKDALIILSGEKYIDKNLSTNKNDNIDLLNKDVIQNNNIFNSNNIDIGIENNISHGDINNDSKNQKGVSFL
PGYNNVDNKNNKYINEYIDDKNIINNKSSHTNNEKGYFHNNILLQSIDNNNNNNLIQENNINIPNVYDLNNNL
IVAEKMKVFIIKCNQISHLYLSILYGVWATGKNNTRKFVNLFKDNYTIIFLFSVNESGGFQGYAKMVTMPIKN
LYENLWGPITSRLGGNFRIQWIKMAKIDFDNFKHIVNPYNDNLPLKKSRDGTELPLNVASILCNKMNDMPNED
FLTGTIYEFKRRINHSSFFINLHKQNILNTNTRWDILIFNLNQKSDCTNITLIDGTEQNIT*

>N.PF14_0194
MFTQHKNQEISHIYERNNEATLYIANLDAQVDEEILCELFMQCGNVKNVHIPRDKINGYHAGYGFVEYEYEYE
CEYAANILNMTKLFGKALRCNKATQDKRSFDVGANLFIGNLDDEVDEKMLFDIFSSFGQIMTVKVMRNEDDTS
KGHGFISYDNFESSDLAIENMNNQFICNKKVHISYAFKKDSKGERHGTAAERFIAANKALNLYPLNENNNNNN
VNPSPFTSSNDIPLTNNTTLNLTNNVYLNNTIITNNIIPKLSNDTISPIMNNFYPPNPHQPTSSNYLQAQVGH
PTNIPMNARGSTSFMIPPKINNRIGNILPSNISSNLPPNIPPNLPPNFPPNFPPNLPPNLPPTLPPTLPPTLP
PNLPPTLPPTLPPNLPPTLPPTLPPNLPPNLPPNFPPTLPPNFPPTLPPNFPPTLPPNFPPTLPPNFPPGFPP
NLPPNFPPTLPPNIPPGFPPNIPPNLPPNIPSYSENNLNTKGENKENTSD*
>N.PF14_0195
MNNIINKIDPNLQKKEKGYININFNFNETKNDMEKISYTQKNEISDKIVHNQDLSDNIHYINNYLTSIFDSGK
RKESWDNLFYRNNIIKEEKEMEDDKNSTLLLRGPTKNYNLNTELKLDDTYEFEISEHENEKKKFDSINNTLNT
KHSENIDNVENIENMNNMGNVQNDGFLYNEFIFSGHQNIELKKANTTVVNNLNKEDVLKQGNMYHDFHSFNNN
NFVDGWWFRRNQEIDQIPKNEYLEHSIKDTYEKTDYPFFNENYIKNEGNYTNHTIDNIVEENENDNMLFLLNE
KSNIQEIIDSSNKCKNMIHMKSEKFSKKDPNVYDNFPTYNKNIIDTNSNMNISNGQNLNESPKMDMQNEDIKL
IQLMNTMLQENEKTDTNFKSAFNYANGSKLNVDNKKELDMQNGKLYQNNCNSNNNNNNNNMDIHDNMGEYNNV
GIYNTNFNIDKRNGDILKYSNTDIVWEENIKILDSISRDYSKNVMNCPSNNTEVKQIYKNDDNGGNILLETLK
NNIKLLLENEKDDNVLSEHIIRTLDDYNKKKDAYMDKNNSSDYKNNNCLNINSRNIRKDYISNVNSNMMKENI
TNIQHDIELNHNYEEKIMNQKGNDNNILSRGNMYESLTFHNNSDKTNMHNNNNSNGNNLKGSLLDAYVIDDNV
QLKNRILGNQNNMEYFPKCAEECMDMEKLKKLKLAELWKYNSGVEKNETVLNINNVPIDSNNINNNNNNNNNN
NNNNNGDGSVVILGQEKKHEPTMDMYKIGKPFYDIDKRAHLKEENIKLLGNEKVYETKKNSNYNVFKQTENVP
KVRNAKGYNDLFMKIQNINELNLDTYYKDEENRYADRKKLECMNENNSEEFIRIRKGNSSPYDEAFSFLCGYI
GKNINEYKKDDDDFNNMDIYNNNNNNINRNNNINNYSNNYSNNYSKNYSNNYSNNYINNNSNNNSNNYSNNNS
YYYHHHHLNDSIKNEEKKEVMFDENINKNESWGFDKDGSYKYLKNNKFESEKYNGYIIKREEEDSCLKSKSLC
FNKYKREHGNYHKDSDLFLYKNESGLGKRVDNNNNNCSSSSSSSAFNFSSRKKDSPFLSFHKGGKDTDDKDSI
SNVSSMNMYKNNTFFVKGRYSNNYENTKVVERAKEEICKGREKNGHNKNDVDNHLKYKSMISNLKMKKKQSTD
IIKELSSEEVKSLLTVQDENSLEILLESLYEDRILPLLFNIKGRADEYEYREILRNNIKTAYSLYPEKYIIKP
NESGDDYIIYFTNKKVSEDYFMSINNLKDIYSSNLWKQFEEYLEEISKSEDATLYTFSGGRYGMAKELKKRNL
PFFQGLFLGQLCHIVHISTNKKIIAYENNYLKPISQCHKYTSAKKGIINGGEKNVENYIMTITELRHYMNKLL
KYYKGGFNISTLKKKLKNRFNKQICESVFHCIKLIEVLQLEELKDICVVDMETKIVKSVTDSM*
>N.PF14_0196
MQEFVMNILSKEKIGKIEKLKNDGNELYRNKKYKEALCIYDNAVCEFCGGSGDSLKIKEMVKEFSKNDETNNI
NKLSDDNNNNNMIDENNKKCDFSYICEIQNLFIKICHNISLCYYFLEDFEKSIEYCLYINEMNNNHYKSYHTL
GLCYEKLKDYQKSIHYFDRCKIVLLKNQNNKNKDNNNKSEINRINEKLRDIMKIIDQNKNDPYKNISNIKKYL
LDENTNNINDIEENNKKIKLLHSIYNQKFYILLKENIFLFLFDFIKKNNDISNYDDCNNNNNNNLYSHNINSL
LLEKTAIYVIYKILSKLDNEHIIIENSKDDNYNKNRNICINKLDNSKLQYYYDLDYIKMILSFNEYFTKDWIY
NYIKKKINILENLKFSKDETLYKEHVDILIYIINIMKYVYVINNDYILNIINSYYLNSDNSNINNSGINALTF
LCKKKQFLTQNSKNNRKKKNDLLEMLKKENYLNIQNNIQNNNNNNNYYFYKNEFIEFHFSDSHKYPLCINSEI
KKIIQNVIGMYEHFSSSIEYTLILIFTLLHDPQRPKEKDIEMNDVIYDCIDNYFHHNENILIEWFVCIKCLFL
VDKNIILNYLIGKTEYIVKILHFITNCIGRKTKEELSIYIDVLLLLLNISEIRFMFTNYIDMYINIMKSLNYD
QCFLKLLLGTFKLYMHNIDFKQQIQDNVDLFFYAKEILKQFLLTYDNDADGKNNTNNDKREENEEQTSHLNYS
NLNSYTCCVKKCDDNDTKKKDIINQKKDEKNKKHCELVDKKKKDHTYIHSNMSCEKTLKDLIEMLFYLSLHIE
FKKQLLEEKNNYILFFLIKVGHDINKKKLDNTYKYIYCNTINNLILTKNDEKIKRREINKTNLSNFDNEQIEA
LEQFYDKLPKEARPKTDPLYDYGDEETSNKLIDLLLYNEKYQMNHINDKNKNNNNNNNINNGNVSPLSSKCSY
TNGTIINIIYNFINSNFFTTNIAESVCEIISKFVKNTNNIGIVLVNNGLKTLLLASKHITNKKNCALALSEIF
IYTNPKLIHFYEAYDSLPLLIEQLKSDEELLIFKTLMAITNILTIDENVAIKAMQLNLWYKCFDILSTENEYI
KSASLECICNLCSQSHVHQYIYDKYQTIMKSKNESDKDILFVDIQIIYSFTMEYQNYKCVFAATGALGMLSSD
LRLPYYLVRTKGIDHIFSSFNNTTDQNILLRILTFFNNIMTCDDIPDDILKKIKTYVEKKKDLNEENTQMANF
ILQ*
>N.PF14_0197
MFQKIGRKLIERRTFSRIILTKDINFKKEKKNFSSLFQRYNINNLSSKNDNHFCIVPFNSTNNFIISKKSFVT
KNERVQNEKIIQDDKFEKSNDKVIYDNKSKHLSDKIICDDKIIELNENIITNTINNNNNNNNNSNNNNNNSN
NNNNNNEEIINTSSNILDIENNKIIQINNGEESEILDKNLNEDLVDKEIDQKKKEYLVLMFTCNICEKKSAKK
FSKQAYYNGVVIVRCPSCENLHLISDQLGWFQDGKTNIEKILEEKGEKVVKKFSYNNLLEVDDLLNAYK*
>N.PF14_0198

MIIIVIFLLSMFFFENKTYCFISKINIKNLRHNKNNNNKYHLFASRELRKISSFVLDNRIVFNKQKIRKRKFN
FFNVYSNKMSDMDNIKEQKENDGCGSLEELIERYVCEISSLIERNECIKGIDDEDLKKEFMENEKLLQDKKMK
LKDLYGKPSEELKLVENRTKLENLVKRKFFYTNSFEIYGGASGLFDYGPSGCLLKSELENLWRCHFIYYDEML
EISGSCVTPYQVLKTSGHVDRFTDLMIRDVVTNDCYRADKYLGDFLKAKIEELRKKNTKGDEHVDEKEKKQKR
DDDINENKENNKNNENNKNDENNENNKNDENNKNNDNNNNNNSCNYNCDMVGGLHNGNERLIQKIELILRKLD
GMNEEEIKEIIKEYNIKSPLKNDLSEPFPFNLMFQTKIGPKDDNTEDKNKEKCKNVSNNKNLNSTNTNNSMNN
VAFLRPETAQGIFVNFKKLLEYNGGKTPFAGAQLGLGFRNEISPRNGLLRVREFQMAEIEYFVNPKKKNHEKY
YLFKYLMLPLYPRDNQLTDGVVIKNMTLEEAVEKNIIANEALAYFLARTYLFLLKCGINKDGIRFRQHLKTEM
AHYANDCWDAEILTSFGFIEVVGHADRSAYDLQHHMKYTGANLYACEKYNEPIEVEYVKMIPNKAKIGHTFKS
EQNKIYACLNEKSNEELLIIDEELSKNNKYILNISNNDSINNECKYELTRDMLSFQKYKKKVQEENFIPNVIE
PSFGIGRLIFCILEHSFRIRRFTDDKEERQYLSLPYKLAPIKCSILSISNNKAFYPYIKQIQMLLNQYNISSK
IDNSSVSIGKKYARTDEIGIPFAVTIDFQTLKDKTITLRERDSMLQIRISMSHLVDIINSMLHAKKNWNEYIA
QYGLFTQQVLDS*
>N.PF14_0199
MINSNIIFLFFYHVSLCILGCLFFENFLFKDFDNSFSLVKIIFCCSFVLCLSLLSMILFEITGFIATRLQLFI
WYIDISLMILFIYIIIPISLIYTYVTYEDEKTSNSLFKFQYFYTTLIKINNKKKKKFHSPNEEDKKEREREK
NQDKGQEKNQDKGQEKNQDKGQEKCHDKGQEKCHDKGQEKYHDKEQEKTHFMKRLLYVSEDLIEKMKNYNYKI
KYKNKQKLYKFIFGCIFLLPIIWFTFYKISMLTLKNNKDIYDKSDIYNIGSILLDKFNEINKEFEEEYNINNH
KNNYFFFFNFMKNLLTYMSVTGMTIVSALSAFTSLYSPYSNISNFFFFVNIKKVKVIEKKIKFIKQELSSKKK
ILLLYEYHPHLLKPFYENQKREYNNIHNNMSSYKYINHSNNYNNYRNDIELQNINECRNVYHNDDNTTNCMTS
SLRSSTSRGNDNIESNNNNNNNNNNNNNNNNNNNYYYYYVNVNQNLNHTNNLNTRYFQNNKISINNSKNSYEQYY
NNVNSFNMKNTILCNNEKDGIHRTYKNHQVMKNEDYENKDNNKINRENKKEEIFFTSFKSYKKCNLYLGNYKS
IYKDIYETFIKRENSFISLFNITELSQRKSNNNLLYITEKINCENYSMDDIIIVNGIKNKCKKQNNKQIKSYK
NNDLEQKDTSCIDEYNQDDCFINIFSNNHIKNYSYIRNSKQNSFNQKCVLSSFNQNINSNNSLYKRNIKSSND
LLDDTHMEYQKKFHFFKTKEQNISLENKVTDDYRREYKYENGKYKTFFLFNYPIFSFKRLFKGSKNNMGGKGY
QISDSIHMNREANYTINKNSCDNNNKNSCDNNNKNNCDNNNKNNCDNNNKNNCDNNNTNNCDNNNKNNCDNNN
IHSFNYKWEGFIRKSVISSFFYGFQKPYKTLQSVKEFFTRRKDNVNKKSKQVLIQDIKSLEYMIKKLYFLLDD
VIKEQIRINQSTTFCGILLYILGIIMSILCVYKIIKTCYIIYMIEIHYKFISTYSSGHVLMLFYSKNMNISII
NELKNVLHIVHININLDNYVISITSILLLCFIFTNLKTFMEKIIKLRYSTKSSLYSNLAILLMCEIMDLYFSA
YCIQLFDYLPIKEKIKMLYIFFNNNLLNLFKLKYHFDFVYVISLFISLILLKFHHKNKSDQFKEIENYLYKCI
YK*INIQTYIQIYIMYC*NL*YFFKCLNEYIFLSKKKKKKYTYIHTYINICIYITYGCLILLMFIFYISRVHI
YFYSSIIF*
>N.PF14_0200
MRKYKNELNISNVLEKKDDCCSLDIGGTLIKVVYVNHKYIHDDNIKENTEHLMIKMNGNKNIYLTFFDISKLD
DTLYFLLRNNLIKKKITLTGGGAHKYFYHVLEKALYHKLGMEINKGDNKIYVSKYLYDEKLSIFSIFVCSTKS
NDQNKNDDINNFNSKIIEGQVLFDKKFVDKFPSDTIKIYVEKRYFDKNGNNDDNNNDDDDNNDDDNNNDDDNN
NDDNNNDNYMILTCSRKDEMNCIMNGIHTLFSVDKSFFRYERFLNVKVPVKITSPFHPFIIANIGSGISILKS
NGYDSYQRIAGTAIGGGTLMGLAKIILDNISFEELIKCAEDKNKNISFDLKMKHIIGDAPVDGCTHANTLASC
FGCLKNILKEIKENNGHNKTIHHEVAKGLIQMVSYNIGYMVYLLSKMHNVKRIFFSGKYISNNEYIMESLTHG
VYYYYLHFNSKMNGVDKINDINLKNNIAKNTIKRELYNVKDIHFNDKDMNSYLSYHYKLQDKEILPQVLFPKH
DGFLGALGCFFLA*
>N.PF14_0201
MKIPFFILHILLLQFLLCLIRCYVHNDVIKFGEENSLKCSQGNLYVLHCEVQCLNGNNEIIHKRCNDDIEKKC
NGNNKCIYFFEYELRKKTQSFRNKNSIEISECVESEQNEVKTSTTCLLSNSFILDEAFIQYFFFIKNKNEEPV
ICKDGNINIKSALLHSPFCEIKLKDISEYIRKKCDNNKECLIDPLDVQKNLLNEEDPCYINNSYVSVNVVCNK
EEEIGDESTDSSSMKIQDSTSNEQDENVKGMSSSQEMNSNNDENKNQDNESDDDVNNNNNNNNDDQDEQGNDG
DVTSSMNKNEDNKDLEHGSSNDVNNNTDTLVNNKENKEFVLKEKSSLTSKINKELAHRTALFNKLADNISLLL
NKKYDSFEIKDVLEDRYNEMKRDANPDVYYIYLMDTLDIEKIEDINLEEVKMSLLASLKETMNKIDTIEKKIE
EFKNKYISLYNKVKTTMPELFDLNEDLVLLYNDFPFDNGMISSDIFFKYNPSENIMDHQEMVKKGSITEDELR
IVNDLEPLDNYRRRKRITELRKILVEKLRILYLEKNNLFNTQASCIKSYCYKNPLNLKTLEVLLKKNYYRLKE
NKDYDVVSSIIQHLDNVDANKKKKWLTHERILKKLQVLIAEGYKRINEKEKDIDRRMAVYNALYEKAQSYNLQ
KLFNDSNDFLKKYAIMGNSFDDGDEVFGSQSSNFNIFDSNNTDQNNEQEQPKQDDQLLNNNNDDVLSESNNEN
KEKTSDDATHKETQEKSDQEPSQNIQEDNSDEKHAENEENVEQIETDSNVSEEANDENKDNMQTTTDEGTEEL
QQNDEDAESLTKENSKSEEQENEDSTDAEAIDKEEVETEEKGKDEQKKDEQKEQDEEEDGEKENKHKSSETTN

ETVTDIEENKNEVKGEEHLQGSEQSIEASESSQKDETKETEDKEEYVNANDDESSEEDTTPNETNKTDNGSSF
FFAMSNALLVILLLLFIEFL*
>N.PF14_0202
MKDYLGEYNKYLKYENPIIIEKEDEKNVHSKDDKTMKDKNIVRVIEEIRKNLKSNVLYKNDSKSVLFNDPIYY
IFPSKCIKVKKAEHISYVSRVTNDEDIVLCLKRIYEVLSNMYSKNMIIKEEYLNELLNILTELCRQISVTCFQ
RGLLLKQLFNYNIMLLFEYHKLVKSSLAFNLKKQMKQNDILNNFHKEIEKKNNSINSLTKEIINTEEMMEQEK
INSEKEIAEVNIIYQNKIDKLKKNNQRKKDEFTRLLQL*
>N.PF14_0774
MNALILVGGYGTRLRPLTLTTPKPLINFCNKPIIEHQILHLAKCGIKEIILAIAYKPTNITNFVKEMEKKYNV
QIIFSVEDEPLGTGGPLKLAENYLNKYDDFFVFNSDIICTFPLIEMMNFHKQNKAPLTILVKEVEDPRAFGVV
ITEDKMITKFEEKPLVPKSSLINAGIYILNKQILNFIPQRNCSLEKEIFPKLASDNMLYFYQLNNFWADIGKP
LDFLKGQSLYMENLEERKYEKHMLLDHLLIYYSLNENHTKNVVHKNLFVSFENIEELNKFNENEENSFIKDIF
LHTKIEGNVLISSTTIIKQNCVLGDNVVLGEHVIIGEGCRIKNSCVMSQSTINSYSYIENSIIGSKSRVGNWS
RIEGLCVLGESVILKPEIFVNNAFILPFKEVNNSIYDKGAIIM*
>N.PF14_0775
MSKPTVVFYHIINDKEDKNSQNVFYILKPIGSITLKDIKHEFPLMGTYHFRFKILHNNIPAWVDVTDESSPVP
SLNSCIYAKVLRLSWLDNKTGKEITNDKNLHDRDMMKSYKNDKMTYHQGNETNHIGDHMEESQKTKSNIDMLL
FETAPNKSNQTNINKKNDDSKDQNYFDLMFN*
>N.PF14_0204
MKDIKVLLKKLHINKEKEFEKDVRLLFDMKKYIECLTKRSSNNNNNDNNNDKTNCCIINNNSNRLNSEDMFKD
SIKIKEDNLLKIKHSYNNPIEYIERLYKELKGRTPFEYGYYKNVLNNYNKYDYYNYIMNNNHNLLQENISNEL
YHNNNNNNNSCDKNYLNNIKEKCENLMIFFHMYLIRKYCEKNYYECLHNKFIRSITYNYLDKEIFYNYINSDL
YFLLYSYIQTFAYIILKNEKQNIYLMKLCSDLISRTHEELSTLRKEGTNKMNTYLYDKNFFSPYNNSLIKYVH
FIVEYLSKNKNYNYMNILYGIYSCVYVFSNVFYSCSLSYNSKKVEQYILTENIGKKYKNIQCDHVSSSNQKED
YTNGMTTQDVDNISDRKFNDLLPDNNNNNNNNNNNDNDNNDNDNNDNDNDNNDNDNNDNDNNNNDNNNNDNN
NNDNNNHNNDEKGEKDMLRLYYKYINKYANTEFMKLNEFLLYYISKETVIALLRDNINYNDVIKDLNIIKYST
YLEKYIFLEICTNNMNKEQYSDIIFRDIKMDIFKSICNISENSPLYIELKDEDKKECFEIIKRKWLNENNIKC
TYENGFNKNMETQDHMNKSQKKGIVCPFLHGMNNEKKDNTNDNIKTNINCNNYQNYHNYYYEHIYNSYNIICP
RKNKNSFIHPQYEHLFFEKTKYNIDDTFFKQSLKNTNTLEGYEKKNISCDNYKSKFSGCPAHFHNINYNLCDE
EKEKKKKILNLLNNNDNIINDHINNLKCIDEFFHISIDFDKTIIKKDSYSFFYKLLCKHYNIKKPKDIELTQE
ETDFFENINIENIHKENKTNYTYEERINYIYKISKWYIIKENKLLKELQEKEDISDVYSDSYYYYLKKFDQIN
IAHSVLISYYDILKDVDINAINCLIYDHYDNFQLNDYFLDVFLRFIQYKQHNNNTFFFDIITLNFKKQIVLYT
LQNNIMKLENEHPFNVNTNEATYNNDNNNNDNNNDNYCKDNHSDHNNNNDNYCKDNHSDHNNNNNDNYCKDNG
RDSHNDQVKHKYYQTFKKYFNVYYSKMYSYDKEKNVYTGEFEYDKLKIKHKNYNGSSNKVDYVTILSLFDKTV
VRKKVSTIIKNTNHKLSCFIGDSIIDLDAMLSTDIPILLGCNQLVLTFCKKHNIVIKPLIFAAAKIELLKMKK
NKTESKSNTKGERTVHIHKNEQHESNSIIDQREKELSEIYDENKKVIYATESWLEIGTFFFGKM*
>N.PF14_0205
MFFFFFFFFIVVPPKERKTKEQIAAAAAASGRTKKKKWGKGKNKEKLNHAVFIDKSLHSKILECKNMKVITPS
AIAEKYKVNLSVARAVINHLADKKLIAEVCVQSHSQKLYTKVA*
>N.PF14_0206
MKEIIYVKNKERKNIISNVRIKAQKLDQDEYEISRNDCKEFIRIVKRHTIECMFEELFIDKNTKEKEITADDL
ISIISSKKKRCFYIPIAIYIGKRNELDKYEKYLMKEQVMNEEIKKKDKENLRKQYEIDKKRKQENEMKRYVSI
DSIYGLHEINKIDKEEVCDKEKVCDKEEKKSSYDKIKNDINKINVNNNDGDFFVSTDNNIVKNVNNIPQEEKK
IEIDLDIKQNGKADYKNEQGTVSKIKEKIDEITKDQLCDNENFKMENNKKHKNKKNKKKNNNNNNNNKIKDQK
EDHNEKMINDPKNFISNTKKNNNNNNNEKFCYKTEITNIHTTNDNNVSEEKKNYYNTLLKKVADRIERNSMNKN
IEKTKYLKSFHLVSYYKLNELIYVSFLIRKYYNHYYEKELYDIVDIYEDIQNNKKENIHDKNIEERKIHKTNF
TNDYVKNGPNNDIDNNSNVMIHFNKNDNIIYKDIFKKLDEERKQKKDNIYNDYAHKYIHSDMLYNYRVVQNDL
YKIIQPVLYNLNIFFNQNVIKDINKIMEKYDTVIQDVQQVGRKKEYNKRINEGLEKREGHKSTYEKENEVKQN
NVTQNGDKKLQNNVQTYEQEYIQTNLHNDDKNINTKKIQNSKNYHVNEKRNDKNVDNKNKDKEECINYTELDK
GDKHIMNTLKIPNNKMQKEEKRKENVLFKKEEETHYINKEKDECDNVRNTFKMMGYSSKNITNNNLNNLSKSS
PRAVYPLKCIETESFKIMILKKKKKKDSYQSINYNSDSYISSTMCDNLEDDNDKVQSDNYINYLNIKYSKEEL
KSLNNRNFIYYITNDIETLGIYKKIYFYDFFFIYLLRKLFWNIFSLYYIYLQKYNDRREEKRKNKIQQNINNI
NLPSISSSSSNDETNISKKYIHINNNNNNNYKIKKIYCDHLLLYESDVSVNNSQINKEYLYNTNKLLHDDLQI
LFNLIRKQKILIYFRYDQNKKIKCKNKTQYVHRDIWIYLILKALKKKNDNTIFNEFIFFKEKQ*

>N.PF14_0207
MNNINKQLVKDIYQIGLEHKEAISIDSLEEIYEKKKKGKLKRNEIVYALNILENARACSIKNENNTIITRMRN
EQVTKKLKELSDIDFLIFTKVENSQNNGIWTADLRKQTKLLIHQVQKGVKLLCENKLIKQVNNIHVKNRKMYI
LYDLEASEKVIGGSFYTDGEFNKKVVDYIRENICFYLYNNNNSSVPSVINYIKKLNNSVDYFSENDIYRVIKT
LSYEERINIYKSNNDEELIYYYNNEKKNFLYNFPCFSCNLFNKCNSDINTTINPRSCMYLNFYLNLEVTNVYV
CIYVCVYFYIYLLYCIYDS*
>N.PF14_0208
MENKAKEENLDNFLTQLNTLNKIITSFKETCKISSYCFDKCVSYPEKSLSNTNKKCIWNCTQRYIECEYFIKN
RSKDNQSLSNFELIKNMNSSENLAELKNDNNKTENFVLKD*
>N.PF14_0209
MDENKYFKENFIYVDVEKEKEEKKKVYVEYIKRCFNYLLRQDDNFILFTLKKNVSLLSYFFSYLLKSDRNYDH
PFELLGDDKKYNAELNLCILNFYLKLVDIYLNKNMCDVLFLKNVFIKIHIMMDIIMMCYTVDKKNKRIKTYIY
NIYNKTQFNCFYIFKYIKHLYEELKNMKKKLEHYITYKNKITQYIVQLNELLFTIYCFCKFFKKYHYFDIKEN
KLSNELIIVKLMIYTDDKNNIDDDRNNHTSQDMLLKGDTSFLYIFIQTYVEMINHVYDSQLNDGHMKDILFLR
YRYMCILNILIKYNLKYGDSKNSVFYFSLIVDMLKEKSNKTNIQFIKNDIQLCNWLFLDKYILEENVIDLYFF
SYINNFMNLKNKKQVEQIIHKKYKNEIDQIKEITNVNNNNFILKILKKYNFNISQSIEYIFMNQLNQDNNQND
EKSDDDNDEDNNDEDNNDEDNNDEDNNNDEDNNNDDDNNNDDDNNNDDNNNAQNVQHTDNIFYKKKMNKILPG
NEKYNNIDKDINEDILDMKNKKLNNVIELLKKRNIIKSTQKKKTHKYKYINNTLDEENKNKILNISESSSNDE
ESSDMSLDNFRNEIINSSKYRKNEVQQDEENEQPANIKKGENTQLYEKNPLSFKSSRNNLNTIKNIKDEHNIQ
SDNKKRYYYKKTVNKGRSHRNNFDKKMSKGMF*
>N.PF14_0210
MNVKVEEGHPYNSYAEIKKRKTKKKKYIQNGRAGNNVEDNVDDNVEDNVGNNVDDNVEDNVGNNVDNNVDDNV
GNNVDDNVDNNVDDNVKNRLSPKNNEKSNPRHKKKNTSKSPICDGRKKDIEFHTLYESDEKNNMVSRRSSVES
IGEEKDMKADVKKNDINYKVKLNTLMLQSYLHKSYDKVSNILKKTSQTFMKKSLSYETEKVDDKTGNNIQNNN
NEKNETHGNINYDQDDFVQNFNKIFNNMIENPTNDLSEEEKKFFEQNSQCINKMLHENASCQKNKKEKILKKY
KKKIIENKDISSLYDKFKELLDERTLLFKLIIYYKNINYNYKQHIDKIQHSYDILISENEQLNLNNITLKKHI
DLFRKGDNIKRDDNEYVK*
>N.PF14_0211
MQNIYKSYLKYYTLAIFCLTFTFDFVSSSCCHSKNDDGVMLPMYFSNNGNIKMLFDIFQVKNRYQFIFCNILC
IIMGFFSVYIKVLKKRLHHNVQKVADGGDGSYVNMSPCQNVNYGFLSFLHYTIDYLLMLIVMTFNPYIFLSIM
TGLSSAYLFYGHLI*
>N.PF14_0787
MGVKFEFFRFFKLNYYAFYVKKEKFYTFLHTTTAYSLVGLTIYLGYTFIHMWNDAVHYSYKHYIRKEKQRKEL
YEKIRNARENGLIPKSEVLN*
>N.PF14_0212
MMGKHRKRKINPPVIPIHPNEEKKKNLNNFITYKDLKIRWRNTSKNYRKKVTIARKWKNLHCLLPMNKSTCMF
VLHNNLPYTRLTKKSYKSQEKNEIVLNESYEEAKDESKEYCEGVKFNNNNKCDNKNDADIYNASLGRRKIYNW
TKKEEDKKEEEEEEKKNKSNNNPCNMNEGNYYEKPPKNLEYLLKKDKNDLFCIFKSNVTNEHKVVIGDIVQTE
RLHRKKAGDVIYFGTILFVGSPNFSIIGKPTIPYCKVKAVIEQITLSKEILSFRYKKVRRSSRFLRIKHWITI
LKIQDIIIELNQHTIHDERKKPLQILDLWANRWLYQKELNFMKFDEYNKPLADQIFNLIEHQPNTLHRRGLNK
CYRYPPDPHVPLTY*
>N.PF14_0213
MKRGKEDIRFGDVVSRYEKYSIYDELNKNTIDKNIIKKISILYDIFKHINNVQNNNTNDFIYTNDIIYCLRYD
NFVRIRFHIISMERNKINNIDKDNYNFIENLNNIISNIYNPSYYDKFLNNKIICDKEKLNIMLKYIREIENAY
IYKYRSDHNIKLTFDVFSHTFYIFLSYDYKKKLSIKNFKNKYNQIENNNIYEYTPAQKMLQFNSLGTFLTTNL
AYQTNYKNPTYNYNSFEHETLERITPFIYESEHIINHLHKKKIYPIKNLEIENDFFIKNKNYEQNMTNLIKLE
KIKDEHKRLKNNIQHNFLF*
>N.PF14_0213a
MDLSFIPKELRGKDIYKILGLHINDCNNENAKNIIRKKYLKAALILHPDKKEVHHEQKEEKKVYAENFATLKS
AYEFLLNENLRKKYNLYLEKKGKVVNNTPVNNTSLKRYLDKKKFLSFNLEKLHLKKKLEEKEKIAQRINENNS
ISKKKKVIINKNKNDNHYNHEQHHLKDIKKQNESFMKKNKSKHFYKKQREIVDQNEKIIEVYLKNYQQNVKLL
QSYIETKNILKFFINFNFKRYSLNFINDEQHDTNLDKNGDNNNKNGDKNDDGNKNDDKVKVGYLIFSHRFETI
RAYLHYKNNMNEINKNFILKLRVPCDQKKNKNCKKKNGNEDEDEKDNIDKIMNEMVNELDKIFTT*
>N.PF14_0214

MNCFSDEKISLASNSSFDYNKVDEENIRRELGSITKDYDYNNVNIDTILNYLNYYKKNITNINILCSIKDITI
CFIKNIQDDIRTFMENDTSNFLNYYLKGFILQNFNVLGSYKYNMYLNNYINIDIGIEYNILNIQEAEHCNYQN
FYIYHNKYINILKSFFYNKNFAQYIKEANIHYNLDLSIGVRPFINLYKSILVINISKQNKYIGSIHLILYPSN
ELSHSLYNVYHELCTSRLKKNSNDKRNVNNKDNIYNKDNIYNDDNIYNNDKRNVNNKDNIYNDDNIYNKDNIH
NNNHGDLYNKELKNKSNGKNSTKNFKENLNKNEKEDKNIAQHTILLNFLSTEIFIVPCNNIINECIKKYKNIR
NAVKLLKLWCINKKLLYTLPTYENNKYIKFKKKEKYKFKNFFYYNNINSFDLTLLCSYVCYTNNLEDTSALDI
FHKTIDFISSININDHFYSYDNGVFKINNKNYDKKSKNELFEKVNNEMNNMNTFLINSTYDIFKNKIYAFIEL
IEESKKAIYYLNKKNYFYLFITNNDSYPLNYEEELFFPFLGNNYILNYNFIIGHIKKTLIKALGDRLDDITYR
LVSYEFMEKKNNKRNKQMSTLKNKYIQPNNTNHYNIHNDCNDNLKSDVHNNKKNKYSLLNTFQDVLINKDAIT
NKIKHDVIKKKMFLGVSFFIKTNNVIRYMDVSKYLYKPEKISTFKKFWKDKVTIRRFENNTIFEVLLWDTPGE
HKQKENSTNEYSSKDINSLEEIKQCDDKKKKKKKKKQLIQNIQDEKKNYNTSNFVFDKLCSELYEDKNSSVH
VQIIKYILNKMKFEEEKDLKKCFYKHIKNMDIQNNNDFFCYEFIKDKTPYIYFANPIFVKDIYFDYYECIWEC
YNEIKSILYNISEPYFTICNINSSNDFLKMTDLGYKKKGIQIIDIIIDIKFNNNIYKNGMQYFKNYEIVKNII
KHKLRNESSKNISNVEQKNFYIDITFKQFIFRLHIFTSIIIQKYLIQITDMDNLNIETVHYLDKIKEYVYKPL
ISSFIYYYIMKFSSFHISLKICKLWCISNGIYCYQDFIENILFYIYRQEFKKHCRMNNFYHQKGLHMNNESLS
KFCEKYKYWDSTNVMGSMDKEVDNTKRNLNNRCDNRCDNRCDNRCDNRCDNTCDNTCDNICANICDNTCDNIC
DNICDNRCDNTCDNTCDNRCDNICANICDNICDNLFDLISDDDNISEEDQFHEKEKKKDGSSILNIEKDASGK
HNNRKNKYSENMKNKSSILNLEKIEKDEDSFLDSFSFSSKNILIKFLRFILNFDWLNNPLIIEYDECDISEEY
KTKLINSFITRKKNNKENKKKFWISSIYDPHCILITLPQQSFDIIMNAARITLQNIKEFHRSYEKQNWISLFF
MNKKNYDIILQFNKPKDSISMLKEEIAKASNLGKQEECVDVTLYDDNNNDDNNNDDNNNDDNNNDDSSNMGDD
QSVDNSVYNMEDQEYSLDYINNIKKNKNVDISNMVHTLKDYELLHVYKTHLQNFIKDLENKFTSDILILYNPL
CFDEVLNINKFKNKMKNKMKNNINKNIHVLNSWAPYIFISLNPKIIKNLDITHNHKNEEYKNELMNKEYIKDI
YNTSLSLNNINLLIYYIKNNVKDLLKTVKFTTI*
>N.PF14_0215
MELNMRLYILLSHLGLAFILMYAFMKYDEFYLAVVYLSTEKFPRTIIYNFFLMVFIVLCKLLLNVFIGELRYL
EVEQLIDNARAFIMDTILFLVLSKPTINGKEVSSIILIKYLSIIVILKAYHLVLYSRVSNIFELGVPRTRVLV
KLFIFMCLLSIANLSLFTYFYKNSLKNSTMYLWLFFECLSIFESCQISICKFFVNVIDLRSPNGLSSKATILF
FLDILHDIMSLIIFLVFILVFVLNNFSNLPLHMTADIIHVVKTLISRFKSFQKYRELTKNIETKFANATEEEL
KEAGTCIICRDDLKEGSKKLSCSHIFHVDCLKSWFIQQQTCPICRTEIKPYAKNEQNKSENDTTQKEKQEEKI
EQPTIQEIITKPNFLINDQHLYLNDTNVMGTLNINQQYYPPINKNINTKEQIKILMDLCNYYRELSLVCLKEI
DKINHSSIPANVMLRNIYGSIHYNILNNMEDDEVQKYLNKISHIPQMKGLKEYIEKVLITDMKYTKDICSSL*
>N.PF14_0216
MTKITISANNEKGENHYNNNNNNNNNNNECKYNNKRRVVDECLEESIENEYNRNIKKTYPSKNHLDENTLPDFL
IKYYEWEELPSFYNLPYGLINKKNEHFFFKNKKECFNEYIKEKNTSLINIKNNDKKDHIHIKNCDNFNLNKGE
NDICVEEKQQEDVCIKNNHDDFVLSLIYDDIVTYFFKVQEKEMKVKGTPNNQERGNNNDQNNHDMRNVEGNIA
KLSYTDQQKILKINFKKEDTITKQEFRNGDCCENAQNCNYCEKGKSCANENKCTTNDKCSGENKHVAKKECSG
ENKHVAKNERSGENKCVAKNECSGENKYTAKNECSGENKYTAKNECSAENKRVAKNDCSGEEVPNEINWMNDF
ISLTNMQILSKNSNKFKEWNNLEIVIPHIINYCCSVRSCICKNSFLTITYICKSLKEEKDILCKFFVYIFPFI
IKKLDIKNNFLNKCVTNAIEEFMLHSSNMNDYEMLRLICSYSNDKNSCISKKMSYFVYLFLKNLPQKELINFR
ISDFASPFLDFINAKLEDTKKYIKQALQILLEYHDEDNIIDNFKVGLEKHEQNNQVHNFFLISKQIRNILRSN
NTNTLRKKYATFHDFKNTKIVKNVNKPKPSSFIF*
>N.PF14_0217
MSIQENQKVLRSLIRTVGRISQFFDEYDMWKCFLSNIYRYENEKCDKYNDFMKYYLNNNTHYCPITSNNKKLR
GELLKYMRRNINDEQVYNIGFTAIRHLGRIKNLLTNVSSNNNNNNNMNIQNNVEKNIMKMVYNKNIWASKEEE
FLFKKNLTMIHFNNNDERRKNSMDRYNDTLYENEKNKKSKKGKKSYGLKKKYTMNRRLHKASFEEISHVRDNI
NMVDNTNDGHIINNDNITNNDDITNNNNITNNNNITNNNNITNNQHNNDALVNNNNFTCNDKSTSHVLFNKIN
LLNRNKNNAFKKSIHKNEIKKGTILISHPLTSNPLWNRAVILITNKEKNNIVCGLILNKHPLYNDYMKMSNNK
EINKILNKIYLKKKKFIQDIIGDYISNTKERNKVKDNINNNIINDENNVRGICNNVNVDILNNHEEQLKEEEK
EKNTMKNKCDTLEDSNILYNEENIKDNIHNEKNLPIKNNISEICNENTYTHSNEQDNVEGEMKYEEKHDISNN
HNIINNSKNNEVLKSKNKKNNNYLLEQKKYEIEKKKDLKYNNQTNNSDDFPTLKDLQEMFLRIQFRSSTKHHM
NKKVKKKNNFFVMMDEHLLDIITHYIIKLNKVPLYLRFLPSYNIGSMTDIKNEIKKLQFLNEFYKIYFEKYNK
WKVVIINNRIHIFDKEKNKNKINALDNNSTETKNNQENKKLTEEEKELIHKKIQKFKETNQLSFDPKEKDKKK
KNKKHKNYNKNNTHYEKSENNTTVNNTNKGICDKIDENETNTQMKHQENFYDSQQEEDQDEEDNDEDEGEDDY
DDEDDDDEDDEDDDDEDDEDDDDEDEEDSYDDDEDEEDSYDEDEQEEDSYDHIDEANDKLVHQNGEYNHAIKE

NNYKTLQNQEIIHNNDTNQKNNKVSSINKINTNKIHANYLLGNPVNLFWLGGPLPGLTILHNMKKYSKNVVVN
DLIYEGYNEGVNESVSENINQNINQNVNESINTNEKINMLNNVSMNNSVNVLNNVNMNDNINIIHNNNVMLND
NSTNNFTCSTMSNMNEEQYNIKHCNIINNNNFNDVHNNMNDGHNNMNKGHKKLIKRFIGKATWDMNQLVDELN
NDYWIAINCDNKELLSNIIFNASNETDSNNINNNFDNSACGNVSSNSYKGECLWEKILASLNNDYEQISKIPQ
TVIDNFLKDFKSIENE*
>N.PF14_0218
MKLKNDENVNFLCGGEDISSLVVDLGFYSCKIGHNQEDTPRIFLNSICGEREMNDEYENYMDGSLKNINYKEL
KYPLNLYNRNENIRIKPLFYKDDNKDDIKLNADVFEKILEYSIEGIEIPKVFECTDDIIMDPIKLGGLNLNFE
EHPILLTEFNMHNHKIREQMTEILFEKYNIPALYFAKKAKLTSFSLGRSTSLVIDIGYNSLDINPVYEGYVLQ
KNSLEYNIGGQYFDKLIYEQLIKDNVNIIPYFVYCNGYDNMHTCFNDNINGSIHNNNHNNIGHNNNIDNINHH
GNSNTYQENSLENSVNPFYNIHHSYKEECILDVIRYMKESVCRTRIINDDSKCEEKKNSIQNITNLNEQNNIH
ISNENNIKDACTKEEFFELPDGYKINIHKYKYDIAEHLFHSIPSLNNFKGLPQSIINCIISTDVDIRKELLQS
IVITGGSSLFPGLIERLHNSLKEKECFTQSNKFKILNMTSLVEQKYSSWLGGSILASLGTFQQMWVSKKEYHE
SGHNIILDRCF*
>N.PF14_0220
MNYDHNNNLMRKKIQVNKIKSPKDFVHFRKAKTVEGNVKIKNHVLFNDTKKNGKEKIKNLSRTYQTIDDRTLN
GSKKNKKKKIHYNNLINYPKKTNLPLEVHTYSDEDNIISEKSEKETEEKIKYIKDKILNKYKDLFETYNNIIN
VLINSNEEIKVELNRETIYNFLKMSYKYADYNYSLACAILLLIDRLNYPDIELNENEKEGLENIFRSYSYHTI
LSYKYAFKAYHHNDKDTNNNNDHKNNHNNNNNCITFQVKKNICKSIRHKIKNQFLLNSEAIINLINCIFYTIK
EERNKFFYTYLNANQYKIISDMTETGVKYKYEQLAERTFKRALDLGMIYLKPTDINFLNLASSYIYFLYFNLG
YEQKALNICIDIFDKSSNILDEIEDKDHAHKCLEILSKMRHHIKRWSKKLNKNSILFLTF*
>N.PF14_0221
MIKKGSLKIKNLSVAKNRVNNVKENGRLINKGNGQEKKEAEKLKRLKMYRTKANLKKMNQKINEVRRIEPSIK
WFNNTRTISQNKLEIFRNKLEETTHDPFSIVIKRSKIPVELLKDEKNVVTKKYKNENFLRIEKYHDIYSKKKK
RKKPKLNVGTLEEYAKRAEQNLSTYETKNDNSLIEKRNEIEKKFSKDQLLKIGQSKRIWAELYKVIDSSDILL
QVLDARDPIGTRCKKLEETLKKDRPNKHMILILNKIDLIPVSVAEKWIKILSKDYPTIAYHANINKPFGKSDL
FNIIRQYTDFFKSQKKKHIHIGLIGYPNVGKSAIINSLKKKVVCISACIPGQTKYWQFIKLTNKIYLIDCPGI
VPYDIEDSEKILRCTMRLEKITNPHFYIDDIFKMVNLSYILKIYKLPEDLTFKNSEEFLEILAKKMGKLLKGG
EPDITSVSKVIINDWIKGKIPYFVNPDKYLTEQDKTQKDEKEDKNETDK*
>N.PF14_0222
MNKSYNNFKTITIKKIHDHNKNKYNYTFNRFLIPLNKLKLTYYKIYQKKTIKNIKQTKHIKKCKNIHKYDEKN
NKSQIKTKSIMKKIHIKKQKMGQIKANTIRAINVQTNNKPILNICTNIPVVTHGILKNKCEGKMKKENDNIKK
KVRINIYSSENYTDKYFLKNRVDHERLIVENEGKTLEGYITNLCRSNNFKKHKNGDENNIDNDEMFYIHNNNN
NNNNNTYYNNYIMNMHEESYYNYDSSYSALDVYMEEYRDTLENSSNIDDISDLQPIHLASKEGNIELIKNFLK
EGIYINSKTKIRKFTPLHLSASKGDIESVKFLIENNADINALSSDNETPLWCASLSNHFDVCKYFIEKGALLN
LNVEKRYDSPLHAASMMGNYEIVKLLIEHGADITCLDLNMLEPVHYASFEGHKGIVKYLIFKQIEQSLKKKMN
DIVYIMKKYNVYTPNLEKHYYLKYKSILKKRITSKILCCAITSGNYKIVNIILKKGADPNYFDVRLQLFPIHA
ASITGNIKIFKSLVKKGANIFMKTICNNLPIDLTEDREIKQFILKCSRKINLRNAWIIRLKKKNSIIARLPYD
TFYYLCTFF*
>N.PF14_0223
MSEVYSIEPKTHGKVIIYTSLGELEIHLFSNECPEACRNFIQHCLNNYYNKNEFFRVIPRFLIQTGDHTNTGL
HNEYAFSSPFKNEYNSRLKFLYTGCLSFANLNIDKPSNGSQFFITLDKAEYLNNKNTLFGKVAKHSIYNLLKF
NNIKTNKHDKPIEDIPYIEYVKIIENPFHHLVPSINYEYNTKNQNVKDKNEYLNKTKKKKEQNLLSFDYDQEI
SDDSETFFRGDKDQTVKQKNVSSYDKFGNVEDNKGEDDKGEDDKGEDDKGEDNKGEDNKGEDNKGEDDKGQDD
KGEDIKGEDIKGEDIKRDEKNNKTTEDKINTLKKRTCDIENSNRKKNKTSSSIKDIINMDEEYLKNMKKYNKM
SKKEREKLSLKKLQDFDNRLKDLFSRENNEESTKHNWLNKSGLKFHIDSSNAYEYEDVKKNMVDTMEREKKNS
YHSKYKTEKYLKQKNEFK*
>N.PF14_0224
MENYNIEDVVMIYGHKAIIESIQNVENVKSEIQPNKIYVGKYVNKKGYSDGTYRKKKIYTPQNDELVILCSYN
SIKPYNEEESACTMIQKMFRGYQGRKSFHSFVCCTVWRKFDHIHEYITLNNHDEIYKPLIKTIKRDIKKGIIQ
FPSKCFSSNSNQFSRVSTTSYESSNYNENVPRLKDKIDRTFATEMFHFFLTSKEIILPYNLVHKVLIKTKKML
EENIKSSVINLDMSKKSKDTKLIILGDVHGQLHDVLWLFNRFGIPSSTNIYIFNGDIADRGENATEIFILLFI
FKLSCNDSVIINRGNHECSYMNEVYGFYNEVLSKYDNTIFDLFQNIFELLGLAVNVQNQIFVVHGGLSRYQDI
TLKEIDELDRKKQEILHPEQYEDIVIFDLLWSDPQKKEGIGGNARGNNCITFGPDVTEMFLKNNNLDILIRSH

QVPKTLKGIESHHEGKCITLFSASNYCNKIKNLGAAIIFNQDLTFEVQEYMSPSLEVIRETFEENQKLREKVL
HCSKIVELEKNEQKNNNKLSTEGLMNDIINCLSTIICNEKNSLWNNLYKQDKDKKGVVHINIWKEELGKLSKA
KKVPWIYLCRKLKMIEDYHVNYNNILSRFKINYAPNEKFLNTEWKNECFEHLYEALLKADLSLRETLMVFDKN
LDGKVSFAEFEQVLRDLNIDLSNEQIRILVRLINSNSLCNNTNLQENDKIDVAEFIGKMRVCYRLSINKDYVN
NEKIQKLIETIGKHILSDSADTANYHYKFYEENNERHNSERRKRSSVIKSVALFQKFKNYDNFGNGYLDYNDF
VKAIKNFDMNKISKEVEFEVDDDILMEXXXXXXXXXXXXXXXXXXXXXXXXXXXXXXXXXXXXXXXXXXX
XXXXXXXXXXXXXXXXXXXXXXXXXXXXXXXXXXXXXXXXXXXXXXXXXIYIYIYIYVFFFFFFFFFFLY
ISS*PNR*T*QNQNKPLTDEQIDLLAYTVETDDKVDYVEFFNSFKPTYIYSNN*
>N.PF14_0225
MSRIIDELKLFLTNLSKGSDTVDTNDLMDSGIADKTVILNIKEKLGDSPWNVQNLINLGIDVIEENNNKGDTY
DDNNNNYDNNNNNYDNNNNNYDNNNNNYDNNNNNYDNNNNNYDYNDDYELEVKHDSSDNIISDVEWEKICSDY
EKFTSGNVEDIIHKDVHDIMKYEKKKNDNNNDINNDINNDINNDINNDINNDINNDINNDINNDYNNDYNNDY
NNDYNNDYNNNDYYNNHRGYCDKDNNNNIYHDYNIININDDENFYVKYVKYKKCKAIEELLKNMVSDKKMFSN
YPIIKEFILLLIFLFQRIRAENEYKNVDNINNIKNGDGTNFSCLEKKKNKKIKLTKLRSNSCMTQKIENTKNS
YDMKNVGKEIKHINNIMSDETCFPVYNNLKLKEDHVHKYNTSDHDHMLLKYIDKEEKENMRYCMNRDSCFNYS
SNDEKERIYKYHNNDNIKNNKNYKMPHNMSHNKFYKIDRYLEEPFCTFPIYNNIFSNLTHKKKSMYPYNIDDI
KHALNNENINKVCETLFKYMHYIRRSKNEEMKKRSINMIIILLYLTYISFGIYFLNTYNIYIRLYYLYKEFQI
FKKTNNDIQKKLNKKIQQHLHQHKLSEQYTQEYDKLKTSFLHLKKENEKLKNYNKVIQKLKNKIDIMQIEKTI
IMKKKEELDDLIKQKNILLNQNNITNKTNKNKTFNNIIKKTSPVINKKDYLKNNTLQSKNTKIYFKNKHIQLS
QGLNTPIESTSQNNSTHETYSSDERKNIKINCSKINDEQINIKHNEITNKWYYTSTENDDKNILTYSNICKNE
QTLQSINNHYEENIKQMVTRKKNQNNIKKIYTNAYTQYDIKTNTTECANCINNIFKNNKLTPKQETQTYIYNN
NNNNNINMYILKNMNSTIIHTTFYQNKFNKITGILNYDDNYFMPLYIFYMKKINYKELIKNYFLYNKYFYIND
LFQLKINTNYDDHHIISKNRNHPNGYHHLYYYDYYLYYLYYYDCMVSQHIFQNINGQEKQITLKNEQNKNTYK
DFNIKNKKEFILFENKINMLHITKPMFNIKYINNILNLYDYDKVFQEYLSLTNSCYNNVHDLLPMFHTHYHYY
YDYSIYSEKKKCSQEIIYLYNKMDKINKNKVFIEKEKNICIKNKKNYKLPNINQCYQKSTFNIYFNLLKQTQH
IRKEIYISNVKLCNIVSSDDNIIIINNKNIHKHKQTTNNKYNHNNYNNNIYYYSYFKLHQLFLKKKNIHFPTD
KYKTHLYCSHFGYIYKTIISFKKKYKQYLLFNKYINNLNKFKHKYLIMNTYKIKKKKKTKRKRKRKRKRKTHI
HYIPYKNNLNTKNSISNKLSKYKKKIYKDKIVNTNFSYHNILIKSLYIFNLIFFLTFLLLLDVYILDHTLLSG
LIYILCILLFLLTSLFRSIVYLINIPLNKKIIPQHFLDTFSVHFVKIVNYMQLYFYENNDDI*
>N.PF14_0226
MSVPNNSFFVVKSNEQIPGVIKNYAEERKKKKKKMEQEKYSTILVGGYSKKVVEKDNDIYKENNICRSNNIYK
ENNIYDNNHVYNHSNKNEKKKKMVRNSFISEKEKLNILENLRRINITKDEDISEISPKKNNLFPYHSFYINHM
NKNEIQYCDINKDNMKHVHSCHNKQQEKNMKDMKDMKDMKNMKNMKNMKNMKDMKDMKDMKNMKDMKNMKDMK
NMKNMKDMKDMKNMKDMKDMKNMKDMKNMKDIQNNNKINIMYKNYNNNNVIEKEEYINNENLFFKEDILNDQK
DNVQNKEYIKKKKMNIIYEKKIYDDNLNKKKQISNKYMCNTEVMYNNKKDHIKKLSPHNNNNKYIKTFSEKDT
FYSTKTQHDSKKNKNYWSHMNLKRSEKKTLKEKKNEMNVSTLKEKKNEMNVSTLKEKKNEMNVWTLKEKKNET
YISTLKDINKGNQRGYFKTEENKIYKNNKNNKNDKNDKTHINNIDNIDNINNIDNVNMRDHFYNHHKKDNINN
SSSTQYGNIIKKQNCYTNNIFKLNNSSFLKNNKDKYSDDNIFLSNDFINSSNIFINNYSSNNSLKLTVSSSSS
NIISSVSSYETPQKEKKKTKKKKNGNTLDIIKETGNHNNLHQEIEYNKNHSEDIPKINSIGKDMYCEEYCMDK
NEYIKKETTYNNNNNNNKNINHNNNNNKNINHNNNHNNNHNNNHNNNHNNNHNNNHNHNNDNYFPISNNLTTY
IYKNNMNKDIYNTCNKENKNIPIIKLDISDNIIKYTKVVKKLKRNILCIINKFIQTNNGKKKNPHFYTHKHFI
KFINKIKKILKNNKTKNDIFYKQSKNKKRNIYHLFLEYNQNVLLVQKYLKKKKILIKYINQLFEKHVLPSYNS
SNENGINQAIFEQMKKEKKLFLQNIFTLKKKSLNKNTDILQFLPKNKKQEINYTNHKDENINKTNDICPKPNV
CEHFSYFLNNKNEQEEHKKHSSSLKQINRYYSEKHKKNYNDYKKNYHKNGKQNYWKKYPRRSSSLVYSNAKKS
ININNNDNNNNDDNNDNNNDNNNDNNNFINSYANSYFSKNTKLINTLSDEKKKKKFPHKQTNSNRQINIHNEK
KGNQINTYEEDKKKNTLIVETIQKVVGLMYNENDKMASQTSQTNIKDNFKNEMNTTKMLEKLSNVNMSSEKLS
NVNMSGEKLSNVNMSGEKLSNINMSGEKLSNVNMSGEKLSNVNMSGEKLSNVNMSGEKLSNVNMSGEKLPNIC
STNEVQIDHLELNKIRKKKVVSHDNLNNISSRYTSINKSIVENNKLYHNLIDHQDRCVNLKNQDDLYCPYNNV
NAANNNNNNNIMNFSNIDNIKINIINNSIQDNLNEKNYYIINENGKNYKGMNINNMDIQQTSFNKHNEHLINN
KIKIIKNNKKFLCDTDTLFYENNVRPVKIIQKRGNNNNIINKIKKHIYKNYNNALVFLKKQIYKTNWLIHKNK
KKLWESPYVHRFQQKIKYHLANFKSMSHSFSSNIKQNYFNNIQPNKERIHKINHFICNGPYINSKVCHLSPPR
VTNVGSPIIFRRRKI*
>N.PF14_0227

MGNALNKLLKHYRNVEKKKNEYKFGKILGCGSFGVVRECINKMTKEVYAVKIIKKKKKHKKSYNFEKMVKNEI
KYLSIMSHENIIKFKDFFEDKNKFYIVLEKCEGGELFYKVVKNKCLMESESALIVRQICCALQYLHSNNIIHR
DIKAENFLFKNKNTKNIKLIDFGMAKRVNCEYLTELCGSPHYISPELIRKKYTMSSDIWALGVMVFFMLTGKY
PFEGKNTPKVVDEILNKNINWKGKEFSSLSIEAVDFLKRLLERNEKKRLTAYQALHHPWITSQVG*
>N.PF14_0228
MNKQSDSKPYTKSKSKFHMFFYKNKYSSSKYNNNNVNENNVNENNVIENNVIENNVIDNNMKGNNVNDHKVND
HKVNDHNVNNHNMNDHNMNNVTQGGHHEKGDDINSVNLNVFPQNCNSNDKNKKNTTLNNHNNHHLNSSGNVPY
GKKKNSTNPRSSVILKNGEHNVLSTYNEDMYNNIGKPNEEMNSTCDINMNNYNNVNIDEEYNNVGTGSYNKYH
DPNNNNNNNNNSSSSNIHHSVSHVSNYNQHNQGYNKNVVVPYNDIGNKNEEKHNTNEDYFNFGKDMGNPSNNQ
HAMNNVYRNYKNYNNVDHTNESYLHMSKNSNVHYNNYQSYYNNHNVQKYGEIHDNLLNSNDQGQKKISTYFNS
TQGYNNNNNNNNNNNNISNHNNNYYSTHANITQFNADMNNSNNQNNTTAPCSNEHMSSNNNNNNNNNKNKNSN
MLDDLYNMEDNKNVYNNGTRKDSILNNMINHAGDKNKKHENKIQKSGEQNKRDERKEDTYKKYEKRDNMPMNS
SSNNNININNINVNNNNNNNYNNNKKDDKRIDSRGSHNKSNNSNYMKGMENCNMNIKNVDIKNDNIKYDNTDD
DNYNYNNINNTSNDESEHMDDVCNMNNMKDINCKNLNSSNGNAYNISYMFIYQYYYVLHTKKEMMYNFYSEKA
ILLVSFNDESKIINTKVHNNSAHIMNNENYNTDKQTHVDDIKMIKNKNNNNNNNNNNNNNNNNNNNNDDDDVD
NNTCRNQFFNLNEINIEGCHKSDNMIKLESRNMIYEYYKNLDVNECTVHIISIEVVNLHDEIYIYIYGKIKKS
KHTNVYYYYFIQNIHLHKYNVYQYYVDVFVHYYNAPVNKETQAQLLQSKSVREAGQHNMKNNEDDKKKKKNKV
SYNLNTTNENTTTNITTTQNKMNEKKKKKNETQLLGEHTNVSTRNIQNRYKDKQSVLNNEKKELRNEEILYYE
DEHEDNLYYDDANEEMLYYNNNDHNKIECDDDDVYYEDDDDDDHDHHDHDHYHDNDNEDIYYEEENDDIYYDE
YHDEYNQMLDNQNEVAEIYKHNNNMNDIKKNKGNEQYKNQKKNKLHLTKNNMNDQNGNNNNNNNNILKDDHHV
ISNEVNKTKHHKNINEEKEELSPEVIKGSELYTNDKQKKKDETNKDIYKNNDKKNIPRNSKNNNENYHDHNNC
DDRKMLTQQDQKQMNNSYEDNVEGQNGQEKILNMNNNEKKGKKNKHEKNVQKKNTHEHEQKSTEVNTVLKKEQ
NNSNDILSKKKKKEKKKVDQDSWVSRVMKNNTTNIIPENNKNDDNNKGDNKKNDHLLNNNNNKNNNNINNDNN
NNNNNNNKGDDQVKKDKEIKEYDELKQDDNQEYITEENNKKENEEINVNDKKIIIHNIHKSMDKKKINDCIID
RLKKYNDGHAVQVDIYQRSFKKAYQTNFNYKNTYDNNKNQTNDYSYAIAELDCRQSQQLLLDLGLYCNGIKLN
IEHFKEKKKFIDNKKFTKKFSTYSNNNNNNNNNNNNNNNNNNGSVSFKTKDDKYKNSYNNRPSTSIHSATVVS
SGGNNNTSSSFKNKKNNVFVK*
>N.PF14_0229
MDLFKRESVNLNLNYEITFSCCIKDILYVGSKDGIIYEYELIKEDPRCDDIKKEYMKNERSPNYNNLTDLNSL
KGSLKVKPVGSHLIKKNKIINNIIIVEEKENIFIMLILVDDILYYGKNNNLEVLNIIYKNVLLYTINENNINE
LLIYTKKKKLYIYKYENGLYKYFKEITNPLNDLISCLVWINDSLFLTINKEYYFLNIKNNQKILLYSHEYEQT
YKKITLINMNEIFIVCDLNIGVFYDVDTSMPSRKNTIILSPNIKQIISFRFFLFCLNFKGIINIYNIKNQQHI
QEITLNYIPDIVINYTNITKYSTFLCLFNDDDQDNGEKILSTQKNKNKWTSSDIGIKFYEEKIKKNRNLLKME
DEGILSKSTMENDFFSGFSPKGIYDDDDDNNNNNNNSNILFNKYLYENNEMLTKKNRNLYNSLYNNLYFINKD
CLQILTCLEIYQYLPQCIEQGNTKKGFVLIDNFVCESKKEKYDIFCEYNKACAYYFFKNLNFSISFIFFQKVN
INIFFLLFFWIDYLPSSHKNILQNMIENKRIEENINKQFKCFVPFPCSIKELIDRNYENYIKDKPDYYISNNM
FDDFYNEDDDNDKNSLIYSSLQRDIEIKKHLLHTANKCLVKFLLIKRDHFLQNKDMYKLSYKKENDDSIHVNN
NIHIIDELIDTLLIKLLTINNYNKQFFHFIMQTSKLHVDLEECVQFLKKEGKFVEIIFLYIRLGYFEEAIEIC
SYFFHYYNEKYNVIQSTANIGVYKYEIGQEGNNEKKTKNDDKKKKKSDDEKKKKSDDEKKKKSDDEKKKKSDD
EKKKKSDDKKNDDDNNNNSDNNFCNNNIYNNNIYNDVRKKVEENNQYSFFNIKELNKEKYLTWIKIFEKENAH
KNNVLDKKNNNFQYSLEKIYNILIIILNDHIHLLNVHQEKIKKLFEYAFPFLIKYNEHFFFHFLKKKNILISPE
EIITIFKKMIQIDKKNDKIKYYLQKYIIHYLKHDKYNKNINTTLIELYINNVQTKNNKTHFQKKLITFMLYEY
PIHINYLTGIIKNTSFYLFKVLLYAKLHRHYESLQILADQNLRVCEKYCLYHNFILKKEVSKIKEEKYQELYN
GIYNNDKNIYYNIFQKFIDRQINSAEKKNWNKTVNNKTKNTGFIKYILNEYHKYIYFSMNKNIPSKFLFHKKN
ETLNKAYVQEMDQRNIIKKNKKIAQENKNILVQNEYDYMLHLIKDHQEDEEVDNENDQLDDDILNYYETCSDE
SGECYTSDIICDATISSDDHKNYDNKSDMITSDNSTSSNNKTKKIIKTSAQDKLLKGKHRNKDPDPLAHFHVY
NNCKKNTGGLFFLLIKVYLDKYYNEEMNLMKKKQYKNNILYILNKYANHNDLDNIYIFNIIPKYWNISEISQF
INFNLKKKRNTHMNLQIYHNLIKSNYLNVSYEKIKKKEQKIIIKDKIFCKVCNNPILEKSFAFFSENITVHIH
CIEKYDE*
>N.PF14_0230
MAYVKVVKNKAYFKRYQVKYRRRREGKTDYRARKALILQDKNKYNAQKLRFVVRKTNSQVICQIACAHIEGDK
ILAEAKSKELIRYGIPVGLKNYAAAYATGLLCARRFLKSLNLDTQFLGVEKVTGEIENNEDKEDEEGRKPIKA
FLDVGITRTTTGNRVFAALKGACDGGLNIPHGNNRFPGSKNEFNPEQLRKNILGIHVAEYMKTLQEEDNDKYK

THFNDYLKNNIGADDIEQMYLNAHEKIRQNPEKESKDTKNVKKFVSKHEKPKKLNAKLRKKRVKEKLALYVEK
LQ*
>N.PF14_0231
MAKDKKQQKKPSKKTPAPCPLSTKVTVKKEIKKEKKTGIKDNPLIFEKKKRSNIIGVGVRPKKDLSKYVKWPR
YIRIQRKKKILLQRLKVPPSINQFNHTLPKSQTQDLLNFLKAYKPESKTDKKQRLLNKAKEALNKNITKDKKP
LFLKYGINHITKLVENKKANLVVIANDVSPIELVLFLPALCRLKEVPYCIVKDKATLGKLVHKKTATAVCLES
VKKEDQEKLDYFAKVCKENFNDNVDLRRKWGGQKMSAKSMLLKKMKDKARKIEEAKKKEISAKL*
>N.PF14_0232
MHVNCKKKKTCSYFIKRYINTPRDSDIASSLPSEKEKKFNEEKSVHKENASEGENILACEEERQYFKNHKYVD
FFRDESRSIGLITFKNMNEKKNIFYNFLEELKNVLEHVNNIISNEENNTFYIKEFKNKENYLIKNIKNCIPYY
DNKLKILIIHSVMDNNIFLNSLDYNSYLKNDEDTNVEISNIFRFLCNTIQQLPIITISNINGLCLNSGIDLIL
STDFKISKENSLFGFDKMHIGLYPYGGSCQKLFRHIPLNYAKYLLLTNKIINAQDALKINLIDICIKQNENFF
IQNSNIYFEQNLSNEQIFSIIKENILNYFKDIFHHHLFQKKINDDSFIFTLFYSFQFLFIPTYILQNIKVGIN
EGLNFNDVNSYLDYDRHMFEKCINSIPRLDILNYIKKKKNQ*
>N.PF14_0233
MARGEHNESINEKEIQNFFEEIDNLVNEKEKEDDNYMDVEKQLHEKCLKLINKKKETCNLCIKSKIKLFLDEI
NFINNSLDIFENEKRQKQIKQENKKIQKNGTKELDEILKDVIFLELGLKESKSKNETNHLLLKISENLHKIED
KEFLLTTKKSLDIRIDDFQKKQICEEYFAEKIKEIYENTVDKLSSQHNEKENQLKENLRDSTKRKVENLIVEN
NVLSSRIISKDTDEGKKHIIGIKHENDNVKDEKSEEIKPMEKKTSKKNKKEIRIDSSNYFHKKKLKLVERWNR
VAQESKLLSDYDSNS*
>N.PF14_0234
MNKNWIKNLENIIDKNKIFEWDEKKNEKLKDVCKNFIKKDYSIHHYVTNPIIIDNYKKEKIKELYEWQDECLR
ELRKVNWEKGENFICVAPTSGGKTLVTELFIFEEIKNGKKQIFFLFPLKSLINEKMTYLKNICKGTNIRIGVE
IEENDIILCTYEKFNNYLNKRKLYDDNKSDKYNIKSNISSNCTNNNNNNNNLENLKNDNIIVIIDEFHHINEK
GRGIYIENIVSKILYMNKKRSRIKIICMSGTLNNISILKKWMKAKFYISTYRPQEIKEHYVCNFNVYKKVQDG
KFYNTCNVFSFNDTCYDKNVQKNESASFQNQKFYETNMMNTSECSLQYLNKDNIYNNNNNIEMVGMNSSHVFM
AGGQEERKQCSGHNKRSSYNRGRSYSYEPNNNNNNKKRINCNHSSKYSSSNNNTVSYSYFSSIEESDTINNYN
SLNSMNSMNNVCPSILNNNRSYSNYNNMHSSNISLTYSCNKNNYPNNMKSSAHNNMHHNINHQENYRNQNSYS
HFSGHGSYIDNQHVNNNDKCNYEGILNNKNQINNPIYSCDNDSISNIKDSQYLSQNRYVHTEKKDSLDDHINY
ILKEKEKLICETNSKDSIMCFLNKKNQNKNKYNTLNTSLINSLLYFALHSYINNLNTLIFCCTKKMCEFYINL
INEYLNTLKGLPIPQYIDMKRKELKEKIYNIDKCIYEKMHKLISNGLCYYYSDISHSVKRLLEVAYKEKTLFL
LTCTSTLSVGLNLFVDRVIISSPFVAQNFLTYTQYKQMIGRAARLKKGDSFIFVDKEYEKKMLNIFKETFTNI
TSTMHANTFEELEKYVIEFLCLYNENEYISFYDVIHMFSFSLYYTETILNTLKQEMNSSPSIYTYKEGKDQKH
APTISVKKILDSLHRDSTSTEETATKRKNHNKEDNYMGGNIYGDNNKNDDKCDDKCDDKYVENCYTLYNNNQR
GFIHGENVTPINKTEEVKKEYKNEILKENINTSYSFYFNINLNYFTCDEILFYHNKKNEIYKVLDNLLKHKCI
EIHNEKIKITNFCRSLCISNLNLSIGIDLINEIRMYDKIYLYNKFHLCYICSSYNINIVSFNYELSHIKNVLS
LISDNYTKNIIFQTLKFDSDLINMLNLNSQHNQKKKTYFSNSQLERKYSKLYLSILLFLYLNDEDIQIICSIY
KITPYVLKSIVKHTYMYINILISFFDKLNEWILVCLLKKFLQNFKSSKQLSIYAGKFKKGKSYDQMK*
>N.PF14_0235
MRRFNTSQNKNERNLFNDYIKNEDELYDERDDEGINKEPISLKADVNNNFFPIDQIHSTYFLCNNLNNDNTEK
EINEHISFEKIQDHEKEEKKKIFLFNLSDNQKKQIENLKINKIIQNEMYLKKNKILKYIIHIFISDILKEKPN
DVYDYASNYFTQPQFKMYILEKLKLRINK*
>N.PF14_0236
MNEELNKMINSFQIKEKEGKEVNKNNNIEKNQNIDLNIYPNMSNYVDIGSNIYVEQIKNISKEEVTKKKSILN
SKYISSKNNEFVVAQLYELNNYNENNIYEDRNLFSNSTNIYSNDNNMKKYLIQKCGKKNIKKRMDILNQENNN
MGIHKNIVYDDNNNNKNVTYDDNNKNVTYDNNNKNVTYDNNNKNVTYDNNNKNVTYDNNNNNSCSIIKYELRK
TSICKYWIKGICANVECNFAHGEHELKYTFGVYKTTICKHWKKNGMCSSGIHCRHAHGESELQPKNLPLHLLK
KKNNLKNKNQTKSFHTNKELTINEYNDRSANNRNVEHMYKNKVDPLKNNNNNNDNIYYYGNEENQKDVNIFRM
DTFYNNIFDSRNHMDKPPPHNINNNNSNNNNNNNIVSVEGKPINHNTPNILNDGNYTNHLNHSNYIYNNEKEE
NEKRNFNYYDTCKNIWNYQICKDDNNLLNNNEKTFFFFSNVNNNKMVECNNMNNIFNDIHKKENTITLNNNSN
NVINIKKNIIDDADISKVTNVHIYKDDHLKNTPINNKKKETRLSQGKKNTYLKVNFFNNKNKDNNYNNNIIVD
TNNNNNNNNVIKNDHNKINNNNLIFQNSRFMDHTGACDTIKSGDTTKSGDHIKSGDHIKSGDHIKSGDTIKNV
ENFVNYTNSNNISNINISINCNNYEKYINNMSFINNKESSNINKDDVYNGNMDNHNHHVNNNNTLCNTSLSDL
CSNNSSESKKQEAVCLNKNDTHDIIKNVSNNMKRFSLYMNPINNNNNNNNNDDTSNNVQFINNYTNDYFYYDE

KKDEEQHNPYDNKNNKIKGFRNINIRIIKKEDEQEHTNEKNNTIFNKNVNEIMYSKEITNMNNINRSSDEYIT
NNNMDNDNNIMNNTLYPWKENKFKNVDMLNIYKINKDDYLHTDIVKNIDCVISPYKDPNIIMDRINDDNNINM
DNLLFTYNEQMNNHHNNKKWNVFNNSIILEKNEKITNSKKKNNYKIHQRQNINKNVSDNNENINNKNVISKDK
FKIINSYIDYKLNYHKNNKYSYNNMEHNIKNVNEQSSINNNNNNNNNNILYTTTKDLRNNIHTINFNDTKNII
NSDDYFVDHNYNYNYNYNYNYNYNYNYNYAYDNIELSNKNMKDVINLYTYVVNKKNEKNIYTSTNNIICNDEYIK
KEDCGDCQMVESTQMFDEEINCSPENKSNNNNNINSNNININSSSSSNNNNNNNNYYYNDYHDDDNNNIMNHS
YYNHINDSYYYQFNDLHSKENQQKYTYNINNLIHNMKLLNTEYESPLNSEQEKTILKNIAVDRNNNININNIT
LPTLQDNQMNNYKKYTNDLGSVSEGYTSTYNDTLKMHSETFMDSQNGMYILPQYVTRECINSPYDSSLFTDEN
REEKKDNKEREIIGNMLYDEHICMDDEDLFGRSHLFNIFNNEEEIDINQKDNYYDRDDHNDYHRDDHNDYDRD
DHNDYHRDDHNNYHRDDHNNHHRDDNNNHHRDDHNNHHRDDHNNYHRDDHNNHHRDDNNNHHRDDHNNHHGDD
VIYEETKKTDNIEIPLKDNDIMINNSYNDSLINYNKYFVKEHEYNNINNNNKIEENLKIKNSYDTSSKQNYKE
NNMFHDVDNFTSLLLHINNYNEKDFMNFKNEDYTLNKEIYFNECKYVKEIKNIDQDNTKELGIVLQNDDQISE
SDMRTKKMIYSIFIKEEETKKNKNLENICYTNEQEKYNNLSIINQKQNITMDIIKNVDELSFDNMEQMNIKIN
DNQMYNEQVMDNMEDRIEKINILTNDNIQNGIHNNNNNIIEEKQSLKDNNYEEKKKKKGIQ*
>N.PF14_0237
MPAKSKKDIQEEKCDRIKEDNKNENDSNNNLFLKEEKEIYTYKKLDEQNKEKECNDNKDQEINNKKKKINDNK
KEDMDKQNTTQNEEKKDEDSVFFKRIINVEITPNIKNFIELFLLSREDILNWCSKNNSMYSYKINEKIWNIIF
SDSKNTLENFLKEKSINIDMLSVLVYIYNLYCTNNNKDKINFQDIMNSSCITYRGIYENIDENSKTFKNLLRE
EREYKRIVGDTNNQTNDFFSNYKKSYPYGINLIIGIFLTFLGGYYGSLILGYTKFTTRLICGILFSYVTLILE
VIIFIIINEKIENVKKNQKKMGSNYELYERVHIKDDNEDKKKKKIMENVELIEETTKPSTVKQRKKT*
>N.PF14_0238
MNNDIKNELINNLPFKFSDISLNSFEKKKEKDKDYIPDEDGGHTWGDFSRDKIGNVKKKEKQKKKKDNEDYSD
DNNDNNNNYSSDHNNNNNNSSSDHNNNKRLVLLKEKLKYNHYYYAGKLAEKEWEENYDNLKKKSQLFKDVLEK
NKGKNFSTFNITKNEKICNIKEKAQKKKQNKNQKNLKKKNFKKEHNDISFNDTYTKYSSSLNDFNDISDSLNL
KNDIINNEEEYNLTNSLFQSFPMEQNLPLFKYKKENKQDYDNNVKCREYDEKKVPKGIIHEIIEGEEEEGENK
KKKKKKKKKNTLDIVHNVINKKISYDDNVNIPYFEDDDNKTNILGENMSNIFEEDKELDLFSSFKDIFNNHKK
KIKLIDYNKILNKSLYDNVENVDSLIAQEKRKKGKKEERKPQKKKKKKRNNDQNSAHNEENMKQIKNNDNFNN
MQSLNEYNEKVRGNFFEKKKTENDVKEIIKNIKMRLGFYSNEESDSHDIKCLENDDDNDDDDNNDDNDDNNDD
NDDNNDDDDNNNDGDDDNNNNNDDDNNKNDIYNNLIINEEEEKQNLENLKDNINIPYTSKELYTYKEYSKKLD
CNDLNKLSDEKETTKNISNDLELIKKEKKKKNDDTFFFISHNEKDKNYLFPYDHKGDDNYNDEDKNYLFTYDH
ICDDNNNDDDKNNLFPYDHICDDNYNDEDKNNLLPCDHICDDNYNDEDLFSLSMSSITFDKRKGIKNMECKKS
IECVDDKNKLSINYDENNNKNILSYNINVQDENDKLYKNEQKIKQDIYNDNENXXXXXXXXXXXXXXXXXXXX
XXXXXXXXXXXXXXXXXXXXXXXXXXXXXXXXXXXXXXXXXXXXXXXXXXXXXXXXXXXXXXXXXXXXXXXXX
XXXXXXXXKIKQDIYNDNENDKLYKNEQKIKQDIYNDNENDMLINSMSPENTKYIKNIKENDKLLKNVMKKEE
IDKKEKSNICICKYIDKYDMKNLIDEIKKKKKKNDVENSNNSYSEDDVCFYELYKFICSNNIEGNNENIFDDT
NINVENVSSNIDNYMQSIFNNQIKYLDNLISQIFS*
>N.PF14_0239
MEEQNEEIEKTVEESEEINEEVYEELFLFASSDNDLVKKESLRIILSLLEEKEFIDHIIKNNKKYLKTLISSL
NSRCKLLTLECLLNLSAQLPKELTERNLIEILFDMMKEEEKIEDNCIDLYIMIISNLTRCKEGVYKVLDINND
SNINIKEDNFKVSFFLNKLLYFFFLPIKPSINKNLSDKYIYVSHVLINISSIKESIVFFKNVAFLNKISDQIL
NVERFRAILPFIINLCLNEVIHPYIFHDDCYLFPYVLSYLYTNDYNITKSGSYNNSNNNEEINTQNIHHIIMN
KSSILVSCSVIKSRILIILFYLCNRDYSREKLLSYGISDILKNWKSCEKNAEFINDIENVTNKLIEGSNSREA
IAT*
>N.PF14_0240
MGGKSRGKRSGTRYKFSKKFRKHGECTANKYLEKLNYGDYVDIVCDSTQQKGMPFNYYHGKTGKIFHITKRGV
GVLVNKRVKHRIEQKKVCVRIEHVRKSRCNEDFLLRKIKNAELIKEAKLKNEHINIKRKTEGPKPAAMIKVPP
SKIITIEPLPFYEEY*
>N.PF14_0241
MEKISPEILAARAKLKEKMGGNLRQIGGKGSARRKIKKVHKNSISNEKKINIILKKIGASYFGDVDEICVYRT
GDTFLEFKKPKLCASLQSNTYIVTGKFNEHKIDINKLFEGLKGNKNLDMNLLEKIKNDPNIKNILNKESGDTP
KREEDEQEANDVPDLVENFEEVSKE*
>N.PF14_0242
MTNKFNKINKNNYETREAVYYDKGNRINSNKEKEMKEFKEEELKEFFNKWNPFYKEKLNSEIEKKNFINFDGE
KKMENGNNEYFNSYNYIHIHEDMIKDEVRTRTYYDSIRKNEHLIKDKIVLDVGCGTGILSFFAATHGAKHVYS

IEKSDIIYTAIKIRDENNLTDKVTFLKGLAEEIELPVDKVDIIISEWMGYCLLYENMLDTVLYCRDKWLKEGG
LIFPDKAHMYIAGIEDSLYREEKFDFWKNCYDLNFSSVLPIIKEEVVIDYVDRNFVVTDTCCILTLDLNTCTP
DQLSFVSPFQLKMIRKDYLHALVIWFDISFSACHTEVNFTTGPYGAHTHWKQIVLYTDHIITAERNETLKGIF
ALKRNQKNKRHLDMKLHYIFDGVHTKAKSTQLFNIS*
>N.PF14_0243a
MIDYYYCYSKNKSDIGKKCLFKKSKSQIIGRIEANEELKKKYIYKENIYYNDDNIKKYSEQYVNIESLKLENK
FFYHNEGGWTKDVDISEQQNKIKYIKRLDKDINLINSLKILMNETTKILNKNNSINIYEEYFKNDLQNEENFK
IKSILVIKDTMRKYQRFITSIKWSTDNTYKLAISYCVNDYQKILHNSPKNCLLYNLNQINNPYQTLYSKTFIK
CMRFNHKNSDLLLAGSYDGTVNLWDLRKKNTLCESSSINVSHKNTVHDIIWLQTKTNNHCLSISSDGLSLIWD
IRNFAEPIESFYLNKDPSEICELVETANNELHDLSKISNDINNNNNNNNNILYNNNTNINTMNKSLHNNTTAT
KNLYYSPNCLEWNLEAGPSKILIGTDEGYILSLSKRQAKNLELLQKYGASNEKHLSSITSIKRIPINLKYFLS
TDKWGFNIWSEDIKFPIISNYYNECVINKGLWIYDSLFFMIIRKDGYLDFWNLLYNFNQPIIKHKICNCSITE
IDSHANNKYVAIGNEMGDIHILKLGDSFCKNSSEEKNALDELFERESKREKNLEYIRKQLNCVKKTKEYLNVQ
DIEILEDVLRETEREYESIA*
>N.PF14_0243b
MSRIIYSDEKKTKGKYIYANGNVYEGEFKNEKFHGEGMLIFKDQGKYKGKWENGKFVDGKYYFSDGLQYEDNW
TYLIDSPYFYNEKINNNEIIYTEEKKNSYLDDIYDIGDGYCDIDKQIVYDFKDNKEIRKINEREKNWIKNHCS
KY*
>N.PF14_0244
MDLKGWISKKSSNVSYEKIKYEFSNVKYSVDHGRLEILHGIKGILLPKTITVIMGPSGSGKTTLLNILSMKVT
EGVEGDFLINDMSRMKNIKRHMGYVLQDDYFFSRLTVYETIEFTAKLKLDIRDKKKLNELVHSVLDIMSLTHV
KDTIVGDAFIRGISGGQRKRLSIANEILSNPPLLLMDEPTSGLDSSSALSLVECIQRIAQTSNTTIISSLHQP
SSQVFSKFDRLIAITNGYIIYHGKTTDLNKYLKKVGFICPYGWNVADYLMDILSNKKFEAILIDNYNKYLHFD
SDVGYYMNVKAIDSKIIHDDYDSILEKTNQSISKNSQTNNKSSTNLSSINKSPILSTQENKTREHDILKFQAV
ELLISLQENKTPYIRQNFFLLIRGLKKIITDEITIIKVIDLVVILTLFGVLWLHTFKEETEEGIIDTIGAIFF
ILSYWTFYPAYLSLYSFPSEREVIAKERNMKTFQVSNYFLSKLLAEFIYFFMIIAFWILVTHLILYGTLKIGI
YLSYAFITLLNALISSSLGYFISTLFDNFSKAVSFLSVVLLTMTLTNGFYVEISKLDVPFRYLQWLSYQTYSA
SVLAKIKFDDALIKCSPDNNSLPCKNNGFFPGEIIVQKRFATLHISISILILICFYSVIKTFTYVSLRWSQAL
KMK*
>N.PF14_0245
MMYFKKDKKLFDEKLNEELTQLKDYRIIHDVLVSLKDEKRKKQKKKRGSLFNLIELKYINKILYELYISQKKK
CIRNFVNIYDHISLHCNIQKIIQIEKGKKYVALVRLGQNVHMNGSLHFLKDKNNNFFIFHKINYYMNKQLFEI
KNDLRHVSTSSCIVIFYNESSYSRRKKYMNCNYEKDECSNISYKTYYKKCMDEKNNILTYMKENQLNGINKNN
KEKKNIYICSSKNKENNNYTYDNLIHSDNHTFNSVPLLYNSFIKNNDKQVKKNEDTKKFVDHYMLFINYKNKS
ISHMSFIIKFKQVLYELLHNEENRHTFIINQNNNNVYMNHTKDNTNGFEKCPFGNNNMKNSLVYDMNLRDVVN
FIRDRKKYNSYNIFPVDYLKYIFNNKKKSKCLKEGGLKQSLESIASLLLPRGVGQNINENIYKHINKNIYKSK
IKKGSNINCNENMNHNNTICNYEIYIYHSDILKIEKNIFVTFNNMNQCFYIFIYPFIIYFNKYKYVLSNIIPN
LKEKKKKKKNNNNDFDFDLILKFVVELFIIFRIMYKKKKLINKLKKYLSNRTLKGFINYNNLNKMKKNNNKKN
MDTCNYIYIYTFYSIMDNFCLKEKNYNIHNTLFHKIKKVLMKYANIIHSNNIYIKFVQNKLIRICKNVTDLLS
WNYMLENMILSENQYIYVYFFLLKKNTKEQFNILNKDNIISYNNKYDYYNKRKDKNEKKKKKKKHNFHLINI
FKIITTCKRKILNITKNICSCIYFFFYKYNTFTHLERISILNNFQYILKKFPYLLLLLHFKIIRKIDNIFYTR
FLQNYNNMFFIKLFKDKYQSQLQIIKKKKKKLLKNICYSNDVEKKRFLFLFKMNKWLLYFFLYLMNIYFSKDN
FYTTTTKKKKDIHIDIQEIIKNIYITNNKKKQLSNNLIYMHKCIFYNFKKFIQNIKKQKKSIYKKFSENINKN
YNNIKTEKKLNYIYIHDNKDIIKNNHNHDFFQTKKNYKHISSSIQPITSSKYISKIISLEHILNLKCPNIMYH
RQNKKSININTNQNRIDQEKKFINTNFLKNFFTLKNMKYIVSSKYINNLIHKNKEKEQFTKRTNILYFLNVHN
IIQKSKLIKIIQLFDDNMKNMLLHLLHIKNNHINKNDASYNNDKIFSLNKNDVDQILRYKYNPIKVNKKLCIF
NNIENIILTHKLKYQKKKKYLYVQPNIIYNIFHYNEKQSFSKIMKNKQIESYKITLNYFIYFVNAHISYSVNI
FNHNYEKVSDSNGIHILNIYKNDIIKNMFIEKTMNFLEIILFLYLRKYVPFVQKYNMTQGKQEKEKEKEKKQE
KEKEKKQEKEQEKEQYNNINKYPLNIYQIFCYDIVQNHFQIKQIFDVEKEGQFNLNIFIYHEENLLFFYSSRK
NKKQNDTTNKSFYNPFFVKFLENDEYYDVSLDSSMIRRERN*
>N.PF14_0246
MSNNHNNFIYCSGTEDDHVNNAETGGKEDEKLKFLIEGKMIEKKGCVDFIKPLKDDIKALDFLLFDMLKDNLP
NNLFEILCTIHDLSETYSENPNDDNFLSLKNCIYNLKDEYLGTIVNAFGHMCVISNFAEWAHRGRRRKAFDKS
FIPNDKIYGSVNETLKGTFNILIQNGFQLNDIYGQLCNQTIEFVLTTHPTQAIRTSLLKNYIRLGELLLKLDN

TDKELYKKKLLYDNLKTNLLSSWKTDVIRRIKPTPIDEAISLVDIVENCIFYRIPNIIRYIDNVLFEYNLPPV
KLNSKLCIFSSWAGGDRDGNPFVLPETTKYVCYMNKIRGCELFIPMIEILIRDLTLHHCTQHFRSYVKLLEDE
VSDYIFDKDHKYLAKKFQWFSPFSKSNKKEIYRRALLVVWAKLKSVVEVYKSLISNQRVDEDFKKLMFHNSDE
FEEILLECYKSLVESGNTLIAEGYLKDVIRNVKIFGLHLMKLDIRQESEKHISTMNYICQKLNMKKYSLLNEE
QKINFLTDILNSNRPLIPKNIEEEEDVPNDFINVIKTFDVCSQIEDSALGAYIVSMCTNASDILLVEVFQKEM
KKGTQRKTQRVVPLLETIQSLQNSSIILENLLKNPWYRKHLTTNFEDKQEIMIGYSDSGKDGGRLTSAWELFK
AQEKLVHIGKKYSVEIRFFHGRGGSVSRGGGPQHLAILSQPINTIKNYLRVTIQGEVITQDFCLKGMALRSVE
IYMSALLKCSLLKNTLVIKKEWRDLMDEISEISTKEYRKVVYENKDFVKYFRCATPEIEIGKLNLGSRPSKRK
EGNVESLRAIPWVFSWTQNRMHLSVWLGIEKIYDYLINNNKLHIIQDMYTHWPFCTSFFNLISMVMAKASIQI
SQEYDILVPEELKYIGVLLREKLKKSMELTFLVTNEKKFCDNDQLTKRSIECRTKWVTVCNLIQIQALKRLRE
KESKQKNENKDQLDNKDNSSYTNITHNYKIYENTLVTPTSEYTNVENIKQDNNQLNNSMENHNINDLNTLKIN
YTENYNFNKQNELFNNDEGKHMESINKNSTVRINSYLSRKKKKLIKNPKFHPLIELHPYVFPQSETENESFFD
YVKTIDSNFERIPSRNNVSTYDEATNKFIDYTSLNDALIVSIKAIAAGMQNTG*
>N.PF14_0247
MNYIERNKIETIYTDDYTDFTRLSDFEIFTKDILVSLKKLNILETKRKYEEKYAFINNEKREKPLAPLSLIIT
VDDTCNFLKKSIKKEYGVKLNYVYYPYNKCQKIIPFDNNNNNNNNNNNNNNNNNISNNINNSNNSGNNNDAS
TSFYNKNVSIVNSFYCLHGNLNCSEITLFPARTFPIQRFFFVNEYVELEITDKSNVEEEQCINIDQFSISNLI
LNSLKLALSELDISLPTFVIIDKNAHVYQGHFYTSDYTYYKCINPFKPNHKYSIMSDDYKEVNKSLHTFTSNS
NYEKENNYISIYKGVGFEVNYHSFKYYNYFKSFTFDHLVALFCLFLHLSNIKGRNIYNINVHLKNIYLIDKPH
VCYNNWKGFNIKNKNYFILKNKVRGKEKNNCNHHKNNNHKMKKKTNIFKKYYLFHNKKNNQEASIYFLNHIYN
FIKKNKYYGNKVHLCYYTNSVKDNYDMMINSMEKKEYNDFEQKKKIDEDREKRNNHTKNTKEIHNEKEIIREE
IGTVTHYLYNIKNNMCKPTYEKNERTKIILTNILSDDEEDNMSDNIFYYSSPYNNYPLSNNEHIKMKKKRTTY
EDINRLYNNLRSHKNIPKNESNLFLSIYVLNFISNIQHKILDTNENEKKNIMNTWKIRFINNDKYKGRNSYLL
KNILKLYHNELVDLKKKIHDKYSNGYNSDQHNEQNNDHSSEQNNEQNYERNNERNNERNNGKNNEQNNERNNE
RNNERNNEQNNEHNNEKYNEQNNEQNNEQNNNNNNNGDNNYDNIYDNDMSFQNYLVQNICMKNSEWLRDSSIS
SFSCFNVEDDSTRYYIDKILNNMFSNNKKNIYEWNDNNNNNHFHNHNNNNKPWKNTKWSKGKFIYDHELNNIM
NNSYLKKNNYHESFYTHEILAHNNIGINDVSNVHKNIFDASHKYDNVYSLSQNNHGYNNGYNNGYNNGYNNVY
KNSGCSTVITNNNNNNNNNIYNNNINNVNNNIYNNSSIYNNSNIYNNSSIYNNSNIYNNSNIYNNNIYNNNIY
NNNIYNNNIYNNNLYNNNLYNNNVYNNNLYNNNIYNSNNNSSIYNNTHNNDTYSMYDGFYFFLSNNKSPHFLR
KNLLYRKPRNKKEYLDFKKKYWKKKKAEEKLQQIIKDFLMIKEKYFLCHKEEKIVILFFYLISHSSNIKTVYY
IWNQFFDNLRMKYENGEYIFNDYLYLSYGPLNIYSYSCSLLSQYIKALNISTQQFKINEMKNQLKNKNSLSCS
YLESDDNSSLINEYSNMDSYSNINDNETNTSISHNNNINNNNSNNSNNNNNSNNNNNSNNNNNNSNNSNNNSN
NNNNNNNNNNNNNNNNHYSNNNIISKSCSSSTIARRNISDENIFYSCELNDAAWKKNINKNDSSSNHEMEYMK
GSTDSLVKNQVYVNVDHDNESNHNSSCTNNNIDNNNNNNNNNNNSMCGAVYNDSSHNINTINHIKNCKDNNNV
SNNTPLTIRNHKKRKLLISNFFPYPLNNMELFISKNNVGTRNVNDQDDMITLIYEILQIKNKDTYHFNDFLKW
YKINIGKLYTSEQVLKYCYNCIKYSHFINIDIYYIKEKYKEIKRKYYSYDDFLKNMFNYVKDNFDQIKKRKCP
FNTFFVGELNLDNLLNMSILDILENSFKVFFISYIDSIVNSKLIYVRKIQEVLSKMFHIIKKFHRKKKKKDNK
FLKSLMAANNKCTNSPLGNSTMNNNDINNDVYINFREGNNTIYICPMKDYFKNKYKSNDDSNNNDNSNNNDDS
NNNEDSNNEDSTNNNNTEERHPFNKVEPICPEHKSTSELNKKEYFHSWKKSIHTLKNNSDKNSSDDNNIDINS
NERRSKYSHSFNINKEENYFKKFCVNPERKQKKIPYTDMNFSTAIKKIYKRRYQMIFNKKLFYLFQKCEELFN
KGIWLFKIFNNTLNEKKIFKIINMILESEEKEILIKPKYEKYFYNFIKNVSLNDNKNMYDKKLNNASKFQRLN
KTISARNFHRTIKSKHNKNIINNAYHPNFGTSLKSKKNETTLLSSQPNQEYNMLNKDKLSLLLHSNNKKQTIQ
ECIIDYVDYNFHSQMYNSPTRMYCSVNQLDTTVSFIKTPSFL*
>N.PF14_0248
MSYPYYTEFFVRYPKFKERDEKDRTVDPRIELEKKCAVKCVRPVNEYQNCVSRVRARTDNKGNCLGQYEELYI
CIDHCVAKDLFNYLA*
>N.PF14_0249
MVLGFKLITQKKFWFFIYFIFHINGIHYYQSVYKYNEHVKKRPIVPFNSKSVLLNNKINEKKKNIYIYNVKYN
NCCYFNITGVLNGFPKKISKNDLISKKKVEEIILNNLRYKKNLFFFLPLLKLKVKCKKNDGQWNEYFCPLSKI
RNIYKFKKSIIHFVENNSDKQNIEEQKKKKLIEKKKELFLNDNIEKKYLSIDYIDEKNETSDILLKVHIKSKL
REYYYDLCVKEYKEKKLLENKYEYSYLNDVPINNLINYIDKNEYMKIFLKYVNDDIIKVYKSVKGLHLIGSPR
LLNNVDHLCITKFDDILLKFSIDKYPTIIFQKPYINLDIKVKIPPYEKGTAFKEFLKIVENKNDVIEETDKNH
KVEWNNDVQVNVSRGWVYNFSNVYYDEYCKMDTQNRDTLFDGIRNNENVEKKKDKEKKENKQESSRKINNLEK
ERKETNGLSTNEEDKKENEKNKDQNNNTNKEQIIISNYEKNFLQNNKINNMIDIDKDEENFINNSDKYIEQNL

TNDDNDEKLFNDSYENKSHYEFLKNADFTNYFRQGYQLPNDVISMNNETISVKENFNPLGFNESLLGMSKNEK
KNITVYIPVNLFKEYFQKEHKGFEKRKKTKEEDFDEKIINLKEIDKDSITKFKEIIYTEIKKLKNNRFLQKIE
KILKSNENKFFDSHTLEKSLKRNENEQYENNDDNKDEQKKNDLNSNDYFYLKNNEQSIDSILSDHNITYDDEE
EEDDIKENANDGINKIEEETKEDVVEEQNEEIHFNKNFDKYLKELLKDDINSLDEKLYNQKEDKTKEIFQNLY
DFNEEYSKDQNVHVNDNENENKDSNLDTNLHDYILGESDLIKCVLEIEVLDIKIRKKGNEDVKGYVLKKYNKT
IDELYEDIESRAMKDIQSKCVQERRMEAYKKLMEITSLNIPMTLFHAQGKRLYSSYLNKMKMKNDENKENKIL
CYEEYIKRSEKEIYDQIKFSFIVKSIFQNSKLQLNYEEIIKDVIKTLIKTPTNNIRSLIKRIYTIHQAQYVLD
FVSLNSNISFHTDDKSAVNFSVSFKKGSEYSKDDYGNHDEQLTYTTNQITNDNKQTDDNNKHLSNFNSLENNA
KINQDKNFDILNNNDENIKEENKIFHFKDESNFVVEKKKENNENIQYEYFTFKKGANYTKYFQDNNVHNNPKK
*
>N.PF14_0250
MLPCVIISFLYLFYLTYIFKVVIIYCDSSFNRKNIKGHTFDSSSEYIHILRGSQPFFSKKILNKKILKTPGDK
RGNYEKKHNAAQETKNDHLKKERKVVQTIKGKQDGQAINEKRDGQAINERQDGQIINEKKDGQIINEKKDGQI
INEKQDGQIINEKQDGQTTNENNDSISIMYESKNNIEENSKESISLTPYGQESVVKKNELIKEEENNEEKTGA
IYVDILNNNNNNNEYEHNSVIINNEQQHENVVDTEKYNNYNSDNNNDNINDNHNNDNINDNHNNDNINDNYNN
DNHDNNNNNNNNNNNNNNNNNNNNNNNNNDNNNNNNDNNNNNDNNNNNNDNNNNNNDNNNNNNDNVDNNNNNN
NNNNNNNVDNNQENKEEIINKKETYAQKDEKIDELNNDKTEEEKHIPTESVNNIKKESDINEQIDDTSNNDNN
SDNKKDTDEGKQKENDTNVQEECVQENVINYDKEREQKYEIAINNLIKYHKELTDDDDDDDEEEEEEEENENE
NNEINGVYRKIDESNENEKERDSYSKRNYPNIYVKYKDDLEFYNAKLKENDLDEFYDYIIGRRNLINITYKNE
YALKLKYHKGYTTSLYNLYVVNELKLSDNMYSNFTSPLEEDDNPTLQEASYYADLSKIDIDPTVFKEYFLKVN
QCDGDYLPLKNTLVEKICHLSHHLRKHPYVNKIKITNQKSMLYRIFLKVMKSFKNLSVQQCIRNVDESNLLDI
LFLFNEEIVKESKKELNLGKCVNNMIARNKDWYNLINLITLALLGCVTYYKEHYYLNKEEVEEMKATKKVVDL
PIFDFFNEKVMDIFVPVDLETKIDIDLVEDVTKPTERRRKIFQTWYMAYLFIHKIYLISKMWNIVKNWEAADF
VSNPWYVEHIGSALIPHIMNLHKIEDDEYTFFRYIDQVQFFMSLKRSKNFPISNYDKKNYHIVEQIITFQGTS
SPSIWLTNLIYELTPYPFLSKGRLHKGYLFMFEQTVKPYLDGLKKVLLNELKNKSKYTSETPYAIIFTGHSFG
AALAQISSFYFSKIMDLKNRTNLKLYSITFGLPTYQDSTFYEDFRNSGVIINNININHDPVRVIMAVPGINDF
VNHDEERKLMINFEVEQLKKLNYDFGKSVFNAVEFFNVKNHQRSMLYVFAKYIFNKNVFLELGELHILQTHYY
FYYVFLTLLSGWVNEADWGTYFMVPFFLFENIDFSHNELMNKCKEFHKKYNGEMVKKLPNNENCDDN*
>N.PF14_0251
MDTNENMKNVKDMMSFTTKNIIYIFIGISLLIFIYKILKKNKKDAEVKRNNEISIKMKLSREKQLQELDKEMM
INKEKMKEQNIKKNEEKKKDADQAKPKLGSKDNSSFNHLNDYSNYYRPSLKNRYNNRKS*
>N.PF14_0252
MLKRDSLSNLHSKETRRFSIHNKDIFKYDYPEYLNLCLEEYRNNIYDLDLINDNEECTREVVNYYNYIYGNYY
ENVLNVFENNPNEKTIVNESYLLKLDLLNKRHIESLYNISILSISDKLKYYMLRGEVKKNKDDIIEDDKLIQE
KNNENRTLKKNLFTNDSLKTNMNLNDFNNNKNKFLDKNSKLYTNDELSSTDMKREEEFDIIKYLFYIIRLVEY
SLSSENGNLSSFYNRLKIYFFRDNVNENYYNYIYLLTLCLHCLENITSNIVTFDIKDIENEELNLANEKNNSV
YSSINKMNNSQNSIKRKYSTSNFRNYTSSNENNNNFHQNNLVTNNNNNNNNNNNNNFLRAASKECNSFKIYSN
HMYFYKIFFYLLSFLNFYLEDMIKDGSLLYSDMMKMHSENNARRNRKDQMLNSSNVINGTTNNSNMSSFIDGK
EELFFLGKNKVSYCNILLKSLNIINNLMNLDALREKIMNSSNDLNELKIFLSKFYKYLNEYDDIEVYKNFYIS
LYKTYIFIFPEELEKENYFSPMLLFENIVNNGNLIRMRTIVDTLIFCFNDKKFVYFFEENINITKVLFIFVTY
SSRLLSNKQYELITNISFLFYIILIKFPNVSITIFHILNENNDYIMKYNEDKKFNSIFSNINEHINMLFLSTI
HKNKNLATIISLIFSKLINLYCQFSAKKNIDNNNNINNNYPYGTHLNTNNYGLRNLNSSYLNANGQNVSNVPG
TTLIKNANVNNLNYSYASNNENEGNKLYKEIFCKIFDNIKNNERNSVIKAILINLHIIPDVYSSNNLFQIEEN
SEKTRYIIVIINFLFTLMKTKFYDAIHENTFEYIDYFIDELKKSVHMKNIKLVCGYISLLLNYSLNKKTNNLN
ETADLKSSIFIKNIHYNNQPNIIKIHKALYESGALTFLISSLYESKDSLIIYNCVYSISFLLYKKSILDYDKN
IELSNMKIAILNIVRHYYNLDMYLENYTENFIDYKGFLKGNSKKNLSLHEDDNYNTLLNKLNKRNSYECDDLN
KKYLYEEENKFLLGEKNDNYLLNHMNTNYGSNIKKIGSNENIGNKNVFPSLKYQYTSANYENSFYLNFDKIFF
ETVKNRKSSFNFYFDTIYNTQYLFNFLLHNFLFYSKDADEYSKKYLLNILLHNNITRSSDINFYSNKIKNLNS
QFNESQSRLEIYKQDLERYKDVINVLNKEIEEKQNAHNVLIQKIRKENEMEIDRKLQENNNLENIISQKNDII
DELQRKYEEVYLQKKETEEENVNIKNKTVSMESLLKSLQSKYTHMQSQNKTLNNKLIDQKNRINEGISIINSL
TSENEKLKTIEENQNNELEGTFKKLIVVVKELSDKNKELDEKEKKIKTYNGDIQELKTIINKSNNDLKEQALI
IENFTGLNKNLNEELNNANKNLELSKRYIMNYEKNEEEMKNRIMKLEKELYEKSEECEIKTQKLIMKEKELSD
KDIQLKKIASVIIN*
>N.PF14_0253.1

MILMGLNSDYNARINLKIKFIVKSIVKVITKHLLLISILNLIISLCYNYIINRDSNHQLNIVAHLLRGTFVSS
IPSCLTLSLLIFFKEHSLNNYKYNEWNILNIFCVILTFYSIFYTKTVFSKDYGNNIYDIFYVASISYILQKRF
NMRKSSIHDFQHCMNTETKNIIELIQKGIILNIIPLIFSYVLSFIVDILMTIINSVSFNIIVSILTHPFDVIL
SLPKIYNLSNIYVITNFSYCFSIFSAIWITAVLEYHFIIFNFIFNKYDSKVLTSLNNFPMHNQCSYHDEFFVV
SCIYNYLKDISYRIKGNNIEITNMDKKTKLKLLLFKKLILMNASLKYKDEINILSHLCNPIHTILFEDNKFWN
AYIDSCILCIDDLISQLKKYINILNHDQTVYSFEEKLEIKNVLETSRINKLKDTIILTCIYLKGVSLWSIAIS
IICKNVIVSVIRKLSSIIVSLSYVLFDFLNFIKINELYDPLHHLLYEMNSIVDIFKFYKEDILDNKYQYSYNL
YKGLSYLFRNRSLNY*
>N.PF14_0253.2
MILMGLNSDYNARINLKIKFIVKSIVKVITKHLLLISILNLIISLCYNYIINRDNQLNIVAHLLRGTFVSSIP
SCLTLSLLIFFKEHSLNNYKYNEWNILNIFCVILTFYSIFYTKTVFSKDYGNNIYDIFYVASISYILQKRFNM
RKSSIHDFQHCMNTETKNIIELIQKGIILNIIPLIFSYVLSFIVDILMTIINSVSFNIIVSILTHPFDVILSL
PKIYNLSNIYVITNFSYCFSIFSAIWITAVLEYHFIIFNFIFNKYDSKVLTSLNNFPMHNQCSYHDEFFVVSC
IYNYLKDISYRIKGNNIEITNMDKKTKLKLLLFKKLILMNASLKYKDEINILSHLCNPIHTILFEDNKFWNAY
IDSCILCIDDLISQLKKYINILNHDQTVYSFEEKLEIKNVLETSRINKLKDTIILTCIYLKGVSLWSIAISII
CKNVIVSVIRKLSSIIVSLSYVLFDFLNFIKINELYDPLHHLLYEMNSIVDIFKFYKEDILDNKYQYSYNLYK
GLSYLFRNRSLNY*
>N.PF14_0254
MENHEVEEVNEDQILCLYIDTKKYQKSLGVCFYNYLKYEFLMTEFIDNGHFTALESLFIQKRPHKCFFNSTND
LVDDERLLNLFKICNVQAIPLEKKKYDATNLKDELKLIISHNDDVRNYDKHLELENACKCLMVLINYLKLKEN
QDIHNQCKINIHNMDLYMRLDKAAISALNILPNKKNIHSYNNNTSLLKFLDKCNTTIGSKKLVSWLTQPLTNV
AEINKRLNIVEFFIKEDDARNVIFCNYLKRIPELDKLNHYLKEINQNNEIRVNSKYNEEMILKDIVKMYYSIL
DFKQIYFTLKPIQGKNKETIDEIIINPLRDILNKFSKLLDMIEITIDLEEVQENKVYLISTSFDNELEIIANE
KNALMKKIKKHKDDVEKDIFADKYDRTYKRANREDIRLVDCNTNVFLFRVTKKDCGLVQQDKKKYMTVRMNKN
EFLFTTNTLKNLCKQYDHCLNIYNTLQSEIINKTICAVSTYTPVIEKFIDLVSTLDVLISFSVVCHNSPFPYV
RPVIVDHGENVIMRKSRHPLLELQYNLNNFIPNDIHMNKKNSRLIIVTGPNMGGKSTYIRQTAIICILAQIGM
FVPCDFCEVPIFTQIMCRVGASDFQLKGISTFLSEMIEAAAIVKNADQNSFIIVDELGRGTSTYEGLGISWSI
GKYILDNIKCFCLFATHFHEMSNIAYQCEGVINRHVETTIDKEKKKICFLYEIKDGASNKSYGVNVAEIAKLP
KEVIQKAYEKVEELESAENKYYLKEKLNIDTSASADENYKMKISNYMKIKDEIHYLFSSTNENEFMERFVSKK
NYLKELAI*
>N.PF14_0255
MSDEGKLKKPLLNINEKNEELKEYKKTEDYHSKLSILSNYNENHDVKNLMQRNEKNFKDESSYMNYIKRGFKR
NSSEEYRIHNNISDEEHIKDFKETKGLRESFLYSNKNIFSYMKDNREEYMKSDDFKIRIKCFNKYLKHEVHKI
YNGEDTPYTLQSHELLKMVHKIKGETTPENSSGLAQYRDIRQLLSNNTTTRFDISPKRNCVLINLPYRKCIIF
KDFLLYIPTFTNSTIPEIAEKEEKMCKYFIENSKVISLIKDSLPFEILILEAIFVDISEELKNEIEPVICEAE
KLFEIISNNLSIYKCINKLTDMRRKLKIIDEKVQSVYKSIHGVLNNDDDVRRLEVSYFWDKPELWEKSDPTPN
NEDTEMLLEYYCHEIEEFLKIIHRTDQSLDDVLQMVELNLDDARNNVLKLELGLKIYGIIITVVGTIAAIFGM
NLKNGFESDQYVFWSLAFSLMFITIMCLIYVMVSFKKVKI*
>N.PF14_0255a
MNDDYASKPPTYIWNINIGLNALSENILDSLLFFGFIISILGSFYFLYWLQSGEMRNMCNKSS*
>N.PF14_0256
MSIVQYINDEGYRIDGRKNDECRLIKISLGNGNELIDVDGFSFFEIGNTKLFAYIQGPNEYRRPDEKCLVKCN
VFLSPFNILEKKRKKSKDNVTREISSYIRNICNHIILLDLYKNSEINIFLYIIERDGGLKAAAVNTCILALID
AGIAIKYFISASSVLYLQNNIIVDGNQFEVNSGSPELTLAIDMSSNNIVLLEFDAEVPIDIFQSMLKTCAQAC
IHVGNIMKLSIKENAIKLLSLNSILNT*
>N.PF14_0257
MEKAIRDRVEKWKKEHTIDGKDATNEHAYESLNELILDGKKLTSIKNEEKELLKNFKNLERLCLNQTGIQTLE
NIPSIATLNVLELTDNHLSSVEVLKYIVQNFPNIKTLEIGGNHFKNINDFETLKELKNLVRLGVQFNPFADNP
NYRKELFEFLPNVKIIDCYNKEGMEVLSSDEEEEEEYEEDNTLKNFYEADFKDEDDEDEEFVPNDNEDDDEDD
ELDDDLEDEDMEDLDKEDLDKEDYDIDTKETEGVNKDEKSNKRKQDALDNTNDMDLKKTKLE*
>N.PF14_0258
MESLNIPKNVSMDLLEPFITICVIFICLYMIISIESPVYYLIRSFLFKSKIFLHEIKDIFTKINYFALIFSSF
FFVSYYILNILNYKNVKSLFCVNFDYYDTILNYNLKYINIQSIFQTAISENYLVNFLYSFKFLFHIFFLNNSN
LIKTICTSSLVYLMLSLYAKRVNRIMFISSIFSIIILSGFVIVYFFKKFLNIKYNHCGHEIFSFVFLLFFYIK

NPYLSVYQYNDRSLHPYHGTEIRIYIHILFFIFYIINNEKFYEIKLLVAIIIYFIIFLRNTIDSFKMYTFLKV
IWLVAYYVTNNYLPFTFSQHFNISTIFNSNIMGILSKEIKNNISLYHFNTISKIPFNYLISKTSFFWIPYMLL
NKDNKNFKYVIMLLMVVNIIATCTYLPHNIYPGIPLNLLLLYYLNKIKI*
>N.PF14_0259
MNHHKLSKKHVKKSVLPTDAYRKQQKKKEKKKKKKEKALQLENIISKKNPHRVKEKLDYLRTKEKQGKLLPVE
KNKLKEYENLWNLIKEKVKNNEYVYNNNGYIFNLNEENKNEEISNSEKKSVSSKDDINTSSSLYSSSDDDEDI
DDDLKEIQNDDGKKEDDDFFLESLPSLPKGLPKEVIKLIENSKLPQPDNIKKMMENMYQTYGTTQLPVQNHNI
LLNNYTYNQVYYNLQNYYNNANVYNIYPTVPYNKNYEKNLKNESVKNDNTLLSYPNSYLINGANNVSMINQNY
YTNNSNINTTVNKNIINNNTNMNEKLNINKDNEKLYNIEKTHNNNINHDDNKNGDNKNNDDNNINDDNNKNDD
NKNNDDNNINDDDNKNNDDNNINDDNNINDDDIINNDPFLKDKSKHKDIIQQSSLSNDNPNFLKDKADINNSY
NYMNYYYNYANNYINSYNYNPLNHGPYNPMYYYNYNNIKGNNQTTNLTNNNNSLISSYNHSNMNMMNYYGTNS
NNGNVKNRGNFMRPKNIKGKDNHSDNNNKHNNNFNNHNNNNKTNYDSHTKKFINEEKNHKRNSINTKDEKQSS
VQYFVPINLRLKNKLNDVCETKINTIDQKNKNVDENINIDQEYNKFIKEVNFN*
>N.PF14_0260
MEKVDTANNDGEKCKNNMFIIKRIFCDLKNLFPKSKVQYTYVNVLLLSLFFTFIHKYLDQTLPSLYKSIENDF
NVDVKSLYYMNTLYKLAYSASNFFFALFFDYTFKRIVCAKYYDKEKEEDRSTNNTTNYEVSVKNDSDYKETTK
LIDDMSIQTNYKEEDISCINISKEEKEEEIIFHSVDDSDDLFKKAEDVTISEYGYILNILIISSIIYIIVILG
IMISNSIFNFFFFMFVMGINNSCIYILIQKIYTNNVFSENRSTIFGFLHFFSSVSHMLSISINTNLSNKLYYG
FTGWRICYFIISFFPVFVSIFIFKLIRNHKLKKNKTKENFMMNYVLSFDKLTDVFNEPKKKKKKLDKLEELEE
QYPSLYYKKDKMDTKCSSKKKKEQENHIFREDNEYNKKKISPNVKVNTYEDNFNFNEEKSPKKKNEYRNLENKE
MSEIINGTTKMNDDKNVNSKNITNFKMDSNNITRRSYSRSYIKENQLKIQNNNNNNNESEDNYVNNIYQTSNV
LLKSSSIPYENYKKKLYENKNPFSIFKNNNKYEKNDKNDNRGEMDELLGNEKQNTSKYEFSYLYEIKYVFKNY
SFWLMITMGMLNGIPKHVLSLMIYFFQYCNISDFKSGFIISVSWLCASLISPFIGIISDYIYKLNKDINRQRI
GMYTHCFRIFLMFTLFYFIPKEATSFIYFVIISLFMGILSGWINIGTHKPIIIDIVKQRHTAFIMALMNAFEN
IGSSIIGTFLLSHLLNKYDYIDKKKIHTVVNVNVNKHNVNVLSDVLLMLTCFPWLLSFCLLYALKYTYKKDKL
YNNII*
>N.PF14_0261
MEPTTETKTEEIDLEKYTHSGSIANTTLKKVIEKCVHGAKIGELCEYGEKFMKDELDKVYTKKEKGNKVEKGI
SFPVTINVNEICNNYAPSLDCVETIKNGDIVKISLGCHIDGHISIVGHTIYIGAENESIEGVKGEVLKNAHIL
SQLFLKSLKVGINASDITKNIQKACEELKCNVISNCVTYQIKKYILEGSKYIFLKDNIENKIDDFQIEADDIY
IIDVMVTSGDGKIKESDHKTTIYKREVQKNYQLKTNLGRSFINELNKKFPVLPFHVKHLEDQRAALIGIPEAL
RHNLIKPYSVYTEKKKEVVAEFKYTVMVKEDGVKQFTGLKFAQLNNCKTDNSIQDETLKNILSLPLSGKKKKN
KAKNNEEANQEN*
>N.PF14_0262
MKYNSHQNDNVYNDIIKLKYNKNYSNYIHQNVQSNKFFISRLKNTMTLNGHTSFVNRLKWNDDNHLLASCGSD
RKVLIWNINSYNYKPKPKCIVNTNHVSSIFGVSFIDNDFVITGAMDKQVHVYNIHDDSYKVIHNCHLKTVRHI
ATIPKENKNIFWSSSDDGTVRQYDTREKHICVSPNCRNVIINLNRIGDQYYTQRNPEERLYHRMKFQSHYTKN
TYELIRAMNEQKFCNVNVPIQARCIAVNPIFNNYIGVCSNDMLSRVYDRRMLGKFSLNEKMTLRSCIPSDTYY
PKHLWNYIDEESDFRINYHLFYSTDLCWSNNGKYLGVTYNTEHVYLYDFLNKEEERSLNYNIIDNDTKFDYNK
SFDILFPPYDNKDVYMYWNNKKNDRNRMKEYFDYNNNMSLIQDIEKSAILAYDNKEYYKAEYLYNNCLNLSRN
KNIRKILYCNLAMVLIQRKARNDGYLAEQYALEALKLDPNYYKAVYRRIQANIINNRLINAYRITLSACSHFK
GVVEFIYLRKKIRRTLYHIYKDKIYSYWRNQNNDTEDSKQPYYYSTIKKQTSKVQQKDEKERIQDNCSEFLDI
SDNTAYSYDSIRTDDSDFTDDPYGSDYLYCSDKTRKMSTLGEKEIKQREKNLNMESKNVESVYIDSVNVESVY
IDSVNVESVNMDSLNVENANMDSINVESVYIDSVNVDRVNLINDKRKYSNPQNSDHVNIENDRSETTFKEIKE
DNDLGKKKEETNKYNENYKINKKNIEKNDTCNNNNDNNNDNNYDNVLMISKMKKIKDKKKKERELKKALSLKR
RKKYTNIIDIIASEELYSLPNSYPFFLIYYVSFCYRNSINAWNGWPSFNDLYKTYNICEKILKEKKRKDKKGK
NNKSIKRINCNYEYILKHKEAQNISYFNKKDKKNILKNNYSNKFIKNNMLKDSSKRKKIHKSSQSDIYEEDKT
IKKNKKKFHLNNSDDHNIEHIYGHIDETDILDNTNIYDDDNNNNTNDITKYNKKGKNKISHTQKKKNTNTNTH
IQFNENKEKEIKTSNNILKVNERNKDIYEDVKSDEPKGRYAWLEEKCFTGKKKRKYGKKQNTTNIKRNAYLKK
RRKRKNLTDDDNYNDDNYNDDNYNDDNYNDDNYNDGDKDNDSNNDSNNDSNNDNNNNNNQNNYYLHYGFISDL
YNRTHSHDNDKERNRKENLKKEINYILEELTKKNSPKNLVRINIYHEAYHTVTMNQVYGYNESSFDMNDVSLN
VSVNNERNSVINEETYENNGNYEYADTSIYNESNVYIMNSWDNMNYSENFVSTNISDNTLSIGHYSNTDVTNI
TDNVNSNNDDTNDNNNDDTNDNNNDDNNDDNNDDNNDNNNDNNNDNNNDNNNDNNNYQNNYQNNDNNQNVEEN
KIIGKVESVSQELYNNYKIDDIQFGNYIDTLLVDNNYKINNNMLASCYGTSIRSNNTSNLGINMNQNTPYDDS

SIIVDSPITKNYYLYINNFYDFVYNYNNNNIIRKNINHSNNNNNNNMYRLQMNTTTYHNSSSYYSDISDWNNN
QIPKNKLHKKGYKYYSNDCKVCSKKYNKVKEQKENKRKKKKVKKEEERKFSDNEKKNFSDEEKQWNDVKKKMS
SYYKDENDMRYYEKYRTGKKKKQQNKDCFYKKDKTNSDGDNSSNDKIGDDVNHNIMGFFEKSSGRNEDILNIK
KKKKKEKKKKDSNKKSDTLEYSKMNIENYNLKRNIKKKEEESHDMSGIYKRYFDKSTNNISQHKEYIIHDSDN
LVYKINVEDIDKNNMNKIIASNEKYLLNNGEIANFKFDPYSNIMETDESNSSNLSNVSYKSSSSYEHQFYDSF
YNNIQYEDRKKHSNIYVFKERKKIKNYVEKIQDPLWVPKGTCKRFLGHSNTAWEMKELAFWNDDVILAASDNG
EVYFWSIKDGKLLNVIRSQARHVNCVQVHPQGTCLATSGLENYIKIWKPHDTVEFVFVIKNEQNIYIKMNTLV
KEFNI*
>N.PF14_0264
MDNDNNKRKKTKKKKVRNVNNKNSNENKKKGIQENQDEQNYTEQYADQIEEILALHNIFFPMYVDNPGHVKKS
DLPKDLMKRNEYIKNSKDLSENIIVDINDEINKNTCLTFSMFLNIDNIMEKYKVTFMYEKIYPYSYPNVVIHM
NTKLNEEQKNDVTLNIRKICAKNYGRITLFEICLFINEYLNKIFNNDFQNLWEEMNYRIDDTSFKKDRDNEIY
NDLHNEIEDGRKLDNYKNVQNDNNDDDYKKNNEHMNNQSDGIYEHMKNHQCDQKGYYDNGACLNDQIESKKKK
TQILYEHGIQYDEIEINNKNKHIDNYIKDTYNFHSQPYNKKGYYNNEYLKEMYLSKNNDNETINDEKNLDHNS
TNTLENYNYLQNNIINEQKYFLNTYNFNSISGFSSINSCLINQQQYEINRKKEEWETKIKTNFLENKLNSKNI
MNNSENYDMIIQCKDSNFKNFNIIKNISINNYRNTFLVRHSIDTNVYIIHSYVLLNISYFLTFFFNHMVFCNR
DFNLFCNILNDKKGIQKNKKNKKNEELFKEYLNDIINLRNSKKKKNFLLKNYQDEEKNKQNYYIPAVHFFCSE
YQFYANKNCVITQNKNNKTLHNILNEINHNRLKKVIHHYKLVRKINIKKIICELAKLTKIHHKYLARYHFSWF
EKEKIINKEQKNNQKINQVNDLMKNVIINRLDHNVHYMEEHYKTEVEDNINNDKQKIKIKIKNNNNNNNKKGI
SYIQNKENINNGHVLKYNANKIEEDFQCVQSFDSYCTDCACYYCCCSSSSCPLENDDTILQTNEDKNGNYKDS
CENIKNEINHLEKEKPTKNNEEDEEDEEEEKKNHEVLDEKKNKLIGAFSNNIERKGSQSKNSINIMEGSKNKD
KDKHIHHHNIINNNNNRNDKECCNNMKNKKNENYITPFKNDINYIKNVAYIKSILNKKEYNKKTLYIQCEHCK
GQSLEKEIENNFFQKNKYLIWNIFRQVLESLSYLHKQKIYIKNLNSQNIYLDNDKYGAHIKIINYSICNMIDY
FYFYHYSYKNNSSYFQQFMQELYNDLKNIGKPLNKQDIENMIHTERIHDKESKEEDIFVKKCNSFEIKNDSSC
KEYINDEKINKEKNVPIDEYDRNNKCVNYNDYNNDDYDDDKEKTIYTYPSSNEYKDFYVHSYNQYFYKCLNKK
KYDYEELDLFSLGLLLYELWHTPFKSTEEKLINITTMIKEKTFSESFIKNIDNDSLKVLKFILISTINYDTNE
KKITKLEYVDILNLNIKKDKEDYEHLNNSMKTKTIKDMSDDYIIKEEGIYNKNVNIDIENEIKMNKIKKNINN
YKDHMNNEMLLNMNKKNLNVPEKPNLYCINNSISSILAKYNENDDYISNQKKNKDSEIYLDVKGDIRLLLMNS
MESPSSHHIASENLKKADHSNTYSEQLKKIYNFFNQDINVLEDGKHISMKKDNIQKENVVEKNDIFEDNEKNN
KINQNDKINKDDENIKYTNEDKYKINENKKNNNNNNNNNNNNNNNNNNNNFQMNHIVEEAIYSTHEHANNKEEY
IYKKNYTNKLPKITAEKLLNYPLIPVVIQRDIFKYFLQKLKHNSLNECMNVLNILLYNNKDLSFPFERNRNAY
VYTHTSTYEYDVIKSYIFEYFHWYLSKKLCTYNQPHVFQLIKNKTDTSNEDVHNDVISKIPNNFLKKINLYKK
EIKTKKMDMENDSDNNENKYEDINNSIEHLDILEKNKSNKKNKKMKKKEIKNNSRYYSFDSLPEQNSNNLKNL
ENRQDLKKKKSYKKKENDNSTTSSKDKNEKDNISKYEKKKKKSDDEKKKDNKKRDFPILGKFSMFKKFPLIKR
SNTYNVYRTNDMNTNFLINYDDKNGEHEKGDKADDTSSSYNNNNINNMQNSSRMIEQLKTENEENKRNILNRF
KDKLQLIDKNNKLVYMPFYLTESFLNLIPHYNLNKSFMNSFCFFQNYFFENNQYKIIKRENSIIYNNIITIND
KKDFFYGNNINGNNKTEVNISFCIYQLIILCNIMEIFEKFEGYLNNLIIKWCYTDLIIILIRDMLSLNCNKKI
YFLYKIFEDGNISFKNLKKLLLYLDITYDDKILKILYSVLYDQTDNLYNKMKKIIKMYDIKNHKNTKFINYIT
STIIYLNNLFQYFNKSNHIFTWDFFMNHFQNTFGFAFAFNIYSNKNTKYLLSFGGVSTQVFTNYDKVPQNGTY
NIIFEIFVDSISNIIFQDVKKDNFSNLLIDFHSPSVVITAKAYKLLMYAFSLYNSLIQNGIKCECRITPLMET
SKFEKSLLKYKNINIHVQINQKISSSTSINIEDYENSAENISSSNIINSEENVNIIYSTHIIRLNIKKNFENE
ASLINYIKQFYLKK*
>N.PF14_0265
MKKLLFFFYCYIWFDLFVHVSNFYIKPKNYYTHKAERTKKSVLKRYSFQLYNKKQTDLYNDNKNFIRIEFRPG
VGGEEAFTWSKELLNTYKLFAERNKCKVEKIQDISDSLVISSKENLRIIKEDKQIEINLYDLFKNESGIHQVK
RIPKNDSKNRIHSSTATVAIFFHKKEEPKYEINLKDLKISTFRSSKPGGQNVNKIESGVCILHKPTGIQTECQ
EERTQELNKKLAMKRLIQKIQHMESKEKENMVQHERAKLIKESNRSNRIRTYNFFRGYITDHINKKKIDLIYF
EKGKLEYILNIF*
>N.PF14_0266
MNDIKEEIQVHKKENISFENFELFENVAIIKKELYDEEDMSSNLCKEKINKYIDYNDKDNKNYSIGTLSNISL
NVDIENKHHNNYEKYCNACIHCDDVCFSQICNLCIIKRKNLYLKYKKFKKHNLRIREKHFKKIMESNKEMHDN
NLNILKYQKNVQNGNEPNMLQNDTLLKEHKYYKDNDSNDNNKDNDNNNNNNKNNNTFDNNNNNYISHLCNGND
NYLNDSPNHNINNCNTQKEIISNHNNTNDTKFKIIISKEDKYTNTNNNSINNIDLYNNIRLNNNHELTSTLNE

TNYINYNNNILNEKYEKQKQVLKYNFVKYKEDFTLCEIKRHYKINDCWIIANGYVYDVSTFINHHPGGINSIL
KKGGGKIDATVDYSFHSKYAQTNFWEPLKIGQVVKCKKEFLEEKIKNQNTPKKRNKCNFM*
>N.PF14_0267
MYPPCKKKKLNNNEVTNIFLKNENNMSVHNISMNAVLCSSLNLDNIYKYFSNCIYNPREFKCMRIDVPVTLST
VQKYIKYLNNKKEKQNDDICKMKNEQVNQTKHSNNNIKEEIKTNGSNKLSDNKLLDSSNNSSSNKILTNNKSH
ENLINISKNNSYLDDNVNKNNFFINDNNSDNKKIDTSDIKNMDPFIESEHIINKKLIINVSIFSNGKIICTGN
NSIEACKIAMKKIEKKLKQLNFKNIKLKKITITNILAVYNVGFSIVLPLFAQYYKSVDYDPNVFPACKVKIAL
MNDENKSNDNKENDNNFAWCNAKNTIDKDKSKVDIVSASIFSTGNITLTGGKSYENLQKCINILLPYLIKSKS
QH*
>N.PF14_0268
MALKNFKSSSINNNNKSMSEFLKGPQIKECSNTRNKRKKRKCQEKLPDFSCLKKLIYEQKRKENLGNLFSFND
PSIKERIDKFNKIIEKNRQEDSNLGYNEDDEIAGNFCETSKFGYTFINKIKNNQNKGDDIEDLIQHDEEEEID
EDLDINETDTRNKDLSTYNNHDDNNNNNNNNIENYNDNINDANNNMNMKNNNNNNIFNNIHSNIHNNNHNNNH
NNKIVNNSDIKNKDILLDNLKLRFDEFKIDTLTIPQKPINIIDENGKEKKKLFIFNINNNTTKGKNEKKRIEI
IYDDKENDTEESSMEHQTNYLYVDKEEENIKDDNKYDKRDNKKGYLQDESKNNKINDSSIIGSISHTNNNYIY
SSNKKNSSIYECNKLSNDISNNNFNYLSNERIRKTTYEKIYPYTNEKENIIQHVNKYCIGNVYHSNNKMRVER
KENILELVNKIKDQKESYLYFAVKDNTLLLNENNDYFSIMMHYLCERTNDWNIQMLYYLIMKPANMDLLLYLI
LTYYKFPFLDIIEQYQNISINNIIQSTYEEISLYDYYYYVFHILEISHEQYYMSLELFNGMKFLLDKYNFFLS
RMHKNSIFSKNKFVLGLGDIQNVKHIFLNDSICNSIYQKKQKLESKDYIDNEDIVDGEESTTDDPGERIQEKR
RTKKKKKKKKEIKGKNICQERPCLMRATSMEDIHDGCIGKYKHDDDNNNNKCDDDNNNKHDDNNNNNKCDDNN
NNNKCDDNNNNNNKCDDNAKNDGEHNNNNNNNNFNTLKRKNGTLQYILNTKLDFINYQQKNFFNETHWNEMENYY
HLNNLDKLVYFTKDLYYKFTKKNIYHKLNEITCEQKHFDKIQIIEHCKKKIPKYDKDVKFMNFLGVYSHSFIW
QVYCLELEQFCVLKFKRINLGFLYMHDDKQIENKNKKKTHHQNDDNNREEGHNGIVKSKKIYLEHMSSKVPYE
KRHRINTIPKYYMNGREQQVNQNYNEDDGDGDDNNNDDDNNNDDDNNNDDGDNNNDDDNNNDDNNNDDDNNNN
DGDNNNNNLYIDYNNNIQNINNFYNDDIQPLKENCDYIMKGKHIIEMLYHQMDEKKKNEMNKKKRKGYILNLR
LIENHITIALKTFKFLVKEYKIENIFFPISVEIKKKIIEKSKYINGTPLNEFIYSEFLSGIDLKLRVFKLKCI
MLKIMKILITFLKFDILIILKCSRIFLKEDNLIINVGIPLGLLPNRTVLFLLNNIEQTIATKSYSKTIFHYIP
PEIKNKLKIVDCEQYIMDYTNNKQTVQENMDPLDYDTEKNCFYFDDKLKLQKAYSYMIGKIFEETLIDTFTGD
KLDYLNFDQTIKSFLYSCLQEDVEKRDPLQNLLNHKCFHDCFDLYINIYDDNINSYKNDKEKAVRLSDLNYIN
NYDYNIFSVPSDHSSTSSSFSTDREITTSSRYSTSSS*
>N.PF14_0269
MSNPFSKKEIISLPQRNRRKVNKSYISEDENEEEENKHIFNGDNNTSDRMDDHINDYVDEHINDHVDEHINDH
VDDHINDYVDDHINKDIYNKFSNKTYEYKYLDHTADIILHSSGKNLRECFESICICMFDYMCNLNSVELKKRR
KITVSGANIEDLLFNFLTEFHFLYGSEYFICKHIEIIYFNMNLFLIEAYGYGDIFNISIHESGTEIKAVTKHE
LKIISNQDACEMFVLVDI*
>N.PF14_0270
MNYFLFFLFCSWILIKGYIINKNEVKYFNPLSNKNRIKEKYKHTYIPAKYQNKLYIFKEEEVEEKYNTIDTIN
SLLGKIKRQFNINFDLENERKKIEEEKKKKKKMERDNQSEHEKNELKNFFGTMLDGSFKIGRANTMFKELKEE
KKLVDLKYKRLISKKLKLRREQLKKIEEEINNGTYKSPYNEEQREFLQNVIKEMEQAIEPINKEIEQIEKNRF
LVFDEKNDLLVKLREQIEKKIDEVNMKYQEQAIKLGIKKIMDDYYEKTKTPLVWDLTQMDWPKAIYPLFNNMK
LKADIYWESTVVGGNREKNEYFFSPFNLPSLGEKKKRRKGRGVGSKRGGSCGRGMKGQKSRSGGSIPIGFEGG
QTPLYRKLPKFVAAPLGPGHRFNRYDYELISLNLINLAYSRSGGKKYLEVDWNVIDKMGLRIGKYKRKHPIKV
VGCNLKKFKMKNGYDFEFFAKNVIVKAHAFTVKAAREIIKLGGKCLLLKKNTQDIVYQEYNPDDENLNRIPRR
IVFSGKPSKYERKLYWLKKVKLEKEQEMQQGNEK*
>N.PF14_0271
MTQYFENFDEFLKEIDLENYASFVKENVSNEAKKYDENICAQDLRNLAKCLPRVSGVWYDLHKNSWEARWTDR
GKSARKYYSVQKFGFHEARKLAIKTIQTKEINYLCNSINENYDRPLKWNLTSDFLEITRDPTINLKKKSRSKN
CRIKAKKGTEKNTLLIKSTSNNNNNNNNNMNGKCSDNENEYKNKNKNKNENENKNENKNKNENKNDIGYIYDN
INSKKTKYERKCIHTTNSKKKNSNDVLKINYYEDFQNENISQENDEKKRSIGKKTCDNFSIKNIENIKYSYEE
NFEENNNREENLNLFTSIETEKNETNGSSYIFISDSVSYSNKKENDKSEYAHHMDIDSRSNRKNDTSERLNEK
YINENNYSDEWNYMNDTHLNNIFNIKRMDNGNNVNEYKRDDDSNGSEDKLIQNIINKCDDNNDEDDASCGGNH
NGNNNRNGDSEDDDDVCIIKNVKDSHKNEQLYYNTEYNQGTKEFPREEIFNYMFNQQNVFSKEDFYNKQINLL
KYYMKKDNDEIHENKMGTCNEFNSMRNFEMAQNWSSNEAHNCVLNDLTCNSHMHNEHMSDGYMNNDNMNNEYM
YNSCEKKSSINNEYIRSYCKENNCKKDFYGKNTYRNSFCMNNKCMNDICLGENCENQFCMNKCCMNRCCMNKY

CMNKYCMNKYCMDKYCTNKYCMNKYCMDKYCMDKYCTNKYCTNKYCMDKYCTNKYCMDYCCMNNVCMHNNCML
HNYVRNNCSCIKNTMNDMNGSSYQIFDENKMVSFYEDLKNIGLNKLNGNNSSLLNDTICKVSDSSNSNNYYNN
SNFCTSYKNDSYSFSNIFKIIQKNTDDFLREKKDRNMLTEHNARNNILRLLTSQINRKDYSPNGEMEDGHEEH
KTEEAKEDMEDSKCREVTGRYYKYNELEGGGKEEEDGKRGQSEVKEEEKNKKEQKEKKNNICDESEYGQEDYN
CGKLEREEKKGEDKIDESKCEEYKYEKLERRRECKDETEKSNEKNILNEENILNENEKSNEKSNEKEKSNEKN
ILNEENILNEENKSNEENKSNEENKSNEKNVSNEESDEHNPIKQPFIIHNSLDSLNSPDNLLIYKNAIVLLLN
DIKLKCLPQLGKQFSDVGRIIDNYLIYVNSVLNENFLCTFVNLFDMCVTNNTLPSQMDTKIQHIFCNALIAFH
VVFLNLLHNNNNHLLDN*
>N.PF14_0272
MFSPNFFFFCFVFSQIKSVISQKSTFCQKDYSKNCAEQWTKLSTSEECVSPLSYRGPCPRYLQAEHETKKKKL
LERECNIFWPCLDNCEKDYSSQCPTNWVPEDEKYCRPLSIYEGTCLLPYDFSNMTNEQKEIWSNKCSSSWPCK
EKCKKDYSKICPKGWIKESDGVCSAPKNYNGPCLSRASLITLDKDMKVAFEKLCKLNFPCLKECKMDDNDPCP
KDWILKSDEFGTPQNCLPPDYYNGSCDEHTKFIGLNSTLKESLAYECDIKWPCEIDTTKLINYEEYCPEYWTQ
SEKYCIAPQSYMGPCSKKKSFQSFKKEIKKAYAEECDIEWPLYKNSREEYLPKISTLRKGKYSYGSVEPITGN
IISTMK*
>N.PF14_0273
MVMNSVDVLNLLVNFSNSNNIKEYDDELTRNFFSHIMLLLIKDEDKYELCKDVNNYLFDEVIKKEEEYKGDIK
NVKMNDGKKKKKECDKKNDGKYGNNLEIIILFIDCLFKKCWNESFFNNNNNNIDISWVYKLFQLKGGEKNKYS
RILLNLLKNILIKIQEENPDFSIIIFLLKHIQIKIQNHIKENQDKNMYLFYEKWKNKLTELSSKGLYMYSYKH
LLDNIYVKNQVLSECNEDEKKNIIYNNNNDDNNINHNNSEGINNIILTNKQKRYKNLLSTFDLNFEHLCALFV
LYLEEVNGNQNYKTSTFQNEVVGLLKNEISKEHKINIDYDDESIIMELINYILYHIILINDKDMRNILIKDFS
FAVKEYIYPTHVFTNVCLNIIDLMYFFLTNDMLEKNMVNGNVPIIKTDKMRNGNHNNINNNVDMKKDDNVEET
IQSNDKKKVFIYNNENECILDIYYKYMINMYNTDINIDEYLLNDIYNFIILYIDYIYNEEFVKNGHINDIRYY
FLLFNKGYMDENVLKKLLYIFNTLINICRKTTTKNGNNEMSLFIKVWEHFECIVRCIENFSVHLLKSNWFKIV
ELINIVSSIKVMYEKKYEGDKNISDVYDEYNKDTYIHRNHFGKNIEKDIDNNYINTIIHNNEKILFFDYLDFY
IFQTCVKRSYNKMNTYMMKDKKMRKEKNDMSSEINFVIYIDYIILKLNEYLIWLLILHKNDSVIRYSLCGLLD
YENKKNLEISKILSNETNAIKYTDDPLLCNDKKESDEKKIHILTFINSICDHFLFNIFFMHIVKPTLFIKGDM
LYYESRIKNFIMSKILYKHKYAYNYLFKFIISLGNINYFYINLRIILETLLQSINIHNNNNNNNNIYHHTFNN
IFTFYNMIIMNNENTQTYQNYLYKLNKENINKNYFNTFYYPYNIYYNQNIHSIIINIIKNVKNISFSRRNNIL
FIILEIIIKMNDALNIFTNIKNYCIFLNLFEDSFFIEHLNIFQDILSINENHFLKDILRLKEQKNQINNDLIN
KTYNIYNNEHTDQCNDVIRYQETSGKITKFLFIENRKKELLYNFIDLLNEHIYFNKYIHYYGYLRLFHLILIN
EGQDNKSELREGIIHLIEYYNEKCNMKSNNDDDNNNNNNNHYHYNNHCNNHNDDDNHCTGLISILICYIIKNN
LFYYFNKSCVVKIIHNNIKKLKDIIFENNSICNEDIDTHNNNNNDNNNITSCYKFYKYIESNFLYMDTLEYIN
INEQYISSQNLIHYYNSDYPHKQHRTYRNADTYVKNKMNHVVYNDDDDDNNNYLVNNKVLTYISRDLENQEWY
CCFFNNVKELYDTCCDYLLLTRNKDITKYNNNNNDNNNNDNNNNDNKNGCDNIKLVLVLSFLSNIYNIYNPGK
WDKELNFKIQELSLKRLHNVKFIHSNKYSLHNYYDKFNYYKYKYFYNCFFCCGSYISLNEEEMFMFCEKIIND
IEICKDELVYIYLIIKNYMIPYIYTKSNDNMSTSIYNINQMNEKKMIIFKLLFKQSVILVDRFSVCKYPIHLL
ELIFLTLFHPLLIRFEINNFIDKRNYDSHIYNVGENDIRENKKEEIYNTKNGIICTKENFKKDQESISNDKYC
PDERYQKCCSNIKKEKYTHGDKKNNDKKDNDKKDNDKKNNDKKDSDKKNNDNNNDNNNDNNNDNSNDNINDNS
NDNINDNNNDDNNIYNDDTYLLLNYVRKILENGKTKLSITRNIVIPMLTSFFYNLINYDDIIYVKKEYLNNFI
ELIVDILMYKEYVLLDGKRNFTNESFKIKKNNNLLLNNFINENICNYLINKLETNEEIWFPPELYEIHHTPIF
LRILTLIFLINLFNILDSTYLSSIKLCTTREDKYEKVSFNKKKKMILYIYETLIIYILKKIDNKNIYHMNNKS
PNIINSSNDNKSFFNIQQNKEKEKIPPLPSSHGHNIQLRGLQALCMLSKYFKHLKKKKRKEIISYINIILIYD
HLNNTRQYLEYFCLAISHSSFVLMYPYLIKYLSHDNVNNQLTITSLIICAYLFLKSKFSYYSFEQLYINSYAQ
ICCTKEKKKKKKSVLTKNGPILNKKESKLKRRAQGKKVKHIINELVKKNKNDINGIVQGVKKMKNMSTHKNDK
KGYNDYVTNNNIVHNFDNKKYDNKKYDNKKGKDHNYNMIKVEMVRRKDSFKNNDLKINEDKNGDICNISNEKK
ERRKDNIQNDYFYSEHIFKCLLKLLYGIVSLCSSHSAIIRSMAQFILYKFIKIKGTSHLNPFFSSLFSYIKYN
KDCKRIRKKIKNQFNYWNVSFFDNIRFLLPTENYSYNHFDEDRNYNNVNTFAHILYNNELVMSYTFLDKFKQI
VQQEMSFMIFNIDLEKEKQTDKNFKLNSEQNVSLNMINRIYEHNIRTQIERVHDKEGSSKNVYSDSTEETFMS
YDNSVDIKSDDVNKKSDSVNKKSDGVNKKSDGVNKKSDGVHKKSDDVHKKSDDVHKKGDDIDKKSDDIDKKSD
DIDKKSDDIDKKSNIADEHIPINYNEGNKKNITNISSEINDDIIILENILEKNKNNYQKKFDPISNIIEIHNE
FRKNYLNNKKKNDLIVIASLIDKLPNLAGLCRTCEIFNVKKLLIDNINIIKDYQFQKISSTANKWMNIKELKK
SNIINYIIKKKKKYSIIGLEQTHNSQKLNHFVFPKKCILILGDEKEGLPSSILMFLHHCIEIPGQGIIRSLNV
HVSAAITIYEYFKQQL*

>N.PF14_0274
MHDIVERYELKLLINIILNNNYKNVAIQLPDCMLKDSLCITNLLKNEFQKYNKEIIFNESIKNNGSINRNDSE
CSPYCCKEKKNGEDLHTNEKKKNNCNDNKMDDDKGNIKSSVHVDVVENNNNNNEHNNEHNNEHNNEHNNEHNI
NHNNNNNNNNKDKHVNLYILGDTTLNECCEDYVSAEHVEADFLVHYGISCQSFITPFIPSIYIFNKQNIEDNF
FHNIKQYLKNENVFKQNKTSIILCDASFAHVLPELVRCFFDKIEKNNNICTDLKNEEKKEINDSLSFYNYNNK
VLYNVDDKMYVNKTFYEKGEDNYNECNITDLKNDNNRNNDDYYDNSEDDMIFTNIIVCLYRIANNINGKVCYG
FFNNYVEFDIKDKIEDKYLFMCGRLVFKVFYNMKKEMIVYKIVEKKKEERQIYEENGDDELINNKNMNLFIFS
SDNKNFINRCLLEYNNCNNIYIYDSKDIFIKDTRKIIDKVLLKRYSLIEKCKQVNNVGILISNVNLEKNKEIR
ILINYILRKNKKKCFTVVTNKLNTAKLENFYDIEIYILLTCPENNLLELKEFSKKIINPYEFFIAYNYIEWDG
KYVFDFFELLNISSIKKEYQAIHNNKYFLWNMDADFFTIKNHHKDNKDNNDENVNVIIQQNNLVLEKYDHLID
TNLSISVEDQKKFITRFQEDSQMCTYFLNILKENNKREYKGVDINYNTDHVPHIVKGLYGIAQKYDTDIKHLK
EQ*
>N.PF14_0275
MKDIALFFLPLGIILSFLLLSGIAMFQRLAIFLQKKSINFQLSSHKINISISSIISFYALLRLLCTIIELNNI
IYEKEIYENITSLNYNRSYLKKIRLQRNFWILLLCSITWIFYIRFTYLLIYYREKVKESDEQYEQVLETRREE
NEDHTNKNDDIQEISKQMKQDDDDVTFLKNYNDMSTNNDGTPRSAASEECVHFKEKSYLLDKDLTKSCIRQRK
*
>N.PF14_0276
MIKNGNLLKRNLLRNGLCSDFFFFFFKSNCYINHNKKGNFISINYFSNEVKRNKENEIQVAKHFREIFHDGSE
KNFECHPFNRRFAYSKKSFFPIEARNLRSLGLNRQKKKKRGRGDKCSGKGIRDKHKHRKSGRPNSRTFESGRT
PLYRRLPKWPEAWLSRQKKNYDCLNLSKLRYFIEKGRLDTRFPITQRHLNDSKCVKVKNGVKLFNINDYPFPY
KIDIEVSHADQSSIDVIKKVGGSVTIIYMERVNLRAHIKPYKFDILPKTARPNLDMIHFLEKMKSKGCIVKYI
KPLWLIEEEKRIINELIEIQENEKLSHLMEKNVHNNNNLHVNDLTMEDSQEHDHVKDKLLQKYRLQYTKLGHH
P*
>N.PF14_0277
MSNFEIDNNCTLYICTDNCEVPTNNEIQKKLESQNVDKKIEGMENLIFNIIQGVSYENLLMCVIRYIVPHKDH
RLKKMCHIFFEIVDKCNSDGSLKEEMILVCNALRNDIISPNEYVRGSTLRLLSKIKYLKILDPLMETITKNLG
HRHSYVRKNAITCIHTIIKHHGVDIIPNAVKEVEKILFLEGDISTKRSALAMLTDIDPLTTLKYILSLNDQLY
DTADVILLEVIHLFKKLYIPHVFDDSYLLINDEDKDDDDENNDENNDKESDEEDDHGDDHNDYNINSNLKEEE
KYKYNYNDHIYDENKNNKENNLLDYKKINNIHNITSNERYSQSTNHIYTQNNKIGDTYKYISNKSNNVIHHKN
EYKELGEELNLLSEDINFKQKKLNENNYNQYKNHVIKILLNMLSKNVSYSVLYEGACCLLYMSTSALSIKTAC
ECFIKLLINQHDNNIKLIVIDKLYYIMCKWKNILENYAMDLLRALNFPSRDIKVKILNLVLHVLTKRNVHLVL
NVLKKELLKLNDQVVYTKHIITTTNNNNNNNTNMNMSNNNNDNNDNNNNNNNNNNNNNNNNNSNNNSSNNNNN
NLSTNYQEVTSYKKILIKSLQHICNLYSSECLHIVDLLLIYVNHNEQDINYEAAVCIRKLVKNNNFQNNILEK
MIDSIFDIKKATILRIFFWVIGQYMFNEHMIINFLNNLYDNLSPLLNNSLENDMINKIQNERFKKNGNINFNL
SNSNIQTKTVILEDGTYATEAFLKNQNICNKNNDKNKENTFVYNILYENDDILLSVICVSLTKLYLKLLSKMN
NSFDFILNQNEIICSKEKKEMKHSLIKHEHFLNHVTNNNTLDTEECVISKDPNRISTNSENKITEHDINEYRN
KCIYILASIIKYIGEKNSKPDSNNIYEYDSNVIRINQCLKIFFYLTANKKELDEQSKKLIKIFIDGDYYYNKF
LEKEEEKYCSIYGSNYKYMKRNNMYANKTNGGNKTNGGNKTNGGNKTNGDNNTFDGNNTFDGNNTFDGNNTFD
GNNTFDGNNTFDGNNTFDGNNILEKSTYSRNKLFLNDQGEKENVDDDIYFRVLKEKKNILNMLDEETSSVCIN
ENLEKLKLKYTLNNDMLFEENEFQYPSIKYNYSSLFLAKLYNSQILTGTDDDIFIEALPIISNVNLIIEIYVY
NQSNVYLQNIYINLSTHGNLKPIDKIPEFNLAPNEKKKFKISVKVHTTEAGIIFGYVFYERKNDNHKNYIVLN
ELHINMTDYINASFISSHLFRIMWSEFEWENKINIHTSIRDAFELLKLIIKNTNMTIVERFMPLEYYDMEIKN
NVNQVNISPIDIYISYISTLEDLKCLVNNSAFFSVNLFSRSIFGEDSLANLSVQKSADGKLSGSIRVRSRTQX
XXXXXXXXXXXXXXXXXXXXXXXXXXXXXXXXXXXXXXXXXXXXXXXXXXXXXXXXXXXXXXXXXXXXXXXXX
XXXXXXXXXXXIYIYIYIYIYIYIYLFIYLFLFIYFIGYRS*
>N.PF14_0278
MLKFLNVKSPVVSSDVDEITDDSDKGDVSYTLEELRMKMEITQKKHFGYKNLKDFQVEAVHATFHKKDSFIVM
ATGMGKSLCYQIPSLMDVCRKKFTIVISPLISLMKDQVDNLNRKRISSVFLGSGQKMNNNKILNEIKHGIYKI
VYCSPEYALNNKDVFILLKNRILLIAIDEVHCMSEWGHDFRPSYRKLNELRIILKEIPIMCLTATCTKNVQSD
ILKNLNFDLQNCLIKRSSVNKKNLFYSVREKTDIYHDLKDILDIPLKESIERTKKFIDNSKICSYNSTLIYVN
SKKECESVYSFLKEKGLLVLMYHADLTNDQKKEAHEKFLKDEIQIIVATVAFGMGIDKPDIRRIIHYGFARSL
EAYVQQVGRAGRDNSDAEAILFFHINDESKIKNIILRENTANNLIETNFQRVEHIVHIFTQASDYAYSTACRR
KKIYEYFDEAPLTSYDIDIFNDKRNEGICYYIKKYDIYLCGKCDNCVYCLNLIKKKNGIKTNKINNNNNNINI

YSNSNNNNINSNNNSREYYLDNDIKKETSLKSLTCYYISSSSNSSFSSYIFDNVVDLTNELKTLLNCISSLKG
KTGLSTVCKILVKSKESSIIKKNYHNIKEYGKGAHKSTNWWSSFMKVVRNDKFIKETLNCGKEMCYISVGVTD
KGEEFIQSKEKYIIALPFFLNTSPKSSNTRNKKTKKDTYGKYSNNKETKYENITYEYKDKYNDNDNDNDKDND
NDNDYSKDIYNVDDYKYDDHHISSKIKQQQEQHNNNIRDIYYNDKDVEYDKSIHNINKNTNKNLSYITIESGD
EKGNTNCIINKENINRDNNFEKTHKNNIEKYEYIESYQKQKNNNKNNNIQINNKKYDDNKYEYFNIDKNDFIM
KENRYTKEEKILSDEQINDSIMKILLRTRMLEARKQNIPPFQLISDQPLKDICHKRLTSVELIRKHVYNISPI
CPNTFLEKIVSGIRGFCLLHDLKTDILNLNIYNPNHNNSPIPINAKTSTLKNFENLISSYNYYDRNKYAPREE
SKSNETYSYEHYTGTERKCKHFVTQEDEYNHADISKMSRHTNIYEHKKNMNDHTYDLHNYNNNITYDYGDIPK
NTLIQYKNSLTDQNTISMSDINSDNKNMVYNNHIFNKNHSIITNYNSSAENNSSLQNEFYRKKTNIIDDNNIG
HHTYNINDNYHTNDVLLTNAIIKDRPKKKHDDIFSNFCYKRDESETGVQGNVTHVKKDINKGYQENKNDITFL
QNNISQHTNNMIHNNGKDVKNNLSALDDDDDDDFKFINQIQTLGLHEQNCKEENNYQNSLTNEHVHIPNYNLY
QEENTRNENEKRKININLKQEDQNIYTKRHQYLTNPNEHSVNNMHNIHLKENNIPKYDYTNQNVHESNQKKND
FYEEKKKKFNFIESFSYNNNQEDKKRVNNLDILDQFVYRDVKKRKT*
>N.PF14_0279
MDSFIDDEINLIDENEPILIDIQDLINVGKVPKKKGNGHVVKNYEKNNEKLNKNKINEYSNINNKINNYKMNH
EYGNNYNTYDSEIENYNDKDFLIYKEGNLKKGKDKKLKEYSEYSISHISNNIYNSFKNNYEKIFDKLKNIYIL
RKKKKKKPDDKFMYYSDDISDDFFHILASRLYYSKITTYIYFLVIILNLIILIYTIFTNVLNKYVVCAEIFVI
FMLFFEIFLRLLTEGTSYFYHFDGLFDVTVSFICLLLLLNSGDFKIFFLENKIVKTNKKEIEEIISQSLTVLR
FSFQLFRTITFFMHYKRTKLPTDNIDFSLLNLPRDDI*
>N.PF14_0280
MGDVNSLSYKIINDESIIRFTKSSIYNDIIEFITNLNKSVVGVEMKPLEDFKLCNENDMINNDNFLFLSKNVY
NIFQLIKNMNKCIDSCPPINQSSRFGNRGFQHFCDAYYKEVDEYLPHALSESNIPNISEHTYQISYYLKNSIG
NKKRIDYGTGHELNFLLFLFCLNKINFFIPSDYKHLVLVLYRQYLEGVRRVQIIYTVEPAGSRGAWGLDDFQF
LVFLFGAAQLSYNRKIKTDDIEKKELLELWAPKYLYFDALKYISMIKHAPFHESSQMLYDISGVETWEKICNG
LLKMYQAEIIQKRQILQHILFGNLIDF*
>N.PF14_0281
MFFINFKKIKKKQFPIYLTQHRIITVFLIFIYFINLKDCFHINNSRILSDVDKHRGLYYNIPKCNVCHKCSIC
THENGEAQNVIPMVAIPSKRKHIQDINKEREENKYPLHIFEEKDIYNNKDNVVKKEDIYKLRKKKKQKKNCLN
FLEKDTMFLSPSHDKETFHINHMNKIKDEKYKQEYEEEKEIYDNTNTSQEKNETNNEQNLNINLINNDKVTLP
LQQLEDSQYVGYIQIGTPPQTIRPIFDTGSTNIWIVSTKCKDETCLKVHRYNHKLSSSFKYYEPHTNLDIMFG
TGIIQGVIGVETFKIGPFEIKNQSFGLVKREKASDNKSNVFERINFEGIVGLAFPEMLSTGKSTLYENLMSSY
KLQHNEFSIYIGKDSKYSALIFGGVDKNFFEGDIYMFPVVKEYYWEIHFDGLYIDHQKFCCGVNSIVYDLKKK
DQENNKLFFTRKYFRKNKFKTHLRKYLLKKIKHQKKQKHSNHKKKKLNKKKNYLIFDSGTSFNSVPKDEIEYF
FRVVPSKVKYKTWHIICNIYKIYNYHAYIYVYMYICIYVYMYICIYIYIYIXXXXXXXXXXXXXXXXXXXXXX
XXXXXXXXXXXXXXXXXXXXXXXXXXXXXXXXXXXXXXXXXXXXXXXXXXXXXXXXXXXXXXXXXXXXXXXXX
XXXIYIYIYIYIYIYIYIHIHIYIYTYTYIHIYIYIYTYIHIHMYPYLWPIFCRTKCRSR*PPHKWVLLKLFI
QRKTKNISVPYIIK*
>N.PF14_0282
MDEGKKKSKSRKHITSLDEEKLFDNISKDDCGIIKKFTLGVCAMESKVESAPMECILKRLAKSGDFHIIKFKE
DMILNHDIDCWPIVDCLIAFYSTGFPLKKAIEYVKKYKPITLNNLEKQMILRSRLQIYEELKKWRVPHANYVV
VDHDTVKRGEHIFEEYYDYIVYDNIRLNKPFIEKPINADNHNNWIYYPKNTGGGCKKLFRKIKDRSSEYCPEI
HKVRTNGTYIYEEFLSTFGTDIKVYTVGQMFAHAEARKSPALDGKVCRTSDGKEVRYAVILSEAEKIIAYRIV
EAFQQTVCGFDILRTTMGPFVCDVNGWSFVKGNIKYYNDCAHILRAMFLAKLEEKYNIIPRDLADNWYNIENE
EEVLRKTFRQPDDLHCSHHEELCSVIIVMRHGDRKPKQKMKFFTTKPLILDYFNSEENLYNVISNRYAHDDTI
KINNNELHTTNYNTQDVLKYNAKLDHATNQYQGTHKNHSNSKTFNYNCENNCSSKCNGHINDTQKDIKIPCAH
HEPLCNNSTNTNKENINENNKSCENNKSCENSKSCENNKSCENSKSCENNNDNQNNCTCNDKNKPIKDLNNQD
KTDTFLLSDNKTMNEKNSSNNKNINPDTCNTSTQTDEVKQANLYKSYTKKEIKFKSPEELEDLFLRNNVILND
IEKEFKLIKEQLYNIQQKLEKDDINEKNGQKLDAPSKKEDLTRSTMKDKDNENVDDKKEIPHRKKLGHDHDEN
NNNTSGICNDTSDTCNNTSSTCNNNNTSGICNDNTCNNNTCNSSCSNIKLNVHCKNKPSEEERCTTCSGEDAL
NLMTKEELEIKRGEYEVMIENHKTLQKILERGDGFTGINRKIQLKPVDFIIVNDKVIVTKILVVAKWGGELTR
MGRRQSENLGKRFRATLYPGDSDGLLRLHSTFRHDFKIFTSDEGRCQITSAAFTKGFLDLDGELTPILVAMVI
RNSKAHSLLDDNNPCLERSECKEYIDEILNKNNDIDEDLLKKLTPGKNARGFRESLRKISNFYELMDKVRTTI
YEFLKSLNQEVQKWLNLFPYDEYALYVIDILHEIQVRWKSLTKMWYKKNKNKYDTSKIPDIVDNVRFDLIHHH
SYLGSGLDKAFEIYNQIEPLANFISQAEYGITPQDKVKIGVHIVGKLLRKLIHDVTYYRDEEERNKKNNKGNN

VLKNALHISYMNPFYFKKTDELQKKDKDQHQRDTSKNFNMCKYDTKQNFLDNFLFKRDSNEINKKLETNNKSN
IINDKADMLYNANVKKEKTINDVVEETTVQNIHDEKGDNMYKYEHGKNKEDTIKPVDININGDNNINGDNNIN
GDNNINGDNKKNGDNNNNNMEEGDETKELVIEKEEKTFNNEKNKHLDKSYLRCASICSENCKRNLITQNEYQR
KDKLEKCSSLTEKYVPDISTNIIKNRKECICKNNLYDNEKKNNSLYRCDDLICNMKYSETINGTNSEPSNSKR
VLNKALCIGGYSNSINNNNVNGNHKNINDDNNINDNNINDNNINDNNINDNNINDNNNNNNNNNNNNNNNNNY
CNNNYNNNSTSTFLDTNIGESYNELKNYKSTVTYNTNVKDIYTPKLINKKEALVINSSDLWAHQNKYRKENNE
KQKMKKIKNMIDNDFNEKKEDSNDRGVSYKYSENFLKKESGSYFMRTVEDSDKRHKTKIDEDQMDSTSNVHEK
TKELKNSGSLENKENKENKQNKQNKQNKEYTQYTQYTQNKQNKEYNDKHGDNLINDVKGDHMKGTNVDEEDKE
KTKNGCSELDTNNIKEDTDVNINGKENPNDENVNGQVDVEAEADAEADIDVDEDEEEHEHDDDEDIIRLKETD
ARRLGIRSPWRMVRSRYYVTSASHMISLLNILIHAKKADNTISQNIIDNDSFKSVSDVTDLHYLSHLVFRVWE
RKHLKRDDSNRFRIEILFSSGAKDGFGQNYELLEKDAKAQQQKYERHFNKYFDDTKRINADKSLGATTGGVNA
ADTQNGTTKSTGNNSVIDTQNGTTKRTGNNSMIDTQNGTTKRTGNNSVIETGNVQENYKNMNNHLNNNNNLEE
GDTNRSHVPAEEYINRTTTRENENVKNKNNSNNMKDINRNGTKIGDTCEEKYEVENLENKDKSIINDKNNINC
VITTNNCYSDREEKGNFVSAEGVSNNSIINKKEIRIEHDKNIRSGHFSNSVDEIILNNNKKKNTMNLNILNKE
IKKSSLHSISNSSVNPFVFDSLNVFRRENEGKSPHIINYNKREASYGFGCLNEKKMDSINNNLSRAGSYMVRS
ISSNLRKKSKQKDILKLDYDKKEKEDITKENLLTYENEYINNSSNEINSKSYRSYSCISDRSYYRNKKEFEIG
NEEHNRKNKLNAFNHIMDNDKLKNQFIRKHTLEINHIDNNDNNNENNNNNNNENNNNNISNNNNNISNNNNNN
ISNNNNNNSSNNNNNNCSNNNNSSSNNNNSSSNNKNNNLNSNLNSNNNNNIYQDKKKVNLFSHVYEHFNENKKN
STSSLKFFYKTYMPDYEKIIENDKKAETFDVPPYCELAPLIVLTKNCQLSTFENILTKLLNNYSKNTKNKDKN
KMDKVSKNKSK*
>N.PF14_0283
MEIGYMEVPNIKDINSNIEKSGLVLCVSLNQKESESLISPSYYYIYVHRYMYLSNIIPKCLEFFKSFILPFYG
NKFGVYFECIKKEQKKSSNNNNNNNNNNNNNNNITYTSTNNYEEKIVLDWRLPIGVLFDIYCDLDKSQKEYIKT
YEKFNDTNINMNNKKNITICSNNELFINHINIVKLKGTDKNYDKKSNERQNQKNIYDANRNISTNTNNSSNVN
HQQNCDRTDDDTLHKGDDILHKGDDILHKGDDILHKGDDILHKGDDILYKGNDILHKGDDILHKGDDILHKGD
DILHTDAFINEKETKEGTDKKKQINRNIKNEERERGKEVKNIIVENYYEENYMKEKKIIVENGKGSTKNDVEE
NKKDINTHYDDIKLNNVDIYDDIKLNNVDIYDDTADFQYIQFIQNEKINNEWYNKQFVNISNKNIPWMLIVHF
KGEEEYPLSIINKKYDEKNTHFKGDINILPYNNYIPLYKGFNNFEEYIINQLKKANYILNKNNRALEILPQRI
QKDILYSLKHFHIEKICSLYREYIDYNMLNFINYFNNSYIKKVQQVCDLNRHKDNSIQINGKSSGKQNEENLC
LLKMDLHEEESPVKITHCKDILNNDNENYDNENNNNENNNEHNCHRNSYDVHLNENFINDQSQKNHISNIRTS
YNNDHVSDSLQTDIQNDFSSIILENTKEQGDHINFKNNLHNNEKYPNVYDILKDEKVLKDCPIILHIYGPPYN
QILTKYPFLKLIHSNNNNNNNNNNNNDNKDNNNNSNKYDGCKKYIQNVHLNTLGDFLHEQLPSFVRKIINKDEK
NETSHILNKYDKDKYLNSETIYYFIEDDYLIFSPYMFIIVNGIQIPLNTPLYWLAANFSQFDHFLHITIRIPP
Y*
>N.PF14_0284
MSERLYKKIKSMECESNLYISNNKVLFESIYNLINKGVPVPSIYYTNNKIVDKETDRYVVTKYIYDVDDIIYK
LEAHSKINLEKFKNIKIKCLEEEYLKDNEVEDSYKDDKYNQLDSLQIYVSHTHFDYIWNEYTMQYFLSCYHDE
DNLKIHTHILEKEEFGYDLQIELYEIPTNPMQAFQIASHLSKIREALQCGPLMHFFLTKTNITENIFEKFIIR
KNEVIFILKKNDYLLVLISIHFVDSYDQFIILGLCKNIHLTNKSMDLSGNLDCSFYTDFPSHLITSEFFIYNY
EDDMHNYEQNKREGTEDNEDIDMDHNKNVDMERMHSMNSSGNIIINSSSINIEKNITNEENIETKKYQKETWK
DYINNSFDENKIDDEIKLKVPNIGFISLKMDIKLFNACKDFSELIQISSRISHIIVSFRDFLKNSLILHRIKT
RRMF*
>N.PF14_0285
MYIYIYIYMYIFLFYLYIIFFFFFFFFFFLMEKIGLASWNVNGWKKSCEIIKRNDDDLVQFLKKLDIDILCLQ
ETKTNESVIENDCNLLEADSNMYESYWTCCKKKKGDKTHKGYSGLATYVKNENKIICSTNNVFDDFSFFFNDY
IKKEDLLIKKKSEIDKTSISFFLLNDNKKIYNDQNIKCDKNDEHNKKKNKTNISVSEFFNEGRILITMHKHFI
IVNIYAPYSGHNYERLYYKVRFFHAVRAKIIQLRIVTGLPIILLGDFNISYRNKDIYYLNNIINLDILLKNIH
NIDLKEDIKMKIIKNIPLIINTLKDEHNFIIKKYKIQNNELYNLYLKFDTHVKFIGNNFSSVEEILYFFSLDP
VYVHDKYKDYPYEYYFVVNNVSDDFHDVFKSPTDSNINVSNNKNDHPLSSTNKDDHHGNNHHNNKDDHHGNNH
HNNKDDHHGNNHHNNKDNHHGNNHHNNKDNHHGNNHHNNKDDHHNSSNNKCCNFKGQNENKENTPKMSYHNTY
MKNNMNKNEDSIQKKNREKEDDEFRSTHKNKYNFEHDELLKRYENFSINNEKIFESITNNNNNNNNNNEDFDL
GFFLKKDKNIKYNNDDMNKNERNKKILNCKKILCKYKYKNNINGQYMVKNPNCIYLKHLNDIFISLNINLSEH
DLLNIANSIGEPSSPPCCVDIIKNLIYEDNMIDTFSFFHPNVNGKFTCWDTYRQCRVHNEGSRIDYIFMDYIL
YEHFIKRNTYLYESPVILNHELYEMLKQKLKKKNSLNYHDMNSFQTNQYYANYFNKIKTKQKYLLSNDNINDT

SRKKKNKQEKDEEQDEHKLEDQIQDAEFYKFQFKISTYIGFIYTSPKLSDHIAVKCTFIKNYNRINYNNKNKI
IIKLCSSRFNIILNYLCTSINQYIMYLPLFLINPSLFSLTTYTLLDHVHLYDGLCPNILNTQPHKKTNKITQY
FTLKKKK*
>N.PF14_0286
MILYSCVVCFIVFVFHVKAYSKNKVLKYAKPGFITNEIDIGAYAKRRGKSRLGSLHNYGYTSTKSVDNQIEEL
REKVVSKNKNEPEFLQAFEEVLSCLKPVFKKDNVYIGVLENIAEPERVIQFRVPWINDKGEHKMNRGFRVQYN
SVLGPYKGGLRFHPTVNLSVIKFLGFEQIFKNSLTTLPMGGGKGGSDFDPKGKSENEILKFCQSFMTNLFRYI
GPNTDVPAGDIGVGGREIGYLFGQYKKLKNSFEGVLTGKNIKWGGSNIRAEATGYGVVYFAENVLKDLNDNLE
NKKCLVSGSGNVAQYLVEKLIEKGAIVLTMSDSNGYILEPNGFTKEQLNYIMDIKNNQRLRLKEYLKYSKTAK
YFENQKPWNIPCDIAFPCATQNEINENDADLFIQNKCKMIVEGANMPTHIKALHKLKQNNIILCPSKAANAGG
VAVSGLEMSQNSMRLQWTHQETDMKLQNIMKSIYEQCHNTSKIYLNESDLVAGANIAGFLKVADSFLEQGGL*
>N.PF14_0287
MAESSYRDTQLFKPNGVNIFDSNLGMNNKISENISAEELFQSKNIYRDVKSKVIPGPIKTTTVEKVTKIPKII
FRERQKDIVKKEKKIIHKEVEIEKIVEVIENKDEIVYNEVKVPKYIDVPILQPRQEVHYQQISKNVPRGVELV
ITQTLEVPRIKPKYVEIPVPIYVPCYIEVPIPAQYIPIEQNDDKEHFTSGISNASKLSKHPHMQNNSMEFPHH
YNNPYVMNPNLNMNDDKSVCGSSKASIQNISSEQKNVEKMVIAGNL*
>N.PF14_0288
MDKIKSWLGFNKKIVFTENKFLLRNVNDQESLQAENVRAEDYPIPEKYLEDPDKIPKYYVFQSNMVTDEDLQP
GMLRQLEVDKRLTLPTRTHISFLVTATDVIHSWSIPSLGIKADAIPGRLHKITTFILREGVYYGQCSEMCGTL
HGFMPIVVEAVSPEAYAAHAKKYYKE*
>N.PF14_0289
MGYTSTLKFVNLGVRRRLFRRAHKQPHHKWDSIKNQLNELLKYGRIETTLTKAKELQGYAEELIYLAKKDNVE
NNLKVESMLRTAQGRRRLYEFYVVPLYRHRPFFFTRIINQWKLRLRDSAPLAYIEFIDRPGELRPAKPVGYNKI
KFIYEEMKKNRRNFRKYFNIAKRFHLLDENDHLLPDFADTGHVPEKSWYDEENEEDDDEIIINPELIQKYKKF
NNPMLKGPKKGREPFYVDLPLPSVTERTFLNYRKKFKP*
>N.PF14_0290
MDKDKDKSKNTPLLYQDTMNDLGDGLQNMDTLRLNRNGNTGNGNKNKTNGSKGVKGEYEKKKETNGHDDYVKE
SLNRMLESHIDQYFKGLDYYKNNPKCKIDPKIHLHGSEEIENYIVKEKIFVTTYFQYIDVYENAINVDEDLEK
EEIVYVEVPKYVTKYKPKIVTNIIEKTIEIPSGEEIKQPKYNTVNVPYVIPNIVENEILVVLKKIIQPEIEIT
NEELEIEVEKYIPRLVPVNVYVPRYFGISAKAKGEPEESVRYVDLTQDQIDELMKELNPHLNELKVFNETQLK
RMDEYMRESQMQARAHNFEPPQPQLITYDESGHCQSYDYSEFHRFKETCIKELTH*
>N.PF14_0291
MGYSGIDIKEINVKRKNSVYFDNVDVCNILKENNTYKQKKHISININRKCASYNNIYYINNDHPGLGKNISYY
QNKDNMQLKHFFNSNKINIHDNKIKTTQSYSYYEPLRYPAFKMSDKIKSETNELKKMDTKKDVHMKDIHPKNH
KISKNDDLGNNNIDNNNNNNNNNNNNNNDNNNSNNNNNNNNIKCVSNRSTSNKHINRRNMCIFHNKINKKEKNI
NEQGEKNEHSKNDHKHFGNHILKDVKNKKKSNNIIPLLYEENKNNININSKNGNSNNLEHEHVQEKPARVHKK
KRKKKENKLAGNKIKNNGKNEEVKQSSVIEMEKINYLDDKVNGNVEEKKKKKKKNKNKDKDKKRDEEKEEDKN
KDKDKDNNNNNNNNNNNNNNNNNKNNNKNKNKKKKNKINNNNINKNKDKDMSKNKRKNKNKNEVVEDNKNKHYL
EKKENNINEIPKEVMYIPIEERCKSIVSSSDEENLYYEKPYEEVENYFEFIENKHLINPSDITNEVKFILHMT
LLTLYKDQIKPSYGKIKKRLTCFNENLEIKYNFLNIYASLRNEYIVVRTKRNNIFVLLRETPKWFLGWVKTRC
FKNSYPKKVWKKLIEYFLNMTKSNMNNNLYVSMYIPFIKKFYDKRFIFYLNEKDNEKNKCYEKIYNFSFLSFD
MNEQKKKRNNFNVLFYIYNMYHNNFSYFSQCNDYYIKNVEKNFLLYYTYIFFNYDKNDLNNNNSNIDLSKKNY
LCEDKNKDTTTTSNNNNNNDNNNNNNNNNDNNNNSSSCSNNNNNNNSSSSSSYNNNCNNYTSLYVEHLFNDKK
ENILQTDEIIKYDITKNLINEENNIDTTNMFDIFNNDIYEVADILKKKNFPFLKDYSLGKIAHIIYLCLYNGL
LLEENQKIIPACSSKNIISSIFYIKNKNSYLYDNYSHLNQNFYCDDNNISTYGYDYNESTSINLMTKEYDDKM
DSFLNVYENFLKNEEGLFFSKKKNNKCDVNVSLNKCTEEFHIPAITNLEEAKFKIERLLKSSYKKCIYLLFFR
EKFLKKYKQNINPLIFGYNSLIEFLFYGCREVCKIYILNNNLLIVHLSYDIAKHINNNNEKEKDKEKEKEKEK
ENVIEEFYYSDYCYNKTENNNNKFNNSSLEVCTIMKDNAKKKNSFFITYSYWKYMSKKEKQNDILDNVSFLKG
EQNYIFSDDIWKINKCSFDKTNPIQQSGKDIPLYYKNMKNINTGIFNMPNLVQINNYDFEFFSTC*
>N.PF14_0292
MAVLNKNRRKVDNKFMGRSLMRNKLRHKEISDNILYSQIGNDNEVVKKNKKLSILNKDSLDDFLDNQLIINNV
EVSKVFLKKYEIKEKKNYGTNKLNTKDNIQNIVLPIPGRALFLKKDDKIHILMKKKEEKEMEKKRKKKKNYKN
KISFLMSGKSLIPLNVRVPKGDSSEKNKTKKIKKKKLDGINGKGGDDNDIEDKEVEDNEVDDDNDGDDDVDVD
VDVDVDDDVDIDDDNDVDVDNDVDVEDNDDMDNIDDMDNIDDMDNIDDMDNIDDMDNNYDIDNNDDINNNDDI

NNIDDINNINDNINDYENDYNYKYLYMYERLGEKYKRLNTNNSRLNKESIDKYEHEYFIEWRKLLSEIEEKEG
YYVTPYEKNIEYWKQLWRVIEKSHVLFYILDVRNPLFFYCPGLEYYIKKVDKRKKLILILNKADFLTYEERKI
WAEYFEKKKVPFVFFSALRELYHQNKITIENMPCDNIPKREDFMGDIYDNNNMNGKQNDDDDDDDDDDDDDDD
EDYIIHNKTNINNVFDLYNNIDEEKKDIINVGFGNLSYEQKKNDNTDILSVNDLINLIKKIKKEIKEFYHDIE
IETFSSIPKFMIGFIGFPNVGKSSIINCLIGKKKVSVSRQPGKTKHFQTITLKHFPFSLCDCPGLIFPSLVFN
KNDLIINGVFSIDHFKGDVVTLVQIICNIIPFKLCNNYKIDTNIIHQYLNEKGHISYFLDASEFLKKFCTFRK
FVSGGKGGQLNFSHATRIIIHDFISGKLLYNFLPDYFKKTSHIYCKNYIHQTAMDNILDNELLTESNANEEIL
TTKRKFRYMQKKMMKGKNVIKCDKV*
>N.PF14_0293
MFLSSKKRRVFVLFMYFFVQNVNSNKSTNVSNNFSSNIATNLASSLATPLPTNVASHLIPNLVAAQQNNTAHV
DSHKTISTNKHTCEAAGCSSYKDIIKNNSSFHTSNGHQSNDGDCLNGFICKKCKKTHAKSPNICFYSSLDTYE
NLYEGILEDFKQTSYDSFKIPLDKSSKNIKSKGKHKNHNTNKEDNDNNDNNNDNNNDNNNNNNNKDNHNKKDT
NENEEDDEDDDNKNKNKGDKDDDRDDEEDYKSSTKENNLKKKKKKSDEDNEETEDIDDEGEEDDQNEEDDEDN
NNKKKKFNNKTNNETDSFLEKYSKISFHTFLTPNKLTCKDIKEKKKSKKMKQRIYINNNKKNPACPLEPLEPS
LIQYNIINLKTNKNETIDKNKRIDINNKGDTYTYSFPKYSFLKEEERIQEMVSNEKNDKKEEHVLYRRLKINI
NKYEEYLKSKLNKCHISDDGIATIFIKLILQIVKNKSEIYTDPSKRSILNNNSSINTSTTKNNNNNNNSNKEK
HKYSDEDDEDNEDNEDNENDGYNSYGQKNKNNLSEDDYDNENDNDEYGNKNKNSNQSYNYMSSQNNNDHNNQV
NKYAYIQLLDKNYQNIYNTNNPINKIHQKNYTYEKKYNQDRKNKILSTQVPNTTKYNFFEYHNVDELEGDSME
NYYKSKRGFFKNIFKKVFKRKKGKEGDEGEGEGEEEFHNEEVESKGKEKKKKKLFSFFRRKNKKNKSKNEGDD
SYNESNQGEDNEDNYEESGEDNYEKKKNKRYDMDNDEKEKENDDEEDNDNNNNNSYSYISNNKKKKKNDKKKK
KKFNKHKLKIKNFFTKLKQKVLPKKQKLHIEAFFNNIIVKSCKNSLKWKGKMFKKRSLIEMTLKVPAKIKYIE
DEPLNFFRSGYEVILTCKNCEEILFNSCVQKTNKHRCDYSKFYYHGFCCKFCSFAYNYQQLYPLNSNWSLSDY
FSCDTHTYIKYSLVFLLMIFISIY*
>N.PF14_0294
MPKEDCKTEKSIDSIDENVLKKYDILKKVGKGAYGVVFKGRCKKNKNIVAVKKIFGAFQNCTDAQRTFREIIF
LYELNGHDNIIKLMDVIKAKNDNDIYLIFDFMETDLHEVIKADLLEEIHKKYIIYQLLRALKYIHSGGLLHRD
IKPSNILVNSECHIKVADFGLARSISTHVNENKVPILTDYVATRWYRAPEILLGSTHYTEDVDMWSLGCIMGE
LLCGKPLFTGNSTMNQLEKIIQVIGKPNKKDIEDIRSPFAEKIISSFVDLKKKNLKDICYKASNESLDLLEKL
LQFNPSKRISAENALKHKYVEEFHSIIDEPTCRHIITIPINDNTKYRVNFYRNVVYFVIMRRNKFHSNVLNQG
ESKKEEKKDRYYRRDKDKKKCICENNKVHMEDKEYKNKIIFSQGDKPEDIKEDHKKCKICDEIHVDTSKQIDS
INLEHVVLNNNDVSYVDKKKKTKYEPVYKERGKMKKDKMGNVYCKDRERRVFYEARKKESITNFTTATTISKS
DDTEVEMSQMEINEIESNEMKGKIKEQIKEQIKEQIKEQIKEQIKKTQNNISKISIGSNTMSSTISKTEPNSR
NYFINKKSVESFYTKERKNNDILFHANNKKVIFFKDKNKIKNHSSEKKKIKYKVFYGFKKSYENEQNVPHYLE
TKCSNTPLGKNYYYKKYVDGSNKKYVDGGNKKYVDGGNKKYVDGSNKKYVDGSSKKYVDGGNKKYVDGRDKKY
NHNYNHFNNNYYYYSRRGSLKSVNKEEENCSQRTWKNTAKLHLPLVVNKSTKPLNTLSHDYYNKDEENKYMNN
SMLKKEVKKKIWRHLIDNENKKNQDMTDYYYNPIINKTEKNNKRINFKCGEKKKTEEKCVSNE*
>N.PF14_0295
MYQRNTVCIKKKKKKNEQIIQE*INQLRVIYIYIYIYI*YIYIIFYFFYLFFFFAMARFKSQFNFMLKFKRHI
GNKIIWGRTNNFFYKKEYIFSSFRNNIGKRYLSIHEYLSVDLLRSHNVPCPEGYAAKTAEEAEEKALLLQNVC
GDNDLVIKAQVLSGGRGVGYFKENNFEGGVHVCRNSMEVKEIATKMLNNTLITKQSGPEGKKCNTVFICERFY
IRKERYIAFLLDRNSDGIILLGSSIGGSSIEDIIKKNPDAIYKLNIDINNGLTTGQAREFSENIGFKNDQLNI
VTDMIVNLYKVFKKYDCTLLEINPLSELNDGRVLCCDAKLNFDDNAEYRQKNIFEKRDLTQENKEELLAKKYN
LNYVSLNGNIACMVNGAGLAMATLDLIILHKGNPSNFLDVGGGATEEEITEALKIINNNENAKICFINILGGI
MRCDIIANGIINAFKQVKFNKPIIIRLEGTNQEQAHEIIKNSNIKCIVCQDMNIAAQKSVAMAKIIEIAEREN
IDISFN*
>N.PF14_0296
MPRVELTEEEKLYISKKNLLFKRFVEPGRLCLIEYGPYAGKLCFIVDIVTITRVIVDGAFITGVPRMVIPLKR
LKLLKERIKINKNCKSGFLRKTVNSTKVLEEFNNSKLGKKMIIKKKRDEATDFERFQVYFAKRELKKKMTTIK
NKKNGENKKVKKNVQKVKA*
>N.PF14_0297
MENLIGTPLIEKKKKESHISIRKDKKLNDIEGEKKEKKIKERLFLCLICLLVIIWLIYFCYKNHIFLSYLQNE
KEYGIIIDAGSNGTRIHLFEWKKREYELSNKKEENNLIELKEIFNAKVKKSISTISYNEIKDILIYLINKVID
HLIEKKIYVYNKQKWKSYPFYFQATGGMRNLKQEDRDLRMKYIKNILSNDNYNPFYFLNEYARILSGEEEGIY
GWLAVNNLLNSIFSKPNNTYGAIDLGGSSTQITFYPMDHNILENYNSILLNNILIRLYSHSFIGYGWIDSLFR

VNIYLCLEIIKKLSTRNIKNIYQLENYYDDYINDYFYNNLPNYNWFFYSTKKIINNNKENYNHHNYENINYDV
NKRNYPYNNFQQKYIHTNPKKGNYTKGNINNSNKSYKNLSNILLQEIVKYIQHSQNDYPYSSLTDIYDYTYNI
YDEKPYDIENFIKTIKNFNRHSKEDTIMLVQNPCLPYPYEMKITFPTYNLVTSQFVILKKNSRNEIGNINDIK
NDGNNYTQNDDNNYTQNDGNYYRQNVGNYYRHNHNINDVYELNSSESYDNFISNFVLKLKIINNNMSLMDNIY
EDFKNKGYLNNVLKKVNDNKISNTLKNEIIKRLFEKIINEKIKKLYINITVRIVGSNDFKKCLENTKKLFYEQ
PCFLSSCSFNGIYQPNLENNKFVLHGQFKKVITYLGFKKYVDLNQMKIYIQKLCNMNLYELTYNMSNKIMHNQ
IPTFCWKSIWSYSLLFYGFKFKETTKLLIINDNTNISYDSSSTSQASKQFYKRVENKEQNNYLHDKTNYNNEY
NLNNKIDNISWTHGSMIYQINIFQERFNSRKYKLYSEVFFVLFLIIFIILIILGGYIFKFKEILNKKYRFL*
>N.PF14_0298
MNYIEMEEREYKPLIEEVDNGNNIIINNKEYYNMYENNNINNDNINIHERVPLGTPLTYISGNPGDYIQHIDM
YNVEEEKKGKKRLATDIKYFNYFVTYFMISLIIIFYYFYYGKYSRILYGINYNGKICGKDLHKYPYLYFPLTP
KNPKPEILSTYAKCLESCPSSNIVSKDIADLNKDKNKDKNKYFVESYFFSYKKNNNNNNNIVIDKRGNSATNI
VYSDYTKSPNNNLYVEYSLNSPYYDTVNIMNICYPKDKLLREKVAKIIFTDRYKTFVNLFSLHNSFIFIFLFV
FTTIVLCFLYLLFLYISSTPTFHLFLISFVSLMFFLPIYFIHKHLYMIYNPVKTSLFSHQYFISIFICIIIFI
QSFFFLSIFFIYKSTYKYTSQIIGITLNFIYKMTNIIYSPIIVSSISFVWFFVWLYIYIMIMTAGGVHEKRLR
MELDSNGFSEIMPLQKFFYYFKSSSFFSILWVYSYFFICEILQSLNQFTINYLGTVWYFSDKSNFPKQNNVWK
VMKTIINYHLGSLVLSSFINLLFKPLRVIFFWTNSTLSLPFFYNHIIHKIKHNFYIFLKPISKIIDSYTSAAY
CEMSISSYNYLFACDTSCKKLINSTSPAAALHGITYILNIIFPCFTTLIITFLSFNIFNNFQRYNDLYSSNFI
PNPFFASLIIGTLCGIISSYFITLISSLSDCILYCFVCECYKNQMIDEDPMRNIFTPPMLRNFILEIYDEYNS
NL*
>N.PF14_0300
MEDILNEIISLSEKKRKEEQLKDMEEKNKKNKNDHIVDIKKIREEKSEYDFKIRKENKNTIKDIISNKKKKSS
DSIKDDNRSIYCDASTICTIDDDINIEKANIFSKIIHDKKMNEDNYSNISNINNNNNNNNNNYYYYYDDDILY
FKDLENNIDENTPLKVNGFNNNLNSYLLHVNDINTHISSIYKNIDKINVIKKKIDLNIYDNEKLYNKVNVIIT
NSEDIVKYIKLKINELNNENNEFERNSNMVSEIKLRINIFIDVVNKYKSCINKYKNICNQYYEYVNKNIIKHY
KLIHPNLKDHTIHKLLKQNNNNVEDFLNSNKNSFYENNIFCTNVEQIEIEKIKKKYNELKNLENNILSLNELY
IELAYVIKKRKNLINNIENNVFQVKEYTQDALNNIVDAKKYNAMIKQRILYFSIFLLIVAFIILFPVFFNYTI
F*
>N.PF14_0301
MDFPKLLNKPSLDLLKNDFPLSSKFELEHSSISKHPFLKSGFTFSNNTYSIYTNFKNNIYNSKNEIKFDNSGI
SLLDIKYEPGFIKNLNLCGKYTKTNGKEDDTFEVYSEYTTDDMNIFSSVNVRNFAFKYIHVSSHPKYRNFKFG
GLLEGNMDINNLQYSFGGAYTKHHKDNLYIFSLRSIPSNKHFYGSLALNLFFQNKSINDNAISVEVIQNIMEK
KANINVASIWYLDNKNTFVKTKISNDTKVALSLTHKYNEFVTITLGSQVDISKMSLPDNTKFGMKLYLKS*
>N.PF14_0303
MKEKYVLIKKILNKNNDIISNDTTVSCCDNILCTSSCSSLDEKCDEEEINKYDDFLKGTSRLSSLLKSPYFSS
ISSYRPCKGLSNNASFDLSSHKSSFDVSSHKSSFDVSSHKSSFDLSSQKSSFDLSSQKSSFDLSSHKSSFSSS
SCPPSSTFSYASSVTLSSRKERKKWDIRTHGSLYKKWSCNKNDDKDMWIHRMNFHKHPNHIVKKKKHDYNKEH
KSAYCKNGDDVNDEEYLKRMTKNIENYIRKMKDMIYYKRQIKKTKKRKEKKRKEKKIKEKKRKKKIKRSVNIK
SICNDNNIKEYDNMNYNYINYKDHLNYENNNINDYNIHHYKKDIQKLYEHLNYEQNNLSTSLLENHYINKNET
DHNLFWTCCSEENVEKPREMVFQKNCDLKKKSNEIFISHTQNTFPYNNNLSCICCARIYLIDKIIKVTSNKIK
PLCICCFDILLNLIGINTIICISCFKCYFPFEFVNKCVICNSFDMINLKKEDKRNMNIVKKELHIFMELYKYK
FSDHFNSDIFLNYFINMNYCFKYLYYYFTIHQQIVISNNFVLARYTFPFSQLLQIIQKDKHKNKMNMEKNMRN
EFHDDKHEFDDEEKYKNEIQLKIVNTKENDHVINHMNSHLSDHVNNNIKDNINHNMCVNTQNELHDEYTNKVN
EYYQIKNFEGKNKKHIGEQTFEELNSIQNKDAQEEYNIIQEIKEDIMMGERNNKKENYIMKNVETNKEKEQII
KEKEMKHIENDTIIKDDKVVEKEVNDKINVVNVKDIVDKINVVNVKDIVDEINVVNVKDIVDKINNLDNPNKN
DKENNINLNTVHINNKKKEDKKEEICSEKRNKDILIVEKKNKDIFNKKKSINIRNKLYKNKDKGNSKDSVENF
FYTFSKTIISHIEKLKNKKIKFSFNEFKNTHIKLNELNFNNTIRKYYANGDLILYYIKYLQRQEKEIHKLYQN
YKKTENDVEHFKRRFRIHYGKEANEYDLNKNIYLKFKLLNKVQLLLEQKIKNFDNMPKNMEVYKIINEFGELI
EEVSLQNNTYEKNNNILINYYLKNNIFRNDKSLLRQKKKRNISINKLNENYELLKENINSVLNILLFLYDTFN
KNKNLNQLDLFHILKKIKMFLYDKEIKKLYNHFNDDQYNHNKNNKTTKSNEITKWKEKNVEKCDHILSRNDIR
PEIICILNIRKKNMNNFLINNNNKVQIINKNCLQLFNEPYNFDLIYINNIYFKHFISNKIINKYIHNFVNKNN
VVLFNFSINYLKNKKKISFFNSFHDYFMNVDKTKINTYIKHQLHKKNEEKKNFINNNEENHQHINEYKINSVQ
HINKCSQKCMNRKKKKKKKYSDKKGKNILSSKNRKNQEKNIKYKKKTLFKKIINELNNKLNQKYSNEKYNLTL
NIYAQKKKKIYNINKCLFRNETNLYYQKNDIHKKEHKVEIMLINNYDNNTNHKINEKIIQKEDMNKCKENEDS

FQDRKNNLYDENNIDHTYYDEDKTNPFLPSYGMDIKNIKNINKYINFNNMNKFKKSSIIFLIQLELFLTSDEN
INIPKKKILHFTLVDINIPILYILKKKNNVNYFNEFKKKQNFDNIFINYIQSYFFNTNVNHYSHNIITHLFQK
NPYICFLLYIHDDFIFTNNSEYNKLHQIHNVKKENYSRNMILQLLYIYYMCNICECFNFIKQHC*
>N.PF14_0304
MEEIEEKYKIIIDALYDTWSNDHDKRKESEKILYEIEKDEKFIICLFDIYTSKSIHYNIRKLGIIYCKNLIVR
YWNNRDGFHYSDNTKKIIKKKILDILNNVEYLNNYREFSILLKRISRYELVHNYPELLDCLLYNINIHKTNIN
NIYIYIYLLYKILREQYSKKLFKDKKETFDISEKFIKSLEFFWNNSINLYFQNNATNIFCMCNDEKNCNKCKN
LNSLINLYEKQLFVSNVDDTIRNEGTANENIPEDENYYIHDKKIHMMKYLDSIILNLIINRNDIKKVNNYVNS
KMSKENKGDQENKVDQEYKVDQEYKVDQEYKVDAEYKVDQEYKVDAEYKVDPEFKVDQEYKVDAEYKVDSEYK
ECKENYKNKESPNSSNNTTTTTNNNNNNNNNNNVNVNVNDTDLNIRTNFLNAFINKIIYYLKFINNNSASYYL
KYLKFLLTSFFLMIEFHTSFHVYLKKDITEKLICLFLKKNLSLNNYTNDYEHKFIEIVNLCISILKSIFYHFC
LNQYNLIKQKKVRENYINVKNIMCNKNINIENYNVNTLESKIINNFMINVKSMENENNILFNKNLLLKDILTY
IREEVKQALSNDEFILIHNEKALEIFYFLRIYCINISSEQIIEVTLNIRDDYETEENIFYNNAKDCIIELSSE
PFLFSIFHHFFEPLINSFHNYAHFLKGLPVHLKNIQIPEFSSAIIQLDGYLNIYYILYPSFHKKIKIEHILCM
IRFFMDFLNIKYTHPLISYRIALIIKIWTKSYKTSFPFIEDLILLIYENIRFLYIQLLQKNVVSLITENIPSY
VNDMTFQHYAIKSVNFLPVLFFKFISLFKYFFKYEYVYKNYDYINEFLVSCLINVLTKITYPKNIQKILKILS
HIVFMSNKEKDITIFKDNYNFLLDLYMNSNISIKEYILNILVQILNKNYEYVGSLVEYMNKENYHNDNNKVGV
NEKVCDDIVLFYFSYDIILYTLSAKIEKQPFKDNIIFNHPHIPSEYKANHNINNDITSDINSDVNNNINNFQF
NNIYNNLINFPYIKEINLDIDKTISDNFYSLWICILKLIYKMFHQKKEEIVKKICSFYIATINYLYEYFKNQT
LGKISYELSNLCLDVVIEYMCLFIIHETHNFYLTYESINIKMLTNDIIKSTILKVVEFNFILNDEIKIEKCIY
ILHLCLSIYKNDMKNEKSIIYTYIAQFVIIYLLKVLLKYFSKYFNFYNYIQQNKNKRAERNDANKNYNLHSNN
NNNFNDDVQKNGNVIFDEYKTIGEEKSNRNKNNIPDISFDLQGNNTNNYYLNHFKNDVNNNDNDDNNNNKNND
NNNDDNNNNNNNSSSNSEQINNKLNQCNKNHIKYIYDNIINYQHDENFLNEFTHFQNDVKKLFNINYINDYDIT
KTFLNFNFSTINEDNYSYINKHSVFTLISILSLYEPNYLNYILITFLTYFKINIPIFIFSFFYQSQYIHNKNI
LNSLLIFIYILTQNYILTPFPSIFLQTNFPSITNTQSFEKLMYDTNKNNHLFTCNKDEHTLLIIEILKLINNI
LNTNKDIYIEKKNILHLSTISTNLNGTKIFHSYNYNYILRKVLKYDDFASICSRALDNILTYLIDTNNVEDNN
RKNIIVNYLKDNIDDMNSAIVDMYKKYKNFF*
>N.PF14_0305
METLKDEENYIKLIKVSNNVEDKKNEEIEKIEVCMEKYINLNIFEYFKNLKELYLVKNNITDITPLFNCINLT
ILFLQINKIKSILGIEKLRYLEKLNLFNNELNEQFINIEHNKYLVHIDLSDNQIERIEFFYNTNVFLYINLAN
NSIRNIEPLKNNFHLEYLNISGNKIEKFEDIQVLHKLKKIKELYLNSIYYKNNILTDNLFYKHYFFNNFPNLQ
VLDHEIITDKERKDTMSDIEALKCIIDFKINFIREKYKKEKYYILFINNKNISYLNEVLKPYHFYLNVVQEYN
FLENKEKENISNIKEQIKFYLSAYTARFNYIMKRLKEERNLQIRYLTTSINSYFNIFFKSITKEQEEYKFIEN
FIKLNFNSESLKNYYIDDIKIENIIKVKKLNHNVLDTLIEEQTNSLIYKKNILFLHPYNYCKLDDNFDFDEKE
YTVDDSEKKKFFEKNYICSCYNINHILKTLIKLFLEKDQLHELVHLIYYKNKQNDDILNYKNDINIKKKILRN
KKFDFPFFSIYIVENYFFEKEYKEVTAYSTQDNKINENDENENDENKNDENKNDENKNDENEYKNKNGENKRT
NNEIKFKIYYTLPKHTNLKYIVNLSLLNKNKEKNNEKNNEKNNEKNDEKNELNKIHSSIIKTCAPKKIKRDDF
LKFIGIKNKTNTIFFNENLIDDFFHYILTFEHISFFHITKYFIKLSKIICEIKKNIYLLILKNNLNCSEKDTS
LIENGEIRVYEDMEIVKKEKEKIINNLKNKNVDINYYNDHSNGKNNDVTIIYMNSLHLSKIYVHVFKNHYNNL
KELYLRNNNINNLKTFFHFDSNDIESLEVLDLSFNCISDLTPLFKYFPNIINIDLSFNYIYDYQQIQLFSVHH
QHIEYLSILNNSLYIKKIYYENIHLLFPQIKIFNDITITNKTNFDIKNYAFICKNKERVYDEYGMDDDDEDGH
DKNYIKIKNINNEQCLKVYEDINIHNYEMHIQMINLSNLYMKLTYLNFEIFKYLKVLDLSNNGIEHLTNLTLP
KQLKVLNLRNNKIFCIDFLHDKLEIEMLILDNNELKNINKVNVLKKLKILRCSHNKLSTIPLFQKCSLKEMDI
HNNFIKDITHIVLIKNKQSLICINIYNNKINFLNLDLYLIHIFPNLLILNNSCVERRQNSEKYFKNIYTLDVF
FDIYNMYPPYTSLQSLEINNLKIKNILFDINNDNFYNLKFLNISNNYINNIHNIGPLDNLKALILNNNKHINE
NSFMGEHERNVLNSFKSLEDLDISFCLLSKTNFFKKCPNLKNLKTLNLEGNNINSIKYLENLERLKVLNLSNN
KISKISPDSFPKSLENLNISNNLIRNLSPFDNLKNVETLDLRVNRIDNIDEFKYLKNLEKLKTLYLNGNRKIK
EHFIIIKNMLTQIENFDNKIMKEQNNLTSNLEEKREEFKLHKDISKKNILITSNNDNNNNNNYYNVTHNYSNI
KSPAKNKTKMTKLKGHIKKEPYEDRLKKDSFTVIGKKVNSSGNY*
>N.PF14_0307
MGDDEKYPFIDKVSKKHINKSNKKKKNNISNEENEKLNINNIEEKHKIDNTKESNVEQFNVSLNDDIKNSITK
RIPNISRDLKLDVCVAIPSTIINNKSDVIKSYLTSFLARIFTIFSISKIYIYDDYLEQEHLINKQKNYGDYQN
EKYKKDIKNNSNNVKNMHMNLKKDSNDNDHNTFDNIVNNEKNKEESPKYSYLCKYMHYNLQYLETPQYLRKH
LFPITNFLKNSGIMNPVDAPHHLRSDEWLPFREGVVLQKSSNGIIVDVGLFSNVLIENVNYINIGTRVTVLFQ

SNSYELFQKKNPNVLFTGKLIHPSVPKLYNLYWGYSIEILKNLSDIFKLQVDYLIGTSERGKFIDDSMVSFRD
AKSVLIVFGNKDGLEDLLIKEKEKKKNKVFSPEKKIKVLNKMLKKFDLFINTCPCQTSRTIRTEEAITITLSL
LHNILK*
>N.PF14_0308
MEKKKRKKDIYDEKSDDIDSWNEIDRELFGEDELKILEKGDKEVSENKEEDNDYYIDKEILNVTNDEDFIHLD
DIYESDNTTTNEDKSSSSDVEIIEIDSEELSNEKDMNKNKKRNLKNDHKNKKGNIDKNKKGNSNKKKNNTNDD
NIKNNYSVDKNSDDDISNESKLFSLDELRKFKDMKYKNIQKRIEENKLITFLLCWICHLYFLNNICNNKLVQG
LIYSVYVNYKSTKKLYSDPIYLFLFIKNYFELTIDRKAWIIGDKILSKYFKGSIIFRLVRCIHRRKGNNVLLN
LIFVSLCRCLNIPSRICMSLPMLKDEIYTIDNNDHFIKKCYHKYSEKFNKNIFFNDFINNIETYSSDQERLLN
NEERGIDNYMNNEDNNDNNSYKHNESNNNSYHNNNSYHNNNSYHNNNSYHNDDVIIIEKEDFILNKKKKSSKN
YKTHETTQYHIFTECYSASFNKWVSFFFPFNVYYFNFFNFSKNDNTNNYESKIFHILLPLPKTSEEIKIYKNE
KTKSNLNKFPHSLFYGNNKKIKEYISESSSDPTTLLSIYSYDENDKKEKHPKDKYINNSIENNVYIEKDDNMF
KNTKTCSNKNIEPTEDNNYIINEDLIRKKKKTNNINYASSNILKENKCHITSHIYFNHDLKLNKLDNKKYSHK
NFHILCENYKGKMYVEKNNKFFIFDKKKKNILRIIYLQYNIKKSEDTKRKNKNYIKDNLKFDSSQNVDDDIFD
FEEDEEHMNIKNSLADIYSTNDTNKFLLNQIIELKNANKKLKKKEYNFNISNHLIEYDEIIYDKFGDISKVNS
YENKYIKVFINSNVYKYFYNLDHYNLYLSINKYNILKDISVRHKLRCSTNSNKSNTINAIIKGDKQCNIIRES
FRKLCFYTNKKNYKNSELVIDKRDDDYLYNLYVTNMIPKEKTDFLKSNYYILKSMLKKNQVIYPNKPVGLFKG
ENVYLKENFYNLTRREYLENRHYYLSDSEEPLNYEYDEYNKIRIPLYAPFQLKKRINRNRKNENCLLSLGPND
GITNDTIQNNNDINNNLLGIDEQHFDYIYDKKDKLDILKNKLFAHIKANNIIELNEDDICIYNIQLKYILKHI
KGIIPYKLIYNNSYFLYNKFRKRQNVKVDVNKIIIRKKDFIPFQNLYIPPKKTQDEFLIRDKTKQIKNLWKVL
FKSIMYEQKNKELSIQKKKAKNIYKMKDFNMDVDRYFEI*
>N.PF14_0309
MYKLSENNHKSLLENLKRRGIIDDDDVYNTMLQVDRGKYIKEIPYIDTPVYISHGVTISAPHMHALSLKRLIN
VLKPGSRAIDVGSGSGYLTVCMAIKMNVLENKNSYVIGLERVKDLVNFSLENIKRDKPELLKIDNFKIIHKNI
YQVNEEEKKELGLFDAIHVGASASELPEILVDLLAENGKLIIPIEEDYTQVLYEITKKNGKIIKDRLFDVCFV
SLKKN*
>N.PF14_0310
MTICSGCGVVTEVTICCPICLKNNKKIFYCSQECFEQNYKEHKKIHYLINIVSREEENQNKICENKLNAVKDD
LPIIVLTNEHGTHINNSDVSQNFSNNMNGNVCNDIYETSYYNNLSDSSTHNGTNNNNINVDKVDDNKMINGIM
NDNKINDDDDDDNNNKSSSNKCLNNNNNNNSSSKSYSNNHISNNIIILQNEDDRSNDTSFLHLHKSDNTKNLE
DTHNYNNRDYKNTRSLTFEKLHRKNNYLKYKNIKDDHLLNNTNNEDKKQQENEENEYNNKNNLHSKNSFVMKV
SYYLSYICSYIFSNKYHMLLPYYDDVSLTNEKKKKKNFKSFKLKNKLSEHKIVELKKKIRIKKFVQLTLLLLL
ISIIVFISTYGFSYILEISQKKLQINAEQKMMFNEENKKNQEMQQIKSYINVIEDLRKEISEMKEILYVHNVI
LNKNFHLNNSYTLFNNKMKSANSSYNKKGSDNLGALTSHYYYDDTSSSYRDININNNNNGNNNNDNINYMDAV
NINYVPTITQNEYDIKELYPKNQNSDQITLEKEKNYFDKKLSKEETKETMSDIHSIPHNNRNKEDIYNNDDNN
NNIKDNTEKILNSTISQLNEHSNIEIVKTNNQNVNENINNYIKNHKQSISHNAEYAQVSNAIVNEEDNKYVDN
KIILENMNNIKNTGTVSMDINNVSNNNFIHSHQIKDKYLNTSSKMEESDKPENGDRKVTTNKMNKKKQNTI*
>N.PF14_0311
MTINSDSSTVSDIHFDDQNDGDKNKINDMNLFNDSDFFDEGRNKKKNSCSNDNFYEEMKYDILRIERNINSEV
KKRLDANKNIQLLIEQMANNMINNVLNKITTKIESISSDLDKIIHKCEELEKVISQIKVDIPTKIQTDMISLK
REIADFQLVINKYLNNKKKRDNILYGKIENISAYLTGKIQSEIAFKQHDLSYLKNESDRLLNYDNDDDVNFKN
VFLQEVEEIKDALNLTVKAREQSDDDIIQAMNKYTSVLQKALQSAIINTN*
>N.PF14_0784
MYMLRFYLDENGKRVYTVKPVVNGKVTFSAHPCRFSPDDKFSSHRINIKKRFNLL*
>N.PF14_0312
MGNGNSKVVKIFLLIQNERDFKTKEEKEIYLQMLLTLDNDDIFKNDGNFYAIKNIIDDEANISVLFKFVMNTL
RKLIHNETGEIFVQECKICVKIFRNIFPIIQYNYKKLNFFSLLWKEDNIPCFILQNYKCKNILILHIFNFLLI
LLFTENICINKKENVNEKINEKYIDNNNNNITYIRPLVKNYVDIYKLWSSKMIYSCLYNKYCVNNNNNEIVNR
CFKNIMKNEKYRHSLFQKEYNDDITNKDKIIKTFYSSDYYHKNKIYHNSNVEKYEFINDVKIVSYNCNDKNHF
NNINNGHLNNVNKDTAPSSSLPLYPQINSNNNNNDIINLEYIKNRTDILKCLLILLSSYMYYNNENFLKEKNF
PLFLFTSGEVYFSANFFLSLLTIIYENEFNYFNFYFYNDTYLEFYNLCIHILNILIDFVPLVLKKNKKKRVHY
MNGALKYFHKCKETINDDEKKNDEKKNDDKINDDKINDDKINDDKINDDKINDDDKINDDEMINDNDTYNHNN
NNHSCFKSSKQNYHINIKNKEEKNVKIERDINIDDTNYSSSCSSDESIIYNDFSNDSCSSSCSSSKKSDQSSY
NNYIENKRSINTKYKYENDIYYSNVNNVLKNKKLFFNLKKGNSNYYIYLKKKRDVETYCRNNVFLQMLRGLDR

NNIKYIYRGSLNMLISYQLYYENYNESLLFLDNYLCLLWNIMNNNKQFIKYMEKCSTVGGINENKNKHINKNI
NINKNKKNDNYNDNNNNMEYNNIYNNSHNNYRNDCSTSSSNLFVFYILYILLSFNNERKKEKQELVTIKKQQI
NKEHTNINNKITKDSVFYNNEEFCKTISREDYNIRKVDGIIYICLFIILKLSSNKNICKNLNEKFDKKKKIKY
NNILKSSYEFDNYIDFVVCALNTLINDNIFFLKFERIIDMSITILTNISVYIKSMNTYSCECIINILNKVLKK
EWLLSSQHHYYSLFLLLDFINNILSHNLIDNYNLVYMIIKNKHVFFKIHNLNQILKSNYFVGSSTKDYWIPTE
SWLVSWMNKLPLHFINKIIYDLANMIEVECDEKEILDYIEVVDLIRSHCSKIENKQIPYIIRKYEKNILLSKW
LTNYIYFLIFLHMYKKNLFINNGLTFIF*
>N.PF14_0313
MNEKKKLDIIRNMLGELLGNKEDKNNKGNEKKDKLKKKYIEKSKKVLHFYRNYMNILDKEELDKFENFYNMVL
NNSFDYSKKENIDIYMYCNIYPILKRTFESFVSYLSNLLEHKDDDNYKKIINNFDPILLFSQYIIRLTNDDDD
DNDVNVVDVVDVDGFSLNEIDNNMKICMRNTHETETNYINYNMYSTIKKEEHYLQDGYYTNDEKYIKCTTMKN
KDILGNNKIKNEIKDFYNTYDNLIEENKLKEQQKRKEIRKNIYEKKNILTHDEISLYENYYEENIKIKTSIRY
RKKTNLNSTSYIDINNSNKWSNHFPNTFNISLDLKESIESVICQDGIINTENKIHIKSSDNIEMKELKNNNDN
IGCKKFTNNSYIKKEDDVNVEEYKNNEKEDNFDNIIKLIESQENSYIEKRIYLLLNKWVKEENGRKFILHNKS
KIEPLFNEYKKKNYIVTDRDIIPLLFFIDKKLHLNYSLVHPYNYNYFYYIFHLNSSFYCADTNSITFEEFWNF
LVSNLPLNNYLYKEDILIGLEKYEKKKQLHKNYLFTVTFIKNFLLFILIDIFFMLSYIIKTHYYKCIFDYIYL
EKNIHDVEKTDHYVKEDMKETYKDEEIKTKNKNNNNNNNDNSNNNDNNNDNNNNNNSNNNDNSNNSNNNDNFF
LYITTSFSSFLSSSSSASFTSDISSTTTLTYKGVENNGDVKNNEHIIKESIQEKQDNNDAIFHHHQVFQKDTE
CACDENGIWNNTESKILNTNCQCEKNENNIKDIKKKDNINESNIKEDKKEEYFLSNQLNTFINFLLLLWGINI
SFDHINHEEFLITEDLSTDTDDNFINVDTNYSTNKFTTTTTTNYNNSNNYKDDDEKLKCINQTNKNVKCKKED
IRNNYSPYDTNDEDKEEKITNTWKMKTQNLYKKNYQSESNMNISIKKYRKNTQKKNKNEERTKSLIIINRKGN
NEKKKNKNKINNNNNNNYNKINKNLLYHNTDQQNINTFDDNQNYVKNQEYLNDENGIHDYKAYYEFKEMYQQKN
YEHIINNFIHSIVKQLKPYKKNYYNKIIKIIKNNKLQNVFINIFRKQTSYLQNKNVQTYKKLEELPKCDENKI
KNNINENSVNNNYNNDNMAHINNYNDNNINNYLQDQKIKRSRNSSCNKQTHMNCVQENYMTQNSSFDILSTEK
SISYIIYFTETYIKKKTKKKVQDKKISSINILKNEQKKNTFIDSILTLCNFKLYKKKFLNKFNSENIYILLFF
VYYVNKQIDDLLTYNILPKKKKKKN*
>N.PF14_0314
IYIYIYIYNIFILFCDRNDEEYAYQDLILGTYTTEKNNYILILEVIKKKKKKKIYIYIYIYIYIYXXXXXXXX
XXXXXXXXXXXXXXXXXXXXVTLPSEEFSYSSFYYEKINDYRHNFCNDTSKNFRIKNKIYHESEINKISCYPE
NADIVACFCSDGNINIFNINDYYNKDDEDEIKNDNILTFDKTLKGHLYQGWGLEWDDKNNYISSCGDDSYLCI
WDMNTSDKIMHPIVKYFNHNIPLQDCCWNDNNVLSVSENGHINIYDIRNKTVVNSIRATNCTLNSIDVNPHNK
NIFATAGTNKEIDLWDMRFTNKSLHRIISQKETIIKLKWDKFQPGILSSSTSDKFIYFFDTNKIGIEQTYEDS
QDGPPELIFIHGGHSSNILDYSLNNSYSMMISSISEDNTLHIWQPSKQAYEDASDTYDDTEVE*
>N.PF14_0315
MDYVHPVDVSKVIHGNESFFTFIDFLRTKKVCNRRNVHIENSFKKLNNITFDNGYNKENKILLSLKEIYKLMV
DIQNKNKIYRDIKGNYNSNNNNNNKKNIHEMKKNYYNNLSSWNNYLLYVLKNDIMENSYNVSYIILIFILINY
QKKIFYIINIYYKDINCQYKKKKKKESYQKDYSHNNNYSLHLINNIVLLKNKFSNNEISLTSKNFNYNLKIKK
KYIYKKKKKKKKKKRVPKRFWLYIQKLFLYKCYLINKTVNYKCENNILYNQKINYLYNIKKKKNFLFKNFILQ
KLFWVYKINKHNIQIKQNNNNNEPFLFTNILYDSSIKYICNKLLFIYFCLKNYHHYNDSFDIMRTKHIFMMLW
NYIYNTDYYIKSKQVNNSLEYIMNLYIFHNLLKLQVYIIIQISQMLIQFILKIITLWIINMMNNIIMYLSIIF
RMITILKMFYSTFIINIFVLHKDIILCDQYEKYNDNKQIVHNDNYYINRSIILYNLCNIKQYTIKNYNIINQC
IHKLYNIMVIKQKNTYNYITKSYLCVSFLNQLYYSKTQCIMIYEKNRYSISLDHLWKSNINIYFKKILLFLLL
LLLLLLLLFSSYNNLNNTNVLQLYDCYKYNVHNNYLFIYFNTLIVASILNLLYIFYYVCINKFCIYLYRDCII
SFFLLYIFILYLYKYYNHIIKIRDISRLSYIIYVVVLLIHIMMYIKCLYNLIRIISLVMFFFVFINTSLSYPN
MKINNIIAYYNNTYNSICKFYLLYIPTIFNMYKYSSLLICIYFIHTINVFVNCVTFILLHFIMILSFSLFMDM
YICSAPILYDTHLSKKKIMNFEKDRRYTFVSYSSSYALMRTINLIHLNKLYIYDIFIKRNLVGCHGFTLFIHE
KHETNDYIVNNNYNIVIVLVLILVGRRNRTIQIEDRRIIYKLPVIILNLPSRKNKIYTNIYSCEKHFNTKLYC
FFIFKQRNNNEIVDGVHNEKSKNYYMNDHVKFVKNKKSFLIQTYSDLYHFKNYNDNKIFNNISLCYNINLEHR
MNNVQKYIHADMSIHFKKNYKRDKGINQYIYNYKHNFYLRVFNFLLNFIKRTKNVINIVLCIFKLLILTKEIL
VYYILNLFIYYYIWLKCYFVLYNIIYEYIERKDYMLMGLYNNSKRFKDNDKKDLFNKNINEEKERKAKFVSCA
TNNEYNDNNNNNDNNNDNNNNNNDNNNDNNNNNDNNNDNNNDNNNNNYNNNNNNNNNNNNNYNNNNNGGGDRN
DENNDKDKKEDNDNDNINNMLENDDGKKKRTRRRKTSDKSKTINKMTTGRRGRKSTSMLNAHIDANNILKNNL
YNINNGNYMNNINNSILLNNNMQENYMNDNKSSNMLMNNIPNKMNMKMNGQINNEIMNDMINYNMNNNMGNHM
NNNMGNHMNNNMGNHMNNHISNHMNNHISNHMNNQLNNNIYVNNRVGIGYMSNSVNQHNHMSPHMNNTINVHN

NNNNNNNSNNNNNNNNNNNNNNNNSSSGSNCVNNTLRNNLMKNNFNQFNRMVNNNSINIHTSSSNINNNMNNYNI
HLTHQKINSNNNNINNNNNNINNNNLNMNGKLNYHQMSNKVHGTVPIPMNVHMGGTSISNINHMNNINNINNM
NNMNNINNMGSSNRMGTSPINHPIPHKKGGTHMSNQITRNAMNKMLSNPINVDNKMNVLNRKNSCTVNFNMNN
PMVSNMNNYMNSNNMNRNSLPNNICSGGTIMPNVMSKGKIKDTAINNNSFRNNNNINNMNNMNSMNHMNSMNH
MNSMNHMNNVNHMNNMNNVNSMMNNRMSEQNIIANNMIKTEMRNNNLMNMKLVNRNQHMNMKYVSEKYPMNTK
MIQNNNMVTNRDMNIINNNNINGINNMGFINYMNKNNTLNKNMKNIKTENYTYDHHLLNHDPLSPMDTYDIQH
MNNNINNMNNINNVNNINNINNINSINNINNMNSIHSVNTKFMLNKQEDDKTLHPNYLNNQNVQSNTYYHQTK
EEEINTYNNTHSSTTINMKKKHKNSLKNSKEDDLNNNQNLRSVKINWNKYDNLFKRIPLERNYEGFILEQKNI
KDEDTINDEKTFMLENNEDKEDDAIYCDLSNINFKTFFKDYNDILNKLNLIKLNDKTNFKNNCYSQLYEEIFL
IIEKFLDISHLKYHNNFTHTNFYNNCLKYAIYQNKENTNLHNIKEENVDNKNIHGLDIHLTHENNETNKEDHN
QNDNNENNTKKNNNNNNDHNNDDHNNNDIKYLIFKGKSSIFDSVSKSSNNSIFFKTLSKKKKEFNELCEKNNNF
DFVPRNVNLIQANESNINMYPSNASSLTISKNNTLKNSVVRSPSKDVNVETPTTFENNIVNNNNNNNNDSNNN
NNYNNNLFSSKRSLSESKLIFNQNVLIEEILSKKYRTFSINEFLDVELNNNLKELVRVYKKNLCYNIKTFLKK
KMKGSKKMYHQKSYYDTLSHLFKHRMTRKLFKRIKRKFLKTTKKRINKMKKKKCKKNMKNEEISKQKNGNIVD
DSLFLETTDQIDKCDTRNKSTVDEKNGMNEYLDEEEKPLFSFCSGSSRKKKHMDDDRDLIERGIIRIKGEFSE
DSNIIHSNNITSDSTQKGRKNTTSKMNKFVEGKLNSNICSKDNNYILYQVKMEEDQMDPLKINNDDNKNDDNN
NNDNNNNNNNNDCNNNNNNDCNNNNNNDCNNNNNNDCLSLDERKVSNACLESGQQKEFNHINISNNNNNNNNI
NNNNNSNNNSNSNESPFSSNVSSNKTSGNYDINVYIEKESMKIKYSMTKDKDTTEDKKKNKNKEDTQINEKLF
KYSFLNLSLYRYMKNLENQRNKNTCDKIKKQQKKKQTNVKYKTNAIIDFGEKIVWVSFEFKNLHMLKKIKNNF
LCSNYLNNIQNITKEPEEKFEKYITDEMKKYKNIKIKFYKFLTYYDFVFLKNRLNFIKITYKNDNDYMLQRAG
EETKSAMGINNGSTNFQDENKELKFNDSTDCLLNGYKMDECNNISNNNNNNILINKNTNNEMSEMNNVTLKSK
VKKESKRLLKRNKLFNDNISLYPLIDKKKLNSYIDLNLNVDVKKEELENGSEEKLNDKVLSFINKYLYKGLKI
DTKNECSKMGIYENYDKIKNMTIGMDKSNDSIIIDNFLWYNNPALFTIFEKYYLLLKMQKYILQYKHYSKNNL
KKKISSESNFEMYSSSTSINCTSLNKLKGKRRCIRVKTANFGEDGCLLPKHNRRSNVSNSHRYMNNINEKKKK
KGSITKQVTENDDNNNMNKNNYNNNCNADAPSTGLDINNENATTLIDESQIADINNIKSEPILEKNDKEEKKG
NESITQEKNKEHGSMEPNINMDINVDINRDINMDKNNSGLVRKLCHADENVENVENAENAENAENVENAENAE
NVENAENAENAENAENAENAENAENVDEQKKEKGDEGKANELIVEKKQKEEAEEEEEEEEDGDENSVSSSSTVK
QLDNNLNINGEVSNNIKKELQKGRNTRLGNNKVNTKRYCSSQGTTPRGEMNFLEDTDDENLKRKRGRKKGKKR
GRKKGVKNEIKLLNENIDSKKNKVLFEGLIEESNAKGYNKLGNKILMQDGGEMLDNSLIGIDEKENELLLNEL
VPIDKDIIIDLLCEEEKLIESYIKNSRFVDKNLICLLIYMNNIQKKLSSICNNLLKKIMKENKMPTRLCDNIE
KSNEITYKYIMFERWNQLRYSLFYTISLMNSSIQYNHNHNNNNNNNNHHHFHNNDKNNKNNKNQLLIYNKNINI
NSIFDLHEYSCKHKKYKIPPFEYSVFLKYKIYKKNKTSTKKDDLNENIKDSTIKNISDDINDSIDEKPKEDII
QNLEDKKNNFANDEEKFCGFSNNNDLNITPAFSYCCICFNDDYNTMQINEQNENHNNSSRNNSLQNITNDKKN
ENLNNKIKSGKQDEQKEQEEKEEKEEKKENIKIKENNNLHFKDNVEKIERKENQNKENTNNKDISPNNAKIYN
NNNNDKNNLYNYCQNKKNSNCSSSSNDVDMKLIERNIKNTHNLMLKCSRCYMHVHKFCYISTKKSDENILSNI
QNSASINSNEWLCQRCEFEKKSLGNNFLYIFDTNIKCYICIERGGAFIQIKNNIFVHTFCATFCVPDLLVNNN
VNLEIFNEYLQLNNMSLSQEIFSAFQKKKKQKINQKKKQRKDYISVKDSNIKQLYHNNNNNNTECNDAEHVYF
KKIKESDKEINEKDDHAHTNDKIHDVLQHDNECENEKEAHSCKSVFSSNNKNEEICEQKCEQKCEQKCKEVMK
KKKENKIYDFFSFIKKDDKKRGNECASTVENIQKRVKLDNMDEKKNEDYTNDNLNDNKNSNTNNKEKEDTNNV
MCDNNNNINKMESPQSCNVKYDINLDDDHDNESYTSEASSLSVVSRYSINSISTLSDKNNMKLCNKGKEKKDK
IKIEDTYNDINKYIKKDYILNGYLNEDVRCDVCLKNKGIFIKCHNMNCGKYIHPLCAYMCGLFIKCKNSNKKF
LKFQNKIDYCFPRIFFYVKCILHSIKKYGVVNIYDQIITRRTKYLNRDLYPNIYENNKKERKPKNSLKYNKIR
NSNNNNNNNNSSSNNYYYFNVNINKKNDIYEILAYNFNHLDSYQYNFFLLPTIDYMKNNICLVCFTSYKKSEL
VHCKYCNMCVHKNCYLVDSSYLEYQFYRKKKNTCTLLVHNKEHKIVNLISQMKARKESVLSFLYDQNVGRINN
YTTINNTTNSYINNQSSYSQVVDSEGLFMSPNYYERKKKKKNHSFGDNIISYSLNENESNNDKIKINKNGNSF
PKEDLYDNGQINNSCDNFVLNKEDKDIIIDNNNNNNNNNNNEEIFLKNCKSEKYNCDNDNCHNNNNESIINPCV
DKNMDANCNIDKLLNKNDSYDNFGKGNDFNSGNNIHGDYMNSKDNLIYHDENVSFDIDDNNDNNMKYRKELEN
SEIFKELIHNEINNCLFTKENEEKVDFVNKLRDFFRNDKTYESLKKCTSEEEKCVTDIDDVYEKKLFVCDLCA
NNYNYENVNCILCSRKGGALKMVKINDYIKIDKTKNKKNSFMKNERDYVFVHMQCALYTPQVVIQDINEEYYQ
IYNYDNINNLNEKELMKKKHYESDTELQYLNVEYNQDEKQSLLNEFYYKQFCNNIGQMKNIKKGIDKRNSL
PIRNTHKLYDLPEYEQIYPLDNMNNMNNMNNMNNNYNVSSCNNIYKKVFFNFKRKKSDNKEKEEEEEVANIKI
NDFNNDQGENKNKTEFNLVNNHEINTDQNDNNQVYDNQNNDESKNSNHDHILENNQGNDITNSVALTTVEDIC
EKGNQTLNNNLEIKKEESSFSNKVTNENIKIINVNSYNKWKSGRFSVYNNICFNNKMKINKRGTIGTTTGISG
YHGNHLNVNKFINKKLKKNKEYISSTYNDNKNNYKNLNNINKKILYNRRNTNLYYYNLNRKKNNYKIIIKDRI

KKYLNKNTCYICNLSYGYTQKCVENTCTHYFHISCARIHKLFFEFDYNFLKTHPDSTTTDLSKIPNSMIFCED
HSKIRRRVKPSIKLFSKLRSFLELARIIVDQMKKREDIKNSWITDKYKNALYNNDMLYDEENDITSSALYNDD
NNMEIVNLHNDSNYYNYDDDDDDNNNDDSIDHNNEDYEYEENDPYCEQKYAYTTSKDHYNNDDDISLVRNNKR
RNRRLLNNVPNDHIKNELNINYTNEMSSHNMREKSHNLMFPSNSNDKDKISFLVSNEGGAVNNDEHEKENDFI
NFKTEQESMNENNENNEENENNEENEENENNENNENNEEIKEKEPNTFYNSVQDDNVKAEDYKDENNDNDGDI
*
>N.PF14_0316
MAKNKTIEERYQKKSQIEHILLRPDTYIGSVEMHTQLLWVWNKEKNRMVQKNITYVPGLYKIFDEIIVNAADV
KAREKEKSENPMTCIKIEINKENKRISVYNDGEGIPVDIHKEMNIYVPHMIFGELLTSDNYDDAEDRITGGRN
GFGAKLTNIFSKEFIVQCGDSSRKKEFKMTWSDNMSKFSEPHIKNYNGKDYVKVTFKPDLNKFGMTEMDDDIE
SLLFKRVYDLAGTCSVRVYLNGQRLAVKDFKSYVDLYLKDNSNDNKNNKGQNDNNNNNNNNNDENANQNNDNL
DVSLSNEPADGTPTKNNNNNNNNNDEDEIVKIHEKQHRWEIVVSKSDGSQFQQVSFVNSICTTKGGSHVNYIV
EQLLSSLSKKANAKNKGGMEIKSGHIRNHLWVFVNCLIVNPTFDSQTKETLTTKPVKFGSKCILSDKTINNVL
KSPILSNILLWAQAKAQVELKKKMKAGSSKARERIIGIPKLEDANDAGSKYSQECTLILTEGDSAKTSCLAGL
SIVGRDKYGVFPLKGKLLNVRDASFKQLMDNKEIQNIFRIMGLDITDKNKDDIKGLRYGSLMIMTDQDYDGSH
IKGLLINMIHKFWPSLLKHKGFLSEFVTPIVKVQKGSQEYSFFTIAEYEQWKENTNLLGWKIKYYKGLGTSTD
REFKQYFSDIKNHKIMFLWTGDRDGDSIDMAFSKKRIEDRKLWLQNFILGSYVDHKEKDLSYYDFVNKELIYY
SRYDTERSIPNIMDGWKPGQRKVLYGCFKRNLRNECKVAQLVGYIAEHSAYHHGESSLQQTIINMAQTFVGSN
NINFLEPCGQFGSRKEGGKDASAARYIFTKLASSTRSIFNEYDDPILKYLNEEGQKIEPQYYIPVIPTILVNG
CEGIGTGYSSFIPNYNYKDIIDNIKRYINKEPLIPMVPWYKDFKGRIESNGKTGYETIGIINKIDNDTLEITE
LPIKKWTQDYKEFLEELLTDEKHQLILDYIDNSSHEDICFTIKMDPAKLQKAEEEGLEKVFKLKSTLTTTNMT
LFDPNLKLQRYSTELDILKEFCYQRLKAYENRKSYLISKLEKEKRIISNKTKFILAIVNNELIVNKKKKKVLV
EELYRKGYDPYKDINKIKKEEIFEQELLDAADNPEDNEEIIAGITVKDYDYLLSMPIFSLTLEKVEDLLTQLK
EKERELEILRNITVETMWLKDIEKVEEAIEFQRNVELSNREESNKFKVARKQGPSSMKKKKKKKKLSSDEESE
GGDTSDSSEFLVNTLNIKKNTNKKTTTSSNNVNNSKKRLRKADDLNSNELDNTLSVSKTFDDNNNLTDNTPLI
NRLNDENNEFSSNNVDNKSTNKNSRKKKPKIADSTNDNNSELNSSIQINDNVNDDINITISPNKTINVNEFSS
IKNKLLELENKKKPRLTLSEKVKMKNNDKKDTPNKGSAGKRTPSKKLKGTLIGDDSKSRQDSNFDSDASSKNN
ESYGDSDSSYNI*
>N.PF14_0317
MDVLNYFISIIKHTFVCLKSNQMDYCGQNLAYTIRNIIFGISTIISIIVGYYSQSLALSTYIILGGTALASIL
ILPTWPMYNRNNIEWEKSYSSSNDKKRK*
>N.PF14_0318
MIYIFLFIIFLNVYHNIICQFCFLKKRKSVKNLLFSVCPNGNNYKRFNIYKRSTIKYDKKRKNIYLLLPNNNT
KFPIIKNGNYCILNKCTYSRRWNLNDFLYNYINILKYKKNIKIGRLLKCIGYQNEYVYRKKKKKEKIRLFYIL
NKISLINLKKSIRYKNNYKYNNINIYNSNNKLCYIPINHFNIWNKETFLYNNIGENMCNRSKKLFKNVLYYFH
TLDDIKNAHQKNRNLFCLFNNYIKEEENLCNNSVLIYNIFKASQIYNYAYNESIKKILKEIIFLFHKIKKNNF
IKTNHNKNIYEHIDVLEKDVYSNDNMLYRGRENINKILSEDDCLLKNENLDLYRNLSNDEKNIQLRNKKLSDS
QNVRDKKNIGDNQHILDNQHIWDNNNFNIRVKNILRPTHLVFIKDWIDKFTKYMFQENISFTNLLSNNILIKS
DYIFQEKFLLFFHIQCLHFLYTKRNEDIKKDIVSINNERTCDEEKRNKYDMIKYNSHNNYENIIIYLYKENNN
NMEEFYEYLKNIYGEEKICFFNYEKYINNDKSFFIILLSYEELFNIYRYSNNTSYNVFQEYMESKIRNTQDNI
RMYKILSYLWNNSSKDSNIQRDDTLNHTIQRYQFKIFLHEFNVIYNYKKSIIEKNLYEDLFCLIPLNNEKRIT
FYFLSNTHFNETIFYIWIDSIYEKTKKLCSLDWTNKNTEETKKKFFLLHNKGIIQLNKNNEHCSNLKYSSNFI
NEENTHKKQEDVLINIVNKKKKNVYKNDKLINEYMVLFNNIRKEIIINYLKQNNLYNINKLKKKDKKIKRIFN
IAKRILNKRIKKKKKKKKKKKKIYIYKTHIKNNPLHISDYINFIINKDKKGNILLSKNIISPSYIEKEENLQI
HDKIKLLSNTLNICYDNNYVQTLSKQQFNNNFSHTNMLHNLRYSNTNVINKNQNFLKNNKPSLSLPCEDKNCT
KKKVDENICIQKKDDISLITQNEKNSSHYLSKYFYLLNKKNISKPNTFGSTEKLDIYPCIYYIFNEKIYFLKF
SKELYEHLSVLDEFSKKRMDDIIQKFKGKINITENMYIYLNKGIYLINDNINRYCERFIKELLKKNIIKIILS
SVDISSGGYKVKSVFIEDVHIQDKNYIYEYNNIIDNINKLHKDIYIYNNSSKNNSSNNNNSNNNNSNNNNSNN
NNNNNCSSSSKNKFVEHNKNDPYLSYVNQPDDIYRLHFIKYFMFLNIRNIFKKYTLSNNHLLNLTRNTTNIFL
IPKKYEDIQIMLNILNDNYFNFSNMHDYNISDFYFNLYYNSIHKGINIKLFVTKMDNLVSVNGLSNNKNNNLD
INTKNIYKQNNILKEEKKNNYEDYTNNSIQYDKIRDDMYYNNNLFKIKNLFFLKSFFSHIHNNVDILPPIVHL
SNLLKFCKNIHSIYKYRQTNEYVFIKNSVNIYNNVCYSLNYFDFLYYYINNKNKLENMDKALKNHFVNFFLLK
RKQYEKKFIKHNKELKLNYYHNKIKKLKKYFDEQYLYMYYNTYNNYNKKKKKLNDIMIMLYKEKYYRLHKYIT
KNYKNKVFNTIDRNKCIILDIYKDKQNFLNDKFICLNNINELIVCNIYFFLNIYENSKIKNKKNKVNNKSNDF

LFFSNNIINKNFNYFDYLFKERQIITYNIYLEHYDFLFDIYNKGDHISTIRKIKRNISKFSVNKIMINHTNDM
NDMNDLNNSKQISKYININKIFSNSYHHEGIYTLYNNVLLFLKKLDNMKKKLYDQYFEKKKYLLLQKRKENRN
YFIHNFMTGNEEDNNLYDKNIDNSNIINMNINREHVDNEENNVLSSHFLSKEYYNNNHKEIKKKFYDHHNSHN
NKPTYEIVKNEEISKSHVRNDINNNINKKILSFKKMELLNKYKKKKNTGNRNNYINELNKINRIFGNKYHTFN
KEFQNIFHFLYNINMLDYLQNYINPYIKNSLWLYIITLYVKNSYSLNKNQFISQPEFLIIIFYISFYEHENNN
YLTNYSHSFEIPKDSFLQHIVTDIFVYKELLQSLQNRFKVHIAIPFNLSDILQVLDGLRKLRRKDFGKINENI
ISKLTNLSVILHSASLYFDKEINETIFEYIGYINMLKGYKQIE*
>N.PF14_0319
MYIIKRPLF*IVLSFH*SNIYKMTLYSKKKRKCNINFIDIELEAFIFGCFLYSWNDILTNNIIYKKCNNNKDE
KIINNNNNNNKINYNVVNNKKKKLTTLKSSGVNKRNCSSIKNNKKNITTNHIFYNCSAKNKIINKSNSKNYVN
NCNKYKEHISCSINTDFQIKHNIDINALNDKLLYFVKINFCLNNKDFNIFCNLYHFYKHIKEDKKNEETKLLN
SFFKHKVVKCGKKFKNCCTNKEDIKGHSQSEKNDNMYDGIANKCDDHKYDKYDDHKYDKYDDHKYDKYDDHKY
NIHDNHKYNIHDDHKYDKYDDHMYNKYDDHMYNKYDDHVYNKRDHLTYDKDDNILNGPYNNSYEERRKLHRNS
KKNKNGRTKKHVNCFFCDIYINKNNFDDSTKMEKFIHSCMNKVYANKKCNLMNKCIINIKTFDDNFIHSTTIE
RLFCFIYTHMDKNLIFFWFNFLKVLLNVTIDKHLMIKIFFFFCKKNFLLSSFFHSEENIINHEFFSKNISINK
PSNNFIIDIMKPYKCQNITNLILNNNFVLLQNLLDYFYMICSTRNMSFYDNFFLNIQMINFIYPVQLNVFHFY
YMKLYKKFNHQRHVYHQRINFKPILFYSNTSNIIILMKKKKQNITKHNYNEKKQKKTKLIPSLDNSAIHNSDK
SKNIYINNTKEFTNNYTNSILSISKNNLNNQMSYLNKKMITNEGNYQNRATSHSRYNYEKIYTRYTNFIIGKN
CNMMNTHFNHFNITYLNKKGKIIYDRFNIKGFITEQINKYFYKDYKPNYITTTTKKKKKKKKKGIPAQVRKQ
YINRYFYEQLFSRKEKSLIKNVVAYFKRTYEILNKYIGMNRLEKQSNMFYFLFDALPTLKRKSSIKNILEPYI
CTIFSSYEFMDIYNFIENLKRLYTLKNNKKGKNSIRIKEQHPCLRFLNDLQGLNYIYNNLRGDYLMLMQNVYQ
EIKGENNKKMNNNMFYDNGLNYNMLDNNTFNGNILNNNQIIKCNKSTIHKLIRNEKTDIYKHVSIYKFSSNLI
NIQHNLFNIYLECNLLNRAYELVMSDFDFSFSLTEEIIFLYKIYLIELKKKNILHSFEILNIAFDKCYVLLKK
YLCNPTSFKLLSVISIHFSHMLFNYPYLSKFLTFLPCQKIKFIKNNILMNENIKMEHYNNMFYEKNYFNYFNM
YDNNNNNNNNNNSNNNSNNNNNNNNNNSSNQNNYLDDKNVKKINESKEDNNYYQPKEFVNRNNFNLYPTYMEH
THNYLQLLGNKEKKRGVHDYYKDVNKEPYNDISMDCYKHMDVDNYNHIDIDNYNHVDIDNYNHVDIDNYKHIN
KESYNDTYNYYKELRTRHNSYNNIQSDDHYDNNLKRVDTNNEVLLLSNDILKYDLTNDNNIDKNKTNSYNDMK
KRNDSTGNDNDNDNDNNDYIYFLTNKSDYNFLNKYDYFHMNKILKVFNFIFNLLECTLHNNDVSIFNYNKDTT
YTFDERVYKNELILDNNNYENKNITNIRNYIKEIVYKIELYFTSIKNKYVQRFENIQDIQNMENAPDEQYIKN
VVKDIDRSRFRNLLHDTKLNKSLKYNDQYNSFFSFEFLYLAYICSNSFPDNISKNYKNLLFKNFNFNEIWLTS
HQIKIKIFISLMYMSLYEINSMNLGEFFMEILKIILHRFYNNLDYNDIQIIQYIFLNISNDTLFSCYNMIDDI
KDIIRKIPQISNREFFYVYNLYETKNILQNYQNNLDKGDTKDKFINRLLNYLIKKKHEIENKKQNLKEIHNVV
NNNINEKKKLSNTLGTNGKINNNMTNEMDEYTIKDHMHDISNGSYDTQWKETMLQVDILKEEQIIIHNQTKDD
NINLYIDGNILCHNLSEHDYANETYQEFKEIEEFEKFEDFQELEDFEEFEDFEELFANNNTYTFDFFLTEKCL
EKNDFVFFKKCNKGKHQNNYSDQHPCGSSFFFNMLNHLKECDTDGKKKNKKKKLKREGEKKLKMKGEKKLKMK
GEKKLKMEGEKKLKMEGEKKLKMEGEKKLKMKGEKKLKMKGEKKNNTLDEHIVTTKKKKKREKNILHIPMNNT
NDEFTLTPKKNKYLRNVDDDINKKYKLIWNYFKINKEIYIDNFIDEKVESIINIQKYGYKLNYYENTKVEYYQ
HYSFQKHFNIPEEISETYSVLIALNRSIRDNNLFLSFENNNYVNTCLNLLYIIDLYMEFKSNIFNKPFYFIKN
LIIMCANISFSKELTIIYINALIRFCLFELRMDNLFISSNMLIEILNNFEKIKNYKNLLGIIFYLCGYILLQH
LNYNIQEQCYEENKNALIDEYNYYVLMKKNYINTFQKIKMEKSHLKKKKNYFEQFLKHTRDELYNLNNTPLFF
QENGKNVISSKKEKVKDKMDHYAKGKLNEKLDEHLNEKVDDHLNEKVDDHLNEHLNEKLDEHLDEHLNEHLNE
HLDEYSYSNQQSNKNKSNLGTYIYLPNVDMDDCIKKNNENCYLRTKGVEKITDDFLMNEKKNNTFVKKKSARK
LIPLIKNQIKITDYFSNIKNEKSFDMTYDNMYVSDNIRMNNTSYMHVNKKRKKSSYILCEDVISDKKTYYSEK
STSTKRNEYSSYLYDQINKKQFFLFLICFYINQALYYWSNDGDAIGIANGVQQKKRINDSFFFLMSSYKYFYK
SSLYLSKQINIHEYKSPFFNYSLFNTYYQFYIYYKTIFMKCNNENVINLVKYNNYY*
>N.PF14_0320
MENAFFFKYKDEQLLEHILINFIYKKKDCLKNIFITNGNDKDLFFERNIFNYIYSYLFKTSKIKKTRIKKNDS
YLLNNKRTKKLLYLDCSKNDYQRKRNIYIGNVKRRGIYNNIPSSFYKLFRNKKKMKYIQSLYVIIKKLFLIIM
NKKGNFNPTIRYLFLLKNLMNIFYKYFSIYIIRYVYIKYNNNNDNNNNDIIIKSYNSKRKKCSIKNFLFLYFK
NMKSSNKLTDEYMFMKNYLLFIFIINEIFYIYIWLLYVCLHKYTYDTKKIYLLLKIIGDDNYGSFIFSSKQIC
TYKRKDTRHISFFLFLSRKNYMNEREKLKYKNYKRIICFVWIYKIIKNIHFLVSLYNVICNEKEHTISLTMYR
KYILLLFILKILKIIKIYSFQSAHPFFFFTTVKRKDILNNNNYYYNNKSMIKRKYYLKVLHICHIIYEQFIFLL
SKKKLYLYFELFFILNSKRYNKKEFNSYRFSRTIIYNIINNNNYYNYTNVNIVDRTYYKHNLKNKQNKYTIL
LLYLYYKIYEHFLELYKKDNEWYPKKKKDNYYIMKCKKCVKCMKCKKCLKCMKCVKCKKCLKCTKYAKYKKNI

SLLSQKNSFFVHLIHKYFVLFILLYSINRKTNITLHFIKTYIQILFLISQRKNLFAQFYFYKINLLTYFLYCI
NQIIISKQNNYPNYCIELKKQRKQNYNIRRLYEKEKNNSHITYNKKSTTLGIPSLKLKKIEDMLQKKSNVKEN
NDKEYYYKNKTKKDFFFKTIFKCTHCIFSFKRNIIYPTLFSTKMVVDYLNKNVKEKTKKKKKKKITLIIFTRK
KKMKTPLCMNHSNLHMNEEKIVSSFFSTQINDDVIFEWIIYLIISLITSYNKKDINFLYFNENYYDDNYHNKI
LYSKLEEKLFTSKKLKIIIYKHIKTLQDNFSFFKDFKKVEDVNIMNIVQTFVFINKKIKNKIKNKIKNIINNK
SSLHLHILYNLSINIYNKSKFSKHFMIQKKVGQGAYGKIYLCHYPSNIKTKQNTCVVKFINVPENMNQNYIFR
NIYNEIKALVILNKVYNNIKNNKIRNYGLILNDEDNKYDFSYYILMHYYISSLKEFINKQHDNYIKQLKRVQL
KNVVNGFVSFKNINDIKKKKKKKKMKKNIYIYLSNNKPYIFNKSYLYHINLCKHKYRILYYDLLKKISHIKCI
KYTYVLFILHLFIQIMDKLKNIHKKGIAHFDINTNNIMINYNKKFLLDSFISTNTDYIYHHKDQMIRNKPILT
INNINKQHMNHIYNEPIKKCKHDDQHLYVPSVNIYDFGECKFFFNNTDFIFLRTNRGNEIFSAPELLINTHKN
INKYKSNLSNNKRIKSKNKINNIIQNNKNHIRKCKIFIKCREKQKSKLLFYVFYKKKRKKSIYPFNYVQHLIF
KMKRKLKNLYILKKKKKKIFLTDIWLLGLLLYEMITKKQVFNLSNFLYIKLYKRKDLLKNIINNNIDQHFKYI
KYLLYNTINFNIKNRKSLNDLRRQTYALYNLYMNILRKHQIVLQILNQVE*
>N.PF14_0321
MNQIVINNLNYNYYNGIQRTRIKALQNVNLCFERGMRILVCGKNGAGKSTLLSIIAGKKLIKENEVLIFNKQA
FHDTDISNKIGYVGEWWSEEYAMNISIKDFCFNYEYTKRYKNLLKLFELDENKIISNLSKGERKKVQIMVNII
VRKDIYIFDEATESLDLVSRKLLLEFLKKECIKYNSIVIYSTHIFDHMDKWSTHVLYLSEGIVTFFSNIYNIT
NLENYSSLAEYICDHMVEEIKKKENVDKLDDLLLIDSD*
>N.PF14_0322
MKVVSCNYRVKNVKDIETPTYTILTNNITPEYINLELLRKIDKKFIINCSLLEIYNNLDVFEKVKEYFSSNKT
NEVSGHYLHEFCDFQDSYRYLNIRNVLVDDYNINVHKFISLKYDNSTAVFSIDDYIKCLKIFEPDIFCIPCEE
IKINEQVGKKKKNRIINLMNEFLEKVQIIKNTNLSNSLCILSIPCTVDIDTVITETINKYDSIIDGILLSGLG
YDESNETRTNAFKNILNILPNNKLKFIQLSNGNPIEILHAIYHGIDVIEPNFPYYLAKNGKAINMNLKMDNLQ
DGNEYNLQDKNNDNIYDINLLDFKNDVNFIIDLNNPKYVLDHSTITCNSPRKESKSYIHHLLKCHELTAHVIL
TYHNLYIYRSFFQEIQLHIKENNFLSYINWFIEKNELNKKEE*
>N.PF14_0323
MADKLTEEQISEFKEAFSLFDKDGDGTITTKELGTVMRSLGQNPTEAELQDMINEIDTDGNGTIDFPEFLTLM
ARKLKDTDTEEELIEAFRVFDRDGDGYISADELRHVMTNLGEKLTNEEVDEMIREADIDGDGQINYEEFVKMM
IAK*
>N.PF14_0324
MVNKEEAQRLKELGNKCFQEGKYEEAVKYFSDAITNDPLDHVLYSNLSGAFASLGRFYEALESANKCISIKKD
WPKGYIRKGCAEHGLRQLSNAEKTYLEGLKIDPNNKSLQDALSKVRNENMLENAQLIAHLNNIIENDPQLKSY
KEENSNYPHELLNTIKSINSNPMNIRIILSTCHPKISEGVEKFFGFKFTGEGNDAEERQRQQREEEERRKKKE
EEERKKKEEEEMKKQNRTPEQIQGDEHKLKGNEFYKQKKFDEALKEYEEAIQINPNDIMYHYNKAAVHIEMKN
YDKAVETCLYAIENRYNFKAEFIQVAKLYNRLAISYINMKKYDLAIEAYRKSLVEDNNRATRNALKELERRKE
KEEKEAYIDPDKAEEHKNKGNEYFKNNDFPNAKKEYDEAIRRNPNDAKLYSNRAAALTKLIEYPSALEDVMKA
IELDPTFVKAYSRKGNLHFFMKDYYKALQAYNKGLELDPNNKECLEGYQRCAFKIDEMSKSEKVDEEQFKKSM
ADPEIQQIISDPQFQIILQKLNENPNSISEYIKDPKIFNGLQKLIAAGILKVR*
>N.PF14_0325
MKLSHLIIILNYLFFDNLNGVRSNNISHKEDDCGASELAIKKLQELKKFELDILKDFIKKDTDHTDMYKRYHCI
SSDFLKNTKQEDKNGSFYMIFGARNTDYPWACSCDTLQLEEYKEKKRSYVLCSNQLDMSIQNSDLFCNPKNGA
HYYYLSYFILFFLFITYIDNIYEKKKKNIYIYIYIYIYIYEIIEFSFKGSHK*
>N.PF14_0326
MKRTDDKIILLWCGEIVKNIKNKKITKYSLRNKKNEDISLKEENYKDAPYVVKSSCVDNENIYCPNNMCLINY
IYKKCRLLQKCLFYKIYISQLKNKKLKQLKEYIYLAKDTNQKKMKKKKKKNGQDNKVEEEEEEKKKIKKSTHK
KTNIYKKAYNINMIFTKLLILILFDTQLLKISLKYFNHMIVSIINQLLEEAFLLSQNNYDFMNESYNKNCKSN
NTSTINYNKNILSYKRKKLRPSLHLYNIIKKCNINIEIYFCMICWNNILFCKESLFKDTINYFFKQVLPKSYM
ACNYFKMVIIKYQKYNKEKKNENSLRYDKDILYYNYCIKILLICYYLSKNNIMDNRYMDLLTHMLQFQDPLFE
SLIYKIYLNYYKIGDTPKYANNQNNQNIQYNQNIQYNKNIQNNEYSEQNNNNIFFNYFINKTNANANANANAN
LNIHQSVFINYEDLNSCLDSYDKIYEMYLKENIKDFEHIYIDKFPRINILKNNKFYNFYIHVENKILYPTFCI
KRGKNIVDKLSKSKFDFIITERLKRICELLILNIYNKKSIVLIGNYGCGKTGIINYMYEKIYGKRKDKQNNII
SVYLDDVSDSKSILGVWESNENYFEYKYGILSKAMMDGKWIVFENINTISNSIIEKLEEVNSKGYIYISEKGE
YIYPHKDFRLFATITLCNRDHKNNNKNNNNNNNNNNNEINDNICDKMINSQDNNLHNVHIYDNTINNTHSSQR
HRAHNSHYNHFDVNENVRKKEEIEEIIHMNYRPKYLGPLFNKWHSIYIGNYNKNEIKEILFKNLYDVNDNYKK

ILLNCYNIIKITLEKYHILRNMNLHDLIKIIKRINKKKIFVYENEKTKLILFQTIKSILISHIPIEALRNYFL
SIISNIFQLNHHVIIKYLKSYSVDIFIKQFNETLKKKYQKISINIEKIYNYSFLLTSVHKSILYDIIEAVYNK
EPILLIGDTGVGKTALIDYVAKIFQKKLYVFVFSEMAEASDLIGNYYPFNISVKTNDLFENMKRLNKQISDIL
CKKEMELFYIKLRILLSEKKFITFLNTCSNFANSLIQNVKQNVQSFDMKIIRRYKNFMNTCKNIRDFWYDNNT
TIKGKNYTLPIDYQKLFEKKICDKNQINIDDITSCNHYDNKYKSSDHNINTEIHLDVVQDEEERNTEMNELIF
KFHDGILIDCIKNGHWILLDEINLAQTEILQRLQGLLDSSNKYFDIIEKGNEQIKVHKNFRLFACMNPPIIPN
IRMKKDKNIVKEEFYDNKDVVNNDICLYDHNNNNNNKIHKIHKQIDFQNDIDLYNSTMSSGKKELAPIIRNKF
TEIFVDEILEYNDIHMIVKDLLKNTTSDNTLIGNITNCYLEIKKKSLNNIFNTENKPISFSTRNLVRAIKYAI
YVNKRKFKSVQLQNAVRNGFICNFMSCLNSNNQKLMENIFKKYFKYSYEKDMDNNKNKNKISLVLNNNENDLY
SQHNINHNNHIINNINNFNSNSMHIQKDGIINYDILDYYYEYKYYNKKKGEEENIQTIENNKVNHHSNINNKT
NLCLSDKNYMNMLFLHKNKKDIEKYACIENSWILCGNEEINLKRILSNFIITKNVKDNIKKLALCLTSIKTPI
LLEGNTSVGKTSLVKFFADITGHKFIRINNHMNTDINEYFGQFVNDKKSGNLIFEEGVFVKAVKYGHWVVLDE
LNLAPSEVLESLNRILDDNKELYIPELKSYVKAHKDFMLFATQNPANNNSYLGRKELSKAFRSRFIEFQINDF
DIEELEIILHRRCTISPNISKKMINVYTDLKNIKSNYNYFNDNIMTLRDLIKWANRYPNNNMEACLNGYFIIA
EKLRNNKDKESVKKILAKYFLPKGEELKIDYENDSDVRILKDAYFKKIQQMKNHKKIKNKTKHKGDHFVNIDD
NNNISSSNKFVMNYSDNNFSEKNSNKKNRLDDIQRYEYLKYMHFGKTTSRLICLLLKCFKYKESALLIGETGC
GKTTCCELLSFVENIKLNILNCNESTDIYDMIGSLKFVKNTKEHLEKLKKNCIELYNIINENYEMDNYASAYL
SAIIYEQISEIKKEDFLVFTLYIQKKYNINNDIQSKLCKIERCINNLKSLFTWYDGILVSSLKKGHIFLMDEI
SLVESSVIERLNSVLEYERTLLLTEKGGKHVKYIKAHDNFYFIGTMNPSGDFGKKELSHTLKSRFTEIYVDSF
APDTDDYYFLIKNQIKFTNNEYIKSVIAKQLCKLFDQMCKKELHHINKENNQNNESNQNNESNQSNQNNHSIN
NNHDSNNYGEYKFVRTHLVLSIRDAVKWISFMNTYINNKKITYKRKKNMNIKRNNNCNPYNICENNNTKQIFA
FPIKKLKFYCLRSFYHSGCLILVDSNESVIKNNDLLKNILLQHLEELRMSIKYNVSRLIQEKKKDKNMLRHMK
YFDETLDFKFKKRYLKINDFYITFMKKLKCDINTFSKNSNFVFDTPNIKQNLYKIVRAMQLPNAILLEGSPGV
GKTCIIDILAKITNNKLIRINLSECTDIYDFIGSYFPIKEKNKKTHETLNGDNKNDTCDDNLYNNNVDNIFDK
KNKSFQYAWEDGKLIDCMKKGYWILIDEINLANQQTLEGLNSILDHRKEIFIPEKNEVVKCHKNFRLFCCQNP
YKEGGGRKGLPKSFLNRFSKIYFEDLDDFDYFCIVEKLYGDIISIDIIKKIIKIIFTIKKINFLLNHSESWVW
NLRDILRICKVLKYSKTNDQFFNICDMIICNRLVCKQDKYIIRSIILNIYKDNENFIENILSILLNSQAYQSD
LQMSTKEKLIHFINSNVDNYSWENYMCMDIQIERNEEIVDIKGKKNKNEKIKKNNINYTKNIYNIYNIINIYK
NYSSYYCSDLCSYNNFHNDTSSFILCKDEKIYSAFLSAIRLNMPILLNGKNNSGKSSFIRNISQLFNKKLFEF
SLTDDIDTFDLFGSYEHNSKDNFINRFMNKIKKLNKLIIKRLIKKNKKNNLHNVIKKYFGFENNKPKNDCNNK
LYIYVKQYNKDPLKNQNNKYFILHIMNNLNELSVQFIDCLKNEHMKNMLKEVQKKYLELIQKCKKIIIGKNIM
LKKKMNNIISNILYLSKLISTNQINENVYIFKESNFIKAIKKGYWIMIKHLHYSTPSLLDRLNSLFEENGYIL
LHEFGKKKKIRPHKNFQIFLTLSSQEHYKISKALRNRCFEIFFDNNNVEYIVGRDIHTSQNHKENISQSTTTK
NNDMNSSNNYNPFFDTLDNIYHPIHAGDNTCNRVDVHGTNIKKYESPFCVYPFINIKNKENIHINDCNIQGEW
SDNRFYDLINVGFKKDNLSNLQNKGLGNFSLCNNIFNIIKEDNIFFYIVNNFLKNVEKTIEKEKKKNDIHMKN
INKFVKFFFYDDDKIRKNKQSSNIKINNIKILDYINYCYNSFCKQYSITYNNVDIIYFSIIISVFIYIIVEYM
FDENIQNKRKNKINTKLNIKKINKNNTRNENMNEGNDIYEKKRTNIHINSFYDNYNEERKNGLYMNLNKLLSN
KFDNTLYCIDNYINIICIHFLRAENRKKHIKKNYNLKKYVNLFSQYFIYFYCQLFSNLSQDVQIFFYPFFYFL
NNMIIITIYKMKHKLSRCYIKEETNKNKKIKKIKKIENIKNNNNMLLNSLIEKYINSSSSNKNNVCVHKLSPN
ILNCLYLHIFIMYIYYYNKLYTKDTPFFMRNTIIEDENIIYLDKHKCIDSKIHVDNNILKEISLINTFYYLKL
NKFFCFLICDFMERFSLYDIFYRYQYLLTMKNKIYLRRTKQKEGKENCSLEYNNYLYNIEMEKYLDFITNKII
CKKLEYFYIISKFIYNNIIKNILNEKNKITNNTLYYEHSLYFKSPLIEMKKYITKKESTNLFFYYFFDFVLSF
FKFNFSYLYYKKREHYYKQNKNNTHNYNNNNNNNNGVVENHFYIFQANKETKREEERVIFHCLNKFVNILEGF
IFEGIQKDDIKKRNINNSENNEILMQTFFSYYVFLYIYTFETICNFNYDTIYYKIIYIYDSIKYILKTNKCFE
KKENKKIKILIKCIQKAKWIIENNYRVSNKKSINNIRNVEYDIEKKEKERNYYMDTLFNKENMILNIFQKKKN
LDGKMYNIYNKYNIYNVYNNINQIHFDNKWIQNIFQVINNIYISYFIYIRNEYNYRHILLNNYTNNLQHNYCD
TLTNMKKTLYENNIITNDNIEIFLIQLKSFTYTLNFLHMQKDLKKKKNYIENIITSYFIKNNKYIENILQIKL
LQNKEIYLNNKNNMYRNYNFEYEQLLYPSNNNNNNNNNNNNNNNNNNNDNHNNDNTFDICDYKNNVLLLNYEY
ILYNINLKYIYNACVYLSSIYIYLQYVNIEKKIGMENIEKYSNTLINRSYEKNALNRFFFYSYLNNIKNNNNL
SSVSSTYEQIRCIIRNTYEYLNNIDNTIFIFEPKIDLLSLLKERYDMLEYVYTNIYKNEEDTQEVYLLNTLFG
IIEELLYLIYCILINIYMIRDQHYEYYFKDNNNNNNINNNNNNINNNNNNINNNNNNINNNNNNYYYFNEHVG
ETSMYNNYLTKPKENYCTKLNNKNIVIHKQCDEIPNIVTYEEYGIFKNIYRNKNINDIHISMDDNYSYINTSI
YNKTTILDSFINMYKQINEKSKNKKGAIITLQHLNATHLIFQNMKQNYFHYLIKYPYINYNYSILKEKENKKE
KEMEQMKYTYDSAFTKCSNENILCHYFAYHVFIIFCNIMQNDIAYEFVLYKNGIEKAARDFNITNVNQNQHQN

ENQNIICCSNISYFCIFLKFFQIYQVIMFKKDASTDLLSFLLCLYKYFHNLQKQNKQHRYGQNDGLQYILVFI
FLLYKIIRMNIEKNNTNIIMNNEKKKKKKKKKKKKMKNYYYNYYYCNNYNCYINFEYDKTSSEEDTTNISHVK
NMLNDEPNYWHDITNDLFVENKILNNLINFFKDDEYFIYYLGSLYISHYFLNIVCTQIKKQVKDIFKKKGMKK
YFKEVLNSIDEEIYMNAQLNNLYIDKINLSESNSLNYNTYKLNKKRYIKNKINNLERDIPLFPNILKNISKSK
KKKLFKILNINIDNKNDHNINSNYVDNYSNNFHCIIIDYIKNYIYKNNTVNVESLYINLKKDFKNFMKNILSY
HNLNSIMLSITNNNTNVDTYNLSPQYIHFSIVNSCVSFIHHIKEKYGLILKFTHGIIYSLSAFIHSIHNINFS
IYQIRNKEKNGIFNMCTINRENDLCDEICKYIKFPLNFNKIINHKRDGFYNNNNNNSDCDGNNYNNNMVTVHK
KKKKKKLNALSELTYCEFVIRVLKNPKSIIKEYDINNLLNCLKYFCKNIILFNEVDDTNDMNEFFKIEQFKNK
HLKNKLDYMEDKENENLNKNIEKELDNIFYSQIYLYKNQEVAISNISSTIINEKYSIEREDDDHYNYDEERIY
KNCKKQNNDSNIDDCERLQNEDNNVEDMNTKEHIENECNTNEEVLKNKDIDNSSVIKESKEKINEVLFNKIIY
KLVKLFKRKLKEKIEDKNNNIKNYNTCSEYIKQKTYINRIQKNINHLFEMLIFNINNKEKMNSFISNNKNENF
DLNIWLNKANEENKFLIFTLNILNYFNDFTNYNDEEFENYKKDISEFLLKNKKRNHAYINKYMNLLEYNNNIE
KYIFSALKHLCTFLNNKKNDLLYIYNNSNNYKILNIINSINHILNIPLRNVKNQIEKIISKLENIITLINALK
KDNFLNFDEDTMNKFKDIMKNNILENFLVYTIYFRLYKFKEIKNIRKKLYHKIVKNKTLNLFAYLFYLCYEDD
KDEKVSMEVYSKDMDDNKLVSHHMTNNTDGCIKQGGDNKGDDDDDGDNNGDNNDDDDDNDDDNNDDDDNNNNN
NDDIIFQMNNFLIDFSKYHLTNHKEEKNKNVPLSDLFKLKIIKTFECLIIFLRDSMIGEFPVRLNLIYFIAII
FKNSSNMNERIMSNVFFYVYNYMYIYLPLIRKKIRMSKNYFDLQLKKSLQKINFDLIDIDSYKSSIIKFKNKI
VYFTKLFFQQINISVDKFLLENRRSLFNSEMQKEEKKNITLERNINVCYKNGVHNDPQYVFDEKNKKKLLNME
NSVEKGNSNDIMSDDNIDHDDDNIDHDDDNIDHDDNNIDHDDNNIDHDDDNINHDDDNINHDDDNIDHDDNNI
DHDDNNIDHDDNNIDHDEDKSYLLSDVSSEMSDQNINVKIPIDGKENYIKNNLCELQKCFINLENKIERNISI
ITKMKKDSSNIINLTMRKNLHNFIKNIDNYYSFEDMSNNYSKEIQNILNVQCVNANTNQKKRWIYILKHFLTK
KLNIPILNDLSNITFFSNVFKDCVYFENVNNKGDVIQEYNNCKEILDELKSALLYMSQKKSEHVRRKNKEEKY
MDMKREPIINNNNNNNENNNENINENINENNNNNNYCYNNNYCYNNNYRYNNNFVDITSTHRILEINEELSYL
EDNLKKYIKDKRIYNIDFINEMNYILKENEKKNYKILYLTIFIKSINIHRDMQNEDILYYLFNSINMEYFKLG
KEMYILYEHFKLYYFLIGQNVLLYKKKRNENDIRFIQIKFSTFEKFLYQCNIMKTCLLQFLSIFYEELSKVNI
FKKILFSIYEIIYIIYNSNEFLIFKYFKNIQNVNCLQEFNKTISSNMKLNVNNYLLEYIYKCIQYILDNINII
YNLKVPVYVKDQLYYRIQTFVLSFDKIKKNSIYMKDILEKKVNKKIINNNRNNNNNNNKNNNYNNNYNNNNLS
SKKENIFDENIKNMNKHIYKFIQHIINAKSIICKKEYPDLQENKNICDIYCMKNIVHFYLNTNDIITLLYNKN
TTSHFCNDHNLQRNKYVDIFFYLSKNLFYFVKYVFLYFADIYIGFSNISLYFLKVLKILYTKGLCRDVDHKND
QEENMEGDNKNEQKKKDEDFHKVDFLKGIGLGEGKGIKNIVDEVDNEDIDNIYDENEQKVSSDEEQDFNEEAI
EASYNFEKFCEKEFKDINNFDNEKKLENEKDNINMDPEKKNENDHKKDDKYDHKDENMKNDPTAQNNQNEEND
LTNIDDVFERNKSNDFNISHDNITKDLNQNNKDDVNQNNEDDVNQNNENDVNQNNEDDVNKNNEKDVNQNNKE
DVNQNNKDDVNQNNENDVNQNNENDVNQNNENDVNKNNENDVNQNNNSNIVNEDMKINKDDEYNMNDMEPLEE
NNNEDVLNYDDENNKNRMNLDNLTSQLDNDKFDETYDNLSNINDDDSYNFDSNFEIESSDFSKDFSDKSYSDN
DGEKEDKDDAGQDNQKWEDDIKNENKDKHDKNEKNDKNDDDVQLDDNNMLKENYKQEISEGSINYEDELFEEE
EKDNVENNEDRENKYEREEQLNEDKKEEDHLEIVDEKGEGCVDNNDGNNNDGDNNDGDNNDGNSNDDNNNDDN
NNIDNNDGNSNDDNNNDDNNNIDNNDDNNDDNNNEKGKSSNCNDETMDEQANQYDNKLANQKDDKNVEEKNNA
ENRITDNIYDNEDLNNDPMKKSKQNNININTQKDKGNGKDNMEGEQNVNGGDNNENIQDDQLSRNDFSYMINK
CNMYMEKINDISKFLDKIQDRIRNMDENNINDNNINDNNMDENNINDNNINDNNINDNNINDNNINDNNINDN
NINDNNINDDNMFDNNHINERAMNKEDSKLDDEEDAYKGQEKNKNGEMNNVLSMEEREMKKNITNRFDDVNKD
ESFNMEEKEKKNILNDNKDEVLKTNFDNKEEENLNNVDCDNVKEEDYDNTNDIIKDGYNKVHSNIKIFENKID
DNNNIIEEEEEEDYIININEMKDKEKNNFMEHINNNDEYEDIYEKINNETEILSSQLCEELKMILEPTIRNKY
EGDYKSGKKLNIKKLVNYFASDFRNNKIWKRKTKLDKRDYNIIIAIDNTKSMKINNIQKTTLNTIFLVAKAFE
KLNVGKISICSFGENEKINSNNIVCSMTNNLNKQDFLKILNHFQFNYDTETSFDNAMLNALKICNYIFKNTYN
HKNNIINHLMLIISDGRFNKNSVKSEILKCIQNNFIPILMIIDTPASNNNNKKTQSIFDLKQTFYKNNKLQIV
PYLHDFPFPYFVVVDDLNNIPSLTCDIIRQWFEILNNK*
>N.PF14_0327
MNKNNNKGSVKKNNNSKKKTSTNINNVNNLSSIKNIKNEQNVKNGDVNDEIATTTSSNHVNMVLNGNDEGIKD
VKVVNNLNIDNKREDNNVRINVQGEETLNNDSKNKEAENKSNIKNNKSNQSNQSNKKTKNNNNKNNNNNNNNN
NNNNNNNNNNKNSTSNKKNSKNKKKVDKLDLILNEFKNDIKNEKGENNQNDNKLLNGAIETNNNNNNNNNNSG
NNRIHFDKEEIKEIKKKIILKEHVLEEQDNSSIRLLKNWPQLEKTYQTNPPCIPIDMIYGKDGKYPVGEILEY
NNYNLSGQSLVEKKEREKLSIDYYEDLRKAAECHRQVRKHMQAFIKPGKKMIDIAQETERKTKELILAEKLKC
GWGFPTGCSLNHCAAHYTPNYGDETVLKYDDVCKLDFGVHVNGYIIDCAFTIAFNEKYDNLIKATQDGTNTGI
KEAGIDARMCDIGEAIQEAIESYEIELNQKIYPIKAISNLRGHSINKYIIHGGKCVPIVRQKEKNEIMEEGEL

FAIETFASTGKGYVNHENECSHYMRNPEKQFVPIRLNSAKTLLKVINDNFDTLPFCNRWLDDLGQTRHFMALK
TLIDLNIVEPYPPLCDIKNSFTSQMEHTILLRPTCKEVLSRGPDF*
>N.PF14_0328
MLQERDLAREPCPDRIIEDMGGAFGMGCIGGYIWHFLKGARNSPKGDVLSGALYSSRMRAPILGGNFAVWGGT
FSCFDCAFQYMRKKEDHWNAIGSGFCTGGVLAMRGGWRSASRNAIVGGVLLAIIEIVSIVLTRKTTPTPRQQF
QQQMELEKRMATKNNR*
>N.PF14_0329
MNSLLRNTLNSSKGKIFTKYLNNSFNKVANLNSRKVSNFTKYGKCVQRVDKNFNTINKRSFSSSEAQKLSEVV
KAEVQHEKSNYEAPDNIKKFLQTSGWKFEEQEGDVNMVLTKNVDGMKIIIDFQLVSPFQAEGENEAQAEMTDF
SVTVEKPNQNGGITFYCTTLQNDEKFRYMIGNVKYYKNEEGKNSVSAYNGPEFEDLDDSLQTSLDEWLANLGV
DSELCDFIDSCSIDKEQREYMSWLQNISNFIEA*
>N.PF14_0330
MENINKKDVLVNYLKLCGKHDMVNAVIKYCDEDHGIVFNKHPARNEKKFGTIYDYGVSSKLRGCENLEKLQNS
KVFHKEKNISNHLNDIVLESSKCVDDIPRVKCYGRDESSLEGMEERRTEKPVKSYYNQRKKKTNLLNKQDNAI
SEILDDICEVIAPEGKGKFLKFIGENYVYILLENGTYHCYPIHLLKLNRSFLKNNSMFTLKFDYKDMALKHAR
TLIKSKNKKNYKRPNLIAQDNMFHDNFVDFPKELDYIKDYIIKNDNKYLTSCIKENMVYDVLDNGKCSYNKEV
LNCLQNCNDEIKQNIKIKTLNNIYKLENSKNIYIS*
>N.PF14_0331
MGFNKIFPNIFVGNILKHGYKKRLIYIDKRRFNTFNKLSRSEESYVIRKAEKCMNYVKEGYELKVGMWLSTCS
LLILGMISIGGYTRLTESGLSITHWKFRGIKYPRNEEAWIKEFDKYKNTPEYKEVHYNMTLDEYKKIFFNEWL
HRTFGRAIGLFFLSGASFFLYKNALKKNIIKKLSLVFLLGGFQGVVGWWMVKSGFDKPTTENKTPRVSPYRLV
FHLFCASAMYSYIFLNSLTLIELGKMRKLFAKSQIPTWPEFLRNELMHEMYEKRNVTKNMKFGIFAFTLLVLS
NIMYGGFVAGNDAGYAYNTWPKMIDKFVPDDVMNFIKSKKKKYSQLFENTAIVQFNHRMLGYLIVLNSFLLYY
YSKSLALSKKTKRLFSLLPLITTTQLITGIHVLVHHVPIHVALVHQFGGFCILSTLLHLIKRTFK*
>N.PF14_0332
MNDDKNVFLFHLFCSSVSEINKKTDGKVTIKNYFFIKLPSISEPSKTVLYHYNEETNELFLLERNYYNPKIET
IRYESEKINKNYISLFVNNYTIENDCGYYCYPIDALFVFISIIYNNYTHNTYTSLEDYLIFIIKDNIKLRKDV
TKNLLLILKNNVNNIKERLKYVCDELHENNIYYYKPNINKVRKFYNLKCIILFNYIIENKIVFPDYSQCIERD
LLDKYKSYQNNGNNNYISSTNFIYTLMKENKPQIHKYNEYKKYVDNYLIEFNKKHYKFNAHNLRTFVWQIIKG
FICISLCEQITPQDISDKLKKMKEEETNKKIIQQNETFAKGKKHTLQQTPRNQLMIDSFFKKKVKLK*
>N.PF14_0333
MGNITSEQKKKPLMLHELSTKIGDYRTIYEGDPEMLEDPDFPHKMMTEALNNNNTIDGTSVHGTFVYYPNKRK
AKMWIGNYKILNPEDILCANVAEKIGITEMEWKKYIKRKKKNENDEVMKKNGNIGYNENPFEEPSVSLNDIIE
KDNNNYNMDTNNNITNNLNRYMKTFKQNKNHPYNISNKNNSNIYASTHESNTKDDILVNQLQNNILSNEKDKE
EKKKKSESRDSYIDKLNDEITNIREDQEKIIKQECKNIYKYTKSNYPQMIIPQKDGSFLIENVDPIKYNNKKK
LIIQELLNEHENRFQSQVSNLKKKINKKGYTNLSSMSTKDSLGLPHLKNKDTNISKRNIYLNRTLVK*
>N.PF14_0334
MGKENIDNKGLYDSRNEKDACGVGVVADINGKFSRNVIQKALQILLRLSHRGGVQGNNGDGAGILVGIPHEYF
QMLSDTCQLPFLLPEKGEYAVAQIFLPQNEERRLFLYHEIEKEVYNHGLVVLGWRSPIPVRRDVISCSVKKSE
PFIAQMFVCKRSVFKNYDDFNIYPFSDINFTDDSNLKLNELPDDIELLSFFIRKKLVRHNDMYFCSFSSRTIV
YKGLLTPSQIYLYFEDLLSENFTSYLAMVHVRFSTNTSPTWYAAHPFRRICHNGEINTISVNSILFQERMESA
KTPECFKSVSIKDLRPINEENYIIYDNDDEIIQKRDTFEDIMLTQNRLKKKSRRNLLLKLKKDYTYNSSAIHY
NIKREYMNSDINSNSEFLSNVNDDSKSSSDVDKISEENLMNHLKEKNNKAFRKSMKNQIKHGESQKTKGAISY
PSDSSIFDNAIDFLTMTGREIEHAIMMLMPRAYQKDPNISPLEKAFYEYHTCIMEPWDGPALIIFTDGYKVGA
SLDRNGLRPARYSITNYGLFVLSSETGVVDLSYDKIVHKGSVYPGKVVLVDFKEKKFLRHEEVLSKFLRERPY
KKWIDHYSIHMDNIIRPVTNMENIRMNAYKYGAILNKDNYYHNNNTNINNSNNNNNNNMIKSNSNALSINNNK
TCLNDIDYYDESLIKKLKAFGYTYDAFNMIISPMVKHAADGLGSMGNDTAFPFLSYLHRNLLYYFQQTFAQVT
NPAIDPIREENIMSLMSNLGKEGDLLQYSHVNCHRIFINGPILNEALYNMLLQLPDFPHIIIDMTIDLDKLQS
MMINVNSNFSPNKDGDPSQNIHNNIVSNKNSEDPSKLDVCKTKQAKEEHTKKKHISSKYLIKFIDSVNSKAEN
AIKKGCQLIILSHNNVNEKRVPIFSILIVGALHKYLLSKNLRTKCSLIVKAGDCFEIHHLAVLLSFGADCIYP
YILYESLQFIKFADNEARLSNNQMISNYRHAANYGILKIMSKNGISTLPSYKGCGLMQPLGISDEILKKCFIN
VCDSIIGGVTFEFVEKEILNIFYLAYPKRILTLNLKDANEELDDYGEYHFRNIGKSQIHMNHPETISLLQKAT
RSGNKDTYKKYSELQNELINHCEIRGQLEINYKKCPSYNKIKKKHEPINIHLVEPVNKILLRFCTGAMSFGSL
SEEAHTTVATAMNSMGLKSNTGEGGEAEERIKTANNEINTNSAVKQIASARFGVTAYSLVNAQELQIKVAQGS

KPGEGGELPGYKVSSKIASIRRSTPGVGLISPPPHHDMYSIEDVGQLVYDLKNINKEAKISVKLVSKLGIGVI
TSGVVKGNCEQILISGTSGGTGASKWTSIKHAGLPWEIGLAEAHQTLCKSKLRKRVILQIDGQLKTGRDVILG
ALLGAESFSFSTQPLISLGCIMMRKCHLNICPVGICTQDPELRKKFAGKPEYIVNFFFMLAEEVREYLASMGF
RKFSQIIGRSDLLKKKDHLKYDEKRKLLDFSKLLFPGWKYYAEDEMKDDQNKRYKNISNKKKKYNTNSLYSAS
KHKKKNTKAGLHSIKKKMKNRKQVLINESFNTIKKYKINSKQADTIERVNGQNEYFINVECDEADAENESDQN
DNNEEEETDKSDVEENIDNDENEDMDQIDDIDEEDDEYYNDGSDDSDDSDDSDGNDDSDDNDDDIDDLDDIDD
LDDVDDVDDLDDVDDLDDLDDVDDIDDVDDVHNADNVNCRKDNHTDHDYDDNNNVKDEYPNNNDKKGQNSREK
RNKKKSMNRNIIYKYQYKEKGSYNINKKNHNNNNINKKNTKKIKKKIHLKKKKVKPIPIFCCETQNHKLSDVI
DRELIRRSKIALLNCTPVNIKMPIKNTDRAVGAMLSYHIIQKYGEEGLPIDTINVKFRGTGGLSFGVFLTSGV
NFELEGDANDFVGKGLSGGIISVHFPKNALFINECQKNVIAGNSVLYGATKGRAFFAGRAGERFAVRNSGAIA
VVEGVGSHGCEYMTKGIVVVLGEIGCNFAAGMSGGIAYVLDINEKNVNHQMVDLKVCKTMDEKMTLEKLLHEH
YERTNSYTAKMLLQNFDENLQRFTKIIPRDIKRVLTEACIEFVKTGNEEAAAILDDWASLLKSVDQPEHMYKK
AMQFFTTITDDISGLSKSLALRSVDGLIIYDILQEQRSRNLLSIKNSSNDFNSLNNEEQNIKKKKFFDFESFI
HNLELCRSNNRKWRRFSEIPFKCNAFSDFKNTFPEKLDDKSKEMIKKYILNEQYLLDLHLKSLNSNKFIEKLI
IRETDDYIHMDQKQLSPNIYTLKKYLYPSDNYDVSNISNSMTAYNKINGVHLMNKQNENKQNAEVPNSYDFSG
YTLGARRKTADMVNIQKDDENDIRIANNTKSISNTNDMNNVSNMNNMNNVNNVNNVNNVSNMNNMNNMNNINN
MNNINNMNNINKDDNNNINGDDTNNTVISEVTNTTYANKSSNYSQSQYAKTSGDGKIKKYSVDTEISDMTRVS
SDYGNASSISSTKPFLVANPNKVTGFKEYERLSHPLKDISSRVLDYSEIIVPINVKSKLHNQLLKTQASRCVD
CGTPTCHYPNSSGGGCPLGNRIFDWNNLVYENEWKKALERLLDTNNFPEITGRVCPAPCEDACVLSINEKAVS
IKFIELSIIEYGFIKKWIQPMIPISRTGKKVAIVGSGPAGLTAAQQLNKAGHEVTVFEKDEYFGGLLMNGIPN
VRLDKKILERRLILMKKEGILMKNNVNVGVDITLSDLVKEYDAILLATGYKIPRKLDIPGANLNNIYFAMDFL
TSCQISLTKSDMTDDNYIDVSEKHVIILGGGKTAVDCISLAIRMGASSVLQFTRQEILPMNTSEYWWPGLKNI
FKVEYSHEEAIIIQGRDPREFCVRSLEFIPSNKDPKRVSGIRAIRVKLKKNLKSEMKQNNKMLYNNYMDNHRN
NSSKIDLKKHLSKTNKLKSIKSISKGPTQETTTASSYSSSSPAENDIIQNYQIIKDHNEYFDIPFTERIYKAD
VVILALGFSKTDDSIWENDNIKIELNNYNCIKTNNRIYQTNHKNIFACGDCRIGPSTVVQAIAEGRDVASKID
EYLTNSVSILPPCNTYYYYPKNYKNVPFGSSNWG*
>N.PF14_0335
MSRWDFSLKKNEQKNENILEPFGYKFVNEAKNIYGNGDSYLKTEKNINTLNKLNKSNNKKSDTVEINKKLLHK
AAWGICVNSFKGLLMNLFVMFMSGGASGIFGIIFIVYSIYNILKSLININDVFKTVENNKTEKFLLQKICFFI
MNCLVLLYIMSVCSNSGLLPIRSGDYFYFIPHQKIKQKGVASMF*
>N.PF14_0336
MSELKEFDEFNFKNYDNSNNNNEKENDEGPSNIFYEENNEVGTSLDKINYMNPIENVDYNNMNMSSNNFYNEL
DKRESSSSLLYNNNNNNNMNNINNNIHKKYNGSDIKNNDSFYKSNLSSPIKENRMSFENSYETFFEKESDISD
TEVSDVWEKERLQRIKERKEYEEKEKKEIKKKAAQDLKKWYEEIAIVIEEKKKLSNQKLSEDKKKEQNMDNKT
WLKVSQYLDMEKGEYFKENSRMKQVLLKLIQKESS*
>N.PF14_0337
MKDIIAEYEIVGKIEEPKEGHSNDIVENEKYKNDENDKNDGNDKNDKNNGKDDRIHVEINNEGKSLGVSNMKN
EKEEEKKTMLNSTYNKCTHLENKNEMDNIIEEESEYNIINNDEGKKMKECQIIGNGTIIKDISTEEKNKIKCM
SINNNECDIHKEDDNKNNIRNINKMKSIIINKNENEYRNDDKQDNTNSFGNNNNYNCNIVNKRTSHVSINMSD
KCSNMKHYKVLIHAPACWKGEKFNGTPFIFVYDKSINFYTYSESPLEIEKYIIDTNDENFENRVLTIIFYDSL
FACSTSSLLINNNKNELQHLPKPLSVFYLPLCKLDLENDSVKYRLGLKTNSILCNININEAIGLFNNSTKLSG
QNINKFSLHFFLKNNNYLNNNNNNNCNFPYNYLNYNTKIYGIPRSNLSSCVQKHTLYENNKYKNNNNNNNNKN
NSNNNNNNSNNNKNNSNNSCSSTIGKRHILNSSKSQVHYFKTNNSGDQNEYISLDKIKSKWEQYAHMNNTGNQ
NNIHLIKNDKVLQHMMRNEKKNKNNNNNNNKFINLNKYDSTYENICIDNESFTSKMKSDIYLNNGQNMYHNMN
KTELKKNIFLERDILTKENNKNKEAHNNNCNNCNNCNNCNNTFKKENTNIMSRDKSDYHIYEQEEKYLEELDI
KDDVLPDDSASMIHIRDKDSIISGRSKKMHEKSKLTTNFNVAHNIFSTVKQIIQKKNDHRKVEKNNIEENTNS
QMCNENRNNINNINNNNNICCDNKREECGYDRINEITQEKNKTRKEPNFINDDKSVCYSQVSNLKYFNTNHSI
CSSYGKNDEHLILAIKEIKDWMEKIEDKVSTISACEKSDVNVTTNGISRCHEDKYNRLLKFLIKSVKNNIKLK
NKSIYKRCYLNIRNLYLVKKNDKIKFENKLLERNYNKIKTKYRNVLVNICKKIFLLKYYSAKDKLHFNKKYEN
MMRHLQLEKNDLLSIIEEKKYELENNVNLNHILSEQLKKNQEEKEDIINKNFYYEQQVDNVTKKYEHLHNDVI
TLKEEIEQLQKDNHALKKNKEQLLNENDIMKKENDMLKKELGETKAKYFNLTGILEGEEKMYSQKVEELEEKL
QKVTYEKNNMINDIKDEIDKFTILINEKDKENMRIKDKLKELKNVEEKYKNTQKNFDSLKKEFEESFEQVQII
LNEMIQKEKEYTKKYNNSITSLEKEHNEIIQKIIEDKNLIIKELQYFKDLCKNQCNKIITFDESLITAEKLLE
HVLSQYPQIFNEFKNSSRNNLLNKSFGKMHYENIHAVRDNIKLIRKSLSHFKMTCKNNSFQFFHNSDSTKKYT

SNHNNNKNNNHNNNHNNYHNNNHNNNHNINYYHHNYNSANNIQAYTSRNKRVTSLLRNYEKTDSQKEYKNKNI
HVLHTGRKRTVSSNSTRNNSILSDCAKKNIDLRKKIDSYNSLFQKNFIETTSKQNTTTEKNKKINDDIMNTYE
ENTNMDIKKEINNLQNEHHNKNIYNNGNEFHVTSKKHVNTENETIQNSYDNITKIINDITNYDEIKYNQIDIL
NEDNLSKDINELNNIDIVNMYEKNNSSNNMNDSEHVYTNRKKQSNGNMDLPENNKNEMFDVVKVNKLHINKPH
EDNSSNIDIDIDNNNNNNSSSVKYVQEFHKNNFKEVISFIEKNVIKNIPAVKCSDKNTKEINKLNVNDVPTKL
STNLIKHSRQVSSSVNSSKSLNVHKGYETNKNEKKIIRTLSDNSPTKKGVNNIYTQTKVYTTHNKCKNIDEVE
NDKKIEIMNSKSPKASLRKKIIDK*
>N.PF14_0338.2
MNKMKKTENINSSGAHKNINEDRLSYVMTCLLGNEVNVYMKDGKEIRGLFHAYNLTAKNDKKEIDISLNHTRM
IPKNENTNGPINKSMIITDNLYTTIIGENINMNLKDPDNSIYSKDIFRIDADISENKKGKLNGHTNNRELKRW
VGDNDIVDETKLKLDDKLNEPWDQFEHNRKLYGVTSTYKEEIYTTNLDINKIPHHVKIQADKIAKELEKRGMH
LDPEDQEKNNNEIDEEDLFGAVRQNKDKFFKNNKFKKEDNKYKNYQGKYHHVNVKDLKEKLQLVKRENEKKYP
STANKQKQINFTVSSSIEENICANKKNKIPSKKSVSKNSEFIGINALNLEPALPKLDEKTRTEWIMFKNQTKN
KALHKKDKTTEKQEFITASKEFNEKLTYKMNLNKKDANIRTIKDHSGMNQPYIDSQKNISHIAEKTKHNNAND
PDENKIQAKENVLNNNMNINNNNYNIMLNNSNIRPYDGYIMNMNNYINAKNNMIGPNIEYYPNVPIFPESQNQ
MFPYADPNFCKNGRMRPIYQNINIDMMLYKFQNSVNQTTKDPNFLKLSNELCQFNVRKEEANPVGLNTFMGNI
LRCSTTEDCTLSPFKFDCYQNLSYKNILGELRANNVSNNYQSSNNISKNMPAQNISNTLVNLPYIPMIPPNIT
TVTNHNNNMNNNNNVNNNNNINNNNNINNSNNINNNNNINNNNIISSNNNISSNNIISSNNNISSNNNISSNN
NNNITNSGYIENPIYYNPYMRNYFSPHNNNSSLHISSNNPYFFNLPSTNMDTNFTSNKNMNIYPMRNPHVVQN
NHMNFPHMHSYMHSNINYAINNNSINLMANPNIANTNMNVPGTFPPDFLLMNAHKYVNSQPVPMPFFPQVPYP
NYYASSHGMSPT*
>N.PF14_0338.1
MNKMKKTENINSSGAHKNINEDRLSYVMTCLLGNEVNVYMKDGKEIRGLFHAYNLTAKNDKKEIDISLNHTRM
IPKNENTNGPINKSMIITDNLYTTIIGENINMNLKDPDNSIYSKDIFRIDADISENKKGKLNGHTNNRELKRW
VGDNDIVDETKLKLDDKLNEPWDQFEHNRKLYGVTSTYKEEIYTTNLDINKIPHHVKIQADKIAKELEKRGMH
LDPEDQEKNNNEIDEEDLFGAVRQNKDKFFKNNKFKKEDNKYKNYQGKYHHVNVKDLKEKLQLVKRENEKKYP
STANKQKQINFTVSSSIEENICANKKNKIPSKKSVSKNSEFIGINALNLEPALPKLDEKTRTEWIMFKNQTKN
KALHKKDKTTEKQEFITASKEFNEKLTYKMNLNKKDANIRTIKDHSGMNQPYIDSQKNISHIAEKTKHNNAND
PDENKIQAKENVLNNNMNINNNNYNIMLNNSNIRPYDGYIMNMNNYINAKNNMIGPNIEYYPNVPIFPESQNQ
MFPYADPNFCKNGRMRPIYPHNHMENFKSCYRINPPFFPQNINIDMMLYKFQNSVNQTTKDPNFLKLSNELCQ
FNVRKEEANPVGLNTFMGNILRCSTTEDCTLSPFKFDCYQNLSYKNILGELRANNVSNNYQSSNNISKNMPAQ
NISNTLVNLPYIPMIPPNITTVTNHNNNMNNNNNVNNNNNINNNNNINNSNNINNNNNINNNNIISSNNNISS
NNIISSNNNISSNNNISSNNNNNITNSGYIENPIYYNPYMRNYFSPHNNNSSLHISSNNPYFFNLPSTNMDTN
FTSNKNMNIYPMRNPHVVQNNHMNFPHMHSYMHSNINYAINNNSINLMANPNIANTNMNVPGTFPPDFLLMNA
HKYVNSQPVPMPFFPQVPYPNYYASSHGMSPT*
>N.PF14_0339
MLKGIYLREHLCGQYKKTNYFCVRYFSILQRYIKARPHSHELKVNETDINEEKKEEDSKRKRGESKYYAPNIT
RGIIPKCAYMNTWEMPEQPENAQFLKIALIGAPNAGKSSLLNSILNKTISAVSPKINTTKYDIKGIYSKDNVQ
LIFIDSPGIIPSHKKKKFCKELVTYAWKGYEEADLVLFIADAVKRPTHDILNIVRMLAPKNVEPYDSESDDES
FQNNKINDNRQNIINTESSNDPLDYMNNIDNKSEDENNISHIKYDEMNREEKFIKYFSKEMDKDLQYSDKSIK
ENLFKSRKVIRNSYSDNIKNNITIDHNNEQEKLHKTNKKNDFILPVILVLNKVDLCTHNKWANARAKEYMNNG
IFDNIFFISAKYNKGIEELLNYIIKYKTKNQYWVYPKDAITTLSKVQIVEQLINTYIYCWFNKDVPYKVKHHM
LSWCVNLDNSLIIEYQIIVKSEKVAKMICGVHNKLIINMRKNVSYKLTKLWNKNVYVHIHVKSIST*
>N.PF14_0340
MYKYLRITNYKHRNFKCVKRFKCIIDNTLMDNKLKWKTTKESYCEYILNEEEEKEIRKNHEYIMSNKNINIDN
NLIYECIKKYNQNNVSDYYKKLNSNLNNLYCNNSIIAYYYLNFVNKFMKKIKNISIVNYFTNIINSKYKINIH
IFISPIYERINNQVLLSNFHLNKNDVREIMYFLCMHVWIYCVKLNMINNNHLKVLLWEKIWDYYRGLIIKSKI
PEFSFNTYLINMQEYSLGFCVGLDDCITKELYAGHISNLLFNHVYNENENYKSSKELSNLTIYCIRMYNFICH
LPEDNFVQAKFIWPDLEYGLV*
>N.PF14_0341
MNMEITNLKSYKELVTLSAEEKTKDLKDYLNDKNRSESLIKKFKNFYMDLSRQRYSEKTLNKLVEYAEEVELK
KKVEKTFMGEKVNMTENRSVLHTALRIPIEKINTHKIIIDNKNVLEDVHGVLKKIEKYSDDIRNGVIKTCKNT
KFKNVICIGIGGSYLGTEFVYEAMKYYYYNMELNKNEKDQVNNFNNNYDQDNVFNVRFLANVDPNDVNRAIQN
LDQYDTLVIIISKTFTTAETMLNARSIKKWLSLKIKDDENLSKHMVAVSTNLKLTDEFGISRDNVFEFWDWVG

GRFSVTSSVGILPLSIAFGYKNMRNFLNGCHDMDEHFLHADLKENIPVLLALTSFYNSHFFDYKNVAILPYFQ
NLLKFSAHIQQLSMESNGKSVDRNNQPIHYNTCQVYFGEPGTNGQHSFYQLIHQGQVIPVELIGFKHSHFPIK
FDKEVVSNHDELMTNFFAQADALAIGKTYEQVKEENEKNKMSPELLTHKVFNGNRPSTLLLFDELNFYTCGLL
LSLYESRIVAEGFLLNINSFDQWGVELGKVLAKEVRNYFNDTRNQKKSDNTYNFNESTKILLNYYLSK*
>N.PF14_0342
MIRTRIVFERIFFLIIFSGIVYFFLDFMTVYESSIRYLSFLLLLFIYGVSYILAIIPDIANSINYISFYKTRK
KKVLSYSFKKDIFCEKKKKKKISNKDLEIHNLIDTNFFYINPGVIEKEYYEKKNEFNDAYMYYLKHIKYMQKR
DRKHGDQTNTNNYINSKEGMNDEDFNSNINNRVHDNINYKKDRKEDIYKNDDNYSDNNMYGMNKKENKEHKKY
IIDNCEEFEKICFNDQGKKIKIKDKIIRYIELSNNNNKNNNSNNNNNNNNNNNNNNNNNNNNNNNNNNNNNNN
NNNNNNNSNSVNVKKIDEKENSHVNQNNYVLEEGNKKTKKNVSYKSYAYDEKTRKVQNIYEIHNEGLSIYNEM
IGKHDNSLMHYKKKDTKKMIGSFLHEKIYLDDENDLSSDEDIEYINKKQKYVYLLLNKILCKNKNKRNNSYLL
SGYGSNINSYTLDSFYDRNKRINKNTNFNKWHNLYLKCIINIKINFMEFCKNSAFIIADIIITLLLNILWLNV
HKYIYRNNDDLDRGMFNWNTSEIPKIYNSIEGYLQYFILYDMCIKLFLFFSSNYILSFWFLLNLLNTPFFYIL
VSYFNNIKYKKMHWLYLSCPLRFLNFLRIEDLFGKNNNYNKLNILLIKLSVQIFVMIYTYACLNYLIEQPCRG
EYELYDYVFSGMQTVTTAALGKGTCFPFSFKGRFAHILYIFMTFTYIHYKIRSLKNYIIEEKKIYGKIPNIGS
CYFVIIGHMKPIQLYVIINELQSTYNNLDEIIILTSLPIKFYWNVIRLLNKKNMCQISVCLYDLNKPMPLKIK
KIISYSSGIFICNNIINTHYNINNDMETLKRYNEINTFGPYDKHISIFLNNMCNRNILLQRYNRHVICLNDLK
MKLFAKTVDDCHGMFLLILLFFMNTPQKIKCKHTYILTKYFDKQIKRHNKKKKKYKKLRKYNKLKKYTHHNNN
VNIFFKINMKLKSLFYPTKTKRIKTNIQNQTNNKPRYHFLKNMFFIFYTKRFIRNGNNKNMYINQKKHILNEK
QKNKTHMDDQENYYKINIGNQNIENNDSSSYNINHKNVKETNSDDRWLYKNQIYGKKKKNKKKRKRVFFVNEN
IKNNDEKKNKNKNNIYNNIKSNKTIRNQYYEKNFKKSLCKTFLSYKFDEDTTFNSFTNYLNYMKGIKYNIYKI
KFPAAFLNIHFVTIVQYMYMNYNTFVIGIMDEDNEVKLNPVDYIYNNPDIYFIILTDSFNLLHKITHIKKIEL
DWLDKIDQIKIKRTQKFVRNEKKDNLPSQGTVEGYSTYGRKNHQIRNDNNEKKKKINNNNNNNNSDYNNNNNN
NNSDYNNNNNNNNNSDNNNDYNNDDNNDDDNNNYCSYIKWNNYFEYYNKELSQKHREISKLRQEDNIYFNPVL
NIFKVENYLEAYKIFKLKNKNKNESISQNKKQSINQNKKQSISQNKKQSISQNKNQSISQNKKQSISQNKKLN
LNVGRNHSISDHKNCTPYNEQNIYNKNEEILLTNDKENKKKKKLKQKKEHLSNDSMNNHNVDDNYNNCNFIIL
IYWPQSLNTFLKILFQKREHNIIILSDQIPSYIYNNNLFPYRYNICYIQKSPLVLFNLILSGILVCEKCIIFK
NYLKLDSYQNIVSYNEKTKNINFLEYMCEYNDSDLIIIYNNIENIFRRKDINTNFINYYIKNYNEKYKYHDYL
KNKLSIHYRDNPSVINNFYTHHDNINNDKKKKNTKKTSQNQYYTNNIKPINESNIKEKKKYKNNKKKKLYLLI
ELNNALSIQYLNNDVLTNVNILKEEIDNINKNKSHNLLFVENYKKQIQFFNYGNLLVENIYKKIEHIFIDNYF
FYLYFLQFTSASIFIDELMYHLIGYTFPIKKNSLDISTINSFIHGTYVDKKKKIKTDLILKNIHPKYHCRNFF
FLFQNI*RKEEL**EFIVAMKIII*L*LSHVHRKIF*CIKTTRFMYYKVNMKVNKLK*
>N.PF14_0343
MSNIKKSIYFYKDWNDEIESEFYSEENNVSVNENSKKSSANLINEEIIEKEKSIENSVHLEHNNTYKNSGDSI
FYDVENSQENKHSSFEHEKTKSSKKSFYSIYDDTDEDEDSDYHILKDNRKEKMLSLCNKKTMDIYEKEGFFNE
TPLNNNDNMLNENNNFNNVDNQINNNNNNNDNNNNDIYSNNFMHIYDTEALFSNNTEKEKYDSDDSHKYTSSN
NLSEKIKRDIIIKNVLRKINTLITNFKMNKSEINVSYIGIFTILKKYLYIIKNKKMIFIKKKRYTKKMHSCNN
KDMKKKFCRNKTFYCLKRKFLYNVDIKFLIKEKHFLKYLYLCTLKKKIQAHYNKQKRKSEKVLNKLIKYIINN
EIKKKSKNKINKTTSNDDINDIKNDKYDMDRKIKNYIFKNKRSLLNYKYYNSKDYSVKKYLTEFFVNMNKSDD
HKMSDQSYVEHPNIDNNSFDKDITNMCTIYEDKNDQNGENDDIRKNDENDEIRKNDENGDNDEIRKNDENGDN
DEIRKNDEIRKNDEIRKNDEDCKNSKNNNYEEINNIEKEKTNEDVHNDEIYMTYIKDETKLMNSESFATSQDM
NNSIKENNNNKNCHNMDNGTDSVDNLSVCKEYVNDMKREHDIHNDINYKDKNNDTYEEKYYNSISYIDESVQN
VSYDNEIQKSYSDEIIQNGSDEIVQNGSDEIIQNGSDEIIQNGSDEIVQNGSDEIIQNGSDEIIQNDDDIVIP
NDGDKIIQNDGDKIIQNDDDIIIPNDGDIVIPNDGDIIIPNDGDKIIQNDGDKIIQNDDDIIIPNDGDKIIPN
NSDEHLSSDYDQKSLNYHSSKSYISDSEENIHYESNIKDVNNYSSEHYLSDYCYDKNSSNYNNSEGYINNNVE
KENDQYDEKRSIQYNGNSYDKSYSYYSDRAQKDIYGYKSICSEDNSKNMNYSKDSIAIIKSITSANSEEKKEM
KYNKNIIYNNDCNDCNDYNVNSFYDLYDQEQFDEISQKNKIITRIGSFVTNKSVYQYEKYKKDQSSTSVVIPS
KYDTHFNDIENKMEKEIRRSFEQFFNIPIQYNDLKNTEEMKHTNEYIKQSKEEIKNMENQQGINIIKQSNYSS
SMQNIMNINEEEYIPLNQNMRVESIPNLYQSFENINSNYNINTNKEEIEDEKDKNNKNINKNIKNYPYNDDSD
VQLNEEDIITEKHDDTTKMIRKQEQIYKIYSRLQNRILNDFKKGCLKKMKKIFNKKCKKIEKDISQNLQSVVQ
NYNLSDINCSEFVNIYKKKSKKKKKTKNVKSKKWGNNMHLFKNEHNDMKDEQCVKSIHNDEKKMTDRFYEIDN
IKNSDQNIMNNNTCYDKKCDIINNYTYNIIQYDDEKYKICDPSKVYPYYYVTKNEEATYNNPYYYNEYNYEDF
NKLDNEHSKDKYGENALINYVYLNKGNVYKNVEKNEKNKKNKERTHVNTEIKLNIYQRKESSDSNSKENIDMN
NLECTSLYFTKNDLQNKNKLFDLLRNIDKKNLRDVIFKIFAVINSKRFNGDMIGEKGKFKNNEITKYIKNKNV

FNKIKPYKKNTNYKNRKENKKENKKENKKENKNNKKKLHSFFIRNKLIYNNNNNSNNNNICKNKNNIQKISKQ
KKINKIFYPTNIYKRMYQKIKENNKNMEGNQKIIETYNTKYKLINVPNKEIYFHKNENNNIGVKKTLPVYIYR
MTMKQKNKYIPIKKFNYSYLFIEKKKKNYNSSTRNNKILPYYYFFDLNNFTQNMKQNLYFKPMVTNKKSVYNE
YIPTLENNKNIYVTNNMINLYNNNNDNNNNNSNNNNKYYNMSTNVDGKNIFFDGNKTCMYYYSKDYISNEDIH
TKYIMDKCSEYKCSLSMEKKTNDNFDNHKPILNDIQQKNITEKEDDHKVSLNKEIHIDVDLKEKINDNKVVSN
IVYVDHKNEIMNEKMVCVDDNIKDTYIVNENNELVKMDNYDSNLQTILSTDKKLNDDKIFSYAIRQYYYNLNK
CTLCDKENGKKCHPLIYNNNNNMISENAHQNKGEKSQNYNIQNNKVVKSDDHYINDDSLSKGTKKTTLKKSNN
IKKDIINNINKTNNSSNRNINYACNSVVQVKNNVINVETQKKQKQNDNKEIYNLSFTNEKVIPSYYTNLVKKY
KNDVGKCILKNIYNGSEYFKNSNIKYTCMNNIYMNKIYQKKKNIYTNKTHMMHNKYVNQVYQKEYDKPNVTKY
QLKVKKITFNSKNQSKQINNTIFNNLDLYNNKNYDYFYYNKNCNLESSIKLKNNNIESFQICSINSPINHKAT
NTNYKHNVENICIFKDF*
>N.PF14_0344
MRIIILALLIVCTIINYYCAVQNNGNKSLNVMPTCSKPGNDSDSNDNETGDVDNDKNNELGNANDNNEMNNEN
AESKNMQGENSNNQEQLNENVHANDDAMYEGTPSSDNPPQENVDANNNEQEYGPPQEEPVSENNVENVEVATD
DSGNDNINNNDNFNNNDNYNDNDNFNEEPPSDDGNKNEDELTEGNQSDDKPMNEEEATINEMGKITNPFEDML
KGKVDDMDFGKMMNKDNLQSFLSSLTGNKDGSGKNPLSDMMNIFGVPQTGKEGAEGGVNKENQMKQINELKDK
LETMLKGAGVNVDKIKDSIKNNDLLKNKQLLKEAISKLTLDPSMMNMLNNKDGANGKPFDINPDSMMKMFNAL
SNENGNLDDLKMKPTDGSFDSFNDGVDNNLVPSNPKGQNNNEEDDEEGGDDDDYDDKSFVVNSKYADNSFEDK
FNTFDEKDDDVKYELFGENEEAEELNNNTTTASSKGDANNSVNTQEGEGEEESFSANEENINNNNNHNNKNYN
NYNTSQQEEDDNSFNENDEPLISSSQFDNNKKNKMSVSTHNKKSKNLMDSLDLESTNYGSNSSSSMSNNYNSK
NKNSKKNNKKKSSQKDYIRTDGKVSFDMATLQKTIKNFGGADNEIVQNILKKYVTIDNDDDNDADEDEDEDDD
DDDDLDEDEFSVKDIKKLIEEGILDYEDLTENELRKLAKPDDNFYELSPYASDEKDLSLNETSGLTNEQLKNF
LGQNGTYHMSYDSKSIDYAKQKKSEKKEDQQEDDDGFYDAYKQIKNSYDGIPNNFNHEAPQLIGNNYVFTSIY
DTKENLIKFLKKNSEYDLYDDDDKEGGNFKSPLYDKYGGKLQKFKRQRAFNILKQWRAKEKKLKEKKKKEEME
ENKEFDFSKNYNFSSKNDGGVTMFSKDQLEDMVKNFGGKPSAHVTDSFSRKENPFVPTNTKNNSNDDDDMDNG
YVTFDGKNKVSENDDDEKGNNNDDENDNDDSNDEEELDEEEDDN*
>N.PF14_0345a
MILKNILKGQVNYKNASYINIEKDINNKINKILNLIVYDVNKKKTTSCSYITDYIKYMNKHVYKLRDEQDLIP
DSKDNLYIQWKYYNQITVNVLKEYVKKCHRNNIDGNYNYEKKKRTEPYILTANDTCALFPFHMIDNYAKTKEK
KKKHDVYYDMCNILGNKNVSCEKNIINLQKCEEEITCEIQNDKSIDNHIISKRNNIEEHIFENYVQKDKNILN
NSSYIYYNIPNLNYIKYMWLHHSLVIFKLLIDTNLKVDIKRQFNMTKIIVSLLKQLNNNNSNNNNNINSSSNN
INSSSNNINSSSNNINSSSNKYYYTNIIDIDLYLSYFYLLSKINMIDKHMVIQLCTLFKKDYKKLSDLNKFYL
LRSIYNFDYSSKHFQQMLNYLQKYFYTLIKKHYLDNNTYHNYDACISSIYGKKEISSNDMCYNIGRTQKGEKN
EITSLRKYTKEIDVNENVNANVNVNENINNINYNNSDDAYNNTELYNTFLPSSYNMYNTDMCYNVLSKKINKN
NDKLFFHNYLLEIFEVLFKRKKHISLLDKTIFKCVVNQEISIYNKINLDLFKNIVKLCDEELIYFLFNKIIQD
IENYDNLEIYQLLTHLKKYEENNSLCYFYNFFLILKEKQFEYMNIKHVIYIYIYINRYLTKEFKKQQKKKMMK
DLISINITEKINEIHNVDNMIDMLHKKSNFTSQYEYIYTFLNFSIYNINNINKYDNKQIIKNKTDDNHNVEIF
NKLLNEHINEKDKDVKKDDFFSSENKDKMDMKMKIKNEFMCIELLNDINKNITIVLSQKINYINIKNIITLLY
NTCQIRYEYQMSFVMNLFFHLYEEYSMLINFLHLYKILFQNFLFYYYNGITEYIKEIYDENKGEYIYMDKRNN
YMIYTYENKMKHIYNYINRRYYINEHRLYDINKFTILLNIYEKYLFNFITYNIYKCSNIQNEKINEKINEKIK
ENEILKNSFFIIYIYTHLQYIIFNKKKKYNHNIPLNNVELIFKYSKQIIKKINIYLKSNLWALGYIQNMNDFK
CNDDNHKNINNFEMSKHTKYKFNNEVECNVLSNHFYEKNKFDILLFEKLEDKKFAPIINISTFINSYNNKEYN
EFTNDIIPYNINNSILNFEYNVVPLFYHILQYINYICTYIYFNTNTNENINIKESLIFISLTCLKNNFNISNH
NILLMNNIYTILKSIHINEINKNILFIISLDYIYKYCNVKNIIRKLFRSSKNRKFNYNFNINDIHENTRCKIL
SRYIKYLKSVHIS*
>N.PF14_0345b
MDSSRISVWKKEIGPSLNDVFLKKDLDKIKHGDDENKNNIYLNTINVNKKNINNNINNNNNNNISSSCSSSEL
VEKKEKYSDFICRDKFIFDMNITCFPNYMHKSSLNIKKKFDISDLIIEVRDARLPFTCTNDFIIDDLKKKLAI
NYNNNNNKKKRIIILNKVDLIPRRLALKAQSLIEEKTNTMCLLTSSKYNKNISKIRDICKEMKPKFKSFGLFA
MLIGLPNVGKSSIINSFKDITYNLSKYGYKNNKIAFEVNRKRVKTNVLAGTTKMIDTYKVSNNPLLYLIDTPG
IFLPKLEDKEISLKLSSIGNTLDYKYDHMYVGDYILFTLNKHKHYNYVKILGLDSPSNDIRFISNVISTKLNL
CRNYKYLDINGGCRFFIDMFRLGLLGHVCLEPVPDKQDFITYLDFKSDEPLSINSKKYPTPFRGLL*
>N.PF14_0346

MEEDDNLKKGNERNKKKAIFSNDDFTGEDSLMEDHLELREKLSEDIDMIKTSLKNNLVCSTLNDNEILTLSNY
MQFFVFKSGNLVIKQGEKGSYFFIINSGKFDVYVNDKKVKTMGKGSSFGEAALIHNTQRSATIIAETDGTLWG
VQRSTFRATLKQLSNRNFNENRTFIDSVSVFDMLTEAQKNMITNACVIQNFKSGETIVKQGDYGDVLYILKEG
KATVYINDEEIRVLEKGSYFGERALLYDEPRSATIIAKEPTACASICRKLLNIVLGNLQVVLFRNIMTEALQQ
SEIFKQFSGDQLNDLADTAIVRDYPANYNILHKDKVKSVKYIIVLEGKVELFLDDTSIGILSRGMSFGDQYVL
NQKQPFKHTIKSLEVCKIALITETCLADCLGNNNIDASIDYNNKKSIIKKMYIFRYLTDKQCNLLIEAFRTTR
YEEGDYIIQEGEVGSRFYIIKNGEVEIVKNKKRLRTLGKNDYFGERALLYDEPRTASVISKVNNVECWFVDKS
VFLQIIQGPMLAHLEERIKMQDTKVEMDELETERIIGRGTFGTVKLVHHKPTKIRYALKCVSKRSIINLNQQN
NIKLEREITAENDHPFIIRLVRTFKDSKYFYFLTELVTGGELYDAIRKLGLLSKSQAQFYLGSIILAIEYLHE
RNIVYRDLKPENILLDKQGYVKLIDFGCAKKVQGRAYTLVGTPHYMAPEVILGKGYGCTVDIWALGICLYEFI
CGPLPFGNDEEDQLEIFRDILTGQLTFPDYVTDTDSINLMKRLLCRLPQGRIGCSINGFKDIKDHPFFSNFNW
DKLAGRLLDPPLVSKSETYAEDIDIKQIEEEDAEDDEDDEEPLNDEDNWDIDF*
>N.PF14_0347
MNNKMLTRRILFSRYFHGPSLYKRYILIKNDIPEFIKRVNFNSMQKNNNDKDDKVKNLNIQNNKNEEYAHTKE
NCDNIKSPFFNDILKKHDKKNPDVIPNFIIDFFTKESTDDKKNIYINEYKNVLLEFIQYKIEFSKDDSNKHQI
KFVDIFSMLYFCYMERIYDLDFLTELSKQFDNIYKSKNTKDLLNNININYLMMMFYYFSFLKVSNGTLDNILR
DFIINNEEVEPVLIYKYFECLSLLSDVHDNNKIVPIIEIFIENFYNFNNYILLNILQFLHKMNFFDKNIFSLL
TRKINKNVYEQNANLFEVCMLSRIYALYKNENITFNNYLLDDLIQTITRHEDNFQNDELYEEEDKEHYNKVDK
ENDSSNIKQLNNNNNNNDHIEQFNNPDSININSNNDIDKNNETNDNDIHNLYIQEIKNINRDNYTLFKNSLYN
DGLNFYSFFLNSKSTNKNLFKKKENIHIEINNDKKKSTSKMLHMSQWDKLFFYEDYKTKDIKNEPFINMHKNK
NGNNNNNNNNNNNKNKNIDNKENLHIDKYINMYNDFIRENLYYEFNYQKMIDVFKNICIHLDNTKIAIYINKK
DNMLSCNNNKICRYRKKLLFIDNYYIGHIFYIIDSMLSLNMHHNSSNFNKLQNKVLNLIKNNETYIIDNFDSK
EIKSVLIFLVHTDRYYKESFIYSITHRIIDLYINNLCDAKTLSIYLYNLLAFTKQTIVKKNRFNHTIKNVVYN
TYLWLNNNKNVSNEQGQNNKILFYNNIKVTNYTLLQILSIYICKNVYFISLPILASLLRSFSYLSFNDINFYN
VFIPLFLKHIKYLKNVDILNITQAYNRQKINNKYFYYLLSKQYQNANSKDNKKSSVEVKLIG*
>N.PF14_0348
MQGLLLFMLICINFCKCTKIITRYNFITNKINIKRYKKKNNNKSPLYYSDNEHHKYNINIPSLLLSKRIIFLS
SPIYPHISEQIISQLLYLEYESKRKPIHLYINSTGDIDNNKIINLNGITDVISIVDVINYISSDVYTYCLGKA
YGIACILASSGKKGYRFSLKNSSFCLNQSYSIIPFNQATNIEIQNKEIMNTKKKVIEIISKNTEKDTNVISNV
LERDKYFNADEAVDFKLIDHILEKE*
>N.PF14_0349
MEKYKNILEKLEWYKNKSSEKYEFGIYEIDKREVFITTKYSYGFVNNKPLLPGHILLTTLKKKKHYNDLDIEE
IIDINLLCNFMCYIMGNLFNTTDFSIAIQDGKEAGQTVDHVHIHIIPRKINDYKNNDNIYNDMNKINLGYGKN
IICNSCNNTINVCSQNEIERNFKLEEFNTSIRSIEQMEEEANLIKSYINEKFSS*
>N.PF14_0350
MNIFKQKENTPNTISHVLMNKHKGKFPEAIVSIRQLEERDINEVRQLLYDHFNSLTLPAVIYWSIQHIYDLLV
IIVINYIFFLDLKKIFYFLIIFLIYLYIRAKLEFLNHIRKDCPDLENLYKSYINVEGCNFWVAEVYDNNFGSA
SRTTCSDIHEREKIILEQEEQEKLLSNNNKGNNNNTLDEKGGEVDQKLFKHNLENNEYNNETQNKVLDHVKEN
KEININNNKEDNKKDNKKDNKKDNKKDNKKDNNNNNNNNKDNKKEITSNFTSDNNNSNYNNLYYKSNDQINNN
SENQKDNVKENLKDTSSTQNVCEEKNNNDTCNEQNKDNNNNNNNNNNNNNNQLSKFNIFKNKKSSNDDKDNNE
NCSNEKKNKLLFNISEISNRHFMETKRNVSSGFNEIRRNIFNCKVQDEEDFNKNILNKKIVGCVGIVPFKGDN
TIAQLVRMVVKKDNRRMRIGSRLLTQLENFAHEQNYNELKVFTNNLNTDSLYFVKQNGFNLSQIVRRGLMRGD
LLIWSKILNKDDFYKFNSPEIQSYTNNILD*
>N.PF14_0351
MYIFIVIIIYTVSSIKIKRNIYYINNNILKNLYSNTNILKSHNKINITKEENYLKKLHSNISKIGNNIYKNIN
PFNIAVTIFTFCSLIGLLFKDLIKGKKDNIKSNQSNDENLFKYMCAKCNLVIYPAKGREQKFLKDDYICPNCG
KSNMDKHNLIKK*
>N.PF14_0352
MGDNVKRLPLPSENGEIKKSTSGRLSDDGIKRTPSGKPIQTMYVLNRKGEEEDISFDQILKRIQRLSYGLHEL
VDPARVTQGVINGMYSGIKTCELDELAAQTCAYMATTHPDFSILAARITTDNLHKNTSDDVAEVAEALYTYKD
VRGRPASLISKEVYDFILLHKDRLNKEIDYTRDFNYDYFGFKTLERSYLLRINNKIIERPQHLLMRVSIGIHI
DDIDKALETYHLMSQKYFTHATPTLFNSGTPRPQMSSCFLLSMKADSIEGIFETLKQCALISKTAGGIGVAVQ
DIRGQNSYIRGTNGISNGLVPMLRVFNDTARYVDQGGGKRKGSFAVYIEPWHSDIFEFLDLRKNHGKEELRAR
DLFYAVWVPDLFMKRVKENKNWTLMCPNECPGLSETWGEEFEKLYTKYEEENMGKKTVLAQDLWFAILQSQIE

TGVPYMLYKDSCNAKSNQKNLGTIKCSNLCCEIIEYTSPDEVAVCNLASIALCKFVDLEKKEFNFKKLYEITK
IITRNLDKIIERNYYPVKEAKTSNTRHRPIGIGVQGLADTFMLLRYPYESDAAKELNKRIFETMYYAALEMSV
ELASIHGPYESYQGSPASQGILQFDMWNAKVDNKYWDWDELKAKIRKHGLRNSLLLAPMPTASTSQILGNNES
FEPYTSNIYYRRVLSGEFFVVNPHLLKDLFDRGLWDEDMKQQLIAHNGSIQYISEIPDDLKELYKTVWEIKQK
NIIDMAADRGIFIDQSQSLNIYIQKPTFAKLSSMHFYGWEKGLKTGAYYLRTQAATDAIKFTVDTHVAKNAVK
LKNADGVQITREVSRETISTESTVTQNVCPLRRNNDEQCLMCSG*
>N.PF14_0353
XXXXXXXXINATLKKCSEMDDMCHLYNMELMQQRERIENLKRKLHSYNKNIEEQESIFKNVVKYTDGFINNII
TFLERFSNSKELNVLSNCAKYCLADDVDELNLINNFNKVNNISSHKKKEIKTTKLVNNKYKNDYSNKISIYSD
ENKVNKKIPKKKKTKNKYKNDMKKKNVQLNENIYNLHNPYDTVEIRDLYNIYRKKSTTSYENTYIN*
>N.PF14_0354
MGNTLGIECSFNYVHVTTVLINKKLIKESNNDSKNEKDIREEKEKKLPKNVSIPSNDNKLNMNVHLSWLKEKY
KKEYINLEEDVSKSDEYNNCDDDYIKMKKNTFSYILDMQHIIDSDVQVFSLKKNSEKNVHTHISINELYKCFH
EDDNLEKKFIKYLNFVKYHKLDISQIDTVNIQHLYDDEIAEIYFWSFKLKYLDECILKYYKKNINYMFINVTG
KNKNLIKKKFLQITGKNNIFQHNEIKCINNSICFLKRFMPTNLYYFTKYNEENERASCTSQNDEDDEKKKKKK
NLLYQSFINKEEVSEIKSYVIVNMKRAVCYHLVNEQNLIERIGTLYVGFRTVMGLFLLITGRPCSLQRICQLA
KNGTNRTFDMTVQDIYGTSYSNAGLCKDLTASFFGNAQHIENVKNILNTYDEEKNINIKEEDDKLMNVYEYEN
ESSCYENVSSCMSTEISECQEIFETEECIGFEVEKNNINYYRNKYSFLSKDKTKKLLVSNKNFNVYDNCKIPI
FNFNSDSYCNYMNSFKSKEKLFKHNIEEQQKQEEGYKQKEDDNFLMNDIDNYLSIKHSLSDNEINMYEYHRKN
YNLKKSFLKKLFKKQYLNENGKIIHNINLSIQKEVSSFILKNKKRFSYKTYDKQIVKKACKSITLKNVCSETN
KNKMDSNLKDKTIVKYNTKMCDLSRSLLSMAIFTTVYLSYIHCNLYNADHIFFTGYNFEDDVCKELFQIIIKF
LSHNRQKIYFAKISKYISSLGSAIELINWERIHIDN*
>N.PF14_0355
MPKVNTVKIDNGESDEYNSTNQSPRKLNDSSGLSKKKSGNISRKASSFIYKISKNDKKGVSFVNKKGEYIIGD
EDASKITFRDFLNCPLEYHTNVMGTVLFILGSAFFLYPPLYISGCVTFAIACALCLYSNFIGAINKEQRKKNE
YYGFILYIIGCAIFILGSIYSCFENDIYSIITFIIGSLFFLWGSIMFLFSIKFTQIKNVDINILVVFFSNFIG
GILFTVGSVFFFYPQLYDAACYLFIIGSIIFTLAVYFDYIIYINNNFTLDIV*
>N.PF14_0356
MLLYNKRIGKIAIQGRRKCYSWLYFSSYRSNNIIYKEYEDFINIGKKNPNQIISMLNRLSKEKRCDIELIKNI
TNHIYDFNEDYFCPQLIEVLKNYVKLKYSDETLLGILSNRIDDLLSIKSCLRTKDMINIYKQLNFHHPVVKDP
LLTKFNNNINNYKNEMVEIVRNLSYLYIDKDLCEHIFNHIIINYDYYHKDIFSIFEGVSRFDYNDNQTFIQLI
LKKKIKELEQQNICCNMKDFFKFLSASERLGLNNNTYLHNEFEKKINHIKNISPNNISYILLSMLSTKYRNDT
LFELIIMNIENYVNNQNDNNKLLNEPINNIYYNHISSDQYKNNESKLNLSNTINGNNNNKYVLNNKRDIITTE
QNDYYNFFNSYILNYLPFHLLLLILLNYDNKNILLYLLNICINEYTYLYNTSNLIKLLYSNTLLMLPSKIKKY
QDKEKVHLEKNVLKIFSALQNVYQSCNINDYKILHQCYLFHINMIQNNDTLKHVYKDFLNKECFATLPSSYSK
LNFEEMQVIRYASMSFLKNKKGHISYYLNKNDFYSSNHNNYDNALLSVKFKVELLRKYFDKSEFNVIYNSEKM
KI*
>N.PF14_0357
MISKRTNSTFLIIKNYYNVWVGYNKNVQHNSYFKNSLDYIFKPRSIGIIGATERKGSVGNSIVDNLIKGESNY
KLYFVNSKGSKIYNRQSYKSLKDIKEDSIDLAVIAVPRNNVVNVMHELKIKNVRGVVIITAGFKETGAEGLRL
ENEIINIGKTNNMRIIGPNCLGIIHSYHNMNASFASSDILKGHFSLLSQSGAICSAALDLSLQHNIGFSHFIS
VGSMCDVQFYELVEYLFYDVNTKYILLYVESIGDMNRFVAVCKKVCLYKPIILLKSGKTAKAAEAAISHTGSM
VGNYEIFYATMKKLGVLVVDNFEELFNMCKVLNSSKYPETNEVCVVTNAGGPGVLLVDNIIKNDGVLSNLNDK
LKNNLNSFLPDSWSKANPIDILGDASPVLYKKTIDAIIKDDQYKNIIVLLSPQSVTEPLDTANEIINLKKQAN
EQGRLILCNYLGGTALEESANMLNKNYIPTFVHPEHSVQNLLKLYENIKHIQGIYEEIPEGLSNSVENHYLNK
LIEEYYYMKNKSDIHMSPKKKKSIDIIKKAIQNKSYILNEFDSKVILQNYDIPIVQTRIFENVDDIKDYDHFA
FPCAMKIYSDKITHKKDIGGVILNINNKEELIESYKKIYNNVKAHSLEKYFKGVTIQDMVNINDGIEVILGYY
YDNNFGPVLLFGSGGSYVEIFKDSVLLIPPLNFSYTHHTIKNTKIYKALAGNYSRFTKCDMSKLITTIVKFSE
LILDLLPYINECDINPLYVCGDKIIALDARFTLRKNNITDKQENIYYDTFSKIYPIDFISFNDKKKNDSNFLE
GSNNINVSNNIPNETTLNSSLDKIDSITRCIHSYDFKLIHKFCIDNYKELHNSTFFYTDVDSIKSELFSYELC
NCDYDLYNVILYFNNKQINGLMKIEKRNDNNHCYIYAKDNSTFYILIQKLNILAKKQNNKHNYVYLKTNSPYI
QDLQSMKYNLEHTYKDVSCYKSNN*
>N.PF14_0358

MDKSKSSIEKELNRIKQDVSLSAFSILFSEMVQYCLYKSKRGYRIEDCLHEMGLRVGYKLNEYLTYKNKVKRS
INIINILTFISKHVWKYLFQHSSDLLKSQDSIYEYMICDKNILLNKFINVPKDYGNINCAAFAAGIVEGFLCS
SEFQADVTAHTIHEGDDNYNTTIFIKFYPEVVEREKNH*
>N.PF14_0359
MFFSSGFPFDSMGGQQARRKREVNNNKFYEVLNLKKNCTTDEVKKAYRKLAIIHHPDKGGDPEKFKEISRAYE
VLSDEEKRKLYDEYGEEGLENGEQPADATDLFDFILNAGKGKKKRGEDIVSEVKVTLEQLYNGATKKLAISKD
IICTNCEGHGGPKDAKVDCKQCNGRGTKTYMRYHSSVLHQTEVTCNTCRGKGKIFNEKDKCANCKGMCVLKTR
KIIEVYIPKGAPNKHKIVFNGEADEKPNVITGNLVVILNEKQHPVFRREGIDLFMNYKISLYESLTGFVAEVT
HLDERKILVNCTNSGFIRHGDIREVLDEGMPTYKDPFKKGNLYITFEVEYPMDLIITNENKEVLKILKKQNEV
EKKYDLENSELEVVSCSPVDKEYIKVRVTKQQQQQQEAYDDEDHQPEMEGGRVACAQQ*
>N.PF14_0360
MIPDNNENNNNLYFLAFSKNKVTIYEYDVDLSKNIVDDINDEIKRCEEEDEKNKINENTVPMTNDEKKVDMNH
IRNNNIINNNTNNTNQIKKSTNKFNNDFVLNRGIKIYKEYDDVELATCTNDYKKIILVRKSDLNVAEIIDIKS
ENKNVTCIKTKTQIKRIITSPRDNYIVLHCQYKPDITNTNLYVYKLNVKSSKKKKKKDKKNSECENIKGEENS
VGKENDNYEEEKKNINMNNNNVPIHNNNNNSDNNESTNDNIIYNENLIIELTLKSYSNKNWPFFKWSDSESIC
TCLINNQVYIYKDNNFNTTVNKLKLDNIEFFDISPELNNKKGILIATYEQGSKGNPSIFKIFHIDNLNKHIYS
KNFFNSDEIKLKWNKNGSSLLLQIHTQVDKEKQSYYGSSNLYFIDTVTLKDVNVMTNKGLIYDTIWSNNQNKF
YVCKGEIPAEIVLHDKNGNVSHSYGRHKFNTLKLNYNEKLLLTGGFGNLSGDISIWNTSTKKEITKTKSSCAV
ICEFFNDGKHFLTATTHPRLRVDNNLKIFKYNGLIVSRINFEELYNVIILPFNKIKFQQTEEPVDINLEHTTL
QPYINKQLGIDSKKTGIYRAPRSTGLVNLNGYLSSKLPDREKSSLPPGCNFVNENKNYNKKKKKKRSSKSKQK
RETFNNKKKKKKKKNIYIYIYIYIYIYXXXXXXXXXXXXXXXXXXXXXXXXXXXXXXXXXXXXXXXXXXXXXX
XXXXXXXXXXXXXXXXXXXXXXXXXXXXXXXXXXXXXXXXXXXXXXXXXXXXXKTKKKKKKKKKKKKKIYI
YIYIYIYMFIHILLHIHS*
>N.PF14_0361
MSNRMEELDPDMLGVLKCAFNAGVKVKSAAEVGKRAVEYFRGDDFVNFLSTNGEMLKKKFPNLFINRNLSDMK
EIEEFADMFIQKGYIYKAQYKPIKGINEKDENGVYKRPKWPKRLIMTSKQNFDKTSFYILVHERNKKLQYFML
ISLISIVLICCMFPVWPLRLKLALWHISVAFLTLISIIIVGRLVAFIFFWFFGVDYWIFPNLFDEECNVVESF
LPLQSWVYRNDTWFLFVARMCTAVLLAIAIQQLGKTHSIADIRNFATQSYIDIIEWGNKKLAASPENVSMYKS
IDSKATFEGNVDDDDTVYDENEENYDCLKKCGFTSFEELVRKCFLKCECMEKVIKSECYKKKCSKVTKAVLDE
AHKEACFDKSDN*
>N.PF14_0363
MNSMNTYSPLYSSYENINIYKKNRKFEEDNYETSTANISCDSKGRILVKESENDRNESIQKKQMLSSMDNRKT
NKSEKHNISSYYKMRKKASNHLKDKEKLNNNIIYAKNPHANRYDKYYSLNMNIMNNMNNMNNMNNVNNMNNVN
NMNNVNNMNNMNNVNNMNNMNNVNNVKSMYNNNNNNNNNNSNVYYRISRQNINGKERLLNRYNIMRPQRSADM
LIRNYLYNELPYEINQQNLNKNINDNNAGSFDNLNKTVFENYNKNILDEYNRNIMQYYNSNLKNYRNSSFDNI
YTRYNNNNNNNNNNNVYSNNNVYSNNNIYNNNNNDNINNSFQFSKQEGLMKIYSNNNRNMNDYVVKTSNITRA
ADNTCAQEQINIPTYNKTVLLKSSPNTENTFNKTLSNILKEQPKRSSMFSFNSSDFFKDFGKLRKNFVGKKKN
KINKDIVINGNIFNNKIIDNNTSNAKSEGNSTLHKIYNFIFPSSVKKLQKKKQKLQVIEDKEKNSNRAVVMPL
KNKIIYINDANITNDKFKHSFENYNYHQTNIPFVSVTNYMNKDSTKNVRSFSSFDVKERISPRVIRNGNINNN
KNNNINNNNNNINNNNNNINNNNNINNNNNINNNNNINNNNINNINNDFVNDSNINKTVNIHNNNNNLNNYSN
TMKSVAKNTQKLSAQKNSLVNDKHHTYHEKLITNIYQFTGGQSQKSCNIKNQNVKEEKINNTQISYINNTAYN
DYTNLPNVETYVLSEKSNNLENKEDNNINEKFKNDNNVDKKNNDENNIIKHNVNQNNNEVIVMNNQTEEQIND
NKFIKPLNNITGIDPKKKNIYNTVMHINDKMDLNKHDYNYNYNNIIPHNYINSSDKMTNNSLHINKKADDIFQ
NMISNNDTNVPCVYVNKYINNIFQNNNPSNVTNYINTINMNKAPITIDENSNKLDNNINYENVTKNMIYNNYT
NNNVLYITKEKSDNKLNNSTNHLNDLKNNYYVINPSLLYIHNNNNNIEWNKQYTNNFSKPNFLDKFFYEKKVE
SIQPHHLNQYYYNNQNVKYNPSHINILENKYVNQFTNQNENSLYNFTTHHDNIKNNNIDNILLLDKAIRNQSI
YYNNNKGDDETKSAEHTTNPSINLLKNNYEDKNKSNYVHSIVSITDKDNSIYAKNKYAEVVTKNNEKREDAIH
KKDEQYIFNNNDNNNDNIHNNNIHNNIHNNIHNNIHNNIHNNIHNNIHNNIDVSSRENFFQHDIQNTNQINNK
TILDITQNEKPNATNEKCLDINNKEKKKKKIKSVHYDMDEILSLKCKVYDELDTCLKCINNLTKNKKLYEQLK
KYKHKLDKDTKTKKLYDECTSEKTGSYYTMKDSYISSLLCNEYKLNHILNKSNNKVSDDKHILSYNHHHNKNN
NINDINNIKHISKFVNKDNEKTLNNINILNHNNKDPNSGVFLPSDNTESVIYNMNRNRKLCDNNFNVSKHVLY
KDSKGTDSTRTNFYDDYSMSYVKSNVDNVEKKEKIKNNINISDKYFLSETDSPYIGKKKALMITLNYNGLLEG
CVNDTVDMCDHLMQRFGFNDFILLNDCNLCYRNFVTQKANKKNILSNLHNFIVNSNNGDILFFYFCGYSIKLI
DSKFTENYNFALLPQDHSKNNYIYSNEIFNIIKKLQGGKQLCIIFDTTYTSYFVPVPTSITYNKNMNTTEIYK

YNNFSSNQKYLKSLKTFGKIRDRNVDSIFVENIKKPLLYEIYKKENDTNTNDKIILVPSIFFFSPDCNDRNDF
EFSIKNKVRGLLTYCLGKAIELLKNDFSYHDLFVAASQILIDIKKEYNLKYVKFKLSFLNEYSPDDIKFLSHE
SLFLKKKLQLDEPLWKPSLKLNNLNQYIQDICNMDERKMLKSSKKKCLLIFIKDIKFYTYKNIDTKNEYFVSC
FIKNKNVNILCVRRNNTKEQRIVQDKIFFLEYITLNVTHMENANIYVELFKKKKKNYFVARSIFNIRNVNGKF
SLSDEKKNIIGIIDLNIKCVS*
>N.PF14_0364
MSNINIVCLGGASEVGRSCVIIECDKTSVMLDCGIHPAFMGIGCLPIYDAYDISKVDLCLITHFHMDHSGALP
YLINKTRFKGRIFMTEATKSICYLLWNDYARIEKYMNVVNKNKLSKNKKGGEDENGLNNGNMLLSNEYSSDEN
IDDNGDVYENNDNGDGNSNVLYDENDIDKTMDLIETLNFHQNFEFPNVKFTAYRAGHVIGACMFLVEINNIRF
LYTGDYSREIDRHIPIAEIPNIDVHVLICEGTYGIKVHDDRKKREIRFLNILTSMIHNKGKVLLPVFALGRAQ
ELLLILEEHWDKNKHLQNIPIFYISSMATKSLCIYETFINLCGEFVKKVVNEGKNPFNFKYVKYAKSLESISS
YLYQDNNPCVIMASPGMLQNGISKNIFNIIASDKKSGVILTGYTVKGTLADELKTEPEFVTINDKVVKRKCRF
EQISFSAHSDFNQTKTFIEKLKCPNVVLVHGDKNELNRLKNKLIEEKQYLSVFTPELLQKLSFHFEQNDSLIS
LGKLSEHIKKINKKIKLEGLKMKKEKMIANDEHISVKNEMGDINNDEENLQISDKKKNKVDEHDKHNINNNIS
NEKHNVNNQIEGIIITEPQNVPILIYPNDIYEYTNLKTAMIDQTINISFPYRFDLLYNVIINVYEETHMDDNL
IIVKDIKIIYCKDDKMIKINWLSSPLNDLIADSINFLILEFLETMNTNQNSIPIADVLTDHNIYEMIISYVEE
NFTNVERISKEILKEKTLQMIKKKEQLKLKNNDNENSQNFKDDNVCEDQNVEDSNTNNIYLDEQINLFSYDQI
LFDYISKIIAVDLKQHDEINEILKFNAKDNNNNDVQVFVDMDNREIICKDQNILTKVREILRNIEESLLPMCF
*
>N.PF14_0365
MKKFKFSKEKLAKEKIENGLHYEAHQLIKSIINRKLQKNQISNCISVIFYFSLKFATAEQYVLLCDLMYQCCL
ILTQHDIEFNLEYANKIIEIFQKCPPNSTEEKYKFMNKSILWSKSEENVYGYLEFHKVIGYAYLEDKQYSLAH
NHFIYLDDNDILYEIICLWRKEAYPSESHFFVLRTILCLLVLDKFDQALNLINMFETNLNRQDVPLPIQLAYL
ITSSCIYKSLALYEDVKYKYRLILNYDPDFQKYINIIDAAVFNKKKHNLFSMFQNIFA*
>N.PF14_0366
MKMEIVGDIKDSIVNENDLIFYYRSLCPINDLYNWLNYKNDIKGKYTKLNDPHFFSKREFSFTCKKSDQGKEE
IYIRWLSFSNPEEFKNKLLSDLVPIKFDIGAIYNFPVSQKDQKGDIFLPVQKELIFDIDMNDYDDIRTCCTDK
KVCKLCWKFLTVAIVLLDTALREDFSFEHILWVYSGRRGIHCWVADESCRYYTTDARAALADYLNILSGSDTK
KKKVSIWGKDKYPMFERAFDICYKYFDVLMEEQDFFKKGSPHVQKLIDYLPYASGKVTDPLKAMKLNELKEYI
NNNNFNSREIFEKFSSIYNFLTPSNYFKRKNVSGNINMPSFVKEIVFHFTYPRLDINVSKEINHLLKSPFCIH
NSTGRVCVPLDIKNINNFNPQSVPTLKLLREQFDDPKNSHIEAENRTSLKPYIDYFRRHFIENILLSCVEKKK
RLNENSKYVDYNNI*
>N.PF14_0367
MADLNNYKEMNSYAEGTYIMPNKIENVYCRNMDTTPNFGDKYYDTEYVFPNNMTMPVWNNYNMNIPNYNSAAQ
ISEELMPNQLYSTMDDPSARNTNYGTLNVRLCNKDRVFYANNERDGNVHENNVIRYPRFDMNAAPRVNRRSDF
PRNTCHARRNLRRNIMHERYNRIRREAPFNMDMYGKNQNRNYSCNCLYIYRNIYKNRYDVVDMKQVHTELIYL
IIKYLYYERILPEANEIKRKINKYFPDCPILNNNFINICREDILNIFDIYKANSQEKEDKKSKNYYKKTYNNE
NICIYLRGIPRDYFINPNDDNENISSYIPLIFIHIIDRFRLQSNDNNHKGGRYILAETLKHTGPYIFRTMKLG
RIIHILQKCIDLNILSYFNNNIIPIFTSMSISKTYVSKLHVNETPALKNHQENSINLIKSRISYLLYSFSEDK
TKSKGFSLSRLPLIYKNVYKENINIDQIGYSKLTEFINNEMSDICYISTQHKFQCILLPVMEDEQKHRDKNAK
LRKETELKKSLDYIKNYRWERCISYLHYSNCLKDKNIEFELEYNYCNPTLEELKKDMHFYDEEDDNEEESTQN
DNPNDKTDDVPLLLPLDVFNLPSTYDMYDSYYKSSTYYSKDEKEDKAKIKFPDNSQNKFDIMCDFKKCENTNI
SVEESINLNHMYYNFPSSYYVFNYLFNDYVEDLSVVDQIRIQ*
>N.PF14_0368
MASYVGREAPYFKAEAVFADNTFGEVNLHDFIGKKYVLLYFYPLDFTFVCPSEIIALDKALDAFKERNVELIG
CSVDSKYTHLAWKKTPLTKGGIGNIQHTLISDITKSISRSYNVLFGDSVSLRAFVLIDKQGVVQHLLVNNLAI
GRSVEEVLRIIDAVQHHEQHGDVCPANWKKGKVAMKPSEEGVSEYLSKL*
>N.PF14_0369
MCKNIYFYIILILKLFIVLADKSDNSICKPSVKISCAHNCCKNKLSFMYNCWKHYDKYSNIIKDNLQKEEDTV
VQLQDHDNIDIVEHVETMPMSFQLTTHTIILFNKWETKSALSYYISLVLCFFFGIISVGFKVVRLNVEQALPK
TEDTNIFKSLVLFKNNSYRMLLSFVIYSWDYLLMLIVMTFNVGLFVAVVLGLSFGFFIFGNKFVSSKKCSPDD
LDVHKEFTADPACCGC*
>N.PF14_0370

MKERINEKFKINSYQDYSDKNNEWNIDDAINYIKRNAFFKKYKDINDVNDICDINVDDHINDDDKFYQNFENI
LIEEKKYLKKLLRENRWNDNILKEYVDGKVLENEENEESGLMYVYNTCYNILSSKNEITDINNKDNSIINHSN
YNEGYIIINKNNTMKKIFINEINELLIEIIYITLTRENLENSLVDLLGDRFLDFIIQIIKNKEQIEKDIKLLS
KQIVIKENTLVSNFFITNKSSKKIKNQQILYKKENIEKIIDHFLYLLTDDSYNQMKKIYIPNDQDKLEEIHVP
KNTIYSYTDNITKVKISRLENLQFHKKELVSVNVLPFWHKYIFDFEYFNYVQSKVFNSAFRSNKNLLVCAPTG
CGKTNIALLVILQQIILFCEQNKIKLEKIVKSYLIKNGLLSCDEDIKKRNDDNERTRDELKSYGYNEPNNYVD
NNNNNNSDNDDNNNININSEGEDEGDIIHLSDNGDKNFDDDNNFDDYDDDVDGDGENYCGYINSKEFKIIYIA
PMKSLVFEITNLFRKKLKIFNLNVCEYTKEYSLSSKELEQVHIIVTVPEKLDILLRNSSYSTTVSDESLIKYI
KCLILDEVHLLNTDRGDVIETIVSRFLQYSETSQSIRRIMAMSATLPNYKDVRDFLKVENDMCYYFNEKYRSI
QLDKTLYGIHEENNNKLYIAKNIYTYNEIINSLKKDKQCIIFVCSRNETNKTIEFLINHALKNNEIEYFVNNV
YTDNDIKKKIKKSNNLYIKQFYEYGCTIHHAGMSRSDKILVESLFKKKVFNVLCCTSTLAWGVNLPVHTVIIK
GTNYFSSESGKLEDMDILDINQIFGRCGRPQYESHGHAILITERTKLYKYIKLLTNNTVIESNFLKNIENHLN
AEISIGTIKNIEDGIKWLEYTYLFIRMKKNPYLYDVDINNDLNLYNKRKDIIMKAIQNLSENKLVRRVLLTND
FIGTFYGQIAAKYYVDYKTIGMFAENVQNNNYIEIIQVISKAKEFENIQIRNEDMKDFLYLKDKCDIKEEYDE
SKNMTLRILIEVYLRRLQINNFSIICEINYIVQNIIRILYAYYDICLNILKNISNLIMNTHNLIVAILRRLPI
NCGIFRHFCYKNEMLEKNKKAILTNKDRLKNIKKDNEESDKKNNISDRNVGVHLIDINEENEYSNTYNNYSNN
NRRNQNYTVYLKEAAVNILEKKNLTYESIEHLSKKELLFFMRNEIYTNQILYYRNIIPNLDIDGYIQPITQTI
MKINLNVKLTNTIWSDQWNDIIENFHIFLLNTLNNDILYFQKFSIHKKDRKKIHDISFEFPLSNQIPPQITVQ
FLSMNWCNLSFVHIFNTNNLFINQKINVFSEILPLTPLSTNVLNIPNYIKFFSFKYFNPIQTQMFHATFHTDE
NILLGAPTGSGKTVIGELCILRNLLRCEGQKSVYICPMKAIVNERYKSWKSKFKSLFNKNVIELTGDKNENKE
NIDESNIIICTPEKLDVITRNWKNKKFVKNINLIIFDEIHLLGQENRGGVIEILVNRFKNMQNELNKKIRLIG
LTTVITSVDDLILWLDVKENYLFNFPSSCRIVPCKTHILGFTQKAYCNRMSVMNKNVFDAINQYAQTKNVLIF
VSSRRQTRLTAYDIISLNLSSHNLNFLHVENLLNDKNHIDFLLNTQKKNKKNKINNKDMNEGDNNIAENINTF
EYTEYKMNINRKDNIASYNNMSYYHKSSVTDEHNINQCNNNDDNNKGFFGYMKKKTLGNDKIKKEYKQYNNED
DNKIHNNYYNNNFCDNEIYDYNLLFNNSKLSEEERKNVANILFQNYLNLIENEHLKEILKYGIGIHHAGLNEN
DKTIVEYLFLNKIIQILICTSTLAWGINLPAYLVIIKGNEFYDAKTKKYKDIPYTDLLQMIGRAGRPQFDDKA
LAILLVQEKRKNAIKNFLYHPMNIESNIMENFNEHINAEICSNVINNKEDIFNYLTKSYYFKRLFSNPSYYIK
EVQYVQFFENSKLSTHAKKIIYDHLNDVIENGIKFLVQNKCLEVVQENYVLNYYATPLGHIASMYYIKCETVY
FFYTSIQAGKKHKNDKMNNMNSTKIDDNTMDHVDSINGDDDRENHVDSLNVEAIKMNDMDNTNGNNNNNNNT
NAVNNNINGDIDKLNDNMQTDFCTLKNDSLEFYDIFELVVQAKEFDDIPLRHNEDKYNVKLRNQIPLDIDMNM
PNVKTYLLLLSRFYECTYETVDYHIDLKLVMDQIARVINAFIDICLLFHNYNYIKKLILIFNCINQKVKPNTN
SLYIIKDITEYQIYKLKQLDIYNINQLIKFDKSYLYSLNIFDITQINFILQLPVFNMNVKLFYKDIHSENENE
KEKEKENKNEEKNRNTTNSYVNIPYAQHFRNQHKYSFKINSYYSKEIVIKLFFNFMNRTGKEASSQNVQWFAI
LHDTEQDESISIKRFNNNNLKKVSVISFTLEDMEKGKYNFVIYIHNDTYYGIEHEVHIDLCIE*
>N.PF14_0371
MELNWKEDFEEIEITINKEKEEGKKLFYNFFASGDFFFVENYDYFELGNTQDILITEAYIKIVDKNDAINIDL
YDRISIKKGDIKIRKNKKIIILNLKKKEKKLWGYLHFFSIFVIYKNEICPNLLKCTKEEKNNIMSKEHIFKAF
MNKRRMESLEHLKKMMKKEKSLNREFLNKLEDEAQKIQCKLEEVKYEQFKMQKENIKKRAIDFIYDDKTEGWK
DEKDISNNKENCKHKEYNKDDINNKNNVPLNNNNNQHVEYNLHNNNECDVNKNNLIFCKNNIKTEKAVIGNYH
NEKKVIELKFTQLKKNELPARESRNLKKSLSNYSSTKNFFLIILIEKAKKMFFRNLDFNSSLEVLKSITECIS
KGNKLIKEEHIKVSANISLLYLLTNNLDKCIEECDKCVDLINEEIKTYNVQHKHCEIIHKEEDVLKIFYSLEE
IKSENYIKYLYIIYVMVILRKIYAKIKKEKWIDVESLFISIEKVQEYLPTSIYKSIKMDMNNIHFFEVIKEHL
LHTNVLNIKDIKNNLYTLNLEILKNKEINDNFPLNIYITLKRSYYLKNLNLFYKNVINIFLCLYNILYRQNKI
YSFLNNIESLNLSKFLLDIYILIMLLKHESVFYEKIMEIEKCKNCIENKNISQEILNILLSYIMDDSQNEFNF
VVQNIDLLKRIIYNANINMNDKNVNEKLLDDISEKRKYVYIVNLCFSDILLKYMDVLRKKNKNGIQKKKFVRE
IICSEQNKIINDMSIKFFSNNEYYKNIKENCDLLKLKITFQIIKTMIYVFNLIQKIYKNKDKEKIYLRNIISI
LLLDLSYSISNLNHTSYMLNYENLIISHLFLYFCFSFLYNFKYNEFIIDIYKKTHTSQTCNNFMSIENIKSEI
TKKKKKSIIKDMLNEDILLNNFYADYKNYMQKKKREKQMNGHHSMLRNNYINLMIAKICYLIKKIKHMKHNNK
KNHICSNNNDNNISNSLMLFSLINDIYSNQISFNSPYFLKDKIYYLLCIQYNLILLNKKYKSSEIINFSYVTL
IKQNFNYSFFLNNDKQKNMEHLYYRTNNMTIINNEYINKYFNYFTNDIITFHNIERNHFLKHVSNNNIETLLY
PYIQCVVWKHKRIKIFSNINICLLKLKVNQGILIKFRNMIIKYMENLLIDFHILYFVFHLKKLNLSLHFFDPN
SGIPTDLDHILLPIKELNKKFFKNKLEYQNIFKYYFNKFLKIKIELLSNLYGSMLYIY*
>N.PF14_0372

MIWWFLISVNFLFFLIKSFFTPKATIYRNDLNTNYTNSSTEKYILGENYNLTSLKLKIDFGSLDTGTKIIEYS
KGIINIRSIQQYDYDSYMLTPCNSDIWWIYSFSDIIHIEKIGLVSLEHYASNFKVIEILGSDVYPATKWKKLG
KISTNFTKSFELFNIYEYCKNYDEDNCWVKYLKFHVLSHHNLENNYYCTLTHLQIFASSGVDMLSDKIYSDEN
INKIEKDLQDNYMDQNNEQLYDQEIIENLEELYENNESLDDTLSNTSNEKKSKTKTSTNDDNDKIHGSNIYYD
NIKKNDEKKKKKKEKKSTRTQAKSLDTKLIDKDLMNTKQIEKELLDTKLIENEFIHNKLFDTDMIEKELMDTE
LIENELMNYELFDKDTFFKENYFNDEQQRTDESNVDQQNDMYVIKNNKDSMKGDYYIKKKKKKLVTDNTKDLN
KCSSYKSSKRDKFFENIKRENHMDDQHNENIYINIKNNKSTHTYKKKNNHIFHKNVYYNILIVLYYLFNQHIK
KELYHFNMLKNKMQSSFFMNRFYITTRYKYLNKKYINFINFIKVLKENHEQKLSEYYDNDIYQKLYIKQEEQK
KYIYNLIMNTQNKYEALIKLLPFSSIQFLLKRKKWIPLLYTMYNKKEFVKNPYFSIINRGERKKRKKTYTIMN
DDYIPLNTFYKKNHIIILHDLIYSLFFSNILCNGDLVCLFENKILLSKVWISRSNIKHFITTKYIFDNYYYND
KGINKLKNIYLSPHFYFFRNIFISHKFQNKIYLKFPYYSYYDKKGVLKYMHNNINMNDFLYTTTDVDINEFIP
YSRYKNYEYEKERVHMIEREKKKKKMYKRKPYENYMILYVIYRLKANNIYHFEFIKYCTNLINNWSKKEIKKN
KHLTLLYNLERKYLSTFYNKMKCVDIYKSYKILKKKRVFVGAHHHIFNNEYNNNDNNSSNTCRRDKLINDIYK
YLQKRNKLNYIPILHNNIKKKNEIQKIKKQQIKMRENIFNRIILFFHKFVFRKLPYIYFLNFWIYNKNDMIFQ
MNDCLDILFNYIFNNIKRIIIIKKKKKKNLYIFLEKLVTSYIKQNCFLKKNFSSLFLKKRNKKHNRHNNKRGY
INMNKNYIILYLHYYMEKSKLRPFHKYLNNIEMSYYIKTANNKNYMSLSDIYFFSKNKEYYLTNYITWYITNM
IFLLLNRKEKNWKVDINKEDIYKNEKYFYDNEGVDEKEKINAYRKMNMIPNYLYNNDSYNDEYFYYDDDDDND
NDNIGNNNFYNNNVLYRDHYQYDNDNKNSYLNYYLTNRIIINHKKNFLENVCLTIGDMENIIYSNYYIDKFEK
KDSKYDKCMKNKIKENIIYDEDKDSHDSSSIKILNEIKKNEESNNKLIHDVYDIISEYDDNVSNKYRDDDKNV
ENNHKNTYKRNKQTSKNISVITGKKERNENIKVNQKRKEQIKKTKKINFIEEFDNKIIDENLNFPNEENCIRE
EKIEEKSKNTRGHALLTLVDKIKTIETKNNYLLTKLRDIIKITNNKTKIIYHMLSNFKILQNTISLLLKYIMI
NEKHMKDLNMNKKNSDTFYKILKEICLEQINDKSKNMDSLKYLCKYLQNFLYDEFERKYLFEKIRPGNYPMCD
DDQNILLHNKNGYHQKNTDFIIKEKKKSLFNFLYYENHCHNDIFKTPLIYYNSQVDRLQTVYLNIFNFIVNLK
VVKFLIYKFKYWKNIFKRYIINGLSYNINPHTFMNYSPKDNHTTNNNMNNTNNVMNNINNNNSNYHYENWYQI
FTNNFYVIFFYILFIIFIVNNFLCFMFYKHLSNKLNAYVKTCTCHNK*
>N.PF14_0373
MNNIKYVELFYKCKIFRKNGLNRIIRRNGGTFNHNIKENERIPPASEDPSYKNLFDHAEDIKLWEIEEKQNVS
HKKVEDLSELVEPSNHPHQYEGIFARTRYAHYNQTAEPVFPRKPDLEKGELASGANVTRTDVWHNPKEPAIVS
IGKFEPRNFRPAGYAENCPNPESINSDHHPDFREYRLRSGNEDRRSFMYFISASYFFIMSSIMRSAICKSVHF
FWISKDLVAGGTTELDMRTVNPGEHVVIKWRGKPVFVKHRTPEDIQRAKEDDKLIQTMRDPQLDSDRTIKPEW
LVNIGICTHLGCVPAQGGNYSGYFCPCHGSHYDNSGRIRQGPAPSNLEVPPYEFVDENTIKIG*
>N.PF14_0374
MNLKNNENDDTNNFWKEQLFEICNMSPEDLKIHNLPISRIKKIMKEDDEIKSNQMVSADTPVLLAKACELFIM
ELTSNAWKYTEEGKRRTLQRQDVVSAACKKDTFDFLIDLIPLEDRMKFINLHMKMNSRKSQTNMNYDMMQQYY
NLYSTCNDNKNSMNVINGMNIINGLNNMENLNGNNSNNMNSQIMNTLNNSTNDLNLLYRSSNNNNNNNNNNT
YNNINNNNVDDFNNSSINLQNNIYMNQNKYFNNKDFNISNNNYMPPNGIISNNIYPSNNFSNRHMSMYSNRGM
NTTQGNNNSNNNNNNNNNSSFNNSSSFNNSSSNNSSSNNNNNSSNYNSSCSNNNSSSNNNSSSTSSNNNGNNNK
NNYNNNYNNSLSTNENFTNNLVNPINYKKVSSKKRSSKKDFSNNTLTEMNNNINNDMKDITTNSFQSNDFVNS
YTSHNSKINQSKNNYSSYNNNNNNNNNNFNNVCAQNGVNTYNGINTEDIKNNNFNGNYCPMQDMNMMYDVNGI
MYGYSYVKNDDKNMNDIQSQKDYVYNYYNGTNKTMNNTTIIGNAVSNNNYDSSSRNVSSCSSFELKNNMITND
SRKNISSVITNDMKNNNYNRNIHNNNNNNMIKQNNIIINDYSPINNSVQGNISQELINRNSLSVKSNIELNKD
VLNNFFLRNNNSATTTSTTTTTATTHNNVMSNNSMSSGNDGKAKNNKSNNKNKNGTNTSTNNNNNVNNSNHN
NKHSNNSNSNSKNNNNNNNNNNNRISINNQMSNNNKRMSNTQYVLNNQSNKNKTLKNRHNNNNNNNLLNDHMN
NPIQNNYQLSMQQNNLMATPMNQQMSSLMNDPNMSDISINNMNNVNNMNNVNNVSNMNNVNNMNNMNNVNNMN
NMNNMNNMNSMDSMNNINNMNNIIMNNIIMNENIINDTMLNDNMMNDTMINDTMLNDTMLNDTMMNDTMINDN
IININNISDHIIMNDTIINDIINNSENNITRKNSSINIINQNIDNSVVHNHLNNIIPHINNNVQQNMKLNMNS
NMYMNNNTQNQIYQPTAQYSIPENNYICNYNTQTYNNKAKKKIYTYS*
>N.PF14_0375
MLIFQNAQHYFNFFRNFNGCYTDNKLLKFFFKKSYNNWFNDPSQYNYYFISYYAVALSMCIILRNLLFNPDVH
FRRQDKRRNIIDRYQHHAYSLPYFNHWLRNFSQSFKSSLIDNEPDYQDVDPWSFRPNRIQFYGRFPFFFEIPK
YHIDDPTFDKNSYKYMQNYYEEIGYVQKPENMRV*
>N.PF14_0376
MNSSKSSKHNVQNTTYEAASSKLSAVKLGYYDDPFLKHFVKRIETRSPLINRGYYARVAALRLYIELFFKSID
NKQSIQIVNIGSGLDTTFFWINQKYQDVKYYEIDFYDLLKEKTDIIKKYTEMKNFLKYEKDNEEKDEDLINCL

NYKMVPLDLNDSSSFEKILLSYNFDFNKPTIFICECVLIYLETESSDNLIKKLSELMKNTSCIIVYEQVNPNT
AFGKVMIDNFSHRGINLKSIYKYYNLQTQLERYKSLGWVNVYINDMNEIYDYHINMEEKKKIELIEMFDEFEE
WRLLQNHYFILVAFNCYNNINLEELKQFLTCQKEK*
>N.PF14_0377
MKLLRVTPEKNIEFPLVHFQAVTQVVKLENVSDKKVAFKIKTTAPNNYLVRPSFGLISVRETIEIQIILQPLS
DKDNISNDKFQVQCLNVDDNTTVDKQFWITVNKNEIQDHKLIVVLNDENNSKLNHSYIPSNNVPLSEMNNKNI
HNMGYVDNNNINQDDPNLADGLKGGLPGMQRKYHELLNYCVFVDKQKAALEKENESLKNQLKAYNSNSNKFLI
DNKLIPIIIVMLAIITKYMGYW*
>N.PF14_0378
MARKYFVAANWKCNGTLESIKSLTNSFNNLDFDPSKLDVVVFPVSVHYDHTRKLLQSKFSTGIQNVSKFGNGS
YTGEVSAEIAKDLNIEYVIIGHFERRKYFHETDEDVREKLQASLKNNLKAVVCFGESLEQREQNKTIEVITKQ
VKAFVDLIDNFDNVILAYEPLWAIGTGKTATPEQAQLVHKEIRKIVKDTCGEKQANQIRILYGGSVNTENCSS
LIQQEDIDGFLVGNASLKESFVDIIKSAM*
>N.PF14_0379
MSDDDFFHIKSDCSLQYIKCKTSTKIDDILKRRRNQKKKLKDSSNYKSDEFLSLDNSEIEDQYISNDNKKVYN
EDDKIYYNNNKVNIREGQTCESIHMFNGINKNNIDIINDDNIKKDNINNDNINNDNINNDNINNDNINNNNYY
KQIYNYNKSGNFNNFSRGYFSNTYEKGNTYLLKNSSEEMLYNKDHIRKRSLNNLCFQSNLLNRRSNINTFNDL
SSFENSKKSSLYAKCSDILQKYDSNKFNRKTNEDIYTRQNRSSIIYQNSSKEFTLNKLISNNDSKDELFSNLN
RIYSNDIDLNKTPSKSLSKGYIEYLKYETAGKMNLSELETPLNMKEDEGNNTPFITYFLAGKTESKTINEESN
DDNKYGNNKYDNNDNINNNDNNDNHINNNNNNIIDTLLNRHIHTNSSIKNNEGNTNSSPIHINKEHNKSEHIY
NSKVSSTVFEELKNLCHEKEKKVNDVSSSHINNQKTILLTSYIKEQNSNNNQDNVMPYNSSNTLFDKIKNSQK
ISFIKTEEEYINMLKNDEKDNVPINTLNKNSNNNNNNNNKICSSNNNVSFADNIFSNNKSYIDNTKIYSEDYI
NIEKQKSEIFLQDQEKENIMNHVRKKESIPNKINDDIVKSDYQNVISNNYEHLISSISKKENENNQNKYMDND
NMGNNTLHSETFTDIRKDKHSIYHNNTCDNIVNTEKNNDIILHNNYNRVLNEENIPQIYFNNHPFINVNMKRS
MSLCNLKDTQVDDIKYDDLIQNVNNHNIYDMYINQKKNIKGNSDILFDHTKGMLFENIKENGFYIGTPTTGQH
INPKNNNDINNIYSNNNICEYRKCTSNAQNSICYNDQHKLNYVDMGQLNYNPTSKNNMKFTNKENDTNKINID
INNNVDNKKSCDDIYYVNEMKKCNILDNNQMNNSQMLDIPSCNINVKNNNNIYDNNLYDNNIYDNNIYDNKLY
DNNGSGVILSHNVLSKNNNSNNQENYENYKKMNEKKKNIYIDMYDVQKENLNEGKKVAEKLILSTLNKNINHK
LSETADFGYKENDLMNESKTSDNTIKIVNSNLYNDNDCSLINDINLKQYWTDYIMNNMNNVNNKNNVNNMNNV
NNMNSVNNENNVNNKNNVNNTYSLNNIKNIDNTQNDSKLKSVMSFNTFCKDMKDENFFNQFLREHIKNSINNV
VEEILNDKQNEILKNKIIISDINNKEVYNKDNENNNNNLSVKHYEKGIHINNNYNINHDPIIKEDKIISSENS
TKLEDNGIIHKNVTSSTYNNGTNNTNISQMKEENITNNDIICNDENIYNDENICNDNIYCNNKKLHKDEKSFN
HSCSDIQFNQDDLQEIQKLNNLLKSRKVNKLIQIKKDDINIKKVSWKNISYNCIDIIYYLIHENNKKNNKMNA
LINDINNIHEYEKKIEYLNKENEKLKIEMKQKNDNEKKKELKEKANLNNKILEQSKKITNFEKDIYIYKNKLN
KLNNKVINKDIEINKLKKQYQIILEEIENCKNDANIVLKKVLNKKNTNLIDKQCVDISKYYQILINSLNDEIK
NLKHLIKSEAKDKIILKKKISQLTQSNQNESNKNSYIEKNKTDNKCTNDNYISQYTNDDEHINDIDNDSYANN
SNIQKNIEYSNSSYNSNIIKKDINKSSNCSNNNNNNNNNNTCSISSSSCCRDIYSDSLNEEKKYKKMYEDLIK
QMNDIKKDHENEKIIYNKKIEQLAKHQEIQNIKNQLDTSEKIVEVYQHIFKENINNIRNDNIHSTQNNELKSM
DYYNLKNNIYNKPTSLTYINDNKNGSNEATNKINSGHIFNSNINRSNIFNNNHNNYDICNQDNNVISHILSNG
DIIKQDKNYTLKDTKLINVLPNSICNNNFMEGINRQSQNNYLNQFLELYIKNNSINNEAFNIYMKNLNKEKQN
QEIKEIKEIKQNVQESNILFEDNNLNNKLQNEHNKKYLLNTLMKYNKIDLINIFVDICNHLKTDNISIVIQFI
KFMSFIVYEQFPLFTSFYYTITSLISLDNIKFDEYINVIKKWKSFYTSGQKYYLFRRKVLLIFQSDLKDIQKS
QIDKTCLDLIQNNYDRNKELSTYTEGSFEKAEYILNKHSKEIISKIIKTYMNIYNIDKINNIIGHMNSLNSKI
KTQSYFIRSISSCLNLNKVDNFEEIEKKVKELITTREIGEKIKNKLK*
>N.PF14_0380
MEVLSTADVLRPRIKRSRSIYEREETNQENSPNHHQIVLYGGSIRNHSLNYAWNAYFDKNKVKKNIMVCCDLN
KICNDILKGVENNSLTIKTFSFLLIGVCNIYKKKVYFIGQDYDFLKRKILSLYKNKEDNLNELINNKETKKRK
GGKDNVEDSNNNNNNNNNNSGTNKRRMSRTSLHIKRGSIGMNELLPSVIDDFTSRRSRFSLNADEISIAGTQQ
NSYDIFNENEINNQMMLDMSGYNYDNMHGEHVMEKVLDASYISRNSIPINNLEGLHFNRALSESFNHMDNINL
DNSILNNMIPKNMFSNIDASLNGKSVMGDMNNLELNSLHRSELYSPVNDIYNIQDNNKNINDNNMNNMNNINI
SPFSNVVRTNEYTNDNVYNNISNEFSNMQNINSSNFMGNFNSINNSMNNRNDLYNLNPFMSMQSMNQDMNNRN
NYPFEPRGSNYGNIDARSDVVVGSIFSPIKKKNIPKKNDYANMLNFETYQNFDYFDQKDDDAFIKKLNTLLGL
DEAEEIILNDKNNDDNNKYNDNNNKNDDDNNQNNDDNNQNNDGNNKNNDRSDTNDVLNNSNSLASEKNNQLAL
HENDKGKEIIIEEQENQNMKKRKLEKHIIDRNYIIKDSVIRSMFRDERVDYKYFLIDTINNEIYEKIEKEYPG

YQCFFLQTSSDKIKPVHKLEINFGYESFDHEKKKKELENYLISLGNNKSILCENKNVVFDNFNIKKCLDNIDE
QMTYYNENDSLSNNFLSFEDNNEEYIEGSDNNIPSDGKNNNDNDDVYDDMHFEQVREEIGALDFHTSHNIHAN
DNIFNKDRFSIDPMLTQNEQLDLNLNDLNFKDDIESRINIESQMSKSLHMYRDGDSITEKNDNITSDINFVND
KLNSEFNSSPLNLSISDNYDFSNIFSKDNQSSLYSILEDKNIEIDINLNQDFDVIARELLEVYKNLSKKINYI
FFDMITRNRKSKDEVAILFYVTLHLANLGYIYIMQKPILPEQINYYEEHFSRPILIQYIEKKKS*
>N.PF14_0381
MLKSDGVLLLYILIINLICCLNGNSKKRAYILNTPKSSNCKRSSFRRWNNPVNNNNSQILSKNEGSIEDVYNK
KISGRSNIKNFSKDINNNIYIETNRRERRIKRNKYLLSLYNNTNIKTSNFIYPLFIHEEDVEKKHTQLEGIYT
YNVDGIIKEIEECIKLNIHHFMFFPVIREENKTVYCEESYNENSYFCKTISRIKEKFSDDIIVYTDVALDPYN
IYGHDGIYDDNKKEILNDITVHTLVKQSLCLAKSGADVVCPSDSMDKRIELIRKNLDFHNFRDILILSYTCKY
SSSMYKPFRSILNSNILKNFVKNKQSYQHDFNSYMDLNNVDKHIIEGADIIMVKPSMFYLDIIHKIKNRIKDD
VQIPIAVYNVSGEYMMIKNYVKYLNEDINYENEIITELFKSYLRAGANIIITYFAKQYGLYMKKLYDKNIIID
DNSNNNFNIELTL*
>N.PF14_0382
MLKSDGVLLLYILIINLICCLNGNSKKRAYILNTPKSSNCKRSSFRRWNNPVNNNINELKEDEDLIKYELQNG
LSNIIYKKRKSVNKKKYILNNENHVILPTYKISDEYKCYINPIDYNYEELHVYMEVNTGSVNEKKNQQGISHL
CEHVSYMGSKNRKNIVDKNIRTNAYTDFHHIVFYISVSLNNEIYKENFYSDFKCDQFIKNIREEDIEDYDIYT
VDEFNYKHAILSQCIDTMVEVLKGETQFNKERITKEKKAIFSEYSIINNIEYKMNSDIIKVLHKENRLSHRLP
IGKLELLKRYGEKDVKEYFNLFFRPENVNFFVYGDVNVDIAQKLISSKLENIKGKDLKEQDKIYLNILNDRCT
LRSRNKNLPAVFHMFGNNSDIKVKEEEEEEVKKNIQLEKNEKNIETKDEKFHINNNENITFKDTDENFQEDDY
NNKKENDCNTYDDDNIIDLEKYKEFKRNENDIVCELKFRNYLKNKYGVNMEEEKLLNKNEMKNINTSFEIIKY
SLNNVNINILLKEEIKSVRTLEDLKISVIKEVIFYCLSFRFNIHRNNLFNSIDINEYTNINEGATIRTIEIKT
TIKAFEKTIECFYNFIKSLLKYGFSEDELLNYKMNEIDYEDDLKDVEEKKSGQIIDHQNISHDVSEAYTNSNK
HSSKNMKLIHPHDDDYDDNHNDAEKDMNNKMNEQDEEYILDSTKINETYTDEIQKIIDYNTCQHIYLNEKREK
KIKREIFEKLKLQEINNFAKQYFQYLFNIFNPYSNMKPHCVVIHVPSKNENLFHKNKIRKLFFNNIYSMNDVQ
NYSINIQNKLLSPHYVYENITKKLSQARYIIPQKRENKYSEDIFNFIVNKMEDHKNENIFDVNNMCLSLPFYG
LSTNPDLNMFKKCYCIKENKDDHINNVNDHMNETKILPQITESSVQPSLPTSSKKSNNNLCVDMKEKPKTMEI
NNKYNNMHDDVVCDNRRNNLSEKGVTFLNLKNYVLSLKNQKEIENYELLNGIKVNLYKTKVDKKNIYMRLIIP
HNDIIKKKKENVHLLLFSIICLFEGGEIENVTREHVEIHCSNKSINIYIDINDEYFFIDIYTHNKYENISSAF
SILNNIILETKIENTALPRVVDKLKKDFFEYKNSLQSLLLGQTISYVTGGKIGYQNFEVSDTENITMDDVQRM
LKHIFSDLSLFELTIVGDFSDFIHYYILHYLGTLQGKIIEQEGEKKRSDVCLKNNKDNIIIEKHRTNNLSNES
YPINEKKKKNEDNGCKSNSEILLNDYYSMLCPFADFEEKSKNTTYVYIKEKEEHGIFLLVGKGANMYGFLSNG
IHISFYLMEYLKRIINGEEKNKNCENIYKDEYICNELDKIKSQVLEDFHFEDKHNINEYLKKKKKLYTSPLFF
NAVSYIIQYILNSKLFHYLREKKELTYDSSFEFISYEKYFAGFFTILVQTNPRDLELIKKEVLSCIELFTTNY
NNFSDYLIENSKLSYLNKKNKDLKYFVDKISGMQLTHFPLKYKNKHILKDNILLNKIQKIDVLLVLFILFNQT
KNYHISYGISAPHDIWKETYKNINKLY*
>N.PF14_0383
MMDKNYGVEGETFQNAYQKIVLKDRILNEKEKEIRKNHLFEISKIKDYNLITHYEYILKLHYFNSNINVLNIY
RLVTHDYEKKFEEIAYSLKSNILLSLLDINEIKQTDNSMINYFIHNYNDMKNFTYVIGNQKYPMGFQKNSNET
FQIYVFKIIPNKTLLLNKKNVQDVLSSAEIPNGYDSIAIEEKLYYENVEIKGNEQEKVCYKENKNDVDRSGCN
KREGDNNINNNNNNNNNIYNNNYYNNSGLKNNYSYIYKVRNSEQILPLYLIEFEFKCLRVDISIPVCEYCYST
KAITYCYNDKVHLCDICDIKHHEKNKILKNHKRIHISESPYQFGKCPYHINELIESVCMKCFCTLCPSCILIG
THSKCFDQDDDNNNHPIMNIKDAFILSNQRKSLSDISLQNRKTRILQLLKKKHKLLSEIYSNYSSLQKRIDML
YQYIINEIKFLKKKKINFLMSLKRAVLSELLIIEWMEAFFFHTKLSLNISNFIIYQKKHQLLMNYLINNKNNK
HHHHHHLLKYIPQWLMDKISVHSNLYIFQDHFNNILNINNSDKNENDKKYRSLNNITKNKINGSHLIHQNSYT
HTKKLDSIYNINNMFDNNNRNAFSLYDQANVNDNQVLNKICKMKSNHILFDNNNHDENIVYSKLVDKTKLVQN
NNLQDNKNNYHTDVNRNYNKNNTTHFMNNTFYEDNYFMDELNKDNNNIISEEHGVTSDYTSIINISQDYNNST
TNILYNIFNNNNNNNSNSCNCCNSFDYSMYCTQHDTNMKTLKNTYIYKELWKNLMNYKYINVIHILKAQNKKY
SFQLISSFLNISNYYKSLEDFVKHIIKHEVYTLLNKNIECHTKMKITYLLDNITTLLCINNLKLAFIIDTHIT
TCYSEIQQNEKQFLIDVENIKQNDLVLYKTYLQQKNNYNQRFIDKDVKNEEHVMDAQNEETIHDDQKMEINIE
EKINQNEFIMQQTLNSKKVSFTESENKEKQSVIEDTKDNVHYDNTIMDEEQVKDINAVKKYDISKSIDYNNIF
NNDNDICIDKLISDKEKNELATLKIIKDYVYIYLEKVINDIINISHKDLNDSIRFLFYTIHDEIDGINKQIIY
EKKFSIHTLTLCLDLFINSILYPYIFYIHELNVQNKNMTENNIYQKVLIIFGQTLREISIYIFQIYNLGMYDN

NLKVFVNNIKNNNMLKKRITNENMFFLDVAQKLFNWIIKNLESPRYYSPLKWYYGESIEKSYKNVVQEIINID
KSSSIKCVNENVDYNILFSTTNFKDILTLCYSINKEWKLNI*
>N.PF14_0384
MKESSSKEEGGIYNPLCNMNDNGTCKKRKLNEGDISNDINEENNNFNRKKQRKDMKNIRQIMNIFNGIFNWIV
SKGMNKIPCREIENINDVDFFNFNNVLSKHYGSRIIFVTDDSISKSENILVEDDKVGWITRRRRDVGHEWLII
KLKHPCIIYGIELNFDNIAEDVCPHLSIEVSNNNAIDDIIKEEEFILNERDKVGNFTTFTRNEIEDNAGDNIK
MEKNKDMENLKNADILKDTENTKNEQTIKRVDNIKNMHGTQTIVNYEKNTKRKSYNFLDSYKIDKSISDLLEQ
QNTPWVELLQADCVDFLKTVGKKKTYYFKINNEDLIKKPWTHIRVNLYPDGGINKINFYGEFISLFKKHTIIS
KQKIFLNKPENGCTLIYYHCDHIYKGHPKYIIDSCKTDGFCTRRLINRPPIILRTLTNNCILNSSIFKFGVRG
IVENFTIDIGNYKYDHPECIQIYLLDCIDILTLNLLEQKKIFEEDEKLLKKKMEWIQLPPFKITTNQKKTFFH
FNLLEYNFTLMERTATHFKISIHPDGGISQINVIGTVLSTLL*
>N.PF14_0385
MKNQNTDKLTNLKDLVPFYKKDNEKLNENTEDIVYSVKKKFNCIKEIGESTFNKYEHTRKNLELLNRELKLNK
ISLGHQKNEYEEIKIKNEEILNKFENLKHSKCTYQIMLQRIKKEKKLLYFYLNSLERTVASLKNNEKQLHNNI
QKITSDNKNLKKSIEEKKMDIIKAKNKNEEILKYMNVNKEHSNILRIRREMINEYKTNKLNDSNIALMIEEKK
KYKKLLIYYLLYNNYLKLSASNIFENASSIYATISKLREATGVTDIYDINQKFMDIENKKNLLQKEEELSQKR
LEDAIQEYQALNNEILNTFSEDKNILKKKIQNEKEKISDDIYESYKLVFASERELEDINIKLDKIKKFLEKQN
SYFHSINMEEKMEFHSNEDMLQYIKNLKGVVETLMKITQQNRENGNISRSYKSLEFLEIMNLYRNVDFHKNMC
RVDDTQELENTIKMGTS*
>N.PF14_0386
MLDLFCIYSSNGKLLIEQCFNKKRYKRNIHNVIKRILYNKSNNNNNNNNNNYNYYDYFNNNVDNNYYLILNNT
SYDHTNDKFHLLNGRSLDNKKFVYIIKSDDLYFVTLIEGENNPILIVEMMQEIIKIIKKYFDIDKLKENILIH
NYTIINFLINEILVQNGKPSLFIHTILKELINNDNNNNNNNYYNFNYNTSNNFLNETLKIGPIPNNIYNMITQ
NKYININVGNSNNNMNNISNNNNNSNLLMLHINNEKNINYSYSNGSYLHGKHSIYNYWRSYNNSCTSSNEIYI
DVEENVNCIVNKNNKIIHYYIQGNVYINSNINGSPYIKLYFNQLDVQKCNFHYTINANKLFNKNKDEHTLNNK
NVIIHFVPLHEKYCLLHYYDKILLHPGPSPLYNNNEYYNKNNIKSNTHFNDEQQMNNTLTNVLKLSSVEDAIN
ENAKKNEHDIKNEWDIKNEHQNVMKRNHCNILEKQNSSNSFQTLSRSISYQENMNIQNNNIFSSNNYLEYNND
FLFSNLLNLWNDEHMNMPQNYFDIPNDNCKNSYDNDLFSHPKYKLPILIKGNIHFDSTENMYKIKLNVLLNNI
NTNKNNSNILLNRYENILIKIPIHHFIKSVNFHTSLGNINYNEKKKCIYWFINYVTDMNTQMYAHISLYVPTH
EQNDMNQMKNTYQISPLNNFNLYPLFNFVAYASLKINGVSITDHKIEKIEVQNLKNVEIRKGCRYSTIYNNIE
FRL*
>N.PF14_0387
MTMFKDDENNFNYNKEYEKVKKRTLFKILLSVFIKTLFESFLQPLLPFYILNYYDIEVKELGILLSFYSFSQC
VMCLIIGLFSSINRKHLLVFLLMFNLVGIYLFYMKLNFTLLIINRIICGSSSVFIVVVNTIINDLVDNNVCIY
YTYINIFNAIGIILGPLLSSFFLTIFNFQIILNFNTFGLLVSLCIVLTISSDLLVQNKIKNQPTESTLHSNLP
QTNEDKKNITKNNNFNSNKNNINNINNSNNINNNNNINNVDDIKHDEDSEGNTKAFNEENKTEDNHSILQIKS
TEHLRSNDFIISSYNEEENNTNNYIVNKNEKNSSDGIFNNDKIFKYYSEEFSEISSYQQYLKTKDNYYISRFI
YFLKQKLNLLIKAMFTFKFKCLLSICFFRFTSAFASNLMSNIFFVFYNDNVSSXXXXXXXXXXXXXXXXXXXX
XXXXXXXXXXXXXXXXXYIYIYIYVYILYTYSMSTIIYYVIYIYIFIYLDNKQIQISIFVSLSGIIMIFYQYF
SFSYIVNNFGYNGKEKSSPYMRNHAK*
>N.PF14_0388
MNIGIKNINKSDEKNYSNMTNEEKINFKRRIILDGINFSQNFSYSNFAYYENLSSVYAGCLEELNNIEVMVKD
INLDIKKKKELLELARQEIPLFYFPLNNMYSESELRYFENCILPYVNKQKEINEIKKLLFNSYQNYVIYNDIY
ELWTEELDTLHKFIIFETFRNKNKHLTNKTQSDKKEQVHLNKIEKIKRRIKMHISKDNNVVLNEENTNVNMNR
NEKIKCPPINFDDWI*
>N.PF14_0389
MNEENIYLENTATKYRDMEKIKITPQCYKCKKNYAVVFTREKSCKECFLQLVEYNFKNTLREKCLFKSKTKFN
QSNKRHVIQNNVKQNEELNTQNEKRFINEQGDNKNKIPVVINNNSDIVSSHLDTLGKNSADSKKCIPNNMEKN
SVGEKIFNDFNICKEKKKRTSIAFSGGIYSSFLLYAFVKYLKNVKNRQSDLYISNEHAIFNEIIFVDIYDDIN
YIYKLINIITNIFNMLDVEQNKSNININKIDIKNENENRIIKPKEKICFVNGVYKKQINKIHFVVIKNNYFIN
DIYKDAFILNYHNIKTKQNNYYMQYINELIIYNNIFTYCHNENIHYVIIGDNANNIANKTFLYTIYGYGINIP
THTSYIDSRHNDILFIRPLKDLLSKEIYIYSYYKNIEYLNKNISNNNILYKTVNDMLLNLDNNNYNVTSIINN
TTNNLINIMHIFNTCQYNINNTNNKKQDYNQVTQLLSSDEYSNKNYNNSNTSCNITKNMCLICYGNKENMEQK
KLIIKLNKVQLDNIKSMKCKDFICSTCLCIFSSNNHFVKLYNCFF*

>N.PF14_0390
MGSKKNSNTVDSSENVEEVVDNLTSEKNKESLKKDKRKKKEKKNNDVDDINEEEDEEGNDEDTMKKFSVDTSE
NEDDKEDDDDDEDDDDDDDDDDDEDDDDEDDDDDDDDDDDDDDDDDEDDDDDDDDDEDDDDEDDEDDDDDDED
DDDDFDDMDEDDDDDDDDDEDDDDEEDYDDDDDDDDDDDEDDDEDDDEDDDDENDQNEYAGDDKKDEDGDAKK
GSDDEGFD*
>N.PF14_0391
MSKLNQDLLKKAISDVFEGTKQKKRKFVETIELQIGLKDYDTQRDKRFSGTVRLSNEVRKKLKVCILGDAVHV
EEAQKLELDYMDIEAMKKLNKDKTLVKKLAKKYDAFLASQVILPQIPKLLGPGLNKAGKFPSLITHNDKINDK
ILELKSSIKFQLKKVLCMGVPVGHANLKEEELRSNIVHAINFLVSLLKKNWQNIRTLHIKSTMGKPQRIYG*
>N.PF14_0392
MTTTDTWKVEWDNELRQLPNVESVTCFDLQLTENVFLIWQFLKLYLSTSPQVRSIIDFIAIKKAQSLQWGDHI
DSVMSSGLRKGQELFHVFLSNGKKIQTTYSKKKLCDRLEYYHIKNYIILEKINTGSVGQVHVALDKSTDTFVA
AKAIDKSTVQGDIGLFEKLKDEIKISCMMNHPNVVKTLNILETKDKIIQIMEYCDAGDLISYVRNKLYLDEVS
AQYFFRKIVEGLKYMHKNNIAHRDLKPENIFLCKIQISQKEKTLIRVGKLPSCIEYDLKIGDFGACCINEKNK
LHHDIVGTLSYAAPEVLNCNNNNGYNSEKADIWSLGIILYAMLFGLLPYDSEDKDVKEAYNEIIKKKIVFPKN
RVNKFSTSVRSLLLAMLNINPQNRLSLDEVMKHEWLAGTAKNRLEMSNINKKINFPISSTVGYPIYSNKNIMD
YNIYKRLALIKNDHNSSTNSLYSNGNSTTISNSNTTNMNNNVNNNVNSNVNNNVNNNVNNNVNNNMHNNIMNN
VYNNMSNNISAILKVNNLRDKNNIAENTYKLDSITNSLYGVHNNNVNSNNLKEVLYNHMKYYLKGDNKYNNTP
GEHKNDYNLETLLKKKEDQVVGDLKVINKKVGHYLSSKNTKEININNKIANLVVGNKNGNNNIIAKKNIMINN
NNNDNNNISSDNIPNKMCKVDNNIVSYNKFNVEHYEYNNKGCMQSKCNQNKKDEEVVHMNKLHSICSSNDHKL
YEMYMYQKNDNNMNEHVNFINKSSNKEISRLENKYVEKIYYLKNNDMHINDDYIKDKAIKNVEKYNFINKDKN
SSTYKIKDYINNNTLNRTKYNIRADKFNNIIALNNHIISNDTNRHLYINNKLSINKQKYNNTPYYNYYYYYDD
DDDDRKKEYNKYVYKKNKKCGTNNSELISYNSSVYEKGSTPSSYNINNVPISYIKHDSIYNGSLSQYVNNSSY
DKKILSSSYIKDNESHVNKMNIEKSTIWYCSTNELPNDTIKKDSVHKKESLSQEFPVNNNDDVLDIKNDEQSK
CAYNNSVVDEKENNNINVGEFKIISPNNLDSNNISSTKHITVRDTYNDNINKTKEAYILIKKDVNNAKCDNIF
ELSKMNEHSENGNNSKHTNSDIEINDRAKNLNDFVSMHEMNNFNKVDDTQLYKKKISELSNNNEKCCNINEDK
NKENKNIHMHVIKEDPNNNNNDNNNTNISADNNYSYKYVDGDIKRECVYSTDDINYVYVKPSVCSLNADGNEN
TEKIIKNMNFYGMDKIYSTIVQEKNEETKEDINMDHIKYNINGNIKEDIINSNNVFNTHNEQPFIDNKIMMSN
INSKDNTKMYGNIFQNRNSNSIYEIKRKEKIYIINVDTPDECNEVKCDFSYNDHKIEANKDGDNKEHNNKDKD
GDNDEHNNKDKDGDNDEHNNKDKYGDNNEHNYIDNNEDNYIDKDEDVNYYHDYNNPENIDHNKYEQISGLNIK
EYDINEKGNYNNKKKKKQKNEHKKEANFTKHKMFILEDQKMLNTRRGNNHMKLMDKDYINEYIKIETSNYFNK
EHIKKYSSLYGKNEKKNDIINIRNFNDCISDYKNSICDGFQDIKNNSVKNIFNQDKKIYHDLSNNCVLYNNIF
TQTNLEHFSSNKLEYNKNCKYPYNNLMKNYSYSEYSFDIQNKTSTYTNKVDINKADLKEAYSDKKIKYKISNI
TKNEQINIYDDIKKCSSNNNNTFYIDDEASCLDITNLSDKEIKNKKERAKKKNILNNKKCILSNGSNIFIGKE
YKKNQHMDYMNKIKKNENDVLYLNNSVSLKRSFSMLHFNRNIKNHVNYYYDDHNKDRREKDILLVDHMNNYVI
EHKNKINIYNINLNNSNLNNINKNENNKIDSITSIAKKCHSSDIFLIRDNILNNLSNKEFKNIIARNVSLSHE
FPLIHTNEELNKKTKKQIINNNIIYDKIGISNNKKCNIPTKKYVNNMYNLKYKKCEPNQQFNDNVKNYKVDNV
VSDFILTSEESLMTKKNNKNYKNDKNDKNYFDNSNIISYNEMKKKVTMENINMDCVVKNKTYDNMNKSNMKKI
NFNKLNISHNTQNNNNHIYNKINTYDNNLMNIKDTLGTYSVTEKHYCRQKNIMNEKEIFHYDLISSNNWDNHL
RDYMLYSLSKNHYTFLRKNTLSKDIPMHSDINLFNNKKEDTTSKNKNEKNFKINHNEKNISEYPNLSNNSISH
ISHTNIKSKKVKQNNDQDNHFHKKIINEQSNVFQPKLKWLNIFTRNFNKDQLGTKYK*
>N.PF14_0393
MGDNNTSSGSSPVLSVGNIRGFGGSDFGSFRMSNEFLGWKNKKTNNVYQYKCSDIDEGCWIKTSYNNNRLHLK
LGESKENIIIYFDGFPDRNVNEITQHFQKYFNIRLNNRKIATKGWNWGEFKLENSNLCFDIDNKYAFNLPTNN
INQLNVQIKTDIAMEFKNDENNNKGNEDFLAEIRFYYPHENDENQNFQNLKNDLLEKVNIGDTKSESIASLSN
IPLLVPRGRYDIEMYSSTFKLHGKSYDFNIQYTNINKMILVPKSNSNQYVLIFSLSNKMKQGQTEYPFILIQL
NNDDDMELDISASDEVMTKYKLEKTISGKAHDVVTKLFTALVNKNVIVPGDYRTSKNQHGITCSYRAASGQLY
PLNKYFLFIVKPVILISFDDIVTLSFQRTGNINQHRFFSLIIKHKRGMSYEYTNIDKSEYNPLLTFLKSKNIN
IQDDANDLEKKQDFHNELDESDEEEYVADDDDDEEDYVAEEEDEDDDGDDDDDDEEEEEEEEEEDDDK*
>N.PF14_0394
MDITEFDIPLFSPNITEINFSIFDLWLRGYNEEEVLYVLQNSFYFDLKKSKKGEKNKNEEEFLIKEITNEEKK
GGKEKMPIGRAKDNVFYVKNRILNIFNTDNNDNNYNSRNIFFQHFLCLYIKQQFYIFNILSYHLMNLDLRFSN
FYVPISKHKALKLIRIYYDIDFNLEKEIIKYKNINNINKYTSELVKVTSIHKISIKRQIENIKNMWKYIINIY
EDRNINIGKLQKKRRISLKKIVKYIKTRFRFSSCFSKRESFIDNDDGNCRNGSFLFKKKKKKKKGKGKKKGKI

EDRYVQYNVIKTLENLIGIYLAKRYFRLYWIIVYHIEIPKKINIAYPYFNNIILLLLEKLEINDFILSKEYIN
ISKNIHATYKNSKNMDTLKGKACVIADINNTLKSKVLIRLKCILNLLCCFRNSYELTKFISIFFELQKNEPLT
NEKTFIKQNIGNNNFVNRIMCFIKEAENYSKIKKEEYEKIHEKNINV*
>N.PF14_0395
MELEKLSMHEGYKKFCSKYPLKKLSMPKSDLVWCYYDINSKKENIVIFLHGVCGTAGCYFYQLDNLSTLGFRV
ISLQYPCYNNLKDWMKNMCNILEYLNIKKAHFFASDLGGYLIQLYAKLYPSKVESLILCNSYRKTDNFASIAS
LRNIYGKFYSFLPHVLLKKIILENYIYLNYEHVDLKEMNSLEFMSNEIDLISASDLGGRISLQLSSDNIDKIY
MNDKCITILQTLNNIYPDSLNDDMKKAYPLAKYAIMKSGGNFPYLSRHEEVNMYILVHLRNNCNTNFIKQQIT
TMSTINLNKYKKEQTNQYNHEHKYYDKGSCKINYKKDHNEYYTNPDEQKNKDSYNEHYTNDYIHKQHFSNNKI
NKNSKENYMPHDIKYDTSKNTYQDITDDDLSYKYSNYNSSENMNDYINDKISKIQNMENNYSESNTNDKNFYQ
PKIYSYNQNKQYNYIKDNYSNNNNNNNYNNDELDDGIYNINPNDNVNYQNEYYNRIASSSDEQHYEKMNNQYK
FKSANNDNNTSQSSKQNIQSSYESYNNNSINYEEKNINPNDSNYRNSNFDNKDIYCQF*
>N.PF14_0396
MEEISKKLEELKENIRRFNHENKIFDLIKNVENYFFSNKPNQKDNLNDMLFLIQYSLKYKGICKWPNYSNLFV
INYNLNENFKSMVQNNKEFFEEYVKMNINKYNTKNKEENINRDDNKNHTKYMDSKENMLNNDHNNNNINNNNN
NLFTKNDIELNEDNMKKKIKEHNQISNNDYKKMERKINTYFNDDISLFNKIKLQIFMYMILNNYRIKILVNSL
SALNRPINVIYINCPNNKDQKKKIYEKFTNFFSSYNKFNNTYINNTKKNNYQGIIKDDNCSCSELSSLNQNIK
TINNNSYNKKSDKYVGGYNPINNTIWLCSNNITNYYKLKYILTHELIHAFDFARANIDMYNCKHIACSEIRAY
NLSNQCNYFNSKYFVGHKDVFNSSKSSVIENTSKNKCIYNNVYSSLYQYKPCSTDTHTYINNVFDKCLHDYWP
FMCPPEQDSKYKPSKIFKKD*
>N.PF14_0397
MKFFKGKKDKTLDEAYDTLEKSVKGIDENIEKYNKELNVIKQKIEEEKKKNPINQHIINSLRNKAANIIQKKK
VYENNKESTMGIQFNIDQIKYANDNVQLSIDTYKALKNTSKVLKKNIKKVNINKIEKLQDDLFDYIEDTKEIG
NILSSSYDIPLNLDEQEIDAELALIEDSILDENVEQDNIASYIEDDKKEEDKNLLQQIPVEEKNFDAIQQKTK
KETYFAQKES*
>N.PF14_0398
MVTAIKGVLVKCDEPTMQIILMLNEEKNFLIEKISDTVCLCKENVHDFLEKEVIKQLEYSERHETED*
>N.PF14_0399
MVLLKILKKIKDNKRNLRILILGLDNAGKTTIIKRLLGEDIYSVSPTFGFNIETLEFGNNILNIWDIGGQKSI
RHFWKNYYEDVDGIIFVVDSTDLFRLQLCSFELKQILKEERLYGSTLLILSNKVDIDKSLTINQIVEILKLNE
MNMDRHWCINECSAFSGKGLLKSFMWLIDDITDRIDS*
>N.PF14_0783
MEHFVAPRVNKKYLNKFYNKNVRLVGKVLKKDGNELTLLTCDNAEIKCYLNEEQADDSFETYVEVLGRVNEDD
TLSNIVYVQNGGNSMNLNELNNLINLTFLEELEEVF*
>N.PF14_0400
MNLMNYALRRYSSVQNLMRVENLRRNVNMINDEMLNYNGYMSKRKREKCLKLDLNIYEQLKRKMIGKPLNKLQ
KKYMNEKRPNFAPIFLDQLKPRMVLYKTAIQVNELPLPKYPEIAFIGRSNCGKSTLINELCGRTNKAKVSKIP
GCTKEIHFYKIGKPCLMCLVDLPGYGYAESKEELRLQWNEFTLFYLKNRKNLKKVFVLIDCRVGLKTSDKELL
HFFDRYNIKYQIVLSKCDLLNTKDLAIKIQIINQDILPFKNLEKPLIPLSSIKNQNLSELRNEIAKYQLNKTI
VKNNIVMKINDLIEQKKLKKLKNTKDKSINMNLNINNHTCTNNRSHINDNVGENNLSVTLNNSNIKEDTQTHV
DIHNTNIKNPINGYREKSILISDHTIFEALNRWNNIDTNKSNYNYNYNYIFNFNTHMNSHIKHLISSLHKKFI
SQCLREYNINDLQIIDNIIHQEIIEHDKSEEKLAPIINKNENITTSNKYYPNDYKINTYLYRKQNKFRNNKQQ
GECYNIYDKENVINDSYKCNYNSLSKSLNEDHKNSEQLDENIILKNDLCINENDEILYKENYINNKEKNNIKN
NNSQNIAEKKKNLYNLAENCIDKKNENVDILCKSIIKDIMSNQGKMEDTKETYEEYKSNNFINDKNNIEKELL
EKSLFPFDLSQSKNQDNNICSENIQMNNLFNDQRQTNQWNNLFKKKDYNKINDDILLQEEDTYECEDYISGFK
RPSKENSNSSLYNYSFNINDKSTHYIYEKSKSDSYKIYKSRQLEDIHDQLKDNSIDTPKRKVKENGINANLMS
AEDVSSKNHFNKRNTNVDALCKINKDESINEEKEEYTKKKIYIGLKTRSKIIKGTKKLKLFGKKKKHDIVNTP
IDLATDYFKLNNNSTFYDKKKNSWNYINAKYNKWIKKSNRKNIASEIPLSPIKKEEVMKRYVQKQETKYTKEK
NKLLRQKKNLGMISKPPNHKNKKRISKNYSLSDEQKIFDREAFFKYRDVQK*
>N.PF14_0401
MCVLTLVKDDIKSDILKLVLDYIKVTVVQDNENVKLPEICYDKKITLQYKNKTYKDLFCTLYALIDIYDCYSE
LFNEDEGKVSENEEFIFHLASDKYILKQSDMKHLNDLLCEKSYIISNKHASIVDIFYFCAIHKLLDEMAVKER
IEFSYIYRWYLHIQETLLANFSTLKKLIVKDSLENLLNNKTTNNAPEHKNNFVSKESKENKSQNNESPKNKKK
DVQNKNNAPNKKVEETKKLDDISRLNVLVGYVEQVEIHPDADTLYCLKINLGEDKPRDICSGLRNKKNAEDLL

NKYVLVLANLKEKSLRGKKSHGMVLCGSFDEKVELLVPPNGVKIGERILFHNMDPNVIPDKNLSSDKEKNPFF
HIQPHLILKDGVAHYKDTKWISSQGDITCVLNQGTIS*
>N.PF14_0402
MQNNNAVQYSIENIMKKNRNENKYVDLCDIKTNNNHIKVNKYITCDNITFEGKNNNNYYHKSNSHINNYFCYG
SINKRVDENLLSYNGRIDDLRSGKKCVYSEKPGEDKNTLLGMNKEDNKFFRSEFIKDYNMNYNDCGLINMMNN
HYNNIVKNTNKGIEDNMNYNCDSLLEGYNYNSETKKEKKKVLIDKNYIYNNDYYLYDDDMENDKLMIHNSDMK
KEYSLYDKNVCMNKNCDNIKFQSEIMRANPNQNQNKNQNKNQNKNQNNINNNINNSHHILNNSHHINNNNPNN
YNIINNDINSLAITNEEELENRMYNLDFEEEKSESNNLLLNYPHEEKESTHKLNIYNLFNKSNKNKNNNANTG
IINFLRNKRLNKYSYDNKGLKYDSTEIDSNVNMKKNYMIKGSYDTSPNLKGYIYENNDIMKKKINTINNLNRF
NNGNENVIHLKENNEYKSMMNSFETLKIYEQDENNKQNNIYLNKMNEHSKYKDDNQFVNDNINDKNIHNNNNN
NNNNIKDSINIHSNNNNNNAADIKKCSQKKISERNPFSIHHYVKQEHMSATNLGNINKNNILLNNINNNSFEK
RNEKVYIKQRRMMEKKGMDIFKTLSEEQSVQNMNQNSFYENDFKNNATNKMKMKENDDNHNNINNIDNNNNNN
NNHYYYKNEKSEVGLKKRKYEINYKGLNNSKVGNINNDDKINTNEKKEYYMNIKSDYPNNVKRNDIKNNDCLK
DFDEDSSSSKYISSHSMNVSSCESYKSLCKNKKKKSKSNCRDKGYNNKSYKIKNNKKLKCSKKEQNMEENNSQ
NKICRNSLIGCLSCMYEGTKMYCHCHNINNKNLNTLITNVKNFDDYSKCSPNKQNKNNKILTEQNIITNKSIN
ISNINKNNNNNNNDNCNVNCSNVFFDNINENINPKREDSSFNLNQGNNKSIYDYQNNAPHHEMVQTNGNIVIN
NNNHDKKIEKTYLLNKIVESKNVLKMDVNVVGDMVKKTNQPNITNTKLLKNNNINNNMIKKNIKNETLSIDNN
EQVNKQDDGSTKKKIMEVNQKMNKFNLMENLIYNGNNCRKNMEQNNIKLFKRNIKNNTTKYINKDNFIKIDYI
KQHRILENNQKGIIFDINEEKNNNCMNKSNFIYHNNNINNMNNMNNMNNINNMNNMNNMSNNNNNNFIYQNNS
NMPIYLNEKKKNMEKKVYNMNEGNIILNPSNKLVNYKNGLGGKNNNICIHTHNLKLKKVEQDDGKYNKSIGTI
MKCKLYNLKKNVYKNDSKVVDIPMKLNDLNNNSSRICTISNLLNENSSMYNKNECECKRDILQNQEFLSTKRC
DKKVEIKTILNSNINLSNKKTKKKKNEDVSLIKIKKDELIIKQVKKLNKNCLLKNIQNVGNIKSYINRDICKA
LENVKRSNYQVNNEDISNNTTYEKEEAVLYVKNKLNTQRKWRSDNIITTKGKEDKEYIIKWNKKTGVSIYYNK
NLNESNSILNANNYYIEKDHVEMEQDKCHVDMECFSKNDNTQDMYINKNDIYNNNDNNNNDNNNNNSDHNNNF
NSFSNDKNNIHNSTLLEASPNEKNINMEKYNEDQNNIYSNNIKVENQDDVNNSCYYNDLYESNLKEYVQRITC
NNNLEKKNDHKEELEKKKINNGSNIYNFNNDEERNLLIMKNKMEQNNCVNYINNKRNLDTENNTILLNDIILS
NDINNVEGIVNYDKENINHLNNNINKYHENFLSNVRKHYKKKSINYMNKKYFDDLNNIYFLLEDFTNTYKTCS
NNLKCILQLLKYELDKEKKASVYFSNNQKFLYFAVPLMSQDKSCDYNINDEEKNKEIKKNEKIGEEAPNNSML
NGKEYILEGTTYNILGSYKKSHAVNNIKNDTKEYIETKNDNIQSAINNSNDNLDSISNKNSKLNVKKIYRNMN
KWVKKNKKYPFSNKKINFKYFIMNDKKKDKSCKCCIKKKIINDEKILLRMNIFTIRHKNHFYKLKFNNNFVIK
RNKVFNKKNGKLYKFYPFYNELLLKYENDPNGKVETNEKLKCLDKTISNNTNDQEDHNTNDKNHINNMNNNHIS
NKIDDVAHTNINTNDNHNMNNNVNVSNNNNILDDLFISDDDINLIYMYNTNTNIEKQYSLEKININ*
>N.PF14_0403
MHGRRANRAQEEEQARLQKVKELIPLVNNLIKKKKTNNYDKEYIDRTSLILMKCPYLQTLWNYRREYFEYIKP
EYLKGKKEIEDEKCDMTKTSEELKKILKDENNMVEDILNTFNKCNELWFHKLWIIKYGLKDNLIDMKDLLNEL
EYCKRSFYKDDRNYHCWNYRSYIISCIHIIHKKLNNMNKENKENKEKEKNLDETNKTHQTHQTIQSNQSNQSN
GCFNFDVYETNYKLSKMLIENNFSNFSAWFLKYSLKKSIIDTNNELNLIKNAIFTDPFDQSLWEFYKWFLFQN
GNDKEEILFILLNNNTIYLFFPNLINLNISKCICYDNNNKEIKGSWDKHIIKINNSNNINDSNNKESFVFSFT
FDKNYLLLGNIPYIKFVLYYYKYNIYVPHQIHYEQNILKDIYRLYNNSSKENNKYEHSIIYQIDITKISKCQL
FNILLDNNQYNRKGIYQFSNNIDDNNDQNKIYYNFYKYINSSNILLNISTNMDLHLLNLELEQINELLLLEND
CKFALYTKFEILKRIEHFEEALKVLDYLKQVDFLRLEYYKDQETEILIKKRIHDYYETSEKGNNNEMLDLTHM
NIKSIIYPLMIEAFFIPKIDLSNNSISEGYNGKGTLNFLYNLRELCLNNNKLKNFIFLMKNLYNLKLLEKLDV
SNNHLVDMEQDLDQYEFVILPSLKYINISNTNIALLLNEKYKHKNILNTYNIVRHNNTVVLSKL*
>N.PF14_0404
MNFFSIFIILNFFMLSLTSSLGNGFLHSSITFSNDFCKGMNLHFLISAEEIDSKFFLNKMLLLILGGSSYMRY
SKENYFSVSIYDKKLVYKNIKKIENIQGASYYEQLQSAFKKHIKPSARRTNLYESLKEYYEKYIIGNKNIKKS
KNIIIIVKKFTSDDLSEYHENLNRYIKEIRSKDVGIYFFSDDSIYSKNIILNISESGYNMEVQYPIAHFVSES
YRANDLFGVKKFCYQLLHGATCKSYNEWSEWSGPCEFERRQRIIPLKITNIESTNFRSHYDPSCRNVFDYSMS
IKEDFRVNCKNSIYECRGICDKGYKFKPYAKSNEIVESYEECNDLPPCTRDQRKNGDHTLYADVILNSLLSES
SIQQIRDRDDRALIEKDMPGDTEEMSNERRIKDMHLFLDNKMKYMIDKQHKLIEQLQKDIQDRKNKKDGIIED
KKKEVEEKKEQVEEKKEEMEEKKEQVEEKENQEQKENTNLQETKTINESPIIDMKNNENTLQHTQKEEEIKRT
RKPIDNFENDGTNKLHVINTGNTNTQSSPKDLDGDHYPNKQNVLNNELNKLEDQNEKDNANVFSIPINGENIK
KKDIYEHPKGVESAEDVHIIHNPTDQSIENGNNIINMNNKSGSENSSKNMNSETHNISDPFNLKHLKKNNDHD
GSSEANGNTSSYNDKKEDVEINLERKVVEQTNEHKETGQSDLNHEKKEESIYINKENKEGDIIRNNGENVEIH

LGNNRKKIVDLKKEDIEKNVNVKHLSVDDYGYELEGKRIIGANKNEDKEIENKNAEGKKIVDKEIENEFVGGK
QSGTKEIENELIGGKQIEDKKISGEQNQGEYVKGEKDNGVHVKVQHIDGQHIEDHDNEDHHIKDHQTGGKQGT
DEKNEYNNTQDSSLTYGKDKDEEFTTESSNINYTPYIEKNNLSLKTDKIYEPHVVEEINEEDKLSKEKTERFS
NNQEIIRKYEQENHKINEHEEEEPDKIKYKNLNIEPQVGHKIREKNIDDDVEKKKYEFTNTEETYKNNEKNHN
LIGKNEVSDETMNEYIISPKSEIINEQENTPQYEEVLSKNINNDKDNGKRINNNDQLESPILSNEKKTDDIHI
IEEKEKIDKPIHNNDLENQNHEKKHIQDDKLEEYSTLPNSKEMKNISGDNIKNNLLSNKKEENHKIIHENIEE
DTTSKDNEKNMQNYIIHDDLGKKDISRENTGEDISPNKYLKNKENYGILDDKGKIKIYEENNEEYVTDVGSGE
NKVSEGNSNDNIMPEEYGKNKNSQENSEDNIMTEKNGKNKSSEENTDDNIMTEKNGKDKSSEENANDNIMTEK
NGKNKSSQGNIDDNIMTEKNGKNNNSQENSDDNIMTEEYEKNKNSQENTDDNIMTEKNGKNKSSEENTDDNIM
TEKNGKDKSSEENANDNVMTEKNGKNKSSEENADDNIIREENGKNKSSQENTDDNIMTEKNGKNNNSQENSDD
NIMTEEYGENKNSHENSEDNIMTEEYGKNKSPQENIDDNIIPEKNGENKNSQQNSDHNIMTEEYEKNKNSQEN
IDDNIMTEKNGGNKNSQENTDDNIMTEGYGKNKNSEKNKEEDIASYEIDKNRISHENDQEHFTPYESRINKEF
HTNVDYNIVSGDNEEKGISVKNISEDIIPDGKGKNIQDDIILEENGENKNFEENIEEDKISDKTQKSKISHEN
AEGHFTPYESGKNKISDENDVEYNISDINTNKDQEEVESKRIFETNDNINKHISSSDNNKINKMKQNNILINE
SQDKNIDVHNKLDKILKNEHVTSDESLEKIKEENGNTRKNKGSINNEEKIEEEKENVKNDETIIGKKEENTES
DDLKIQKISNENINKNILYTDNYNKDKSYNAQGGTHGENDETTNGTNISNDGLDKNVKIDQYISKGENILQEN
KEDIIPSVTINNSLGDKYVKENLSPEDIKKMEVAHKNIQNITSEDELGTQKKDNERNKEDKSPNGVEENHQEN
DKIIGEVNLSNMNMNESNIGNSDTINQHLLNEGKNIHHKGNVNSETNEMTNNSGTQNIISNEQFEKNIIRGDD
IKDKMNENVKIEDETGNNIKINKYNDNAKILNELIIKNQGTQDSDADDISTNGSDKMDQIENKNENIHKINNV
NVEKDKISNDKEDNIVPPEHKEHDIISDNKKKEFDNVLEIPIKGHNILDDKETITEQVEEKSIGQDKSMENNN
VSTNDGKDIHIQEEDIKGNIINNVNDKHSESKKNNLHIDEPNKYVEEKEIKKHEIADHDIKKEFKEIQENDSN
KNEPSNENILVDVNAQDDKNISKLTNDLHDQEKGTNNDSVVEHNVSDKTIGKHKFKNEKIGDIESFETNINEN
NEKIVDSINGNGIRQKIVQNENKNNLNNQDNKEYNEHITIPNEKIQESGYINYKKENEKDVQNQFNGNEQISS
NVSQTEGERKENYIIDGENHNSTDQINNIKMEGDISSNEEHKKINSNFNDEKEIKNNTREIPIMEHNFNTNMD
SVNDDKIKEEMSHIKNMEYVVDSFDKIDNAPNEQNIIDHNGKHSVIKGEIKEDTKHEKENAKQMNEEIKNVKE
QEIEHERKNETKEEKEQSINEGKNKEFEEGRNKEFEEGRNKEFEEGRNKEFEDGRNKEFEEGRNKEFEERRNK
EFEEGRNKEFEEGKNKEIKEVNDKKFEEGKNKEIKEGNKKKVQEQNDKKFNEEIEKLIEEGNEKEIKEGRNKE
IRERNDKEINEGNDKNFEKEIEKQSEEANEKEIKEGNDKIFEEGNYKEINEGKNKEFEEGNNKDVKEENEKEI
NEGNEEEINEGNEKEIKEGNEKEIKEGNDKKFEEGNDKEIKEGNDKEIKEGDYKKFEEEIEKQIEEGNDKEIE
EEIEKKIEEANDKKFEEGNDKEIKEGNEKEINEGNEKEINEGNDKEINEGEYKKFEEETEKQIEEGNDKEIKE
GDHKKFEEKIEKQVEEGMNKVIKEGNEKEINEGNDKEIKEGEYKKFEEEIEKQIEKENNKEIKELNDKKIKEA
NDKEINEGNNNTIEVGTEKENKDKKEDKLAPQEFSSDMVKGNEKNVEISESNNNINNKIDSEYTKFPDNLNNN
SNASEDDKKKEKVINDIIDNVMHDYDNSMKDDKKASDINDKIDKLGDIVENTKNISEGKNSFPYKYYTDTKSV
HKIIYKKSKDGEMERKKFIIVGQNNFIIDSDDMKNPYPKKLSDEVNKHLEEQINKDISNTLSKDEKEKKEKKY
AYSNTFKYAGATVLAGLFVLLASVLVFKKVKGFTYKNNDYTNDLQYHFNSVILLDEEKGKDTDCGTISYMDES
WS*
>N.PF14_0405
MLLQKHFKLVPNQDNNLEELINELALSKKNINEIRKYGTKLKRHNDDIHKGYDNNIEDKENYNNLSINKDNII
CENDNNLIYKEDNITYKKNNKNIINNNFQVRTKNGSISNLLLNKKRREEKMKEQTNISKKQDNVYNHENCNDK
SMNKIEYSKYPIDKNTSEYNLLCSSNVLNLSNFKDNALFQRIFKSIEEEKMKSKYEKIKNKKEQWQKRKLKNE
HIRNEHIRNEYIRNEHIRNEHIRNEHIKNEYIKNEHIRNEHIRNEHIKNEHIKNENINNEKIKNENIKNEKIK
NENIKNKKIKNEKTIYERSHNKSSDNISDNIIQLEELSKDEKKKKNEILKAKRKCSYNNTFRSFSPLVRTSEY
TDNISFNTNNENKNNKFMKHNVTNYNEDVKRKRYIYYNDMNKKETKNIKYVDNNIKPLYHIWNKKNYTTDCDK
KKKKKKEDNKKGKDVLYNFVMEKRGHIENCKKKIINNNNINENFLKSYKNKKKHKRKCKSMNNICDVKNNGDN
VIDYKKMCTTFKNLKHIKKKNILRKKQEYICDNIINKYNNSSEHYLNITTSNESVKSLKSNSAYIFKYKNSYI
PNSLLIKNQINNIKENKKNKSIKPSSKQDIFCKSGDINLYVHKYQYLVLKKRKRKRKFIHVRSIIEQKGGRRK
CIHMFKKWIHEKNVKLSNCIINDNFNNNIKYYINNKSPSKLNKNEVNWIIDNDIYMNSTECSDKREDIMLTSH
IISSNDNNGYIPDEEIHIENANTSREEVTQIDIINNKEQNISSNSVIKGKLHNSDTNDMKDTHMLNNDISINL
LNNEENIEDKNNMMKSFNDSFLEMNNENNKNLNENNGNMIVCTNIDIEEQNNTQKDNIKEQSDISYTIYSQQD
EKKDGTLSNNYTNNYTNNCLNSYIDLENKNIPFPNNDKIYKDVLSFYNMYKDKVKFLLLEECNYLKELRNVIE
ENSKNMSENLNNNISKNEYYSNSIFEKNQSLFDELIKTLKSNKMSENNTHSDLFNMNRNSENEKNMKKNIIES
NKNDLINFYKDPINFLFICNQTEKRIKEIERILNINDNDNHNHNGKVDNINDEYNMEKTKNIFEKKETNCNKD
ENKKNMCQKNNFLCNNNNYNNDIINIINPSQLHQNKIEETNFRSKNPFLSVFNFNSLILANTITDIQKEKNDS
VKKKKKTNKYIKVRKNKYFKFSHLYTMYIQNNFDYLYKKNSSIYYIINLKSVDMMCHNVDIRYIVLNNIRRKN

FTYSFPIIKRNYYIKRHLNCNNKINPKRKNYIFLFKYNKFFYKMLENHREERNLIRMKYKCTPQSIMIPILDK
DNFLNSKICDNNLKYVHNTTINKFNDLTNKEEKNMVYFPSIDNIKKEKNIDIVYGNMKNVLYRQEDDKNVYMH
VHNNNIHLNNNSEYIKSNSLEKNDSDLFTSNILNNNLMYQEENDNNPLRKNKLNHSYNSIKSLDIKNDIKLEE
NNKEIKIKEMKINYNKPCIVHLKKKNDIPKQTENNKINMENKNMLSNSKEYITNSIKYEHSNNYYNNMINNEK
YKYHTNNNIYSLNMDNTCDNYSSIYLQKDEKNKNGLNGNKKKKEYFFNLYVSNYNPFHSKDKVRSMYKLNNSY
DNIVNTLCSSVGGGESFHLSNNKNYKFNQNKSSNITRTSEHINLNSQRNKDKSKIINQRNSNEHYTKNDMIHN
LNVREKTYPYDDTNILENKNFDNINKLRNKRNTAYKKKDNLFFNNDINKNIITNVNDRDYVNVSFEDTKNEGN
LINNKKLQKNNFNSNSIYKCVRKNMNEDNLFLYNNKISSDLNIDSKFLYSNNITSDNNKTQNECHVDNYNFMK
KEGKNNYSYVKPIMNTLINNNINNKSKIFIKEEDILGKNKKENFKLCESNKNSFYFSNENMNSNKLLNVETNK
HYNIKALSYNINRNINQKKLNESKRYDSSFNTNNEEGNFIDNENWECNYKDELKTNCGNSIKYNNEYLDNYYI
NSLLSHSFNIYDNKSIYDEGSLLNGLSSSIMDTDNLDEIIQKIRTLKTINERNNNYISCLYCDKYLFKLDNYH
EDFFFVCSNKEELRKEKYTCSICKHKLRVLNKYKKDNVQKYINIVGNDTIRERSTNMIIKNMDNNIKVKRDTI
NENYMEDEYMKHYNTHDINYVNNNNYNDGDRNYEENHSDYTKGDDTIIQSNMIYLNKDFQDGFSYNIDKKKSI
YSRKKKFCDFKELNNFGTKNLYSDVFNVPKECNKSSTYTSHKSNIFDEKNELSNINYYDNNYANDIYYICNNK
SNNDMNRTFNHIHKNYKSYSDDEINKIYNKNDHMVKFDKSFSLDMCLLKLRRKNLRQNFIPNGRLHNNNKKKN
SLYDSNGSSLIIDRMTNVNVTNMNTYEKEKSIYNINIKEINENKNITCERNFINRTIENTGKCYNHMNDYISE
NNNNNNNIFENQIEKNYMLNEDKYKGVDNKHVTNLNIENMYLKEEKKKQIKEIRTSNNESYPLINPKHNFFKS
LLINGDKNMLNISNDENNCFKKYYSTYIN*
>N.PF14_0406
MLLLLNLRKYLSRTPFQKCNFNTSSSSLLSNEKLNKIKEFRKITNLSIAICKNILNKNDYNIVNSINYIFQNF
NTNYEKKDKKLTEGFYCFTQHENVIGLTELNCYNDLISENIHFKELLINLCNNLTLYYINKKDIQREDDKKTQ
NDNFLHKNVIDNYMPLYYKSNNIINVDKKLENVIDINIFKEIYFNRSFKQLINYTSFILNDFIFFRKFFLIYN
MDKLLSNRKNIHIIKKYYYHKEMNIGNFTLCKGFAFTFLFIESYKNLDEFTKLILKKLSYLICINILIYKSNC
CSKGQNKFDIHSCLENKLCYLSKNDINDDNNNLNKENHVQNEEYVNDIMKKKIESFEILNQCFNDSNNKNFKF
HNNITFIELIHLLENELHIKIFPKIIYSMINEDIVIL*
>N.PF14_0407
MEYKYYDKKMDKCISFNEAYDLNNDEKKVGTSYEDFLMNSSSCMNDEYMKLNIFTVIKNEIINVLSVIKKHEY
NKNLDSSIVDALFILYNNINNLNNNLKEEIDIHDYNNKLDIYHIAPFLNIIRNNNIFYKIKLIALHSLDHILK
YNSSYFNDKCTNEHINRKQISYLCDDYDHDNDHDHDVKSNISDNLCKRDIINESIEKHWTKEFDNKHDGVSTK
MLNEKVRKKKKYHFFSKNYKLHKKYHMEKEEAEENFTFLINKGNNIINISIETLLNAPINYNNMNYEEIILYD
TMILFNNILMNSIKVVDKNNIIKIICYIFKIYKNTKYSLLFKGNCESILINIFHVVMKSYVNNIDDDFIHDIL
TIILILINNNKNKFVLDLLNKKDYINLYNELFENEMSHENLENINILSFSLLNILMEIYGYSLINKNFDSISN
IIRCLFIHITSSSYVLICKALRSYINIFILYKKHFFIYNEIFINILLNTLKKGSSKNITETALLTLSHFCLEN
VLFEIYYNYDVNLYASDLLQNFIQSILELCVNFVNNTAIINEVIFKMIKNILYILVPYAHHTKQKKNSDHNCH
SHHNYHSHHNYHCHHNYHNHHNYHCHHNYHNHHNEHNNNHNEHNHNNEHSHHNEHNHHNEHNHHNHHNHHNEHN
HHNHHNHHNHHNHHNHHNHHNHHNHHNHHNHHNHHNHHNHHNHHNEHNHLNSIHLHSNHSYYNHNSINEMNHI
NHDIGYINQLIIEFKNDIYSDTYNKVFVSRKRKRGRKRMSILHEFNIKERDKEDGHDNENKDEDKNKKHSNSN
NNNNDGNDYDNDGSLHQHNTDNRRRTSFCKREAINELIDRYNDNKNELKKKKHYLNELYSLNFLSSSTKDNRF
VTFCALLLFEHFREYVKVKYIKKKKHIMRKKKRRKEILKKSATIFNTLKGKSIDELIKMRIIQTQETYNNIDT
NNAIIDSEAMHKDDDIIEYSSSLYENRSFLTSENAAERGSSIKSNSYNPDTHDNKMQMNNSNIDMNLSNISYN
HDELENNKSLINIHSNFTTEESCDDMNENEKDIKKKKKHTKGHTSKKEGKFGKMDEDEIDVEQENNNDNCNDV
DVMKQENKMLNGIVDEKISANDQKKNNKNSSNIHKNISDDHSVLNNNSKNSVAHYERNDEGKKIDKKDNTENN
IINNIDDDNIHNNYTNEEISKKESDLHKIDNHHKKKKHHHHMKASEIISSVAHNFSKKHFKHSTKNNLMEDEY
FVKSMAKFVRYNPFLDKEFVGEYISHRKNVNLLKRYVRLFDFCNLSLLSSLRLFLRCFKLPGEAQLIERILEH
FSLCFFYSNPIHGDLSNIYKVENDKVVCLVNDEELANKKRYILIDFLNENSNKNNVTHDDSVLPNGNETDAKK
NVDENNNENDSNLNENSTNLKKNNVDNNKDNVNDLHIEEKINLKKNVDINDYMHKNVYYLSKKIESMSEEEIQ
KKYVLVENSDVIFILTYSIIMLNTDLHNNQVKNKMKLEEFIKNNRGINNGKNIDRIYLENLYNCILNEEIKLF
SNTQNTYTNDDQYWKLLDQKKEEYKYYHYFKANEIYFYRYDINKLLIRNNFLPIFFELFKRTNDYNLIENCTF
MFKMVINNLAYYHDLGNINKICYIFKYINFYLTQKCQSLLYLFFHFIKKCYNSFRNCWSIYINIIFKLVTIDL
LPIFFYPHIFINNTQFNIDKDVLNRKSKKGNTLETYNINKSITETYQHPFLVFKKNVKKLNKSKWIDDFSSMF
FSRHHTTENNNLSIIFKDNGNYNEKIEDIKNQTKEKEKEKKKKKKTDKDDKDKEHENEHDNLKNDKKKNNENL
KHEDRENENHSDGHNAERNDIDEEDSDEDDDDDDDDDDDVDEDDNDYDDDFDDDDYIDYDCHNENYHYDNIIN
NINNDNNDELEYIYVNIKVDPKNIDNSAIIDNVNIYKKLKLDIYNFFTFNDFHNNMITNLNISSLIYLIKILI
IKCSIKKENEIHNVSAHHTNKSDVHHHSFLKSHTLSHHHNNNYLTNEKSGNTFNVNDYINDTNPIIVNSESSF

DQFYHSKEKLLSTQMFFHIMCYKINYTYILYNILCKLKLKSYRTRNRFIDKGEGHELNLYMKQIHEARKCKNI
IQEERNKKNYFDLTLNNIYDTDNTSDAGLSDSDYSDISNDSFFVRRKERILVDIYMHSHEARGEKHRDERNRN
SHHDDDNNHHNHHHHHDDNFDNHKYVDEDDLYDSYYNHHENKHKRLQEEQNEECDEYKEMYNEFDKIYSYADS
TDDEREDGHDEDHSYEHAHHNHDEDEDSTYLSDDKENADVFNSINYNKHKSGHNIKSGFHTTNDGNVNKNLKR
SKQYLIDKIKMDLYMKTYIMHIKIIVFTFEHYFNLLNRYLLINYDASIFINIYNSIFKNYKMENVKVMTEEDI
SNDKSYNLYDTNFEDINKKINYKEEEEEEDEEKRININDIENNLFTVKMGKAETEEGDWLFIEQLIMSIMNFS
YLCFNIYKENKVKISKKLLKKMNMNADTVRRICLELQRKNKKFFFLCGIYLIYILQFLNKNILYRFIDKIIYV
LEKISKNVYVNSCIINIYLNMLQLITPNNILYKNTNNTNISLNDKDIYIYIEKATIYTESINNIINNNNVLLK
LNNFNIENIILSLLPYLLYFNNKKEEINSHIASINSECLHIISNIYYKSIYMYMNNNVNHRDNDKYIKMKKKS
AAVLLNSEYDMEKLHDIPFEKIIELKKMYIFLLTCFVLSLACSFSSKRTRSEAYIKLQQFLFNESYIFKRVNK
KEDNNNNNNNDNNSNNNNSNSGKNDKNCKEEKYVYRDEKLIDLISNFIILPLITYNYYFPFICKNKYGGISSN
DGEDGNIKQSDNEIKDSEPSSENLLTLDQNNYCKEALLNYNLFHKKNYVSIMSEEMWKNNEYENCGCIINYKN
IENIEKDNIVLNNILNYANDYYIHTILHKQYNYYFSYLYAKKMLTYDNVCYRKSMSISFVSHIMLSFLYSLLN
CSDDIYDDEKKQTNNLLNNELKNNKTKETHNNDEDNIINIYSLLCLNNEAMYNKIVTKGKCENNCIYYFLKHF
YHSLLTIKEEANKILNIYKETFIENMKNIIYVSSSYAYCLKDHRISCFSHIKNGHFFLREQEKDIYIKLLNVQ
NINDKEYPQELDNDVVKNIKKLQLNVRISIVIVYYILYMDNNSNEQFKATFEELLNVLLTKYSDNEHDENVEE
KKQNDVKEVQSNDNKDKSVVTSKNEDNTEKNEDNVNITNDKKEDNVENLTEEKQNNVNVSTQDKEDNVEDLTE
EKQNNIKDSTQEKEDNIKDSTQDIEDNVEDLIEEKQNNIKDSTQEKEDNIKDSTQEVEDNIKDSIEGNKDDNA
NISNDDEEHEKTQELKEEGKEDDAHSSDEKDGKNN*
>N.PF14_0408
MSKTEYDILSLVNAVEEFSKYCSEYKKRHLTNYIQSCSVKNQINNNEEVIFKTTLQDFMNKVNMLGNNILQIE
KINKCQIPENILYEKNGNKMIYDPKHYNEDKLNNENILEDKNKRNICEHIKSKINDKNFVQNNDYGKNIKIIN
NINEQNLLMKDKNDYTNISENLYMRKISTEYTSSDRSSEDNVLLHNINNVLVKYVSGLDYTLNIRRISRKDLI
EEIKQFYIIHNNNIQLNDYSTYLSDEVINILKQKKDNIKCLDKNNLFIIEKNVDFYKNNFLPFFYIPCNYLFI
KNYCNDNERCLETTYDTNDEKNSTTENSYILSSPNHMYNTNTNSYNSHSNSIIARNTSSNKCNSPIFGINERN
SSPNFGIFNESIKNHKDQYNMDPNYLIMRYNLNEEVHIKDDRVINSPNNIKSMNDNRYNSPNFNIEHIHINNN
DTQINNYDIYDINNMDGKKNNLNSNTSDFYQSISDFYKNNEKNLICQEQNNNNTYIQKCAFFLNSNETNVLTN
NKNHHKSGKNLISLCFDDELSSAITINNDSSLNKAIYNEFNNSEKDIVEDICNTTNYKDINMEHMNTYQNDER
KWMDEYCNGDYNLFRSKQLKKKKELLLSCISYNEKENKYNDQDNMINVEINNDTKVKNILYDTGNISFDENIG
INNHYNTINLKVIYEANKSEYGNNERLNFIKGQIILNKYKVVKVLSKTQFSTTLKCLNLLYKKVKTDTQVFLP
YCHKYMKDDSEITHDKKKNNYDKFVNLNTIKKKKNENYNRQHDIKNNLHDNKHQIINNKKKVEPKYVCLKVMK
NGKQFLDQGLLELMVLNILCNANTNNNLSNKNIIQLYDSFYYKEHLIIVTEYMQSDLYNYFIRKGKLGTLGQL
QILTKNLLEGLAYIHSKNLIHCDLKPENIMINMKKNKKNHEKGKYNKVNQNGVNIYNDTIEPHILNSSNINNS
NLEKKNIIAYPSFDQTFIENKDAQYDNNEKTSNVLYDSDKSYNNNVKNMIDNNLYCNNIKNIDNNSDNNNNNN
NNNNNNNNNNFPHNNINIYNTKQFDKIKIIDFNSCIYESDKLEMYIQTRSYRSPEVLLQQNYDRKIDIWSLGC
ILFEFLTKKILFDYQNIYRFIYSIVSYIGPFPFYMINNCRIPHIFTKHGLIILKKFTTDNMYENYIKEEQLNQ
EDDEEIVFNSNDFFRLNKKDNILTKDLLKNKNPNTSTPRKRNNNNNEIYYDVCYPSDNLLKNNFQISDTLFVD
FLSSLLQIDPSKRCNAMEALKHPWLQPNLYKDGL*
>N.PF14_0409
MFNVFSAAFLDNNNINKNDNVLGKTILTNANNENERTKNLPLKDRLATVRNVQNILNKKNINKTDENEEKKTL
NNNFKTNTGIENTKPTIINESTFNRFKNYSSVVNEKQNEELEKKLKKRKRRKKHVILMERMKQKEREKRKQRK
MEQDQNGEEQYDDEEHEEEGEDDNDDECDRQNNEQEDHFEDEDYYAENDHNNNNNNNNNNINNRSFKFNRSSK
NKYDENDFNNHKKDKGDDEEYEDDDEENDDGYDEDGGGSYKYNNNNSKDDNTFNKKNYHSSGNVNNTFSHTTS
SNNNHMNDNSNSQQNMKPKRKRRKKKEMEEYRARLLKEKMHQQNSLGSILNKTNSFNKNDNNSTQEEIKVKRR
RGRPKASEVAAINIMNDKLKQNDIRKYYSKNSDNGFMKKNSMNEKNKEQQNEEANTNVFKIIKSSIQENSSFM
KKSPNEIVELEKNYKNNIKKGVTCIPLNYQSKGGAMAIILIGTDTTYGPVKNSYGFMTFLVLDCHTNNFFIDT
GIKNNVIECEKHMQLLISPGDMYMFKNQSHEMEARLLLIVCNKMNQPFDVKMHIKDPQMNNEK*
>N.PF14_0410
MRKLLSTAIMDLKKIKGNNFESKHCNDNLKSLIIKNIHKFENDEILKIIEKYNEKNYKDTKILKCSYDVFKNN
VHRYSFDQINKIIRLYNISEIYDIHFNTSVFNYIIKRLDAIPPYVVVNVFHNLIRSGLRDYNNINLIKEYFIK
NIDKFNNLDLTIILSSFTILQEELLKKIPNVNNKDVSFNIYDTKKDHSLCSEALYDYMDIFKIILNKIQKDKN
IHENLSVVNSVLILNMLSRINFCNYEIFKFFTKNYYKNLNDKDLEPHHFTLLLNSFAKCNIHINIMKYILKYM
NNKNFINNLSYVNITNAVHYMAKFNYRNATFLNHLKDKVIEIIDIIPQREFSNIMWSLAKLHIKDDYFYYIAF
QKIKQIIDVMDMMSVAQILDAMRRRKNVSNGQDIGATIKEKKNLENDISCPLRDIKDTTLNGQNIDYKNNIEH

NKYHNNEEKVKEKEIITTQTTTQNKDLNNNIQSTYKTPENNIISIEKNINTYNIISDDLEKNILHLLVNKYIK
HIQHCSLHVLTQVPFCCLQLNYINSDIYYKSLEILRKKRNDMTTLNLIYAKYFLRIFIEKQEAHFQKLPRSLK
QFAKEILNSDNN*
>N.PF14_0411
MATISGSETFLPLALMDKCIGK*NMDNDERR*RNCR*IGWIR*ICKYGIRRCYRIHVRK*
>N.PF14_0412
MNDCDTKKFRDISEKIHGKYVPIIKFGGSKKYIKNDYKNEDADINNISYASEFLNNNVDIYYDNKYGINKVCF
SPQGKYVAGCTSNGFIYLYDLYENKLLSKICTRIDNIQDIAISDNDKYIYACGENRLIEIFDIYEENRKRCNN
DIGKYVDVSIPSIIKNTKLKMKNCSESDNMKHNNNILSKNALDNKYIPYHKTKYKNISNGKSIYIPIYNYEDL
IKSNINMIDLNYVKLNKVNEIINKSTFLIKESHEYITSCIAIPNICNFLVYSGGGDGVLKIWDLRMNLFNISK
KSSRSNKSDIIFIDNKNPFASICSHEDTVTSITFNKTIDHEGSYKLKKEQRKKKKIFNSKKECIDNKNVYSSS
LDCENMNDSTSDYEEDRESDNSSVSSPHSSICYSSLTFDLSKDKFNNILMTSGYDGYIRLYDISNNIIKSFYD
EEKSITHCMFSNNNKYIISTNKSKFGKVFDFMYMNNKMNSKNMATYLNNYKTYCLNKPLIRKENLENENYHDT
INEDDIFSSQLYNDKEYALCKIGNIFEDEELEERIKIVNELNRKINEQENKHFEPNKNDNNNNNNNKDNNNKD
KNKNNYKKYDNNKYYNNNDNNITVEHDDYPESNYSNSDSSIYDENTIKSNTFYEHVNNKLEPTWSLFVDNLKY
MDLVPYDNINDSLKYNYNYVKSYFNHNNFLNTNYHSLNIDEKEPSLYYMLSEIISNEDDIPKFSSCIAGDKCI
IPSIDTYAHVYDLYTGFHVNSIKNLYLPNYYVDNHSYYSHSIDTNKKRISFLTSVDTYPKNQNIIATSNGYPD
GSIVLWAFVAF*
>N.PF14_0413
MSSVFNYFFKNFVLKEKRNLFFKYNILERGYSKITSVNLNNWNNIHKEIIQKINNSDFVSIDVEYTGLHLKDE
RYISLDSSYEAHCYGAKSFFPCQIGISIAKKKDIVEDLYQTDEENNKIKKKSSVHTRKVIYENGNMNKICNIE
KKKQEWHISPYCIYVFPKENKYFSVSTSTLSFLKENNFDFNEWIFNGVGYLRPNEEEEKKKNIVEKINELNNL
LSKCNTNKKNIIPGEKIHIDKIIQEIENYKIVDDEDKEAIISIIKKIGKWINNTNNDKNYCKHEEDKKKLNMN
KDTNINYINNNKDKANGNDIHIHIPTTNIDNNNIYNITNNIVKEKTFASLNNNFIQKQKNELTNNNVYNVKQT
FSSINSNIKNIPNNYITNEDVYDNNEKIINKSTKENDNKCYNNIHNNDTIGHIECDINNMDDISNYPLYLEIE
NPYLRLLAHTLITKYFDSIFCISVKVNDRKHLAVYRSEKDSYKEQIKNLEQEIEKINQIIGVRLLFDEIIKNK
KIIIGHNCFYDILHIYQTFYHDLPKSINVFKKKWTELFPHTFDTKYINETNEYLYALNGPATLKGLCEYMASL
ISSSNDFDFFFNFMNDPTDLPQCFIPFINPKKMTKYNMTQNFIHQKNNMLYSNDGNNMENDKIQQELLYGTKG
DNIIPNRSIELNSENNTLKNNNNNNDNYNENNMIYVNNTNDKHILNSDEHNAGYDSLLTCLLFIFQCHYILKK
NNLMWKDIYFSESNKANNTNSKYFFDIFLNMSNKIKIVKTQPNVISLCSAENYEMSRHFYMYDYPRYFKKWEI
MKIWSPIWITLSKVDDQSCWIIAKSDDDAKNIKMIYKMLQNPQFKLCTYEEYIHKFKSN*
>N.PF14_0414
MIHEVILSLIGQTGDIIVLVHKNKKTTINGNINIDDYRFEVNNNIHIFLNSEIKIINEIVELGYYFYIINIFF
LLVKKNTVYKNITHIHKYNKLNQERKNNKNLKNCNIYDKVNNGYYSSGNDSFSSISSSSDDEEESDDESDSDN
DNEEEKNESASGYMTRKEHNKKNSYDNNNKNKMDTYFGVILKNVKSINNASPYGYYANGICNEIKKFIKKYLK
KISDVEEYINNNSNTPLTQIVTMLEKKREELLVIINIIKNFLNFQKREEENVIEGECRNKTKEILDYLYEHSL
NGNSRIKKIYHKYLKNVGKIFLHQLFSWILYGQLIDPYNEFFIQKRQFIYSDEKKIYLTPEELYENLTLSNVQ
SLNFEWNYLFFQSLNNLPQCCIDKIAGRKILFIGKSIRILIRSNKWNTSDILKLFPIIKILSKAFDESSKFSL
IHSSYYYKDDPNFMSNDPSFVKDGNEDENSNYSNSSSDNSSSYADLSDSSNDNINLYCKEIFDITIEKIRSII
AYKLWKYIVKDINLIKIFDLFKDIYLLNNGDFYDYFLEKSWSLMHIPPNMKNEILLKSMAWKNSSVLVEEYCK
SKYENKKDKLIIDNYDNSLFTHIYSNNHDSKEEQSNFFFHNNANSSYYTFDTDTSENFISKYFYPRISYKKFS
YDTFEGEKFDNLILGGMCYIYDNKIVLNDFYKGIQTLFKKKYYDYDNIFSVCINNYRQQILRGFKHGFDFSVN
FNNFFDNQLYDQDITRNNINEQNFVKDNNISNKNYDGKNNEDHFLVGSCFALVIHSVNNPLLYKTDILNSSQG
YWGTLGDCLSVEIRVKFYEKKKTINHPNINNNIQNNISDVILGDVEIEVSLYIGGKGISSFVHNSNSYSIDYN
KECILNNKLLEDSLNQERERTYQEDDHENDLKNIKKKGRIYPMGISQEISNTSENMRYSNNYYKDDNNSSYNI
NNSSNDREQYPVLKIQSNKQTFYNINKNTITKFRVRINCLKHTFSVYIQKLDEYNLNFNNTKNKIKPIIHIRA
LDMTQAFSLDIGNAYIGLYTCPILFKHKLLKKKSKLNEWFKNFYNVNMSDDMSTIYEKNKNINIHNENANGTI
ENEIRNRDNLALNRNYQDTTKNNNNNNNNIKYNREIDSEDDIFPYKKRNIKNSENRLLFSCYDCSVEIYKWFH
QSYKSAIEIPEIDIDNETLIFYDKQINKNIKIHSGLNLWNNIEIHFKLTWPIALIINTNTIYTYNSIFQFLFL
LSRIHYNLKILCYHNRNLYKYLYYGKGSSLFSYLFSIRYKMQFFFSHVIRYLQEDIINYEYKLMITQIHKSKD
FEYTKSVHDLYISQIATKCFLRIQDLTYPLMELIDTSFKFCYFFQCLVENELFTDILNNEISNDKDNHEHMKN
EKKKKKDHSNIDTFIIDEDDKNEDLKQVIEKLLQYNDIFNDKLIVVINEMIGISSNNNHAYIIHLLTILDFNS
YITKIKDSRKKYNDLNSNKNSKNDEIKNLNKNIIEDDNVMKETHIDKTSHLQRNNFNINNSNDEKMEYQDIEN
IHIIDNYDVDKNHSYNPNISNLENNSTNILSQINIINGINELNEKNTPLKSRIVNNFNNLLYNNIDLTSNIKP

NDLSPYNQHMIMPQTNEGFLNGNINIGSETMLNNNKYNNYNYSYNNIMNSQNNIINSHNNIMDSHNNIINNHS
NIMNSYNNTANNNYNSLIDKYSNILNSYMNNTSSIYNSPKNFINNSNINSYNSILNTSKLRNINNISSVSNNI
SNDYNTNNINKSSHNILHYDDNDDNNNISNDIGSIPKQIKLSNINKSIYDPNVLNNNNTTTDNIATNNLNNLM
DIKVKNQFNVQNIIDQYNYNTLSKNNISLNNTYNINKISTLSPLNKVENTLNYSNIPLHIYHSENNTPKKSAH
P*
>N.PF14_0415
MFLKFFLDKCILCFLALGSIPIGYINRKNKFRKIENQKYALFSSIVMNSFLIYLNKFFGILGIFNFFLGNYLC
LIGITGGIAVGKSTFCNFLKKKDVVVINADEITSKIYTKDSICYKKIVKHFGENILNNDKSINRTLLRKIVFN
NEENVKYINKITHTYIILQIIKECLKYKFLYFKYNVAIEAPLLIETKLYLLTSPVILLKSSVRNQIKRILSRD
KNCTYDTAMGIIKNQLPTDEKIKYADIIINNDGDILDLQMKCDVVYNKYLKNFFF*
>N.PF14_0416
MFKKRFVGNVKNRKKTEDIKDGEDKEKQEEEEKKTNEENTKKENDNSNDSSLMDANDMFNKKKRVLEEDHEIS
DDDVVCKYFFKKKLKKMNEENSLLLRKKQNKLIHCKSEDITQSNTLTEDRIYKGDFSGSKNYGSYEIDQDIKN
DHRSIMERNIKIGEEILKGNLKDNIYRGKDAHEKAIMIKKDSLAKNKYTGLYGPVRSSGSNVRVTLRIDYEPC
ICKDYKETGYCGFGDTCIYLHDRSDYKSGWKIEQEYEQKRKRDEALRKEKMEKWNEKMLKKLKEKEERLNNHH
NNLNDNNINNDQYSDEHENNSNQSNLSSCSHNDNNSDTNSVDSDTSLPFACIKCKKKWKIEMNPSVTECMHYF
CEKCFIDMFQKNKKCFKCGLQLNGIMNTAQNIIDILNKRKTVK*
>N.PF14_0417
MQNVYYVGNKIKFIILYFFCVLFLKDYERSEAFNLARTTEKLNYILNYKTPNRYDLNNNVNKLFFEKQKKKIEF
SRKPLNSFNEDVKTIREDISSDSSPVEKYNFKAEVNKVMDIIVNSLYTDKDVFLRELISNASDACDKKRIILE
NNKLIKDAEVVTNEEIKNETEKEKTENVNESTDKKENVEEEKNDIKKLIIKIKPDKEKKTLTITDNGIGMDKS
ELINNLGTIAQSGTAKFLKQIEEGKADSNLIGQFGVGFYSSFLVSNRVEVYTKKEDQIYRWSSDLKGSFSVNE
IKKYDQEYDDIKGSGTKIILHLKEECDEYLEDYKLKELIKKYSEFIKFPIEIWSEKIDYERVPDDSVSLKDGD
KMKMKTITKRYHEWEKINVQLPIWKQDEKSLTENDYYSFYKNTFKAYDDPLAYVHFNVEGQISFNSILYIPGS
LPWELSKNMFDEESRGIRLYVKRVFINDKFSESIPRWLTFLRGIVDSENLPLNVGREILQKSKMLSIINKRIV
LKSISMMKGLKETGGDKWTKFLNTFGKYLKIGVVEDKENQEEIASLVEFYSINSGDKKTDLDSYIENMKEDQK
CIYYISGENKKTAQNSPSLEKLKALNYDVLFSLEPIDEFCLSSLTVNKYKGYEVLDVNKADLKLKKENDQNKS
DSLDKQKMEYEILCRWLHNKFSHKVHEVRISDRLINSPALLVQGEMGMSPSMQKYMKQQATAQGISENEMFGG
QSANQPVLEINPNHFIIKQLNHLIQIDKMNLQNSEIAEQIFDVASMQGGYTIDDTGLFAKRVIGMMEKNAEQY
LMNVQSNISNNTLNNNTSGSEMPQNNSPNELQSEMKSTNGIDDNSNISENKINESSSNQNNIGENSIAEENNI
KNIAESDVNKINLGENDVSQNTMHKQDSGLFNLDPSILNSNMLSGSDKTLL*
>N.PF14_0418
MKELIDKVNFQNLEENYNAKKIYVTKKIPFNVILNKQKQEQDKKEDNKLFKAVKTYYFTKLKRKTFFFLLNEY
YKSQSILYFFKLRDIDRKRKWVFYSFLKNKLQGKIKRCVMNSLNNFTDAATRFYIKLKCFNILKKNFLNTKRI
YLRIKKKRINIIIINIFYKWLDLSVMHLHKKYEKAKDYYILRKKSRIFNLWKRIINDNKFFKKIDIVFLKLEE
IANSWEQK*
>N.PF14_0419
MEDNNNNETHLENVKNGEPFYILNPSEFIRNKEKVLKEFITKKYDDIIKQNKLSADIRCTYFQCNPKIVIFTT
YTPFQKYESVVSLKNVDTAARRLNIQMHEDNTFKVIYISDIKKKIAPGMLFNFKIEFYPKSHANYEYDLEVHS
EGKYFLLPIRCMNDQIILNVQDEVIIDDTPIYMKSEKNISIKNIGQYENKFEVIINPPFYLSVSSSIHSLKIG
EMEHFKIMFVPVHNKCYEDYMIINYENGLSTYTKIKSKGIIADVFIKDKEIIAHDIYINKSKEINIFLKNLSF
FLLSYKVIKYQVYEYKYDNDSEDIKNTNFNVNHKVIQKSFINKQELSDENIVKNELYGNEDLYDYETDEYYVN
ENNDNNDLSDNQSSQSDFYEEKFPEDMVNINDEKIKNELCTYSLNNSNKFLHSNSNYVSRKKEDDLQIKKNKI
IKIYPKYQKLYNDTDTPISILINPQFASFFKIELFLFIKGYSKEEKVVIYLKSITPEIYISNSIHDIDTILIN
TCKTLHFDLSNNHPIDASITIENSDDYYKEYKIETKKFIIPANSKYLLIIVYLPTRHGPFEKTFIIHQKYTNV
KKNIKLLGTCILPDLKFDIDEINFNEVSHSFLYEKSFHIINNSHLTLNYSFKYSNELNEEIQIIKPFGELQKY
EKEQVVIKFCPKYIKTYFYNIEFFIEEIKTYKIKLPFHAICSKPLVLCEPLFLNLEHIIIDQIYTEKIKIINE
SQETDIKYEFIIEEKIKDICSFQVCENEGIIQRNSFKSIGISLKSNSIGNILIVIKIKIPGLDDFLFFNIKAF
CTCPYIKIIPNIIDFEKCNCLDVKQKIIEIKNESPISTFITLSNENSVFTYSDNNFYVQPNKSIFINVYAQCL
ETITYNDILIVTVHRKDDIHIPIRAQGIGSPIFIHEEVINFDIVNTSKKYFHELIIFNKGMNERNIYFVFESE
KNRKRKSEQVQIFNITQKNLSIKGGSNIVCILNCFNIKEEKCEDILYIYDDIAKNKKKALHNYKKINIVATFV
HPEVELSEVENFVYNFNDYHDIDEIGRIEKRYKKDINKKKKYINDKINENVNDKIDENVNDKINENVNDKINE
NMYDKINENMYDKINNDIYNLNNKNSQQKKMILIKDKKQLMQEVEIRNLKNANIKFALSIKDPFFVQSDIIEL
KGKEKKKVPIYFDINYMNNKLNTTYQENLIINYFENSRRQKYELVGQTIYPNIIMNKSCIDFQYILQNLYAQE

FVEITNVCDYKIYFKWFFEDNSLYKDFNEVFDINPNRGYLLPHEKKIIKITYNSINENYYNITILCQIKNGPM
YPLKIIAGYSEIKYSINKKILEYNIHYKTIGEDIITLQNIGKIKLFIEIIYNIKFPSLLYTNINNFFIEPNNS
RDIIFYFIPGIPIDIIDNILIKICNFEEIKIKLKSTNSCNILYLNINQFDNHQSIIKDEIYLLQDVDNMQDLD
TTFHSKMKNDSINKKNNFKNINNLLKNNFHLYDNVQNDYYEKERSCLIKKLKNMYSDIFSSLRKYIQEILDIY
YSIIRNNKNSDDHNDDDKDKMEDINLMNKKDNTKNEKMFIRSSIIHTNEKDSKNNMKCKYHLIPILKNYTLKN
NSNCAIFLDLLKALILNHKDDNYILELFTKLKKLSPFKLFCHGYTTNEFILKYLKRIILERNMELRNICLNVG
NIIINEKKEYSITIKNVSNKIVTLNYILKNSETNDIKLVHEHKEIDCNNSIIFRIIIQKSSGIEYIYMEKVYI
YINDNNYYSINLKYHFIIPDIFLYYKQLRFEGVELKTCCCKVNRLINTKNVNIKFKIKNITFYNKKLEYYHLK
KKKQFFIYPNKGIIKKNSFLDMKIFFEPSNIYTDAKIQIEIFIYNSNFVKNIIIYLSSIKHNMNIDPCDIIIK
PLLYNSGHSYQMIKINNLNNYSKYLFIYKLDIYYKYFQSFIYDLLNIHKHIYIEKNHENDFFYTNLLYYLMDI
YINKMEYIDNQYYHNQQFYNKDIYDGNLCQTNQNMILCKDKCNENNVIHNGDIKYGNQKENKTKNKISNNINE
KQNKKLKRQNDYLNIPNNIKNIQKDNINEATFQQQCEQKKENIISKNNIKDTNALKKKIHILEDIIKKNQTKY
NYFHEYLNIIKVKKKKFKDNYIIVSPKYLDHKKIASYLSEYKNNQEFKEKNGIQINFNNIETDNNHNMNSGDI
HSNHFNNYIEEYKSIACNNKQYSQQNVNFNNNLLTIDKIFHWCVELNEKENYPLKTKSNFIHYLIHEIDLKNN
KKNLGMSNINEHMKNSNKTNNENKTTFGNHQFYYISDILNMNDKRKEKEYLELNTNITINNISKNVPTHIYNE
KDTCNIKDTSDDMKNIDNINDTSCVSINKNNNFMEKKKKFINIYKNKIKKKFHIKNVKSKIDNNKHITYINKK
ENKYENDTMKKKKKKHYEVDSFLSDEKTFFYKYNLKYKYEYLKHFFHFALLNDDEFMNNIKSFLNETILSEKK
KNKNDNVIKKKNNMVHLLEIILKKLLLSPNYINGVVFFFKKNEYINSEMFFNIFLNIHIDQNIFIIHLNPIEN
YEEYININLTGDHVLKEASEMKTKAVKDVVYKDEKEMKKKSESFEVQRIKLFYEKLIKQFQEKIKSKLNEKEK
IEKIIESIDLNKGLLIIEKDKGSRDVEDKSVYDKKNESLSNLDTDCHNVNEYKEEKKKISEIYKEINTEECKN
MLDNNNNNNNNYTNNNNSNNNCTNVADTICYEENDEHVSINNMDIQNNSIHKYSPVEQLYYEIVIKKKDINIH
LNNKYINELCLECLMRKDKIDSQIIKYNKKINKINDLIKTNNYIDHVSFYKEIKETLQKLWNEYTKRDNVNIS
IFHEQSNNMNHMPNMNNKENENENDTLFIDETHHNMFDTKTIKTDQNKYYFIKQINLQLSENYVDKLKDEIIE
NKDIYGDINNMNNILYSNHNNHINDRINVSNNMLKQLNQTKSYNDHTTYLKEENVHIFNENISNDPKRNLNII
NDKNNMIVKNGNVNVNKKKEESQEYVDNENINTLTVIYTNDELMKYMQKNSVQINFCYFFINNDIDFGKKNIA
NKILKCSQEKYKDIKTSAYFFDIPLQRIFDSFDMKHFVKGKDNLEEYEDNFYFYSEEDEEYECEKIEEEKRKD
VWGIHKNGDINNDMQINEEKEEKFYKDKKKEKNNKKKEKGKKRNQENEEKEMNKEKKKEKEKEKEKEKEKENK
KERNKEKKKEKEKEKEKEKEKENKKERNKEKEKNKKIEKKNRKEENYTENKYEEEKDKKKMEKDNIKKDKHKK
NDNHKKSDKDIVKEYKEKEQKEENNYMNENIDEKKRKRKKKDKMKYDKREETEEIDLEGTIKTYNEKEGNTND
KLNEKGNEKSNEKSNEKSNEKINDKSNEKINEKSNEKSNEKSNEKSNDKRVDKYRGDKGNEYIKSDQQTENQK
EKMILKEEKISGKSKGNYKKNLYNSYDKNTNTHINKYSTENIKEYSNIDEKNICRNEKEHLDEYNHEFLEKRK
YDIKCEYDYMFYNNANINNNNKMYDPNNDNNNNNNNNNDNNSNNNDNDNNDNHININKKCYNSQENIIEFQQI
DTSYDLKDNIRYKNITHLMLEKNSIEEIYLKINTCKIINIKKKICLIDILGNTFFINIHIIIDKPRIYTNPYF
LFNNNVSIFPKNNSFVLSNKSYHFDRVLIGKNIHLFKDIIQIELKEEFEENIIKFIRHFEGIYTPKLKILSKL
KTKNVVPKIDTIEKGNKNQVNNIDIIRNKDILKNLYKHIQIINFFNYSHFDCFVELSFESKLNQGNKDILSLN
EMNESELFLSFNDFYVYPSKFFLLSKKCKKIILLFLPKNVQSYEERLFLYNYSIKDEPNESMEYTNNKQLYIK
GKKELNKNIDKSPNNNVLSNDNIFCNNINNILSNKIINNYIYDMFSFVIKGEGIQCYLKIEPEYINFHKVLLN
SVQKKNIEIKNRSYCDIIWYIDSNSIKDNSFYINPTRGTLNKNERKKIITIGLNTNTVKIIKKEIKIYYSEAK
VNKNDKVNVNNLCTAICNIEADICISFIQVNFEIIDEKILYDHNHTLIEDVIKMKENNFLKEYVTILKNRKKD
KWYPSKELLLLNNVIENVIQMKYVPVNEAKICLFKIKNTGKVGIHYFVEINDETFLQYITINDIYNIINMNEEK
EIKLKIKSYKKIKFQNIDLFIHICDSIENNYIQSYKYILSVSFDYNYLKIVPHVINYDSVEIKKEETKIFKII
NDGYFDFKYDIKLLKGKIKNNKYQNENFVYHNKENNKNFNKIIKNDNEANKNPFKINPMSGLIKSKEEQVITV
VFYSEEENYYKYIYIVQVENLISSIGHNNINTNITNNATYNKEYKKHMKDTINDTDKYNEIIKLFACSVRPKI
SCDIKNLFEEMFILNNLNNYNHFVHYFLTKNSYYNVEENTLYYKYGYVNEYINERIKISNLTDVNTNIKIEMK
EIDFSSLTNKSKKNKEAEKNKKNETNISPNSAIKYNLKIQNYNEEKYNWNCSYIKNDNLSNNINEQFSNKNNI
KTCASDHIIIYPYQHKFIDICFHSTQIACKKFLFNIYMMKPEHILCYSFYINIEFFLPSAQLIIPSYLLKLTN
NVHDKVIDIGNIHINSVLSFKIQLMNSFKYPVMVKVIFHRNENNHDNIKNEFIRNLPYINQNEENNIILDKKE
NVSIPHNNLYRRQKEDENIFTQQNKKLLQSEIKKKNILTSDVYQNIYPNEKNIANESFNNNIGYPRVEEKKEL
YNNITNNDTILFNIHNMKIKKKNNITYFNYENVKDIIYNHRCWDRTNNMYSIGYLCFIGNINNLLRENEVAYF
KIKLNKNKLKYIESNESQNETNPEQIHVSEKNPDISYLDNLNKKENKYKNDIDITNVNGKNKTNNINKNKEDY
KNNNDNEKKNLDDNSSNNYINQIEKTNDLSNDEKYKKKKKKDKNVHNNNNIYNKNEKKTLGNIQIKILNNNYE
CYNLKFIGNIIERKNYWNLKHLKHHIKKIYNNNFYIFKNHINFDIVPLNFKHTYKLLYINENNHTLFFHIEVD
KKIKNIIDIKPINGSIPSSSSLYFYFTIDVKELVNFMDRPILCKFSQHEISINKKNIKLNNDNLKYDEDDLYI
SLRSEEIKVSYKQKKIHFKNIPLFNYSKYKFTLENTSNVKLFAELSITPNNYMDDILSTYSFEPFKEKHMLTI

ITNKATNTFTYNTQQVDNKSFSSSYRNDHEKKNSLMDNLIKTNIVEDNCVKENRESIDEFYLTKYDLNISDLN
NMNKSDIGNIYNDTLHKEQSTKKNTEMDNNTITKYITINAKSNKYIYIYCFHNIYKQKLESILHIKIKYYEDI
KIPIKMTFDNYDKEKKNINDFFLLSKFKYSKQVIDKKENNKLDNLYKNKHHILVISRGILSKNYYKIYMINMS
NKHIEYCFEKYKENENSSMIKYPNITNNDKNKFIKNNSTNNSIECVNNKGNISENNLYFAKFLFQTFNSIPYR
QNYNLFINNEKEENVTLDLRVIEPIIFFNTSFIHSNNLIVGKSYCFIFYLINFDFIDIHFEFDKNSYNSFNSK
KETIKIIPSEGLVKSCYRKNKYINNYINKLNKHEEAVNDFDNKNRKTNNLRSLLWLSSQDEGDSISHNSYNDN
MTFDDNNKMKLLEKMKGNKVNVKQKGAEKYSFAHIHDKSNSRNNSSNSSFCTLSNMSYSESSHCNKIRNNKNN
VSRQGYDDNTDIYFPTNHMLNYRNDNTRKENNYYYDDDNNSYSSETNESYIDNISSDEYEYEYDNNIMNKFFL
LNYYLKNIWKTNNTMLKKEIQHIIGKGGIKKIKLFFKPHMEQFYNFSLLCKIDSKEEPLKINFKMQVNKLNIK
CYIENYDKEHITLNLDNSLSIKKKKNKYDMIHIYEVINFKETIIGKKKMAKFVLENLCYFDLSYKYYLQKKDD
EISITCSNNIIKKNSFFYINIEYTSLIHHIYENHLTININDYYLINLKITAMSTNVLIHFSFYNYDFGNLILF
PNVDKNENTKVICMEENYRDSFSSSDTSLSSLISTYKINELNEKKMKKKKEMDLKIEEKRVQKGREKESEQKC
DKEEKKKKRQKKEKKHKVKKKNEKNEKNEKYKSENICGEQENINKDNLDVKNINKDNYDNNNNNNNNNNHNK
KNNIDNKVLEKNKQCNKKQNKERNDKDGNINNLLNIQHDEKKKNNIKTYVNKDENVATKQINRTNYNDTNNKH
TNSFDINNINYNINTVETKLFIHNNNDEDCDIEYEINESCLKINNDGKKLHIKGKSKTFLLISFTPNEEKEYN
FKIPFKINNDSQKIVYIYLKGSASHFKLPFSCNNLNEINFKDIHVDKEVINKLVLHNKNMEDISFNILNYKNL
CKYYLSFINFDNNTSHILKKKEKKTYEIKFAPKKYIKLQIPLYLNIYYKGKCIAYYLTHININSISYKVIIKD
NLLTFNKIDKLHEEHIINDNNSISEINHIREQYFKHLSNKNGVRGKKIQLHNTGDMNAQFQIIYPQKYEKYLF
LYPKKGIIFSFNLVNIYIYIDTDYINKDIYINDVYIQLYPKFNKINSKLALKLFVAVPSNSIKYTKSSYKNFA
KLNSENNDHGKYNNYDDGEDNNNNNKKEQIVKGEQQLTKKNKNIEQKDNVSNEEKNIKLFVLKNETIITFITD
IRKEICKIIEIYNETSSNFKIKYKFLYNEQNFFSIEKCEEIIKPNDYGSFQIVYNPLFIEKNKKNSKNYHNNL
CEKYVKYCSNIHHISKLIIYYPNETAKNYTLLGYSNNVVYEKNVIYNFNSKHLNNTSIRIKNWLNENQNLTIS
YQTCDKEGNPSNINYFYF*ILNQIDLCNKEEKNIAFKVLSLKDGVFYVMLILSNKKYEDVYKILLQFEISRSE
ILSIKNIHRSKKDIINEHIVIYNPLDENVEMEPLFDYNYIFVQKPIILEKRKNNIINIYFCIVKEEEEKHVII
SFTNKILGSYSFKFILNINMYDFEENFYFNTDLGSIQMNEVSFTNVCNEKINYDVMIEDFDENNKNAKQIFQC
IDKISVKPIDTSYFLKNGMLNNVTDKINLTIKYNPSDIKINKAIIKLISKEGIVYKGLIMGKSVSPKPKGPIF
CSSQKTTFLTFTNPFLHIKEFFIKTDDYFSVSIENIKIEGRETVQIPIKCQTTNATSENFLLNPKMILYGCII
*
>N.PF14_0420
MNEKPYIPIIEKLNNEKNPNFYICGNGYIAPLDIKNLKKISNTEKKNLQRVFSMMDKDNTGKISVCNLHDILH
RYNYKISKSEVERMIWEFDDNMDNCLDYDEIYFLYLRCVNDKKKQVPSDLYNIIQFFMFDYEMNGYITVEKTL
QILYVRFGREKMDQEVQEIFGDKYEDESGVEKQIFLKEYLENEKKRIRKYRSENPKKGGKS*
>N.PF14_0421
MEIDVKKKYKHSNILIRLILTIYIYIVMFLTLSFSLICQLSSLVIFFPLFLYSKKAKLYILGLCIQFGAYLLC
SFINPFWKLVIIRKSKKKYEPTNTILFINHLSSVDPWVVNATTFPWPIKFVFKSSLLKVPIGGQALYFSGSIP
LHFTKDKGGWGVKQGEVQKIMNICKEYQDMNIPLAIFPEGTRSVQGHLQLFKSGFFRFAIENNCEILPCALHG
TNKVWPLKSSLFDKGTIYFSFGEPFKPTPGMTYEELRDKTRMIMYEMIKEFPDYDPEVDTLAKEFSQARGHGF
*
>N.PF14_0422
MSVEEPYSEINIIKKYYNFKNVERKKLDDDEEDKIINLIVFFKDKIEIDVNEKYEYNIDDENIIKNILINYVK
TLKILIFYIPYNDSIKKKITEKGIYKLLFLGNYNKHVDIYQNKLRNNKCQNVEDGNNIFSLYKEEIDKYELIF
LINLITHNENNIEIFKYLYPFYFYYIMVFEKQEKDTIFMLLNNFIINEETYIYISKKEDFSFLLFLSVLDSLK
NIKNKAIICNYFYVFIENCLKNECKLFIHIYETLRILYFNFYSYSSKLEDLNFPLKKIKLKILTKKDNKYYLL
KYISLMCKLLLETLYNILDSKNLDLEKIDGYKNLYKMIMNEIKENINYINTVNYFDKYDDTHILKYYKNVLFM
DVFIYHKKKFVIENFKLILFISNIFSDRYEQLDDFSCNDKISFINIIFTYLKNIFEKRNDSIEQKKQIIYHNF
VLNRYIISLVANFSLDNTVSSYIRRNNGLDLLRKFMYIDDKDPCLSEYSILAIKHIKENENLDDL*
>N.PF14_0423
MTSEDKTALCAENILNDIYILTINRNIDIDIIQEGLLRKVMDEEESNSILFLEDSSFSKNDISILNVHLKIED
GKNKYNNYYEFKYADINVTIDFYQKECKYSIEKSNMHKCHYFKMMNKINYITEQKMDFKDSILYLYNTINDKN
FFKCPCWLNDNKNIKLNNIINDHQQKKKKKNLYDKSVQVTNDVTNIMVNEIQEQKMKKPIFFDNKNDLNNSDD
EFNSAHSSLIKINKLLNVEENDIFHNLKGHVYEEYFDKHEDNKNQKDNIESNIIVKDKKDRANNIISEQMKED
YIKNVEEVLSKHINNNKNNNVEDSVSLYSLDEEISEQIDKQHFFSIDKRQDEISNFIQMEENINDHMNDNINK
MGKKKHGKENRVVEDEKYIQDDKKKKRFYRNVNDNIPLTVQLANKGNEICKEIIEMNSRYYRDFFEEKILGCG
GFGYVMKVKNKKFNITYALKIIRLSNSKYNTQTNNKHINEKDNNSYIMEEAIMIAKLQHENIVRYYDAWVEEN

VDFFLYKELQNEFKNIKIKIKNEKMKNEKIISGKIKNENIKSENIKSENIKSENIKSENIKSEKVKNLYIEEI
KYFWNHYKKNKDPNVNDKYLYILMEYCPGKTLREAIDCGFICRNEKLIWELIKQILKGISYIHDMKIMHRDIK
PSNIFLQITDNILIAKIGDFGLTTRIGDTQINPSAGTIHYISPEQLNGEPFNEKADIFSLGVVFFEMFHEPFS
TSMERSITLSNLLKGIYPEYMKADNKKFQFLSSLLAINPQERCCAYNLLHESVLFTFEKDFTDIYNLIDNKRN
CEEVHTIISTLFDKFEYLNSDKILKKEDFSTFQNAKIFTDDLEMRKKQKIIKKKILISLKKRGAIFILTPIIL
LNKYYINLENTYMEEYNIYYNINKKKENKINNIYINTNKNKNIDNLIYMLDIYGNSITLRNSFFLSFAEYIYE
SIDSYNKYNEDNLFYKFYTYGYTYKNQIIKSNKHIKKDTINNNINTTINITTTTCNTTTGTSTNNNNNNNNNM
GNNNIGNNMGNNVSNIISNNNINNHMISNNPFYFYNIYPDENEKMFYCILSSSKNVFGNEELNYLSIFSNADI
MVSIYTLYNHINYFNKLLFVWSYIDLLEIILKECLDIPNDISYHLSIDLKKNSTLLVNKSFVMSLLQKYKIKD
MSKISDHILSLFYIKCESNKVDDYLNNIYNFVDDLLNKKSSLQLKRNTSITPKEVPYVRSRSNNISTSASKII
KYDIQKNVGTKEKGNQNDNEKKKMQIYSILDRIKKINNFICTNTVINNTCFDLFLNYEESIFCNEVIFYVICE
SKNKEIIAYGGRFDEIIRNMANEVKYKNHANYKYVYNNIKDIYNNNNNNNNNIMNIKAYGVEIYLDKIYSKVI
ESNEKISIHLQYNPSTDKSQLFKNFVTSCQQNDFACTSHIIQQPLLNDDYLNYSSTKILIQVYEISNLLIAYD
LSKKLLTKNISSYTHLSINNVNIKKKIKNFKPHKIQFFITIKSNNTDNSFDALKPINFDNVVYKIVNYDDQDC
NFMDQAELINYLIKYF*
>N.PF14_0781
MIEEENNTVHMDELERLVKNLPNEFNEKLSKCNNNLTSTFSQEFYEEKKRAITDILNKEKKKAYDDIENIKNY
VLKNINIINEKNEGIRKQTDILNNERKQNLTCIIDLENKIENIKRIIM*
>N.PF14_0424
MNDIMCEENKIKCKNIVSIYTIRMYLNGFIIGYIFRDDIYNKYYKHKKSNIFFHYIKQNILDINKNLHLKNLV
YIPKAYSFETYISKRLNCNKNRHLVGSTTKTSRLYILNKNLQVSNFVGFRLSFWTCFCTYSCWQLNNILENNI
LSPIISGCVASAFSKGIFRESKKSLIPCWMGCMLSYIIFT*
>N.PF14_0425
MAHCTEYMNAPKKLPADVAEELATTAQKLVQAGKGILAADESTQTIKKRFDNIKLENTIENRASYRDLLFGTK
GLGKFISGAILFEETLFQKNEAGVPMVNLLHNENIIPGIKVDKGLVNIPCTDEEKSTQGLDGLAERCKEYYKA
GARFAKWRTVLVIDTAKGKPTDLSIHETAWGLARYASICQQNRLVPIVEPEILADGPHSIEVCAVVTQKVLSC
VFKALQENGVLLEGALLKPNMVTAGYECTAKTTTQDVGFLTVRTLRRTVPPALPGVVFLSGGQSEEEASVNLN
SINALGPHPWALTFSYGRALQASVLNTWQGKKENVAKAREVLLQRAEANSLATYGKYKGGAGGENAGASLYEK
KYVY*
>N.PF14_0426
MIKKINGIFLTSKYYYKKYGRVNYSSLQLFQKNVSKYTDIIKMFTVNKNEKNMKCSYDNKNIYYIPLCNSFIY
HKEKDACSYIKKIKNRNNDNYKNDNNYYDDHKCIQNYCNDKNVGMKNDKVLLFSWLKNVRKKQYYNIIGKIGI
KKKNAGMYWSFYINKRKKIRKKRRTI*
>N.PF14_0427
MDIVWDKDTVDKFYNYLYDVINYEKCVELERNIRIETGTEDKLNLKNCLCYNNENCSEECNKINISSYKFKKD
EDDILGEIIDNEKEKENIECNIGLITQRVFSIYTNVIKKAKLEGTYNINLETDNFIRRDIFRLVFHKYILQNT
RNKIKQIQCKDTKNIISLANPLHIKDVELNKINSDTIANLMNNYIGIQKNFLGKQYMNLIYNELIFIEYNNQF
NEFNTEYKNIRTDLFCWAYITSLDREKQKGLYKLLKELSYLPYELNKKANLSLQLSTLFQFLYFSPNHSFLKN
HSDGGYDNLDNGKKITCIYIPSEYEHNQVLIKLYKNINTIQNNQSRNDTKIKNINDNISSNLENNKPIQVIKA
EGDSLILLQTRNVSYEISMSKEKFFMVNLWIPGPVSVDKHM*
>N.PF14_0428
MSISIYKSKVFNSKDIVDISIHEKNLKVEPSSFKDCFYRLNDKRINIDELENKIEYHLLNDEISNNNNNNYDN
SVKKEYIVEKDQNDEKCNNKKTNVFDIKLNNDLNYNMHLTSNNLEEIKCLYFFFLINILRFKNNLDLNVIRSI
CTHINNISNNDKNNNQYMCIDKIKENCLYSLKLKDFFVSVLKQCGKNEYNLNDFNRSIHIVIEKSSVIFLNTY
KIVSSCKYLTCCFANLLEIFNLNYDILFMNSFNNNINNSNIKSINNIVSNLKWMLHDSKIEKNSNLSKKFSSN
ILLYIYTQSKLTDECEYFMNLINQNFKNSIESFTHEYNEEMTSIDTYINILCNNMNQTFYIYIDNLLKMCKNI
MPLYIEKLNEFLLSEEKIITHNVNFKKPPVDGIILKDMEKQEDEHKGDIKLNLVNIYEKNILDKIELMFKQIS
EQVQKNNLPNSDIICNQKENDNTPKNQYRYLNNSPVEMLKKLNCVFINVTHLITDIIMHLNILNDIRAYNKAI
SQHMKQKKVSSCVHNVGVGCNEFKNFLYSFLSSENNILLNKSYVNKDKKLVTSHFYDFLEKYFSPYKYEEELN
EILLPKNINFNLKTAKGCKDFTGEDMQLRNIFFEYIKKKFLLHGAVEIDTPIFELKETLMNKYGEDSKLIFDL
KDQGGESLSLRYDLTVPLYRFINTNNVSHLKRFHIGKVYRRDEPSMNRGRFREFYQCDFDIVGKFDTLRTDFH
ILYIFWDILYNLRNVLGNFKCKLNHRKILEYMLLSCNIHKDKVKTVSSSIDKLDKITFQQFRDELLNDKGIHI
EAIDKIEQYISKTLSLSPFLVIEFLRNDLKESTLNEEYKLEIQNCINHLEQIFDLLKHFNMLNQFSFDLSLAR
GLDYYTGIIFEFVLLSETNVGSVAAGGRYDFLIRNKRREYLPSVGASIGIERIITIAEEFIRTKNLFCKTKDE

TTNKDNKNNHQNHVNVQIDENVQNKKKTNISNKEIKPKSSNNKNNSNNSNNNNSENSNKLNMKDNAVDVLVCN
IKKDCFKEIVELCTKLWENDISTEFTYVKDQKIQKQLVYALEKQIPLVLIIGDEIEQGIVKLREITLDKDKST
GEKEININDCIQEIKKYFTHNCKWKQNITNILFEKKQ*
>N.PF14_0429
MKQTSNMNFKPLKIKPSLLKELKKEGLISIEEAEGNNVKILSADNLNSHNIHYGNKCKNVSKNNESKKKLSLK
RKKDSTKRENQDDTTVGCISREDKKIKIEESLQNDRLEHFDINDNNFIEKRKRKRKRGKKKKKIINSNMQINT
TDNNLKKEKDDEEEEEREQEREQENEEKRGEKKSTENVNISNINSSSNQNSNKKKELFDIERIIKNEEELEKD
NNIKYKIHCNNWNNGGKIFILHSVMKSLFDNAFFKPTEIQSKTLEKSINDKNDIVVISKTGTGKTLTFCLPIL
NNILINKLKEYKKKLKNIQKFRCLILVPTRELALQILKHFNYINKYINLFISTIIGGLNLNKQKRILMKKPEI
LICTPGRLKYFLHLENPIKYIYQMKNIRYLVCDEIDKMIEISFMKDISYIAKHIYKSVGDKKKKLIQTFLLSA
TLSLTVQLQNDNMTKLLNSIIIRKDKSFIINLSNEQNVYNDNSNILPELLTLYIVKLNERDIVCKLFYLIKSY
FSYDINNNNHNDHHDNNKLEHPQDTHQNDEINKIIIFVNTIKSAKQLNAIFKHLFLHNNLESSIPKKYRSNLY
IKNKVNIYSIHSKQKLKERLENINKFSQQNHKAILFCTDVLSRGIDLDKCDLIIQLNCPISDITFVHRSGRTA
RNFKKGKCICFITETEIYKWKTSLEKVGINIQDLQELDYLKSINEEDYAKINKAIECANKMIEFQDKLILKKK
DSLLKKLAREAELDDEDEEEEERRRNKFYSDSENEYEHLNNQTIYKNILHLKKELYNTLYKQ*
>N.PF14_0430
MPFKSHQLIMKNCLTIFRSFNTYNIPSISNSSRVHRSYRESNKTHNDDFSKIFNKELVAYPYEEERYKKLLNI
EDNKVKRNIANFFFNRNMYINVTNDNKIRKEMYSSDFCFKNVDKYFIFEKEYLDNLFPEGLAGELPKDILMHT
DNDENFHIFMNELNKNSNNNNNNNKSNISNVKGVGLLYRKQTYEIIKELKYCQDVYKLKEERESLGLDNYFLK
KGDNVSCYYDRYRECESVQNDTSDLIEKYITNNIKENDDEINMNNSNNRNRKCTSLYQSRSIFIDGKRGTGKS
CILNSVVLWAKLNKWFVIFIPDVKKYKFDINNIVRSNTNLYIQPELSRQFLEDIVKINETFLKEILVNKNIIN
NTCLDGTHLLYNKRIYENIIQKAIETEILNDENYKNLNENEKFKCKQKLYKFYYDNIKIPNLLDKFALPTNLL
ELSKIGINNSAYSNLCIYALFEHLKKQNQFPLLICLDQFNYNLSVSEYLSINFENTKYNGYIPTYYFTIPKLL
LQWDTSKYKRCFKIASTCWDRENRRNFQPELLGIHKKQIKIIRNFTIREFKNYLAHLYNQNVIYNFDINKLEY
FYMLTGGNGFQARKLLTTLY*
>N.PF14_0431
MTELNIIDNNEKHSKYKLYKYCRNYINDFKNKYMMGYSSNLYKNSPNKYYYDTRNNSNNNNNGYYYHNYNNGY
QIRNKRNRSFETVSNSKNKHKISKKYKYNNEPHESFRDYDYKSKPSYSSVKKIYDREQKRYNKMFVESRYNEN
HKNKYINNYNLSRKRNYYSYKKKIYTDRRNFDTHFGQGNIIYNNEYYLINDGSSLNKKRLHYAKGSFRSRSRS
KNYNTYNLEKSKKMKYSSRHNIYRYHNGNKNKSISRWEDNYTYKDIYQPSSSLHKYKRRSNFTRDDNSIKSKM
YYENKKTDHLYSYEDKYYNKYDDKYDDKYDDKYDDKYDDKYDDKYDDKYDDKYDDKYDDNYDDKYDYGEEEHR
QEYYEKKKISFNNSTNNKSSINYDDVKRELKKRKKKKYSNESKVYDSLKRDETNHHTNNNSYLNEINKKNSNL
SNNYVAVRNKKRDKEYISDSNKSGFSNKGSYYMQKKKLHVDKYDNDSHNRTISRTSSDNYSRKKYTHKRNRTK
TSDTEDKKERKKKKKKKENNESDDEIVHFSWKKGMLLNNAFLVIRKMGDGTFGRVLLCQHIDNKKYYAVKVVR
NIKKYTRSAKIEADILKKIQNDDINNNNIVKYHGKFMYYDHMCLIFEPLGPSLYEIITRNNYNGFHIEDIKLY
CIEILKALNYLRKMSLTHTDLKPENILLDDPYFEKSLITVRRVTDGKKIQIYRTKSTGIKLIDFGCATFKSDY
HGSIINTRQYRAPEVILNLGWDVSSDMWSFGCVLAELYTGSLLFRTHEHMEHLAMMESIIQPIPKNMLYEATK
TNGSKYVNKDELKLAWPENASSINSIKHVKKCLPLYKIIKHELFCDFLYSILQIDPTLRPSPAELLKHKFLEE
NYEYY*
>N.PF14_0432
MEEITKNKTVDKEESIETKTKKDVGTESVKSIDLPIKEFQKSFRLLNENFNIKDLENVLTPIEYAEYNSFLAY
SICSIFYSYLKLSGDVLTNHPIKNELKNVQALMKEIKEKAKTNVEEKRSLTINKEASKRIIESSISYNKHIKK
NKN*
>N.PF14_0433
MTDIQIQNQTSNFNTSKKEFENRFDDKALRNLCVKNIPKETKENELLEIFQPFGLIESIKLKVNKNVGPYAIY
AHVLFSTPEEAKRCLKQMNGKILNGRALRIDYKRKKNPGDNPENINFNNYNNNKFHRKINRTNQFHNNRYNNN
KRNIRNTNMV**MMI*IVNMIRKIVTL*IMRK*TRDLGLEI*TT*KVVI*IRPLS*IN**KIILRKKC*ISYL
TTIQQMKLNKLYKHY*II**IINQQG*NYIYVII*EHVQNMYLIGLQYI*VIITEWKIVQIKICIKIIIIIII
IIIIVMVNHYYIIHQIK*IIMIYH*IIKIFFLFLKKKVYIIINLMYQQITMNIFGKVS*V*RIKKI*ILLGMH
YKEM*TFF*MLTLQIL**VIGKG*KVYLKWKLYIILKSKINKMKIFLILIKIILIRKIELD*YQQMKIGIYI*
YFQEVRYLINSVIIII*IIYL*VLYVIILKS*TREVLYLLRDKIWMHQTIITKEIFLAIII*
>N.PF14_0434
MATKIRYSVHTTNKFSVFDSDDNSEEEIENKDLNESPKNEPDIVYEEKTIDNSNEVNKIIEVHDNIPSPIIKD
YYNKRVNVHYNDRYKYPVTSINDVNENTYRSYRDNYHIRGNNLRNKFTRKRGGGYSNASTNILGGSNFYRGSR

NYDYKMNMDRRNYNPSYNYNKFDFREKMYNDYNDNTSKYDSMKRDNLIDFKNKNYEDSSKAGRKYKSVTIDYD
LYRRQQEKKLNSNKSAESNDKKKKKNEENNTEKVNKVTTNVPKRNDTLEEDLKSDHPKRKAINVYQYILEEGG
RVDRLPGFRRSYRSFKKVDEGNWNNKYEKSKMQIDEKIFKKRDPPNINDTRAFPSLTSK*
>N.PF14_0435
MFIHSCNFYLFLLFYFIFINCSYIHFRKEKSNMNNHKNYCYDGSIKDACIHVKENPSSYDNNNIEHTNQIWNN
SPTNIINTKKVSNVEQNLQKDTKKYDLHDRNYPNIIKEEENIFLLTCKKFVNFTIQKRFFVVLSILIGFLLLM
LVSIPLYETMISNKIETSFVTFDNSLKYIRNIYPLTFEIKKAGDVLTQSSDKLGNNTNHFINIYKKDKTATLM
FFSENNNNKENHILDYNTLKDIFFLLQYFKQITILKDQKEIYWKDICKKYDTPLSNPKCFVLGLFTISELTNI
NYNNIEKWNVFFDKIIKEDTKYIKRFFSQALYFLPNFLYIPNHFIYKIEEQQNMHNIINKIYTNIKGLLFVYT
FDDNISNELLDNWYNKLNEYIQLINNNKLSYINIKNPDGTIYTHILKYNKMWNVLTINDKLLQDEEQNSILLG
FQSNYLFIILSLLFIFFYININLSTFVTYTKKIVLLICVYLLTFFSLSSTFFIYLIFKLYIMRIFLLNYFVLF
FLSVLFCCVNIFYYNQYCTSPGKDSHNNNIYENLNNHIYNSSYPISDNHMETFYYLQATYKSLYFNGKITLVL
ISIYTIGLFCSYTITKWFCLNTIFSLISLYIYYVFFFNNIFSYLLYVKGKNNNINYNPNDQIKVIDYSKVNNE
EDKKEMIIFFNAQQNVNNNNCNNIEENNYPFTNNNIHTNNDILKNNDILKNNDILKNNDMLKNNDMLKNNDTH
MYTKKCVTNHFDKMNTFQNNTQYTEHKMFNKPKKYNEKPFHIFKKISLLLLLILSFLILYLYIFINNKTQFSI
FRYMNKNSNVRMFIEIFEGIANHVIEPGYLVLPESFNYEKEDNLINVVKLIEDLKKEKCIYNPIISWISTFEL
LKNDCTNIDFFDTQYELDNNSEECINYNLQNVGKKNKKLYLELLSAQFCKNIQKPLCDTFNKIIYNWIHHKND
DIYESHMFQIKTSYNDNIDQILPQYHTISPHIFYDKYIKMDENYNILNSRIGSIFHNYPSSYNKNIETIEKIN
NVIKNSNIENIYFYSETYVLYQQAMKFLKEFKFMFFFYIFIYIISIYIFNKMGVPIIFPFLLFNSLSMLYFTY
FFSINTDAITIILLKITTAISLSNYLYSTLYFNKTKNGTLHFDNTIKKSLPYLLFLILYLISFSAGDYISNVV
RLYILNHVLWYILYSLTIFCIHRIMYK*
>N.PF14_0437
MRGFNYNNNSNNNNRYSAYPDYNNSYGNYQAYGQQYRANYGTGYVPNYNNNSNNYGKNLAPIDWKTINLVPFE
KNFYKEHEDISKLSTKEVKEIRDKHKITILEGENVPKPVVSINKIGFPDYVIKSLKNNNIVAPTPIQIQGWPI
ALSGKDMIGKAETGSGKTLAFILPAFVHILAQPNLKYGDGPIVLVLAPTRELAEQIRQECIKFSTESKIRNTC
AYGGVPKSGQIYALKQGVHILIACPGRLIDLLEQNVTNLMRVTYLVLDEADKMLDMGFELQIRKIVDQIRPDR
QTLMWSATWPKEVQALAKDLCKEQPIQVNVGSLTLTACRSIKQEIYLLEEHEKIGNLKSLLQRIFKDNDRIIV
FVETKKNADFITKALRLDGMPALCIHGDKKQEERRWVLNEFKTGKSPIMIATDVASRGLDIKNVKYVINFDFP
NQIEDYVHRIGRTGRAGSHGASFTFLTADKYRLAKDLVKILRESEQPVPPQLEKISYSMGNNQRRNPYYSSGR
SNNVNNIPLRGNNRFY*
>N.PF14_0438
MENESQYKNINNVDKTIIPFQNTTFLNIIESVWNLLNVEKENSIDLFNEQIYTYLNKKAKVIFENKLLPHFIT
NAHNENILQNILFEKRINIRNKTHKNINELKRITNENDLNLLIRNNQGMHSKYHMDRKKCRDILLISKQAFFK
IITNIVCNNKKVITSSNLYNNTIYKTLLYILKNTNNEINKKIYTYSFYEENGIIQKKTYMNKHQAKGILNTIH
KNSTYENIEKSKHFIKNDYLLKEDYDHIKNSYTLNDIIHNSIIQLSKGKDLHYEHTREKNNKQIKDYLDKNNS
IKYAKENNISGTQLHERRTLYKDPVYILKKFLERDRNILKYEDMWKDIAQYTFQPNIHKYIDIEYNQGIKKKE
NEKIKDKQLNLKNIFKNNEHIMHNNILTYMGNDEFSLLEKNEKKKISDEKYLELIYQFYGTHQANNNITMIRK
KWDDESKPIHRISRYISRKNKILNNDVVKNNVLYNSPLGIPKEKFVNSLLSNCEKKKKWQNELRNGYHRNNFN
FSLHDEQEIKKECDERKKRINSYDWNKQIRLYERGLKTKVTQNYELYNNIISTNMNNTNNYMPKNMNNTYNYM
QKNMNNTNNYMTTNIHQYENNYKASNNTLQKKKNNNFDKINMCPLIISYNRSIPTPKVVTIKSTQDDILQNIK
MEILSLPKSVQLL*
>N.PF14_0439
MYFSSLCKFLPISEKEKIYLNIVKKRFCKSNIYYNNNNNNNNIINYNKRGLKFYPFCNNLKKNINFVNINNKK
GINFHSINKERKMASEVPQVVSLDPTSIPIEYNTPIHDIKVQVYDIKGGCNVEEGLTIFLVNNPGKENGPVKI
SSKVNDKNVSEFLKDENMEKFNVKLGTSKHFYMFNDNKNSVAVGYVGCGSVADLSEADMKRVVLSLVTMLHDN
KLSKLTVVFEINVDKNLFRFFLETLFYEYMTDERFKSTDKNVNMEYIKHLGVYINNADTYKEEVEKARVYYFG
TYYASQLIAAPSNYCNPVSLSNAAVELAQKLNLEYKILGVKELEELKMGAYLSVGKGSMYPNKFIHLTYKSKG
DVKKKIALVGKGITFDSGGYNLKAAPGSMIDLMKFDMSGCAAVLGCAYCVGTLKPENVEIHFLSAVCENMVSK
NSYRPGDIITASNGKTIEVGNTDAEGRLTLADALVYAEKLGVDYIVDIATLTGAMLYSLGTSYAGVFGNNEEL
INKILNSSKTSNEPVWWLPIINEYRATLNSKYADINNISSSVKASSIVASLFLKEFVQNTAWAHIDIAGVSWN
FKARKPKGFGVRLLTEFVLNDAL*
>N.PF14_0440
MKVLILFFIFSFFHIIYVLFVYASLNIKTRNVFPFDVVENNYPHFPQNISFVEENVDYKNYCFTIYYNNKTNE
QKVTKNITKESITRVNFISYNTVQDIQEKKQYKRNIREQKKKNEKEKNMNNSVKTKPNILIWKIKQNMNKIYS

FYYLRMFFENHINYNNIYFGQYNIYPFNNDAMFIQNINSNNIYCKNGYHFIYKTNKIENTIENSPCGTHKNFF
LVYELYEHQIPKYLEKIYTFKKILRTPKENIPFINTYEIYISSCDKIKECDLSNENYCSSYHEKIYNNIHMKY
NNINLYKNSKRRKKKKKGDKENDLLDNKKVKNKEKSTSDKELTNSLPSRYPCVFCMKKGIKYDNKYINIISKY
VKYKQLYNENYIHKNNKSSITTCSFPTKYKCTFSTLFMYTSYEKYNKPSEQINNTQGIKNINEKLKKNKFTKK
NINKKEKVLYIYDHLKKEVIIYLIVSYSKKKEFDYIKEIYDKNNTNDAYENYTIKSEPEFVSPKKQNINGMNN
IRENNNNNNMKHMINMNKVKNMNDTNYNIYNNPKNTHGYIFYNNYYPYNKEIYTNKCHISDLHNCTDIYETRD
GMCYYHNINNSFMYEKNDEKLVCPINTKSEIMKNRIINFEIINGNIRNELHYKKDLYEMDIENEKGLYRKYKN
NKRRLDILSPLHMYIDLFLKVLNNLYFWLFGRINRYKTVLYEKYIKKIQKNKNTYIYEIQDKMSSQVMLEDTC
TCNNDDSYMYDDKINNKQIASNNNNNNNKSIASHCLKKCNEENIKNKRSANDNKNEKSYYYYYINYNNSNYVF
EVSSNLGLLGDVYMSEEIVYPFYPIPNDILINNMKKYIKEGKSFSVYVDQLRPDIFNCNKFNKIKWALILFFI
PAWLIINIVYIILVQKMWRQMLNPLHRLLISPSLIRLSFYILLLMFCMQCPYVNNNCVEYTLMGYMALNTMFS
TIFHGNLLLISKGYMITRGNFNKKESLYLTLIISIIYIITSINQLNIINSTPILISLYIILLFILVYNILSII
NFLKLKLSFVRDVGIDAWEESIRIKLNMYKVYLSVIIIFFCFEIILHTLNMLFKSSSGNLTLVEYTIELVMWC
CVLYIFRYRGDVLYFSLLYDNITLNVIPLYIANSTKIIDFTTDKDTKKKDKHLPFNIPIFILNPIEKIDQYFL
SRMSVGWPINMTLQLEKVSISK*
>N.PF14_0441
MGRKRNNIVNIRYNFFLIYFWVIFMYPCETNKGNVKPLNFIKGKNIFLNITKNKVKHLNTINGIETISNVENK
NILNDTNYINEMKNIKVRRNISEALHMAIYEEMKKDKGVYVLGEDVGLYGGSYKVTKNLAHFFGFSRVLDTPI
CENAFMGLGIGSAINDLRPIIEGMNLSFLILAFNQISNNACMMRYMCDGQFNIPIVIRGPGGIGKQLGPEHSQ
RIESYLMSIPGIKIVSCSTPFNARGLLKSAIRDNNPILFIEHVLLYNYEQEIPLLPYTLPIDKAEVVKNGKDL
TVLSYGITRHLASEAAKELTKFNIDIEVIDLISLKPFDMETIEKSLKKTKKCLILDESAGFGGIGAELYTQVI
EMFSSYLITKPIRLCTKDIPIAYSNKYEDACIIKKEDIVYMSTYLHSLSS*
>N.PF14_0442
MILHNRKRKNYDRKNDERNEEEKKNIYDRDIIYEQIYSLPKKNKPRCLVISDYKNNLLRYFSNIVKVENYGSK
DFRSKWYIHFYKICLYNEKIYGGVTGGNKKNIDKLIMEYNVEDVLRKLQTLVVRRLDCENLYYQKILVITCLL
FDFFQNKKHVNIKSLKHLEGEEDDTKNTENDPNVEDDEEENDKKNNIDDKNIEYLNGKHIKDKYNKKVSKKRS
RKNSSRSNYYDEYDDSIYSSNKDEDSSEYMMANRRKDHKIEIRKDHPGDINYHKNYYIIEFKNILYNEKKKFN
NLEIFYHISNFIIHRYWYSQEYSTYNIFIWFLNYMHSKLEIRKSIQKINEQLEMFINIHPKIFNISHAKKKRK
KNNNNSYNTNSTGRYFENGVLDYPYIKNRQKQIGGGFSDMADVEKKNKQNLPNLNTFGNNVMNNHSFFNNTMN
NINNKNITLTNNMNNNGTTNGLSLNSFNSPFKSFDQSNNIFSSKNGENSLFKKTITLTNNLSNNNNNNNINSN
NLSSSSLFNNSFGNNTSTGNNTAGSGLSKFNLGFSNIQNNNNNNNNMNAFNVGMNKTNIMTPGSSLFGDKSMLS
NNTSNVINMNNANNINSMNNNLLLNSTQNKQVNLFENNSNNNNNNMSNVNKSTNGLFGFEKNNFNSIFGNNSN
KEKTNIFSFNSNQSQNTLFENKQINNISNTMSNNKDILGNTQNNSVSNNLFGSNITNVNSNMSLNSKSLFDNS
MNNQKSNIFSSVKPNESLFSSNNNNNNSNSSTTVASGPSNIFGAPLSNTQQSPFSKTTNNTFGNLNIDSNNNN
NNNNMIITGNGANNNTGLNFNSSNITNSVTQMNVNNQTFNNPLNNVVQNDNNLINLFNNENGSKELFKDLKIN
NDSGPKFTVNKKPLFSRRATKNSTLSTNTLNQENNIFSSSYMNKDNLLNNQEKPLKVIFGQNNTSNENVTNVN
TTINSSSVNNTTLFNAPNNNNNNNNILSTNVEKNLFGQTNNLLKGSSSIMFNSASISGTTNTINNNIDNNNNN
NSSSLNNNSSGGISGGLFGNKNLFGNTTNLFGNNNNNNNNNITNNNSDNVENNMLFNCNDPNNNTPNLSIPVK
KLTLEERKKKTSKNSMLSLFSNNNNNTNENQSADNTLNDLSKNFINNNTTNLNNTSSLFNFKSNNENNNDNTT
QTTSTIFGFNTKDKQTTWGDNKITFGLSNNNLLANQENKETNKNTLDNVSSTNTTENKPAKKINIFGSSGSLF
KTTEKTDEKTDEKEDDKEKQKTSEENKIKNIFQFNTKDEKNDNKGETKDNLLSKSDVNINKESDKEKNNKEEN
VVKGTIFGFSKDKDSLFGSSINNDDKSKINLFGSTMNDGDKNKTNLFGSLNKDDKDKPSIFGFSSNKDDKDKA
PIFGSPSNKDDKDKATIFGFSSNKDDKDKAPIFGSPSNKDDKDKATIFGSPSNKDDKDKAPNFGFSSNKDDKD
KAPIFGFSSNKDDKDKAPIFGSPSNKDDKDKAPIFGSPSNKDDKDKTPIFGSPSNKDDKDKTPIFGSPSNKDD
KDKTAIFGGSTFGNNKSPMFGQGILNKGDNVSVPVFGNTVTSTDNDKSKLKLFNGNKDKEETKENVFISKDNI
NNNIFNNDNMKYVLSVTKVDDENKSKPNINYDDVLNRKRRRNVDDKNETNSNADERDKDDDRYGRKQVGKGFN
VNSSNVSNDKNNLKNKRNENNMFNNDEDLFLKKSKVGNALNATKNMLETLKYDNIPSKDILNESIVNKNMDTS
ENVIVEHNVLNASATFLEDGKNNNMINMNNYSDDKDGFFSSSQIGNKISPIKDATIKEVNINNRDMNENNSNT
YLTHNNNNNNNMMVMMNNVKNDEGNKMHLINKQYIKDKGLYDSLYDNNDYENLNVREYLMDINNEHNNYNDNI
RNNKKGINNNNNNNNSTYVNKKEMDRIVQYNKKISFDDIVVDLDKDMIDLNELRQNLFLCAINFHLNYWAEKV
ILFYPHNEKINKYCTKLIRIKNEFDDTYDFSLFEYIHEISRKLLKTLDRNSCIYESVVFLSAENLYVLKNAQL
SLFEAFIIYHFWYQKNSVDIKKNFRNFLYTNKIYPCYVNYYMNMYKNYKSFNGKSAEFNKNMSKRIRDNDHMV
SNMNHMEDANHMKDLNHNNNGDIKTYQGNFLNVENNNSSRHSSKGGSFLLPPSSASNGNSKYSVVNLKHCSSA
SNRTHASDYSNNRSEESDYSSSDKKGYKNGNMNTNSNSSYDDYTSDCNKSQSNDYSEDSQRGRENKYDVYSDR

RNRNKDDNDDNDDNTYDEYNDSRNTYSYESNSDESNSLNKRRRKRKMNNNRNLKKNGTKYNKYKKKNVNENSL
IDILNSNSSSSENSSDILDNNSNMDEPLNMNIKEKYIYFSKEKKKKKKKLILPGHIKIKKLSACECVLLELLL
KNDILNVLRKYKIFKTEKYEYLYVNLLAMLKHYNYFSTIESDENINENKEKYHKNMTNMKQNNNNNNNYNNNN
NRNLKRISEHLNSNCIINTKNDKINILKKICDKNLLYYINMQLKNISHLISYKEIEIGCTYLNNIKNNLYVMF
QLPIILYNVLFEKTINSFNDYIILDNFFSNSCVYKDFRYEYIFKTKIAYMLIKKFYAADDIENTIRFAIYFEE
CKNKTQKNNIYNDYIFHKNLINSYIKVHEHIVPSEWFHLQNYNTGHKNSGSVSNSRSDSISGSNNLNDMNDSE
YLDYYSFFFKDISKYIKFHIIEKIINSNIYGLHKEDYSINGCNKSDDSSMINNKPYNIITSNNYNNNNNNDNI
HYEERHFSCYFLYMVNKSILDELVNIYSLKGEYFYKRISTCLIKSSLVKDYEKSQNKHFHMYVQLANINHFMK
NHFFEYINFFKKKELYHIEKTKNEDEIMENLTNVIKANGKKIILVINTLVEIINKIEREIIKDINIFVTINFR
NLIIDTLYLFKYIFNNKEFSLSMVDDLYRNVIIFNDVINSTWKDDVHFYPKEQIKELYTHIRHKFMNQNMIN*
>N.PF14_0443
MTDNTAVRRRYEKSFTERPGFTEDEIEEIREAFNLLDTDGTGTIDPKEIKCAMQSLGLDAKNPMIFRMIADLE
KDGYSSIDFEEFMEVITSKLGNKDTREGIQRIFNLFDDDKTGSISLKNLKRVAKELGETLTDEELRDMIDRAD
SKGEGEISFEDFYTIMTKKSFL*
>N.PF14_0444
MENISVLTNNNMSVKNLNPYNNNDEEKQNSDVLNQERNSTNDLFSSFYEKFNDIFLFIEDTTKNNNCLEDEVD
DNLIKTLYNLCELFLNYMKNINDFANFKISQQDQELIIHSYENFVRQCKEYNDKPKVKQLENEISNLLDLLFN
NTSEAVNTLFSADSFFMDHVENLKLKMSEKNQKIQFENERLKKLELEKQVEQFDESFEANKKKNNEMDETINK
LKKDINKLNEKIEKYDNFMKMKRKEIDIDFSDTLDCKERIKLLENCIKTTERNMKTTQLAPLKIVENCADYII
FEFMTTRNLMRFKVSNYESDNVHIEINPYEENILAYINKNVKKCKDISEISYLIKEAIYLNFLDL*
>N.PF14_0446
MKNRFYYNLIIKRLYTRSGGLRKPQKVTNDPESINRKVYWCFEHKPVKRTIINLIYSHNELKIFSNLLNHPTV
GSSLIHELSLDGPYTAFLPSNEAMQLINIESFNKLYNDENKLSEFVLNHVTKEYWLYRDLYGSSYQPWLMYNE
KREAPEKLRNLLNNDLIVKIEGEFKHCNHSIYLNGSKIIRPNMKCHNGVVHIVDKPIIF*
>N.PF14_0447
MENDKLINKKKDSKEETLDKIEKSFRFIRKALVLTIVIVLLVIVASVFLILHFINSAKEGNIFPYGIGIYTYK
VLNTYDHIHDPLVGKHIKNENILNIRRDDKHFPFTQGLFFSNNETLIESTGLYNASYLREFDLLSGKTIRFHN
LESKYFGEGTTLVIEPSTFRKFLFVLTYKEKKILVFNYETFELYHTYYNELDGYGLTSNVDPLQSKEDLRQNN
FPLYQKLWATSGDEYLYELDIPPNLKDTDKLSVVNKKKIKCAGFHIYRVNELEYHPKTKSIFANIFLTDLILQ
IDVDTATCLKIIDLKGLIERCSNYKQIKNKLETVLNGIAIRPESRQDELPTLLVTGKLWSNIFEITFVRNKNA
FAPNVFLKEYYKTIGNKI*
>N.PF14_0448
MEDRGGFSRGFGRGVRGTRGRGGRGARGRGRGSAEDDLKNWVPVTKLGRLVKEGKIVSIEEIYLHSLPIKEYQ
IIDYFFQPNECSHPLKDDVVKIMPVQKQTRAGQRTRFKAFVAIGDGNGHCGLGVKCAKEVATAIRGAIISAKL
SLIPVRRGYWGNKIGDPHTVPMKVSGKCGSVRIRLVPAPRGTQIVGAPTTKKMLNFAGIKDCFSSSCGKTKTK
GNFLRAIFNALSKTYGYLTPDLWKVTNFDKSPYEEWSDFLETYQNLKGIKGTV*
>N.PF14_0449
XXXXXXXXXXXIYNII*YNII*YNII*YNIIIDAYIYYLYV*YYIYIKKKKKKFIKQNDYTYIYIFIYTLLYF
YISCLIFF*NNITSFNLLLKKRIKQMNKQTENTQDKEDDRYSFQDEIADFGKSWYEEKLVNDIPRKLTIF*
>N.PF14_0450
MNEASSDALKYSDFTLSKYGNKRIRINKPEDGTKYFVSRFSDNKELNKLTSPITFICKDEDTDNGSSLNKNNN
NSTSWVLKNASIEKMRINHKSYVSKKCNLDTDVFFVLIENEEYSDIYPVSSWQVFEPNLSSKTLNKFNVDNSE
EKLRTQMDNKRIDDIIERLKNKEEQNKLNIKAKEEKKKKKKKKNILPSDSSEDLDDDDDDLGLKRQEKRRIKN
LIKLKRGENKEEIEYANSALSITSLRKSKVNWDYNNDGRKSDDEDLNEEVSGQEDFDNDNDDNDDSENNSDND
QYKLTSYGQAMRSLLKQQVNDEEDDELNQYSDDDDEEDEEDEDEEDEEDDDDDDEDDDEEDSDDDKPSNKKQL
KNNKKIQNEREKDKYKLEKNKKNNNKHFYEEDEQEDQENSDDNKKNTHDDHNEMKKDTPKINQEKTQEELKRR
LSTNDKFNKFNEYIKEKIKNKEIQIKEDNCAKLIVKIVEKNNGKMNIMTLLDTLNIREKNDNFLIVQKYIKNL
CNISSETVNDKKIKTITIKPKYLQKK*
>N.PF14_0451
MAARDPGPYLNQVPLGFPSYNRRVFRDILARRAFMESEVNTRVYKNIFENLGFKGHIRINNKVGINRIRMRCI
QGGYSRGIYKFTRMAKMAFFQTAREGWLKKYGYRPDLFR*
>N.PF14_0790
MVVPPQKLIVHYHHCSIKDIGDIYINYLNVQLFFLKNVLNCSFLLLVEEIHPYSNYGSYPYAFNTLEGNTLND
VEIIDYMKNIYLFDLVEYDLYAGIINELKIILTYYIWEDDKIFNNFTKKIYEDKFFYIYYLYLIRKLKKENRK

ICQERGLDNHKFNISRLKTILHILDKAVMNSNNSDIKSDNVSYFHSLCFSILSIFYSIPSQFNNELQDILLSS
PKLIEFVKNMNDKYKIWKNEKSFLMGIRNAYHNR*
>N.PF14_0452
MNLFKKSTCSACNSCNCCCERDEVKITERTYTYNDLDNLYDNEGSSSFPYIPTERIILNSYNNPDLYYHQIDT
EFYTKNILYDVPLVSYAQGPYYERIPDMKLNEKYRLPTLSYVSDPVYIRRREKCTISNFPRTMCWAKCRSS*
>N.PF14_0452a
MKGIAFRLFVFVLFFYITNYNVIYGQMCTSDICQKCCHPTNDGCENNFYKTIRGNYYTDRTYCQQCDCKNPET
GCGWIAQKYEKVNCTSCSFLKHASIKVKYFDTCGCGHKLKNGTLRINI*
>N.PF14_0453
MEKYREFADASTGINPFLPVWVNKKLSFQEKLLKLLLFPIVVMRLCFLSLTLIFMFFLNSLLKILIFQCIKDF
FYQIIQTIYCRLLLFYLGFLYLDEQYANNKRVKIKCTKQKIPFTYDTYGHIFLSNFTSFVDILYLSFRLNPLF
IVINKNGSLSPVCFYDLIKLSLKFSIPNKMGIFKNIEQIHNYARTHKIKNVVIFPEAMKSNGSCILLWKNDIF
QNSDLVLRNKCNIITFIYDEINIINKKLNHFYTSPHTVFHPFIHITFLCFNIYNKIKIVWISEKDISQAIKEQ
SFKNNDEFVYYLRNIMGQMKPTGGTLVNVKSEMLEKFVNYWNMTRKKAYL*
>N.PF14_0454
MADETKIGKEELRVWIEDTLKRKDFSFNCLKDGDIYLQLFEYIWPKVMKKYKGRIIMYPSSDNERKENWKVIN
IVLKKVQLEEDFIKYNDIVKNNFKPCYESLIILYFLYSLVRYHECDFILAHPIDQKLTDFMSSEKPLTCLVKA
GSVNLPKNVFENFGNMSQWEDLHVIDDDKGENDDRGKNDDGGKNDDKEKNDDQEKNYDGGKNDDKEKNDDKGK
NDDKEKNDDKEKNDDKEKNDDIYNEMYNQFNNILSTSHKKLDMQNNVSFKKDDIKKDSSMEHLNLIYPINSHM
ENTFDKYIKNCETYTIDHNKEEQQNMMDPIDSNSNIKMEEVSKEYKKKKFFLEMNKENSEELDTRVNRNYTKE
EIKYDKKYDQSDTVVGYEKCVNDDTISYNKHFSDNILSEVTWNRFSSNISCYDDNKLYNKGLHNNMSNIYNDN
MDIKTKNSKEFHINGIKRKSVSHNLSNICTSPCSEFFKYFRLDDEIFQDNKTKNNVDMINSKEDLLKNDLGLY
KKEKYNMRTYKYNSNNNNNNNNKNNNNNNKNNNNNNNNNNNNKNNNNNNNNKNNNNNNNNNNDCFGRRPIHFF
KHLNKHKVDVPSQTDNNLEDIFLNNIIHHENIKNLEIIWNNNEQKLKGESLILFLKNQIKMYKKELSIKNDEI
KSIQNIHKKNIDDIISSHILEISKLKEKHQADIFFIKNQHLENIVSVRKNFESKLNHIEEDLFLDLDILYNED
NGYVINEQINQIIIGEQNEREKYGTDEHIDNFLNNKEIKKCDMNNNVNNSNDHINNNNNNNNNDDDKINNNNN
DDDNINNNNNNDDNINNNNNNNNYCYYYNNTLKCSERANNIRDVNYLEIKDNNNLMIYEKDKIKEHTPNNDILY
NNISNEFIRNKDIPFKHEEHNLRNNIIIPHGLDDNPLKKLKFLWSYKNKEHEDNNKYIKEEMTHLKKIYNQVF
LEKTKEYVELYVNNKIFYDILQYIEKDNNVVKEDILKKNNEHYGIEEIKKRIYDIILKNNNESNTYMNGKKKE
ENEIVKELDENWLIKLLIYMNSLLQLERLRLEDMKIKQERNNGINNYIKNKNMKKKELIIDEEEEEEELEEDI
SSNEIHRLEKEMDTNEKIKMLKEKNRKLLSLNEFYKKKLEHIQVWKTKLEIMKKNKCNEVIHNENNMDNKETI
FFRKYKCEDDKGVSNKVEYTHNNNNNYSSDNNNNYSSSNNINYSSDNNNNNNYSNNLRNGFTSIWFPLIYLKP
VEEENKRDAELMYLLKMLGRYNNENKKNDIWSSKYEDNDSNTDYTKLINDTYNNDDNIFNFAYDENEYISCIK
TENKKSTHKCYNDNENTNYKNINVEDRKDNFLYAPYNLLNVLKKKLTYIFWQLLGDIYKYRNVIVETCNYIYN
IHNLFNEYINKYEKSEKKNEECYNKYMNDKEDDFLNEKYNLRKIIGVTKLKLEICTNEYMELKENYDKEKKRL
IMLTNTCYENECMKYKNTIQQFKNVDKQLKIYKAREKKWINLVKLLILELKNSSKTNQEDIKNIWIEIISTKK
EIINHNNIYMENNNTNDYFTIHKEDQGKGNNLLNDEKLFIQNINRHIDENNFPFEYNHDTSTIYNNNNIVKNN
DDLIVKNNKTNKKENRNKKNIQMKEKQNDKINFYKKDFHSFLDDIVKSELFHFYSYDEPNNFITNKINSHIDM
TSININTNEYYRHGDELYIDKDNMEDYNLYYNKKINHLYNNYILRNYNNNDNNDKNNNAHSNNIHNNNKHNNN
AHNNNTHNNNAHNNNTHNNNTNNTTKHLSNQIKDKLYEGNDFFSLVSSIAKESCLIFQILLKIKEDDIKEKTE
KITSLEKKLYLLKQYNINEKNKNKLLLAKQEILTNKINTLNIDKHKLQTHISSINKEKLHLKSHNLTKQQELN
LIITYYKNIIISIQKHINKYSTVLHLLDNIPQHDKDIILNLTNNHDEKCNNLIFSLHKQQKDDLKTKNEFSDQ
GSHDSSIQQTNLQLLKSKSTNENMITGQD*
>N.PF14_0455
MDVSNYEYLRSYGIKNELKRKRTHKKIIIYHLLDIIIFFLLFFSCYNFNLELCYKYEKAIFYNFFKSSVDLFL
LNVIRIIYTVILFRLHKKLTELNTLGKVYVLSRHITGILVILNVIKMINYSYVIKSENPLYNTNMYLITLKVL
FMVYSMISSIYYYFIQFKLYNIKKKYIIARVELEKILINDIKSKKYNIYKSDENSGLLGTDNNSTIMNNEYLN
LDYKNLLDMNISYNKLNEKINNDIINNTSDVQEKNMDYNDIHNFQKKKKSSNFAYLNFFHKESKDNKIDVKES
FLNKRYGSNKRSSKIYDNNNNNNNNNINSKIDYLENNITYTEFKKILLPYLWPSKRIDMKGNSSILRTYIVLI
FLFILVSKVFSVISPIYLGWASNEVLKKSLSSSVYYLGLYVTFFFISKFLKEVCGVLYSQVQQSAFIELQESI
FQTFHNLSYEWYSSKNSGGIMRIVDRGTESANNLMRSVLMYIIPATIEGLITCIIFIFKYKNSLLGSVLFIGL
TLYIYSTIKITKWRKKIRTKANEMDNVYHDIAHDSLTNYENVKYFSNEKFEIKKFCNALSNYHRYNLKILNSL
GILNTVQQFILNGTLFFTLLCVIYMIVKEGSDPGTFISVVVYTSNVFAPLSILGTLYATIIKSFTDISDLIDI
LRDKIDISNDKNLKNFDLTSQEKKFGVSIEFNNVHFNYPTQPLHTSLKDINIYIKPGTTCALVGHTGSGKTTI

SKLLYRFYDSKGEIKIGGRNINEYTRNSIRNIIGIVPQDTILFNESIKYNILYGKLDATEEELIQAVKSAQLY
DFIQSLPKKWDTLVGDKGVKLSGGERQRISIARCLLKDPKIVIFDEATSSLDSRTEYLFQKAVEDLRKNRTII
IIAHKLCTITTAELIILLNKGKIIERGTHLDLLKCNGEYTEMWNMQSKSNEPHTETNSSIDKDDVNKNNNKNN
DVILNTCKNDITTSFRSNSEKSSQEFSDASNHIKQSKTSNDHNNNINVHKKNEQEQLFLTNDKTDMDDNMNNK
KK*
>N.PF14_0456
MVKSYDRYEYEDCLGIVNSKSSNCVFLNNNNIISGHDECVGIWNINEKEIKKKLSVPFLPPYYFFTYHVTYIC
LNDVNKNIVAVGYTNGSIRLFDMEKNKILSTFSGHTSGICKLKFNENGNYLCSCSKDTNIILWDIINDKGVFK
LEGHTNVVTDIEFLQRKNDYLDDFLDNNILISVSKDCLIKVWELNIQTCVQTIVDCEEEITSLIINQTNTRLI
VACNSFLKIYKINLYSNHIIKDTYNTNSKIYITFLANIKRPTNCRIQNMKMIFFIDNEDIYNYPFELEDEKSD
NLLYSNPNNQKVNNLFISSSNGDNIKQNEEILTTDHLSNPNLLLTNNNNLNTSIKYCERARYSVDGDNSGLLI
CCTNLKKIEFYKINSIKNQKKSEKNKKKRYIEKLKKKKIKILNEKKKIEKFKGKESIDYNFLNQKLEDLEKEL
FIYNNYDIHTANDEIKYLFNYNYKFKLQNIDVMKKKKKDNYIYLLVSFMSNRLSVYQVNLYDILHNKDNFVVM
DNENKEEREDKDNMDRESDMKDHKSGNNIDSDIESGSDNDSDNKSDSNIDSDNESGNNIDSNIDSDNKSGSNI
DSGEDDETHNKKKVKIVQKKIYDYSKCFKEISEINKGHNSSVDFLNLSSNNELLVSICKKYVKIWNMNNLQNI
ITINLEGCTNAIFCNNDESLIISDECGYLYLYELKNIELKYTYKAHANKIINLSKKCNQKNNNNNYNNDNYYY
DHNDSSRNNDKGFLSVGEENYLKIFEYTMGVVTNESSDSEKDDNNNNNNNNNDNTYYNNNNDNTYYNNNNERE
KKSFIHNKRSNYKKNSSRDELNNDTYNKYYDDSNDVRKIENCEIFMFKEIDCYHLSDKVSCAMYSPDGKYICI
GYLNNLIEILYSDTLKLHLTLYGHSLPITCMDISKDNKILASSGADKFIFLWNMEYGNVNKRIHTECDVVNKI
QFFNKNNNLISISRDGYIKMWDAIKFQCICTVDGNFGILKCLVINHNDDFFLTSGTHKSIRIWKKGEDLIFLE
EERDKELNLQIEKEAIRNDLAYPSSVEKNVLLNKATIKTIETIKSSEKLIEYLDIIEEEIILLDTYYKNLTAY
EEAKGKNELPDFVQPPTKPLGRPELLNKDPHEFIIEIMCNIKNNILNEVLISLPFSYAYKLLDYIKTYLISFH
FFQKIQDNYKKYLSCGNFNFYVEYSINIVLTIINIYRNQFLFDNKFRFLLYELQQLILPHLKKSVDQCAFNQT
TLNFLMYSMDDDELNLDHILQNNSFINKETKKIDQQQGHAKGQ*
>N.PF14_0457
MKRKKSQKVSKETDDNIGGKKENVIDEEKDEISLLNNKGNEYIIYNPLNEYTINTELNHDEIFEQLNDRIYNK
LNENINYDLLEDEIFKELNEKIYEEIKENIQNDDKKYKEDIFNDNIFKELKCDNSEELTTNEKNKNINENVIP
FLLKKKLYEKNIAQIEQQIQNDKLNKMMKTKEIILDMLTIIKNRKLNIL*
>N.PF14_0458
MNKDILDNIKELPQFKLLCKKKSDLLKYFESCNRNKYDEIWMTLHTSLVEYISADNLIYDEEKSTLLFKEENN
RQYLLTSILFVSIYLQYLHNKTQKKGITFEDDFQILFCKLIEIQFMLSDKEVRLSFGKCLLTICELNIKENEF
TNNVKINLLLYLLWKCCHIEGKGTDITKLKKFKDFCKYINWGISERTTDSFYILCSYTLNLPKFYETSDGKIF
LSHVWSQNESIAYHLFNKFVHNTAVLAHNHICHYSEIIYSTWRNCEEDMKEILYTQIEYLVNFSLKCPIKIAA
RFRDVLSIFHTNKGDKDINRLIFKIYDPVIWRSLMCPNWKIRFNATCIFQLIYPVVDPGIKDINYLQEMEKAY
NALLDLSEDKNTYVLQATAKCICYILSELWEIISHDKRMKLIDILINKFLKEKCNECVRVEVVLGFCEMSKNP
MIRKIILKIFDRIKYLINDNSLRVRKNFIMLILDLQDYLKDTFSYDIDFNELIKKLTKDFITFNVQSCIKKMS
YMKYEYHDKLKNKEMYEYLKLSTNLISYSIWKCDIKEQAMKCINLLNEYPVLMICISKFSTNVNLIDRYKLSS
VLFEITNSKLKEENNFIMLNKTNQDIEDKKDKYDKHDKQDKKDKYDKHDKHDKQDKQDKYLIMDKNDNKNIYI
NNNINIILESDSNLKKRYIRYSTLLICIANFLKPRNEEEIELCYSEEIEEFLKIKFKELYFVESINTIMQPFY
FKVLKNIYLDYDNYYMNIHTYSTNELNNLYTMKNLYLCKTIIIPLFYKWKLLNIYIIDHLKFLNISMECIIYN
INKCISNVDSHYYHLNIDKHTFQVIEQNVIELINTHKGYNDNTNNNVNTQKRHIHHMSNIMLDYNELKDASIN
KQKELNCLIFLSLVVKKKKYHHILFKPFPYVIYNIIHKFNNFLLHIFYNISLHDFELPSHFLSAYYKGGNKKN
DTENLILLILYNKKEIKLYIHIYVSFFFLFHSYCTHNNILYEWTDLIQNTSKCIEHISNIKFKNEIQLKLYNV
TSREDIHNKDEQQNKMKWANYTSSIIYLITYFLDMVEYTISMKMISIDEVDINIMTYNLFLFYKCYEIVCDTE
NEIKLIYFTVWYRIYSFIICMLNLGYFEKKTEIKLAVLNTFFLFTYEFVPNIYIENFMKKFACIIKEKDIFQN
FLSNFKNNSDVLNYMSNNQRSKIQQVIDKIIFQKEKIKNKT*
>N.PF14_0461
MATIEKNKNVESEISVFYKNEKKNKSTKVREHSSIVNDEKKEVEFNSLNENIKEQGYGNVFNMNSQVSGVVNI
SDEVDKIGKVSKAGKNIKAGKSSKGGKNDENKNSNSNNNNNNNINNMNDGDDKDCANYVHNMNDRNNLNYINE
PCDNKKNTEGYDENILNDEGNLSDEGEEVEEENVDERDLILQRYKHGINISLNFSDIRQRNKIIDFYSECLDK
YKNECNFVYYQNYNNTNNVIINDTIDMSPINTKYFMNEQLDPINNLSNSNDKCVNSKNKNLIKYANNNMTIIN
NNGNTNENMNTNINANINTNINMNTNINMNTNINNNNNNNNNNNNNNNNNNNNNNNNNNNGIGDNNNNINNVNIS
NNAYPNDPFNNANNSLPYVDDFKNFDLQKKKQESLDPLLKCINSLSDRINLQHLVGDPTTYFKIGGGDMFYDI
NDPFLDDEEMYNELNKTKNEIILTRQIEDEYSVWSGDMSDDYIDINPDYFISFYSSKCKYVFTSDDEEKLEKK

QTQIQKQKQNINDKCAVPSRGTCDDDIINIDDDDNNNNNNNNNNNNNNNNNNNNKNNSYANTKNKKYYDNYNNP
NDDIISSSSYSGYDSTDDGTLNKYNFKGNFEKYKYDFIIYSSDGDLDSCSSSSSEDEHSDESDVSEEEIIFNP
FAWKKFEKHIPCEFIELFEKMEKQLDMLPLEADISEIKHIIKKYIYNIFSQVVNKCKNVIHHFNKPLNDFLEI
DIKLLRWLTTIMIKTSNSLNDIDICRMWFEQVFSYNTRVYIEYEKEFIARIKTSKIFEKNNKQHMNDLLNNIT
LWRKYQEDKEKESLSKDMSEINNIPDSNNNTTTSNNNNNNNNNNNKIVKKNKNNNNNNHESNEENQNSFIKDE
DNYDNNNNKNNTETNSHTYNNNYSGNSIIKNNNGNVTNLENKSNHSNGIMNIVKKGDNSGEQSKSKFMNIKKE
SDNNDNTSLYLSNHSTMAQMDNDKFVSDEGTAANNINVANNNNNNHNNNNNNYSQKDSHTNKYCNDLSGDMAD
YQYDDSNKNKNKIEDKRKDNKSNNNQHNNLIIRNEYNKMDDNKNNSSKDEEDTNNEDDTLEPDNSIHDNNNNN
NNNNNINNNINNNNNNNEYNIELEKYFDILSDISLDILNYIKMYTKQKSLLKEMISAGFEALVEKQYKFFMKL
PYITVSEMLTNLFNFRYKRNITIAYDYVESLIGFYQDKYKIDISYERGKNKKVFLFDTTVMQKLNNLYYKNKY
NPVKDKNVSNNVSKDENKGKNNNVTNNKPNIDERNNINRKKYIHNFKNDIEGTTDIQSNKNYLTTNKKHGLIK
NNDVTNKKKLNDNYKSNSISHHMEGNQHNQYRNNNKMDKTKCVNGNEIIICSDDESYEYDDEEGADYDDDGDD
DGDDDGDDDDDDDDDNDDDDDNNNDDEDNYYDDNNFEEIQRTKQKKKHNHEKSRNFNHNIGNNSNVTTDITNF
FDNLSKDEKKIYNHHNYNSTASSIISSNATTKYNMKNISNSVISQYIYHNTNSADIVKSIHTSNNIYHNKYKK
KNKYDDDQNFIVSDDKPHINSSYNLNEQNNLFKKRKLKNECLPNIYNKNNKYLEKNKTKSMDSNNKIQKTHNF
ICIISSEEENGC*
>N.PF14_0462
MYTFSSYFKIYYIYIILIILCFEKKVCTDDNVRNLNEIKDQFVEAYDLLDKAEYEKSFSILKNCVHYDIRCLT
LSGLFYYLGLKPVNRNVCNALYIWKVCADYGSSDAQFYLGVMYSNYFSLPNLYSYYYKENEIENNMILFRTYL
FLKAKNLEKQISDCLHNQKDEYDNYDDDDNYYYNDSIYDKDGEVYKKFRNVPIIHFNRNNELILKIKNIRYNI
DELENYFKNLNNFPSKNKNDNSINNLNNMNFMRNHNLSLLYFYSSSLGNHPASTLTLGVKYMNGYGVEKSCEA
ASRYYLKLTNEIFNFNEKNEFEIIDLIKLNVPYYDRYNMNNKKIKSIEIFLESSLHENHKILTMIARRYLIGI
DGIEKDYKKAHNYLIKASKYNNSEAISLLGYIYLLGLGVPINYDKAMEYFIKGNKLNDSLSHNGLGYMHFFGL
GSFRKNTNLAFYYFELASKNNLSSAQFNLACMYLSGVGVLQSFQHAFFWFYKSLNNGNLLAAYVIGFMHYNGI
IASRNCKLAVSLLSKVAESNSFVLNTTNKIIRYTEKGRNKEALFLMALLAETGNLQSQVNLSYQIQTSDFPLF
LPSNNIHTKKIYASRYLAMASEHNDFKSLYTLGDYAYKGYGLYVHIHPKNNLPPNQFYDLTTNTNIYQRQTSP
QKKKNKIKKEENVKDEKKQQNYGNVINNNNNNNNNNNDHVNKENNSPYIHQTNNAHQKNLPAIHNFDKIISTD
FVDEQGNIFNDKWRFNYYFKFSFNQVDYELAYHHYKSIISYYPSNFHVIQTISRACYNLGYMHYYGIGVSKNI
EKALIYFNSSIKIYPSYKVPSVLFVLYINFHKYLYHFKKKINNFKKYFSFF*
>N.PF14_0463
MEKKWIREKCVEYNFNKMQNEETINEKDDEIMNKNSWHVIEERDEKYKKKENKIIINNNNGFVNKGNNIKINK
HNECINNNVNNIQSNKLQSNNIQSNYIQSNNIQSNNTFNISSEDFIDDTKIFNDDLSLKSEAHFNYIPNNIYK
DQDNQENCNTFHNNNNNNVDNSPVMYKEEIKSLKKENKNLINSNMSLNKSISINNINEQEEDIKKDLIQNEIS
TEININNNYDDYNNNYFSYNYVNNNDQLVSLQNGCDYKDRCIIENNVEDENLKLQTNKKRNLTNEYINKSYYE
NIKNLDSNNSMSFKGNIYQLPTRAPSICPSMVPNTSNYFMEKDKIKHKQNEKQIDNEDIKYKDHNEKECVYDH
RDDNNNNNNNNNNNNSKKDIYYYKDNKETYNYQEMSNNIVENQYTIGNMNNINNNTYKPINTHMLYDNKNDML
ITRQKKNLKLKKKKEKNKDKITINYKEKIIINDKEKEKIMKKIDKINKKKRLKLTNKYNNNYKLFKSFVFSLN
NPINFSPSSKEQDSNYMKYKTLKNEKYDFMNKNSTYKKFDQIFFSEDFITYDFQWVETDGVVSSYAYDFSFFD
ESDKCMVSLDIIDKDEEQDQEDNEKYNTCYNSHNEQAILHSNIPKTIEDSPNNENEIKYNIATNINNDMSNKI
HMNDYQKKEQNNKNNIDVKKESKLSKEYNNVDEEKKKNLYVENENDFVIMYSKIINIRFIYSTDMLKHYNHPS
INLFNTNNTHGTKGKDKSTNIISSNKNDKLMGKKIYLTNDDTTIKCNNNMRNDIFDEQSSQMVSNNFLDSINE
YHIETSNNKRNIMEKNGEDIFISDIKEDKDNNILIKQEDFEKKKKKKILNNKIKKHLNSMNVRIIIKEWYIDY
VLYPNSIPYIYVISINNNRYRLDKPLAQYFHIFTKVRQKFEITTRILKTLQVYPNMSYNRLISYLTLYNSIKK
KMKLINEEDQDVPLGYTKSEENESSEEIEKCDKNINNDVHIKTSNFFNLHDSNKINKLYSINDDEIYHQNIRN
EDNLIRNNNEFLSDNLSYYESCTSDFKERNIHHDEFDNIIIKQDTALTTKRDKYDVIQKNINLSEKNGTGVYI
MPDGSKSKKCSWGAPYAVFECDKKKIINLYNFIKKEIKLLECFNINHLLDTPFMKTLEEKYHAHYDKRSEQKN
KNVQHNDMNIIGSNNMVDVKDINFNADEKGSNIHHKDETNLYKMEKMESGEIDNNVFDKKDSNIHHVYNNNNN
NNNNKNNNEYSNDRCTLKHAEDVKIDIKRSGKKKKKMINEEHINRVKKTKEKEFNIKRQNEISNITFNHNINQ
IYELPLHIIVPLEHENILKIWSFFNNFKDDIFHTNISLKKIEDIFLFKRNIFNKETIYYSIINSTYLKDYVEK
HNLLYRLFLKISNNINKKKEKTFPNINIKDNILKSLLILLHYSLIRKKNASDIQNSDSKKKRKQEKYIIKNED
FNIYQMMDEYEFPLLNNFCNLELKKNIEMNTENINTDDMDDTNNNNNKNNDDNNNDDNKNDDNRVDNNHNNDD
NNNNVEEKKKMIPNDKMCTEEYKMKVLLLEDIKRQCIRYHNKNKKKFIKKYEMDRYSLYNTNYEENIYRINTK
RRKINKPLRKIRPIRQLRQLRKIRQIRQIKQINEKRKMRKKKRRRRKKKIIISRRRKINEQNDICSVNDNK
NIKIFSNKISKDYIYDDYTYSHINKMNYNNYISDTLSCINSEDSLLPLTRKRRRRKKKKKKRTRTRIRIRRR

SETISRIRRRSETISTIRRRNGTISRTRNRTLFNALYNSQNEYMFKELNNIQPNQILINESMNNYTPAENIYY
NNSTEPYINNNDIACNDNMNYDNNTFDLFNYEYTSFDNTYNSYQNEDYYMDDNTDIIFYSKDTAKYYMDTLSN
KQNDDVGKDEQILNDNKMLNENKMLNENKMLNENKILNENKMLNENKILNENKILNENKILNENKILNENKIL
NENKILNENDVTNKNQSNEENNLLIEQDNEQICKNEKNSNHINVIQFEENKEYNKEDVKEKLHENESFHNEDE
TYTSSICEEMPSDLNPYNERQEYEEEITNDNYKNILSNVFLHRNKKKMIFEKYDDIRILDNISWTILLKKYIF
IILYTMKHCIVFNDKEEKKADDKLGVDKGDLINKCNIMDKCEPVDVYDKKRENIKNDIECVVTKNDENITLSD
DIIKNEENTHKNLLHNNLNPKDVINNNIEKEEVLYDTFIKTNDNTEIVDDRLSFFNDNLDVEDDKYYHFNMSH
CLNGEKNKKNVFNNLNSLYSINLDNINLDSINLKKYKLMWLLNVNKTYLNYNEIIDLIHIWNFLCKENNTYEM
LNETDKMKIIMFFINIIRDSNICNEFVKKNIDTNKTFLNLDQLNNSVNHVLINEKNDSNMCATNKEIDTNKLL
LVKYEMKNLFTKGEEANIVSNVNNNKGINNYEPVSINQKECEKIYMPMESSLLKETSDHTNYKNGGNVKSDSN
NNNNNNNSSSCSNNNICIMQNEYLGNDRYDNKYYYLADIFGYINRIYVLYNNNKNIINDENKKLHTEHNNNNN
NNNNNNNNNNCCNISVEIMNSFVFCPIHQNANTTKLSKYNINIGYIEGIQNIDLFINNFDTSIYNETNLKKKF
IQIKEIIHNEQFKVLKKNFKKEKCNIKKKVNDKNYNCNDLEGTIKDIDDEESLKDTTHFILDHPICENKKNFD
KDYKEKELAKLPTKRSNGDNFTPVIKGKENKTNHNKENNVIKKSNVKSKTQSTQSKTHSTQSKTQSTQSKTHS
SQSKTHSSQSKTHSSQSKTQSTQSKTQSTQSKTQSTQRKTQSTQSKTHSTQSKTQSTQSKTHSTQSKTKSTQS
KSDKKDLKENIKTKEKIIYFTENEKLFEQVFYICEKILHFRKEINNLLSVTYERQIDRKIYNFINYSMHHIIN
GTVTSNTIKLIFNKYIHILYIIEKCMCSNTPCYFIKKKWITTFHKQWIDDIELINNQIDDYMDTLNIFDILPL
LNIYFNFFYIYGINVKALYKWKTYNIEHRILTKYKEESIIWTNEVKYLEKFFMKDYNKNYEKMMARRNKKNQT
EQKLKDQQEDKVYNNGNHEQQNDAEHKKNDDSIIQGDQKICHDDHLNDDQKICHDDHLNDDQKICHDDHLNDD
QKMSDDTKINDDQKKCDDTKKDDDESESEKSTDEEDTEDEIIIQKRKKMNVLRKNLYEYMPFQKNEKYFYFKE
GHMEHINKFKDYHTIYKKEDNIDEFASVEQLHVHDIKIFFIPKIYKENEEDNKNKQKKKKNDTTIQQQNILLH
THQKEENPLHGKIIRNNNYYHNYNNCWKCTKGQYPIHNMTCTKIKKKKKKNADIRKDYINNNNIEYTFQELQL
TQNMKKKKKQKIKNKKHETFHIINNDMINDHIYHKLYNDIFEIKNKIQNNHNIMNEKKVNIYNSKNLTYLGNL
TNTIDLRINDNLSTLYNDLIHNDQNIIQHNDINNNQNYTNQNNNNITNQNQSCLLLCQLHCTIVSRQHEIADS
LYKKLNFGNKNVTLPNNIKKNTNIYLFNLYFDENMKTLLYIIPSKK*NNLKKIKIYIKTDIFFFLYINTYNYN
AYSARIRRVKIKKIIKIMKI*THICFNMNI*CIIYDPQHLWKCVTVKKDTQKFNKNSINQKVNFWELTKKKNV
*
>N.PF14_0464
MSDIYFYSEEINNLLEDFKRILQELEKEEVKNEKTEKYCNDIHFINERIKTAKDAYFIEIRNLSEEEQIENIS
KIKNKMIILENLNIQFDFKKSKLIYEEDKQAKENKKLLKVRYVSAKDIETKGDIIQDQTDDAIFRMKMMVDES
ENITKDAADKLNIQNEKLQKARDKIEDVDINVYSAKQTLKEIAKEAVTDRFVRLMSILIFIVVSVLITVIIS
RKQTPVVIHDVMTENEHIRKNKSVRRQEMSPKLT*
>N.PF14_0465
MSTVINYEKFEKIDISSSDDDNKKKLNPHLTKLDNKDKVVIGPNGLTILKNNNDEKIKHDKYNNTSKNVEEIK
DKNDEYKTIYNETNNEILNNKNDNIHVQNMNNYVADDKKYKDMFINGAVVPYEYIWNQSIHYINAFICLPLNC
KAKNLIVDIKEDKLIVKIKNYKCGINYNNNNNNNNNNNNNNKTNQHNTQSNSFHDNVEILVDKKFPFKINTDE
DTQIWEIKNMQINWYTLFNLSNEINNQNVHYLFEDYNMLDKQETFLYISVKKINEIENAYIWWSTLFKNEHPI
KIENLPSRSSTNKAKKTMNSFKTAWDEAHEIFKKNISMKQLPFRIDSS*
>N.PF14_0466
MLFNKFFKHDTFHITLSKIVIRNYKTSKKINIGKLITKNKIKNHEKHSIEDIELLLHEKHHDVLESHPTIKNV
NKIIDVNYLPVFKKNENQFNILNKIVLHFGDLSYLKGDAVVNGTNKVFELMKEGNGYDCSGNFLKTCGDELLN
DMKNAKEQNKGKSIILTKGYNSSYKHIIHVVEPYYNQTQKLKNCYENILLTAKDNNFKNIVFPLLGSGISLFK
KHDVVISCLEGIYEFMKKRDNFNAIEKIILCTITDSYWMLLRDSIYLYLDINI*
>N.PF14_0815
MEKLDTVTNESPKMNKLRRSLTMSLYFIGISTVSWFICTTSRRKEWADIMLDYVHHKRCSFLSNSWASFLKRL
KS*
>N.PF14_0467
MNKFTYCITLLLIALVLPIRSIQLHSVNYAPLNYIEIGDTLSNKVGELWHSHLNSFIDIVSTKVVNKLEDDLSN
GNNLEKFMILLEDDADIFDASAYEGKNLALIQTEFIRKLKDKFKNSKFGQKLKKLGSKAKEKLLSLYYKHKGK
LRHFFSILLKSLVIPIAVQFIRKNLNKWKQRTLEATQKLDEQSKNIAQPIINRLYNSFEDKIEEYSQENKINV
GDELDALGQLDKDKQDIQKLEEQEKALLQ*
>N.PF14_0468
[truncated: 443,461 more chars]
